# Reconciling Mining with the Conservation of Cave Biodiversity: A Quantitative Baseline to Help Establish Conservation Priorities

**DOI:** 10.1371/journal.pone.0168348

**Published:** 2016-12-20

**Authors:** Rodolfo Jaffé, Xavier Prous, Robson Zampaulo, Tereza C. Giannini, Vera L. Imperatriz-Fonseca, Clóvis Maurity, Guilherme Oliveira, Iuri V. Brandi, José O. Siqueira

**Affiliations:** 1 Vale Institute of Technology - Sustainable Development, Belém, Pará, Brazil; 2 Environmental Licensing and Speleology, Vale, Nova Lima, Minas Gerais, Brazil; University of Waikato, NEW ZEALAND

## Abstract

Caves pose significant challenges for mining projects, since they harbor many endemic and threatened species, and must therefore be protected. Recent discussions between academia, environmental protection agencies, and industry partners, have highlighted problems with the current Brazilian legislation for the protection of caves. While the licensing process is long, complex and cumbersome, the criteria used to assign caves into conservation relevance categories are often subjective, with relevance being mainly determined by the presence of obligate cave dwellers (troglobites) and their presumed rarity. However, the rarity of these troglobitic species is questionable, as most remain unidentified to the species level and their habitats and distribution ranges are poorly known. Using data from 844 iron caves retrieved from different speleology reports for the Carajás region (South-Eastern Amazon, Brazil), one of the world's largest deposits of high-grade iron ore, we assess the influence of different cave characteristics on four biodiversity proxies (species richness, presence of troglobites, presence of rare troglobites, and presence of resident bat populations). We then examine how the current relevance classification scheme ranks caves with different biodiversity indicators. Large caves were found to be important reservoirs of biodiversity, so they should be prioritized in conservation programs. Our results also reveal spatial autocorrelation in all the biodiversity proxies assessed, indicating that iron caves should be treated as components of a cave network immersed in the karst landscape. Finally, we show that by prioritizing the conservation of rare troglobites, the current relevance classification scheme is undermining overall cave biodiversity and leaving ecologically important caves unprotected. We argue that conservation efforts should target subterranean habitats as a whole and propose an alternative relevance ranking scheme, which could help simplify the assessment process and channel more resources to the effective protection of overall cave biodiversity.

## Introduction

Decisions about which lands to allocate for conservation often involve conflicting interests between industry, local communities, and environmental protection agencies [1]. Such conflicts can be particularly acute in the mining sector, given that mineral resources are not uniformly distributed but rather spatially clustered, implying that the allocation of conservation areas may be constrained [2]. A common alternative to solve these conflicts has been the use of offsets (conservation areas located outside development areas) to compensate for the residual and unavoidable impacts of development projects on biodiversity [3,4]. Offsets, however, are more difficult to apply in cases where endemic or threatened species occur inside areas containing rich mineral resources.

Caves pose significant challenges for mining projects, since they harbor many such endemic or threatened species, also known as *short range endemics* because of their small distribution area and assumed restricted dispersal [5]. For instance, different troglobitic species (obligate subterranean dwellers which must complete their entire life cycle in caves [6]) have halted mining projects worldwide [7], resulting in economic losses rising to billions of dollars [8]. Brazil has one of the most stringent cave protection regimes in the world, requiring extensive speleological surveys prior to the implementation of any development project [8]. The consulting companies performing these surveys must then assign caves into one of four relevance categories (maximal, high, mid, or low), based on a complex set of biological, geological, and cultural attributes. Caves containing rare troglobitic species, for instance, are always defined as *maximal relevance* caves, which must be protected along with a buffer area of 250m [8]. *High relevance* caves, on the other hand, can be impacted if appropriate compensation offsets are provided (i.e. preserving two similar caves).

Recent discussions between academia, environmental protection agencies, and industry partners [9], have identified problems with the current Brazilian legislation for the protection of caves (Federal Decree 6640/2008), highlighting the need for novel quantitative approaches to determine conservation priorities [8–11]. First, the criteria used to assign caves into relevance categories are often subjective, given that the factors influencing cave biodiversity remain largely unknown [12–16]. Second, the relevance classification process is long, complex and cumbersome [8]. Any cavity with over 5m of extension must be considered in speleology surveys, so these take between one and two years to complete, and involve intensive field work and considerable resources. Third, identifying caves for compensation has become increasingly difficult, because most caves must be compensated [8]. The great majority of caves are classified as *high relevance* caves, since caves exhibiting any one of a long list of criteria must be classified so. Finally, there are few taxonomists specialized in identifying cave fauna in the country, and they are asked to analyze thousands of specimens collected in areas of high biodiversity. For instance, most troglobites remain unidentified to the species level, some represent new undescribed taxa, and their distribution ranges are usually unknown [10]. In consequence, the characterization of threatened troglobitic species is often questionable.

Here we aim to help optimize the current Brazilian cave protection regime by: 1) Filling part of the knowledge gap regarding the main drivers of cave biodiversity; 2) Examining how the current relevance classification scheme ranks caves with different biodiversity indicators; and 3) Proposing a new scheme to establish conservation priorities. We first perform a large-scale quantitative study relating iron cave biodiversity proxies to different cave characteristics. We analyzed data for 844 iron caves, which contain higher richness of troglomorphic species than caves of other lithologies [14]. The data was retrieved from different speleology reports for the Carajás region (South-Eastern Amazon, Brazil), one of the world's largest deposits of high-grade iron ore [17]. Several mines are already operating in the region and the world's largest iron-ore mine (project S11D) is about to begin operating, so there is now a pressing need to achieve a compromise between mining and the conservation of cave biodiversity in the area. We then examine how the current relevance classification scheme ranks caves with different biodiversity indicators, and discuss the implications of these results for the conservation of cave biodiversity. Finally, we propose a new scheme to establish conservation priorities and estimate environmental offsets, which could help simplify the assessment process and channel more resources to the effective protection of cave biodiversity.

## Materials and Methods

### Dataset

**Fig 1:**
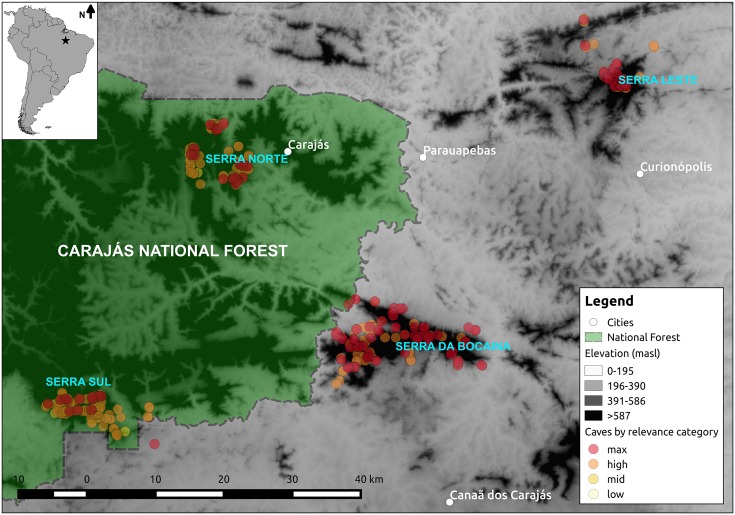
Location of the study region (upper left corner) and zoom of our study area showing the spatial distribution of the caves included in our analyses over an elevation layer. Caves are colored according to their relevance category (N = 843 caves), and the Carajás National Forest is shown in green. The digital elevation raster (SRTM, 1 arc-second) was obtained from USGS Earth Explorer, the National Forest shapefile from ICMBIO, and the South America shapefile from Thematicmapping.org. All layers are copyright-free.

We retrieved data contained in eight speleology reports for the region of Carajás, State of Pará, Brazil (see Table A in S1 File). Together, these reports surveyed a total of 844 caves distributed across Serra Norte, Serra Sul, Serra Leste and Serra da Bocaina (Fig 1). All surveys employed similar methods, and reports assigned cave relevance based on the same set of cave attributes (those specified in the current Brazilian legislation for the protection of caves). We gathered all relevant data available on cave attributes and biodiversity indicators, including cave ID, coordinates, altitude, horizontal projection (length), slope, area, volume, presence of percolating water and water reservoirs, cave lithology, presence of plant material, presence of plant detritus, presence of roots, presence of guano, presence of other feces, presence of regurgitation balls, presence of carcases, presence of troglobites and rare troglobites, presence of resident bat populations, and species richness. Cave attributes were surveyed twice a year, during the dry and wet seasons, and total species richness was calculated as the total number of species found in both seasons. Specialist taxonomists classified species as troglobites if they exhibited troglomorphic traits [6] unknown in phylogenetically related taxa occurring in above-ground habitats. While troglobites found in three or less caves were considered rare, only groups of 100 or more bats were considered resident populations. Stygofauna was not considered in any of the speleology reports. In addition we retrieved from the reports the overall cave relevance classification, determined considering a complex set of biological, geological, and cultural attributes [8], as well as the biological relevance classification of each cave, determined using biological attributes only. Information on the richness of troglobitic species (for caves containing troglobites) was unavailable in some reports, and in others was either vaguely mentioned in the text or presented in tables with varying formats and nomenclatures. We thus only retrieved data on the richness of troglobitic species when this information was clear and explicitly made available (in 329 caves). Detailed descriptions of the survey methods and taxa inventories for all caves are presented in the original reports (Table A in S1 File and S1 Dataset).

We selected four proxies of cave biodiversity: Total species richness, presence of troglobites, presence of rare troglobites, and presence of resident bat populations. For each one, we constructed a complete-cases dataset (excluding observations containing missing data), to avoid fitting models containing a different number of observations. A principal component analysis showed that length, area, and volume were highly correlated (the first component explaining 95% of total variance). We therefore chose to use cave area as a proxy for cave size, because it reflects cave size more accurately than length alone, and its estimation is less susceptible to measurement errors than volume. All statistical analyses were implemented in R [18]. The full dataset, including the geographic coordinates for the locations of all caves, can be found along with all R scripts as S2 Dataset.

### Modeling species richness

To test if species richness was spatially aggregated [19], we quantified spatial autocorrelation in species richness. We used the R package *spdep* [20] to compute Moran's I, a standard measure of spatial autocorrelation ranging from -1 (indicating perfect dispersion) to +1 (perfect correlation, with zero indicating a random spatial pattern). Moran's I is affected by the spatial scale and the coding scheme style chosen to assign weights to neighbors. We thus quantified spatial autocorrelation across the full range of spatial scales of our data. To do so we computed Moran's I in networks of neighboring caves located within increasing distances, until we reached the maximal extent of our study region. We chose the “W” style, which standardizes rows and thus favors observations with few neighbors, but results did not change substantially when using different styles.

Species richness was modeled using a linear mixed model (LMM) with total species richness as response variable, all meaningful cave attributes as predictors (Table B in S1 File), and the report containing the original data as a random effect. This allowed us to account for variation between the different reports (arising due to different teams, timing, and geographic locations). We then followed a model-selection protocol, based on likelihood ratio tests (henceforth LRT), to find the best model describing species richness. We used the *lme4* package [21] to compare a full model (containing all meaningful predictors) to reduced models where each predictor was eliminated one by one. A predictor was excluded if the reduced model (lacking that particular predictor) was not significantly different from the full model (using LRT, α = 0.05). Predictors were eliminated sequentially, beginning with those showing the highest *p*-values. Likelihood ratio tests were also used to compare the resulting model to models containing all possible combinations of first-degree interactions between the remaining predictors. We then used the *nlme* package [22] and restricted maximum likelihood estimation to test whether including a constant variance function to account for variance heterogeneity improved our model. Finally, we tested for spatial autocorrelation in the residuals of our final model, calculating Moran's I across different spatial scales as described above. The final model was validated by plotting residual vs. fitted values, residual vs. predictors, by looking at the distribution of residuals, and by checking for multicollinearity. To evaluate the relationship between total species richness and the richness of troglobitic species, we included the richness of troglobitic species as an additional predictor of our final model, using a data subset for which information on the richness of troglobitic species was available.

### Modeling the presence of troglobites, rare troglobites, and resident bat populations

We first tested for spatial autocorrelation in the presence of troglobites, the presence of rare troglobites, and the presence of resident bat populations. We employed the Join Count Test of the *spdep* package to assess spatial autocorrelation across the full range of spatial scales of our data. We computed the Single Color Statistic for presence-presence in networks of neighboring caves located within increasing distances, until we reached the maximal extent of our study region. As in the case of species richness we chose the “W” style, but results did not change substantially when using different styles.

We then modeled these variables using generalized linear mixed models (GLMM) with Bernoulli distributed responses (logistic regressions). Presence/absence was thus set as response variable, all meaningful cave attributes as predictors (Table B in S1 File), and report as a random effect. As in the case of species richness we kept report as a random effect in all models to account for variation between the different speleology reports. Likewise, we used the *lme4* package to compare full models (containing all meaningful predictors) to reduced models where each predictor was eliminated one by one, using likelihood ratio tests. We compared the resulting models to models containing all possible combinations of first degree interactions between the remaining predictors, and also tested for spatial autocorrelation in the residuals of our final models, calculating Moran's I across different spatial scales as described above. Best models were finally validated by plotting residual vs. fitted values, residual vs. predictors, by looking at the distribution of residuals, and by checking for multicollinearity. To evaluate the effect of species richness on the presence of troglobites, the presence of rare troglobites, and the presence of resident bat populations, we included species richness as an additional predictor of our final models.

### Reassessing cave relevance for conservation

In order to quantitatively assess how the current relevance classification scheme ranks caves with different biological characteristics, we scored caves according to the number of biodiversity indicators they contained. These indicators comprised high species richness (higher than the upper quartile of species richness per cave, across all analyzed caves), presence of rare troglobites, presence of troglobites, and presence of resident bat populations. We assigned a value of 1 or zero for the presence or absence of each indicator, and then added all values to obtain a total score, representing the number of biodiversity indicators found in each cave.

## Results

**Fig 2:**
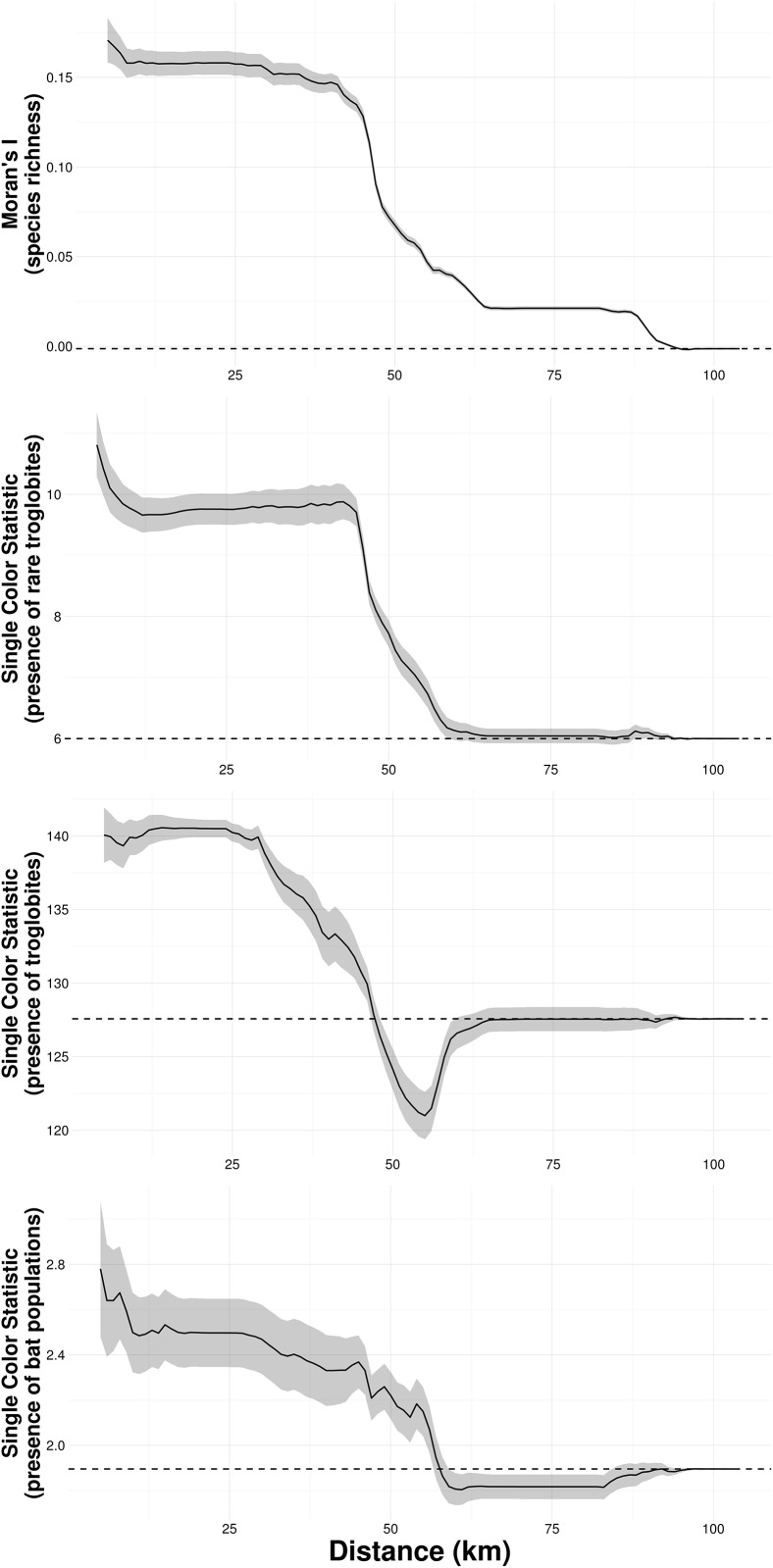
Spatial autocorrelation of species richness, presence of rare troglobites, presence of troglobites, and presence of resident bat populations, across different spatial scales. While the solid lines show the value of estimates (Moran's I and Single Color Statistic), the gray area depict 95% confidence intervals. Dashed lines represent expected values under a null model of no spatial autocorrelation.

While we found significant spatial autocorrelation in species richness across most of the analyzed spatial scales, the presence of rare troglobites, presence of troglobites, and presence of bat populations only showed significant spatial autocorrelation at small and intermediate spatial scales (Fig 2). In all cases spatial autocorrelation decreased with increasing spatial scale, and did not differ from the random expectation when reaching the maximal extent of our study area (Fig 2). No spatial autocorrelation was detected in the residuals of our final models (Fig A in S1 File).

**Fig 3:**
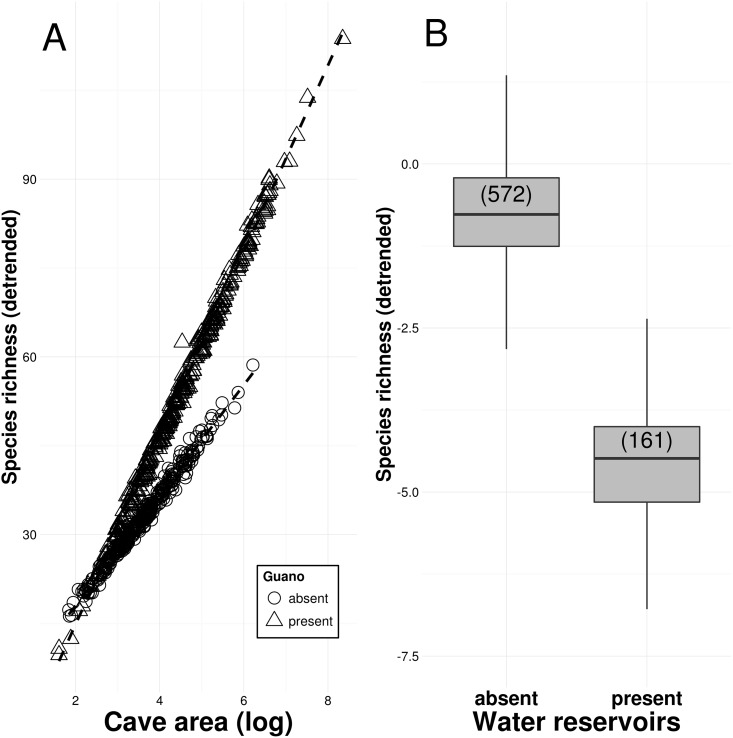
A: Relationship between species richness and cave area in caves with guano (triangles) and caves without guano (circles). B: Species richness in caves with and without water reservoirs (sample sizes are given in parentheses). Species richness is detrended for the effect of other predictors.

Cave area and the presence of organic material and water reservoirs were found to be key predictors of cave biodiversity (Table 1). Specifically, species richness increased with cave area, and this increase was more pronounced in caves containing guano (Table 2, Fig 3A). Species richness was also higher in caves containing detritus, roots, and resident bat populations, but lower in caves with water reservoirs than in caves without them (Fig 3B). Larger caves were also more likely to contain troglobitic species, rare troglobites, and resident bat populations (Table 2). However, caves with water reservoirs were less likely to contain rare troglobites. Species richness was positively associated to the presence of troglobites, rare troglobites, and bat populations (Table C in S1 File). Moreover, total species richness was positively associated to the richness of troglobitic species in a data subset for which this information was available (Table C in S1 File).

**Table 1:** Summary of the best models describing cave biodiversity.

Response	Model^a^	N^b^	Predictor	*X*^2^	*p*-value
Species richness	LMM	733	Interaction Area (log)—Guano	18.45	<0.001
			Water Reservoirs	4.42	0.04
			Detritus	12.89	<0.001
			Roots	19.63	<0.001
			Bat population	14.62	<0.001
Rare troglobites	GLMM	833	Area (log)	57.99	<0.001
			Water Reservoirs	6.25	0.01
Troglobites	GLMM	833	Area (log)	79.20	<0.001
Bat populations	GLMM	833	Area (log)	118.59	<0.001

Response variables are shown followed by the type of model employed (Model), the number of observations included in each model (N), the predictors included in each model, *X*^2^ values from likelihood ratio tests (LRT, in which the full model was compared with a reduced model without each of the predictor variables), and *p*-values of the LRT.

^a^ Linear mixed models (LMM) or Generalized Linear Mixed Models (GLMM). All GLMM had Bernoulli distributed response variables (logistic regressions). All models accounted for possible variations between reports by keeping report as a random effect.

^b^ Sample sizes vary between models because a different set of parameters were assessed in each report.

**Table 2:** Parameter estimates and hypothesis tests for the best models describing cave biodiversity.

Response	Predictor	Estimate	SE	*t/z*-value	*p*-value
Species richness	Area (log)	9.32	1.19	7.83	<0.001
	Guano (present)	-15.99	6.01	-2.66	0.01
	Interaction Area (log)—Guano	6.41	1.49	4.3	<0.001
	Water Reservoirs (present)	-3.88	1.85	-2.10	0.04
	Detritus (present)	5.61	1.57	3.58	<0.001
	Roots	16.70	3.76	4.45	<0.001
	Bat population (present)	22.26	5.52	4.03	<0.001
Rare troglobites	Area (log)	0.85	0.12	7.28	<0.001
	Water Reservoirs	-0.81	0.34	-2.41	0.02
Troglobites	Area (log)	0.69	0.084	8.26	<0.001
Bat populations	Area (log)	1.65	0.19	8.77	<0.001

Response variables are shown followed by the predictors included in each model, estimates, standard errors (SE), *t* or *z*-values and *p*-values.

**Fig 4:**
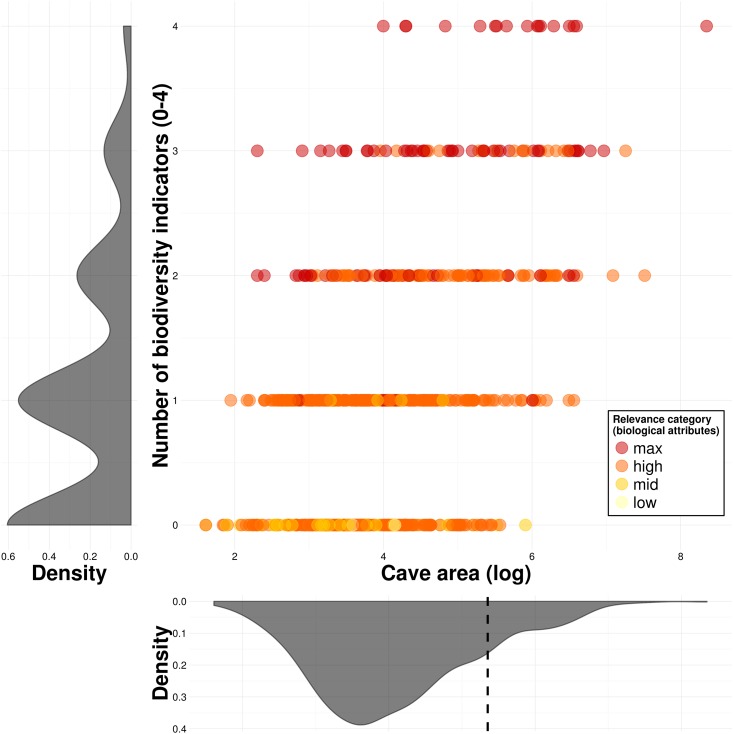
Relevance classification of caves with different numbers of biodiversity indicators (high species richness, presence of rare troglobites, presence of troglobites, and presence of resident bat populations). While variation in cave area is represented in the X axis, density plots are shown besides each variable (the vertical dashed line represents the median area of caves containing three or more biodiversity indicators). The relevance categories shown are based on biological attributes only, following the current Brazilian legislation.

Large caves containing high species richness, were often classified as *high relevance* caves in the speleology reports (Fig B in S1 File), as were caves with a high total species richness and a high richness of troglobitic species (Fig C in S1 File). All caves of *maximal relevance* contained troglobites, and all caves containing rare troglobites were classified with *maximal relevance* (Fig D in S1 File), as stipulated in the current Brazilian legislation for cave protection. When assessing how the current relevance classification scheme ranks caves with different biological characteristics, we found that caves containing zero or one biodiversity indicator are often classified as *high relevance* caves (Fig 4). Moreover, many caves with two biodiversity indicators were classified as *maximal relevance* caves, while not all caves with three biodiversity indicators were (Fig 4).

## Discussion

Our results reveal spatial autocorrelation in the four cave biodiversity proxies analyzed (species richness, presence of troglobites, presence of rare troglobites, and presence of resident bat populations), and underscore the importance of cave area and the presence of organic material as key predictors of cave biodiversity. Larger caves had higher species richness and were more likely to contain troglobitic species, rare troglobites, and bat populations. Additionally, our data shows that the current relevance classification scheme does not prioritizes the conservation of overall cave biodiversity.

Cave biodiversity is thought to be determined by an island biogeography dynamic [23], whereby small and isolated caves have higher extinction, lower colonization rates, and fewer available habitats than larger and inter-connected caves [12,24–26]. Indeed, species richness was found to be spatially autocorrelated, suggesting that caves containing many species facilitate the colonization of nearby caves. Previous studies have shown that cave size is a key predictor of cave biodiversity, because larger caves not only have higher colonization rates, but can also host larger and more diverse communities. Analyzing seven caves from Slovenia, Culver et al. 2004 [12] found a higher richness of troglobitic species in larger caves, whereas Brunet and Medellín 2001 [25] found a significant association between the number of bat species and cave area in 20 caves from central Mexico. Studying 55 limestone caves located in the Brazilian Savannah, Simões et al. (2015) [16] found a positive association between species richness, width of entrances and linear development of the caves. A similar relationship between cave size and species richness was found in a study analyzing 91 caves of different lithologies from Southern Brazil, with iron caves showing the strongest effect [14]. Preliminary studies on iron caves also found higher species richness and higher richness of troglobitic species in larger caves [11,27]. Our results go in line with these previous findings, reaffirming the importance of cave area as a key predictor of species richness, the presence of troglobites, rare troglobites, and bat populations.

Our data also supports the idea that a higher availability of trophic resources facilitates colonization of the cave's interior [11,13,26,28], as species richness was higher in caves with detritus, roots, and resident bat populations, and the relationship between cave area and species richness was found to be stronger in caves containing guano. For instance, the cave's deep interior has been compared to a desert [29], since it is largely deprived of trophic resources [6,13,30]. The fauna that inhabit the cave's interior thus rely on external material that is washed into the cave or brought in by mobile species [13,28,31,32]. In our case, detritus might have been brought inside the caves by bats, as guano, or may have been washed in by water. Interestingly, our results reveal that roots also seem to constitute an important food resource for the fauna of iron caves. Indeed, cave root communities are known to include many rare and highly specialized species [33], and they are considered one of the main food resources in iron caves [11,14,34].

The importance of bat colonies for cave macro-invertebrate communities has been well documented. Bats provide nutrients and water resources through guano, respiration and urination, which can support diverse cave arthropod communities [13,35–38]. These resources are especially important in iron caves, which rarely receive additional resources from permanent streams [14,39]. Our findings highlight the importance of bat colonies as pillars of iron cave biodiversity. Additionally, bats are important providers of ecosystem services. As mobile agents [40] bats contribute to pollination [41,42] and seed dispersal [43–45], thus mediating plant reproductive success and enhancing the restoration of degraded habitats [46,47]. As predators of pest insects, bats provide biological pest control services to many commercial crops, including cocoa [48], coffee [49], cotton [50], and corn [51]. Bats thus have a key ecological importance both inside and outside caves, so conservation efforts to preserve bat populations should not be undermined by the focus on rare troglobitic species.

We found lower species richness in caves containing water reservoirs than in caves without them. This result was unexpected, and contradicts earlier findings showing higher species richness in caves containing permanent water bodies [16,27]. One reason for this apparent discrepancy, could be that the water effect found by these previous studies was driven by the effect of cave size (i.e. caves containing water bodies were usually larger than caves without them). Indeed, the effect of water reservoirs on species richness was not found to be significant when we ran a simple mixed model (containing the presence of water reservoirs as the only predictor), although it was significant in our final model (which contained six other predictors, Table 2). One potential explanation for the observed effect of water reservoirs, is the fact that some can occupy a relatively large area inside the caves, thus reducing the effective cave area. As the speleology reports analyzed here focused exclusively in troglofauna (ignoring stygofauna), large water reservoirs are expected to undermine the effect of cave area on species richness. Alternatively, water bodies that are subject to flood pulses can eventually be detrimental for the establishment of troglobitic species, and thus imply a reduction in species richness [16].

The occurrence of troglobites and rare troglobites was found to be spatially aggregated (i.e. neighboring caves tended to either have or lack troglobites). Analyzing over 3000 records from more than 450 troglobitic species occurring across the eastern United States, Christman et al. 2005 [52] also found significant spatial autocorrelation in the total number of troglobitic species, the number of non-endemics, and the number and occurrence frequency of single-cave endemics. Our results thus support these earlier findings, and suggests that troglobitic species can move between caves separated by up to 40 Km (Fig 2), although the dispersal mechanisms remain unknown. The presence of water reservoirs was negatively associated to the occurrence of rare troglobites, which suggests water is enhancing connectivity between caves. Indeed, Simões et al. (2015) found that the community of troglophile species was more similar between caves containing streams than between dry caves or caves with puddles. Similarly, Pipan and Culver 2007 [53] identified lateral epikarst connections based on the distribution of copepods, indicating that water in shallow subterranean habitats facilitates dispersal in these arthropods.

The banded ironstone formations known as *Canga*, where our study caves occur, are constituted by highly porous rocks that form many micro-cavities and cracks [14,34,54]. Preliminary evidence indicates these are potential subterranean habitats that could serve as dispersal corridors to some troglobitic species [55]. Alternatively, these cracks and crevices might actually represent the primary habitat for troglobitic species, with caves serving as convenient sampling sites. For instance, troglobites were also found in small caves (Fig D in S1 File), which were common in our dataset (median cave length = 16.40m; mean cave length = 31.01m; see distribution of cave area in Fig 4). Since small caves may not comprise suitable stable habitats for obligate dwellers of the dark zone, our results suggest that troglobites are using subterranean habitats other than caves. Our findings thus provide additional evidence for the permeable nature of banded ironstone formations, and indicate that iron caves should not be viewed as isolated entities but rather as components of a cave network immersed in the karst landscape [34,52]. Such broader perspective would imply shifting conservation efforts from individual caves to subterranean habitats as a whole.

We propose a simple but effective scheme to establish cave conservation priorities and estimate compensation offsets. Using a set of cave biodiversity indicators, including high species richness (in our case higher than the upper quartile of species richness per cave, across all analyzed caves), presence of troglobitic species, presence of rare troglobites, and presence of resident bat populations, we ranked caves according to the number of indicators they exhibited. We decided to assign an equal weight to all biodiversity indicators, but weights could be modified if particular indicators need to be prioritized. Conservation priorities and compensation offsets can then be established based on these ranks. In our case (Fig 4), caves containing three or four indicators are considered the most important caves for conservation (maximal relevance, 10% of all caves), followed by caves with two (high relevance, 17%), one (mid relevance, 35%), and no indicator (low relevance, 38%).

Our proposed scheme is appealing for three reasons. First, it constitutes a shift from a reactive approach focused on rare troglobites to one that supports a more complete suite of conservation priorities, as stressed out in the precautionary principle [56], and recommended by recent analyses of current conservation initiatives [4,57]. For instance, preserving ecosystem function is also important, so a cave containing a rare troglobite may be as important for conservation as a cave lacking rare troglobites but containing high species richness, many troglobitic species, and a resident bat population. Second, because it involves few biodiversity indicators, which are already measured during speleology surveys and constitute proxies of cave biodiversity, it implies a more efficient allocation of resources towards the preservation of the most relevant caves. Even though our approach is considerably less complex than some reserve selection algorithms [58], we believe that simple indicators may be more appropriate when the taxonomic framework (and hence species range information) is so poorly developed. Finally, it represents a better compromise between cave protection and the extraction of mineral resources than the current cave protection regime, by which an average of 70% of the surveyed caves are assigned high relevance (according to our dataset), and must then be compensated by offsets [8,9]. Following our scheme only 17% of caves would need to be compensated by other high relevance caves, a more realistic requirement that should be possible to fulfill.

Our relevance ranking scheme demonstrates that the current Brazilian cave protection regime does not allocate maximal conservation priority to caves exhibiting more biodiversity indicators (Fig 4). The strong emphasis on rare troglobites is leaving ecologically important caves unprotected, while protecting less relevant caves that contain rare troglobites (Fig D in S1 File). This is further aggravated by the fact that the rarity of these troglobitic species is questionable, as most remain unidentified to the species level, and their distribution ranges are poorly known [10,11]. For instance, many troglobites are considered rare in the absence of extensive surveys in the area, although sampling intensification often expands the distribution range of troglobitic species [7,59]. Our work thus suggests that the great emphasis on rare troglobitic species may be a poor allocation of scarce conservation resources, as shown for many other surrogate species [60]. These findings highlight the need to develop more human resources to aid the taxonomic identification of cave fauna, unify databases containing the occurrences of these organisms [52], and implement the use of molecular bar coding techniques to increase the speed and accuracy of taxonomic identifications [61].

Our results also show that the most relevant caves for conservation are larger caves (see the vertical dashed line in Fig 4). For instance, larger caves not only exhibit a high overall species richness, but are also more likely to contain more troglobitic species, rare troglobites, and bat populations (Table 2, Table C in S1 File). A similar effect was reported by Christman et al. (2005), who found that more diverse communities usually contain a higher fraction of endemic troglobites. Large caves with high species richness should thus be viewed as key potential reservoirs of endemic troglobitic species, so they should be considered of maximal conservation priority.

## Conclusions

Overall, our findings have important implications for the conservation of iron cave biodiversity. First, large caves were found to be important reservoirs of biodiversity, so they should be prioritized in conservation programs. Second, we found that species richness and the presence of troglobites and bat populations were spatially aggregated, implying that there are delimited areas containing groups of caves with high (or low) relevance for conservation. Third, spatial autocorrelation and the presence of troglobitic species in small caves suggest that troglobites can move between caves, and thus that iron caves should be treated as components of a cave network immersed in the karst landscape. This implies that conservation efforts should target subterranean habitats as a whole. Finally, our relevance ranking scheme demonstrates that the current Brazilian cave protection regime does not prioritizes the conservation of overall cave biodiversity. We believe that our proposed approach could help simplify the assessment process and channel more resources to the effective protection of cave biodiversity.

## Supporting Information

S1 File This file contains three Tables (A-C) and four figures (A-D). (PDF)

S1 Dataset Additional reports for Serra Leste and full taxa inventories for all caves. (ZIP)

S2 Dataset Full dataset and R scripts. (ZIP)
