# Supplementary material for: Reconciling Mining with the Conservation of Cave Biodiversity: A Quantitative Baseline to Help Establish Conservation Priorities
Source: PLoS One. 2016 Dec 20;11(12):e0168348. doi: 10.1371/journal.pone.0168348 (PMC5173368; doi:10.1371/journal.pone.0168348)
Supplement: S1 File — (PDF) [file pone.0168348.s001.pdf]

Supporting Information

This file contains Supporting Information for the manuscript "*Reconciling mining with the conservation of cave biodiversity: A quantitative baseline to help establish conservation priorities*" by by Rodolfo Jaffé, Xavier Prous, Robson Zampaulo, Tereza C. Giannini, Vera L. Imperatriz-Fonseca, Clóvis Maurity, Guilherme Oliveira, Iuri V. Brandi, and José O. Siqueira. It includes three Tables (A-C) and four Figures (A-D). Separately, we include two compressed folders.

Table A: List of the speleology reports considered in this assessment.

Area	Year	Company	Number of Caves	Repository link
Serra da Bocaina	2014	CARSTE	235	http://licenciamento.ibama.gov.br/Mineracao/Projeto%20Ferro%20Carajas%20S11D/Espeleologia/Serra_Bocaina/BOCAINA_235/BOCAINA_235.pdf
Serra Norte	2013	CARSTE	252	http://licenciamento.ibama.gov.br/Mineracao/VALE_Ferro%20Caraj%C3%A1s%20Norte/Espeleologia/2013_SERRA%20NORTE_ESPELEOLOGIA_EIA%20GLOBAL/Espeleologia_EIA_Global_Serra%20Norte_Print_Final.pdf
Serra Norte	2007	PRCZ	9	http://licenciamento.ibama.gov.br/Mineracao/VALE_Ferro%20Caraj%C3%A1s%20Norte/Espeleologia/2013_SERRA%20NORTE_ESPELEOLOGIA_EIA%20GLOBAL/ANEXOS/ANEXO%20IX_RELAT%C3%93RIO%20N5E.pdf
Serra Norte	2011	CARSTE	44	http://licenciamento.ibama.gov.br/Mineracao/VALE_Ferro%20Caraj%C3%A1s%20Norte/Espeleologia/2013_SERRA%20NORTE_ESPELEOLOGIA_EIA%20GLOBAL/ANEXOS/ANEXO%20XI_RELAT%C3%93RIO%20N5SM1.pdf
Serra Sul	2012	CARSTE	14	http://licenciamento.ibama.gov.br/Mineracao/Projeto%20Ferro%20Carajas%20S11D/Espeleologia/Relevancia/14%20cavidades/Relatorio_espeleo_S11D_14_cavernas_print.doc
Serra Sul	2010	CARSTE	173	http://licenciamento.ibama.gov.br/Mineracao/Projeto%20Ferro%20Carajas%20S11D/Espeleologia/Relevancia/Espeleologia%20S11D_Final_PRINT.pdf
Serra Leste [a]	2013	CARSTE	21	See file Serra_Leste_2013.pdf in Data S1
Serra Leste [a]	2011	CARSTE	96	See file Serra_Leste_2011.pdf in Data S1

The area surveyed by each report is shown followed by the report's publication year, the consulting company undertaking the work, the number of caves surveyed, and the url link of the official repository (see Data S1 for full taxa inventories).

[a] These report were submitted to the Brazilian Environmental Protection Agency (IBAMA) on July 2016, but they are still not available online.

Table B: Response and predictor variables included in the full models.

Response	Predictors included in the full models
Species richness	Altitude, cave slope, cave area, presence of percolating water, presence of water reservoirs, presence of plant material, presence of detritus, presence of roots, presence of guano, presence of other feces, presence of regurgitation balls, presence of carcases, presence of resident bat populations, and cave lithology.
Presence of troglobites	Altitude, cave slope, cave area, presence of percolating water, presence of water reservoirs, presence of plant material, presence of detritus, presence of roots, presence of guano, presence of other feces, presence of regurgitation balls, presence of carcases, presence of resident bat populations, and cave lithology.
Presence of rare troglobites	Altitude, cave slope, cave area, presence of percolating water, presence of water reservoirs, presence of plant material, presence of detritus, presence of roots, presence of guano, presence of other feces, presence of regurgitation balls, presence of carcases, presence of resident bat populations, and cave lithology.
Presence of bat populations	Altitude, cave slope, cave area, presence of percolating water, presence of water reservoirs, presence of plant material, presence of detritus, presence of roots, presence of guano, presence of other feces, presence of regurgitation balls, presence of carcases, and cave lithology.

Table C: Parameter estimates and hypothesis tests for the best models describing cave biodiversity including total species richness and the richness of troglobitic species as additional predictors.

**Response**	**Predictor**	**Estimate**	**SE**	***t/z*-value**	***p*-value**
Species richness (total) [a]	Area (log)	6.99	1.59	4.38	<0.001
	Guano (present)	-13.87	8.34	-1.66	0.1
	Interaction Area (log) - Guano	4.71	2	2.36	0.02
	Water Reservoirs (present)	0.95	2.34	0.41	0.68
	Detritus (present)	8.95	1.87	4.78	<0.001
	Roots	13.31	6.42	2.07	0.04
	Bat population (present)	29.22	7.05	4.15	<0.001
	Species richness (troglobites)	2.21	0.53	4.18	<0.001
Rare troglobites [b]	Area (log)	0.37	0.16	2.29	0.02
	Water Reservoirs	-0.48	0.36	-1.35	0.18
	Species richness (total)	0.02	0.01	3.73	<0.001
Troglobites [b]	Area (log)	0.04	0.12	0.30	0.76
	Species richness (total)	0.05	0.01	8.27	<0.001
Bat populations [b]	Area (log)	1.10	0.25	4.43	<0.001
	Species richness (total)	0.03	0.01	3.55	<0.001

Response variables are shown followed by the predictors included in each model, estimates, standard errors (SE), *t* or *z*-values and *p*-values.

[a] This model was ran using a data subset for which information on the richness of troglobitic species was available (N = 276 caves). Parameter estimates thus differ from those of the best model presented in Table 2 (which employed the full dataset).

[b] Parameter estimates differ from those of the best models presented in Table 2 because total species richness was included as an additional predictor (N = 733 caves).

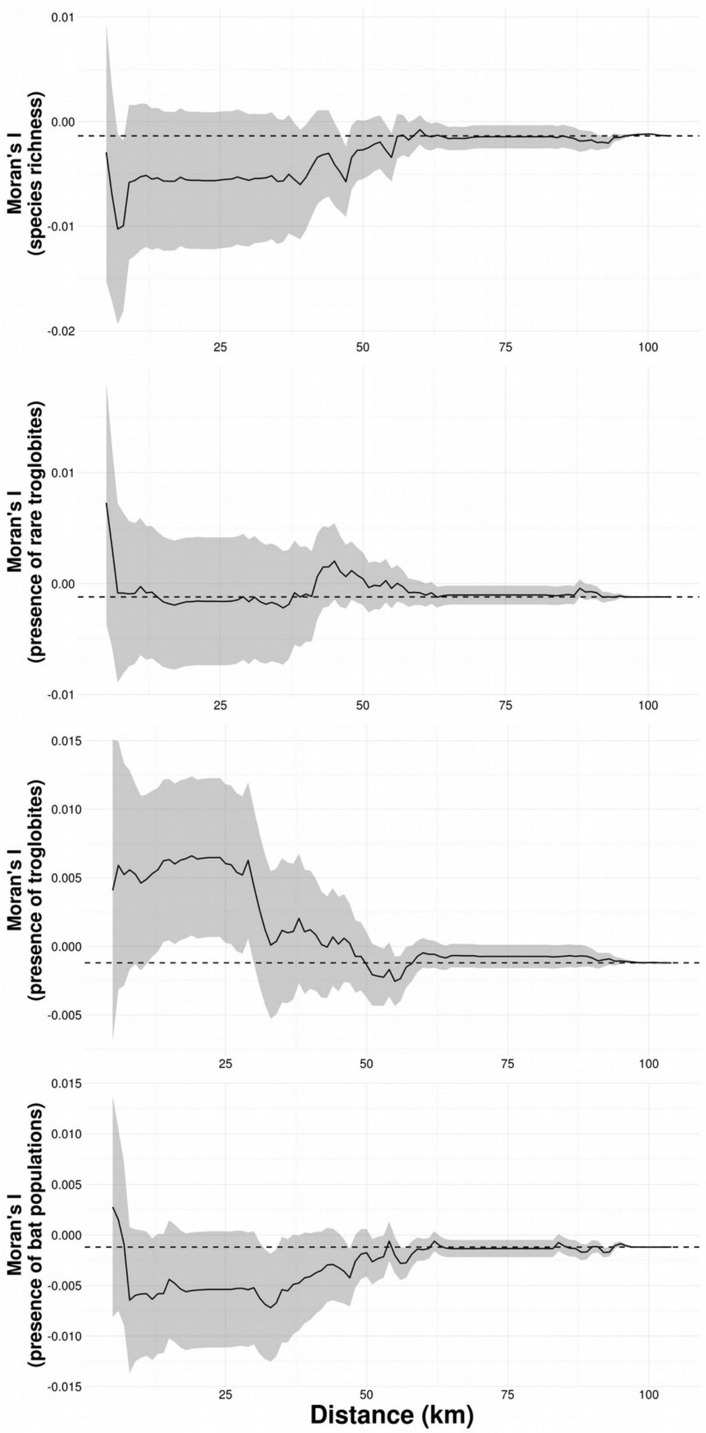

Figure A: Spatial autocorrelation in the residuals of our final models for species richness, presence of rare troglobites, presence of troglobites, and presence of resident bat populations, across different spatial scales. While the solid lines show the value of Moran's I, the gray area depict 95% confidence intervals. Dashed lines represent expected values under a null model of no spatial autocorrelation. Morans' I values were not significantly different from the null model, at any spatial scale.

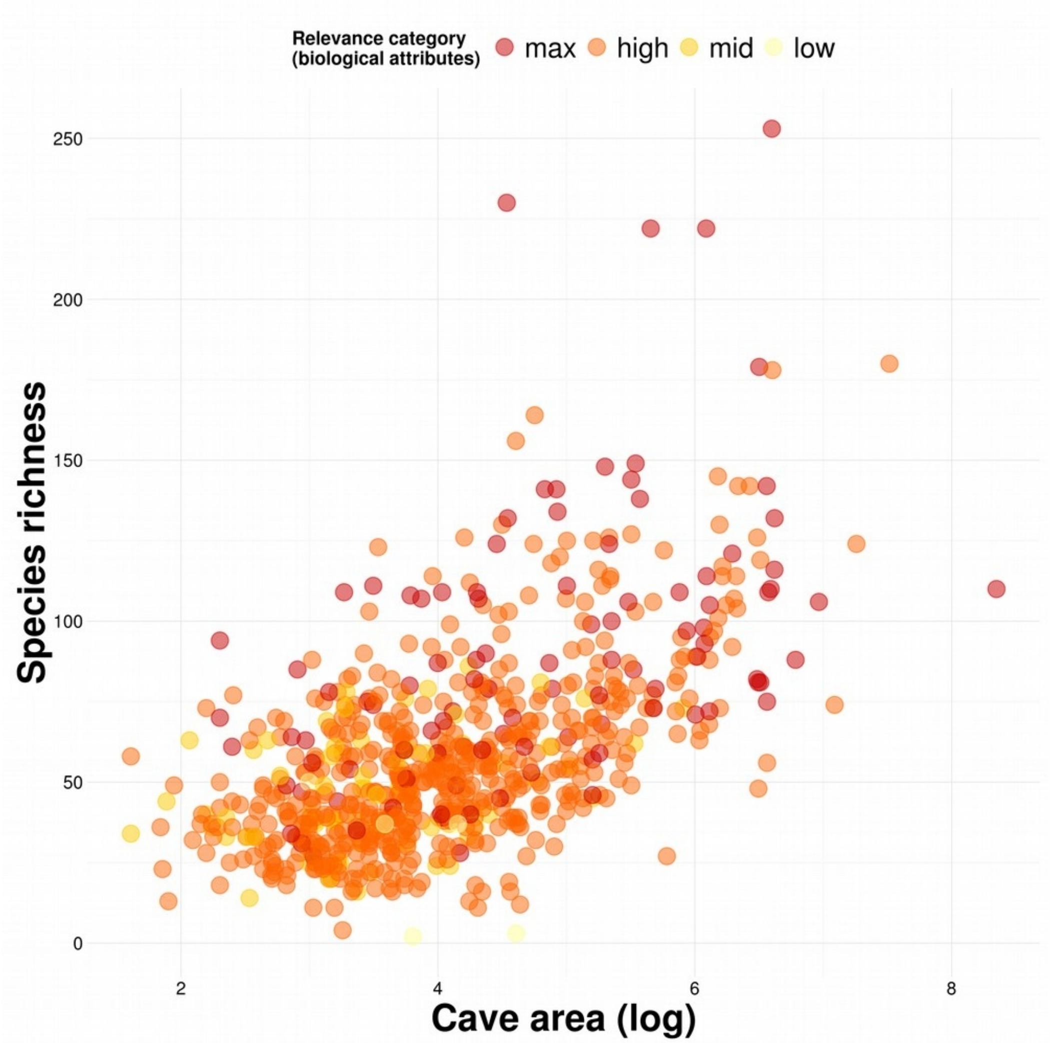

Figure B: Relationship between species richness and cave area across all analyzed caves. The relevance categories shown are based on biological attributes only, following the current Brazilian legislation.

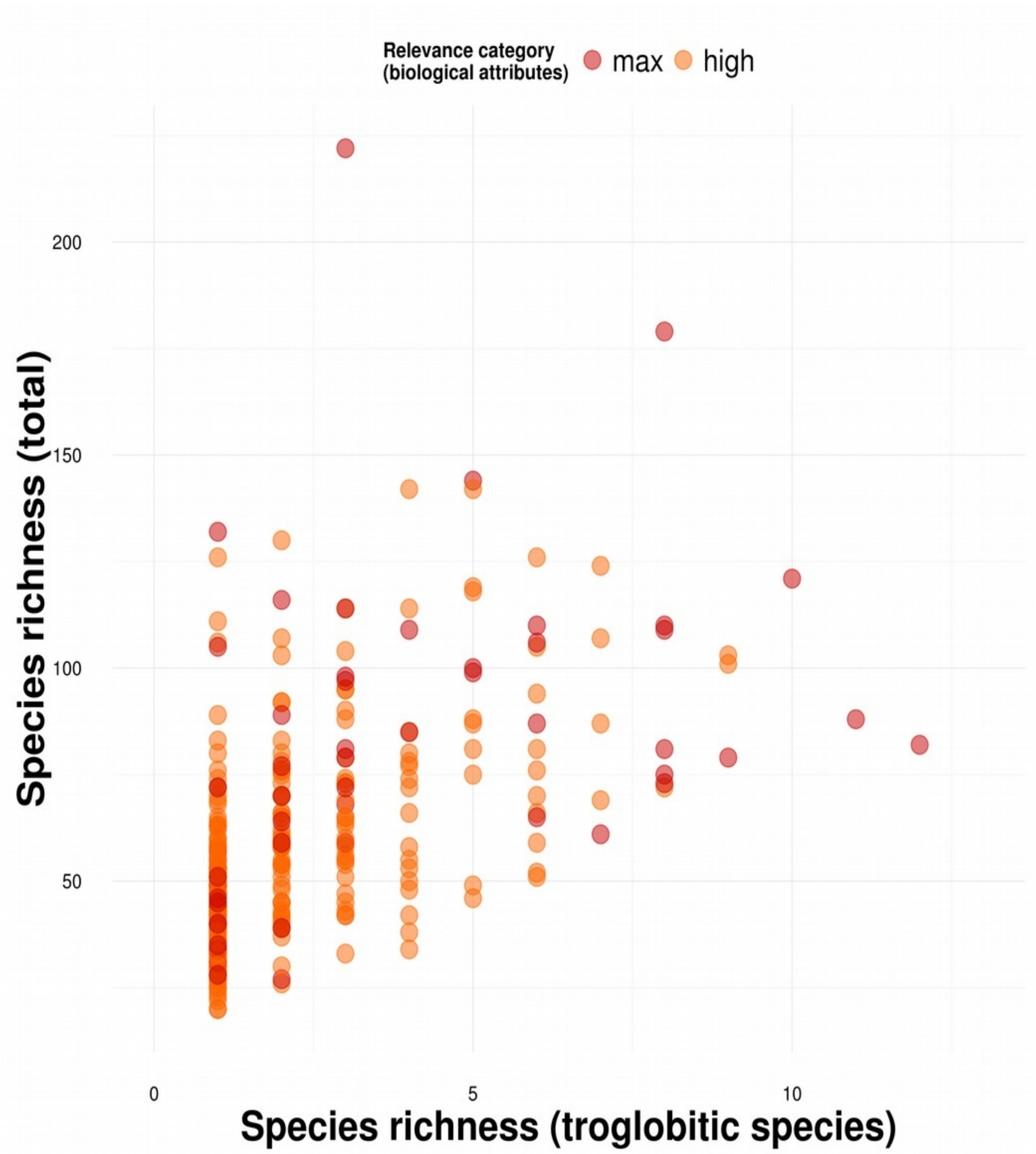

Figure C: Relationship between total species richness and the richness of troglobitic species across a data subset for which this information was available (N = 276 caves). The relevance categories shown are based on biological attributes only, following the current Brazilian legislation.

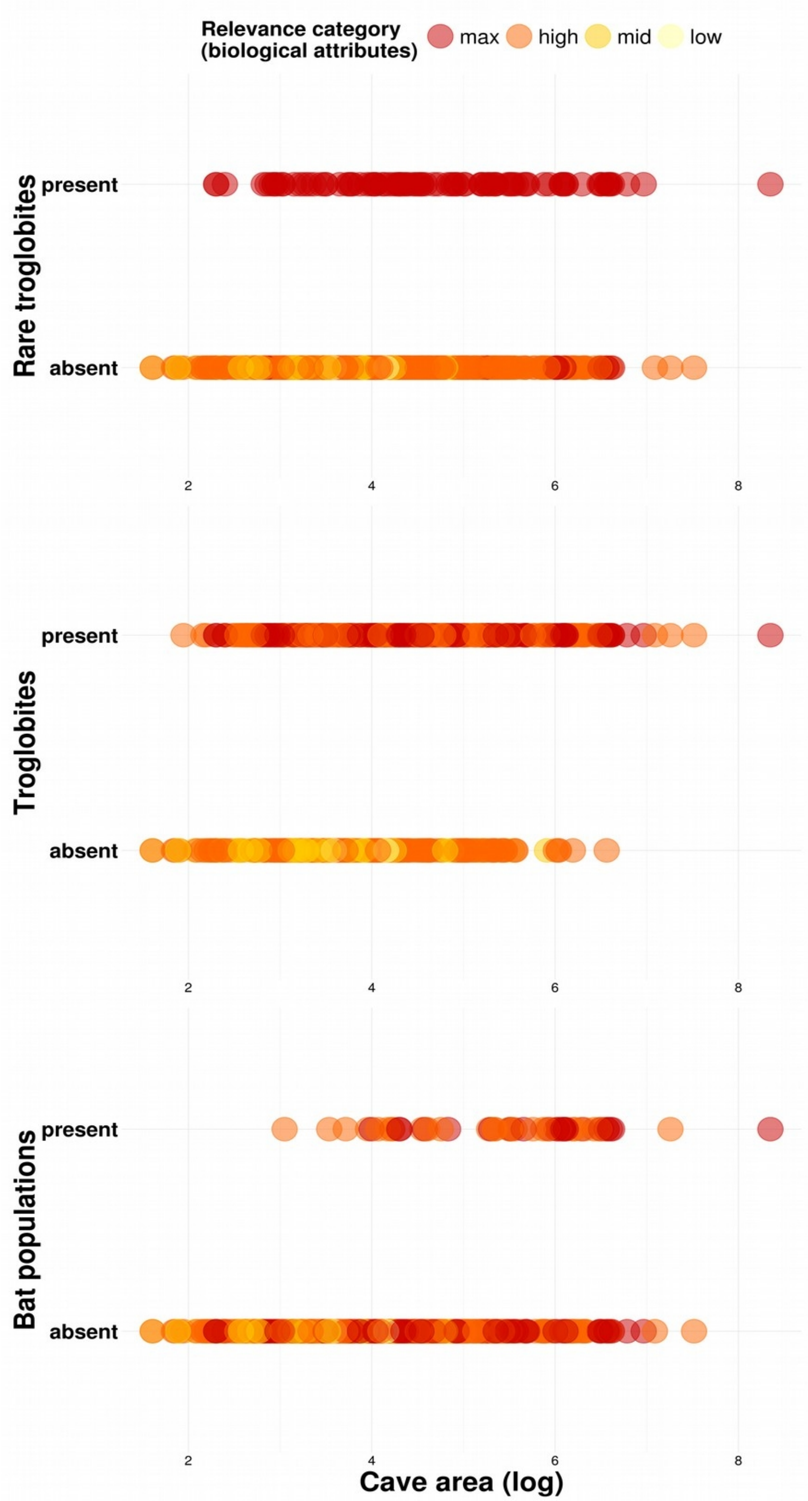

Figure D: Relevance classification of caves containing rare troglobites, troglobites, and resident bat populations. Variation in cave area is represented in the X axis, and the relevance categories shown are based on biological attributes only, following the current Brazilian legislation.
