# Supplementary material for: Reconciling Mining with the Conservation of Cave Biodiversity: A Quantitative Baseline to Help Establish Conservation Priorities
Source: PLoS One. 2016 Dec 20;11(12):e0168348. doi: 10.1371/journal.pone.0168348 (PMC5173368; doi:10.1371/journal.pone.0168348)
Supplement: S1 Dataset — (ZIP) [file pone.0168348.s002.zip › Serra Leste Reports/Serra_Leste_2011.pdf]

SERRA LESTE - ESPELEOLOGIA

Diagnóstico Geoespeleológico
Diagnóstico Bioespeleológico
Análise de Relevância

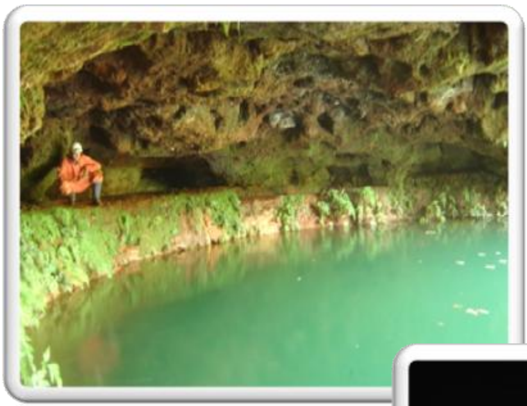

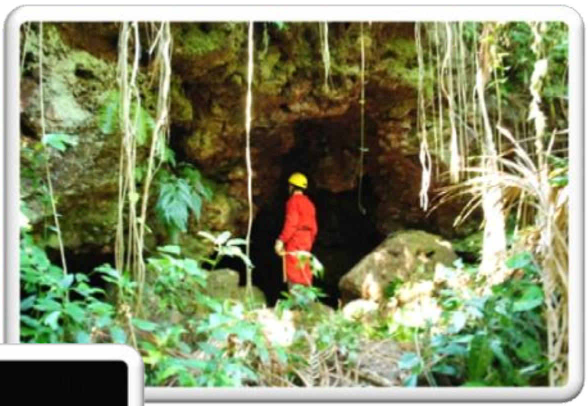

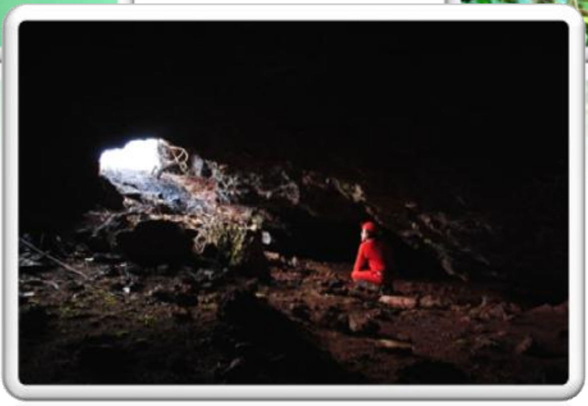

CARSTE
CONSULTORES ASSOCIADOS

SERRA LESTE - ESPELEOLOGIA

Diagnóstico Geoespeleológico
Diagnóstico Bioespeleológico
Análise de Relevância

GEOESPELEOLOGIA E ANÁLISE DE RELEVÂNCIA

Allan Calux

Augusto Auler, PhD

BIOESPELEOLOGIA E ANÁLISE DE RELEVÂNCIA

Dr. Rodrigo Lopes Ferreira

Dr. Marconi Souza Silva

Belo Horizonte, Setembro de 2011

EMPRESA E EQUIPE TÉCNICA

**Carste Consultores Associados Ltda**<br>**CNPJ 08.000.418/0001-00**<br>**Rua Brasópolis, 139. Bairro Floresta.**<br>**CEP. 30150-170. Fone: (31) 25529976**<br>**www.carste.com.br**				
**Nome**	**Responsabilidade**	**Formação**	**Função**	**Conselho de Classe**
Augusto Sarreiro Auler	Coordenador Geral; Geoespeleologia	Geólogo, PhD	Análise e relatório final	CREA MG 72.076/D
Allan Silas Calux	Coordenador Técnico; Geoespeleologia	Geógrafo	Campo, análise e relatório final	CREA 5062840039 SP
Dr. Rodrigo Lopes Ferreira	Bioespeleologia	Biólogo	Análise e relatório final	CRB 13.978/4-D
Dr. Marconi Souza Silva	Bioespeleologia	Biólogo	Análise e relatório final	CRB 16.755/4-D
Msc. Robson de Almeida Zampaulo	Coordenador de Campo e Laboratório; Bioespeleologia	Biólogo	Campo, análises e relatório final	CRB 56.210/01-D

SUMÁRIO

1. INTRODUÇÃO 40

2. METODOLOGIA 42
- **2.1. Levantamento bibliográfico 42**
- **2.2. Prospecção Espeleológica 42**
- **2.3. Espeleotopografia 42**
- **2.4. Geoespeleologia 45**
  - 2.4.1. Campanhas de campo 45
  - 2.4.2. Análise litológica e das estruturas 45
  - 2.4.3. Análise morfológica 46
  - 2.4.4. Hidrologia 46
  - 2.4.5. Depósitos clásticos, orgânicos e químicos. 46
- **2.5. Bioespeleologia 46**
  - 2.5.1 Procedimentos de coleta 46
  - 2.5.2. Triagem e identificação dos taxa 50
  - 2.5.3. Determinação de troglomorfismos 50
  - 2.5.4. Determinação de raridade 51
  - 2.5.5. Análises 52
    - 2.5.5.1. Análises ecológicas das cavidades 52
    - 2.5.5.2. Determinação do grau de relevância 53

3. ESTUDOS ANTERIORES E ESPELEOLOGIA REGIONAL 54

4. INSERÇÃO DAS CAVERNAS NO CONTEXTO GEOLÓGICO, GEOMORFOLÓGICO e FITOGEOGRáFICO 58
- **4.1 Geologia 58**
  - 4.1.1. Rochas vulcânicas máficas 63
  - 4.1.2. Anfibolitos 63
  - 4.1.3. Formações Ferríferas 64
- **4.2 Geomorfologia 65**
- **4.3 Fitogeografia 66**

5. APRESENTAÇÃO DOS RESULTADOS 67
- **5.1. Prospecção espeleológica 67**

5.2. Espeleotopografia 69
5.2.2. Espeleometria 71
5.3 Geoespeleologia 73
5.3.1. Inserção das cavernas na paisagem 73
5.3.2. Morfologia 78
5.3.3. Litologia 87
5.3.4. Estruturas 90
5.3.5. Hidrologia 95
5.3.6. Depósitos clásticos 97
5.3.7. Depósitos químicos (espeleotemas) 98
5.3.8. Aspectos espeleogenéticos e cronológicos 102
5.3.9. Aspectos sócio econômicos, culturais e estado de conservação 107
5.4. Bioespeleologia 107
5.4.1. Recursos tróficos e condições ambientais 107
5.4.2. Caracterização faunística geral das cavidades 109
5.4.2.1. Caracterização realizada no período seco 109
5.4.2.2 Caracterização realizada no período chuvoso 111
5.4.3. Fauna geral 114
5.4.3.1 Espécies troglomórficas 132
5.4.4. Descrição específica de cada cavidade 160
5.4.4.1. SL-001 160
5.4.4.1.1 Caracterização trófica 160
5.4.4.1.2. Caracterização faunística no período de seca 161
5.4.4.1.3. Caracterização faunística no período de chuva 162
5.4.4.1.4. Caracterização geral da fauna da cavidade 163
5.4.4.2. SL-002 164
5.4.4.2.1. Caracterização trófica 164
5.4.4.2.2. Caracterização faunística no período de seca 166
5.4.4.2.3. Caracterização faunística no período de chuva 166
5.4.4.2.4. Caracterização geral da fauna da cavidade 167
5.4.4.3. SL-003 168
5.4.4.3.1. Caracterização trófica 168
5.4.4.3.2. Caracterização faunística no período de seca 169
5.4.4.3.3. Caracterização faunística no período de chuva 170
5.4.4.3.4. Caracterização geral da fauna da cavidade 170
5.4.4.4. SL-004 172
5.4.4.4.1. Caracterização trófica 172
5.4.4.4.2. Caracterização faunística no período de seca 174
5.4.4.4.3. Caracterização faunística no período de chuva 174
5.4.4.4.4. Caracterização geral da fauna da cavidade 175

5.4.4.5. SL-005 177
5.4.4.5.1. Caracterização trófica 177
5.4.4.5.2. Caracterização faunística no período de seca 177
5.4.4.5.3. Caracterização faunística no período de chuva 178
5.4.4.5.4. Caracterização geral da fauna da cavidade 179
5.4.4.6. SL-006 180
5.4.4.6.1. Caracterização trófica 180
5.4.4.6.2. Caracterização faunística no período de seca 181
5.4.4.6.3. Caracterização faunística no período de chuva 182
5.4.4.6.4. Caracterização geral da fauna da cavidade 182
5.4.4.7. SL-007 183
5.4.4.7.1. Caracterização trófica 183
5.4.4.7.2. Caracterização faunística no período de seca 184
5.4.4.7.3. Caracterização faunística no período de chuva 184
5.4.4.7.4. Caracterização geral da fauna da cavidade 185
5.4.4.8 SL-008 185
5.4.4.8.1. Caracterização trófica 185
5.4.4.8.2. Caracterização faunística no período de seca 187
5.4.4.8.3. Caracterização faunística no período de chuva 187
5.4.4.8.4. Caracterização geral da fauna da cavidade 188
5.4.4.9. SL-009 189
5.4.4.9.1. Caracterização trófica 189
5.4.4.9.2. Caracterização faunística no período de seca 190
5.4.4.9.3. Caracterização faunística no período de chuva 191
5.4.4.9.4 Caracterização geral da fauna da cavidade 191
5.4.4.10. SL-011 192
5.4.4.10.1. Caracterização trófica 192
5.4.4.10.2. Caracterização faunística no período de seca 193
5.4.4.10.3. Caracterização faunística no período de chuva 194
5.4.4.10.4. Caracterização geral da fauna da cavidade 194
5.4.4.11. SL-012 195
5.4.4.11.1. Caracterização trófica 195
5.4.4.11.2. Caracterização faunística no período de seca 196
5.4.4.11.3. Caracterização faunística no período de chuva 197
5.4.4.11.4. Caracterização geral da fauna da cavidade 197
5.4.4.12. SL-013 198
5.4.4.12.1. Caracterização trófica 198
5.4.4.12.2. Caracterização faunística no período de seca 199
5.4.4.12.3. Caracterização faunística no período de chuva 199
5.4.4.12.4. Caracterização geral da fauna da cavidade 200

5.4.4.13. SL-014 201
5.4.4.13.1. Caracterização trófica 201
5.4.4.13.2. Caracterização faunística no período de seca 201
5.4.4.13.3. Caracterização faunística no período de chuva....202
5.4.4.13.4. Caracterização geral da fauna da cavidade 203
5.4.4.14. SL-015 203
5.4.4.14.1. Caracterização trófica 203
5.4.4.14.2. Caracterização faunística no período de seca 204
5.4.4.14.3. Caracterização faunística no período de chuva....204
5.4.4.14.4. Caracterização geral da fauna da cavidade 205
5.4.4.15. SL-016 206
5.4.4.15.1. Caracterização trófica 206
5.4.4.15.2. Caracterização faunística no período de seca 207
5.4.4.15.3. Caracterização faunística no período de chuva....208
5.4.4.15.4. Caracterização geral da fauna da cavidade 209
5.4.4.16. SL-017 211
5.4.4.16.1. Caracterização trófica 211
5.4.4.16.2. Caracterização faunística no período de seca 212
5.4.4.16.3. Caracterização faunística no período de chuva....212
5.4.4.16.4. Caracterização geral da fauna da cavidade 212
5.4.4.17. SL-018 213
5.4.4.17.1. Caracterização trófica 213
5.4.4.17.2. Caracterização faunística no período de seca 214
5.4.4.17.3. Caracterização faunística no período de chuva....214
5.4.4.17.4. Caracterização geral da fauna da cavidade 215
5.4.4.18. SL-019 216
5.4.4.18.1. Caracterização trófica 216
5.4.4.18.2. Caracterização faunística no período de seca 217
5.4.4.18.3. Caracterização faunística no período de chuva....218
5.4.4.18.4. Caracterização geral da fauna da cavidade 218
5.4.4.19. SL-020 218
5.4.4.19.1. Caracterização trófica 218
5.4.4.19.2. Caracterização faunística no período de seca 219
5.4.4.19.3. Caracterização faunística no período de chuva....219
5.4.4.19.4. Caracterização geral da fauna da cavidade 220
5.4.4.20. SL-022 220
5.4.4.20.1. Caracterização trófica 220
5.4.4.20.2. Caracterização faunística no período de seca 221
5.4.4.20.3. Caracterização faunística no período de chuva....221
5.4.4.20.4. Caracterização geral da fauna da cavidade 222

5.4.4.21. SL-023 223
5.4.4.21.1. Caracterização trófica 223
5.4.4.21.2. Caracterização faunística no período de seca 224
5.4.4.21.3. Caracterização faunística no período de chuva..................................224
5.4.4.21.4. Caracterização geral da fauna da cavidade 224
5.4.4.22. SL-024 225
5.4.4.22.1. Caracterização trófica 225
5.4.4.22.2. Caracterização faunística no período de seca 226
5.4.4.22.3. Caracterização faunística no período de chuva..................................227
5.4.4.22.4. Caracterização geral da fauna da cavidade 227
5.4.4.23. SL-025 228
5.4.4.23.1. Caracterização trófica 228
5.4.4.23.2. Caracterização faunística no período de seca 229
5.4.4.23.3. Caracterização faunística no período de chuva..................................230
5.4.4.23.4. Caracterização geral da fauna da cavidade 231
5.4.4.24. SL-026 231
5.4.4.24.1. Caracterização trófica 231
5.4.4.24.2. Caracterização faunística no período de seca 233
5.4.4.24.3. Caracterização faunística no período de chuva..................................233
5.4.4.24.4. Caracterização geral da fauna da cavidade 234
5.4.4.25. SL-027 234
5.4.4.25.1. Caracterização trófica 234
5.4.4.25.2. Caracterização faunística no período de seca 235
5.4.4.25.3. Caracterização faunística no período de chuva..................................236
5.4.4.25.4. Caracterização geral da fauna da cavidade 237
5.4.4.26. SL-028 237
5.4.4.26.1. Caracterização trófica 237
5.4.4.26.2. Caracterização faunística no período de seca 238
5.4.4.26.3. Caracterização faunística no período de chuva..................................238
5.4.4.26.4. Caracterização geral da fauna da cavidade 239
5.4.4.27. SL-029 239
5.4.4.27.1. Caracterização trófica 239
5.4.4.27.2. Caracterização faunística no período de seca 241
5.4.4.27.3. Caracterização faunística no período de chuva..................................241
5.4.4.27.4. Caracterização geral da fauna da cavidade 242
5.4.4.28. SL-030 244
5.4.4.28.1. Caracterização trófica 244
5.4.4.28.2. Caracterização faunística no período de seca 246
5.4.4.28.3. Caracterização faunística no período de chuva..................................246
5.4.4.28.4. Caracterização geral da fauna da cavidade 247

5.4.4.29 SL-031 250
5.4.4.29.1. Caracterização trófica 250
5.4.4.29.2. Caracterização faunística no período de seca 251
5.4.4.29.3. Caracterização faunística no período de chuva....................................251
5.4.4.29.4. Caracterização geral da fauna da cavidade 252
5.4.4.30. SL-032 254
5.4.4.30.1. Caracterização trófica 254
5.4.4.30.2. Caracterização faunística no período de seca 254
5.4.4.30.3. Caracterização faunística no período de chuva....................................255
5.4.4.30.4. Caracterização geral da fauna da cavidade 255
5.4.4.31. SL-033 256
5.4.4.31.1. Caracterização trófica 256
5.4.4.31.2. Caracterização faunística no período de seca 257
5.4.4.31.3. Caracterização faunística no período de chuva....................................258
5.4.4.31.4. Caracterização geral da fauna da cavidade 258
5.4.4.32. SL-035 260
5.4.4.32.1. Caracterização trófica 260
5.4.4.32.2. Caracterização faunística no período de seca 261
5.4.4.32.3. Caracterização faunística no período de chuva....................................262
5.4.4.32.4. Caracterização geral da fauna da cavidade 263
5.4.4.33. SL-036 264
5.4.4.33.1. Caracterização trófica 264
5.4.4.33.2. Caracterização faunística no período de seca 265
5.4.4.33.3. Caracterização faunística no período de chuva....................................265
5.4.4.33.4. Caracterização geral da fauna da cavidade 266
5.4.4.34. SL-037 266
5.4.4.34.1. Caracterização trófica 266
5.4.4.34.2. Caracterização faunística no período de seca 267
5.4.4.34.3. Caracterização faunística no período de chuva....................................268
5.4.4.34.4. Caracterização geral da fauna da cavidade 268
5.4.4.35. SL-038 270
5.4.4.35.1. Caracterização trófica 270
5..4.4.35.2. Caracterização faunística no período de seca 270
5.4.4.35.3. Caracterização faunística no período de chuva....................................271
5.4.4.35.4. Caracterização geral da fauna da cavidade 271
5.4.4.36. SL-039 272
5.4.4.36.1. Caracterização trófica 272
5.4.4.36.2. Caracterização faunística no período de seca 273
5.4.4.36.3. Caracterização faunística no período de chuva....................................273
5.4.4.36.4. Caracterização geral da fauna da cavidade 273

5.4.4.37. SL-040 274
5.4.4.37.1. Caracterização trófica 274
5.4.4.37.2. Caracterização faunística no período de seca 275
5.4.4.37.3. Caracterização faunística no período de chuva....................................275
5.4.4.37.4. Caracterização geral da fauna da cavidade 275
5.4.4.38. SL-041 276
5..4.4.38.1. Caracterização trófica 276
5.4.4.38.2. Caracterização faunística no período de seca 277
5.4.4.38.3. Caracterização faunística no período de chuva....................................277
5.4.4.38.4. Caracterização geral da fauna da cavidade 278
5.4.4.39. SL-042 278
5.4.4.39.1. Caracterização trófica 278
5.4.4.39.2. Caracterização faunística no período de seca 279
5.4.4.39.3. Caracterização faunística no período de chuva....................................280
5.4.4.39.4. Caracterização geral da fauna da cavidade 281
5.4.4.40. SL-043 282
5.4.4.40.1. Caracterização trófica 282
5.4.4.40.2. Caracterização faunística no período de seca 283
5.4.4.40.3. Caracterização faunística no período de chuva....................................284
5.4.4.40.4. Caracterização geral da fauna da cavidade 284
5.4.4.41. SL-044 285
5.4.4.41.1. Caracterização trófica 285
5.4.4.41.2. Caracterização faunística no período de seca 287
5.4.4.41.3. Caracterização faunística no período de chuva....................................287
5.4.4.41.4. Caracterização geral da fauna da cavidade 288
5.4.4.42. SL-045 290
5.4.4.42.1. Caracterização trófica 290
5.4.4.42.2. Caracterização faunística no período de seca 290
5.4.4.42.3. Caracterização faunística no período de chuva....................................291
5.4.4.42.4. Caracterização geral da fauna da cavidade 291
5..4.4.43. SL-046 292
5.4.4.43.1. Caracterização trófica 292
5.4.4.43.2. Caracterização faunística no período de seca 294
5.4.4.43.3. Caracterização faunística no período de chuva....................................294
5.4.4.43.4. Caracterização geral da fauna da cavidade 295
5.4.4.44. SL-047 295
5.4.4.44.1. Caracterização trófica 295
5.4.4.44.2. Caracterização faunística no período de seca 296
5.4.4.44.3. Caracterização faunística no período de chuva....................................297
5.4.4.44.4. Caracterização geral da fauna da cavidade 297

5.4.4.45. SL-048 298
5.4.4.45.1. Caracterização trófica 298
5.4.4.45.2. Caracterização faunística no período de seca 299
5.4.4.45.3. Caracterização faunística no período de chuva 299
5.4.4.45.4. Caracterização geral da fauna da cavidade 300
5.4.4.46. SL-049 300
5.4.4.46.1. Caracterização trófica 300
5.4.4.46.2. Caracterização faunística no período de seca 301
5.4.4.46.3. Caracterização faunística no período de chuva 302
5.4.4.46.4. Caracterização geral da fauna da cavidade 302
5.4.4.47. SL-050 303
5.4.4.47.1. Caracterização trófica 303
5.4.4.47.2. Caracterização faunística no período de seca 304
5.4.4.47.3. Caracterização faunística no período de chuva 305
5.4.4.47.4. Caracterização geral da fauna da cavidade 305
5.4.4.48. SL-051 306
5.4.4.48.1. Caracterização trófica 306
5.4.4.48.2. Caracterização faunística no período de seca 307
5.4.4.48.3. Caracterização faunística no período de chuva 308
5.4.4.48.4. Caracterização geral da fauna da cavidade 309
5.4.4.49. SL-052 309
5.4.4.49.1. Caracterização trófica 309
5.4.4.49.2. Caracterização faunística no período de seca 311
5.4.4.49.3. Caracterização faunística no período de chuva 311
5.4.4.49.4. Caracterização geral da fauna da cavidade 311
5.4.4.50. SL-053 313
5.4.4.50.1. Caracterização trófica 313
5.4.4.50.2. Caracterização faunística no período de seca 315
5.4.4.50.3. Caracterização faunística no período de chuva 315
5.4.4.50.4. Caracterização geral da fauna da cavidade 316
5.4.4.51. SL-054 316
5.4.4.51.1. Caracterização trófica 316
5.4.4.51.2. Caracterização faunística no período de seca 317
5.4.4.51.3. Caracterização faunística no período de chuva 318
5.4.4.51.4. Caracterização geral da fauna da cavidade 318
5.4.4.52. SL-055 319
5.4.4.52.1. Caracterização trófica 319
5.4.4.52.2. Caracterização faunística no período de seca 320
5.4.4.52.3. Caracterização faunística no período de chuva 320
5.4.4.52.4. Caracterização geral da fauna da cavidade 321

5.4.4.53. SL-056 321
5.4.4.53.1. Caracterização trófica 321
5.4.4.53.2. Caracterização faunística no período de seca 323
5.4.4.53.3. Caracterização faunística no período de chuva....................................323
5.4.4.53.4. Caracterização geral da fauna da cavidade 323
5.4.4.54. SL-057 325
5.4.4.54.1. Caracterização trófica 325
5.4.4.54.2. Caracterização faunística no período de seca 327
5.4.4.54.3. Caracterização faunística no período de chuva....................................327
5.4.4.54.4. Caracterização geral da fauna da cavidade 328
5.4.4.55. SL-058 328
5.4.4.55.1. Caracterização trófica 328
5.4.4.55.2. Caracterização faunística no período de seca 331
5.4.4.55.3. Caracterização faunística no período de chuva....................................331
5.4.4.55.4. Caracterização geral da fauna da cavidade 332
5.4.4.56. SL-059 334
5.4.4.56.1. Caracterização trófica 334
5.4.4.56.2. Caracterização faunística no período de seca 335
5.4.4.56.3. Caracterização faunística no período de chuva....................................335
5.4.4.56.4. Caracterização geral da fauna da cavidade 336
5.4.4.57. SL-060 337
5.4.4.57.1. Caracterização trófica 337
5.4.4.57.2. Caracterização faunística no período de seca 339
5.4.4.57.3. Caracterização faunística no período de chuva....................................339
5.4.4.57.4. Caracterização geral da fauna da cavidade 340
5.4.4.58. SL-061 342
5.4.4.58.1. Caracterização trófica 342
5.4.4.58.2. Caracterização faunística no período de seca 344
5.4.4.58.3. Caracterização faunística no período de chuva....................................344
5.4.4.58.4. Caracterização geral da fauna da cavidade 345
5.4.4.59. SL-062 346
5.4.4.59.1. Caracterização trófica 346
5.4.4.59.2. Caracterização faunística no período de seca 347
5.4.4.59.3. Caracterização faunística no período de chuva....................................348
5.4.4.59.4. Caracterização geral da fauna da cavidade 348
5.4.4.60. SL-063 349
5.4.4.60.1. Caracterização trófica 349
5.4.4.60.2. Caracterização faunística no período de seca 350
5.4.4.60.3. Caracterização faunística no período de chuva....................................350
5.4.4.60.4. Caracterização geral da fauna da cavidade 350

5.4.4.61. SL-064 351
5.4.4.61.1. Caracterização trófica 351
5.4.4.61.2. Caracterização faunística no período de seca 353
5.4.4.61.3. Caracterização faunística no período de chuva 353
5.4.4.61.4. Caracterização geral da fauna da cavidade 354
5.4.4.62. SL-065 354
5.4.4.62.1. Caracterização trófica 354
5.4.4.62.2. Caracterização faunística no período de seca 356
5.4.4.62.3. Caracterização faunística no período de chuva 356
5.4.4.62.4. Caracterização geral da fauna da cavidade 357
5.4.4.63. SL-066 358
5.4.4.63.1. Caracterização trófica 358
5.4.4.63.2. Caracterização faunística no período de seca 359
5.4.4.63.3. Caracterização faunística no período de chuva 360
5.4.4.63.4. Caracterização geral da fauna da cavidade 360
5.4.4.64. SL-067 361
5.4.4.64.1. Caracterização trófica 361
5.4.4.64.2. Caracterização faunística no período de seca 361
5.4.4.64.3. Caracterização faunística no período de chuva 362
5.4.4.64.4. Caracterização geral da fauna da cavidade 362
5.4.4.65. SL-068 364
5.4.4.65.1. Caracterização trófica 364
5.4.4.65.2. Caracterização faunística no período de seca 364
5.4.4.65.3. Caracterização faunística no período de chuva 365
5.4.4.65.4. Caracterização geral da fauna da cavidade 365
5.4.4.66. SL-069 365
5.4.4.66.1. Caracterização trófica 365
5.4.4.66.2. Caracterização faunística no período de seca 368
5.4.4.66.3. Caracterização faunística no período de chuva 368
5.4.4.66.4. Caracterização geral da fauna da cavidade 369
5.4.4.67. SL-070 372
5.4.4.67.1. Caracterização trófica 372
5.4.4.67.2. Caracterização faunística no período de seca 372
5.4.4.67.3. Caracterização faunística no período de chuva 373
5.4.4.67.4. Caracterização geral da fauna da cavidade 373
5.4.4.68. SL-071 374
5.4.4.68.1. Caracterização trófica 374
5.4.4.68.2. Caracterização faunística no período de seca 375
5.4.4.68.3. Caracterização faunística no período de chuva 375
5.4.4.68.4. Caracterização geral da fauna da cavidade 375

5.4.4.69. SL-072 376
5.4.4.69.1. Caracterização trófica 376
5.4.4.69.2. Caracterização faunística no período de seca 378
5.4.4.69.3. Caracterização faunística no período de chuva 378
5.4.4.69.4. Caracterização geral da fauna da cavidade 379
5.4.4.70. SL-073 380
5.4.4.70.1. Caracterização trófica 380
5.4.4.70.2. Caracterização faunística no período de seca 383
5.4.4.70.3. Caracterização faunística no período de chuva 383
5.4.4.70.4. Caracterização geral da fauna da cavidade 384
5.4.4.71. SL-074 386
5.4.4.71.1. Caracterização trófica 386
5.4.4.71.2. Caracterização faunística no período de seca 388
5.4.4.71.3. Caracterização faunística no período de chuva 389
5.4.4.71.4. Caracterização geral da fauna da cavidade 390
5.4.4.72. SL-075 392
5.4.4.72.1. Caracterização trófica 392
5.4.4.72.2. Caracterização faunística no período de seca 393
5.4.4.72.3. Caracterização faunística no período de chuva 393
5.4.4.72.4. Caracterização geral da fauna da cavidade 394
5.4.4.73. SL-076 396
5.4.4.73.1. Caracterização trófica 396
5.4.4.73.2. Caracterização faunística no período de seca 396
5.4.4.73.3. Caracterização faunística no período de chuva 397
5.4.4.73.4. Caracterização geral da fauna da cavidade 398
5.4.4.74. SL-077 398
5.4.4.74.1. Caracterização trófica 398
5.4.4.74.2. Caracterização faunística no período de seca 400
5.4.4.74.3. Caracterização faunística no período de chuva 400
5.4.4.74.4. Caracterização geral da fauna da cavidade 401
5.4.4.75. SL-078 402
5.4.4.75.1. Caracterização trófica 402
5.4.4.75.2. Caracterização faunística no período de seca 404
5.4.4.75.3. Caracterização faunística no período de chuva 405
5.4.4.75.4. Caracterização geral da fauna da cavidade 405
5.4.4.76. SL-079 406
5.4.4.76.1. Caracterização trófica 406
5.4.4.76.2. Caracterização faunística no período de seca 408
5.4.4.76.3. Caracterização faunística no período de chuva 408
5.4.4.76.4. Caracterização geral da fauna da cavidade 409

5.4.4.77. SL-080 411
5.4.4.77.1. Caracterização trófica 411
5.4.4.77.2. Caracterização faunística no período de seca 411
5.4.4.77.3. Caracterização faunística no período de chuva 412
5.4.4.77.4. Caracterização geral da fauna da cavidade 412
5.4.4.78. SL-081 413
5.4.4.78.1. Caracterização trófica 413
5.4.4.78.2. Caracterização faunística no período de seca 414
5.4.4.78.3. Caracterização faunística no período de chuva 414
5.4.4.78.4. Caracterização geral da fauna da cavidade 415
5.4.4.79. SL-082 416
5.4.4.79.1. Caracterização trófica 416
5.4.4.79.2. Caracterização faunística no período de seca 417
5.4.4.79.3. Caracterização faunística no período de chuva 418
5.4.4.79.4. Caracterização geral da fauna da cavidade 419
5.4.4.80. SL-083 420
5.4.4.80.1. Caracterização trófica 420
5.4.4.82.2. Caracterização faunística no período de seca 421
5.4.4.80.3. Caracterização faunística no período de chuva 422
5.4.4.80.4. Caracterização geral da fauna da cavidade 422
5.4.4.81. SL-084 423
5.4.4.81.1. Caracterização trófica 423
5.4.4.81.2. Caracterização faunística no período de seca 424
5.4.4.81.3. Caracterização faunística no período de chuva 424
5.4.4.81.4. Caracterização geral da fauna da cavidade 425
5.4.4.82. SL-085 425
5.4.4.82.1. Caracterização trófica 425
5.4.4.82.2. Caracterização faunística no período de seca 426
5.4.4.82.3. Caracterização faunística no período de chuva 427
5.4.4.82.4. Caracterização geral da fauna da cavidade 427
5.4.4.83. SL-086 428
5.4.4.83.1. Caracterização trófica 428
5.4.4.85.2. Caracterização faunística no período de seca 429
5.4.4.83.3. Caracterização faunística no período de chuva 429
5.4.83.4. Caracterização geral da fauna da cavidade 429
5.4.4.84. SL-087 430
5.4.4.84.1. Caracterização trófica 430
5.4.4.84.2. Caracterização faunística no período de seca 431
5.4.4.84.3. Caracterização faunística no período de chuva 432
5.4.4.84.4. Caracterização geral da fauna da cavidade 433

5.4.4.85. SL-088 434
5.4.4.85.1. Caracterização trófica 434
5.4.4.85.2. Caracterização faunística no período de seca 435
5.4.4.85.3. Caracterização faunística no período de chuva....435
5.4.4.85.4. Caracterização geral da fauna da cavidade 436
5.4.4.86. SL-089 436
5.4.4.86.1. Caracterização trófica 436
5.4.4.86.2. Caracterização faunística no período de seca 438
5.4.4.86.3. Caracterização faunística no período de chuva....438
5.4.4.86.4. Caracterização geral da fauna da cavidade 439
5.4.4.87. SL-090 440
5.4.4.87.1. Caracterização trófica 440
5.4.4.87.2. Caracterização faunística no período de seca 441
5.4.4.87.3. Caracterização faunística no período de chuva....441
5.4.4.87.4. Caracterização geral da fauna da cavidade 442
5.4.4.88. SL-091 444
5.4.4.88.1. Caracterização trófica 444
5.4.4.88.2. Caracterização faunística no período de seca 444
5.4.4.88.3. Caracterização faunística no período de chuva....445
5.4.4.88.4. Caracterização geral da fauna da cavidade 445
5.4.4.89. SL-092 446
5.4.4.89.1. Caracterização trófica 446
5.4.4.89.2. Caracterização faunística no período de seca 447
5.4.4.89.3. Caracterização faunística no período de chuva....447
5.4.4.89.4. Caracterização geral da fauna da cavidade 448
5.4.4.90. SL-093 449
5.4.4.90.1. Caracterização trófica 449
5.4.4.90.2. Caracterização faunística no período de seca 450
5.4.4.90.3. Caracterização faunística no período de chuva....451
5.4.4.90.4. Caracterização geral da fauna da cavidade 452
5.4.4.91. SL-094 454
5.4.4.91.1. Caracterização trófica 454
5.4.4.91.2. Caracterização faunística no período de seca 454
5.4.4.91.3. Caracterização faunística no período de chuva....455
5.4.4.91.4. Caracterização geral da fauna da cavidade 455
5.4.4.92. SL-095 456
5.4.4.92.1. Caracterização trófica 456
5.4.4.92.2. Caracterização faunística no período de seca 457
5.4.4.92.3. Caracterização faunística no período de chuva....457
5.4.4.92.4. Caracterização geral da fauna da cavidade 458

5.4.4.93. SL-096 460
5.4.4.93.1. Caracterização trófica 460
5.4.4.93.2. Caracterização faunística no período de seca 460
5.4.4.93.3. Caracterização faunística no período de chuva 461
5.4.4.93.4. Caracterização geral da fauna da cavidade 461
5.4.4.94. SL-097 462
5.4.4.94.1. Caracterização trófica 462
5.4.4.94.2. Caracterização faunística no período de seca 462
5.4.4.94.3. Caracterização faunística no período de chuva 463
5.4.4.94.4. Caracterização geral da fauna da cavidade 464
5.4.4.95. SL-099 465
5.4.4.95.1. Caracterização trófica 465
5.4.4.95.2. Caracterização faunística no período de seca 466
5.4.4.95.3. Caracterização faunística no período de chuva 466
5.4.4.95.4. Caracterização geral da fauna da cavidade 466
5.4.4.96. SL-100 468
5.4.4.96.1. Caracterização trófica 468
5.4.4.96.2. Caracterização faunística no período de seca 469
5.4.4.96.3. Caracterização faunística no período de chuva 470
5.4.4.96.4. Caracterização geral da fauna da cavidade 470
5.4.5. Análises Ecológicas 472
5.4.5.1. Padrões gerais de riqueza e diversidade 472
5.4.5.2. Estimadores de riqueza 476
5.4.5.3. Padrões gerais de similaridade da fauna 480
5.4.5.4. Relações entre variáveis bióticas e abióticas 484
5.4.5.5. “Turnover” de espécies (β diversidade) 487

6. ANÁLISE DE RELEVÂNCIA DAS CAVERNAS DE SERRA LESTE 494
6.1. Metodologia da análise de relevância 494
6.1.1. Discriminação litológica 495
6.1.2. Escala de análise 495
6.1.3. Graus de relevância 495
6.2.1. Discriminação litológica 496
6.2.2. Definição das escalas local e regional 496
6.2.3. Análise das cavernas de Serra Leste 498
6.2.3.1. Relevância máxima dos atributos físicos e histórico-cultural 498
6.2.3.2. Relevância alta, média e baixa dos atributos físicos e histórico-cultural 499
6.2.3.2. Análise dos atributos biológicos 506
6.2.3.2.1. Considerando a utilização direta da Instrução Normativa (análise legalmente válida) 509
6.2.3.2.2. Considerando “adequações” na Instrução Normativa 526

6.2.3.3. Considerações finais sobre a relevância, os impactos potenciais e a conservação de cavernas no Projeto Serra Leste. 529

7. SUGESTÕES DE ENCAMINHAMENTO 537

8. REFERÊNCIAS BIBLIOGRÁFICAS..540

Geoespeleologia 540

Bioespeleologia...543

Anexo I – mapas das cavernas 549

Anexo II – fichas de geoespeleologia...549

ANEXO III – FICHAS DE BIOESPELEOLOGIA..549

Anexo IV – AMOSTRA LOCAL E REGIONAL...549

ÍNDICE DE TABELAS

Tabela 1 - Localização das cavidades inventariadas e respectivas datas de amostragem. 47

Tabela 2 - Principais troglomorfismos observados em espécies troglóbias (retirado de Christiansen, K. Morphological Adaptations, em Enciclopedia of Caves, Culver & White 2004)...51

Tabela 3 - Coluna crono-litoestratigráfica do Cinturão de Cisalhamento Itacaiúnas. Adaptado de Nunes (2002). 59

Tabela 4 - Coordenadas e espeleometria das cavidades estudadas em Serra Leste. 69

Tabela 5 – Frequência e percentual de cavernas segundo intervalo hipsométrico. 77

Tabela 6 - Frequência de cavernas em função do padrão planimétrico. 78

Tabela 7 - Frequência de cavernas segundo litotipo encaixante. 88

Tabela 8 - Frequência de cavernas por feição hidrológica observada. 97

Tabela 9 - Frequência de cavernas por tipo de espeleotema. 99

Tabela 10 - Número absoluto e relativo (número de espécies dividido pelo número de cavernas amostradas) de espécies troglóbias em algumas regiões do país..................133

Tabela 11 - Valores de riqueza, dominância, diversidade e equitabilidade para as cavernas do estudo (estação seca)..473

Tabela 12 - Valores de riqueza por estação, riqueza média, riqueza total e turnover para cada caverna inventariada..487

Tabela 13 - Classificação de relevância máxima na área de Serra Leste: atributos físicos e histórico-culturais..498

Tabela 14 - Classificação em termos de importância acentuada sob enfoque local e regional: Atributos físicos e histórico-econômicos. 499
Tabela 15 - Parâmetros espeleométricos da projeção horizontal sob enfoque regional. 500
Tabela 16 - Parâmetros espeleométricos da área sob enfoque regional. 500
Tabela 17 - Parâmetros espeleométricos do volume sob enfoque regional. 501
Tabela 18 - Síntese da classificação dos atributos que conferem importância acentuada sob enfoque local. 502
Tabela 19 - Atributos físicos que conferem importância significativa sob enfoque local e regional das cavernas de Serra Leste. 503
Tabela 20 - Cavernas com média projeção horizontal classificadas como de importância significativa sob enfoque local e regional. 505
Tabela 21 - Graus de relevância das cavernas da área de acordo com metodologia estabelecida na Instrução Normativa N0 2, de agosto de 2009. Relevância máxima (RM), importância acentuada regional (IAR), enfoque regional (ER), importância acentuada local (IAL), enfoque local (EL). Máxima (Máx.), Alta (alt.), Média (méd.) e Baixa (Baix.), Alta (A), média (M) e baixa (B). 1 presença, 0 ausência. A legenda referente a cada atributo encontra-se logo abaixo da tabela. 510
Tabela 22 - Relação de cavidades que apresentam espécies troglóbias com raridade tipo I, II, (I+II) e III, em Serra Leste. 514
Tabela 23 - Modalidades de abundância relativa, considerando-se somente as espécies com indivíduos maiores que 1 cm (IN 02), usando métodos de divisão do valor absoluto por três e raiz quadrada do valor absoluto dividido por três. A sobreposição refere-se ao resultado final entre as estimativas nas estações seca e estação úmida das duas metodologias. Mudanças de categoria em consequência do uso da metodologia de raiz quadrada dividido por três são destacadas em verde. 526
Tabela 24 - Síntese da relevância das cavidades estudadas em Serra Leste, de acordo com a configuração de importância dos atributos físicos e biológicos. 530
Tabela 25 – Cavernas com sobreposição entre o perímetro de proteção (250 m) e estruturas projetadas do Plano Diretor do projeto Serra Leste. 537
Tabela 26 – Sugestões de encaminhamento para o tema bioespeleologia no projeto Serra Leste. 538

ÍNDICE DE FIGURAS

Figura 1 - Localização da área de estudo...41
Figura 2 - Medição da projeção horizontal de acordo com o método da descontinuidade....43
Figura 3 - Medição do desnível da caverna. 44
Figura 4 - Medição da área da caverna descontando a área de pilares. 44

Figura 5 - Cálculo do volume de uma caverna a partir da altura média das seções verticais. 45
Figura 6 - Principais serras da região de Carajás, onde ocorrem as cavidades ferríferas. 55
Figura 7 - Mapa geológico simplificado de Serra Leste. Adaptado de DOCEGEO, 1988; Nunes, 2004; e Costa, 2007. 62
Figura 8 - Rochas vulcânicas máficas. Fonte: Costa (2007). 63
Figura 9 - Anfibolitos. Fonte: COSTA, 2007. 64
Figura 10 - Formação ferrífera de Serra Leste. Fonte: COSTA, 2007........ 65
Figura 11 - Mapa de caminhamento realizado na área do Projeto Serra Leste. Fonte: GEM/FCCM, 2005. 68
Figura 12 - Percentual de cavidades de Serra Leste em função da projeção horizontal. 71
Figura 13 - Percentual de cavidades de Serra Leste em função do desnível. 72
Figura 14 - Percentual de cavidades de Serra Leste em função da área........ 72
Figura 15 - Percentual de cavidades de Serra Leste em função do volume........ 73
Figura 16 – Modelo Digital de Elevação (MDE) de Serra Leste: compartimentos da paisagem. Exagero vertical: 3 vezes. 74
Figura 17 - Superfícies erosivas de Serra Leste. 75
Figura 18 - Visão geral das rupturas de relevo na borda dos platôs. 75
Figura 19 - Visão parcial do sistema fluvial interiorano, com destaque para as drenagens encaixadas em carapaças laterizadas. 76
Figura 20 - Dispersão das cavernas segundo intervalo hipsométrico. 77
Figura 21 - Percentual de cavernas segundo intervalo hipsométrico. 77
Figura 22 - Frequência de cavernas em função do padrão planimétrico........ 78
Figura 23 - Representação dos padrões planimétricos observados nas cavernas estudadas: espongiforme (SL-029); globular (SL-035); retilíneo (SL-081); e reticulado (SL-072). As linhas em vermelho representam as regularidades/irregularidades e “controles” das galerias. 80
Figura 24 – Pilares observados na caverna SL-001 (a e b)........ 81
Figura 25 - Modelo de evolução dos pilares diagenéticos. Fonte: dados dos autores. 82
Figura 26 - Modelo de evolução dos pilares biogênicos. Fonte: dados dos autores. 82
Figura 27 - Conjunto de pendentes observados ao longo de toda a caverna SL-001 (a, b, c e d)........ 83
Figura 28 - Pontão estrutural observado na caverna SL-042 (destacado em amarelo)........ 83
Figura 29 - Paleopiso observado na caverna SL-029: registro de ciclos de deposição e capeamento. Em a), visão geral, em b), visão em detalhe do capeamento. 84
Figura 30 - Piso capeado observado na caverna (a) SL-079 e (b) SL-089. 84
Figura 31 - *Bell holes* observados na caverna SL-001. 85
Figura 32 – a) Canalículo observado na junção piso/parede da caverna SL-002; b) canalículo presente diretamente na parede da caverna SL-093. 86
Figura 33 - Clarabóia observada na caverna SL-001 (a) e SL-006 (b). 86
Figura 34 - Frequência de cavernas por feição interna observada. 87

Figura 35 – Percentual de cavernas segundo litotipo encaixante...88
Figura 36 - Variação granulométrica da canga detrítica na caverna SL-008 (a e b): clastos subarredondados com tamanho seixo a calhau com predomínio do primeiro. 89
Figura 37 - Jaspilito alterado observado na caverna SL-078. 89
Figura 38 – Formação ferrífera bandada com relictos do bandamento de jaspe lixiviado (SL-019)...90
Figura 39 - Gráfico de roseta de fraturas medidas nas cavernas de Serra Leste (n=64). Medidas podem ser observadas no Anexo II (Fichas de Geoespeleogia).........................91
Figura 40 - Gráfico de roseta das principais direções dos condutos das cavernas de Serra Leste (n=17). (Medidas feitas sobre os mapas topográficos). 92
Figura 41 - Juntas de alívio sub-verticais (a) e sub-horizontais (b) observadas na caverna SL-026...92
Figura 42 – Dobras observadas nas cavernas (a) SL-038, (b) SL-083, (c) SL-085 e (d) SL-089..93
Figura 43 - Contato geológico erosivo entre cangas de diferentes texturas na entrada da caverna SL-047. 94
Figura 44 - Frequência de cavernas por feição estrutural observada nas cavernas de Serra Leste. 94
Figura 45 - Surgência observada na caverna SL-078: baixa vazão mesmo no período úmido.95
Figura 46 - Drenagem perene entrecortando a caverna SL-001. 96
Figura 47 - Drenagem externa da caverna SL-074 durante o período seco (a) e úmido (b) após evento de elevada pluviosidade...96
Figura 48 - Frequência de cavernas por feição hidrológica observada. 97
Figura 49 - Frequência de cavernas segundo granulometria dos sedimentos clásticos. 98
Figura 50 - Freqüência de cavernas por tipo de espeleotema. 99
Figura 51 - Freqüência de cavernas por tipo de espeleotema. 100
Figura 52 - Crostas observadas nas cavernas de Serra Leste: a) crosta branca, delgada, milimétrica (SL-061); b) crosta amarela (SL-073); c) crosta vermelha (SL-089), d) crosta cinza escuro (SL-035)..100
Figura 53 - Coralóides milimétricos registrados na caverna SL-006. A esquerda, afilados, de coloração amarela a cinza, tonalidade clara. A direita, microcoralóides recobertos por uma encrustração branca. 101
Figura 54 - Cortina da caverna (a) SL-030 e (b) SL-051...102
Figura 55 - Microtravertino observado na caverna SL-001. 102
Figura 56 - Modelo de evolução endógena/exógena do tipo dissolução/erosão. Adaptado de Pinheiro & Maurity (1988). 104
Figura 57 - Modelo de evolução exógena do tipo coluvionar. Fonte: dados dos autores.....105
Figura 58 - Modelo de evolução exógena do “tipo fluvial”. Fonte: dados dos autores.........106
Figura 59 - Distribuição das cavernas inventariadas nas diferentes fitofisionomias. 109
Figura 60 - a) Ricinulei: Ricinoididae (*Cryptocellus tarsilae*); b) Opilioacarida: Opilioacaridae (*Neoacarus* spn.); c) Acari: Trombidiforme; d) Araneae: Theraphosidae (*Nhandu*

coloratovillosus); e) Araneae: Pholcidae (*Mesabolivar* sp.); f) Araneae: Theraphosidae (*Acanthoscurria* sp.); g) Opiliones: Gonyleptidae; h) Opiliones: *Manaosbiidae*; i) Acari; j) Amblypygi: Phrynidae (*Heterophrynus longicornis*); K) Opiliones: Scadabiidae; l) Araneae: Pholcidae (*Mesabolivar* sp.). 118

Figura 61 - a) Lepidoptera: Noctuidae (*Latebraria* sp.) b) Collembola: Hypogastruridae; c) Homoptera: Kinnaridae; d) Hemiptera: Reduviidae (*Panstrongylus* sp.); e) Orthoptera: Aclodidae (*Aclodes* sp.); f) Larva de Diptera: Culicidae (*Toxorhynchites* sp.); g) Hemiptera: Nabidae; h) Coleoptera: Tenebrionidae; i) Hemiptera: Cercopidae; j) Orthoptera: Tettigoniidae; K) Blattodea: Blaberidae (*Blaberus* sp.); l) Hymenoptera: Formicidae (*Odontomachus* sp.); m) Coleoptera: Carabidae. 119

Figura 62 - a) Onychophora: Peripatidae; b) Glomeridesmida: Glomeridesmidae (*Glomeridesmus* sp.); c) Polydesmida: Chelodesmidae; d) Siphonophorida: Siphonophorida; e) Decapoda: Trichodactylidae; f) Gastropoda; g) Scolopendromorpha; h) Scutigeromorpha: Scutigeridae 120

Figura 63 - Ordens encontradas e suas respectivas porcentagens em relação à riqueza total de invertebrados registrada nas cavernas. 121

Figura 64 - Ordens encontradas e suas respectivas porcentagens em relação à riqueza total de vertebrados registrada nas cavernas. 122

Figura 65 - a) Rodentia: Cricetidae (*Riphydomis* sp.); b) Chiroptera: Emballonuridae (*Peropteryx kappleri*); c) Chiroptera: Phyllostomidae (*Carollia* sp.); d) Chiroptera: Phyllostomidae; e) Chiroptera: Emballonuridae (*Peropteryx* sp.); f) Rodentia: Cricetidae (*Riphydomis* sp.). 123

Figura 66 - Aves encontradas em cavernas inventariadas na área do Projeto Serra Leste. a) Urubu de cabeça preta (*Coragyps atratus*); b) Ninho com filhotes não identificados. ..124

Figura 67 - a) Squamata: Colubridae (*Chironius* sp.); b) Squamata: Colubridae (*Leptodeira annulata*); c) Squamata: Colubridae (*Spilotes pullatus*); d) Squamata: Boidae (*Epicrates* sp.); e) Squamata: Colubridae; f) Squamata: Gekkonidae (*Thecadactylus rapicauda*); Squamata: Iguanidae. 126

Figura 68 - *Lutzomyia* sp. (Diptera: Psychodidae) realizando repasto sanguíneo em Anuro (Bufonidae indet.). 127

Figura 69 - a) Anura: Dendrobatidae (*Ameerega* sp.); b) Anura: Leptodactylidae (*Eleutherodactylus* sp.); c) Anura: Dendrobatidae; d); e) Anura: Leptodactylidae (*Leptodactylus* cf. *labyrinthicus*); f) Anura: Leptodactylidae (*Pristimantis* cf. *fenestratus*). 128

Figura 70 - *Diplothyrus schubarti* (Holothyrida): macho (à esquerda) e fêmea (à direita). ...130

Figura 71 - *Cryptocellus tarsilae* (Ricinulei): (A) Detalhe de um espécime vivo; (B) detalhe de um espécime fixado (vista ventral); (C) Ilustração das modificações do terceiro par de patas de um macho relacionada à transferência de espermatóforo. Estas estruturas são fundamentais para a identificação da espécie (ilustração retirada do artigo de descrição

da espécie); (D) Fotografia da terceira perna de um macho coletado em serra leste. Comparar com a ilustração da descrição. 131
Figura 72 - a) Glomeridesmida: Glomeridesmidae (*Glomeridesmus* sp.); b) Polyxenidae: Lophoproctidae; c) Isopoda: Styloniscidae; d) Spirostreptida: Pseudonannolenidae; e) Diplopoda: Balloniscidae; f) Polydesmida: Pyrgodesmidae; g) Isopoda: Platyarthridae; h) Araneae: Prodidomidae; i) Coleoptera: Carabidae (*Coarazuphium* sp.); j) Schizomida: Hubbardiidae; k) Planaria; l) Amblypygi: Charinidae (*Charinus* sp.); m) Gastropoda: Systrophiidae (*Happia* sp.); n) Acari: Trombidiforme. 134
Figura 73 - Trombidiformes encontrados em cavernas, exibindo diferentes níveis de troglomorfismos: A) Trombidiforme não troglomórfico encontrado em uma caverna quartzítica de Ibitipoca, MG; B) Trombidiforme troglomórfico encontrado em uma caverna calcária em Cordisburgo, MG; C) Trombidiforme troglomórfico encontrado em uma caverna arenítica em Altinópolis, SP; D) Trombidiforme sp1 (Acari: Prostigmata) encontrado em cavernas de Serra Leste. Reparar, nos três últimos, a pronunciada despigmentação (comparar com a espécie A) e tendência ao alongamento corporal. De todas as espécies troglomórficas ilustradas na figura, a espécie encontrada no presente estudo compreende a mais modificada (reparar o alongamento corporal e do primeiro par de pernas). 136
Figura 74 - Espécies troglmórficas de *Charinus* encontradas em Serra Leste: (A, A´) Prossoma em vista dorsal e porção ventral mostrando as placas do Sternum da espécie A; (B, B´) Prossoma em vista dorsal e porção ventral mostrando as placas do Sternum da espécie B; (C, C´) Prossoma em vista dorsal e porção ventral mostrando as placas do Sternum da espécie C; reparar, nas três espécies, a pronunciada redução da pigmentação e a regressão dos olhos laterais. 137
Figura 75 - Espécies troglmórficas de *Charinus* encontradas em Serra Leste: (A) espécie A; (B) espécie B; (C) espécie C. 138
Figura 76 - Espécies do gênero *Charinu*s encontradas em cavernas: (A) *Charinus acaraje* (não troglóbio) de cavernas da Bahia (reparar no pequeno tamanho corporal da prole e no número de filhotes); (B) *Charinus* spnA (troglomórfico), encontrado em Serra Leste (reparar no grande tamanho corporal da prole e no número reduzido de filhotes). 139
Figura 77 - Opiliões Escadabiidae encontrados em cavernas de Serra Leste: (A) espécie não troglomórfica (Escadabiidae sp2), com olhos e pigmentação bem desenvolvidos; (B) espécie troglomórfica (Escadabiidae sp5); (C) espécie troglomórfica (Escadabiidae sp6); (D) espécie troglomórfica (Escadabiidae? sp). Nos últimos, observar pronunciada redução da pigmentação e dos olhos (ausentes em (C) e (D). 140
Figura 78 - (A) oonopidae sp9; (B) Prodidomidae sp1. 141
Figura 79 - Aranhas Ochyroceratidae encontradas em Serra Leste: (A) Ochyroceratidae troglóbia (B) Ochyroceratidae não troglóbia. As imagens estão em mesma escala. Reparar no tamanho dos ovos de ambas as espécies que, embora sejam equivalentes

em volume, foram produzidos por aranhas de grande diferença de tamanho, o que indica o considerável aumento no tamanho corporal da prole na espécie troglóbia. ...141
Figura 80 - Schizomida Hubardiidae troglomórfico encontrado em cavernas de Serra Leste. 143
Figura 81 - Diplópodes Glomeridesmida encontrados em cavernas de Serra Leste: (A) Espécie troglóbia (reparar a total despigmentação e alongamento corporal e de antenas); (B) Espécie não troglóbia, fortemente pigmentada e com antenas comparativamente mais curtas. 144
Figura 82 - (A) Polydesmida sp5 (detalhe da região cefálica em vista lateral); (B) Polydesmida sp6 (detalhe da região cefálica em vista lateral); (C) Pyrgodesmidae sp6 (vista dorsal); (D) Pyrgodesmidae sp7 (detalhe da região cefálica em vista ventral); (E) Pyrgodesmidae sp6 (vista lateral). 145
Figura 83 - Spirostreptida sp2: (A) Região anterior do corpo; (B) Detalhe da região cefálica em vista lateral, onde percebe-se a ausência de olhos; (C) Organismo vivo (reparar pronunciada despigmentação). 146
Figura 84 - Formigas do gênero *Hypoponera*: (A) *Hypoponera* sp4 (troglomórfica); (B) Detalhe da região cefálica de *Hypoponera* sp4 (reparar a despigmentação e anoftalmia); (C) *Hypoponera* sp6 (não troglomórfica); (D) Detalhe da região cefálica de *Hypoponera* sp6 (reparar a pigmentação e presença de olhos). 148
Figura 85 - Formigas do gênero *Solenopsis*: (A) *Solenopsis* sp3 (não troglomórfica); (B) Detalhe da região cefálica de *Solenopsis* sp3 (reparar a pigmentação e presença de olhos); (C) *Solenopsis* sp7 (troglomórfica); (D) Detalhe da região cefálica de *Solenopsis* sp7 (troglomórfica); (D) Detalhe da região cefálica de *Solenopsis* sp7 (reparar a despigmentação e anoftalmia). 149
Figura 86 - Hemípteros Scutelleridae: (A) Scutelleridae sp1 (reparar pronunciada redução da pigmentação e anoftalmia). (B) Imagem de Scutelleridae (não troglóbio) retirada da internet. Reparar a pronunciada pigmentação (geralmente as espécies desta família são fortemente pigmentadas) e os grande olhos compostos. 150
Figura 87 - *Coarazuphium* sp. n: (A) espécime vivo; (B) espécime fixado; (C) maxila; (D) labrum; (E) porção distal da mandíbula esquerda; (F) fungo fixado à tíbia de um dos exemplares. Em detalhe, à direita, o aspecto do fungo; (G) Tíbia do primeiro par de pernas mostrando a estrutura tibial de limpeza de antenas. 152
Figura 88 - Coleópteros Dyticidae: (A) Dyticidae sp4 (troglomórfico); (B) Detalhe da região cefálica de Dyticidae sp4 (reparar a despigmentação e anoftalmia); (C) Dyticidae sp3 (não troglomórfico); (D) Detalhe da região cefálica de Dyticidae sp3 (reparar a pigmentação e presença de olhos). 153
Figura 89 - Coleópteros Eucnemidae: (A) Eucnemidae sp3 (troglomórfico); (C) Detalhe da região cefálica de Eucnemidae sp3 (reparar a despigmentação e pronunciada recução ocular); (B) Eucnemidae sp1 (não troglomórfico); (D) Detalhe da região cefálica de Eucnemidae sp1 (reparar a pigmentação e presença de grandes olhos). 154

Figura 90 - Coleópteros Pselaphidae: (A) Pselaphidae sp12 (não troglomórfico); (B) Detalhe da região cefálica de Pselaphidae sp12 (reparar a pigmentação e presença de grandes olhos); (C) Pselaphidae sp6 (troglomórfico); (D) Detalhe da região cefálica de Pselaphidae sp6 (reparar a anoftalmia). 155
Figura 91 - Coleópteros Scydmaenidae: (A) *Scydmaenidae* sp20 (troglomórfico) em vista dorsal e lateral; (B,C) Detalhes da região cefálica de *Scydmaenidae* sp20 (reparar a pronunciada recução ocular); (D) *Scydmaenidae* sp15 (não troglomórfico) em vista dorsal e e lateral; (E,F) Detalhe da região cefálica de *Scydmaenidae* sp15 (reparar a pigmentação e presença de grandes olhos). 156
Figura 92 - Coleópteros Scydmaenidae: (A) Scydmaenidae sp3 (não troglomórfico); (B) Detalhe da região cefálica de Scydmaenidae sp3 (reparar a pigmentação e presença de grandes olhos); (C) Scydmaenidae sp22 (troglomórfico); (D) Detalhe da região cefálica de de Scydmaenidae sp22 (reparar a redução das estruturas oculares). 156
Figura 93 - Coleópteros Scydmaenidae: (A) Scydmaenidae sp2 (não troglomórfico); (B) Detalhe da região cefálica de Scydmaenidae sp2 (reparar a pigmentação e presença de grandes olhos); (C) Scydmaenidae sp21 (troglomórfico); (D) Detalhe da região cefálica de Scydmaenidae sp21 (reparar a redução da pigmentação tegumentar e ocular). 157
Figura 94 - Isópodes troglomórficos encontrados em Serra Leste: (A) Styloniscidae sp1; (B) *Trichorhina* sp4. 158
Figura 95 - Isópode troglomórfico da família Baloniscidae encontrado em Serra Leste: (A) Aspecto do organismos vivo; (B) Detalhe da cavbeça em vista ventral; (C) Espécime fixado – vista ventral; (D) Espécime fixado – vista dorsal. 159
Figura 96 - a) Zona eufótica onde observa-se os líquens e musgos revestindo as paredes; b) Pórtico de entrada da cavidade em área de mata ciliar com piso alagado; c) Depósito de guano frugívoro produzido por *G. soricina* com muitas plântulas mortas junto ao substrato; d) Antigo cupinzeiro abandonado no interior da cavidade. 161
Figura 97 - a) Bufonidae indet.; b) Chiroptera: Mormopidae (P. parnellii); c) Theraphosidae. 164
Figura 98 - a) Pórtico de entrada da cavidade em área de mata ciliar; b) Piso da cavidade composto por sedimento granulado com guano misturado ao mesmo e com alguns isópodes (indicado pelo círculo amarelo) associados a este recurso; c) Depósito de guano (guanomite) com inúmeros caules secos de plântulas que germinaram ao seu redor. 165
Figura 99 - Leptodactylidae (*Eleutherodactylus* sp.); b) Amblypygi: *Heterophrynus longicornis*; c) Diplopoda: Chelodesmidae. 167
Figura 100 - a) Pórtico de entrada da cavidade em área de mata ciliar; b) Depósito de guano de morcegos frugívoros com intenso desenvolvimento de plântulas; c) Guano esparso misturado ao solo e pequenas guanomites no interior da cavidade; d) Detalhe da porção superior de uma guanomite produzida por morcegos frugívoros. 169

Figura 101 - a) Gekkonidae (*Thecadactylus rapicauda*); b) Roedor (Cricetidae: *Rhipidomys* sp.); c) Dendrobatidae (*Colostethus* sp.); d) Acari; e) Coleoptera imaturo (Dermestidae); f) Theraphosidae; g) Phyllostomidae (*G. soricina*); h) Lepidoptera (Arctiidae); i) Vespidae. 171
Figura 102 - a) Pórtico de entrada da cavidade em área de mata ciliar; b) Salão com piso revestido de guano onde se observam diversas guanomites; c) Detalhe das guanomites; d) Conjunto de guanomites encontradas na zona afótica; e) Rizotema com gastrópodes associados em zona afótica; f) Detalhe do rizotema com gastrópodes associados; g) Inúmeras plântulas germinadas em guano de morcegos frugívoros; h) resto de foqueira observada na cavidade; i) restos de pequenas manilhas; j) rolo de arame farpado deixado no interior da caverna. 173
Figura 103 - a) Leptodactylidae (*Pristimantis cf. fenestratus*); b) Phyllostomidae (*Carollia* sp); c) Blaberidae (*Blaberus* sp.).; d) Formicidae (*Gigantiops* sp.); e) Formicidae (*Odontomachus* sp.); f) Coleoptera (Cetoniidae: *Gymnets* sp.) 176
Figura 104 -) Pórtico de entrada da cavidade em área de mata ciliar com muitas raízes e serrapilheira esparsa pelo piso da cavidade; b) Parte interna da cavidade, onde existe um sistema radicular superficial bem desenvolvido. 177
Figura 105 - a) Anfíbio anuro; b) Leptodactylidae (*Pristimantis cf. fenestratus*); 180
Figura 106 - a) Porção externa à cavidade vista do interior da entrada; b) Interior da caverna onde se observa a condição topográfica do conduto que se segue à entrada; c) Guano envelhecido de morcegos frugívoros presente no piso da caverna; d) Grande quantidade de serrapilheira acumulada junto à linha d'água. 181
Figura 107 - a) Leptodactylidae (indet.); b) Sphaerodactylidae (*Coleodactylus cf. amazonicus*); Ctenidae (*Ctenus* sp.); d) Lepidoptera (Noctuidae: *Latebraria* sp.). 183
Figura 108 - a) Aspecto geral da entrada com muitas angiospermas; b) Aspecto geral do teto da cavidade completamente revestido por Actinomicetos. 184
Figura 109 - a) Aspecto geral da entrada com muitas plantas, briófitas e pteridófitas; b) Vista da porção interna da cavidade; c) Rejeito de matéria orgânica descartado por formigas (Attini); d) Lixo despejado no interior da cavidade. 186
Figura 110 -) Onychophora (Peripatidae); b) Opiliones (Cosmetidae: *Anduzeia* sp.); Collembola (Hypogastruridae); d) Opiliones (Escadabiidae); e) Gekkonidae (*Thecadactylus rapicauda*). 189
Figura 111 - a) Aspecto geral da entrada da cavidade onde se observa a presença de uma área alagada; b) Grande quantidade de algas, briófitas e pteridófitas nas paredes da entrada; c) Depósito de guano de morcegos frugívoros submerso onde podem ser observados vários caules de plântulas que germinaram nesta condição; d) Mandíbula de capivara encontrada junto ao lago no interior da cavidade. 190
Figura 112 - a) Decapoda (Palaemonidae: *Macrobrachium* sp.); b) Pseudothelphusidae (*Microthelphusa somanni*). 192

Figura 113 - a) Aspecto geral da entrada com a presença de muita serrapilheira acumulada junto à linda d'água; b) Morfologia geral predominante na cavidade e aspecto do piso com blocos esparsos. 193
Figura 114 - a)Aspecto geral da entrada da cavidade; b) Cano utilizado pela comunidade de Serra Pelada para captação de água; c) Morfologia geral de uma das galerias da cavidade (reparar nas paredes revestidas por Actinomicetos); d) Parede intensamente revestida por briófitas junto à entrada principal da cavidade. 196
Figura 115 - a) Opiliones (Cosmetidae); b) Reduviidae (*Panstrongylus* sp.); c) Scutigeromorpha (Sphendononema sp.); d) Diplopoda (Chelodesmidae); e) Opiliones (Cosmetidae); f) Coleoptera (Chrysomelidae). 198
Figura 116 - a) Aspecto geral da entrada em teto baixo com muita serrapilheira no entorno; b) Porção mais interior da cavidade. 199
Figura 117 - a) Aspecto geral da entrada, onde se observa a serrapilheira acumulada junto a linha d'água;b) Pequena depressão alagada da cavidade, no interior da qual existem raízes em desenvolvimento. 201
Figura 118 - . a) Aspecto geral do conduto e da entrada principal da cavidade; b) Depósito fresco de guano frugívoro com restos de plântulas. 204
Figura 119 - a) Aspecto geral da entrada principal da cavidade; b) Entrada secundária onde é possível observar a vegetação do entorno composta basicamente por samambaias; c) Blocos abatidos próximo a linha d'água; d) Depósito fresco de guano frugívoro com restos de plântulas e um sistema radicular desenvolvido na região periférica; e) Detalhe de um sistema radicular bem desenvolvido com o piso composto por sedimento granulado; f) Ninfa de barata (Blaberidae) associada a um conjunto de raízes. 207
Figura 120 - a) Emballonuridae (*Peropteryx kappleri*); b) Leptodactylidae (*Pristimantis cf. fenestratus*); c) Trombidiforme; d) Onychophora (Peripatidae). 210
Figura 121 - a) Aspecto geral da entrada da cavidade onde é possível observar vegetação abundante em virtude da elevada taxa de luminosidade; b) Depósito de guano de morcegos insetívoros presente na zona disfótica da caverna; c) Conduto artificial localizado na margem esquerda da cavidade 211
Figura 122 - a) Aspecto geral da entrada da caverna onde é possível observar a vegetação na região superior e um pequeno arbusto germinado junto à linha d'água; b) Zona eufótica com paredes e piso da cavidade revestida por líquens, fungos e briófitas. 214
Figura 123 - a) Leptodactylidae (*Pristimantis cf. fenestratus*); b) Diplopoda (Siphonophoridae); c) Ricinulei (*Cryptocellus tarsilae*); d) Lepidoptera (Noctuidae: *Latebraria* sp.). 216
Figura 124 - a) Aspecto geral da entrada onde é possível observar a vegetação desenvolvida no período de chuva do entorno (após queimada); b) Detalhe do afloramento ferruginoso e parte da entrada; c) Vista interna da cavidade onde é possível observar a influência da luminosidade do ambiente epígeo, o acúmulo de serrapilheira junto à linha

d’água e os blocos dispostos junto ao piso predominantemente plano; d) porção interna da cavidade. 217
Figura 125 - a) Vegetação predominante na região de entorno ao abrigo que encontra-se localizado no afloramento que pode ser observado na parte superior direita da imagem. 219
Figura 126 - a) Abrigo de entrada da cavidade, associado à quebra na canga; b) Porção interna da cavidade com detalhes das paredes e piso onde existe uma forte influência das condições ambientais epígea. 221
Figura 127 - a) Vegetação de entorno do abrigo localizado na encosta; b) Região de entrada da cavidade com detalhes das paredes e piso; c) Aspecto geral da entrada; d) Folhiço na entrada da caverna. 223
Figura 128 - a) Meio externo que circunda a região de entrada da caverna; b) Detalhe do piso da cavidade que é plano neste setor e composto por sedimento granulado com alguns pequenos seixos; c Vista interna da entrada da cavidade com detalhes da vegetação epígea; d) Grande quantidade de colônias de Actinomicetos crescendo sobre as paredes 226
Figura 129 - a) Aspecto geral da entrada da cavidade; b) Parede completamente revestida por Actinomicetos no interior da caverna; c) Fungo se desenvolvendo sobre substrato orgânico no interior da caverna. 229
Figura 130 - a) Phyllostomidae (*Carollia* sp.); b) Leptodactylidae (*Pristimantis cf. fenestratus*); b) Diplopoda (Siphonophoridae); c) Ricinulei (*Cryptocellus tarsilae*) 231
Figura 131 - a) Aspecto geral da entrada da caverna onde é possível observar a vegetação associada à zona eufótica; b) Aspecto geral da entrada vista por dentro da cavidade; c) Grande quantidade de colônias de Actinomicetos crescendo nas paredes; d) Área escavada na zona mais profunda da cavidade. No canto direito inferior da imagem observe uma ferramenta utilizada na escavação e posteriormente abandonada. 232
Figura 132 - a) Aspecto geral da entrada principal da cavidade onde é possível observar a vegetação associada na área do entorno; b) Aspecto geral da entrada secundária da cavidade onde é possível observar a vegetação associada ao entorno bem como as paredes internas revestidas por fungos. 235
Figura 133 - a) Aspecto geral da entrada da cavidade onde é possível observar a vegetação associada na área epígea; b) Aspecto geral da porção mais profunda da cavidade onde é possível observar a forte influência das condições ambientais epígeas. 238
Figura 134 - a) Aspecto geral da entrada da cavidade onde é possível observar a vegetação da área de entorno; b) Depósito de guano envelhecido de morcegos hematófagos; c) Detalhe da galeria de entrada da cavidade; d) Cupinzeiro presente no interior da cavidade; e) Algas e briófitas se desenvolvendo nas proximidades da entrada; f) Guano fresco de morcegos frugívoros. 240
Figura 135 - a) Colubridae (Chironius sp.); b) Dendrobatidae (Ameerega sp.); c) Leptodactylidae (Eleutherodactylus sp.); d) Phyllostomidae (Carollia sp.); e) Opiliones

(Manaosbiidae); f) Araneae; g) Coleoptera (Carabidae); Nematomorpha (Gordioidea). 243
Figura 136 -) Aspecto geral da entrada da cavidade vista por fora; b) Vista interna com detalhes da área empoçada e da vegetação externa; c) Grande cupinzeiro presente no interior da caverna; d,e) Detalhe da colônia; f) Depósito fresco de guano de morcegos frugívoros; g) Depósito velho de guano de morcegos frugívoros. 245
Figura 137 - a) Anura (Bufonidae); b) Leptodactylidae (*Leptodactylus labyrinthicus*); c) Dendrobatidae (*Ameerega* sp.); d) Phyllostomidae (*Carollia* sp.); e) Amblypygi (*Heterophrynus longicornis*); f) Phalangopsidae: (*Phalangopsis* sp.); g) Opilioacaridae (*Neoacarus* sp.); h) Theridiosomatidae (*Plato* sp.); i) Ricinulei (*Cryptocellus tarsilae* - ninfa); j) Carabidae (*Coarazuphium* sp.); k) Polydesmida; l) Araneae (Oonopidae). 249
Figura 138 - a) Aspecto geral da entrada da cavidade vista por fora; b) Aspecto geral da porção interna da entrada, onde é possível observar a serrapilheira acumulada junto à linha d'água; c) Depósito fresco de guano de morcegos frugívoros; d) Fezes de anfíbio Bufonidae com cupins (Nasutitermes sp.) associados. 250
Figura 139 - a) Leptodactylidae (Pristimantis cf. fenestratus); b) Amblypygi (Heterophrynus longicornis); c) Phalangopsidae (Aclodes sp.); d) Glomeridesmida (Glomeridesmus sp.); e) Schizomida (Hubbardiinae); f) Isopoda (Trichorhina sp.); g) Diplopoda (Pyrgodesmidae). 253
Figura 140 - a) Aspecto geral da cavidade onde é possível observar a serrapilheira esparsa pelo piso do abrigo; b) Lixeira (descarte) de matéria orgânica vegetal produzida por formigas da tribo Attini. 254
Figura 141 - a) Hemiptera (Nabidae); b) Opiliones (Gonyleptidae); c) Ctenidae (*Ctenus* sp.). 256
Figura 142 - a) Aspecto geral da entrada do abrigo; b) Vista interna da entrada onde é possível observar a vegetação do entorno; c) Grande quantidade de colônias de Actinomicetos crescendo nas paredes; d) Guano fresco de morcegos frugívoros no interior da cavidade. 257
Figura 143 - a) Gekkonidae (Thecadactylus rapicauda); b) Colubridae (*Leptodeira annulata*); c) Decapoda (Pseudothelphusidae: *Microthelphusa somanni*); d) Ctenidae; e) Scolopendromorpha (*Otostigmus* sp.); f) Isopoda (*Trichorhina* sp.). 259
Figura 144 - a) Aspecto geral da entrada da cavidade onde é possível observar a vegetação externa; b) Paredes e piso revestidos de líquens, briófitas e Actinomicetos; c) Sistema radicular apresentando-se de forma superficial nesta cavidade; d) Grande quantidade de algas, briófitas e pteridófitas nas proximidades de uma das entradas. 261
Figura 145 - a) Ricinulei (*Cryptocellus tarsilae*); b) Opiliones (Escadabiidae); c) Pseudoscorpiones; d) Carabidae (*Coarazuphium* sp.); e) Ochyroceratidae; f) Diplura (Anajapygidae). 264
Figura 146 - a) Aspecto geral da cavidade onde é possível observar fungos revestindo as paredes da mesma; b) Paredes e piso revestidos de líquens, briófitas e pteridófitas. ..265

Figura 147 - a) Aspecto geral da entrada da cavidade; b) Escavação realizada por garimpeiros; c) Dente de anta (Tapirus terrestris) encontrado no interior da caverna; d) Cadáver de barata sendo consumido por formigas. 267
Figura 148 - a) Phyllostomidae (*Carollia* sp.); b) Phyllostomidae (*Carollia* sp.); c) Anura (Bufonidae indet.); d) Leptodactylidae (*Pristimantis cf. fenestratus*); e) Scytodidae*:* *Scytodes eleonora*; f) Formicidae (*Cyphomyrmex* sp). 269
Figura 149 - a) Aspecto geral da entrada da cavidade onde é possível observar a vegetação externa; b) Cadáver de anuro em elevado estado de decomposição. Reparar o intenso crescimento fúngico. 270
Figura 150 - a) Aspecto geral da entrada da cavidade onde é possível observar a vegetação externa; b) Vista interna da cavidade com blocos abatidos na porção central do salão; c) Detalhe do salão interno; d) Cupinzeiro presente no interior da caverna. 272
Figura 151 - a) Aspecto geral da entrada da cavidade onde é possível observar a vegetação associada a zona de entrada; b) Detalhe da porção interior da cavidade. 274
Figura 152 - a) Cavidade com inúmeros pontos de gotejamentos durante a estação úmida; b) Poça de água formada no interior de um “quadrat” de escavação arqueológica durante o período chuvoso. 276
Figura 153 - a) Aspecto geral da vegetação do lado externo da cavidade; b) Aspecto geral da entrada da caverna. Reparar na grande quantidade de colônias de Actinomicetos junto ao teto da cavidade; c) Depósito de guano de morcegos frugívoros com sistema radicular associado; d) Piso da cavidade com sistema radicular superficial bem desenvolvido e com uma intensa associação fúngica..279
Figura 154 - a) Leptodactylidae (*Eleutherodactylus* sp.); b) Leptodactylidae (*Pristimantis cf. fenestratus*); d) Opiliones (Stygnidae); d) Polyxenida (Lophoproctidae). 282
Figura 155 - a) Entrada da cavidade; b) Conduto com piso predominantemente plano composto por sedimento de granulometria fina. 283
Figura 156 - a) Entrada principal da cavidade onde existem grandes blocos abatidos e muita vegetação recobrindo o piso. b) Vista da entrada pela porção interior da caverna; c) Resíduos sólidos abandonados no interior da cavidade; c) Regurgito de coruja Suindara (Tyto alba); d) Uma das bolotas regurgitadas depois de desfeita por ação do tempo. .286
Figura 157 - a) Diplopoda (Chelodesmidae); b) Hemiptera (Cydnidae); c) Gastropoda (Systrophiidae); d) Ricinulei (*Cryptocellus tarsilae*); e) Opiliones (Stygnidae: *Protimesius aff. gracilis*); f) Gekkonidae (*Thecadactylus rapicauda*)..289
Figura 158 - a) Entrada principal da cavidade com piso descendente e onde existem grandes blocos abatidos; b) Aspecto geral do salão principal da cavidade com muitos blocos junto ao piso e com fungos revestindo as paredes. 290
Figura 159 - a) Araneae (Ctenidae); b) Diplopoda (Pyrgodesmidae); c) Ricinulei (*Cryptocellus tarsilae*). 292
Figura 160 - a) Entrada principal da cavidade onde é possível observar a vegetação externa; b) Vista geral do salão principal da cavidade; c) Grande quantidade de Actinomicetos se

desenvolvendo em profusão pelas paredes da cavidade; d) Guano fresco de morcegos frugívoros; e) Regurgito de coruja (Tyto alba); f) “Quadrat” de escavação arqueológica, deixado aberto após escavação. 293
Figura 161 - a) Aspecto geral da entrada da caverna com vegetação epígea vista do interior da cavidade; b) Vista geral do salão principal da cavidade com líquens, fungos e briófitas revestindo as paredes. 296
Figura 162 - a) Hemiptera (Lygaeidae) ; b) Diplopoda (Pseudonannolenidae); c) Geophilomorpha. 298
Figura 163 - a) Salão principal da cavidade e piso com blocos e muita serrapilheira acumulada; b) Vista geral do salão principal da cavidade. 299
Figura 164 - a) Aspecto geral da cavidade; b) Entrada visualizada pela porção interna da caverna. 301
Figura 165 - a) Vista externa da entrada da cavidade onde se observa o lago artificial; b) Detalhe da situação do nível do mesmo lago durante a estação úmida; c) Colônias de Actinomicetos crescendo nas paredes da caverna; d) Fezes de um vertebrado encontradas na caverna. 304
Figura 166 - a) Vista externa da entrada da cavidade onde se observa a matriz da paisagem composta principalmente por pastagem (estação seca); b) Vista externa da cavidade na estação chuvosa; C) Vista interna da entrada da cavidade com vegetação muito desenvolvida na estação chuvosa; E) Rizotema presente no interior da cavidade; F) Porção mais interior da cavidade, mostrando os substratos de piso mais ressequidos (estação chuvosa). 307
Figura 167 - a) Vista externa da entrada da cavidade onde se observa a matriz da paisagem composta principalmente por pastagem; b) Pequeno salão principal da cavidade, onde se observa a presença de vegetais superiores, domosntrando a condição fótica observada na caverna; c) Outra vista do salão principal, onde se observa opiso composto por sedimento granulado com muitos seixos e calhaus; d) Ninho de cupins abandonado, presente no interior da cavidade. 310
Figura 168 - a) Phyllostomidae (G. soricina); b) Emballonuridae (Peropteryx kappleri); c) Diplopoda (Pseudonannolenidae); d) Gastropoda (Systrophiidae). 313
Figura 169 - . a) Vista externa da entrada da cavidade onde se observa a matriz da paisagem composta principalmente por pastagem; b) Detalhe da entrada; c) Salão próximo à entrada da caverna; d) Pequeno salão na zona mais profunda da cavidade com muitas briófitas e blocos esparsos pelo piso; e) Fungo Basidiomiceto se desenvolvendo no piso da cavidade; f) Teto da cavidade com uma estrutura fúngica cultivada por formigas cortadeiras (Apterostigma sp.). 314
Figura 170 - a) Vista externa da entrada da cavidade onde se observa a matriz da paisagem composta principalmente por pastagem; b) Depósito recente de guano de morcegos frugívoros; c) porção interna da cavidade na qual se observa as paredes revestidas por Actinomicetos; d) detalhe das paredes internas revestidas por Actinomicetos. 317

Figura 171 - a) Vista externa da entrada da cavidade onde se observa a matriz da paisagem composta principalmente por pastagem; b) Trecho em teto baixo sob alta influência das condições ambientais epígeas. 319

Figura 172 - a) Vista externa da entrada da cavidade onde se observa a drenagem e a área de mata ciliar em estado avançado de degradação; b) Vista da entrada da cavidade; c) Interior da cavidade com piso encharcado e sob forte influência das condições ambientais epígeas; d) detalhe da entrada, onde se observa grande quantidade de material orgânico de origem vegetal que penetra na caverna; e) Interior da cavidade com piso erodido sob efeito de enxurradas. 322

Figura 173 - a) Anura (Dendrobatidae: *Colostethus sp* .); b) Opiliones (Cosmetidae); c) Diplopoda (Siphonophoridae); d) Homoptera (indet.). 324

Figura 174 - a) Vista externa da entrada da cavidade onde se observa a área de mata com baixa densidade arbórea; b) Vista do pequeno salõa de entrada da cavidade, onde se observa uma considerável quantidade de serrapilheira importada do meio externo; c) quadrat de escavação arqueológica, aberto no piso da caverna; d) Rizotema formado em área de gotejamento próximo a zona de entrada; e) Fezes de um vertebrado bastante antigas, parcialmente decompostas e com considerável crescimento radicular. 326

Figura 175 - a) Vista externa da entrada principal da cavidade; b) vista interna de uma das estradas da cavidade, onde se observa o piso com disposição descendente em direção ao ambiente hipógeo; c) Material orgânico de origem vegetal (serrapilheira) penetrando na cavidade pos uma das entradas; d) Salão principal da cavidade que apresenta dimensões muito amplas; e) Colônia abandonada de cupins; f) guano de morcegos frugívoros observado na caverna; g) Detalhe do depósito de guano, onde se percebem sementes indigeridas. 330

Figura 176 - a) Phyllostomidae (*Carollia* sp.); b) Phyllostomidae (*Carollia* sp.); c) Anura (Leptodactylidae: *Eleutherodactylus* sp.); d) Theraphosidae; e) Isopoda (Armadillidae; f) Amblypygi (*H. longicornis*); g) Scytodidae*: Scytodes eleonora*; h) Orthoptera (Phalangopsidae)..333

Figura 177 - a) Vista externa da entrada principal da cavidade; b) Detalhe da porção lateral próxima à entrada, onde ocorrem pequenos arbustos; c) Zona eufótica com piso composto por sedimento granulado e com muita vegetação associada; d) Porção interna da cavidade. 334

Figura 178 - a) Vista interna da cavidade onde se observa grande quantidade de Actinomicetos desenvolvendo-se nas paredes; b) Depósito de guano de morcegos frugívoros em deposição; c) Depósito de guano de morcegos frugívoros envelhecido; d) Restos alimentares de morcegos Glossophaginae; e) Restos alimentares de morcegos Glossophaginae; f) Depósito de guano de morcegos frugívoros com muitas plântulas secas; g) Escavação realizada para estudos arqueológicos. 338

Figura 179 - a) Phyllostomidae (*Carollia sp*.); b) Anura (indet.).; c) Iguanidae; d) Diplopoda (Siphonophoridae); e) Hemiptera (Ploiariidae); f) Diplopoda (Polydesmida); g) Diptera (Tipulidae); h) Araneae (Thomisidae).....342
Figura 180 - a) Vista interna da entrada da cavidade; b) Vista interna do conduto que se segue à entrada da cavidade; c) Escavação realizada para estudos arqueológicos. 343
Figura 181 - a) Theraphosidae (*Acanthoscurria sp*.); b) Opiliones (Cosmetidae: *Anduzeia sp*.); c) Orthoptera (Phalangopsidae: *Aclodes sp*.).....346
Figura 182 - a) Vista interna da entrada da cavidade com mata ciliar no entorno; b) Aspecto geral do piso da cavidade com muitos blocos; c) vista interna da cavidade; d) restos esqueletais de uma capivara (*Hydrochaerus hydrochaeris*). 347
Figura 183 - a) Vista externa da entrada da cavidade com mata ciliar no entorno; b) Vista interna da entrada da cavidade com mata ciliar no entorno. 349
Figura 184 - a) Aspecto geral da entrada da cavidade com vegetação bem desenvolvida; b) Vista interna da entrada da cavidade localizada em frente à represa de Serra Leste; c) Interior da caverna (notar a grande quantidade de vegetais e serrapilheira); d) Entrada do conduto inferior que tornou-se acessível após o rebaixamento do nível da represa durante a estação úmida; e) Interior da caverna.....352
Figura 185 - a) Vista externa da entrada da cavidade localizada em pequeno fragmento de mata na margem direita da represa de Serra Leste; b) Restos de uma fogueira no interior da cavidade; c) Grande quantidade de colônias de Actinomicetos nas paredes da caverna; d) Tronco apodrecido de aproximadamente 80 cm encontrado no fundo da cavidade. 355
Figura 186 - a) Anura (indet.); b) Colubridae; c) Araneae (Corinidae); d) Planaria (Geoplanidae); e) Isopoda (Armadillidae); f) Orthoptera (Phalangopsidae: Phalangopsis sp.); g) Diplopoda (Polydesmida); h) Scutigeromorpha (Sphendononema sp.); i) Nematomorpha (Gordioidea). 358
Figura 187 - a) Vista externa da entrada da cavidade localizada em pequeno fragmento de mata na margem direita da represa de Serra Leste; b) Vista da porção interna da cavidade. 359
Figura 188 - a) Vista externa da entrada da cavidade localizada na margem direita da represa de Serra Leste; b) vista interna da entrada. 361
Figura 189 - a) Amblypygi (H. longicornis); b) Diplopoda (Paradoxosomatidae); c) Diplopoda (Polydesmida); d) Coleoptera (Curculionidae). 363
Figura 190 - a) Vista externa da entrada da cavidade localizada em uma pequena drenagem na margem direita da represa de Serra Leste; b) Vista interna da cavidade com detalhes do piso alagado com muitas raízes; c) Grande quantidade de colônias de Actinomicetos se desenvolvendo sobre as paredes; d) Detalhe dos tufos radiculares se desenvolvendo no piso alagado da cavidade. 364
Figura 191 - a) Salão principal da cavidade muito iluminado e com muita vegetação associada junto ao piso e as paredes; b) Vista interna da entrada da cavidade localizada em mata

ciliar próxima a uma pequena drenagem de Serra Leste. No detalhe a vegetação bem desenvolvida próximo a linha d'água; c) Depósito de guano de morcegos frugívoros com muitas plântulas recém germinadas; d) Detalhe das plântulas germinadas no guano; e) Cadáver de grilo (*Phalangopsis sp*.) com grande desenvolvimento fúngico; f) Sistema radicular bem desenvolvido associado a uma grande mancha de guano de morcegos frugívoros. 367

Figura 192 - a) Anura (Leptodactylidae: *Eleutherodactylus* sp.); b) Phyllostomidae (*Carollia* sp.); Hemiptera (Cercopidae); d) Diplopoda (*Glomeridesmus sp*.); e) Diplopoda (Polydesmida); f) Diplopoda (Chelodesmidae). 371

Figura 193 - Vista externa da entrada da cavidade localizada em mata ciliar próxima a uma pequena drenagem de Serra Leste. No detalhe a vegetação bem desenvolvida próximo a linha d'água; b) Detalhe de uma porção interior da caverna. 372

Figura 194 - a) Vista externa da entrada da cavidade onde é possível observar uma grande quantidade de serrapilheira; b) Porção interna da cavidade, com paredes e teto repletos de Actinomicetos; c) Sistemas radiculares junto ao piso; d) Teto da cavidade com uma estrutura fúngica cultivada por formigas cortadeiras (Apterostigma sp.). 374

Figura 195 - a) Anura (*Pristimantis cf. fenestratus*) predando um grilo Phalangopsidae. 376

Figura 196 - a) Vista interna da entrada da cavidade localizada em mata ciliar; b) Porção interior da cavidade, onde se observa a serrapilheira esparsa e sistemas radiculares; c) Raízes de pequeno calibre crescendo sobre a parede (em detalhe, colônias de Actinomicetos); d) Colônias de Actinomicetos se desenvolvendo em profusão nas paredes da cavidade; e) Rizotema encontrado no interior da cavidade. 377

Figura 197 - a) Opiliones (Stygnidae: *Protimesius sp*.); b) Araneae (indet.); c) Homoptera (Cixiidae); d) Onychophora (Peripatidae); e) Formicidae (*Odontomachus sp*.); f) Araneae (Ctenidae); g) Amblypygi (*Heterophrynus longicornis*); h) Vespidae; i) Colubridae (*Spilotes pullatus*). 380

Figura 198 - a) Vista interna da entrada da cavidade localizada em pequeno fragmento de mata; b) Piso da cavidade completamente revestido por guano; c) Fezes de um vertebrado; d) Guano de morcegos frugívoros encontrado no interior da caverna; e) Rizotemas crescendo na base e sobre um bloco de rocha; f) Vísceras sendo consumidas por formiga *Camponotus sp*. g) Imagem anterior agora em detalhe; h) Fragmento de madeira observado no interior da cavidade; i) Ferramenta de madeira utilizada na extração de guano; j) garrafa plástica deixada no interior da caverna. 382

Figura 199 - a) Anura (indet.); b) Phyllostomidae (*Carollia sp*.); c) Theraphosidae (*Nhandu coloratovillosus*); d) Corinidae; e) Coleoptera (Tenebrionidae); f) Geophilomorpha. (As letras da legendas das fotos estavam erradas). 385

Figura 200 - a) Vista interna da entrada da cavidade localizada em pequeno fragmento de mata ciliar; b,c) Acúmulo de serrapilheira transportada durante o período de chuvas; d) Depósito de guano de morcegos frugívoros; e) Detalhe do depósito anterior; f)

Actinomicetos se desenvolvendo nas paredes; g) porção alagada da cavidade durante o período de chuvas. 387
Figura 201 - a) Ricinulei (*Cryptocellus tarsilae*); b) Araneae (Palpimanidae); c) Diplopoda (Chelodesmidae); d) Scutigeromorpha (*Sphendononema sp*.); e) Amblypygi (Charinidae: *Charinus sp.)* com filhotes; f) Ninfas de Ricinulei (*Cryptocellus tarsilae*). 391
Figura 202 - a) Vista geral da vegetação onde a cavidade encontra-se inserida; b) Vista interna da cavidade, onde visualiza-se a entrada; c) porção alagada da cavidade durante o período de chuvas; d) Actinomicetos se desenvolvendo nas paredes. 392
Figura 203 - a) Cadáver de morcego sendo consumido por grilos Phalangopsidae; b) Cadáver de morcego sendo consumido por grilos Phalangopsidae; c) Trombidiforme; d) Homoptera (Kinnaridae); e) Hemiptera (Ploiariidae); f) Boidae (Epicrates sp). 395
Figura 204 - a) Aspecto geral do salão principal da cavidade com piso composto por sedimento fino com alguns blocos esparsos; c) Depósito de matéria orgânica associado ao conduto descendente. 396
Figura 205 - . a) Vista da área externa onde observa-se a entrada da cavidade e a vegetação epígea associada; b) galeria principal da cavidade; c) Actinomicetos se desenvolvendo em profusão nas paredes; d) Ninhos de vespas presentes no interior da caverna; e) Rizotema encontrado associado a um ponto de gotejamento ativo; f) detalhe de agregações de raízes no piso da caverna. 399
Figura 206 - a) Phyllostomidae (*Anoura sp*.) com filhotes; b) Lagarto Gekkonidae (*Thecadactylus rapicauda*); c) Anura (Dendrobatidae); d) Gastropoda (Systrophiidae). 402
Figura 207 - a) Vista interna da entrada da cavidade com detalhes da vegetação epígea; b) Vista o salão da entrada da cavidade; c) Pequena drenagem ativa presente na cavidade; d) Sistema radicular bem desenvolvido associado ao teto da cavidade; e) Cadáver recente de morcego insetívoro (*Pteronotus sp*.) encontrado durante a estação úmida; f) Cadáveres de morcegos em avançado estado de decomposição. 404
Figura 208 - Vista interna da entrada da cavidade com detalhes da vegetação epígea associada; b) Musgos associados à zona de entrada da cavidade; c) Depósito de guano de morcegos frugívoros; d) "Pellet" de fezes frescas de um anfíbio; e) "Pellet" de fezes antigas de um anfíbio, onde se percebe o crescimento fúngico; f) Ninhos abandonados de vespas nas paredes da caverna. 407
Figura 209 - a) Phyllostomidae (*Trachops cirrhosus*); b) Anura (Leptodactylidae: *Pristimantis cf. fenestratus*); c) Araneae (Scytodidae*: Scytodes eleonora*); d) Araneae (Pholcidae); e) Amblypygi (*Heterophrynus longicornis*); f) Orthoptera (Gryllidae). 410
Figura 210 - a) Vista interna da entrada da cavidade com detalhes da vegetação epígea associada; b) Salão principal da caverna onde o ambiente é um pouco mais confinado. 411
Figura 211 - a) Vista externa da entrada da cavidade com detalhes da vegetação epígea associada; b) Vista interna da cavidade. 414

Figura 212 - a) Lepidoptera (Tineioidea); b) Araneae (Filistatidae) predando uma lepidóptera (Tineidae); d) Microcoryphia (Meinertellidae). 415
Figura 213 - a) Vista interna da entrada da cavidade com blocos revestidos por briófitas; b) Depósito de guano de morcegos frugívoros; c) Depósito de guano de morcegos hematófagos; d) Poça de água misturada ao guano de morcegos hematófagos; e) Sistema radicular bem desenvolvido nas paredes da caverna; f) Detalhe das raízes, algumas das quais eliminando exudatos. 417
Figura 214 - a) Emballonuridae (*Peropteryx kappleri*); b) Diptera (Culicidae*:* Sabethini); c) Araneae (Ochyroceratidae); d) Larva díptera Culicidae (Toxorhynchites sp.) ; e) Amblypygi (*Charinus sp*.); f) Pseudoscorpiones (Chernetidae); g) Orthoptera (indet.); h) Araneae (Ctenidae); i) Planaria. 420
Figura 215 - a) Vista interna da entrada da cavidade evidenciando a vegetação externa; b) Aspecto interno da cavidade onde se observa o piso e os blocos abatidos. 421
Figura 216 - a) Vista da porção interna da cavidade; b) Rizotema junto ao piso. 424
Figura 217 - a) Vista interna da entrada da cavidade; b) Serrapilheira presente nas proximidades da entrada da caverna; c) Raízes de pequeno calibre crescendo sobre as paredes; d) detalhe das raízes mostradas na foto anterior. 426
Figura 218 - a) Vista da porção interna da cavidade; b) presença de grande quantidade de musgos, líquens e Actinomicetos nas paredes da caverna. 428
Figura 219 - a)Vista externa da entrada da cavidade; b) Aspecto geral de um dos salões principais da cavidade; c) Depósito de guano de morcegos hematófagos em zona afótica; d) Fungos cultivados por colônia de formigas (*Apterostigma sp*.); e) Restos de frutos abandonados no interior da cavidade; f) Cabeça de um lagarto em avançado estágio de decomposição (reparar no crescimento fúngico). 431
Figura 220 - a) Araneae (Pholcidae); b) Amblypygi (*Heterophrynus longicornis*); c) Amblypygi (*Charinus sp*.); d) Araneae (Corinidae); e) Ricinulei (*Cryptocellus tarsilae*); f) Gastropoda (Systrophiidae). 434
Figura 221 – a) Aspecto geral da entrada e da vegetação associada; b) Pteridófitas associadas à zona de entrada da cavidade. 435
Figura 222 - a) Vista externa da entrada da cavidade, onde se observa grande quantidade de pteridófitas no piso externo; b) Vista interna da entrada da cavidade; b) Detalhe de um conduto que conecta à entrada; d) Rizotema em zona afótica da cavidade; e) Depósito de guano de morcegos frugívoros com heterópteros associados. 437
Figura 223 - . a) Gastropoda; b) Opiliones (Escadabiidae); c) Ricinulei (*Cryptocellus tarsilae*); d) Amblypygi (*Charinus sp*.); e) Hemiptera (Ploiariidae); f) Araneae (Ochyroceratidae). 440
Figura 224 - a) Vista externa da entrada da cavidade; b) Vista interna da entrada da cavidade; c) Aspecto geral do piso no interior da cavidade (notar a presença de rizotemas); d) Rizotema associado a um ponto de gotejamento inativo; e) Ninhos abandonados de

vespas presos às paredes da caverna; f) Teto completamente revestido por Actinomicetos...443
Figura 225 - a) Vista interna da entrada da cavidade; b) Aspecto geral do piso da cavidade na zona distal da cavidade. 444
Figura 226 - a) Vista interna da entrada da cavidade (reparar a grande quantidade de Actinomicetos nas paredes); b) Rizotema se desenvolvendo no piso; c) Fezes de um vertebrado frugívoro. 447
Figura 227 - a) Sistema radicular superficial bem desenvolvido; b) Detalhe de sistema radicular superficial bem desenvolvido; c) Depósitos de guano de morcegos frugívoros; d) Detalhe de depósitos de guano; e) Grilo com intenso crescimento fúngico; f) restos de madeira em decomposição na porção mais interna da caverna; g) Poça de água formada durante a estação úmida...450
Figura 228 - a) Morcego Phyllostomidae (*G. soricina*); b) Anura (Leptodactylidae: *Leptodactylus labyrinthicus*); c) Hemiptera (Reduviidae: Panstrongylus sp.); d) Amblypygi (*Heterophrynus longicornis*); e) Amblypygi (*Heterophrynus longicornis*) vista ventral com ovos; g) Gastropoda (Systrophiidae); h) Araneae (indet.); i) Gastropoda (Subulinidae); i) Isopoda (Balloniscidae); k) Diplopoda (Pyrgodesmidae); l) Diplopoda (Pyrgodesmidae). 453
Figura 229 - a) Vista externa da entrada da cavidade com detalhes da vegetação associada; b) Vista interna do pórtico de entrada da cavidade. 454
Figura 230 - a) Vista externa da entrada da cavidade com detalhes da vegetação associada; b) Depósito de guano de morcegos frugívoros localizado no interior da cavidade. 456
Figura 231 - a) Anura (Leptodactylidae: *Pristimantis cf. fenestratus*); b) Anura (Leptodactylidae: *Pristimantis cf. fenestratus*); c) Scutigeromorpha (*Sphendononema sp.);* d) Gastropoda (Glomeridesmidae: *Glomeridesmus sp.*) 459
Figura 232 - a) Vista externa da entrada da cavidade com detalhes da vegetação associada; b) Vista interna do pórtico de entrada da cavidade com muita vegetação associada. ..460
Figura 233 - a) Vista externa da entrada da cavidade; b) Vista interna da entrada, onde se observam blocos abatidos e serrapilheira proveniente da vegetação externa.............462
Figura 234 - a) Boidae (*Epicrates* sp.); b) Araneae (Corinidae); c) Gastropoda (Systrophiidae). 464
Figura 235 - a) Vista interna da entrada da cavidade; b) Aspecto geral do piso da caverna; c) Vegetação associada às paredes da cavidade...465
Figura 236 - a) Araneae (Pholcidae); b) Opiliones (Manaosbiidae); c) Araneae (indet.); d) Scolopendromorpha (Otostigmidae: *Otostigmus sp*.). 468
Figura 237 - a) Vista interna da entrada da cavidade; b) Fungos cultivados por colônia de formigas (Apterostigma sp.); c) Grande quantidade de raízes se desenvolvendo no piso da cavidade; d) depósito antigo de guano de morcegos. 469
Figura 238 - a) Anura (Leptodactylidae: *Pristimantis* cf. *fenestratus*); b) Roedor (Cricetidae: *Rhipidomys sp*.); c) Pholcidae; d) Orthoptera (Tettigoniidae: Listroscelidinae). 472

Figura 239 - Classes de riqueza total das cavernas da área. Os valores no eixo vertical indicam o número total de espécies, e no eixo horizontal, o número de cavernas em cada categoria...475
Figura 240 - Curvas de coletor construídas para as cavernas da área. O primeiro gráfico refere-se ao período de seca, o segundo ao período chuvoso e o último ao somatório do total de espécies. Nenhuma das curvas atingiu a assíntota. 477
Figura 241 - Curvas de coletor construídas para as cavernas da área, a partir da exclusão das espécies “acidentais”. O primeiro gráfico refere-se ao período de seca, o segundo ao período chuvoso e o último ao somatório do total de espécies. Percebe-se que a curva que representa o somatório de espécies tendeu à assíntota.......................................478
Figura 242 - A) Curvas acumulativas de espécies, indicando o número observado de espécies nas cavernas da área (em vermelho) e o número estimado pelo modelo Jack-Knife 1 considerando-se todas as espécies (em azul) e o número estimado pelo modelo Jack-Knife 1 excluindo-se as espécies “acidentais” (em verde); B) Curvas acumulativas de espécies construídas para o período de seca; (C) Curvas acumulativas de espécies construídas para o período de chuva...480
Figura 243 - Dendrogramas de similaridade (Bray-Curtis), evidenciando os padrões de similaridade entre as cavernas. A figura da esquerda representa o dendrograma referente à estação de seca e a da direita representa o dendrograma referente à estação chuvosa. Os pontos vermelhos representam valores de similaridade entre cavernas superiores a 60% e os pontos verdes valores superiores a 50%. 482
Figura 244 - Sobreposição do gráfico da análise de n-MDS e da distribuição espacial das cavernas da área. A elipse azul contorna a nuvem de dispersão que representa a quase totalidade das cavernas da área. A forma verde contorna 4 cavernas que se mostraram altamente dissimilares em relação às demais (SL-001, SL-002, SL-003 e SL-004) 483
Figura 245 - Sobreposição do gráfico da análise de n-MDS e da distribuição espacial das cavernas da área. A elipse azul contorna a nuvem de dispersão que representa a quase totalidade das cavernas da área. A forma verde contorna 4 cavernas que, nesta estação, se sobrepuseram à nuvem geral de dispersão de similaridade (SL-01, SL-02, SL-03 e SL-04)..484
Figura 246 - (A) Relação entre o tamanho das cavernas e a riqueza de espécies na estação seca; (B) Relação entre o tamanho das cavernas e a riqueza de espécies na estação chuvosa; (C) Relação entre o tamanho das cavernas e a riqueza total de espécies; (D) Relação entre o tamanho das cavernas e o número de espécies troglomórficas (troglóbias) presentes. 485
Figura 247 - Relação entre a riqueza total de espécies e o número de espécies troglomórficas (troglóbias) presentes nas cavernas de Serra Leste...487
Figura 248 - Histograma evidenciando intervalos de valores de “turnover” e o número de cavernas em cada intervalo. 490

Figura 249 - Relação entre a o “turnover” de espécies e o tamanho das cavernas de Serra Leste. 490
Figura 250 - Relação entre o número de espécies troglomórficas (troglóbias) e o “turnover” de espécies nas cavernas de Serra Leste. 493
Figura 251 - Fluxograma de classificação do grau de relevância de cavidades naturais subterrâneas, segundo a Instrução Normativa N. 2, do MMA. 6.2. Resultados da análise de relevância das cavernas de Serra Leste...496
Figura 252 - Mapa da localização da unidade espeleológica de Carajás, em destaque (seta amarela), a unidade Serra Leste. Fonte: Valentim & Olivito, 2011. 497
Figura 253 - Cavidades que apresentam espécies troglóbias com raridade tipo IV (Uniques – um único indivíduo foi observado durante o estudo). 516
Figura 254 - Cavidades que apresentam espécies troglóbias com raridade tipo III. Tal raridade resulta da sobreposição da raridade tipo I (ocorrência em até 3 cavernas) com a raridade tipo II (um único indivíduo observado por caverna, independentemente do número de cavernas). 518
Figura 255 - Cavidades que apresentam espécies troglóbias com raridade tipo II (um único indivíduo observado por caverna, independentemente do número de cavernas). 519
Figura 256 - Cavidades que apresentam espécies troglóbias com raridade I (ocorrência em até 3 cavernas). Tais cavidades compreendem aquelas consideradas como de relevância máxima. 520
Figura 257. Cavidades que apresentam as maiores populações de espécies troglóbias consideradas não raras. Destaca-se a caverna SL-031 (em vermelho), que apresenta as maiores populações de cinco espécies de troglóbios não raros. 522
Figura 258 - Cavidades aqui consideradas como “hotspots” para a conservação. Nelas, se sobrepõem todos os critérios anteriormente considerados (todos os tipos de raridade e maiores populações de troglóbios não raros). Estas cavernas correspondem a SL-031, SL-035, SL-074 e SL-096...523
Figura 259 - Riqueza de espécies troglóbias nas cavernas da área (são destacadas somente as cavernas com riqueza de troglóbios superior a 4 espécies). As estrelas verdes compreendem cavernas com riqueza entre 4 e 6 espécies; as estrelas brancas compreendem cavernas com riqueza entre 7 e 10 espécies; as estrelas amarelas compreendem cavernas com riqueza superior a 10 espécies 525
Figura 260 - Gráfico dos graus de relevância final das cavernas de Serra Leste...................536
Figura 261 - Cenário de interferências entre o Plano Diretor e as cavernas de Serra Leste. 534

1. INTRODUÇÃO

As cavernas são cavidades naturais subterrâneas formadas frequentemente pela ação da água que atua dissolvendo e/ou erodindo diferentes tipos de rochas (Gilbert et al 1994). A maioria das cavernas localiza-se em rochas carbonáticas (calcários e dolomitos), as mais favoráveis aos processos de dissolução. Entretanto, arenitos e quartzitos são também muito susceptíveis a formar cavernas, aparentemente devido a fatores geomorfológicos e climáticos mais erosivos (Auler, 2006). A ocorrência de cavernas em granito, gnaisse, micaxistos, filitos e até mesmo solo também são registradas, entretanto em escala menor que os carbonatos, quartzitos e arenitos (Auler, 2006). Recentemente, constatou-se, em áreas de minério de ferro, a existência de um número expressivo de cavernas, fato que adicionou mais uma potencialidade de ocorrência de cavernas ao já variado cenário espeleológico Brasileiro (Auler, 2006).

No Brasil há aproximadamente 9.000 cavidades naturais subterrâneas cadastradas, mas estima-se que o potencial brasileiro seja superior a 100 mil cavernas (Auler, 2006). Destas, são conhecidas aproximadamente 1.000 cavernas ferruginosas (30% do potencial) (Auler, 2006). Entretanto, estes números refletem apenas o total de cavernas visitadas por espeleólogos que se preocuparam em cadastrar os dados, podendo existir um número maior de cavernas conhecidas e ainda não cadastras (Auler, 2006; Souza-Silva, 2008).

O presente documento tem como objetivo apresentar os resultados dos estudos espeleológicos realizados em 96 cavernas localizadas na área do Projeto Serra Leste, empreendimento da empresa VALE, situado na região de Carajás, Estado do Pará (Figura 1). Tais estudos estão em consonância com o Decreto Federal Nr. 6.640 de 2008, e foram conduzidos segundo as metodologias estabelecidas na Instrução Normativa Ministério do Meio Ambiente Nr. 02 de 2009.

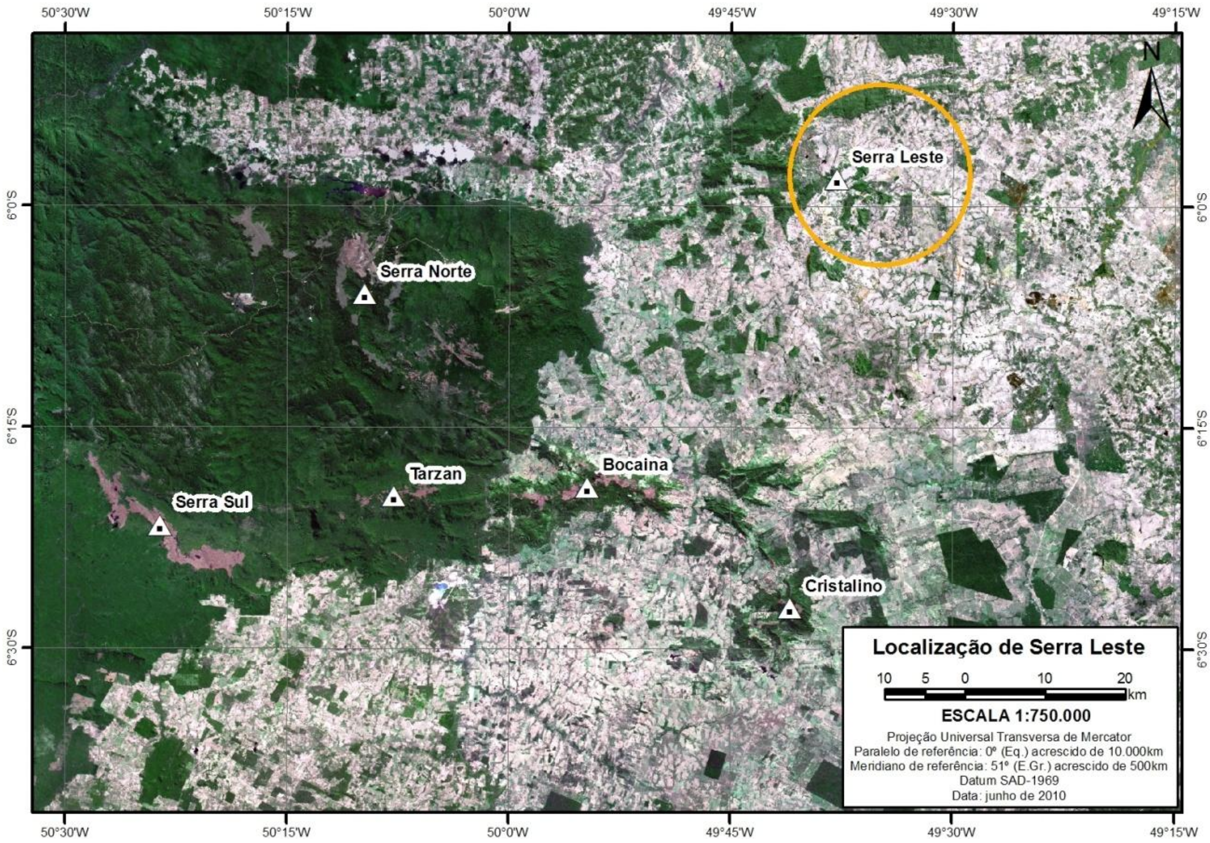

Figura 1 - Localização da área de estudo.

Especificamente, pretende-se:

- Analisar os aspectos físicos das cavernas inseridas na área do projeto, incluindo sua morfologia e seus aspectos espeleométricos; a litologia e suas estruturas; os processos hidrológicos reconhecidos através das feições observadas; e, por fim os seus depósitos clásticos, químicos e orgânicos;
- Analisar questões sócio-econômicas e culturais envolvendo estas cavidades;
- Caracterizar o ecossistema de cada uma das cavidades inventariadas, a partir da descrição de sua fauna, bem como da avaliação do sistema trófico;
- Avaliar o estado de conservação das cavernas e de seus respectivos entornos;
- Efetuar análise de relevância das cavidades, conforme determina o Decreto Federal Nr. 6.640 e a Instrução Normativa Nr. 2, do Ministério do Meio Ambiente – MMA;
- Identificar e avaliar os impactos ambientais (reais e potenciais) considerando, inicialmente, a não implantação do empreendimento e, em seguida, a instalação e operação das atividades minerárias.

2. METODOLOGIA

2.1. Levantamento bibliográfico

Consistiu em um levantamento bibliográfico sobre cavernas em rochas ferríferas no Brasil, com destaque para a região de Carajás, no Pará. Com o objetivo de melhor caracterizar o ambiente onde essas cavernas estão inseridas, também se realizou uma pesquisa envolvendo os temas geologia (lito-estruturas), geomorfologia, hidrografia, hidrologia e pedologia.

2.2. Prospecção Espeleológica

A prospecção espeleológica na região de Serra Leste foi realizada pela equipe do Grupo Espeleológico de Marabá (GEM), através da Fundação Casa de Cultura de Marabá (FCCM), em duas fases. A primeira, financiada pela própria instituição, ocorreu entre setembro de 1997 e outubro de 1998. Posteriormente, em função de um convênio firmado entre a VALE e FCCM, realizou-se uma segunda etapa de campo em setembro de 2005. Ambos os levantamentos foram realizados de maneira sistemática, no entanto, devido a tecnologia empregada na época, não existe o registro linear dos caminhamentos executados, apenas uma "nuvem" de pontos coletados quando da mudança de trajetória das equipes de campo.

Os caminhamentos realizados seguiram a seguinte metologia:

- Caminhamentos diários feitos em subgrupos percorrendo a área em linhas paralelas em distâncias de 15 a 20 m entre si;
- Feições morfológicas com maior probabilidade de ocorrência de cavernas, tais quais drenagens, bordas de platôs, desníveis, afloramentos rochosos e áreas com vegetação distinta das de seu entorno, foram percorridas com mais atenção;
- Registro dos pontos de caminhamento foram coletados através do GPS de navegação de marca Garmin, modelo Garmin V;
- Os grupos mantiveram comunicação visual ou através de apitos e rádio comunicadores;
- Nos terrenos de maior declividade ou com vários níveis de paredões paralelos, foram feitas de duas a seis linhas paralelas, de forma a garantir um levantamento mais seguro.

2.3. Espeleotopografia

O levantamento espeleotopográfico das cavernas de Serra Leste foi realizado pelo Grupo Espeleológico de Marabá (GEM/FCCM) e pela empresa Carste Consultores Associados

(CARSTE) que atuaram em campanhas de campo distintas. A campanha de campo da CARSTE ocorreu entre os dias 19 de julho e 02 de agosto de 2008, e a do GEM/FCCM entre os dias 16 de outubro e 06 de novembro de 2008.

As cavernas foram topografadas com bússola Suunto KB-14 e clinômetro Suunto PM-5, além de trena laser Leica Disto A3 ou trena de fita (30 m). O grau de precisão dos mapas atingiu 5D, com base no sistema British Cave Research Association – BCRA. Esse grau prevê medidas de ângulo na linha central de topografia com precisão de ± 1°, medidas de distância com precisão de 1 cm e posicionamento das bases com erro menor do que 10 cm. Medidas entre as bases topográficas devem ocorrer sempre que houver alterações na morfologia da caverna. O caminhamento da topografia seguiu o método de bases fixas.

Os dados da topografia foram tratados no software OnStation (2000), permitindo assim a visualização dos dados da linha de trena de forma tridimensional. A declinação magnética foi obtida no site do Observatório Nacional (www.on.br).

A cartografia final foi realizada em software de plataforma CAD (AutoCAD 2008). As linhas de trena foram devidamente declinadas e georrefenciadas. Nesse programa foram finalizados os itens da planta baixa como contorno de paredes, curvas de nível, linha d’água, entre outros. As seções foram anexadas contendo escala gráfica e numérica. Nesta etapa, foram calculados os atributos espeleométricos das cavernas, incluindo projeção horizontal (método da descontinuidade), desnível, área e volume estimado.

Para o cálculo da ***projeção horizontal*** utilizou-se o princípio da descontinuidade (Rubbioli & Moura, 2005), que desconsidera a largura dos condutos no cálculo final. Deste modo, a soma do comprimento de um conduto é feita em seu eixo central. No cruzamento entre dois condutos esta medição é interrompida de modo a não incluir o comprimento lateral do novo conduto (Figura 2).

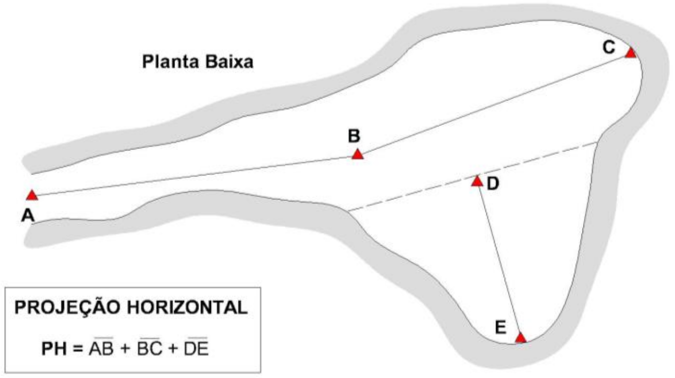

Figura 2 - Medição da projeção horizontal de acordo com o método da descontinuidade.

O ***desnível*** é o resultado da diferença altimétrica das bases topográficas (Figura 3). Importante salientar que, na etapa de campo, a altura das bases visadas acima do nível do piso tiveram sua altura registrada, para que posteriormente esta medida fosse subtraída no momento da construção das curvas de nível. Em casos de patamar, paleopiso ou nível superior sua altura foi considerada no cálculo do desnível.

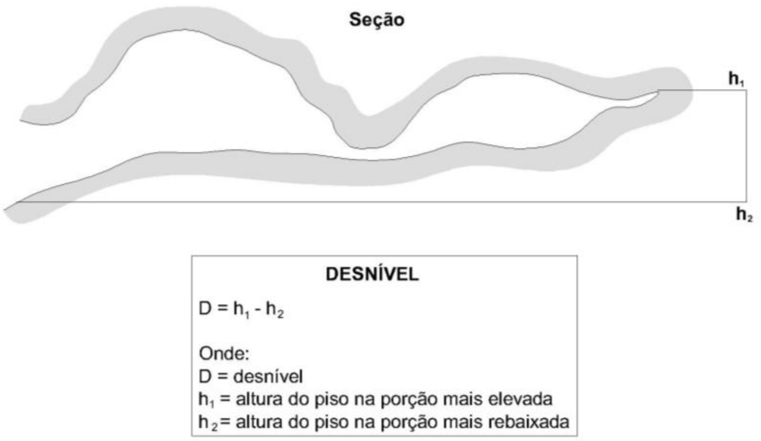

Figura 3 - Medição do desnível da caverna.

A ***área*** foi calculada no software AutoCAD a partir da planta baixa das cavernas. Em caso de ocorrência de pilares, as suas áreas foram calculadas individualmente e subtraídas do valor total calculado. Em planta baixa, o nível superior é apenas indicado, sendo seu detalhamento e cálculo espeleométrico feito separadamente. A área total é o resultado da soma da área da planta baixa de todos os níveis de piso, descontadas as áreas dos pilares (Figura 4).

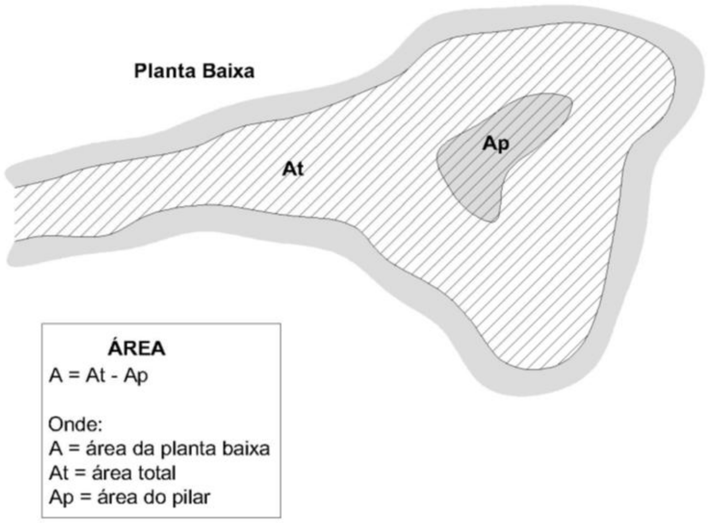

Figura 4 - Medição da área da caverna descontando a área de pilares.

O ***volume*** é o produto entre a área total da caverna e a altura média de seus condutos e salões. Para a obtenção da altura média, é necessária que sejam feitas seções em pontos representativos da cavidade onde existam mudanças morfológicas do piso, teto e paredes. Para que se calcule um valor mais próximo da realidade são feitas seções longitudinais sempre no eixo central do conduto e o maior número possível de seções transversais. Quanto mais seções forem consideradas, maior será a precisão no cálculo do volume (Figura 5).

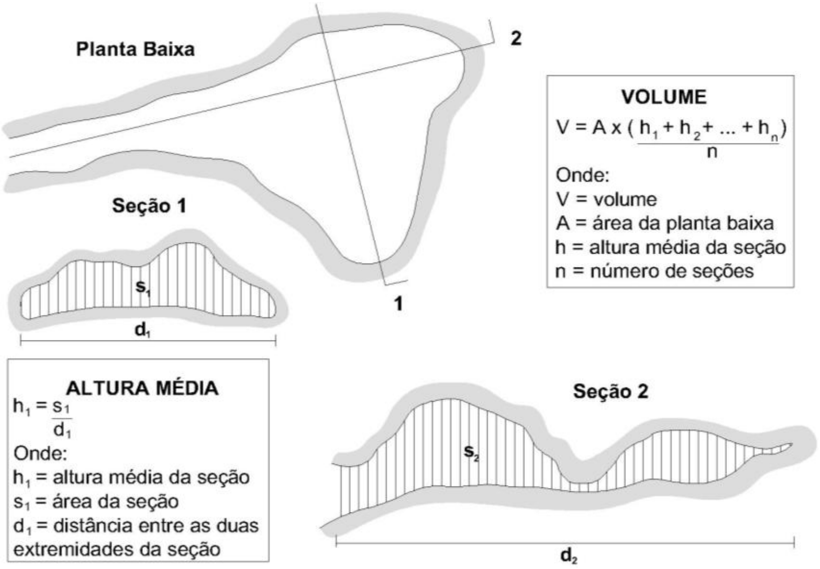

Figura 5 - Cálculo do volume de uma caverna a partir da altura média das seções verticais.

2.4. Geoespeleologia

2.4.1. Campanhas de campo

O estudo geoespeleológico de Serra Leste foi realizado em quatro campanhas de campo. A primeira entre os dias 20 de maio e 10 de junho de 2010, a segunda entre 05 e 19 de agosto de 2010, a terceira entre 9 e 23 de setembro de 2010 e a quarta entre 1 e 8 de fevereiro de 2011.

2.4.2. Análise litológica e das estruturas

A análise da litologia buscou caracterizar os litotipos em que as cavernas estão inseridas. Estruturas como bandamento, foliação, fraturas, juntas de alívio, dobras e falhas presentes foram descritas e, quando possível, medidas.

Essas estruturas representam descontinuidades importantes que condicionam a circulação de fluidos no interior do maciço rochoso, além de intervir direta ou indiretamente no processo de gênese e desenvolvimento das cavernas. Muitas vezes tais estruturas controlam

a morfologia das cavernas, além de condicionarem processos de abatimentos em paredes e teto. A medição das estruturas foi realizada com bússola de marca Brunton, modelo DF5008.

2.4.3. Análise morfológica

Na análise morfológica buscou-se interpretar o padrão planimétrico da caverna, com o objetivo de correlacioná-lo a dados estruturais e litológicos. Para tanto foi utilizada a planta baixa e seções transversais e longitudinais das cavidades, uma vez que suas formas estão diretamente relacionadas aos processos de gênese e evolução das cavernas.

2.4.4. Hidrologia

Com o objetivo de se determinar a dinâmica hidrológica das cavernas foi avaliada a presença de águas de percolação e condensação, além da presença de corpos hídricos.

2.4.5. Depósitos clásticos, orgânicos e químicos.

Depósitos de sedimentos clásticos foram classificados conforme sua granulometria e descritos de modo a fornecer pistas sobre sua origem e sedimentação. Os depósitos orgânicos também foram descritos de forma breve, quando existentes. Os depósitos químicos (espeleotemas) foram identificados, fotografados e descritos individualmente ou em conjunto.

2.5. Bioespeleologia

2.5.1 Procedimentos de coleta

O inventário de fauna foi realizado em dois períodos, sendo um correspondente à estação chuvosa, e o outro, à estação seca na região. As datas das amostragens realizadas em cada caverna em cada estação, são mostradas na Tabela 1.

A coleta de invertebrados foi feita através de captura manual (com o auxílio de pinças e pincéis) em quaisquer biótopos potenciais no interior de cada caverna (em cada estação).

Foram coletados somente espécimes não identificados *in loco*, o que corresponde a um percentual reduzido da fauna de cada cavidade. Tal procedimento minimiza enormemente os impactos em relação a uma coleta exaustiva, não recomendada em casos onde se faz necessária uma nova coleta na estação subsequente. Desta forma, inventários que requerem a coleta de todos os organismos observados certamente têm grande potencial de desestruturar as comunidades de uma dada caverna, o que certamente pode interferir nas análises de relevância das cavidades. Reitera-se, ainda, que as licenças de coleta para organismos cavernícolas (salvo exceções vinculadas a projetos específicos) geralmente não permitem a coleta de mais de 10 espécimes (de cada espécie) por caverna.

Não foram utilizadas armadilhas de queda pela baixa eficiência que tais armadilhas demonstraram em muitos trabalhos (Ferreira, 2004). Além disso, armadilhas de queda notoriamente causam distúrbios em populações cavernícolas (Weinstein & Slaney, 1995; Sharrat *et al*., 2000; Souza-Silva *et al*., 2011). Para a amostragem de invertebrados, buscas diretas são bastante efetivas, desde que realizadas por bioespeleólogos experientes (Weinstein & Slaney, 1995).

Todos os organismos coletados foram fixados em álcool 70% e encontram-se depositados na coleção de invertebrados subterrâneos (ISLA) do laboratório de Ecologia Subterrânea do Departamento de Biologia da Universidade Federal de Lavras. Algumas das espécies inventariadas neste projeto já se encontram em processo de descrição.

As coletas dos organismos do presente trabalho foram realizadas com o auxílio de pinças e pincéis, através de uma procura visual detalhada priorizando micro-habitats, como depósitos de matéria orgânica, espaços sob rochas, tetos e paredes, bem como locais com maior umidade para determinar a composição da fauna de artrópodes nas cavernas. Os organismos coletados foram fixados em álcool 70% para uma análise posterior com o intuito de separá-los em morfoespécies. Espécies previamente conhecidas tiveram suas abundâncias estimadas através de contagem dos organismos em campo, com anotação do seu local de captura no croqui da caverna, para minimizar o impacto da coleta. Eventuais comportamentos e interações ecológicas observadas durante o inventário, bem como vestígios e presença de vertebrados também foram registradas.

Tabela 1 - Localização das cavidades inventariadas e respectivas datas de amostragem.

CAVERNA	ESTAÇÃO SECA	ESTAÇÃO ÚMIDA
SL-001	04/07/2010	14/01/2011
SL-002	03/07/2010	13/01/2011
SL-003	03/07/2010	13/01/2011
SL-004	03/07/2010	13/01/2011
SL-005	28/06/2010	13/01/2011
SL-006	28/06/2010	13/01/2011
SL-007	04/07/2010	14/01/2011
SL-008	05/07/2010	14/01/2011
SL-009	05/07/2010	14/01/2011
SL-011	05/07/2010	14/01/2011
SL-012	04/07/2010	14/01/2011
SL-013	28/06/2010	13/01/2011
SL-014	28/06/2010	13/01/2011
SL-015	28/06/2010	13/01/2011
SL-016	06/06/2010	12/01/2011

SL-017	09/06/2010	08/01/2011
SL-018	11/06/2010	12/01/2011
SL-019	09/06/2010	12/01/2011
SL-020	06/06/2010	12/01/2011
SL-022	09/06/2010	29/01/2011
SL-023	11/06/2010	12/01/2011
SL-024	09/06/2010	02/02/2011
SL-025	08/06/2010	08/01/2011
SL-026	09/06/2010	08/01/2011
SL-027	08/06/2010	06/01/2011
SL-028	09/06/2010	06/01/2011
SL-029	08/06/2010	06/01/2011
SL-030	08/06/2010	06/01/2011
SL-031	08/06/2010	04/12/2011
SL-032	27/07/2010	29/01/2011
SL-033	27/07/2010	29/01/2011
SL-035	29/07/2010	31/01/2011
SL-036	23/07/2010	31/01/2011
SL-037	08/07/2010	27/01/2011
SL-038	29/07/2010	27/01/2011
SL-039	08/07/2010	27/01/2011
SL-040	29/07/2010	27/01/2011
SL-041	09/07/2010	27/01/2011
SL-042	08/07/2010	27/01/2011
SL-043	09/07/2010	29/01/2011
SL-044	30/06/2010	28/01/2011
SL-045	01/07/2010	28/01/2011
SL-046	01/07/2010	28/01/2011
SL-047	30/06/2010	28/01/2011
SL-048	27/06/2010	29/01/2011
SL-049	27/06/2010	29/01/2011
SL-050	27/06/2010	17/01/2011
SL-051	03/06/2010	01/12/2011
SL-052	03/06/2010	01/12/2011
SL-053	03/06/2010	01/12/2011
SL-054	04/06/2010	01/12/2011
SL-055	04/06/2010	01/12/2011
SL-056	04/06/2010	02/12/2011
SL-057	05/06/2010	17/01/2011
SL-058	27/06/2010	29/01/2011
SL-059	04/06/2010	02/12/2011
SL-060	10/06/2010	10/01/2011

SL-061	10/06/2010	10/01/2011
SL-062	10/06/2010	10/01/2011
SL-063	10/06/2010	10/01/2011
SL-064	10/06/2010	10/01/2011
SL-065	05/06/2010	02/12/2011
SL-066	05/06/2010	02/12/2011
SL-067	05/06/2010	03/12/2011
SL-068	27/06/2010	17/01/2011
SL-069	11/06/2010	03/12/2011
SL-070	11/06/2010	03/12/2011
SL-071	23/07/2010	07/01/2011
SL-072	23/07/2010	07/01/2011
SL-073	26/06/2010	07/01/2011
SL-074	24/06/2010	11/01/2011
SL-075	26/06/2010	12/01/2011
SL-076	29/06/2010	31/01/2011
SL-077	29/06/2010	31/01/2011
SL-078	29/06/2010	31/01/2011
SL-079	28/07/2010	01/02/2011
SL-080	28/07/2010	01/02/2011
SL-081	22/07/2010	18/01/2011
SL-082	07/07/2010	18/01/2011
SL-083	07/07/2010	18/01/2011
SL-084	21/07/2010	18/01/2011
SL-085	21/07/2010	18/01/2011
SL-086	21/07/2010	18/01/2011
SL-087	21/07/2010	02/02/2011
SL-088	22/07/2010	18/01/2011
SL-089	22/07/2010	02/02/2011
SL-090	22/07/2010	02/02/2011
SL-091	22/07/2010	02/02/2011
SL-092	22/07/2010	02/02/2011
SL-093	06/07/2010	17/01/2011
SL-094	06/07/2010	03/02/2011
SL-095	06/07/2010	03/02/2011
SL-096	06/07/2010	03/02/2011
SL-097	28/07/2010	04/12/2011
SL-099	27/07/2010	07/01/2011
SL-100	27/07/2010	10/01/2011

Os recursos visíveis na cavidade foram examinados (*in situ*), para a determinação do seu status trófico. A caracterização trófica restringiu-se ao aspecto qualitativo.

2.5.2. Triagem e identificação dos taxa

Todos os invertebrados coletados foram identificados até o nível taxonômico possível, fazendo-se uso de estereomicroscópios (Zeiss Stemi DV-4 e Zeiss Stemi 2000). Tal identificação resultou nos dados de riqueza de espécies (número de espécies), que puderam ser comparadas, entre diferentes cavernas.

2.5.3. Determinação de troglomorfismos

A determinação de espécies potencialmente troglóbias foi realizada através da identificação, nos espécimes, de características morfológicas denominadas troglomorfismos. Tais características, como redução da pigmentação melânica, redução das estruturas oculares, alongamento de apêndices, dentre outras, são utilizadas freqüentemente para a maioria dos grupos, uma vez que resultam de processos evolutivos ocorrentes após o isolamento de populações em cavernas. Os principais troglomorfismos encontrados em diferentes grupos de organismos troglóbios no mundo encontram-se sumarizados na Tabela 2. As características a serem utilizadas para estes diagnósticos, no entanto, diferem no caso de organismos pertencentes à taxa distintos. Certos grupos, por exemplo, possuem espécies sempre despigmentadas e anoftálmicas, mesmo no ambiente epígeo (e.g. Palpigradi). Nestes casos, os troglomorfismos são mais específicos (para Palpigradi: alongamento dos flagelômeros, aumento no número de órgãos laterais, razão entre os comprimentos do propeltídio e do basitarso IV, razão entre os comprimentos da tíbia e do basitarso IV, dentre outros). Desta forma, é necessário se conhecer a biologia de cada grupo no intuito de se diagnosticar efetivamente a existência ou não de troglomorfismos. Despigmentação e anoftalmia muitas vezes não são diagnósticas de troglomorfismos.

Muitas vezes, entretanto, existe a necessidade de consulta a especialista em certos grupos taxonômicos. Muitos destes especialistas infelizmente não existem no Brasil. Desta forma, a determinação da real categoria a qual pertence certa espécie pode demandar um longo tempo.

Tabela 2 - Principais troglomorfismos observados em espécies troglóbias (retirado de Christiansen, K. Morphological Adaptations, em Enciclopedia of Caves, Culver & White, 2004).

Morphological characteristic	Ref.
Specialization of sensory organs (touch, chemoreceptor, hygroreceptor, thermoreceptor, pressure receptors)	Vandel, 1964; Sbordoni, 1980; Weber; 2000
Elongation of appendages	Sbordoni, 1980; Coineau & Boutin, 1992; Weber, 2000; Christiansen, 1961 Harvey, Shear, & Hoch, 2000; Vandel 1964
Pseudophysogastry	Vandel 1964, Sbordoni 1980; Accordi et al., 1980
Reduction of eyes, pigment, wings	Vandel 1964, Sbordoni 1980, Coineau & Boutin,1992; Weber, 2000
Compressed or depressed body form (hexapods)	Harvey, Shear, & Hoch, 2000
Increased egg volume	Vandel, 1964; Sbordoni, 1980; Poulson, 1963
Increased size (Collembola, Arachnida)	Christiansen, 1961; Harvey, Shear, & Hoch, 2000
Unguis elongation (Collembola)	Christiansen, 1961
Foot modification (Collembola, planthoppers)	Christiansen,1961; Howarth et al., 1990
Scale reduction or loss (Fish)	Wilkens, 1988; Weber, 2000; Ercolini et al., 1982
Loss of pigment cells and deposits	Numerous
Cuticle thinning (terrestrial arthropods)	Numerous
Elongate body form (Teleost fishes, Arachnids)	Weber, 2000; Coineau & Boutin, 1992.
Depressed, shovellike heads (Teleost fishes, salamanders)	Weber, 2000
Reduction or loss of swim bladder	Romero & Paulson, 2001
Decreasing Hind femur length/crop empty Live weight, ratio (crickets)	Studier, E., Lavoie, K., & Howarth, F., 2002

2.5.4. Determinação de raridade

No presente relatório, foram mostrados diferentes conceitos de “raridade” atribuídos às espécies troglóbias. Tais atribuições foram primordialmente ilustrativas, tendo sido realizadas no intuito de exemplificar a possibilidade de aplicação de diferentes abordagens, desde aquelas mais “restritivas ” até as mais amplas.

No entanto, para efeito da legislação vigente, utilizou-se, neste relatório, as duas abordagens propostas no workshop técnico científico **“Troglóbios raros”: incertezas e encaminhamentos**, realizado em Belo Horizonte nos dias 03 e 04 de Março de 2011. Tal encontro contou com a participação de diversos especialistas em biologia subterrânea, dentre os quais: Prof. Dr. Antonio Brescovit, Dra. Flávia Pellegatti Franco, Prof. Dr. Marconi Souza Silva, Dra. Regina Bessi Pascoaloto, Dra. Renata Andrade, MSc. Robson Zampaulo e Prof. Dr. Rodrigo Lopes Ferreira.

Levando em consideração o atributo de distribuição geográfica, foi sugerido, no referido workshop, que uma espécie troglóbia encontrada em até três cavidades seja considerada rara. Neste relatório, este tipo de raridade é chamado de raridade **tipo I**. Com relação ao atributo de abundância, foi sugerido pelos Profs. Dr. Marconi Souza Silva e Dr. Rodrigo Lopes Ferreira que deve ser considerada rara a espécie troglóbia com um exemplar por cavidade amostrada, não importando o número de cavidades em que ocorra, independentemente da distribuição geográfica. Esta abordagem também foi adotada no presente relatório, sendo aqui chamada de raridade **tipo II**.

Além das abordagens utilizadas para a definição de troglóbios raros (aplicadas para a obtenção do grau de relevância das cavernas – raridades dos tipos I e II), foram também exemplificadas outras abordagens, a saber: espécies que compreenderam *uniques* (com a ocorrência de um único espécie em todo o estudo – raridade **tipo III**); e espécies que corresponderam à sobreposição dos critérios de distribuição (até três cavernas) e abundância (um exemplar por cavidade amostrada, não importando o número de cavidades em que ocorra), aqui chamadas de raridade **tipo IV**.

2.5.5. Análises

2.5.5.1. Análises ecológicas das cavidades

Com base na presença das espécies de invertebrados de cada cavidade, foram realizadas algumas análises, descritas a seguir.

Para cada caverna, em cada estação, foram determinadas a riqueza (número total de espécies), diversidade (Shannon-Wiener), dominância, e equitabilidade. Além disso, foi determinada também a riqueza total de espécies encontrada em cada caverna (por meio do somatório das espécies observadas no período seco e no período chuvoso).

Foi calculado o índice de similaridade de Bray-Curtis (Wolda, 1981) entre cada uma das cavernas amostradas. A partir dos dados de similaridade foi construído um dendrograma para evidenciar a situação geral de similaridade das cavernas da área. Foi feita também uma análise de Escalonamento Multidimensional não métrico (n-MDS) utilizando o índice de Bray-Curtis. Tal análise permite a observação da similaridade faunística através da plotagem das amostras (no caso, as cavernas) em um espaço bi ou tridimensional, no qual a distância entre cada ponto corresponde à sua similaridade. Optou-se, neste relatório, pela análise bi-dimensional.

Foram construídas curvas do "coletor", que consistem na plotagem do número acumulativo de espécies em cada caverna amostrada. Tais análises pretenderam evidenciar se a amostragem foi ou não satisfatória, indicando quanto do total de espécies presentes na área foram amostradas na coleta destas cavernas. A curva acumulativa de espécies foi comparada às tendências esperadas, segundo os dados gerados pelos modelos Jack-Knife 1 e Jack-Knife 2. Para tal, foram realizadas análises considerando-se toda a fauna e excluindo-se as espécies "acidentais".

Finalmente, foi determinado o "turnover" de espécies em cada caverna (β diversidade). Tal parâmetro determina a porcentagem de substituição de espécies em um dado sistema ao longo do tempo. Desta forma, comparando-se as comunidades observadas em cada uma das

estações em cada caverna, determinou-se o “turnover” (β diversidade) a partir de dados de presença e ausência através do índice de Harrison (1992), modificado por Whittaker (1960), no intuito de se comparar amostras de diferentes tamanhos. O “turnover” corresponde a: **β Harrison = {[(S/a) - 1]/(N - 1)}*100)**; onde: S = riqueza total observada, a = riqueza média por caverna e N = número de amostragens (no caso, N=2, o que corresponde às coletas de seca e chuva, para cada caverna). Estas medidas variam de 0 (nenhum turnover de espécies) a 100 (cada estação possui um conjunto único de espécies, isto é, a comunidade foi completamente substituída de uma estação para a outra) (Koleff *et al*., 2003). Assume-se aqui que sistemas subterrâneos mais “estáveis” do ponto de vista ecológico apresentam menores trocas de espécies ao longo do tempo.

Por fim, foram realizados testes de regressão simples e múltipla entre diferentes variáveis bióticas e abióticas. As variáveis abióticas testadas foram: a extensão total das cavernas, o número de entradas de cada caverna e a incidência de inundações. As variáveis bióticas testadas foram a riqueza de espécies (da seca, da chuva e total), a riqueza de espécies troglóbias (troglomórficas) e o “turnover” (β diversidade) de espécies.

Reitera-se aqui que neste relatório, as espécies troglóbias foram assim definidas em função dos seus troglomorfismos. Desta forma, os termos **Troglóbios** e **Troglomórficos**, para efeito deste relatório, são coincidentes.

2.5.5.2. Determinação do grau de relevância

A determinação dos graus de relevância das cavernas da área foi realizada por meio da aplicação da metodologia explicitada na Instrução Normativa Nr. 02 referente ao Decreto Federal 6.640.

Foram considerados todos os atributos biológicos mencionados na referida Instrução Normativa. No entanto, o presente relatório foi elaborado considerando-se dois “panoramas”: o primeiro deles utilizou-se da aplicação direta de todos os atributos da IN Nr. 02, que resultou em uma análise de relevância elaborada inteiramente em conformidade com a legislação vigente.

No entanto, foi realizada uma segunda análise de relevância considerando-se a adequação de alguns atributos da IN, visando uma análise mais refinada ou real das relevâncias das cavidades. Reitera-se aqui que esta segunda análise está, no presente momento, em desconformidade com a legislação vigente, já que utiliza alguns parâmetros modificados em relação à normatização. No entanto, optou-se aqui pela realização desta análise diferencial com o objetivo básico de que esta possa fornecer subsídios para futuras adequações na referida Instrução Normativa quando de sua re-avaliação.

A atual Instrução Normativa apresenta falhas claras, sejam relativas à falta de definição objetiva de critérios, sejam relativos a erros em estabelecimentos de categorias referentes aos níveis de relevância. Tais erros serão apontados à frente, no item “Análises de Relevância”.

3. ESTUDOS ANTERIORES E ESPELEOLOGIA REGIONAL

O Brasil, possuidor de algumas das maiores reservas de minério de ferro do mundo é, portanto, candidato natural a apresentar um grande potencial espeleológico em cavidades nessa litologia. De fato, as duas maiores áreas de explotação de minério de ferro no Brasil (Quadrilátero Ferrífero/MG e Serra dos Carajás/PA) têm revelado uma série de cavernas encaixadas nestes litotipos. No entanto, outras regiões do país possuem reservas de ferro apresentando razoável potencial espeleológico, como Mato Grosso do Sul (Corumbá) e Bahia (Caetité). Uma introdução às ocorrências espeleológicas em minério de ferro e canga é apresentada por Auler & Piló (2005).

As cavernas ferríferas têm merecido alguma atenção na literatura especializada, sendo a maior parte das referências provindas do Quadrilátero Ferrífero (MG) e Serra dos Carajás e arredores (PA). Provavelmente a primeira menção a cavidades em minério de ferro no Brasil seja devida ao francês Noël Aimé Pissis que, em 1842, descreveu cavidades e estalactites no itabirito do Quadrilátero Ferrífero (Pissis, 1842). Em 1871 o mineralogista inglês William Jory Henwood (1871) descreveu a existência de estreitas fendas em itabirito na região de Água Quente, Quadrilátero Ferrífero. Após essas referências pioneiras, importantes descrições de cavernas foram feitas no Quadrilátero Ferrífero, dentre as quais: Tassini (1947), Guild (1957), Simmons (1963) e Dorr (1969).

Recentemente houve uma retomada dos estudos espeleológicos nas cavernas em minério de ferro e canga do Quadrilátero Ferrífero. Piló & Auler (2005) apresentaram uma revisão sobre o tema, com ênfase nas cavidades da Mina de Capão Xavier. Novamente, Piló & Auler (2006) elaboraram uma pesquisa em quatro áreas do Quadrilátero ferrífero: Mina do Pico, Parque Estadual Serra do Rola Moça, Capão Xavier e Serra da Moeda.

Na região da Serra dos Carajás (PA), a primeira referência a cavernas deve-se a Tolbert *et al*. (1971), que comenta sobre as grandes cavernas encontradas sob o manto de canga. Os estudos espeleológicos na Serra dos Carajás tiveram um grande avanço a partir dos trabalhos de integrantes do Grupo Espeleológico Paraense (GEP), notadamente Clóvis W. Maurity e Roberto Vizeu L. Pinheiro. Em seu primeiro relatório Pinheiro *et al*. (1985) detalham quatro cavernas, além de discorrerem sobre as feições encontradas e sua possível gênese. Este trabalho foi detalhado em Pinheiro & Maurity (1988) onde a interpretação

espeleogenética é aprimorada. Maurity & Kotschoubey (1995) posteriormente produziram um detalhado estudo não só sobre a gênese das cavidades, como também sua mineralogia, trabalho este reproduzido recentemente (Maurity & Kotschoubey, 2005). Nos arredores da Serra dos Carajás, Atzingen & Crescêncio (1999) apresentaram uma descrição com mapas de algumas cavernas na região do garimpo de Serra Pelada.

Piló & Auler (2009) apresentaram, mais recentemente, uma caracterização das cavernas ferríferas da região de Carajás. Segundo os autores, essas cavidades já foram identificadas nas serras Norte, Sul, Leste, Tarzan e Bocaina (Figura 6).

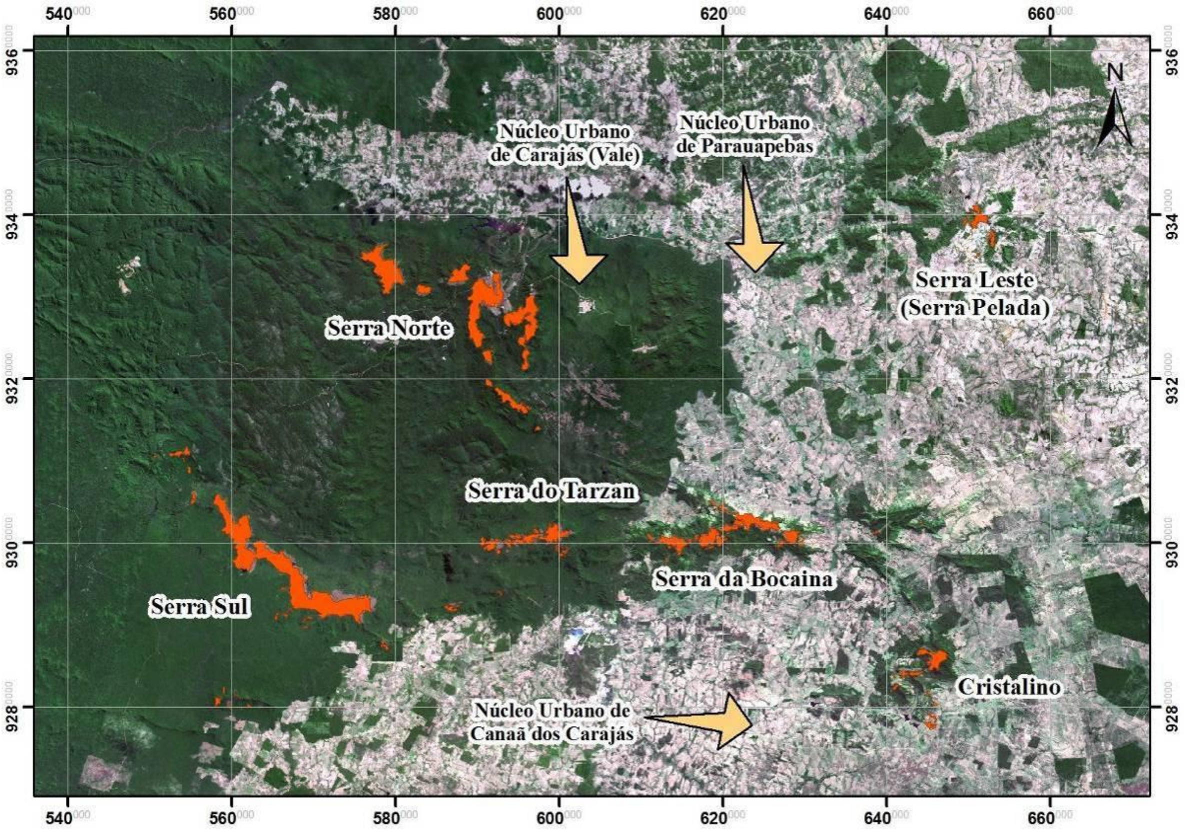

Figura 6 - Principais serras da região de Carajás, onde ocorrem as cavidades ferríferas.

Essas cavernas encontram-se inseridas na base de pequenas escarpas rochosas posicionadas, predominantemente, no terço superior/médio das vertentes inclinadas, morfologia que faz a conexão entre o topo dos planaltos dissecados, localmente denominados de platôs, e as planícies aluviais.

A maioria das cavernas já registradas na região de Carajás, até o momento, é constituída por dois litotipos: a formação ferrífera (minério de ferro) e a canga detrítica. Mas já foram registradas cavernas inseridas exclusivamente na formação ferrífera, na canga detrítica e em rochas máficas.

As superfícies do bandamento da formação ferrífera, muitas vezes, controlam o desenvolvimento da direção preferencial de cavernas. As fraturas também respondem como estruturas de controle na direção preferencial de condutos. Flexuras do tipo *kink bands* também foram registradas nas cavernas, mas não foi constatado nenhum tipo de controle na morfologia ocasionado por essas estruturas.

Fraturas atectônicas, de alívio de pressão, foram registradas na grande maioria das cavernas, tanto na canga como no minério de ferro, influenciando localmente a ocorrência de movimentos de massa do tipo abatimentos. Importantes frentes de alteração da rocha também são guiadas por essas juntas.

O padrão planimétrico das cavernas de Carajás pode ser dividido preliminarmente em três grupos, ou seja, cavernas espongiformes, cavernas retilíneas e cavernas mistas.

As cavernas espongiformes se caracterizam por condutos de tamanho variado que se interconectam de forma irregular (globular), como os poros de uma esponja. Normalmente a conexão entre câmaras maiores é feita por meio de condutos estreitos.

As cavernas retilíneas são formadas por condutos simples, seguindo uma direção preferencial, condicionada por junta ou pela superfície inclinada do bandamento da rocha ferrífera.

As cavernas em minério de ferro e canga são em geral de pequenas dimensões. A grande maioria possui desenvolvimento inferior a 30 m. Cavernas com projeções horizontais superiores a 100 m são menos freqüentes. A maior caverna de minério constatada na região, localizada na Serra Norte, possui 372 m de projeção horizontal.

A grande maioria das cavernas estudadas na região de Carajás é seca. Ou seja, os processos hidrológicos associados a cursos d’água perenes são muito restritos no interior das mesmas, só ocorrendo em poucas cavernas localizadas nas proximidades de igarapés. Surgências e canais de drenagem temporários podem ocorrer, gerados pela concentração do gotejamento ou percolação de águas infiltradas via canalículos ou juntas. A atividade hidrológica mais freqüente nas cavernas, portanto, consiste em gotejamentos devido a águas de percolação que se infiltram até a caverna e gotas de condensação nas paredes.

Os depósitos clásticos das cavernas de Carajás podem ser divididos em dois tipos. Depósitos formados por sedimentos predominantemente de caráter autogênico, gerados a partir de material do próprio substrato encaixante (formação ferrífera e/ou canga); depósitos formados por sedimentos mistos: autogênicos e alogênicos, ou seja, gerados tanto no interior da caverna como vindos de fora.

Os depósitos predominantemente autogênicos são constituídos por clastos de hematita originados do minério de ferro, da canga e, por vezes, de rochas máficas. Esses clastos são originados principalmente a partir de processos gravitacionais do tipo abatimentos de porções do teto e das paredes. Diante disso, os depósitos apresentam uma estreita relação com os constituintes do substrato sobrejacente.

Os depósitos mistos também são muito freqüentes nas cavernas. Os depósitos alogênicos são originados principalmente dos canalículos e juntas alargadas existentes na grande maioria das cavernas, como também de clarabóia e cones sedimentares de entrada.

Depósitos formados por sedimentos orgânicos também já foram identificados. Destaca-se, nesse contexto, a serrapilheira, que ocorre principalmente nas entradas e sob as clarabóias. Raízes também pendem do teto de diversas cavernas, como também podem aflorar frequentemente no piso. Poças de guano também foram identificadas, além de fezes de mamíferos.

Os depósitos químicos (espeleotemas) podem ser identificados em praticamente todas as cavernas da região de Carajás. Os mais freqüentes são os depósitos de cobertura (crostas) de blocos abatidos, pisos e paredes. Os coralóides, também são freqüentes nas cavernas, constituindo depósitos de exudação de grande diversidade nas cavernas de Carajás. Microtravertinos, escorrimentos, pingentes e cortinas também já foram identificados.

Dados já obtidos de Difração de Raios X – DRX (Maurity & Kotschoubey, 2005; Piló & Andrade, 2006) evidenciaram uma grande diversidade de minerais, bem superior daquela encontrada nas cavernas carbonáticas. O óxido de ferro do tipo hematita (Fe_2O_3) é o mineral mais abundante nos espeleotemas analisados, juntamente com a goethita [FeO(OH)], sendo também freqüentes em cavernas ao redor do mundo (Hill & Forti, 1997). Em função da estabilidade termodinâmica similar, os minerais goethita e hematita frequentemente ocorrem juntos. Já a gibbsita ($AlOH_3$) foi identificada em um número menor de amostras até o momento.

Quanto aos fosfatos, a leucofosfita [$KFe_2(PO_4)_2(OH).2(H_2O)$] já foi identificada em vários espeleotemas de cavernas da Serra Norte. Esse mineral também foi identificado por Maurity & Kotschoubey (1995) na Serra dos Carajás.

A estrengita [$(Fe)PO_4.2(H_2O)$], mineral fosfático de ferro e alumínio, foi detectado em algumas cavernas, tendo sido também identificado por Maurity & Kotschoubey (1995) como revestimentos de pisos, blocos, bem como cimento de paleopavimentos em cavernas da serra Norte.

Três sulfatos já foram registrados: a gipsita ($CaSO_4.2H_2O$), a alunita [$KAl_3(SO_4)2(OH)_6$] e a basaluminita [$Al_4(SO_4(OH)10.5(H_2O)$)].

Poucos estudos faunísticos foram realizados em cavidades subterrâneas naturais presentes em minério de ferro e canga no mundo como um todo, em grande parte devido à sua pequena dimensão, não despertando a atenção de pesquisadores. No Brasil, a maioria dos estudos bioespeleológicos foi conduzida em cavernas carbonáticas, e, em menor quantidade, em rochas areníticas e quartzíticas (Pinto-da-Rocha, 1995). Levantamentos faunísticos em cavidades presentes em minério de ferro foram efetuados inicialmente no Pará (Moreira & Paiva, 1988; Paiva & Moreira, 1988; Pinheiro *et al.*, 2001), entretanto, com pouco refinamento nas identificações e sem menção aos aspectos ecológico-evolutivos dos organismos registrados.

Levantamentos mais recentes, efetuados em cavidades de canga e minério de ferro na região do Quadrilátero Ferrífero (MG) (Ferreira, 2005) e Serra dos Carajás (PA) (Andrade, 2007), têm revelado uma fauna diversificada e interessante, inclusive com organismos troglomórficos. Inventários estão sendo conduzidos em cavidades de diferentes regiões na Serra dos Carajás, sendo que os resultados poderão facilitar a compreensão da distribuição de comunidades subterrâneas na região. Estudos bioespeleológicos anteriores na área de S11D foram efetuados dentro de um projeto mais amplo, denominado Área Mínima de Canga (Golder Associates, 2009), e indicaram uma fauna diversificada, além da presença de troglomórficos não restritos às cavidades da área.

4. INSERÇÃO DAS CAVERNAS NO CONTEXTO GEOLÓGICO, GEOMORFOLÓGICO E FITOGEOGRÁFICO

4.1 Geologia

A Província Mineral de Carajás (PMC) está situada na porção sudeste do estado do Pará. É limitada a leste pelos rios Araguaia-Tocantins, a oeste pelo rio Xingu, a norte pela Serra do Bacajá e a sul pela serra dos Gradaús (DOCEGEO, 1988). É designada como província devido a sua enorme riqueza mineral que inclui depósitos de ferro, níquel, cobre e ouro. A área coberta por esses depósitos é constituída por rochas metavulcanossedimentares, tida como uma bacia neoarqueana, sobreposta a um embasamento mesoarqueano e neoarqueano contendo rochas de complexos ígneos e metamórficos (Tassinari *et al.*, 2000).

Do ponto de vista estratigráfico, a PMC é definida por sequências vulcanossedimentares que sofreram os efeitos de dobramento regional, falhamento transcorrente, metamorfismo de

contato e/ou metassomatismo ao redor de domos graníticos , além de alteração hidrotermal relacionada a diversos eventos de mineralização (Lobato *et al.*, 2005).

Segundo Beisegel *et al.* (1973), a estrutura dominante da Serra dos Carajás é um sinclinório de aproximadamente 1.000 km de comprimento e 100 km de largura, com eixo WNW-ESE. Araújo & Maia (1991) definiram o Cinturão de Cisalhamento Itacaiúnas, de orientação E-W, representado na PMC pelas zonas de cisalhamento Carajás e Cinzento. Tais zonas de cisalhamento apresentam movimentação transcorrente e cinemática predominante sinistral, de caráter rúptil-dúctil, comumente acompanhadas de silicificação das rochas por ela cortadas. Apresentam forma sigmoidal e orientação variando entre E-W e ESE-NNW, sub-paralelas ao plano axial de dobras de escala regional (Rosière *et al.*, 2005). Uma xistosidade penetrativa ocorre associada e restrita as zonas de cisalhamento, principalmente na do Cinzento, com orientação subparalela as falhas e identificada principalmente nas rochas metavulcânicas e metapelíticas.

A região de Serra Leste, localizada na porção leste da PMC, insere-se no Cinturão de Cisalhamento Itacaiúnas (Costa & Siqueira, 1990), na zona de cisalhamento do Cinzento. Segundo Pinheiro & Holdsworth (2000), Tassinari *et al*. (2000), Villas & Santos (2001), Nunes (2002) e Veneziani *et al*. (2004), o embasamento é dominantemente composto por rochas graníticas (granodioritos e tonalitos da fácie anfibolito) arqueanas do Complexo Xingu (2.872 +/- 10 Ma). O arcabouço geológico é ainda composto por rochas supracrustais da sequência metavulcanossedimentar do Grupo Rio Novo (2.757 +/- 2 Ma), além de sequências sedimentares (DOCEGEO, 1995) crono-correlatas ao Grupo Rio Fresco (2.580 a 2.500 Ma).

Tabela 3 - Coluna crono-litoestratigráfica do Cinturão de Cisalhamento Itacaiúnas. Adaptado de Nunes (2002).

Éon	Era	Idade (Ma)	Unidades Litoestratigráficas
Proterozóico	Neo	550	Diques e sills máficos
	Meso		Fm. Gorotire
	Paleo	1880	Suíte Granítica Anorogênica
			Metagrabo Sta. Inês Metaultramáfica (Vermelho, Puma e Onça)
		1900?	
Arqueano	Neo	2525	Complexo Granítico Estrela

		2570	
		2645	
			Fm. Águas Claras
		2680	
		2749	Suíte Plaquê
			Gr. Buritrama
			Gr. Igarapé Bahia
		2760	Gr. Gão-Pará
		2763	Complexo Luanga
			Gr. Pojuca
		2770?	Gr. Salobo
	Meso	2859	Complexo Xingu
		3002	Complexos Granulíticos Pium e Bacajá)

O Grupo Rio Novo (Araújo & Maia, 1991) encontra-se em contato discordante com rochas do complexo Xingu. É compreendido como uma sequência supracrustral de natureza vulcanossedimentar, metamorfizada na fácie xisto-verde, contendo formações ferríferas, xistos, anfibolito, rochas máficas e ultramáficas. Os corpos ultramáficos ocorrem associados às rochas máficas, apresentando como mineralogia principal tremolita e clorita, além de actinolita em menor proporção.

Costa (2007) dividiu o Grupo Rio Novo em três unidades informais:

- ***Arqueano Grupo Rio Novo porção sedimentar (Arns)*** - caracterizada pela predominância de rochas metassedimentares clasto-químicas. Essa unidade ocorre principalmente na Serra do Sereno em uma faixa que se estende na direção E-W com aproximadamente 8 km de largura, composta principalmente por quartzitos e metassiltitos intercalados com camadas manganesíferas. Os metassiltitos apresentam granulação fina e textura granolepdoblástica, exibindo uma fina clivagem de crenulação. Em escala mesoscópica, o acamamento da rocha é

reconhecido por variação composicional registrada pela alternância de camadas de espessura centimétricas de cor marrom clara e escura. Os quartzitos no geral apresentam coloração branca e podem variar de moderadamente a bem recristalizados, quando ocorrem próximo a granitos intrusivos (e.g. Granito Cigano). Essa unidade é interpretada como a porção sedimentar de topo da sequência vulcanossedimentar Rio Novo;

- ***Arqueano Grupo Rio Novo Indiviso (Arni)*** - região indivisa do Grupo Rio Novo onde predominam rochas metavulcanossedimentares;

- ***Arqueano Grupo Rio Novo porção vulcanossedimentar (Arnv) -*** constituída essencialmente por rocha vulcânica máfica e subordinadamente por FFs e anfibolitos. Representa a porção vulcânica e sedimentar-química da seqüência Rio Novo. Essa unidade hospeda corpos de minério de ferro de alto teor nos depósitos SL1 e SL2, localizados na região de Serra Leste.

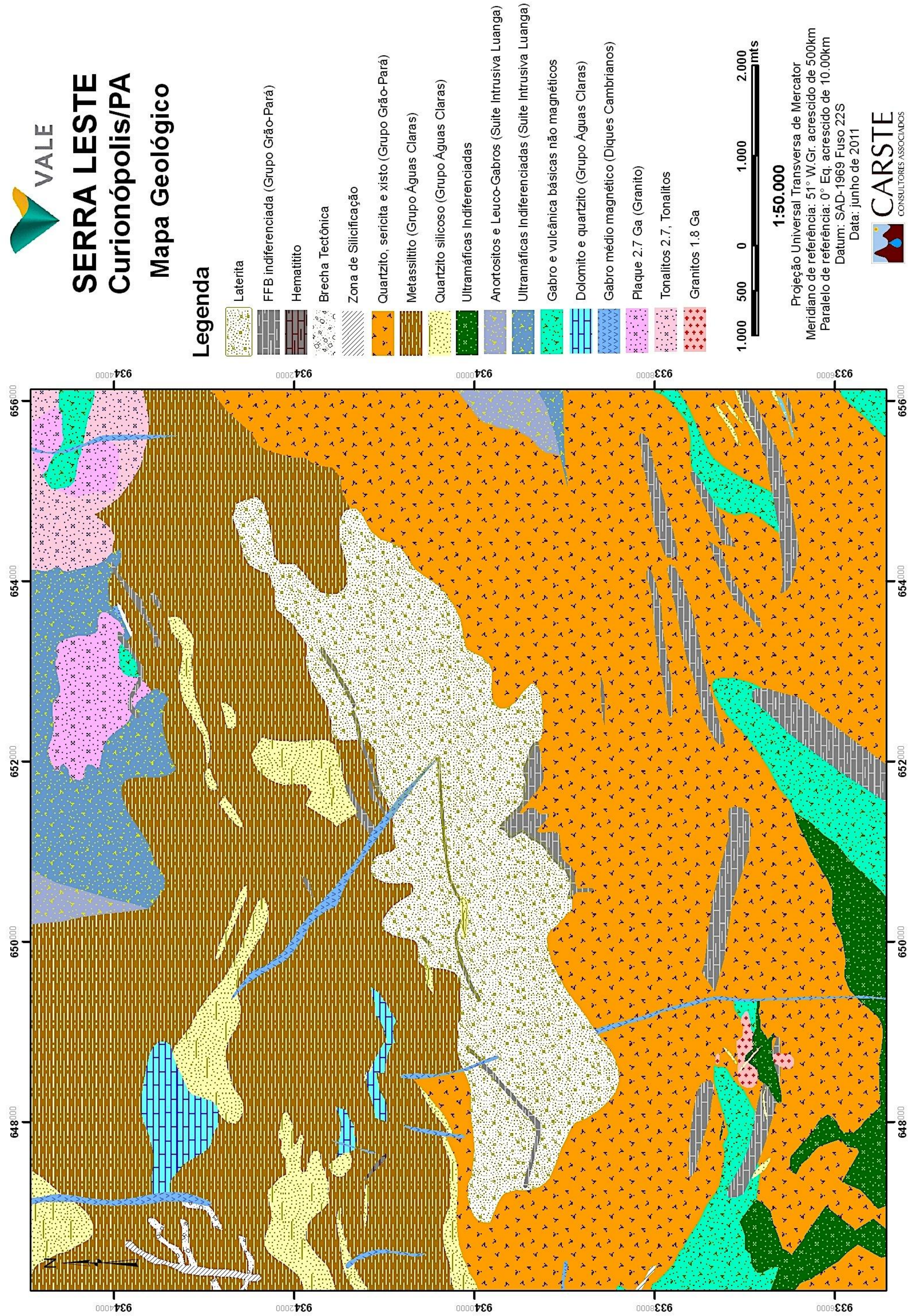

Figura 7 - Mapa geológico simplificado de Serra Leste. Adaptado de DOCEGEO, 1988; Nunes, 2004; e Costa, 2007.

4.1.1. Rochas vulcânicas máficas

As rochas vulcânicas máficas apresentam maior distribuição espacial em relação às formações ferríferas e anfibolitos (Costa, 2007). Quando expostas a superfície, são mais susceptíveis ao intemperismo. Apresentam coloração esverdeada e, de forma geral, composição basáltica, com granulação variando entre fina e média, sugerindo texturas vulcânicas e sub-vulcânicas. Apresentam texturas ofítica e granular hipidiomórfica com cristais de até 4 mm de comprimento. Os constituintes primários são plagioclásio, piroxênio, quartzo e ilmenita, como mineral acessório ocorre cobre nativo (Figura 8), os cristais são subédricos com até 0,1 mm de diâmetro, apresentando coloração alaranjada típica (Costa, 2007).

Localmente, à semelhança do que ocorre nos depósitos de minério de ferro de Serra Norte (Lobato *et al.*, 2005), ocorrem porções hematitizadas da rocha máfica. Estão associadas a presença de foliação na rocha (xistosidade ou clivagem espaçada), mudança na coloração da rocha máfica, que passa a adquirir o tom marrom, e a mudança parcial da mineralogia. A intensidade e o estilo das feições associadas à hematitização variam desde esteiras de óxido de ferro com média de 0,1 mm de espessura, até a presença de bolsões centimétricos de hematita compacta.

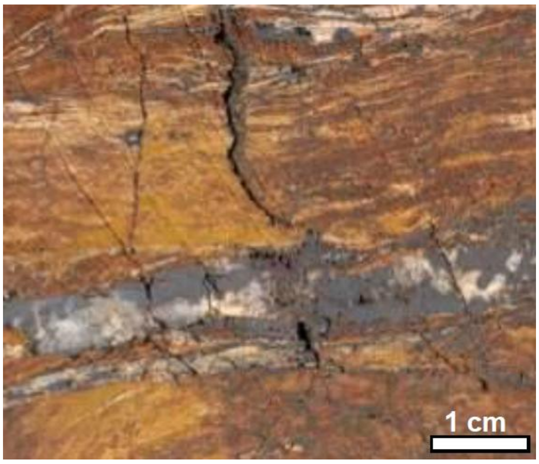

Figura 8 - Rochas vulcânicas máficas. Fonte: Costa (2007).

4.1.2. Anfibolitos

Os anfibolitos ocorrem na porção vulcanossedimentar do Grupo Rio Novo (Figura 9). Em escala mesoscópica, a rocha apresenta coloração esverdeada e granulação média com cristais de anfibólio visíveis a olho nú. São caracterizados por serem rochas duras e resistentes quando frescas. A textura pode variar entre granoblástica, na presença de pequena quantidade de quartzo e plagioclásio, e nematoblástica onde os anfibólios somam quantidade igual ou superior a 90% da composição mineralógica. De forma geral, apresentam cristais de plagioclásio anédricos, que podem chegar a 30% da rocha. É possível

identificar porções contínuas de anfibólio com aproximadamente 1 cm de espessura, intercaladas com quartzo (Costa, 2007).

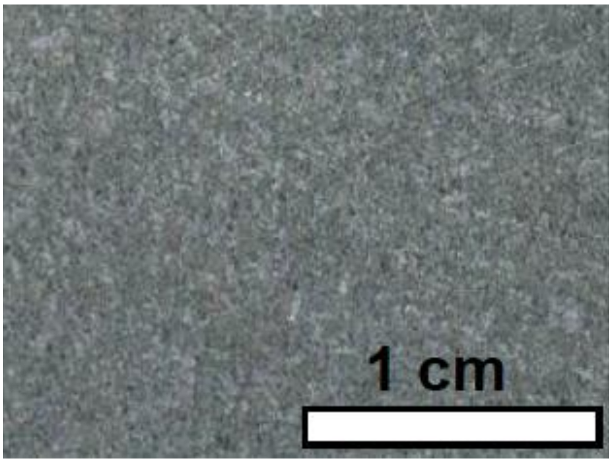

Figura 9 - Anfibolitos. Fonte: COSTA, 2007.

4.1.3. Formações Ferríferas

As formações ferríferas apresentam distribuição e características variadas na área de estudo. Segundo Costa (2007), são raras as exposições de formação ferrífera na região de Serra Leste (Figura 10). O autor identificou e descreveu três ocorrências na área, duas em afloramentos e uma amostra de furo de sondagem.

Nas proximidades do platô do depósito SL1, na escarpa sul de Serra Leste, foram identificadas rochas que, em escala mesoscópica, apresentam coloração cinza e foliação de transposição semelhante a uma laminação, com dobras de escala milimétrica isoclinais e intrafoliais, localmente com charneira rompida. Em escala microscópica, apresentam microbandamento registrado pela alternância de lâminas de martita com relictos de kenomagnetita e lâminas de quartzo e chert. A martita apresenta granulação grossa em relação à outras fases e comumente apresenta relictos de kenomagnetita no interior dos cristais. Localmente ocorrem porções contendo microbrechas e quartzo recristalizado; segundo o autor, é provável que essas feições estejam associadas à transposição que obliterou todas as estruturas primárias da rocha, incluindo bandamento e laminação.

Na amostra de formação ferrífera do furo de sondagem, apresenta bandamento registrado pela alternância de bandas de quartzo e bandas de óxido de ferro com em média 0,5 cm de espessura. Em escala microscópica é possível observar uma fina laminação com aproximadamente 0,5 mm de espessura, alternando porções de composição similar ao bandamento. O quartzo apresenta textura granoblástica com contato entre grãos com forma lobada, o diâmetro dos cristais é em média de 25 µm. Os óxidos de ferro são principalmente hematita e martita, na forma microlamelar, com até 10 µm de comprimento e anédricas.

Cortando a trama da rocha ocorrem venulações subparalelas com espaçamento aproximado de 2 cm, contendo principalmente quartzo e goethita.

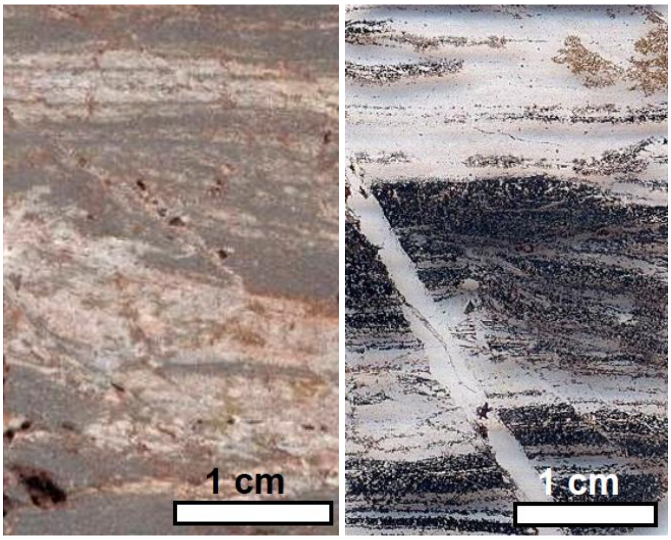

Figura 10 - Formação ferrífera de Serra Leste. Fonte: COSTA, 2007.

4.2 Geomorfologia

A Serra dos Carajás encontra-se inserida no domínio do Planalto Dissecado do Sul do Pará, caracterizado por maciços residuais de topo aplainado ou ondulado, além de cristas e picos interpenetrados por faixas de terrenos rebaixados (Radambrasil, 1974). Essa unidade apresenta-se intensamente dissecada por vales encaixados, adaptados às redes de fraturas em rochas arqueanas, além de algumas estruturas proterozóicas.

Destacam-se, nesse domínio, serras com amplitude de 300 m, com altitude média de 700 m, chegando a 900 m nas partes mais elevadas. Entre as principais serras tem-se: Serra Norte, Serra Leste, Serra da Bocaina, Serra do Tarzan e a Serra Sul. Alguns autores colocam essas elevações como testemunhos de uma superfície de aplainamento resultante de uma pediplanação terciária que reelaborou um pediplano cretácico ou pré-cretácico (Radambrasil, 1974).

Nas porções mais altas das serras, muitas vezes ocorrem coberturas lateríticas e concrecionárias que recobrem as rochas ferríferas arqueanas, pertencentes à Formação Carajás (Grupo Grão-Pará). Esses platôs de canga ferrífera apresentam-se como fragmentos de dimensões variadas, apresentando uma evolução morfodinâmica singular no contexto regional.

Possuem encostas com predomínio de feições côncavas portadoras de depósitos de tálus grosseiros originários da erosão e solapamento da cobertura de canga que reveste e mantém os platôs.

A dissolução química da base dos saprolitos ferríferos, abaixo da canga cimentada, possibilita o desenvolvimento de feições "doliniformes" e cavernas.

4.3 Fitogeografia

A Serra Leste localiza-se na borda sudeste da grande Região Amazônica, que tem a maior e mais rica formação florestal do planeta, tanto em extensão como em variedade de espécies vegetais e animais, condicionada pelo clima equatorial úmido.

A localidade está coberta por floresta ombrófila densa, formação caracterizada por fanerófitos, justamente pelas formas de vida macro e mesofanerófitos, além de lianas lenhosas e epífitas em abundância que os diferenciam das outras classes de formações. Porém, sua característica ecológica principal reside nos ambientes ombrófilos que marcam muito bem a "região florística florestal". Assim, a característica ombrotérmica da Floresta Ombrófila Densa está presa aos fatores climáticos tropicais de elevadas temperaturas (médias de 25°C) de alta precipitação bem distribuída durante o ano (0 a 60 dias secos), o que determina uma situação bioecológica praticamente sem período seco. Dominam nos ambiente dessas florestas os latossolos com características distróficas e raramente eutróficas, originados de vários tipos de rocha desde a cratônicas (granitos e gnaisses) até os arenitos com derrames vulcânicos de variados períodos geológicos.

A região em específico apresenta uma variação no sub-tipo: floresta ombrófila densa submontana. Apresenta formação florestal com fanerófitos com alturas aproximadamente uniformes. A submata é integrada por plântulas de regeneração natural, poucos nanofanerófitos e caméfitos, além da presença de palmeiras de pequeno porte e lianas herbáceas em maior quantidade. Sua principal característica fica por conta dos fanerófitos de alto porte, alguns ultrapassando os 50 m.

Esta formação é caracterizada por ecótipos que variam influenciados pelo posicionamento dos ambientes de acordo com a latitude, ressaltando-se também a importância do fator tempo nesta variação ambiental.

Uma importante ocorrência de vegetação para Serra Leste é o campo rupestre. Os campos rupestres são formações que ocorrem exclusivamente no alto das serras. São em geral campos abertos e atravessados por inúmeros riachos e rios permanentes. O solo é pedregoso, possui baixa capacidade de retenção de água e as formações rochosas são muito

comuns, crescendo a maior parte das plantas nas pequenas frestas eroditas. Como após as chuvas as águas escoam rapidamente por sobre as rochas, não há formação de lençol freático. O ambiente, portanto, é seco, e as plantas desenvolveram adaptações diversas para resolver o problema da falta de água. A biodiversidade deste ecossistema é grande, variando inclusive de uma região para outra. As plantas são quase todas rasteiras, encontrando-se, arbustos baixos.

Diversos tipos de líquens, orquídeas e sempre-vivas são encontrados na região, além de inúmeras outras plantas de grande valor ornamental, como o Paepalanthus, por exemplo. A fauna dos campos rupestres é rica em espécies de anfíbios, répteis, aves e pequenos mamíferos, além de uma infinidade de insetos.

5. APRESENTAÇÃO DOS RESULTADOS

Neste item serão apresentados os resultados dos estudos espeleológicos, incluindo a prospecção espeleológica e os diagnósticos geoespeleológicos e bioespeleológicos. A análise de relevância das cavernas será apresentado no item 6.

5.1. Prospecção espeleológica

Como dito anteriormente, a prospecção espeleológica na área do Projeto Serra Leste foi realizada pelo Grupo Espeleológico de Marabá (GEM/FCCM) através de um convênio firmado entre a VALE e a Fundação Casa de Cultura de Marabá (FCCM). O trabalho foi realizado em duas etapas. A primeira e mais longa ocorreu entre setembro de 1997 e outubro de 1998. A segunda campanha ocorreu em setembro de 2005. Apesar de terem sido realizados de forma sistmática, registros lineares foram coletados apenas nas vias de acesso. A prospecção foi evidenciada através de uma “nuvem” de pontos coletados quando da mudança de trajetória das equipes em campo e expresso no relatório em uma tabela por nós espacializada na Figura 11.

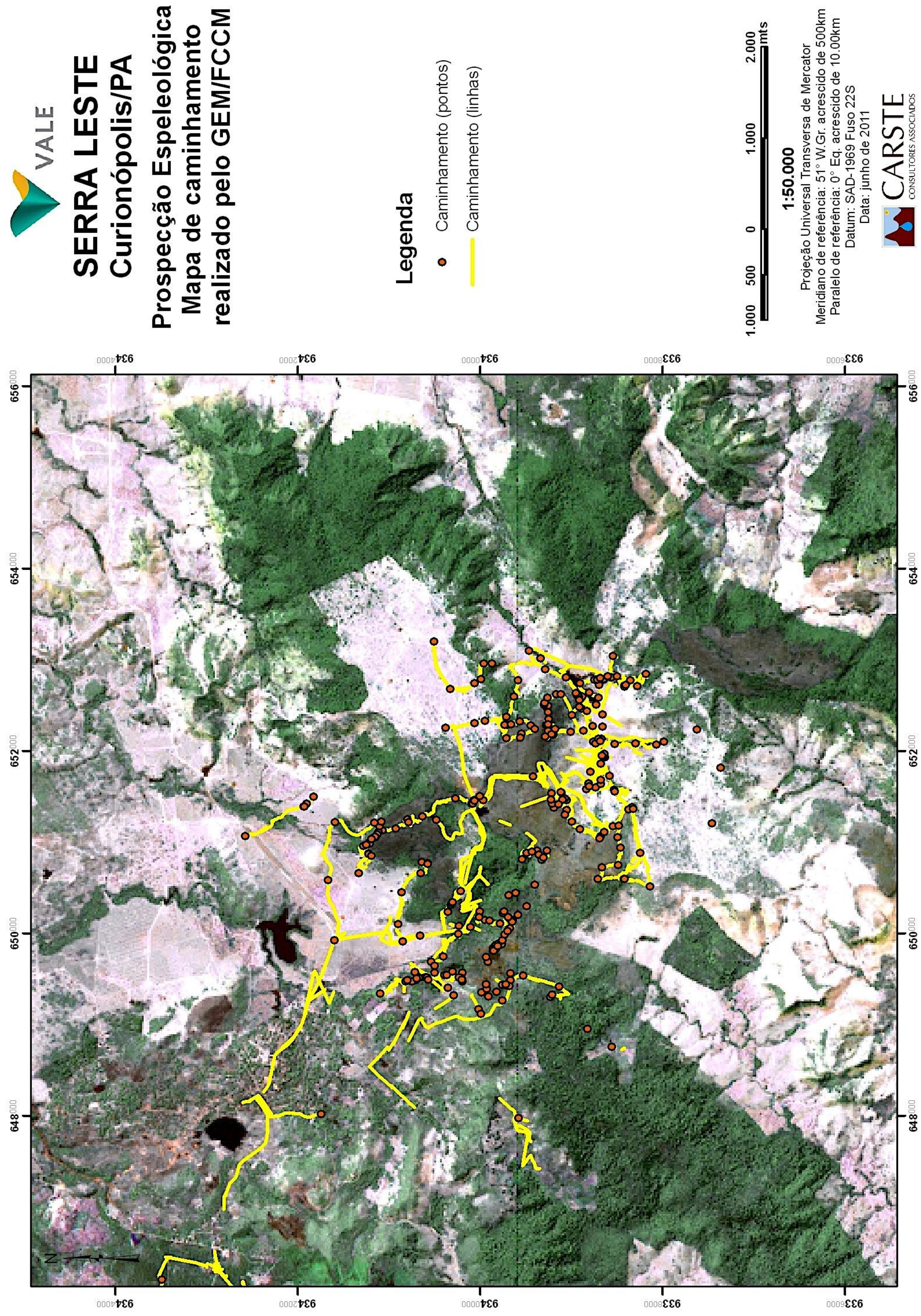

Figura 11 - Mapa de caminhamento realizado na área do Projeto Serra Leste. Fonte: GEM/FCCM, 2005.

5.2. Espeleotopografia

O levantamento espeleotopográfico das cavernas de Serra Leste foi em parte realizado pelo Grupo Espeleológico de Marabá (GEM/FCCM) e em parte pela empresa Carste Consultores Associados (CARSTE) que atuaram em campanhas de campo distintas nos anos de 2008 e 2009. A Tabela 4 apresenta a síntese dos atributos espeleométricos das cavernas de Serra Leste. Os mapas topográficos de cada caverna podem ser vistos no Anexo I.

As cavidades SL-010, SL-021, SL-034 não foram topografadas pois, durante a campanha de campo, constatou-se que tais cavernas possuíam menos de cinco metros de desenvolvimento (projeção horizontal). A cavidade SL-098 foi topografada, no entanto, foi desconsiderada quando dos estudos geoespeleológicos por tratar-se integralmente de uma galeria de mina.

Tabela 4 - Coordenadas e espeleometria das cavidades estudadas em Serra Leste.

Cavidade	UTM leste	UTM norte	Altitude (m)	Projeção Horizontal (m)	Desnível (m)	Área (m^2)	Volume (m^3)
SL-001	649535	9340391	345	83,1	2,6	286,2	657,8
SL-002	649508	9340593	334	29,8	2,7	52,4	59,5
SL-003	649504	9340606	330	21,8	0,4	34,3	54,4
SL-004	649501	9340664	330	25,3	1,8	93,3	187,5
SL-005	649504	9340670	320	15,5	1,8	26,3	44,7
SL-006	649509	9340698	317	11,6	2,0	31,5	60,3
SL-007	649532	9340368	352	5,5	0,6	11,0	12,0
SL-008	649504	9340220	352	20,4	2,0	67,1	97,1
SL-009	649503	9340212	336	19,1	2,1	68,2	150,1
SL-011	649484	9340212	342	26,0	1,6	121,5	79,0
SL-012	649487	9340570	325	15,6	2,9	18,3	23,8
SL-013	649472	9340714	330	11,5	3,3	28,5	26,0
SL-014	649465	9340754	321	8,1	1,1	11,1	18,7
SL-015	649460	9340822	335	8,5	0,7	13,4	11,5
SL-016	649731	9339934	456	45,5	7,9	200,5	460,0
SL-017	649832	9339827	488	20,5	2,5	62,5	92,5
SL-018	649850	9339808	486	9,0	1,4	17,5	13,0
SL-019	649863	9339839	477	8,5	1,4	24,5	25,0
SL-020	649877	9339836	477	18,5	2,9	53,5	63,5
SL-022	649854	9339835	479	14,5	1,3	33,0	34,0
SL-023	649877	9339774	495	8,0	0,8	10,0	12,5
SL-024	650026	9339703	500	17,0	1,6	54,5	67,0
SL-025	650041	9339682	488	11,5	1,9	27,5	34,0
SL-026	650050	9339678	449	25,0	1,2	107,0	231,0
SL-027	650134	9339654	493	14,0	1,4	58,5	103,5
SL-028	650140	9339651	497	12,0	2,9	43,5	81,5
SL-029	650175	9339688	494	33,0	2,7	246,0	505,0
SL-030	650181	9339696	494	32,0	0,8	125,5	221,5
SL-031	650201	9339709	496	25,5	3,2	74,0	80,0

SL-032	650073	9340094	387	7,0	1,1	10,0	15,0
SL-033	650164	9340027	405	6,5	1,9	10,0	11,0
SL-035	650799	9339330	610	39,9	6,5	86,3	198,3
SL-036	650865	9339358	618	7,2	0,9	16,8	18,9
SL-037	651480	9339079	614	23,1	3,6	65,1	113,7
SL-038	651421	9339134	659	9,7	3,2	22,5	33,7
SL-039	651481	9339076	610	17,0	1,0	49,5	59,4
SL-040	651415	9339145	659	8,1	2,6	14,5	18,4
SL-041	651372	9339196	647	10,1	1,3	30,4	41,4
SL-042	651412	9339102	657	10,0	0,5	44,1	66,6
SL-043	651470	9339222	652	15,8	1,6	34,1	69,5
SL-044	651071	9338693	660	38,3	3,8	263,1	973,1
SL-045	651043	9338690	657	20,5	1,8	52,0	105,5
SL-046	651104	9338630	684	28,3	1,2	94,8	293,8
SL-047	651165	9338550	650	15,2	3,6	50,5	86,6
SL-048	651562	9338525	542	13,8	1,6	69,4	138,5
SL-049	651571	9338538	529	8,5	1,2	15,5	40,5
SL-050	651607	9338810	528	27,0	5,0	226,2	665,0
SL-051	651924	9338655	500	11,3	1,6	56,4	71,2
SL-052	651945	9338641	503	10,0	1,1	43,8	69,4
SL-053	651960	9338643	500	12,7	2,4	34,6	52,3
SL-054	651992	9338639	500	8,7	0,9	23,4	22,8
SL-055	651998	9338630	490	5,2	0,4	12,6	8,9
SL-056	652131	9338703	484	8,0	1,2	30,1	48,0
SL-057	652175	9339217	593	23,8	1,2	79,2	152,4
SL-058	652290	9339260	643	37,5	12,0	208,5	520,0
SL-059	652266	9338764	473	14,4	2,4	26,4	18,3
SL-060	652489	9338910	479	25,2	2,5	100,2	223,1
SL-061	652555	9338912	483	23,5	2,3	43,6	71,9
SL-062	652596	9338926	480	16,0	1,4	42,0	24,5
SL-063	652609	9338941	484	12,2	0,7	36,1	90,5
SL-064	652628	9338951	478	9,9	1,6	40,8	51,3
SL-065	652793	9338700	489	19,1	0,3	74,9	122,5
SL-066	652775	9338719	483	15,2	1,1	26,8	41,8
SL-067	652780	9338744	485	20,5	1,4	72,5	42,5
SL-068	652778	9338642	480	13,1	0,4	63,8	56,2
SL-069	652718	9338409	516	34,6	1,6	116,0	157,7
SL-070	652710	9338411	516	11,3	0,9	25,5	48,3
SL-071	652286	9339736	640	16,0	0,9	41,0	30,5
SL-072	652300	9339652	647	30,1	2,0	33,0	56,2
SL-073	651342	9339791	611	26,1	2,0	209,1	408,7
SL-074	651442	9340402	558	127,2	10,0	734,9	1081,2
SL-075	651435	9340470	554	88,8	6,5	240,5	433,0
SL-076	650329	9340298	448	15,5	3,2	90,0	91,0
SL-077	650300	9340324	434	8,0	1,0	48,0	77,0
SL-078	650320	9340322	444	13,5	2,3	58,5	93,0
SL-079	650762	9340580	458	60,0	4,0	317,2	576,5
SL-080	650747	9340586	463	18,5	3,9	57,0	154,5
SL-081	650867	9341232	330	9,8	0,4	6,6	13,1

SL-082	650866	9341226	330	50,1	3,2	137,4	54,4
SL-083	650980	9341249	369	29,0	3,8	119,0	202,0
SL-084	650976	9341228	360	11,6	1,2	13,1	9,2
SL-085	650985	9341219	366	8,9	1,2	33,3	56,6
SL-086	651013	9341211	373	13,2	2,0	33,9	54,4
SL-087	651034	9341194	381	36,0	2,1	169,0	235,0
SL-088	651034	9341200	358	7,6	0,8	23,7	62,8
SL-089	651056	9341167	370	44,0	0,4	183,0	347,0
SL-090	651051	9341170	380	12,5	0,5	60,0	159,0
SL-091	651066	9341155	393	5,5	2,3	16,0	24,0
SL-092	651080	9341122	383	15,0	7,1	77,0	339,0
SL-093	651388	9341930	298	49,0	3,8	211,0	228,0
SL-094	651393	9341944	286	6,5	1,4	19,5	23,5
SL-095	651413	9341912	288	21,5	3,3	94,0	88,5
SL-096	651387	9341948	283	9,5	2,2	20,5	22,0
SL-097	650190	9339722	493	24,5	4,0	73,6	101,5
SL-099	652288	9339282	651	9,0	1,6	28,0	34,5
SL-100	652517	9338932	609	7,5	3,0	20,5	38,0

5.2.2. Espeleometria

As cavernas de Serra Leste desenvolvem-se sob contexto de rochas ferríferas e, portanto, foram analisadas em um único conjunto no que diz respeito aos seus atributos espeleométricos.

A média da projeção horizontal (PH) das cavidades foi de 20,9 m, com valor máximo e mínimo de 127,2 (SL-074) e 5,2 metros (SL-055), respectivamente. A maior parte das cavernas da área do Projeto Serra Leste apresenta projeção horizontal igual ou inferior a 15 metros (47,9%), 35,4% apresentam entre 15 e 30 metros, 11,5% entre 30 e 50 metros, 4,2% entre 50 e 100 metros e apenas 1% mais de 100 metros de projeção horizontal (Figura 12).

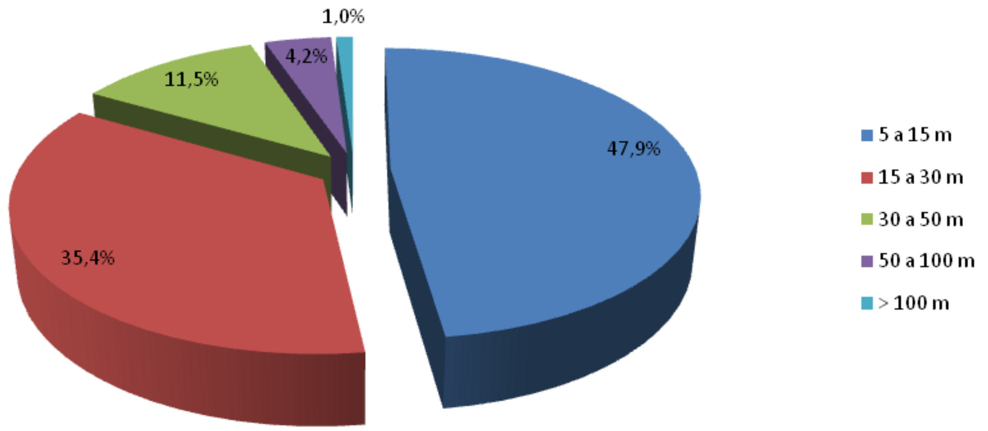

Figura 12 - Percentual de cavidades de Serra Leste em função da projeção horizontal.

O desnível médio das cavernas foi de 2,3 metros, com valor máximo de 12 metros (SL-058) e mínimo de 0,3 metros (SL-065), sendo que a maior parte delas (38,5%) possui entre 1 e 2 metros de desnível (Figura 13).

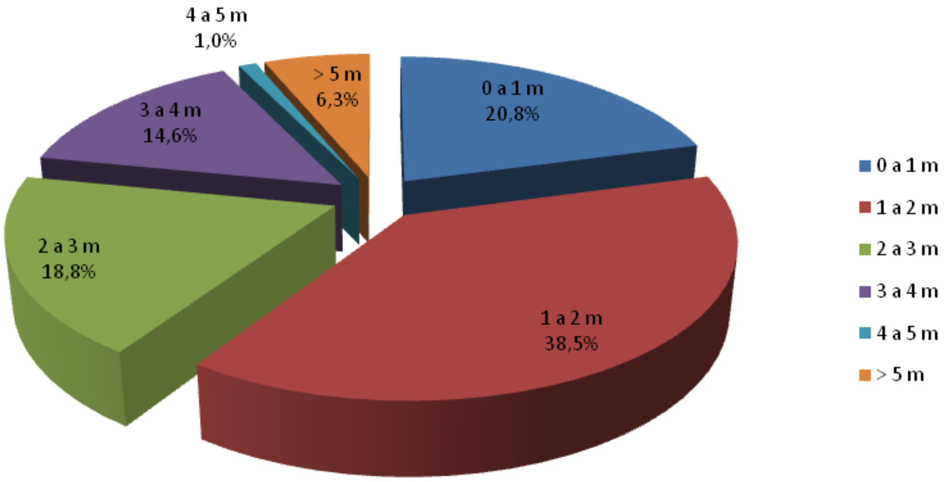

Figura 13 - Percentual de cavidades de Serra Leste em função do desnível.

Em relação à área, as cavernas apresentaram média de 77,9 m² para a área. O valor máximo para esse atributo foi de 734,9 m² (SL-074) e o mínimo de 6,6 m² (SL-081). No universo de cavernas estudadas, 22,9% têm área igual ou inferior a 25 m²; 28,1% tem área entre 25 e 50 m^2; 28,1% entre 50 e 100 m^2; 7,3% entre 100 e 150 m^2; 2,1% entre 150 e 200 m^2; 7,3% entre 200 e 250 m^2; e, por fim, 4,2% tem área superior a 250 m^2 (Figura 14).

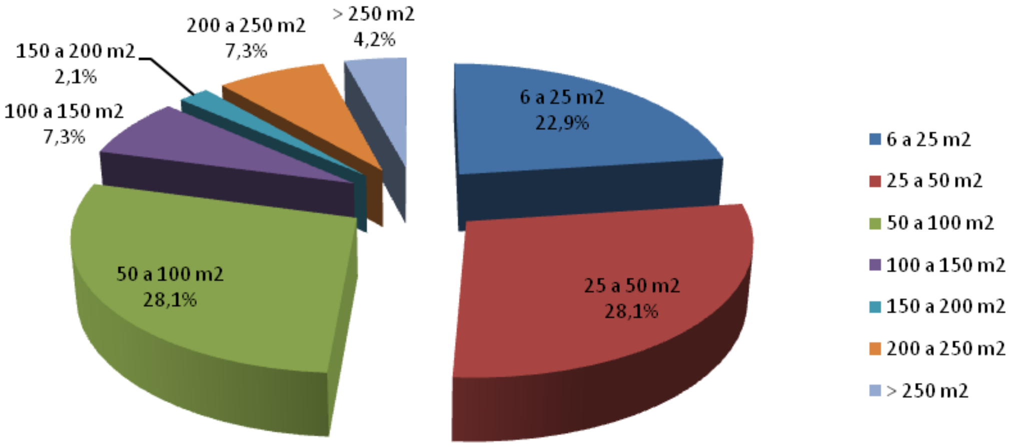

Figura 14 - Percentual de cavidades de Serra Leste em função da área.

A média para volume foi de 140 m³, com valor máximo e mínimo de 1081,2 m³ (SL-074) e 8,9 m³ (SL-055) respectivamente. A maior parte das cavernas (66,7%) apresenta volume inferior a 100 m^3 (Figura 15).

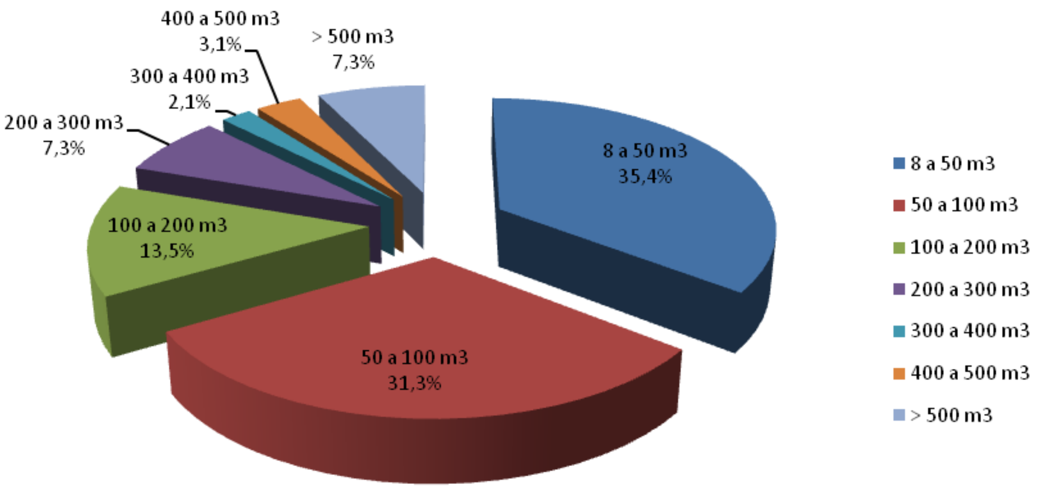

Figura 15 - Percentual de cavidades de Serra Leste em função do volume.

5.3 Geoespeleologia

A seguir serão apresentados os resultados dos estudos geoespeleológicos realizados em 96 cavernas do Projeto Serra Leste. As fichas de geoespeleologia de cada caverna estudada encontram-se no Anexo II.

5.3.1. Inserção das cavernas na paisagem

As cavernas do Projeto Serra Leste estão distribuídas, em escala de análise local, em três principais compartimentos (Figura 16):

i) cavernas inseridas no topo dos platôs e rupturas de relevo na borda de platô;
ii) cavernas inseridas nas vertentes inclinadas da serra;
iii) cavernas inseridas nos sistemas fluviais no sopé da serra;

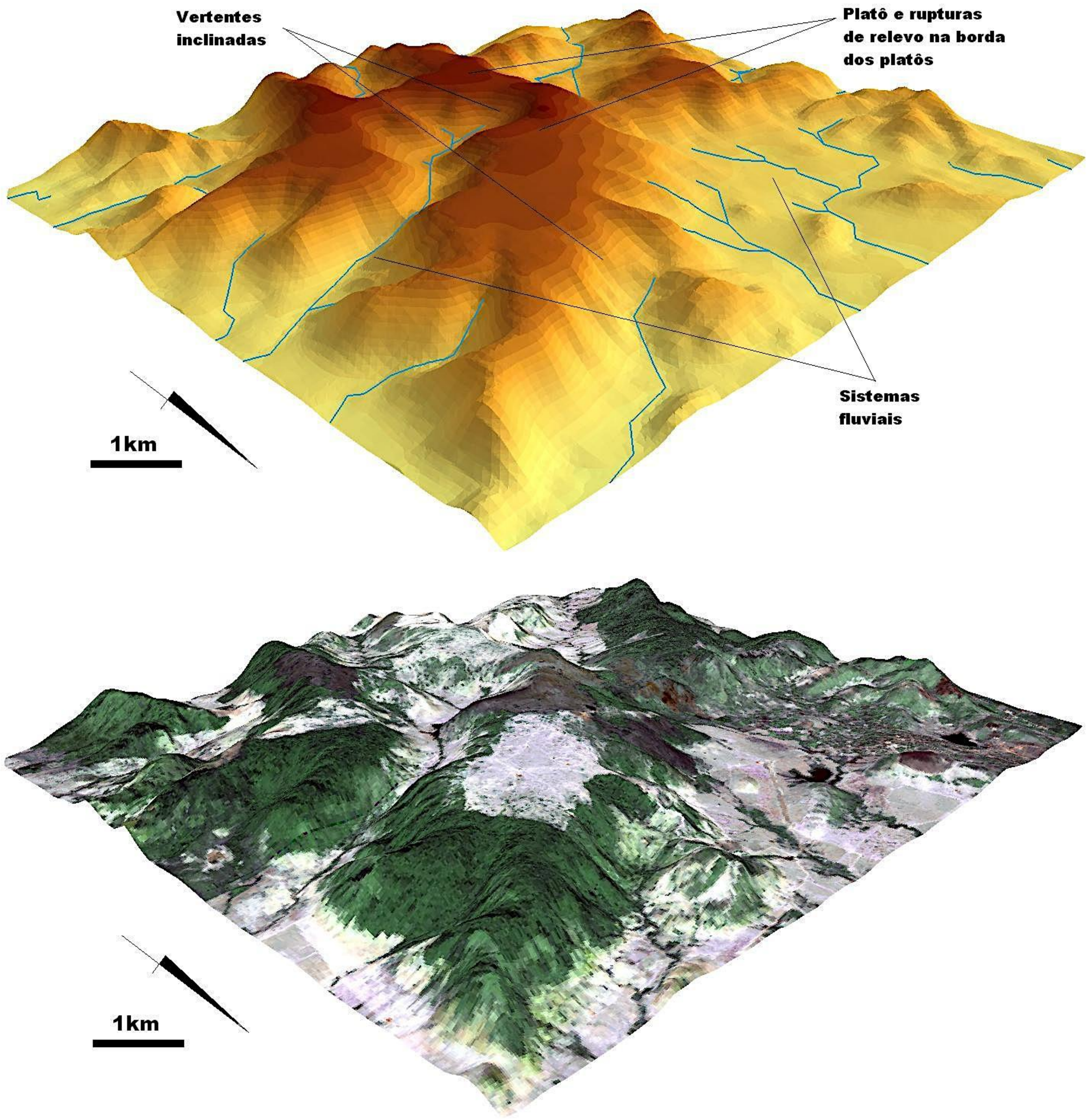

Figura 16 – Modelo Digital de Elevação (MDE) de Serra Leste: compartimentos da paisagem. Exagero vertical: 3 vezes.

A ***superfície do topo dos platôs*** possui relevo ondulado, com colinas de vertentes côncavas a convexas, sustentadas de um modo geral pela cobertura laterítica (canga). As superfícies erosivas ocupam porções variadas no contexto hipsométrico da área e estão arranjadas de maneira escalonada, no entanto, encontram-se sempre acima dos 450 m (Figura 17). Duas cavernas (SL-023 e SL-073) estão inseridas neste compartimento da paisagem, correspondendo a aproximadamente 2,1% das cavernas estudadas.

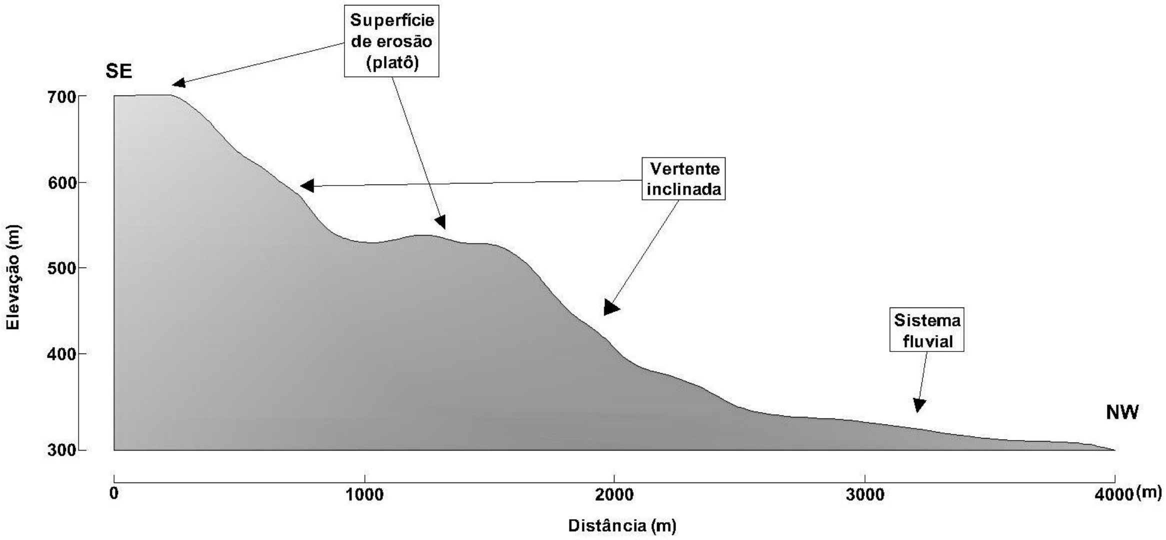

Figura 17 - Superfícies erosivas de Serra Leste.

As ***rupturas de relevo na borda dos platôs*** são comuns nas encostas interiores, mas ocorrem de modo mais pronunciado nas bordas do platô, gerando, nesse compartimento, escarpas de altura variável, podendo atingir até 10 m (Figura 18). A ausência de estudos geomorfológicos e pedológicos na área não permite afirmar se a gênese e evolução dessas rupturas escarpadas estão associadas a uma dinâmica remontante de regressão da vertente (*backwearing*) ou a processos de erosão diferencial. Independentemente dessa questão, observa-se que as cavernas inseridas nesse compartimento da paisagem desenvolvem-se a partir da base das escarpas. Duas cavernas (SL-071 e SL-072) foram identificadas neste compartimento, correspondendo a aproximadamente 2,1% das cavernas estudadas.

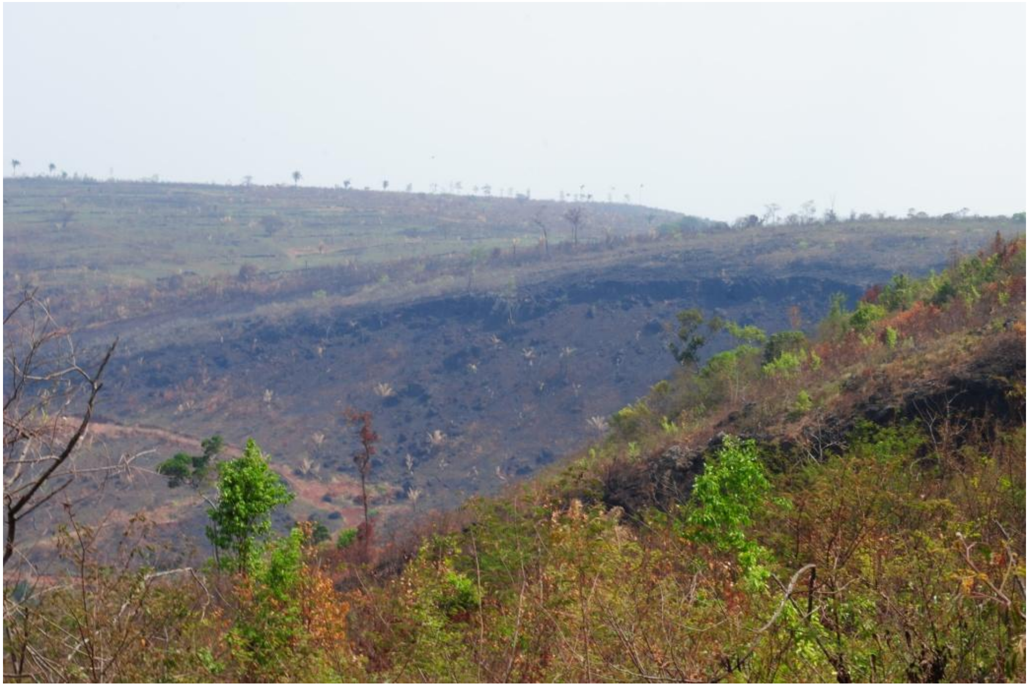

Figura 18 - Visão geral das rupturas de relevo na borda dos platôs.

Ao longo das ***vertentes inclinadas da serra*** foram registradas 53 cavernas, correspondendo a 55,2% do total estudado. As cavidades estão distribuídas ao longo deste compartimento de forma fortuita, sendo 42 posicionadas na alta vertente (43,7%) e 11 (11,5%) na média vertente. O intervalo altimétrico para essas cavidades variou entre 684 e 330 metros.

Os ***sistemas fluviais*** *no sopé da* serra podem ser divididos em dois: a) *sistema fluvial norte,* localizado na porção norte, externa a serra; e b) *sistema fluvial depressão interiorana,* localizado na depressão interna a serra (Figura 19). Juntos, os sistemas fluviais concentram 42 (43,7%) das ocorrências de cavernas de Serra Leste, sendo que 20 estão inseridas no *sistema fluvial norte* e 22 no *sistema fluvial depressão interiorana*.

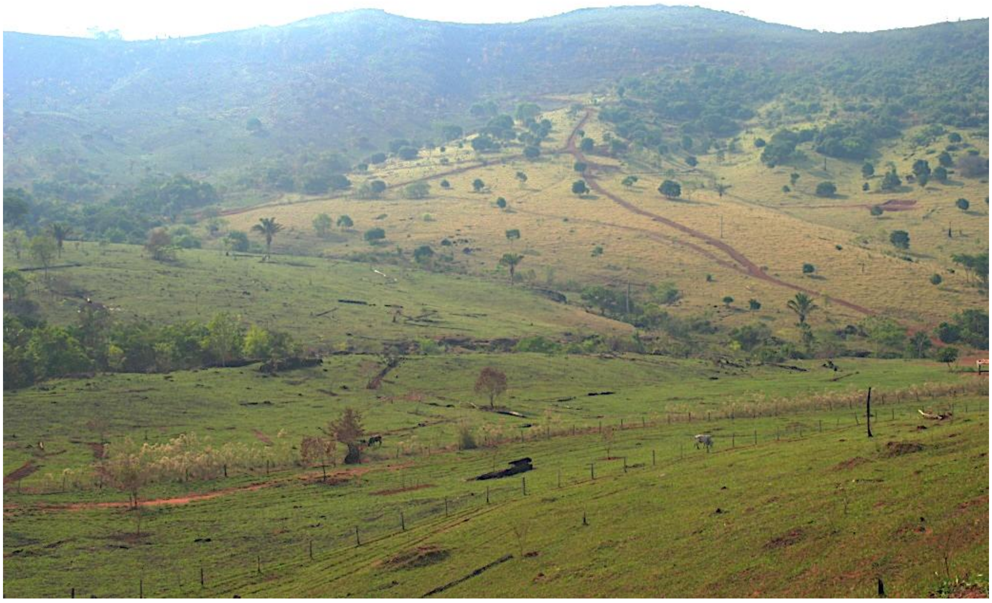

Figura 19 - Visão parcial do sistema fluvial interiorano, com destaque para as drenagens encaixadas em carapaças laterizadas.

Nos compartimentos de topo e vertente inclinada as cavernas ocorrem associadas a paredões rochosos, distribuídos de forma escalonada ao longo da vertente inclinada, nas bordas ou mesmo no interior do platô. Estes paredões são muitas vezes representados por afloramentos da formação ferrífera bandada encimados por uma cobertura de canga.

Pinheiro *et al.* (1985), coloca o intervalo 650-600 m, como o mais marcante para a ocorrência de cavernas na região de Carajás. Esta tendência foi observada em outros estudos realizados em cavernas de N4E, N4WS e N5S em Serra Norte (relatórios inéditos). No entanto, no caso de Serra Leste, essa tendência não foi corroborada. Como podemos observar na Tabela 5 e Figuras 20 e 21, as cavernas estudadas ocupam todos os compartimentos da paisagem e, embora estejam concentradas em determinados intervalos, não foi possível definir nenhum padrão de distribuição.

Tabela 5 – Frequência e percentual de cavernas segundo intervalo hipsométrico.

Classe (m)	Frequência	%
283 - 300	4	4,2%
300 - 350	14	14,6%
350 - 400	13	13,5%
400 - 450	5	5,2%
450 - 500	32	33,3%
500 - 550	6	6,3%
550 - 600	3	3,1%
600 - 650	11	11,5%
650 - 700	8	8,3%
Total	96	100,0%

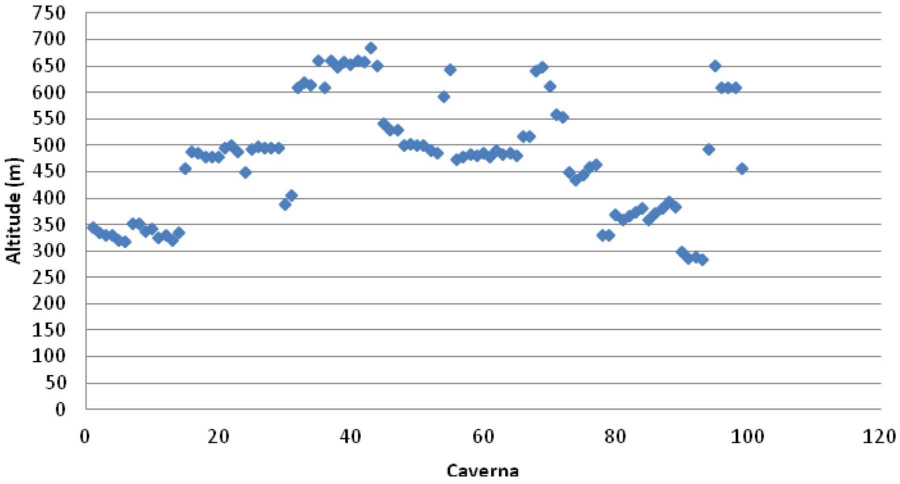

Figura 20 - Dispersão das cavernas segundo intervalo hipsométrico.

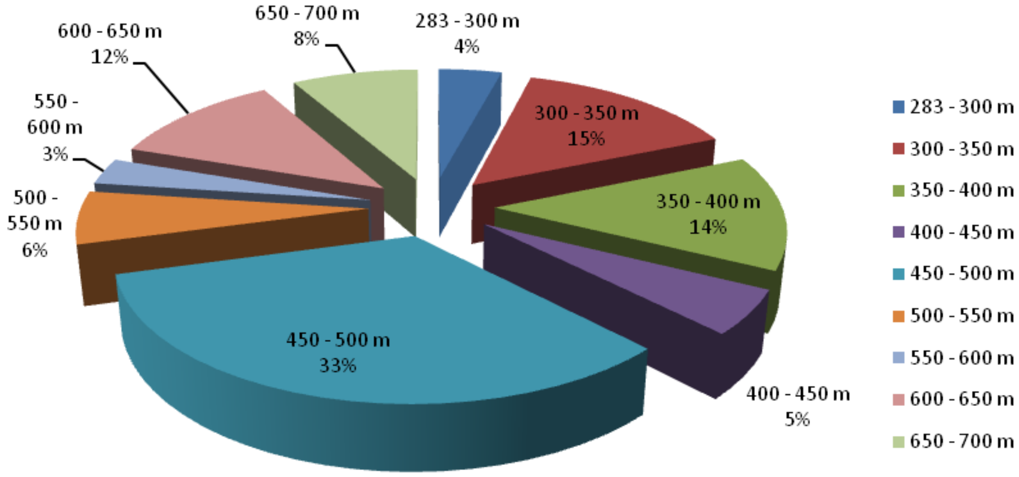

Figura 21 - Percentual de cavernas segundo intervalo hipsométrico.

5.3.2. Morfologia

Foram identificados nas cavernas estudadas quatro tipos de padrão planimétrico: espongiforme, globular, retilíneo e reticular (Tabela 6). O padrão mais recorrente é o espongiforme, presente em 46 (47,9%) cavernas. O segundo padrão mais frequente foi o globular, com 10 (10,4%) cavidades (Figura 22). O padrão retilíneo foi observado em cinco (5,2%) cavidades e o reticular também em cinco (5,2%). Devido ao seu reduzido tamanho, 33 cavidades (31,3%) não apresentaram padrão morfológico definido.

Tabela 6 - Frequência de cavernas em função do padrão planimétrico.

Padrão planimétrico	Frequência	%
Espongiforme	46	47,9%
Globular	10	10,4%
Retilíneo	5	5,2%
Reticulado	5	5,2%
Não definido	30	31,3%
Total	96	100,0%

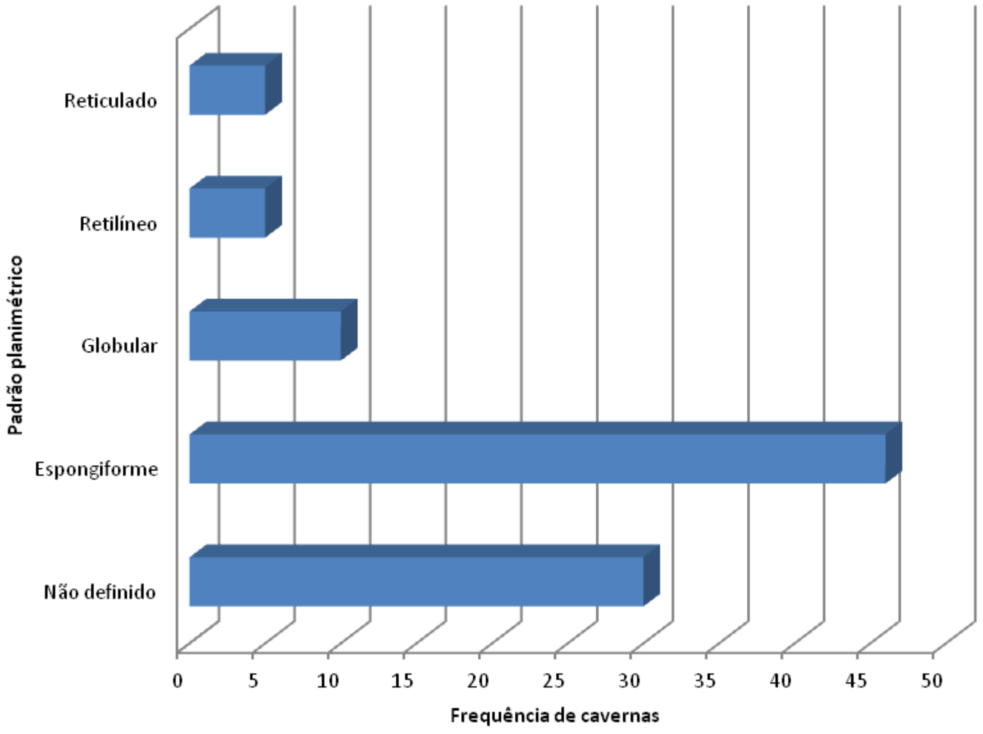

Figura 22 - Frequência de cavernas em função do padrão planimétrico.

Padrão Espongiforme

Caracteriza-se por apresentar condutos de tamanho variado com aspecto de "esponja". É mais facilmente reconhecido em cavernas com maior desenvolvimento, no entanto, cavidades menores também podem apresentar feições típicas desse tipo de padrão planimétrico. Trata-se de um padrão bastante frequente nas maiores cavernas do Quadrilátero Ferrífero e Carajás. Em Serra Leste, as cavernas que apresentaram esse padrão foram SL-001, SL-003, SL-005, SL-007, SL-008, SL-009, SL-011, SL-014, SL-016, SL-017, SL-018, SL-020, SL-023, SL-024, SL-025, SL-026, SL-027, SL-028, SL-029, SL-030, SL-037, SL-041, SL-045, SL-046, SL-047, SL-049, SL-051, SL-052, SL-062, SL-065, SL-066, SL-073, SL-075, SL-076, SL-077, SL-078, SL-079, SL-082, SL-083, SL-087, SL-089, SL-090, SL-092, SL-095, SL-099 e SL-100.

Padrão Globular

O padrão globular, muito semelhante ao padrão espongiforme, apresenta salões bastante porosos, aproximadamente circulares ou semi-circulares, que se interconectam através de passagens estreitas em teto rebaixado. No caso de Serra Leste, foi possível identificar cavernas com padrão globular "interrompido" onde a evolução remontante da vertente, através de abatimentos, interceptou os "glóbulos". Exemplo notável desta interceptação pode ser observada na caverna SL-48. Apresentaram esse padrão as cavernas SL-002, SL-004, SL-022, SL-031, SL-035, SL-042, SL-048, SL-071, SL-093 e SL-097.

Padrão retilíneo

Constituído por conduto simples, seguindo uma única direção, geralmente condicionado por fratura, junta de alívio ou pelo plano inclinado do bandamento ou foliação da rocha. Esse tipo de padrão morfológico foi observado nas cavernas SL-044, SL-057, SL-059, SL-068 e SL-081.

Padrão reticulado

O padrão reticulado, tal qual o retilíneo, é condicionado por fratura, junta-de-alívio, bandamento ou até mesmo foliação, cujos planos se entrecruzam formando uma rede que se alarga gerando condutos interconectados em ângulos variados. Apresentaram esse padrão as cavernas SL-043, SL-069, SL-072, SL-074 e SL-080.

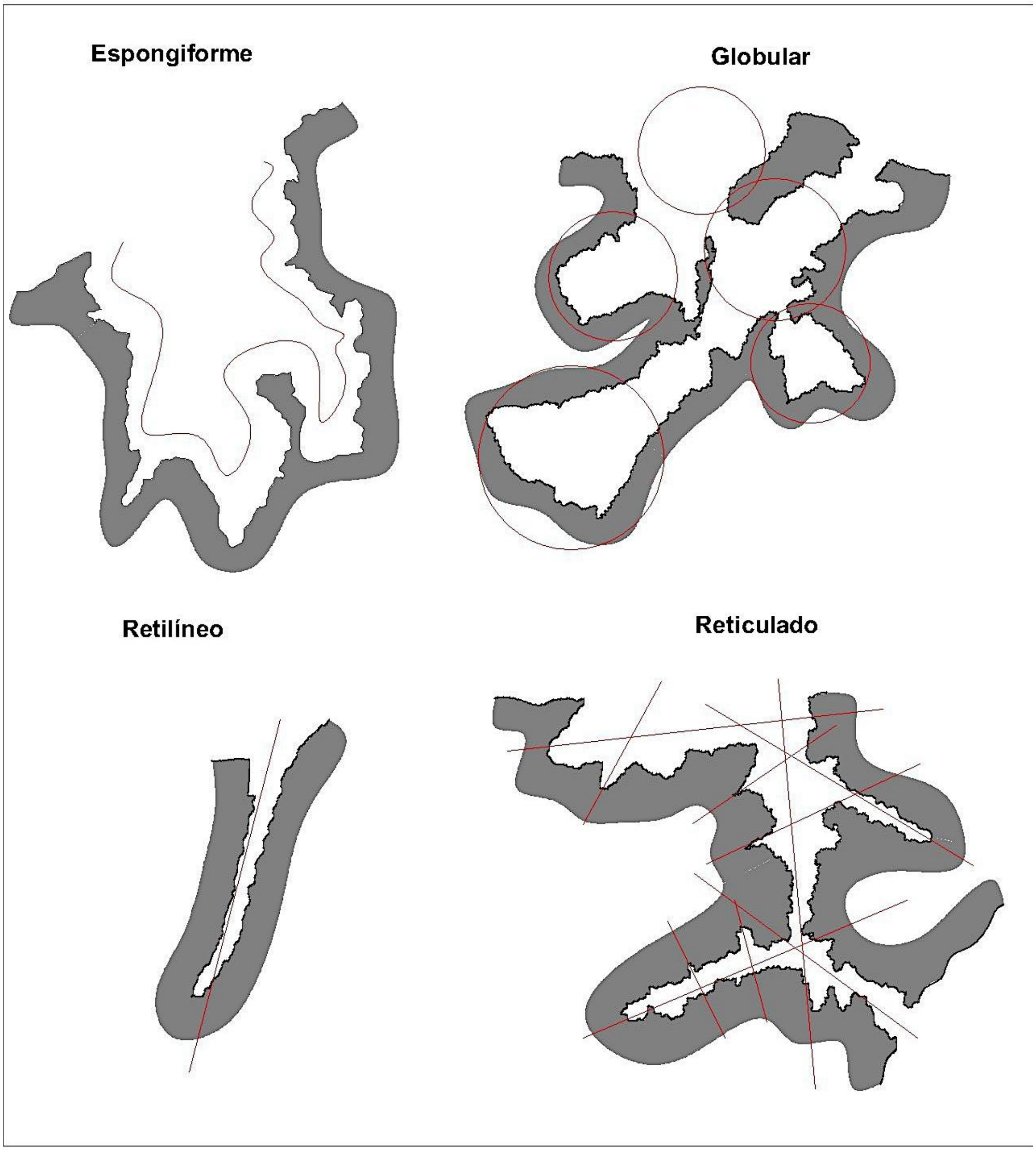

Figura 23 - Representação dos padrões planimétricos observados nas cavernas estudadas: espongiforme (SL-029); globular (SL-035); retilíneo (SL-081); e reticulado (SL-072). As linhas em vermelho representam as regularidades/irregularidades e "controles" das galerias.

É importante salientar que os padrões planimétricos foram definidos para cavernas calcárias e estão associados à gênese e desenvolvimento das cavernas nesta litologia. A classificação de cavidades ferríferas segundo esse sistema resulta em incongruências uma vez que nem sempre se observa correlação entre as formas e os processos inicialmente preconizados.

As cavernas estudadas apresentam padrão de desenvolvimento predominantemente horizontal, pisos irregulares (82,5%), planos (42,6%) a suavemente inclinados (57,4%). As cavidades cujo piso apresentou regularidade são essencialmente planas (85,7%). As paredes

são irregulares em praticamente todas as cavernas estudadas. Essa característica, no caso das cavernas inseridas em canga e laterita, está associada ao arranjo caótico do arcabouço litológico, independentemente da participação de matriz. No caso das cavernas inseridas em formação ferrífera bandada, essa característica está associada ao nível de alteração do substrato rochoso. Apenas a caverna SL-068 apresentou paredes relativamente regulares, controladas pelo bandamento da rocha em que está encaixada. O teto de todas as cavernas é irregular, pelos mesmos motivos apresentados para as paredes. Em algumas cavidades, foi possível identificar setores com arredondamento (quatro cavernas ou 4,2%) e significativa inclinação (sete cavernas ou 7,3%), mas constitui exceção.

Em relação às feições internas, foram observados pilares, pendentes, pontões estruturais, pisos suspensos, paleopisos, pisos capeados, canalículos, *bell holes* (cúpulas) e clarabóias.

Os ***pilares*** são feições residuais resultantes de processos de erosão diferencial do substrato rochoso em que está encaixada a caverna, feição relativamente comum nas cavernas ferríferas. Foram observadas em 51 (53,1%) cavernas de Serra Leste. Deste conjunto, destaca-se a caverna SL-001 (Figura 24), que apresentou um conjunto único de pilares cuja controversa gênese não foi completamente desvendada. Foram aventadas duas hipóteses para a gênese destas feições aqui denominados de *pilar diagenético* e *pilar biogênico*.

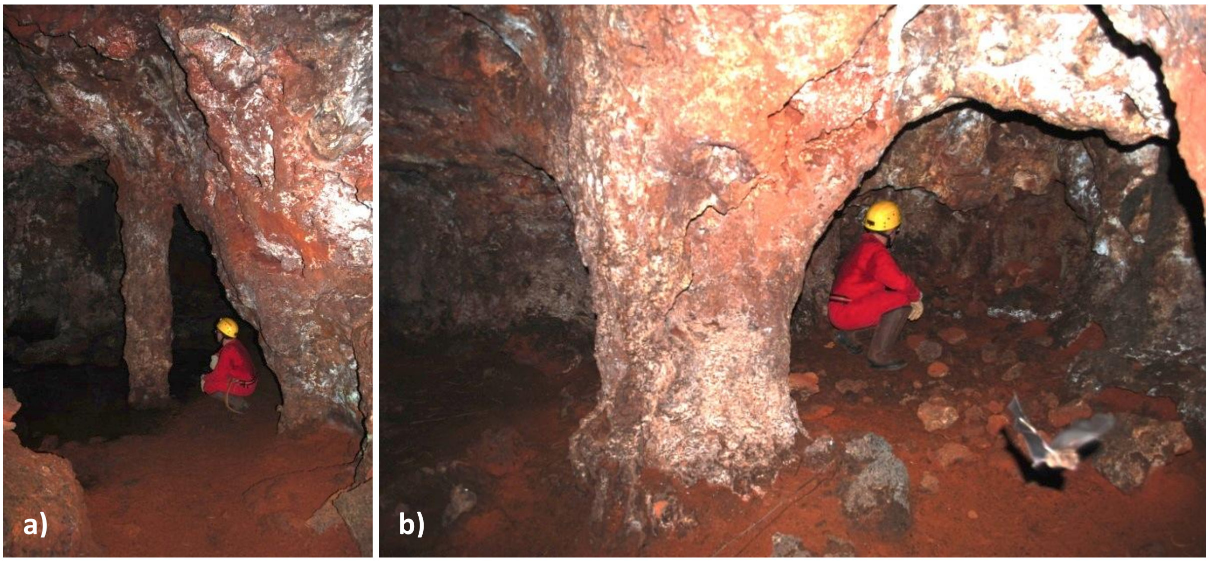

Figura 24 – Pilares observados na caverna SL-001 (a e b).

A hipótese do ***pilar diagenético*** sugere o pilar e a rocha foram formados conjuntamente, no entanto, com textura e estrutura diferentes. Posteriormente, através da ação erosiva em ambiente vadoso, os pilares, mais resistentes, foram preservados, ao passo que a rocha encaixante foi erodida. A existência de sulcos de percolação vertical corroboram com essa hipótese, sendo responsáveis, inclusive, por permitir circulação na porção interna do pilar gerando os vazios internos (Figura 25).

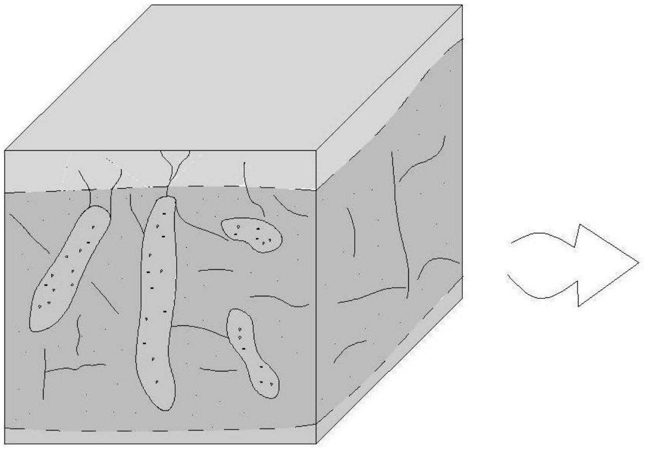

Figura 25 - Modelo de evolução dos pilares diagenéticos. Fonte: dados dos autores.

A hipótese do ***pilar biogênico*** sugere que processos de bioturbação e "crotovinamentos" (processo de preenchimento de vazios originados por bioturbação) anteriores a existência da caverna produziram sulcos verticais cuja percolação de fluidos ferruginizados foram responsáveis pelo enrijecimento periférico destes sulcos, produzindo uma auréola mais resistente. A lentidão da percolação favoreceu a circulação a partir de vetores verticais e horizontal, possibilitando a formação de auréolas com diâmetros superiores a um metro em alguns casos. Com a formação da caverna e erosão do substrato rochoso menos resistente, restaram os pilares ocos (Figura 26).

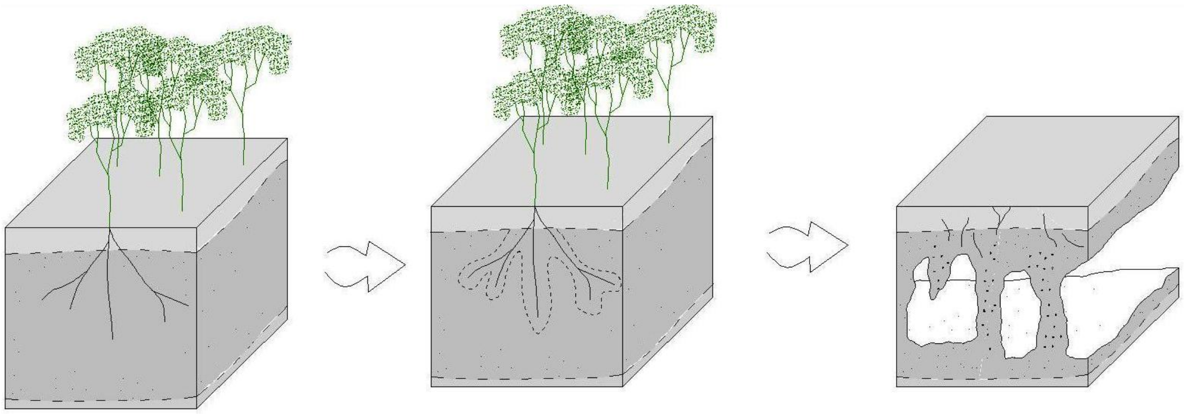

Figura 26 - Modelo de evolução dos pilares biogênicos. Fonte: dados dos autores.

Os ***pendentes*** correspondem a feições residuais normalmente associadas ao solapamento de um pilar ou rebaixamento do piso. Caracterizam-se por projeções rochosas de formato aproximadamente cônico invertido a partir do teto. Essas feições foram observadas em oito (7,8%) cavernas, SL-001, SL-002, SL-009, SL-051, SL-087, SL-089, SL-092 e SL-093. Cabe destacar as ocorrências observadas na caverna SL-001 (Figura 27).

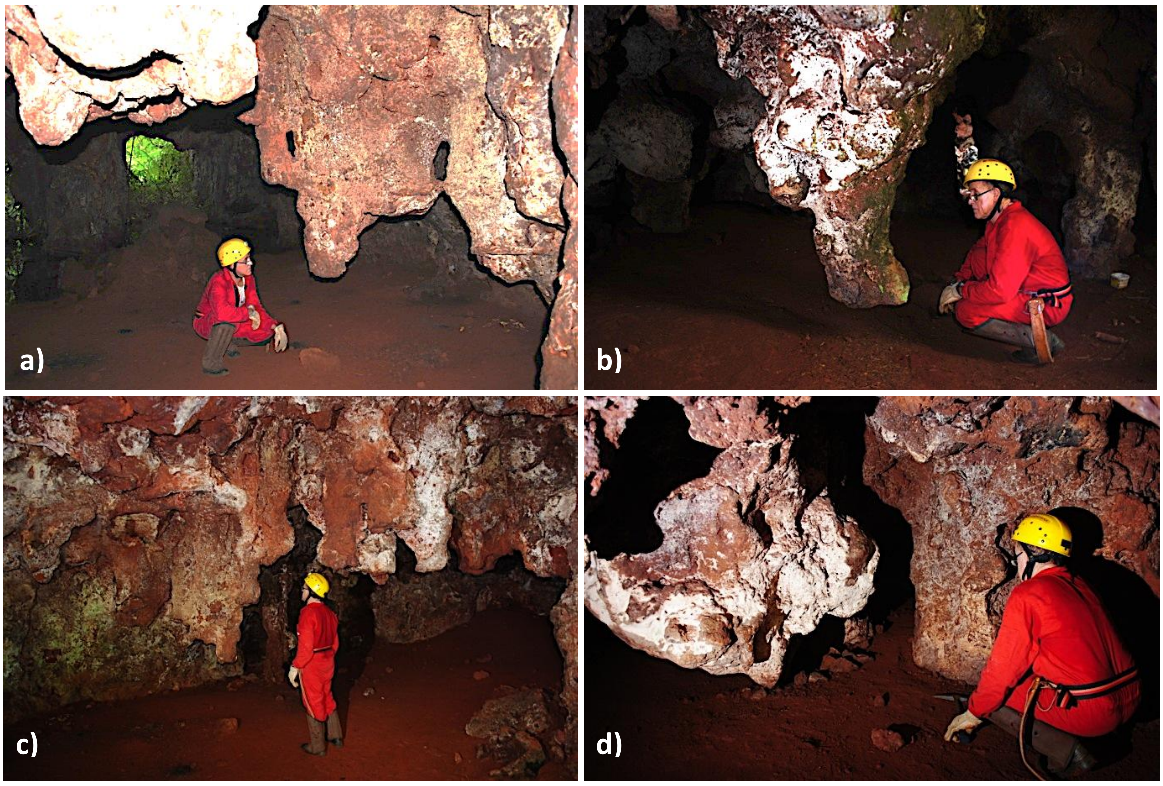

Figura 27 - Conjunto de pendentes observados ao longo de toda a caverna SL-001 (a, b, c e d).

As seções de algumas das cavidades inseridas na formação ferrífera bandada apresentaram irregularidades no teto e paredes denominadas de pontões estruturais. Essa feição, registrada em cinco cavernas (5,2%), é caracterizada por pontas retangulares (dissimétricas), sub-retangulares, que se projetam do teto de forma escalonada (Figura 28). Ocorrem mais claramente quando o bandamento, com mergulho concordante com o maior prolongamento do conduto, é interceptado por um plano de fratura perpendicular. Essas feições, presentes em diversas cavernas de Carajás, também foram observadas em cavidades no Quadrilátero Ferrífero.

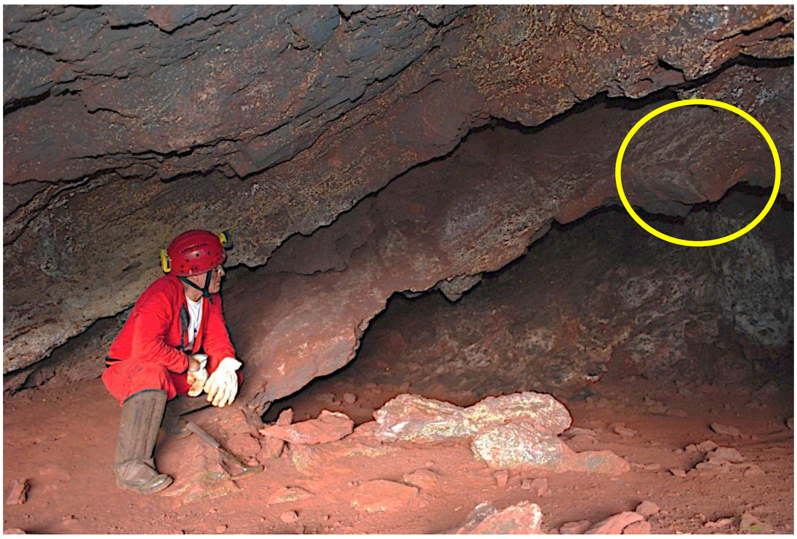

Figura 28 - Pontão estrutural observado na caverna SL-042 (destacado em amarelo).

Pisos suspensos e paleopisos são feições caracterizadas por estarem localizadas em posições distintas em relação ao nível atual do piso da caverna (Figura 29). Em cavernas ferríferas sua individualização e conservação está associada, muitas vezes, ao capeamento de crostas que o recobre, protegendo-o dos processos erosivos. É comum estarem sobrepostos a canalículos, condutos ou a pontos da parede onde tenha ocorrido solapamento. Foram observados em quatro (4,2%) cavernas, SL-001, SL-029, SL-051 e SL-061, merecendo destaque a caverna SL-029. Nesta caverna o piso preserva ciclos de deposição e capeamento de grande importância para a interpretação da gênese e desenvolvimento do conjunto de cavernas inseridas no contexto da SL-029.

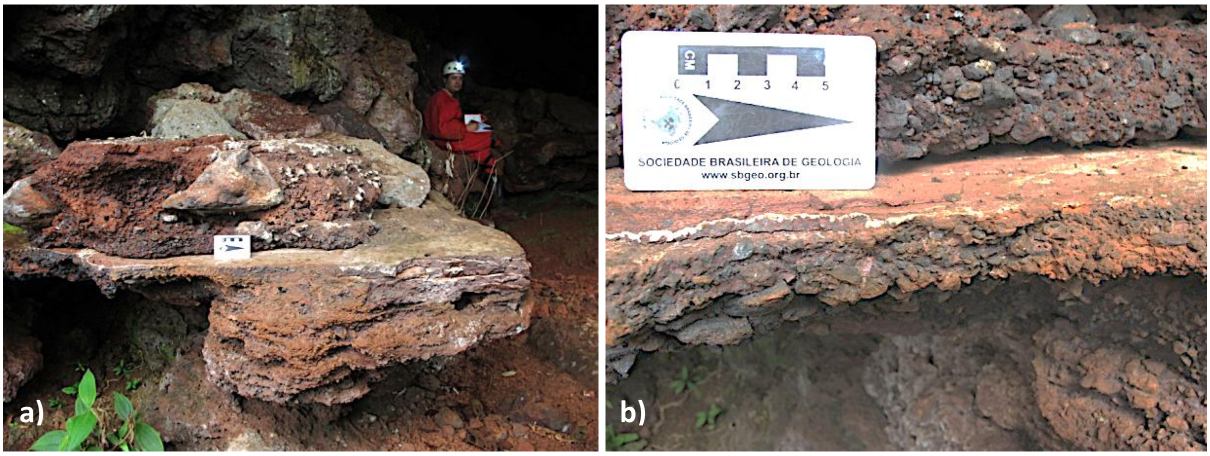

Figura 29 - Paleopiso observado na caverna SL-029: registro de ciclos de deposição e capeamento. Em a), visão geral, em b), visão em detalhe do capeamento.

Pisos capeados foram observados em sete (7,3%) cavernas, SL-029, SL-057, SL-073, SL-079, SL-083, SL-087 e SL-089. A espessura do capeamento varia de poucos milímetros a até 5 cm. Merecem destaque as feições observadas nas cavernas SL-029, SL-073, SL-079 e SL-089, dada sua espessura e abundância (Figura 30).

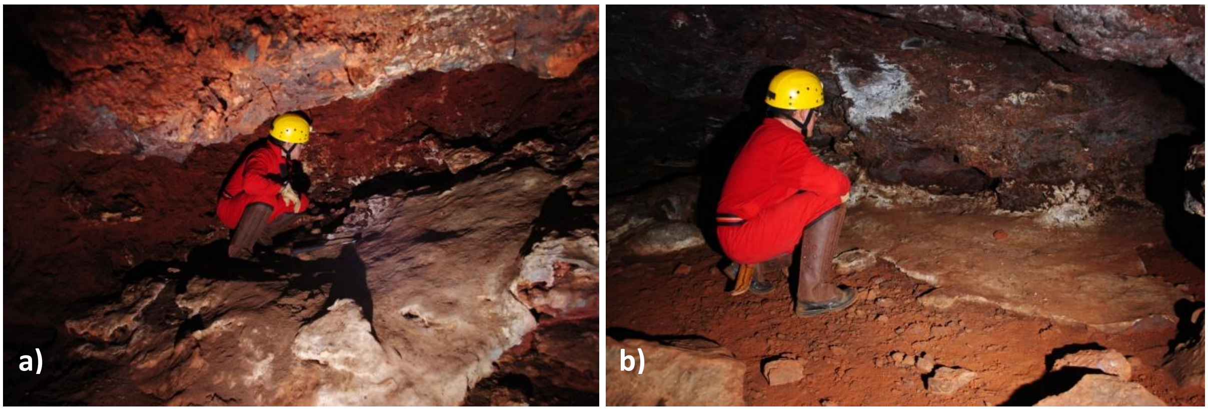

Figura 30 - Piso capeado observado na caverna (a) SL-079 e (b) SL-089.

As feições do tipo *bell holes* (cúpula) foram observadas na caverna SL-001 (Figura 31). Estão associadas a abatimentos de porções do teto onde, sob as cúpulas, é possível observar deposição de cone de sedimentos clásticos.

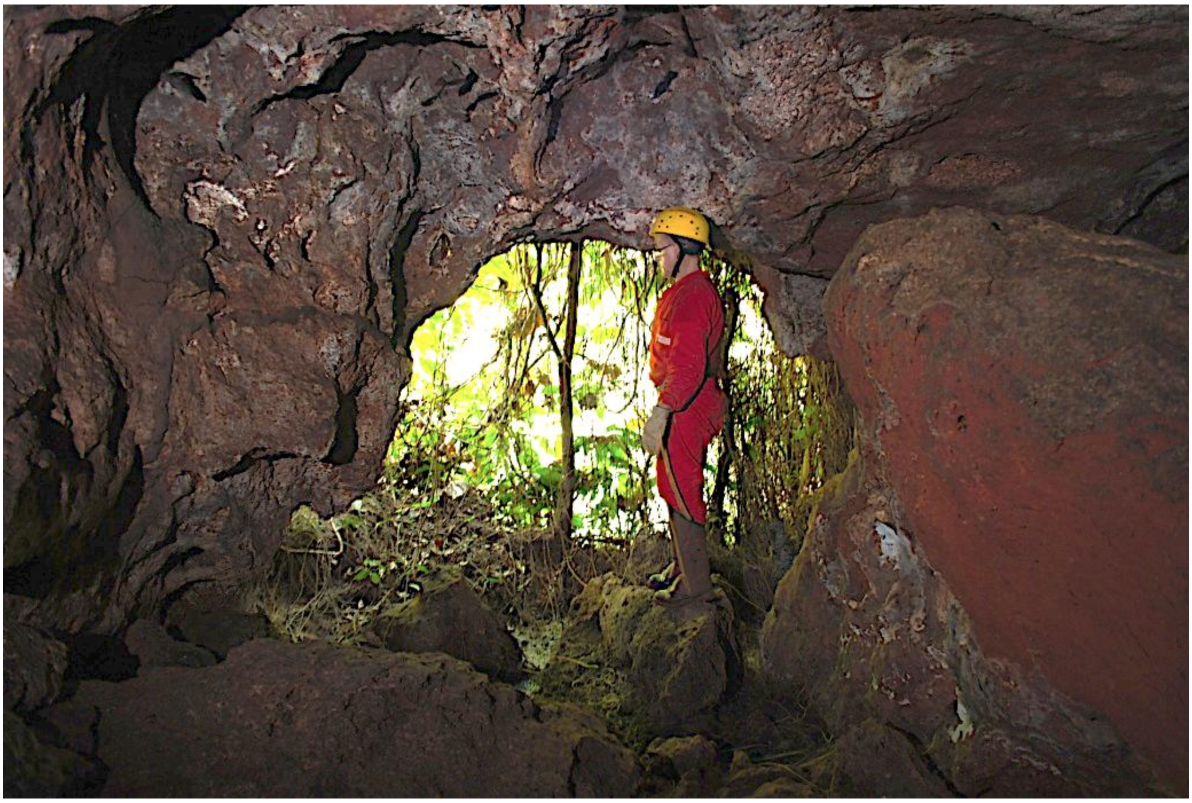

Figura 31 - *Bell holes* observados na caverna SL-001.

Os ***canalículos*** constituem-se como canais de pequena dimensão, geralmente de ordem centimétrica. Podem desenvolver-se subhorizontalmente ou subverticalmente, sendo ambos muito comuns nas cavernas ferríferas. Normalmente estão localizados no contato entre o piso e as paredes da caverna ou no teto. Raramente ocupam porções intermediárias da parede, embora esta situação tenha sido também observada. Eles afetam a morfologia das cavernas, pois sua evolução gera apêndices ou expansões laterais que podem eventualmente evoluir para galerias laterais. Em Serra Leste essa feição foi observada em 92 (95,8%) cavidades (Figura 32).

Segundo Piló & Auler (2005), de uma forma geral, há uma relação diretamente proporcional entre o perímetro da caverna e o número de canalículos, mostrando que a presença dos mesmos causa um "alongamento" do perímetro da caverna. A mesma relação é observada em relação à área que tende a ser maior diante da presença de canalículos. Estas duas relações demonstram a importância dos canalículos na geração da morfologia final da caverna.

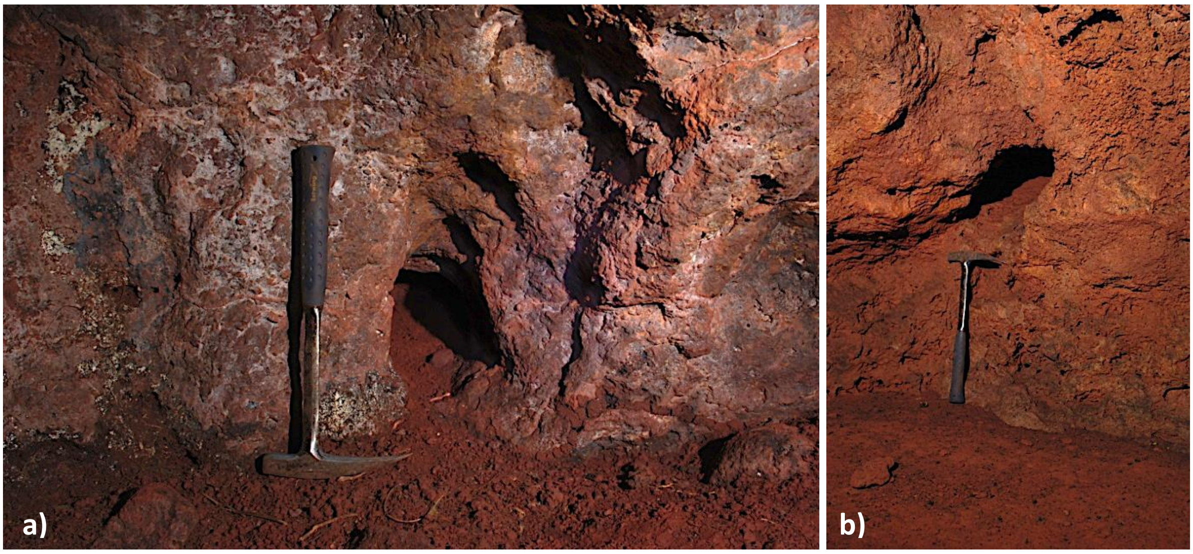

Figura 32 – a) Canalículo observado na junção piso/parede da caverna SL-002; b) canalículo presente diretamente na parede da caverna SL-093.

As ***clarabóias*** são feições resultantes do abatimento de porções do teto da cavidade, interceptando a superfície do terreno. Ocorrem geralmente onde o pacote rochoso é pouco espesso (Figura 33). A primeira referência a clarabóias em cavernas ferríferas foi feita por Simmons (1963) ao descrever cavidades no Quadrilátero Ferrífero. Em cavernas de Carajás, clarabóias já foram descritas por Maurity & Kotschoubey (1995) e Piló & Auler (2007). Esse tipo de feição pode estar associado a processos doliniformes e, em alguns casos, representar o único acesso ao interior da cavidade. Essas feições foram observadas em dez (10,4%) cavernas, a saber: SL-001, SL-006, SL-040, SL-048, SL-056, SL-057, SL-058, SL-066, SL-091 e SL-093.

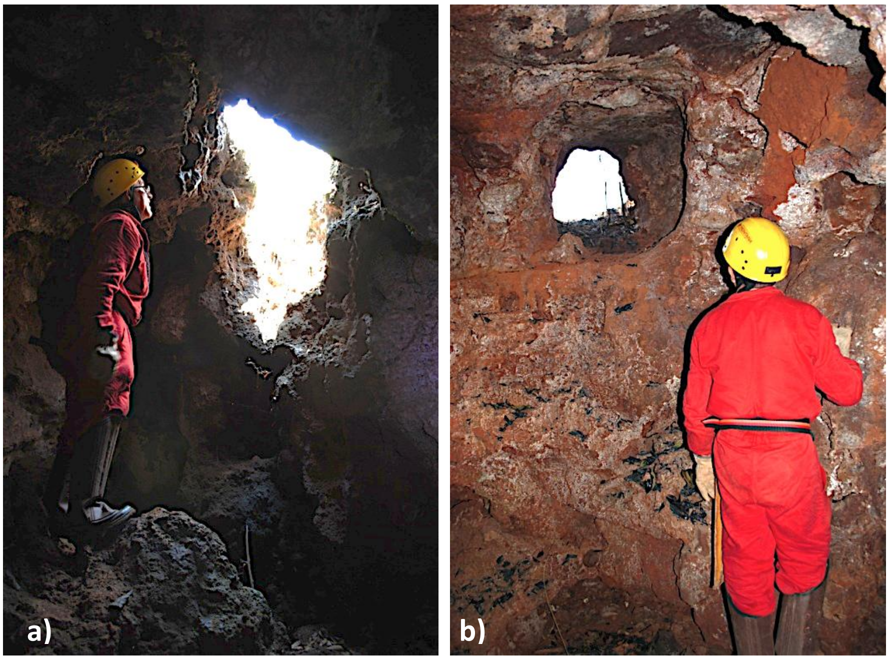

Figura 33 - Clarabóia observada na caverna SL-001 (a) e SL-006 (b).

A Figura 34 apresenta a frequência de cavernas segundo feições internas observadas.

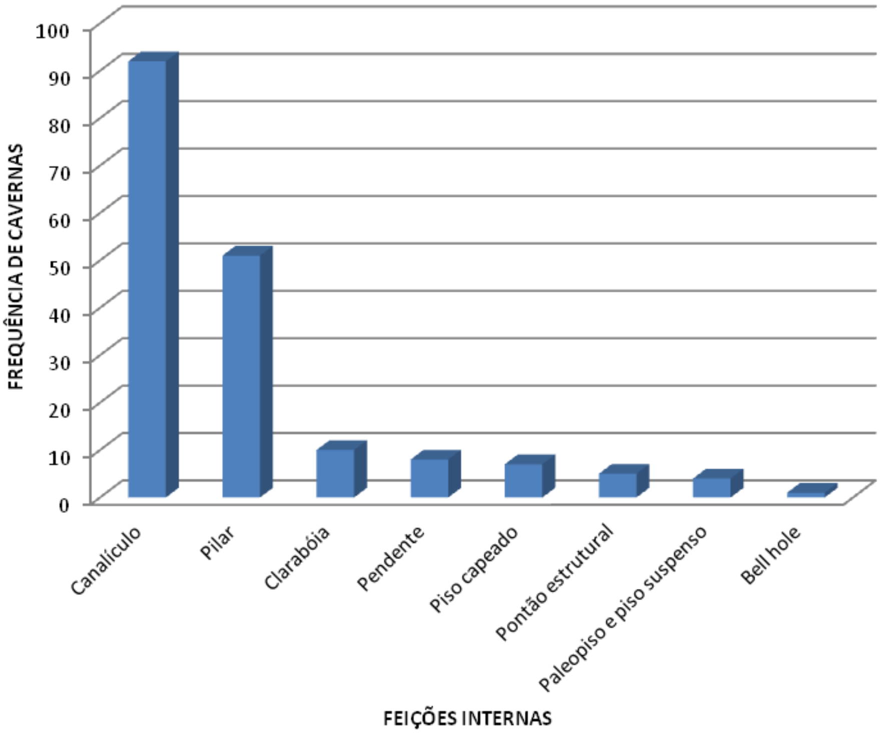

Figura 34 - Frequência de cavernas por feição interna observada.

5.3.3. Litologia

As cavernas de Serra Leste desenvolveram-se sob rochas ferríferas, incluindo formação ferrífera bandada (jaspilito) e canga (química e detrítica). A textura e estrutura do substrato rochoso das cavernas estudadas possuem relação direta com as formas das cavides, uma vez que influenciam de maneira decisiva a gênese, e sobretudo o desenvolvimento dessas feições geológicas.

A literatura apresenta diversas classificações para os litotipos ferruginosos. No presente trabalho, optou-se por tratar genericamente como ***formação ferrífera bandada*** todo e qualquer substrato ferruginoso bandado, em qualquer grau de deformação ou alteração. A classe ***canga***, por sua vez, englobou todo substrato, transportado ou não, onde fragmentos rochosos, arredondados ou angulosos, bem ou mal selecionados, encontram-se cimentados por uma matriz ferruginosa, em qualquer proporção matriz/arcabouço. Vale salientar que quando da realização dos trabalhos de campo, os litotipos foram estudados detalhadamente e a importância das características texturais/estruturais foram consideradas e estão detalhadas nas fichas de campo (Anexo II). Foram aqui agrupados apenas para fins estatísticos por apresentarem semelhanças em termos do condicionamento das formas e processos espeleogenéticos.

Entre as 96 cavernas estudadas, 88 (91,6%) estão encaixadas em uma mescla de canga e formação ferrífera bandada, normalmente este sob aquele. Em algumas situações, pelitos e conglomerados ocorrem conjuntamente. Cavernas desenvolvendo-se exclusivamente em formação ferrífera bandada foram registradas quatro (4,2%) vezes. Cavidades com desenvolvimento exclusivo em canga foi observado em quatro (4,2%) outros casos (Tabela 7 e Figura 35).

Tabela 7 - Frequência de cavernas segundo litotipo encaixante.

Litologia	Frequência	%
Litotipo misto (canga + FFB, pelitos, conglomerados)	88	91,6%
Formação ferrífera bandada (jaspilito, hematititos)	4	4,2%
Canga (detrítica, química, lateritas e ferricretes)	4	4,2%
Total	96	100,0%

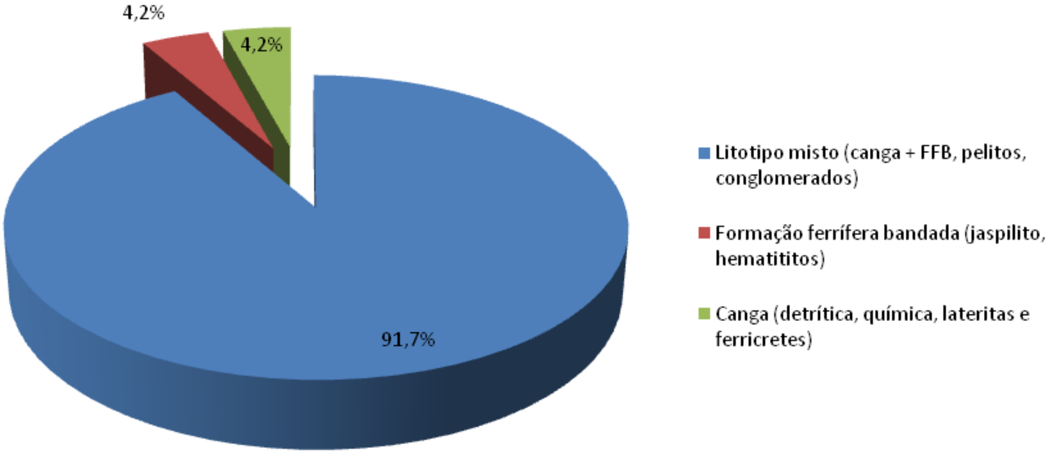

Figura 35 – Percentual de cavernas segundo litotipo encaixante.

A ***canga*** representa o litotipo com o maior número de ocorrência de cavernas e, como vimos acima, ocorre na maior parte das vezes associada à formação ferrífera bandada e outras litologias. Foram observados dois tipos de canga na área de estudo: a canga detrítica e a canga química. A primeira se caracteriza pela ocorrência de fragmentos clásticos da formação ferrífera cimentados por matriz limonítica. A dimensão do material clástico observado varia entre grânulo e matacão, com predomínio de seixos (Figura 36). A forma dos clastos varia entre arredondada e angulosa, predominando subarredondada e subangulosa.

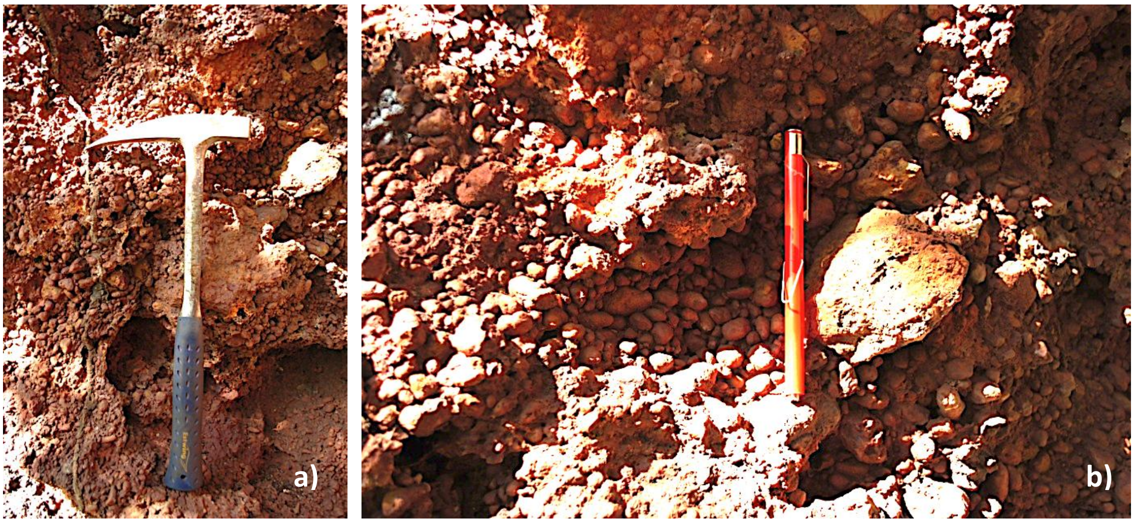

Figura 36 - Variação granulométrica da canga detrítica na caverna SL-008 (a e b): clastos subarredondados com tamanho seixo a calhau com predomínio do primeiro.

De forma geral, este litotipo apresenta má seleção e arranjo caótico dos clastos que compõe o arcabouço litológico. A participação de matriz cimentante variou bastante. Foram observadas canga matriz-suportada a clasto-suportada e com ocorrência independente de sua inserção na paisagem, ou seja, do topo a baixa vertente, não sendo observado qualquer tipo de padrão em sua distribuição.

A canga química, por sua vez, consiste de um substrato em geral homogêneo onde a participação do arcabouço é bastante incipiente. Quando são observados clastos, sua granulometria máxima é de grânulo.

A formação ferrífera banda, neste trabalho, é composto por jaspilitos e outros litotipos ferruginosos bandados. O jaspilito foi observado em apenas três (3%) cavidades de Serra Leste. A rocha se caracteriza pela ocorrência de bandas alternadas de hematita, jaspe e localmente quartzo (Figura 37). A espessura dessas camadas é milimétrica podendo atingir um ou dois centímetros no caso da hematita. As bandas de jaspe e sílica são milimétricas.

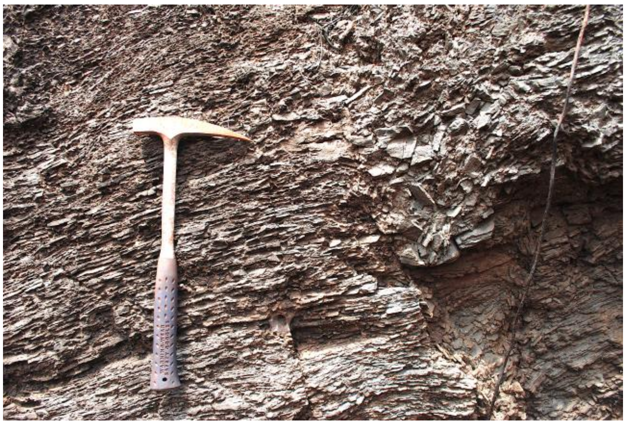

Figura 37 - Jaspilito alterado observado na caverna SL-078.

A formação ferrífera bandada em diversos casos apresenta um enriquecimentos relativo, ou seja, com suas bandas de jaspe total ou parcialmente lixiviadas. Nas caveras de Serra Leste, este litotipo é constituído predominantemente por bandas de hematita com espessura variando entre poucos milímetros e cerca de dois centímetros. Entre as bandas é comum a ocorrência de vazios milimétricos a centimétricos, outrora preenchidos por jaspe. No entanto, a ocorrência de vazios não é uma regra, uma vez que as camadas hematíticas podem por vezes se apresentar bem compactas.

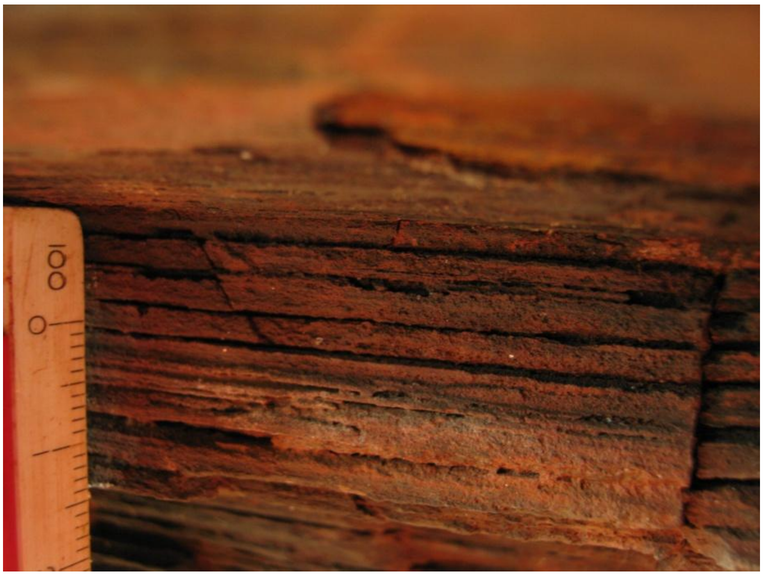

Figura 38 – Formação ferrífera bandada com relictos do bandamento de jaspe lixiviado (SL-019).

5.3.4. Estruturas

Como visto anteriormente, a Serra Leste está inserida no contexto do Cinturão de Cisalhamento Itacaiúnas, de forma que a falha no Cinzento perpassa a porção norte da referida serra. Uma grande quantidade de feições estruturais tais quais fraturas, juntas-de-alívio, brechações, dobras e contatos geológicos erosivos foram observados no interior das cavernas. Por outro lado, toda a paisagem parece evoluir segundo essas estruturas herdadas, as vertentes tendem a ser "desmontadas" segundo esses alinhamentos, as drenagens, retilíneas, ocupam antigos diques de gabros cambrianos (DOCEGEO, 2005).

As feições estruturais mais frequêntes foram as fraturas, presentes em 60 (62,5%) cavernas exercendo em nove (9,3%) delas controle determinante sobre as formas e direção das galerias. Devido a interferência magnética, foi possível medi-las em apenas 13 (13,5%) cavernas. Embora o universo amostral seja reduzido (n=64) (Figura 39), constatou-se uma tendência de concentração na direção NE e NW.

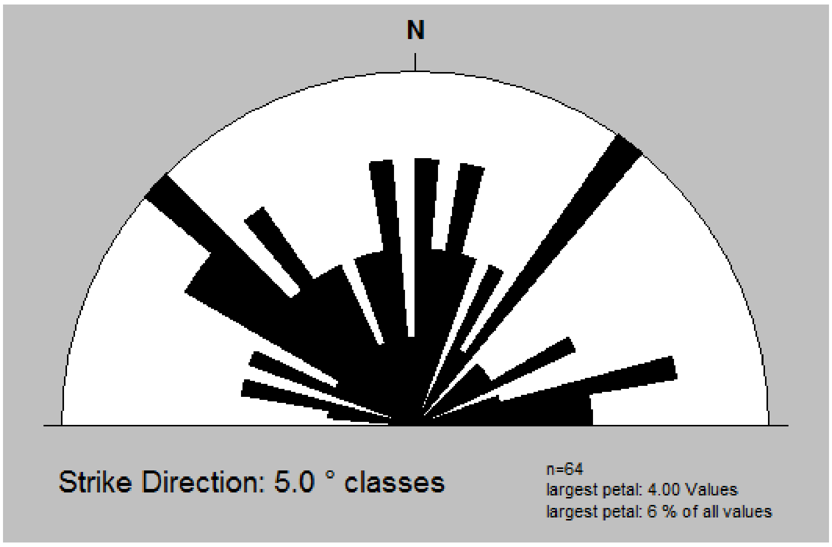

Figura 39 - Gráfico de roseta de fraturas medidas nas cavernas de Serra Leste (n=64). Medidas podem ser observadas no Anexo II (Fichas de Geoespeleogia).

Fraturas muitas vezes podem exercer influência na espeleogênese da cavidade, pois consistem de descontinuidades que facilitam o fluxo da água no interior do maciço rochoso. Importantes frentes de alteração também são guiadas por essas estruturas.

Na formação ferrífera o bandamento foi observado em 12 cavidades, perfazendo 12,5% das cavernas amostradas. As bandas são compostas principalmente por hematita, intercaladas por jaspe, sílica, ou por vazios reliquiares. No presente trabalho essas estruturas foram avaliadas do ponto de vista qualitativo. Em diversas cavernas tais feições exercem influência determinante na conformação das galerias, condutos e salões.

Uma análise das direções principais dos condutos foi realizada com base nos mapas disponíveis. Foram consideradas apenas cavernas desenvolvidas em jaspilito. A Figura 40 apresenta a direção preferencial identificada nas galerias. Apesar do pequeno universo de análise (n=17), é possível assumir uma tendência de concentração nos eixos NNW a WNW e NE, o que pode, em alguma medida, ser correlacionado com a tendência de direção das fraturas (Figura 39), demonstrando controle estrutural da direção de desenvolvimento dos condutos.

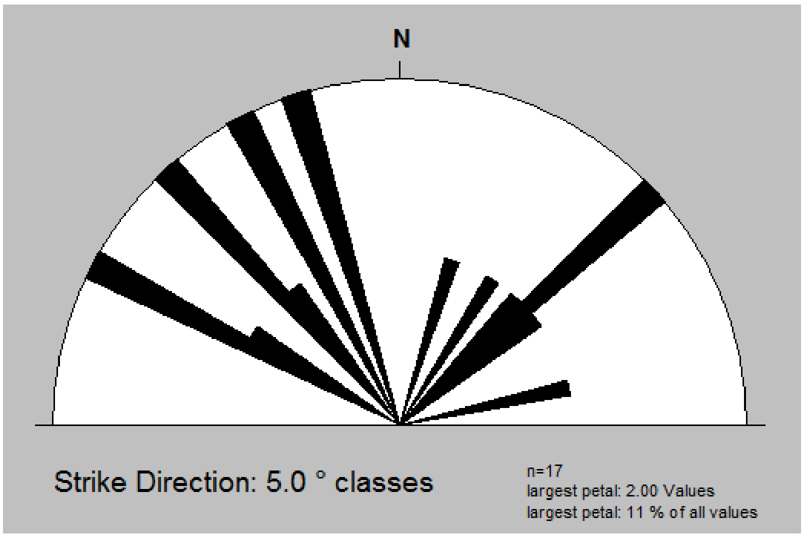

Figura 40 - Gráfico de roseta das principais direções dos condutos das cavernas de Serra Leste (n=17). (Medidas feitas sobre os mapas topográficos).

Juntas de alívio ocorrem em 22,9% das cavernas estudadas, estando presentes em 22 cavidades. São caracterizadas por descontinuidades irregulares, dispostas de forma subhorizontal, subvertical ou mesmo oblíqua. A espessura varia entre poucos milímetros a poucos centímetros (Figura 41). Assim como as fraturas, as juntas de alívio podem influenciar no desenvolvimento de condutos ou alterar sua morfologia.

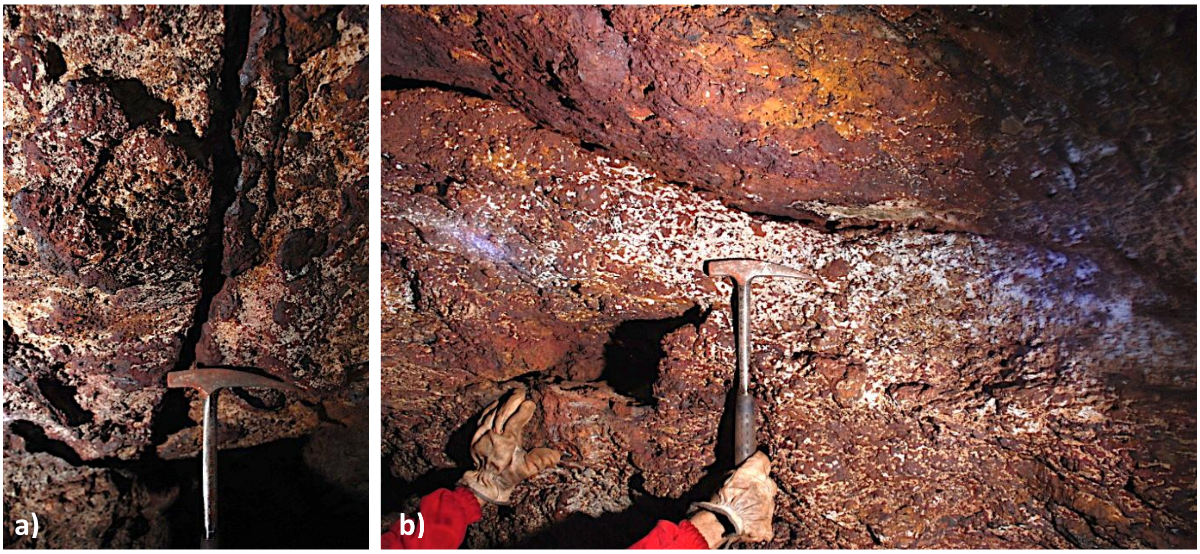

Figura 41 - Juntas de alívio sub-verticais (a) e sub-horizontais (b) observadas na caverna SL-026.

Dobras ou flexuras foram observadas em apenas cinco (5%) cavidades. A dimensão das dobras observadas variou de milimétrica a decimétrica. Segundo Ribeiro (2003), estruturas deste tipo podem, localmente, estar relacionadas à deformação por colapso gravitacional,

em função da lixiviação de minerais no processo supergênico. Nota-se que a maior friabilidade do substrato não está associada apenas a um processo químico, mas também à movimentação do maciço e à quebra das partículas.

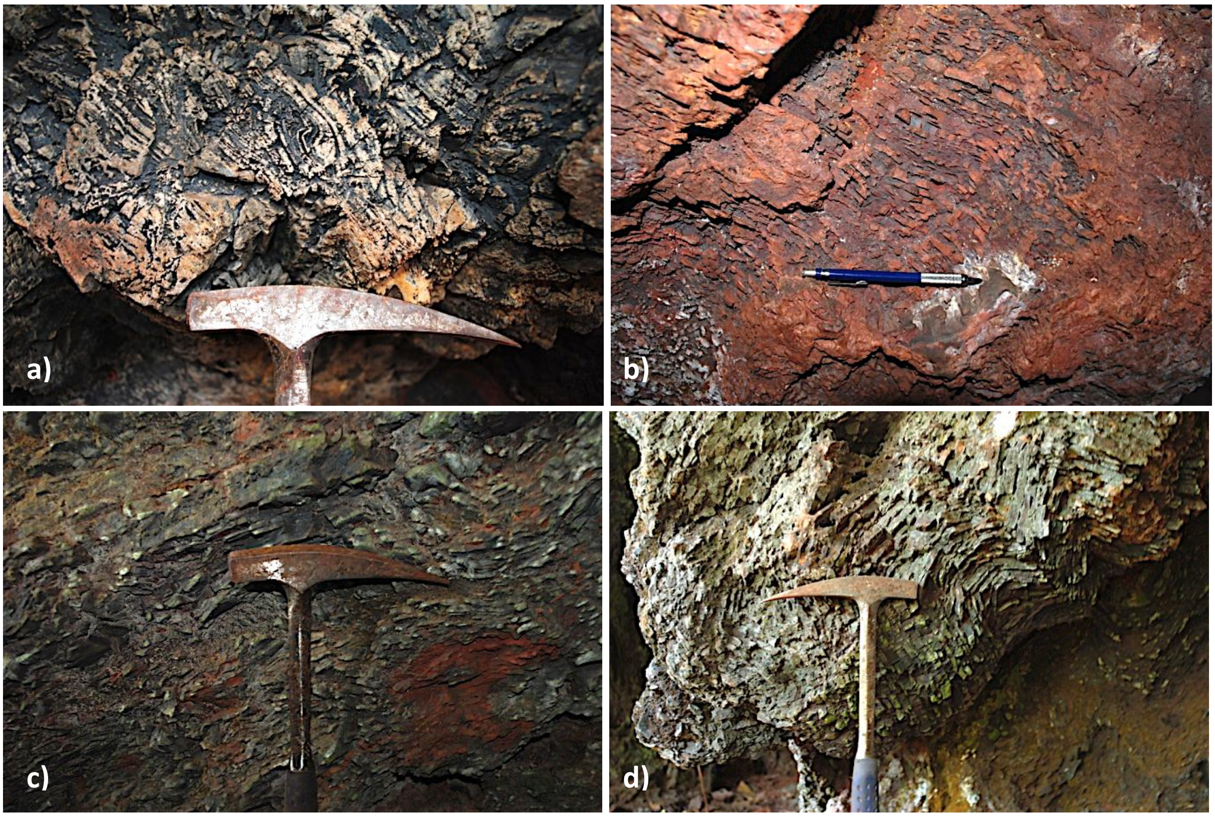

Figura 42 – Dobras observadas nas cavernas (a) SL-038, (b) SL-083, (c) SL-085 e (d) SL-089.

Contato geológico foi observado em 12 (12,5%) cavernas. Apresentaram contato erosivo, concordante e discordante (Figura 43).

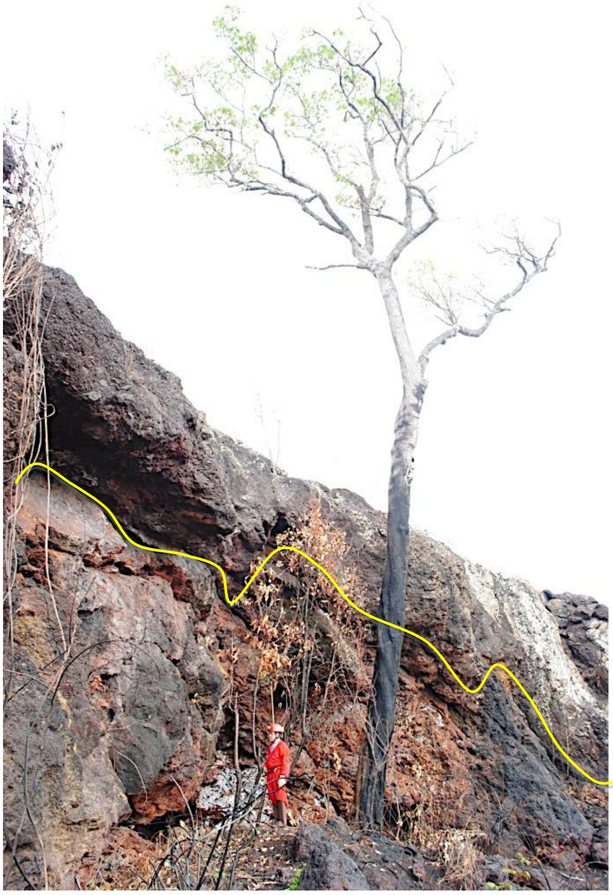

Figura 43 - Contato geológico erosivo entre cangas de diferentes texturas na entrada da caverna SL-047.

A Figura 44 apresenta uma síntese das feições estruturais observadas nas cavernas de Serra Leste.

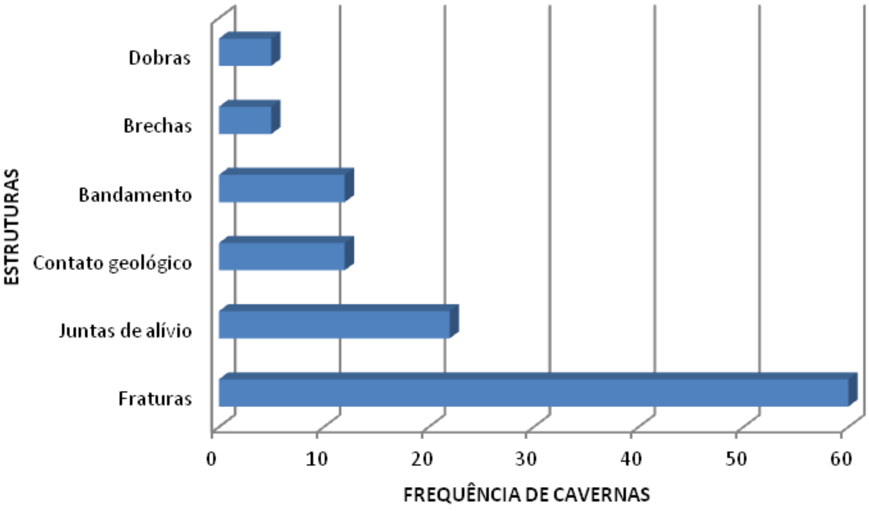

Figura 44 - Frequência de cavernas por feição estrutural observada nas cavernas de Serra Leste.

5.3.5. Hidrologia

Embora não tenham sido realizados estudos hidrogeológicos na área, é possível afirmar, dada a configuração geomorfológica e a inserção na paisagem, que as cavernas ferríferas de Serra Leste estão "desconectadas" entre si do ponto de vista hidrológico, ou seja, não constituem clássicos sistemas observados no carste carbonático, quartzítico e arenítico. Mesmo nas cavernas inseridas nos sistemas fluviais, o que se observa é uma captura da caverna pela drenagem e não o inverso, ou seja, uma captura da drenagem pelo sistema subterrâneo.

Feições do tipo surgência foram observadas em quatro (4,2%) cavernas, SL-050, SL-063, SL-068 e SL-078. Mesmo durante os períodos úmidos, o volume de água observado é mínimo, com vazões que não ultrapassam os 0,25 $L.seg^{-1}$ (Figura 45).

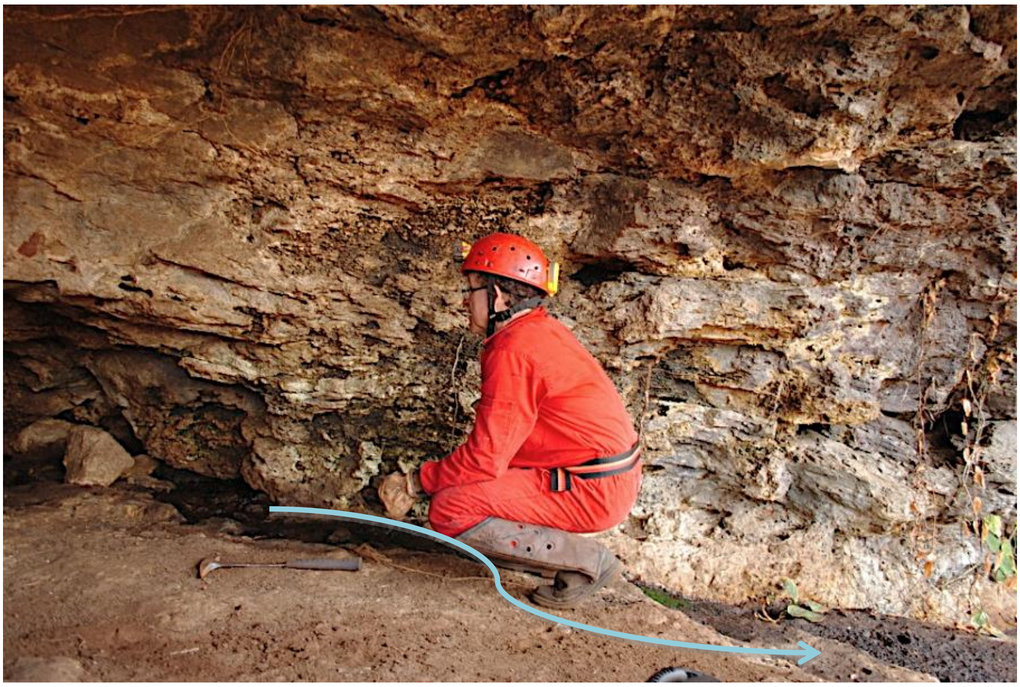

Figura 45 - Surgência observada na caverna SL-078: baixa vazão mesmo no período úmido.

Drenagens perenes foram registradas em seis (6,2%) cavidades, incluindo as três onde foram observadas surgências perenes (SL-050, SL-063 e SL-068 e SL-078). As cavernas SL-001 e SL-056 são entrecortadas por drenagem externa, por isso apresentam perenidade.

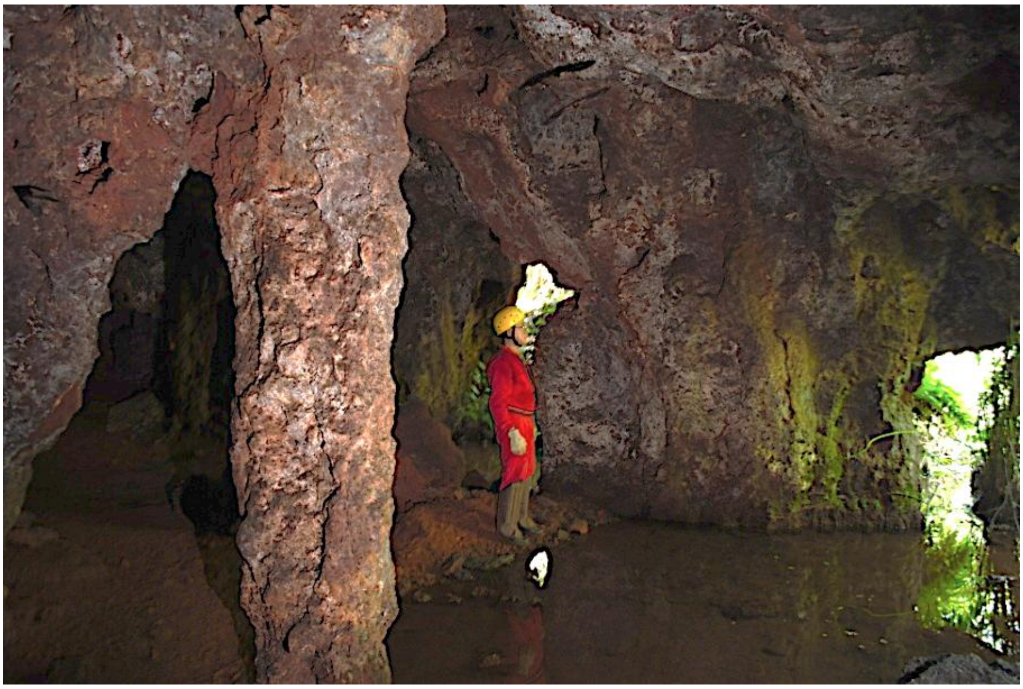

Figura 46 - Drenagem perene entrecortando a caverna SL-001.

A caverna SL-074 apresenta uma drenagem temporária cuja atividade está associada a eventos de grande pluviosidade, quando então o canal de drenagem atual transborda, sendo capturado pelo antigo canal de escoamento localizado no interior da caverna. Foram realizadas visitas técnicas nas duas condições, ou seja, com drenagem seca e ativa (Figura 47).

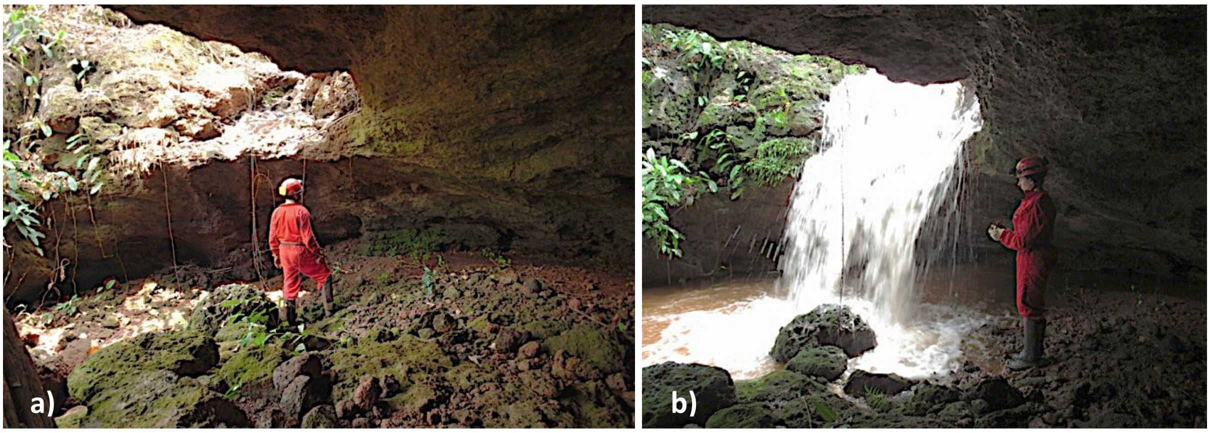

Figura 47 - Drenagem externa da caverna SL-074 durante o período seco (a) e úmido (b) após evento de elevada pluviosidade.

Feições pontuais como condensação, exudação, percolação entre outros, também estão presentes. A Tabela 8 e a Figura 48 apresentam uma síntese das principais feições hidrológicas observadas quando das visitas técnicas nas cavernas de Serra Leste.

Tabela 8 - Frequência de cavernas por feição hidrológica observada.

Feição	Frequência	%
Gotejamento	33	34,4%
Condensação	16	16,7%
Infiltração	14	14,6%
Drenagem perene	6	6,3%
Empoçamento	6	6,3%
Lago perene	5	5,2%
Exudação perene	3	3,1%
Surgência perene	3	3,1%
Escoamento temporário	2	2,1%
Drenagem temporária	1	1,0%
Sumidouro temporário	1	1,0%
Percolação	1	1,0%

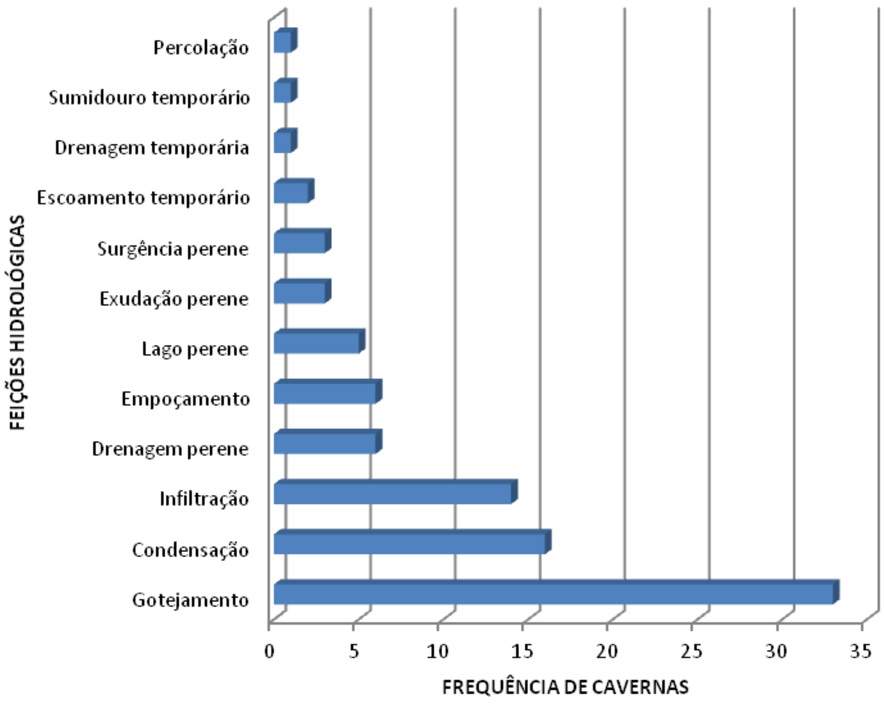

Figura 48 - Frequência de cavernas por feição hidrológica observada.

5.3.6. Depósitos clásticos

Os sedimentos clásticos compreendem todos os detritos transportados e depositados ao longo dos condutos e salões das cavernas. Estes depósitos estão presentes em todas as cavernas de Serra Leste (Figura 49) e não apresentaram diferenças significativas quando comparados a outras cavidades estudadas na região de Carajás. Foram observados sedimentos em todo o intervalo granulométrico, ou seja, de silte/argila a matacões, com

origem alóctone, autóctone e mista, e arredondamento variando de arredondado a anguloso.

De maneira geral, é possível afirmar que em Serra Leste os depósitos clásticos com granulometria seixo, calhau e matacão, tem origem autóctone, associada a processos de desmonte (abatimentos) da rocha encaixante. Calhaus e matacões são frequentemente observados ao longo da linha d'água das cavernas e tem sua origem associada a processos de abatimento resultantes da evolução remontante das frentes das escarpas. Os depósitos com granulometria mais fina, por sua vez, parecem ter origem alogênica e, em geral, tais sedimentos são carreados para o interior das cavernas através da própria entrada da cavidade, por canalículos, clarabóias ou outros interstícios capazes de transportar sedimentos em suspensão.

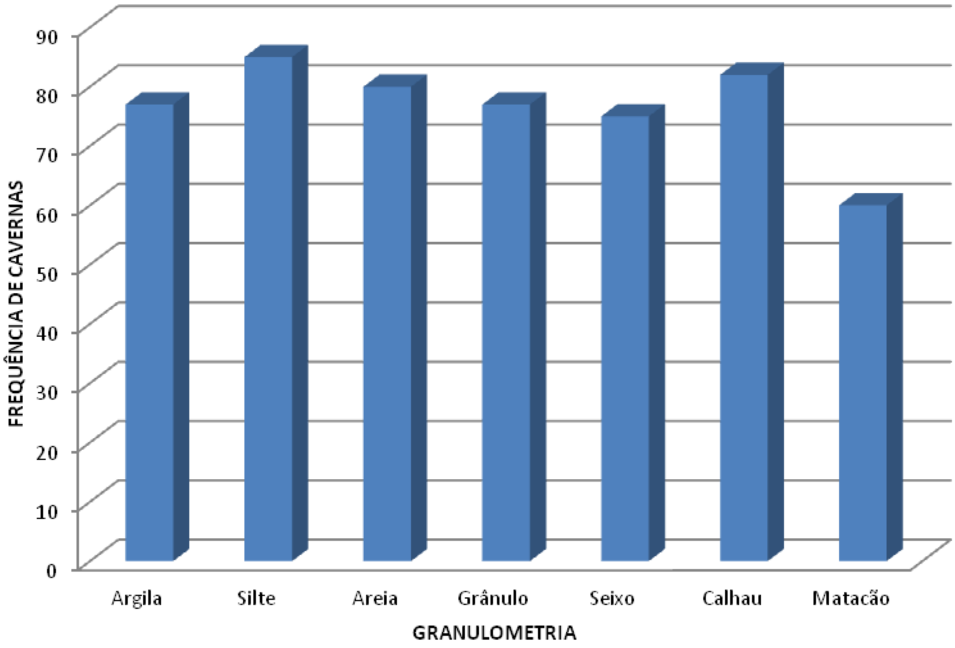

Figura 49 - Frequência de cavernas segundo granulometria dos sedimentos clásticos.

5.3.7. Depósitos químicos (espeleotemas)

Os depósitos químicos (espeleotemas) das cavernas ferríferas compreendem, em geral, feições de pequeno porte com mineralogia limitada a óxidos/hidróxidos de ferro, sulfatos e fosfatos (Piló & Auler, 2009). A variedade tipológica é restrita, estando limitada, até o momento, a uma ou duas dezenas de tipologias. Foram identificados espeleotemas em quase todas as cavernas de Serra Leste. A Tabela 9 e Figura 50 apresentam os espeleotemas mais freqüentes.

Tabela 9 - Frequência de cavernas por tipo de espeleotema.

Espeleotema	Frequência	%
Crosta branca	84	87,5%
Crosta laranja	55	57,3%
Crosta vermelha	36	37,5%
Coralóide	32	33,3%
Crosta amarela	32	33,3%
Crosta cinza	23	24,0%
Crosta marrom	12	12,5%
Crosta preta	11	11,5%
Micro-travertino	7	7,3%
Crosta ocre	4	4,2%
Travertino	3	3,1%
Cortina	3	3,1%
Crosta verde	2	2,1%
Estalactite	2	2,1%
Pingente	1	1,0%
Escorrimento	1	1,0%
Coluna	1	1,0%

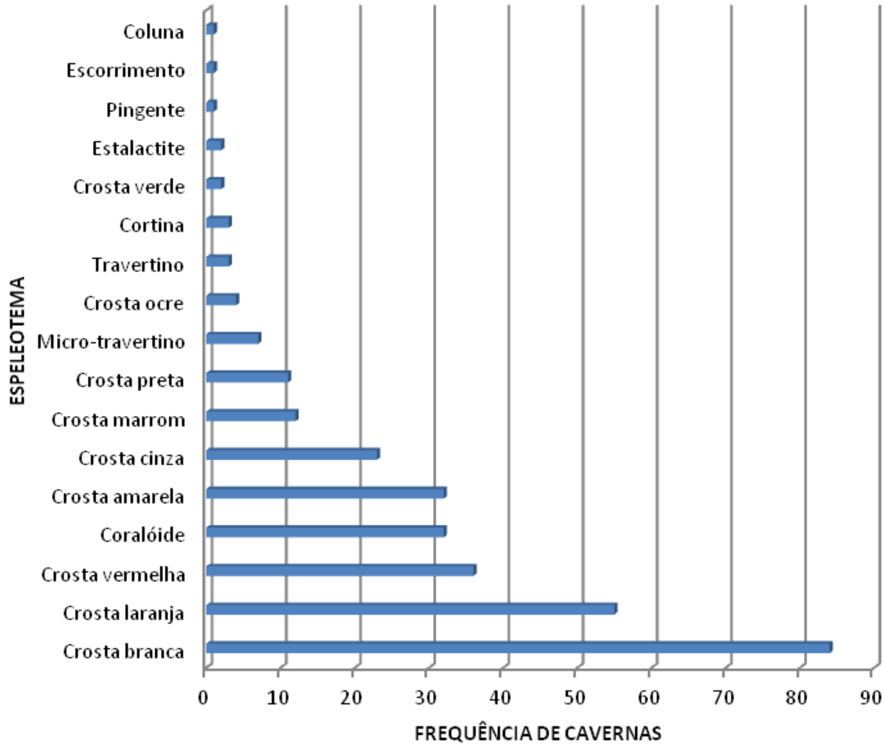

Figura 50 - Freqüência de cavernas por tipo de espeleotema.

Como se pode observar (Figura 50 – acima), as crostas delgadas e os coralóides são as feições mais frequentes nas cavernas estudadas. Se agruparmos os espeleotemas segundo o processo de formação, isto se tornará ainda mais evidente (Figura 51).

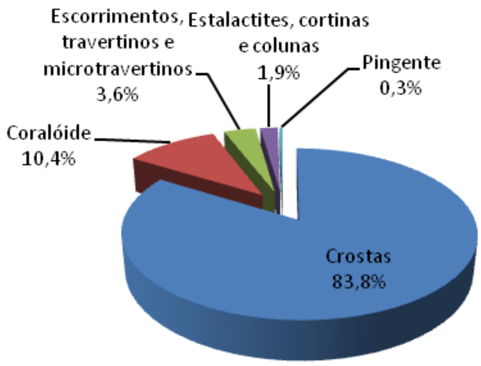

Figura 51 - Freqüência de cavernas por tipo de espeleotema.

As crostas ferruginosas constituem, em geral, películas de espessura milimétrica a centimétrica que recobrem blocos, piso, paredes e teto das cavernas. Sua cor pode variar do amarelo ao vermelho escuro e o brilho varia de opaco a vítreo (Figura 52). Estudos em andamento tem revelado uma grande diversidade de minerais fosfáticos e sulfáticos associados a esses encrustramentos, no entanto, os estudos são ainda incipientes. Não foi possível, dado o reduzido universo de espeleotemas analisados, conceber modelos genéticos e/ou padrões morfológicos a cada um desses espeleotemas. Por essa razão, foram aqui designados genericamente como crostas ferruginosas.

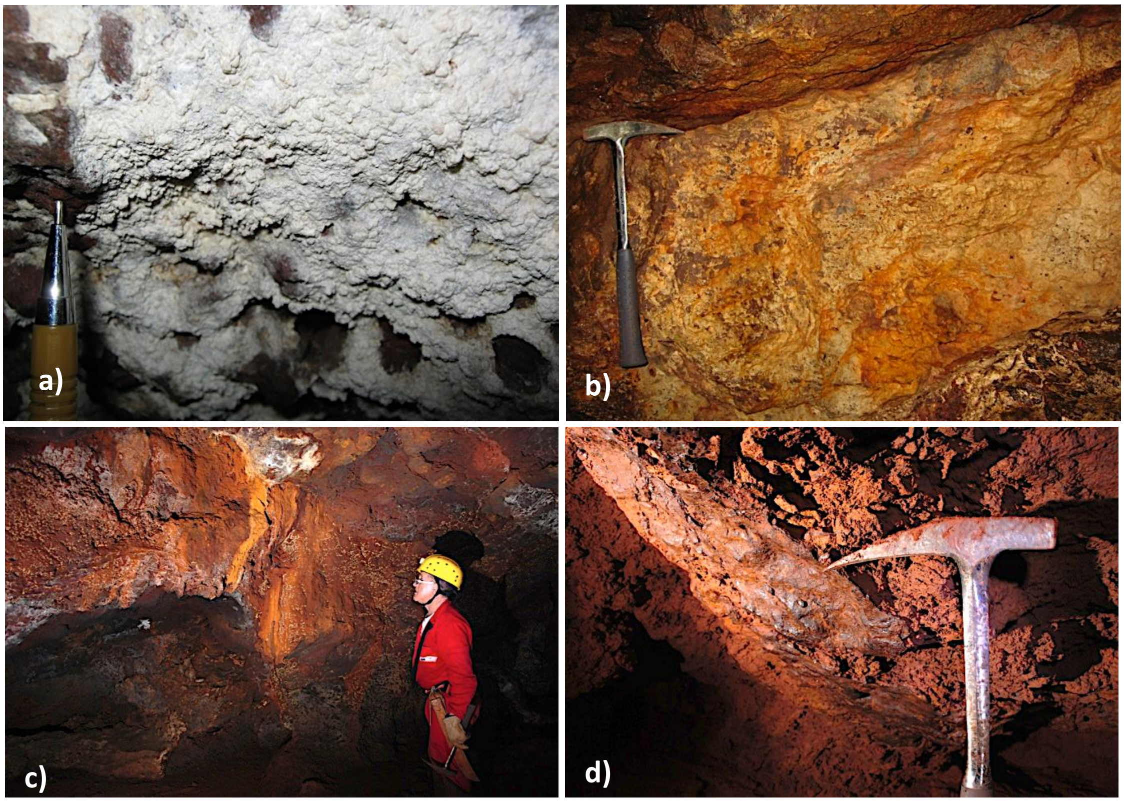

Figura 52 - Crostas observadas nas cavernas de Serra Leste: a) crosta branca, delgada, milimétrica (SL-061); b) crosta amarela (SL-073); c) crosta vermelha (SL-089), d) crosta cinza escuro (SL-035).

Coralóides foram registrados em 32 cavernas, correspondendo a 32,3% da amostragem de cavernas. Em geral apresentam dimensões milimétricas a até 1 cm, com formatos variados, podendo ser alongados (tipo agulha), na forma de couve-flor ou rombudos (morfologia mais arredondada). Segundo Piló & Auler (2009), há maior incidência de coralóides do tipo agulha em locais onde o fluxo de ar é mais acentuado, ao passo que os coralóides de topo arredondado (rombudos) são mais recorrentes em ambientes onde a atmosfera é mais estável e com maior umidade. É comum que estes espeleotemas estejam associados a crostas. A coloração desses espeleotemas dependerá dos minerais envolvidos na cristalização. Já foram registrados coralóides nas cores branca, branco-amarelada, marrom, cinza e preta. Ocorrem em paredes, sobre blocos abatidos, sobre pisos ou nos tetos das cavernas (Figura 53).

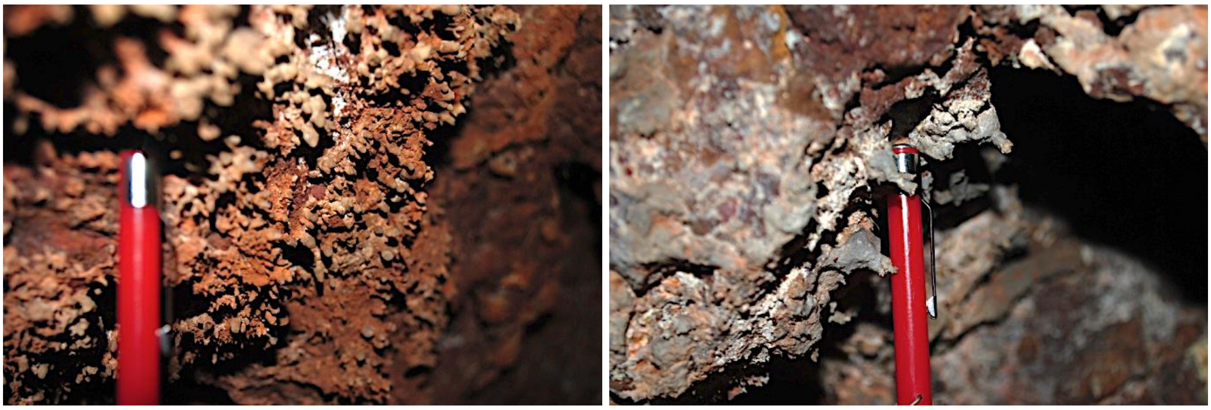

Figura 53 - Coralóides milimétricos registrados na caverna SL-006. A esquerda, afilados, de coloração amarela a cinza, tonalidade clara. A direita, microcoralóides recobertos por uma encrustração branca.

Cortinas foram observadas nas cavernas SL-029, SL-030 e SL-051. Esses espeleotemas são relativamente incomuns em cavernas ferríferas. Embora tenham ampla distribuição (ocorrem em praticamente todas as unidades geomorfológicas de Carajás – Serra Norte, Serra Sul, Serra da Bocaina e Serra do Rabo-Estrela), poucas são as cavernas onde são registrados. Dentre essas três cavidades, cabe destaque a caverna SL-051 onde a cortina desenvolve-se em textura microtravertínica com extremidades serrilhadas (Figura 54 – esquerda).

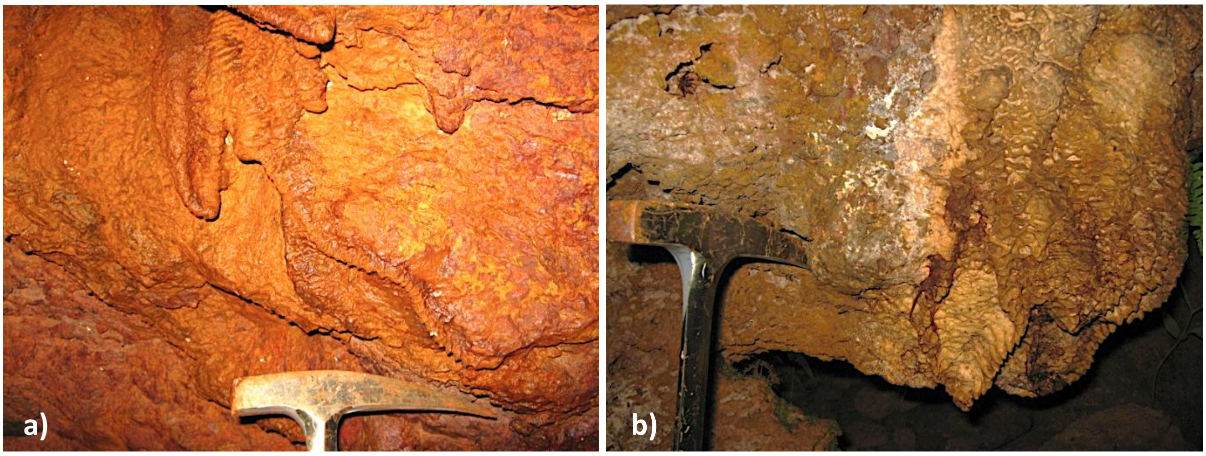

Figura 54 - Cortina da caverna (a) SL-030 e (b) SL-051.

Os depósitos de microtravertinos ocorrem de forma incipiente. Foram observados em três cavernas: SL-001, SL-051 e SL-061. Detalhes desses espeleotemas podem ser observados na Figura 55.

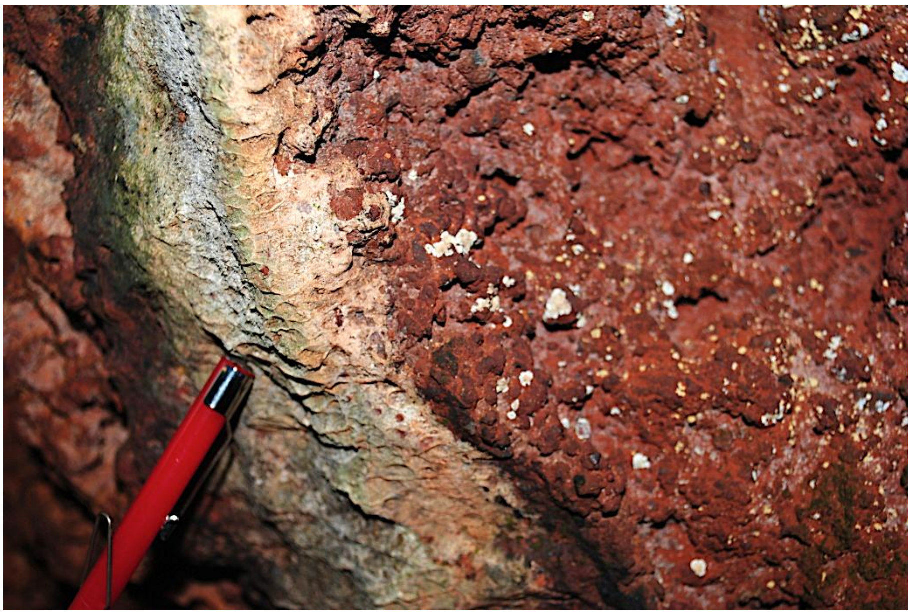

Figura 55 - Microtravertino observado na caverna SL-001.

5.3.8. Aspectos espeleogenéticos e cronológicos

O trabalho de Simmons (1963) em áreas com presença de minério de ferro dolomítico no Quadrilátero Ferrífero foi o pioneiro em atribuir a gênese de cavernas ferríferas a processos de dissolução. Segundo ele, a dissolução do dolomito, mas também de quartzo e hematita, leva à formação de uma zona de minério de ferro alterado de alta porosidade que chega a atingir 50% do volume da rocha. Em regiões de minério de ferro silicoso, a dissolução da sílica também exerceria um papel importante.

Uma vez que o enriquecimento supergênico consiste na retirada de matéria do protominério (lixiviação), além do aumento residual do teor em ferro, o processo promove também um significativo aumento da porosidade e permeabilidade do corpo mineral. Ribeiro (2003), trabalhando no Quadrilátero Ferrífero, demonstrou que pode haver uma redução de até 40% do volume da rocha, resultando em minério friável com alta porosidade. A remoção de sílica e/ou dolomito resulta em uma concentração relativa e conseqüente geração de zonas com minérios de ferro de alto teor (processo de mineralização).

Na Serra dos Carajás, duas fases espeleogenéticas foram propostas por Pinheiro & Maurity (1988). Durante a primeira fase, inteiramente na zona freática, ocorre a formação de complexos alumino-ferrosos e argilo-minerais instáveis de ferro, alumínio e sílica que preenchem os vazios da canga e da formação ferrífera bandada. A remoção deste material residual instável leva à formação de orifícios irregulares que podem ser observados nas paredes das cavernas (Pinheiro & Maurity, 1988). A segunda etapa, ainda na zona freática, envolve processos erosivos (*piping*), que basicamente expandem as cavidades geradas na primeira etapa, levando então à formação de condutos e salões. Esses processos erosivos serão intensificados quando a caverna passar a ser exposta à atuação de processos vadosos, propiciando também a atuação de processos de abatimento (Pinheiro & Maurity, 1988).

Piló & Auler (2005) consideram a existência de duas etapas distintas na formação das cavernas de minério de ferro e canga. A primeira delas com predominância de processos dissolutivos (químicos) e a segunda sob a ação de processos erosivos (físicos). A primeira etapa, endógena, envolve reações químicas no interior da massa rochosa na zona freática, gerando zonas de alta porosidade. Posteriormente o material friável resultante será lixiviado para o exterior através de processos similares ao *piping*, descrito para cavidades em rochas siliciclásticas. Essa segunda etapa, iniciada na zona freática, pode ter continuidade na zona vadosa, quando os processos de piping podem vir a ser favorecidos.

Iniciada a etapa erosiva das cavidades e sendo estabelecida uma saída para o exterior, os processos físicos serão incrementados. Material de granulometria fina será aportado para o interior das cavernas, principalmente, via canalículos, de onde será evacuado para o exterior.

Como podemos observar, os modelos propostos por Pinheiro & Maurity (1988) e Piló & Auler (2005) possuem bastante semelhança e foram aqui denominados de **modelo endógeno/exógeno do tipo dissolução/erosão** (Figura 56). Nas cavernas de Serra Leste esse modelo pode ser aplicado a praticamente todas as cavernas inseridas no topo dos platôs ou em alta vertente, nas rupturas de cobertura de relevo implantadas na frente de evolução das escarpas.

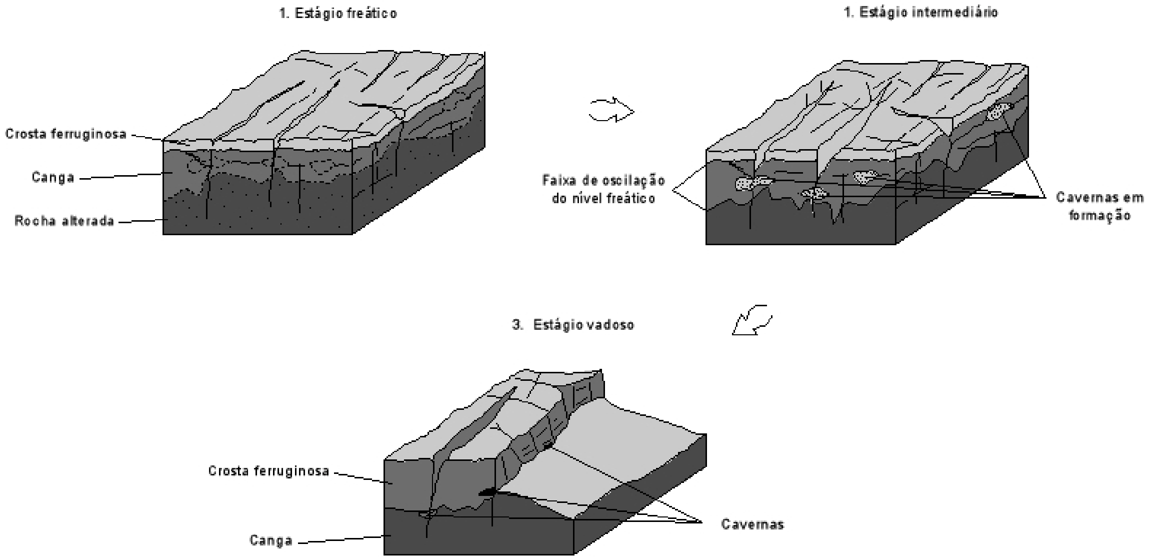

Figura 56 - Modelo de evolução endógena/exógena do tipo dissolução/erosão. Adaptado de Pinheiro & Maurity (1988).

Na porção intermediária da paisagem, ou seja, entre as frentes de evolução da escarpa e a baixa vertente, foram identificadas cavernas cujo modelo genético está associado a formação de rampas de colúvio que foram posteriormente cimentadas por fluidos ferruginosos (Fe^{2+}). Este modelo espeleogenético, está bastante associado à morfodinâmica da paisagem, e por isso foi definido como **modelo exógeno do tipo coluvionar**. Formam-se neste contexto paisagístico dois tipos de cavidades, as cavernas em talus e as cavernas sob *duricrust* (Figura 57).

As cavernas em talus estão em geral inseridas nas porções hipsométricas mais elevadas. Consistem de um empilhamento de blocos caoticamente arranjados, cimentados ou não. Em alguns casos estão recobertos por sedimentos inconsolidados. Sob estes blocos desenvolvem-se as cavernas.

Cavernas em *duricrust*, por sua vez, ocupam normalmente as porções mais rebaixadas da paisagem, onde os fluidos ferruginizados se acumularam por tempo suficiente para que fosse formada uma crosta bastante enrijecida. Após a formação desta “carapaça”, o rebaixamento do nível de base favorece a formação de protocondutos (*pipes*) no contato entre a crosta rígida e o substrato subjacente, onde descontinuidades texturais e estruturais facilitam o fluxo subterrâneo de fluidos coloidais e soluções. A ampliação desses protocondutos é responsável pela formação das primeiras galerias subterrâneas, cuja

"eclosão" se dá de duas formas, através de abatimentos doliniformes ou, mais comumente, através da interceptação resultante da evolução da paisagem (ravinamentos).

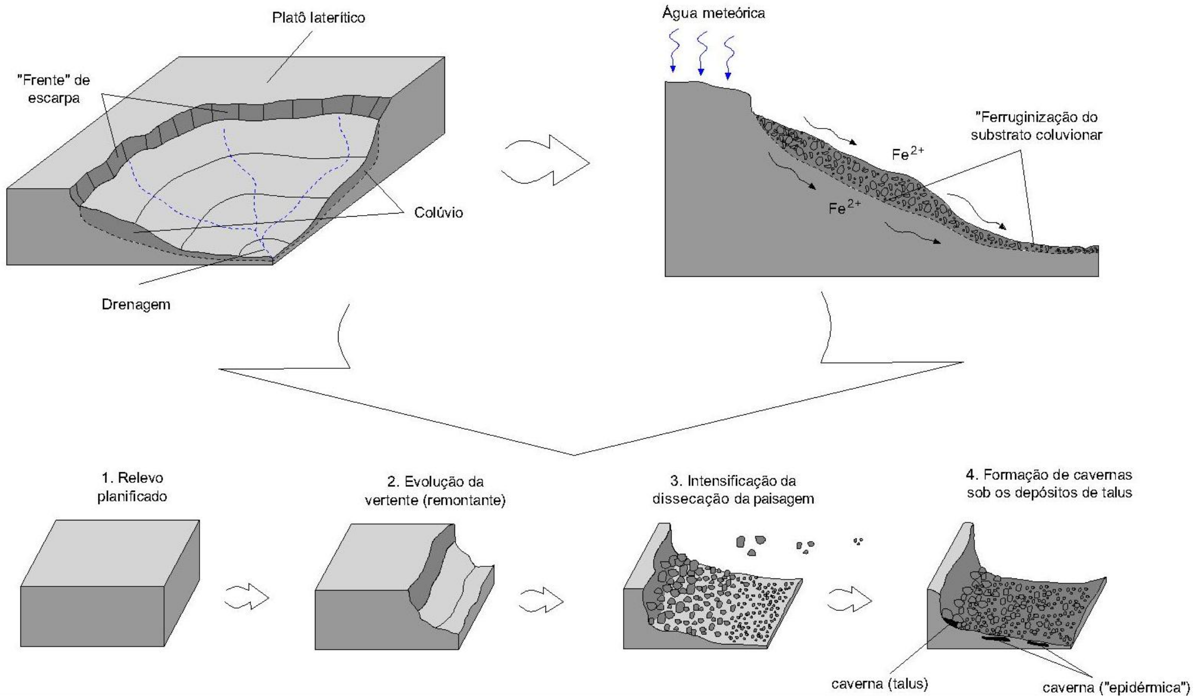

Figura 57 - Modelo de evolução exógena do tipo coluvionar. Fonte: dados dos autores.

Em Serra Leste foram identificadas diversas cavidades nos sistemas de drenagem de baixa vertente. Nestes compartimentos a energia potencial foi praticamente transformada em energia cinética de forma que a estabilidade crítica está associada a variação sazonal do fluxo (laminar/turbulento), permitindo a conformação de meandros. Por outro lado, estas drenagens encontram-se localmente controladas por intrusões ígneas (diques), ou seja, os meandros estão circunscritos às bordas do contato com essas intrusões.

A gênese e evolução destas cavernas fluviais está basicamente associada a processos de erosão lateral (Figura 58), hipótese corroborada pela morfologia destas cavidades, cuja entrada é comumente mais extensa do que a sua profundidade. Tal qual no modelo exógeno tipo coluvionar, as cavernas fluviais tem sua gênese e desenvolvimento intrinsecamente associados aos processos morfodinâmicos que esculturam a paisagem, sendo aqui definido como **modelo exógeno do tipo fluvial** (Figura 58).

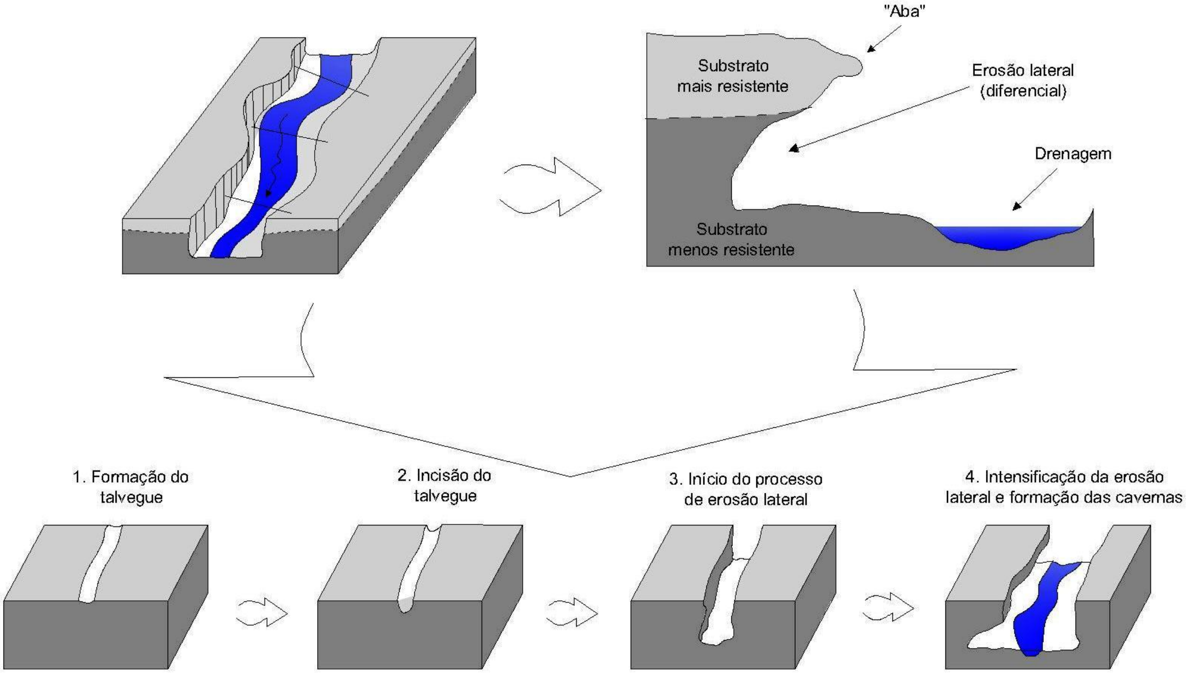

Figura 58 - Modelo de evolução exógena do "tipo fluvial". Fonte: dados dos autores.

Poucas inferências têm sido feitas a cerca da idade das cavernas ferríferas. Em Carajás, Pinheiro *et al.* (1985) e Pinheiro & Maurity (1988) teceram breves considerações sobre o tema. Esses autores associam o início da formação das cavidades ao processo de geração da canga, atrelando a idade das cavernas à idade da canga. No entanto, existem grandes incertezas com relação à idade do início da formação da canga. Uma idade aproximada, final do Cretáceo/início do Terciário e desenvolvimento durante o Pleistoceno foi aventada por Pinheiro et al. (1985) e Pinheiro & Maurity (1988).

Segundo Spier (2005), os intensos processos de intemperismo químico que atuaram nas formações ferríferas do Quadrilátero Ferrífero permitiram a completa dissolução da dolomita e a formação de minerais de manganês a partir das FFBs. Datações, pelo método $^{40}Ar/^{39}Ar$, forneceram idades entre 61,5 Ma a 14,2 Ma, sugerindo uma longa história de intemperismo na região. No entanto, a maior parte dos óxidos de manganês precipitou no intervalo entre 51 e 41 Ma, com pico por volta de 46,7 Ma. Os dados cronológicos também confirmaram que o perfil de intemperismo das formações ferríferas já tinha atingido o atual estágio de desenvolvimento há cerca de 50 Ma, sugerindo que a frente intempérica avançou muito pouco durante o Neógeno. Esses dados indicam que a partir do Eoceno as formações ferríferas já apresentam boas condições para a espeleogênese. Apesar da falta de dados demonstrativos e cronológicos absolutos, acredita-se que as cavernas desenvolvidas no minério de ferro são realmente muito antigas (acima de 2 milhões de anos), com idades bem superiores às cavernas carbonáticas.

5.3.9. Aspectos sócio econômicos, culturais e estado de conservação

Nenhuma das cavernas estudadas na área do Projeto Serra Leste apresentou valor cultural ou sócio econômico. Com relação ao seu estado de conservação, 10 (10,1%) cavidades apresentaram algum tipo de interferência (impacto) em seu interior. As principais alterações estão associadas a presença de dejetos (lixo doméstico), garimpo e compactação dos sedimentos do piso. Em relação ao entorno (250 metros), praticamente todas as cavernas tiveram seu perímetro de proteção impactado pois a área de Serra Leste foi inteiramente queimada entre os meses de junho a setembro de 2010. Além das queimadas, estradas de sondagem estão largamente presentes de forma que é difícil encontrar uma caverna a mais de 250 metros das estradas. Outra alteração bastante comum é a substituição da cobertura vegetal natural por pastagens. Tanto as queimadas quanto a substituição por pastagens são, dado o elevado estágio de antropização do meio, uma prática bastante antiga.

5.4. Bioespeleologia

5.4.1. Recursos tróficos e condições ambientais

Em relação aos recursos tróficos observados, estes se constituem basicamente de matéria orgânica vegetal alóctone depositada principalmente nas zonas de entrada (serrapilheira e partes vegetais), depósitos de guano provenientes principalmente de morcegos de hábitos alimentares frugívoros e nectarívoros como os Phyllostominae (*Carollia perspicillata*) e Glossophaginae (*Glossophaga soricina*), sistemas radiculares que atingem as macro-cavernas e, em alguns casos, descarte de materiais orgânicos oriundos de formigueiros principalmente de formigas da tribo Attini (*Apterostigma* sp.). Além destes, uma série de outros recursos alimentares podem ser observados esporadicamente nas cavernas da região, dentre os quais, regurgitos de corujas, cadáveres de vertebrados (principalmente morcegos) e de invertebrados (aranhas e amblipígeos), fezes de vertebrados não voadores (principalmente roedores e anfíbios) e também de invertebrados (principalmente baratas), restos alimentares como frutos abandonados por animais, além de uma grande quantidade de microrganismos (fungos e bactérias) que podem ser utilizados como recurso por uma grande diversidade de espécies de invertebrados cavernícolas.

Além destes, é importante ressaltar que o transporte de material orgânico dissolvido carreado pela ação da água através de gotejamentos e de percolação também deve ser considerado importante no fluxo energético entre os ambientes epígeo e hipógeo principalmente durante a estação úmida. Entretanto, são raros os casos onde o transporte eólico e gravitacional contribuem de maneira efetiva para a importação de recursos orgânicos para o meio subterrâneo uma vez que a maioria das cavidades apresentam entradas de pequeno porte e são raros os casos onde existem clarabóias.

De forma geral, a maioria das cavernas estudadas pode ser considerada como ambientes oligotróficos, sendo exceções as grutas SL-001, SL-004, SL-031, SL-069, SL-073, SL-074, SL-079 e SL-082 que apresentam grandes depósitos de guano produzidos por morcegos de hábitos alimentares frugívoros e ou insetívoros. Dentre estas, a gruta SL-073 e SL-079 se destacam por apresentarem uma grande área do piso recoberta por guano de morcegos insetívoros, que em alguns pontos chega a atingir até 30 cm de profundidade, além de inúmeros depósitos recentes de guano produzido por morcegos frugívoros. Nestas cavernas, observou-se comunidades extremamente complexas com uma elevada riqueza e diversidade. Nestes casos, existem grandes populações de baratas (Blaberidae e Polyphagidae), hemípteros (Cydnidae), colêmbolos (Entomobryidae), traças (Nicoletiidae) e isópodes (Platyarthridae). Entretanto, é preciso ressaltar que de maneira geral, na maioria das cavernas estudadas o guano de morcegos frugívoros é predominante, sendo este um dos principais recursos existentes nas cavernas da região.

Quanto às condições ambientais, grande parte das cavernas apresentam apenas zona de incidência direta de luz e áreas de penumbra, sendo raros os caso onde existem zonas completamente afóticas. Em geral, as cavernas estudadas no sistema ferruginoso da Serra Leste encontram-se sob forte influência das condições ambientais epígeas e, portanto, apresentam uma baixa estabilidade ambiental. Entretanto, algumas se destacam por apresentarem um maior grau de confinamento, uma elevada estabilidade ambiental, grande diversidade de recursos tróficos e de micro-hábitats. Dentre estas, destacam-se as grutas SL-004, SL-030, SL-031, SL-035, SL-072, SL-074, SL-079, SL-087, SL-093 e SL-097.

De forma geral, as cavernas da região encontram-se de maneira superficial estando muitas vezes em contato com solos rasos ou inseridas diretamente na canga aflorada. Desta forma, em muitas oportunidades é possível encontrar em muitas cavidades a presença de sistemas radiculares bem desenvolvidos, bem como estas podem apresentar temperaturas elevadas em virtude da influência das condições ambientais epígeas. Em relação à umidade, esta se apresenta moderada em grande parte das cavernas inventariadas, sendo raros os casos onde a atmosfera cavernícola encontra-se saturada ou com baixas porcentagens de umidade. De forma geral, a maioria das cavernas não apresenta pontos de gotejamento ativos durante a estação seca. Esta condição se modifica completamente durante a estação úmida, uma vez que a superficialidade das cavidades favorece a penetração das águas pluviais nas macro-cavernas formando inúmeros pontos de gotejamento, de percolação e inúmeras oportunidades, represamentos.

Quanto à distribuição das cavernas na paisagem, na Figura 59 é possível observar a distribuição das mesmas de acordo com a fitofisionomia do entorno. Apesar de estas terem sido escolhidas em função de agrupamentos durante os inventários biológicos, a maioria das

cavernas conhecidas na região encontram-se inseridas em áreas de encosta ou associadas a áreas de drenagem junto à mata ciliar.

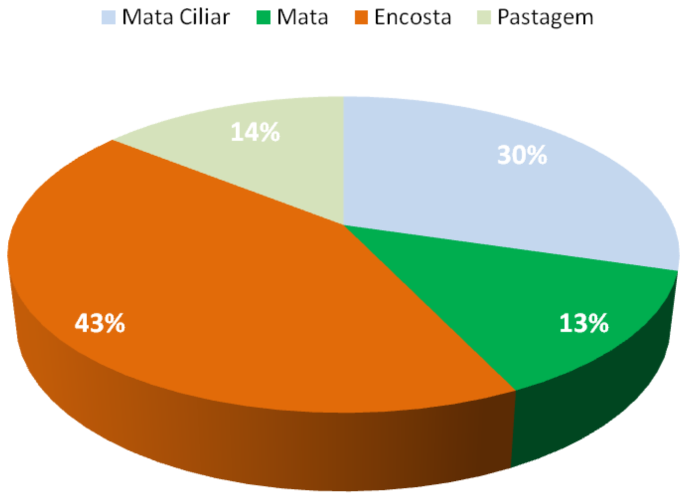

Figura 59 - Distribuição das cavernas inventariadas nas diferentes fitofisionomias.

5.4.2. Caracterização faunística geral das cavidades

5.4.2.1. Caracterização realizada no período seco

Foi observado nas cavernas amostradas, um total de 903 morfoespécies de invertebrados de pelo menos 259 famílias das Ordens: Gordioidea, Onychophora (Peripatidae), Oligochaeta, Gastropoda (Subulinidae, Systrophiidae, Valloniidae), Turbellaria (Dugesiidae, Geoplanidae, Temnocephalidae), Isopoda (Armadillidae, Balloniscidae, Dubioniscidae, Philosciidae, Plathyarthridae: *Trichorhina* sp.; Scleropactidae, Styloniscidae), Decapoda (Pseudothelphusidae: *Microthelphusa somanni*; Palaemonidae: *Macrobrachium* sp., Trichodactylidae: *Valdivia serrata*), Acari (Neothyridae: *Diplothyrus schubarti*, Argasidae: *Antricola* sp., *Ornithodoros* sp.; Ixodidae: *Amblyomma cajennense*; Diploginiidae; Laelapidae: *Stratiolaelaps* sp.; Macrochelidae: *Macrocheles* sp.; Macronyssidae, Ologamasidae, Otopheidomenidae, Podocinidae: *Podocinum* sp.; Uropodina, Opilioacaridae: *Neoacarus* sp., Acaridae, Anoetidae, Astigmatina (Hypopus), Oribatida, Anystidae: *Erythracarus* sp., Cunaxidae, Erythraeidae, Eupodidae : *Linopodes* sp., Labdostomatida, Rhagidiidae, Teneriffiidae, Tydeidae), Amblypygi (Phrynidae: *Heterophrynus longicornis*; Charinidae: *Charinus* sp.), Ricinulei (Ricinoididae : *Cryptocellus tarsilae*), Schizomida (Hubbardiidae: Hubbardiinae), Scorpiones (Buthidae: Ananteris luciae), Pseudoscorpiones (Chernetidae, Chtoniidae), Opiliones (Neogoveidae: *Canga renatae*; Cosmetidae: *Anduzeia* sp.; Escadabiidae, Fissiphaliidae, Kimmulidae, Manaosbiidae, Sclerosomatidae: *Prionostemma* sp.; Stygnidae: *Eutimesius* sp., *Protimesius aff. gracilis*), Opiliones (Amaurobiidae; Anapidae, Araneidae: *Alpaida* sp.; Anyphaenidae, Corinidae: *Tupirina* sp.; Ctenidae: *Ctenus* sp.;

Dipluridae, Filistatidae, Gnaphosidae, Linyphiidae, Mimetidae, Ochyroceratidae, Oonopidae: Oonopinae, Gamasomorphinae, *Coxapopha* sp., Salticidae, Segestridae, Drymusidae: Sicariidae: *Drymusa* sp.; Senoculidae, Scytodidae*: Scytodes eleonora*, Palpimanidae, Paratropidae, Psauridae, Pholcidae: *Mesabolivar* sp., *Metagonia* sp.; Prodidomidae, Tetrablemidae, Theraphosidae Theridiidae, Theridiosomatidae, Thomisidae, Uloboridae), Thysanura (Nicoletiidae: Nicoletiinae, Atelurinae), Microcoryphia (Meinertellidae), Diplura (Anajapygidae, Campodeidae, Japygidae, Parajapygidae, Procampodeidae), Collembola (Sminthuridae, Cyphoderidae, Entomobryomorpha: Entomobryidae; Hypogastruridae, Isotomidae, Katiannidae?, Neanuridae, Paronellidae, Tomoceridae), Odonata (Aeshnidae: *Castoraeschna* sp., Coenagrionidae), Neuroptera (Mantispidae: *Plega* sp.; Myrmelionthidae), Orthoptera (Mogoplistinae; Gryllidae; Nemobiinae, Gryllinae, Pentacentrinae; Phalangopsidae: *Aclodes* sp., *Phalangopsis* sp.; Tetrigidae), Mantodea (Mantinae), Blattodea (Blaberidae: *Blaberus* sp., Blattellidae, Blattidae, Polyphagidae), Embiidina, Isoptera (Rhinotermitidae: *Heterotermes* sp.; Termitidae: *Armitermes* sp., *Cornitermes* sp., *Diversitermes* sp., *Embiratermes* sp., *Nasutitermes* sp., *Subulitermes*? sp., *Termes* sp., *Velocitermes* sp.), Dermaptera (Labiidae, Forficulidae: Neolobophorinae), Psocoptera (Archipsocidae, Cladiopsocidae; *Cladiopsocus* sp.; Epipsocidae, Lepidopsocidae, Myopsocidae: *Lichenomina* sp., Pachytroctidae, Pseudococcidae, Psyllipsocidae: *Psyllipsocus* sp.; Ptiloneuridae: *Ptiloneura* sp.), Hemiptera (Alydidae, Cydnidae, Dipsocoridae, Enicocephalidae, Gerridae, Lygaeidae, Mesoveliidae, Nabidae, Ochteridae, Reduviidae: *Panstrongylus* sp.; Pentatomidae, Ploiariidae, Pyrrhocoridae, Veliidae), Homoptera (Aphididae, Cicadellidae, Cixiidae: *Cixius* sp.; Delphacidae, Derbidae, Dictyopharidae, Membracidae, Coccoidea), Lepidoptera (Arctiidae: Arctiinae; Drepanidae: Thyatirinae, Hesperiidae, Heterocera; Noctuidae: *Latebraria* sp.; Agaristinae, Geometridae, Limacodidae, Lycaenidae, Tineioidea: Tineidae; Thyrididae, Zygaenidae), Diptera (Agromyzidae, Anthomyiidae, Bibionidae, Calliphoridae, Cecidomyiidae, Cecidomyiidae, Ceratopogonidae, Chaoboridae, Chironomidae, Chloropidae, Clusiidae, Conopidae, Culicidae: *Anopheles* sp., *Culex* sp., Dixidae, Dolichopodidae, Drosophilidae: *Drosophila* sp.; Empididae, Fanniidae, Lauxaniidae, Milichiidae, Muscidae, Mycetophilidae, Phoridae, Psychodidae: *Lutzomyia* sp.; Sciaridae, Simulidae, Stratiomyidae, Streblidae, Syrphidae, Tephritidae, Tipulidae), Hymenoptera (Formicidae: *Acanthognatus* sp., *Acromyrmex* sp., *Anochetus* sp., *Apterostigma* sp., *Atta* sp., *Azteca* sp., *Camponotus* sp., *Carebara* sp., *Cephalotes* sp., *Crematogaster* sp., *Cyphomyrmex* sp., *Dolichoderus* sp., *Ectatomma* sp., *Gigantiops* sp., *Gnamptogenys* sp., *Hypoponera* sp., *Labidus* sp., *Linepthema* sp.; Myrmicinae; *Neivamyrmex* sp., *Octostruma* sp., *Odontomachus* sp., *Pachycondyla* sp., *Pseudomyrmex* sp., *Pheidole* sp., *Rogeria* sp., *Solenopsis* sp., *Strumigenys* sp., *Tapinoma* sp., *Trachymyrmex* sp., *Zacryptocerus* sp.; Apidae, Bethylidae, Braconidae; Chalcidoidea; Cynipidae, Diapriidae, Elasmidae, Eulophidae, Ichneumonidae, Mutillidae, Pompilidae, Pteromalidae, Scelionidae, Trichogrammatidae, Vespidae), Thysanoptera (Phaleothripinae), Coleoptera (Byrrhidae, Carabidae: Harpalinae, Cerambycidae, Coccinellidae, Erotylidae,

Cetoniidae: *Gymnets* sp.; Chrysomelidae, Curculionidae: Scotylinae; Dermestidae, Dytiscidae, Elateridae: Agryphinae, Cardiophorinae, Elaterinae; Eucnemidae, Hysteridae, Leiodidae, Nitidulidae, Phalacridae Pselaphidae, Ptylidae, Rhizophagidae, Scarabeidae, Scydmaenidae, Staphylinidae, Tenebrionidae: Coelometropinae), Diplopoda (Chelodesmidae, Oniscodesmidae, Cyrtodesmidae, Paradoxosomatidae, Pyrgodesmidae, Polydesmidae, Glomeridesmidae: *Glomeridesmus* sp.; Lophoproctidae, Polyxenidae, Siphonophoridae, Pseudonannolenidae, Stemmiulidae), Geophilomorpha (Ballophilidae: *Ballophililus* sp., *Taeniolinum* sp., Geophilidae; Schendylidae), Lithobiomorpha (Henicopiidae: *Lamyctes* sp.), Scolopendromorpha (Scolopendridae: *Cormocephalus* sp.; Cryptopidae: *Cryptops* sp.; Otostigmidae: *Otostigmus* sp.; Scolopocryptopidae: *Dinocryptops* sp., *Newportia* sp., *Scolopocryptops* sp., *Tidops* sp.), Scutigeromorpha (Scutigeridae: *Sphendononema* sp.), Symphyla (Scutigerellidae: *Hanseniella* sp., *Scutigerella* sp.).

Dentre os vertebrados, foram encontradas 32 espécies das ordens Chiroptera (Emballonuridae: *Peropteryx kappleri*; Phyllostomidae: *Anoura* sp., *Glossophaga soricina; Diphylla ecaudata*; *Trachops cirrhosus*. Furipteridae: *Furipterus horrens*; Mormopidae: *Pteronotus parnellii*), Squamata (Colubridae: *Leptodeira annulata*, *Spilotes pullatus*, *Chironius* sp., *Oxybelis* sp.; Boidae: *Epicrates* sp.; Gekkonidae: *Thecadactylus rapicauda*; Sphaerodactylidae: *Coleodactylus cf. amazonicus*; Iguanidae), Anura (Leptodactylidae: *Pristimantis cf. fenestratus*, *Eleutherodactylus* sp., *Adenomera* sp., *Leptodactylus labyrinthicus*; Bufonidae, Dendrobatidae: *Ameerega* sp., *Colostethus* sp.), Rodentia (Cricetidae: *Rhipidomys* sp.), Siluriformes (*Trichomicterus* sp.), Characiformes, Passeriformes (Trochilidae sp.), Cathartiformes (Cathartidae: *Coragypis atratus*).

Desta forma, no total foram encontrados 935 morfoespécies. Entre estas 40 espécies de invertebrados foram consideradas troglomórficas: Gordioidea, Gastropoda (Systrophiidae), Amblypygi (Charinidae: *Charinus* 3 spp.), Isopoda (Balloniscidae 2spp.; Plathyarthridae: *Trichorhina* 3 spp.; Styloniscidae), Acari (Rhagidiidae), Schizomida (Hubbardiidae: Hubbardiinae), Opiliones (Escadabiidae 2spp., indet. sp.), Araneae (Ochyroceratidae 3 spp., Oonopidae: Oonopinae 2 spp., Prodidomidae 2 spp.), Thysanura (Nicoletiidae: Atelurinae), Collembola (Cyphoderidae 2 spp., Isotomidae 2spp.), Hemiptera (Thyreocoridae), Hymenoptera (Formicidae: *Hypoponera* 2 spp.), Coleoptera (Carabidae: *Coarazuphium* 2 spp.; Pselaphidae; Ptylidae, Scydmaenidae 2 spp.), Diplopoda (Pyrgodesmidae; Glomeridesmidae: *Glomeridesmus* sp.; Lophoproctidae; Pseudonannolenidae).

5.4.2.2 Caracterização realizada no período chuvoso

Foi observado nas cavernas amostradas, um total de 1.049 morfoespécies de invertebrados de pelo menos 250 famílias das Ordens: Gordioidea, Onychophora (Peripatidae), Oligochaeta, Gastropoda (Subulinidae, Systrophiidae, Valloniidae, Verocinellidae, Streptaxidae), Turbellaria (Geoplanidae), Isopoda (Armadillidae, Balloniscidae,

Dubioniscidae, Philosciidae, Plathyarthridae: *Trichorhina* sp., Scleropactidae), Decapoda (Pseudothelphusidae: *Microthelphusa somanni*; Palaemonidae: *Macrobrachium* sp.), Acari (Neothyridae: *Diplothyrus schubarti*; Argasidae*: Antricola* sp., *Carios rondoniensis, Ornithodoros marinkellei*; Ixodida: *Amblyomma cajennense;* Laelapidae: *Stratiolaelaps* sp.; Macrochelidae: *Macrocheles* sp.; Macronyssidae, Ologamasidae, Otopheidomenidae Podocinidae: *Podocinum* sp.; Mesostigmata: Uropodina; Ologamasidade; Opioacaridae: *Neoacarus* sp.; Acaridae; Astigmatina, Oribatida, Anystidae: *Erythracarus* sp.; Bdellidae, Cunaxidae, Eupodidae: *Linopodes* sp., Labdostomatida, Rhagidiidae, Teneriffiidae, Tydeidae), Amblypygi (Phrynidae: *Heterophrynus longicornis),* Palpigradi (Eukoenennidae: *Eukoenennia* sp.), Ricinulei (Ricinoididae: *Cryptocellus tarsilae*), Scorpiones (Buthidae: *Ananteris luciae*), Pseudoscorpiones (Chernetidae, Chtoniidae), Opiliones (Neogoveidae: *Canga renatae;* Cosmetidae: *Anduzeia* sp.; Escadabiidae, Fissiphaliidae, Gonyleptidae, Kimmulidae, Manaosbiidae, Sclerosomatidae: *Prionostemma* sp.; Stygnidae: *Eutimesius* sp., *Protimesius aff. gracilis*; Phalangiidae), Araneae (Amaurobiidae, Anapidae, Araneidae: *Alpaida* sp.; Anyphaenidae, Corinidae: *Tupirina* sp.; Ctenidae: *Ctenus* sp.; Dipluridae, Filistatidae, Gnaphosidae, Linyphiidae, Ochyroceratidae, Oonopidae: Oonopinae, Gamasomorphinae, *Coxapopha* sp.; Salticidae Segestridae, Drymusidae: *Drymusa* sp.; Senoculidae, Scytodidae*: Scytodes eleonora*, Palpimanidae, Paratropidae, Psauridae, Pholcidae: *Mesabolivar* sp., *Metagonia* sp.; Prodidomidae, Tetrablemidae, Theraphosidae: *Acanthoscurria* sp., *Nhandu coloratovillosus*; Theridiidae, Theridiosomatidae), Thysanura (Nicoletiidae: Nicoletiinae, Atelurinae), Microcoryphia (Meinertellidae), Diplura (Anajapygidae, Campodeidae, Japygidae, Parajapygidae), Collembola (Sminthuridae, Cyphoderidae, Dicyrtomidae, Entomobryomorpha: Entomobryidae; Hypogastruridae, Katiannidae?, Onychiuridae, Paronellidae, Poduridae, Tomoceridae), Neuroptera (Chrysopidae, Myrmelionthidae), Orthoptera (Gryllidae: Gryllinae; Phalangopsidae: *Aclodes* sp., *Phalangopsis* sp.; Tetrigidae; Tettigoniidae: Copiphorinae, Listroscelidinae), Mantodea (Mantinae), Phasmatodea (Pseudophasmatidae), Blattodea (Blaberidae: *Blaberus* sp.; Blattellidae, Blattidae, Polyphagidae), Embiidina, Isoptera (Rhinotermitidae: *Heterotermes* sp., *Rhinotermes* sp.; Termitidae: *Cortaritermes* sp., *Diversitermes* sp., *Embiratermes* sp., *Nasutitermes* sp., *Subulitermes*? sp., *Termes* sp., *Velocitermes* sp.), Dermaptera (Labiidae; Forficulidae: Neolobophorinae), Psocoptera (Archipsocidae, Ectopsocidae, Epipsocidae, Lepidopsocidae, Myopsocidae: *Lichenomina* sp., Pachytroctidae: *Tapinella* sp.; Psyllipsocidae: *Psyllipsocus* sp.; Ptiloneuridae: *Ptiloneura* sp.; Trogiidae), Hemiptera (Alydidae, Aradidae, Cydnidae, Dipsocoridae, Enicocephalidae, Gerridae, Hebridae, Lygaeidae, Mesoveliidae, Miridae, Nabidae, Ochteridae, Reduviidae: *Panstrongylus* sp.; Ploiariidae, Pseudococcidae, Pyrrhocoridae, Veliidae, Homoptera (Cercopidae, Cicadellidae, Cixiidae: *Cixius* sp.; Derbidae, Fulgoridae, Kinnaridae; Coccoidea; Membracidae), Lepidoptera (Geometridae, Heliozelidae, Hesperiidae, Noctuidae: *Latebraria* sp., Agaristinae; Notodontidae; Tineioidea: Tineidae, Tortricidae, Zygaenidae), Diptera (Agromyzidae, Calliphoridae, Cecidomyiidae, Ceratopogonidae, Chaoboridae, Chironomidae, Chloropidae,Clusiidae, Conopidae, Culicidae:

Aedes sp., *Anopheles* sp., *Culex* sp., *Toxorhynchites* sp.; Dolichopodidae, Drosophilidae: *Drosophila* sp.; Empididae, Fanniidae, Lauxaniidae, Micropezidae, Milichiidae, Muscidae, Mycetophilidae, Otitidae, Phoridae, Psychodidae: *Lutzomyia* sp.; Sciaridae, Simulidae, Streblidae, Syrphidae: *Ornidia obesa*; Tephritidae, Tipulidae), Hymenoptera (Formicidae: *Acanthognatus* sp., *Acanthostichus* sp., *Acromyrmex* sp., *Anochetus* sp., *Apterostigma* sp., *Atta* sp., *Azteca* sp., *Basiceros* sp., *Camponotus* sp., *Carebara* sp., *Cephalotes* sp., *Crematogaster* sp., *Cyphomyrmex* sp., *Dolichoderus* sp., *Ectatomma* sp., Gnamptogenys sp., *Hypoponera* sp., *Labidus* sp., *Leptogenys* sp.; Myrmicinae; *Myrmicocrypta* sp., *Neivamyrmex* sp., *Octostruma* sp., *Odontomachus* sp., *Pachycondyla* sp., *Platythyria* sp., *Pseudomyrmex* sp., *Pheidole* sp., *Prionopeuta* sp., *Rogeria* sp., *Solenopsis* sp., *Stegomyrmex* sp., *Strumigenys* sp., *Tapinoma* sp., *Tetramorium* sp., *Trachymyrmex* sp., *Zacryptocerus* sp., *Wasmannia* sp.; Apidae, Bethylidae, Braconidae; Chalcidoide; Diapriidae, Elasmidae, Eulophidae, Figitidae, Pteromalidae, Scelionidae, Tiphiidae, Vespidae), Thysanoptera (Phaleothripidae: Phaleothripinae), Coleoptero (Bostrichidae, Carabidae, Coccinellidae: Cetoniidae: *Gymnets* sp.; Chrysomelidae, Curculionidae: Entiminae?, Scotylinae; Dermestidae, Dytiscidae, Elateridae: Agryphinae, Cardiophorinae, Elaterinae; Eucnemidae, Elmidae, Hysteridae, Lampiridae Leiodidae, Nitidulidae, Phalacridae, Pselaphidae, Psephenidae Ptilodactylidae, Ptylidae, Rhizophagidae, Staphylinidae, Tenebrionidae: Coelometropinae), Diplopoda (Chelodesmidae, Oniscodesmidae, Cyrtodesmidae, Paradoxosomatidae, Pyrgodesmidae, Glomeridesmidae: *Glomeridesmus* sp.; Lophoproctidae, Polyxenidae, Siphonophoridae, Pseudonannolenidae, Rhinocricidae, Stemmiulidae), Geophilomorpha: *Ballophililus* sp., *Taeniolinum* sp.; Geophilidae), Macronicophilidae: *Macronicophilus* sp., Schendylidae), Lithobiomorpha (Henicopiidae: *Lamyctes* sp.), Scolopendromorpha (Cryptopidae: *Cryptops* sp., Cryptopinae; Otostigmidae: *Otostigmus* sp.; Scolopendridae: *Cormocephalus* sp., Scolopocryptopidae : *Dinocryptops* sp., Newportinae: *Newportia* sp., *Scolopocryptops* sp., *Tidops* sp.), Scutigeromorpha (Scutigeridae: *Sphendononema* sp.), Symphyla (Scutigerellidae: *Hanseniella* sp., *Scutigerella* sp., Scolopendrellidae).

Dentre os vertebrados, foram encontradas 23 espécies das ordens Chiroptera (Emballonuridae: *Peropteryx kappleri*; Phyllostomidae: *Anoura* sp., *Glossophaga soricina; Diphylla ecaudata*; Furipteridae: *Furipterus horrens*; Mormopidae: *Pteronotus parnellii*), Squamata (Colubridae; Boidae: *Epicrates* sp.; Gekkonidae: *Thecadactylus rapicauda*; Sphaerodactylidae: *Coleodactylus* cf. *amazonicus*), Anura (Leptodactylidae: *Pristimantis* cf. *fenestratus*, *Eleutherodactylus* sp., *Leptodactylus labyrinthicus*; Bufonidae, Dendrobatidae: *Ameerega* sp., *Colostethus* sp.), Rodentia (Cricetidae: *Rhipidomys* sp.; Cuniculidae), Siluriformes (*Trichomicterus* sp.), Characiformes.

Desta forma, no total foram encontrados 1072 morfoespécies. Entre estas 38 espécies de invertebrados, foram consideradas troglomórficas: Gastropoda (Systrophiidae), Turbellaria (Geoplanidae), Amblypygi (Charinidae: *Charinus* sp.), Isopoda (Balloniscidae 2 spp.;

Plathyarthridae: *Trichorhina* sp.), Acari (Trombidiforme; Rhagidiidae), Araneae (Ochyroceratidae sp., Oonopidae: Oonopinae, Prodidomidae), Thysanura (Nicoletiidae: Atelurinae 2 spp.), Diplura (Anajapygidae), Collembola (Cyphoderidae, Isotomidae 2 spp.), Hemiptera (Dipsocoridae), Hymenoptera (Formicidae: *Hypoponera* sp., *Solenopsis* sp.), Coleoptera (Carabidae: *Coarazuphium* sp.; Dytiscidae; Eucnemidae; Pselaphidae; Ptylidae; Scydmaenidae 3 spp.; Staphylinidae), Diplopoda (Pyrgodesmidae 3 spp.; Polydesmida 3spp., Glomeridesmidae: *Glomeridesmus* sp.; Lophoproctidae; Pseudonannolenidae).

5.4.3. Fauna geral

Foi observado nas cavernas amostradas, um total de 1399 morfoespécies de invertebrados de pelo menos 300 famílias das Ordens: Gordioidea, Onychophora (Peripatidae), Oligochaeta, Gastropoda (Subulinidae, Systrophiidae, Valloniidae, Streptaxidae), Turbellaria (Dugesiidae, Geoplanidae, Temnocephalidae), Isopoda (Armadillidae, Balloniscidae, Dubioniscidae, Philosciidae, Plathyarthridae: *Trichorhina* sp., Scleropactidae, Styloniscidae), Decapoda (Pseudothelphusidae: *Microthelphusa somanni*, Palaemonidae: *Macrobrachium* sp., Trichodactylidae: *Valdivia serrata*), Acari (Neothyridae: *Diplothyrus schubarti*; Argasidae: *Antricola* sp., *Carios rondoniensis, Ornithodoros marinkellei, Ornithodoros* spp.; Ixodidae: *Amblyomma cajennense*, *Amblyomma* spp.; Diploginiidae; Laelapidae: *Stratiolaelaps* sp.; Macrochelidae: *Macrocheles* sp.; Macronyssidae; Ologamasidae; Otopheidomenidae; Podocinidae: *Podocinum* sp.; Mesostigmata; Uropodina; Ologamasidade; Opilioacaridae: *Neoacarus* spn.; Acaridae; Anoetidae; Astigmatina; Oribatida; Anystidae: *Erythracarus* sp.; Bdellidae; Cunaxidae; Erythraeidae; Eupodidae: *Linopodes* sp.; Labdostomatidae: *Labdostomatida* sp.; Rhagidiidae; Teneriffiidae; Tydeidae; Trombidiforme), Amblypygi (Phrynidae: *Heterophrynus longicornis*, Charinidae: *Charinus* sp.), Palpigradi (Eukoenennidae: *Eukoenennia* sp.), Ricinulei (Picinoididae: *Cryptocellus tarsilae*), Schizomida (Hubbardiidae: Hubbardiinae), Scorpiones (Buthidae: *Ananteris luciae*), Pseudoscorpiones (Chernetidae, Chtoniidae), Opiliones (Neogoveidae : *Canga renatae*, Cosmetidae: *Anduzeia* sp., Escadabiidae, Fissiphaliidae, Gonyleptidae, Kimmulidae, Manaosbiidae, Sclerosomatidae: *Prionostemma* sp.; Stygnidae: *Eutimesius* sp., *Protimesius* aff. *gracilis;* Stygnomatidae, Phalangiidae), Araneae (Amaurobiidae; Anapidae; Araneidae: *Alpaida* sp; Anyphaenidae; Prodidomidae; Corinidae; Corinidae: *Tupirina* sp.; Ctenidae: *Ctenus* sp.; Dipluridae; Filistatidae; Gnaphosidae; Linyphiidae; Mimetidae; Ochyroceratidae; Oonopidae: Oonopinae, Gamasomorphinae, *Coxapopha* sp.; Salticidae; Segestridae; Drymusidae: *Drymusa* sp.; Senoculidae; Scytodidae*: Scytodes eleonora*; Palpimanidae; Paratropidae; Psauridae; Pholcidae: *Mesabolivar* sp., *Metagonia* sp.; Prodidomidae; Tetrablemidae; Theraphosidae; Theridiidae; Theridiosomatidae; Thomisidae; Uloboridae), Thysanura (Nicoletiidae: Nicoletiinae, Atelurinae), Microcoryphia (Meinertellidae), Diplura (Anajapygidae, Campodeidae, Japygidae, Parajapygidae, Procampodeidae), Collembola (Sminthuridae, Cyphoderidae, Dicyrtomidae, Entomobryidae, Entomobryomorpha, Hypogastruridae, Isotomidae, Katiannidae?, Neanuridae, Onychiuridae, Paronellidae,

Poduridae, Tomoceridae), Odonata (Aeshnidae: *Castoraeschna* sp., Coenagrionidae), Neuroptera (Chrysopidae, Mantispidae: *Plega* sp., Myrmelionthidae), Orthoptera (Gryllidae: Mogoplistinae, Nemobiinae, Pentacentrinae; Phalangopsidae: *Aclodes* sp., *Phalangopsis* sp.; Tetrigidae; Tettigoniidae: Copiphorinae, Tettigoniinae, Listroscelidinae), Mantodea (Mantidae: Mantinae), Phasmatodea (Pseudophasmatidae), Blattodea (Blaberidae: *Blaberus* sp., Blattellidae, Blattidae, Polyphagidae), Embiidina, Isoptera (Rhinotermitidae: *Heterotermes* sp., *Rhinotermes* sp; Termitidae: *Heterotermes* sp., *Armitermes* sp., *Cornitermes* sp., *Cortaritermes* sp., *Diversitermes* sp., *Embiratermes* sp., *Nasutitermes* sp., *Planicapritermes* sp., *Subulitermes* sp., *Termes* sp., *Velocitermes* sp.), Dermaptera (Labiidae, Forficulidae: Neolobophorinae), Psocoptera (Archipsocidae, Cladiopsocidae: *Cladiopsocus* sp., Ectopsocidae, Lepidopsocidae, Myopsocidae: *Lichenomina* sp., Pachytroctidae: *Tapinella* sp., Psyllipsocidae: *Psyllipsocus* sp., Ptiloneuridae: *Ptiloneura* sp., Trogiidae), Hemiptera (Alydidae, Aphididae, Aradidae, Cydnidae, Dipsocoridae, Enicocephalidae, Gerridae, Hebridae, Lygaeidae, Mesoveliidae, Miridae, Nabidae, Ochteridae, Reduviidae: *Panstrongylus* sp., Pentatomidae, Ploiariidae, Pyrrhocoridae, Thyreocoridae, Veliidae), Homoptera (Achilidae, Cercopidae, Cicadellidae, Cixiidae: *Cixius* sp., Delphacidae, Derbidae, Dictyopharidae, Fulgoridae, Kinnaridae, Membracidae, Coccoidea, Pseudococcidae), Lepidoptera (Arctiidae: Arctiinae, Ctenuchinae; Drepanidae: Thyatirinae; Geometridae; Heliozelidae; Hesperiidae; Heterocera; Notodontidae; Noctuidae: *Latebraria* sp., Agaristinae; Limacodidae; Lycaenidae; Tineioidea; Tineidae; Thyrididae; Tortricidae; Zygaenidae), Diptera (Agromyzidae, Anthomyiidae, Asilidae: *Leptogaster* sp., Bibionidae, Calliphoridae, Cecidomyiidae, Cecidomyiidae, Ceratopogonidae, Chaoboridae, Chironomidae, Chloropidae, Clusiidae, Conopidae; Culicidae: *Aedes* sp., *Anopheles* sp., *Toxorhynchites* sp., *Culex* sp., *Anopheles* sp.; Dixidae, Dolichopodidae, Drosophilidae: *Drosophila* sp., Empididae, Fanniidae, Keroplatidae, Lauxaniidae, Micropezidae, Milichiidae, Muscidae, Mycetophilidae, Otitidae, Phoridae, Psychodidae: *Lutzomyia* sp., Sciaridae, Simulidae, Stratiomyidae, Streblidae, Syrphidae: *Ornidia obesa*, Tephritidae, Tipulidae), Hymenoptera (Formicidae: *Acanthognatus* sp., *Acanthostichus* sp., *Acromyrmex* sp., *Anochetus* sp., *Apterostigma* sp., *Atta* sp., *Azteca* sp., *Basiceros* sp., *Camponotus* sp., *Carebara* sp., *Cephalotes* sp., *Crematogaster* sp., *Cyphomyrmex* sp., *Dolichoderus* sp., *Ectatomma* sp., *Gigantiops* sp., *Gnamptogenys* sp., *Hypoponera* sp., *Labidus* sp., *Leptogenys* sp., *Linepthema* sp., Myrmicinae, *Myrmicocrypta* sp., *Neivamyrmex* sp., *Octostruma* sp., *Odontomachus* sp., *Pachycondyla* sp., *Platythyria* sp., *Pseudomyrmex* sp., *Pheidole* sp., *Prionopeuta* sp., *Rogeria* sp., *Solenopsis* sp., *Stegomyrmex* sp., *Strumigenys* sp., *Tapinoma* sp., *Tetramorium* sp., *Trachymyrmex* sp., *Zacryptocerus* sp., *Wasmannia* sp.; Apidae; Bethylidae; Braconidae; Chalcidoidea; Cynipidae; Diapriidae; Elateridae: Cardiophorinae, Elaterinae; Elasmidae; Eulophidae; Figitidae; Ichneumonidae; Mutillidae; Pompilidae; Pteromalidae; Scelionidae; Tiphiidae; Trichogrammatidae; Vespidae), Thysanoptera (Phaleothripidae: Phaleothripinae), Coleoptera (Byrrhidae, Bostrichidae, Carabidae: *Coarazuphium* sp., Harpalinae; Cerambycidae, Coccinellidae, Cholevidae, Erotylidae,

Cetoniidae: *Gymnets* sp., Chrysomelidae; Curculionidae: Entiminae?, Scotylinae; Dytiscidae, Elateridae, Eucnemidae, Elmidae, Hysteridae, Lampiridae, Leiodidae, Nitidulidae, Phalacridae, Pselaphidae, Psephenidae, Ptilodactylidae, Ptylidae, Rhizophagidae, Scarabeidae, Scydmaenidae, Staphylinidae, Tenebrionidae: Coelometropinae), Diplopoda (Chelodesmidae, Oniscodesmidae, Cyrtodesmidae, Paradoxosomatidae, Pyrgodesmidae, Polydesmida, Polydesmidae, Glomeridesmidae: *Glomeridesmus* sp., Lophoproctidae, Polyxenidae, Siphonophoridae, Pseudonannolenidae, Rhinocricidae, Stemmiulidae), Geophilomorpha (Ballophilidae: *Ballophililus* sp., *Taeniolinum* sp; Geophilidae; Macronicophilidae: *Macronicophilus* sp., Schendylidae), Lithobiomorpha (Henicopiidae: *Lamyctes* sp.), Scolopendromorpha (Scolopendridae: *Cormocephalus* sp. , Cryptopidae: *Cryptops* sp., Otostigmidae: *Otostigmus* sp., Scolopocryptopidae: *Dinocryptops* sp., *Newportia* sp., *Scolopocryptops* sp., *Tidops* sp.), Scutigeromorpha (Scutigeridae: *Sphendononema* sp.) e Symphyla (Scutigerellidae: *Hanseniella* sp., *Scutigerella* sp.).

Alguns representantes desta grande diversidade de invertebrados são mostrados nas Figuras 60, 61 e 62.

Dentre os vertebrados foram encontrados 36 espécies das Ordens: Chiroptera (Emballonuridae: *Peropteryx kappleri*; Phyllostomidae: *Anoura* sp., *Glossophaga soricina*, *Diphylla ecaudata*, *Trachops cirrhosus*; Furipteridae: *Furipterus horrens*; Mormopidae: *Pteronotus parnellii*, *Pteronotus* sp.), Squamata (Colubridae: *Leptodeira annulata*, *Spilotes pullatus*, *Chironius* sp., *Oxybelis* sp.; Boidae: *Epicrates* sp.; Gekkonidae: *Thecadactylus rapicauda*; Sphaerodactylidae: *Coleodactylus* cf. *amazonicus*; Iguanidae), Anura (Leptodactylidae: *Pristimantis* cf. *fenestratus*, *Eleutherodactylus* sp., *Adenomera* sp., *Leptodactylus labyrinthicus*; Bufonidae; Dendrobatidae: *Ameerega* sp., *Colostethus* sp.), Rodentia (Cricetidae: *Rhipidomys* sp., Cuniculidae), Siluriformes (Trichomicteridae: *Trichomicterus* sp.), Characiformes, Passeriformes (Trochilidae) e Cathartiformes (Cathartidae: *Coragypis atratus*).

Desta forma, no total foram encontrados 1.435 morfoespécies. Entre estas, 60 espécies de invertebrados foram consideradas troglomórficos: Gordioidea, Gastropoda (Systrophiidae), Turbellaria (Geoplanidae), Isopoda (Balloniscidae, 3spp.; Plathyarthridae: *Trichorhina* sp., 3spp.; Styloniscidae), Acari (Rhagidiidae, Trombidiforme), Amblypygi (Charinidae: *Charinus* sp., 3spp.), Schizomida (Hubbardiidae: Hubbardiinae), Opiliones (Escadabiidae, 2spp.; sp. indet.), Araneae (Prodidomidae; Ochyroceratidae, 4spp.; Oonopidae: Oonopinae, 2spp.), Thysanura (Nicoletiidae: Atelurinae, 2spp.), Diplura (Anajapygidae), Collembola (Cyphoderidae, 2spp.; Isotomidae, 2spp.), Hemiptera (Dipsocoridae, Thyreocoridae), Hymenoptera (Formicidae: *Hypoponera* sp., 2spp.; *Solenopsis* sp.), Coleoptera (Carabidae: *Coarazuphium* sp., 2spp.; Dytiscidae; Eucnemidae; Pselaphidae, 2spp.; Ptylidae;

Scydmaenidae, 4spp.; Staphylinidae) e Diplopoda (Pyrgodesmidae, 3spp.; Polydesmida, 3spp.; Glomeridesmidae: *Glomeridesmus* sp.;Lophoproctidae; Pseudonannolenidae).

Em geral, nas cavernas inventariadas durante a estação seca, os grupos mais diversos e comumente encontrados são representados por organismos que apresentam hábitos alimentares detritívoros ou predadores generalistas, capazes de encontrar alimento em ambientes com recursos escassos e pouco diversificados. Além disso, tais grupos apresentam ampla distribuição epígea, o que favorece a colonização do meio subterrâneo. Dentre estes, vale destacar a ocorrência de isópodes (Armadillidae, Dubioniscidae, Philosciidae), pseudoescorpiões (Chernetidae e Chtoniidae), amblipígeos (*Charinus* sp., *Heterophrynus* sp.), aranhas (Pholcidae, Theraphosidae, Theridiidae, Theridiosomatidae, Salticidae e Scytodidae*: Scytodes eleonora*), grilos (Phalangopsidae: *Aclodes* sp., *Phalangopsis* sp.), colêmbolos (Entomobryidae), dípteros (Ceratopogonidae, Culicidae, Drosophilidae, Psychodidae: *Lutzomyia* sp., Tipulidae), coleópteros (Carabidae, Staphylinidae), lepidópteros (Noctuidae e Tineidae), hemípteros (Cydnidae, Dipsocoridae, Lygaeidae), himenópteros (Formicidae: *Camponotus* sp., *Dolychoderus* sp., *Pachycondyla* sp., *Pheidole* sp., *Solenopsis* sp.) e diplópodes (Pyrgodesmidae). Entretanto, alguns táxons comuns em outras áreas cársticas brasileiras foram raros ou pouco comuns. Destes, vale destacar os opiliões Gonyleptidae, as aranhas Ctenidae, os diplópodes Pseudonannolenidae, os hemípteros e Reduviidae, sendo estes, encontrados apenas em algumas poucas cavernas estudadas.

As Ordens de invertebrados mais ricas em espécies compreenderam Coleoptera (238 espécies – 17,2% da riqueza total observada), Hymenoptera (187 espécies – 13,5% da riqueza total observada), Aranae (171 espécies – 12,4% da riqueza total observada) e Diptera (143 espécies – 10,3% da riqueza total observada) (Figura 5). Dentre os vertebrados, as Ordens mais ricas em espécies compreenderam Anura (11 espécies – 31,4% da riqueza total observada), Chiroptera (9 espécies – 25,7% da riqueza total observada) e Squamata (9 espécies – 25,7% da riqueza total observada) (Figura 64). Exemplos de diferentes espécies encontradas no presente estudo podem ser visualizados na Figura 60 (Arachnida), Figura 61 (Hexapoda), Figura 62 (Invertebrados em geral), Figura 63 (Mammalia), Figura 64 (Squamata), Figura 65 e Figura 66 (Amphibia).

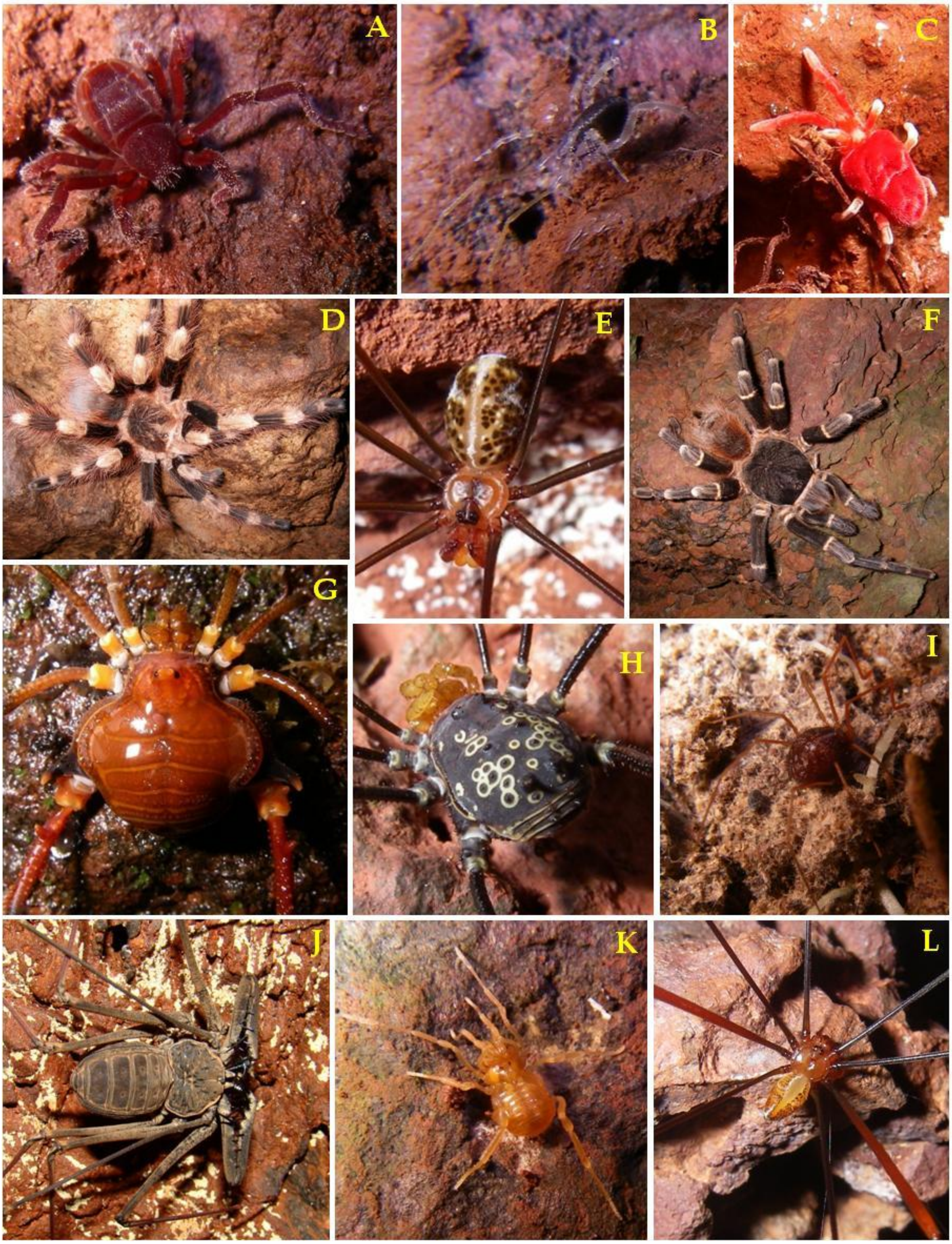

Figura 60 - a) Ricinulei: Ricinoididae (*Cryptocellus tarsilae*); b) Opilioacarida: Opilioacaridae (*Neoacarus* spn.); c) Acari: Trombidiforme; d) Araneae: Theraphosidae (*Nhandu coloratovillosus*); e) Araneae: Pholcidae (*Mesabolivar* sp.); f) Araneae: Theraphosidae (*Acanthoscurria* sp.); g) Opiliones: Gonyleptidae; h) Opiliones: *Manaosbiidae*; i) Acari; j) Amblypygi: Phrynidae (*Heterophrynus longicornis*); K) Opiliones: Scadabiidae; l) Araneae: Pholcidae (*Mesabolivar* sp.).

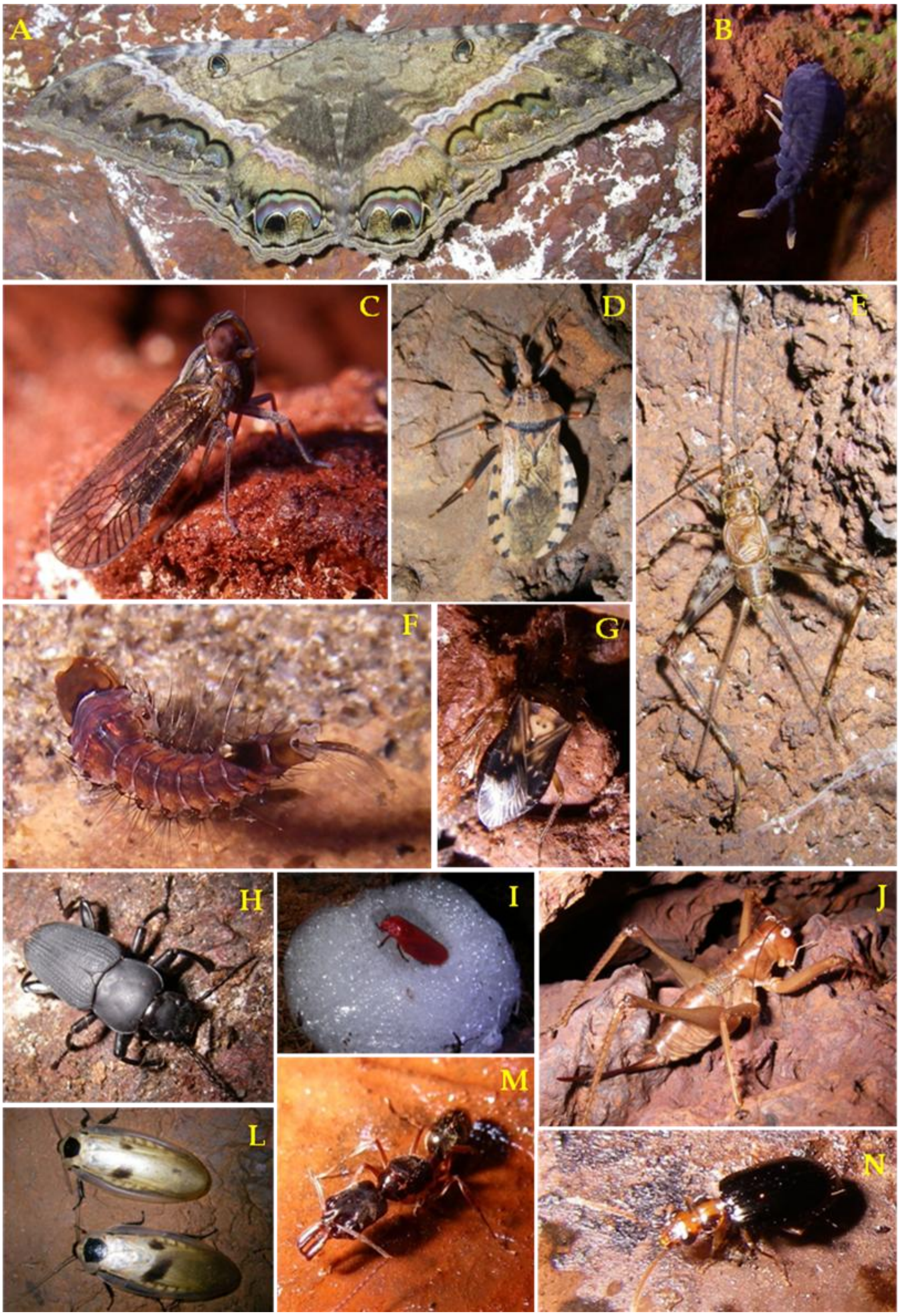

Figura 61 - a) Lepidoptera: Noctuidae (*Latebraria* sp.) b) Collembola: Hypogastruridae; c) Homoptera: Kinnaridae; d) Hemiptera: Reduviidae (*Panstrongylus* sp.); e) Orthoptera: Aclodidae (*Aclodes* sp.); f) Larva de Diptera: Culicidae (*Toxorhynchites* sp.); g) Hemiptera: Nabidae; h) Coleoptera: Tenebrionidae; i) Hemiptera: Cercopidae; j) Orthoptera: Tettigoniidae; K) Blattodea: Blaberidae (*Blaberus* sp.); l) Hymenoptera: Formicidae (*Odontomachus* sp.); m) Coleoptera: Carabidae.

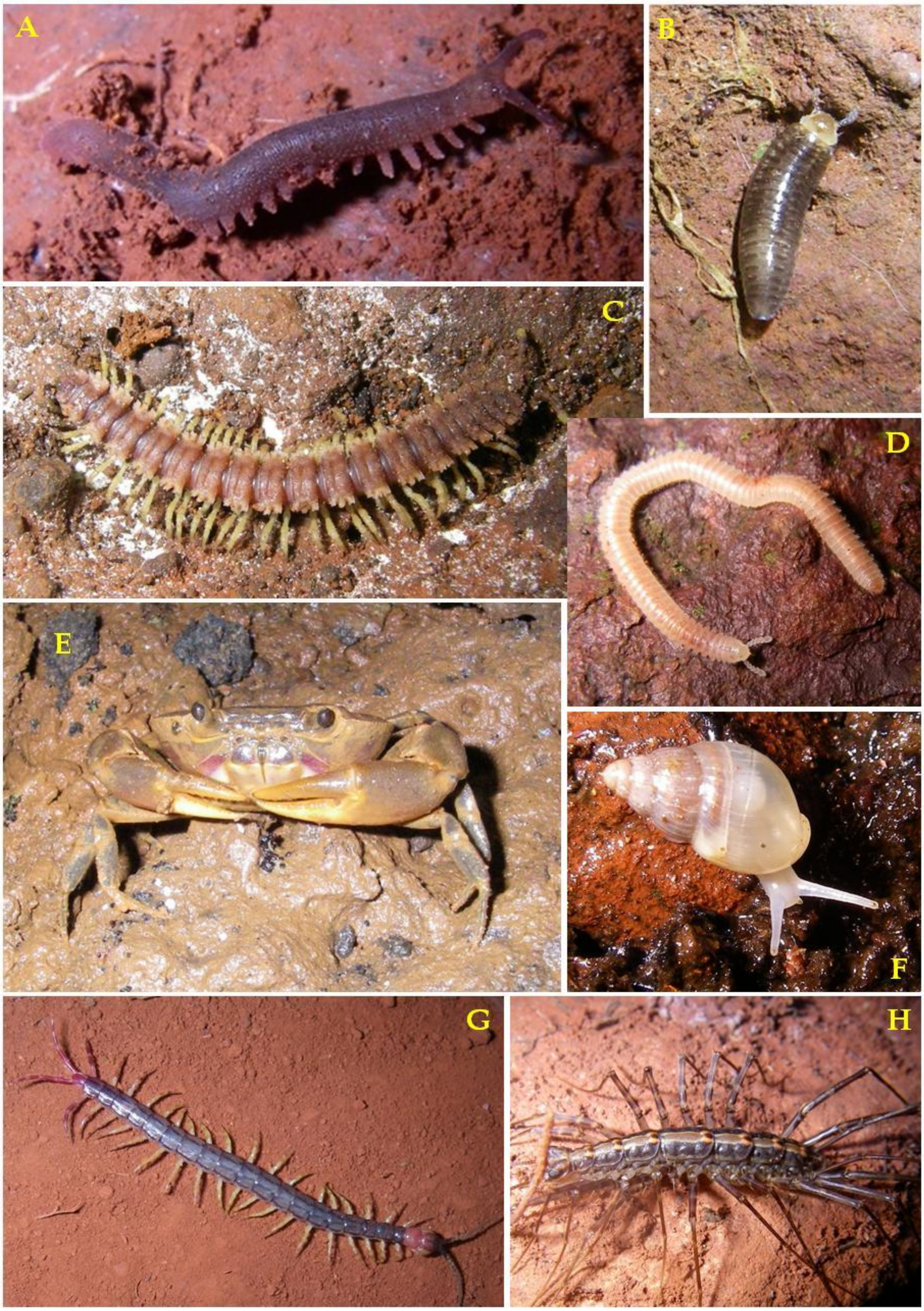

Figura 62 - a) Onychophora: Peripatidae; b) Glomeridesmida: Glomeridesmidae (*Glomeridesmus* sp.); c) Polydesmida: Chelodesmidae; d) Siphonophorida: Siphonophorida; e) Decapoda: Trichodactylidae; f) Gastropoda; g) Scolopendromorpha; h) Scutigeromorpha: Scutigeridae

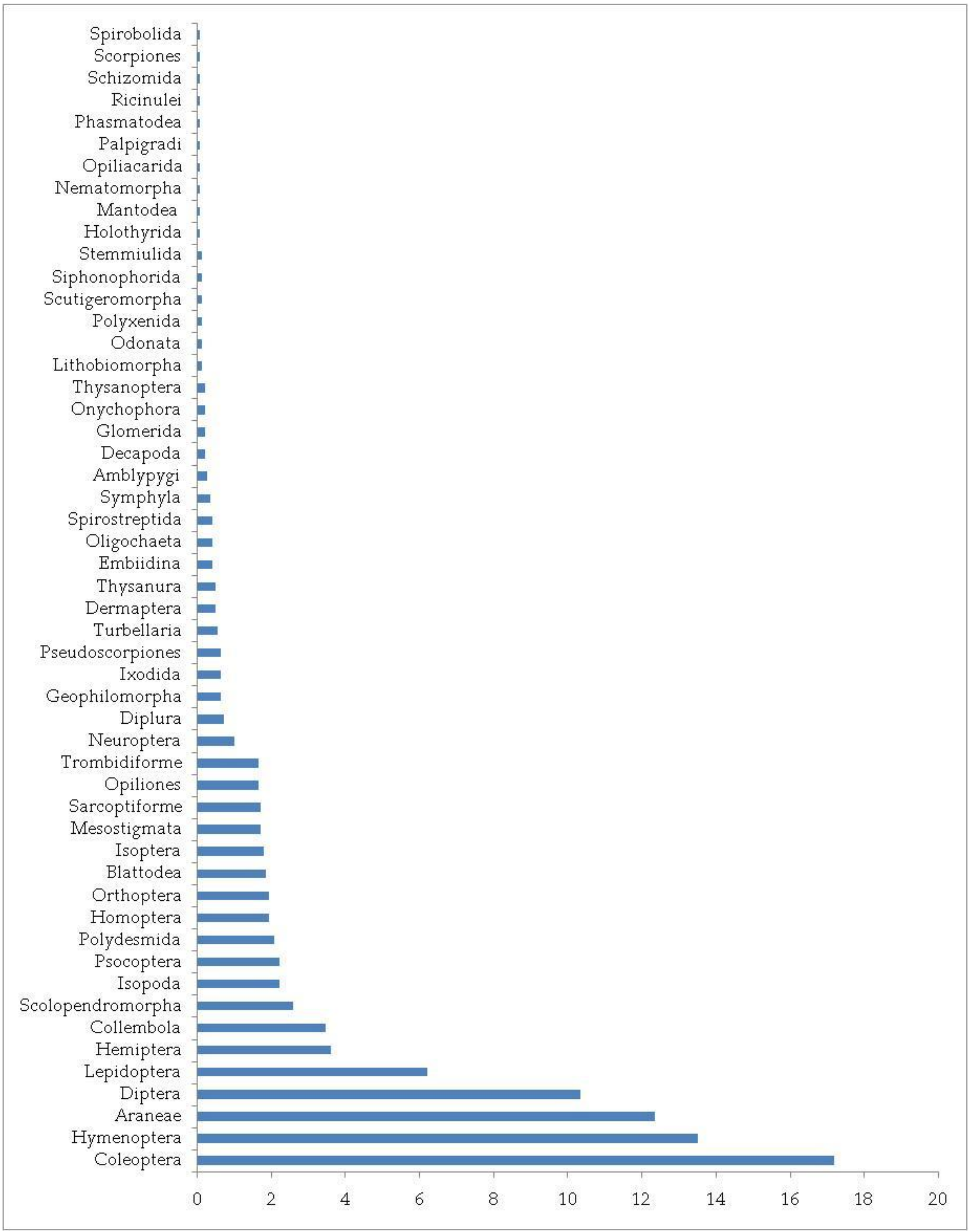

Figura 63 - Ordens encontradas e suas respectivas porcentagens em relação à riqueza total de invertebrados registrada nas cavernas.

Durante o inventário bioespeleológico das cavernas de Serra Leste foram identificadas 38 espécies de vertebrados. Apesar dos morcegos representarem o grupo mais especializado ao ambiente subterrâneo, os anfíbios representaram o grupo com maior riqueza, como pode ser observado na Figura 64 e mais amplamente distribuído entre as cavernas da região.

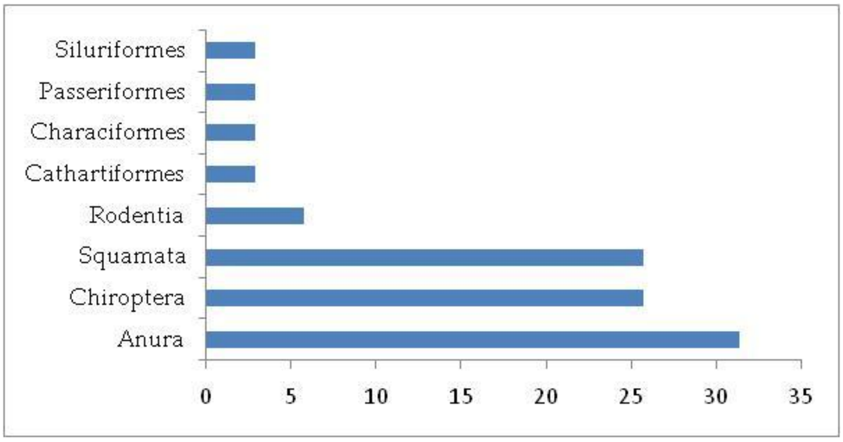

Figura 64 - Ordens encontradas e suas respectivas porcentagens em relação à riqueza total de vertebrados registrada nas cavernas.

Em relação à quiropterofauna, em 70% das cavernas estudadas foi possível observar pelo menos uma espécie de morcego. Ao todo foram identificadas nove espécies de morcegos e em quatro ocorrências não foi possível identificar a espécie. *Glossophaga soricina e Carollia perspicillata* (Phyllostomidae) e *Peropteryx kappleri* (Emballonuridae) foram às espécies mais recorrentes. Em geral, as duas primeiras espécies são encontradas formando grandes colônias, sendo estas extremamente importantes na produção de guano nas cavidades pesquisadas. Já a segunda espécie é observada sempre próxima as zonas de entrada e sempre restrita a poucos indivíduos.

Anoura sp. (Phyllostomidae: Glossophaginae) é a terceira espécie recorrente nas cavernas da região estando esta em muitas oportunidades associadas às colônias de *G. soricina*, mas sempre com abundâncias inferiores. Ainda em relação à quiropterofauna, vale destacar o registro de *Pteronotus parnellii* (Mormopidae) na gruta SL-001 e *Pteronotus gymnonotus* encontrada na gruta SL-078 sendo estes morcegos de hábitos alimentares insetívoros. Ambas as espécies formam colônias grandes sendo estas compostas por centenas de indivíduos e responsáveis por uma elevada produtividade de guano em ambas as cavidades. Além destes, algumas ocorrências formam consideradas raras nas cavernas de Serra Leste como os registros de *Furipterus horrens* (Furipteridae) nas Grutas SL-022, SL-072 e SL-074, *Diphylla ecaudata* (Phyllostomidae) nas grutas SL-029 e SL-082 e *Trachops cirrhosus* (Phyllostomidae) na gruta SL-079. Em relação à riqueza, vale destacar as grutas SL-001 onde foram registradas quatro espécies simpátricas.

Entre os mamíferos, vale destacar o registro de uma espécie de roedor da família Cricetidae encontrada frequentemente utilizando as cavernas da região como abrigo e sítio de reprodução. Em várias cavidades observamos vestígios de seus ninhos e em seis oportunidades pudemos observá-los sozinhos, com ninhos e filhotes inclusive em zonas

afóticas. Trata-se de pelo menos uma espécie do gênero *Rhipidomys* sendo esta considerada arborícola noturnos e solitários e que apresenta-se amplamente distribuído por todos os biomas brasileiros. Até momento não existem registros deste gênero em cavidades naturais sendo de extrema importância o investimento em estudos que busquem elucidar a relação destes mamíferos com o ambiente subterrâneo da região. Alguns mamíferos encontrados em cavernas de Serra Leste são mostrados na Figura 65.

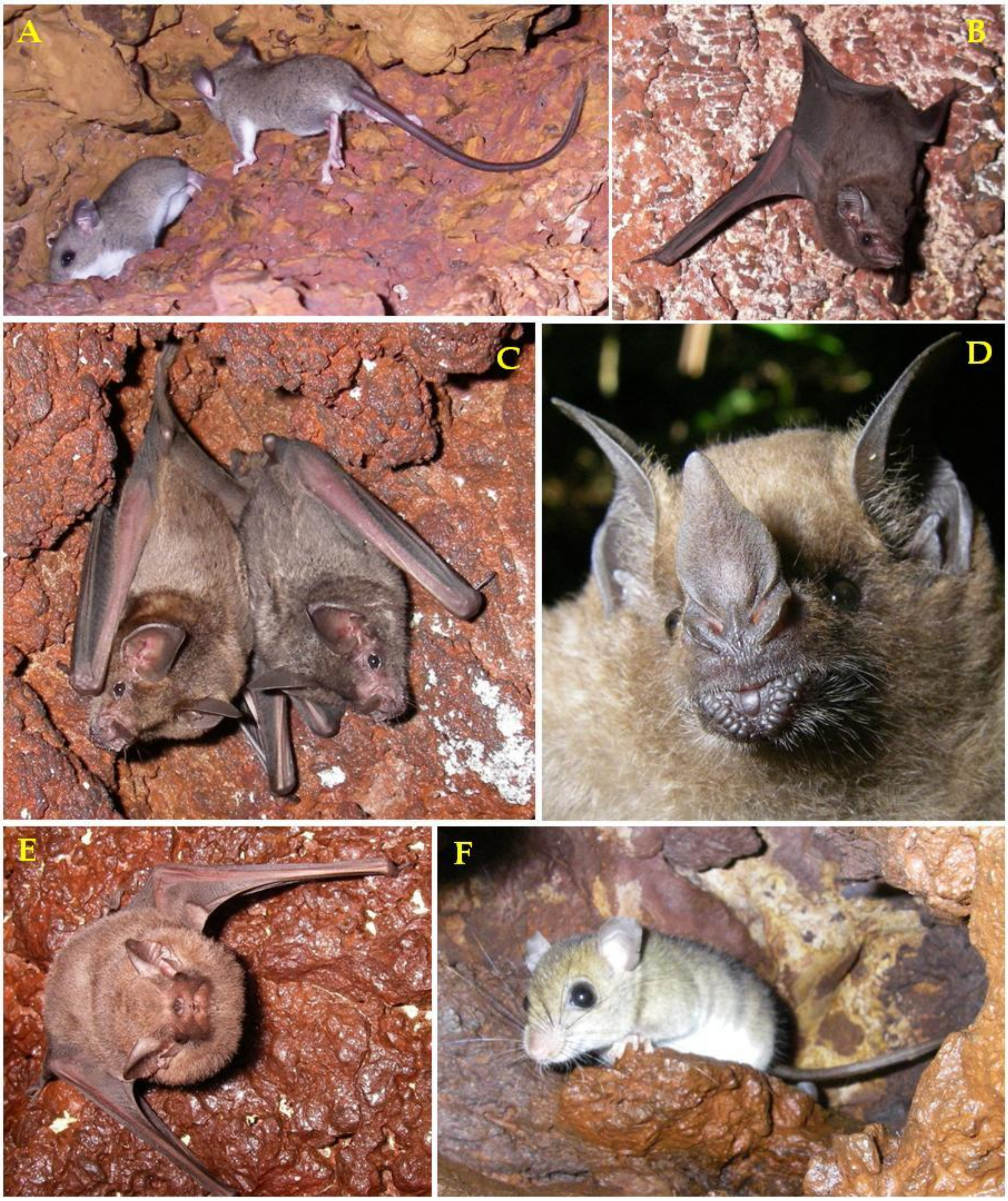

Figura 65 - a) Rodentia: Cricetidae (*Riphydomis* sp.); b) Chiroptera: Emballonuridae (*Peropteryx kappleri*); c) Chiroptera: Phyllostomidae (*Carollia* sp.); d) Chiroptera: Phyllostomidae; e) Chiroptera: Emballonuridae (*Peropteryx* sp.); f) Rodentia: Cricetidae (*Riphydomis* sp.).

Entre as aves, vale destacar o registro de dois indivíduos de urubus de cabeça preta (*Coragyps atratus*) na gruta SL-073 e vestígios recentes de *Tito alba* (coruja Suindara) na gruta SL-083, mas nenhum indício que estas espécies estejam utilizando as cavidades como sítios de reprodução foram observados. Entretanto, na gruta SL-077 foi observado um ninho com filhotes de uma terceira provavelmente de da família Trochilidae (Figura 66).

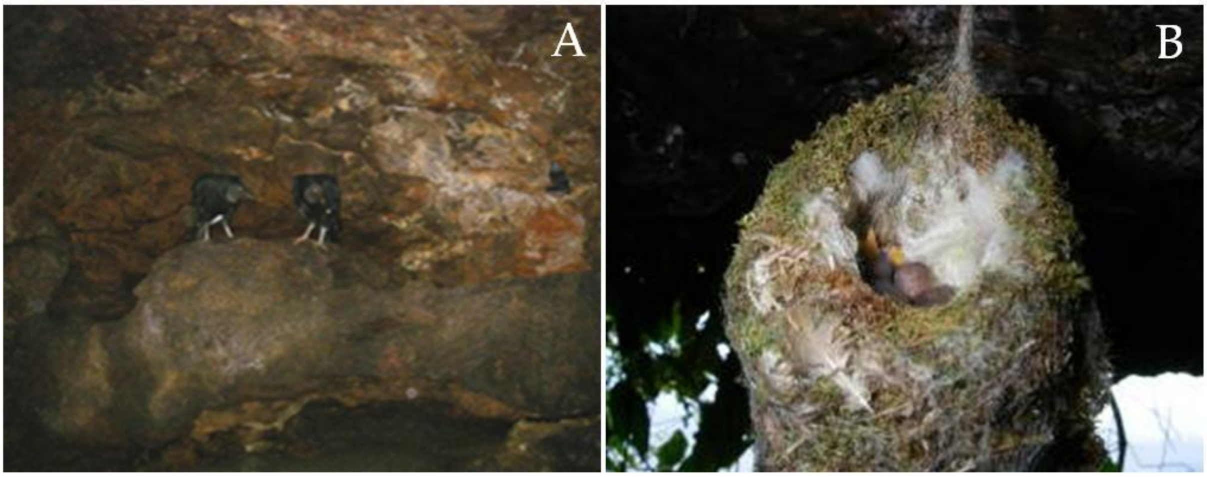

Figura 66 - Aves encontradas em cavernas inventariadas na área do Projeto Serra Leste. a) Urubu de cabeça preta (*Coragyps atratus*); b) Ninho com filhotes não identificados.

Dentre os outros táxons de vertebrados, foram observadas seis espécies de serpentes: *Spilotes pullatus* (SL-072), *Leptodeira annulata* (SL-033), *Oxybelis* sp. (SL-038), *Epicrates* sp. (SL-060 e SL-097), *Chironius* sp. (SL-016 e SL-029) e uma espécie da família Colubridae não determinada (SL-065 e SL-082). Dentre estas, apesar de terem ocorrido apenas dois registros, *Epicrates* sp. apresenta uma maior associação com o ambiente subterrâneo utilizando as cavernas da região como abrigo e sítio de forrageio. Já as outras espécies, a princípio podem ser consideradas como acidentais uma vez que foram encontradas apenas em um evento (exceto *Chironius* sp.) durante os inventários bioespeleológicos na região. Entretanto, cabe ressaltar que algumas espécies se alimentam basicamente de anfíbios anuros (e. g. *L. annulata*), sendo estes recorrentes nas cavernas da Serra Leste. Tal condição pode favorecer a ocorrência destas serpentes no ambiente subterrâneo, como observado na gruta SL-029 onde *Chironius* sp. (espécie de hábito arborícola) foi encontrada alimentando-se de *Pristimantis* cf. *fenestratus* (perereca da família Leptodactylidae) na zona de entrada de uma das cavidades inventariadas.

Ainda entre os répteis pelo menos três espécies de lagartos foram encontradas nas cavidades estudadas, sendo uma espécie de Gekkonidae (*Thecadactylus rapicauda*), uma espécie de Sphaerodactylidae (*Coleodactylus* cf. *amazonicus*) e uma espécie de Iguanidae não determinada.

Thecadactylus rapicauda é a espécie mais recorrente tendo sido encontrada em 19 das 96 cavidades estudadas. Esta espécie considerada arborícola é o maior geconídeo do mundo, possui hábito noturno e aparentemente utiliza as cavernas da região como abrigo diurno principalmente durante a estação seca onde a umidade relativa do ar do ambiente epígeo é, de forma geral, inferior. Já as outras duas espécies a princípio são consideradas acidentais uma vez que o número de ocorrências foi esporádico. Entretanto trata-se de uma conclusão preliminar sendo necessário o desenvolvimento de estudos que visem elucidar a relação destes organismos com o ambiente subterrâneo. Os lagartos do gênero *Coleodactylus* estão entre os lacertílios mais bem representados no Brasil sendo estes animais de hábitos diurnos associados que se associam a serrapilheira e podem ser encontrados em ambientes florestais ou fitofisionomias abertas.

Ao todo, os lagartos estiveram presentes em 22% das cavernas inventariadas. Em nenhuma oportunidade foram observados comportamentos de forrageio ou reprodutivo, mas certamente tais espécies podem se alimentar de uma grande variedade de invertebrados cavernícolas principalmente na zona de ecótono entre os ambientes epígeo e hipógeo, bem como podem utilizar as cavidades para reprodução. Em algumas oportunidades foram observadas cascas de ovos de lagartos geconídeos na entrada das cavernas estudas, mas em nenhuma delas foi possível correlacioná-las com as espécies identificadas. Alguns répteis encontrados em cavernas de Serra Leste são mostrados na Figura 67.

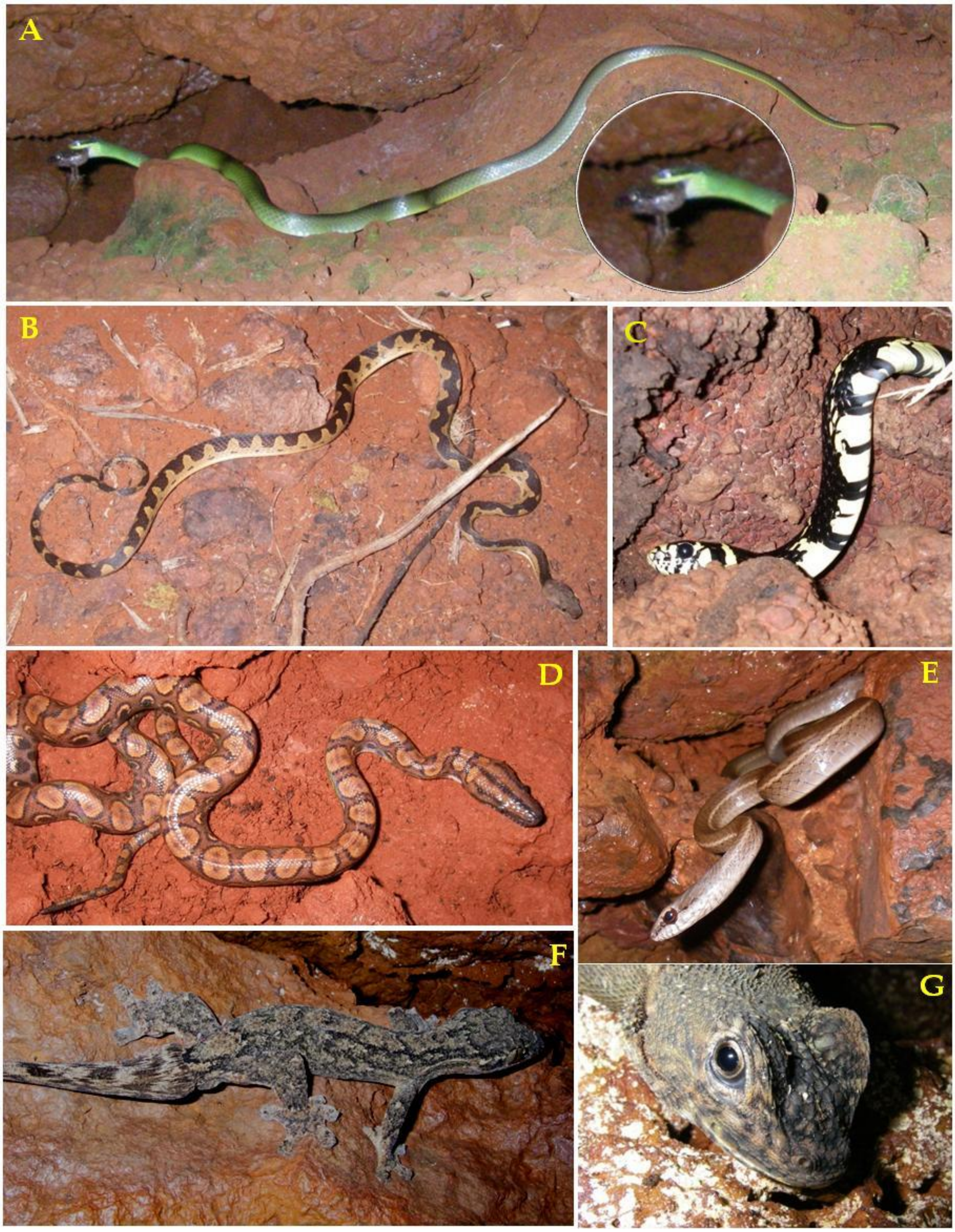

Figura 67 - a) Squamata: Colubridae (*Chironius* sp.); b) Squamata: Colubridae (*Leptodeira annulata*); c) Squamata: Colubridae (*Spilotes pullatus*); d) Squamata: Boidae (*Epicrates* sp.); e) Squamata: Colubridae; f) Squamata: Gekkonidae (*Thecadactylus rapicauda*); Squamata: Iguanidae.

Em relação aos anfíbios, estes representam o grupo de vertebrados não voadores mais recorrentes nas cavernas de Serra Leste tendo sido encontrados em quase todas as cavidades estudas (Figuras 68 e 69). Pelo menos 14 espécies foram encontradas, sendo que estas podem utilizar as cavernas como abrigos, sítios de reprodução e forrageio. *Leptodactylidae é a família mais recorrente e com melhor distribuição tendo sido*

encontradas seis espécies distintas (*Pristimantis* cf. *fenestratus,* Eleutherodactylus sp., Adenomera sp. e Leptodactylus labyrinthicus e duas espécies não determinadas), seguida da família Dendrobatidae (*Ameerega sp. e* Colostethus sp. e uma espécie indet.) e Bufonidae (2 spp.), além de mais três espécies não determinadas.

Dentre estas, *Pristimantis* cf. *fenestratus* (Leptodactylidae) é a espécie mais recorrente sendo encontrada em 81 das 96 cavernas inventariadas. Em geral, os espécimes encontram-se distribuídos ao longo de toda a cavidade incluindo zonas afóticas, sempre em repouso nas paredes e tetos, sozinhos ou em grupos de até seis indivíduos. Em diversas oportunidades *Lutzomyia* sp. (Psychodidae) foi observado realizado repasto sanguíneo em exemplares de *T. Rapicauda* e *P. fenestratus* bem como outras espécies de répteis e anfíbios (Figura 68).

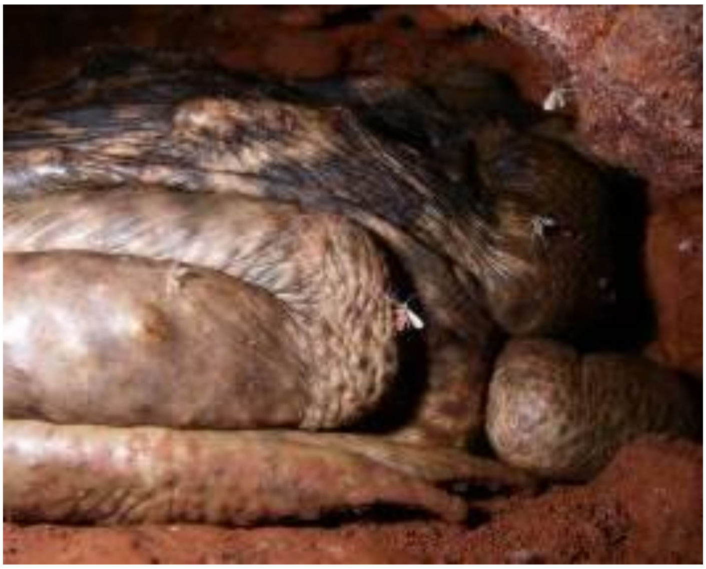

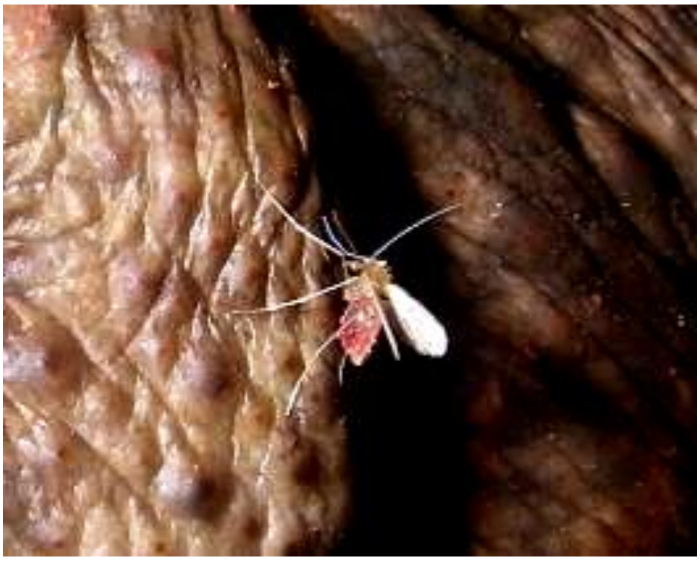

Figura 68 - *Lutzomyia* sp. (Diptera: Psychodidae) realizando repasto sanguíneo em Anuro (Bufonidae indet.). Zoom na figura abaixo.

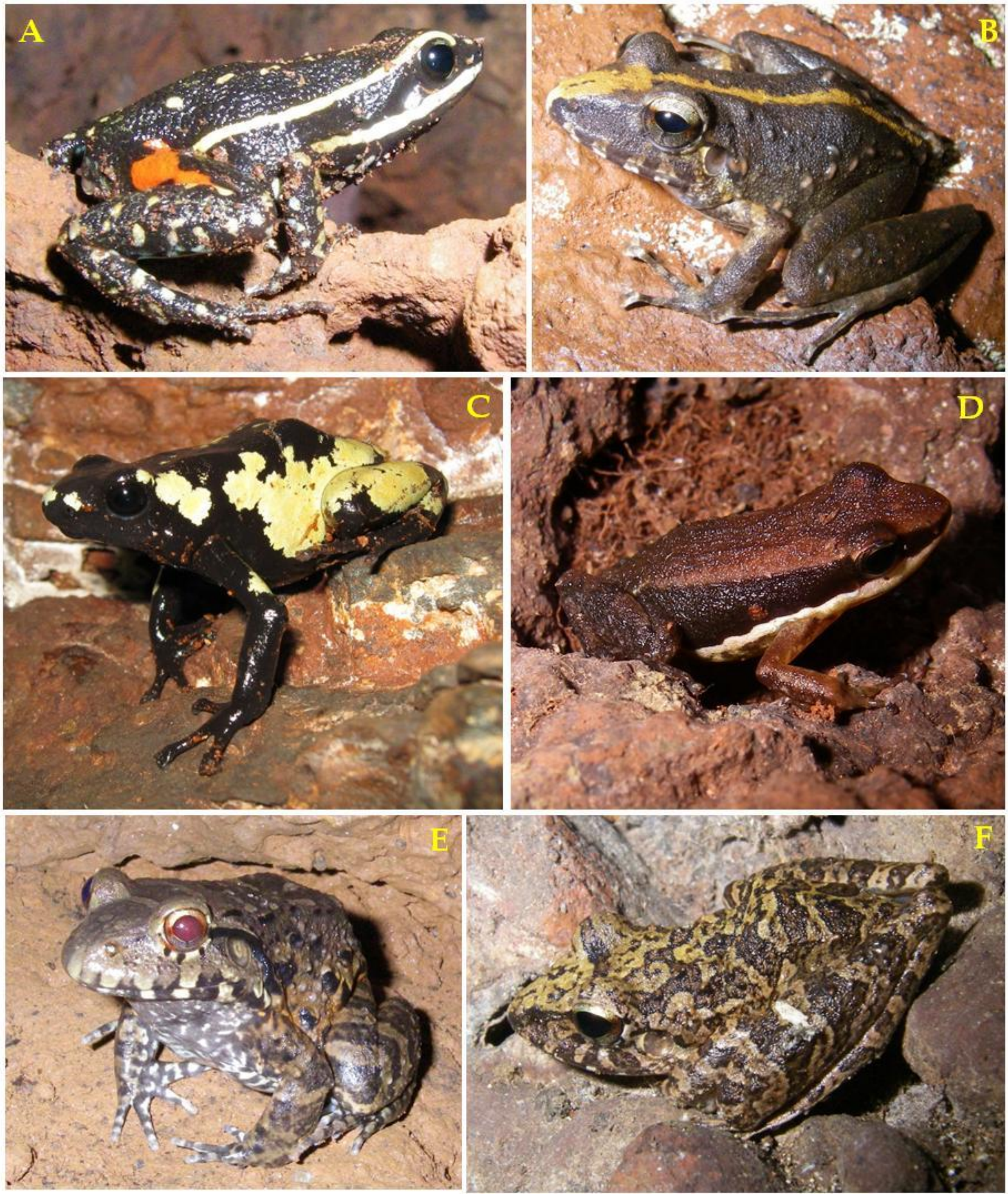

Figura 69 - a) Anura: Dendrobatidae (*Ameerega* sp.); b) Anura: Leptodactylidae (*Eleutherodactylus* sp.); c) Anura: Dendrobatidae; d); e) Anura: Leptodactylidae (*Leptodactylus* cf. *labyrinthicus*); f) Anura: Leptodactylidae (*Pristimantis* cf. *fenestratus*).

Vários grupos troglóxenos e troglófilos podem ser destacados, seja pela riqueza incomum observada ou pela raridade em sistemas subterrâneos. Dentre estes, optou-se por exemplificar apenas algumas espécies.

Freqüentemente, as Ordens mais ricas em espécies em inventários de fauna subterrânea brasileira compreendem Coleoptera, Diptera e Araneae. Surpreendentemente, no presente

trabalho, a Ordem Hymenoptera destacou-se como a segunda Ordem mais rica, com 187 espécies coletadas. Tal número “inesperado” deveu-se à participação da família Formicidae, que, sozinha, compreendeu 122 espécies (65,2% do total de himenópteros coletados). Embora formicídeos não sejam incomuns em cavernas brasileiras, este elevado número de espécies é certamente surpreendente. É prematuro tentar estabelecer qualquer hipótese para explicar a elevada riqueza desta família nas cavernas da área. No entanto, estudos futuros que versem sobre esta questão certamente merecem ser desenvolvidos.

Os onicóforos (Filo Onycophora) compreendem um grupo de organismos muito pouco estudados no país. Durante décadas, acreditou-se que compreendessem um grupo extremamente raro, o que vem sendo desacreditado nos últimos anos. Aparentemente, a suposta raridade do grupo decorria da falta de coletas e mesmo de especialistas no grupo. Tais organismos são predadores noturnos, alimentando-se de outros invertebrados. Passam o dia abrigados em troncos, espaços sob rochas e galerias subterrâneas (como frestas), o que faz com que seja difícil visualizá-los. Embora sejam pouco comuns na maioria das regiões brasileiras, na região Norte, são relativamente frequentes. Das 24 espécies que ocorrem no Brasil, 12 encontram-se na região amazônica (Sampaio-Costa, *et al*., 2009). Existem registros de somente duas espécies troglóbias deste grupo: *Spelaeoperipatus spelaeus* da Jamaica e *Paripatopsis alba* da África do Sul. Recentemente, foi relatada a ocorrência de uma espécie com características troglomórficas em uma caverna no Mato Grosso do Sul. Tal espécie encontra-se em trabalho de descrição.

Outro grupo interessante encontrado em algumas cavernas compreende os Opilioacarida. Organismos desta Ordem conservam muitos aspectos morfológicos que fazem deles o grupo mais primitivo dentro dos ácaros que são encontrados, atualmente, vivendo sobre a terra (Grandjean, 1936; Krantz & Walter, 2009). Tal ordem encontra-se largamente distribuída ao longo de todas as zonas tropicais e temperadas do mundo. Oito de seus dez gêneros conhecidos estão presentes no Velho mundo, e apenas dois, os gêneros *Neoacarus* e *Caribeacarus,* são encontrados nas Ámericas do Norte, Central e Sul (Vázquez & Klompen, 2002; Vázquez & Klompen, 2009).

Para a América do Sul, são conhecidas as espécies *Neoacarus ojastii* Lehtinen 1980 com ocorrência para a Venezuela, e *Neoacarus platensis* Silvestri 1905 que ocorre na região sudeste do Brasil, Uruguai e Norte da Argentina (Silvestri, 1905; Van der Hammen, 1969; Lehtinen, 1980; Leclerc, 1989). Entretanto, a ocorrência de *Neoacarus* no Brasil é representada por inúmeras outras espécies ainda não descritas, distribuídas por todas as regiões do país, com a exceção da região sul (Vázquez & Klompen, 2002; Bernardi *et al*., 2009). Aparente, o número de espécies não descritas para o Brasil deve chegar se aproximar de nove espécies até o momento (Bernardi, *comunicação pessoal*).

Outro grupo interessante encontrado em algumas cavernas compreende os Holothyrida (Figura 70). Esta Ordem de ácaros se caracteriza por apresentar espécies de grande tamanho corporal (2 a 7 mm), por serem fortemente esclerotizados e terem, como habitat, o folhiço presente no solo. Estes ácaros ainda são muito pouco conhecidos, mas sabe-se que suas espécies compõem uma das ordens mais primitivas dentro do grupo dos Anactinotrichidas (Krantz and Walter, 2009).

O primeiro táxon, *Holothyrus coccinella,* foi descrito por Gervais (1842). Atualmente, a ordem é composta por 3 famílias (Allothridae, Holothyridae e Neothyridae), totalizando 13 gêneros e 23 espécies (Krantz and Walter, 2009; Klompen, 2010).

A distribuição geográfica da ordem Holothyrida está restrita às Ilhas do Mar do Caribe e do Oceano Indico, a região da Australásia e as América Central e do Sul. Dentre as espécies descritas para a Região Neotropical, três foram encontradas na porção norte da América do Sul (Krantz and Walter, 2009; Komplen, 2010). O primeiro táxon descrito foi *Neothyrus ana* Lehtinen (1981), presente na Amazônia Peruana. A segunda espécie, *Diplothyrus schubarti*, foi descrita a partir de exemplares encontrados no Brasil em uma floresta primária na Reserva Florestal Adolpho Ducke, localizada no município de Manaus, estado do Amazonas, região norte do país (Lehtinen, 1981)). A terceira, recentemente descrita, é a espécie *D. lecorrei*, encontrada na Amazônia da Guiana francesa, região norte do continente sul americano (Komplen, 2010).

Até o presente momento, a distribuição conhecida da espécie brasileira (*Diplothyrus schubarti*) restringia-se ao estado do Amazonas. Desta forma, os registros deste relatório ampliam consideravelmente a distribuição desta espécie.

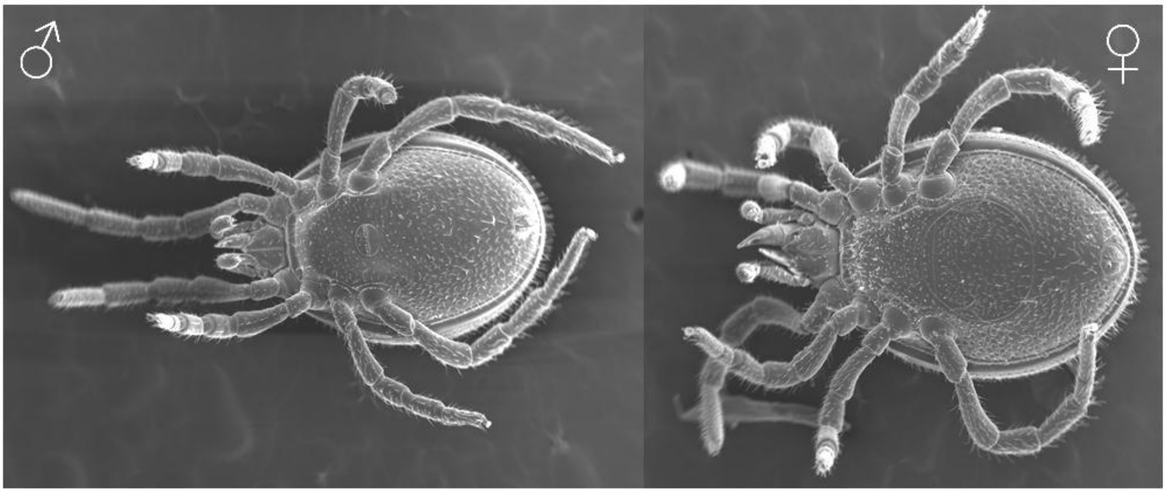

Figura 70 - *Diplothyrus schubarti* (Holothyrida): macho (à esquerda) e fêmea (à direita).

Outro interessante registro compreende a espécie *Cryptocellus tarsilae*, encontrada em algumas cavernas da área (Figura 71). Esta espécie pertence à pequena Ordem de

aracnídeos denominada Ricinulei. Esta Ordem compreende organismos pouco estudados, apresentando, atualmente, cerca de 60 espécies descritas, todas elas pertencentes a uma única família, Ricinoididae. Mesmo sendo pouco diversificados em termos de número de espécies, os ricinulídeos podem ser bastante abundantes em algumas áreas da Amazônia (Adis *et al*., 1989; Barreiros, et *al*., 2005). Na América do Sul ocorrem cerca de 20 espécies, poucas delas distribuídas na Amazônia Oriental. A espécie *Cryptocellus tarsilae* foi descrita a partir de poucos exemplares coletados em uma pequena caverna ferruginosa (N5Sul-07) localizada na Flona Carajás. Esta espécie não possui quaisquer caracteres troglomórficos, fato que fez com que os autores de sua descrição questionassem sua real afinidade com o meio subterrâneo. No entanto, sua ocorrência relativamente freqüente nas cavernas ferruginosas de Serra Leste mostra que a espécie possui pouca probabilidade de ser acidental. No entanto, estudos específicos devem ser conduzidos com esta interessante espécie no intuito de desvendar suas reais relações com as cavernas da área.

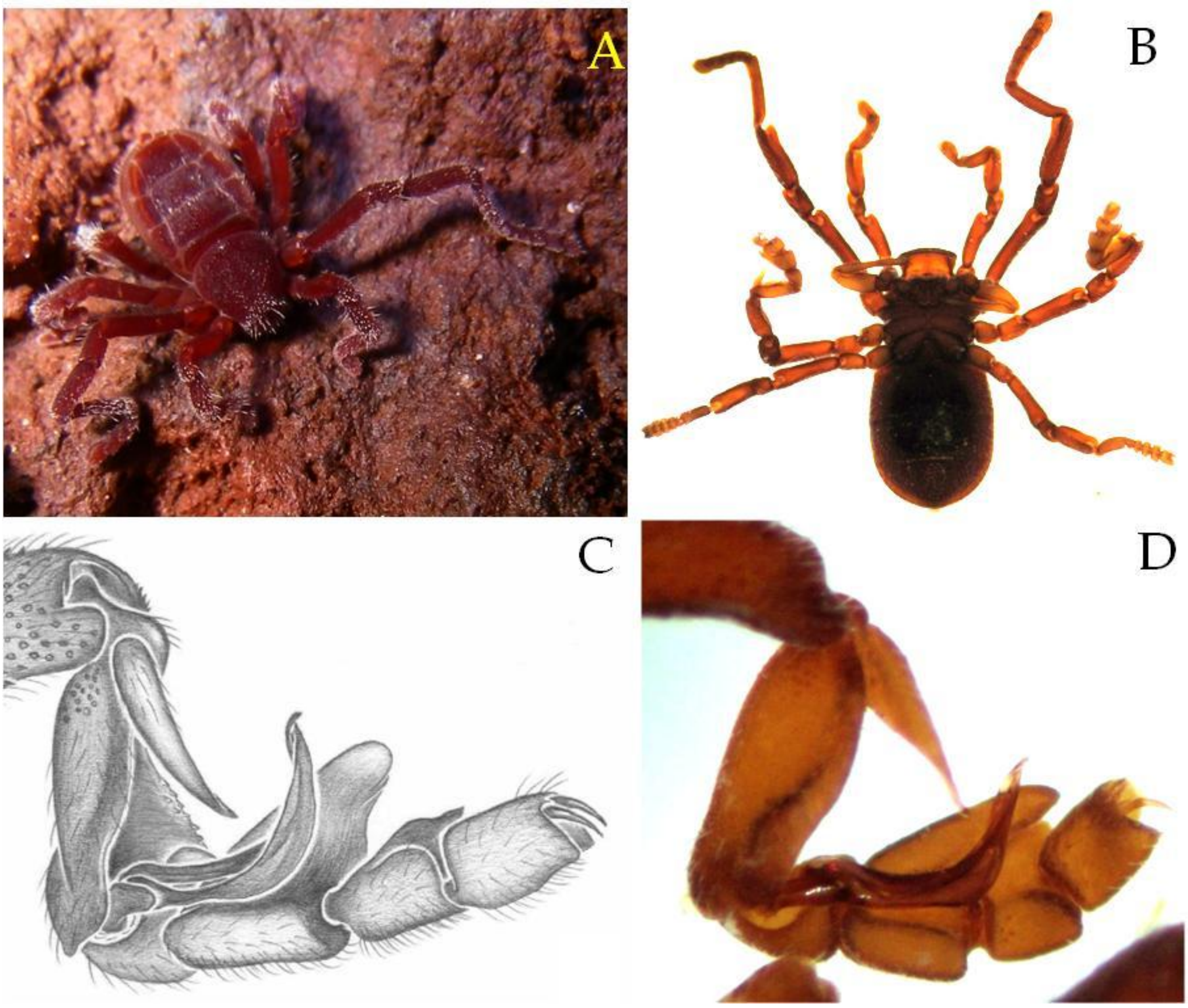

Figura 71 - *Cryptocellus tarsilae* (Ricinulei): (A) Detalhe de um espécime vivo; (B) detalhe de um espécime fixado (vista ventral); (C) Ilustração das modificações do terceiro par de patas de um macho relacionada à transferência de espermatóforo. Estas estruturas são fundamentais para a identificação da espécie (ilustração retirada do artigo de descrição da espécie); (D) Fotografia da terceira perna de um macho coletado em serra leste. Comparar com a ilustração da descrição.

5.4.3.1 Espécies troglomórficas

Foram encontradas 60 espécies troglomórficas, todas muito provavelmente troglóbias. Tais organismos pertencem aos seguintes taxa: Nematomorpha (Gordioidea), Gastropoda (Systrophiidae), Turbellaria (Geoplanidae), Isopoda (Balloniscidae, Plathyarthridae, Styloniscidae), Trombidiforme (Rhagidiidae), Amblypygi (Charinidae), Schizomida (Hubbardiidae), Opiliones (Escadabiidae), Aranae (Ochyroceratidae, Oonopidae, Prodidomidae), Thysanura (Nicolletiidae), Diplura (Anajapygidae), Collembola (Cyphoderidae, Isotomidae, Tomoceridae), Hemiptera (Dipsocoridae, Thyreocoridae), Hymenoptera (Formicidae), Coleoptera (Carabidae, Dytiscidae, Eucnemidae, Pselaphidae, Ptylidae, Scydmaenidae, Staphylinidae), Polydesmida (Pyrgodesmidae), Glomeridesmida (Glomeridesmidae), Polyxenida (Lophoproctidae) e Spirostreptida (Pseudonannolenidae). O número de espécies é variável dentro de cada táxon apresentado acima. A listagem de espécies troglóbias encontradas em Serra Leste é mostrada na Tabela 22 presente no tópico "Análises de relevância", presente no final deste relatório.

Uma vez que o número de espécies troglóbias é extremamente elevado, o mesmo deve ser contextualizado em relação a outras áreas no país que apresentam grande concentração de espécies troglóbias.

Trajano & Bichuette (2010) em uma recente revisão da biodiversidade subterrânea de invertebrados no Brasil publicaram uma lista com aproximadamente 120 espécies troglóbias para o país, dos quais apenas 51 (42%) estão oficialmente descritas.

No entanto, tais autoras ignoraram completamente inúmeros registros de espécies troglóbias presentes em diversas regiões do país. No Estado de Minas Gerais, existem pelo menos 165 espécies conhecidas de troglóbios (Ferreira *et al*, 2009). Tal número encontra-se desatualizado, já se aproximando de 175 espécies (Ferreira, dados não publicados). Considerando-se os biomas brasileiros, para cavernas presentes na Mata Atlântica, existem pelo menos 180 espécies trglóbias conhecidas e para o Cerrado, 110 espécies troglóbias já foram encontradas até o momento (Ferreira *et al.*, 2009; Souza-Silva *et al.*, 2011).

De acordo com Trajano & Bichuette (2010), as áreas cársticas do Alto Ribeira (São Paulo) com 39 espécies, Chapada Diamantina (Bahia) com 15 espécies, Serra do Ramalho (Bahia) com 11 espécies e Serra da Bodoquena (Mato Grosso do Sul) com 14 espécies, representam as maiores concentrações de invertebrados troglóbios conhecidas para o País. No entanto, tais autoras mais uma vez ignoraram inúmeras áreas de extrema relevância, como o quadrilátero ferrífero (Minas Gerais - cerca de 60 espécies troglomórficas), o carste de Felipe Guerra (Rio Grande do Norte - com cerca de 55 espécies troglomórficas), e a região de Arcos-Pains-Doresópolis, que representa, atualmente, a maior concentração de troglóbios no país, com 79 espécies nesta categoria, das quais 78 ainda não descritas (Zampaulo, 2010).

Desta forma, percebe-se que a região de Serra Leste compreende, na atualidade, a segunda maior concentração de espécies troglóbias no Brasil. No entanto, reitera-se que inúmeras formações ferríferas não foram sequer inventariadas, podendo exibir um número bem maior de registros.

No entanto, merece menção a relação entre número de espécies troglóbias e o número de cavernas inventariadas em cada uma destas regiões. Obtendo-se esta razão, percebe-se que as importâncias contextuais se alteram significativamente (Tabela 10).

Tabela 10 - Número absoluto e relativo (número de espécies dividido pelo número de cavernas amostradas) de espécies troglóbias em algumas regiões do país.

Região	Troglóbios	Cavernas amostradas	Troglóbios/caverna
Arcos-Pains-Doresópolis	79	282	**0,28**
Serra Leste	60	98	**0,61**
Felipe Guerra	56	34	**1,65**
Quadrilátero Ferrífero	60	35	**1,71**

Comparando-se algumas das áreas com maior riqueza de espécies troglóbias no país, destacam-se claramente a região do quadrilátero ferrífero (MG) e a região de Felipe Guerra (RN). No entanto, estas razões foram baseadas em dados disponíveis na literatura (incluindo dissertações). Especificamente para a região do quadrilátero ferrífero, vários inventários já foram realizados em outras cavernas, mas infelizmente não tivemos acesso aos dados. Estas razões, desta forma, podem ser alteradas com a inclusão de dados referentes a mais cavidades em cada uma destas áreas. Algumas das espécies troglóbias encontradas nas cavernas de Serra Leste são mostradas na Figura 72.

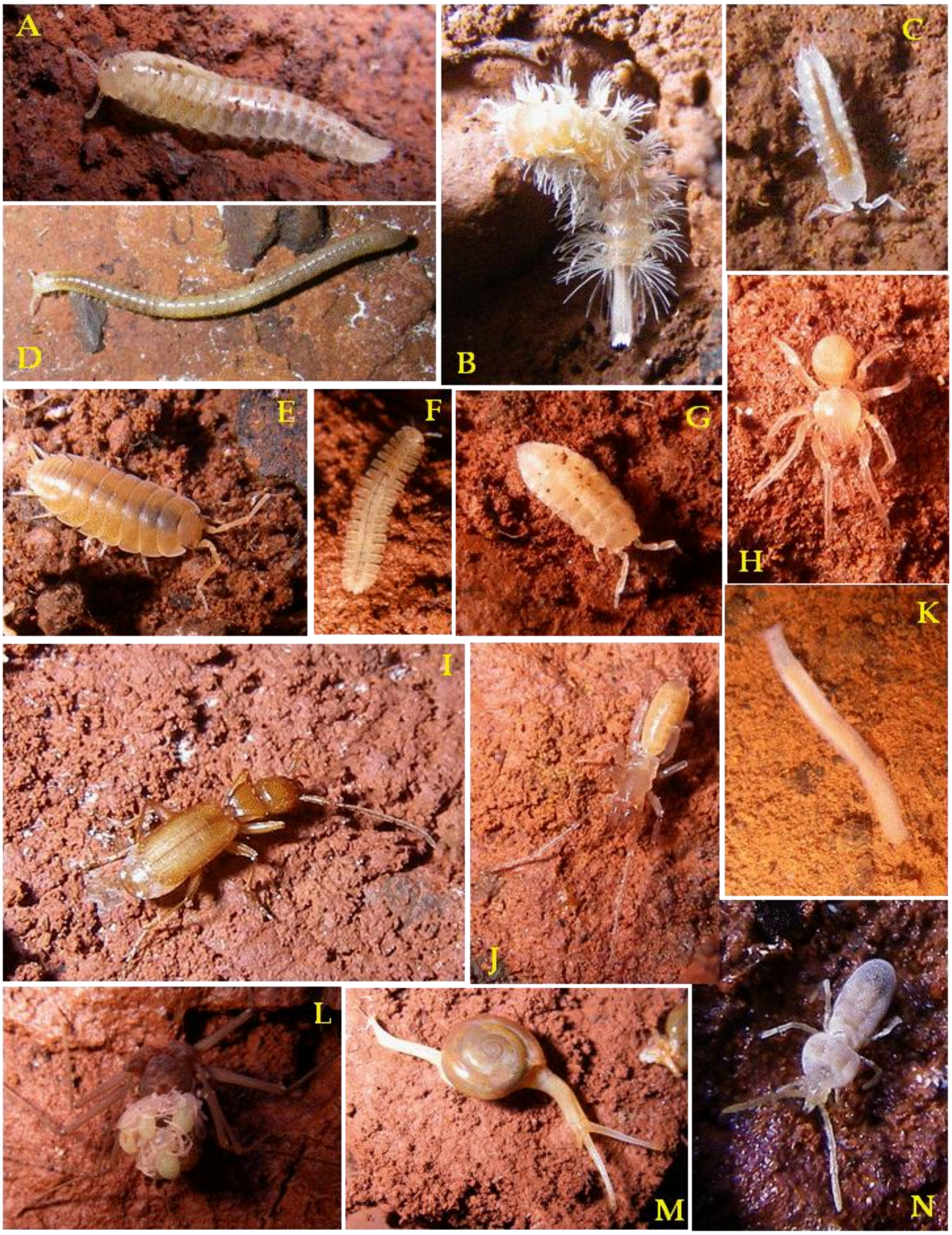

Figura 72 - a) Glomeridesmida: Glomeridesmidae (*Glomeridesmus* sp.); b) Polyxenidae: Lophoproctidae; c) Isopoda: Styloniscidae; d) Spirostreptida: Pseudonannolenidae; e) Diplopoda: Balloniscidae; f) Polydesmida: Pyrgodesmidae; g) Isopoda: Platyarthridae; h) Araneae: Prodidomidae; i) Coleoptera: Carabidae (*Coarazuphium* sp.); j) Schizomida: Hubbardiidae; k) Planaria; l) Amblypygi: Charinidae (*Charinus* sp.); m) Gastropoda: Systrophiidae (*Happia* sp.); n) Acari: Trombidiforme.

Algumas espécies troglóbias serão destacadas a seguir, seja pela riqueza incomum observada ou pela raridade em sistemas subterrâneos.

Os ácaros Prostigmata compreendem uma das espécies mais troglomórficas desta Ordem já encontrada em cavernas do país. Embora os troglomorfismos específicos para este grupo não sejam bem definidos, as características troglomórficas reconhecidas genericamente para artrópodes são bem nítidas no espécime encontrado (Tabela 2).

Para outros aracnídeos (como Palpigradi), o alongamento corporal e dos apêndices, a redução da pigmentação e a redução no número e tamanho de cerdas de cobertura compreendem troglomorfismos bem definidos. Todos estes caracteres foram observados no espécime encontrado na gruta do Rio Preto.

Além disso, comparando-se o indivíduo encontrado com outras espécies também troglomórficas já observadas em outras cavernas do país, confirma-se claramente a condição de troglomorfismo mais avançado na espécie de Serra Leste (Figura 73 D). As espécies deste grupo de ácaros são frequentemente bem pigmentadas, não apresentando corpos ou apêndices demasiadamente alongados (Figura 73 A).

Além dos ácaros Trombidiformes, foram também observados ácaros da família Rhagidiidae com caracteres troglomórficos (não ilustrados). Tal família é relativamente comum em cavernas brasileiras, compreendendo ácaros diminutos, anoftálmicos e despigmentados. Os troglomorfismos mais comuns para este grupo compreendem o alongamento dos apêndices locomotores, bem como o aumento do tamanho (e provavelmente função) do órgão ragidial, presente no primeiro par de pernas.

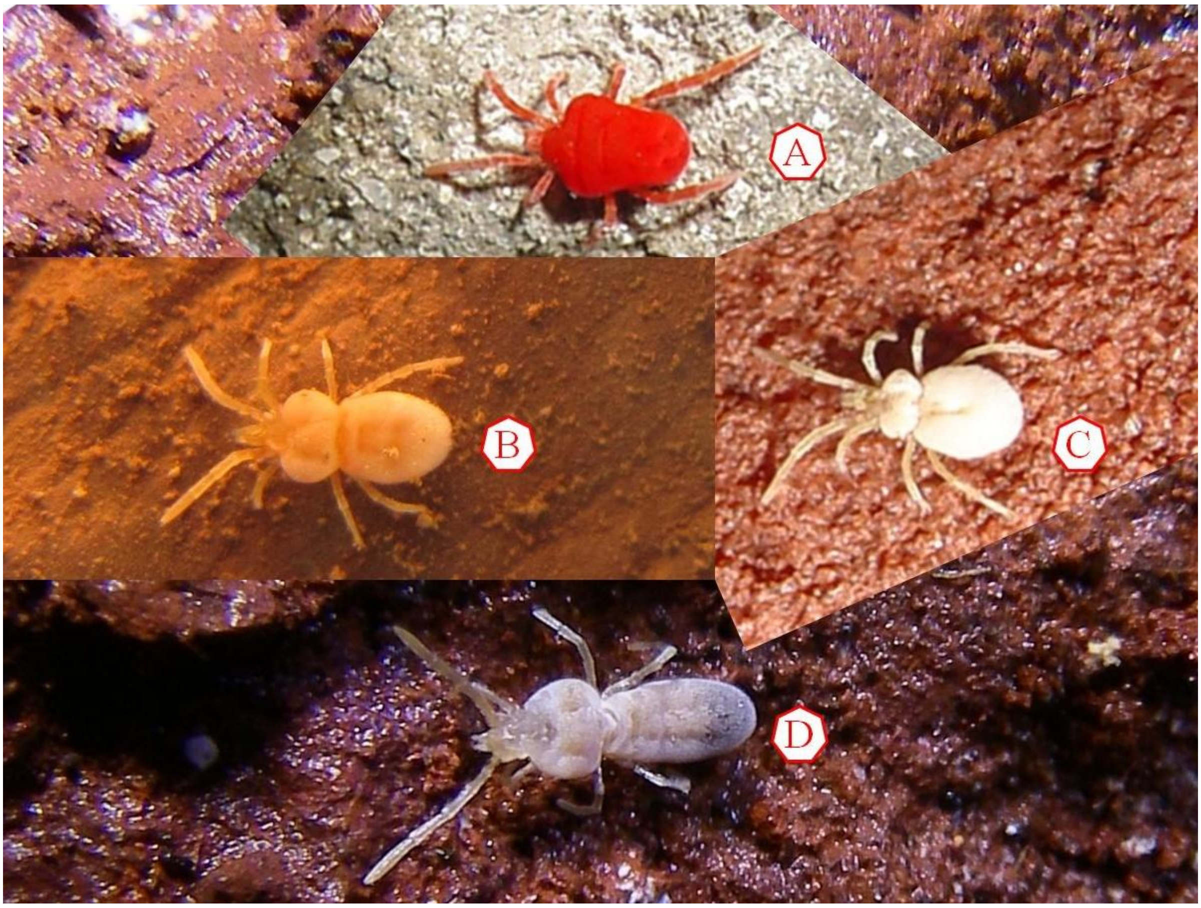

Figura 73 - Trombidiformes encontrados em cavernas, exibindo diferentes níveis de troglomorfismos: A) Trombidiforme não troglomórfico encontrado em uma caverna quartzítica de Ibitipoca, MG; B) Trombidiforme troglomórfico encontrado em uma caverna calcária em Cordisburgo, MG; C) Trombidiforme troglomórfico encontrado em uma caverna arenítica em Altinópolis, SP; D) Trombidiforme sp1 (Acari: Prostigmata) encontrado em cavernas de Serra Leste. Reparar, nos três últimos, a pronunciada despigmentação (comparar com a espécie A) e tendência ao alongamento corporal. De todas as espécies troglomórficas ilustradas na figura, a espécie encontrada no presente estudo compreende a mais modificada (reparar o alongamento corporal e do primeiro par de pernas).

Outro interessante registro compreende as espécies troglomórficas do gênero *Charinus*, encontradas em algumas cavernas da área. O gênero *Charinus* Simon, 1892 atualmente inclui 23 espécies descritas na região Neotropical, das quais sete são conhecidas para o Brasil (Weygoldt, 1972, 2000; Quintero, 1983; Giupponi & Kury, 2002; Baptista & Giupponi, 2002). Na América do Sul, estes organismos são dificilmente observados, em função de seus hábitos crípticos. No Brasil, a maioria das espécies abriga-se em espaços sob rochas ou troncos nas florestas tropicais, sendo ativos à noite. No Brasil, conhecem-se oficialmente somente duas espécies troglóbias: *Charinus troglobius* (Baptista & Giupponi, 2002) (da Bahia), e *Charinus eleonorae* (Baptista & Giupponi, 2003) (de Minas Gerais). Desta forma, percebe-se claramente a importância das espécies troglomórficas presentes em Serra Leste (certamente ainda não descritas pela ciência), seja pela condição em que se apresentam (três espécies em uma área relativamente reduzida) e também pelo seu "*status*" - no caso, troglomórficas. Além disso, destaca-se que nas outras duas espécies troglóbias brasileiras, os

ocelos mesiais sofreram regressão estrutural (apresentando-se reduzidos em *Charinus eleonorae* e ausentes em *Charinus troglobius*). Nas espécies troglóbias de Serra Leste, as séries oculares que sofreram regressão foram as laterais, diferentemente do que ocorreu com as previamente citadas. As ilustrações referentes a estas espécies compreendem as Figuras 74 e 75.

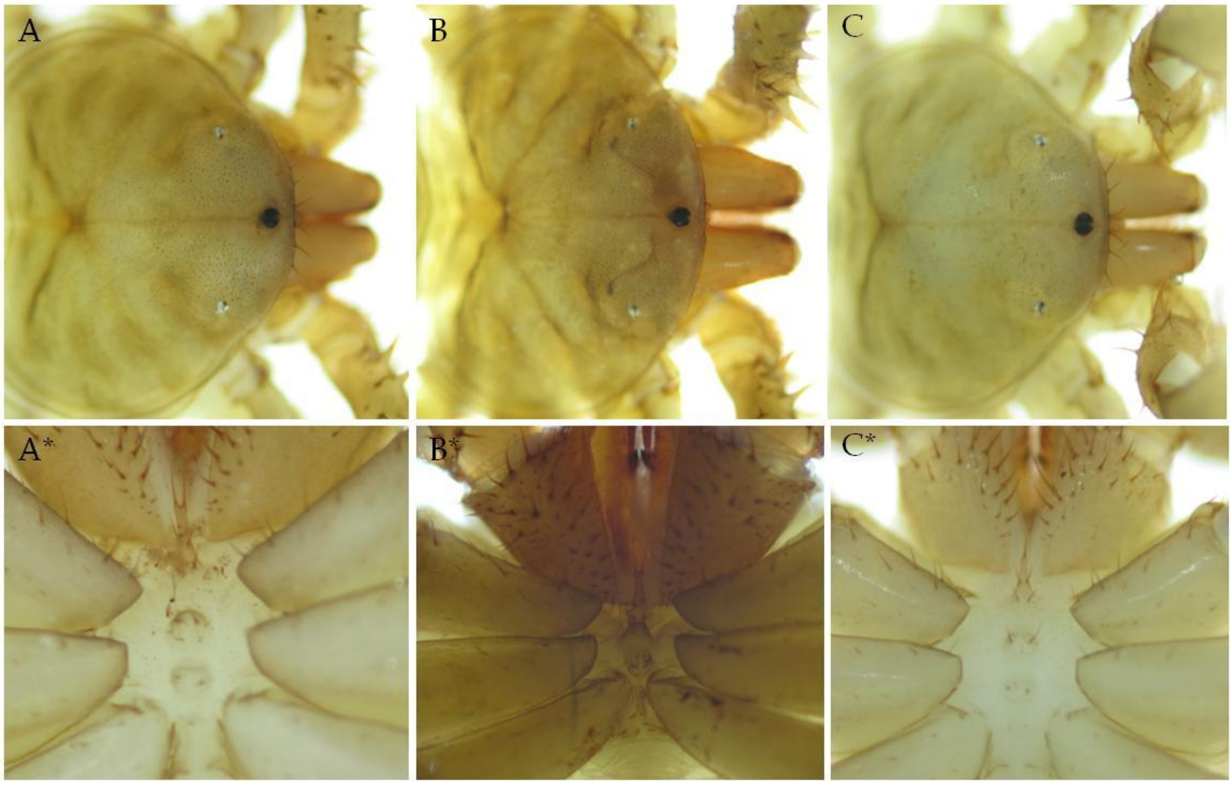

Figura 74 - Espécies troglmórficas de *Charinus* encontradas em Serra Leste: (A, A´) Prossoma em vista dorsal e porção ventral mostrando as placas do Sternum da espécie A; (B, B´) Prossoma em vista dorsal e porção ventral mostrando as placas do Sternum da espécie B; (C, C´) Prossoma em vista dorsal e porção ventral mostrando as placas do Sternum da espécie C; reparar, nas três espécies, a pronunciada redução da pigmentação e a regressão dos olhos laterais.

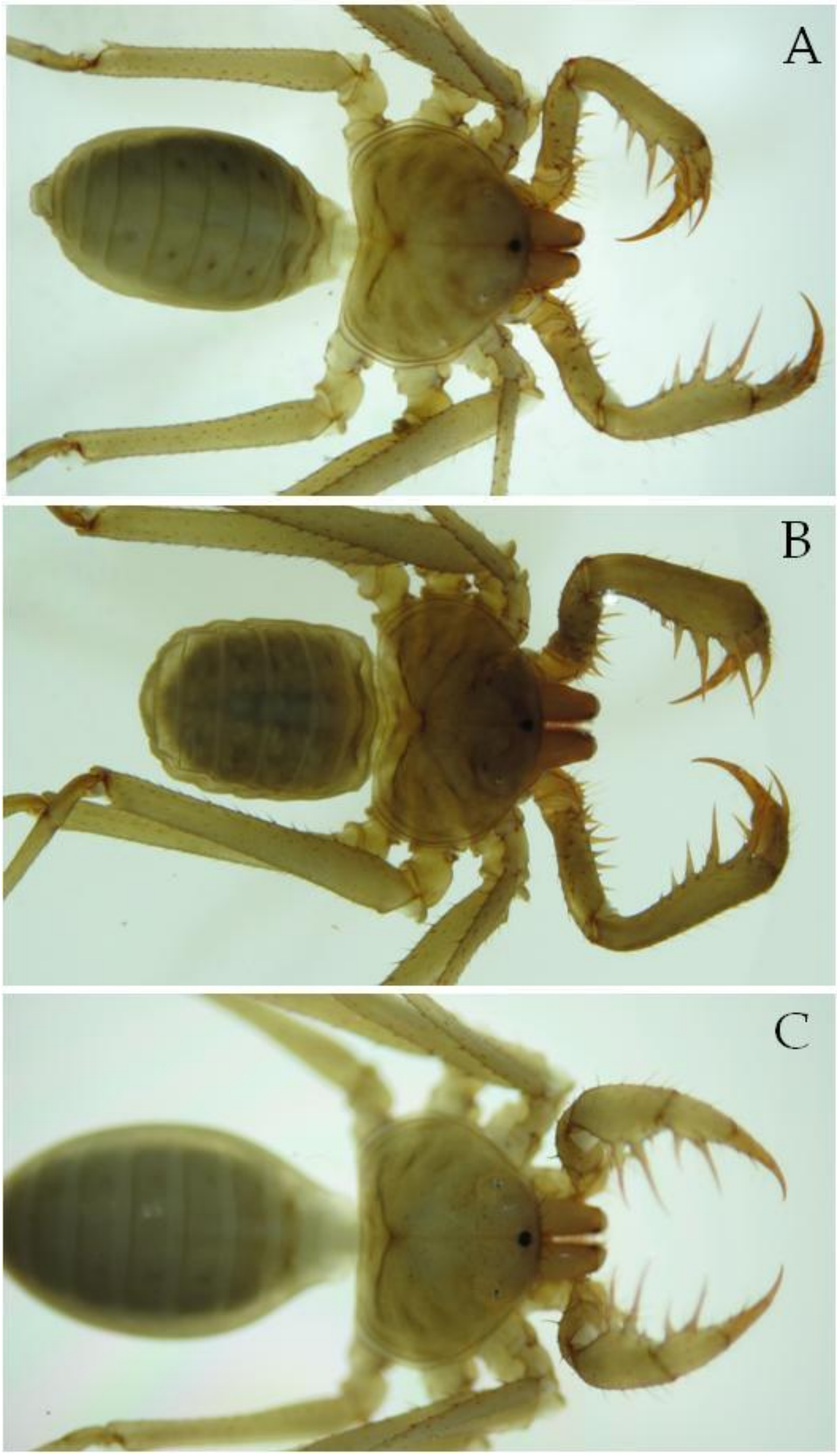

Figura 75 - Espécies troglmórficas de *Charinus* encontradas em Serra Leste: (A) espécie A; (B) espécie B; (C) espécie C.

Outro aspecto que merece menção é o tamanho reduzido da prole aliado ao grande tamanho dos filhotes como observado em algumas fêmeas de espécies troglomórficas de

Charinus de Serra Leste (Figura 76). Estas modoficações estão claramente ligadas à estratégia ecológica do tipo K, que em geral ocorre em espécies associadas a habitats mais estáveis. Os troglóbios são frequentemente K-estrategistas, e, do ponto de vista reprodutivo, esta estratégia envolve: i) redução do tamanho da prole; ii) aumento do tamanho corporal da prole; iii) sobreposição de geraçõpes e iiii) cuidado parental. Todas estas características podem ser observadas na espécie troglomórfica mostrada na Figura 76.

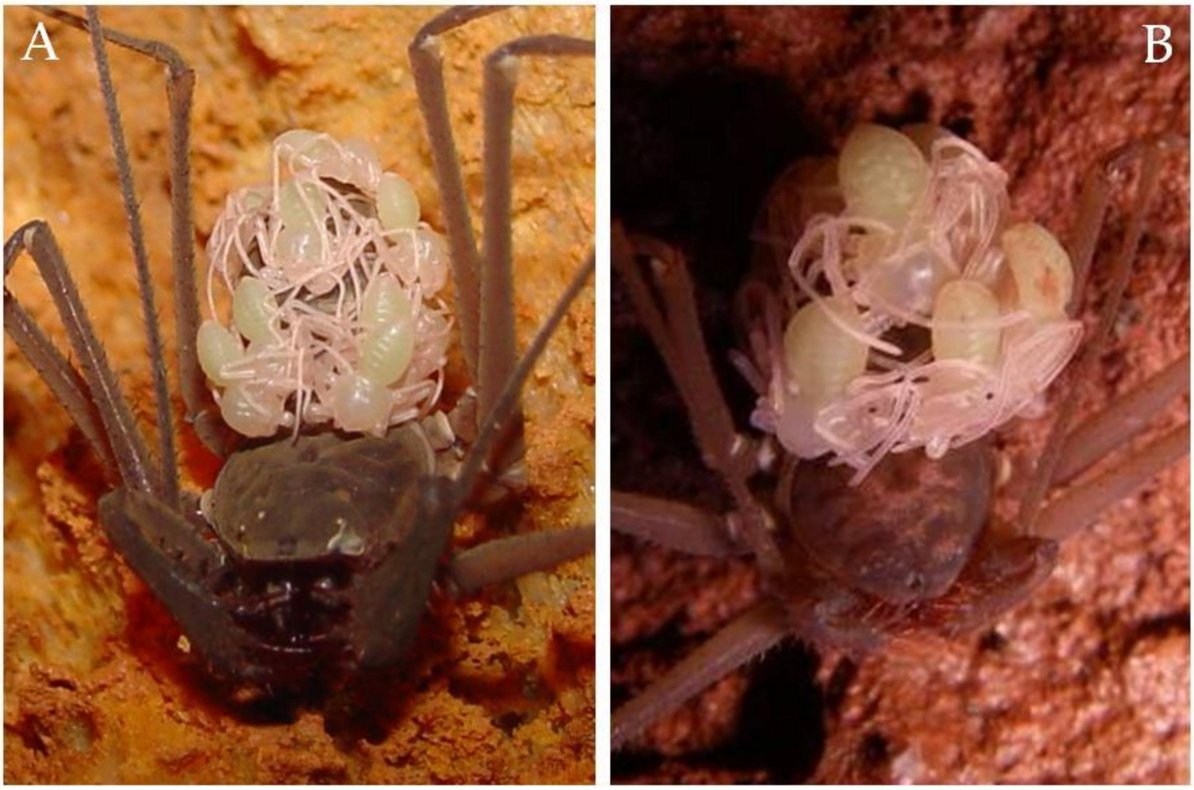

Figura 76 - Espécies do gênero *Charinus* encontradas em cavernas: (A) *Charinus acaraje* (não troglóbio) de cavernas da Bahia (reparar no pequeno tamanho corporal da prole e no número de filhotes); (B) *Charinus* spnA (troglomórfico), encontrado em Serra Leste (reparar no grande tamanho corporal da prole e no número reduzido de filhotes).

Dentre os opiliões, destacam-se algumas espécies troglomórficas da família Escadabiidae. Todas as espécies desta família são endêmicas do Brasil. Existem muitas espécies ainda não descritas desta família, que possui distribuição que vai deste a região costeira do Ceará até áreas mais secas no centro de Minas Gerais, onde algumas espécies cavernícolas podem representar exemplos de distribuição relicta. Dentre os troglomorfismos observados estão a redução da pigmentação tegumentar e a redução das estruturas oculares, que, em algumas espécies, foram completamente perdidas (Figura 77).

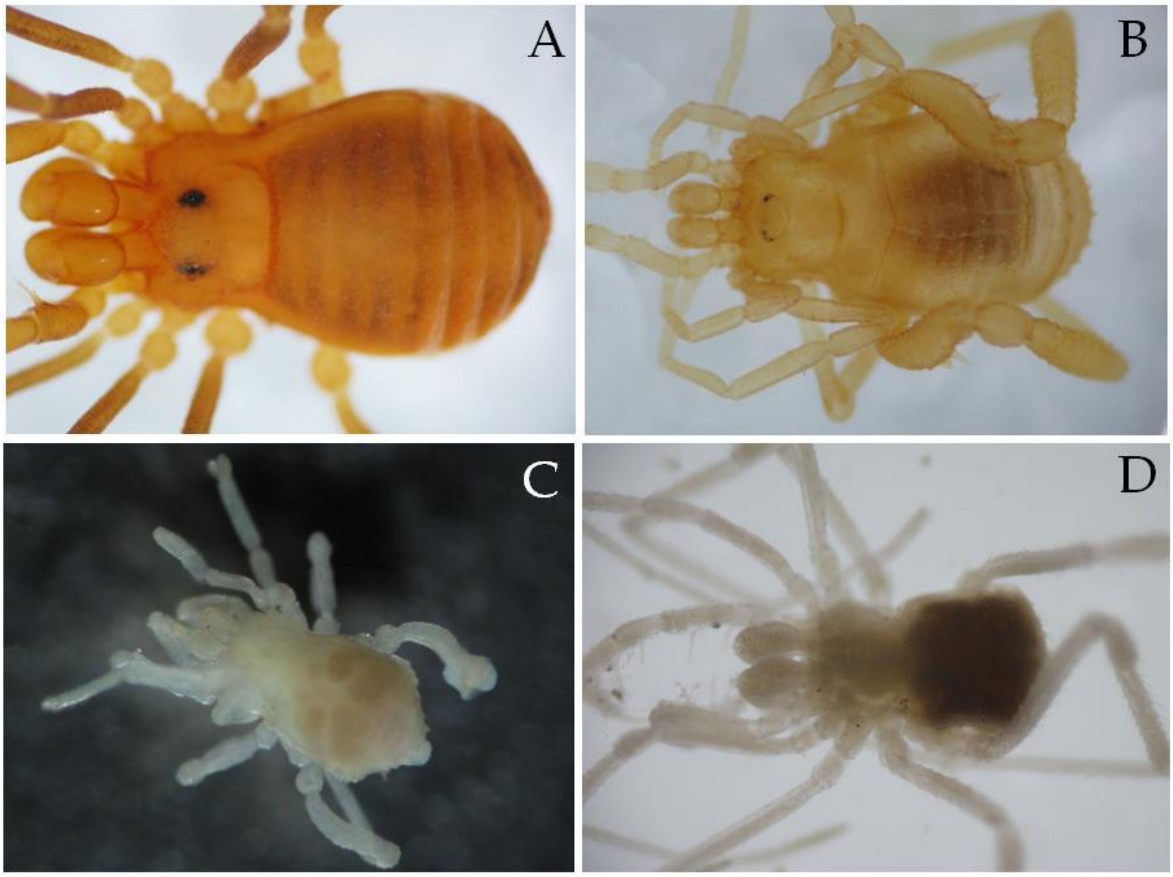

Figura 77 - Opiliões Escadabiidae encontrados em cavernas de Serra Leste: (A) espécie não troglomórfica (Escadabiidae sp2), com olhos e pigmentação bem desenvolvidos; (B) espécie troglomórfica (Escadabiidae sp5); (C) espécie troglomórfica (Escadabiidae sp6); (D) espécie troglomórfica (Escadabiidae? sp). Nos últimos, observar pronunciada redução da pigmentação e dos olhos (ausentes em (C) e (D).

Embora não represente a espécie mais troglomórfica da família Prodidomidae já encontrada no país, a aranha encontrada em Serra Leste também exibe troglomorfismos óbvios, como a despigmentação e anoftalmia (Figura 78).

Outras aranhas troglóbias desta família já foram descobertas em cavernas carbonáticas (na província de Arcos/Pains/Doresópolis e na gruta de Maquiné - *Lygromma ybiguara*), quartzíticas e ferruginosas (um novo gênero, em descrição). Todas estas espécies são também anoftálmicas, algumas delas apresentando uma condição mais avançada de troglomorfismo quando comparadas à encontrada neste estudo. O espécime coletado em Serra Leste é imaturo, o que infelizmente inviabiliza a descrição da nova espécie. Desta forma, é fundamental a execução de novos inventários na caverna para a coleta de espécimes adultos, para que a espécie possa ser formalmente descrita.

Além disso, foram observadas também aranhas troglomórficas da família Oonopidae (Figura 78 A) e Ochyroceratidae (Figura 79 A).

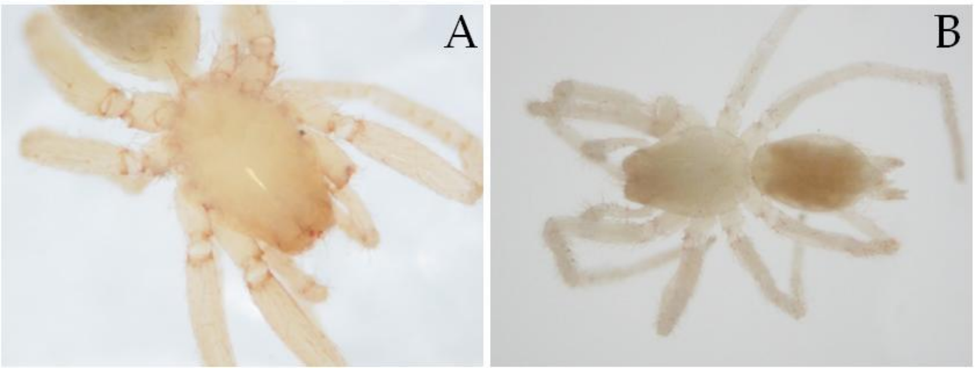

Figura 78 - (A) oonopidae sp9; (B) Prodidomidae sp1.

As aranhas Ochyroceratidae foram as mais representativas dentre as aracnhas troglóbias observadas em Serra Leste. Em alguns casos, além dos óbvios troglomorfismos, foram observadas especializações reprodutivas, como a redução da prole acompanhado do aumento corporal dos imaturos (Figura 79).

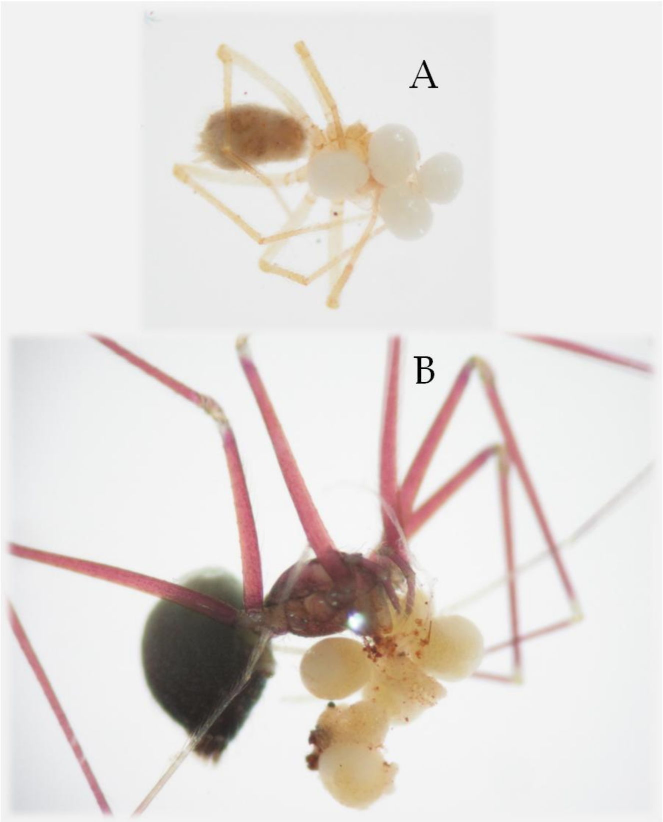

Figura 79 - Aranhas Ochyroceratidae encontradas em Serra Leste: (A) Ochyroceratidae troglóbia (B) Ochyroceratidae não troglóbia. As imagens estão em mesma escala. Reparar no tamanho dos ovos de ambas as espécies que, embora sejam equivalentes em volume, foram produzidos por aranhas de grande diferença de tamanho, o que indica o considerável aumento no tamanho corporal da prole na espécie troglóbia.

A ordem Schizomida é representada por aracnídeos pequenos, ágeis, de cutícula fina, coloração acinzentada, anoftálmicos, com pedipalpos preênseis e primeiro par de pernas sensorial (Armas, 2004). Atualmente, conhecem-se 258 espécies distribuídas em 46 gêneros (Harvey, 2007). No Brasil, A fauna de schizomidas é relativamente pouco conhecida (Santos *et al*., 2008). Apenas nove espécies foram registradas para o país (Harvey, 2007; Bonaldo & Pinto-da-Rocha, 2007), sendo uma introduzida na costa do Rio de Janeiro (Tourinho & Kury, 1999) e as restantes restritas a localidades de floresta amazônica (Cokendolpher & Reddell 2000; Reddell & Cokendolpher, 2002). Entretanto, Harvey (2007) sugere que a maior parte da diversidade do grupo está ainda a ser descoberta. O número de espécies brasileiras vem aumentando nos últimos anos, isto sugere que a baixa amostragem seja resultado da falta de estudos. Como as espécies de schizomidas geralmente apresentam distribuição restrita, espera-se que várias novas espécies da Amazônia sejam descobertas e descritas (Santos *et al*., 2008).

No presente estudo foram encontrados nove espécimes pertencentes à família Hubbardiidae, coletados em quatro cavidades. Com exceção da cavidade SL-031, onde seis indivíduos foram coletados, as ocorrências destes aracnídeos foram pontuais nas cavidades SL-022, SL-064 e SL-090.

A família Hubbardiidae é a mais amplamente distribuída no mundo, ocorrendo na Ásia, África, Américas e Oceania (Reddell & Cokendolpher, 1995). E assim como todos os Schizomida brasileiros, este pertence à subfamília Hubbardiinae Cook, 1899. As características não sexuais dos espécimes coletados (uma setae no processo anterior e metapeltídio não dividido) remetem ao gênero *Artacarus* Cook, que ocorre apenas na Libéria e possivelmente na Costa do Marfim. Entretanto, devido ao endemismo do grupo e pela falta da análise de caracteres sexuais tal identificação não pôde ser confirmada. De acordo com Santos (comunicação pessoal) o espécime talvez possa pertencer ao gênero *Surazomus*, mas a impossibilidade de realizar estudos mais detalhados não permitiu um diagnóstico preciso do material. No entanto, há a probabilidade de ser uma nova espécie e, talvez, um novo gênero.

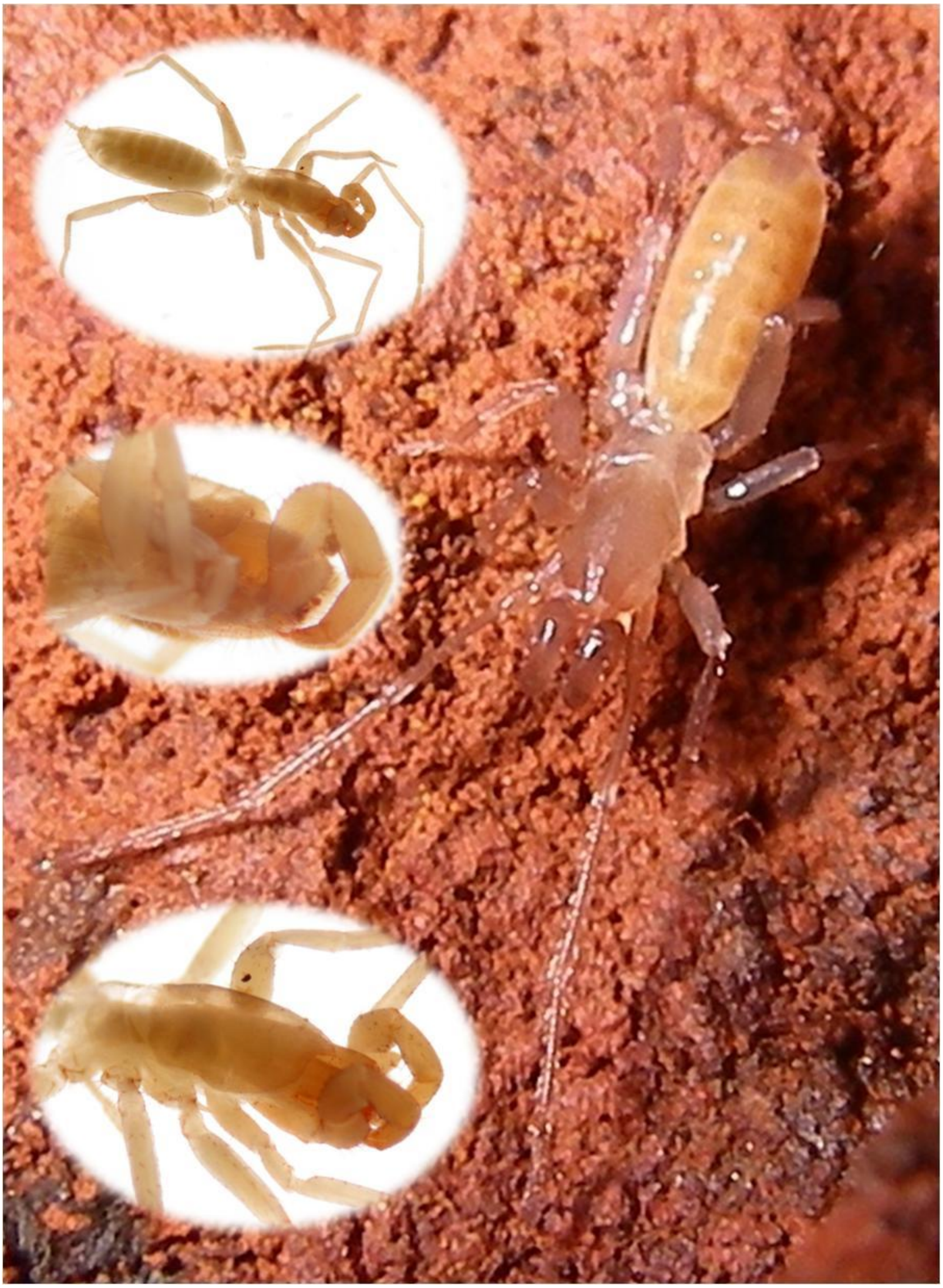

Figura 80 - Schizomida Hubardiidae troglomórfico encontrado em cavernas de Serra Leste.

A Ordem Glomeridesmida compreende diplópodes de reduzido tamanho, encontrados em regiões intertropicais. O único gênero observado no Brasil, *Glomeridesmus*, possui cerca de 30 espécies descritas. No entanto, muitas destas espécies foram descritas na primeira metade do século XX, e tais descrições são muito deficitárias. Os organismos desta Ordem são sempre anoftálmicos, embora sejam fortemente pigmentados (Figura 81 B). Estes organismos possuem órgãos de Tomosvary, com função sensorial. Duas espécies troglomórficas foram encontradas em cavernas de Serra Leste, uma delas mostrada na

Figura 81 A. Os troglomorfismos destas espécies incluem a total despigmentação do corpo, o alongamento corporal e de antenas e um aumento do tamanho dos órgãos de Tomosvary. A única espécie troglóbia conhecida para esta Ordem corresponde a *Glomeridesmus sbordonii*, de cavernas do México.

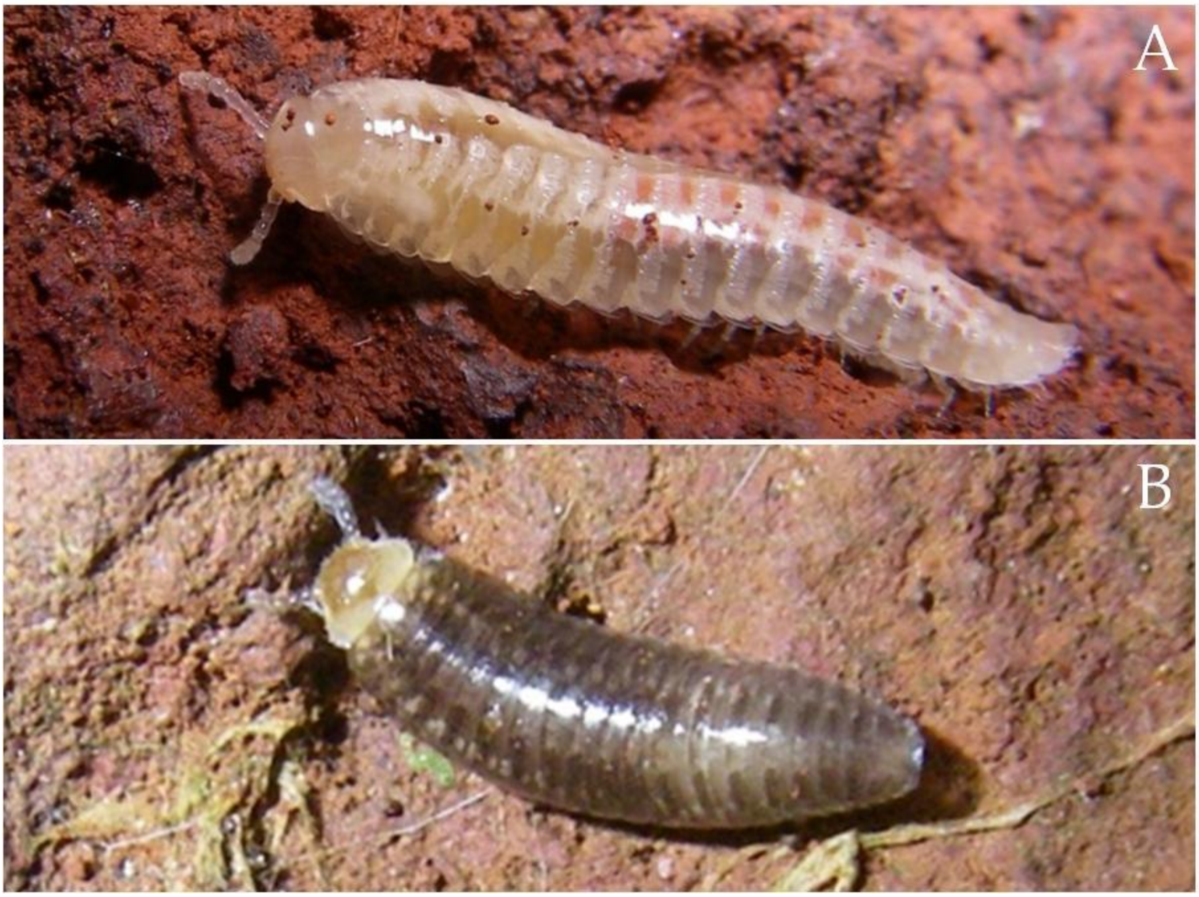

Figura 81 - Diplópodes Glomeridesmida encontrados em cavernas de Serra Leste: (A) Espécie troglóbia (reparar a total despigmentação e alongamento corporal e de antenas); (B) Espécie não troglóbia, fortemente pigmentada e com antenas comparativamente mais curtas.

Diplópodes são muitas vezes os componentes mais abundantes de comunidades terrestres detritívoras em cavernas. Polydesmida é a ordem de Diplopoda com maior número de famílias (cerca de 30 famílias). São notáveis por seus segmentos, geralmente em número aproximado de 20, com extensões laterais no lado dorsal do corpo e contendo glândulas que, muitas vezes, secretam cianeto (Gunn, 2004). Todas as espécies da ordem não possuem olhos. Algumas espécies troglóbias de Polydesmida têm sido descritas nas Américas, Europa, Japão, México e Brasil. Os troglomorfismos mais evidentes são a despigmentação do tegumento, descalcificação da cutícula e o alongamento das antenas e apêndices locomotores (Shear 1969; Holsinger & Culver 1988, Golovatch & Wytwer 2004; Lewis 2005; Golovatch *et al*., 2006).

Embora a sinopse da fauna de cavernas brasileiras (Pinto-da-Rocha, 1995) inclua um grande número de referências a diplópodos, muito pouco tem sido registrado em níveis inferiores a ordem ou família (Trajano *et al.*, 2000).

Pyrgodesmidae é uma família de Polydesmida pouco registrada em cavernas brasileiras. Diplópodos Pyrgodesmidae foram coletados em cavernas de Altamira/Itaiatuba, Bambuí, Vale do Ribeira e áreas graníticas. Alguns deles podem ser considerados troglóbios, apesar de que a maioria dos espécimes coletados mostra-se bem pigmentada. Existe atualmente, somente uma espécie troglombia descrita (*Yporangiella stygius* - Schubart, 1946) (Trajano *et al.*, 2000). Alguns Polydesmida troglomórficos de Serra Leste podem ser visualizados na Figura 82.

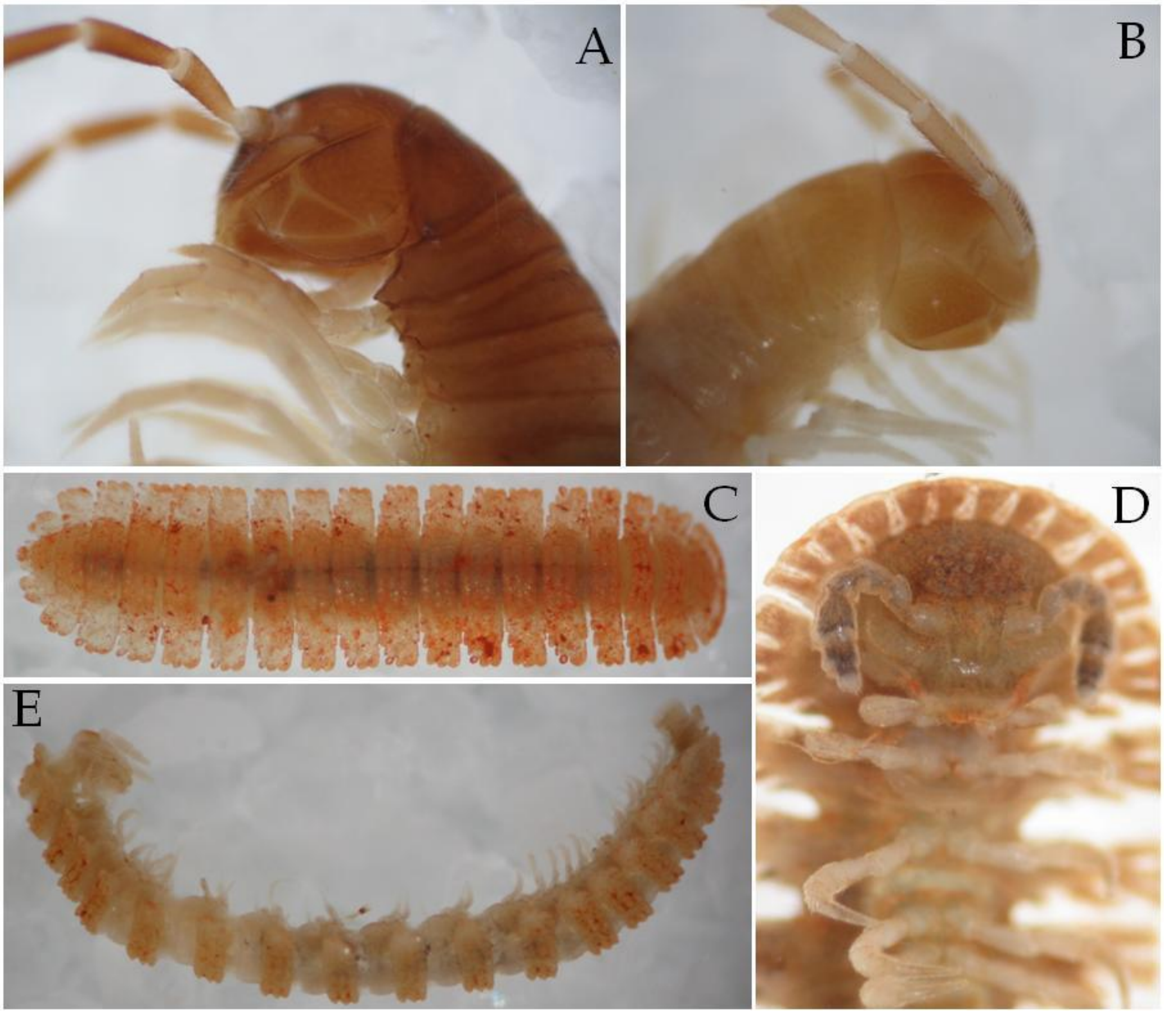

Figura 82 - (A) Polydesmida sp5 (detalhe da região cefálica em vista lateral); (B) Polydesmida sp6 (detalhe da região cefálica em vista lateral); (C) Pyrgodesmidae sp6 (vista dorsal); (D) Pyrgodesmidae sp7 (detalhe da região cefálica em vista ventral); (E) Pyrgodesmidae sp6 (vista lateral).

A ordem Spirostreptida (Diplopoda) possui artrópodes, terrestres detritívoros, muito diversificados. Entre as aproximadamente 1700 espécies descritas muitas vivem em

florestas, vegetações campestres, desertos e cavrenas. Os membros desta oedem possuem dois pares de pernas por segmentos, exceto nos segmentos de 1-4 onde ocorre somente 1 par por segmento. Os gonópodos, quando ocorrem, estão no sétimo segmento e são rudimentares. Os estipes da gnatoquilária são separados, mas as lâminas linguais são contíguas. Este grupo é predominantemente tropical.

No Brasil existe uma dominância na ocorrência de espécies de *Pseudonannolene* (Spirostreptida) em cavernas. Este gênero possui a maior riqueza de espécies troglófias (até 10 espécies, de um total de cerca de 45 espécies do gênero). No entanto, deve ressaltar que *Pseudonannolene* sp., são diplópodos relativamente grandes, portanto, facilmente encontrados em oposição aos grupos de menor tamanho corporal como o Oniscodesmidae, Pyrgodesmidae e Cryptodesmidae (Trajano *et al.*, 2000).

Nâo existem espécies troglóbias de Spirostreptida descritas para o Brasil. Nos Estados Unidos existe uma espécie de Spirostreptida descrita (Shear, 1969).

A espécie troglomórfica encontrada em Serra Leste pode ser visualizada na figura 83.

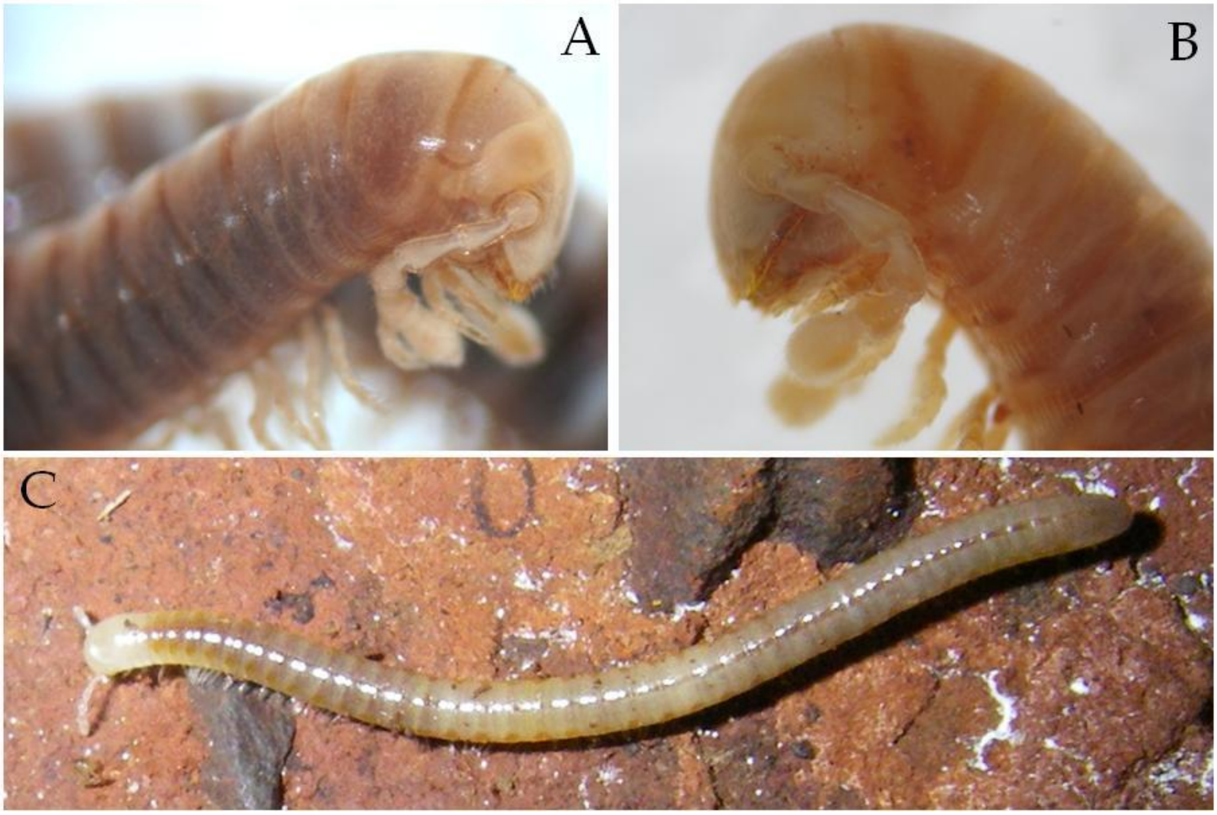

Figura 83 - Spirostreptida sp2: (A) Região anterior do corpo; (B) Detalhe da região cefálica em vista lateral, onde percebe-se a ausência de olhos; (C) Organismo vivo (reparar pronunciada despigmentação).

Outro grupo que se destaca compreende as formigas troglomórficas. Nesta família, ocorreram dois gêneros com espécies troglomórficas: *Hypoponera* e *Solenopsi*s.

O gênero *Hypoponera* sp. é caracterizado por apresentar olhos compostos presentes, atrofiados ou reduzidos a um único omatídeo; pernas relativamente curtas; cápsula cefálica geralmente mais longa que larga; mandíbulas mais ou menos triangulares ou semitriangulares e pigmentação corporal variável, sendo composto por formigas relativamente pequenas. As morfoespécies deste gênero consideradas troglomórficas no presente trabalho apresentam como principais caracteres a ausência das estruturas oculares; alongamento dos apêndices locomotores e sensoriais, apresentando escapo antenal largo e longo, o qual ultrapassa a cápsula cefálica; despigmentação melânica, alongamento e estreitamento corporal; e alongamentos cefálico e mandibular. A Figura 84 demonstra a espécie troglomórfica comparada a outra espécie do mesmo gênero sem características troglomórficas. É perceptível, na primeira, a pronunciada despigmentação e anoftalmia. Além disso, os apêndices (pernas e antenas) são consideravelmente longos quando comparadas a outras espécies não troglomórficas.

O gênero *Solenopsis* sp. é um gênero amplamente distribuído e altamente diverso por todo o mundo, o qual é composto por formigas pequenas, de coloração variável, olhos compostos usualmente presentes ou ocasionalmente reduzidos, mandíbulas triangulares a subtriangulares. Dentre os principais troglomorfismos observados na espécie encontrada neste trabalho, foram considerados a acentuada despigmentação melânica corporal, anoftalmia e alongamento cefálico. A Figura 85 demonstra a espécie troglomórfica comparada a outra espécie do mesmo gênero sem características troglomórficas. É perceptível, na primeira, a pronunciada despigmentação e anoftalmia.

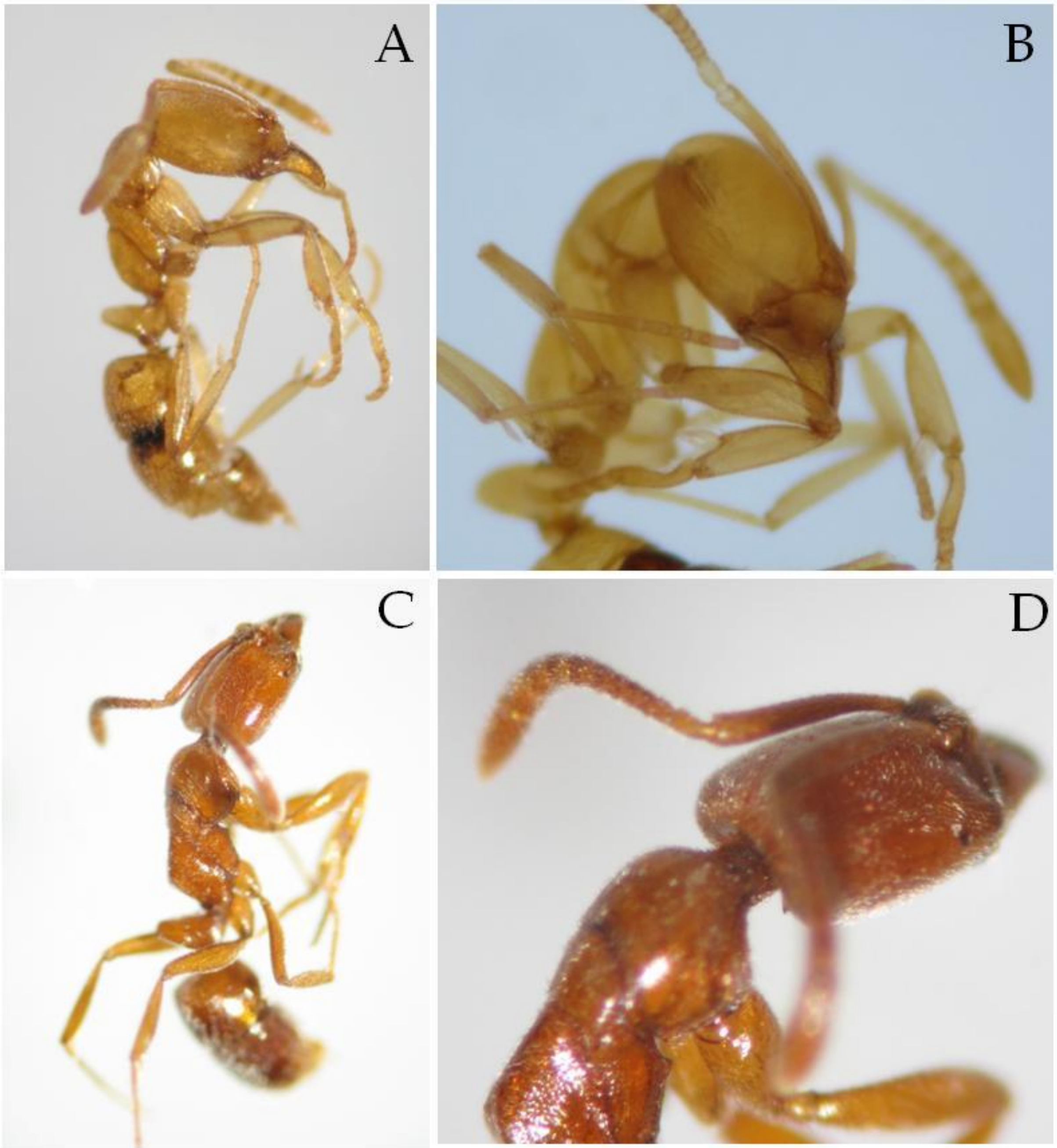

Figura 84 - Formigas do gênero *Hypoponera*: (A) *Hypoponera* sp4 (troglomórfica); (B) Detalhe da região cefálica de *Hypoponera* sp4 (reparar a despigmentação e anoftalmia); (C) *Hypoponera* sp6 (não troglomórfica); (D) Detalhe da região cefálica de *Hypoponera* sp6 (reparar a pigmentação e presença de olhos).

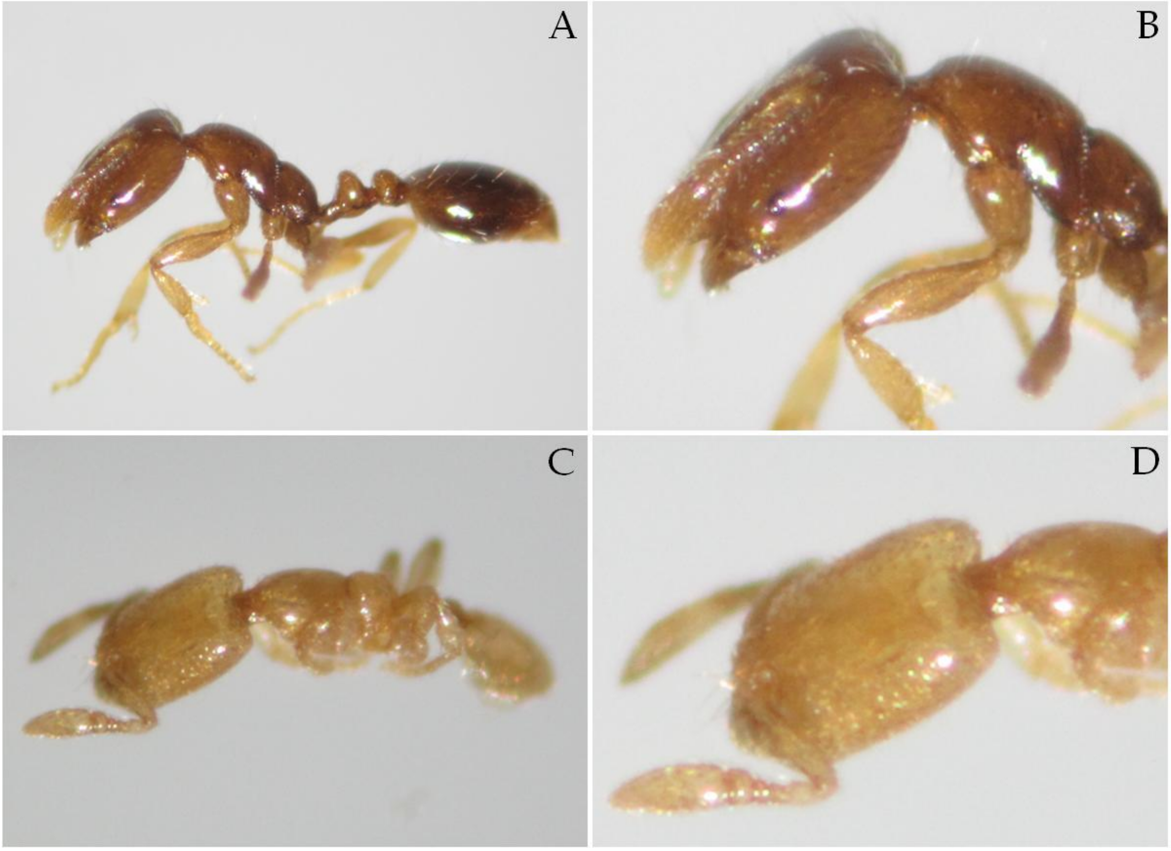

Figura 85 - Formigas do gênero *Solenopsis*: (A) *Solenopsis* sp3 (não troglomórfica); (B) Detalhe da região cefálica de *Solenopsis* sp3 (reparar a pigmentação e presença de olhos); (C) *Solenopsis* sp7 (troglomórfica); (D) Detalhe da região cefálica de *Solenopsis* sp7 (troglomórfica); (D) Detalhe da região cefálica de *Solenopsis* sp7 (reparar a despigmentação e anoftalmia).

Uma espécie que merece atenção compreende um organismo troglomórfico aparentemente pertencente à família Scutelleridae. Organismos desta família lembram, em um primeiro momento, coleópteros, mas suas características de Hemiptera são claramente notadas em exames mais minuciosos. Compreendem percevejos pequenos de coloração viva e brilhante e de formato elipsóide. Estes insetos são geralmente encontrados em gramados ou arbustos, se alimentando preferencialmente de néctar ou sementes. O único exemplar encontrado em Serra Leste compreende o primeiro registro de organismos desta família com caracteres troglomórficos, que correspondem a: i) completa anoftalmia; ii) redução marcante da pigmentação tegumentar. Uma imagem do exemplar de Serra Leste comparado a um típico Scutelleridae é mostrada na Figura 86. O fato surpreendente corresponde aos hábitos alimentares destes organismos, que são quase sempre fitófagos. Desta forma, acredita-se que esta espécie possa ter modificado seus hábitos alimentares, estando consumindo fluidos de raízes, bastante comuns nas cavernas da região.

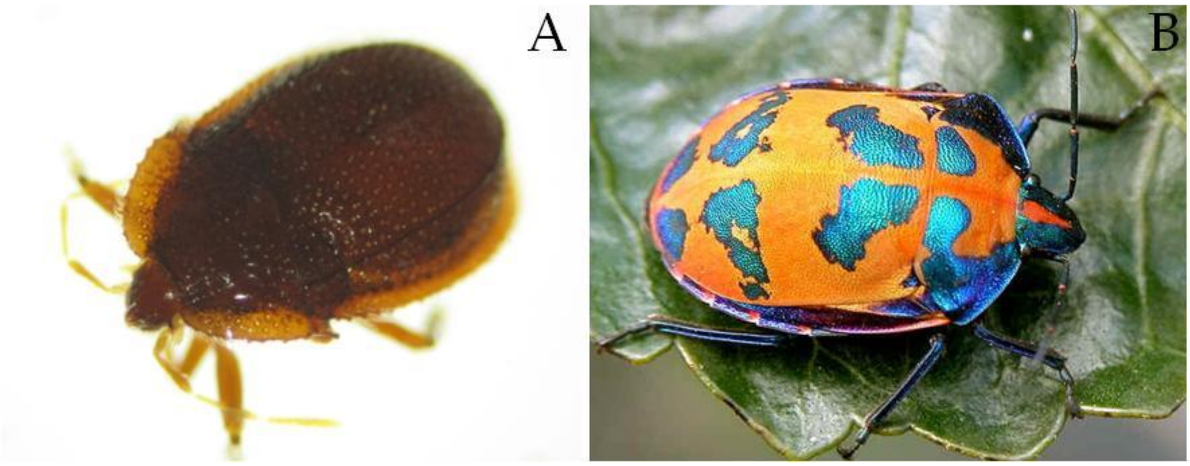

Figura 86 - Hemípteros Scutelleridae: (A) Scutelleridae sp1 (reparar pronunciada redução da pigmentação e anoftalmia). (B) Imagem de Scutelleridae (não troglóbio) retirada da internet. Reparar a pronunciada pigmentação (geralmente as espécies desta família são fortemente pigmentadas) e os grande olhos compostos.

Das aproximadamente 40 familias da ordem Coleoptera, 15 têm espécies exclusivamente subterrâneas. Dryopidae, Dytiscidae, Elmidae, Hydrophilidae e Noteridae aquáticos, bem como Carabidae, Cholevidae, Curculionidae, Histeridae, Pselaphidae, Staphylinidae, Merophysidae, Ptiliidae, Scydmaenidae e Tenebrionidae terrestres (Moldovan, 2004).

A ordem Coleoptera é incrivelmente bem sucedida em cavernas com aproximadamente 261 espécies em 21 gêneros (Splanger & Decu, 1998; Culver & White, 2004). Os troglomorfismos mais evidentes para a ordem são a despigmentação do tegumento, potencialização de órgãos sensoriais, redução de olhos, redução de asas e perda da capacidade de voar, mudança de hábitos locomotores e de trocas gasosas e mudanças de ambiente usado para empupar (Splanger & Decu, 1998).

Uma das primeiras mudanças morfológicas que ocorrem entre estes insetos durante a colonização das cavernas é a perda de pigmentação. A cutícula se torna mais fina, e a cor muda para um vermelho-amarronzado. A característica morfológica mais evidente é a falta ou redução dos olhos. Alongamento do corpo e antenas. As asas são completamente perdidas em algumas espécies, e os élitros se fundem. Além disso, em espécies altamente especializadas, sob os élitros está localizado um compartimento contendo ar para a regulação da umidade, causando uma pseudofisograstria. As alterações anatômicas internas são principalmente devido à escassez de alimentos. Espécies mais especializadas desenvolveram um corpo alargado contendo vesículas de gordura, proteínas e glicogênio que permitem a sobrevivência durante vários meses de jejum (Moldovan, 2004).

Dentre os coleópteros, um importante grupo associado às cavernas de Serra Leste compreendem espécies troglóbias de besouros Carabídeos pertencentes à tribo Zuphiini. Os

membros desta tribo são frequentemente alados e epígeos (Casale, 1998). O primeiro registro de um Zuphiini troglóbio foi o da espécie *Parazuphium tessai* (Godoy & Vanin, 1990), encontrada na gruta do Padre (município de Santana, Bahia). Esta espécie possui características comuns com os outros gêneros da tribo, *Parazuphium* Jeannel, 1942 e *Zuphium* Latreille, 1806. Alguns anos mais tarde, Moore (1994) descreveu dois novos generous de Zuphiini troglóbios: *Speozuphium* e *Speothalpius*, cada uma com uma nova espécie: *Speozuphium poulteri* e *Speothalpius grayi*, respectivamente. Ambas foram encontradas em cavernas de Nullarbor Plain, Austrália. Gnaspini e colaboradores (1998), então, propuseram um novo gênero, *Coarazuphium,* que incluía *Parazuphium tessai* e duas novas espécies, *C. cessaima* e *C. bezerra.* Estas três species diferem dos demais Zuphiini em relação aos seus primeiros antenômeros relativamente, margens cefálicas arredondadas e um par de setas laterais à região ocular (ao invés de anterior aos olhos). Outra importante característica do gênero é a presença de típicos troglomorfismos, como a redução ou ausência de pigmentação, olhos, asas e alongamento de apêndices locomotores e sensoriais.

Atualmente, existem cinco espécies descritas neste gênero: *C. tessai* encontrado na Gruta do Padre (Santana, BA), *C. cessaima* encontrado na Lapa do Bode (Itaetê, BA), *C. formoso* encontrado na Toca da Barriguda (Campo Formoso, BA); *C. bezerra* encontrado na Lapa do Bezerra (São Domingos, GO) e *C. pains* encontrado na gruta Tabocas III (Pains, MG).

Foram encontradas duas espécies deste gênero em cavernas de Serra Leste. Uma delas foi representada por um único indivíduo, o que demonstra sua raridade. Embora fosse mais despigmentada, esta espécie apresentava apêndices mais curtos (especialmente as antenas) quando comparada à espécie mais abundante. Esta última encontra-se em descrição pela equipe do Laboratório de Ecologia Subterrânea da Universidade Federal de Lavras (Figura 87). Um aspecto que merece menção é a ocorrência de uma espécie de fungo observada em associação com alguns exemplares da espécie mais abundante (Figura 87F). Como os organismos foram coletados e imediatamente imersos em álcool 70%, foi impossível a indução de crescimento deste fungo para proceder à sua identificação. Alguns indivíduos de *Coarazuphium* foram observados com estruturas (provavelmente hifas) agarradas a diferentes partes do corpo, como pernas e élitros. Eventualmente esta espécie pode ser entomopatogênica, o que é preocupante.

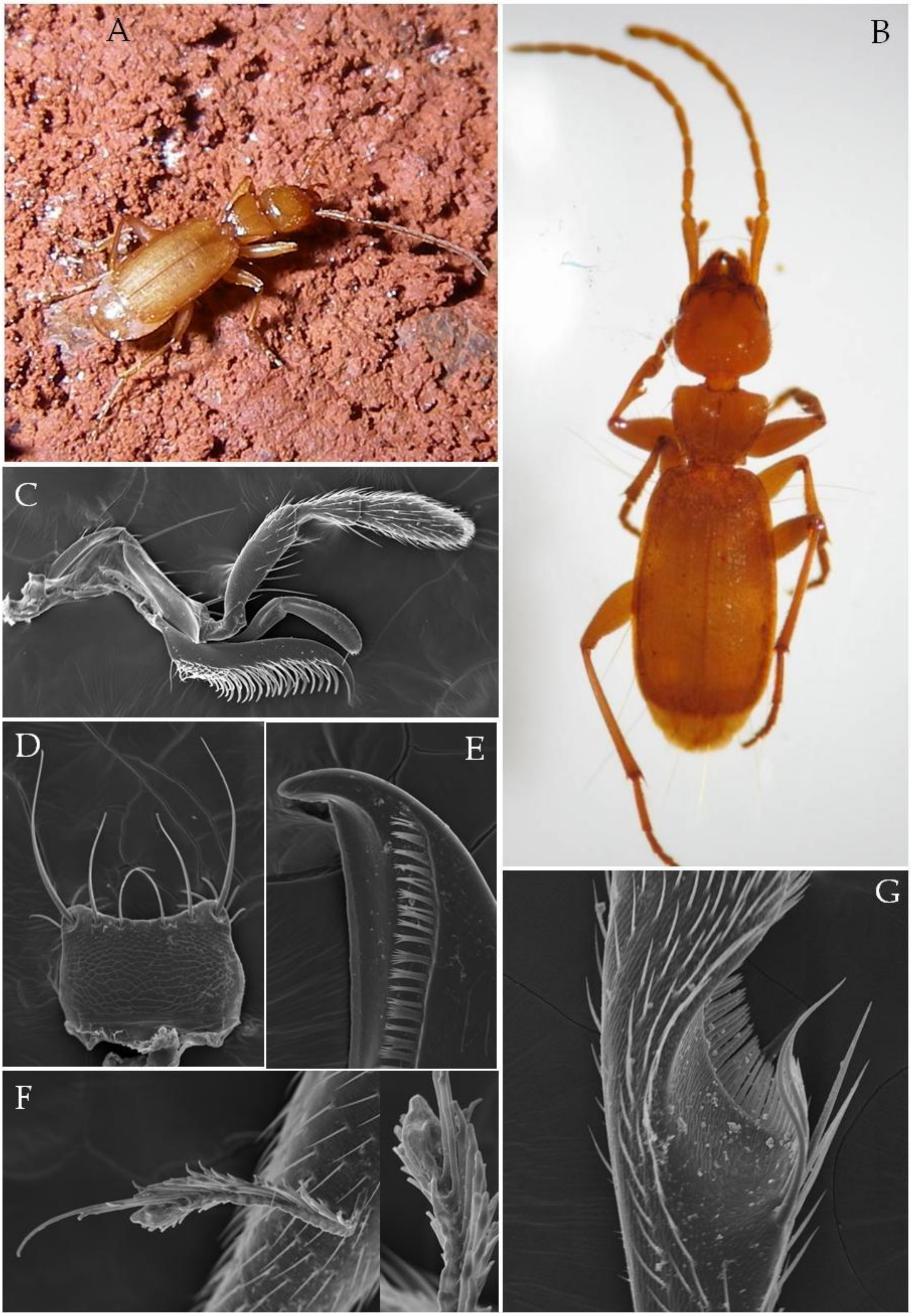

Figura 87 - *Coarazuphium* sp. n: (A) espécime vivo; (B) espécime fixado; (C) maxila; (D) labrum; (E) porção distal da mandíbula esquerda; (F) fungo fixado à tíbia de um dos exemplares. Em detalhe, à direita, o aspecto do fungo; (G) Tíbia do primeiro par de pernas mostrando a estrutura tibial de limpeza de antenas.

Dytiscidae é uma família de coleópteros com cerca de 160 gêneros e 4.000 espécies distribuidas em todas as regiões do mundo. É um dos maiores e mais comuns grupos de besouros aquáticos. Tanto os adultos quanto as larvas são predadores, atacando uma grande variedade de pequenos organismos. Embora a maioria das espécies sejam pequenas e de médio porte, alguns adultos podem atingir um comprimento de 35 mm.

Maior biodiversidade do mundo de coleópteros troglóbios aquáticos é de Dytiscidae. Tem sido relatadas recentemente para águas subterrâneas na zona árida do interior da Austrália 54 espécies (Leyset *et al.*, 2003). Para o Brasil ainda não existem espécies troglóbias de Dytiscidae descritas.

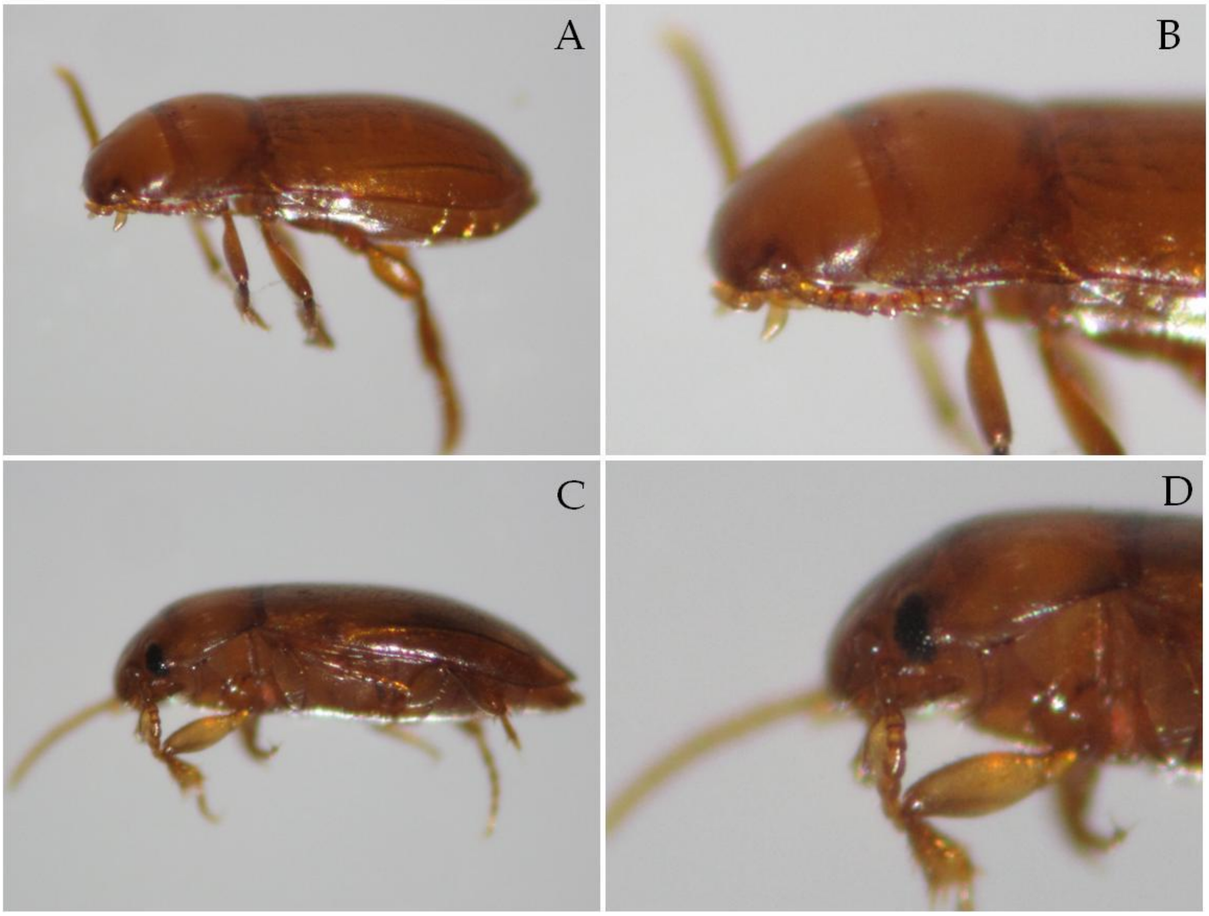

Figura 88 - Coleópteros Dyticidae: (A) Dyticidae sp4 (troglomórfico); (B) Detalhe da região cefálica de Dyticidae sp4 (reparar a despigmentação e anoftalmia); (C) Dyticidae sp3 (não troglomórfico); (D) Detalhe da região cefálica de Dyticidae sp3 (reparar a pigmentação e presença de olhos).

Eucnemidae é uma família de Coleoptera pertencente à superfamília Elateroidea, cujos representantes são conhecidos como falsos vaga-lumes.

São besouros alongados que possuem pequenas projeções para trás da cabeça, nos cantos laterais do escudo cefálico (pronoto). Eles são um pouco achatados e variam em tamanho e

cor. As espécies variam de 0,4 a 1 cm de comprimento. A maioria das espécies é marrom ao preto na cor, embora alguns tenham cores avermelhadas e amareladas. Quando colocados de costas no chão, esses besouros estalam os segmentos do tórax (protórax e mesotórax) para fazer com que seus corpos sejam lançados e assumam a posição de ventre para o chão. Não existem espécies troglóbias descritas no mundo.

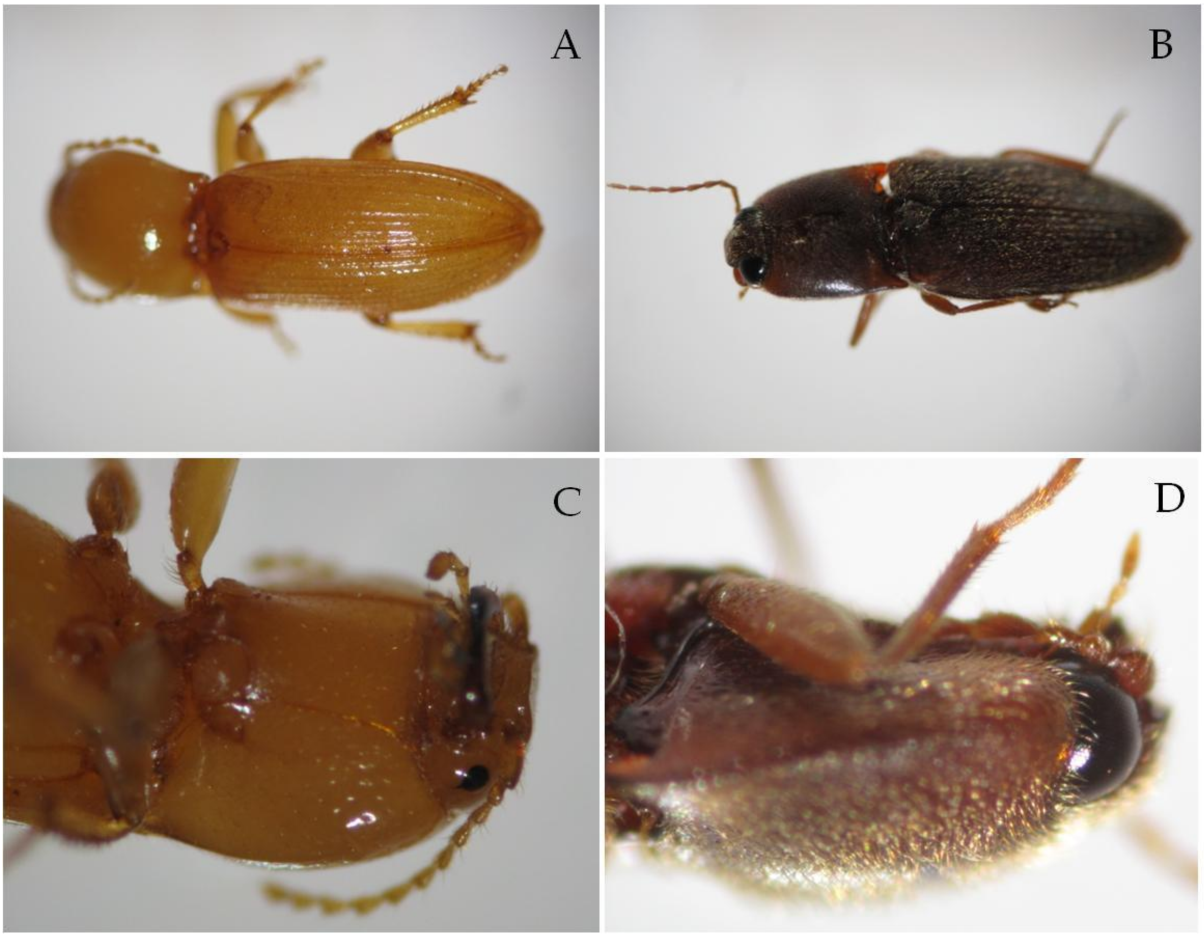

Figura 89 - Coleópteros Eucnemidae: (A) Eucnemidae sp3 (troglomórfico); (C) Detalhe da região cefálica de Eucnemidae sp3 (reparar a despigmentação e pronunciada recução ocular); (B) Eucnemidae sp1 (não troglomórfico); (D) Detalhe da região cefálica de Eucnemidae sp1 (reparar a pigmentação e presença de grandes olhos).

Pselaphidae são coleópteros terrestres com antenas mais ou menos alongadas, palpos maxilares e labiais bem desenvolvidos élitros truncados, muito curtos, cobrindo apenas os 2 primeiros tergitos; os demais, descobertos, mais ou menos fundidos. Encontram-se os Pselafideos sob pedras ou sob a casca e ai se alimentam principalmente de ácaros. Há também espécies cavernicolas.

Aproximadamente Trinta gêneros de Pselaphidae têm representantes troglóbios no mundo (Park, 1960; Hamilton-Smith, 1965; Poggi *et al*., 1998; Moldovam 2004). Existe a citaçao de uma espécie de Pselaphidae troglomorfico para a caverna Santana em Iporanga, São Paulo

(Pinto-da-Rocha, 1995). Entretanto, ainda não existem espécies troglóbias descritas para o Brasil.

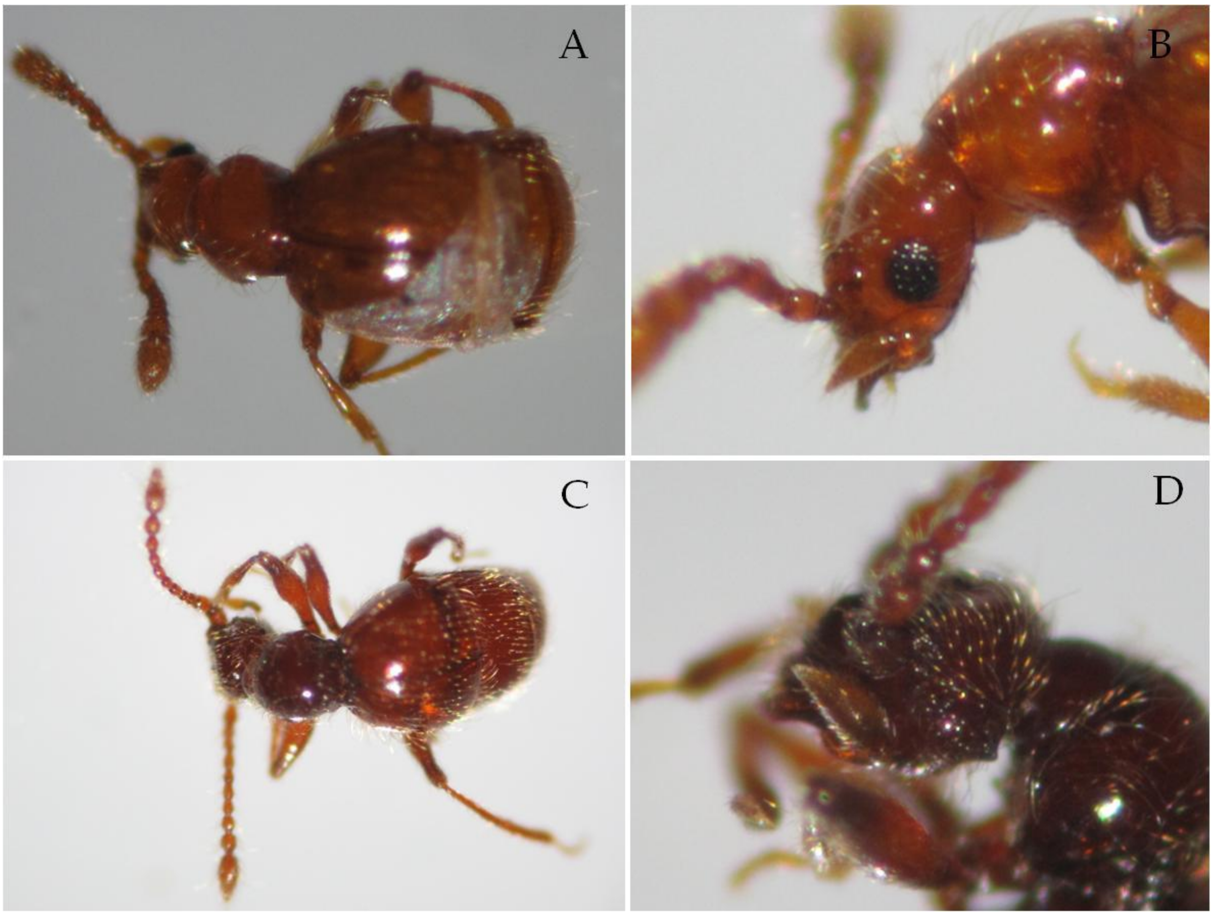

Figura 90 - Coleópteros Pselaphidae: (A) Pselaphidae sp12 (não troglomórfico); (B) Detalhe da região cefálica de Pselaphidae sp12 (reparar a pigmentação e presença de grandes olhos); (C) Pselaphidae sp6 (troglomórfico); (D) Detalhe da região cefálica de Pselaphidae sp6 (reparar a anoftalmia).

Scydmaenidae é uma família de pequenos besouros que ocorrem no mundo inteiro com cerca de 4.500 espécies em cerca de 80 gêneros. Muitas espécies têm um estreitamento entre a cabeça e o tórax e tórax e abdômen, resultando em uma semelhança passageira com formigas que inspira o seu nome comum. Os maiores indivíduos medem apenas 3 mm de comprimento, enquanto algumas espécies muito pequenas só atingem meio milímetro de comprimento. Scydmaenidae geralmente vivem na serapilheira e troncos apodrecidos em florestas, preferindo habitats úmidos. Alguns tipos são conhecidos para se alimentar de ácaros oribatídeos (Decu *et al.*, 1998). Poucas são as espécies de Scydmaenidae troglóbias ao longo do mundo (Vít & Hlavá, 2005). Não existem espécies troglóbias descritas para o Brasil.

Euconnus (Tetramelus) bazgoviensis uma espécies troglóbia da Croácia apresenta anoftalmia, apterismo e corpo delgado e antenas delgados (Vít & Hlavá, 2005).

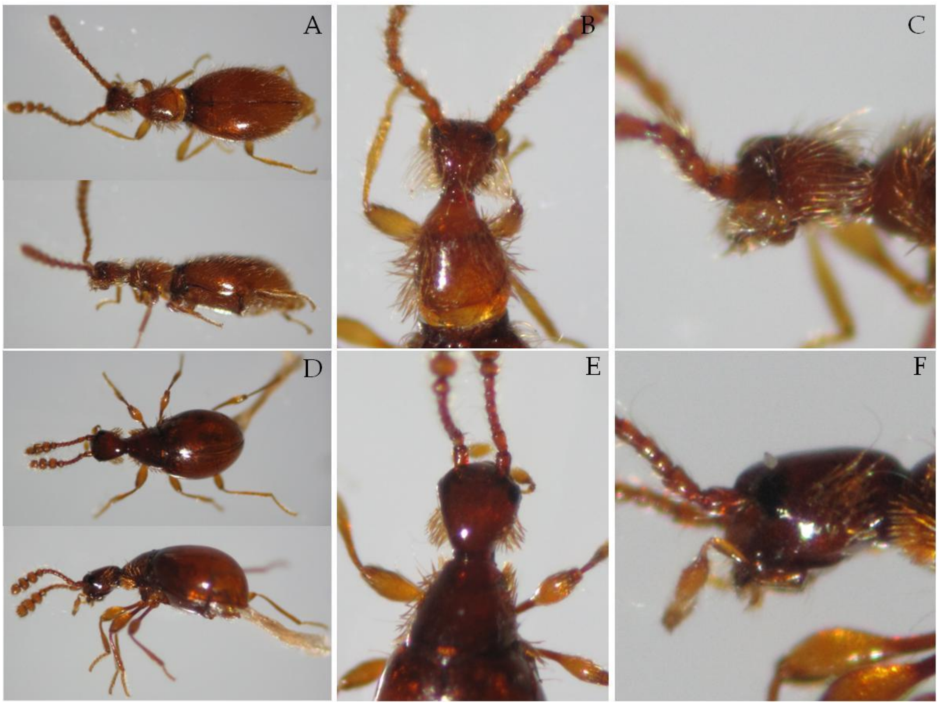

Figura 91 - Coleópteros Scydmaenidae: (A) *Scydmaenidae* sp20 (troglomórfico) em vista dorsal e lateral; (B,C) Detalhes da região cefálica de *Scydmaenidae* sp20 (reparar a pronunciada recução ocular); (D) *Scydmaenidae* sp15 (não troglomórfico) em vista dorsal e e lateral; (E,F) Detalhe da região cefálica de *Scydmaenidae* sp15 (reparar a pigmentação e presença de grandes olhos).

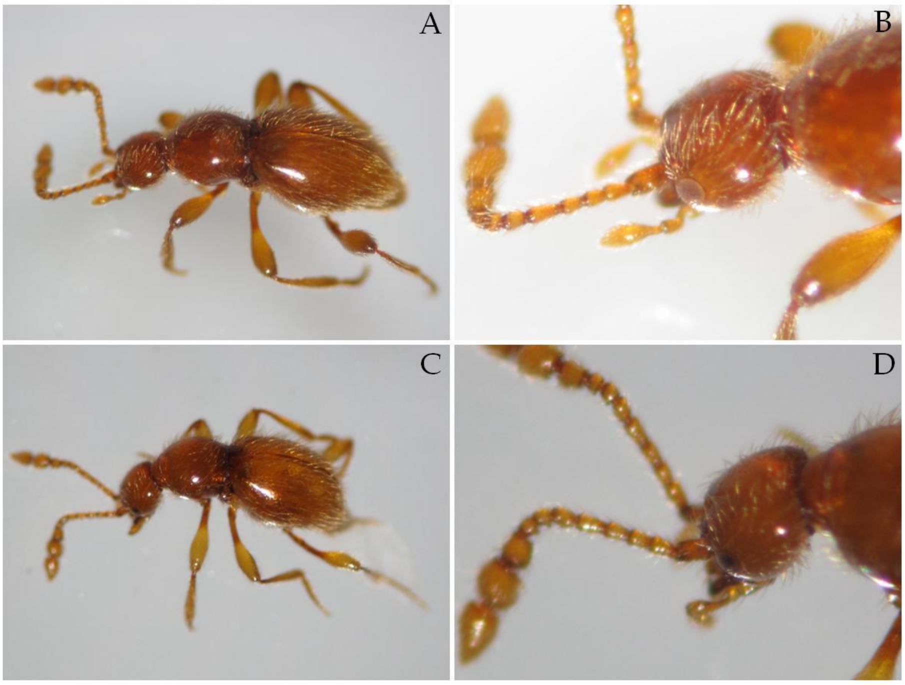

Figura 92 - Coleópteros Scydmaenidae: (A) Scydmaenidae sp3 (não troglomórfico); (B) Detalhe da região cefálica de Scydmaenidae sp3 (reparar a pigmentação e presença de grandes olhos); (C) Scydmaenidae sp22 (troglomórfico); (D) Detalhe da região cefálica de de Scydmaenidae sp22 (reparar a redução das estruturas oculares).

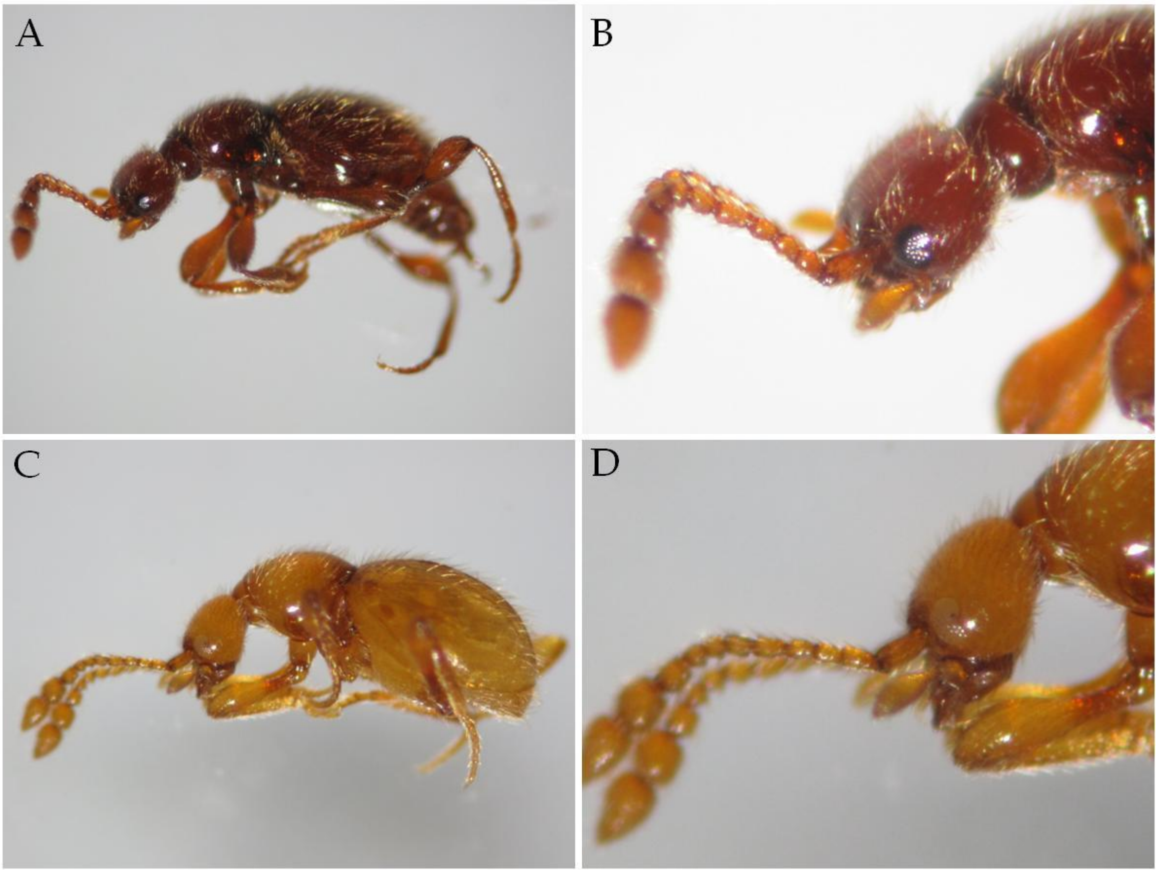

Figura 93 - Coleópteros Scydmaenidae: (A) Scydmaenidae sp2 (não troglomórfico); (B) Detalhe da região cefálica de Scydmaenidae sp2 (reparar a pigmentação e presença de grandes olhos); (C) Scydmaenidae sp21 (troglomórfico); (D) Detalhe da região cefálica de Scydmaenidae sp21 (reparar a redução da pigmentação tegumentar e ocular).

Isópodes são crustáceos relativamente pequenos, com sete pares de pernas do mesmo tamanho e forma, que variam em tamanho corporal desde 300 micrómetros a cerca de 50 cm. Eles são tipicamente achatados dorso-ventralmente, embora muitas espécies especializadas a vida em águas marinhas profundas ou águas subterrâneas se afastem deste plano. Em isópodes terrestres, as estruturas respiratórias se assemelham a pulmões, e são facilmente visíveis na parte de baixo ao final do corpo. Os olhos, quando presentes, são sempre sésseis, nunca em hastes.

Os isópodos terrestres (Crustacea, Oniscidea) são habitantes do solo difundidos através de uma grande variedade de habitats (Schmalfuss, 2003). Cerca de 5.000 espécies são da subordem Oniscidea, representam o grupo de crustáceos de maior sucesso nos ambientes terrestres (Schotte, 2010).

São conhecidas seis subordens de isópodos subterrâneos terrestres e aquáticos com distribuição global (Phreatoicidea, Anthuridea, Microcerberidea, Flabellifera, Calabozoidea,

Asellota, e Oniscidea). Na subordem Oniscidea são conhecidas aproximadamente 276 espécies troglóbias aquáticas e terrestres (Culver & White, 2004).

Isópodos terrestres (Oniscidea) são bastante comums em cavernas brasileiras, ocorrendo principalmente em depósitos úmidos de sedimentos, detritos vegetais e guano.

Várias famílias foram registradas em todo o país, muitas delas incluindo espécies troglomórficas. Algumas famílias estão disseminadas em diferentes áreas cársticas (por exemplo, Platyarthridae e Styloniscidae, ambos encontrados em cavernas da Bahia, Minas Gerais e Vale do Ribeira), outros parecem ser mais frequentes e diversas em determinadas áreas (por exemplo, Phylosciidae, típico da Vale do Ribeira; Armadillidae, mais abundante nos municípios de Bodoquena e Rio Pardo; Scleropactidae, no Pará) (Trajano & Bichuette, 2010). Fotografias de alguns isópodes troglomórficos encontrados em Serra Leste podem ser visualizadas nas Figuras 94 e 95.

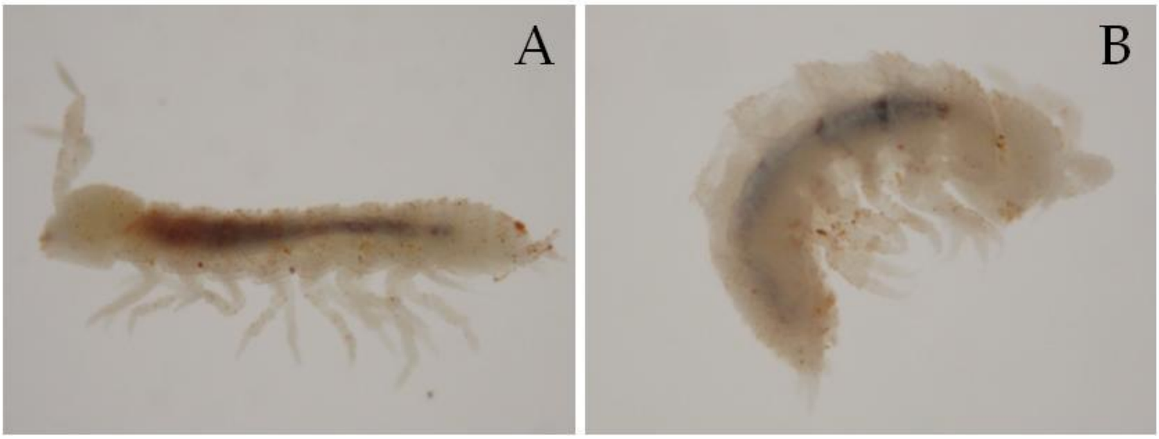

Figura 94 - Isópodes troglomórficos encontrados em Serra Leste: (A) Styloniscidae sp1; (B) *Trichorhina* sp4.

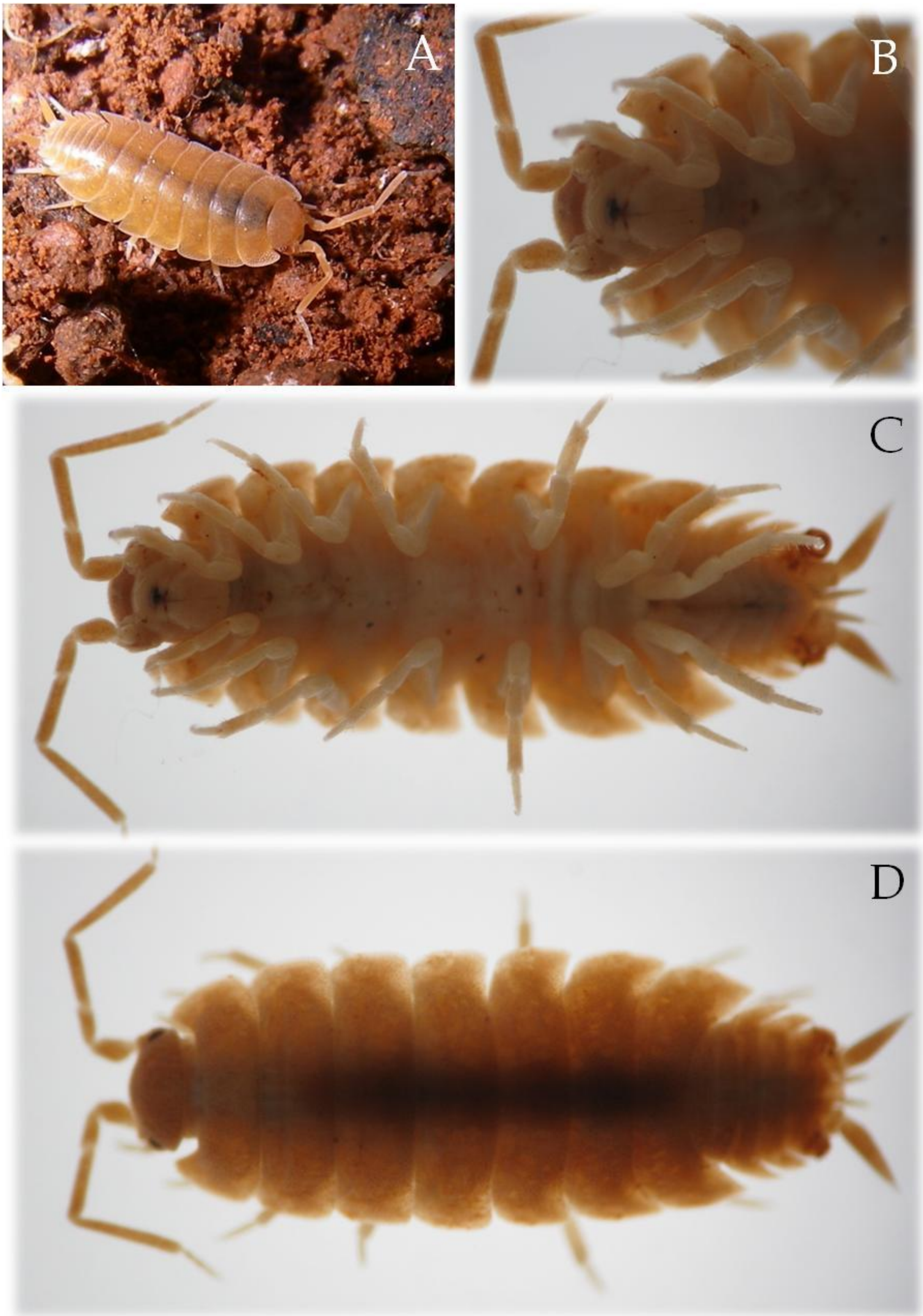

Figura 95 - Isópode troglomórfico da família Baloniscidae encontrado em Serra Leste: (A) Aspecto do organismos vivo; (B) Detalhe da cavbeça em vista ventral; (C) Espécime fixado – vista ventral; (D) Espécime fixado – vista dorsal.

5.4.4. Descrição específica de cada cavidade

5.4.4.1. SL-001

5.4.4.1.1 Caracterização trófica

Grande cavidade com 83,1 m de projeção horizontal localizada em área de mata ciliar na margem direita do rio que abastece o vilarejo de Serra Pelada. Com topografia predominantemente plana, a cavidade possui cinco entradas amplas por onde se dá grande parte da importação de recursos orgânicos (serrapilheira). Tais materiais são carreados através de transporte eólico ou gravitacional e pela água, através de inundações durante os períodos de cheia do rio. Aparentemente, durante o período de cheia, grande parte do piso da cavidade fica submerso. As entradas são sombreadas, e nelas se observam muitos líquens, briófitas e pteridófitas revestindo as paredes e pisos. Na porção mais profunda da cavidade, boa parte das paredes é revestida por Actinomicetos (bactérias) que apresentam coloração esbranquiçada. Uma grande colônia de morcegos insetívoros (*Pteronotus parnellii*), formada por centenas de indivíduos, produz uma considerável quantidade de guano, sendo este, um importante recurso trófico no sistema. Entretanto, além destes, também existem grandes depósitos de guano de morcegos frugívoros (G. soricina). A caverna não apresenta zona afótica, uma vez que seu desenvolvimento preferencial ocorre paralelo à linha externa do afloramento e ao curso do rio. Observam-se somente zonas de penumbra escura em algumas áreas da cavidade. Ainda em relação às características físicas e tróficas, a caverna pode ser dividida em três áreas principais: a área topograficamente mais rebaixada encontra-se parcialmente inundada mesmo durante a estação de seca. Nesta região, o piso emerso é composto de sedimento fino e úmido, sendo coberto por grandes depósitos de guano. Na área intermediária, o sedimento apresenta maior granulomentria, estando mesclado ao guano. Existem, ainda, muitas pequenas raízes associadas. Na terceira área, existe uma clarabóia, sob a qual se observa pequenos depósitos de serrapilheira e o piso é mais elevado com padrão ascendente. Neste local o piso é composto por sedimento fino e seco, e existe uma maior quantidade de micro-hábitats formados por blocos e praticamente não existem depósitos de guano. Em boa parte da cavidade existe um sistema radicular superficial bem desenvolvido e o sistema de canalículos ou alvéolos apresenta-se de maneira heterogênea quanto à sua distribuição. Tais estruturas se concentram em algumas áreas da cavidade. Durante a estação úmida a principal alteração observada foi uma redução na área alagada da cavidade, uma vez que o nível da represa encontrava-se mais baixo quando comparado à estação seca. Neste período, os depósitos de guano fresco de morcegos nectarívoros também se faziam presentes, já que a colônia de morcegos Glossophaginae continuava utilizando a cavidade como abrigo (Figura 96). Entretanto, a colônia de *Pteronotus* encontrava-se muito reduzida, o que resultava em uma menor quantidade de guano de insetívoros disponível.

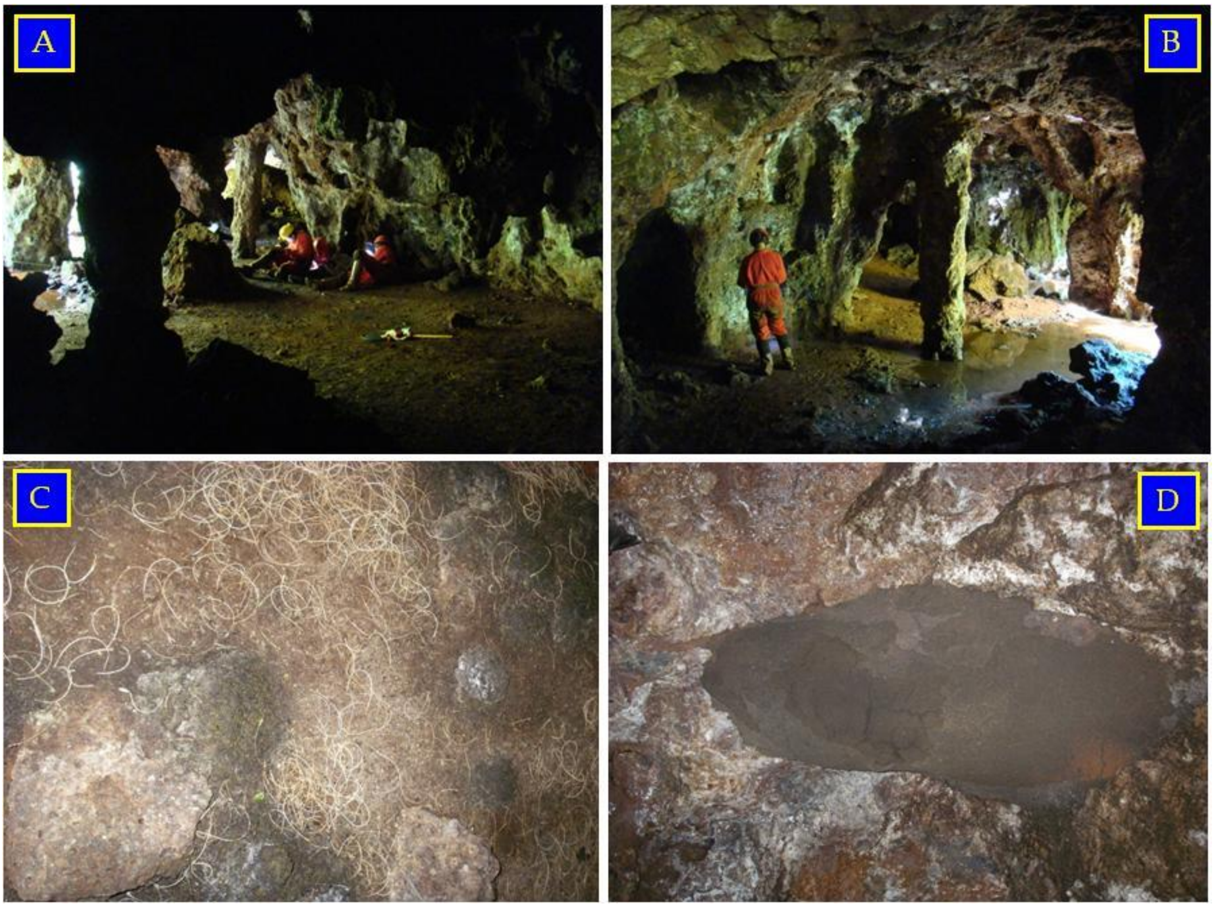

Figura 96 - a) Zona eufótica onde observa-se os líquens e musgos revestindo as paredes; b) Pórtico de entrada da cavidade em área de mata ciliar com piso alagado; c) Depósito de guano frugívoro produzido por *G. soricina* com muitas plântulas mortas junto ao substrato; d) Antigo cupinzeiro abandonado no interior da cavidade.

5.4.4.1.2. Caracterização faunística no período de seca

Foi observado na caverna, um total de 125 morfoespécies de invertebrados de pelo menos 91 famílias das Ordens: Oligochaeta, Gastropoda (Subulinidae, Systrophiidae), Turbellaria (Geoplanidae), Isopoda (Armadillidae, Philosciidae, Scleropactidae), Decapoda (Pseudothelphusidae: *Microthelphusa somanni*, Trichodactylidae: *Valdivia serrata*), Acari (Laelapidae: *Stratiolaelaps* sp., Macronyssidae, Ologamasidae, Astigmatina, Anystidae: *Erythracarus* sp., Cunaxidae, Rhagidiidae, Trombidiforme), Amblypygi (Phrynidae: *Heterophrynus longicornis*), Pseudoscorpiones (Chernetidae, Chtoniidae), Opiliones (Escadabiidae, Sclerosomatidae: *Prionostemma* sp.), Araneae (Amaurobiidae; Araneidae; Ctenidae; Gnaphosidae; Oonopidae: Oonopinae; Scytodidae*: Scytodes eleonora*; Paratropidae; Pholcidae: *Mesabolivar* sp.; Theraphosidae; Theridiidae), Thysanura (Nicoletiidae: Nicoletiinae), Diplura (Campodeidae), Collembola (Sminthuridae, Cyphoderidae, Entomobryidae, Hypogastruridae, Paronellidae), Odonata (Coenagrionidae), Orthoptera (Gryllidae: Nemobiinae, Phalangopsidae: *Aclodes* sp. e *Phalangopsis* sp.),

Blattodea (Blaberidae: *Blaberus* sp., Blattidae, Polyphagidae), Isoptera (Rhinotermitidae: *Heterotermes* sp., Termitidae: *Nasutitermes* sp.), Psocoptera (Epipsocidae), Hemiptera (Cydnidae, Dipsocoridae, Gerridae, Nabidae, Reduviidae), Homoptera (Cixiidae: *Cixius* sp.), Lepidoptera (Tineidae), Diptera (Anthomyiidae, Cecidomyiidae, Culicidae, Drosophilidae, Empididae, Fanniidae, Phoridae, Psychodidae: *Lutzomyia* sp., Streblidae, Sciaridae, Tephritidae, Tipulidae), Hymenoptera (Formicidae: *Camponotus* sp., *Cephalotes* sp., *Crematogaster* sp., *Dolichoderus* sp., *Odontomachus* sp., *Pachycondyla* sp.; Braconidae, Scelionidae, Vespidae), Coleoptera (Carabidae, Chrysomelidae, Elateridae: Cardiophorinae, Hysteridae, Leiodidae, Pselaphidae, Scydmaenidae, Staphylinidae, Tenebrionidae), Diplopoda (Chelodesmidae, Oniscodesmidae, Glomeridesmidae: *Glomeridesmus* sp.), Scutigeromorpha (Scutigeridae: *Sphendononema* sp.) e Symphyla (Scutigerellidae: *Hanseniella* sp.).

Dentre os vertebrados foram encontrados sete espécies das Ordens: Chiroptera (Emballonuridae: *Peropteryx kappleri*, Phyllostomidae: *Anoura* sp., *Glossophaga soricina*; Mormopitdae: *Pteronotus parnellii*), Anura (Lepdoctylidae: *Pristimantis* cf. *fenestratus*, *Eleutherodactylus* sp.; e Bufonidae).

Desta forma, no total foram encontrados 132 morfoespécies. Entre estas, quatro espécies de invertebrados foram consideradas troglomórficas: Gastropoda (Systrophiidae), Acari (Rhagidiidae), Collembola (Cyphoderidae) e Coleoptera (Scydmaenidae).

5.4.4.1.3. Caracterização faunística no período de chuva

Foi observado na caverna, um total de 102 morfoespécies de invertebrados de pelo menos 78 famílias das Ordens: Oligochaeta, Gastropoda (Subulinidae), Turbellaria (Geoplanidae), Isopoda (Balloniscidae, Dubioniscidae, Philosciidae), Acari (Ixodidae: *Amblyomma cajennense*, Laelapidae: *Stratiolaelaps* sp., Macrochelidae: *Macrocheles* sp.; Podocinidae: *Podocinum* sp.; Oribatida, Cunaxidae, Eupodidae, Trombidiforme), Ricinulei (Ricinoididae: *Cryptocellus tarsilae*), Schizomida (Hubbardiidae: Hubbardiinae), Opiliones (Kimmulidae), Araneae (Amaurobiidae; Anyphaenidae; Dipluridae; Filistatidae; Gnaphosidae; Linyphiidae; Onopidae: Oonopinae, Gamasomorphinae; Senoculidae; Scytodidae*: Scytodes eleonora*; Paratropidae; Psauridae; Pholcidae: *Mesabolivar* sp.; Prodidomidae; Theraphosidae: *Acanthoscurria* sp.; Theridiidae), Collembola (Entomobryidae), Orthoptera (Gryllidae: Gryllinae; Phalangopsidae; Tetrigidae), Phasmatodea (Pseudophasmatidae), Blattodea (Blatellidae, Blattidae), Isoptera (Termitidae: *Cortaritermes* sp., *Nasutitermes* sp., *Termes* sp.), Dermaptera (Labiidae), Psocoptera (Archipsocidae, Lepidopsocidae, Myopsocidae: *Lichenomina* sp., Pachytroctidae, Ptilioneuridae: *Ptiloneura* sp., Trogiidae), Hemiptera (Enichocephalidae, Gerridae, Reduviidae), Homoptera (Cercopidae), Lepidoptera (Noctuidae, Tineidae), Diptera (Culicidae, Dolichopodidae, Drosophilidae, Lauxaniidae, Muscidae, Phoridae, Sciaridae, Tipulidae), Hymenoptera (Formicidae: *Achantostichus* sp., *Camponotus*

sp., *Leptogenys* sp., *Pseudomyrmex* sp., Myrmicinae; Apidae, Bethylidae, Braconidae), Thysanoptera (Phaleotripidae: Phaleotripinae), Coleoptera (Elateridae: Cardiophorinae, Hysteridae, Leiodidae, Nitidulidae, Pselaphidae, Scydmaenidae, Staphylinidae), Diplopoda (Chelodesmidae, Oniscodesmidae, Pyrgodesmidae), Scolopendromorpha (Cryptopidae: *Cryptops* sp; Scolopocryptopidae: *Dinocryptops* sp., *Newportia* sp.) e Neuroptera (Myrmeleontidae).

Dentre os vertebrados foram encontrados duas espécies das Ordens: Chiroptera (Mormopidae: *Pteronotus parnellii*) e Anura (Bufonidae).

Desta forma, no total foram encontrados 104 morfoespécies. Entre estas, três espécies de invertebrados foram consideradas troglomórficas: Schizomida (Hubbardiidae: Hubbardiinae), Coleoptera (Scydmaenidae) e Diplopoda (Pyrgodesmidae).

5.4.4.1.4. Caracterização geral da fauna da cavidade

Foi observado na caverna, um total de 215 morfoespécies de invertebrados de pelo menos 122 famílias das Ordens: Oligochaeta, Gastropoda (Subulinidae, Systrophiidae), Turbellaria (Geoplanidae), Isopoda (Armadillidae, Balloniscidae, Dubioniscidae, Philosciidae, Scleropactidae), Decapoda (Pseudothelphusidae: *Microthelphusa somanni*, Trichodactylidae: *Valdivia serrata*), Acari (Eupodidae, Ixodidae: *Amblyomma cajennense,* Laelapidae: *Stratiolaelaps* sp., Macrochelidae: *Macrocheles* sp.; Macronyssidae, Ologamasidae, Astigmatina, Anystidae: *Erythracarus* sp., Cunaxidae, Oribatida, Podocinidae: *Podocinum* sp.; Rhagidiidae, Trombidiforme), Amblypygi (Phrynidae: *Heterophrynus longicornis*), Ricinulei (Ricinoididae: *Cryptocellus tarsilae*), Schizomida (Hubbardiidae: Hubbardiinae), Pseudoscorpiones (Chernetidae, Chtoniidae), Opiliones (Escadabiidae, Kimmulidae, Sclerosomatidae: *Prionostemma* sp.), Araneae (Amaurobiidae; Anyphaenidae; Araneidae; Ctenidae; Dipluridae; Filistatidae; Gnaphosidae; Linyphiidae; Oonopidae: Oonopinae, Gamasomorphinae; Senoculidae; Scytodidae*: Scytodes eleonora*; Paratropidae; Pholcidae: *Mesabolivar* sp.; Psauridae; Prodidomidae; Theraphosidae: *Acanthoscurria* sp.; Theridiidae), Thysanura (Nicoletiidae: Nicoletiinae), Diplura (Campodeidae), Collembola (Sminthuridae, Cyphoderidae, Entomobryidae, Hypogastruridae, Paronellidae), Odonata (Coenagrionidae), Orthoptera (Gryllidae: Gryllinae, Nemobiinae; Phalangopsidae: *Aclodes* sp. e *Phalangopsis* sp.; Tetrigidae), Phasmatodea (Pseudophasmatidae), Blattodea (Blaberidae: *Blaberus* sp., Blatellidae, Blattidae, Polyphagidae), Isoptera (Termitidae: *Cortaritermes* sp., *Nasutitermes* sp., *Termes* sp.; Rhinotermitidae: *Heterotermes* sp.), Dermaptera (Labiidae), Psocoptera (Archipsocidae, Epipsocidae, Lepidopsocidae, Myopsocidae: *Lichenomina* sp., Pachytroctidae, Ptilioneuridae: *Ptiloneura* sp., Trogiidae), Hemiptera (Cydnidae, Dipsocoridae, Enichocephalidae, Gerridae, Nabidae, Reduviidae), Homoptera (Cixiidae: *Cixius* sp.; Cercopidae), Lepidoptera (Noctuidae, Tineidae), Diptera (Anthomyiidae, Cecidomyiidae, Culicidae, Dolichopodidae, Drosophilidae, Empididae, Fanniidae, Lauxaniidae, Muscidae,

Phoridae, Psychodidae: *Lutzomyia* sp., Streblidae, Sciaridae, Tephritidae, Tipulidae), Hymenoptera (Formicidae: *Achantostichus* sp., *Camponotus* sp., *Cephalotes* sp., *Crematogaster* sp., *Dolichoderus* sp., *Leptogenys* sp., *Odontomachus* sp., *Pachycondyla* sp., *Pseudomyrmex* sp., Myrmicinae; Apidae, Bethylidae, Braconidae, Scelionidae, Vespidae), Thysanoptera (Phaleotripidae: Phaleotripinae), Coleoptera (Carabidae, Chrysomelidae, Elateridae: Cardiophorinae, Hysteridae, Leiodidae, Nitidulidae, Pselaphidae, Scydmaenidae, Staphylinidae, Tenebrionidae), Diplopoda (Chelodesmidae; Oniscodesmidae; Glomeridesmidae: *Glomeridesmus* sp.; Pyrgodesmidae), Scolopendromorpha (Cryptopidae: *Cryptops* sp; Scolopocryptopidae: *Dinocryptops* sp., *Newportia* sp.), Scutigeromorpha (Scutigeridae: *Sphendononema* sp.), Symphyla (Scutigerellidae: *Hanseniella* sp.) e Neuroptera (Myrmeleontidae).

Dentre os vertebrados foram encontrados sete espécies das Ordens: Chiroptera (Emballonuridae: *Peropteryx kappleri*, Phyllostomidae: *Anoura* sp., *Glossophaga soricina*; Mormopidae: *Pteronotus parnellii*), Anura (Lepdoctylidae: *Pristimantis* cf. *fenestratus*, *Eleutherodactylus* sp.; e Bufonidae).

Desta forma, no total foram encontrados 222 morfoespécies. Entre estas, seis espécies de invertebrados foram consideradas troglomórficas: Gastropoda (Systrophiidae), Acari (Rhagidiidae), Schizomida (Hubbardiidae: Hubbardiinae), Collembola (Cyphoderidae) e Coleoptera (Scydmaenidae) e Diplopoda (Pyrgodesmidae). Alguns organismos encontrados nesta caverna são mostrados na Figura 97.

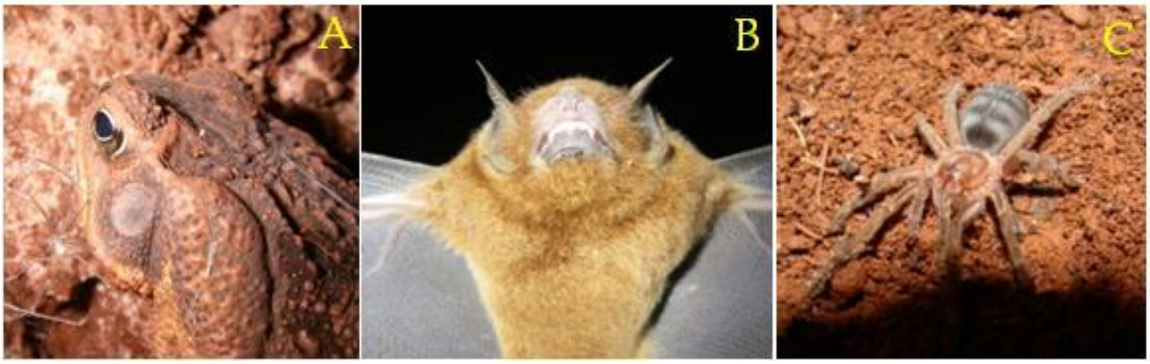

Figura 97 - a) Bufonidae indet.; b) Chiroptera: Mormopidae (P. parnellii); c) Theraphosidae.

5.4.4.2. SL-002

5.4.4.2.1. Caracterização trófica

Caverna com projeção horizontal de 29,8 m, localizada em área de mata ciliar na margem direita do rio e sujeita à inundação em períodos de chuva. Possui entrada ampla e sombreada, com piso plano, formado de sedimento granulado e com poucos musgos nas

paredes, pisos e teto. A zona de entrada é o principal meio de importação de recursos tróficos, onde existe uma pequena drenagem ativa que provavelmente invade todo o piso da cavidade durante a época de chuvas. O conduto principal da cavidade possui teto baixo. Neste conduto, existe uma colônia de morcegos (*G. soricina*) formada por dezenas de indivíduos. A zona eufótica se restringe à entrada e a afótica aos condutos laterais, os quais são predominantemente secos e com poucos recursos alimentares, excetuando-se os salões mais profundos onde existem guanomites com muitas plântulas germinadas a partir de sementes presentes no guano. A cavidade apresenta poucos canalículos e o sistema radicular é pouco desenvolvido, apesar de a cavidade ser bastante superficial (Figura 98). Durante a estação úmida, além de um pequeno aumento na taxa de umidade da cavidade, foram observados pontos de gotejamento ativos próximos à linha d’água.

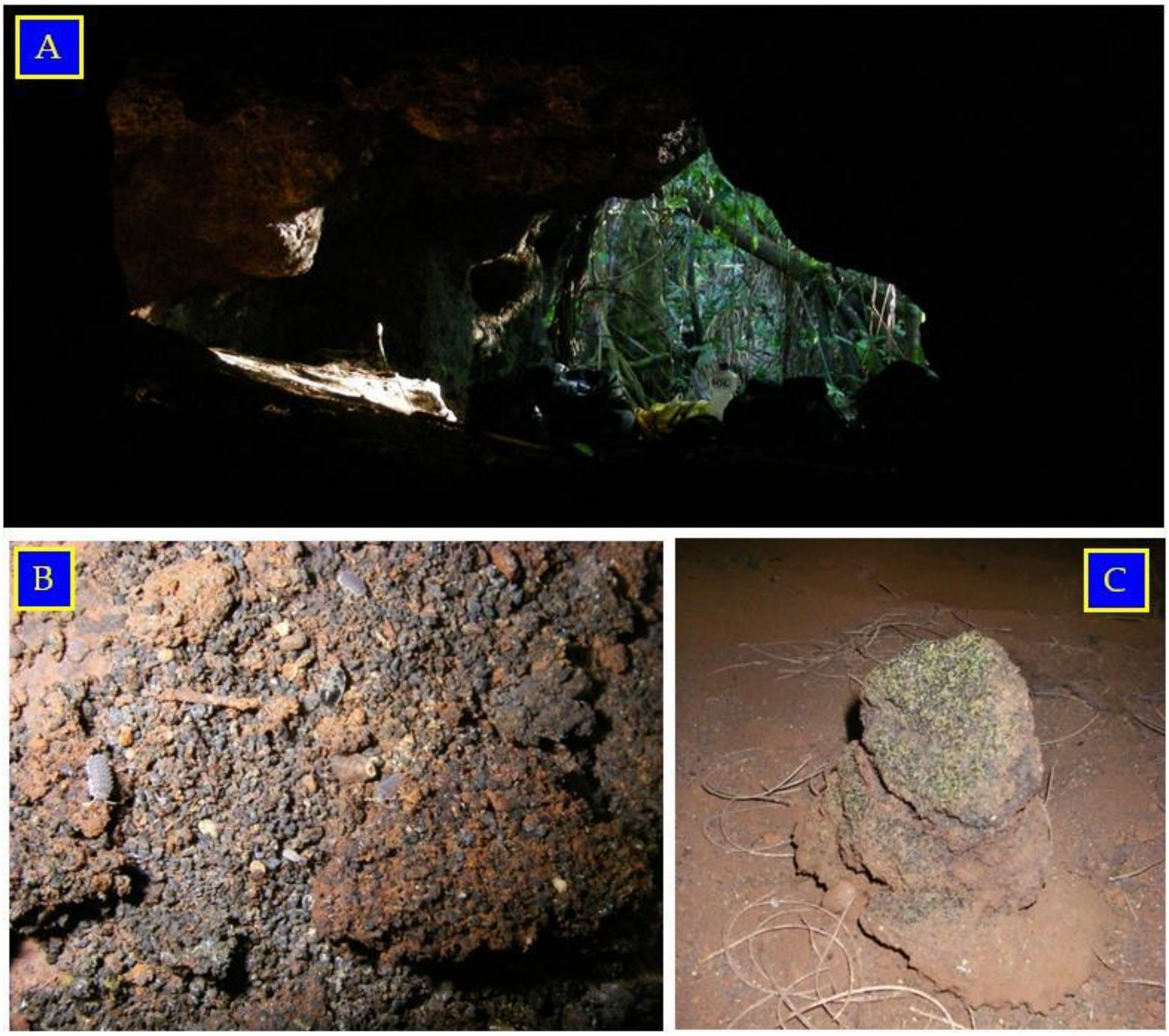

Figura 98 - a) Pórtico de entrada da cavidade em área de mata ciliar; b) Piso da cavidade composto por sedimento granulado com guano misturado ao mesmo e com alguns isópodes (indicado pelo círculo amarelo) associados a este recurso; c) Depósito de guano (guanomite) com inúmeros caules secos de plântulas que germinaram ao seu redor.

5.4.4.2.2. Caracterização faunística no período de seca

Foi observado na caverna, um total de 53 morfoespécies de invertebrados de pelo menos 39 famílias das Ordens: Oligochaeta, Isopoda (Armadillidae, Philosciidae), Acari (Laelapidae: *Stratiolaelaps* sp.), Amblypygi (Phrynidae: *Heterophrynus longicornis*), Pseudoscorpiones (Chernetidae, Chtoniidae), Opiliones (Cosmetidae; Sclerosomatidae: *Prionostemma* sp.), Araneae (Gnaphosidae, Ochyroceratidae, Pholcidae: *Mesabolivar* sp., Theridiidae, Theridiosomatidae), Collembola (Cyphoderidae, Entomobryidae, Paronellidae), Orthoptera (Phalangopsidae: *Aclodes* sp., *Phalangopsis* sp.), Blattodea (Blaberidae: *Blaberus* sp.; Blattidae; Polyphagidae), Isoptera (Rhinotermitidae: *Heterotermes* sp.; Termitidae: *Nasutitermes* sp.), Hemiptera (Cydnidae, Dipsocoridae, Reduviidae), Homoptera (Pseudococcidae), Lepidoptera (Tineidae), Diptera (Ceratopogonidae, Drosophilidae, Fanniidae), Hymenoptera (Formicidae: *Camponotus* sp., *Gnamptogenys* sp., *Odontomachus* sp., *Pachycondyla* sp.; Vespidae), Coleoptera (Carabidae, Elateridae, Eucnemidae, Scydmaenidae, Staphylinidae), Diplopoda (Chelodesmidae), Scolopendromorpha (Cryptopidae: *Cryptops* sp.) e Symphyla (Scutigerellidae: *Hanseniella* sp.).
Dentre os vertebrados foram encontrados três espécies das Ordens: Chiroptera (Phyllostomidae: *Glossophaga soricina*), Anura (Lepdoctylidae: *Pristimantis* cf. *fenestratus* e *Eleutherodactylus* sp.).

Desta forma, no total foram encontrados 56 morfoespécies. Entre estas, uma espécie de Collembola (Cyphoderidae) foi considerada troglomórfica.

5.4.4.2.3. Caracterização faunística no período de chuva

Foi observado na caverna, um total de 60 morfoespécies de invertebrados de pelo menos 42 famílias das Ordens: Oligochaeta, Turbellaria (Geoplanidae), Isopoda (Dubioniscidae), Acari (Macrochelidae: *Macrocheles* sp.; Ologamasidae; Opiliacaridae: *Neoacarus* spn.; Oribatida; Teneriffiidae; Trombidiforme), Ricinulei (Ricinoididae: *Cryptocellus tarsilae*), Pseudoscorpiones, Araneae (Ctenidae, Filistatidae, Oonopinae, Scytodidae*: Scytodes eleonora*, Phoilcidae: *Mesabolivar* sp., Prodidomidae, Theridiidae), Collembola (Sminthuridae, Isotomidae), Orthoptera (Phalangopsidae, Tetrigidae), Blattodea (Polyphagidae), Psocoptera (Lepidopsocidae, Ptilioneuridae: *Ptiloneura*), Homoptera (Cercopidae, Derbidae), Lepidoptera (Tineidae), Diptera (Culicidae: *Aedes* sp., Chironomidae, Clusiidae, Dolichopodidae, Muscidae, Phoridae, Tipulidae), Hymenoptera (Formicidae: *Acanthostichus* sp., *Camponotus* sp., *Cyphomyrmex* sp., Myrmicinae, *Tetramorium* sp.; Vespidae), Thysanoptera (Phaleotripidae: Phaleothripinae), Coleoptera (Pselaphidae, Ptylidae, Rhizophagidae, Scydmaenidae, Staphylinidae), Diplopoda (Chelodesmidae) e Scolopendromorpha (Cryptopidae: *Cryptops* sp.; Scolocryptopidae: *Dinocryptops* sp., *Newportia* sp.).

5.4.4.2.4. Caracterização geral da fauna da cavidade

Foi observado na caverna, um total de 111 morfoespécies de invertebrados de pelo menos 72 famílias das Ordens: Oligochaeta, Turbellaria (Geoplanidae), Isopoda (Armadillidae, Dubioniscidae, Philosciidae), Acari (Laelapidae: *Stratiolaelaps* sp.; Macrochelidae: *Macrocheles* sp.; Ologamasidae; Opiliacaridae: *Neoacarus* spn.; Oribatida; Teneriffiidae; Trombidiforme), Amblypygi (Phrynidae: *Heterophrynus longicornis*), Ricinulei (Ricinoididae: *Cryptocellus tarsilae*), Pseudoscorpiones(Chernetidae, Chtoniidae), Opiliones (Cosmetidae; Sclerosomatidae: *Prionostemma* sp.), Araneae (Ctenidae, Filistatidae, Gnaphosidae, Ochyroceratidae, Oonopinae, Scytodidae*: Scytodes eleonora*, Phoilcidae: *Mesabolivar* sp., Prodidomidae, Theridiidae, Theridiosomatidae), Collembola (Sminthuridae, Cyphoderidae, Entomobryidae, Isotomidae, Paronellidae), Orthoptera (Phalangopsidae: *Aclodes* sp., *Phalangopsis* sp.; Tetrigidae), Blattodea (Blaberidae: *Blaberus* sp.; Blattidae; Polyphagidae), Isoptera (Rhinotermitidae: *Heterotermes* sp.; Termitidae: *Nasutitermes* sp.), Psocoptera (Lepidopsocidae, Ptilioneuridae: *Ptiloneura*), Hemiptera (Cydnidae, Dipsocoridae, Reduviidae), Homoptera (Cercopidae, Derbidae, Pseudococcidae), Lepidoptera (Tineidae), Diptera (Culicidae: *Aedes* sp., Chironomidae, Ceratopogonidae, Clusiidae, Dolichopodidae, Drosophilidae, Fanniidae, Muscidae, Phoridae, Tipulidae), Hymenoptera (Formicidae: *Acanthostichus* sp., *Camponotus* sp., *Cyphomyrmex* sp., *Gnamptogenys* sp., Myrmicinae, *Odontomachus* sp., *Pachycondyla* sp., *Tetramorium* sp.; Vespidae), Thysanoptera (Phaleotripidae: Phaleothripinae), Coleoptera (Carabidae, Elateridae, Eucnemidae, Pselaphidae, Ptylidae, Rhizophagidae, Scydmaenidae, Staphylinidae), Diplopoda (Chelodesmidae), Scolopendromorpha (Cryptopidae: *Cryptops* sp.; Scolocryptopidae: *Dinocryptops* sp., *Newportia* sp.) e Symphyla (Scutigerellidae: *Hanseniella* sp.).

Dentre os vertebrados foram encontrados três espécies das Ordens: Chiroptera (Phyllostomidae: *Glossophaga soricina*), Anura (Lepdoctylidae: *Pristimantis* cf. *fenestratus* e *Eleutherodactylus* sp.). Desta forma, no total foram encontrados 114 morfoespécies. Entre estas, uma espécie de Collembola (Cyphoderidae) foi considerada troglomórfica. Alguns organismos encontrados nesta caverna são mostrados na Figura 99.

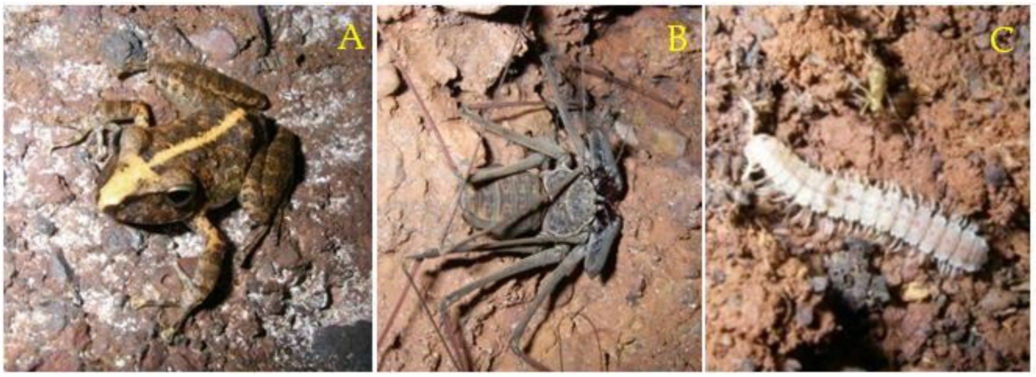

Figura 99 - Leptodactylidae (*Eleutherodactylus* sp.); b) Amblypygi: *Heterophrynus longicornis*; c) Diplopoda: Chelodesmidae.

5.4.4.3. SL-003

5.4.4.3.1. Caracterização trófica

Pequena cavidade sem zona afótica com projeção horizontal de 21,8 m localizada em área de mata ciliar na margem direita do rio que abastece o vilarejo da Serra Pelada. Desenvolvida em canga possui uma entrada sombreada e ampla com muitos líquens, poucas briófitas e muita serrapilheira acumulada junto à linha d'água. De maneira geral, o piso é descendente a partir da entrada na porção inicial da cavidade, sendo composto por sedimento granulado mesclado ao guano onde existem inúmeras plântulas germinadas, muitas das quais já se encontram mortas. Ainda em relação ao sedimento, podem ser observados alguns seixos e calhaus concentrados na zona mais profunda da cavidade. No piso também é possível observar um sistema radicular muito desenvolvido onde existe um predomínio de raízes de diâmetro reduzido além de algumas poucas raízes que chegam a atingir até 3 cm de diâmetro. De maneira geral, esta cavidade pode ser considerada rica em recursos alimentares. As principais vias de importação destes recursos são transporte eólico ou gravitacional, a água de enchentes durante o período de chuva e a produção de guano por morcegos Glossophaginae (*G. soricina* e *Anoura* sp.) que formam uma colônia mista composta por aproximadamente trinta indivíduos. Junto a esta colônia existem grandes depósitos de guano de morcegos frugívoros que chegam a formar pequenos conjuntos de guanomites em alguns pontos da cavidade (Figura 100). De maneira geral, o sistema de canalículos é pouco desenvolvido sendo possível observar nas paredes e teto grandes áreas revestidas por Actinomicetos. Nenhuma alteração significativa foi observada durante a estação úmida nas condições ambientais da cavidade. Entretanto, os depósitos de guano de morcegos frugívoros encontravam-se maiores e mais frescos uma vez que a colônia de morcegos glossofagíneos era maior do que na estação seca. Em alguns casos, observou-se formigas do gênero *Camponotus* e hemípteros (Lygaeidae) se alimentando deste recurso.

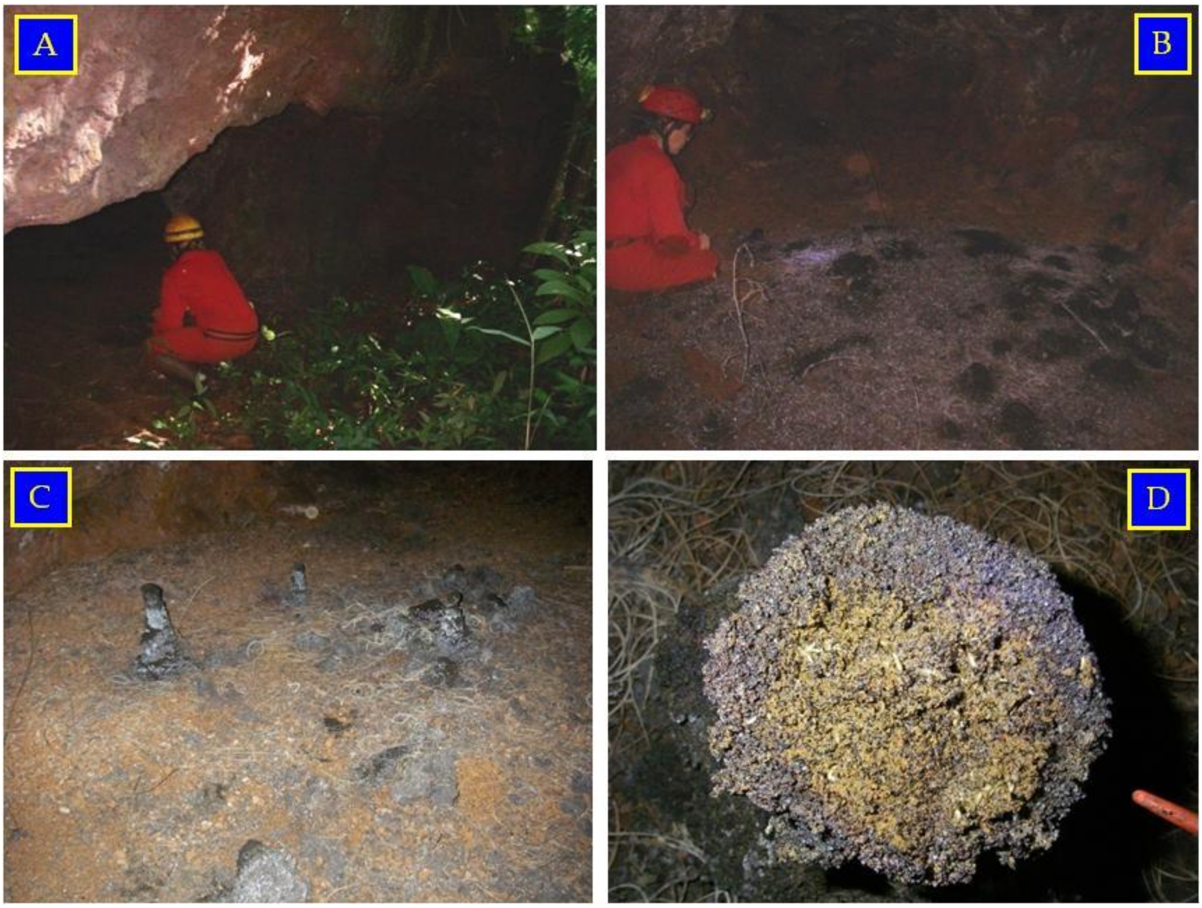

Figura 100 - a) Pórtico de entrada da cavidade em área de mata ciliar; b) Depósito de guano de morcegos frugívoros com intenso desenvolvimento de plântulas; c) Guano esparso misturado ao solo e pequenas guanomites no interior da cavidade; d) Detalhe da porção superior de uma guanomite produzida por morcegos frugívoros.

5.4.4.3.2. Caracterização faunística no período de seca

Foi observado na caverna, um total de 49 morfoespécies de invertebrados de pelo menos 38 famílias das Ordens: Isopoda (Armadillidae, Balloniscidae), Acari (Laelapidae: *Stratiolaelaps* sp., Macrochelidae: *Macrocheles* sp., Oribatida), Pseudoscorpiones (Chernetidae), Opiliones (Stygnidae: *Protimesius* aff. *gracilis*), Araneae (Gnaphosidae, Scytodidae*: Scytodes eleonora*, Pholcidae: *Mesabolivar* sp., Theridiidae), Thysanura (Nicoletiidae: Nicoletiinae), Collembola (Entomobryidae), Orthoptera (Phalangopsidae), Blattodea (Blaberidae: *Blaberus* sp., Blattidae, Polyphagidae), Isoptera (Termitidae: *Nasutitermes* sp.), Dermaptera (Forficulidae: Neolobophorinae), Psocoptera, Hemiptera (Cydnidae), Lepidoptera (Noctuidae, Tineidae), Diptera (Cecidomyiidae, Ceratopogonidae, Culicidae, Drosophilidae, Phoridae, Psychodidae, Streblidae, Stratiomyidae), Hymenoptera (Formicidae: *Azteca* sp., *Camponotus* sp., *Crematogaster* sp., *Dolichoderus* sp., *Odontomachus* sp., *Pachycondyla* sp., *Pheidole* sp.), Coleoptera (Cetoniidae: *Gymnets* sp., Elateridae) e Scolopendromorpha (Cryptopidae: *Cryptops* sp.).

Dentre os vertebrados foram encontrados cinco espécies das Ordens: Chiroptera (Phyllostomidae: *Anoura* sp., *Glossophaga soricina*), Squamata (Gekkonidae: *Thecadactylus rapicauda*), Anura (Lepdoctylidae: *Pristimantis* cf. *fenestratus*), Rodentia (Cricetidae: *Rhipidomys* sp.). Desta forma, no total foram encontrados 54 morfoespécies.

5.4.4.3.3. Caracterização faunística no período de chuva

Foi observado na caverna, um total de 74 morfoespécies de invertebrados de pelo menos 58 famílias das Ordens: Isopoda (Dubioniscidae), Acari (Ixodidae, Laelapidae: *Stratiolaelaps* sp., Macrochelidae: *Macrocheles* sp., Mesostigmata), Opiliones (Kimmulidae, Stygnidae), Araneae (Corinidae, Filistatidae, Gnaphosidae, Oonopidae: Gamasomorphinae, Scytodidae*:* *Scytodes eleonora*, Psauridae, Pholcidae: *Mesabolivar* sp., Prodidomidae, Theraphosidae, Theridiidae), Diplura (Parajapygidae), Collembola (Sminthuridae, Katiannidae?), Neuroptera (Chrysopidae), Orthoptera (Gryllidae: Gryllinae, Phalangopsidae, Tetrigidae), Blattodea (Polyphagidae), Isoptera (Termitidae: *Subulitermes* sp.?), Dermaptera (Forficulidae: Neolobophorinae), Psocoptera (Ectopsocidae, Lepidopsocidae, Ptilioneuridae: *Ptiloneura* sp.), Hemiptera (Cydnidae, Dipsocoridae, Gerridae), Lepidoptera (Heliozelidae, Noctuidae, Tineidae, Zygaenidae), Diptera (Culicidae: *Anopheles* sp., Chironomidae, Dolichopodidae, Milichiidae, Muscidae, Tipulidae), Hymenoptera (Formicidae: *Acanthostichus* sp., *Camponotus* sp., *Gnamptogenys* sp., *Leptogenys* sp.; Bethylidae, Diapriidae, Vespidae), Thysanoptera (Phaleothripidae: Phaleothripinae), Coleoptera (Carabidae, Curculionidae: Scotylinae, Pselaphidae, Scydmaenidae, Staphylinidae, Tenebrionidae) e Scolopendromorpha (Scolopocryptopidae: *Newportia* sp.).

Dentre os vertebrados foram encontrados uma espécie de Chiroptera (Phyllostomidae: *Glossophaga soricina*). Desta forma, no total foram encontrados 75 morfoespécies.

5.4.4.3.4. Caracterização geral da fauna da cavidade

Foi observado na caverna, um total de 118 morfoespécies de invertebrados de pelo menos 79 famílias das Ordens: Isopoda (Armadillidae, Balloniscidae, Dubioniscidae), Acari (Ixodidae, Laelapidae: *Stratiolaelaps* sp., Macrochelidae: *Macrocheles* sp., Mesostigmata, Astigmatina, Oribatida, Bdellidae, Trombidiforme), Pseudoscorpiones (Chernetidae), Opiliones (Kimmulidae, Stygnidae: *Protimesius* aff. *gracilis*), Araneae (Corinidae, Filistatidae, Gnaphosidae, Oonopidae: Gamasomorphinae, Scytodidae*:* *Scytodes eleonora*, Psauridae, Pholcidae: *Mesabolivar* sp., Prodidomidae, Theraphosidae, Theridiidae), Thysanura (Nicoletiidae: Nicoletiinae), Diplura (Parajapygidae), Collembola (Sminthuridae, Entomobryidae, Katiannidae?), Neuroptera (Chrysopidae, Myrmeleontidae), Orthoptera (Gryllidae: Gryllinae, Phalangopsidae: *Aclodes* sp., Tetrigidae), Blattodea (Blaberidae: *Blaberus* sp., Blattidae, Polyphagidae), Isoptera (Termitidae: *Nasutitermes* sp., *Subulitermes* sp.?), Dermaptera (Forficulidae: Neolobophorinae), Psocoptera (Ectopsocidae, Lepidopsocidae, Ptilioneuridae: *Ptiloneura* sp.), Hemiptera (Cydnidae, Dipsocoridae,

Gerridae), Lepidoptera (Heliozelidae, Noctuidae, Tineidae, Zygaenidae), Diptera (Cecidomyiidae, Ceratopogonidae, Culicidae: *Anopheles* sp., Chironomidae, Dolichopodidae, Drosophilidae, Milichiidae, Muscidae, Phoridae, Psychodidae, Streblidae, Stratiomyidae, Tipulidae), Hymenoptera (Formicidae: *Acanthostichus* sp., *Azteca* sp., *Camponotus* sp., *Crematogaster* sp., *Dolichoderus* sp., *Gnamptogenys* sp., *Leptogenys* sp., *Odontomachus* sp., *Pachycondyla* sp., *Pheidole* sp.; Bethylidae, Diapriidae, Vespidae), Thysanoptera (Phaleothripidae: Phaleothripinae), Coleoptera (Carabidae, Curculionidae: Scotylinae, Cetoniidae: *Gymnets* sp., Coccinellidae, Elateridae, Pselaphidae, Scydmaenidae, Staphylinidae, Tenebrionidae) e Scolopendromorpha (Cryptopidae: *Cryptops* sp., Scolopocryptopidae: *Newportia* sp.).

Dentre os vertebrados foram encontrados cinco espécies das Ordens: Chiroptera (Phyllostomidae: *Anoura* sp., *Glossophaga soricina*), Squamata (Gekkonidae: *Thecadactylus rapicauda*), Anura (Lepdoctylidae: *Pristimantis* cf. *fenestratus*), Rodentia (Cricetidae: *Rhipidomys* sp.). Desta forma, no total foram encontrados 123 morfoespécies. Alguns organismos encontrados nesta caverna são mostrados na Figura 101.

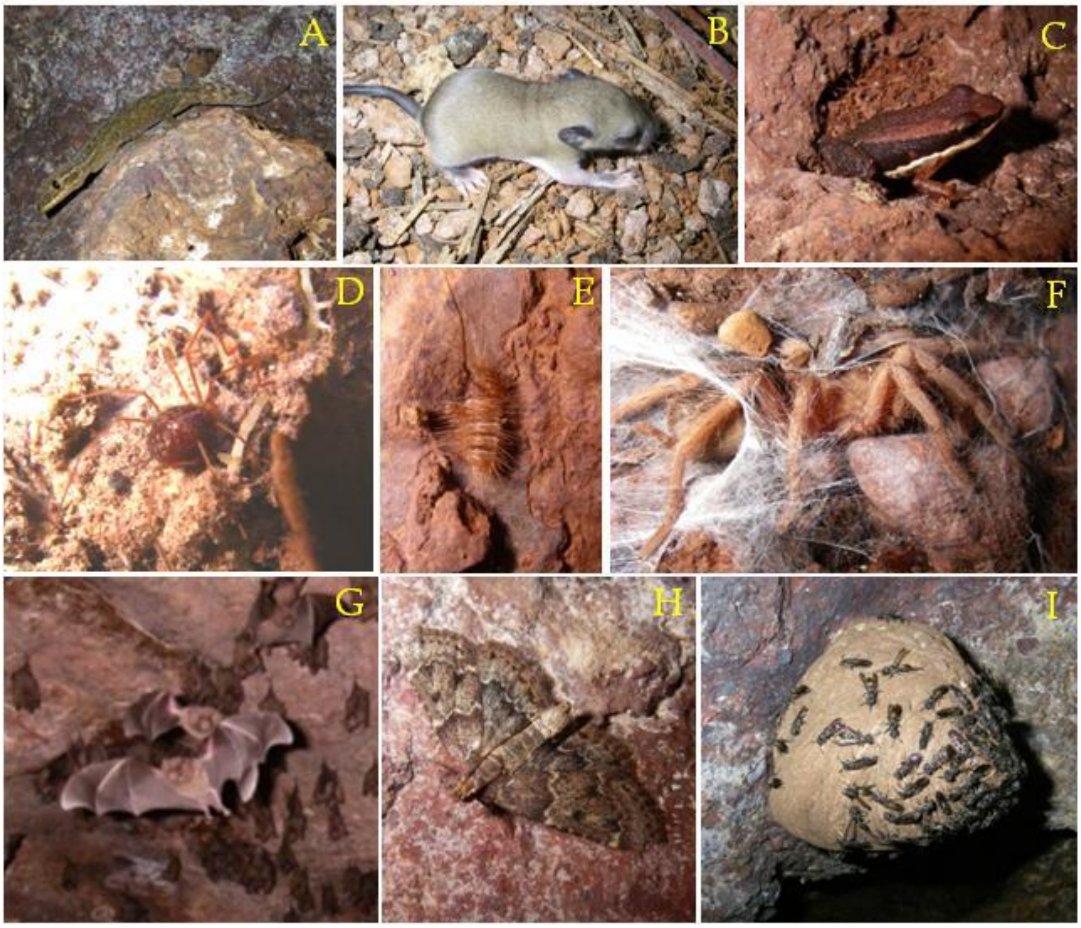

Figura 101 - a) Gekkonidae (*Thecadactylus rapicauda*); b) Roedor (Cricetidae: *Rhipidomys* sp.); c) Dendrobatidae (*Colostethus* sp.); d) Acari; e) Coleoptera imaturo (Dermestidae); f) Theraphosidae; g) Phyllostomidae (*G. soricina*); h) Lepidoptera (Arctiidae); i) Vespidae.

5.4.4.4. SL-004

5.4.4.4.1. Caracterização trófica

Caverna desenvolvida na canga localizada na margem direita do rio, dentro da mata ciliar e sem sinais de inundação. Esta apresenta 25,3 m de projeção horizontal e dois setores bem distintos separados por um trecho de teto baixo. O primeiro setor corresponde à área eufótica, onde existe uma entrada ampla e sombreada com líquens, briófitas e Actinomicetos revestindo trechos do piso e paredes além de uma grande quantidade de serrapilheira acumulada junto à linha d'água. Nesta área, existe uma grande quantidade de guano esparso ou em depósitos concentrados além de muitas plântulas germinadas junto à linha d'água ou a partir de sementes presentes no guano. Pode-se ainda observar algumas folhas esparsas, trazidas pelo vento ou por força gravitacional. Após um pequeno trecho de rastejo que conecta os dois salões é possível observar uma zona afótica que corresponde a aproximadamente 50% da área da cavidade. Neste setor existem depósitos de guano de morcegos frugívoros além de um grande conjunto de guanomites observados apenas nesta cavidade com estas proporções. Junto a estes depósitos de guano foi observada uma grande quantidade de baratas (*Blaberus* sp.) sendo raro o registro de muitos indivíduos desta espécie em uma única cavidade na região. De maneira geral o piso da caverna é seco e plano composto por sedimento granulado mesclado ao guano de morcegos frugívoros que apresenta-se de maneira predominante em relação a pequenos depósitos antigos de guano de morcegos insetívoros. O guano é produzido por morcegos Glossophaginae (*G. soricina* e *Anoura* sp.) que formam uma grande colônia composta por dezenas de indivíduos e distribuídos ao longo de toda a cavidade. O sistema radicular é muito desenvolvido chegando a formar alguns rizotemas associados a pontos de gotejamentos, onde foram observados inúmeros gastrópodes e homópteros (Cixiidae) (Figura 102). Apesar de encontra-se formada na canga, esta cavidade não apresenta um sistema de canalículos bem desenvolvido e existem poucos abrigos (blocos) no piso da cavidade. Quanto ao estado de conservação da cavidade, vale ressaltar que durante o estudo foram encontrados resíduos de fogueiras, sinais de pisoteamento além do abando de lixo no interior desta cavidade. Nenhuma alteração significativa foi observada durante a estação úmida além das alterações normais na umidade relativa do ar.

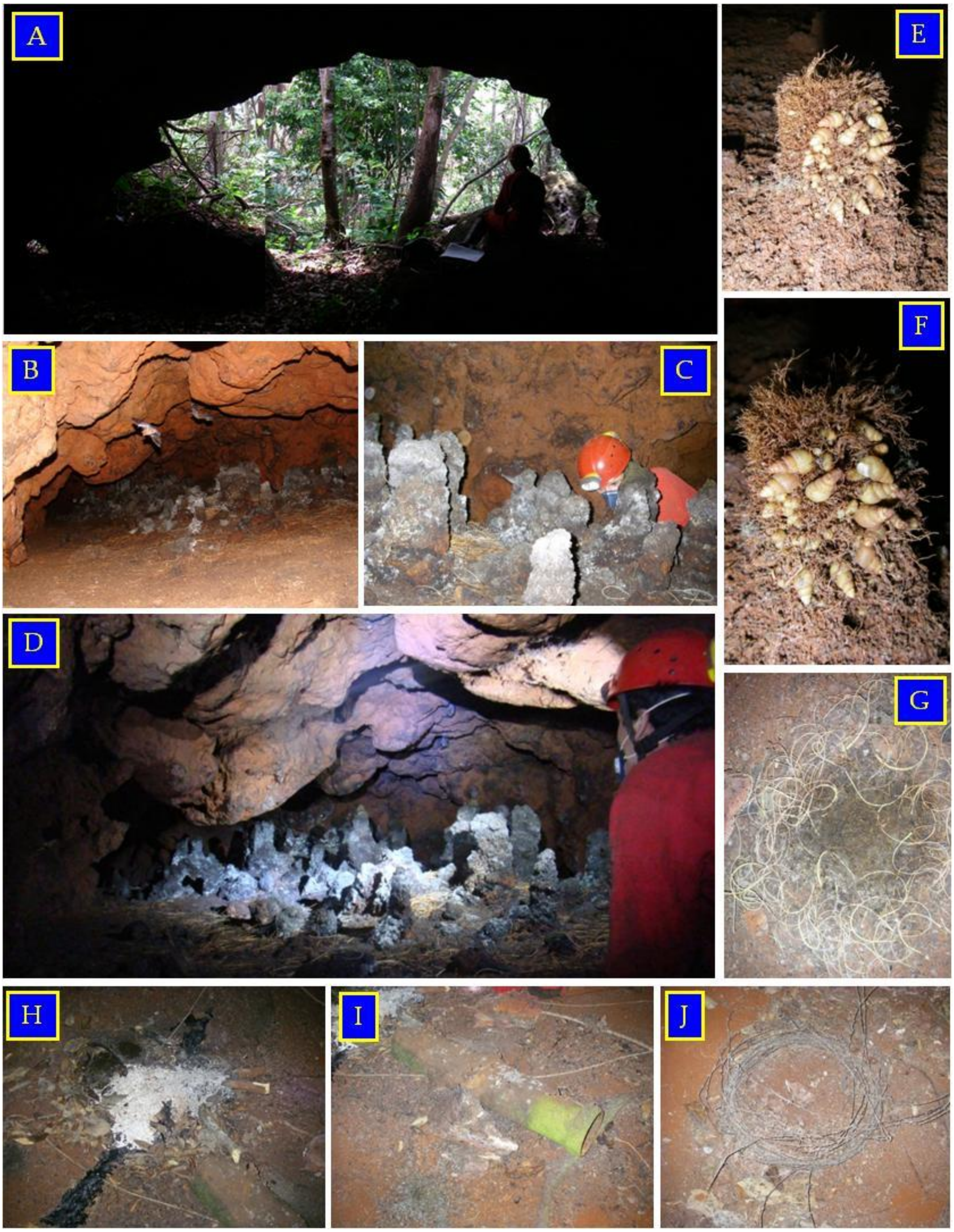

Figura 102 - a) Pórtico de entrada da cavidade em área de mata ciliar; b) Salão com piso revestido de guano onde se observam diversas guanomites; c) Detalhe das guanomites; d) Conjunto de guanomites encontradas na zona afótica; e) Rizotema com gastrópodes associados em zona afótica; f) Detalhe do rizotema com gastrópodes associados; g) Inúmeras plântulas germinadas em guano de morcegos frugívoros; h) resto de foqueira observada na cavidade; i) restos de pequenas manilhas; j) rolo de arame farpado deixado no interior da caverna.

5.4.4.4.2. Caracterização faunística no período de seca

Foi observado na caverna, um total de 118 morfoespécies de invertebrados de pelo menos 70 famílias das Ordens: Gastropoda (Subulinidae, Systrophiidae, Valloniidae), Isopoda (Armadillidae, Balloniscidae, Philosciidae, Plathyarthridae: *Trichorhina* sp.), Acari (Laelapidae: *Stratiolaelaps* sp., Macronyssidae, Otopheidomenidae, Uropodina, Astigmatina, Oribatida, Anystidae: *Erythracarus* sp., Labdostomatida, Rhagidiidae, Trombidiforme), Amblypygi (Phrynidae: *Heterophrynus longicornis*), Pseudoscopiones (Chernetidae, Chtoniidae), Opiliones (Sclerosomatidae: *Prionostemma* sp.) Araneae (Dipluridae, Gnaphosidae, Oonopidae: Oonopinae, Salticidae, Scytodidae*: Scytodes eleonora*, Pholcidae, Theraphosidae, Theridiidae, Theridiosomatidae), Thysanura (Nicoletiidae: Nicoletiinae, Atelurinae), Diplura (Campodeidae), Collembola (Entomobryidae), Orthoptera (Phalangopsidae: *Aclodes* sp., *Phalangopsis* sp.), Blattodea (Blaberidae: *Blaberus* sp., Blattidae, Polyphagidae), Embiidina, Isoptera (Termitidae: *Cornitermes* sp., *Nasutitermes* sp.), Psocoptera (Epipsocidae, Myopsocidae: *Lichenomina* sp.), Hemiptera (Aphididae, Cydnidae, Dipsocoridae, Nabidae), Homoptera (Cixiidae: *Cixius* sp.), Lepidoptera (Arctiidae: Arctiinae, Tineidae), Diptera (Culicidae, Dolichopodidae, Drosophilidae, Milichiidae, Psychodidae: *Lutzomyia* sp., Sciaridae, Streblidae), Hymenoptera (Formicidae: *Camponotus* sp., *Dolichoderus* sp., *Ectatomma* sp., *Gigantiops* sp., Myrmicinae, *Pachycondyla* sp., *Pheidole* sp., *Solenopsis* sp., *Strumigenys* sp; Bethylidae; Vespidae), Coleoptera (Carabidae, Cetoniidae: *Gymnets* sp., Chrysomelidae, Curculionidae: Scotylinae, Elateridae: Cardiophorinae, Leiodidae, Pselaphidae, Scydmaenidae, Staphylinidae), Geophilomorpha (Geophilidae), Lithobiomorpha (Henicopiidae: *Lamyctes* sp.), Scolopendromorpha (Scolopocryptopidae: *Newportia* sp., *Tidops* sp.), Scutigeromorpha (Scutigeridae: *Sphendononema* sp.) e Symphyla (Scutigerellidae: *Hanseniella* sp.).

Dentre os vertebrados foram encontrados duas espécies de Anura (Lepdoctylidae: *Pristimantis* cf. *fenestratus*, *Eleutherodactylus* sp).

Desta forma, no total foram encontrados 120 morfoespécies. Entre estas, três espécies de invertebrados foram consideradas troglomórficas: Gastropoda (Systrophiidae), Isopoda (Plathyarthridae: *Trichorhina* sp.) e Coleoptera (Scydmaenidae).

5.4.4.4.3. Caracterização faunística no período de chuva

Foi observado na caverna, um total de 117 morfoespécies de invertebrados de pelo menos 71 famílias das Ordens: Oligochaeta, Gastropoda (Subulinidae), Isopoda (Balloniscidae, Philosciidae), Acari (Argasidae: *Ornithodoros marinkellei*, Laelapidae, Ologamasidae, Mesostigmata, Oribatida, Cunaxidae, Teneriffiidae, Trombidiforme), Ricinulei (Ricinoididae: *Cryptocellus tarsilae*), Pseudoscopiones, Opiliones (Kimmulidae, Manaosbiidae, Sclerosomatidae: *Prionostemma* sp., Stygnidae), Araneae (Filistatidae, Gnaphosidae,

Linyphiidae, Ochyroceratidae, Oonopidae: Oonopinae, Salticidae, Scytodidae*: Scytodes eleonora*, Pholcidae: *Mesabolivar* sp., Theridiidae), Thysanura (Nicoletiidae: Atelurinae), Diplura (Campodeidae, Japygidae), Collembola (Sminthuridae, Hypogastruridae, Isotomidae), Orthoptera (Gryllidae: Gryllinae; Phalangopsidae; Tetrigidae; Tettigoniidae: Copiphorinae), Blattodea (Blattidae), Embiidina, Isoptera (Rhinotermitidae: *Heterotermes* sp., Termitidae: *Nasutitermes* sp.), Psocoptera (Ptiloneuridae: *Ptiloneura* sp., Trogiidae), Lepidoptera (Tineidae), Hemiptera (Alydidae, Dipsocoridae, Gerridae, Pyrrhocoridae, Veliidae), Homoptera (Fulgoridae), Diptera (Agromyzidae, Chironomidae, Conopidae, Culicidae, Muscidae, Tipulidae), Hymenoptera (Formicidae: *Acanthostichus* sp., *Azteca* sp., *Camponotus* sp., *Crematogaster* sp., *Leptogenys* sp., Myrmicinae, *Pachycondyla* sp., *Pheidole* sp., *Rogeria* sp; Apidae; Bethylidae; Braconidae; Eulophidae), Thysanoptera (Phaleothripidae: Phaleothripinae), Coleoptera (Carabidae, Curculionidae: Scotylinae, Elmidae, Lampiridae, Phalacridae, Pselaphidae, Ptylidae, Rhizophagidae, Staphylinidae), Diplopoda (Chelodesmidae), Geophilomorpha (Schendylidae), Lithobiomorpha (Henicopiidae: *Lamyctes* sp.), Scolopendromorpha (Scolopocryptopidae: *Dinocryptops* sp.) e Neuroptera (Myrmeleontidae).

Dentre estas, seis espécies de invertebrados foram consideradas troglomórficas: Acari (Trombidiforme), Opiliones, Araneae (Ochyroceratidae spp.) e Thysanura (Nicoletiidae: Atelurinae).

5.4.4.4.4. Caracterização geral da fauna da cavidade

Foi observado na caverna, um total de 228 morfoespécies de invertebrados de pelo menos 116 famílias das Ordens: Oligochaeta, Gastropoda (Subulinidae, Systrophiidae, Valloniidae), Isopoda (Armadillidae, Balloniscidae, Philosciidae, Plathyarthridae: *Trichorhina* sp.), Acari (Argasidae: *Ornithodoros marinkellei*, Laelapidae: *Stratiolaelaps* sp., Macronyssidae, Ologamasidae, Otopheidomenidae, Mesostigmata, Uropodina, Astigmatina, Oribatida, Anystidae: *Erythracarus* sp., Cunaxidae, Labdostomatida, Rhagidiidae, Teneriffiidae, Trombidiforme), Amblypygi (Phrynidae: *Heterophrynus longicornis*), Ricinulei (Ricinoididae: *Cryptocellus tarsilae*), Pseudoscopiones (Chernetidae, Chtoniidae), Opiliones (Kimmulidae, Manaosbiidae, Sclerosomatidae: *Prionostemma* sp., Stygnidae), Araneae (Dipluridae, Filistatidae, Gnaphosidae, Linyphiidae, Ochyroceratidae, Oonopidae: Oonopinae, Salticidae, Scytodidae*: Scytodes eleonora*, Pholcidae: *Mesabolivar* sp., Theraphosidae, Theridiidae, Theridiosomatidae), Thysanura (Nicoletiidae: Nicoletiinae, Atelurinae), Diplura (Campodeidae, Japygidae), Collembola (Sminthuridae, Entomobryidae, Hypogastruridae, Isotomidae), Orthoptera (Gryllidae: Gryllinae; Phalangopsidae: *Aclodes* sp., *Phalangopsis* sp.; Tetrigidae; Tettigoniidae: Copiphorinae), Blattodea (Blaberidae: *Blaberus* sp., Blattidae, Polyphagidae), Embiidina, Isoptera (Rhinotermitidae: *Heterotermes* sp., Termitidae: *Cornitermes* sp., *Nasutitermes* sp.), Psocoptera (Epipsocidae, Myopsocidae: *Lichenomina* sp., Ptiloneuridae: *Ptiloneura* sp., Trogiidae), Hemiptera (Alydidae, Aphididae, Cydnidae,

Dipsocoridae, Gerridae, Nabidae, , Pyrrhocoridae, Veliidae), Homoptera (Cixiidae: *Cixius* sp., Fulgoridae), Lepidoptera (Arctiidae: Arctiinae, Tineidae), Diptera (Agromyzidae, Chironomidae, Conopidae, Culicidae, Dolichopodidae, Drosophilidae, Fanniidae, Milichiidae, Muscidae, Psychodidae: *Lutzomyia* sp., Sciaridae, Streblidae, Tipulidae), Hymenoptera (Formicidae: *Acanthostichus* sp., *Azteca* sp., *Camponotus* sp., *Crematogaster* sp., *Dolichoderus* sp., *Ectatomma* sp., *Gigantiops* sp., *Leptogenys* sp., Myrmicinae, *Pachycondyla* sp., *Pheidole* sp., *Rogeria* sp., *Solenopsis* sp., *Strumigenys* sp; Apidae; Bethylidae; Braconidae; Eulophidae; Vespidae), Thysanoptera (Phaleothripidae: Phaleothripinae), Coleoptera (Carabidae, Cetoniidae: *Gymnets* sp., Chrysomelidae, Curculionidae: Scotylinae, Elateridae: Cardiophorinae, Elmidae, Lampiridae, Leiodidae, Phalacridae, Pselaphidae, Ptylidae, Rhizophagidae, Scydmaenidae, Staphylinidae), Diplopoda (Chelodesmidae), Geophilomorpha (Geophilidae, Schendylidae), Lithobiomorpha (Henicopiidae: *Lamyctes* sp.), Scolopendromorpha (Scolopocryptopidae: *Dinocryptops* sp., *Newportia* sp., *Tidops* sp.), Scutigeromorpha (Scutigeridae: *Sphendononema* sp.), Symphyla (Scutigerellidae: *Hanseniella* sp.) e Neuroptera (Myrmeleontidae).

Dentre os vertebrados foram encontrados duas espécies de Anura (Lepdoctylidae: *Pristimantis* cf. *fenestratus*, *Eleutherodactylus* sp).
Desta forma, no total foram encontrados 230 morfoespécies. Entre estas, nove espécies de invertebrados foram consideradas troglomórficas: Gastropoda (Systrophiidae), Isopoda (Plathyarthridae: *Trichorhina* sp.), Acari (Trombidiforme), Opiliones, Araneae (Ochyroceratidae), Thysanura (Nicoletiidae: Atelurinae), Coleoptera (Scydmaenidae). Alguns organismos encontrados nesta caverna são mostrados na Figura 103.

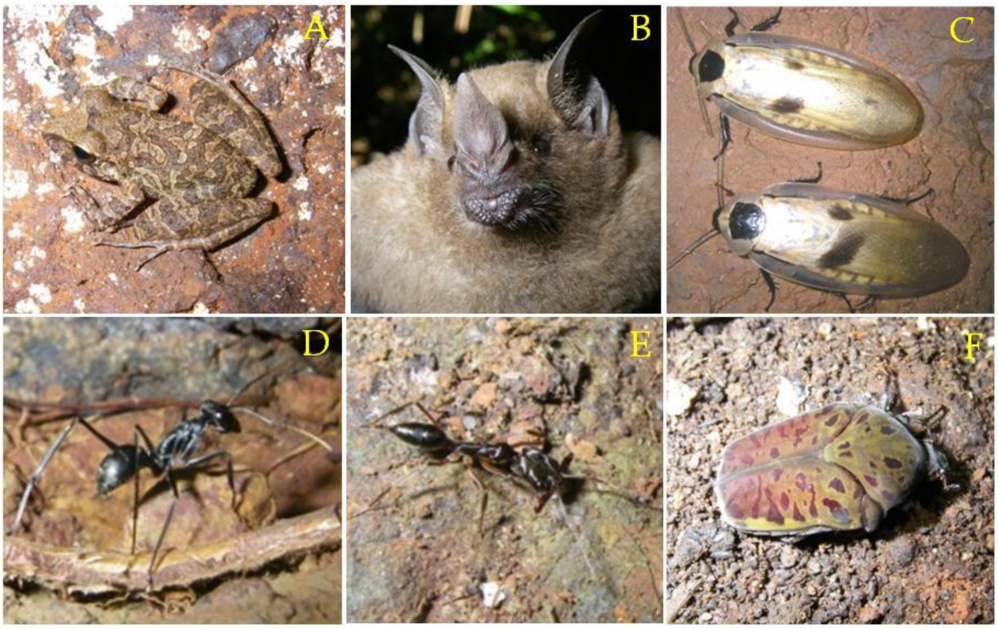

Figura 103 - a) Leptodactylidae (*Pristimantis cf. fenestratus*); b) Phyllostomidae (*Carollia* sp); c) Blaberidae (*Blaberus* sp.).; d) Formicidae (*Gigantiops* sp.); e) Formicidae (*Odontomachus* sp.); f) Coleoptera (Cetoniidae: *Gymnets* sp.)

5.4.4.5. SL-005

5.4.4.5.1. Caracterização trófica

Pequena cavidade com projeção horizontal de 15,5 m sem zona afótica localizada em área de mata ciliar na margem direita do rio e sujeita a inundação em períodos de chuva. Entrada ampla e sombreada pela mata, com líquens e briófitas associadas ao piso e paredes além de muita serrapilheira restrita a linha d'água. Existem grandes blocos abatidos junto à porção direita da entrada e trechos de teto baixo onde os recursos alimentares tornam-se aparentemente mais escassos. De maneira geral, toda sua extensão é composta por um piso predominantemente plano e seco formado por sedimento granulado com poucos blocos (seixos e calhaus) esparsos. O sistema radicular é bem desenvolvido com inúmeras raízes apresentando-se de forma superficial e sub-superficial. Apesar de não terem sido observados morcegos frugívoros existem dois depósitos de guano desta natureza na zona de entrada (Figura 104). O sistema de canalículos é pouco desenvolvido e cavidade apresenta uma baixa estabilidade ambiental. As principais alterações observadas durante a estação úmida foi a existência de vários pontos de gotejamento ativos ao longo de toda a extensão da cavidade e um aumento na quantidade de lianas associadas ao piso da cavidade.

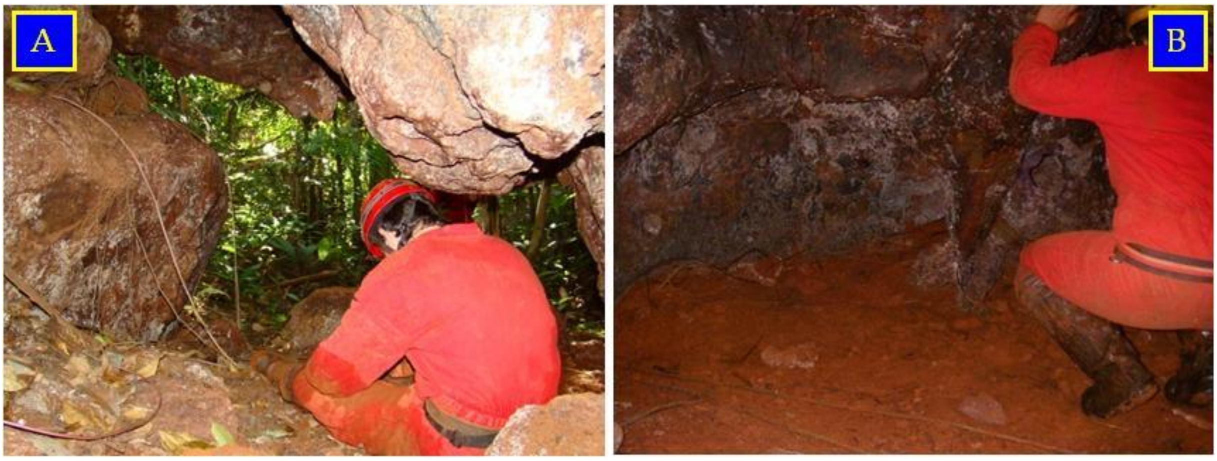

Figura 104 -) Pórtico de entrada da cavidade em área de mata ciliar com muitas raízes e serrapilheira esparsa pelo piso da cavidade; b) Parte interna da cavidade, onde existe um sistema radicular superficial bem desenvolvido.

5.4.4.5.2. Caracterização faunística no período de seca

Foi observado na caverna, um total de 45 morfoespécies de invertebrados de pelo menos 32 famílias das Ordens: Isopoda (Plathyarthridae: *Trichorhina* sp.), Acari (Mesostigmata), Amblypygi (Phrynidae: *Heterophrynus longicornis*), Pseudoscorpiones (Chernetidae), Opiliones (Escadabiidae, Sclerosomatidae: *Prionostemma* sp.), Araneae (Oonopidae: Oonopinae, Salticidae, Pholcidae: *Mesabolivar* sp., Theraphosidae), Diplura (Campodeidae), Collembola (Cyphoderidae, Entomobryidae), Orthoptera (Gryllidae: Nemobiinae), Isoptera

(Termitidae: *Diversitermes* sp., *Nasutitermes* sp.), Psocoptera (Epipsocidae), Hemiptera (Cydnidae), Homoptera (Cixiidae, Membracidae), Diptera (Drosophilidae, Mycetophilidae, Phoridae, Psychodidae: *Lutzomyia* sp.), Hymenoptera (Formicidae: *Apterostigma* sp., *Azteca* sp., *Dolychoderus* sp., *Pachycondyla* sp.; Eulophidae), Coleoptera (Carabidae, Pselaphidae, Scydmaenidae, Staphylinidae), Diplopoda (Pseudonannolenidae), Scolopendromorpha (Scolopocryptopidae: *Newportia* sp., *Tidops* sp.) e Symphyla (Scutigerellidae: *Hanseniella* sp.).

Dentre os vertebrados foram encontrados seis espécies das Ordens: Chiroptera (Emballonuridae: *Peropteryx kappleri*), Squamata (Gekkonidae: *Thecadactylus rapicauda*, Sphaerodactylidae: *Coleodactylus* cf. *amazonicus*), Anura (Leptodactylidae: *Pristimantis* cf. *fenestratus, Eleutherodactylus* sp.; Bufonidae).

Desta forma, no total foram encontrados 51 morfoespécies. Entre estas, duas espécies de invertebrados foram consideradas troglomórficas: Isopoda (Plathyarthridae: *Trichorhina* sp.) e Opiliones (Escadabiidae).

5.4.4.5.3. Caracterização faunística no período de chuva

Foi observado na caverna, um total de 70 morfoespécies de invertebrados de pelo menos 53 famílias das Ordens: Gastropoda (Subulinidae, Systrophiidae), Turbellaria (Geoplanidae), Isopoda (Armadillidae, Balloniscidae, Plathyarthridae: *Trichorhina* sp.), Acari (Ologamasidae, Oribatida, Eupodidae: *Linopodes* sp.), Amblypygi (Prhynidae: *Heterophrynus longicornis*), Pseudoscorpiones (Chernetidae, Chtoniidae), Opiliones (Neogoveidae: *Canga renatae*, Cosmetidae: *Anduzeia* sp., Escadabiidae, Sclerosomatidae: *Prionostemma* sp., Phalangiidae), Araneae (Ctenidae, Gnaphosidae, Linyphiidae, Salticidae, Scytodidae*: Scytodes eleonora*, Paratropidae, Psauridae, Pholcidae: *Mesabolivar* sp., Theridiidae, Theridiosomatidae), Diplura (Campodeidae), Collembola (Sminthuridae, Entomobryidae, Paronellidae), Orthoptera (Gryllidae: Nemobiinae, Phalangopsidae: *Phalangopsis* sp.), Blattodea (Blattidae), Isoptera (Termitidae: *Nasutitermes* sp.), Psocoptera (Epipsocidae, Lepidopsocidae), Hemiptera (Cydnidae, Ochteridae, Pyrrhocoridae), Homoptera (Cixiidae), Lepidoptera (Tineidae), Diptera (Ceratopogonidae, Chironomidae, Drosophilidae, Psychodidae: *Lutzomyia* sp., Sciaridae), Hymenoptera (Formicidae: *Basiceros* sp., *Carebara* sp., *Cyphomyrmex* sp., *Dolichoderus* sp., *Myrmicocrypta* sp., *Odontomachus* sp., *Pachycondyla* sp., *Pheidole* sp., *Solenopsis* sp.; Chalcidoidea), Coleoptera (Curculionidae: Scotylinae, Scydmaenidae, Staphylinidae) e Diplopoda (Chelodesmidae).

Dentre os vertebrados foi encontrada uma espécie de Chiroptera (Emballonuridae: *Peropteryx kappleri*).

Desta forma, no total foram encontrados 71 morfoespécies. Entre estas, duas espécies de invertebrados foram consideradas troglomórficas: Gastropoda (Systrophiidae) e Isopoda (Plathyarthridae: *Trichorhina* sp.).

5.4.4.5.4. Caracterização geral da fauna da cavidade

Foi observado na caverna, um total de 103 morfoespécies de invertebrados de pelo menos 64 famílias das Ordens: Gastropoda (Subulinidae, Systrophiidae), Turbellaria (Geoplanidae), Isopoda (Armadillidae, Balloniscidae, Plathyarthridae: *Trichorhina* sp.), Acari (Mesostigmata, Ologamasidae, Oribatida, Eupodidae: *Linopodes* sp.), Amblypygi (Phrynidae: *Heterophrynus longicornis*), Pseudoscorpiones (Chernetidae, Chtoniidae), Opiliones (Neogoveidae: *Canga renatae*, Cosmetidae: *Anduzeia* sp., Escadabiidae, Sclerosomatidae: *Prionostemma* sp., Phalangiidae), Araneae (Ctenidae, Gnaphosidae, Linyphiidae, Oonopidae: Oonopinae, Salticidae, Scytodidae*: Scytodes eleonora*, Paratropidae, Psauridae, Pholcidae: *Mesabolivar* sp., Theraphosidae, Theridiidae, Theridiosomatidae), Diplura (Campodeidae), Collembola (Sminthuridae, Cyphoderidae, Entomobryidae, Paronellidae), Orthoptera (Gryllidae: Nemobiinae, Phalangopsidae: *Phalangopsis* sp.), Blattodea (Blattidae), Isoptera (Termitidae: *Diversitermes* sp., *Nasutitermes* sp.), Psocoptera (Epipsocidae, Lepidopsocidae), Hemiptera (Cydnidae, Ochteridae, Pyrrhocoridae), Homoptera (Cixiidae, Membracidae), Lepidoptera (Tineidae), Diptera (Ceratopogonidae, Chironomidae, Drosophilidae, Mycetophilidae, Phoridae, Psychodidae: *Lutzomyia* sp., Sciaridae), Hymenoptera (Formicidae: *Apterostigma* sp., *Azteca* sp., *Basiceros* sp., *Carebara* sp., *Cyphomyrmex* sp., *Dolichoderus* sp., *Myrmicocrypta* sp., *Odontomachus* sp., *Pachycondyla* sp., *Pheidole* sp., *Solenopsis* sp.; Chalcidoidea; Eulophidae), Coleoptera (Carabidae, Curculionidae:Scotylinae, Pselaphidae, Scydmaenidae, Staphylinidae), Diplopoda (Chelodesmidae, Pseudonannolenidae), Scolopendromorpha (Scolopocryptopidae: *Newportia* sp., *Tidops* sp.) e Symphyla (Scutigerellidae: *Hanseniella* sp.).

Dentre os vertebrados foram encontrados seis espécies das Ordens: Chiroptera (Emballonuridae: *Peropteryx kappleri*), Squamata (Gekkonidae: *Thecadactylus rapicauda*, Sphaerodactylidae: *Coleodactylus* cf. *amazonicus*), Anura (Leptodactylidae: *Pristimantis* cf. *fenestratus, Eleutherodactylus* sp.; Bufonidae).
Desta forma, no total foram encontrados 109 morfoespécies. Entre estas, três espécies de invertebrados foram consideradas troglomórficas: Gastropoda (Systrophiidae), Isopoda (Plathyarthridae: *Trichorhina* sp.) e Opiliones (Escadabiidae). Alguns organismos encontrados nesta caverna são mostrados na figura 105.

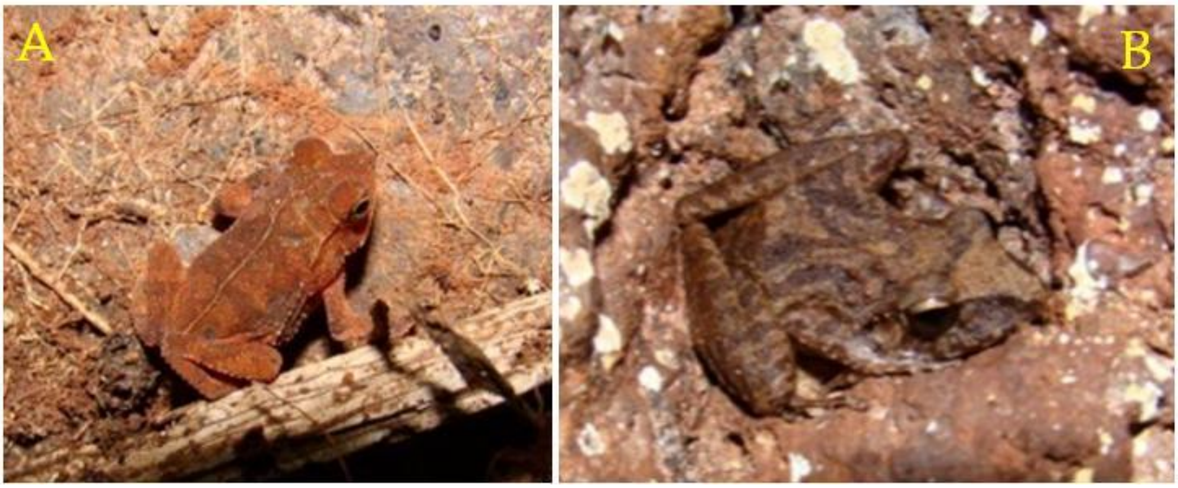

Figura 105 - a) Anfíbio anuro; b) Leptodactylidae (*Pristimantis cf. fenestratus*);

5.4.4.6. SL-006

5.4.4.6.1. Caracterização trófica

Pequena cavidade (11,6 m) formada na canga localizada em área de mata ciliar na margem direita do rio que abastece a vila de Serra Pelada. Apresenta uma entrada larga e um conduto pouco profundo o que não permite a existência de zonas completamente afóticas. A entrada é sombreada, apresentando muitos líquens e briófitas associadas, além de grandes depósitos de serrapilheira restritos à linha d'água. De forma geral o piso é plano e seco composto por sedimento granulado com muitos blocos (calhaus e matacões) abatidos além de algumas raízes de pequeno calibre concentradas em alguns setores da cavidade. A água e o vento apresentam importante função na importação de recursos orgânicos para o interior da cavidade, sendo esta muito influenciada pelas condições ambientais epígeas o que resulta em uma baixa estabilidade ambiental. Junto à entrada ainda existem depósitos antigos de guano de morcegos frugívoros e o sistema de canalículos pode ser considerado pouco desenvolvido (Figura 106). Durante a estação úmida a principal alteração observada foi que a vegetação de entorno havia sido queimada. Sendo assim, a mata ciliar encontrava-se pouco densa e a entrada da caverna encontrava-se completamente ensolarada. Além disso, existia um pequeno gotejamento ativo na zona mais distal da cavidade e muitas cinzas espalhadas pelo substrato.

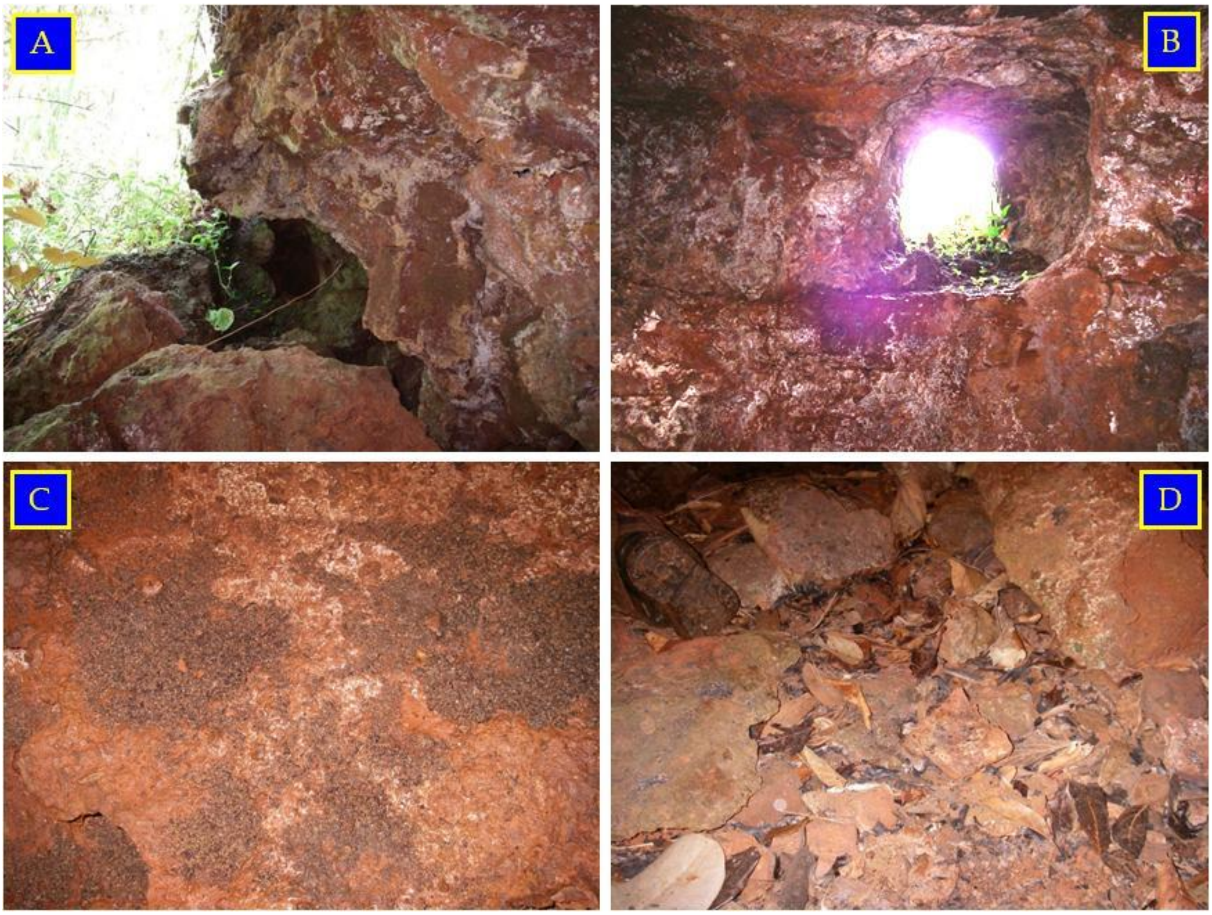

Figura 106 - a) Porção externa à cavidade vista do interior da entrada; b) Interior da caverna onde se observa a condição topográfica do conduto que se segue à entrada; c) Guano envelhecido de morcegos frugívoros presente no piso da caverna; d) Grande quantidade de serrapilheira acumulada junto à linha d'água.

5.4.4.6.2. Caracterização faunística no período de seca

Foi observado na caverna, um total de 28 morfoespécies de invertebrados de pelo menos 20 famílias das Ordens: Isopoda (Scleropactidae), Acari (Laelapidae: *Stratiolaelaps* sp., Astigmatina, Oribatida), Amblypygi (Phrynidae: *Heterophrynus longicornis*), Pseudoscorpiones (Chernetidae, Chtoniidae), Araneae (Theraphosidae), Diplura (Anajapygidae, Campodeidae), Collembola (Entomobryidae), Blattodea (Blattidae, Polyphagidae), Isoptera (Rhinotermitidae: *Heterotermes* sp.), Hemiptera (Cydnidae), Lepidoptera (Tineidae), Diptera (Bibionidae), Hymenoptera (Formicidae: *Cyphomyrmex* sp., *Odontomachus* sp., *Pachycondyla* sp., *Rogeria* sp., *Zacryptocerus* sp.; Bethylidae), Coleoptera (Pselaphidae) e Scutigeromorpha (Scutigeridae: *Sphendononema* sp.).

Dentre os vertebrados foram encontrados quatro espécies das Ordens: Squamata (Gekkonidae: *Thecadactylus rapicauda*, Sphaerodactylidae: *Coleodactylus* cf. *amazonicus*), Anura (Leptodactylidae: *Pristimantis* cf. *fenestratus*, sp. indet.). Desta forma, no total foram encontrados 32 morfoespécies. Entre estas, uma espécie de Coleoptera (Pselaphidae) foi considerada troglomórfica.

5.4.4.6.3. Caracterização faunística no período de chuva

Foi observado na caverna, um total de 48 morfoespécies de invertebrados de pelo menos 33 famílias das Ordens: Gastropoda (Systrophiidae), Isopoda (Armadillidae, Dubioniscidae, Philosciidae, Plathyarthridae: *Trichorhina* sp.), Acari (Oribatida, Trombidiforme), Amblypygi (Charinidae: *Charinus* sp.), Pseudoscorpiones (Chernetidae), Opiliones (Cosmetidae: *Anduzeia* sp. Phalangiidae), Araneae (Ctenidae: *Ctenus* sp., Salticidae, Scytodidae*: Scytodes eleonora*, Psauridae, Pholcidae: *Metagonia* sp.), Blattodea (Polyphagidae), Isoptera (Termitidae: *Nasutitermes* sp.), Psocoptera (Psyllipsocidae: *Psyllipsocus* sp.), Homoptera (Cixiidae: *Cixius* sp.), Lepidoptera (Noctuidae, Tineidae), Diptera (Cecidomyiidae, Ceratopogonidae, Chironomidae, Micropezidae, Phoridae, Psychodidae: *Lutzomyia* sp.), Hymenoptera (Formicidae: *Myrmicocrypta* sp., *Pachycondyla* sp., *Pseudomyrmex* sp., *Rogeria* sp.; Braconidae, Diapriidae), Coleoptera (Leiodidae, Pselaphidae, Scydmaenidae) e Diplopoda (Siphonophoridae). Dentre os vertebrados foram encontrados uma espécie de Anura (Leptodactylidae: *Pristimantis* cf. *fenestratus*).

Desta forma, no total foram encontrados 49 morfoespécies. Entre estas, três espécies de invertebrados foram consideradas troglomórficas: Gastropoda (Systrophiidae), Isopoda (Plathyarthridae: *Trichorhina* sp.) e Amblypygi (Charinidae: *Charinus* sp.).

5.4.4.6.4. Caracterização geral da fauna da cavidade

Foi observado na caverna, um total de 71 morfoespécies de invertebrados de pelo menos 48 famílias das Ordens: Gastropoda (Systrophiidae), Isopoda (Armadillidae, Dubioniscidae, Philosciidae, Plathyarthridae: *Trichorhina* sp., Scleropactidae), Acari (Laelapidae: *Stratiolaelaps* sp., Astigmatina, Oribatida, Labdostomatidae: *Labdostomatida* sp., Trombidiforme), Amblypygi (Phrynidae: *Heterophrynus longicornis*, Charinidae: *Charinus* sp.), Pseudoscorpiones (Chernetidae, Chtoniidae), Opiliones (Cosmetidae: *Anduzeia* sp., Phalangiidae), Araneae (Ctenidae: *Ctenus* sp., Salticidae, Scytodidae*: Scytodes eleonora*, Psauridae, Pholcidae: *Metagonia* sp., Theraphosidae), Diplura (Anajapygidae, Campodeidae), Collembola (Entomobryidae), Blattodea (Blattidae, Polyphagidae), Isoptera (Rhinotermitidae: *Heterotermes* sp., Termitidae: *Nasutitermes* sp.), Psocoptera (Psyllipsocidae: *Psyllipsocus* sp.), Hemiptera (Cydnidae), Homoptera (Cixiidae: *Cixius* sp.), Lepidoptera (Noctuidae, Tineidae), Diptera (Bibionidae, Cecidomyiidae, Ceratopogonidae, Chironomidae, Micropezidae, Phoridae, Psychodidae: *Lutzomyia* sp.), Hymenoptera (Formicidae: *Cyphomyrmex* sp., *Myrmicocrypta* sp., *Odontomachus* sp., *Pachycondyla* sp., *Pseudomyrmex* sp., *Rogeria* sp., *Zacryptocerus* sp.; Bethylidae, Braconidae, Diapriidae), Coleoptera (Leiodidae, Pselaphidae, Scydmaenidae), Diplopoda (Siphonophoridae) e Scutigeromorpha (Scutigeridae: *Sphendononema* sp.).

Dentre os vertebrados foram encontrados quatro espécies das Ordens: Squamata (Gekkonidae: *Thecadactylus rapicauda*, Sphaerodactylidae: *Coleodactylus* cf. *amazonicus*), Anura (Leptodactylidae: *Pristimantis* cf. *fenestratus*, sp. indet.).

Desta forma, no total foram encontrados 75 morfoespécies. Entre estas, quatro espécies de invertebrados foram consideradas troglomórficas: Gastropoda (Systrophiidae), Isopoda (Plathyarthridae: *Trichorhina* sp.), Amblypygi (Charinidae: *Charinus* sp.) e Coleoptera (Pselaphidae). Alguns organismos encontrados nesta caverna são mostrados na Figura 107.

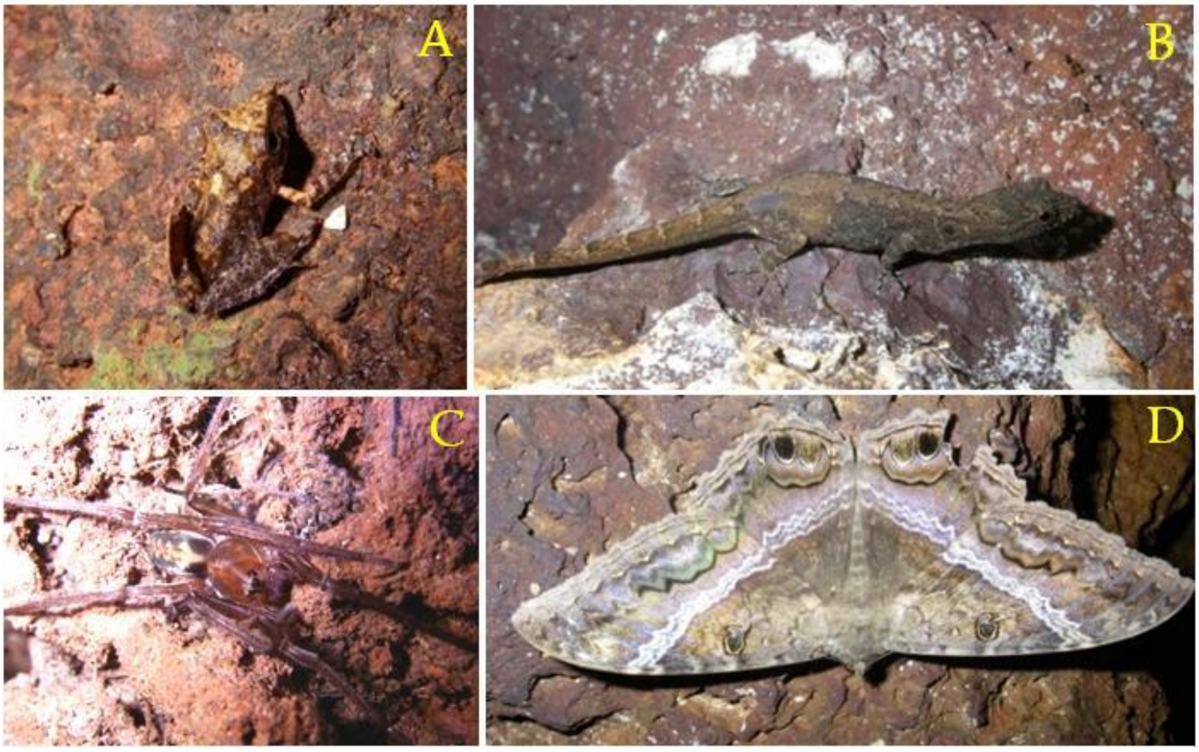

Figura 107 - a) Leptodactylidae (indet.); b) Sphaerodactylidae (*Coleodactylus cf. amazonicus*); Ctenidae (*Ctenus* sp.); d) Lepidoptera (Noctuidae: *Latebraria* sp.).

5.4.4.7. SL-007

5.4.4.7.1. Caracterização trófica

Pequeno abrigo localizado em área de mata ciliar na margem esquerda do rio com projeção horizontal de 5,5 metros. Trata-se de uma cavidade predominantemente seca que não apresenta sinais de inundações uma vez que esta se encontra em um nível mais elevado do que a situação atual do leito do rio. Sua entrada é sombreada e de pequenas dimensões com líquens, fungos, briófitas, pteridófitas e plântulas. A cavidade apresenta somente zona eufótica e seu piso é plano composto por sedimento granulado com alguns seixos e calhaus distribuídos de forma esparsa. O sistema radicular é pouco desenvolvido, sendo este composto apenas por poucas raízes muito finas e sub-superficiais, esparsas pelo piso da

cavidade. O teto encontra-se praticamente todo revestido por Actinomicetos e não existem depósitos de guano no interior da cavidade (Figura 108). Apesar de encontrar-se inserida na canga o sistema de canalículos é pouco desenvolvido e a cavidade apresenta uma baixa estabilidade ambiental. Nenhuma alteração significativa foi observada durante a estação úmida além das alterações normais na umidade relativa do ar.

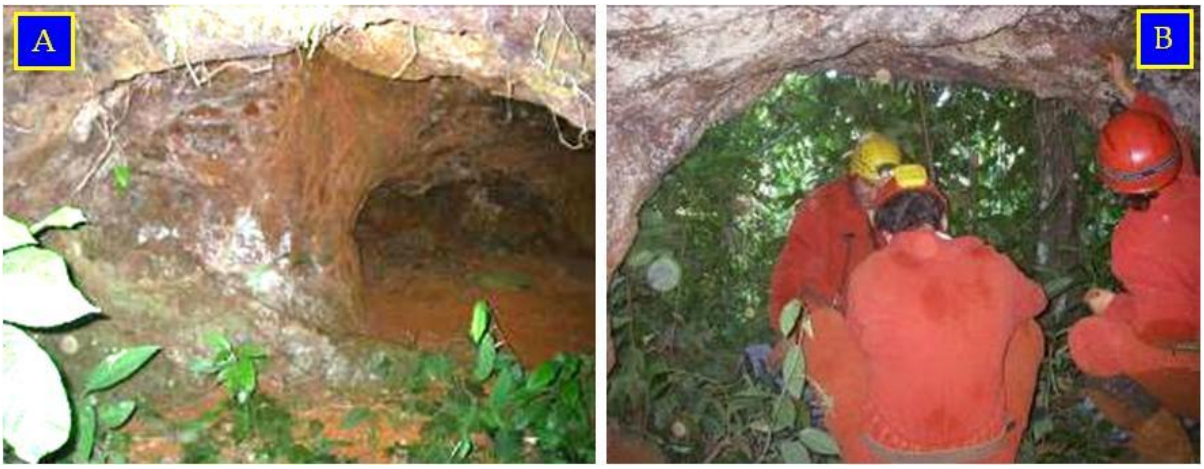

Figura 108 - a) Aspecto geral da entrada com muitas angiospermas; b) Aspecto geral do teto da cavidade completamente revestido por Actinomicetos.

5.4.4.7.2. Caracterização faunística no período de seca

Foi observado na caverna, um total de 31 morfoespécies de invertebrados de pelo menos 25 famílias das Ordens: Isopoda (Armadillidae, Styloniscidae), Acari (Rhagidiidae), Amblypygi (Phrynidae: *Heterophrynus longicornis*), Pseudoscorpiones (Chernetidae, Chtoniidae), Opiliones (Cosmetidae: *Anduzeia* sp.), Araneae (Araneidae: *Alpaida* sp., Dipluridae, Gnaphosidae, Scytodidae: *Scytodes eleonora*), Diplura (Campodeidae), Collembola (Entomobryidae), Orthoptera (Phalangopsidae: *Aclodes* sp.), Blattodea (Blaberidae: *Blaberus* sp., Blattidae, Polyphagidae), Isoptera (Termitidae: *Nasutitermes* sp.), Hemiptera (Cydnidae, Reduviidae), Lepidoptera (Noctuidae), Diptera (Simulidae), Hymenoptera (Formicidae: *Camponotus* sp., *Dolichoderus* sp., *Pachycondyla* sp.; Vespidae) e Symphyla (Scutigerellidae: *Hanseniella* sp.).

Dentre os vertebrados foram encontradas duas espécies das Ordens: Chiroptera (Phyllostomidae: *Glossophaga soricina*) e Anura (Leptodactylidae: *Pristimantis* cf. *fenestratus*). Desta forma, no total foram encontradas 33 morfoespécies. Entre estas, uma espécie de Isopoda (Styloniscidae) foi considerada troglomórfica.

5.4.4.7.3. Caracterização faunística no período de chuva

Foi observado na caverna, um total de 38 morfoespécies de invertebrados de pelo menos 26 famílias das Ordens: Isopoda (Armadillidae), Acari (Mesostigmata, Oribatida), Amblypygi

(Phrynidae: *Heterophrynus longicornis*), Pseudoscorpiones (Chernetidae, Chtoniidae), Araneae (Araneidae: *Alpaida* sp., Gnaphosidae, Oonopidae: Oonopinae, Salticidae, Scytodidae*: Scytodes eleonora*, Pholcidae: *Mesabolivar* sp., Theraphosidae), Thysanura (Nicoletiidae: Nicoletiinae), Collembola (Entomobryidae), Orthoptera (Phalangopsidae: *Aclodes* sp.), Blattodea (Blattidae, Polyphagidae), Isoptera (Termitidae: *Nasutitermes* sp.), Hemiptera (Cydnidae, Lygaeidae), Homoptera (Cercopidae, Kinnaridae), Lepidoptera (Noctuidae, Tineidae), Diptera (Milichiidae, Psychodidae: *Lutzomyia* sp.), Hymenoptera (Formicidae: *Apterostigma* sp., *Myrmicocrypta* sp., *Pheidole* sp., *Rogeria* sp., *Trachymyrmex* sp.), Coleoptera (Carabidae, Staphylinidae) e Symphyla (Scutigerellidae: *Scutigerella* sp.).

5.4.4.7.4. Caracterização geral da fauna da cavidade

Foi observado na caverna, um total de 59 morfoespécies de invertebrados de pelo menos 38 famílias das Ordens: Isopoda (Armadillidae, Styloniscidae), Acari (Mesostigmata, Oribatida, Rhagidiidae), Amblypygi (Phrynidae: *Heterophrynus longicornis*), Pseudoscorpiones (Chernetidae, Chtoniidae), Opiliones (Cosmetidae: *Anduzeia* sp.), Araneae (Araneidae: *Alpaida* sp., Dipluridae, Gnaphosidae, Oonopidae: Oonopinae, Salticidae, Scytodidae*: Scytodes eleonora*, Pholcidae: *Mesabolivar* sp., Theraphosidae), Thysanura (Nicoletiidae: Nicoletiinae), Diplura (Campodeidae), Collembola (Entomobryidae), Orthoptera (Phalangopsidae: *Aclodes* sp.), Blattodea (Blaberidae: *Blaberus* sp., Blattidae, Polyphagidae), Isoptera (Termitidae: *Nasutitermes* sp.), Hemiptera (Cydnidae, Lygaeidae, Reduviidae), Homoptera (Cercopidae, Kinnaridae), Lepidoptera (Noctuidae, Tineidae), Diptera (Milichiidae, Psychodidae: *Lutzomyia* sp., Simulidae), Hymenoptera (Formicidae: *Apterostigma* sp., *Camponotus* sp., *Dolichoderus* sp., *Myrmicocrypta* sp., *Pachycondyla* sp., *Pheidole* sp., *Rogeria* sp., *Trachymyrmex* sp.; Vespidae), Coleoptera (Carabidae, Staphylinidae) e Symphyla (Scutigerellidae: *Hanseniella* sp., *Scutigerella* sp.).

Dentre os vertebrados foram encontradas duas espécies das Ordens: Chiroptera (Phyllostomidae: *Glossophaga soricina*) e Anura (Leptodactylidae: *Pristimantis* cf. *fenestratus*). Desta forma, no total foram encontradas 61 morfoespécies. Entre estas, uma espécie de Isopoda (Styloniscidae) foi considerada troglomórfica.

5.4.4.8 SL-008

5.4.4.8.1. Caracterização trófica

Caverna formada na canga com 20,4 m de projeção horizontal localizada em área de mata ciliar. Esta se encontra um pouco acima do nível atual da drenagem, embora não esteja suscetível a inundações. Possui duas entradas opostas entre as quais existe um trecho de teto baixo na parte central da cavidade. Ambas as entradas apresentam-se em condição descendente em relação ao meio epígeo. As entradas são sombreadas com muitos líquens,

fungos, musgos, pteridófitas e plantas distribuídas de maneira esparsa. O trecho da entrada norte é seco, descendente, com blocos abatidos e áreas de teto baixo. Nesta foram encontrados resíduos sólidos antigos (latas, arames, madeiramento) provavelmente abandonados por moradores da região. De forma geral o piso é descendente composto por sedimento granulado com poucos blocos (seixos e calhaus) distribuídos de maneira esparsa. O teto é completamente revestido por Actinomicetos e no piso não existem depósitos de guano. As raízes apresentam grosso calibre e ocorre apenas pouca serrapilheira. A cavidade não apresenta zona afótica, mas existem trechos de penumbra escura (Figura 109). A entrada sul é ampla e sombreada, sendo o piso e as paredes cobertos por musgo e muitas pteridófitas. O conduto que segue é descendente, apresentando muita serrapilheira transportada pela chuva e pelo vento onde existe um grande tronco caído além de rejeitos de formigueiros produzidos por formigas da tribo Attini. De forma geral, o sistema de canalículos é bem desenvolvido e a estabilidade ambiental é baixa existindo uma circulação intensa de ar entre as duas entradas. Durante a estação úmida foi observado um aumento na umidade do solo em ambas as entradas, além de um crescimento da vegetação epígea gerando um maior sombreamento sobre o pórtico da cavidade. Além disso, a quantidade de serrapilheira carreado para o interior da caverna apresentou um aumento significativo durante este período.

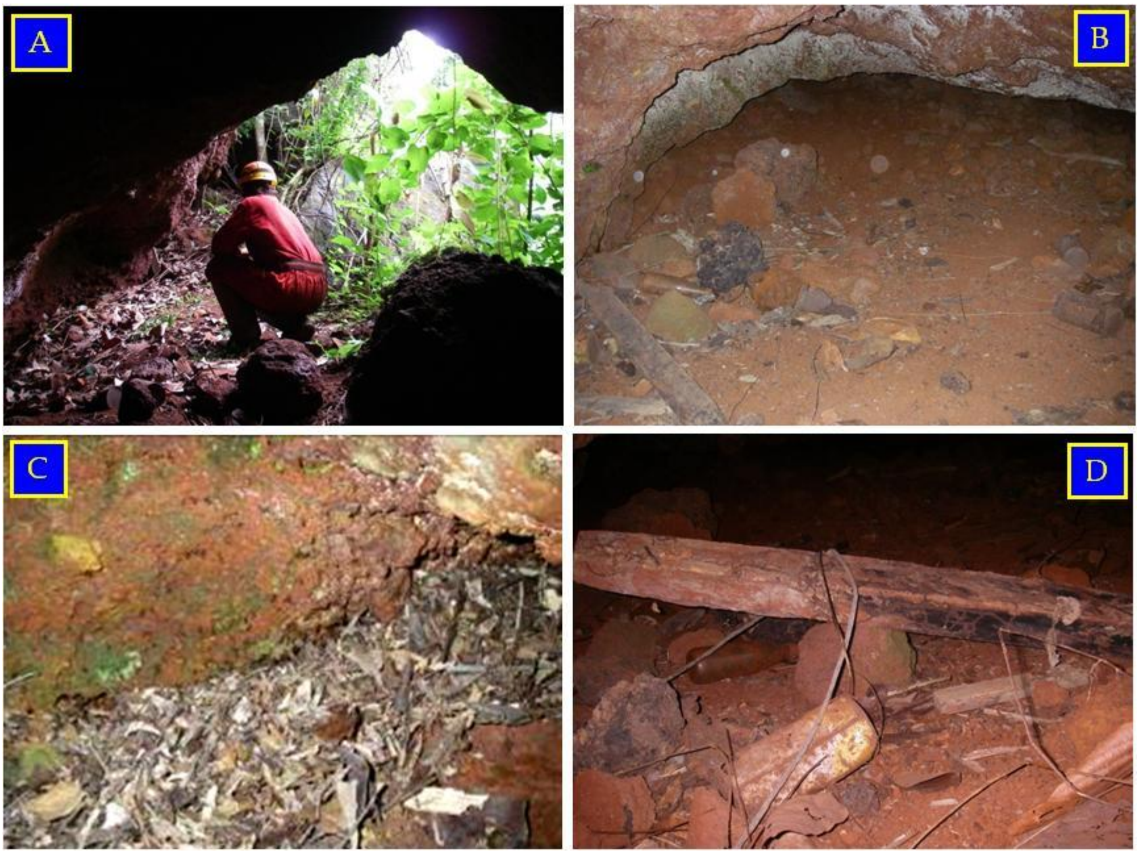

Figura 109 - a) Aspecto geral da entrada com muitas plantas, briófitas e pteridófitas; b) Vista da porção interna da cavidade; c) Rejeito de matéria orgânica descartado por formigas (Attini); d) Lixo despejado no interior da cavidade.

5.4.4.8.2. Caracterização faunística no período de seca

Foi observado na caverna, um total de 62 morfoespécies de invertebrados de pelo menos 42 famílias das Ordens: Onychophora (Peripatidae), Oligochaeta, Gastropoda (Systrophiidae), Isopoda (Armadillidae, Balloniscidae, Philosciidae, Scleropactidae), Acari (Neothyridae: *Diplothyrus schubarti*, Mesostigmata, Oribatida, Labdostomatidae: *Labdostomatida* sp.), Amblypygi (Phrynidae: *Heterophrynus longicornis*), Pseudoscorpiones (Chernetidae, Chtoniidae), Opiliones (Cosmetidae: *Anduzeia* sp., Sclerosomatidae: *Prionostemma* sp., Stygnidae: *Protimesius* aff. *gracilis*), Araneae (Araneidae: *Alpaida* sp., Oonopidae: Oonopinae, Pholcidae: *Mesabolivar* sp., Theraphosidae), Thysanura (Nicoletiidae: Nicoletiinae), Collembola (Entomobryidae, Isotomidae), Orthoptera (Phalangopsidae: *Aclodes* sp., *Phalangopsis* sp.), Blattodea (Blaberidae: *Blaberus* sp., Polyphagidae), Isoptera (Termitidae: *Diversitermes* sp., *Nasutitermes* sp., *Termes* sp.), Psocoptera (Epipsocidae), Hemiptera (Reduviidae), Homoptera (Cixiidae: *Cixius* sp.), Diptera (Ceratopogonidae, Culicidae, Dolichopodidae, Drosophilidae, Psychodidae: *Lutzomyia* sp., Tipulidae), Hymenoptera (Formicidae: *Acromyrmex* sp., *Camponotus* sp., *Dolichoderus* sp., *Gnamptogenys* sp., *Pachycondyla* sp., *Rogeria* sp; Chalcidoidea; Eulophidae; Vespidae), Coleoptera (Carabidae: Harpalinae, Scydmaenidae), Diplopoda (Polydesmida, Pseudonannolenidae) e Scutigeromorpha (Scutigeridae: *Sphendononema* sp.).

Dentre os vertebrados foram encontradas duas espécies das Ordens: Squamata (Gekkonidae: *Thecadactylus rapicauda*) e Anura (Leptodactylidae: *Pristimantis* cf. *fenestratus*). Desta forma, no total foram encontradas 64 morfoespécies.

5.4.4.8.3. Caracterização faunística no período de chuva

Foi observado na caverna, um total de 81 morfoespécies de invertebrados de pelo menos 57 famílias das Ordens: Onychophora (Peripatidae), Oligochaeta, Gastropoda (Subulinidae, Systrophiidae), Isopoda (Armadillidae, Balloniscidae), Acari (Opilioacaridae: *Neoacarus* spn., Oribatida, Rhagidiidae, Trombidiforme), Amblypygi (Phrynidae: *Heterophrynus longicornis*), Pseudoscorpiones (Chernetidae, Chtoniidae), Opiliones (Cosmetidae: *Anduzeia* sp., Escadabiidae, Fissiphaliidae, Sclerosomatidae: *Prionostemma* sp., Stygnidae: *Protimesius* aff. *gracilis*, Phalangiidae), Araneae (Araneidae: *Alpaida* sp., Ctenidae: *Ctenus* sp., Gnaphosidae, Ochyroceratidae, Oonopidae: Oonopinae, Salticidae, Scytodidae*: Scytodes eleonora*, Psauridae, Pholcidae: *Mesabolivar* sp., Theraphosidae, Theridiidae, Theridiosomatidae), Thysanura (Nicoletiidae: Atelurinae), Microcoryphia (Meinertellidae), Diplura (Campodeidae), Collembola (Isotomidae, Poduridae), Orthoptera (Phalangopsidae: *Aclodes* sp.), Blattodea (Blaberidae: *Blaberus* sp., Blattellidae), Isoptera, Psocoptera (Epipsocidae, Myopsocidae: *Lichenomina* sp.), Hemiptera (Cydnidae, Hebridae, Reduviidae), Lepidoptera (Tineidae), Diptera (Cecidomyiidae, Ceratopogonidae, Chloropidae, Culicidae, Sciaridae), Hymenoptera (Formicidae: *Camponotus* sp., *Cyphomyrmex* sp., *Pachycondyla* sp., *Pheidole*

sp., *Rogeria* sp; Vespidae), Coleoptera (Carabidae, Pselaphidae), Diplopoda (Chelodesmidae, Cyrtodesmidae), Geophilomorpha (Macronicophilidae: *Macronicophilus* sp.), Scolopendromorpha (Cryptopidae: *Cryptops* sp., Scolopocryptopidae: *Tidops* sp.), Symphyla (Scutigerellidae: *Scutigerella* sp.) e Neuroptera (Myrmeleontidae).

Dentre estas, uma espécie de Gastropoda (Systrophiidae) foi considerada troglomórfica.

5.4.4.8.4. Caracterização geral da fauna da cavidade

Foi observado na caverna, um total de 124 morfoespécies de invertebrados de pelo menos 76 famílias das Ordens: Onychophora (Peripatidae), Oligochaeta, Gastropoda (Subulinidae, Systrophiidae), Isopoda (Armadillidae, Balloniscidae, Philosciidae, Scleropactidae), Acari (Neothyridae: *Diplothyrus schubarti*, Mesostigmata, Opilioacaridae: *Neoacarus* spn., Oribatida, Labdostomatidae: *Labdostomatida* sp., Rhagidiidae, Trombidiforme), Amblypygi (Phrynidae: *Heterophrynus longicornis*), Pseudoscorpiones (Chernetidae, Chtoniidae), Opiliones (Cosmetidae: *Anduzeia* sp., Escadabiidae, Fissiphaliidae, Sclerosomatidae: *Prionostemma* sp., Stygnidae: *Protimesius* aff. *gracilis*, Phalangiidae), Araneae (Araneidae: *Alpaida* sp., Ctenidae: *Ctenus* sp., Gnaphosidae, Ochyroceratidae, Oonopidae: Oonopinae, Salticidae, Scytodidae*: Scytodes eleonora*, Psauridae, Pholcidae: *Mesabolivar* sp., Theraphosidae, Theridiidae, Theridiosomatidae), Thysanura (Nicoletiidae: Nicoletiinae, Atelurinae), Microcoryphia (Meinertellidae), Diplura (Campodeidae), Collembola (Entomobryidae, Isotomidae, Poduridae), Orthoptera (Phalangopsidae: *Aclodes* sp., *Phalangopsis* sp.), Blattodea (Blaberidae: *Blaberus* sp., Blattellidae, Polyphagidae), Isoptera (Termitidae: *Diversitermes* sp., *Nasutitermes* sp., *Termes* sp.), Psocoptera (Epipsocidae, Myopsocidae: *Lichenomina* sp.), Hemiptera (Cydnidae, Hebridae, Reduviidae), Homoptera (Cixiidae: *Cixius* sp.), Lepidoptera (Tineidae), Diptera (Cecidomyiidae, Ceratopogonidae, Chloropidae, Culicidae, Dolichopodidae, Drosophilidae, Psychodidae: *Lutzomyia* sp., Sciaridae, Tipulidae), Hymenoptera (Formicidae: *Acromyrmex* sp., *Camponotus* sp., *Cyphomyrmex* sp., *Dolichoderus* sp., *Gnamptogenys* sp., *Pachycondyla* sp., *Pheidole* sp., *Rogeria* sp; Chalcidoidea; Eulophidae; Vespidae), Coleoptera (Carabidae: Harpalinae, Pselaphidae, Scydmaenidae), Diplopoda (Chelodesmidae, Cyrtodesmidae, Polydesmida, Pseudonannolenidae), Geophilomorpha (Macronicophilidae: *Macronicophilus* sp.), Scolopendromorpha (Cryptopidae: *Cryptops* sp., Scolopocryptopidae: *Tidops* sp.), Scutigeromorpha (Scutigeridae: *Sphendononema* sp.), Symphyla (Scutigerellidae: *Scutigerella* sp.) e Neuroptera (Myrmeleontidae).

Dentre os vertebrados foram encontradas duas espécies das Ordens: Squamata (Gekkonidae: *Thecadactylus rapicauda*) e Anura (Leptodactylidae: *Pristimantis* cf. *fenestratus*). Desta forma, no total foram encontradas 126 morfoespécies. Entre estas, uma espécie de Gastropoda (Systrophiidae) foi considerada troglomórfica. Alguns organismos encontrados nesta caverna são mostrados na Figura 110.

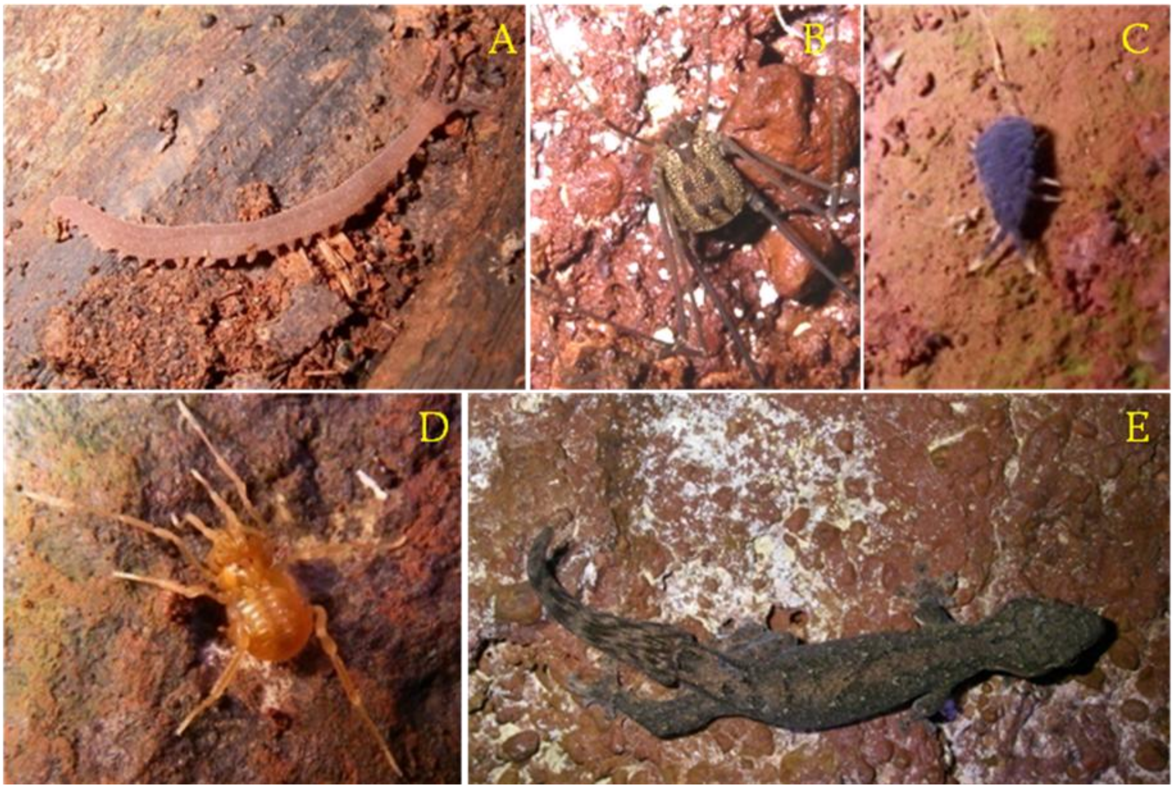

Figura 110 -) Onychophora (Peripatidae); b) Opiliones (Cosmetidae: *Anduzeia* sp.); Collembola (Hypogastruridae); d) Opiliones (Escadabiidae); e) Gekkonidae (*Thecadactylus rapicauda*).

5.4.4.9. SL-009

5.4.4.9.1. Caracterização trófica

Caverna alagada com projeção horizontal de 19,1 m localizada em área de mata ciliar na margem direita do rio. Nesta existe uma nascente à esquerda da projeção da linha d'água da cavidade que forma um pequeno lago em seu salão principal. Formada na canga, possui uma única entrada ampla e sombreada com muitos líquens, briófitas, pteridófitas e brotos de angiospermas. O principal recurso trófico é constituído por depósitos de guano de morcegos frugívoros produzidos por uma grande colônia de *G. soricina* e pela grande quantidade de matéria orgânica importada pela água. Grande parte deste guano encontra-se submerso pelo lago que constitui cerca de 80% do piso da cavidade. Nestes depósitos existem muitas plântulas germinadas, que também representam um importante recurso alimentar no sistema aquático (Figura 111). Nesta cavidade também foram observados exemplares de *P. kappleri* na zona de entrada e de *Anoura sp*. entremeados a outros Glossophaginae. Dentro do lago foram encontrados alguns esqueletos de vertebrados, que podem ter morrido no interior da cavidade ou terem sido carreados para a mesma. O setor da caverna que não se encontra alagado localiza-se em um nível superior e corresponde a uma área muito úmida onde existe uma intensa percolação de água no teto, paredes e piso, dificultando a

deposição de recursos alimentares. Ao longo da cavidade existem alguns blocos (calhaus e matacões) esparsos de diferentes dimensões sendo o solo na região não alagada constituída por um sedimento muito argiloso. De forma geral, o sistema de canalículos é bem desenvolvido em toda a extensão da cavidade. Por fim, vale destacar que durante o estudo, alguns exemplares de *Anoura sp*. foram encontrados em período reprodutivo. Durante a estação úmida foi possível observar um aumento da vegetação associada à entrada da cavidade favorecendo um incremento no sombreamento da mesma. Os depósitos de guano submersos no lago subterrâneo encontravam-se reduzidos, uma vez que a colônia de morcegos também havia diminuído.

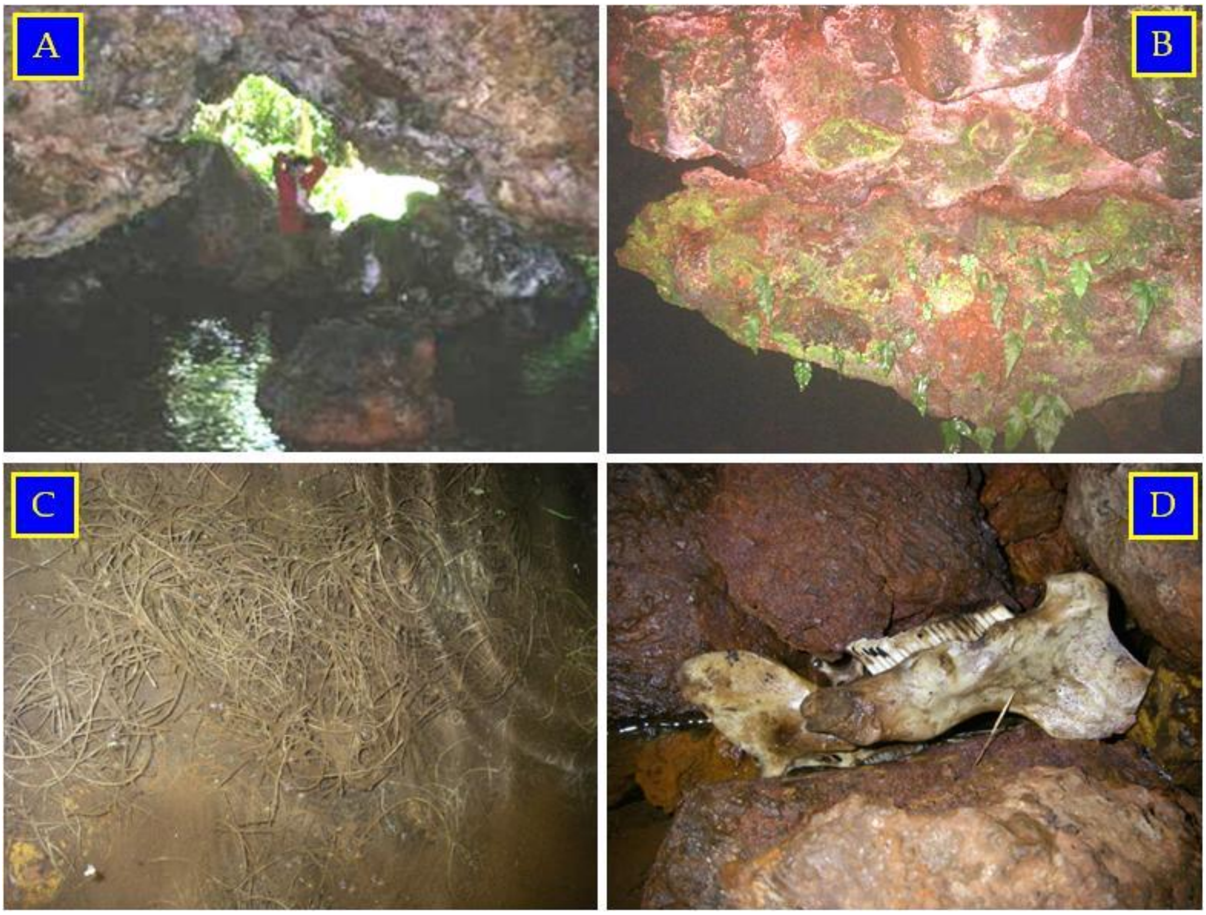

Figura 111 - a) Aspecto geral da entrada da cavidade onde se observa a presença de uma área alagada; b) Grande quantidade de algas, briófitas e pteridófitas nas paredes da entrada; c) Depósito de guano de morcegos frugívoros submerso onde podem ser observados vários caules de plântulas que germinaram nesta condição; d) Mandíbula de capivara encontrada junto ao lago no interior da cavidade.

5.4.4.9.2. Caracterização faunística no período de seca

Foi observado na caverna, um total de 29 morfoespécies de invertebrados de pelo menos 26 famílias das Ordens: Turbellaria (Dugesiidae, Temnocephalidae), Decapoda (Pseudothelphusidae: *Microthelphusa somanni*, Palaemonidae: *Macrobrachium* sp.), Acari

(Oribatida), Amblypygi (Charinidae: *Charinus* sp.), Opiliones (Escadabiidae), Araneae (Psauridae, Pholcidae: *Mesabolivar* sp., Theraphosidae, Theridiosomatidae), Collembola (Entomobryidae), Odonata (Aeshnidae: *Castoraeschna* sp.), Orthoptera (Phalangopsidae: *Aclodes* sp.), Isoptera (Termitidae: *Nasutitermes* sp.), Psocoptera (Epipsocidae), Hemiptera (Pyrrhocoridae), Diptera (Culicidae, Chironomidae, Dolichopodidae, Drosophilidae, Milichiidae, Streblidae, Tipulidae), Hymenoptera (Vespidae), Coleoptera (Dytiscidae) e Diplopoda (Pseudonannolenidae).

Dentre os vertebrados foram encontradas quatro espécies das Ordens: Chiroptera (Emballonuridae: *Peropteryx kappleri*, Phyllostomidae: *Anoura* sp., *Glossophaga soricina*) e Siluriforme (Trichomicteridae: *Trichomicterus* sp.).

Desta forma, no total foram encontradas 33 morfoespécies. Entre estas, uma espécie de Amblypygi (Charinidae: *Charinus* sp.) foi considerada troglomórfica.

5.4.4.9.3. Caracterização faunística no período de chuva

Foi observado na caverna, um total de 28 morfoespécies de invertebrados de pelo menos 23 famílias das Ordens: Decapoda (Pseudothelphusidae: *Microthelphusa somanni*, Palaemonidae: *Macrobrachium* sp.), Acari (Laelapidae, Oribatida), Amblypygi (Phrynidae: *Heterophrynus longicornis*, Charinidae: *Charinus* sp.), Opiliones (Sclerosomatidae: *Prionostemma* sp.), Araneae (Amaurobiidae, Scytodidae*: Scytodes eleonora*, Psauridae, Pholcidae: *Mesabolivar* sp., Theridiosomatidae), Collembola (Entomobryidae, Paronellidae), Orthoptera (Phalangopsidae: *Aclodes* sp., *Phalangopsis* sp.), Blattodea (Blattellidae), Hemiptera (Cydnidae, Pyrrhocoridae, Veliidae), Diptera (Chaoboridae, Culicidae), Hymenoptera (Formicidae: *Azteca* sp.), Coleoptera (Pselaphidae) e Scutigeromorpha (Scutigeridae: *Sphendononema* sp.).

Dentre os vertebrados foram encontradas quatro espécies das Ordens: Chiroptera (Emballonuridae: *Peropteryx kappleri*, Phyllostomidae: *Glossophaga soricina*), Squamata (Sphaerodactylidae: *Coleodactylus* cf. *amazonicus*) e Siluriforme (Trichomicteridae: *Trichomicterus* sp.).

Desta forma, no total foram encontradas 32 morfoespécies. Entre estas, uma espécie de Amblypygi (Charinidae: *Charinus* sp.) foi considerada troglomórfica.

5.4.4.9.4 Caracterização geral da fauna da cavidade

Foi observado na caverna, um total de 51 morfoespécies de invertebrados de pelo menos 40 famílias das Ordens: Turbellaria (Dugesiidae, Temnocephalidae), Decapoda (Pseudothelphusidae: *Microthelphusa somanni*, Palaemonidae: *Macrobrachium* sp.), Acari (Laelapidae, Oribatida), Amblypygi (Phrynidae: *Heterophrynus longicornis*, Charinidae:

Charinus sp.), Opiliones (Escadabiidae, Sclerosomatidae: *Prionostemma* sp.), Araneae (Amaurobiidae, Scytodidae*: Scytodes eleonora*, Psauridae, Pholcidae: *Mesabolivar* sp., Theraphosidae, Theridiosomatidae), Collembola (Entomobryidae, Paronellidae), Odonata (Aeshnidae: *Castoraeschna* sp.), Orthoptera (Phalangopsidae: *Aclodes* sp., *Phalangopsis* sp.), Blattodea (Blattellidae), Isoptera (Termitidae: *Nasutitermes* sp.), Psocoptera (Epipsocidae), Hemiptera (Cydnidae, Pyrrhocoridae, Veliidae), Diptera (Culicidae: *Anopheles* sp., Chaoboridae, Chironomidae, Dolichopodidae, Drosophilidae, Milichiidae, Streblidae, Tipulidae), Hymenoptera (Formicidae: *Azteca* sp.; Vespidae), Coleoptera (Dytiscidae, Pselaphidae), Diplopoda (Pseudonannolenidae) e Scutigeromorpha (Scutigeridae: *Sphendononema* sp.).

Dentre os vertebrados foram encontradas cinco espécies das Ordens: Chiroptera (Emballonuridae: *Peropteryx kappleri*, Phyllostomidae: *Anoura* sp., *Glossophaga soricina*), Squamata (Sphaerodactylidae: *Coleodactylus* cf. *amazonicus*) e Siluriforme (Trichomicteridae: *Trichomicterus* sp.). Desta forma, no total foram encontradas 56 morfoespécies. Entre estas, uma espécie de Amblypygi (Charinidae: *Charinus* sp.) foi considerada troglomórfica. Alguns organismos encontrados nesta caverna são mostrados na Figura 112.

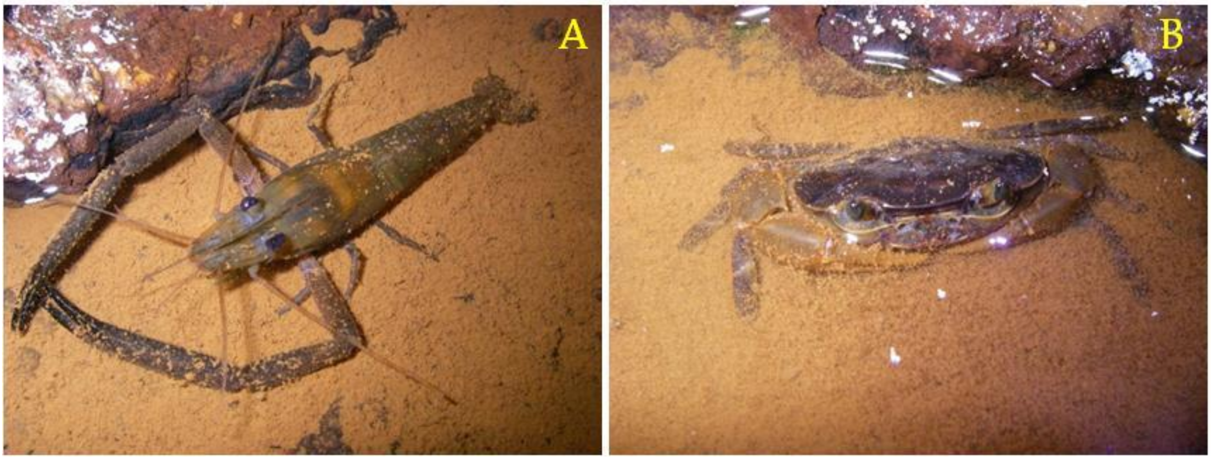

Figura 112 - a) Decapoda (Palaemonidae: *Macrobrachium* sp.); b) Pseudothelphusidae (*Microthelphusa somanni*).

5.4.4.10. SL-011

5.4.4.10.1. Caracterização trófica

Caverna formada na canga com 26 m de desenvolvimento localizada em área de mata ciliar na margem direita do rio. Possui uma entrada larga, mas em teto baixo e composta por piso predominantemente plano e inclinado lateralmente. A entrada é sombreada, apresentando muita serrapilheira acumulada junto à linha d'água e com as paredes revestidas por líquens

e Actinomicetos. O piso é composto por sedimento granulado no trecho inicial com seixos, calhaus e matacões e pela rocha matriz mais ao fundo. Também existe serrapilheira esparsa por todo o piso sendo esta carreada principalmente pela ação eólica. No piso também podem ser observadas algumas plântulas e no teto existe uma grande área revestida por Actinomicetos. O sistema de canalículos é pouco desenvolvido e não foram observados depósitos de guano no interior da cavidade apesar de terem sido encontrados morcegos Glossophaginae. Quando à iluminação, a caverna apresenta pequenas zonas afóticas em suas áreas mais profundas. Durante a estação úmida a entrada encontrava-se totalmente coberta em virtude do crescimento da vegetação epígea e havia um incremento significativo de serrapilheira no interior da cavidade (Figura 113).

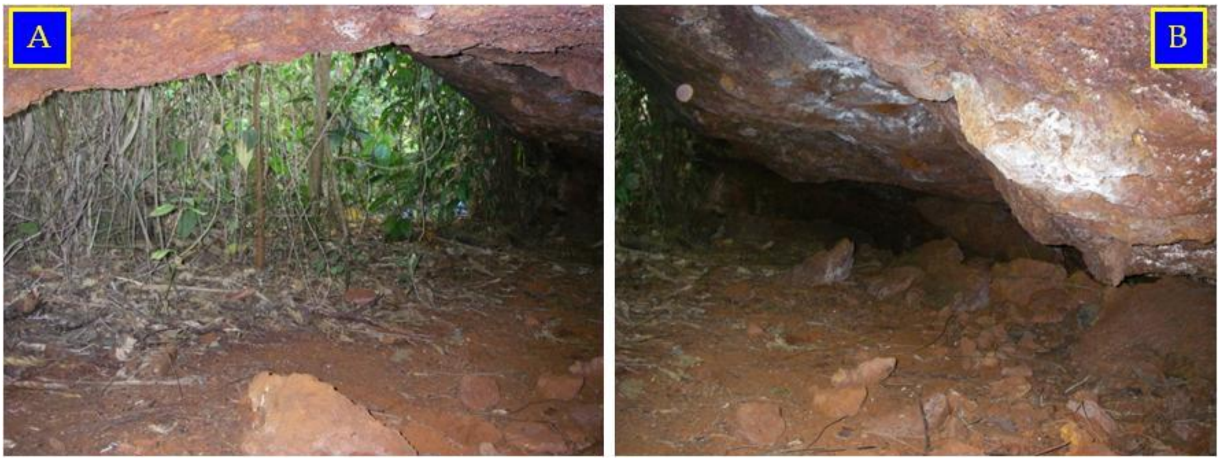

Figura 113 - a) Aspecto geral da entrada com a presença de muita serrapilheira acumulada junto à linda d'água; b) Morfologia geral predominante na cavidade e aspecto do piso com blocos esparsos.

5.4.4.10.2. Caracterização faunística no período de seca

Foi observado na caverna, um total de 32 morfoespécies de invertebrados de pelo menos 24 famílias das Ordens: Isopoda (Balloniscidae), Amblypygi (Phrynidae: *Heterophrynus longicornis*), Pseudoscorpiones (Chernetidae, Chtoniidae), Opiliones (Cosmetidae: *Anduzeia* sp.), Araneae (Gnaphosidae, Ochyroceratidae, Oonopidae, Scytodidae*: Scytodes eleonora*, Pholcidae: *Mesabolivar* sp.), Diplura (Campodeidae), Collembola (Entomobryidae), Orthoptera (Phalangopsidae: *Aclodes* sp.), Blattodea (Blaberidae: *Blaberus* sp.), Isoptera (Termitidae: *Nasutitermes* sp.), Psocoptera (Lepidopsocidae), Homoptera (Cixiidae), Lepidoptera (Tineidae), Diptera (Psychodidae: *Lutzomyia* sp.), Hymenoptera (Formicidae: *Camponotus* sp., *Pachycondyla* sp.; Vespidae), Coleoptera (Scydmaenidae, Dermestidae) e Polyxenida (Polyxenidae).

Dentre os vertebrados foram encontradas cinco espécies das Ordens: Chiroptera (Phyllostomidae: *Anoura* sp., *Glossophaga soricina*), Gekkonidae (Squamata: *Thecadactylus rapicauda*) e Anura (Leptodactylidae: *Pristimantis* cf. *fenestratus*, *Eleutherodactylus* sp.). Desta forma, no total foram encontradas 37 morfoespécies.

5.4.4.10.3. Caracterização faunística no período de chuva

Foi observado na caverna, um total de 58 morfoespécies de invertebrados de pelo menos 45 famílias das Ordens: Gastropoda (Subulinidae), Turbellaria (Geoplanidae), Isopoda (Armadillidae, Balloniscidae), (Acari (Ixodidae: *Amblyomma* sp., Opilioacaridae: *Neoacarus* sp., Oribatida, Trombidiforme), Amblypygi (Phrynidae: *Heterophrynus longicornis*), Pseudoscorpiones (Chernetidae, Chtoniidae), Opiliones (Sclerosomatidae: *Prionostemma* sp.), Araneae (Gnaphosidae; Oonopidae; Salticidae; Scytodidae*: Scytodes eleonora*; Psauridae; Pholcidae: *Mesabolivar* sp., *Metagonia* sp.; Theraphosidae), Thysanura (Nicoletiidae), Microcoryphia (Meinertellidae), Collembola (Entomobryidae, Hypogastruridae, Isotomidae), Blattodea (Blaberidae: *Blaberus* sp.), Isoptera (Termitidae: *Nasutitermes* sp.), Psocoptera, Hemiptera (Reduviidae: *Panstrongylus* sp., Ploiariidae), Homoptera (Cixiidae), Lepidoptera (Tineidae), Diptera (Ceratopogonidae, Chloropidae, Psychodidae: *Lutzomyia* sp., Sciaridae), Hymenoptera (Formicidae: *Camponotus* sp., *Cephalotes* sp., *Hypoponera* sp., *Pachycondyla* sp., *Pheidole* sp., *Rogeria* sp.), Coleoptera (Carabidae, Pselaphidae, Ptylidae, Scydmaenidae, Staphylinidae), Scutigeromorpha (Scutigeridae).

Dentre os vertebrados foi encontrada uma espécie de Chiroptera (Phyllostomidae: *Glossophaga soricina*). Desta forma, no total foram encontradas 59 morfoespécies.

5.4.4.10.4. Caracterização geral da fauna da cavidade

Foi observado na caverna, um total de 76 morfoespécies de invertebrados de pelo menos 54 famílias das Ordens: Gastropoda (Subulinidae), Turbellaria (Geoplanidae), Isopoda (Armadillidae, Balloniscidae), Acari (Ixodidae: *Amblyomma* sp., Opilioacaridae: *Neoacarus* sp., Oribatida, Trombidiforme), Amblypygi (Phrynidae: *Heterophrynus longicornis*), Pseudoscorpiones (Chernetidae, Chtoniidae), Opiliones (Cosmetidae: *Anduzeia* sp.*)*, Sclerosomatidae: *Prionostemma* sp.), Araneae (Gnaphosidae; Ochyroceratidae; Oonopidae; Salticidae; Scytodidae*: Scytodes eleonora*; Psauridae; Pholcidae: *Mesabolivar* sp., *Metagonia* sp.; Theraphosidae), Thysanura (Nicoletiidae), Microcoryphia (Meinertellidae), Diplura (Campodeidae), Collembola (Entomobryidae, Hypogastruridae, Isotomidae), Orthoptera (Phalangopsidae: *Aclodes* sp.), Blattodea (Blaberidae: *Blaberus* sp.), Isoptera (Termitidae: *Nasutitermes* sp.), Psocoptera (Lepidopsocidae), Hemiptera (Reduviidae: *Panstrongylus* sp., Ploiariidae), Homoptera (Cixiidae), Lepidoptera (Tineidae), Diptera (Ceratopogonidae, Chloropidae, Sciaridae, Psychodidae: *Lutzomyia* sp.), Hymenoptera (Formicidae: *Camponotus* sp., *Cephalotes* sp., *Hypoponera* sp., *Pachycondyla* sp., *Pheidole* sp., *Rogeria* sp.; Vespidae), Coleoptera (Carabidae, Pselaphidae, Ptylidae, Scydmaenidae, Staphylinidae, Dermestidae), Polyxenida (Polyxenidae) e Scutigeromorpha (Scutigeridae).

Dentre os vertebrados foram encontradas cinco espécies das Ordens: Chiroptera (Phyllostomidae: *Anoura* sp.; *Glossophaga soricina*), Gekkonidae (Squamata: *Thecadactylus rapicauda*) e Anura (Leptodactylidae: *Pristimantis cf. fenestratus*, *Eleutherodactylus* sp.). Desta forma, no total foram encontradas 81 morfoespécies.

5.4.4.11. SL-012

5.4.4.11.1. Caracterização trófica

Pequena caverna formada na canga com 15,6 m de desenvolvimento localizada na margem esquerda do rio em área de mata ciliar. No entorno existem grandes árvores, muitas palmeiras e lianas, além de uma trilha utilizada pelos moradores da Vila de Serra Pelada para manutenção do sistema de captação de água. Com duas entradas em teto baixo (aproximadamente 1 metros de altura), estas são sombreadas, apresentando umidade elevada e muitos líquens, briófitas, pteridófitas e plântulas revestindo o piso e as paredes. Em geral o piso é plano composto por sedimento fino e seco com seixos e calhaus concentrados junto à entrada principal da cavidade. A caverna pode ser dividida em dois pequenos salões conectados por um trecho de teto baixo nos quais existem zonas completamente afóticas. Não existem depósitos de guano, apenas poucas raízes de pequeno calibre e a serrapilheira está restrita a linha d’água (Figura 114). Apesar de formada na canga o sistema de canalículos é pouco desenvolvido. Nenhuma alteração significativa foi observada durante a estação úmida, além das alterações normais na umidade relativa do ar.

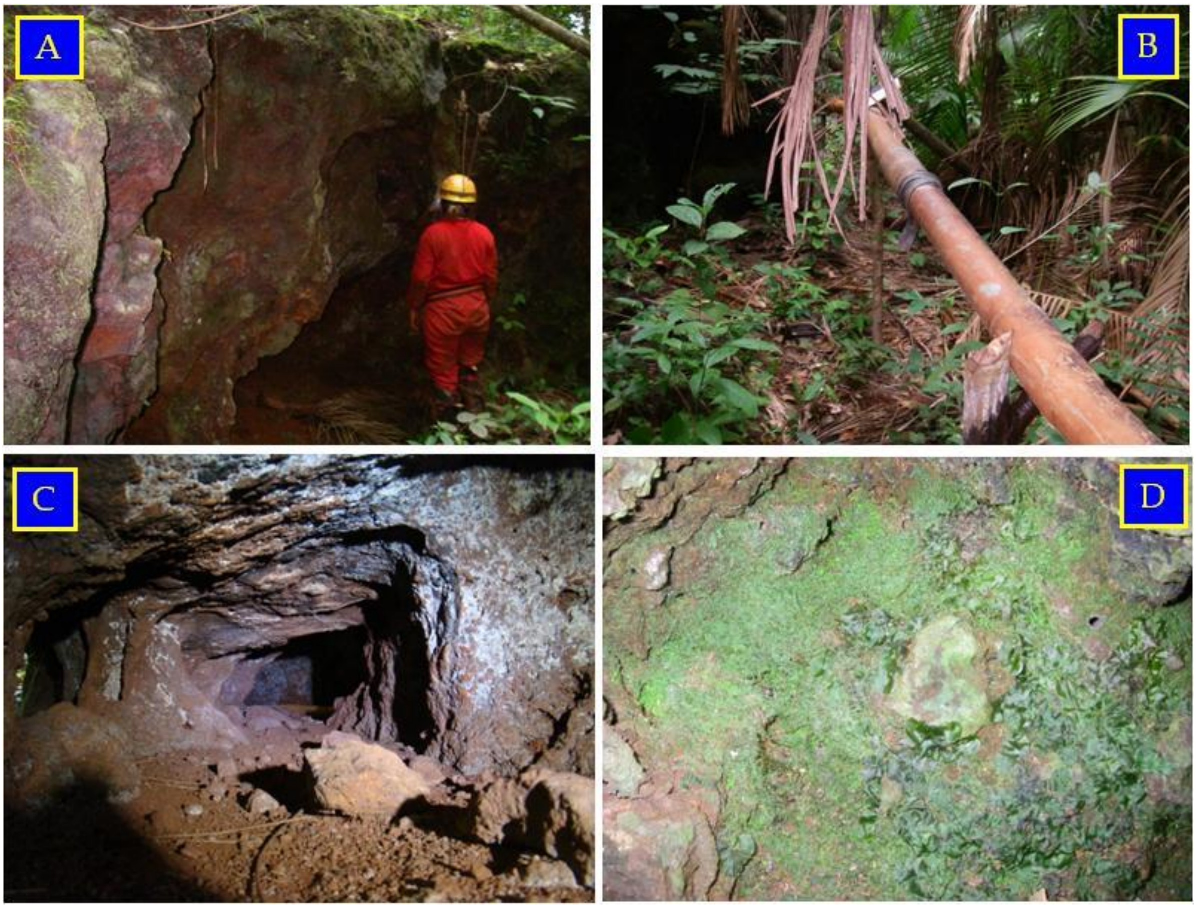

Figura 114 - a)Aspecto geral da entrada da cavidade; b) Cano utilizado pela comunidade de Serra Pelada para captação de água; c) Morfologia geral de uma das galerias da cavidade (reparar nas paredes revestidas por Actinomicetos); d) Parede intensamente revestida por briófitas junto à entrada principal da cavidade.

5.4.4.11.2. Caracterização faunística no período de seca

Foi observado na caverna, um total de 39 morfoespécies de invertebrados de pelo menos 33 famílias das Ordens: Isopoda (Armadillidae, Philosciidae, Scleropactidae), Acari (Labdostomatidae: *Labdostomatida* sp., *Astigmatina* sp.), Amblypygi (Phrynidae: *Heterophrynus longicornis*), Pseudoscorpiones (Chernetidae, Chtoniidae), Opiliones (Cosmetidae: *Anduzeia* sp., Escadabiidae), Araneae (Gnaphosidae, Ochyroceratidae, Scytodidae*: Scytodes eleonora*, Theridiidae), Thysanura (Nicoletiidae: Nicoletiinae), Collembola (Entomobryidae), Orthoptera (Phalangopsidae: *Aclodes* sp.), Isoptera (Termitidae: *Nasutitermes* sp.), Hemiptera (Dipsocoridae, Reduviidae: *Panstrongylus* sp.), Homoptera (Fulgoridae), Lepidoptera (Noctuidae), Diptera (Ceratopogonidae, Culicidae, Dolichopodidae, Lauxaniidae, Psychodidae: *Lutzomyia* sp.) Hymenoptera (Formicidae: *Azteca* sp., *Camponotus* sp., *Pachycondyla* sp.), Coleoptera (Curculionidae, Elateridae: *Cardiophorinae* sp.), Diplopoda (Chelodesmidae, Pyrgodesmidae, Polydesmida), Siphonophorida (Siphonophoridae), Scutigeromorpha (Scutigeridae: *Sphendononema* sp.) e Symphyla (Scutigerellidae: *Hanseniella* sp., *Scutigerella* sp.).

Dentre os vertebrados foi encontrada uma espécie de Anura (Leptodactylidae: *Pristimantis* cf. *fenestratus*). Desta forma, no total foram encontradas 39 morfoespécies.

5.4.4.11.3. Caracterização faunística no período de chuva

Foi observado na caverna, um total de 59 morfoespécies de invertebrados de pelo menos 28 famílias das Ordens: Gastropoda (Subulinidae) Isopoda (Philosciidae, Scleropactidae), Acari (Ixodidae: *Amblyomma* sp.) Amblypygi (Phrynidae: *Heterophrynus longicornis*), Pseudoscorpiones (Chernetidae, Chtoniidae), Opiliones (Cosmetidae: *Anduzeia* sp., Phalangiidae), Araneae (Ctenidae, Gnaphosidae, Linyphiidae, Ochyroceratidae, Oonopidae, Salticidae, Scytodidae*: Scytodes eleonora*, Psauridae, Pholcidae: *Metagonia* sp., Prodidomidae, Theraphosidae, Theridiidae), Thysanura (Nicoletiidae: Nicoletiinae, Atelurinae), Microcoryphia (Meinertellidae), Collembola (Paronellidae), Orthoptera (Phalangopsidae: *Phalangopsis* sp.), Blattodea (Blaberidae: *Blaberus* sp.), Isoptera (Termitidae: *Nasutitermes* sp.), Psocoptera (Epipsocidae), Hemiptera (Lygaeidae), Homoptera (Fulgoridae), Lepidoptera (Noctuidae), Diptera (Cecidomyiidae, Psychodidae: *Lutzomyia* sp.) Hymenoptera (Formicidae: *Camponotus* sp., *Myrmicocrypta* sp., *Pachycondyla* sp., *Solenopsis* sp.), Coleoptera (Chrysomelidae, Curculionidae: Scotylinae, Elateridae: Elaterinae, Staphylinidae), Diplopoda (Chelodesmidae, Pyrgodesmidae, Polydesmida), Siphonophorida (Siphonophoridae) e Scutigeromorpha (Scutigeridae: *Sphendononema* sp.).

5.4.4.11.4. Caracterização geral da fauna da cavidade

Foi observado na caverna, um total de 84 morfoespécies de invertebrados de pelo menos 58 famílias das Ordens: Gastropoda (Subulinidae) Isopoda (Armadillidae, Philosciidae, Scleropactidae), Acari (Labdostomatidae: *Labdostomatida* sp. *Astigmatina* sp. Ixodidae: *Amblyomma* sp.), Amblypygi (Phrynidae: *Heterophrynus longicornis*), Pseudoscorpiones (Chernetidae, Chtoniidae), Opiliones (Cosmetidae: *Anduzeia* sp*.,* Escadabiidae, Phalangiidae), Araneae (Araneidae: *Alpaida* sp., Ctenidae, Gnaphosidae, Linyphiidae, Ochyroceratidae, Oonopidae, Salticidae, Scytodidae*: Scytodes eleonora*, Psauridae, Pholcidae: *Metagonia* sp., Prodidomidae, Theraphosidae, Theridiidae), Thysanura (Nicoletiidae: Nicoletiinae, Atelurinae), Microcoryphia (Meinertellidae), Collembola (Entomobryidae, Paronellidae), Orthoptera (Phalangopsidae: *Aclodes* sp., *Phalangopsis* sp.), Blattodea (Blaberidae: *Blaberus* sp.), Isoptera (Termitidae: *Nasutitermes* sp.), Psocoptera (Epipsocidae), Hemiptera (Dipsocoridae, Lygaeidae, Reduviidae: *Panstrongylus* sp.), Homoptera (Fulgoridae), Lepidoptera (Noctuidae), Diptera (Cecidomyiidae, Ceratopogonidae, Culicidae, Chironomidae, Dolichopodidae, Lauxaniidae, Psychodidae: *Lutzomyia* sp.) Hymenoptera (Formicidae: *Azteca* sp., *Camponotus* sp., *Myrmicocrypta* sp., *Pachycondyla* sp., *Solenopsis* sp.), Coleoptera (Chrysomelidae, Curculionidae: Scotylinae, Elateridae: Elaterinae, Staphylinidae), Diplopoda (Chelodesmidae, Pyrgodesmidae, Polydesmida), Siphonophorida

(Siphonophoridae), Scutigeromorpha (Scutigeridae: *Sphendononema* sp.), Symphyla (Scutigerellidae: *Hanseniella* sp., *Scutigerella* sp.).

Dentre os vertebrados foi encontrada uma espécie de Anura (Leptodactylidae: *Pristimantis* cf. *fenestratus*). Desta forma, no total foram encontradas 85 morfoespécies. Alguns organismos encontrados nesta caverna são mostrados na Figura 115.

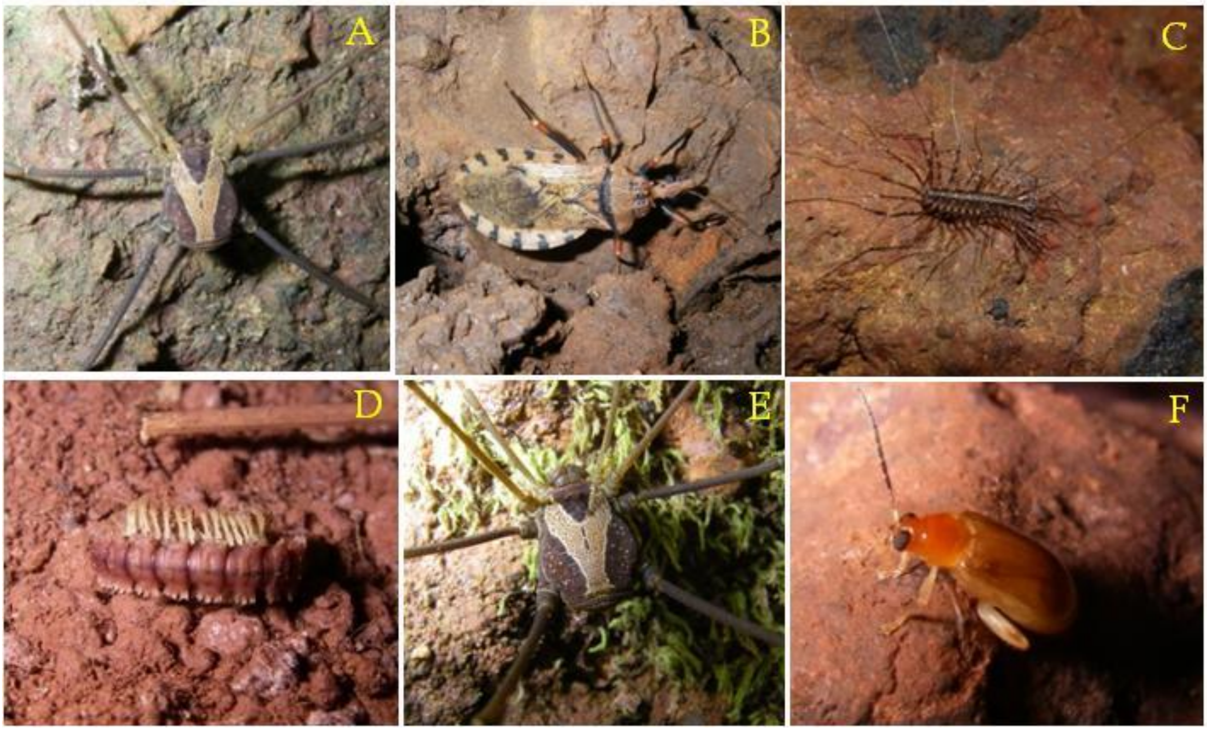

Figura 115 - a) Opiliones (Cosmetidae); b) Reduviidae (*Panstrongylus* sp.); c) Scutigeromorpha (Sphendononema sp.); d) Diplopoda (Chelodesmidae); e) Opiliones (Cosmetidae); f) Coleoptera (Chrysomelidae).

5.4.4.12. SL-013

5.4.4.12.1. Caracterização trófica

Pequeno abrigo (11,5 m) formado na canga localizado em área de mata ciliar na margem esquerda do rio. Não apresenta zona afótica, possui duas entradas principais em teto baixo, sendo uma delas muito estreita. Junto à entrada existem muitos líquens, Actinomicetos, briófitas e plântulas além de muita serrapilheira restrita a linha d'água. Esta serrapilheira também se apresenta de maneira esparsa pelo piso da cavidade sendo transportada de forma gravitacional ou pelo vento. O piso é levemente descendente e irregular estando sujeito a inundações em épocas de chuva. No momento da primeira coleta apresentava-se seco e composto por sedimento granulado com poucos seixos e calhaus esparsos. Não foram observados depósitos de guano apesar de terem sido avistados morcegos no interior da

cavidade, mas existem poucas raízes de reduzido calibre (Figura 116). O sistema de canalículos é pouco desenvolvido e a caverna apresenta uma forte influência das condições ambientais epígeas. Por fim, vale destacar que apesar desta gruta estar localizada em área de mata ciliar e junto ao curso d'água, uma grande população de anfíbios (Leptodactylidae: *Pristimantis cf. fenestratus*) foi observada no interior da cavidade. Durante a estação úmida observou-se a existência de alguns pequenos gotejamentos esparsos ao longo de toda a cavidade.

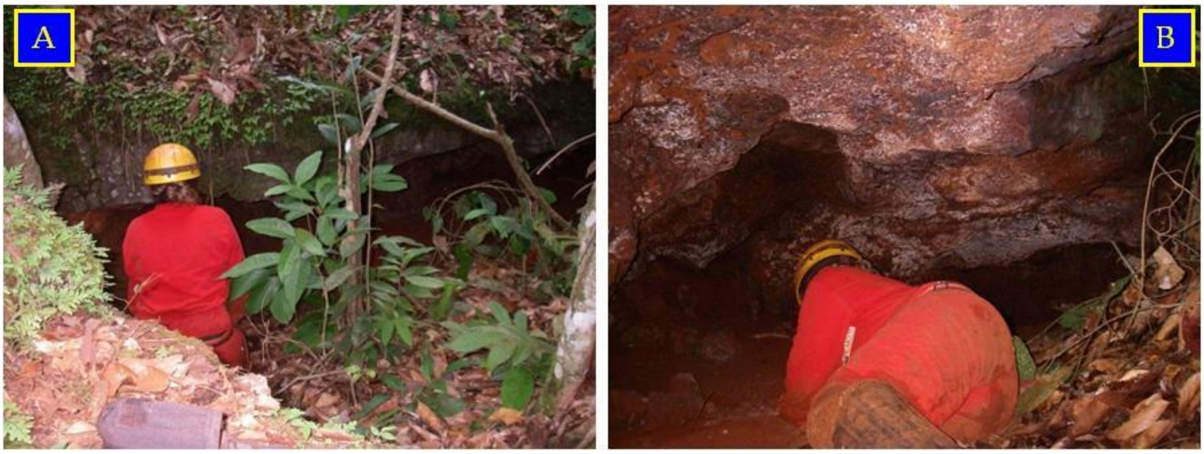

Figura 116 - a) Aspecto geral da entrada em teto baixo com muita serrapilheira no entorno; b) Porção mais interior da cavidade.

5.4.4.12.2. Caracterização faunística no período de seca

Foi observado na caverna, um total de 35 morfoespécies de invertebrados de pelo menos 28 famílias das Ordens: Acari (Trombidiforme), Pseudoscorpiones (Chernetidae), Opiliones (Cosmetidae: *Anduzeia* sp., Stygnidae: *Protimesius* aff. *gracilis*), Araneae (Corinidae, Ctenidae, Gnaphosidae, Oonopidae, Scytodidae: *Scytodes eleonora*, Pholcidae: *Mesobolivar* sp., Theridiidae), Thysanura (Nicoletiidae: Nicoletiinae), Diplura (Campodeidae), Orthoptera (Phalangopsidae: *Aclodes* sp.), Blattodea (Blaberidae, Polyphagidae), Isoptera (Termitidae: *Nasutitermes* sp.), Psocoptera (Epipsocidae), Hemiptera (Reduviidae), Lepidoptera (Noctuidae), Diptera (Bibionidae, Cecidomyiidae, Chaoboridae, Culicidae, Mycetophilidae, Psychodidae: *Lutzomyia* sp., Tipulidae) e Hymenoptera (Formicidae: *Dolichoderus* sp., *Trachymyrmex* sp., *Pachycondyla* sp.; Braconidae).

Dentre os vertebrados foram encontradas duas espécies das Ordens: Chiroptera (Phyllostomidae: *Glossophaga soricina*) e Anura (Lepdoctylidae: *Pristimantis* cf. *fenestratus*). Desta forma, no total foram encontradas 37 morfoespécies.

5.4.4.12.3. Caracterização faunística no período de chuva

Foi observado na caverna, um total de 43 morfoespécies de invertebrados de pelo menos 33 famílias das Ordens: Gastropoda (Subulinidae, Systrophiidae) Isopoda (Armadillidae,

Dubioniscidae), Acari (Trombidiforme), Amblypygi (Phrynidae: *Heterophrynus longicornis*), Pseudoscorpiones (Chernetidae, Chtoniidae), Opiliones (Phalangiidae), Araneae (Salticidae, Scytodidae*: Scytodes eleonora*, Psauridae, Pholcidae: *Metagonia* sp.), Thysanura (Nicoletiidae: Nicoletiinae), Collembola (Cyphoderidae, Entomobryidae), Blattodea (Polyphagidae), Embiidina, Isoptera (Termitidae: *Nasutitermes* sp.), Psocoptera (Myopsocidae: *Lichenomina* sp.), Hemiptera (Cydnidae, Lygaeidae), Homoptera (Cixiidae), Diptera (Calliphoridae, Ceratopogonidae, Sciaride, Psychodidae: *Lutzomyia* sp.), Lepidoptera (Tineidae), Hymenoptera (Formicidae: *Dolichoderus* sp. *Odontomachus* sp., *Pheidole* sp., *Trachymyrmex* sp., *Camponotus* sp.), Coleoptera (Carabidae, Pselaphidae), Diplopoda (Pyrgodesmidae) e Symphyla (Scutigerellidae: *Hanseniella* sp.).

Dentre os vertebrados foi encontrada uma espécie de Chiroptera (Phyllostomidae: *Glossophaga soricina*). Desta forma, no total foram encontradas 44 morfoespécies. Entre estas, uma espécie de invertebrado foi considerada troglomórfica: Gastropoda (Systrophiidae).

5.4.4.12.4. Caracterização geral da fauna da cavidade

Foi observado na caverna, um total de 72 morfoespécies de invertebrados de pelo menos 53 famílias das Ordens: Gastropoda (Subulinidae, Systrophiidae) Isopoda (Armadillidae, Dubioniscidae), Acari (Trombidiforme), Amblypygi (Phrynidae: *Heterophrynus longicornis*), Pseudoscorpiones (Chernetidae, Chtoniidae), Opiliones (Cosmetidae: *Anduzeia* sp., Stygnidae: *Protimesius* aff. *gracilis*, Phalangiidae), Araneae (Corinidae, Ctenidae, Gnaphosidae, Oonopidae, Salticidae, Scytodidae*: Scytodes eleonora*, Psauridae, Pholcidae: *Metagonia* sp., *Mesobolivar* sp., Theridiidae), Thysanura (Nicoletiidae: Nicoletiinae), Diplura (Campodeidae), Collembola (Cyphoderidae, Entomobryidae), Orthoptera (Phalangopsidae: *Aclodes* sp.), Blattodea (Blaberidae, Polyphagidae), Isoptera (Termitidae: *Nasutitermes* sp.), Psocoptera (Epipsocidae, Myopsocidae: *Lichenomina* sp.), Hemiptera (Cydnidae, Lygaeidae, Reduviidae), Homoptera (Cixiidae), Lepidoptera (Noctuidae), Diptera (Bibionidae, Tineidae, Calliphoridae, Cecidomyiidae, Ceratopogonidae, Chaoboridae, Culicidae, Mycetophilidae, Tipulidae Psychodidae: *Lutzomyia* sp.) Hymenoptera (Formicidae: *Cyphomyrmex* sp., *Dolichoderus* sp. *Odontomachus* sp., *Pheidole* sp., *Trachymyrmex* sp., *Braconidae* sp.*Camponotus* sp., *Pachycondyla* sp.), Coleoptera (Carabidae, Pselaphidae), Diplopoda (Polydesmida) e Symphyla (Scutigerellidae: *Hanseniella* sp.).

Dentre os vertebrados foram encontradas duas espécies das Ordens: Chiroptera (Phyllostomidae: *Glossophaga soricina*) e Anura (Lepdoctylidae: *Pristimantis* cf. *fenestratus*). Desta forma, no total foram encontradas 74 morfoespécies, das quais apenas uma considerada troglomórfica: Gastropoda (Systrophiidae).

5.4.4.13. SL-014

5.4.4.13.1. Caracterização trófica

Pequena cavidade com 8,1 m de desenvolvimento, formada na canga localizada em área de mata ciliar na margem esquerda do rio. Possui apenas uma entrada, sendo esta iluminada e em teto baixo com muitos líquens, briófitas, plântulas e micro-raízes revestindo as paredes e o piso. Além disso, existe grande quantidade de serrapilheira acumulada junto à linha d'água. Esta cavidade encontra-se suscetível à inundação em épocas de cheia e apresenta uma forte influência das condições ambientais epígeas. O piso é levemente descendente a partir da entrada, sendo este composto por sedimento granulado e com muitos seixos, calhaus e alguns matacões. A cavidade é completamente eufótica e possui um sistema de canalículos bem desenvolvido. As paredes e teto são completamente revestidos por Actinomicetos em suas zonas mais profundas e não foram observados depósitos de guano. Em relação aos vertebrados, durante o inventário observou-se um ninho de roedor no interior da cavidade. Durante a estação úmida observou-se um pequeno aumento na umidade relativa do ar, além de uma maior quantidade de matéria orgânica disponível junto ao substrato. Na margem esquerda da entrada principal existe uma pequena depressão onde acorre um pequeno acúmulo de água da chuva (Figura 117).

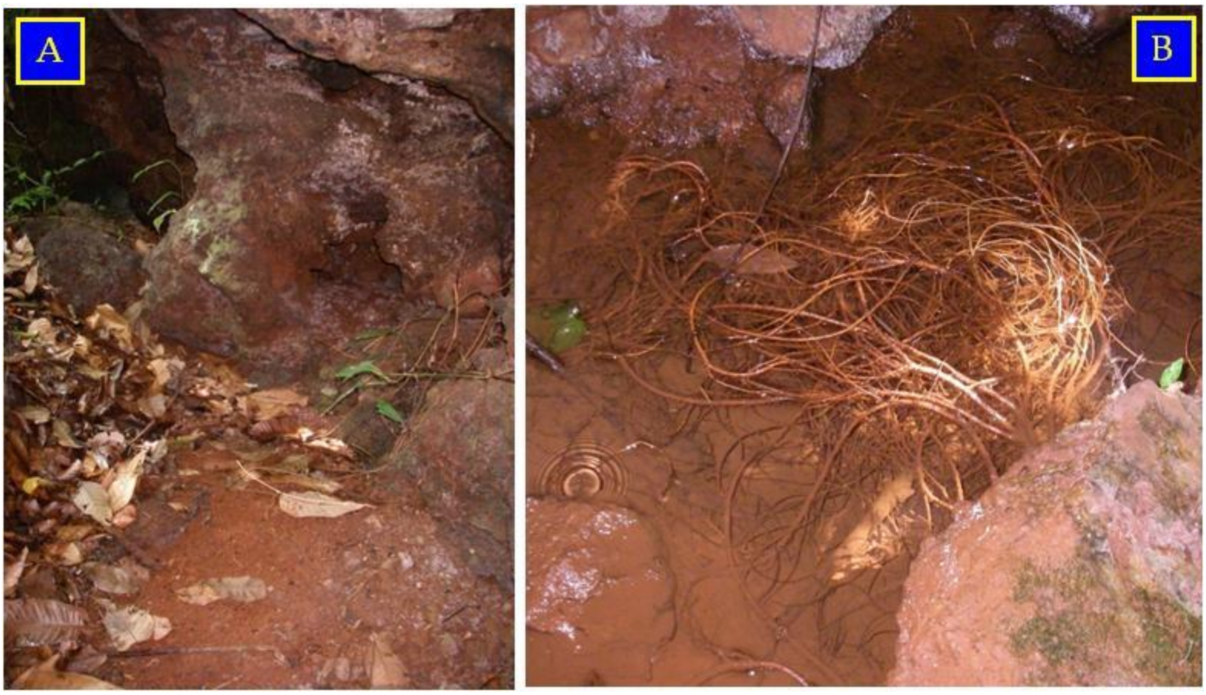

Figura 117 - a) Aspecto geral da entrada, onde se observa a serrapilheira acumulada junto a linha d'água;b) Pequena depressão alagada da cavidade, no interior da qual existem raízes em desenvolvimento.

5.4.4.13.2. Caracterização faunística no período de seca

Foi observado na caverna, um total de 33 morfoespécies de invertebrados de pelo menos 25 famílias das Ordens: Isopoda (Armadillidae), Opiliones (Cosmetidae: *Anduzeia* sp., Stygnidae:

Eutimesius sp. *Protimesius* aff. *gracilis*), Araneae (Araneidae, Salticidae, Psauridae, Pholcidae: *Metagonia* sp., *Mesabolivar* sp., Theraphosidae), Orthoptera (Phalangopsidae: *Aclodes* sp.), Isoptera (Termitidae: *Nasutitermes* sp.), Psocoptera (Epipsocidae, Archipsocidae), Hemiptera (Veliidae), Homoptera (Cixiidae: *Cixius* sp.), Lepidoptera (Noctuidae), Diptera (Culicidae, Clusiidae, Drosophilidae: *Drosophila* sp., Muscidae, Psychodidae: *Lutzomyia* sp.), Hymenoptera (Formicidae: *Azteca* sp., *Pachycondyla* sp., *Dolichoderus* sp., *Gigantiops* sp., *Rogeria* sp.), Coleoptera (Carabidae, Elateridae, Staphylinidae), Scolopendromorpha (Scolopendridae) e Scutigeromorpha (Scutigeridae: *Sphendononema* sp.).

Dentre os vertebrados foram encontradas três espécies das Ordens: Gekkonidae (Squamata: *Thecadactylus rapicauda*) e Anura (Leptodactylidae: *Pristimantis cf. fenestratus*, *Eleutherodactylus* sp.). Desta forma, no total foram encontradas 36 morfoespécies.

5.4.4.13.3. Caracterização faunística no período de chuva

Foi observado na caverna, um total de 47 morfoespécies de invertebrados de pelo menos 38 famílias das Ordens: Isopoda (Armadillidae), Acari (Oribatida, Trombidiforme), Amblypygi (Charinidae: *Charinus* sp.), Opiliones (Cosmetidae: *Anduzeia* sp., Sclerosomatidae: *Prionostemma* sp., Phalangiidae), Araneae (Ctenidae, Ochyroceratidae, Oonopidae, Scytodidae*: Scytodes eleonora*, Psauridae, Pholcidae: *Mesabolivar* sp., Theraphosidae, Theridiidae, Theridiosomatidae), Microcoryphia (Meinertellidae), Diplura (Campodeidae), Collembola (Entomobryidae, Isotomidae), Orthoptera (Phalangopsidae: *Aclodes* sp., *Phalangopsis* sp.), Blattodea (Blatellidae), Isoptera (Termitidae: *Nasutitermes* sp.), Psocoptera (Ptiloneuridae: *Ptiloneura* sp.) Hemiptera (Cydnidae, Veliidae), Homoptera (Cixiidae: *Cixius* sp.), Lepidoptera (Noctuidae), Diptera (Cecidomyiidae, Culicidae, Phoridae, Psychodidae: *Lutzomyia* sp.), Hymenoptera (Formicidae: *Azteca* sp., *Camponotus* sp., *Pachycondyla* sp., *Cyphomyrmex* sp.), Coleoptera (Carabidae, Pselaphidae), Diplopoda (Polydesmida), Spirostreptida (Pseudonannolenidae), Symphyla (Scutigerellidae: *Hanseniella* sp.) e Geophilomorpha (Ballophilidae: *Ballophililus* sp.).

Dentre os vertebrados foi encontrada uma espécie de Chiroptera (Phyllostomidae: *Glossophaga soricina*). Desta forma, no total foram encontradas 48 morfoespécies. Entre estas, uma espécie de invertebrado foi considerada troglomórfica: Amblypygi (Charinidae: *Charinus* sp.).

5.4.4.13.4. Caracterização geral da fauna da cavidade

Foi observado na caverna, um total de 73 morfoespécies de invertebrados de pelo menos 50 famílias das Ordens: Isopoda (Armadillidae), Acari (Oribatida), Amblypygi (Charinidae: *Charinus* sp.), Opiliones (Cosmetidae: *Anduzeia* sp., Sclerosomatidae: *Prionostemma* sp., Stygnidae: *Eutimesius* sp. *Protimesius* aff. *gracilis,* Phalangiidae), Araneae (Araneidae, Ctenidae: *Ctenus* sp., Ochyroceratidae, Oonopidae, Scytodidae*: Scytodes eleonora*, Salticidae, Psauridae, Pholcidae: *Metagonia* sp., *Mesabolivar* sp., Prodidomidae, Theraphosidae, Theridiidae, Theridiosomatidae), Microcoryphia (Meinertellidae), Diplura (Campodeidae), Collembola (Entomobryidae, Isotomidae), Orthoptera (Phalangopsidae: *Aclodes* sp., *Phalangopsis* sp.), Blattodea (Blatellidae), Isoptera (Termitidae: *Nasutitermes* sp.), Psocoptera (Ptiloneuridae: *Ptiloneura* sp., Epipsocidae, Archipsocidae), Hemiptera (Cydnidae, Veliidae), Homoptera (Cixiidae: *Cixius* sp.), Lepidoptera (Noctuidae), Diptera (Cecidomyiidae, Culicidae, Clusiidae, Drosophilidae: *Drosophila* sp., Muscidae, Phoridae, Psychodidae: *Lutzomyia* sp.) Hymenoptera (Formicidae: *Azteca* sp., *Camponotus* sp., *Pachycondyla* sp., *Cyphomyrmex* sp., *Dolichoderus* sp., *Gigantiops* sp., *Rogeria* sp.), Coleoptera (Carabidae, Elateridae: Elaterinae, Pselaphidae, Staphylinidae), Diplopoda (Polydesmida), Spirostreptida (Pseudonannolenidae), Scutigeromorpha (Scutigeridae: *Sphendononema* sp.), Symphyla (Scutigerellidae: *Hanseniella* sp.) Geophilomorpha (Ballophilidae: *Ballophililus* sp.), Scolopendromorpha (Scolopendridae) e Scutigeromorpha (Scutigeridae: *Sphendononema* sp.).

Dentre os vertebrados foram encontradas quatro espécies das Ordens: Chiroptera (Phyllostomidae: *Glossophaga soricina*), Gekkonidae (Squamata: *Thecadactylus rapicauda*) e Anura (Leptodactylidae: *Pristimantis cf. fenestratus*, *Eleutherodactylus* sp.). Desta forma, no total foram encontradas 77 morfoespécies. Entre estas, uma espécie de invertebrado foi considerada troglomórfica: Amblypygi (Charinidae: *Charinus* sp.).

5.4.4.14. SL-015

5.4.4.14.1. Caracterização trófica

Pequena cavidade com 8,5 m de desenvolvimento, formada na canga localizada em área de mata ciliar na margem esquerda do rio. A cavidade possui duas entradas amplas e sombreadas com líquens, briófitas e muita serrapilheira acumulada junto à linha d’água. Os condutos são uniformes com passagens amplas e não apresentam zonas afóticas. O piso é predominantemente plano e seco composto por sedimento granulado com alguns blocos abatidos de diferentes tamanhos (calhaus e matacões). Neste, existem depósitos de guano de morcegos frugívoros frescos com restos de plântulas e poucas raízes de pequeno calibre além de áreas com serrapilheira esparsa transportada por ação eólica (Figura 118). O sistema de canalículos é bem desenvolvido e as paredes e o teto são completamente

revestidos por Actinomicetos nas zonas mais profundas da cavidade. Nenhuma alteração significativa foi observada durante a estação úmida, além das alterações normais na umidade relativa do ar.

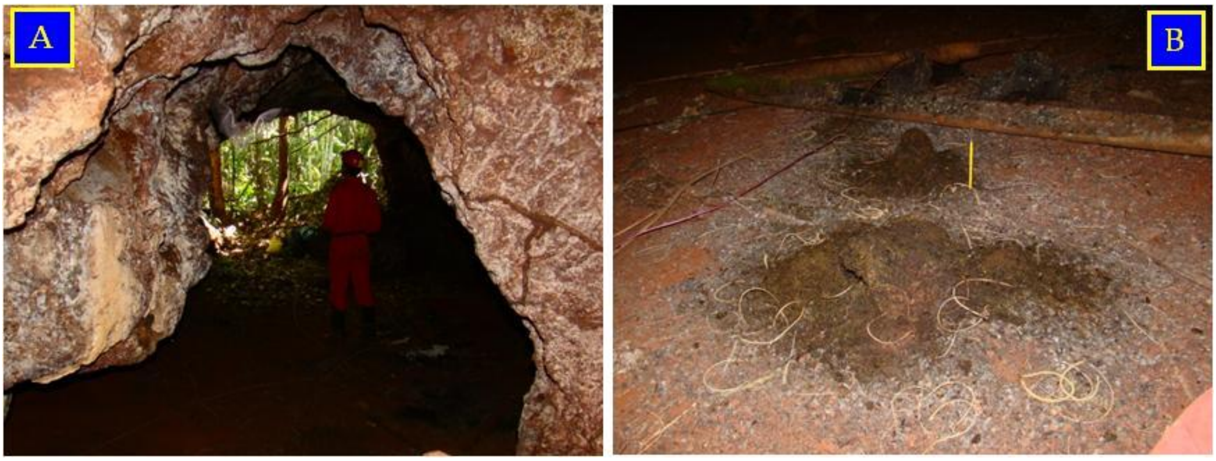

Figura 118 - . a) Aspecto geral do conduto e da entrada principal da cavidade; b) Depósito fresco de guano frugívoro com restos de plântulas.

5.4.4.14.2. Caracterização faunística no período de seca

Foi observado na caverna, um total de 33 morfoespécies de invertebrados de pelo menos 22 famílias das Ordens: Acari (Neothyridae: *Diplothyrus schubarti*), Pseudoscorpiones (Chernetidae), Opiliones (Cosmetidae: *Anduzeia sp.*, Manaosbiidae, Sclerosomatidae: *Prionostemma* sp., Stygnidae: *Eutimesius* sp.), Araneae (Linyphiidae, Salticidae, Psauridae, Pholcidae: *Metagonia* sp., Theridiidae), Thysanura (Nicoletiidae: Nicoletiinae), Neuroptera (Mantispidae: *Plega* sp.), Orthoptera (Phalangopsidae: *Aclodes* sp.), Isoptera (Termitidae: *Nasutitermes* sp.), Hemiptera (Dipsocoridae, Pentatomidae), Lepidoptera (Noctuidae), Diptera (Culicidae, Tipulidae), Hymenoptera (Formicidae: *Acromyrmex* sp., *Dolichoderus* sp., *Pachycondyla* sp., *Pseudomyrmex* sp., *Rogeria* sp., *Solenopsis* sp.; Mutillidae) e Coleoptera (Staphylinidae).

Dentre os vertebrados foi encontrada uma espécie de Anura (Leptodactylidae: *Pristimantis* cf. *fenestratus*). Desta forma, no total foram encontradas 34 morfoespécies.

5.4.4.14.3. Caracterização faunística no período de chuva

Foi observado na caverna, um total de 41 morfoespécies de invertebrados de pelo menos 33 famílias das Ordens: Gastropoda (Systrophiidae), Isopoda (Armadillidae, Balloniscidae, Plathyarthridae: *Trichorhina* sp.), Amblypygi (Phrynidae: *Heterophrynus longicornis*), Pseudoscorpiones (Chernetidae, Chtoniidae), Opiliones (Cosmetidae: *Anduzeia* sp., Sclerosomatidae: *Prionostemma* sp., Stygnidae), Araneae (Ochyroceratidae, Psauridae, Pholcidae), Microcoryphia (Meinertellidae), Diplura (Parajapygidae), Collembola

(Entomobryidae, Hypogastruridae, Isotomidae), Neuroptera (Chrysopidae), Mantodea (Mantidae: Mantinae), Isoptera (Termitidae: *Nasutitermes* sp., *Velocitermes* sp.), Psocoptera (Myopsocidae: *Lichenomina* sp.), Hemiptera (Cydnidae, Lygaeidae), Lepidoptera (Noctuidae), Diptera (Cecidomyiidae, Sciaridae), Hymenoptera (Formicidae: *Acanthostichus* sp., *Acromyrmex* sp., *Crematogaster* sp., *Dolichoderus* sp., *Ectatomma* sp., *Pachycondyla* sp.; Braconidae), Coleoptera (Scydmaenidae, Staphylinidae), Diplopoda (Chelodesmidae) e Symphyla (Scutigerellidae: *Hanseniella* sp.).

Entre estas, duas espécies de invertebrados foram consideradas troglomórficas: Gastropoda (Systrophiidae) e Isopoda (Plathyarthridae: *Trichorhina* sp.).

5.4.4.14.4. Caracterização geral da fauna da cavidade

Foi observado na caverna, um total de 66 morfoespécies de invertebrados de pelo menos 46 famílias das Ordens: Gastropoda (Systrophiidae), Isopoda (Armadillidae, Balloniscidae, Plathyarthridae: *Trichorhina* sp.), Acari (Neothyridae: *Diplothyrus schubarti*), Amblypygi (Phrynidae: *Heterophrynus longicornis*), Pseudoscorpiones (Chernetidae, Chtoniidae), Opiliones (Cosmetidae: *Anduzeia sp.*, Manaosbiidae, Sclerosomatidae: *Prionostemma* sp., Stygnidae: *Eutimesius* sp.), Araneae (Linyphiidae, Ochyroceratidae, Salticidae, Psauridae, Pholcidae: *Metagonia* sp., Theridiidae), Thysanura (Nicoletiidae: Nicoletiinae), Microcoryphia (Meinertellidae), Diplura (Parajapygidae), Collembola (Entomobryidae, Hypogastruridae, Isotomidae), Neuroptera (Chrysopidae, Mantispidae: *Plega* sp.), Orthoptera (Phalangopsidae: *Aclodes* sp.), Mantodea (Mantidae: Mantinae), Isoptera (Termitidae: *Nasutitermes* sp., *Velocitermes* sp.), Psocoptera (Myopsocidae: *Lichenomina* sp.), Hemiptera (Cydnidae, Dipsocoridae, Lygaeidae, Pentatomidae), Lepidoptera (Noctuidae), Diptera (Cecidomyiidae, Culicidae, Sciaridae, Tipulidae), Hymenoptera (Formicidae: *Acanthostichus* sp., *Acromyrmex* sp., *Crematogaster* sp., *Dolichoderus* sp., *Ectatomma* sp., *Pachycondyla* sp., *Pseudomyrmex* sp., *Rogeria* sp., *Solenopsis* sp.; Braconidae; Mutillidae), Coleoptera (Scydmaenidae, Staphylinidae), Diplopoda (Chelodesmidae) e Symphyla (Scutigerellidae: *Hanseniella* sp.).

Dentre os vertebrados foi encontrada uma espécie de Anura (Leptodactylidae: *Pristimantis* cf. *fenestratus*). Desta forma, no total foram encontradas 67 morfoespécies. Entre estas, duas espécies de invertebrados foram consideradas troglomórficas: Gastropoda (Systrophiidae) e Isopoda (Plathyarthridae: *Trichorhina* sp.).

5.4.4.15. SL-016

5.4.4.15.1. Caracterização trófica

Grande cavidade com 45,5 m de desenvolvimento, formada na canga em área de encosta. Possui salões e condutos amplos com muitos blocos abatidos de diferentes dimensões (calhaus e matacões). A vegetação de entorno é composta predominantemente por samambaias e poucas angiospermas estando esta muito alterada e suscetível a frequentes queimadas. A caverna possui duas entradas principais, sendo ambas ensolaradas e com muitos líquens e briófitas além de muita serrapilheira restrita à linha d'água. Em uma parte da caverna o piso é predominantemente plano e seco, sem gotejamentos ativos, sendo composto por sedimento fino e coberto por muitos blocos abatidos. Nesta região foram observadas fezes de aves, pouca serrapilheira e pequenas raízes esparsas. Outras regiões da caverna, no entanto, podem ser consideradas eutróficas em virtude da grande quantidade de matéria orgânica representada principalmente pelos depósitos de guano frugívoro produzidos por uma grande colônia de morcegos Glossophaginae (*G. soricina*). Nestas áreas, o piso pode ser considerado irregular e ascendente onde existem muitos blocos abatidos (seixos, calhaus e matacões) além de muitas micro-raízes que chegam a formar rizotemas em algumas áreas. Não existem pontos de gotejamento e nem zonas afóticas (Figura 119). De forma geral o sistema de canalículos é pouco desenvolvido e a estabilidade ambiental da cavidade é mediana. Após o inventário realizado durante a estação seca, a vegetação do entorno das cavidades desta área foi incendiada. Apesar de a vegetação encontrar-se em regeneração durante a estação úmida, uma das entradas desta cavidade estava totalmente recoberta por espécies vegetais pioneiras e oportunistas. Ao longo da cavidade foram observados vários pontos de gotejamentos ativos, estando estes concentrados próximos à entrada. O guano de morcegos frugívoros apresentou-se constante também durante este período.

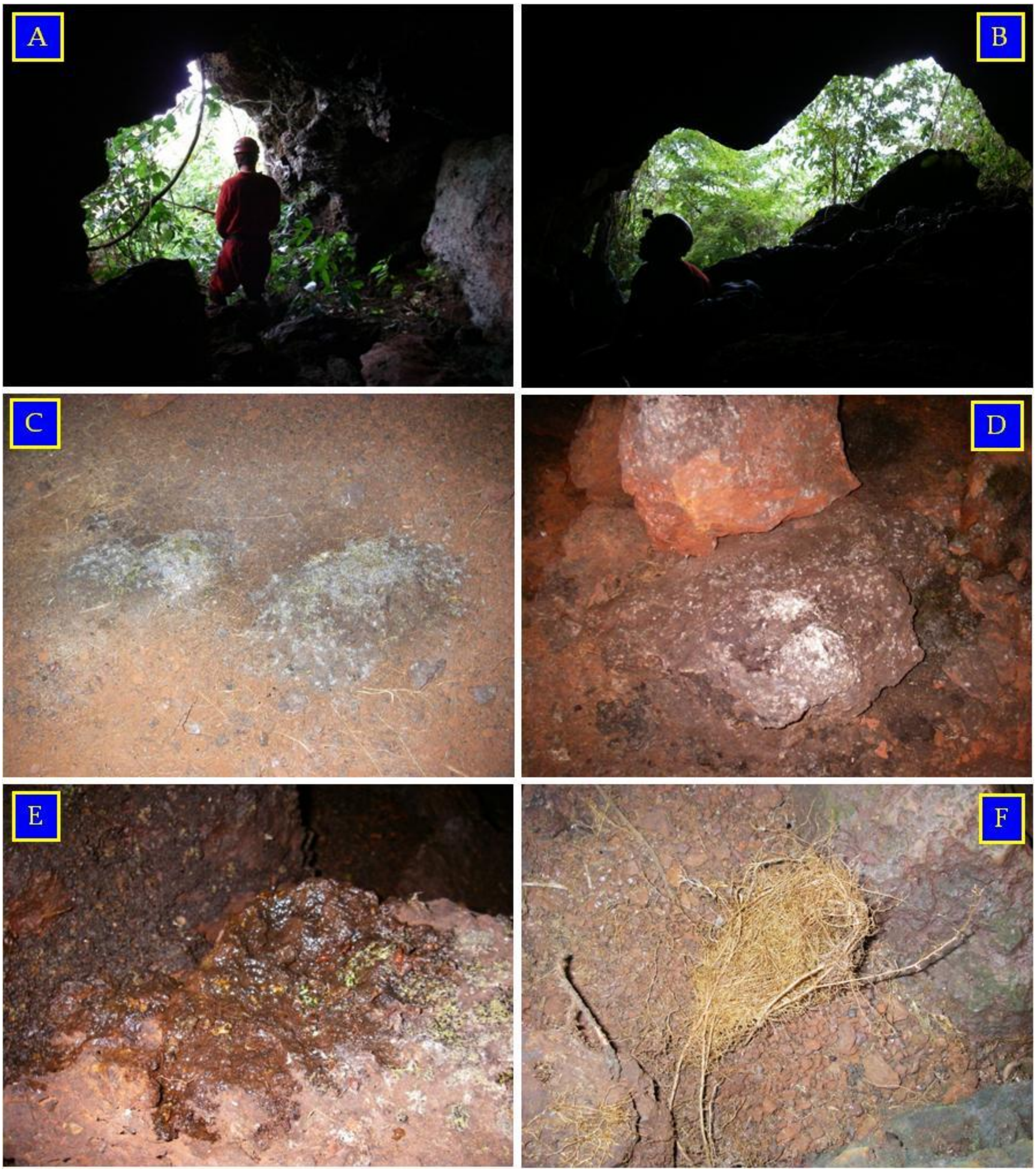

Figura 119 - a) Aspecto geral da entrada principal da cavidade; b) Entrada secundária onde é possível observar a vegetação do entorno composta basicamente por samambaias; c) Blocos abatidos próximo a linha d'água; d) Depósito fresco de guano frugívoro com restos de plântulas e um sistema radicular desenvolvido na região periférica; e) Detalhe de um sistema radicular bem desenvolvido com o piso composto por sedimento granulado; f) Ninfa de barata (Blaberidae) associada a um conjunto de raízes.

5.4.4.15.2. Caracterização faunística no período de seca

Foi observado na caverna, um total de 51 morfoespécies de invertebrados de pelo menos 40 famílias das Ordens: Onychophora (Peripatidae), Oligochaeta, Gastropoda (Subulinidae, Systrophiidae, Valloniidae), Isopoda (Armadillidae, Philosciidae, Plathyarthridae: *Trichorhina*

sp.), Acari (Macronyssidae, Oribatida), Pseudoscorpiones (Chernetidae, Chtoniidae), Opiliones (Escadabiidae), Araneae (Araneidae: *Alpaida* sp.; Gnaphosidae; Ochyroceratidae; Oonopidae: Oonopinae; Segestridae; Scytodidae*: Scytodes eleonora*; Pholcidae: *Mesabolivar* sp.; Theridiidae; Theridiosomatidae), Thysanura (Nicoletiidae: Nicoletiinae), Diplura (Campodeidae), Collembola (Entomobryidae), Orthoptera (Phalangopsidae: *Aclodes* sp., *Phalangopsis* sp.), Blatttodea (Blaberidae: *Blaberus* sp., Blattidae), Isoptera (Termitidae: *Nasutitermes* sp.), Hemiptera (Cydnidae, Dipsocoridae, Enicocephalidae), Lepidoptera (Noctuidae, Tineidae), Diptera (Dolichopodidae, Phoridae, Psychodidae: *Lutzomyia* sp., Tipulidae), Hymenoptera (Formicidae: *Camponotus* sp., *Cyphomyrmex* sp., *Pachycondyla* sp., *Tapinoma* sp., *Zacryptocerus* sp.), Coleoptera (Carabidae), Diplopoda (Pyrgodesmidae), Scolopendromorpha (Cryptopidae: *Cryptops* sp.) e Symphyla (Scutigerellidae: *Hanseniella* sp.).

Dentre os vertebrados foram encontradas quatro espécies das Ordens: Chiroptera (Phyllostomidae: *Glossophaga soricina*), Squamata (Colubridae: *Chironius* sp.), Anura (Leptodactylidae: *Pristimantis* cf. *fenestratus*; Bufonidae). Desta forma, no total foram encontradas 55 morfoespécies. Entre estas, uma espécie de Isopoda (Plathyarthridae: *Trichorhina* sp.) foi considerada troglomórfica.

5.4.4.15.3. Caracterização faunística no período de chuva

Foi observado na caverna, um total de 118 morfoespécies de invertebrados de pelo menos 77 famílias das Ordens: Onychophora (Peripatidae), Oligochaeta, Gastropoda (Subulinidae, Systrophiidae, Valloniidae), Isopoda (Armadillidae, Balloniscidae, Plathyarthridae: *Trichorhina* sp.), Acari (Argasidae: *Ornithodoros* sp., Ixodidae: *Amblyomma* sp., Laelapidae: *Stratiolaelaps* sp., Podocinidae: *Podocinum* sp., Opilioacaridae: *Neoacarus* spn., Oribatida, Anystidae: *Erythracarus* sp., Rhagidiidae, Trombidiforme), Amblypygi (Phrynidae: *Heterophrynus longicornis*), Ricinulei (Ricinoididae: *Cryptocellus tarsilae*), Pseudoscorpiones (Chernetidae, Chtoniidae), Opiliones (Escadabiidae, Sclerosomatidae: *Prionostemma* sp., Phalangiidae), Araneae (Araneidae: *Alpaida* sp.; Dipluridae; Filistatidae; Gnaphosidae; Oonopidae: Oonopinae; Salticidae; Segestridae; Scytodidae*: Scytodes eleonora*; Pholcidae: *Mesabolivar* sp., *Metagonia* sp.; Theraphosidae; Theridiidae; Theridiosomatidae), Thysanura (Nicoletiidae: Nicoletiinae), Microcoryphia (Meinertellidae), Diplura (Anajapygidae, Campodeidae), Collembola (Cyphoderidae, Entomobryidae), Orthoptera (Phalangopsidae: *Phalangopsis* sp., Tettigoniidae: Copiphorinae), Blatttodea (Blaberidae: *Blaberus* sp., Blattidae, Polyphagidae), Isoptera (Termitidae: *Nasutitermes* sp., *Subulitermes* sp.?), Psocoptera (Archipsocidae, Epipsocidae, Psyllipsocidae: *Psyllipsocus* sp., Trogiidae), Hemiptera (Cydnidae, Lygaeidae, Reduviidae), Homoptera (Cixiidae), Lepidoptera (Noctuidae, Tineidae), Diptera (Cecidomyiidae, Ceratopogonidae, Chloropidae, Chironomidae, Culicidae, Drosophilidae: *Drosophila* sp., Milichiidae, Psychodidae: *Lutzomyia* sp., Sciaridae, Syrphidae: *Ornidia obesa*), Hymenoptera (Formicidae: *Apterostigma* sp.,

Camponotus sp., *Cyphomyrmex* sp., *Pachycondyla* sp., *Pseudomyrmex* sp., *Pheidole* sp., *Tapinoma* sp.), Coleoptera (Carabidae, Scydmaenidae, Staphylinidae), Diplopoda (Pseudonannolenidae), Scutigeromorpha (Scutigeridae: *Sphendononema* sp.) e Symphyla (Scutigerellidae: *Hanseniella* sp.).

Dentre os vertebrados foram encontradas três espécies das Ordens: Chiroptera (Emballonuridae: *Peropteryx kappleri*, Phyllostomidae: *Glossophaga soricina*) e Anura (Leptodactylidae). Desta forma, no total foram encontradas 121 morfoespécies. Entre estas, quatro espécies de invertebrados foram consideradas troglomórficas: Gastropoda (Systrophiidae), Isopoda (Plathyarthridae: *Trichorhina* sp.), Acari (Trombidiforme) e Diplopoda (Psedonannolenidae).

5.4.4.15.4. Caracterização geral da fauna da cavidade

Foi observado na caverna, um total de 142 morfoespécies de invertebrados de pelo menos 82 famílias das Ordens: Onychophora (Peripatidae), Oligochaeta, Gastropoda (Subulinidae, Systrophiidae, Valloniidae), Isopoda (Armadillidae, Balloniscidae, Philosciidae, Plathyarthridae: *Trichorhina* sp.), Acari (Argasidae: *Ornithodoros* sp., Ixodidae: *Amblyomma* sp., Laelapidae: *Stratiolaelaps* sp., Macronyssidae, Podocinidae: *Podocinum* sp., Opilioacaridae: *Neoacarus* spn., Oribatida, Anystidae: *Erythracarus* sp., Rhagidiidae, Trombidiforme), Amblypygi (Phrynidae: *Heterophrynus longicornis*), Ricinulei (Ricinoididae: *Cryptocellus tarsilae*), Pseudoscorpiones (Chernetidae, Chtoniidae), Opiliones (Escadabiidae, Sclerosomatidae: *Prionostemma* sp., Phalangiidae), Araneae (Araneidae: *Alpaida* sp.; Dipluridae; Filistatidae; Gnaphosidae; Ochyroceratidae; Oonopidae: Oonopinae; Salticidae; Segestridae; Scytodidae*: Scytodes eleonora*; Pholcidae: *Mesabolivar* sp., *Metagonia* sp.; Theraphosidae; Theridiidae; Theridiosomatidae), Thysanura (Nicoletiidae: Nicoletiinae), Microcoryphia (Meinertellidae), Diplura (Anajapygidae, Campodeidae), Collembola (Cyphoderidae, Entomobryidae), Orthoptera (Phalangopsidae: *Aclodes* sp., *Phalangopsis* sp., Tettigoniidae: Copiphorinae), Blatttodea (Blaberidae: *Blaberus* sp., Blattidae, Polyphagidae), Isoptera (Termitidae: *Nasutitermes* sp., *Subulitermes* sp.?), Psocoptera (Archipsocidae, Epipsocidae, Psyllipsocidae: *Psyllipsocus* sp., Trogiidae), Hemiptera (Cydnidae, Dipsocoridae, Enicocephalidae, Lygaeidae, Reduviidae), Homoptera (Cixiidae), Lepidoptera (Noctuidae, Tineidae), Diptera (Cecidomyiidae, Ceratopogonidae, Chloropidae, Chironomidae, Culicidae, Dolichopodidae, Drosophilidae: *Drosophila* sp., Milichiidae, Phoridae, Psychodidae: *Lutzomyia* sp., Sciaridae, Syrphidae: *Ornidia obesa*, Tipulidae), Hymenoptera (Formicidae: *Apterostigma* sp., *Camponotus* sp., *Cyphomyrmex* sp., *Pachycondyla* sp., *Pseudomyrmex* sp., *Pheidole* sp., *Tapinoma* sp., *Zacryptocerus* sp.), Coleoptera (Carabidae, Scydmaenidae, Staphylinidae), Diplopoda (Pyrgodesmidae, Polydesmida, Pseudonannolenidae), Scolopendromorpha (Cryptopidae: *Cryptops* sp.), Scutigeromorpha (Scutigeridae: *Sphendononema* sp.) e Symphyla (Scutigerellidae: *Hanseniella* sp.).

Dentre os vertebrados foram encontradas seis espécies das Ordens: Chiroptera (Emballonuridae: *Peropteryx kappleri*, Phyllostomidae: *Glossophaga soricina*), Squamata (Colubridae: *Chironius* sp.) e Anura (Leptodactylidae: *Pristimantis* cf. *fenestratus,* sp. indet.; Bufonidae).

Desta forma, no total foram encontradas 148 morfoespécies. Entre estas, quatro espécies de invertebrados foram consideradas troglomórficas: Gastropoda (Systrophiidae), Isopoda (Plathyarthridae: *Trichorhina* sp.), Acari (Trombidiforme) e Diplopoda (Psedonannolenidae). Alguns organismos encontrados nesta caverna são mostrados na Figura 120.

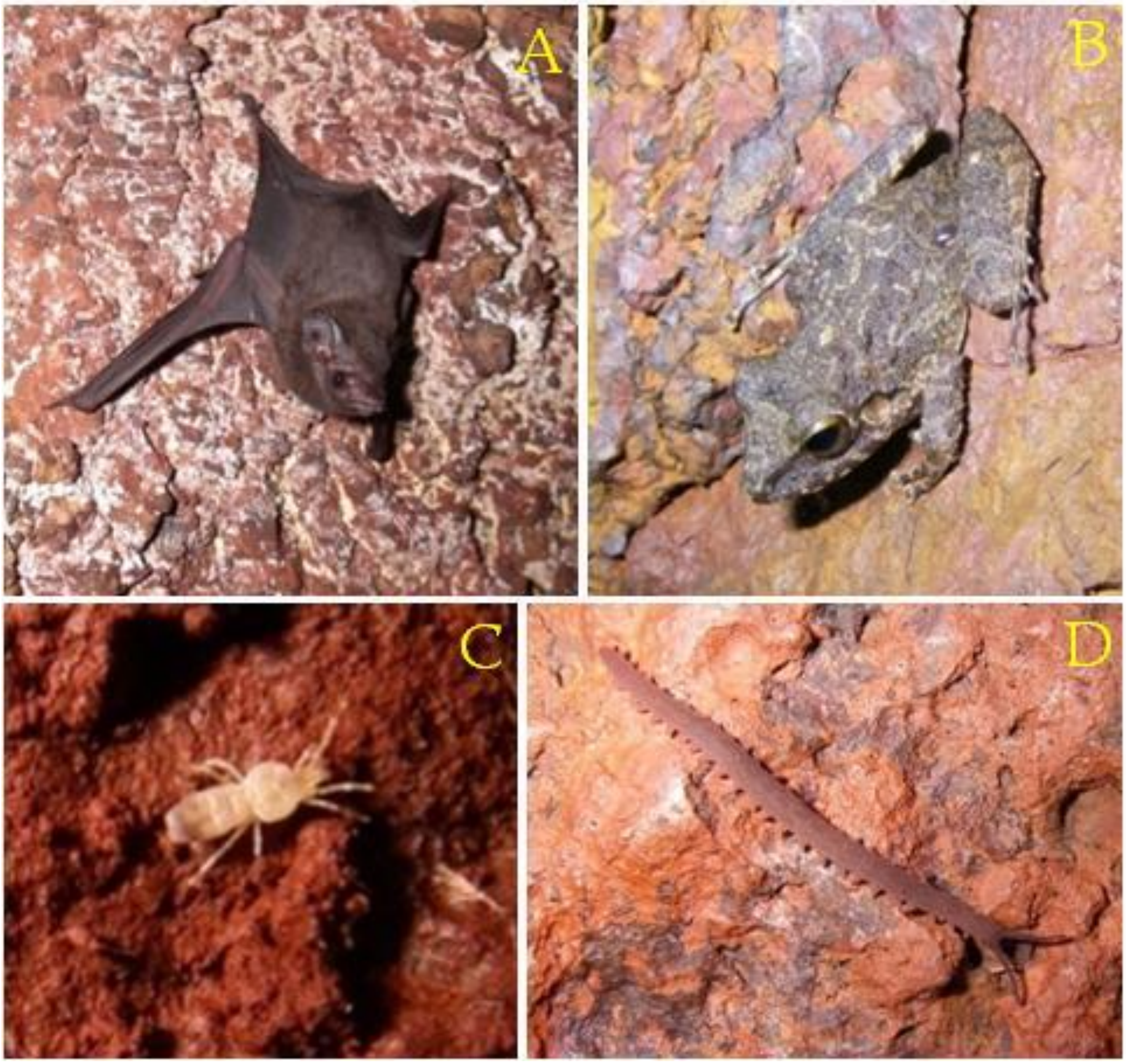

Figura 120 - a) Emballonuridae (*Peropteryx kappleri*); b) Leptodactylidae (*Pristimantis cf. fenestratus*); c) Trombidiforme; d) Onychophora (Peripatidae).

5.4.4.16. SL-017

5.4.4.16.1. Caracterização trófica

Grande abrigo com projeção horizontal de 20,5 m formado na canga em área de topo de encosta. A vegetação de entorno é composta predominantemente por samambaias e poucas angiospermas estando esta muito alterada e suscetível a frequentes queimadas. Somente com zona eufótica, a cavidade possui uma entrada ampla e ensolarada com muitas plantas (principalmente Melastomatáceas), pteridófitas, briófitas e líquens. O piso é predominantemente plano composto por sedimento fino com poucos blocos de pequenas dimensões (seixos e calhaus) e revestido por líquens e briófitas e plântulas de angiospermas. Na margem esquerda da caverna existe um conduto artificial descendente com umidade elevada, o qual apresenta piso composto por sedimento fino com zona disfótica e afótica e com muitas micro-raízes (Figura 121). Esta zona artificial da cavidade pode ser considerada um hábitat significativo para populações de grilos Phalangopsidae (*Phalangopsis* sp.) e anfíbios (*P. fenestratus*) em virtude da grande quantidade de indivíduos destes táxons encontrados nesta área. De forma geral, a cavidade pode ser considerada de baixa estabilidade ambiental e a mesma apresenta um sistema de canalículos pouco desenvolvido. Durante a estação úmida observamos alguns pontos de gotejamento ativos no interior da cavidade e um aumento significativo na quantidade de serrapilheira importada para o interior da mesma. Na zona disfótica ainda foram encontrados alguns pequenos depósitos de guano de morcegos insetívoros.

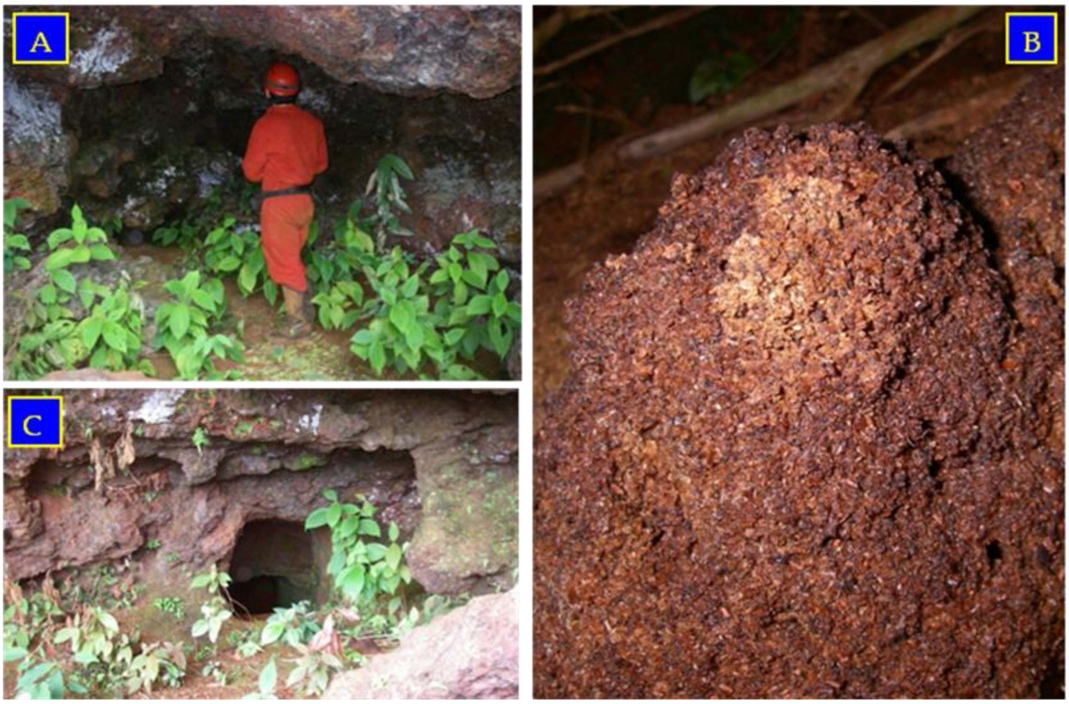

Figura 121 - a) Aspecto geral da entrada da cavidade onde é possível observar vegetação abundante em virtude da elevada taxa de luminosidade; b) Depósito de guano de morcegos insetívoros presente na zona disfótica da caverna; c) Conduto artificial localizado na margem esquerda da cavidade

5.4.4.16.2. Caracterização faunística no período de seca

Foi observado na caverna, um total de 33 morfoespécies de invertebrados de pelo menos 24 famílias das Ordens: Isopoda (Philosciidae), Acari (Laelapidae: *Stratiolaelaps* sp., Astigmatina, Trombidiforme), Amblypygi (Phrynidae: *Heterophrynus longicornis*), Pseudoscorpiones (Chernetidae), Opiliones (Manaosbiidae, Sclerosomatidae: *Prionostemma* sp.), Araneae (Salticidae, Pholcidae: *Mesabolivar* sp., Theridiosomatidae), Microcoryphia (Meinertellidae), Diplura (Campodeidae), Collembola (Entomobryidae), Neuroptera (Mantispidae: *Plega* sp.), Orthoptera (Phalangopsidae: *Aclodes* sp., *Phalangopsis* sp.), Isoptera (Termitidae: *Nasutitermes* sp.), Psocoptera (Epipsocidae), Hemiptera (Cydnidae), Homoptera (Cixiidae: *Cixius* sp.), Lepidoptera (Noctuidae, Tineidae), Diptera (Dolichopodidae, Psychodidae: *Lutzomyia* sp., Tipulidae) e Hymenoptera (Formicidae: *Acromyrmex* sp., *Pachycondyla* sp.).

Dentre os vertebrados foram encontradas duas espécies de Anura (Dendrobatidae: *Ameerega* sp., Leptodactylidae: *Pristimantis* cf. *fenestratus*). Desta forma, no total foram encontradas 35 morfoespécies.

5.4.4.16.3. Caracterização faunística no período de chuva

Foi observado na caverna, um total de 45 morfoespécies de invertebrados de pelo menos 33 famílias das Ordens: Isopoda (Armadillidae, Philosciidae), Acari (Argasidae: *Ornithodoros* sp., Laelapidae: *Stratiolaelaps* sp., Oribatida, Eupodidae: *Linopodes* sp., Trombidiforme), Amblypygi (Phrynidae: *Heterophrynus longicornis*), Pseudoscorpiones (Chernetidae), Opiliones (Cosmetidae: *Anduzeia* sp., Phalangiidae), Araneae (Pholcidae: *Mesabolivar* sp., *Metagonia* sp., Theridiosomatidae), Collembola (Entomobryidae, Isotomidae), Orthoptera (Phalangopsidae: *Aclodes* sp., *Phalangopsis* sp.), Isoptera (Termitidae: *Nasutitermes* sp.), Psocoptera (Ptiloneuridae: *Ptiloneura* sp.), Hemiptera (Cydnidae), Homoptera (Cercopidae, Cixiidae: *Cixius* sp.), Lepidoptera (Noctuidae, Tineidae), Diptera (Cecidomyiidae, Ceratopogonidae, Chloropidae, Culicidae, Psychodidae: *Lutzomyia* sp., Tipulidae), Hymenoptera (Formicidae: *Apterostigma* sp., *Camponotus* sp., *Cyphomyrmex* sp., *Pachycondyla* sp., *Pheidole* sp.; Apidae; Vespidae) e Diplopoda (Pyrgodesmidae, Stemmiulidae).

Dentre os vertebrados foram encontradas duas espécies das Ordens: Chiroptera (Phyllostomidae: *Glossophaga soricina*) e Anura (Leptodactylidae: *Pristimantis* cf. *fenestratus*). Desta forma, no total foram encontradas 47 morfoespécies.

5.4.4.16.4. Caracterização geral da fauna da cavidade

Foi observado na caverna, um total de 68 morfoespécies de invertebrados de pelo menos 41 famílias das Ordens: Isopoda (Armadillidae, Philosciidae), Acari (Argasidae: *Ornithodoros* sp., Laelapidae: *Stratiolaelaps* sp., Astigmatina, Oribatida, Eupodidae: *Linopodes* sp., Trombidiforme), Amblypygi (Phrynidae: *Heterophrynus longicornis*), Pseudoscorpiones

(Chernetidae), Opiliones (Cosmetidae: *Anduzeia sp.*, Manaosbiidae, Sclerosomatidae: *Prionostemma* sp., Phalangiidae), Araneae (Salticidae, Pholcidae: *Mesabolivar* sp., *Metagonia* sp., Theridiosomatidae), Microcoryphia (Meinertellidae), Diplura (Campodeidae), Collembola (Entomobryidae, Isotomidae), Neuroptera (Mantispidae: *Plega* sp.), Orthoptera (Phalangopsidae: *Aclodes* sp., *Phalangopsis* sp.), Isoptera (Termitidae: *Nasutitermes* sp.), Psocoptera (Epipsocidae, Ptiloneuridae: *Ptiloneura* sp.), Hemiptera (Cydnidae), Homoptera (Cercopidae, Cixiidae: *Cixius* sp.), Lepidoptera (Noctuidae, Tineidae), Diptera (Cecidomyiidae, Ceratopogonidae, Chloropidae, Culicidae, Dolichopodidae, Psychodidae: *Lutzomyia* sp., Tipulidae), Hymenoptera (Formicidae: *Acromyrmex* sp., *Apterostigma* sp., *Camponotus* sp., *Cyphomyrmex* sp., *Pachycondyla* sp., *Pheidole* sp.; Apidae; Vespidae) e Diplopoda (Pyrgodesmidae, Stemmiulidae).

Dentre os vertebrados foram encontradas três espécies das Ordens: Chiroptera (Phyllostomidae: *Glossophaga soricina*) e Anura (Dendrobatidae: *Ameerega* sp., Leptodactylidae: *Pristimantis* cf. *fenestratus*). Desta forma, no total foram encontradas 71 morfoespécies.

5.4.4.17. SL-018

5.4.4.17.1. Caracterização trófica

Pequeno abrigo com 9 m de desenvolvimento formado na quebra da canga e localizado na parte superior da encosta. A vegetação do entorno é composta basicamente por samambaias e não existe sombreamento no pórtico de entrada (Figura 122). Acima da cavidade a vegetação é rasteira composta basicamente por capim. A caverna possui somente zona eufótica. Sua entrada é pequena e o teto é baixo em toda sua extensão, com muitos líquens, briófitas e poucas pteridófitas nas paredes e piso na zona de entrada. O piso é seco e ascendente a partir da entrada, composto por sedimento fino com alguns poucos blocos de diferentes tamanhos (seixos, calhaus e matacões) e com muitas raízes de pequeno tamanho em toda sua extensão. Na linha d'água existe um pequeno arbusto que pode contribuir com a importação de matéria orgânica vegetal para o interior da cavidade, sendo esta transportada de forma eólica ou gravitacional. Não existem depósitos de guano e, de forma geral, a caverna é pobre em recursos orgânicos e extremamente suscetível a influência das condições ambientais epígeas. O sistema de canalículos é pouco desenvolvido, as paredes e teto são revestidos por Actinomicetos e não existem pontos de gotejamento ativos durante a estação seca. As principais alterações observadas durante a estação úmida foram a existência de vários pontos de gotejamento ativos ao longo de toda a extensão da cavidade, além de um aumento na quantidade de lianas associadas ao piso.

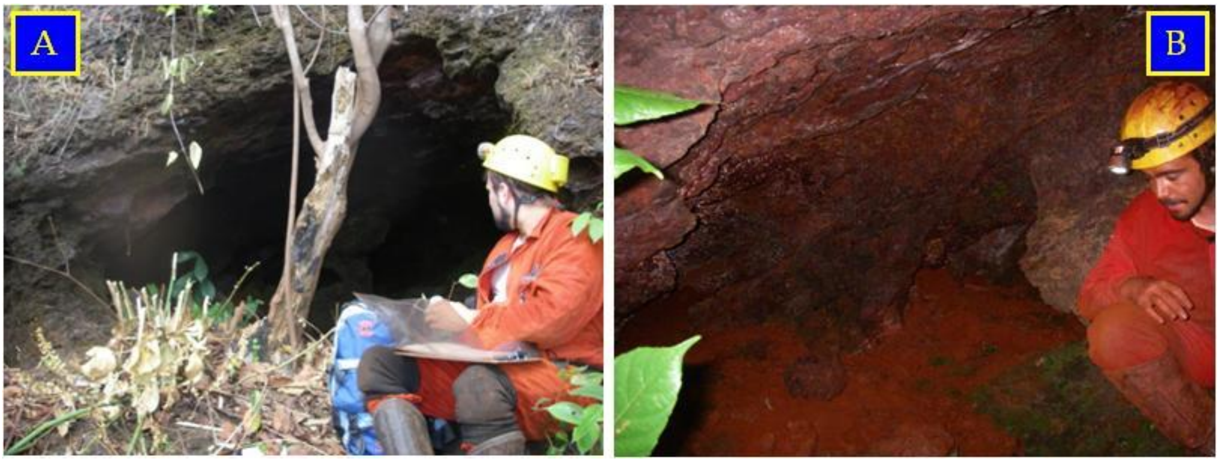

Figura 122 - a) Aspecto geral da entrada da caverna onde é possível observar a vegetação na região superior e um pequeno arbusto germinado junto à linha d'água; b) Zona eufótica com paredes e piso da cavidade revestida por líquens, fungos e briófitas.

5.4.4.17.2. Caracterização faunística no período de seca

Foi observado na caverna, um total de 29 morfoespécies de invertebrados de pelo menos 24 famílias das Ordens: Isopoda (Armadillidae), Acari (Oribatida), Amblypygi (Phrynidae: *Heterophrynus longicornis*), Ricinulei (Ricinoididae: *Cryptocellus tarsilae*), Scorpiones (Buthidae: *Ananteris luciae*), Pseudoscorpiones (Chernetidae, Chtoniidae), Opiliones (Escadabiidae), Araneae (Ochyroceratidae, Oonopidae: Oonopinae, Salticidae, Pholcidae: *Mesabolivar* sp., Theridiidae, Theridiosomatidae), Thysanura (Nicoletiidae: Nicoletiinae), Diplura (Anajapygidae), Collembola (Entomobryidae), Psocoptera (Epipsocidae), Hemiptera (Cydnidae, Scutelleridae), Homoptera (Cixiidae: *Cixius* sp.), Lepidoptera (Noctuidae: *Latebraria* sp.), Diptera (Dolichopodidae) e Hymenoptera (Formicidae: *Camponotus* sp., Pteromalidae).

Dentre os vertebrados foram encontradas duas espécies das Ordens: Chiroptera (Phyllostomidae: *Glossophaga soricina*) e Anura (Leptodactylidae: *Pristimantis* cf. *fenestratus*).

Desta forma, no total foram encontradas 31 morfoespécies. Entre estas, três espécies de invertebrados foram consideradas troglomórficas: Araneae (Ochyroceratidae), Opiliones (Escadabiidae) e Hemiptera (Scutelleridae).

5.4.4.17.3. Caracterização faunística no período de chuva

Foi observado na caverna, um total de 40 morfoespécies de invertebrados de pelo menos 35 famílias das Ordens: Gastropoda (Systrophiidae), Isopoda (Scleropactidae), Acari (Neothyridae: *Diplothyrus schubarti*, Opilioacaridae: *Neoacarus* spn., Oribatida), Pseudoscorpiones (Chernetidae, Chtoniidae), Opiliones (Escadabiidae, Phalangiidae),

Araneae (Linyphiidae, Ochyroceratidae, Salticidae, Pholcidae: *Mesabolivar* sp., Prodidomidae, Theridiidae), Microcoryphia (Meinertellidae), Diplura (Anajapygidae), Collembola (Entomobryidae), Orthoptera (Phalangopsidae: *Phalangopsis* sp.), Blattodea (Blattellidae), Psocoptera (Myopsocidae: *Lichenomina* sp.), Hemiptera (Cydnidae, Ploiariidae), Homoptera (Derbidae), Lepidoptera (Noctuidae, Tineidae), Diptera (Chloropidae, Culicidae, Phoridae, Psychodidae: *Lutzomyia* sp.), Hymenoptera (Formicidae: *Azteca* sp., *Pheidole* sp.), Coleoptera (Curculionidae: Scotylinae), Diplopoda (Siphonophoridae), Geophilomorpha (Macronicophilidae: *Macronicophilus* sp.), Scolopendromorpha (Cryptopidae: *Cryptops* sp.) e Scutigeromorpha (Scutigeridae: *Sphendononema* sp.).

Dentre os vertebrados foi encontrada uma espécie de Chiroptera (Phyllostomidae: *Glossophaga soricina*). Desta forma, no total foram encontradas 41 morfoespécies.

5.4.4.17.4. Caracterização geral da fauna da cavidade

Foi observado na caverna, um total de 62 morfoespécies de invertebrados de pelo menos 47 famílias das Ordens: Gastropoda (Systrophiidae), Isopoda (Armadillidae, Scleropactidae), Acari (Neothyridae: *Diplothyrus schubarti*, Opilioacaridae: *Neoacarus* spn., Oribatida), Amblypygi (Phrynidae: *Heterophrynus longicornis*), Ricinulei (Ricinoididae: *Cryptocellus tarsilae*), Scorpiones (Buthidae: *Ananteris luciae*), Pseudoscorpiones (Chernetidae, Chtoniidae), Opiliones (Escadabiidae, Phalangiidae), Araneae (Linyphiidae, Ochyroceratidae, Oonopidae: Oonopinae, Salticidae, Pholcidae: *Mesabolivar* sp., Prodidomidae, Theridiidae, Theridiosomatidae), Thysanura (Nicoletiidae: Nicoletiinae), Microcoryphia (Meinertellidae), Diplura (Anajapygidae), Collembola (Entomobryidae), Orthoptera (Phalangopsidae: *Phalangopsis* sp.), Blattodea (Blattellidae), Psocoptera (Epipsocidae, Myopsocidae: *Lichenomina* sp.), Hemiptera (Cydnidae, Ploiariidae, Scutelleridae), Homoptera (Cixiidae: *Cixius* sp., Derbidae), Lepidoptera (Noctuidae: *Latebraria* sp., Tineidae), Diptera (Chloropidae, Culicidae, Dolichopodidae, Phoridae, Psychodidae: *Lutzomyia* sp.), Hymenoptera (Formicidae: *Azteca* sp., *Camponotus* sp., *Pheidole* sp.; Pteromalidae), Coleoptera (Curculionidae: Scotylinae), Diplopoda (Siphonophoridae), Geophilomorpha (Macronicophilidae: *Macronicophilus* sp.), Scolopendromorpha (Cryptopidae: *Cryptops* sp.) e Scutigeromorpha (Scutigeridae: *Sphendononema* sp.).

Dentre os vertebrados foram encontradas duas espécies das Ordens: Chiroptera (Phyllostomidae: *Glossophaga soricina*) e Anura (Leptodactylidae: *Pristimantis* cf. *fenestratus*).

Desta forma, no total foram encontradas 64 morfoespécies. Entre estas, três espécies de invertebrados foram consideradas troglomórficas: Araneae (Ochyroceratidae), Opiliones

(Escadabiidae) e Hemiptera (Scutelleridae). Alguns organismos encontrados nesta caverna são mostrados na Figura 123.

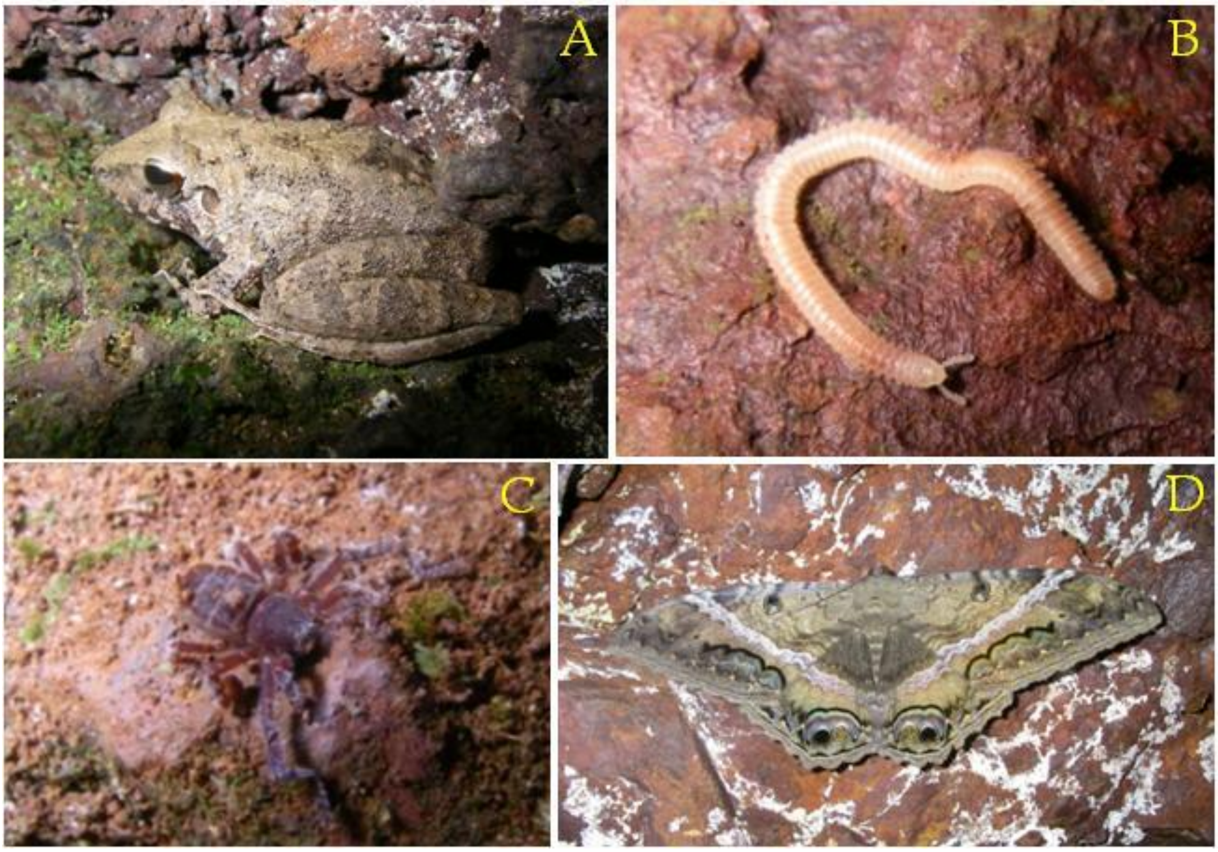

Figura 123 - a) Leptodactylidae (*Pristimantis cf. fenestratus*); b) Diplopoda (Siphonophoridae); c) Ricinulei (*Cryptocellus tarsilae*); d) Lepidoptera (Noctuidae: *Latebraria* sp.).

5.4.4.18. SL-019

5.4.4.18.1. Caracterização trófica

Pequeno abrigo com 8,5 m de desenvolvimento formado no minério de ferro localizado na parte superior da encosta. A vegetação do entorno é composta basicamente por samambaias e na região superior da cavidade existe um predomínio de vegetação rasteira. Cavidade pouco profunda com duas entradas, sendo estas largas, mas em teto baixo com aproximadamente 1,5 metros de altura. Não existe sombreamento no seu pórtico de entrada, as paredes são revestidas de líquens e briófitas e existem algumas pequenas plantas e muita serrapilheira acumulada junto à linha d’água. A cavidade não apresenta zona afótica com teto baixo em toda sua extensão e com muitos blocos abatidos. O piso é seco e predominantemente plano, composto por sedimento fino com muitos blocos de diferentes tamanhos (seixos, calhaus e matacões) e o sistema de canalículos é pouco desenvolvido (Figura 124). Não existem depósitos de guano sendo, de forma geral, uma caverna pobre em recursos alimentares e extremamente suscetível a influência das condições ambientais

epígeas. Após o inventário realizado durante a estação seca, a vegetação do entorno das cavidades desta área foi incendiada. Durante a estação úmida sua entrada encontrava-se completamente ensolarada e havia reduzido a quantidade de serrapilheira no interior da mesma. Junto ao piso, havia uma grande quantidade de lianas.

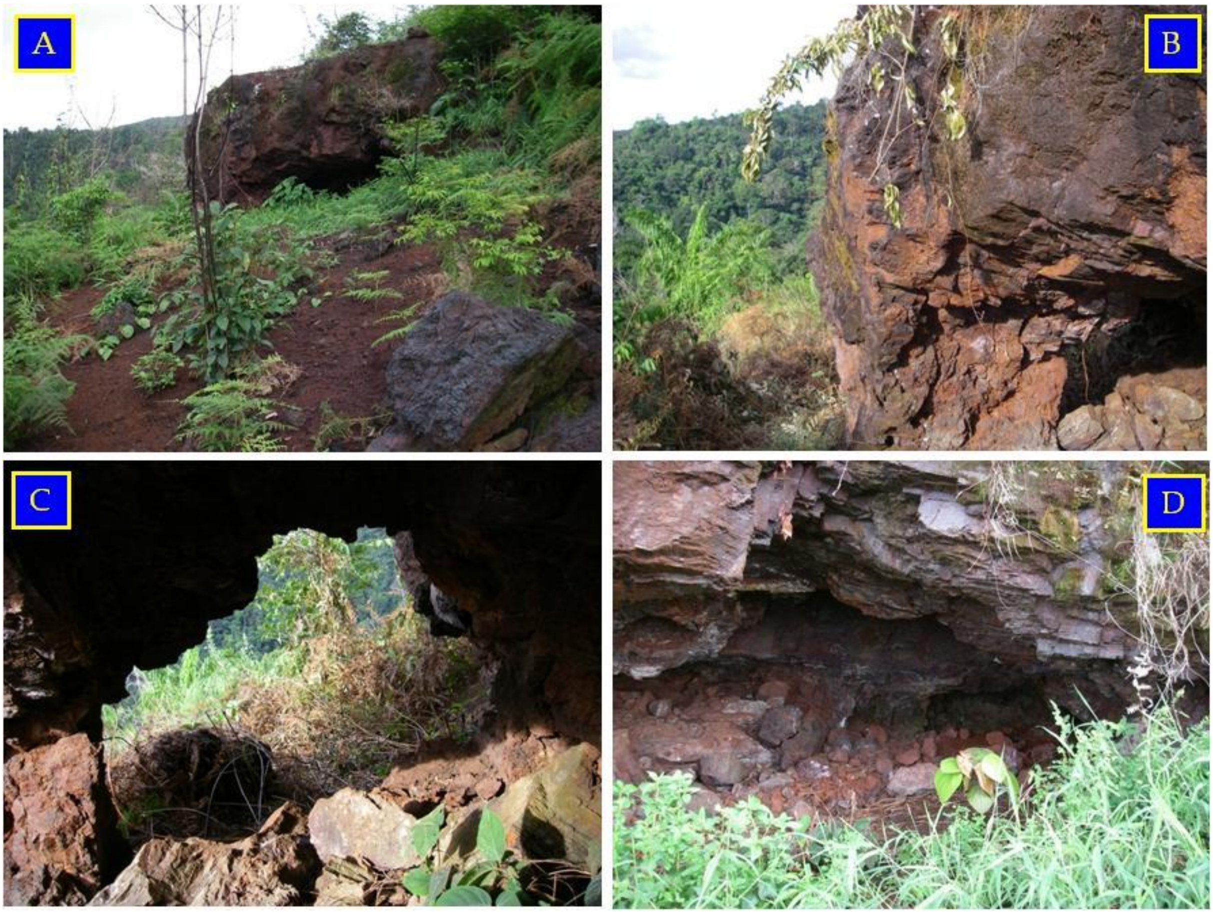

Figura 124 - a) Aspecto geral da entrada onde é possível observar a vegetação desenvolvida no período de chuva do entorno (após queimada); b) Detalhe do afloramento ferruginoso e parte da entrada; c) Vista interna da cavidade onde é possível observar a influência da luminosidade do ambiente epígeo, o acúmulo de serrapilheira junto à linha d'água e os blocos dispostos junto ao piso predominantemente plano; d) porção interna da cavidade.

5.4.4.18.2. Caracterização faunística no período de seca

Foi observado na caverna, um total de 30 morfoespécies de invertebrados de pelo menos 22 famílias das Ordens: Isopoda (Philosciidae), Pseudoscorpiones (Chernetidae, Chtoniidae), Opiliones (Escadabiidae, Kimmulidae), Araneae (Oonopidae: Oonopinae, Salticidae, Scytodidae*: Scytodes eleonora*, Psauridae, Pholcidae: *Mesabolivar* sp., Theridiosomatidae), Diplura (Anajapygidae), Collembola (Entomobryidae), Orthoptera (Gryllidae: Pentacentrinae), Blattodea (Polyphagidae), Isoptera (Termitidae: *Nasutitermes* sp.), Lepidoptera (Tineidae), Diptera (Chloropidae, Psychodidae: *Lutzomyia* sp.), Hymenoptera

(Formicidae: *Acromyrmex* sp., *Dolichoderus* sp., *Pachycondyla* sp.; Vespidae) e Neuroptera (Myrmeleontidae).

5.4.4.18.3. Caracterização faunística no período de chuva

Foi observado na caverna, um total de 38 morfoespécies de invertebrados de pelo menos 28 famílias das Ordens: Isopoda (Armadillidae), Acari (Ixodidae: *Amblyomma* sp., Oribatida), Pseudoscorpiones (Chernetidae, Chtoniidae), Opiliones (Phalangiidae), Araneae (Salticidae, Scytodidae*: Scytodes eleonora*, Pholcidae: *Mesabolivar* sp., *Metagonia* sp., Theridiidae), Orthoptera (Phalangopsidae), Blattodea (Blattellidae, Polyphagidae), Embiidina, Isoptera (Termitidae: *Nasutitermes* sp.), Psocoptera, Hemiptera (Ploiariidae), Lepidoptera (Noctuidae, Tineidae), Diptera (Ceratopogonidae, Chloropidae, Culicidae, Milichiidae, Simulidae), Hymenoptera (Formicidae: *Camponotus* sp., *Pheidole* sp.; Apidae; Bethylidae), Coleoptera (Staphylinidae, Tenebrionidae: Coelometropinae), Scutigeromorpha (Scutigeridae: *Sphendononema* sp.) e Neuroptera (Myrmeleontidae).

5.4.4.18.4. Caracterização geral da fauna da cavidade

Foi observado na caverna, um total de 61 morfoespécies de invertebrados de pelo menos 48 famílias das Ordens: Isopoda (Armadillidae, Philosciidae), Acari (Ixodidae: *Amblyomma* sp., Oribatida), Pseudoscorpiones (Chernetidae, Chtoniidae), Opiliones (Escadabiidae, Kimmulidae, Phalangiidae), Araneae (Oonopidae: Oonopinae, Salticidae, Scytodidae*: Scytodes eleonora*, Psauridae, Pholcidae: *Mesabolivar* sp., *Metagonia* sp., Theridiidae, Theridiosomatidae), Diplura (Anajapygidae), Collembola (Entomobryidae), Orthoptera (Gryllidae: Pentacentrinae; Phalangopsidae), Blattodea (Blattellidae, Polyphagidae), Embiidina, Isoptera (Termitidae: *Nasutitermes* sp.), Psocoptera, Hemiptera (Ploiariidae), Lepidoptera (Noctuidae, Tineidae), Diptera (Ceratopogonidae, Chloropidae, Culicidae, Milichiidae, Psychodidae: *Lutzomyia* sp., Simulidae), Hymenoptera (Formicidae: *Acromyrmex* sp., *Camponotus* sp., *Dolichoderus* sp., *Pachycondyla* sp., *Pheidole* sp.; Apidae; Bethylidae; Vespidae), Coleoptera (Staphylinidae, Tenebrionidae: Coelometropinae), Scutigeromorpha (Scutigeridae: *Sphendononema* sp.) e Neuroptera (Myrmeleontidae).

5.4.4.19. SL-020

5.4.4.19.1. Caracterização trófica

Pequeno abrigo com 18,5 m de desenvolvimento formado na quebra da canga e localizado na parte superior da encosta. A vegetação do entorno é composta basicamente por samambaias e não existe sombreamento no seu pórtico de entrada. Acima da cavidade a vegetação é rasteira composta basicamente por Poaceae (capim) (Figura 125). Cavidade pouco profunda com entrada larga e em teto baixo com aproximadamente 1,5 m de altura. As paredes da região de entrada são revestidas de líquens e briófitas e muitas plântulas de

angiospermas (principalmente Melastomatáceas) além de muita serrapilheira acumulada junto à linha d'água. Abrigo seco e somente com zona eufótica, sendo o piso predominantemente plano e composto por sedimento fino com poucos seixos e calhaus. Não existem depósitos de guano no interior da cavidade, os sistemas radiculares são pouco desenvolvidos apresentando-se forma sub-superficial e a quantidade de canalículos disponíveis é aparentemente reduzida. Cavidade com baixa estabilidade ambiental sendo extremamente influenciada pelas condições ambientais epígeas. Durante a estação úmida observou-se a existência de uma lixeira de formigueiro, além de uma grande quantidade de cinzas (derivadas da queima da vegetação epígea) depositas junto ao substrato.

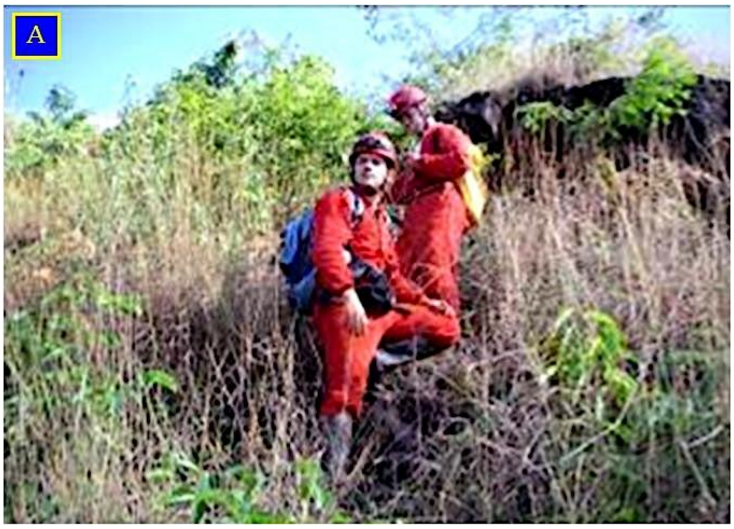

Figura 125 - a) Vegetação predominante na região de entorno ao abrigo que encontra-se localizado no afloramento que pode ser observado na parte superior direita da imagem.

5.4.4.19.2. Caracterização faunística no período de seca

Foi observado na caverna, um total de 26 morfoespécies de invertebrados de pelo menos 19 famílias das Ordens: Acari (Oribatida), Pseudoscorpiones (Chernetidae, Chtoniidae), Opiliones (Phalangiidae), Araneae (Ctenidae; Oonopidae: Oonopinae; Salticidae; Scytodidae*: Scytodes eleonora*; Psauridae; Pholcidae: *Mesabolivar* sp.), Collembola (Entomobryidae), Psocoptera (Archipsocidae, Pachytroctidae), Homoptera (Cixiidae: *Cixius* sp.), Lepidoptera (Noctuidae), Diptera (Chloropidae, Dolichopodidae, Psychodidae: *Lutzomyia* sp.), Hymenoptera (Formicidae: *Camponotus* sp., *Dolichoderus* sp., *Tapinoma* sp.), Geophilomorpha (Schendylidae) e Neuroptera (Myrmeleontidae).

5.4.4.19.3. Caracterização faunística no período de chuva

Foi observado na caverna, um total de 40 morfoespécies de invertebrados de pelo menos 28 famílias das Ordens: Isopoda (Armadillidae, Dubioniscidae), Acari (Oribatida), Pseudoscorpiones (Chernetidae, Chtoniidae), Opiliones (Phalangiidae), Araneae (Dipluridae; Gnaphosidae; Linyphiidae; Salticidae; Scytodidae*: Scytodes eleonora*; Pholcidae: *Mesabolivar*

sp., *Metagonia* sp.; Theridiosomatidae), Collembola (Entomobryidae), Isoptera (Termitidae: *Nasutitermes* sp.), Psocoptera (Epipsocidae, Pachytroctidae), Homoptera (Cixiidae: *Cixius* sp.), Lepidoptera (Noctuidae, Tineidae), Diptera (Chloropidae, Culicidae, Psychodidae: *Lutzomyia* sp.), Hymenoptera (Formicidae: *Acromyrmex* sp., *Pheidole* sp., *Solenopsis* sp.), Coleoptera (Pselaphidae, Staphylinidae), Scutigeromorpha (Scutigeridae: *Sphendononema* sp.) e Neuroptera (Myrmeleontidae).

5.4.4.19.4. Caracterização geral da fauna da cavidade

Foi observado na caverna, um total de 56 morfoespécies de invertebrados de pelo menos 34 famílias das Ordens: Isopoda (Armadillidae, Dubioniscidae), Acari (Oribatida), Pseudoscorpiones (Chernetidae, Chtoniidae), Opiliones (Phalangiidae), Araneae (Ctenidae; Dipluridae; Gnaphosidae; Linyphiidae; Oonopidae: Oonopinae; Salticidae; Scytodidae*:* *Scytodes eleonora*; Psauridae; Pholcidae: *Mesabolivar* sp., *Metagonia* sp.; Theridiosomatidae), Collembola (Entomobryidae), Isoptera (Termitidae: *Nasutitermes* sp.), Psocoptera (Archipsocidae, Epipsocidae, Pachytroctidae), Homoptera (Cixiidae: *Cixius* sp.), Lepidoptera (Noctuidae, Tineidae), Diptera (Chloropidae, Culicidae, Dolichopodidae, Psychodidae: *Lutzomyia* sp., Sciaridae), Hymenoptera (Formicidae: *Acromyrmex* sp., *Camponotus* sp., *Dolichoderus* sp., *Pheidole* sp., *Solenopsis* sp., *Tapinoma* sp.), Coleoptera (Pselaphidae, Staphylinidae), Geophilomorpha (Schendylidae), Scutigeromorpha (Scutigeridae: *Sphendononema* sp.) e Neuroptera (Myrmeleontidae).

5.4.4.20. SL-022

5.4.4.20.1. Caracterização trófica

Pequeno abrigo formado na canga com projeção horizontal de 14,5 m localizado na parte mais alta da encosta. A vegetação do entorno é composta basicamente por samambaias e na região superior existe um predomínio de vegetação rasteira (capim). Cavidade pouco profunda com uma entrada principal e duas pequenas conexões com o meio epígeo na margem esquerda do seu desenvolvimento. A entrada principal é composta basicamente por uma depressão na rocha que depois segue por um conduto ascendente a meia altura em rastejo que corresponde ao restante da cavidade (Figura 126). Não existe sombreamento no seu pórtico de entrada, as paredes são revestidas por fungos, líquens e briófitas e existem muitas plântulas de angiospermas (basicamente Melastomatáceas) e muita serrapilheira acumulada junto à linha d’água. Cavidade sem zona afótica, sendo a região de teto baixo representada por uma zona de penumbra escura e com muitos blocos abatidos. O piso é seco, composto por sedimento fino com muitos blocos de diferentes tamanhos (seixos, calhaus e matacões) e o sistema de canalículos é pouco desenvolvido. Não existem depósitos de guano e a caverna, de forma geral, é pobre em recursos alimentares e extremamente suscetível as condições ambientais epígeas. Nenhuma alteração significativa

foi observada durante a estação úmida, além das alterações normais na umidade relativa do ar.

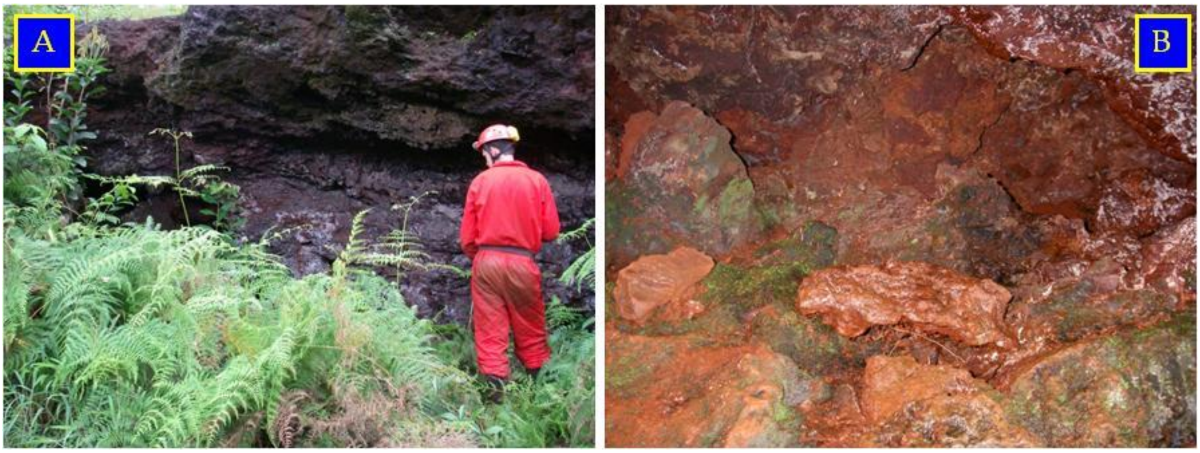

Figura 126 - a) Abrigo de entrada da cavidade, associado à quebra na canga; b) Porção interna da cavidade com detalhes das paredes e piso onde existe uma forte influência das condições ambientais epígea.

5.4.4.20.2. Caracterização faunística no período de seca

Foi observado na caverna, um total de 34 morfoespécies de invertebrados de pelo menos 25 famílias das ordens: Oligochaeta, Gastropoda (Systrophiidae), Acari (Oribatida), Amblypygi (Phrynidae: *Heterophrynus longicornis,* Charinidae: *Charinus* sp.), Pseudoscorpiones (Chernetidae, Chtoniidae), Araneae (Corinidae, Gnaphosidae, Oonopidae, Salticidae, Scytodidae*: Scytodes eleonora*, Pholcidae*: Mesabolivar* sp.*,* Theridiidae), Diplura (Campodeidae), Collembola (Entomobryidae), Orthoptera (Tetrigidae), Isoptera (Termitidae: *Nasutitermes* sp.), Psocoptera (Lepidopsocidae), Homoptera (Cixiidae: *Cixius* sp.), Diptera (Dolichopodidae), Hymenoptera (Formicidae: *Camponotus* sp., *Hypoponera* sp.*, Tapinoma* sp.; Mutillidae; Vespidae), Symphyla (Scutigerellidae: *Hanseniella* sp.), Coleoptera (Elateridae, Tenebrionidae: Coelometropinae) e Lepidoptera.

Dentre os vertebrados foi encontrada uma espécie de Chiroptera (Furipteridae: *Furipterus horrens.*)

Desta forma, no total foram encontradas 35 morfoespécies. Entre estas, três espécies de invertebrados foram consideradas troglomórficas: Gastropoda (Systrophiidae), Amblypygi (Phrynidae: *Charinus* sp.) e Hymenoptera (Formicidae: *Hypoponera* sp.)

5.4.4.20.3. Caracterização faunística no período de chuva

Foi observado na caverna, um total de 44 morfoespécies de invertebrados de pelo menos 35 famílias das ordens: Gastropoda (Systrophiidae), Isopoda (Armadillidae, Dubioniscidae, Philosciidae), Acari (Teneriffiidae), Schizomida (Hubbardiidae: Hubbardiinae),

Pseudoscorpiones (Chernetidae), Opiliones (Cosmetidae: *Anduzeia* sp., Phalangiidae), Araneae (Oonopinae, Salticidae, Scytodidae*: Scytodes eleonora*, Pholcidae: *Metagonia* sp.), Diplura (Campodeidae), Collembola (Entomobryidae), Orthoptera (Phalangopsidae: *Phalangopsis* sp.) Blattodea (Blattellidae), Isoptera *(Nasutitermes* sp.), Psocoptera, Hemiptera (Cydnidae, Miridae, Reduviidae), Homoptera (Cixiidae), Lepidoptera (Geometridae, Noctuidae), Diptera (Psychodidae*: Lutzomyia* sp.), Hymenoptera (Formicidae*: Pachycondyla* sp., *Pheidole* sp*., Solenopsis* sp.; Braconidae), Thysanoptera (Phaleothripidae: Phaleothripinae), Coleoptera (Curculionidae: Scotylinae; Leiodidae; Staphylinidae), Diplopoda (Polydesmida, Glomeridesmidae: *Glomeridesmus* sp., Stemmiulidae) e Lepidoptera (Tineidae).

Desta forma, no total foram encontradas 35 morfoespécies. Entre estas, uma espécie de Gastropoda (Systrophiidae) foi considerada troglomórfica.

5.4.4.20.4. Caracterização geral da fauna da cavidade

Foi observado na caverna, um total de 73 morfoespécies de invertebrados de pelo menos 49 famílias das ordens: Gastropoda (Systrophiidae), Isopoda (Armadillidae; Dubioniscidae; Philosciidae), Acari (Oribatida, Teneriffiidae), Amblypygi (Phrynidae: *Heterophrynus longicornis,* Charinidae*: Charinus* sp.), Schizomida (Hubbardiidae: Hubbardiinae), Pseudoscorpiones (Chernetidae, Chtoniidae), Opiliones (Cosmetidae: *Anduzeia* sp., Phalangiidae), Araneae (Corinidae; Gnaphosidae; Oonopidae: Salticidae; Scytodidae*: Scytodes eleonora*; Pholcidae: *Mesabolivar* sp., *Metagonia* sp.; Theridiidae), Diplura (Campodeidae), Collembola (Entomobryidae), Orthoptera (Phalangopsidae: *Phalangopsis* sp., Tetrigidae), Blattodea (Blattellidae), Isoptera (Termitidae: *Nasutitermes* sp.). Psocoptera (Lepidopsocidae). Hemiptera (Cydnidae, Miridae, Reduviidae), Homoptera (Cixiidae: *Cixius* sp.), Lepidoptera (Geometridae; Noctuidae; Tineidae), Diptera (Dolichopodidae, Psychodidae: *Lutzomyia* sp.), Hymenoptera (Formicidae*: Camponotus* sp., *Hypoponera* sp., *Pachycondyla* sp*., Pheidole* sp., *Solenopsis* sp*., Tapinoma* sp., Braconidae; Mutillidae; Vespidae), Thysanoptera (Phaleothripinae), Coleoptera (Curculionidae: Scotylinae; Leiodidae; Staphylinidae; Elateridae; Tenebrionidae: Coelometropinae), Diplopoda (Polydesmida, Glomeridesmidae: *Glomeridesmus* sp.). Stemmiulida (Stemmiulidae) e Symphyla (Scutigerellidae*: Hanseniella* sp.),

Dentre os vertebrados foi encontrada uma espécie de Chiroptera (Furipteridae: *Furipterus horrens.*).

Desta forma, no total foram encontradas 73 morfoespécies. Entre estas, quatro espécies de invertebrados foram consideradas troglomórficas: Gastropoda (Systrophiidae), Amblypygi (Charinidae*: Charinus* sp.), Schizomida (Hubbardiidae: Hubbardiinae) e Hymenoptera (Formicidae: *Hypoponera* sp.).

5.4.4.21. SL-023

5.4.4.21.1. Caracterização trófica

Pequeno abrigo com 8 m de desenvolvimento formado na canga e localizado na parte superior da encosta. A vegetação do entorno é composta basicamente por samambaias e por vegetação rasteira na parte superior da cavidade. Não existe sombreamento no seu pórtico de entrada e a cavidade não possui zona afótica. Sua entrada é pequena e o teto é baixo em toda sua extensão sendo esta representada por uma pequena fenda. Existem muitas briófitas, líquens e pteridófitas nas paredes e pouca serrapilheira acumulada junto à linha d'água. O piso é levemente ascendente o que dificulta a importação de recursos orgânicos, sendo este muito seco e composto por sedimento fino com alguns poucos blocos de diferentes tamanhos e com muitas raízes de pequeno tamanho em toda sua extensão (Figura 127). O sistema de canalículos é pouco desenvolvido e não existem depósitos de guano no seu interior. De forma geral, trata-se de uma cavidade pobre em recursos alimentares e extremamente suscetível as condições ambientais epígeas. Nenhuma alteração significativa foi observada durante a estação úmida, além das alterações normais na umidade relativa do ar.

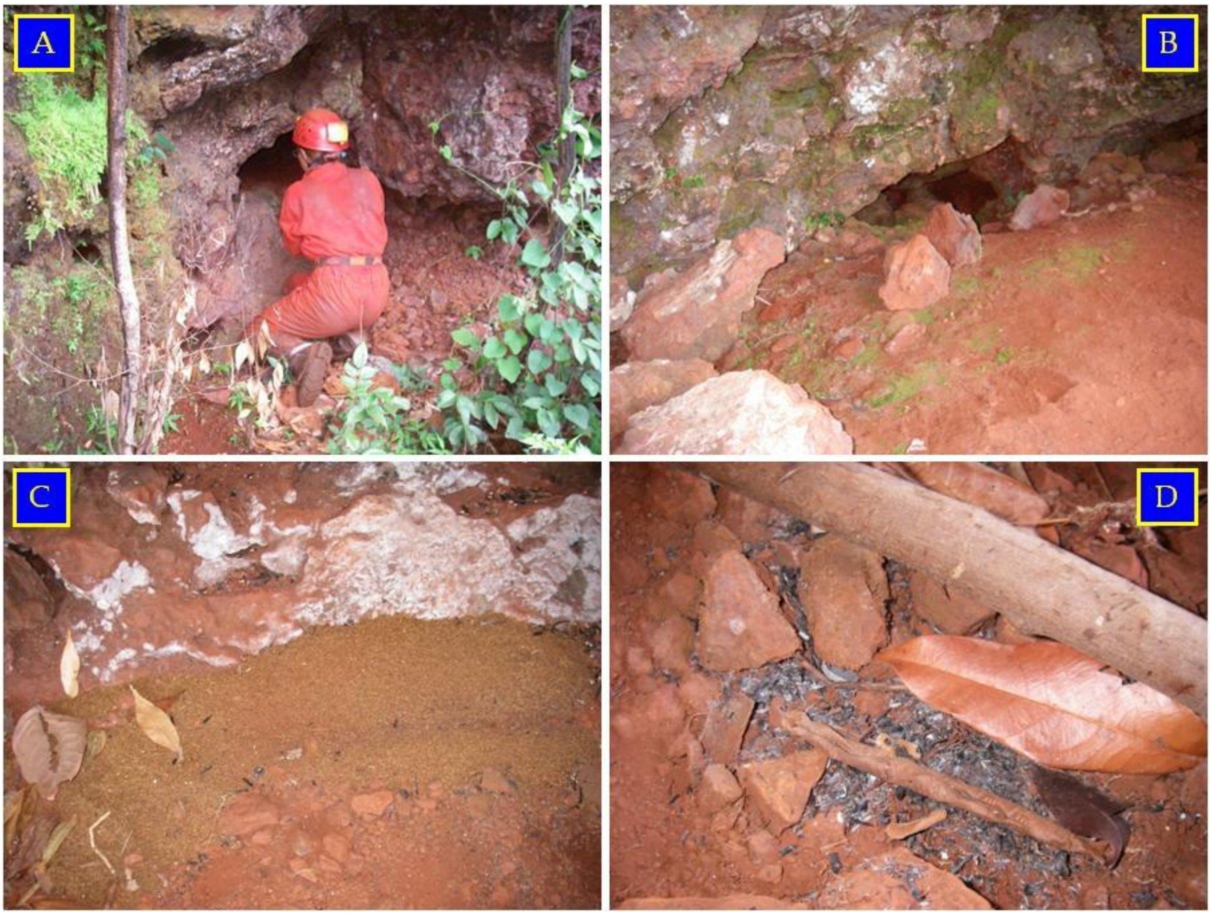

Figura 127 - a) Vegetação de entorno do abrigo localizado na encosta; b) Região de entrada da cavidade com detalhes das paredes e piso; c) Aspecto geral da entrada; d) Folhiço na entrada da caverna.

5.4.4.21.2. Caracterização faunística no período de seca

Foi observado na caverna, um total de 19 morfoespécies de invertebrados de pelo menos 18 famílias das ordens: Amblypygi (Phrynidae: *Heterophrynus longicornis;* Charinidae: *Charinus* sp.), Pseudoscorpiones (Chernetidae; Chtoniidae), Araneae (Scytodidae*: Scytodes eleonora*; Psauridae; Pholcidae: *Mesabolivar* sp. Theridiosomatidae), Collembola (Sminthuridae), Orthoptera (Phalangopsidae), Blattodea (Blaberidae*: Blaberus* sp.), Isoptera (Termitidae: *Diversitermes* sp.), Homoptera (Cixiidae*: Cixius* sp.), Lepidoptera (Noctuidae), Diptera (Ceratopogonidae; Dolichopodidae; Tipulidae), Hymenoptera (Formicidae: *Camponotus* sp., *Pachycondyla* sp.).

Desta forma, no total foram encontradas 19 morfoespécies. Entre estas, uma espécie de invertebrado foi considerada troglomórfica: Amblypygi (Charinidae: *Charinus* sp.).

5.4.4.21.3. Caracterização faunística no período de chuva

Foi observado na caverna, um total de 35 morfoespécies de invertebrados de pelo menos 30 famílias das ordens: Isopoda (Armadillidae). Acari (Oribatida). Amblypygi (Phrynidae: *Heterophrynus longicornis*; Charinidae: *Charinus* sp.). Pseudoscorpiones (Chernetidae). Opiliones (Cosmetidae: *Anduzeia* sp.; Phalangiidae). Araneae (Araneidae: *Alpaida* sp. Corinidae; Ctenidae: *Ctenus* sp. Filistatidae; Ochyroceratidae; Salticidae; Theridiidae). Collembola (Isotomidae). Orthoptera (Phalangopsidae: *Phalangopsis* sp.). Blattodea (Blaberidae: *Blaberus* sp. Polyphagidae). Isoptera. Hemiptera (Cydnidae; Lygaeidae; Ploiariidae). Lepidoptera (Noctuidae: Agaristinae). Diptera (Cecidomyiidae; Ceratopogonidae; Culicidae; Milichiidae Mycetophilidae;Psychodidae *Lutzomyia* sp.). Hymenoptera (Formicidae: *Pheidole* sp. *Rogeria* sp.) Coleoptera (Scydmaenidae; Tenebrionidae: Coelometropinae).

Desta forma, no total foram encontradas 35 morfoespécies. Entre estas, uma espécie de invertebrado foi considerada troglomórfica: Amblypygi (Charinidae: *Charinus* sp.).

5.4.4.21.4. Caracterização geral da fauna da cavidade

Foi observado na caverna, um total de 50 morfoespécies de invertebrados de pelo menos 40 famílias das ordens: Isopoda (Armadillidae). Acari (Oribatida). Amblypygi (Phrynidae*: Heterophrynus longicornis,* Charinidae: *Charinus* sp.), Pseudoscorpiones (Chernetidae, Chtoniidae), Opiliones (Cosmetidae: *Anduzeia* sp.*;* Phalangiidae), Araneae (Araneidae: *Alpaida* sp., Corinidae; Ctenidae: *Ctenus* sp., Filistatidae; Ochyroceratidae; Salticidae; Scytodidae*: Scytodes eleonora*; Psauridae; Pholcidae: *Mesabolivar* sp., Theridiidae; Theridiosomatidae), Collembola (Sminthuridae; Isotomidae), Orthoptera (Phalangopsidae*: Phalangopsis* sp.), Blattodea (Blaberidae: *Blaberus* sp., Polyphagidae), Isoptera (Termitidae: *Diversitermes* sp.), Hemiptera (Cydnidae; Lygaeidae; Ploiariidae), Homoptera (Cixiidae: *Cixius*

sp.) Lepidoptera (Noctuidae: Agaristinae), Diptera (Cecidomyiidae; Ceratopogonidae; Culicidae; Dolichopodidae; Milichiidae; Mycetophilidae; Psychodidae: *Lutzomyia* sp.; Tipulidae). Hymenoptera (Formicidae: *Camponotus* sp.; *Pachycondyla* sp.; *Pheidole* sp.; *Rogeria* sp.), Coleoptera (Scydmaenidae; Tenebrionidae: Coelometropinae).

Desta forma, no total foram encontradas 50 morfoespécies. Entre estas, uma espécie de invertebrado foi considerada troglomórfica: Amblypygi (Charinidae: *Charinus* sp.).

5.4.4.22. SL-024

5.4.4.22.1. Caracterização trófica

Cavidade com 17 m de desenvolvimento, formada na quebra da canga e localizada em na encosta. A vegetação do entorno é composta basicamente por samambaias e não existe sombreamento em sua entrada. Cavidade com padrão retilíneo e com entrada de pequenas dimensões (cerca de $1m^2$) onde existem poucas briófitas e líquens. O teto é baixo apenas na porção inicial, e existe um pequeno salão na zona mais profunda da cavidade que aparentemente chega a ficar afótico. O piso é levemente ascendente, o que dificulta a importação de recursos orgânicos pela entrada, sendo basicamente seco composto por sedimento granulado com muitos blocos de diferentes tamanhos (seixos, calhaus e matacões). A serrapilheira está restrita à zona de entrada e o sistema radicular é bem desenvolvido com raízes de diferentes calibres principalmente associados a alguns pontos de gotejamento ainda ativos durante a estação seca. Existem pequenos depósitos de guano envelhecido de morcegos insetívoros, além de Actinomicetos revestindo as paredes e o teto onde existem poucos canalículos (Figura 128). Nenhuma alteração significativa foi observada durante a estação úmida, além das alterações normais na umidade relativa do ar.

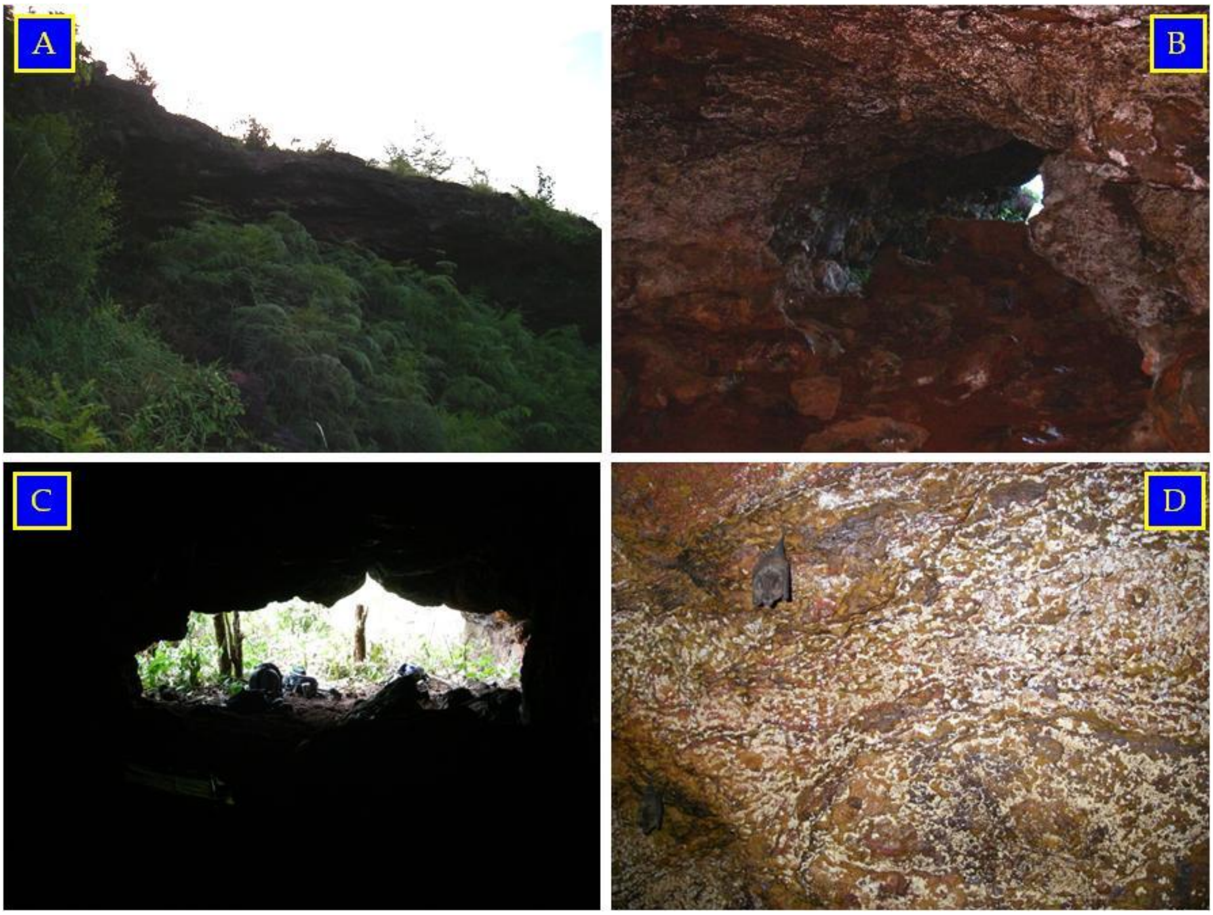

Figura 128 - a) Meio externo que circunda a região de entrada da caverna; b) Detalhe do piso da cavidade que é plano neste setor e composto por sedimento granulado com alguns pequenos seixos; c Vista interna da entrada da cavidade com detalhes da vegetação epígea; d) Grande quantidade de colônias de Actinomicetos crescendo sobre as paredes .

5.4.4.22.2. Caracterização faunística no período de seca

Foi observado na caverna, um total de 41 morfoespécies de invertebrados de pelo menos 34 famílias das ordens: Gastropoda (Systrophiidae), Turbellaria (Geoplanidae), Isopoda (Armadillidae), Acari (Oribatida, Labdostomatidae: *Labdostomatida* sp.), Amblypygi (Charinidae: *Charinus* sp.), Pseudoscorpiones (Chernetidae, Chtoniidae), Opiliones (Escadabiidae), Araneae (Gnaphosidae; Ochyroceratidae; Oonopinae; Scytodidae*: Scytodes eleonora*; Pholcidae: *Mesabolivar* sp., *Metagonia* sp., Theridiosomatidae), Thysanura (Nicoletiidae: Nicoletiinae), Diplura (Anajapygidae, Campodeidae), Collembola (Entomobryidae), Orthoptera (Phalangopsidae: *Phalangopsis* sp.), Isoptera (Rhinotermitidae: *Heterotermes* sp., Termitidae: *Nasutitermes* sp.), Psocoptera (Myopsocidae: *Lichenomina* sp.), Hemiptera (Cydnidae), Lepidoptera (Noctuidae), Diptera (Dolichopodidae, Psychodidae: *Lutzomyia* sp., Tipulidae, Sciaridae), Hymenoptera (Formicidae: *Camponotus* sp.; *Pachycondyla* sp.), Coleoptera (Scydmaenidae, Elateridae), Polydesmida (Pyrgodesmidae),

Spirostreptida (Pseudonannolenidae), Scolopendromorpha (Scolopocryptopidae: *Dinocryptops* sp.)

Dentre os vertebrados foi encontrada uma espécie da ordem Chiroptera (Phyllostomidae: *Glossophaga soricina*) e uma espécie da ordem Anura (Leptodactylidae: *Pristimantis cf. fenestratus*).

Desta forma, no total foram encontradas 41 morfoespécies. Entre estas, duas espécies de invertebrados foram consideradas troglomórficas: Amblypygi (Charinidae: *Charinus* sp.) e Gastropoda (Systrophiidae).

5.4.4.22.3. Caracterização faunística no período de chuva

Foi observado na caverna, um total de 58 morfoespécies de invertebrados de pelo menos 45 famílias das ordens: Gastropoda (Systrophiidae), Isopoda (Armadillidae), Acari (Argasidae: *Ornithodoros* sp., Ixodidae: *Amblyomma* sp., Laelapidae: *Stratiolaelaps* sp., Opilioacaridae: *Neoacarus* spn., Trombidiforme), Amblypygi (Phrynidae: *Heterophrynus longicornis*), Pseudoscorpiones (Chernetidae, Chtoniidae), Opiliones (Cosmetidae: *Anduzeia* sp.; Sclerosomatidae: *Prionostemma* sp.; Phalangiidae), Araneae (Gnaphosidae, Ochyroceratidae, Scytodidae*: Scytodes eleonora*, Palpimanidae, Psauridae, Theridiosomatidae), Thysanura (Nicoletiidae: Nicoletiinae; Meinertellidae), Diplura (Anajapygidae). Collembola (Isotomidae), Orthoptera (Phalangopsidae: *Phalangopsis* sp.), Blattodea (Blattellidae, Blattidae), Isoptera. Psocoptera (Ectopsocidae, Psyllipsocidae: *Psyllipsocus* sp.; Trogiidae), Hemiptera (Cydnidae), Lepidoptera (Noctuidae; Tineidae), Diptera (Cecidomyiidae; Ceratopogonidae; Chloropidae; Culicidae; Phoridae; Psychodidae: *Lutzomyia* sp.; Drosophilidae; Muscidae; Sciaridae), Hymenoptera (Formicidae: *Acromyrmex* sp., *Camponotus* sp., *Labidus* sp., *Odontomachus* sp., *Pachycondyla* sp.). Coleoptera (Scydmaenidae, Staphylinidae). Diplopoda (Stemmiulidae) e Scutigeromorpha (Scutigeridae: *Sphendononema* sp.).

Dentre os vertebrados foram encontradas duas espécies de Chiroptera (Phyllostomidae: *Glossophaga soricina*; Emballonuridae: *Peropteryx kappleri*).

Desta forma, no total foram encontradas 58 morfoespécies. Entre estas, duas espécies de invertebrados foram consideradas troglomórficas: Gastropoda (Systrophiidae) e Araneae (Ochyroceratidae).

5.4.4.22.4. Caracterização geral da fauna da cavidade

Foi observado na caverna, um total de 83 morfoespécies de invertebrados de pelo menos 64 famílias das ordens: Gastropoda (Systrophiidae), Turbellaria (Geoplanidae), Isopoda (Armadillidae), Acari (Argasidae: *Ornithodoros* sp., Ixodidae: *Amblyomma* sp., Laelapidae: *Stratiolaelaps* sp., Opilioacaridae: *Neoacarus* spn1., Oribatida., Labdostomatidae:

Labdostomatida sp.), Amblypygi (Phrynidae: Heterophrynus longicornis; Charinidae: *Charinus* sp.), Pseudoscorpiones (Chernetidae, Chtoniidae), Opiliones (Cosmetidae: *Anduzeia* sp.; Escadabiidae; Sclerosomatidae: *Prionostemma* sp.; Phalangiidae), Araneae (Gnaphosidae, Ochyroceratidae, Oonopinae, Scytodidae*: Scytodes eleonora*, Palpimanidae, Psauridae, Pholcidae: *Mesabolivar* sp., *Metagonia* sp1.; Theridiosomatidae), Thysanura (Nicoletiidae: Nicoletiinae), Microcoryphia (Meinertellidae), Diplura (Anajapygidae, Campodeidae), Collembola (Entomobryidae, Isotomidae), Orthoptera (Phalangopsidae: *Phalangopsis* sp.). Blattodea (Blattellidae, Blattidae), Isoptera (Rhinotermitidae: *Heterotermes* sp., Termitidae: *Nasutitermes* sp.), Psocoptera (Ectopsocidae, Myopsocidae: *Lichenomina* sp., Psyllipsocidae: *Psyllipsocus* sp., Trogiidae), Hemiptera (Cydnidae), Lepidoptera (Noctuidae, Tineidae), Diptera (Cecidomyiidae; Ceratopogonidae; Chloropidae; Culicidae; Dolichopodidae; Phoridae; Psychodidae: *Lutzomyia* sp., Tipulidae; Drosophilidae; Sciaridae; Muscidae), Hymenoptera (Formicidae: *Acromyrmex* sp.; *Camponotus* sp.; *Labidus* sp.; *Odontomachus* sp.; *Pachycondyla* sp.). Coleoptera (Scydmaenidae, Staphylinidae, Elateridae). Diplopoda (Pyrgodesmidae, Pseudonannolenidae, Stemmiulidae). Chilopoda (Scolopocryptopidae: *Dinocryptops* sp.; Scutigeridae: *Sphendononema* sp.).

Dentre os vertebrados foram encontradas duas espécies de Chiroptera (Phyllostomidae: *Glossophaga soricina*; Emballonuridae: *Peropteryx kappleri*). E uma espécie de Anura (Leptodactylidae: *Pristimantis cf. fenestratus*).

Desta forma, no total foram encontradas 58 morfoespécies. Entre estas, três espécies de invertebrados foram consideradas troglomórficas: Gastropoda (Systrophiidae), Araneae (Ochyroceratidae) e Amblypygi (Charinidae: *Charinus* sp.).

5.4.4.23. SL-025

5.4.4.23.1. Caracterização trófica

Pequeno abrigo com 11,5 m de desenvolvimento formado na quebra da canga e localizado a meia encosta. A vegetação do entorno é composta basicamente por samambaias e não existe sombreamento no pórtico de entrada da cavidade. Acima da cavidade a vegetação é rasteira composta basicamente por Capim (Poaceae). Cavidade pouco profunda com entrada estreita e em teto baixo com aproximadamente 1 m de altura. Na entrada, as paredes são revestidas por líquens, briófitas e pteridófitas, além de pouca serrapilheira acumulada junto à linha d’água. Abrigo seco somente com zona eufótica, onde o piso é levemente ascendente e composto por sedimento fino com poucos seixos e calhaus. Não existem depósitos de guano no interior da cavidade, o sistema radicular é pouco desenvolvido apresentando-se sub-superficial e a quantidade de canalículos ou alvéolos disponíveis é elevada. O teto e as paredes são revestidos por Actinomicetos e a cavidade apresenta uma baixa estabilidade

ambiental sendo extremamente influenciada pelas condições ambientais epígeas (Figura 129). Durante a estação úmida a caverna encontrava-se muito úmida e com vários pontos de gotejamentos ativos ao longo de toda sua extensão. Em sua porção mais distal observamos alguns pequenos depósitos frescos de guano de morcegos frugívoros.

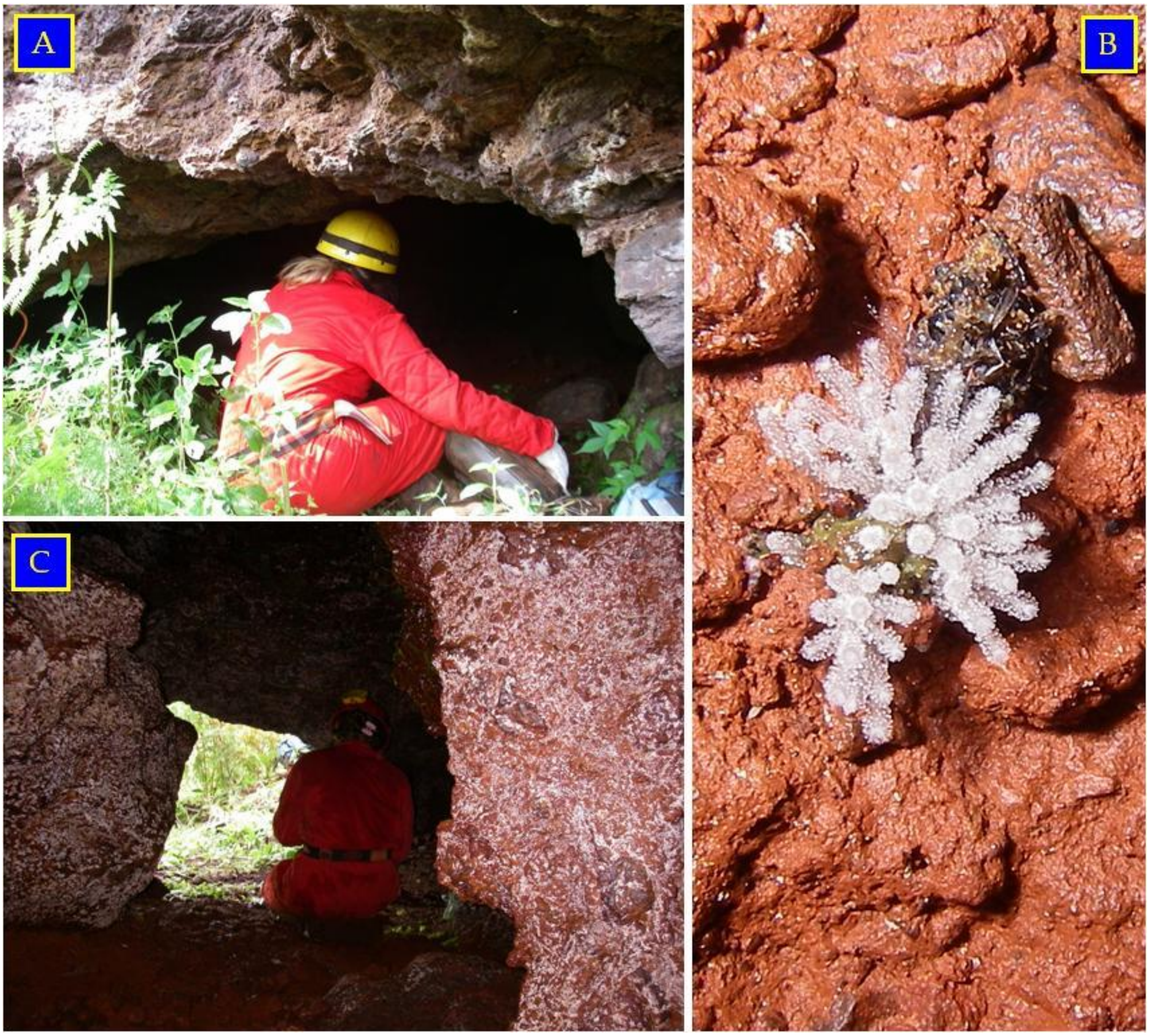

Figura 129 - a) Aspecto geral da entrada da cavidade; b) Parede completamente revestida por Actinomicetos no interior da caverna; c) Fungo se desenvolvendo sobre substrato orgânico no interior da caverna.

5.4.4.23.2. Caracterização faunística no período de seca

Foi observado na caverna, um total de 24 morfoespécies de invertebrados de pelo menos 19 famílias das ordens: Isopoda (Philosciidae, Scleropactidae), Acari (Trombidiforme), Amblypygi (*Heterophrynus longicornis*), Pseudoscorpiones (Chernetidae), Opiliones (Cosmetidae: *Anduzeia* sp.*;* Escadabiidae; Kimmulidae; Stygnidae: *Protimesius aff. Gracilis*), Araneae (Prodidomidae, Ochyroceratidae, Scytodidae*: Scytodes eleonora*, Theridiosomatidae). Collembola (Sminthuridae), Orthoptera (Phalangopsidae: *Phalangopsis*

sp.), Homoptera (Cixiidae: *Cixius* sp), Lepidoptera (Noctuidae), Diptera (Tipulidae), Hymenoptera (Formicidae: *Apterostigma* sp., *Azteca* sp., *Pachycondyla* sp.)

Dentre os vertebrados foi encontrada uma espécie de Anura (Leptodactylidae: *Pristimantis cf. fenestratus*).

Desta forma, no total foram encontradas 24 morfoespécies. Entre estas, uma espécie de invertebrado foi considerada troglomórfica: Araneae (Prodidomidae).

5.4.4.23.3. Caracterização faunística no período de chuva

Foi observado na caverna, um total de 34 morfoespécies de invertebrados de pelo menos 31 famílias das ordens: Isopoda (Philosciidae, Scleropactidae), Acari (Macronyssidae; Sarcoptiforme: Oribatida; Eupodidae: *Linopodes* sp.), Pseudoscorpiones (Chernetidae, Chtoniidae), Opiliones (Cosmetidae: *Anduzeia* sp.; Escadabiidae, Phalangiidae), Araneae (Gnaphosidae, Linyphiidae, Ochyroceratidae, Pholcidae), Diplura (Anajapygidae), Collembola (Entomobryidae, Isotomidae, Paronellidae), Orthoptera (Phalangopsidae: *Phalangopsis* sp.), Hemiptera (Cydnidae), Diptera (Cecidomyiidae, Culicidae, Milichiidae, Psychodidae: *Lutzomyia* sp.; Tipulidae), Hymenoptera (Formicidae: *Pachycondyla* sp.), Coleoptera (Pselaphidae, Staphylinidae), Diplopoda (Pyrgodesmidae) e Chilopoda (Scutigeridae: *Sphendononema* sp., Scutigerellidae: *Hanseniella* sp.).

Dentre os vertebrados foi encontrada uma espécie de Chiroptera (Phyllostomidae: *Glossophaga soricina*).

Desta forma, no total foram encontradas 34 morfoespécies. Entre estas, duas espécies de invertebrados foram consideradas troglomórficas: Araneae (Ochyroceratidae) e Collembola (Isotomidae).

5.4.4.23.4. Caracterização geral da fauna da cavidade

Foi observado na caverna, um total de 52 morfoespécies de invertebrados de pelo menos 41 famílias das ordens: Isopoda (Philosciidae, Scleropactidae), Acari (Macronyssidae; Trombidiforme: Oribatida, Eupodidae: *Linopodes* sp.), Amblypygi (Phrynidae: *Heterophrynus longicornis*), Pseudoscorpiones (Chernetidae, Chtoniidae), Opiliones (Cosmetidae: *Anduzeia* sp.*;* Escadabiidae; Kimmulidae; Stygnidae: *Protimesius aff. Gracilis;* Phalangiidae), Araneae (Prodidomidae, Gnaphosidae, Linyphiidae, Ochyroceratidae, Scytodidae*: Scytodes eleonora*, Pholcidae, Theridiosomatidae), Diplura (Anajapygidae), Collembola (Sminthuridae, Entomobryidae, Isotomidae, Paronellidae), Orthoptera (Phalangopsidae: *Phalangopsis* sp.), Blattodea (Polyphagidae), Hemiptera (Cydnidae), Homoptera (Cixiidae: *Cixius* sp.), Lepidoptera (Noctuidae), Diptera (Cecidomyiidae, Culicidae, Milichiidae, Psychodidae: *Lutzomyia* sp.; Tipulidae), Hymenoptera (Formicidae: *Apterostigma* sp, *Azteca* sp., *Pachycondyla* sp.), Coleoptera (Pselaphidae, Staphylinidae), Diplopoda (Pyrgodesmidae), Chilopoda (Scutigeridae: S*phendononema* sp.), Symphyla (Scutigerellidae: *Hanseniella* sp.).

Dentre os vertebrados foram encontradas uma espécie de Chiroptera (Phyllostomidae: Glossophaga soricina) e uma de Anura (Leptodactylidae: *Pristimantis cf. fenestratus*).

Desta forma, no total foram encontradas 52 morfoespécies. Entre estas, três espécies de invertebrados foram consideradas troglomórficas: Araneae (Ochyroceratidae, Prodidomidae) e Collembola (Isotomidae). Alguns organismos encontrados nesta caverna são mostrados na Figura 130.

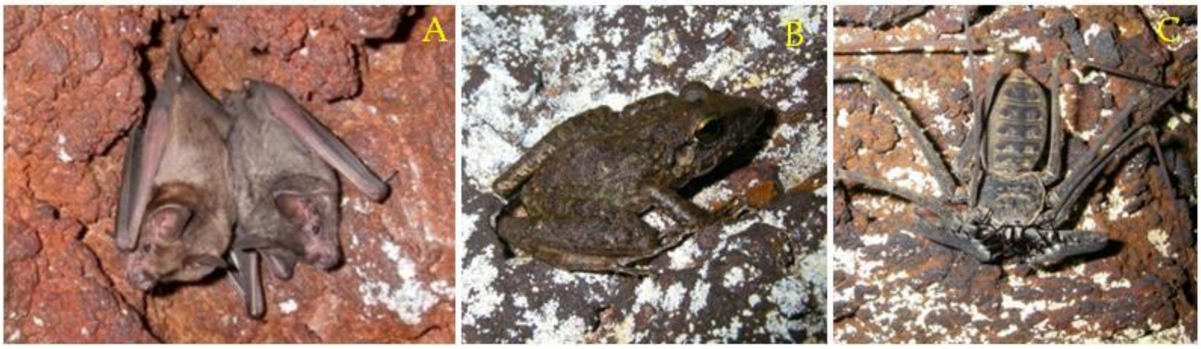

Figura 130 - a) Phyllostomidae (*Carollia* sp.); b) Leptodactylidae (*Pristimantis cf. fenestratus*); b) Diplopoda (Siphonophoridae); c) Ricinulei (*Cryptocellus tarsilae*)

5.4.4.24. SL-026

5.4.4.24.1. Caracterização trófica

Cavidade em meia encosta com 25 m de projeção horizontal formada na canga e com padrão de desenvolvimento retilíneo. A vegetação do entorno é composta basicamente por samambaias e não existe sombreamento no pórtico de entrada da cavidade. Na região acima

da cavidade a vegetação é rasteira composta basicamente por capim. A entrada é ampla, muito iluminada e em condição ascendente com muitos líquens, briófitas e angiospermas distribuídas pelas paredes e piso. As briófitas revestem toda a parede na margem direita da cavidade que não possui zona afótica, mas apresenta uma grande área de penumbra escura nas zonas mais profundas. O piso é seco e irregular, sendo este descendente a partir da porção intermediária. Este é composto por sedimento fino com muitos seixos e calhaus além de grandes matacões abatidos principalmente na porção central da cavidade. A serrapilheira está restrita a entrada, uma vez que sua importação é limitada em virtude da irregularidade do terreno, sendo a matéria orgânica vegetal transportada principalmente por ação eólica e ou gravitacional. Existem alguns pequenos depósitos de guano envelhecidos de morcegos insetívoros nas zonas mais profundas da cavidade além de poucas raízes de pequeno tamanho. As paredes e teto são revestidos por Actinomicetos onde o sistema de canalículos é bem desenvolvido e não foram observados pontos de gotejamentos ativos na cavidade (Figura 131). Em relação ao estado de conservação, em sua zona mais profunda existe uma pequena área escavada onde foram abandonadas ferramentas de trabalho utilizado nos processos de garimpo (Figura 131). Durante a estação úmida, a umidade relativa do ar encontrava-se extremamente elevada, com inúmeros pontos de gotejamento ativos e a parte escavada encontrava-se cheia de água.

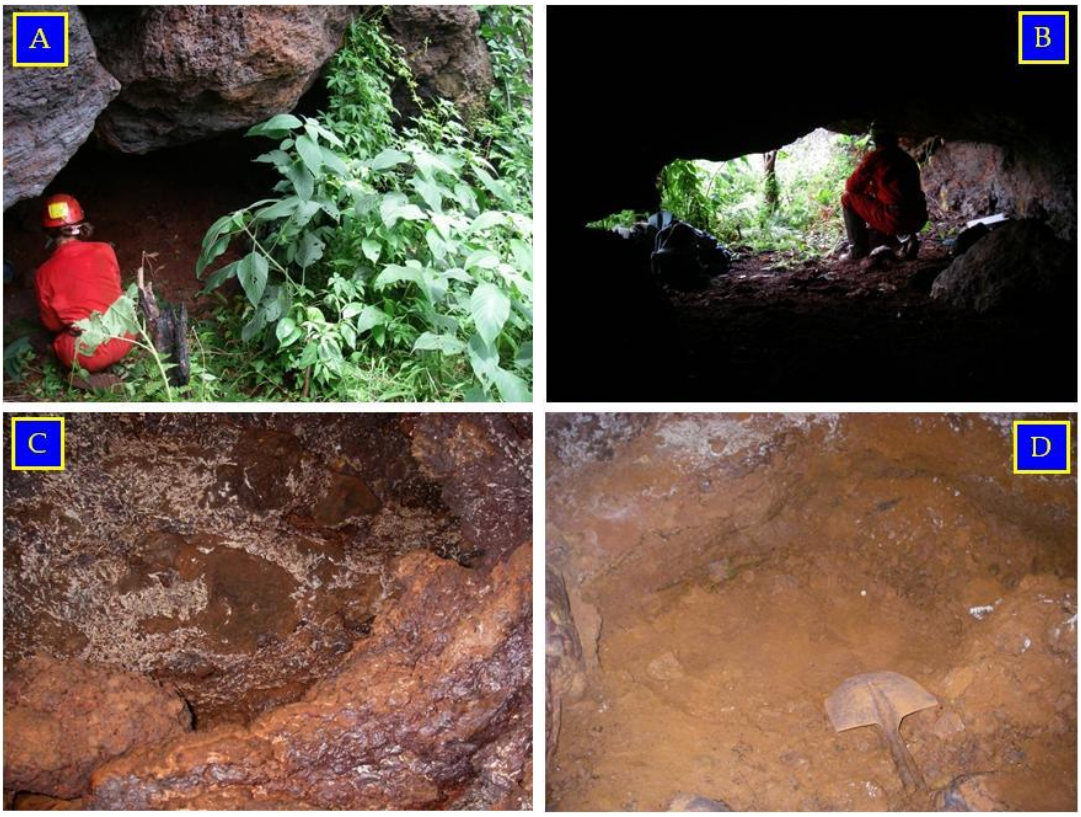

Figura 131 - a) Aspecto geral da entrada da caverna onde é possível observar a vegetação associada à zona eufótica; b) Aspecto geral da entrada vista por dentro da cavidade; c) Grande quantidade de colônias de Actinomicetos crescendo nas paredes; d) Área escavada na zona mais profunda da cavidade. No canto direito inferior da imagem observe uma ferramenta utilizada na escavação e posteriormente abandonada.

5.4.4.24.2. Caracterização faunística no período de seca

Foi observado na caverna, um total de 40 morfoespécies de invertebrados de pelo menos 32 famílias das Ordens: Isopoda (Armadillidae), Acari (*Ornithodoros* sp., Macronyssidae), Amblypygi (*Heterophrynus longicornis*), Pseudoscorpiones (Chernetidae, Chtoniidae), Araneae (Oonopidae, Oonopinae, Salticidae, Scytodidae*: Scytodes eleonora*, Pholcidae: *Mesabolivar* sp. Theridiosomatidae), Thysanura (Nicoletiidae: Nicoletiinae), Diplura (Campodeidae), Collembola (Entomobryidae, Paronellidae, Gryllidae: Mogoplistinae, Gryllinae, Pentacentrinae, Phalangopsidae: *Aclodes* sp.), Blattodea (Blaberidae: *Blaberus* sp.), Hemiptera (Reduviidae), Lepidoptera (Noctuidae, Tineidae), Diptera (Ceratopogonidae, Culicidae, Dolichopodidae, Psychodidae: *Lutzomyia* sp.), Hymenoptera (Formicidae: *Azteca* sp., *Camponotus* sp.; *Pachycondyla* sp.), Coleoptera (Scydmaenidae, Staphylinidae).

Dentre os vertebrados foram encontradas quatro espécies das ordens Chiroptera (*Anoura* sp., *G. soricina*), Squamata (*T. rapicauda*), Anura (*P.* cf. *frenestatus*). Desta forma, no total foram encontradas 44 morfoespécies.

5.4.4.24.3. Caracterização faunística no período de chuva

Foi observado na caverna, um total de 30 morfoespécies, das quais 28 eram invertebrados de pelo menos 41 famílias das Ordens: Gastropoda (Systrophiidae), Turbellaria (Geoplanidae), Acari (Trombidiforme), Amblypygi (*Heterophrynus longicornis*), Pseudoscorpiones (Chernetidae), Araneae (Ochyroceratidae, Salticidae, Scytodidae*: Scytodes eleonora*, Pholcidae, Theridiidae, Theridiosomatidae), Thysanura (Nicoletiidae: Nicoletiinae), Diplura (Campodeidae), Collembola (Paronellidae), Orthoptera (*Phalangopsis* sp.), Blattodea (*Blaberus* sp., Polyphagidae), Psocoptera: (*Psyllipsocus* sp.), Hemiptera (Cydnidae), Lepidoptera (Noctuidae, Tineidae), Diptera (Ceratopogonidae, Culicidae, *Lutzomyia* sp.), Hymenoptera (*Pachycondyla* sp.), Coleoptera (Staphylinidae), Diplopoda (Polydesmida: Chelodesmidae), Symphyla (Scutigerellidae: *Hanseniella* sp.). Destes, duas morfoespécies de invertebrados foram considerados troglomórficos (Gastropoda: Systrophiidae; Turbellaria: Geoplanidae).

Dentre os vertebrados foi encontrada apenas uma espécie da Ordem Chiroptera (*G. soricina*). Desta forma, no total foram encontradas 30 morfoespécies, dos quais Além destes, duas morfoespécies de invertebrados foram consideradas troglomórficas (Gastropoda: Systrophiidae; Turbellaria: Geoplanidae).

5.4.4.24.4. Caracterização geral da fauna da cavidade

Foi observado na caverna, um total de 57 morfoespécies de invertebrados de pelo menos 42 famílias das Ordens: Gastropoda (Systrophiidae), Turbellaria (Geoplanidae), Isopoda (Armadillidae), Acari (*Ornithodoros* sp., Macronyssidae, Trombidiforme), Amblypygi (*Heterophrynus longicornis*), Pseudoscorpiones (Chernetidae, Chtoniidae), Araneae (Ochyroceratidae, Oonopidae: Oonopinae, Salticidae, Scytodidae*: Scytodes eleonora*, Pholcidae: *Mesabolivar* sp., Theridiidae, Theridiosomatidae), Thysanura (Nicoletiidae: Nicoletiinae), Diplura (Campodeidae), Collembola (Entomobryidae, Paronellidae), Orthoptera (Gryllidae: Mogoplistinae, Gryllinae, Pentacentrinae, Phalangopsidae: *Phalangopsis* sp.), Blattodea (Blaberidae: *Blaberus* sp., Polyphagidae), Psocoptera: (*Psyllipsocus* sp.), Homoptera (Cydnidae, Reduviidae), Lepidoptera (Noctuidae, Tineidae), Diptera (Ceratopogonidae, Chironomidae, Culicidae, Dolichopodidae, Psychodidae: Lutzomyia), Hymenoptera (*Azteca* sp., *Camponotus* sp., *Pachycondyla* sp.) Coleoptera (Scydmaenidae, Staphylinidae), Diplopoda (Polydesmida: Chelodesmidae), Symphila (Scutigerellidae: *Hanseniella* sp.).

Dentre os vertebrados foram encontradas quatro espécies das ordens Chiroptera (*Anoura* sp., *G. soricina*), Squamata (*T. rapicauda*), Anura (*P.* cf. *frenestatus*).

Desta forma, no total foram encontradas 61 morfoespécies, dos quais duas morfoespécies de invertebrados foram consideradas troglomórficos (Gastropoda: Systrophiidae; Turbellaria: Geoplanidae).

5.4.4.25. SL-027

5.4.4.25.1. Caracterização trófica

Abrigo desenvolvido na canga com 14 m de projeção horizontal. Localiza-se na vertente em área de mata com vegetação arbórea muito densa. A caverna possui duas entradas amplas e sombreadas com muitos líquens, briófitas e Actinomicetos revestindo as paredes e pisos. A serrapilheira está restrita a zona de entrada e acumulada principalmente na linha d'água, mas também esparsa pelo piso da cavidade sendo esta transportada pelo vento ou de forma gravitacional. O piso é predominantemente seco e plano, composto por sedimento granulado com poucos seixos e calhaus distribuídos de forma esparsa (Figura 132). O sistema radicular é bem desenvolvido com raízes de diferentes calibres distribuídas pelas paredes e piso apresentando-se muitas vezes de forma superficial. Não foram observados depósitos de guano apesar de terem sido observados seis indivíduos de morcegos frugívoros (G. soricina). O teto e as paredes da cavidade são completamente revestidos por Actinomicetos e o sistema de canalículos é pouco desenvolvido. Cavidade sob forte influência das condições

ambientais epígeas e, portanto, com baixa estabilidade ambiental. Durante a estação úmida foram observados vários pontos de gotejamento ao longo de toda a extensão da cavidade.

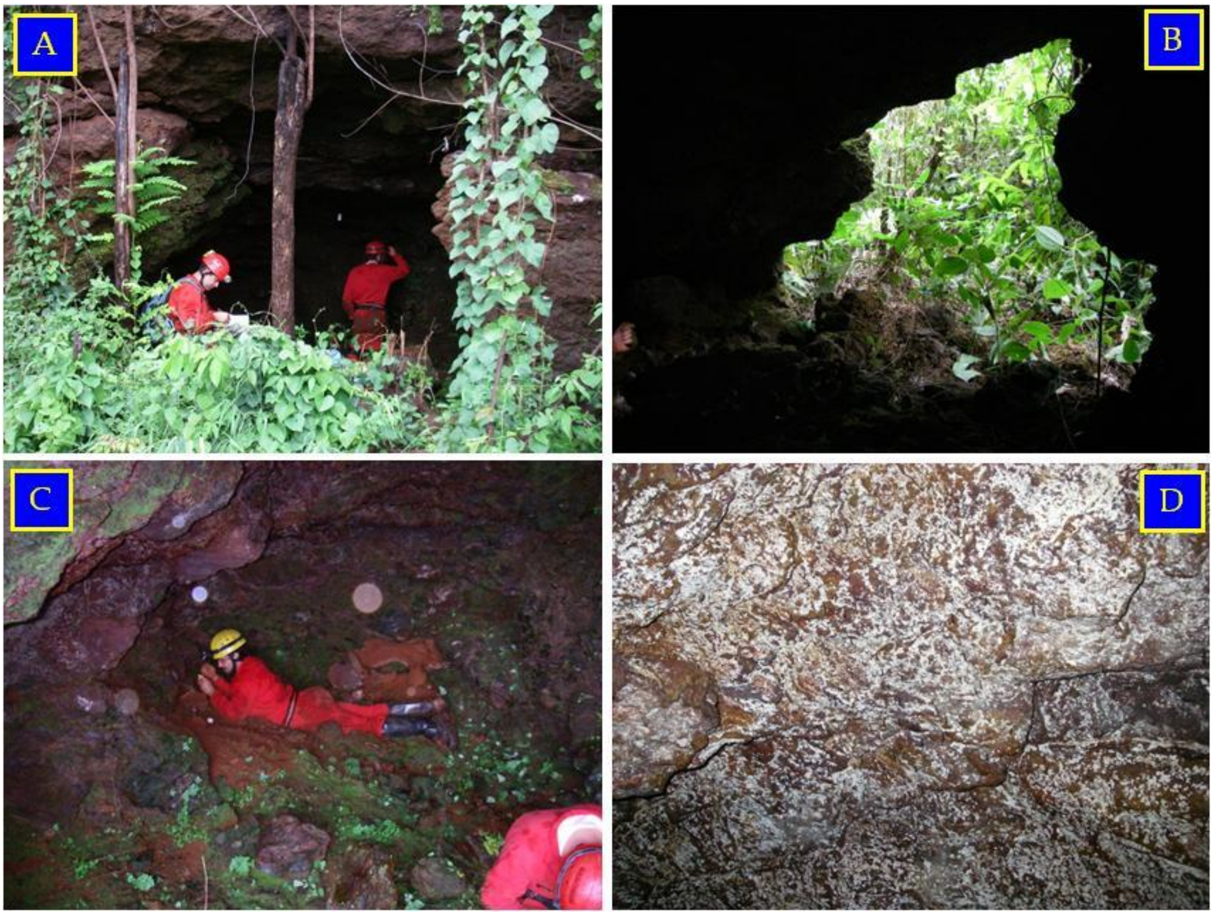

Figura 132 - a) Aspecto geral da entrada principal da cavidade onde é possível observar a vegetação associada na área do entorno; b) Aspecto geral da entrada secundária da cavidade onde é possível observar a vegetação associada ao entorno bem como as paredes internas revestidas por fungos.

5.4.4.25.2. Caracterização faunística no período de seca

Foi observado na caverna, um total de 34 morfoespécies de invertebrados de pelo menos 32 famílias das Ordens: Isopoda (Philosciidae, Scleropactidae), Acari (Mesostigmata, Oribatida), Amblypygi (*Heterophrynus longicornis, Charinus* sp.), Pseudoscorpiones (Chernetidae, Chtoniidae), Araneae (*Alpaida* sp., Ochyroceratidae, Oonopidae: Oonopinae, Salticidae, Scytodidae*: Scytodes eleonora*, Psauridae, Pholcidae: *Metagonia* sp., Theraphosidae), Thysanura (Atelurinae), Diplura (Anajapygidae), Collembola (Cyphoderidae, Isotomidae, Paronellidae), Orthoptera (Phalangopsidae: *Aclodes* sp.), Blattodea (Polyphagidae), Psocoptera (Epipsocidae), Lepidoptera (Tineidae), Diptera (Chironomidae, Psychodidae: *Lutzomyia* sp., Tipulidae sp.), Hymenoptera (*Pachycondyla* sp.), Diplopoda (Pseudonannolenidae), Scolopendromorpha (Scolopendridae: *Cormocephalus* sp.). Destes,

quatro espécies foram consideradas troglomórficas: Amblypygi (*Charinus* sp.), Araneae (Ochyroceratidae, Oonopidae) e Collembola (Cyphoderidae).

Dentre os vertebrados foram encontradas três espécies das Ordens: Chiroptera (*Glossophaga soricina)*, Squamata (*Thecadactylus rapicauda*) e Anura *(Pristimantis* cf. *fenestratus*). Desta forma, no total foram encontradas 37 morfoespécies.

5.4.4.25.3. Caracterização faunística no período de chuva

Foi observado na caverna, um total de 38 morfoespécies de invertebrados de pelo menos 32 famílias das Ordens: Gastropoda (Systrophiidae), Isopoda (Philosciidae), Acari (Oribatida, Bdellidae, Cunaxidae, Eupodidae: *Linopodes* sp., Labdostomatida, Teneriffiidae), Ricinulei (Ricinoididae: *Cryptocellus tarsilae*), Pseudoscorpiones (Chernetidae, Chtoniidae), Opiliones (Kimmulidae, Sclerosomatidae: *Prionostemma* sp., Stygnidae), Araneae (Ochyroceratidae, Salticidae, Scytodidae*: Scytodes eleonora*, Pholcidae, Prodidomidae, Theraphosidae, Theridiidae), Microcoryphia (Meinertellidae), Collembola (Entomobryidae, Tomoceridae), Orthoptera (Phalangopsidae), Blattodea (Blattellidae), Psocoptera (Epipsocidae), Hemiptera (Cydnidae), Lepidoptera (Geometridae), Diptera (Cecidomyiidae, Chironomidae, Milichiidae), Hymenoptera (*Camponotus* sp., *Pheidole* sp.), Diplopoda (Pseudonannolenidae), Lithobiomorpha (Henicopiidae: *Lamyctes* sp.). Destas, duas morfoespécies foram consideradas troglomórficas: Gastropoda (Systrophiidae) e Araneae (Ochyroceratidae).

Dentre os vertebrados foram duas espécies da Ordem Chiroptera (*Peropteryx kappleri*, *Glossophaga soricina*). Desta forma, no total foram encontradas 40 morfoespécies.

5.4.4.25.4. Caracterização geral da fauna da cavidade

Foi observado na caverna, um total de 63 morfoespécies de invertebrados de pelo menos 56 famílias das Ordens: Gastropoda (Systrophiidae), Isopoda (Philosciidae, Scleropactidae), Acari (Mesostigmata, Oribatida, Bdellidae, Cunaxidae, Eupodidae: *Linopodes* sp., Labdostomatidae: *Labdostomatida* sp., Teneriffiidae), Amblypygi (*Heterophrynus longicornis*, *Charinus* sp.), Ricinulei (Ricinoididae: *Cryptocellus tarsilae*), Pseudoscorpiones (Chernetidae, Chtoniidae), Opiliones (Kimmulidae, Sclerosomatidae: *Prionostemma* sp., Stygnidae), Araneae (*Alpaida* sp., Ochyroceratidae, Oonopidae: Oonopinae, Salticidae, Scytodidae*:* *Scytodes eleonora*, Psauridae, Pholcidae: *Metagonia* sp., Prodidomidae, Theraphosidae, Theridiidae), Thysanura (Nicoletiidae: Atelurinae), Microcoryphia (Meinertellidae), Diplura (Anajapygidae), Collembola (Cyphoderidae, Entomobryidae, Isotomidae, Paronellidae, Tomoceridae), Orthoptera (Phalangopsidae: *Aclodes* sp.), Blattodea (Blattellidae, Polyphagidae sp.), Psocoptera (Epipsocidae), Hemiptera (Cydnidae), Lepidoptera (Geometridae), Diptera (Cecidomyiidae, Chironomidae, Milichiidae, Psychodidae: *Lutzomyia* sp., Tipulidae), Hymenoptera (*Camponotus* sp., *Pachycondyla* sp., *Pheidole* sp.), Diplopoda (Pseudonannolenidae), Lithobiomorpha (Henicopiidae: *Lamyctes* sp.), Scolopendromorpha (Scolopendridae: *Cormocephalus* sp.).

Dentre os vertebrados foram encontradas quatro espécies das Ordens Chiroptera (*Peropteryx kappleri*, *Glossophaga soricina*) e Squamata (*Thecadactylus rapicauda*) e Anura (*Pristimantis* cf. *fenestratus*).

Desta forma, no total foram encontradas 67 morfoespécies, das quais duas morfoespécies foram consideradas troglomórficas: Gastropoda (Systrophiidae) e Araneae (Ochyroceratidae).

5.4.4.26. SL-028

5.4.4.26.1. Caracterização trófica

Abrigo desenvolvido na canga com 12 m de projeção horizontal. Localizado na vertente a meia encosta em área de mata com vegetação arbórea muito densa. A caverna possui uma entrada ampla e sombreada com muitos líquens, briófitas e Actinomicetos revestindo as paredes e pisos até a porção mais profunda da cavidade, além de muitas plântulas de Melastomatáceas junto à linha d'água (Figura 133). A serrapilheira está restrita a zona de entrada, mas também esparsa pelo piso da cavidade sendo esta transportada pelo vento ou de forma gravitacional. O piso é predominantemente seco e levemente descendente estando este suscetível ao transporte de matéria orgânica pela água da chuva. O piso é composto por sedimento granulado com muitos seixos e calhaus distribuídos de forma esparsa. A cavidade não apresenta zona afótica e o sistema radicular é pouco desenvolvido.

Não foram observados depósitos de guano e o teto e as paredes da cavidade são completamente revestidos por Actinomicetos e apresentam sistema de canalículos pouco desenvolvido. Cavidade sob forte influência das condições ambientais epígeas e, portanto, com baixa estabilidade ambiental. Durante a estação úmida observamos um pequeno gotejamento na porção mais distal da cavidade.

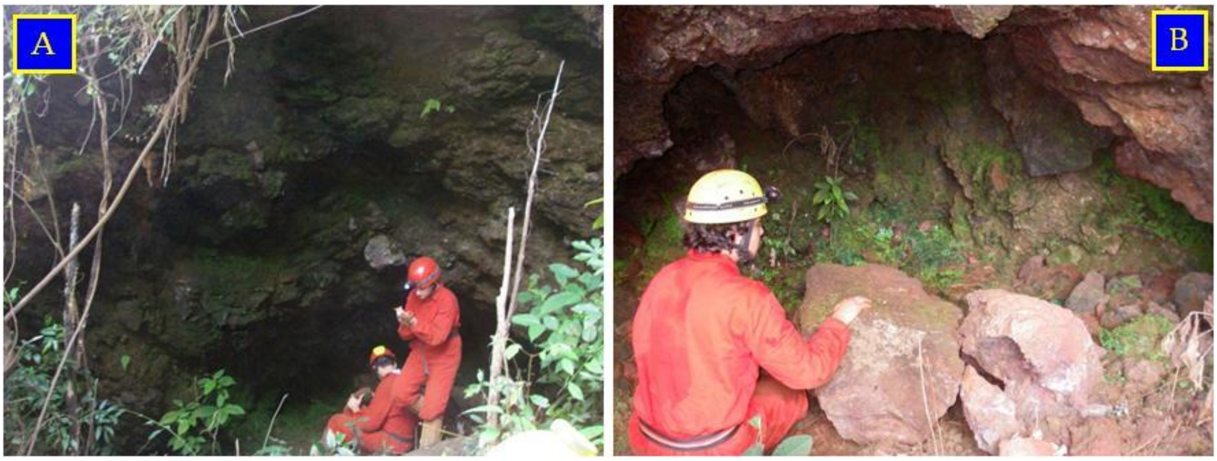

Figura 133 - a) Aspecto geral da entrada da cavidade onde é possível observar a vegetação associada na área epígea; b) Aspecto geral da porção mais profunda da cavidade onde é possível observar a forte influência das condições ambientais epígeas.

5.4.4.26.2. Caracterização faunística no período de seca

Foi observado na caverna, um total de 30 morfoespécies de invertebrados de pelo menos 23 famílias das Ordens: Isopoda (Philosciidae), Acari (Astigmatina, Oribatida, Trombidiforme), Amblypygi (*Heterophrynus longicornis*), Pseudoscorpiones (Chernetidae, Chtoniidae), Opiliones (Sclerosomatidae: *Prionostemma* sp., Stygnidae: *Protimesius* aff. *gracilis, Alpaida* sp., Salticidae, Scytodidae*: Scytodes eleonora*, Psauridae, Pholcidae: *Metagonia* sp., Theridiosomatidae), Thysanura: Nicoletiidae: Nicoletiinae), Collembola (Katiannidae?), Orthoptera (Phalangopsidae: *Aclodes* sp.), Blattodea (Polyphagidae), Psocoptera (Epipsocidae), Lepidoptera (Hesperiidae), Diptera (Dolichopodidae, Mycetophilidae, Tipulidae), Hymenoptera (*Pachycondyla* sp.) e Scolopendromorpha (Scolopocryptopidae: *Newportia* sp.).

5.4.4.26.3. Caracterização faunística no período de chuva

Foi observado na caverna, um total de 37 morfoespécies de invertebrados de pelo menos 23 famílias das Ordens: Isopoda (Armadillidae), Acari (Neothyridae: *Diplothyrus schubarti*; Oribatida, Eupodidae: *Linopodes* sp.), Ricinulei (Ricinoididae: *Cryptocellus tarsilae*), Scorpiones (*Ananteris luciae*), Pseudoscorpiones (Chernetidae, Chtoniidae), Opiliones (Sclerosomatidae: *Prionostemma* sp., Stygnidae, Phalangiidae), Araneae (Araneidae: *Alpaida* sp., Salticidae, Scytodidae*: Scytodes eleonora*, Pholcidae, Theridiidae), Microcoryphia

(Meinertellidae), Diplura (Campodeidae), Collembola (Entomobryidae), Psocoptera (Myopsocidae: *Lichenomina* sp., Psyllipsocidae: *Psyllipsocus* sp.), Hemiptera (Cydnidae), Diptera (Cecidomyiidae, Chironomidae, Culicidae), Hymenoptera (*Camponotus* sp., *Odontomachus* sp.), Coleoptera (Pselaphidae, Staphylinidae), Diplopoda (Stemmiulidae), Scutigeromorpha (Scutigeridae: *Sphendononema* sp.).

5.4.4.26.4. Caracterização geral da fauna da cavidade

Foi observado na caverna, um total de 60 morfoespécies de invertebrados de pelo menos 43 famílias das Ordens: Isopoda (Armadillidae, Philosciidae), Acari (Neothyridae: *Diplothyrus schubarti*, Astigmatina, Oribatida, Eupodidae: *Linopodes* sp., Trombidiforme), Amblypygi (*Heterophrynus longicornis*), Ricinulei (Ricinoididae: *Cryptocellus tarsilae*), Scorpiones (*Ananteris luciae*), Pseudoscorpiones (Chernetidae, Chtoniidae), Opiliones (Esclerosomatidae: *Prionostemma* sp., Stygnidae: *Protimesius aff. gracilis*, Phalangiidae), Araneae (Araneidae: *Alpaida* sp., Salticidae, Scytodidae: *Scytodes eleonora*, Psauridae, Pholcidae: *Metagonia*, Theridiidae, Theridiosomatidae), Thysanura (Nicoletiidae: Nicoletiinae), Microcoryphia (Meinertellidae), Diplura (Campodeidae), Collembola (Entomobryidae, Katiannidae?), Orthoptera (Phalangopsidae: *Aclodes* sp.), Blattodea (Polyphagidae), Psocoptera (Epipsocidae, Myopsocidae: *Lichenomina* sp., Psyllipsocidae: *Psyllipsocus* sp.), Hemiptera (Cydnidae), Lepidoptera (Hesperiidae), Diptera (Cecidomyiidae, Chironomidae, Culicidae, Dolichopodidae, Mycetophilidae, Tipulidae), Hymenoptera (*Camponotus* sp., *Odontomachus* sp., *Pachycondyla* sp.), Coleoptera (Pselaphidae, Staphylinidae sp.), Diplopoda (Stemmiulidae), Scolopendromorpha (Scolopocryptopidae: *Newportia* sp.), Scutigeromorpha (Scutigeridae: *Sphendononema* sp.).

5.4.4.27. SL-029

5.4.4.27.1. Caracterização trófica

Caverna desenvolvida na canga com 33 m de projeção horizontal e localizada na vertente a meia encosta com vegetação arbórea muito densa. Possui duas entradas amplas e sombreadas com muitos líquens, briófitas e Actinomicetos revestindo as paredes e pisos e com muita serrapilheira acumulada junto à linha d’água. De forma geral, o piso é úmido e ascendente composto por sedimento fino com muitos seixos, calhaus e matacões esparsos pela cavidade. Desta forma, os recursos alimentares são importados principalmente através de transporte eólico ou gravitacional e através de organismos trogloxenos, especialmente morcegos. A cavidade possui um padrão retilíneo que se bifurca em sua porção mais distal, sendo o conduto da direita úmido com trechos com gotejamento e água de percolação que formam grandes poças em áreas de penumbra escura. Os gotejamentos estão presentes também na zona intermediária da cavidade mesmo durante a estação seca caracterizando um ambiente de elevada umidade em sua zona mais profunda. O conduto da direita é muito

amplo com grandes blocos abatidos e grandes depósitos de guano de morcegos frugívoros com plântulas germinadas. Tais depósitos são produzidos por uma grande colônia de morcegos Glossophaginae (*G. soricina*). Existe um grande cupinzeiro na zona central da cavidade com galerias distribuídas ao longo de toda a caverna. As paredes e o teto da cavidade são revestidos por Actinomicetos ao longo de toda extensão e apresentam uma morfologia irregular favorecendo a existência de micro-hábitats e possuem um sistema de canalículos muito desenvolvido. A cavidade não apresenta uma zona completamente afótica, mas existe uma grande área de penumbra escura, com elevada estabilidade ambiental (Figura 134). Nenhuma alteração significativa foi observada durante a estação úmida, além das alterações normais na umidade relativa do ar. Algumas pequenas manchas de guano hematófago foram encontradas na porção intermediária da cavidade.

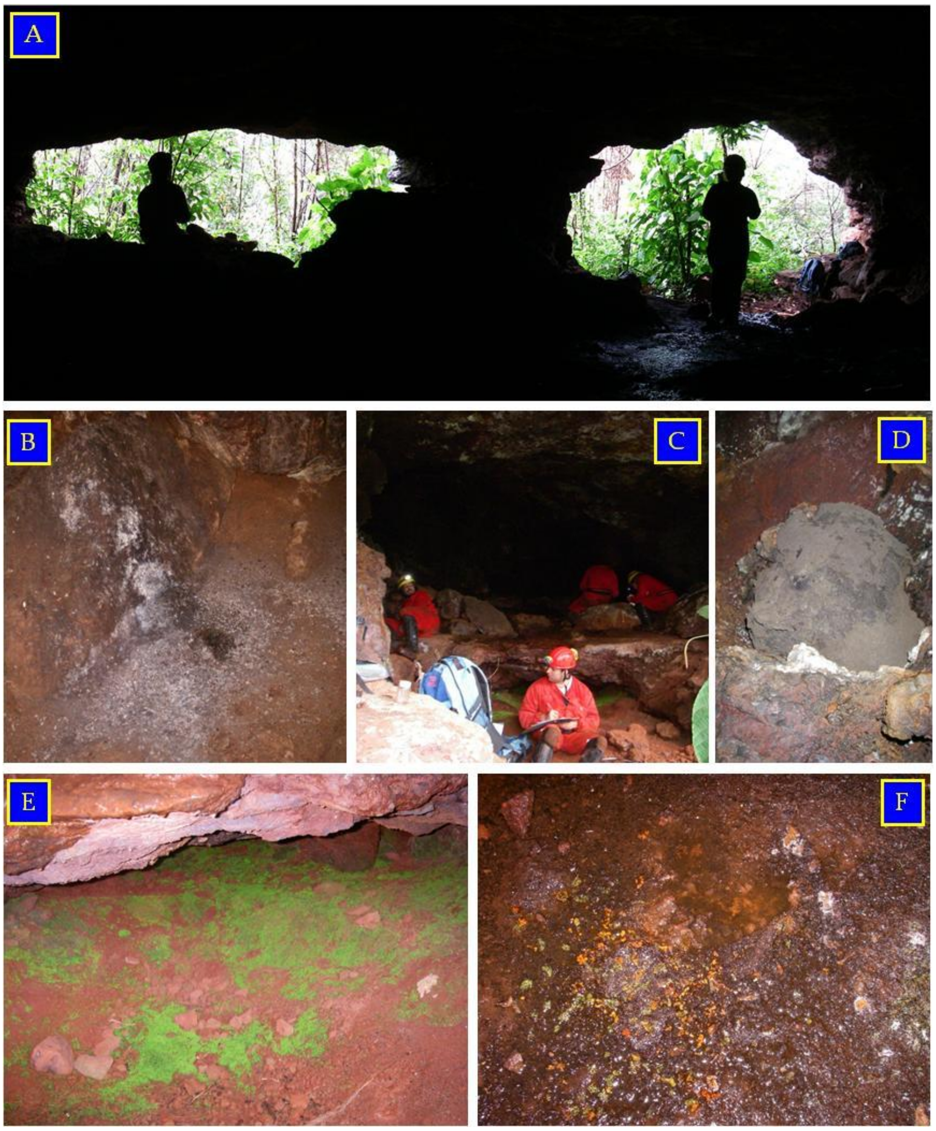

Figura 134 - a) Aspecto geral da entrada da cavidade onde é possível observar a vegetação da área de entorno; b) Depósito de guano envelhecido de morcegos hematófagos; c) Detalhe da galeria de entrada da cavidade; d) Cupinzeiro presente no interior da cavidade; e) Algas e briófitas se desenvolvendo nas proximidades da entrada; f) Guano fresco de morcegos frugívoros.

5.4.4.27.2. Caracterização faunística no período de seca

Foi observado na caverna, um total de 61 morfoespécies de invertebrados de pelo menos 44 famílias das Ordens: Gordioidea, Gastropoda (Systrophiidae), Isopoda (Philosciidae), Acari (Laelapidae: *Stratiolaelaps* sp., Macronyssidae, Mesostigmata, Astigmatina, Oribatida), Amblypygi (Phrynidae: *Heterophrynus longicornis*), Pseudoscorpiones (Chernetidae, Chtoniidae), Opiliones (Cosmetidae: *Anduzeia* sp., Manaosbiidae), Araneae (Araneidae: *Alpaida* sp., Gnaphosidae, Ochyroceratidae, Salticidae, Segestridae, Scytodidae*: Scytodes eleonora*, Psauridae, Pholcidae: *Metagonia* sp., Theridiidae, Theridiosomatidae), Thysanura (Nicoletiidae: Nicoletiinae, Atelurinae), Diplura (Campodeidae), Collembola (Entomobryidae), Orthoptera (Phalangopsidae: *Aclodes* sp., *Phalangopsis* sp.), Orthoptera (Blattidae, Polyphagidae), Isoptera (Termitidae: *Nasutitermes* sp.), Hemiptera (Cydnidae, Lygaeidae, Veliidae), Lepidoptera (Noctuidae), Diptera (Chironomidae, Culicidae: *Anopheles* sp., Milichiidae, Psychodidae, Tipulidae), Hymenoptera (*Camponotus* sp., *Gnamptogenys* sp., *Pachycondyla* sp., *Solenopsis* sp., *Tapinoma* sp.; Scelionidae), Coleoptera (Carabidae, Curculionidae: Scotylinae, Elateridae: Cardiophorinae, Scydmaenidae), Diplopoda (Chelodesmidae).

Dentre os vertebrados foram encontradas seis espécies das seguintes ordens: Chiroptera (Phyllostomidae: *Glossophaga soricina*), Squamata (Colubridae: *Chironius* sp.), Anura (Leptodactylidae: *Pristimantis* cf. *fenestratus*, *Eleutherodactylus* sp., *Adenomera* sp.).

Desta forma, no total foram encontradas 67 morfoespécies das quais duas foram consideradas troglomórficas: Gordioidea, Gastropoda (Systrophiidae).

5.4.4.27.3. Caracterização faunística no período de chuva

Foi observado na caverna, um total de 85 morfoespécies de invertebrados de pelo menos 54 famílias das Ordens: Isopoda (Dubioniscidae, Philosciidae), Acari (Argasidae: *Ornithodoros* sp., Laelapidae: *Stratiolaelaps* sp., Mesostigmata, Astigmatina, Oribatida, Trombidiforme) Amblypygi (Phrynidae: *Heterophrynus longicornis*) Pseudoscorpiones (Chernetidae sp., Chtoniidae), Araneae (*Alpaida* sp., Corinidae: *Tupirina* sp., Gnaphosidae sp., Ochyroceratidae, Oonopidae: Oonopinae, Salticidae, Scytodidae*: Scytodes eleonora*, Theridiidae sp., Theridiosomatidae), Thysanura (Nicoletiidae: Nicoletiinae), Diplura (Campodeidae), Collembola (Entomobryidae, Katiannidae?), Orthoptera (Phalangopsidae: *Aclodes* sp., *Phalangopsis* sp.), Blattodea (Polyphagidae), Isoptera (Termitidae: *Nasutitermes* sp.), Psocoptera (Archipsocidae, Myopsocidae: *Lichenomina* sp., Psyllipsocidae: *Psyllipsocus* sp.), Hemiptera (Cydnidae, Lygaeidae, Veliidae), Neuroptera (Myrmelionthydae), Lepidoptera (Noctuidae, Tineidae), Diptera (Cecidomyiidae, Chloropidae, Culicidae: *Culex* sp., Drosophilidae, Empididae, Faniidae, Milichiidae, Mycetophilidae, Otitidae, Psychodidae: *Lutzomyia* sp., Sciaridae), Hymenoptera (*Camponotus* sp., *Gnamptogenys* sp.,

Odontomachus sp., *Pachycondyla* sp., *Pheidole* sp., *Rogeria* sp., Braconidae, Pteromalidae), Coleoptera (Carabidae, Pselaphidae, Ptylidae, Scydmaenidae, Staphylinidae), Geophilomorpha (Ballophilidae: *Ballophililus* sp.), Scolopendromorpha (Scolopocryptopidae: *Dinocryptops* sp.), Scutigeromorpha (Scutigeridae: *Sphendononema* sp.).

Dentre os vertebrados foram encontradas três espécies das seguintes ordens: Chiroptera (*Glossophaga soricina*, *Diphylla ecaudata*) e Anura (*Pristimantis* cf. *fenestratus*). Desta forma, no total foram encontradas 88 morfoespécies, das quais uma foi considerada troglomórfica (Araneae: Ochyroceratidae).

5.4.4.27.4. Caracterização geral da fauna da cavidade

Foi observado na caverna, um total de 120 morfoespécies de invertebrados de pelo menos 69 famílias das Ordens: Gordioidea, Gastropoda (Systrophiidae), Isopoda (Dubioniscidae, Philosciidae), Acari (Argasidae: *Ornithodoros* sp., Laelapidae: *Stratiolaelaps* sp., Macronyssidae, Mesostigmata, Astigmatina, Oribatida, Trombidiforme), Amblypygi (Phrynidae: *Heterophrynus longicornis*), Pseudoscorpiones (Chernetidae, Chtoniidae), Opiliones (Cosmetidae: *Anduzeia* sp., Manaosbiidae, Araneidae: *Alpaida* sp., Corinidae: *Tupirina* sp., Gnaphosidae, Ochyroceratidae, Oonopidae: Oonopinae, Salticidae, Segestridae, Scytodidae*: Scytodes eleonora*, Psauridae, Pholcidae: *Metagonia* sp., Theridiidae, Theridiosomatidae), Thysanura (Nicoletiidae: Nicoletiinae, Atelurinae), Diplura (Campodeidae sp.), Collembola (Entomobryidae, Katiannidae?), Orthoptera (Phalangopsidae: *Aclodes* sp., *Phalangopsis* sp.), Blattodea (Blattidae, Polyphagidae), Isoptera (Termitidae: *Nasutitermes* sp.), Psocoptera (Archipsocidae, Myopsocidae: *Lichenomina* sp., Psyllipsocidae: *Psyllipsocus* sp.), Hemiptera (Cydnidae, Lygaeidae, Veliidae), Neuroptera (Myrmeleontidae), Lepidoptera (Noctuidae, Tineidae), Diptera (Cecidomyiidae, Chironomidae, Chloropidae, Culicidae: (*Culex* sp., *Anopheles* sp.), Drosophilidae, Empididae, Faniidae, Milichiidae, Mycetophilidae, Otitidae, Psychodidae: *Lutzomyia* sp., Sciaridae, Tipulidae), Hymenoptera (Formicidae: *Camponotus* sp., *Gnamptogenys* sp., *Odontomachus* sp., *Pachycondyla* sp., *Pheidole* sp., *Rogeria* sp., *Solenopsis* sp., *Tapinoma* sp.; Braconidae, Pteromalidae, Scelionidae), Coleoptera (Carabidae, Curculionidae: Scotylinae, Elateridae: Cardiophorinae, Pselaphidae sp., Ptylidae sp., Scydmaenidae sp., Staphylinidae sp.), Diplopoda (Chelodesmidae), Geophilomorpha (Ballophilidae: *Ballophililus* sp.), Scolopendromorpha (Scolopocryptopidae: *Dinocryptops* sp.), Scutigeromorpha (Scutigeridae: *Sphendononema* sp.).

Dentre os vertebrados, foram encontradas sete espécies das seguintes ordens: Chiroptera (Phyllostomidae: *Glossophaga soricina*, *Diphylla ecaudata*), Squamata (Colubridae: *Chironius* sp.), Anura (Leptodactylidae: *Pristimantis cf. fenestratus, Eleutherodactylus* sp., *Adenomera* sp.).

Desta forma, no total foram encontradas 127 morfoespécies, das quais três foram consideradas troglomórficas: Gordioidea, Gastropoda (Systrophiidae) e Araneae (Ochyroceratidae).

Por fim vale ressaltar o registro de uma serpente do gênero *Chironius* sp. (Colubridae) que foi observada alimentando-se de uma perereca (*P. fenestratus*) próximo a zona de entrada. Apesar de tratar-se apenas de uma hipótese, e plausível esperar que algumas espécies de serpentes utilizem as zonas de entrada das cavidades para forrageio de anfíbios uma vez que estes são comuns e muitas vezes abundantes em muitas cavidades da região. Alguns organismos encontrados nesta caverna são mostrados na Figura 135.

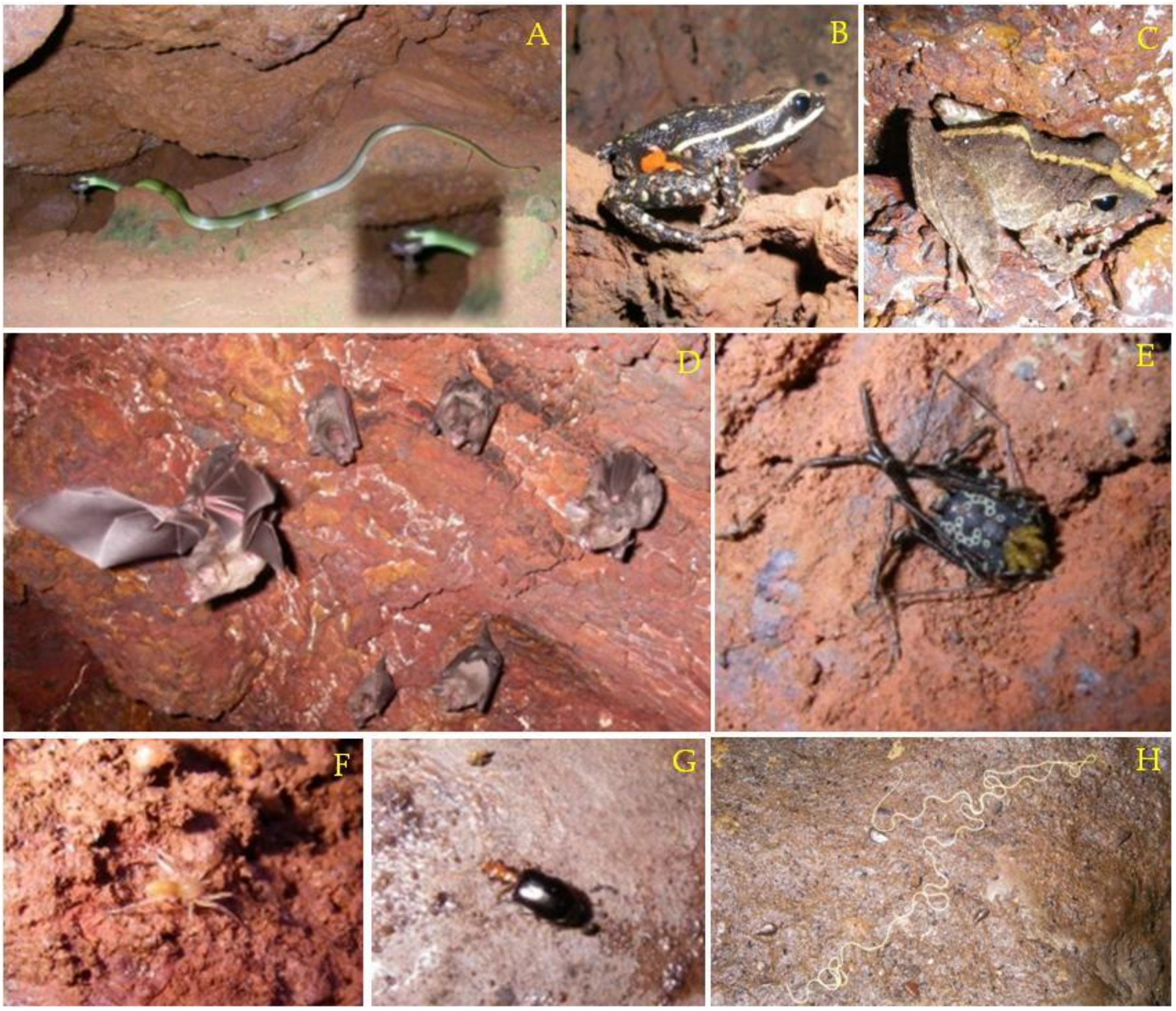

Figura 135 - a) Colubridae (Chironius sp.); b) Dendrobatidae (Ameerega sp.); c) Leptodactylidae (Eleutherodactylus sp.); d) Phyllostomidae (Carollia sp.); e) Opiliones (Manaosbiidae); f) Araneae; g) Coleoptera (Carabidae); Nematomorpha (Gordioidea).

5.4.4.28. SL-030

5.4.4.28.1. Caracterização trófica

Caverna desenvolvida na canga com 32 m de projeção horizontal e localizada na vertente a meia encosta em área de mata com vegetação arbórea muito densa. Possui uma única entrada ampla e sombreada com muitos líquens, briófitas e Actinomicetos revestindo as paredes e pisos, além de muitas angiospermas e serrapilheira acumulada junto à linha d’água. Sua entrada é larga e em teto baixo, onde existe uma área empoçada junto à linha d’água produzida através de uma percolação que ocorre na porção mais distal da cavidade sendo esta provocada por uma sondagem localizada na área acima da cavidade. De forma geral, o piso é úmido e ascendente composto por sedimento fino com muitos seixos, calhaus e matacões esparsos. O sistema radicular é bem desenvolvido sendo formado principalmente por raízes de fino calibre distribuídas por toda a cavidade. Existem grandes depósitos de guano de morcegos frugívoros produzidos pela espécie *G. soricina*, além de uma grande população de anfíbios que produzem muitas fezes observadas principalmente nas laterais da cavidade (Figura 136). O sistema de canalículos é pouco desenvolvido e as paredes e o teto são revestidos por Actinomicetos. A cavidade apresenta uma elevada estabilidade ambiental sendo a umidade e temperatura do ar elevadas. Não existem zonas afóticas, mas apenas uma grande zona de penumbra escura. Durante a estação úmida, a área empoçada junto à entrada da cavidade encontrava-se completamente seca e havia inúmeras rachaduras que funcionavam como abrigos para alguns invertebrados, como por exemplo, coleópteros estafilinídeos. No interior da cavidade existiam inúmeros gotejamentos ao longo de toda sua extensão e pontos de percolação junto às paredes da cavidade. Outro aspecto a ser destacado é que algumas fêmeas de *G. soricina* estavam com filhotes durante o inventário.

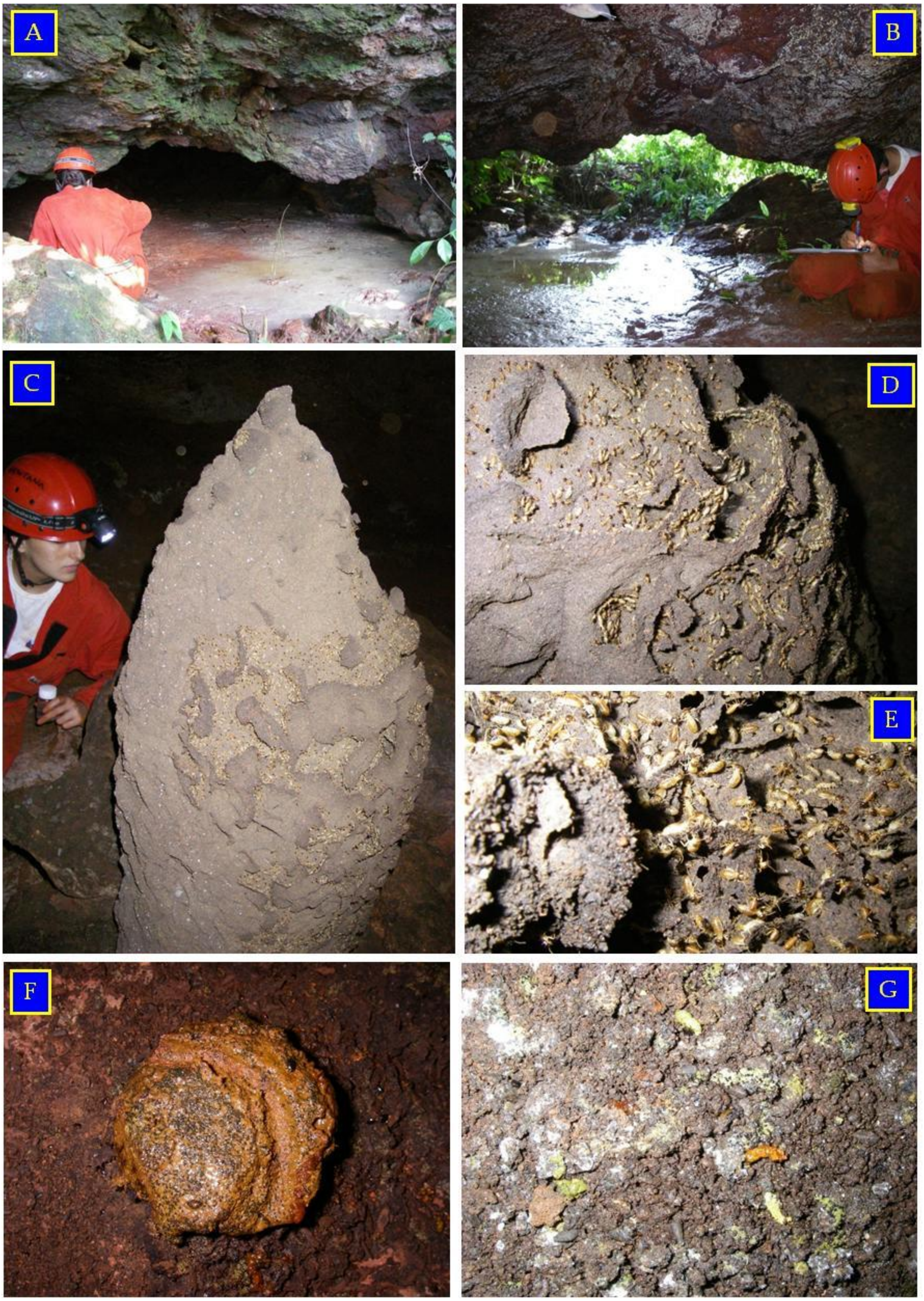

Figura 136 -) Aspecto geral da entrada da cavidade vista por fora; b) Vista interna com detalhes da área empoçada e da vegetação externa; c) Grande cupinzeiro presente no interior da caverna; d,e) Detalhe da colônia; f) Depósito fresco de guano de morcegos frugívoros; g) Depósito velho de guano de morcegos frugívoros.

5.4.4.28.2. Caracterização faunística no período de seca

Foi observado na caverna, um total de 64 morfoespécies de invertebrados de pelo menos 43 famílias das Ordens: Gordioidea, Gastropoda (Systrophiidae), Isopoda (Armadillidae), Acari (Argasidae: *Ornithodoros* sp., Laelapidae: *Stratiolaelaps* sp., Macronyssidae, Mesostigmata, Astigmatina), Amblypygi (Phrynidae: *Heterophrynus longicornis*), Pseudoscorpiones (Chernetidae, Chtoniidae), Opiliones (Escadabiidae), Araneae (Gnaphosidae, Ochyroceratidae, Oonopidae: Oonopinae, Pholcidae, Theridiidae, Theridiosomatidae), Thysanura (Nicoletiidae: Nicoletiinae, Atelurinae), Diplura (Campodeidae), Collembola Entomobryidae), Blattodea (Polyphagidae), Isoptera (*Nasutitermes* sp.), Dermaptera (Labiidae), Hemiptera (Cydnidae, Nabidae), Diptera (Ceratopogonidae, Drosophilidae, Milichiidae, Phoridae, Psychodidae: *Lutzomyia* sp., Sciaridae, Tipulidae), Hymenoptera (*Apterostigma* sp., *Camponotus* sp., *Pachycondyla* sp., *Pheidole* sp., *Rogeria* sp., *Strumigenys* sp., Bethylidae, Scelionidae) Coleoptera (Carabidae: *Coarazuphium* sp., Curculionidae: Scotylinae, Elateridae: Cardiophorinae, Pselaphidae, Scydmaenidae, Staphylinidae), Diplopoda (Pseudonannolenidae), Scolopendromorpha (Scolopocryptopidae: *Newportia* sp.), Symphyla (Scutigerellidae: *Hanseniella* sp.). Dentre os vertebrados foram encontradas duas espécies das seguintes Ordens: Anura (Leptodactylidae: *Pristimantis cf. fenestratus*, *Ameerega* sp.).

Desta forma, no total foram encontradas 66 morfoespécies das quais cinco morfoespécies foram consideradas troglomórficas: Gordioidea, Gastropoda (Systrophiidae), Thysanura (Nicoletiidae: Atelurinae), Coleoptera (Carabidae: *Coarazuphium* sp.) e Diplopoda (Pseudonannolenidae).

5.4.4.28.3. Caracterização faunística no período de chuva

Foi observado na caverna, um total de 101 morfoespécies de invertebrados de pelo menos 59 famílias das Ordens: Gastropoda (Systrophiidae), Isopoda (Armadillidae, Dubioniscidae, Philosciidae, Scleropactidae), Acari (Argasidae: *Ornithodoros* sp., Macronyssidae, Opilioacaridae: *Neoacarus* spn., Astigmatina, Trombidiforme), Amblypygi (*Heterophrynus longicornis*), Ricinulei (Ricinoididae: *Cryptocellus tarsilae*), Pseudoscorpiones (Chernetidae, Chtoniidae), Opiliones (*Anduzeia* sp., *Prionostemma* sp., Phalangiidae), Araneae (Ctenidae: *Ctenus* sp., Gnaphosidae, Ochyroceratidae, Oonopidae: Oonopinae, Gamasomorphinae, Salticidae, Scytodidae*: Scytodes eleonora*, Psauridae, Pholcidae, Theridiidae sp., Theridiosomatidae sp., Thysanura (Nicoletiidae: Nicoletiinae, Atelurinae), Microcoryphia (Meinertellidae), Collembola (Cyphoderidae, Entomobryidae, Isotomidae), Orthoptera (Phalangopsidae: *Aclodes* sp., *Phalangopsis* sp.,) Blattodea (*Blaberus* sp., Polyphagidae), Psocoptera (Myopsocidae (*Lichenomina* sp.,) Hemiptera (Cydnidae, Lygaeidae, Nabidae), Lepidoptera (Tineidae), Diptera (Ceratopogonidae, Chironomidae, Drosophilidae, Phoridae, Psychodidae: *Lutzomyia* sp., Streblidae sp.), Formicidae (*Apterostigma* sp., *Camponotus* sp.,

Pachycondyla sp., *Pheidole* sp., *Prionopeuta* sp., *Rogeria* sp., *Solenopsis* sp., *Strumigenys* sp., Bethylidae, Braconidae), Coleoptera (Carabidae sp., Cetoniidae: *Gymnets* sp., Eucnemidae, Hysteridae., Ptylidae, Scydmaenidae, Staphylinidae, Diplopoda (Polydesmida: Chelodesmidae), Scolopendromorpha (Scolopocryptopidae: *Dinocryptops* sp., *Scolopocryptops* sp.).

Dentre os vertebrados foram encontradas quatro espécies das seguintes Ordens: Chiroptera (*Peropteryx kappleri*, *Glossophaga soricina*), Squamata (Gekkonidae: *Thecadactylus rapicauda*) e Anura (Leptodactylidae: *Leptodactylus labyrinthicus*).

Desta forma, no total foram encontradas 105 morfoespécies dos quais cinco foram consideradas troglomórficas: Gastropoda (Systrophiidae), Araneae (Ochyroceratidae), Collembola (Cyphoderidae, Isotomidae), Coleoptera (Scydmaenidae).

5.4.4.28.4. Caracterização geral da fauna da cavidade

Foi observado na caverna, um total de 135 morfoespécies de invertebrados de pelo menos 59 famílias das Ordens: Gordioidea, Gastropoda (Systrophiidae), Isopoda (Armadillidae, Dubioniscidae, Philosciidae, Scleropactidae), Acari (Argasidae: *Ornithodoros* sp., Laelapidae: *Stratiolaelaps* sp., Macronyssidae, Mesostigmata, Opilioacaridae: *Neoacarus* spn., Astigmatina, Trombidiforme), Amblypygi (Phrynidae: *Heterophrynus longicornis*), Ricinulei (Ricinoididae: *Cryptocellus tarsilae*), Pseudoscorpiones (Chernetidae, Chtoniidae), Opiliones (Cosmetidae: *Anduzeia* sp., Escadabiidae, Esclerosomatidae: *Prionostemma* sp., Phalangiidae sp.), Araneae (Ctenidae: *Ctenus* sp., Gnaphosidae, Ochyroceratidae, Oonopidae: Oonopinae, Gamasomorphinae, Salticidae, Scytodidae*:* *Scytodes eleonora*, Psauridae, Pholcidae, Theridiidae, Theridiosomatidae), Thysanura (Nicoletiidae: Nicoletiinae, Atelurinae), Microcoryphia (Meinertellidae), Diplura (Campodeidae), Collembola (Cyphoderidae, Entomobryidae, Isotomidae), Orthoptera (Phalangopsidae: *Aclodes* sp., *Phalangopsis* sp.), Blattodea (Blaberidae: *Blaberus* sp., Polyphagidae), Isoptera (*Nasutitermes* sp.), Dermaptera (Labiidae), Psocoptera (Myopsocidae: *Lichenomina* sp.), Hemiptera (Cydnidae, Lygaeidae, Nabidae), Lepidoptera (Tineidae), Diptera (Ceratopogonidae, Chironomidae, Drosophilidae, Milichiidae, Phoridae, Psychodidae: *Lutzomyia* sp., Sciaridae, Streblidae, Tipulidae), Hymenoptera (Formicidae: *Apterostigma* sp., *Camponotus* sp., *Pachycondyla* sp., *Pheidole* sp., *Prionopeuta* sp., *Rogeria* sp., *Solenopsis* sp., *Strumigenys* sp., Bethylidae, Braconidae, Scelionidae), Coleoptera (Carabidae: *Coarazuphium* sp., Cetoniidae: *Gymnets* sp., Curculionidae: Scotylinae, Elateridae: Cardiophorinae, Eucnemidae, Hysteridae, Pselaphidae, Ptylidae, Scydmaenidae, Staphylinidae, Diplopoda (Polydesmida: Chelodesmidae), Spirostreptida (Pseudonannolenidae), Scolopendromorpha (Scolopocryptopidae: *Dinocryptops* sp., *Newportia* sp., *Scolopocryptops* sp.), Symphyla (Scutigerellidae: *Hanseniella* sp.).

Dentre os vertebrados foram encontradas seis espécies das seguintes ordens: Chiroptera (Emballonuridae: *Peropteryx kappleri*, Phyllostomidae: *Glossophaga soricina*), Squamata (Gekkonidae: *Thecadactylus rapicauda*), Anura (Leptodactylidae: *Pristimantis* cf. *fenestratus*, *Leptodactylus labyrinthicus, Ameerega* sp.).

Desta forma, no total foram encontradas 141 morfoespécies das quais nove foram consideradas troglomórficas: Gordioidea, Gastropoda (Systrophiidae), Araneae (Ochyroceratidae), Thysanura (Nicoletiidae: Atelurinae), Collembola (Cyphoderidae, Isotomidae), Coleoptera (Carabidae: *Coarazuphium* sp., Scydmaenidae) e Diplopoda (Pseudonannolenidae). Alguns organismos encontrados nesta caverna são mostrados na Figura 137.

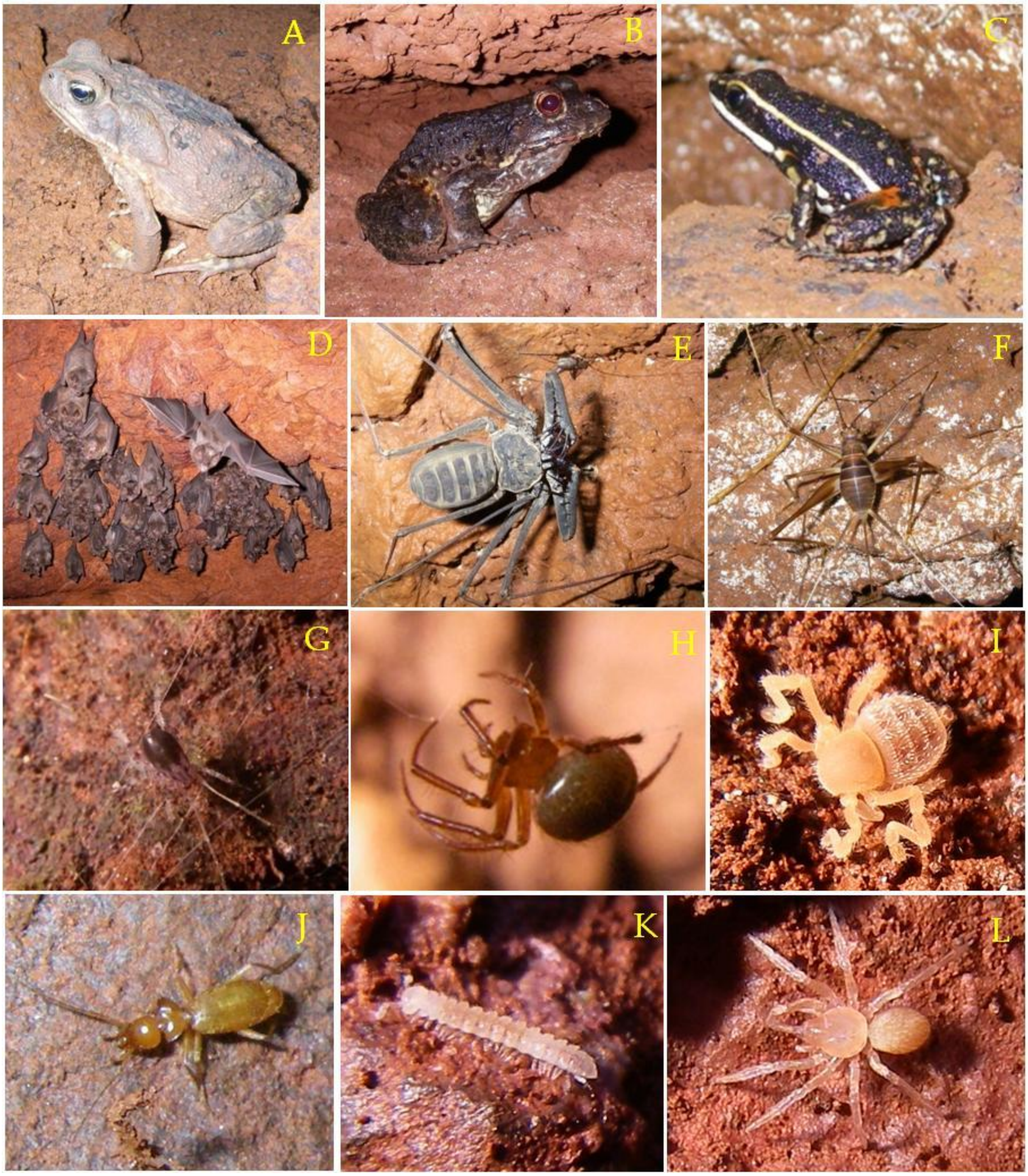

Figura 137 - a) Anura (Bufonidae); b) Leptodactylidae (*Leptodactylus labyrinthicus*); c) Dendrobatidae (*Ameerega* sp.); d) Phyllostomidae (*Carollia* sp.); e) Amblypygi (*Heterophrynus longicornis*); f) Phalangopsidae: (*Phalangopsis* sp.); g) Opilioacaridae (*Neoacarus* sp.); h) Theridiosomatidae (*Plato* sp.); i) Ricinulei (*Cryptocellus tarsilae* - ninfa); j) Carabidae (*Coarazuphium* sp.); k) Polydesmida; l) Araneae (Oonopidae).

5.4.4.29 SL-031

5.4.4.29.1. Caracterização trófica

Caverna desenvolvida na canga com 25,5 m de projeção horizontal e localizada em área de vegetação arbustiva em zona próxima ao topo da encosta. A cavidade apresenta-se de forma muito superficial sendo a vegetação predominante na área superior da cavidade composta pela Savana Metalófila. Sua entrada é muito iluminada, descendente e muito estreita, seguindo em teto baixo por aproximadamente dez metros em um conduto apertado, seco e com muitos blocos. Na zona eufótica existem poucos líquens e briófitas e alguns pequenos depósitos de serrapilheira restritos a linha d'água. Após o teto baixo abre-se um pequeno salão que apresenta elevada umidade e temperatura sendo este completamente afótico e onde existem muitos recursos alimentares disponíveis. Neste, o guano fresco de morcegos frugívoros é predominante e disposto por todo o piso da cavidade onde também existem algumas plântulas germinadas a partir das sementes presentes neste recurso. No teto e no piso existem muitas raízes de diferentes calibres além de Actinomicetos revestindo as paredes. Uma grande colônia de cupins foi observada associada ao guano de morcegos frugívoros e à fezes de anfíbios (Figura 138). A cavidade apresenta um elevado grau de confinamento com sistema de canalículos muito desenvolvido e elevada estabilidade ambiental. Nenhuma alteração significativa foi observada durante a estação úmida, além das alterações normais na umidade relativa do ar.

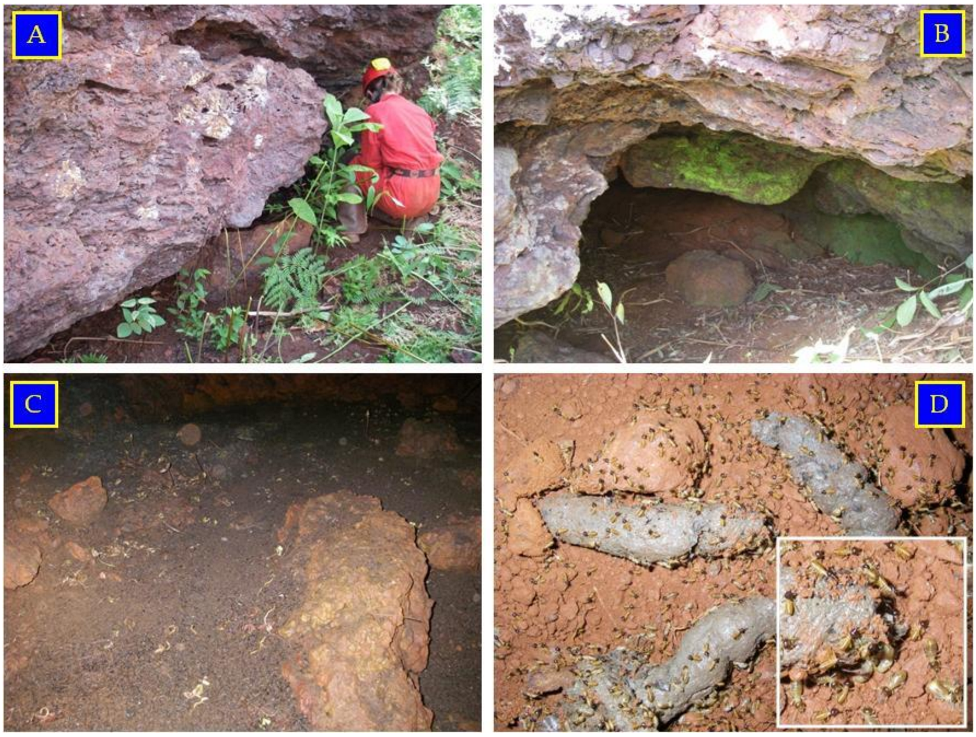

Figura 138 - a) Aspecto geral da entrada da cavidade vista por fora; b) Aspecto geral da porção interna da entrada, onde é possível observar a serrapilheira acumulada junto à linha d'água; c) Depósito fresco de guano de morcegos frugívoros; d) Fezes de anfíbio Bufonidae com cupins (Nasutitermes sp.) associados.

5.4.4.29.2. Caracterização faunística no período de seca

Foi observado na caverna, um total de 58 morfoespécies de invertebrados de pelo menos 40 famílias das Ordens: Oligochaeta, Gastropoda (Systrophiidae), Isopoda (Platyarthridae: *Trichorhina* sp.), Acari (Laelapidae: *Stratiolaelaps* sp., Anoetidae, Astigmatina, Oribatida, Eupodidae: *Linopodes* sp, Trombidiforme), Amblypygi (Phrynidae: *Heterophrynus longicornis*, Charinidae: *Charinus* sp.), Schizomida (Hubbardiidae: Hubbardiinae), Pseudoscorpiones (Chernetidae, Chtoniidae), Opiliones (Escadabiidae), Araneae (Gnaphosidae, Ochyroceratidae, Prodidomidae, Theridiosomatidae), Thysanura (Nicoletiidae: Nicoletiinae, Atelurinae), Collembola (Entomobryidae), Orthoptera (Phalangopsidae: *Phalangopsis* sp.), Blattodea (Blaberidae: *Blaberus* sp., Blattidae), Isoptera (*Nasutitermes* sp.), Hemiptera (Cydnidae, Reduviidae), Homoptera (Cixiidae: *Cixius* sp.), Lepidoptera (Tineidae), Diptera (Dolichopodidae, Psychodidae: *Lutzomyia* sp., Sciaridae, Tipulidae), Hymenoptera (Formicidae: *Camponotus* sp., *Pachycondyla* sp., *Pheidole* sp., *Solenopsis* sp., *Strumigenys* sp.), Coleoptera (Carabidae: *Coarazuphium* sp.; Chrysomelidae, Curculionidae: Scotylinae; Scydmaenidae, Staphylinidae), Diplopoda (Chelodesmidae, Pyrgodesmidae, Glomeridesmidae: *Glomeridesmus* sp., Pseudonannolenidae), Scolopendromorpha (Scolopocryptopidae: *Newportia* sp.), Symphyla (Scutigerellidae: *Hanseniella* sp.).

Dentre os vertebrados foram encontradas duas espécies das seguintes Ordens: Chiroptera (Phyllostomidae: *Glossophaga soricina*) e Anura (Leptodactylidae: *Pristimantis* cf. *fenestratus*).

Desta forma, no total foram encontradas 60 morfoespécies das quais nove foram consideradas troglomórficas: Gastropoda (Systrophiidae), Isopoda (Platyarthridae: *Trichorhina* sp.), Amblypygi (Charinidae: Charinus sp.), Schizomida (Hubbardiidae), Araneae (Prodidomidae), Thysanura (Nicoletiidae: Atelurinae), Coleoptera (Carabidae: *Coarazuphium* sp.), Diplopoda (Glomeridesmidae: *Glomeridesmus* sp., Pyrgodesmidae).

5.4.4.29.3. Caracterização faunística no período de chuva

Foi observado na caverna, um total de 77 morfoespécies de invertebrados de pelo menos 49 famílias das Ordens: Oligochaeta, Gastropoda (Subulinidae, Systrophiidae), Isopoda (Armadillidae, Platyarthridae: *Trichorhina* sp., Laelapidae: *Stratiolaelaps* sp., Mesostigmata, Uropodina, Astigmatina, Oribatida, Eupodidae: *Linopodes* sp., Trombidiforme) Amblypygi (Phrynidae: *Heterophrynus longicornis*, Charinidae: *Charinus* sp.), Ricinulei (Ricinoididae: *Cryptocellus tarsilae*), Schizomida (Hubbardiidae: Hubbardiinae), Pseudoscorpiones (Chernetidae, Chtoniidae), Opiliones (Cosmetidae: *Anduzeia* sp., Escadabiidae, Phalangiidae), Araneae (Gnaphosidae, Ochyroceratidae, Oonopidae: Oonopinae, Psauridae, Pholcidae, Theraphosidae, Theridiosomatidae), Thysanura (Nicoletiidae: Nicoletiinae, Atelurinae), Collembola (Sminthuridae, Entomobryidae, Isotomidae), Blattodea (Blaberidae: *Blaberus* sp.,

Polyphagidae), Psocoptera (Epipsocidae), Hemiptera (Cydnidae, Dipsocoridae, Reduviidae), Lepidoptera (Noctuidae: *Latebraria* sp., Tineidae), Diptera (Culicidae, Drosophilidae, Lampiridae, Psychodidae: *Lutzomyia* sp., Sciaridae), Hymenoptera (Formicidae: *Apterostigma* sp., *Camponotus* sp., *Pachycondyla* sp., *Pheidole* sp., *Solenopsis* sp.) Thysanoptera (Phaleothripidae: Phaleothripinae), Coleoptera (Carabidae: *Coarazuphium* sp., Scydmaenidae, Staphylinidae), Diplopoda (Pyrgodesmidae, Glomeridesmidae: *Glomeridesmus* sp., Rhinocricidae), Scolopendromorpha (Cryptopidae: *Cryptops* sp.), Symphyla (Scutigerellidae: *Hanseniella* sp.)

Dentre os vertebrados foi encontrada apenas uma espécie da Ordem Chiroptera (Phyllostomidae: *Glossophaga soricina*).

Desta forma, no total foram encontradas 78 morfoespécies das quais 11 foram consideradas troglomórficas: Gastropoda (Systrophiidae), Isopoda (Platyarthridae: *Trichorhina* sp.), Amblypygi (Charinidae: *Charinus* sp.), Schizomida (Hubbardiidae), Araneae (Ochyroceratidae spp.), Collembola (Isotomidae), Hemiptera (Dipsocoridae), Coleoptera (Carabidae: *Coarazuphium* sp.), Diplopoda (Pyrgodesmidae, Glomeridesmidae: *Glomeridesmus* sp.).

5.4.4.29.4. Caracterização geral da fauna da cavidade

Foi observado na caverna, um total de 107 morfoespécies de invertebrados de pelo menos 63 famílias das Ordens: Oligochaeta, Gastropoda (Subulinidae, Systrophiidae), Isopoda (Armadillidae, Plathyarthridae: Trichorhina sp.), Acari (Stratiolaelaps sp., Mesostigmata, Uropodina, Anoetidae, Astigmatina, Oribatida, Eupodidae: *Linopodes* sp., Trombidiforme), Amblypygi (Phrynidae: *Heterophrynus longicornis*, Charinidae: *Charinus* sp.), Ricinulei (Ricinoididae: *Cryptocellus tarsilae*), Schizomida (Hubbardiidae), Pseudoscorpiones (Chernetidae, Chtoniidae), Opiliones (Cosmetidae: *Anduzeia* sp., Escadabiidae, Phalangiidae), Araneae (Gnaphosidae, Ochyroceratidae, Oonopidae: Oonopinae, Psauridae, Pholcidae, Prodidomidae, Theraphosidae sp., Theridiosomatidae), Thysanura (Nicoletiidae: Nicoletiinae, Atelurinae), Collembola (Sminthuridae, Entomobryidae, Isotomidae), Orthoptera (*Phalangopsis* sp.), Blattodea (Blaberidae: *Blaberus* sp., Blattidae, Polyphagidae), Isoptera (Termitidae: *Nasutitermes* sp.), Psocoptera (Epipsocidae), Hemiptera (Cydnidae, Dipsocoridae, Reduviidae, Reduviidae), Homoptera (Cixiidae: *Cixius* sp.), Lepidoptera (Noctuidae: *Latebraria* sp., Tineidae), Diptera (Culicidae sp., Dolichopodidae sp., Drosophilidae sp., Psychodidae: *Lutzomyia* sp., Sciaridae, Tipulidae), Hymenoptera (Formicidae: *Apterostigma* sp., *Camponotus* sp., *Pachycondyla* sp., *Pheidole* sp., *Solenopsis* sp., *Strumigenys* sp.), Thysanoptera (Phaleothripinae), Coleoptera (Carabidae: *Coarazuphium* sp., Chrysomelidae sp., Curculionidae: Scotylinae, Lampiridae, Scydmaenidae, Staphylinidae), Diplopoda (Chelodesmidae, Pyrgodesmidae, Glomeridesmidae: *Glomeridesmus* sp., Pseudonannolenidae sp., Rhinocricidae), Scolopendromorpha (Cryptopidae: *Cryptops* sp., Scolopocryptopidae: *Newportia* sp.), Symphila (Scutigerellidae: *Hanseniella* sp.).

Dentre os vertebrados foram encontradas duas espécies das Ordens: Chiroptera (Phyllostomidae: *Glossophaga soricina*) e Anura (Leptodactylidae: *Pristimantis* cf. *fenestratus*).

Desta forma, no total foram encontradas 109 morfoespécies das quais 13 espécies foram consideradas troglomórficas: Gastropoda (Systrophiidae), Isopoda (Platyarthridae: *Trichorhina* sp.), Amblypygi (Charinidae: *Charinus* sp.), Schizomida (Hubbardiidae), Araneae (Ochyroceratidae spp., Prodidomidae), Thysanura (Nicoletiidae: Atelurinae), Collembola (Isotomidae), Hemiptera (Dipsocoridae), Coleoptera (Carabidae: *Coarazuphium* sp.), Diplopoda (Pyrgodesmidae, Glomeridesmidae: *Glomeridesmus* sp.). Alguns organismos encontrados nesta caverna são mostrados na Figura 139.

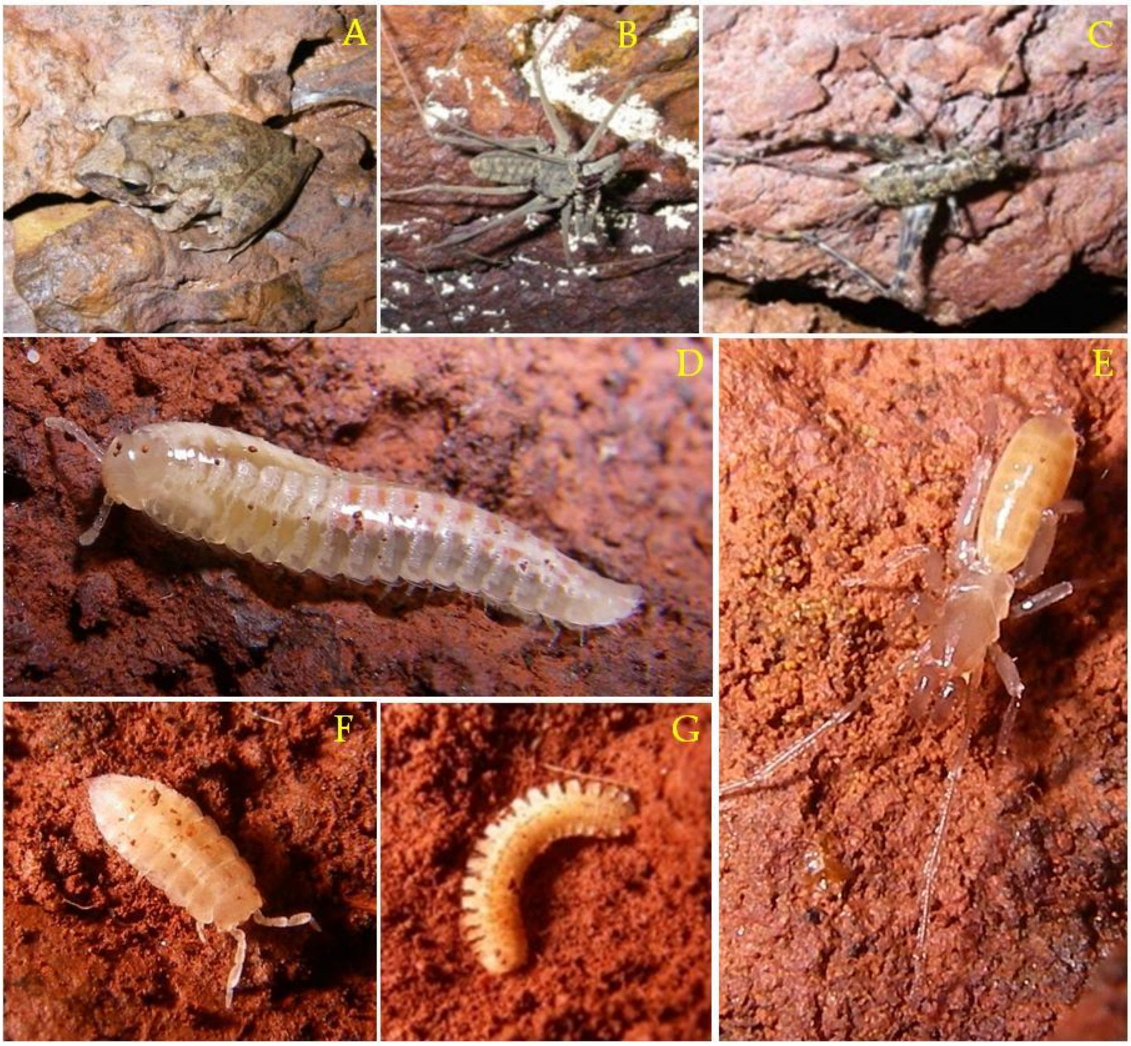

Figura 139 - a) Leptodactylidae (Pristimantis cf. fenestratus); b) Amblypygi (Heterophrynus longicornis); c) Phalangopsidae (Aclodes sp.); d) Glomeridesmida (Glomeridesmus sp.); e) Schizomida (Hubbardiinae); f) Isopoda (Trichorhina sp.); g) Diplopoda (Pyrgodesmidae).

5.4.4.30. SL-032

5.4.4.30.1. Caracterização trófica

Pequena cavidade com 7 m de desenvolvimento, formada na canga e localizada em área de mata ciliar na margem esquerda do rio. A região de entorno é composta por vegetação densa com árvores de grande porte, lianas, cipós, bambus e palmeiras. A entrada é ampla e sombreada com umidade elevada onde existem muitos líquens, briófitas, Actinomicetos e angiospermas na zona eufótica além de muita serrapilheira acumulada junto à linha d'água e esparsa pelo piso da cavidade. Trata-se de uma pequena reentrância na canga com teto alto e piso plano, sendo este seco e composto por sedimento fino e com blocos de diferentes tamanhos (seixos e calhaus) esparsos. A caverna não apresenta zona afótica, não existem depósitos de guano, mas observou-se a presença de muitas raízes de fino calibre distribuídas de forma superficial. Nas porções mais profundas existe uma grande quantidade de rejeitos (lixeira) de formigas cortadeiras (*Atta* sp.) e o sistema de canalículos é bem desenvolvido (Figura 140). Caverna com baixa estabilidade ambiental estando muito suscetível a influência das condições ambientais epígeas. Nenhuma alteração significativa foi observada durante a estação úmida, além das alterações normais na umidade relativa do ar.

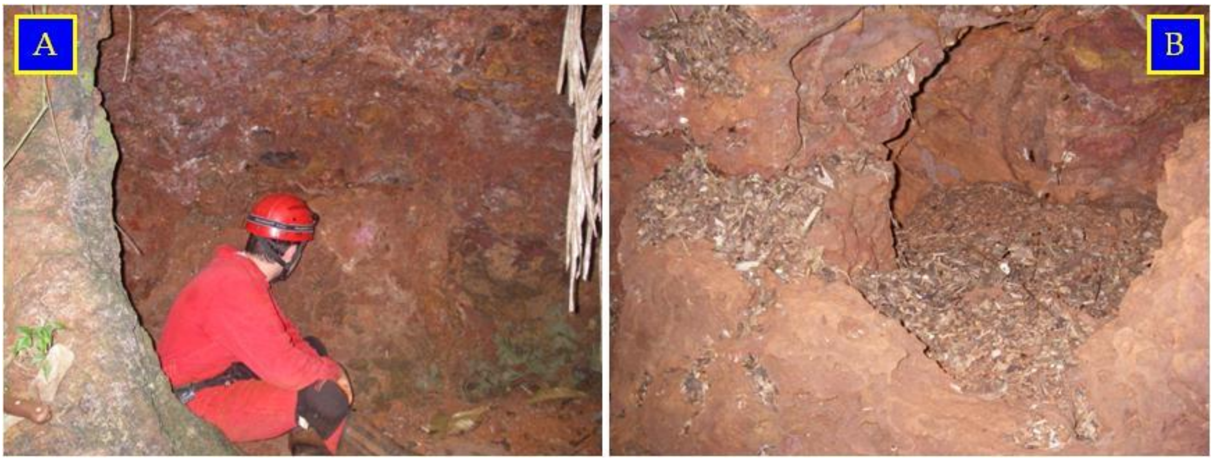

Figura 140 - a) Aspecto geral da cavidade onde é possível observar a serrapilheira esparsa pelo piso do abrigo; b) Lixeira (descarte) de matéria orgânica vegetal produzida por formigas da tribo Attini.

5.4.4.30.2. Caracterização faunística no período de seca

Foi observado na caverna, um total de 33 morfoespécies de invertebrados de pelo menos 24 famílias das Ordens: Isopoda (Armadillidae), Acari (Mesostigmata, Trombidiforme), Amblypygi (Phrynidae: *Heterophrynus longicornis*), Pseudoscorpiones (Chernetidae), Opiliones (*Protimesius* aff. *gracilis*), Araneae (Pholcidae: *Mesabolivar* sp., Theridiidae), Neuroptera (Mantispidae: *Plega* sp., Myrmelionthydae), Orthoptera (Phalangopsidae: *Aclodes* sp., *Phalangopsis* sp.), Isoptera (Termitidae: *Nasutitermes* sp.), Hemiptera (Cydnidae, Reduviidae), Lepidoptera (Noctuidae), Diptera (Ceratopogonidae, Conopidae, Culicidae, Simulidae, Tipulidae), Hymenoptera (Formicidae: *Acromyrmex* sp., *Azteca* sp.,

Solenopsis sp.; Apidae, Scelionidae), Coleoptera (Elateridae: Cardiophorinae, Pselaphidae), Diplopoda (Pyrgodesmidae).

5.4.4.30.3. Caracterização faunística no período de chuva

Foi observado na caverna, um total de 45 morfoespécies de invertebrados de pelo menos 36 famílias das Ordens: Isopoda (Armadillidae, Philosciidae), Acari (Laelapidae, Oribatida), Amblypygi (*Heterophrynus longicornis*), Pseudoscorpiones (Chernetidae), Opiliones (Gonyleptidae), Araneae (Ctenidae: *Ctenus* sp., Gnaphosidae, Linyphiidae, Ochyroceratidae, Salticidae, Drymusidae: *Drymusa* sp., Scytodidae*: Scytodes eleonora*, Paratropidae, Pholcidae: *Mesabolivar* sp., Theridiidae, Theridiosomatidae), Collembola (Entomobryidae, Katiannidae?), Orthoptera (Phalangopsidae: *Aclodes* sp., *Phalangopsis* sp.), Phasmatodea (Pseudophasmatidae), Blattodea (Polyphagidae), Isoptera, Hemiptera (Cydnidae, Lygaeidae, Nabidae, Cicadellidae), Homoptera (Cixiidae), Lepidoptera (Tineidae), Diptera (Cecidomyiidae, Chironomidae, Psychodidae: *Lutzomyia* sp.), Hymenoptera (Formicidae: *Odontomachus* sp., *Pachycondyla* sp., *Pheidole* sp., *Solenopsis* sp., Vespidae), Coleoptera (Scydmaenidae, Staphylinidae, Dermestidae). Destes apenas uma morfoespécie foi considerada troglomórfica (Scydmaenidae).

5.4.4.30.4. Caracterização geral da fauna da cavidade

Foi observado na caverna, um total de 70 morfoespécies de invertebrados de pelo menos 52 famílias das Ordens: Isopoda (Armadillidae, Philosciidae), Acari (Laelapidae, Mesostigmata, Oribatida, Trombidiforme), Amblypygi (*Heterophrynus longicornis*), Pseudoscorpiones (Chernetidae), Opiliones (Gonyleptidae, Stygnidae: *Protimesius* aff. *gracilis*), Araneae (Ctenidae: *Ctenus* sp., Gnaphosidae, Linyphiidae, Ochyroceratidae, Salticidae, Drymusidae: *Drymusa* sp., Scytodidae*: Scytodes eleonora*, Paratropidae, Pholcidae: *Mesabolivar* sp., Theridiidae, Theridiosomatidae), Collembola (Entomobryidae, Katiannidae?), Neuroptera (Mantispidae: *Plega* sp., Myrmelionthydae), Orthoptera (Phalangopsidae: *Aclodes* sp., *Phalangopsis* sp.), Phasmatodea (Pseudophasmatidae), Blattodea (Polyphagidae), Isoptera (Termitidae: *Nasutitermes* sp.), Hemiptera (Cydnidae, Lygaeidae, Nabidae, Reduviidae, Cicadellidae), Homoptera (Cixiidae), Lepidoptera (Noctuidae, Tineidae), Diptera (Cecidomyiidae, Ceratopogonidae, Conopidae, Chironomidae, Culicidae: *Lutzomyia* sp., Simulidae, Tipulidae), Diptera (Formicidae: *Acromyrmex* sp., *Azteca* sp., *Odontomachus* sp., *Pachycondyla* sp., *Pheidole* sp., *Solenopsis* sp., Apidae, Scelionidae, Vespidae), Coleoptera (Dermestidae, Elateridae: Cardiophorinae, Pselaphidae, Scydmaenidae, Staphylinidae), Diplopoda (Pyrgodesmidae). Destes apenas uma morfoespécie foi considerada troglomórfica (Scydmaenidae). Alguns organismos encontrados nesta caverna são mostrados na Figura 141.

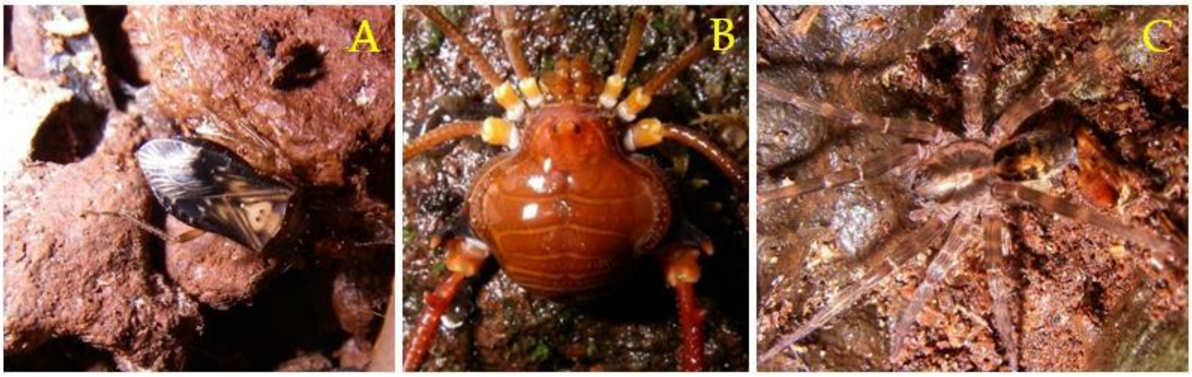

Figura 141 - a) Hemiptera (Nabidae); b) Opiliones (Gonyleptidae); c) Ctenidae (*Ctenus* sp.).

5.4.4.31. SL-033

5.4.4.31.1. Caracterização trófica

Pequeno abrigo formado na canga com 6,5 m de projeção horizontal localizado em área de mata ciliar na margem esquerda do rio. A região de entorno é composta por vegetação densa com árvores de grande porte, lianas, cipós, bambus e palmeiras. A entrada é ampla e sombreada com umidade elevada onde existem muitos líquens, briófitas, Actinomicetos e angiospermas na zona eufótica além de muita serrapilheira acumulada junto à linha d'água e também esparsa pelo piso da cavidade. Trata-se de uma pequena reentrância na canga com teto alto e piso plano e seco composto por sedimento fino e com blocos de diferentes tamanhos (seixos e calhaus) esparsos. A caverna não apresenta zona afótica, mas existe uma pequena zona de penumbra. Existem alguns pequenos depósitos frescos de guano de morcegos frugívoros, muitas raízes de diferentes calibres distribuídas de forma superficial além de Actinomicetos revestindo as paredes e o teto. Nas porções mais profundas existe uma grande quantidade de cupinzeiros abandonados e o sistema de canalículos é bem desenvolvido (Figura 142). Caverna com baixa estabilidade ambiental estando muito suscetível a influência das condições ambientais epígeas. Durante a estação úmida foram observados alguns pontos de gotejamento, áreas de percolação e pontos de represamento do interior da cavidade.

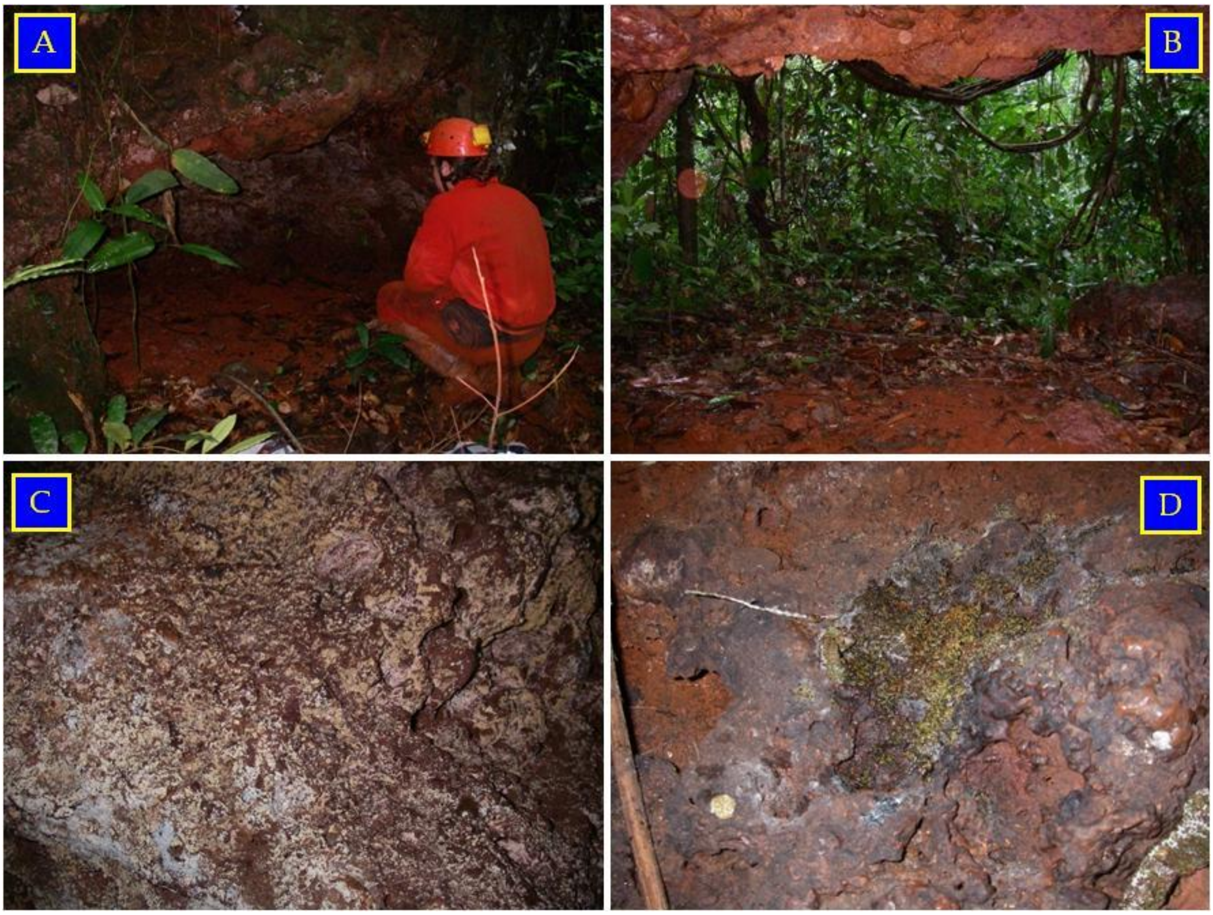

Figura 142 - a) Aspecto geral da entrada do abrigo; b) Vista interna da entrada onde é possível observar a vegetação do entorno; c) Grande quantidade de colônias de Actinomicetos crescendo nas paredes; d) Guano fresco de morcegos frugívoros no interior da cavidade.

5.4.4.31.2. Caracterização faunística no período de seca

Foi observado na caverna, um total de 53 morfoespécies de invertebrados de pelo menos 36 famílias das Ordens: Isopoda (Philosciidae, Plathyarthridae: *Trichorhina* sp.), Acari (Mesostigmata, Oribatida, Anystidae: *Erythracarus* sp., Labdostomatidae: Labdostomatida sp., Trombidiforme) Amblypygi (Phrynidae: *Heterophrynus longicornis*), Pseudoscorpiones (Chernetidae, Chtoniidae), Opiliones (Cosmetidae: *Anduzeia* sp., Stygnidae: *Protimesius* aff. *gracilis*), Araneae (Corinidae: *Tupirina* sp., Gnaphosidae, Scytodidae*: Scytodes eleonora*, Pholcidae: *Mesabolivar* sp, Theraphosidae), Thysanura (Nicoletiidae: Atelurinae), Collembola (Entomobryidae), Orthoptera (Phalangopsidae: *Aclodes* sp., *Phalangopsis* sp.), Blattodea (Blattidae sp.), Psocoptera (Archipsocidae, Epipsocidae), Hemiptera (Dipsocoridae, Coccoidea), Lepidoptera (Noctuidae), Diptera (Culicidae, Drosophilidae, Psychodidae: *Lutzomyia* sp., Simulidae, Tipulidae), Hymenoptera (*Camponotus* sp., *Odontomachus* sp., *Pachycondyla* sp., *Pheidole* sp., *Solenopsis* sp., *Strumigenys* sp.; Bethylidae, Cynipidae, Vespidae), Coleoptera (Pselaphidae, Staphylinidae), Diplopoda (Chelodesmidae, Cyrtodesmidae).

Dentre os vertebrados foram encontradas quatro espécies das seguintes Ordens: Squamata (Colubridae: *Leptodeira annulata*, Gekkonidae: *Thecadactylus rapicauda*), Anura (Leptodactylidae: *Pristimantis* cf. *fenestratus* e Leptodactylidae sp.). Desta forma, no total foram encontradas 57 morfoespécies das quais duas foram consideradas troglomórficas: Isopoda (Plathyarthridae: *Trichorhina* sp.), Thysanura (Nicoletiidae: Atelurinae).

5.4.4.31.3. Caracterização faunística no período de chuva

Foi observado na caverna, um total de 48 morfoespécies de invertebrados de pelo menos 37 famílias das Ordens: Gastropoda (Subulinidae, Valloniidae, Streptaxidae), Isopoda (Plathyarthridae: *Trichorhina* sp.), Decapoda (Pseudothelphusidae: *Microthelphusa somanni*), Acari (Laelapidae: *Stratiolaelaps* sp., Mesostigmata, Oribatida, Anystidae: *Erythracarus* sp., Trombidiforme), Amblypygi (Phrynidae: *Heterophrynus longicornis*), Pseudoscorpiones (Chernetidae, Chtoniidae), Opiliones (Escadabiidae, Sclerosomatidae: *Prionostemma* sp.), Araneae (Araneidae: *Alpaida* sp., Ctenidae: *Ctenus* sp., Gnaphosidae, Linyphiidae, Oonopidae: Oonopinae, Pholcidae: *Mesabolivar* sp., Theridiosomatidae), Thysanura (Nicoletiidae: Atelurinae), Diplura (Campodeidae), Collembola (Dicyrtomidae, Paronellidae), Orthoptera (Phalangopsidae), Hemiptera (Cydnidae), Diptera (Tipulidae, Culicidae: *Culex* sp.), Lepidoptera (Tineidae), Hymenoptera (*Camponotus* sp., *Pachycondyla* sp., *Pheidole* sp.) Coleoptera (Indet., Elateridae: Elaterinae, Staphylinidae), Diplopoda (Chelodesmidae, Pyrgodesmidae, Glomeridesmidae: *Glomeridesmus* sp., Stemmiulidae), Scolopendromorpha (Otostigmidae: *Otostigmus* sp.).

5.4.4.31.4. Caracterização geral da fauna da cavidade

Foi observado na caverna, um total de 90 morfoespécies de invertebrados de pelo menos 62 famílias das Ordens: Gastropoda (Subulinidae, Valloniidae, Streptaxidae), Isopoda (Philosciidae, Plathyarthridae: *Trichorhina* sp.), Decapoda (Pseudothelphusidae: *Microthelphusa somanni*), Acari (Laelapidae: *Stratiolaelaps* sp., Mesostigmata, Oribatida, Anystidae: *Erythracarus* sp., Labdostomatidae: *Labdostomatida* sp., Trombidiforme), Amblypygi (Phrynidae: *Heterophrynus longicornis*), Pseudoscorpiones (Chernetidae, Chtoniidae), Opiliones (Cosmetidae: *Anduzeia* sp., Escadabiidae, Esclerosomatidae: *Prionostemma* sp., Stygnidae: *Protimesius* aff. *gracilis*), Araneae (Araneidae: *Alpaida* sp., Corinidae: *Tupirina* sp., Ctenidae: *Ctenus* sp., Gnaphosidae, Linyphiidae, Oonopinae: Oonopinae, Scytodidae*: Scytodes eleonora*, Pholcidae: *Mesabolivar* sp., Theraphosidae, Theridiosomatidae), Thysanura (Nicoletiidae: Atelurinae), Diplura (Campodeidae), Collembola (Dicyrtomidae, Entomobryidae, Paronellidae), Orthoptera (Phalangopsidae: *Aclodes* sp., *Phalangopsis* sp.), Blattellidae (Blattidae sp.), Psocoptera (Archipsocidae, Epipsocidae), Hemiptera (Cydnidae, Dipsocoridae, Coccoidea), Lepidoptera (Noctuidae, Tineidae), Diptera (Culicidae: *Culex* sp., Drosophilidae, Psychodidae: *Lutzomyia* sp., Simulidae, Tipulidae), Hymenoptera (*Camponotus* sp., *Odontomachus* sp., *Pachycondyla* sp., *Pheidole* sp., *Solenopsis* sp., *Strumigenys* sp., Bethylidae, Cynipidae, Vespidae), Coleoptera

(Elateridae: Elaterinae, Pselaphidae, Staphylinidae), Diplopoda (Chelodesmidae, Cyrtodesmidae, Pyrgodesmidae, Polydesmida, Glomeridesmidae: *Glomeridesmus* sp., Stemmiulidae), Scolopendromorpha (Otostigmidae: *Otostigmus* sp.).

Dentre os vertebrados foram encontradas quatro espécies das seguintes Ordens: Squamata (Colubridae: *Leptodeira annulata*, Gekkonidae: *Thecadactylus rapicauda*), Anura (Leptodactylidae: *Pristimantis* cf. *fenestratus* e Leptodactylidae sp.). Desta forma, no total foram encontradas 94 morfoespécies das quais duas foram consideradas troglomórficas: Isopoda (Plathyarthridae: *Trichorhina* sp.), Thysanura (Nicoletiidae: Atelurinae). Alguns organismos encontrados nesta caverna são mostrados na Figura 143.

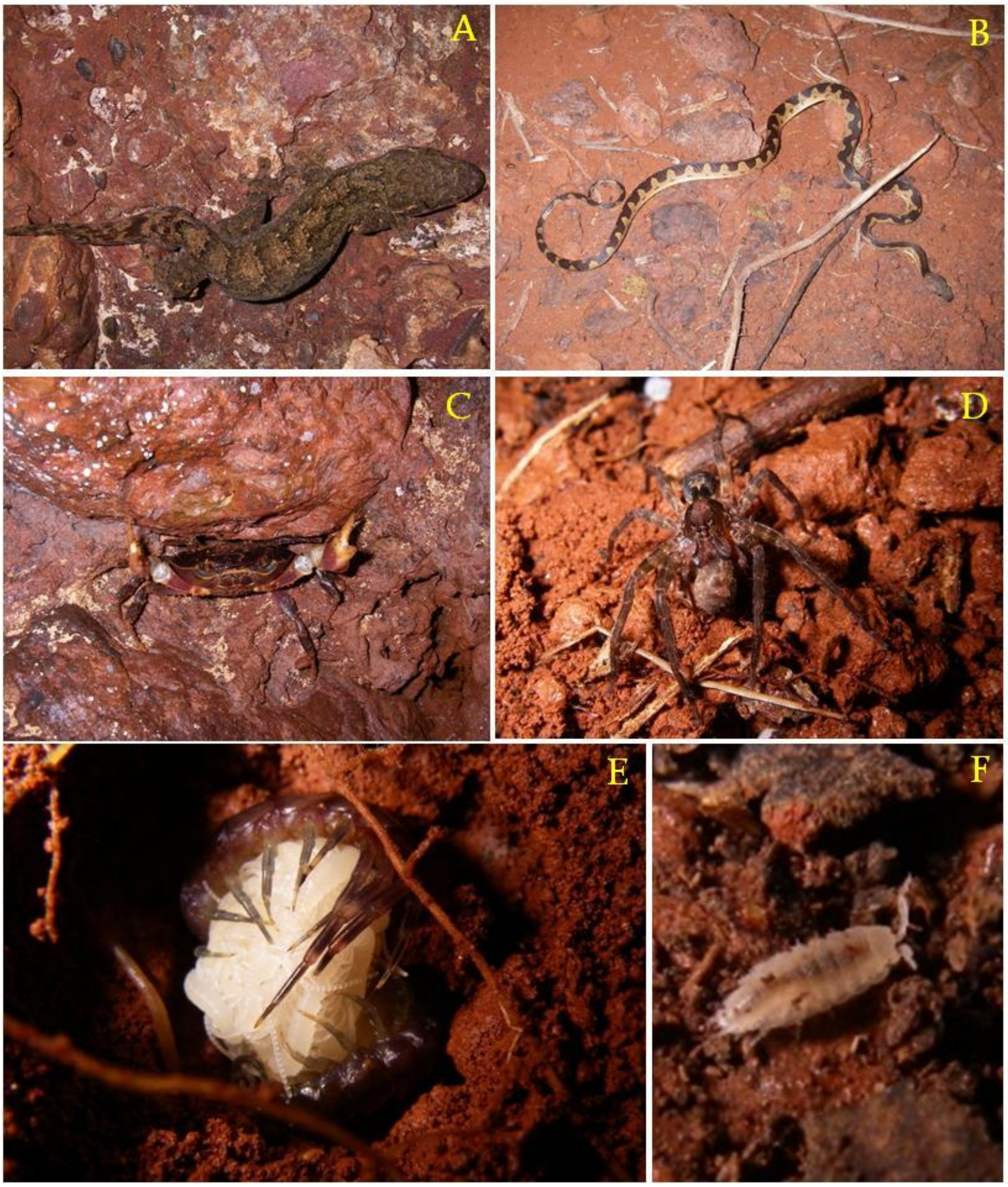

Figura 143 - a) Gekkonidae (Thecadactylus rapicauda); b) Colubridae (*Leptodeira annulata*); c) Decapoda (Pseudothelphusidae: *Microthelphusa somanni*); d) Ctenidae; e) Scolopendromorpha (*Otostigmus* sp.); f) Isopoda (*Trichorhina* sp.).

5.4.4.32. SL-035

5.4.4.32.1. Caracterização trófica

Caverna formada em minério de ferro na parte mais alta da encosta, com projeção horizontal de 39,9 m. Vegetação de entorno composta principalmente por samambaias além de alguns poucos arbustos distribuídos de maneira esparsa e por capim na região superior da cavidade. A caverna possui duas entradas estreitas e muito ensolaradas com paredes e pisos totalmente cobertos por líquens, briófitas, pteridófitas e brotos de melastomatáceas. Esta possui piso irregular ascendente, com baixa umidade na zona de entrada, sendo este composto por sedimentos granulados e por pequenos blocos representados principalmente por seixos e calhaus. Ainda na entrada existem pequenos depósitos de guano de morcegos frugívoros produzidos por Glossophaginae. O sistema radicular é bem desenvolvido sendo composto por raízes de fino calibre que encontram-se distribuídas por todo o piso da cavidade de maneira superficial e sub-superficial. A cavidade apresenta uma grande zona afótica que é acessada por um conduto à direita da entrada após um pequeno trecho de rastejo. O sistema de alvéolos ou canalículos é muito bem desenvolvido fornecendo inúmeros abrigos para os organismos cavernícolas além da possibilidade de funcionarem como vias de colonização e dispersão. Existem depósitos de guano fresco de pequenas dimensões de morcegos frugívoros em diversos trechos da cavidade. Este conduto confinado apresenta o piso ascendente composto por sedimentos finos, com poucos blocos e muitas raízes (Figura 88). Este setor apresenta uma elevada umidade além de alguns pequenos depósitos de guano de morcegos frugívoros. De forma geral, o teto é revestido por Actinomicetos e a cavidade apresenta uma elevada estabilidade ambiental. Durante a estação úmida foram observados inúmeros pontos de gotejamentos, além de um aumento significativo na umidade relativa do ar. Além disso, próximo a uma das entradas da cavidade, observamos uma escavação realizada para estudos arqueológicos onde foram abandonados alguns equipamentos de trabalho (Figura 144).

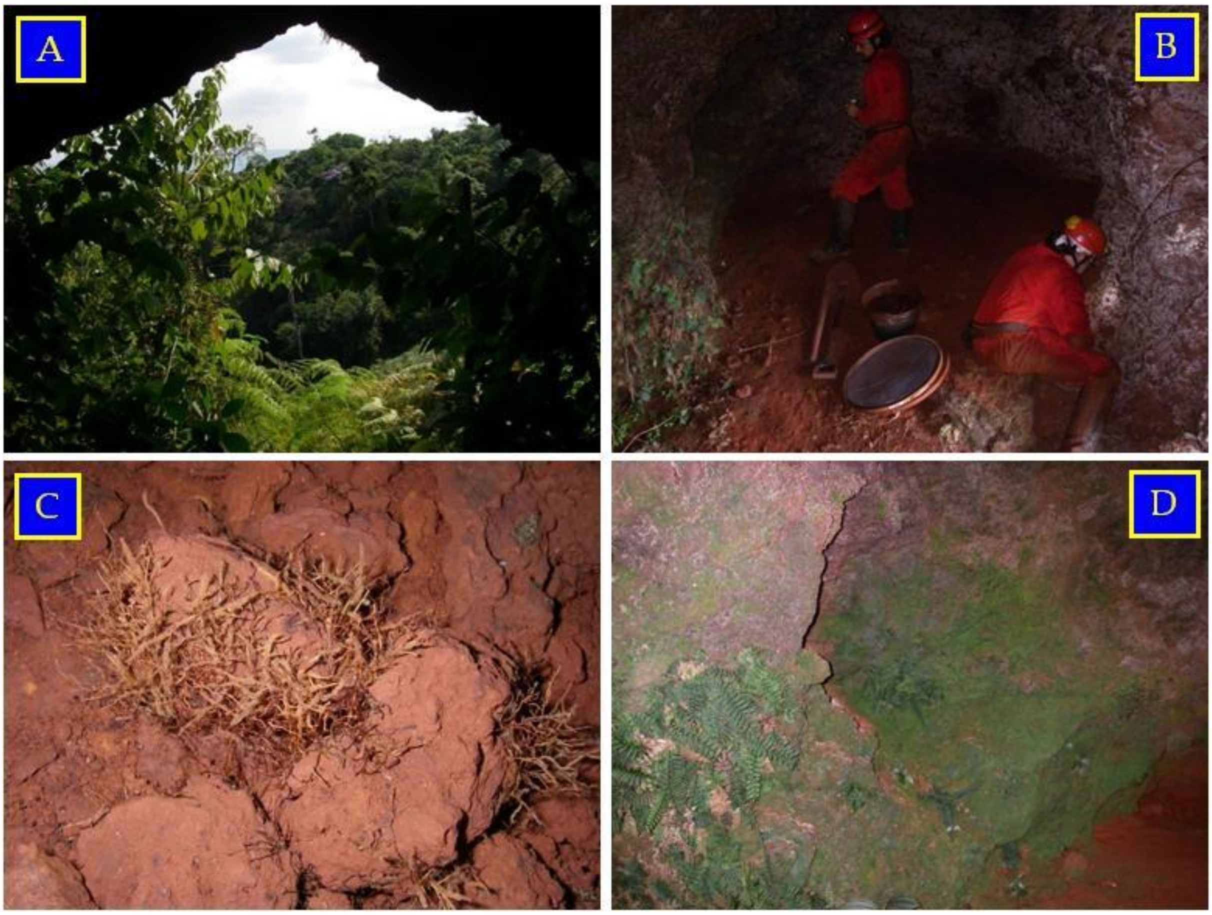

Figura 144 - a) Aspecto geral da entrada da cavidade onde é possível observar a vegetação externa; b) Paredes e piso revestidos de líquens, briófitas e Actinomicetos; c) Sistema radicular apresentando-se de forma superficial nesta cavidade; d) Grande quantidade de algas, briófitas e pteridófitas nas proximidades de uma das entradas.

5.4.4.32.2. Caracterização faunística no período de seca

Foi observado na caverna, um total de 71 morfoespécies de invertebrados de pelo menos 51 famílias das Ordens: Gastropoda (Subulinidae, Systrophiidae), Isopoda (Philosciidae), Acari (Ixodidae: *Amblyomma* sp.; Laelapidae: *Stratiolaelaps* sp., Macronyssidae sp., Oribatida sp., Eupodidae: *Linopodes* sp., Labdostomatidae: *Labdostomatida* sp., Trombidiforme), Amblypygi (Phrynidae: *Heterophrynus longicornis*, Charinidae: *Charinus* sp.), Ricinulei (Ricinoididae: *Cryptocellus tarsilae*), Pseudoscorpiones (Chernetidae sp., Chtoniidae), Opiliones (Escadabiidae), Araneae (Gnaphosidae, Ochyroceratidae, Salticidae, Scytodidae: *Scytodes eleonora*, Pholcidae: *Mesabolivar* sp., Theridiidae sp., Theridiosomatidae), Thysanura (Nicoletiidae: Atelurinae), Diplura (Campodeidae, Parajapygidae), Collembola (Sminthuridae, Cyphoderidae, Entomobryidae, Paronellidae), Orthoptera (Phalangopsidae: *Phalangopsis* sp.), Blattodea (Blattidae, Polyphagidae), Isoptera, Psocoptera (Ptiloneuridae: *Ptiloneura* sp.), Hemiptera (Cydnidae, Dipsocoridae, Ochteridae), Homoptera (Cixiidae: *Cixius* sp.), Lepidoptera (Drepanidae: Thyatirinae, Noctuidae, Tineidae), Diptera (Ceratopogonidae, Dolichopodidae, Faniidae, Psychodidae: *Lutzomyia* sp., Sciaridae), Hymenoptera (Formicidae:

Camponotus sp., *Carebara* sp., *Gnamptogenys* sp., *Pachycondyla* sp.), Coleoptera (Carabidae: *Coarazuphium* sp.; Curculionidae: Scotylinae; Scydmaenidae), Diplopoda (Pyrgodesmidae), Scolopendromorpha (Cryptopidae; *Cryptops* sp.).

Dentre os vertebrado foi encontradas quatro espécies das Ordens: Chiroptera (Emballonuridae: *Peropteryx kappleri*, Phyllostomidae: *Glossophaga soricina*), Anura (Leptodactylidae: *Pristimantis* cf. *fenestratus*, Bufonidae).

Desta forma, no total foram encontradas 75 morfoespécies, das quais oito espécies foram consideradas troglomórficas: Gastropoda (Systrophiidae), Amblypygi (Charinidae: *Charinus* spp.), Araneae (Ochyroceratidae), Thysanura (Nicoletiidae: Atelurinae), Collembola (Cyphoderidae), Coleoptera (Carabidae: *Coarazuphium* sp.), Diplopoda (Pyrgodesmidae).

5.4.4.32.3. Caracterização faunística no período de chuva

Foi observado na caverna, um total de 76 morfoespécies de invertebrados de pelo menos 55 famílias das Ordens: Oligochaeta, Gastropoda (Subulinidae, Systrophiidae), Isopoda (Armadillidae, Balloniscidae, Philosciidae), Acari (Laelapidae: Stratiolaelaps sp., Macronyssidae, Oribatida, Eupodidae: Linopodes sp., Labdostomatidae: Labdostomatida sp., Rhagidiidae, Trombidiforme), Amblypygi (Phrynidae: Heterophrynus longicornis, Charinidae: Charinus sp.), Pseudoscorpiones (Chernetidae, Chtoniidae), Opiliones (Cosmetidae: Anduzeia sp., Escadabiidae, Phalangiidae), Araneae (Gnaphosidae, Linyphiidae, Ochyroceratidae, Oonopidae: Oonopinae, Salticidae, Scytodidae*: Scytodes eleonora*, Pholcidae: Mesabolivar sp., Theridiosomatidae), Diplura (Anajapygidae, Campodeidae), Collembola (Sminthuridae, Cyphoderidae, Entomobryidae), Orthoptera (Phalangopsidae: Phalangopsis sp.), Orthoptera (Blattellidae, Polyphagidae), Psocoptera (Epipsocidae, Myopsocidae: Lichenomina sp.), Hemiptera (Cydnidae, Lygaeidae), Lepidoptera (Noctuidae, Tineidae, Geometridae), Diptera (Psychodidae), Hymenoptera (Apterostigma sp., Pachycondyla sp., Braconidae, Elasmidae), Coleoptera (Carabidae: Coarazuphium sp., Pselaphidae, Scydmaenidae, Staphylinidae), Diplopoda (Pyrgodesmidae, Polydesmidae), Geophilomorpha (Ballophilidae: Taeniolinum sp.), Scolopendromorpha (Cryptopidae: Cryptopinae, Scolopocryptopidae: *Dinocryptops* sp., Newportia sp.), Coleoptera (Elateridae, Staphylinidae).

Dentre os vertebrado foi encontradas duas espécies de Chiroptera (Phyllostomidae: *Anoura* sp., *Glossophaga soricina*).

Desta forma, no total foram encontradas 78 morfoespécies, das quais oito espécies foram consideradas troglomórficas: Gastropoda (Systrophiidae), Acari (Rhagidiidae, Trombidiforme), Amblypygi (Charinidae: *Charinus* spp.), Araneae (Ochyroceratidae), Collembola (Cyphoderidae), Coleoptera (Carabidae: *Coarazuphium* sp.) e Diplopoda (Pyrgodesmidae).

5.4.4.32.4. Caracterização geral da fauna da cavidade

Foi observado na caverna, um total de 119 morfoespécies de invertebrados de pelo menos 72 famílias das Ordens: Oligochaeta, Gastropoda (Subulinidae, Systrophiidae), Isopoda (Armadillidae, Balloniscidae, Philosciidae), Acari (Ixodidae: *Amblyomma* sp., Laelapidae: *Stratiolaelaps* sp., Macronyssidae, Oribatida, Eupodidae: *Linopodes* sp., Labdostomatidae: *Labdostomatida* sp., Rhagidiidae, Trombidiforme), Amblypygi (Phrynidae: *Heterophrynus longicornis*, Charinidae: *Charinus* sp.), Ricinulei (Ricinoididae: *Cryptocellus tarsilae*), Pseudoscorpiones (Chernetidae, Chtoniidae), Opiliones (Cosmetidae: *Anduzeia* sp., Escadabiidae, Phalangiidae), Araneae (Gnaphosidae, Linyphiidae, Ochyroceratidae, Oonopidae: Oonopinae, Salticidae, Scytodidae*: Scytodes eleonora*, Pholcidae: *Mesabolivar* sp., Theridiidae, Theridiosomatidae), Thysanura (Nicoletiidae: Atelurinae), Diplura (Anajapygidae, Campodeidae, Parajapygidae), Collembola (Sminthuridae, Cyphoderidae, Entomobryidae, Paronellidae), Orthoptera (Phalangopsidae: *Phalangopsis* sp.), Blattodea (Blattellidae, Blattidae, Polyphagidae), Isoptera, Psocoptera (Epipsocidae, Myopsocidae: *Lichenomina* sp., Ptiloneuridae: *Ptiloneura* sp.), Hemiptera (Cydnidae, Dipsocoridae, Lygaeidae, Ochteridae), Homoptera (Cixiidae: *Cixius* sp.), Lepidoptera (Drepanidae: Thyatirinae, Geometridae, Noctuidae, Tineidae), Diptera (Ceratopogonidae, Dolichopodidae, Fanniidae, Psychodidae: *Lutzomyia* sp., Sciaridae), Hymenoptera (Formicidae: *Apterostigma* sp., *Camponotus* sp., *Carebara* sp., *Gnamptogenys* sp., *Pachycondyla* sp.; Braconidae; Elasmidae), Coleoptera (Carabidae: *Coarazuphium* sp., Curculionidae: Scotylinae, Elateridae, Pselaphidae, Scydmaenidae, Staphylinidae), Diplopoda (Pyrgodesmidae, Polydesmidae), Geophilomorpha (Ballophilidae: *Taeniolinum* sp.) e Scolopendromorpha (Cryptopidae: Cryptopinae, *Cryptops* sp.; Scolopocryptopidae: *Dinocryptops* sp., *Newportia* sp.)

Dentre os vertebrados, foram encontradas cinco morfoespécies das seguintes ordens: Chiroptera (Emballonuridae: *Peropteryx kappleri*; Phyllostomidae: *Anoura* sp., *Glossophaga soricina),* e Anura (Leptodactylidae: *Pristimantis cf. fenestratus*; Bufonidae*).*

Desta forma, no total foram encontradas 124 morfoespécies, das quais 11 espécies foram consideradas troglomórficas: Gastropoda (Systrophiidae), Acari (Rhagidiidae, Trombidiforme), Amblypygi (Charinidae: *Charinus* spp.), Araneae (Ochyroceratidae spp.), Thysanura (Nicoletiidae: Atelurinae), Collembola (Cyphoderidae), Coleoptera (Carabidae: *Coarazuphium* sp.) e Diplopoda (Pyrgodesmidae). Alguns organismos encontrados nesta caverna são mostrados na Figura 145.

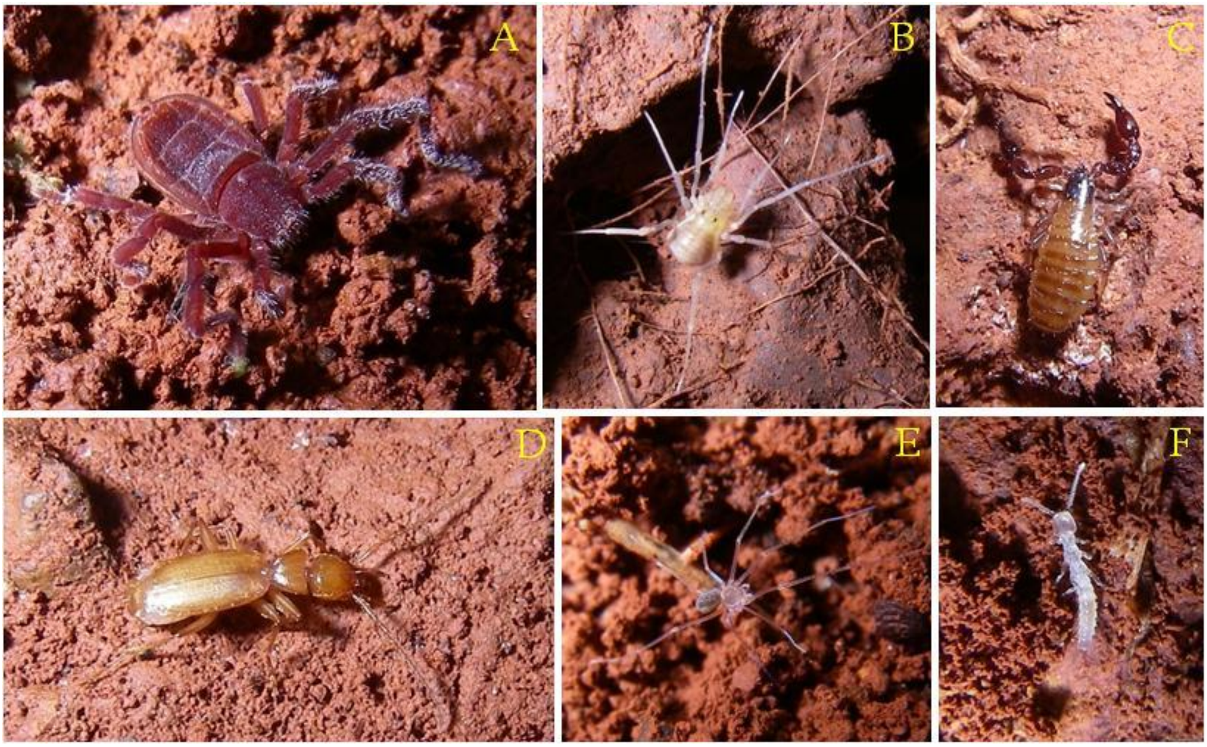

Figura 145 - a) Ricinulei (*Cryptocellus tarsilae*); b) Opiliones (Escadabiidae); c) Pseudoscorpiones; d) Carabidae (*Coarazuphium* sp.); e) Ochyroceratidae; f) Diplura (Anajapygidae).

5.4.4.33. SL-036

5.4.4.33.1. Caracterização trófica

Pequena cavidade formada em canga com 7,2 m de projeção horizontal localizada em área de mata. A vegetação do entorno é muito densa com árvores, lianas e cipós e na parte superior da cavidade existe um predomínio de arbustos associados a uma vegetação rasteira. O acesso a esta cavidade se dá através de uma pequena escalada de aproximadamente 4 m de altura. Sua entrada é ampla e sombreada, com piso ascendente e com muitos líquens, briófitas e brotos de angiospermas distribuídos nas paredes e piso da cavidade. Pouco profunda, não apresenta zona afótica e nem disfótica. Seu piso é composto por sedimento granulado com poucos blocos (seixos e calhaus) esparsos. As paredes são revestidas por Actinomicetos e o sistema de canalículos é pouco desenvolvido. Existem poucas raízes e nenhum depósito de guano foi observado apesar da presença de morcegos da família Emballonuridae e Phyllostomidae (Glossophaginae). Cavidade com baixa estabilidade ambiental, muito suscetível as condições ambientais do meio externo. Nenhuma alteração significativa foi observada durante a estação úmida, além das alterações normais na umidade relativa do ar (Figura 146).

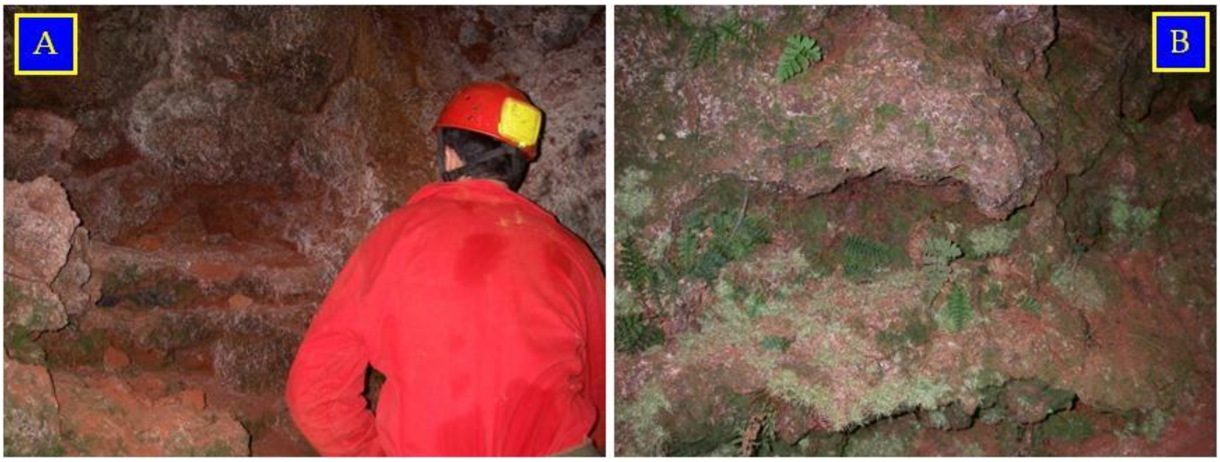

Figura 146 - a) Aspecto geral da cavidade onde é possível observar fungos revestindo as paredes da mesma; b) Paredes e piso revestidos de líquens, briófitas e pteridófitas.

5.4.4.33.2. Caracterização faunística no período de seca

Foi observado na caverna, um total de 27 morfoespécies de invertebrados de pelo menos 25 famílias das Ordens: Isopoda (Armadillidae, Philosciidae), Pseudoscorpiones (Chernetidae, Chtoniidae), Opiliones (Sclerosomatidae: *Prionostemma* sp.), Araneae (Corinidae, Ochyroceratidae, Salticidae, Scytodidae*: Scytodes eleonora*: Pholcidae: *Mesabolivar* sp., Theraphosidae, Theridiosomatidae) Thysanura (Nicoletiidae: Nicoletiinae), Diplura (Anajapygidae), Collembola (Entomobryidae), Blattodea (Polyphagidae), Psocoptera (Myopsocidae: *Lichenomina* sp.), Hemiptera (Cydnidae), Homoptera (Cixiidae: *Cixius* sp.), Lepidoptera (Noctuidae), Diptera (Dolichopodidae, Psychodidae: *Lutzomyia* sp.) Hymenoptera (Formicidae: *Pachycondyla* sp.), Scutigeromorpha (Scutigeridae: *Sphendononema* sp.), Coleoptera (Tenebrionidae: Coelometropinae).

Dentre os vertebrados, foram encontradas duas espécies da ordem Chiroptera (Emballonuridae: *Peropteryx kappleri;* Phyllostomidae*: Carollia* sp.). Desta forma, no total foram encontradas 29 morfoespécies das quais uma foi considerada troglomórfica: Araneae (Ochyroceratidae).

5.4.4.33.3. Caracterização faunística no período de chuva

Foi observado na caverna, um total de 31 morfoespécies de invertebrados de pelo menos 27 famílias das Ordens: Isopoda (Philosciidae), Pseudoscorpiones (Chernetidae, Chtoniidae) Opiliones (Cosmetide: *Anduzeia* sp.*;* Escadabiidae, Stygnidae) Araneae (Araneidae: *Alpaida* sp., Ctenidae, Ochyroceratidae, Oonopidae: Oonopinae, Scytodidae*: Scytodes eleonora*, Pholcidae: *Mesabolivar* sp., Theraphosidae: *Theraphosa blondi*, Theridiosomatidae), Thysanura (Nicoletiidae: Nicoletiinae), Collembola (Entomobryidae, Paronellidae), Orthoptera (Phalangopsidae: *Aclodes* sp.), Blattodea (Blattellidae), Psocoptera (Epipsocidae, Myopsocidae: *Lichenomina* sp.), Hemiptera (Cydnidae, Enicocephalidae), Homoptera

(Cixiidae), Lepidoptera (Noctuidae), Hymenoptera (Formicidae: *Solenopsis* sp.), Coleoptera (Pselaphidae), Geophilomorpha (Geophilidae).

5.4.4.33.4. Caracterização geral da fauna da cavidade

Foi observado na caverna, um total de 47 morfoespécies de invertebrados de pelo menos 25 famílias das Ordens: Isopoda (Armadillidae, Philosciidae), Pseudoscorpiones (Chernetidae, Chtoniidae), Opiliones (Cosmetidae: *Anduzeia* sp.; Escadabiidae, Sclerosomatidae: *Prionostemma* sp. Stygnidae.), Araneae (Araneidae: *Alpaida* sp.; Corinidae, Ctenidae, Ochyroceratidae, Oonopidae: Oonopinae, Salticidae, Scytodidae*: Scytodes eleonora*, Pholcidae: *Mesabolivar* sp., Theraphosidae, Theridiosomatidae), Thysanura (Nicoletiidae: Nicoletiinae), Diplura (Anajapygidae), Collembola (*Entomobryidae*, Paronellidae), Orthoptera (Phalangopsidae: *Aclodes* sp.), Blattodea (Blattelidae, Polyphagidae), Psocoptera (Epipsocidae, Myopsicidade: *Lichenomina* sp.), Hemiptera (Cydnidae, Enicocephalidae), Homoptera (Cixiidae: *Cixius* sp.), Lepidoptera (Noctuidae), Diptera (Dolichopodidae, Psychodidae: *Lutzomyia* sp.), Hymenoptera (Formicidae: *Pachycondyla* sp., *Solenopsis* sp.), Coleoptera (Pselaphidae, Tenebrionidae: Coelometropinae). Geophilomorpha (Geophilidae), Scutigeromorpha (Scutigeridae, *Sphendononema* sp.)

Dentre os vertebrados, foram encontradas duas espécies da ordem Chiroptera (Emballonuridae: *Peropteryx kappleri;* Phyllostomidae: *Carollia* sp.)*.* Desta forma, foram encontradas 49 morfoespécies no total. Das quais, uma foi considerada troglomórfica: Araneae (Ochyroceratidae).

5.4.4.34. SL-037

5.4.4.34.1. Caracterização trófica

Caverna formada em minério de ferro com 23,1 m de desenvolvimento, localizada no topo da encosta, onde existe uma vegetação composta principalmente por samambaias. Na região superior da cavidade existem muitos arbustos associados a uma vegetação rasteira. A cavidade possui duas entradas, estando uma em nível inferior a outra. Ambas são ensolaradas com líquens, briófitas e brotos associados além de muita serrapilheira que se acumula junto à linha d'água e em uma depressão artificial existente logo no início da cavidade. Esta depressão na realidade é uma escavação realizada por garimpeiros onde inclusive foram abandonadas algumas ferramentas de trabalho (Figura 147). O piso é seco e irregular sendo predominantemente ascendente e composto por sedimento com muitos blocos abatidos (seixos e calhaus). Existe alguns pequenos depósitos de guano de morcegos frugívoros na porção mais distal da cavidade produzidos por uma colônia mista de morcegos Glossophaginae (*G. soricina* e *Anoura* sp.) e poucas raízes distribuídas de forma sub-superficial e esparsa. O sistema de canalículos é pouco desenvolvido e a cavidade apresenta

uma baixa estabilidade ambiental uma vez que sua morfologia favorece a uma intensa circulação de ar entre os ambientes epígeo e hipógeo. Durante a estação úmida foram observados alguns pontos de gotejamento o que não alterou a condição de umidade da cavidade que continuava seca mesmo durante o período de chuva.

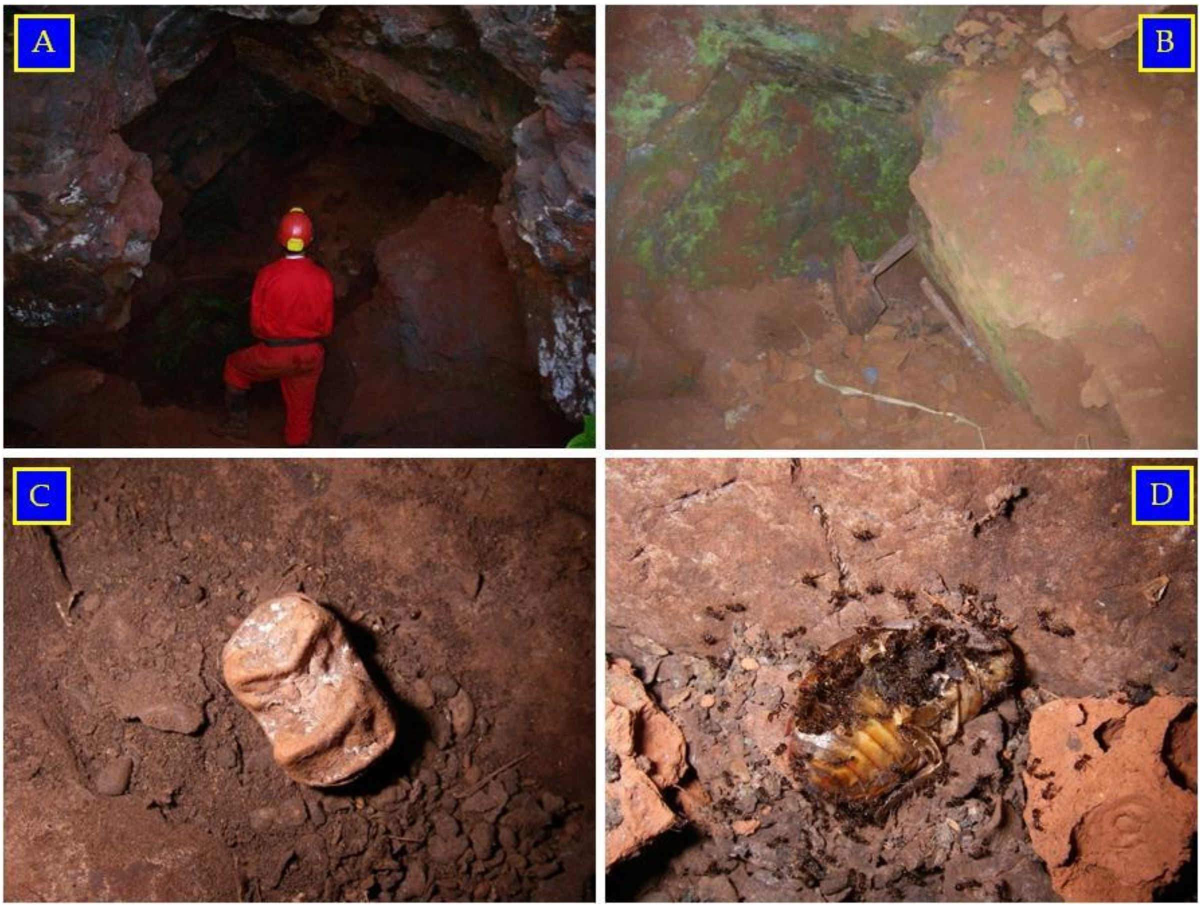

Figura 147 - a) Aspecto geral da entrada da cavidade; b) Escavação realizada por garimpeiros; c) Dente de anta (Tapirus terrestris) encontrado no interior da caverna; d) Cadáver de barata sendo consumido por formigas.

5.4.4.34.2. Caracterização faunística no período de seca

Foi observado na caverna, um total de 65 morfoespécies de invertebrados de pelo menos 45 famílias dos grupos: Nematomorpha (Gordioidea), Isopoda (Armadillidae), Acari (Laelapidae: *Stratiolaelaps* sp.; Macrochelidae: *Macrocheles* sp.; Otopheidomenidae, Uropodina, Oribatida), Amblypygi (Phrynidae: *Heterophrynus longicornis*), Pseudoscorpiones (Chernetidae, Chtoniidae), Opiliones (Cosmetidae: *Anduzeia* sp.; Manaosbiidae) Araneae (Anyphaenidae, Oonopidae: Oonopinae, Salticidae, Scytodidae*: Scytodes eleonora*, Pholcidae: *Metagonia* sp.; Theridiidae), Thysanura (Nicoletiidae: Nicoletiinae), Diplura (Campodeidae), Collembola (Cyphoderidae, Entomobryidae), Orthoptera (Phalangopsidae : *Aclodes* sp., *Phalangopsis* sp.) Blattodea (Blaberidae: *Blaberus* sp.; Blattidae, Polyphagidae), Isoptera (Termitidae: *Nasutitermes* sp.), Psocoptera (Epipsocidae, Psyllipsocidae: *Psyllipsocus*

sp.), Hemiptera (Cydnidae), Homoptera (Cixiidae: *Cixius* sp.), Lepidoptera (Noctuidae, Tineidae), Diptera (Culicidae, Dolichopodidae, Drosophilidae, Milichiidae, Psychodidae: *Lutzomyia* sp., Tipulidae, Fanniidae), Hymenoptera (Formicidae: *Acromyrmex* sp., *Camponotus* sp., *Solenopsis* sp., *Tapinoma* sp., Diapriidae, Vespidae), Coleoptera (Carabidae, Chrysomelidae, Staphylinidae), Neuroptera (Myrmelionthidae).

Dentre os vertebrados, foram encontradas cinco morfoespécies das seguintes ordens: Chiroptera (Phyllostomidae: *Anoura* sp., *Glossophaga soricina),* Squamata *(*Gekkonidae: *Thecadactylus rapicauda*), Anura (Leptodactylidae: *Pristimantis cf. fenestratus).* Desta forma, no total foram encontradas 70 morfoespécies das quais, duas foram consideradas trogomórficas: Nematomorpha (Gordioidea), Collembola (Cyphoderidae).

5.4.4.34.3. Caracterização faunística no período de chuva

Foi observado na caverna, um total de 41 morfoespécies de invertebrados de pelo menos 34 famílias das ordens: Isopoda (Armadillidae), Acari (Laelapidae: *Stratiolaelaps* sp., Macrochelidae: *Macrocheles* sp.), Pseudoscorpiones (Chernetidae, Chtoniidae), Opiliones (Kimmulidae), Araneae (Gnaphosidae, Salticidae, Scytodidae*: Scytodes eleonora*, Pholcidae: *Metagonia* sp., Prodidomidae, Theridiidae), Thysanura (Meinertellidae), Collembola (Cyphoderidae, Entomobryomorpha, Entomobryidae), Orthoptera (Phalangopsidae: *Phalangopsis* sp.), Blattodea (Blaberidae: *Blaberus* sp., Blattidae, Polyphagidae), Psocoptera (Psyllipsocidae: *Psyllipsocus* sp.), Hemiptera (Cydnidae, Lygaeidae), Lepidoptera (Noctuoidea, Noctuidae, Tineidae), Diptera (Culicidae, Drosophilidae, Psychodidae: *Lutzomyia* sp., Ceratopogonidae), Hymenoptera (Formicidae: *Cyphomyrmex* sp., *Solenopsis* sp., Apidae), Coleoptera (Carabidae, Dermestidae, Elateridae: Elaterinae).

Dentre os vertebrados, foram encontradas quatro morfoespécies das seguintes ordens: Chiroptera (Emballonuridae: *Peropteryx kappleri;* Phyllostomidae: *Glossophaga soricina),* Anura *(*Leptodactylidae: *Pristimantis cf. fenestratus,* Bufonidae). Totalizando, portanto, 45 morfoespécies.

5.4.4.34.4. Caracterização geral da fauna da cavidade

Foi observado na caverna, um total de 85 morfoespécies de invertebrados de pelo menos 54 famílias dos grupos: Nematomorpha (Gordioidea), Isopoda (Armadillidae), Acari (Laelapidae: *Stratiolaelaps* sp.; Macrochelidae: *Macrocheles* sp.; Otopheidomenidae, Uropodina, Oribatida), Amblypygi (Phrynidae: *Heterophrynus longicornis*), Pseudoscorpiones (Chernetidae, Chtoniidae), Opiliones (Cosmetidae: *Anduzeia* sp.; Kimmulidae, Manaosbiidae), Araneae (Anyphaenidae, Gnaphosidae, Oonopinae, Salticidae, Scytodidae*: Scytodes eleonora*, Pholcidae: *Metagonia* sp.; Prodidomidae, Theridiidae), Thysanura (Nicoletiidae: Nicoletiinae, Meinertellidae), Diplura (Campodeidae), Collembola (Cyphoderidae, Entomobryomorpha, Entomobryidae), Orthoptera (Phalangopsidae: *Aclodes*

sp., *Phalangopsis* sp.), Blattodea (Blaberidae: *Blaberus* sp.; Blattidae, Polyphagidae), Isoptera (Termitidae: *Nasutitermes* sp.), Psocoptera (Epipsocidae, Psyllipsocidae: *Psyllipsocus* sp.), Hemiptera (Cydnidae, Lygaeidae), Homoptera (Cixiidae: *Cixius* sp.), Lepidoptera (Noctuoidea, Noctuidae, Tineidae), Diptera (Ceratopogonidae, Culicidae, Dolichopodidae, Drosophilidae, Fanniidae, Milichiidae, Psychodidae: *Lutzomyia* sp.; Tipulidae), Hymenoptera (Formicidae: *Acromyrmex* sp., *Camponotus* sp., *Cyphomyrmex* sp., *Solenopsis* sp., *Tapinoma* sp.; Apidae, Diapriidae, Vespidae), Coleoptera (Carabidae, Chrysomelidae, Dermestidae, Elateridae: Elaterinae; Staphylinidae), Neuroptera (Myrmelionthidae).

Dentre os vertebrados, foram encontrados seis morfoespécies: Chiroptera (Emballonuridae: *Peropteryx kappleri;* Phyllostomidae: *Anoura* sp., *Glossophaga soricina),* Squamata (Gekkonidae: *Thecadactylus rapicauda),* Anura (Leptodactylidae: *Pristimantis cf. fenestratus;* Bufonidae). Desta forma, no total foram encontradas 92 morfoespécies das quais, duas foram consideradas troglomórficas: Nematomorpha (Gordioidea), Collembola (Cyphoderidae). Alguns organismos encontrados nesta caverna são mostrados na Figura 148.

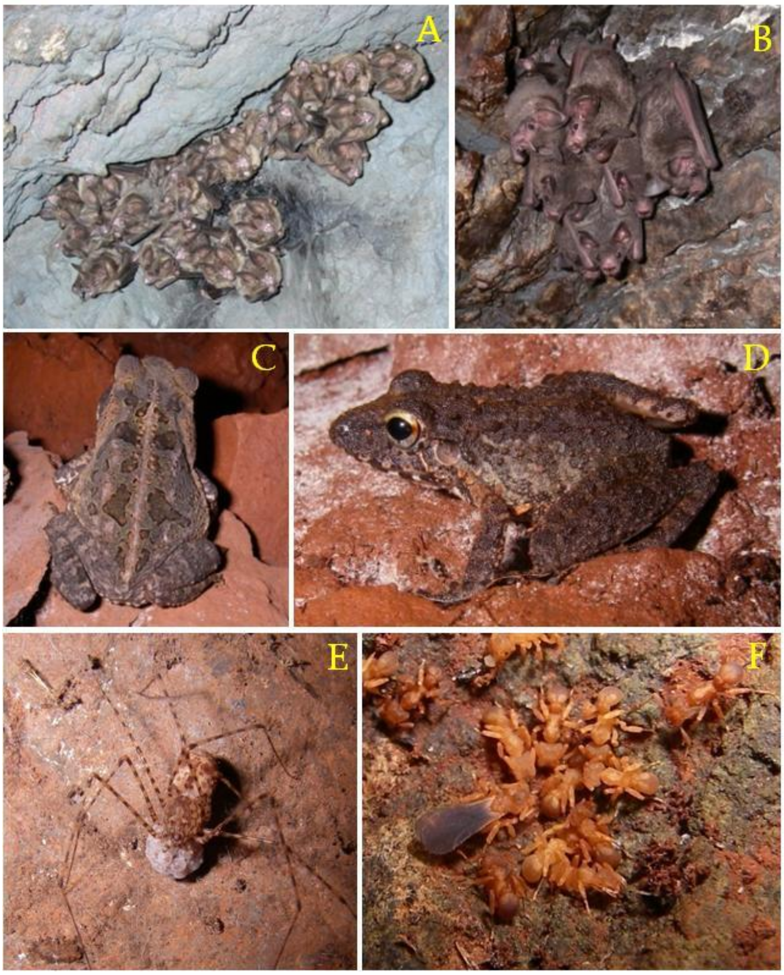

Figura 148 - a) Phyllostomidae (*Carollia* sp.); b) Phyllostomidae (*Carollia* sp.); c) Anura (Bufonidae indet.); d) Leptodactylidae (*Pristimantis cf. fenestratus*); e) Scytodidae*: Scytodes eleonora*; f) Formicidae (*Cyphomyrmex* sp).

5.4.4.35. SL-038

5.4.4.35.1. Caracterização trófica

Pequena caverna formada em minério de ferro com 9,7 m de desenvolvimento, localizada no topo da encosta, onde existe uma vegetação composta principalmente por samambaias. Na região superior da cavidade existem muitos arbustos associados a uma vegetação rasteira. Caverna composta basicamente por dois condutos retilíneos e paralelos que se conectam em uma das extremidades. Com três entradas, sendo uma em situação oposta as outras, a cavidade não apresenta zona afótica e possui uma baixa estabilidade ambiental sendo muito influenciada pelas condições ambientais epígeas. Sua entrada principal apresenta teto baixo onde existem muitos líquens, briófitas e pouca serrapilheira acumulada junto à linha d'água e esparsa pelo piso da cavidade (Figura 149). O piso é descendente e composto por sedimento granulado com alguns blocos arrastados e empilhados pela água da chuva. O sistema radicular é bem desenvolvido, sendo este composto por muitas raízes que em conjunto com a serrapilheira são as principais fontes de recurso deste sistema. O sistema de canalículos é pouco desenvolvido, não existem pontos de gotejamento e nem depósitos de guano. Nenhuma alteração significativa foi observada durante a estação úmida, além das alterações normais na umidade relativa do ar.

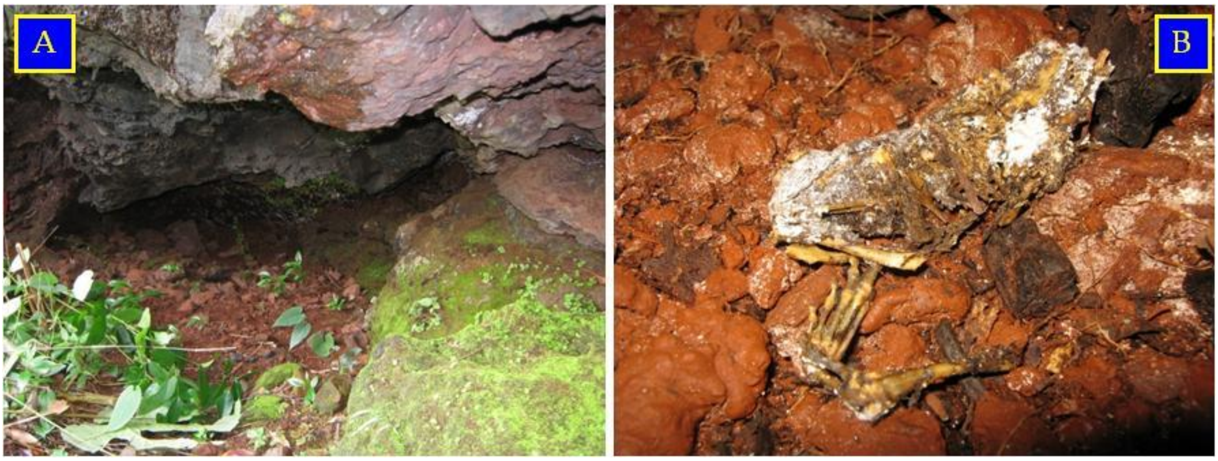

Figura 149 - a) Aspecto geral da entrada da cavidade onde é possível observar a vegetação externa; b) Cadáver de anuro em elevado estado de decomposição. Reparar o intenso crescimento fúngico.

5..4.4.35.2. Caracterização faunística no período de seca

Foi observado na caverna, um total de 15 morfoespécies de invertebrados de pelo menos 09 famílias das Ordens: Pseudoscorpiones, Araneae (Corinidae: *Tupirina* sp.; Salticidae, Scytodidae*: Scytodes eleonora*, Pholcidae: *Metagonia* sp.; Theridiidae), Orthoptera (Phalangopsidae: *Aclodes* sp.), Psocoptera, Hemiptera (Cydnidae), Homoptera (Cixiidae: *Cixius* sp.), Hymenoptera (Formicidae: *Gnamptogenys* sp., *Odontomachus* sp., *Tapinoma* sp.).

Dentre os vertebrados, foram encontradas duas morfoespécies: Chiroptera (Emballonuridae: *Peropteryx kappleri*), Squamata (Colubridae: *Oxybelis* sp.). Totalizando, desta forma, 17 morfoespécies.

5.4.4.35.3. Caracterização faunística no período de chuva

Foi observado na caverna, um total de 37 morfoespécies de invertebrados de pelo menos 27 famílias das Ordens: Oligochaeta, Acari (Oribatida), Palpigradi (Eukoenennidae: *Eukoenennia* sp.), Pseudoscorpiones, Opiliones (Stygnidae, Phalangiidae), Araneae (Ochyroceratidae, Scytodidae*: Scytodes eleonora*, Pholcidae), Diplura (Campodeidae), Colembolla (Entomobryidae), Isoptera, Psocoptera (Epipsocidae), Hemiptera (Cydnidae, Lygaeidae, Pyrrhocoridae), Homoptera (Cixiidae: *Cixius* sp.), Lepidoptera (Noctuidae, Tineidae), Diptera (Cecidomyiidae, Psychodidae: *Lutzomyia* sp.), Hymenoptera (Formicidae: *Camponotus* sp., *Pheidole* sp., *Tapinoma* sp.; Apidae, Tiphiidae), Glomeridesmida (Glomeridesmidae: *Glomeridesmus* sp.), Stemmiulida (*Stemmiulidae* sp.), Scolopendromorpha (Cryptopidae: *Cryptops* sp.; Scolopocryptopidae: *Newportia* sp.), Scutigeromorpha (Scutigeridae: *Sphendononema* sp.), Symphyla (Scutigerellidae: *Hanseniella* sp.).

Dentre os vertebrados, foi encontrada uma espécie de Chiroptera (Phyllostomidae*: Carollia* sp.).

5.4.4.35.4. Caracterização geral da fauna da cavidade

Foi observado na caverna, um total de 46 morfoespécies de invertebrados de pelo menos 30 famílias das Ordens: Oligochaeta, Acari (Oribatida), Palpigradi (Eukoenennidae: *Eukoenennia* sp.), Pseudoscorpiones, Opiliones (Stygnidae, Phalangiidae), Araneae (Corinidae: *Tupirina* sp.; Ochyroceratidae, Salticidae, Scytodidae*: Scytodes eleonora*, Pholcidae: *Metagonia* sp.; Theridiidae), Diplura (Campodeidae), Collembola (Entomobryidae), Orthoptera (Phalangopsidae: *Aclodes* sp.), Isoptera, Psocoptera (Epipsocidae), Hemiptera (Cydnidae, Lygaeidae, Pyrrhocoridae), Homoptera (Cixiidae: *Cixius* sp.), Lepidoptera (Noctuidae, Tineidae), Diptera (Cecidomyiidae, Psychodidae: *Lutzomyia* sp.), Hymenoptera (Formicidae: *Camponotus* sp., *Gnamptogenys* sp., *Odontomachus* sp., *Pheidole* sp., *Tapinoma* sp.; Apidae, Tiphiidae), Glomeridesmida (Glomeridesmidae: *Glomeridesmus* sp.), Stemmiulida (Stemmiulidae), Scolopendromorpha (Cryptopidae: *Cryptops* sp.; Scolopocryptopidae: *Newportia* sp.), Scutigeromorpha (Scutigeridae: *Sphendononema* sp.), Symphyla (Scutigerellidae: *Hanseniella* sp.).

Dentre os vertebrados, foram encontradas três morfoespécies: Chiroptera (Emballonuridae: *Peropteryx kappleri*; Phyllostomidae*: Glossophaga soricina),* Squamata (Colubridae: *Oxybelis* sp.). Totalizando, desta forma, 49 morfoespécies.

5.4.4.36. SL-039

5.4.4.36.1. Caracterização trófica

Caverna formada em minério de ferro com 17 m de desenvolvimento, localizada no topo da encosta, onde existe uma vegetação composta principalmente por samambaias. Na região superior da cavidade existem muitos arbustos associados a uma vegetação rasteira. A cavidade possui duas entradas estreitas sendo estas ensolaradas e com líquens, briófitas e brotos associados além de muita serrapilheira cumulada junto à linha d'água. O piso é seco e irregular com predomínio ascendente e composto por sedimento granulado com muitos blocos abatidos (seixos, calhaus e matacões) dispostos principalmente na região central da cavidade. Não existem depósitos de guano e apenas poucas raízes distribuídas de forma sub-superficial e esparsa. Um grande cupinzeiro associa-se ao salão da caverna (Figura 150). O sistema de canalículos é pouco desenvolvido e a cavidade apresenta uma baixa estabilidade ambiental uma vez que sua morfologia favorece a uma intensa circulação de ar entre os ambientes epígeo e hipógeo. Durante a estação úmida foram observados alguns pontos de gotejamento, mas estes não alteraram a condição de umidade da cavidade.

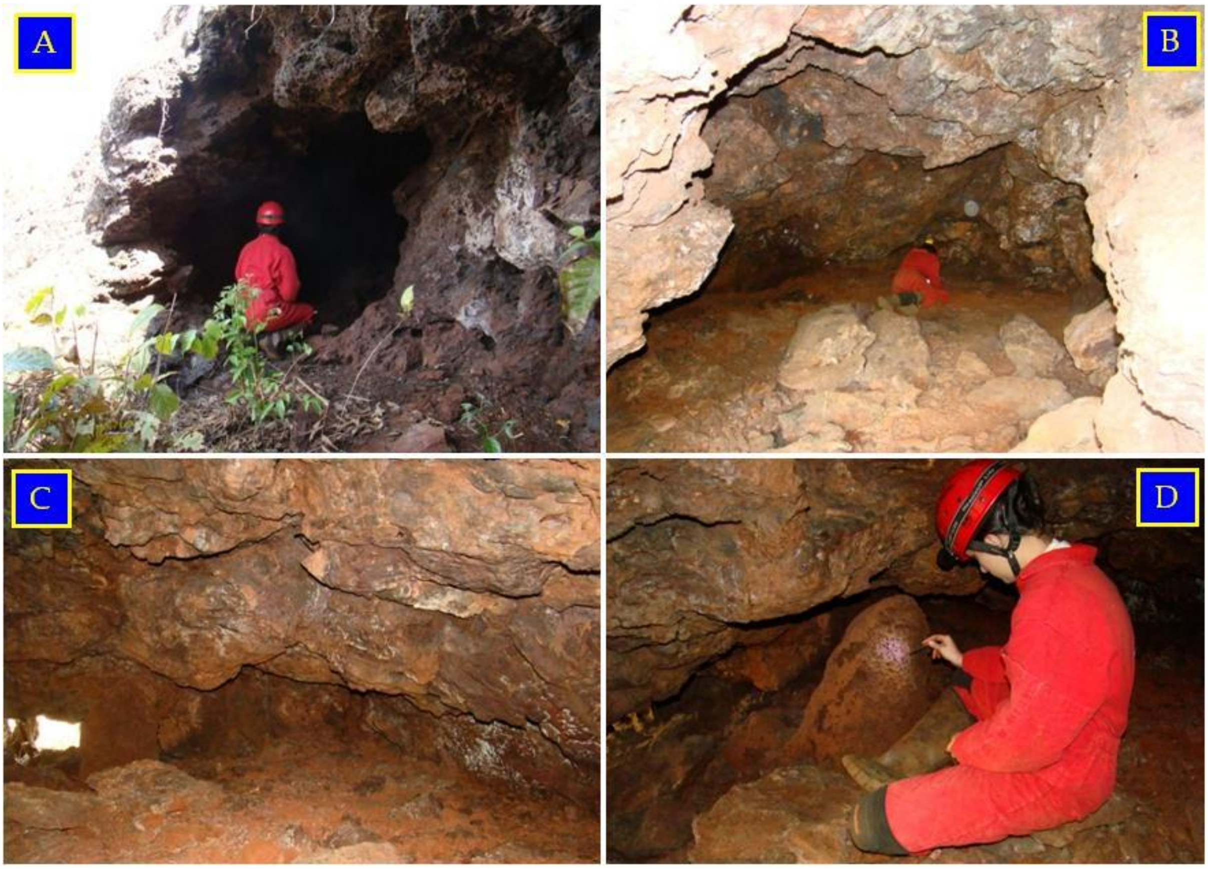

Figura 150 - a) Aspecto geral da entrada da cavidade onde é possível observar a vegetação externa; b) Vista interna da cavidade com blocos abatidos na porção central do salão; c) Detalhe do salão interno; d) Cupinzeiro presente no interior da caverna.

5.4.4.36.2. Caracterização faunística no período de seca

Foi observado na caverna, um total de 23 morfoespécies de invertebrados de pelo menos 19 famílias das Ordens: Isopoda (Armadillidae, Balloniscidae), Acari (Oribatida), Pseudoscorpiones (Chernetidae), Araneae (Salticidae, Scytodidae*: Scytodes eleonora*, Palpimanidae, Pholcidae: *Metagonia* sp.), Thysanura (Nicoletiidae: Nicoletiinae), Collembola (Paronellidae), Blattodea (Blaberidae: *Blaberus* sp.; Blattidae), Isoptera (Termitidae: *Nasutitermes* sp.), Psocoptera (Psyllipsocidae: *Psyllipsocus* sp.), Hemiptera (Cydnidae), Homoptera (Membracidae), Lepidoptera (Noctuidae), Diptera (Dolichopodidae), Hymenoptera (Formicidae: *Tapinoma* sp.), Scolopendromorpha (Scolopocryptopidae: *Newportia* sp.).

Dentre os vertebrados, foi encontrada uma espécie da ordem Anura (Leptodactylidae: *Pristimantis cf. fenestratus).* Totalizando, portanto, 24 morfoespécies.

5.4.4.36.3. Caracterização faunística no período de chuva

Foi observado na caverna, um total de 39 morfoespécies de invertebrados de pelo menos 32 famílias das Ordens: Isopoda (Balloniscidae), Acari (Ixodidae: *Amblyomma* sp.; Trombidiforme), Pseudoscorpiones (Chernetidae, Chtoniidae), Opiliones (Escadabiidae), Araneae (Salticidae, Scytodidae*: Scytodes eleonora*, Pholcidae: *Metagonia* sp.; Theridiidae), Collembola (Entomobryidae), Blattodea (Blaberidae: *Blaberus* sp.; Polyphagidae), Isoptera (Termitidae: *Nasutitermes* sp.), Psocoptera (Epipsocidae, Pachytroctidae: *Tapinella* sp.; Psyllipsocidae: *Psyllipsocus* sp.; Trogiidae), Hemiptera (Cydnidae, Lygaeidae, Reduviidae), Lepidoptera (Noctuidae, Tineidae), Diptera (Culicidae, Milichiidae, Psychodidae: *Lutzomyia* sp.), Hymenoptera (Formicidae: *Pachycondyla* sp., *Pheidole* sp.; Elasmidae, Eulophidae), Coleoptera (Dermestidae, Staphylinidae), Neuroptera (Myrmelionthidae), Scolopendromorpha (Cryptopidae: *Cryptops* sp.)

Dentre os vertebrados, foram encontradas duas espécies de Chiroptera (Emballonuridae*: Peropteryx kappleri*; Phyllostomidae: *Glossophaga soricina).* Desta forma, totalizando 41 morfoespécies.

5.4.4.36.4. Caracterização geral da fauna da cavidade

Foi observado na caverna, um total de 56 morfoespécies de invertebrados de pelo menos 40 famílias das Ordens: Isopoda (Armadillidae, Balloniscidae), Acari (Ixodidae: *Amblyomma* sp.; Oribatida, Trombidiforme), Pseudoscorpiones (Chernetidae, Chtoniidae), Opiliones (Escadabiidae), Araneae (Salticidae, Scytodidae*: Scytodes eleonora*, Palpimanidae, Pholcidae: *Metagonia* sp.; Theridiidae), Thysanura (Nicoletiidae: Nicoletiinae), Collembola (Entomobryidae, Paronellidae), Blattodea (Blaberidae: *Blaberus* sp.; Blattidae, Polyphagidae), Isoptera (Termitidae: *Nasutitermes* sp.), Psocoptera (Epipsocidae, Pachytroctidae: *Tapinella*

sp.; Psyllipsocidae: *Psyllipsocus* sp.; Trogiidae), Hemiptera (Cydnidae, Lygaeidae, Reduviidae), Homoptera (Membracidae), Lepidoptera (Noctuidae, Tineidae), Diptera (Culicidae, Dolichopodidae, Milichiidae, Psychodidae: *Lutzomyia* sp.), Hymenoptera (Formicidae: *Pachycondyla* sp., *Pheidole* sp., *Tapinoma* sp.; Elasmidae, Eulophidae), Coleoptera (Dermestidae, Staphylinidae), Neuroptera (Myrmelionthidae), Scolopendromorpha (Cryptopidae: *Cryptops* sp.; Scolopocryptopidae: *Newportia* sp.)

Dentre os vertebrados, foram encontradas três espécies das ordens: Chiroptera (Emballonuridae*: Peropteryx kappleri*; Phyllostomidae: *Glossophaga soricina), Anura (*Leptodactylidae: *Pristimantis cf. fenestratus*). Desta forma, totalizando 41 morfoespécies.

5.4.4.37. SL-040

5.4.4.37.1. Caracterização trófica

Caverna formada em minério de ferro com 8,1 m de desenvolvimento, localizada no topo da encosta, onde existe uma vegetação composta principalmente por samambaias de grande porte. Na região superior da cavidade existem muitos arbustos associados a uma vegetação rasteira com predomínio de poáceas. Cavidade completamente eufótica em condição muito superficial com apenas uma entrada e composta por dois pequenos condutos retilíneos e paralelos. A entrada é ensolarada e em rastejo com piso descendente com líquens, briófitas e brotos de angiospermas (Melastomatáceas). O piso é seco e inclinado, sendo este composto por sedimento granulado com poucos blocos (seixos e calhaus) esparsos (Figura 151). Toda a extensão da cavidade apresenta musgos no piso e nas paredes e um pouco de serrapilheira esparsa no chão. O sistema de canalículos é pouco desenvolvido e existem raízes no teto da cavidade. A cavidade apresenta uma baixa estabilidade ambiental uma vez que sua morfologia favorece a uma intensa circulação de ar entre os ambientes epígeo e hipógeo. Nenhuma alteração significativa foi observada durante a estação úmida, além das alterações normais na umidade relativa do ar.

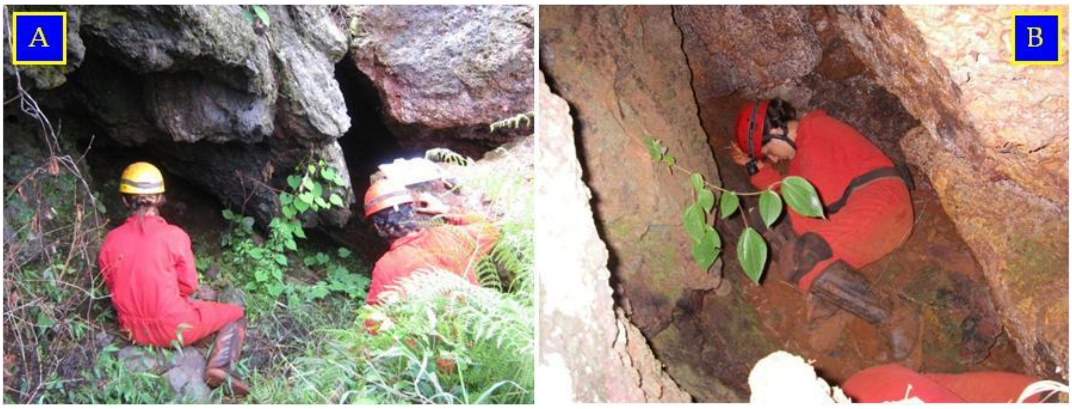

Figura 151 - a) Aspecto geral da entrada da cavidade onde é possível observar a vegetação associada a zona de entrada; b) Detalhe da porção interior da cavidade.

5.4.4.37.2. Caracterização faunística no período de seca

Foi observado na caverna, um total de 36 morfoespécies de invertebrados de pelo menos 25 Famílias distribuídas nas seguintes Ordens: Isopoda (Philosciidae), Acari (Oribatida), Pseudoscorpiones (Chernetidae), Opiliones (Kimmulidae, Manaosbiidae), Araneae (Ochyroceratidae, Salticidae, Scytodidae: *Scytodes eleonora*, Pholcidae: *Mesabolivar* sp.; *Metagonia* sp.), Thysanura (Nicoletiidae: Nicoletiinae), Collembola (Entomobryidae), Orthoptera (Phalangopsidae: *Aclodes* sp.), Isoptera (Termitidae: *Nasutitermes* sp.) Psocoptera (Epipsocidae), Hemiptera (Cydnidae), Homoptera (Cixiidae: *Cixius* sp.), Lepidoptera (Noctuidae: *Latebraria* sp., Tineidae), Diptera (Dolichopodidae, Psychodidae: *Lutzomyia* sp., Tipulidae), Hymenoptera (Formicidae: *Acromyrmex* sp.; *Camponotus* sp.; *Pheidole* sp.; Braconidae, Scelionidae, Vespidae), Coleoptera (Dermestidae, Dytiscidae, Tenebrionidae, Staphylinidae).

Dentre os vertebrados foi encontrada uma espécie de Chiroptera (Emballonuridae: *Peropteryx kappleri*). Desta forma, no total foram encontradas 37 morfoespécies.

5.4.4.37.3. Caracterização faunística no período de chuva

Foi observado na caverna, um total de 31 morfoespécies de invertebrados de pelo menos 24 Famílias distribuídas nas seguintes Ordens: Isopoda (Balloniscidae, Philosciidae, Scleropactidae), Acari (Ixodidae: *Amblyomma* sp., Oribatida, Trombidiforme), Ricinulei (Ricinoididae: *Cryptocellus tarsilae*), Pseudoscorpiones (Chernetidae), Opiliones (Sclerosomatidae: *Prionostemma* sp., Stygnidae: *Eutimesius* sp.), Araneae (Ochyroceratidae, Salticidae, Scytodidae: *Scytodes eleonora*, Pholcidae, Theridiosomatidae), Thysanura (Nicoletiidae: Nicoletiinae), Diplura (Anajapygidae), Collembola (Entomobryidae), Orthoptera (Gryllidae), Lepidoptera (Noctuidae), Diptera (Psychodidae: *Lutzomyia* sp), Hymenoptera (Formicidae: *Cyphomyrmex* sp., *Pachycondyla* sp.), Coleoptera (Pselaphidae, Staphylinidae), Scolopendromorpha (Scolopocryptopidae: *Tidops* sp.).

5.4.4.37.4. Caracterização geral da fauna da cavidade

Foi observado na caverna, um total de 63 morfoespécies de invertebrados de pelo menos 36 Famílias distribuídas nas seguintes Ordens: Isopoda (Balloniscidae, Philosciidae, Scleropactidae), Acari (Ixodidae: *Amblyomma* sp., Oribatida, Trombidiforme), Ricinulei (Ricinoididae: *Cryptocellus tarsilae*), Pseudoscorpiones (Chernetidae), Opiliones (Kimmulidae, Manaosbiidae, Sclerosomatidae: *Prionostemma* sp., Stygnidae: *Eutimesius* sp.), Araneae (Ochyroceratidae, Salticidae, Scytodidae: *Scytodes eleonora*; Pholcidae: *Mesabolivar* sp., *Metagonia* sp.; Theridiosomatidae), Thysanura (Nicoletiidae: Nicoletiinae), Diplura (Anajapygidae), Collembola (Entomobryidae), Orthoptera (Gryllidae, Phalangopsidae: *Aclodes* sp.), Isoptera (Termitidae: *Nasutitermes* sp.), Psocoptera (Epipsocidae), Hemiptera (Cydnidae), Homoptera (Cixiidae: *Cixius* sp.) Lepidoptera (Noctuidae: *Latebraria* sp.,

Tineidae), Diptera (Dolichopodidae, Psychodidae: *Lutzomyia* sp.; Tipulidae), Hymenoptera (Formicidae: *Acromyrmex* sp., *Camponotus* sp., *Cyphomyrmex* sp., *Pachycondyla* sp., *Pheidole* sp.) Coleoptera (Dermestidae, Dytiscidae Pselaphidae, Staphylinidae, Tenebrionidae: Coelometropinae) e Scolopendromorpha (Scolopocryptopidae: *Tidops* sp.).

Dentre os vertebrados foi encontrada uma espécie de Chiroptera (Emballonuridae: *Peropteryx kappleri*). Desta forma, no total foram encontradas 64 morfoespécies na caverna.

5.4.4.38. SL-041

5..4.4.38.1. Caracterização trófica

Caverna formada na canga com 10,1 m de desenvolvimento, localizada no topo da encosta, onde existe uma vegetação composta principalmente por samambaias de grande porte. Na região superior da cavidade existem muitos arbustos associados a uma vegetação rasteira com predomínio de poáceas. Cavidade completamente eufótica em condição muito superficial com apenas uma entrada e composta por um pequeno conduto retilíneo. A entrada é ensolarada e em teto baixo com piso plano e com muitos líquens, briófitas e brotos de angiospermas (Melastomatáceas) revestindo as paredes e o piso. O piso é úmido e inclinado, sendo este composto por sedimento granulado com muitos blocos (seixos e calhaus) esparsos (Figura 152). Toda a extensão da cavidade apresenta musgos e líquens no piso e nas paredes e um pouco de serrapilheira esparsa no chão. O sistema de canalículos é pouco desenvolvido e existem poucas raízes dispostas de forma esparsa. A cavidade apresenta uma baixa estabilidade ambiental uma vez que sua morfologia favorece a uma intensa circulação de ar entre os ambientes epígeo e hipógeo. Durante a estação úmida foram observados inúmeros pontos de gotejamento e percolação, sendo que o piso encontrava-se muito encharcado formando uma grande poça de água em uma cavidade deixada aberta pela equipe de arqueologia.

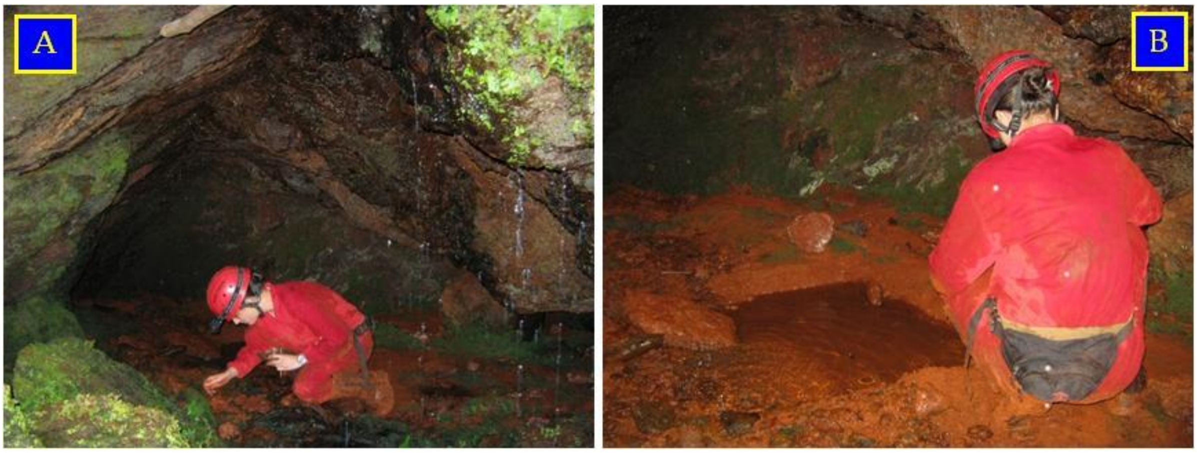

Figura 152 - a) Cavidade com inúmeros pontos de gotejamentos durante a estação úmida; b) Poça de água formada no interior de um "quadrat" de escavação arqueológica durante o período chuvoso.

5.4.4.38.2. Caracterização faunística no período de seca

Foi observado na caverna, um total de 63 morfoespécies de invertebrados de pelo menos 36 Famílias distribuídas nas seguintes Ordens: Isopoda (Balloniscidae, Philosciidae, Scleropactidae), Acari (Ixodidae: *Amblyomma* sp., Oribatida, Trombidiforme), Ricinulei (Ricinoididae: *Cryptocellus tarsilae*), Pseudoscorpiones (Chernetidae), Opiliones (Kimmulidae, Manaosbiidae, Sclerosomatidae: *Prionostemma* sp., Stygnidae: *Eutimesius* sp.), Araneae (Ochyroceratidae, Salticidae, Scytodidae*: Scytodes eleonora*; Pholcidae: *Mesabolivar* sp., *Metagonia* sp.; Theridiosomatidae), Thysanura (Nicoletiidae: Nicoletiinae), Diplura (Anajapygidae), Collembola (Entomobryidae), Orthoptera (Gryllidae, Phalangopsidae: *Aclodes* sp.), Isoptera (Termitidae: *Nasutitermes* sp.), Psocoptera (Epipsocidae), Hemiptera (Cydnidae), Homoptera (Cixiidae: *Cixius* sp.) Lepidoptera (Noctuidae: *Latebraria* sp., Tineidae), Diptera (Dolichopodidae, Psychodidae: *Lutzomyia* sp.; Tipulidae), Hymenoptera (Formicidae: *Acromyrmex* sp., *Camponotus* sp., *Cyphomyrmex* sp., *Pachycondyla* sp., *Pheidole* sp.) Coleoptera (Dermestidae, Dytiscidae Pselaphidae, Staphylinidae, Tenebrionidae: Coelometropinae) e Scolopendromorpha (Scolopocryptopidae: *Tidops* sp.).

Dentre os vertebrados foi encontrada uma espécie de Chiroptera (Emballonuridae: *Peropteryx kappleri*). Desta forma, no total foram encontradas 64 morfoespécies na caverna.

5.4.4.38.3. Caracterização faunística no período de chuva

Foi observado na caverna, um total de 28 morfoespécies de invertebrados de pelo menos 22 Famílias distribuídas nas seguintes Ordens: Acari (Oribatida), Amblypygi (Phrynidae: *Heterophrynus longicornis),* Pseudoscorpiones (Chernetidae), Araneae (Araneidae: *Alpaida* sp.; Dipluridae, Ochyroceratidae, Scytodidae*: Scytodes eleonora*, Theridiosomatidae), Collembola (Isotomidae), Orthoptera (Phalangopsidae: *Aclodes* sp.), Dermaptera (Labiidae), Psocoptera, Hemiptera (Hebridae, Veliidae), Homoptera (Cixiidae: *Cixius* sp.), Lepidoptera (Noctuidae), Diptera (Sciaridae), Hymenoptera (Formicidae: *Acromyrmex* sp., *Gnamptogenys* sp., *Pachycondyla* sp.) Coleoptera (Carabidae, Dytiscidae, Nitidulidae), Diplopoda (Chelodesmidae, Pyrgodesmidae), Glomeridesmida (Glomeridesmidae: *Glomeridesmus* sp).

Dentre os vertebrados foram encontradas duas espécies das Ordens: Chiroptera (Emballonuridae: *Peropteryx kappleri)* e Anura (Leptodactylidae: *Pristimantis cf. fenestratus*). Desta forma foram encontradas, no total, 30 morfoespécies nesta caverna.

5.4.4.38.4. Caracterização geral da fauna da cavidade

Foi observado na caverna, um total de 48 morfoespécies de invertebrados de pelo menos 37 Famílias distribuídas nas seguintes Ordens: Isopoda (Philosciidae), Acari (Oribatida, Eupodidae: *Linopodes* sp.), Amblypygi (Phrynidae: *Heterophrynus longicornis*), Pseudoscorpiones (Chernetidae), Araneae (Araneidae: *Alpaida* sp.; Dipluridae, Ochyroceratidae, Oonopidae: Oonopinae, Salticidae, Scytodidae*: Scytodes eleonora*, Theridiosomatidae), Collembola (Sminthuridae, Entomobryomorpha, Isotomidae), Orthoptera (Phalangopsidae: *Aclodes* sp.), Dermaptera (Labiidae), Psocoptera, Hemiptera (Hebridae, Veliidae), Homoptera (Cixiidae: *Cixius* sp.), Lepidoptera (Noctuidae), Diptera (Calliphoridae, Dolichopodidae, Psychodidae: *Lutzomyia* sp., Sciaridae, Tipulidae), Hymenoptera (Formicidae: *Acromyrmex* sp., *Camponotus* sp., *Dolichoderus* sp., *Gnamptogenys* sp.; Myrmicinae, *Pachycondyla* sp.) Coleoptera (Carabidae, Dytiscidae, Nitidulidae, Pselaphidae, Staphylinidae), Diplopoda (Chelodesmidae, Pyrgodesmidae), Diplopoda (Glomeridesmidae: *Glomeridesmus* sp.) e Symphyla (Scutigerellidae: *Hanseniella* sp.).

Dentre os vertebrados foram encontradas quatro morfoespécies das Ordens: Chiroptera (Emballonuridae: *Peropteryx kappleri)* e Anura (Leptodactylidae: *Pristimantis cf. fenestratus* e *Eleutherodactylus sp.*). Desta forma, foram encontradas, no total, 52 morfoespécies nesta caverna.

5.4.4.39. SL-042

5.4.4.39.1. Caracterização trófica

Caverna formada na canga com 10 m de desenvolvimento, localizada no topo da encosta, onde existe uma vegetação composta principalmente por samambaias de grande porte. Na região superior da cavidade existem muitos arbustos associados a uma vegetação rasteira. Sua entrada é larga e em teto baixo e completamente sombreada por um conjunto denso de samambaias. O piso da entrada é descendente, com muitos blocos abatidos de diferentes tamanhos e com muitos líquens, briófitas e brotos de angiospermas dispostos no piso e paredes da cavidade. Existe muita serrapilheira acumulada junto à linda d’água e também esparsa pelo piso da cavidade sendo esta transportada principalmente pela água durante os períodos de chuva. Caverna com umidade elevada, mas sem pontos de gotejamento durante a estação seca e com o sistema de canalículos bem desenvolvido. O sistema radicular é composto por raízes de diferentes calibres que se encontram distribuídas ao longo de todo o piso da cavidade. As paredes e o teto são revestidos por Actinomicetos e existem pequenos depósitos de guano de morcegos frugívoros na zona de penumbra além de alguns fungos associados à matéria orgânica em decomposição (Figura 153). A cavidade não apresenta zona afótica e possui uma relativa estabilidade ambiental. Durante a estação chuvosa a

cavidade estava muito úmida com inúmeros pontos de gotejamento e percolação, além de algumas pequenas áreas com poças de água.

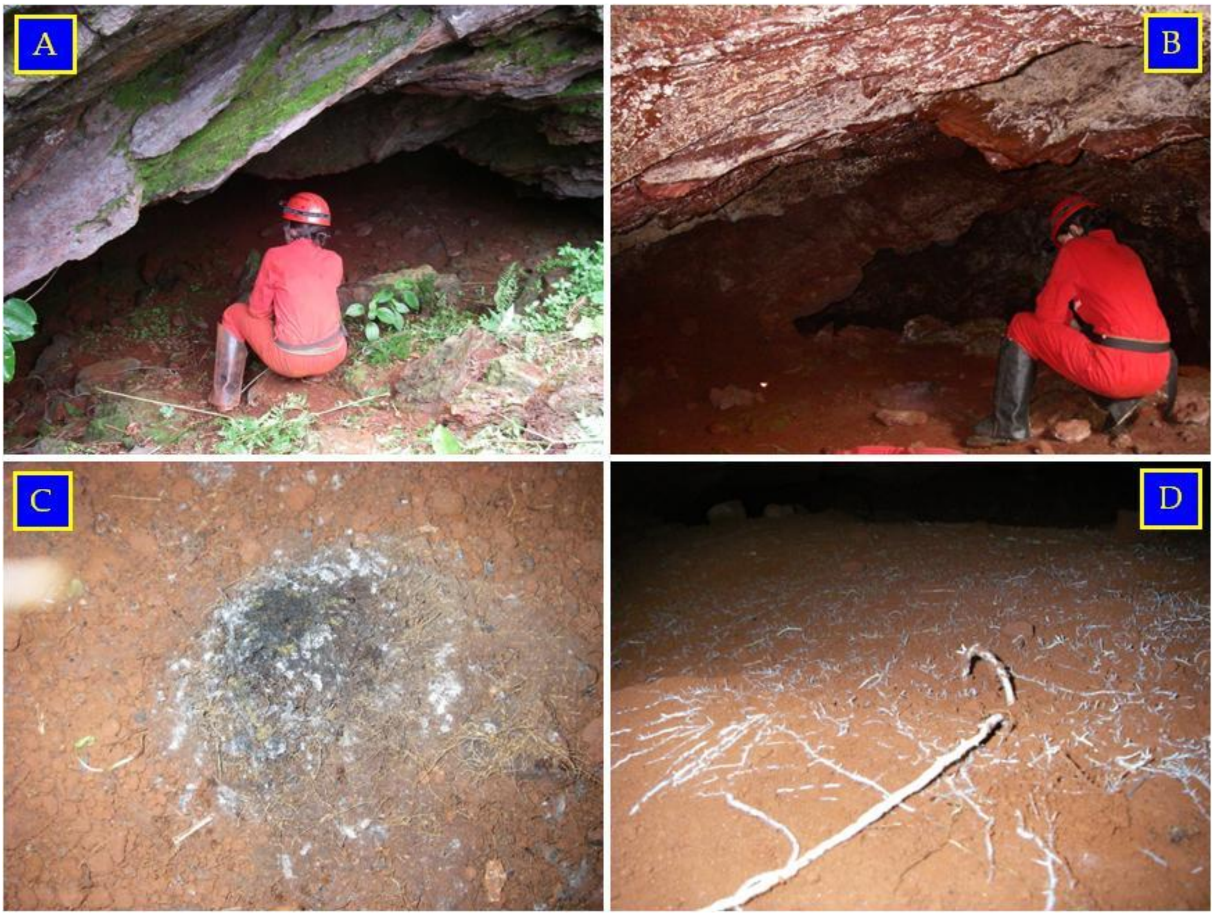

Figura 153 - a) Aspecto geral da vegetação do lado externo da cavidade; b) Aspecto geral da entrada da caverna. Reparar na grande quantidade de colônias de Actinomicetos junto ao teto da cavidade; c) Depósito de guano de morcegos frugívoros com sistema radicular associado; d) Piso da cavidade com sistema radicular superficial bem desenvolvido e com uma intensa associação fúngica.

5.4.4.39.2. Caracterização faunística no período de seca

Foi observado na caverna, um total de 59 morfoespécies de invertebrados de pelo menos 44 Famílias distribuídas nas seguintes Ordens: Gastropoda (Subulinidae, Systrophiidae), Isopoda (Armadillidae, Philosciidae), Acari (Ixodidae: *Amblyomma* sp., Laelapidae: *Stratiolaelaps* sp.; Macronyssidae, Mesostigmata, Uropodina, Oribatida, Trombidiforme), Amblypygi (Phrynidae: *Heterophrynus longicornis*; Charinidae: *Charinus* sp), Pseudoscorpiones (Chernetidae, Chtoniidae), Opiliones (Escadabiidae, Stygnidae: *Protimesius aff. gracilis*), Araneae (Araneidae: *Alpaida* sp.; Gnaphosidae, Ochyroceratidae, Salticidae, Theridiosomatidae), Thysanura (Nicoletiidae: Atelurinae), Diplura (Campodeidae), Collembola (Sminthuridae, Entomobryidae, Entomobryomorpha, Hypogastruridae), Orthoptera (Phalangopsidae: *Aclodes* sp., *Phalangopsis* sp.) Blattodea (Polyphagidae), Isoptera (Termitidae: Nasutitermes), Psocoptera (Epipsocidae), Hemiptera (Cydnidae),

Homoptera (Cixiidae: *Cixius* sp.) Diptera (Ceratopogonidae, Faniidae, Psychodidae: *Lutzomyia* sp.), Hymenoptera (Formicidae: *Acromyrmex* sp., *Camponotus* sp., *Pachycondyla* sp., *Rogeria* sp., *Solenopsis* sp.; Apidae), Coleoptera (Elateridae: Cardiophorinae, Pselaphidae, Ptylidae, Scydmaenidae, Staphylinidae), Diplopoda (Lophoproctidae, Pseudonannolenidae), Scolopendromorpha (Scolopocryptopidae: *Newportia* sp.), Lepidoptera (Tineidae).

Dentre os vertebrados, foram observadas sete morfoespécies das Ordens: Chiroptera (Emballonuridae: *Peropteryx kappleri*, *Anoura* sp.; Phyllostomidae: *Glossophaga soricina*), Anura (*Pristimantis cf. fenestratus*, *Eleutherodactylus* sp.).

No total, foram encontradas 66 morfoespécies sendo que destas, três são consideradas troglomórficas: Amblypygi (Charinidae: *Charinus* sp.) e Diplopoda (Lophoproctidae e Pseudonannolenidae).

5.4.4.39.3. Caracterização faunística no período de chuva

Foi observado na caverna, um total de 71 morfoespécies de invertebrados de pelo menos 52 Famílias distribuídas nas seguintes Ordens: Nematomorpha: (Gordioidea), Oligochaeta, Gastropoda (Subulinidae, Systrophiidae), Isopoda (Armadillidae, Philosciidae), Acari (Neothyridae: *Diplothyrus schubarti*; Laelapidae: *Stratiolaelaps* sp.; Macronyssidae, Oribatida, Anystidae: *Erythracarus* sp.; Trombidiforme), Amblypygi (Phrynidae: *Heterophrynus longicornis*; Charinidae: *Charinus* sp.), Palpigradi (Eukoeneniidae: *Eukoenenia* sp.) Pseudoscorpiones (Chernetidae, Chtoniidae), Opiliones (Escadabiidae, Stygnidae: *Eutimesius* sp.), Araneae (Gnaphosidae, Linyphiidae, Oonopinae: Oonopinae, Scytodidae*:* *Scytodes eleonora*, Pholcidae: *Metagonia* sp.; Theridiosomatidae), Diplura (Anajapygidae, Campodeidae), Collembola (Sminthuridae, Cyphoderidae, Entomobryidae, Hypogastruridae, Tomoceridae), Orthoptera (Phalangopsidae: *Aclodes* sp., *Phalangopsis* sp.), Blattodea (Blattellidae), Isoptera (Termitidae: *Nasutitermes* sp.), Psocoptera (Myopsocidae: *Lichenomina* sp.) Hemiptera (Cydnidae), Homoptera (Cixiidae, Derbidae), Lepidoptera (Noctuidae, Tineidae), Diptera (Ceratopogonidae, Chironomidae, Drosophilidae, Empididae, Psychodidae: *Lutzomyia* sp.; Tephritidae), Coleoptera (Dytiscidae, Elateridae: Cardiophorinae, Staphylinidae), Hymenoptera (Formicidae: *Apterostigma* sp., *Camponotus* sp., *Pachycondyla* sp., *Pheidole* sp.; Apidae, Chalcidoidea), Diplopoda (Chelodesmidae, Glomeridesmidae: *Glomeridesmus* sp.) e Symphyla (Scutigerellidae: *Hanseniella* sp.).

Dentre os vertebrados, foram encontradas três espécies divididas nas seguintes Ordens: Chiroptera (Emballonuridae: *Peropteryx kappleri*, Phyllostomidae: *Glossophaga soricina*) e Anura (*Pristimantis cf. fenestratus*).

No total, foram encontradas 74 morfoespécies sendo que destas, três são consideradas troglomórficas: Nematomorpha (Gordioidea), Gastropoda (Systrophiidae) e Amblypygi (Charinidae: *Charinus* sp.).

5.4.4.39.4. Caracterização geral da fauna da cavidade

Foi observado na caverna, um total de 101 morfoespécies de invertebrados de pelo menos 68 Famílias distribuídas nas seguintes Ordens: Nematomorpha (Gordioidea), Oligochaeta, Gastropoda (Subulinidae, Systrophiidae), Isopoda (Armadillidae, Philosciidae), Acari (Neothyridae: *Diplothyrus schubarti*; Ixodidae: *Amblyomma* sp., Laelapidae: *Stratiolaelaps* sp.; Macronyssidae, Uropodina, Oribatida, Anystidae: *Erythracarus* sp.; Trombidiforme), Amblypygi (Phrynidae: *Heterophrynus longicornis*; Charinidae: *Charinus* sp.), Palpigradi (Eukoeneniidae: *Eukoenenia* sp.), Pseudoscorpiones (Chernetidae, Chtoniidae), Opiliones (Escadabiidae, Stygnidae: *Eutimesius* sp. e *Protimesius aff. gracilis*), Araneae (Araneidae: *Alpaida* sp.; Gnaphosidae, Linyphiidae, Ochyroceratidae, Oonopinae: Oonopinae, Salticidae, Scytodidae*: Scytodes eleonora*, Pholcidae: *Metagonia* sp.; Theridiosomatidae), Thysanura (Nicoletiidae: Atelurinae), Diplura (Anajapygidae, Campodeidae), Collembola (Sminthuridae, Cyphoderidae, Entomobryidae, Entomobryomorpha, Hypogastruridae, Tomoceridae), Orthoptera (Phalangopsidae: *Aclodes* sp., *Phalangopsis* sp.), Blattodea (Blattellidae, Polyphagidae), Isoptera (Termitidae: *Nasutitermes* sp.), Psocoptera (Epipsocidae, Myopsocidae: *Lichenomina* sp.) Hemiptera (Cydnidae), Homoptera (Cixiidae: *Cixius* sp.; Derbidae), Lepidoptera (Noctuidae, Tineidae), Diptera (Ceratopogonidae, Chironomidae Drosophilidae, Empididae, Faniidae, Psychodidae: *Lutzomyia* sp.; Tephritidae), Hymenoptera (Formicidae: *Acromyrmex* sp., *Apterostigma* sp., *Camponotus* sp., *Pachycondyla* sp., *Pheidole* sp., *Rogeria* sp., *Solenopsis* sp.; Apidae, Chalcidoidea) Coleoptera (Dytiscidae, Elateridae: Cardiophorinae, Staphylinidae, Pselaphidae, Ptylidae, Scydmaenidae), Diplopoda (Chelodesmidae, Glomeridesmidae: *Glomeridesmus* sp.; Lophoproctidae, Pseudonannolenidae), Scolopendromorpha (Scolopocryptopidae: *Newportia* sp.) e Symphyla (Scutigerellidae: *Hanseniella* sp.).

Dentre os vertebrados, foram observadas sete espécies das Ordens: Chiroptera (Emballonuridae: *Peropteryx kappleri* e Phyllostomidae: *Anoura* sp., *Glossophaga soricina*) e Anura (Leptodactylidae: *Pristimantis cf. fenestratus*, *Eleutherodactylus* sp., *Leptodactylus labyrinthicus*) sendo que uma espécie de Leptodactylidae não foi identificada.

No total, foram encontradas 108 morfoespécies sendo que destas, cinco são consideradas troglomórficas: Nematomorpha (Gordioidea), Gastropoda (Systrophiidae), Amblypygi (Charinidae: *Charinus* sp.) e Diplopoda (Lophoproctidae e Pseudonannolenidae). Alguns organismos encontrados nesta caverna são mostrados na Figura 154.

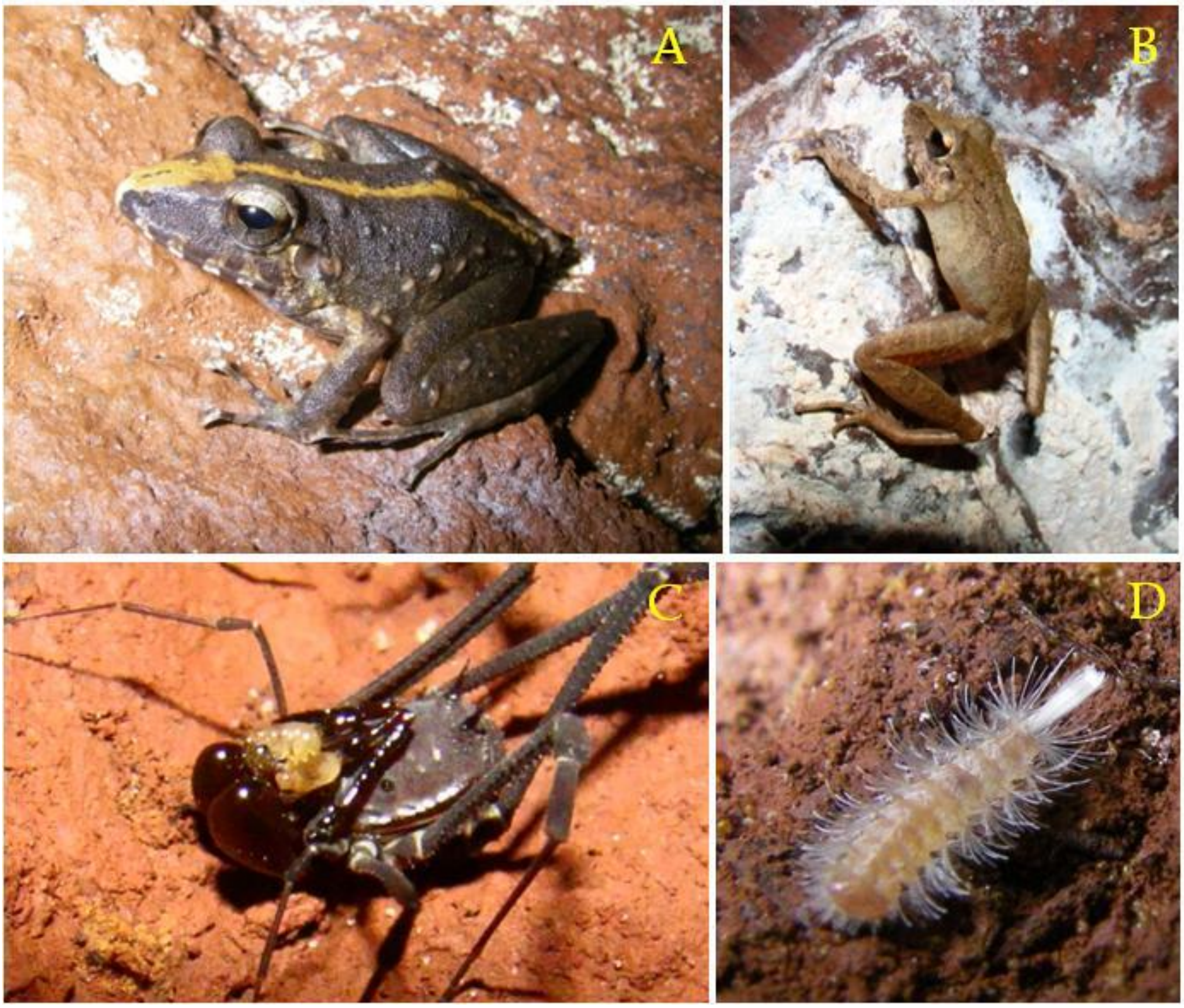

Figura 154 - a) Leptodactylidae (*Eleutherodactylus* sp.); b) Leptodactylidae (*Pristimantis cf. fenestratus*); d) Opiliones (Stygnidae); d) Polyxenida (Lophoproctidae).

5.4.4.40. SL-043

5.4.4.40.1. Caracterização trófica

Caverna formada em minério de ferro com 15,8 metros de desenvolvimento, localizada no topo da encosta, onde existe o predomínio de uma vegetação composta principalmente por arbustos associados a uma vegetação rasteira. Sua entrada é ampla e iluminada com muitos líquens, fungos, briófitas, pteridófitas e brotos de angiospermas sendo estes representados principalmente por Melastomatáceas. Na entrada o piso é levemente ascendente o que dificulta o transporte de matéria orgânica pela água da chuva para o interior da cavidade (Figura 155). A serrapilheira encontra-se acumulada junto à linha d’água e esparsa pelo conduto principal onde é transportada de forma gravitacional e/ou eólica. Entretanto, existe pequeno conduto localizado na margem direita da cavidade onde existem acúmulos de serrapilheira uma vez que o piso é descendente. De forma geral o piso é úmido sendo este composto por sedimento fino com muitos blocos (seixos, calhaus e matacões) abatidos.

Apesar de terem sido observadas duas espécies de morcegos Glossophaginae em um pequeno salão com iluminação disfótica, não foram observados depósitos de guano no interior da cavidade. A cavidade não apresenta zona afótica e nem pontos de gotejamento, mas possui um sistema de canalículos bem desenvolvido além de poucas raízes de diferentes calibres distribuídas de maneira esparsa. Durante a estação chuvosa a cavidade estava muito úmida com inúmeros pontos de gotejamento e percolação.

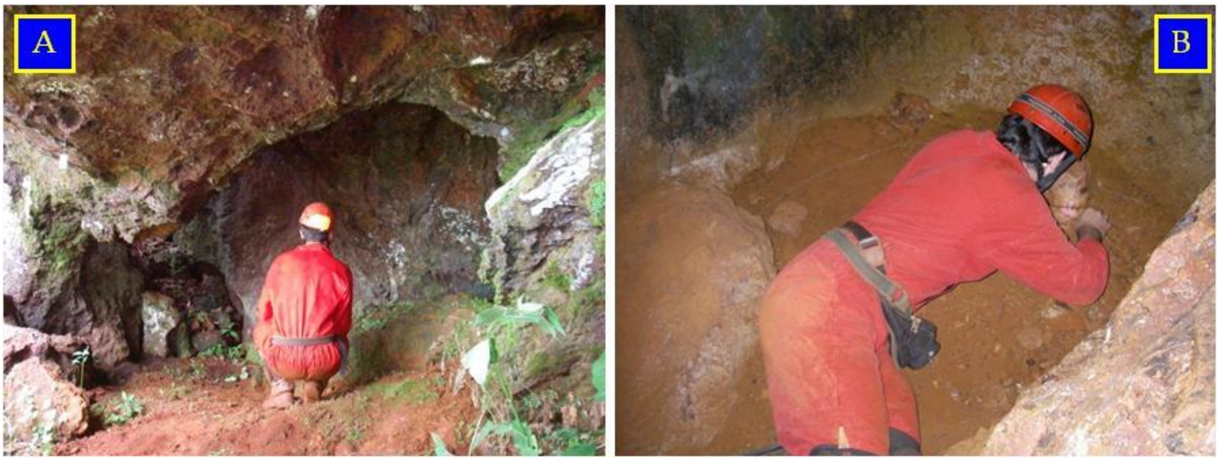

Figura 155 - a) Entrada da cavidade; b) Conduto com piso predominantemente plano composto por sedimento de granulometria fina.

5.4.4.40.2. Caracterização faunística no período de seca

Foi observado na caverna, um total de 52 morfoespécies de invertebrados de pelo menos 44 Famílias distribuídas nas seguintes Ordens: Isopoda (Philosciidae), Acari (Oribatida, Trombidiforme), Amblypygi (Phrynidae: *Heterophrynus longicornis*), Pseudoscorpiones (Chernetidae), Opiliones (Escadabiidae) Araneae (Araneidae: *Alpaida* sp.; Ochyroceratidae, Salticidae, Scytodidae*: Scytodes eleonora*, Pholcidae: *Metagonia* sp.; Theridiidae, Theridiosomatidae, Thomisidae), Thysanura (Nicoletiidae: Nicoletiinae), Collembola (Sminthuridae, Entomobryidae, Tomoceridae), Orthoptera (Phalangopsidae: *Aclodes* sp., *Phalangopsis* sp.) Blattodea (Polyphagidae), Isoptera (Termitidae *Nasutitermes* sp.) Psocoptera (Epipsocidae), Homoptera (Cixiidae: *Cixius* sp.), Lepidoptera (Limacodidae, Noctuidae, Tineidae), Diptera (Ceratopogonidae, Dolichopodidae, Drosophila, Psychodidae: *Lutzomyia* sp.; Tipulidae), Hymenoptera (Formicidae: *Acromyrmex* sp., *Camponotus* sp., *Gnamptogenys* sp., *Pachycondyla* sp.) e Scutigeromorpha (Scutigeridae: *Sphendononema* sp.).

Dentre os vertebrados foram observadas seis espécies das seguintes Ordens: Chiroptera (Emballonuridae: *Peropteryx kappleri*, Phyllostomidae: *Anoura* sp. e *Glossophaga soricina*) e Anura (*Pristimantis cf. fenestratus*, *Eleutherodactylus* sp.) sendo que uma espécie de

Leptodactylidae não foi identificada. No total, foram encontradas 58 morfoespécies sendo que destas, uma foi considerada troglomórfica: Araneae (Ochyroceratidae).

5.4.4.40.3. Caracterização faunística no período de chuva

Foi observado na caverna, um total de 40 morfoespécies de invertebrados de pelo menos 28 Famílias distribuídas nas seguintes Ordens: Oligochaeta, Gastropoda (Subulinidae), Isopoda (Philosciidae), Acari (Oribatida, Trombidiforme), Amblypygi (Phrynidae: *Heterophrynus longicornis*), Pseudoscorpiones (Chernetidae), Opiliones (Kimmulidae), Araneae (Gnaphosidae, Oonopidae: Oonopinae, Theridiosomatidae), Diplura (Campodeidae), Collembola (Sminthuridae, Entomobryidae), Orthoptera (Gryllidae, Phalangopsidae: *Aclodes* sp., Phalangopsis sp.), Psocoptera (Epipsocidae), Hemiptera (Cydnidae), Homoptera (Derbidae), Lepidoptera (Noctuidae, Tineidae), Diptera (Culicidae, Psychodidae: *Lutzomyia* sp.), Hymenoptera (Formicidae: *Camponotus* sp., *Gnamptogenys* sp.), Coleoptera (Ptylidae, Staphylinidae), Diplopoda (Polydesmida, Glomeridesmidae: *Glomeridesmus* sp.), Lithobiomorpha (Henicopiidae: *Lamyctes* sp.), Scolopendromorpha (Cryptopidae: Cryptopinae) e Symphyla (Scutigerellidae: *Hanseniella* sp.).

Dentre os vertebrados foram observadas duas espécies de: Chiroptera (Emballonuridae: *Peropteryx kappleri*) e Anura (Leptodactylidae *Pristimantis cf. fenestratus*). No total, foram encontradas 42 morfoespécies nesta cavidade.

5.4.4.40.4. Caracterização geral da fauna da cavidade

Foi observado na caverna, um total de 78 morfoespécies de invertebrados de pelo menos 45 Famílias distribuídas nas seguintes Ordens: Oligochaeta, Gastropoda (Subulinidae), Isopoda (Philosciidae), Acari (Oribatida, Trombidiforme), Amblypygi (Phrynidae: *Heterophrynus longicornis*), Pseudoscorpiones (Chernetidae), Opiliones (Escadabiidae, Kimmulidae), Araneae (Araneidae: *Alpaida* sp., Gnaphosidae, Ochyroceratidae, Oonopidae: Oonopinae, Salticidae, Scytodidae*: Scytodes eleonora*, Pholcidae: *Metagonia* sp.; Theridiidae, Theridiosomatidae, Thomisidae), Thysanura (Nicoletiidae: Nicoletiinae), Diplura (Campodeidae), Collembola (Sminthuridae, Entomobryidae, Tomoceridae), Orthoptera (Gryllidae, Phalangopsidae: *Aclodes* sp., *Phalangopsis* sp.), Blattodea (Polyphagidae), Isoptera (Termitidae: *Nasutitermes* sp.), Psocoptera (Epipsocidae), Hemiptera (Cydnidae), Homoptera (Cixiidae: *Cixius* sp., Derbidae), Lepidoptera (Limacodidae, Noctuidae, Tineidae), Diptera (Ceratopogonidae, Culicidae, Dolichopodidae, Drosophila, Psychodidae: *Lutzomyia* sp.; Tipulidae), Hymenoptera (Formicidae: *Acromyrmex* sp., *Camponotus* sp., *Gnamptogenys* sp., *Pachycondyla* sp.), Coleoptera (Ptylidae, Staphylinidae), Diplopoda (Polydesmida, Glomeridesmidae: *Glomeridesmus* sp.) Lithobiomorpha (Henicopiidae: *Lamyctes* sp.), Scolopendromorpha (Cryptopidae: Cryptopinae), Scutigeromorpha (Scutigeridae: *Sphendononema* sp.) e Symphyla (Scutigerellidae: *Hanseniella* sp.).

Dentre os vertebrados, foram observadas seis espécies de: Chiroptera (Emballonuridae: *Peropteryx kappleri*, Phyllostomidae: *Anoura* sp., *Glossophaga soricina*) e Anura (Leptodactylidae: *Pristimantis cf. fenestratus* e *Eleutherodactylus* sp.), sendo que uma espécie de Leptodactylidae não foi identificada. No total, foram encontradas 84 morfoespécies, sendo que uma Araneae (Ochyroceratidae) foi considerada troglomórfica.

5.4.4.41. SL-044

5.4.4.41.1. Caracterização trófica

Caverna formada em minério de ferro com 38,3 m de desenvolvimento, localizada de forma muito superficial no topo da encosta, onde existe uma vegetação composta principalmente por samambaias. Na região superior da cavidade existem muitos arbustos associados a uma vegetação rasteira com predomínio de poáceas. A cavidade possui duas entradas sendo uma delas muito ampla e completamente ensolarada onde existem muitos líquens, fungos, briófitas, pteridófitas e brotos de angiospermas (principalmente Melastomatáceas). A outra entrada corresponde a uma pequena clarabóia, localizada em posição oposta à entrada principal, sendo muito estreita e situada a aproximadamente 1,5 m de altura em relação ao solo. A cavidade apresenta um padrão retilíneo representado por um único conduto, sendo este muito amplo e volumoso. O piso é predominantemente plano, úmido, com aproximadamente 50% de sua área coberta por líquens e briófitas e composto por sedimento granulado com blocos (seixos, calhaus e matacões) distribuídos de maneira esparsa. O teto é revestido por Actinomicetos e apresenta uma grande quantidade de canalículos disponíveis para abrigo e dispersão dos organismos cavernícolas (Figura 156). Na zona de entrada existem inúmeros regurgitos de corujas (*Tito Alba*) e nas porções mais profundas observaram-se pequenos depósitos de guano de morcegos frugívoros produzidos por morcegos Glossophaginae (*G. soricina* e *Anoura* sp.). A cavidade não apresenta zona afótica, mas possui uma elevada umidade apesar de não terem sido observados pontos de gotejamento. Em relação ao seu estado de conservação, apesar de seu difícil acesso, existem sinais de acampamento evidenciado pela presença de restos de fogueira e de resíduos sólidos abandonados. Durante a estação chuvosa a cavidade estava muito úmida com inúmeros pontos de gotejamento e percolação, além de algumas pequenas áreas com poças de água.

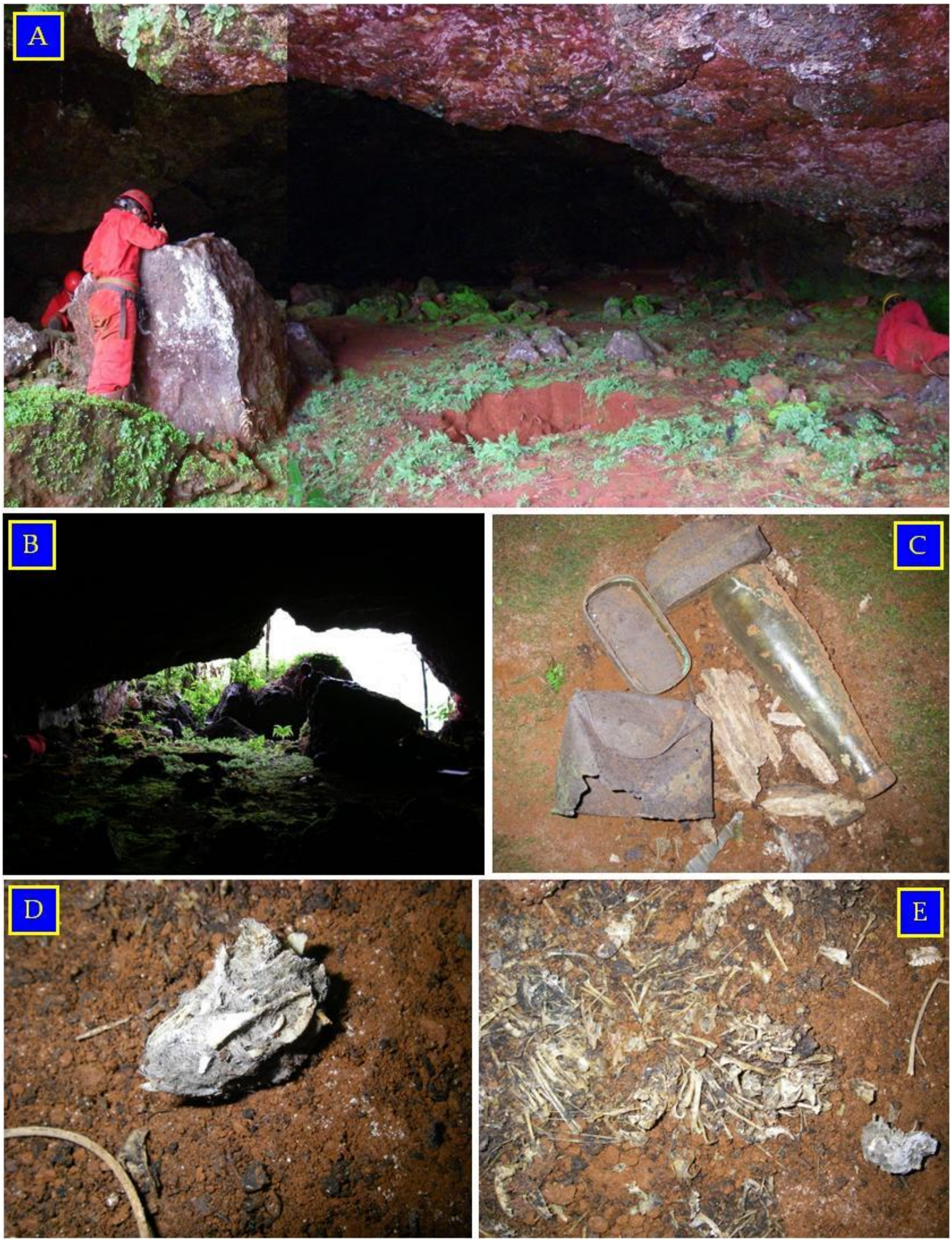

Figura 156 - a) Entrada principal da cavidade onde existem grandes blocos abatidos e muita vegetação recobrindo o piso. b) Vista da entrada pela porção interior da caverna; c) Resíduos sólidos abandonados no interior da cavidade; c) Regurgito de coruja Suindara (Tyto alba); d) Uma das bolotas regurgitadas depois de desfeita por ação do tempo.

5.4.4.41.2. Caracterização faunística no período de seca

Foi observado na caverna, um total de 78 morfoespécies de invertebrados de pelo menos 45 Famílias distribuídas nas seguintes Ordens: Oligochaeta, Gastropoda (Systrophiidae), Isopoda (Armadillidae, Balloniscidae, Philosciidae), Acari (Laelapidae: *Stratiolaelaps* sp., Astigmatina, Labostomatidae: *Labdostomatida* sp., Tenerifidae), Amblypygi (Phrynidae: *Heterophrynus longicornis*), Pseudoscorpiones (Chernetidae, Chtoniidae), Opiliones (Stygnidae: *Protimesius aff. gracilis*), Araneae (Gnaphosidae, Linyphiidae, Oonopidae: Oonopinae, Salticidae, Scytodidae*: Scytodes eleonora*, Pholcidae, Theridiosomatidae), Thysanura (Nicoletiidae: Nicoletiinae), Diplura (Anajapygidae, Campodeidae), Collembola (Sminthuridae, Entomobryidae) Orthoptera (Phalangopsidae: *Aclodes* sp., *Phalangopsis* sp.) Blattodea (Blattidae, Polyphagidae), Psocoptera (Epipsocidae, Myopsocidae: *Lichenomina* sp.), Hemiptera (Cydnidae), Lepidoptera (Noctuidae), Diptera (Bibionidae, Ceratopogonidae, Culicidae, Fanniidae, Phoridae, Psychodidae: *Lutzomyia* sp.; Sciaridae, Tipulidae), Hymenoptera (Formicidae: *Camponotus* sp., *Gnamptogenys* sp., *Labidus* sp., *Pachycondyla* sp.), Coleoptera (Carabidae, Elateridae: Cardiophorinae e Elaterinae, Ptylidae, Scydmaenidae, Staphylinidae), Diplopoda (Chelodesmidae) e Scolopendromorpha (Cryptopidae: *Cryptops* sp.).

Dentre os vertebrados, foram observadas cinco espécies de: Chiroptera (Emballonuridae: *Peropteryx kappleri*, Phyllostomidae: *Glossophaga soricina*), Squamata (Gekkonidae: *Thecadactylus rapicauda*), Anura (Leptodactylidae: *Pristimantis cf. fenestratus*, *Adenomera* sp.). No total, foram encontradas 77 morfoespécies, sendo que uma forma de Coleoptera (Ptylidae) foi considerada troglomórfica.

5.4.4.41.3. Caracterização faunística no período de chuva

Foi observado na caverna, um total de 87 morfoespécies de invertebrados de pelo menos 60 Famílias distribuídas nas seguintes Ordens: Oligochaeta, Gastropoda (Systrophiidae), Isopoda (Dubioniscidae, Philosciidae, Argasidae: *Ornithodoros* sp.; Laelapidae: *Stratiolaelaps* sp.; Oribatida, Trombidiforme), Amblypygi (Phrynidae: *Heterophrynus longicornis*, Charinidae: *Charinus* sp.), Palpigradi (Eukoenennidae: *Eukoenennia* sp.), Ricinulei (Ricinoididae: *Cryptocellus tarsilae*), Pseuscorpiones (Chernetidae, Chtoniidae), Opiliones (Kimmulidae, Stygnidae, Phalangiidae), Araneae (Araneidae: *Alpaida* sp., Gnaphosidae, Linyphiidae, Oonopidae: Oonopinae, Salticidae, Scytodidae*: Scytodes eleonora*, Pholcidae: *Mesabolivar* sp., *Metagonia* sp.; Theraphosidae, Theridiidae, Theridiosomatidae), Diplura (Anajapygidae, Campodeidae), Collembola (Sminthuridae, Entomobryidae), Orthoptera (Phalangopsidae: *Phalangopsis* sp), Blattodea (Blattellidae, Polyphagidae), Psocoptera (Archipsocidae, Epipsocidae, Myopsocidae: *Lichenomina* sp.), Hemiptera (Cydnidae, Lygaeidae, Pyrrhocoridae), Lepidoptera (Noctuidae, Notodontidae, Tineidae), Diptera (Culicidae, Drosophilidae), Hymenoptera (Formicidae: *Apterostigma* sp., *Camponotus* sp.,

Gnamptogenys sp., *Hypoponera* sp., *Pachycondyla* sp.; Braconidae, Scelionidae, Vespidae), Coleoptera (Carabidae, Elateridae: Agryphinae; Pselaphidae, Ptylidae, Staphylinidae, Tenebrionidae: Coelometropinae), Diplopoda (Chelodesmidae, Pyrgodesmidae, Polydesmida), Lithobiomorpha (Henicopiidae: *Lamyctes* sp.), Scolopendromorpha (Cryptopidae: *Cryptops* sp.; Scolopocryptopidae: *Dinocryptops* sp.) e Scutigeromorpha (Scutigeridae: *Sphendononema* sp.).

Dentre os vertebrados foram observadas três espécies de: Chiroptera (Emballonuridae: *Peropteryx kappleri*, Phyllostomidae: *Glossophaga soricina*) e Anura (*Leptodactylus labyrinthicus*).

No total foram encontradas 90 morfoespécies sendo que, destas, cinco foram consideradas troglomórficas: Gastropoda (Systrophiidae), Amblypygi (Charinidae: *Charinus* sp.), Collembola (Isotomidae), Hymenoptera (Formicidae: *Hypoponera* sp.) e Diplopoda (Pyrgodesmidae).

5.4.4.41.4. Caracterização geral da fauna da cavidade

Foi observado na caverna, um total de 132 morfoespécies de invertebrados de pelo menos 74 Famílias distribuídas nas seguintes Ordens: Oligochaeta, Gastropoda (Systrophiidae), Isopoda (Armadillidae, Balloniscidae, Dubioniscidae, Philosciidae), Acari (Argasidae: *Ornithodoros* sp.; Lelapidae: *Stratiolaelaps* sp.; Astigmatina, Oribatida, Labdostomatidae: *Labdostomatida* sp. Teneriffiidae, Trombidiforme), Amblypygi (Phrinidae: *Heterophrynus longicornis*; Charinidae: *Charinus* sp.), Palpigradi (Eukoenennidae: *Eukoenennia* sp.), Ricinulei (Ricinoididae: *Cryptocellus tarsilae*), Pseudoscorpiones (Chernetidae, Chtoniidae), Opiliones (Kimmulidae, Stygnidae: *Protimesius aff. gracilis*; Phalangiidae), Araneae (Araneidae: *Alpaida* sp., Gnaphosidae, Linyphiidae, Oonopidae: Oonopinae, Oonopinae, Salticidae, Scytodidae*:* *Scytodes eleonora*, Pholcidae: *Mesabolivar* sp., *Metagonia* sp.; Theraphosidae, Theridiidae, Theridiosomatidae), Thysanura (Nicoletiidae: Nicoletiinae), Diplura (Anajapygidae, Campodeidae), Collembola (Sminthuridae, Entomobryidae, Isotomidae,), Orthoptera (Phalangopsidae: *Aclodes* sp., *Phalangopsis* sp.), Blattodea (Blattellidae, Blattidae, Polyphagidae), Pscoptera (Archipsocidae, Epipsocidae, Myopsocidae: *Lichenomina* sp.), Hemiptera (Cydnidae, Lygaeidae, Pyrrhocoridae), Lepidoptera (Noctuidae, Notodontidae, Tineidae), Diptera (Bibionidae, Ceratopogonidae, Culicidae, Drosophilidae, Fanniidae, Phoridae, Psychodidae: *Lutzomyia* sp.; Sciaridae, Tipulidae), Hymenoptera (*Apterostigma* sp., *Camponotus* sp., *Gnamptogenys* sp., *Hypoponera* sp., *Labidus* sp., *Pachycondyla* sp.; Braconidae, Scelionidae, Vespidae), Coleoptera (Carabidae, Elateridae: Agryphinae, Cardiophorinae, Elaterinae, Pselaphidae, Ptylidae, Scydmaenidae, Staphylinidae, Tenebrionidae: Coelometropinae), Diplopoda (Chelodesmidae, Pyrgodesmidae, Polydesmida), Lithobiomorpha (Henicopiidae: *Lamyctes* sp.), Scolopendromorpha

(Cryptopidae: *Cryptops* sp.; Scolopocryptopidae: *Dinocryptops* sp.) e Scutigeromorpha (Scutigeridae: *Sphendononema* sp.).

Dentre as espécies de vertebrados, foram observadas seis espécies de: Chiroptera (Emballonuridae: *Peropteryx kappleri* e Phyllostomidae: *Glossophaga soricina*), Squamata (Gekkonidae: *Thecadactylus rapicauda*) e Anura (Leptodactylidae: *Pristimantis cf. fenestratus*, *Adenomera* sp., *Leptodactylus labyrinthicus*).

Do total, 138 morfoespécies foram encontradas sendo que destas, seis foram consideradas troglomórficas: Gastropoda (Systrophiidae), Amblypygi (Charinidae: *Charinus* sp.), Collembola (Isotomidae), Hymenoptera (Formicidae: *Hypoponera* sp.), Coleoptera (Ptylidae) e Diplopoda (Pyrgodesmidae). Alguns organismos encontrados nesta caverna são mostrados na Figura 157.

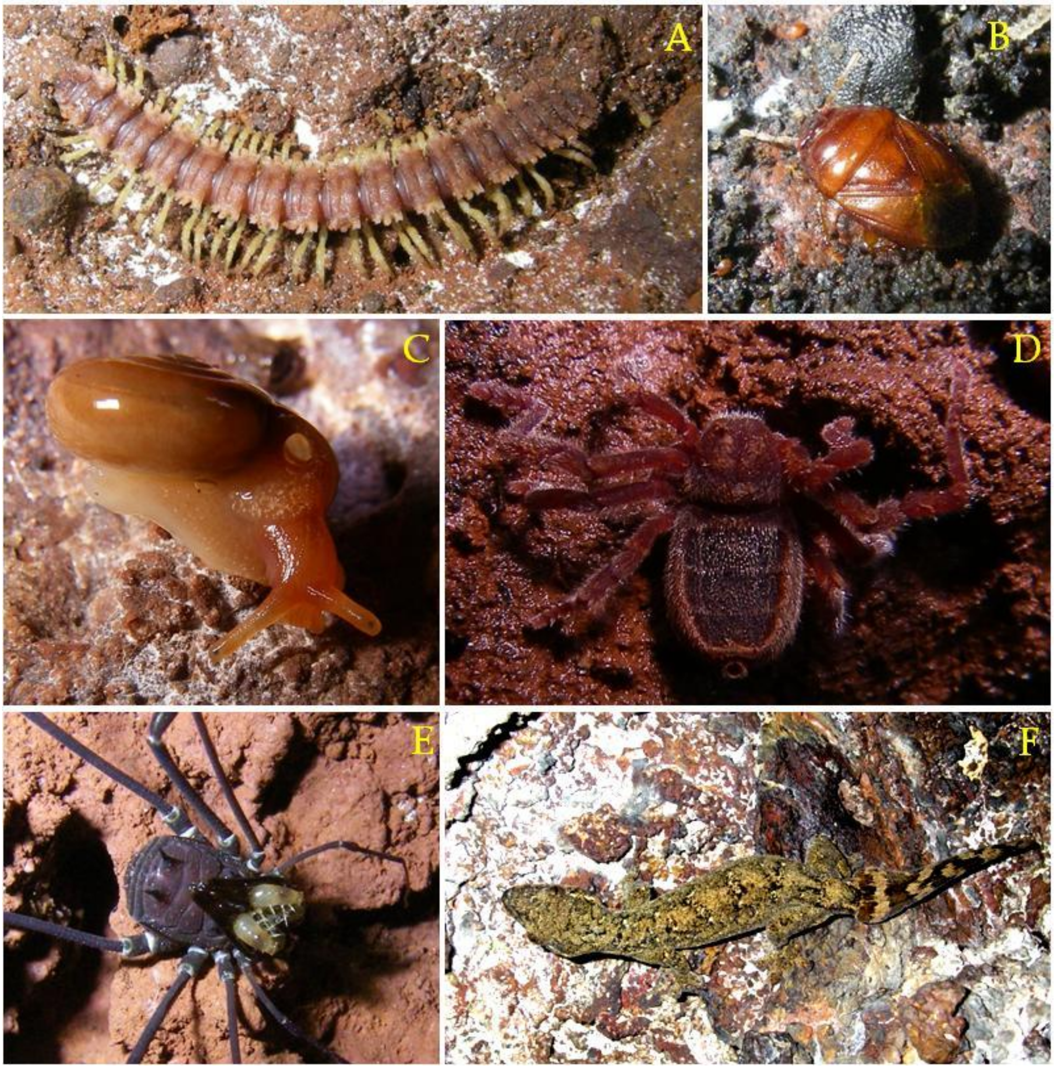

Figura 157 - a) Diplopoda (Chelodesmidae); b) Hemiptera (Cydnidae); c) Gastropoda (Systrophiidae); d) Ricinulei (*Cryptocellus tarsilae*); e) Opiliones (Stygnidae: *Protimesius aff. gracilis*); f) Gekkonidae (*Thecadactylus rapicauda*).

5.4.4.42. SL-045

5.4.4.42.1. Caracterização trófica

Caverna formada na canga com 20,5 m de desenvolvimento localizada de forma muito superficial no topo da encosta onde existe uma vegetação composta principalmente por samambaias de grande porte. Na região superior da cavidade existem muitos arbustos associados a uma vegetação rasteira com predomínio de poáceas. A cavidade possui uma entrada principal pequena e estreita e uma conexão secundária com o meio epígeo que não permite a passagem de uma pessoa. Na entrada principal o piso é descendente composto por sedimento granulado com muitos blocos batidos, sendo esta iluminada e com muitos líquens, briófitas e brotos de angiospermas. A cavidade é composta por um único conduto retilíneo e não apresenta zona afótica. A serrapilheira encontra-se acumulada junto à linha d'água e de forma esparsa pelo restante da cavidade. De forma geral, o piso é predominantemente plano com muitos blocos de diferentes tamanhos distribuídos de forma esparsa (Figura 158). As paredes são úmidas apesar de não terem sido observados pontos de gotejamento e percolação e existem pequenos depósitos de guano de morcegos frugívoros. O sistema de canalículos é pouco desenvolvido e as paredes e o teto são revestidos por fungos. Durante a estação chuvosa a cavidade estava muito úmida com inúmeros pontos de gotejamento e percolação, além de algumas pequenas áreas com poças de água.

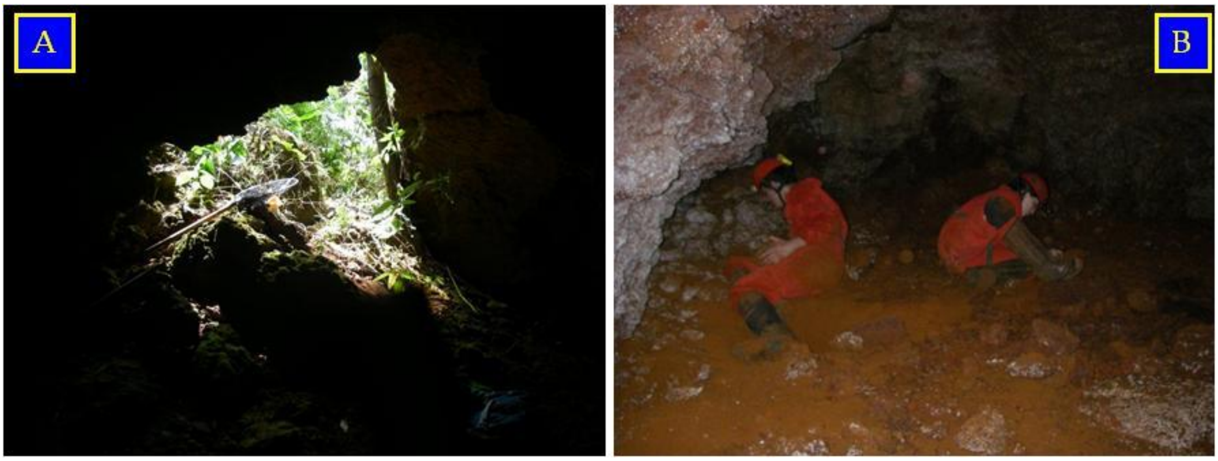

Figura 158 - a) Entrada principal da cavidade com piso descendente e onde existem grandes blocos abatidos; b) Aspecto geral do salão principal da cavidade com muitos blocos junto ao piso e com fungos revestindo as paredes.

5.4.4.42.2. Caracterização faunística no período de seca

Foi observado na caverna, um total de 37 morfoespécies de invertebrados de pelo menos 28 Famílias distribuídas nas seguintes Ordens: Acari (Ixodidae: *Amblyomma* sp.; Podocinidae: *Podocinum* sp.; Astigmatina, Rhagidiidae, Trombidiforme), Amblypygi (*Heterophrynus longicornis* e Charinidae: *Charinus* sp.), Scorpiones (Buthidae: *Ananteris luciae*), Opiliones (Cosmetidae: *Roquettea* sp.), Araneae (Ctenidae, Ochyroceratidae, Salticidae, Prodidomidae,

Theridiosomatidae), Diplura (Campodeidae), Collembola (Sminthuridae, Entomobryidae, Entomobryomorpha), Orthoptera (Phalangopsidae: *Phalangopsis* sp.), Psocoptera (Epipsocidae) Hemiptera (Cydnidae) Homoptera (Cixiidae: *Cixius* sp.), Lepidoptera (Noctuidae), Diptera (Bibionidae, Ceratopogonidae, Dolichopodidae, Psychodidae: *Lutzomyia* sp.; Tipulidae), Hymenoptera (Formicidae: *Dolichoderus* sp., *Pachycondyla* sp.), Coleoptera e Diplopoda (Pyrgodesmidae).

Dentre os vertebrados, foram observadas duas espécies de Anura (Leptodactyllidae: *Pristimantis cf. fenestratus,* Dendrobatidae: *Ameerega* sp.).

Do total, 39 morfoespécies foram encontradas sendo que destas, duas foram consideradas troglomórficas: Amblypygi (Charinidae: *Charinus* sp.) e Diplopoda (Pyrgodesmidae).

5.4.4.42.3. Caracterização faunística no período de chuva

Foi observado na caverna, um total de 33 morfoespécies de invertebrados de pelo menos 26 Famílias distribuídas nas seguintes Ordens: Acari (Macronyssidae, Oribatida), Ricinulei (Ricinoididae: *Cryptocellus tarsilae*), Araneae (Theraphosidae, Theridiosomatidae), Diplura (Anajapygidae), Collembola (Sminthuridae, Entomobryidae) Orthoptera (Phalangopsidae: *Aclodes* sp., *Phalangopsis* sp.), Hemiptera (Cydnidae), Lepidoptera (Geometridae, Noctuidae), Diptera (Ceratopogonidae, Chloropidae, Culicidae, Drosophilidae, Empididae, Milichiidae, Muscidae, Phoridae, Psychodidae: *Lutzomyia* sp.; Streblidae), Hymenoptera (Formicidae: *Cyphomyrmex* sp., *Pheidole* sp.) Coleoptera (Carabidae, Dytiscidae, Pselaphidae) e Diplopoda (Pyrgodesmidae).

Dentre os vertebrados, foi observada uma espécie de Chiroptera (Phyllostomidae: *Glossophaga soricina*).

Do total, 34 morfoespécies foram observadas sendo que, destas, duas são consideradas troglomórficas: Coleoptera (Dytiscidae) e Diplopoda (Pyrgodesmidae).

5.4.4.42.4. Caracterização geral da fauna da cavidade

Foi observado na caverna, um total de 63 morfoespécies de invertebrados de pelo menos 46 Famílias distribuídas nas seguintes Ordens: Acari (Ixodidae: *Amblyomma* sp.; Macronyssidae, Podocinidae: *Podocinum* sp.; Astigmatina, Oribatida, Rhagidiidae), Amblypygi (Phrinidae: *Heterophrynus longicornis,* Charinidae: *Charinus* sp.), Ricinulei (Ricinoididae: *Cryptocellus tarsilae*), Scorpiones (Buthidae: *Ananteris luciae*), Opiliones (Cosmetidae: *Roquettea* sp.), Araneae (Ctenidae, Ochyroceratidae, Salticidae, Prodidomidae, Theraphosidae, Theridiosomatidae), Diplura (Anajapygidae, Campodeidae), Collembola (Sminthuridae, Entomobryidae, Entomobryomorpha), Orthoptera (Phalangopsidae: *Aclodes* sp., *Phalangopsis* sp.), Psocoptera (Epipsocidae), Hemiptera (Cydnidae), Homoptera (Cixiidae:

Cixius sp.), Lepidoptera (Geometridae, Noctuidae), Diptera (Bibionidae, Ceratopogonidae, Chloropidae, Culicidae, Dolichopodidae, Drosophilidae, Empididae, Milichiidae, Muscidae, Phoridae Psychodidae: *Lutzomyia* sp.; Streblidae, Tipulidae), Hymenoptera (Formicidae: *Cyphomyrmex* sp., *Dolichoderus* sp., *Dolichoderus* sp., *Pachycondyla* sp., *Pheidole* sp.) Coleoptera (Carabidae, Dytiscidae, Pselaphidae) e Diplopoda (Pyrgodesmidae).

Dentre os vertebrados foram observadas três espécies de: Chiroptera (Phyllostomidae: *Glossophaga soricina*) e Anura (Leptodactyllidae: *Pristimantis cf. fenestratus,* Dendrobatidae: *Ameerega* sp.).

Do total, foram observadas 47 morfoespécies, da quais três foram consideradas troglomórficas: Amblypygi (Charinidae: *Charinus* sp), Coleoptera (Dytiscidae) e Diplopoda (Pyrgodesmidae). Alguns organismos encontrados nesta caverna são mostrados na Figura 159.

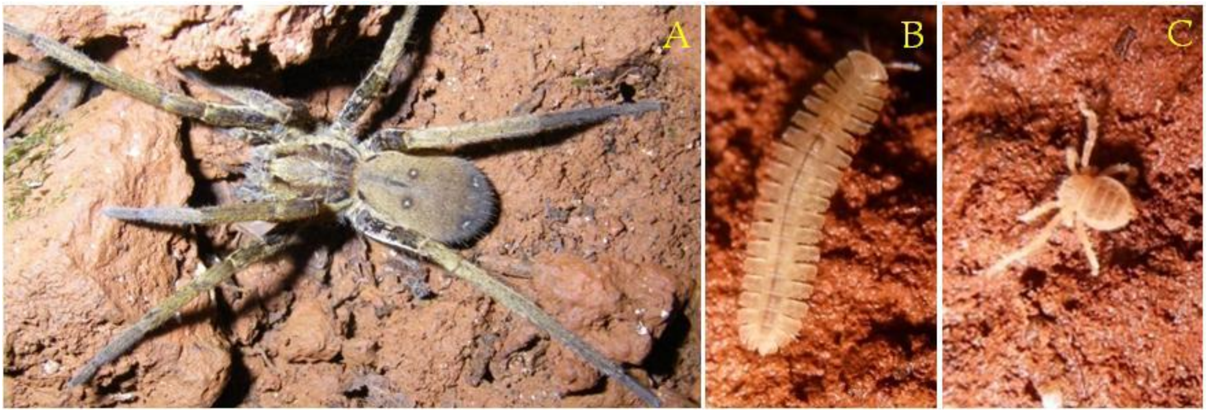

Figura 159 - a) Araneae (Ctenidae); b) Diplopoda (Pyrgodesmidae); c) Ricinulei (*Cryptocellus tarsilae*).

5..4.4.43. SL-046

5.4.4.43.1. Caracterização trófica

Caverna formada em minério de ferro com 28,3 m de desenvolvimento, localizada de forma muito superficial no topo da encosta, onde existe uma vegetação composta principalmente por samambaias de grande porte. Na região superior da cavidade existem muitos arbustos associados a uma vegetação rasteira. Possui apenas uma entrada sendo esta ampla e pouco sombreada e com muitos líquens, fungos, briófitas e muitas angiospermas principalmente Melastomatáceas. O piso é levemente ascendente o que dificulta a importação de recursos orgânicos pela água durante os períodos de chuva. O piso é úmido, completamente revestido por briófitas na região eufótica e composto por sedimento fino com muitos blocos (calhaus e matacões). Existe pouca serrapilheira acumulada junto à entrada e de forma esparsa pelo restante da cavidade. Nas zonas disfóticas existem pequenos depósitos de

guano de morcegos frugívoros e o sistema radicular é pouco desenvolvido. Junto à entrada foram observou-se alguns regurgitos de coruja apesar de nenhum indivíduo ter sido avistado (Figura 160). As paredes e o teto são revestidos por Actinomicetos e existem poucos canalículos visíveis na macro-caverna. Um “quadrante” de escavação arqueológica mostrava-se presente na cavidade. Durante a estação chuvosa a cavidade estava muito úmida com inúmeros pontos de gotejamento.

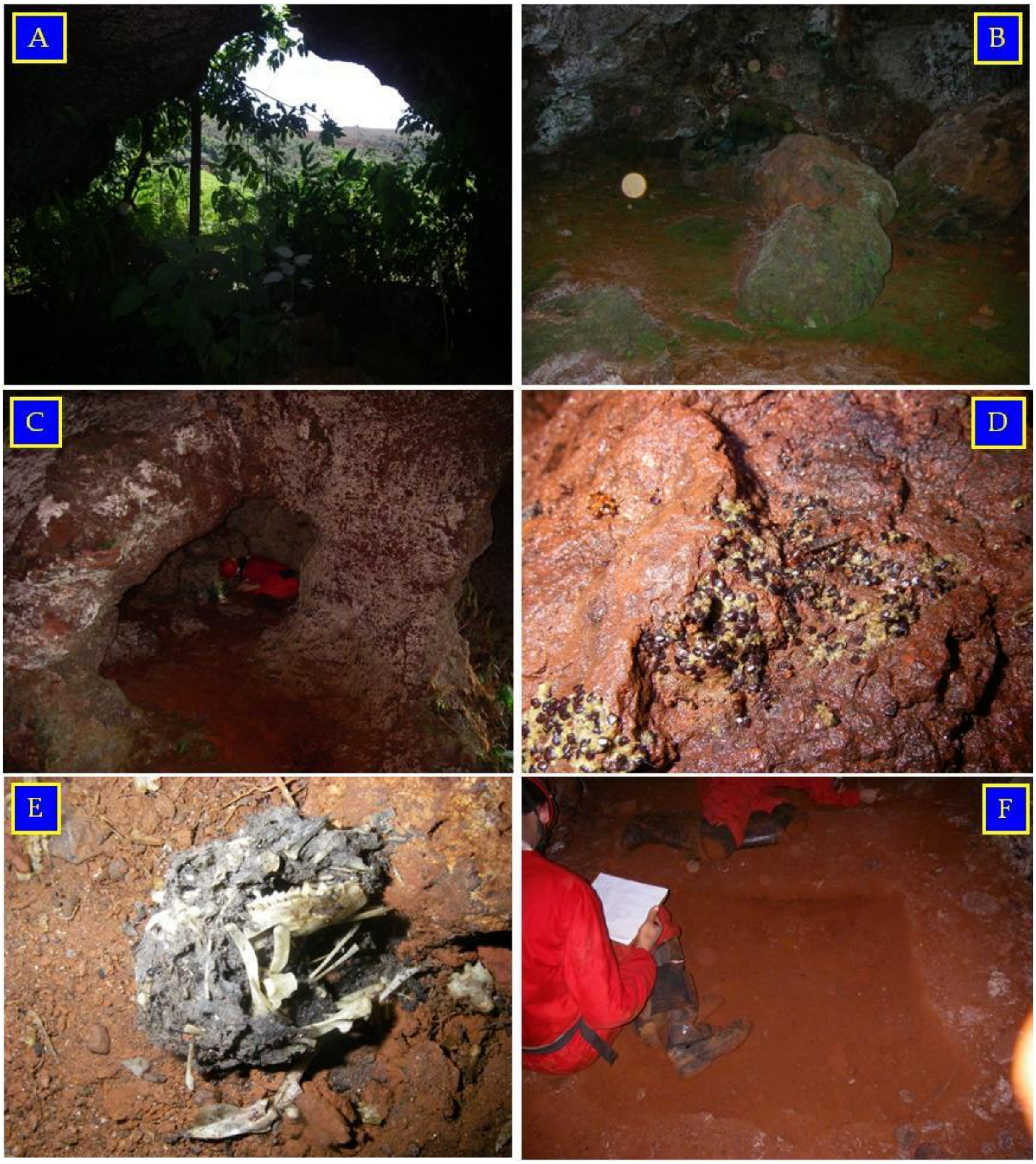

Figura 160 - a) Entrada principal da cavidade onde é possível observar a vegetação externa; b) Vista geral do salão principal da cavidade; c) Grande quantidade de Actinomicetos se desenvolvendo em profusão pelas paredes da cavidade; d) Guano fresco de morcegos frugívoros; e) Regurgito de coruja (Tyto alba); f) “Quadrat” de escavação arqueológica, deixado aberto após escavação.

5.4.4.43.2. Caracterização faunística no período de seca

Foi observado na caverna, um total de 47 morfoespécies de invertebrados de pelo menos 36 famílias das ordens: Oligochaeta, Isopoda (Philosciidae), Acari (Argasidae: *Ornithodoros* sp.; Trombidiforme; Labdostomatidae: *Labdostomatida* sp.), Amblypygi (*Heterophrynus longicornis*), Pseudoscorpiones (Chernetidae, Chtoniidae), Araneae (Corinidae: *Tupirina* sp.; Ochyroceratidae; Salticidae; Scytodidae*: Scytodes eleonora*; Pholcidae; Theridiosomatidae), Diplura (Anajapygidae, Campodeidae, Procampodeidae), Collembola (Entomobryidae), Orthoptera (Phalangopsidae: *Aclodes* sp.; *Phalangopsis* sp.), Psocoptera (Epipsocidae; Psyllipsocidae: *Psyllipsocus* sp.), Hemiptera (Cydnidae, Enicocephalidae), Homoptera (Cixiidae: *Cixius* sp.), Lepidoptera (Noctuidae, Geometridae), Diptera (Ceratopogonidae; Dolichopodidae; Muscidae; Psychodidae: *Lutzomyia* sp.), Hymenoptera (Formicidae: *Camponotus* sp., *Labidus* sp., *Pachycondyla* sp.; Vespidae), Coleoptera (Carabidae, Pselaphidae, Ptylidae), Diplopoda (Chelodesmidae, Pyrgodesmidae) e Chilopoda (Scutigeridae: *Sphendononema* sp.)

Dentre os vertebrados foram encontradas uma espécie de Chiroptera (Phyllostomidae: *Glossophaga soricina*) e quatro de Anura (Leptodactylidae: *Pristimantis cf. fenestratus*; *Eleutherodactylus* sp.; Bufonidae; Dendrobatidae: *Ameerega* sp.).

Desta forma, no total foram encontrados 47 morfoespécies. Entre estas, uma espécie de invertebrado foi considerada troglomórfica: Diplopoda (Pyrgodesmidae).

5.4.4.43.3. Caracterização faunística no período de chuva

Foi observado na caverna, um total de 55 morfoespécies de invertebrados de pelo menos 41 famílias das ordens: Gastropoda (Systrophiidae), Isopoda (Balloniscidae, Philosciidae), Acari (Mesostigmata; Sarcoptiforme; Oribatida; Rhagidiidae; Trombidiforme), Amblypygi (Phrynidae: *Heterophrynus longicornis*), Pseudoscorpiones (Chernetidae, Chtoniidae), Opiliones (Phalangiidae), Araneae (Corinidae: *Tupirina* sp.; Linyphiidae; Ochyroceratidae; Oonopidae: Oonopinae; Scytodidae*: Scytodes eleonora*; Pholcidae: *Metagonia* sp.; Theridiosomatidae), Diplura (Campodeidae), Collembola (Sminthuridae, Entomobryidae, Isotomidae, Paronellidae), Orthoptera (Phalangopsidae: *Aclodes* sp.; *Phalangopsis* sp.), Blattodea (Blattellidae), Psocoptera (Epipsocidae; Myopsocidae: *Lichenomina* sp.; Psylliopsocidae: *Psyllipsocus* sp.), Hemiptera (Cydnidae, Reduviidae), Lepidoptera (Noctuidae), Diptera (Chloropidae, Drosophilidae, Psychodidae, Chironomidae, Muscidae), Hymenoptera (Formicidae: *Camponotus* sp.; *Pachycondyla* sp.; Scelionidae), Coleoptera (Ptylidae, Scydmaenidae, Staphylinidae, Tineidae) e Diplopoda (Pyrgodesmidae).

Dentre os vertebrados foram encontradas duas espécies de Chiroptera (Phyllostomidae: *Glossophaga soricina*; Emballonuridae: *Peropteryx Kappleri*).

Desta forma, no total foram encontrados 57 morfoespécies. Entre estas, duas espécies de invertebrados foram consideradas troglomórficas: Diplopoda (Pyrgodesmidae) e Collembola (Isotomidae).

5.4.4.43.4. Caracterização geral da fauna da cavidade

Foi observado na caverna, um total de 81 morfoespécies de invertebrados de pelo menos 57 famílias das ordens: Oligochaeta, Gastropoda (Systrophiidae), Isopoda (Balloniscidae, Philosciidae), Acari (Argasidae*:* *Ornithodoros* sp.; Mesostigmata; Oribatida; Labdostomatidae: *Labdostomatida* sp.; Rhagidiidae; Trombidiforme), Amblypygi (Phrynidae: *Heterophrynus longicornis*), Pseudoscorpiones (Chernetidae, Chtoniidae), Opiliones (Phalangiidae), Araneae (Corinidae: *Tupirina* sp.; Linyphiidae; Ochyroceratidae; Oonopinae; Salticidae; Scytodidae*: Scytodes eleonora* ; Pholcidae : *Metagonia* sp.; Theridiosomatidae) Diplura (Anajapygidae, Campodeidae, Procampodeidae), Collembola (Sminthuridae, Entomobryidae, Isotomidae, Paronellidae), Orthoptera (Phalangopsidae: *Aclodes* sp.; *Phalangopsis* sp.), Blattodea (Blattellidae), Psocoptera (Epipsocidae, Myopsocidae: *Lichenomina* sp.; Psyllipsocidae: *Psyllipsocus* sp.), Hemiptera (Cydnidae, Enicocephalidae, Reduviidae), Homoptera (Cixiidae: *Cixius* sp), Lepidoptera (Noctuidae, Geometridae, Tineidae), Diptera (Ceratopogonidae, Chloropidae, Dolichopodidae, Drosophilidae, Muscidae, Psychodidae: *Lutzomyia* sp.; Chironomidae), Hymenoptera (Formicidae: *Camponotus* sp., *Labidus* sp., *Pachycondyla* sp.; Scelionidae; Vespidae), Coleoptera (Carabidae, Pselaphidae, Ptylidae, Scydmaenidae, Staphylinidae), Diplopoda (Chelodesmidae, Pyrgodesmidae) e Chilopoda (Scutigeridae: *Sphendononema* sp.)

Dentre os vertebrados foram encontradas duas espécies de Chiroptera (Phyllostomidae: *Glossophaga soricina*; Emballonuridae: *Peropteryx Kappleri*) e quatro espécies de Anura (Leptodactylidae: *Pristimantis cf. fenestratus*; *Eleutherodactylus* sp.; Bufonidae; Dendrobatidae: *Ameerega* sp.).

Desta forma, no total foram encontrados 87 morfoespécies. Entre estas, duas espécies de invertebrados foram consideradas troglomórficas: Diplopoda (Pyrgodesmidae) e Collembola (Isotomidae).

5.4.4.44. SL-047

5.4.4.44.1. Caracterização trófica

Caverna formada na canga com 15,2 m de desenvolvimento localizada de forma muito superficial no topo da encosta onde existe uma vegetação composta principalmente por samambaias de grande porte (Figura 161). Na região superior da cavidade existem muitos arbustos associados a uma vegetação rasteira com predomínio de poáceas. Cavidade pouco

profunda com entrada ampla, sombreada por algumas árvores germinadas próximas a linha d'água e com muitos líquens, briófitas, pteridófitas e brotos de angiospermas principalmente Melastomatáceas distribuídos pelo piso e paredes da cavidade. O piso é predominantemente plano e seco, sendo este composto por sedimento granulado com muitos blocos (seixos, calhaus e matacões) distribuídos de maneira esparsa. Existe muita serrapilheira esparsa transportada pelo vento e também acumulada junto à linha d'água. Não foram observados depósitos de guano e nem sinais de gotejamento no interior da cavidade (Figura 161). As paredes apresentam áreas revestidas por líquens, fungos e briófitas mesmo nas zonas mais profundas. Caverna completamente eufótica, com sistema de canalículos bem desenvolvido e com baixa estabilidade ambiental. Mesmo durante a estação úmida a cavidade continuava seca e sem pontos de gotejamentos ativos.

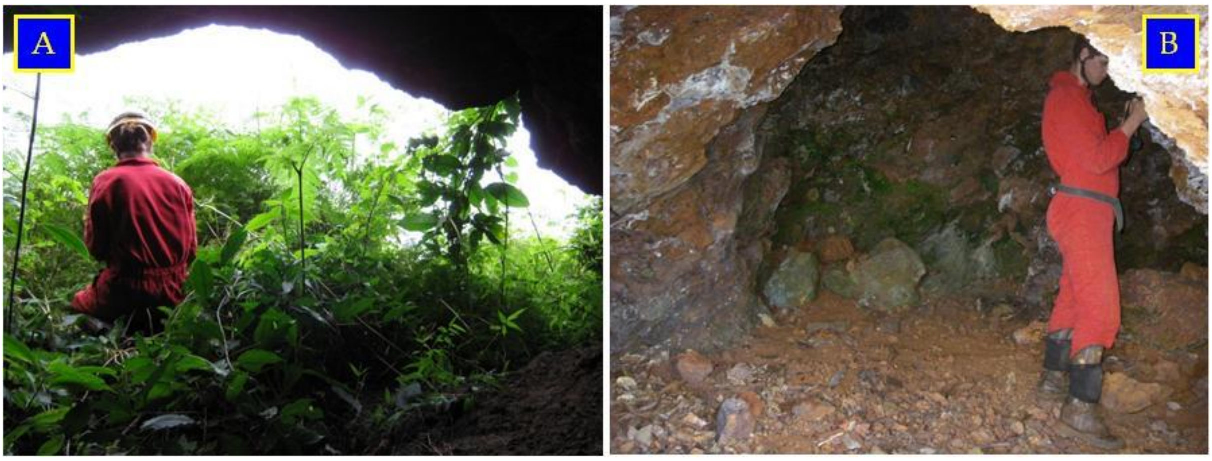

Figura 161 - a) Aspecto geral da entrada da caverna com vegetação epígea vista do interior da cavidade; b) Vista geral do salão principal da cavidade com líquens, fungos e briófitas revestindo as paredes.

5.4.4.44.2. Caracterização faunística no período de seca

Foi observado na caverna, um total de 52 morfoespécies de invertebrados de pelo menos 36 famílias das ordens: Isopoda (Armadillidae, Balloniscidae), Acari (Laelapidae: *Stratiolaelaps* sp., Otopheidomenidae), Pseudoscorpiones (Chernetidae, Chtoniidae), Opiliones (Escadabiidae, Kimmulidae, Manaosbiidae), Araneae (Corinidae: *Tupirina* sp.; Ctenidae: *Ctenus* sp.; Filistatidae; Gnaphosidae; Oonopinae; Salticidae; Scytodidae*: Scytodes eleonora*; Pholcidae: *Metagonia* sp.; Prodidomidae; Theridiosomatidae), Thysanura (Nicoletiidae: Nicoletiinae), Collembola (Entomobryidae), Blattodea (Blattidae, Polyphagidae), Isoptera (Termitidae: *Embiratermes* sp., *Nasutitermes* sp.), Dermaptera (Labiidae), Psocoptera (Myopsocidae: *Lichenomina* sp.; Pachytroctidae; Psyllipsocidae: *Psyllipsocus* sp.; Pyrrhocoridae), Homoptera (Cixiidae: *Cixius* sp.), Lepidoptera (Noctuidae), Diptera (Dolichopodidae, Psychodidae: *Lutzomyia* sp., Syrphidae), Hymenoptera (Formicidae: *Camponotus* sp., *Pheidole* sp.; Vespidae), Coleoptera (Erotylidae), Chilopoda

(Geophilomorpha). Dentre os vertebrados foram encontradas duas espécies de Anura (Leptodactylidae: *Pristimantis cf. fenestratus*; *Eleutherodactylus*).

5.4.4.44.3. Caracterização faunística no período de chuva

Foi observado na caverna, um total de 42 morfoespécies de invertebrados de pelo menos 33 famílias das ordens: Isopoda (Balloniscidae), Acari (Ixodidae: *Amblyomma* sp.), Pseudoscorpiones (Chernetidae, Chtoniidae), Opiliones (Cosmetidae: *Roquettea* sp.*;* Kimmulidae; Phalangiidae), Araneae (Dipluridae, Filistatidae, Salticidae, Scytodidae*: Scytodes eleonora*, Pholcidae : *Metagonia* sp.; Theraphosidae), Diplura (Parajapygidae), Collembola (Entomobryidae), Orthoptera (Gryllidae), Blattodea (Polyphagidae), Isoptera (Termitidae: *Embiratermes* sp., *Nasutitermes* sp.), Psocoptera (Epipsocidae, Myopsocidae: *Lichenomina* sp.; Pachytroctidae; Psyllipsocidae: *Psyllipsocus* sp.), Hemiptera (Cydnidae, Lygaeidae), Lepidoptera (Noctuidae), Diptera (Chloropidae, Muscidae, Sciaridae), Hymenoptera (Formicidae: *Acromyrmex* sp., *Apterostigma* sp., *Camponotus* sp., *Pachycondyla* sp., *Pheidole* sp., *Trachymyrmex* sp.; Eulophidae),Diplopoda (Polydesmida), Chilopoda (Scutigeridae: *Sphendononema* sp.) e Neuroptera (Myrmeleontidae).

5.4.4.44.4. Caracterização geral da fauna da cavidade

Foi observado na caverna, um total de 77 morfoespécies de invertebrados de pelo menos 56 famílias das ordens: Isopoda (Armadillidae, Balloniscidae), Acari (Ixodidae: *Amblyomma* sp.; Laelapidae: *Stratiolaelaps* sp.; Otopheidomenidae), Pseudoscorpiones (Chernetidae, Chtoniidae), Opiliones (Cosmetidae: *Roquettea* sp.*;* Escadabiidae; Kimmulidae; Manaosbiidae; Phalangiidae) Araneae (Corinidae: *Tupirina* sp.; Ctenidae: *Ctenus* sp.; Dipluridae; Filistatidae; Gnaphosidae; Oonopinae; Salticidae; Scytodidae*: Scytodes eleonora*; Pholcidae: *Metagonia* sp.; Prodidomidae; Theraphosidae; Theridiosomatidae), Thysanura (Nicoletiidae: Nicoletiinae), Diplura (Parajapygidae), Collembola (Entomobryidae), Orthoptera (Gryllidae), Blattodea (Blattidae, Polyphagidae) Isoptera (Termitidae*: Embiratermes* sp., *Nasutitermes* sp.), Dermaptera (Labiidae), Psocoptera (Epipsocidae, Myopsocidae: *Lichenomina* sp.; Pachytroctidae; Psyllipsocidae: *Psyllipsocus* sp.), Hemiptera (Cydnidae, Lygaeidae, Pyrrhocoridae), Homoptera (Cixiidae: *Cixius* sp.), Lepidoptera (Noctuidae), Diptera (Chloropidae, Dolichopodidae, Muscidae, Psychodidae : *Lutzomyia* sp.; Syrphidae, Sciaridae), Hymenoptera (Formicidae: *Acromyrmex* sp., *Apterostigma* sp., *Camponotus* sp., *Pachycondyla* sp., *Pheidole* sp., *Trachymyrmex* sp.; Eulophidae; Vespidae), Coleoptera (Erotylidae, Tenebrionidae: Coelometropinae), Diplopoda (Polidesmida), Chilopoda (Geophilomorpha; Scutigeridae: *Sphendononema* sp.) e Neuroptera (Myrmeleontidae).

Dentre os vertebrados foram encontradas duas espécies de Anura (Leptodactylidae: *Pristimantis cf. fenestratus*; *Eleutherodactylus*). Desta forma, no total foram encontradas 79 morfoespécies. Alguns organismos encontrados nesta caverna são mostrados na Figura 162.

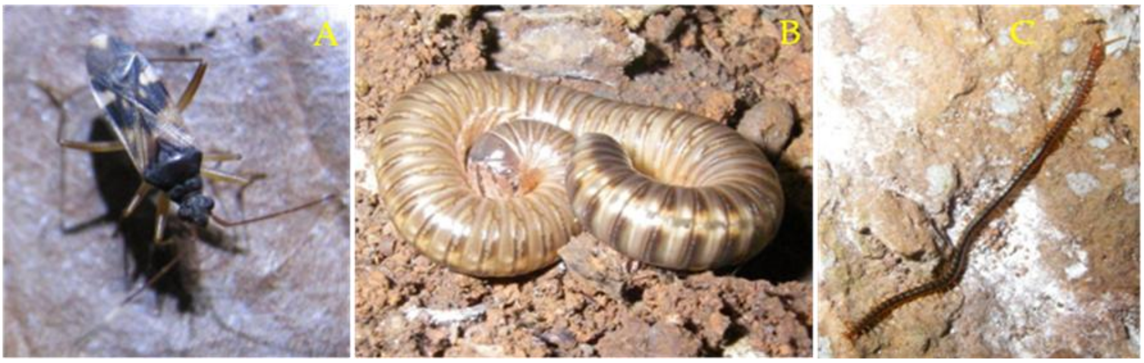

Figura 162 - a) Hemiptera (Lygaeidae) ; b) Diplopoda (Pseudonannolenidae); c) Geophilomorpha.

5.4.4.45. SL-048

5.4.4.45.1. Caracterização trófica

Pequena cavidade com 13,8 m de projeção horizontal localizada em área de mata próximo a uma drenagem. Formada em minério de ferro, trata-se de uma cavidade pouco profunda com duas entradas paralelas muito amplas. O entorno é composto por mata ciliar com muitas árvores de grande porte, lianas, cipós e palmeiras e com muita serrapilheira depositada no solo. As entradas são sombreadas e úmidas com muitos líquens, briófitas, pteridófitas e brotos de angiospermas. O piso é levemente ascendente o que dificulta a importação de matéria orgânica pela água em períodos de chuva. Entretanto, a matéria orgânica é ativamente transportada pelo vento em virtude da dimensão das aberturas e de forma gravitacional por uma pequena clarabóia localizada próxima a uma das entradas. O piso é seco é predominantemente plano, sendo este composto por sedimento granulado com blocos de diferentes tamanhos alternados com áreas onde a própria rocha constitui o piso da cavidade (Figura 163). Existe muita serrapilheira esparsa, muitas raízes de diferentes calibres e não foram observados depósitos de guano no interior da cavidade apesar de terem sido encontrados exemplares de morcegos Glossophaginae. As paredes e o teto apresentam uma morfologia irregular com sistema de canalículos pouco desenvolvido e com muitos Actinomicetos revestindo algumas de suas áreas. Cavidade com baixa estabilidade ambiental sendo esta muito influenciada pelas condições ambientais epígeas. Durante a estação úmida a cavidade continuava seca, mas com alguns pequenos pontos de gotejamento esparsos ao longo de sua extensão.

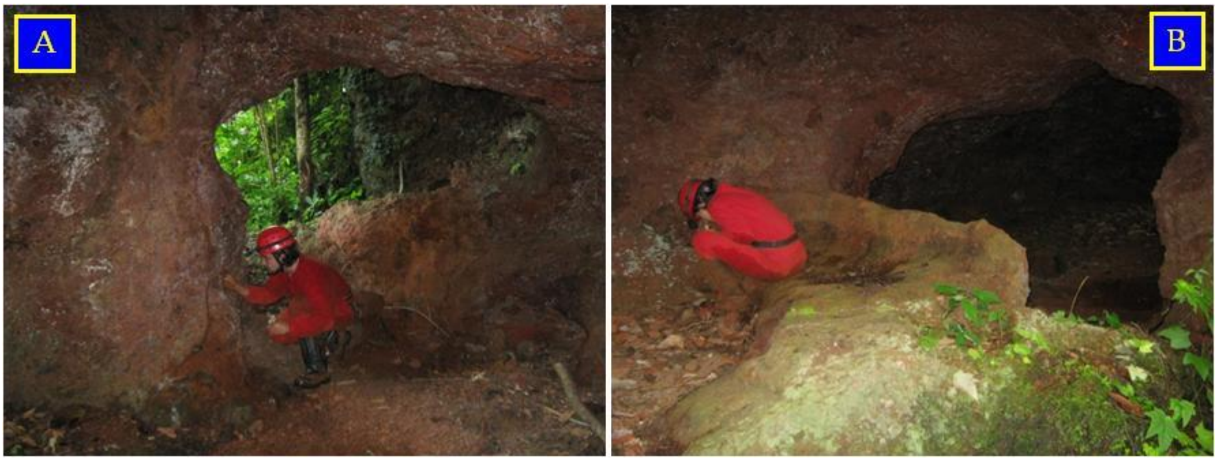

Figura 163 - a) Salão principal da cavidade e piso com blocos e muita serrapilheira acumulada; b) Vista geral do salão principal da cavidade.

5.4.4.45.2. Caracterização faunística no período de seca

Foi observado na caverna, um total de 44 morfoespécies de invertebrados de pelo menos 23 famílias das ordens: Acari (Mesostigmata, Astigmatina, Trombodiforme), Amblypygi (Phrynidae: *Heterophrynus longicornis*), Pseudoscorpiones, Opiliones (Cosmetidae: *Roquettea* sp.*;* Manaosbiidae), Areneae (Salticidae, Scytodidae*: Scytodes eleonora*, Psauridae, Pholcidae: *Mesabolivar* sp., Theridiidae; Theridiosomatidae), Thysanura (Nicoletiidae: Nicoletiinae), Collembola (Entomobryidae), Blattodea (Polyphagidae), Isoptera (Nasutitermes), Psocoptera (Archipsocidae, Myopsocidae*: Lichenomina* sp.), Hemiptera (Alydidae), Homoptera (Cixiidae: *Cixius* sp.), Diptera (Psychodidae: *Lutzomyia* sp., Tipulidae), Hymenoptera (Formicidae: *Acromyrmex* sp., *Apterostigma* sp., *Camponotus* sp., *Cyphomyrmex* sp., *Pachycondyla* sp., *Solenopsis* sp.), Coleoptera (Rhizophagidae), Chilopoda (Otostigmidae: *Otostigmus* sp.), Neuroptera (Myrmeleontidae).

Dentre os vertebrados foram encontradas uma espécie de Squamata (Gekkonidae: *Thecadactylus rapicauda*) e uma espécie de Anura (Leptodactylidae: *Eleutherodactylus* sp.).

5.4.4.45.3. Caracterização faunística no período de chuva

Foi observado na caverna, um total de 49 morfoespécies de invertebrados de pelo menos 30 famílias das ordens: Isopoda (Armadillidae) Acari (Uropodina, Oribatida, Trombidiforme), Amblypygi (Phrynidae*: Heterophrynus longicornis*), Opiliones (Cosmetidae: *Roquettea* sp.; Stygnidae: *Eutimesius* sp., *Protimesius aff. gracilis*), Areneae (Araneidae*: Alpaida* sp.; Ochyroceratidae; Oonopidae: Oonopinae; Salticidae, Scytodidae*: Scytodes eleonora*, Pholcidae: *Mesabolivar* sp., *Metagonia* sp.), Diplura (Campodeidae), Collembola (Dicyrtomidae, Entomobryidae), Orthoptera (Phalangopsidae: *Aclodes* sp.), Isoptera (Termitidae: *Nasutitermes* sp., *Subulitermes* Sp.; Rhinotermitidae: *Rhinotermes* sp.,), Hempitera (Cydnidae, Lygaeidae, Ploiariidae), Homoptera (Cixiidae: *Cixius* sp.; Fulgoridae),

Lepidoptera (Tineidae), Diptera (Cecidomyiidae, Psychodidae: *Lutzomyia* sp.; Tipulidae; Sciaridae), Hymenoptera (Formicidae: *Camponotus* sp., *Cyphomyrmex* sp., *Pachycondyla* sp., *Pheidole* sp., *Solenopsis* sp.), Coleoptera (Carabidae, Staphylinidae), Diplopoda (Polydesmida), Chilopoda (Cryptopidae: *Cryptops* sp.)

Dentre os vertebrados foi encontrada uma espécie de Chiroptera (Phyllostomidae: *Glossophaga soricina*).

5.4.4.45.4. Caracterização geral da fauna da cavidade

Foi observado na caverna, um total de 83 morfoespécies de invertebrados de pelo menos 42 famílias das ordens: Isopoda (Armadillidae), Acari (Mesostigmata, Uropodina, Astigmatina, Oribatida, Trombidiforme), Amblypygi (Phrynidae: *Heterophrynus longicornis*), Pseudoscorpiones, Opiliones (Cosmetidae: *Roquettea* sp.; Manaosbiidae; Stygnidae: *Eutimesius* sp., *Protimesius aff. gracilis*), Araneae (Araneidae: *Alpaida* sp.; Ochyroceratidae; Oonopinae; Salticidae; Scytodidae*: Scytodes eleonora*; Psauridae; Pholcidae: *Mesabolivar* sp., *Metagonia* sp.; Theridiidae; Theridiosomatidae), Thysanura (Nicoletiidae: Nicoletiinae), Diplura (Campodeidae), Collembola (Dicyrtomidae, Entomobryidae), Orthoptera (Phalangopsidae: *Aclodes* sp.), Blattodea (Polyphagidae), Isoptera (Termitidae: *Nasutitermes* sp.,*Subulitermes* sp.; Rhinotermitidae: *Rhinotermes* sp., Psocoptera (Archipsocidae; Myopsocidae: *Lichenomina* sp.), Hempitera (Alydidae, Cydnidae, Lygaeidae, Ploiariidae), Homoptera (Cixiidae: *Cixius* sp.; Fulgoridae), Lepidoptera (Tineidae), Diptera (Cecidomyiidae; Psychodidae: *Lutzomyia* sp.; Tipulidae), Hymenoptera (Formicidae: *Acromyrmex* sp., *Apterostigma* sp., *Camponotus* sp., *Cyphomyrmex* sp., *Pachycondyla* sp., *Pheidole* sp., *Solenopsis* sp.), Coleoptera (Carabidae, Rhizophagidae, Staphylinidae), Diplopoda (Polydesmida), Chilopoda (Cryptopidae: *Cryptops* sp.; Otostigmidae*: Otostigmus* sp.), Neuroptera (Myrmeleontidae).

Dentre os vertebrados foi encontrada uma espécie de Chiroptera (Phyllostomidae: *Glossophaga soricina*), uma espécie de Squamata (Gekkonidae: *Thecadactylus rapicauda*) e uma espécie de Anura (Leptodactylidae: *Eleutherodactylus* sp.).

5.4.4.46. SL-049

5.4.4.46.1. Caracterização trófica

Pequena cavidade (8,5 m de desenvolvimento) formada em minério de ferro localizada em uma área um pouco acima da margem esquerda de uma pequena drenagem. Na parte superior da cavidade a vegetação predominante é composta por pastagem e junto à entrada existem poucos arbustos uma vez que a mata ciliar neste ponto encontra-se muito depauperada. A caverna possui duas entradas pouco sombreadas, sendo uma entrada

principal (2 m^2) e uma pequena entrada paralela onde existem poucos líquens e briófitas além de serrapilheira acumulada junto a linha d'água. O piso é predominantemente plano e seco, sendo este composto por sedimento fino com poucos seixos e calhaus e com muitas raízes de diferentes calibres. As paredes e o teto são revestidos por Actinomicetos, a cavidade não possui zona afótica e o sistema de canalículos é muito desenvolvido formando uma grande quantidade de micro-hábitats (Figura 164). Apesar de terem sido observados três exemplares de morcegos frugívoros (*Anoura sp.*) não foram observados depósitos de guano no interior da cavidade. Durante a estação úmida a caverna continuava seca, mas apresentava alguns pequenos pontos de gotejamento.

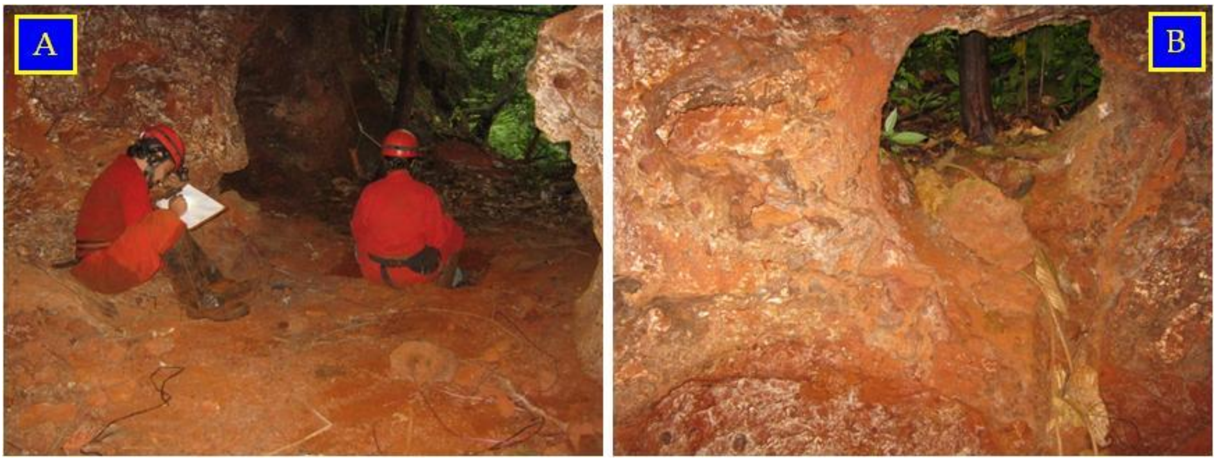

Figura 164 - a) Aspecto geral da cavidade; b) Entrada visualizada pela porção interna da caverna.

5.4.4.46.2. Caracterização faunística no período de seca

Foi observado na caverna, um total de 35 morfoespécies de invertebrados de pelo menos 25 Famílias distribuídas nas seguintes Ordens: Isopoda (Armadillidae, Philosciidae), Acari (Trombidiforme), Amblypygi (Phrynidae: *Heterophrynus longicornis*), Pseudoscorpiones (Chernetidae, Chtoniidae), Opiliones (Cosmetidae: *Roquettea* sp.*;* Sclerosomatidae: *Prionostemma* sp.), Araneae (Scytodidae*: Scytodes eleonora*, Pholcidae: *Metagonia* sp., Theridiidae, Theridiosomatidae), Thysanura (Nicoletiidae: Atelurinae), Diplura (Campodeidae), Collembola (Entomobryidae), Orthoptera (Gryllidae: Nemobiinae, Phalangopsidae: *Aclodes* sp.), Isoptera (Termitidae: *Nasutitermes* sp.), Psocoptera, Hemiptera (Cydnidae, Nabidae), Diptera (Culicidae, Psychodidae: *Lutzomyia* sp.), Hymenoptera (Formicidae: *Acromyrmex* sp., *Apterostigma* sp., *Camponotus* sp., *Pachycondyla* sp.) Coleoptera (Carabidae, Staphylinidae) e Scutigeromorpha (Scutigeridae: *Sphendononema* sp.).

Dentre os vertebrados, foram observadas duas espécies de: Chiroptera (Phyllostomidae: *Glossophaga soricina*) e Anura (Leptodactylidae: *Pristimantis cf. fenestratus*). No total, foram encotradas 37 morfoespécies nesta caverna.

5.4.4.46.3. Caracterização faunística no período de chuva

Foi observado na caverna, um total de 45 morfoespécies de invertebrados de pelo menos 32 Famílias distribuídas nas seguintes Ordens: Isopoda (Armadillidae), Acari (Laelapidae: *Stratiolaelaps* sp., Ologamasidae, Opilioacaridae: *Neoacarus* sp., Astigmatina sp., Trombidiforme), Pseudoscorpiones (Chtoniidae), Opiliones (Cosmetidae: *Roquettea* sp., Sclerosomatidae: *Prionostemma* sp.; Phalangiidae), Araneae (Ctenidae: *Ctenus* sp.; Gnaphosidae, Oonopidae: Oonopinae, Scytodidae*: Scytodes eleonora*, Pholcidae: *Mesabolivar* sp.; Theridiidae, Theridiosomatidae), Diplura (Campodeidae), Isoptera (Termitidae: *Termes* sp.), Psocoptera (Myopsocidae: *Lichenomina* sp.), Hemiptera (Alydidae, Lygaeidae), Homoptera (Cixiidae: *Cixius* sp.), Lepidoptera (Noctuidae), Diptera (Cecidomyiidae, Chironomidae, Psychodidae: *Lutzomyia* sp.; Sciaridae, Tipulidae), Hymenoptera (Formicidae: *Acromyrmex* sp., *Camponotus* sp., *Gnamptogenys* sp., *Odontomachus* sp., *Pachycondyla* sp., *Pheidole* sp.), Coleoptera (Curculionidae: Scotylinae, Pselaphidae) e Geophilomorpha (Ballophilidae: *Taeniolinum* sp.).

Dente os vertebrados, foi observada uma espécie de Chiroptera (Phyllostomidae: *Glossophaga soricina*). No total, foram encontradas 45 morfoespécies nesta caverna sendo que, destas, uma Araneae (Oonopidae) foi considerada troglomórfica.

5.4.4.46.4. Caracterização geral da fauna da cavidade

Foi observado na caverna, um total de 68 morfoespécies de invertebrados de pelo menos 44 Famílias distribuídas nas seguintes Ordens: Isopoda (Armadillidae, Philosciidae), Acari (Laelapidae: *Stratiolaelaps* sp.; Ologamasidae, Opilioacaridae: *Neoacarus* sp.; Astigmatina, Oribatida, Trombidiforme), Amblypygi (Phrynidae: *Heterophrynus longicornis*), Pseudoscorpiones (Chernetidae, Chtoniidae), Opiliones (Cosmetidae: *Roquettea* sp., Sclerosomatidae: *Prionostemma* sp.; Phalangiidae), Araneae (Ctenidae: *Ctenus* sp.; Gnaphosidae, Oonopidae: Oonopinae, Scytodidae*: Scytodes eleonora*, Pholcidae: *Mesabolivar* sp., *Metagonia* sp1., Theridiidae, Theridiosomatidae), Thysanura (Nicoletiidae: Atelurinae) Diplura (Campodeidae), Colleoptera (Entomobryidae), Orthoptera (Grylidae: Nemobiinae, Phalangopsidae: *Aclodes* sp.), Isopoda (Termitidae: *Nasutitermes* sp., *Termes* sp.), Psocoptera (Myopsocidae: *Lichenomina* sp.), Hemiptera (Alydidae, Cydnidae, Lygaeidae, Nabidae), Homoptera (Cixiidae: *Cixius* sp.), Lepidoptera (Noctuidae), Diptera (Cecidomyiidae, Chironomidae, Culicidae, Psychodidae: *Lutzomyia* sp.; Sciaridae, Tipulidae), Hymenoptera (Formicidae: *Acromyrmex* sp., *Apterostigma* sp., *Camponotus* sp., *Gnamptogenys* sp., *Odontomachus* sp., *Pachycondyla* sp1., *Pheidole* sp.), Coleoptera (Carabidae, Curculionidae: Scotylinae, Pselaphidae, Staphylinidae), Geophilomorpha (Ballophilidae: *Taeniolinum* sp.) e Scutigeromorpha (Scutigeridae: *Sphendononema* sp.).

Dentre os vertebrados foram observadas duas espécies de: Chiroptera (Phyllostomidae: *Glossophaga soricina*) e Anura (Leptodactylidae: *Pristimantis cf. fenestratus*). No total foram encontradas 70 morfoespécies sendo que, destas, uma Araneae (Oonopidae) foi considerada troglomórfica.

5.4.4.47. SL-050

5.4.4.47.1. Caracterização trófica

Caverna formada na canga com 27 m de projeção horizontal localizada em área de pastagem. Na região próxima à entrada existem muitas árvores e arbustos que se limitam a uma pequena mancha de vegetação. Esta cavidade sofreu grande alteração pelos processos de sondagem onde sua entrada foi escavada, tendo sido formado um lago artificial para extração de água. Mesmo durante o inventário, ainda existiam máquinas de sondagem em atividade a apenas algumas dezenas de metros de sua entrada, além de inúmeras estradas abertas na região de entorno (Figura 165). Desta forma, na situação atual, a cavidade apresenta uma entrada ampla e iluminada onde existe um lago com água barrenta (de pouca visibilidade) além de uma grande quantidade de líquens, briófitas e pteridófitas revestindo as paredes e piso e muitas angiospermas, principalmente Melastomatáceas, formando pequenas manchas na zona de entrada. O acesso a cavidade é realizado por uma pequena passagem estreita junto à parede esquerda onde logo em seguida se conecta com um conduto em teto baixo que leva a uma pequena área localizada na região disfótica da cavidade. O piso da caverna é predominantemente plano e úmido, composto por sedimento fino com alguns seixos, calhaus e matacões esparsos. Existem restos de serrapilheira espalhados em diferentes áreas da cavidade que podem indicar uma elevação no nível do lago durante o período de chuvas intensas. Existem pequenos depósitos de guano de morcegos frugívoros na zona disfótica produzidos por Glossophaginae (*Anoura sp.*). As paredes e o teto são revestidos por Actinomicetos e o sistema de canalículos é bem desenvolvido. Durante a estação úmida a cavidade encontrava-se muito úmida com inúmeros pontos de gotejamentos. Entretanto, o nível do lago encontrava-se rebaixado (cerca de 1 m) quando comparado com a estação seca. Tal condição provavelmente é resultante da extração de água utilizada nos processos de sondagem.

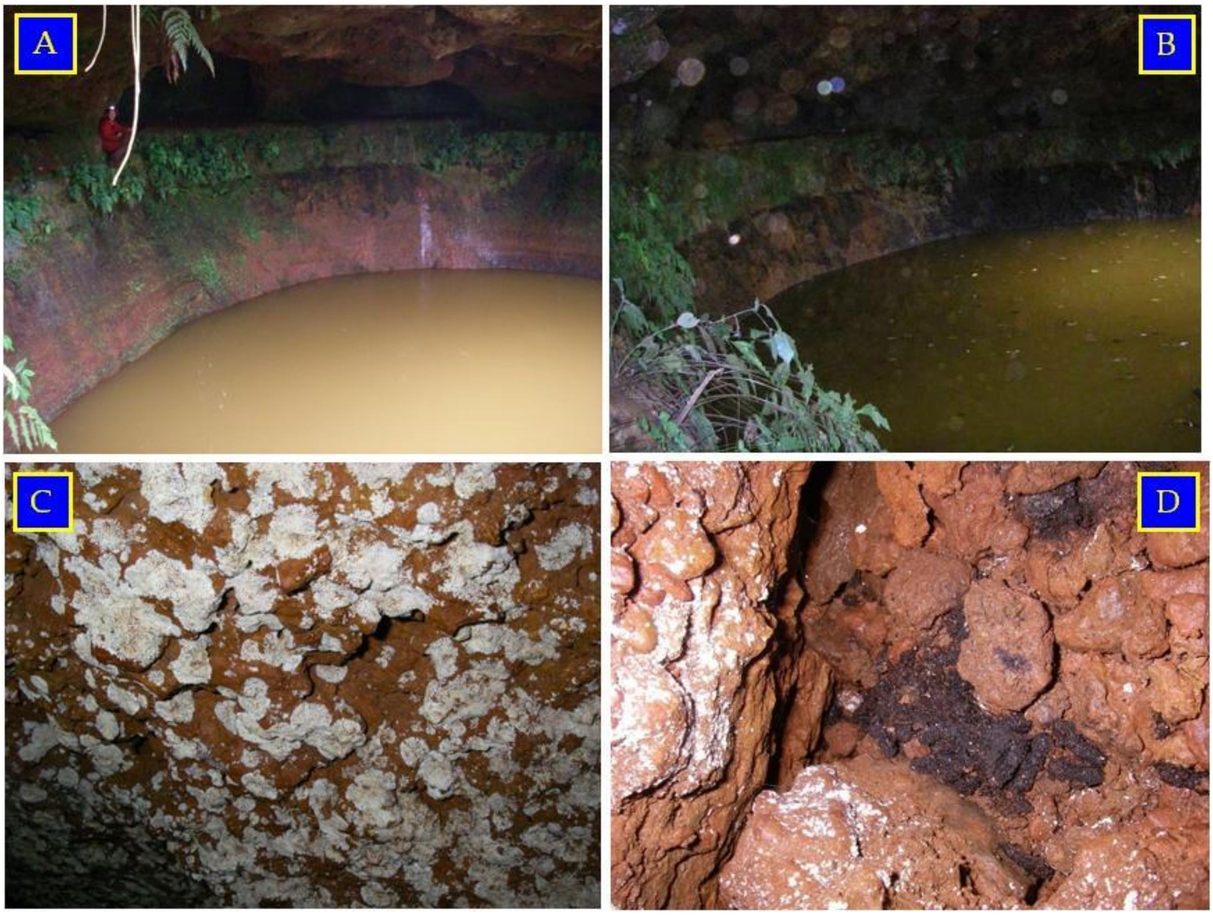

Figura 165 - a) Vista externa da entrada da cavidade onde se observa o lago artificial; b) Detalhe da situação do nível do mesmo lago durante a estação úmida; c) Colônias de Actinomicetos crescendo nas paredes da caverna; d) Fezes de um vertebrado encontradas na caverna.

5.4.4.47.2. Caracterização faunística no período de seca

Foi observado na caverna, um total de 41 morfoespécies de invertebrados de pelo menos 31 famílias das Ordens: Oligochaeta, Decapoda (Pseudothelphusidae: *Microthelphusa somanni*), Acari (Podocinidae: *Podocinum* sp., Oribatida, Trombidiforme), Amblypygi (Charinidae: *Charinus* sp.), Pseudoscorpiones (Chtoniidae), Opiliones (Sclerosomatidae: *Prionostemma* sp.), Araneae (Ochyroceratidae, Scytodidae*: Scytodes eleonora*, Pholcidae: *Mesabolivar* sp., Theridiidae, Theridiosomatidae), Thysanura (Nicoletiidae: Nicoletiinae), Collembola (Sminthuridae, Entomobryidae, Isotomidae), Blattodea (Blattellidae), Psocoptera (Epipsocidae), Homoptera (Cixiidae: *Cixius* sp.), Diptera (Cecidomyiidae, Ceratopogonidae, Culicidae, Drosophilidae, Phoridae, Psychodidae: *Lutzomyia* sp., Sciaridae), Hymenoptera (Formicidae: *Crematogaster* sp., *Linepthema* sp., *Pseudomyrmex* sp., *Solenopsis* sp., *Tapinoma* sp.), Coleoptera (Carabidae, Pselaphidae, Scydmaenidae, Staphylinidae), Diplopoda (Chelodesmidae), Scolopendromorpha (Ototigmidae: *Otostigmus* sp.), Scutigeromorpha (Scutigeridae: *Sphendononema* sp.) e Symphyla (Scutigerellidae: *Hanseniella* sp., *Scutigerella* sp.).

Dentre os vertebrados, foi encontrada uma espécie de Anura (Leptodactylidae: *Pristimantis* cf. *fenestratus*). Desta forma, no total foram encontradas 42 morfoespécies. Entre estas, duas espécies de invertebrados foram consideradas troglomórficas: Amblypygi (Charinidae: *Charinus* sp.) e Collembola (Isotomidae).

5.4.4.47.3. Caracterização faunística no período de chuva

Foi observado na caverna, um total de 49 morfoespécies de invertebrados de pelo menos 36 famílias das Ordens: Gastropoda (Subulinidae), Turbellaria (Geoplanidae), Isopoda (Philosciidae, Scleropactidae), Decapoda (Pseudothelphusidae: *Microthelphusa somanni*), Amblypygi (Charinidae: *Charinus* sp.), Pseudoscorpiones (Chtoniidae), Opiliones (Escadabiidae, Sclerosomatidae: *Prionostemma* sp.), Araneae (Ctenidae, Oonopidae: Oonopinae, Scytodidae*: Scytodes eleonora*, Pholcidae: *Mesabolivar* sp., Theridiosomatidae), Thysanura (Nicoletiidae: Nicoletiinae), Collembola (Entomobryidae), Orthoptera (Phalangopsidae: *Aclodes* sp., *Phalangopsis* sp.), Blattodea (Blattellidae), Psocoptera (Myopsocidae: *Lichenomina* sp.), Hemiptera (Cydnidae, Enicocephalidae), Lepidoptera (Tineidae), Diptera (Drosophilidae, Psychodidae: *Lutzomyia* sp., Sciaridae), Hymenoptera (Formicidae: *Azteca* sp., *Pachycondyla* sp., *Solenopsis* sp., *Tapinoma* sp.), Coleoptera (Carabidae, Pselaphidae, Scydmaenidae, Staphylinidae), Diplopoda (Chelodesmidae, Polydesmida, Siphonophoridae, Pseudonannolenidae), Scolopendromorpha (Scolopocryptopidae: *Dinocryptops* sp.), Scutigeromorpha (Scutigeridae: *Sphendononema* sp.) e Symphyla (Scutigerellidae: *Hanseniella* sp., *Scutigerella* sp.).

Dentre os vertebrados foi encontrada uma espécie de Chiroptera (sp. indet.). Desta forma, no total foram encontradas 50 morfoespécies. Entre estas, uma espécie de Amblypygi (Charinidae: *Charinus* sp.) foi considerada troglomórfica.

5.4.4.47.4. Caracterização geral da fauna da cavidade

Foi observado na caverna, um total de 76 morfoespécies de invertebrados de pelo menos 50 famílias das Ordens: Oligochaeta, Gastropoda (Subulinidae), Turbellaria (Geoplanidae), Isopoda (Philosciidae, Scleropactidae), Decapoda (Pseudothelphusidae: *Microthelphusa somanni*), Acari (Podocinidae: *Podocinum* sp., Oribatida, Trombidiforme), Amblypygi (Charinidae: *Charinus* sp.), Pseudoscorpiones (Chtoniidae), Opiliones (Escadabiidae, Sclerosomatidae: *Prionostemma* sp.), Araneae (Ctenidae, Ochyroceratidae, Oonopidae: Oonopinae, Scytodidae*: Scytodes eleonora*, Pholcidae: *Mesabolivar* sp., Theridiidae, Theridiosomatidae), Thysanura (Nicoletiidae: Nicoletiinae), Collembola (Sminthuridae, Entomobryidae, Isotomidae), Orthoptera (Phalangopsidae: *Aclodes* sp., *Phalangopsis* sp.), Blattodea (Blattellidae), Psocoptera (Epipsocidae, Myopsocidae: *Lichenomina* sp.), Hemiptera (Cydnidae, Enicocephalidae), Homoptera (Cixiidae: *Cixius* sp.), Lepidoptera (Tineidae), Diptera (Cecidomyiidae, Ceratopogonidae, Culicidae, Drosophilidae, Phoridae, Psychodidae: *Lutzomyia* sp., Sciaridae), Hymenoptera (Formicidae: *Azteca* sp.,

Crematogaster sp., *Linepthema* sp., *Pachycondyla* sp., *Pseudomyrmex* sp., *Solenopsis* sp., *Tapinoma* sp.), Coleoptera (Carabidae, Pselaphidae, Scydmaenidae, Staphylinidae), Diplopoda (Chelodesmidae, Polydesmida, Siphonophoridae, Pseudonannolenidae), Scolopendromorpha (Ototigmidae: *Otostigmus* sp., Scolopocryptopidae: *Dinocryptops* sp.), Scutigeromorpha (Scutigeridae: *Sphendononema* sp.) e Symphyla (Scutigerellidae: *Hanseniella* sp., *Scutigerella* sp.).

Dentre os vertebrados foram encontradas duas espécies das Ordens: Chiroptera e Anura (Leptodactylidae: *Pristimantis* cf. *fenestratus*). Desta forma, no total foram encontradas 78 morfoespécies. Entre estas, duas espécies de invertebrados foram consideradas troglomórficas: Amblypygi (Charinidae: *Charinus* sp.) e Collembola (Isotomidae).

5.4.4.48. SL-051

5.4.4.48.1. Caracterização trófica

Pequena cavidade com 11,3 m de projeção horizontal localizada em área de pastagem na margem direita de um pequeno rio. Formada em canga em condição bem superficial, possui entrada ampla e apresenta teto baixo ao longo de toda sua extensão. Sua entrada é ensolarada com paredes e pisos revestidos por líquens, briófitas, pteridófitas e plântulas. Não apresenta zona afótica e seu piso é descendente e seco, sendo este composto por sedimento granulado com vários blocos esparsos (seixos e calhaus). Existem alguns depósitos frescos de guano de morcegos frugívoros na porção direita da caverna. Tais depósitos apresentam-se mesclados ao substrato. Tais depósitos são produzidos por uma colônia de morcegos Glossophaginae (*G. soricina*) composta por aproximadamente 20 indivíduos. O sistema radicular é bem desenvolvido com alguns rizotemas distribuídos em pontos de gotejamentos, apesar destes encontrarem-se inativos durante a estação seca. A condição de piso descendente a partir da entrada da caverna também favorece a importação de matéria orgânica proveniente do maio epígeo, apesar de grande parte da serrapilheira encontrar-se acumulada próxima à linha d'água (Figura 166). Em relação aos vertebrados, foram encontrados dois ninhos abandonados de aves próximos à zona de entrada. O sistema de canalículos é pouco desenvolvido e as paredes e o teto são revestidos por Actinomicetos (bactérias). Durante a estação úmida a cavidade apresentava maior umidade relativa do ar e apenas um ponto de gotejamento ativo em sua porção mais interna. Além disto, o depósito de guano de morcegos frugívoros ocupava uma área maior, tendo se mostrado fresco nesta estação.

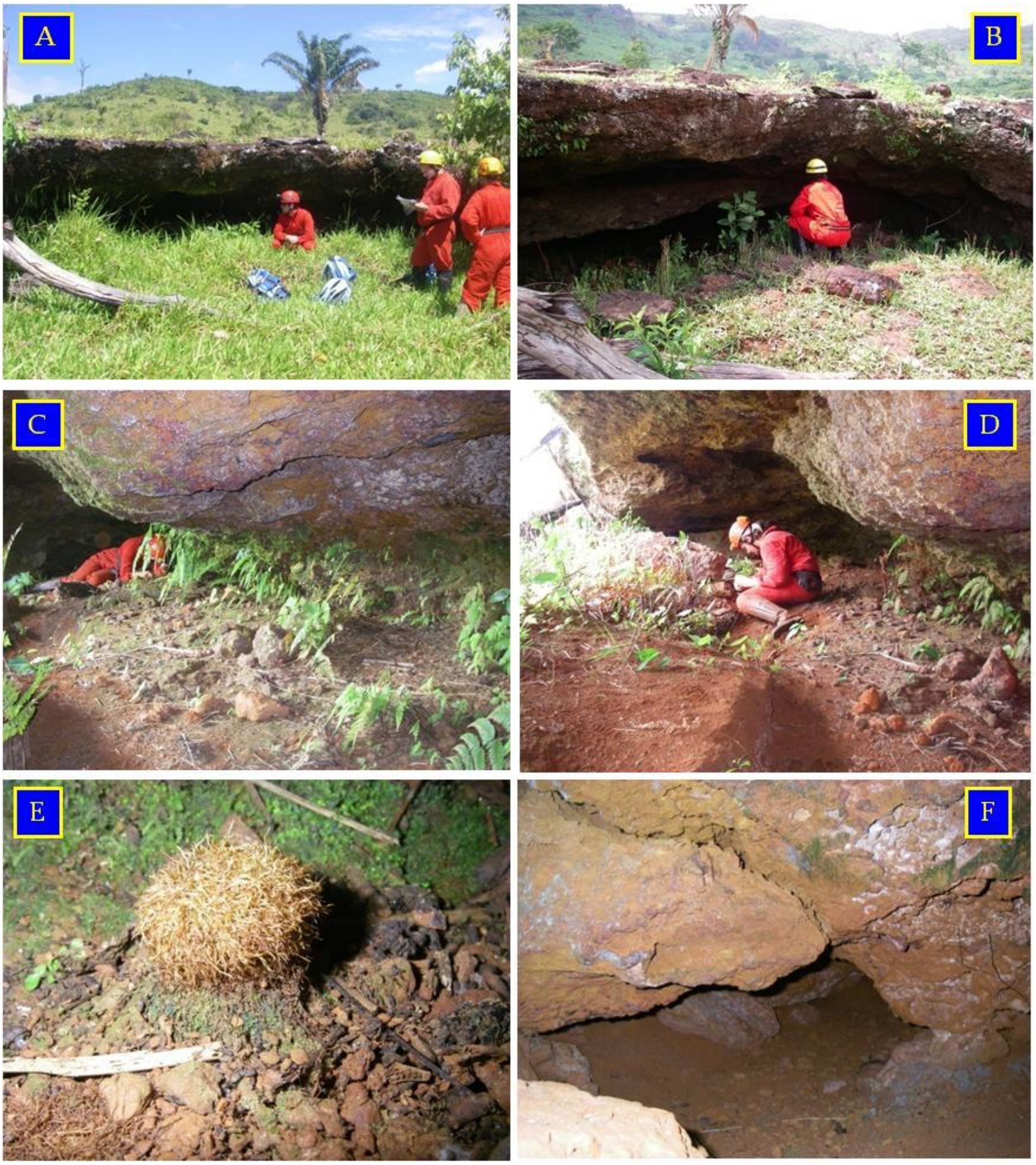

Figura 166 - a) Vista externa da entrada da cavidade onde se observa a matriz da paisagem composta principalmente por pastagem (estação seca); b) Vista externa da cavidade na estação chuvosa; C) Vista interna da entrada da cavidade com vegetação muito desenvolvida na estação chuvosa; E) Rizotema presente no interior da cavidade; F) Porção mais interior da cavidade, mostrando os substratos de piso mais ressequidos (estação chuvosa).

5.4.4.48.2. Caracterização faunística no período de seca

Foi observado na caverna, um total de 41 morfoespécies de invertebrados de pelo menos 31 famílias das Ordens: Oligochaeta, Decapoda (Pseudothelphusidae: *Microthelphusa somanni*), Acari (Podocinidae: *Podocinum* sp., Oribatida, Trombidiforme), Amblypygi (Charinidae:

Charinus sp.), Pseudoscorpiones (Chtoniidae), Opiliones (Sclerosomatidae: *Prionostemma* sp.), Araneae (Ochyroceratidae, Scytodidae*: Scytodes eleonora*, Pholcidae: *Mesabolivar* sp., Theridiidae, Theridiosomatidae), Thysanura (Nicoletiidae: Nicoletiinae), Collembola (Sminthuridae, Entomobryidae, Isotomidae), Blattodea (Blattellidae), Psocoptera (Epipsocidae), Homoptera (Cixiidae: *Cixius* sp.), Diptera (Cecidomyiidae, Ceratopogonidae, Culicidae, Drosophilidae, Phoridae, Psychodidae: *Lutzomyia* sp., Sciaridae), Hymenoptera (Formicidae: *Crematogaster* sp., *Linepthema* sp., *Pseudomyrmex* sp., *Solenopsis* sp., *Tapinoma* sp.), Coleoptera (Carabidae, Pselaphidae, Scydmaenidae, Staphylinidae), Diplopoda (Chelodesmidae), Scolopendromorpha (Ototigmidae: *Otostigmus* sp.), Scutigeromorpha (Scutigeridae: *Sphendononema* sp.) e Symphyla (Scutigerellidae: *Hanseniella* sp., *Scutigerella* sp.).

Dentre os vertebrados, foi encontrada uma espécie de Anura (Leptodactylidae: *Pristimantis* cf. *fenestratus*). Desta forma, no total foram encontradas 42 morfoespécies. Entre estas, duas espécies de invertebrados foram consideradas troglomórficas: Amblypygi (Charinidae: *Charinus* sp.) e Collembola (Isotomidae).

5.4.4.48.3. Caracterização faunística no período de chuva

Foi observado na caverna, um total de 49 morfoespécies de invertebrados de pelo menos 36 famílias das Ordens: Gastropoda (Subulinidae), Turbellaria (Geoplanidae), Isopoda (Philosciidae, Scleropactidae), Decapoda (Pseudothelphusidae: *Microthelphusa somanni*), Amblypygi (Charinidae: *Charinus* sp.), Pseudoscorpiones (Chtoniidae), Opiliones (Escadabiidae, Sclerosomatidae: *Prionostemma* sp.), Araneae (Ctenidae, Oonopidae: Oonopinae, Scytodidae*: Scytodes eleonora*, Pholcidae: *Mesabolivar* sp., Theridiosomatidae), Thysanura (Nicoletiidae: Nicoletiinae), Collembola (Entomobryidae), Orthoptera (Phalangopsidae: *Aclodes* sp., *Phalangopsis* sp.), Blattodea (Blattellidae), Psocoptera (Myopsocidae: *Lichenomina* sp.), Hemiptera (Cydnidae, Enicocephalidae), Lepidoptera (Tineidae), Diptera (Drosophilidae, Psychodidae: *Lutzomyia* sp., Sciaridae), Hymenoptera (Formicidae: *Azteca* sp., *Pachycondyla* sp., *Solenopsis* sp., *Tapinoma* sp.), Coleoptera (Carabidae, Pselaphidae, Scydmaenidae, Staphylinidae), Diplopoda (Chelodesmidae, Polydesmida, Siphonophoridae, Pseudonannolenidae), Scolopendromorpha (Scolopocryptopidae: *Dinocryptops* sp.), Scutigeromorpha (Scutigeridae: *Sphendononema* sp.) e Symphyla (Scutigerellidae: *Hanseniella* sp., *Scutigerella* sp.).

Dentre os vertebrados foi encontrada uma espécie de Chiroptera (sp. indet.). Desta forma, no total foram encontradas 50 morfoespécies. Entre estas, uma espécie de Amblypygi (Charinidae: *Charinus* sp.) foi considerada troglomórfica.

5.4.4.48.4. Caracterização geral da fauna da cavidade

Foi observado na caverna, um total de 76 morfoespécies de invertebrados de pelo menos 50 famílias das Ordens: Oligochaeta, Gastropoda (Subulinidae), Turbellaria (Geoplanidae), Isopoda (Philosciidae, Scleropactidae), Decapoda (Pseudothelphusidae: *Microthelphusa somanni*), Acari (Podocinidae: *Podocinum* sp., Oribatida, Trombidiforme), Amblypygi (Charinidae: *Charinus* sp.), Pseudoscorpiones (Chtoniidae), Opiliones (Escadabiidae, Sclerosomatidae: *Prionostemma* sp.), Araneae (Ctenidae, Ochyroceratidae, Oonopidae: Oonopinae, Scytodidae*: Scytodes eleonora*, Pholcidae: *Mesabolivar* sp., Theridiidae, Theridiosomatidae), Thysanura (Nicoletiidae: Nicoletiinae), Collembola (Sminthuridae, Entomobryidae, Isotomidae), Orthoptera (Phalangopsidae: *Aclodes* sp., *Phalangopsis* sp.), Blattodea (Blattellidae), Psocoptera (Epipsocidae, Myopsocidae: *Lichenomina* sp.), Hemiptera (Cydnidae, Enicocephalidae), Homoptera (Cixiidae: *Cixius* sp.), Lepidoptera (Tineidae), Diptera (Cecidomyiidae, Ceratopogonidae, Culicidae, Drosophilidae, Phoridae, Psychodidae: *Lutzomyia* sp., Sciaridae), Hymenoptera (Formicidae: *Azteca* sp., *Crematogaster* sp., *Linepthema* sp., *Pachycondyla* sp., *Pseudomyrmex* sp., *Solenopsis* sp., *Tapinoma* sp.), Coleoptera (Carabidae, Pselaphidae, Scydmaenidae, Staphylinidae), Diplopoda (Chelodesmidae, Polydesmida, Siphonophoridae, Pseudonannolenidae), Scolopendromorpha (Ototigmidae: *Otostigmus* sp., Scolopocryptopidae: *Dinocryptops* sp.), Scutigeromorpha (Scutigeridae: *Sphendononema* sp.) e Symphyla (Scutigerellidae: *Hanseniella* sp., *Scutigerella* sp.).

Dentre os vertebrados foram encontradas duas espécies das Ordens: Chiroptera e Anura (Leptodactylidae: *Pristimantis* cf. *fenestratus*). Desta forma, no total foram encontradas 78 morfoespécies. Entre estas, duas espécies de invertebrados foram consideradas troglomórficas: Amblypygi (Charinidae: *Charinus* sp.) e Collembola (Isotomidae).

5.4.4.49. SL-052

5.4.4.49.1. Caracterização trófica

Pequena cavidade com 10 m de projeção horizontal localizada em área de pastagem na margem direita de um pequeno rio. Formada em canga em condição bem superficial, possui entrada ampla e apresenta teto baixo ao longo de toda sua extensão. A região de entrada é eufótica, apresentando paredes e pisos revestidos por líquens, briófitas, pteridófitas e plântulas. Não apresenta zona afótica e seu piso é descendente e seco composto por sedimento de granulometria variável, com blocos de diferentes tamanhos (seixos, calhaus e matacões). Existem alguns depósitos frescos de guano de morcegos frugívoros, sendo estes produzidos por uma pequena colônia de Glossophaginae. O piso é predominantemente descendente, o que favorece a importação de matéria orgânica externa para o interior da cavidade pela água, especialmente nos períodos chuvosos. Desta forma, material orgânico

de origem externa encontra-se distribuído de maneira esparsa pela cavidade. Junto à linha d'água existe uma árvore de médio porte que contribui de maneira significativa para a produção de serrapilheira, que é importada para a cavidade. De forma geral, existem poucas raízes superficiais ou sub-superficiais, o sistema de canalículos é pouco desenvolvido e as paredes e teto são revestidos por microorganismos de coloração branca. Foi observado um ninho de cupins em uma porção interior da caverna. Tais ninhos, mesmo depois de abandonados pela colônia de cupins, podem fornecer recursos orgânicos a certos organismos cavernícolas (Figura 167).

A caverna apresenta baixa estabilidade ambiental, estando sob alta influência do sistema externo. Durante a estação úmida, a cavidade apresentava maior umidade relativa do ar e apenas um ponto de gotejamento ativo em sua porção mais distal.

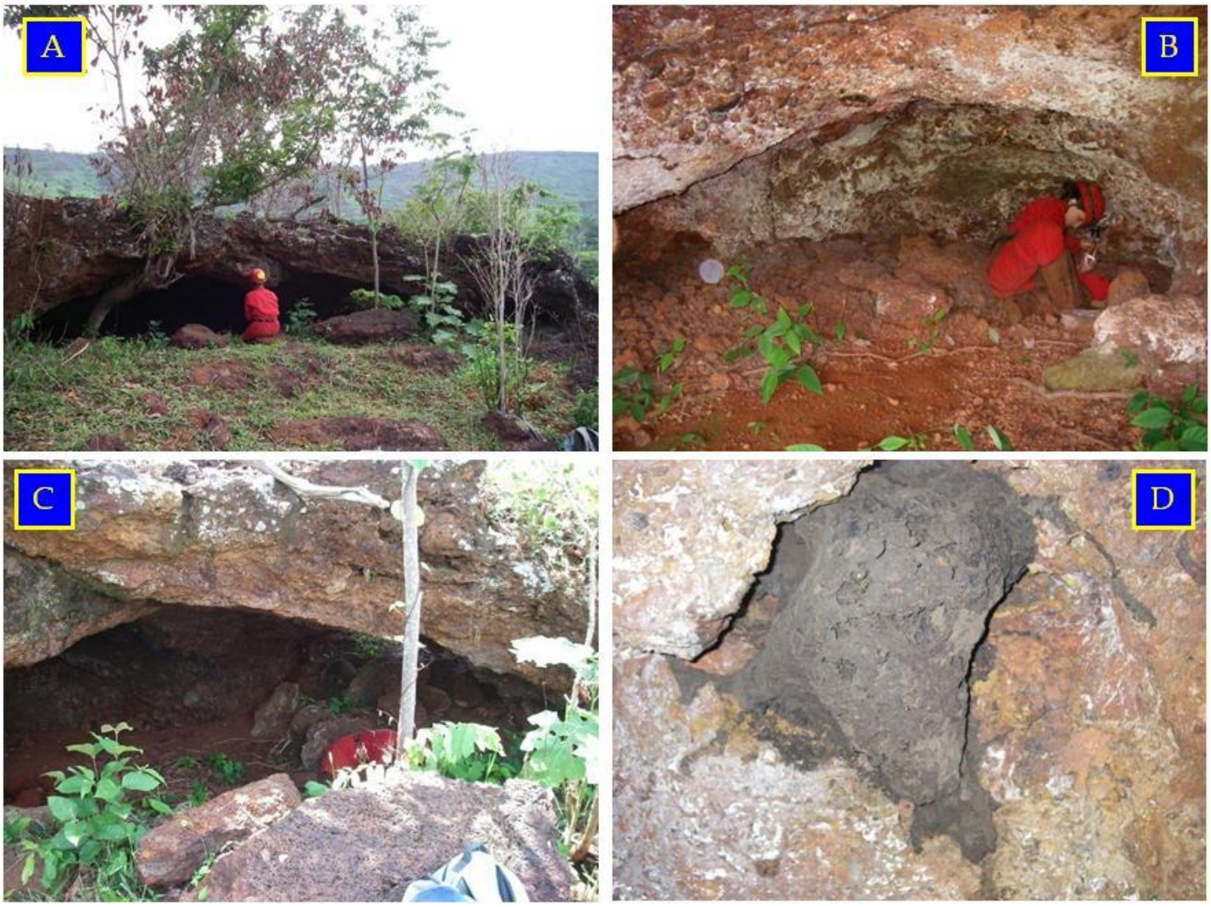

Figura 167 - a) Vista externa da entrada da cavidade onde se observa a matriz da paisagem composta principalmente por pastagem; b) Pequeno salão principal da cavidade, onde se observa a presença de vegetais superiores, domosntrando a condição fótica observada na caverna; c) Outra vista do salão principal, onde se observa opiso composto por sedimento granulado com muitos seixos e calhaus; d) Ninho de cupins abandonado, presente no interior da cavidade.

5.4.4.49.2. Caracterização faunística no período de seca

Foi observado na caverna, um total de 41 morfoespécies de invertebrados de pelo menos 25 famílias das Ordens: Isopoda (Balloniscidae, Phalacridae, Plathyarthridae: *Trichorhina* sp.), Acari (Neothyridae: *Diplothyrus schubarti*), Pseudoescorpiones (Chernetidae), Opiliones (Escadabiidae), Araneae (Gnaphosidae, Salticidae, Scytodidae*: Scytodes eleonora*, Pholcidae, Theraphosidae), Diplura (Campodeidae), Collembola (Entomobryidae), Orthoptera (Phalangopsidae: *Aclodes* sp. *Phalangopsis* sp.), Blattodea (Polyphagidae), Psocoptera (Archipsocidae), Hemiptera (Cydnidae, Reduviidae) Diptera: (Psychodidae: *Lutzomyia* sp.; Streblidae Tipulidae), Hymenoptera (Formicidae: *Atta* sp., *Azteca* sp., *Camponotus* sp., *Crematogaster* sp., *Pachycondyla* sp., *Pheidole* sp., *Solenopsis* sp., *Tapinoma* sp.), Coleptera (Ptylidae), Diplopoda (Chelodesmidae), Scolopendromorpha (Cryptopidae: *Cryptops* sp.), Symphyla (Scutigerellidae: *Hanseniella* sp., *Scutigerella* sp.).

Dentre os vertebrados, foram encontradas duas espécies das ordens Chiroptera (Phyllostomidae: *Glossophaga soricina*) e Anura (Leptodactylidae: *Pristimantis cf. fenestratus*).

5.4.4.49.3. Caracterização faunística no período de chuva

Foi observado na caverna, um total de 44 morfoespécies de invertebrados de pelo menos 33 famílias das Ordens: Oligochaeta, Acari (Mesostigmata: Uropodina), Palpigradi (Eukoenennidae: *Eukoenennia* sp.), Pseudoescorpiones (Chernetidae, Chtoniidae), Araneae (Gnaphosidae, Salticidae, Scytodidae*: Scytodes eleonora*, Pholcidae, Theridiidae), Microcrocoryphia (Meinertellidae), Diplura (Campodeidae), Collembola (Entomobryidae), Neuroptera (Myrmeleontidae), Blattodea (Blattellidae) Isoptera (Termitidae: *Subulitermes* sp., *Termes* sp.), Psocoptera(Ectopsocidae), Hemiptera (Cydnidae, Lygaeidae) Lepidoptera (Noctuidae, Tineidae), Diptera (Cecidomyiidae, Drosophilidae, Milichiidae, Psychodidae: *Lutzomyia* sp.) Hymenoptera (Formicidae: *Apterostigma* sp., *Azteca* sp., *Crematogaster* sp., *Pseudomyrmex* sp., *Pheidole* sp.; Apidae), Coleoptera (Dermestidae, Tenebrionidae: Coelometropinae), Diplopoda (Chelodesmidae, Pyrgodesmidae, Pseudonannolenidae), Scolopendromorpha (Cryptopidae: *Cryptops* sp.) Symphyla (*Hanseniella* sp.).

Dentre os vertebrados, foram encontradas três espécies das ordens Chiroptera (Emballonuridae: *Peropteryx kappleri*; Phyllostomidae: *Glossophaga soricina*) e Anura (Leptodactylidae: *Pristimantis cf. fenestratus*). Além destes, duas espécies de invertebrados foram considerados troglomórficos (Gastropoda: Systrophiidae; Polydesmida).

5.4.4.49.4. Caracterização geral da fauna da cavidade

Foi observado na caverna, um total de 77 morfoespécies de invertebrados de pelo menos 45 famílias das Ordens: Oligochaeta, Isopoda (Balloniscidae, Phalacridae, Plathyarthridae:

Trichorhina sp.), Acari (Mesostigmata: Uropodina; Neothyridae: *Diplothyrus schubarti*), Palpigradi (Eukoenennidae: *Eukoenennia* sp.), Pseudoescorpiones (Chernetidae, Chtoniidae), Opiliones (Escadabiidae), Araneae (Gnaphosidae, Salticidae, Scytodidae*: Scytodes eleonora*, Pholcidae, Theraphosidae, Theridiidae), Microcrocoryphia (Meinertellidae), Diplura (Campodeidae), Collembola (Entomobryidae), Neuroptera (Myrmeleontidae), Orthoptera (Phalangopsidae: *Aclodes* sp. *Phalangopsis* sp.), Blattodea (Blattellidae , Polyphagidae), Psocoptera (Archipsocidae), Isoptera (Termitidae: *Subulitermes* sp., *Termes* sp.), Psocoptera(Ectopsocidae), Hemiptera (Cydnidae, Lygaeidae, Reduviidae), Diptera: (Cecidomyiidae, Drosophilidae, Milichiidae, Psychodidae: *Lutzomyia* sp.; Streblidae Tipulidae), Lepidoptera (Noctuidae, Tineidae), Hymenoptera (Formicidae: *Apterostigma* sp., *Atta* sp., *Azteca* sp., *Camponotus* sp., *Crematogaster* sp., *Pseudomyrmex* sp., *Pachycondyla* sp., *Pheidole* sp.; *Solenopsis* sp., *Tapinoma* sp., Apidae), Coleoptera (Dermestidae, Ptylidae, Tenebrionidae: Coelometropinae), Diplopoda (Chelodesmidae, Pyrgodesmidae; Pseudonannolenidae), Scolopendromorpha (Cryptopidae: *Cryptops* sp.), Symphyla (*Hanseniella* sp., *Scutigerella* sp.).

Dentre os vertebrados, foram encontradas três espécies das ordens Chiroptera (Emballonuridae: *Peropteryx kappleri*; Phyllostomidae: *Glossophaga soricina*) e Anura (Leptodactylidae: *Pristimantis cf. fenestratus*).

Desta forma, no total foram encontradas 80 morfoespécies. Entre estas duas espécies de invertebrados, foram consideradas troglomórficos (Gastropoda: Systrophiidae; Polydesmida). Alguns organismos encontrados nesta caverna são mostrados na Figura 168.

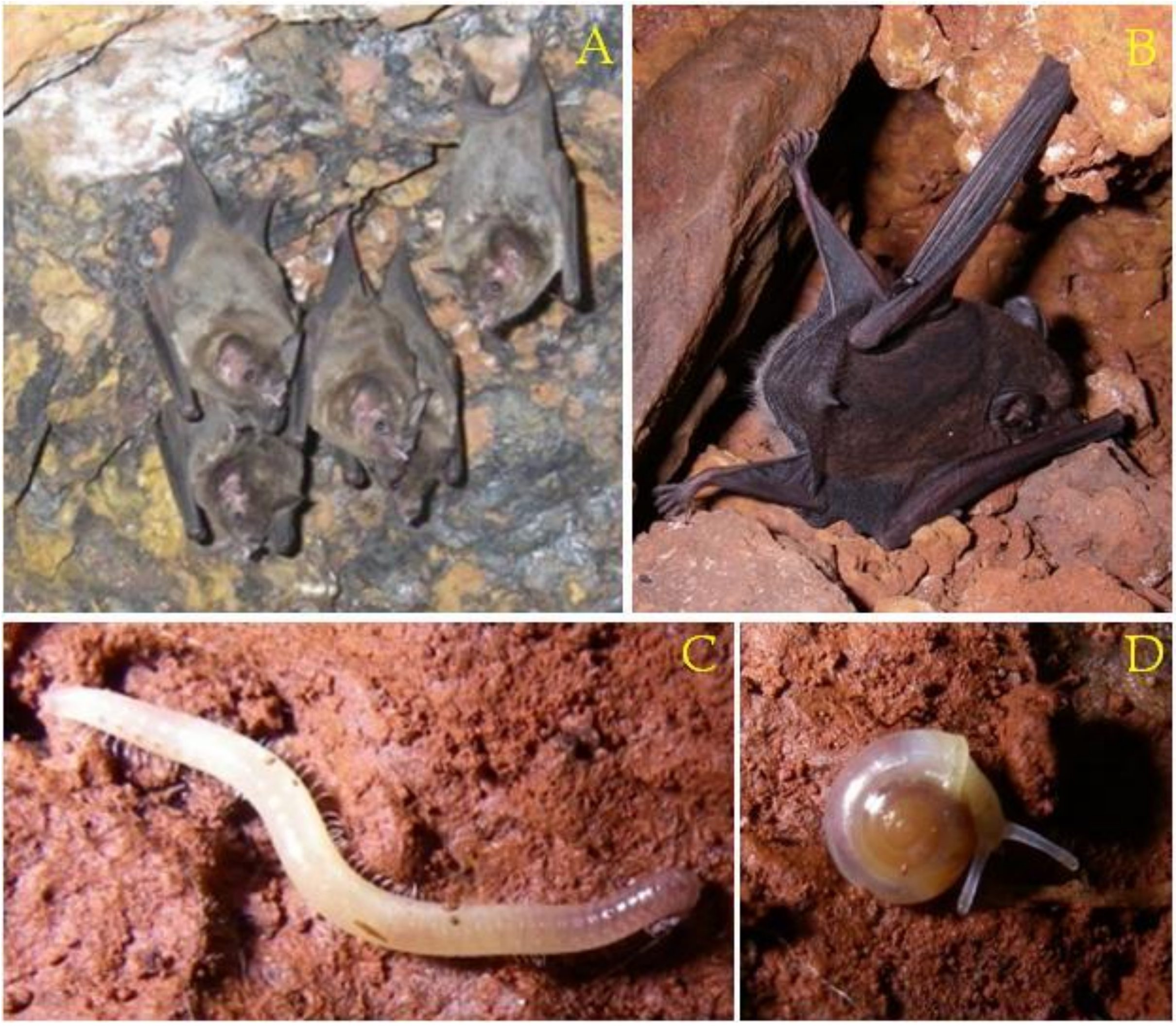

Figura 168 - a) Phyllostomidae (G. soricina); b) Emballonuridae (Peropteryx kappleri); c) Diplopoda (Pseudonannolenidae); d) Gastropoda (Systrophiidae).

5.4.4.50. SL-053

5.4.4.50.1. Caracterização trófica

Pequena cavidade com 12,7 m de projeção horizontal localizada em área de pastagem na margem direita de um pequeno rio. Formada em canga em condição bem superficial, possui entrada ampla e apresenta teto baixo ao longo de todo seu desenvolvimento. A entrada é eufótica com paredes e pisos revestidos por líquens, briófitas, pteridófitas e angiospermas. Não apresenta zona afótica e seu piso é descendente a partir da entrada e predominantemente plano no restante da cavidade, sendo seco e composto por sedimento de granulometria variável, com vários blocos esparsos (seixos e calhaus). Existem alguns pontos de gotejamento ativos, além de depósitos de guano de morcegos frugívoros. A serrapilheira é importada pela ação da água durante os períodos de chuva e pelo vento e está limitada à zona de entrada. As áreas mais profundas da cavidade também apresentam

muitas briófitas e pteridófitas além de um sistema radicular bem desenvolvido (Figura 169). O sistema de canalículos é pouco desenvolvido e as paredes são revestidas por Actinomicetos. Nenhuma alteração significativa foi observada durante a estação úmida além das alterações normais na umidade relativa do ar e do solo.

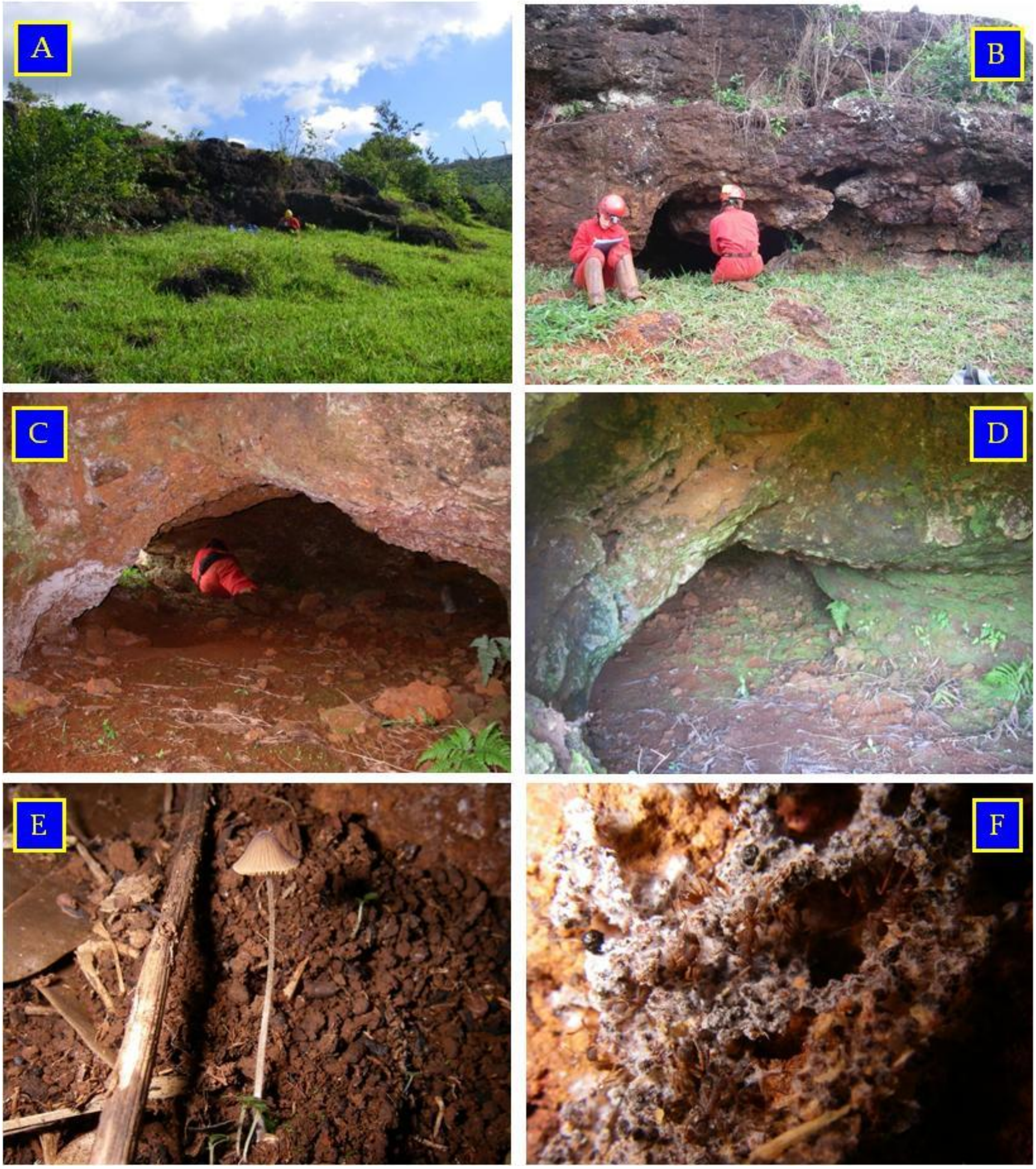

Figura 169 - . a) Vista externa da entrada da cavidade onde se observa a matriz da paisagem composta principalmente por pastagem; b) Detalhe da entrada; c) Salão próximo à entrada da caverna; d) Pequeno salão na zona mais profunda da cavidade com muitas briófitas e blocos esparsos pelo piso; e) Fungo Basidiomiceto se desenvolvendo no piso da cavidade; f) Teto da cavidade com uma estrutura fúngica cultivada por formigas cortadeiras (Apterostigma sp.).

5.4.4.50.2. Caracterização faunística no período de seca

Foi observado na caverna, um total de 35 morfoespécies de invertebrados de pelo menos 27 famílias das Ordens: Acari (Neothyridae: *Diplothyrus schubarti*; Ixodidae: *Amblyomma* sp., Laelapidae: *Stratiolaelaps* sp., Oribatida), Pseudoescorpiones (Chernetidae), Opiliones (Escadabiidae), Araneae (Dipluridae, Oonopinae: Gamasomorphinae Salticidae, Scytodidae*: Scytodes eleonora*, Pholcidae, Theridiidae), Diplura (Campodeidae), Collembola (Cyphoderidae, Entomobryidae, Tomoceridae), Blattodea (Polyphagidae), Hemiptera (Cydnidae), Homoptera (Delphacidae), Lepidoptera (Tineidae), Hymenoptera (Formicidae: *Atta* sp., *Pachycondyla* sp., *Solenopsis* sp.), Coleptera (Carabidae, Scydmaenidae), Diplopoda (Chelodesmidae), Geophilomorpha (Geophilidae), Scolopendromorpha (Cryptopidae: *Cryptops* sp., Scolopocryptopidae: *Newportia* sp.), Symphyla (Scutigerellidae: *Hanseniella* sp.).

Dentre os vertebrados, foram encontradas três espécies das ordens Chiroptera (Emballonuridae: *Peropteryx kappleri*; Phyllostomidae: *Glossophaga soricina*) e Anura (Leptodactylidae: *Pristimantis cf. fenestratus*).

5.4.4.50.3. Caracterização faunística no período de chuva

Foi observado na caverna, um total de 52 morfoespécies de invertebrados de pelo menos 37 famílias das Ordens: Isopoda (Dubioniscidae), Acari (Neothyridae: *Diplothyrus schubarti*; Ixodidae*: Amblyomma* sp., Laelapidae: *Stratiolaelaps* sp.; Oribatida), Palpigradi (Eukoenennidae: *Eukoenennia* sp.), Scorpiones (*Ananteris luciae*), Pseudoescorpiones (Chernetidae), Opiliones (Escadabiidae, Kimmulidae, Phalangiidae), Araneae (Oonopidae: Oonopinae, Gamasomorphinae; Scytodidae*: Scytodes eleonora*, Pholcidae), Microcoryphia (Meinertellidae), Collembola (Entomobryidae Paronellidae), Neuroptera (Chrysopidae), Orthoptera (Phalangopsidae: *Aclodes* sp., *Phalangopsis* sp.), Blattodea (Polyphagidae), Psocoptera (Archipsocidae, Epipsocidae, Myopsocidae: *Lichenomina* sp.), Hemiptera (Cydnidae, Lygaeidae, Ploiariidae, Pyrrhocoridae), Homoptera (Cecidomyiidae), Diptera (Drosophilidae, Mycetophilidae, Psychodidae: *Lutzomyia* sp., Sciaridae), Hymenoptera (Formicidae: *Acromyrmex* sp., *Apterostigma* sp., *Atta* sp., *Gnamptogenys* sp., *Pachycondyla* sp. *Pheidole* sp.), Coleoptera (Pselaphidae, Scydmaenidae), Diplopoda (Polydesmida; Glomeridesmidae: *Glomeridesmus* sp.), Scolopendromorpha (Cryptopidae: *Cryptops* sp.).

Dentre os vertebrados, foram encontradas três espécies das ordens Chiroptera (Emballonuridae: *Peropteryx kappleri*; Phyllostomidae: *Glossophaga soricina*) e Anura (Leptodactylidae: *Pristimantis cf. fenestratus*). Além destes, apenas uma espécie foi considerada troglomórfica (Gastropoda: Systrophiidae).

5.4.4.50.4. Caracterização geral da fauna da cavidade

Foi observado na caverna, um total de 74 morfoespécies de invertebrados de pelo menos 50 famílias das Ordens: Isopoda (Dubioniscidae), Acari (Neothyridae: *Diplothyrus schubarti*; Ixodidae*: Amblyomma* sp., Laelapidae: *Stratiolaelaps* sp.; Oribatida), Palpigradi (Eukoenennidae: *Eukoenennia* sp.), Scorpiones (*Ananteris luciae*), Pseudoescorpiones (Chernetidae), Opiliones (Escadabiidae, Kimmulidae, Phalangiidae), Araneae (Dipluridae, Oonopidae: Oonopinae, Gamasomorphinae; Salticidae, Scytodidae*: Scytodes eleonora*, Pholcidae, Theridiidae), Microcoryphia (Meinertellidae), Diplura (Campodeidae), Collembola (Cyphoderidae, Entomobryidae, Paronellidae, Tomoceridae), Neuroptera (Chrysopidae), Orthoptera (Phalangopsidae: *Aclodes* sp., *Phalangopsis* sp.), Blattodea (Polyphagidae), Psocoptera (Archipsocidae, Epipsocidae, Myopsocidae: *Lichenomina* sp.), Hemiptera (Cydnidae, Lygaeidae, Ploiariidae, Pyrrhocoridae), Homoptera (Cecidomyiidae, Delphacidae), Diptera (Drosophilidae, Mycetophilidae, Psychodidae: *Lutzomyia* sp., Sciaridae), Lepidoptera (Tineidae), Hymenoptera (Formicidae: *Acromyrmex* sp., *Apterostigma* sp., *Atta* sp., *Gnamptogenys* sp., *Pachycondyla* sp. *Pheidole* sp., *Solenopsis* sp.), Coleoptera (Carabidae, Pselaphidae, Scydmaenidae), Diplopoda (Chelodesmidae; Glomeridesmidae: *Glomeridesmus* sp.), Geophilomorpha (Geophilidae), Scolopendromorpha (Cryptopidae: *Cryptops* sp.; Scolopocryptopidae: *Newportia* sp.), Symphyla (Scutigerellidae: *Hanseniella* sp.).

Dentre os vertebrados, foram encontradas três espécies das ordens Chiroptera (Emballonuridae: *Peropteryx kappleri*; Phyllostomidae: *Glossophaga soricina*) e Anura (Leptodactylidae: *Pristimantis cf. fenestratus*).

Desta forma, no total foram encontradas 77 morfoespécies. Entre estas apenas uma espécie de invertebrado foi considerado troglomórfico (Gastropoda: Systrophiidae).

5.4.4.51. SL-054

5.4.4.51.1. Caracterização trófica

Pequena cavidade com 8,7 metros de projeção horizontal localizada em área de pastagem na margem direita de uma pequena drenagem. Formada em canga em condição bem superficial, a caverna apresenta trechos de teto baixo e rastejo ao longo de toda sua extensão. A entrada é eufótica com paredes e pisos revestidos por líquens, briófitas, pteridófitas e plântulas além de uma pequena quantidade de serrapilheira junto à linha d'água. Esta cavidade não apresenta zona afótica e seu piso é predominantemente plano, mas levemente descendente a partir da entrada, sendo este composto por sedimento de granulometria variável. Existem dois pequenos depósitos frescos de guano de morcegos frugívoros produzidos por uma pequena colônia de Glossophaga soricina. A caverna é seca, sob alta influência das condições epígeas, com uma grande quantidade de microorganismos

(Actinomicetos) revestindo as paredes e o teto e com sistema de canalículos pouco desenvolvido (Figura 170). Nenhuma alteração significativa foi observada durante a estação úmida, além das alterações normais na umidade relativa do ar.

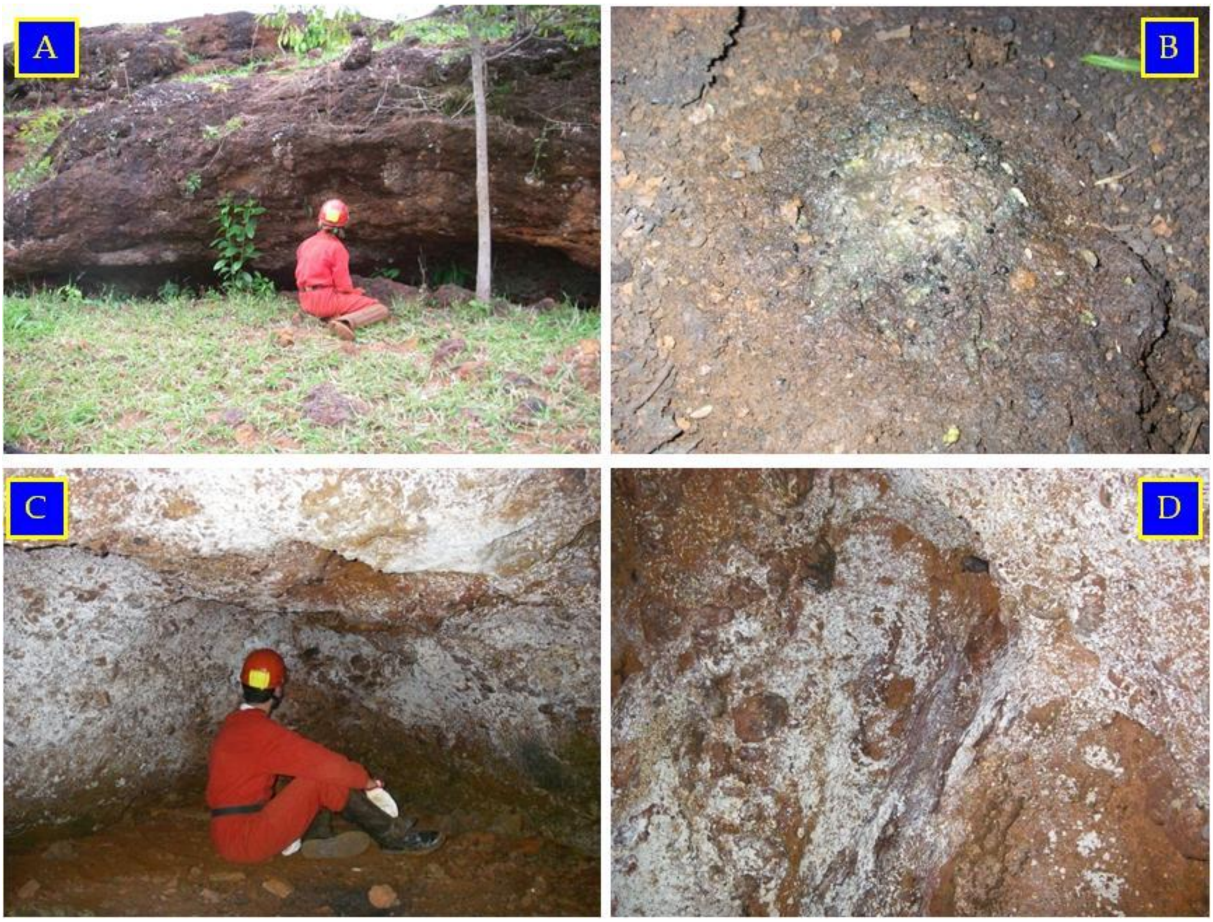

Figura 170 - a) Vista externa da entrada da cavidade onde se observa a matriz da paisagem composta principalmente por pastagem; b) Depósito recente de guano de morcegos frugívoros; c) porção interna da cavidade na qual se observa as paredes revestidas por Actinomicetos; d) detalhe das paredes internas revestidas por Actinomicetos.

5.4.4.51.2. Caracterização faunística no período de seca

Foi observado na caverna, um total de 47 morfoespécies de invertebrados de pelo menos 31 famílias das Ordens: Isopoda (Dubioniscidae), Acari (Laelapidae: *Stratiolaelaps* sp.; Astigmatina; Oribatida), Amblypygi (*Heterophrynus longicornis*), Pseudoescorpiones (Chernetidae), Araneae (Gnaphosidae, Oonopidae: Oonopinae; Salticidae, Scytodidae*:* *Scytodes eleonora*, Pholcidae, Theraphosidae), Thysanura (Nicoletiidae: Nicolletiinae), Diplura (Campodeidae), Collembola (Isotomidae), Orthoptera (Phalangopsidae: *Aclodes* sp., *Phalangopsis* sp.), Blattodea (Polyphagidae), Psocoptera (Archipsocidae, Epipsocidae), Hemiptera (Cydnidae, Reduviidae), Lepidoptera (Tineidae), Diptera (Ceratopogonidae, Drosophilidae, Streblidae), Hymenoptera (Formicidae: *Gnamptogenys* sp., *Octostruma* sp.,

Pachycondyla sp., *Pheidole* sp., *Solenopsis* sp.), Coleoptera (Leiodidae), Diplopoda (Chelodesmidae), Scolopendromorpha (Cryptopidae: *Cryptops* sp.; Scolopocryptopidae: *Tidops* sp.) Scutigeromorpha (Scutigeridae: *Sphendononema* sp., *Hanseniella* sp.).

Dentre os vertebrados, foram encontradas duas espécies das ordens Chiroptera (Phyllostomidae: *Glossophaga soricina*) e Anura (Leptodactylidae: *Pristimantis cf. fenestratus*).

Além destes, duas espécies de invertebrados, foram considerados troglomórficos (Isopoda: Plathyarthridae: *Trichorhina* sp.; Oonopidae: Oonopinae; Ptylidae).

5.4.4.51.3. Caracterização faunística no período de chuva

Foi observado na caverna, um total de 33 morfoespécies de invertebrados de pelo menos 25 famílias das Ordens: Isopoda (Dubioniscidae), Acari (Oribatida, Anystidae: *Erythracarus* sp.), Pseudoescorpiones (Chernetidae), Opiliones (Escadabiidae, Fissiphaliidae, Kimmulidae), Araneae (Corinidae, Oonopidae: Oonopinae; Salticidae, Scytodidae*: Scytodes eleonora*, Pholcidae, Theridiosomatidae), Diplura (Campodeidae), Collembola (Entomobryidae), Neuroptera (Chrysopidae), Orthoptera (Phalangopsidae: *Aclodes* sp., *Phalangopsis* sp.), Blattodea (Blattellidae), Psocoptera (Archipsocidae, Epipsocidae), Hemiptera (Cydnidae, Reduviidae), Homoptera, Lepidoptera (Tineidae), Diptera (Psychodidae: *Lutzomyia* sp.), Hymenoptera (Formicidae: *Camponotus* sp., *Pseudomyrmex* sp., *Pheidole* sp.), Coleoptera (Elateridae, Phalacridae, Tenebrionidae), Scolopendromorpha (Cryptopidae: Cryptopinae; Scolopocryptopidae: *Dinocryptops* sp.). Além destes, duas espécies de invertebrados, foram considerados troglomórficos (Gastropoda: Systrophiidae; Polydesmida).

5.4.4.51.4. Caracterização geral da fauna da cavidade

Foi observado na caverna, um total de 76 morfoespécies de invertebrados de pelo menos 57 famílias das Ordens: Gastropoda (Systrophiidae), Isopoda (Dubioniscidae, Plathyarthridae: *Trichorhina* sp.), Acari (Laelapidae: *Stratiolaelaps* sp.; Astigmatina; Oribatida, Anystidae: *Erythracarus* sp.), Amblypygi (*Heterophrynus longicornis*), Pseudoescorpiones (Chernetidae), Opiliones (Escadabiidae, Fissiphaliidae, Kimmulidae), Araneae (Corinidae, Gnaphosidae, Oonopidae: Oonopinae; Salticidae, Scytodidae*: Scytodes eleonora*, Pholcidae, Theraphosidae, Theridiosomatidae), Thysanura (Nicoletiidae: Nicolletiinae), Diplura (Campodeidae), Collembola (Entomobryidae, Isotomidae), Neuroptera (Chrysopidae), Orthoptera (Phalangopsidae: *Aclodes* sp., *Phalangopsis* sp.), Blattodea (Blattellidae, Polyphagidae), Psocoptera (Archipsocidae, Epipsocidae), Hemiptera (Cydnidae, Reduviidae), Homoptera, Lepidoptera (Tineidae), Diptera (Ceratopogonidae, Drosophilidae, Psychodidae: *Lutzomyia* sp.; Streblidae), Hymenoptera (Formicidae: *Camponotus* sp., *Gnamptogenys* sp., *Octostruma* sp., *Pachycondyla* sp., *Pseudomyrmex* sp., *Pheidole* sp., *Solenopsis* sp.), Coleoptera (Elateridae, Leiodidae, Tenebrionidae, Phalacridae, Ptylidae), Diplopoda

(Chelodesmidae), Scolopendromorpha (Cryptopidae: Cryptopinae, *Cryptops* sp.; Scolopocryptopidae: *Dinocryptops* sp., *Tidops* sp.) Scutigeromorpha (Scutigeridae: *Sphendononema* sp., *Hanseniella* sp.).

Dentre os vertebrados, foram encontradas duas espécies das ordens Chiroptera (Phyllostomidae: *Glossophaga soricina*) e Anura (Leptodactylidae: *Pristimantis cf. fenestratus*).

Desta forma, no total foram encontradas 78 morfoespécies. Entre estas duas espécies de invertebrados, foram consideradas troglomórficas (Gastropoda: Systrophiidae; Isopoda: Plathyarthridae: *Trichorhina* sp.; Oonopidae: Oonopinae; Ptylidae; Polydesmida).

5.4.4.52. SL-055

5.4.4.52.1. Caracterização trófica

Pequena caverna com 5,2 m de extensão localizada em área de pastagem na margem direita de uma pequena drenagem. Formada em canga em condição bem superficial, apresenta teto baixo ao longo de todo seu desenvolvimento e sua entrada é repleta de líquens, briófitas, pteridófitas e plântulas. Não possui zona afótica e seu piso é plano e seco com vários blocos de diferentes tamanhos (seixos, calhaus e matacões). A serrapilheira encontra-se restrita à zona de entrada (Figura 171) e foram observados pequenos depósitos de guano de morcegos frugívoros na porção mais interior da cavidade somente na estação úmida. A caverna apresenta um sistema de canalículos pouco desenvolvido e apresenta-se sob alta influência das condições epígeas. As paredes das porções internas são revestidas por Actinomicetos.

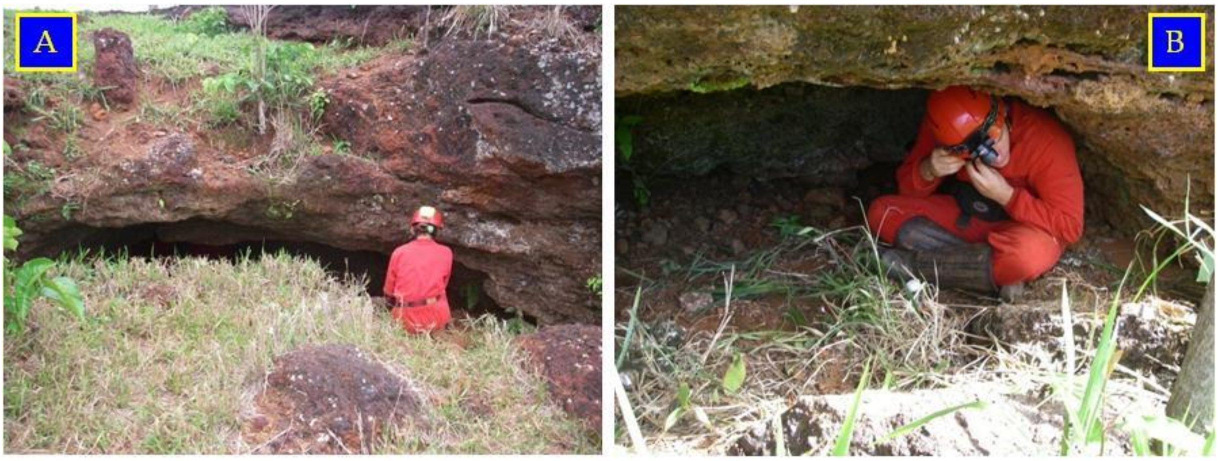

Figura 171 - a) Vista externa da entrada da cavidade onde se observa a matriz da paisagem composta principalmente por pastagem; b) Trecho em teto baixo sob alta influência das condições ambientais epígeas.

5.4.4.52.2. Caracterização faunística no período de seca

Foi observado na caverna, um total de 25 morfoespécies de invertebrados de pelo menos 16 famílias das Ordens: Oligochaeta, Acari (Laelapidae: *Stratiolaelaps* sp., Oribatida), Pseudoescorpiones (Chernetidae, Chtoniidae), Opiliones (Fissiphaliidae, Kimmulidae), Araneae (Oonopidae: Ooonopinae; Scytodidae*: Scytodes eleonora*, Theridiidae), Thysanura (Nicoletiidae: NIcoletiinae), Diplura (Campodeidae), Collembola (Cyphoderidae, Entomobryidae, Hypogastruridae), Orthoptera (Gryllidae: Nemobiinae), Lepidoptera (Geometridae), Blattodea (Polyphagidae), Embiidina, Hymenoptera (Formicidae: *Hypoponera* sp., *Pheidole* sp., *Solenopsis* sp., *Zacryptocerus* sp.), Coleoptera (Scydmaenidae), Diplopoda (Pyrgodesmidae), Scolopendromorpha (Cryptopidae: *Cryptops* sp.).

Dentre os vertebrados, foi encontrada uma espécie da ordem Chiroptera (Phyllostomidae: *Glossophaga soricina*).

Além destes, uma espécie de invertebrado, foi considerado troglomórfico (Polydesmida: Pyrgodesmidae).

5.4.4.52.3. Caracterização faunística no período de chuva

Foi observado na caverna, um total de 44 morfoespécies de invertebrados de pelo menos 37 famílias das Ordens: Oligochaeta, Isopoda (Dubioniscidae, Plathyarthridae: *Trichorhina* sp.), Acari (Laelapidae: *Stratiolaelaps* sp.), Pseudoescorpiones (Chernetidae, Chtoniidae), Opiliones (Stygnidae), Araneae (Dipluridae, Linyphiidae, Ochyroceratidae, Oonopidae: Ooonopinae; Salticidae, Scytodidae*: Scytodes eleonora*, Pholcidae), Diplura (Campodeidae), Collembola (Cyphoderidae, Entomobryidae, Hypogastruridae), Blattodea (Blattidae), Embiidina, Isoptera (Termitidae: *Velocitermes* sp.), Psocoptera: Epipsocidae, Myopsocidae: *Lichenomina* sp.; Psyllipsocidae: *Psyllipsocus* sp.), Hemiptera (Cydnidae, Lygaeidae), Diptera (Cecidomyiidae, Drosophilidae, Mycetophilidae), Hymenoptera (Formicidae: *Ectatomma* sp., *Hypoponera* sp., *Octostruma* sp., *Pachycondyla* sp., *Pheidole* sp., *Solenopsis* sp.) Coleoptera (Carabidae, Staphylinidae), Diplopoda (Oniscodesmidae, Stemmiulidae), Lithobiomorpha (*Lamyctes* sp.), Scolopendromorpha (Cryptopidae: *Cryptops* sp.), Scutigeromorpha (Scutigeridae: *Sphendononema* sp.), Neuroptera (Myrmeleontidae).

Dentre os vertebrados, foi encontrada uma espécie da ordem Chiroptera (Phyllostomidae: *Glossophaga soricina*).

Além destes, uma espécie de invertebrado, foi considerado troglomórfico (Isopoda - Plathyarthridae: *Trichorhina* sp.Scolopendromorpha - Cryptopidae: *Cryptops* sp.).

5.4.4.52.4. Caracterização geral da fauna da cavidade

Foi observado na caverna, um total de 63 morfoespécies de invertebrados de pelo menos 47 famílias das Ordens: Oligochaeta, Isopoda (Dubioniscidae, Plathyarthridae: *Trichorhina* sp.), Acari (Laelapidae: *Stratiolaelaps* sp., Oribatida), Pseudoescorpiones (Chernetidae, Chtoniidae), Opiliones (Fissiphaliidae, Kimmulidae, Stygnidae), Araneae (Dipluridae, Linyphiidae, Ochyroceratidae, Oonopidae: Ooonopinae; Salticidae, Scytodidae*: Scytodes eleonora*, Pholcidae, Theridiidae), Thysanura (Nicoletiidae: NIcoletiinae), Diplura (Campodeidae), Collembola (Cyphoderidae, Entomobryidae, Hypogastruridae), Orthoptera (Gryllidae: Nemobiinae), Blattodea (Blattidae, Polyphagidae), Embiidina, Isoptera (Termitidae: *Velocitermes* sp.), Psocoptera: Epipsocidae, Myopsocidae: *Lichenomina* sp.; Psyllipsocidae: *Psyllipsocus* sp.), Hemiptera (Cydnidae, Lygaeidae), Homoptera (Cecidomyiidae), Lepidoptera (Geometridae), Diptera (Cecidomyiidae, Drosophilidae, Mycetophilidae, Sciaridae), Hymenoptera (Formicidae: *Ectatomma* sp., *Hypoponera* sp., *Octostruma* sp., *Pachycondyla* sp., *Pheidole* sp., *Solenopsis* sp., *Zacryptocerus* sp.) Coleoptera (Carabidae, Staphylinidae, Scydmaenidae) Diplopoda (Oniscodesmidae, Pyrgodesmidae, Stemmiulidae), Lithobiomorpha (*Lamyctes* sp.), Scolopendromorpha (Cryptopidae: *Cryptops* sp.), Scutigeromorpha (Scutigeridae: *Sphendononema* sp.), Neuroptera (Myrmeleontidae).

Dentre os vertebrados, foi encontrado uma espécie da ordem Chiroptera (Phyllostomidae: *Glossophaga soricina*).
Desta forma, no total foram encontradas 78 morfoespécies. Entre estas duas espécies de invertebrados, foram consideradas troglomórficas (Isopoda: Plathyarthridae: *Trichorhina* sp.; Polydesmida: Pyrgodesmidae).

5.4.4.53. SL-056

5.4.4.53.1. Caracterização trófica

Pequena cavidade com 8 m de projeção horizontal formada em canga e localizada na margem esquerda de uma pequena drenagem. Vegetação do entorno formada por um pequeno remanescente de mata ciliar, estando esta inserida em uma matriz composta por pastagem. A caverna é pouco profunda e apresenta uma entrada ampla e com muitos líquens, briófitas além de alguns brotos de angiospermas e duas pequenas clarabóias por onde são importados recursos orgânicos para o interior da cavidade. Na entrada existem grandes blocos abatidos além de grandes árvores junto à linha d’água. A cavidade encontra-se praticamente no mesmo nível da drenagem intermitente o que permite o carreamento de serrapilheira para seu interior por meio de pulsos de inundação (Figura 172). Não existem depósitos de guano no interior da cavidade e as paredes e o teto são revestidos por Actinomicetos. O piso é irregular e muito encharcado, apresentando muitos depósitos de serrapilheira e raízes de fino calibre. A caverna não apresenta zona afótica, o sistema de

canalículos é pouco desenvolvido e esta se encontra sob forte influência das condições ambientais epígeas. A principal alteração durante a estação úmida foi uma redução na vazão do pequeno riacho localizado próximo à cavidade que, por consequência, promoveu uma redução na mata ciliar presente no entorno da cavidade.

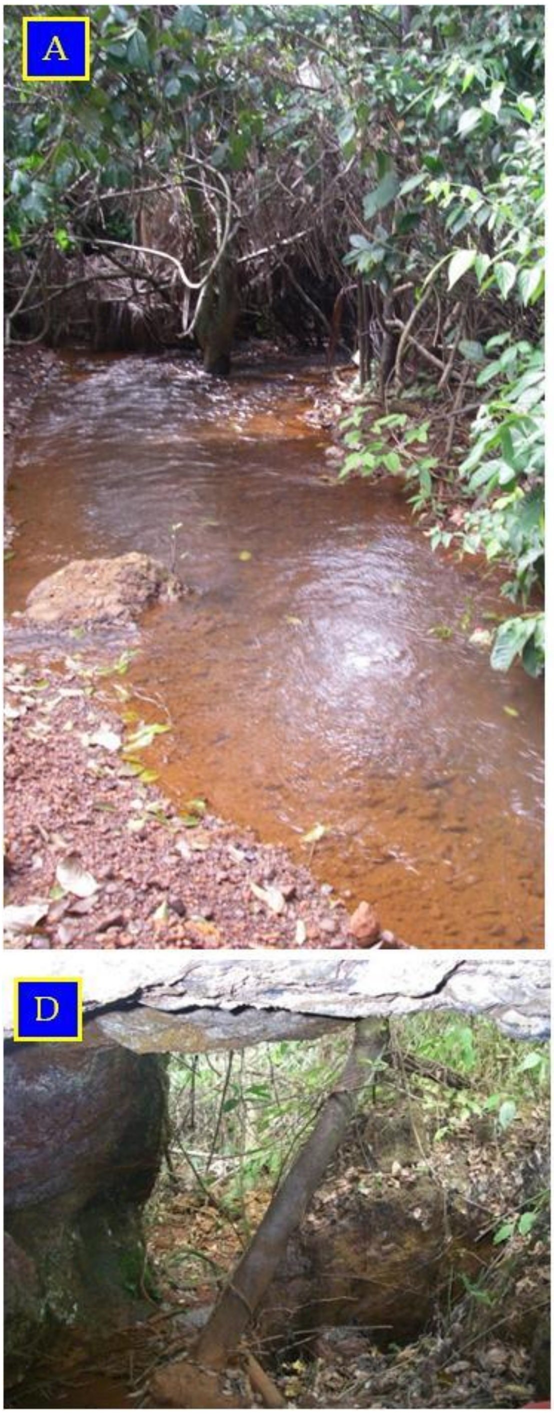

Figura 172 - a) Vista externa da entrada da cavidade onde se observa a drenagem e a área de mata ciliar em estado avançado de degradação; b) Vista da entrada da cavidade; c) Interior da cavidade com piso encharcado e sob forte influência das condições ambientais epígeas; d) detalhe da entrada, onde se observa grande quantidade de material orgânico de origem vegetal que penetra na caverna; e) Interior da cavidade com piso erodido sob efeito de enxurradas.

5.4.4.53.2. Caracterização faunística no período de seca

Foi observado na caverna, um total de 31 morfoespécies de invertebrados de pelo menos 20 famílias das Ordens: Oligochaeta, Acari (Oribatida, Eupodidae: *Linopodes* sp.), Amblypygi (*Heterophrynus longicornis*), Opiliones (Neogoveidae: *Canga renatae*; Sclerosomatidae: *Prionostemma* sp.), Araneae (Pholcidae: *Mesabolivar* sp., Theridiidae), Thysanura (Nicoletiidae: Nicoletiinae), Diplura (Campodeidae), Collembola (Entomobryidae, Tomoceridae), Orthoptera (Phalangopsidae: *Aclodes* sp.), Isoptera (Rhinotermitidae: *Heterotermes* sp.; Termitidae: *Nasutitermes* sp.), Psocoptera (Ptiloneuridae: *Ptiloneura* sp.), Hemiptera (Cydnidae), Diptera (Culicidae, Psychodidae), Hymenoptera (Formicidae: *Atta* sp., *Azteca* sp., *Camponotus* sp., *Dolichoderus* sp., *Hypoponera* sp., *Pachycondyla* sp.), Coleoptera (Pselaphidae, Staphylinidae), Geophilomorpha (Ballophilidae: *Taeniolinum* sp.), Scutigeromorpha (Scutigeridae: *Sphendononema* sp.), Symphyla (Scutigerellidae: *Hanseniella* sp.).

Dentre os vertebrados, foram encontradas três espécies das ordens Chiroptera (Emballonuridae: *Peropteryx kappleri*) e Anura (Dendrobatidae: *Colostethus* sp.; Leptodactylidae: *Pristimantis cf. fenestratus*).

5.4.4.53.3. Caracterização faunística no período de chuva

Foi observado na caverna, um total de 48 morfoespécies de invertebrados de pelo menos 32 famílias das Ordens: Oligochaeta, Acari (Oribatida), Amblypygi (*Heterophrynus longicornis*), Opiliones (Cosmetidae: *Roquettea* sp*.,* Sclerosomatidae: *Prionostemma* sp., Phalangiidae), Araneae (Araneidae, Salticidae, Scytodidae*: Scytodes eleonora*, Pholcidae: *Mesabolivar* sp., Theridiidae, Theridiosomatidae), Collembola (Entomobryidae), Isoptera (Termitidae: *Nasutitermes* sp.), Psocoptera (Ptiloneuridae: *Ptiloneura* sp.), Hemiptera (Cydnidae, Gerridae, Ochteridae, Pseudococcidae), Homoptera (Cixiidae: *Cixius* sp.), Lepidoptera (Noctuidae), Diptera (Cecidomyiidae, Chironomidae, Culicidae, Mycetophilidae, Psychodidae, Sciaridae), Hymenoptera (Formicidae: *Apterostigma* sp., *Atta* sp., *Azteca* sp., *Camponotus* sp., *Dolichoderus* sp., *Pachycondyla* sp., *Prionopeuta* sp., *Rogeria* sp.), Coleoptera (Staphylinidae), Diplopoda (Siphonophoridae), Lithobiomorpha (Henicopiidae: *Lamyctes* sp.), Scutigeromorpha (Scutigeridae: *Sphendononema* sp).

Além destes, duas espécies de invertebrados, foram considerados troglomórficos (Gastropoda (Systrophiidae); Polydesmida: Pyrgodesmidae).

5.4.4.53.4. Caracterização geral da fauna da cavidade

Foi observado na caverna, um total de 65 morfoespécies de invertebrados de pelo menos 47 famílias das Ordens: Oligochaeta, Acari (Oribatida, Eupodidae: *Linopodes* sp.), Amblypygi (*Heterophrynus longicornis*), Opiliones (Neogoveidae: *Canga renatae*; Cosmetidae: Roquettea

sp., Sclerosomatidae: *Prionostemma* sp., Phalangiidae), Araneae (Araneidae, Salticidae, Scytodidae: *Scytodes eleonora*, Pholcidae: *Mesabolivar* sp., Theridiidae, Theridiosomatidae), Thysanura (Nicoletiidae: Nicoletiinae), Diplura (Campodeidae), Collembola (Entomobryidae, Tomoceridae), Orthoptera (Phalangopsidae: *Aclodes* sp.), Isoptera (Rhinotermitidae: *Heterotermes* sp.; Termitidae: *Nasutitermes* sp.), Psocoptera (Ptiloneuridae: *Ptiloneura* sp.), Hemiptera (Cydnidae, Gerridae, Ochteridae, Pseudococcidae), Homoptera (Cixiidae: *Cixius* sp.), Lepidoptera (Noctuidae), Diptera (Cecidomyiidae, Chironomidae, Culicidae, Mycetophilidae, Psychodidae, Sciaridae), Hymenoptera (Formicidae: *Apterostigma* sp., *Atta* sp., *Azteca* sp., *Camponotus* sp., *Dolichoderus* sp., *Hypoponera* sp., *Pachycondyla* sp., *Prionopeuta* sp., *Rogeria* sp.), Coleoptera (Pselaphidae, Staphylinidae), Diplopoda (Siphonophoridae), Geophilomorpha (Ballophilidae: *Taeniolinum* sp.), Lithobiomorpha (Henicopiidae: *Lamyctes* sp.), Scutigeromorpha (Scutigeridae: *Sphendononema* sp.), Symphyla (Scutigerellidae: *Hanseniella* sp.).

Dentre os vertebrados, foi encontrado três espécies das ordens Chiroptera (Emballonuridae: *Peropteryx kappleri*) e Anura (Dendrobatidae: *Colostethus* sp.; Leptodactylidae: *Pristimantis cf. fenestratus*).
Desta forma, no total foram encontradas 68 morfoespécies. Entre estas duas espécies de invertebrados, foram consideradas troglomórficas (Gastropoda (Systrophiidae); Polydesmida: Pyrgodesmidae). Alguns organismos encontrados nesta caverna são mostrados na Figura 173.

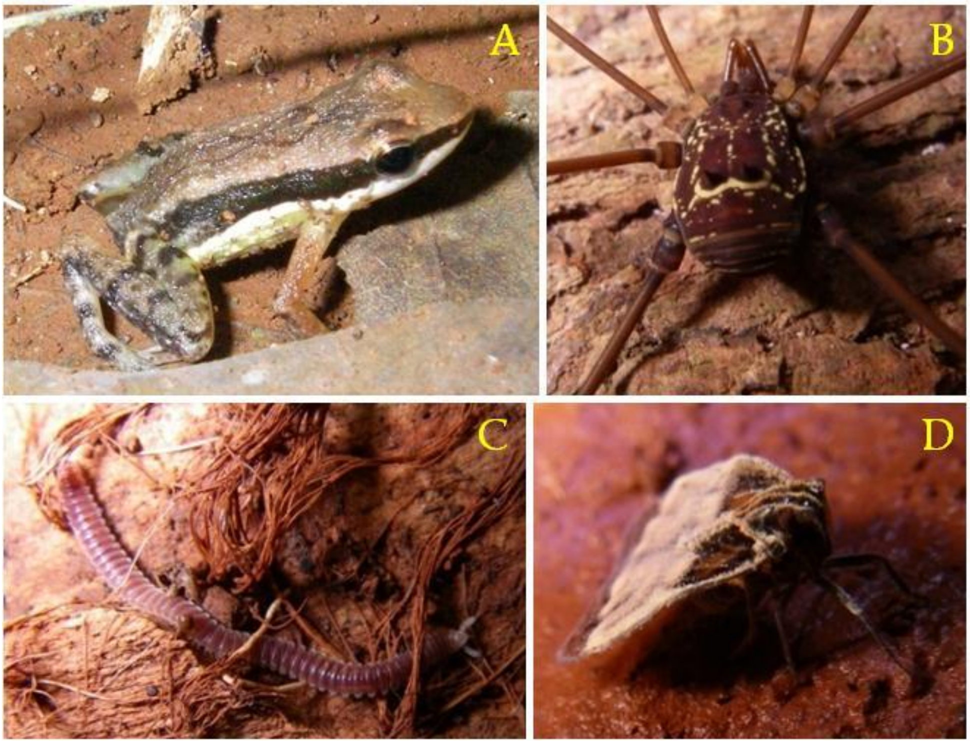

Figura 173 - a) Anura (Dendrobatidae: *Colostethus sp* .); b) Opiliones (Cosmetidae); c) Diplopoda (Siphonophoridae); d) Homoptera (indet.).

5.4.4.54. SL-057

5.4.4.54.1. Caracterização trófica

Caverna confinada, com projeção horizontal de 23,8 m, formada em minério de ferro. Tal cavidade localiza-se próxima ao topo da encosta em uma área de mata pouco densa, apresentando vegetação do tipo savana metalófila na porção logo acima da caverna. Possui uma entrada pequena e seca formada na quebra da canga. O piso que segue-se à entrada é descendente em relação ao meio epígeo, o que favorece a importação de material orgânico pela água em períodos de chuva. A zona de entrada apresenta poucos líquens e muitas briófitas, além de muita serrapilheira acumulada junto à linha d'água. O piso da cavidade é predominantemente plano, sendo composto por sedimentos granulados com seixos, calhaus e matacões. O piso apresenta, ainda, trechos úmidos onde existem gotejamentos ativos alternados com áreas onde o substrato é mais seco. A cavidade apresenta um padrão retilíneo, sem zona afótica, mas com uma extensa área de penumbra escura. O sistema radicular é bem desenvolvido, sendo composto por pequenas raízes de diversos calibres espalhadas pelo piso e paredes. Além disso, as paredes e o teto são revestidos por Actinomicetos. A região mais profunda da cavidade é disfótica e úmida, com pontos de gotejamento e de percolação onde existe uma grande quantidade de ferrobactérias. Nesta região, existem ainda depósitos de guano de morcegos frugívoros e, de forma geral, o sistema de canalículos é pouco desenvolvido. A cavidade apresenta, aparentemente, alta estabilidade ambiental em função do elevado grau de confinamento, o que favorece uma temperatura mais elevada além de uma umidade relativa do ar mais acentuada. Em relação aos vertebrados, na zona intermediária da cavidade foi observado um ninho de roedor (*Rhipidomys sp*.) com filhotes. A única alteração significativa observada durante a estação úmida foi a presença de um "quadrat" de escavação arqueológica em uma porção interior da caverna (Figura 174). É recomendável que estas escavações sejam novamente soterradas (ou ao menos recobertas e protegidas) após os términos dos trabalhos de escavação. Escavações certamente alteram o piso e conseqüentemente os microhabitats das regiões onde são desenvolvidas (especialmente entradas e abrigos, no caso de cavernas). Tais alterações podem causar diferentes impactos sobre a fauna das entradas, que compõe um importante gradiente ecotonal entre sistemas epígeo e hipógeo. Deixar tais escavações abertas, além dos potenciais prejuízos que podem ser causados ao próprio sedimento arqueológico, certamente representa um grande descaso para com a fauna atual residente nas cavernas.

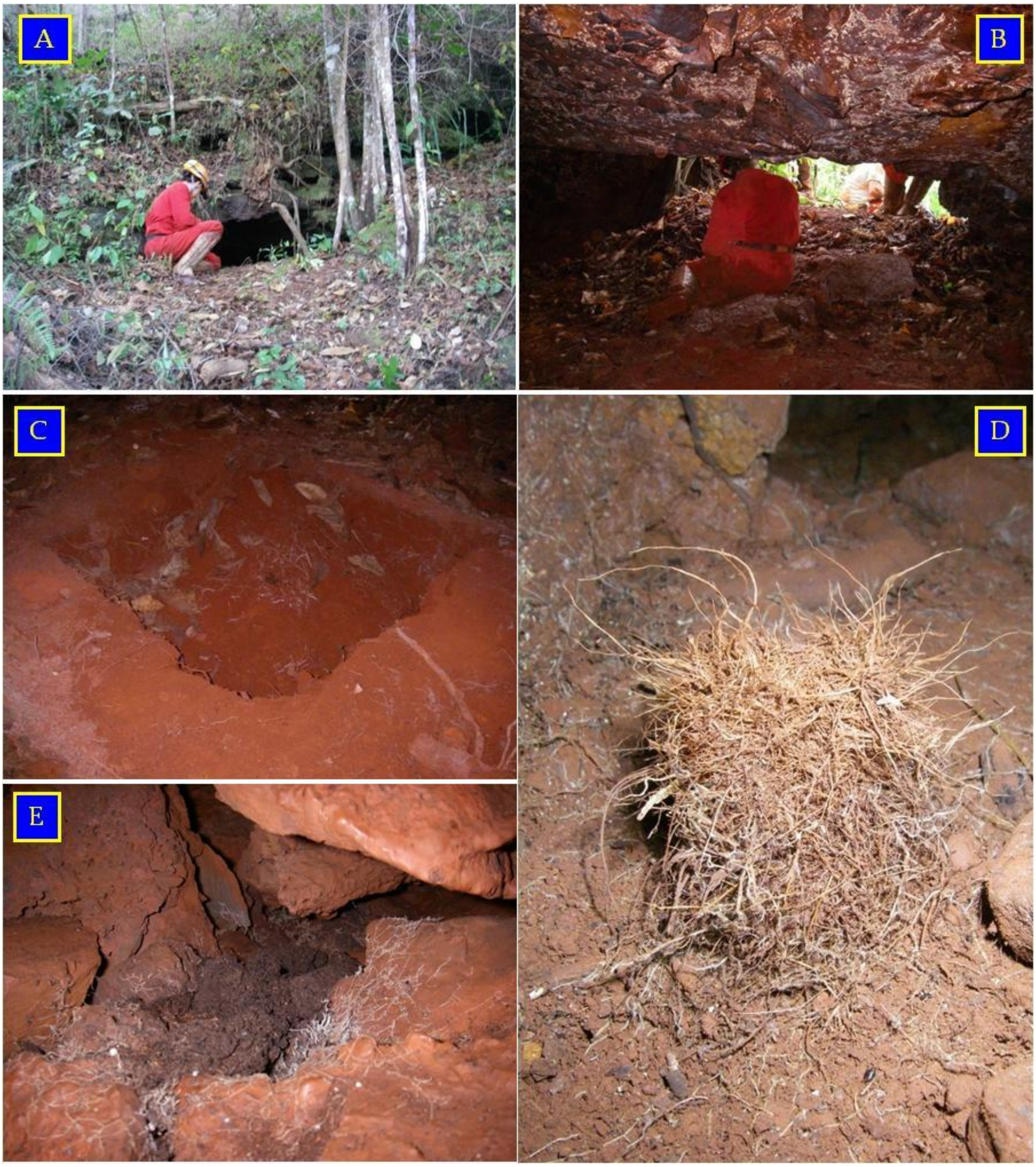

Figura 174 - a) Vista externa da entrada da cavidade onde se observa a área de mata com baixa densidade arbórea; b) Vista do pequeno salõa de entrada da cavidade, onde se observa uma considerável quantidade de serrapilheira importada do meio externo; c) quadrat de escavação arqueológica, aberto no piso da caverna; d) Rizotema formado em área de gotejamento próximo a zona de entrada; e) Fezes de um vertebrado bastante antigas, parcialmente decompostas e com considerável crescimento radicular.

5.4.4.54.2. Caracterização faunística no período de seca

Foi observado na caverna, um total de 31 morfoespécies de invertebrados de pelo menos 24 famílias das Ordens: Acari (Ixodidae: *Amblyomma* sp., Trombidiforme), Amblypygi (Phrynidae: *Heterophrynus longicornis*), Pseudoescorpiones (Chernetidae, Chtoniidae), Opiliones (Escadabiidae), Araneae (Araneidae: *Alpaida* sp.; Ochyroceratidae, Oonopidae: Oonopinae, Theridiosomatidae), Diplura (Campodeidae), Collembola (Entomobryidae), Orthoptera (Phalangopsidae: *Phalangopsis* sp.), Isoptera (Termitidae: *Nasutitermes* sp.), Hemiptera (Cydnidae, Reduviidae: Panstrongylus sp., Pseudococcidae, Pyrrhocoridae), Lepidoptera (Noctuidae), Diptera (Psychodidae: *Lutzomyia*), Hymenoptera (Formicidae: *Dolichoderus* sp.,*Pachycondyla* sp., *Rogeria* sp.), Coleoptera (Elateridae: Cardiophorinae), Diplopoda (Chelodesmidae), Scolopendromorpha (Scolopocryptopidae: *Newportia* sp.).

Dentre os vertebrados, foram encontradas cinco espécies das ordens Chiroptera (Emballonuridae: *Peropteryx kappleri*), Anura (Leptodactylidae: *Pristimantis cf. fenestratus, Eleutherodactylus* sp.) e Rodentia (Cricetidae: *Rhipidomys* sp.). Além destes, duas espécies de invertebrados, foram considerados troglomórficos (Gordioidea; Amblypygi: *Charinus* sp.).

5.4.4.54.3. Caracterização faunística no período de chuva

Foi observado na caverna, um total de 70 morfoespécies de invertebrados de pelo menos 51 famílias das Ordens: Oligochaeta, Isopoda (Armadillidae, Philosciidae), Acari (Ixodidae: *Ornithodoros* sp., *Amblyomma* sp., Laelapidae: *Stratiolaelaps* sp.; Uropodina, Oribatida, Anystidae: *Erythracarus* sp.; Trombidiforme), Amblypygi (Phrynidae: *Heterophrynus longicornis*), Ricinulei (*Cryptocellus tarsilae*), Pseudoescorpiones (Chernetidae, Chtoniidae), Opiliones (Escadabiidae, Phalangiidae), Araneae (Ctenidae: *Ctenus* sp.; Gnaphosidae, Ochyroceratidae, Oonopidae: Oonopinae, Salticidae, Scytodidae*: Scytodes eleonora*, Psauridae, Pholcidae: *Mesabolivar* sp., *Metagonia* sp.; Prodidomidae, Theraphosidae, Theridiosomatidae), Thysanura (Nicoletiidae: Nicoletiinae), Diplura (Campodeidae), Collembola (Sminthuridae, Entomobryidae Onychiuridae, Paronellidae), Orthoptera (Phalangopsidae: *Phalangopsis* sp.), Blattodea (Blaberidae, Polyphagidae), Isoptera (Termitidae: *Nasutitermes* sp.), Psocoptera (Epipsocidae), Hemiptera (Cydnidae, Reduviidae: Panstrongylus sp., Pseudococcidae, Pyrrhocoridae), Homoptera (Cixiidae: *Cixius* sp., Derbidae sp.), Lepidoptera (Noctuidae), Diptera (Cecidomyiidae, Psychodidae: *Lutzomyia*), Hymenoptera (Formicidae: *Camponotus* sp., *Dolichoderus* sp.,*Pachycondyla* sp., *Pseudomyrmex* sp., *Tapinoma* sp.), Coleoptera (Elateridae: Cardiophorinae, Psephenidae, Staphylinidae, Scydmaenidae, Tenebrionidae).

Dentre os vertebrados, foram encontradas duas espécies da ordem Chiroptera (Emballonuridae: *Peropteryx kappleri*, Phyllostomidae: *Glossophaga soricina*). Além destes,

duas espécies de invertebrados, foram considerados troglomórficos (Aranaea: Oonopinae; Amblypygi: *Charinus* sp.).

5.4.4.54.4. Caracterização geral da fauna da cavidade

Foi observado na caverna, um total de 85 morfoespécies de invertebrados de pelo menos 60 famílias das Ordens: Oligochaeta, Isopoda (Armadillidae, Philosciidae), Acari (Ixodidae: *Ornithodoros* sp., *Amblyomma* sp., Laelapidae: *Stratiolaelaps* sp.; Uropodina, Oribatida, Anystidae: *Erythracarus* sp.; Trombidiforme), Amblypygi (Phrynidae: *Heterophrynus longicornis*), Ricinulei (*Cryptocellus tarsilae*), Pseudoescorpiones (Chernetidae, Chtoniidae), Opiliones (Escadabiidae, Phalangiidae), Araneae (Araneidae: *Alpaida* sp.; Ctenidae: *Ctenus* sp.; Gnaphosidae, Ochyroceratidae, Oonopidae: Oonopinae, Salticidae, Scytodidae*: Scytodes eleonora*, Psauridae, Pholcidae: *Mesabolivar* sp., *Metagonia* sp.; Prodidomidae, Theraphosidae, Theridiosomatidae), Thysanura (Nicoletiidae: Nicoletiinae), Diplura (Campodeidae), Collembola (Sminthuridae, Entomobryidae Onychiuridae, Paronellidae), Orthoptera (Phalangopsidae: *Phalangopsis* sp.), Blattodea (Blaberidae, Polyphagidae), Isoptera (Termitidae: *Nasutitermes* sp.), Dermaptera (Labiidae), Psocoptera (Epipsocidae), Hemiptera (Cydnidae, Reduviidae: Panstrongylus sp., Pseudococcidae, Pyrrhocoridae), Homoptera (Cixiidae: *Cixius* sp., Derbidae sp.), Lepidoptera (Noctuidae), Diptera (Cecidomyiidae, Psychodidae: *Lutzomyia*), Hymenoptera (Formicidae: *Camponotus* sp., *Dolichoderus* sp.,*Pachycondyla* sp., *Pseudomyrmex* sp., *Rogeria* sp., *Tapinoma* sp.), Coleoptera (Elateridae: Cardiophorinae, Psephenidae, Staphylinidae, Scydmaenidae, Tenebrionidae), Diplopoda (Chelodesmidae), Scolopendromorpha (Scolopocryptopidae: *Newportia*).

Dentre os vertebrados, foram encontradas cinco espécies das ordens Chiroptera (Emballonuridae: *Peropteryx kappleri*, Phyllostomidae: *Glossophaga soricina*), Anura (Leptodactylidae: *Pristimantis cf. fenestratus, Eleutherodactylus* sp.) e Rodentia (Cricetidae: *Rhipidomys* sp.)

Desta forma, no total foram encontradas 90 morfoespécies. Entre estas duas espécies de invertebrados, foram consideradas troglomórficas (Gordioidea; Amblypygi: *Charinus* sp.; Aranaea: Oonopinae).

5.4.4.55. SL-058

5.4.4.55.1. Caracterização trófica

Caverna formada em canga com 37,5 m de projeção horizontal localizada próxima ao topo da encosta. A área de entorno apresenta vegetação arbustiva e savana metalófila na porção logo acima da caverna. A caverna é formada por um amplo salão principal e por um pequeno

conduto meândrico e estreito em sua zona mais profunda. Existem duas entradas de pequeno porte por onde ocorre uma intensa circulação de ar entre os ambientes epígeo e hipógeo. Tais entradas, bastante iluminadas, apresentam líquens e briófitas revestindo as paredes e piso. De forma geral, trata-se de uma cavidade muito seca com piso composto por sedimento granulado e blocos de diversos tamanhos, como seixos, calhaus e matacões distribuídos de maneira esparsa. O piso na região das entradas é descendente com grande quantidade de serrapilheira acumulada através de transporte eólico, gravitacional e pela ação da água em períodos de chuva. No piso, existe ainda um sistema radicular muito desenvolvido com uma grande quantidade de raízes de fino calibre. As paredes e o teto da cavidade são completamente revestidos por uma camada de Actinomicetos e existem pequenos depósitos de guano de morcegos frugívoros com muitas plântulas secas germinadas a partir das sementes presentes no mesmo. Foi observado um ninho de cupins em uma porção interior da caverna. Tais ninhos, mesmo depois de abandonados pela colônia de cupins, podem fornecer recursos orgânicos a certos organismos cavernícolas (Figura 175). O sistema de canalículos é pouco desenvolvido e cavidade não apresenta zona afótica. Foram observados vestígios de aves (ninho), mas nenhum indivíduo foi observado. Durante a estação úmida observou-se um aumento significativo na umidade da cavidade e na quantidade de matéria orgânica importada para o interior da mesma. O solo encontrava-se um pouco mais úmido e apenas alguns pequenos pontos de gotejamento foram observados.

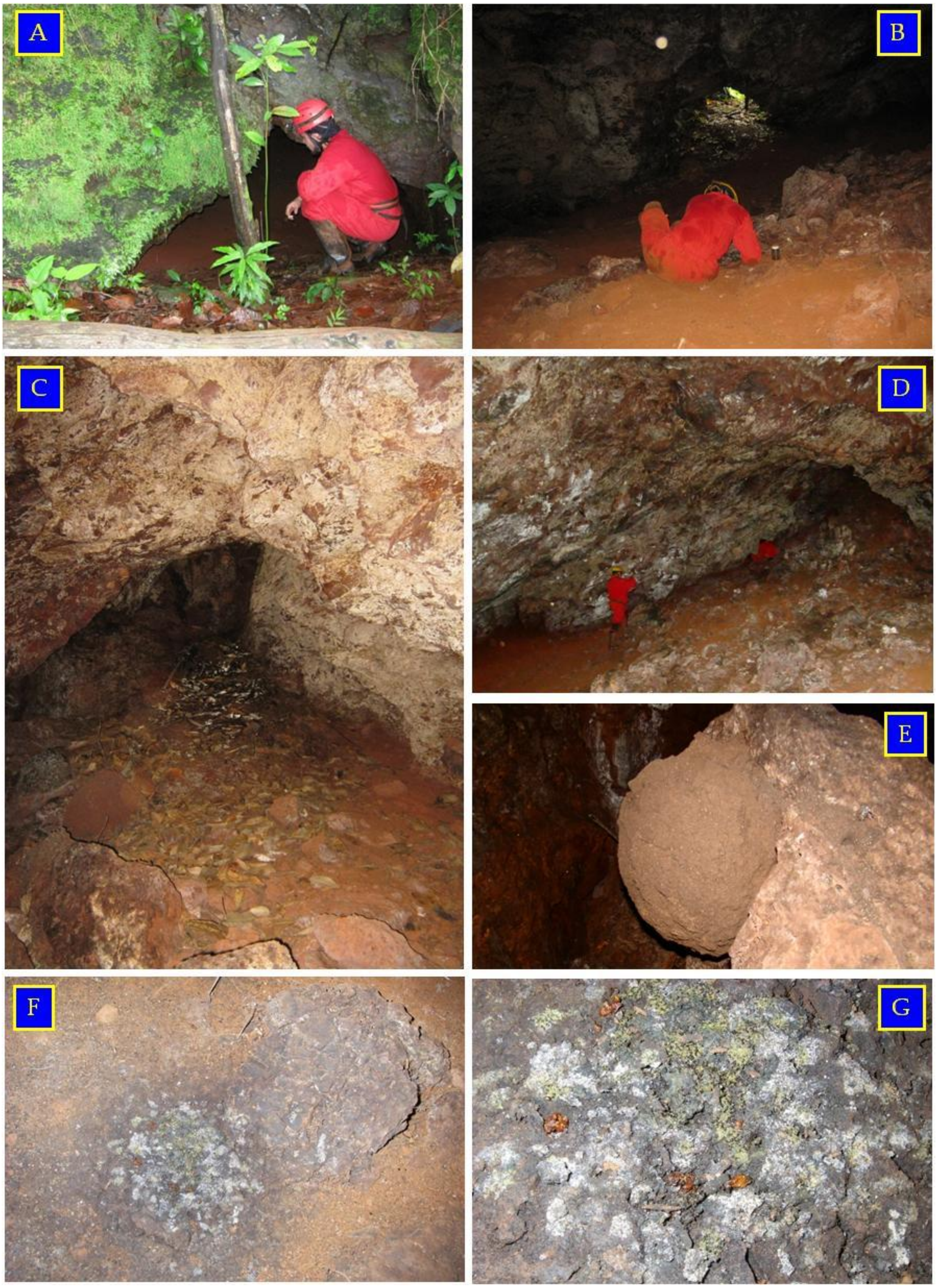

Figura 175 - a) Vista externa da entrada principal da cavidade; b) vista interna de uma das estradas da cavidade, onde se observa o piso com disposição descendente em direção ao ambiente hipógeo; c) Material orgânico de origem vegetal (serrapilheira) penetrando na cavidade pos uma das entradas; d) Salão principal da cavidade que apresenta dimensões muito amplas; e) Colônia abandonada de cupins; f) guano de morcegos frugívoros observado na caverna; g) Detalhe do depósito de guano, onde se percebem sementes indigeridas.

5.4.4.55.2. Caracterização faunística no período de seca

Foi observado na caverna, um total de 65 morfoespécies de invertebrados de pelo menos 47 famílias das Ordens: Isopoda (Armadillidae, Balloniscidae, Philosciidae), Acari (Ixodidae: *Amblyomma* sp.; Laelapidae: *Stratiolaelaps* sp.; Otopheidomenidae, Oribatida, Anystidae: *Erythracarus* sp.), Amblypygi (Phrynidae: *Heterophrynus longicornis*), Scorpiones (Buthidae: *Ananteris luciae*), Pseudoescorpiones (Chernetidae, Chtoniidae), Opiliones (Cosmetidae: Roquettea sp., Escadabiidae), Araneae (Araneidae: *Alpaida* sp.; Ctenidae: *Ctenus* sp., Gnaphosidae, Ochyroceratidae, Oonopidae: Oonopinae, Scytodidae*: Scytodes eleonora*, Pholcidae: *Mesabolivar* sp., Theraphosidae, Theridiidae, Theridiosomatidae), Diplura (Campodeidae), Collembola (Cyphoderidae, Entomobryidae) Orthoptera (Phalangopsidae: *Aclodes* sp., *Phalangopsis* sp.), Blattodea (Polyphagidae), Isoptera (Termitidae: *Nasutitermes* sp.), Psocoptera (Archipsocidae, Psyllipsocidae: *Psyllipsocus* sp.), Hemiptera (Cydnidae, Lygaeidae, Reduviidae), Lepidoptera (Noctuoidea, Tineidae), Diptera (Ceratopogonidae, Dolichopodidae, Psychodidae: *Lutzomyia* sp.), Hymenoptera (Formicidae: *Camponotus* sp., *Pachycondyla* sp., *Solenopsis* sp., *Strumigenys* sp.), Thysanoptera (Phaleothripinae), Coleoptera (Curculionidae: Scotylinae; Dermestidae, Staphylinidae), Diplopoda (Chelodesmidae), Geophilomorpha (Geophilidae).

Dentre os vertebrados, foram encontradas quatro espécies das ordens Chiroptera (Emballonuridae: *Peropteryx kappleri*, Phyllostomidae: *Glossophaga soricina*) e Anura (Leptodactylidae: *Pristimantis cf. fenestratus, Eleutherodactylus* sp.).

Além destes, duas espécies de invertebrados, foram considerados troglomórficos (Gastropoda: Systrophiidae; Araneae: Ochyroceratidae; Collembola: Cyphoderidae).

5.4.4.55.3. Caracterização faunística no período de chuva

Foi observado na caverna, um total de 70 morfoespécies de invertebrados de pelo menos 52 famílias das Ordens: Gastropoda (Subulinidae), Turbellaria (Geoplanidae), Isopoda (Armadillidae, Balloniscidae, Dubioniscidae, Philosciidae), Acari (Laelapidae: *Stratiolaelaps* sp.; Teneriffiidae, Trombidiforme), Scorpiones (Buthidae: *Ananteris luciae*), Pseudoescorpiones (Chernetidae, Chtoniidae), Opiliones (Phalangiidae), Araneae (Araneidae: *Alpaida* sp.; Ctenidae: *Ctenus* sp., Gnaphosidae, Ochyroceratidae, Oonopidae: Oonopinae, Salticidae, Scytodidae*: Scytodes eleonora*, Theraphosidae, Theridiidae, Theridiosomatidae), Diplura (Campodeidae), Collembola (Sminthuridae, Cyphoderidae, Entomobryidae), Orthoptera (Phalangopsidae: *Aclodes* sp., *Phalangopsis* sp.), Blattodea (Blattellidae, Polyphagidae) Isoptera (Rhinotermitidae: *Heterotermes* sp.; Termitidae: *Diversitermes* sp. *Nasutitermes* sp.), Psocoptera (Myopsocidae: *Lichenomina* sp.; Psyllipsocidae: *Psyllipsocus* sp.), Hemiptera (Cydnidae, Lygaeidae, Reduviidae), Homoptera (Cixiidae, Derbidae), Lepidoptera (Noctuoidea, Tineidae), Diptera (Ceratopogonidae, Drosophilidae,

Mycetophilidae, Psychodidae: *Lutzomyia* sp.), Hymenoptera (Formicidae: *Camponotus* sp., *Pachycondyla* sp., *Solenopsis* sp., *Strumigenys* sp.), Thysanoptera (Phaleothripinae), Coleoptera (Elateridae: Elaterinae, Agryphinae; Scydmaenidae, Staphylinidae), Diplopoda (Chelodesmidae).

Dentre os vertebrados, foram encontradas duas espécies das ordens Chiroptera (Phyllostomidae: *Glossophaga soricina*) e Anura (Leptodactylidae: *Leptodactylus labyrinthicus*).

Além destes, quatro espécies de invertebrados, foram considerados troglomórficos (Gastropoda: Systrophiidae; Amblypygi: *Charinus* sp.; Collembola: Cyphoderidae; Diplopoda - Glomeridesmidae: *Glomeridesmus* sp.).

5.4.4.55.4. Caracterização geral da fauna da cavidade

Foi observado na caverna, um total de 108 morfoespécies de invertebrados de pelo menos 66 famílias das Ordens: Gastropoda (Subulinidae), Turbellaria (Geoplanidae), Isopoda (Armadillidae, Balloniscidae, Dubioniscidae, Philosciidae), Acari (Ixodidae: *Amblyomma* sp.; Laelapidae: *Stratiolaelaps* sp.; Otopheidomenidae, Oribatida, Anystidae: *Erythracarus* sp.; Teneriffiidae, Trombidiforme), Amblypygi (Phrynidae: *Heterophrynus longicornis*), Scorpiones (Buthidae: *Ananteris luciae*), Pseudoescorpiones (Chernetidae, Chtoniidae), Opiliones (Cosmetidae: *Roquettea* sp., Escadabiidae, Phalangiidae), Araneae (Araneidae: *Alpaida* sp.; Ctenidae: *Ctenus* sp., Gnaphosidae, Ochyroceratidae, Oonopidae: Oonopinae, Salticidae, Scytodidae*: Scytodes eleonora*, Pholcidae: *Mesabolivar* sp., Theraphosidae, Theridiidae, Theridiosomatidae), Diplura (Campodeidae), Collembola (Sminthuridae, Cyphoderidae, Entomobryidae), Orthoptera (Phalangopsidae: *Aclodes* sp., *Phalangopsis* sp.), Blattodea (Blattellidae, Polyphagidae) Isoptera (Rhinotermitidae: *Heterotermes* sp.; Termitidae: *Diversitermes* sp. *Nasutitermes* sp.), Psocoptera (Archipsocidae, Myopsocidae: *Lichenomina* sp.; Psyllipsocidae: *Psyllipsocus* sp.), Hemiptera (Cydnidae, Lygaeidae, Reduviidae), Homoptera (Cixiidae, Derbidae), Lepidoptera (Noctuoidea, Tineidae), Diptera (Ceratopogonidae, Dolichopodidae, Drosophilidae, Mycetophilidae, Psychodidae: *Lutzomyia* sp.; Sciaridae), Hymenoptera (Formicidae: *Camponotus* sp., *Pachycondyla* sp., *Solenopsis* sp., *Strumigenys* sp.), Thysanoptera (Phaleothripinae), Coleoptera (Curculionidae: Scotylinae; Dermestidae, Elateridae: Elaterinae, Agryphinae; Scydmaenidae, Staphylinidae), Diplopoda (Chelodesmidae), Geophilomorpha (Geophilidae).

Dentre os vertebrados, foram encontradas cinco espécies das ordens Chiroptera (Emballonuridae: *Peropteryx kappleri*, Phyllostomidae: *Glossophaga soricina*) e Anura (Leptodactylidae: *Pristimantis cf. fenestratus, Eleutherodactylus* sp., *Leptodactylus labyrinthicus*).

Desta forma, no total foram encontradas 113 morfoespécies. Entre estas cinco espécies de invertebrados, foram consideradas troglomórficas (Gastropoda: Systrophiidae; Amblypygi: *Charinus* sp.; Araneae: Ochyroceratidae; Collembola: Cyphoderidae; Diplopoda - Glomeridesmidae: *Glomeridesmus* sp.). Alguns organismos encontrados nesta caverna são mostrados na Figura 176.

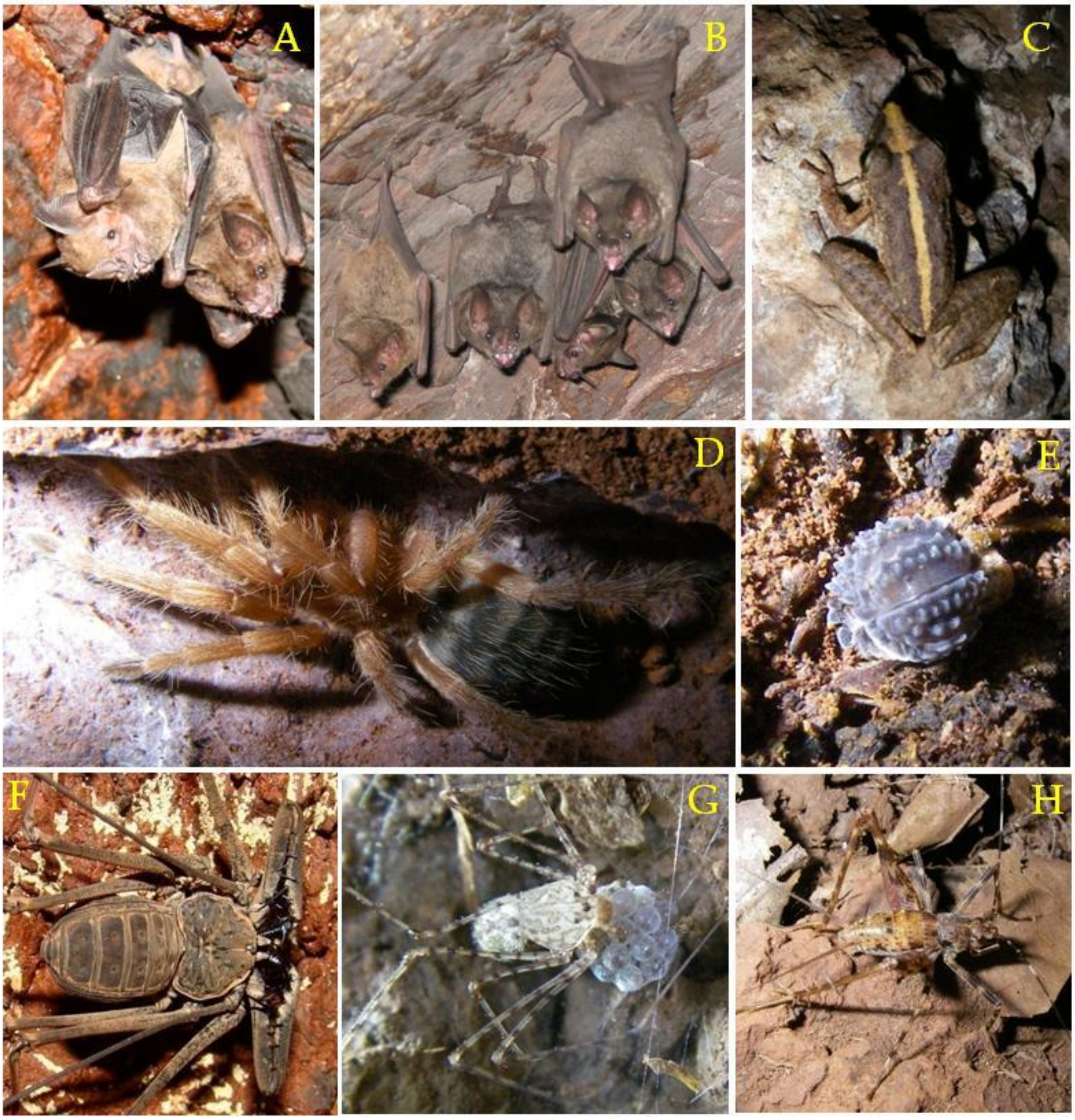

Figura 176 - a) Phyllostomidae (*Carollia* sp.); b) Phyllostomidae (*Carollia* sp.); c) Anura (Leptodactylidae: *Eleutherodactylus* sp.); d) Theraphosidae; e) Isopoda (Armadillidae; f) Amblypygi (*H. longicornis*); g) Scytodidae*: Scytodes eleonora*; h) Orthoptera (Phalangopsidae).

5.4.4.56. SL-059

5.4.4.56.1. Caracterização trófica

Pequena cavidade formada na canga com 14,4 m de projeção horizontal localizada na margem direita de uma drenagem. A vegetação do entorno é composta por mata ciliar em estado avançado de degradação localizada em uma matriz depastagens. A caverna é pouco profunda e apresenta duas entradas em situação paralela à linha do afloramento. Tais entradas são conectadas por um único conduto transversal. As entradas possuem pequenas dimensões, sendo sombreadas e, em conjunto com as áreas eufóticas, apresentam muitos líquens, briófitas e pteridófitas, além de muitas plântulas de angiospermas germinadas próximas à linha d'água. A cavidade não apresenta zona afótica e possui o sistema de canalículos pouco desenvolvido. O piso é predominantemente plano e seco, sendo este composto por sedimento granulado com seixos e calhaus distribuídos de maneira irregular e com pouca serrapilheira acumulada junto à linha d'água. Não existem depósitos de guano no interior da cavidade e não foram observados morcegos durante o estudo (Figura 177). Nenhuma alteração significativa foi observada durante a estação úmida, além das alterações normais na umidade relativa do ar.

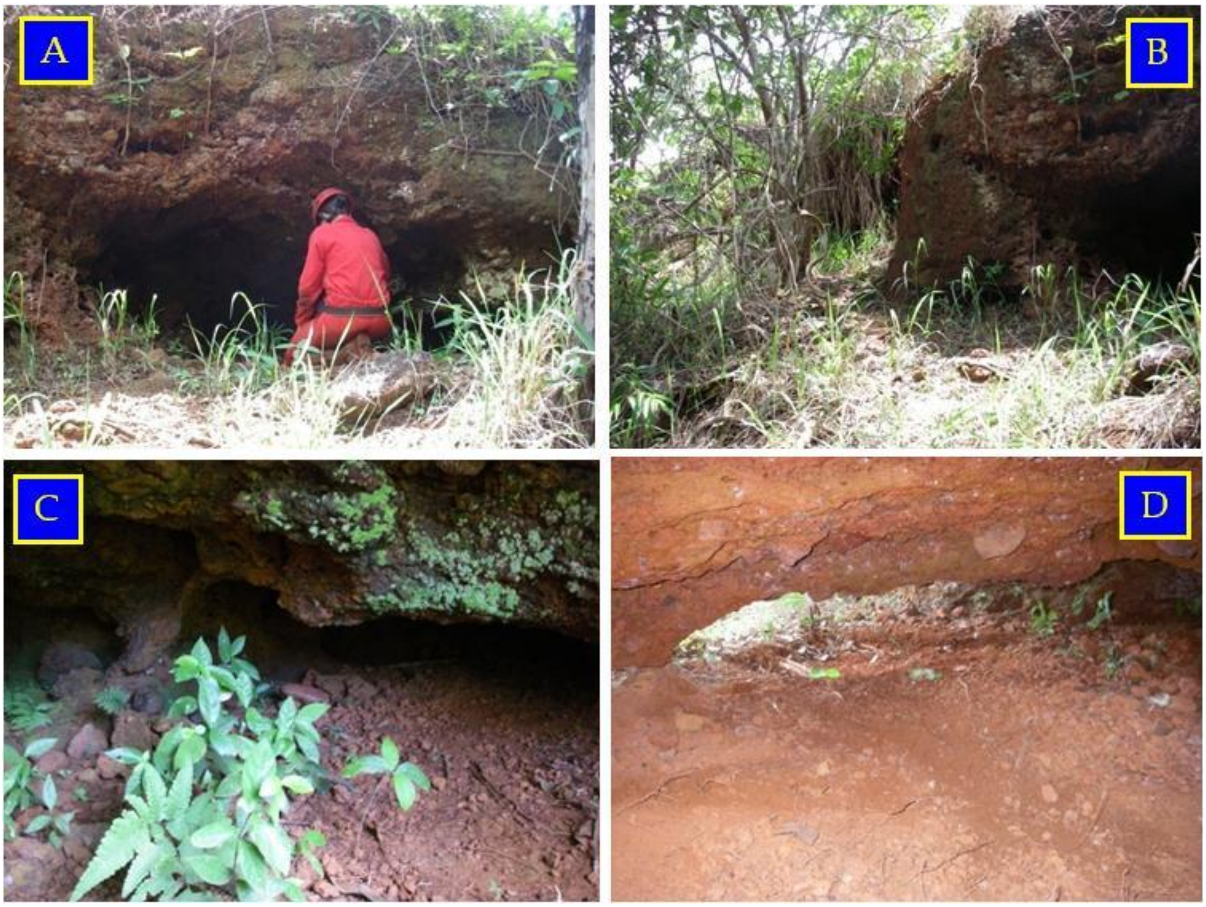

Figura 177 - a) Vista externa da entrada principal da cavidade; b) Detalhe da porção lateral próxima à entrada, onde ocorrem pequenos arbustos; c) Zona eufótica com piso composto por sedimento granulado e com muita vegetação associada; d) Porção interna da cavidade.

5.4.4.56.2. Caracterização faunística no período de seca

Foi observado na caverna, um total de 65 morfoespécies de invertebrados de pelo menos 47 famílias das Ordens: Isopoda (Armadillidae, Balloniscidae, Philosciidae), Acari (Ixodidae: *Amblyomma* sp.; Laelapidae: *Stratiolaelaps* sp.; Otopheidomenidae, Oribatida, Anystidae: *Erythracarus* sp.), Amblypygi (Phrynidae: *Heterophrynus longicornis*), Scorpiones (Buthidae: *Ananteris luciae*), Pseudoescorpiones (Chernetidae, Chtoniidae), Opiliones (Cosmetidae: *Roquettea* sp., Escadabiidae), Araneae (Araneidae: *Alpaida* sp.; Ctenidae: *Ctenus* sp., Gnaphosidae, Ochyroceratidae, Oonopidae: Oonopinae, Scytodidae*: Scytodes eleonora*, Pholcidae: *Mesabolivar* sp., Theraphosidae, Theridiidae, Theridiosomatidae), Diplura (Campodeidae), Collembola (Cyphoderidae, Entomobryidae) Orthoptera (Phalangopsidae: *Aclodes* sp., *Phalangopsis* sp.), Blattodea (Polyphagidae), Isoptera (Termitidae: *Nasutitermes* sp.), Psocoptera (Archipsocidae, Psyllipsocidae: *Psyllipsocus* sp.), Hemiptera (Cydnidae, Lygaeidae, Reduviidae), Lepidoptera (Noctuoidea, Tineidae), Diptera (Ceratopogonidae, Dolichopodidae, Psychodidae: *Lutzomyia* sp.), Hymenoptera (Formicidae: *Camponotus* sp., *Pachycondyla* sp., *Solenopsis* sp., *Strumigenys* sp.), Thysanoptera (Phaleothripinae), Coleoptera (Curculionidae: Scotylinae; Dermestidae, Staphylinidae), Diplopoda (Chelodesmidae), Geophilomorpha (Geophilidae).

Dentre os vertebrados, foram encontradas quatro espécies das ordens Chiroptera (Emballonuridae: *Peropteryx kappleri*, Phyllostomidae: *Glossophaga soricina*) e Anura (Leptodactylidae: *Pristimantis cf. fenestratus, Eleutherodactylus* sp.).

Além destes, duas espécies de invertebrados, foram considerados troglomórficos (Gastropoda: Systrophiidae; Araneae: Ochyroceratidae; Collembola: Cyphoderidae).

5.4.4.56.3. Caracterização faunística no período de chuva

Foi observado na caverna, um total de 70 morfoespécies de invertebrados de pelo menos 52 famílias das Ordens: Gastropoda (Subulinidae), Turbellaria (Geoplanidae), Isopoda (Armadillidae, Balloniscidae, Dubioniscidae, Philosciidae), Acari (Laelapidae: *Stratiolaelaps* sp.; Teneriffiidae, Trombidiforme), Scorpiones (Buthidae: *Ananteris luciae*), Pseudoescorpiones (Chernetidae, Chtoniidae), Opiliones (Phalangiidae), Araneae (Araneidae: *Alpaida* sp.; Ctenidae: *Ctenus* sp., Gnaphosidae, Ochyroceratidae, Oonopidae: Oonopinae, Salticidae, Scytodidae*: Scytodes eleonora*, Theraphosidae, Theridiidae, Theridiosomatidae), Diplura (Campodeidae), Collembola (Sminthuridae, Cyphoderidae, Entomobryidae), Orthoptera (Phalangopsidae: *Aclodes* sp., *Phalangopsis* sp.), Blattodea (Blattellidae, Polyphagidae) Isoptera (Rhinotermitidae: *Heterotermes* sp.; Termitidae: *Diversitermes* sp. *Nasutitermes* sp.), Psocoptera (Myopsocidae: *Lichenomina* sp.; Psyllipsocidae: *Psyllipsocus* sp.), Hemiptera (Cydnidae, Lygaeidae, Reduviidae), Homoptera (Cixiidae, Derbidae), Lepidoptera (Noctuoidea, Tineidae), Diptera (Ceratopogonidae, Drosophilidae,

Mycetophilidae, Psychodidae: *Lutzomyia* sp.), Hymenoptera (Formicidae: *Camponotus* sp., *Pachycondyla* sp., *Solenopsis* sp., *Strumigenys* sp.), Thysanoptera (Phaleothripinae), Coleoptera (Elateridae: Elaterinae, Agryphinae; Scydmaenidae, Staphylinidae), Diplopoda (Chelodesmidae).

Dentre os vertebrados, foram encontradas duas espécies das ordens Chiroptera (Phyllostomidae: *Glossophaga soricina*) e Anura (Leptodactylidae: *Leptodactylus labyrinthicus*).

Além destes, quatro espécies de invertebrados, foram considerados troglomórficos (Gastropoda: Systrophiidae; Amblypygi: *Charinus* sp.; Collembola: Cyphoderidae; Diplopoda - Glomeridesmidae: *Glomeridesmus* sp.).

5.4.4.56.4. Caracterização geral da fauna da cavidade

Foi observado na caverna, um total de 108 morfoespécies de invertebrados de pelo menos 66 famílias das Ordens: Gastropoda (Subulinidae), Turbellaria (Geoplanidae), Isopoda (Armadillidae, Balloniscidae, Dubioniscidae, Philosciidae), Acari (Ixodidae: *Amblyomma* sp.; Laelapidae: *Stratiolaelaps* sp.; Otopheidomenidae, Oribatida, Anystidae: *Erythracarus* sp.; Teneriffiidae, Trombidiforme), Amblypygi (Phrynidae: *Heterophrynus longicornis*), Scorpiones (Buthidae: *Ananteris luciae*), Pseudoescorpiones (Chernetidae, Chtoniidae), Opiliones (Cosmetidae: *Roquettea* sp., Escadabiidae, Phalangiidae), Araneae (Araneidae: *Alpaida* sp.; Ctenidae: *Ctenus* sp., Gnaphosidae, Ochyroceratidae, Oonopidae: Oonopinae, Salticidae, Scytodidae*:* *Scytodes eleonora*, Pholcidae: *Mesabolivar* sp., Theraphosidae, Theridiidae, Theridiosomatidae), Diplura (Campodeidae), Collembola (Sminthuridae, Cyphoderidae, Entomobryidae), Orthoptera (Phalangopsidae: *Aclodes* sp., *Phalangopsis* sp.), Blattodea (Blattellidae, Polyphagidae) Isoptera (Rhinotermitidae: *Heterotermes* sp.; Termitidae: *Diversitermes* sp. *Nasutitermes* sp.), Psocoptera (Archipsocidae, Myopsocidae: *Lichenomina* sp.; Psyllipsocidae: *Psyllipsocus* sp.), Hemiptera (Cydnidae, Lygaeidae, Reduviidae), Homoptera (Cixiidae, Derbidae), Lepidoptera (Noctuoidea, Tineidae), Diptera (Ceratopogonidae, Dolichopodidae, Drosophilidae, Mycetophilidae, Psychodidae: *Lutzomyia* sp.; Sciaridae), Hymenoptera (Formicidae: *Camponotus* sp., *Pachycondyla* sp., *Solenopsis* sp., *Strumigenys* sp.), Thysanoptera (Phaleothripinae), Coleoptera (Curculionidae: Scotylinae; Dermestidae, Elateridae: Elaterinae, Agryphinae; Scydmaenidae, Staphylinidae), Diplopoda (Chelodesmidae), Geophilomorpha (Geophilidae).

Dentre os vertebrados, foram encontradas cinco espécies das ordens Chiroptera (Emballonuridae: *Peropteryx kappleri*, Phyllostomidae: *Glossophaga soricina*) e Anura (Leptodactylidae: *Pristimantis cf. fenestratus, Eleutherodactylus* sp., *Leptodactylus labyrinthicus*).

Desta forma, no total foram encontradas 113 morfoespécies. Entre estas cinco espécies de invertebrados, foram consideradas troglomórficas (Gastropoda: Systrophiidae; Amblypygi: *Charinus* sp.; Araneae: Ochyroceratidae; Collembola: Cyphoderidae; Diplopoda - Glomeridesmidae: *Glomeridesmus* sp.).

5.4.4.57. SL-060

5.4.4.57.1. Caracterização trófica

Caverna formada na canga com 25,2 m de desenvolvimento, localizada na margem esquerda de uma drenagem ativa em área de mata ciliar. A vegetação do entorno é composta por uma floresta densa com dossel superior a dez metros de altura e com uma elevada quantidade de serrapilheira junto ao piso. A cavidade possui uma entrada ampla e larga, com altura superior a três metros. Tal entrada apresenta-se sombreada, possuindo muitos líquens, briófitas e plântulas de angiospermas, além de algumas árvores germinadas junto à linha d'água. O piso na entrada é descendente, o que facilita a importação de matéria orgânica para o interior da cavidade pela ação da água em períodos de chuva. Esta matéria orgânica de origem vegetal acumula-se nas proximidades das entradas, sendo pouco abundantes em regiões mais interiores. Basicamente podem ser identificadas apenas duas zonas fóticas: a zona de entrada (eufótica) e a porção mais distal da cavidade (penumbra escura). A caverna é formada por um único conduto com padrão retilíneo composta por um pequeno salão em sua área mais profunda. O piso é predominantemente plano, composto por sedimento granulado com alguns blocos (seixos, calhaus e matacões) distribuídos de maneira esparsa. O piso encontrava-se seco durante o inventário, não tendo sido observados pontos de gotejamento. Além disso, o piso da porção mais central do salão apresenta um sistema radicular bem desenvolvido formado por raízes de pequeno calibre. Neste salão também existe um grande depósito de guano de morcegos frugívoros com muitas plântulas germinadas. Este depósito encontra-se ativo, sendo produzido por uma grande colônia de morcegos Glossophaginae. Em virtude da grande quantidade de recursos alimentares disponíveis nesta região da cavidade, algumas populações de invertebrados apresentam-se extremamente abundantes, com algumas populações formadas por milhares de indivíduos. Dentre estas, destacam-se as traças (Nicoletiidae), os colêmbolos (Entomobryiidae), os isópodes e as baratas (Polyphagidae). Quanto à morfologia das paredes e teto, estes apresentavam um padrão irregular, com muitos canalículos e depressões de diferentes tamanhos e encontravam-se revestidos por uma grande quantidade de Actinomicetos. Apesar de ser uma cavidade relativamente pequena, a mesma apresenta uma considerável estabilidade ambiental, possuindo também uma elevada diversidade de recursos alimentares e grande quantidade de micro-hábitats. Durante a estação úmida não foi observada nenhuma alteração significativa nas condições tróficas da cavidade, exceto por alguns restos de plantas (flores e frutos) deixados pelos morcegos juntos aos depósitos de

guano. Na porção inicial da caverna foram observados alguns pontos de gotejamentos ativos e uma pequena área foi escavada para os estudos arqueológicos. Novamente, o "quadrat" de escavação arqueológica foi deixado aberto (Figura 178). Escavações certamente alteram o piso e conseqüentemente os microhabitats das regiões onde são desenvolvidas (especialmente entradas e abrigos, no caso de cavernas). Tais alterações podem causar diferentes impactos sobre a fauna das entradas, que compõe um importante gradiente ecotonal entre sistemas epígeo e hipógeo.

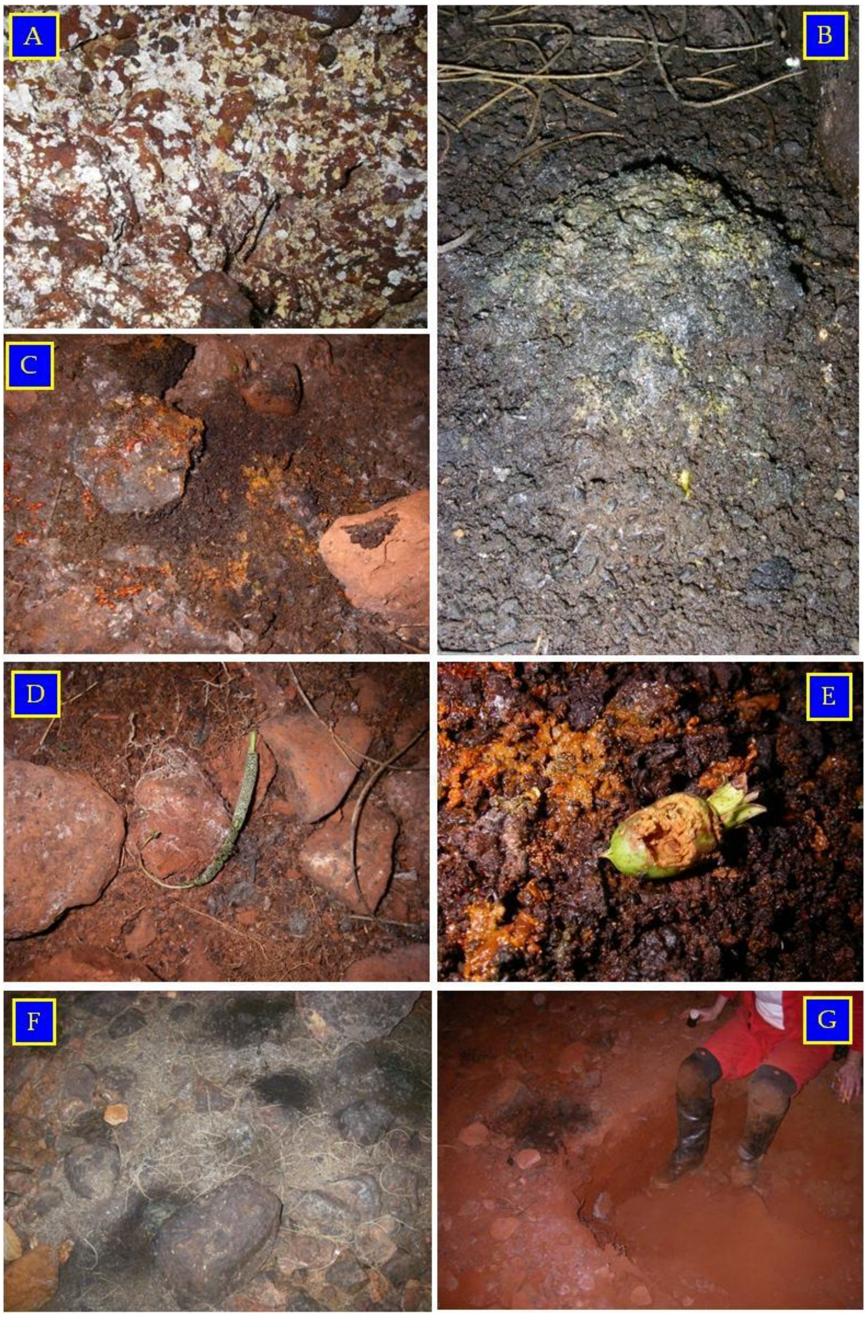

Figura 178 - a) Vista interna da cavidade onde se observa grande quantidade de Actinomicetos desenvolvendo-se nas paredes; b) Depósito de guano de morcegos frugívoros em deposição; c) Depósito de guano de morcegos frugívoros envelhecido; d) Restos alimentares de morcegos Glossophaginae;

e) Restos alimentares de morcegos Glossophaginae; f) Depósito de guano de morcegos frugívoros com muitas plântulas secas; g) Escavação realizada para estudos arqueológicos.

5.4.4.57.2. Caracterização faunística no período de seca

Foi observado na caverna, um total de 82 morfoespécies de invertebrados de pelo menos 58 famílias das Ordens: Gastropoda (Subulinidae), Isopoda (Armadillidae, Philosciidae, Scleropactidae), Acari (Ixodidae: *Amblyomma cajennense;* Laelapidae: *Stratiolaelaps* sp., Ologamasidae, Mesostigmata, Uropodina, Opilioacaridae: *Neoacarus* sp., Anoetidae, Oribatida, Labdostomatidae: *Labdostomatida* sp., Trombidiforme), Pseudoscorpiones (Chernetidae, Chtoniidae), Opiliones (Cosmetidae: *Anduzeia* sp*.,* Escadabiidae), Araneae (Araneidae: *Alpaida* sp.; Ctenidae: *Ctenus* sp., Gnaphosidae, Linyphiidae, Oonopidae: Oonopinae, Scytodidae*: Scytodes eleonora*, Pholcidae: *Mesabolivar* sp., *Metagonia* sp., Theridiidae, Theridiosomatidae), Diplura (Campodeidae), Collembola (Sminthuridae, Cyphoderidae, Entomobryidae, Paronellidae), Orthoptera (Phalangopsidae: *Aclodes* sp1, *Phalangopsis* sp.) Blattodea (Blattellidae, Polyphagidae), Isoptera (Termitidae: *Nasutitermes* sp.), Psocoptera (Epipsocidae), Hemiptera (Cydnidae, Reduviidae: *Panstrongylus* sp.), Homoptera (Derbidae), Lepidoptera (Noctuidae), Diptera (Dixidae, Dolichopodidae, Faniidae, Muscidae, Psychodidae: *Lutzomyia* sp., Sciaridae, Tipulidae), Hymenoptera (Formicidae: *Camponotus* sp., *Hypoponera* sp., *Pachycondyla* sp., *Pheidole* sp., *Solenopsis* sp.) Coleoptera (Carabidae, Curculioniodae: Scotylinae; Elateridae; Scydmaenidae, Staphylinidae), Diplura (Chelodesmidae, Cyrtodesmidae , Pyrgodesmidae, Siphonophoridae), Scolopendromorpha (Scolopocryptopidae: *Dinocryptops* sp., *Newportia* sp., *Scolopocryptops* sp.), Symphila (Scutigerellidae: *Hanseniella* sp.), Neuroptera (Myrmeleontidae).

Dentre os vertebrados, foram encontradas seis espécies das ordens Chiroptera (Phyllostomidae: *Glossophaga soricina*), Squamata (Boidae: *Epicrates* sp.; Iguanidae), Anura (Leptodactylidae, Bufonidae) e Rodentia (Cricetidae: *Rhipidomys* sp.) Uma espécie de invertebrado, foi considerada troglomórfica: Gastropoda (Systrophiidae).

5.4.4.57.3. Caracterização faunística no período de chuva

Foi observado na caverna, um total de 105 morfoespécies de invertebrados de pelo menos 62 famílias das Ordens: Gastropoda (Subulinidae, Valloniidae) Acari (Ologamasidae, Opilioacaridae: *Neoacarus* sp., Acaridae, Oribatida, Labdostomatidae: *Labdostomatida* sp.), Amblypygi (*Heterophrynus longicornis*), Pseudoscorpiones (Chernetidae, Chtoniidae), Opiliones (Cosmetidae: *Anduzeia* sp., Sclerosomatidae: *Prionostemma* sp., Phalangiidae), Araneae (Araneidae: *Alpaida* sp.; Ctenidae: *Ctenus* sp., Gnaphosidae, Linyphiidae, Ochyroceratidae, Oonopidae: Oonopinae, Salticidae, Scytodidae*: Scytodes eleonora*, Pholcidae: *Mesabolivar* sp., *Metagonia* sp., Theraphosidae, Theridiidae, Theridiosomatidae,

Thomisidae), Thysanura (Nicoletiidae: Nicoletiinae), Microcoryphia (Meinertellidae), Diplura (Campodeidae), Collembola (Sminthuridae, Cyphoderidae, Entomobryidae), Orthoptera (Phalangopsidae: *Aclodes* sp., *Phalangopsis* sp.) Blattodea (Blattellidae, Polyphagidae), Isoptera (Termitidae: *Nasutitermes* sp.), Psocoptera (Archipsocidae, Ptiloneuridae: Ptiloneura), Hemiptera (Cydnidae, Dipsocoridae, Lygaeidae, Nabidae, Reduviidae, Ploiariidae), Homoptera (Cixiidae), Lepidoptera (Noctuidae, Tineidae), Diptera (Cecidomyiidae, Culicidae, Dolichopodidae, Drosophilidae, Mycetophilidae, Psychodidae: *Lutzomyia* sp., Tipulidae), Hymenoptera (Formicidae: *Camponotus* sp., *Crematogaster* sp., *Dolichoderus* sp., *Gnamptogenys* sp., *Hypoponera* sp., *Pachycondyla* sp., *Pheidole* sp., *Wasmannia* sp.) Coleoptera (Elateridae: Cardiophorinae, Elaterinae; Pselaphidae, Scydmaenidae, Staphylinidae), Diplura (Chelodesmidae, Oniscodesmidae), Scolopendromorpha (Cryptopidae: *Cryptops* sp.; Scolopendridae: *Cormocephalus* sp.; Scolopocryptopidae: *Dinocryptops* sp.), Symphila (Scutigerellidae: *Hanseniella* sp.), Neuroptera (Myrmeleontidae).

Dentre os vertebrados, foram encontradas três espécies das ordens Chiroptera (Emballonuridae: *Peropteryx kappleri*, Phyllostomidae: *Glossophaga soricina*) e Anura (Leptodactylidae). Duas espécies de invertebrados foram consideradas troglomórficas: Gastropoda (Systrophiidae), Araneae (Ochyroceratidae).

5.4.4.57.4. Caracterização geral da fauna da cavidade

Foi observado na caverna, um total de 149 morfoespécies de invertebrados de pelo menos 89 famílias das Ordens: Gastropoda (Subulinidae, Systrophiidae, Valloniidae) Isopoda (Armadillidae, Philosciidae, Scleropactidae), Acari (Ixodidae: *Amblyomma cajennense;* Laelapidae: *Stratiolaelaps* sp., Ologamasidae, Mesostigmata, Uropodina, Opilioacaridae: *Neoacarus* sp., Acaridae, Anoetidae, Oribatida, Labdostomatidae: *Labdostomatida* sp., Trombidiforme), Amblypygi (*Heterophrynus longicornis*), Pseudoscorpiones (Chernetidae, Chtoniidae), Opiliones (Cosmetidae: *Anduzeia* sp*.,* Escadabiidae, Sclerosomatidae: *Prionostemma* sp., Phalangiidae), Araneae (Araneidae: *Alpaida* sp.; Ctenidae: *Ctenus* sp., Gnaphosidae, Linyphiidae, Ochyroceratidae, Oonopidae: Oonopinae, Salticidae, Scytodidae*: Scytodes eleonora*, Pholcidae: *Mesabolivar* sp., *Metagonia* sp., Theraphosidae, Theridiidae, Theridiosomatidae, Thomisidae), Thysanura (Nicoletiidae: Nicoletiinae), Microcoryphia (Meinertellidae), Diplura (Campodeidae), Collembola (Sminthuridae, Cyphoderidae, Entomobryidae, Paronellidae), Orthoptera (Phalangopsidae: *Aclodes* sp1, *Phalangopsis* sp.) Blattodea (Blattellidae, Polyphagidae), Isoptera (Termitidae: *Nasutitermes* sp.), Psocoptera (Archipsocidae, Epipsocidae, Ptiloneuridae: Ptiloneura), Hemiptera (Cydnidae, Dipsocoridae, Lygaeidae, Nabidae, Reduviidae: *Panstrongylus* sp., Ploiariidae), Homoptera (Cixiidae, Derbidae), Lepidoptera (Noctuidae, Tineidae), Diptera (Cecidomyiidae, Chironomidae, Culicidae, Dixidae, Dolichopodidae, Drosophilidae, Faniidae, Muscidae, Mycetophilidae, Psychodidae: *Lutzomyia* sp., Sciaridae, Tipulidae), Hymenoptera (Formicidae:

Camponotus sp., *Crematogaster* sp., *Dolichoderus* sp., *Gnamptogenys* sp., *Hypoponera* sp., *Pachycondyla* sp., *Pheidole* sp., *Solenopsis* sp., *Wasmannia* sp.) Coleoptera (Carabidae, Curculioniodae: Scotylinae; Elateridae: Cardiophorinae, Elaterinae; Pselaphidae, Scydmaenidae, Staphylinidae), Diplura (Chelodesmidae, Oniscodesmidae, Cyrtodesmidae, Pyrgodesmidae, Siphonophoridae), Scolopendromorpha (Cryptopidae: *Cryptops* sp.; Scolopendridae: *Cormocephalus* sp.; Scolopocryptopidae: *Dinocryptops* sp., *Newportia* sp., *Scolopocryptops* sp.), Symphila (Scutigerellidae: *Hanseniella* sp.), Neuroptera (Myrmeleontidae).

Dentre os vertebrados, foram encontradas sete espécies das ordens Chiroptera (Emballonuridae: *Peropteryx kappleri*, Phyllostomidae: *Glossophaga soricina*), Squamata (Boidae: *Epicrates* sp.; Iguanidae), Anura (Leptodactylidae, Bufonidae) e Rodentia (Cricetidae: *Rhipidomys* sp.)

Desta forma, no total foram encontrados 156 morfoespécies. Entre estas duas espécies de invertebrados foram consideradas troglomórficas: Gastropoda (Systrophiidae) e Araneae (Ochyroceratidae). Alguns organismos encontrados nesta caverna são mostrados na Figura 179.

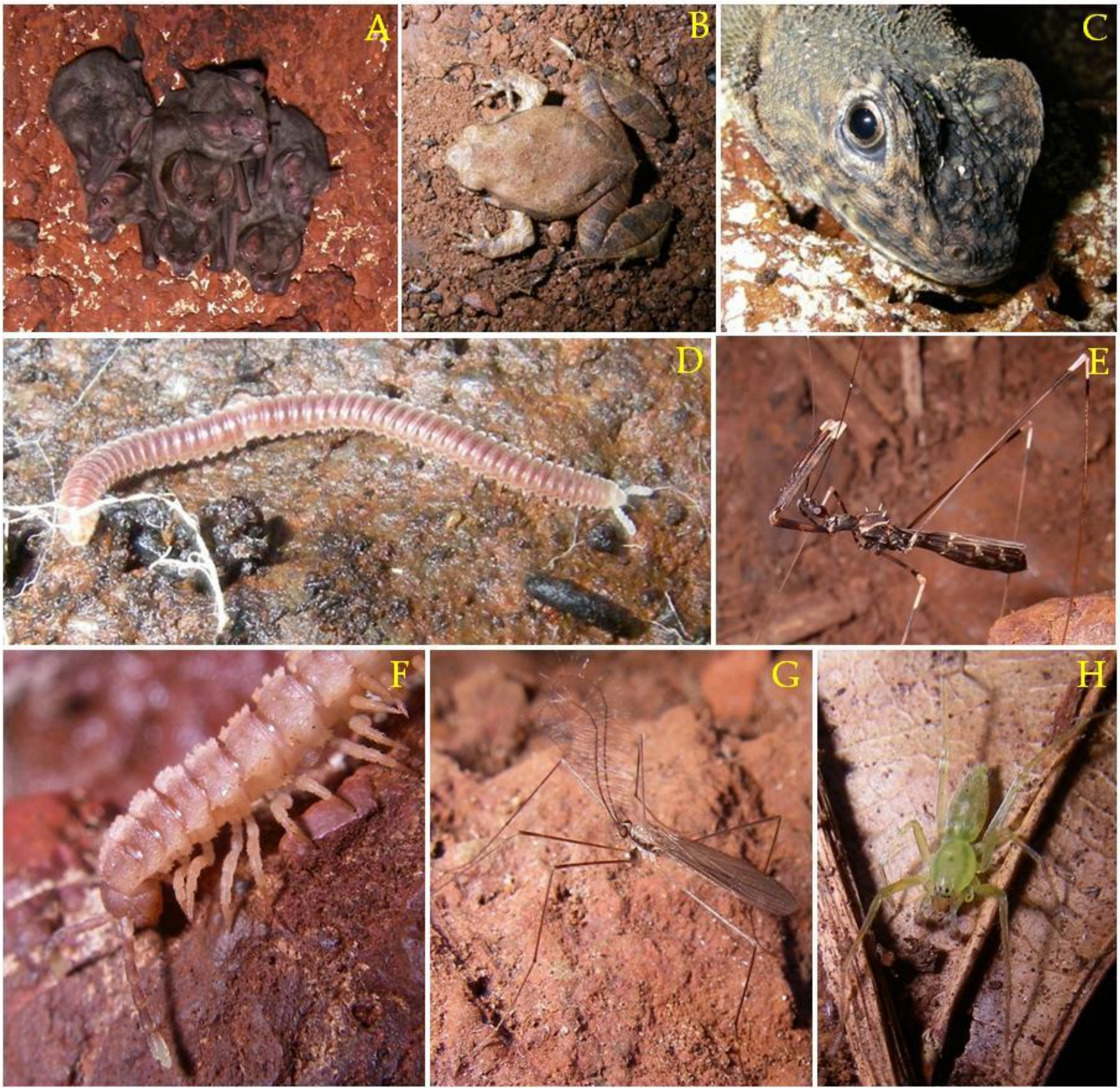

Figura 179 - a) Phyllostomidae (*Carollia sp.*); b) Anura (indet.).; c) Iguanidae; d) Diplopoda (Siphonophoridae); e) Hemiptera (Ploiariidae); f) Diplopoda (Polydesmida); g) Diptera (Tipulidae); h) Araneae (Thomisidae).

5.4.4.58. SL-061

5.4.4.58.1. Caracterização trófica

Caverna formada na canga com projeção horizontal de 23,5 metros localizada na margem esquerda de uma drenagem ativa que abastece uma represa localizada em uma área de pastagem de Serra Leste. O entorno da cavidade é formado por mata ciliar com dossel superior a dez metros de altura, sendo o piso repleto de serrapilheira. A cavidade é pouco profunda, apresentando o conduto principal paralelo à quebra da canga. A entrada é ampla, o que faz com que a cavidade sofra grande influênciado ambiente epígeo. O piso que se segue à entrada é descendente, no qual se observa grande quantidadede serrapilheira,

especialmente junto à linha d’água. A caverna é praticamente toda iluminada, com exceção de um conduto interior descendente que aparenta ser completamente afótico. O piso da cavidade é formado, em grande parte, pela rocha matriz. No entanto, existem trechos de sedimento clástico. De forma geral, existem poucos musgos, líquens e fungos em toda a extensão da cavidade (Figura 123). Na zona de entrada observou-se pequenos depósitos de guano envelhecidos de morcegos insetívoros, além de e fezes de anfíbios ao fundo da cavidade. O sistema de canalículos é bem desenvolvido e a caverna é suscetível a inundações em períodos de chuva intensa. Durante a estação úmida, foram observados alguns pontos de gotejamento ativos em sua porção inicial e uma pequena área foi escavada para os estudos arqueológicos. Mais uma vez, o “quadrat” foi deixado aberto (Figura 180).

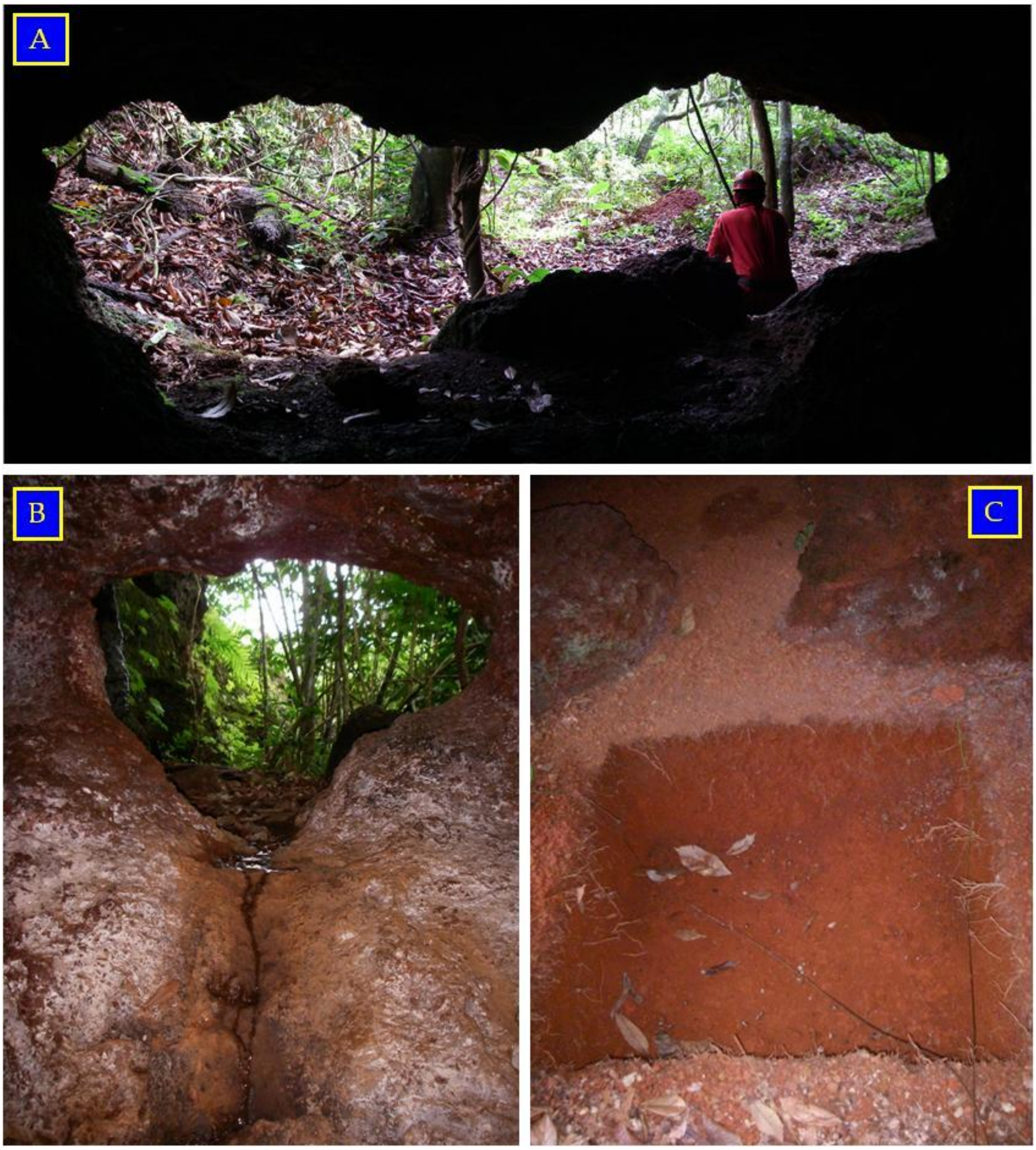

Figura 180 - a) Vista interna da entrada da cavidade; b) Vista interna do conduto que se segue à entrada da cavidade; c) Escavação realizada para estudos arqueológicos.

5.4.4.58.2. Caracterização faunística no período de seca

Foi observado na caverna, um total de 47 morfoespécies de invertebrados de pelo menos 36 famílias das Ordens: Acari (Mesostigmata: Laelapidae, Trombidiforme), Amblypygi (Phrynidae: *Heterophrynus longicornis*), Pseudoescorpiones (Chernetidae, Chtoniidae), Opiliones (Cosmetidae: *Anduzeia sp.*; Stygnidae: *Protimesius aff. gracilis*; Phalangiidae), Araneae (Araneidae: *Alpaida* sp.; Dipluridae, Gnaphosidae, Ochyroceratidae, Oonopidae: Oonopinae; Salticidae, Pholcidae: *Mesabolivar* sp., *Metagonia* sp., Theraphosidae, Theridiidae, Uloboridae), Thysanura (Nicoletiidae: Nicoletiinae), Collembola (Entomobryidae), Orthoptera (Phalangopsidae: *Aclodes* sp.), Blattodea (Blaberidae: *Blaberus* sp.; Blattidae, Polyphagidae), Isoptera (Termitidae: *Embiratermes* sp., *Nasutitermes* sp.), Psocoptera (Epipsocidae), Hemiptera (Cydnidae), Lepidoptera (Noctuidae, Tineidae), Diptera (Culicidae, Psychodidae: *Lutzomyia* sp., Sciaridae, Tipulidae), Hymenoptera (Formicidae: *Apterostigma* sp., *Dolichoderus* sp., *Solenopsis* sp.), Scutigeromorpha (Scutigeridae: *Sphendononema* sp.), Neuroptera (Myrmeleontidae).

Dentre os vertebrados, foi encontrada uma espécie da ordem Anura (Leptodactylidae: *Pristimantis cf. fenestratus*). Desta forma, no total foram encontradas 48 morfoespécies. Entre estas, duas espécies de invertebrados foram consideradas troglomórficas: Gastropoda (Systrophiidae), Amblypygi (Charinidae: *Charinus* sp.).

5.4.4.58.3. Caracterização faunística no período de chuva

Foi observado na caverna, um total de 56 morfoespécies de invertebrados de pelo menos 39 famílias das Ordens: Isopoda (Philosciidae), Acari (Ixodidae: *Amblyomma* sp.; Oribatida), Amblypygi (Phrynidae: *Heterophrynus longicornis*), Ricinulei (*Cryptocellus tarsilae*), Pseudoescorpiones (Chernetidae), Opiliones (Sclerosomatidae; *Prionostemma* sp.; Phalangiidae), Araneae (Gnaphosidae, Ochyroceratidae, Oonopidae: Oonopinae; Salticidae, Psauridae, Pholcidae: *Mesabolivar* sp., *Metagonia* sp., Theraphosidae, Theridiidae, Theridiosomatidae), Collembola (Entomobryidae), Orthoptera (Phalangopsidae: *Aclodes* sp., *Phalangopsis* sp.), Blattodea (Blaberidae), Isoptera (Termitidae: *Nasutitermes* sp.), Psocoptera (Myopsocidae: *Lichenomina* sp.; Pachytroctidae, Psyllipsocidae: *Psyllipsocus* sp.; Plitoneuridae: *Ptiloneura* sp.), Hemiptera (Cydnidae, Reduviidae, Pyrrhocoridae), Lepidoptera (Noctuidae, Tineidae), Diptera (Cecidomyiidae, Ceratopogonidae, Phoridae, Psychodidae: *Lutzomyia* sp., Sciaridae, Tipulidae), Hymenoptera (Formicidae: *Dolichoderus* sp., *Gnamptogenys* sp., *Rogeria* sp., *Solenopsis* sp.; Eulophidae), Coleoptera (Bostrichidae, Chrysomelidae), Neuroptera (Myrmeleontidae).

Dentre os vertebrados, foi encontrada uma espécie da ordem Chiroptera (Phyllostomidae: *Glossophaga soricina*). Desta forma, no total foram encontradas 57 morfoespécies. Entre estas, três espécies de invertebrados, foram consideradas troglomórficas: Gastropoda

(Systrophiidae), Araneae (Ochyroceratidae), Diplopoda (Glomeridesmidae: *Glomeridesmus* sp.).

5.4.4.58.4. Caracterização geral da fauna da cavidade

Foi observado na caverna, um total de 91 morfoespécies de invertebrados de pelo menos 54 famílias das Ordens: Isopoda (Philosciidae), Acari (Ixodidae: *Amblyomma* sp.; Mesostigmata: Laelapidae, Oribatida, Trombidiforme), Amblypygi (Phrynidae: *Heterophrynus longicornis*), Ricinulei (*Cryptocellus tarsilae*), Pseudoescorpiones (Chernetidae, Chtoniidae), Opiliones (Cosmetidae: *Anduzeia sp.*; Sclerosomatidae; *Prionostemma* sp.; Stygnidae: *Protimesius aff. gracilis*; Phalangiidae), Araneae (Araneidae: *Alpaida* sp.; Dipluridae, Gnaphosidae, Oonopidae: Oonopinae; Salticidae, Psauridae, Pholcidae: *Mesabolivar* sp., *Metagonia* sp., Theraphosidae, Theridiidae, Theridiosomatidae, Uloboridae), Thysanura (Nicoletiidae: Nicoletiinae), Collembola (Entomobryidae), Orthoptera (Phalangopsidae: *Aclodes* sp., *Phalangopsis* sp.), Blattodea (Blaberidae: *Blaberus* sp.; Blattidae, Polyphagidae), Isoptera (Termitidae: *Embiratermes* sp., *Nasutitermes* sp.), Psocoptera (Epipsocidae, Myopsocidae: *Lichenomina* sp.; Pachytroctidae, Psyllipsocidae: *Psyllipsocus* sp.; Plitoneuridae: *Ptiloneura* sp.), Hemiptera (Cydnidae, Reduviidae, Pyrrhocoridae), Lepidoptera (Noctuidae, Tineidae), Diptera (Cecidomyiidae, Ceratopogonidae, Culicidae, Phoridae, Psychodidae: *Lutzomyia* sp., Sciaridae, Tipulidae), Hymenoptera (Formicidae: *Apterostigma* sp., *Dolichoderus* sp., *Gnamptogenys* sp., *Rogeria* sp., *Solenopsis* sp.; Eulophidae), Coleoptera (Bostrichidae, Chrysomelidae), Scutigeromorpha (Scutigeridae: *Sphendononema* sp.), Neuroptera (Myrmeleontidae).

Dentre os vertebrados, foram encontradas duas espécies das ordens Chiroptera (Phyllostomidae: *Glossophaga soricina*), e Anura (Leptodactylidae: *Pristimantis cf. fenestratus*).
Desta forma, no total foram encontradas 93 morfoespécies. Entre estas quatro espécies de invertebrados, foram consideradas troglomórficas Gastropoda (Systrophiidae), Amblypygi (Charinidae: *Charinus* sp.), Araneae (Ochyroceratidae), Diplopoda (Glomeridesmidae: *Glomeridesmus* sp.). Alguns organismos encontrados nesta caverna são mostrados na Figura 181.

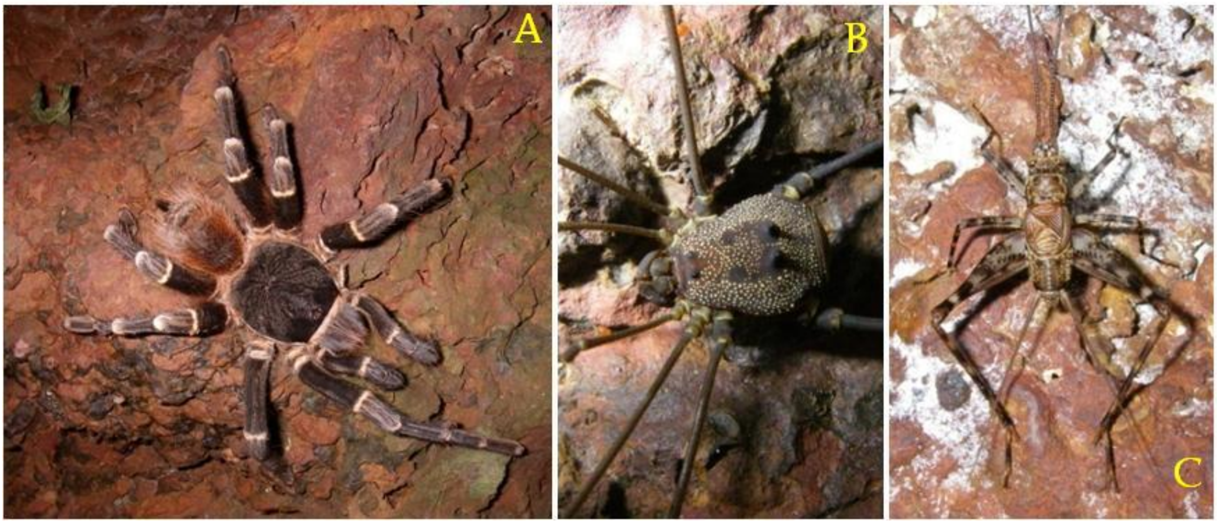

Figura 181 - a) Theraphosidae (*Acanthoscurria sp.*); b) Opiliones (Cosmetidae: *Anduzeia sp.*); c) Orthoptera (Phalangopsidae: *Aclodes sp.*).

5.4.4.59. SL-062

5.4.4.59.1. Caracterização trófica

Pequeno abrigo formado na canga, com 16 m de projeção horizontal. Tal cavidade localiza-se na margem esquerda de uma represa em área de mata ciliar. A vegetação do entorno é composta por uma floresta densa com dossel superior a dez metros de altura, sendo o piso repleto de serrapilheira. A entrada possui pequenas dimensões, a partir da qual desenvolve-se um conduto com piso ascendente a partir da entrada. A área sobre a cavidade é composta por uma vegetação arbustiva característica dos afloramentos ferruginosos, onde existe uma pequena quantidade de solo disponível. Ainda na entrada, existem muitos líquens, briófitas e pteridófitas uma vez que a umidade é elevada pela proximidade da represa e por uma pequena nascente que percola junto à rocha na porção acima da cavidade (Figura 182). Desta forma, mesmo durante a estação seca, existem muitos pontos de gotejamento junto à entrada. A caverna não apresenta zona afótica, mas existem alguns trechos de teto baixo que levam à zonas de penumbra escura. O piso é irregular e ascendente composto por sedimento fino com muitos blocos sobrepostos. Este é encharcado junto à entrada e seco na zona mais profunda. O sistema de canalículos é bem desenvolvido e existem poucos Actinomicetos associados às paredes e ao teto. Nesta cavidade foi encontrado um esqueleto de uma capivara (*Hydrochaerus hydrochaeris*) e não foram observados morcegos ou depósito de guano. Durante a estação úmida não foram observadas alterações significativas nas condições tróficas da cavidade e os praticamente não existiam pontos de gotejamento ativos.

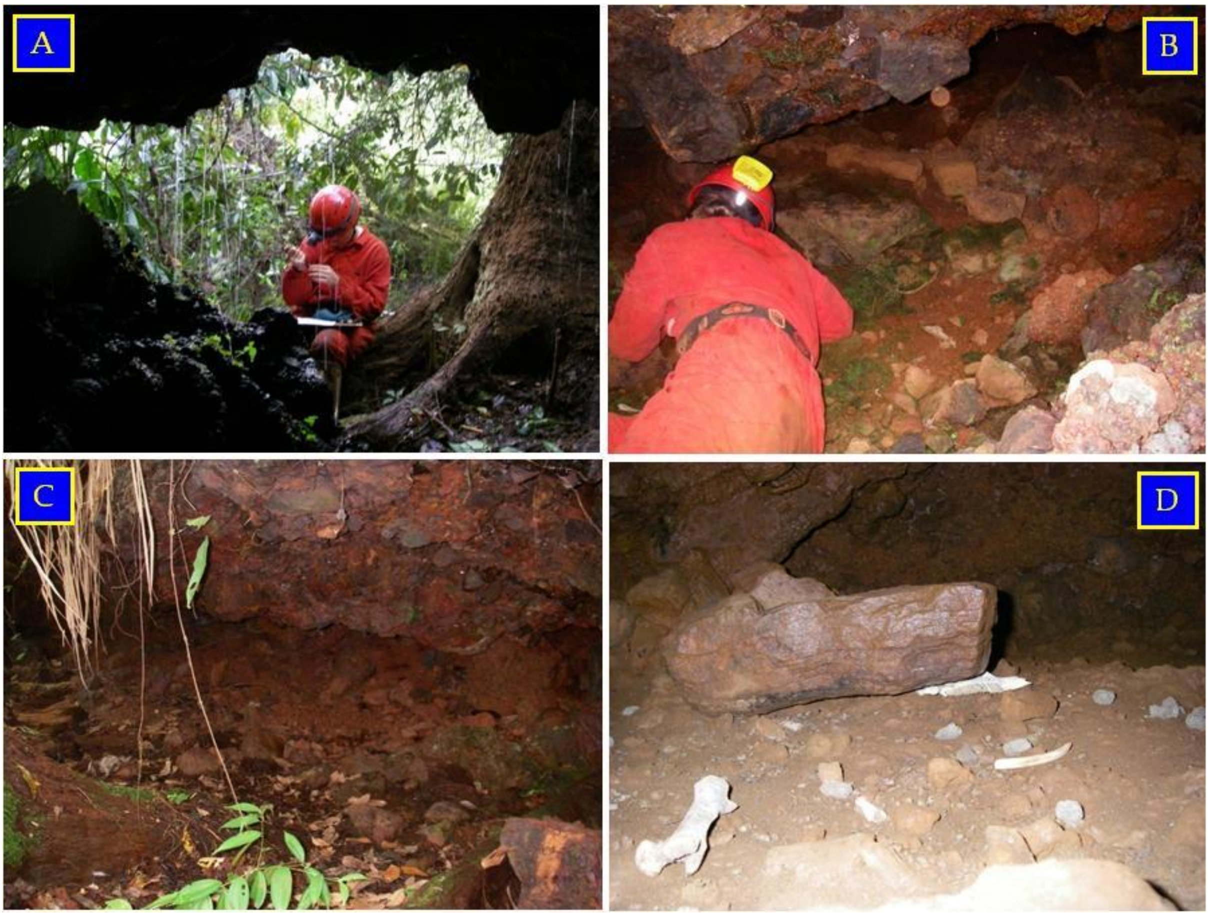

Figura 182 - a) Vista interna da entrada da cavidade com mata ciliar no entorno; b) Aspecto geral do piso da cavidade com muitos blocos; c) vista interna da cavidade; d) restos esqueletais de uma capivara (*Hydrochaerus hydrochaeris*).

5.4.4.59.2. Caracterização faunística no período de seca

Foi observado na caverna, um total de 32 morfoespécies de invertebrados de pelo menos 30 famílias das Ordens: Acari (Neothyridae: *Diplothyrus schubarti*; Ixodidae: *Amblyomma* sp.), Scorpiones (Buthidae: *Ananteris luciae*), Pseudoescorpiones (Chernetidae, Chtoniidae), Opiliones (Neogoveidae: *Canga renatae*; Cosmetidae: *Anduzeia sp.;* Sclerosomatidae: *Prionostemma* sp.), Araneae (Araneidae: *Alpaida* sp., Scytodidae*: Scytodes eleonora*, Psauridae, Pholcidae, Prodidomidae, Theridiidae, Theridiosomatidae), Collembola (Paronellidae), Orthoptera (Phalangopsidae: *Phalangopsis* sp.), Blattodea (Blattellidae), Isoptera (Termitidae: *Nasutitermes),* Psocoptera (Epipsocidae), Hemiptera (Reduviidae), Homoptera (Cixiidae), Lepidoptera (Tineidae), Diptera (Cecidomyiidae, Psychodidae: *Lutzomyia* sp., Tipulidae), Hymenoptera (Formicidae: *Azteca* sp., *Crematogaster* sp., *Pachycondyla* sp.), Coleoptera (Tenebrionidae: Coelometropinae), Diplopoda (Chelodesmidae), Scolopendromorpha (Scolopocryptopidae: *Tidops* sp.).

Dentre os vertebrados, foi encontrada uma espécie da ordem Anura (Leptodactylidae: *Pristimantis cf. fenestratus*). Desta forma, no total foram encontradas 33 morfoespécies. Entre estas, três espécies de invertebrados foram consideradas troglomórficas: Gastropoda (Systrophiidae), Amblypygi (Charinidae: *Charinus* sp.), Hymenoptera (Formicidae: *Hypoponera* sp.).

5.4.4.59.3. Caracterização faunística no período de chuva

Foi observado na caverna, um total de 36 morfoespécies de invertebrados de pelo menos 27 famílias das Ordens: Oligochaeta, Acari (Oribatida), Amblypygi (Phrynidae: *Heterophrynus longicornis*), Pseudoescorpiones (Chernetidae, Chtoniidae), Opiliones (Cosmetidae: *Anduzeia sp.;* Escadabiidae, Phalangiidae), Araneae (Gnaphosidae, Oonopidae: Oonopinae, Salticidae, Scytodidae*: Scytodes eleonora*, Psauridae, Pholcidae, Theridiosomatidae), Collembola (Cyphoderidae, Entomobryidae, Isotomidae), Orthoptera (Phalangopsidae: *Phalangopsis* sp.), Blattodea (Polyphagidae), Isoptera*,* Psocoptera, Hemiptera (Cydnidae, Reduviidae), Diptera (Phoridae, Psychodidae: *Lutzomyia* sp.), Hymenoptera (Formicidae: *Azteca* sp., *Gnamptogenys* sp., *Pachycondyla* sp.), Coleoptera (Phalacridae, Pselaphidae), Symphyla (Scutigerellidae: *Hanseniella* sp.).

Dentre os vertebrados, foi encontrada uma espécie da ordem Chiroptera (Phyllostomidae: *Glossophaga soricina*). Desta forma, no total foram encontradas 37 morfoespécies. Entre estas, uma espécie de invertebrado, foi considerada troglomórfica: Amblypygi (Charinidae: *Charinus* sp.).

5.4.4.59.4. Caracterização geral da fauna da cavidade

Foi observado na caverna, um total de 58 morfoespécies de invertebrados de pelo menos 47 famílias das Ordens: Oligochaeta, Acari (Neothyridae: *Diplothyrus schubarti*; Ixodidae: *Amblyomma* sp.; Oribatida), Amblypygi (Phrynidae: *Heterophrynus longicornis*), Scorpiones (Buthidae: *Ananteris luciae*), Pseudoescorpiones (Chernetidae, Chtoniidae), Opiliones (Neogoveidae: *Canga renatae*; Cosmetidae: *Anduzeia sp.;* Escadabiidae, Sclerosomatidae: *Prionostemma* sp.; Phalangiidae), Araneae (Araneidae: *Alpaida* sp., Gnaphosidae, Oonopidae: Oonopinae, Salticidae, Scytodidae*: Scytodes eleonora*, Psauridae, Pholcidae, Prodidomidae, Theridiidae, Theridiosomatidae), Collembola (Cyphoderidae, Entomobryidae, Isotomidae, Paronellidae), Orthoptera (Phalangopsidae: *Phalangopsis* sp.), Blattodea (Blattellidae, Polyphagidae), Isoptera (Termitidae: *Nasutitermes),* Psocoptera (Epipsocidae), Hemiptera (Cydnidae, Reduviidae), Homoptera (Cixiidae), Lepidoptera (Tineidae), Diptera (Cecidomyiidae, Phoridae, Psychodidae: *Lutzomyia* sp., Tipulidae), Hymenoptera (Formicidae: *Azteca* sp., *Crematogaster* sp., *Gnamptogenys* sp., *Pachycondyla* sp.), Coleoptera (Phalacridae, Pselaphidae, Tenebrionidae: Coelometropinae), Diplopoda (Chelodesmidae), Scolopendromorpha (Scolopocryptopidae: *Tidops* sp.), Symphyla (Scutigerellidae: *Hanseniella* sp.).

Dentre os vertebrados, foram encontradas duas espécies das ordens Chiroptera (Phyllostomidae: *Glossophaga soricina*) e Anura (Leptodactylidae: *Pristimantis cf. fenestratus*).

Desta forma, no total foram encontradas 60 morfoespécies. Entre estas três espécies de invertebrados foram consideradas troglomórficas: Gastropoda (Systrophiidae), Amblypygi (Charinidae: *Charinus* sp.), Hymenoptera (Formicidae: *Hypoponera* sp.).

5.4.4.60. SL-063

5.4.4.60.1. Caracterização trófica

Caverna alagada mesmo durante a estação seca, formada na canga com 12,2 metros de projeção horizontal. Esta cavidade localiza-se na margem esquerda de uma represa. A matriz na região é formada por pastagens, mas a vegetação do entorno da cavidade é composta por uma mata ciliar, sendo esta formada por uma floresta densa com dossel superior a dez metros de altura e com uma elevada quantidade de serrapilheira acumulada junto ao substrato de piso (Figura 183). Sua entrada apresenta altura superior a três metros, sendo sombreada pela mata. A área existente acima da cavidade é composta por uma vegetação arbustiva característica da canga aflorada, onde existe uma pequena quantidade de solo disponível. O piso é totalmente alagado pela água da represa desde a entrada até a zona mais profunda. A cavidade apresenta apenas zonas de penumbra clara com muitos líquens, briófitas e pteridófitas. As paredes e o teto são revestidos por Actinomicetos, além de apresentarem um sistema de canalículos bem desenvolvido. O substrato na parte alagada é composto por sedimento fino com muitos seixos e poucos blocos, onde existe uma grande quantidade de matéria orgânica e raízes. Não foram observados depósitos de guano e nenhum outro vestígio de outros vertebrados. Nenhuma alteração significativa foi observada durante a estação úmida, além das alterações normais na umidade relativa do ar.

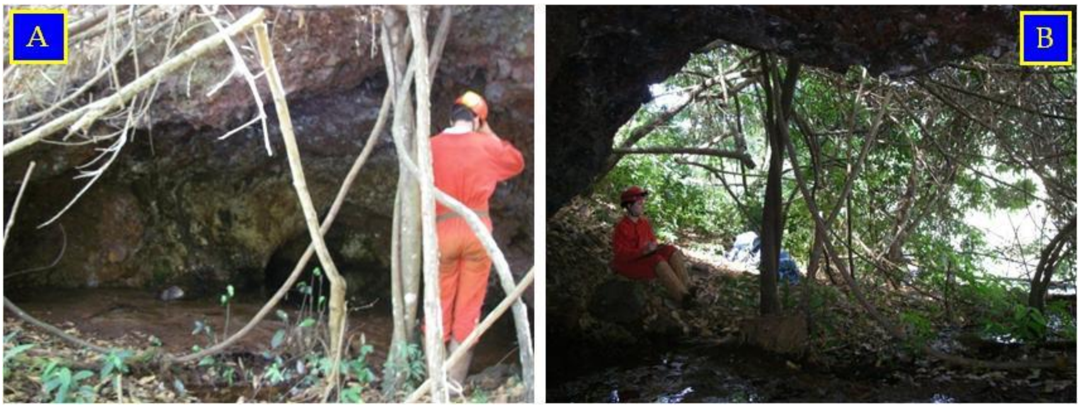

Figura 183 - a) Vista externa da entrada da cavidade com mata ciliar no entorno; b) Vista interna da entrada da cavidade com mata ciliar no entorno.

5.4.4.60.2. Caracterização faunística no período de seca

Foi observado na caverna, um total de 12 morfoespécies de invertebrados de pelo menos nove famílias das Ordens: Opiliones (Sclerosomatidae: *Prionostemma* sp.), Araneae (Ctenidae, Scytodidae*: Scytodes eleonora*, Pholcidae: *Mesabolivar* sp.), Orthoptera (Phalangopsidae: *Aclodes* sp., *Phalangopsis* sp.), Lepidoptera (Tineioidea), Diptera (Drosophilidae, Tipulidae), Hymenoptera (Ichneumonidae), Coleoptera (Dytiscidae).

Dentre os vertebrados, foram encontradas três espécies das ordens Chiroptera (Phyllostomidae: *Glossophaga soricina*), Anura (Leptodactylidae) e Characiformes. Desta forma, no total foram encontradas 15 morfoespécies.

5.4.4.60.3. Caracterização faunística no período de chuva

Foi observado na caverna, um total de 26 morfoespécies de invertebrados de pelo menos 23 famílias das Ordens: Acari (Laelapidae), Amblypygi (Phrynidae: *Heterophrynus longicornis*), Opiliones (Cosmetidae: *Anduzeia sp.*; Sclerosomatidae: *Prionostemma* sp.), Araneae (Filistatidae, Salticidae, Scytodidae*: Scytodes eleonora*, Pholcidae: *Mesabolivar* sp.; Theridiidae, Theridiosomatidae), Collembola (Entomobryidae), Orthoptera (Phalangopsidae: *Aclodes* sp.), Psocoptera (Trogiidae), Hemiptera (Cydnidae, Gerridae, Veliidae), Lepidoptera (Tineidae), Diptera (Cecidomyiidae, Ceratopogonidae, Psychodidae: *Lutzomyia* sp., Tipulidae), Hymenoptera (Formicidae: *Azteca* sp., *Dolichoderus* sp., *Tetramorium* sp.), Coleoptera (Dytiscidae).

Dentre os vertebrados, foram encontradas três espécies das ordens Chiroptera (Phyllostomidae: *Glossophaga soricina*), Siluriformes (Trichomicteridae: *Trichomicterus* sp.) e Characiformes. Desta forma, no total foram encontradas 29 morfoespécies.

5.4.4.60.4. Caracterização geral da fauna da cavidade

Foi observado na caverna, um total de 33 morfoespécies de invertebrados de pelo menos 26 famílias das Ordens: Acari (Laelapidae), Amblypygi (Phrynidae: *Heterophrynus longicornis*), Opiliones (Cosmetidae: *Anduzeia sp.*; Sclerosomatidae: *Prionostemma* sp.), Araneae (Ctenidae, Filistatidae, Salticidae, Scytodidae*: Scytodes eleonora*, Pholcidae: *Mesabolivar* sp.; Theridiidae, Theridiosomatidae), Collembola (Entomobryidae), Orthoptera (Phalangopsidae: *Aclodes* sp., *Phalangopsis* sp.), Psocoptera (Trogiidae), Hemiptera (Cydnidae, Gerridae, Veliidae), Lepidoptera (Tineioidea: Tineidae), Diptera (Cecidomyiidae, Ceratopogonidae, Drosophilidae, Psychodidae: *Lutzomyia* sp., Tipulidae), Hymenoptera (Formicidae: *Azteca* sp., *Dolichoderus* sp., *Tetramorium* sp.; Ichneumonidae), Coleoptera (Dytiscidae).

Dentre os vertebrados foram encontradas quatro espécies das ordens Chiroptera (Phyllostomidae: *Glossophaga soricina*), Anura (Leptodactylidae), Siluriformes

(Trichomicteridae: *Trichomicterus* sp.) e Characiformes. Desta forma, no total foram encontradas 37 morfoespécies.

5.4.4.61. SL-064

5.4.4.61.1. Caracterização trófica

Pequena cavidade formada na canga com 9,9 m de projeção horizontal, localizada na margem esquerda de uma represa. A matriz na região é formada por pastagem, mas a vegetação do entorno da cavidade é composta por mata ciliar, sendo esta formada por uma floresta densa com dossel superior a dez metros de altura. A caverna é pouco profunda, apresentando desenvolvimento preferencial paralelo à sua abertura. A entrada é ampla e larga, com altura superior a dois metros de altura, sendo parcialmente sombreada, o que favorece o desenvolvimento de uma vegetação muito densa nesta região. A cavidade é amplamente iluminada sendo o piso, paredes e teto revestido por líquens, fungos, briófitas, pteridófitas, plântulas de angiospermas, lianas e filodendros. O piso é ascendente a partir da entrada, o que dificulta a importação de matéria orgânica externa pela água da chuva, sendo a serrapilheira restrita à linha d'água ou encontrada de forma esparsa transportada pelo vento. Praticamente todo o piso é formado pela rocha matriz com pouco sedimento argiloso depositado em pontos isolados. Uma pequena área da cavidade encontrava-se alagada durante o inventário e o teto e paredes apresentam uma morfologia irregular com sistema de canalículos bem desenvolvido (Figura 184). Não foram observados depósitos de guano, apesar de terem sido encontrados alguns exemplares de morcegos insetívoros no interior da cavidade. Nenhuma alteração significativa foi observada durante a estação úmida, além das alterações normais na umidade relativa do ar. Entretanto, como o nível de água da represa encontrava-se mais baixo quando comparado ao observado na estação seca, um conduto inferior tornou-se acessível permitindo a coleta nesta nova área. Este conduto consiste de uma área linear estreita com piso encharcado e com muitos blocos. Existem poucos recursos orgânicos e a iluminação é composta por uma penumbra escura.

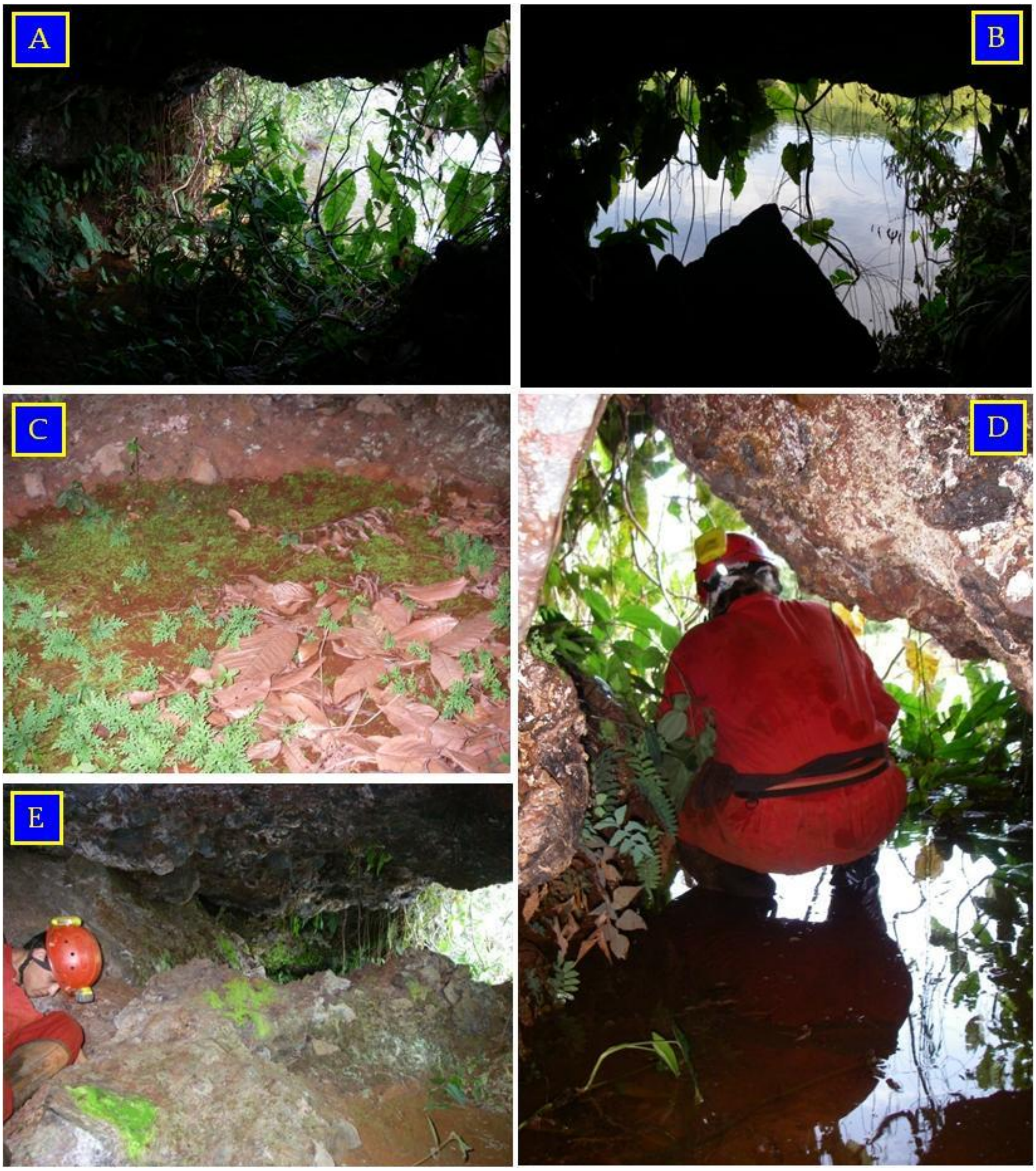

Figura 184 - a) Aspecto geral da entrada da cavidade com vegetação bem desenvolvida; b) Vista interna da entrada da cavidade localizada em frente à represa de Serra Leste; c) Interior da caverna (notar a grande quantidade de vegetais e serrapilheira); d) Entrada do conduto inferior que tornou-se acessível após o rebaixamento do nível da represa durante a estação úmida; e) Interior da caverna.

5.4.4.61.2. Caracterização faunística no período de seca

Foi observado na caverna, um total de 39 morfoespécies de invertebrados de pelo menos 24 famílias das Ordens: Isopoda (Armadillidae), Acari (Mesostigmata, Astigmatina, Oribatida, Trombidforme), Amblypygi (Phrynidae: *Heterophrynus longicornis*), Pseudoscorpiones (Chtoniidae), Opiliones (Cosmetidae: *Anduzeia sp.*; Escadabiidae), Araneae (Mimetidae, Oonopidae: Oonopinae, Gamasomorphinae; Salticidae, Scytodidae*: Scytodes eleonora*, Pholcidae: *Mesabolivar* sp.; Theridiosomatidae), Collembola (Isotomidae), Orthoptera (Gryllidae: Gryllinae:; Phalangopsidae: *Phalangopsis* sp.), Blattodea (Polyphagidae), Embiidina, Isoptera (Termitidae: *Nasutitermes* sp.), Psocoptera (Epipsocidae), Lepidoptera (Tineidae), Diptera (Culicidae, Dixidae, Psychodidae: *Lutzomyia* sp., Tipulidae), Hymenoptera (Formicidae: *Azteca* sp., *Dolichoderus* sp.; *Odontomachus* sp., *Pachycondyla* sp., *Strumigenys* sp.; Vespidae), Scolopendromorpha (Cryptopidae: *Cryptops* sp.), Scutigeromorpha (Scutigeridae: *Sphendononema* sp.).

Dentre os vertebrados, foram encontradas três espécies das ordens Chiroptera (Emballonuridae: *Peropteryx kappleri*), Anura (Leptodactylidae: *Pristimantis cf. fenestratus*) e Passeriformes (Trochilidae). Desta forma, no total foram encontradas 42 morfoespécies.

5.4.4.61.3. Caracterização faunística no período de chuva

Foi observado na caverna, um total de 49 morfoespécies de invertebrados de pelo menos 37 famílias das Ordens: Acari (Neothyridae: *Diplothyrus schubarti*; Laelapidae: *Stratiolaelaps* sp.; Mesostigmata, Astigmatina, Anystidae: *Erythracarus* sp.), Amblypygi (Phrynidae: *Heterophrynus longicornis*), Pseudoscorpiones (Chtoniidae), Opiliones (Phalangiidae), Araneae (Ochyroceratidae, Oonopidae: Oonopinae; Scytodidae*: Scytodes eleonora*, Pholcidae: *Mesabolivar* sp., *Metagonia* sp.; Theridiosomatidae), Thysanura (Nicoletiidae: Nicoletiinae), Diplura (Campodeidae, Parajapygidae), Collembola (Entomobryidae, Isotomidae), Orthoptera (Phalangopsidae: *Aclodes* sp., *Phalangopsis* sp.), Isoptera (Termitidae: *Nasutitermes* sp.), Psocoptera (Epipsocidae, Lepidopsocidae, Myopsocidae: *Lichenomina* sp.; Psyllipsocidae: *Psyllipsocus* sp.), Hemiptera (Cydnidae, Lygaeidae), Lepidoptera (Noctuidae, Tineidae), Diptera (Chironomidae, Muscidae, Psychodidae: *Lutzomyia* sp., Sciaridae), Hymenoptera (Formicidae: *Dolichoderus* sp.; Myrmicinae; *Odontomachus* sp., *Pachycondyla* sp., *Solenopsis* sp., Vespidae), Coleoptera (Carabidae), Diplopoda (Chelodesmidae), Neuroptera (Myrmeleontidae).

Desta forma, no total foram encontradas 49 morfoespécies. Entre estas, duas espécies de invertebrados foram consideradas troglomórficas: Amblypygi (Charinidae: *Charinus* sp.), Schizomida (Hubbardiinae.).

5.4.4.61.4. Caracterização geral da fauna da cavidade

Foi observado na caverna, um total de 75 morfoespécies de invertebrados de pelo menos 49 famílias das Ordens: Isopoda (Armadillidae), Acari (Neothyridae: *Diplothyrus schubarti*; Laelapidae: *Stratiolaelaps* sp.; Mesostigmata, Astigmatina, Oribatida, Anystidae: *Erythracarus* sp.), Amblypygi (Phrynidae: *Heterophrynus longicornis*), Pseudoscorpiones (Chtoniidae), Opiliones (Cosmetidae: *Anduzeia sp.*; Escadabiidae, Phalangiidae), Araneae (Mimetidae, Ochyroceratidae, Oonopidae: Oonopinae, Gamasomorphinae; Salticidae, Scytodidae*:* *Scytodes eleonora*, Pholcidae: *Mesabolivar* sp., *Metagonia* sp.; Theridiosomatidae), Thysanura (Nicoletiidae: Nicoletiinae), Diplura (Campodeidae, Parajapygidae), Collembola (Entomobryidae, Isotomidae), Orthoptera (Gryllidae: Gryllinae:; Phalangopsidae: *Aclodes* sp., *Phalangopsis* sp.), Blattodea (Polyphagidae), Embiidina, Isoptera (Termitidae: *Nasutitermes* sp.), Psocoptera (Epipsocidae, Lepidopsocidae, Myopsocidae: *Lichenomina* sp.; Psyllipsocidae: *Psyllipsocus* sp.), Hemiptera (Cydnidae, Lygaeidae), Lepidoptera (Noctuidae, Tineidae), Diptera (Chironomidae, Culicidae, Dixidae, Muscidae, Psychodidae: *Lutzomyia* sp., Sciaridae, Tipulidae), Hymenoptera (Formicidae: *Azteca* sp., *Dolichoderus* sp.; Myrmicinae; *Odontomachus* sp., *Pachycondyla* sp., *Solenopsis* sp., *Strumigenys* sp.; Vespidae), Coleoptera (Carabidae), Diplopoda (Chelodesmidae), Scolopendromorpha (Cryptopidae: *Cryptops* sp.), Scutigeromorpha (Scutigeridae: *Sphendononema* sp.), Neuroptera (Myrmeleontidae).

Dentre os vertebrados, foram encontradas três espécies das ordens Chiroptera (Emballonuridae: *Peropteryx kappleri*), Anura (Leptodactylidae: *Pristimantis cf. fenestratus*) e Passeriformes (Trochilidae).

Desta forma, no total foram encontradas 78 morfoespécies. Entre estas duas espécies de invertebrados, foram consideradas troglomórficas: Amblypygi (Charinidae: *Charinus* sp.), Schizomida (Hubbardiinae).

5.4.4.62. SL-065

5.4.4.62.1. Caracterização trófica

Cavidade com 19,1 m de projeção horizontal localizada em área de pastagem na margem direita de uma represa. Na parte superior da cavidade a vegetação é composta por pastagem e junto à entrada existe um pequeno fragmento de mata ciliar. Sua entrada é ampla e seu desenvolvimento é predominantemente retilíneo e não existem áreas afóticas. De maneira geral, o piso da cavidade é plano sendo este composto principalmente por sedimento granulado e praticamente não existem blocos de grandes dimensões. Na entrada existem muitas briófitas, líquens, pteridófitas e plântulas de diferentes espécies. A pouca serrapilheira encontra-se praticamente restrita à linha d’água, sendo esta muito limitada,

exceto por um grande fragmento de tronco apodrecido encontrado na porção mais distal da cavidade. Na zona entrada existem ainda muitas fezes de gado nas quais existe um grande número de dípteros Dolichopodidae e larvas de besouros escarabeídeos associados. Na zona mais distal da cavidade existe um ponto de gotejamento onde o piso fica completamente encharcado onde existe uma grande quantidade de ferrobactérias. Neste setor, também existe uma grande quantidade de Actinomicetos associados às paredes e ao teto da cavidade (Figura 185). O sistema de canalículos é pouco desenvolvido e não foram observados depósitos de guano no interior da cavidade apesar de terem sido encontrados alguns exemplares de G. soricina. Foi observado um resto de fogueira na cavidade, o que denota seu eventual uso humano. Nenhuma alteração significativa foi observada durante a estação úmida, além das alterações normais na umidade relativa do ar.

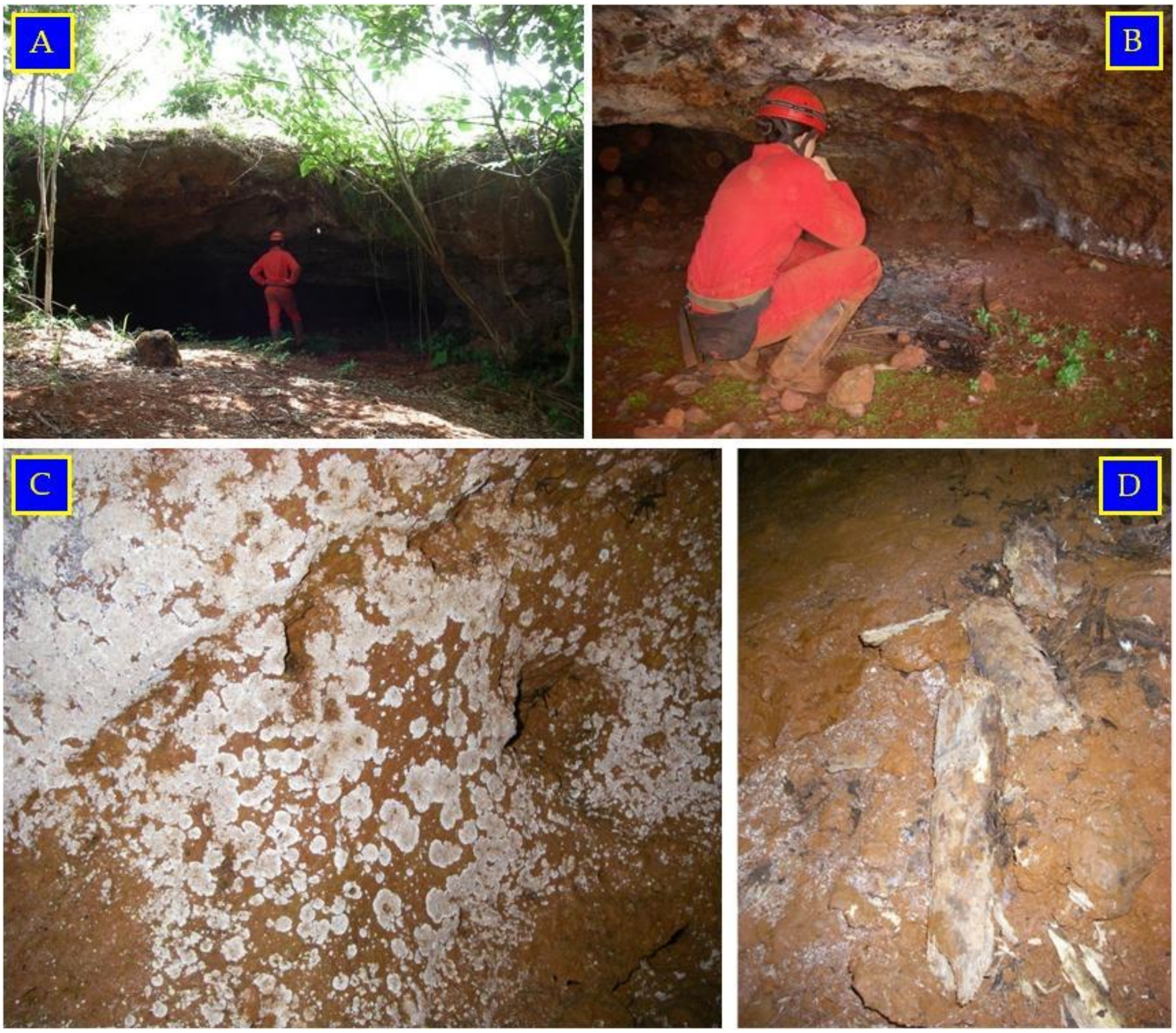

Figura 185 - a) Vista externa da entrada da cavidade localizada em pequeno fragmento de mata na margem direita da represa de Serra Leste; b) Restos de uma fogueira no interior da cavidade; c) Grande quantidade de colônias de Actinomicetos nas paredes da caverna; d) Tronco apodrecido de aproximadamente 80 cm encontrado no fundo da cavidade.

5.4.4.62.2. Caracterização faunística no período de seca

Foi observado na caverna, um total de 72 morfoespécies de invertebrados de pelo menos 49 famílias das Ordens: Oligochaeta, Isopoda (Armadillidae, Balloniscidae), Acari (Laelapidae: *Stratiolaelaps* sp., Macrochelidae: *Macrocheles* sp.; Macronyssidae, Ologamasidae, Trombidiforme), Amblypygi (Phrynidae: *Heterophrynus longicornis*), Pseudoscorpiones (Chernetidae, Chtoniidae), Opiliones (Kimmulidae, Sclerosomatidae: *Prionostemma* sp.), Araneae (Gnaphosidae, Linyphiidae, Ochyroceratidae, Oonopidae: Oonopinae, Gamasomorphinae; Scytodidae: *Scytodes eleonora*, Psauridae, Pholcidae: *Mesabolivar* sp.), Thysanura (Nicoletiidae: Nicoletiinae), Diplura (Campodeidae), Collembola (Entomobryidae, Isotomidae, Paronellidae), Orthoptera (Phalangopsidae: *Aclodes* sp., *Phalangopsis* sp.), Blattodea (Polyphagidae), Psocoptera, Hemiptera (Cydnidae, Dipsocoridae), Lepidoptera (Noctuidae: Agaristinae), Diptera (Chironomidae, Culicidae, Psychodidae: *Lutzomyia* sp., Sciaridae, Tipulidae), Hymenoptera (Formicidae: *Atta* sp., *Gnamptogenys* sp., *Octostruma* sp., *Odontomachus* sp., *Pachycondyla* sp., *Pheidole* sp., *Solenopsis* sp.; Diapriidae, Scelionidae), Coleoptera (Byrrhidae, Carabidae, Pselaphidae, Scarabeidae, Staphylinidae), Diplopoda (Chelodesmidae, Cyrtodesmidae, Paradoxosomatidae, Pyrgodesmidae), Lithobiomorpha (Henicopiidae: *Lamyctes* sp.), Scutigeromorpha (Scutigeridae: *Sphendononema* sp.), Symphyla (Scutigerellidae: *Hanseniella* sp.).

Dentre os vertebrados, foram encontradas duas espécies das ordens Chiroptera (Phyllostomidae: *Glossophaga soricina*), Anura (Leptodactylidae: *Pristimantis cf. fenestratus*).

Desta forma, no total foram encontradas 74 morfoespécies. Entre estas, duas espécies de invertebrados, foram consideradas troglomórficas: Gordeoidea, Collembola (Cyphoderidae).

5.4.4.62.3. Caracterização faunística no período de chuva

Foi observado na caverna, um total de 50 morfoespécies de invertebrados de pelo menos 34 famílias das Ordens: Turbellaria (Geoplanidae), Isopoda (Armadillidae, Balloniscidae), Acari (Oribatida, Trombidiforme), Amblypygi (Phrynidae: *Heterophrynus longicornis*), Pseudoscorpiones (Chernetidae, Chtoniidae), Opiliones (Escadabiidae, Phalangiidae), Araneae (Ctenidae: *Ctenus* sp.; Gnaphosidae, Linyphiidae, Oonopidae: Oonopinae, Gamasomorphinae; Salticidae, Scytodidae: *Scytodes eleonora*, Psauridae, Pholcidae: *Mesabolivar* sp.; Theraphosidae, Theridiosomatidae), Collembola (Entomobryidae, Hypogastruridae), Orthoptera (Phalangopsidae: *Aclodes* sp., *Phalangopsis* sp.), Isoptera (Rhinotermitidae: *Heterotermes* sp.), Psocoptera, Hemiptera (Cydnidae, Dipsocoridae, Lygaeidae, Reduviidae), Lepidoptera (Noctuidae: Agaristinae), Diptera (Ceratopogonidae, Mycetophilidae, Psychodidae: *Lutzomyia* sp.), Hymenoptera (Formicidae: *Atta* sp., *Octostruma* sp., *Odontomachus* sp., *Pachycondyla* sp., *Solenopsis* sp.), Coleoptera

(Carabidae, Elateridae: Elaterinae; Scydmaenidae, Staphylinidae), Diplopoda (Cyrtodesmidae), Lithobiomorpha (Henicopiidae: *Lamyctes* sp.).

Dentre os vertebrados, foram encontradas três espécies das ordens Chiroptera (Phyllostomidae: *Glossophaga soricina*), Squamata (Colubridae) e Anura (Dendrobatidae: *Colostethus sp.*).

Desta forma, no total foram encontradas 53 morfoespécies. Entre estas, uma espécie de invertebrado foi considerada troglomórfica: Diplopoda (Polydesmida).

5.4.4.62.4. Caracterização geral da fauna da cavidade

Foi observado na caverna, um total de 103 morfoespécies de invertebrados de pelo menos 63 famílias das Ordens: Oligochaeta, Turbellaria (Geoplanidae), Isopoda (Armadillidae, Balloniscidae), Acari (Laelapidae: *Stratiolaelaps* sp., Macrochelidae: *Macrocheles* sp.; Macronyssidae, Ologamasidae, Oribatida, Trombidiforme), Amblypygi (Phrynidae: *Heterophrynus longicornis*), Pseudoscorpiones (Chernetidae, Chtoniidae), Opiliones (Escadabiidae, Kimmulidae, Sclerosomatidae: *Prionostemma* sp.; Phalangiidae), Araneae (Ctenidae: *Ctenus* sp.; Gnaphosidae, Linyphiidae, Ochyroceratidae, Oonopidae: Oonopinae, Gamasomorphinae; Salticidae, Scytodidae: *Scytodes eleonora*, Psauridae, Pholcidae: *Mesabolivar* sp.; Theraphosidae, Theridiosomatidae), Thysanura (Nicoletiidae: Nicoletiinae), Diplura (Campodeidae), Collembola (Entomobryidae, Hypogastruridae, Isotomidae, Paronellidae), Orthoptera (Phalangopsidae: *Aclodes* sp., *Phalangopsis* sp.), Blattodea (Polyphagidae), Isoptera (Rhinotermitidae: *Heterotermes* sp.), Psocoptera, Hemiptera (Cydnidae, Dipsocoridae, Lygaeidae, Reduviidae), Lepidoptera (Noctuidae: Agaristinae), Diptera (Ceratopogonidae, Chironomidae, Culicidae, Mycetophilidae, Psychodidae: *Lutzomyia* sp., Sciaridae, Tipulidae), Hymenoptera (Formicidae: *Atta* sp., *Gnamptogenys* sp., *Octostruma* sp., *Odontomachus* sp., *Pachycondyla* sp., *Pheidole* sp., *Solenopsis* sp.; Diapriidae, Scelionidae), Coleoptera (Byrrhidae, Carabidae, Elateridae: Elaterinae; Pselaphidae, Scarabeidae, Scydmaenidae, Staphylinidae), Diplopoda (Chelodesmidae, Cyrtodesmidae, Paradoxosomatidae, Pyrgodesmidae), Lithobiomorpha (Henicopiidae: *Lamyctes* sp.), Scutigeromorpha (Scutigeridae: *Sphendononema* sp.), Symphyla (Scutigerellidae: *Hanseniella* sp.).

Dentre os vertebrados, foram encontradas quatro espécies das ordens Chiroptera (Phyllostomidae: *Glossophaga soricina*), Squamata (Colubridae) e Anura (Leptodactylidae: *Pristimantis cf. fenestratus;* Dendrobatidae: *Colostethus sp.*).

Desta forma, no total foram encontradas 107 morfoespécies. Entre estas, três espécies de invertebrados foram consideradas troglomórficas: Gordeoidea, Collembola (Cyphoderidae), Diplopoda (Polydesmida). Alguns organismos encontrados nesta caverna são mostrados na Figura 186.

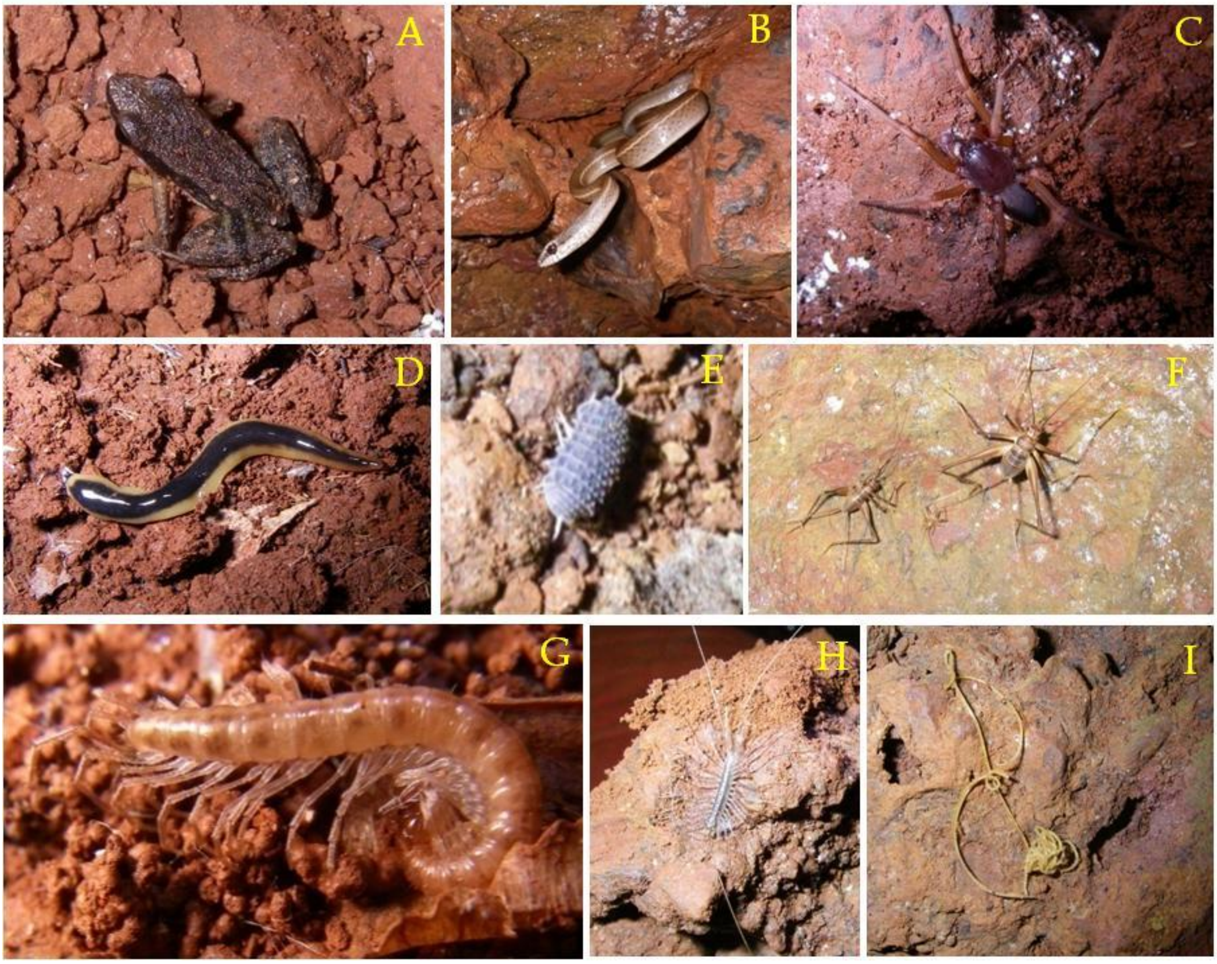

Figura 186 - a) Anura (indet.); b) Colubridae; c) Araneae (Corinidae); d) Planaria (Geoplanidae); e) Isopoda (Armadillidae); f) Orthoptera (Phalangopsidae: Phalangopsis sp.); g) Diplopoda (Polydesmida); h) Scutigeromorpha (Sphendononema sp.); i) Nematomorpha (Gordioidea).

5.4.4.63. SL-066

5.4.4.63.1. Caracterização trófica

Pequena cavidade com 15,2 m de projeção horizontal localizada em área de pastagem na margem direita de uma represa. Na parte superior da cavidade a vegetação é composta por pastagem e junto à entrada existe um pequeno fragmento de mata ciliar. Caverna pouco profunda sem zona afótica com desenvolvimento preferencial paralelo à abertura da entrada. Entrada ampla (com altura superior a dois metros) e muito larga, sendo esta, parcialmente sombreada, o que favorece o desenvolvimento de líquens e briófitas nesta

área. Piso predominantemente plano e seco, composto por sedimento granulado com alguns seixos e poucos blocos de grandes dimensões (calhaus e matacões). A serrapilheira encontra-se acumulada junto à entrada, mas também ocorre de forma esparsa pelo piso da cavidade (Figura 187). Ainda junto ao piso, existem algumas plântulas de angiospermas e o sistema radicular é bem desenvolvido com raízes de fino calibre além de pequenos depósitos de guano de morcegos frugívoros produzidos por morcegos Glossophaginae. No interior da cavidade existe um grande cupinzeiro, as paredes e o teto são revestidos por fungos, o sistema de canalículos é bem desenvolvido e, de forma geral, a cavidade apresenta uma baixa estabilidade ambiental. Nenhuma alteração significativa foi observada durante a estação úmida, além das alterações normais na umidade relativa do ar.

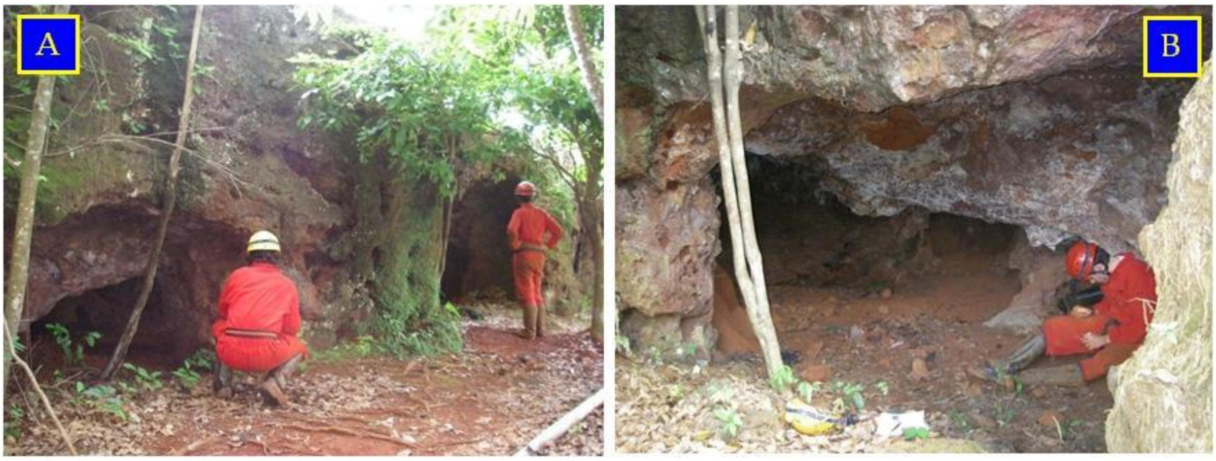

Figura 187 - a) Vista externa da entrada da cavidade localizada em pequeno fragmento de mata na margem direita da represa de Serra Leste; b) Vista da porção interna da cavidade.

5.4.4.63.2. Caracterização faunística no período de seca

Foi observado na caverna, um total de 44 morfoespécies de invertebrados de pelo menos 28 famílias das Ordens: Isopoda (Armadillidae), Acari (Uropodina, Oribatida, Trombidiforme), Pseudoscorpiones (Chernetidae), Opiliones (Cosmetidae: *Anduzeia sp.*), Araneae (Gnaphosidae, Scytodidae*: Scytodes eleonora*, Pholcidae: *Mesabolivar* sp., Theridiidae), Thysanura (Nicoletiidae: Nicoletiinae), Orthoptera (Phalangopsidae: *Phalangopsis* sp.), Blattodea (Polyphagidae), Psocoptera (Epipsocidae), Hemiptera (Cydnidae, Lygaeidae), Diptera (Cecidomyiidae, Ceratopogonidae, Chironomidae, Culicidae, Drosophilidae, Psychodidae: *Lutzomyia* sp.; Sciaridae, Tipulidae), Hymenoptera (Formicidae: *Acanthognatus* sp., *Apterostigma* sp., *Atta* sp., *Pachycondyla* sp., *Pheidole* sp., *Solenopsis* sp., Chalcidoidea), Coleoptera (Carabidae, Curculionidae: Scotylinae; Dermestidae, Elateridae, Scydmaenidae, Staphylinidae).

Dentre os vertebrados, foram encontradas duas espécies das ordens Chiroptera (Emballonuridae: *Peropteryx kappleri*) e Anura (Leptodactylidae: *Pristimantis cf. fenestratus).* Desta forma, no total foram encontradas 46 morfoespécies.

5.4.4.63.3. Caracterização faunística no período de chuva

Foi observado na caverna, um total de 44 morfoespécies de invertebrados de pelo menos 35 famílias das Ordens: Isopoda (Armadillidae, Balloniscidae), Acari (Opilioacaridae: *Neoacarus* sp.), Pseudoscorpiones (Chernetidae, Chtoniidae), Araneae (Araneidae: *Alpaida* sp., Gnaphosidae, Salticidae, Scytodidae*: Scytodes eleonora*, Pholcidae: *Mesabolivar* sp., *Metagonia* sp., Theridiidae, Theridiosomatidae), Collembola (Hypogastruridae), Orthoptera (Phalangopsidae: *Phalangopsis* sp.), Blattodea (Polyphagidae), Psocoptera (Epipsocidae, Ectopsocidae), Hemiptera (Alydidae, Cydnidae, Lygaeidae), Homoptera (Cixiidae: *Cixius* sp.), Diptera (Ceratopogonidae, Chironomidae, Drosophilidae, Mycetophilidae, Phoridae, Psychodidae: *Lutzomyia* sp.; Sciaridae, Tipulidae), Hymenoptera (Formicidae: *Atta* sp., *Pachycondyla* sp., *Pheidole* sp., *Wasmannia* sp.; Figitidae, Tiphiidae), Coleoptera (Elateridae: Cardiophorinae), Scolopendromorpha (Cryptopidae: *Cryptops* sp.), Neuroptera (Myrmeleontidae).

Dentre os vertebrados, foram encontradas quatro espécies das ordens Chiroptera (Emballonuridae: *Peropteryx kappleri,* Phyllostomidae: *Glossophaga soricina*) e Anura (Leptodactylidae: *Pristimantis cf. fenestratus;* Dendrobatidae: *Colostethus* sp.). Desta forma, no total foram encontradas 48 morfoespécies.

5.4.4.63.4. Caracterização geral da fauna da cavidade

Foi observado na caverna, um total de 74 morfoespécies de invertebrados de pelo menos 44 famílias das Ordens: Isopoda (Armadillidae, Balloniscidae), Acari (Uropodina, Opilioacaridae: *Neoacarus* sp.; Oribatida, Trombidiforme), Pseudoscorpiones (Chernetidae, Chtoniidae), Opiliones (Cosmetidae: *Anduzeia sp.*), Araneae (Araneidae: *Alpaida* sp., Gnaphosidae, Salticidae, Scytodidae*: Scytodes eleonora*, Pholcidae: *Mesabolivar* sp., *Metagonia* sp., Theridiidae, Theridiosomatidae), Thysanura (Nicoletiidae: Nicoletiinae), Collembola (Hypogastruridae), Orthoptera (Phalangopsidae: *Phalangopsis* sp.), Blattodea (Polyphagidae), Psocoptera (Epipsocidae, Ectopsocidae), Hemiptera (Alydidae, Cydnidae, Lygaeidae), Homoptera (Cixiidae: *Cixius* sp.), Diptera (Cecidomyiidae, Ceratopogonidae, Chironomidae, Culicidae, Drosophilidae, Mycetophilidae, Phoridae, Psychodidae: *Lutzomyia* sp.; Sciaridae, Tipulidae), Hymenoptera (Formicidae: *Acanthognatus* sp., *Apterostigma* sp., *Atta* sp., *Pachycondyla* sp., *Pheidole* sp., *Solenopsis* sp., *Wasmannia* sp.; Chalcidoidea; Figitidae, Tiphiidae), Coleoptera (Carabidae, Curculionidae: Scotylinae; Dermestidae, Elateridae: Cardiophorinae; Scydmaenidae, Staphylinidae), Scolopendromorpha (Cryptopidae: *Cryptops* sp.), Neuroptera (Myrmeleontidae).

Dentre os vertebrados, foram encontradas quatro espécies das ordens Chiroptera (Emballonuridae: *Peropteryx kappleri,* Phyllostomidae: *Glossophaga soricina*) e Anura

(Leptodactylidae: *Pristimantis cf. fenestratus;* Dendrobatidae: *Colostethus* sp.). Desta forma, no total foram encontradas 78 morfoespécies.

5.4.4.64. SL-067

5.4.4.64.1. Caracterização trófica

Cavidade com 20,5 m de projeção horizontal, localizada em área de pastagem na margem direita de uma represa. Trata-se de uma caverna com teto baixo, com desenvolvimento predominantemente retilíneo e sem zonas afóticas. De maneira geral o piso da cavidade é plano e seco, sendo este composto principalmente por sedimento fino com poucos grânulos, seixos e calhaus distribuídos de maneira esparsa. A entrada é iluminada onde existem muitos líquens e briófitas além de uma árvore de pequeno porte, sendo que, de maneira geral, existe pouca matéria orgânica associada ao substrato (Figura 188). O sistema radicular é pouco desenvolvido e não foram observados depósitos de guano no interior da cavidade. Não existem pontos de gotejamento e o sistema de canalículos é bem desenvolvido. Nenhuma alteração significativa foi observada durante a estação úmida, além das alterações normais na umidade relativa do ar.

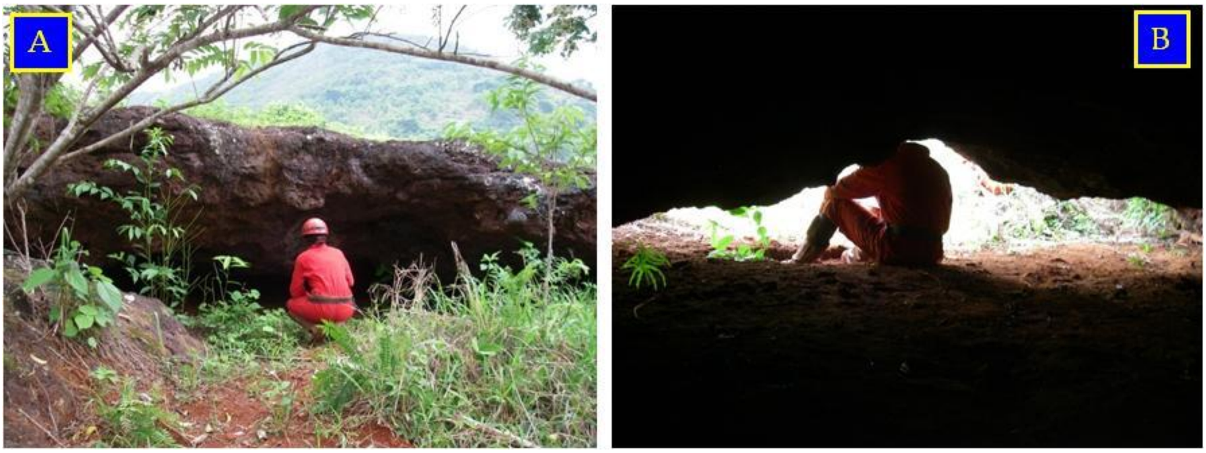

Figura 188 - a) Vista externa da entrada da cavidade localizada na margem direita da represa de Serra Leste; b) vista interna da entrada.

5.4.4.64.2. Caracterização faunística no período de seca

Foi observado na caverna, um total de 35 morfoespécies de invertebrados de pelo menos 25 famílias das Ordens: Isopoda (Armadillidae, Philosciidae), Acari (Mesostigmata, Anystidae: *Erythracarus* sp.), Amblypygi (Phrynidae: *Heterophrynus longicornis*), Pseudoscorpiones (Chernetidae), Araneae (Ochyroceratidae, Salticidae, Senoculidae, Scytodidae*: Scytodes eleonora*, Psauridae, Pholcidae, Theridiosomatidae), Orthoptera (Phalangopsidae: *Phalangopsis* sp.), Dermaptera (Labiidae), Hemiptera (Cydnidae, Reduviidae, Pyrrhocoridae), Homoptera (Cixiidae), Diptera (Culicidae), Hymenoptera (Formicidae: *Anochetus* sp., *Apterostigma* sp., *Atta* sp., *Crematogaster* sp., *Pachycondyla* sp., *Pheidole* sp., *Zacryptocerus*

sp.; Elasmidae, Vespidae), Coleoptera (Cerambycidae), Diplopoda (Paradoxosomatidae), Lithobiomorpha, Symphyla (Scutigerellidae: *Hanseniella* sp.), Neuroptera (Myrmeleontidae).

Dentre os vertebrados, foi encontrada uma espécie da ordem Anura (Leptodactylidae: *Pristimantis cf. fenestratus).* Desta forma, no total foram encontradas 36 morfoespécies.

5.4.4.64.3. Caracterização faunística no período de chuva

Foi observado na caverna, um total de 58 morfoespécies de invertebrados de pelo menos 42 famílias das Ordens: Isopoda (Armadillidae, Balloniscidae, Dubioniscidae), Acari (Laelapidae: *Stratiolaelaps* sp., Opioacaridae: *Neoacarus* sp., Oribatida, Anystidae: *Erythracarus* sp.; Eupodidae: *Linopodes* sp.), Pseudoscorpiones (Chernetidae), Opiliones (Stygnidae), Araneae (Gnaphosidae, Linyphiidae, Oonopidae: Oonopinae, Salticidae, Scytodidae*: Scytodes eleonora*, Pholcidae, Tetrablemidae, Theridiidae), Collembola (Entomobryidae, Tomoceridae), Orthoptera (Phalangopsidae: *Phalangopsis* sp.), Psocoptera (Archipsocidae, Ectopsocidae, Myopsocidae: *Lichenomina* sp.), Hemiptera (Cydnidae, Lygaeidae, Pyrrhocoridae), Homoptera (Cixiidae), Diptera (Cecidomyiidae, Psychodidae: *Lutzomyia* sp.), Hymenoptera (Formicidae: *Apterostigma* sp., *Atta* sp., *Cyphomyrmex* sp., *Pachycondyla* sp., *Pheidole* sp., *Tapinoma* sp.; Elasmidae, Vespidae), Coleoptera (Curculionidae, Lampiridae, Phalacridae, Pselaphidae, Scydmaenidae), Diplopoda (Paradoxosomatidae), Lithobiomorpha (Henicopiidae: *Lamyctes* sp.), Scolopendromorpha (Cryptopidae: *Cryptops* sp.), Symphyla (Scutigerellidae: *Hanseniella* sp.), Neuroptera (Myrmeleontidae).

Dentre os vertebrados, foi encontrada uma espécie da ordem Anura (Leptodactylidae: *Pristimantis cf. fenestratus).*

Desta forma, no total foram encontradas 59 morfoespécies. Entre estas, uma espécie de invertebrado foi considerada troglomórfica: Diplopoda (Lophoproctidae).

5.4.4.64.4. Caracterização geral da fauna da cavidade

Foi observado na caverna, um total de 81 morfoespécies de invertebrados de pelo menos 52 famílias das Ordens: Isopoda (Armadillidae, Balloniscidae, Dubioniscidae, Philosciidae), Acari (Laelapidae: *Stratiolaelaps* sp., Mesostigmata, Opioacaridae: *Neoacarus* sp., Oribatida, Anystidae: *Erythracarus* sp.; Eupodidae: *Linopodes* sp.), Amblypygi (Phrynidae: *Heterophrynus longicornis*), Pseudoscorpiones (Chernetidae), Opiliones (Stygnidae), Araneae (Gnaphosidae, Linyphiidae, Ochyroceratidae, Oonopidae: Oonopinae, Salticidae, Senoculidae, Scytodidae*: Scytodes eleonora*, Psauridae, Pholcidae, Tetrablemidae, Theridiidae, Theridiosomatidae), Collembola (Entomobryidae, Tomoceridae), Orthoptera (Phalangopsidae: *Phalangopsis* sp.), Dermaptera (Labiidae), Psocoptera (Archipsocidae, Ectopsocidae, Myopsocidae: *Lichenomina* sp.), Hemiptera (Cydnidae, Lygaeidae, Reduviidae, Pyrrhocoridae), Homoptera (Cixiidae), Diptera (Cecidomyiidae, Culicidae, Psychodidae:

Lutzomyia sp.), Hymenoptera (Formicidae: *Anochetus* sp., *Apterostigma* sp., *Atta* sp., *Crematogaster* sp., *Cyphomyrmex* sp., *Pachycondyla* sp., *Pheidole* sp., *Tapinoma* sp., *Zacryptocerus* sp.; Elasmidae, Vespidae), Coleoptera (Cerambycidae, Curculionidae, Lampiridae, Phalacridae, Pselaphidae, Scydmaenidae), Diplopoda (Paradoxosomatidae), Lithobiomorpha (Henicopiidae: *Lamyctes* sp.), Scolopendromorpha (Cryptopidae: *Cryptops* sp.), Symphyla (Scutigerellidae: *Hanseniella* sp.), Neuroptera (Myrmeleontidae).

Dentre os vertebrados, foi encontrada uma espécie da ordem Anura (Leptodactylidae: *Pristimantis cf. fenestratus).*

Desta forma, no total foram encontradas 82 morfoespécies. Entre estas, uma espécie de invertebrado foi considerada troglomórfica: Diplopoda (Lophoproctidae). Alguns organismos encontrados nesta caverna são mostrados na Figura 189.

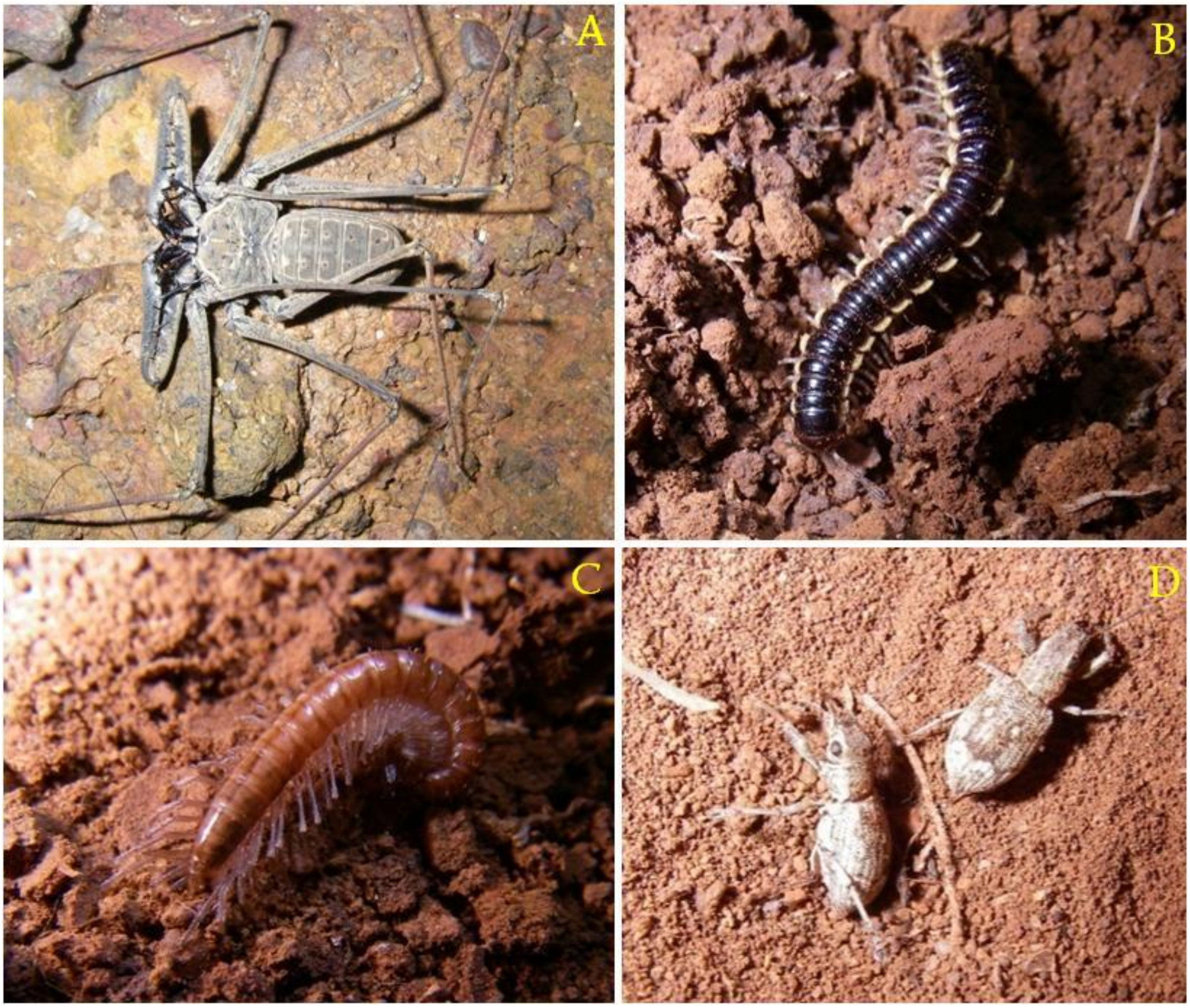

Figura 189 - a) Amblypygi (H. longicornis); b) Diplopoda (Paradoxosomatidae); c) Diplopoda (Polydesmida); d) Coleoptera (Curculionidae).

5.4.4.65. SL-068

5.4.4.65.1. Caracterização trófica

Pequena cavidade com 13,1 m de desenvolvimento, formada na canga e localizada na margem direita de uma pequena drenagem. A matriz do entorno é formada por pastagem e a cavidade corresponde a uma fenda estreita e retilínea com pouco mais de dois metros de altura. A entrada é iluminada e apresenta líquens, briófitas e plântulas de angiospermas restritas à linha d'água além de pouca serrapilheira. O piso é plano e alagado com muita matéria orgânica e raízes submersas, com porções emersas (Figura 190). A caverna não apresenta zona afótica e o sistema de canalículos é pouco desenvolvido. Nenhuma alteração significativa foi observada durante a estação úmida, além das alterações normais na umidade relativa do ar.

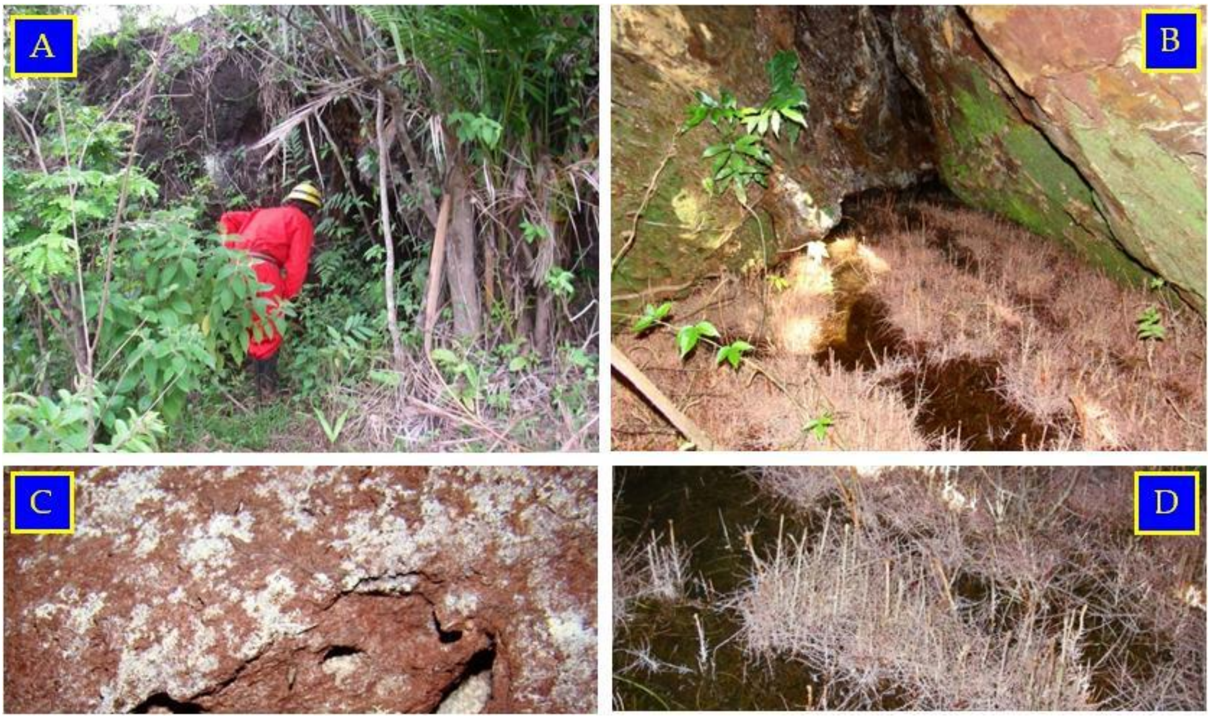

Figura 190 - a) Vista externa da entrada da cavidade localizada em uma pequena drenagem na margem direita da represa de Serra Leste; b) Vista interna da cavidade com detalhes do piso alagado com muitas raízes; c) Grande quantidade de colônias de Actinomicetos se desenvolvendo sobre as paredes; d) Detalhe dos tufos radiculares se desenvolvendo no piso alagado da cavidade.

5.4.4.65.2. Caracterização faunística no período de seca

Foi observado na caverna, um total de 17 morfoespécies de invertebrados de pelo menos 14 famílias das Ordens: Amblypygi (Phrynidae: *Heterophrynus longicornis*), Opiliones (Sclerosomatidae: *Prionostemma* sp.), Araneae (Gnaphosidae, Pholcidae: *Mesabolivar* sp., Theridiidae), Orthoptera (Phalangopsidae: *Aclodes* sp.), Psocoptera (Epipsocidae), Lepidoptera (Noctuidae), Diptera (Culicidae: *Anopheles* sp., Drosophilidae, Tipulidae), Hymenoptera (Formicidae: *Acromyrmex* sp., *Ectatomma* sp.), Coleoptera (Pselaphidae).

Dentre os vertebrados, foram encontradas duas espécies das ordens Chiroptera (Phyllostomidae: *Glossophaga soricina*), Anura (Leptodactylidae: *Pristimantis cf. fenestratus*). Desta forma, no total foram encontradas 19 morfoespécies.

5.4.4.65.3. Caracterização faunística no período de chuva

Foi observado na caverna, um total de 20 morfoespécies de invertebrados de pelo menos 14 famílias das Ordens: Decapoda (*Microthelphusa somanni*), Acari (Mesostigmata), Amblypygi (Phrynidae: *Heterophrynus longicornis*), Opiliones (Sclerosomatidae: *Prionostemma* sp.), Araneae (Salticidae, Scytodidae*: Scytodes eleonora*, Pholcidae: *Mesabolivar* sp.), Collembola (Isotomidae), Diptera (Culicidae, Drosophilidae, Psychodidae: *Lutzomyia* sp.; Simulidae), Hymenoptera (Formicidae: *Apterostigma* sp., *Camponotus* sp., *Pheidole* sp., *Solenopsis* sp., Vespidae), Coleoptera (Scydmaenidae).

Dentre os vertebrados, foi encontrada uma espécie da ordem Chiroptera Anura (Dendrobatidae: *Ameerega* sp.). Desta forma, no total foram encontradas 21 morfoespécies.

5.4.4.65.4. Caracterização geral da fauna da cavidade

Foi observado na caverna, um total de 34 morfoespécies de invertebrados de pelo menos 21 famílias das Ordens: Decapoda (*Microthelphusa somanni*), Acari (Mesostigmata), Amblypygi (Phrynidae: *Heterophrynus longicornis*), Opiliones (Sclerosomatidae: *Prionostemma* sp.), Araneae (Gnaphosidae, Salticidae, Scytodidae*: Scytodes eleonora*, Pholcidae: *Mesabolivar* sp., Theridiidae), Collembola (Isotomidae), Orthoptera (Phalangopsidae: *Aclodes* sp.), Psocoptera (Epipsocidae), Lepidoptera (Noctuidae), Diptera (Culicidae: *Anopheles* sp.; Drosophilidae, Psychodidae: *Lutzomyia* sp.; Simulidae, Tipulidae), Hymenoptera (Formicidae: *Acromyrmex* sp., *Apterostigma* sp., *Camponotus* sp., *Ectatomma* sp., *Pheidole* sp., *Solenopsis* sp., Vespidae), Coleoptera (Pselaphidae, Scydmaenidae).

Dentre os vertebrados, foram encontradas três espécies das ordens Chiroptera (Phyllostomidae: *Glossophaga soricina*), Anura (Leptodactylidae: *Pristimantis cf. fenestratus*; Dendrobatidae: *Ameerega* sp.). Desta forma, no total foram encontradas 37 morfoespécies.

5.4.4.66. SL-069

5.4.4.66.1. Caracterização trófica

Cavidade formada na canga, com 34,6 m de projeção horizontal localizada junto à margem esquerda de uma drenagem ativa, mas em um nível mais elevado que a drenagem. A vegetação de entorno é composta por uma mata ciliar localizada em uma matriz formada por pastagens. Trata-se uma cavidade ampla que apresenta uma morfologia em “T” com uma entrada principal e uma pequena clarabóia no conduto localizado à esquerda da

mesma. Sua entrada é ampla e sombreada com muitos líquens, briófitas, pteridófitas e angiospermas. Seu piso é relativamente plano e seco, composto por sedimento granulado com poucos blocos esparsos e uma rede superficial de raízes de pequeno calibre bem desenvolvida. A cavidade não apresenta zona afótica e apenas algumas áreas de penumbra escura. Nas porções mais ao fundo da cavidade existem grandes depósitos de guano de morcegos frugívoros produzido por uma grande colônia de *G. soricina* formada por dezenas de indivíduos. Estes depósitos apresentam muitas sementes e plântulas recém germinadas. A serrapilheira encontra-se concentrada na zona de entrada e junto às clarabóias (Figura 191). O sistema de canalículos é bem desenvolvido e as paredes são revestidas por uma grande quantidade de Actinomicetos. Trata-se de uma cavidade com muitos recursos alimentares o que favorece a existência de algumas grandes populações de invertebrados. Durante a estação úmida a cavidade apresentou uma pequena elevação na umidade do solo, mas não foram observados pontos de gotejamento e percolação.

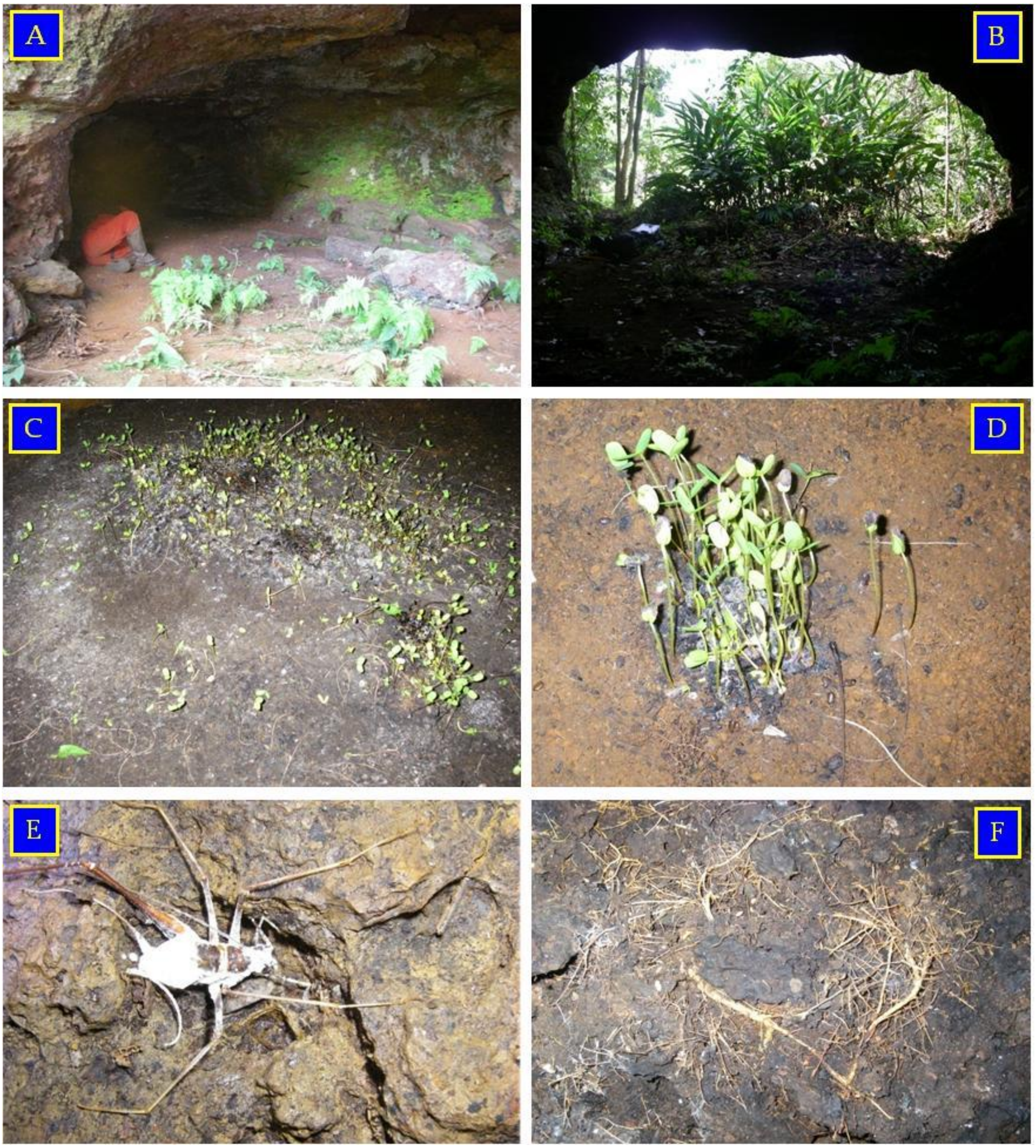

Figura 191 - a) Salão principal da cavidade muito iluminado e com muita vegetação associada junto ao piso e as paredes; b) Vista interna da entrada da cavidade localizada em mata ciliar próxima a uma pequena drenagem de Serra Leste. No detalhe a vegetação bem desenvolvida próximo a linha d'água; c) Depósito de guano de morcegos frugívoros com muitas plântulas recém germinadas; d) Detalhe das plântulas germinadas no guano; e) Cadáver de grilo (*Phalangopsis sp*.) com grande desenvolvimento fúngico; f) Sistema radicular bem desenvolvido associado a uma grande mancha de guano de morcegos frugívoros.

5.4.4.66.2. Caracterização faunística no período de seca

Foi observado na caverna, um total de 77 morfoespécies de invertebrados de pelo menos 53 famílias das Ordens: Gastropoda (Valloniidae), Isopoda (Armadillidae, Balloniscidae, Philosciidae), Acari (Ixodidae, Macronyssidae, Ologamasidae, Rhagidiidae, Tydeidae), Amblypygi (Phrynidae: *Heterophrynus longicornis*), Pseudoscorpiones (Chernetidae), Opiliones (Sclerosomatidae: *Prionostemma* sp.), Araneae (Araneidae: *Alpaida* sp., Ctenidae, Gnaphosidae, Ochyroceratidae, Salticidae, Scytodidae*: Scytodes eleonora*, Pholcidae: *Mesabolivar* sp.; Theridiidae, Theridiosomatidae), Thysanura (Nicoletiidae: Nicoletiinae), Diplura (Campodeidae), Collembola (Sminthuridae, Entomobryomorpha: Entomobryidae), Orthoptera (Phalangopsidae: *Phalangopsis* sp.), Mantodea (Mantinae), Blattodea (Polyphagidae), Isoptera (Termitidae: *Nasutitermes* sp.), Dermaptera (Labiidae), Psocoptera (Epipsocidae), Hemiptera (Cydnidae, Dipsocoridae, Lygaeidae, Reduviidae), Homoptera (Cixiidae: *Cixius* sp.), Diptera (Ceratopogonidae, Drosophilidae, Psychodidae: *Lutzomyia* sp., Sciaridae), Hymenoptera (Formicidae: *Anochetus* sp., *Apterostigma* sp., *Camponotus* sp., *Crematogaster* sp., *Pachycondyla* sp., *Pheidole* sp., *Rogeria* sp.; Vespidae), Thysanoptera (Phaleothripinae), Coleoptera (Carabidae, Curculionidae: Scotylinae, Elateridae: Agryphinae, Cardiophorinae; Nitidulidae, Ptylidae, Scydmaenidae, Staphylinidae), Symphyla (Scutigerellidae: *Hanseniella* sp., *Scutigerella* sp.).

Dentre os vertebrados, foram encontradas cinco espécies das ordens: Chiroptera (Emballonuridae: *Peropteryx kappleri*; Phyllostomidae: *Glossophaga soricina*), Anura (Leptodactylidae: *Pristimantis cf. fenestratus, Eleutherodactylus* sp.), Rodentia (Cricetidae: *Rhipidomys* sp.).

Desta forma, no total foram encontradas 82 morfoespécies. Entre estas, uma espécie de invertebrado foi considerada troglomórfica: Gastropoda (Systrophiidae).

5.4.4.66.3. Caracterização faunística no período de chuva

Foi observado na caverna, um total de 114 morfoespécies de invertebrados de pelo menos 63 famílias das Ordens: Oligochaeta, Gastropoda (Subulinidae, Systrophiidae), Isopoda (Armadillidae, Balloniscidae, Philosciidae, Scleropactidae), Acari (Laelapidae: *Stratiolaelaps* sp.; Macrochelidae, Ologamasidae, Oribatida, Tydeidae), Amblypygi (Phrynidae: *Heterophrynus longicornis*), Pseudoscorpiones (Chernetidae), Opiliones (Cosmetidae: *Anduzeia sp.;* Sclerosomatidae: *Prionostemma* sp.), Araneae (Oonopidae: Oonopinae; Salticidae, Scytodidae*: Scytodes eleonora*, Pholcidae: *Mesabolivar* sp.; Theridiidae), Thysanura (Nicoletiidae: Nicoletiinae), Diplura (Campodeidae), Collembola (Sminthuridae, Cyphoderidae, Entomobryomorpha: Entomobryidae; Isotomidae, Paronellidae), Orthoptera (Phalangopsidae: *Aclodes* sp., *Phalangopsis* sp.), Blattodea (Polyphagidae), Isoptera (Termitidae: *Nasutitermes* sp.), Dermaptera (Labiidae), Psocoptera (Epipsocidae,

Lepidopsocidae, Myopsocidae: *Lichenomina* sp.), Hemiptera (Cydnidae, Dipsocoridae, Lygaeidae, Nabidae, Reduviidae), Homoptera (Cercopidae, Cicadellidae, Cixiidae: *Cixius* sp.; Coccoidea), Lepidoptera (Tineidae), Diptera (Cecidomyiidae, Drosophilidae, Milichiidae, Phoridae, Psychodidae: *Lutzomyia* sp., Sciaridae, Tipulidae), Hymenoptera (Formicidae: *Anochetus* sp., *Camponotus* sp., *Cyphomyrmex* sp., *Gnamptogenys* sp., *Hypoponera* sp., *Pachycondyla* sp., *Pheidole* sp., *Solenopsis* sp.), Coleoptera (Carabidae, Curculionidae: Scotylinae, Dermestidae, Elateridae; Elmidae, Pselaphidae, Scydmaenidae, Staphylinidae, Tenebrionidae: Coelometropinae), Diplopoda (Chelodesmidae; Glomeridesmidae: *Glomeridesmus* sp.; Stemmiulidae), Scolopendromorpha (Cryptopidae: *Cryptops* sp.), Symphyla (Scutigerellidae: *Hanseniella* sp.).

Dentre os vertebrados, foram encontradas duas espécies das ordens: Chiroptera (Emballonuridae: *Peropteryx kappleri*; Phyllostomidae: *Glossophaga soricina*).

Desta forma, no total foram encontradas 116 morfoespécies. Entre estas, três espécies de invertebrados foram consideradas troglomórficas: Isopoda (*Trichorhina* sp.), Collembola (Cyphoderidae), Diplopoda (Pseudonannolenidae).

5.4.4.66.4. Caracterização geral da fauna da cavidade

Foi observado na caverna, um total de 159 morfoespécies de invertebrados de pelo menos 82 famílias das Ordens: Oligochaeta, Gastropoda (Subulinidae, Systrophiidae, Valloniidae), Isopoda (Armadillidae, Balloniscidae, Philosciidae, Scleropactidae), Acari (Ixodidae, Laelapidae: *Stratiolaelaps* sp.; Macrochelidae, Macronyssidae, Ologamasidae, Oribatida, Rhagidiidae, Tydeidae), Amblypygi (Phrynidae: *Heterophrynus longicornis*), Pseudoscorpiones (Chernetidae), Opiliones (Cosmetidae: *Anduzeia sp.;* Sclerosomatidae: *Prionostemma* sp.), Araneae (Araneidae: *Alpaida* sp., Ctenidae, Gnaphosidae, Ochyroceratidae, Oonopidae: Oonopinae; Salticidae, Scytodidae*: Scytodes eleonora*, Pholcidae: *Mesabolivar* sp.; Theridiidae, Theridiosomatidae), Thysanura (Nicoletiidae: Nicoletiinae), Diplura (Campodeidae), Collembola (Sminthuridae, Cyphoderidae, Entomobryomorpha: Entomobryidae; Isotomidae, Paronellidae), Orthoptera (Phalangopsidae: *Aclodes* sp., *Phalangopsis* sp.), Mantodea (Mantinae), Blattodea (Polyphagidae), Isoptera (Termitidae: *Nasutitermes* sp.), Dermaptera (Labiidae), Psocoptera (Epipsocidae, Lepidopsocidae, Myopsocidae: *Lichenomina* sp.), Hemiptera (Cydnidae, Dipsocoridae, Lygaeidae, Nabidae, Reduviidae), Homoptera (Cercopidae, Cicadellidae, Cixiidae: *Cixius* sp.; Coccoidea), Lepidoptera (Tineidae), Diptera (Cecidomyiidae, Ceratopogonidae, Drosophilidae, Milichiidae, Phoridae, Psychodidae: *Lutzomyia* sp., Sciaridae, Tipulidae), Hymenoptera (Formicidae: *Anochetus* sp., *Apterostigma* sp., *Camponotus* sp., *Crematogaster* sp., *Cyphomyrmex* sp., *Gnamptogenys* sp., *Hypoponera* sp., *Pachycondyla* sp., *Pheidole* sp., *Rogeria* sp., *Solenopsis* sp.; Vespidae), Thysanoptera (Phaleothripinae), Coleoptera (Carabidae, Curculionidae: Scotylinae, Dermestidae,

Elateridae: Agryphinae, Cardiophorinae; Elmidae, Nitidulidae, Pselaphidae, Ptylidae, Scydmaenidae, Staphylinidae, Tenebrionidae: Coelometropinae), Diplopoda (Chelodesmidae; Glomeridesmidae: *Glomeridesmus* sp.; Stemmiulidae), Scolopendromorpha (Cryptopidae: *Cryptops* sp.), Symphyla (Scutigerellidae: *Hanseniella* sp., *Scutigerella* sp.).

Dentre os vertebrados, foram encontradas cinco espécies das ordens: Chiroptera (Emballonuridae: *Peropteryx kappleri*; Phyllostomidae: *Glossophaga soricina*), Anura (Leptodactylidae: *Pristimantis cf. fenestratus, Eleutherodactylus* sp.), Rodentia (Cricetidae: *Rhipidomys* sp.).

Desta forma, no total foram encontradas 164 morfoespécies. Entre estas, quatro espécies de invertebrados foram consideradas troglomórficas: Gastropoda (Systrophiidae), Isopoda (*Trichorhina* sp.), Collembola (Cyphoderidae), Diplopoda (Pseudonannolenidae). Alguns organismos encontrados nesta caverna são mostrados na Figura 192.

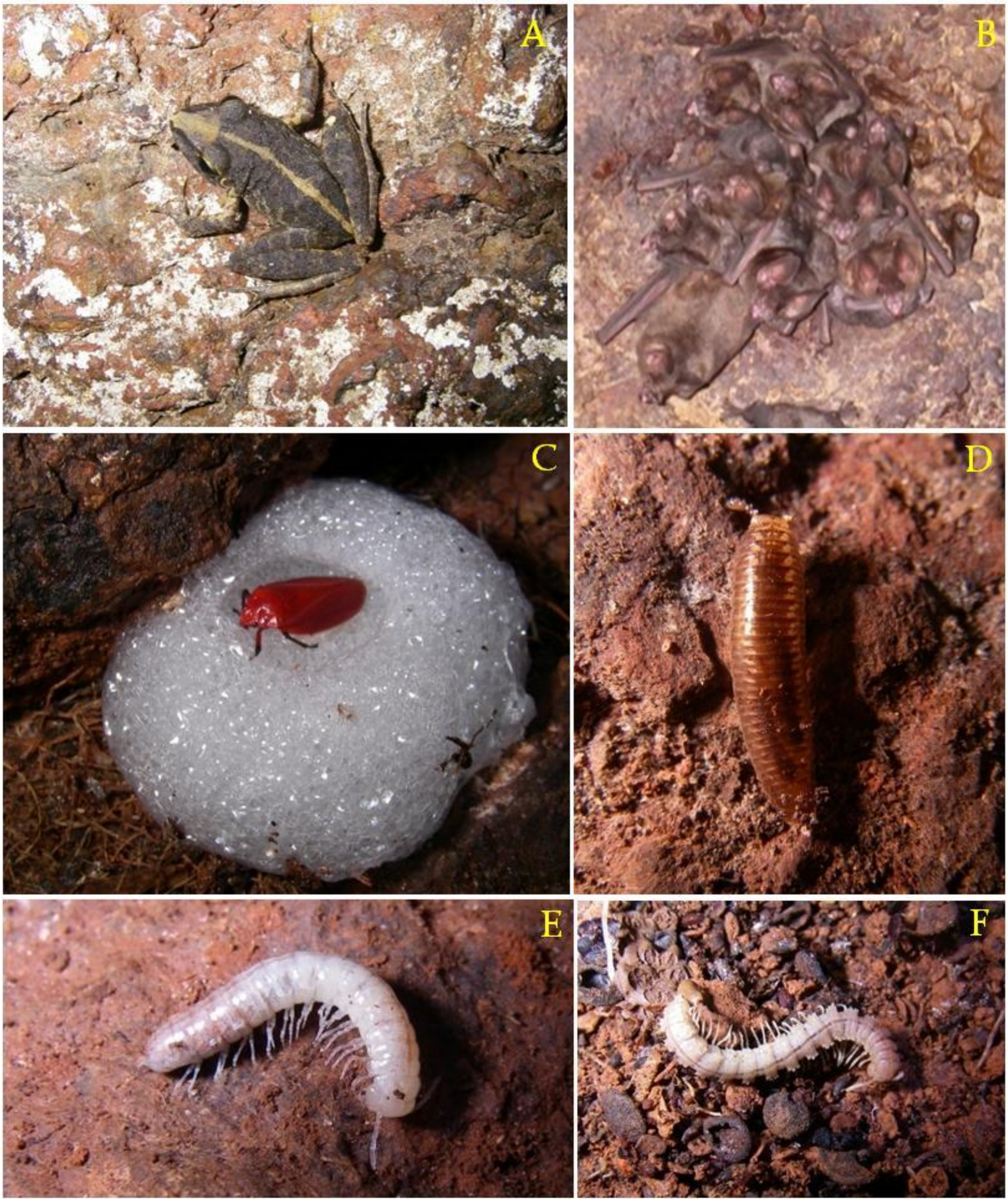

Figura 192 - a) Anura (Leptodactylidae: *Eleutherodactylus* sp.); b) Phyllostomidae (*Carollia* sp.); Hemiptera (Cercopidae); d) Diplopoda (*Glomeridesmus sp*.); e) Diplopoda (Polydesmida); f) Diplopoda (Chelodesmidae).

5.4.4.67. SL-070

5.4.4.67.1. Caracterização trófica

Caverna formada na canga com 11,3 m de projeção horizontal localizada na margem esquerda de uma drenagem ativa, em um nível mais elevado que a mesma. A vegetação de entorno é composta por uma mata ciliar localizada em uma matriz formada por pastagens. Sua entrada tem aproximadamente dois metros de altura, sendo esta sombreada com muitas plantas, líquens, briófitas e pteridófitas. O piso da cavidade é predominantemente plano, composto por sedimento fino com poucos blocos abatidos sendo estes cobertos por plantas e trepadeiras na região próxima a entrada onde existe muita serrapilheira acumulada junto à linha d'água. Caverna totalmente iluminada, exceto no conduto à esquerda onde existe um quebra corpo com muitos blocos e uma zona afótica (Figura 193). O sistema radicular é bem desenvolvido e não existem depósitos de guano apesar de terem sido encontrados exemplares de G. soricina. Nenhuma alteração significativa foi observada durante a estação úmida, além das alterações normais na umidade relativa do ar.

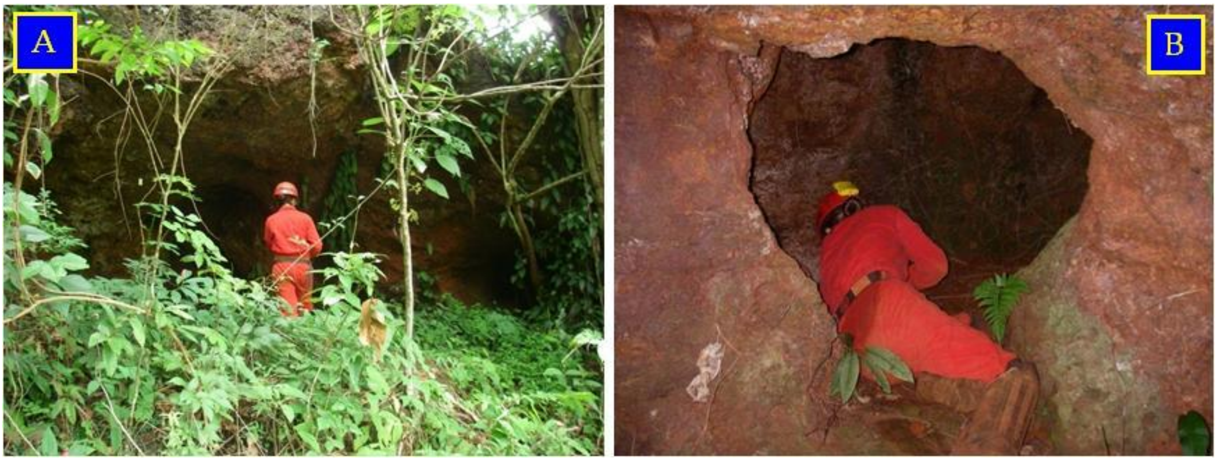

Figura 193 - Vista externa da entrada da cavidade localizada em mata ciliar próxima a uma pequena drenagem de Serra Leste. No detalhe a vegetação bem desenvolvida próximo a linha d'água; b) Detalhe de uma porção interior da caverna.

5.4.4.67.2. Caracterização faunística no período de seca

Foi observado na caverna, um total de 17 morfoespécies de invertebrados de pelo menos 15 famílias das Ordens: Acari (Astigmatina), Amblypygi (Phrynidae: *Heterophrynus longicornis*), Pseudoscorpiones (Chernetidae, Chtoniidae), Araneae (Araneidae: *Alpaida* sp., Corinidae: *Tupirina* sp., Pholcidae: *Mesabolivar* sp.; Theridiidae), Diplura (Campodeidae), Lepidoptera (Noctuidae), Diptera (Ceratopogonidae, Culicidae, Psychodidae: *Lutzomyia* sp.; Tipulidae), Hymenoptera (Formicidae: *Linepthema* sp.; Vespidae).

Dentre os vertebrados foram encontradas duas espécies das ordens Chiroptera (Emballonuridae: *Peropteryx kappleri*) e Anura (Leptodactylidae: *Pristimantis* cf. *fenestratus*).

Desta forma, no total foram encontradas 19 morfoespécies. Entre estas, uma espécie de invertebrado foi considerada troglomórfica: Collembola (Isotomidae).

5.4.4.67.3. Caracterização faunística no período de chuva

Foi observado na caverna, um total de 37 morfoespécies de invertebrados de pelo menos 27 famílias das Ordens: Oligochaeta, Isopoda (Scleropactidae), Acari (Opilioacaridae: *Neoacarus* sp.), Amblypygi (Phrynidae: *Heterophrynus longicornis*), Pseudoscorpiones (Chernetidae, Chtoniidae), Opiliones (Cosmetidae: *Anduzeia sp.*; Sclerosomatidae: *Prionostemma* sp.; Phalangiidae), Araneae (Salticidae, Scytodidae*: Scytodes eleonora*, Pholcidae: *Mesabolivar* sp.), Collembola (Entomobryidae), Orthoptera (Phalangopsidae: *Phalangopsis* sp.), Blattodea (Blaberidae: *Blaberus* sp.), Psocoptera (Psyllipsocidae: *Psyllipsocus* sp.), Hemiptera (Cydnidae, Lygaeidae, Reduviidae), Lepidoptera (Tineidae), Diptera (Ceratopogonidae, Psychodidae: *Lutzomyia* sp.; Tipulidae), Hymenoptera (Formicidae: *Acanthognatus* sp., *Camponotus* sp., *Cyphomyrmex* sp., *Gnamptogenys* sp., *Pachycondyla* sp., *Rogeria* sp.; Vespidae), Coleoptera (Nitidulidae, Pselaphidae, Scydmaenidae), Diplopoda (Polydesmida).

Dentre os vertebrados, foi encontrada uma espécie da ordem Chiroptera (Phyllostomidae: *Glossophaga soricina*). Desta forma, no total foram encontradas 38 morfoespécies.

5.4.4.67.4. Caracterização geral da fauna da cavidade

Foi observado na caverna, um total de 50 morfoespécies de invertebrados de pelo menos 35 famílias das Ordens: Oligochaeta, Isopoda (Scleropactidae), Acari (Opilioacaridae: *Neoacarus* sp., Astigmatina), Amblypygi (Phrynidae: *Heterophrynus longicornis*), Pseudoscorpiones (Chernetidae, Chtoniidae), Opiliones (Cosmetidae: *Anduzeia sp.*; Sclerosomatidae: *Prionostemma* sp.; Phalangiidae), Araneae (Araneidae: *Alpaida* sp., Corinidae: *Tupirina* sp., Salticidae, Scytodidae*: Scytodes eleonora*, Pholcidae: *Mesabolivar* sp.; Theridiidae), Diplura (Campodeidae), Collembola (Entomobryidae), Orthoptera (Phalangopsidae: *Phalangopsis* sp.), Blattodea (Blaberidae: *Blaberus* sp.), Psocoptera (Psyllipsocidae: *Psyllipsocus* sp.), Hemiptera (Cydnidae, Lygaeidae, Reduviidae), Lepidoptera (Noctuidae, Tineidae), Diptera (Ceratopogonidae, Culicidae, Psychodidae: *Lutzomyia* sp.; Tipulidae), Hymenoptera (Formicidae: *Acanthognatus* sp., *Camponotus* sp., *Cyphomyrmex* sp., *Gnamptogenys* sp., *Linepthema* sp., *Pachycondyla* sp., *Rogeria* sp.; Vespidae), Coleoptera (Nitidulidae, Pselaphidae, Scydmaenidae), Diplopoda (Polydesmida).

Dentre os vertebrados, foram encontradas três espécies das ordens Chiroptera (Emballonuridae: *Peropteryx kappleri*; Phyllostomidae: *Glossophaga soricina*), Anura (Leptodactylidae: *Pristimantis cf. fenestratus*).

Desta forma, no total foram encontradas 53 morfoespécies. Entre estas, uma espécie de invertebrado foi considerada troglomórfica: Collembola (Isotomidae).

5.4.4.68. SL-071

5.4.4.68.1. Caracterização trófica

Caverna com 16 m de projeção horizontal, formada na canga e localizada em área de mata ciliar, sendo a vegetação na parte superior da cavidade composta por savana metalófila. A cavidade apresenta teto baixo ao longo de toda sua extensão e sua entrada é larga e sombreada com grande acúmulo de serrapilheira e blocos abatidos. Nesta área existem poucos locais com líquens, briófitas e algumas plântulas distribuídas de forma esparsa. O piso é predominantemente plano e seco, sendo este composto por sedimento granulado com poucos blocos (calhaus e seixos) e com raízes de pequeno calibre bem distribuídas e formando pequenos rizotemas junto aos pontos de gotejamento (que encontravam-se inativos durante a estação seca). A cavidade não apresenta zona afótica e ao final de um dos condutos foi observada uma estrutura fúngica cultivada por formigas cortadeiras (*Apterostigma sp*.) (Figura 194). O sistema de canalículos é pouco desenvolvido e as paredes são revestidas por Actinomicetos. Não foram observados depósitos de guano no interior da cavidade. Durante a estação úmida a cavidade estava muito úmida, com piso encharcado e com vários pontos de gotejamentos e de percolação ativos.

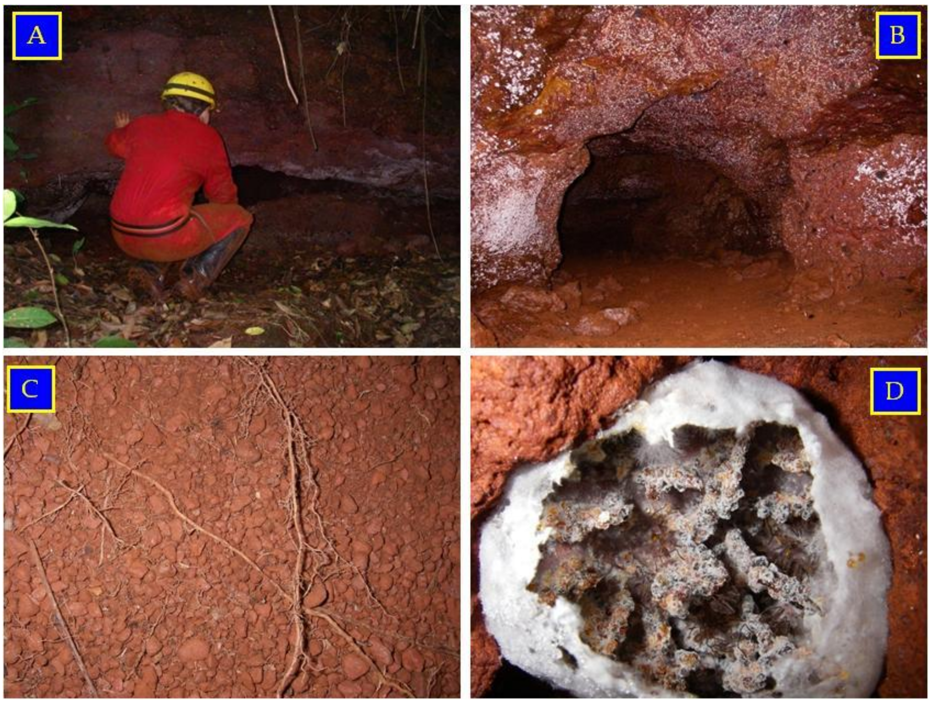

Figura 194 - a) Vista externa da entrada da cavidade onde é possível observar uma grande quantidade de serrapilheira; b) Porção interna da cavidade, com paredes e teto repletos de Actinomicetos; c) Sistemas radiculares junto ao piso; d) Teto da cavidade com uma estrutura fúngica cultivada por formigas cortadeiras (Apterostigma sp.).

5.4.4.68.2. Caracterização faunística no período de seca

Foi observado na caverna, um total de 34 morfoespécies de invertebrados de pelo menos 26 famílias das Ordens: Acari (Ixodidae), Amblypygi (Phrynidae: *Heterophrynus longicornis*), Pseudoscorpiones (Chernetidae, Chtoniidae), Opiliones (Cosmetidae: *Anduzeia sp.*; Stygnidae: *Protimesius aff. gracilis*), Araneae (Araneidae: *Alpaida* sp.; Corinidae: *Tupirina* sp.; Ochyroceratidae, Oonopidae: Oonopinae, Drymusidae: *Drymusa* sp., Theridiosomatidae), Diplura (Campodeidae), Collembola (Entomobryidae), Orthoptera (Phalangopsidae: *Aclodes* sp., *Phalangopsis* sp.), Blattodea (Polyphagidae), Isoptera (Termitidae: *Nasutitermes* sp.), Psocoptera (Epipsocidae), Homoptera (Cixiidae: *Cixius* sp.), Lepidoptera (Noctuidae), Diptera (Dolichopodidae, Drosophilidae, Psychodidae: *Lutzomyia* sp.; Tipulidae), Hymenoptera (Formicidae: *Apterostigma* sp., *Camponotus* sp., *Dolichoderus* sp., *Hypoponera* sp., *Pachycondyla* sp.), Symphyla (Scutigerellidae: *Hanseniella* sp., *Scutigerella* sp.).

Dentre os vertebrados foram encontradas duas espécies da ordem Anura (Leptodactylidae: *Pristimantis cf. fenestratus; Eleutherodactylus* sp.). Desta forma, no total foram encontradas 36 morfoespécies.

5.4.4.68.3. Caracterização faunística no período de chuva

Foi observado na caverna, um total de 34 morfoespécies de invertebrados de pelo menos 26 famílias das Ordens: Oligochaeta, Acari (Oribatida), Amblypygi (Phrynidae: *Heterophrynus longicornis*), Pseudoscorpiones (Chernetidae), Araneae (Ctenidae: *Ctenus* sp.; Gnaphosidae, Linyphiidae, Paratropidae, Theraphosidae, Theridiosomatidae), Diplura (Anajapygidae, Campodeidae), Collembola (Entomobryidae, Isotomidae, Paronellidae), Orthoptera (Phalangopsidae: *Aclodes* sp.), Hemiptera (Cydnidae, Dipsocoridae), Homoptera (Derbidae), Lepidoptera (Tineidae), Diptera (Phoridae, Psychodidae: *Lutzomyia* sp.), Hymenoptera (Formicidae: *Apterostigma* sp., *Pachycondyla* sp.), Coleoptera (Carabidae, Curculionidae: Scotylinae), Symphyla (Scutigerellidae: *Hanseniella* sp., *Scutigerella* sp.). Desta forma, no total foram encontradas 34 morfoespécies.

5.4.4.68.4. Caracterização geral da fauna da cavidade

Foi observado na caverna, um total de 60 morfoespécies de invertebrados de pelo menos 44 famílias das Ordens: Oligochaeta, Acari (Ixodidae, Oribatida), Amblypygi (Phrynidae: *Heterophrynus longicornis*), Pseudoscorpiones (Chernetidae, Chtoniidae), Opiliones (Cosmetidae: *Anduzeia* sp.; Stygnidae: *Protimesius* aff. *gracilis*), Araneae (Araneidae: *Alpaida* sp.; Corinidae: *Tupirina* sp.; Ctenidae: *Ctenus* sp.; Gnaphosidae, Linyphiidae, Ochyroceratidae, Oonopidae: Oonopinae, Salticidae, Drymusidae: *Drymusa* sp., Paratropidae, Pholcidae: *Mesabolivar* sp., Theraphosidae, Theridiosomatidae), Diplura (Anajapygidae, Campodeidae), Collembola (Entomobryidae, Isotomidae, Paronellidae), Orthoptera (Phalangopsidae: *Aclodes* sp., *Phalangopsis* sp.), Blattodea (Polyphagidae),

Isoptera (Termitidae: *Nasutitermes* sp.), Psocoptera (Epipsocidae), Hemiptera (Cydnidae, Dipsocoridae), Homoptera (Cixiidae: *Cixius* sp.; Derbidae), Lepidoptera (Noctuidae, Tineidae), Diptera (Dolichopodidae, Drosophilidae, Phoridae, Psychodidae: *Lutzomyia* sp.; Tipulidae), Hymenoptera (Formicidae: *Apterostigma* sp., *Camponotus* sp., *Dolichoderus* sp., *Hypoponera* sp., *Pachycondyla* sp.), Coleoptera (Carabidae, Curculionidae: Scotylinae), Symphyla (Scutigerellidae: *Hanseniella* sp., *Scutigerella* sp.).

Dentre os vertebrados, foram encontradas duas espécies da ordem Anura (Leptodactylidae: *Pristimantis cf. fenestratus; Eleutherodactylus* sp.). Desta forma, no total foram encontradas 62 morfoespécies. A Figura 195 mostra um anuro se alimentando de um grilo no interior da cavidade.

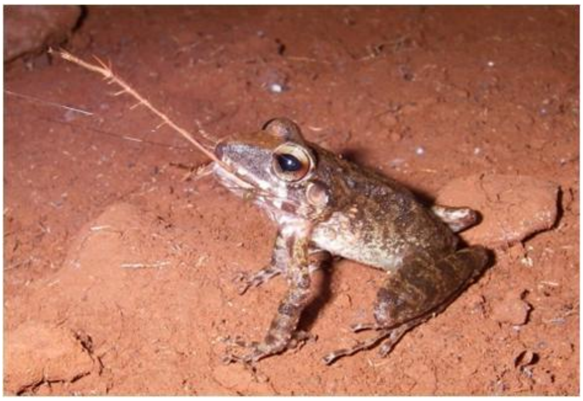

Figura 195 - a) Anura (*Pristimantis cf. fenestratus*) predando um grilo Phalangopsidae.

5.4.4.69. SL-072

5.4.4.69.1. Caracterização trófica

Caverna formada na canga em condição muito superficial com 30,1 m de projeção horizontal e localizada em área de mata ciliar com vegetação na região superior da cavidade composta por savana metalófila. A entrada é alta e larga, correspondendo a um grande abrigo. Esta parte é eufótica e seca, com muitos blocos e paredes revestidas de musgos e líquens. As porções medianas e finais da cavidade correspondem a condutos em rastejo em condição ascendente com zonas afóticas. Nestas áreas, o piso é composto por sedimento granulado com poucos seixos e calhaus e onde predomina uma baixa estabilidade ambiental. Entretanto, existe ainda um piso inferior com uma entrada secundária de pequeno porte ($1m^2$) seguida por um conduto estreito em quebra-corpo que leva até uma outra pequena entrada lateral da caverna. Esta área é afótica, um pouco mais úmida, e o piso também é composto por sedimento granulado com poucos seixos e calhaus. Neste, o sistema radicular é bem desenvolvido sendo formado por raízes de médio e fino calibre (Figura 196). O sistema de canalículos é pouco desenvolvido e nas paredes podem ser observados Actinomicetos distribuídos de forma esparsa. Durante a estação úmida o conduto inferior encontrava-se encharcado com vários pontos de gotejamento e de percolação.

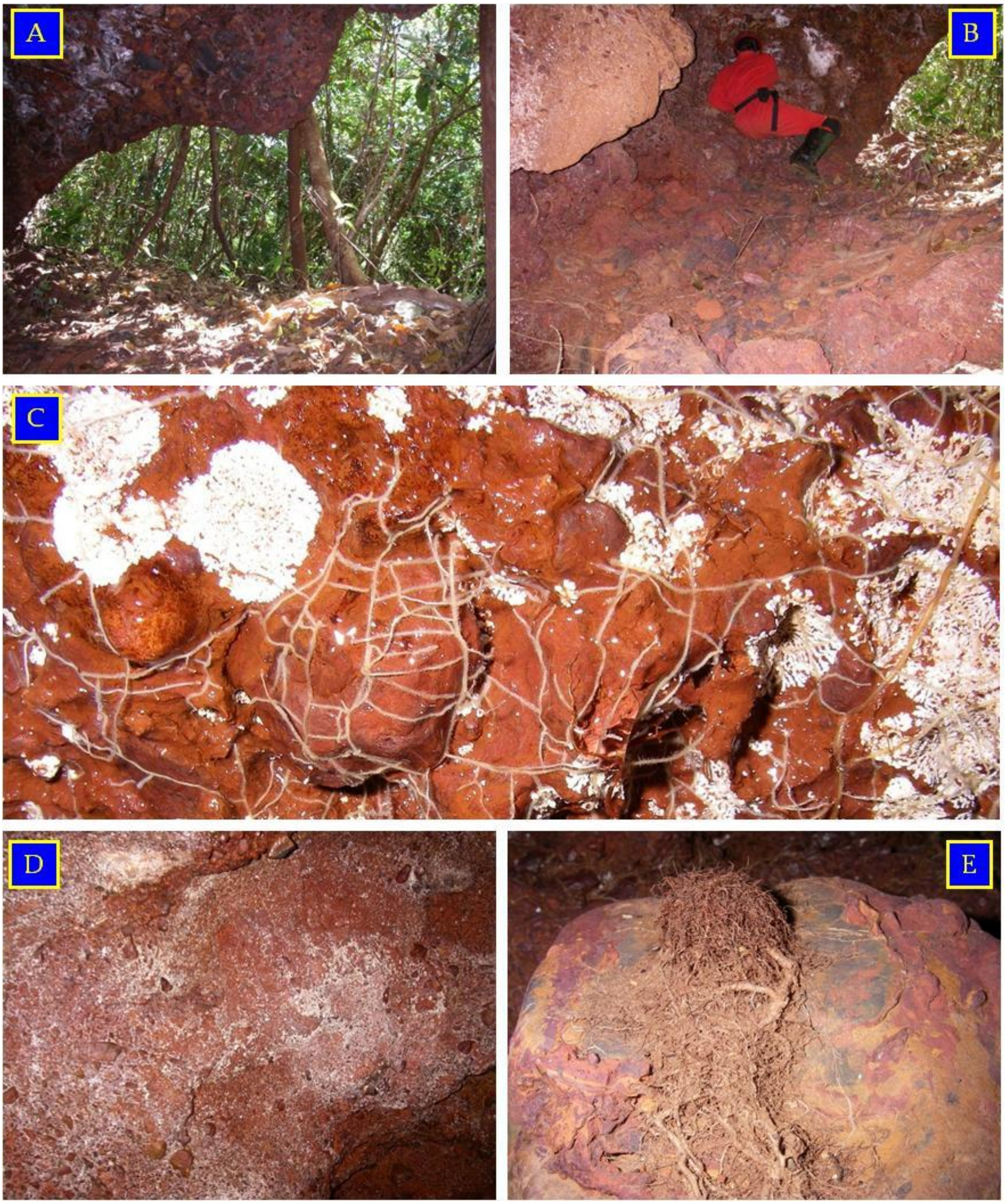

Figura 196 - a) Vista interna da entrada da cavidade localizada em mata ciliar; b) Porção interior da cavidade, onde se observa a serrapilheira esparsa e sistemas radiculares; c) Raízes de pequeno calibre crescendo sobre a parede (em detalhe, colônias de Actinomicetos); d) Colônias de Actinomicetos se desenvolvendo em profusão nas paredes da cavidade; e) Rizotema encontrado no interior da cavidade.

5.4.4.69.2. Caracterização faunística no período de seca

Foi observado na caverna, um total de 56 morfoespécies de invertebrados de pelo menos 40 famílias das Ordens: Onychophora (Peripatidae), Isopoda (Armadillidae), Acari (Mesostigmata, Oribatida), Amblypygi (Phrynidae: *Heterophrynus longicornis*), Pseudoscorpiones (Chernetidae, Chtoniidae), Opiliones (Stygnidae: *Protimesius aff. gracilis*), Araneae (Araneidae: *Alpaida* sp., Gnaphosidae, Ochyroceratidae , Oonopidae: Oonopinae, Salticidae, Drymusidae: *Drymusa* sp., Scytodidae*: Scytodes eleonora*, Pholcidae: *Mesabolivar* sp., *Metagonia* sp.; Theridiidae, Theridiosomatidae), Diplura (Anajapygidae, Campodeidae), Collembola (Entomobryidae), Neuroptera (Mantispidae: *Plega* sp.), Orthoptera (Phalangopsidae: *Aclodes* sp.), Blattodea (Blattellidae, Polyphagidae), Isoptera (Termitidae: *Nasutitermes* sp.), Psocoptera (Epipsocidae, Ptiloneuridae: *Ptiloneura* sp.), Hemiptera (Cydnidae, Nabidae, Pseudococcidae, Reduviidae: *Panstrongylus* sp.), Homoptera (Cixiidae: *Cixius* sp.), Lepidoptera (Noctuidae, Tineidae), Diptera (Dolichopodidae, Drosophilidae, Mycetophilidae, Psychodidae: *Lutzomyia* sp., Tipulidae), Hymenoptera (Formicidae: *Apterostigma* sp., *Camponotus* sp., *Odontomachus* sp., *Pachycondyla* sp., *Pheidole* sp), Scolopendromorpha (Cryptopidae: *Cryptops* sp.; Scolopocryptopidae: *Newportia* sp.).

Dentre os vertebrados, foram encontradas cinco espécies das ordens Chiroptera (Phyllostomidae: *Glossophaga soricina*), Squamata (Colubridae: *Spilotes pullatus*), Anura (Leptodactylidae: *Pristimantis cf. fenestratus, Eleutherodactylus* sp.), Rodentia (Cricetidae: *Rhipidomys* sp.). Desta forma, no total foram encontradas 61 morfoespécies.

5.4.4.69.3. Caracterização faunística no período de chuva

Foi observado na caverna, um total de 71 morfoespécies de invertebrados de pelo menos 43 famílias das Ordens: Oligochaeta, Isopoda (Armadillidae, Philosciidae), Acari (Oribatida), Amblypygi (Phrynidae: *Heterophrynus longicornis*), Pseudoscorpiones (Chernetidae, Chtoniidae), Opiliones (Cosmetidae, Stygnidae: *Protimesius aff. gracilis*, Phalangiidae), Araneae (Ctenidae: *Ctenus* sp., Ochyroceratidae , Oonopidae: Oonopinae, Salticidae, Drymusidae: *Drymusa* sp., Scytodidae*: Scytodes eleonora*, Pholcidae: *Mesabolivar* sp., *Metagonia* sp.; Theridiosomatidae), Diplura (Anajapygidae, Campodeidae), Collembola (Entomobryidae, Isotomidae), Neuroptera (Mantispidae: *Plega* sp.), Orthoptera (Phalangopsidae: *Aclodes* sp.), Blattodea (Blattellidae), Isoptera, Psocoptera (Epipsocidae), Hemiptera (Alydidae, Cydnidae), Homoptera (Cixiidae: *Cixius* sp.), Lepidoptera (Noctuidae), Diptera (Ceratopogonidae, Chironomidae, Phoridae, Psychodidae: *Lutzomyia* sp., Sciaridae), Hymenoptera (Formicidae: *Acanthognatus* sp., *Anochetus* sp., *Apterostigma* sp., *Camponotus* sp., *Gnamptogenys* sp., *Neivamyrmex* sp., *Odontomachus* sp., *Pachycondyla* sp., *Platythyria* sp., *Pheidole* sp., *Rogeria* sp., *Trachymyrmex* sp., *Zacryptocerus* sp., *Wasmannia* sp.), Coleoptera (Dytiscidae, Elateridae, Lampiridae, Pselaphidae, Scydmaenidae), Diplopoda

(Stemmiulidae), Scolopendromorpha (Scolopocryptopidae: *Newportia* sp.), Symphyla (Scutigerellidae: *Hanseniella* sp.).

Desta forma, no total foram encontradas 71 morfoespécies. Entre estas, quatro espécies de invertebrados foram consideradas troglomórficas: Araneae (Ochyroceratidae), Collembola (Isotomidae), Coleoptera (Staphylinidae), Diplopoda (Glomeridesmidae: *Glomeridesmus* sp.).

5.4.4.69.4. Caracterização geral da fauna da cavidade

Foi observado na caverna, um total de 106 morfoespécies de invertebrados de pelo menos 60 famílias das Ordens: Onychophora (Peripatidae), Oligochaeta, Isopoda (Armadillidae, Philosciidae), Acari (Mesostigmata, Oribatida), Amblypygi (Phrynidae: *Heterophrynus longicornis*), Pseudoscorpiones (Chernetidae, Chtoniidae), Opiliones (Cosmetidae, Stygnidae: *Protimesius aff. gracilis*, Phalangiidae), Araneae (Araneidae: *Alpaida* sp., Ctenidae: *Ctenus* sp., Gnaphosidae, Ochyroceratidae , Oonopidae: Oonopinae, Salticidae, Drymusidae: *Drymusa* sp., Scytodidae*: Scytodes eleonora*, Pholcidae: *Mesabolivar* sp., *Metagonia* sp.; Theridiidae, Theridiosomatidae), Diplura (Anajapygidae, Campodeidae), Collembola (Entomobryidae, Isotomidae), Neuroptera (Mantispidae: *Plega* sp.), Orthoptera (Phalangopsidae: *Aclodes* sp.), Blattodea (Blattellidae, Polyphagidae), Isoptera (Termitidae: *Nasutitermes* sp.), Psocoptera (Epipsocidae, Ptiloneuridae: *Ptiloneura* sp.), Hemiptera (Alydidae, Cydnidae, Nabidae, Pseudococcidae, Reduviidae: *Panstrongylus* sp.), Homoptera (Cixiidae: *Cixius* sp.), Lepidoptera (Noctuidae, Tineidae), Diptera (Ceratopogonidae, Chironomidae, Dolichopodidae, Drosophilidae, Mycetophilidae, Phoridae, Psychodidae: *Lutzomyia* sp., Sciaridae, Tipulidae), Hymenoptera (Formicidae: *Acanthognatus* sp., *Anochetus* sp., *Apterostigma* sp., *Camponotus* sp., *Gnamptogenys* sp., *Neivamyrmex* sp., *Odontomachus* sp., *Pachycondyla* sp., *Platythyria* sp., *Pheidole* sp., *Rogeria* sp., *Trachymyrmex* sp., *Zacryptocerus* sp., *Wasmannia* sp.), Coleoptera (Dytiscidae, Elateridae, Lampiridae, Pselaphidae, Scydmaenidae), Diplopoda (Stemmiulidae), Scolopendromorpha (Cryptopidae: *Cryptops* sp.; Scolopocryptopidae: *Newportia* sp.), Symphyla (Scutigerellidae: *Hanseniella* sp.).

Dentre os vertebrados, foram encontradas cinco espécies das ordens Chiroptera (Phyllostomidae: *Glossophaga soricina*), Squamata (Colubridae: *Spilotes pullatus*), Anura (Leptodactylidae: *Pristimantis* cf. *fenestratus, Eleutherodactylus* sp.), Rodentia (Cricetidae: *Rhipidomys* sp.).

Desta forma, no total foram encontradas 111 morfoespécies. Entre estas, quatro espécies de invertebrados foram consideradas troglomórficas: Araneae (Ochyroceratidae), Collembola (Isotomidae), Coleoptera (Staphylinidae), Diplopoda (Glomeridesmidae: *Glomeridesmus* sp.). Alguns organismos encontrados nesta caverna são mostrados na Figura 197.

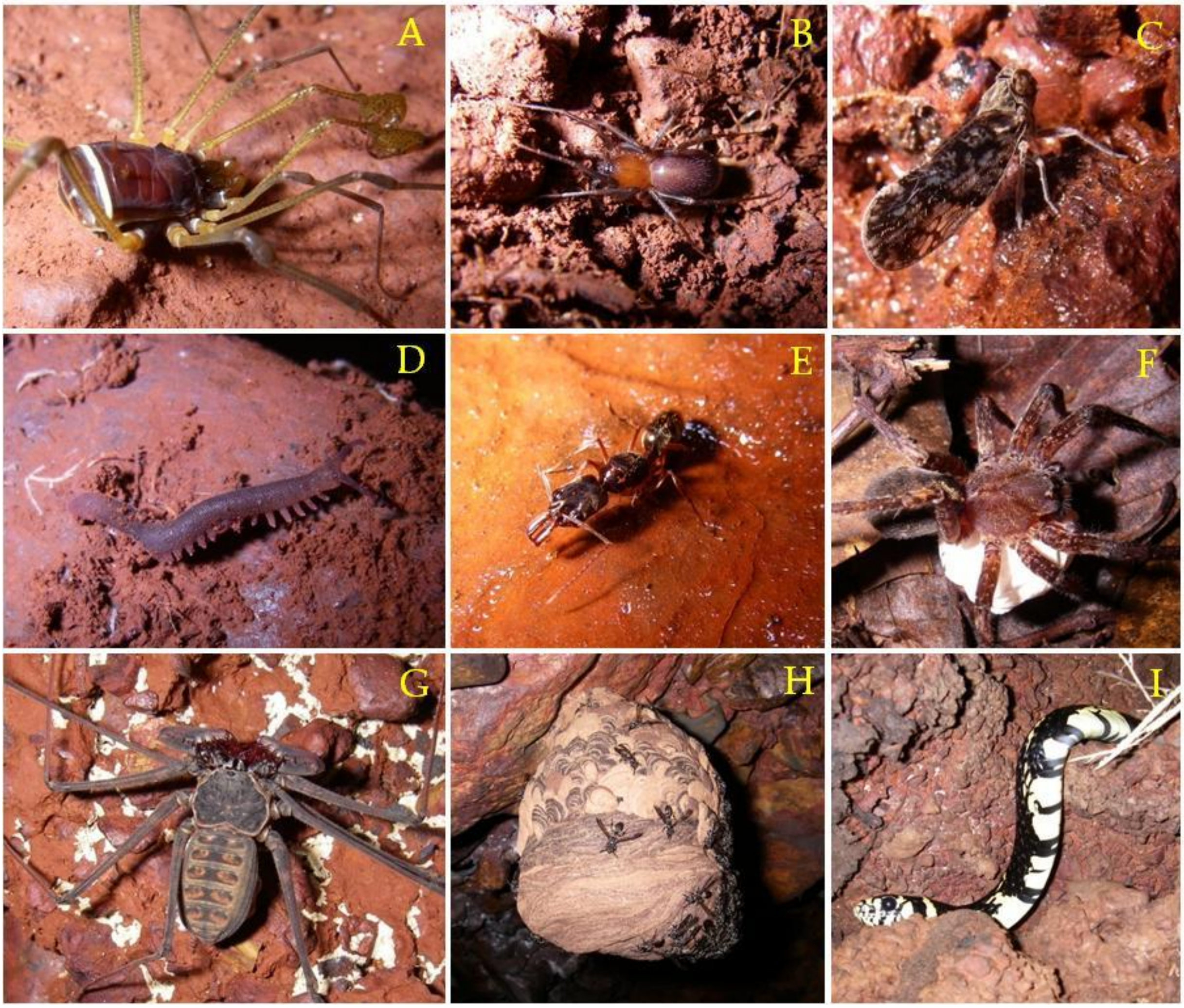

Figura 197 - a) Opiliones (Stygnidae: *Protimesius sp*.); b) Araneae (indet.); c) Homoptera (Cixiidae); d) Onychophora (Peripatidae); e) Formicidae (*Odontomachus sp*.); f) Araneae (Ctenidae); g) Amblypygi (*Heterophrynus longicornis*); h) Vespidae; i) Colubridae (*Spilotes pullatus*).

5.4.4.70. SL-073

5.4.4.70.1. Caracterização trófica

Caverna muito volumosa formada na canga com padrão retilíneo e 26,1 m de desenvolvimento. Localizada em área de savana metalófila próxima ao ponto de encontro (base de trabalho) de Serra Leste e a 50 m da estrada principal de acesso ao sistema Serra Leste. Sua entrada é ampla com muitos musgos e líquens sendo parcialmente sombreada por um pequeno fragmento de mata. Não apresenta zona afótica, mas existe uma grande área de penumbra com uma grande quantidade de morcegos frugívoros (*G. soricina*). Trata-se de uma *bat-cave*, onde existe uma grande quantidade de guano antigo de morcegos insetívoros e com poucos depósitos de guano de morcegos frugívoros recentes. O guano de morcegos insetívoros é distribuído de forma generalizada, com mais de 15 cm de profundidade em alguns pontos e cobrindo cerca de 70% do piso da cavidade. O piso é seco

e irregular com alguns trechos planos, sendo este formado por áreas que se alternam entre a rocha nua e sedimento granulado com muitos calhaus e matacões esparsos. A serrapilheira está restrita a zona de entrada e o sistema radicular é bem desenvolvido em alguns pontos chegando a formar pequenos rizotemas. Em geral, o sistema de canalículos é pouco desenvolvido. No momento da coleta foram observados dois exemplares de urubus de cabeça preta (*Coragyps atratus*), os quais haviam transportado pequenos pedaços de vísceras para a parte mais profunda da cavidade. Estas vísceras estavam sendo consumidas por uma grande quantidade de formigas do gênero Camponotus (Figura 198). Em relação aos impactos, existem sinais de fogueira na zona de entrada e indícios antigos de extração de guano. A caverna apresenta uma baixa umidade e uma elevada temperatura. Nenhuma alteração significativa foi observada durante a estação úmida, além das alterações normais na umidade relativa do ar.

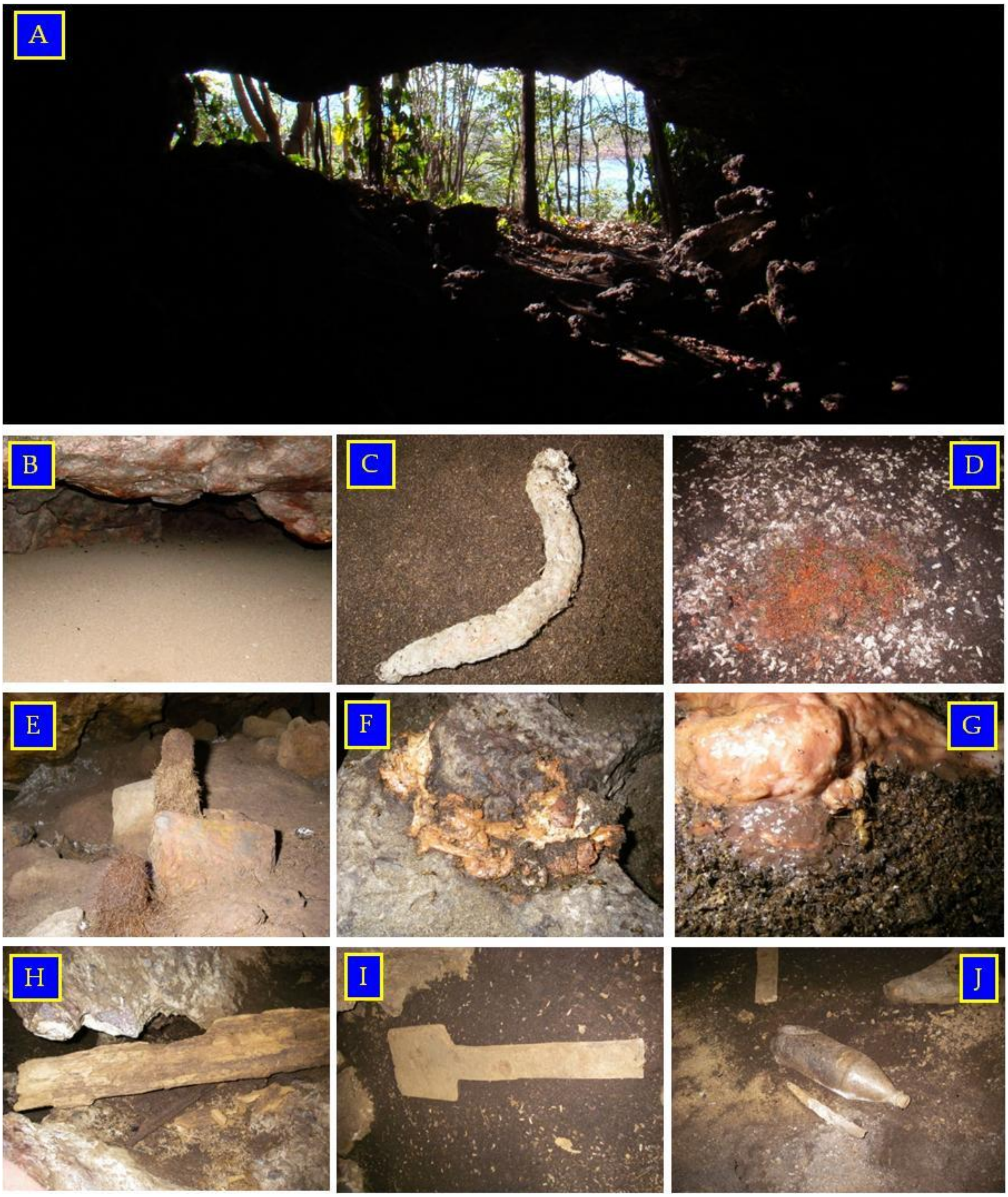

Figura 198 - a) Vista interna da entrada da cavidade localizada em pequeno fragmento de mata; b) Piso da cavidade completamente revestido por guano; c) Fezes de um vertebrado; d) Guano de morcegos frugívoros encontrado no interior da caverna; e) Rizotemas crescendo na base e sobre um bloco de rocha; f) Vísceras sendo consumidas por formiga *Camponotus sp*. g) Imagem anterior agora em detalhe; h) Fragmento de madeira observado no interior da cavidade; i) Ferramenta de madeira utilizada na extração de guano; j) garrafa plástica deixada no interior da caverna.

5.4.4.70.2. Caracterização faunística no período de seca

Foi observado na caverna, um total de 41 morfoespécies de invertebrados de pelo menos 28 famílias das Ordens: Acari (Argasidae: *Antricola* sp., Diploginiidae, Laelapidae, Anoetidae, Oribatida), Pseudoescorpiones (Chernetidae), Araneae (Gnaphosidae, Scytodidae*: Scytodes eleonora*, Pholcidae: *Mesabolivar* sp., Theridiidae), Thysanura (Nicoletiidae: Nicoletiinae, Atelurinae), Collembola (Entomobryidae), Orthoptera (Phalangopsidae: *Phalangopsis* sp.), Blattodea (Blaberidae: *Blaberus* sp.), Isoptera (Termitidae: *Cortaritermes* sp.), Lepidoptera (Noctuidae, Tineidae), Diptera (Dolichopodidae, Drosophilidae, Milichiidae, Psychodidae: *Lutzomyia* sp.), Hymenoptera (Formicidae: *Camponotus* sp., *Gnamptogenys* sp., *Pachycondyla* sp., *Solenopsis* sp.; Braconidae), Coleoptera (Carabidae, Elateridae: Elaterinae, Hysteridae, Leiodidae, Tenebrionidae: Coelometropinae), Scolopendromorpha (Scolopocryptopidae: *Dinocryptops* sp.).

Dentre os vertebrados, foram encontradas quatro espécies das ordens Chiroptera (Emballonuridae: *Peropteryx kappleri*; Phyllostomidae: *Glossophaga soricina*), Anura (Leptodactylidae: *Pristimantis cf. fenestratus*), Cathartiformes (Cathartidae: *Coragypis atratus*). Desta forma, no total foram encontradas 45 morfoespécies.

5.4.4.70.3. Caracterização faunística no período de chuva

Foi observado na caverna, um total de 85 morfoespécies de invertebrados de pelo menos 53 famílias das Ordens: Isopoda (Balloniscidae, Dubioniscidae, Scleropactidae), Acari (Argasidae: *Antricola* sp., *Ornithodoros* sp.; Ixodidae: *Amblyomma* sp.; Diploginiidae, Laelapidae: *Stratiolaelaps* sp.; Opilioacaridae: *Neoacarus* sp., Oribatida), Amblypygi (Phrynidae: *Heterophrynus longicornis*), Pseudoescorpiones (Chernetidae, Chtoniidae), Araneae (Corinidae: *Tupirina* sp.; Gnaphosidae, Salticidae, Scytodidae*: Scytodes eleonora*, Pholcidae: *Mesabolivar* sp., *Metagonia* sp.; Theraphosidae: *Nhandu coloratovillosus*, Theridiidae), Thysanura (Nicoletiidae: Nicoletiinae), Diplura (Campodeidae), Collembola (Sminthuridae, Entomobryidae), Orthoptera (Phalangopsidae: *Aclodes* sp., *Phalangopsis* sp.), Blattodea (Blaberidae: *Blaberus* sp.), Psocoptera, Hemiptera (Cydnidae, Lygaeidae, Nabidae, Pyrrhocoridae), Homoptera (Cixiidae: *Cixius* sp.; Derbidae), Lepidoptera (Noctuidae: Agaristinae; Tineidae), Diptera (Ceratopogonidae, Culicidae, Drosophilidae: *Drosophila* sp.; Empididae, Fanniidae, Milichiidae, Psychodidae: *Lutzomyia* sp.; Streblidae), Hymenoptera (Formicidae: *Acromyrmex* sp., *Camponotus* sp., *Cyphomyrmex* sp., *Gnamptogenys* sp., *Pheidole* sp., *Solenopsis* sp.; Bethylidae, Scelionidae), Coleoptera (Carabidae, Chrysomelidae, Elateridae: Cardiophorinae; Hysteridae, Ptylidae, Scydmaenidae, Staphylinidae, Tenebrionidae), Geophilomorpha (Geophilidae sp.), Scolopendromorpha (Scolopocryptopidae - Newportinae: *Newportia* sp.).

Dentre os vertebrados, foram encontradas três espécies das ordens Chiroptera (Phyllostomidae: *Glossophaga soricina*), Squamata (Gekkonidae: *Thecadactylus rapicauda*) e Anura (Leptodactylidae: *Pristimantis cf. fenestratus*).

Desta forma, no total foram encontradas 88 morfoespécies. Entre estas, uma espécie de invertebrado foi considerada troglomórfica: Amblypygi (Charinidae: *Charinus* sp.).

5.4.4.70.4. Caracterização geral da fauna da cavidade

Foi observado na caverna, um total de 109 morfoespécies de invertebrados de pelo menos 59 famílias das Ordens: Isopoda (Balloniscidae, Dubioniscidae, Scleropactidae), Acari (Argasidae: *Antricola* sp., *Ornithodoros* sp.; Ixodidae: *Amblyomma* sp.; Diploginiidae, Laelapidae: *Stratiolaelaps* sp.; Opilioacaridae: *Neoacarus* sp., Anoetidae, Oribatida), Amblypygi (Phrynidae: *Heterophrynus longicornis*), Pseudoescorpiones (Chernetidae, Chtoniidae), Araneae (Corinidae: *Tupirina* sp.; Gnaphosidae, Salticidae, Scytodidae*: Scytodes eleonora*, Pholcidae: *Mesabolivar* sp., *Metagonia* sp.; Theraphosidae: *Nhandu coloratovillosus*, Theridiidae), Thysanura (Nicoletiidae: Nicoletiinae, Atelurinae), Diplura (Campodeidae), Collembola (Sminthuridae, Entomobryidae), Orthoptera (Phalangopsidae: *Aclodes* sp., *Phalangopsis* sp.), Blattodea (Blaberidae: *Blaberus* sp.), Isoptera (Termitidae: *Cortaritermes* sp.), Psocoptera, Hemiptera (Cydnidae, Lygaeidae, Nabidae, Pyrrhocoridae), Homoptera (Cixiidae: *Cixius* sp.; Derbidae), Lepidoptera (Noctuidae: Agaristinae; Tineidae), Diptera (Ceratopogonidae, Culicidae, Dolichopodidae, Drosophilidae: *Drosophila* sp.; Empididae, Fanniidae, Milichiidae, Psychodidae: *Lutzomyia* sp.; Streblidae), Hymenoptera (Formicidae: *Acromyrmex* sp., *Camponotus* sp., *Cyphomyrmex* sp., *Gnamptogenys* sp., *Pachycondyla* sp., *Pheidole* sp., *Solenopsis* sp.; Bethylidae, Braconidae, Scelionidae), Coleoptera (Carabidae, Chrysomelidae, Elateridae: Cardiophorinae, Elaterinae, Hysteridae, Leiodidae, Ptylidae, Scydmaenidae, Staphylinidae, Tenebrionidae: Coelometropinae), Geophilomorpha (Geophilidae sp.), Scolopendromorpha (Scolopocryptopidae: *Dinocryptops* sp., Newportinae: *Newportia* sp.).

Dentre os vertebrados, foram encontradas cinco espécies das ordens Chiroptera (Emballonuridae: *Peropteryx kappleri*; Phyllostomidae: *Glossophaga soricina*), Squamata (Gekkonidae: *Thecadactylus rapicauda*), Anura (Leptodactylidae: *Pristimantis cf. fenestratus*), Cathartiformes (Cathartidae: *Coragypis atratus*).

Desta forma, no total foram encontradas 114 morfoespécies. Entre estas, uma espécie de invertebrado foi considerada troglomórfica: Amblypygi (Charinidae: *Charinus* sp.). Alguns organismos encontrados nesta caverna são mostrados na Figura 199.

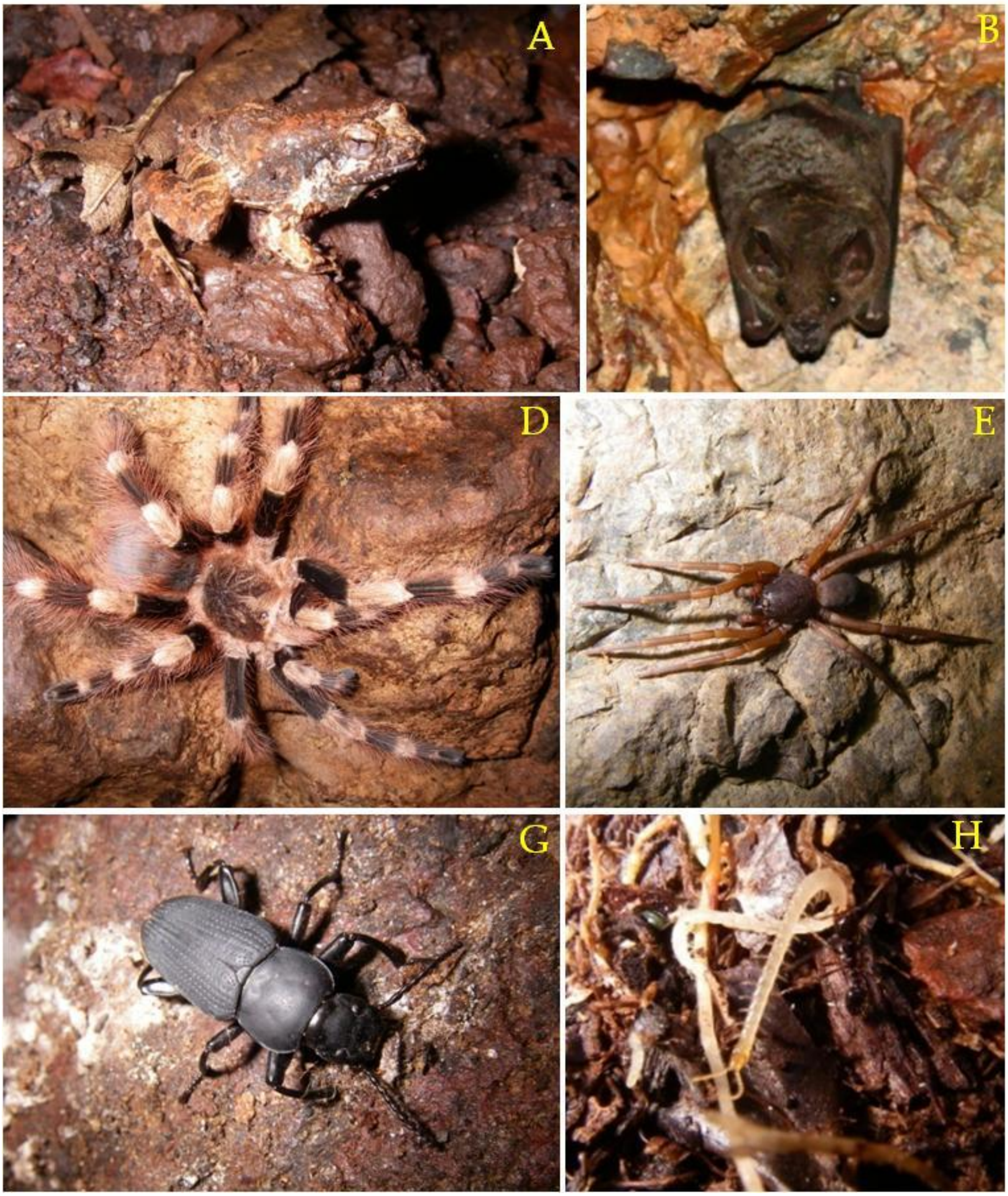

Figura 199 - a) Anura (indet.); b) Phyllostomidae (*Carollia sp*.); c) Theraphosidae (*Nhandu coloratovillosus*); d) Corinidae; e) Coleoptera (Tenebrionidae); f) Geophilomorpha. (As letras da legendas das fotos estavam erradas).

5.4.4.71. SL-074

5.4.4.71.1. Caracterização trófica

Grande cavidade com 127,2 m de projeção horizontal formada na canga e inserida em uma drenagem intermitente. Entrada principal com mais de três metros de altura, sujeita a inundação em épocas de chuvas, onde se acumulam grandes depósitos de serrapilheira que chegam a cobrir o piso em alguns trechos da região de entrada. Neste setor existem muitas plântulas de angiospermas (Melastomataceae). Esta cavidade possui três entradas. A principal é ampla (como supracitado) e o conduto que se segue apresenta piso ascendente e paredes e tetos cobertos por uma grande quantidade de briófitas, líquens e bactérias. A segunda é paralela à primeira, possuindo menores dimensões e também inserida dentro da drenagem. A terceira corresponde a uma pequena clarabóia na porção final da cavidade pela qual penetram pequenas quantidades de serrapilheira que se depositam logo abaixo desta clarabóia. A cavidade é volumosa, com trechos de teto alto e grandes blocos abatidos em sua porção mediana. Existem três áreas distintas que apresentam condições afóticas e alta estabilidade ambiental, com umidade e temperatura elevada e com alguns pontos de gotejamento e percolação. Existe uma grande área de penumbra sendo as zonas eufóticas restritas às entradas. Em geral, existe pouco sedimento clástico, sendo que o piso da cavidade, na maioria das vezes, formado pela rocha matriz ou por grandes blocos sobrepostos. Nas zonas mais profundas existe grande quantidade de depósitos de guano de morcegos frugívoros, além de um conduto lateral onde o piso também é completamente coberto por guano (Figura 200). Neste setor, o sedimento clástico encontra-se muitas vezes revolvido e mesclado ao guano. Esta área possui ainda muitas raízes de pequeno calibre. Entretanto, de forma geral, existem poucas raízes na cavidade, sendo observados também alguns pequenos depósitos velhos de guano de morcegos insetívoros. *G. soricina* é a espécie dominante na cavidade, formando uma grande população de centenas de indivíduos. Durante a estação úmida existiam mais pontos de gotejamento e de percolação. Como a cavidade encontra-se inserida junto a uma drenagem, neste período o conduto inferior que leva uma entrada secundária encontrava-se completamente alagado após um dia de chuva intensa (Figura 203).

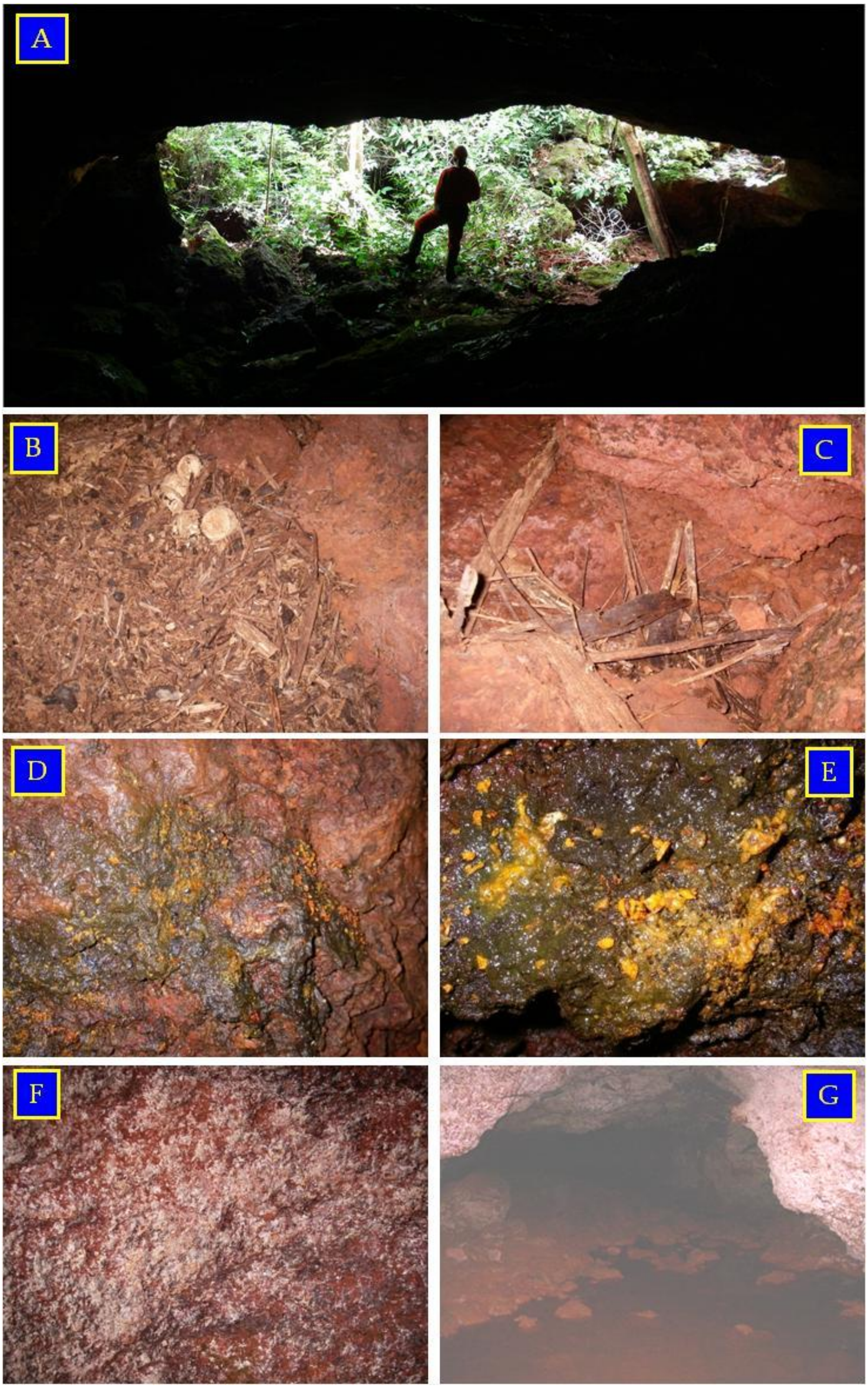

Figura 200 - a) Vista interna da entrada da cavidade localizada em pequeno fragmento de mata ciliar; b,c) Acúmulo de serrapilheira transportada durante o período de chuvas; d) Depósito de guano de morcegos frugívoros; e) Detalhe do depósito anterior; f) Actinomicetos se desenvolvendo nas paredes; g) porção alagada da cavidade durante o período de chuvas.

5.4.4.71.2. Caracterização faunística no período de seca

Foi observado na caverna, um total de 140 morfoespécies de invertebrados de pelo menos 87 famílias das Ordens: Oligochaeta, Gastropoda (Subulinidae), Turbellaria (Geoplanidae), Isopoda (Armadillidae, Philosciidae, Scleropactidae), Acari (Ixodidae: *Amblyomma cajennense*; Laelapidae: *Stratiolaelaps* sp.; Macrochelidae: *Macrocheles* sp.; Macronyssidae, Uropodina, Astigmatina, Oribatida, Anystidae: *Erythracarus* sp.; Cunaxidae, Eupodidae: *Linopodes* sp.; Rhagidiidae), Amblypygi (Phrynidae: *Heterophrynus longicornis*), Scorpiones (Buthidae: *Ananteris luciae*), Pseudoescorpiones (Chernetidae, Chtoniidae), Opiliones (Cosmetidae: *Anduzeia sp.*; Escadabiidae, Sclerosomatidae: *Prionostemma* sp.), Araneae (Araneidae: *Alpaida* sp.; Filistatidae, Gnaphosidae, Linyphiidae, Oonopidae: Oonopinae, Salticidae, Segestridae, Scytodidae*: Scytodes eleonora*, Pholcidae: *Mesabolivar* sp., *Metagonia* sp.; Theridiidae, Theridiosomatidae), Thysanura (Nicoletiidae: Nicoletiinae, Atelurinae), Diplura (Campodeidae, Japygidae, Parajapygidae), Collembola (Cyphoderidae, Entomobryomorpha, Hypogastruridae, Paronellidae), Orthoptera (Gryllidae: Nemobiinae; Phalangopsidae: *Aclodes* sp., *Phalangopsis* sp.; Tetrigidae), Blattodea (Blaberidae: *Blaberus* sp., Blattellidae, Blattidae, Polyphagidae), Isoptera (Termitidae: *Nasutitermes* sp.), Psocoptera (Epipsocidae, Pachytroctidae), Hemiptera (Cydnidae, Mesoveliidae, Reduviidae, Ploiariidae), Homoptera (Cicadellidae, Dictyopharidae), Lepidoptera (Lycaenidae, Noctuidae, Tineidae), Diptera (Ceratopogonidae, Culicidae: *Culex* sp., Dolichopodidae, Drosophilidae, Fanniidae, Psychodidae: *Lutzomyia* sp.; Sciaridae, Streblidae, Tipulidae), Hymenoptera (Formicidae: *Acanthognatus* sp., *Camponotus* sp., *Dolichoderus* sp., *Octostruma* sp., *Pachycondyla* sp., *Solenopsis* sp.; Diapriidae), Thysanoptera (Phaleothripinae), Coleoptera (Byrrhidae, Carabidae, Coccinellidae, Curculionidae: Scotylinae, Leiodidae, Scydmaenidae, Staphylinidae, Tenebrionidae), Diplopoda (Chelodesmidae, Pyrgodesmidae, Pseudonannolenidae), Geophilomorpha (Ballophilidae: *Ballophililus* sp.), Lithobiomorpha (Henicopiidae: *Lamyctes* sp.), Scolopendromorpha (Cryptopidae: *Cryptops* sp.), Scutigeromorpha (Scutigeridae: *Sphendononema* sp.), Neuroptera (Myrmeleontidae).

Dentre os vertebrados, foram encontradas 11 espécies das ordens Chiroptera (Phyllostomidae: *Anoura* sp., *Glossophaga soricina;* Furipteridae: *Furipterus horrens*), Squamata (Sphaerodactylidae: *Coleodactylus cf. amazonicus*), Anura (Leptodactylidae: *Pristimantis cf. fenestratus*, *Eleutherodactylus* sp., Bufonidae, Dendrobatidae: *Ameerega* sp., *Colostethus* sp.), Rodentia (Cricetidae: *Rhipidomys* sp.).

Desta forma, no total foram encontradas 151 morfoespécies. Entre estas, sete espécies de invertebrados foram consideradas troglomórficas: Gordioidea, Gastropoda (Systrophiidae), Amblypygi (Charinidae: *Charinus* sp.), Araneae (Oonopidae: Oonopinae), Hymenoptera (Formicidae: *Hypoponera* sp.), Coleoptera (Carabidae: *Coarazuphium* sp.), Diplopoda (Pyrgodesmidae).

5.4.4.71.3. Caracterização faunística no período de chuva

Foi observado na caverna, um total de 159 morfoespécies de invertebrados de pelo menos 89 famílias das Ordens: Oligochaeta, Gastropoda (Subulinidae, Valloniidae), Turbellaria (Geoplanidae), Isopoda (Armadillidae, Philosciidae, Scleropactidae), Acari (Ixodidae: *Amblyomma cajennense*; Macronyssidae, Oribatida, Anystidae: *Erythracarus* sp.), Amblypygi (Phrynidae: *Heterophrynus longicornis*), Ricinulei (Ricinoididae: *Cryptocellus tarsilae*), Pseudoescorpiones (Chernetidae, Chtoniidae), Opiliones (Escadabiidae, Sclerosomatidae: *Prionostemma* sp.; Stygnidae, Phalangiidae), Araneae (Araneidae: *Alpaida* sp.; Corinidae: *Tupirina* sp, Ctenidae: *Ctenus* sp., Filistatidae, Gnaphosidae, Linyphiidae, Ochyroceratidae, Oonopidae: Oonopinae, Salticidae, Scytodidae*: Scytodes eleonora*, Palpimanidae, Psauridae, Pholcidae: *Mesabolivar* sp., *Metagonia* sp.; Theraphosidae, Theridiosomatidae), Thysanura (Nicoletiidae: Nicoletiinae), Microcoryphia (Meinertellidae), Diplura (Anajapygidae, Campodeidae, Parajapygidae), Collembola (Sminthuridae, Cyphoderidae, Entomobryomorpha, Hypogastruridae, Isotomidae, Onychiuridae, Paronellidae), Orthoptera (Phalangopsidae: *Aclodes* sp., *Phalangopsis* sp.), Blattodea (Blaberidae: *Blaberus* sp., Blattellidae, Blattidae), Embiidina, Isoptera (Termitidae: *Nasutitermes* sp.), Dermaptera (Labiidae), Psocoptera (Myopsocidae: *Lichenomina* sp.; Pachytroctidae, Psyllipsocidae: *Psyllipsocus* sp.), Hemiptera (Cydnidae, Dipsocoridae, Enicocephalidae, Hebridae, Mesoveliidae, Nabidae, Reduviidae, Veliidae), Homoptera (Derbidae), Lepidoptera (Tineidae), Diptera (Chironomidae, Drosophilidae, Psychodidae: *Lutzomyia* sp.; Sciaridae, Streblidae, Tipulidae), Hymenoptera (Formicidae: *Acanthognatus* sp., *Apterostigma* sp., *Camponotus* sp., *Dolichoderus* sp., *Labidus* sp., *Pachycondyla* sp., *Pheidole* sp., *Solenopsis* sp.), Thysanoptera (Phaleothripinae), Coleoptera (Carabidae, Elateridae: Cardiophorinae; Leiodidae, Ptylidae, Scydmaenidae, Staphylinidae), Diplopoda (Pyrgodesmidae, Glomeridesmidae: *Glomeridesmus* sp.; Siphonophoridae), Geophilomorpha (Ballophilidae: *Ballophililus* sp., *Taeniolinum* sp.; Geophilidae), Lithobiomorpha (Henicopiidae: *Lamyctes* sp.), Scolopendromorpha (Otostigmidae: *Otostigmus* sp.; Cryptopidae: *Cryptops* sp.; Scolopocryptopidae: *Dinocryptops* sp.), Scutigeromorpha (Scutigeridae: *Sphendononema* sp.), Neuroptera (Myrmeleontidae).

Dentre os vertebrados, foram encontradas seis espécies das ordens Chiroptera (Emballonuridae: *Peropteryx kappleri;* Phyllostomidae: *Anoura* sp., *Glossophaga soricina*), Squamata (Gekkonidae: *Thecadactylus rapicauda*), Anura (Leptodactylidae: *Pristimantis cf. fenestratus*) e Rodentia (Cuniculidae).

Desta forma, no total foram encontradas 165 morfoespécies. Entre estas, nove espécies de invertebrados foram consideradas troglomórficas: Gastropoda (Systrophiidae), Amblypygi (Charinidae: *Charinus* sp.), Araneae (Prodidomidae), Thysanura (Atellurinae), Collembola (Cyphoderidae, Isotomidae), Hymenoptera (Formicidae: *Hypoponera* sp.), Coleoptera (Carabidae: *Coarazuphium* sp.), Diplopoda (Pyrgodesmidae).

5.4.4.71.4. Caracterização geral da fauna da cavidade

Foi observado na caverna, um total de 240 morfoespécies de invertebrados de pelo menos 129 famílias das Ordens: Oligochaeta, Gastropoda (Subulinidae, Valloniidae), Turbellaria (Geoplanidae), Isopoda (Armadillidae, Philosciidae, Scleropactidae), Acari (Ixodidae: *Amblyomma cajennense*; Laelapidae: *Stratiolaelaps* sp.; Macrochelidae: *Macrocheles* sp.; Macronyssidae, Uropodina, Astigmatina, Oribatida, Anystidae: *Erythracarus* sp.; Cunaxidae, Eupodidae: *Linopodes* sp.; Rhagidiidae), Amblypygi (Phrynidae: *Heterophrynus longicornis*), Ricinulei (Ricinoididae: *Cryptocellus tarsilae*), Scorpiones (Buthidae: *Ananteris luciae*), Pseudoescorpiones (Chernetidae, Chtoniidae), Opiliones (Cosmetidae: *Anduzeia sp.*; Escadabiidae, Sclerosomatidae: *Prionostemma* sp.; Stygnidae, Phalangiidae), Araneae (Araneidae: *Alpaida* sp.; Corinidae: *Tupirina* sp, Ctenidae: *Ctenus* sp., Filistatidae, Gnaphosidae, Linyphiidae, Ochyroceratidae, Oonopidae: Oonopinae, Salticidae, Segestridae, Scytodidae*: Scytodes eleonora*, Palpimanidae, Psauridae, Pholcidae: *Mesabolivar* sp., *Metagonia* sp.; Theraphosidae, Theridiidae, Theridiosomatidae), Thysanura (Nicoletiidae: Nicoletiinae, Atelurinae), Microcoryphia (Meinertellidae), Diplura (Anajapygidae, Campodeidae, Japygidae, Parajapygidae), Collembola (Sminthuridae, Cyphoderidae, Entomobryomorpha, Hypogastruridae, Isotomidae, Onychiuridae, Paronellidae), Orthoptera (Gryllidae: Nemobiinae; Phalangopsidae: *Aclodes* sp., *Phalangopsis* sp.; Tetrigidae), Blattodea (Blaberidae: *Blaberus* sp., Blattellidae, Blattidae, Polyphagidae), Embiidina, Isoptera (Termitidae: *Nasutitermes* sp.), Dermaptera (Labiidae), Psocoptera (Epipsocidae, Myopsocidae: *Lichenomina* sp.; Pachytroctidae, Psyllipsocidae: *Psyllipsocus* sp.), Hemiptera (Cydnidae, Dipsocoridae, Enicocephalidae, Hebridae, Mesoveliidae, Nabidae, Reduviidae, Ploiariidae, Veliidae), Homoptera (Cicadellidae, Derbidae, Dictyopharidae), Lepidoptera (Lycaenidae, Noctuidae, Tineidae), Diptera (Ceratopogonidae, Chironomidae, Culicidae: *Culex* sp., Dolichopodidae, Drosophilidae, Fanniidae, Psychodidae: *Lutzomyia* sp.; Sciaridae, Streblidae, Tipulidae), Hymenoptera (Formicidae: *Acanthognatus* sp., *Apterostigma* sp., *Camponotus* sp., *Dolichoderus* sp., *Labidus* sp., *Octostruma* sp., *Pachycondyla* sp., *Pheidole* sp., *Solenopsis* sp.; Diapriidae), Thysanoptera (Phaleothripinae), Coleoptera (Byrrhidae, Carabidae, Coccinellidae, Curculionidae: Scotylinae, Elateridae: Cardiophorinae; Leiodidae, Ptylidae, Scydmaenidae, Staphylinidae, Tenebrionidae), Diplopoda (Chelodesmidae, Pyrgodesmidae, Glomeridesmidae: *Glomeridesmus* sp.; Siphonophoridae, Pseudonannolenidae), Geophilomorpha (Ballophilidae: *Ballophililus* sp., *Taeniolinum* sp.; Geophilidae), Lithobiomorpha (Henicopiidae: *Lamyctes* sp.), Scolopendromorpha (Otostigmidae: *Otostigmus* sp.; Cryptopidae: *Cryptops* sp.; Scolopocryptopidae: *Dinocryptops* sp.), Scutigeromorpha (Scutigeridae: *Sphendononema* sp.), Neuroptera (Myrmeleontidae).

Dentre os vertebrados, foram encontradas 13 espécies das ordens Chiroptera (Emballonuridae: *Peropteryx kappleri*; Phyllostomidae: *Anoura* sp., *Glossophaga soricina;* Furipteridae: *Furipterus horrens*), Squamata (Gekkonidae: *Thecadactylus rapicauda*; Sphaerodactylidae: *Coleodactylus cf. amazonicus*), Anura (Leptodactylidae: *Pristimantis cf.*

fenestratus, *Eleutherodactylus* sp., Bufonidae, Dendrobatidae: *Ameerega* sp., *Colostethus* sp.), Rodentia (Cricetidae: *Rhipidomys* sp.; Cuniculidae).

Desta forma, no total foram encontradas 253 morfoespécies. Entre estas, quatorze espécies de invertebrados foram consideradas troglomórficas: Gordioidea, Gastropoda (Systrophiidae), Amblypygi (Charinidae: *Charinus* sp.), Araneae (Ochyroceratidae, Oonopidae: Oonopinae, Prodidomidae), Thysanura (Atellurinae), Collembola (Cyphoderidae, Isotomidae), Hymenoptera (Formicidae: *Hypoponera* sp.), Coleoptera (Carabidae: *Coarazuphium* sp.), Diplopoda (Pyrgodesmidae). Alguns organismos encontrados nesta caverna são mostrados na Figura 201.

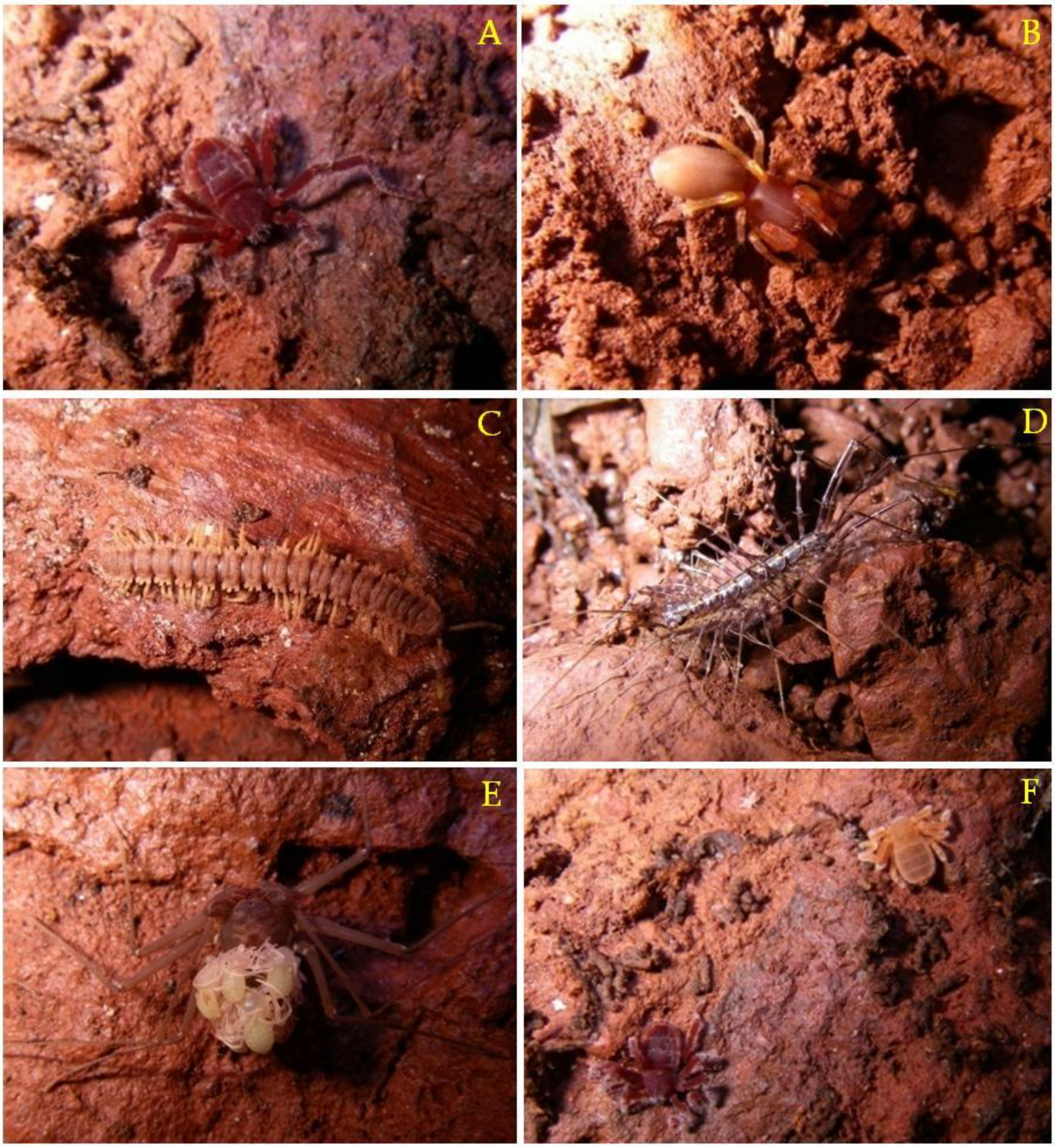

Figura 201 - a) Ricinulei (*Cryptocellus tarsilae*); b) Araneae (Palpimanidae); c) Diplopoda (Chelodesmidae); d) Scutigeromorpha (*Sphendononema sp.*); e) Amblypygi (Charinidae: *Charinus sp.)* com filhotes; f) Ninfas de Ricinulei (*Cryptocellus tarsilae*).

5.4.4.72. SL-075

5.4.4.72.1. Caracterização trófica

Caverna com 88,8 m de projeção horizontal, ao lado direito de uma drenagem intermitente. A caverna encontra-se inserida em área de mata ciliar sendo a matriz composta por savana metalófila. Apresenta um padrão labiríntico com dois níveis predominantes, um descendente à esquerda e outro ascendente à direita da entrada da cavidade. Sua entrada é ampla, com muitos líquens, briófitas e plântulas de angiospermas além de muitos blocos abatidos. O piso é extremamente irregular, composto por sedimento argiloso com muitos seixos, calhaus e matacões. Este se encontrava úmido durante o inventário, sendo possível observar pontos de percolação de água e gotejamento. Existem muitas raízes de pequeno calibre nas paredes laterais na zona eufótica, além de briófitas, pteridófitas, lianas, líquens e bactérias. O conduto principal da cavidade é predominantemente ascendente, composto por piso argiloso com áreas de gotejamento e pequenas poças d'água junto a trechos de rastejo (Figura 202). Este setor encontra-se muito "lavado", onde praticamente não existiam recursos orgânicos. Foram observados alguns depósitos de guano de morcegos frugívoros em alguns pontos da cavidade. Nestes, observou-se algumas plântulas germinadas a partir de sementes presentes no guano, além de muitos Actinomicetos associados às paredes e ao teto da cavidade. A caverna apresenta uma elevada estabilidade ambiental, com zonas afóticas em suas porções mais distais e possui um sistema de canalículos muito desenvolvido. Nenhuma alteração significativa foi observada durante a estação úmida, além das alterações normais na umidade relativa do ar.

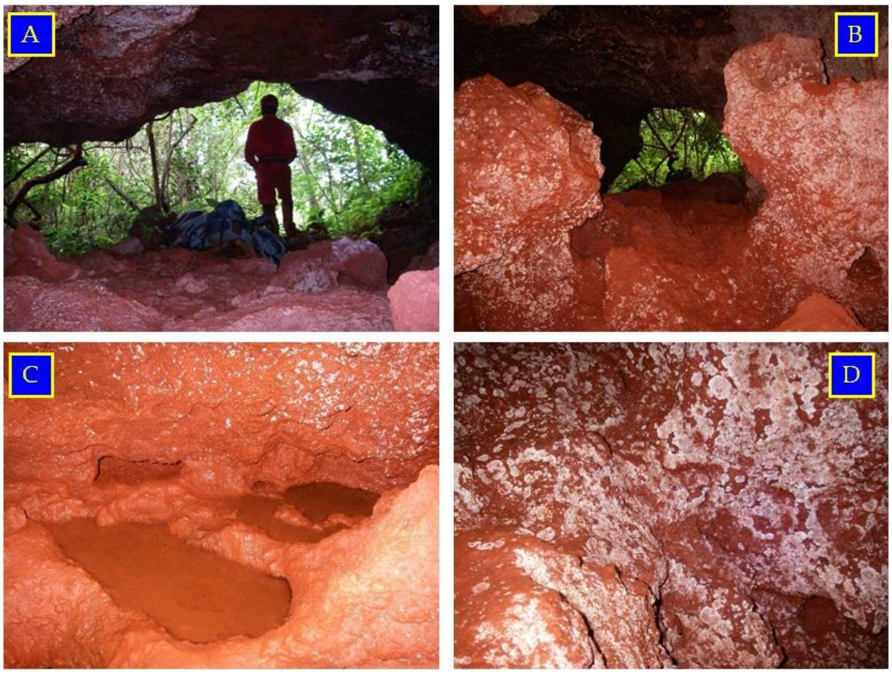

Figura 202 - a) Vista geral da vegetação onde a cavidade encontra-se inserida; b) Vista interna da cavidade, onde visualiza-se a entrada; c) porção alagada da cavidade durante o período de chuvas; d) Actinomicetos se desenvolvendo nas paredes.

5.4.4.72.2. Caracterização faunística no período de seca

Foram encontradas na caverna, 65 morfoespécies de invertebrados em pelo menos 50 famílias dos táxons: Gastropoda (Subulinidae), Isopoda (Philosciidae), Acari (Laelapidae: *Stratiolaelaps* sp.; Podocinidae: *Podocinum* sp.; Uropodina; Opiliacaridae: *Neoacarus* spn.; Astigmatina; Oribatida; Cunaxidae; Eupodidae: *Linopodes* sp.; Labdostomatidae: Labdostomatida;, Rhagidiidae; Trombidiforme), Amblypygi (Phrynidae: *Heterophrynus longicornis*), Scorpiones (Buthidae: *Ananteris luciae*), Pseudoscorpiones (Chernetidae; Chtoniidae), Opiliones (Cosmetidae: *Anduzeia* sp.; Sclerosomatidae: *Prionostemma* sp.; Stygnidae: *Protimesius aff. gracilis)*, Araneae (Araneidae: *Alpaida* sp.; Ctenidae; Oonopidae: Oonopinae; Pholcidae: *Mesabolivar* sp.; Theridiosomatidae), Thysanura (Nicolletiidae: Nicolletiinae), Diplura (Campodeidae), Collembola (Sminthuridae; Entomobryidae), Neuroptera (Mantispidae: *Plega* sp.), Orthoptera (Phalangopsidae: *Aclodes* sp.; *Phalangopsis* sp.), Isoptera (Termitidae: *Nasutitermes* sp. e *Velocitermes* sp.) Psocoptera (Epipsocidae), Hemiptera (Cydnidae), Homoptera (Cixidae: *Cixius* sp.), Lepidoptera (Tineidae), Diptera (Ceratopogonidae, Culicidae, Dolichopodidae, Drosophilidae, Psychodidae: *Lutzomyia* sp., Tipulidae e Sciaridae), Hymenoptera (Formicidae: *Camponotus* sp., *Pachycondyla* sp., *Pheidole* sp., *Zacryptocerus* sp. e Scelionidae), Coleoptera (Carabidae, Ptylidae e Staphylinidae), Diplopoda (Chelodesmidae e Pyrgodesmidae), Scutigeromorpha (Scutigeridae: *Sphendononema* sp.), Symphyla (Scutigerellidae: *Hanseniella* sp.)

Dentre os vertebrados foram encontrados dois morfótipos pertencentes às ordens Chiroptera (*Glossophaga soricina*) e Anura (Leptodactylidae: *Pristimantis cf. fenestratus*). Dessa forma, foram encontradas um total de 67 morfoespécies. Dentre essas, uma morfoespécie foi considerada troglóbia, a qual pertence ao táxon Diplopoda (Pyrgodesmidae).

5.4.4.72.3. Caracterização faunística no período de chuva

Foram encontradas na caverna, 61 morfoespécies de invertebrados em pelo menos 49 famílias dos táxons: Isopoda (Armadillidae e Philosciidae), Acari (Argasidae: *Ornithodoros* sp., Oribatida, Eupodidae: *Linopodes* sp., Trombidiforme), Amblypygi (Phrynidae: *Heterophrynus longicornis*, Charinidae: *Charinus* sp.), Pseudoscorpiones (Chernetidae e Chtoniidae), Opiliones (Escadabiidae; Sclerosomatidae: *Prionostemma* sp., Stygnidae: *Protimesius aff. gracilis*; Phalangiidae), Araneae (Araneidae: *Alpaida* sp.; Ctenidae: *Ctenus* sp.; Gnaphosidae sp2; Oonopidae: Oonopinae; Salticidae; Scytodidae*: Scytodes eleonora*; Pholcidae: *Mesabolivar* sp.; Theridiosomatidae), Thysanura (Nicolletiidae: Nicolletiinae; Meinertellidae), Collembola (Entomobryidae; Paronellidae), Orthoptera (Phalangopsidae: *Phalangopsis* sp.), Isoptera (Termitidae: *Nasutitermes* sp), Psocoptera (Epipsocidae; Myopsocidae: *Lichenomina* sp.), Hemiptera (Cydnidae; Enicocephalidae; Lygaeidae; Reduviidae; Ploiariidae), Homoptera (Cixidae; Kinnaridae; Coccoidea), Lepidoptera (Noctuidae; Tineidae), Diptera (Psychodidae:

Lutzomyia sp.; Drosophilidae), Hymenoptera (Formicidae: *Apterostigma* sp., *Pachycondyla* sp., *Tetramorium* sp.; Scelionidae), Coleoptera (Ptylidae e Pselaphidae), Diplopoda (Chelodesmidae; Pyrgodesmidae; Polydesmida), Scutigeromorpha (Scutigeridae: *Sphendononema* sp.).

Dentre os vertebrados foram encontrados dois morfótipos pertencentes às ordens Chiroptera (*Glossophaga soricina*) e Squamata (Boidae: *Epicrates* sp.)

Dessa forma, foram encontrados um total de 63 morfoespécies. Dentre essas, três foram consideradas troglóbias, as quais pertencem aos táxons Amblypygi (Charinidae: *Charinus* sp.), Coleoptera (Pselaphidae), Diplopoda (Pyrgodesmidae).

5.4.4.72.4. Caracterização geral da fauna da cavidade

Foram encontrados na caverna, 103 morfoespécies de invertebrados em pelo menos 71 famílias dos táxons: Gastropoda (Subulinidae), Isopoda (Armadillidae; Philosciidae), Acari (Argasidaae: *Ornithodoros* sp.; Laelapidae: *Stratiolaelaps* sp.; Podocinidae: *Podocinum* sp.; Uropodina; Opiliacaridae: *Neoacarus* spn.; Astigmatina; Oribatida; Cunaxidae; Eupodidae: *Linopodes* sp.; Labdostomatidae: Labdostomatida; Rhagidiidae; Trombidiforme), Amblypygi (Phrynidae: *Heterophrynus longicornis*; Charinidae: *Charinus* sp), Scorpiones (Buthidae: *Ananteris luciae*), Pseudoscorpiones (Chernetidae; Chtoniidae), Opiliones (Cosmetidae: *Anduzeia* sp.; Escadabiidae; Sclerosomatidae: *Prionostemma* sp.; Stygnidae: *Protimesius aff. gracilis*; Phalangiidae*)*, Araneae (Araneidae: *Alpaida* sp.; Ctenidae: *Ctenus* sp.; Gnaphosidae; Oonopidae: Oonopinae; Scytodidae*: Scytodes eleonora*; Pholcidae: *Mesabolivar* sp.; Theridiosomatidae), Thysanura (Nicolletiidae: Nicolletiinae; Meinertellidae), Diplura (Campodeidae), Collembola (Sminthuridae; Entomobryidae; Paronellidae), Neuroptera (Mantispidae: *Plega* sp.), Orthoptera (Phalangopsidae: *Aclodes* sp.; *Phalangopsis* sp.), Isoptera (Termitidae: *Nasutitermes* sp. e *Velocitermes* sp.) Psocoptera (Epipsocidae; Myopsocidae: *Lichenomina* sp.), Hemiptera (Cydnidae; Enicocephalidae; Lygaeidae; Reduviidae; Ploiariidae), Homoptera (Cixidae: *Cixius* sp.; Kinnaridae; Coccoidea), Lepidoptera (Noctuidae; Tineidae), Diptera (Ceratopogonidae, Culicidae, Dolichopodidae, Drosophilidae, Psychodidae: *Lutzomyia* sp., Tipulidae e Sciaridae), Hymenoptera (Formicidae: *Apterostigma* sp., *Camponotus* sp., *Pachycondyla* sp., *Tetramorium* sp., *Pheidole* sp., *Zacryptocerus* sp. e Scelionidae), Coleoptera (Carabidae,; Ptylidae; Pselaphidae e Staphylinidae), Diplopoda (Chelodesmidae; Pyrgodesmidae; Polydesmida), Scutigeromorpha (Scutigeridae: *Sphendononema* sp.), Symphyla (Scutigerellidae: *Hanseniella* sp.)

Dentre os vertebrados foram encontrados três morfótipos pertencentes às ordens Chiroptera (*Glossophaga soricina*), Squamata (Boidae: *Epicrates* sp.) e Anura (Leptodactylidae: *Pristimantis cf. fenestratus*).

Dessa forma, foram encontradas 106 morfoespécies. Dentre essas, três foram consideradas troglóbias, as quais pertencem aos táxons Amblypygi (Charinidae: *Charinus* sp.), Coleoptera (Pselaphidae) e Diplopoda (Pyrgodesmidae). Alguns organismos encontrados nesta caverna, e algumas relações tróficas, são mostrados na Figura 203.

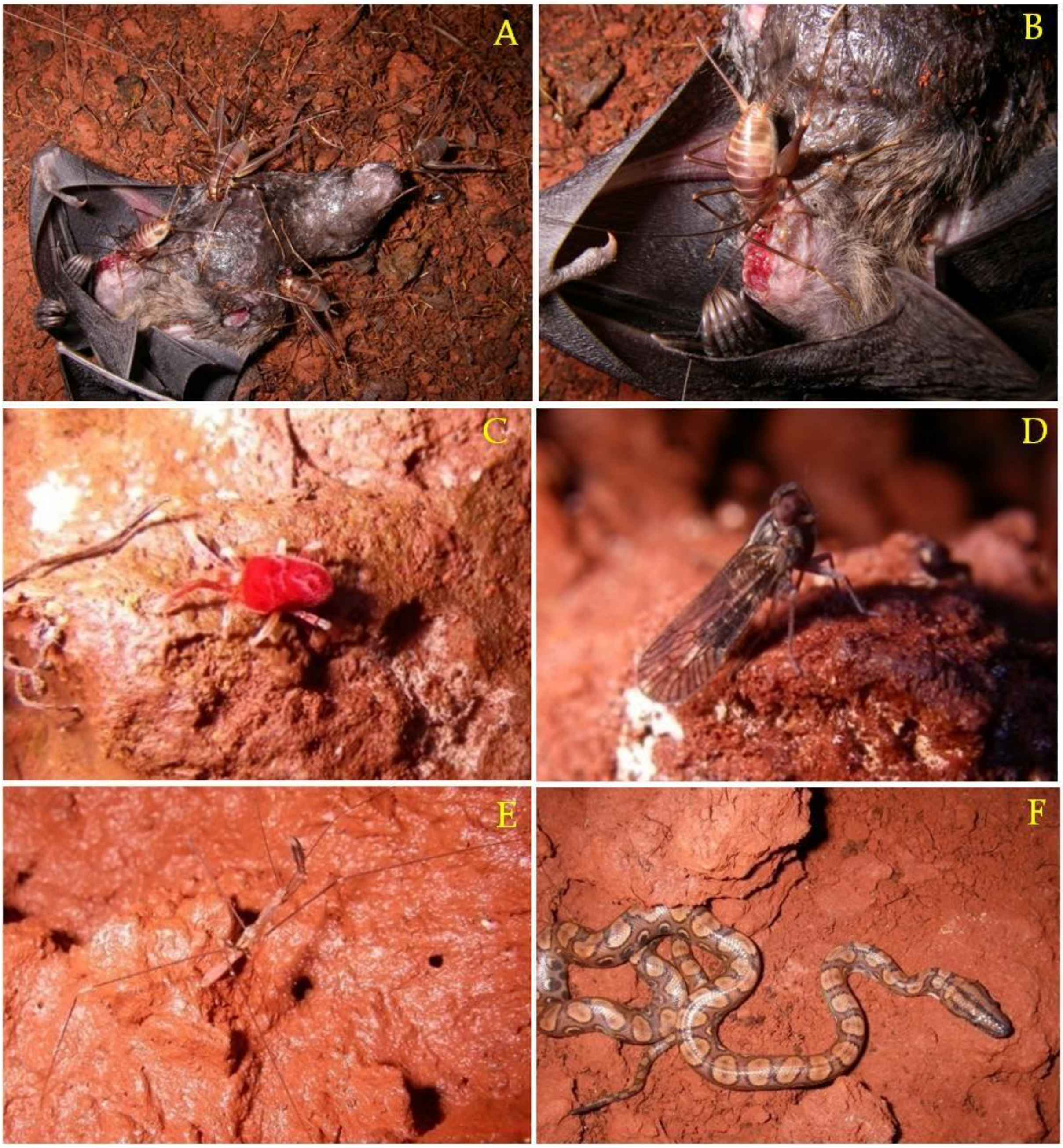

Figura 203 - a) Cadáver de morcego sendo consumido por grilos Phalangopsidae; b) Cadáver de morcego sendo consumido por grilos Phalangopsidae; c) Trombidiforme; d) Homoptera (Kinnaridae); e) Hemiptera (Ploiariidae); f) Boidae (Epicrates sp).

5.4.4.73. SL-076

5.4.4.73.1. Caracterização trófica

Abrigo formado na canga com 15,5 m de projeção horizontal e sem zona afótica. A área de entorno é composta por vegetação densa e sua entrada principal é bem sombreada. Possui duas entradas, sendo uma com piso plano onde a serrapilheira fica restrita à linha d'água e outra com piso descendente que permite a importação de matéria orgânica para o interior da cavidade pela ação da água em períodos de chuva. De forma geral, o piso é predominantemente plano e com baixa umidade, composto por sedimento argiloso em alguns trechos e granulado em outros com inúmeros blocos (seixos e calhaus) esparsos (Figura 204). O sistema de canalículos é pouco desenvolvido e existem poucas raízes de pequeno calibre distribuídas de forma pontual. A cavidade possui uma alta influência das condições ambientais epígeas e as paredes e o teto são revestidos por líquens, briófitas e por colônias de bactérias. Não foram encontrados depósitos de guano e nenhum morcego foi observado. Nenhuma alteração significativa foi observada durante a estação úmida, além das alterações normais na umidade relativa do ar.

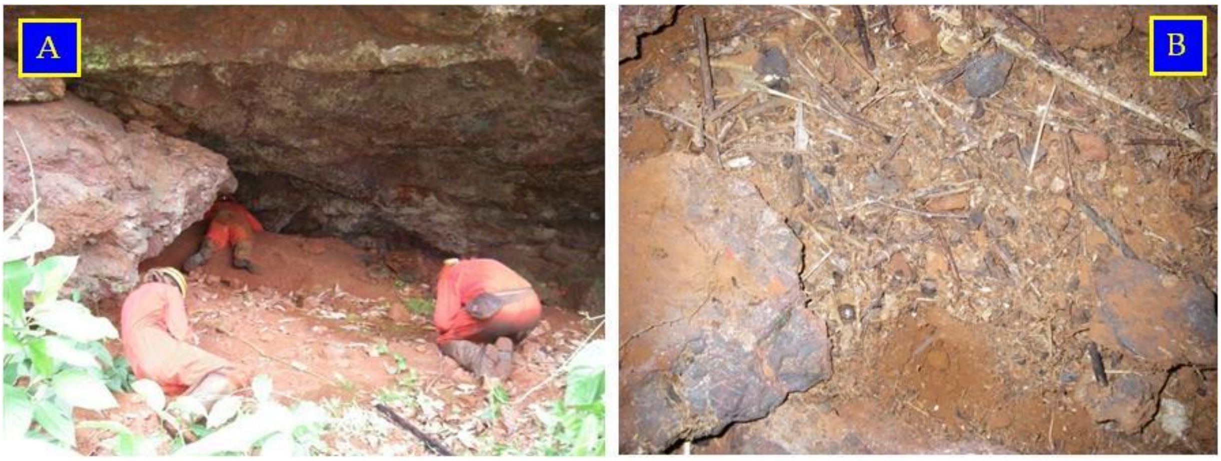

Figura 204 - a) Aspecto geral do salão principal da cavidade com piso composto por sedimento fino com alguns blocos esparsos; c) Depósito de matéria orgânica associado ao conduto descendente.

5.4.4.73.2. Caracterização faunística no período de seca

Foram encontradas na caverna, 59 morfoespécies de invertebrados em pelo menos 48 famílias dos táxons: Acari (Astigmatina), Amplypygi (Phrynidae: *Heterophrynus longicornis*), Pseudoscorpiones (Chernetidae; Chtoniidae), Opiliones (Cosmetidae: *Anduzeia* sp.; Escadabiidae; Manaosbiidae; Sclerosomatidae: *Prionostemma* sp.), Araneae (Araneidae: *Alpaida* sp.; Corinidae: *Tupirina* sp.; Ochyroceratidae; Oonopidae: Oonopinae; Salticidae; Scytodidae*: Scytodes eleonora*; Psauridae; Pholcidae: *Mesabolivar* sp.; Theraphosidae; Theridiidae), Thysanura (Nicolletiidae: Nicolletiinae), Diplura (Campodeidae), Collembola (Entomobryidae), Orthoptera (Phalangopsidae: *Aclodes* sp.; Tetrigidae), Blattodea (Blaberidae: *Blaberus* sp.; Blattidae; Polyphagidae), Isoptera (Termitidae: *Diversitermes* sp),

Dermaptera (Labiidae), Psocoptera (Epipsocidae; Myopsocidae: *Lichenomina* sp.), Hemiptera (Cydnidae; Ochteridae; Reduviidae; Ploiariidae), Diptera (Agromyzidae; Dolichopodidae; Psychodidae: *Lutzomyia* sp.; Tipulidae), Hymenoptera (Formicidae: *Camponotus* sp.; *Pachycondyla* sp.; *Pheidole* sp.; Vespidae), Coleoptera (Pselaphidae; Scydmaenidae), Scolopendromorpha (Scolopocryptopidae: *Newportia* sp.), Scutigeromorpha (Scutigeridae: *Sphendononema* sp), Neuroptera (Myrmeleontidae).

Dentre os vertebrados foram encontrados três morfótipos pertencentes às ordens Chiroptera (*Anoura* sp.), Anura (Leptodactylidae: *Pristimantis cf. fenestratus*) e Squamata (Gekkonidae: *Thecadactylus rapicauda*). Dessa forma, foram encontrados um total de 62 morfoespécies.

5.4.4.73.3. Caracterização faunística no período de chuva

Foram encontradas na caverna, 85 morfoespécies de invertebrados em pelo menos 58 famílias dos táxons: Gastropoda (Systrophiidae), Isopoda (Armadillidae), Acari (Neothyridae: *Diplothyrus schubarti*; Ixodidae: *Amblyomma* sp.; Laelapidae: *Stratiolaelaps* sp.; Macronyssidae; Podocinidae: *Podocinum* sp.; Uropodina; Oribatida; Anystidae: *Erythracarus* sp.; Eupodidae: *Linopodes* sp.; Labdostomatidae: *Labdostomatida* sp.; Rhagidiidae; Trombidiforme), Amblypygi (Charinidae: *Charinus* sp.), Pseudoscorpiones (Chernetidae; Chtoniidae), Opiliones (Cosmetidae: *Anduzeia* sp.; Stygnidae; Phalangiidae), Araneae (Araneidae: *Alpaida* sp.; Corinidae; Ctenidae: *Ctenus* sp.; Gnaphosidae; Linyphiidae; Ochyroceratidae; Oonopidae: Oonopinae; Salticidae; Scytodidae*: Scytodes eleonora*; Psauridae; Pholcidae: *Mesabolivar* sp.; *Metagonia* sp.; Theraphosidae; Theridiidae; Theridiosomatidae), Thysanura (Meinertellidae), Collembola (Entomobryidae; Hypogastruridae; Paronellidae), Orthoptera (Phalangopsidae: *Aclodes* sp., *Phalangopsis* sp.), Psocoptera (Epipsocidae; Lepidopsocidae; Psyllipsocidae: *Psyllipsocus* sp.), Hemiptera (Cydnidae; Reduviidae; Ploiariidae), Lepidoptera (Hesperiidae; Noctuidae: Agaristinae; Tineidae), Diptera (Psychodidae: *Lutzomyia* sp.; Chironomidae), hymenoptera (Formicidae: *Camponotus* sp., *Pachycondyla* sp.), Coleoptera (Carabidae; Elateridae; Pselaphidae; Scydmaenidae; Staphylinidae), Diplopoda (Chelodesmidae; Glomeridesmidae: *Glomeridesmus* sp.), Scutigeromorpha (Scutigeridae: *Sphendononema* sp.), Neuroptera (Myrmeleontidae).

Dentre os vertebrados foi encontrado um morfótipo pertencente à ordem Chiroptera (*Furipterus horrens*).

Dessa forma, foram encontradas 86 morfoespécies. Dentre essas, três foram consideradas troglóbias, as quais pertencem aos táxons Amblypygi (Charinidae: *Charinus* sp.) , Acari (Rhagidiidae) e Gastropoda (Systrophiidae).

5.4.4.73.4. Caracterização geral da fauna da cavidade

Foram encontradas na caverna, 126 morfoespécies de invertebrados em pelo menos 80 famílias dos táxons: Gastropoda (Systrophiidae), Isopoda (Armadillidae), Acari (Neothyridae: *Diplothyrus schubarti*; Ixodidae: *Amblyomma* sp.; Laelapidae: *Stratiolaelaps* sp.; Macronyssidae; Podocinidae: *Podocinum* sp.; Uropodina; Astigmatina; Oribatida; Anystidae: *Erythracarus* sp.; Eupodidae: *Linopodes* sp.; Labdostomatidae: *Labdostomatida* sp.; Rhagidiidae; Trombidiforme), Amblypygi (Phrynidae: *Heterophrynus longicornis*; Charinidae: *Charinus* sp.), Pseudoscorpiones(Chernetidae; Chtoniidae), Opiliones (Cosmetidae: *Anduzeia* sp.; Escadabiidae; Manaosbiidae; Sclerosomatidae: *Prionostemma* sp.; Stygnidae; Phalangiidae), Araneae (Araneidae: *Alpaida* sp.; Corinidae: *Tupirina* sp.; Ctenidae: *Ctenus* sp.; Gnaphosidae; Linyphiidae; Ochyroceratidae; Oonopidae: Oonopinae; Salticidae; Scytodidae: *Scytodes eleonora*; Psauridae; Pholcidae: *Mesabolivar* sp., *Metagonia* sp.; Theraphosidae; Theridiidae; Theridiosomatidae), Thysanura (Nicolletiidae: Nicolletiinae; Meinertellidae), Diplura (Campodeidae), Collembola (Entomobryidae; Hypogastruridae; Paronellidae), Orthoptera (Phalangopsidae: *Aclodes* sp., *Phalangopsis* sp.; Tetrigidae), Blattodea (Blaberidae: *Blaberus* sp.; Blattidae; Polyphagidae), Isoptera (Termitidae: *Diversitermes* sp.), Dermaptera (Labiidae), Psocoptera (Epipsocidae; Lepidopsocidae; Myopsocidae: *Lichenomina* sp.; Psyllipsocidae: *Psyllipsocus* sp.), Hemiptera (Cydnidae; Ochteridae; Reduviidae; Ploiariidae), Lepidoptera (Hesperiidae; Noctuidae: Agaristinae; Tineidae), Diptera (Agromyzidae; Chironomidae; Dolichopodidae; Psychodidae: *Lutzomyia* sp.; Tipulidae), Hymenoptera (Formicidae: *Camponotus* sp., *Pachycondyla* sp., *Pheidole* sp.; Vespidae), Coleoptera (Carabidae; Elateridae: Elaterinae; Pselaphidae; Scydmaenidae; Staphylinidae), Diplopoda (Chelodesmidae; Glomeridesmidae: *Glomeridesmus* sp.), Scolopendromorpha (Scolopocryptopidae: *Newportia* sp.), Scutigeromorpha (Scutigeridae: Sphendononema sp.), Neuroptera (Myrmeleontidae).

Dentre os vertebrados foram encontrados quatro morfótipos pertencentes às ordens Chiroptera (*Furipterus horrens* e *Anoura* sp.), Anura (Leptodactylidae: *Pristimantis cf. fenestratus*) e Squamata (Gekkonidae: *Thecadactylus rapicauda*).

Dessa forma, foram encontrados um total de 130 morfoespécies. Dentre essas, três foram consideradas troglóbias, as quais pertencem aos táxons Amblypygi (Charinidae: *Charinus* sp.), Acari (Rhagidiidae) e Gastropoda (Systrophiidae).

5.4.4.74. SL-077

5.4.4.74.1. Caracterização trófica

Pequeno abrigo formado na canga com 8 m de projeção horizontal e localizada em um pequeno fragmento de mata inserido dentro de uma matriz composta por savana metalófila.

A cavidade não apresenta zona afótica e seu piso é predominantemente plano e composto por sedimento fino em alguns trechos e granulado em outros com alguns blocos grandes (calhaus e matacões) esparsos cobertos por muitos líquens e briófitas. A entrada é ampla e sombreada, com grandes acúmulos de serrapilheira próximos à linha d'água (Figura 205). Nesta região, existem ainda algumas plântulas de angiospermas. O sistema de canalículos é pouco desenvolvido com poucos microorganismos associados às paredes e ao teto. Existem ainda pequenos depósitos de guano de morcegos frugívoros próximos à zona de entrada e ao fundo da cavidade, além de um sistema radicular muito desenvolvido com muitas raízes de fino calibre chegando a formar pequenos rizotemas em locais onde existem gotejamentos ativos mesmo durante a estação seca. Durante a estação úmida a caverna apresentava maior umidade com alguns pontos de gotejamento ativos e com zonas de percolação.

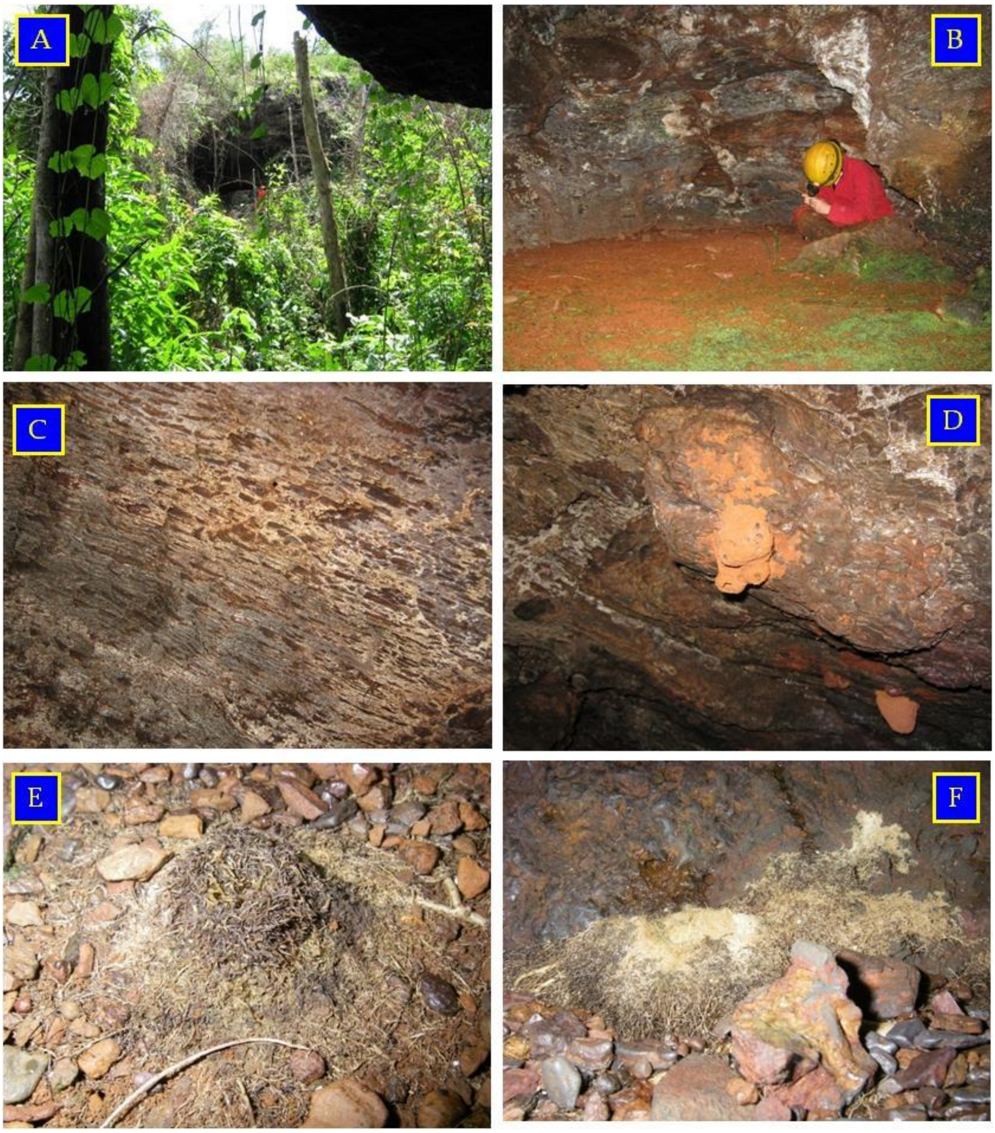

Figura 205 - . a) Vista da área externa onde observa-se a entrada da cavidade e a vegetação epígea associada; b) galeria principal da cavidade; c) Actinomicetos se desenvolvendo em profusão nas paredes; d) Ninhos de vespas presentes no interior da caverna; e) Rizotema encontrado associado a um ponto de gotejamento ativo; f) detalhe de agregações de raízes no piso da caverna.

5.4.4.74.2. Caracterização faunística no período de seca

Foram encontradas na caverna, 60 morfoespécies de invertebrados em pelo menos 44 famílias dos táxons: Isopoda (Armadillidae; Philosciidae), Acari (Laelapidae: *Stratiolaelaps* sp.; Oribatida; Eupodidae: *Linopodes* sp.; Labdostomatidae: *Labdostomatida* sp.; Trombidiforme), Amblypygi (Phrynidae: *Heterophrynus longicornis*), Pseudoscorpiones (Chernetidae; Chtoniidae), Opiliones (Cosmetidae: *Anduzeia* sp.; Manaosbiidae), Araneae (Araneidae*: Alpaida* sp.; Corinidae: *Tupirina* sp.; Filistatidae; Gnaphosidae; Oonopidae: Oonopinae, *Coxapopha* sp.; Salticidae; Psauridae; Pholcidae: *Mesabolivar* sp.), Thysanura (Nicolletiidae: Nicolletiinae; Atelurinae), Diplura (Campodeidae), Collembola (Entomobryidae; Paronellidae), Orthoptera (Phalangopsidae: *Aclodes* sp.), Blattodea (Blaberidae: *Blaberus* sp.), Isoptera (Termitidae: *Nasutitermes* sp.), Hemiptera (Cydnidae; Reduviidae), Lepidoptera (Tineidae), Diptera (Culicidae; Dolichopodidae; Empididae; Psychodidae: *Lutzomyia* sp.; Tipulidae), Hymenoptera (Formicidae: *Camponotus* sp., *Dolichoderus* sp., Myrmicinae, *Pachycondyla* sp., *Pseudomyrmex* sp., *Pheidole* sp., *Rogeria* sp., *Solenopsis* sp.), Coleoptera (Carabidae; Elateridae; Staphylinidae), Geophilomorpha (Ballophilidae: *Ballophililus* sp.), Scolopendromorpha (Cryptopidae: *Cryptops* sp.), Scutigeromorpha (Scutigeridae: *Sphendononema* sp.)

Dentre os vertebrados foram encontrados cinco morfótipos pertencentes às ordens Chiroptera (*Peropteryx kappleri* e *Anoura* sp.), Anura (Leptodactylidae: *Pristimantis cf. fenestratus* e *Eleutherodactylus* sp.) e Squamata (Gekkonidae: *Thecadactylus rapicauda*). Dessa forma, foram encontradas 65 morfoespécies. Dentre essas, uma foi considerada troglóbia, a qual pertence ao táxon Araneae (Oonopidae: *Coxapopha* sp.)

5.4.4.74.3. Caracterização faunística no período de chuva

Foram encontradas na caverna, 60 morfoespécies de invertebrados em pelo menos 43 famílias dos táxons: Oligochaeta, Gastropoda (Systrophiidae), Isopoda (Armadillidae; Philosciidae), Acari (Laelapidae: *Stratiolaelaps* sp.; Uropodina; Oribatida; Eupodidae: *Linopodes* sp.; Trombidiforme), Amblypygi (Phrynidae: *Heterophrynus longicornis*; Charinidae: *Charinus* sp.), Ricinulei (Ricinoididae: *Cryptocellus tarsilae*), Pseudoscorpiones (Chernetidae; Chtoniidae), Opiliones (Stygnidae: *Protimesius aff. gracilis*; Phalangiidae), Araneae (Dipluridae; Filistatidae; Gnaphosidae; Oonopidae: *Coxapopha* sp.; Salticidae; Psauridae; Pholcidae: *Mesabolivar* sp., *Metagonia* sp.; Theridiidae; Theridiosomatidae), Thysanura (Meinertellidae), Diplura (Anajapygidae; Campodeidae), Collembola (Entomobryidae; Isotomidae), Orthoptera (Phalangopsidae: *Aclodes* sp., *Phalangopsis* sp.), Blattodea (Blaberidae: *Blaberus* sp.), Isoptera (Termitidae: *Nasutitermes* sp.), Psocoptera, Hemiptera (Cydnidae), Lepidoptera (Noctuoidea; Tineidae), Diptera (Ceratopogonidae; Chironomidae; Chloropidae; Psychodidae: *Lutzomyia* sp.; Sciaridae), Hymenoptera

(Formicidae: *Apterostigma* sp., *Dolichoderus* sp., *Pachycondyla* sp., *Prionopeuta* sp., *Strumigenys* sp., *Tapinoma* sp.), Coleoptera (Carabidae; Scydmaenidae; Staphylinidae).

Dentre os vertebrados foram encontrados dois morfótipos pertencentes à ordem Anura (Leptodactylidae: *Pristimantis cf. fenestratus* e Dendrobatidae)

Dessa forma, foram encontrados um total de 62 morfoespécies. Dentre essas, três foram consideradas troglóbias, as quais pertencem aos táxons Gastropoda (Systrophiidae), Amblypygi (Charinidae: *Charinus* sp.), Araneae (Oonopidae: *Coxapopha* sp.), Coleoptera (Scydmaenidae).

5.4.4.74.4. Caracterização geral da fauna da cavidade

Foram encontradas na caverna, 101 morfoespécies de invertebrados em pelo menos 62 famílias dos táxons: Oligochaeta, Gastropoda (Systrophiidae), Isopoda (Armadillidae; Philosciidae), Acari (Laelapidae: *Stratiolaelaps* sp.; Uropodina; Oribatida; Eupodidae: *Linopodes* sp.; Labdostomatidae: Labdostomatida sp.; Trombidiforme), Amblypygi (Phrynidae: *Heterophrynus longicornis*; Charinidae: *Charinus* sp.), Ricinulei (Ricinoididae: *Cryptocellus tarsilae*), Pseudoscorpiones (Chernetidae; Chtoniidae), Opiliones (Cosmetidae: *Anduzeia* sp.; Manaosbiidae; Stygnidae: *Protimesius aff. gracilis;* Phalangiidae), Araneae (Araneidae: *Alpaida* sp.; Corinidae: *Tupirina* sp.; Dipluridae; Filistatidae; Gnaphosidae; Oonopidae: Oonopinae, *Coxapopha* sp.; Salticidae; Psauridae; Pholcidae: *Mesabolivar* sp., *Metagonia* sp.; Theridiidae; Theridiosomatidae), Thysanura (Nicolletiidae: Nicolletiinae; Atelurinae; Meinertellidae), Diplura (Anajapygidae; Campodeidae), Collembola (Entomobryidae; Isotomidae; Paronellidae), Orthoptera (Phalangopsidae: *Aclodes* sp., *Phalangopsis* sp.), Blattodea (Blaberidae: *Blaberus* sp.), Isoptera (Termitidae: *Nasutitermes* sp.), Psocoptera; Hemiptera (Cydnidae; Reduviidae), Lepidoptera (Noctuoidea; Tineidae), Diptera (Ceratopogonidae; Chironomidae; Chloropidae; Culicidae; Dolichopodidae; Empididae; Psychodidae: *Lutzomyia* sp.; Tipulidae; Sciaridae), Hymenoptera (Formicidae: *Apterostigma* sp., *Camponotus* sp., *Dolichoderus* sp., Myrmicinae, *Pachycondyla* sp., *Pseudomyrmex* sp., *Pheidole* sp., *Prionopeuta* sp., *Rogeria* sp., *Solenopsis* sp., *Strumigenys* sp., *Tapinoma* sp.), Coleoptera (Carabidae; Elateridae; Scydmaenidae; Staphylinidae), Geophilomorpha (Ballophilidae: *Ballophililus* sp.), Scolopendromorpha (Cryptopidae: *Cryptops* sp.), Scutigeromorpha (Scutigeridae: *Sphendononema* sp.)

Dentre os vertebrados foram encontrados seis morfótipos pertencentes às ordens Chiroptera (*Peropteryx kappleri*, *Anoura* sp.), Anura (Leptodactylidae: *Pristimantis cf. fenestratus, Eleutherodactylus* sp.; Dendrobatidae), Squamata (Gekkonidae: *Thecadactylus rapicauda*)

Dessa forma, foram encontrados um total de 107 morfoespécies. Dentre essas, três foram consideradas troglóbias, as quais pertencem aos táxons Gastropoda (Systrophiidae), Amblypygi (Charinidae: *Charinus* sp.), Araneae (Oonopidae: *Coxapopha* sp.), Coleoptera (Scydmaenidae). Alguns organismos encontrados nesta caverna são mostrados na Figura 206.

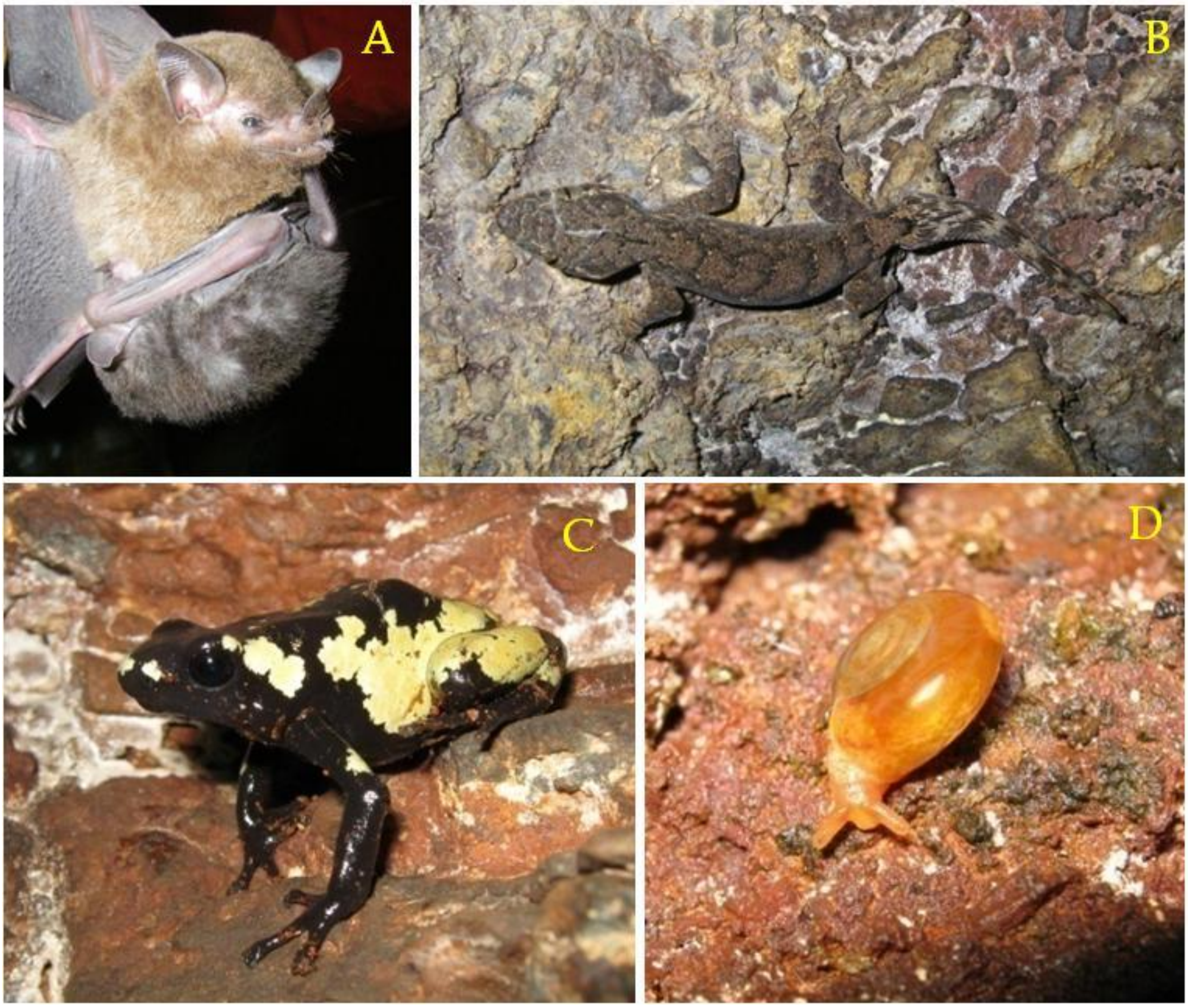

Figura 206 - a) Phyllostomidae (*Anoura sp.*) com filhotes; b) Lagarto Gekkonidae (*Thecadactylus rapicauda*); c) Anura (Dendrobatidae); d) Gastropoda (Systrophiidae).

5.4.4.75. SL-078

5.4.4.75.1. Caracterização trófica

Abrigo formado na canga com 13,5 m de projeção horizontal inserido próximo a um fragmento de mata no topo de encosta. Entrada ampla, muito iluminada e com piso ascendente o que dificulta a importação de matéria orgânica pela ação da água em períodos de chuva. Na entrada, existem líquens e briófitas nas paredes, muitas lianas e cipós e pouca serrapilheira. Basicamente a cavidade apresenta dois setores muito distintos, sendo o

primeiro correspondente à zona de entrada, eufótica, e onde ocorre uma forte influência das condições ambientais epígeas. Nesta área existe um grande depósito de guano (ressecado e envelhecido) de morcegos insetívoros, além de alguns cadáveres de morcegos em avançado estado de decomposição. O segundo setor corresponde a um pequeno salão muito confinado acessado após um trecho de rastejo completamente afótico. Neste salão, a temperatura é elevada e a umidade é alta em virtude de uma pequena drenagem ativa mesmo durante a estação seca. Nesta área, existe uma grande colônia de morcegos insetívoros (*Pteronotus sp*.) que produz uma considerável quantidade de guano. Apesar da disponibilidade de recurso, este guano encontra-se encharcado pela pequena drenagem, sendo lixiviado lentamente para o ambiente epígeo. O piso da cavidade é composto, em grande parte, pela própria rocha matriz e por sedimento clástico refinado depositado em algumas regiões da cavidade. Existem muitas raízes de diferentes calibres distribuídas de forma superficial pelo piso, paredes e teto e o sistema de canalículos é pouco desenvolvido (Figura 210). Durante a estação úmida o salão com guano encontrava-se com muitos pontos de gotejamento e percolação resultando em uma maior lixiviação deste recurso para o ambiente epígeo. Além disso, cadáveres recentes de morcegos insetívoros (*Pteronotus sp*.) foram encontrados durante esta campanha, o que indica que este pode ser um recurso alimentar constante neste sistema (Figura 207).

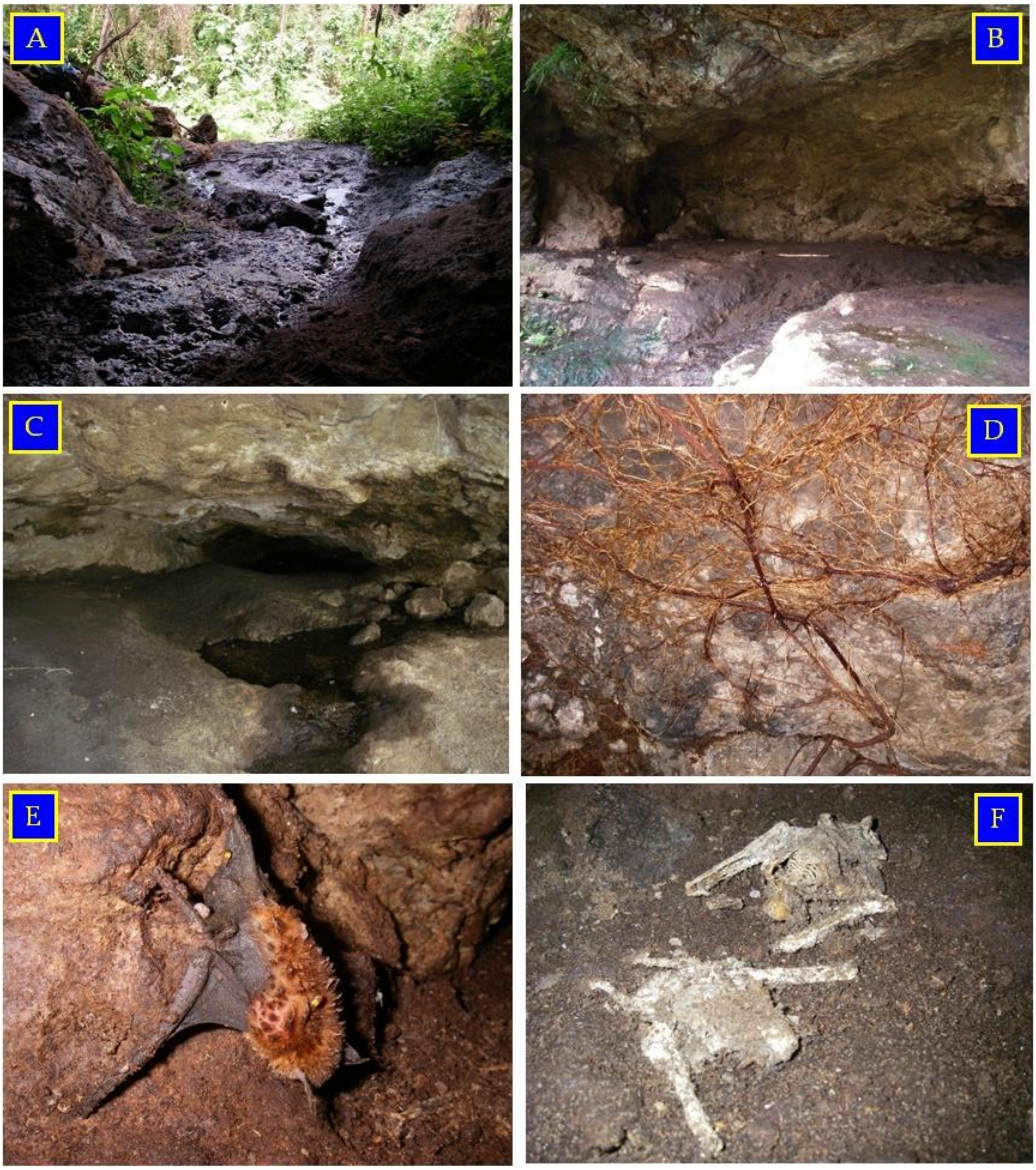

Figura 207 - a) Vista interna da entrada da cavidade com detalhes da vegetação epígea; b) Vista o salão da entrada da cavidade; c) Pequena drenagem ativa presente na cavidade; d) Sistema radicular bem desenvolvido associado ao teto da cavidade; e) Cadáver recente de morcego insetívoro (*Pteronotus sp*.) encontrado durante a estação úmida; f) Cadáveres de morcegos em avançado estado de decomposição.

5.4.4.75.2. Caracterização faunística no período de seca

Foram encontradas na caverna, 23 morfoespécies de invertebrados em pelo menos 20 famílias dos táxons: Acari (Laelapidae: *Stratiolaelaps* sp.), Araneae (Araneidae: *Alpaida* sp.; Filistatidae; Salticidae; Scytodidae*: Scytodes eleonora*; Theridiidae), Thysanura (Nicolletiidae: Nicolletiinae), Isoptera (Termitidae: *Nasutitermes* sp.), Hemiptera (Dipsocoridae),

Homoptera (Cixidae: Cixius sp.), Lepidoptera (Tineidae; Zygaenidae), Diptera (Dolichopodidae; Drosophilidae: *Drosophila* sp.; Psychodidae), Hymenoptera (Formicidae: *Azteca* sp., *Camponotus* sp., *Pachycondyla* sp., *Pheidole* sp.; Braconidae), Coleoptera (Curculionidae: Scotylinae), Lepidoptera (Tineidae).

Dentre os vertebrados foi encontrado um morfótipo pertencente à ordem Chiroptera (Mormopidae: *Pteronotus* sp.). Dessa forma, foram encontrados um total de 24 morfoespécies.

5.4.4.75.3. Caracterização faunística no período de chuva

Foram encontradas na caverna, 28 morfoespécies de invertebrados em pelo menos 22 famílias dos táxons: Acari (Argasidae: *Carios rondoniensis*, *Ornithodoros marinkellei;* Otopheidomenidae; Mesostigmata), Araneae (Gnaphosidae; Linyphiidae; Salticidae; Scytodidae*: Scytodes eleonora*; Pholcidae: *Metagonia* sp.; Theridiidae), Orthoptera (Phalangopsidae: *Aclodes* sp.), Lepidoptera (Noctuidae; Tineidae), Diptera (Ceratopogonidae; Milichiidae; Psychodidae: *Lutzomyia* sp.), Hymenoptera (Formicidae: *Pheidole* sp.; Apidae; Braconidae), Coleoptera (Leiodidae; Dermestidae).

Dentre os vertebrados foram encontrados dois morfótipos pertencentes às ordens Chiroptera (*Pteronotus* sp.) e Squamata (Sphaerodactylidae: *Coleodactylus cf. amazonicus).* Dessa forma, foram encontradas 30 morfoespécies.

5.4.4.75.4. Caracterização geral da fauna da cavidade

Foram encontradas na caverna, 46 morfoespécies de invertebrados em pelo menos 32 famílias dos táxons: Acari (Argasidae: *Carios rondoniensis*, *Ornithodoros marinkellei;* Laelapidae: *Stratiolaelaps* sp.; Otopheidomenidae; Mesostigmata), Araneae (Anapidae; Araneidae: *Alpaida* sp.; Filistatidae; Gnaphosidae; Linyphiidae; Salticidae; Scytodidae*: Scytodes eleonora*; Pholcidae: *Metagonia* sp.; Theridiidae), Thysanura (Nicolletiidae: Nicolletiinae), Orthoptera (Phalangopsidae: *Aclodes* sp.); Isoptera (Termitidae: *Nasutitermes* sp.), Hemiptera (Dipsocoridae), Homoptera (Cixidae: *Cixius* sp.), Lepidoptera (Noctuidae; Tineidae; Zygaenidae), Diptera (Ceratopogonidae; Dolichopodidae; Drosophilidae: *Drosophila* sp.; Milichiidae; Psychodidae: *Lutzomyia* sp.), Hymenoptera (Formicidae: *Azteca* sp., *Camponotus* sp., *Pachycondyla* sp., *Pheidole* sp.; Apidae; Braconidae), Coleoptera (Curculionidae: Scotylinae; Leiodidae; Dermestidae).

Dentre os vertebrados foram encontrados dois morfótipos pertencentes às ordens Chiroptera (*Pteronotus* sp.) e Squamata (Sphaerodactylidae: *Coleodactylus cf. amazonicus).* Dessa forma, foram encontradas 48 morfoespécies.

5.4.4.76. SL-079

5.4.4.76.1. Caracterização trófica

Caverna inserida na formação ferrífera com 60 m de projeção horizontal localizada em uma grande depressão em área de encosta. A matriz de entorno é composta por savana metalófila. No entanto, junto à entrada da cavidade, existe o predomínio de uma vegetação arbórea com árvores de baixa estatura. Sua entrada é muito ampla, sombreada, com piso ascendente e grandes blocos abatidos. Nas paredes, piso e teto existe uma vegetação muito desenvolvida com muitos líquens, briófitas, pteridófitas e uma área com grande quantidade de plântulas de melastomatáceas (Figura 208). A serrapilheira encontra-se restrita à entrada uma vez que a condição ascendente do piso impossibilita a importação de recursos orgânicos pela água da chuva e não existem clarabóias na cavidade. De forma geral, o piso é irregular e seco em quase toda sua área e composto por sedimento fino com grandes blocos abatidos. A cavidade apresenta uma morfologia retilínea seguida por dois condutos bifurcados em sua porção mais distal. O primeiro conduto à esquerda é seco, disfótico e coberto por guano velho de morcegos insetívoros, que chega a atingir 20 cm de profundidade em alguns pontos. O segundo conduto à direita é mais confinado, sendo acessado após um trecho de rastejo, sendo este completamente afótico com umidade elevada em função da existência de pontos de gotejamento e de percolação, mesmo durante a estação seca. Neste conduto, o piso é coberto por guano velho de morcegos insetívoros em sua porção inicial e de morcegos frugívoro recente na sua zona mais profunda, chegando a formar pequenas guanomites. Este último depósito é produzido por uma grande colônia de morcegos Glossophaginae. Ainda em relação à quiropterofauna também foram observados cinco exemplares de morcegos carnívoros no conduto mais confinado, mas não foram observados depósitos de fezes destes no interior da cavidade. A temperatura na zona mais profunda desta cavidade é elevada existindo pouca ventilação. O sistema de canalículos é pouco desenvolvido e existem poucas raízes distribuídas de maneira esparsa em algumas áreas da cavidade. Nenhuma alteração significativa foi observada durante a estação úmida, além das alterações normais na umidade relativa do ar. Dentre os recursos orgânicos, neste período foram encontrados algumas fezes recentes e antigas de anfíbios (Figura 208).

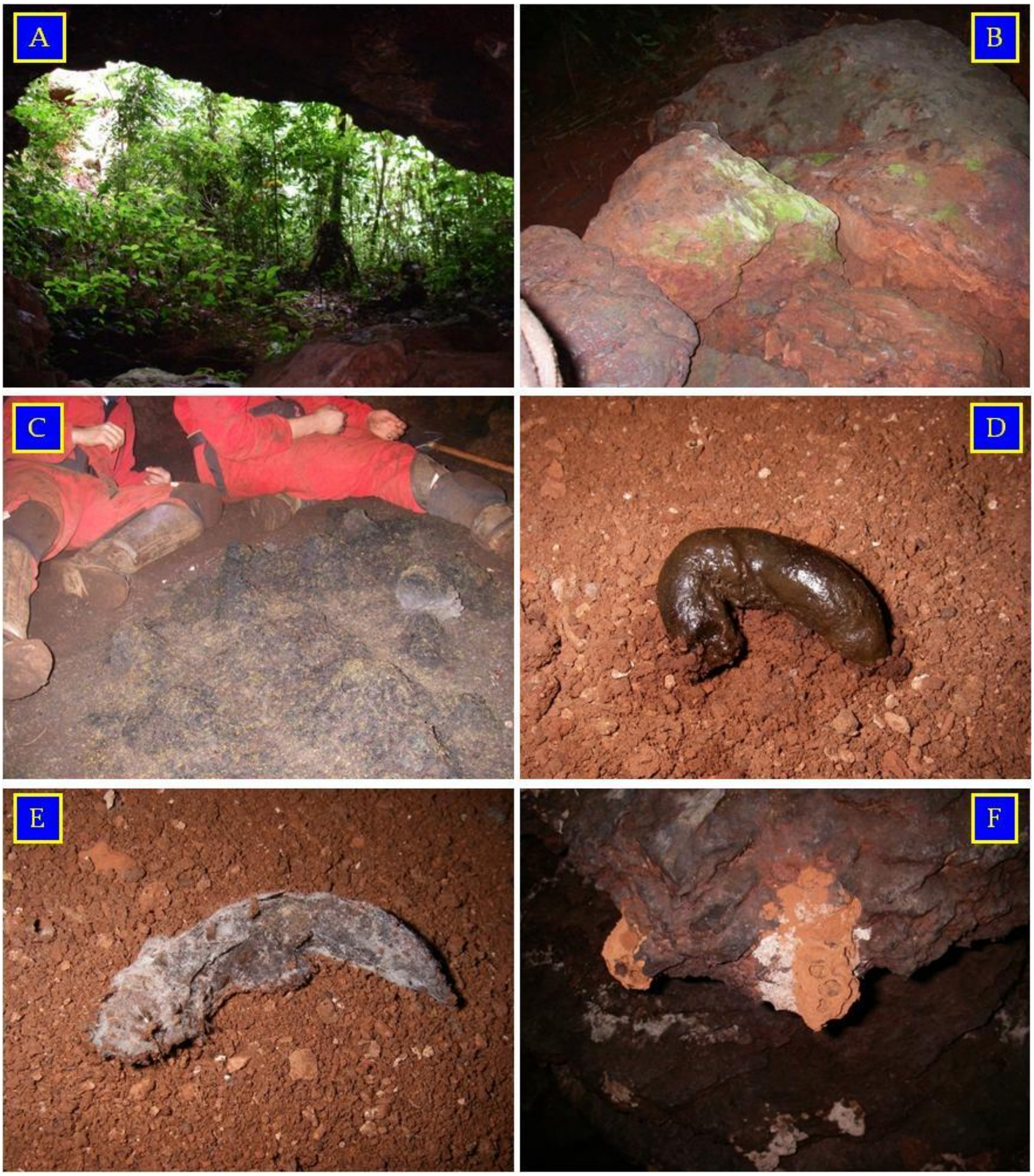

Figura 208 - Vista interna da entrada da cavidade com detalhes da vegetação epígea associada; b) Musgos associados à zona de entrada da cavidade; c) Depósito de guano de morcegos frugívoros; d) "Pellet" de fezes frescas de um anfíbio; e) "Pellet" de fezes antigas de um anfíbio, onde se percebe o crescimento fúngico; f) Ninhos abandonados de vespas nas paredes da caverna.

5.4.4.76.2. Caracterização faunística no período de seca

Foram encontradas na caverna, 71 morfoespécies de invertebrados em pelo menos 53 famílias dos táxons: Isopoda (Dubioniscidae), Acari (Argasidae: *Ornithodoros* sp.; Laelapidae: *Stratiolaelaps* sp.; Macrochelidae: *Macrocheles* sp.; Macronyssidae; Uropodina; Astigmatina; Oribatida), Amblypygi (Phrynidae: *Heterophrynus longicornis*), Pseudoscorpiones (Chernetidae; Chtoniidae), Opiliones (Escadabiidae), Araneae (Filistatidae; Ochyroceratidae; Oonopidae: Oonopinae; Salticidae; Scytodidae*: Scytodes eleonora*; Psauridae; Pholcidae: *Mesabolivar* sp.; Theridiidae; Theridiosomatidae), Thysanura (Nicolletiidae: Nicolletiinae), Collembola (Sminthuridae; Cyphoderidae; Entomobryidae), Orthoptera (Phalangopsidae: *Aclodes* sp.), Blattodea (Blaberidae: *Blaberus* sp.), Isoptera (Termitidae: *Nsutitermes* sp., *Velocitermes* sp.), Dermaptera (Labiidae), Psocoptera (Epipsocidae; Pachytroctidae), Hemiptera (Cydnidae; Reduviidae), Homoptera (Cixidae: *Cixius* sp.), Lepidoptera (Noctuidae; Tineidae), Diptera (Ceratopogonidae; Culicidae; Dolichopodidae; Drosophilidae; Fanniidae; Milichiidae; Muscidae; Psychodidae: *Lutzomyia* sp.), Hymenoptera (Formicidae: *Apterostigma* sp., *Camponotus* sp., *Gnamptogenys* sp., *Pachycondyla* sp., *Solenopsis* sp.; Bethylidae; Diapriidae; Pompilidae), Coleoptera (Carabidae; Hysteridae; Staphylinidae; Tenebrionidae: Coelometropinae), Diplopoda (Chelodesmidae), Neuroptera (Myrmeleontidae).

Dentre os vertebrados foram encontrados três morfótipos pertencentes às ordens Chiroptera (Phyllostomidae: *Glossophaga soricina*, *Trachops cirrhosus*) e Anura (Leptodactylidae: *Pristimantis cf. fenestratus*).

Dessa forma, foram encontrados um total de 74 morfoespécies. Dentre essas, uma foi considerada troglóbia, a qual pertence ao táxon Collembola (Cyphoderidae).

5.4.4.76.3. Caracterização faunística no período de chuva

Foram encontradas na caverna, 75 morfoespécies de invertebrados em pelo menos 51 famílias dos táxons: Isopoda (Armadillidae; Dubioniscidae; Philosciidae; Scleropactidae), Acari (Argasidae: *Ornithodoros* sp.; Laelapidae: *Stratiolaelaps* sp.; Macrochelidae: *Macrocheles* sp.; Macronyssidae; Oribatida; Trombidiforme), Amblypygi (Phrynidae: *Heterophrynus longicornis*), Pseudoscorpiones (Chernetidae), Araneae (Araneidae: *Alpaida* sp.; Corinidae: *Tupirina* sp.; Ctenidae; Gnaphosidae; Ochyroceratidae; Oonopidae: Oonopinae; Salticidae; Scytodidae*: Scytodes eleonora*; Psauridae; Pholcidae: *Mesabolivar* sp., *Metagonia* sp.; Theridiidae; Theridiosomatidae), Thysanura (Nicolletiidae: Nicolletiinae), Collembola (Sminthuridae; Entomobryidae), Orthoptera (Phalangopsidae: *Aclodes* sp., *Phalangopsis* sp.), Blattodea (Blaberidae: *Blaberus* sp.), Isoptera (Termitidae: *Nasutitermes* sp., *Velocitermes* sp.), Psocoptera (Archipsocidae; Pachytroctidae), Hemiptera (Cydnidae), Lepidoptera (Noctuidae; Tineidae), Diptera (Cecidomyiidae; Ceratopogonidae;

Chironomidae; Drosophilidae; Milichiidae; Psychodidae: *Lutzomyia* sp.; Streblidae; Tipulidae), Hymenoptera (Formicidae: *Apterostigma* sp., *Dolichoderus* sp., *Gnamptogenys* sp.; Diapriidae), Coleoptera (Carabidae; Chrysomelidae; Hysteridae; Scydmaenidae; Staphylinidae).

Dentre os vertebrados foram encontrados três morfótipos pertencentes às ordens Chiroptera (Phyllostomidae: *Glossophaga soricina*), Squamata (Boidae: *Epicrates* sp.) e Anura (Bufonidae). Dessa forma, foram encontradas 78 morfoespécies.

5.4.4.76.4. Caracterização geral da fauna da cavidade

Foram encontradas na caverna, 117 morfoespécies de invertebrados em pelo menos 68 famílias dos táxons: Isopoda (Armadillidae; Dubioniscidae; Philosciidae; Scleropactidae), Acari (Argasidae: *Ornithodoros* sp.; Laelapidae: *Stratiolaelaps* sp.; Macrochelidae: *Macrocheles* sp.; Macronyssidae; Uropodina; Astigmatina; Oribatida; Trombidiforme), Amblypygi (Phrynidae: *Heterophrynus longicornis*), Pseudoscorpiones (Chernetidae; Chtoniidae), Opiliones (Escadabiidae), Araneae (Araneidae: *Alpaida* sp.; Corinidae: *Tupirina* sp.; Ctenidae; Filistatidae; Gnaphosidae; Ochyroceratidae; Oonopidae: Oonopinae; Salticidae; Scytodidae*: Scytodes eleonora*; Psauridae; Pholcidae: *Mesabolivar* sp., *Metagonia* sp.; Theridiidae; Theridiosomatidae), Thysanura (Nicolletiidae: Nicolletiinae), Collembola (Sminthuridae; Cyphoderidae; Entomobryidae), Orthoptera (Phalangopsidae: *Aclodes* sp., *Phalangopsis* sp.), Blattodea (Blaberidae: *Blaberus* sp.), Isoptera (Termitidae: *Nasutitermes* sp., *Velocitermes* sp.), Dermaptera (Labiidae), Psocoptera (Archipsocidae; Epipsocidae; Pachytroctidae), Hemiptera (Cydnidae; Reduviidae), Homoptera (Cixidae: *Cixius* sp.), Lepidoptera (Noctuidae; Tineidae), Diptera (Cecidomyiidae; Ceratopogonidae; Chironomidae; Culicidae; Dolichopodidae; Drosophilidae; Fanniidae; Milichiidae; Muscidae; Psychodidae: *Lutzomyia* sp.; Streblidae; Tipulidae), Hymenoptera (Formicidae: *Apterostigma* sp., *Camponotus* sp., *Dolichoderus* sp., *Gnamptogenys* sp., *Pachycondyla* sp., *Solenopsis* sp.; Bethylidae; Diapriidae; Pompilidae), Coleoptera (Carabidae; Chrysomelidae; Hysteridae; Scydmaenidae; Staphylinidae; Tenebrionidae: Coelometropinae); Diplopoda (Chelodesmidae), Neuroptera (Myrmeleontidae).

Dentre os vertebrados foram encontrados cinco morfótipos pertencentes às ordens Chiroptera (Phyllostomidae: *Glossophaga soricina* e *Trachops cirrhosus*), Squamata (Boidae: *Epicrates* sp.) e Anura (Bufonidae; Leptodactylidae: *Pristimantis cf. fenestratus*).

Dessa forma, foram encontradas 122 morfoespécies. Dentre essas, uma foi considerada troglóbia, a qual pertence ao táxon Collembola (Cyphoderidae).

Alguns organismos encontrados nesta caverna são mostrados na Figura 209.

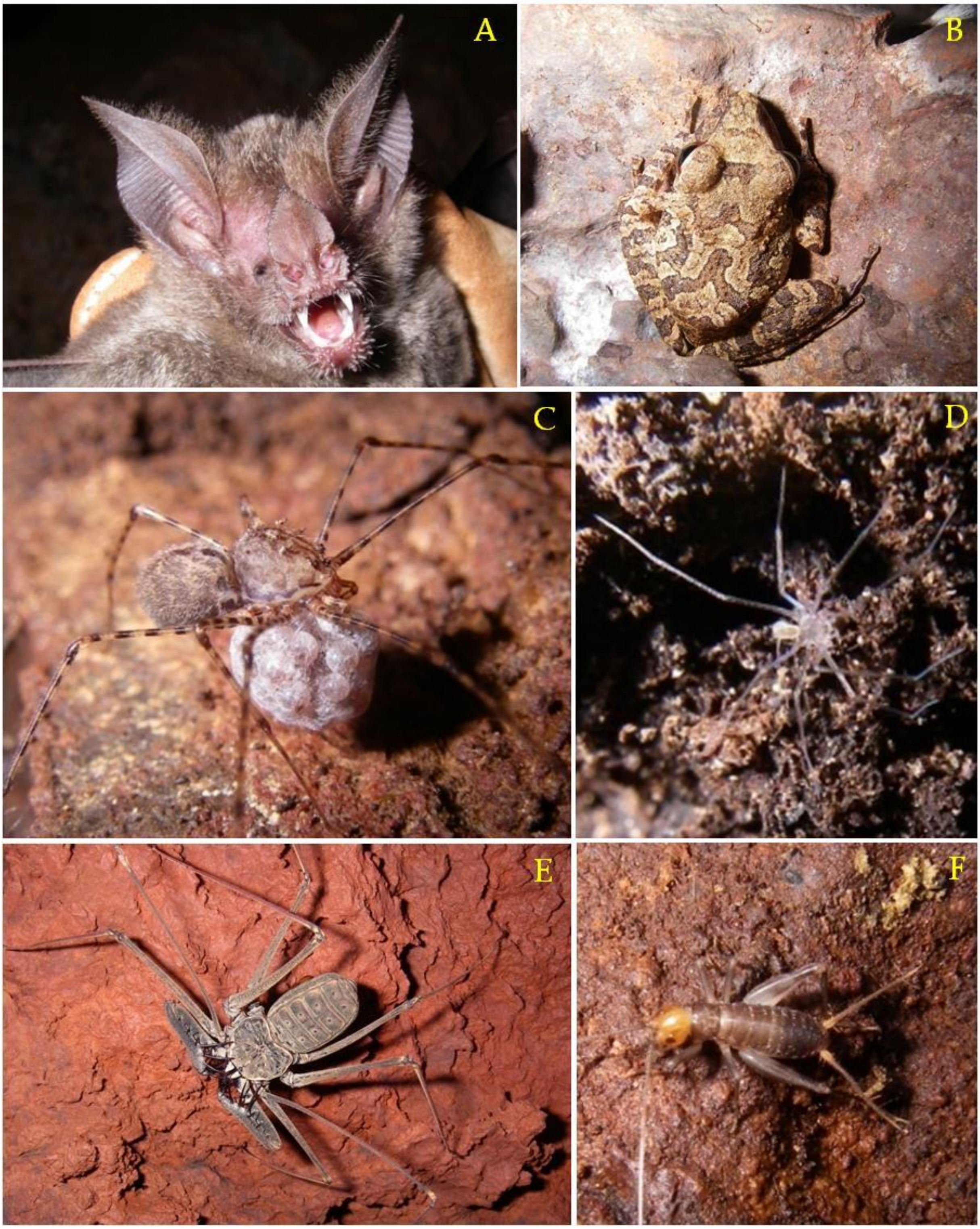

Figura 209 - a) Phyllostomidae (*Trachops cirrhosus*); b) Anura (Leptodactylidae: *Pristimantis cf. fenestratus*); c) Araneae (Scytodidae*: Scytodes eleonora*); d) Araneae (Pholcidae); e) Amblypygi (*Heterophrynus longicornis*); f) Orthoptera (Gryllidae).

5.4.4.77. SL-080

5.4.4.77.1. Caracterização trófica

Abrigo formado na canga com 18,5 m de projeção horizontal localizado em uma grande depressão em área de encosta. A matriz de entorno é composta por savana metalófila, mas junto à entrada da cavidade, existe o predomínio de uma vegetação arbórea com árvores de baixa estatura. Sua entrada é muito ampla, sombreada, com piso descendente e com grandes blocos abatidos. Nesta existem muitos líquens e briófitas associados ao piso e paredes (Figura 210). Na lateral direita da cavidade existe uma passagem em rastejo que leva um pequeno salão com iluminação disfótica onde a umidade do solo é maior e existem muitas raízes e pequenos depósitos de guano de morcegos frugívoros com muitas plântulas germinadas a partir de sementes presentes no mesmo. De forma geral, o piso da cavidade é plano (exceto na entrada) e seco, sendo composto por sedimento clástico e por alguns grandes blocos (calhaus e matacões) abatidos. O sistema de canalículos é pouco desenvolvido e as paredes e teto são revestidos por Actinomicetos, sendo estas mais abundantes no salão mais confinado. Durante a estação úmida havia alguns pequenos pontos de gotejamento ativos.

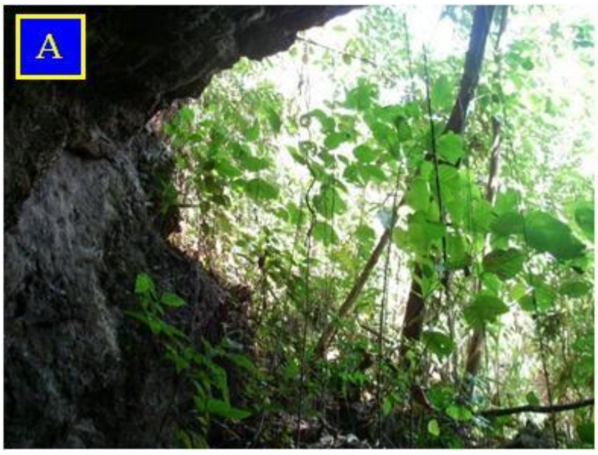

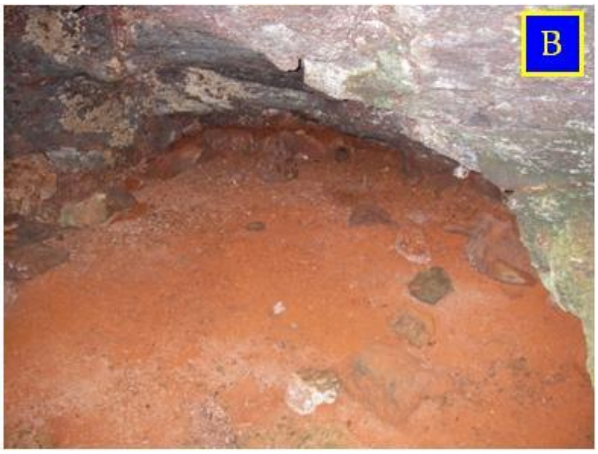

Figura 210 - a) Vista interna da entrada da cavidade com detalhes da vegetação epígea associada; b) Salão principal da caverna onde o ambiente é um pouco mais confinado.

5.4.4.77.2. Caracterização faunística no período de seca

Foram encontradas na caverna, 41 morfoespécies de invertebrados em pelo menos 31 famílias dos táxons: Gastropoda (Subulinidae), Isopoda (Armadillidae; Philosciidae), Acari (Macronyssidae; Trombidiforme), Amblypygi (Phrynidae: *Heterophrynus longicornis),* Pseudoscorpiones (Chernetidae; Chtoniidae), Araneae (Araneidae: *Alpaida* sp.; Corinidae: *Tupirina* sp.; Salticidae; Pholcidae: *Mesabolivar* sp.; Theridiidae), Collembola (Cyphoderidae; Entomobryidae), Orthoptera (Phalangopsidae: *Aclodes* sp., *Phalangopsis* sp.), Blattodea (Blaberidae: *Blaberus* sp.), Isoptera (Termitidae: *Nasutitermes* sp.), Psocoptera (Epipsocidae),

Homoptera (Cixidae: *Cixius* sp.), Lepidoptera (Hesperiidae; Noctuidae; Tineidae), Diptera (Ceratopogonidae; Culicidae; Dolichopodidae; Drosophilidae; Psychodidae: *Lutzomyia* sp.; Tipulidae), Hymenoptera (Formicidae: *Camponotus* sp., *Dolichoderus* sp., *Neivamyrmex* sp., *Pachycondyla* sp.), Coleoptera (Staphylinidae), Scutigeromorpha (Scutigeridae: *Sphendononema* sp.).

Dentre os vertebrados foram encontrados três morfótipos pertencentes às ordens Chiroptera (Phyllostomidae: *Glossophaga soricina*, Emballonuridae: *Peropteryx kappleri*) e Anura (Leptodactylidae: *Pristimantis cf. fenestratus*). Dessa forma, foram encontradas 44 morfoespécies.

5.4.4.77.3. Caracterização faunística no período de chuva

Foram encontradas na caverna, 41 morfoespécies de invertebrados em pelo menos 25 famílias dos táxons: Acari (Oribatida; Trombidiforme), Amblypygi (Phrynidae: *Heterophrynus longicornis*), Pseudoscorpiones (Chernetidae, Chtoniidae), Araneae (Araneidae: Alpaida sp.; Gnaphosidae; Ochyroceratidae; Salticidae; Pholcidae: *Mesabolivar* sp., *Metagonia* sp.), Thysanura (Nicolletiidae: Atelurinae), Diplura (Campodeidae), Collembola (Entomobryidae), Orthoptera (Phalangopsidae: *Aclodes* sp.), Blattodea (Blaberidae: *Blaberus* sp.; Polyphagidae), Isoptera (Termitidae: *Nasutitermes* sp.), Psocoptera (Psyllipsocidae: *Psyllipsocus* sp.), Hemiptera (Cydnidae; Reduviidae), Lepidoptera (Noctuidae; Tineidae), Diptera (Psychodidae: *Lutzomyia* sp.), Hymenoptera (Formicidae: *Camponotus* sp., *Cyphomyrmex* sp., *Dolichoderus* sp., *Pachycondyla* sp.), Coleoptera (Scydmaenidae; Staphylinidae), Diplopoda (Stemmiulidae), Scolopendromorpha (Scolopocryptopidae: *Dinocryptops* sp.).

Dessa forma, foram encontradas 41 morfoespécies. Dentre essas, uma foi considerada troglóbia, a qual pertence ao táxon Thysanura (Nicolletiidae: Atelurinae).

5.4.4.77.4. Caracterização geral da fauna da cavidade

Foram encontradas na caverna, 66 morfoespécies de invertebrados em pelo menos 42 famílias dos táxons: Gastropoda (Subulinidae), Isopoda (Armadillidae; Philosciidae), Acari (Macronyssidae; Oribatida; Trombidiforme), Amblypygi (Phrynidae: *Heterophrynus longicornis*), Pseudoscorpiones (Chernetidae; Chtoniidae), Araneae (Araneidae: *Alpaida* sp.; Corinidae: *Tupirina* sp.; Gnaphosidae; Ochyroceratidae; Salticidae; Pholcidae: *Mesabolivar* sp., *Metagonia* sp.; Theridiidae), Thysanura (Nicolletiidae: Atelurinae), Diplura (Campodeidae), Collembola (Cyphoderidae; Entomobryidae), Orthoptera (Phalangopsidae: *Aclodes* sp., *Phalangopsis* sp.), Blattodea (Blaberidae: *Blaberus* sp.; Polyphagidae), Isoptera (Termitidae: *Nasutitermes* sp.), Psocoptera (Epipsocidae; Psyllipsocidae: *Psyllipsocus* sp.), Hemiptera (Cydnidae; Reduviidae), Homoptera (Cixidae: *Cixius* sp.), Lepidoptera (Hesperiidae; Noctuidae; Tineidae), Diptera (Ceratopogonidae; Culicidae; Dolichopodidae;

Drosophilidae; Psychodidae: *Lutzomyia* sp.; Tipulidae), Hymenoptera (Formicidae: *Camponotus* sp., *Cyphomyrmex* sp., *Dolichoderus* sp., *Neivamyrmex* sp., *Pachycondyla* sp.), Coleoptera (Scydmaenidae; Staphylinidae), Diplopoda (Stemmiulidae), Scolopendromorpha (Scolopocryptopidae: *Dinocryptops* sp.), Scutigeromorpha (Scutigeridae: *Sphendononema* sp.).

Dentre os vertebrados foram encontrados três morfótipos pertencentes às ordens Chiroptera (Phyllostomidae: *Glossophaga soricina*, Emballonuridae: *Peropteryx kappleri*) e Anura (Leptodactylidae: *Pristimantis cf. fenestratus*).

Dessa forma, foram encontrados um total de 69 morfoespécies. Dentre essas, uma foi considerada troglóbia, a qual pertence ao táxon Thysanura (Nicolletiidae: Atelurinae).

5.4.4.78. SL-081

5.4.4.78.1. Caracterização trófica

Pequena cavidade formada no minério de ferro com 9,8 m de projeção horizontal composta por uma fenda estreita e retilínea localizada no topo da encosta próxima a uma pequena drenagem perene. A matriz do entorno é composta por savana metalófila com uma vegetação arbórea associada à drenagem. Sua entrada é estreita e muito iluminada existindo apenas poucos arbustos e muito capim no seu entorno. Apenas com zona eufótica, a cavidade é muito seca e o piso é composto por sedimento clástico refinado com poucos blocos (seixos e calhaus) esparsos. Apesar da baixa umidade no interior da cavidade, as paredes na porção inicial da caverna são revestidas por liquens (Figura 211). Nas paredes e no teto foram observados, ainda, poucas colônias de Actinomicetos. O piso é ascendente a partir da entrada e existem muitas raízes de pequeno calibre distribuídas de forma superficial. Apesar de terem sido observados alguns morcegos insetívoros durante o inventário, não existiam depósitos de guano no interior da cavidade. De forma geral, o sistema de canalículos é pouco desenvolvido e a cavidade encontra-se muito suscetível a uma intensa circulação de ar. Durante a estação úmida a cavidade apresentava inúmeros pontos de gotejamento e percolação. A vegetação epígea cresceu durante o intervalo das campanhas e na última coleta propiciava um maior sombreamento da entrada da cavidade.

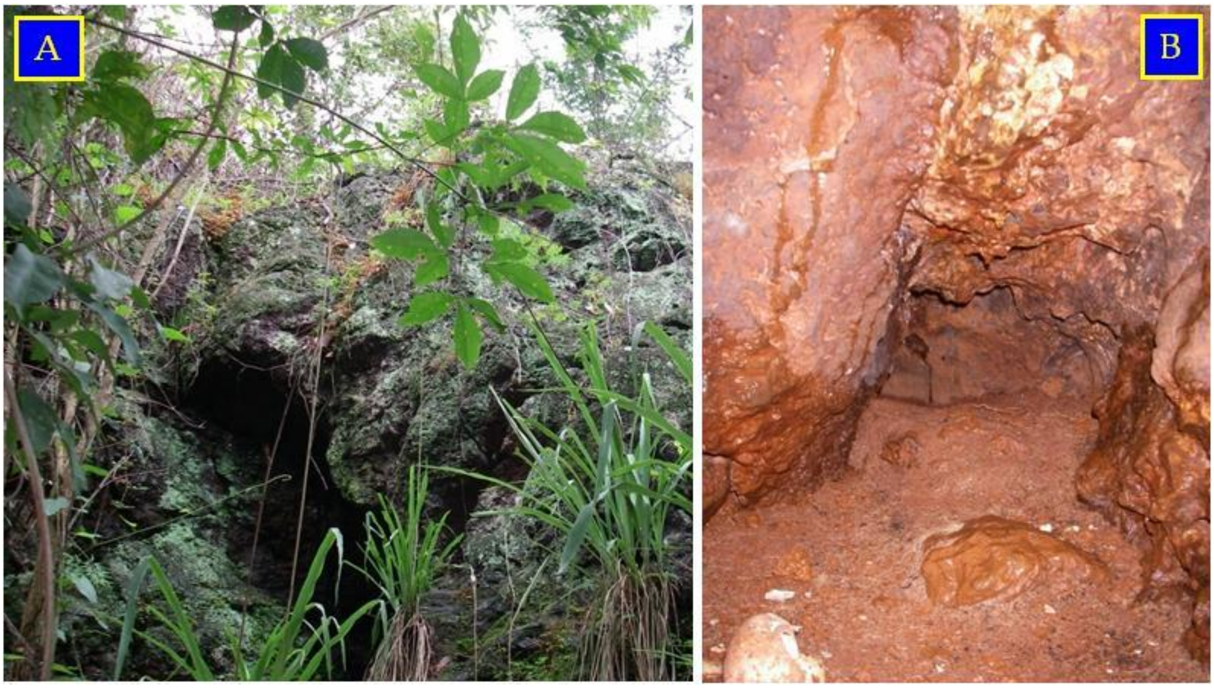

Figura 211 - a) Vista externa da entrada da cavidade com detalhes da vegetação epígea associada; b) Vista interna da cavidade.

5.4.4.78.2. Caracterização faunística no período de seca

Foram encontradas na caverna, 19 morfoespécies de invertebrados em pelo menos 16 famílias dos táxons: Acari (Argasidae: *Ornithodoros* sp.), Araneae (Ctenidae: *Ctenus* sp.; Filistatidae; Gnaphosidae; Salticidae; Pholcidae; Theridiidae), Orthoptera (Phalangopsidae: *Aclodes* sp.), Isoptera (*Nasutitermes* sp.), Homoptera (Cixidae: *Cixius* sp.), Lepidoptera (Noctuidae; Tineidae), Diptera (Dolichopodidae; Drosophilidae; Psychodidae: *Lutzomyia* sp.), Hymenoptera (Formicidae: Pachycondyla sp., *Pheidole* sp.).

Dentre os vertebrados foi encontrado um morfótipo pertencente à ordem Chiroptera (Emballonuridae: *Peropteryx kappleri*). Dessa forma, foram encontradas 20 morfoespécies.

5.4.4.78.3. Caracterização faunística no período de chuva

Foram encontradas na caverna, 27 morfoespécies de invertebrados em pelo menos 19 famílias dos táxons: Oligochaeta; Isopoda (Armadillidae; Dubioniscidae), Acari (Argasidae: *Ornithodoros* sp.; Trombidiforme), Araneae (Filistatidae; Gnaphosidae; Psauridae; Pholcidae: *Metagonia* sp.), Thysanura (Meinertellidae), Diplura (Campodeidae), Collembola (Entomobryidae; Entomobryomorpha), Orthoptera (Phalangopsidae: *Aclodes* sp.), Isoptera (Termitidae: *Nasutitermes* sp.), Lepidoptera (Noctuidae), Diptera (Ceratopogonidae; Muscidae; Psychodidae: *Lutzomyia* sp.), Hymenoptera (Formicidae: *Camponotus* sp., *Dolichoderus* sp., *Odontomachus* sp., *Pheidole* sp., *Solenopsis* sp.), Coleoptera (Chrysomelidae).

Dentre os vertebrados foram encontrados três morfótipos pertencentes às ordens Chiroptera (Emballonuridae: *Peropteryx kappleri*), Squamata (Gekkonidae: *Thecadactylus rapicauda*) e Anura (Leptodactylidae: *Pristimantis cf. fenestratus*).Dessa forma, foram encontradas 30 morfoespécies.

5.4.4.78.4. Caracterização geral da fauna da cavidade

Foram encontradas na caverna, 41 morfoespécies de invertebrados em pelo menos 26 famílias dos táxons: Oligochaeta; Isopoda (Armadillidae; Dubioniscidae), Acari (Argasidae: *Ornithodoros* sp.; Trombidiforme), Araneae (Ctenidae: *Ctenus* sp.; Filistatidae; Gnaphosidae; Salticidae; Psauridae; Pholcidae: *Metagonia* sp.; Theridiidae), Thysanura (Meinertellidae), Diplura (Campodeidae), Collembola (Entomobryidae; Entomobryomorpha), Orthoptera (Phalangopsidae: *Aclodes* sp.), Isoptera (Termitidae: *Nasutitermes* sp.), Homoptera (Cixidae: *Cixius* sp.), Lepidoptera (Noctuidae; Tineidae), Diptera (Ceratopogonidae; Dolichopodidae; Drosophilidae; Muscidae; Psychodidae: *Lutzomyia* sp.), Hymenoptera (Formicidae: *Camponotus* sp., *Dolichoderus* sp., *Odontomachus* sp., *Pachycondyla* sp., *Pheidole* sp., *Solenopsis* sp.), Coleoptera (Chrysomelidae).

Dentre os vertebrados foram encontrados três morfótipos pertencentes às ordens Chiroptera (Emballonuridae: *Peropteryx kappleri*), Squamata (Gekkonidae: *Thecadactylus rapicauda*) e Anura (Leptodactylidae: *Pristimantis cf. fenestratus*). Alguns organismos encontrados nesta caverna são mostrados na Figura 213.

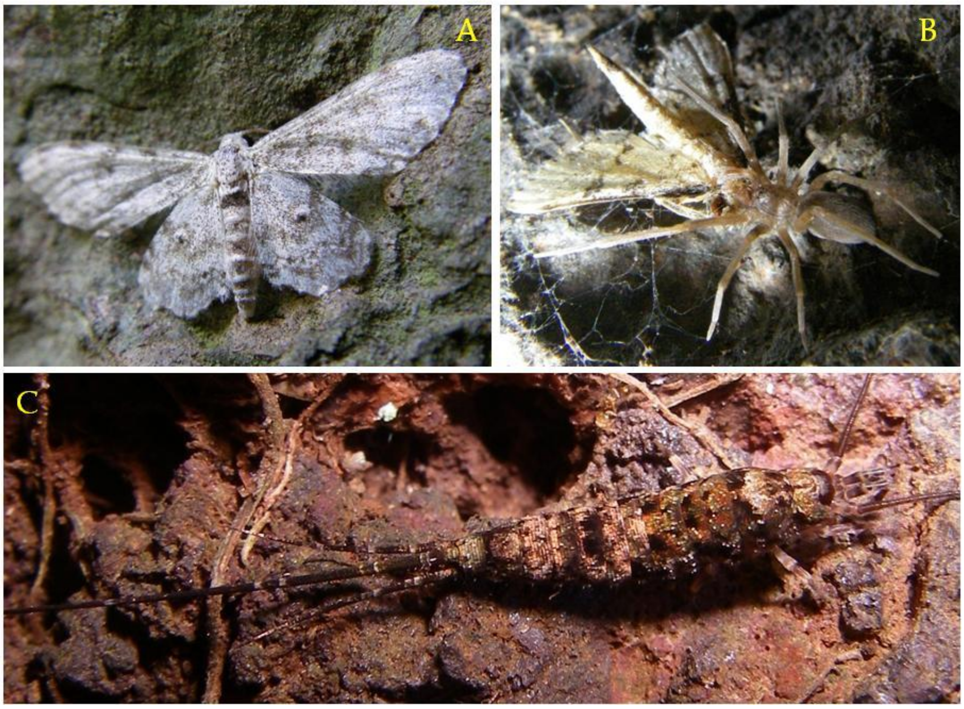

Figura 212 - a) Lepidoptera (Tineioidea); b) Araneae (Filistatidae) predando uma lepidóptera (Tineidae); d) Microcoryphia (Meinertellidae).

5.4.4.79. SL-082

5.4.4.79.1. Caracterização trófica

Caverna formada no minério de ferro com 50,1 m de projeção horizontal localizada no topo da encosta próxima a uma pequena drenagem perene. A matriz do entorno é composta por savana metalófila com um fragmento de mata associado à área de drenagem. A cavidade possui uma entrada ampla, sombreada e com muita vegetação associada ao seu entorno, além de líquens e musgos associados às paredes e piso. Na entrada, o piso é plano composto por sedimento fino com blocos de diferentes tamanhos e muita serrapilheira depositada junto à linha d'água. De forma geral, o piso é irregular com muitos blocos abatidos, sendo que aproximadamente metade se sua área é formada pela própria rocha matriz. A caverna apresenta um padrão labiríntico, possuindo salões amplos com grandes blocos abatidos conectados por trechos de rastejo e quebra-corpos. O sistema de canalículos é pouco desenvolvido, mas existem fraturas grandes de até dez centímetros de largura. A caverna possui uma grande área afótica e existem inúmeros pontos de gotejamento espalhados pela cavidade, os quais formam pequenas poças d'água. O guano mais abundante na caverna é produzido por morcegos frugívoros, mas existem depósitos de guano produzidos por morcegos insetívoros e hematófagos. Estes últimos, no entanto, estão restritos às zonas afóticas (Figura 213). Tais áreas estão localizadas nos salões mais interiores e são compostas por piso argiloso com muitos blocos de pequenas dimensões (seixos e calhaus). Estas áreas apresentam um sistema radicular bem desenvolvido, com raízes de fino calibre distribuídas de maneira heterogênea. Durante a estação úmida foram observados inúmeros pontos de gotejamentos e o piso encontrava-se alagado em algumas áreas próximo a entrada da cavidade.

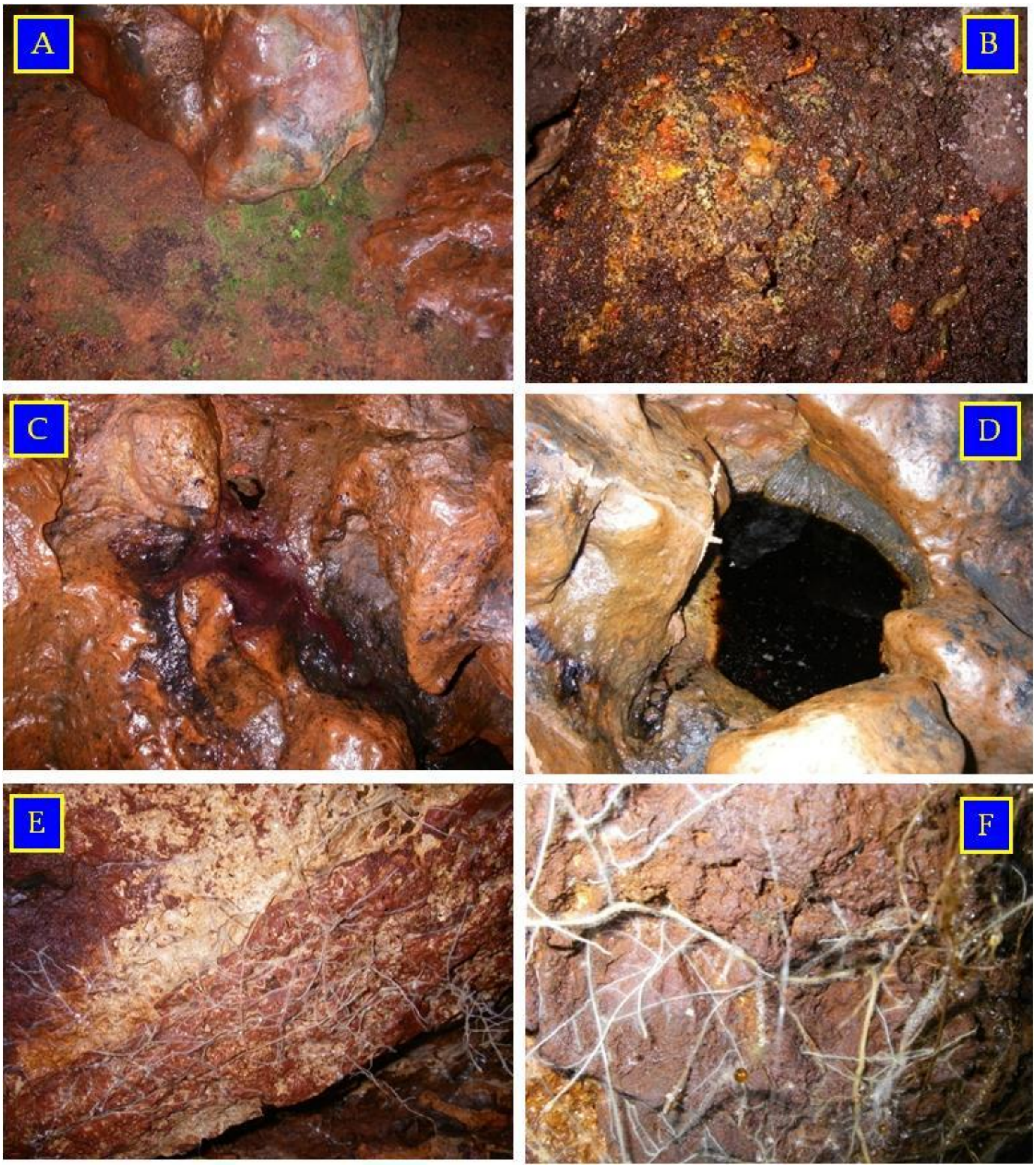

Figura 213 - a) Vista interna da entrada da cavidade com blocos revestidos por briófitas; b) Depósito de guano de morcegos frugívoros; c) Depósito de guano de morcegos hematófagos; d) Poça de água misturada ao guano de morcegos hematófagos; e) Sistema radicular bem desenvolvido nas paredes da caverna; f) Detalhe das raízes, algumas das quais eliminando exudatos.

5.4.4.79.2. Caracterização faunística no período de seca

Foi encontrado na caverna um total de 77 morfoespécies de invertebrados em pelo menos 47 famílias dos táxons: Oligochaeta; Isopoda (Armadillidae), Acari (Argasidae: *Ornithodoros* sp.; Laelapidae; Macronyssidae; Mesostigmata; Uropodina; Acaridae; Oribatida; Cunaxidae;

Trombidiforme), Amblypygi (Phrynidae: *Heterophrynus longicornis*; Charinidae: *Charinus* sp.), Pseudoscorpiones (Chernetidae; Chtoniidae), Opiliones (Escadabiidae), Araneae (Corinidae: *Tupirina* sp.; Ochyroceratidae; Salticidae; Scytodidae*: Scytodes eleonora*; Pholcidae: *Mesabolivar* sp., *Metagonia* sp.; Theridiidae; Theridiosomatidae), Thysanura (Nicoletiidae: Nicoletiinae), Collembola (Sminthuridae; Entomobryidae), Orthoptera (Phalangopsidae: *Aclodes* sp., *Phalangopsis* sp.), Blattodea (Blattidae), Isoptera (Rhinotermitidae: *Heterotermes* sp.), Psocoptera (Epipsocidae), Hemiptera (Cydnidae; Dipsocoridae; Enicocephalidae; Pyrrhocoridae), Homoptera (Cixidae: *Cixius* sp.; Derbidae), Lepidoptera (Noctuidae; Tineidae), Diptera (Ceratopogonidae; Chironomidae; Culicidae: Culex sp.; Dolichopodidae; Drosophilidae; Fanniidae; Psychodidae: Lutzomyia sp.; Tipulidae), Hymenoptera (Formicidae: *Azteca* sp., *Camponotus* sp., *Pachycondyla* sp., *Solenopsis* sp.), Coleoptera (Carabidae; Ptylidae; Staphylinidae), Diplopoda (Pyrgodesmidae), Scolopendromorpha (Cryptopidae: *Cryptops* sp.).

Dentre os vertebrados foram encontrados oito morfoespécies pertencentes às ordens Chiroptera (Phyllostomidae: *Glossophaga soricina*, *Diphylla ecaudata*; Emballonuridae: *Peropteryx kappleri*), Squamata (Colubridae) e Anura (Leptodactylidae: *Pristimantis* cf. *fenestratus*; *Leptodactylus labyrinthicus*; Bufonidae).

Dessa forma, foram encontradas 85 morfoespécies. Dentre essas, três foram consideradas troglomórficas, as quais pertencem aos táxons Amblypygi (Charinidae: *Charinus* sp.), Araneae (Ochyroceratidae), Diplopoda (Pyrgodesmidae).

5.4.4.79.3. Caracterização faunística no período de chuva

Foi encontrado na caverna, um total de 86 morfoespécies de invertebrados em pelo menos 50 famílias dos táxons: Gordioidea; Oligochaeta; Gastropoda (Subulinidae), Tubellaria (Geoplanidae), Acari (Laelapidae: *Stratiolaelaps* sp.; Laelapidae; Macronyssidae; Oribatida; Trombidiforme), Amblypygi (Phrynidae: *Heterophrynus longicornis*; Charinidae: *Charinus* sp), Ricinulei (Ricinoididae: *Cryptocellus tarsilae*), Pseudoscorpiones (Chernetidae; Chtoniidae), Opiliones (Escadabiidae), Araneae (Gnaphosidae; Ochyroceratidae; Salticidae; Scytodidae*: Scytodes eleonora*; Psauridae; Pholcidae: Mesabolivar sp., *Metagonia* sp.; Theridiidae), Thysanura (Nicoletiidae: Nicoletiinae, Atelurinae), Diplura (Campodeidae), Collembola (Sminthuridae; Entomobryidae; Tomoceridae), Orthoptera (Phalangopsidae: *Aclodes* sp., *Phalangopsis* sp.), Psocoptera (Archipsocidae), Hemiptera (Cydnidae; Dipsocoridae; Lygaeidae; Ploiariidae), Homoptera (Cercopidae; Derbidae; Coccoidea), Lepidoptera (Noctuidae; Tineidae), Diptera (Cecidomyiidae; Chironomidae; Culicidae: *Toxorhynchites* sp.; Drosophilidae; Fanniidae; Milichiidae; Muscidae; Phoridae; Psychodidae: *Lutzomyia* sp.; Sciaridae), Hymenoptera (Formicidae: *Cyphomyrmex* sp., *Dolichoderus* sp., *Pachycondyla* sp., *Rogeria* sp., *Solenopsis* sp.), Coleoptera (Carabidae; Curculionidae: Scotylinae; Dytiscidae; Staphylinidae), Diplopoda (Chelodesmidae), Lithobiomorpha (Henicopiidae: *Lamyctes* sp.).

Dentre os vertebrados foi encontrado dois morfoespécies pertencentes às ordens Chiroptera (Phyllostomidae: *Glossophaga soricina*; Emballonuridae: *Peropteryx kappleri*).

Dessa forma, foram encontrados um total de 88 morfoespécies. Dentre essas, seis foram consideradas troglóbias, as quais pertencem aos táxons Gordioidea; Turbellaria (Geoplanidae), Amblypygi (Charinidae: Charinus sp.), Araneae (Ochyroceratidae), Hemiptera (Dipsocoridae), Coleoptera (Dytiscidae).

5.4.4.79.4. Caracterização geral da fauna da cavidade

Foi encontrado na caverna, um total de 133 morfoespécies de invertebrados em pelo menos 68 famílias dos táxons: Gordioidea; Oligochaeta; Gastropoda (Subulinidae), Tubellaria (Geoplanidae), Isopoda (Armadillidae), Acari (Argasidae: *Ornithodoros* sp.; Laelapidae: *Stratiolaelaps* sp.; Macronyssidae; Mesostigmata; Uropodina; Acaridae; Oribatida; Cunaxidae; Trombidiforme), Amblypygi (Phrynidae: *Heterophrynus longicornis*; Charinidae: *Charinus* sp.), Ricinulei (Ricinoididae: *Cryptocellus tarsilae*), Pseudoscorpiones (Chernetidae; Chtoniidae), Opiliones (Escadabiidae), Araneae (Corinidae: *Tupirina* sp.; Gnaphosidae; Ochyroceratidae; Salticidae; Scytodidae*: Scytodes eleonora*; Psauridae; Pholcidae: *Mesabolivar* sp., *Metagonia* sp.; Theridiidae; Theridiosomatidae), Thysanura (Nicoletiidae: Nicoletiinae, Atelurinae), Diplura (Campodeidae), Collembola (Sminthuridae; Entomobryidae; Tomoceridae), Orthoptera (Phalangopsidae: *Aclodes* sp., *Phalangopsis* sp.), Blattodea (Blattidae), Isoptera (Rhinotermitidae: Heterotermes sp.), Psocoptera (Archipsocidae; Epipsocida), Hemiptera (Cydnidae; Dipsocoridae; Enicocephalidae; Lygaeidae; Ploiariidae; Pyrrhocoridae), Homoptera (Cercopidae; Cixidae: *Cixius* sp.; Derbidae; Coccoidea), Lepidoptera (Noctuidae; Tineidae), Diptera (Cecidomyiidae; Ceratopogonidae; Chironomidae; Culicidae: *Toxorhynchites* sp., *Culex* sp. ; Dolichopodidae; Drosophilidae; Fanniidae; Muscidae; Milichiidae; Phoridae; Psychodidae: *Lutzomyia* sp.; Sciaridae; Tipulidae), Hymenoptera (Formicidae: *Azteca* sp., *Camponotus* sp., *Cyphomyrmex* sp., *Dolichoderus* sp., *Pachycondyla* sp., *Rogeria* sp., *Solenopsis* sp.), Coleoptera (Carabidae; Curculionidae: Scotylinae; Dytiscidae; Ptylidae; Staphylinidae), Diplopoda (Chelodesmidae; Pyrgodesmidae), Lithobiomorpha (Henicopiidae: *Lamyctes* sp.), Scolopendromorpha (Cryptopidae: *Cryptops* sp.).

Dentre os vertebrados foram encontrados oito morfoespécies pertencentes às ordens Chiroptera (Phyllostomidae: *Glossophaga soricina*, *Diphylla ecaudata*; Emballonuridae: *Peropteryx kappleri*), Squamata (Colubridae) e Anura (Leptodactylidae: *Pristimantis* cf. *fenestratus*; *Leptodactylus labyrinthicus*; Bufonidae).

Dessa forma foi encontrado um total de 141 morfoespécies. Dentre essas, sete foram consideradas troglomórficas, as quais pertencem aos táxons Gordioidea; Turbellaria

(Geoplanidae), Amblypygi (Charinidae: *Charinus* sp.), Araneae (Ochyroceratidae), Hemiptera (Dipsocoridae), Coleoptera (Dytiscidae), Diplopoda (Pyrgodesmidae). Alguns organismos encontrados nesta caverna são mostrados na Figura 214.

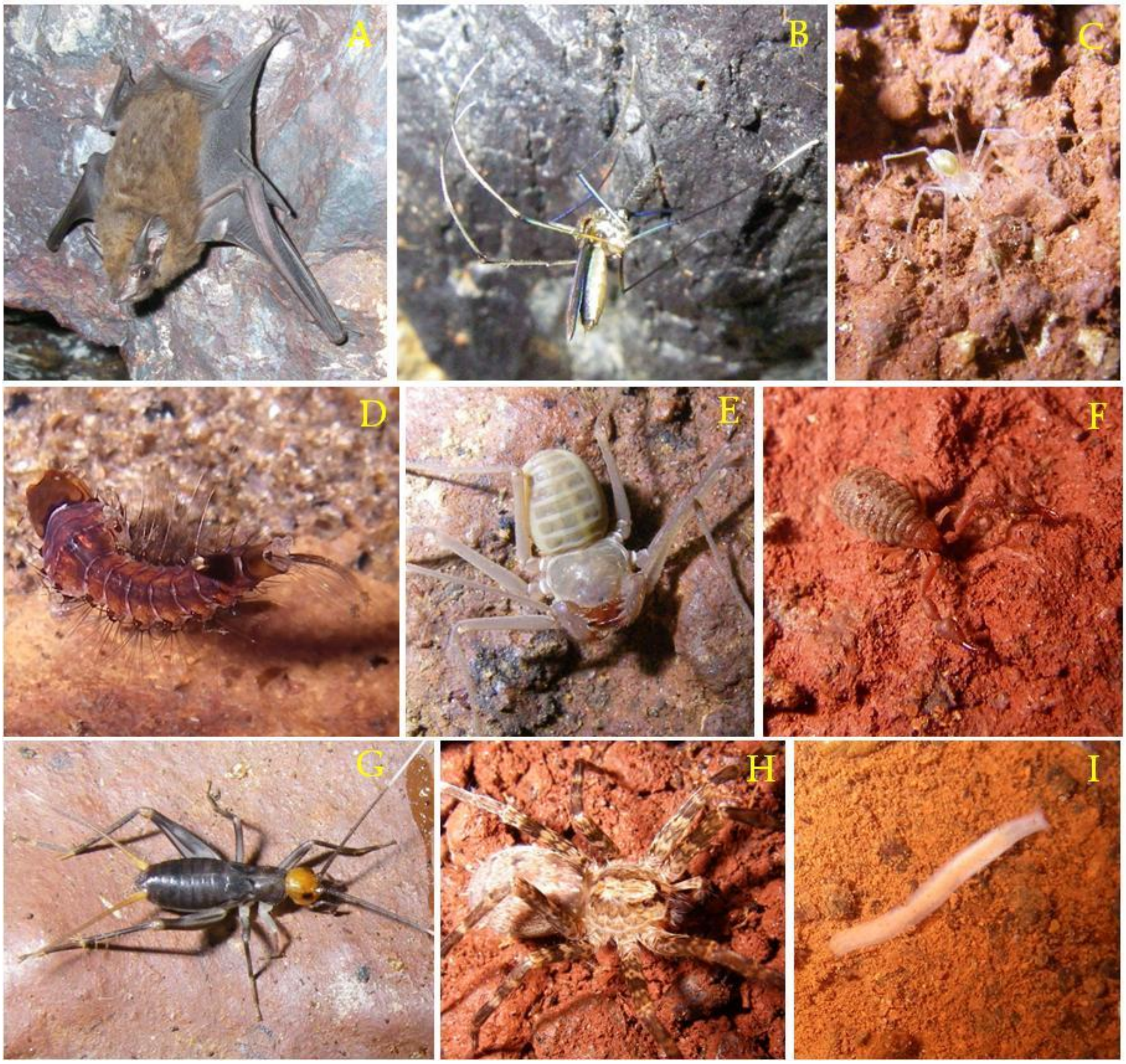

Figura 214 - a) Emballonuridae (*Peropteryx kappleri*); b) Diptera (Culicidae*:* Sabethini); c) Araneae (Ochyroceratidae); d) Larva díptera Culicidae (Toxorhynchites sp.) ; e) Amblypygi (*Charinus sp*.); f) Pseudoscorpiones (Chernetidae); g) Orthoptera (indet.); h) Araneae (Ctenidae); i) Planaria.

5.4.4.80. SL-083

5.4.4.80.1. Caracterização trófica

Caverna parcialmente formada em canga e parcialmente formada no minério de ferro. Apresenta 29 m de projeção horizontal, estando localizada em área de encosta. A vegetação da área de entorno é composta por mata com árvores de troncos finos com muitas lianas e cipós, e grande quantidade de serrapilheira acumulada junto ao substrato. A cavidade possui

uma entrada ampla, sombreada, com líquens, briófitas e plântulas de angiospermas associados à linha d'água, onde também existem inúmeros blocos abatidos. O piso é descendente a partir da entrada e plano no restante da cavidade, sendo este seco e composto por sedimento fino com muitos seixos, calhaus e alguns matacões distribuídos de forma esparsa (Figura 215). De maneira geral, a cavidade é oligotrófica, sem depósitos de guano e serrapilheira nas zonas mais profundas. No fundo da cavidade existe um ponto de gotejamento ativo, mas este é insuficiente para aumentar a umidade relativa da cavidade. Existem poucas raízes visíveis na macro-caverna e o sistema de canalículos é pouco desenvolvido. A caverna não apresenta zona afótica e existem poucos microorganismos associados às paredes e teto da cavidade. Durante a estação úmida a cavidade encontrava-se totalmente seca uma vez que os pontos de gotejamento e percolação haviam cessado. No ambiente externo a vegetação foi queimada em algumas áreas diminuindo o sombreamento nas entradas das cavidades desta região e por conseqüência a quantidade de matéria orgânica disponível no ambiente hipógeo.

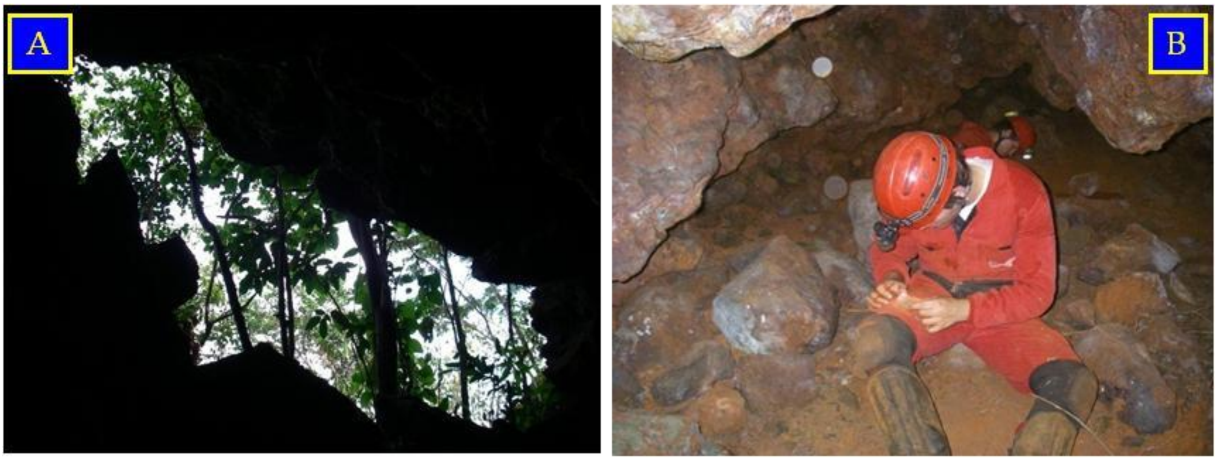

Figura 215 - a) Vista interna da entrada da cavidade evidenciando a vegetação externa; b) Aspecto interno da cavidade onde se observa o piso e os blocos abatidos.

5.4.4.82.2. Caracterização faunística no período de seca

Foi encontrado na caverna, um total de 45 morfoespécies de invertebrados em pelo menos 31 famílias dos táxons: Isopoda (Armadillidae), Acari (Argasidae: *Ornithodoros* sp.; Mesostigmata), Amblypygi (Phrynidae: *Heterophrynus longicornis*; Charinidae: *Charinus* sp.), Pseudoscorpiones (Chernetidae; Chtoniidae), Araneae (Dipluridae; Filistatidae; Ochyroceratidae; Scytodidae*: Scytodes eleonora*; Psauridae; Pholcidae: *Mesabolivar* sp., *Metagonia* sp.; Theridiosomatidae), Thysanura (Nicoletiidae: Nicoletiinae; Meinertellidae), Orthoptera (Phalangopsidae: *Aclodes* sp., *Phalangopsis* sp.), Blattodea (Polyphagidae), Isoptera (Termitidae: *Nasutitermes* sp., *Subulitermes*? sp., *Termes* sp.), Psocoptera (Pachytroctidae), Homoptera (Cixidae: *Cixius* sp.), Lepidoptera (Noctuidae), Diptera (Culicidae; Dolichopodidae; Milichiidae; Psychodidae: Lutzomyia sp.), Hymenoptera

(Formicidae: *Azteca* sp., *Camponotus* sp., *Pachycondyla* sp.; Vespidae sp.), Diplopoda (Pseudonannolenidae), Geophilomorpha (Schendylidae), Neuroptera (Myrmeleontidae), Coleoptera (Dermestidae).

Dentre os vertebrados foram encontrados duas morfoespécies pertencentes às Ordens Anura (Leptodactylidae: *Pristimantis* cf. *fenestratus*) e Rodentia (Cricetidae: *Rhipidomys* sp.).

Dessa forma foi encontrado um total de 47 morfoespécies. Dentre essas, duas foram consideradas troglomórficas, as quais pertencem aos táxons Amblypygi (Charinidae: *Charinus* sp.), Diplopoda (Pseudonannolenidae).

5.4.4.80.3. Caracterização faunística no período de chuva

Foi encontrado na caverna, um total de 43 morfoespécies de invertebrados em pelo menos 31 famílias dos táxons: Gordioidea; Acari (Argasidae: *Ornithodoros* sp.; Eupodidae: *Linopodes* sp.; Trombidiforme), Amblypygi (Phrynidae: *Heterophrynus longicornis*; Charinidae: *Charinus* sp.), Pseudoscorpiones (Chernetidae; Chtoniidae), Opiliones (Escadabiidae), Araneae (Araneidae: *Alpaida* sp.; Scytodidae*: Scytodes eleonora*; Pholcidae: *Mesabolivar* sp.), Thysanura (Nicoletiidae: Nicoletiinae), Collembola (Entomobryidae; Isotomidae), Orthoptera (Phalangopsidae: *Phalangopsis* sp.), Blattodea (Blaberidae: *Blaberus* sp.; Polyphagidae), Isoptera (Termitidae: *Termes* sp.), Psocoptera (Pachytroctidae; Psyllipsocidae: *Psyllipsocus* sp.), Lepidoptera (Noctuidae; Tineidae), Diptera (Ceratopogonidae; Culicidae; Psychodidae: *Lutzomyia* sp.), Hymenoptera (Formicidae: *Azteca* sp., *Camponotus* sp., *Solenopsis* sp., *Zacryptocerus* sp.), Thysanoptera (Phaleothripidae: Phaleothripinae), Coleoptera (Tenebrionidae, Dermestidae, Staphylinidae), Diplopoda (Siphonophoridae), Neuroptera (Myrmeleontidae).

Dentre os vertebrados foi encontrada uma morfoespécie pertencente à ordem Chiroptera (Emballonuridae: *Peropteryx kappleri*).

Dessa forma foi encontrado um total de 44 morfoespécies. Dentre essas, duas foram consideradas troglomórficas, as quais pertencem aos táxons Gordioidea e Amblypygi (Charinidae: *Charinus* sp.).

5.4.4.80.4. Caracterização geral da fauna da cavidade

Foi encontrado na caverna, um total de 72 morfoespécies de invertebrados em pelo menos 44 famílias dos táxons: Gordioidea; Isopoda (Armadillidae), Acari (Argasidae: *Ornithodoros* sp.; Mesostigmata; Eupodidae: *Linopodes* sp.; Trombidiforme), Amblypygi (Phrynidae: *Heterophrynus longicornis*; Charinidae: *Charinus* sp.), Pseudoscorpiones (Chernetidae; Chtoniidae), Opiliones (Escadabiidae), Araneae (Araneidae: *Alpaida* sp.; Dipluridae; Filistatidae; Ochyroceratidae; Scytodidae*: Scytodes eleonora*; Psauridae; Pholcidae:

Mesabolivar sp., *Metagonia* sp.; Theridiosomatidae), Thysanura (Nicoletiidae: Nicoletiinae; Meinertellidae), Collembola (Entomobryidae; Isotomidae), Orthoptera (Phalangopsidae: *Aclodes* sp., *Phalangopsis* sp.), Blattodea (Blaberidae: *Blaberus* sp.; Polyphagidae), Isoptera (Termitidae: Nasutitermes sp., *Subulitermes*? sp., *Termes* sp.), Psocoptera (Pachytroctidae; Psyllipsocidae: *Psyllipsocus* sp.), Homoptera (Cixidae: *Cixius* sp.), Lepidoptera (Noctuidae; Tineidae), Diptera (Ceratopogonidae; Culicidae; Dolichopodidae; Milichiidae; Psychodidae: *Lutzomyia* sp.;), Hymenoptera (Formicidae: *Azteca* sp., *Camponotus* sp., *Pachycondyla* sp., *Solenopsis* sp., *Zacryptocerus* sp.; Vespidae sp.), Thysanoptera (Phaleothripidae: Phaleothripinae), Coleoptera (Tenebrionidae; Dermestidae; Staphylinidae), Diplopoda (Siphonophoridae; Pseudonannolenidae), Geophilomorpha (Schendylidae), Neuroptera (Myrmeleontidae).

Dentre os vertebrados foram encontrados três morfoespécies pertencentes às ordens: Anura (Leptodactylidae: *Pristimantis cf. fenestratus*), Rodentia (Cricetidae: *Rhipidomys* sp.) e Chiroptera (Emballonuridae: *Peropteryx kappleri*)

Dessa forma, foi encontrado um total de 75 morfoespécies. Dentre essas, duas foram consideradas troglomórficas, as quais pertencem aos táxons Gordioidea; Amblypygi (Charinidae: *Charinus* sp.), Diplopoda (Pseudonannolenidae).

5.4.4.81. SL-084

5.4.4.81.1. Caracterização trófica

Pequena cavidade formada no minério de ferro com 11,6 m de projeção horizontal e localizada em área de encosta. A vegetação da área de entorno é composta por mata com árvores de troncos finos com muitas lianas e cipós e grande quantidade de serrapilheira acumulada junto ao substrato. A caverna possui apenas uma entrada em teto baixo com piso descendente e muitos blocos abatidos e com muita serrapilheira acumulada junto à linha d’água. A mesma apresenta teto baixo ao longo de toda sua extensão, não existem zonas afóticas e o piso é descendente e seco, sendo completamente coberto por seixos e calhaus. Existem poucas raízes na macro-caverna onde é possível observar alguns pequenos rizotemas distribuídos de forma esparsa e não foram observados depósitos de guano (Figura 216). O sistema de canalículos é pouco desenvolvido, mas existem muitas frestas nas paredes e teto. Não foram observados gotejamentos ativos na cavidade durante a estação seca e praticamente não existem microorganismos associados às paredes e teto. Nenhuma alteração significativa foi observada durante a estação úmida, além das alterações normais na umidade relativa do ar. No ambiente externo a vegetação foi queimada em algumas áreas diminuindo o sombreamento nas entradas das cavidades desta região e por conseqüência a quantidade de matéria orgânica disponível no ambiente hipógeo.

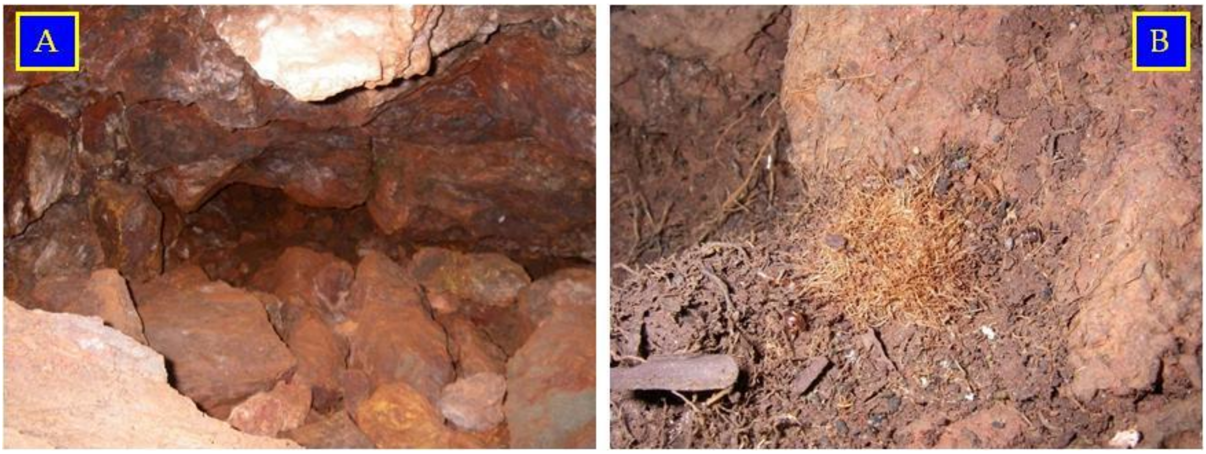

Figura 216 - a) Vista da porção interna da cavidade; b) Rizotema junto ao piso.

5.4.4.81.2. Caracterização faunística no período de seca

Foi encontrado na caverna, um total de 13 morfoespécies de invertebrados em pelo menos 12 famílias dos táxons: Amblypygi (Phrynidae: *Heterophrynus longicornis*), Pseudoscorpiones (Chernetidae*),* Araneae *(*Filistatidae; Ochyroceratidae; Pholcidae: *Metagonia* sp.), Orthoptera (Phalangopsidae: *Aclodes* sp.), Blattodea (Polyphagidae), Isoptera (Termitidae: *Nasutitermes* sp.), Psocoptera, Lepidoptera (Noctuidae), Diptera (Dolichopodidae; Psychodidae: *Lutzomyia* sp.), Hymenoptera (Formicidae: *Pheidole* sp.).

Dentre os vertebrados foram encontradas duas morfoespécies pertencentes à ordem Anura (Leptodactylidae: *Pristimantis cf. fenestratus*; Dendrobatidae: *Ameerega* sp.). Dessa forma, foi encontrado um total de 15 morfoespécies.

5.4.4.81.3. Caracterização faunística no período de chuva

Foi encontrado na caverna, um total de 20 morfoespécies de invertebrados em pelo menos 15 famílias dos táxons: Acari (Oribatida; Anystidae: *Erythracarus* sp.), Amblypygi (Phrynidae: *Heterophrynus longicornis*), Pseudoscorpiones, Opiliones (Phalangiidae), Araneae (Filistatidae; Salticidae; Scytodidae*: Scytodes eleonora*), Collembola (Entomobryidae), Isoptera (Termitidae: *Nasutitermes* sp.), Psocoptera (Lepidopsocidae), Hemiptera (Lygaeidae), Lepidoptera (Noctuidae; Tineidae), Diptera (Culicidae; Psychodidae: *Lutzomyia* sp.), Hymenoptera (Formicidae: *Dolichoderus* sp., *Pheidole* sp.).

Dentre os vertebrados foi encontrada uma morfoespécie pertencente à ordem Chiroptera (Emballonuridae: *Peropteryx kappleri*). Dessa forma, foi encontrado um total de 21 morfoespécies.

5.4.4.81.4. Caracterização geral da fauna da cavidade

Foi encontrado na caverna, um total de 30 morfoespécies de invertebrados em pelo menos 22 famílias dos táxons: Acari (Oribatida; Anystidae: *Erythracarus* sp.), Amblypygi (Phrynidae: *Heterophrynus longicornis*), Pseudoscorpiones (Chernetidae), Opiliones (Phalangiidae), Araneae (Filistatidae; Ochyroceratidae; Salticidae; Scytodidae*: Scytodes eleonora*; Pholcidae: *Metagonia* sp.), Collembola (Entomobryidae), Orthoptera (Phalangopsidae: *Aclodes* sp.), Blattodea (Polyphagidae), Isoptera (Termitidae: *Nasutitermes* sp.), Psocoptera (Lepidopsocidae), Hemiptera (Lygaeidae), Lepidoptera (Noctuidae; Tineidae), Diptera (Culicidae; Dolichopodidae; Psychodidae: *Lutzomyia* sp.), Hymenoptera (*Dolichoderus* sp., *Pheidole* sp.).

Dentre os vertebrados foram encontradas três morfoespécies pertencentes às ordens: Anura (Leptodactylidae: *Pristimantis cf. fenestratus*; Dendrobatidae: *Ameerega* sp.), Chiroptera (Emballonuridae: *Peropteryx kappleri*). Dessa forma, foi encontrado um total de 33 morfoespécies.

5.4.4.82. SL-085

5.4.4.82.1. Caracterização trófica

Pequena cavidade formada no minério de ferro com 8,9 m de projeção horizontal localizada em área de encosta. A vegetação do entorno é composta basicamente por samambaias de grande porte. Existe pouca serrapilheira acumulada junto ao substrato. A caverna possui apenas uma entrada em teto baixo com piso plano e muitos blocos abatidos e com pouca serrapilheira acumulada junto à linha d'água. Sua entrada é bastante iluminada, apresentando muitos líquens, briófitas, pteridófitas e plântulas de angiospermas (Melastomatáceas). A cavidade apresenta teto baixo ao longo de toda sua extensão e um pequeno trecho de rastejo. Não existe zona afótica e o piso é predominantemente plano e seco sendo composto por sedimento clástico com poucos seixos e calhaus distribuídos de forma esparsa. Existem poucas raízes e na zona mais distal da cavidade existem alguns pequenos depósitos de guano envelhecidos distribuídos de maneira esparsa. Em geral, a cavidade apresenta forte influência das condições epígeas e o sistema de canalículos é pouco desenvolvido, mas existem muitas frestas nas paredes e teto (Figura 217). Não foram observados gotejamentos ativos na cavidade durante a estação seca e praticamente não existem microorganismos associados às paredes e teto. Nenhuma alteração significativa foi observada durante a estação úmida, além das alterações normais na umidade relativa do ar. No ambiente externo a vegetação foi queimada em algumas áreas diminuindo o sombreamento nas entradas das cavidades desta região e por conseqüência a quantidade de matéria orgânica disponível no ambiente hipógeo.

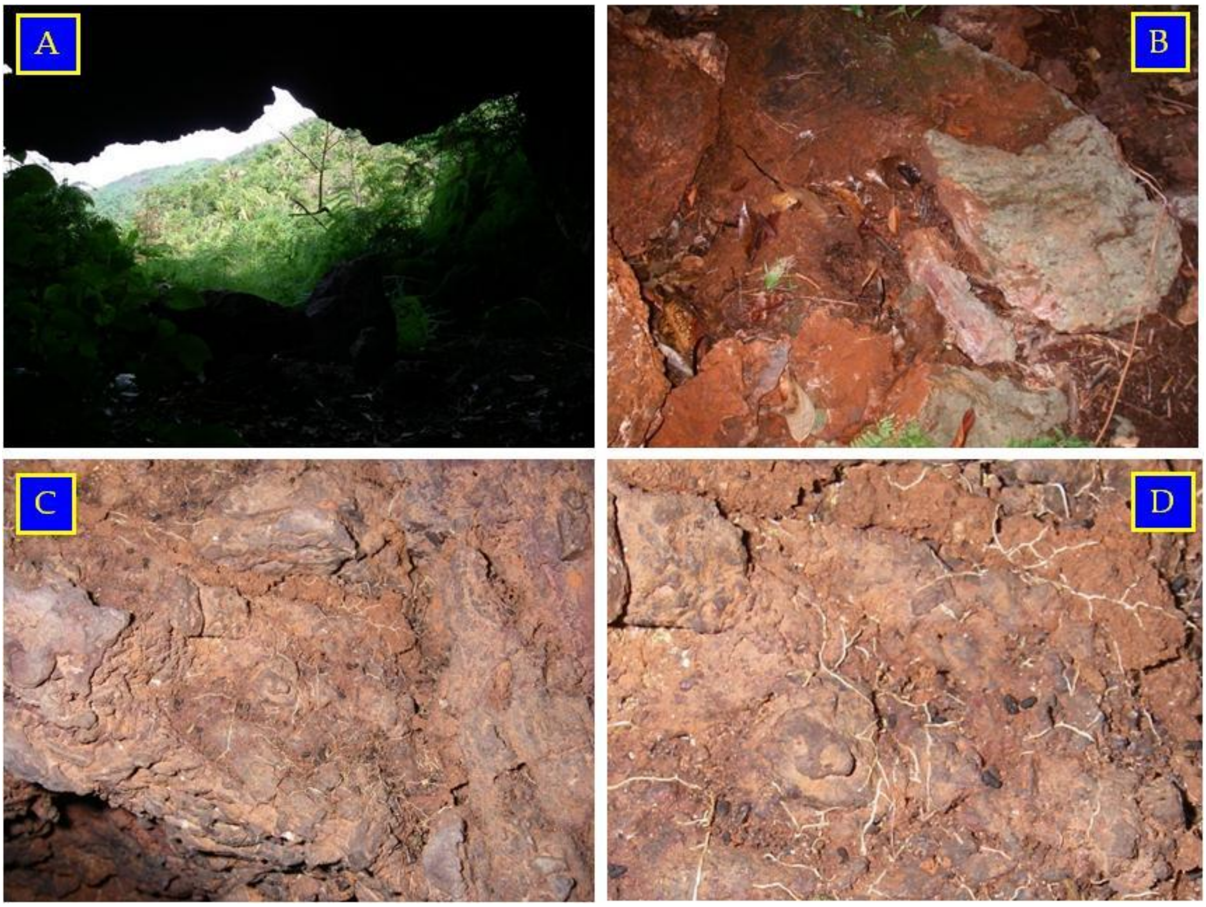

Figura 217 - a) Vista interna da entrada da cavidade; b) Serrapilheira presente nas proximidades da entrada da caverna; c) Raízes de pequeno calibre crescendo sobre as paredes; d) detalhe das raízes mostradas na foto anterior.

5.4.4.82.2. Caracterização faunística no período de seca

Foi encontrado na caverna um total de 33 morfoespécies de invertebrados em pelo menos 26 famílias dos táxons: Isopoda (Scleropactidae), Amblypygi (Phrynidae: *Heterophrynus longicornis*), Pseudoscorpiones (Chernetidae; Chtoniidae), Araneae (Filistatidae; Salticidae; Scytodidae*: Scytodes eleonora*; Psauridae; Pholcidae: *Metagonia* sp.; Theridiosomatidae), Thysanura (Nicoletiidae: Nicoletiinae), Diplura (Campodeidae), Collembola (Entomobryidae), Orthoptera (Phalangopsidae: *Phalangopsis* sp.), Blattodea (Polyphagidae), Isoptera (Termitidae: *Armitermes* sp., *Nasutitermes* sp.), Psocoptera (Archipsocidae; Epipsocidae), Hemiptera (Cydnidae; Ploiariidae), Homoptera (Cixidae: *Cixius* sp.), Lepidoptera (Noctuidae), Diptera (Dolichopodidae; Psychodidae: *Lutzomyia* sp.), Hymenoptera (Formicidae: *Anochetus* sp., *Pachycondyla* sp., *Zacryptocerus* sp.), Neuroptera (Myrmeleontidae).

Dentre os vertebrados foram encontrados quatro morfoespécies pertencentes às ordens: Anura (Leptodactylidae: *Pristimantis cf. fenestratus*), Chiroptera (Emballonuridae: *Peropteryx kappleri*; Phyllostomidae: *Glossophaga soricina*), Squamata (Gekkonidae: *Thecadactylus rapicauda*). Dessa forma, foi encontrado um total de 37 morfoespécies.

5.4.4.82.3. Caracterização faunística no período de chuva

Foi encontrado na caverna, um total de 52 morfoespécies de invertebrados em pelo menos 36 famílias dos táxons: Gastropoda (Subulinidae; Systrophiidae), Isopoda (Armadillidae; Balloniscidae), Acari (Oribatida; Trombidiforme), Amblypygi (Phrynidae: *Heterophrynus longicornis*), Pseudoscorpiones (Chernetidae; Chtoniidae), Araneae (Ctenidae: *Ctenus* sp.; Filistatidae; Gnaphosidae; Oonopidae: Oonopinae; Salticidae; Scytodidae*: Scytodes eleonora*; Pholcidae: *Mesabolivar* sp., *Metagonia* sp.; Theridiosomatidae), Thysanura (Meinertellidae), Diplura (Campodeidae), Collembola (Entomobryidae; Isotomidae), Isoptera (Termitidae: *Nasutitermes* sp.), Psocoptera (Pachytroctidae), Hemiptera (Aradidae; Cydnidae; Lygaeidae; Ploiariidae), Homoptera (Derbidae), Lepidoptera (Noctuidae; Tineidae), Diptera (Cecidomyiidae; Chironomidae; Drosophilidae; Psychodidae: *Lutzomyia* sp.), Hymenoptera (Formicidae: *Acromyrmex* sp., *Camponotus* sp., *Dolichoderus* sp., *Pachycondyla* sp., *Rogeria* sp., *Solenopsis* sp., *Strumigenys* sp.), Neuroptera (Myrmeleontidae), Coleoptera (Dermestidae; Staphylinidae).

Dentre os vertebrados foram encontrados duas morfoespécies pertencentes à ordem Chiroptera (Emballonuridae: *Peropteryx kappleri*; Phyllostomidae: *Glossophaga soricina*).

Dessa forma, foi encontrado um total de 54 morfoespécies. Dentre essas, uma foi considerada troglóbia, a qual pertence ao táxon Gastropoda (Systrophiidae).

5.4.4.82.4. Caracterização geral da fauna da cavidade

Foi encontrado na caverna, um total de 71 morfoespécies de invertebrados em pelo menos 45 famílias dos táxons: Gastropoda (Subulinidae; Systrophiidae), Isopoda (Armadillidae; Balloniscidae; Scleropactidae), Acari (Oribatida; Trombidiforme), Amblypygi (Phrynidae: *Heterophrynus longicornis*), Pseudoscorpiones (Chernetidae; Chtoniidae), Araneae (Ctenidae: *Ctenus* sp.; Filistatidae; Gnaphosidae; Oonopidae: Oonopidae; Salticidae; Scytodidae*: Scytodes eleonora*; Psauridae; Pholcidae: *Mesabolivar* sp., *Metagonia* sp.; Theridiosomatidae), Thysanura (Nicoletiidae: Nicoletiinae; Meinertellidae), Diplura (Campodeidae), Collembola (Entomobryidae; Isotomidae), Orthoptera (Phalangopsidae: *Phalangopsis* sp.), Blattodea (Polyphagidae), Isoptera (Termitidae: *Armitermes* sp., *Nasutitermes* sp.), Psocoptera (Archipsocidae; Epipsocidae; Pachytroctidae), Hemiptera (Aradidae; Cydnidae; Lygaeidae; Ploiariidae; Ploiariidae), Homoptera (Cixidae: *Cixius* sp.; Derbidae), Lepidoptera (Noctuidae; Tineidae), Diptera (Cecidomyiidae; Chironomidae; Dolichopodidae; Drosophilidae; Psychodidae: *Lutzomyia* sp.), Hymenoptera (Formicidae: *Acromyrmex* sp., *Anochetus* sp., *Camponotus* sp., *Dolichoderus* sp., *Pachycondyla* sp., *Rogeria* sp., *Solenopsis* sp., *Strumigenys* sp., *Zacryptocerus* sp.), Neuroptera (Myrmeleontidae), Coleoptera (Dermestidae; Staphylinidae).

Dentre os vertebrados foram encontrados quatro morfoespécies pertencentes às ordens: Anura (Leptodactylidae: *Pristimantis cf. fenestratus*), Chiroptera (Emballonuridae: *Peropteryx kappleri*; Phyllostomidae: *Glossophaga soricina*), Squamata (Gekkonidae: *Thecadactylus rapicauda*).

Dessa forma, foi encontrado um total de 75 morfoespécies. Dentre essas, uma foi considerada troglóbia, a qual pertence ao táxon Gastropoda (Systrophiidae).

5.4.4.83. SL-086

5.4.4.83.1. Caracterização trófica

Pequena cavidade formada no minério de ferro com 13,2 m de projeção horizontal localizada em área de encosta. A vegetação do entorno é composta basicamente por samambaias de grande porte e pequenos arbustos distribuídos de forma esparsa. A caverna possui apenas uma entrada ampla, com piso descendente e muitos blocos abatidos. Esta área apresenta pouca serrapilheira acumulada junto à linha d'água. A entrada é, ainda, parcialmente ensolarada com muitos líquens e briófitas que se distribuem até as zonas mais profundas da cavidade revestindo as paredes e o teto. A cavidade apresenta trechos em teto baixo ao longo de sua extensão, não possui zona afótica e o piso é descendente e seco, com alguns blocos abatidos (calhaus e matacões) na região próxima à entrada. Após os blocos, o piso é plano composto por sedimento fino, úmido e sem blocos (Figura 218). Existem poucas raízes na macro-caverna e não foram observados depósitos de guano. O sistema de canalículos é pouco desenvolvido, mas existem muitas frestas nas paredes e teto. Não foram observados gotejamentos ativos na cavidade durante a estação seca e existem muitos Actinomicetos associados às paredes e teto. Durante a estação úmida, observou-se um aumento da umidade no solo e a presença de alguns pontos de gotejamento nas zonas mais internas da cavidade. No ambiente externo a vegetação foi queimada em algumas áreas diminuindo o sombreamento nas entradas das cavidades desta região e por conseqüência a quantidade de matéria orgânica disponível no ambiente hipógeo.

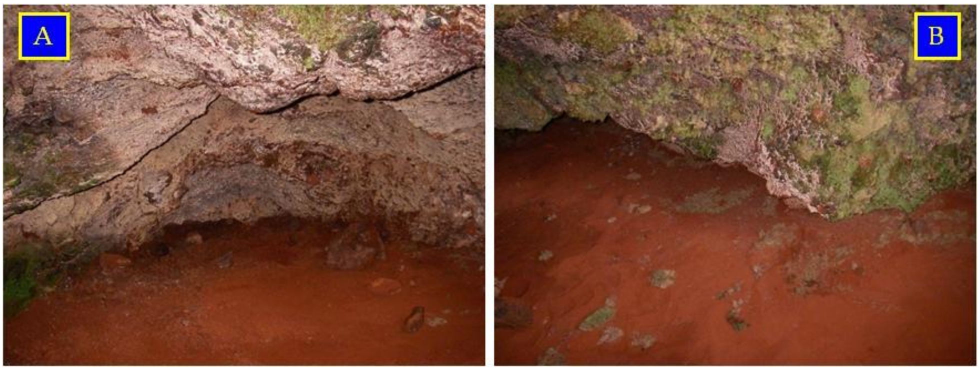

Figura 218 - a) Vista da porção interna da cavidade; b) presença de grande quantidade de musgos, líquens e Actinomicetos nas paredes da caverna.

5.4.4.85.2. Caracterização faunística no período de seca

Foi encontrado na caverna, um total de 17 morfoespécies de invertebrados em pelo menos 15 famílias dos táxons: Isopoda (Balloniscidae indet.), Acari (Ixodidae: *Amblyomma* sp.), Amblypygi (Phrynidae: *Heterophrynus longicornis*), Pseudoscorpiones (Chernetidae; Chtoniidae), Araneae (Pholcidae; Theridiosomatidae), Thysanura (Nicoletiidae: Nicoletiinae), Orthoptera (Phalangopsidae: *Phalangopsis* sp.), Psocoptera (Epipsocidae), Lepidoptera (Noctuidae; Tineidae), Diptera (Dolichopodidae; Psychodidae: *Lutzomyia* sp.), Hymenoptera (Formicidae: *Camponotus* sp., *Pachycondyla* sp.).

Dentre os vertebrados foi encontrada uma morfoespécie pertencente à ordem Anura (Leptodactylidae: *Pristimantis cf. fenestratus*). Dessa forma, foi encontrado um total de 18 morfoespécies.

5.4.4.83.3. Caracterização faunística no período de chuva

Foi encontrado na caverna, um total de 32 morfoespécies de invertebrados em pelo menos 23 famílias dos táxons: Acari (Argasidae: *Ornithodoros* sp.; Oribatida), Pseudoscorpiones (Chtoniidae), Opiliones (Phalangiidae), Araneae (Salticidae; Scytodidae*: Scytodes eleonora*; Pholcidae; Prodidomidae; Theridiosomatidae), Thysanura (Meinertellidae), Collembola (Sminthuridae; Entomobryidae), Orthoptera (Phalangopsidae: *Aclodes* sp., *Phalangopsis* sp.), Blattodea (Polyphagidae), Psocoptera (Ptiloneuridae: *Ptiloneura* sp.), Lepidoptera (Noctuidae; Tineidae), Diptera (Ceratopogonidae; Chironomidae; Culicidae; Milichiidae; Psychodidae: *Lutzomyia* sp.), Hymenoptera (Formicidae: *Pachycondyla* sp.), Coleoptera (Staphylinidae).

Dentre os vertebrados foi encontrada uma morfoespécie pertencente à ordem Chiroptera (Emballonuridae: *Peropteryx kappleri*). Dessa forma, foi encontrado um total de 33 morfoespécies.

5.4.83.4. Caracterização geral da fauna da cavidade

Foi encontrado na caverna, um total de 44 morfoespécies de invertebrados em pelo menos 30 famílias dos táxons: Isopoda (Balloniscidae indet.), Acari (Argasidae: *Ornithodoros* sp.; Ixodidae: Amblyomma sp.; Oribatida), Amblypygi (Phrynidae: *Heterophrynus longicornis*), Pseudoscorpiones (Chernetidae; Chtoniidae), Opiliones (Phalangiidae), Araneae (Salticidae; Scytodidae*: Scytodes eleonora*; Pholcidae; Prodidomidae; Theridiosomatidae), Thysanura (Nicoletiidae: Nicoletiinae; Meinertellidae), Collembola (Sminthuridae; Entomobryidae), Orthoptera (Phalangopsidae: *Aclodes* sp., *Phalangopsis* sp.), Blattodea (Polyphagidae), Psocoptera (Epipsocidae; Ptiloneuridae: *Ptiloneura* sp.), Lepidoptera (Noctuidae; Tineidae), Diptera (Ceratopogonidae; Chironomidae; Culicidae; Dolichopodidae; Milichiidae;

Psychodidae: *Lutzomyia* sp.), Hymenoptera (Formicidae: *Camponotus* sp., *Pachycondyla* sp.), Coleoptera (Staphylinidae).

Dentre os vertebrados foram encontradas duas morfoespécies pertencentes às ordens Anura (Leptodactylidae: *Pristimantis cf. fenestratus*) e Chiroptera (Emballonuridae: *Peropteryx kappleri*). Dessa forma, foi encontrado um total de 46 morfoespécies.

5.4.4.84. SL-087

5.4.4.84.1. Caracterização trófica

Caverna formada no minério de ferro com 36 m de projeção horizontal e localizada em área de encosta. A vegetação do entorno é composta por mata arbórea com árvores de troncos finos e dossel inferior a dez metros de altura com muitas lianas e cipós. A caverna possui uma entrada ampla e sombreada com piso irregular formado por muitos blocos abatidos. Nesta área existem muitos líquens, briófitas além de plântulas de angiospermas e muita serrapilheira acumulada junto à linha d'água. Existe uma pequena área com teto baixo, mas, de forma geral, trata-se de uma caverna ampla com dois condutos principais contendo pequenas zonas afóticas. O piso da cavidade é predominantemente plano, sendo seco na entrada e úmido nas zonas mais profundas da cavidade. Este piso é composto por sedimento fino e granulado com alguns blocos de grande porte (calhaus e matacões) distribuídos de forma esparsa. O sistema radicular é bem desenvolvido com muitas raízes de fino calibre no conduto à direita da entrada da cavidade, onde existem grandes depósitos de guano produzidos por morcegos Glossophaginae e uma estrutura fúngica cultivada por formigas cortadeiras (*Apterostigma sp.*). Foi observada uma cabeça de um lagarto em avançado estágio de decomposição, repleta de fungos (Figura 219). Tal resto orgânico frequentemente serve de alimento para inúmeros invertebrados saprófagos. Existem alguns pontos de gotejamento ativos mesmo durante a estação seca nas zonas mais internas da cavidade, além de pequenas manchas envelhecidas de guano de morcegos hematófagos. O sistema de canalículos é pouco desenvolvido e a cavidade apresenta uma elevada estabilidade ambiental em suas zonas mais interiores. Durante a estação úmida algumas áreas mais distais da cavidade estavam encharcadas em virtude da presença de pontos de gotejamento e percolação. No ambiente externo a vegetação foi queimada em algumas áreas diminuindo o sombreamento nas entradas das cavidades desta região e por conseqüência a quantidade de matéria orgânica disponível no ambiente hipógeo.

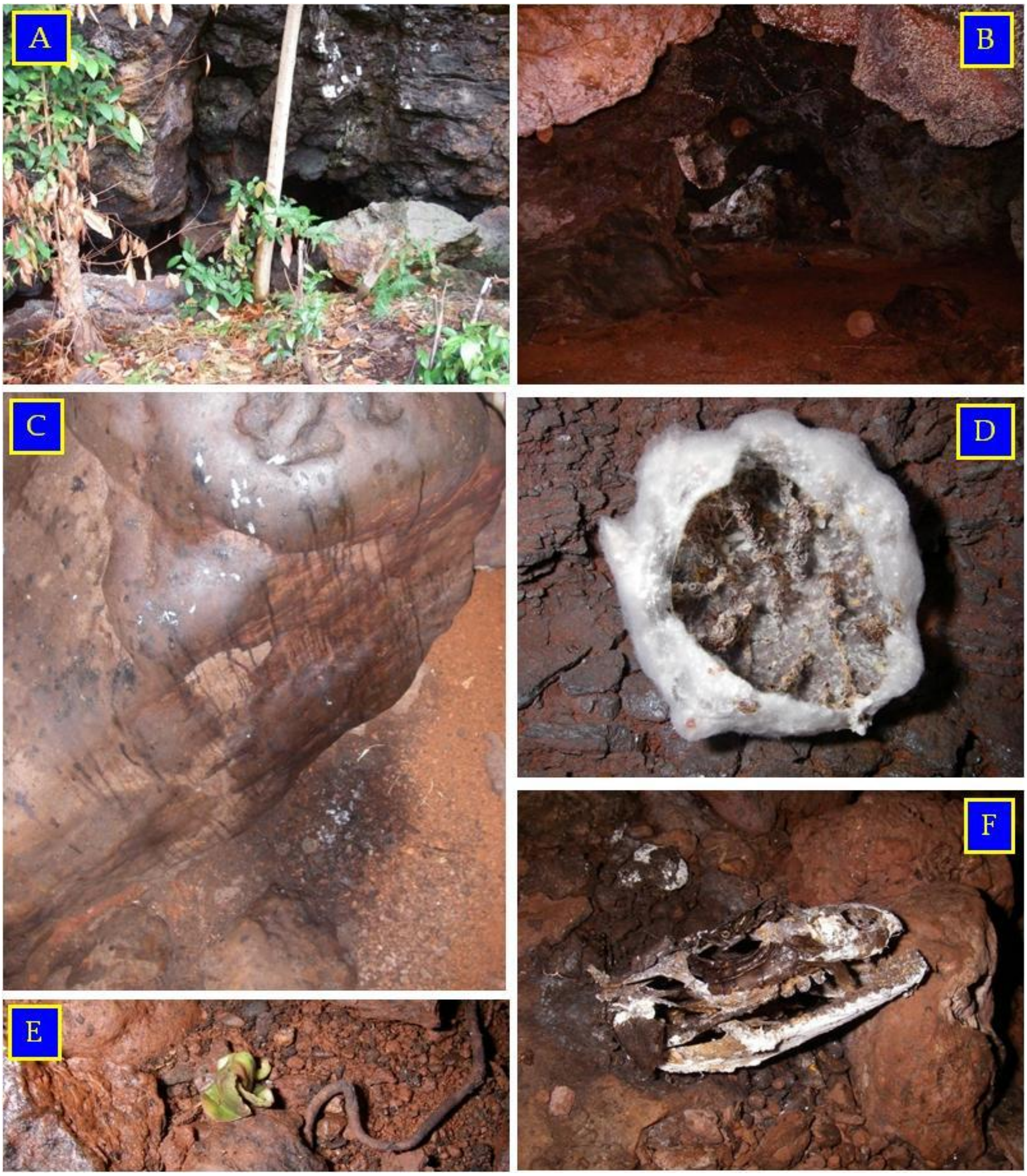

Figura 219 - a)Vista externa da entrada da cavidade; b) Aspecto geral de um dos salões principais da cavidade; c) Depósito de guano de morcegos hematófagos em zona afótica; d) Fungos cultivados por colônia de formigas (*Apterostigma sp*.); e) Restos de frutos abandonados no interior da cavidade; f) Cabeça de um lagarto em avançado estágio de decomposição (reparar no crescimento fúngico).

5.4.4.84.2. Caracterização faunística no período de seca

Foi encontrado na caverna, um total de 61 morfoespécies de invertebrados em pelo menos 44 famílias dos táxons: Gordioidea; Gastropoda (Systrophiidae), Isopoda (Armadillidae), Acari (Argasidae: *Ornithodoros* sp.; Mesostigmata; Oribatida), Amblypygi (Phrynidae:

Heterophrynus longicornis; Charinidae: *Charinus* sp.), Pseudoscorpiones (Chernetidae; Chtoniidae), Araneae (Gnaphosidae; Ochyroceratidae; Oonopidae: Oonopinae; Salticidae; Scytodidae*: Scytodes eleonora*; Pholcidae: *Mesabolivar* sp.; Theraphosidae; Theridiidae; Theridiosomatidae), Thysanura (Nicoletiidae: Nicoletiinae), Collembola (Sminthuridae; Entomobryidae), Orthoptera (Phalangopsidae: *Aclodes* sp., *Phalangopsis* sp.), Blattodea (Blaberidae: *Blaberus* sp.; Polyphagidae), Isoptera (Rhinotermitidae: *Heterotermes* sp.; Termitidae: *Nasutitermes* sp.), Psocoptera (Pachytroctidae), Hemiptera (Cydnidae; Dipsocoridae, Enicocephalidae), Homoptera (Cixidae: *Cixius* sp.), Lepidoptera (Noctuidae; Tineidae), Diptera (Dolichopodidae; Drosophilidae; Milichiidae; Psychodidae: *Lutzomyia* sp.; Streblidae; Fanniidae), Hymenoptera (Formicidae: *Apterostigma* sp., *Camponotus* sp., *Cyphomyrmex* sp., *Pachycondyla* sp.; Vespidae), Coleoptera (Ptylidae; Scydmaenidae; Elateridae: Cardiophorinae), Diplopoda (Pyrgodesmidae), Lithobiomorpha (Henicopiidae: *Lamyctes* sp.).

Dentre os vertebrados foram encontradas duas morfoespécies pertencentes à ordem Chiroptera (Emballonuridae: *Peropteryx kappleri*; Phyllostomidae: *Glossophaga soricina*).

Dessa forma, foi encontrado um total de 63 morfoespécies. Dentre essas, cinco foram consideradas troglomórficas, as quaisl pertencem aos táxons: Gordioidea, Gastropoda (Systrophiidae), Amblypygi (Charinidae: *Charinus* sp.), Araneae (Oonopidae: Oonopinae), Diplopoda (Pyrgodesmidae).

5.4.4.84.3. Caracterização faunística no período de chuva

Foi encontrado na caverna, um total de 56 morfoespécies de invertebrados em pelo menos 40 famílias dos táxons: Gastropoda (Subulinidae), Isopoda (Armadillidae), Acari (Argasidae: *Ornithodoros* sp.; Ixodidae: *Amblyomma* sp.; Trombidiforme), Amblypygi (Phrynidae: *Heterophrynus longicornis*; Charinidae: *Charinus* sp.), Ricinulei (Ricinoididae: *Cryptocellus tarsilae*), Pseudoscorpiones (Chtoniidae), Opiliones (Escadabiidae), Araneae (Ctenidae: *Ctenus* sp.; Filistatidae; Gnaphosidae; Ochyroceratidae; Salticidae; Pholcidae: *Mesabolivar* sp.; Theridiidae; Theridiosomatidae), Thysanura (Nicoletiidae: Nicoletiinae), Diplura (Anajapygidae; Campodeidae), Collembola (Cyphoderidae; Entomobryidae), Blattodea (Blaberidae: *Blaberus* sp.; Polyphagidae), Psocoptera (Pachytroctidae; Psyllipsocidae: *Psyllipsocus* sp.; Ptiloneuridae: *Ptiloneura* sp.), Hemiptera (Cydnidae), Lepidoptera (Noctuidae; Tineidae), Diptera (Chironomidae; Culicidae; Milichiidae; Psychodidae: *Lutzomyia* sp.), Hymenoptera (Formicidae: *Apterostigma* sp., *Odontomachus* sp., *Pachycondyla* sp., *Solenopsis* sp.), Diplopoda (Pyrgodesmidae), Scolopendromorpha (Cryptopidae: *Cryptops* sp.), Neuroptera (Myrmeleontidae), Coleoptera (Elateridae; Tenebrionidae: Coelometropinae).

Dentre os vertebrados foram encontradas duas morfoespécies pertencentes à ordem Chiroptera (Emballonuridae: *Peropteryx kappleri*; Phyllostomidae: *Glossophaga soricina*).

Dessa forma, foi encontrado um total de 58 morfoespécies. Dentre essas, cinco foram consideradas troglomórficas, as quaisl pertencem aos táxons: Amblypygi (Charinidae: *Charinus* sp.), Collembola (Cyphoderidae), Diplopoda (Pyrgodesmidae).

5.4.4.84.4. Caracterização geral da fauna da cavidade

Foi encontrado na caverna, um total de 98 morfoespécies de invertebrados em pelo menos 60 famílias dos táxons: Gordioidea, Gastropoda (Subulinidae; Systrophiidae), Isopoda (Armadillidae), Acari (Argasidae: *Ornithodoros* sp.; Ixodidae: *Amblyomma* sp.; Mesostigmata; Oribatida; Trombidiforme), Amblypygi (Phrynidae: *Heterophrynus longicornis*; Charinidae: *Charinus* sp.), Ricinulei (Ricinoididae: *Cryptocellus tarsilae*),Pseudoscorpiones (Chernetidae; Chtoniidae), Opiliones (Escadabiidae), Araneae (Ctenidae: *Ctenus* sp.; Filistatidae; Gnaphosidae; Ochyroceratidae; Oonopidae: Oonopinae; Salticidae; Scytodidae*: Scytodes eleonora*; Pholcidae: *Mesabolivar* sp.; Theraphosidae; Theridiidae; Theridiosomatidae), Thysanura (Nicoletiidae: Nicoletiinae), Diplura (Anajapygidae; Campodeidae), Collembola (Sminthuridae; Cyphoderidae; Entomobryidae), Orthoptera (Phalangopsidae: *Aclodes* sp., *Phalangopsis* sp.), Blattodea (Blaberidae: *Blaberus* sp.; Polyphagidae), Isoptera (Rhinotermitidae: *Heterotermes* sp.; Termitidae: *Nasutitermes* sp.), Psocoptera (Pachytroctidae; Psyllipsocidae: *Psyllipsocus* sp.; Ptiloneuridae: *Ptiloneura* sp.), Hemiptera (Cydnidae; Dipsocoridae; Enicocephalidae), Homoptera (Cixidae: *Cixius* sp.), Lepidoptera (Noctuidae; Tineidae), Diptera (Chironomidae; Culicidae; Dolichopodidae; Drosophilidae; Milichiidae; Fanniidae; Psychodidae: *Lutzomyia* sp.; Streblidae), Hymenoptera (Formicidae: *Apterostigma* sp., *Camponotus* sp., *Cyphomyrmex* sp., *Odontomachus* sp., *Pachycondyla* sp., *Solenopsis* sp.; Vespidae), Coleoptera (Ptylidae; Scydmaenidae; Elateridae: Cardiophorinae; Tenebrionidae: Coelometropinae), Diplopoda (Pyrgodesmidae), Lithobiomorpha (Henicopiidae: *Lamyctes* sp.), Scolopendromorpha (Cryptopidae: *Cryptops* sp.), Neuroptera (Myrmeleontidae).

Dentre os vertebrados foram encontradas duas morfoespécies pertencentes à ordem Chiroptera (Emballonuridae: *Peropteryx kappleri*; Phyllostomidae: *Glossophaga soricina*).

Dessa forma, foi encontrado um total de 100 morfoespécies. Dentre essas, seis foram consideradas troglomórficas, pertencendo aos táxons: Gordioidea, Gastropoda (Systrophiidae), Amblypygi (Charinidae: *Charinus* sp.), Araneae (Oonopidae: Oonopinae), Collembola (Cyphoderidae), Diplopoda (Pyrgodesmidae). Alguns organismos encontrados nesta caverna são mostrados na Figura 220.

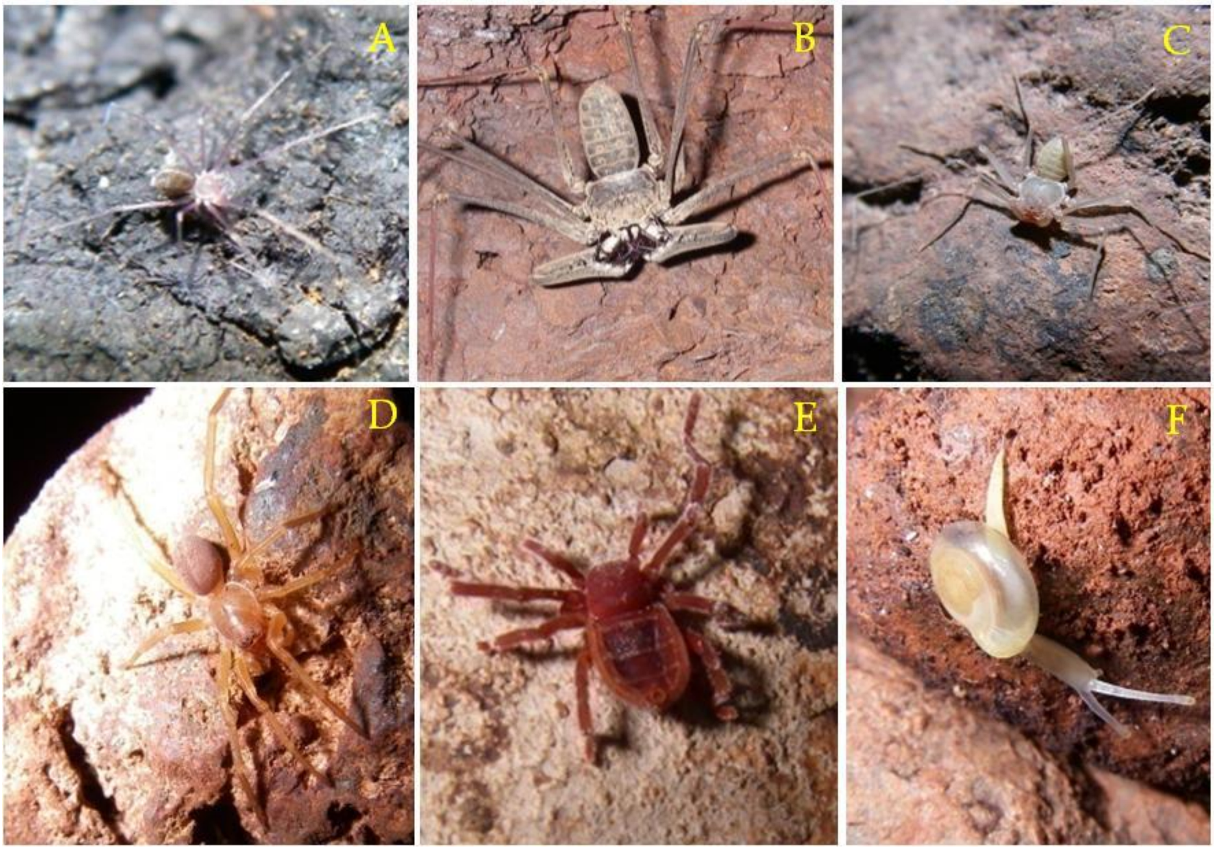

Figura 220 - a) Araneae (Pholcidae); b) Amblypygi (*Heterophrynus longicornis*); c) Amblypygi (*Charinus sp*.); d) Araneae (Corinidae); e) Ricinulei (*Cryptocellus tarsilae*); f) Gastropoda (Systrophiidae).

5.4.4.85. SL-088

5.4.4.85.1. Caracterização trófica

Pequena cavidade desenvolvida em minério de ferro com 7,6 m de projeção horizontal localizada no topo da encosta. A vegetação do entorno é composta por mata arbórea com árvores de troncos finos e dossel inferior a dez metros de altura com muitas lianas, cipós e samambaias. Possui uma entrada ampla e muito iluminada com muitos líquens, briófitas, pteridófitas, filodendros e plântulas de angiospermas distribuídas pelo piso, paredes e teto. Na entrada, o piso é descendente, apresentando muitos blocos abatidos e grande quantidade de serrapilheira acumulada junto à linha d'água. Trata-se de uma pequena cavidade completamente eufótica e com forte influência das condições ambientais epígeas. O piso da porção interna da cavidade é plano e seco, sendo composto por sedimento granulado e com alguns blocos maiores (calhaus) restritos à zona de entrada (Figura 221). Existe um sistema radicular bem desenvolvido com raízes de diferentes calibres distribuídas pelo piso e paredes. Não foram observados depósitos de guano e pontos de gotejamento e o sistema de canalículos é pouco desenvolvido. Nenhuma alteração significativa foi observada durante a estação úmida, além das alterações normais na umidade relativa do ar. No ambiente externo a vegetação foi queimada em algumas áreas diminuindo o sombreamento

nas entradas cavidades desta região e por conseqüência a quantidade de matéria orgânica disponível no ambiente hipógeo.

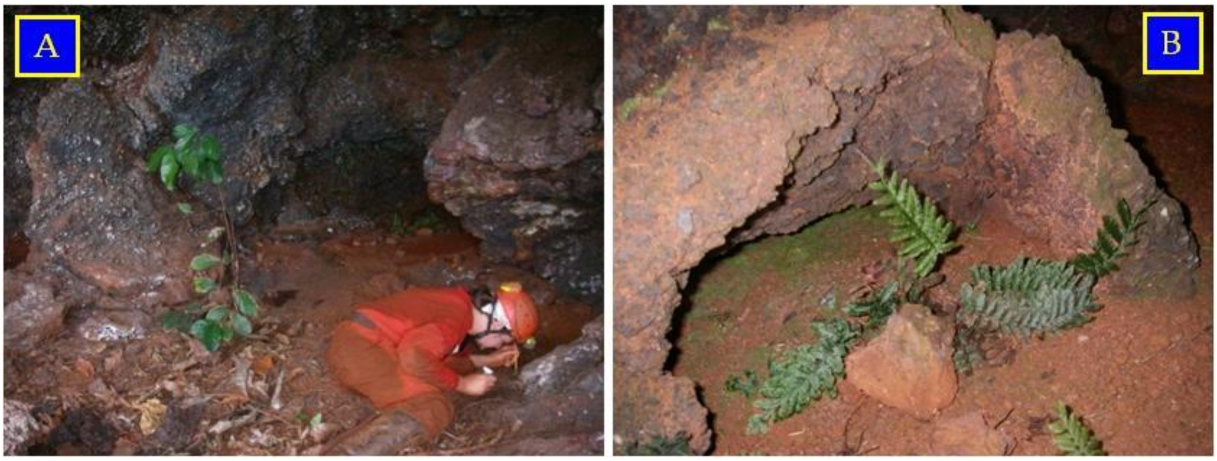

Figura 221 – a) Aspecto geral da entrada e da vegetação associada; b) Pteridófitas associadas à zona de entrada da cavidade.

5.4.4.85.2. Caracterização faunística no período de seca

Foi encontrado na caverna, um total de 36 morfoespécies de invertebrados em pelo menos 30 famílias dos táxons: Isopoda (Armadillidae; Dubioniscidae), Acari (Anystidae: *Erythracarus* sp.; Trombidiforme), Pseudoscorpiones (Chtoniidae), Araneae (Filistatidae; Oonopidae: Oonopinae; Salticidae; Scytodidae*: Scytodes eleonora*; Pholcidae: *Mesabolivar* sp.; Theridiosomatidae), Orthoptera (Phalangopsidae: *Aclodes* sp.), Blattodea (Blattellidae; Polyphagidae), Isoptera (Termitidae: *Nasutitermes* sp.), Psocoptera (Archipsocidae; Pachytroctidae; Psyllipsocidae: *Psyllipsocus* sp.), Hemiptera (Enicocephalidae), Homoptera (Cixidae: *Cixius* sp.), Diptera (Culicidae; Dolichopodidae; Drosophilidae; Milichiidae; Psychodidae: *Lutzomyia* sp.), Hymenoptera (Formicidae: *Camponotus* sp., *Pachycondyla* sp., *Tapinoma* sp.; Vespidae), Coleoptera (Staphylinidae; Dermestidae), Scolopendromorpha (Scolopocryptopidae: *Newportia* sp.), Neuroptera (Myrmeleontidae).

Dentre os vertebrados foram encontradas três morfoespécies pertencentes às ordens Chiroptera (Emballonuridae: *Peropteryx kappleri*), Anura (Leptodactylidae: *Pristimantis cf. fenestratus*; Dendrobatidae: *Ameerega* sp.). Dessa forma, foi encontrado um total de 39 morfoespécies.

5.4.4.85.3. Caracterização faunística no período de chuva

Foi encontrado na caverna, um total de 28 morfoespécies de invertebrados em pelo menos 23 famílias dos táxons: Acari (Trombidiforme), Pseudoscorpiones (Chernetidae; Chtoniidae), Araneae (Filistatidae; Salticidae; Scytodidae*: Scytodes eleonora*; Pholcidae: *Mesabolivar* sp.; Theridiidae), Blattodea (Polyphagidae), Isoptera (Termitidae: *Nasutitermes* sp.), Psocoptera

(Pachytroctidae), Hemiptera (Lygaeidae; Reduviidae), Homoptera (Cixiidae), Lepidoptera (Noctuidae; Tineidae; Tortricidae), Diptera (Ceratopogonidae; Culicidae; Milichiidae; Psychodidae: *Lutzomyia* sp.), Hymenoptera (Formicidae: *Pachycondyla* sp.), Coleoptera (Elmidae), Neuroptera (Myrmeleontidae).

5.4.4.85.4. Caracterização geral da fauna da cavidade

Foi encontrado na caverna, um total de 55 morfoespécies de invertebrados em pelo menos 39 famílias dos táxons: Isopoda (Armadillidae; Dubioniscidae), Acari (Anystidae: *Erythracarus* sp.; Trombidiforme), Pseudoscorpiones (Chernetidae; Chtoniidae), Araneae (Filistatidae; Oonopidae: Oonopinae; Salticidae; Scytodidae*: Scytodes eleonora*; Pholcidae: *Mesabolivar* sp.; Theridiidae; Theridiosomatidae), Orthoptera (Phalangopsidae: *Aclodes* sp.), Blattodea (Blattellidae; Polyphagidae), Isoptera (Termitidae: *Nasutitermes* sp.), Psocoptera (Archipsocidae; Pachytroctidae; Psyllipsocidae: *Psyllipsocus* sp.), Hemiptera (Enicocephalidae; Lygaeidae; Reduviidae0, Homoptera (Cixidae: *Cixius* sp.), Lepidoptera (Noctuidae; Tineidae; Tortricidae), Diptera (Ceratopogonidae; Culicidae; Dolichopodidae; Drosophilidae; Milichiidae; Psychodidae: *Lutzomyia* sp.), Hymenoptera (Formicidae: Camponotus sp., Pachycondyla sp., Tapinoma sp.; Vespidae), Coleoptera (Elmidae; Staphylinidae; Dermestidae), Scolopendromorpha (Scolopocryptopidae: *Newportia* sp.), Neuroptera (Myrmeleontidae).

Dentre os vertebrados foram encontradas três morfoespécies pertencentes às ordens Chiroptera (Emballonuridae: *Peropteryx kappleri*), Anura (Leptodactylidae: *Pristimantis cf. fenestratus*; Dendrobatidae: *Ameerega* sp.). Dessa forma, foi encontrado um total de 58 morfoespécies.

5.4.4.86. SL-089

5.4.4.86.1. Caracterização trófica

Caverna de grande volume e dimensões com 44 m de projeção horizontal formada no minério de ferro e localizada em área de encosta. A vegetação do entorno é composta por mata arbórea com árvores de troncos finos e dossel inferior a dez metros de altura com muitas lianas, cipós e samambaias. Possui uma entrada ampla e sombreada pela mata e a região acima da cavidade é ocupada por uma vegetação do tipo arbustiva (savana metalófila). Na entrada existem muitos líquens, briófitas e angiospermas além de muita serrapilheira acumulada junto à linha d’água. Apesar da cavidade apresentar uma morfologia linear, é possível identificar dois compartimentos bem distintos. O primeiro corresponde à região de entrada (eufótico) onde a cavidade é mais seca. O piso neste compartimento é ascendente e composto por sedimento clástico no qual se observam muitas raízes, algumas das quais se emaranhando na forma de rizotemas (Figura 222). Nesta região, o sistema de

canalículos é pouco desenvolvido e as paredes e o teto são revestidos por Actinomicetos. O segundo compartimento corresponde à porção mais distal da cavidade, onde existe uma maior estabilidade ambiental. Esta região é disfótica, embora apresente um pequeno salão completamente afótico. As galerias possuem o teto alto e o conduto é amplo. O piso é composto por sedimento fino argiloso, muito úmido, com poucos blocos e poucas raízes. Neste setor existem depósitos de guano de morcegos frugívoros lixiviados por processos de gotejamento. Nesta área o sistema de canalículos também é pouco desenvolvido. Durante a estação úmida algumas áreas apresentaram muitos pontos de gotejamento deixando os depósitos de guano muito úmidos. No ambiente externo a vegetação foi queimada em algumas áreas diminuindo o sombreamento nas entradas das cavidades desta região e por conseqüência a quantidade de matéria orgânica disponível no ambiente hipógeo.

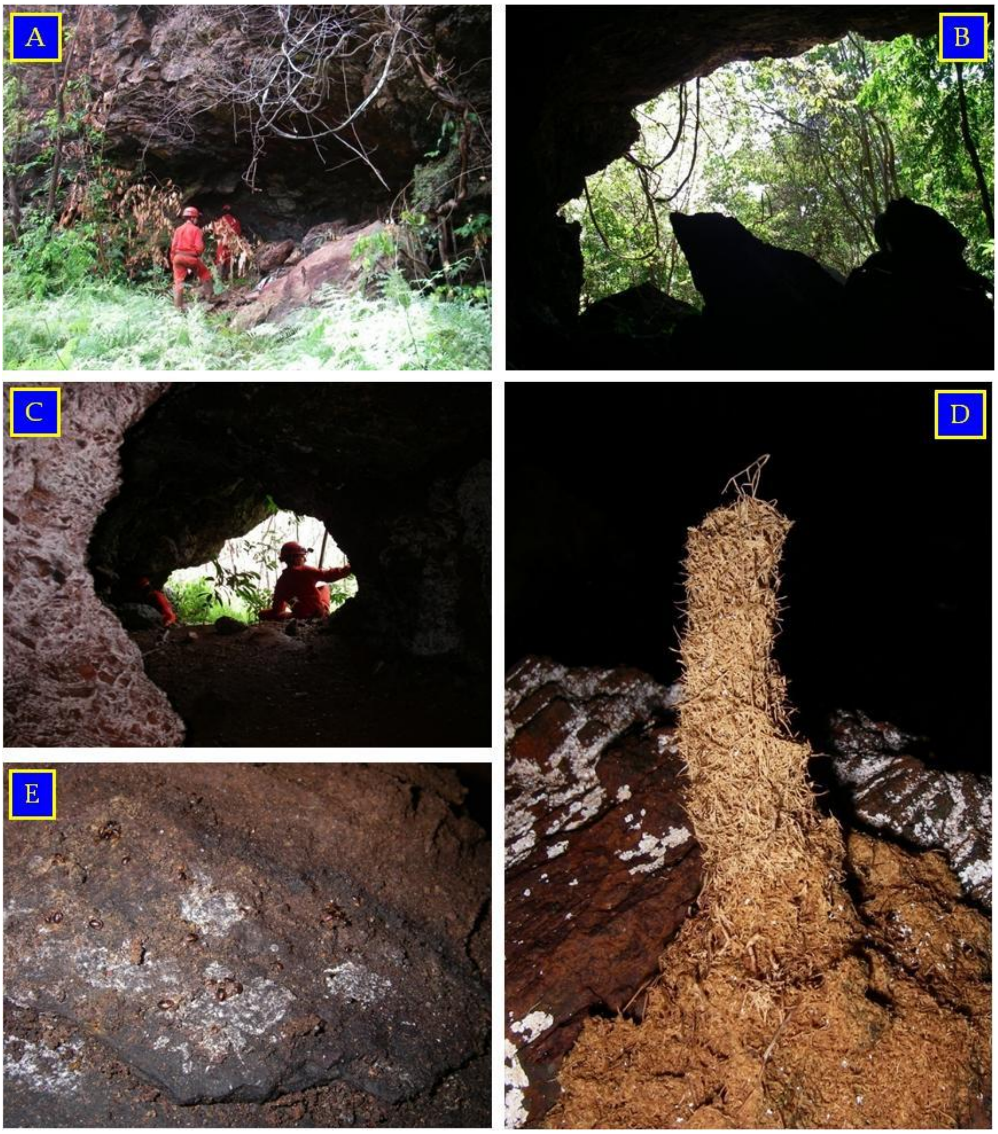

Figura 222 - a) Vista externa da entrada da cavidade, onde se observa grande quantidade de pteridófitas no piso externo; b) Vista interna da entrada da cavidade; b) Detalhe de um conduto que conecta à entrada; d) Rizotema em zona afótica da cavidade; e) Depósito de guano de morcegos frugívoros com heterópteros associados.

5.4.4.86.2. Caracterização faunística no período de seca

Foi encontrado na caverna, um total de 73 morfoespécies de invertebrados em pelo menos 52 famílias dos táxons: Gordioidea; Gastropoda (Subulinidae; Systrophiidae), Isopoda (Armadillidae), Acari (Argasidae: *Ornithodoros* sp.; Ixodidae: *Amblyomma cajennense*; Laelapidae: *Stratiolaelaps* sp.; Macronyssidae; Oribatida; Erythraeidae; Trombidiforme), Amblypygi (Charinidae: *Charinus* sp.), Ricinulei (Ricinoididae: *Cryptocellus tarsilae*), Pseudoscorpiones (Chtoniidae), Opiliones (Escadabiidae), Araneae (Ochyroceratidae; Oonopidae: Oonopinae; Salticidae; Scytodidae*: Scytodes eleonora*; Pholcidae: *Mesabolivar* sp.; Theridiidae; Theridiosomatidae), Thysanura (Nicoletiidae: Nicoletiinae, Atelurinae), Diplura (Campodeidae), Collembola (Sminthuridae; Entomobryidae; Isotomidae), Orthoptera (Gryllidae; Phalangopsidae: *Phalangopsis* sp.), Blattodea (Polyphagidae), Isoptera (Rhinotermitidae: *Heterotermes* sp.), Psocoptera (Cladiopsocidae: *Cladiopsocus* sp.; Myopsocidae: *Lichenomina* sp.; Pachytroctidae; Psyllipsocidae: *Psyllipsocus* sp.), Hemiptera (Cydnidae; Enicocephalidae; Reduviidae; Ploiariidae), Homoptera (Cixiidae: *Cixius* sp.), Lepidoptera (Noctuidae; Tineidae), Diptera (Culicidae; Dolichopodidae; Drosophilidae; Psychodidae: *Lutzomyia* sp.), Hymenoptera (Formicidae: *Anochetus* sp., *Gnamptogenys* sp., *Pachycondyla* sp., *Rogeria* sp., *Tapinoma* sp.; Vespidae), Coleoptera (Curculionidae: Scotylinae), Diplopoda (Pyrgodesmidae), Lithobiomorpha (Henicopiidae: *Lamyctes* sp.), Scolopendromorpha (Cryptopidae: *Cryptops* sp.; Scolopocryptopidae: *Dinocryptops* sp.), Neuroptera (Myrmeleontidae).

Dentre os vertebrados foram encontradas seis morfoespécies pertencentes às ordens Chiroptera (Emballonuridae: *Peropteryx kappleri;* Phyllostomidae: *Glossophaga soricina*), Anura (Leptodactylidae: *Pristimantis cf. fenestratus*, *Leptodactylus labyrinthicus*, Leptodactylidae indet.; Bufonidae).

Dessa forma, foi encontrado um total de 79 morfoespécies. Dentre essas, seis foram consideradas troglomórficas, as quais pertencem aos táxons: Gordioidea, Gastropoda (Systrophiidae), Amblypygi (Charinidae: *Charinus* sp.), Araneae (Ochyroceratidae), Collembola (Isotomidae), Diplopoda (Pyrgodesmidae).

5.4.4.86.3. Caracterização faunística no período de chuva

Foi encontrado na caverna, um total de 78 morfoespécies de invertebrados em pelo menos 55 famílias dos táxons: Gordioidea; Gastropoda (Subulinidae; Systrophiidae), Isopoda (Armadillidae; Balloniscidae; Philosciidae), Acari (Argasidae: *Ornithodoros* sp.; Macronyssidae; Trombidiforme), Amblypygi (Phrynidae: *Heterophrynus longicornis*; Charinidae: *Charinus* sp.), Ricinulei (Ricinoididae: *Cryptocellus tarsilae*), Pseudoscorpiones (Chernetidae; Chtoniidae), Opiliones (Cosmetidae: *Anduzeia* sp.; Escadabiidae; Phalangiidae), Araneae (Ochyroceratidae; Salticidae; Scytodidae*: Scytodes eleonora*; Psauridae; Pholcidae:

Mesabolivar sp., *Metagonia* sp.; Theridiidae; Theridiosomatidae), Thysanura (Nicoletiidae: Nicoletiinae), Diplura (Anajapygidae; Campodeidae), Collembola (Sminthuridae; Cyphoderidae; Entomobryidae; Isotomidae), Orthoptera (Phalangopsidae: *Aclodes* sp., *Phalangopsis* sp.), Blattodea (Polyphagidae), Psocoptera, Hemiptera (Cydnidae; Enicocephalidae; Lygaeidae; Ploiariidae), Homoptera (Derbidae), Lepidoptera (Noctuoidea; Noctuidae; Tineidae), Diptera (Cecidomyiidae; Ceratopogonidae; Chironomidae; Drosophilidae; Milichiidae; Psychodidae: *Lutzomyia* sp.; Sciaridae), Hymenoptera (Formicidae: *Gnamptogenys* sp., *Pachycondyla* sp.), Coleoptera (Elateridae: Elaterinae; Pselaphidae; Ptylidae; Scydmaenidae; Staphylinidae), Diplopoda (Pyrgodesmidae), Lithobiomorpha (Henicopiidae: *Lamyctes* sp.), Scolopendromorpha (Cryptopidae: *Cryptops* sp.; Scolopendrellidae), Neuroptera (Myrmeleontidae).

Dentre os vertebrados foram encontradas três morfoespécies pertencentes às ordens Chiroptera (Emballonuridae: *Peropteryx kappleri;* Phyllostomidae: *Glossophaga soricina*), Anura (Leptodactylidae: *Pristimantis cf. fenestratus*).

Dessa forma, foi encontrado um total de 81 morfoespécies. Dentre essas, seis foram consideradas troglomórficas, as quais pertencem aos táxons: Gordioidea, Amblypygi (Charinidae: *Charinus* sp.), Collembola (Isotomidae), Diplopoda (Pyrgodesmidae).

5.4.4.86.4. Caracterização geral da fauna da cavidade

Foi encontrado na caverna, um total de 119 morfoespécies de invertebrados em pelo menos 74 famílias dos táxons: Gordioidea, Gastropoda (Subulinidae; Systrophiidae), Isopoda (Armadillidae; Balloniscidae; Philosciidae), Acari (Argasidae: *Ornithodoros* sp.; Ixodidae: *Amblyomma cajennense;* Laelapidae: *Stratiolaelaps* sp.; Macronyssidae; Oribatida; Erythraeidae; Trombidiforme), Amblypygi (Phynidae: *Heterophrynus longicornis*; Charinidae: *Charinus* sp.), Ricinulei (Ricinoididae: *Cryptocellus tarsilae*),Pseudoscorpiones (Chernetidae; Chtoniidae), Opiliones (Cosmetidae: *Anduzeia* sp.; Escadabiidae; Phalangiidae), Araneae (Ochyroceratidae; Oonopidae: Oonopinae; Salticidae; Scytodidae*: Scytodes eleonora*; Psauridae; Pholcidae: *Mesabolivar* sp., *Metagonia* sp.; Theridiidae; Theridiosomatidae), Thysanura (Nicoletiidae: Nicoletiinae, Atelurinae), Diplura (Anajapygidae; Campodeidae), Collembola (Sminthuridae; Cyphoderidae; Entomobryidae; Isotomidae), Orthoptera (Gryllidae; Phalangopsidae: *Aclodes* sp., *Phalangopsis* sp.), Blattodea (Polyphagidae), Isoptera (Rhinotermitidae: *Heterotermes* sp.), Psocoptera (Cladiopsocidae: Cladiopsocus sp.; Myopsocidae: Lichenomina sp.; Pachytroctidae; Psyllipsocidae: Psyllipsocus sp.), Hemiptera (Cydnidae; Enicocephalidae; Lygaeidae; Reduviidae; Ploiariidae), Homoptera (Cixiidae: *Cixius* sp.; Derbidae), Lepidoptera (Noctuoidea; Noctuidae; Tineidae), Diptera (Cecidomyiidae; Ceratopogonidae; Chironomidae; Culicidae; Dolichopodidae; Drosophilidae; Milichiidae; Psychodidae: *Lutzomyia* sp.; Sciaridae), Hymenoptera (Formicidae: *Anochetus* sp., *Gnamptogenys* sp., *Pachycondyla* sp., *Rogeria* sp., *Tapinoma* sp.; Vespidae), Coleoptera

(Elateridae: Elaterinae; Curculionidae: Scotylinae; Pselaphidae; Ptylidae; Scydmaenidae; Staphylinidae), Diplopoda (Pyrgodesmidae), Lithobiomorpha (Henicopiidae: *Lamyctes* sp.), Scolopendromorpha (Cryptopidae: *Cryptops* sp.; Scolopendrellidae; Scolopocryptopidae: *Dinocryptops* sp.), Neuroptera (Myrmeleontidae).

Dentre os vertebrados foram encontradas seis morfoespécies pertencentes às ordens Chiroptera (Emballonuridae: *Peropteryx kappleri;* Phyllostomidae: *Glossophaga soricina*), Anura (Leptodactylidae: *Pristimantis cf. fenestratus*, *Leptodactylus labyrinthicus*, Leptodactylidae indet.; Bufonidae).

Dessa forma, foi encontrado um total de 125 morfoespécies. Dentre essas, seis foram consideradas troglomórficas, as quais pertencem aos táxons: Gordioidea, Gastropoda (Systrophiidae), Amblypygi (Charinidae: *Charinus* sp.), Araneae (Ochyroceratidae), Collembola (Isotomidae), Diplopoda (Pyrgodesmidae). Alguns organismos encontrados nesta caverna são mostrados na Figura 223.

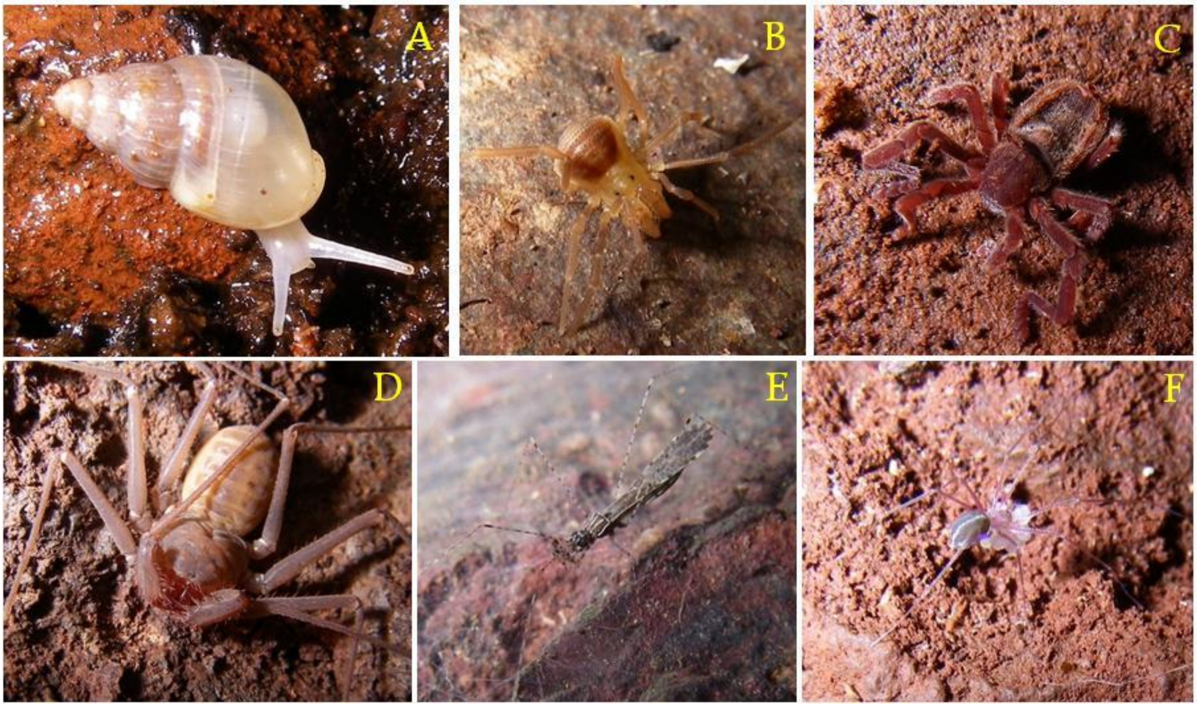

Figura 223 - . a) Gastropoda; b) Opiliones (Escadabiidae); c) Ricinulei (*Cryptocellus tarsilae*); d) Amblypygi (*Charinus sp*.); e) Hemiptera (Ploiariidae); f) Araneae (Ochyroceratidae).

5.4.4.87. SL-090

5.4.4.87.1. Caracterização trófica

Pequena cavidade formada na canga com 12,5 m de desenvolvimento localizada no topo da encosta. A vegetação do entorno é composta por mata arbórea com árvores de troncos finos

e dossel inferior a dez metros de altura com muitas lianas, cipós e samambaias. Possui uma entrada ampla, mas parcialmente obstruída por um grande bloco abatido, sendo sombreada pela mata. A região acima da cavidade é ocupada por savana metalófila. Na região de entrada existem muitos líquens, briófitas e angiospermas além de muita serrapilheira acumulada junto à linha d'água. A cavidade é formada basicamente por um conduto retilíneo e amplo com piso ascendente a partir da entrada e plano mais ao fundo, composto por sedimento fino com seixos e calhaus esparsos. O sistema radicular é bem desenvolvido, sendo composto por raízes de fino calibre e alguns rizotemas esparsos associados a pontos de gotejamentos inativos (Figura 224). A caverna é completamente eufótica com algumas plântulas germinadas em sua zona mais distal e com as paredes e teto completamente revestidas por Actinomicetos. Além disso, apresenta um sistema de canalículos pouco desenvolvido e apenas pequenos depósitos de guano antigos de morcegos frugívoros, apesar de terem sido observadas dezenas de morcegos Glossophaginae (*G. soricina*) no interior da cavidade. Durante a estação úmida algumas áreas apresentaram muitos pontos de gotejamento deixando os depósitos de guano muito úmidos. No ambiente externo a vegetação foi queimada em algumas áreas diminuindo o sombreamento nas entradas das cavidades desta região e por conseqüência a quantidade de matéria orgânica disponível no ambiente hipógeo.

5.4.4.87.2. Caracterização faunística no período de seca

Foi encontrado na caverna, um total de 23 morfoespécies de invertebrados em pelo menos 19 famílias dos táxons: Acari (Ixodidae: *Amblyomma* sp.), Araneae (Oonopidae: Oonopinae; Senoculidae; Scytodidae*: Scytodes eleonora*; Pholcidae; Tetrablemidae), Diplura (Campodeidae), Orthoptera (Phalangopsidae), Blattodea (Blaberidae: *Blaberus* sp.), Psocoptera (Epipsocidae), Homoptera (Cixiidae: *Cixius* sp.), Lepidoptera (Noctuidae; Tineidae), Diptera (Ceratopogonidae), Hymenoptera (Formicidae: *Gnamptogenys* sp., *Pachycondyla* sp.), Coleoptera (Carabidae; Phalacridae INDET.), Scolopendromorpha (Cryptopidae: *Cryptops* sp.), Neuroptera (Myrmeleontidae).

Dentre os vertebrados foram encontradas quatro morfoespécies pertencentes às ordens Chiroptera (Emballonuridae: *Peropteryx kappleri;* Phyllostomidae: *Glossophaga soricina, Anoura* sp.), Anura (Leptodactylidae: *Pristimantis cf. fenestratus*). Dessa forma, foi encontrado um total de 27 morfoespécies.

5.4.4.87.3. Caracterização faunística no período de chuva

Foi encontrado na caverna, um total de 34 morfoespécies de invertebrados em pelo menos 26 famílias dos táxons: Gastropoda (Subulinidae), Acari (Trombidiforme), Amblypygi (*Heterophrynus longicornis*; Charinidae: *Charinus* sp.), Schizomida (Hubbardiidae: Hubbardiinae), Pseudoscorpiones (Chernetidae; Chtoniidae), Araneae (Araneidae: *Alpaida* sp.; Filistatidae; Oonopidae: Oonopinae; Salticidae; Scytodidae*: Scytodes eleonora*;

Pholcidae: *Metagonia* sp.; Theridiosomatidae), Diplura (Campodeidae; Japygidae), Collembola (Sminthuridae; Entomobryidae), Orthoptera (Gryllidae; Phalangopsidae: *Phalangopsis* sp.), Psocoptera, Hemiptera (Cydnidae), Homoptera (Cixiidae), Lepidoptera (Noctuoidea; Tineidae), Hymenoptera (Formicidae: *Gnamptogenys* sp.; Elasmidae), Coleoptera (Carabidae), Neuroptera.

Dentre os vertebrados foi encontrada uma morfoespécie pertencente à ordem Chiroptera (Phyllostomidae: *Glossophaga soricina).* Dessa forma, foi encontrado um total de 35 morfoespécies. Dentre estas, duas foram consideradas troglomórficas, as quais pertencem aos táxons: Amblypygi (Charinidae: *Charinus* sp.) e Schizomida (Hubbardiidae: Hubbardiinae).

5.4.4.87.4. Caracterização geral da fauna da cavidade

Foi encontrado na caverna, um total de 52 morfoespécies de invertebrados em pelo menos 36 famílias dos táxons: Gastropoda (Subulinidae), Acari (Ixodidae: *Amblyomma* sp.; Trombidiforme), Amblypygi (Phrynidae: *Heterophrynus longicornis*; Charinidae: *Charinus* sp.), Schizomida (Hubbardiidae: Hubbardiinae), Pseudoscorpiones (Chernetidae; Chtoniidae), Araneae (Araneidae: *Alpaida* sp.; Filistatidae; Oonopidae: Oonopinae; Salticidae; Senoculidae; Scytodidae*: Scytodes eleonora*; Pholcidae: *Metagonia* sp.; Tetrablemidae; Theridiosomatidae), Diplura (Campodeidae; Japygidae), Collembola (Sminthuridae; Entomobryidae), Orthoptera (Gryllidae; Phalangopsidae: *Phalangopsis* sp.), Blattodea (Blaberidae: *Blaberus* sp.), Psocoptera (Epipsocidae), Hemiptera (Cydnidae), Homoptera (Cixiidae: *Cixius* sp.), Lepidoptera (Noctuoidea; Noctuidae; Tineidae), Diptera (Ceratopogonidae), Hymenoptera (Formicidae: *Gnamptogenys* sp., *Pachycondyla* sp.; Elasmidae), Coleoptera (Carabidae; Phalacridae), Scolopendromorpha (Cryptopidae: *Cryptops* sp.), Neuroptera (Myrmeleontidae).

Dentre os vertebrados foram encontrados quatro morfoespécies pertencentes às ordens Chiroptera (Emballonuridae: *Peropteryx kappleri;* Phyllostomidae: *Glossophaga soricina, Anoura* sp.), Anura (Leptodactylidae: *Pristimantis cf. fenestratus*).

Dessa forma, foi encontrado um total de 56 morfoespécies. Dentre estas, duas foram consideradas troglomórficas, as quais pertencem aos táxons: Amblypygi (Charinidae: *Charinus* sp.) e Schizomida (Hubbardiidae: Hubbardiinae).

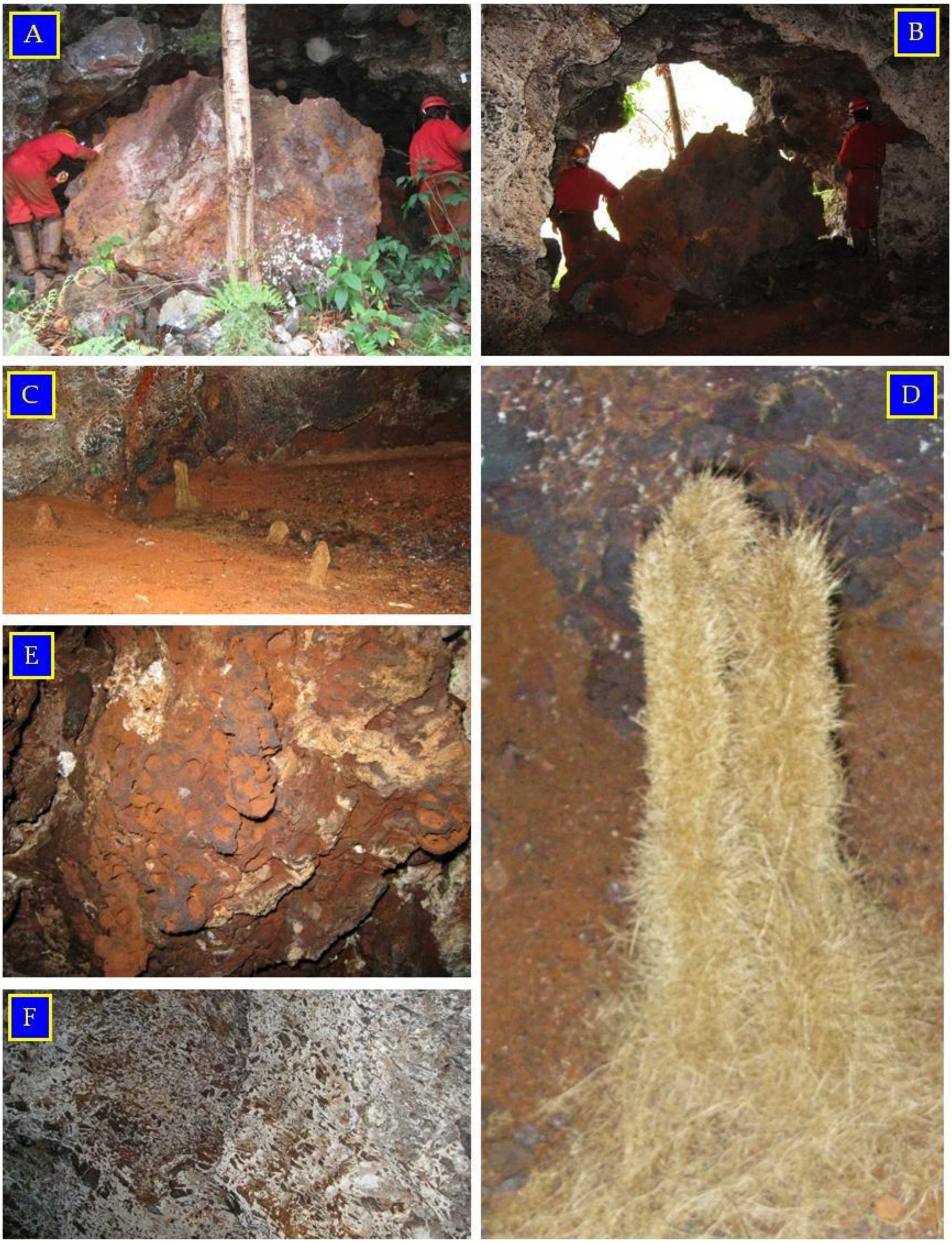

Figura 224 - a) Vista externa da entrada da cavidade; b) Vista interna da entrada da cavidade; c) Aspecto geral do piso no interior da cavidade (notar a presença de rizotemas); d) Rizotema associado a um ponto de gotejamento inativo; e) Ninhos abandonados de vespas presos às paredes da caverna; f) Teto completamente revestido por Actinomicetos.

5.4.4.88. SL-091

5.4.4.88.1. Caracterização trófica

Pequena cavidade formada por grandes blocos abatidos sobrepostos com projeção horizontal de 5,5 m. A vegetação do entorno é composta por mata arbórea com árvores de troncos finos e dossel inferior a dez metros de altura com muitas lianas, cipós e samambaias. A região acima da cavidade é ocupada por uma vegetação do tipo arbustiva (savana metalófila). Sua entrada é ampla e ensolarada com muitos líquens, briófitas além de muita serrapilheira acumulada junto à linha d'água. Trata-se de uma cavidade seca, sem pontos de gotejamento, com muitas raízes de pequeno calibre e depósitos de serrapilheira junto aos blocos. O piso é ascendente com muitas briófitas secas, sendo este completamente formado pelos próprios blocos abatidos (Figura 225). Não existem depósitos de guano no interior da cavidade e não existem zonas afóticas. O sistema de canalículos é pouco desenvolvido e cavidade apresenta uma baixa estabilidade ambiental. Nenhuma alteração significativa foi observada durante a estação úmida, além das alterações normais na umidade relativa do ar. No ambiente externo a vegetação foi queimada em algumas áreas diminuindo o sombreamento nas entradas das cavidades desta região e por conseqüência a quantidade de matéria orgânica disponível no ambiente hipógeo.

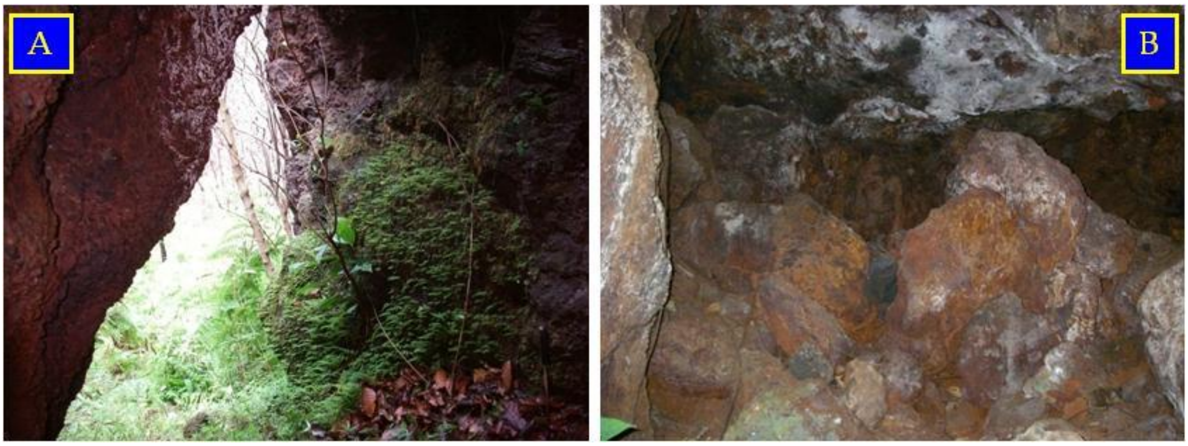

Figura 225 - a) Vista interna da entrada da cavidade; b) Aspecto geral do piso da cavidade na zona distal da cavidade.

5.4.4.88.2. Caracterização faunística no período de seca

Foi encontrado na caverna, um total de 21 morfoespécies de invertebrados em pelo menos 14 famílias dos táxons: Isopoda (Armadillidae), Pseudoscorpiones; Araneae (Salticidae; Scytodidae*: Scytodes eleonora*; Pholcidae: *Mesabolivar* sp., *Metagonia* sp.; Theraphosidae; Theridiosomatidae), Collembola (Entomobryidae), Psocoptera (Pachytroctidae), Homoptera (Cixiidae: *Cixius* sp.), Lepidoptera (Noctuidae), Diptera (Psychodidae: *Lutzomyia* sp.), Hymenoptera (Formicidae: *Gnamptogenys* sp., *Pachycondyla* sp., *Pheidole* sp., *Zacryptocerus* sp.), Coleoptera (Staphylinidae), Neuroptera (Myrmeleontidae).

Dentre os vertebrados foram encontradas duas morfoespécies pertencentes às ordens Chiroptera (Emballonuridae: *Peropteryx kappleri),* Anura (Leptodactylidae: *Pristimantis cf. fenestratus*). Dessa forma, foi encontrado um total de 23 morfoespécies.

5.4.4.88.3. Caracterização faunística no período de chuva

Foi encontrado na caverna, um total de 33 morfoespécies de invertebrados em pelo menos 23 famílias dos táxons: Isopoda (Scleropactidae), Acari (Argasidae: *Ornithodoros* sp.; Astigmatina), Pseudoscorpiones (Chtoniidae), Opiliones (Escadabiidae), Araneae (Ochyroceratidae; Salticidae; Scytodidae*: Scytodes eleonora*; Pholcidae: *Mesabolivar* sp., *Metagonia* sp.; Theridiidae), Collembola (Entomobryidae), Blattodea (Polyphagidae), Psocoptera, Hemiptera (Lygaeidae), Homoptera (Derbidae), Lepidoptera (Noctuidae; Tineidae), Hymenoptera (Formicidae: *Camponotus* sp., *Pachycondyla* sp., *Tapinoma* sp.; *Braconidae*), Coleoptera (Scydmaenidae; Dermestidae; Tenebrionidae: Coelometropinae), Diplopoda (Polydesmida), Neuroptera (Myrmeleontidae), Diptera (Chironomidae).

Dentre os vertebrados foram encontrados duas morfoespécies pertencentes às ordens Chiroptera (Emballonuridae: *Peropteryx kappleri),* Anura (Leptodactylidae: *Pristimantis cf. fenestratus*). Dessa forma, foi encontrado um total de 35 morfoespécies.

5.4.4.88.4. Caracterização geral da fauna da cavidade

Foi encontrado na caverna, um total de 50 morfoespécies de invertebrados em pelo menos 30 famílias dos táxons: Isopoda (Armadillidae; Scleropactidae), Acari (Argasidae: *Ornithodoros* sp.; Astigmatina), Pseudoscorpiones (Chtoniidae), Opiliones (Escadabiidae), Araneae (Ochyroceratidae; Salticidae; Scytodidae*: Scytodes eleonora*; Pholcidae: *Mesabolivar* sp., *Metagonia* sp.; Theraphosidae; Theridiidae; Theridiosomatidae), Collembola (Entomobryidae), Blattodea (Polyphagidae), Psocoptera (Pachytroctidae), Hemiptera (Lygaeidae), Homoptera (Cixiidae: *Cixius* sp.; Derbidae), Lepidoptera (Noctuidae; Tineidae), Diptera (Chironomidae; Psychodidae: *Lutzomyia* sp.), Hymenoptera (Formicidae: *Camponotus* sp., *Gnamptogenys* sp., *Pachycondyla* sp., *Pheidole* sp., *Tapinoma* sp., *Zacryptocerus* sp.; Braconidae), Coleoptera (Scydmaenidae; Staphylinidae; Dermestidae; Tenebrionidae: Coelometropinae), Diplopoda (Polydesmida), Neuroptera (Myrmeleontidae).

Dentre os vertebrados foram encontradas duas morfoespécies pertencentes às ordens Chiroptera (Emballonuridae: *Peropteryx kappleri),* Anura (Leptodactylidae: *Pristimantis cf. fenestratus*). Dessa forma, foi encontrado um total de 52 morfoespécies.

5.4.4.89. SL-092

5.4.4.89.1. Caracterização trófica

Pequena cavidade formada em minério de ferro com 15 m de projeção horizontal localizada em área de encosta. A vegetação do entorno é composta por mata arbórea com árvores de troncos finos e dossel inferior a dez metros de altura com muitas lianas, cipós e samambaias. A região acima da cavidade é ocupada por savana metalófila. Inserida em um paredão com aproximadamente 15 m de altura, possui uma entrada ampla com muitos líquens, briófitas, plântulas de angiospermas e uma árvore de grande porte germinada junto à linha d'água, além de muita serrapilheira associada ao piso. Na entrada existem muitos blocos abatidos e o piso é ascendente o que dificulta a importação de matéria orgânica pela ação da água em períodos de chuva, sendo o transporte destes recursos para o interior da cavidade realizado de forma eólica e ou gravitacional. Na parte mais distal da cavidade existe uma constrição, a partir da qual a cavidade apresenta uma maior estabilidade ambiental. Nesta área, o sistema de canalículos é bem desenvolvido, o piso é inclinado e extremamente seco, sendo composto por sedimento granulado com alguns blocos (seixos e calhaus) esparsos. Não existem zonas afóticas e nem depósitos de guano apesar de terem sido observados alguns indivíduos de morcegos Glossophaginae. O sistema radicular é bem desenvolvido formado por raízes de fino calibre e não foram observados pontos de gotejamentos ativos (Figura 226). No fundo da cavidade, próximo ao trecho de rastejo, foram observadas fezes antigas de algum animal herbívoro. As paredes e o teto da cavidade são completamente revestidos por Actinomicetos (Figura 226). Nenhuma alteração significativa foi observada durante a estação úmida, além das alterações normais na umidade relativa do ar. No ambiente externo a vegetação foi queimada em algumas áreas diminuindo o sombreamento nas entradas das cavidades desta região e por conseqüência a quantidade de matéria orgânica disponível no ambiente hipógeo.

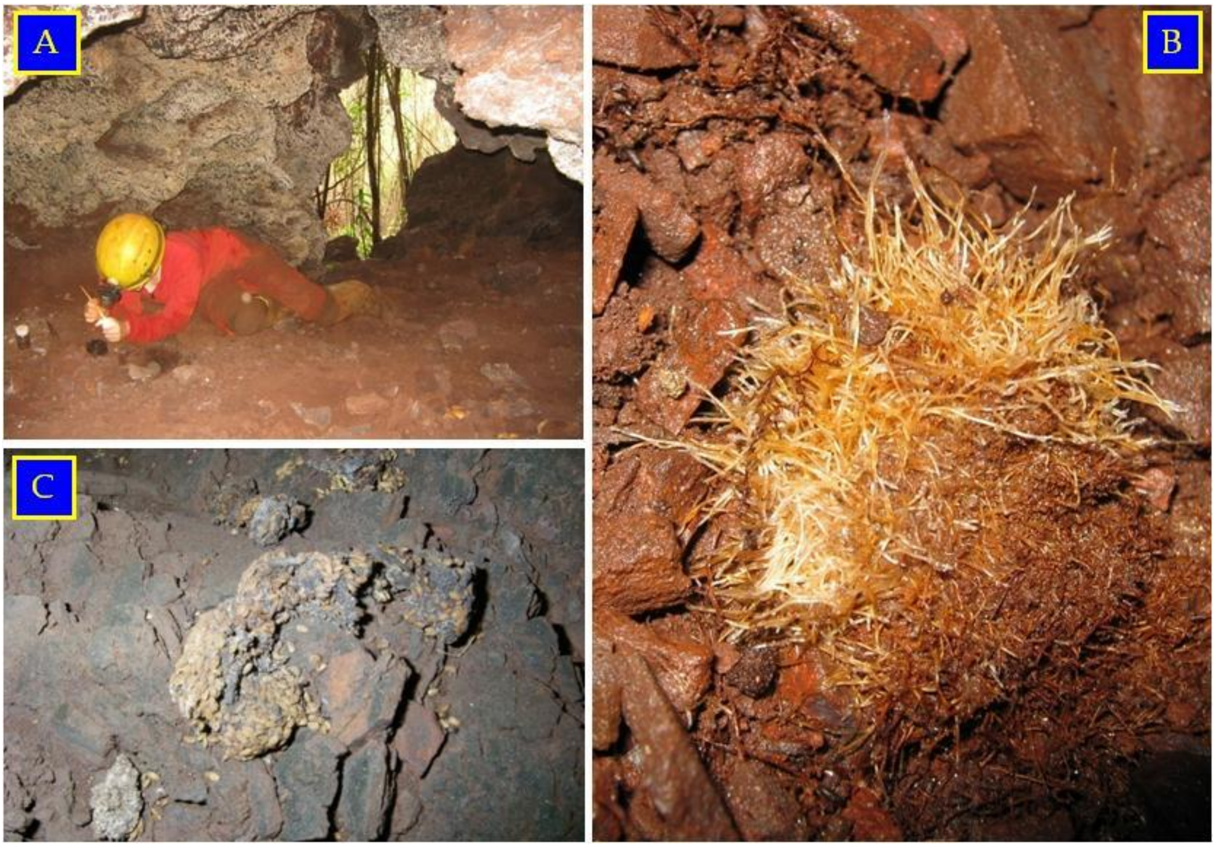

Figura 226 - a) Vista interna da entrada da cavidade (reparar a grande quantidade de Actinomicetos nas paredes); b) Rizotema se desenvolvendo no piso; c) Fezes de um vertebrado frugívoro.

5.4.4.89.2. Caracterização faunística no período de seca

Foi encontrado na caverna, um total de 25 morfoespécies de invertebrados em pelo menos 19 famílias dos táxons: Isopoda (Armadillidae), Amblypygi (*Heterophrynus longicornis*), Pseudoscorpiones (Chernetidae; Chtoniidae), Opiliones (Sclerosomatidae: *Prionostemma* sp.), Araneae (Scytodidae*: Scytodes eleonora*; Pholcidae: *Mesabolivar* sp.; Theridiidae), Thysanura (Nicoletiidae: Nicoletiinae), Diplura (Anajapygidae), Orthoptera (Phalangopsidae: *Aclodes* sp., *Phalangopsis* sp.), Hemiptera (Cydnidae), Lepidoptera (Noctuidae; Tineidae), Diptera (Dolichopodidae; Milichiidae; Psychodidae: *Lutzomyia* sp.), Hymenoptera (Formicidae: *Camponotus* sp., *Pachycondyla* sp.), Neuroptera (Myrmeleontidae).

Dentre os vertebrados foram encontradas três morfoespécies pertencentes às ordens Chiroptera (Phyllostomidae: *Glossophaga soricina),* Anura (Leptodactylidae: *Pristimantis cf. fenestratus;* Leptodactylidae Indet.). Dessa forma, foi encontrado um total de 28 morfoespécies.

5.4.4.89.3. Caracterização faunística no período de chuva

Foi encontrado na caverna, um total de 42 morfoespécies de invertebrados em pelo menos 32 famílias dos táxons: Isopoda (Armadillidae), Acari (Argasidae: *Ornithodoros* sp.;

Macronyssidae; Otopheidomenidae; Anystidae: *Erythracarus* sp.; Cunaxidae; Trombidiforme), Amblypygi (Phrynidae: *Heterophrynus longicornis*), Pseudoscorpiones (Chernetidae; Chtoniidae), Araneae (Filistatidae; Gnaphosidae; Ochyroceratidae; Scytodidae*:* *Scytodes eleonora*; Pholcidae: *Mesabolivar* sp., *Metagonia* sp.; Theridiosomatidae), Diplura (Anajapygidae), Collembola (Entomobryidae), Orthoptera (Phalangopsidae: *Phalangopsis* sp.), Dermaptera (Labiidae), Psocoptera (Pachytroctidae), Hemiptera (Cydnidae), Lepidoptera (Noctuidae; Tineidae), Diptera (Ceratopogonidae; Drosophilidae; Muscidae; Psychodidae: *Lutzomyia* sp.), Hymenoptera (Formicidae: *Apterostigma* sp.), Coleoptera (Elateridae: Cardiophorinae; Curculionidae: Scotylinae), Symphyla (Scolopendrellidae), Neuroptera (Myrmeleontidae).

Dentre os vertebrados foi encontrada uma morfoespécie pertencente à ordem Chiroptera (Phyllostomidae: *Glossophaga soricina).* Dessa forma, foi encontrado um total de 43 morfoespécies. Dentre estas, uma foi considerada troglóbia, a qual pertence ao táxon: Diplura (Anajapygidae).

5.4.4.89.4. Caracterização geral da fauna da cavidade

Foi encontrado na caverna, um total de 57 morfoespécies de invertebrados em pelo menos 38 famílias dos táxons: Isopoda (Armadillidae), Acari (Argasidae: *Ornithodoros* sp.; Macronyssidae; Otopheidomenidae; Anystidae (*Erythracarus* sp.; Cunaxidae; Trombidiforme), Amblypygi (*Heterophrynus longicornis*), Pseudoscorpiones (Chernetidae; Chtoniidae), Opiliones (Sclerosomatidae: *Prionostemma* sp.), Araneae (Filistatidae; Gnaphosidae; Ochyroceratidae; Scytodidae*: Scytodes eleonora*; Pholcidae: *Mesabolivar* sp., *Metagonia* sp.; Theridiidae; Theridiosomatidae), Thysanura (Nicoletiidae: Nicoletiinae), Diplura (Anajapygidae), Collembola (Entomobryidae), Orthoptera (Phalangopsidae: *Aclodes* sp., *Phalangopsis* sp.), Dermaptera (Labiidae), Psocoptera (Pachytroctidae), Hemiptera (Cydnidae), Lepidoptera (Noctuidae; Tineidae), Diptera (Ceratopogonidae; Dolichopodidae; Drosophilidae; Milichiidae; Muscidae; Psychodidae: *Lutzomyia* sp.), Hymenoptera (Formicidae: *Apterostigma* sp., *Camponotus* sp., *Pachycondyla* sp.), Coleoptera (Elateridae: Cardiophorinae; Curculionidae: Scotylinae), Symphyla (Scolopendrellidae), Neuroptera (Myrmeleontidae).

Dentre os vertebrados foram encontradas três morfoespécies pertencentes às ordens Chiroptera (Phyllostomidae: *Glossophaga soricina),* Anura (Leptodactylidae: *Pristimantis cf. fenestratus;* Leptodactylidae Indet.).

Dessa forma, foi encontrado um total de 60 morfoespécies. Dentre estas, uma foi considerada troglóbia, a qual pertence ao táxon: Diplura (Anajapygidae).

5.4.4.90. SL-093

5.4.4.90.1. Caracterização trófica

Grande cavidade formada na canga com 49 m de projeção horizontal e localizada em área de pastagem, possuindo situação muito superficial. Caverna extremamente confinada com apenas duas pequenas entradas, ambas ensolaradas, sendo uma formada por uma pequena clarabóia associada a alguns arbustos no meio da pastagem e outra em rastejo localizada em uma pequena quebra da canga. Sua entrada principal (clarabóia) permite o acesso a um pequeno patamar predominantemente plano, disfótico e com piso composto por sedimento argiloso, úmido e com pouca serrapilheira. Após este patamar, é possível acessar o conduto principal da cavidade após um novo desnível. Este conduto é completamente afótico com piso plano composto também por sedimento argiloso e úmido com alguns blocos esparsos (calhaus e matacões). O sistema radicular é bem desenvolvido sendo formado por raízes de fino calibre. Existem pequenos depósitos recentes de guano de morcegos frugívoros produzidos por morcegos Glossophaginae esparsos pela cavidade. Durante o inventário biológico foram observados pontos de gotejamento mesmo durante a estação seca. Foram observados, ainda, restos de madeira em decomposição e alguns cadáveres de grilos com crescimento fúngico (Figura 227). Em situação oposta a este conduto principal existe um conduto em teto baixo acessado após um trecho em rastejo que se conecta à entrada secundária da cavidade localizada na quebra da canga. Este conduto é muito diferente da região anteriormente descrita, sendo extremamente seco, com piso composto por sedimento granulado e com poucos blocos. Trata-se de uma área disfótica com sistema radicular bem desenvolvido e com pouca serrapilheira esparsa. De forma geral, o sistema de canalículos é bem desenvolvido e a cavidade apresenta uma elevada estabilidade ambiental. Durante a estação úmida a cavidade apresentava umidade elevada com inúmeros pontos de gotejamento, poças e depósitos maiores de guano fresco de morcegos frugívoros. A entrada em clarabóia encontrava-se encoberta pela vegetação e havia uma maior quantidade de matéria orgânica acumulada nesta área.

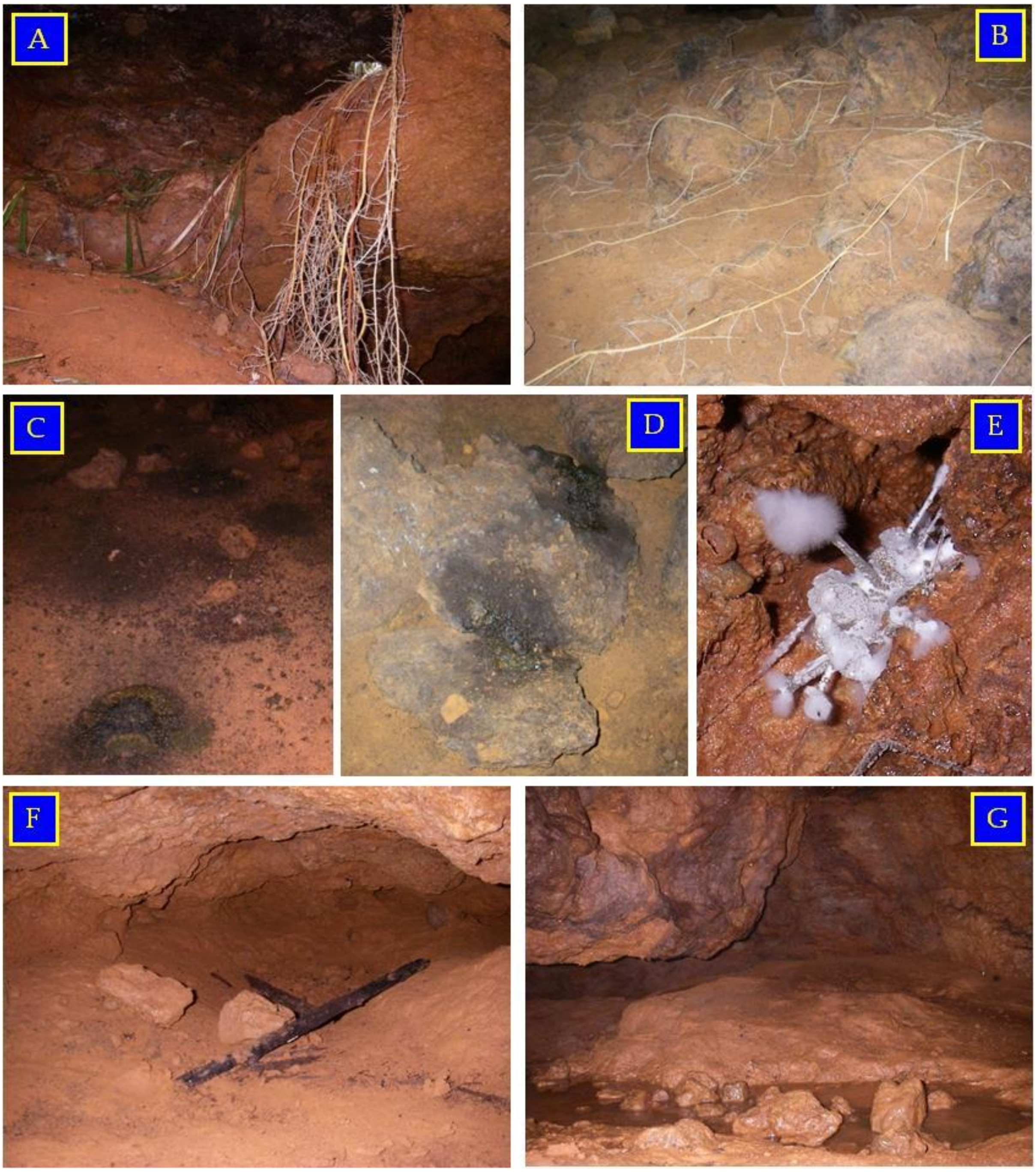

Figura 227 - a) Sistema radicular superficial bem desenvolvido; b) Detalhe de sistema radicular superficial bem desenvolvido; c) Depósitos de guano de morcegos frugívoros; d) Detalhe de depósitos de guano; e) Grilo com intenso crescimento fúngico; f) restos de madeira em decomposição na porção mais interna da caverna; g) Poça de água formada durante a estação úmida.

5.4.4.90.2. Caracterização faunística no período de seca

Foi encontrado na caverna, um total de 52 morfoespécies de invertebrados em pelo menos 39 famílias dos táxons: Oligochaeta; Gastropoda (Subulinidae; Systrophiidae), Isopoda (Balloniscidae; Dubioniscidae; Plathyarthridae: *Trichorhina* sp.; Scleropactidae), Acari

(Laelapidae: *Stratiolaelaps* sp.; Uropodina; Oribatida; Trombidiforme), Amblypygi (Phrynidae: *Heterophrynus longicornis*; Charinidae: *Charinus* sp.), Pseudoscorpiones (Chernetidae; Chtoniidae), opiliones (Escadabiidae), Araneae (Linyphiidae; Pholcidae: *Mesabolivar* sp.; Theridiosomatidae), Thysanura (Nicoletiidae: Atelurinae), Diplura (Campodeidae; Parajapygidae), Collembola (Entomobryidae; Isotomidae; Paronellidae), Orthoptera (Phalangopsidae: *Phalangopsis* sp.), Hemiptera (Cydnidae; Dipsocoridae), Diptera (Ceratopogonidae; Chironomidae; Drosophilidae; Empididae; Fanniidae; Psychodidae: *Lutzomyia* sp.; Sciaridae; Tipulidae), Hymenoptera (Formicidae: *Pachycondyla* sp., *Solenopsis* sp.), Coleoptera (Elateridae; Scydmaenidae), Diplopoda (Chelodesmidae; Pyrgodesmidae), Symphyla (Scutigerellidae: *Hanseniella* sp.), Lepidoptera (Tineidae).

Dentre os vertebrados foram encontradas quatro morfoespécies pertencentes às ordens Chiroptera (Phyllostomidae: *Glossophaga soricina, Anoura* sp.*)* e Anura (Leptodactylidae: *Leptodactylus labyrinthicus*; *Eleutherodactylus* sp.).

Dessa forma, foi encontrado um total de 56 morfoespécies. Dentre estas, seis foram consideradas troglomórficas, as quais pertencem aos táxons: Gastropoda (Systrophiidae), Isopoda (Balloniscidae; Plathyarthridae: *Trichorhina* sp), Amblypygi (Charinidae: *Charinus* sp.), Collembola (Isotomidae), Diplopoda (Pyrgodesmidae).

5.4.4.90.3. Caracterização faunística no período de chuva

Foi encontrado na caverna, um total de 62 morfoespécies de invertebrados em pelo menos 41 famílias dos táxons: Oligochaeta; Gastropoda (Subulinidae; Systrophiidae), Turbellaria (Geoplanidae), Isopoda (Balloniscidae; Plathyarthridae: *Trichorhina* sp.), Acari (Argasidae: *Antricola* sp.; Laelapidae: *Stratiolaelaps* sp.; Uropodina; Oribatida; Trombidiforme), Amblypygi (Phrynidae: *Heterophrynus longicornis*), Pseudoscorpiones (Chernetidae; Chtoniidae), Opiliones (Escadabiidae), Araneae (Gnaphosidae; Theraphosidae; Theridiidae; Theridiosomatidae), Thysanura (Nicoletiidae: Atelurinae), Diplura (Campodeidae; Japygidae), Collembola (Sminthuridae; Entomobryidae; Paronellidae), Orthoptera (Phalangopsidae: *Phalangopsis* sp.), Hemiptera (Cydnidae; Reduviidae: *Panstrongylus* sp.), Diptera (Ceratopogonidae; Chironomidae; Drosophilidae; Phoridae; Psychodidae: *Lutzomyia* sp.; Sciaridae), Hymenoptera (Formicidae: *Hypoponera* sp., *Labidus* sp., *Pachycondyla* sp., *Solenopsis* sp.; *Diapriidae* sp.), Coleoptera (Elateridae ; cydmaenidae; Staphylinidae), Diplopoda (Pyrgodesmidae; Polydesmida; Glomeridesmidae: *Glomeridesmus* sp.), Lithobiomorpha (Henicopiidae: *Lamyctes* sp.), Scolopendromorpha (Scolopocryptopidae: *Newportia* sp.), Symphyla (Scutigerellidae: *Hanseniella* sp., *Scutigerella* sp.), Lepidoptera (Tineidae).

Dentre os vertebrados foram encontradas duas morfoespécies pertencentes às ordens Chiroptera (Phyllostomidae: *Glossophaga soricina),* Anura (Leptodactylidae: *Leptodactylus labyrinthicus*.).

Dessa forma, foi encontrado um total de 64 morfoespécies. Dentre estas, seis foram consideradas troglomórficas, as quais pertencem aos táxons: Gastropoda (Systrophiidae), Isopoda (Balloniscidae; Plathyarthridae: *Trichorhina* sp.), Diplopoda (Pyrgodesmidae; Glomeridesmidae: *Glomeridesmus* sp.).

5.4.4.90.4. Caracterização geral da fauna da cavidade

Foi encontrado na caverna, um total de 84 morfoespécies de invertebrados em pelo menos 53 famílias dos táxons: Oligochaeta; Gastropoda (Subulinidae; Systrophiidae), Turbellaria (Geoplanidae), Isopoda (Balloniscidae; Dubioniscidae; Plathyarthridae: *Trichorhina* sp.; Scleropactidae), Acari (Argasidae: *Antricola* sp.; Laelapidae: *Stratiolaelaps* sp.; Uropodina; Oribatida; Trombidiforme), Amblypygi (Phrynidae: *Heterophrynus longicornis*; Charinidae: *Charinus* sp.), Pseudoscorpiones (Chernetidae; Chtoniidae), Opiliones (Escadabiidae), Araneae (Gnaphosidae; Linyphiidae; Pholcidae: *Mesabolivar* sp.; Theraphosidae; Theridiidae; Theridiosomatidae), Thysanura (Nicoletiidae: Atelurinae), Diplura (Campodeidae; Japygidae; Parajapygidae), Collembola (Sminthuridae; Entomobryidae; Isotomidae; Paronellidae), Orthoptera (Phalangopsidae: *Phalangopsis* sp.), Hemiptera (Cydnidae; Dipsocoridae; Reduviidae: *Panstrongylus* sp.), Diptera (Ceratopogonidae; Chironomidae; Drosophilidae; Empididae; Fanniidae; Phoridae; Psychodidae: *Lutzomyia* sp.; Sciaridae; Tipulidae), Hymenoptera (Formicidae: *Hypoponera* sp., *Labidus* sp., *Pachycondyla* sp., *Solenopsis* sp.; Diapriidae), Coleoptera (Elateridae; Scydmaenidae; Staphylinidae), Diplopoda (Chelodesmidae; Pyrgodesmidae; Polydesmida; Glomeridesmidae: *Glomeridesmus* sp.), Lithobiomorpha (Henicopiidae: *Lamyctes* sp.), Scolopendromorpha (Scolopocryptopidae: *Newportia* sp.), Symphyla (Scutigerellidae: *Hanseniella* sp., *Scutigerella* sp.),Lepidoptera (Tineidae).

Dentre os vertebrados foram encontradas quatro morfoespécies pertencentes às ordens Chiroptera (Phyllostomidae: *Glossophaga soricina, Anoura* sp.*)* e Anura (Leptodactylidae: *Leptodactylus labyrinthicus*; *Eleutherodactylus* sp.).

Dessa forma, foi encontrado um total de 88 morfoespécies. Dentre estas, oito foram consideradas troglomórficas, as quais pertencem aos táxons: Gastropoda (Systrophiidae), Isopoda (Balloniscidae; Plathyarthridae: *Trichorhina* sp), Amblypygi (Charinidae: *Charinus* sp.), Collembola (Isotomidae), Diplopoda (Pyrgodesmidae; Glomeridesmidae: *Glomeridesmus* sp.). Alguns organismos encontrados nesta caverna são mostrados na Figura 228.

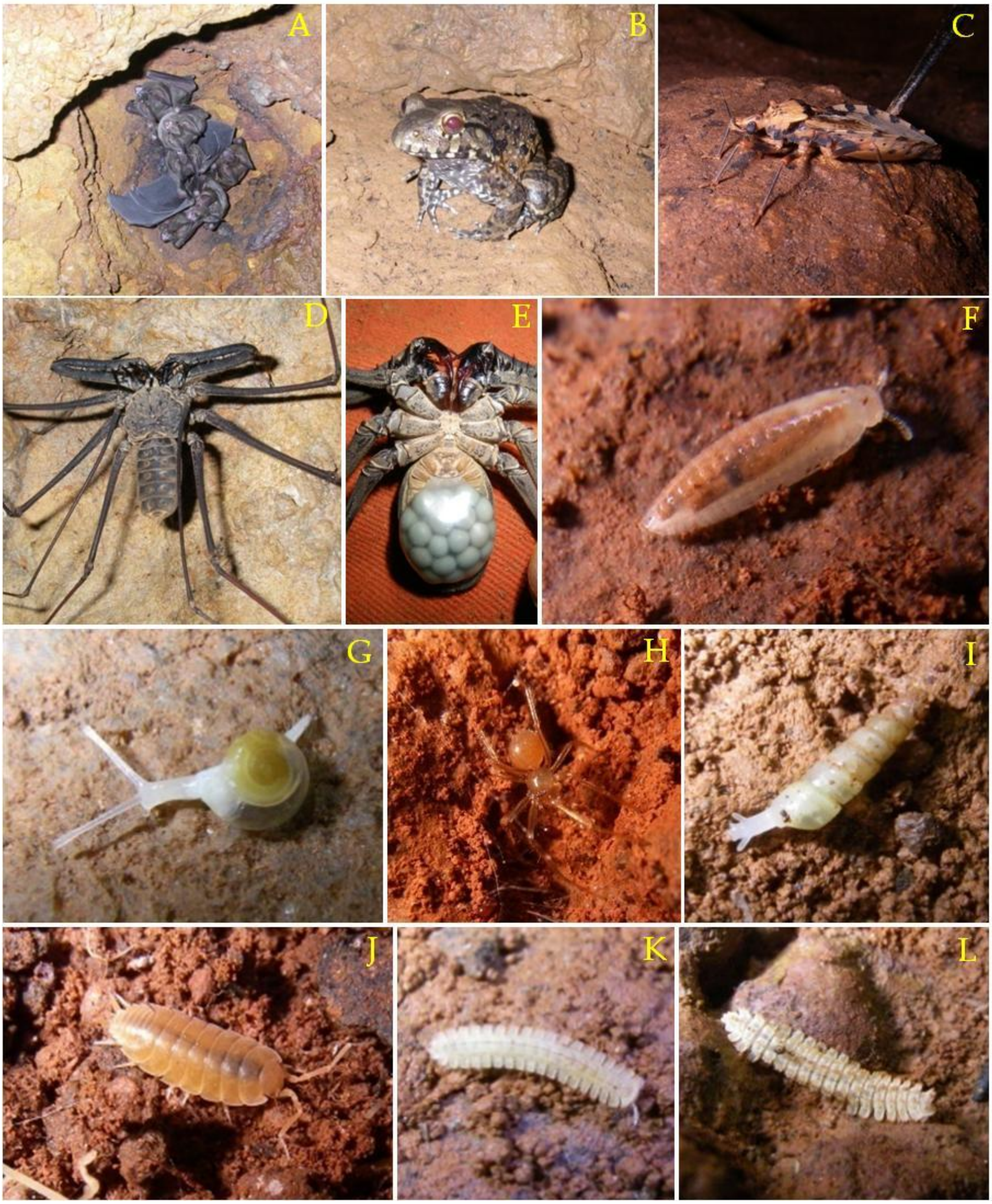

Figura 228 - a) Morcego Phyllostomidae (*G. soricina*); b) Anura (Leptodactylidae: *Leptodactylus labyrinthicus*); c) Hemiptera (Reduviidae: Panstrongylus sp.); d) Amblypygi (*Heterophrynus longicornis*); e) Amblypygi (*Heterophrynus longicornis*) vista ventral com ovos; g) Gastropoda (Systrophiidae); h) Araneae (indet.); i) Gastropoda (Subulinidae); i) Isopoda (Balloniscidae); k) Diplopoda (Pyrgodesmidae); l) Diplopoda (Pyrgodesmidae).

5.4.4.91. SL-094

5.4.4.91.1. Caracterização trófica

Pequeno abrigo com 6,5 m de projeção horizontal, desenvolvido na canga e localizado em área de pastagem. Possui uma entrada em teto baixo, completamente ensolarada, com muitos líquens e briófitas, além de plântulas de angiospermas germinadas junto à linha d'água. Esta cavidade não apresenta zona afótica e possui uma baixa estabilidade ambiental. Seu piso é predominantemente plano e seco e levemente descendente na porção mais distal da cavidade, sendo composto por sedimento granulado com muitos blocos (seixos e calhaus). Existe muita serrapilheira acumulada na linha d'água e alguns pontos esparsos ao longo da cavidade. O abrigo apresenta teto baixo ao longo de toda sua extensão e o sistema de canalículos é pouco desenvolvido (Figura 229). Não existem depósitos de guano no interior da cavidade, nem pontos de gotejamento e o sistema radicular é pouco desenvolvido. Nenhuma alteração significativa foi observada durante a estação úmida, além das alterações normais na umidade relativa do ar.

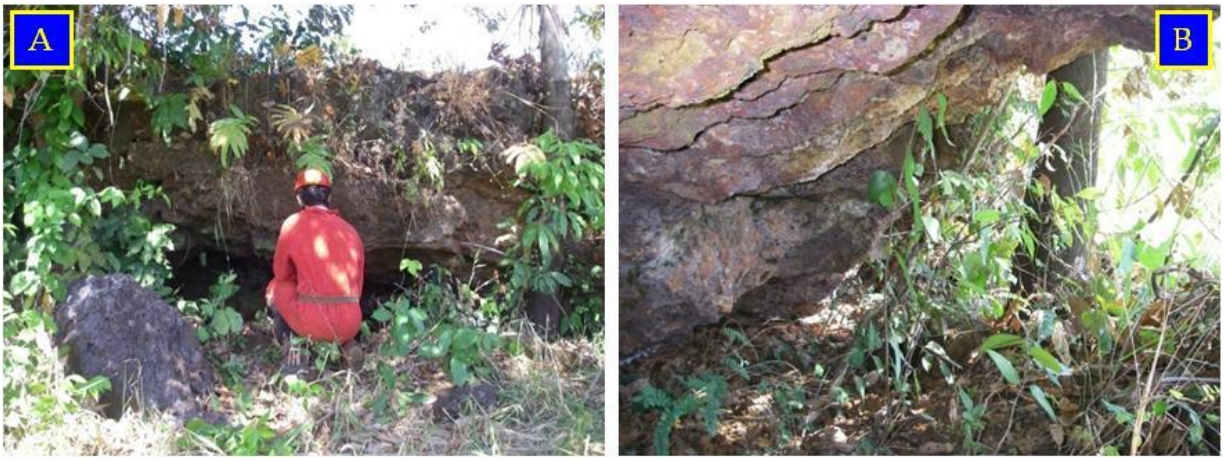

Figura 229 - a) Vista externa da entrada da cavidade com detalhes da vegetação associada; b) Vista interna do pórtico de entrada da cavidade.

5.4.4.91.2. Caracterização faunística no período de seca

Foi encontrado na caverna, um total de 32 morfoespécies de invertebrados em pelo menos 13 famílias dos táxons: Isopoda (Balloniscidae), Acari (Oribatida), Pseudoscorpiones (Chernetidae; Chtoniidae), Araneae (Oonopidae: Oonopinae; Salticidae; Scytodidae*: Scytodes eleonora*; Pholcidae; Theridiidae), Collembola (Entomobryidae), Orthoptera (Gryllidae: Nemobiinae; Phalangopsidae: *Aclodes* sp.), Blattodea (Polyphagidae), Isoptera, Psocoptera (Pachytroctidae; Ptiloneuridae: *Ptiloneura* sp.), Hemiptera (Cydnidae), Homoptera (Cixiidae: *Cixius* sp.), Lepidoptera (Tineidae), Diptera (Simulidae), Hymenoptera (Formicidae: *Cyphomyrmex* sp., *Hypoponera* sp., *Pachycondyla* sp., *Pseudomyrmex* sp., *Pheidole* sp.,

Solenopsis sp.; Trichogrammatidae; Vespidae), Coleoptera (Ptylidae), Scolopendromorpha (Cryptopidae: *Cryptops* sp.).

Dentre as morfoespécies encontradas, duas foram consideradas troglomórficas, as quais pertencem aos táxons: Isopoda (Balloniscidae), Coleoptera (Ptylidae).

5.4.4.91.3. Caracterização faunística no período de chuva

Foi encontrado na caverna, um total de 38 morfoespécies de invertebrados em pelo menos 27 famílias dos táxons: Isopoda (Balloniscidae), Acari (Oribatida), Pseudoscorpiones (Chernetidae; Chtoniidae), Opiliones (Cosmetidae: *Anduzeia* sp.; Escadabiidae), Araneae (Gnaphosidae; Salticidae; Scytodidae*: Scytodes eleonora*; Psauridae; Theridiosomatidae), Blattodea (Polyphagidae), Psocoptera (Epipsocidae), Myopsocidae (*Lichenomina* sp.), Hemiptera (Cydnidae; Lygaeidae; Ploiariidae), Lepidoptera (Noctuidae; Tineidae), Diptera (Cecidomyiidae; Tipulidae; Sciaridae), Hymenoptera (Formicidae: *Cyphomyrmex* sp., *Pachycondyla* sp., *Tapinoma* sp.), Diplopoda (Pyrgodesmidae), Scolopendromorpha (Cryptopidae: Cryptopinae), Symphyla (Scutigerellidae: *Hanseniella* sp.), Neuroptera (Myrmeleontidae), Coleoptera (Elateridae: Cardiophorinae).

Dentre os vertebrados foi encontrada uma morfoespécie pertencente à ordem Chiroptera (Phyllostomidae: *Glossophaga soricina).* Dessa forma, foi encontrado um total de 39 morfoespécies. Dentre estas, uma foi considerada troglóbia, a qual pertence ao táxon: Isopoda (Balloniscidae).

5.4.4.91.4. Caracterização geral da fauna da cavidade

Foi encontrado na caverna, um total de 62 morfoespécies de invertebrados em pelo menos 40 famílias dos táxons: Isopoda (Balloniscidae), Acari (Oribatida), Pseudoscorpiones (Chernetidae; Chtoniidae), Opiliones (Cosmetidae: *Anduzeia* sp.; Escadabiidae), Araneae (Gnaphosidae; Oonopidae: Oonopinae; Salticidae; Scytodidae*: Scytodes eleonora*; Psauridae; Pholcidae; Theridiidae; Theridiosomatidae), Collembola (Entomobryidae), Orthoptera (Gryllidae: Nemobiinae; Phalangopsidae: *Aclodes* sp.), Blattodea (Polyphagidae), Isoptera; Psocoptera (Epipsocidae; Myopsocidae: *Lichenomina* sp.; Pachytroctidae; Ptiloneuridae: *Ptiloneura* sp.), Hemiptera (Cydnidae; Lygaeidae; Ploiariidae), Homoptera (Cixiidae: *Cixius* sp.), Lepidoptera (Noctuidae; Tineidae), Diptera (Cecidomyiidae; Simulidae; Tipulidae; Sciaridae), Hymenoptera (Formicidae: *Cyphomyrmex* sp., *Hypoponera* sp., *Pachycondyla* sp., *Pseudomyrmex* sp., *Pheidole* sp., *Solenopsis* sp., *Tapinoma* sp.; Trichogrammatidae; Vespidae), Coleoptera (Ptylidae; Elateridae: Cardiophorinae), Diplopoda (Pyrgodesmidae), Scolopendromorpha (Cryptopidae: Cryptopinae, *Cryptops* sp.), Symphyla (Scutigerellidae: *Hanseniella* sp.), Neuroptera (Myrmeleontidae).

Dentre os vertebrados foi encontrada uma morfoespécie pertencente à ordem Chiroptera (Phyllostomidae: *Glossophaga soricina).*

Dessa forma, foi encontrado um total de 63 morfoespécies. Dentre estas, duas foram consideradas troglomórficas, as quais pertencem aos táxons: Isopoda (Balloniscidae) e Coleoptera (Ptylidae).

5.4.4.92. SL-095

5.4.4.92.1. Caracterização trófica

Caverna com 21,5 m de desenvolvimento, formada na canga, inserida de maneira muito superficial e localizada em área de pastagem. Possui uma entrada larga com teto baixo, sendo esta, completamente iluminada. O sistema de canalículos é pouco desenvolvido e o piso é descendente e composto por sedimento granulado com muitos seixos de tamanhos variados, além de poucos calhaus e matacões. Na entrada existem muitos líquens, briófitas e pteridófitas, além de muitas plântulas e uma pequena árvore que germinou junto à linha d'água. Atualmente, sua copa encontra-se acima da altura da quebra da canga. Na região central do salão de entrada o sedimento encontra-se mesclado a um depósito de guano envelhecido de morcegos frugívoros. Neste, existe, ainda, serrapilheira em pequenas quantidades e raízes de diferentes calibres (inclusive rizotemas) na zona de penumbra. No lado esquerdo da cavidade o piso encontra-se muito revolvido e escavado por algum animal de pequeno porte. A cavidade não apresenta zona afótica e não existem pontos de gotejamento durante a estação seca. Durante a estação úmida a caverna apresentava umidade elevada e inúmeros pontos de gotejamento, além de depósitos recentes de guano (Figura 230).

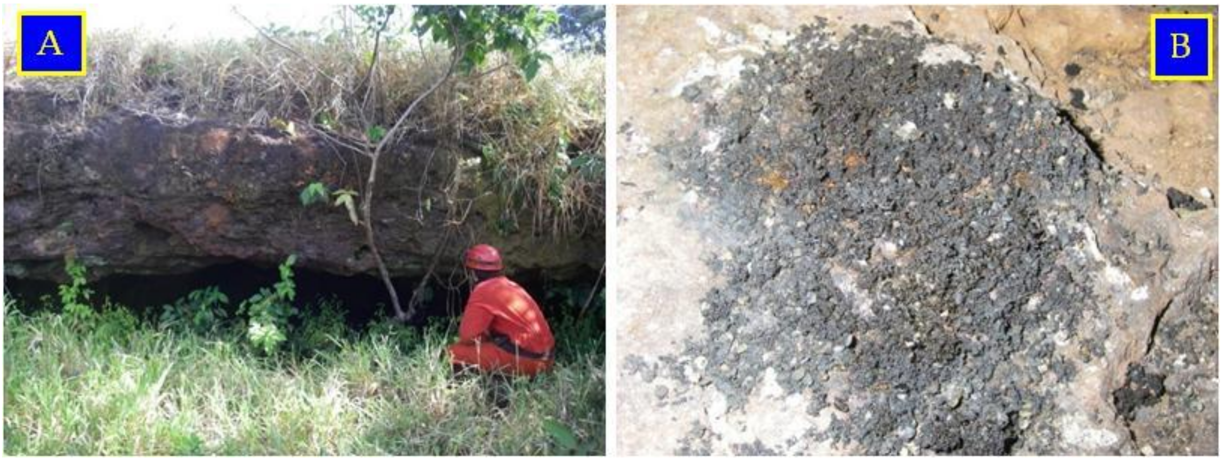

Figura 230 - a) Vista externa da entrada da cavidade com detalhes da vegetação associada; b) Depósito de guano de morcegos frugívoros localizado no interior da cavidade.

5.4.4.92.2. Caracterização faunística no período de seca

Foi encontrado na caverna, um total de 50 morfoespécies de invertebrados em pelo menos 35 famílias dos táxons: Isopoda (Subulinidae; Systrophiidae; Balloniscidae), Acari (Laelapidae: *Stratiolaelaps* sp.; Oribatida), Amblypygi (Phrynidae: *Heterophrynus longicornis*), Pseudoscorpiones (Chernetidae; Chtoniidae), Araneae (Araneidae; Oonopidae: Oonopinae; Scytodidae*: Scytodes eleonora*; Pholcidae; Theridiidae), Thysanura (Nicoletiidae: Nicoletiinae), Diplura (Campodeidae), Collembola (Sminthuridae; Paronellidae), Orthoptera (Phalangopsidae: *Phalangopsis* sp.), Blattodea (Blaberidae), Isoptera, Psocoptera (Epipsocidae; Myopsocidae: *Lichenomina* sp.; Psyllipsocidae: *Psyllipsocus* sp.), Hemiptera (Cydnidae; Lygaeidae; Pyrrhocoridae), Homoptera (Cixiidae), Lepidoptera (Tineidae), Diptera (Drosophilidae; Psychodidae: *Lutzomyia* sp.), Hymenoptera (Formicidae: *Ectatomma* sp., *Hypoponera* sp., *Pachycondyla* sp., *Solenopsis* sp., *Strumigenys*.; Vespidae), Coleoptera (Elateridae: Cardiophorinae; Phalacridae; Tenebrionidae: Coelometropinae; Staphylinidae), Diplopoda (Pyrgodesmidae), Scolopendromorpha (Cryptopidae: *Cryptops* sp.).

Dentre os vertebrados foram encontradas três morfoespécies pertencentes às ordens Chiroptera (Phyllostomidae: *Glossophaga soricina)*, Anura (Leptodactylidae: *Pristimantis* cf. *fenestratus*; Leptodactylidae Indet.).

Dessa forma, foi encontrado um total de 53 morfoespécies. Dentre estas, duas foram consideradas troglomórficas, as quais pertencem aos táxons: Gastropoda (Systrophiidae) e Diplopoda (Pyrgodesmidae).

5.4.4.92.3. Caracterização faunística no período de chuva

Foi encontrado na caverna, um total de 102 morfoespécies de invertebrados em pelo menos 60 famílias dos táxons: Gastropoda (Subulinidae; Systrophiidae; Verocinellidae), Turbellaria (Geoplanidae), Isopoda (Armadillidae; Balloniscidae; Philosciidae; Plathyarthridae: *Trichorhina* sp.), Acari (Laelapidae: *Stratiolaelaps* sp.; Macrochelidae; Oribatida; Anystidae: *Erythracarus* sp.; Trombidiforme), Pseudoscorpiones (Chernetidae; Chtoniidae), Araneae (Gnaphosidae; Linyphiidae; Ochyroceratidae; Oonopidae: Oonopinae; Salticidae; Scytodidae*: Scytodes eleonora*; Psauridae), Thysanura (Nicoletiidae: Nicoletiinae; Atelurinae), Diplura (Campodeidae), Collembola (Entomobryidae; Hypogastruridae; Paronellidae), Orthoptera (Phalangopsidae: *Aclodes* sp., *Phalangopsis* sp.), Blattodea (Blaberidae: *Blaberus* sp.), Isoptera (Rhinotermitidae: *Heterotermes* sp.), Psocoptera (Epipsocidae; Myopsocidae: *Lichenomina* sp.; Psyllipsocidae: *Psyllipsocus* sp.), Hemiptera (Cydnidae; Dipsocoridae; Hebridae; Lygaeidae; Nabidae; Ploiariidae), Homoptera (Cercopidae; Cixiidae), Lepidoptera (Tineidae), Diptera (Chironomidae; Cecidomyiidae; Ceratopogonidae; Drosophilidae; Psychodidae: *Lutzomyia* sp.; Sciaridae), Hymenoptera (Formicidae: *Carebara* sp., *Ectatomma* sp., *Hypoponera* sp., *Leptogenys* sp., *Odontomachus* sp., *Pachycondyla* sp., *Pheidole* sp.,

Solenopsis sp., *Strumigenys* sp.; Diapriidae), Coleoptera (Elateridae; Eucnemidae; Pselaphidae; Staphylinidae), Diplopoda (Glomeridesmidae: *Glomeridesmus* sp.; Siphonophoridae), Lithobiomorpha (Henicopiidae: *Lamyctes* sp.), Scolopendromorpha (Cryptopidae: *Cryptops* sp.; Scolopocryptopidae: *Dinocryptops* sp., *Tidops* sp.), Scutigeromorpha (Scutigeridae: Sphendononema sp.), Symphyla (Scutigerellidae: *Hanseniella* sp.), Neuroptera (Myrmeleontidae).

Dentre os vertebrados foram encontradas duas morfoespécies pertencentes às ordens Chiroptera (Phyllostomidae: *Glossophaga soricina)*, Anura (Leptodactylidae: *Pristimantis* cf. *fenestratus*).

Dessa forma, foi encontrado um total de 104 morfoespécies. Dentre estas, seis foram consideradas troglomórficas, as quais pertencem aos táxons: Gastropoda (Systrophiidae), Isopoda (Balloniscidae; Plathyarthridae: *Trichorhina* sp.), Hemiptera (Dipsocoridae), Coleoptera (Eucnemidae), Diplopoda (Glomeridesmidae: *Glomeridesmus* sp.).

5.4.4.92.4. Caracterização geral da fauna da cavidade

Foi encontrado na caverna, um total de 129 morfoespécies de invertebrados em pelo menos 70 famílias dos táxons: Gastropoda (Subulinidae; Systrophiidae; Verocinellidae), Turbellaria (Geoplanidae), Isopoda (Armadillidae; Balloniscidae; Philosciidae; Plathyarthridae: *Trichorhina* sp.), Acari (Laelapidae: *Stratiolaelaps* sp.; Macrochelidae; Oribatida; Anystidae: *Erythracarus* sp.; Trombidiforme), Amblypygi (Phrynidae: *Heterophrynus longicornis*), Pseudoscorpiones (Chernetidae; Chtoniidae), Araneae (Araneidae; Gnaphosidae; Linyphiidae; Ochyroceratidae; Oonopidae: Oonopinae; Salticidae; Scytodidae*: Scytodes eleonora: Scytodes eleonora*; Psauridae; Pholcidae; Theridiidae), Thysanura (Nicoletiidae: Nicoletiinae; Atelurinae), Diplura (Campodeidae), Collembola (Sminthuridae; Entomobryidae; Hypogastruridae; Paronellidae), Orthoptera (Phalangopsidae: *Aclodes* sp., *Phalangopsis* sp.), Blattodea (Blaberidae: *Blaberus* sp.), Isoptera (Rhinotermitidae: *Heterotermes* sp.), Psocoptera (Epipsocidae; Myopsocidae: *Lichenomina* sp.; Psyllipsocidae: *Psyllipsocus* sp.), Hemiptera (Cydnidae; Dipsocoridae; Hebridae; Lygaeidae; Nabidae; Ploiariidae; Pyrrhocoridae), Homoptera (Cercopidae; Cixiidae), Lepidoptera (Tineidae), Diptera (Cecidomyiidae; Chironomidae; Ceratopogonidae; Drosophilidae; Psychodidae: *Lutzomyia* sp.; Sciaridae), Hymenoptera (Formicidae: *Carebara* sp., *Ectatomma* sp., *Hypoponera* sp., *Leptogenys* sp., *Odontomachus* sp., *Pachycondyla* sp., *Pheidole* sp., *Solenopsis* sp., *Strumigenys* sp.; Diapriidae; Vespidae), Coleoptera (Eucnemidae; Phalacridae; Pselaphidae; Staphylinidae; Elateridae: Cardiophorinae; Tenebrionidae: Coelometropinae), Diplopoda (Pyrgodesmidae; Glomeridesmidae: *Glomeridesmus* sp.; Siphonophoridae), Lithobiomorpha (Henicopiidae: *Lamyctes* sp.), Scolopendromorpha (Cryptopidae: *Cryptops* sp.; Scolopocryptopidae: *Dinocryptops* sp., *Tidops* sp.), Scutigeromorpha (Scutigeridae:

Sphendononema sp.), Symphyla (Scutigerellidae: *Hanseniella* sp.), Neuroptera (Myrmeleontidae).

Dentre os vertebrados foram encontradas três morfoespécies pertencentes às ordens Chiroptera (Phyllostomidae: *Glossophaga soricina)*, Anura (Leptodactylidae: *Pristimantis* cf. *fenestratus*; Leptodactylidae INDET).

Dessa forma, foi encontrado um total de 132 morfoespécies. Dentre estas, sete foram consideradas troglomórficas, as quais pertencem aos táxons: Gastropoda (Systrophiidae), Isopoda (Balloniscidae; Plathyarthridae: *Trichorhina* sp.), Hemiptera (Dipsocoridae), Coleoptera (Eucnemidae), Diplopoda (Pyrgodesmidae; Glomeridesmidae: *Glomeridesmus* sp.). Alguns organismos encontrados nesta caverna são mostrados na Figura 231.

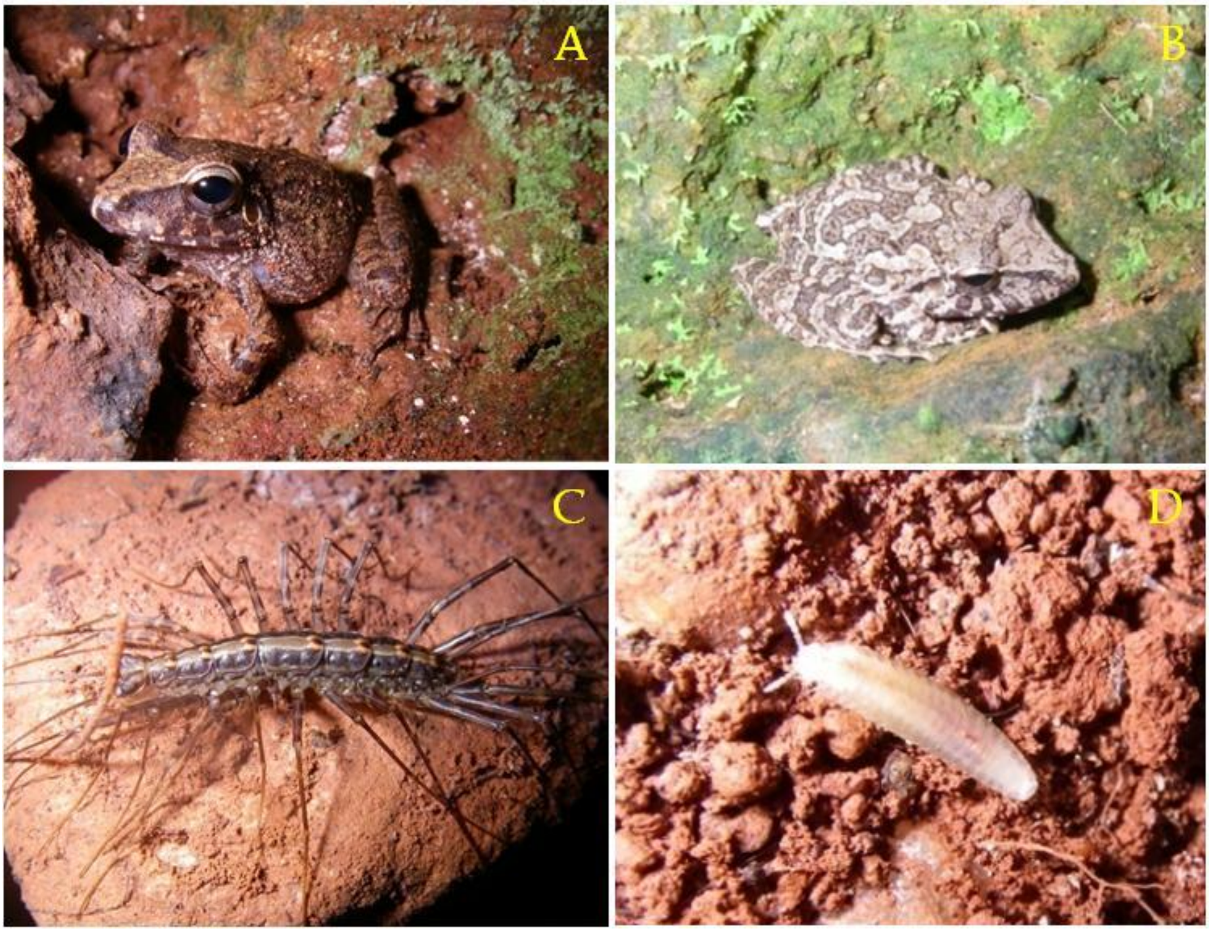

Figura 231 - a) Anura (Leptodactylidae: *Pristimantis cf. fenestratus*); b) Anura (Leptodactylidae: *Pristimantis cf. fenestratus*); c) Scutigeromorpha (*Sphendononema sp.);* d) Gastropoda (Glomeridesmidae: *Glomeridesmus sp*.) .

5.4.4.93. SL-096

5.4.4.93.1. Caracterização trófica

Abrigo com 9,5 m de projeção horizontal, formado na canga e localizado em área de pastagem. Possui duas entradas, sendo uma em teto baixo, ensolarada e com muitos líquens, briófitas e pteridófitas associadas ao piso, paredes e teto, e a outra compreende uma pequena conexão com o meio epígeo. O abrigo é completamente eufótico, exceto por um conduto à esquerda, o qual é disfótico (Figura 232). A cavidade apresenta piso descendente a partir da entrada, composto por sedimento granulado com muitos seixos esparsos e com sistema de canalículos pouco desenvolvido. Existe muita serrapilheira acumulada e plântulas de angiospermas junto à linha d'água, além de alguns pequenos depósitos de guano de morcegos frugívoros esparsos e pequenos acúmulos de fezes de roedores. A cavidade apresenta baixa estabilidade ambiental e elevada influência das condições ambientais epígeas. Nenhuma alteração significativa foi observada durante a estação úmida, além das alterações normais na umidade relativa do ar.

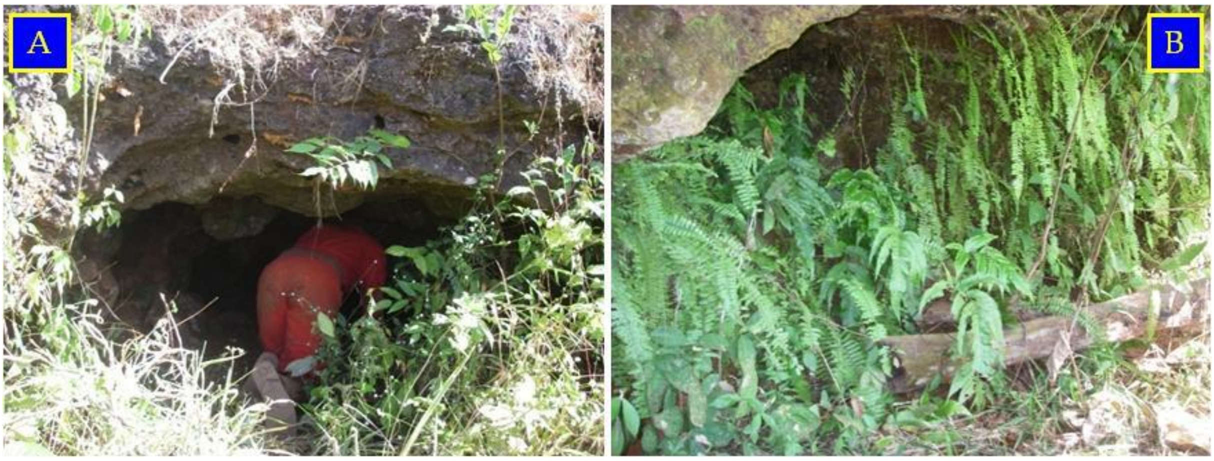

Figura 232 - a) Vista externa da entrada da cavidade com detalhes da vegetação associada; b) Vista interna do pórtico de entrada da cavidade com muita vegetação associada.

5.4.4.93.2. Caracterização faunística no período de seca

Foi encontrado na caverna, um total de 32 morfoespécies de invertebrados em pelo menos 24 famílias dos táxons: Isopoda (Balloniscidae; Plathyarthridae: *Trichorhina* sp.), Acari (Laelapidae), Amblypygi (Charinidae: *Charinus* sp.), Pseudoscorpiones (Chernetidae; Chtoniidae), Araneae (Fissiphaliidae; Ochyroceratidae; Scytodidae*: Scytodes eleonora: Scytodes eleonora*; Pholcidae: *Mesabolivar* sp.), Collembola (Entomobryidae), Orthoptera (Gryllidae: Nemobiinae; Phalangopsidae: *Aclodes* sp., *Phalangopsis* sp.), Blattodea (Blaberidae: *Blaberus* sp.; Polyphagidae), Isoptera , Psocoptera (Epipsocidae), Hemiptera (Cydnidae; Reduviidae; Pyrrhocoridae), Diptera (Dixidae; Sciaridae), Hymenoptera (Formicidae: *Pachycondyla* sp., *Solenopsis* sp., *Zacryptocerus* sp.; Apidae), Coleoptera (Scarabaeoideae), Lepidoptera (Tineidae).

Dentre os vertebrados foram encontradas duas morfoespécies pertencentes à ordem Chiroptera (Phyllostomidae: *Glossophaga* soricina; *Anoura* sp.). Dessa forma, foi encontrado um total de 34 morfoespécies. Dentre estas, três foram consideradas troglomórficas, as quais pertencem aos táxons: Isopoda (Balloniscidae; Plathyarthridae: *Trichorhina* sp.), Amblypygi (Charinidae: *Charinus* sp.)

5.4.4.93.3. Caracterização faunística no período de chuva

Foi encontrado na caverna, um total de 30 morfoespécies de invertebrados em pelo menos 24 famílias dos táxons: Isopoda (Armadillidae; Balloniscidae), Acari (Oribatida), Pseudoscorpiones (Chernetidae; Chtoniidae), Araneae (Ctenidae: *Ctenus* sp.; Gnaphosidae; Ochyroceratidae; Oonopidae: Oonopinae; Salticidae; Scytodidae*: Scytodes eleonora: Scytodes eleonora*; Pholcidae; Thomisidae), Diplura (Campodeidae), Collembola (Paronellidae), Orthoptera (Phalangopsidae), Blattodea (Blaberidae: *Blaberus* sp.), Isoptera (Termitidae: *Subulitermes*? sp.), Hemiptera (Alydidae), Homoptera (Cercopidae), Hymenoptera (Formicidae: *Ectatomma* sp., *Pachycondyla* sp.; Apidae), Coleoptera (Curculionidae: Entiminae?; Tenebrionidae), Lepidoptera (Tineidae).

5.4.4.93.4. Caracterização geral da fauna da cavidade

Foi encontrado na caverna, um total de 54 morfoespécies de invertebrados em pelo menos 37 famílias dos táxons: Isopoda (Armadillidae; Balloniscidae; Plathyarthridae: *Trichorhina* sp.), Acari (Laelapidae; Oribatida), Amblypygi (Charinidae: *Charinus* sp.), Pseudoscorpiones (Chernetidae; Chtoniidae), Araneae (Fissiphaliidae; Ctenidae: *Ctenus* sp.; Gnaphosidae; Ochyroceratidae; Oonopinae; Salticidae; Scytodidae*: Scytodes eleonora: Scytodes eleonora*; Pholcidae: *Mesabolivar* sp.; Thomisidae), Diplura (Campodeidae), Collembola (Entomobryidae; Paronellidae), Orthoptera (Gryllidae: Nemobiinae; Phalangopsidae: Aclodes sp., Phalangopsis sp.), Blattodea (Blaberidae: *Blaberus* sp.; Polyphagidae), Isoptera (Termitidae: *Subulitermes*? sp.), Psocoptera (Epipsocidae), Hemiptera (Alydidae; Cydnidae; Reduviidae; Pyrrhocoridae), Homoptera (Cercopidae), Diptera (Dixidae; Sciaridae), Hymenoptera (Formicidae: *Ectatomma* sp., *Pachycondyla* sp., *Solenopsis* sp., *Zacryptocerus* sp.; Apidae), Coleoptera (Curculionidae: Entiminae?; Tenebrionidae; Scarabaeoideae), Lepidoptera (Tineidae).

Dentre os vertebrados foram encontradas duas morfoespécies pertencentes à ordem Chiroptera (Phyllostomidae: *Glossophaga* soricina; *Anoura* sp.). Dessa forma, foi encontrado um total de 56 morfoespécies. Dentre estas, três foram consideradas troglomórficas, as quais pertencem aos táxons: Isopoda (Balloniscidae; Plathyarthridae: *Trichorhina* sp.), Amblypygi (Charinidae: *Charinus* sp.).

5.4.4.94. SL-097

5.4.4.94.1. Caracterização trófica

Caverna formada na canga com 24,5 m de projeção horizontal localizada em topo de encosta. Vegetação do entorno composta principalmente por samambaias, sendo a região acima da cavidade dominada por savana metalófila. Possui uma entrada estreita, sendo completamente iluminada em sua porção mais externa. Apresenta muitos blocos abatidos, líquens, briófitas e serrapilheira acumulada junto à linha d'água (Figura 233). O piso é descendente a partir da entrada e plano no salão principal, sendo este composto por sedimento granulado com alguns calhaus e matacões esparsos. Trata-se de uma caverna seca sem pontos de gotejamento (durante as amostragens), com sistema de canalículos bem desenvolvido e com elevada estabilidade ambiental em virtude do seu grau de confinamento. Seu salão principal é completamente afótico onde existem pequenos de depósitos de guano envelhecidos de morcegos frugívoros. O sistema radicular é pouco desenvolvido com raízes de pequeno calibre distribuídas de maneira esparsa. Nenhuma alteração significativa foi observada durante a estação úmida, além das alterações normais na umidade relativa do ar.

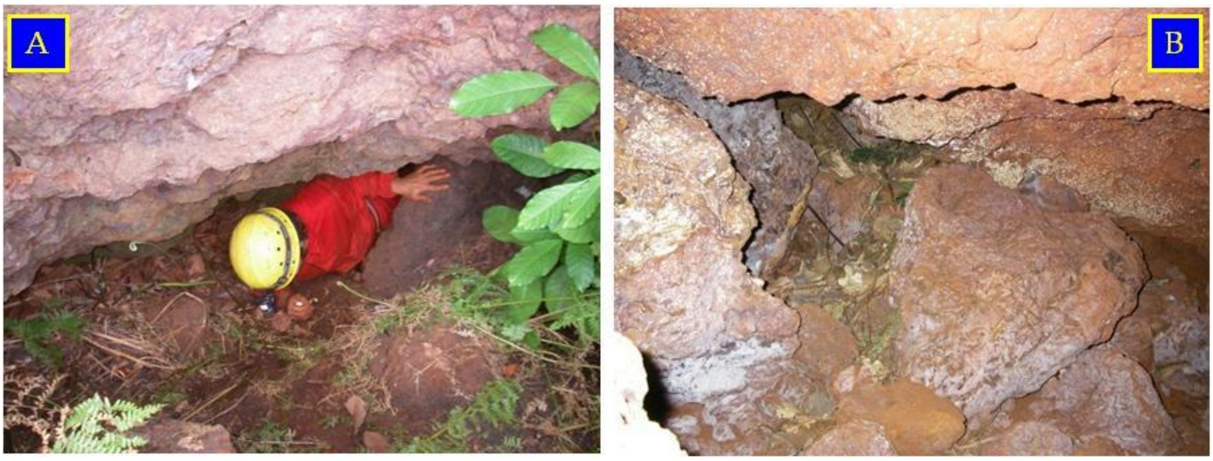

Figura 233 - a) Vista externa da entrada da cavidade; b) Vista interna da entrada, onde se observam blocos abatidos e serrapilheira proveniente da vegetação externa.

5.4.4.94.2. Caracterização faunística no período de seca

Foi encontrado na caverna, um total de 55 morfoespécies de invertebrados em pelo menos 36 famílias dos táxons: Gastropoda (Systrophiidae), Isopoda (Philosciidae; Plathyarthridae: *Trichorhina* sp.), Acari (Laelapidae: *Stratiolaelaps* sp.; Macronyssidae; Astigmatina; Oribatida; Trombidiforme), Amblypygi (Phrynidae: *Heterophrynus longicornis*; Charinidae: *Charinus* sp.), Pseudoscorpiones (Chernetidae; Chtoniidae), Opiliones (Cosmetidae: *Anduzeia* sp.), Araneae (Gnaphosidae; Ochyroceratidae; Oonopidae: Oonopinae; Pholcidae: *Mesabolivar* sp.; Theraphosidae; Theridiosomatidae), Diplura (Anajapygidae; Campodeidae), Collembola

(Sminthuridae; Cyphoderidae; Entomobryomorpha; Paronellidae), Orthoptera (Phalangopsidae: *Aclodes* sp., *Phalangopsis* sp.), Blattodea (Blaberidae: *Blaberus* sp.), Isoptera, Hemiptera (Cydnidae; Dipsocoridae; Reduviidae; Ploiariidae), Homoptera (Cixiidae), Lepidoptera (Tineidae), Diptera (Psychodidae: *Lutzomyia* sp.; Tipulidae), Hymenoptera (Formicidae: *Camponotus* sp., Myrmicinae; *Pachycondyla* sp., *Strumigenys* sp.), Coleoptera (Carabidae; Scydmaenidae; Staphylinidae).

Dentre os vertebrados foram encontradas quatro morfoespécies pertencentes às ordens Chiroptera (Phyllostomidae: *Glossophaga* soricina), Anura (Leptodactylidae: *Pristimantis* cf. *fenestratus*, *Eleutherodactylus* sp.), Squamata (Boidae: *Epicrates* sp.).

Dessa forma, foi encontrado um total de 59 morfoespécies. Dentre estas, cinco foram consideradas troglomórficas, as quais pertencem aos táxons: Gastropoda (Systrophiidae), Isopoda (Plathyarthridae: *Trichorhina* sp.), Amblypygi (Charinidae: *Charinus* sp.), Araneae (Ochyroceratidae), Collembola (Cyphoderidae).

5.4.4.94.3. Caracterização faunística no período de chuva

Foi encontrado na caverna, um total de 45 morfoespécies de invertebrados em pelo menos 30 famílias dos táxons: Oligochaeta, Gastropoda (Systrophiidae), Isopoda (Philosciidae; Plathyarthridae: *Trichorhina* sp.), Acari (Macronyssidae; Mesostigmata; Oribatida; Trombidiforme), Amblypygi (Phrynidae: *Heterophrynus longicornis*; Charinidae: Charinus sp.), Pseudoscorpiones (Chernetidae; Chtoniidae), Opiliones (Phalangiidae), Diplura (Anajapygidae; Campodeidae), Collembola (Sminthuridae; Paronellidae), Orthoptera (Phalangopsidae: *Aclodes* sp., *Phalangopsis* sp.), Isopoda (Termitidae: *Nasutitermes* sp.), Psocoptera (Epipsocidae), Hemiptera (Cydnidae; Dipsocoridae; Pyrrhocoridae), Homoptera (Cixiidae), Diptera (Chironomidae; Drosophilidae; Psychodidae: *Lutzomyia* sp.), Hymenoptera (Formicidae: *Camponotus* sp., *Pachycondyla* sp., *Pheidole* sp., *Solenopsis* sp., *Stegomyrmex* sp., *Strumigenys* sp.), Coleoptera (Carabidae; Scydmaenidae), Diplopoda (Pyrgodesmidae; Glomeridesmidae: *Glomeridesmus* sp.; Polydesmida), Scutigeromorpha (Scutigeridae: *Sphendononema* sp.), Lepidoptera (Tineidae).

Dentre os vertebrados foram encontradas duas morfoespécies pertencentes às ordens Chiroptera (Phyllostomidae: *Glossophaga* soricina), Rodentia (Cricetidae: *Rhipidomys* sp.).

Dessa forma, foi encontrado um total de 47 morfoespécies. Dentre estas, sete foram consideradas troglomórficas, as quais pertencem aos táxons: Gastropoda (Systrophiidae), Isopoda (Plathyarthridae: *Trichorhina* sp.), Amblypygi (Charinidae: *Charinus* sp.), Hemiptera (Dipsocoridae), Hymenoptera (Formicidae: *Solenopsis* sp.), Diplopoda (Pyrgodesmidae; Glomeridesmidae: *Glomeridesmus* sp.).

5.4.4.94.4. Caracterização geral da fauna da cavidade

Foi encontrado na caverna, um total de 83 morfoespécies de invertebrados em pelo menos 44 famílias dos táxons: Oligochaeta, Gastropoda (Systrophiidae), Isopoda (Philosciidae; Plathyarthridae: *Trichorhina* sp.), Acari (Laelapidae: *Stratiolaelaps* sp.; Macronyssidae; Mesostigmata; Astigmatina; Oribatida; Trombidiforme), Amblypygi (Phrynidae: *Heterophrynus longicornis*; Charinidae: Charinus sp.), Pseudoscorpiones (Chernetidae; Chtoniidae), Opiliones (Cosmetidae: *Anduzeia* sp.; Phalangiidae), Araneae (Gnaphosidae; Ochyroceratidae; Oonopidae: Oonopinae; Pholcidae: *Mesabolivar* sp.; Theraphosidae; Theridiosomatidae), Diplura (Anajapygidae; Campodeidae), Collembola (Sminthuridae; Cyphoderidae; Entomobryomorpha; Paronellidae), Orthoptera (Phalangopsidae: *Aclodes* sp., *Phalangopsis* sp.), Blattodea (Blaberidae: *Blaberus* sp.), Isoptera (Termitidae: *Nasutitermes* sp.), Psocoptera (Epipsocidae), Hemiptera (Cydnidae; Dipsocoridae; Reduviidae; Ploiariidae; Pyrrhocoridae), Homoptera (Cixiidae), Lepidoptera (Tineidae), Diptera (Chironomidae; Drosophilidae; Psychodidae: *Lutzomyia* sp.; Tipulidae), Hymenoptera (Formicidae: *Camponotus* sp., Myrmicinae, *Pachycondyla* sp., *Pheidole* sp., *Solenopsis* sp., *Stegomyrmex* sp., *Strumigenys* sp.), Coleoptera (Carabidae; Scydmaenidae; Staphylinidae), Diplopoda (Pyrgodesmidae; Polydesmida; Glomeridesmidae: *Glomeridesmus* sp.), Scutigeromorpha (Scutigeridae: *Sphendononema* sp.).

Dentre os vertebrados foram encontradas cinco morfoespécies pertencentes às ordens Chiroptera (Phyllostomidae: *Glossophaga* soricina), Anura (Leptodactylidae: *Pristimantis* cf. *fenestratus*, *Eleutherodactylus* sp.), Squamata (Boidae: *Epicrates* sp.), Rodentia (Cricetidae: *Rhipidomys* sp.).

Dessa forma, foi encontrado um total de 88 morfoespécies. Dentre estas, cinco foram consideradas troglomórficas, as quais pertencem aos táxons: Gastropoda (Systrophiidae), Isopoda (Plathyarthridae: *Trichorhina* sp.), Amblypygi (Charinidae: *Charinus* sp.), Araneae (Ochyroceratidae), Collembola (Cyphoderidae), Hemiptera (Dipsocoridae), Hymenoptera (Formicidae: *Solenopsis* sp.), Diplopoda (Pyrgodesmidae; Glomeridesmidae: *Glomeridesmus* sp.). Alguns organismos encontrados nesta caverna são mostrados na Figura 234.

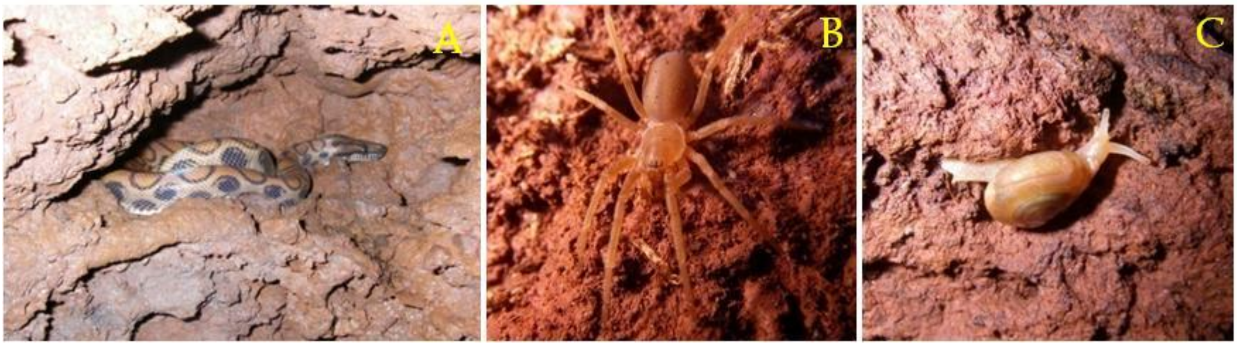

Figura 234 - a) Boidae (*Epicrates* sp.); b) Araneae (Corinidae); c) Gastropoda (Systrophiidae).

5.4.4.95. SL-099

5.4.4.95.1. Caracterização trófica

Abrigo formado na canga com 9 m de projeção horizontal localizado no topo da encosta. Vegetação do entorno composta principalmente por samambaias e região acima da cavidade dominada pela savana metalófila. A cavidade possui apenas uma entrada em teto baixo, levemente sombreada, com piso descendente e com muitas briófitas, líquens, filodendros e plântulas de angiospermas associadas à linha d'água. Na entrada existe muita serrapilheira acumulada além de alguns blocos abatidos (Figura 235). A cavidade é completamente eufótica, com piso úmido, composto por sedimento fino e com alguns blocos esparsos na zona mais distal. O sistema de canalículos é pouco desenvolvido, não existem depósitos de guano e nem raízes superficiais ou sub-superficiais. O teto e as paredes são revestidos por Actinomicetos e a cavidade apresenta uma baixa estabilidade ambiental. Durante a estação úmida a caverna apresentava uma maior umidade, alguns pontos de gotejamento ativos e uma pequena área escavada para estudos arqueológicos.

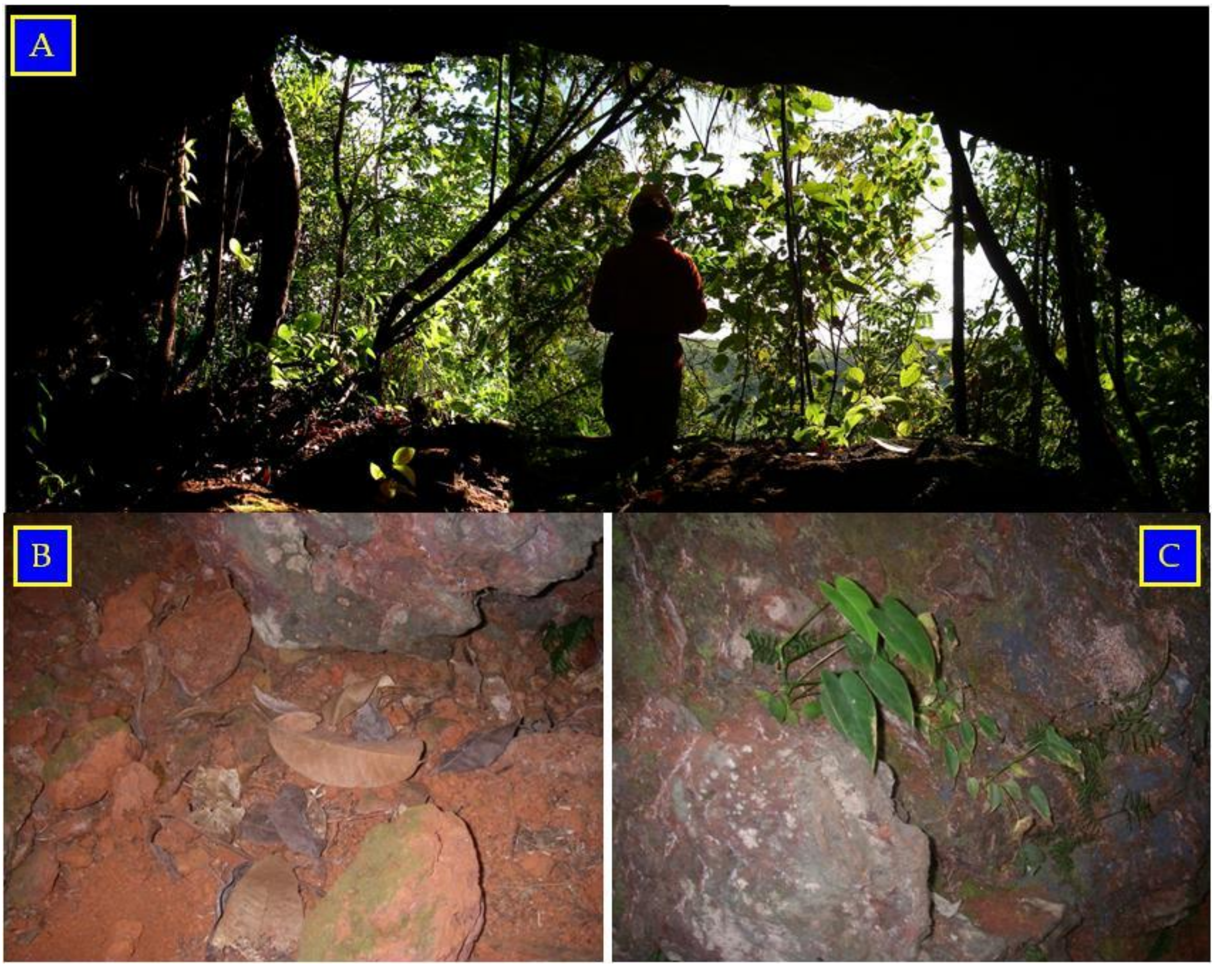

Figura 235 - a) Vista interna da entrada da cavidade; b) Aspecto geral do piso da caverna; c) Vegetação associada às paredes da cavidade.

5.4.4.95.2. Caracterização faunística no período de seca

Foi observado na caverna, um total de 43 morfoespécies de invertebrados de pelo menos 28 famílias das Ordens: Acari (Ixodidae: *Amblyomma cajennense*), Amblypygi (*Heterophrynus longicornis*), Pseudoscorpiones (Chernetidae), Opiliones (Cosmetidae: *Anduzeia* sp., Escadabiidae), Araneae (Araneidae: *Alpaida* sp., Corinidae: *Tupirina* sp., Salticidae, Scytodidae*: Scytodes eleonora*, Psauridae, Pholcidae: *Mesabolivar* sp.), Collembola (Entomobryidae), Orthoptera (Phalangopsidae: *Aclodes* sp.), Blattodea (Polyphagidae), Isoptera (Termitidae: *Nasutitermes* sp.), Psocoptera (Epipsocidae), Hemiptera (Reduviidae), Homoptera (Cixiidae: *Cixius* sp.), Lepidoptera (Noctuidae), Diptera (Ceratopogonidae, Drosophilidae, Phoridae, Psychodidae: *Lutzomyia* sp.), Hymenoptera (Formicidae: *Azteca* sp., *Camponotus* sp., *Gnamptogenys* sp., *Pachycondyla* sp.), Scolopendromorpha (Otostigmidae: *Otostigmus* sp.; Scolopocryptopidae: *Newportia* sp.), Scutigeromorpha (Scutigeridae: *Sphendononema* sp.).

Dentre os vertebrados, foram encontradas duas espécies das ordens Chiroptera (Emballonuridae: *Peropteryx kappleri*), Anura (Leptodactylidae: *Pristimantis cf. fenestratus*). Desta forma, no total foram encontradas 45 morfoespécies. Entre estas uma espécie de invertebrado, foi considerada troglomórfica: Araneae (Ochyroceratidae).

5.4.4.95.3. Caracterização faunística no período de chuva

Foi observado na caverna, um total de 52 morfoespécies de invertebrados de pelo menos 35 famílias das Ordens: Gastropoda, Isopoda (Armadillidae, Balloniscidae, Philosciidae), Acari (Ixodidae: *Amblyomma cajennense,* Laelapidae, Opioacaridae: *Neoacarus* sp.; Oribatida, Anystidae: *Erythracarus* sp.), Pseudoscorpiones (Chernetidae), Opiliones (Phalangiidae), Araneae (Araneidae: *Alpaida* sp., Oonopidae: Oonopinae; Salticidae, Pholcidae: *Mesabolivar* sp., Theraphosidae: *Acanthoscurria* sp.), Collembola (Cyphoderidae, Entomobryidae), Orthoptera (Phalangopsidae: *Phalangopsis* sp.), Blattodea (Blattellidae, Polyphagidae), Isoptera (Termitidae: *Nasutitermes* sp.), Psocoptera (Myopsocidae: *Lichenomina* sp.*;* Psyllipsocidae: *Psyllipsocus* sp.), Hemiptera (Lygaeidae, Reduviidae), Homoptera (Cixiidae: *Cixius* sp.), Lepidoptera (Noctuidae), Diptera (Cecidomyiidae, Chloropidae, Psychodidae: *Lutzomyia* sp.), Hymenoptera (Formicidae: *Camponotus* sp., *Gnamptogenys* sp., *Pseudomyrmex* sp., *Pheidole* sp.), Coleoptera (Curculionidae: Scotylinae; Elateridae: Elaterinae; Phalacridae, Pselaphidae), Scutigeromorpha (Scutigeridae: *Sphendononema* sp.), Neuroptera (Myrmeleontidae). Desta forma, no total foram encontradas 52 morfoespécies.

5.4.4.95.4. Caracterização geral da fauna da cavidade

Foi observado na caverna, um total de 81 morfoespécies de invertebrados de pelo menos 49 famílias das Ordens: Gastropoda, Isopoda (Armadillidae, Balloniscidae, Philosciidae), Acari (Ixodidae: *Amblyomma cajennense,* Laelapidae, Opioacaridae: *Neoacarus* sp.; Oribatida,

Anystidae: *Erythracarus* sp.), Amblypygi (*Heterophrynus longicornis*), Pseudoscorpiones (Chernetidae), Opiliones (Cosmetidae: *Anduzeia* sp., Escadabiidae, Phalangiidae), Araneae (Araneidae: *Alpaida* sp., Corinidae: *Tupirina* sp., Oonopidae: Oonopinae; Salticidae, Scytodidae*: Scytodes eleonora*, Psauridae, Pholcidae: *Mesabolivar* sp., Theraphosidae: *Acanthoscurria* sp.), Collembola (Cyphoderidae, Entomobryidae), Orthoptera (Phalangopsidae: *Aclodes* sp., *Phalangopsis* sp.), Blattodea (Blattellidae, Polyphagidae), Isoptera (Termitidae: *Nasutitermes* sp.), Psocoptera (Epipsocidae, Myopsocidae: *Lichenomina* sp.*;* Psyllipsocidae: *Psyllipsocus* sp.), Hemiptera (Lygaeidae, Reduviidae), Homoptera (Cixiidae: *Cixius* sp.), Lepidoptera (Noctuidae), Diptera (Cecidomyiidae, Ceratopogonidae, Chloropidae, Drosophilidae, Phoridae, Psychodidae: *Lutzomyia* sp.), Hymenoptera (Formicidae: *Azteca* sp., *Camponotus* sp., *Gnamptogenys* sp., *Pachycondyla* sp., *Pseudomyrmex* sp., *Pheidole* sp.), Coleoptera (Curculionidae: Scotylinae; Elateridae: Elaterinae; Phalacridae, Pselaphidae), Scolopendromorpha (Otostigmidae: *Otostigmus* sp.; Scolopocryptopidae: *Newportia* sp.), Scutigeromorpha (Scutigeridae: *Sphendononema* sp.), Neuroptera (Myrmelionthidae).

Dentre os vertebrados, foram encontradas duas espécies das ordens Chiroptera (Emballonuridae: *Peropteryx kappleri*), Anura (Leptodactylidae: *Pristimantis cf. fenestratus*).

Desta forma, no total foram encontradas 83 morfoespécies. Entre estas, uma espécie foi considerada troglomórfica: Araneae (Ochyroceratidae). Alguns organismos encontrados nesta caverna são mostrados na Figura 236.

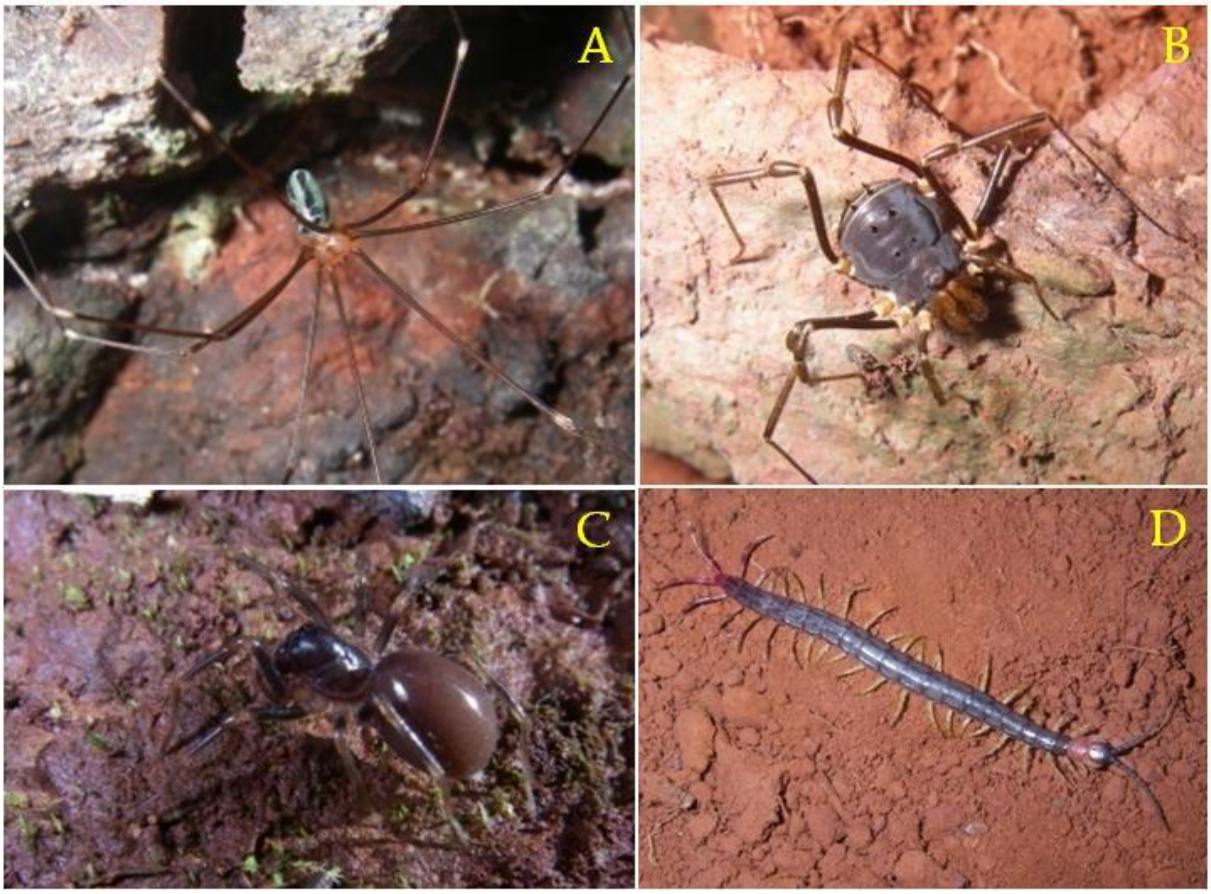

Figura 236 - a) Araneae (Pholcidae); b) Opiliones (Manaosbiidae); c) Araneae (indet.); d) Scolopendromorpha (Otostigmidae: *Otostigmus sp.*).

5.4.4.96. SL-100

5.4.4.96.1. Caracterização trófica

Abrigo localizado um pouco acima da drenagem, formado na canga em área de mata ciliar. Possui 7,5 m de projeção horizontal, sua entrada é pequena e estreita, mas logo em seguida abre-se um pequeno salão de aproximadamente 16 m^2 onde existem poucos líquens e briófitas. O piso é plano no interior da cavidade e ascendente na zona de entrada. Este é composto por sedimento granulado com poucos blocos abatidos sendo estes basicamente representados por calhaus. Existe um sistema radicular bem desenvolvido formados por raízes de diferentes calibres que se apresentam de maneira superficial. A serrapilheira encontra-se restrita à zona de entrada e existem depósitos de rejeitos de formigueiros (lixeiras) na zona afótica. Na porção mais distal da cavidade existem dois pequenos depósitos envelhecidos de guano de morcegos frugívoros, além de muitas raízes de pequeno calibre distribuídas de forma superficial por todo este setor da cavidade (Figura 237). Trata-se de uma caverna seca, sem pontos de gotejamento durante a estação seca e com forte influência das condições epígeas. O sistema de canalículos é pouco desenvolvido e não

existem zonas afóticas. Nenhuma alteração significativa foi observada durante a estação úmida, além das alterações normais na umidade relativa do ar.

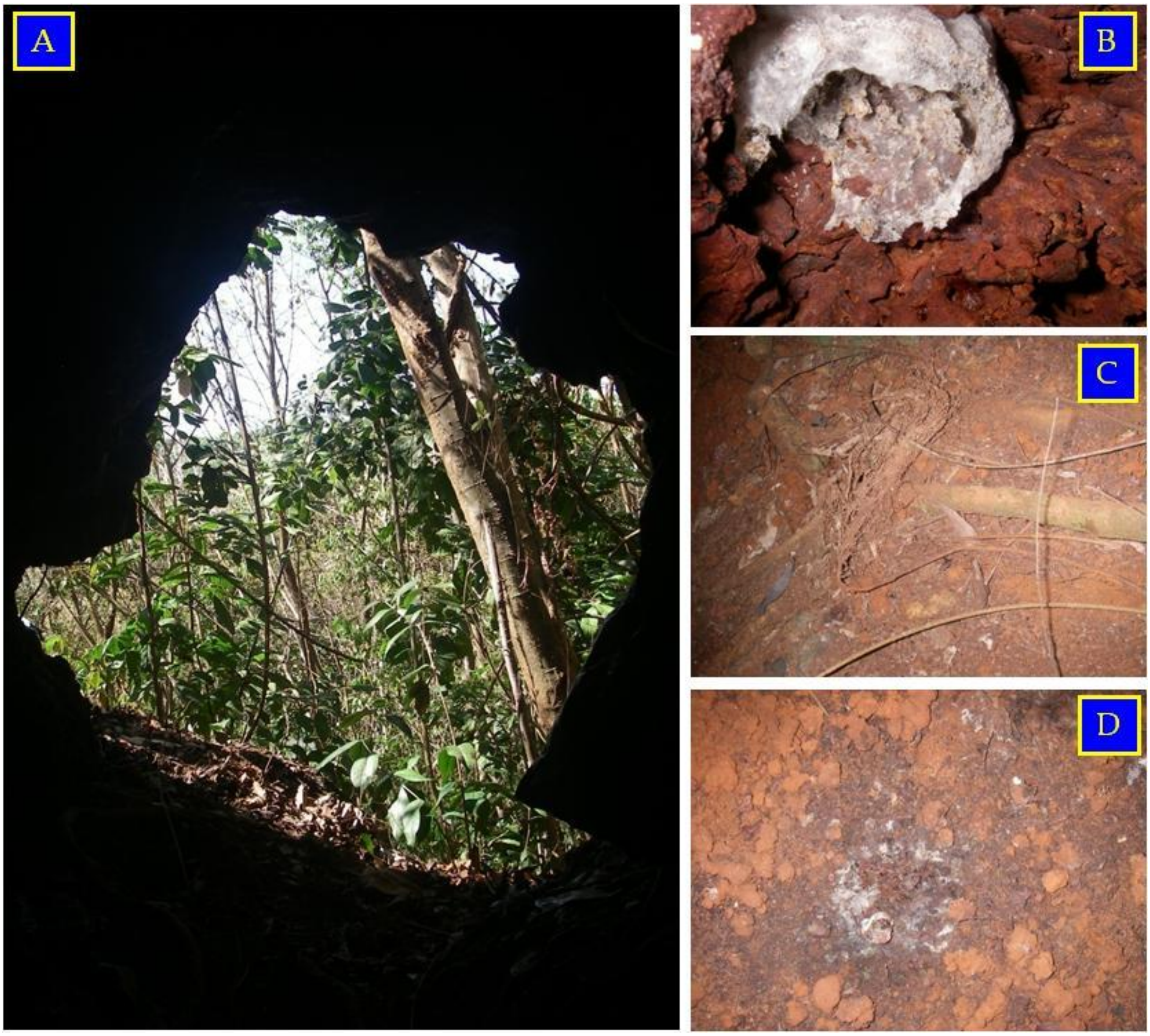

Figura 237 - a) Vista interna da entrada da cavidade; b) Fungos cultivados por colônia de formigas (Apterostigma sp.); c) Grande quantidade de raízes se desenvolvendo no piso da cavidade; d) depósito antigo de guano de morcegos.

5.4.4.96.2. Caracterização faunística no período de seca

Foi observado na caverna, um total de 49 morfoespécies de invertebrados de pelo menos 32 famílias das Ordens: Isopoda (Armadillidae), Acari (Ixodidae: *Amblyomma* sp., Laelapidae: *Stratiolaelaps* sp., Mesostigmata), Amblypygi (Phrynidae: *Heterophrynus longicornis*), Pseudoscorpiones (Chernetidae, Chtoniidae), Opiliones (Cosmetidae: *Anduzeia* sp.), Araneae (Gnaphosidae; Scytodidae*: Scytodes eleonora*; Pholcidae: *Mesabolivar* sp., *Metagonia* sp; Theridiidae), Diplura (Campodeidae), Collembola (Entomobryidae), Orthoptera (Phalangopsidae: *Aclodes* sp.), Blattodea (Blaberidae: *Blaberus* sp., Polyphagidae), Isoptera

(Termitidae: *Nasutitermes* sp.), Psocoptera, Hemiptera (Cydnidae, Reduviidae), Homoptera (Cixiidae: *Cixius* sp.), Lepidoptera (Hesperiidae, Thyrididae), Diptera (Culicidae, Dolichopodidae, Psychodidae: *Lutzomyia* sp., Tipulidae), Hymenoptera (Formicidae: *Apterostigma* sp., *Camponotus* sp., *Cyphomyrmex* sp., *Dolichoderus* sp., *Gnamptogenys* sp., *Hypoponera* sp., *Pachycondyla* sp., *Pseudomyrmex* sp.), Coleoptera (Carabidae, Cetoniidae: *Gymnets* sp.), Scolopendromorpha (Cryptopidae: *Cryptops* sp.), Scutigeromorpha (Scutigeridae: *Sphendononema* sp.) e Symphyla (Scutigerellidae: *Scutigerella* sp.).

Dentre os vertebrados foram encontradas três espécies das Ordens: Chiroptera (Phyllostomidae: *Glossophaga soricina*) e Anura (Leptodactylidae: *Pristimantis* cf. *fenestratus*, *Eleutherodactylus* sp.). Desta forma, no total foram encontradas 52 morfoespécies.

5.4.4.96.3. Caracterização faunística no período de chuva

Foi observado na caverna, um total de 55 morfoespécies de invertebrados de pelo menos 43 famílias das Ordens: Isopoda (Armadillidae, Philosciidae), Acari (Mesostigmata, Ologamasidade, Oribatida, Trombidiforme), Amblypygi (Phrynidae: *Heterophrynus longicornis*, Charinidae: *Charinus* sp.), Pseudoscorpiones (Chernetidae, Chtoniidae), Opiliones (Sclerosomatidae: *Prionostemma* sp., Phalangiidae), Araneae (Dipluridae; Ochyroceratidae; Oonopidae: Oonopinae; Scytodidae*: Scytodes eleonora*; Pholcidae: *Mesabolivar* sp., *Metagonia* sp; Theraphosidae; Theridiidae), Diplura (Campodeidae), Collembola (Entomobryidae), Orthoptera (Phalangopsidae: *Aclodes* sp., Tettigoniidae: Listroscelidinae), Blattodea (Blaberidae, Polyphagidae), Psocoptera (Myopsocidae: *Lichenomina* sp.), Hemiptera (Cydnidae, Reduviidae), Homoptera (Cercopidae, Cixiidae), Lepidoptera (Noctuidae, Tineidae), Diptera (Ceratopogonidae, Culicidae, Psychodidae: *Lutzomyia* sp., Tipulidae), Hymenoptera (Formicidae: *Apterostigma* sp., *Camponotus* sp., *Pachycondyla* sp.; Eulophidae), Coleoptera (Carabidae, Chrysomelidae, Phalacridae, Scydmaenidae, Staphylinidae), Diplopoda (Pyrgodesmidae, Stemmiulidae) e Symphyla (Scutigerellidae: *Hanseniella* sp.).

Dentre os vertebrados foram encontradas três espécies das Ordens: Chiroptera (Phyllostomidae: *Glossophaga soricina*), Anura (Leptodactylidae: *Pristimantis* cf. *fenestratus*) e Rodentia (Cricetidae: *Rhipidomys* sp.).

Desta forma, no total foram encontradas 58 morfoespécies. Entre estas, uma espécie de Amblypygi (Charinidae: *Charinus* sp.) foi considerada troglomórfica.

5.4.4.96.4. Caracterização geral da fauna da cavidade

Foi observado na caverna, um total de 84 morfoespécies de invertebrados de pelo menos 55 famílias das Ordens: Isopoda (Armadillidae, Philosciidae), Acari (Ixodidae: *Amblyomma* sp.,

Laelapidae: *Stratiolaelaps* sp., Mesostigmata, Ologamasidade, Oribatida, Trombidiforme), Amblypygi (Phrynidae: *Heterophrynus longicornis*, Charinidae: *Charinus* sp.), Pseudoscorpiones (Chernetidae, Chtoniidae), Opiliones (Cosmetidae: *Anduzeia* sp., Sclerosomatidae: *Prionostemma* sp., Phalangiidae), Araneae (Dipluridae; Gnaphosidae; Ochyroceratidae; Oonopidae: Oonopinae; Scytodidae: *Scytodes eleonora*; Pholcidae: *Mesabolivar* sp., *Metagonia* sp; Theraphosidae; Theridiidae), Diplura (Campodeidae), Collembola (Entomobryidae), Orthoptera (Phalangopsidae: *Aclodes* sp., Tettigoniidae: Listroscelidinae), Blattodea (Blaberidae: *Blaberus* sp., Polyphagidae), Isoptera (Termitidae: *Nasutitermes* sp.), Psocoptera (Myopsocidae: *Lichenomina* sp.), Hemiptera (Cydnidae, Reduviidae), Homoptera (Cercopidae, Cixiidae: *Cixius* sp.), Lepidoptera (Hesperiidae, Noctuidae, Thyrididae, Tineidae), Diptera (Ceratopogonidae, Culicidae, Dolichopodidae, Psychodidae: *Lutzomyia* sp., Tipulidae), Hymenoptera (Formicidae: *Apterostigma* sp., *Camponotus* sp., *Cyphomyrmex* sp., *Dolichoderus* sp., *Gnamptogenys* sp., *Hypoponera* sp., *Pachycondyla* sp., *Pseudomyrmex* sp.; Eulophidae), Coleoptera (Carabidae, Cetoniidae: *Gymnets* sp., Chrysomelidae, Phalacridae, Scydmaenidae, Staphylinidae), Diplopoda (Pyrgodesmidae, Stemmiulidae), Scolopendromorpha (Cryptopidae: *Cryptops* sp.), Scutigeromorpha (Scutigeridae: *Sphendononema* sp.) e Symphyla (Scutigerellidae: *Hanseniella* sp., *Scutigerella* sp.).

Dentre os vertebrados foram encontradas quatro espécies das Ordens: Chiroptera (Phyllostomidae: *Glossophaga soricina*), Anura (Leptodactylidae: *Pristimantis* cf. *fenestratus*, *Eleutherodactylus* sp.) e Rodentia (Cricetidae: *Rhipidomys* sp.). Desta forma, no total foram encontradas 88 morfoespécies. Entre estas, uma espécie de Amblypygi (Charinidae: *Charinus* sp.) foi considerada troglomórfica. Alguns organismos encontrados nesta caverna são mostrados na Figura 238.

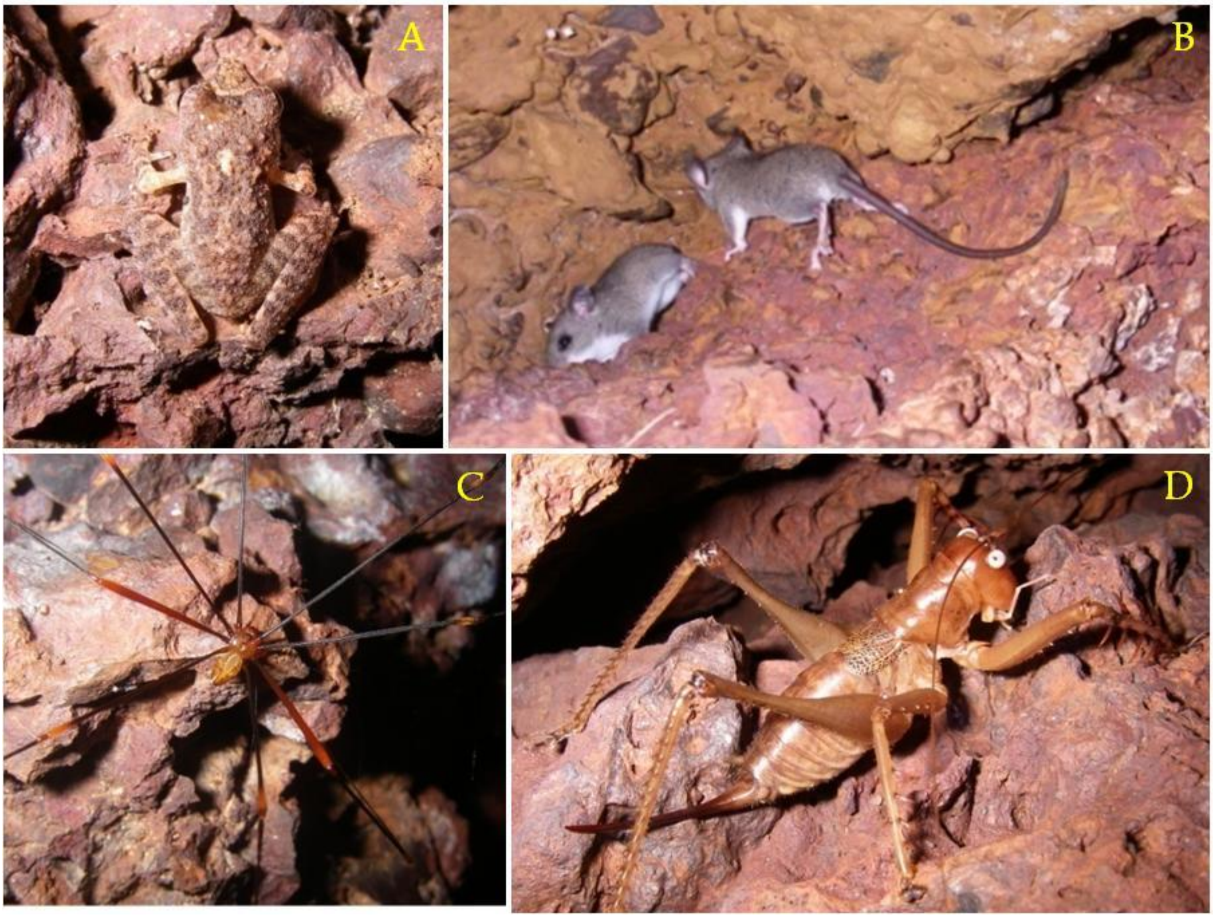

Figura 238 - a) Anura (Leptodactylidae: *Pristimantis* cf. *fenestratus*); b) Roedor (Cricetidae: *Rhipidomys sp.*); c) Pholcidae; d) Orthoptera (Tettigoniidae: Listroscelidinae).

5.4.5. Análises Ecológicas

5.4.5.1. Padrões gerais de riqueza e diversidade

As cavidades mostraram valores de riqueza de espécies bastante variáveis. Além disso, a diversidade, equitabilidade e dominância também se mostraram variáveis entre estas cavernas (Tabela 11). Estas variações são esperadas, e decorrem das diferentes condições físicas, tróficas e dinâmicas de cada um dos sistemas estudados. Por mais parecidas que duas cavidades possam ser, dificilmente possuirão atributos biológicos semelhantes, em função da complexidade (e dinamismo) das interações que estruturam cada comunidade.

Tabela 11 - Valores de riqueza, dominância, diversidade e equitabilidade para as cavernas do estudo (estação seca).

Caverna	Riqueza			Dominância		Diversidade		Equitabilidade	
	Seca	Chuva	Total	Seca	Chuva	Seca	Chuva	Seca	Chuva
SL-001	132	104	222	0,21	0,16	1,87	2,09	0,38	0,45
SL-002	56	60	114	0,07	0,74	3,05	0,73	0,76	0,18
SL-003	54	75	123	0,78	0,45	0,72	1,01	0,18	0,23
SL-004	120	117	230	0,18	0,52	1,91	1,55	0,40	0,33
SL-005	51	71	109	0,94	0,06	0,24	3,57	0,06	0,84
SL-006	32	49	75	0,07	0,05	3,07	3,39	0,89	0,87
SL-007	33	38	61	0,09	0,10	2,87	2,81	0,82	0,77
SL-008	64	81	126	0,05	0,12	3,40	3,31	0,82	0,75
SL-009	33	32	56	0,15	0,16	2,34	2,33	0,67	0,67
SL-011	35	60	81	0,12	0,04	2,69	3,52	0,76	0,86
SL-012	40	59	85	0,08	0,08	3,08	3,33	0,84	0,82
SL-013	37	44	74	0,24	0,07	2,17	3,22	0,60	0,85
SL-014	36	48	77	0,15	0,06	2,49	3,31	0,70	0,85
SL-015	34	41	67	0,11	0,10	2,76	2,99	0,78	0,81
SL-016	55	121	148	0,23	0,18	1,58	1,92	0,39	0,40
SL-017	35	47	71	0,92	0,08	0,28	2,99	0,08	0,78
SL-018	31	41	64	0,69	0,09	0,91	3,07	0,26	0,83
SL-019	30	38	61	0,08	0,12	2,99	2,84	0,88	0,78
SL-020	26	40	56	0,32	0,19	1,80	2,36	0,55	0,64
SL-022	35	44	74	0,09	0,10	2,84	3,04	0,80	0,80
SL-023	19	36	50	0,34	0,15	1,79	2,37	0,61	0,66
SL-024	43	61	87	0,46	0,08	0,95	2,99	0,25	0,73
SL-025	25	35	54	0,11	0,10	2,60	2,79	0,81	0,78
SL-026	44	30	61	0,92	0,21	0,29	2,12	0,08	0,62
SL-027	37	40	67	0,93	0,09	0,26	2,91	0,07	0,79
SL-028	30	37	60	0,38	0,05	1,66	3,34	0,49	0,92
SL-029	67	88	127	0,12	0,59	2,75	1,25	0,65	0,28
SL-030	66	105	141	0,73	0,37	0,84	1,41	0,20	0,30
SL-031	60	78	109	0,12	0,18	2,82	2,51	0,69	0,58
SL-032	33	45	70	0,12	0,07	2,72	3,29	0,78	0,87
SL-033	57	48	94	0,04	0,11	3,58	2,78	0,89	0,72
SL-035	75	78	124	0,61	0,71	1,21	0,92	0,28	0,21
SL-036	29	31	49	0,90	0,11	0,27	2,88	0,08	0,84
SL-037	70	45	92	0,31	0,07	1,36	3,08	0,32	0,81
SL-038	17	38	49	0,11	0,09	2,48	2,89	0,87	0,79
SL-039	24	41	59	0,46	0,13	1,45	2,61	0,46	0,70
SL-040	36	31	63	0,22	0,06	2,37	3,08	0,66	0,90

SL-041	33	30	52	0,16	0,17	2,53	2,54	0,72	0,75
SL-042	66	74	108	0,80	0,09	0,70	3,19	0,17	0,74
SL-043	58	42	84	0,11	0,09	2,86	2,97	0,70	0,79
SL-044	77	90	138	0,10	0,14	2,94	2,78	0,68	0,62
SL-045	39	34	66	0,25	0,11	2,11	2,71	0,58	0,77
SL-046	52	57	87	0,88	0,07	0,45	3,24	0,11	0,80
SL-047	54	42	79	0,25	0,42	2,34	1,81	0,59	0,48
SL-048	46	50	86	0,08	0,16	3,08	2,81	0,80	0,72
SL-049	37	45	70	0,09	0,04	3,08	3,50	0,85	0,92
SL-050	42	50	78	0,16	0,21	2,46	2,28	0,66	0,58
SL-051	58	66	109	0,12	0,15	2,91	2,48	0,72	0,59
SL-052	43	50	80	0,09	0,08	3,02	3,22	0,80	0,82
SL-053	38	55	77	0,06	0,05	3,22	3,45	0,89	0,86
SL-054	49	33	78	0,06	0,11	3,29	2,84	0,84	0,81
SL-055	25	44	63	0,06	0,27	2,98	2,02	0,93	0,53
SL-056	34	48	68	0,08	0,08	3,05	3,10	0,87	0,80
SL-057	35	72	90	0,16	0,73	2,44	0,83	0,69	0,19
SL-058	69	72	113	0,06	0,28	3,27	2,23	0,77	0,52
SL-059	47	41	73	0,07	0,17	3,18	2,51	0,83	0,68
SL-060	87	108	156	0,23	0,28	1,63	1,53	0,36	0,33
SL-061	48	57	93	0,04	0,11	3,49	2,82	0,90	0,70
SL-062	33	37	60	0,05	0,19	3,22	2,52	0,92	0,70
SL-063	15	29	37	0,19	0,14	1,94	2,59	0,72	0,77
SL-064	42	49	78	0,10	0,14	2,94	2,70	0,79	0,69
SL-065	74	53	107	0,06	0,15	3,36	2,59	0,78	0,65
SL-066	46	48	78	0,06	0,26	3,24	2,30	0,85	0,59
SL-067	36	59	82	0,13	0,11	2,63	3,02	0,74	0,74
SL-068	19	21	37	0,12	0,11	2,51	2,61	0,85	0,86
SL-069	82	116	164	0,13	0,32	2,92	2,30	0,66	0,48
SL-070	19	38	53	0,09	0,09	2,65	2,97	0,90	0,82
SL-071	36	34	62	0,13	0,16	2,57	2,48	0,72	0,70
SL-072	61	71	111	0,19	0,07	2,67	3,56	0,65	0,84
SL-073	45	88	114	0,19	0,20	1,75	1,86	0,46	0,42
SL-074	151	165	253	0,26	0,16	1,73	2,27	0,34	0,44
SL-075	67	63	106	0,05	0,14	3,49	2,89	0,83	0,70
SL-076	62	86	130	0,88	0,03	0,42	3,93	0,10	0,88
SL-077	65	62	107	0,06	0,17	3,29	2,48	0,79	0,60
SL-078	24	30	48	0,66	0,22	1,02	2,04	0,32	0,60
SL-079	74	78	122	0,40	0,33	1,27	1,94	0,29	0,45
SL-080	44	41	69	0,29	0,17	1,89	2,45	0,50	0,66
SL-081	20	30	44	0,24	0,12	1,82	2,61	0,61	0,77

SL-082	85	88	141	0,35	0,10	1,56	2,89	0,35	0,64
SL-083	47	44	75	0,83	0,32	0,54	1,81	0,14	0,48
SL-084	15	21	33	0,27	0,13	1,67	2,46	0,62	0,81
SL-085	37	54	75	0,11	0,12	2,77	2,69	0,77	0,67
SL-086	18	33	46	0,37	0,09	1,66	2,82	0,57	0,81
SL-087	63	58	100	0,82	0,05	0,62	3,40	0,15	0,84
SL-088	39	28	58	0,04	0,20	3,35	2,39	0,91	0,72
SL-089	79	81	125	0,11	0,10	3,10	2,97	0,71	0,68
SL-090	27	35	56	0,17	0,07	2,28	3,04	0,69	0,86
SL-091	23	35	52	0,07	0,11	2,91	2,73	0,93	0,77
SL-092	28	43	60	0,28	0,07	1,82	3,14	0,55	0,84
SL-093	56	64	88	0,22	0,18	1,66	2,32	0,41	0,56
SL-094	32	39	63	0,09	0,31	2,92	2,26	0,84	0,62
SL-095	53	104	132	0,89	0,43	0,39	1,61	0,10	0,35
SL-096	34	30	56	0,10	0,25	2,81	2,02	0,80	0,59
SL-097	59	47	88	0,09	0,09	2,95	2,86	0,72	0,74
SL-099	45	52	83	0,10	0,37	2,82	1,95	0,74	0,49
SL-100	52	58	88	0,11	0,09	2,93	2,95	0,74	0,73

Um fato realmente notável diz respeito à elevada riqueza observada nas cavernas da área. Um total de 28 cavidades (29,2% do total inventariado) apresentou mais de 100 espécies, considerando-se as duas amostragens (Figura 239). Além disso, algumas cavernas apresentaram valores surpreendentes, como as grutas SL-074 (253 espécies), SL-004 (230 espécies) e SL-001 (222 espécies).

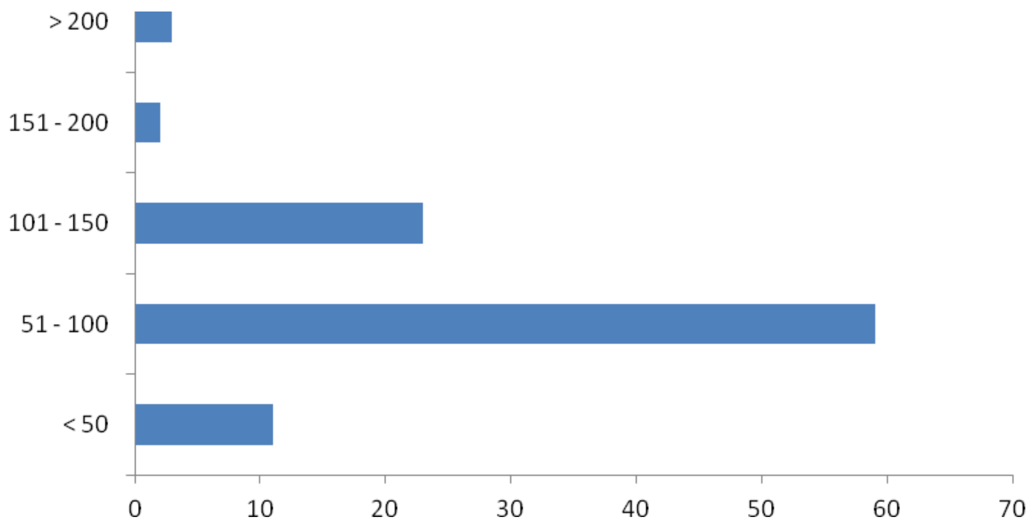

Figura 239 - Classes de riqueza total das cavernas da área. Os valores no eixo vertical indicam o número total de espécies, e no eixo horizontal, o número de cavernas em cada categoria.

Souza-Silva e colaboradores (2011) demonstraram as fortes relações existentes entre o tamanho das cavernas e a riqueza de espécies na Mata Atlântica brasileira, dentre as quais se destacaram as cavernas ferríferas. As cavernas ferríferas inventariadas naquele estudo situam-se no Quadrilátero Ferrífero, em Minas Gerais. Tais autores encontraram uma riqueza média correspondente a 53 espécies (± 26.35) em cavernas carbonáticas, 45,4 espécies (± 22.7) em cavernas siliciclásticas, 39,9 espécies (± 19.27) em cavernas de origem íngea e 37,5 espécies (± 20.96) em cavernas ferríferas. A média de riqueza encontrada nas cavernas de Serra Leste (considerando a média obtida entre os valores médios de riqueza do período de seca e do período chuvoso) correspondeu a 51 espécies, um número consideravelmente maior que o observado por Souza-Silva e colaboradores (2011) para as cavernas do quadrilátero ferrífero. Tal fato pode decorrer do elevado "turnover" de espécies observado nas cavernas da área (ver tópico "Turnover" de espécies ou β diversidade).

5.4.5.2. Estimadores de riqueza

As curvas do coletor construídas (Figuras 240 e 241) indicaram amostragens, a princípio, não satisfatórias do número potencial de espécies presente na área (considerando-se o total de espécies e as coletas referentes a cada estação, separadamente). Caso o total de espécies presente na região tivesse sido amostrado, seria observada uma estabilização nas curvas (assíntota – a linha se curvaria tornando-se paralela ao eixo horizontal do gráfico), indicando que o que existe já foi amostrado. O padrão obtido indica que se a amostragem fosse intensificada (através da inclusão de outras cavidades existentes nos arredores e que não foram amostradas), provavelmente seriam encontradas mais espécies que ainda não foram avistadas nas cavernas já amostradas.

No entanto, destaca-se novamente o elevado "turnover" de espécies observado nas cavernas da área. Tal substituição intensa de espécies entre estações pode resultar em uma impossibilidade de assíntota nas cavernas da área, caso sejam consideradas as espécies "transientes" (ou eventualmente acidentais) nestas na construção das curvas de coletor.

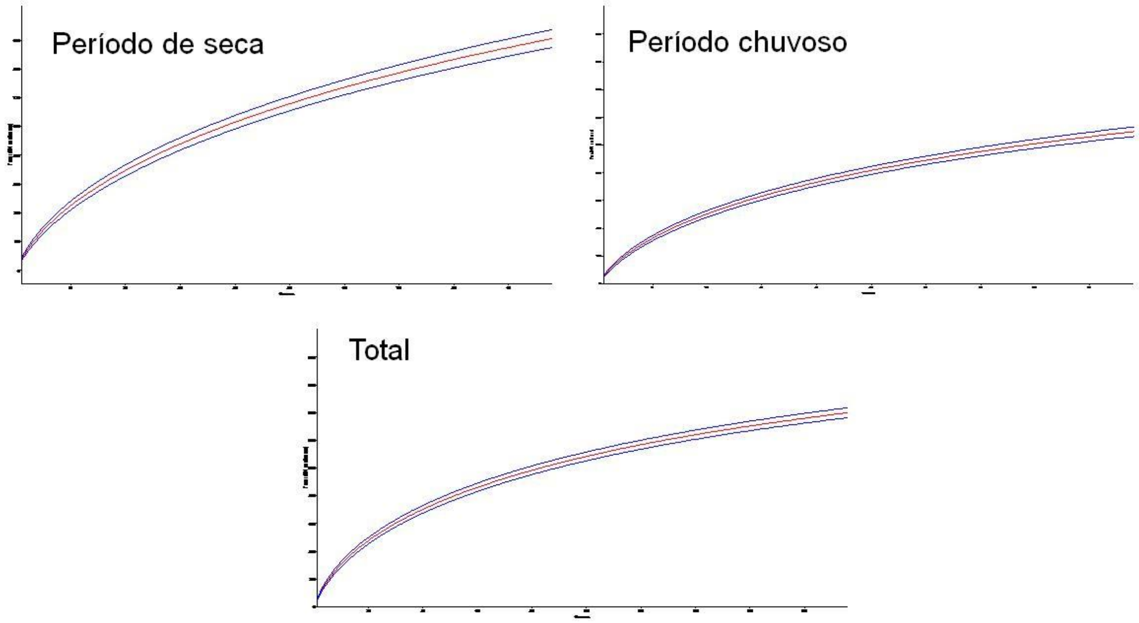

Figura 240 - Curvas de coletor construídas para as cavernas da área. O primeiro gráfico refere-se ao período de seca, o segundo ao período chuvoso e o último ao somatório do total de espécies. Nenhuma das curvas atingiu a assíntota.

Reitera-se que dificilmente atinge-se a assíntota nestas curvas do coletor, a não ser que o número de amostras (no caso, cavernas) seja muito grande. Mesmo assim, tendo em vista a constante contribuição de espécies acidentais ou mesmo “transientes”, é provável que, para certos sistemas, a assíntota nunca seja atingida, já que o “estoque” epígeo de grupos acidentais é inimaginável (especialmente quando se considera a região Neotropical, conhecida pela megadiversiade epígea).

Como exemplo disso pode-se citar o trabalho realizado por Zampaulo (2010) na região de Pains (Minas Gerais). Um total de 296 cavernas foi inventariada na área, o que corresponde a cerca de 25% das cavernas conhecidas para a região. Mesmo com este número extremamente expressivo, a curva do coletor não alcançou a assíntota. Somente quando retiradas as espécies “acidentais” da análise, houve uma estabilização da curva do coletor (Zampaulo, 2010).

Desta forma, certamente com a intensificação de coletas, seriam amostradas mais espécies. No entanto, o real significado destas novas espécies acrescidas à listagem é questionável, pois certamente muitos dos novos registros corresponderão a grupos acidentais.

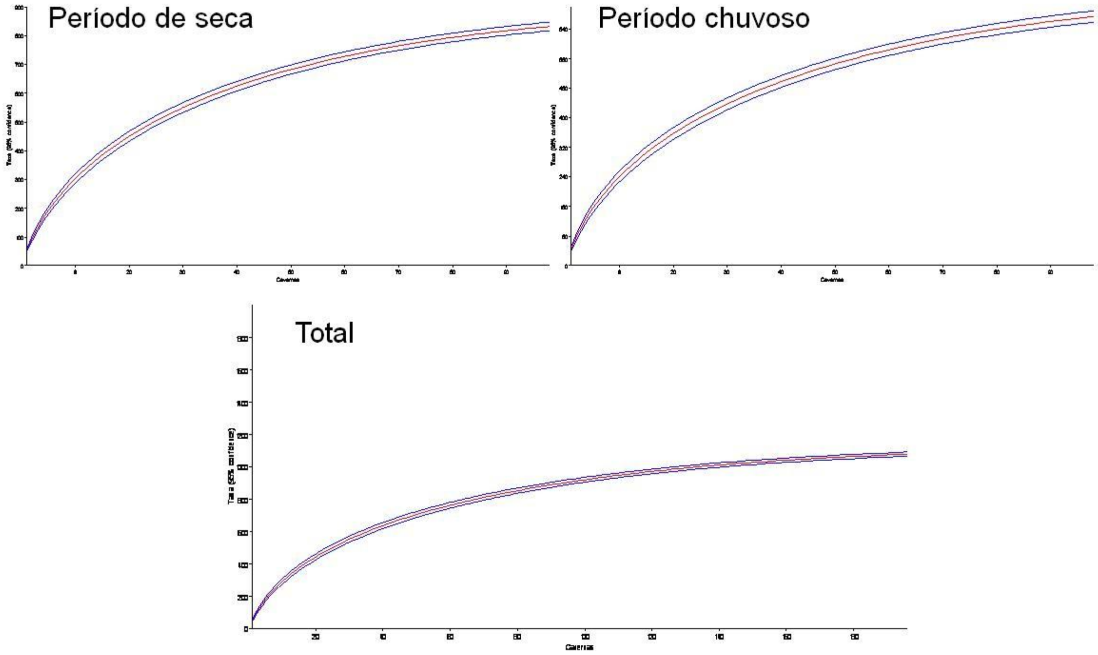

Figura 241 - Curvas de coletor construídas para as cavernas da área, a partir da exclusão das espécies "acidentais". O primeiro gráfico refere-se ao período de seca, o segundo ao período chuvoso e o último ao somatório do total de espécies. Percebe-se que a curva que representa o somatório de espécies tendeu à assíntota.

Aplicando a metodologia proposta por Zampaulo (2010) para a determinação de espécies "acidentais" (no caso, aquelas que foram representadas por um único indivíduo amostrado em uma única cavidade), tem-se que o total de espécies acidentais amostradas nas cavernas da área corresponde a 79 espécies. Este total corresponde a cerca de 35% do total de espécies amostradas nas cavernas da área. No entanto, diferentemente do observado por Zampaulo (2010), a exclusão destas espécies ditas "acidentais", não gerou, no modelo Jack-knife, uma perceptível tendência à assíntota (Figura 242-B). Tal fato certamente decorre da presença de cavernas, copm condições tróficas e de microhabitats bastante distinta que permite atrair um número "infindável" de potenciais colonizadores. Tal característica faz com que a presença desta caverna na amostra dificilmente resulte em uma assíntota. No entanto, percebe-se que a exclusão das supostas espécies acidentais (Figura 242-B) reduziu sensivelmente as diferenças entre o número de espécies observadas e esperadas, ao contrário do mostrado na Figura 242-A. Tal fato é esperado, já que este modelo baseia-se preferencialmente na presença de *singletons*.

O modelo Jackknife 1 gerado (Figura 242) indica um número esperado de espécies superior ao observado nas cavidades. Tal fato reflete a existência de sistemas intersticiais de amostragem inviável (no caso, os canalículos presentes na canga), com um número considerável de espécies potenciais. Além disso, tanto o modelo quanto a riqueza real não atingiram a assíntota.

No entanto, excluindo-se as espécies “acidentais” conforme metodologia sugerida por Zampaulo (2010), percebe-se que o modelo Jack-Knife 1 gerado tende claramente à assíntota, seja em cada uma das estações, seja considerando-se todas as espécies encontradas. A inclusão de espécies acidentais nas estimativas da riqueza de invertebrados pode resultar em uma estimativa “inflada” ou superestimada, uma vez que os principais estimadores (incluindo Jackknife 1) baseiam-se principalmente na ocorrência de espécies raras.

A análise gráfica (Figura 242) permite visualizar uma diferença significativa entre a riqueza esperada para as cavernas de Serra Leste com e sem a inclusão de espécies provavelmente acidentais. Nestes gráficos percebe-se uma tendência à estabilização das curvas do coletor quando o esforço de coleta se aproximou de 70 cavernas, em ambas as estações. Com a inclusão de todas as espécies encontradas, incluindo os prováveis acidentais, as 96 cavernas amostradas não foram suficientes para se conseguir uma estabilização na curva de rarefação. Caso esta condição seja verdadeira, este pode ser um indicativo importante sobre o quanto deve ser ampla uma amostragem para que a biodiversidade de invertebrados associados a ecossistemas em locais com grande concentração de cavernas como Serra Leste seja satisfatoriamente acessada. Ressalta-se que um padrão similar foi observado por Zampaulo (2010) para a província cárstica Arcos-Pains-Doresópolis.

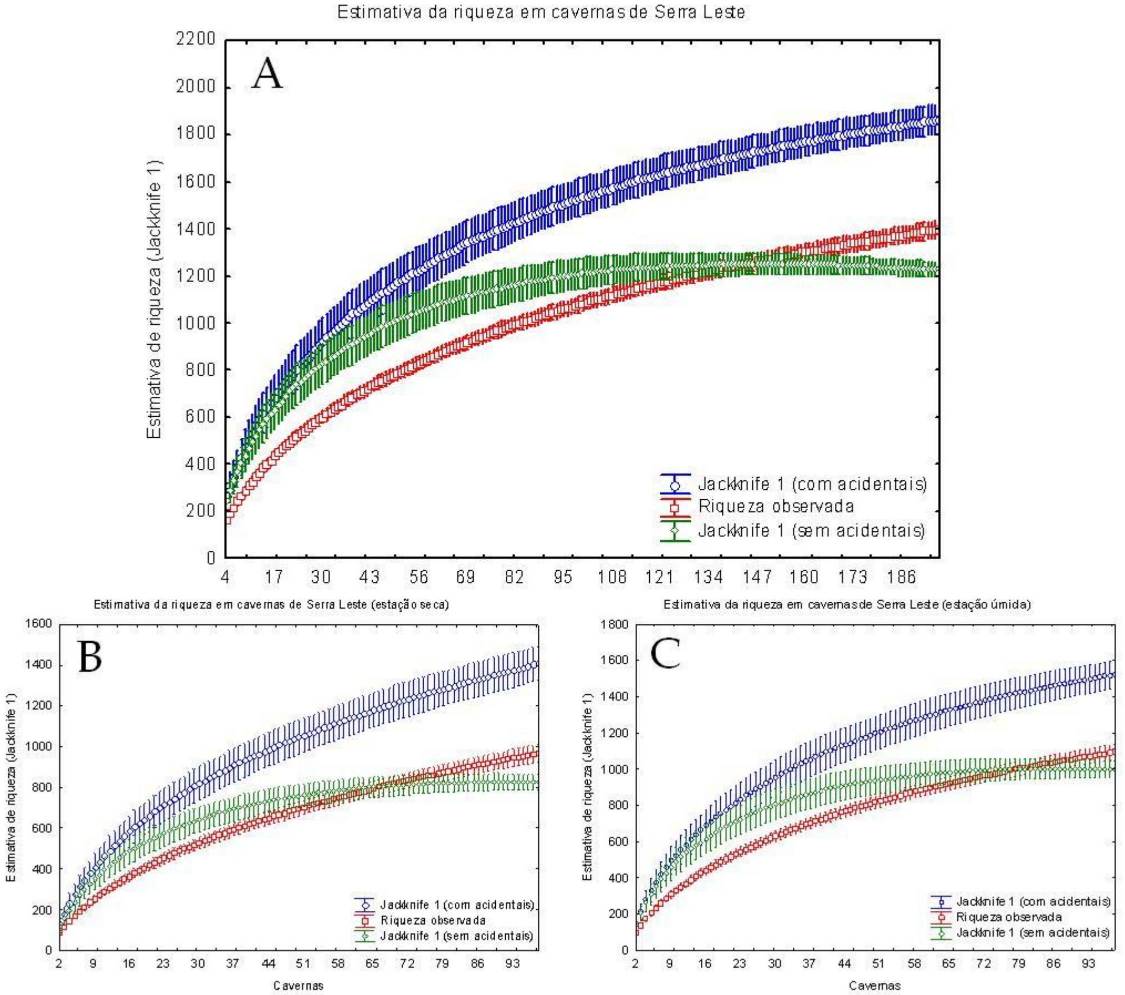

Figura 242 - A) Curvas acumulativas de espécies, indicando o número observado de espécies nas cavernas da área (em vermelho) e o número estimado pelo modelo Jack-Knife 1 considerando-se todas as espécies (em azul) e o número estimado pelo modelo Jack-Knife 1 excluindo-se as espécies "acidentais" (em verde); B) Curvas acumulativas de espécies construídas para o período de seca; (C) Curvas acumulativas de espécies construídas para o período de chuva.

5.4.5.3. Padrões gerais de similaridade da fauna

Os dendrogramas de similaridade entre as diferentes cavernas são mostrados na Figura 243. De forma geral, as cavidades apresentaram baixos valores de similaridade entre as comunidades bióticas, sendo que a grande maioria das cavidades apresentou valores de similaridade inferiores a 0,5 (em geral, consideram-se valores elevados aqueles iguais ou superiores a 70% de similaridade – 0,7). A baixa similaridade entre as comunidades presentes nas diferentes cavernas evidencia a importância da estrutura física e trófica (com particularidades inerentes a cada caverna) para a determinação de quais espécies possuem viabilidade de colonização e permanência em cada cavidade.

Além das cavidades terem exibido baixos valores de similaridade, os mesmos aparentemente não estão relacionados à distribuição espacial das cavernas. Desta forma, as

cavidades geograficamente mais próximas não apresentam necessariamente comunidades mais similares quando comparadas a cavernas mais distantes.

O único "padrão" evidenciado em relação à similaridade entre as cavernas diz respeito à tendência ao aumento de similaridade entre cavernas no período seco quando comparadas ao período chuvoso (Figura 243). No período seco, 11 pares de cavernas exibiram valores de similaridade superiores a 60%, contrapondo com somente 5 pares de cavernas que atingiram valores superiores a 60% na estação chuvosa. Além disso, reduzindo-se o "corte" para valores superiores a 50%, nota-se que 19 pares excederam este valore na estação seca contra somente 8 pares na estação chuvosa.

Desta forma, aparentemente, no período chuvoso a presença de um incremento de espécies acidentais ou transientes reduz a similaridade entre as cavernas da área.

Por fim, tendo em vista a elevada dissimilaridade entre as cavernas da área, acredita-se que a destruição de cada uma das cavernas amostradas dificilmente acarretará em colonizações futuras que culminem com a instalação bem sucedida destas comunidades em outras cavernas da região.

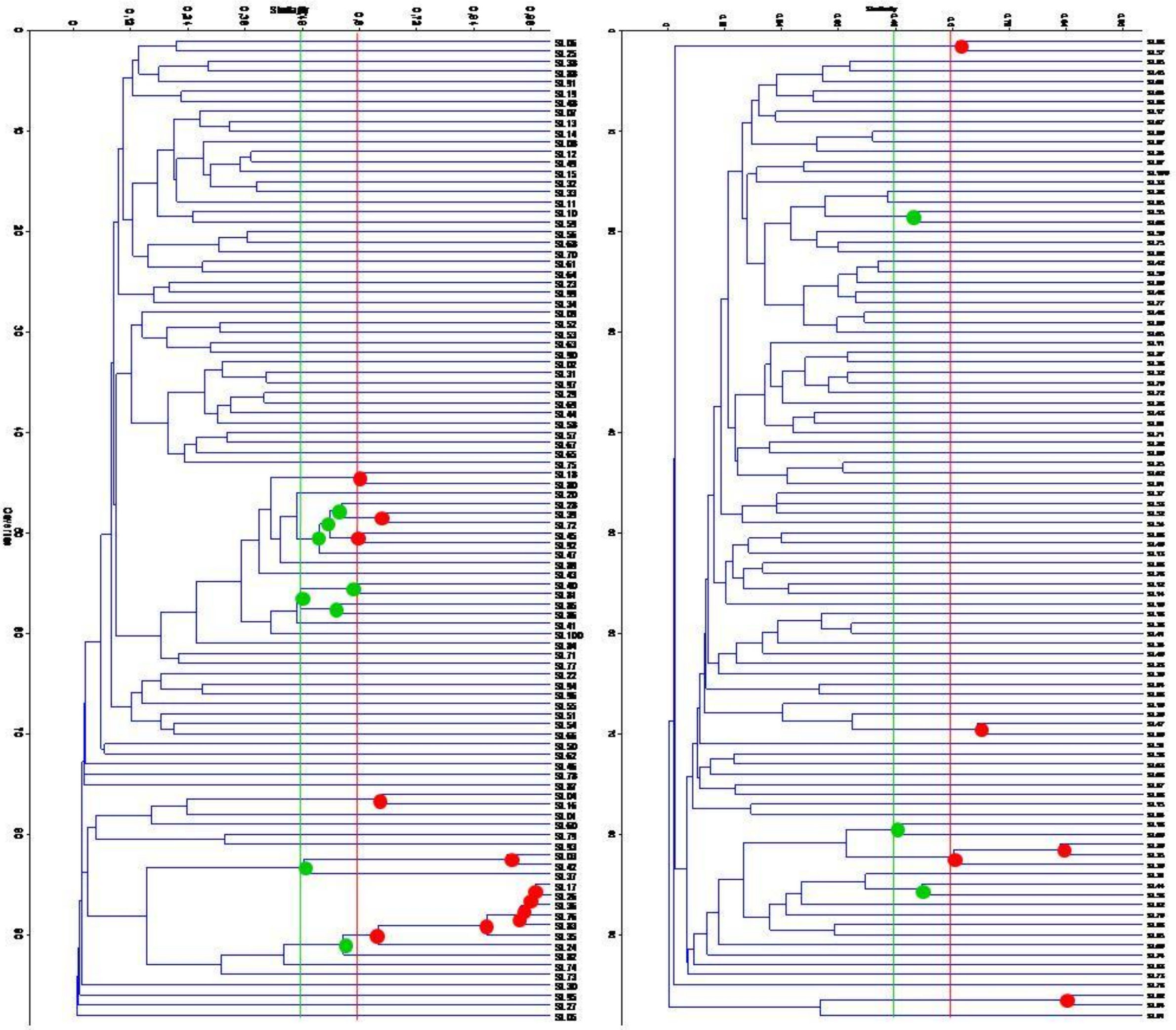

Figura 243 - Dendrogramas de similaridade (Bray-Curtis), evidenciando os padrões de similaridade entre as cavernas. A figura da esquerda representa o dendrograma referente à estação de seca e a da direita representa o dendrograma referente à estação chuvosa. Os pontos vermelhos representam valores de similaridade entre cavernas superiores a 60% e os pontos verdes valores superiores a 50%.

A análise de Escalonamento Multidimensional não-métrico (n-MSD) evidenciou as similaridades através das distâncias em um plano bi-dimensional entre as cavernas. Neste caso, cada ponto na figura representava uma cavidade. O gráfico do n-MDS foi sobreposto a uma fotografia aérea da região, onde a posição de cada caverna pode ser visualizada por meio de pontos vermelhos (Figura 244).

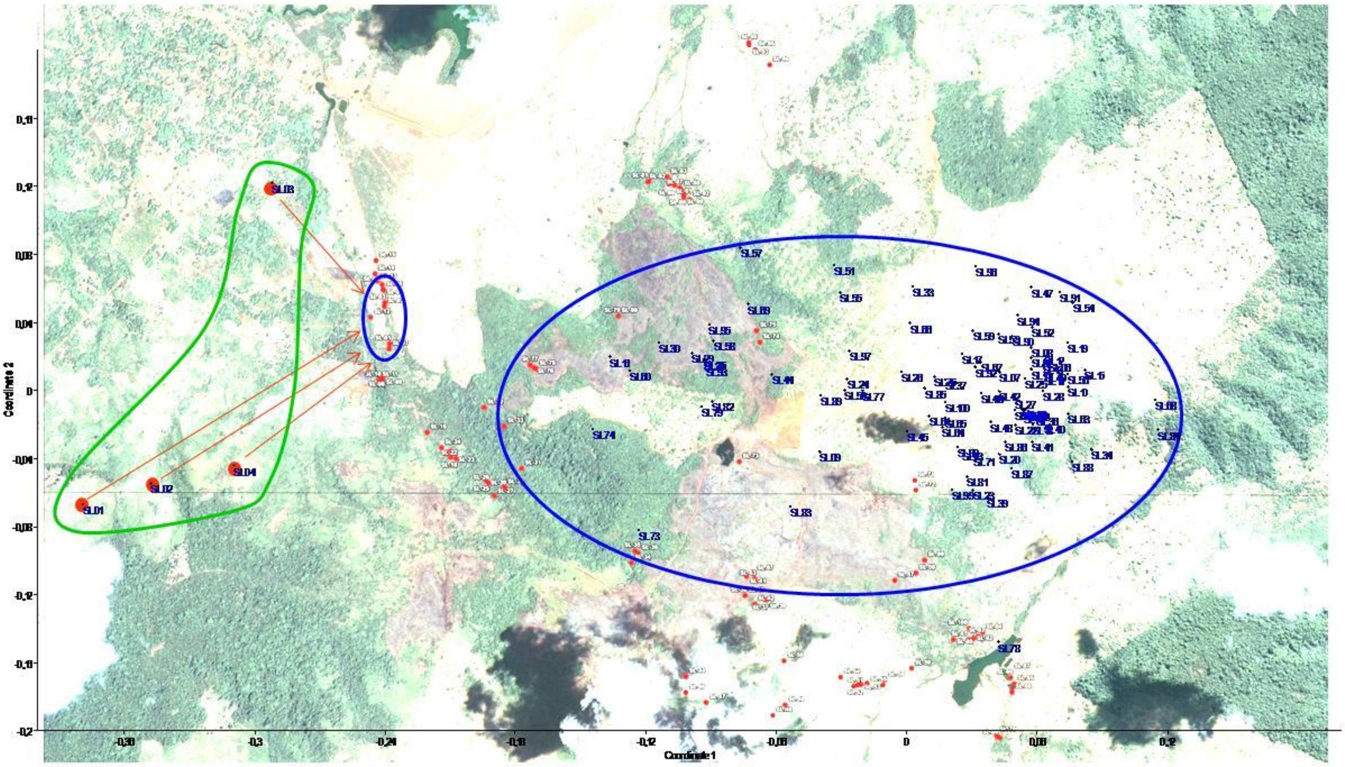

Figura 244 - Sobreposição do gráfico da análise de n-MDS e da distribuição espacial das cavernas da área. A elipse azul contorna a nuvem de dispersão que representa a quase totalidade das cavernas da área. A forma verde contorna 4 cavernas que se mostraram altamente dissimilares em relação às demais (SL-001, SL-002, SL-003 e SL-004)

A análise de n-MDS demonstrou claramente que as similaridades entre as cavernas não estão necessariamente associadas às suas distribuições espaciais (proximidades geográficas).

No entanto, na estação chuvosa, um “padrão” pôde ser evidenciado. Quatro cavernas associadas a uma drenagem (SL-001, SL-002, SL-003 e SL-004) se mostraram altamente dissimilares em relação às demais cavernas da área (Figura 245). É provável que esta grande dissimilaridade exibida por estas 4 cavernas com relação às demais (a grande “nuvem de dispersão” circulada pela elipse azul na figura 245) decorra da grande influência desta drenagem sobre a estruturação e composição das comunidades destas cavernas. No período seco, tais cavidades mostraram-se mescladas à “nuvem de dispersão” que representa a similaridade entre todas as cavernas (Figura 244), indicando que, nesta estação, estas cavernas não se apresentaram distintas em relação à tendência geral das avernas da área. Tal fato corrobora com o anteriormente exposto, isto é, a drenagem que leva à parcial submersão do piso das cavidades SL-001, SL-002, SL-003 e SL-004 no período chuvoso, torna as comunidades destas cavernas bastante distintas do “padrão” geral observado para as cavernas da área.

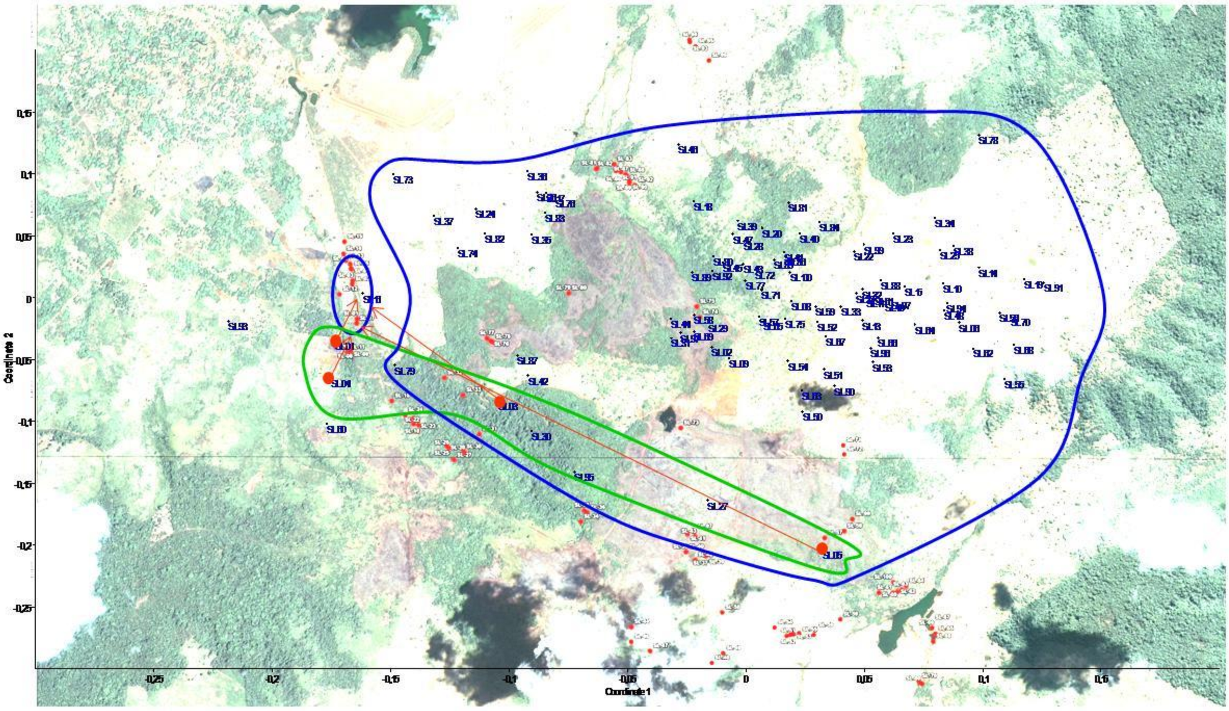

Figura 245 - Sobreposição do gráfico da análise de n-MDS e da distribuição espacial das cavernas da área. A elipse azul contorna a nuvem de dispersão que representa a quase totalidade das cavernas da área. A forma verde contorna 4 cavernas que, nesta estação, se sobrepuseram à nuvem geral de dispersão de similaridade (SL-01, SL-02, SL-03 e SL-04)

5.4.5.4. Relações entre variáveis bióticas e abióticas

O tamanho das cavernas mostrou-se positivamente relacionado à riqueza de espécies na estação seca ($F_{(1,96)}$= 126,14; R =0,75; p<0,000) e na estação chuvosa ($F_{(1,96)}$= 87,96; R =0,69; p<0,000) (Figura 246 A, B). Além disso, o tamanho das cavernas mostrou-se positivamente relacionado ao número total de espécies observado em cada caverna ($F_{(1,96)}$= 103,1439; R =0,72; p<0,000) e ao número de espécies troglomórficas (troglóbias) presentes ($F_{(1,96)}$= 39,16; R =0,54; p<0,000) (Figura 246 C, D).

Por fim, o número de espécies troglomórficas mostrou-se positivamente correlacionado à riqueza total observada em cada caverna ($F_{(1,96)}$= 67,87; R =0,64; p<0,000) (Figura 244).

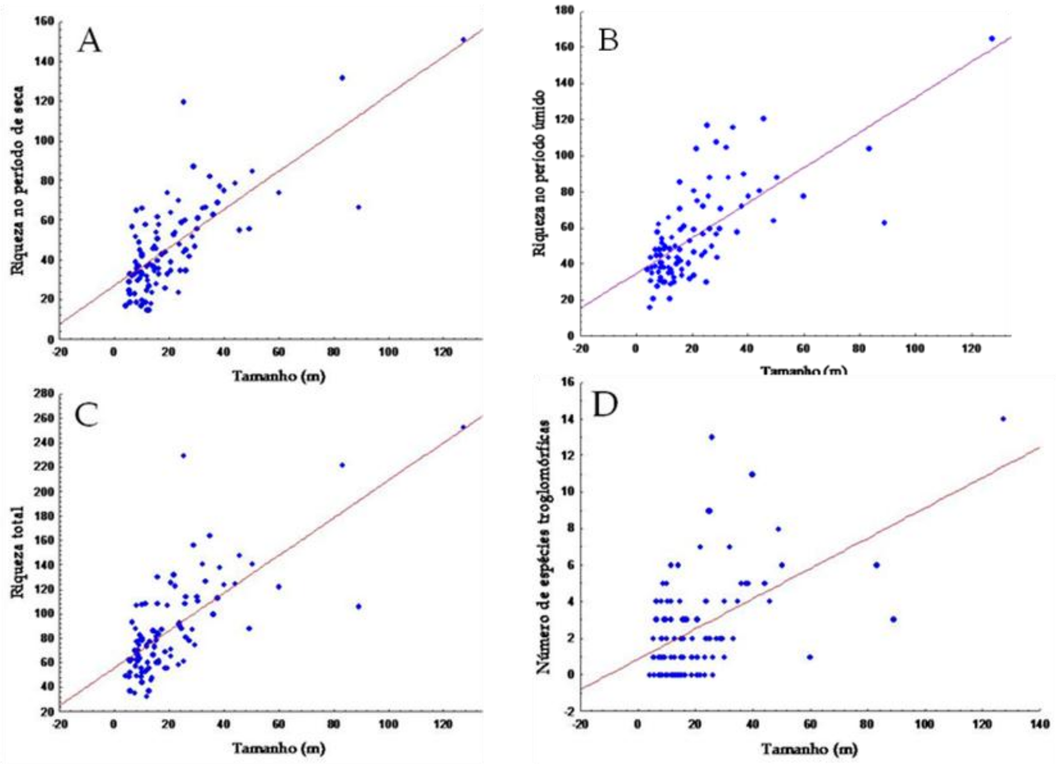

Figura 246 - (A) Relação entre o tamanho das cavernas e a riqueza de espécies na estação seca; (B) Relação entre o tamanho das cavernas e a riqueza de espécies na estação chuvosa; (C) Relação entre o tamanho das cavernas e a riqueza total de espécies; (D) Relação entre o tamanho das cavernas e o número de espécies troglomórficas (troglóbias) presentes.

Embora se tenha especulado durante décadas acerca da relação entre o tamanho de uma caverna e sua riqueza, esta relação foi pela primeira vez demonstrada empiricamente por Ferreira (2004). A relação positiva encontrada entre a projeção horizontal das cavidades e a riqueza das comunidades sugere a importância do espaço para a determinação do número de espécies presente em uma caverna. Segundo aquele autor, cavernas maiores tendem a ser geomorfologicamente mais heterogêneas, o que provavelmente resulta em aumento da quantidade de microhabitats. Consequentemente, um número maior de espécies pode se estabelecer nestas condições. Além disso, a quantidade de recursos orgânicos “estocados” pode ser eventualmente maior, uma vez que há maior quantidade de espaço disponível. Entretanto, a relação entre tamanho da cavidade e quantidade de recursos não deve ser sempre linear, pois também depende de outros fatores relacionados à produção externa de matéria orgânica (de acordo com o bioma em que a caverna se insere) e mesmo da estrutura geral das entradas (que podem possuir maior ou menor propensão a funcionar como “coletoras” de materiais externos).

Nesta perspectiva, novamente menciona-se aqui o trabalho realizado por Souza-Silva e colaboradores (2011), que demonstraram as fortes relações existentes entre o tamanho das

cavernas e a riqueza de espécies para cavernas na Mata Atlântica brasileira. Dentre as diversas litologias testadas, se destacaram as cavernas ferríferas.

Os autores do referido trabalho argumentaram que as cavernas ferríferas mostraram a mais forte tendência de aumento de espécies com o aumento da projeção linear. Tal fato, segundo aqueles autores, pode dever-se às características micro-ambientais heterogêneas destas cavernas associadas à intricada malha de canalículos que são adicionados à extensão disponibilizada aos invertebrados nestas macrocavernas. Provavelmente, as macrocavernas ferruginosas atuam "condensando" ou acumulando uma maior quantidade de recursos orgânicos, além de potencialmente poderem se conectar a uma maior quantidade de canalículos (pelo aumento do volume subterrâneo). Tal condição faz com que as macro-cavernas possam funcionar como "atratores" de fauna (pela quantidade e qualidade de recursos alimentares), sendo que esta atração pode ocorrer de forma exponencial, isto é, pequenos aumentos no desenvolvimento linear podem levar a um aumento exponencial de atratividade, elevando de forma surpreendente a quantidade de espécies presentes, diferentemente do que ocorre para outras litologias, onde tais canalículos não são abundantes.

Outra relação importante evidenciada neste estudo é a observada entre riqueza total e riqueza de espécies troglomórficas (Figura 247). Tal relação demonstra que cavernas mais ricas em espécies (independentemente das categorias a qual pertençam) tendem a possuir uma maior quantidade de troglóbios. Desta forma, é plausível assumir que pelo menos parte das condições que favoreceram a permanência e evolução de grupos no meio subterrâneo também favorece o estabelecimento e permanência atual de várias outras espécies, não troglóbias. Além disso, é notável o fato de que ao se preservar uma caverna rica em espécies troglóbias, preserva-se consequentemente, um número representativo de espécies não troglóbias, também associadas a estes sistemas.

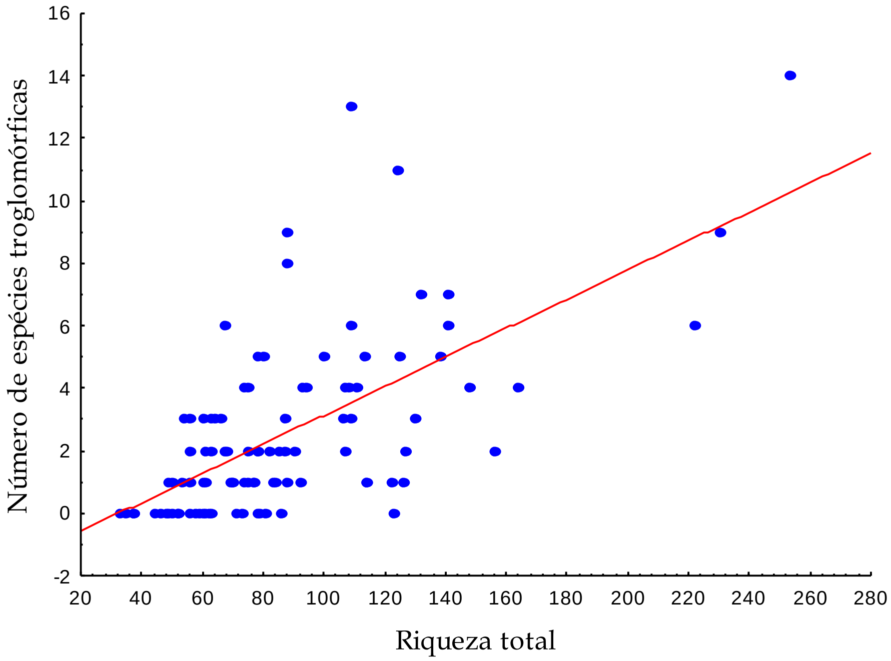

Figura 247 - Relação entre a riqueza total de espécies e o número de espécies troglomórficas (troglóbias) presentes nas cavernas de Serra Leste.

5.4.5.5. "Turnover" de espécies (β diversidade)

Comparando-se as comunidades observadas em cada uma das estações em cada caverna, determinou-se o "turnover" (β diversidade) a partir de dados de presença e ausência através do índice de Harrison (1992), modificado por Whittaker (1960), como já explicitado. O "turnover" das cavernas da área, embora tenham se mostrado variáveis, foram, em geral, bastante elevados (Tabela 12; Figura 248).

Tabela 12 - Valores de riqueza por estação, riqueza média, riqueza total e turnover para cada caverna inventariada.

Caverna	Riqueza Seca	Riqueza Chuva	Riqueza Total	Riqueza Média	"Turnover"
SL-001	132	104	222	118	**88,14**
SL-002	56	60	114	58	**96,55**
SL-003	54	75	123	64,5	**90,70**
SL-004	120	117	230	118,5	**94,09**
SL-005	51	71	109	61	**78,69**
SL-006	32	49	75	40,5	**85,19**
SL-007	33	38	61	35,5	**71,83**
SL-008	64	81	126	72,5	**73,79**
SL-009	33	32	56	32,5	**72,31**
SL-011	35	60	81	47,5	**70,53**
SL-012	40	59	85	49,5	**71,72**
SL-013	37	44	74	40,5	**82,72**
SL-014	36	48	77	42	**83,33**

SL-015	34	41	67	37,5	**78,67**
SL-016	55	121	148	88	**68,18**
SL-017	35	47	71	41	**73,17**
SL-018	31	41	64	36	**77,78**
SL-019	30	38	61	34	**79,41**
SL-020	26	40	56	33	**69,70**
SL-022	35	44	74	39,5	**87,34**
SL-023	19	36	50	27,5	**81,82**
SL-024	43	61	87	52	**67,31**
SL-025	25	35	54	30	**80,00**
SL-026	44	30	61	37	**64,86**
SL-027	37	40	67	38,5	**74,03**
SL-028	30	37	60	33,5	**79,10**
SL-029	67	88	127	77,5	**63,87**
SL-030	66	105	141	85,5	**64,91**
SL-031	60	78	109	69	**57,97**
SL-032	33	45	70	39	**79,49**
SL-033	57	48	94	52,5	**79,05**
SL-035	75	78	124	76,5	**62,09**
SL-036	29	31	49	30	**63,33**
SL-037	70	45	92	57,5	**60,00**
SL-038	17	38	49	27,5	**78,18**
SL-039	24	41	59	32,5	**81,54**
SL-040	36	31	63	33,5	**88,06**
SL-041	33	30	52	31,5	**65,08**
SL-042	66	74	108	70	**54,29**
SL-043	58	42	84	50	**68,00**
SL-044	77	90	138	83,5	**65,27**
SL-045	39	34	66	36,5	**80,82**
SL-046	52	57	87	54,5	**59,63**
SL-047	54	42	79	48	**64,58**
SL-048	46	50	86	48	**79,17**
SL-049	37	45	70	41	**70,73**
SL-050	42	50	78	46	**69,57**
SL-051	58	66	109	62	**75,81**
SL-052	43	50	80	46,5	**72,04**
SL-053	38	55	77	46,5	**65,59**
SL-054	49	33	78	41	**90,24**
SL-055	25	44	63	34,5	**82,61**
SL-056	34	48	68	41	**65,85**
SL-057	35	72	90	53,5	**68,22**
SL-058	69	72	113	70,5	**60,28**
SL-059	47	41	73	44	**65,91**
SL-060	87	108	156	97,5	**60,00**
SL-061	48	57	93	52,5	**77,14**

SL-062	33	37	60	35	**71,43**
SL-063	15	29	37	22	**68,18**
SL-064	42	49	78	45,5	**71,43**
SL-065	74	53	107	63,5	**68,50**
SL-066	46	48	78	47	**65,96**
SL-067	36	59	82	47,5	**72,63**
SL-068	19	21	37	20	**85,00**
SL-069	82	116	164	99	**65,66**
SL-070	19	38	53	28,5	**85,96**
SL-071	36	34	62	35	**77,14**
SL-072	61	71	111	66	**68,18**
SL-073	45	88	114	66,5	**71,43**
SL-074	151	165	253	158	**60,13**
SL-075	67	63	106	65	**63,08**
SL-076	62	86	130	74	**75,68**
SL-077	65	62	107	63,5	**68,50**
SL-078	24	30	48	27	**77,78**
SL-079	74	78	122	76	**60,53**
SL-080	44	41	69	42,5	**62,35**
SL-081	20	30	44	25	**76,00**
SL-082	85	88	141	86,5	**63,01**
SL-083	47	44	75	45,5	**64,84**
SL-084	15	21	33	18	**83,33**
SL-085	37	54	75	45,5	**64,84**
SL-086	18	33	46	25,5	**80,39**
SL-087	63	58	100	60,5	**65,29**
SL-088	39	28	58	33,5	**73,13**
SL-089	79	81	125	80	**56,25**
SL-090	27	35	56	31	**80,65**
SL-091	23	35	52	29	**79,31**
SL-092	28	43	60	35,5	**69,01**
SL-093	56	64	88	60	**46,67**
SL-094	32	39	63	35,5	**77,46**
SL-095	53	104	132	78,5	**68,15**
SL-096	34	30	56	32	**75,00**
SL-097	59	47	88	53	**66,04**
SL-099	45	52	83	48,5	**71,13**
SL-100	52	58	88	55	**60,00**

Os valores de “turnover” encontrados foram muito elevados, como supracitado. Oitenta e oito cavidades (91,6% do total) apresentaram tais valores acima de 60%; 55 cavidades (57,3% do total) apresentaram tais valores acima de 70%; por fim, 19 cavidades (19,8 do total) apresentaram tais valores acima de 80% (Figura 248).

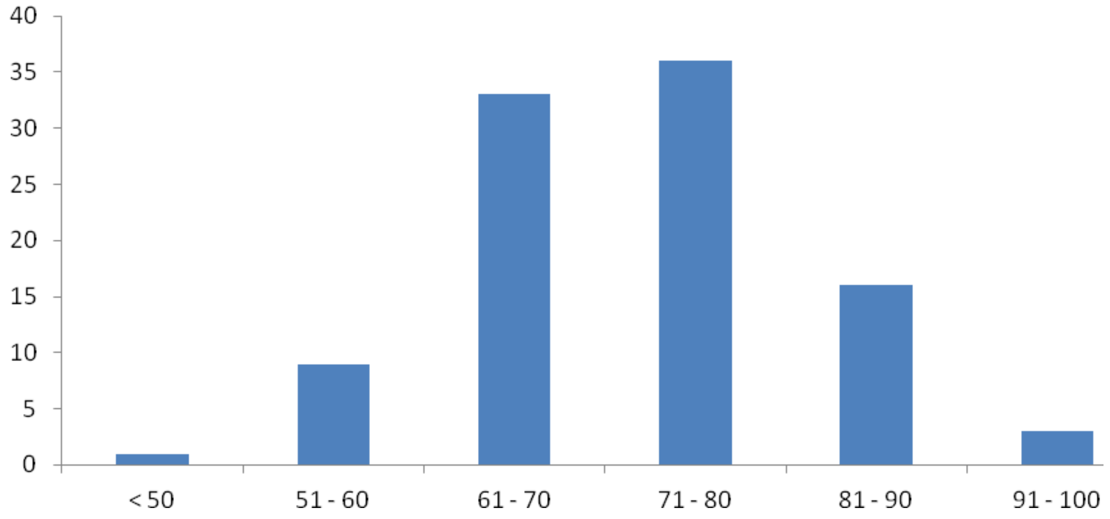

Figura 248 - Histograma evidenciando intervalos de valores de "turnover" e o número de cavernas em cada intervalo.

A análise de regressão múltipla realizada entre o "turnover" de espécies e o tamanho, número de entradas e presença de ciclos de inundação em cada cavidade demonstrou claras relações entre estas variáveis. O "turnover" relacionou-se negativamente com tamanho das cavernas (-0,47), positivamente com número de entradas (0,21) e positivamente com a presença de ciclos de inundação em cavernas (0,33) ($F_{(3,94)}$= 12,24; R =0,53; p<0,000). Desta forma, esta substituição de espécies é maior em cavernas com muitas entradas e sujeitas a ciclos de inundação, sendo, por outro lado, menor em cavernas de maior tamanho (Figura 249).

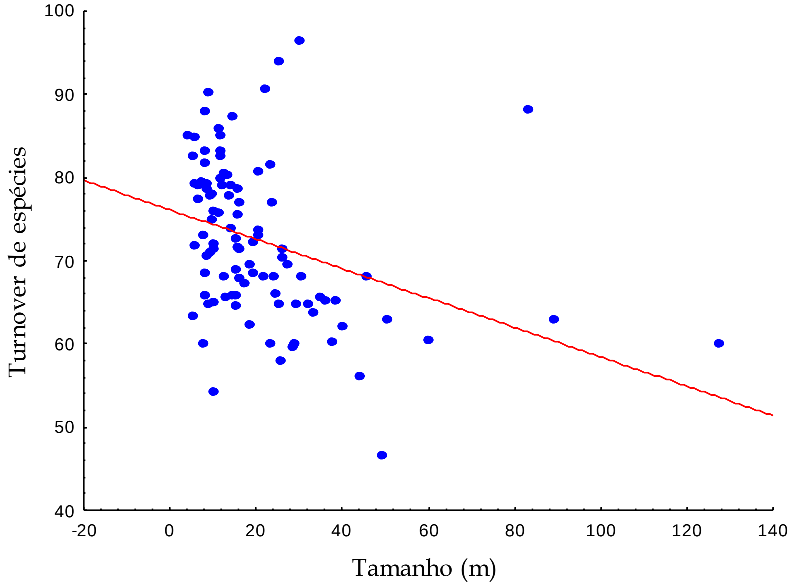

Figura 249 - Relação entre a o "turnover" de espécies e o tamanho das cavernas de Serra Leste.

Tal modelo corrobora com o esperado, já que um grande número de entradas aumenta os contatos com o ambiente externo aumentando as chances de colonização das cavernas por espécies acidentais ou transientes. Os efeitos das entradas de cavernas sobre a composição, distribuição e diversidade da fauna subterrânea já são, há muito, citados na literatura (Peck, 1976; Ferreira & Pompeu, 1997; Ferreira & Martins, 1998; Ferreira *et al.*, 2000). Tais efeitos vão desde a determinação da distribuição da fauna subterrânea geral (Peck, 1976; Prous *et al.*, no prelo) até a distribuição de comunidades específicas, como aquelas associadas a depósitos de guano (Ferreira & Pompeu, 1997; Ferreira & Martins, 1998; Ferreira *et al.*, 2000).

De acordo com Ferreira (2004), cavernas com muitas entradas podem ter áreas maiores de interface com o sistema epígeo circundante, aumentando assim, o estabelecimento de comunidades para-epígeas e mesmo de espécies acidentais (Ferreira & Martins, 2001; Prous *et al.*, 2004).

Por outro lado, os ciclos de inundação certamente atuam gerando distúrbios sobre as comunidades aquáticas e terrestres associadas. Distúrbios oriundos de pulsos de inundação podem ter fortes efeitos sobre as comunidades de invertebrados e ocorrem pelo menos durante parte do ano na maioria dos córregos e rios no mundo inteiro, sendo considerado um dos principais fatores de estruturação de comunidade de ambientes lóticos (Resh *et al.*, 1988; Lake, 1990; Lake, 2000).

Durante eventos de inundação, até mesmo finas camadas de biofilme podem ser afetadas pela movimentação de substratos pelas correntes de água (Scrimgeour *et al.*, 1988). A quantidade de matéria orgânica no sedimento parece não ser afetada por pulsos de inundação, possivelmente porque volumes similares de materiais orgânicos são depositados e lavados (Death & Winterbourn, 1995; Robinson *et al.*, 2003). No entanto, o acúmulo de folhas em riachos de florestas pode ser reduzido por inundações (Angradi, 1997).

A ocorrência de grandes fluxos de inundação geralmente modifica o sedimento de fundo em sistemas lóticos, tendo em vista a forte energia de transporte imposta pela elevação da vazão durante estes fluxos. No entanto, uma vez cessado o fluxo intenso, gradualmente pode haver uma nova fase de deposição de partículas no fundo. Se tais fluxos ocorrerem repetidamente em um curto período de tempo, pode haver um atraso ou impedimento do retorno dos processos de recuperação e acúmulo de recursos e, conseqüentemente, da fauna que dele depende (Gibert *et al*., 1994; Higuti & Takeda, 2002).

Todos os argumentos supracitados corroboram com um maior “turnover” de espécies em cavernas sujeitas à inundação em Serra Leste. Estas cavernas apresentam-se mais sujeitas a

estes distúrbios, certamente apresentando maior propensão à uma elevada e contínua substituição de espécies.

A relação negativa observada entre o tamanho de uma caverna e o "turnover" de espécies demonstra que cavernas maiores tendem a ser mais "estáveis" do ponto de vista ecológico quando comparadas a cavidades de pequeno porte, como abrigos. Como anteriormente exposto, assume-se aqui que a estabilidade ecológica relaciona-se à manutenção temporal dos componentes biológicos (espécies) de uma dada comunidade. Assim, sistemas com elevada substituição de espécies tendem a possuir baixa "manutenção" de um dado conjunto de populações ao longo do tempo, podendo ser considerados pouco "estáveis".

Ferreira (2004) criou o "índice de Estabilidade Ambiental" para cavernas, assumindo que cavernas mais confinadas (que apresentam menores razões entre tamanho da entrada e tamanho total da caverna) são mais estáveis em termos climáticos comparadas a cavernas que apresentam maiores valores desta razão. Além disso, de acordo com Ferreira (2004) quanto maior uma caverna, maiores as chances de a mesma aumentar sua estabilidade ambiental (a não ser que este incremento de tamanho seja acompanhado de um incremento no número e tamanho das entradas).

Desta forma, aparentemente existe uma relação linear que obedece ao seguinte modelo teórico: ↑ Tamanho da caverna → ↑ Estabilidade ambiental → ↓ "Turnover" de espécies → ↑ Estabilidade ecológica.

A partir do anteriormente exposto, percebe-se claramente que as cavernas de Serra Leste tendem a apresentar-se altamente "mutáveis" ao longo do tempo (considerando seus componentes biológicos), especialmente as de reduzido tamanho. No entanto, destaca-se que o número reduzido de coletas (somente duas em cada caverna) tornam estas interpretações parcialmente especulativas. Caso as cavidades tivessem sido amostradas por um maior período de tempo (pelo menos dois ciclos de seca e chuva) seria eventualmente possível definir quais cavernas realmente apresentam-se "instáveis" e quais apresentam um padrão de variação cíclico de grande amplitude. Estas últimas não devem ser consideradas instáveis, já que sua estabilidade apresenta uma dinâmica ligada a grandes flutuações no número de espécies de suas comunidades. No entanto, estes padrões só podem ser diferenciados por meio de estudos de longa duração.

Por fim, após toda esta discussão (em parte especulativa em função do reduzido número de amostras), assume-se como hipótese mais plausível, que as cavernas de reduzido tamanho presentes em Serra Leste realmente apresentem-se como sistemas de baixa estabilidade ecológica, sendo continuamente acessadas por inúmeras espécies provenientes do meio

epígeo. Esta consideração traz consigo duas conseqüências: 1) as cavernas de reduzido tamanho apresentam baixa relevância biológica, em função de sua natureza “mutável”; mas, em contrapartida: 2) as cavernas maiores, com baixos valores de “turnover” tendem a possuir uma elevada relevância contextual, já que se destacam em relação à tendência geral das demais cavernas da área.

Corroborando a idéia do segundo argumento anteriormente apresentado, destaca-se a relação negativa observada entre o número de espécies troglomórficas e o “turnover” de espécies ($F_{(1,96)}$= 7,98; R = -0,28; p<0,006) (Figura 250).

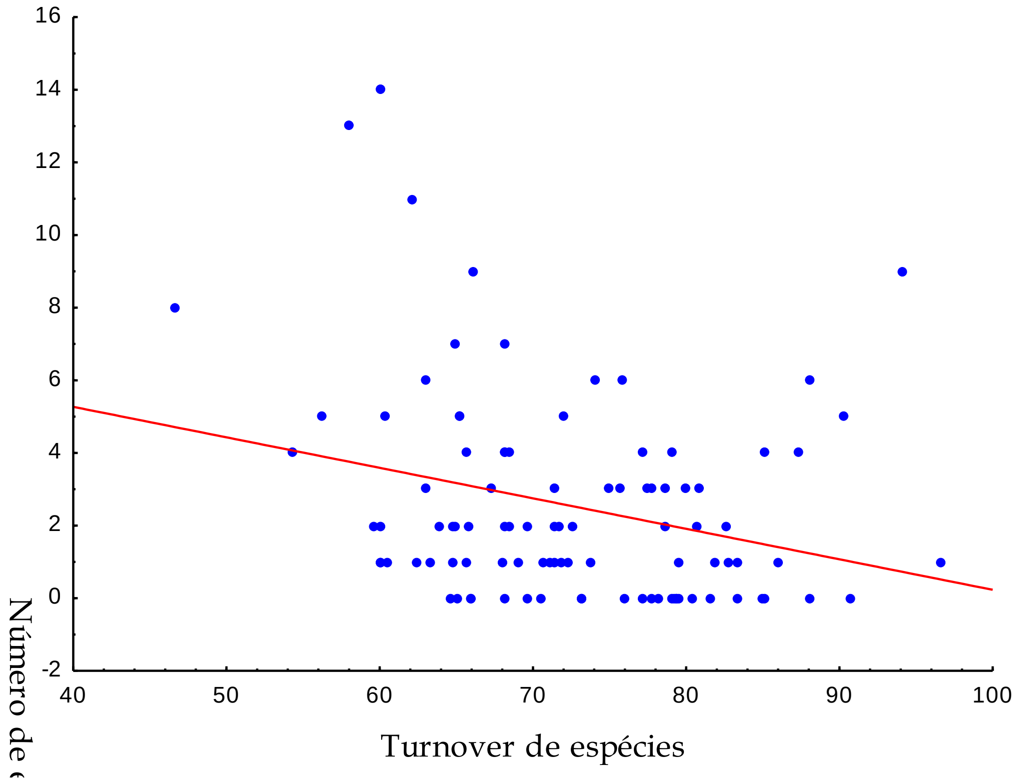

Figura 250 - Relação entre o número de espécies troglomórficas (troglóbias) e o “turnover” de espécies nas cavernas de Serra Leste.

Embora a relação tenha sido fraca, percebe-se claramente que cavernas com mais espécies troglóbias são justamente aquelas com menores valores de “turnover”, e, consequentemente, as mais ecologicamente “estáveis” conforme o anteriormente discutido. Caso esta hipótese seja real, cavernas mais ecologicamente estáveis são justamente aquelas que apresentam condições mais propícias para a evolução (e manutenção) de grupos troglomórficos. Tal fato corrobora com o argumento de que cavernas com menores valores de “turnover” devem possuir uma maior relevância contextual em Serra Leste, merecendo atenção e conservação.

6. ANÁLISE DE RELEVÂNCIA DAS CAVERNAS DE SERRA LESTE

A análise de relevância foi efetuada através dos procedimentos definidos no Decreto Nr. 6.640 de 07 de novembro de 2008. Este decreto alterou significativamente o status jurídico referente à proteção das cavernas brasileiras e prevê a classificação das cavernas segundo quatro graus de relevância: máximo, alto, médio e baixo. A determinação das cavernas de relevância máxima é realizada através de parâmetros definidos pelo próprio decreto. Os parâmetros dos demais graus de relevância foram apenas esboçados, estando remetidos à Instrução Normativa Nr. 2 – IN 2, publicada no dia 20 de agosto de 2009 pelo Ministério do Meio Ambiente - MMA.

No que diz respeito à bioespeleologia, a aplicação das análises de relevância, no presente relatório, foi realizada considerando dois "cenários":

- A aplicação direta da referida IN, incorporando os erros presentes na instrução Normativa Nr. 02, referente ao Decreto 6.640 (análise legalmente válida);

- A aplicação dos critérios da IN acrescidos de mudanças que incorrem na melhoria da avaliação do nível de relevância das cavernas, bem como as sugestões para a melhoria da referida IN (análise sem validação legal no momento).

Embora a segunda análise apresentada neste relatório não possua valor legal, ela certamente irá auxiliar nas futuras revisões da IN, que deverão ocorrer a cada dois anos. Somente por meio da aplicação de critérios "corrigidos" será possível comparar a efetividade e legitimidade dos mesmos, e a verificação das inconsistências decorrerá de comparações diretas, e não de hipóteses, como vem sendo feito.

6.1. Metodologia da análise de relevância

Conforme dito anteriormente, a Análise de Relevância aqui tratada foi realizada com base no Decreto Federal N. 6.640, que prevê que as cavidades naturais subterrâneas sejam classificadas segundo quatro graus de relevância: máximo, alto, médio e baixo, determinados pela análise de atributos físicos, biológicos e socioculturais. Os parâmetros que definem o grau máximo de relevância encontram-se esboçado no referido decreto, que prevê conservação integral da cavidade inserida neste contexto. Os critérios para definição dos graus de relevância alto, médio e baixo, por sua vez, estão definidos na Instrução Normativa N. 2. As cavidades classificadas como de relevância média e alta poderão ser impactadas parcial ou integralmente mediante compensação ambiental a ser definida pelo órgão ambiental. Cavidades de baixa relevância poderão ser impactadas sem compensação.

Qualquer impacto em cavernas somente poderá ocorrer mediante o processo de licenciamento ambiental (Art. 4 do Decreto 6.640).

6.1.1. Discriminação litológica

Após os estudos de campo, as cavidades foram agrupadas segundo seu litotipo, em concordância com o disposto pelo Artigo 1º, §1º, "A análise dos atributos geológicos, para a determinação do grau de relevância, deverá ser realizada comparando cavernas da mesma litologia".

6.1.2. Escala de análise

A determinação da escala de análise foi feita com base na IN. 2, que define que os atributos deverão ser avaliados sob o enfoque regional e local. O enfoque local, segundo a instrução normativa, será limitado à unidade geomorfológica que apresenta continuidade espacial, podendo abranger feições como serras, morrotes ou sistema cárstico, o que for mais restritivo em termos de área, desde que contemplada a área de influência da cavidade.

O enfoque regional deverá compreender uma unidade espeleológica, definida por uma área que apresente homogeneidade fisiográfica. Tal homogeneidade está comumente associada à ocorrência de rochas carbonáticas e suas formas de relevo tipicamente cárstico e pseudocárstico como dolinas, sumidouros, ressurgências, vales cegos, lapiás e cavernas, delimitada por um conjunto de fatores ambientais específicos para sua formação.

6.1.3. Graus de relevância

O grau de relevância máximo é aplicado às cavidades que apresentem ao menos um dos seguintes atributos: gênese única ou rara; morfologia rara; dimensões notáveis em extensão, área ou volume; espeleotemas únicos; isolamento geográfico; cavidade testemunho; ou destacada relevância histórico-cultural ou religiosa. Tais cavidades deverão ser conservadas integralmente, não podendo ser objeto de impactos adversos. A utilização dessas cavidades somente poderá ocorrer caso fique assegurada a manutenção de seu equilíbrio físico e biológico.

As cavidades com grau de relevância alto deverão apresentar atributos de importância acentuada sob enfoque local e regional; ou acentuada sob enfoque local e significativa sob enfoque regional. Essas cavidades poderão sofrer impactos irreversíveis, desde que compensados através da preservação de duas outras cavidades na mesma litologia, com atributos similares e com mesmo grau de relevância da caverna a ser impactada.

As cavidades com grau de relevância médio deverão englobar atributos de importância acentuada sob enfoque local e baixa sob enfoque regional, ou significativa sob enfoque local e regional. Impactos irreversíveis nessas cavidades deverão ser compensados com ações que

contribuam para a conservação e uso adequado do patrimônio espeleológico nacional, a serem definidas pelo órgão ambiental competente.

As cavernas classificadas com grau de relevância baixo deverão conter atributos considerados de importância significativa sob enfoque local e baixa sob enfoque regional, ou baixa sob enfoque local e regional. Tais cavidades poderão sofrer impactos irreversíveis não sendo necessária a compensação ambiental.

O fluxograma para a classificação do grau de relevância das cavidades é apresentado na Figura 251.

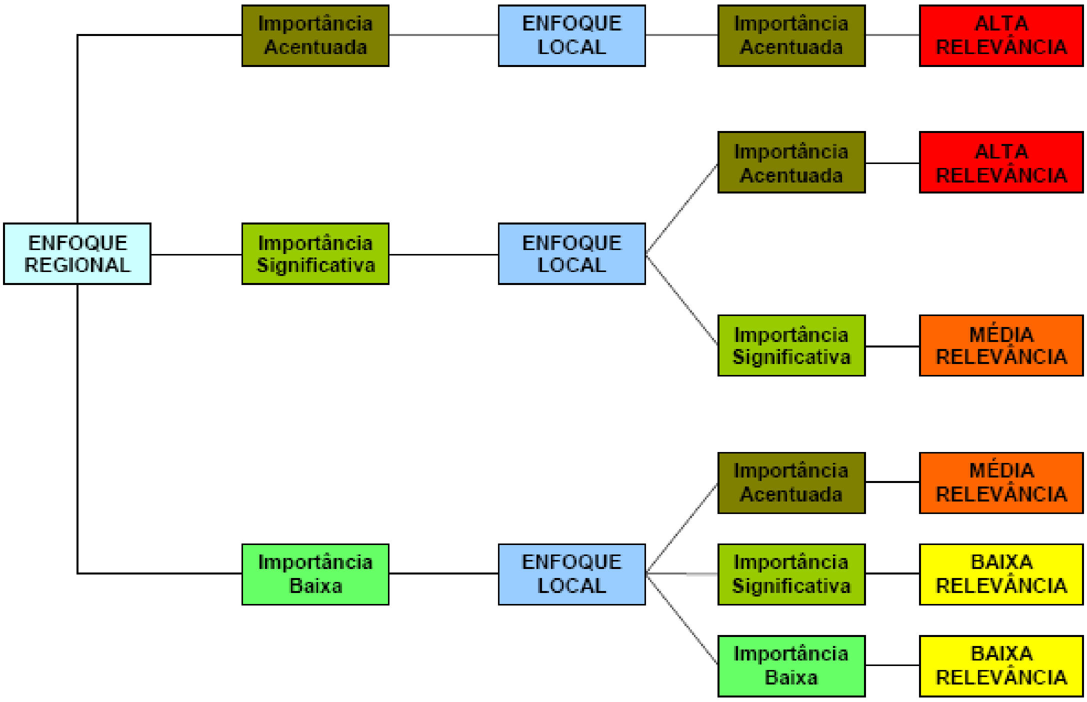

Figura 251 - Fluxograma de classificação do grau de relevância de cavidades naturais subterrâneas, segundo a Instrução Normativa N. 2, do MMA. 6.2. Resultados da análise de relevância das cavernas de Serra Leste.

6.2.1. Discriminação litológica

As cavidades de Serra Leste foram agrupadas em um único grupo litológico, o das rochas ferríferas, incluindo subtipos e litologias derivadas, como a canga.

6.2.2. Definição das escalas local e regional

A escala regional abrange toda a região da Província Mineral de Carajás, Estado do Pará. Nesta unidade espeleológica são oficialmente cadastradas no Cadastro Nacional de

Informações Espeleológicas (CANIE, 2011) 1.046 cavernas, distribuídas em nove unidades geomorfológicas (U.G.) (Figura 255): U. G. Serra Sul com 281 cavernas, U. G. Serra Norte com 501 cavernas, U. G. Serra Leste com 106 cavernas, U.G Serra da Bocaina com 122 cavernas, U. G. Serra do Rabo-Estrela com 36 cavernas, além das U.G.s Serra do Cinzento, Serra do Aquiri, Serra Arqueada e Serra de São Felix, onde prospecções espeleológicas ainda não foram realizadas ou estão em andamento.

A escala local de análise é limitada à Unidade Geomorfológica Serra Leste (Figura 252), e inclui todas as cavernas inseridas no contexto do empreendimento. A definição da escala de análise local foi adotada segundo Valentim & Olivito (2011). A listagem das cavernas que compõem as amostras local e regional encontra-se no Anexo IV.

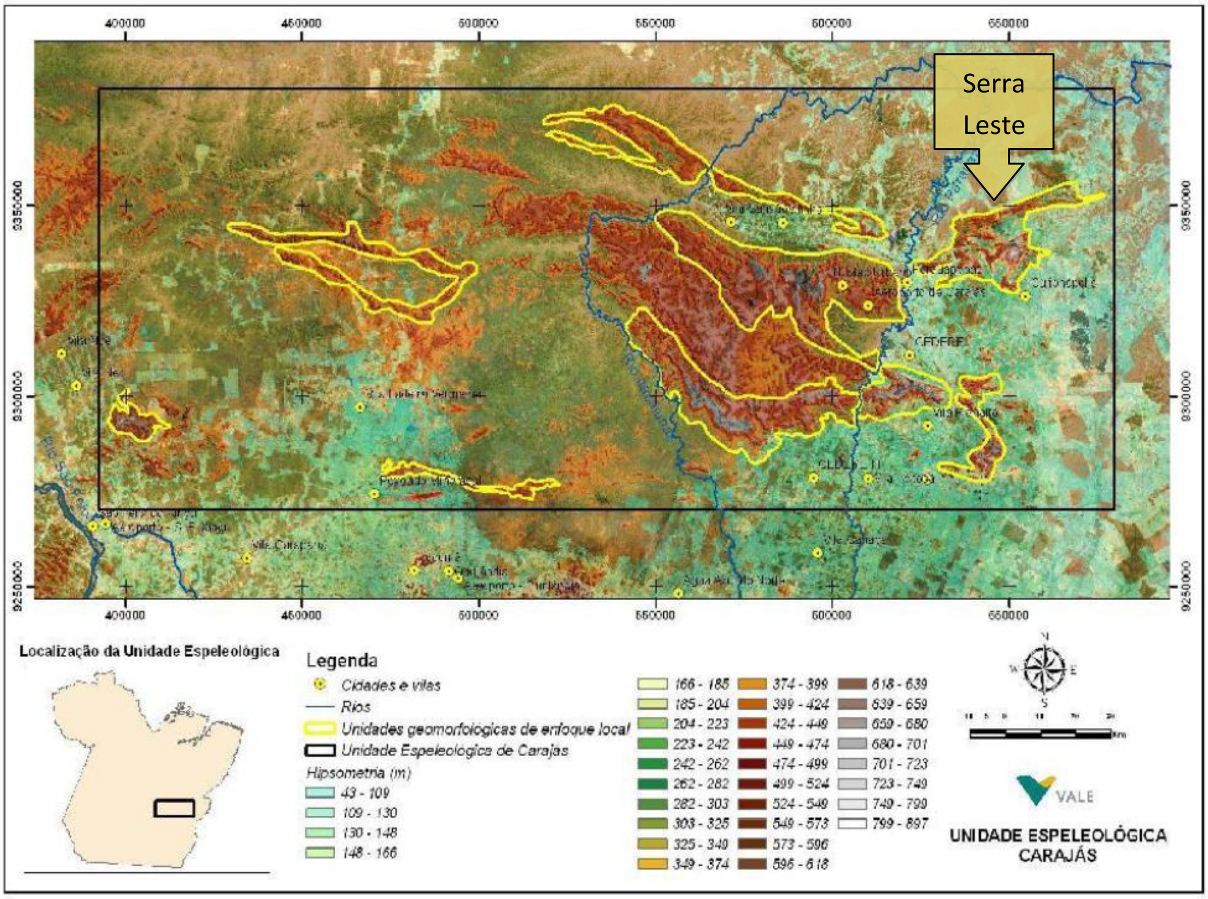

Figura 252 - Mapa da localização da unidade espeleológica de Carajás, em destaque (seta amarela), a unidade Serra Leste. Fonte: Valentim & Olivito, 2011.

6.2.3. Análise das cavernas de Serra Leste

6.2.3.1. Relevância máxima dos atributos físicos e histórico-cultural

Foram incluídas neste estudo, 96 cavernas distribuídas no platô, vertentes inclinadas e sistemas fluviais. Os atributos de ordem física e histórico-cultural de relevância máxima observados estão sintetizado na Tabela 13.

Tabela 13 - Classificação de relevância máxima na área de Serra Leste: atributos físicos e histórico-culturais.

Relevância Máxima	Presente	Ausente
I. Gênese única ou rara;		
II. Morfologia única;	SL-001	
III. Dimensões notáveis em extensão, área ou volume;	SL-074	
IV. Espeleotemas únicos;		
V. Isolamento geográfico;		
X. Cavidade testemunho; ou		
XI. Destacada relevância histórico cultural ou religiosa.		

Nas cavidades analisadas não foi observada nenhuma particularidade quanto aos seus processos genéticos ou evolutivos. Essas cavidades reproduzem os mesmos processos já registrados no conjunto de cavernas da região da Província Mineral de Carajás. Pinheiro & Maurity (1988) consideram a existência de duas etapas distintas na formação das cavernas em rochas ferríferas, a primeira delas com predominância de processos dissolutivos (químicos) e a segunda sob a ação de processos erosivos (físicos).

Esta fase erosiva, aliada aos processos de abatimento de blocos, é responsável pela maior parte da morfologia atualmente observada nas cavernas de Serra Leste. A zona de contato irregular entre os dois litotipos – canga e minério de ferro tem sido considerada muito favorável ao desenvolvimento de cavernas (Piló & Auler, 2005; Piló & Auler, 2009).

Em relação a morfologia, a caverna SL-001 foi classificada como de máxima relevância por apresentar um conjunto de pilares, pendentes e cúpulas bastante significativo dentro do universo de cavernas ferríferas conhecidas em toda a região norte do país.

Para a classificação de relevância máxima das cavernas na formação ferrífera, com base nos valores espeleométricos, o termo “dimensões notáveis” foi aqui definido pelo valor médio da amostra regional multiplicado por cinco. Desse modo, o valor de corte para a projeção

horizontal, área e volume é de 156,01 metros, 636,35 m^2 e 1234,79 m^3, respectivamente. A caverna SL-074, com 734,5 m^2 apresentou relevância máxima para o atributo *área*.

Quanto aos espeleotemas, embora tenha sido observado uma grande profusão de crostas e coralóides em algumas cavernas de Serra Leste, não foi observado nenhum tipo de deposição atípica às cavernas ferríferas.

As cavidades de Serra Leste não se enquadram na definição de isolamento geográfico, tendo em vista a grande quantidade dessas ocorrências na região de Carajás. Não foram registrados geoindicadores testemunhos de processos ambientais ou paleoambientais expressivos.

Nas cavidades analisadas não foram registrados vestígios históricos ou de uso religioso. Na reduzida bibliografia sobre as cavernas de Carajás também não foi registrada nenhuma referência sobre esses aspectos.

6.2.3.2. Relevância alta, média e baixa dos atributos físicos e histórico-cultural

Tabela 14 - Classificação em termos de importância acentuada sob enfoque local e regional: Atributos físicos e histórico-econômicos.

Importância acentuada sob enfoque local e regional	Presente	Ausente
XII - Alta projeção horizontal da cavidade em relação às demais cavidades que se distribuem na mesma unidade espeleológica, conforme definido no § 3o do art. 14, desta Instrução Normativa;	SL-001, SL-074 e SL-075	
XIII - Alta área da projeção horizontal da cavidade em relação às demais cavidades que se distribuem na mesma unidade espeleológica, conforme definido no § 3o do art. 14, desta Instrução Normativa;	SL-074 e SL-079	
XIV - Alto volume da cavidade em relação às demais cavidades que se distribuem na mesma unidade espeleológica, conforme definido no § 3o do art. 14, desta Instrução Normativa;	SL-001, SL-044, SL-050 e SL-074	
XV - Presença significativa de estruturas espeleogenéticas raras;	SL-001	
XVI - Lago ou drenagem subterrânea perene com influência acentuada sobre os atributos da cavidade que tenham as configurações relacionadas nos incisos deste artigo;	SL-001, SL-009, SL-050, SL-056, SL-063, SL-064, SL-068 e SL-078	
XVII - Diversidade da sedimentação química com muitos tipos de espeleotemas e processos de deposição;		
XVIII - Configuração notável dos espeleotemas;	SL-051	
XIX – Alta influência da cavidade sobre o sistema cárstico;		
XX - Presença de inter-relação da cavidade com alguma de relevância máxima;		

XXI - Reconhecimento nacional ou mundial do valor estético/cênico da cavidade;		
XXII - Visitação pública sistemática na cavidade, com abrangência regional ou nacional.		

Alta projeção horizontal

Foram identificadas três cavernas com alta projeção horizontal sob o enfoque regional na área do Projeto Serra Leste: SL-001, com 83,1 metros; SL-074, com 127,2 metros; e SL-075, com 88,8 metros. O valor-limite que determina alta projeção horizontal atinge 69,41 metros e resulta da soma da média da projeção horizontal e o desvio padrão das 451 cavidades que compõem a amostra regional (Tabela 15).

Tabela 15 - Parâmetros espeleométricos da projeção horizontal sob enfoque regional.

Parâmetros	Projeção Horizontal (m)
Média	31,06
Desvio Padrão	38,35
Alto ($> \mu + \sigma$)	**69,41**
Médio - Intervalo entre ($\mu - \sigma$) e ($\mu + \sigma$)	-7,29* e 69,41
Baixo ($< \mu - \sigma$)	-7,29*

* O valor negativo é resultante de um problema estatístico da metodologia.

Alta área

Foram identificadas duas cavidades que apresentaram alta relevância com base no atributo espeleométrico área sob enfoque regional: a caverna SL-074, com 734,9 m^2; e a caverna SL-079, com 317,2m^2 de área. A soma do desvio padrão e a média regional foi de 288,60 m^2 (Tabela 16).

Tabela 16 - Parâmetros espeleométricos da área sob enfoque regional.

Parâmetros	Área da Projeção Horizontal (m^2)
Média	119,18
Desvio Padrão	169,42
Alto ($> \mu + \sigma$)	**288,60**
Médio - Intervalo entre ($\mu - \sigma$) e ($\mu + \sigma$)	-50,23* e 288,60
Baixo ($< \mu - \sigma$)	-50,23*

* O valor negativo é resultante de um problema estatístico da metodologia.

Alto volume

Quatro cavernas, SL-001 (657,8 m^3), SL-044 (973,1 m^3), SL-050 (665,0 m^3) e SL-074 (1081,2 m^3), apresentaram alta relevância sob enfoque regional, com base nos valores de volume. A soma do desvio padrão e a média foi de 649,26 m³ (Tabela 17).

Tabela 17 - Parâmetros espeleométricos do volume sob enfoque regional.

Parâmetros	Volume (m³)
Média	241,20
Desvio Padrão	408,06
Alto (> µ +σ)	**649,26**
Médio - Intervalo entre (µ - σ) e (µ + σ)	-166,86* e 649,26
Baixo (< µ – σ)	-166,86*

* O valor negativo é resultante de um problema estatístico da metodologia.

Estruturas espeleogenéticas raras

Presença significativa de estruturas espeleogenéticas raras foram observadas na caverna SL-001. Tratam-se de pilares cuja gênese está provavelmente ligada a própria gênese e evolução da caverna. Nesta mesma cavidade, *bell holes* (cúpulas) ocorrem com relativa abundância no teto. Esse tipo de estrutura também foi registrado em cavernas de Serra Sul, mas, de maneira geral, sua ocorrência é rara.

Lago ou drenagem subterrânea perene

Drenagens subterrâneas com escoamento perene foram identificadas nas cavernas SL-001, SL-050, SL-056, SL-063, SL-068 e SL-078. As cinco primeiras se inserem em calhas de drenagem, onde parte do fluxo é capturado, ressurgindo no interior dessas cavidades, com influência em sua gênese e evolução. A caverna SL-078 comporta-se como surgência.

As cavernas SL-009, SL-050, SL-063, SL-064 e SL-068 apresentaram lagos perenes em seu interior.

Diversidade da sedimentação química

As cavernas de Serra Leste não apresentaram elevada diversidade de sedimentação química (espeleotemas).

Configuração notável de espeleotemas

A configuração notável está relacionada ao aspecto, maturidade ou abundância dos espeleotemas. A caverna SL-051 apresentou um exemplar de cortina com extremidades serrilhadas. O espeleotema possui cerca de 20 cm de comprimento por 15 cm de diâmetro e coloração amarelada.

Alta influência da cavidade sobre o sistema cárstico

As cavernas de Serra Leste não apresentam influência sobre o sistema cárstico. De modo geral, as cavernas ferríferas encontram-se individualizadas uma das outras, ou seja, não estão conectadas através de sistema hídrico, este é o caso das cavernas de Serra Leste.

Inter-relação da cavidade com caverna de relevância máxima

Não há inter-relação das cavernas classificadas como de relevância máxima, com as demais cavidades da área.

Reconhecimento mundial ou nacional do valor estético ou cênico

As cavernas estudadas em Serra Leste não apresentam esse tipo de reconhecimento.

Visitação pública sistemática

Nenhuma das cavernas estudadas apresentou visitação pública regular.

IMPORTÂNCIA ACENTUADA SOB ENFOQUE LOCAL

A Tabela 18 sintetiza os atributos físicos considerados acentuados sob enfoque local.

Tabela 18 - Síntese da classificação dos atributos que conferem importância acentuada sob enfoque local.

Importância acentuada sob enfoque local	Presente	Ausente
VIII - Presença de estrutura geológica de interesse científico;		
IX - Presença de registros paleontológicos;		
X - Reconhecimento local do valor estético/cênico da cavidade;		
XI - Visitação pública sistemática na cavidade, com abrangência local;		
XII - Presença de água de percolação ou condensação com influência acentuada sobre os atributos da cavidade que tenham as configurações relacionadas nos incisos deste artigo;		

XIII - Lago ou drenagem subterrânea intermitente com influência acentuada sobre os atributos da cavidade que tenham as configurações relacionadas nos incisos deste artigo.		

Estrutura geológica de interesse científico

Não foram observardas estruturas geológicas de interesse científico no interior das cavernas em Serra Leste. No entanto, a Falha do Cinzento (Costa, 2007) parece condicionar a evolução da escarpa onde estão inseridas as cavernas SL-081, SL-082, SL-083, SL-084, SL-085, SL-086, SL-087, SL-088, SL-089, SL-090, SL-091 e SL-092.

Registros paleontológicos

Em nenhuma das cavernas estudadas foram encontrados vestígios paleontológicos em superfície.

Reconhecimento local do valor estético/cênico

Não há reconhecimento local do valor estético/cênico das cavidades.

Visitação pública sistemática na cavidade, com abrangência local

Não foram observados vestígios de visitação pública sistemática nas cavidades do Projeto Serra Leste.

IMPORTÂNCIA SIGNIFICATIVA SOB ENFOQUE LOCAL E REGIONAL

Os atributos que definem importância significativa sob enfoque local e regional encontram-se listados na tabela 19.

Tabela 19 - Atributos físicos que conferem importância significativa sob enfoque local e regional das cavernas de Serra Leste.

Importância significativa sob enfoque local e regional	Presente	Ausente
II - Média projeção horizontal da cavidade em relação às demais cavidades que se distribuem na mesma unidade espeleológica, conforme definido no § 3o do art. 14, desta Instrução Normativa;	SL-002, SL-003, SL-004, SL-005, SL-006, SL-007, SL-008, SL-009, SL-011, SL-012, SL-013, SL-014, SL-015, SL-016, SL-017, SL-018, SL-019, SL-020, SL-022, SL-023, SL-024, SL-025, SL-026, SL-027, SL-028, SL-029, SL-030, SL-031, SL-032, SL-033, SL-	

	035, SL-036, SL-037, SL-038, SL-039, SL-040, SL-041, SL-042, SL-043, SL-044, SL-045, SL-046, SL-047, SL-048, SL-049, SL-050, SL-051, SL-052, SL-053, SL-054, SL-055, SL-056, SL-057, SL-058, SL-059, SL-060, SL-061, SL-602, SL-063, SL-064, SL-065, SL-066, SL-067, SL-068, SL-069, SL-070, SL-071, SL-072, SL-073, SL-076, SL-077, SL-078, SL-079, SL-080, SL-081, SL-082, SL-083, SL-084, SL-085, SL-086, SL-087, SL-088, SL-089, SL-090, SL-091, SL-092, SL-093, SL-094, SL-095, SL-096, SL-097, SL-099 e SL-100.	
III - Média área da cavidade em relação às demais cavidades que se distribuem na mesma unidade espeleológica, conforme definido no § 3o do art. 14, desta Instrução Normativa;	SL-001, SL-002, SL-003, SL-004, SL-005, SL-006, SL-007, SL-008, SL-009, SL-011, SL-012, SL-013, SL-014, SL-015, SL-016, SL-017, SL-018, SL-019, SL-020, SL-022, SL-023, SL-024, SL-025, SL-026, SL-027, SL-028, SL-029, SL-030, SL-031, SL-032, SL-033, SL-035, SL-036, SL-037, SL-038, SL-039, SL-040, SL-041, SL-042, SL-043, SL-044, SL-045, SL-046, SL-047, SL-048, SL0-49, SL-050, SL-051, SL-052, SL-053, SL-054, SL-055, SL-056, SL-057, SL-058, SL-059, SL-060, SL-061, SL-062, SL-063, SL-064, SL-065, SL-066, SL-067, SL-068, SL-069, SL-070, SL-071, SL-072, SL0-73, SL-075, SL-076, SL-077, SL-078, SL-080, SL-081, SL-082, SL-083, SL-084, SL-085, SL-086, SL-087, SL-088, SL-089, SL-090, SL-091, SL-092, SL-093, SL-094, SL-095, SL-096, SL-097, SL-099 e SL-100.	
IV - Alto desnível da cavidade em relação às demais cavidades que se distribuem na mesma unidade espeleológica, conforme definido no § 3o do art. 14, desta Instrução Normativa;		
V - Médio volume da cavidade em relação às demais cavidades que se distribuem na mesma unidade espeleológica, conforme definido no § 3o do art. 14, desta Instrução Normativa;	SL-002, SL-003, SL-004, SL-005, SL-006, SL-007, SL-008, SL-009, SL-011, SL-012, SL-013, SL-014, SL-015, SL-016, SL-017, SL-	

	018, SL-019, SL-020, SL-022, SL-023, SL-024, SL-025, SL-026, SL-027, SL-028, SL-029, SL-030, SL-031, SL-032, SL-033, SL-035, SL-036, SL-037, SL-038, SL-039, SL-040, SL-041, SL-042, SL-043, SL-045, SL-046, SL-047, SL-048, SL-049, SL-051, SL-052, SL-053, SL-054, SL-055, SL-056, SL-057, SL-058, SL-059, SL-060, SL-061, SL-062, SL-063, SL-064, SL-065, SL-066, SL-067, SL-068, SL-069, SL-070, SL-071, SL-072, SL-073, SL-075, SL-076, SL-077, SL-078, SL-079, SL-080, SL-081, SL-082, SL-083, SL-084, SL-085, SL-086, SL-087, SL-088, SL-089, SL-090, SL-091, SL-092, SL-093, SL-094, SL-095, SL-096, SL-097, SL-099 e SL-100.	
VI - Presença de estruturas espeleogenéticas raras;		
VII - Lago ou drenagem subterrânea intermitente com influência significativa sobre os atributos da cavidade que tenham as configurações relacionadas nos incisos deste artigo;		
VIII - Diversidade da sedimentação química com muitos tipos de espeleotemas ou processos de deposição;		
IX - Sedimentação clástica ou química com valor científico;		
X - Reconhecimento regional do valor estético/cênico da cavidade;		
XI - Uso constante, periódico ou sistemático para fins educacionais, recreativos ou esportivos.		

Média projeção horizontal

As cavidades classificadas como de média relevância com base na projeção horizontal possuem valores dimensionais situados entre a média - desvio padrão e a média + desvio padrão. Este intervalo situa-se desde o valor negativo (-7,29 m) a 69,41 m (Tabela 20), o que leva a inclusão de todas as cavidades nesta litologia, com exceção daquelas classificadas como de alta e máxima relevância.

Tabela 20 - Cavernas com média projeção horizontal classificadas como de importância significativa sob enfoque local e regional.

Parâmetros	Projeção Horizontal (m)
Média	31,06

Desvio Padrão	38,35
Alto (> μ +σ)	69,41
Médio - Intervalo entre (μ - σ) e (μ + σ)	-7,29* e 69,41
Baixo (< μ – σ)	-7,29*

*0 valor negativo é resultante de um problema estatístico da metodologia.

6.2.3.2. Análise dos atributos biológicos

No que diz respeito aos parâmetros biológicos, existem inúmeras inconsistências na IN. As mesmas serão listadas abaixo, de acordo com os critérios retirados da própria IN. Visando uma maior clareza no texto, as partes retiradas da IN nº02 serão apresentadas em itálico, seguidas dos comentários em escala de cinza.

Art. 3° *Entende-se por cavidade natural subterrânea com grau de relevância máximo aquela que possui pelo menos um dos atributos listados abaixo:*

VI - abrigo essencial para a preservação de populações geneticamente viáveis de espécies animais em risco de extinção, constantes de listas oficiais;
A amarração feita ao status das populações, no caso, "geneticamente viáveis" confere uma impossibilidade prática. A amostragem dos organismos e consequente coleta para a realização de um estudo de genética populacional será, em grande parte dos casos, impraticável. Tal problema será certamente intensificado no caso de espécies troglóbias, cujas populações são muitas vezes pequenas e a coleta de muitos indivíduos para um estudo de genética contrariaria a própria legislação, que se fundamentou no princípio de preservação destas espécies.

VII - habitat essencial para preservação de populações geneticamente viáveis de espécies de troglóbios endêmicos ou relíctos;
Novamente, a amarração feita ao status das populações confere uma impossibilidade prática. Além disso, não é indicada na IN a abrangência do endemismo. A espécie troglóbia pode ser endêmica a uma caverna, a um afloramento (ocorrendo em algumas cavernas daquele afloramento), a uma área cárstica, a um município, etc... Desta forma, a falta de definição clara da abrangência do conceito de endemismo torna altamente subjetiva a análise de relevância neste quesito.

VIII - habitat de troglóbio raro;
Existem diferentes conceitos de raridade. O clássico trabalho de Rabinowitz e colaboradores (1986), propôs sete tipos de raridade, que incorporam três dimensões reconhecidas em função dos seguintes atributos: i) Amplitude da distribuição geográfica da espécie (ampla ou

restrita); ii) Condição de abundância da população local (comum ou incomum); iii) Grau de especificidade em relação ao hábitat (baixa ou alta). Zampaulo (2010) propôs quatro tipos de raridade para fauna subterrânea a partir de uma adaptação da proposta de Rabinowitz e colaboradores (1986).

A IN somente faz menção à "distribuição geográfica restrita", o que não auxilia muito na definição do conceito de raridade a ser utilizado.

Desta forma, é fundamental que seja definido qual o conceito de raridade deverá ser utilizado par fins de normatização. O não estabelecimento de uma uniformidade conceitual incorre em sérios riscos em relação às análises de relevância. Especialistas que se utilizarem de diferentes conceitos poderão classificar uma mesma caverna em níveis de relevância distintos, o que é inaceitável.

IX - interações ecológicas únicas;
Este critério é bastante subjetivo. Tal análise dependerá exclusivamente da experiência do especialista responsável pelo trabalho. Somente a partir da sua aplicação será possível verificar quais interações ecológicas são efetivamente únicas ou incomuns. Mas o critério, em si, é extremamente válido e importante.

Art. 7° Para efeito de classificação do grau de relevância de uma cavidade serão considerados de importância acentuada, sob enfoque local e regional, os atributos com pelo menos uma das seguintes configurações:

I - Localidade tipo;
Critério claro e objetivo.

II - Presença de populações estabelecidas de espécies com função ecológica importante;
Este critério é bastante subjetivo. O seria efetivamente "função ecológica importante"? Dispersão de sementes, por exemplo, poderia certamente se enquadrar neste atributo. No entanto, como diferenciar uma caverna com uma população de 10 indivíduos de uma espécie de morcego frugívoro de outra, cuja população desta mesma espécie correspondesse a 10.000 indivíduos? Além disso, qual a efetiva capacidade de dispersão de sementes de cada espécie de morcego? O presente atributo menciona somente presença, desconsiderando todas estas questões. Desta forma, novamente este atributo pode levar a interpretações diversas, resultando em análises insatisfatórias.

III - Presença de táxons novos;
Atributo questionável. Se a caverna possui uma espécie ainda não descrita ela será classificada em um nível de relevância. Quando a espécie for descrita, altera-se a relevância

da caverna? Esta é a "abertura" dada pela IN. Estes atributos que definem o grau de relevância de cavernas devem ser mais "sólidos", permitindo uma análise mais robusta e duradoura. Quaisquer atributos muito mutáveis conferem problemas à análise.

IV - Alta riqueza de espécies;

Atributo importantíssimo, mas sem uma definição clara que demonstre como obtê-lo. As categorias de riqueza podem ser obtidas de diferentes formas, dependendo de cada especialista. Novamente, a falta de uniformidade nestas determinações coloca em risco as análises de relevância.

V - Alta abundância relativa de espécies;

Este atributo é o que talvez possua a maior quantidade de inconsistências e erros. O primeiro erro consiste no fato de que a IN indica que este método seja utilizado somente para espécies com tamanho corporal maior que 1 cm. Isto é inadmissível, principalmente quando se considera que a maior parte das espécies cavernícolas é menor que esta dimensão. Além disso, por que 1 cm? Não há quaisquer justificativas técnicas que subsidiem este atributo. Ele é certamente importante, mas deve considerar todas as espécies, não se restringindo somente às de grande tamanho.

Outro problema consiste em um erro nas categorias deste atributo apresentadas na IN. Considera-se uma abundância relativa Alta cavernas nas quais 30% ou mais das espécies apresentam abundância alta. Considera-se uma abundância relativa Média cavernas nas quais de 10% a 20% das espécies apresentam abundância alta. Desta forma, cavernas nas quais 25% das espécies possuem alta abundância se enquadrariam em qual categoria? O intervalo entre 20 e 30% foi simplesmente ignorado na IN.

Finalmente, as categorias de Abundância relativa podem ser obtidas de diferentes formas (como para riqueza), dependendo de cada especialista. Novamente, a falta de uniformidade nestas determinações coloca em risco as análises de relevância.

VI - Presença de composição singular da fauna;

A definição deste atributo segundo a IN seria: "*ocorrência de populações estabelecidas de espécies de grupos pouco comuns ao ambiente cavernícola*". No entanto, este mesmo conceito se aplica, por exemplo, a espécies raras... Desta forma, embora seja um atributo importante, o mesmo acaba sendo extremamente subjetivo, além de depender de uma grande experiência prévia do especialista.

VII - Presença de troglóbios que não sejam considerados raros, endêmicos ou relictos;

Novamente esbarra-se em um sério problema de ordem conceitual. Se os troglóbios são restritos aos ambientes subterrâneos, podem ser considerados endêmicos destes sistemas...

Desta forma, o importante é definir exatamente o que é RARO, ENDÊMICO E RELICTO. Se estes conceitos forem efetivamente definidos, muitos dos problemas concernentes à biologia serão sanados na IN.

VIII - Presença de espécies troglomórficas;
A grande maioria das espécies troglóbias no Brasil é definida apenas pela presença de troglomorfismos, tendo em vista a megadiversidade externa e consequentemente a impossibilidade de real constatação da presença (ou não) de uma dada espécie no meio externo. Uma exceção a esta regra seriam os peixes, já que a ictiofauna Brasileira pode ser considerada relativamente bem conhecida para algumas bacias hidrográficas. Desta forma, o conceito de troglóbio praticamente se equivale ao conceito de troglomórfico em nosso país.

O critério é relevante, mas, tendo em vista a situação de desconhecimento da fauna externa, este acaba por se sobrepor aos critérios referentes à presença de troglóbios.

IX - Presença de trogloxeno obrigatório;
Critério relevante, mas de diagnóstico intangível. É válido lembrar que a IN exige somente dois inventários em cada cavidade. Este número de visitas definitivamente inviabiliza qualquer diagnóstico sobre a eventual "obrigatoriedade" desta relação em uma dada espécie.

X - Presença de população excepcional em tamanho;
Critério importante, mas novamente subjetivo.

XI - Presença de espécie rara;
Ver comentários no item *VIII - habitat de troglóbio raro;*

6.2.3.2.1. Considerando a utilização direta da Instrução Normativa (análise legalmente válida)

A presente análise considerou todos os atributos constantes na referida IN. A riqueza de espécies foi obtida por meio da média e o desvio padrão para o conjunto de dados obtido. As cavernas que excederam o limite superior à média +/- desvio padrão foram consideradas de alta riqueza de espécies. Da mesma forma, as cavernas que obtiveram valores inferiores a este mesmo intervalo foram consideradas de baixa riqueza de espécies. Cavernas com valores de riqueza dentro deste intervalo foram consideradas como de média riqueza de espécies. Tal procedimento foi realizado considerando-se somente as cavernas inventariadas (enfoque local).

A abundância relativa de espécies foi obtida da seguinte forma: primeiramente extraiu-se a raiz quadrada dos valores de abundância de cada população. Este procedimento teve o

objetivo de reduzir as diferenças numéricas existentes entre certas populações. Tal procedimento poderia ter sido feito por meio do logaritmo natural dos valores de abundância, mas como muitas espécies foram representadas por somente um indivíduo, o grande número de “zeros” poderia levar a ruídos na análise. Os valores transformados (pela extração das raízes quadradas) foram então divididos em três categorias, tomando-se como base o maior valor obtido para cada caverna (que foi, desta forma, dividido por 3, criando cada uma das categorias de tamanhos populacionais para cada caverna).

Os resultados das análises de relevância estão sumarizados na Tabela 21.

Tabela 21 - Graus de relevância das cavernas da área de acordo com metodologia estabelecida na Instrução Normativa N0 2, de agosto de 2009. Relevância máxima (RM), importância acentuada regional (IAR), enfoque regional (ER), importância acentuada local (IAL), enfoque local (EL). Máxima (Máx.), Alta (alt.), Média (méd.) e Baixa (Baix.), Alta (A), média (M) e baixa (B). 1 presença, 0 ausência. A legenda referente a cada atributo encontra-se logo abaixo da tabela.

1	2	3	4	5	6	7	8	9	10	11	12	13	14	15	16	17	18	19	20	21	22	23	24	25	26	27	28	29	30	31	32	33	34
**SL-001**	0	0	1	0	0	x	0	x	x	0	1	1	0	0	0	0	0	1	0	A	B	0	1	1	0	0	0	1	0	A	0	1	Max
**SL-002**	0	0	0	0	0	x	0	x	x	0	1	1	0	0	0	0	0	1	0	M	M	0	1	1	0	0	0	1	0	A	0	1	Alt
**SL-003**	0	0	0	0	0	x	0	x	x	0	0	0	0	0	0	0	0	1	0	M	M	0	0	0	0	0	0	1	0	A	0	1	Alt
**SL-004**	0	0	1	0	0	x	0	x	x	0	1	1	0	0	0	0	0	0	0	A	B	0	1	1	0	0	0	0	0	A	0	1	Max
**SL-005**	0	0	1	0	0	x	0	x	x	0	1	1	0	0	0	0	0	0	0	M	B	0	1	1	0	0	0	0	0	A	0	0	Max
**SL-006**	0	0	1	0	0	x	0	x	x	0	1	1	0	0	0	0	0	0	0	M	M	0	1	1	0	0	0	0	0	A	0	0	Max
**SL-007**	0	0	1	0	0	x	0	x	x	0	0	1	0	0	0	0	0	0	0	M	M	0	0	1	0	0	0	0	0	B	0	0	Max
**SL-008**	0	0	0	0	0	x	0	x	x	0	1	1	0	0	0	0	0	0	0	M	B	0	1	1	0	0	0	0	0	B	0	0	Alt
**SL-009**	0	0	0	0	0	x	0	x	x	0	0	1	0	0	0	0	0	1	0	M	M	0	0	1	0	0	0	1	0	A	0	0	Alt
**SL-011**	0	0	0	0	0	x	0	x	x	0	0	0	0	0	0	0	0	0	0	M	M	0	0	0	0	0	0	0	0	B	0	0	Méd
**SL-012**	0	0	1	0	0	x	0	x	x	0	0	1	0	0	0	0	0	0	0	M	B	0	0	1	0	0	0	0	0	B	0	0	Max
**SL-013**	0	0	0	0	0	x	0	x	x	0	1	1	0	0	0	0	0	0	0	M	B	0	1	1	0	0	0	0	0	B	0	0	Alt
**SL-014**	0	0	0	0	0	x	0	x	x	0	0	1	0	0	0	0	0	0	0	M	M	0	0	1	0	0	0	0	0	B	0	0	Alt
**SL-015**	0	0	0	0	0	x	0	x	x	0	1	1	0	0	0	0	0	0	0	M	B	0	1	1	0	0	0	0	0	A	0	0	Alt
**SL-016**	0	0	1	0	0	x	0	x	x	0	1	1	0	0	0	0	0	1	0	A	M	0	1	1	0	0	0	1	0	A	0	0	Max
**SL-017**	0	0	0	0	0	x	0	x	x	0	0	0	0	0	0	0	0	0	0	M	M	0	0	0	0	0	0	0	0	A	0	0	Méd
**SL-018**	0	0	1	0	0	x	0	x	x	0	1	1	0	0	0	0	0	0	0	M	M	0	1	1	0	0	0	0	0	B	0	0	Max
**SL-019**	0	0	0	0	0	x	0	x	x	0	0	0	0	0	0	0	0	0	0	M	M	0	0	0	0	0	0	0	0	B	0	0	Méd
**SL-020**	0	0	0	0	0	x	0	x	x	0	0	0	0	0	0	0	0	0	0	M	A	0	0	0	0	0	0	0	0	B	0	0	Alt
**SL-022**	0	0	1	0	0	x	0	x	x	0	1	1	0	0	0	0	0	0	0	M	M	0	1	1	0	0	0	0	0	B	0	0	Max
**SL-023**	0	0	0	0	0	x	0	x	x	0	0	1	0	0	0	0	0	0	0	M	M	0	0	1	0	0	0	0	0	B	0	0	Alt
**SL-024**	0	0	1	0	0	x	0	x	x	0	1	1	0	0	0	0	0	1	0	M	M	0	1	1	0	0	0	1	0	A	0	0	Max
**SL-025**	0	0	1	0	0	x	0	x	x	0	1	1	0	0	0	0	0	0	0	M	M	0	1	1	0	0	0	0	0	B	0	0	Max
**SL-026**	0	0	1	0	0	x	0	x	x	0	1	1	0	0	0	0	0	0	0	M	M	0	1	1	0	0	0	0	0	A	0	0	Max
**SL-027**	0	0	0	0	0	x	0	x	x	0	1	1	0	0	0	0	0	0	0	M	M	0	1	1	0	0	0	0	0	B	0	0	Alt
**SL-028**	0	0	0	0	0	x	0	x	x	0	0	0	0	0	0	0	0	0	0	M	M	0	0	0	0	0	0	0	0	B	0	0	Méd
**SL-029**	0	0	0	0	0	x	0	x	x	0	1	1	0	0	0	0	0	1	0	A	B	0	1	1	0	0	0	1	0	A	0	0	Alt

**SL-030**	0	0	1	0	0	x	0	x	x	0	1	1	0	0	0	0	0	1	0	A	B	0	1	1	0	0	0	1	0	A	0	0	Max
**SL-031**	0	0	1	0	0	x	0	x	x	0	1	1	0	0	0	0	0	1	0	M	B	0	1	1	0	0	0	1	0	A	0	0	Max
**SL-032**	0	0	1	0	0	x	0	x	x	0	0	1	0	0	0	0	0	0	0	M	A	0	0	1	0	0	0	0	0	B	0	0	Max
**SL-033**	0	0	1	0	0	x	0	x	x	0	1	1	0	0	0	0	0	0	0	M	M	0	1	1	0	0	0	0	0	B	0	0	Max
**SL-035**	0	0	1	0	0	x	0	x	x	0	1	1	0	0	0	0	0	0	0	M	B	1	1	1	0	0	0	0	0	A	0	0	Max
**SL-036**	0	0	1	0	0	x	0	x	x	0	0	1	0	0	0	0	0	0	0	M	M	0	0	1	0	0	0	0	0	A	0	0	Max
**SL-037**	0	0	0	0	0	x	0	x	x	0	1	1	0	0	0	0	0	1	0	M	M	0	1	1	0	0	0	1	0	A	0	0	Alt
**SL-038**	0	0	0	0	0	x	0	x	x	0	0	0	0	0	0	0	0	0	0	M	M	0	0	0	0	0	0	0	0	A	0	0	Méd
**SL-039**	0	0	0	0	0	x	0	x	x	0	0	0	0	0	0	0	0	0	0	M	M	0	0	0	0	0	0	0	0	A	0	0	Méd
**SL-040**	0	0	0	0	0	x	0	x	x	0	0	0	0	0	0	0	0	0	0	M	M	0	0	0	0	0	0	0	0	A	0	0	Méd
**SL-041**	0	0	0	0	0	x	0	x	x	0	0	0	0	0	0	0	0	0	0	M	M	0	0	0	0	0	0	0	0	A	0	0	Méd
**SL-042**	0	0	1	0	0	x	0	x	x	0	1	1	0	0	0	0	0	0	0	M	B	0	1	1	0	0	0	0	0	A	0	0	Max
**SL-043**	0	0	0	0	0	x	0	x	x	0	1	1	0	0	0	0	0	0	0	M	M	0	1	1	0	0	0	0	0	B	0	0	Alt
**SL-044**	0	0	1	0	0	x	0	x	x	0	1	1	0	0	0	0	0	0	0	A	B	0	1	1	0	0	0	0	0	A	0	0	Max
**SL-045**	0	0	1	0	0	x	0	x	x	0	1	1	0	0	0	0	0	0	0	M	M	0	1	1	0	0	0	0	0	B	0	0	Max
**SL-046**	0	0	0	0	0	x	0	x	x	0	1	1	0	0	0	0	0	0	0	M	M	0	1	1	0	0	0	0	0	A	0	0	Alt
**SL-047**	0	0	0	0	0	x	0	x	x	0	0	0	0	0	0	0	0	0	0	M	M	0	0	0	0	0	0	0	0	A	0	0	Méd
**SL-048**	0	0	0	0	0	x	0	x	x	0	0	0	0	0	0	0	0	0	0	M	M	0	0	0	0	0	0	0	0	B	0	0	Méd
**SL-049**	0	0	0	0	0	x	0	x	x	0	1	1	0	0	0	0	0	0	0	M	M	0	1	1	0	0	0	0	0	B	0	0	Alt
**SL-050**	0	0	0	0	0	x	0	x	x	0	1	1	0	0	0	0	0	0	0	M	M	0	1	1	0	0	0	0	0	A	0	0	Alt
**SL-051**	0	0	1	0	0	x	0	x	x	0	1	1	0	0	0	0	0	0	0	M	M	0	1	1	0	0	0	0	0	A	0	0	Max
**SL-052**	0	0	1	0	0	x	0	x	x	0	1	1	0	0	0	0	0	0	0	M	B	0	1	1	0	0	0	0	0	B	0	0	Max
**SL-053**	0	0	0	0	0	x	0	x	x	0	1	1	0	0	0	0	0	0	0	M	M	0	1	1	0	0	0	0	0	A	0	0	Alt
**SL-054**	0	0	1	0	0	x	0	x	x	0	1	1	0	0	0	0	0	0	0	M	M	0	1	1	0	0	0	0	0	B	0	0	Max
**SL-055**	0	0	0	0	0	x	0	x	x	0	1	1	0	0	0	0	0	0	0	M	M	0	1	1	0	0	0	0	0	A	0	0	Alt
**SL-056**	0	0	0	0	0	x	0	x	x	0	1	1	0	0	0	0	0	0	0	M	M	0	1	1	0	0	0	0	0	B	0	0	Alt
**SL-057**	0	0	1	0	0	x	0	x	x	0	1	1	0	0	0	0	0	0	0	M	B	0	1	1	0	0	0	0	0	A	0	0	Max
**SL-058**	0	0	0	0	0	x	0	x	x	0	1	1	0	0	0	0	0	1	0	M	M	0	1	1	0	0	0	1	0	A	0	0	Alt
**SL-059**	0	0	0	0	0	x	0	x	x	0	0	0	0	0	0	0	0	0	0	M	M	0	0	0	0	0	0	0	0	A	0	0	Méd
**SL-060**	0	0	0	0	0	x	0	x	x	0	1	1	0	0	0	0	0	1	0	A	B	0	1	1	0	0	0	1	0	A	0	0	Alt
**SL-061**	0	0	0	0	0	x	0	x	x	0	1	1	0	0	0	0	0	0	0	M	M	0	1	1	0	0	0	0	0	A	0	0	Alt
**SL-062**	0	0	1	0	0	x	0	x	x	0	1	1	0	0	0	0	0	0	0	M	B	0	1	1	0	0	0	0	0	A	0	0	Max
**SL-063**	0	0	0	0	0	x	0	x	x	0	0	0	0	0	0	0	0	0	0	B	B	0	0	0	0	0	0	0	0	B	0	0	Baix
**SL-064**	0	0	0	0	0	x	0	x	x	0	1	1	0	0	0	0	0	0	0	M	B	0	1	1	0	0	0	0	0	B	0	0	Alt
**SL-065**	0	0	1	0	0	x	0	x	x	0	1	1	0	0	0	0	0	0	0	M	B	0	1	1	0	0	0	0	0	A	0	0	Max
**SL-066**	0	0	0	0	0	x	0	x	x	0	0	0	0	0	0	0	0	0	0	M	M	0	0	0	0	0	0	0	0	A	0	0	Méd
**SL-067**	0	0	1	0	0	x	0	x	x	0	0	1	0	0	0	0	0	0	0	M	B	0	0	1	0	0	0	0	0	B	0	0	Max
**SL-068**	0	0	0	0	0	x	0	x	x	0	0	0	0	0	0	0	0	0	0	B	B	0	0	0	0	0	0	0	0	B	0	0	Baix
**SL-069**	0	0	0	0	0	x	0	x	x	0	1	1	0	0	0	0	0	1	0	A	B	0	1	1	0	0	0	1	0	A	0	0	Alt
**SL-070**	0	0	0	0	0	x	0	x	x	0	1	1	0	0	0	0	0	0	0	M	B	0	1	1	0	0	0	0	0	B	0	0	Alt
**SL-071**	0	0	0	0	0	x	0	x	x	0	0	0	0	0	0	0	0	0	0	M	A	0	0	0	0	0	0	0	0	B	0	0	Alt
**SL-072**	0	0	1	0	0	x	0	x	x	0	1	1	0	0	0	0	0	0	0	M	B	0	1	1	0	0	0	0	0	B	0	0	Max
**SL-073**	0	0	0	0	0	x	0	x	x	0	0	1	0	0	0	0	0	1	0	M	M	0	0	1	0	0	0	1	0	A	0	0	Alt

**SL-074**	0	0	1	0	0	x	0	x	x	0	1	1	0	0	0	0	0	1	0	A	B	0	1	1	0	0	0	1	0	A	0	0	Max
**SL-075**	0	0	1	0	0	x	0	x	x	0	1	1	0	0	0	0	0	0	0	M	B	0	1	1	0	0	0	0	0	A	0	0	Max
**SL-076**	0	0	0	0	0	x	0	x	x	0	1	1	0	0	0	0	0	0	0	A	M	0	1	1	0	0	0	0	0	B	0	0	Alt
**SL-077**	0	0	1	0	0	x	0	x	x	0	1	1	0	0	0	0	0	0	0	M	B	0	1	1	0	0	0	0	0	A	0	0	Max
**SL-078**	0	0	0	0	0	x	0	x	x	0	0	0	0	0	0	0	0	1	0	B	M	0	0	0	0	0	0	1	0	A	0	0	Alt
**SL-079**	0	0	0	0	0	x	0	x	x	0	1	1	0	0	0	0	0	1	0	M	M	0	1	1	0	0	0	1	0	A	0	0	Alt
**SL-080**	0	0	1	0	0	x	0	x	x	0	0	1	0	0	0	0	0	0	0	M	B	0	0	1	0	0	0	0	0	B	0	0	Max
**SL-081**	0	0	0	0	0	x	0	x	x	0	0	0	0	0	0	0	0	0	0	B	M	0	0	0	0	0	0	0	0	A	0	0	Méd
**SL-082**	0	0	1	0	0	x	0	x	x	0	1	1	0	0	0	0	0	0	0	A	B	0	1	1	0	0	0	0	0	A	0	0	Max
**SL-083**	0	0	0	0	0	x	0	x	x	0	1	1	0	0	0	0	0	0	0	M	B	0	1	1	0	0	0	0	0	B	0	0	Alt
**SL-084**	0	0	0	0	0	x	0	x	x	0	0	0	0	0	0	0	0	0	0	B	M	0	0	0	0	0	0	0	0	B	0	0	Méd
**SL-085**	0	0	0	0	0	x	0	x	x	0	1	1	0	0	0	0	0	0	0	M	B	0	1	1	0	0	0	0	0	A	0	0	Alt
**SL-086**	0	0	0	0	0	x	0	x	x	0	0	0	0	0	0	0	0	0	0	B	M	0	0	0	0	0	0	0	0	B	0	0	Méd
**SL-087**	0	0	0	0	0	x	0	x	x	0	1	1	0	0	0	0	0	0	0	M	B	0	1	1	0	0	0	0	0	A	0	0	Alt
**SL-088**	0	0	0	0	0	x	0	x	x	0	0	0	0	0	0	0	0	0	0	M	M	0	0	0	0	0	0	0	0	B	0	0	Méd
**SL-089**	0	0	0	0	0	x	0	x	x	0	1	1	0	0	0	0	0	0	0	M	B	0	1	1	0	0	0	0	0	A	0	0	Alt
**SL-090**	0	0	0	0	0	x	0	x	x	0	1	1	0	0	0	0	0	0	0	M	M	0	1	1	0	0	0	0	0	A	0	0	Alt
**SL-091**	0	0	0	0	0	x	0	x	x	0	0	0	0	0	0	0	0	0	0	M	M	0	0	0	0	0	0	0	0	B	0	0	Méd
**SL-092**	0	0	1	0	0	x	0	x	x	0	0	1	0	0	0	0	0	0	0	M	M	0	0	1	0	0	0	0	0	A	0	0	Max
**SL-093**	0	0	1	0	0	x	0	x	x	0	1	1	0	0	0	0	0	0	0	M	M	1	1	1	0	0	0	0	0	A	0	0	Max
**SL-094**	0	0	1	0	0	x	0	x	x	0	0	1	0	0	0	0	0	0	0	M	M	0	0	1	0	0	0	0	0	A	0	0	Max
**SL-095**	0	0	1	0	0	x	0	x	x	0	1	1	0	0	0	0	0	0	0	A	M	0	1	1	0	0	0	0	0	A	0	0	Max
**SL-096**	0	0	1	0	0	x	0	x	x	0	0	1	0	0	0	0	0	0	0	M	B	0	0	1	0	0	0	0	0	B	0	0	Max
**SL-097**	0	0	1	0	0	x	0	x	x	0	1	1	0	0	0	0	0	1	0	M	M	0	1	1	0	0	0	1	0	A	0	0	Max
**SL-099**	0	0	0	0	0	x	0	x	x	0	1	1	0	0	0	0	0	0	0	M	B	0	1	1	0	0	0	0	0	B	0	0	Alt
**SL-100**	0	0	0	0	0	x	0	x	x	0	0	1	0	0	0	0	0	0	0	M	B	0	0	1	0	0	0	0	0	B	0	0	Alt

1		Cavernas
2	RM	Abrigo essencial para a preservação de populações geneticamente viáveis de espécies animais em risco de extinção, constantes de listas oficiais
3	RM	Habitat para a preservação de populações geneticamente viáveis de relictos.
4	RM	Habitat de troglóbio raro
5	RM	Interações ecológicas únicas
6	IAR	Localidade tipo
7	IAR	Espécies com função ecológica importante
8	IAR	Táxons novos
9	IAR	Riqueza de espécies (Alta, Média e Baixa)
10	IAR	Abundância relativa de espécies (Alta, Média e Baixa)
11	IAR	Composição singular da fauna
12	IAR	Presença de Troglóbios, não raros endêmicos ou relictos
13	IAR	Espécies troglomórficas
14	IAR	Trogloxeno obrigatório
15	IAR	População excepcional em tamanho

16	IAR	Espécie rara
17	ER	Singularidade dos elementos faunísticos da cavidade
18	IAL	Localidade tipo
19	IAL	Espécies com função ecológica importante
20	IAL	Táxons novos
21	IAL	Riqueza de espécies (Alta, Média e Baixa)
22	IAL	Abundância relativa de espécies (Alta, Média e Baixa)
23	IAL	Composição singular da fauna
24	IAL	Presença de Troglóbios, não raros endêmicos ou relictos
25	IAL	Espécies troglomórficas
26	IAL	Trogióxeno obrigatório
27	IAL	População excepcional em tamanho
28	IAL	Espécie rara
29	IAL	Populações residentes de Chiroptera
30	IAL	Local de nidificação de aves silvestres
31	IAL	Diversidade de substratos orgânicos (Alta ou baixa)
32	IAL	Espécies migratórias
33	EL	Singularidade dos elementos faunísticos da cavidade
34	-	**RELEVÂNCIA FINAL**

Quarenta cavernas foram consideradas de relevância MÁXIMA (SL-001, SL-004, SL-005, SL-006, SL-007, SL-012, SL-016, SL-018, SL-022, SL-024, SL-025, SL-026, SL-030, SL-031, SL-032, SL-033, SL-035, SL-036, SL-042, SL-044, SL-045, SL-051, SL-052, SL-054, SL-057, SL-062, SL-065, SL-067, SL-072, SL-074, SL-075, SL-077, SL-080, SL-082, SL-092, SL-093, SL-094, SL-095, SL-096, SL-097) em função da presença de troglóbios “raros” (Figuras 253, 254, 255, 256; Tabelas 21 e 22).

Trinta e oito cavernas foram consideradas de relevância ALTA (SL-002, SL-003, SL-008, SL-009, SL-010, SL-013, SL-014, SL-015, SL-020, SL-023, SL-027, SL-029, SL-037, SL-043, SL-046, SL-049, SL-050, SL-053, SL-055, SL-056, SL-058, SL-060, SL-061, SL-064, SL-069, SL-070, SL-071, SL-073, SL-076, SL-078, SL-079, SL-083, SL-085, SL-087, SL-089, SL-090, SL-099, SL-100) por não se enquadrarem na categoria de máxima. Trinta e três cavernas em função da presença de espécies troglomórficas (SL-002, SL-008, SL-009, SL-013, SL-014, SL-015, SL-023, SL-027, SL-029, SL-037, SL-043, SL-046, SL-049, SL-050, SL-053, SL-055, SL-056, SL-058, SL-060, SL-061, SL-064, SL-069, SL-070, SL-073, SL-076, SL-079, SL-083, SL-085, SL-087, SL-089, SL-090, SL-099 e SL-100) (Figuras 257, 258 e 259; Tabelas 21 e 22). Duas cavernas em função da presença de populações estabelecidas com função ecológica importante (morcegos veiculadores de recursos alimentares e produção de guano) (SL-003 e SL-078). Três cavernas em função da presença de populações com alta abundância relativa de espécies (SL-010, SL-020 e SL-071).

Dezessete cavernas foram consideradas de relevância MÉDIA (SL-011, SL-017, SL-019, SL-028, SL-038, SL-039, SL-040, SL-041, SL-047, SL-048, SL-059, SL-066, SL-081, SL-084, SL-086, SL-088 e SL-091) por não se enquadrarem nas categorias de máxima e alta. Nove cavernas em função da alta diversidade de substratos orgânicos (SL-017, SL-038, SL-039, SL-040, SL-041, SL-047, SL-059, SL-066 e SL-081). Seis cavernas em função da média riqueza de espécies (SL-011, SL-019, SL-028, SL-048, SL-088 e SL-091). Três cavernas em função da presença de populações com média abundância relativa de espécies (SL-034, SL-084 e SL-086).

Duas cavernas foram consideradas de relevância BAIXA em função da baixa diversidade de substratos orgânicos. (SL-063, SL-068) e por não se enquadrarem nas categorias de máxima, alta e média (Tabela 22).

Tabela 22 - Relação de cavidades que apresentam espécies troglóbias com raridade tipo I, II, (I+II) e III, em Serra Leste.

		Raridade		Raridade		Raridade		Raridade		
**Ordem**	**Gênero/espécie**	**Tipo I**		**Tipo II**		**Tipo III**		**Tipo IV**		> popul.
Nematomorpha	Gordioidea sp1									SL-028
Gastropoda	Systrophiidae sp1									SL-093
Turbellaria	Geoplanidae sp6	1	26, 82							
Isopoda	Balloniscidae sp8									SL-096
Isopoda	Balloniscidae sp9	1	93, 95							
Isopoda	Balloniscidae sp10	1	94	1	94	1	94	1	94	
Isopoda	*Trichorhina* sp1									
Isopoda	*Trichorhina* sp2	1	96	1	96	1	96	1	96	
Isopoda	*Trichorhina* sp4	1	33							
Isopoda	Styloniscidae sp1	1	7	1	7	1	7	1	7	
Trombidiforme	Rhagidiidae sp2	1	1, 35, 51							
Trombidiforme	Trombidiforme sp1	1	4, 16, 35							
Amblypygi	*Charinus* sp1									
Amblypygi	*Charinus* sp2	1	24	1	24	1	24	1	24	
Amblypygi	*Charinus* sp3	1	35	1	35	1	35	1	35	
Schizomida	Hubbardiinae									SL-031
Opiliones	Escadabiidae sp5	1	5							
Opiliones	Escadabiidae sp6	1	18	1	18	1	18	1	18	
Opiliones	Opiliones Indet.	1	4, 12							
Araneae	Prodidomidae sp1	1	25, 74							
Araneae	Ochiroceratidae sp8									SL-031
Araneae	Ochiroceratidae sp9									SL-035/SL-031
Araneae	Ochiroceratidae sp10	1	4, 35, 36							
Araneae	Ochiroceratidae sp11	1	4, 30							
Araneae	Oonopinae sp9									SL-087
Araneae	Oonopinae sp11	1	57	1	57	1	57	1	57	
Araneae	Prodidomidae sp1	1	31	1	31	1	31	1	31	
Thysanura	Atelurinae sp2									SL-031
Thysanura	Atelurinae sp4	1	4, 80	1	4, 80	1	4, 80			
Diplura	Anajapygidae sp3	1	92	1	92	1	92	1	92	

Collembola	Isotomidae sp2									SL-031
Collembola	Tomoceridae sp3									SL-093
Collembola	Cyphoderidae sp2									SL-074
Collembola	Cyphoderidae sp3	1	65							
Hemiptera	Dipsocoridae sp5									SL-082
Hemiptera	Thyreocoridae sp1	1	18	1	18	1	18	1	18	
Hymenoptera	*Hypoponera* sp4			1	22, 44, 62, 74					
Hymenoptera	*Hypoponera* sp7	1	74							
Hymenoptera	*Solenopsis* sp7	1	97	1	97	1	97	1	97	
Coleoptera	*Coarazuphium* sp1									SL-035
Coleoptera	*Coarazuphium* sp2	1	74	1	74	1	74	1	74	
Coleoptera	Dytiscidae sp4	1	45, 82							
Coleoptera	Eucnemidae sp3	1	96	1	96	1	95	1	95	
Coleoptera	Pselaphidae sp9	1	6	1	6	1	6	1	6	
Coleoptera	Pselaphidae sp17	1	75	1	75	1	75	1	75	
Coleoptera	Ptylidae sp5			1	44, 52, 54, 94					
Coleoptera	Scydmaenidae sp17	1	32							
Coleoptera	Scydmaenidae sp20	1	77	1	77	1	77	1	77	
Coleoptera	Scydmaenidae sp21	1	1, 30							
Coleoptera	Scydmaenidae sp22	1	4	1	4	1	4	1	4	
Coleoptera	Staphylinidae sp62	1	72							
Polydesmida	Pyrgodesmidae sp3									SL-093
Polydesmida	Pyrgodesmidae sp7	1	74							
Polydesmida	Pyrgodesmidae sp8	1	74	1	74	1	74	1	74	
Polydesmida	Polydesmida sp5	1	52, 65, 67							
Polydesmida	Polydesmida sp6			1	12, 51, 52, 74					
Polydesmida	Polydesmida sp7	1	51, 54	1	51, 54	1	51, 54			
Glomerida	*Glomeridesmus* sp2									SL-031
Polyxenida	Lophoproctidae sp1	1	42, 67							
Spirostreptida	Pseudonannolenidae sp2									SL-069
	**Número cavernas**		36		22		18		16	
	**%**		37,50		22,92		18,75		16,67	

Dentre as cavernas consideradas como de relevância máxima, dezesseis se destacaram. Tais cavernas apresentaram espécies troglóbias aqui categorizadas no tipo mais extremo de raridade (tipo IV), isto é, espécies que foram representadas por um único indivíduo amostrado. Foram encontradas 18 espécies troglóbias enquadradas nesta categoria de raridade.

Algumas destas cavernas encontravam-se próximas geograficamente, enquanto outras apresentavam-se mais isoladas das demais, demonstrando que não há um “padrão” regular ou agregado de distribuição dos troglóbios de extrema raridade (Figura 253).

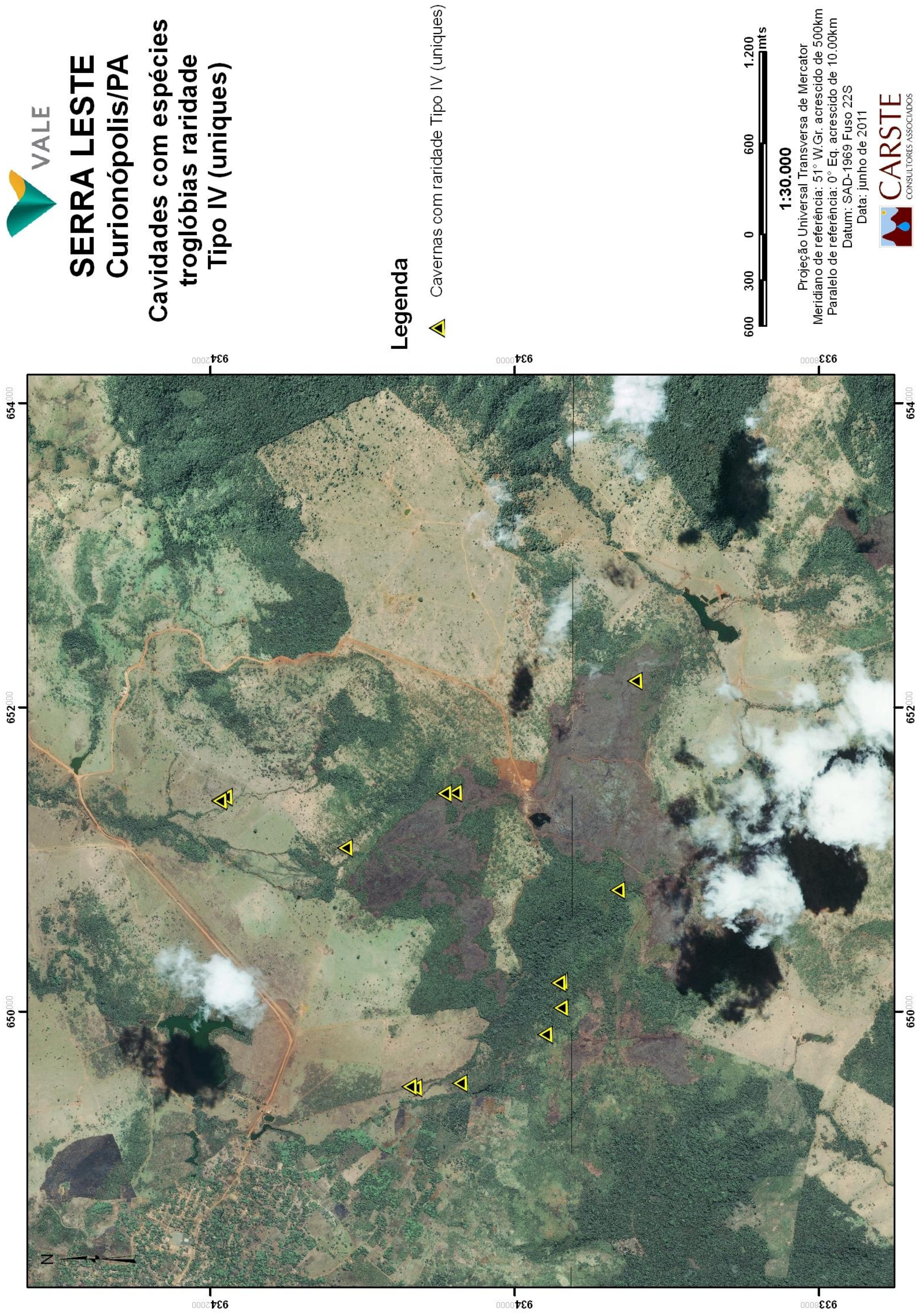

Figura 253 - Cavidades que apresentam espécies troglóbias com raridade tipo IV (Uniques – um único indivíduo foi observado durante o estudo).

Dentre as cavernas consideradas como de relevância máxima, dezoito apresentaram espécies troglóbias aqui categorizadas na raridade tipo III, isto é, espécies para as quais foram sobrepostas as raridades tipo I (distribuídas em até 3 cavernas) e II (um único indivíduo observado por caverna, independentemente do número de cavernas em que a espécie ocorra). Foram encontradas 20 espécies troglóbias enquadradas nesta categoria de raridade.

Novamente, algumas destas cavernas encontravam-se próximas geograficamente, enquanto outras apresentavam-se mais isoladas das demais, demonstrando que não há um “padrão” regular ou agregado de distribuição dos troglóbios de raridade tipo III (Figura 254).

Dentre as cavernas consideradas como de relevância máxima, vinte e duas apresentaram espécies troglóbias aqui categorizadas na raridade tipo II, isto é, espécies nas quais foi observado um único indivíduo por caverna, independentemente do número de cavernas em que a espécie ocorra. Foram encontradas 23 espécies troglóbias enquadradas nesta categoria de raridade.

Novamente, algumas destas cavernas encontravam-se próximas geograficamente, enquanto outras apresentavam-se mais isoladas das demais, demonstrando que não há um “padrão” regular ou agregado de distribuição dos troglóbios de raridade tipo II (Figura 255).

Finalmente, trinta e seis cavernas foram consideradas como de relevância máxima (como anteriormente mencionado), pelo fato de possuírem espécies troglóbias categorizadas na raridade tipo I, isto é, espécies distribuídas em até três cavernas. Foram encontradas 39 espécies troglóbias enquadradas nesta categoria de raridade.

Novamente, algumas destas cavernas encontravam-se próximas geograficamente, enquanto outras apresentavam-se mais isoladas das demais, demonstrando que não há um “padrão” regular ou agregado de distribuição dos troglóbios de raridade tipo III (Figura 256). No entanto, observam-se alguns conjuntos interessantes, dentre os quais se destacam as cavidades associadas à drenagem presente na porção oeste da área.

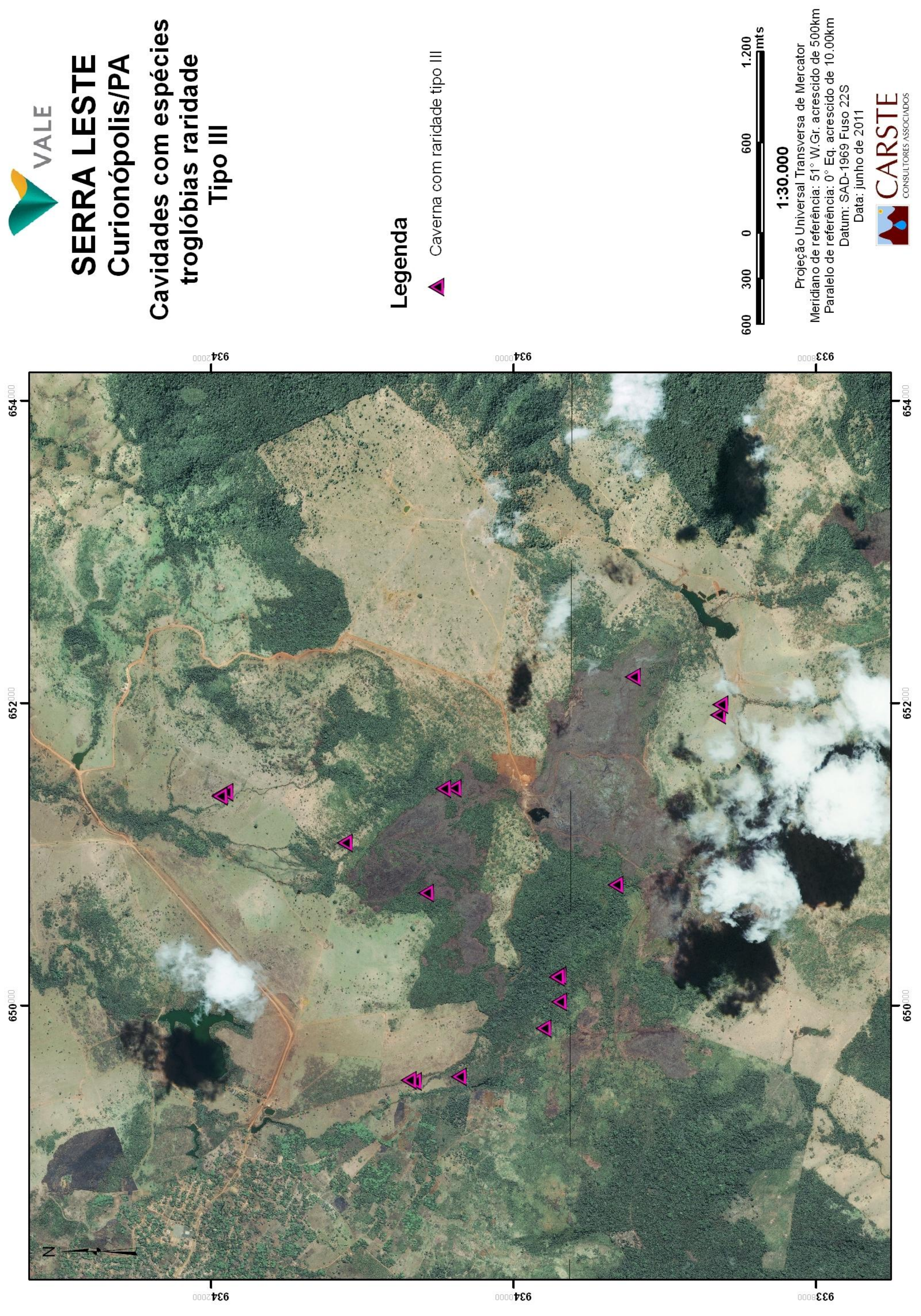

Figura 254 - Cavidades que apresentam espécies troglóbias com raridade tipo III. Tal raridade resulta da sobreposição da raridade tipo I (ocorrência em até 3 cavernas) com a raridade tipo II (um único indivíduo observado por caverna, independentemente do número de cavernas).

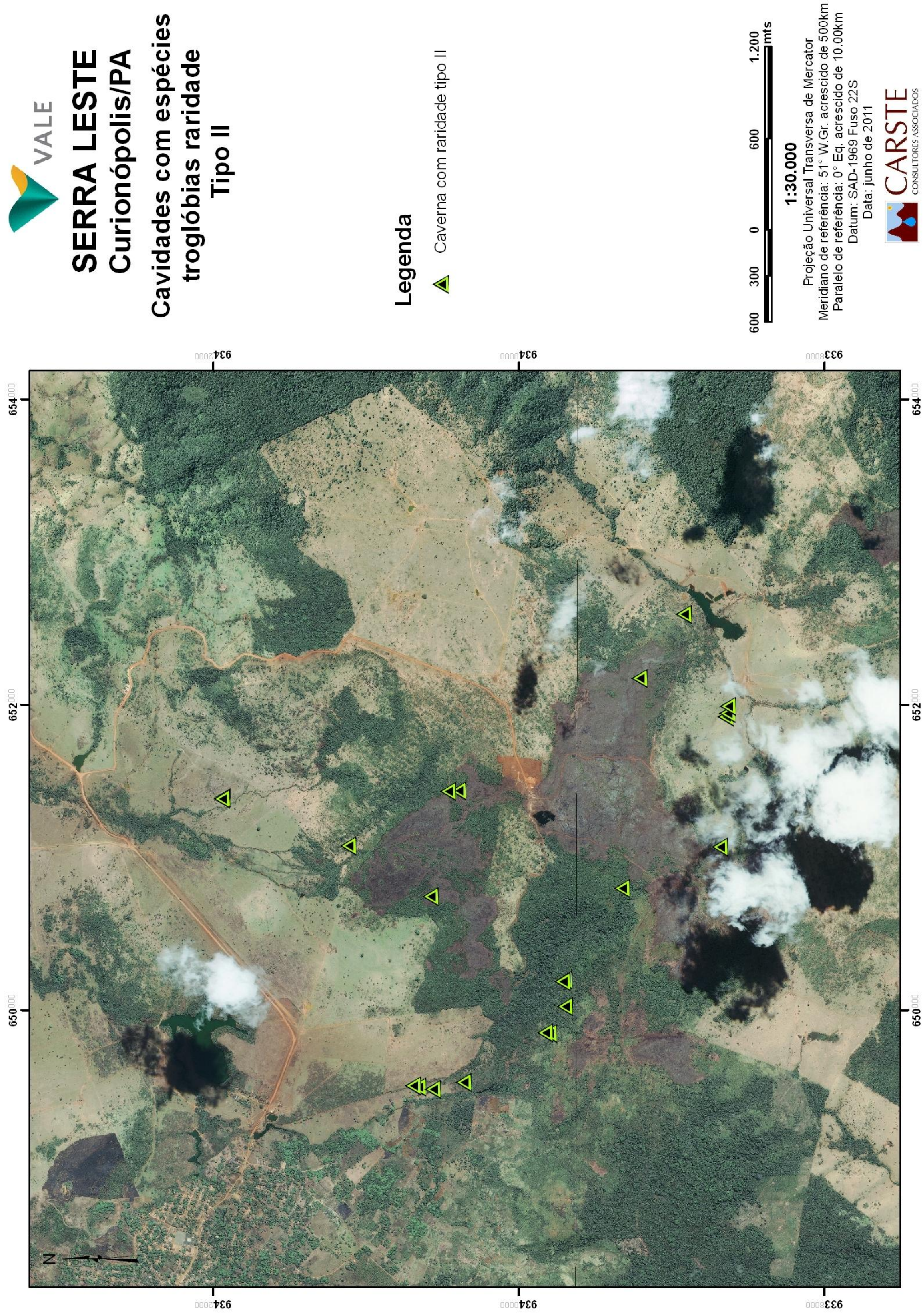

Figura 255 - Cavidades que apresentam espécies troglóbias com raridade tipo II (um único indivíduo observado por caverna, independentemente do número de cavernas).

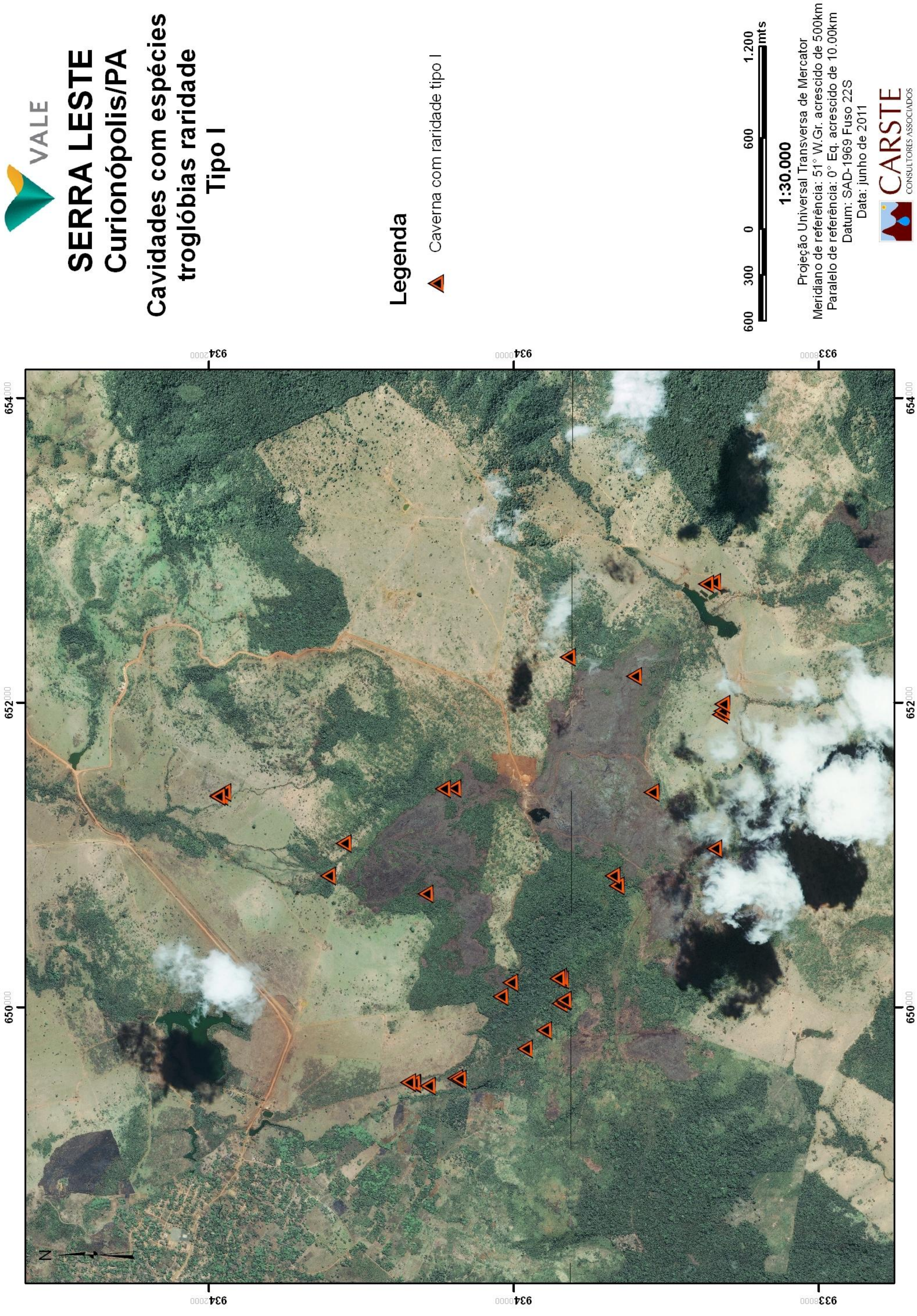

Figura 256 - Cavidades que apresentam espécies troglóbias com raridade I (ocorrência em até 3 cavernas). Tais cavidades compreendem aquelas consideradas como de relevância máxima.

Outro aspecto que merece menção diz respeito à cavernas que abrigam populações de espécies troglóbias “não raras”. A atual legislação não protege tais espécies, já que as cavernas que as abrigam são, desde o ponto de vista biológico, categorizadas como de relevância **alta**. Sendo assim, a atual legislação possui uma enorme falha no que concerne à proteção de algumas espécies troglóbias, já que permite a destruição de cavernas de relevância alta, o que pode vir a causar a completa extinção de espécies de troglóbios “não raros”. Este fato é bastante grave, uma vez que o próprio conceito de “raridade” aplicado a espécies troglóbias é bastante subjetivo.

Tendo em vista o anteriormente exposto, foram assinaladas na área de Serra Leste, as cavernas que apresentaram as maiores populações de espécies de troglóbios “não raros” (Figura 257).

Mais uma vez, algumas destas cavernas encontravam-se próximas geograficamente, enquanto outras apresentavam-se mais isoladas das demais. No entanto, observam-se alguns conjuntos interessantes, dentre os quais se destacam as cavidades associadas à porção norte da área.

Além disso, dentre estas cavidades, certamente merece destaque a caverna SL-031. Esta cavidade, sozinha, concentrou as maiores populações de **seis** espécies troglóbias. Desta forma, sua preservação é essencial no intuito de se garantir a preservação destas espécies na área.

Sobrepondo todos os critérios anteriormente citados (tipos de raridade e cavernas com maiores populações de troglóbios não raros) foram observadas 4 cavernas que certamente representam as mais importantes de Serra Leste desde o ponto de vista biológico, podendo ser consideradas como os “hotspots” de diversidade subterrânea na área (Figura 258). Estas cavernas correspondem a SL-031, SL-035, SL-074 e SL-096. Caso existisse alguma categoria de relevância acima da ”máxima”, certamente estas cavernas nela se enquadrariam.

Desta forma, tais cavernas não devem sofrer quaisquer tipos de impactos irreversíveis (como as demais enquadradas em relevância máxima, obviamente). Além disso, recomenda-se que o entorno de 250 m seja irrestritamente (e minimamente) preservado para estas cavernas.

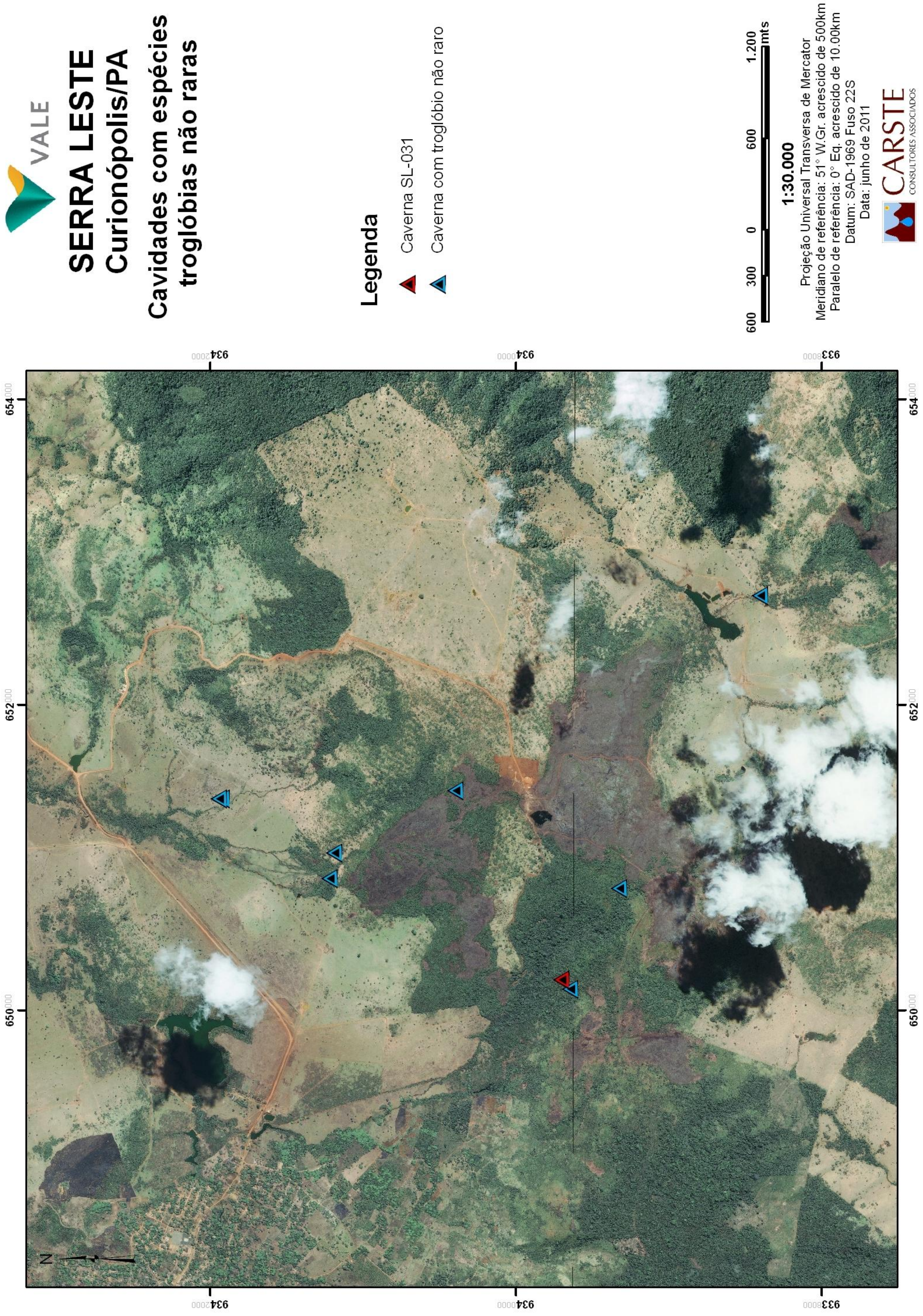

Figura 257. Cavidades que apresentam as maiores populações de espécies troglóbias consideradas não raras. Destaca-se a caverna SL-031 (em vermelho), que apresenta as maiores populações de cinco espécies de troglóbios não raros.

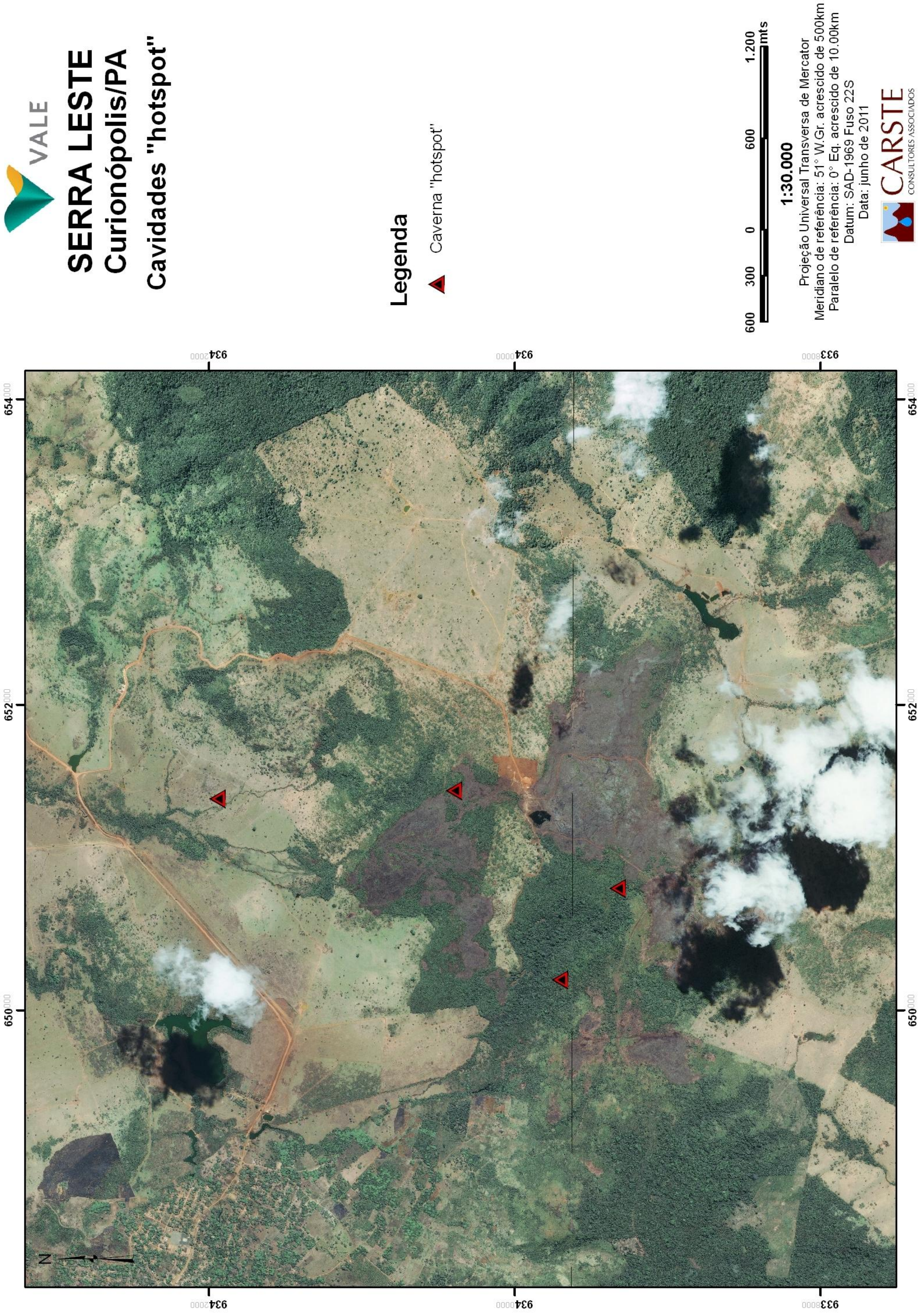

Figura 258 - Cavidades aqui consideradas como “hotspots” para a conservação. Nelas, se sobrepõem todos os critérios anteriormente considerados (todos os tipos de raridade e maiores populações de troglóbios não raros). Estas cavernas correspondem a SL-031, SL-035, SL-074 e SL-096.

Por fim, as cavernas de Serra Leste demonstraram diferentes valores de riqueza de espécies troglóbias (Figura 259). Na referida figura, foram destacadas somente as cavernas que apresentaram mais de 4 espécies troglóbias. Mais uma vez, não observou-se nenhum padrão de distribuição de cavernas mais ricas em espécies troglóbias. Dentre todas as cavernas da área, três se destacaram, por apresentarem mais de 10 espécies troglóbias: SL-031, SL-035, SL-074. Estas três cavernas também foram consideradas “hotspots” de diversidade subterrânea na área.

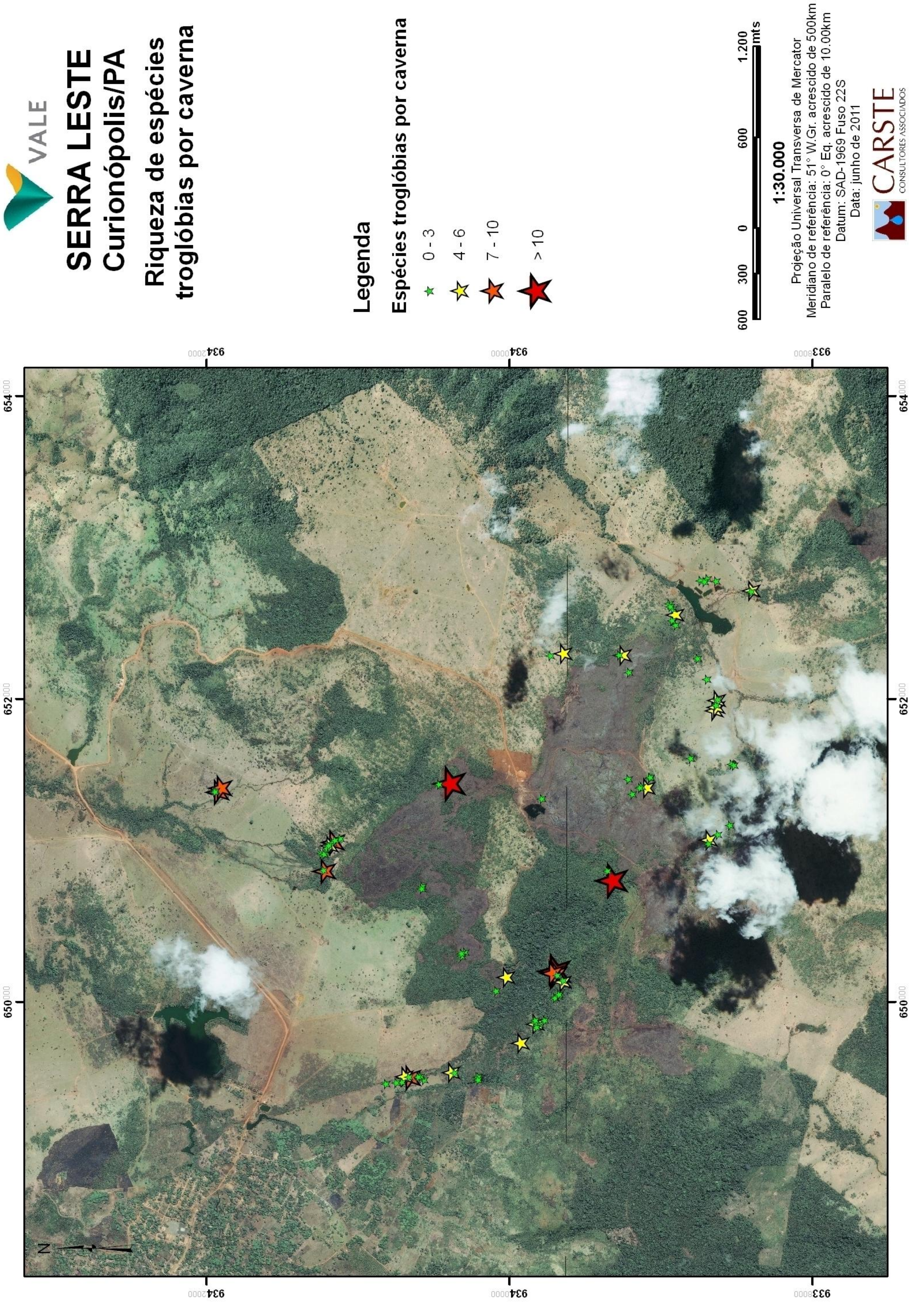

Figura 259 - Riqueza de espécies troglóbias nas cavernas da área (são destacadas somente as cavernas com riqueza de troglóbios superior a 4 espécies). As estrelas verdes compreendem cavernas com riqueza entre 4 e 6 espécies; as estrelas brancas compreendem cavernas com riqueza entre 7 e 10 espécies; as estrelas amarelas compreendem cavernas com riqueza superior a 10 espécies .

6.2.3.2.2. Considerando "adequações" na Instrução Normativa

A Tabela 23 apresenta os resultados da categorização das abundâncias relativas das espécies. Quatro cavernas foram incluídas na categoria de alta abundância relativa de espécies (SL-010, SL-020, SL-032, SL-071), com 30% ou mais das espécies apresentando abundância alta.

Cinquenta e sete cavernas foram incluídas na categoria de média abundância relativa de espécies (SL-002, SL-003, SL-006, SL-007, SL-009, SL-011, SL-014, SL-016, SL-017, SL-018, SL-019, SL-022, SL-023, SL-024, SL-025, SL-026, SL-027, SL-028, SL-033, SL-034, SL-036, SL-037, SL-038, SL-039, SL-040, SL-041, SL-043, SL-045, SL-046, SL-047, SL-048, SL-049, SL-050, SL-051, SL-053, SL-054, SL-055, SL-056, SL-058, SL-059, SL-061, SL-066, SL-073, SL-076, SL-078, SL-079, SL-081, SL-084, SL-086, SL-088, SL-090, SL-091, SL-092, SL-093, SL-094, SL-095, SL-097) com 10% a 20% das espécies apresentando abundância alta.

Trinta e sete cavernas foram incluídas na categoria de baixa abundância relativa de espécies (SL-001, SL-004, SL-005, SL-008, SL-012, SL-013, SL-015, SL-029, SL-030, SL-031, SL-035, SL-042, SL-044, SL-052, SL-057, SL-060, SL-062, SL-063, SL-064, SL-065, SL-067, SL-068, SL-069, SL-070, SL-072, SL-074, SL-075, SL-077, SL-080, SL-082, SL-083, SL-085, SL-087, SL-089, SL-096, SL-099, SL-100) com menos de 10% das espécies apresentam abundância alta.

Mudanças nas categorias de abundância relativas de espécies usando a metodologia de extração da raiz quadrada da abundância de cada população foram observadas de baixa para alta em três cavernas (SL-015, SL-068, SL-070), de baixa para média em vinte e cinco cavernas (SL-001, SL-004, SL-005, SL-008, SL-012, SL-013, SL-030, SL-031, SL-035, SL-042, SL-052, SL-057, SL-062, SL-063, SL-064, SL-065, SL-067, SL-069, SL-072, SL-075, SL-077, SL-082, SL-083, SL-099, SL-100) de média para alta em doze cavernas (SL-007, SL-011, SL-018, SL-024, SL-028, SL-055, SL-056, SL-059, SL-088, SL-091, SL-094, SL-095).

Tabela 23 - Modalidades de abundância relativa, considerando-se somente as espécies com indivíduos maiores que 1 cm (IN 02), usando métodos de divisão do valor absoluto por três e raiz quadrada do valor absoluto dividido por três. A sobreposição refere-se ao resultado final entre as estimativas nas estações seca e estação úmida das duas metodologias. Mudanças de categoria em consequência do uso da metodologia de raiz quadrada dividido por três são destacadas em verde.

Cavernas	Est. seca	Est. úmida	Sobreposição	Est. seca (raiz)	Est. úmida (raiz)	Sobreposição
SL-001	BAIXA	BAIXA	BAIXA	MÉDIA	MÉDIA	MÉDIA
SL-002	MÉDIA	BAIXA	MÉDIA	MÉDIA	BAIXA	MÉDIA
SL-003	MÉDIA	BAIXA	MÉDIA	MÉDIA	BAIXA	MÉDIA
SL-004	BAIXA	BAIXA	BAIXA	MÉDIA	BAIXA	MÉDIA
SL-005	BAIXA	BAIXA	BAIXA	MÉDIA	BAIXA	MÉDIA
SL-006	BAIXA	MÉDIA	MÉDIA	MÉDIA	MÉDIA	MÉDIA

SL-007	MÉDIA	MÉDIA	MÉDIA	MÉDIA	ALTA	ALTA
SL-008	BAIXA	BAIXA	BAIXA	MÉDIA	MÉDIA	MÉDIA
SL-009	MÉDIA	MÉDIA	MÉDIA	MÉDIA	MÉDIA	MÉDIA
SL-011	MÉDIA	MÉDIA	MÉDIA	MÉDIA	ALTA	ALTA
SL-012	BAIXA	BAIXA	BAIXA	MÉDIA	BAIXA	MÉDIA
SL-013	BAIXA	BAIXA	BAIXA	MÉDIA	BAIXA	MÉDIA
SL-014	MÉDIA	BAIXA	MÉDIA	MÉDIA	BAIXA	MÉDIA
SL-015	BAIXA	BAIXA	BAIXA	BAIXA	BAIXA	ALTA
SL-016	BAIXA	MÉDIA	MÉDIA	BAIXA	MÉDIA	MÉDIA
SL-017	BAIXA	MÉDIA	MÉDIA	MÉDIA	MÉDIA	MÉDIA
SL-018	ALTA	MÉDIA	MÉDIA	ALTA	MÉDIA	ALTA
SL-019	BAIXA	MÉDIA	MÉDIA	BAIXA	MÉDIA	MÉDIA
SL-020	ALTA	BAIXA	ALTA	ALTA	ALTA	ALTA
SL-022	MÉDIA	BAIXA	MÉDIA	MÉDIA	BAIXA	MÉDIA
SL-023	MÉDIA	BAIXA	MÉDIA	MÉDIA	BAIXA	MÉDIA
SL-024	MÉDIA	MÉDIA	MÉDIA	ALTA	MÉDIA	ALTA
SL-025	BAIXA	MÉDIA	MÉDIA	MÉDIA	MÉDIA	MÉDIA
SL-026	BAIXA	MÉDIA	MÉDIA	MÉDIA	MÉDIA	MÉDIA
SL-027	MÉDIA	MÉDIA	MÉDIA	MÉDIA	MÉDIA	MÉDIA
SL-028	MÉDIA	MÉDIA	MÉDIA	MÉDIA	ALTA	ALTA
SL-029	BAIXA	BAIXA	BAIXA	BAIXA	BAIXA	BAIXA
SL-030	BAIXA	BAIXA	BAIXA	MÉDIA	BAIXA	MÉDIA
SL-031	BAIXA	BAIXA	BAIXA	MÉDIA	BAIXA	MÉDIA
SL-032	BAIXA	A	ALTA	MÉDIA	ALTA	ALTA
SL-033	BAIXA	MÉDIA	MÉDIA	MÉDIA	MÉDIA	MÉDIA
SL-035	BAIXA	BAIXA	BAIXA	MÉDIA	BAIXA	MÉDIA
SL-036	MÉDIA	BAIXA	MÉDIA	MÉDIA	BAIXA	MÉDIA
SL-037	BAIXA	MÉDIA	MÉDIA	BAIXA	MÉDIA	MÉDIA
SL-038	MÉDIA	BAIXA	MÉDIA	MÉDIA	BAIXA	MÉDIA
SL-039	MÉDIA	MÉDIA	MÉDIA	MÉDIA	MÉDIA	MÉDIA
SL-040	MÉDIA	MÉDIA	MÉDIA	MÉDIA	MÉDIA	MÉDIA
SL-041	MÉDIA	BAIXA	MÉDIA	MÉDIA	BAIXA	MÉDIA
SL-042	BAIXA	BAIXA	BAIXA	MÉDIA	BAIXA	MÉDIA
SL-043	BAIXA	MÉDIA	MÉDIA	MÉDIA	MÉDIA	MÉDIA
SL-044	BAIXA	BAIXA	BAIXA	BAIXA	BAIXA	BAIXA
SL-045	BAIXA	MÉDIA	MÉDIA	BAIXA	MÉDIA	MÉDIA
SL0-46	MÉDIA	BAIXA	MÉDIA	MÉDIA	BAIXA	MÉDIA
SL-047	BAIXA	MÉDIA	MÉDIA	MÉDIA	MÉDIA	MÉDIA
SL-048	MÉDIA	MÉDIA	MÉDIA	MÉDIA	MÉDIA	MÉDIA
SL-049	MÉDIA	MÉDIA	MÉDIA	MÉDIA	MÉDIA	MÉDIA
SL-050	MÉDIA	MÉDIA	MÉDIA	MÉDIA	MÉDIA	MÉDIA

SL-051	BAIXA	MÉDIA	MÉDIA	BAIXA	MÉDIA	MÉDIA
SL-052	BAIXA	BAIXA	BAIXA	MÉDIA	BAIXA	MÉDIA
SL-053	MÉDIA	MÉDIA	MÉDIA	MÉDIA	MÉDIA	MÉDIA
SL-054	BAIXA	MÉDIA	MÉDIA	MÉDIA	MÉDIA	MÉDIA
SL-055	MÉDIA	MÉDIA	MÉDIA	MÉDIA	ALTA	ALTA
SL-056	MÉDIA	MÉDIA	MÉDIA	ALTA	MÉDIA	ALTA
SL-057	BAIXA	BAIXA	BAIXA	MÉDIA	BAIXA	MÉDIA
SL-058	MÉDIA	BAIXA	MÉDIA	MÉDIA	BAIXA	MÉDIA
SL-059	MÉDIA	MÉDIA	MÉDIA	MÉDIA	ALTA	ALTA
SL-060	BAIXA	BAIXA	BAIXA	BAIXA	BAIXA	BAIXA
SL-061	MÉDIA	MÉDIA	MÉDIA	MÉDIA	MÉDIA	MÉDIA
SL-062	MÉDIA	BAIXA	BAIXA	MÉDIA	BAIXA	MÉDIA
SL-063	MÉDIA	MÉDIA	BAIXA	MÉDIA	MÉDIA	MÉDIA
SL-064	MÉDIA	BAIXA	BAIXA	MÉDIA	BAIXA	MÉDIA
SL-065	BAIXA	MÉDIA	BAIXA	MÉDIA	MÉDIA	MÉDIA
SL-066	BAIXA	BAIXA	MÉDIA	MÉDIA	BAIXA	MÉDIA
SL-067	BAIXA	MÉDIA	BAIXA	BAIXA	MÉDIA	MÉDIA
SL-068	MÉDIA	MÉDIA	BAIXA	ALTA	ALTA	ALTA
SL-069	BAIXA	MÉDIA	BAIXA	MÉDIA	MÉDIA	MÉDIA
SL-070	MÉDIA	BAIXA	BAIXA	ALTA	MÉDIA	ALTA
SL-071	BAIXA	A	ALTA	MÉDIA	ALTA	ALTA
SL-072	BAIXA	BAIXA	BAIXA	BAIXA	MÉDIA	MÉDIA
SL-073	MÉDIA	BAIXA	MÉDIA	MÉDIA	BAIXA	MÉDIA
SL-074	BAIXA	BAIXA	BAIXA	BAIXA	BAIXA	BAIXA
SL-075	BAIXA	BAIXA	BAIXA	MÉDIA	BAIXA	MÉDIA
SL-076	BAIXA	MÉDIA	MÉDIA	MÉDIA	MÉDIA	MÉDIA
SL-077	BAIXA	BAIXA	BAIXA	BAIXA	MÉDIA	MÉDIA
SL-078	MÉDIA	MÉDIA	MÉDIA	MÉDIA	MÉDIA	MÉDIA
SL-079	MÉDIA	BAIXA	MÉDIA	MÉDIA	BAIXA	MÉDIA
SL-080	BAIXA	BAIXA	BAIXA	BAIXA	BAIXA	BAIXA
SL-081	MÉDIA	MÉDIA	MÉDIA	MÉDIA	MÉDIA	MÉDIA
SL-082	BAIXA	BAIXA	BAIXA	MÉDIA	BAIXA	MÉDIA
SL-083	BAIXA	BAIXA	BAIXA	MÉDIA	BAIXA	MÉDIA
SL-084	MÉDIA	MÉDIA	MÉDIA	MÉDIA	MÉDIA	MÉDIA
SL-085	BAIXA	BAIXA	BAIXA	BAIXA	BAIXA	BAIXA
SL-086	MÉDIA	MÉDIA	MÉDIA	MÉDIA	MÉDIA	MÉDIA
SL-087	BAIXA	BAIXA	BAIXA	BAIXA	BAIXA	BAIXA
SL-088	MÉDIA	MÉDIA	MÉDIA	MÉDIA	ALTA	ALTA
SL-089	BAIXA	BAIXA	BAIXA	BAIXA	BAIXA	BAIXA
SL-090	BAIXA	MÉDIA	MÉDIA	MÉDIA	MÉDIA	MÉDIA
SL-091	MÉDIA	MÉDIA	MÉDIA	ALTA	MÉDIA	ALTA

SL-092	MÉDIA	BAIXA	MÉDIA	MÉDIA	BAIXA	MÉDIA
SL-093	BAIXA	MÉDIA	MÉDIA	BAIXA	MÉDIA	MÉDIA
SL-094	MÉDIA	MÉDIA	MÉDIA	ALTA	ALTA	ALTA
SL-095	BAIXA	MÉDIA	MÉDIA	BAIXA	ALTA	ALTA
SL-096	BAIXA	BAIXA	BAIXA	BAIXA	BAIXA	BAIXA
SL-097	BAIXA	MÉDIA	MÉDIA	MÉDIA	MÉDIA	MÉDIA
SL-099	BAIXA	BAIXA	BAIXA	MÉDIA	MÉDIA	MÉDIA
SL-100	BAIXA	BAIXA	BAIXA	MÉDIA	BAIXA	MÉDIA

O método de categorização através da divisão da maior abundância, das espécies maiores que 1 cm, encontrada por três (para a criação das categorias de abundância), resulta em maiores discrepâncias quando comparadas às análises realizadas considerando-se o método de extração de raiz quadrada. Desta forma, tal comparação permite inferir que a determinação das categorias de abundância baseada na redução das diferenças entre os tamanhos populacionais (por meio da raiz quadrada) mostra-se mais eficiente que o outro método.

Destaca-se que a aplicação do mesmo método utilizado para a determinação das categorias de riqueza (média +/- desvio padrão) definitivamente não se aplica às análises de abundância. Populações muito numerosas levariam ao surgimento de desvios muito grandes. O resultado disso seria sempre a inexistência de cavernas de baixa abundância populacional média, gerando um sério ruído na análise.

6.2.3.3. Considerações finais sobre a relevância, os impactos potenciais e a conservação de cavernas no Projeto Serra Leste.

Considerando o total de 96 cavidades estudadas no Projeto Serra Leste, duas cavernas (2%) foram classificadas como de relevância máxima diante dos atributos físicos. Onze cavidades (11,3%) foram classificadas como de relevância alta e 85 cavernas (86,7%) com o grau de relevância médio. Não foram classificadas cavernas de relevância baixa.

Em relação aos atributos biológicos, 40 (40,8%) foram classificadas como de relevância máxima. Trinta e oito cavernas foram classificadas com grau de relevância alto, representando 38,8% do total. Dezoito cavernas (18,4%) foram classificadas como de relevância média e somente duas (2%) com grau de relevância baixo.

Integrando os atributos físicos/histórico-culturais e biológicos, 40 cavernas (40,8%) foram classificadas como de relevância máxima. Quarenta cavernas (40,8%) receberam o grau de relevância alto e 18 cavidades (18,4%) o grau de relevância médio. O resumo da classificação

do grau de relevância das cavidades do projeto Serra Leste pode ser observado na Tabela 24 e Figura 260.

Tabela 24 - Síntese da relevância das cavidades estudadas em Serra Leste, de acordo com a configuração de importância dos atributos físicos e biológicos.

Caverna	Grau de relevância e atributos biológicos classificatórios	Grau de relevância e atributos físicos classificatórios	Grau de relevância final
SL-01	Máxima - Habitat de troglóbio raro, presença de Troglóbios, não raros endêmicos ou relictos , espécies troglomórficas, espécies com função ecológica importante, alta riqueza de espécies, baixa abundância relativa de espécies, populações residentes de Chiroptera, alta diversidade de substratos orgânicos e singularidade dos elementos faunísticos da cavidade .	Máxima - morfologia única	Máxima
SL-02	Alta - Presença de Troglóbios, não raros endêmicos ou relictos , espécies troglomórficas, espécies com função ecológica importante, média riqueza de espécies, média abundância relativa de espécies, populações residentes de Chiroptera, alta diversidade de substratos orgânico e singularidade dos elementos faunísticos da cavidade.	Média - média projeção horizontal, média área e médio volume.	Alta
SL-03	Alta - Espécies com função ecológica importante, média riqueza de espécies, média abundância relativa de espécies, populações residentes de Chiroptera, alta diversidade de substratos orgânicos e singularidade dos elementos faunísticos da cavidade.	Média - média projeção horizontal, média área e médio volume.	Alta
SL-04	Máxima - Habitat de troglóbio raro, presença de Troglóbios, não raros endêmicos ou relictos, espécies troglomórficas, alta riqueza de espécies, baixa abundância relativa de espécies, alta diversidade de substratos orgânicos e singularidade dos elementos faunísticos da cavidade.	Média - média projeção horizontal, média área e médio volume.	Máxima
SL-05	Máxima - Habitat de troglóbio raro, presença de Troglóbios, não raros endêmicos ou relictos, espécies troglomórficas, média riqueza de espécies, baixa abundância relativa de espécies e alta diversidade de substratos orgânicos.	Média - média projeção horizontal, média área e médio volume.	Máxima
SL-06	Máxima - Habitat de troglóbio raro, presença de Troglóbios, não raros endêmicos ou relictos, espécies troglomórficas, média riqueza de espécies, média abundância relativa de espécies e alta diversidade de substratos orgânicos.	Média - média projeção horizontal, média área e médio volume.	Máxima
SL-07	Máxima - Habitat de troglóbio raro, espécies troglomórficas, média riqueza de espécies, média abundância relativa de espécies e baixa diversidade de substratos orgânicos.	Média - média projeção horizontal, média área e médio volume.	Máxima
SL-08	Alta - Presença de Troglóbios, não raros endêmicos ou relictos , espécies troglomórficas, média riqueza de espécies, baixa abundância relativa de espécie e baixa diversidade de substratos orgânicos.	Média - média projeção horizontal, média área e médio volume.	Alta
SL-09	Alta - Espécies troglomórficas, espécies com função ecológica importante, média riqueza de espécies, média abundância relativa de espécies, populações residentes de Chiroptera e alta diversidade de substratos orgânicos.	Alta - Lago ou drenagem subterrânea perene com influência acentuada;	Alta
SL-11	Média - Média riqueza de espécies, média abundância relativa de espécies e baixa diversidade de substratos orgânicos.	Média - média projeção horizontal, média área e médio volume.	Média
SL-12	Máxima - Habitat de troglóbio raro, espécies troglomórficas, média riqueza de espécies, baixa abundância relativa de espécies e baixa diversidade de substratos orgânicos.	Média - média projeção horizontal, média área e médio volume.	Máxima
SL-13	Alta - Presença de Troglóbios, não raros endêmicos ou	Média - média projeção horizontal, média área e	Alta

	relictos , espécies troglomórficas, média riqueza de espécies, baixa abundância relativa de espécies e baixa diversidade de substratos orgânicos.	médio volume.	
SL-14	Alta - Espécies troglomórficas, média riqueza de espécies, média abundância relativa de espécies e baixa diversidade de substratos orgânicos.	Média - média projeção horizontal, média área e médio volume.	Alta
SL-15	Alta - Presença de Troglóbios, não raros endêmicos ou relictos , espécies troglomórficas, média riqueza de espécies, baixa abundância relativa de espécies e alta diversidade de substratos orgânicos.	Média - média projeção horizontal, média área e médio volume.	Alta
SL-16	Máxima - Habitat de troglóbio raro, presença de Troglóbios, não raros endêmicos ou relictos , espécies troglomórficas, espécies com função ecológica importante, alta riqueza de espécies, média abundância relativa de espécies, populações residentes de Chiroptera e alta diversidade de substratos orgânicos.	Média - média projeção horizontal, média área e médio volume.	Máxima
SL-17	Média - Média riqueza de espécies, média abundância relativa de espécies e alta diversidade de substratos orgânicos.	Média - média projeção horizontal, média área e médio volume.	Média
SL-18	Máxima - Habitat de troglóbio raro, presença de Troglóbios, não raros endêmicos ou relictos, espécies troglomórficas, média riqueza de espécies, média abundância relativa de espécies e baixa Diversidade de substratos orgânicos.	Média - média projeção horizontal, média área e médio volume.	Máxima
SL-19	Média - Média riqueza de espécies, média abundância relativa de espécies, baixa diversidade de substratos orgânicos .	Média - média projeção horizontal, média área e médio volume.	Média
SL-20	Alta - Média riqueza de espécies , alta abundância relativa de espécies e diversidade de substratos orgânicos.	Média - média projeção horizontal, média área e médio volume.	Alta
SL-22	Máxima - Habitat de troglóbio raro, presença de Troglóbios, não raros endêmicos ou relictos, espécies troglomórficas, média riqueza de espécies, média abundância relativa de espécies e baixa diversidade de substratos orgânicos.	Média - média projeção horizontal, média área e médio volume.	Máxima
SL-23	Alta - Espécies troglomórficas, média riqueza de espécies, média abundância relativa de espécies, baixa diversidade de substratos orgânicos.	Média - média projeção horizontal, média área e médio volume.	Alta
SL-24	Máxima - Habitat de troglóbio raro, presença de Troglóbios, não raros endêmicos ou relictos, espécies troglomórficas, populações residentes de Chiroptera e alta diversidade de substratos orgânicos.	Média - média projeção horizontal, média área e médio volume.	Máxima
SL-25	Máxima - Habitat de troglóbio raro, presença de Troglóbios, não raros endêmicos ou relictos, espécies troglomórficas, média riqueza de espécies, média abundância relativa de espécies e baixa diversidade de substratos orgânicos.	Média - média projeção horizontal, média área e médio volume.	Máxima
SL-26	Máxima - Habitat de troglóbio raro, presença de Troglóbios, não raros endêmicos ou relictos , espécies troglomórficas, média riqueza de espécies, média abundância relativa de espécies e alta diversidade de substratos orgânicos.	Média - média projeção horizontal, média área e médio volume.	Máxima
SL-27	Alta - Presença de Troglóbios, não raros endêmicos ou relictos , espécies troglomórficas, média riqueza de espécies, média abundância relativa de espécies e baixa diversidade de substratos orgânicos.	Média - média projeção horizontal, média área e médio volume.	Alta
SL-28	Média - Média riqueza de espécies, média abundância relativa de espécies e baixa diversidade de substratos orgânicos.	Média - média projeção horizontal, média área e médio volume.	Média
SL-29	Alta - Presença de Troglóbios, não raros endêmicos ou relictos , espécies troglomórficas, espécies com função ecológica importante, alta riqueza de espécies, baixa abundância relativa de espécies, Populações residentes de Chiroptera e alta diversidade de substratos orgânicos.	Média - média projeção horizontal, média área e médio volume.	Alta
SL-30	Máxima - Habitat de troglóbio raro, presença de Troglóbios, não raros endêmicos ou relictos , espécies	Média - média projeção horizontal, média área e médio volume.	Máxima

	troglomórficas, espécies com função ecológica importante, alta riqueza de espécies, baixa abundância relativa de espécies , populações residentes de Chiroptera e alta Diversidade de substratos orgânicos.		
SL-31	Máxima - Habitat de troglóbio raro, presença de Troglóbios, não raros endêmicos ou relictos , espécies troglomórficas, espécies com função ecológica importante, média riqueza de espécies , baixa abundância relativa de espécies, populações residentes de Chiroptera e alta diversidade de substratos orgânicos.	Média - média projeção horizontal, média área e médio volume.	Máxima
SL-32	Máxima - Habitat de troglóbio raro, espécies troglomórficas, média riqueza de espécies , alta abundância relativa de espécies e baixa diversidade de substratos orgânicos.	Média - média projeção horizontal, média área e médio volume.	Máxima
SL-33	Máxima - Habitat de troglóbio raro, presença de Troglóbios, não raros endêmicos ou relictos , espécies troglomórficas, média riqueza de espécies, média abundância relativa de espécies e baixa diversidade de substratos orgânicos.	Média - média projeção horizontal, média área e médio volume.	Máxima
SL-35	Máxima - Habitat de troglóbio raro, presença de Troglóbios, não raros endêmicos ou relictos , espécies troglomórficas, média riqueza de espécies, baixa abundância relativa de espécies, composição singular da fauna e alta diversidade de substratos orgânicos.	Média - média projeção horizontal, média área e médio volume.	Máxima
SL-36	Máxima - Habitat de troglóbio raro, espécies troglomórficas, média riqueza de espécies, média abundância relativa de espécies e alta diversidade de substratos orgânicos.	Média - média projeção horizontal, média área e médio volume.	Máxima
SL-37	Alta - Presença de Troglóbios, não raros endêmicos ou relictos , espécies troglomórficas, espécies com função ecológica importante, média riqueza de espécies, média abundância relativa de espécies, populações residentes de Chiroptera e alta Diversidade de substratos orgânicos.	Média - média projeção horizontal, média área e médio volume.	Alta
SL-38	Média - Média riqueza de espécies , média abundância relativa de espécies e alta diversidade de substratos orgânicos.	Média - média projeção horizontal, média área e médio volume.	Média
SL-39	Média - Média riqueza de espécies , média abundância relativa de espécies e alta diversidade de substratos orgânicos.	Média - média projeção horizontal, média área e médio volume.	Média
SL-40	Média - Média riqueza de espécies , média abundância relativa de espécies e alta diversidade de substratos orgânicos.	Média - média projeção horizontal, média área e médio volume.	Média
SL-41	Média - Média riqueza de espécies , média abundância relativa de espécies e alta diversidade de substratos orgânicos.	Média - média projeção horizontal, média área e médio volume.	Média
SL-42	Máxima - Habitat de troglóbio raro, presença de Troglóbios, não raros endêmicos ou relictos , espécies troglomórficas, média riqueza de espécies , baixa abundância relativa de espécies e alta diversidade de substratos orgânicos.	Média - média projeção horizontal, média área e médio volume.	Máxima
SL-43	Alta - Presença de Troglóbios, não raros endêmicos ou relictos , espécies troglomórficas, média riqueza de espécies, média abundância relativa de espécies e baixa diversidade de substratos orgânicos.	Média - média projeção horizontal, média área e médio volume.	Alta
SL-44	Máxima - Habitat de troglóbio raro, presença de Troglóbios, não raros endêmicos ou relictos , espécies troglomórficas, alta riqueza de espécies, baixa abundância relativa de espécies e alta diversidade de substratos orgânicos .	Alta - alto volume;	Máxima
SL-45	Máxima - Habitat de troglóbio raro, presença de Troglóbios, não raros endêmicos ou relictos , espécies troglomórficas, média riqueza de espécies, média abundância relativa de espécies e baixa diversidade de substratos orgânicos.	Média - média projeção horizontal, média área e médio volume.	Máxima

SL-46	Alta - Presença de Troglóbios, não raros endêmicos ou relictos , espécies troglomórficas, média riqueza de espécies , média abundância relativa de espécies e alta diversidade de substratos orgânicos.	Média - média projeção horizontal, média área e médio volume.	Alta
SL-47	Média - Média riqueza de espécies, média abundância relativa de espécies e alta diversidade de substratos orgânicos .	Média - média projeção horizontal, média área e médio volume.	Média
SL-48	Média - Média riqueza de espécies, média abundância relativa de espécies e baixa diversidade de substratos orgânicos .	Média - média projeção horizontal, média área e médio volume.	Média
SL-49	Alta - Presença de Troglóbios, não raros endêmicos ou relictos , espécies troglomórficas, média riqueza de espécies , média abundância relativa de espécies e baixa diversidade de substratos orgânicos.	Média - média projeção horizontal, média área e médio volume.	Alta
SL-50	Alta - Presença de Troglóbios, não raros endêmicos ou relictos , espécies troglomórficas, média riqueza de espécies , média abundância relativa de espécies e alta diversidade de substratos orgânicos.	Alta - alto volume; Lago ou drenagem subterrânea perene com influência acentuada;	Alta
SL-51	Máxima - Habitat de troglóbio raro, presença de Troglóbios, não raros endêmicos ou relictos , espécies troglomórficas, média riqueza de espécies, média abundância relativa de espécies e alta diversidade de substratos orgânicos.	Alta - configuração notável de espeleotemas;	Máxima
SL-52	Máxima - Habitat de troglóbio raro, presença de Troglóbios, não raros endêmicos ou relictos , espécies troglomórficas, média riqueza de espécies, baixa abundância relativa de espécies e baixa diversidade de substratos orgânicos.	Média - média projeção horizontal, média área e médio volume.	Máxima
SL-53	Alta - Presença de Troglóbios, não raros endêmicos ou relictos , espécies troglomórficas, média riqueza de espécies , média abundância relativa de espécies e alta diversidade de substratos orgânicos.	Média - média projeção horizontal, média área e médio volume.	Alta
SL-54	Máxima - Habitat de troglóbio raro, presença de Troglóbios, não raros endêmicos ou relictos , espécies troglomórficas, média riqueza de espécies, média abundância relativa de espécies e baixa diversidade de substratos orgânicos.	Média - média projeção horizontal, média área e médio volume.	Máxima
SL-55	Alta - Presença de Troglóbios, não raros endêmicos ou relictos , espécies troglomórficas, média riqueza de espécies , média abundância relativa de espécies e alta diversidade de substratos orgânicos.	Média - média projeção horizontal, média área e médio volume.	Alta
SL-56	Alta - Presença de Troglóbios, não raros endêmicos ou relictos , espécies troglomórficas, média riqueza de espécies , média abundância relativa de espécies e baixa diversidade de substratos orgânicos.	Alta - Lago ou drenagem subterrânea perene com influência acentuada;	Alta
SL-57	Máxima - Habitat de troglóbio raro, presença de Troglóbios, não raros endêmicos ou relictos , espécies troglomórficas, média riqueza de espécies, baixa abundância relativa de espécies e alta diversidade de substratos orgânicos.	Média - média projeção horizontal, média área e médio volume.	Máxima
SL-58	Alta - Presença de Troglóbios, não raros endêmicos ou relictos , espécies troglomórficas, média riqueza de espécies , média abundância relativa de espécies, espécies com função ecológica importante e alta diversidade de substratos orgânicos.	Média - média projeção horizontal, média área e médio volume.	Alta
SL-59	Média - Média riqueza de espécies, média abundância relativa de espécies e alta diversidade de substratos orgânicos .	Média - média projeção horizontal, média área e médio volume.	Média
SL-60	Alta - Presença de Troglóbios, não raros endêmicos ou relictos , espécies troglomórficas, alta riqueza de espécies , baixa abundância relativa de espécies, espécies com função ecológica importante, populações residentes de Chiroptera e alta diversidade de substratos orgânicos.	Média - média projeção horizontal, média área e médio volume.	Alta
SL-61	Alta - Presença de Troglóbios, não raros endêmicos ou relictos , espécies troglomórficas, média riqueza de espécies , média abundância relativa de espécies e alta diversidade de substratos orgânicos.	Média - média projeção horizontal, média área e médio volume.	Alta

SL-62	Máxima - Habitat de troglóbio raro, presença de Troglóbios, não raros endêmicos ou relictos , espécies troglomórficas, média riqueza de espécies, baixa abundância relativa de espécies e alta diversidade de substratos orgânicos.	Média - média projeção horizontal, média área e médio volume.	Máxima
SL-63	Baixa - Baixa riqueza de espécies, baixa abundância relativa de espécies e baixa diversidade de substratos orgânicos.	Alta - Lago ou drenagem subterrânea perene com influência acentuada;	Alta
SL-64	Alta - Presença de Troglóbios, não raros endêmicos ou relictos , espécies troglomórficas, média riqueza de espécies , baixa abundância relativa de espécies e baixa diversidade de substratos orgânicos.	Alta - Lago ou drenagem subterrânea perene com influência acentuada;	Alta
SL-65	Máxima - Habitat de troglóbio raro, presença de Troglóbios, não raros endêmicos ou relictos , espécies troglomórficas, média riqueza de espécies, baixa abundância relativa de espécies e alta diversidade de substratos orgânicos.	Média - média projeção horizontal, média área e médio volume.	Máxima
SL-66	Média - Média riqueza de espécies, média abundância relativa de espécies e alta diversidade de substratos orgânicos .	Média - média projeção horizontal, média área e médio volume.	Média
SL-67	Máxima - Habitat de troglóbio raro, espécies troglomórficas, média riqueza de espécies, baixa abundância relativa de espécies e baixa diversidade de substratos orgânicos.	Média - média projeção horizontal, média área e médio volume.	Máxima
SL-68	Baixa - Baixa riqueza de espécies, baixa abundância relativa de espécies e baixa diversidade de substratos orgânicos.	Alta - Lago ou drenagem subterrânea perene com influência acentuada;	Alta
SL-69	Alta - Presença de Troglóbios, não raros endêmicos ou relictos , espécies troglomórficas, espécies com função ecológica importante, alta riqueza de espécies , baixa abundância relativa de espécies, populações residentes de Chiroptera e alta diversidade de substratos orgânicos.	Média - média projeção horizontal, média área e médio volume.	Alta
SL-70	Alta - Presença de Troglóbios, não raros endêmicos ou relictos , espécies troglomórficas, média riqueza de espécies , baixa abundância relativa de espécies e baixa diversidade de substratos orgânicos.	Média - média projeção horizontal, média área e médio volume.	Alta
SL-71	Alta -Média riqueza de espécies , alta abundância relativa de espécies e baixa diversidade de substratos orgânicos.	Média - média projeção horizontal, média área e médio volume.	Alta
SL-72	Máxima - Habitat de troglóbio raro, presença de Troglóbios, não raros endêmicos ou relictos , espécies troglomórficas, média riqueza de espécies, baixa abundância relativa de espécies e baixa diversidade de substratos orgânicos.	Média - média projeção horizontal, média área e médio volume.	Máxima
SL-73	Alta - Espécies troglomórficas, espécies com função ecológica importante, média riqueza de espécies ,média abundância relativa de espécies, populações residentes de Chiroptera e alta diversidade de substratos orgânicos.	Média - média projeção horizontal, média área e médio volume.	Alta
SL-74	Máxima - Habitat de troglóbio raro, presença de Troglóbios, não raros endêmicos ou relictos , espécies troglomórficas, espécies com função ecológica importante, alta riqueza de espécies, baixa abundância relativa de espécies, populações residentes de Chiroptera e alta diversidade de substratos orgânicos.	Máxima - dimensões notáveis	Máxima
SL-75	Máxima - Habitat de troglóbio raro, presença de Troglóbios, não raros endêmicos ou relictos , espécies troglomórficas, média riqueza de espécies, baixa abundância relativa de espécies e alta diversidade de substratos orgânicos.	Alta - alta projeção horizontal.	Máxima
SL-76	Alta - Presença de Troglóbios, não raros endêmicos ou relictos , espécies troglomórficas, alta riqueza de espécies , média abundância relativa de espécies e baixa diversidade de substratos orgânicos.	Média - média projeção horizontal, média área e médio volume.	Alta
SL-77	Máxima - Habitat de troglóbio raro, presença de Troglóbios, não raros endêmicos ou relictos , espécies	Média - média projeção horizontal, média área e médio volume.	Máxima

	troglomórficas, média riqueza de espécies, baixa abundância relativa de espécies e alta diversidade de substratos orgânicos.		
SL-78	Alta - Espécies com função ecológica importante, baixa riqueza de espécies ,média abundância relativa de espécies, populações residentes de Chiroptera e alta diversidade de substratos orgânicos.	Alta - Lago ou drenagem subterrânea perene com influência acentuada;	Alta
SL-79	Alta - Presença de Troglóbios, não raros endêmicos ou relictos , espécies troglomórficas, espécies com função ecológica importante, média riqueza de espécies , média abundância relativa de espécies, populações residentes de Chiroptera e alta diversidade de substratos orgânicos.	Alta - alta área.	Alta
SL-80	Máxima - Habitat de troglóbio raro, espécies troglomórficas, média riqueza de espécies, baixa abundância relativa de espécies e baixa diversidade de substratos orgânicos.	Média - média projeção horizontal, média área e médio volume.	Máxima
SL-81	Média -Baixa riqueza de espécies, média abundância relativa de espécies e alta diversidade de substratos orgânicos .	Média - média projeção horizontal, média área e médio volume.	Média
SL-82	Máxima - Habitat de troglóbio raro, presença de Troglóbios, não raros endêmicos ou relictos , espécies troglomórficas, alta riqueza de espécies, baixa abundância relativa de espécies e alta diversidade de substratos orgânicos.	Média - média projeção horizontal, média área e médio volume.	Máxima
SL-83	Alta - Presença de Troglóbios, não raros endêmicos ou relictos , espécies troglomórficas, média riqueza de espécies , baixa abundância relativa de espécies e baixa diversidade de substratos orgânicos.	Média - média projeção horizontal, média área e médio volume.	Alta
SL-84	Média -Baixa riqueza de espécies, média abundância relativa de espécies e baixa diversidade de substratos orgânicos .	Média - média projeção horizontal, média área e médio volume.	Média
SL-85	Alta - Presença de Troglóbios, não raros endêmicos ou relictos , espécies troglomórficas, média riqueza de espécies , baixa abundância relativa de espécies e alta diversidade de substratos orgânicos.	Média - média projeção horizontal, média área e médio volume.	Alta
SL-86	Média -Baixa riqueza de espécies, média abundância relativa de espécies e baixa diversidade de substratos orgânicos .	Média - média projeção horizontal, média área e médio volume.	Média
SL-87	Alta - Presença de Troglóbios, não raros endêmicos ou relictos , espécies troglomórficas, média riqueza de espécies , baixa abundância relativa de espécies e alta diversidade de substratos orgânicos.	Média - média projeção horizontal, média área e médio volume.	Alta
SL-88	Média -Média riqueza de espécies, média abundância relativa de espécies e baixa diversidade de substratos orgânicos .	Média - média projeção horizontal, média área e médio volume.	Média
SL-89	Alta - Presença de Troglóbios, não raros endêmicos ou relictos , espécies troglomórficas, média riqueza de espécies , baixa abundância relativa de espécies e alta diversidade de substratos orgânicos.	Média - média projeção horizontal, média área e médio volume.	Alta
SL-90	Alta - Presença de Troglóbios, não raros endêmicos ou relictos , espécies troglomórficas, média riqueza de espécies , média abundância relativa de espécies e alta diversidade de substratos orgânicos.	Média - média projeção horizontal, média área e médio volume.	Alta
SL-91	Média -Média riqueza de espécies, média abundância relativa de espécies e baixa diversidade de substratos orgânicos .	Média - média projeção horizontal, média área e médio volume.	Média
SL-92	Máxima - Habitat de troglóbio raro, espécies troglomórficas, média riqueza de espécies, média abundância relativa de espécies e alta diversidade de substratos orgânicos.	Média - média projeção horizontal, média área e médio volume.	Máxima
SL-93	Máxima - Habitat de troglóbio raro, presença de Troglóbios, não raros endêmicos ou relictos , espécies troglomórficas, média riqueza de espécies, média abundância relativa de espécies, composição singular da fauna e alta diversidade de substratos orgânicos.	Média - média projeção horizontal, média área e médio volume.	Máxima
SL-94	Máxima - Habitat de troglóbio raro, espécies	Média - média projeção horizontal, média área e	Máxima

	troglomórficas, média riqueza de espécies, média abundância relativa de espécies e alta diversidade de substratos orgânicos.	médio volume.	
SL-95	Máxima - Habitat de troglóbio raro, presença de Troglóbios, não raros endêmicos ou relictos , espécies troglomórficas, alta riqueza de espécies, média abundância relativa de espécies e alta diversidade de substratos orgânicos.	Média - média projeção horizontal, média área e médio volume.	Máxima
SL-96	Máxima - Habitat de troglóbio raro, espécies troglomórficas, média riqueza de espécies, baixa abundância relativa de espécies e baixa diversidade de substratos orgânicos.	Média - média projeção horizontal, média área e médio volume.	Máxima
SL-97	Máxima - Habitat de troglóbio raro, presença de Troglóbios, não raros endêmicos ou relictos , espécies troglomórficas, média riqueza de espécies, média abundância relativa de espécies, populações residentes de Chiroptera e alta diversidade de substratos orgânicos.	Média - média projeção horizontal, média área e médio volume.	Máxima
SL-99	Alta - Presença de Troglóbios, não raros endêmicos ou relictos , espécies troglomórficas, média riqueza de espécies , baixa abundância relativa de espécies e baixa diversidade de substratos orgânicos.	Média - média projeção horizontal, média área e médio volume.	Alta
SL-100	Alta - Espécies troglomórficas, média riqueza de espécies , baixa abundância relativa de espécies e baixa diversidade de substratos orgânicos.	Média - média projeção horizontal, média área e médio volume.	Alta

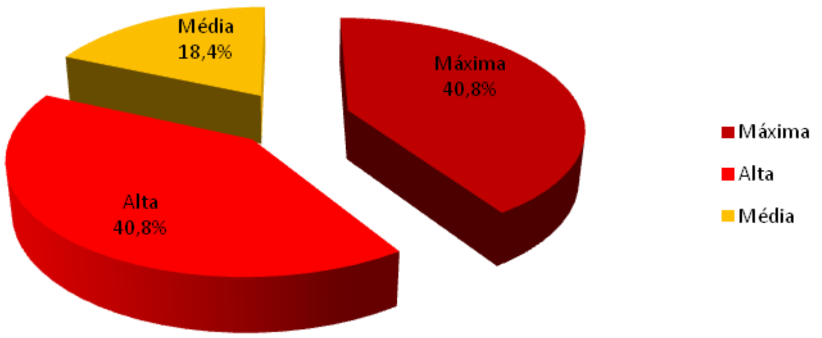

Figura 260 - Gráfico dos graus de relevância final das cavernas de Serra Leste.

Com exceção das quarenta cavernas de relevância máxima, todas as demais poderão, de acordo com a legislação atual, sofrer impactos irreversíveis mediante ações de compensação e anuência do órgão ambiental no processo de licenciamento.

No caso de empreendimento que ocasione impacto negativo irreversível em cavidade natural subterrânea com grau de relevância alto, o empreendedor deverá compensar com duas cavidades naturais subterrâneas, com o mesmo grau de relevância, de mesma litologia e com atributos similares à que sofreu o impacto, que serão consideradas cavidades testemunho (Art. 4o, § 1o do Decreto 6.640).

Conforme previsto no art.4º § 3 o, não havendo, na área do empreendimento, outras cavidades representativas que possam ser preservadas sob a forma de cavidades testemunho, o Instituto Chico Mendes poderá definir, de comum acordo com o empreendedor, outras formas de compensação.

No caso de impactos irreversíveis em cavidades de média relevância, o empreendedor deverá adotar medidas e financiar ações que contribuam para a conservação e o uso adequado do patrimônio espeleológico brasileiro, segundo o Art. 4, § 4o do Decreto Federal N.6.640.

As propostas de compensação estão sendo elaboradas pela Vale e serão oportunamente apresentadas.

De acordo com o Art. 19 da IN Nr. 2, qualquer impacto negativo irreversível nas cavidades deverá ser precedido de registro e armazenamento cartográfico e fotográfico, bem como de inventário e coleta de espeleotemas e elementos geológicos e biológicos representativos do ecossistema cavernícola, compreendendo o resgate, transporte adequado e a destinação a coleções científicas institucionais.

7. SUGESTÕES DE ENCAMINHAMENTO

O Plano Diretor do projeto Serra Leste (2 milhões de toneladas) foi inicialmente proposto de forma a respeitar todas as ocorrências de cavernas conhecidas na área. No entanto, quando da sua concepção, não havia precisão em relação a coordenada de localização de algumas cavidades, dada as condições de sua coleta (vide itens 2.2 e 5.1). Após a realização do diag nóstico geoespeleológico e bioespeleológico, foi possível rever a localização das cavernas com maior acurácia. Uma nova plotagem identificou sobreposições entre o perímetro de proteção de 250 metros de cinco (Tabela 25) cavidades e pilhas e cavas projetadas do Plano Diretor (Figura 261).

Tabela 25 – Cavernas com sobreposição entre o perímetro de proteção (250 m) e estruturas projetadas do Plano Diretor do projeto Serra Leste.

Caverna	Relevância	Área Interferida (ha)
SL-041	Média	0,01
SL-043	Alta	2,15
SL-045	Máxima	0,25
SL-057	Máxima	1,30
SL-071	Alta	0,85

Diante disso, a primeira sugestão é a de realizar, durante a fase de LI, estudos complementares que permitam avaliar o real perímetro de proteção necessário a proteção da integridade física e biológica dessas cinco cavernas. Dados preliminares de diversas cavernas ferríferas localizadas tanto na Serra de Carajás/PA quanto no Quadrilátero Ferrífero/MG, tem indicado que esse perímetro pode ser reduzido. Segere-se testar essa hipótese para as cavernas de Serra Leste.

No entanto, sabe-se que a viabilidade econômica do empreendimento em questão prescinde da ampliação das cavas projetadas. Como podemos observar no diagnóstico apresentado no item 6, os atributos bioespeleológicos são os que mais restringiram em termos da classificação da relevância das cavidades. A Tabela 26 apresenta sugestões de encaminhamento para o projeto Serra Leste no que diz respeito a continuidade dos estudos no tema bioespeleológico.

Tabela 26 – Sugestões de encaminhamento para o tema bioespeleologia no projeto Serra Leste.

Prosposta	Dificuldade	Dedobramentos
Comparar o material de Serra Leste no contexto regional	- Tempo;<br>- Incertea em relação a distribuiçãoo das espécies no ambiente subterrâneo.	- Custo baixo;<br>- Possível “quebra”do *status* de espécie troglóbia para as espécies distribuídas em ambas as áreas.
Paracer de taxonomista especialista	- Tempo;<br>- Incerteza em relação a afirmação ou não da condição de espécie troglóbia	- Possível “quebra”do *status* de espécie troglóbia para as espécies distribuídas em ambas as áreas;<br>- Dificuldade em afirmar ou não o *status* de troglóbio.
Inventário de biodiversidade epígea	Tempo;<br>Demanda de recursos humanos e financeiros.	- Quebra do status de troglóbio para espécies encontradas no ambiente epígeo;<br>- Não encontrar tais espécies no ambiente epígeo.
Novos inventários da fauna cavernícola	- Tempo;<br>- Demanda de recursos humanos e financeiros.	- Quebra de relevância de máxima para alta (troglóbios não raros);<br>- Encontro de novas espécies troglomórficas.

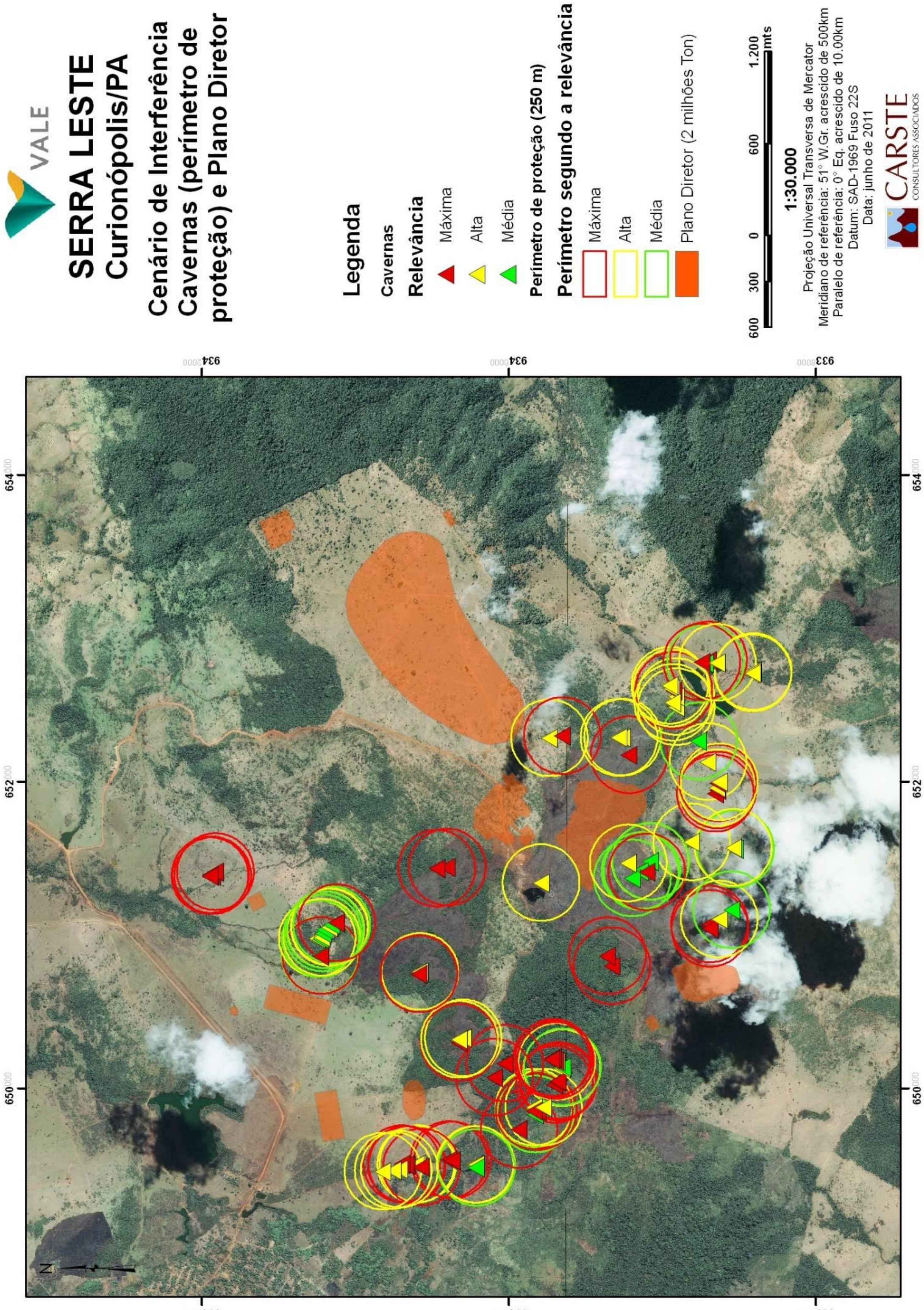

Figura 261 - Cenário de interferências entre o Plano Diretor e as cavernas de Serra Leste.

8. REFERÊNCIAS BIBLIOGRÁFICAS

Geoespeleologia

ARAÚJO O.J.B., MAIA R.G.N. 1991. Programa levantamentos geológicos básicos do Brasil. Projeto especial maps de recursos minerais, de solos e de vegetação para a área do Programa Grande Carajás. Subprojeto Recursos Minerais. Serra dos Carajás, Folha SB.22-Z-A. Brasília: DNPM/Companhia de Pesquisa e Recursos Minerais, 152p.

ARAÚJO O.J.b., MAIA R.G.N., JOÃO X.S.J., COSTA J.B.S. 1988. A megaestruturação arqueana da folha Serra dos Carajás. In: Congresso Latino-Americano de Geologia, 7, Belém, Anais, 1: 324-328.

AULER, A.S; PILÓ, L.B. 2005. Introdução às cavernas em minério de ferro e canga. O Carste 17(3): 70-72.

ATZINGEN, V. N.; CRESCÊNCIO, G. 1999. Estudos espeleológicos em Serra Pelada, Curionópolis – PA. Boletim Informativo da Fundação Casa da Cultura de Marabá, 1: 63-72.

BEISIEGEL V.R., BERNARDELLI A.L., DRUMMOND N.F., RUFF A.W., TREMAINE J.W. 1973. Geologia e recursos minerais da Serra dos Carajás. Revista Brasileira de Geociências, 3(4): 215-242.

BRASIL. INSTRUÇÃO NORMATIVA MMA Nº 2, DE 20 DE AGOSTO DE 2009. Dispões sobre a metodologia e critérios para classificação de cavidades naturais subterrâneas e dá outras providências.

BRASIL. DECRETO Nº 6.640, DE 7 DE NOVEMBRO DE 2008. Dá nova redação aos arts. 1º, 2º, 3º, 4º e 5º e acrescenta os arts. 5-A e 5-B ao Decreto no 99.556, de 1º de outubro de 1990, que dispõe sobre a proteção das cavidades naturais subterrâneas existentes no território nacional. Publicação DOU: Seção 1 - Nº 218, 10/11/2008.

BRASIL. PORTARIA nº 230, de 17 de dezembro de 2002, do Instituto do Patrimônio Histórico e Artístico Nacional – IPHAN, dispõe sobre regras de proteção e preservação do patrimônio arqueológico nacional.

DOCEGEO (Rio Doce Mineração S. A.). Revisão litoestratigráfica da Província Mineral de Carajás. Anexos XXX. Belém: Congresso Brasileiro de Geologia,1988. 11- 54p.

DORR, J.N. 1969. Physiographic, Stratigraphic and Structural Development of the Quadrilátero Ferrífero, Minas Gerais, Brazil. United States Geological Survey Professional Paper 641-A, 110p.

GIBBS A.K., WIRTH K.R., HIRATA W.K., OLSZEWSKI Jr. W.J. 1986. Age and composition of the Grão Pará Group volcanic, Serra dos Carajás. Revista Brasileira de Geociências, 16(2): 201-211.

GRUPO ESPELEOLÓGICO DE MARABÁ/FUNDAÇÃO CASA DE CULTURA DE MARABÁ – GEM/FCCM. 2004. Espeleologia: relatório da 1a etapa de campo. Carajás – Serra Sul/Corpo D. Relatório Inédito. 47p.

GRUPO ESPELEOLÓGICO DE MARABÁ/FUNDAÇÃO CASA DE CULTURA DE MARABÁ – GEM/FCCM. 2005. Espeleologia: relatório da 2a etapa de campo. Carajás – Serra Sul/Corpo D. Relatório Inédito. 51p.

GRUPO ESPELEOLÓGICO DE MARABÁ/FUNDAÇÃO CASA DE CULTURA DE MARABÁ – GEM/FCCM. 2008. Relatório de Caracterização e Documentação Básica das Cavidades Naturais da Área de Instalação das Estruturas de Apoio (Usina, Barragem e Instalações) ao Projeto Serra Sul, Serra dos Carajás, Canaã dos Carajás – PA. Relatório Inédito. 210 p.

GUILD, P.W. 1957. Geology and Mineral Resouces of the Congonhas District. United States Geological Survey Professional Paper 290, 90 p.

HENWOOD, W.J. 1871. On the gold mines of Minas Geraes. – in: Observations on metalliferous deposits. Transactions of the Royal Geological Society of Cornwall 8: 168-370.

MACAMBIRA, M.J.B.; RAMOS, J.F.F.; ASSIS, J.F.P.; FIGUEIRAS, A.J.M. 1990. Projeto Serra Norte e Projeto Pojuca. Convênio SEPLAN/DOCEGEO/UFPA/DNPM. Relatório final. 150 p.

MAURITY, C.W. & KOTSCHOUBEY, B. 1995. Evolução recente da cobertura de alteração no Platô N1 – Serra dos Carajás-PA. Degradação, pseudocarstificação, espeleotemas. Boletim do Museu Paraense Emilio Goeldi. Série Ciências da Terra 7: 331-362.

MAURITY, C.W.; KOTSCHOUBEY, B. 2005. Evolução recente da cobertura de alteração no Platô N1 – Serra dos Carajás-PA. Degradação, pseudocarstificação, espeleotemas. O Carste 17(3): 78-91.

PILÓ, L.B; ANDRADE, R. 2006. Estudos espeleológicos na área da Mina N5S, Serra dos Carajás. CVRD/PRCZ. 170p. (inédito).

PILÓ, L. B. & AULER, 2005. Cavernas de minério de ferro e canga de Capão Xavier, Quadrilátero Ferrífero, MG. O Carste 17(3): 92-105.

PILÓ, L. B. & AULER, 2006. Estudos geoespeleológicos na mina do Pico do Itabirito, MG. Minerações Brasileiras Reunidas - MBR. Relatório Inédito, 380p.

PILÓ, L.B; AULER, A.S. 2007. Mineralogia de espeleotemas das grutas de minério de ferro de Capão Xavier, Quadrilátero Ferrífero, Minas Gerais. II Encontro Brasileiro de Estudos do Carste – 26 a 28 de julho de 2007 – Instituto de Geociências, IG-USP. Caderno de Resumos; pagina 32.

PILÓ, L.B. & AULER, A.S. 2009. Geoespeleologia das cavernas em rochas ferríferas da região de Carajás, PA. XXX Congresso Brasileiro de Espeleologia. Montes Claros. Anais...Montes Claros: SBE, 2009. p. 181-186.

PINHEIRO, R.V.L.; HENRIQUES, A.L.; SILVEIRA, L.T.; MAURITY, C.W. 1985. Considerações Preliminares sobre a Espeleologia da Serra dos Carajás (PA). Grupo Espeleológico Paraense. Relatório inédito, 38p.

PINHEIRO, R.V.L.; MAURITY, C.W. 1988. As cavernas em rochas intempéricas da Serra dos Carajás (PA) – Brasil. Anais 1º Congresso de Espeleologia da América Latina e do Caribe, Belo Horizonte, pp. 179-186.

PINHEIRO R.V.L., HOLDSWORTH R.E. 1997. The structure of the Carajás N-4 ironstone deposit and associated rocks: relationship to the archaean strike-slip tectonics and basement reactivation in the Amazon region, Brazil. Journal of South American Earth Sciences, 10(3-4): 305-319.

PISSIS, N.A. 1842. Mémoire sur la position géologique des terrains de la partie australe du Brésil et sur lês soulèvementes qui, à diverses époques, ont changé le relief de cette contrée. Memoire de L' Institute de France 10: 353-413.

RIBEIRO, D.T. 2003. Enriquecimento Supergênico de Formações Ferríferas Bandadas: Estruturas de Colapso e Desordem. Universidade Federal do Rio de Janeiro, 124 p. (Tese de doutorado).

RUBBIOLI, E. & MOURA, V. 2005. Mapeamento de cavernas: guia prático. São Paulo. Redespeleo Brasil. 92 p.

SIMMONS, G.C. 1963. Canga caves in the Quadrilátero Ferrífero, Minas Gerais, Brazil. The National Speleological Society Bulletin 25: 66-72.

SPIER, C.A. 2005. Geoquímica e gênese das formações ferríferas bandadas e do minério de ferro da mina de Águas Claras, Quadrilátero Ferrífero, MG. Instituto de Geociências, USP. (Tese de Doutoramento).

TASSINI, R. 1947. Verdades Históricas e Pré-históricas de Belo Horizonte. Belo Horizonte, editora do autor.

TOLBERT, G.E.; TREMAINE, J.W.; MELCHER, G.C.; GOMES, C.B. 1971. The recently discovered Serra dos Carajás iron deposits, northern Brazil. Economic Geology 66: 985-994.

TRENDALL A. F., BASEI M.A.S., LAETER J. R., Nelson D. R. 1998. SHRIMP zircon U-Pb constraints on the age of the Carajás Formation, Grão Pará Group, Amazon Craton. Journal of South American Earth Sciences, 11(3): 265-277.

Bioespeleologia

ARECHAVALETA, M., L. L. SALA & P. OROMI, 1999. La fauna invertebada de la Cueva de Felipe Reventón (Icod de los Vinos, Tenerife, Islas Canarias). Vieraea 27:229-244.

AULER A. S. & L. B. PILÓ. 2005. Introdução às cavernas em minério de ferro e canga. O Carste, 17(3):70-72.

AULER A. S. 2006. Relevância de cavidades naturais subterrâneas: contextualização, impactos ambientais e aspectos jurídicos. Relatório técnico, Ministério de Minas e Energia (MME) Brasília, 166 pp. http://www.mme.gov.br

BAHIA, G. R. 2007. Sucessão ecológica em guano de morcegos insetívoros em cavernas. Dissertação apresentada ao Instituto de Ciências Biológicas da Universidade Federal de Minas Gerais, como requisito parcial para a obtenção do título de Mestre em Ecologia, Conservação e Manejo da Vida Silvestre. 117 pp.

CHIVIAN, D., BRODIE, E. L., ALM E. J., CULLEY, D. E., DEHAL. P. S., DESANTIS, T. Z., GIHRING, T. M., LAPIDUS, A., LIN, L.H., LOWRY, S. R., MOSER D. P., RICHARDSON P. M., SOUTHAM , G., WANGER G., PRATT, L. M., ANDERSEN, G. L., HAZEN, T. C., BROCKMAN, F. J., ARKIN, A. P. AND T. C. ONSTOTT. 2008. Environmental Genomics Reveals a Single-Species Ecosystem Deep Within Earth, *Science* 10 (322):275 – 278

CULVER D. C. & W. B. WHITE 2004. Encyclopedia of caves, Publisher ElsevierAcademic Press, 654 pp.

CULVER DC, PIPAN T 2009. The biology of caves and other subterranean habitats. Library of Congress Cataloging in Publication Data, Oxford University Press, Oxford

CULVER, D. C. & B. SKET. 2002 Biological Monitoring in Caves, Acta carsologica, 31(1): 55-64

CULVER, D. C. 1982. Cave Life. Evolution and Ecology. Harvard University Press. Cambridge, Massachussets and London, England. 189 pp.

DECU, V., JUBERTHIE C. & E, NITZU. 1998. Coleoptera (Varia). In: Juberthie, C. &Decu, V. (Org.). Encyclopaedia Biospeologica. 1 ed. França: Moulis: Société de Biospéologie, volume II, 113-1173pp.

FERREIRA R. L. 2004. A medida da complexidade ecológica e suas aplicações na conservação e manejo de ecossistemas subterrâneos. Tese apresentada ao programa de pós-graduação em Ecologia Conservação e Manejo da Vida Silvestre do Instituto de Ciências Biológicas da Universidade Federal de Minas Gerais, Belo Horizonte, Minas Gerais, Brasil, 158pp.

FERREIRA R. L. 2005. A vida subterrânea nos campos ferruginosos. O Carste. 3(17):106-115.

FERREIRA R. L. 2006. Caracterização de ecossistemas subterrâneos do Complexo Mina do Pico (Itabirito, MG), Minerações Brasileiras Reunidas, MBR. Relatório Técnico 123 pp. drops@ufla.br.

FERREIRA, R. L. 2000. Lixeiras de Formigueiros: Recursos Adicionais em Sistemas Cavernícolas ? O Carste, 3(12):154-158

FERREIRA, R. L., & MARTINS, R. P. 2001. Cavernas em risco de ‘extinção’. Ciência Hoje, 29, p.20–28.

FERREIRA, R. L., SOUZA-SILVA, M. E BERNARDI, L. F. O. 2009. Diagnóstico do conhecimento da biodiversidade de invertebrados terrestres em Minas Gerais: contexto Bioespeleológico. Biota Minas: Diagnóstico do conhecimento sobre a biodiversidade no Estado de Minas Gerais - Subsídio ao programa biota minas. Glaucia Moreira Drumond, Cássio Soares Martins e Fábio Vieira Editores, Fundação biodiversitas

FERREIRA, R.L. & MARTINS, R.P. 1998. Diversity and distribution of spiders associated with bat guano piles in Morrinho cave (Bahia State, Brazil). Diversity and Distributions, 4:235-241.

FERREIRA, R.L. & MARTINS, R.P. 1999(a). Guano de morcegos: fonte de vida em cavernas. Ciência Hoje 25(146):34-40

FERREIRA, R.L. & MARTINS, R.P. 1999(b). "Trophic Structure and Natural History of Bat Guano Invertebrate Communities with Special Reference to Brazilian Caves". Tropical Zoology 12(2):231-259.

FERREIRA, R.L. & POMPEU, P.S. 1997. Riqueza e diversidade da fauna associada a depósitos de guano na gruta Taboa, Sete Lagoas, Minas Gerais, Brasil. *O Carste*, 9(2): 30-33.

GILBERT, J., DANIELPOL, D. L. & STANFORD, J. A. 1994. Groundwater Ecology. Academic Press Limited, San Diego, California. 571 pp.

GOMES, F. T. M. C, R. L. FERREIRA & C. M. JACOBI 2000. Comunidade de artrópodes de uma caverna calcária em área de mineração: composição e estrutura, Revista Brasileira de Zoociências, 1(2):77-96.

GUNN J 2004 Encyclopedia of caves and karst science. Taylor & Francis Books Inc, New York

HAMILTON-SMITH. E. 1965. Pselaphidae (Coleoptera) from Australian caves, South Australian Museum, Adelaide, S.A, 70-71

HARRISON S, ROSS SJ, LAWTON JH (1992) Beta diversity on geographic gradients in Britain. J Anim Ecol 61:151–158

HOLSINGER, R. & CULVER, D. C. 1988. The Invertebrate Cave Fauna of Virginia and a Part of Eastern Tennessee: Zoogeography and Ecology. *Brimleyana*, 14. 1-162.

HOSE, L. D., A. N. PALMER, M. V. PALMER, D. E. NORTHUP, P. J. BOSTON, AND H. R. DUCHENE. 2000. Microbiology and geochemistry in a hydrogen-sulphide-rich karst environment. Chemical Geology 169:399-423.

HOWARTH, F. G., JAMES, S. A., MCDOWELL, W., PRESTON D. J. & C. T. IMADA. 2007. Identification of roots in lava tube caves using molecular techniques: implications for conservation of cave arthropod faunas. Journal of Insect Conservation. 3(11): 251-261.

HOWARTH, F.G. 1983. Ecology of cave arthropods. Annual Rewiew of Entomology 28:365-389.

JASINSKA, E.J.; KNOTT, B. & MCCOMB, A.J. 1996. Hot mats in groundwater: a fauna-rich cave habitat. Journal of American Benthological Society 15(4):508-519.

KOLEFF P, GASTON KJ, LENNON JJ (2003) Measuring beta diversity for presence–absence data. J Anim Ecol 72:367–382

LAKE, P.S. 2000. Disturbance, patchiness, and diversity in streams. Journal of the North American Benthological Society 19, 573–592.

LEYS, R., WATTS, C. H. S., COOPER S. J. B., & W. F. 2003. Humphreys. Evolutionofsubterraneandivingbeetles (Coleoptera: Dytiscidae: Hydroporini, Bidessini) in thearid zone ofAustralia, Evolution, 57(12):2819-2834

MARGULES C. R & R. L. PRESSEY. Systematic conservation planning, Nature 405:243-253.

MAURITY, C. W. & B. KOTSCHOUBEY. 2005. Evolução da cobertura de alteração no platô N1 Serra dos Carajás, Pa. Degradação, pseudocarstificação, espeleotemas. O Carste. Vol.17(3):78-91.

MOLDOVAN O. T. 2004. Beetles, In Encyclopedia of caves, Culver D. C. & W. B. White editors Publisher ElsevierAcademic Press, 45- 51pp.

PARK. O. 1960. -Cavernicolous Pselaphid beetles of the United States. Amer. Mid/. Nur. 64: 66-104

PECK S. B. 1976. The effect of cave entrances on the distribution of cave-inhabiting terrestrial arthropods. International Journal of Speleology. 8, 309–21.

PILÓ L. B. & A. S. AULER. 2005. Cavernas em minério de ferro e canga de capão Xavier, quadrilátero ferrífero, MG. O Carste. Vol.17(3):92-105.

PINTO-DA-ROCHA, R. 1995. Sinopse da fauna cavernícola do Brasil (1907 - 1994). Papéis Avulsos de Zoologia, 39(6), 61-163.

POGGI, R., DECU, V. & C. JUBERTHIE 1998. Coleoptera Peselaphidae. In: Juberthie, C. &Decu, V. (Org.). Encyclopaedia Biospeologica. 1 ed. França: Moulis: Société de Biospéologie, volume II, 1138-1146pp.

PROUS, X, FERREIRA, R. L. & R. P. MARTINS. 2004. Ecotone delimitation: epigean-hypogean transition in cave ecosystems Austral Ecology 29, 374–382

ROMERO A. & M. GREEN 2005. The end of regressive evolution: examining and interpreting the evidence from cave fishes. Journal of Fish Biology. 67:3-32.

SARBU, S.M; KANE, T.C. & KINKLE, B.K. 1996. A chemoautotrophically basead cave ecosystem. Science (272):1953-1955.

SCHMALFUSS, H. 2003. World catalog of terrestrial isopods (Isopoda, Oniscidea). Stuttgarter Beiträge zur Naturkunde A 654: 1- 341. Available in the World Wide Web at: http:// www.naturkundemuseum-bw.de/stuttgart/projekte/ oniscidea-catalog/Cat_terr_isop.pdf [Accessed in 20/VI/2007].

SCHNEIDER, K. CHRISTMAN, M. C., & W. F. FAGAN. 2011. The influence of resource subsidies on cave invertebrates: results from an ecosystem-level manipulation experimente, Ecology, 92(3):765–776.

SCHOTTE, C. B. BOYKO, N. L. BRUCE, J. MARKHAM, G. C. B. POORE, S. TAITI & G. D. F. WILSON. 2010 "World List of Marine, Freshwater and Terrestrial Isopod Crustaceans". World Register of Marine Species. http://www.marinespecies.org/isopoda/index.php.

SHARRATT N. J., M. PICKER AND M. SAMWAYS. (2000). The invertebrate fauna of the sandstone of the cave s of the Cape Penisula (South Africa): patterns of endemism and conservation priorities. Biodiversity and Conservation 9: 107-143.

SHEAR, W. A. 1969. A synopsis of the cave millipedes of the United States, with an illustrated key to genera. Psyche 76, 126–143.

SIMMONS G. C. 1963. Canga caves in quadrilátero ferrífero, Minas Gerais, Brasil. The National Speleological Society Bullettin 25: 66-72.

SIMON, K.S., T. PIPAN, & D.C. CULVER . 2007 A conceptual model of the flow and distribution of organic carbon in caves. Journal of Cave and Karst Studies, v. 69, no. 2, p. 279–284.

SOUZA-SILVA, M, MARTINS R. P. & R. L. FERREIRA 2011. Cave lithology determining the structure of the invertebrate communities in the Brazilian Atlantic Rain Forest. Biodiversity and Conservation DOI 10.1007/s10531-011-0057-5

SOUZA-SILVA, M. 2003. Dinâmica de disponibilidade de recursos alimentares em uma caverna calcária. Dissertação de mestrado. Universidade Federal de Minas Gerais/Pós-Graduação em Ecologia Conservação e Manejo da Vida Silvestre.76pp.

SOUZA-SILVA, M. 2008. Ecologia e conservação das comunidades de invertebrados cavernícolas na mata atlântica brasileira. Tese de doutorado. Universidade Federal de Minas Gerais/Pós-Graduação em Ecologia Conservação e Manejo da Vida Silvestre, 217pp.

SPANGLER, P.J. & V. DECU 1998. Coleoptera aquática. In: Juberthie, C. &Decu, V. (Org.). Encyclopaedia Biospeologica. 1 ed. França: Moulis: Société de Biospéologie, volume II, 1030-1046pp.

TRAJANO, E., GOLOVATCH S. I., GEOFFROY, J. J., PINTO-DA-ROCHA R. & C. S. FONTANETTI. 2000. Synopsis of Brazilian cave-dewelling millipides (diplopoda). Papéis Avulsos de Zoologia, 18(41):259-287.

TRAJANO, E. & M. E. BICHUETTE. 2010. Diversity of Brazilian subterranean invertebrates, with a list of troglomorphic taxa. Subterranean Biology 7: 1-16.

TRAJANO, E. & MOREIRA, J.R.A. 1991. Estudo da fauna de cavernas da Província Espeleológica Arenítica Altamira-Itaituba, Pará. Revista Brasileira de Biologia, 51(1):13-29.

VÍT, S. & P. HLAVÁ. 2005. New cavernicolous ant-like beetle of the genus *Euconnus* (subg. *tetramelus*) from Croatia (Coleoptera: Scydmaenidae), Nat. Croat. 1(14): 29-38.

WEINSTEIN, P. & SLANEY, D. 1995. Invertebrate faunal survey of Rope Ladder cave, Northern Queensland: a comparative study of sampling methods. Journal of the Australian Entomological Society 34: 233–236

WOLDA, H. 1981. Similarity indices, sample size and diversity. Oecologia 50(3): 296-302.

ZAMPAULO, R. A. 2010. Diversidade de invertebrados na província espeleológica de Arcos, Pains, Doresópolis (MG): Subsídios para a determinação de áreas prioritárias para a conservação. Dissertação apresentada ao Programa de Pós-graduação em Ecologia Aplicada da Universidade Federal de Lavras, MG 190 pp.

ANEXO I – MAPAS DAS CAVERNAS

ANEXO II – FICHAS DE GEOESPELEOLOGIA

ANEXO III – FICHAS DE BIOESPELEOLOGIA

ANEXO IV – AMOSTRA LOCAL E REGIONAL
