# Supplementary material for: Reconciling Mining with the Conservation of Cave Biodiversity: A Quantitative Baseline to Help Establish Conservation Priorities
Source: PLoS One. 2016 Dec 20;11(12):e0168348. doi: 10.1371/journal.pone.0168348 (PMC5173368; doi:10.1371/journal.pone.0168348)
Supplement: S1 Dataset — (ZIP) [file pone.0168348.s002.zip › Serra Leste Reports/Serra_Leste_2013.pdf]

Estudos Espeleológicos Serra Leste
Geoespeleologia, Bioespeleologia, Análise de Relevância
e Atualização da Relevância

Diagnóstico Geoespeleológico

Diagnóstico Bioespeleológico

Análise de Relevância

Atualização da Análise de Relevância

Projeto Serra Leste

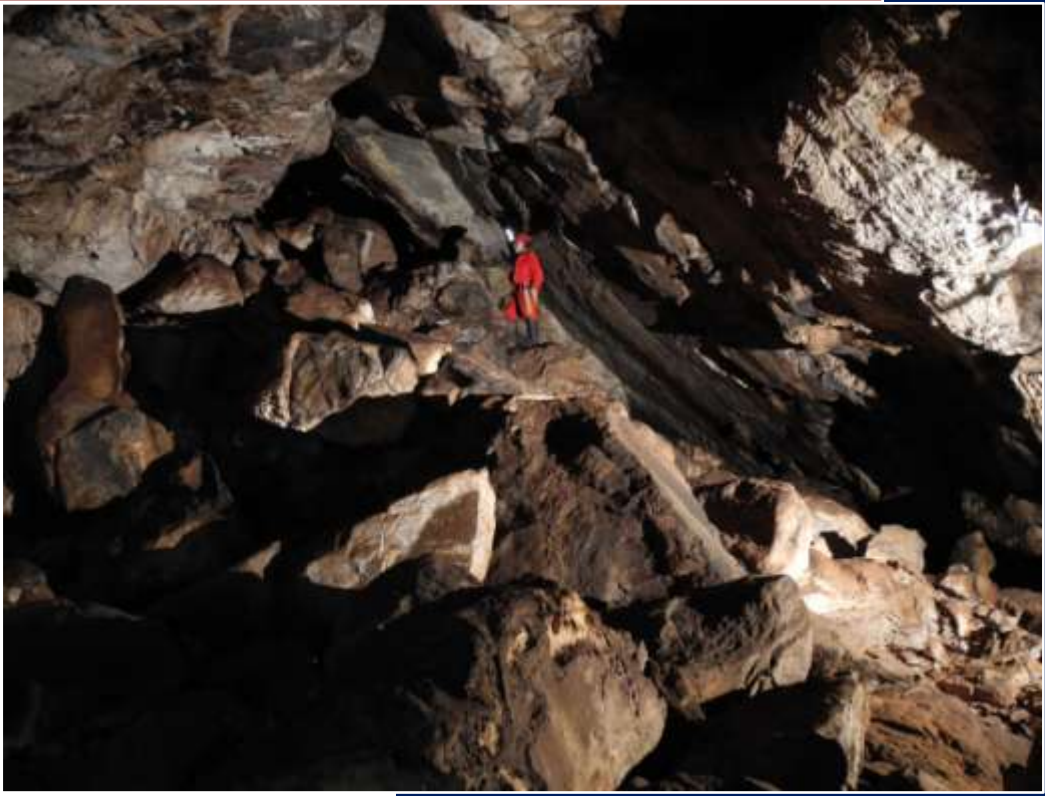

Agosto de 2013

PROJETO SERRA LESTE

Diagnóstico Geoespeleológico
Diagnóstico Bioespeleológico
Análise de Relevância
Atualização da Análise de Relevância

Augusto Auler, PhD
Marina Leão, MSc
Dr Rodrigo Lopes
Dr Marconi Silva
Ana Paula Bueno, MSc

Belo Horizonte, agosto de 2013

EMPRESA RESPONSÁVEL POR ESTE RELATÓRIO	
**Razão social:** Carste Consultores Associados Ltda<br>**CNPJ:** 08.000.418/0001-00<br>**Endereço:** Rua Brasópolis 139, Floresta<br>Belo Horizonte/ MG<br>CEP 30150-170	**http:** www.carste.com.br<br>**Telefone:** (31) 2552-9976

EQUIPE TÉCNICA DA CARSTE CONSULTORES ASSOCIADOS		
**ESTA EQUIPE PARTICIPOU DA ELABORAÇÃO DESTE DOCUMENTO E RESPONSABILIZA-SE TECNICAMENTE POR SUAS ÁREAS**		
**TÉCNICO**	**FORMAÇÃO / REGISTRO PROFISSIONAL**	**RESPONSABILIDADE NO PROJETO**
Augusto Sarreiro Auler	Geólogo<br>MG 72.076/D	Coordenação geral; campo; análise; relatório final.
Marina Ribeiro Leão	Geógrafa<br>MG 144.354/D	Campo; análise; relatório final.
Rodrigo Lopes Ferreira	Biólogo<br>CRBio 13.978/4-D	Análise e relatório final
Marconi Souza Silva	Biólogo<br>CRBio 16.755/4-D	Análise e relatório final
Ana Paula Bueno da Silva	Bióloga<br>CRBio 62.303/4-D	Atualização da Análise de Relevância

EMPRESA RESPONSÁVEL PELO PROJETO	
**Razão social:** Vale S/A -Mina de Carajás<br>**CNPJ:** 33.592.510/0370-74<br>**Endereço:** Área Serra dos Carajás S/N - Serra dos Carajás / PA	**Contato:** Daniele Freitas<br>**Telefone:** (94)88019828 / 33275869<br>**E-mail:** daniele.freitas.goncalves@vale.com

SUMÁRIO

1. INTRODUÇÃO ... 14
2. METODOLOGIA ... 15
2.1. Levantamento bibliográfico ... 15
2.2. Prospecção espeleológica ... 15
2.3. Espeleotopografia ... 15
2.4. Geoespeleologia ... 15
2.4.1. Campanhas de campo ... 15
2.4.2. Análise litológica e das estruturas ... 15
2.4.3. Análise morfológica ... 15
2.4.4. Hidrologia ... 16
2.4.5. Depósitos clásticos, orgânicos e químicos ... 16
2.5. Bioespeleologia ... 16
2.5.1. Procedimentos de coleta ... 16
2.5.2. Triagem e identificação dos taxa ... 17
2.5.3. Determinação de troglomorfismos ... 17
2.5.4. Determinação de raridade ... 18
2.5.5. Análises ... 19
3. ESPELEOGÊNESE EM ROCHAS QUARTZÍTICAS E FERRÍFERAS ... 20
3.1. Cavernas em quartzito ... 20
3.2. Cavernas em formações ferríferas ... 21
3.3. Características ambientais e tróficas das cavernas ... 22
3.4. Relações ecológicas e evolutivas dos animais de cavernas ... 22
3.5. Características ecológicas das cavernas ferruginosas ... 23
3.6. O conhecimento e a conservação da fauna cavernícola ... 24
4. INSERÇÃO DAS CAVERNAS NO CONTEXTO GEOLÓGICO, GEOMORFOLÓGICO E FITOGEOGRÁFICO ... 25
4.1. Geologia Regional ... 25
4.2. Geomorfologia ... 29
4.3. Fitogeografia ... 30
5. APRESENTAÇÃO DOS RESULTADOS ... 31
5.1. Estudos Geoespeleológicos ... 31

5.1.1. Inserção das cavernas na paisagem 75
5.1.2. Litologia e estruturas geológicas 79
5.1.3. Espeleometria 88
5.1.4. Morfologia 92
5.1.5. Hidrologia 100
5.1.6. Depósitos clásticos e orgânicos 103
5.1.7. Depósitos químicos 107
5.1.8. Aspectos espeleogenéticos 112
5.1.9. Aspectos sócio econômicos, culturais e estado de conservação 118
5.1.10. Considerações finais a respeito dos atributos geoespeleológicos 120
5.2. Estudos Bioespeleológicos 121
5.2.1. Caracterização faunística geral das cavidades 121
5.2.2. Espécies troglomórficas 129
5.2.3. Descrição específica de cada cavidade 137
5.2.4. Avaliação preliminar do patrimônio espeleológico 190
5.2.5. Análises ecológicas 190
6. ANÁLISE DE RELEVÂNCIA DAS CAVERNAS DE SERRA LESTE 194
6.1. Metodologia 195
6.1.1. Discriminação litológica 195
6.1.2. Escala de análise 195
6.1.3. Grau de relevância 197
6.2. Resultados da análise de relevância das cavernas de Serra Leste 198
6.3. Síntese da análise de relevância 204
6.4. Considerações finais acerca da análise de relevância 204
7. ATUALIZAÇÃO DA ANÁLISE DE RELEVÂNCIA DAS CAVIDADES DE SERRA LESTE 205
7.1. Metodologia 207
7.1.1. Estudo de Coleta no Entorno de Cavidades 207
7.1.2. Estudo de Pareamento de Espécies Troglomórficas 208
7.2. Novos resultados para os atributos biológicos 210
7.3. Síntese da análise de relevância 215
7.4. Considerações finais acerca da atualização da análise de relevância 217
8. REFERÊNCIAS BIBLIOGRÁFICAS 219
8.1. Geoespeleologia 219

8.2. Bioespeleologia 221

MINUTA

ÍNDICE DE FIGURAS

Figura 1.1 - Localização da área de estudo nas escalas local e regional. 14
Figura 4.1 - Mapa geológico de Serra Leste. Adaptado de DOCEGEO, 1988. 27
Figura 4.2 - Perfil geológico e coluna estratigráfica esquemáticos de Serra Leste. O mergulho e a espessura das camadas foram adaptados de Costa *et al*. 2007.. 28
Figura 4.3 - Bloco diagrama esquemático da Província Mineral de Carajás e arredores. A escala horizontal é aproximada e a vertical exagerada, a charneira da dobra de Carajás apresenta caimento entre 20 e 35º W. Fonte: Rosière et al 2004 e 2005. 29
Figura 5.1 - Localização das cavernas em estudo na Serra Leste. 31
Figura 5.2 - Perfil esquemático de inserção das cavidades na Serra Leste. 75
Figura 5.3 - Modelo digital de elevação de parte da Serra Leste. Exagero vertical: 3 vezes. 75
Figura 5.4 - À esquerda, conjunto de cavidades desenvolvidas em quartzito ao longo da vertente. À direita, caverna SL-114, em rocha ferrífera, situada a jusante de estrada. Fonte: Geoeye, 2012. 76
Figura 5.5 - Cavidades alojadas em rochas ferríferas situadas nas cotas altimétricas mais elevadas. A caverna SL-122 insere-se ao longo de curso fluvial que drena o interior da serra. Fonte: Geoeye, 2012. 76
Figura 5.6 - (A) Visão parcial do sistema fluvial interiorano; (B) Visão parcial do sistema da borda externa da serra.. 77
Figura 5.7 - Conjunto de cavidades inseridas em curso fluvial externo à borda da serra. Fonte: Geoeye, 2012. 77
Figura 5.8 - Dispersão das cavernas anteriormente estudadas segundo intervalo hipsométrico. Extraído de CARSTE, 2011a. 78
Figura 5.9 - Percentual de cavernas segundo intervalo hipsométrico. 78
Figura 5.10 - Dispersão das cavernas em estudo segundo intervalo hipsométrico. 78
Figura 5.11 - Litologia encaixante das cavidades de Serra Leste. 79
Figura 5.12 - Clastos mal selecionados compõem a canga na caverna SL-122: (A) grânulos e seixos angulosos se destacam, além de calhaus e blocos de constituição ferruginosa (B). 80
Figura 5.13 - Canga química: (A) sustenta feições verticalizadas na caverna SL-116; (B) apresenta porções de concentração goethítica na SL-115. 81
Figura 5.14 - Canga detrítica preenche fratura na cavidade SL-121. 82
Figura 5.15 - Ferricrete composto por sedimentos de granulometria fina e apresentando aspecto vugular: (A) na cavidade SL-103 e (B) (C) e (D) na caverna SL-112. 83
Figura 5.16 - Teto predominantemente composto por clastos angulosos de constituição quartzítica: (A) e (B) SL-105 e (C) SL-110. 83
Figura 5.17 - Contato litológico entre rocha ferrífera e rocha quartzítica: (A) SL-103 e (B) SL-111. As linhas amarelas indicam o local aproximado do contato. 84
Figura 5.18 - (A) Quartzito de aspecto vugular e coloração rósea na caverna SL-107.(B) Camada de quartzito xistoso na SL-102. 85
Figura 5.19 - (A) Vermiculações em bloco da cavidade S-104 e (B) no teto da caverna SL-131. 85
Figura 5.20 - Caneluras oblíquas na cavidade SL-104: (A) em visão ampla e (B) em destaque. . 86
Figura 5.21 - Diagrama de roseta para as medidas de fraturas. 87
Figura 5.22 - Diagrama de roseta para as medidas de planos de acamamento. 87

Figura 5.23 - Diagrama de roseta para as medidas de direção de condutos. 87
Figura 5.24 - Diagrama de roseta para medidas de fratura da totalidade das cavernas de Serra Leste. 88
Figura 5.25 - Diagrama de roseta para medidas de planos de acamamento ou bandamento da totalidade das cavernas de Serra Leste. 88
Figura 5.26 - Frequência de cavidades em função da projeção horizontal. 89
Figura 5.27 - Frequência de cavidades em função do desnível. 90
Figura 5.28 - Frequência de cavidades em função da área. 90
Figura 5.29 - Freqüência de cavidades em função do volume. 91
Figura 5.30 - Comparacão entre dados espeleométricos. Em tons claros, a média para as cavidades em estudo. Em tons escuros, a média para cavidades anteriormente estudadas em Serra Leste. 91
Figura 5.31 - Padrões planimétricos: (A) retilíneo (SL-106), (B) curvilíneo (SL-108), (C) reticulado (SL-121), (D) globular (SL-122), (E) triangular (SL-112). 92
Figura 5.32 - Morfologia do piso das cavidades de Serra Leste. 93
Figura 5.33 - Feições morfológicas das cavernas de Serra Leste. 94
Figura 5.34 - Pequena porção de paleopiso capeado observado na caverna SL-121. 94
Figura 5.35 - Morfologia da parede das cavidades de Serra Leste. 95
Figura 5.36 - Caverna SL-101 apresenta trechos com contorno bastante regulares. 95
Figura 5.37 - Canalículo no setor distal da caverna SL-112. 96
Figura 5.38 - Cúpula na cavidade SL-114: (A) visão geral e (B) em detalhe. 97
Figura 5.39 - Morfologia do teto das cavidade de Serra Leste. 98
Figura 5.40 - Pilar de diâmetro métrico na caverna SL-108. 98
Figura 5.41 - Conjunto de pilares na caverna SL-101, visto de dois lados opostos (A) e (B). 99
Figura 5.42 - Pendentes observados nas cavidades SL-108 (A) e SL-122(B). 99
Figura 5.43 - Abatimento de blocos no interior das cavidade de Serra Leste. 100
Figura 5.44 - Frequência de cavidades com feições hidrológicas. 100
Figura 5.45 - Sumidouro temporário na SL-101: curso de água em porção inferior da cavidade. 101
Figura 5.46 - Pequeno estreitamento por onde a água escoa conectando os dois salões. 101
Figura 5.47 - (A) Bloco umidificado pelo gotejamento que percola entre fraturas na cavidade SL-121; (B) Curso fluvial que adentra a caverna SL-111. 102
Figura 5.48 - Cavidade SL-122:(A) Encachoeirado na entrada da cavidade com parte do curso de drenagem adentrando a caverna;(B) Gotejamento formando pingente e curso d'água de baixa vazão no interior da cavidade. 102
Figura 5.49 - Tipo de sedimentação predominante nas cavidades. 103
Figura 5.50 - Granulometria dos sedimentos que recobrem o piso das cavidades ferríferas. 104
Figura 5.51 - Granulometria dos sedimentos que recobrem o piso das cavidades quartzíticas. 105
Figura 5.52 - Matacões e calhaus angulosos de quartzito nas cavernas SL-101 (A) e SL-104 (B). 105
Figura 5.53 - Concentração de clastos tamanho areia em porções de cavernas: (A) Areia branca em porção jusante da SL-117 e (B) Areia marrom em conduto descendente da SL-101. 106

Figura 5.54 - (A) Folhiço observado na zona de entrada da caverna SL-110 e (B) trazido por curso fluvial na cavidade SL-111. 106
Figura 5.55 - Guano seco recobre grande quantidade do piso da cavidade SL-110 (mapa), formando alguns aglomerados de sedimentos orgânicos, em destaque na foto. 107
Figura 5.56 - Tipos de espeleotemas observados nas cavidades alojadas em rochas ferríferas. 108
Figura 5.57 - Tipos de espeleotemas observados nas cavidades desenvolvidas em rochas quartzíticas. 108
Figura 5.58 - Crostas na caverna SL-121: (A) Na parede, apresenta textura lisa e coloração heterogênea; (B) Em pequena porção do teto com coloração predominantemente amarela em tons diversos; (C) Em grande parte do teto próximo a entrada, a crosta tem brilho metálico. 109
Figura 5.59 - Crostas brancas em cavernas ferríferas: (A) Repleta de alvéolos recobrindo porção do piso da caverna SL-109; (B) Em pequena parte da parede da caverna SL-115 associada a coralóides. 109
Figura 5.60 - Coralóides da caverna SL-101: (A) Cinzas com até 1 cm em concentração na parede; (B) Diminutos e pretos em pequena porção de rocha menos alterada. 110
Figura 5.61 - Coralóides centimétricos em porção reclusa da cavidade SL-122: (A) Alongados; (B) Bojudos. 110
Figura 5.62 - Cortina preta em porção média da caverna SL-122. 111
Figura 5.63 - Pingentes da caverna SL-122: (A) Conjunto em porção bastante úmida do teto; (B) Em ponto de exfiltraçao de água. 111
Figura 5.64 - Modelo de evolução endógena/exógena do tipo dissolução/erosão. Adaptado de Pinheiro & Maurity, 1988. Retirado de CARSTE, 2011a. 113
Figura 5.65 - Modelo de evolução exógena do tipo coluvionar. Retirado de CARSTE, 2011a. . 114
Figura 5.66 - Modelo de evolução exógena do “tipo fluvial”. Retirado de CARSTE, 2011a. 115
Figura 5.67 - Fratura preenchida na cavidade SL-121: controle estrutural no desenvolvimento de condutos. Linhas amarelas indicam direção aproximada das fraturas. 115
Figura 5.68 - Interseção de estruturas geológicas na caverna SL-101. 116
Figura 5.69 - Modelo de aprofundamento no nível ativo da caverna pelo contínuo rebaixamento do nível freático. Modificado de Wiegand *et al.,* 2004. 117
Figura 5.70 - Vasilhas plásticas observadas na caverna SL-111. 118
Figura 5.71 - Grande quantidade de sedimentos canalizados para o interior da caverna SL-114. 118
Figura 5.72 - Escavação nas proximidades da entrada da caverna SL-107. 119
Figura 5.73 - Alterações no entorno das cavidades: (A), (B) e (C) Supressão da vegetação para implantação de estruturas minerárias ; (D) Frente de lavra em maciço quartzítico. 120
Figura 5.74 - Ordens encontradas e suas respectivas porcentagens em relação à riqueza total de invertebrados registrada nas cavernas. 127
Figura 5.75 - Ordens encontradas e suas respectivas porcentagens em relação à riqueza total de vertebrados registrada nas cavernas. 128
Figura 5.76 - Espécie troglomórfica do gêmero Charinus encontrada em cavernas da área. .. 130
Figura 5.77 - Espécie troglomórfica da família Oonopidae encontrada em cavernas da área. 131
Figura 5.78 - Espécie troglomórfica do gênero Matta (família Tetrablemmidae) encontrada em cavernas da área. 131

Figura 5.79 - Pyrgodesmidae troglomórfico encontrado em cavernas da área. 132
Figura 5.80 - Pyrgodesmidae troglomórfico encontrado em cavernas da área. 133
Figura 5.81 - Coleóptero troglomórfico da família Dyticidae encontrado em cavernas da área (reparar a despigmentação e pronunciada recução ocular). 134
Figura 5.82 - Coleóptero troglomórfico da família Eucnemidae encontrado em cavernas da área (reparar a despigmentação e pronunciada redução ocular). 135
Figura 5.83 - Coleóptero troglomórfico da família Pselaphidae encontrado em cavernas da área (reparar a despigmentação e pronunciada redução ocular). 135
Figura 5.84 - Coleóptero troglomórfico da família Scydmaenidae encontrado em cavernas da área (reparar a despigmentação e pronunciada redução ocular). 136
Figura 5.85 - Colêmbolos troglomórficos encontrados em cavernas da área. 137
Figura 5.86 - A) Gastropoda: Systrophiidae; B) Formicidae: *Strumigenys* sp.; C) Diptera: Psychodidae; D) Pseudoscorpiones:Olpiidae; E) Coleoptera: Scydmaenidae; F) Hymenoptera: Scelionidae; G) Opiliones: Escadabiidae; H) Orthoptera: Phalangopsidae (*Phalangopsis* sp.); I) Chiroptera: Phyllostomidae (Carollia perspicillata); J) Amblypygi: Phrinidae (*Heterophrynus longicornis*) predando um grilo, *Phalangopsis* sp. 141
Figura 5.87 - A) Isopoda: Platyarthridae (Trichorhina sp.); B) Homoptera: Cercopidae.; C) Polydesmida: Chelodesmidae; D) Gastropoda: Subulinidae; E) Basidiomycota. 145
Figura 5.88 - A) Vista externa da entrada da cavidade; B) Chiroptera: Emballonuridae: Peropteryx sp. 147
Figura 5.89 - A) Orthoptera: Phalangopsidae (*Phalangopsis* sp.); B) Opiliones: Cosmetidae; C) Gastropoda: Systrophiidae; D) Amblypygi: Phrinidae (*Heterophrynus longicornis*). 149
Figura 5.90 - A) Orthoptera: Phalangopsidae (*Phalangopsis* sp.); B) Amblypygi: Phrinidae (*Heterophrynus longicornis*).; C) Isopoda: Platyarthridae (*Trichorhina* sp.)............................ 151
Figura 5.91 - A) Vista interna destacando as manchas de guano associadas ao piso da cavidade; B) Coleoptera: Staphylinidae (Pselaphinae). 154
Figura 5.92 - A) Amblypygi: Phrinidae (*Heterophrynus longicornis*); B) Blattodea: Blaberidae (*Blaberus* sp.); C) Araneae: Theraphosidae. 157
Figura 5.93 - A) Colônia mista de Chiroptera:; B) Araneae: Salticidae; C) Coleoptera: Eucnemidae; D) Pseudoscorpiones: Chthoniidae; E) Decapoda: Pseudothelphusidae (*Microthelphusa somanni*). 162
Figura 5.94 - Decapoda: Pseudothelphusidae (*Microthelphusa somanni*). Em detalhe, filhotes guardados sob o abdômen. 163
Figura 5.95 - Stemmiulida: Stemmiulidae. E, detalhe, um fungo Basidiomyceto. 165
Figura 5.96 - A) Heteroptera: Reduviidae (*Zelurus* sp.); B) Araneae: Ctenidae; C) Coleoptera: Scydmaenidae; D) Ninfas de Cydnidae associadas ao recurso trófico; E) Coleoptera: Carabidae (Clivinina). 168
Figura 5.97 - A) Araneae: Salticidae; B) Lepidoptera: Noctuidae. 170
Figura 5.98 - A) Anura: Strabomantidae (*Pristimantis* sp.); B) Araneae: Theraphosidae; C) Opiliones: Manaosbiidae; D) Colônia de Hymenoptera: Formicidae *(Apterostigma* sp.). 174
Figura 5.99 - A) Opiliones: Cosmetidae e Anura: Strabomantidae (*Pristimantis* sp.); B) Araneae: Pholcidae (*Mesabolivar* sp.); C) Embiidina; D) Polydesmida: Chelodesmidae. 177

Figura 5.100 - A) Anura: Strabomantidae (*Pristimantis* sp.); B) Squamata: Gekkonidae (*Thecadactylus rapicauda*); C) Chiroptera: Phyllostomidae (*Carollia perspicillata*); D) Diptera: Drosophilidae; E) Araneae: Araneidae; F) Orthoptera: Phalangopsidae (Phalangopsinae)...... 182
Figura 5.101 - A) Orthoptera: Phalangopsidae (*Phalangopsis* sp.); B) Heteroptera: Veliidae; C) Araneae: Trechaleidae (*Enna* aff. *paraense*); D) Araneae: Ctenidae. 185
Figura 5.102 - A) Acari: Trombidiforme; B) Palpigradi: Eukoeneniidae (*Leptokoenenia* sp.); C) Geophilomorpha: Geophilidae (*Schizonampa* sp.). 188
Figura 5.103 - Classes de riqueza total das cavernas da área. Os valores no eixo vertical indicam o número total de espécies, e no eixo horizontal, o número de cavernas em cada categoria. 191
Figura 5.104 - Curvas de rarefação construídas para as cavernas da área. 192
Figura 5.105 - Dendrogramas de similaridade (Bray-Curtis), evidenciando os padrões de similaridade entre as cavernas. A figura da esquerda representa o dendrograma referente à estação de seca e, a da direita, a estação chuvosa. Os pontos vermelhos representam valores de similaridade entre cavernas superiores a 60% e, os pontos verdes, valores superiores a 50%. 193
Figura 6.1 - Localização da unidade espeleológica Carajás composta por nove unidades geomorfológicas. 196
Figura 6.2 - Fluxograma de classificação do grau de relevância de cavidades naturais subterrâneas, segundo a Instrução Normativa nº 2, do MMA. 197
Figura 7.1 - Localização pontual dos projetos realizados pela Carste Consultores Associados inseridos nas Unidades Geomorfológicas em Carajás/PA. 210
Figura 7.2 – (A) Matta sp. troglomórfico, semelhante aos capturados nas cavidades e (B) o coleóptero anoftálmico Scydmaenidae troglomórfico, semelhante ao capturado na cavidade de Serra Leste. 211
Figura 7.3 – Localização das morfoespécies troglomórficas Matta sp. e Scydmaenidae spH no ambiente epígeo e a distância em relação às cavidades em que foram originalmente encontradas. 212
Figura 7.4 – (A) Vista dorsal Pselaphinae sp G; (B) vista lateral de Scydmaenidae spH; (C) vista dorsal de Dytiscidae spA: (D) vista lateral de Eucnemidae sp3; 214

ÍNDICE DE QUADROS

Quadro 2.1 - Principais troglomorfismos observados em espécies troglóbias (retirado de Christiansen, K. Morphological Adaptations, em Enciclopedia of Caves, Culver & White 2004). 18
Quadro 4.1 - Coluna estratigráfica e resumo de dados geocronológicos de Província Mineral de Carajás (extraído e adaptado de Tallarico *et al*., 2003). 26
Quadro 5.1 - Síntese das características da caverna SL-101. 33
Quadro 5.2 - Síntese das características da caverna SL-102. 35
Quadro 5.3 - Síntese das características da caverna SL-103. 37
Quadro 5.4 - Síntese das características da caverna SL-104. 39
Quadro 5.5 - Síntese das características da caverna SL-105. 41
Quadro 5.6 - Síntese das características da caverna SL-106. 43

Quadro 5.7 - Síntese das características da caverna SL-107. 45
Quadro 5.8 - Síntese das características da caverna SL-108. 47
Quadro 5.9 - Síntese das características da caverna SL-109. 49
Quadro 5.10 - Síntese das características da caverna SL-110. 51
Quadro 5.11 - Síntese das características da caverna SL-111. 53
Quadro 5.12 - Síntese das características da caverna SL-112. 55
Quadro 5.13 - Síntese das características da caverna SL-113. 57
Quadro 5.14 - Síntese das características da caverna SL-114. 59
Quadro 5.15 - Síntese das características da caverna SL-115. 61
Quadro 5.16 - Síntese das características da caverna SL-116. 63
Quadro 5.17 - Síntese das características da caverna SL-117. 65
Quadro 5.18 - Síntese das características da caverna SL-121. 67
Quadro 5.19 - Síntese das características da caverna SL-122. 69
Quadro 5.20 - Síntese das características da caverna SL-130. 71
Quadro 5.21 - Síntese das características da caverna SL-131. 73
Quadro 5.22 - Estatística dos dados espeleométricos das cavidades inseridas no Projeto Serra Leste. 89
Quadro 6.1 - Classificação de relevância das cavernas em Serra Leste. 201
Quadro 6.2 - Relação das cavidades de Serra Leste (dentre 21 amostradas) que apresentam espécies troglóbias com raridade tipo I, II, III (I+II) e IV. 202
Quadro 6.3 - Relação das cavidades de Serra Leste (dentre 117 amostradas) que apresentam espécies troglóbias com raridade tipo I, II, III (I+II) e IV. 203
Quadro 7.1 - Táxons troglomórficos encontrados nos dois estudos já executados na área de Serra Leste. 206
Quadro 7.2 - Estudos utilizados para subsidiar o pareamento de espécies troglomórficas do Projeto Serra Leste 209
Quadro 7.3 - Relação de morfoespécies caracterizadas anteriormente como “troglóbios raros”, modificação de status e ampliação de distribuição após o pareamento de espécies. 215
Quadro 7.4 - Síntese da relevância das cavidades estudadas na área de Serra Leste, de acordo com a configuração de importância dos atributos físicos e biológicos. 215

ÍNDICE DE TABELAS

Tabela 5.1 - Coordenadas UTM e dados espeleométricos das cavidades estudadas em Serra Leste. 31
Tabela 5.2 - Valores de riqueza, dominância, diversidade e equitabilidade para as cavernas do estudo (estação seca). 190
Tabela 6.1 - Parâmetros espeleométricos do enfoque regional para cavidades em rocha ferrífera de Serra Leste. 198
Tabela 6.2 - Parâmetros espeleométricos do enfoque regional para cavidades em rocha siliciclástica de Serra Leste. 198

Tabela 6.3 - Parâmetros espeleométricos do enfoque local para cavidades em rocha ferrífera de Serra Leste. 198
Tabela 6.4 - Parâmetros espeleométricos do enfoque local para cavidades em rocha siliciclástica de Serra Leste. 199
Tabela 7 - Número de unidades amostrais obtidas para cada área: extrator de winkler; revisão de folhiço do winkler; coleta ativa noturna; coleta aquática 208

MINUTA

1. INTRODUÇÃO

O presente documento tem como objetivo apresentar os resultados dos estudos espeleológicos realizados em 21 cavernas localizadas na área do Projeto Serra Leste, empreendimento da empresa Vale, situado na região de Carajás, Estado do Pará (Figura 1.1). Outras 96 cavernas inseridas na área deste projeto foram analisadas em estudo anterior (CARSTE, 2011a). Os estudos estão em consonância com o Decreto Federal Nº 6.640 de 2008, e foram conduzidos segundo as metodologias estabelecidas na Instrução Normativa MMA Nº 02 de 2009.

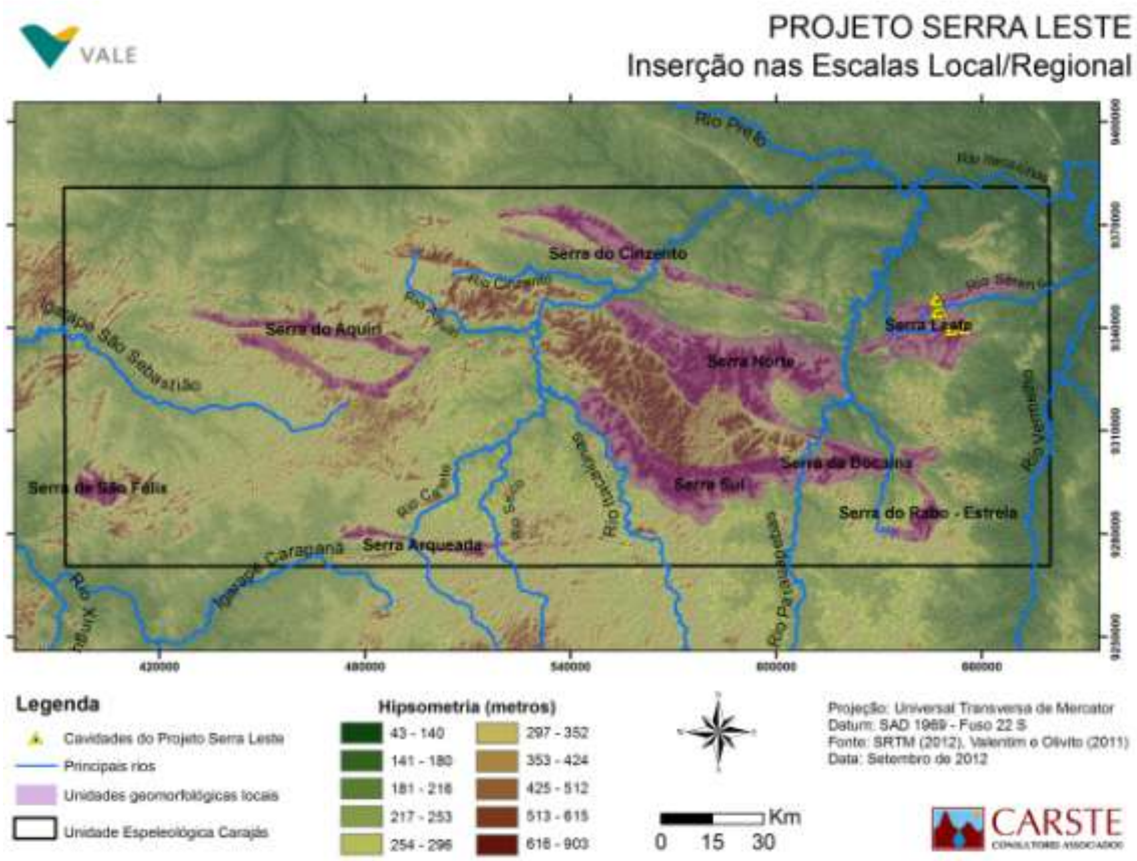

Figura 1.1 - Localização da área de estudo nas escalas local e regional.

Especificamente, pretende-se:

- Analisar os aspectos físicos das cavernas inseridas na área do projeto, incluindo sua morfologia e seus aspectos espeleométricos; a litologia e suas estruturas; os processos hidrológicos reconhecidos através das feições observadas; e, por fim os seus depósitos clásticos, químicos e orgânicos;
- Analisar questões sócio-econômicas e culturais envolvendo estas cavidades;
- Avaliar o estado de conservação das cavernas e de seus respectivos entornos;
- Caracterizar as cavernas da área quanto à composição de espécies;
- Caracterizar as cavernas em relação à sua similaridade faunística;
- Caracterizar tais cavernas quanto ao "status" trófico;

- Efetuar análise de relevância das cavidades, conforme determina o Decreto Federal n° 6.640 e a Instrução Normativa n° 2, do Ministério do Meio Ambiente – MMA;
- Realizar estudos complementares objetivando atualização dos dados de acordo com novas informações e, consequentemente reavaliar as análises apresentadas.

2. METODOLOGIA

2.1. Levantamento bibliográfico

Consistiu em um levantamento bibliográfico sobre cavernas em rochas siliciclásticas e ferríferas no mundo e, especificamente, no Brasil. Com o objetivo de melhor caracterizar o ambiente onde essas cavernas estão inseridas, também se realizou uma pesquisa envolvendo os temas geologia (lito-estruturas), geomorfologia, hidrografia, hidrologia e pedologia.

2.2. Prospecção espeleológica

A prospecção espeleológica na região de Serra Leste foi realizada pela equipe do Grupo Espeleológico de Marabá (GEM), através da Fundação Casa de Cultura de Marabá (FCCM).

2.3. Espeleotopografia

O levantamento espeleotopográfico das cavernas de Serra Leste foi realizado pelo Grupo Espeleológico de Marabá.

2.4. Geoespeleologia

2.4.1. Campanhas de campo

O estudo geoespeleológico de Serra Leste foi realizado por dois espelelógos em uma campanha de campo que ocorreu entre os dias 26/06 e 06/07/12.

2.4.2. Análise litológica e das estruturas

A análise da litologia buscou caracterizar os litotipos em que as cavernas estão inseridas. Estruturas como bandamento, foliação, fraturas, juntas de alívio, dobras e falhas presentes foram descritas e, quando possível, medidas.

Essas estruturas representam descontinuidades importantes que condicionam a circulação de fluidos no interior do maciço rochoso, além de intervir direta ou indiretamente no processo de gênese e desenvolvimento das cavernas. Muitas vezes tais estruturas controlam a morfologia das cavernas, além de condicionarem processos de abatimentos em paredes e teto. A medição das estruturas foi realizada com bússola de marca Brunton, modelo DF5008.

2.4.3. Análise morfológica

Na análise morfológica buscou-se interpretar o padrão planimétrico da caverna, com o objetivo de correlacioná-lo a dados estruturais e litológicos. Para tanto foi utilizada a planta baixa, seções transversais e longitudinais das cavidades, uma vez que suas formas estão diretamente relacionadas aos processos de gênese e evolução das cavernas.

2.4.4. Hidrologia

Com o objetivo de se determinar a dinâmica hidrológica das cavernas foi avaliada a presença de águas de percolação e condensação, além da presença de corpos hídricos.

2.4.5. Depósitos clásticos, orgânicos e químicos

Depósitos de sedimentos clásticos foram classificados conforme sua granulometria e descritos de modo a fornecer pistas sobre sua origem e sedimentação. Os depósitos orgânicos também foram descritos de forma breve, quando existentes. Os depósitos químicos (espeleotemas) foram identificados, fotografados e descritos individualmente ou em conjunto.

2.5.Bioespeleologia

Sendo a espeleologia uma ciência interdisciplinar, a proposição metodológica foi concebida no sentido de inter-relacionar algumas abordagens necessárias para uma melhor identificação e análise global da área e, conseqüentemente, das cavernas inventariadas nas áreas de influência direta e indireta do empreendimento.

Do ponto de vista bioespeleológico, procurou-se caracterizar o ecossistema de cada uma das cavidades inventariadas, a partir da descrição de sua fauna, bem como da avaliação do sistema trófico de cada caverna. Em seguida, as cavernas foram avaliadas dentro da abordagem do patrimônio espeleológico, na busca de possíveis referências de valor existentes no espaço subterrâneo inventariado.

2.5.1. Procedimentos de coleta

O inventário de fauna foi realizado em dois períodos, sendo um correspondente à estação chuvosa e o outro à estação seca na região. A coleta de invertebrados foi feita através de captura manual (com o auxílio de pinças e pincéis) em quaisquer biótopos potenciais no interior de cada caverna (em cada estação).

Foram coletados somente espécimes não identificados *in loco*, o que corresponde a um percentual reduzido da fauna de cada cavidade. Tal procedimento minimiza enormemente os impactos em relação a uma coleta exaustiva, <u>não recomendada</u> em casos onde se faz necessária uma nova coleta na estação subseqüente. Desta forma, inventários que requerem a coleta de todos os organismos observados certamente têm grande potencial de desestruturar as comunidades de uma dada caverna, o que certamente pode interferir nas análises de relevância das cavidades. Reitera-se, ainda, que as licenças de coleta para organismos cavernícolas (salvo exceções vinculadas a projetos específicos) geralmente não permitem a coleta de mais de 10 espécimes (de cada espécie) por caverna.

Não foram utilizadas armadilhas de queda pela baixa eficiência que tais armadilhas vêem demonstrando em muitos trabalhos (FERREIRA, 2004). Além disso, armadilhas de queda notoriamente causam distúrbios em populações cavernícolas (WEINSTEIN & SLANEY, 1995; SHARRAT *et al*., 2000; SOUZA-SILVA *et al*.,2011). Para a amostragem de invertebrados, buscas

diretas são bastante efetivas, desde que realizadas por bioespeleólogos experientes (WEINSTEIN & SLANEY, 1995).

Todos os organismos coletados foram fixados em álcool 70% e encontram-se depositados na coleção de invertebrados subterrâneos (ISLA) do laboratório de Ecologia Subterrânea do Departamento de Biologia da Universidade Federal de Lavras.

As coletas dos organismos do presente trabalho foram realizadas com o auxílio de pinças e pincéis, através de uma procura visual detalhada priorizando micro-habitats, como depósitos de matéria orgânica, espaços sob rochas, tetos e paredes, bem como locais com maior umidade para determinar a composição da fauna de artrópodes nas cavernas. Os organismos coletados foram fixados em álcool 70% para uma análise posterior com o intuito de separá-los em morfoespécies. Espécies previamente conhecidas tiveram suas abundâncias estimadas através de contagem dos organismos em campo, com anotação do seu local de captura no croqui da caverna, para minimizar o impacto da coleta. Eventuais comportamentos e interações ecológicas observadas durante o inventário, bem como vestígios e presença de vertebrados também foram registradas.

Os recursos visíveis na cavidade foram examinados (*in situ*), para a determinação do seu status trófico.

A caracterização topoclimática não foi realizada, tendo em vista o número muito restrito de visitas a cada cavidade, que gerariam dados insuficientes para uma avaliação adequada.

2.5.2. Triagem e identificação dos taxa

Todos os invertebrados coletados foram identificados até o nível taxonômico possível e agrupados em morfótipos fazendo-se uso de estereomicroscópios (Zeiss Stemi DV-4 e Zeiss Stemi 2000). Tal identificação resultou nos dados de riqueza de espécies (número de espécies), das diferentes cavernas.

2.5.3. Determinação de troglomorfismos

A determinação de espécies potencialmente troglóbias foi realizada através da identificação, nos espécimes, de características morfológicas denominadas troglomorfismos. Tais características, como redução da pigmentação melânica, redução das estruturas oculares, alongamento de apêndices, dentre outras, são utilizadas freqüentemente para a maioria dos grupos, uma vez que resultam de processos evolutivos ocorrentes após o isolamento de populações em cavernas. Os principais troglomorfismos encontrados em diferentes grupos de organismos troglóbios no mundo encontram-se sumarizados no Quadro 2.1. As características a serem utilizadas para estes diagnósticos, no entanto, diferem no caso de organismos pertencentes à taxa distintos. Certos grupos, por exemplo, possuem espécies sempre despigmentadas e anoftálmicas, mesmo no ambiente epígeo (e.g. Palpigradi). Nestes casos, os troglomorfismos são mais específicos (para Palpigradi: alongamento dos flagelômeros, aumento no número de órgãos laterais, razão entre os comprimentos do propeltídio e do basitarso IV, razão entre os comprimentos da tíbia e do basitarso IV, dentre outros). Desta forma, é necessário se conhecer a biologia de cada grupo no intuito de se diagnosticar

efetivamente a existência ou não de troglomorfismos. Despigmentação e anoftalmia muitas vezes não são diagnósticas de troglomorfismos.

Muitas vezes, entretanto, existe a necessidade de consulta a especialista em certos grupos taxonômicos. Muitos destes especialistas infelizmente não existem no Brasil. Desta forma, a determinação da real categoria a qual pertence certa espécie pode demandar um longo tempo.

Quadro 2.1 - Principais troglomorfismos observados em espécies troglóbias (retirado de Christiansen, K. Morphological Adaptations, em Enciclopedia of Caves, Culver & White 2004).

Morphological characteristic	Ref.
Specialization of sensory organs (touch, chemoreceptor, hygroreceptor, thermoreceptor, pressure receptors)	Vandel, 1964; Sbordoni, 1980; Weber; 2000
Elongation of appendages	Sbordoni, 1980; Coineau & Boutin, 1992; Weber, 2000; Christiansen, 1961 Harvey, Shear, & Hoch, 2000; Vandel 1964
Pseudophysogastry	Vandel 1964, Sbordoni 1980; Accordi et al., 1980
Reduction of eyes, pigment, wings	Vandel 1964, Sbordoni 1980, Coineau & Boutin,1992; Weber, 2000
Compressed or depressed body form (hexapods)	Harvey, Shear, & Hoch, 2000
Increased egg volume	Vandel, 1964; Sbordoni, 1980; Poulson, 1963
Increased size (Collembola, Arachnida)	Christiansen, 1961; Harvey, Shear, & Hoch, 2000
Unguis elongation (Collembola)	Christiansen, 1961
Foot modification (Collembola, planthoppers)	Christiansen,1961; Howarth et al., 1990
Scale reduction or loss (Fish)	Wilkens, 1988; Weber, 2000; Ercolini et al., 1982
Loss of pigment cells and deposits	Numerous
Cuticle thinning (terrestrial arthropods)	Numerous
Elongate body form (Teleost fishes, Arachnids)	Weber, 2000; Coineau & Boutin, 1992.
Depressed, shovellike heads (Teleost fishes, salamanders)	Weber, 2000
Reduction or loss of swim bladder	Romero & Paulson, 2001
Decreasing Hind femur length/crop empty	Studier, E., Lavoie, K., & Howarth, F., 2002
Live weight, ratio (crickets)	

2.5.4. Determinação de raridade

No presente relatório, foram mostrados diferentes conceitos de "raridade" atribuídos às espécies troglóbias. Tais atribuições foram primordialmente ilustrativas, tendo sido realizadas no intuito de exemplificar a possibilidade de aplicação de diferentes abordagens, desde aquelas mais "restritivas" até as mais amplas.

No entanto, para efeito da legislação vigente, utilizou-se, neste relatório, as duas abordagens propostas no workshop técnico científico **"Troglóbios raros": incertezas e encaminhamentos**, realizado em Belo Horizonte nos dias 03 e 04 de Março de 2011. Tal encontro contou com a participação de diversos especialistas em biologia subterrânea, dentre os quais: Prof. Dr. Antonio Brescovit, Dra. Flávia Pellegatti Franco, Prof. Dr. Marconi Souza Silva, Dra. Renata Andrade, MSc. Robson Zampaulo, Dra. Regina Bessi Pascoaloto e Prof. Dr. Rodrigo Lopes Ferreira.

Levando em consideração o atributo de distribuição geográfica, foi sugerido, no referido workshop, que uma espécie troglóbia encontrada em até três cavidades seja considerada rara. Neste relatório, este tipo de raridade é chamado de raridade **tipo I**. Com relação ao atributo de abundância, foi sugerido pelos Profs. Dr. Marconi Souza Silva e Dr. Rodrigo Lopes Ferreira que deve ser considerada rara a espécie troglóbia com um exemplar por cavidade amostrada, não

importando o número de cavidades em que ocorra, independentemente da distribuição geográfica. Esta abordagem também foi adotada no presente relatório, sendo aqui chamada de raridade **tipo II**.

Além das abordagens utilizadas para a definição de troglóbios raros (aplicadas para a obtenção do grau de relevância das cavernas – raridades dos tipos **I e II**), foram também exemplificadas outras abordagens, a saber: espécies que compreenderam *uniques* (com a ocorrência de uma única espécie em todo o estudo – raridade **tipo III**); e espécies que corresponderam à sobreposição dos critérios de distribuição (até três cavernas) e abundância (um exemplar por cavidade amostrada, não importando o número de cavidades em que ocorra), aqui chamadas de raridade **tipo IV**.

2.5.5. Análises

Análises Ecológicas das Cavidades

Com base na presença das espécies de invertebrados de cada cavidade, foram realizadas algumas análises, descritas a seguir.

Para cada caverna, em cada estação, foram determinadas a riqueza (número total de espécies), diversidade (Shannon-Wiener), dominância, e equitabilidade. Além disso, foi determinada também a riqueza total de espécies encontrada em cada caverna (por meio do somatório das espécies observadas no período seco e no período chuvoso).

Foi feita também uma análise de Escalonamento Multidimensional não métrico (n-MDS) utilizando o índice de Bray-Curtis. Tal análise permite a observação da similaridade faunística através da plotagem das amostras (no caso, as cavernas) em um espaço bi ou tridimensional, no qual a distância entre cada ponto corresponde à sua similaridade. Optou-se, neste relatório, pela análise bi-dimensional.

Foram construídas curvas de rarefação, elaboradas a partir do número acumulativo de espécies em cada caverna amostrada (recalculados por meio de aleatorizações). Tais análises pretenderam evidenciar se a amostragem foi ou não satisfatória, indicando quanto do total de espécies presentes na área foram amostradas na coleta destas cavernas.

Por fim, foram realizados testes de regressão simples e múltipla entre diferentes variáveis bióticas e abióticas. As variáveis abióticas testadas foram: a extensão total das cavernas, o número de entradas de cada caverna e a incidência de inundações. As variáveis bióticas testadas foram a riqueza de espécies (da seca, da chuva e total) e a riqueza de espécies troglóbias (troglomórficas).

Reitera-se aqui que neste relatório, as espécies troglóbias foram assim definidas em função dos seus troglomorfismos. Desta forma, os termos **Troglóbios** e **Troglomórficos**, para efeito deste relatório, são coincidentes.

A raridade das espécies troglomórficas encontrada neste estudo foi definida a partir da comparação com espécies troglomórficas coletadas em cavernas de Serra Leste e Serra Norte (Morro I e Morro II).

Determinação do grau de relevância das cavidades

A determinação dos graus de relevância das cavernas da área foi realizada por meio da aplicação da metodologia explicitada na Instrução Normativa nº 2 referente ao Decreto 6.640.

Foram considerados todos os atributos biológicos mencionados na referida Instrução Normativa. No entanto, o presente relatório foi elaborado considerando-se dois "panoramas": o primeiro deles utilizou-se da aplicação direta de todos os atributos da IN nº. 02, que resultou em uma análise de relevância elaborada inteiramente em conformidade com a legislação vigente.

No entanto, foi realizada uma segunda análise de relevância considerando-se a adequação de alguns atributos da IN, visando uma análise mais refinada ou real das relevâncias das cavidades. Reitera-se aqui que esta segunda análise está, no presente momento, em desconformidade com a legislação vigente, já que utiliza alguns parâmetros modificados em relação à normatização. No entanto, optou-se aqui pela realização desta análise diferencial com o objetivo básico de que esta possa fornecer subsídios para futuras adequações na referida Instrução Normativa quando de sua re-avaliação.

3. ESPELEOGÊNESE EM ROCHAS QUARTZÍTICAS E FERRÍFERAS

As cavernas são cavidades naturais subterrâneas formadas frequentemente pela ação da água que atua dissolvendo e/ou erodindo diferentes tipos de rochas (GILBERT *et al.,* 1994). A maioria das cavernas localiza-se em rochas carbonáticas (calcários e dolomitos), as mais favoráveis aos processos de dissolução. Entretanto, arenitos e quartzitos são também muito susceptíveis a formar cavernas, aparentemente devido a fatores geomorfológicos e climáticos mais erosivos (AULER, 2006). A ocorrência de cavernas em granito, gnaisse, micaxistos, filitos e até mesmo solo também são registradas, entretanto em escala menor que os carbonatos, quartzitos e arenitos (AULER, 2006). Recentemente, constatou-se, em áreas de minério de ferro, a existência de um número expressivo de cavernas, fato que adicionou mais uma potencialidade de ocorrência de cavernas ao já variado cenário espeleológico Brasileiro (AULER, 2006). Estas cavernas ferruginosas podem ocorrer em diferentes rochas ou contatos de rochas (canga, hematita, itabirito) sendo originadas por processos de dissolução e erosão atuando predominantemente em hematita e canga (AULER & PILÓ, 2005; PILÓ & AULER, 2005).

3.1.Cavernas em quartzito

Durante um longo tempo acreditou-se que os quartzitos e arenitos não eram carstificáveis. Por esse fato, ainda hoje existem poucos registros desse fenômeno em nível mundial. As quatro principais regiões geográficas que exibem formações de grande magnitude, consideradas referências na literatura são: os tepuys na Amazônia Venezuelana, onde foram realizadas várias pesquisas (SZCZERBAN *et al.,* 1974; URBANI, 1986, WHITE *et al*., 1966; DOERR, 1999; GALÁN *et al.,* 2004); a península do Cape na África do Sul (MARTINI, 1979); Austrália, sobretudo no noroeste das Kimberleys (JENNINGS, 1983; YOUNG, 1988; WRAY, 1997) e o Brasil.

Diversas hipóteses foram propostas para explicar o desenvolvimento das feições cársticas em rochas quartzíticas e areníticas. O modelo genético postulado por Martini (1979) é o mais aceito pela comunidade científica. Segundo este autor, o desenvolvimento do carste em rochas siliciclásticas abrange essencialmente duas fases, sendo a primeira dominada pelo intemperismo químico, onde a dissolução atua no domínio intergranular, dissolvendo o cimento e promovendo a individualização dos grãos de quartzo. Após esta fase de desagregação química denominada de "arenização", prossegue a etapa controlada pelos processos erosivos, onde os grãos de quartzo podem ser evacuados, principalmente através do processo de "piping" (MARTINI, 1979).

Segundo Galán (1991), o desenvolvimento do carste em quartzitos ou rochas de composição siliciclásticas ocorre em função da dissolução da sílica, mas a fase erosiva via "piping" corresponde pela maior capacidade remoção quantitativa da rocha. "Piping" consiste em remover mecanicamente partículas sólidas, resultando na formação de condutos, que podem evoluir formando galerias subterrâneas em alguma parte do sistema.

3.2. Cavernas em formações ferríferas

O interesse pelo estudo de cavidades em formações ferríferas, no Brasil e no mundo pode ser considerado recente. A partir dos anos 2000, o maior desenvolvimento da atividade minerária voltada à extração do minério de ferro implicou a ampliação da necessidade de se conhecer o patrimônio espeleológico associado (AULER & PILÓ, 2005). Quase a totalidade das cavernas em formações ferríferas cadastradas até hoje no Brasil estão concentradas no Quadrilátero Ferrífero, em Minas Gerais, e em Carajás, no Pará.

A primeira referência a cavernas em minério de ferro no Pará deve- se a Tolbert *et al.* (1971), que relata sobre as grandes cavernas encontradas sob o manto de canga na região de Serra dos Carajás, no sudeste do estado. Os estudos espeleológicos tiveram um grande avanço a partir dos trabalhos do Grupo Espeleológico Paraense - GEP, notadamente Clóvis W. Maurity e Roberto Vizeu L. Pinheiro. Em um primeiro relatório, Pinheiro *et al.* (1985) detalharam aspectos geoespeleológicos de quatro cavernas na Serra Norte, além de discorrer sobre sua possível gênese. Essas revelações foram ampliadas em Pinheiro & Maurity (1988), momento em que a interpretação espeleogenética foi aprimorada. Maurity & Kotschoubey (1995) posteriormente produziram um detalhado estudo não só sobre a gênese das cavidades, como também da geoquímica e da mineralogia dos espeleotemas. Na região de Serra Leste, no leste do Pará, Atzingen & Crescêncio (1999) apresentaram uma descrição, acompanhada de mapas, de algumas cavernas da região (PILÓ & AULER, 2009).

Na região do Quadrilátero Ferrífero, os primeiros estudos devem-se ao geólogo norte americano George Simmons (1963) que afirmava que as cavernas são formadas sob a canga, definida pelo autor como um depósito superficial de brecha rica em hematita e cimentada por limonita. Em relação à gênese, Simmons (1963) classifica-as em cavernas de erosão e dissolução, sendo que algumas outras resultam da ação combinada dos dois processos. Estudos mais recentes foram realizados na Mina de Capão Xavier, no Quadrilátero Ferrífero (PILÓ & AULER, 2005). Neste estudo, os mesmos consideram a ocorrência de cavernas tanto em canga quanto no itabirito. Em relação à gênese dessas cavidades, os autores analisam a ocorrência de processos químicos endógenos seguidos de erosão mecânica. De modo geral,

Piló & Auler (2005) evidenciaram, nas cavernas estudadas, a formação inicial de zonas de alta porosidade no interior das jazidas de minério de ferro. Já a segunda etapa da espeleogênese nestas cavernas estaria relacionada a processos físicos, contanto que haja um posicionamento geomorfológico favorável para sua ocorrência.

3.3.Características ambientais e tróficas das cavernas

Considerações ecológicas referentes ao ambiente de cavernas (sistemas hipógeos) são feitas em comparação com ambientes externos (epígeos). Cavernas são ambientes geralmente oligotróficos com elevada umidade, temperatura constante e ausência permanenete de luz (CULVER, 1982; FERREIRA, 2004). A característica mais marcante dos ambientes subterrâneos é a ausência permanente de luz, sendo que muitas das características bióticas e abióticas destes ambientes são influenciadas pela constância desta pressão ambiental. Exceções ocorrem nas regiões próximas às entradas das cavernas, onde tais aberturas possibilitam acesso de luz solar que podem alterar os efeitos de tal pressão. A total ausência de luz em cavernas exclui a possibilidade de ocorrência de produtores fotossintetizantes. A base da produção primária em algumas cavernas é o quimioautotrofismo, principalmente realizado por bactérias (SARBU *et al.,* 1996; HOSE *et al.,* 2000; CHIVIAN *et al.,* 2008). Entretanto, grande parte (ou a quase totalidade) da produção nos ecossistemas cavernícolas é de origem secundária, baseada em cadeias de detritívoros.

É comum a movimentação de nutrientes e detritos do meio epígeo para as cavernas. Em alguns casos, 100% da matéria orgânica presente no interior das cavernas é importada (HOWARTH, 1983; SCHNEIDER *et al.,* 2011). A matéria orgânica penetra nas cavernas carreada, contínua ou temporariamente, por agentes físicos e biológicos (SOUZA-SILVA, 2003; FERREIRA, 2005; SIMON *et al.,* 2007). A matéria orgânica é carreada por rios, enxurradas e outros cursos d'água que percolam no teto ou parede, através de aberturas ou fraturas que eventualmente existam nas cavernas (GIBERT *et al.,* 1994; SOUZA-SILVA, 2003; FERREIRA, 2005). A veiculação biológica é feita principalmente por meio do crescimento de raízes da vegetação externa, animais que transitam nas cavernas (e.g. morcegos) ou mesmo pelos animais que lá entram casualmente (acidentais). Raízes vegetais são importantes recursos alimentares para os organismos que vivem em tubos de lava vulcânica, em cavernas calcárias e cavernas ferruginosas superficiais (Howarth 1983, Jasinska *et al.* 1996, Arechavaleta *et al.* 1999, Souza Silva 2003, Ferreira 2005, Howarth *et al* 2007). Fezes de morcegos e de animais terrestres (e.g. roedores como *Kerodon rupestris* Wied. 1820) e carcaças de animais são importantes fontes de recursos alimentares para numerosas espécies de microorganismos e artrópodes, principalmente em cavernas permanentemente secas (Ferreira & Martins 1998; Ferreira & Martins 1999, Bahia 2008). Estes recursos alimentares alóctones mantêm populações de organismos em todos os níveis tróficos presentes nas cavernas (Ferreira & Martins 1999).

3.4.Relações ecológicas e evolutivas dos animais de cavernas

Diferentes autores frequentetemente categorizam os organismos de cavernas (cavernícolas) em espécies troglóxenas (trogloxenos), espécies troglófilas (troglófilos) e espécies troglóbias (troglóbios) referindo-se a conceitos que buscam expressar diferentes interações ou relações ecológicas e evolutivas com as cavernas (segundo Holsinger & Culver (1988), modificado do sistema Schiner-Racovitza (Racovitza 1907, Schiner 1954, apud Camacho 1992)).

Os troglóxenos são animais regularmente encontrados no ambiente subterrâneo, mas necessitam sais ao meio externo para completar seu ciclo de vida (em geral deixam as cavernas em busca de alimentos). Muitos destes organismos (e.g. morcegos) são responsáveis pela importação de recursos alimentares provenientes do meio epígeo, sendo muitas vezes os principais responsáveis pelo fluxo energético em cavernas, especialmente naquelas permanentemente secas. Os troglófilos são os organismos que completam seus ciclos de vida no meio hipógeo e/ou epígeo. Nesta categoria encontra-se a maior parte das espécies de invertebrados presentes em cavernas tropicais (Ferreira 2004). Os troglóbios restringem-se ao ambiente cavernícola e podem apresentar diversos tipos de especializações morfológicas, fisiológicas e no comportamento que provavelmente evoluíram em resposta às pressões seletivas presentes em cavernas e\ou à ausência de pressões seletivas típicas do meio epígeo. Freqüentemente nestes organismos há a redução das estruturas oculares, despigmentação e o alongamento de apêndices sensoriais (Romero & Green 2005, Culver & Pipan 2009).

As comunidades de invertebrados terrestres cavernícolas, por sua vez, também podem ser categorozidas em função de relações ecológicas com o ambiente subterrâneo em "comunidades para-epígeas", "comunidades recurso-espaço-dependentes" ou "comunidades recurso-espaço-independentes" (Ferreira & Martins 2001, Prous *et al* 2004).

Comunidades para-epígeas são compostas por um conjunto de espécies que se distribuem preferencialmente em regiões próximas às entradas das cavernas. Nestas, é comum a ocorrência de espécies epígeas e hipógeas, bem como de certas espécies exclusivamente encontradas nestas regiões.

As comunidades recurso-espaço-dependentes são aquelas constituídas por espécies que geralmente ocorrem associadas aos locais onde se encontra o recurso alimentar. Geralmente são predadores e detritívoros de mobilidade limitada, incapazes de percorrer periodicamente grandes extensões em busca de alimento. Tais espécies, em geral, só abandonam o recurso quando este se torna inviável ao consumo (e.g. comunidades associadas ao guano de morcegos).

Comunidades recurso-espaço-independentes são constituídas de espécies geralmente predadoras e detritívoras de alta mobilidade, capazes de se deslocar freqüentemente à procura de alimento. Tais organismos não se limitam à área onde se encontra o recurso alimentar, podendo ocorrer distribuídos em grandes extensões de uma caverna se outros fatores ambientais não limitarem sua distribuição.

3.5.Características ecológicas das cavernas ferruginosas

Grande parte do conhecimento da fauna cavernícola brasileira é oriunda de estudos realizados em cavernas calcárias (Trajano & Moreira, 1991, Gomes *et al* 2000, Ferreira 2004, Ferreira 2005, Souza-Silva et al 2011). Outros estudos realizados em um menor número de cavernas ferruginosas, magmáticas e siliciclásticas mostraram que tais sistemas mantêm ricas e abundantes comunidades de invertebrados (Ferreira 2004, Ferreira 2005, Souza-Silva 2008). Entretato é importante descatar que os estudos relacionados à biodiversidade cavernícola brasileira ainda são incipientes e que poucas cavernas, foram estudadas de forma a avaliar a estrutura das comunidades de forma mais ampla.

Cavernas ferruginosas apresentam como característica marcante, a elevada riqueza e grande variedade de grupos troglomórficos, da fauna de invertebrados (Ferreira 2005, Ferreira 2006, Souza-Silva et al 2011). A singularidade da estrutura das comunidades e a funcionalidade trófica dos ambientes de cavernas ferruginosas foram muito bem descritas por Ferreira (2005). Segundo este autor, cavernas ferruginosas apresentam-se como ambientes variáveis, mas com elevada riqueza de espécies, quando comparados a cavernas de mesma extensão em outros tipos de rochas. A existência de grande quantidade de canalículos que conformam uma extensa rede de espaços intersticiais (meso e microcavernas) conectados às macrocavernas torna os sistemas subterrâneos ferruginosos habitats com extensões que promovem uma ampla disponibilidade e variedade de habitas para manutenção de uma rica fauna (Ferreira 2005). Associadas à superficialidade destas cavernas, as microcavernas são utilizadas por inúmeros organismos que transitam desde a superfície (provenientes de habitats lapidícolas) até regiões mais interiores, acessando, frequentemente, as macrocavernas. As relações tróficas nestas macrocavernas são sustentadas pela produtividade primária de raízes oriundas de poucas árvores externas, manchas de guano e poucos depósitos de matéria orgânica vegetal (Ferreira 2005). Desta forma, a disponibilidade de microhabitas e recursos alimentares atuam na "concentração" da diversidade subterrânea nas macrocavernas ferruginosas (Ferreira 2005, Souza-Silva et al 2011). Assim, nos sistemas ferruginosos a ocorrência de compartimentos sub-superficiais provavelmente podem atuar permitindo uma migração da fauna através de espaços intersticiais entre macrocavernas e atuar aumentando a riqueza através do balanço entre os processos de colonização e extinção.

3.6.O conhecimento e a conservação da fauna cavernícola

O conhecimento da fauna de cavernas apresenta ampla problemática no tocante à acurácia das técnicas utilizadas para inventariar a fauna de invertebrados. Além disto, se observa uma maior atenção voltada aos estudos da fauna de cavernas em rochas carbonáticas em detrimento a outros tipos de rochas (Souza-Silva 2008).

Segundo Ferreira (2005) historicamente, inventários de invertebrados em cavernas têm dado pouca atenção a microhabitats (como espaços sob rochas, fendas, etc.) pelo fato de que as condições prevalentes em sistemas cavernícolas (ausência de luz, temperatura constante e elevada umidade) sugerem uma ausência de necessidade (pelos organismos cavernícolas) de abrigarem-se buscando condições microclimáticas específicas. Entretanto, tal concepção equivocada, tem levado grande parte dos inventários faunísticos desconsideram a importância dos microhabitats como um abrigo para a fauna cavernícola, gerando listagens de fauna que não refletem a real estrutura das comunidades presente em uma dada caverna. Mesmo em regiões mais profundas de cavernas, vários invertebrados abrigam-se em microhabitats em busca de condições microclimáticas ou até mesmo em função da própria determinação comportamental de muitos grupos de invertebrados de solo.

Não é possível propor medidas efetivas de monitoramento e preservação da fauna das cavernas se não compreendermos de forma acurada a composição, abundância e riqueza das comunidades subterrâneas. Deste modo, o planejamento de ações conservacionistas sugere como um dos passos primordiais para a implantação de planos de conservação, a compilação da diversidade, entendendo seus padrões de distribuição, abundância das espécies e ameaças, no intuito de elencar áreas biológicas representativas e temporalmente persistentes (Margules

& Pressey 2000). Desta forma, o mapeamento da biodiversidade subterrânea tem sido uma ferramenta primordial para a conservação, manejo e monitoramento da fauna em regiões naturais ou alteradas pelo homem (Culver & Sket 2000).

O uso de metodologias que integrem aspectos do ambiente físico e seu entorno, com a diversidade, raridade e riqueza de espécies, podem promover uma importante base para iniciar ações de conservação e manejo do ambiente de cavernas.

4. INSERÇÃO DAS CAVERNAS NO CONTEXTO GEOLÓGICO, GEOMORFOLÓGICO E FITOGEOGRÁFICO

4.1.Geologia Regional

A Província Mineral de Carajás (PMC) está situada na porção sudeste do estado do Pará, sendo limitada a leste pelos rios Araguaia-Tocantins, a oeste pelo rio Xingu, a norte pela Serra do Bacajá e a sul pela serra dos Gradaús (DOCEGEO, 1988). Situada na porção leste da Província Mineral de Carajás, a Serra Leste é composta por três unidades litoestratigráficas: Grupo Rio Novo, Grupo Grão Pará e Complexo Estrela (Quadro 4.1).

Os grupos Rio Novo e Grão Pará correspondem às sequências metavulcanossedimentares. De acordo com Araújo & Maia (1991), o Grupo Rio Novo encontra-se em contato discordante com rochas do complexo Xingu, Granito Estrela e Granito Cigano (Figura 4.1 e Figura 4.2). Compreende uma sequência supracrustal de natureza vulcanossedimentar, metamorfisada na fácies xisto-verde, contendo formações ferríferas, xistos, anfibolitos, rochas máficas e ultramáficas. A porção sedimentar é constituída por xistos micáceos, com textura lepidogranoblástica, compostos principalmente por biotita, muscovita, quartzo e feldspato. Os corpos ultramáficos ocorrem associados às rochas máficas, apresentando como mineralogia principal tremolita e clorita e, em menor proporção, actinolita.

Segundo Costa (2007), o grupo Rio Novo pode ser dividido em três unidades informais: (i) Arqueano Grupo Rio Novo, porção sedimentar (Arns), composta principalmente por rochas metassedimentares clasto-químicas; (ii) Arqueano Grupo Rio Novo Indiviso (Arni), em que predominam rochas metavulcanossedimentares; (iii) Arqueano Grupo Rio Novo, porção vulcanossedimentar (Arnv), caracterizada pela predominância de rocha vulcânica máfica e subordinadamente por formações ferríferas e anfibolitos (Figura 4.1 e Figura 4.2). Ainda segundo o autor, o Grupo Grão Pará é composto por formações ferríferas da Formação Carajás, metavulcânicas da Formação Parauapebas e rochas metassedimentares da Formação Águas Claras. A posição desta formação no Grupo Grão Pará é controversa e, alguns autores, como Tallarico *et al.* (2003), discordam, posicionando a Formação Águas Claras como unidade estratigráfica sobreposta ao Grupo Grão Pará. O Complexo Estrela compreende granitos, monzonitos, sienitos e dioritos (COSTA, 2007). A unidade é sin-orogênica e intrusiva em relação às rochas vulcanossedimentares do Grupo Rio Novo.

Quadro 4.1 - Coluna estratigráfica e resumo de dados geocronológicos de Província Mineral de Carajás (extraído e adaptado de Tallarico *et al.*, 2003).

Era	Complexos ou Supergrupos	Grupos ou Formações	Rochas Intrusivas	Idade (Ma)
Proterozóico			Granito Central Carajás	1.820 +- 49
				1.880 +- 2
			Granito Cigano	1.883 +- 3
				1.731 +- 28
			Diques basálticos, andesíticos e riolíticos	1.800
Arqueano			Granito Itacaiúnas	2.560 +- 37
			Sill gabróico Águas Claras	2.645 +- 12
		Águas Claras		2.681 +- 5
			Granito Estrela	2.527 +- 34
				2.763 +- 7
			Suíte granitóide Plaquê	2.736 +- 24
	Supergrupo Itacaiúnas	Igarapé Bahia		2.577 +- 72
				2.747 +- 2
		Grão Pará		2.759 +- 2
				2.758 +- 39
				2.760 +- 11
			Intrusão máfica- ultramáfica Luanga	2.763 +- 6
		Rio Novo		_
	Complexo Xingu			2.859 +- 2
				2.974 +- 15
				2.971 +- 30
				2.798

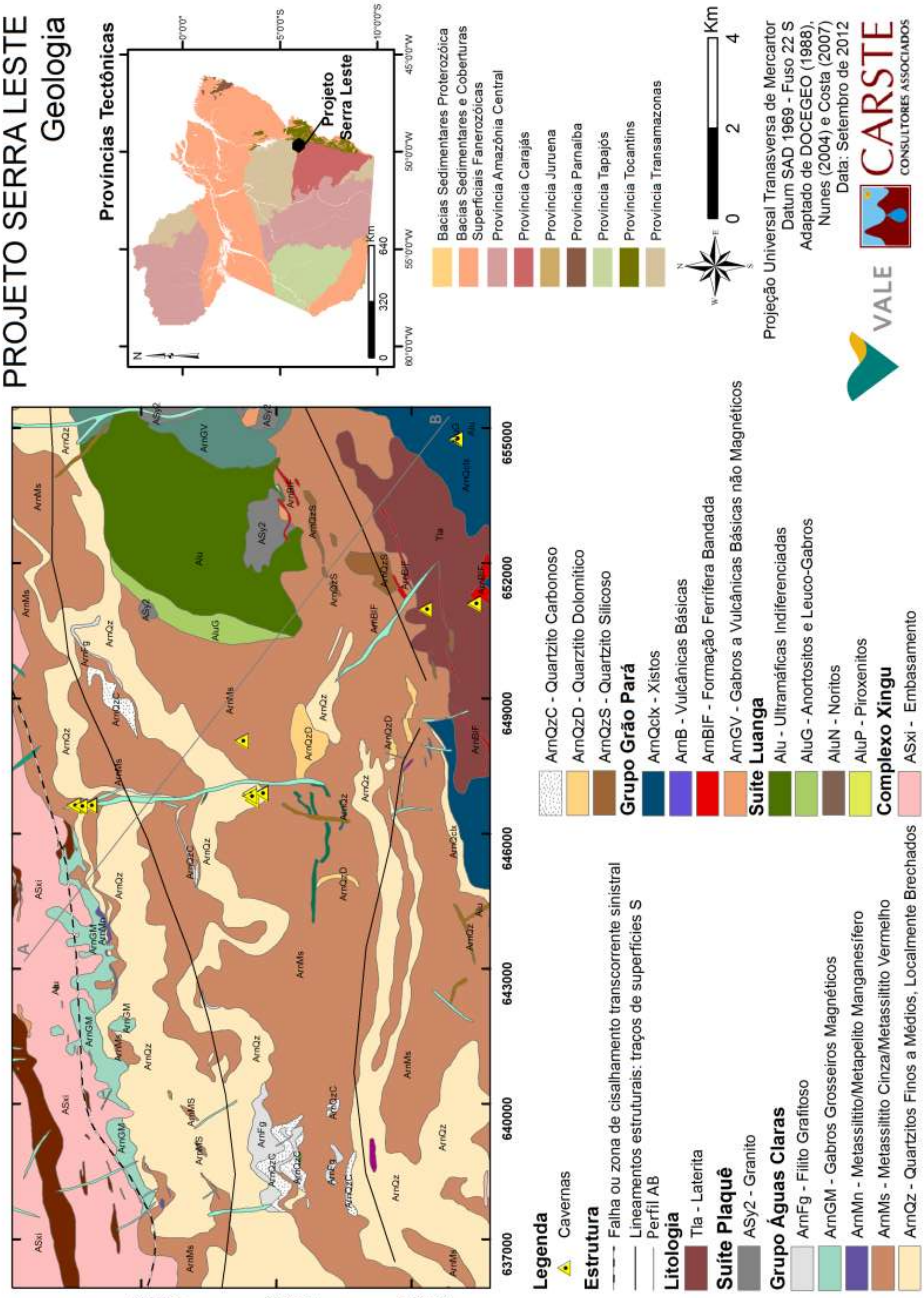

Figura 4.1 - Mapa geológico de Serra Leste. Adaptado de DOCEGEO, 1988.

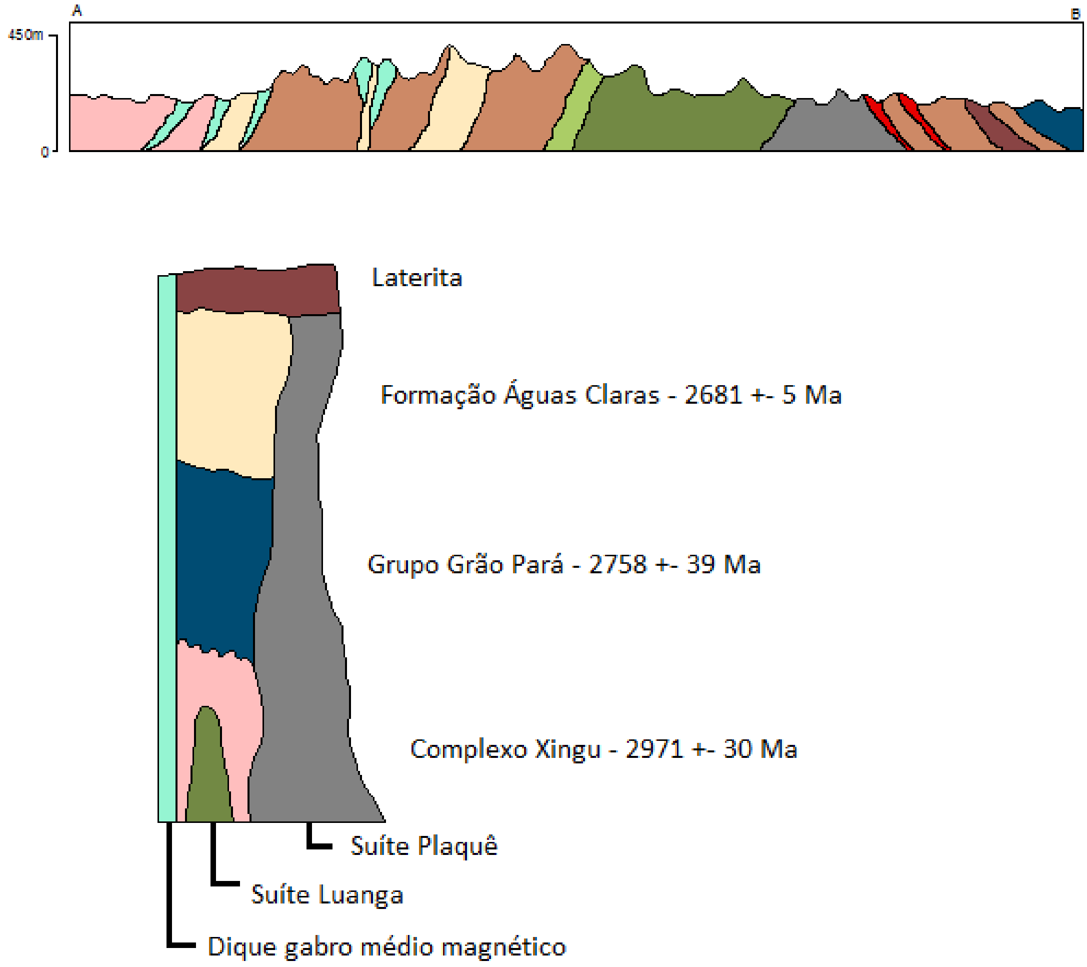

Figura 4.2 - Perfil geológico e coluna estratigráfica esquemáticos de Serra Leste. O mergulho e a espessura das camadas foram adaptados de Costa *et al*. 2007.

A Serra Leste está dentro da área de influência das zonas de cisalhamento regionais Carajás e Cinzento (Figura 4.1), sendo caracterizada pelo desenvolvimento de foliações com orientação subparalela às zonas de cisalhamento. O sistema Cinzento é caracterizado por estruturas penetrativas. Já no sistema Carajás, as estruturas tendem a ser mais discretas com direção sub-paralela a falha Carajás.

Segundo Rosière *et al.* (2005), as características geométricas e cinemáticas das estruturas tectônicas na Província de Carajás indicam uma evolução associada a um encurtamento regional, de direção aproximadamente NE-SW. O primeiro evento principal que afetou a região é associado a tectônica compressiva oblíqua. Esse evento foi responsável por dobramento com sistema de eixo com caimento moderado para WNW das diferentes sequências vulcanossedimentares na província, que originalmente teriam uma estruturação (*strike*) NE-SW. A idade da deformação é associada ao Granito Estrela (BARROS, 1997), plúton que intrudiu as sequências vulcanossedimentares e também mostra condições de deformação por achatamento.

Um segundo evento deformacional é caracterizado por uma tectônica transcorrente, registrado em diversas fraturas, falhas, lineamentos regionais de orientação E-W e em uma clivagem discordante ao bandamento composicional nas rochas metassedimentares, e do bandamento ígneo nos plútons. Este evento é consequência da acomodação da deformação

por cisalhamento puro do primeiro evento por escape lateral. Esse tectonismo seria responsável pelos fortes contrastes no grau metamórfico em escala local a subregional. Durante os dois eventos, possivelmente resultados de uma deformação progressiva, as sequências vulcanossedimentares experimentaram deformação regional em condições dúcteis-rúpteis a rúpteis, com exceção das proximidades dos granitos sintectônicos ou dos domos estruturais, onde a temperatura relativamente mais elevada permitiu o desenvolvimento de estruturas dúcteis.

A Figura 4.3 mostra esquematicamente os principais eventos deformacionais da Província Mineral de Carajás (PMC).

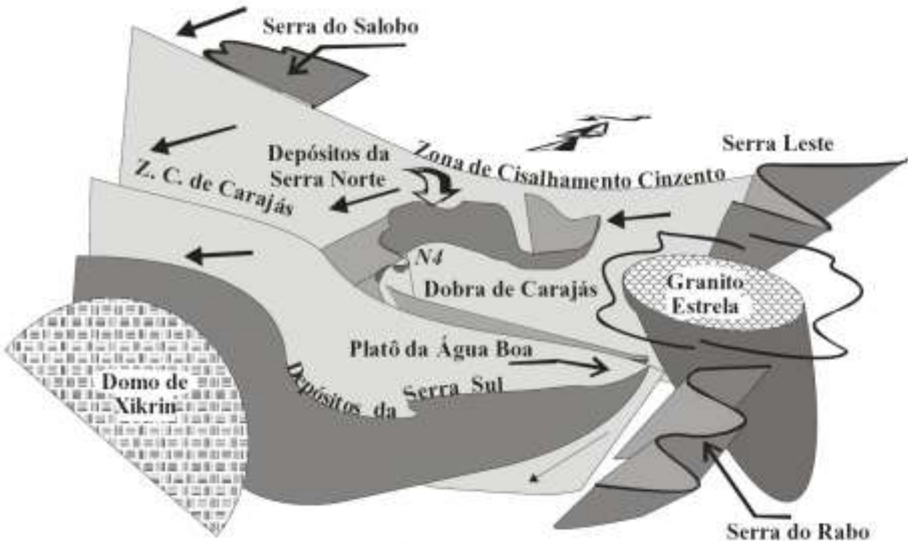

Figura 4.3 - Bloco diagrama esquemático da Província Mineral de Carajás e arredores. A escala horizontal é aproximada e a vertical exagerada, a charneira da dobra de Carajás apresenta caimento entre 20 e 35º W. Fonte: Rosière et al 2004 e 2005.

4.2.Geomorfologia

A Serra dos Carajás encontra-se inserida no domínio do Planalto Dissecado do Sul do Pará, caracterizado por maciços residuais de topo aplainado ou ondulado, além de cristas e picos interpenetrados por faixas de terrenos rebaixados (RADAMBRASIL, 1974). Essa unidade apresenta-se intensamente dissecada por vales encaixados, adaptados às redes de fraturas em rochas arqueanas, além de algumas estruturas proterozóicas.

Entre as principais serras tem-se: Serra Norte, Serra Leste, Serra da Bocaina, Serra do Tarzan e a Serra Sul. Alguns autores colocam essas elevações como testemunhos de uma superfície de aplainamento resultante de uma pediplanação terciária que reelaborou um pediplano cretácico ou pré-cretácico (RADAMBRASIL, 1974).

Nas porções mais altas das serras, muitas vezes ocorrem coberturas lateríticas e concrecionárias que recobrem as rochas ferríferas arqueanas, pertencentes à Formação Carajás (Grupo Grão-Pará). Esses platôs de canga ferrífera apresentam-se como fragmentos de dimensões variadas, apresentando uma evolução morfodinâmica singular no contexto regional. Possuem encostas com predomínio de feições côncavas portadoras de depósitos de

tálus grosseiros originários da erosão e solapamento da cobertura de canga que reveste e mantém os platôs.

A dissolução química da base dos saprolitos ferríferos, abaixo da canga cimentada, possibilita o desenvolvimento de feições “doliniformes” e cavernas.

4.3.Fitogeografia

A Serra Leste localiza-se na borda sudeste da grande Região Amazônica que é coberta por floresta ombrófila densa, formação caracterizada por fanerófitos, além de lianas lenhosas e epífitas em abundância que os diferenciam das outras classes de formações. Porém, sua característica ecológica principal reside nos ambientes ombrófilos que marcam muito bem a “região florística florestal”. Dominam nos ambientes dessas florestas os latossolos com características distróficas e raramente eutróficas, originados de vários tipos de rocha desde as cratônicas (granitos e gnaisses) até os arenitos com derrames vulcânicos de variados períodos geológicos.

A região em específico apresenta uma variação no subtipo: floresta ombrófila densa submontana. Apresenta formação florestal com fanerófitos com alturas aproximadamente uniformes. A submata é integrada por plântulas de regeneração natural, poucos nanofanerófitos e caméfitos, além da presença de palmeiras de pequeno porte e lianas herbáceas em maior quantidade. Sua principal característica fica por conta dos fanerófitos de alto porte. Esta formação é caracterizada por ecótipos que variam influenciados pelo posicionamento dos ambientes de acordo com a latitude, ressaltando-se também a importância do fator tempo nesta variação ambiental.

Uma importante ocorrência de vegetação para Serra Leste é o campo rupestre que recobre exclusivamente, no alto das serras. Em geral, o campo é aberto e atravessado por inúmeros riachos e rios permanentes. O solo é pedregoso, possui baixa capacidade de retenção de água e as formações rochosas são muito comuns, crescendo a maior parte das plantas nas pequenas frestas erodidas. Como após as chuvas as águas escoam rapidamente por sobre as rochas, não há formação de lençol freático. O ambiente, portanto, é seco, e as plantas desenvolveram adaptações diversas para lidar com o problema da falta de água. A biodiversidade deste ecossistema é grande, variando inclusive de uma região para outra. As plantas são quase todas rasteiras, encontrando-se, arbustos baixos.

Diversos tipos de líquens, orquídeas e sempre-vivas são encontrados na região, além de inúmeras outras plantas de grande valor ornamental, como o Paepalanthus, por exemplo. A fauna dos campos rupestres é rica em espécies de anfíbios, répteis, aves e pequenos mamíferos, além de uma infinidade de insetos.

5. APRESENTAÇÃO DOS RESULTADOS

5.1. Estudos Geoespeleológicos

Na presente seção serão apresentados os resultados dos estudos geoespeleológicos realizados nas 21 cavernas de Serra Leste (Figura 5.1). A Tabela 5.1 apresenta as coordenadas UTM, a altitude e os dados espeleométricos das cavidades. Quadros simplificados das cavidades contendo informações gerais, atributos relevantes, algumas fotos e o mapa das cavernas são inseridos neste item (Quadro 5.1 a Quadro 5.21). Os mapas topográficos das cavidades compreendem o Anexo I e, as fichas de campo encontram-se no Anexo II.

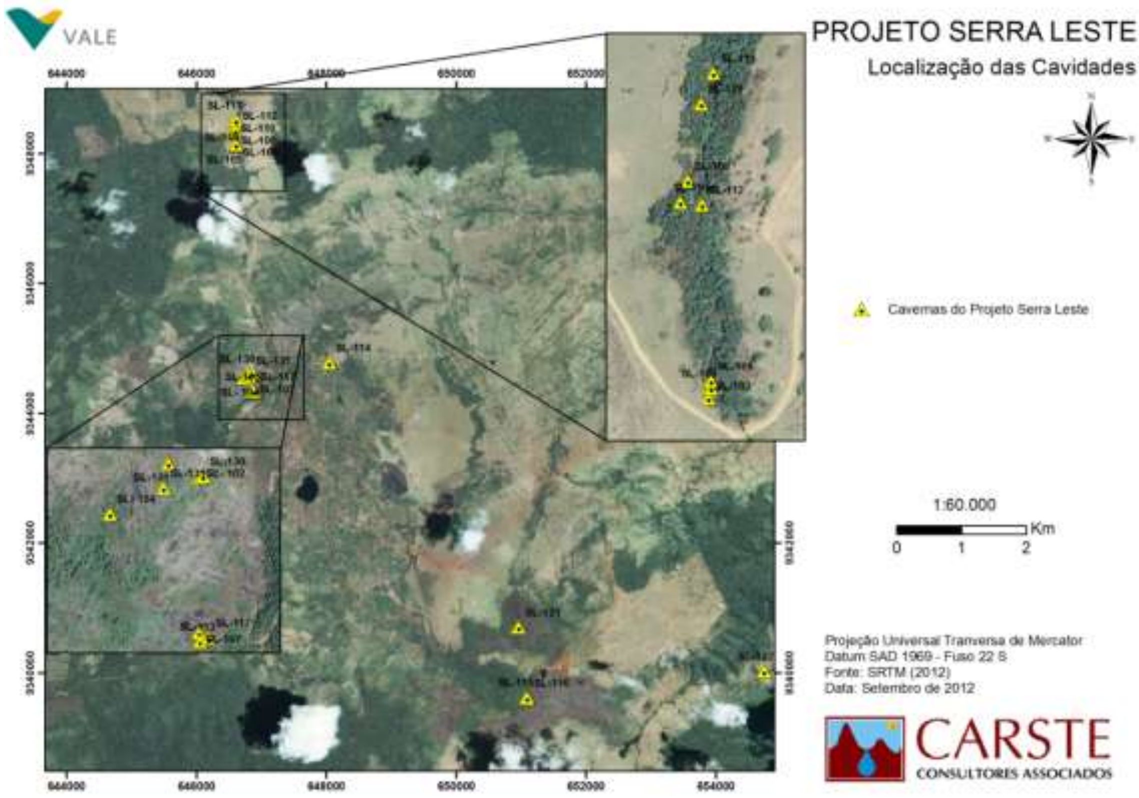

Figura 5.1 - Localização das cavernas em estudo na Serra Leste.

Tabela 5.1 - Coordenadas UTM e dados espeleométricos das cavidades estudadas em Serra Leste.

CAVIDADE	UTM E	UTM N	Altitude (m)	PH (m)	Desnível (m)	Área (m^2)	Volume (m^3)
SL-101	646758	9344588	336	460	32,26	1836	2662
SL-102	646852	9344610	305	20	2,33	43	37,5
SL-103	646590	9348127	245	23	3,43	60	102
SL-104	646718	9344510	351	13	6,33	40,5	78,5
SL-105	646578	9348133	225	23	1,99	38	40
SL-106	646596	9348147	235	8,5	0,71	9,5	5,5
SL-107	646856	9344346	271	39,5	8,47	115	150,5
SL-108	646568	9348388	228	24	1,42	70	91
SL-109	646584	9348481	224	15	1,79	77,5	100
SL-110	646559	9348362	232	32	2,72	254,5	280
SL-111	646599	9348518	240	8	3,7	55,5	138,5

SL-112	646584	9348356	226	21	1,38	149	283
SL-113	656857	9344336	257	5,5	1,02	11	8
SL-114	648031	9344788	250	25,5	1,29	111	89
SL-115	651081	9339629	636	12	1	32	34
SL-116	651081	9339618	629	9	2,18	16,5	18
SL-117	646875	9344347	264	9,5	2,79	29,5	27
SL-121	650952	9340707	466	68,5	1,64	138,5	154
SL-122	654731	9340026	286	34	1,36	171,5	199
SL-130	646864	9344606	299	18,5	3,62	50	38
SL-131	656797	9344587	348	13	5,33	47,5	45,5

MINUTA

Quadro 5.1 - Síntese das características da caverna SL-101.

SL-101			
**UTM E:** 646758	**UTM N**: 9344588	**Datum:** SAD’69	**Altitude:** 336 m
**Projeção Horizontal**: 460 m	**Desnível**: 32,26 m	**Área**: 1.836 m²	**Volume**: 2.662 m³

Litologia: Quartzito

Inserção: Caverna inserida na média vertente em paredão quartzítico escalonado.

Observações Principais: Fraturas condicionam a gênese da caverna que, provavelmente, teve início com fluxo vertical de água percolando entre as diáclases e, posteriormente, houve abatimento de blocos.

Atributo de Relevância: Espeleometria, morfologia e gênese única ou rara.

Fotos: (A) Entrada; (B) Salão amplo com piso recoberto por blocos abatidos; (C) Coralóides.

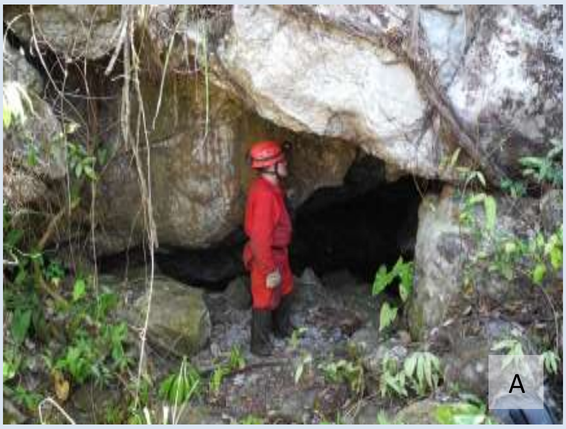

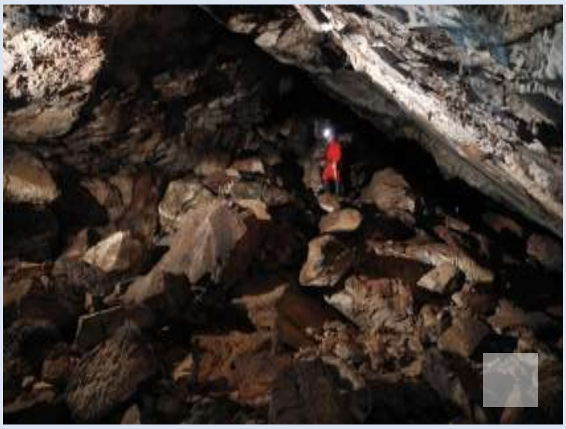

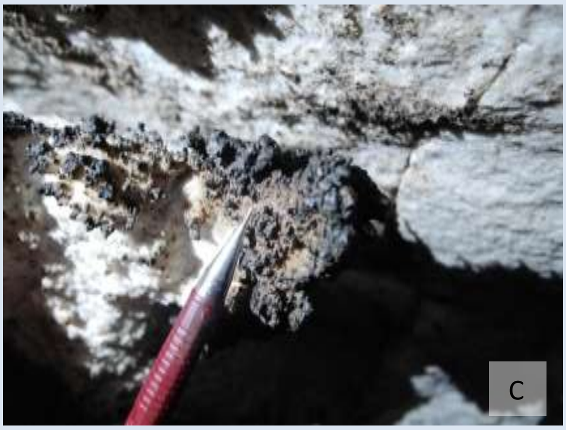

Planta Baixa

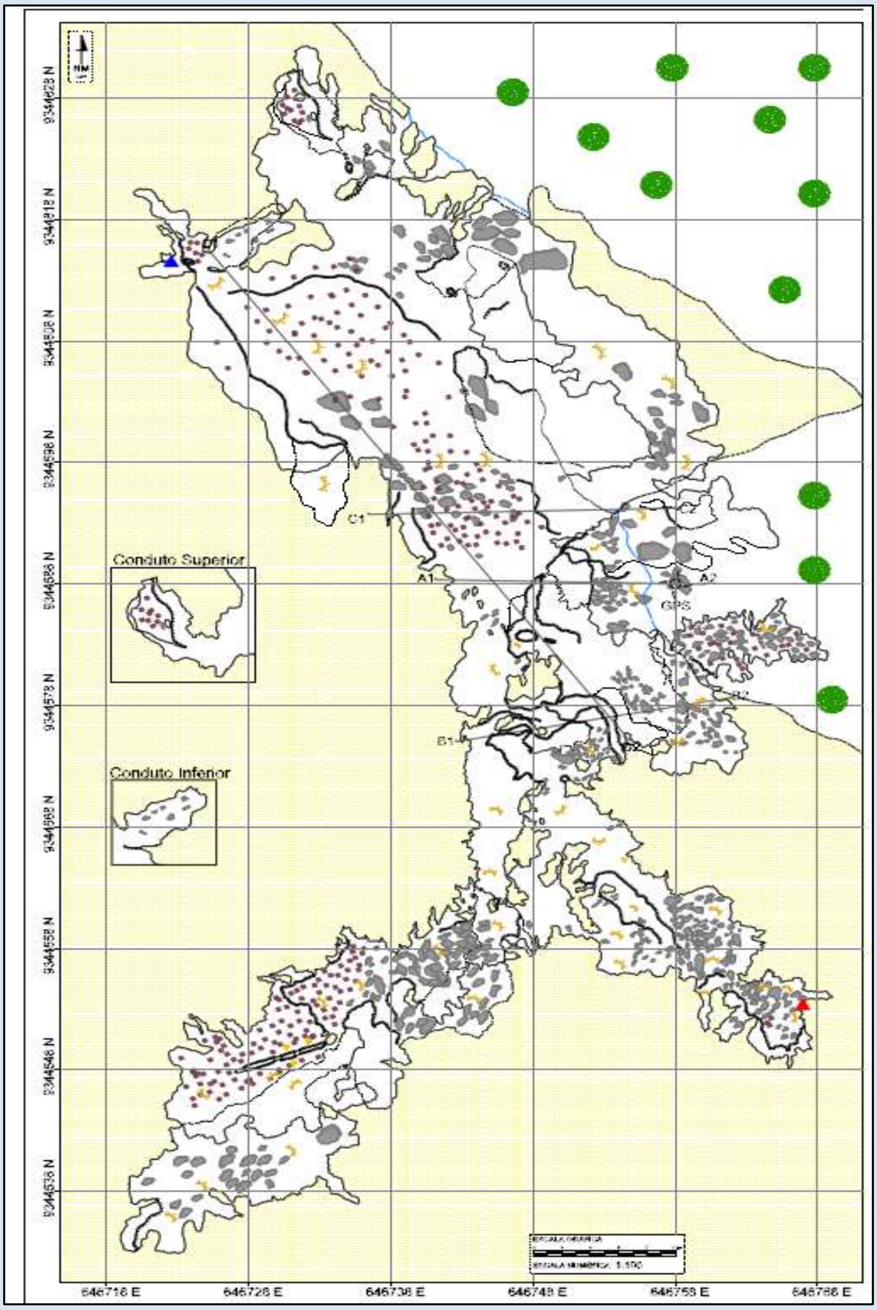

Seções

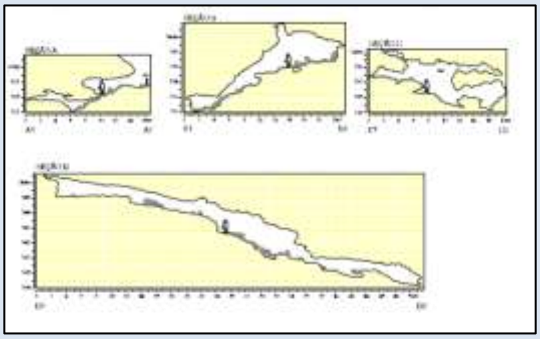

Quadro 5.2 - Síntese das características da caverna SL-102.

SL-102			
**UTM E:** 646852	**UTM N**: 9344610	**Datum:** SAD'69	**Altitude:** 305 m
**Projeção Horizontal**: 20 m	**Desnível**: 2,33 m	**Área**: 43 m²	**Volume**: 37,5 m³

Litologia: Quartzito

Inserção: Caverna inserida em maciço quartzítico, na média vertente.

Observações Principais: Apresenta teto baixo e piso recoberto por blocos e calhaus. Sua gênese provavelmente se iniciou a partir de fluxo de água entre as diáclases que individualizam blocos, culminando com o colapso dos mesmos.

Atributo de Relevância: Não foram observados atributos de relevância.

Fotos: (A) Entrada da caverna; (B) Salão com teto baixo e blocos abatidos; (C) Colônias de microorganismos e invertebrados.

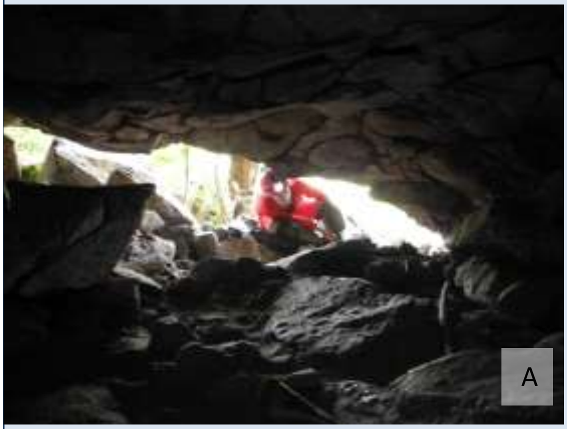
A

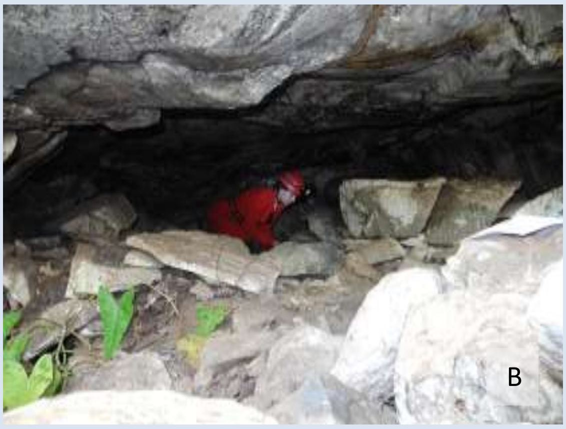
B

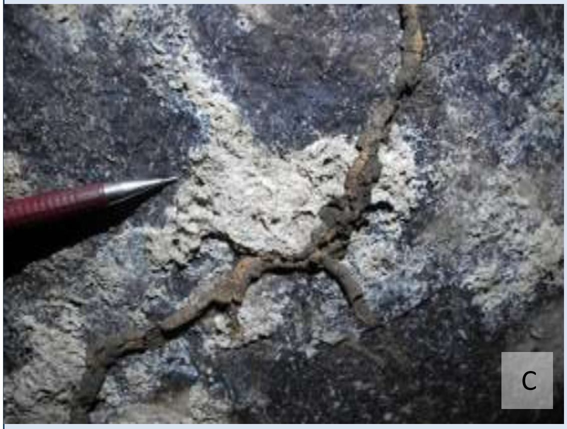
C

Planta Baixa

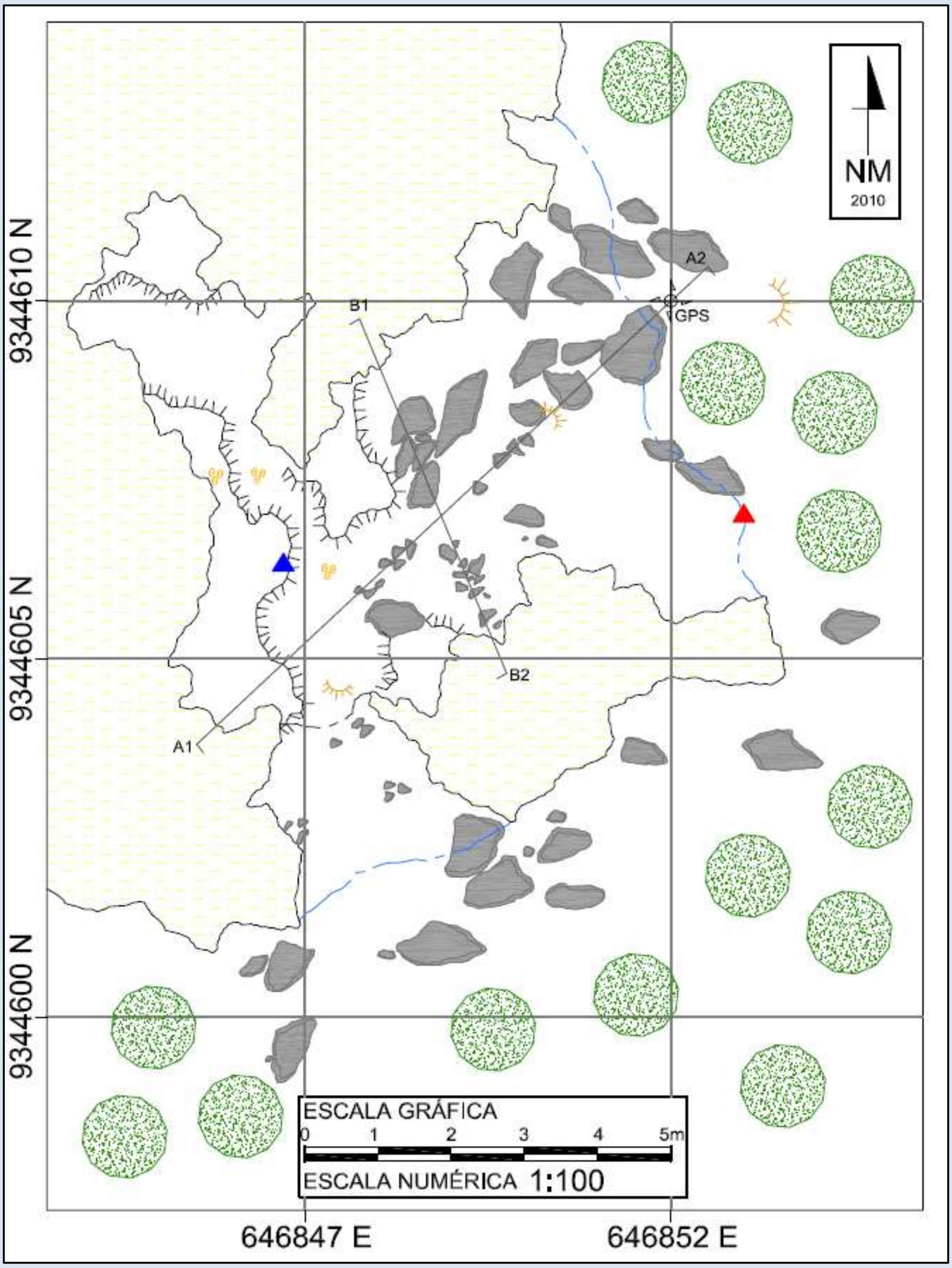

Seções

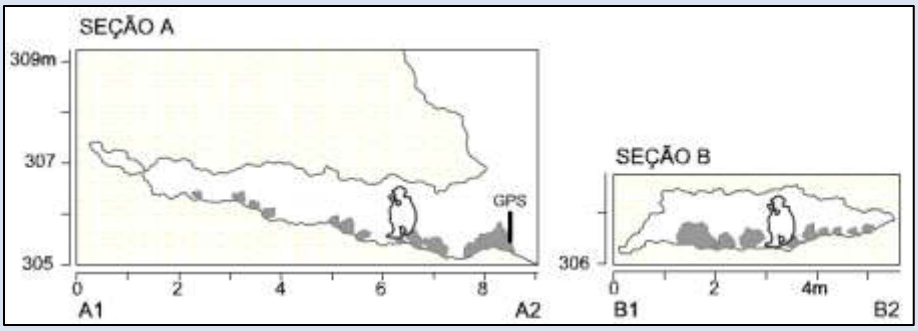

Quadro 5.3 - Síntese das características da caverna SL-103.

SL-103

UTM E: 646590 **UTM N**: 9348127 **Datum:** SAD'69 **Altitude:** 245 m

Projeção Horizontal: 23 m **Desnível**: 3,43 m **Área**: 60 m² **Volume**: 102 m³

Litologia: Contato entre quartzito e ferricrete.

Inserção: Caverna inserida em anfiteatro situado em paredão paralelo à margem direita de curso fluvial.

Observações Principais: Cavidade apresenta gênese associada à diferença na resistência erosiva entre as rochas que compõem as paredes e o teto. Apresenta coralóides orientados segundo a entrada de luminosidade e ar do setor proximal.

Atributo de Relevância: Não foram observados atributos de relevância.

Fotos: (A) Entrada da caverna; (B) Cúpula; (C) Salão amplo com teto formado por clastos angulosos.

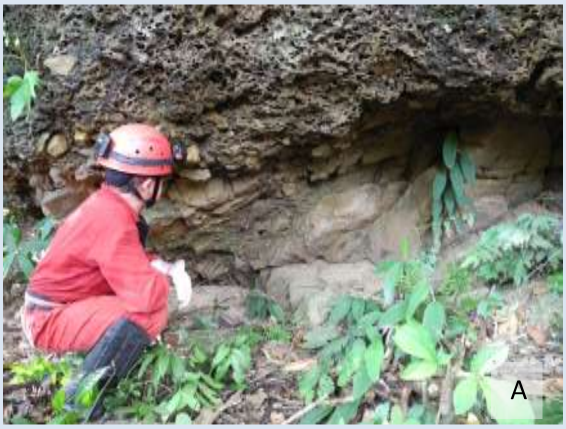
A

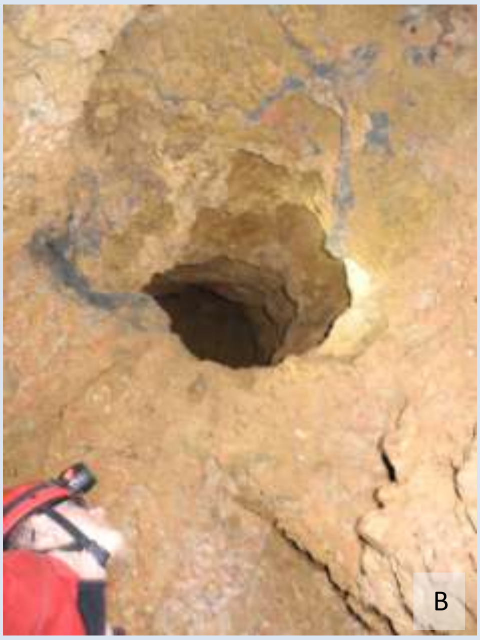
B

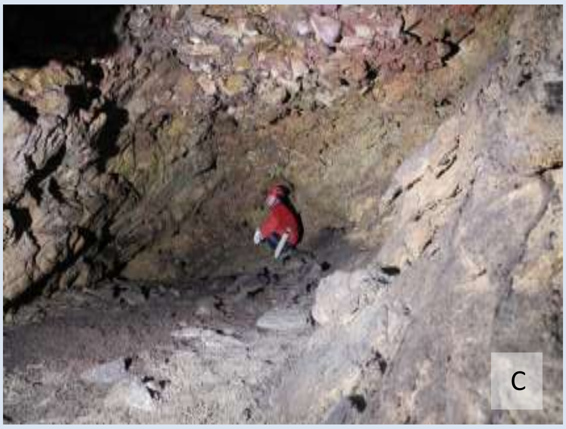
C

Planta Baixa

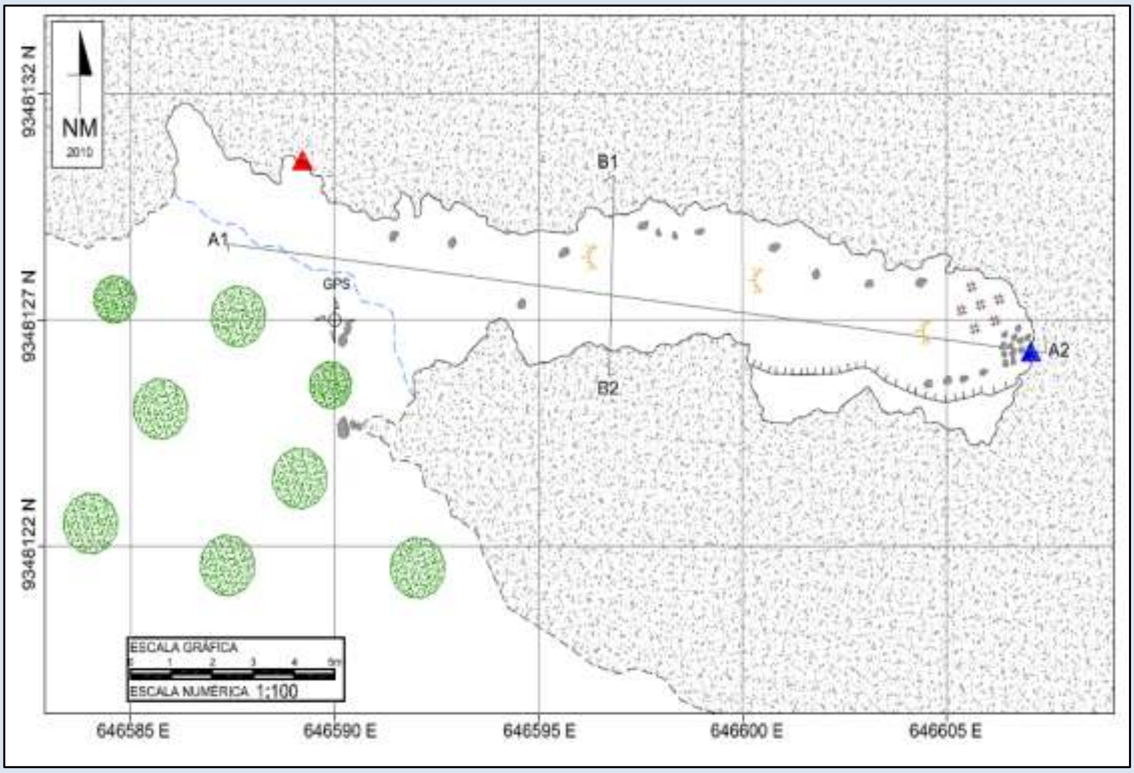

Seções

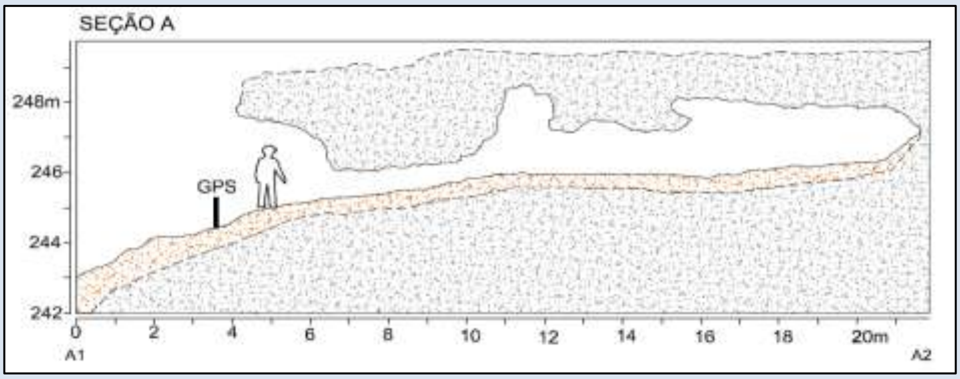

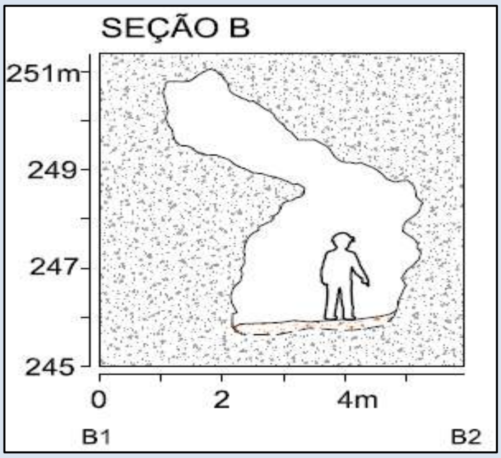

Quadro 5.4 - Síntese das características da caverna SL-104.

SL-104			
**UTM E:** 646718	**UTM N**: 9344510	**Datum:** SAD'69	**Altitude:** 351 m
**Projeção Horizontal**: 13 m	**Desnível**: 6,33 m	**Área**: 40,5 m²	**Volume**: 78,5 m³

Litologia: Quartzito

Inserção: Caverna inserida em paredão quartzítico, escalonado.

Observações Principais: Apresenta abatimentos significativos. A gênese está associada a fluxo de água possivelmente alargando uma fratura que poderia funcionar como um sumidouro na beira do paredão.

Atributo de Relevância: Caneluras oblíquas possivelmente guiadas pelo fluxo de ar.

Fotos: (A) Entrada da caverna; (B) Coralóides; (C) Salão com blocos abatidos.

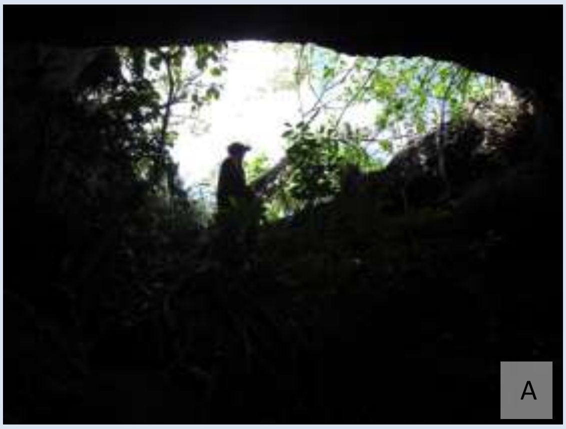

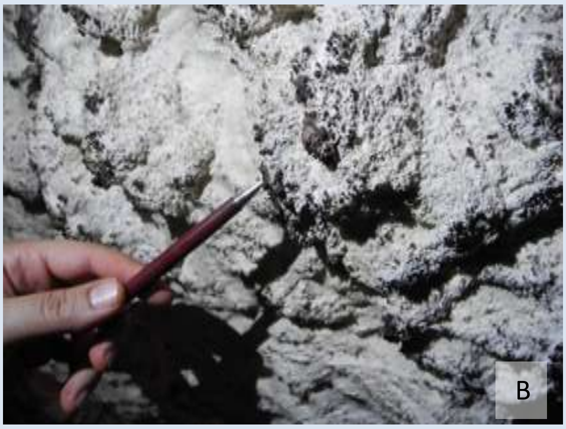

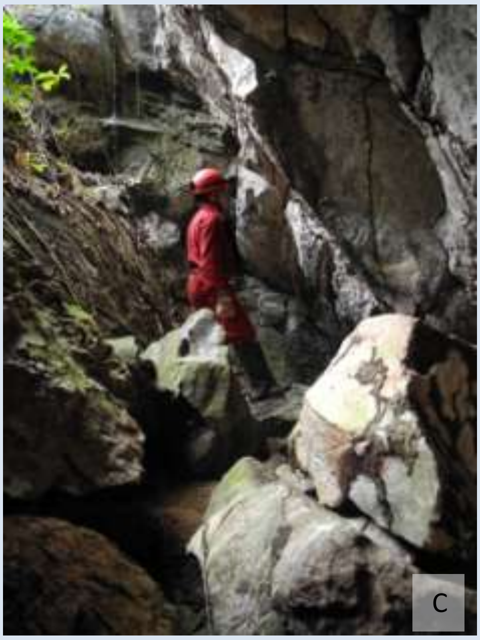

Planta Baixa

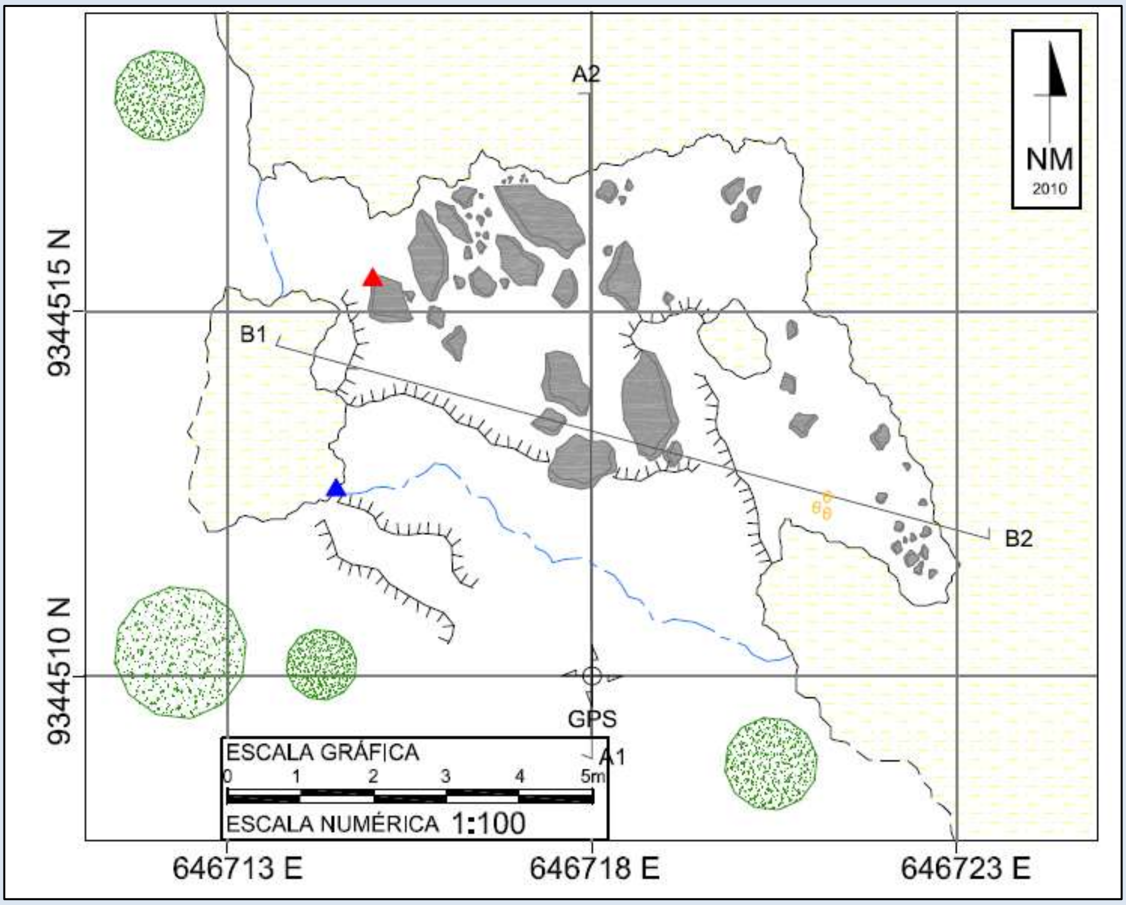

Seções

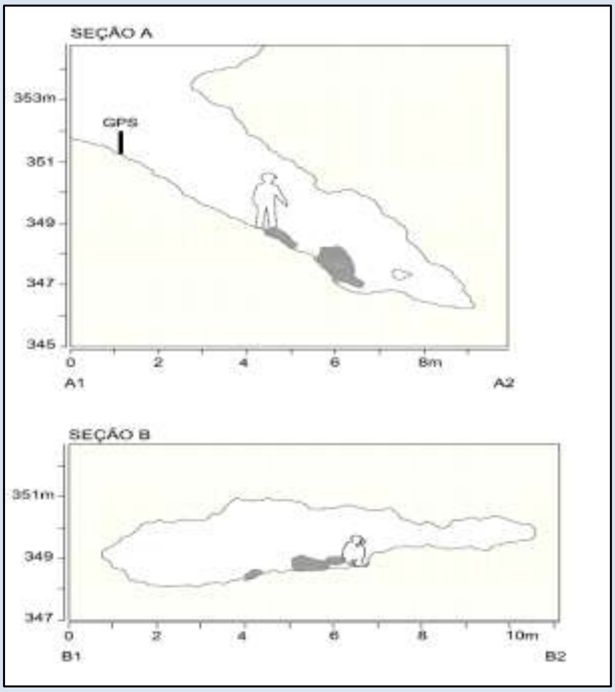

Quadro 5.5 - Síntese das características da caverna SL-105.

SL-105

**UTM E:** 646578	**UTM N**: 9348133	**Datum:** SAD'69	**Altitude:** 225 m
**Projeção Horizontal**: 23 m	**Desnível**: 1,99 m	**Área**: 38 m²	**Volume**: 40 m³

Litologia: Ferricrete.

Inserção: Caverna inserida na borda da calha de drenagem, na margem esquerda do curso d'água.

Observações Principais: Faz parte de um conjunto de cavidades inseridas na borda da calha fluvial, apresentando ferricrete nas paredes e clastos angulosos no teto. A caverna apresenta gênese mista.

Atributo de Relevância: Não foram observados atributos de relevância.

Fotos: (A) Entrada da caverna; (B) Clastos angulosos compõem o teto; (C) Conduto com teto baixo e piso regular.

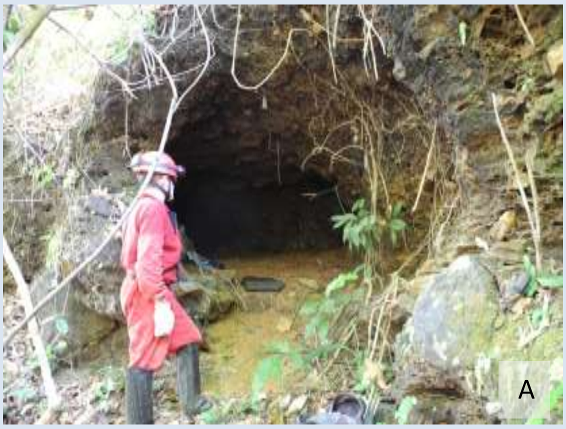

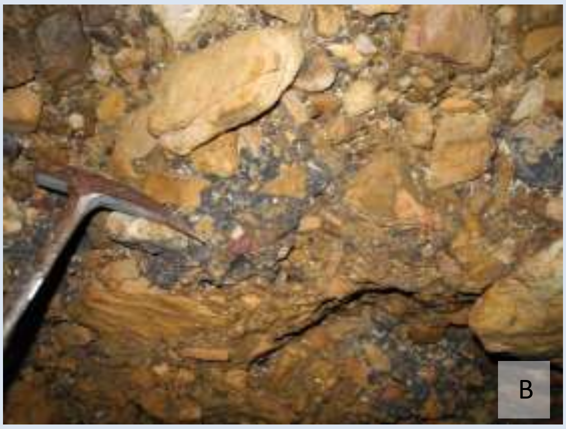

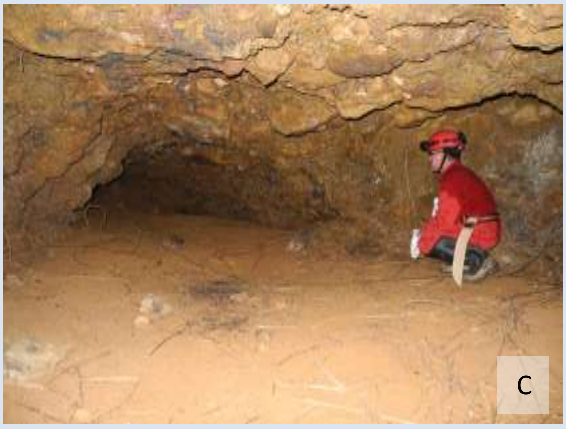

Planta Baixa

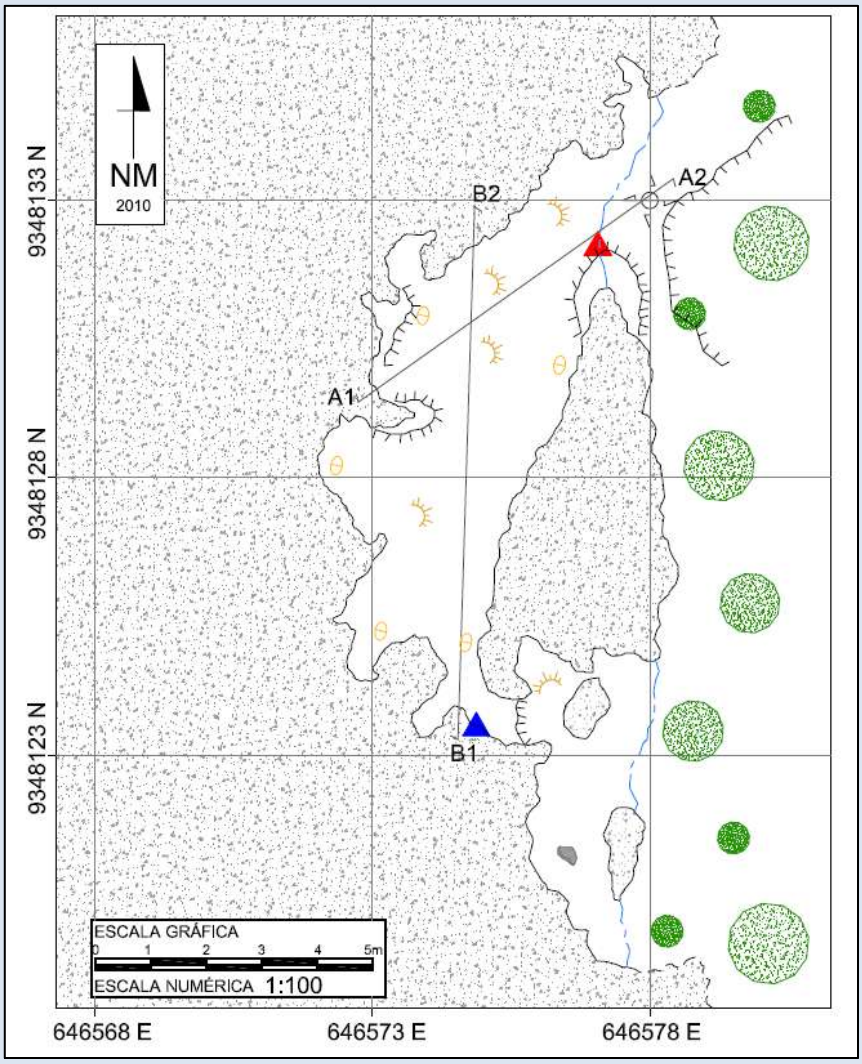

Seções

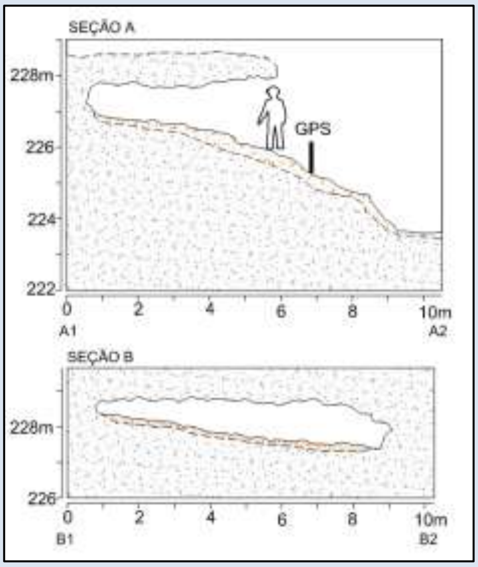

Quadro 5.6 - Síntese das características da caverna SL-106.

SL-106			
**UTM E:** 646596	**UTM N**: 9348147	**Datum:** SAD'69	**Altitude:** 235 m
**Projeção Horizontal**: 8,5 m	**Desnível**: 0,71 m	**Área**: 9,5 m²	**Volume**: 5,5 m³

Litologia: Ferricrete

Inserção: Caverna inserida em média vertente, em paredão irregular e em degraus.

Observações Principais: Cavidade retilínea possivelmente desenvolvida a partir de uma estrutura geológica. Apresenta estreitamento, que indica desenvolvimento ocluso com posterior abertura.

Atributo de Relevância: Não foram observados atributos de relevância.

Fotos: (A) Entrada da caverna; (B) Clastos angulosos dispostos no teto; (C) Conduto com teto baixo.

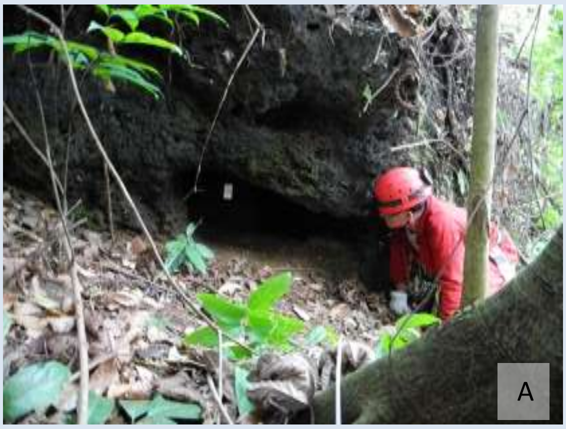

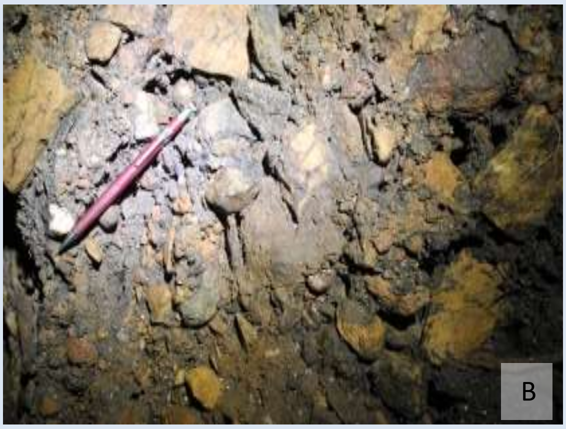

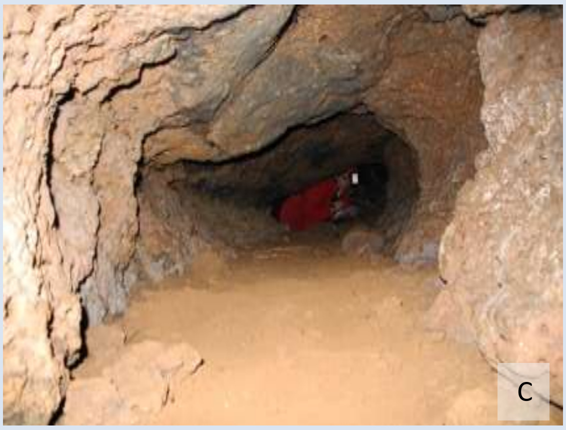

Planta Baixa

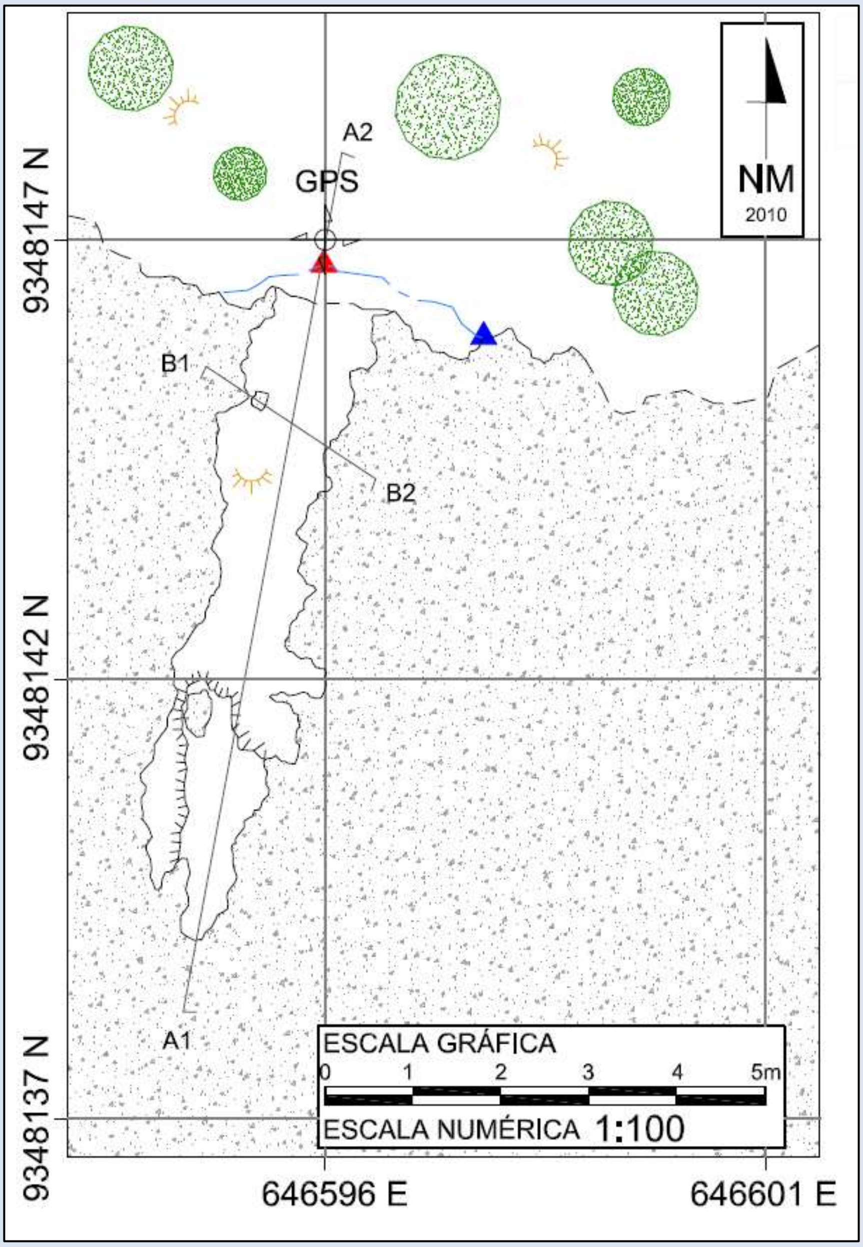

Seções

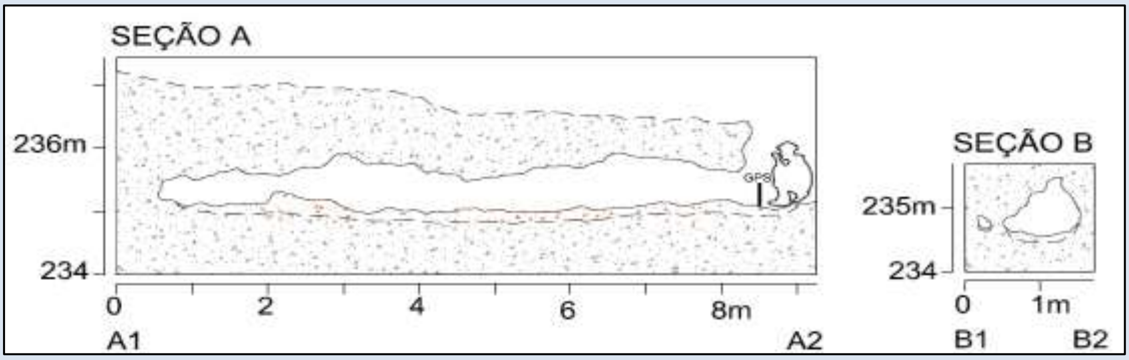

Quadro 5.7 - Síntese das características da caverna SL-107.

SL-107			
**UTM E:** 646856	**UTM N**: 9344346	**Datum:** SAD'69	**Altitude:** 271 m
**Projeção Horizontal**: 39,5 m	**Desnível**: 8,47 m	**Área**: 115 m²	**Volume**: 150,5 m³

Litologia: Quartzito

Inserção: Cavidade inserida em média vertente com duas entradas em oposição, direcionadas segundo a maior inclinação da vertente.

Observações Principais: Caverna rasa com duas entradas em sentidos opostos. Os processos de abatimento são fundamentais para a sua evolução.

Atributo de Relevância: Não foram observados atributos de relevância.

Fotos: (A) Entrada da caverna; (B) Feição de dissolução da rocha; (C) Salão com o piso coberto por blocos abatidos.

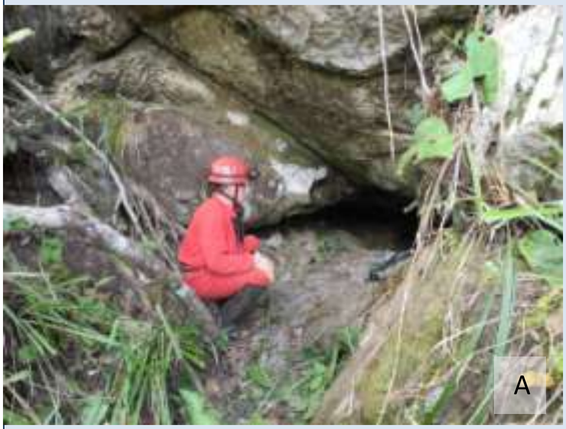

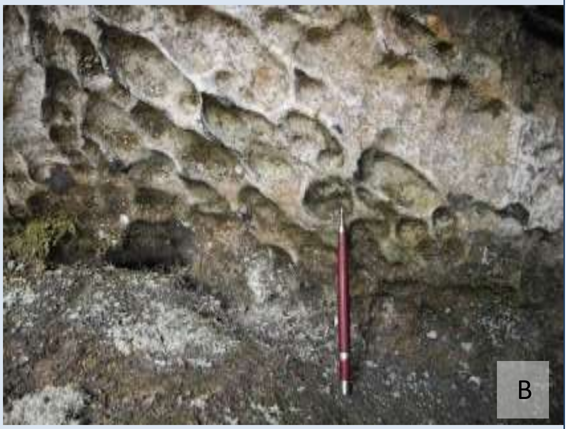

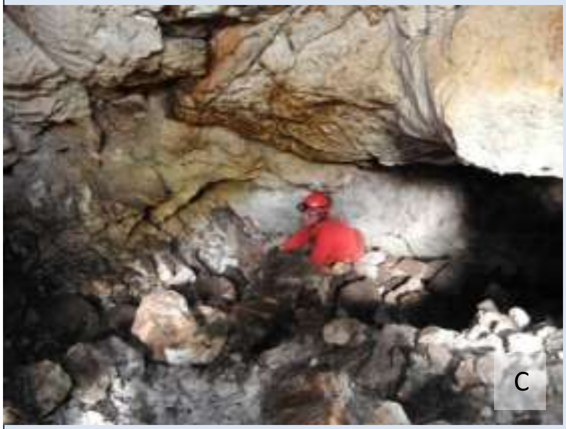

Planta Baixa

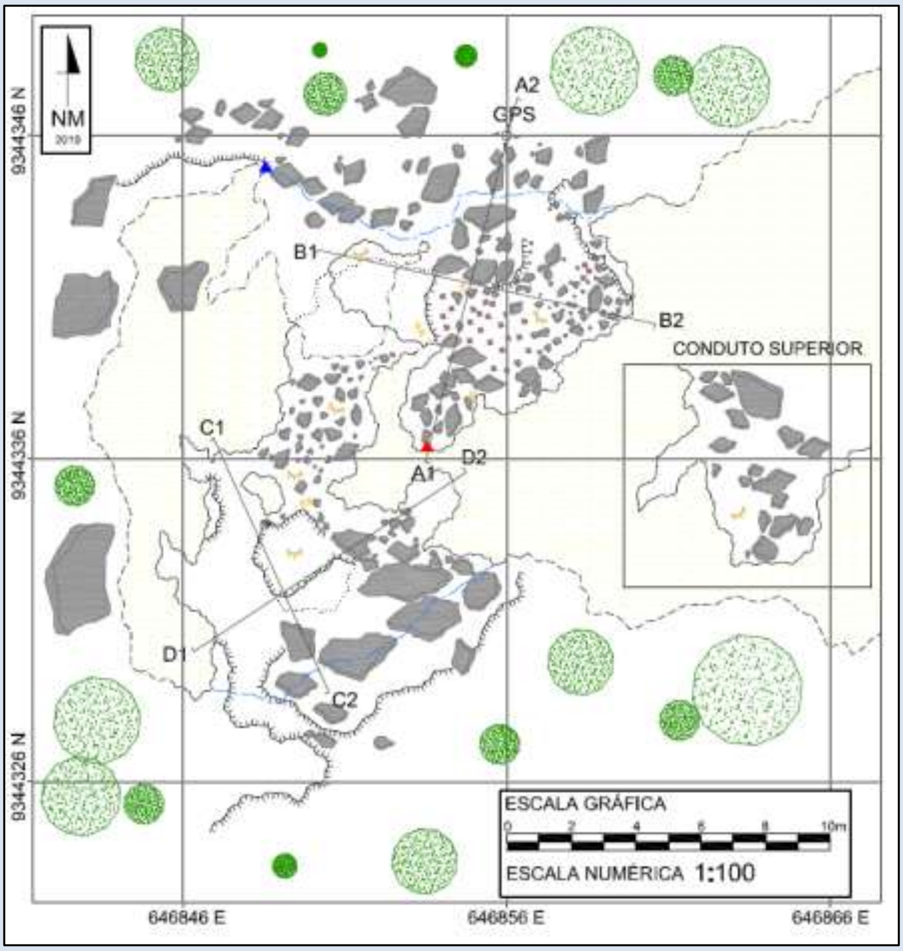

Seções

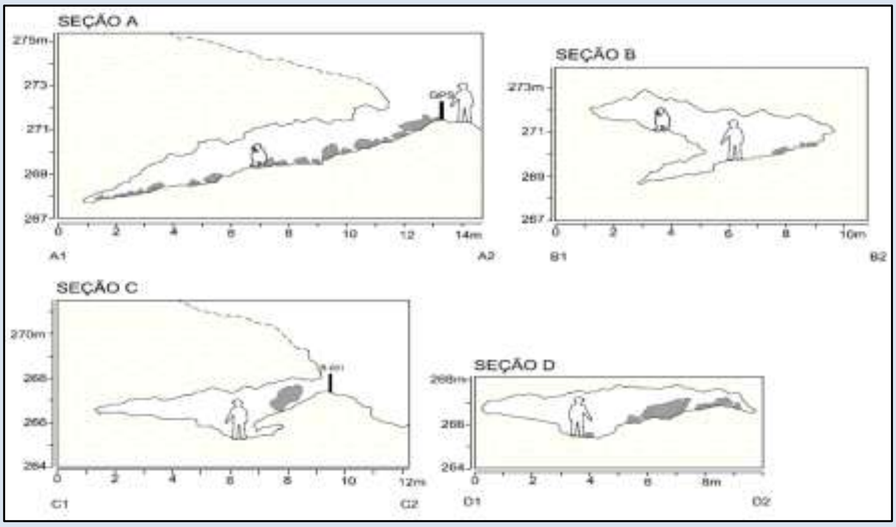

Quadro 5.8 - Síntese das características da caverna SL-108.

SL-108

UTM E: 646568 **UTM N**: 9348388 **Datum:** SAD'69 **Altitude:** 228 m

Projeção Horizontal: 24 m **Desnível**: 1,42 m **Área**: 70 m² **Volume**: 91 m³

Litologia: Ferricrete

Inserção: Caverna inserida em borda de calha de drenagem, na margem esquerda do curso fluvial em paredão paralelo à drenagem.

Observações Principais: Cavidade rasa constituída de conduto único com morfologia curvilínea e feições arredondadas.

Atributo de Relevância: Não foram observados atributos de relevância.

Fotos: (A) Entrada da cavidade; (B) Pilar; (C) Salão com teto baixo

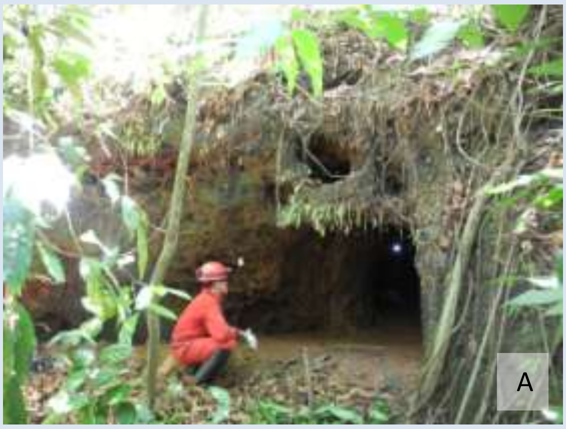
A

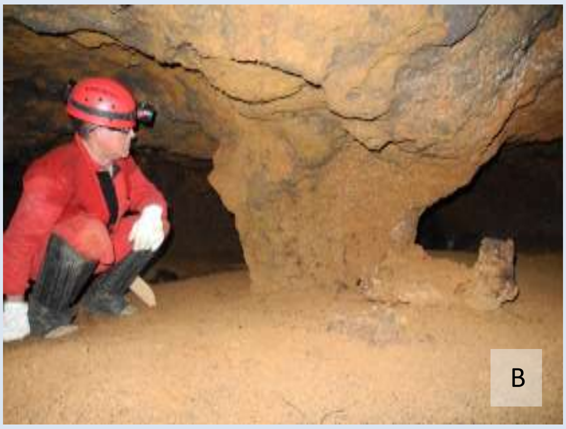
B

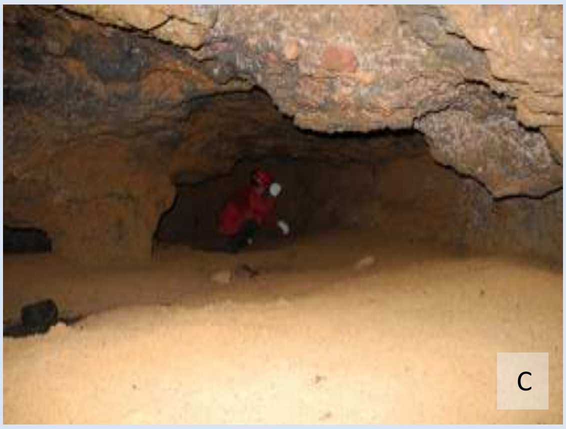
C

Planta Baixa

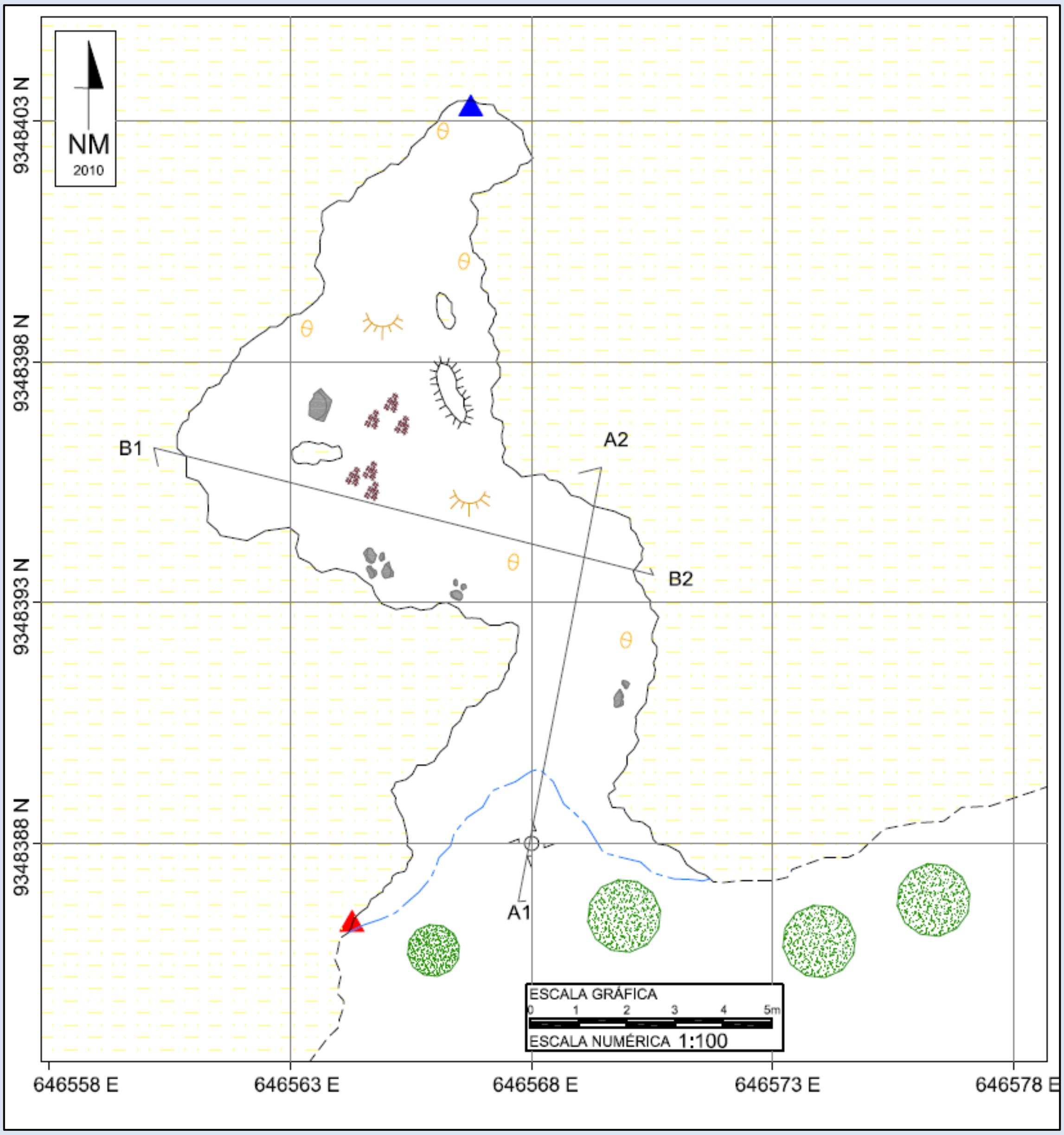

Seções

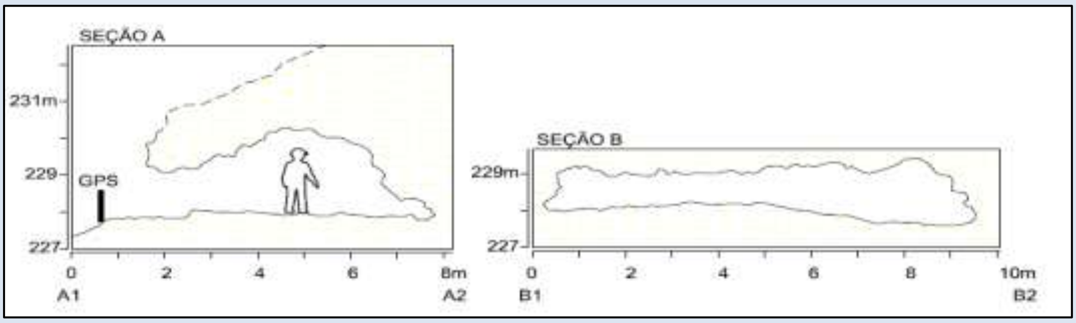

Quadro 5.9 - Síntese das características da caverna SL-109.

SL-109

**UTM E:** 646584	**UTM N**: 9348481	**Datum:** SAD'69	**Altitude:** 224 m
**Projeção Horizontal**: 15 m	**Desnível**: 1,79 m	**Área**: 77,5 m²	**Volume**: 100 m³

Litologia: Ferricrete

Inserção: Caverna está inserida em paredão rochoso de 2,5 m de altura, paralelo à calha de curso fluvial.

Observações Principais: Cavidade rasa com teto irregular e piso em aclive. As paredes apresentam canalículos que promovem o aporte de sedimentos, gerando agradação do piso. Apresenta coralóides maiores e em maior quantidade do que as demais cavidades na área.

Atributo de Relevância: Não foram observados atributos de relevância.

Fotos: (A) Entrada da caverna; (B) Teto formado por clastos angulosos; (C) Coralóides.

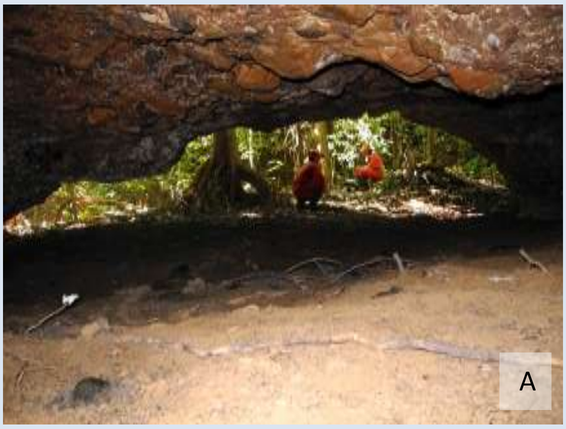

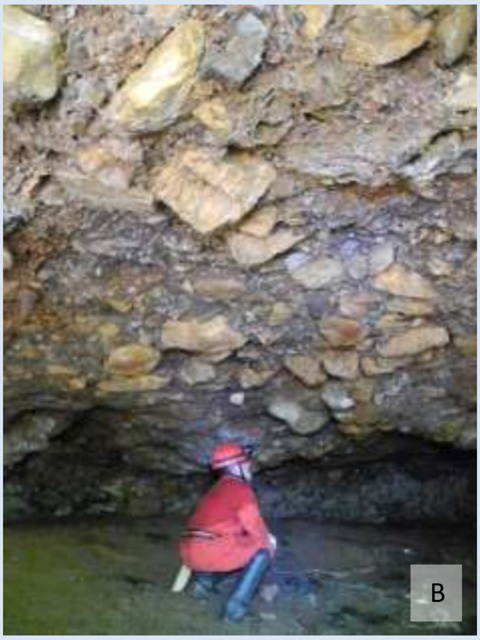

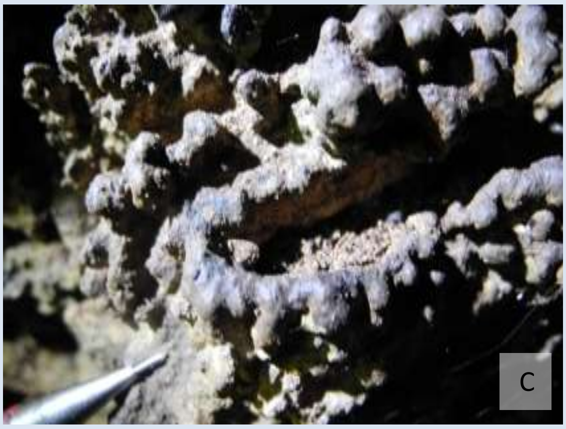

Planta Baixa

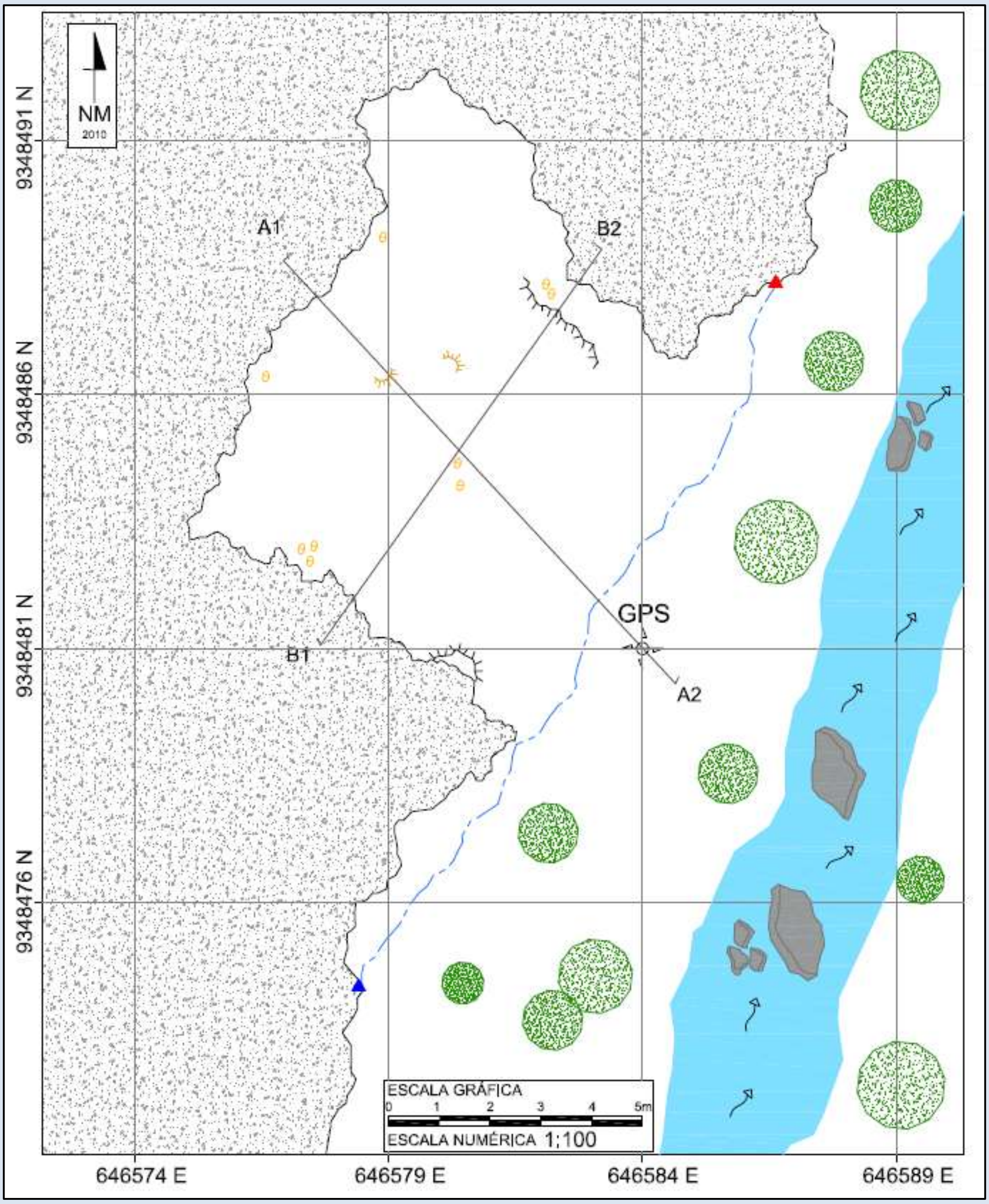

Seções

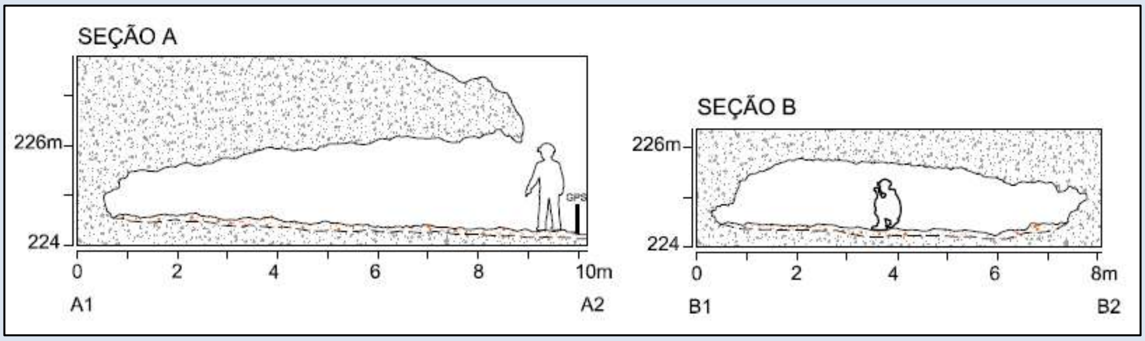

Quadro 5.10 - Síntese das características da caverna SL-110.

SL-110			
**UTM E:** 646559	**UTM N**: 9348362	**Datum:** SAD'69	**Altitude:** 232 m
**Projeção Horizontal**: 32 m	**Desnível**: 2,72 m	**Área**: 254,5 m²	**Volume**: 280 m³

Litologia: Ferricrete

Inserção: Caverna inserida próximo às margens de curso fluvial, em paredão de pequena altura, paralelo à calha da drenagem.

Observações Principais: Cavidade rasa com entrada larga e piso em declive. Apresenta concentração de blocos de abatimento próximo à entrada. Apresenta grande quantidade de guano disposto no piso.

Atributo de Relevância: Não foram observados atributos de relevância.

Fotos: (A) Entrada da caverna; (B) Teto formado por clastos angulosos; (C) Salão com piso coberto por sedimentos orgânicos e clásticos.

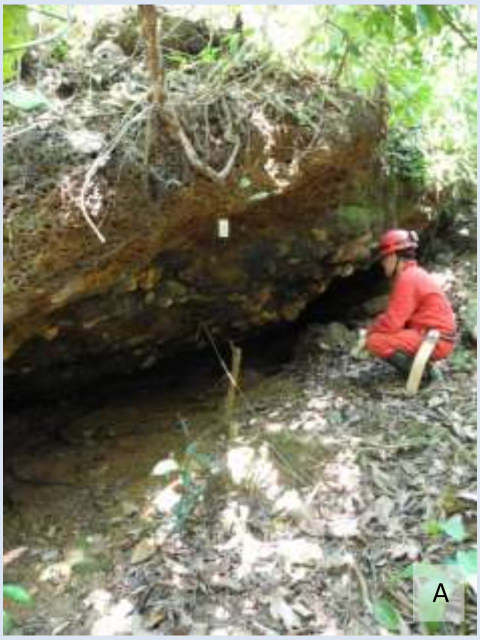

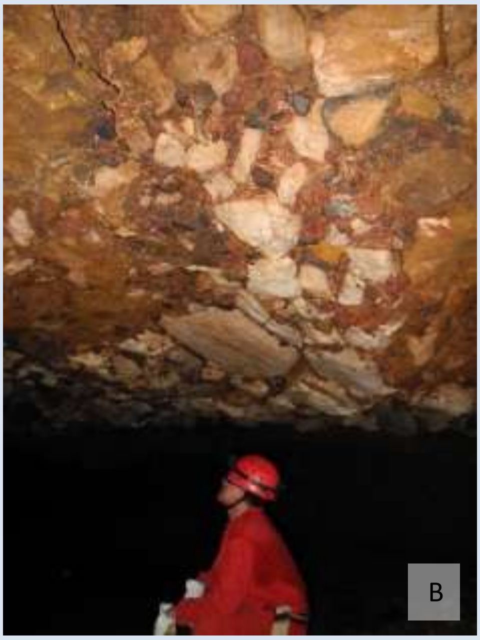

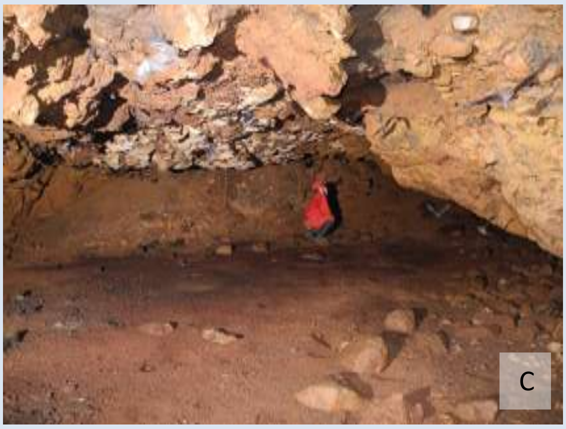

Planta Baixa

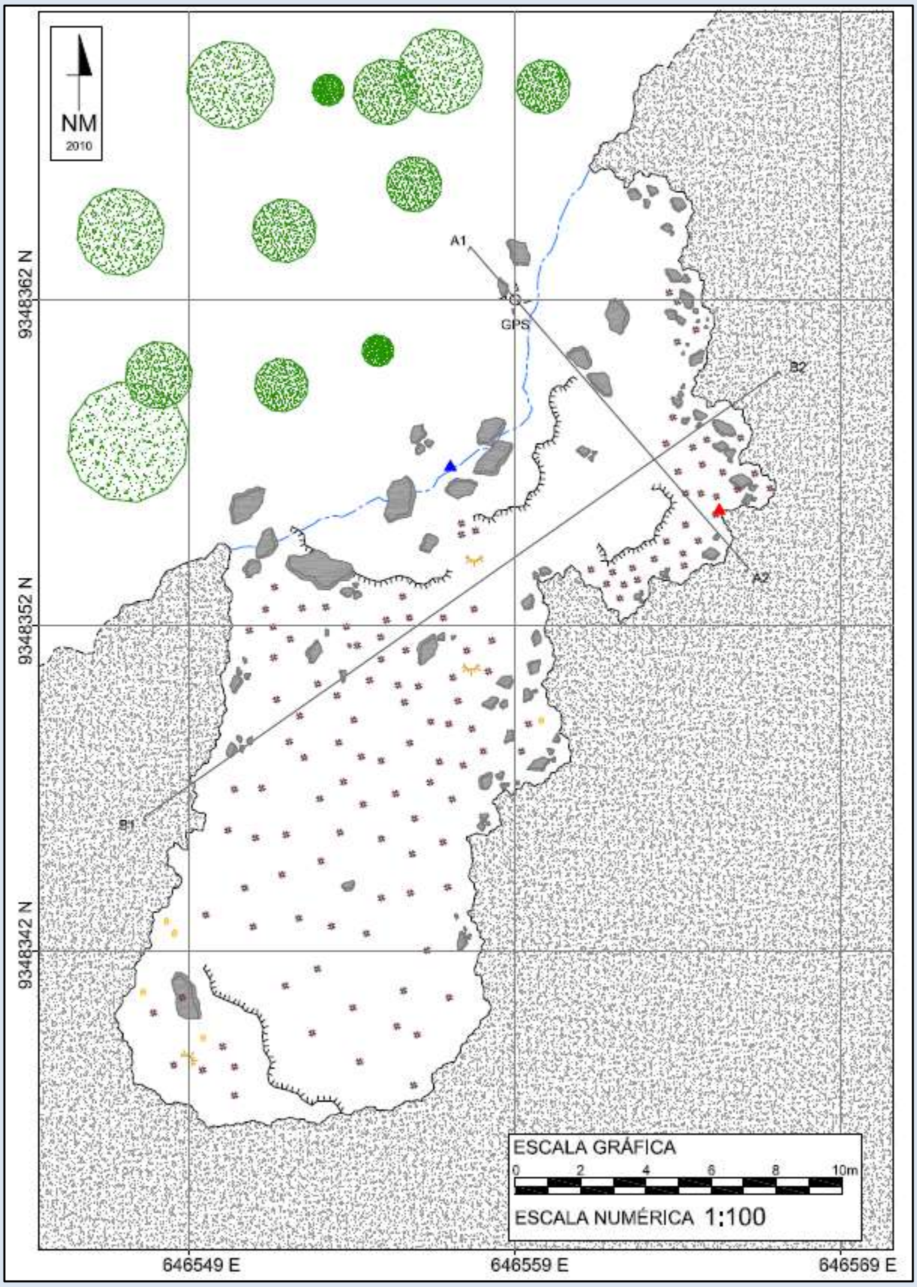

Seções

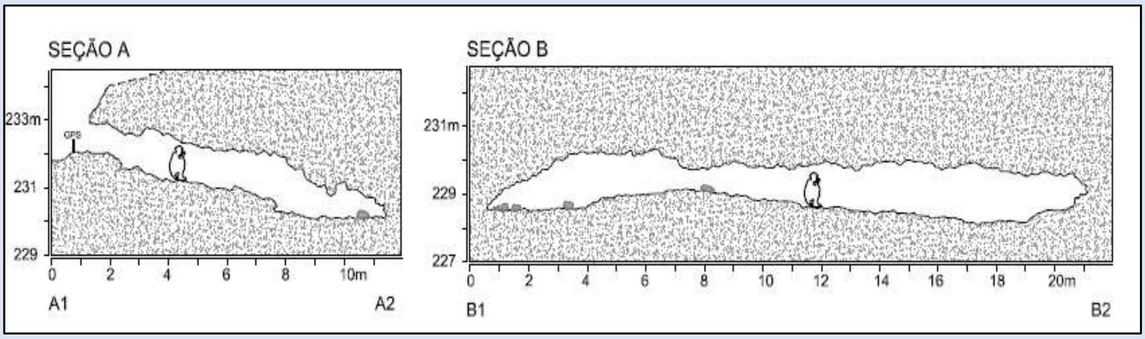

Quadro 5.11 - Síntese das características da caverna SL-111.

SL-111			
**UTM E:** 646599	**UTM N**: 9348518	**Datum:** SAD'69	**Altitude:** 240 m
**Projeção Horizontal**: 8 m	**Desnível**: 3,70 m	**Área**: 55,5 m²	**Volume**: 138,5 m³

Litologia: Contato entre o quartzito e o ferricrete

Inserção: Caverna inserida em paredão rochoso, no nível da calha de drenagem, em trecho encachoeirado.

Observações Principais: Cavidade com entrada ampla e pequena projeção horizontal. Sofre influência externa devido a sua morfologia em forma de abrigo. Em suas paredes, musgos e pteridófitas competem por espaço com os depósitos químicos.

Atributo de Relevância: Não foram observados atributos de relevância.

Fotos: (A) Entrada da caverna; (B) Parede em ferricrete; (C) Cachoeira próxima à entrada da cavidade.

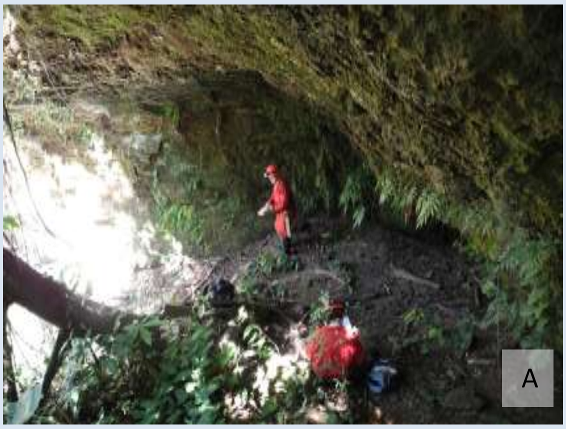
A

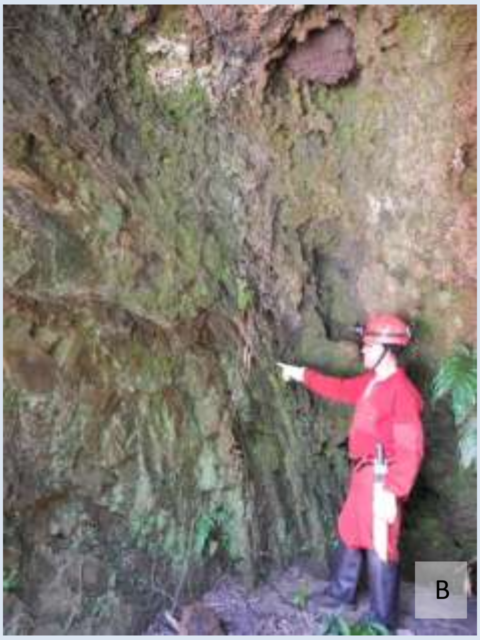
B

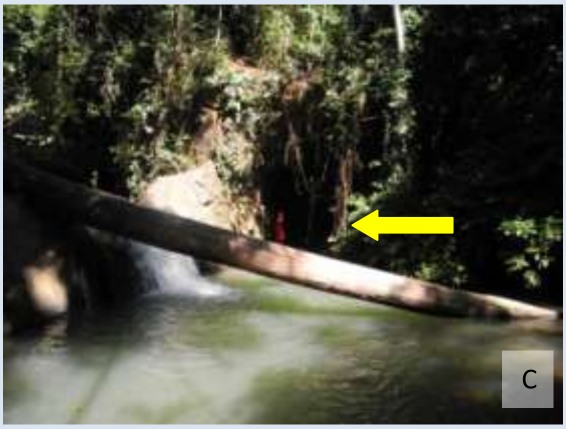
C

Planta Baixa

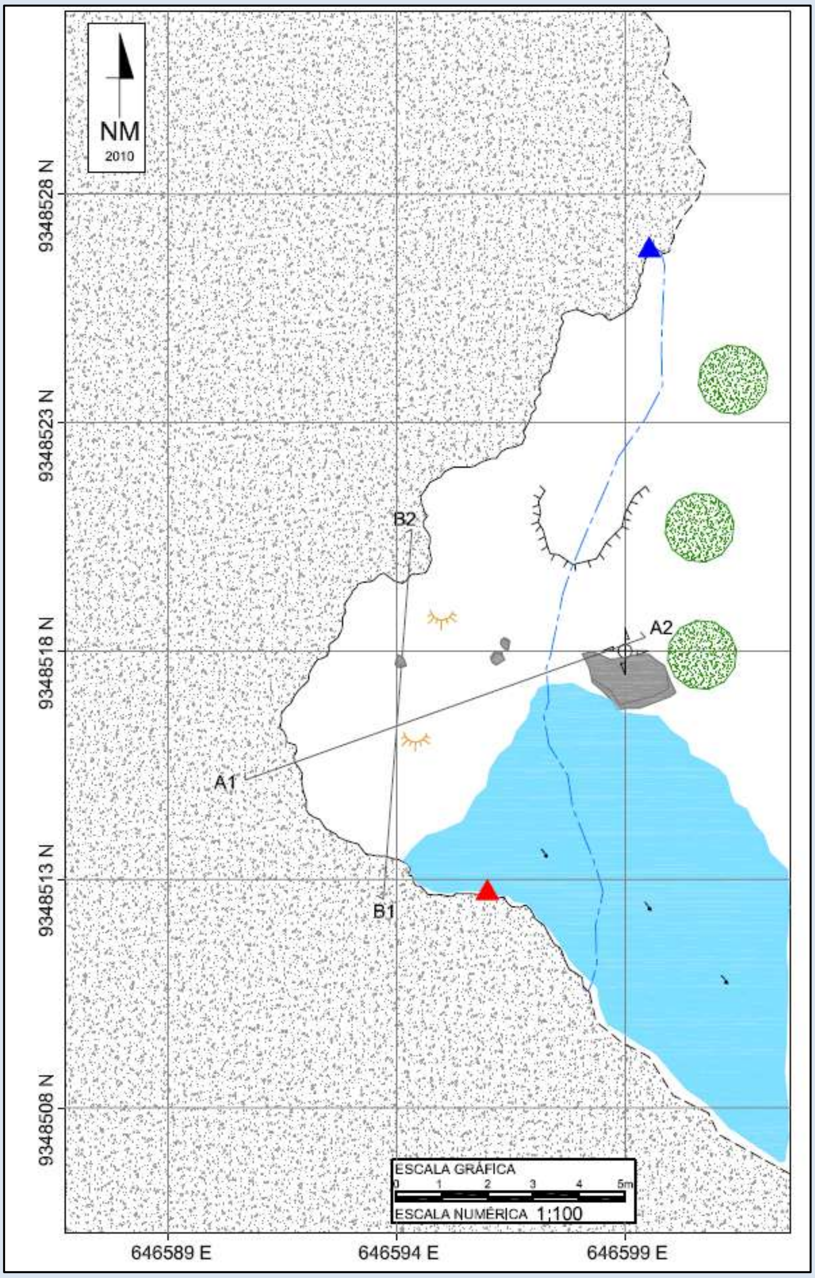

Seções

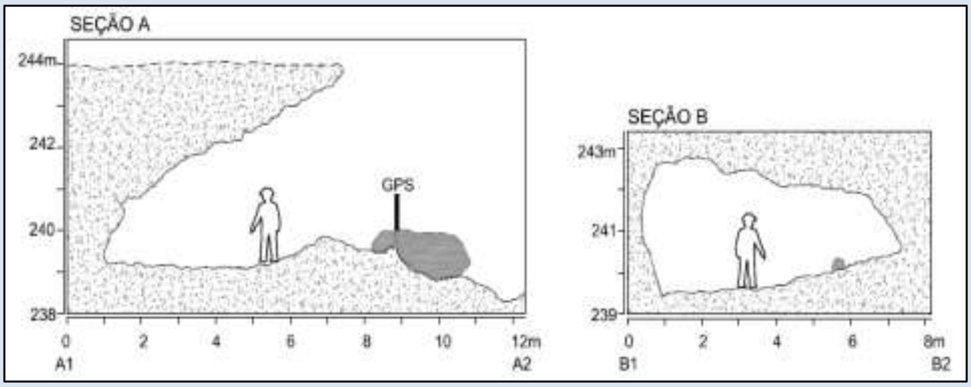

Quadro 5.12 - Síntese das características da caverna SL-112.

SL-112			
**UTM E:** 646584	**UTM N**: 9348356	**Datum:** SAD'69	**Altitude:** 226 m
**Projeção Horizontal**: 21 m	**Desnível**: 1,38 m	**Área**: 149 m²	**Volume**: 283 m³

Litologia: Ferricrete

Inserção: Caverna inserida em paredão rochoso contínuo e irregular, em degraus, paralelo a calha de drenagem, em sua margem esquerda.

Observações Principais: Apresenta entrada ampla e teto abobadado e alto na maior parte da cavidade. Apresenta canalículos ao longo de juntas que, assim como os abatimentos de bloco, promovem o desenvolvimento da cavidade. Podem ser observados espeleotemas do tipo crostas e coralóides.

Atributo de Relevância: Não foram observados atributos de relevância.

Fotos: (A) Entrada da caverna; (B) Salão amplo com teto abobadado; (C) Coralóides.

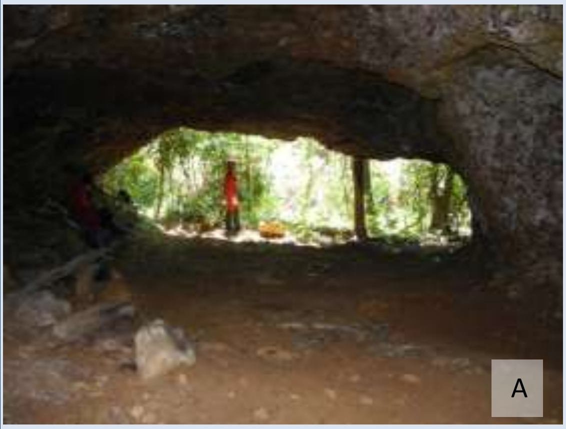

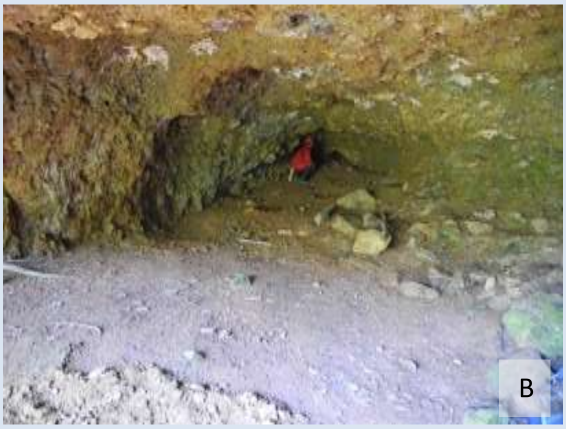

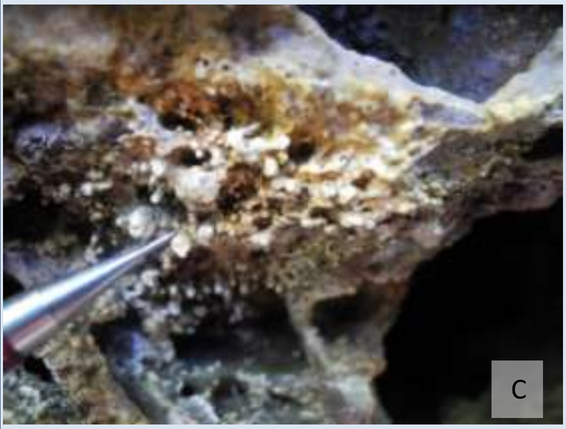

Planta Baixa

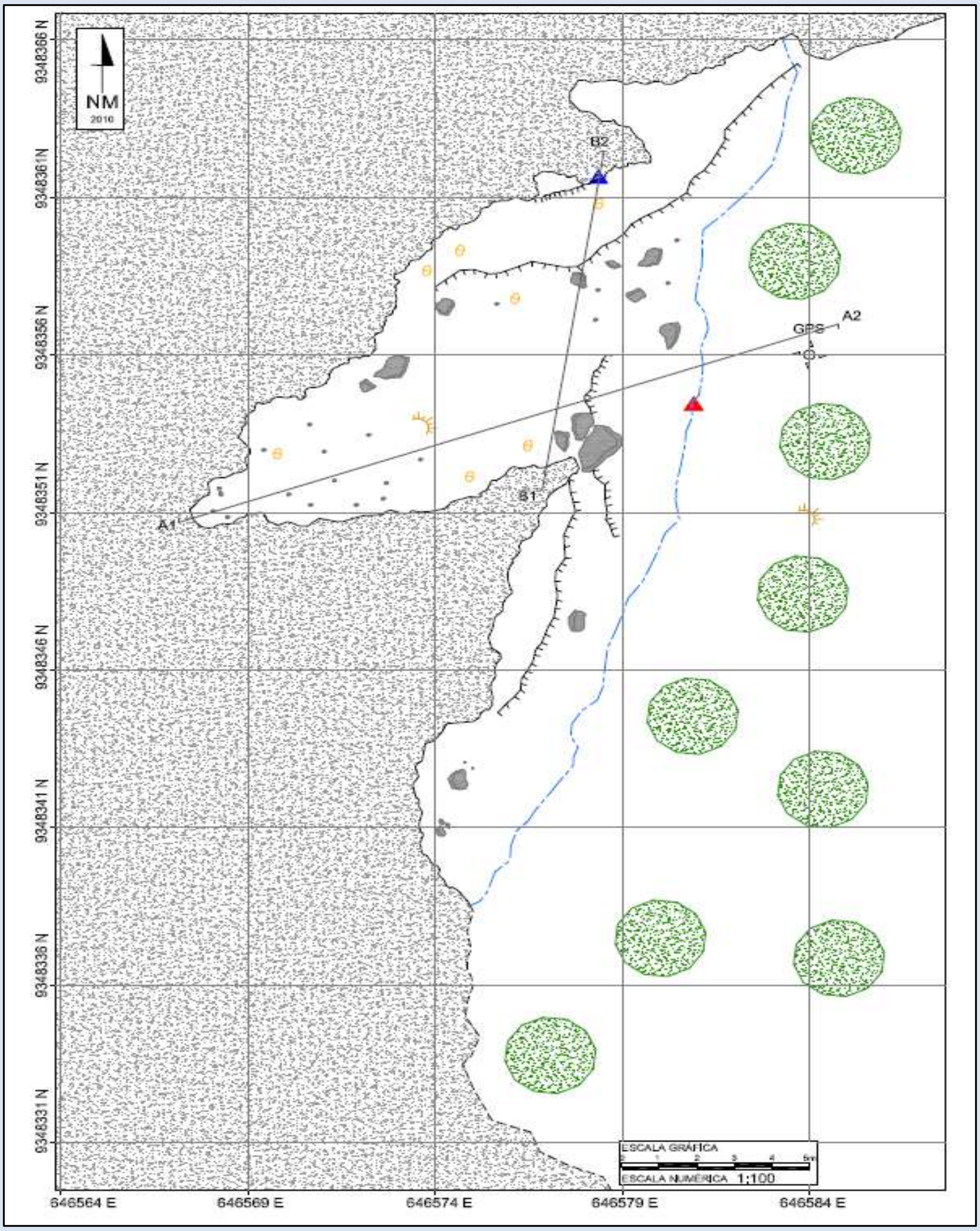

Seções

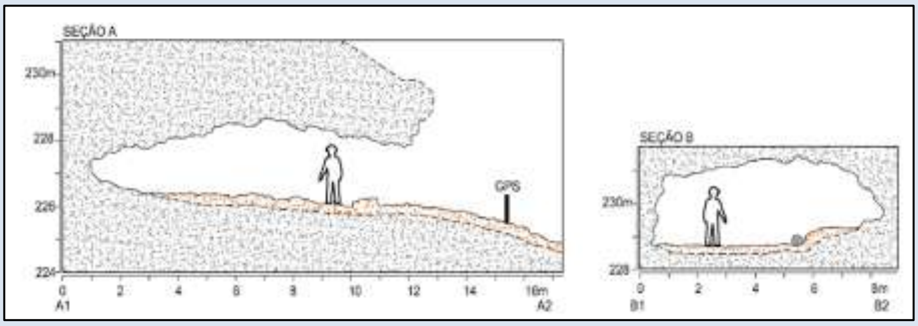

Quadro 5.13 - Síntese das características da caverna SL-113.

SL-113			
**UTM E:** 656857	**UTM N**: 9344336	**Datum:** SAD'69	**Altitude:** 257 m
**Projeção Horizontal**: 5,5 m	**Desnível**: 1,02 m	**Área**: 11 m²	**Volume**: 8 m³

Litologia: Quartzito

Inserção: Caverna ocorre em depósitos de tálus, inseridos na média vertente em porção de afloramentos irregulares e escalonados.

Observações Principais: Cavidade apresenta conduto único que termina em um canalículo que promove o aporte de água e sedimentos oriundos da vertente. Não foram observados espeleotemas no interior da cavidade.

Atributo de Relevância: Não foram observados atributos de relevância.

Fotos: (A) Entrada da caverna; (B) Salão com teto baixo; (C) Crosta branca.

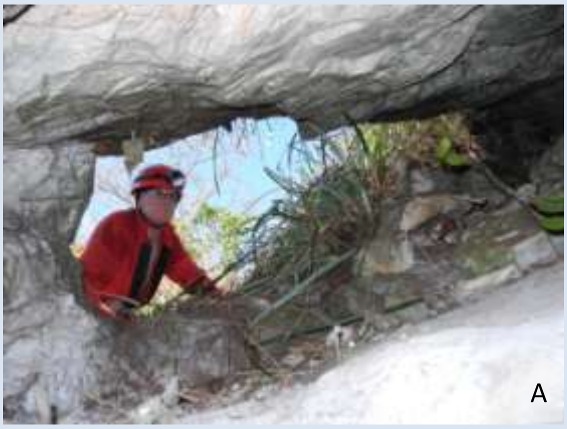
A

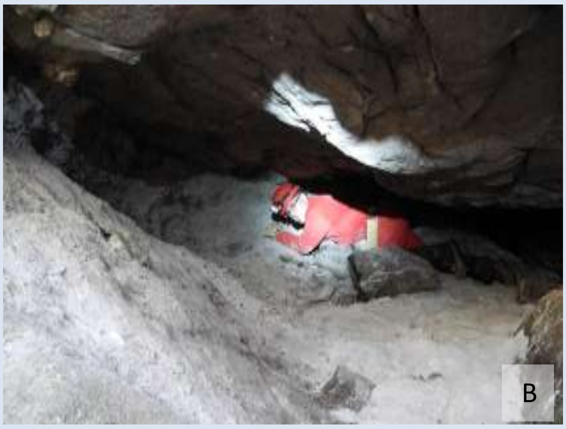
B

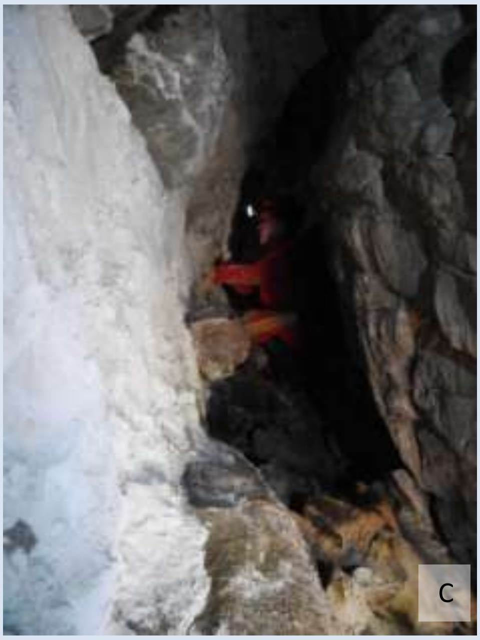
C

Planta Baixa

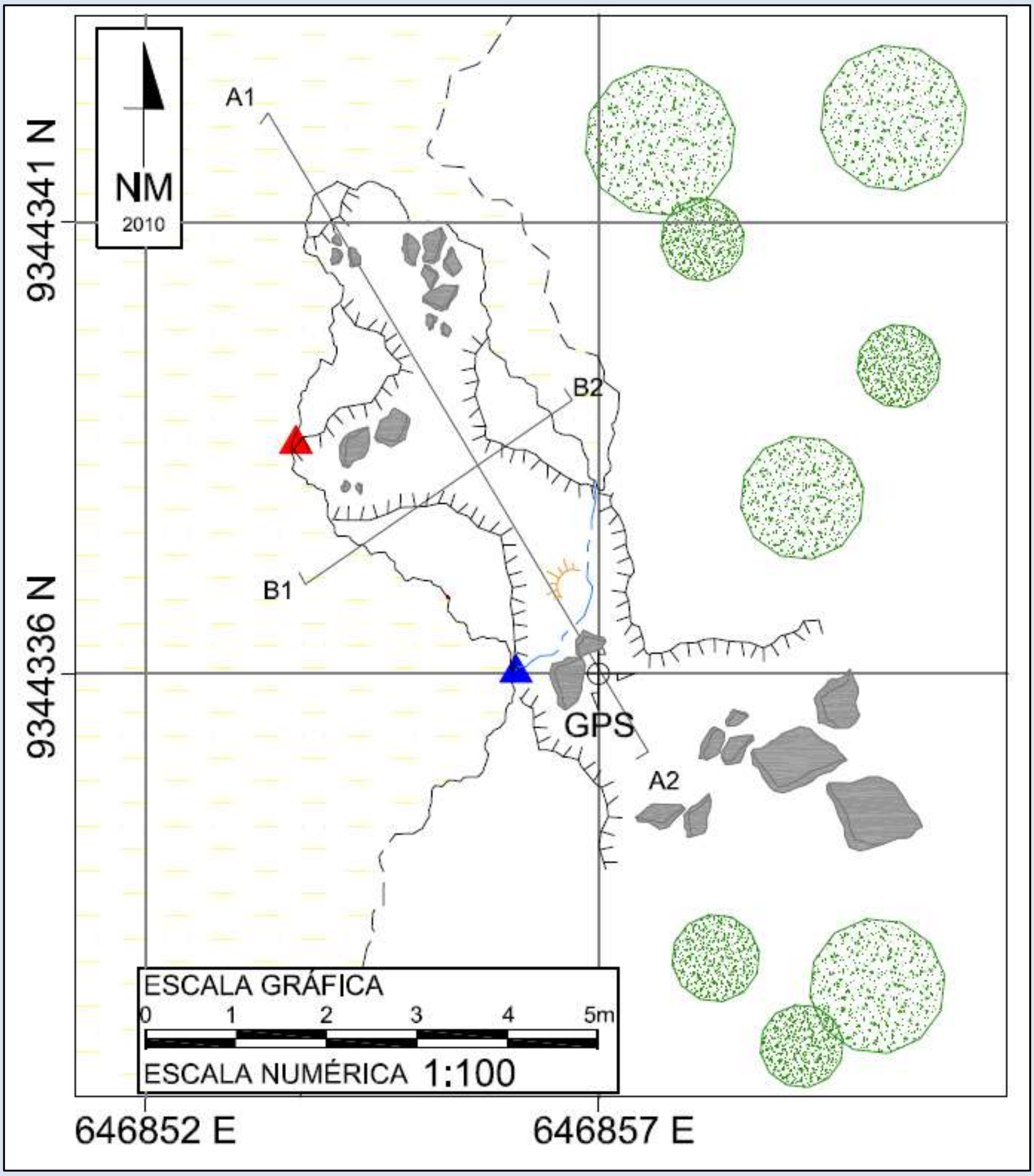

Seções

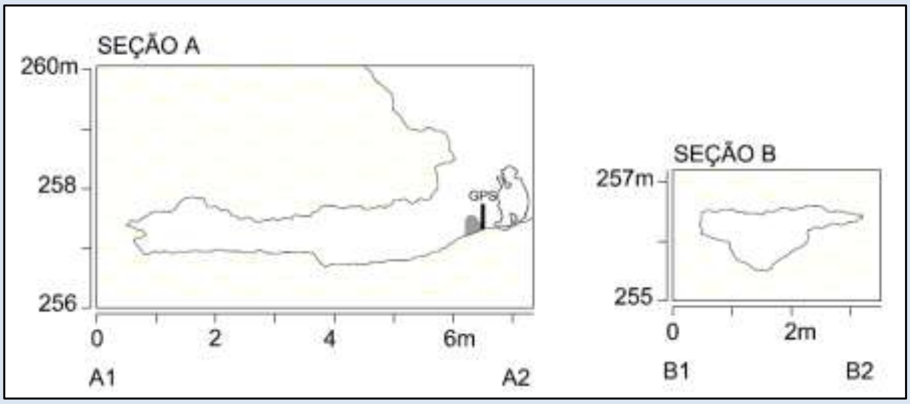

Quadro 5.14 - Síntese das características da caverna SL-114.

SL-114			
**UTM E:** 648031	**UTM N**: 9344788	**Datum:** SAD'69	**Altitude:** 250 m
**Projeção Horizontal**: 25,5 m	**Desnível**: 1,29 m	**Área**: 111 m²	**Volume**: 89 m³
**Litologia:** Ferricrete			
**Inserção:** Caverna inserida na média vertente, em paredão irregular.			
**Observações Principais:** Cavidade impactada, apresentando-se bastante diferente do que foi representado no mapa de junho de 2010. Entupimento de um conduto de teto baixo e a ausência da parte oeste da cavidade, que anteriormente apresentava uma clarabóia. Suspeita-se de ocorrência de colapso desta parte da cavidade. Colônia de bactérias ocupa as paredes e o teto.			
**Atributo de Relevância:** Não foram observados atributos de relevância.			
**Fotos:** (A) Entrada da caverna; (B) Cúpula de teto; (C) Salão com teto baixo			

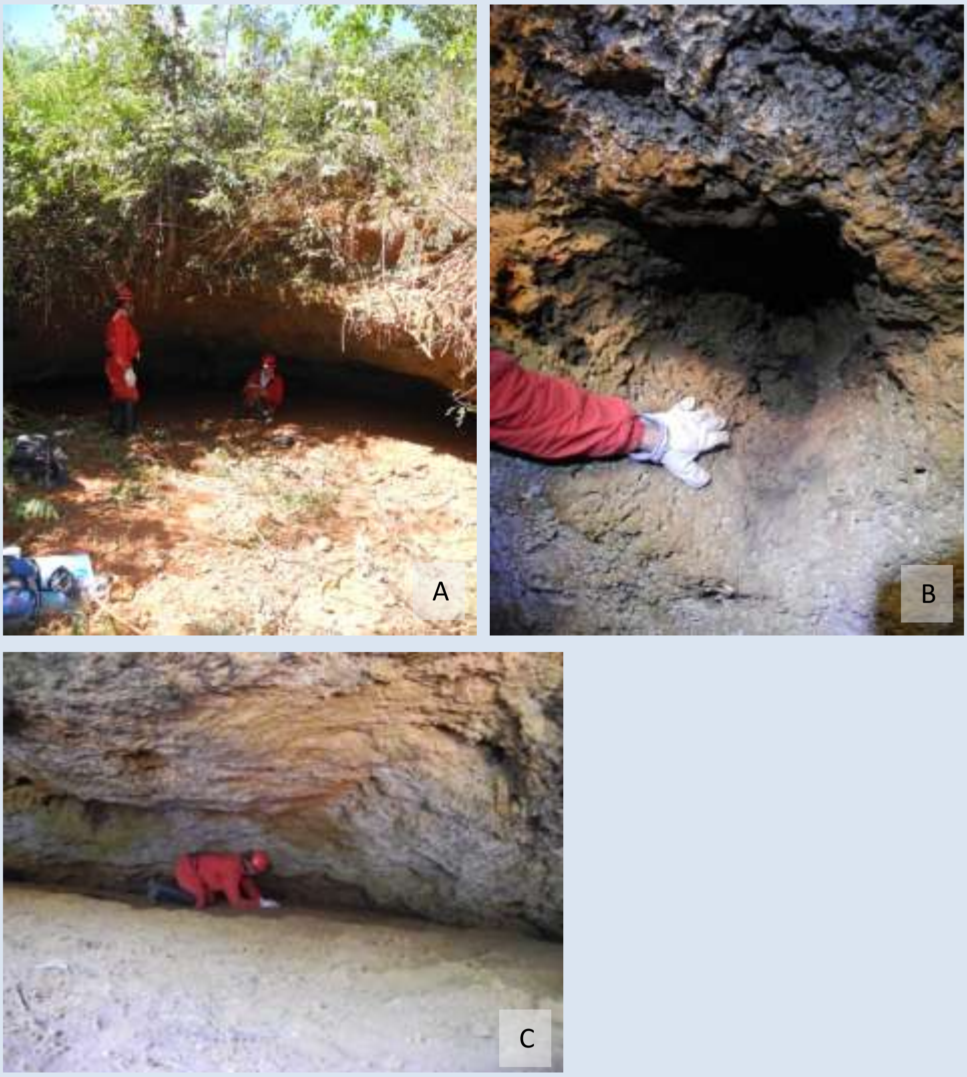

Planta Baixa

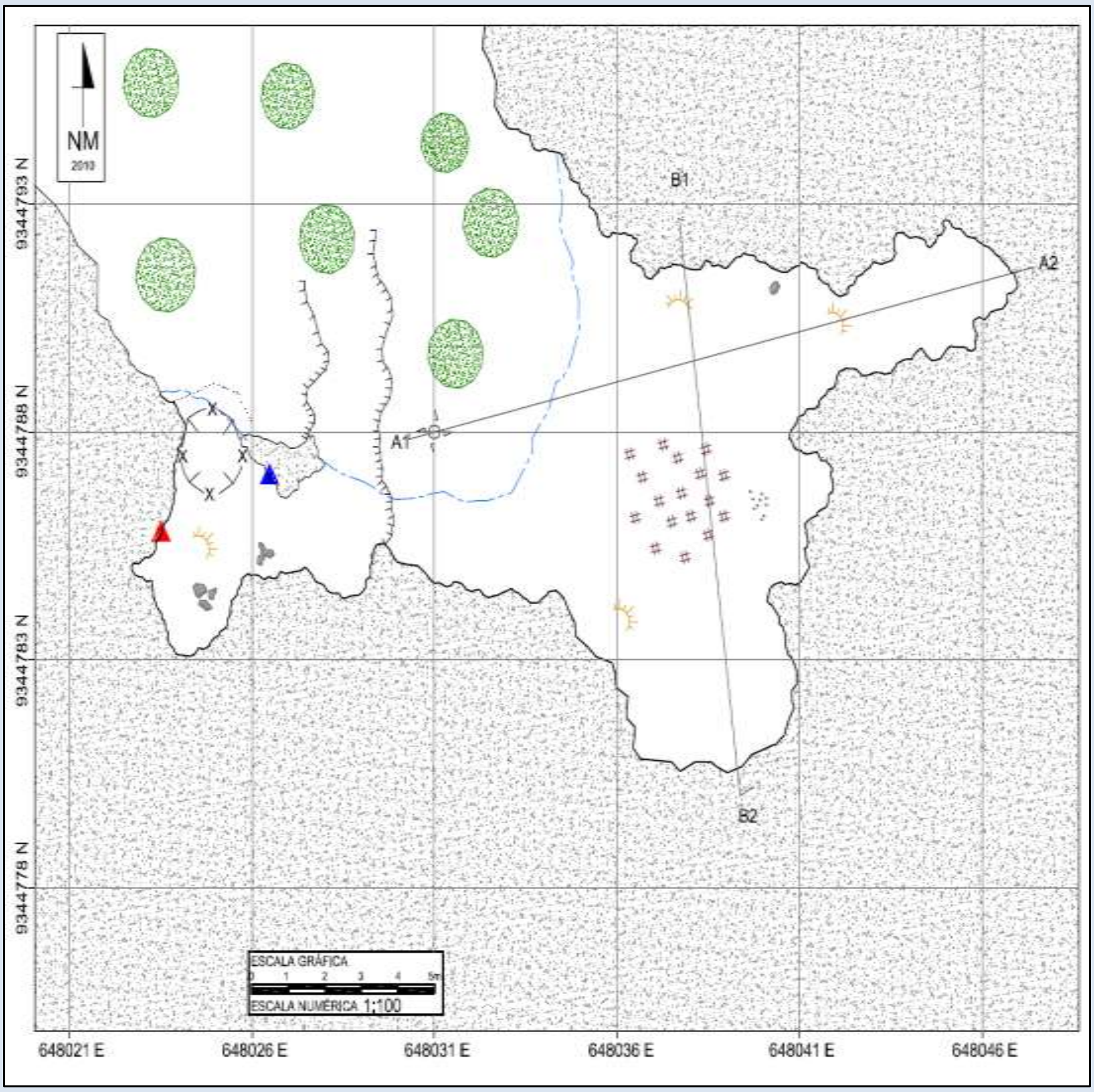

Seções

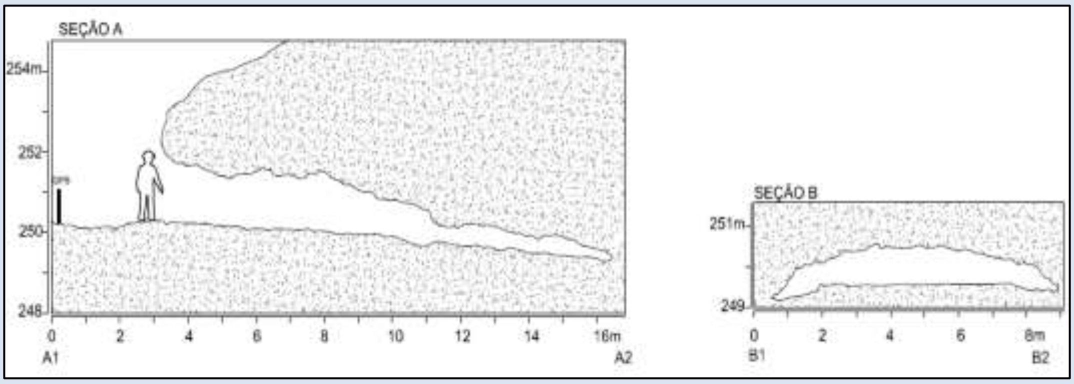

Quadro 5.15 - Síntese das características da caverna SL-115.

SL-115			
**UTM E:** 651081	**UTM N**: 9339629	**Datum:** SAD'69	**Altitude:** 636 m
**Projeção Horizontal**: 12 m	**Desnível**: 1 m	**Área**: 32 m²	**Volume**: 34 m³

Litologia: Canga

Inserção: Caverna inserida em dolina, situada na média vertente.

Observações Principais: Apresenta teto baixo em toda a sua extensão, irregular, com cúpulas onde a incisão vertical é maior.

Atributo de Relevância: Não foram observados atributos de relevância.

Fotos: (A) Entrada da caverna em dolina; (B) Crosta branca; (C) Salão com piso coberto por sedimento cascalhento e teto irregular.

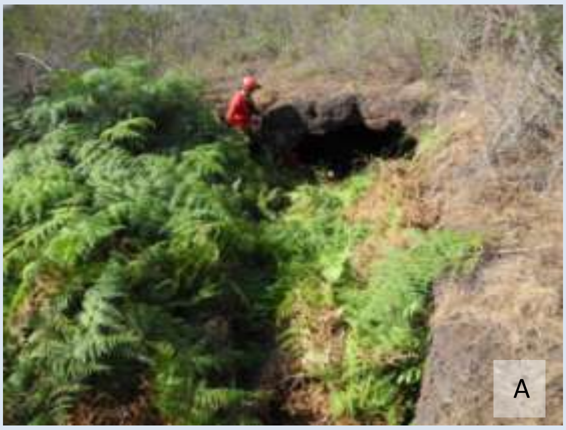

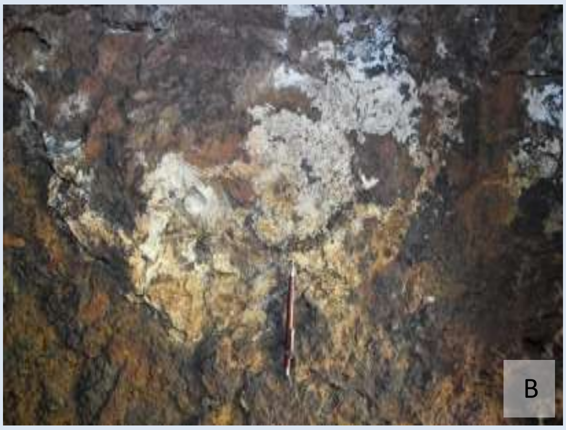

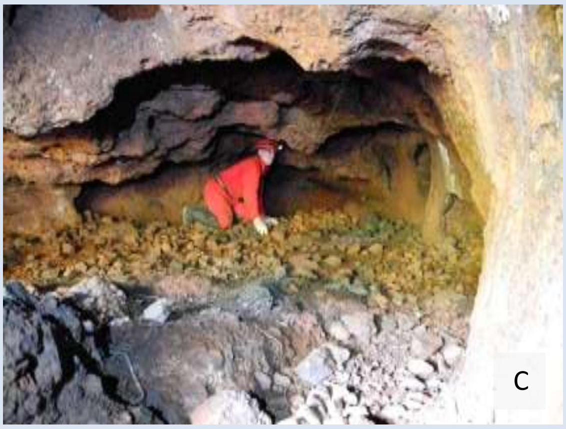

Planta Baixa

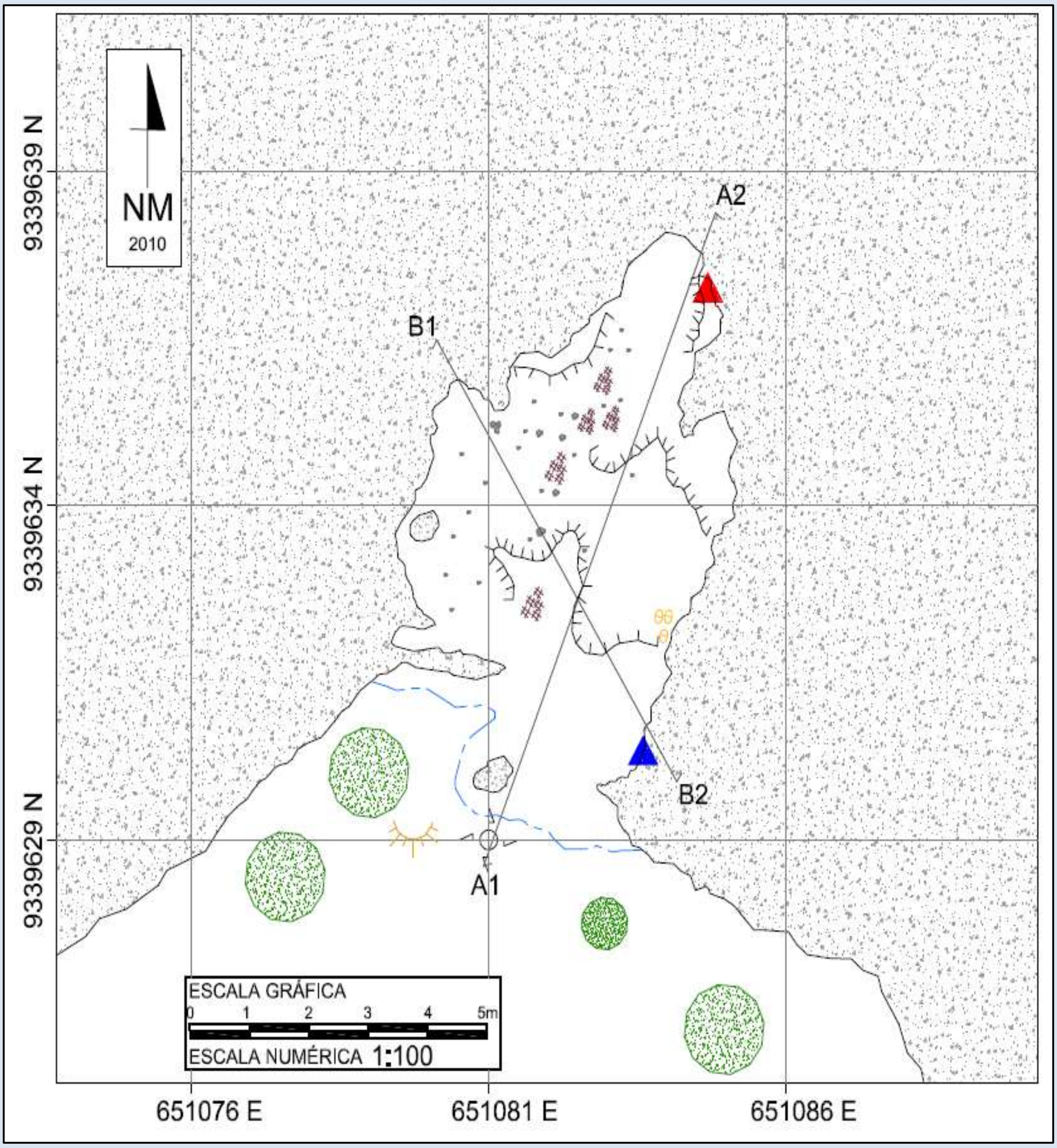

Seções

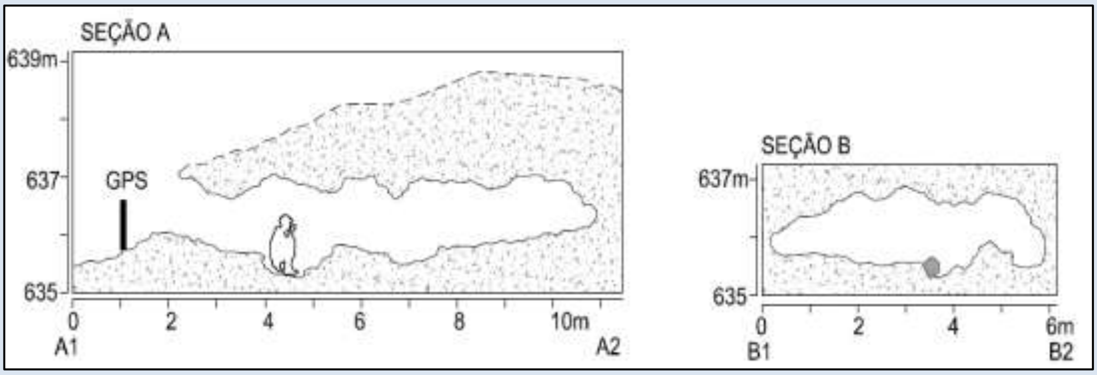

Quadro 5.16 - Síntese das características da caverna SL-116.

SL-116			
**UTM E:** 651081	**UTM N:** 9339618	**Datum:** SAD'69	**Altitude:** 629 m
**Projeção Horizontal:** 9 m	**Desnível:** 2,18 m	**Área:** 16,5 m²	**Volume:** 18 m³

Litologia: Canga

Inserção: Caverna inserida em pequena dolina, situada na média vertente.

Observações Principais: Cavidade apresenta entrada única que dá acesso a um pequeno salão. Piso, teto e paredes são irregulares. O teto é baixo, apresentando grande quantidade de cúpulas.

Atributo de Relevância: Não foram observados atributos de relevância.

Fotos: (A) Entrada da caverna; (B) Canga; (C) Salão com teto baixo e irregular.

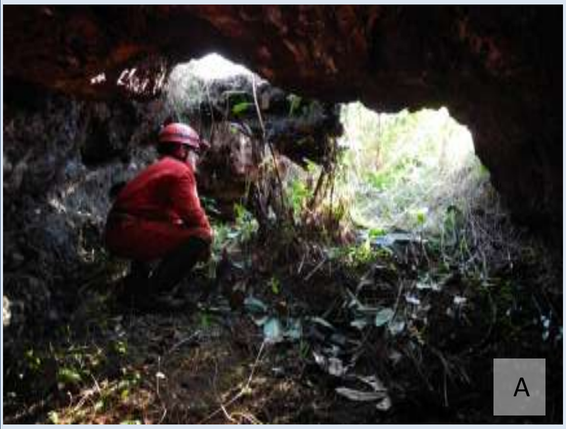

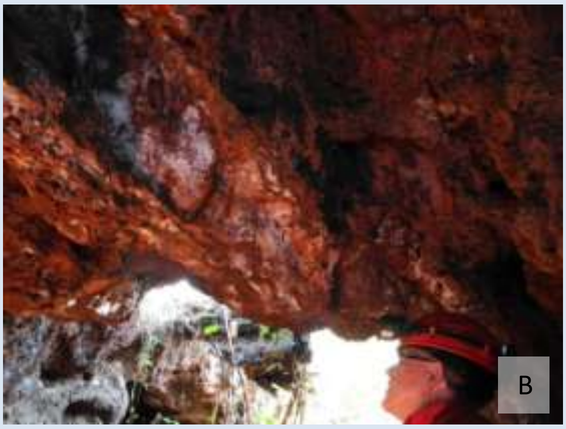

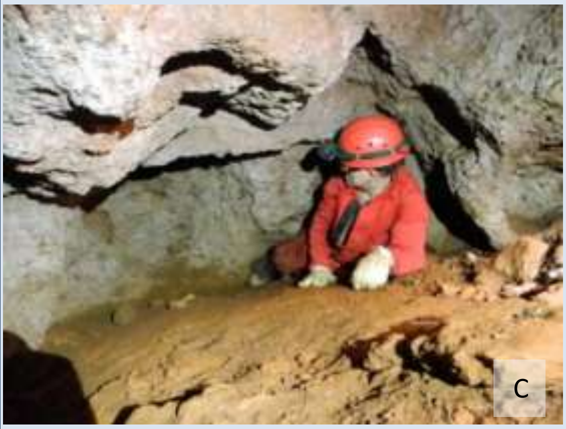

Planta Baixa

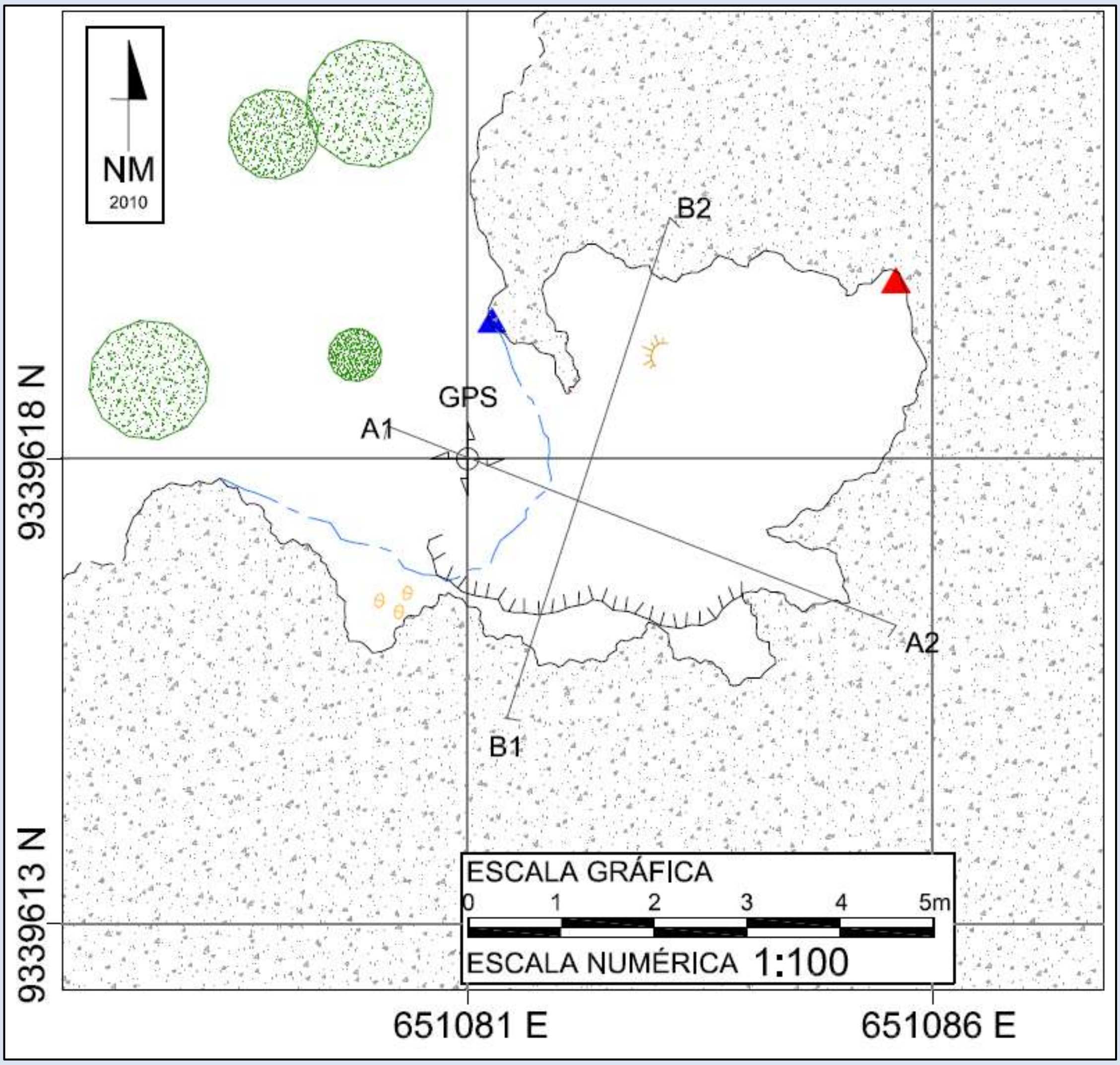

Seções

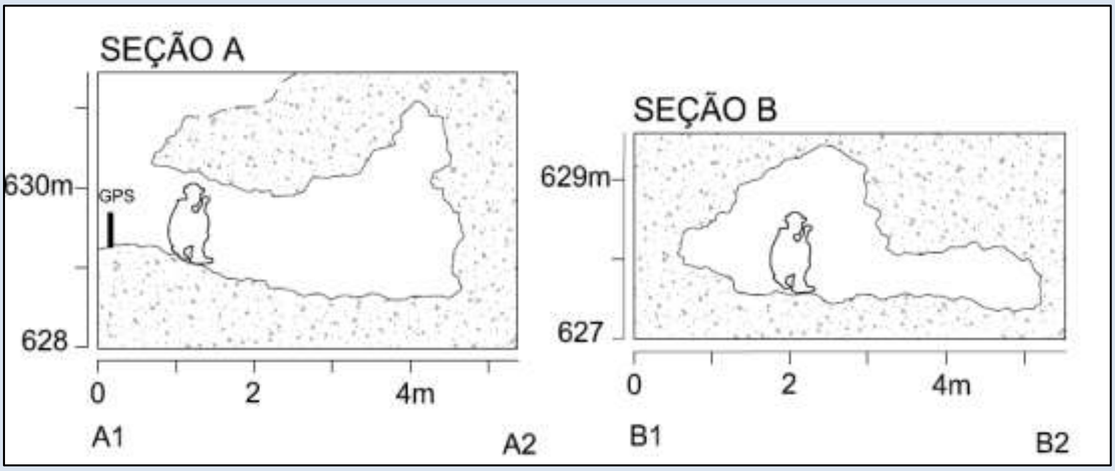

Quadro 5.17 - Síntese das características da caverna SL-117.

SL-117

UTM E: 646875 **UTM N**: 9344347 **Datum:** SAD'69 **Altitude:** 264 m

Projeção Horizontal: 9,5 m **Desnível**: 2,79 m **Área**: 29,5 m² **Volume**: 27 m³

Litologia: Quartzito

Inserção: Caverna inserida em depósito de tálus, na média vertente.

Observações Principais: Cavidade formada em tálus, com morfologia irregular, com blocos cobrindo piso e teto. Apresenta crostas negras com provável contribuição orgânica.

Atributo de Relevância: Não foram observados atributos de relevância.

Fotos: (A) Entrada da caverna; (B) Blocos obstruindo parcialmente uma das entradas; (C) Caverna em tálus com piso coberto por calhaus.

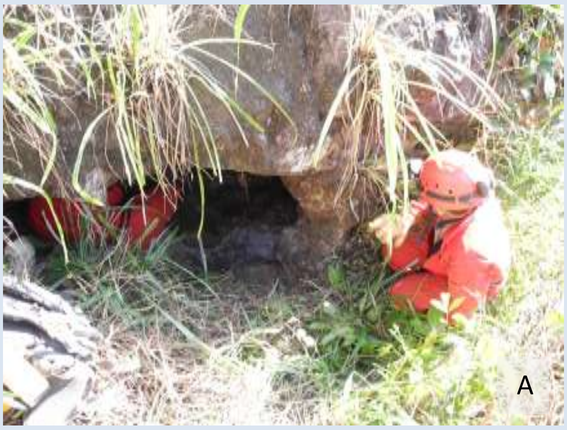
A

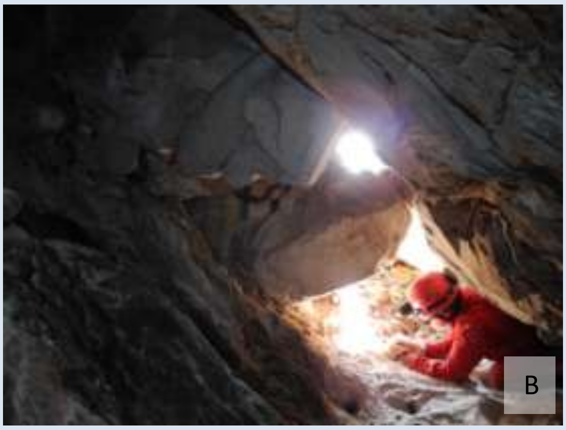
B

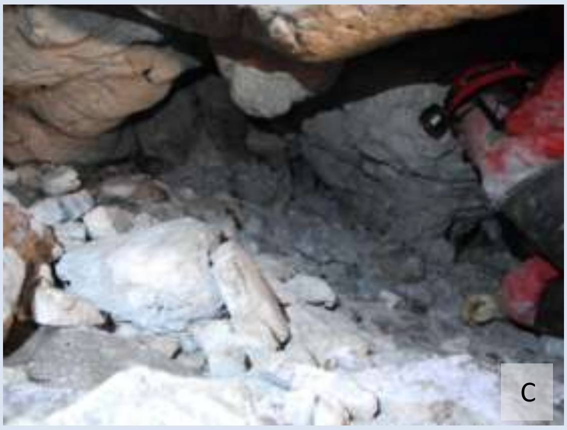
C

Planta Baixa

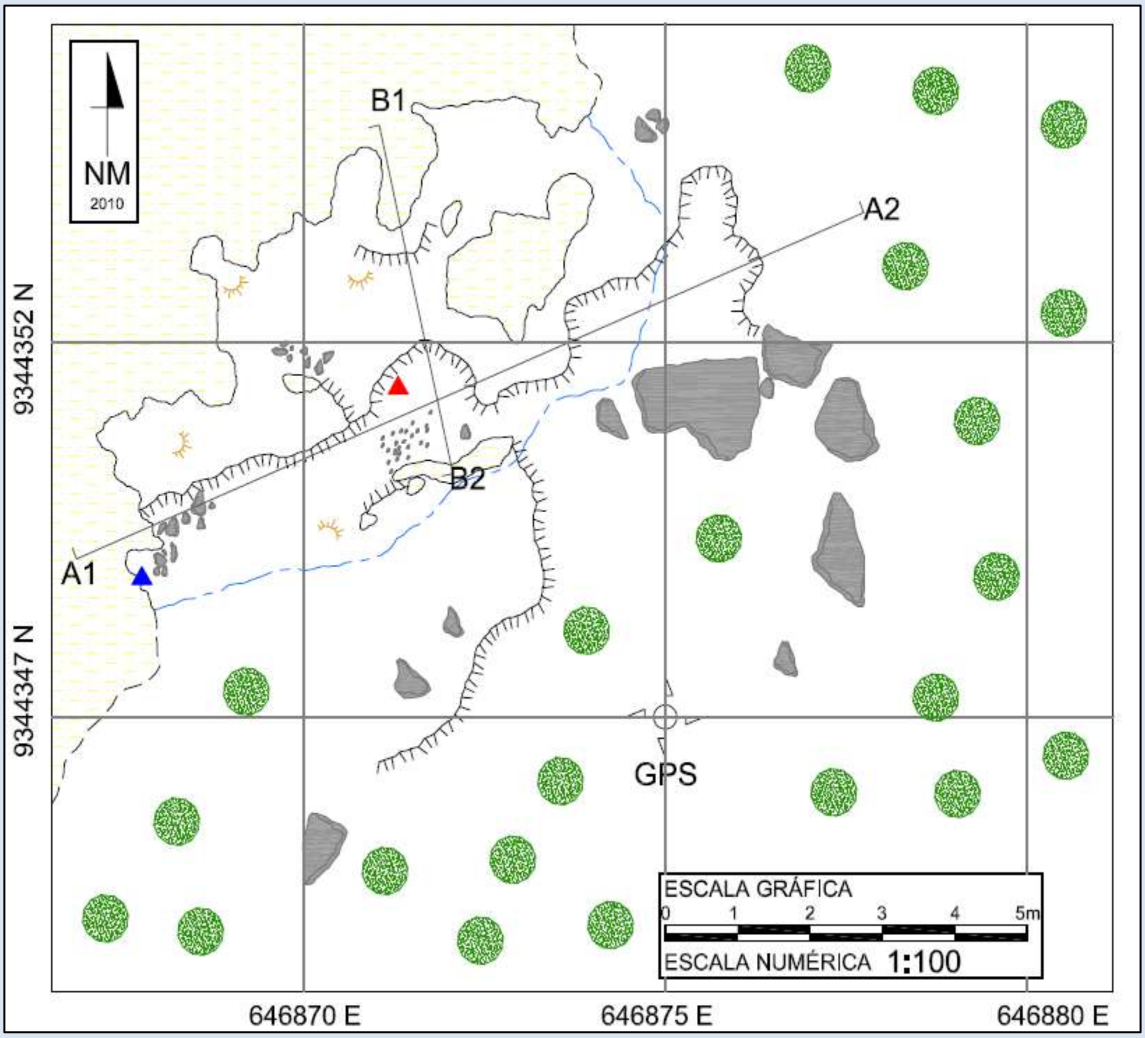

Seções

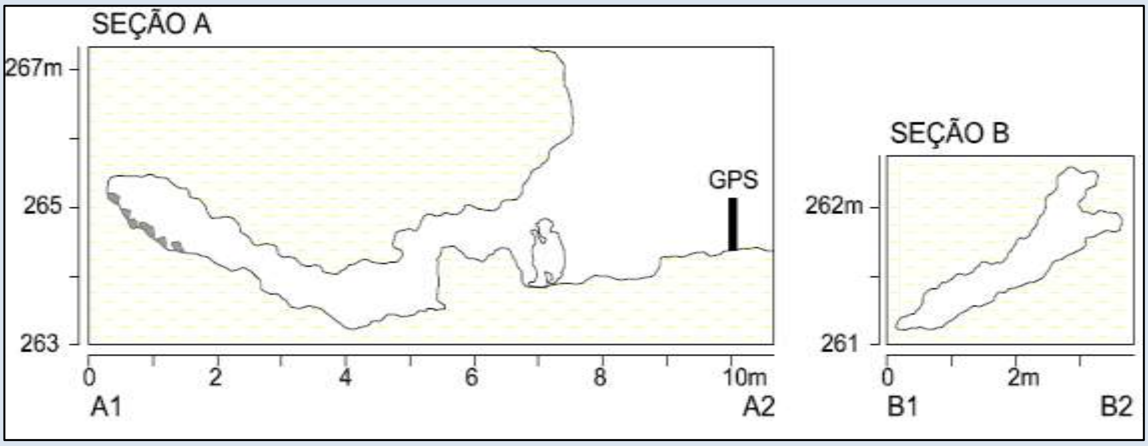

Quadro 5.18 - Síntese das características da caverna SL-121.

SL-121			
**UTM E:** 650952	**UTM N**: 9340707	**Datum:** SAD'69	**Altitude:** 466 m
**Projeção Horizontal**: 68,5 m	**Desnível**: 1,64 m	**Área**: 138,5 m²	**Volume**: 154 m³

Litologia: Canga

Inserção: Caverna inserida em paredão escalonado na alta vertente.

Observações Principais: Crostas ferruginosas são abundantes e ocorrem recobrindo piso, paredes e blocos, preenchendo as fraturas e condicionando a morfologia dos condutos.

Atributo de Relevância: Crostas preenchendo fraturas que condicionam a morfologia dos condutos.

Fotos: (A) Entrada da caverna; (B) Crosta ferruginosa; (C) Salão amplo com blocos abatidos.

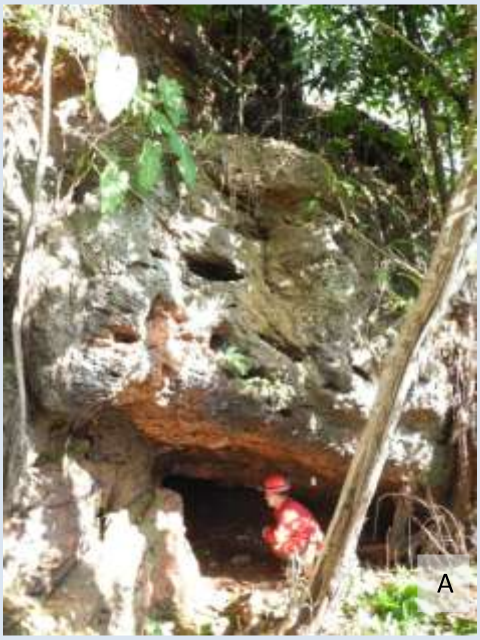

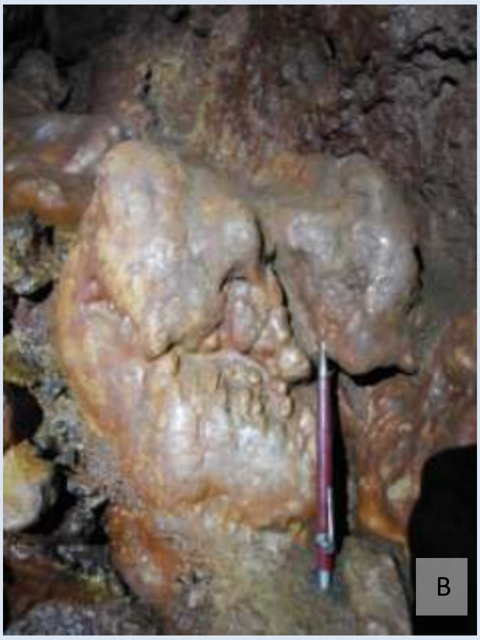

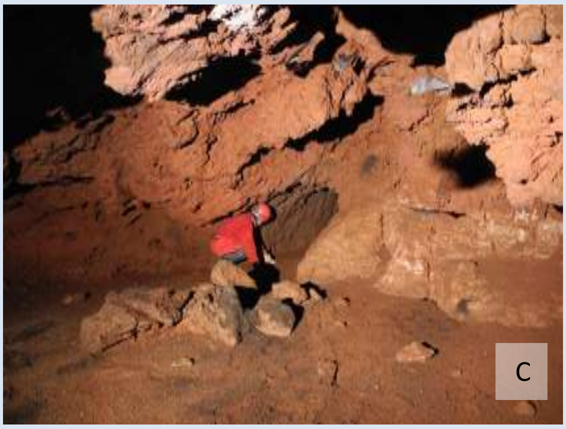

Planta Baixa

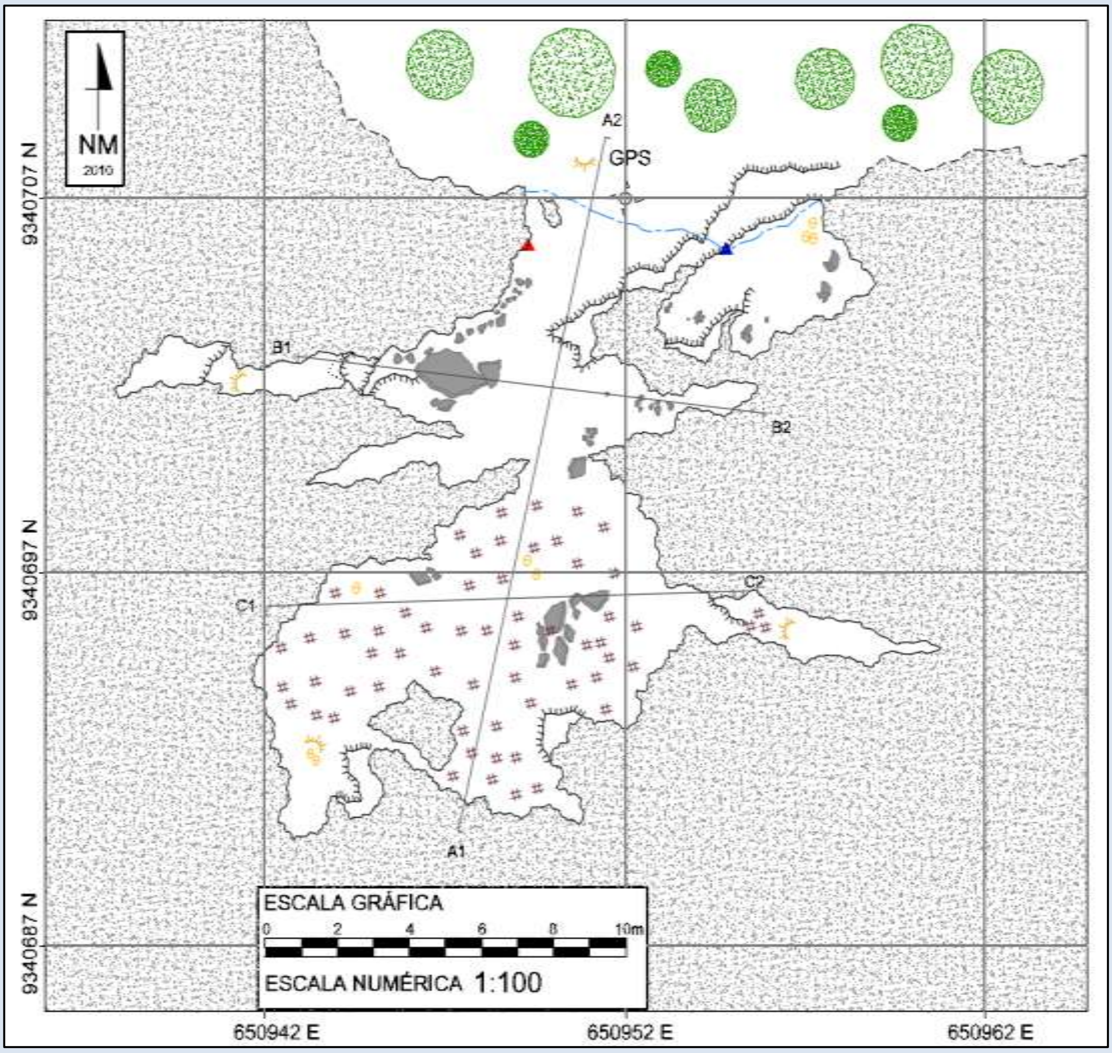

Seções

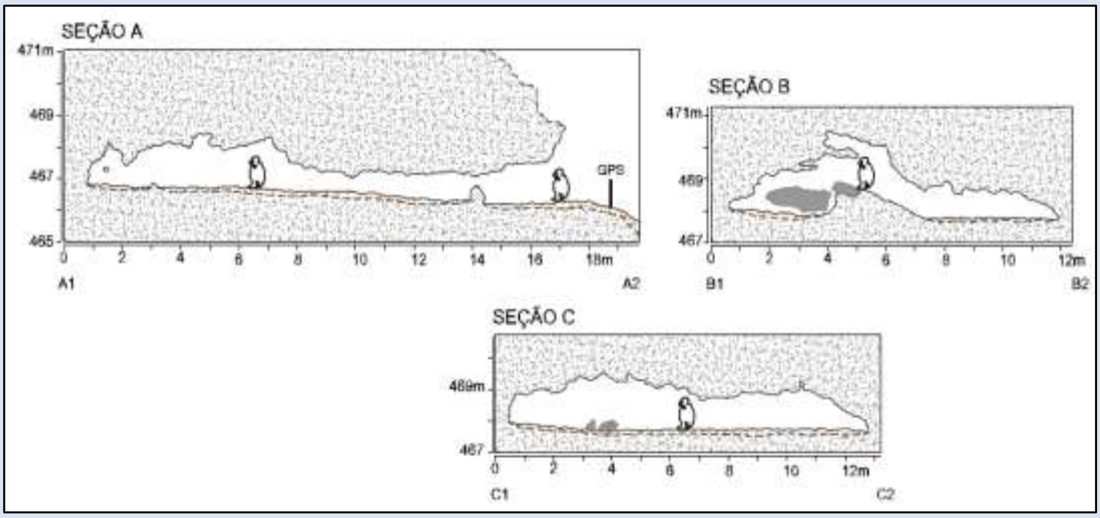

Quadro 5.19 - Síntese das características da caverna SL-122.

SL-122			
**UTM E:** 654731	**UTM N**: 9340026	**Datum:** SAD'69	**Altitude:** 286 m
**Projeção Horizontal**: 34 m	**Desnível**: 1,36 m	**Área**: 171,5 m²	**Volume**: 199 m³

Litologia: Canga

Inserção: Caverna inserida na calha de drenagem da margem esquerda do afluente do córrego principal, em paredão irregular de pequena altura que forma um anfiteatro no ponto de inserção da cavidade.

Observações Principais: Caverna bastante úmida e com várias feições hidrológicas, apresenta a maior variedade de depósitos químicos, que se encontram ativos.

Atributo de Relevância: A presença de água estabelece relação com atributos de deposição química e, possivelmente com atributos relevantes sob o enfoque biológico.

Fotos: (A) Entrada da caverna; (B) Escorrimento; (C) Salão com teto baixo e presença de pilares.

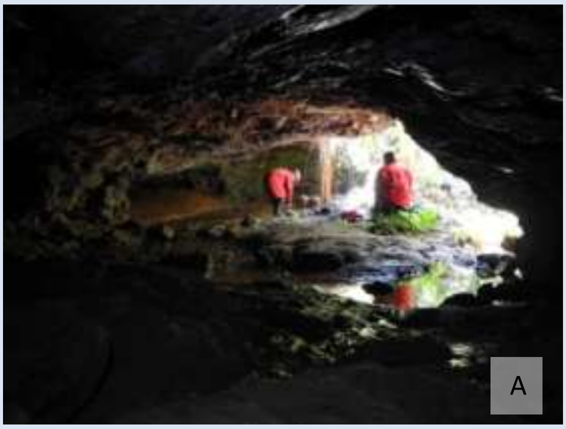

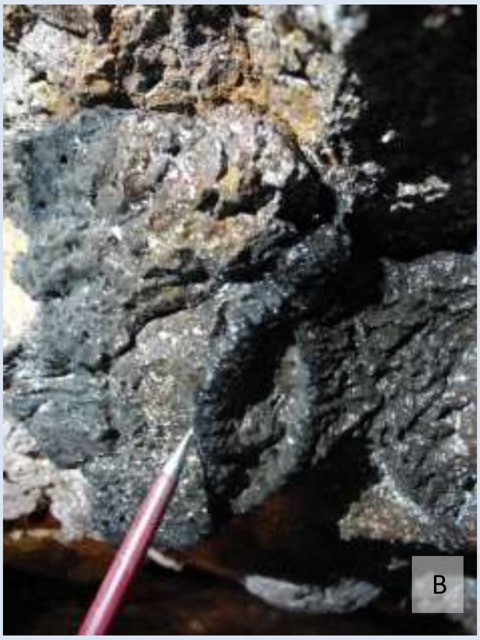

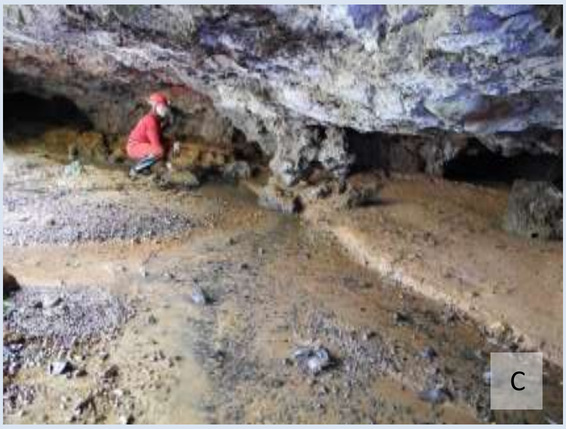

Planta Baixa

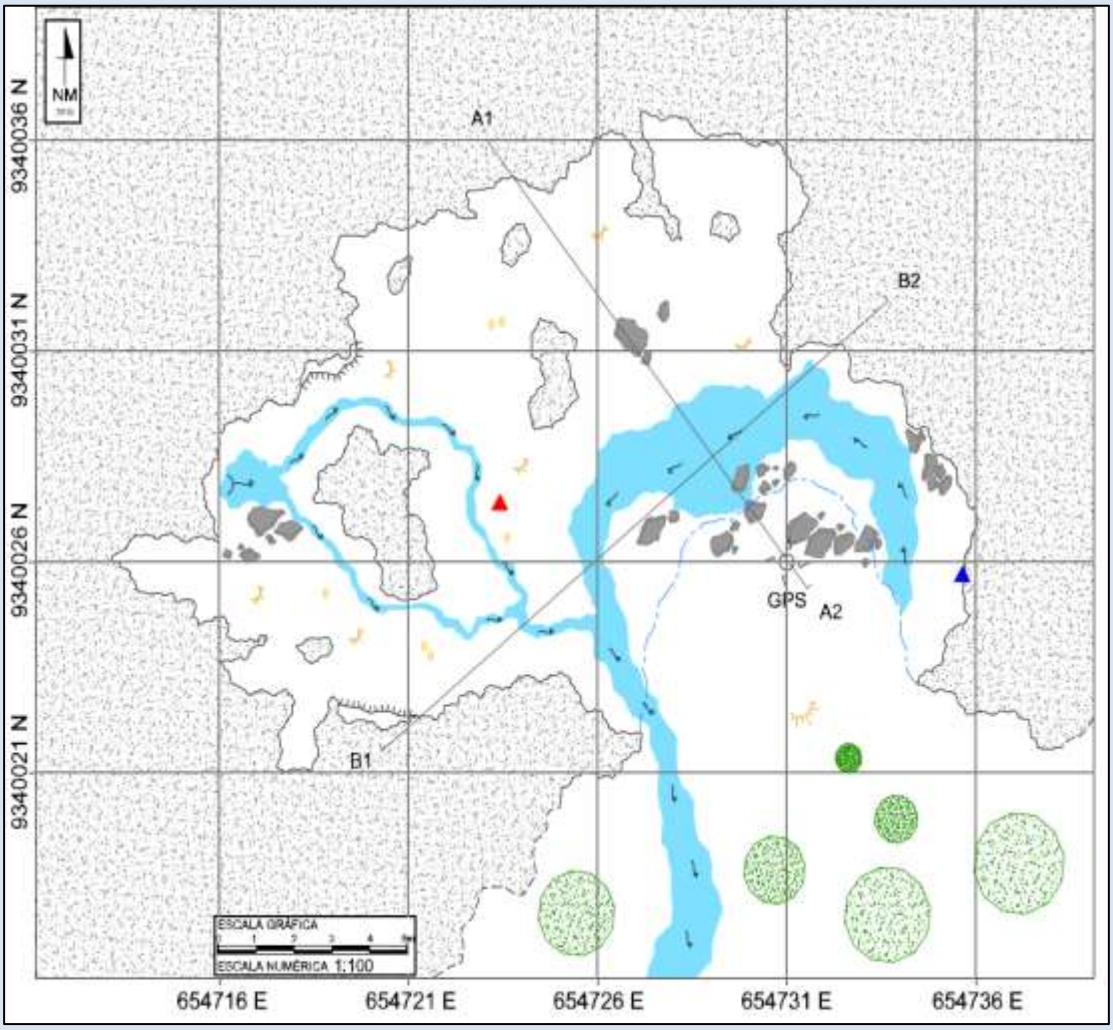

Seções

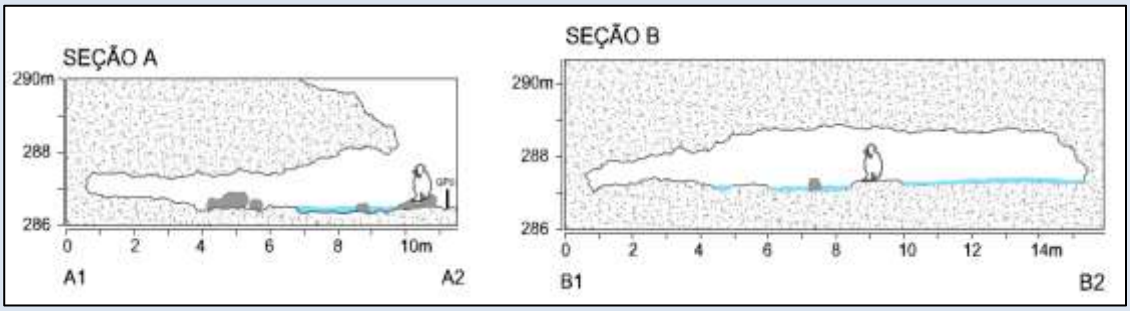

Quadro 5.20 - Síntese das características da caverna SL-130.

SL-130			
**UTM E:** 646864	**UTM N**: 9344606	**Datum:** SAD'69	**Altitude:** 299 m
**Projeção Horizontal**: 18,5 m	**Desnível**: 3,62 m	**Área**: 50 m²	**Volume**: 38 m³

Litologia: Quartzito

Inserção: Caverna inserida em blocos quartzíticos, na média vertente.

Observações Principais: Apresenta grande concentração de coralóides brancos e cinzas nas paredes e teto. Cavidade possivelmente formada nos interstícios de blocos rolados ao longo da vertente.

Atributo de Relevância: Não foram observados atributos de relevância.

Fotos: (A) Entrada da caverna; (B) Salão com teto baixo e piso coberto por calhaus e matacões; (C) Coralóides.

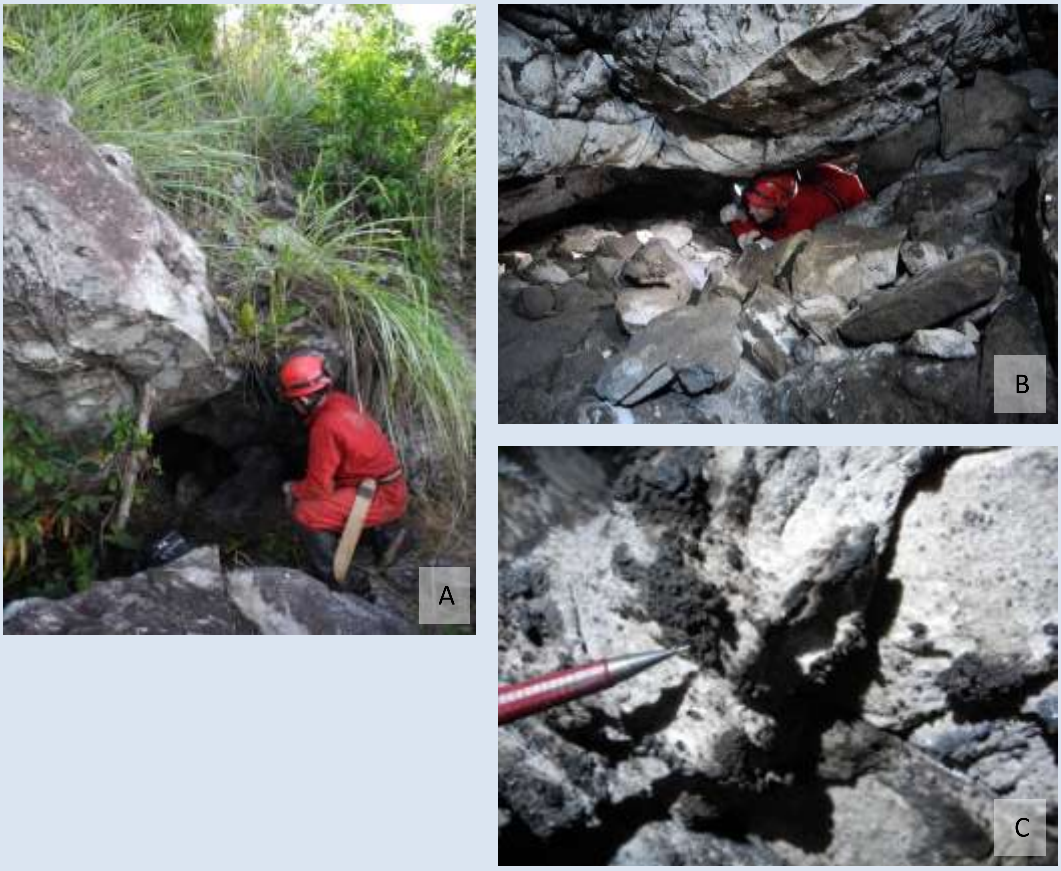

Planta Baixa

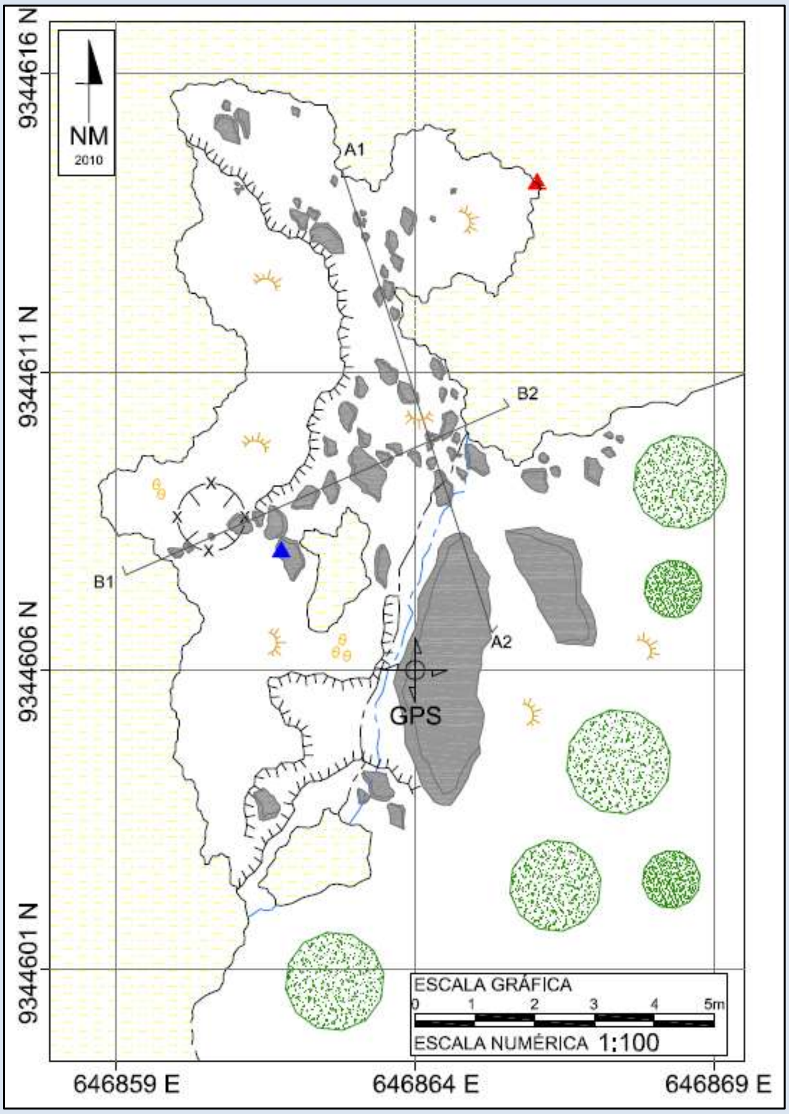

Seções

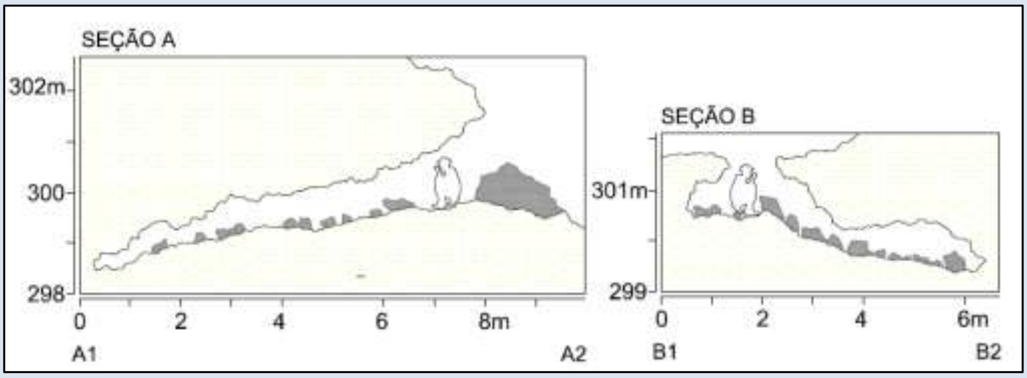

Quadro 5.21 - Síntese das características da caverna SL-131.

SL-131

UTM E: 656797 **UTM N**: 9344587 **Datum:** SAD'69 **Altitude:** 348 m

Projeção Horizontal: 13 m **Desnível**: 5,33 m **Área**: 47,5 m² **Volume**: 45,5 m³

Litologia: Quartzito

Inserção: Caverna inserida em paredão quartzítico escalonado, na alta vertente.

Observações Principais: Apresenta coralóides brancos recobrindo pequenas porções do teto e das paredes. Cavidade formada segundo o mergulho da foliação do quartzito com piso e teto inclinados perpendicularmente à entrada.

Atributo de Relevância: Não foram observados atributos de relevância.

Fotos: (A) Entrada da caverna; (B) e (C) Crostas brancas; (D) Vermiculação.

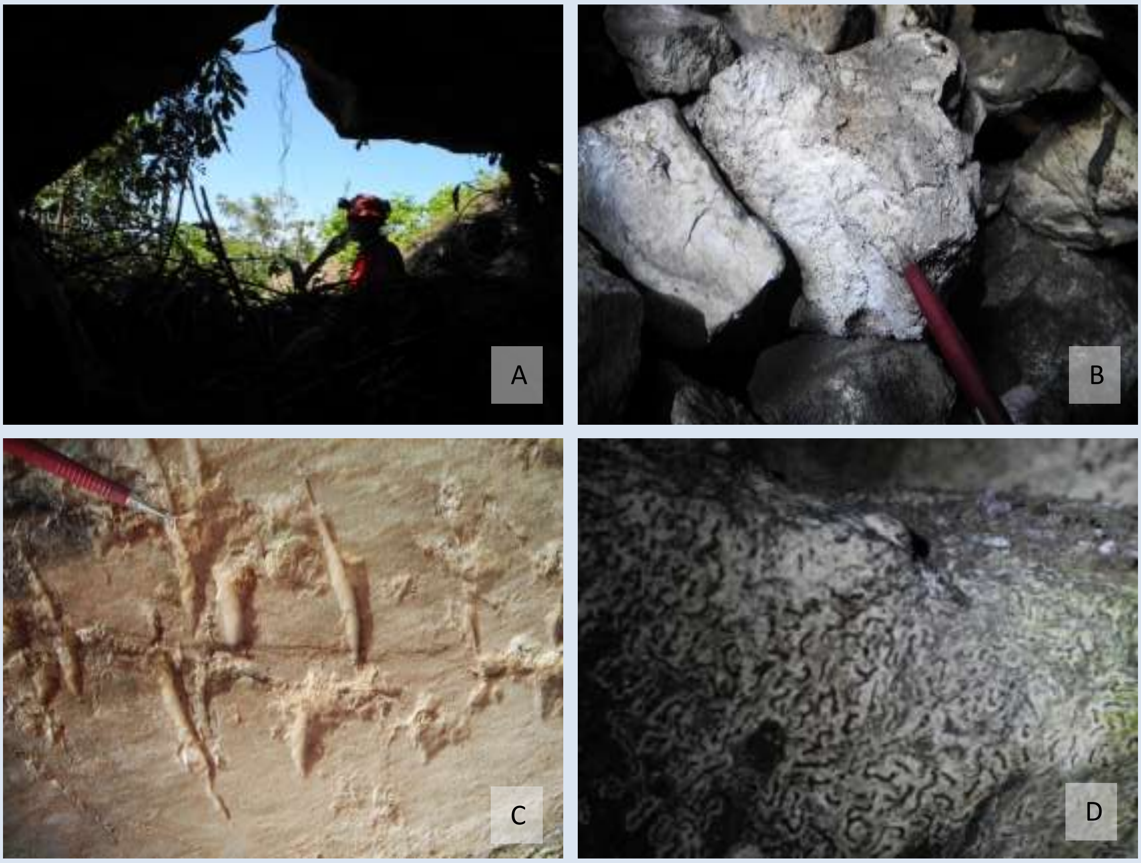

Planta Baixa

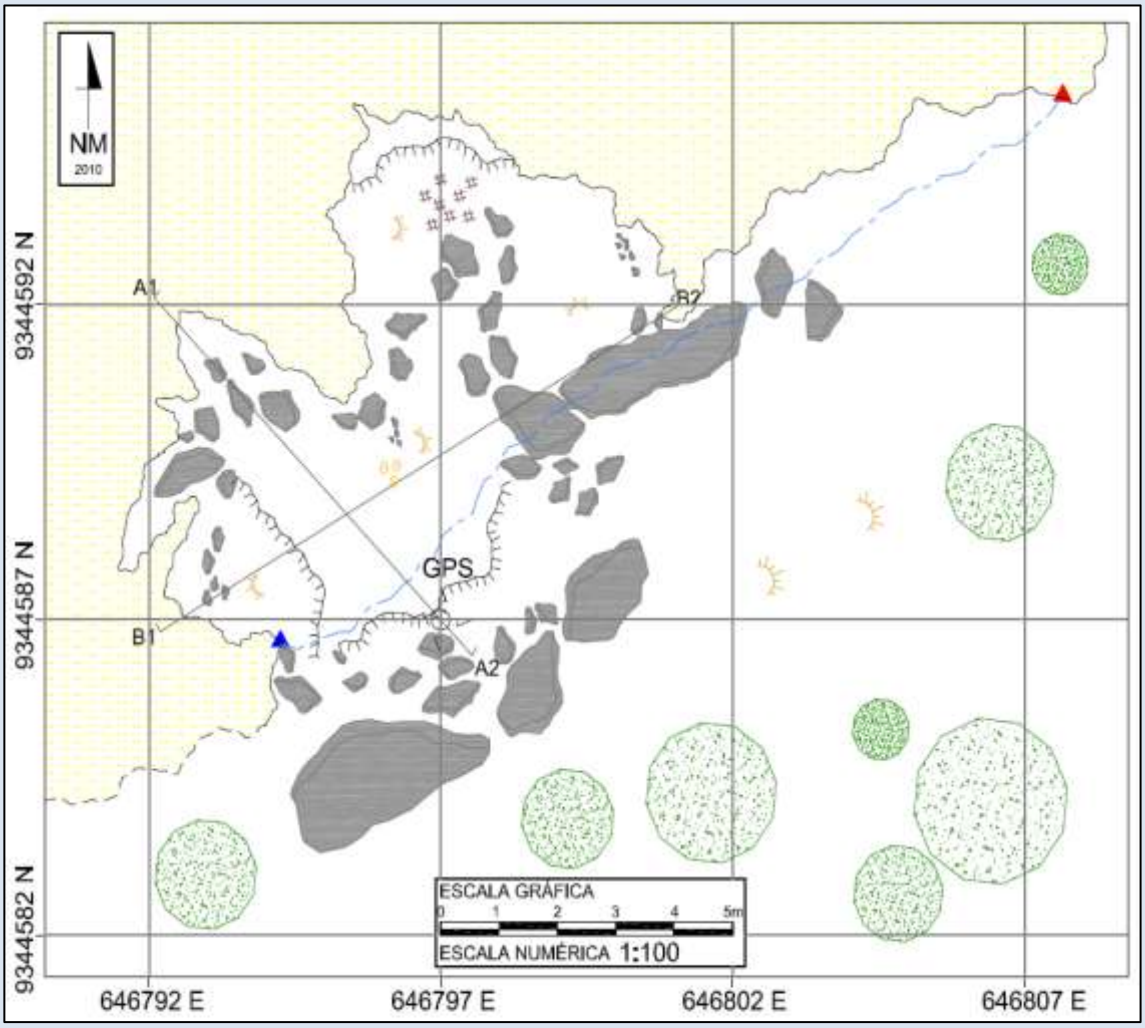

Seções

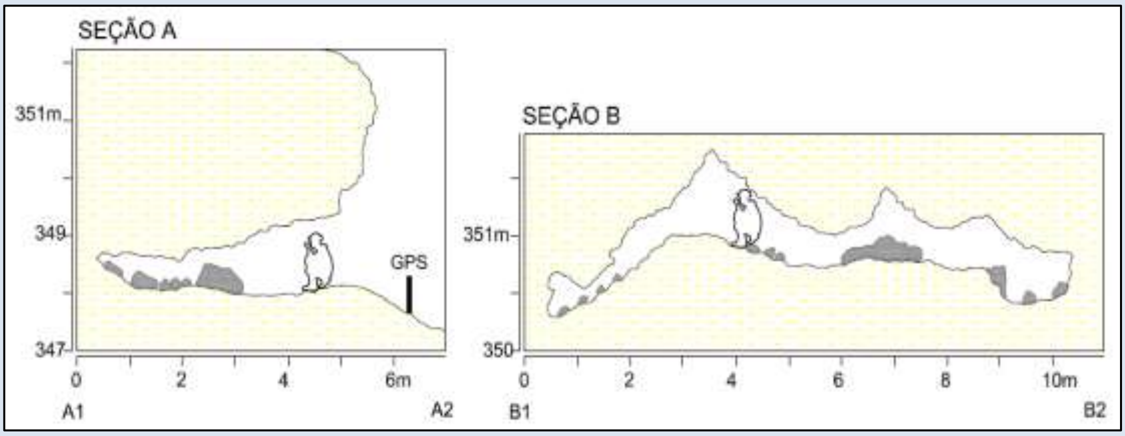

5.1.1. Inserção das cavernas na paisagem

As 21 cavernas analisadas estão inseridas na unidade geomorfológica da Serra Leste, em compartimentos que abarcam (i) as vertentes inclinadas da serra, que conectam o topo dos platôs aos fundos de vale ou (ii) em sistemas fluviais na base da serra (Figura 5.2) (Figura 5.3).

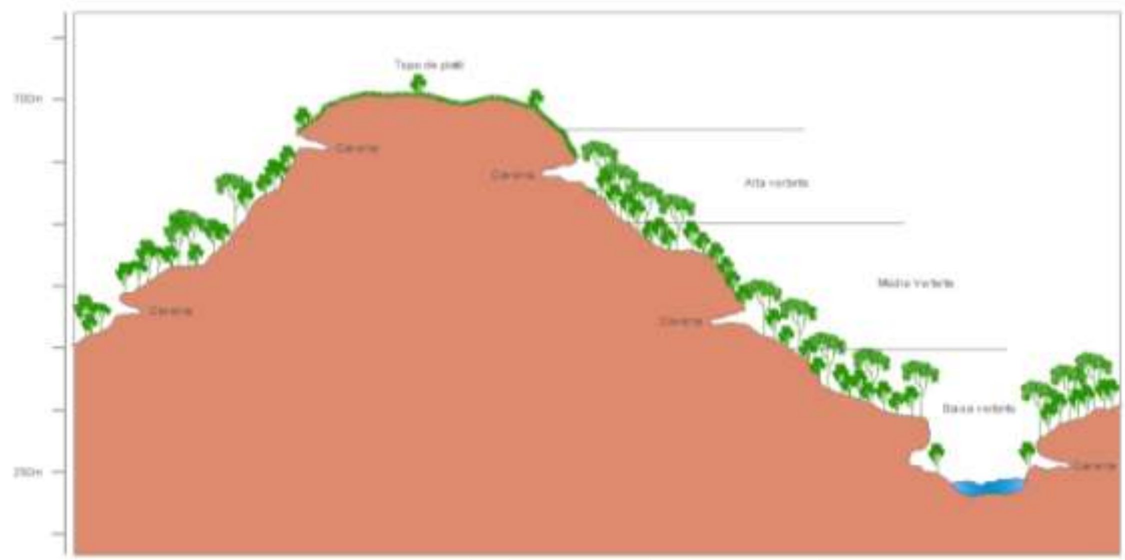

Figura 5.2 - Perfil esquemático de inserção das cavidades na Serra Leste.

Ao longo das ***vertentes inclinadas da serra*** foram registradas 12 cavernas, correspondendo a 57% do total estudado (Figura 5.3). As cavidades estão distribuídas ao longo deste compartimento de forma fortuita, sendo duas posicionadas na alta vertente, seis na média vertente e quatro no terço inferior da vertente.

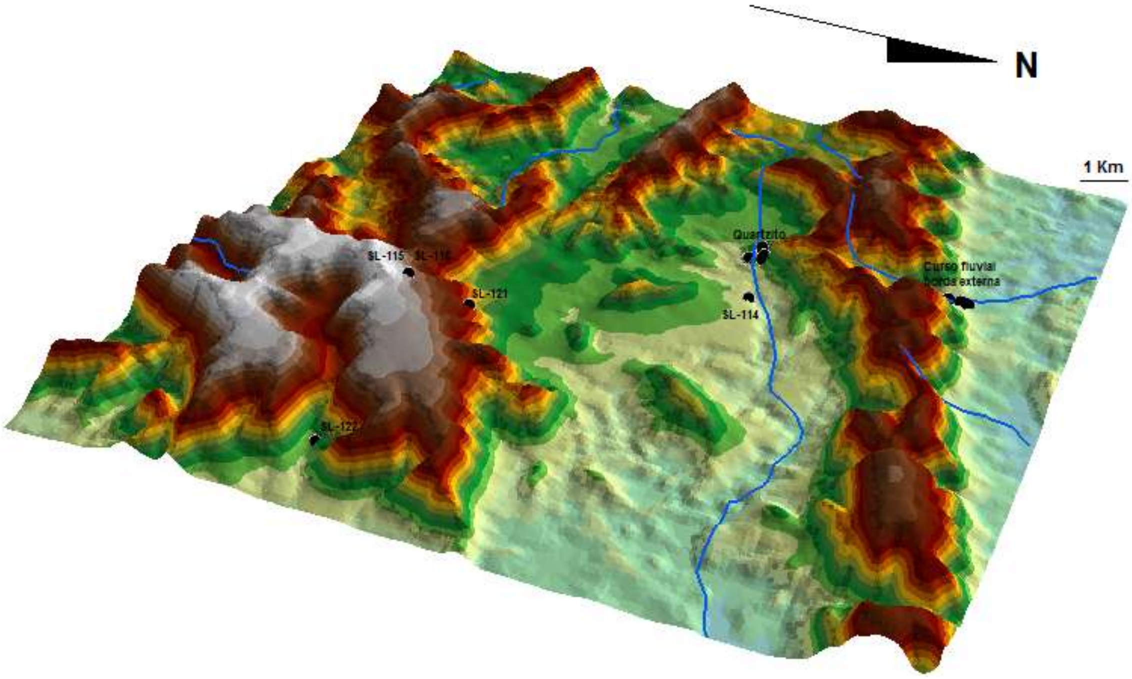

Figura 5.3 - Modelo digital de elevação de parte da Serra Leste. Exagero vertical: 3 vezes.

Dois conjuntos de cavidades são diferenciados ao longo das vertentes de acordo com a litologia: as cavernas SL-101, SL-102, SL-104, SL-107, SL-113, SL-117, SL-130 e SL-131 (Figura 5.4) alojam-se em quartzito (Figura 5.3) e, as cavidades SL-114 (Figura 5.4), SL-115, SL-121 e SL-122 (Figura 5.5) desenvolvem-se em rochas ferríferas.

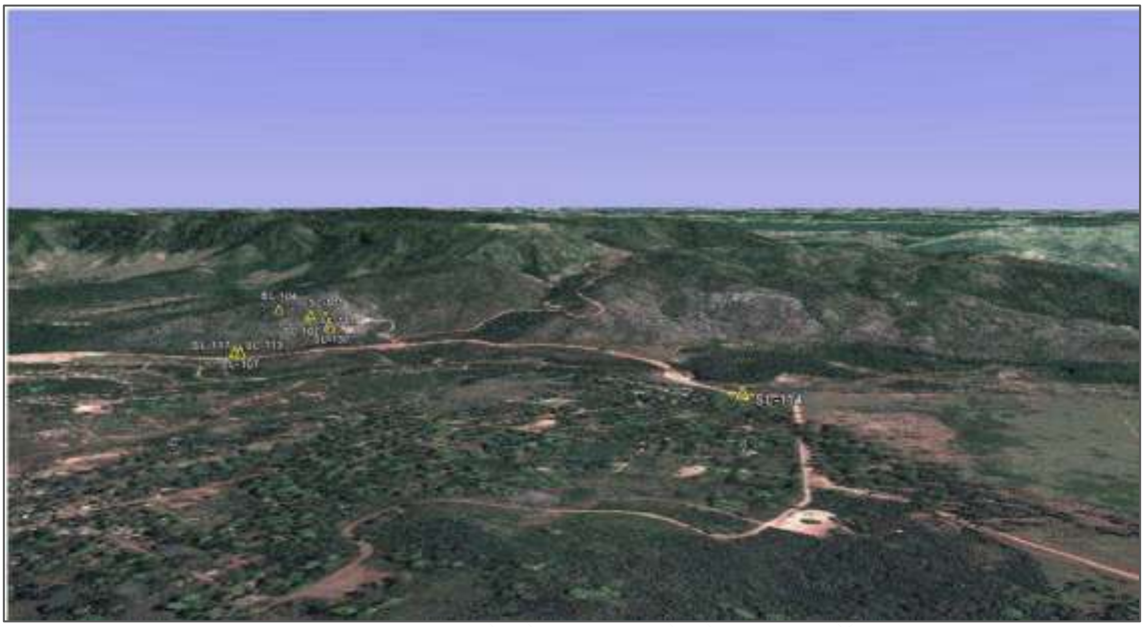

Figura 5.4 - À esquerda, conjunto de cavidades desenvolvidas em quartzito ao longo da vertente. À direita, caverna SL-114, em rocha ferrífera, situada a jusante de estrada. Fonte: Geoeye, 2012.

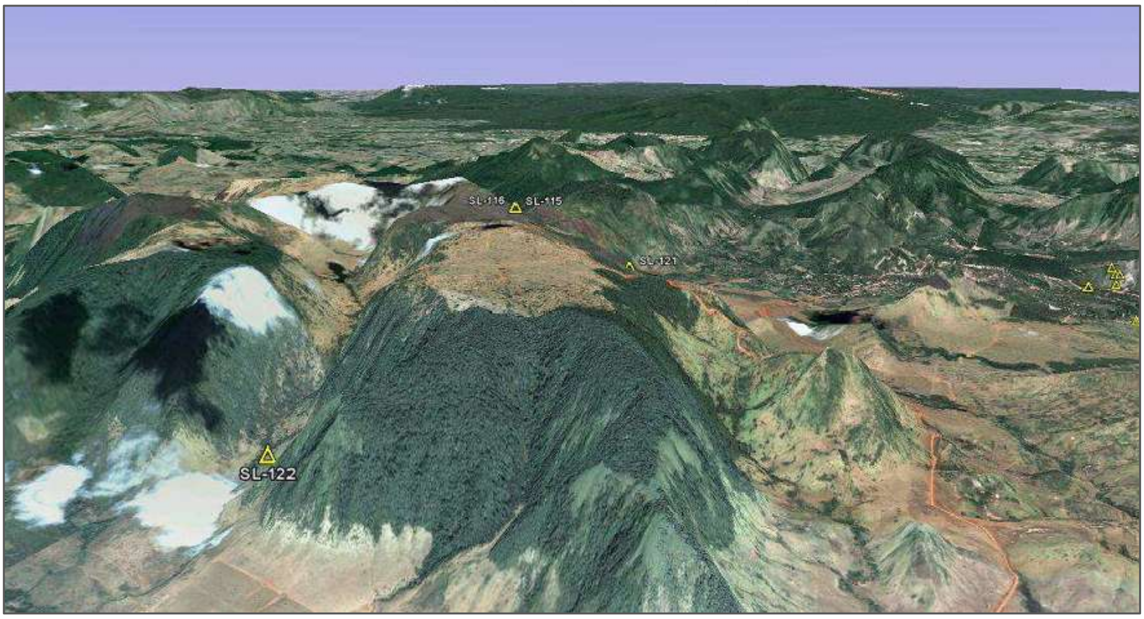

Figura 5.5 - Cavidades alojadas em rochas ferríferas situadas nas cotas altimétricas mais elevadas. A caverna SL-122 insere-se ao longo de curso fluvial que drena o interior da serra. Fonte: Geoeye, 2012.

Os ***sistemas fluviais na base da serra*** estão inseridos (i) em uma depressão interna da Serra Leste ou (ii) na borda desta feição geomorfológica (Figura 5.3 e Figura 5.6). Uma cavidade situada na depressão interiorana está em análise neste documento: SL-122 (Figura 5.5). Outras cavernas deste sistema já foram anteriormente estudadas (CARSTE, 2011a). No sistema fluvial da borda externa da serra, são oito cavidades em estudo (Figura 5.3 e Figura 5.7).

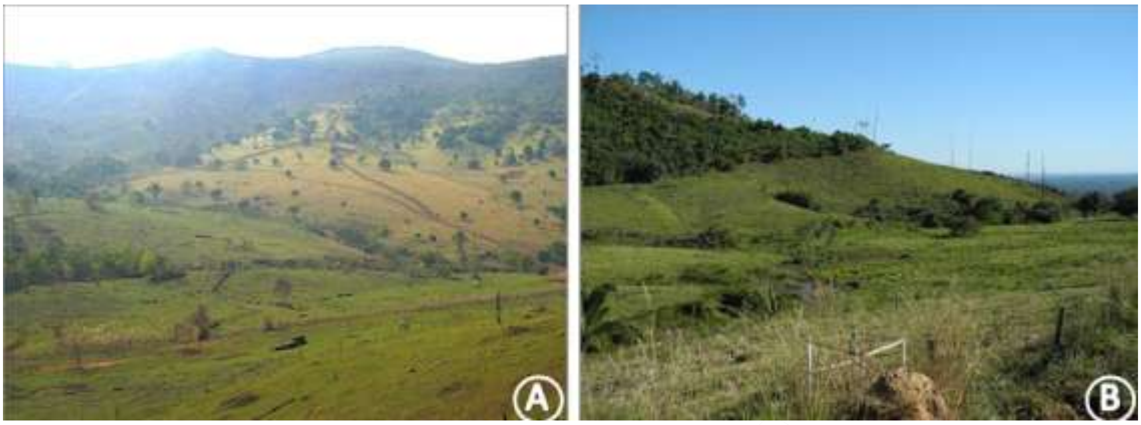

Figura 5.6 - (A) Visão parcial do sistema fluvial interiorano; (B) Visão parcial do sistema da borda externa da serra.

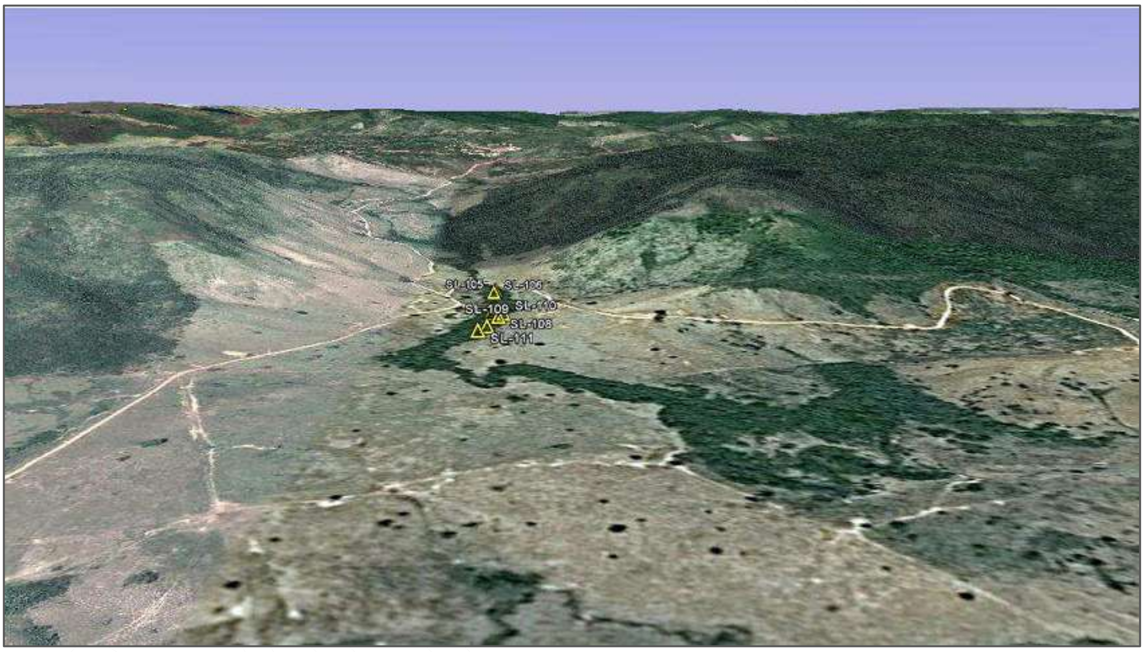

Figura 5.7 - Conjunto de cavidades inseridas em curso fluvial externo à borda da serra. Fonte: Geoeye, 2012.

No compartimento das vertentes, as cavernas ocorrem associadas (i) a paredões rochosos descontínuos, distribuídos de forma escalonada ao longo da vertente inclinada; (ii) a depósitos de tálus, sendo formadas nos interstícios entre os blocos rolados e basculados; (iii) a dolinas, que expõem cavidades possivelmente oclusas anteriormente.

Estudo anterior em cavidades inseridas na Serra Leste (CARSTE, 2011a) atestou que as cavernas estudadas ocupam todos os compartimentos da paisagem e, embora estejam concentradas em determinados intervalos altimétricos, não foi possível definir nenhum padrão de distribuição (Figura 5.8). Dentre as 21 cavernas em análise, nota-se uma predominância de cavernas em altitudes inferiores a 300 metros: oito situam-se entre os 200 e 250 metros de altitude, somando 38% (Figura 5.9). Estas alojam-se no compartimento do sistema fluvial da borda externa da Serra Leste (Figura 5.3). Outras seis (29%) estão localizadas entre os 250 e 300 metros de altitude (Figura 5.9). Duas cavernas destacam-se em cotas bastante elevadas em relação as demais neste estudo (Figura 5.10): SL-115 encontra-se a 636 metros e, SL-116 a

629 metros de altitude (Figura 5.3), muito embora estes valores altimétricos sejam comparáveis aos de diversas cavernas na área anteriormente estudadas (Figura 5.8).

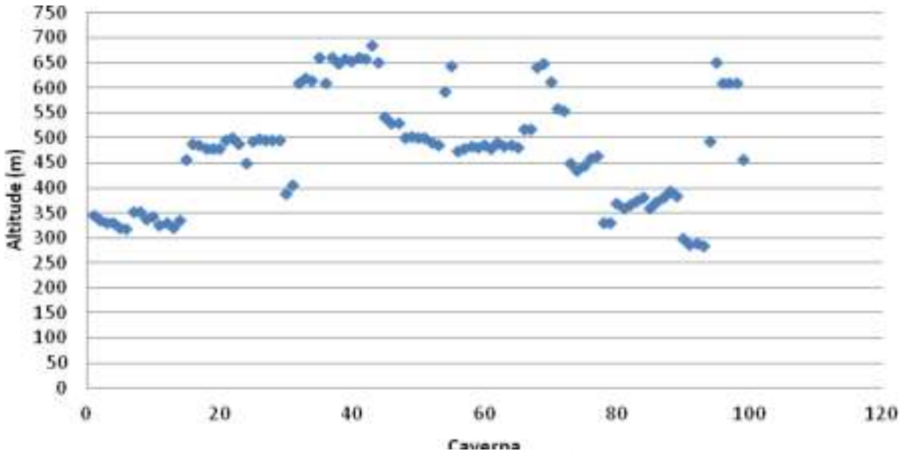

Figura 5.8 - Dispersão das cavernas anteriormente estudadas segundo intervalo hipsométrico. Extraído de CARSTE, 2011a.

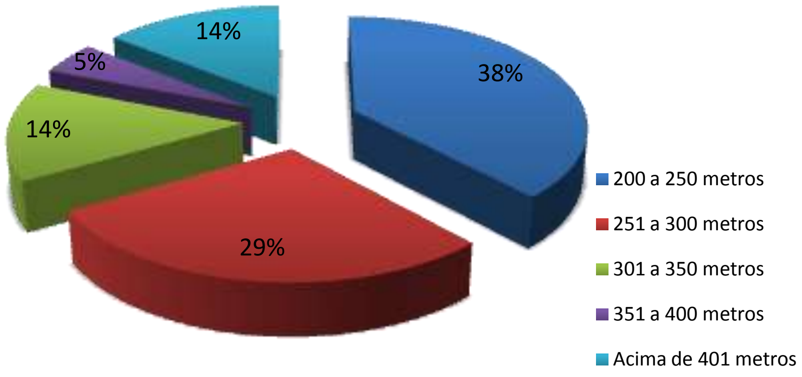

Figura 5.9 - Percentual de cavernas segundo intervalo hipsométrico.

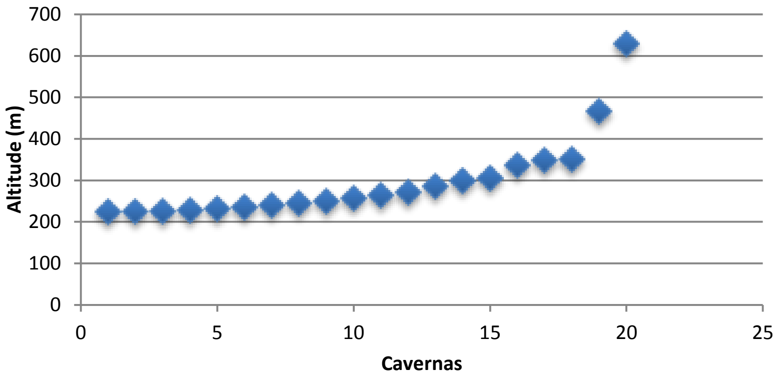

Figura 5.10 - Dispersão das cavernas em estudo segundo intervalo hipsométrico.

As cavernas situam-se na base de afloramentos rochosos, com alturas que normalmente variam de um a até cinco metros, com restrita ou nenhuma continuidade lateral, localizadas principalmente no terço médio de vertentes de média declividade. As escarpas podem estar

localizadas de forma paralela ou perpendicular a maior inclinação da vertente, paralela ou perpendicular à calha de drenagem ou configurando anfiteatros.

5.1.2. Litologia e estruturas geológicas

As cavidades em análise inseridas em Serra Leste desenvolvem-se em rochas ferríferas, em rochas siliciclásticas ou em contato entre estes litotipos (Figura 5.11). Dentre as rochas ferríferas, as crostas (*duricrusts*) formam o substrato em que se inserem as cavernas, sendo elas representadas por ferricrete ou por canga, podendo esta ser detrítica ou química. Estas crostas ferruginosas (*duricrusts)* incorporam material originado dos arredores onde está sendo formada. Em muitos casos, a origem dos materiais (clastos, por exemplo) pode ser facilmente identificada em terrenos vizinhos. O termo deve também ser expandido para incluir materiais cujos constituintes tiveram seus teores elevados pela precipitação ou captura de fluídos derivados da quebra ou mobilização de materiais fora da área onde se deu a formação da crosta. Podem se desenvolver como acumulações ferruginosas na base de escarpas ou no interior de depressões, e normalmente podem ser identificados pelo fato de apresentar uma clara discordância com a litologia situada abaixo. Muitas crostas ferruginosas, vale dizer, repousam por sobre litologias não alteradas e não exibem a típica gradação característica de muitos perfis lateríticos. Na área do Projeto Serra Leste, o ferricrete está assentado sobre quartzitos, enquanto a canga repousa, majoritariamente, sobre formação ferrífera bandada. Essas *duricrusts* são muitas vezes definidas de forma equivocada como lateritas. Vale lembrar que lateritas são crostas ricas em ferro que se formaram diretamente devido à quebra de materiais existentes nos arredores e não apresentam elementos alóctones. Normalmente manifestam-se como camadas superiores, in situ, de perfis de intemperismo. Onde expostos, tipicamente apresentam uma seqüência progressiva desde a rocha não alterada, passando pela frente de intemperismo, saprolito e progressivamente para zonas mais alteradas e ricas em ferro que culminam com a existência de uma rígida laterita no topo do perfil. Esse é o processo de laterização. No entanto, muitas vezes o termo laterita é usado, por alguns autores, somente no sentido petrográfico, sem conotação genética.

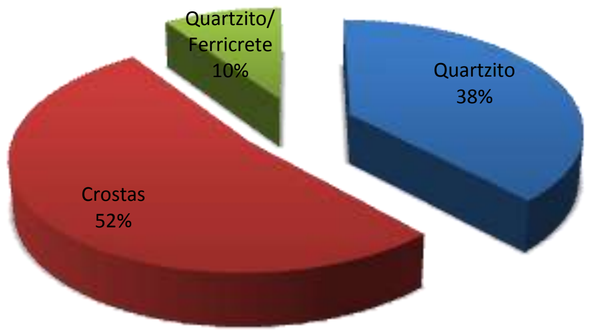

Figura 5.11 - Litologia encaixante das cavidades de Serra Leste.

Alojadas na canga, são quatro cavidades, que correspondem a 19% da amostra. Trinta e três por cento das cavernas em estudo inserem-se exclusivamente em ferricrete, somando sete cavernas. Somadas, as cavidades em crostas (*duricrusts*) totalizam 52%. No contato desta litologia com o quartzito, há duas ocorrências (SL-103 e SL-111) (10%) e, exclusivamente em quartzito, são oito cavidades, totalizando 38% (Figura 5.11).

A canga em que se alojam quatro cavidades em Serra Leste pode ser de dois tipos: (i) canga detrítica: composta por fragmentos de rocha dispostos caoticamente em matriz limonítica ocorre na caverna SL-122. Os clastos, mal selecionados, apresentam variação granulométrica de grânulo a matacão com ocorrência abundante de seixos subarredondados (Figura 5.12), sendo os sedimentos menores compostos por quartzo e os maiores de constituição ferruginosa, denotando maior proximidade da área fonte dos últimos, corroborada ainda pelo menor grau de arredondamento em relação aos primeiros. Esta canga encaixa-se na classificação de Dorr (1969) como canga normal ou rica e, foi descrita por Maurity & Kotschoubey (1995) no platô N1 em Carajás. Os autores a denominaram de hematítica brechóide, sendo originária da alteração das formações ferríferas bandadas (FFB); (ii) canga química: litotipo rico em matriz limonítica, com esparsos fragmentos clásticos de granulometria areia grossa a grânulo estão presentes nas três cavernas situadas nas mais elevadas cotas altimétricas observadas neste estudo (SL-115, SL-116 e SL-121). A massa ferruginosa de aspecto maciço é, aparentemente, bastante resistente aos processos desnudacionais e sustenta feições verticalizadas parcialmente isoladas de porções na parede na cavidade SL-116 (Figura 5.13). Majoritariamente marrom, apresenta pontuais colorações amareladas, sugerindo composição goethítica na caverna SL-115 (Figura 5.13) e brilho metálico na cavidade SL-121. A porosidade neste tipo de canga pode elevar-se devido a processos desnudacionais que promovem vazios, geralmente de aspecto alveolar, nas paredes das cavernas. Neste ponto, a rocha exibe características do ferricrete, litotipo encontrado em outras cavidades da área de estudo.

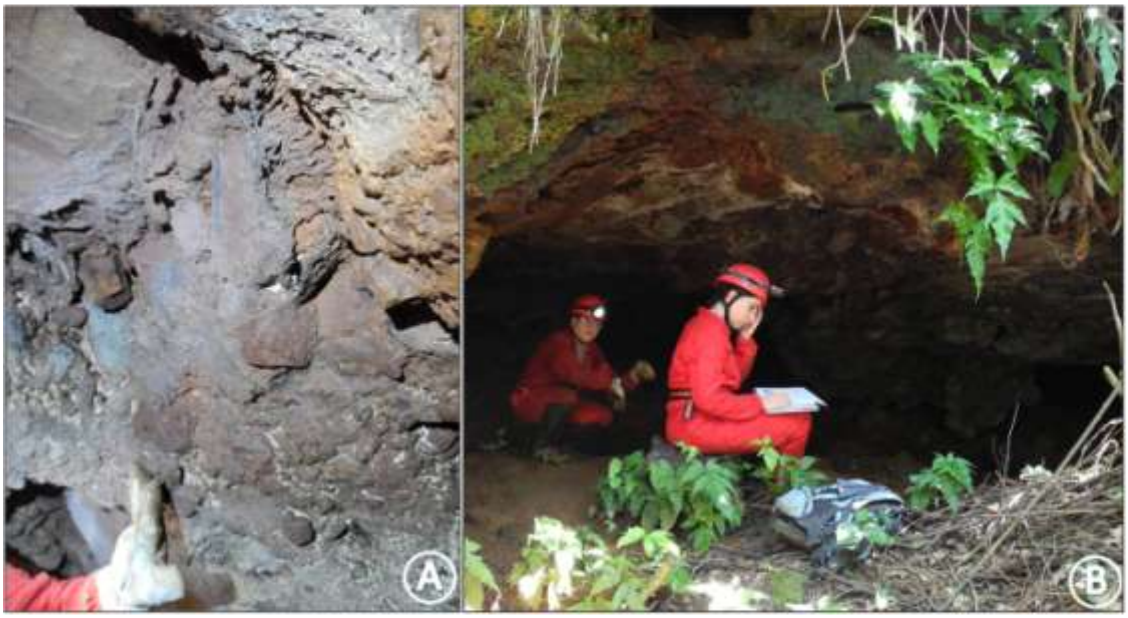

Figura 5.12 - Clastos mal selecionados compõem a canga na caverna SL-122: (A) grânulos e seixos angulosos se destacam, além de calhaus e blocos de constituição ferruginosa (B).

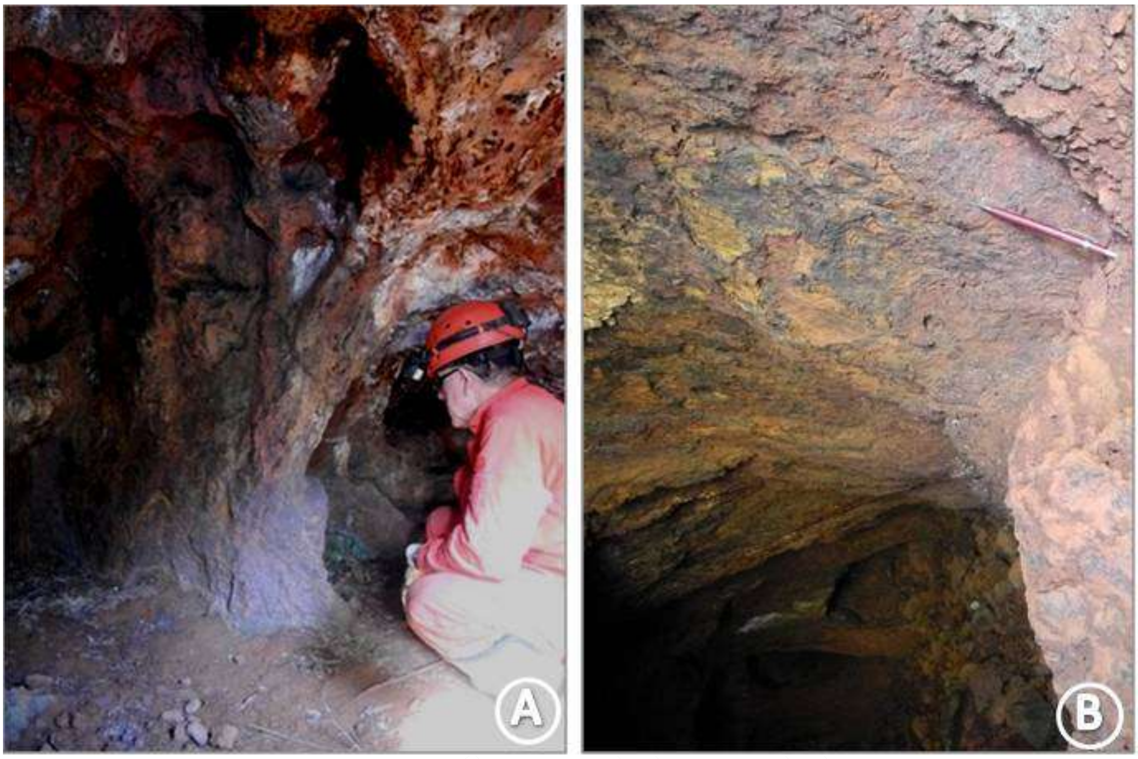

Figura 5.13 - Canga química: (A) sustenta feições verticalizadas na caverna SL-116; (B) apresenta porções de concentração goethítica na SL-115.

Nas três cavidades, há ocorrência também de canga detrítica, composta por seixos angulosos de composição ferruginosa suportados por matriz aparentemente limonítica. De ocorrência restrita, a canga detrítica parece ter entupido as cavidades SL-115 e SL-116, sendo posteriormente removida. Na cavidade SL-121, a canga detrítica preenche fraturas que condicionam a direção de desenvolvimento de condutos, podendo ser esta estrutura geológica de interesse científico (Figura 5.14).

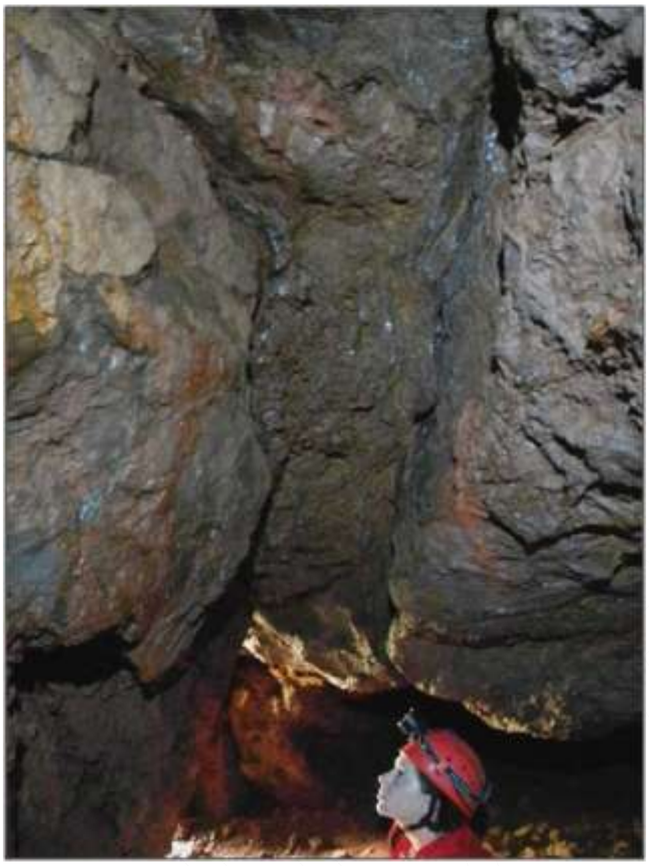

Figura 5.14 - Canga detrítica preenche fratura na cavidade SL-121.

O ferricrete aparece em nove cavidades em análise, sendo em duas observadas também a presença de quartzito. Devido à necessidade de enquadrar as cavernas em um dos tipos litológicos, o critério utilizado foi o de predominância da rocha no interior da cavidade: considera-se que a cavidade SL-103 aloja-se em quartzito e, a SL-111, em ferricrete. Assim, são oito cavernas em ferricrete, sendo uma não exclusivamente nesta litologia. A ocorrência desta rocha dá-se nas cavernas inseridas no sistema fluvial da borda externa da serra e na cavidade SL-114.

A diagênese do ferricrete envolveu a lixiviação do ferro ferroso a montante da paisagem e a sua reprecipitação em porções mais rebaixadas (COELHO *et al.,*2001). De cor avermelhada e altamente poroso, devido à alta concentração de alvéolos que imprimem aspecto vugular à rocha, o ferricrete é composto predominantemente por clastos com granulometria que varia de área a grânulo. Entretanto, há na área, uma contribuição de sedimentos de granulometria mais grossa, possivelmente advindos das vertentes por meio de movimentos de massa.

Nota-se, ao longo das paredes das cavernas inseridas no sistema fluvial da borda externa da serra, a ocorrência do ferricrete de aparência vugular (espongiforme) com grande quantidade de sedimentos de granulometria fina e constituição ferruginosa, possivelmente trazidos em suspensão pelo curso de drenagem (Figura 5.15).

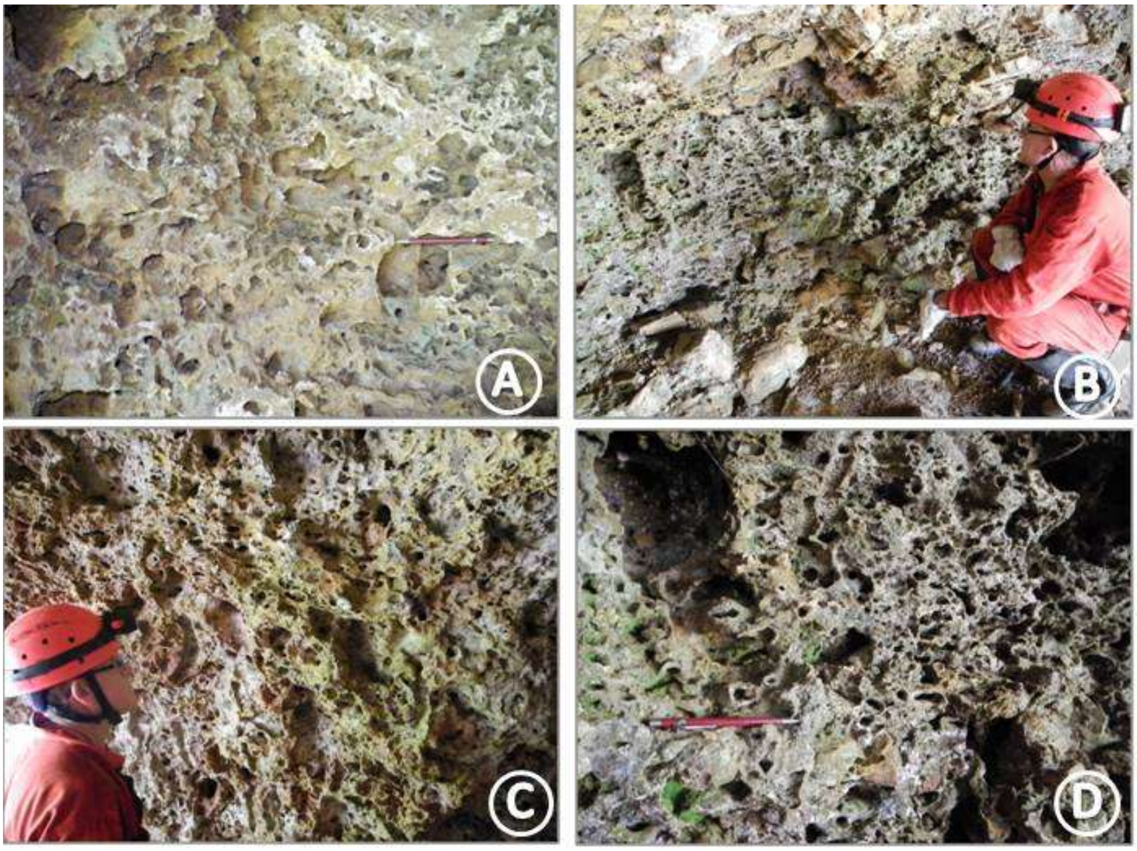

Figura 5.15 - Ferricrete composto por sedimentos de granulometria fina e apresentando aspecto vugular: (A) na cavidade SL-103 e (B) (C) e (D) na caverna SL-112.

No teto, ao contrário do observado nas paredes destas cavidades, a composição predominante é de seixos, calhaus e blocos angulosos a subangulosos de quartzito, sustentados por escassa matriz limonítica (Figura 5.16). O teto cascalhento conta ainda com clastos de constituição ferruginosa de menor granulometria: seixos e grânulos subarredondados.

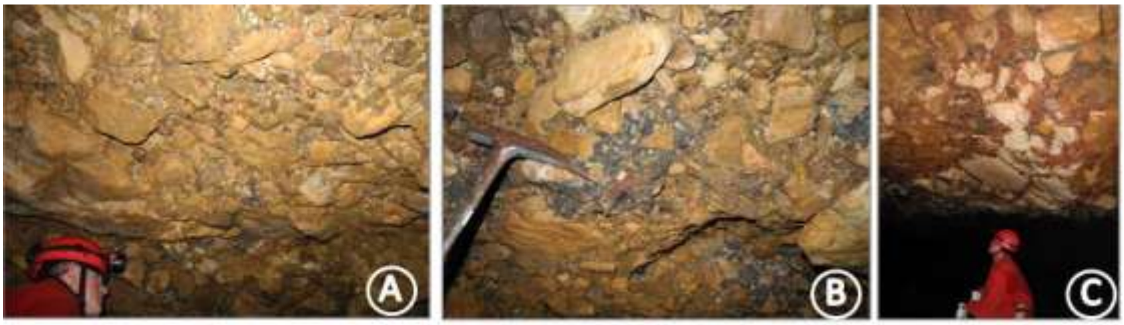

Figura 5.16 - Teto predominantemente composto por clastos angulosos de constituição quartzítica: (A) e (B) SL-105 e (C) SL-110.

Aparentemente, o teto cascalhento é bastante resistente aos processos desnudacionais, promovendo o desenvolvimento de cavidades abaixo deles, em rocha mais friável como o ferricrete. No piso da maior parte das cavernas em análise, a ausência dos grandes clastos quartzíticos que compõem o teto permite inferir que processos de abatimento não ocorrem em larga escala, sendo o teto altamente resistente. Em uma porção no setor proximal da

cavidade SL-110, a ausência destes clastos do teto permite maior incisão na rocha e o teto ganha altura, corroborando esta hipótese.

A cavidade SL-114, única dentre as cavernas alojadas em ferricrete analisadas que não se insere ao longo do curso fluvial, apresenta-se totalmente desenvolvida em ferricrete composto por clastos de granulometria menor que grânulo e, seus alvéolos apresentam-se menores e menos profundos quando comparados aos da litologia encaixante das demais cavernas.

As cavidades SL-103 e SL-111 apresentam-se no contato entre as rochas ferríferas e as rochas siliciclásticas (Figura 5.17). O contato lateral observado na caverna SL-111, dá-se devido ao degrau estabelecido na paisagem: o vale esculpido em quartzito que já apresentava o desnível abrupto provavelmente foi colmatado pelo ferricrete que vem sendo desnudado, voltando a expor a rocha subjacente. O desnível abrupto do quartzito permite seu afloramento na parede sul da caverna. Já a caverna SL-103, a montante da cavidade SL-111, apresenta as litologias em contato horizontal, sendo o ferricrete sobrejacente presente em menor escala no interior da caverna. O quartzito observado em ambas as cavernas é amarelado, maciço e apresenta plano de acamamento de mergulho subvertical de direção aproximadamente N/S, demonstrando-se bastante fraturado.

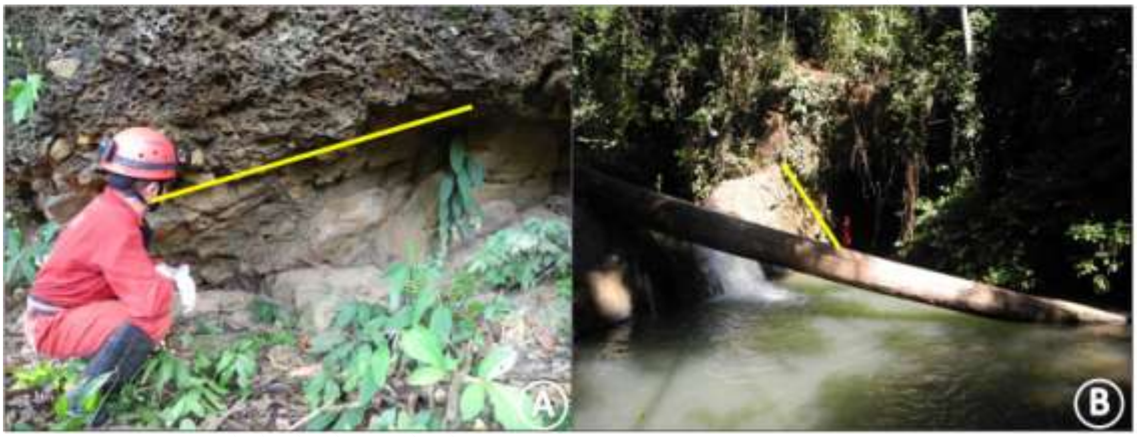

Figura 5.17 - Contato litológico entre rocha ferrífera e rocha quartzítica: (A) SL-103 e (B) SL-111. As linhas amarelas indicam o local aproximado do contato.

O quartzito encaixante das demais cavidades da Serra Leste apresenta algumas diferenças frente ao que ocorre nas cavernas que compõem o sistema fluvial da borda externa da serra. Apesar de também estar bastante fraturado, o quartzito apresenta cor branca quando fresco e cinza claro a escuro quando intemperizado. Aparentemente, há diferença de dureza entre as porções frescas e intemperizadas, com maior friabilidade nas camadas mais interiores da rocha, onde há menor alteração advinda de contato com o meio externo. Nota-se que a rocha apresenta-se bastante friável, uma vez que o quartzito parece ser composto por grãos praticamente soltos, quartzosos, de tamanho areia fina a grossa, sendo escassas a matriz e as impurezas. A falta de coesão entre os grãos que compõem a rocha pode indicar que os demais componentes da rocha tenham sido lixiviados.

Localmente, o quartzito pode apresentar coloração rósea e aspecto vugular, tendo aumentada a sua porosidade (Figura 5.18). Em alguns pontos, nota-se textura vítrea da rocha nas cavernas

SL-102 e SL-131. Também na cavidade SL-102, em porções isoladas, nota-se camada de quartzito xistoso com aproximadamente 15 centímetros de espessura (Figura 5.18).

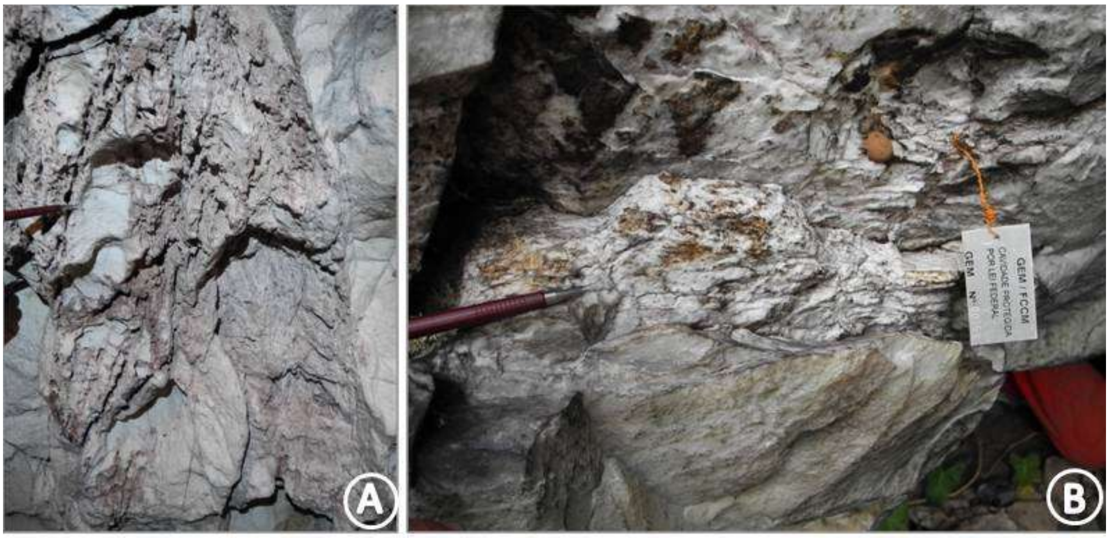

Figura 5.18 - (A) Quartzito de aspecto vugular e coloração rósea na caverna SL-107.(B) Camada de quartzito xistoso na SL-102.

Concentrações bacterianas imprimindo desenhos irregulares no quartzito, denominados vermiculações, são observadas em diversos pontos das cavidades SL-101, SL-104 e SL-131 (Figura 5.19). As vermiculações são depósitos finos, irregulares e descontínuos compostos por materiais como lama ou argila, usualmente encontrados no interior das cavernas, circundados por um halo ligeiramente mais largo que a própria vermiculação. Várias hipóteses para explicar a vermiculações foram teorizadas (ver listagem em HILL & FORTI, 1997). Feições dissolutivas também se apresentam como alterações nas rochas siliciclásticas: em forma de colmeia, ocorrem principalmente nas paredes, como observado nas cavernas SL-130 e SL-131. Outra feição, a canelura, ocorre em pelo menos três cavidades: SL-101, SL-104 e SL-131. Normalmente verticalizada e concordante com o fluxo de água, na cavidade SL-104 ela aparece na parede de forma oblíqua onde há fluxo de ar, entre duas entradas da caverna, sugerindo que sua gênese tenha relação com este fluxo, tratando-se de uma estrutura espeleogenética que pode ser considerada rara (Figura 5.20).

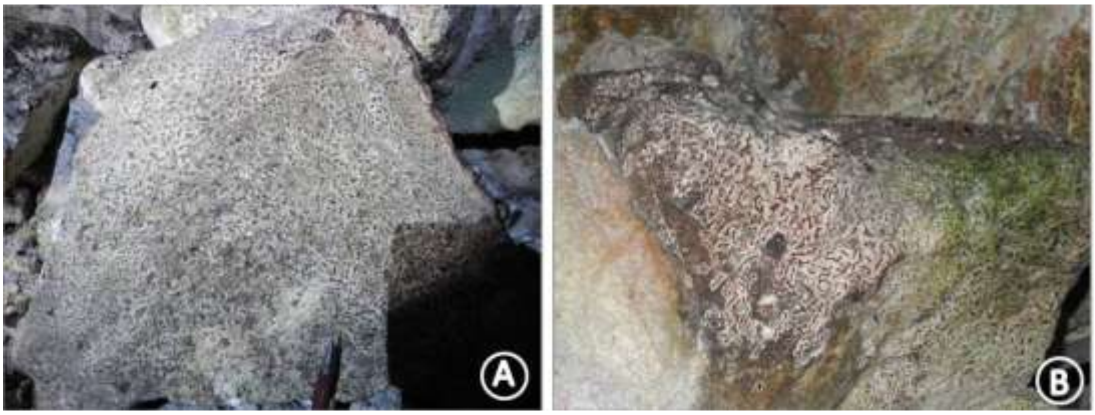

Figura 5.19 - (A) Vermiculações em bloco da cavidade S-104 e (B) no teto da caverna SL-131.

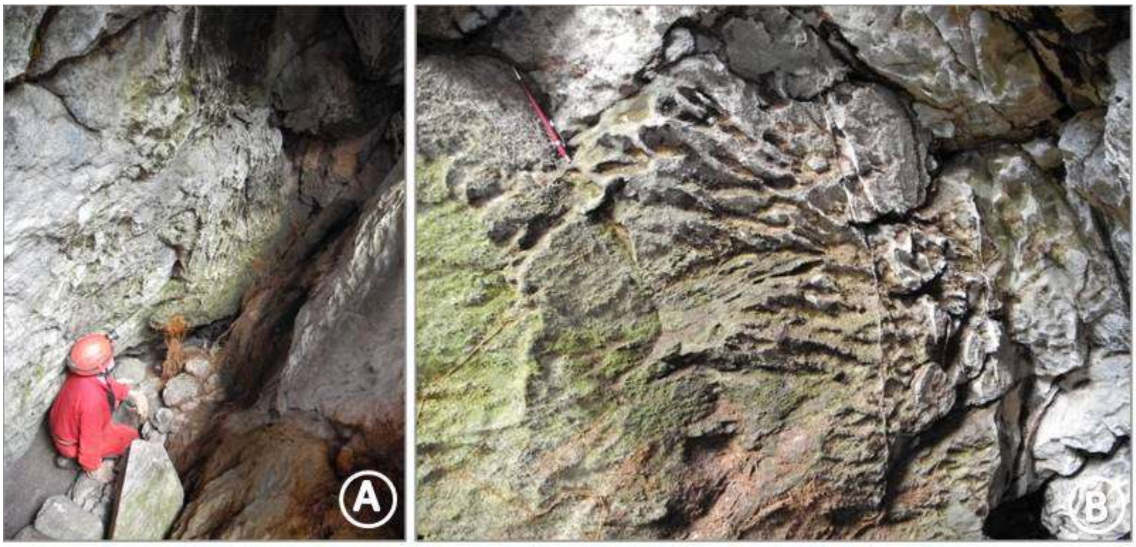

Figura 5.20 - Caneluras oblíquas na cavidade SL-104: (A) em visão ampla e (B) em destaque.

Dentre as estruturas geológicas observadas nas cavidades em análise, as feições mais frequentes foram as juntas de alívio presentes em 12 cavernas (57%). Fraturas ocorrem em sete cavidades (33%), sendo cinco delas em quartzito, em que se pode observar também planos de acamamento (24%).

As juntas de alívio são diáclases irregulares sub-verticais, sub-horizontais ou oblíquas que podem ser visualizadas no teto e paredes das cavidades em análise. A espessura varia de milímetros a poucos centímetros e o comprimento abrange dimensões centimétricas a métricas.

Fraturas e planos de acamamento muitas vezes podem exercer influência na espeleogênese da cavidade, pois constituem descontinuidades que facilitam o fluxo da água no interior do maciço rochoso, sendo consideradas importantes frentes de alteração. Assim, as diáclases mais penetrativas observadas nas cavernas em análise, como as fraturas, e o mergulho do plano de acamamento podem condicionar a morfologia e/ou gênese de pelo menos quatro cavernas: SL-101, SL-103, SL-104 e SL-121.

Foram tomadas medidas de fraturas em oito cavernas (n=24) e, em cinco delas, mediu-se a atitude dos planos de acamamento do quartzito (n=17). A partir dos dados coletados foi possível observar uma tendência de fraturas nas direções ENE e NNE (Figura 5.21) e dos planos de acamamento nas direções NNW e WNW (Figura 5.22).

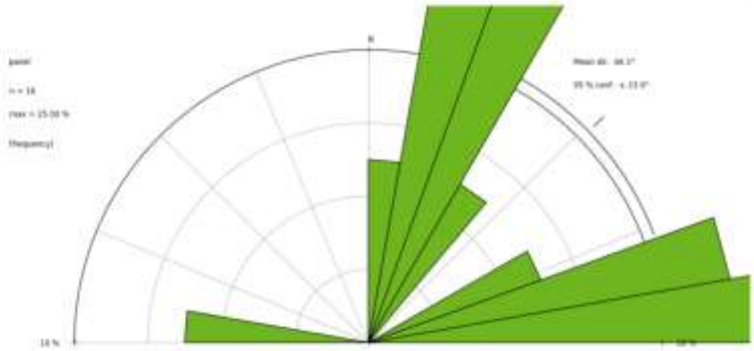

Figura 5.21 - Diagrama de roseta para as medidas de fraturas.

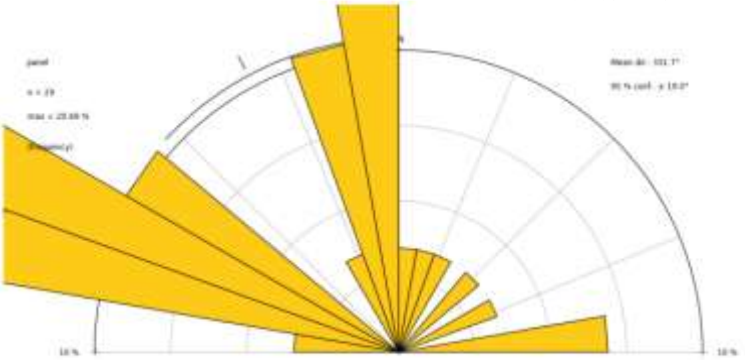

Figura 5.22 - Diagrama de roseta para as medidas de planos de acamamento.

Através dos mapas topográficos das cavidades foi realizada uma análise das direções preferenciais dos condutos em que foram tomadas as atitudes das feições geológicas, sendo uma caverna em canga e sete em quartzito (Figura 5.23). Apesar do reduzido número de medidas, é possível observar uma tendência dos condutos nas direções N, NE, NW e WNW, o que pode ser correlacionado com a tendência de direção das fraturas e planos de acamamento, demonstrando controle estrutural da direção de desenvolvimento destes condutos.

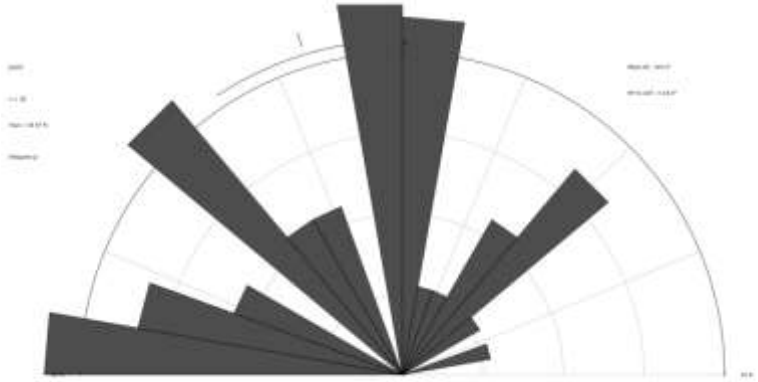

Figura 5.23 - Diagrama de roseta para as medidas de direção de condutos.

Em um estudo realizado anteriormente em 96 cavernas de Serra Leste, o mesmo comportamento foi observado (CARSTE, 2011a). As medidas desse estudo foram realizadas em cavernas de formação ferrífera bandada, jaspilito, canga e laterita. Independente das diferenças litológicas e das diferentes inserções na paisagem, foi possível observar uma mesma tendência direcional em ambos os estudos, indicando um controle regional dessas direções.

Utilizando os dados coletados nas campanhas anteriores e unindo-os aos dados coletados nessa última campanha, podemos analisar a tendência geral de Serra Leste para as estruturas apresentadas. As fraturas tendem a se direcionar para ENE, NW e WNW (Figura 5.24), enquanto os planos de acamamento ou bandamento das rochas apresentam uma tendência menos forte, indicando possível dobramento das litologias (Figura 5.25).

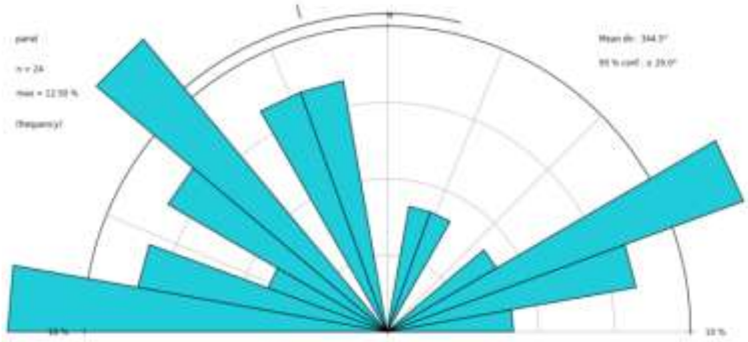

Figura 5.24 - Diagrama de roseta para medidas de fratura da totalidade das cavernas de Serra Leste.

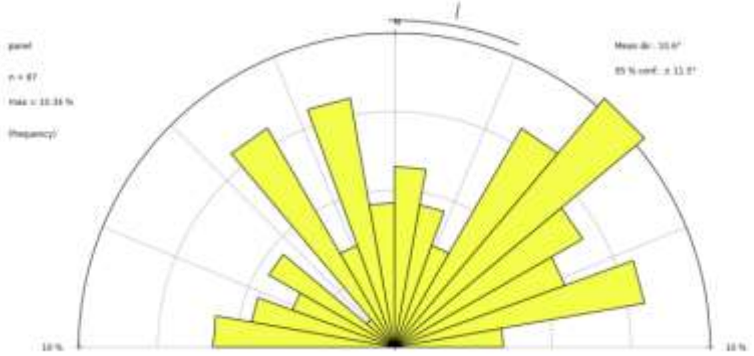

Figura 5.25 - Diagrama de roseta para medidas de planos de acamamento ou bandamento da totalidade das cavernas de Serra Leste.

5.1.3. Espeleometria

As cavernas de Serra Leste desenvolvem-se sob contexto de rochas ferríferas ou rochas quartzíticas, portanto, foram analisadas em conjuntos distintos no que diz respeito aos seus atributos espeleométricos.

A projeção horizontal (PH), calculada segundo o método da descontinuidade, representa a soma do comprimento dos condutos em planta baixa. As cavernas inseridas em rochas siliciclásticas em Serra Leste apresentaram média da projeção horizontal (PH) de 66,9m e as cavernas alojadas em rochas ferríferas têm PH médio de 23,4m (Quadro 5.22). Estes valores, entretanto, não são substanciais, uma vez que o desvio padrão indica que a projeção horizontal das cavidades varia 147,7m a partir da média para as rochas quartzíticas e 16,8m

para as cavernas em rochas ferríferas, com dados dispersos, apontados por um coeficiente de variação de muito alto em ambos os casos (Quadro 5.22). As 12 cavidades inseridas em rochas siliciclásticas têm valor de PH mínimo e máximo de 5,5m e 460m, respectivamente (Quadro 5.22). Em relação às 12 cavernas alojadas em rochas ferríferas, os valores de PH mínimo e máximo são 8m e 68,5m, respectivamente.

Quadro 5.22 - Estatística dos dados espeleométricos das cavidades inseridas no Projeto Serra Leste.

	PH (m)		Desnível (m)		Área (m²)		Volume (m³)	
	Quartzítica	Ferrífera	Quartzítica	Ferrífera	Quartzítica	Ferrífera	Quartzítica	Ferrífera
Mínimo	5,5	8,0	1,0	0,7	11,0	9,5	8,0	5,5
Máximo	460,0	68,5	32,3	3,7	1836,0	254,5	2662,0	283,0
Média	66,9	23,4	7,3	1,8	248,1	93,6	349,9	119,3
Desvio Padrão	147,7	16,8	9,6	0,8	596,2	73,6	868,1	95,1
Coeficiente de Variação (%)	220,9	71,8	132,2	46,0	240,3	78,6	248,1	79,7

Estabelecendo-se limites de classes de frequências semelhantes para ambos os grupos de cavernas, nota-se que as menores cavidades (5 a 10m) apresentam porcentagem semelhante em ambas as litologias (Figura 5.26). Entretanto as cavernas com PH entre 11 e 20m predominam em rochas siliciclásticas (45%), enquanto cavernas um pouco maiores (21 a 30m) são a maioria entre as alojadas em rochas ferríferas (33%) (Figura 5.26).

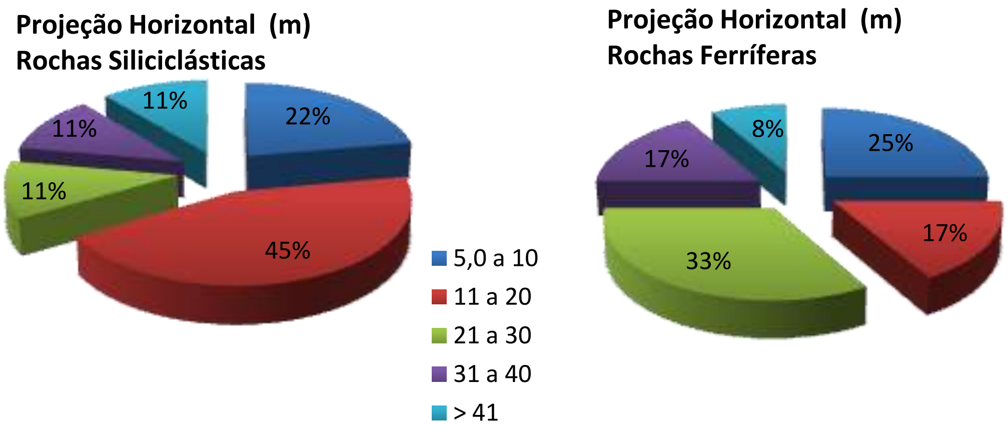

Figura 5.26 - Frequência de cavidades em função da projeção horizontal.

O desnível médio das cavernas inseridas em rochas siliciclásticas em Serra Leste é de 7,3m, com valor mínimo de 1m (SL-113) e máximo de 32,3m (SL-101). Tamanha disparidade entre os dados pode ser percebida pelo elevado coeficiente de variação (132,2%) (Quadro 5.22). Nas rochas ferríferas, os valores são menos díspares, com coeficiente de variação de 46%, apresentando desnível médio de 1,8m, sendo os valores mínimo e máximo de 0,7 e 3,7m (Quadro 5.22). Em rochas ferríferas, os desníveis são majoritariamente pequenos: 50% das cavernas têm menos de 1,5m de desnível (Figura 5.27). Nas rochas siliciclásticas, por outro lado, os maiores desnível predominam: 45% das cavernas apresentam-se com mais de 4,6m de desnível (Figura 5.27).

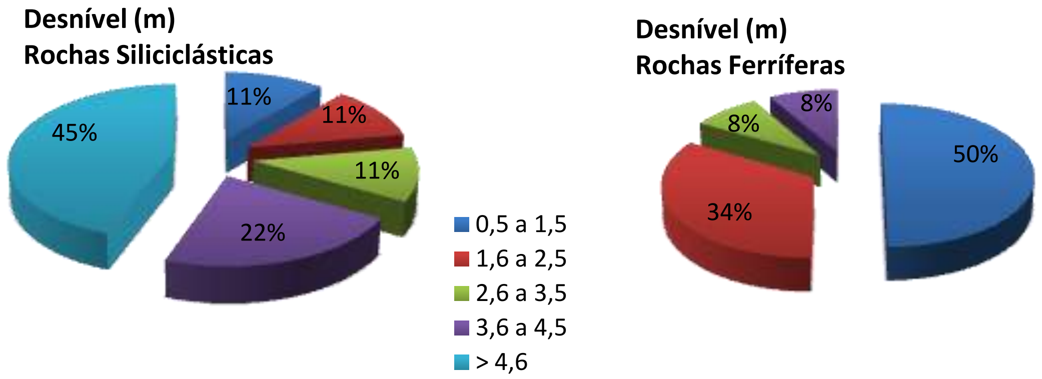

Figura 5.27 - Frequência de cavidades em função do desnível.

Dentre as cavidades desenvolvidas em quartzito, nota-se grande variabilidade entre os valores mínimo ($11m^2$) e máximo ($1836m^2$) de área, em função de a cavidade SL-101 ter tamanho extraordinário em relação às demais (Quadro 5.22). As cavernas alojadas em rochas ferríferas apresentam valores mínimo e máximo de área também elevado: são $9,5m^2$ e $254,5m^2$, o que eleva o coeficiente de variação em ambas as litologias (Quadro 5.22). Há maior quantidade de cavidades em rochas ferríferas maiores em área (entre 100,1 e $1000m^2$ são 42%) quando comparadas às menores (entre 9,5 e $40m^2$ são 33%)(Figura 5.28). Dentre as cavernas em quartzito, nota-se certa equiparidade entre cavernas grandes e pequenas em área, sendo três classes de frequência com aproximadamente 29% de cavernas (Figura 5.28).

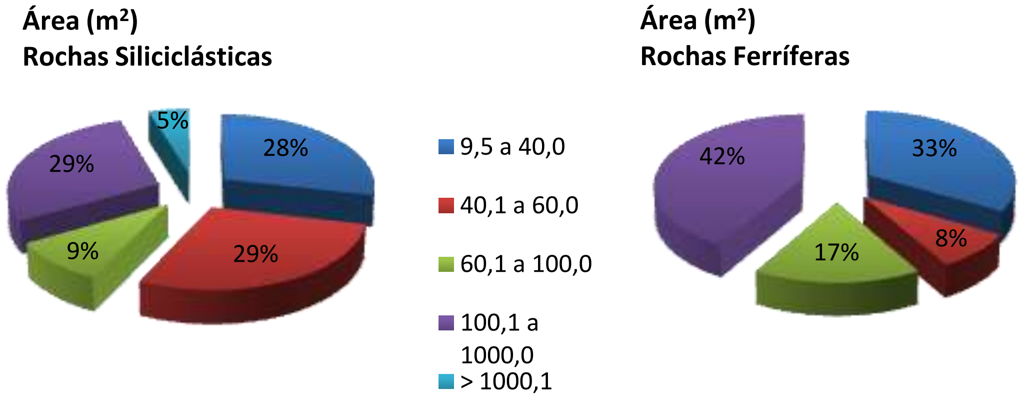

Figura 5.28 - Frequência de cavidades em função da área.

A média de volume para as nove cavernas inseridas em rochas quartzíticas é de $349,9m^3$, sendo o valor mínimo igual a $8m^3$ e o máximo $2662m^3$ (Quadro 5.22). Para as cavidades desenvolvidas em rochas ferríferas, a média é de $119,3m^3$ e, os valores de volume mínimo e máximo são 5,5 e $283m^3$, respectivamente (Quadro 5.22). Entre as cavernas em quartzito, a maior parte (56%) apresenta volume entre 40,1 e $80m^3$, as demais são cavernas de volume muito menor ou muito maior (Figura 5.29). Já entre as cavernas em rochas ferríferas, há uma

distribuição menos heterogênea, com porcentuais mais aproximados de cavernas em cada uma das classes de frequência (Figura 5.29).

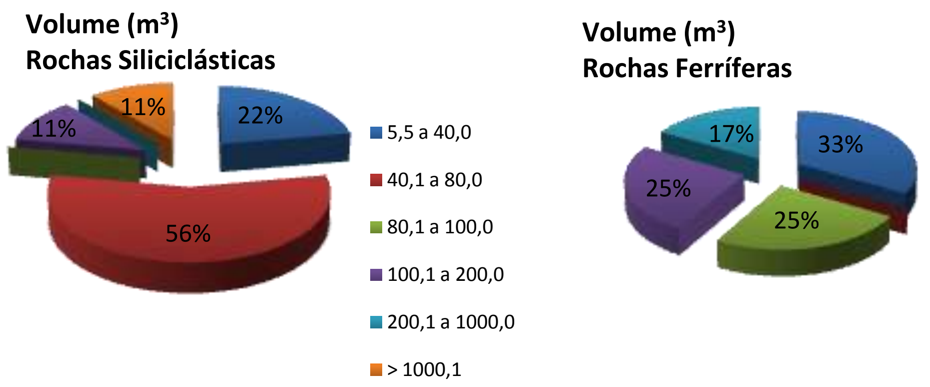

Figura 5.29 - Freqüência de cavidades em função do volume.

Tendo em vista os atributos espeleométricos, nota-se que as cavidades analisadas neste documento apresentam pequenas dimensões e, que devido a uma pequena parcela de cavernas que apresenta dados dimensionais pouco mais altos, a média eleva-se, assim como torna alto o grau de dispersão dos dados, como percebido no Quadro 5.22. Apenas a cavidade SL-101 apresenta dimensões notáveis nas escalas local e regional e, diante disso, sua relevância é elevada. Comparando-se as cavidades desenvolvidas em rochas ferríferas aqui analisadas com as demais cavernas estudadas em Serra Leste (CARSTE, 2011a), nota-se que o conjunto em análise no presente documento apresenta médias semelhantes para projeção horizontal e volume em relação às cavidades analisadas em documento anterior (CARSTE, 2011a) (Figura 5.30). As médias para o desnível e para volume apresentam maior discrepância, sendo as cavernas em análise no momento maiores em área e com menor desnível que as demais cavidades inseridas em Serra Leste (Figura 5.30).

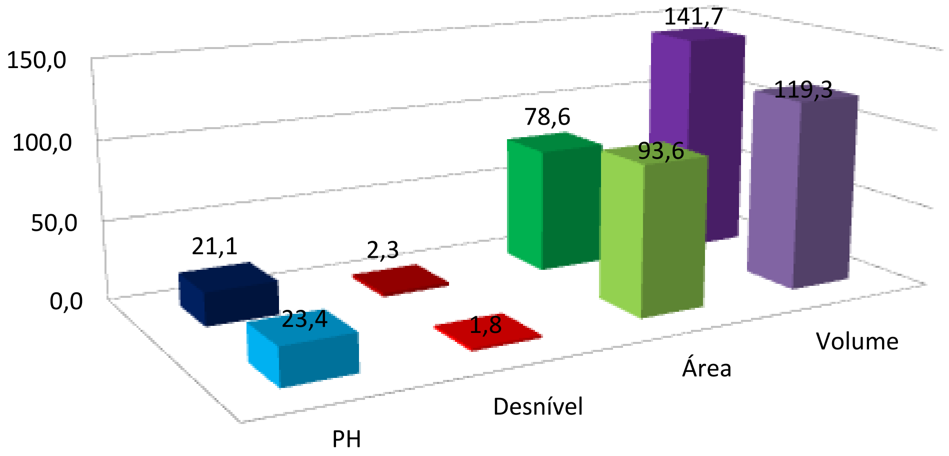

Figura 5.30 - Comparacão entre dados espeleométricos. Em tons claros, a média para as cavidades em estudo. Em tons escuros, a média para cavidades anteriormente estudadas em Serra Leste.

5.1.4. Morfologia

A morfologia das cavernas estudadas foi interpretada em macro, meso e micro escala. A análise da planta baixa das cavidades permite identificar o padrão planimétrico da caverna. Entretanto, os padrões planimétricos foram definidos para cavernas calcárias e estão associados à gênese e desenvolvimento das cavernas nesta litologia. Assim, a classificação de cavidades em outras litologias segundo esse sistema resulta em incongruências uma vez que nem sempre se observa correlação entre as formas e os processos inicialmente preconizados. De qualquer maneira, um padrão planimétrico aproximado pode dar alguns indicativos dos processos genéticos e evolutivos da caverna e, por isso, são adotados neste estudo. A porcentagem de cavernas de Serra Leste que não se enquadrou em nenhum dos padrões de planta baixa (42%) é aceitável e se equipara aos números também em cavernas carbonáticas. Em estudo anterior, dentre as 96 cavidades em análise na área do Projeto Serra Leste, 33,3% também não se enquandram em nenhum padrão planimétrico devido ao reduzido tamanho (CARSTE, 2011a).

Dentre as 12 cavernas desenvolvidas em rochas ferríferas, três não apresentaram um padrão planimétrico definido. O tamanho reduzido destas ocorrências espeleológicas e a configuração em salão único da maior parte das cavernas (66,67%) pode inibir o padrão das cavernas, impossibilitando uma correlação. Dentre as cavernas em rochas siliciclásticas, a dificuldade de se identificar um padrão eleva-se e, seis entre as nove cavernas não tiveram padrão definido.

O padrão planimétrico mais observado dentre as 21 cavernas em análise na área foi o retilíneo (Figura 5.31), presente em cinco cavidades, entre elas as três cavernas de salão único em quartzito, as únicas em que se pode identificar um padrão de planta baixa. Denominadas também “cavernas de passagem única” (PALMER, 2007), as cavernas retilíneas são constituídas por condutos simples, seguindo uma única direção, e, em geral, estão condicionadas por estruturas geológicas. Este padrão planimétrico ocorre, por exemplo, nas cavidades SL-106, SL-113 e SL-117.

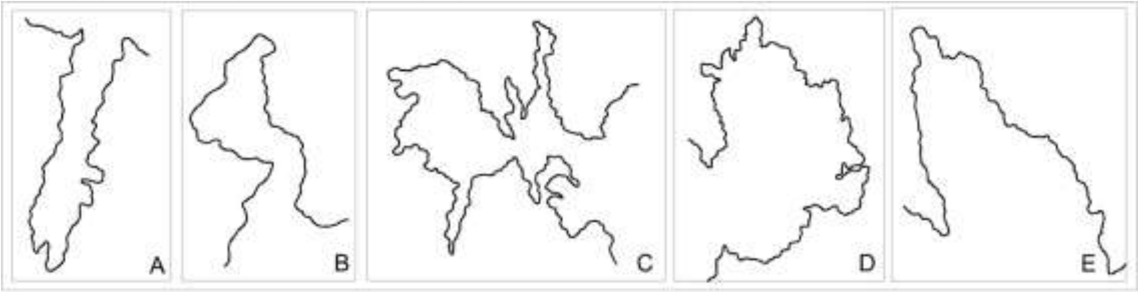

Figura 5.31 - Padrões planimétricos: (A) retilíneo (SL-106), (B) curvilíneo (SL-108), (C) reticulado (SL-121), (D) globular (SL-122), (E) triangular (SL-112).

Com exceção do padrão retilíneo, todos os demais padrões foram observados apenas em cavernas alojadas em rochas ferríferas. O padrão curvilíneo (Figura 5.31), em que há uma inflexão em um conduto também único, foi observado em duas cavernas: SL-105 e SL-108. Já o padrão reticulado, condicionado por fratura, junta-de-alívio, acamamento ou foliação, cujos planos se entrecruzam formando uma rede que se alarga gerando condutos interconectados em ângulos variados pode ser observado apenas na caverna SL-121 (Figura 5.31). Já os padrões

globular e triangular (Figura 5.31), típicos de cavernas ferríferas, foram encontrados em apenas três das 12 cavernas: a SL-122 tem o mapa representando o padrão globular na Figura 5.31, e as cavernas SL-112 e SL-115 apresentam planta baixa com formato triangular (Figura 5.31). O padrão globular apresenta salão bastante poroso, aproximadamente circular ou semicircular e, o triangular apresenta-se afunilado, com entrada mais ampla que a porção distal, em que paredes e teto confluem na mesma direção, diminuindo o volume da caverna neste setor.

Nota-se diferença, em relação aos dois estudos geoespeleológicos realizados na área, a respeito da porcentagem de cavidade que se enquadram nos padrões planimétricos. O padrão mais recorrente dentre as 96 cavernas em rochas ferríferas estudadas anteriormente foi o espongiforme, presente em 46 (46,5%) cavernas. O segundo padrão mais frequente foi o globular, com 10 (10,1%) cavidades e, os padrões retilíneo e reticular foram observados em cinco (5,1%) cada um CARSTE, 2011a).

Diferentemente das 96 cavernas analisadas por CARSTE (2011a) as 21 cavernas analisadas em Serra Leste apresentam padrão de desenvolvimento predominantemente horizontal, com pisos irregulares (34%) e inclinados (59%) (Figura 5.32). As cavidades cujo piso apresentou regularidade (7%) são essencialmente planas. Naquele estudo, cavidades com pisos irregulares atingiam mais de 82% da amostra, pisos planos somavam cerca de 42% e suavemente inclinados, mais de 57% (CARSTE, 2011a).

O piso pode apresentar-se parcialmente capeado, estando recoberto por crostas com espessura que varia de milímetros a até poucos centímetros e imprimem maior resistência aos processos desnudacionais. Protegidos destes processos, pode ocorrer a individualização e conservação de pisos suspensos e paleopisos, feições caracterizadas por estarem localizadas em posições distintas em relação ao nível atual do piso da caverna (Figura 5.33) (Figura 5.34). É comum estarem sobrepostos a canalículos, condutos ou a pontos da parede onde tenha ocorrido solapamento.

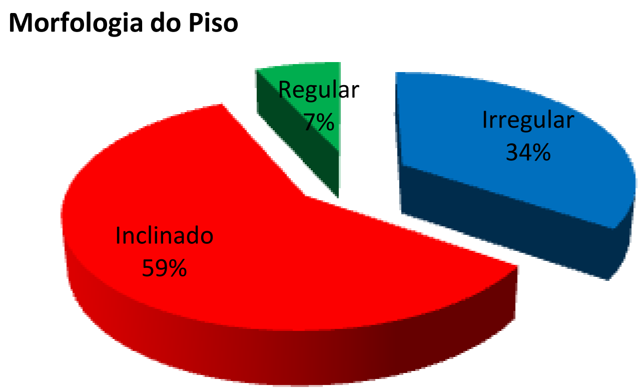

Figura 5.32 - Morfologia do piso das cavidades de Serra Leste.

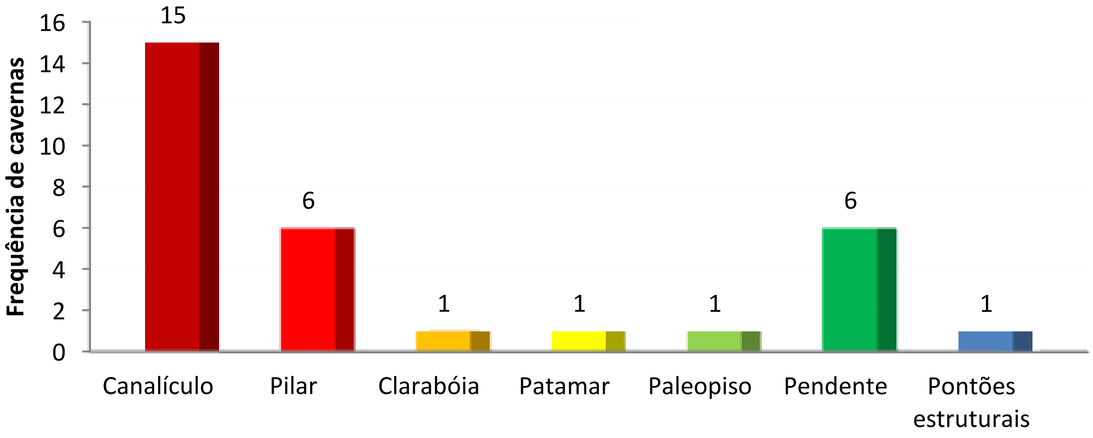

Figura 5.33 - Feições morfológicas das cavernas de Serra Leste.

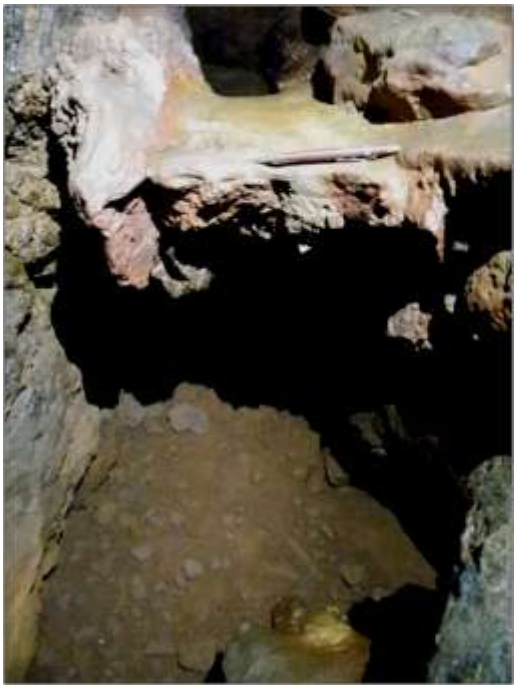

Figura 5.34 - Pequena porção de paleopiso capeado observado na caverna SL-121.

As paredes são irregulares em 86% das cavernas (Figura 5.35). No caso das cavernas inseridas em canga e laterita, esta característica está associada ao arranjo caótico do arcabouço litológico, independentemente da participação de matriz. No caso das cavernas inseridas em quartzito, esta característica está associada ao nível de alteração do substrato rochoso. Apenas três cavernas em quartzito apresentaram pelo menos uma porção das paredes regulares, controladas pelo acamamento da rocha encaixante (Figura 5.35 e Figura 5.36).

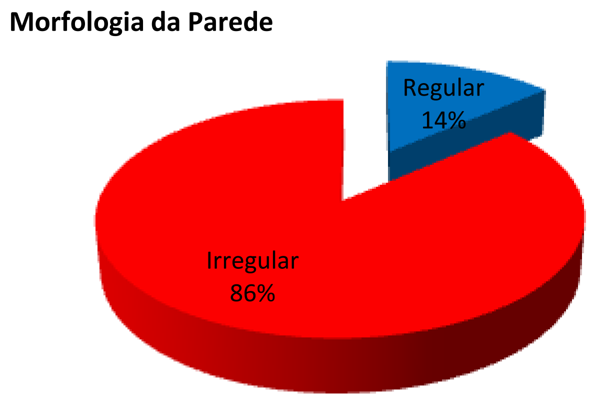

Figura 5.35 - Morfologia da parede das cavidades de Serra Leste.

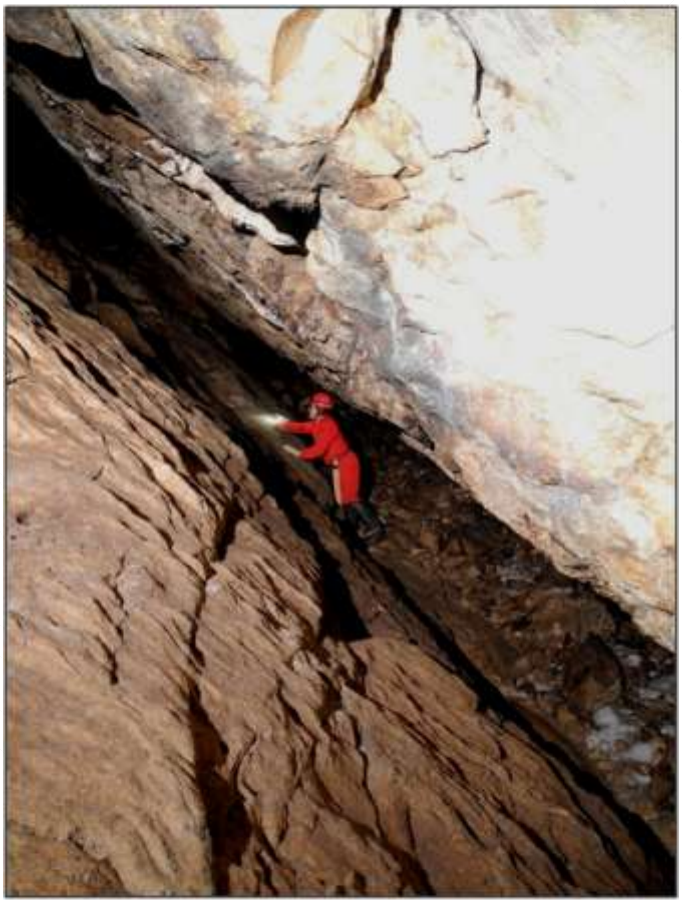

Figura 5.36 - Caverna SL-101 apresenta trechos com contorno bastante regulares.

Canalículos são feições encontradas nas paredes de 15 das 21 cavernas em estudo (Figura 5.33), sendo extremamente comuns nas cavidades inseridas em rochas ferríferas, como anteriormente observado por CARSTE (2011a), em que 93% da amostra das 96 cavernas inseridas na área do Projeto Serra Leste também apresentava esta feição. Os canalículos constituem-se como canais de pequena dimensão, que geralmente apresentam diâmetro

milimétrico a centimétrico e comprimento que varia de poucos centímetros a um ou dois metros, podendo se desenvolver sub-horizontalmente ou subverticalmente. Normalmente, os canalículos estão localizados no contato entre o piso e as paredes da caverna. Raramente eles ocupam porções intermediárias da parede, embora esta situação tenha sido observada. Eles afetam a morfologia das cavernas, pois sua evolução gera apêndices ou expansões laterais que podem eventualmente evoluir para galerias laterais. Segundo Piló & Auler (2005), de uma forma geral, há uma relação diretamente proporcional entre o perímetro da caverna e o número de canalículos, mostrando que a presença dos mesmos causa um "alongamento" do perímetro da caverna. Os canalículos possibilitam o transporte de sedimentos químicos e/ou mecânicos e, a deposição destes no interior da cavidade pode formar cones sedimentares – pequenos leques – na saída dos canalículos. Na cavidade SL-112, um canalículo na porção distal da caverna com seção triangular apresenta abrupta diferença de largura em relação às paredes, com leque de sedimentos a jusante (Figura 5.37).

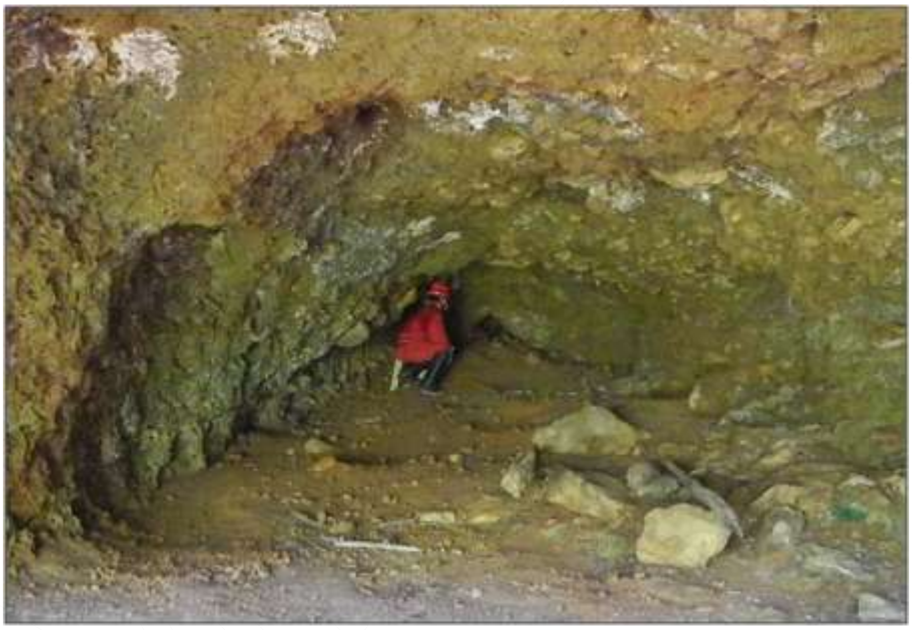

Figura 5.37 - Canalículo no setor distal da caverna SL-112.

A ocorrência de canalículos em posição subvertical no teto pode gerar feições aqui denominadas cúpulas (Figura 5.38), com base alargada e topo mais estreito. O comprimento e o diâmetro podem variar de centimétrico a métrico. A evolução dessas "cúpulas" pode dar origem a clarabóias. As clarabóias são feições resultantes do abatimento de porções do teto da cavidade, interceptando a superfície do terreno, ocorrendo geralmente onde o pacote rochoso é pouco espesso.

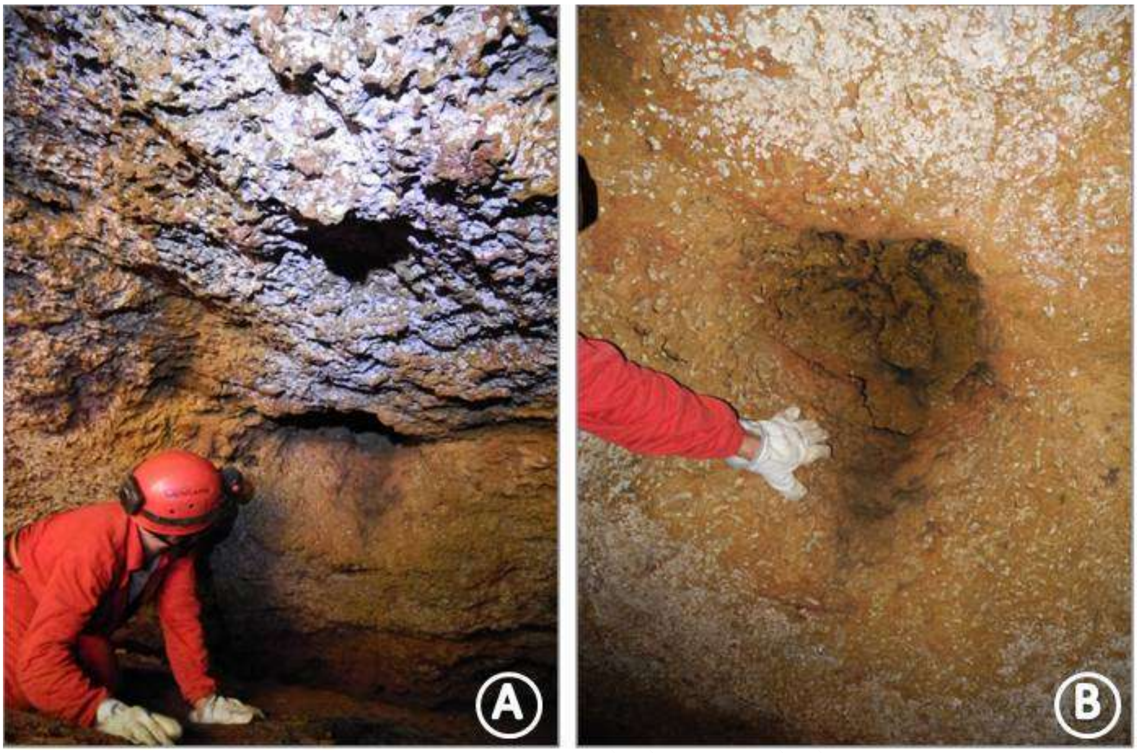

Figura 5.38 - Cúpula na cavidade SL-114: (A) visão geral e (B) em detalhe.

Assim como as cúpulas e clarabóias, feição observada somente na caverna SL-101, outras feições associadas ao teto são pendentes e pilares. Os primeiros imprimem irregularidades ao teto das cavernas que, no caso de Serra Leste, são primordialmente irregulares (Figura 5.39). Tetos inclinados aparecem em 17% das cavernas e, normalmente estão associados a estruturas geológicas, como planos de fratura e mergulho do acamamento (Figura 5.39). Cavidades com teto arredondado são raras entre as estudadas em Serra Leste (Figura 5.39). De maneira geral, a configuração abobadada confere maior estabilidade às paredes e teto.

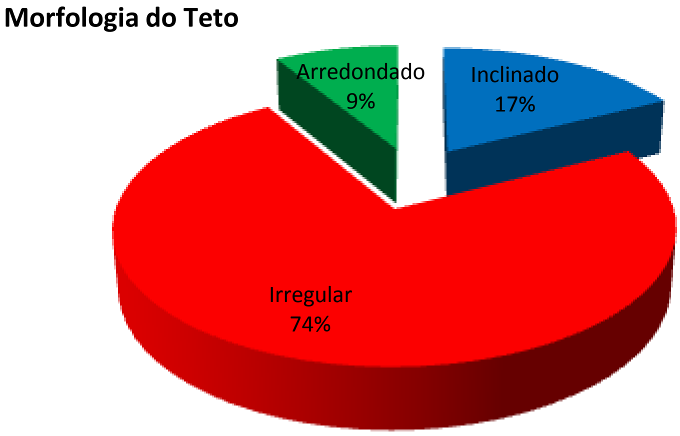

Figura 5.39 - Morfologia do teto das cavidade de Serra Leste.

Os pilares são feições residuais verticalizadas resultantes de processos de erosão diferencial do substrato rochoso em que está encaixada a caverna, conectando o teto ao piso da caverna. Apresentando comprimento e diâmetro variados, a feição relativamente comum nas cavernas ferríferas (Figura 5.40), sendo encontrados também em cavernas quartzíticas. Foram identificados em seis cavernas em análise (Figura 5.33). Deste conjunto, destaca-se a caverna SL-101 (Figura 5.41), que apresentou um conjunto único de pilares.

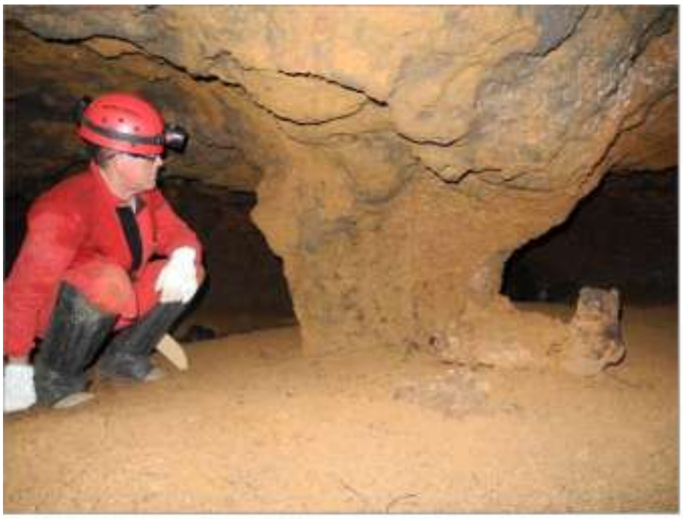

Figura 5.40 - Pilar de diâmetro métrico na caverna SL-108.

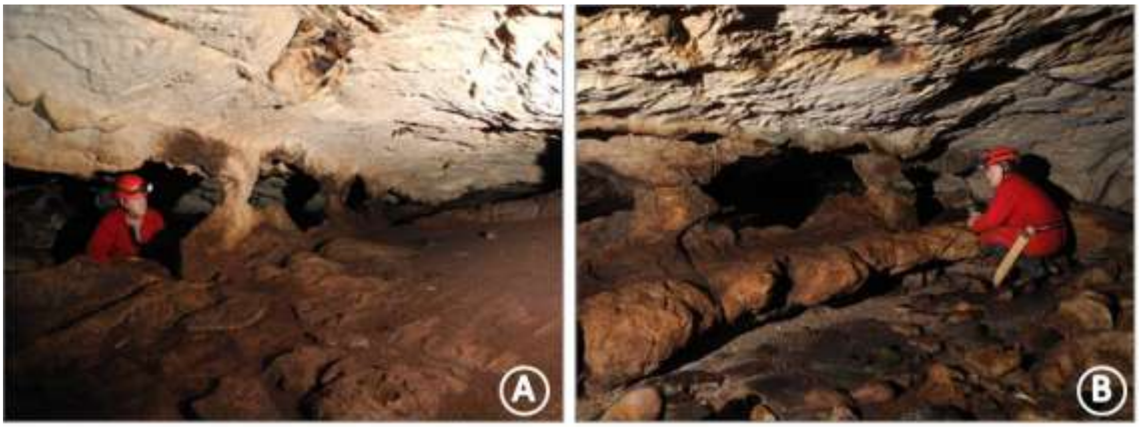

Figura 5.41 - Conjunto de pilares na caverna SL-101, visto de dois lados opostos (A) e (B).

Os pendentes são feições residuais normalmente associadas ao solapamento de um pilar ou rebaixamento do piso. Caracterizam-se por projeções rochosas de formato aproximadamente cônico invertido a partir do teto. Essas feições foram observadas em seis cavernas inseridas em Serra Leste, sendo todas elas desenvolvidas em rochas ferríferas (Figura 5.33). Destaca-se o teto da caverna SL-122, em que quantidade de ocorrência de pendentes é elevada se comparada às demais cavidades em análise (Figura 5.42).

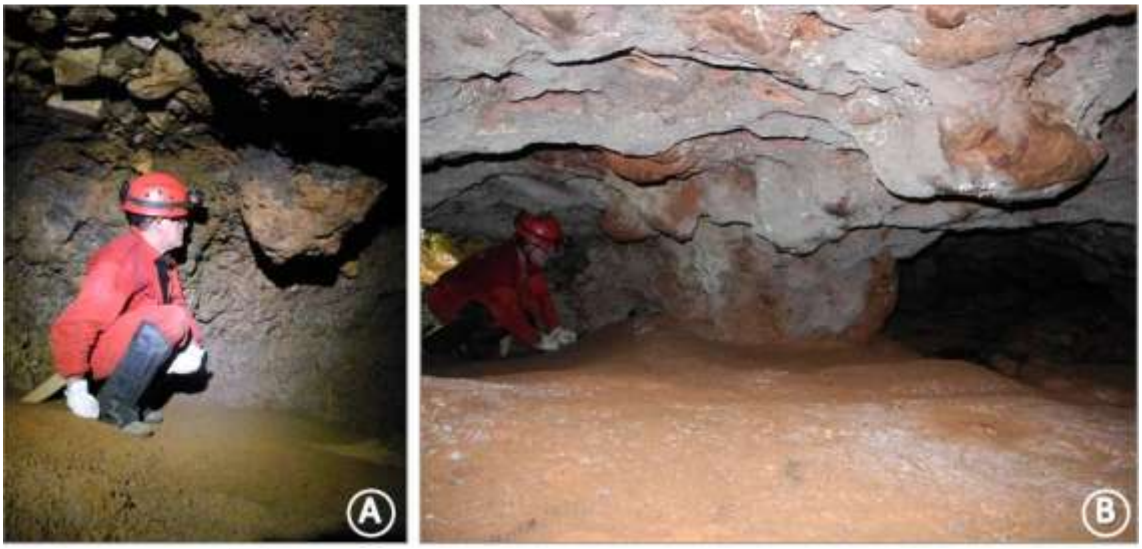

Figura 5.42 - Pendentes observados nas cavidades SL-108 (A) e SL-122(B).

Pontões estruturais são feições que se caracterizam por pontas aproximadamente retangulares que se projetam de forma escalonada no teto. Estão associadas à interrupção da continuidade do plano de bandamento ou acamamento inclinado da rocha devido às fraturas ou juntas. Dentre as cavidades em análise, somente a SL-101 apresenta esta feição morfológica (Figura 5.33).

Como parte do processo de evolução da cavidade, o colapso de blocos do teto mostra-se particularmente importante nas cavidades alojadas em rochas siliciclásticas de Serra Leste. Dentre as 21 cavidades, 38% não apresenta este fenômeno, sendo a maior parte delas

desenvolvidas em rochas ferríferas (sete de oito cavernas) (Figura 5.43). Os outros 62% apresentam abatimento de bloco, sendo que em 43% o volume de blocos abatidos é significativo (Figura 5.43). A alteração da morfologia da caverna é expressiva quando há abatimento de blocos e pode haver perda de volume da cavidade, uma vez que a disposição dos blocos no piso é caótica, formando interstícios inexistentes antes do colapso.

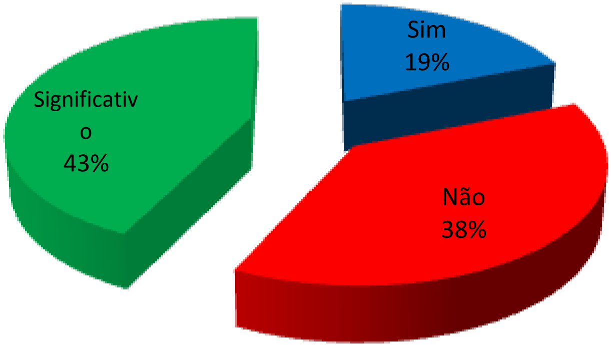

Figura 5.43 - Abatimento de blocos no interior das cavidade de Serra Leste.

5.1.5. Hidrologia

A maior parte das cavernas desenvolvidas em rochas ferríferas descritas na literatura não apresenta drenagem interna. Alguns autores (à exemplo de SIMMONS, 1963; BOWDEN, 1980), no entanto, comentam brevemente sobre a existência de drenagens hipógeas, colocando que cavidades na borda de platôs de canga podem comportar surgências temporárias, em geral, de pequeno porte. Entretanto, já em relação às cavidades desenvolvidas em rochas siliciclásticas, a existência de cursos d'água que permitem sua gênese e evolução é, na maior parte das vezes, destacada.

Dentre as 21 cavidades em estudo, 16 apresentavam-se totalmente secas (76%) (Figura 5.44), quando dos estudos geoespeleológicos, sendo presentes raras marcas de gotejamento em poucas destas cavernas, como na cavidade SL-108. A condensação, embora não tenha sido comprovada, pode ser indicada pelo ambiente extremamente quente e úmido percebido em algumas cavernas na área.

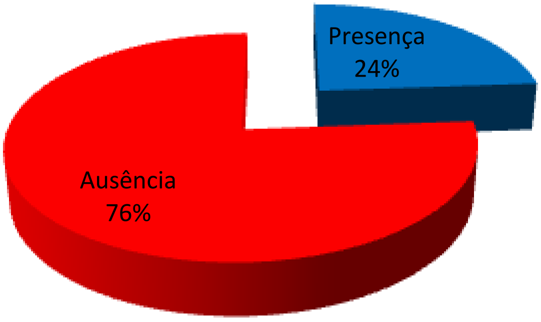

Figura 5.44 - Frequência de cavidades com feições hidrológicas.

Dentre as cinco cavernas que apresentam feições hidrológicas (24%) (Figura 5.44), duas desenvolvem-se em rochas siliciclásticas e três em rochas ferríferas.

A cavidade SL-101, apesar de essencialmente seca, apresenta gotejamento pontual em condutos inferiores, além de marmitas no piso que indicam provável existência de gotejamentos. Nota-se um trecho localizado em conduto inferior, onde o piso aproximadamente plano, recoberto por sedimentos arenosos destoa do restante do piso da cavidade e indica uma possível contribuição fluvial naquela porção. Visita da equipe de bioespeleologia no período úmido comprova a existência de curso de drenagem nesta porção (Figura 5.45).

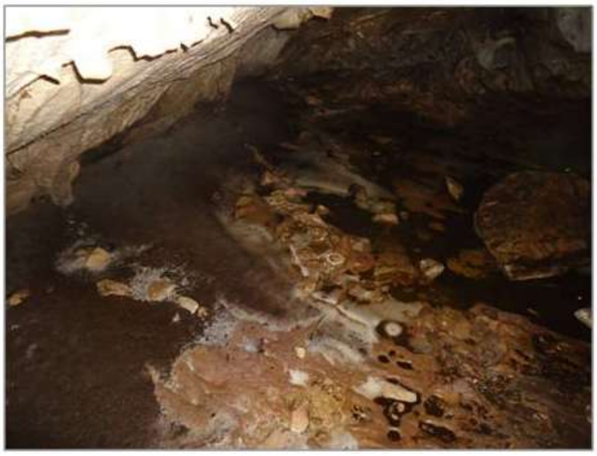

Figura 5.45 - Sumidouro temporário na SL-101: curso de água em porção inferior da cavidade.

A outra cavidade quartzítica que apresenta feição hidrológica, SL-107 conta com pequeno escoamento superficial que percorre parte do conduto montante e conecta-o ao salão jusante (Figura 5.46). Neste último, a água espraia nos sedimentos da porção deprimida do piso, voltando a se infiltrar.

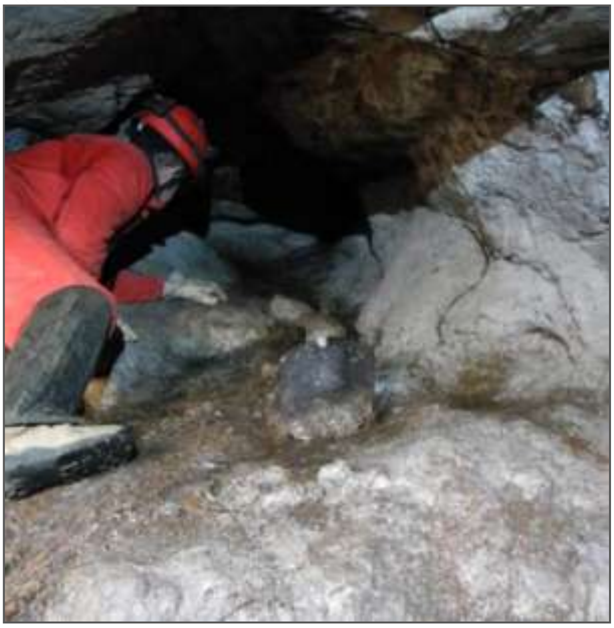

Figura 5.46 - Pequeno estreitamento por onde a água escoa conectando os dois salões.

Dentre as três cavidades alojadas em rochas ferríferas em que estão presentes as feições hidrológicas, a caverna SL-121 pode apresentar condensação em sua porção mais reclusa e se nota que a água percola e permite pontos de gotejamento ao longo de toda a caverna, concentrando-se onde existem fraturas (Figura 5.47). A caverna SL-111 insere-se na calha de curso de drenagem, sendo invadida pelo rio (Figura 5.47). A cavidade SL-122 é bastante úmida e também se insere na calha de drenagem que forma setor encachoeirado na entrada (Figura 5.48). O curso d'água adentra a cavidade e se conecta com outro escoamento de água que se divide em dois cursos de baixa vazão (menos de um litro por minuto) após área empoçada. A água escoa sobre sedimentos argilosos e provoca pequena incisão vertical no piso. Gotejamentos generalizados ao longo de grande parte da caverna, com intensidades diferentes, formam espeleotemas (Figura 5.48).

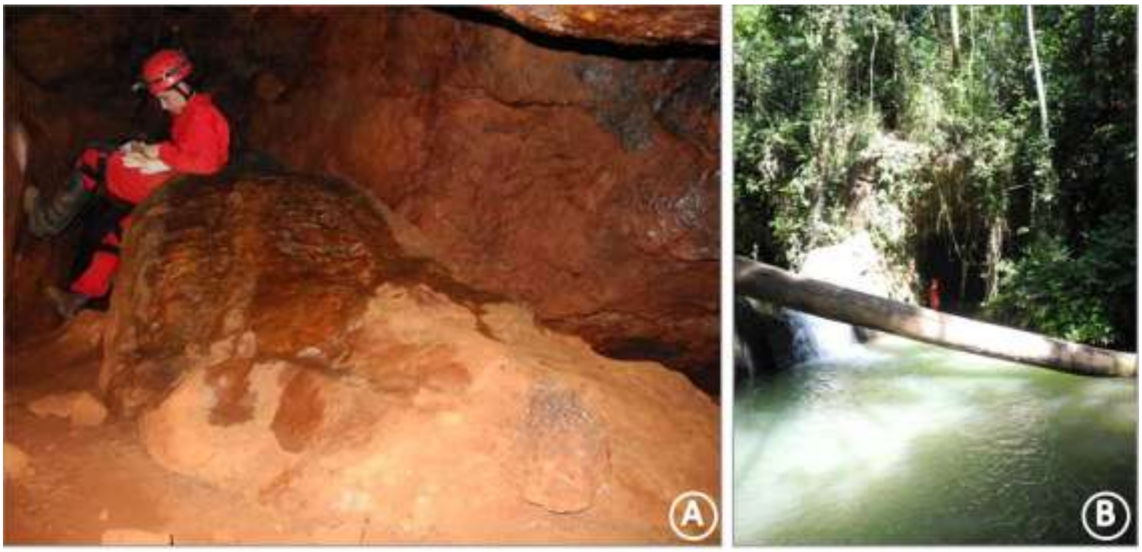

Figura 5.47 - (A) Bloco umidificado pelo gotejamento que percola entre fraturas na cavidade SL-121; (B) Curso fluvial que adentra a caverna SL-111.

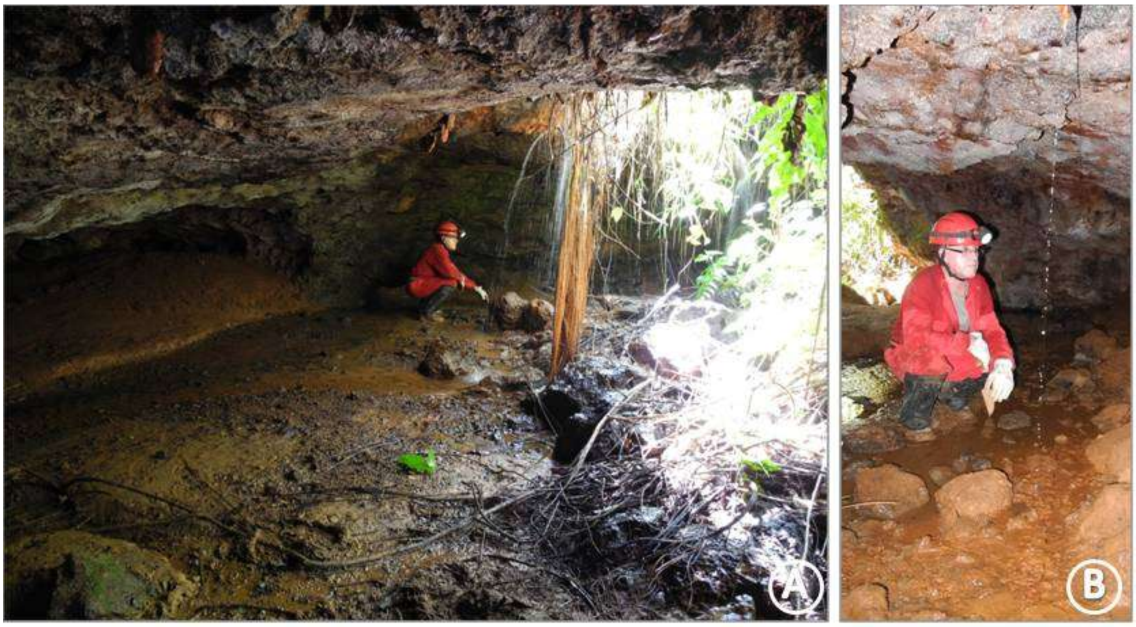

Figura 5.48 - Cavidade SL-122:(A) Encachoeirado na entrada da cavidade com parte do curso de drenagem adentrando a caverna;(B) Gotejamento formando pingente e curso d’água de baixa vazão no interior da cavidade.

Como observado em estudos anteriores na Serra Leste (CARSTE, 2011a):

> *embora não tenham sido realizados estudos hidrogeológicos na área, é possível afirmar, dada a configuração geomorfológica e a inserção na paisagem, que as cavernas de Serra Leste estão "desconectadas" entre si do ponto de vista hidrológico, ou seja, não constituem clássicos sistemas observados no carste carbonático, quartzítico e arenítico. Mesmo nas cavernas inseridas nos sistemas fluviais, o que se observa é uma captura da caverna pela drenagem e não o inverso, ou seja, uma captura da drenagem pelo sistema subterrâneo.*

5.1.6. Depósitos clásticos e orgânicos

Os sedimentos clásticos encontrados no interior das cavidades compreendem todo o detrito sólido depositado sobre o piso da caverna. A deposição pressupõe que houve movimento anterior do material e, no caso de sedimentos clásticos, esta pode ser (i) autogênica, provinda do interior do maciço rochoso ou (ii) alogênica, externa ao maciço rochoso. O mais frequente é uma sedimentação mista, constituída por material autóctone e alóctone, embora a presença do primeiro seja extremamente superior a do segundo, que geralmente concentra-se na porção proximal, perto da entrada da cavidade ou ainda associado às clarabóias. Nas cavidades aqui analisadas, observa-se o predomínio de sedimentos autóctones, sendo comum a sedimentação mista (Figura 5.49). Não foram observadas diferenças significativas quando comparados a outras cavidades estudadas na área do Porjeto Serra Leste ou na região de Carajás.

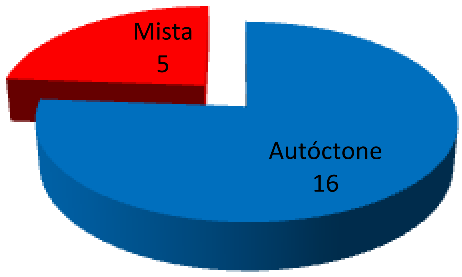

Figura 5.49 - Tipo de sedimentação predominante nas cavidades.

Em cavernas da formação ferrífera, a energia de transporte sedimentar é baixa quando comparada às cavidades em outras litologias. Portanto, não há grande tempo/distância entre a área-fonte dos sedimentos e o depósito sedimentar, sendo pequeno o percurso de transporte. Desta forma, os graus de arredondamento e de seleção dos sedimentos são baixos. Fazem-se comuns clastos angulosos a subangulosos, embora também possam estar presentes clastos subarredondados, que provavelmente apresentam esta esfericidade devido a transporte anterior a formação da própria rocha encaixante da cavidade, quando se trata de canga

detrítica ou ferricrete. De acordo com o grau de seleção, nota-se que a granulometria é bastante variada, podendo os sedimentos ser (i) finos: que incluem argila, silte e areia, sendo os dois primeiros lamosos; (ii) grossos ou cascalhentos: que abarcam grânulos, seixos, calhaus e matacões.

Estes depósitos nas cavernas ferríferas constituem-se majoritariamente por clastos limoníticos originados do desmonte do ferricrete ou da canga. A presença dos sedimentos lamosos deve-se principalmente à matriz limonítica da canga e do ferricrete, sendo o aporte dos sedimentos de granulometria fina bastante comum através de alguns canalículos encontrados nas paredes das cavidades. Argila, silte e areia ocorrem em grande quantidade em um número pouco maior de cavidades se comparados aos sedimentos cascalhentos (Figura 5.50). Sedimentos cascalhentos (seixos, calhaus e matacões) ocorrem igualmente como granulometria predominante em quatro cavidades. Os grânulos, por sua vez, são a granulomentria de maior ocorrência quando se trata de grande quantidade (Figura 5.50).

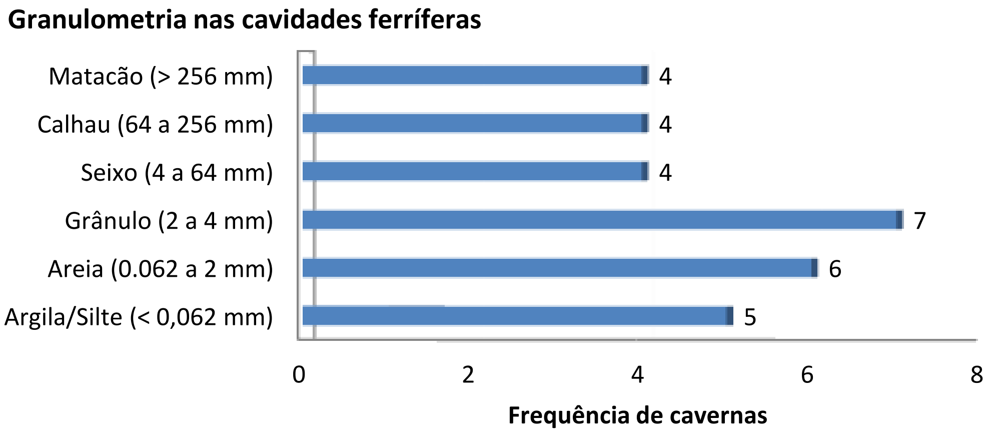

Figura 5.50 - Granulometria dos sedimentos que recobrem o piso das cavidades ferríferas.

As cavernas quartzíticas da área fogem do padrão comumente observado, sendo ausentes os cursos de drenagem que participam da gênese e contribuem com o aporte de sedimentos alóctones. Assim, todas as nove cavernas nesta litologia estudadas em Serra Leste tem sedimentação predominante autóctone, com calhaus e matacões angulosos compondo majoritariamente o piso da caverna (Figura 5.51 e Figura 5.52).

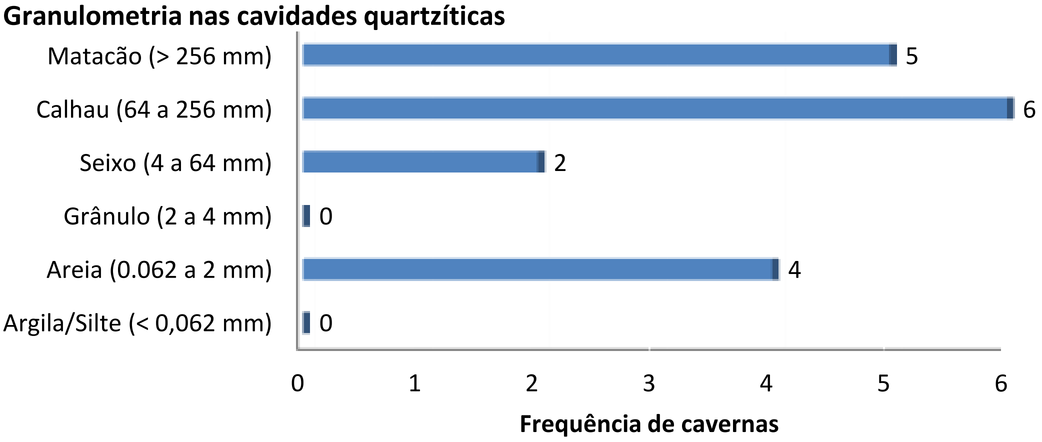

Figura 5.51 - Granulometria dos sedimentos que recobrem o piso das cavidades quartzíticas.

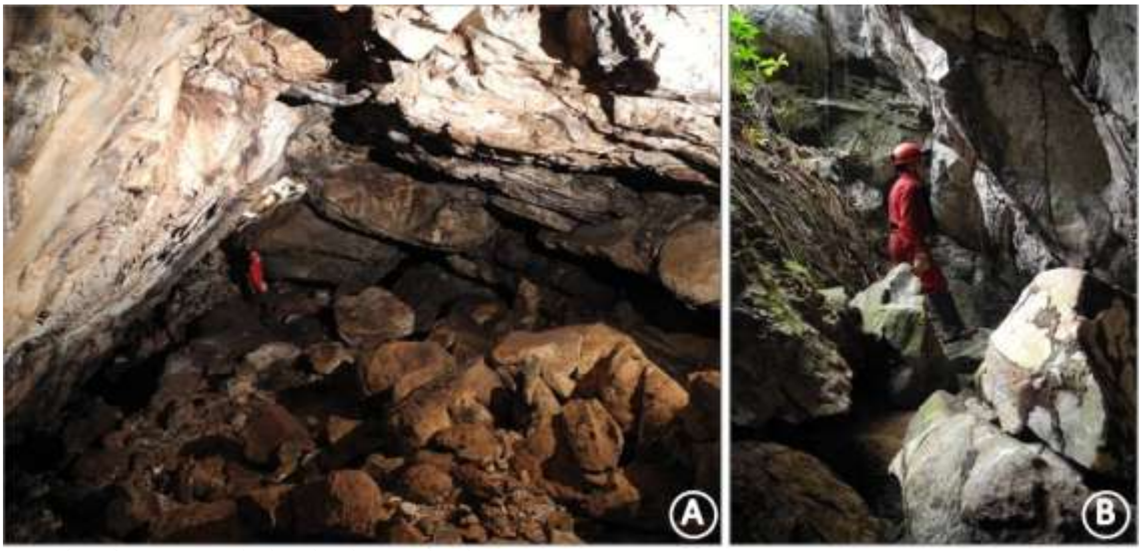

Figura 5.52 - Matacões e calhaus angulosos de quartzito nas cavernas SL-101 (A) e SL-104 (B).

Entre os cascalhos e formando depósitos concentrados em alguns pontos das cavernas, estão os sedimentos arenosos (Figura 5.53). Em sua maioria, os clastos tamanho areia são de coloração branca, podendo algumas vezes, ser escurecidos provavelmente devido à presença de matéria orgânica (Figura 5.53).

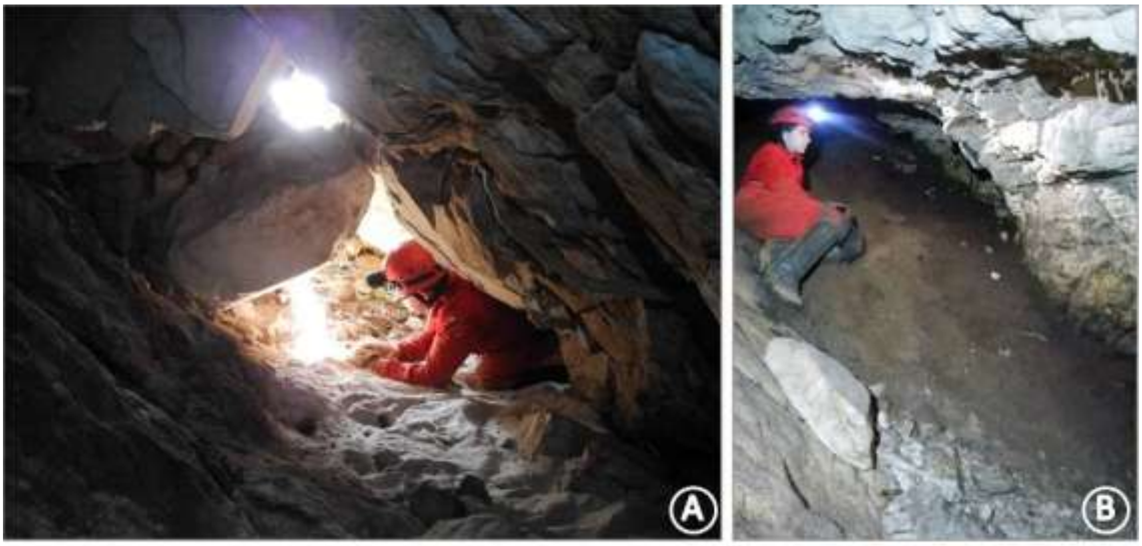

Figura 5.53 - Concentração de clastos tamanho areia em porções de cavernas: (A) Areia branca em porção jusante da SL-117 e (B) Areia marrom em conduto descendente da SL-101.

Sedimentos orgânicos de origem vegetal são identificados em quase todas as cavernas inseridas em Serra Leste, sendo o folhiço, tipo recorrentemente observado, comumente encontrado próximo às entradas das cavidades, podendo ser trazido por curso fluvial como é o caso da caverna SL-111 (Figura 5.54). Raízes de diversos calibres e manchas esparsas e pequenas de guano também podem ser vistas. Na cavidade SL-110, a maior parte do piso é, pelo menos superficialmente, recoberta por sedimentos orgânicos, compostos por guano seco (Figura 5.55).

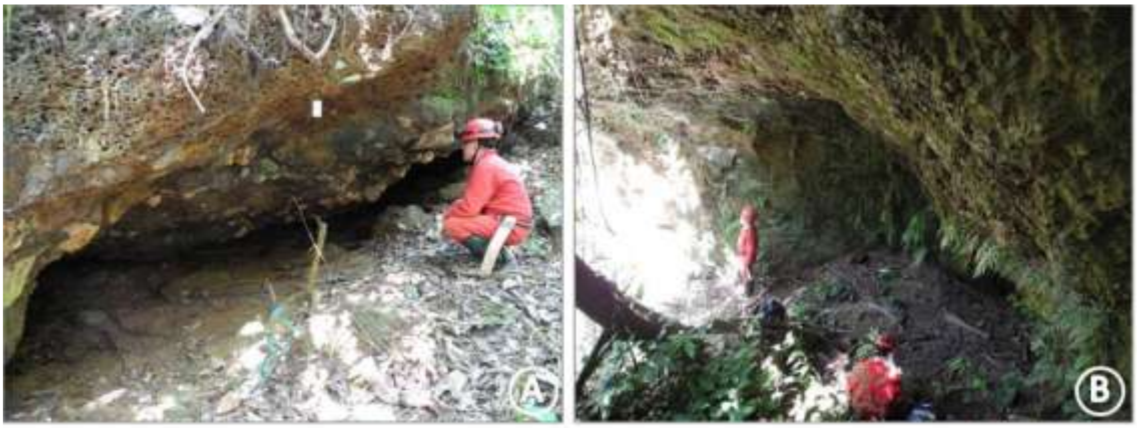

Figura 5.54 - (A) Folhiço observado na zona de entrada da caverna SL-110 e (B) trazido por curso fluvial na cavidade SL-111.

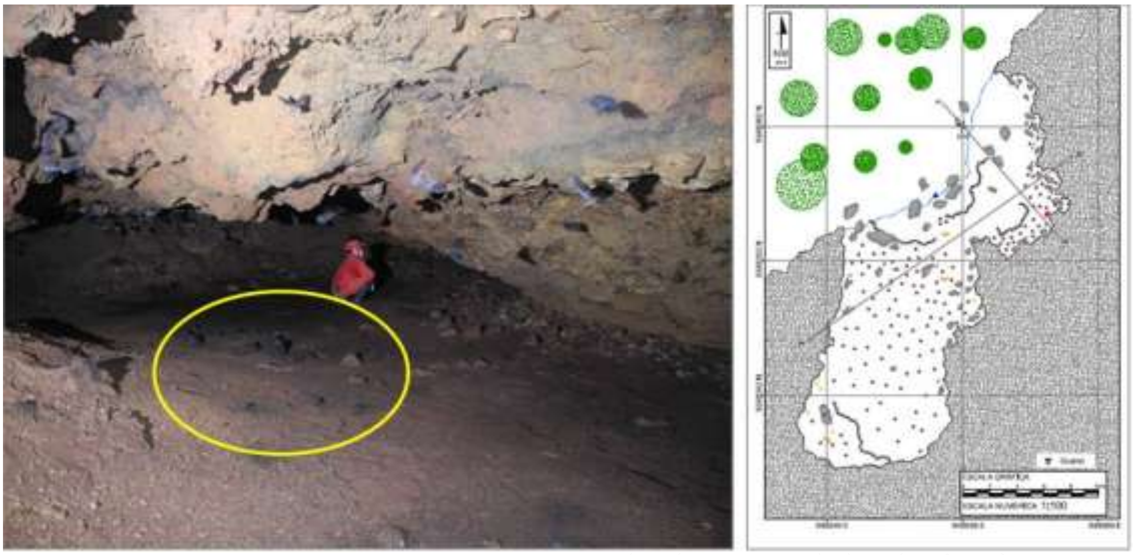

Figura 5.55 - Guano seco recobre grande quantidade do piso da cavidade SL-110 (mapa), formando alguns aglomerados de sedimentos orgânicos, em destaque na foto.

5.1.7. Depósitos químicos

A deposição secundária de parte dos íons transportados quimicamente pela água origina matéria sólida que, no interior das cavidades, é conhecida como espeleotema. Em Serra Leste, estes depósitos podem ser observados em 11 das 12 cavernas desenvolvidas em rochas ferríferas (Figura 5.56). Nas cavernas alojadas em quartzito, apenas uma não apresenta espeleotemas. A variedade tipológica é maior nas cavidades em rochas ferríferas (Figura 5.56), sendo extremamente reduzida nas rochas quartzíticas (Figura 5.57). Não houve algum tipo excepcional ou único de espeleotema entre as cavernas avaliadas, sendo os tipos bastante comuns à formação ferrífera e aos quartzitos. Crostas e coralóides são os depósitos químicos mais recorrentes em ambas as litologias (Figura 5.56 e Figura 5.57), como já atestado em outras cavidades inseridas na área de Serra Leste (CARSTE, 2011a). Segundo este estudo, crostas brancas ocorrem em mais de 84% das cavernas, crostas laranjas em mais de 55% e vermelhas em 36% e, coralóides estão presentes em 32% das 96 cavidades analisadas na área (CARSTE, 2011a).

Em grande parte das 21 cavidades em estudo, as crostas e os coralóides apresentam dimensões diminutas. A pequena quantidade de água retida no maciço devido à porosidade das rochas da área, a intermitência das feições hidrológicas e a pequena dimensão das cavidades, cujo microclima não se difere do ambiente externo, dificultam o desenvolvimento dos espeleotemas.

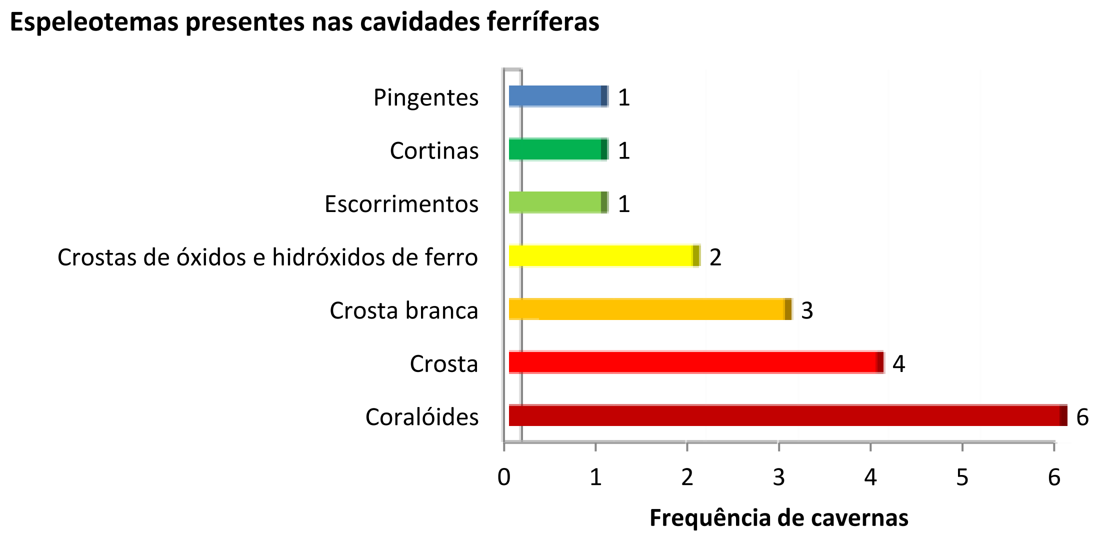

Figura 5.56 - Tipos de espeleotemas observados nas cavidades alojadas em rochas ferríferas.

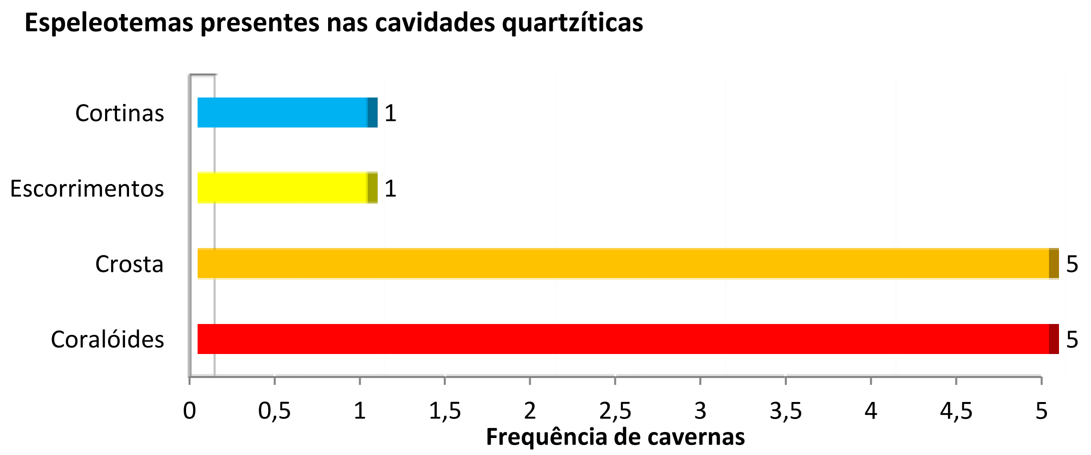

Figura 5.57 - Tipos de espeleotemas observados nas cavidades desenvolvidas em rochas quartzíticas.

As crostas constituem, em geral, películas de espessura milimétrica a centimétrica que recobrem blocos, piso, paredes e teto das cavernas. Sua cor pode variar do amarelo ao vermelho escuro e o brilho varia de opaco a metálico. Normalmente, estas cores estão associadas à presença de ferro na constituição química das crostas, sendo aqui descritas como crostas de óxidos/hidróxidos de ferro, estando presentes somente nas cavernas desenvolvidas em rochas ferríferas (Figura 5.58). Além destas, há as crostas brancas, também diferenciadas das demais (Figura 5.59). Devido ao reduzido universo de espeleotemas avaliados e aos ainda incipientes estudos em espeleotemas nas litologias em análise, não foi possível conceber modelos genéticos e/ou padrões morfológicos a cada um desses espeleotemas, assim algumas crostas foram aqui designadas genericamente.

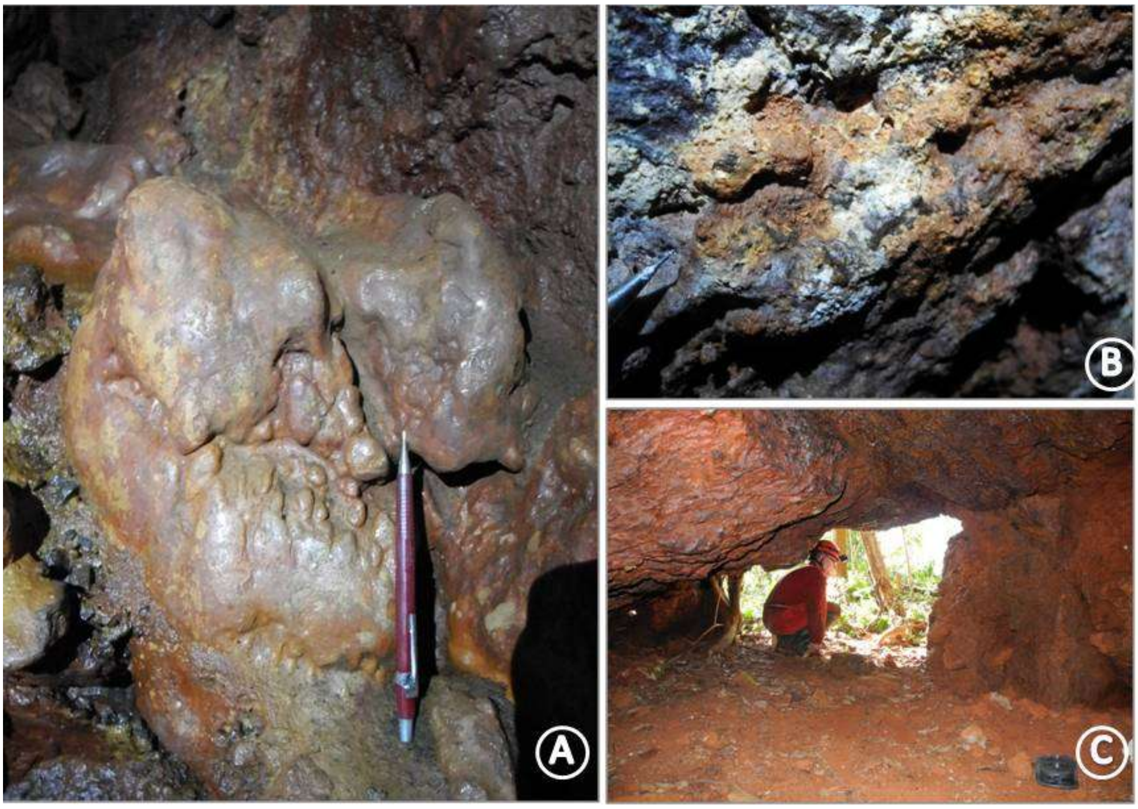

Figura 5.58 - Crostas na caverna SL-121: (A) Na parede, apresenta textura lisa e coloração heterogênea; (B) Em pequena porção do teto com coloração predominantemente amarela em tons diversos; (C) Em grande parte do teto próximo a entrada, a crosta tem brilho metálico.

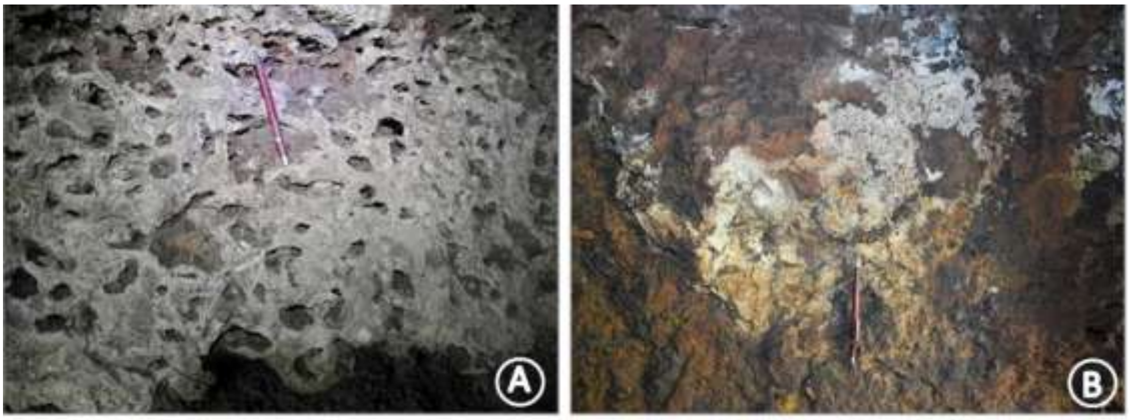

Figura 5.59 - Crostas brancas em cavernas ferríferas: (A) Repleta de alvéolos recobrindo porção do piso da caverna SL-109; (B) Em pequena parte da parede da caverna SL-115 associada a coralóides.

Os coralóides são o tipo mais frequente de espeleotema encontrado nas cavidades inseridas em Serra Leste, ocorrendo em 11 das 21 cavernas (Figura 5.58 e Figura 5.59). Principalmente onde há incidência indireta de luz e fluxo de ar, os coralóides se fazem presentes. Eles ocorrem em variedades de coloração branca, cinza, marrom e preta. Geralmente, estes espeleotemas apresentam dimensões milimétricas a até 1cm, com formatos que variam de alongados ou aciculares (tipo agulha), a couve-flor ou bojudos (morfologia mais arredondada) (Figura 5.60). Excepcionalmente, os coralóides atingem mais de 1cm, como ocorre na caverna SL-122 (Figura

5.61). Segundo Piló & Auler (2009), há maior incidência de coralóides aciculares em locais onde o fluxo de ar é mais acentuado, ao passo que os coralóides de topo arredondado (rombudos ou bojudos) são mais recorrentes em ambientes onde a atmosfera é mais estável e com maior umidade.

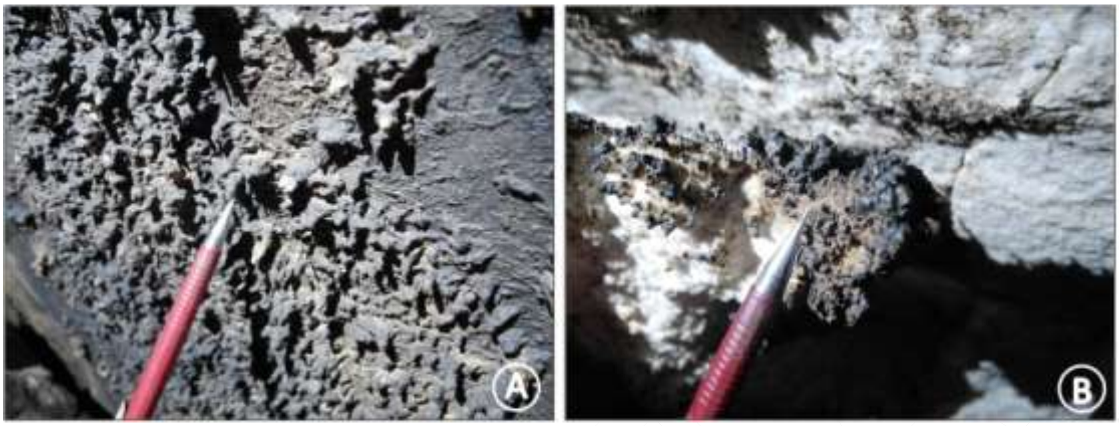

Figura 5.60 - Coralóides da caverna SL-101: (A) Cinzas com até 1 cm em concentração na parede; (B) Diminutos e pretos em pequena porção de rocha menos alterada.

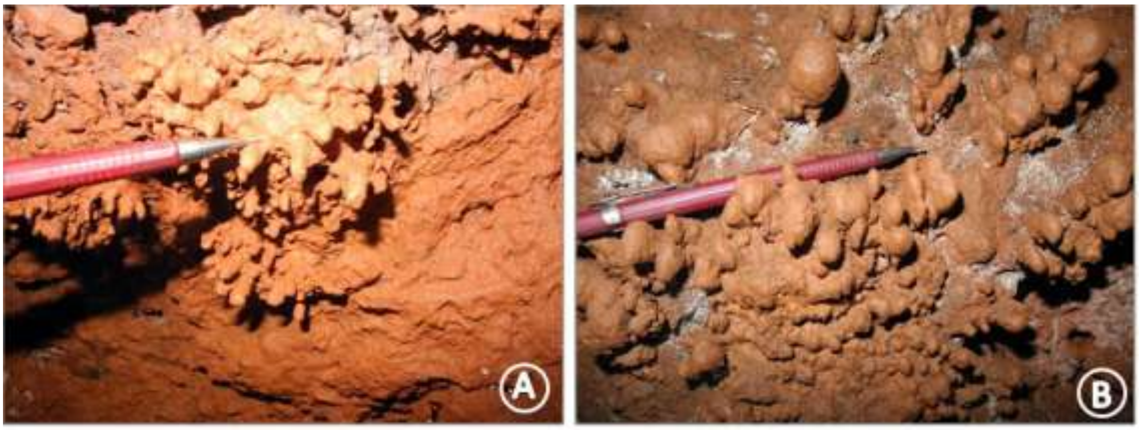

Figura 5.61 - Coralóides centimétricos em porção reclusa da cavidade SL-122: (A) Alongados; (B) Bojudos.

Escorrimento ocorre em duas cavidades, sendo uma alojada em rocha ferrífera (SL-105) e outra em quartzito (SL-104). Em ambos os casos, o escorrimento apresenta pequenas proporções e, na caverna em rocha siliciclástica, associa-se a cortina com textura microtravertínica. Na caverna SL-122, rasa e inserida na calha de drenagem, a presença de água em abundância permite o maior desenvolvimento dos espeleotemas e, comparativamente, maior diversidade de depósitos químicos. Pequenas cortinas serrilhadas ocorrem na porção média da cavidade, apresentando cor preta e marrom semelhante a ferrugem (Figura 5.62) e, pingentes de constituição coloidal ocorrem dispersos em locais onde há gotejamento ou exfiltração de água (Figura 5.63). Os espeleotemas encontram-se ativos nesta caverna.

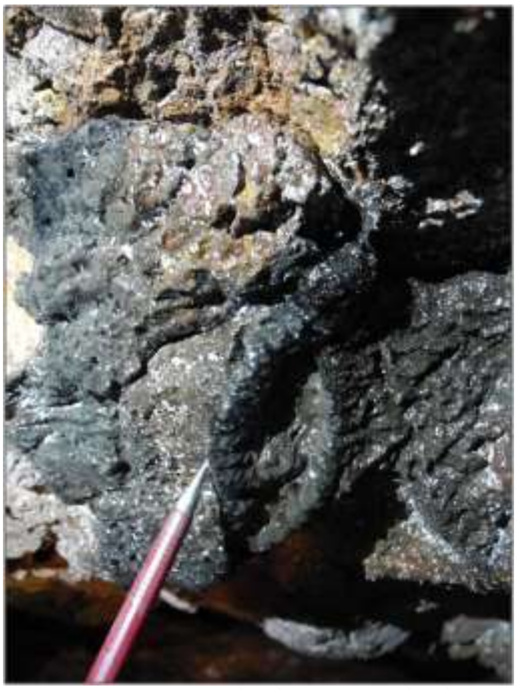

Figura 5.62 - Cortina preta em porção média da caverna SL-122.

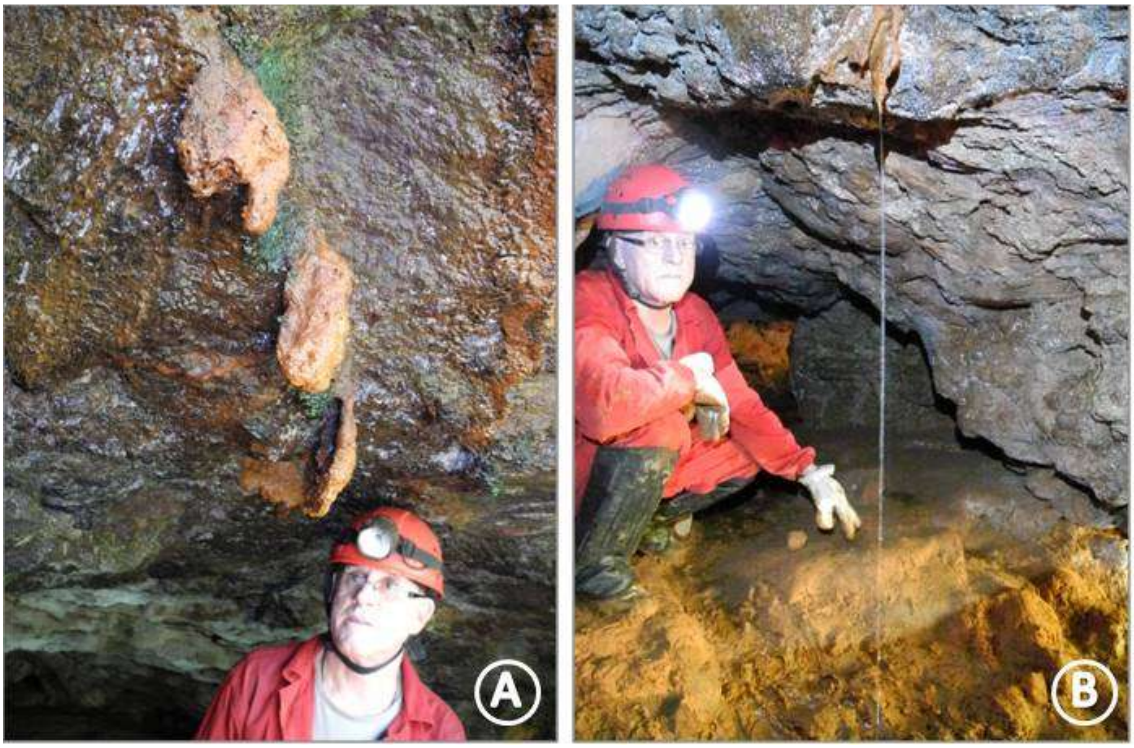

Figura 5.63 - Pingentes da caverna SL-122: (A) Conjunto em porção bastante úmida do teto; (B) Em ponto de exfiltraçao de água.

As cavernas de Serra Leste não apresentam diversidade de espeleotemas. São ausentes também os depósitos químicos com espeleotemas únicos ou com configurações notáveis.

5.1.8. Aspectos espeleogenéticos

A gênese de cavernas guarda relação com uma série de fatores, como litotipo, posição na vertente, relação hidrológica e idade. Informações importantes podem ser obtidas através da análise da planta baixa da caverna, muito embora observações detalhadas no interior da cavidade sejam também essenciais. Cavernas muito pequenas representam a maior parte das cavernas estudadas no Projeto Serra Leste. Nestas cavernas torna-se difícil obter informações sobre a espeleogênese, pois elas tendem a não possuir desenvolvimento suficiente para permitir a manifestação de feições ou padrões diagnósticos. São as maiores cavidades que permitem uma análise mais substanciosa e conclusiva em relação aos mecanismos atuantes.

Em linhas gerais, a gênese de cavernas pode ser dividida em dois grandes ambientes: endógeno e exógeno. Por gênese endógena, entende-se os processos atuantes a partir do interior do maciço rochoso, sem relação direta com o intemperismo externo. As reações químicas envolvidas nos processos de desnudação promovem aumento substancial da porosidade da rocha, que possibilita a gênese das cavidades, que ocorre em pelo menos duas fases: (i) na zona freática, ocorre o esvaziamento geoquímico de materiais mais solúveis, tendo como uma das condicionantes a composição química das rochas que, neste caso, pode determinar a velocidade de evolução das câmaras, uma vez que esta depende do grau de solubilidade dos elementos que compõem o mineral. O ferro e o quartzo, elementos químicos presente em abundância nas rochas da área de estudo, apresentam média solubilidade; (ii) em segunda fase, na zona vadosa, podendo ter início na fase freática, o material friável é removido por processos de *piping.* Este modelo, descrito por Piló & Auler (2005) apresenta grandes semelhanças com o modelo desenvolvido por Pinheiro e Maurity (1988) para as cavidades em rochas ferríferas na região da Serra dos Carajás.

Segundo eles, há duas fases espeleogenéticas: (i) inteiramente na zona freática, ocorre a formação de complexos alumino-ferrosos e argilo-minerais instáveis de ferro, alumínio e sílica que preenchem os vazios da canga e da formação ferrífera bandada. A remoção deste material residual instável leva à formação de orifícios irregulares que podem ser observados nas paredes e cavidades das cavernas (PINHEIRO & MAURITY, 1988); (ii) ainda na zona freática, envolve processos erosivos (*piping*), que basicamente expandem as cavidades geradas na primeira etapa, levando então à formação de condutos e salões. Esses processos erosivos serão intensificados quando a caverna passar a ser exposta à atuação de processos vadosos, propiciando também a atuação de processos de abatimento (PINHEIRO & MAURITY, 1988).

Iniciada a etapa erosiva das cavidades e sendo estabelecida uma saída para o exterior, os processos físicos serão incrementados. Material de granulometria fina será aportado para o interior das cavernas, principalmente, via canalículos, de onde será evacuado para o exterior. As cavernas oclusas que, posteriormente, tiveram suas entradas abertas e/ou de cavernas que evoluem através da expansão de canalículos podem ser evidenciadas pelos padrões globular ou espongiforme que denotam que estas cavernas evoluíram, inicialmente, no interior do maciço rochoso, sem uma conexão com o exterior, a exemplo das cavidades SL-106 e SL-110.

Modelos de espeleogênese foram desenvolvidos para a Serra Leste em trabalho anterior (CARSTE, 2011a). Segundo este documento, os modelos propostos por Pinheiro & Maurity (1988) e Piló & Auler (2005) possuem bastante semelhança e foram denominados de **modelo**

endógeno/exógeno do tipo dissolução/erosão (Figura 5.64). Nas cavernas de Serra Leste, esse modelo pode ser aplicado as cavernas inseridas no topo dos platôs ou em alta vertente, nas rupturas de cobertura de relevo implantadas geralmente na frente de evolução das escarpas. Dentre as cavernas analisadas, SL-115, SL-116 e SL-121 são exemplos deste modelo.

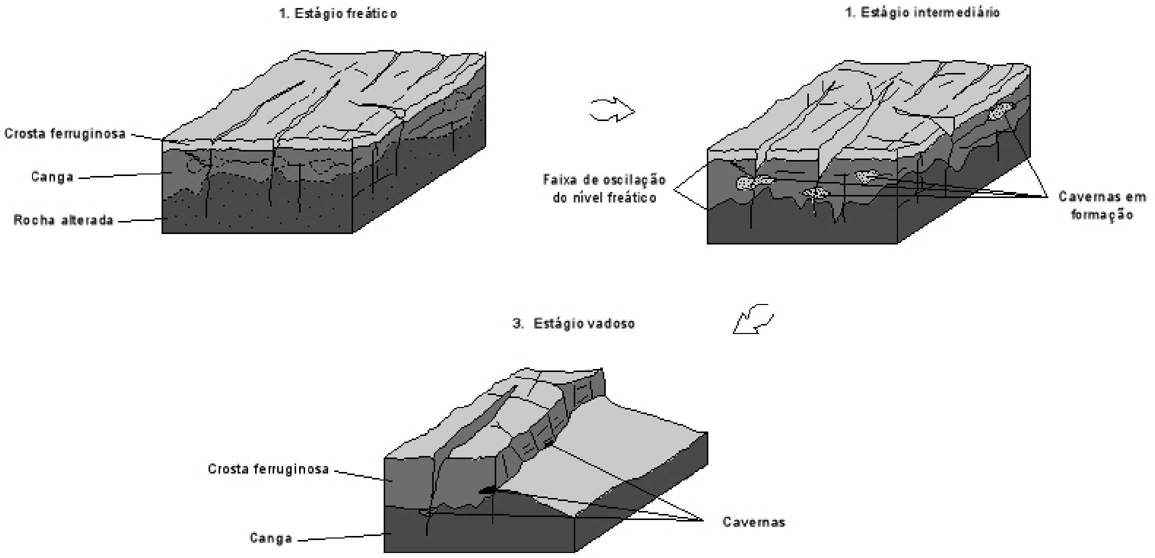

Figura 5.64 - Modelo de evolução endógena/exógena do tipo dissolução/erosão. Adaptado de Pinheiro & Maurity, 1988. Retirado de CARSTE, 2011a.

Na porção intermediária da paisagem, ou seja, entre as frentes de evolução da escarpa e a baixa vertente, foram identificadas cavernas cujo modelo genético está associado a formação de rampas de colúvio que foram posteriormente cimentadas por fluidos ferruginosos (Fe^{2+}). Este modelo espeleogenético, está bastante associado à morfodinâmica da paisagem, e por isso foi definido como **modelo exógeno do tipo coluvionar** (CARSTE, 2011a). Formam-se neste contexto paisagístico dois tipos de cavidades, as cavernas em tálus e as cavernas sob ferricrete (Figura 5.65).

As cavernas em tálus estão em geral inseridas nas porções hipsométricas mais elevadas. Consistem de um empilhamento de blocos caoticamente arranjados, cimentados ou não. Em alguns casos estão recobertos por sedimentos inconsolidados. Sob estes blocos desenvolvem-se as cavernas. Embora este modelo esteja descrito para vertentes desenvolvidas sobre rochas ferríferas e/ou recobertas por sedimentos que tenham esta litologia como origem, algumas cavernas em quartzito analisadas neste documento podem apresentar esta gênese (SL-113, SL-117 e SL-130).

Cavernas em ferricrete, por sua vez, ocupam normalmente as porções mais rebaixadas da paisagem, onde os fluidos ferruginizados se acumularam por tempo suficiente para que fosse formada uma crosta bastante enrijecida. Após a formação desta "carapaça", o rebaixamento do nível de base favorece a formação de protocondutos (*pipes*) no contato entre a crosta

rígida e o substrato subjacente, onde descontinuidades texturais e estruturais facilitam o fluxo subterrâneo de fluidos coloidais e soluções. A ampliação desses protocondutos é responsável pela formação das primeiras galerias subterrâneas, cuja “eclosão” se dá de duas formas, através de abatimentos doliniformes ou, mais comumente, através da interceptação resultante da evolução da paisagem (ravinamentos). As oito cavernas situadas no sistema fluvial da borda externa da serra apresentam parte de sua gênese neste modelo, uma vez que pode haver influência dos processos fluviais em seu desenvolvimento.

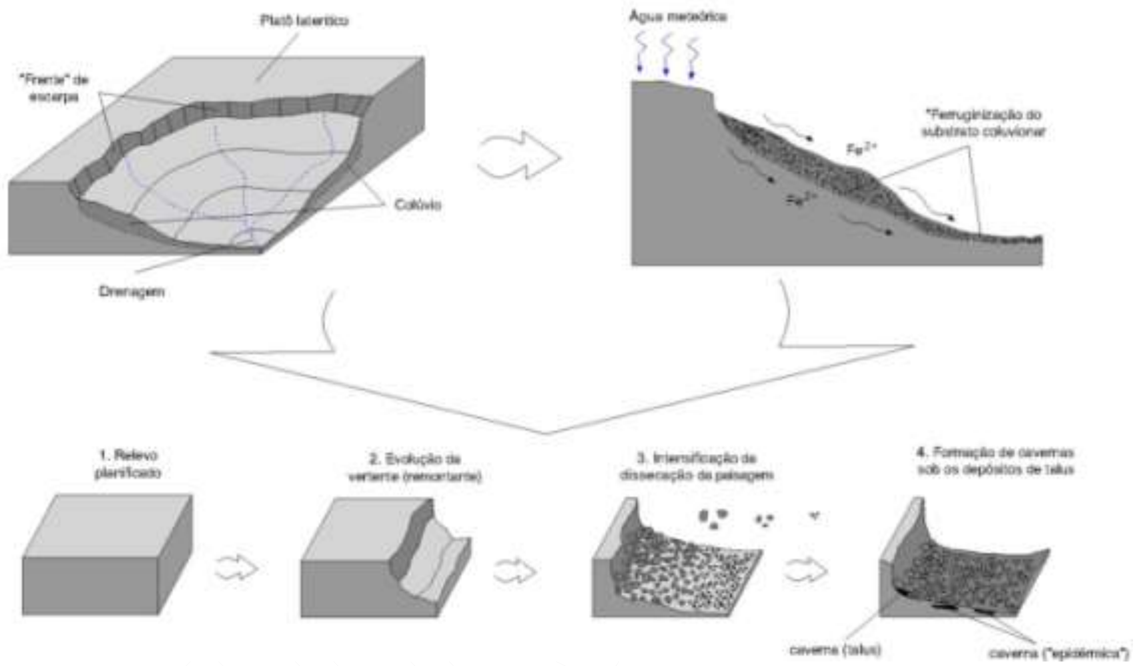

Figura 5.65 - Modelo de evolução exógena do tipo coluvionar. Retirado de CARSTE, 2011a.

Em Serra Leste, algumas das cavidades inseridas nos sistemas de drenagem de baixa vertente têm a gênese e a evolução basicamente associadas a processos de erosão lateral (Figura 5.66), hipótese corroborada pela morfologia destas cavidades, cuja entrada é comumente mais extensa do que a sua profundidade, como as cavernas SL-111 e SL-112. Tal qual no modelo exógeno tipo coluvionar, as cavernas fluviais tem sua gênese e desenvolvimento intrinsecamente associados aos processos morfodinâmicos que esculturam a paisagem, sendo aqui definido como **modelo exógeno do tipo fluvial** (CARSTE, 2011a) (Figura 5.66).

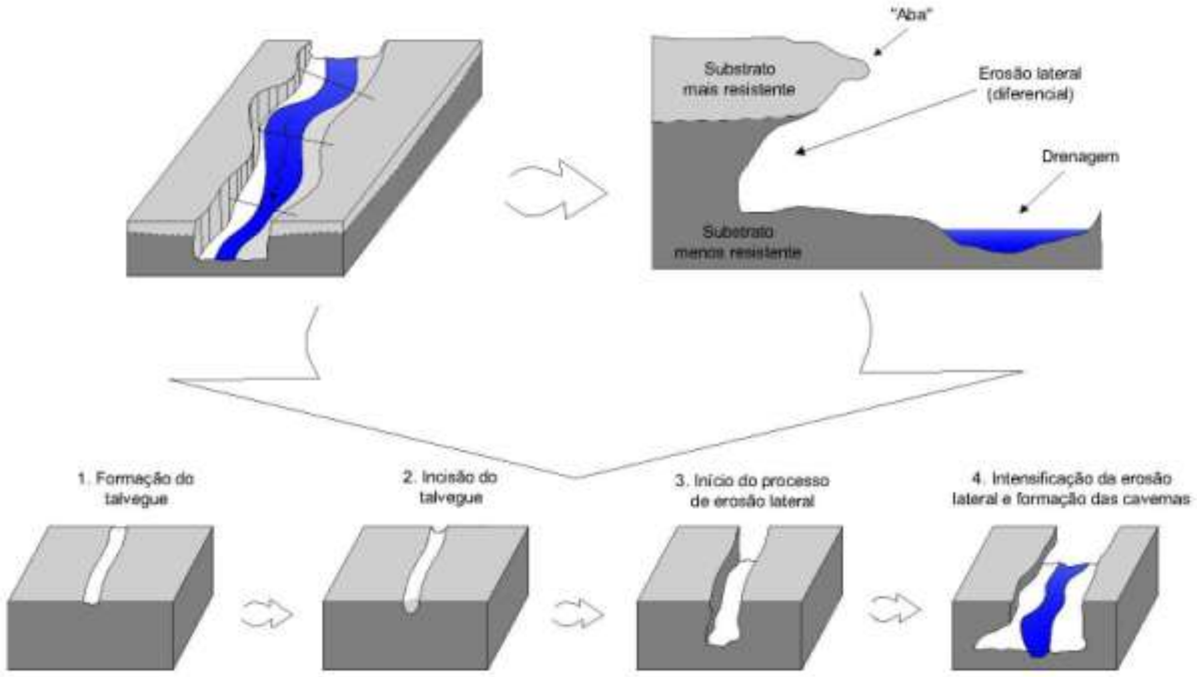

Figura 5.66 - Modelo de evolução exógena do "tipo fluvial". Retirado de CARSTE, 2011a.

Não só o processo gerador, mas também elementos que controlam o direcionamento do desenvolvimento da cavidade devem ser considerados. As cavernas podem apresentar controle por fraturamentos (ou juntas), acamamento, contato litológico, gradiente da vertente, dentre outros. Algumas cavernas apresentam galerias condicionadas por fraturas, como no caso da caverna SL-121 (Figura 5.67). Contato entre litotipos constitui uma zona, muitas vezes, explorada pela espeleogenêse. Algumas cavernas, como a SL-103 e a SL-111, apresentam nítidos contatos litológicos. A gênese de cavernas exclusivamente formadas na canga parece ser controlada pelas variações de fácies (Piló & Auler, 2009). Acredita-se que esta premissa pode ser aplicada nas cavernas analisadas em Serra Leste tanto para aquelas alojadas em canga quando aquelas em ferricrete.

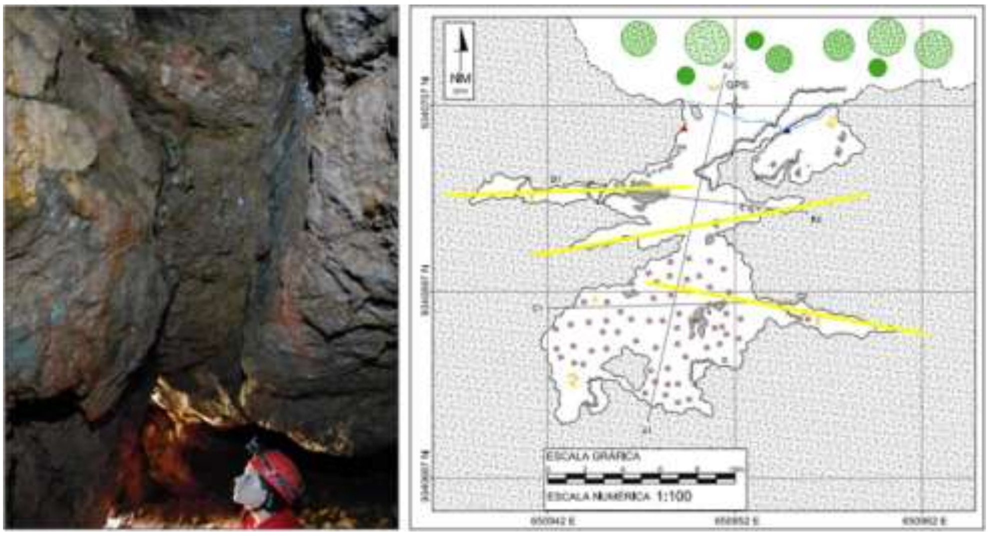

Figura 5.67 - Fratura preenchida na cavidade SL-121: controle estrutural no desenvolvimento de condutos. Linhas amarelas indicam direção aproximada das fraturas.

Processos tardios, como sedimentação e principalmente queda de blocos contribuem para alterar a morfologia original da caverna, além de, em alguns casos, permitirem a conexão da cavidade, até então oclusa, com o meio externo.

Na amostra de rochas ferríferas, não foram encontrados elementos que permitam inferir a idade e taxas de evolução das cavernas. Cavernas em rochas ferríferas que apresentem gênese endógena podem ser muito antigas, da ordem de dezenas de milhões de anos. Segundo Spier (2005), os intensos processos de intemperismo químico que atuaram nas formações ferríferas bandadas do Quadrilátero Ferrífero permitiram a completa dissolução da dolomita e a formação de minerais de manganês a partir das FFB. Datações, pelo método $^{40}Ar/^{39}Ar$, forneceram idades entre 61,5 Ma a 14,2 Ma, sugerindo uma longa história de intemperismo na região. No entanto, a maior parte dos óxidos de Mn precipitou no intervalo entre 51 e 41 Ma, com pico por volta de 46,7 Ma. Os dados cronológicos também confirmaram que o perfil de intemperismo das formações ferríferas já tinha atingido o atual estágio de desenvolvimento há cerca de 50 Ma, sugerindo que a frente intempérica avançou muito pouco durante o Neógeno.

Esses novos dados são de grande importância como balizadores cronológicos da gênese das cavernas, pois indicam que a partir do Eoceno as formações ferríferas já apresentam boas condições para a espeleogênese, tendo em vista que os processos supergênicos já estavam instalados, ocasionando aumento de porosidade e perda de densidade. Apesar da falta de dados demonstrativos e cronológicos absolutos, acreditamos que as cavernas desenvolvidas no minério de ferro são realmente muito antigas (superior a 2 milhões de anos), com idades bem superiores às cavernas carbonáticas.

Morfologias indicadoras de gênese nas cavernas quartzíticas na área nem sempre apresentam elementos usualmente comuns à espeleogênese nesta litologia. O modelo sanding/piping que pressupõe a formação inicial de zonas lineares de porosidade e permeabilidade aumentadas na interseção de fraturas com planos de foliação e/ou acamamento parece estar presente em alguns condutos, na caverna SL-101 (Figura 5.68) (CORRÊA NETO & BAPTISTA FILHO, 1997).

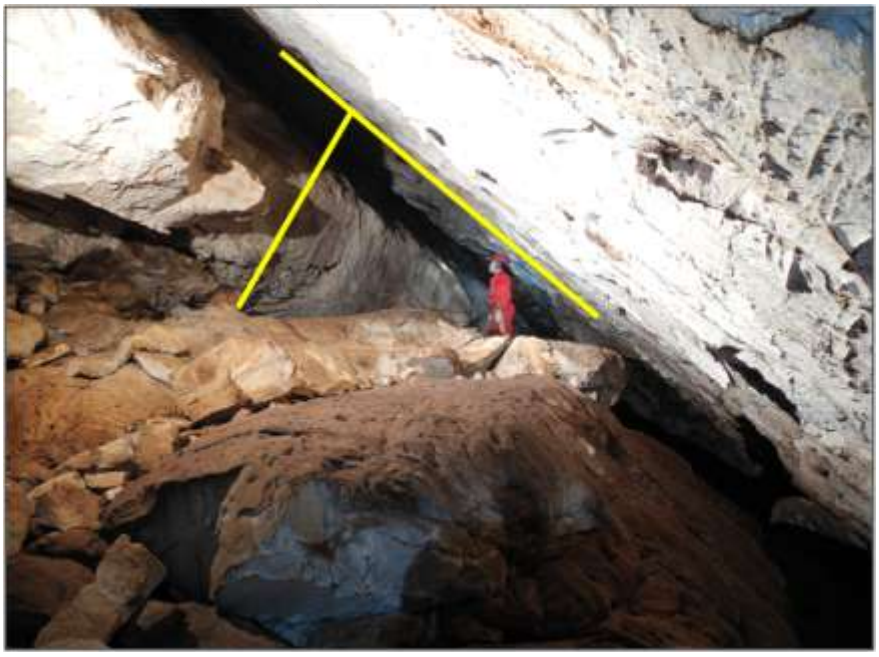

Figura 5.68 - Interseção de estruturas geológicas na caverna SL-101.

Cavernas alojadas em rochas siliciclásticas de elevada projeção horizontal e grande volume, como é caso da cavidade SL-101, usualmente estão associadas aos processos fluviais. Entretanto, evidências morfológicas de contribuições fluviais para a gênese e a evolução desta caverna são muito restritas, ocorrendo em pequena porção de um dos condutos inferiores. A posição atual da cavidade na vertente também não fornece indicativos da pretérita existência de cursos d'água subterrâneos. Assim, um modelo de aprofundamento do nível ativo da caverna causado pelo rebaixamento do nível de água cárstico (Figura 5.69) apresentado por Wiegand *et al.* (2004) não parece se aplicar a esta cavidade, o que a configura como um caso raro, demandando outros e mais aprofundados estudos.

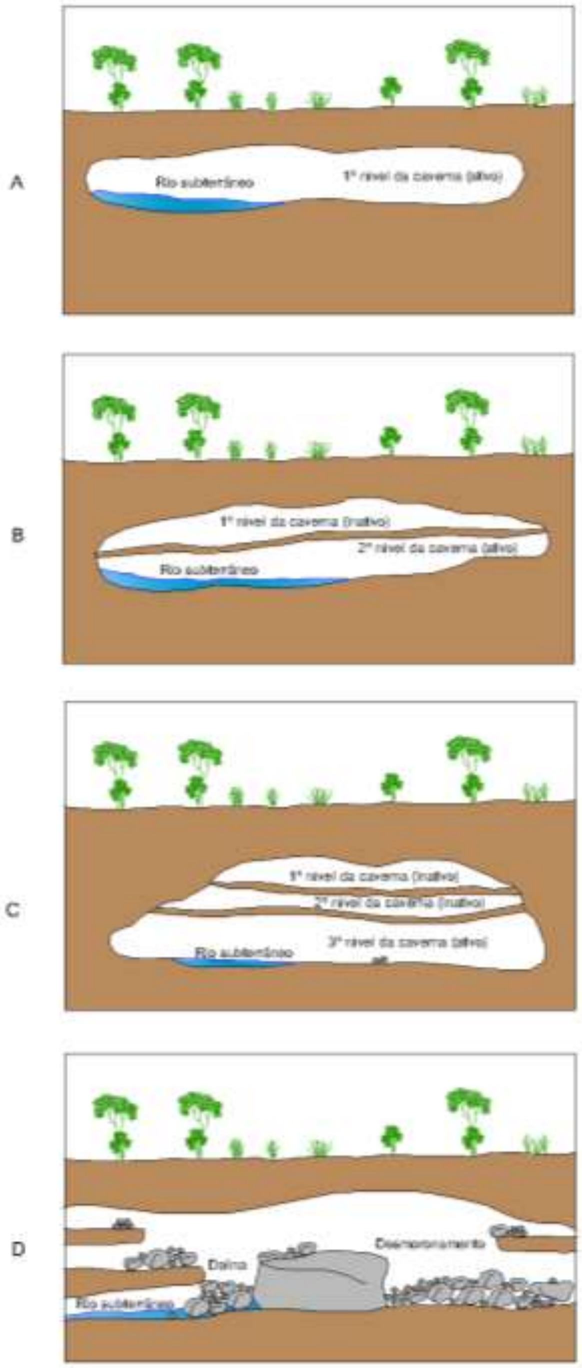

Figura 5.69 - Modelo de aprofundamento no nível ativo da caverna pelo contínuo rebaixamento do nível freático. Modificado de Wiegand *et al.*, 2004.

5.1.9. Aspectos sócio econômicos, culturais e estado de conservação

Nenhuma das cavernas estudadas na área do Projeto Serra Leste apresentou valor cultural ou sócio econômico. Com relação ao seu estado de conservação, três cavidades apresentaram algum tipo de interferência (impacto) em seu interior. A presença de dejetos (lixo doméstico) é comum nas três cavernas impactas: (i) na cavidade SL-111, o lixo leve, constituído principalmente por recipientes plásticos, é transportado pela água fluvial, uma vez que esta caverna insere-se na calha de drenagem, estando sujeita a periódicas inundações (Figura 5.70); (ii) a cavidade SL-112 localiza-se a menos de 50m de uma residência rural e alguns lixos domésticos foram entrados em seu interior; (iii) na caverna SL-114, além de isopor, notam-se alterações possivelmente associadas à estrada não pavimentada situada poucos metros a montante: cicatrizes de abatimento no teto são provavelmente advindas de vibrações do trânsito de veículo na estrada; colapso de uma porção do teto da cavidade pode ser fruto da instalação de uma manilha de escoa águas pluviais da estrada e, entupimento de grande parte da caverna por sedimentos que, possivelmente foram direcionados para este local através do sistema de drenagem da via de acesso (Figura 5.71).

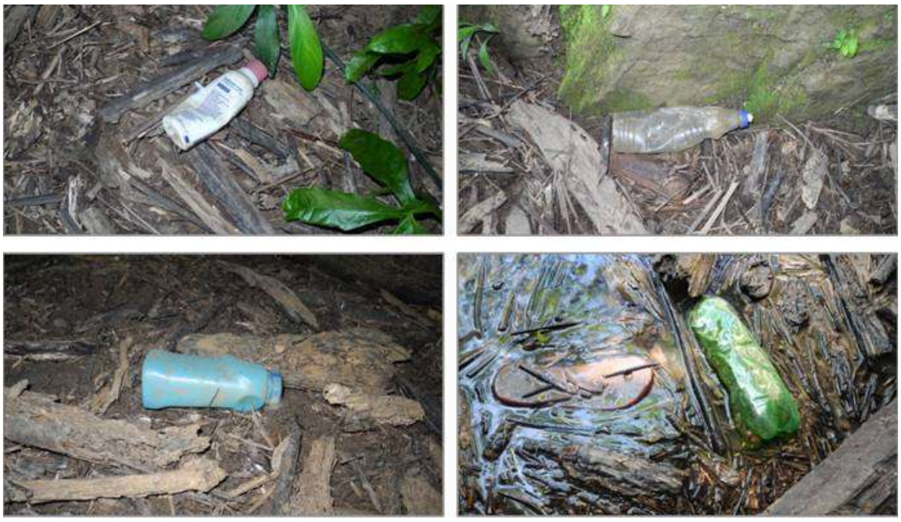

Figura 5.70 - Vasilhas plásticas observadas na caverna SL-111.

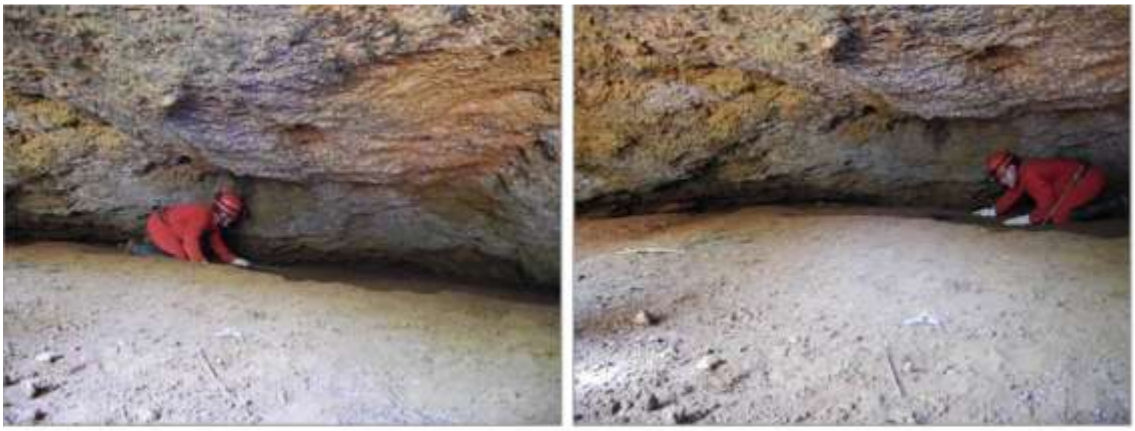

Figura 5.71 - Grande quantidade de sedimentos canalizados para o interior da caverna SL-114.

Além das três cavernas impactadas, é possível que outras três tenham sofrido escavações em seu interior: SL-103, SL-107 e SL-115. A cavidade SL-107 apresenta evidencias desta escavação também em seu entorno imediato (Figura 5.72).

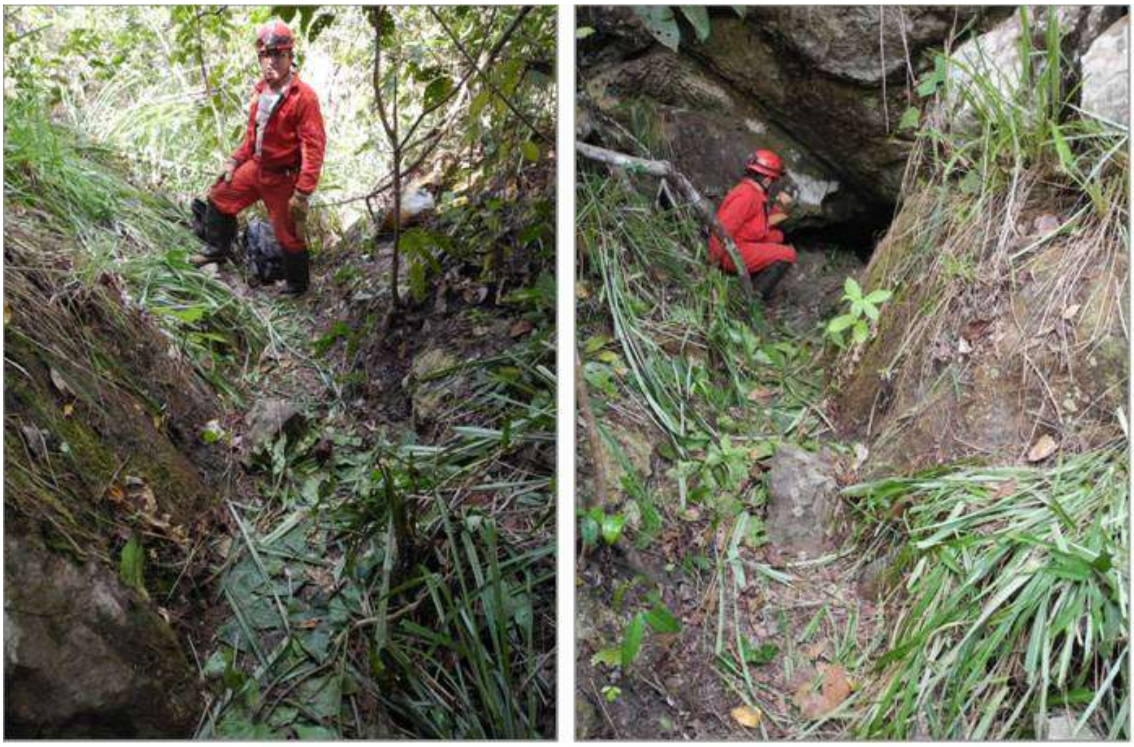

Figura 5.72 - Escavação nas proximidades da entrada da caverna SL-107.

Em relação ao entorno (250 metros), todas as cavernas tiveram seu perímetro de proteção impactado uma vez que a área de Serra Leste encontra-se em estágio avançado de antropização. Estradas de sondagem e vias de acesso estão largamente presentes de forma que é difícil encontrar uma caverna a mais de 250 metros das estradas. Outra alteração bastante comum é a substituição da cobertura vegetal natural por pastagens e a supressão da vegetação para implantação de estruturas minerárias (Figura 5.73). As cavidades inseridas em quartzito contam ainda com alteração devido a atividades exploratórias da rocha, uma vez que uma pedreira esteve em funcionamento em área bastante próxima às cavernas (Figura 5.73).

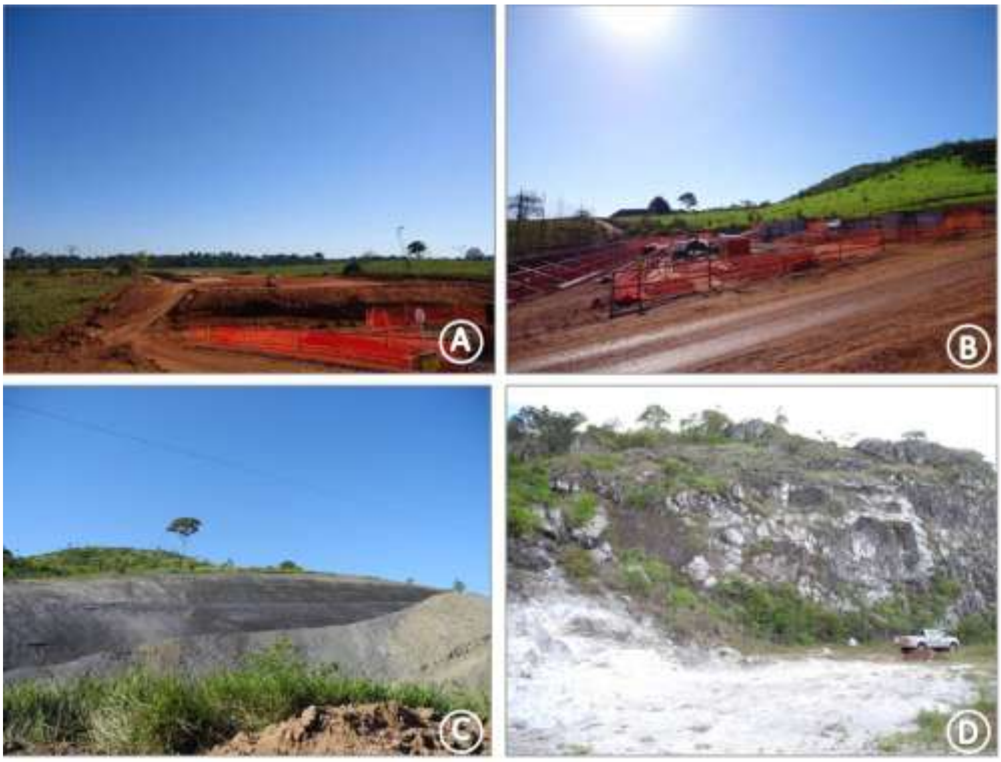

Figura 5.73 - Alterações no entorno das cavidades: (A), (B) e (C) Supressão da vegetação para implantação de estruturas minerárias ; (D) Frente de lavra em maciço quartzítico.

5.1.10. Considerações finais a respeito dos atributos geoespeleológicos

O documento apresenta os estudos geoespeleológicos de 21 cavidades inseridas na área do Projeto Serra Leste. As cavernas alojam-se em litologias siliciclásticas ou em rochas ferríferas. De pequenas dimensões, somente a caverna SL-101 desenvolvida em quartzito apresenta tamanho extraordinário no contexto local e regional. Normalmente com contornos bastante irregulares, as cavernas analisadas apresentam como feição morfológica mais recorrente, os canalículos, por onde aportam sedimentos e a caverna se desenvolve. Pilares e pendentes são também relativamente comuns. Na maior parte dos casos, as cavernas em estudo apresentam um único salão e, dentre os padrões planimétricos predefinidos, algumas cavidades puderam ser classificadas como retilíneas, curvilíneas, reticulares, globulares ou triangulares.

Dentre as 21 cavidades em estudo, 16 apresentavam-se totalmente secas, quando dos estudos geoespeleológicos, sendo presentes raras marcas de gotejamento em poucas destas cavernas. Dentre as cinco cavernas que apresentam feições hidrológicas, duas desenvolvem-se em rochas siliciclásticas e três em rochas ferríferas. Porém, como observado em estudos anteriores na Serra Leste (CARSTE, 2011a), mesmo as cavernas inseridas em sistemas fluviais parecem não formar um sistema hidrogeológico estando, cada feição espeleológica, desconectada uma da outra.

A energia de transporte sedimentar é baixa, portanto não há grande tempo/distância entre a área-fonte dos sedimentos e o depósito sedimentar, sendo pequeno o percurso de transporte. Desta forma, os graus de arredondamento e de seleção dos sedimentos na área são baixos. Fazem-se comuns clastos angulosos a subangulosos, embora também possam estar presentes

clastos subarredondados, que provavelmente apresentam esta esfericidade devido a transporte anterior a formação da própria rocha encaixante da cavidade, quando se trata de canga detrítica ou ferricrete.

Em Serra Leste, depósitos químicos podem ser observados em 11 das 12 cavernas desenvolvidas em rochas ferríferas. Nas cavernas alojadas em quartzito, apenas uma não apresenta espeleotemas. A variedade tipológica é maior nas cavidades em rochas ferríferas, sendo extremamente reduzida nas rochas quartzítica. Não houve algum tipo excepcional ou único de espeleotema entre as cavernas avaliadas, sendo os tipos bastante comuns à formação ferrífera e aos quartzitos. Crostas e coralóides são os depósitos químicos mais recorrentes em ambas as litologias.

Modelos de espeleogênese foram desenvolvidos para a Serra Leste em trabalho anterior (CARSTE, 2011a), sendo perfeitamente aplicados às cavernas em estudo neste documento. Nenhuma das cavernas estudadas na área do Projeto Serra Leste apresentou valor cultural ou sócio econômico.

Com relação ao estado de conservação, três cavidades apresentaram algum tipo de impacto em seu interior: a presença de dejetos (lixo doméstico) é comum nas três cavernas impactas. Além disso, na cavidade SL-114, notam-se cicatrizes de abatimento no teto, provavelmente advindas de vibrações do trânsito de veículo na estrada logo a montante, colapso de uma porção do teto da cavidade e, entupimento de grande parte da caverna por sedimentos que, possivelmente foram direcionados para este local através do sistema de drenagem da via de acesso. Além das três cavernas impactadas, é possível que outras três tenham sofrido escavações em seu interior: SL-103, SL-107 e SL-115. Em relação ao entorno (250 metros), todas as cavernas tiveram seu perímetro de proteção impactado uma vez que a área de Serra Leste encontra-se em estágio avançado de antropização. Estradas de sondagem e vias de acesso estão largamente presentes de forma que é difícil encontrar uma caverna a mais de 250 metros das estradas.

Não foram observados atributos ou diferenças significativas entre as 21 cavidades em análise e as demais cavidades localizadas na área do Projeto Serra Leste, exceto pela inserção de parte das cavernas analisadas neste documento em litologia siliciclástica e dimensões extraordinárias da caverna SL-101. Também não há expressivas discrepâncias quando comparadas a outras cavidades estudadas na região de Carajás.

5.2.Estudos Bioespeleológicos

5.2.1. Caracterização faunística geral das cavidades

- ***Caracterização faunística no período de seca***

Foi observado nas cavernas, um total de 356 morfoespécies de invertebrados de pelo menos 127 famílias dos Taxa: Oligochaeta, Gastropoda (Subulinidae; Systrophiidae), Platyhelminthes (Tricladida), Isopoda (Armadillidae; Dubioniscidae; Platyarthridae: *Trichorhina* sp.; Philosciidae; Scleropactidae), Acari (Actinedida: Rhagidiidae, Parasitengonina; Ixodida; Mesostigmata: Ameroseiidae, Laelapidae, Macrochelidae, Podocinidae, Uropodina; Opiliacarida:

Opiliacaridae; Oribatida; Cunaxidae: *Armascirus* sp.; Acaridae: *Tyrophagus* sp.; Arthronotina; Cheyletidae), Decapoda (Pseudothelphusidae: *Microthelphusa somanni*). Amblypygi (Phrynidae: *Heterophrynus longicornis*; Charinidae: *Charinus* sp.), Pseudoscorpiones (Chernetidae; Chthoniidae), Opiliones (Cosmetidae; Escadabiidae; Manaosbiidae; Sclerosomatidae), Araneae (Araneidae; Corinnidae: *Corinna* sp.; Ctenidae: *Enoploctenus* sp.; Drymusidae; Linyphiidae; Micropholcommatidae; Ochyroceratidae: *Ochyrocera* sp., *Speocera* sp.; Oonopidae: *Oonops* sp.; Palpimanidae; Pholcidae: *Mesabolivar* sp., *Metagonia* sp., Ninetinae; Prodidomidae; Salticidae: *Noegus* sp.; Scytodidae: *Scytodes itapevi*; Theraphosidae; Theridiidae: *Nesticodes rufipes*; Theridiosomatidae: *Plato* sp.; Trechaleidae: *Enna* aff. *paraense*; Uloboridae), Thysanura (Nicoletiidae: Nicoletiinae, Atelurinae), Diplura (Campodeidae), Collembola (Bourletiellidae; Cyphoderidae; Entomobryidae; Hypogastruridae; Isotomidae; Paronellidae; Tomoceridae), Neuroptera (Myrmeleontidae), Orthoptera (Gryllidae; Mogoplistidae; Phalangopsidae: *Phalangopsis* sp., *Uvaroviella* sp., Phalangopsinae), Blattodea (Blaberidae: *Blaberus* spp.; Blattidae; Polyphagidae), Embiidina, Isoptera (Termitidae: *Cornitermes* sp., *Cylindrotermes* sp., *Nasutitermes* spp.), Dermaptera, Siphonaptera (Pulicidae), Psocoptera (Archipsocidae; Epipsocidae; Myopsocidae; Pachytroctidae; Ptiloneuridae), Heteroptera (Cydnidae; Dipsocoridae; Enicocephalidae; Hebridae; Lygaeidae; Nabidae; Reduviidae: *Zelurus* sp., Emesinae; Tingidae; Veliidae: *Paravelia* sp.), Homoptera (Cicadellidae: Typhlocybinae; Cixiidae: *Pintalia* spp.), Lepidoptera (Coleophoridae; Gelechidae; Noctuidae; Tineidae; Tortricidae), Diptera (Cecidomyiidae; Ceratopogonidae; Culicidae: *Anopheles* sp., *Culex* sp.; Dolichopodidae; Drosophilidae; Empididae; Fanniidae; Keroplatidae; Milichiidae; Muscidae; Mycetophilidae; Phoridae; Platypezidae aff.; Psychodidae *: Lutzomyia* sp.; Sciaridae; Streblidae; Tipulidae), Hymenoptera (Formicidae: *Acromyrmex* sp., *Apterostigma* sp., *Atta* sp., *Azteca* spp., *Basiceros* sp., *Camponotus* spp., *Cardiocondyla* sp., *Carebara* spp., *Cremagaster* sp., *Cyphomyrmex* sp., *Dolichoderus* spp., *Ectatomma* spp., *Gnamptogenys* sp., *Hypoponera* spp., *Labidus* sp., *Leptogenys* sp., *Linepithema* sp., *Neivamyrmex* sp., *Odontomachus* sp., *Pachycondyla* spp., *Pheidole* spp., *Proceratium* sp., *Pseudomyrmex* spp., *Rogeria* spp., *Solenopsis* spp., *Strumigenys* sp., *Tapinoma* spp., Myrmicinae; Bethilidae; Braconidae; Chalcididae; Cynipidae; Diapriidae; Scelionidae), Coleoptera (Anthicidae; Carabidae; Chrysomelidae: Alticinae; Curculionidae; Dermestidae; Elateridae: Agrypninae, Cardiophorinae; Histeridae; Hydrophilidae; Leiodidae; Scarabaeidae; Scydmaenidae; Staphylinidae: Pselaphinae), Glomeridesmida (Glomeridesmidae), Polydesmida (Cyrtodesmidae; Pyrgodesmidae), Spirostreptida (Pseudonannolenidae), Geophilomorpha (Geophilidae: *Schizonampa* sp.), Lithobiomorpha (Henicopidae: *Lamyctes* sp.), Scolopendromorpha (Newportiidae: *Dinocryptops* sp.; Scolopendridae: *Cryptops* sp.), Scutigeromorpha (Pselliodidae: *Sphendononema guildingii*) e Symphyla (Scutigerellidae: *Hanseniela* spp.).

Dentre os vertebrados, foram encontradas quatro espécies de Anura (Bufonidae: *Rhinella* sp.; Dendrobatidae: *Ameerega* sp.; Leptodactylidae: *Leptodactylus labyrinthicus*; Strabomantidae: *Pristimantis* sp.) e 12 espécies de Chiroptera (Emballorunidae: *Peropteryx kappleri*; *Peropteryx* sp. ; Furipteridae: *Furipterus horrens*; Mormoopidae: *Pteronotus parnellii*; Phyllostomidae: *Anoura geoffroyi*; *Carollia perspicillata*, *Lionycteris spurrelli, Lonchorhina aurita*; *Diphylla ecaudata*; *Glossophaga soricina,* Glossophaginae).

Desta forma, no total foram encontrados 372 morfoespécies. Dentre essas, sete espécies foram consideradas troglomórficas Gastropoda (Systrophiidae), Amblypygi (Charinidae: *Charinus* sp.), Collembola (Cyphoderidae 2spp.; Isotomidae), Coleoptera (Staphylinidae: Pselaphinae) e Polydesmida (Pyrgodesmidae).

- ***Caracterização faunística no período de chuva***

Foi observado nas cavernas, um total de 539 morfoespécies de invertebrados de pelo menos 167 famílias dos Taxa: Oligochaeta, Gastropoda (Subulinidae; Systrophiidae), Platyhelminthes (Tricladida), Isopoda (Armadillidae; Dubioniscidae; Platyarthridae: *Trichorhina* sp.; Philosciidae; Scleropactidae), Acari (Actinedida: Rhagidiidae, Parasitengonina; Ixodida; Mesostigmata: Ameroseiidae, Podocinidae, Uropodina; Oribatida; Prostigmata: Bdelloidea, Cunaxidae: *Armascirus* sp.; Acaridae: *Tyrophagus* sp.; Arthronotina; Trombidiforme: Anystidae: *Eritracarus* sp., Tydaidae), Decapoda (Pseudothelphusidae: *Microthelphusa somanni*), Amblypygi (Phrynidae: *Heterophrynus longicornis*; Charinidae: *Charinus* sp.), Palpigradi (Eukoeneniidae: *Leptokoenenia* sp.), Scorpiones (Buthidae: *Ananteris luciae*), Pseudoscorpiones (Cheliferidae; Chernetidae; Chthoniidae; Olpiidae; Syarinidae), Opiliones (Cosmetidae; Escadabiidae; Phalangiidae; Sclerosomatidae; Stygnidae; Stygnommatidae), Araneae (Amaurobiidae; Araneidae: *Alpaida* sp.; Ctenidae: *Enoploctenus* sp.; Corinnidae: *Corinna* sp.; Dipluridae; Linyphiidae; Ochyroceratidae: *Ochyrocera* sp., *Speocera* sp.; Oonopidae: Gamasomorphinae, *Oonops* sp.; Palpimanidae; Pholcidae: *Mesabolivar* sp., *Metagonia* sp., Ninetinae; Prodidomidae: *Lygromma* sp.; Salticidae: *Noegus* sp.; Scytodidae: *Scytodes itapevi*, *Scytodes* sp.; Symphytognathidae: *Anapistula* sp.; Tetrablemmidae: *Matta* sp.; Theraphosidae; Theridiidae: *Nesticodes rufipes*; Theridiosomatidae: *Plato* sp.; Trechaleidae: *Enna* aff. *paraense*; Uloboridae), Thysanura (Nicoletiidae: Nicoletiinae, Atelurinae; Meinertellidae), Diplura (Campodeidae; Anajapygidae; Japygidae), Collembola (Bourletiellidae; Cyphoderidae; Entomobryidae; Isotomidae; Paronellidae; Sminthuridae; Tomoceridae), Neuroptera (Myrmeleontidae; Chrysopidae), Orhtoptera (Phalangopsidae: *Phalangopsis* sp., *Uvaroviella* sp., Phalangopsinae), Blattodea (Blaberidae: *Blaberus* spp.; Blattellidae; Blattidae; Polyphagidae), Embiidina; Isoptera (Termitidae: *Nasutitermes* spp., *Velocitermes* sp.; Rhinotermitidae: *Coptotermes* sp., *Heterotermes* sp.), Dermaptera, Ephemeroptera (Euthyplociidae: *Campylocia anceps*), Thysanoptera (Phlaeothripidae), Psocoptera (Archipsocidae; Lepidopsocidae; Myopsocidae; Pachytroctidae; Psyllipsocidae; Psyllipsocidae; Ptiloneuridae; Trogiidae), Coleoptera (Alydidae; Cydnidae; Dipsocoridae; Hebridae; Lygaeidae; Mesoveliidae; Nabidae; Ortheziidae; Pentatomidae; Pyrrhocoridae; Reduviidae: *Zelurus* sp., Emesinae, Triatominae; Veliidae: *Paravelia* sp., *Rhagovelia* sp.), Homoptera (Cercopidae; Cixiidae: *Pintalia* spp.; Derbidae: *Mysidia* sp.), Lepidoptera (Arctiidae; Coleophoridae; Noctuidae; Tineidae; Tortricidae), Diptera (Calliphoridae; Cecidomyiidae; Ceratopogonidae; Chironomidae; Chloropidae; Culicidae: *Anopheles* sp., *Culex* sp.; Drosophilidae; Empididae; Fanniidae; Keroplatidae; Milichiidae; Muscidae; Phoridae; Psychodidae: *Lutzomyia* spp., Sciaridae; Simuliidae; Sphaeroceridae; Stratiomyidae; Streblidae; Syrphidae; Tipulidae), Hymenoptera (Formicidae: *Acanthostichus* sp., *Acromyrmex* sp., *Amblyopone* sp., *Anochetus* sp., *Apterostigma* sp., *Atta* spp., *Azteca* sp., *Camponotus* spp., *Cardiocondyla* sp., *Carebara* spp., *Cephalotes* sp., *Cremagaster* sp., *Cyphomyrmex* spp.,

Ectatomma spp., *Gnamptogenys* sp., *Hypoponera* spp., *Lachnomyrmex* sp., *Leptogenys* spp., *Linepithema* sp., *Octostruma* sp., *Odontomachus* spp., *Pachycondyla* sp., *Pheidole* spp., *Prionopelta* sp., *Pseudomyrmex* spp., *Rogeria* spp., *Solenopsis* spp., *Stegomyrmex* sp., *Strumigenys* spp., *Tapinoma* spp., Myrmicinae; Bethilidae; Braconidae; Chalcididae; Cynipidae; Diapriidae; Dryinidae?; Eulophidae; Scelionidae; Vespidae: Polybiinae), Coleoptera (Alleculidae; Carabidae: Clivinina; Cerambycidae; Chrysomelidae: Alticinae; Curculionidae; Dytiscidae; Elateridae: Agrypninae, Cardiophorinae; Eucnemidae; Histeridae; Hydrophilidae; Lampyridae; Leiodidae; Phalacridae; Ptiliidae; Rhizophagidae; Scarabaeidae; Scydmaenidae; Staphylinidae: Pselaphinae, Scaphidiinae; Tenebrionidae), Glomeridesmida (Glomeridesmidae), Polydesmida (Chelodesmidae; Cyrtodesmidae; Polydesmidae; Pyrgodesmidae), Siphonophorida (Siphonophoridae), Spirostreptida (Pseudonannolenidae), Stemmiulida (Stemmiulidae), Geophilomorpha (Ballophilidae: *Ballophilus* sp.; Geophilidae: *Schizonampa* sp.), Lithobiomorpha (Henicopidae: *Lamyctes* sp.), Scolopendromorpha (Newportiidae: *Dinocryptops* sp., *Newportia* spp.; Scolopendridae: *Cryptops* spp., *Otostigmus* sp.), Scutigeromorpha (Pselliodidae: *Sphendononema guildingii*) e Symphyla (Scutigerellidae: *Hanseniela* spp., *Scutigerella* s., *Symphylella* sp.).

Dentre os vertebrados, foram encontradas quatro espécies de Anura (Bufonidae; Strabomantidae: *Pristimantis* sp.; um indeterminado, e um imaturo), um Apodiformes (Trochilidae), 12 espécies de Chiroptera (Emballorunidae: *Peropteryx kappleri*; *Peropteryx* sp.; Furipteridae: *Furipterus horrens*; Mormoopidae: *Pteronotus parnellii*; Phyllostomidae: *Anoura geoffroyi*; *Carollia perspicillata*, *Carollia* sp., *Lionycteris spurrelli, Lonchorhina* sp*., Diphylla ecaudata*; *Glossophaga soricina,* Glossophaginae), um Rodentia (Cricetidae: *Rhipidomys* sp.) e um Squamata (Gekkonidae: *Thecadactylus rapicauda*).

Desta forma, no total foram encontrados 558 morfoespécies. Dentre essas, 13 espécies foram consideradas troglomórficas Gastropoda (Systrophiidae), Amblypygi (Charinidae: *Charinus* sp.), Araneae (Oonopidae; Tetrablemmidae: *Matta* sp.), Collembola (Cyphoderidae 2spp.; Isotomidae), Coleoptera (Dytiscidae; Eucnemidae; Scydmaenidae; Staphylinidae: Pselaphinae) e Polydesmida (Pyrgodesmidae 2spp.).

- ***Caracterização geral da fauna das cavidades***

Foi observado nas cavernas, um total de 668 morfoespécies de invertebrados de pelo menos 185 famílias dos Taxa: Oligochaeta, Gastropoda (Subulinidae; Systrophiidae), Platyhelminthes (Tricladida), Isopoda (Armadillidae; Dubioniscidae; Platyarthridae: *Trichorhina* sp.; Philosciidae; Scleropactidae), Acari (Actinedida: Rhagidiidae, Ixodida; Mesostigmata: Ameroseiidae, Laelapidae, Macrochelidae, Podocinidae, Uropodina; Opiliacarida: Opiliacaridae; Oribatida; Prostigmata: Bdelloidea, Cunaxidae: *Armascirus* sp.; Sarcoptiformes: Acaridae: *Tyrophagus* sp., Arthronotina; Trombidiforme: Anystidae: *Eritracarus* sp., Cheyletidae, Tydaidae), Decapoda (Pseudothelphusidae: *Microthelphusa somanni*), Amblypygi (Phrynidae: *Heterophrynus longicornis*; Charinidae: *Charinus* sp.), Palpigradi (Eukoeneniidae: *Leptokoenenia* sp.), Scorpiones (Buthidae: *Ananteris luciae*), Pseudoscorpiones (Cheliferidae; Chernetidae; Chthoniidae; Olpiidae; Syarinidae), Opiliones (Cosmetidae; Escadabiidae; Manaosbiidae; Phalangiidae; Sclerosomatidae; Stygnidae; Stygnommatidae), Araneae

(Amaurobiidae; Araneidae: *Alpaida* sp.; Ctenidae: *Enoploctenus* sp.; Corinnidae: *Corinna* spp.; Dipluridae; Linyphiidae; Micropholcommatidae; Ochyroceratidae: *Ochyrocera* sp., *Speocera* sp.; Oonopidae: Gamasomorphinae, *Oonops* sp.; Palpimanidae; Pholcidae: *Mesabolivar* sp., *Metagonia* sp., Ninetinae; Prodidomidae: *Lygromma* sp.; Salticidae: *Noegus* sp.; Scytodidae: *Scytodes itapevi*, *Scytodes* sp.; Symphytognathidae: *Anapistula* sp.; Tetrablemmidae: *Matta* sp.; Theraphosidae; Theridiidae: *Nesticodes rufipes*; Theridiosomatidae: *Plato* sp.; Trechaleidae: *Enna* aff. *paraense*; Uloboridae), Thysanura (Nicoletiidae: Nicoletiinae, Atelurinae; Meinertellidae), Diplura (Campodeidae; Anajapygidae; Japygidae), Collembola (Bourletiellidae; Cyphoderidae; Entomobryidae; Hypogastruridae; Isotomidae; Paronellidae; Sminthuridae; Tomoceridae), Neuroptera (Myrmeleontidae; Chrysopidae), Orthoptera (Gryllidae; Mogoplistidae; Phalangopsidae: *Phalangopsis* sp., *Uvaroviella* sp., Phalangopsinae), Blattodea (Blaberidae: *Blaberus* spp.; Blattellidae; Blattidae; Polyphagidae), Embiidina; Isoptera (Termitidae: *Nasutitermes* spp., *Velocitermes* sp.; Rhinotermitidae: *Coptotermes* sp., *Heterotermes* sp.), Dermaptera, Ephemeroptera (Euthyplociidae: *Campylocia anceps*), Thysanoptera (Phlaeothripidae), Siphonaptera (Pulicidae), Psocoptera (Archipsocidae; Epipsocidae; Lepidopsocidae; Myopsocidae; Pachytroctidae; Psyllipsocidae; Psyllipsocidae; Ptiloneuridae; Trogiidae), Coleoptera (Alydidae; Cydnidae; Dipsocoridae; Enicocephalidae; Hebridae; Lygaeidae; Mesoveliidae; Nabidae; Ortheziidae; Pentatomidae; Pyrrhocoridae; Reduviidae: *Zelurus* sp., Emesinae, Triatominae; Tingidae; Veliidae: *Paravelia* sp., *Rhagovelia* sp.), Homoptera (Cercopidae; Cicadellidae: Typhlocybinae; Cixiidae: *Pintalia* spp.; Derbidae: *Mysidia* sp.), Lepidoptera (Arctiidae; Coleophoridae; Gelechidae; Noctuidae; Tineidae; Tortricidae), Diptera (Calliphoridae; Cecidomyiidae; Ceratopogonidae; Chironomidae; Chloropidae; Culicidae: *Anopheles* sp., *Culex* sp.; Dolichopodidae; Drosophilidae; Empididae; Fanniidae; Keroplatidae; Milichiidae; Muscidae; Mycetophilidae ; Phoridae; Platypezidae aff.; Psychodidae: *Lutzomyia* spp., Sciaridae; Simuliidae; Sphaeroceridae; Stratiomyidae; Streblidae; Syrphidae; Tipulidae), Hymenoptera (Formicidae: *Acanthostichus* sp., *Acromyrmex* sp., *Amblyopone* sp., *Anochetus* sp., *Apterostigma* sp., *Atta* spp., *Azteca* sp., *Basiceros* sp., *Camponotus* spp., *Cardiocondyla* sp., *Carebara* spp., *Cephalotes* sp., *Cremagaster* sp., *Cyphomyrmex* spp., *Dolichoderus* spp., *Ectatomma* spp., *Gnamptogenys* sp., *Hypoponera* spp., *Labidus* sp., *Lachnomyrmex* sp., *Leptogenys* spp., *Linepithema* sp., *Neivamyrmex* sp., *Octostruma* sp., *Odontomachus* spp., *Pachycondyla* spp., *Pheidole* spp., *Proceratium* sp., *Prionopelta* sp., *Pseudomyrmex* spp., *Rogeria* spp., *Solenopsis* spp., *Stegomyrmex* sp., *Strumigenys* spp., *Tapinoma* spp., Myrmicinae; Bethilidae; Braconidae; Chalcididae; Cynipidae; Diapriidae; Dryinidae; Eulophidae; Scelionidae; Vespidae: Polybiinae), Coleoptera (Alleculidae; Anthicidae; Carabidae: Clivinina; Cerambycidae; Chrysomelidae: Alticinae; Curculionidae; Dermestidae; Dytiscidae; Elateridae: Agrypninae, Cardiophorinae; Eucnemidae; Histeridae; Hydrophilidae; Lampyridae; Leiodidae; Phalacridae; Ptiliidae; Rhizophagidae; Scarabaeidae; Scydmaenidae; Staphylinidae: Pselaphinae, Scaphidiinae; Tenebrionidae), Glomeridesmida (Glomeridesmidae), Polydesmida (Chelodesmidae; Cyrtodesmidae; Polydesmidae; Pyrgodesmidae), Siphonophorida (Siphonophoridae), Spirostreptida (Pseudonannolenidae), Stemmiulida (Stemmiulidae), Geophilomorpha (Ballophilidae: *Ballophilus* sp.; Geophilidae: *Schizonampa* sp.), Lithobiomorpha (Henicopidae: *Lamyctes* sp.), Scolopendromorpha (Newportiidae: *Dinocryptops* sp., *Newportia* spp.; Scolopendridae: *Cryptops* spp., *Otostigmus*

sp.), Scutigeromorpha (Pselliodidae: *Sphendononema guildingii*) e Symphyla (Scutigerellidae: *Hanseniela* spp., *Scutigerella* sp., *Symphylella* sp.).

Dentre os vertebrados, foram encontradas sete espécies de Anura (Bufonidae: *Rhinella* sp.; Dendrobatidae: *Ameerega* sp.; Leptodactylidae: *Leptodactylus labyrinthicus*; Strabomantidae: *Pristimantis* sp.; um indeterminado, e um imaturo), um Apodiformes (Trochilidae), 14 espécies de Chiroptera (Emballorunidae: *Peropteryx kappleri*; *Peropteryx* sp. ; Furipteridae: *Furipterus horrens*; Mormoopidae: *Pteronotus parnellii*; Phyllostomidae: *Anoura geoffroyi*; *Carollia perspicillata*, *Carollia* sp., *Lionycteris spurrelli, Lonchorhina aurita*, *Lonchorhina* sp.*, Diphylla ecaudata*; *Glossophaga soricina,* Glossophaginae), um Rodentia (Cricetidae: *Rhipidomys* sp.) e um Squamata (Gekkonidae: *Thecadactylus rapicauda*).

Desta forma, no total foram encontrados 692 morfoespécies. Dentre essas, 13 espécies foram consideradas troglomórficas Gastropoda (Systrophiidae), Amblypygi (Charinidae: *Charinus* sp.), Araneae (Oonopidae; Tetrablemmidae: *Matta* sp.), Collembola (Cyphoderidae 2spp.; Isotomidae), Coleoptera (Dytiscidae; Eucnemidae; Scydmaenidae; Staphylinidae: Pselaphinae) e Polydesmida (Pyrgodesmidae 2spp.).

As Ordens de invertebrados mais ricas em espécies compreenderam Coleoptera (109 espécies – 16,32% da riqueza total observada), Hymenoptera (103 espécies – 15,42% da riqueza total observada), Diptera (87 espécies – 13,02% da riqueza total observada) e Aranae (79 espécies – 11,83% da riqueza total observada) (Figura 5.74). Dentre os vertebrados, as Ordens mais ricas em espécies compreenderam Chiroptera (14 espécies – 58,33% da riqueza total observada) e Anura (7 espécies – 29,17% da riqueza total observada) (Figura 5.75).

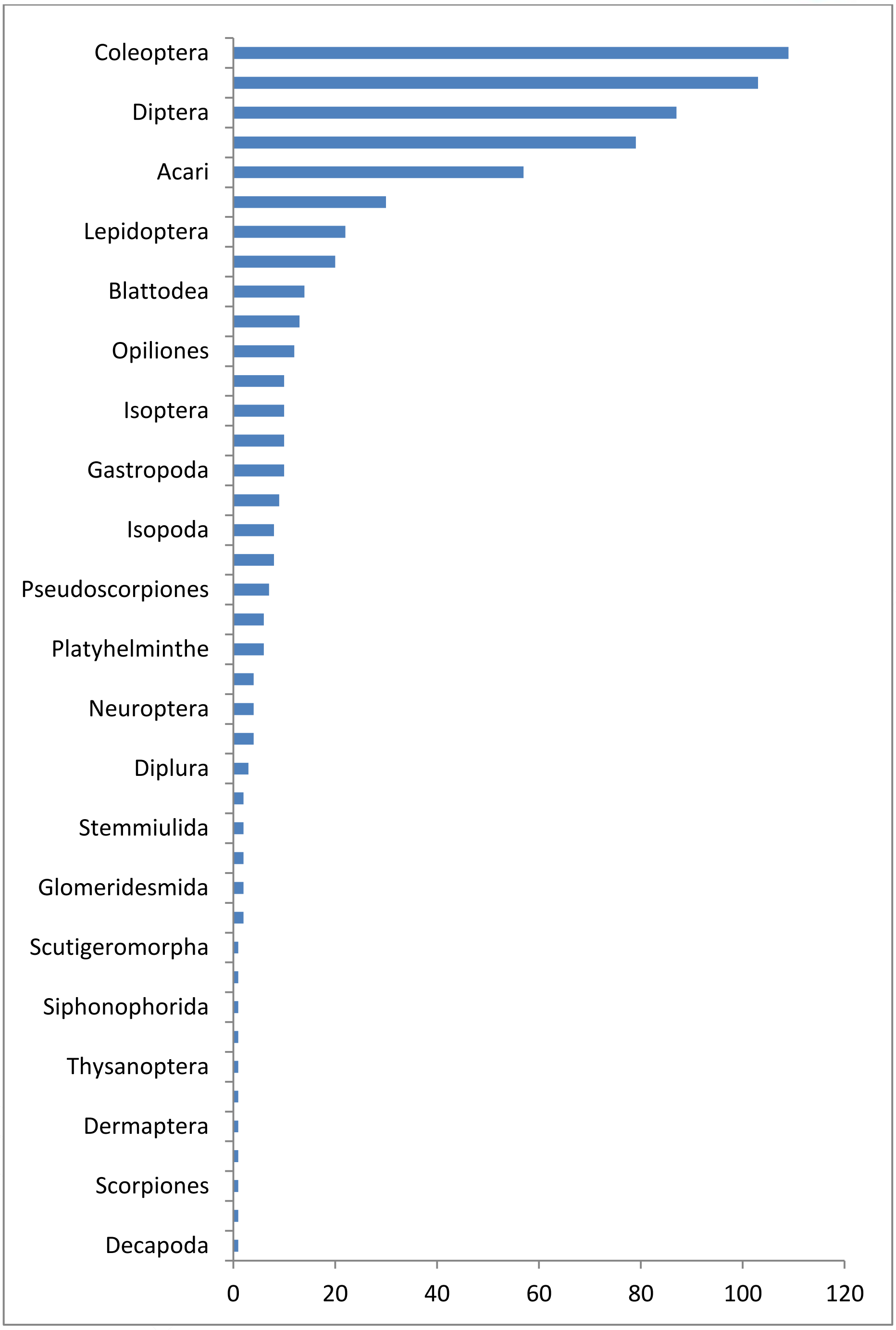

Figura 5.74 - Ordens encontradas e suas respectivas porcentagens em relação à riqueza total de invertebrados registrada nas cavernas.

Durante o inventário bioespeleológico das cavernas de Serra Leste foram identificadas 24 espécies de vertebrados. Diferentemente do observado para outro conjunto de cavernas da região, nas quais os anfíbios representaram o grupo com maior riqueza, os morcegos representaram o grupo mais amplamente distribuído entre as cavernas da região (Figura 5.75).

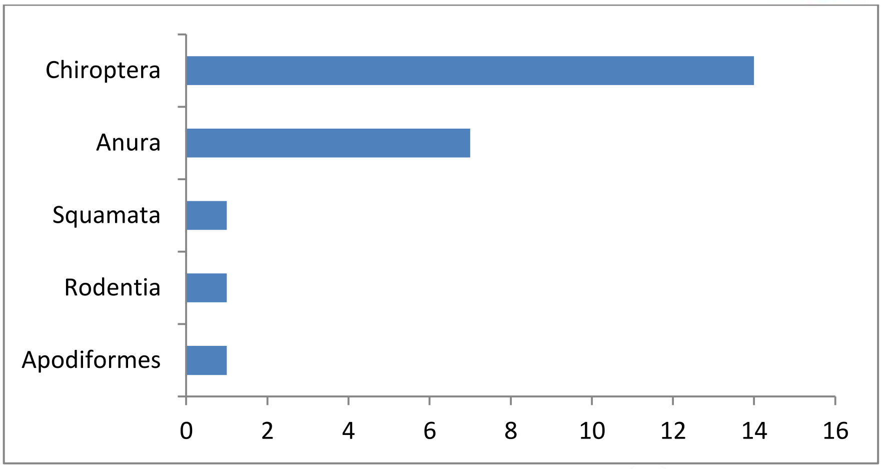

Figura 5.75 - Ordens encontradas e suas respectivas porcentagens em relação à riqueza total de vertebrados registrada nas cavernas.

Vários grupos troglóxenos e troglófilos podem ser destacados, seja pela riqueza incomum observada ou pela raridade em sistemas subterrâneos. Dentre estes, optou-se por exemplificar apenas algumas espécies.

Freqüentemente, as Ordens mais ricas em espécies em inventários de fauna subterrânea brasileira compreendem Coleoptera, Diptera e Araneae. Surpreendentemente, no presente trabalho, a Ordem Hymenoptera destacou-se como a segunda Ordem mais rica, com 103 espécies coletadas. Tal número "inesperado" deveu-se à participação da família Formicidae, que, sozinha, compreendeu 78 espécies (75,7% do total de himenópteros coletados).

Um grupo interessante encontrado em algumas cavernas compreende os Opilioacarida. Organismos desta Ordem conservam muitos aspectos morfológicos que fazem deles o grupo mais primitivo dentro dos ácaros que são encontrados, atualmente, vivendo sobre a terra (GRANDJEAN, 1936; KRANTZ & WALTER, 2009). Tal ordem encontra-se largamente distribuída ao longo de todas as zonas tropicais e temperadas do mundo. Oito de seus dez gêneros conhecidos estão presentes no Velho Mundo, e apenas dois, os gêneros *Neoacarus* e *Caribeacarus,* são encontrados nas Áméricas do Norte, Central e Sul (VÁZQUEZ & KLOMPEN, 2002; VÁZQUEZ & KLOMPEN, 2009).

Para a América do Sul, são conhecidas as espécies *Neoacarus ojastii* Lehtinen 1980 com ocorrência para a Venezuela, *Neoacarus platensis* Silvestri 1905 que ocorre na região sudeste do Brasil, Uruguai e Norte da Argentina (SILVESTRI, 1905; VAN DER HAMMEN, 1969; LEHTINEN, 1980; LECLERC, 1989) e *Neocarus potiguar* Bernardi, Zacarias e Ferreira 2012, encontrado em áreas cársticas do Rio Grande do Norte. Entretanto, a ocorrência de *Neoacarus* no Brasil é representada por inúmeras outras espécies ainda não descritas, distribuídas por todas as regiões do país, com a exceção da região sul (Vázquez & Klompen, 2002; Bernardi et al., 2009). Aparente, o número de espécies não descritas para o Brasil deve se aproximar de nove espécies até o momento (Bernardi, *comunicação pessoal*).

Outro grupo interessante encontrado em algumas cavernas compreende os palpígrados do gênero *Leptokoenenia*. São conhecidas somente duas espécies deste gênero: *Leptokoenenia scurra* e *Leptokoenenia gerlachi,* ambas habitantes de zonas intertidais, vivendo enterradas

nas areias de praias. Este compreende o primeiro registro deste gênero nas Américas, o que denota a extrema importância da área, visto que abriga uma nova espécie com hábitos completamente distintos dos observados nas outras espécies do gênero.

5.2.2. *Espécies troglomórficas*

Foram encontradas 13 espécies consideradas troglomórficas: Gastropoda (Systrophiidae), Amblypygi (Charinidae: *Charinus* sp.), Araneae (Oonopidae; Tetrablemmidae: *Matta* sp.), Collembola (Cyphoderidae 2spp.; Isotomidae), Coleoptera (Dytiscidae; Eucnemidae; Scydmaenidae; Staphylinidae: Pselaphinae) e Polydesmida (Pyrgodesmidae 2spp.). Algumas espécies troglóbias serão destacadas a seguir.

Um interessante registro compreende a espécie troglomórfica do gênero *Charinus*, encontrada em algumas cavernas da área. O gênero *Charinus* Simon, 1892 atualmente inclui 23 espécies descritas na região Neotropical, das quais sete são conhecidas para o Brasil (Weygoldt, 1972, 2000; Quintero, 1983; Giupponi & Kury, 2002; Baptista & Giupponi, 2002). Na América do Sul, estes organismos são dificilmente observados, em função de seus hábitos crípticos. No Brasil, a maioria das espécies abriga-se em espaços sob rochas ou troncos nas florestas tropicais, sendo ativos à noite. No Brasil, conhecem-se oficialmente somente duas espécies troglóbias: *Charinus troglobius* Baptista & Giupponi, 2002 (da Bahia), e *Charinus eleonorae* Baptista & Giupponi, 2003 (de Minas Gerais). Desta forma, percebe-se claramente a importância desta espécie troglomórfica presente em cavernas da área (certamente ainda não descritas pela ciência). Cabe ressaltar que outras espécies troglomórficas deste gênero foram encontradas em um inventário realizado em outras 98 cavernas presentes na mesma região (SL-006, SL-009, SL-010, SL-014, SL-022, SL-023, SL-027, SL-031, SL-035, SL-042, SL-044, SL-045, SL-050, etc) (CARSTE, 2011a). Além disso, destaca-se que nas outras duas espécies troglóbias brasileiras, os ocelos mesiais sofreram regressão estrutural (apresentando-se reduzidos em *Charinus eleonorae* e ausentes em *Charinus troglobius*). Nas espécies troglóbias de Serra Leste, as séries oculares que sofreram regressão foram as laterais, diferentemente do que ocorreu com as previamente citadas. As ilustrações referentes a esta espécie compreendem a Figura 5.76.

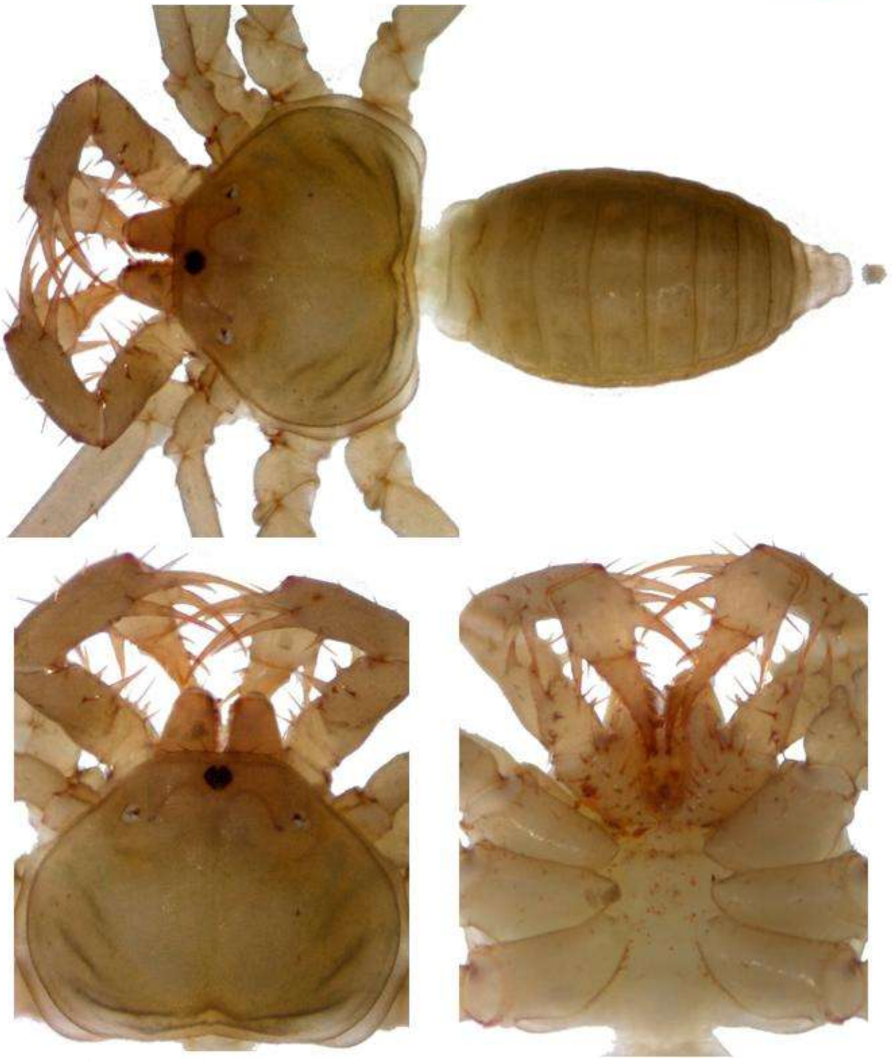

Figura 5.76 - Espécie troglomórfica do gêmero Charinus encontrada em cavernas da área.

Além disso, foram observadas também aranhas troglomórficas da família Oonopidae (Figura 5.77) e Tetrablemmidae (Figura 5.78).

A família Oonopidae é formada por pequenas aranhas haplóginas (1-3 mm), errantes e habitantes de regiões de clima tropical e subtropical (OTT, 2003). São encontradas na camada de serapilheira e embaixo de pedras, mas também constituem um componente significativo da fauna de aranhas que vivem no dossel das florestas tropicais. Há ainda três gêneros Afrotropicais (Anophthalmoonops sp., Caecoonops sp., Termitoonops sp.) exclusivamente encontrados em cupinzeiros, que são anoftálmicos (PLATNICK, 2010). Atualmente, são conhecidas 487 espécies e 72 gêneros, com representantes em todos os continentes (PLATNICK, 2010). Geralmente, apresentam seis olhos, sendo que os olhos anteriores medianos são ausentes. Entretanto, há espécies com quatro (*Opopaea viamao*), dois (e.g. Coxapopha sp., Diblemma sp.) olhos e outras anoftalmicas (Blanioonops patellaris: espécime troglóbia do leste africano; Cousinea keeleyi: Seychelles) (OTT, 2003; PLATNICK 2010). Assim, para esta família, a anoftalmia dos espécimes (que não estavam associados à cupinzeiros), a

redução da pigmentação tegumentar e o alongamento das tricobótrias foram considerados troglomorfismos válidos (Figura 5.77).

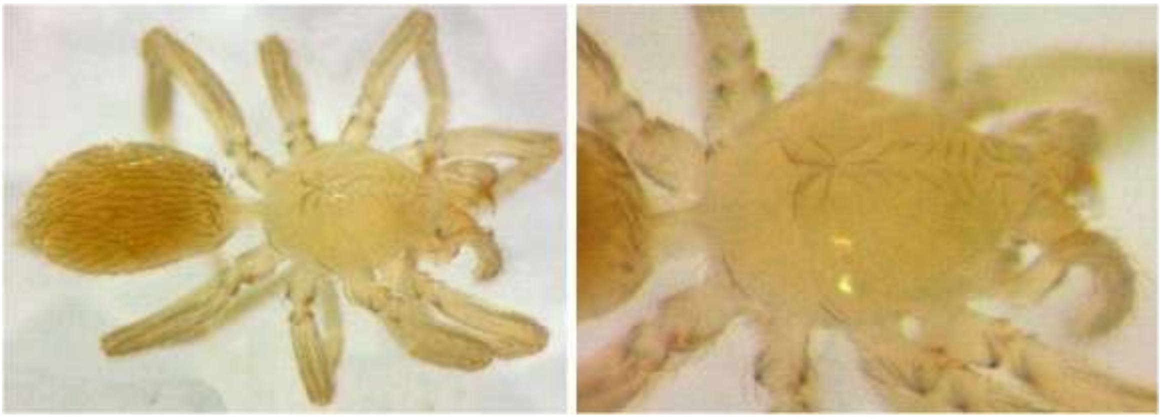

Figura 5.77 - Espécie troglomórfica da família Oonopidae encontrada em cavernas da área.

A família Tetrablemmidae compreende pequenas aranhas que ocorrem em regiões tropicais de todo o mundo. Esta família possui cerca de 127 espécies descritas, distribuídas em 30 gêneros. São vulgarmente conhecidas como *"armored spiders"*, cuja tradução seria "aranhas blindadas". Desta forma, percebe-se que tais organismos frequentemente possuem o exoesqueleto bastante rígido e quitinizado. A família *Pacullidae*, com seu único gênero *Paculla*, foi incorporada à Tetrablemmidae em 1981.

A maioria das espécies de Tetrablemmidae foi coletada em serrapilheira ou solo, embora algumas espécies vivam em cavernas. As espécies cavernícolas frequentemente apresentam típicas modificações ao modo de vida subterrâneo, como perda dos olhos e fraca esclerotização. Tais características, no entanto, já foram observadas em algumas espécies habitantes de solo. As Tetrablemmidae aparentemente não constroem teias. No Brasil, ocorrem pelo menos dois gêneros: Matta Crosby, 1934 (Brazil, México) e Monoblemma Gertsch, 1941 (Brazil, Colombia, Panamá). A espécie encontrada em cavernas de Serra Leste apresenta troglomorfismos claros, como redução da esclerotização do exoesqueleto, redução da pigmentação e ausência de olhos (Figura 5.78).

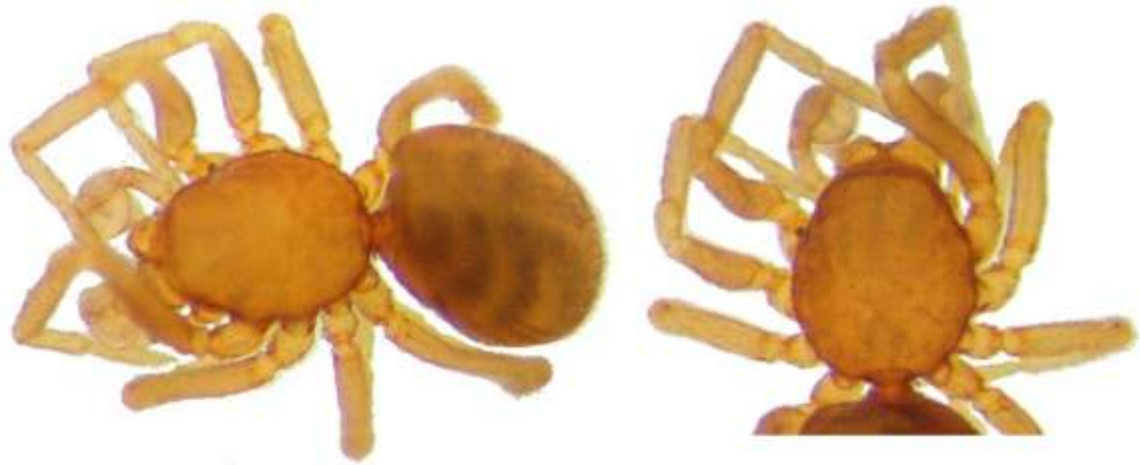

Figura 5.78 - Espécie troglomórfica do gênero Matta (família Tetrablemmidae) encontrada em cavernas da área.

Diplópodes são muitas vezes os componentes mais abundantes de comunidades terrestres detritivoras em cavernas. Polydesmida é a ordem de Diplopoda com maior número de famílias (cerca de 30 famílias). São notáveis por seus segmentos, geralmente em número aproximado de 20, com extensões laterais no lado dorsal do corpo e contendo glândulas que, muitas vezes, secretam cianeto (MAURIÈS, 2004). Todas as espécies da ordem não possuem olhos. Algumas espécies troglóbias de Polydesmida têm sido descritas nas Américas, Europa, Japão, México e Brasil. Os troglomorfismos mais evidentes são a despigmentação do tegumento, descalcificação da cutícula e o alongamento das antenas e apêndices locomotores (SHEAR, 1969; HOLSINGER & CULVER, 1988; GOLOVATCH & WYTWER, 2004; LEWIS, 2005; GOLOVATCH *et al*., 2006).

Embora a sinopse da fauna de cavernas brasileiras (PINTO-DA-ROCHA, 1995) inclua um grande número de referências a diplópodos, muito pouco tem sido registrado em níveis inferiores a ordem ou família (TRAJANO *et al*., 2000).

Pyrgodesmidae é uma família de Polydesmida pouco registrada em cavernas brasileiras. Diplópodos Pyrgodesmidae foram coletados em cavernas de Altamira/Itaiatuba, Bambuí, Vale do Ribeira e áreas graníticas. Alguns deles podem ser considerados troglóbios, apesar de que a maioria dos espécimes coletados mostra-se bem pigmentada. Existe atualmente, somente uma espécie troglombia descrita (Yporangiella stygius Schubart, 1946) (TRAJANO *et al*., 2000). Alguns Pyrgodesmidae (Polydesmida) troglomórficos de Serra Leste podem ser visualizados na Figura 5.79 e na Figura 5.80.

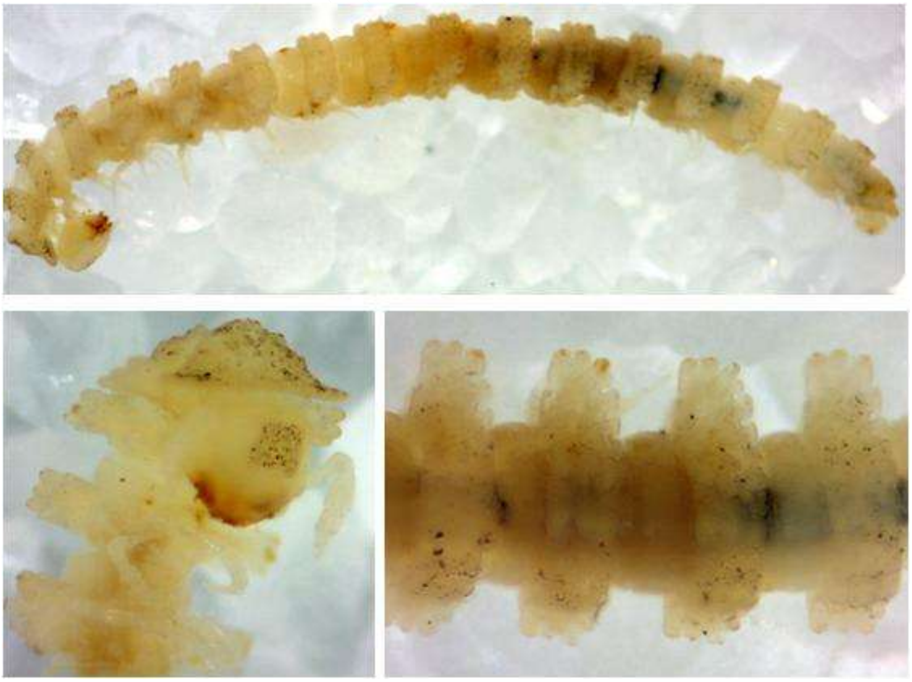

Figura 5.79 - Pyrgodesmidae troglomórfico encontrado em cavernas da área.

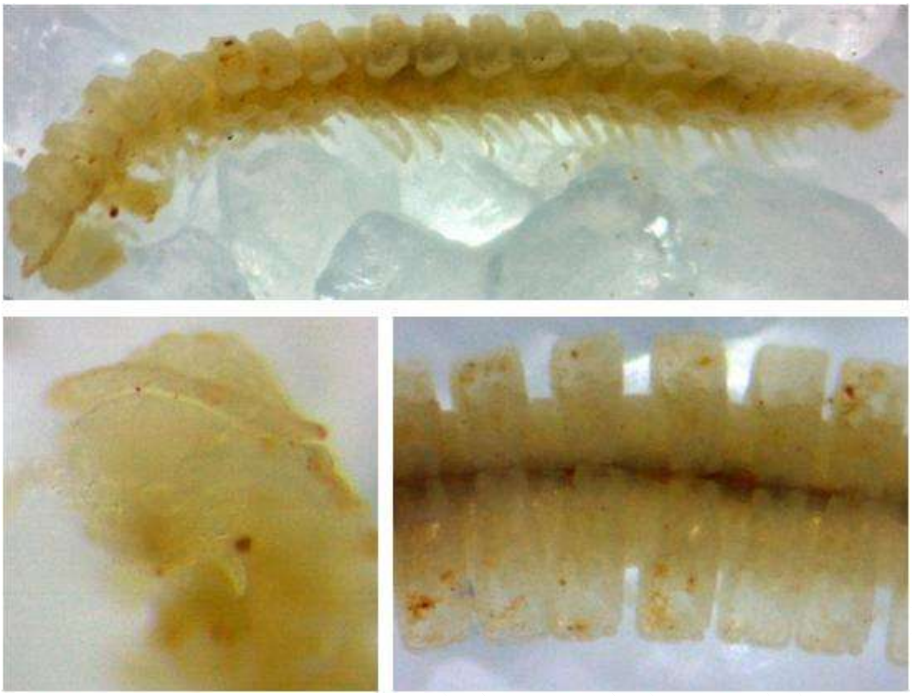

Figura 5.80 - Pyrgodesmidae troglomórfico encontrado em cavernas da área.

Das aproximadamente 40 famílias da ordem Coleoptera, 15 têm espécies exclusivamente subterrâneas. Dryopidae, Dytiscidae, Elmidae, Hydrophilidae e Noteridae aquáticos, bem como Carabidae, Cholevidae, Curculionidae, Histeridae, Pselaphidae, Staphylinidae, Merophysidae, Ptiliidae, Scydmaenidae e Tenebrionidae terrestres (MOLDOVAN, 2004).

A ordem Coleoptera é incrivelmente bem sucedida em cavernas com aproximadamente 261 espécies em 21 gêneros (SPLANGER & DECU, 1998; CULVER & WHITE, 2004). Os troglomorfismos mais evidentes para a ordem são a despigmentação do tegumento, potencialização de orgãos sensoriais, redução de olhos, redução de asas e perda da capacidade de voar, mudança de hábitos locomotores e de trocas gasosas e mudanças de ambiente usado para empupar (SPLANGER & DECU, 1998).

Uma das primeiras mudanças morfológicas que ocorrem entre estes insetos durante a colonização das cavernas é a perda de pigmentação. A cutícula se torna mais fina, e a cor muda para um vermelho-amarronzado. A característica morfológica mais evidente é a falta ou redução dos olhos. Alongamento do corpo e antenas. As asas são completamente perdidas em algumas espécies, e os élitros se fundem. Além disso, em espécies altamente especializadas, sob os élitros está localizado um compartimento contendo ar para a regulação da umidade, causando uma pseudofisograstria. As alterações anatômicas internas são principalmente devido à escassez de alimentos. Espécies mais especializadas desenvolveram um corpo alargado contendo vesículas de gordura, proteínas e glicogênio que permitem a sobrevivência durante vários meses de jejum (MOLDOVAN, 2004).

Dytiscidae é uma família de coleópteros com cerca de 160 géneros e 4000 espécies distribuidas em todas as regiões do mundo. É um dos maiores e mais comuns grupos de besouros aquáticos. Tanto os adultos quanto as larvas são predadores, atacando uma grande

variedade de pequenos organismos. Embora a maioria das espécies sejam pequenas e de médio porte, alguns adultos podem atingir um comprimento de 35 mm.

Maior biodiversidade do mundo de coleópteros troglóbios aquáticos é de Dytiscidae. Tem sido relatadas recentemente para águas subterrâneas na zona árida do interior da Austrália 54 espécies (LEYS *et al*.,2003). Para o Brasil ainda não existem espécies troglóbias de Dytiscidae descritas.

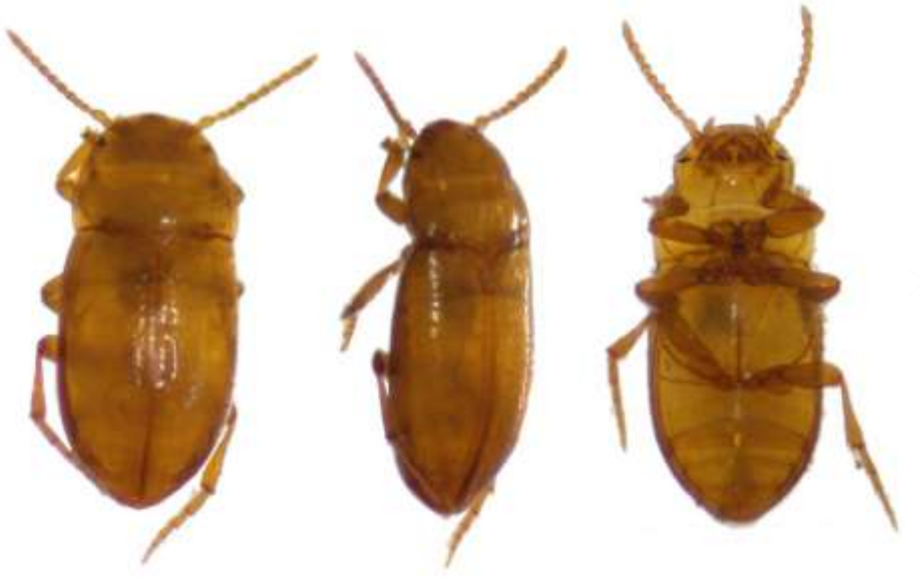

Figura 5.81 - Coleóptero troglomórfico da família Dyticidae encontrado em cavernas da área (reparar a despigmentação e pronunciada recução ocular).

Eucnemidae é uma família de Coleoptera pertencente à superfamília Elateroidea, cujos representantes são conhecidos como falsos vaga-lumes. São besouros alongadas que possuem pequenas projeções para trás da cabeça, nos cantos laterais do escudo cefálico (pronoto). Eles são um pouco achatados e variam em tamanho e cor. As espécies variam de 0,4 a 1 centímetro de comprimento. A maioria das espécies é marrom ao preto na cor, embora alguns tenham cores avermelhadas e amareladas. Quando colocados de costas no chão, esses besouros estalam os segmentos do tórax (protórax e mesotórax) para fazer com que seus corpos sejam lançados e assumam a posição de ventre para o chão. Não existem espécies troglóbias descritas no mundo. As imagens da espécie troglomórfica encontrada em Serra Leste são mostradas na Figura 5.82.

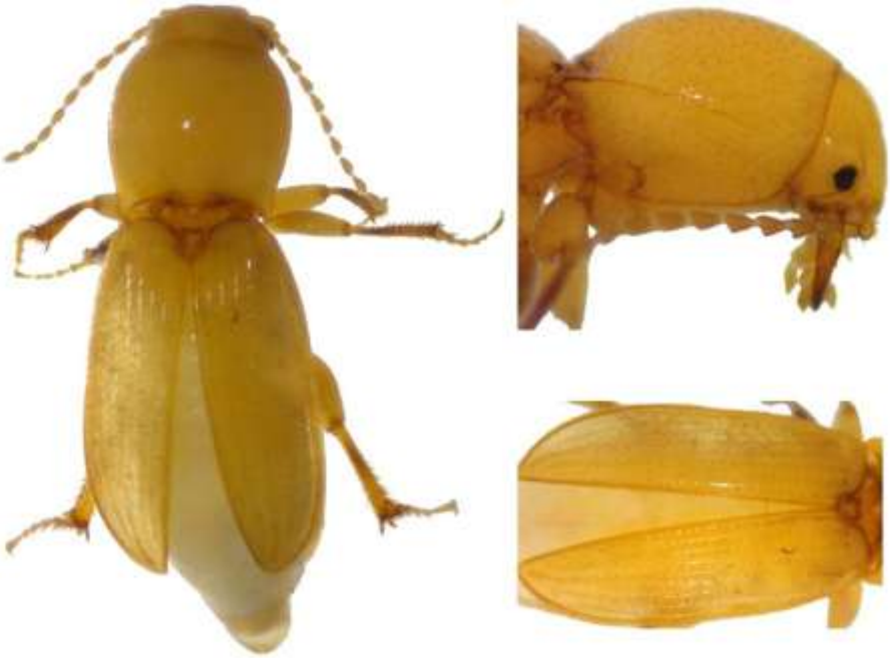

Figura 5.82 - Coleóptero troglomórfico da família Eucnemidae encontrado em cavernas da área (reparar a despigmentação e pronunciada redução ocular).

Pselaphidae são coleópteros terrestres com antenas mais ou menos alongadas, palpos maxilares e labiais bem desenvolvidos élitros truncados, muito curtos, cobrindo apenas os dois primeiros tergitos; os demais, descobertos, mais ou menos fundidos. Encontram-se os Pselafideos sob pedras ou sob a casca e ai se alimentam principalmente de ácaros. Há também espécies cavernicolas. Aproximadamente 30 gêneros de Pselaphidae têm representantes troglóbios no mundo (PARK, 1960; HAMILTON-SMITH, 1965; POGGI *et al.,* 1998; MOLDOVAM, 2004). Existe a citaçao de uma espécie de Pselaphidae troglomorfico para a caverna Santana em Iporanga, São Paulo (PINTO-DA-ROCHA, 1995). Entretanto, ainda não existem espécies troglóbias descritas para o Brasil. As imagens da espécie troglomórfica encontrada em Serra Leste são mostradas na Figura 5.83.

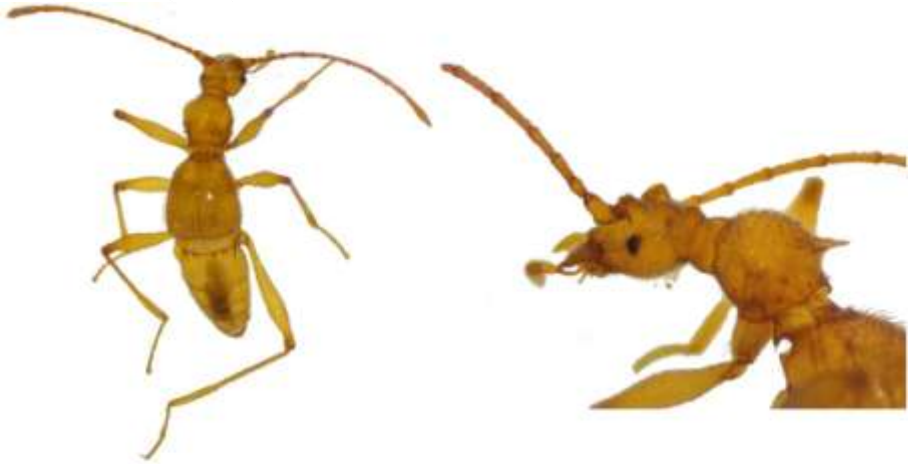

Figura 5.83 - Coleóptero troglomórfico da família Pselaphidae encontrado em cavernas da área (reparar a despigmentação e pronunciada redução ocular).

Scydmaenidae é uma família de pequenos besouros que ocorrem no mundo inteiro com cerca de 4.500 espécies em cerca de 80 gêneros. Muitas espécies têm um estreitamento entre a cabeça e o tórax e tórax e abdômen, resultando em uma semelhança passageira com formigas que inspira o seu nome comum. Os maiores indivíduos medem apenas 3 milímetros de comprimento, enquanto algumas espécies muito pequenas só atingem meio milímetro de comprimento. Scydmaenidae geralmente vivem na serapilheira e troncos apodrecidos em florestas, preferindo habitats úmidos. Alguns tipos são conhecidos para se alimentar de ácaros oribatídeos (DECU *et al.*, 1998). Poucas são as espécies de Scydmaenidae troglóbias ao longo do mundo (VÍT & HLAVÁ, 2005). Não existem espécies troglóbias descritas para o Brasil.

Euconnus (Tetramelus) bazgoviensis uma espécies troglóbia da Croácia apresenta anoftalmia, apterismo e corpo e antenas delgados (VÍT & HLAVÁ, 2005). As imagens da espécie troglomórfica encontrada em Serra Leste são mostradas na Figura 5.84.

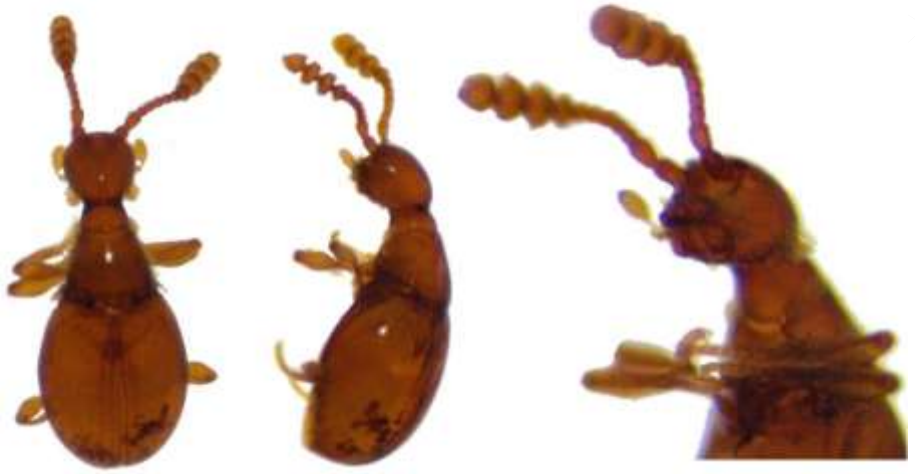

Figura 5.84 - Coleóptero troglomórfico da família Scydmaenidae encontrado em cavernas da área (reparar a despigmentação e pronunciada redução ocular).

Os colêmbolos são pequenos artrópodes (0,2 a 3mm), ápteros e hexápodes, presentes em todo o mundo, em habitats variados, podendo viver no folhiço, na água, no solo, em árvores, em troncos em decomposição, em cavernas, entre outros. Atualmente, são conhecidas mais de 7.500 espécies, em cerca de 600 gêneros de 30 famílias distintas. Para o Brasil, o número total de colêmbolos registrados é de 209 espécies, distribuídas em 19 famílias e 82 gêneros (ZAPPELINI & BELLINI, 2004).

Geralmente apresentam oito pequenas lentes semelhantes a omatídeos, que formam uma mancha ocular, com frequência fortemente pigmentada, em cada lado da cabeça (ZAPPELINI & BELLINI, 2004). Entretanto, existem espécies de colêmbolos anoftálmicas e despigmentadas, dependendo das condições ambientais do local onde habitam. Assim, como muitas espécies deste grupo são habitantes de solo, estas podem apresentar morfologia endogeomórfica.

Desta forma, para Collembola, o mais confiável diagnóstico refere-se ao alongamento da unguis e a redução do *"tenent hair".* Dependendo do comprimento das estruturas

anteriormente citadas considera-se a espécie como troglóbia, assumindo-se que esta mudança no tamanho (especialmente o alongamento da unguis) está ligada à evolução no meio subterrâneo, em função de melhorar a deambulação sobre substratos encharcados (CHRISTIANSEN, 1985).

Apesar de não serem adaptados à vida na água, muitos colêmbolos desenvolveram mecanismos adaptativos para explorar superfície da água ou a película d'gua em ambientes extremamente úmidos (DEHARVENG *et al*., 2008). Algumas características foram essenciais para tal adaptação: Primeiro, os colêmbolos apresentam uma cutícula extremamente repelente a água, o que lhes permite flutuar pela superfície (NOBLE-NESBITT, 1963). Assim, eventos de inundação podem atuar como um agente importante para a dispersão de colêmbolos (DEHARVENG *et al*., 2008). Segundo, garras e mucro são, geralmente, extremamente modificados em colêmbolos hidrófilos. As garras tendem a se tornar finas e alongadas em comparação aos colêmbolos de ambientes secos (DEHARVENG *et al*., 2008). Esta tendência é observada em muitas espécies que vivem em habitats encharcados (ao longo de riachos, em torno das lagoas, em cavernas), independente do grupo a qual pertencem (CHRISTIANSEN, 1961; DEHARVENG, 1988; D'HAESE, 2003). O alongamento da garra é considerado um caráter adaptativo que aparece de forma independente em linhagens filogeneticamente distintas. Christiansen (1965) investigou a sua base funcional, e mostrou que tais adaptações possibilitaram que colêmbolas cavernícolas deslocassem sobre a água. Outra adaptação morfológica (limitada a Symphypleona e Neelipleona) consiste em um considerável alargamento do mucro (DEHARVENG *et al*., 2008). As imagens das espécies troglomórficas encontradas em Serra Leste são mostradas na Figura 5.85.

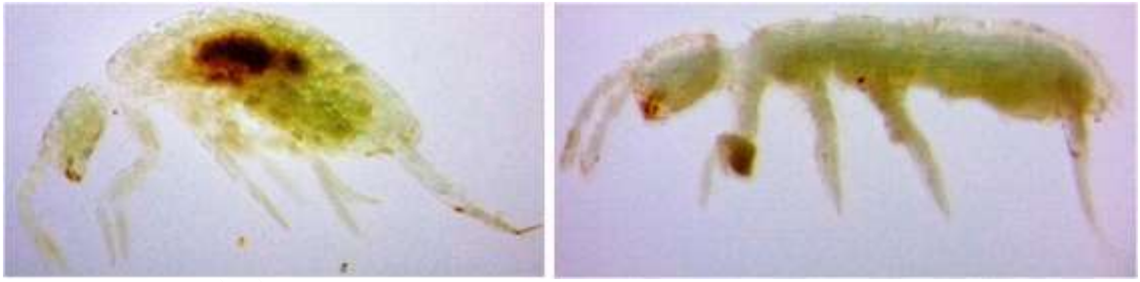

Figura 5.85 - Colêmbolos troglomórficos encontrados em cavernas da área.

5.2.3. Descrição específica de cada cavidade

CAVIDADE SL-101

Grande cavidade em quartzito com 460m de projeção horizontal localizada na parte superior da encosta na Serra do Sereno. O entorno é composto por vegetação arbórea com árvores de médio porte e sub-bosque de baixa densidade com vegetação arbustiva além de muitas gramíneas, lianas e pteridófitas. A caverna possui apenas uma entrada, sendo esta ampla e com piso levemente em declive, por onde ocorre importação de recursos orgânicos (serrapilheira). Estes são carreados através de transporte eólico ou gravitacional e pela água,

através de enxurradas. A entrada é sombreada, com muitos liquens, briófitas e pteridófitas revestindo as paredes e pisos. Trata-se de uma cavidade úmida, com muitos pontos de gotejamento, áreas de percolação e pequenas drenagens ativas durante o período úmido. Já no período seco foram observados apenas alguns pontos de gotejamento e percolações. O piso é irregular, em grande parte coberto por areia e guano, com muitos seixos e blocos abatidos. O sistema radicular é bem desenvolvido, sendo este composto por raízes de médio porte na entrada e muitas micro-raízes amplamente distribuídas pelo restante da cavidade. Em algumas áreas, há grande aglomeração de raízes no teto. A serrapilheira está restrita a linha d'água. Uma grande colônia de morcegos frugívoros/nectarívoros, formada por centenas de indivíduos ainda é visível na campanha seca. Esses morcegos são responsáveis pela produção elevada de guano, sendo este, um importante recurso trófico no sistema, estando presente em quase toda área do piso à esquerda da cavidade. Quase toda a caverna é afótica, com áreas de penumbra clara e escura próxima a região da entrada. De forma geral trata-se de uma cavidade com grande área, alta diversidade de recursos alimentares e com baixa influência das condições ambientais epígeas.

Caracterização faunística no período de seca

Foi observado na caverna, um total de 101 morfoespécies de invertebrados de pelo menos 47 famílias dos Taxa: Oligochaeta, Gastropoda (Systrophiidae), Isopoda (Armadillidae; Scleropactidae), Acari (Mesostigmata: Uropodina; Oribatida; Actinedida: Rhagidiidae), Amblypygi (Phrynidae: *Heterophrynus longicornis*); Pseudoscorpiones (Chernetidae), Opiliones (Escadabiidae), Araneae (Corinnidae; Linyphiidae; Ochyroceratidae: *Ochyrocera* sp.; Oonopidae: *Oonops* sp.; Pholcidae: *Mesabolivar* sp.; Ninetinae; Scytodidae: *Scytodes itapevi*; Theridiosomatidae: *Plato* sp.), Thysanura (Nicoletiidae: Nicoletiinae, Atelurinae), Diplura (Campodeidae), Collembola (Bourletiellidae; Cyphoderidae; Entomobryidae), Orthoptera (Phalangopsidae: *Phalangopsis* sp., *Uvaroviella* sp.), Blattodea (Blattidae; Polyphagidae), Isoptera (Termitidae: *Nasutitermes* sp.), Psocoptera (Pachytroctidae), Heteroptera (Cydnidae), Homoptera (Cixiidae: *Pintalia* spp.), Lepidoptera (Tineidae; Tortricidae), Diptera (Ceratopogonidae; Drosophilidae; Milichiidae; Muscidae;; Sciaridae; Psychodidae: *Lutzomyia* sp.; Streblidae), Hymenoptera (Formicidae: *Camponotus* sp., *Carebara* sp., *Hypoponera* sp., *Leptogenys* sp., *Neivamyrmex* sp., *Pachycondyla* sp., *Rogeria* spp., *Solenopsis* spp., *Strumigenys* sp., *Tapinoma* sp.; Diapriidae; Scelionidae), Coleoptera (Carabidae; Dermestidae; Elateridae: Cardiophorinae; Histeridae; Staphylinidae: Pselaphinae sp.; Scydmaenidae), Polydesmida (Pyrgodesmidae), Geophilomorpha (Geophilidae: *Schizonampa* sp.), Scolopendromorpha (Scolopendridae: *Cryptops* sp.) e Symphyla (Scutigerellidae: *Hanseniella* sp.).

Dentre os vertebrados, foram encontradas duas espécies de Anura (Bufonidae: *Rhinella* sp.; Strabomantidae: *Pristimantis* sp.) e sete espécies de Chiroptera (Emballorunidae: *Peropteryx kappleri*; Mormoopidae: *Pteronotus parnellii*; Phyllostomidae: *Carollia perspicillata, Anoura geoffroyi*, *Lionycteris spurrelli*, *Lonchorhina aurita*, *Diphylla ecaudata*).

Desta forma, no total foram encontrados 110 morfoespécies. Dentre essas, quatro foram consideradas troglomórficas: Gastropoda (Systrophiidae), Collembola (Cyphoderidae), Coleoptera (Staphylinidae: Pselaphinae) e Polydesmida (Pyrgodesmidae).

Caracterização faunística no período de chuva

Foi observado na caverna, um total de 130 morfoespécies de invertebrados de pelo menos 67 famílias dos Taxa: Oligochaeta, Gastropoda (Subulinidae; Systrophiidae), Platyhelminthes (Tricladida, Isopoda (Armadillidae; Philosciidae; Scleropactidae), Acari (Mesostigmata: Uropodina, Ameroseiidae; Cunaxidae: *Armascirus* sp.; Actinedida: Parasitengonina, Rhagidiidae; Sarpoptiforme: *Tyrophagus* sp.), Amblypygi (Phrynidae: *Heterophrynus longicornis*), Pseudoscorpiones (Chernetidae; Chthoniidae; Olpiidae), Opiliones (Escadabiidae), Araneae (Corinnidae; Ctenidae; Ochyroceratidae: *Ochyrocera* sp., *Speocera* sp.; Oonopidae: *Oonops* sp.; Pholcidae: *Mesabolivar* sp., *Metagonia* sp., Ninetinae; Prodidomidae: *Lygromma* sp.; Scytodidae: *Scytodes itapevi, Scytodes* sp.; Tetrablemmidae: *Matta* sp.; Theridiosomatidae: *Plato* sp.), Thysanura (Nicoletiidae: Nicoletiinae, Atelurinae), Diplura (Campodeidae; Anajapygidae), Collembola (Bourletiellidae; Entomobryidae; Isotomidae), Orthoptera (Phalangopsidae: *Phalangopsis* sp., *Uvaroviella* sp.), Blattodea (Blattidae; Polyphagidae), Embiidina; Isoptera (Termitidae: *Nasutitermes* sp.), Dermaptera, Psocoptera (Pachytroctidae), Heteroptera (Cydnidae), Homoptera (Cixiidae: *Pintalia* sp.; Derbidae), Lepidoptera (Coleophoridae; Tineidae), Diptera (Ceratopogonidae; Culicidae; Drosophilidae; Empididae; Keroplatidae; Milichiidae; Muscidae; Sciaridae; Psychodidae: *Lutzomyia* sp.; Streblidae), Hymenoptera (Formicidae: *Amblyopone* sp., *Camponotus* sp., *Carebara* sp., *Cephalotes* sp., *Hypoponera* sp., *Leptogenys* sp., *Odontomachus* sp., *Pachycondyla* sp., *Rogeria* sp., *Solenopsis* spp., *Strumigenys* spp., Myrmicinae; Diapriidae; Eulophidae; Scelionidae), Coleoptera (Alleculidae; Carabidae; Chrysomelidae: Alticinae; Dytiscidae; Elateridae: Cardiophorinae; Histeridae; Ptiliidae; Scarabaeidae; Scydmaenidae; Staphylinidae: Pselaphinae), Polydesmida (Chelodesmidae, Pyrgodesmidae), Spirostreptida (Pseudonannolenidae), Geophilomorpha (Geophilidae: *Schizonampa* sp.) e Scutigeromorpha (Pselliodidae: *Sphendononema guildingii*).

Dentre os vertebrados, foi encontrada uma espécie de Anura (Bufonidae) e sete de Chiroptera (Emballorunidae: *Peropteryx kappleri;* Furipteridae: *Furipterus horrens*; Phyllostomidae: *Carollia perspicillata, Anoura geoffroyi, Lonchorhina* sp., *Diphylla ecaudata, Lionycteris spurrelli).*

Desta forma, no total foram encontrados 138 morfoespécies. Dentre essas, cinco foram consideradas troglomórficas: Gastropoda (Systrophiidae), Araneae (Tetrablemmidae: *Matta* sp.), Collembola (Isotomidae), Coleoptera (Staphylinidae: Pselaphinae) e Polydesmida (Pyrgodesmidae).

Caracterização geral da fauna da cavidade

Foi observado na caverna, um total de 167 morfoespécies de invertebrados de pelo menos 72 famílias dos Taxa: Oligochaeta, Gastropoda (Subulinidae; Systrophiidae), Platyhelminthes (Tricladida), Isopoda (Armadillidae; Philosciidae; Scleropactidae), Acari (Mesostigmata: Uropodina, Ameroseiidae; Cunaxidae: Armascirus sp.; Oribatida; Actinedida: Parasitengonina, Rhagidiidae; Acaridae: Tyrophagus sp.), Amblypygi (Phrynidae: Heterophrynus longicornis), Pseudoscorpiones (Chernetidae; Chthoniidae; Olpiidae), Opiliones (Escadabiidae), Araneae (Corinnidae; Ctenidae; Linyphiidae; Ochyroceratidae: Ochyrocera sp., Speocera sp.;

Oonopidae: *Oonops* sp.; Pholcidae: *Mesabolivar* sp., *Metagonia* sp., Ninetinae; Prodidomidae: *Lygromma* sp.; Scytodidae: *Scytodes itapevi*, *Scytodes* sp.; Tetrablemmidae: *Matta* sp.; Theridiosomatidae: *Plato* sp.), Thysanura (Nicoletiidae: Nicoletiinae, Atelurinae), Diplura (Campodeidae; Anajapygidae), Collembola (Bourletiellidae; Cyphoderidae; Entomobryidae; Isotomidae), Orthoptera (Phalangopsidae: *Phalangopsis* sp., *Uvaroviella* sp.), Blattodea (Blattidae; Polyphagidae), Embiidina, Isoptera (Termitidae: *Nasutitermes* sp.), Dermaptera, Psocoptera (Pachytroctidae), Heteroptera (Cydnidae), Homoptera (Cixiidae: *Pintalia* sp.; Derbidae), Lepidoptera (Coleophoridae; Tineidae; Tortricidae), Diptera (Ceratopogonidae; Culicidae; Drosophilidae; Empididae; Keroplatidae; Milichiidae; Muscidae; Sciaridae; Psychodidae: *Lutzomyia* sp.; Streblidae), Hymenoptera (Formicidae: *Amblyopone* sp., *Camponotus* spp., *Carebara* sp., *Cephalotes* sp., *Hypoponera* sp., *Leptogenys* spp., *Neivamyrmex* spp., *Odontomachus* sp., *Pachycondyla* sp., *Rogeria* spp., *Solenopsis* spp., *Strumigenys* spp., *Tapinoma* sp., Myrmicinae; Diapriidae; Eulophidae; Scelionidae), Coleoptera (Alleculidae; Carabidae; Chrysomelidae: Alticinae; Dermestidae; Dytiscidae; Elateridae: Cardiophorinae; Histeridae; Ptiliidae; Scarabaeidae; Scydmaenidae; Staphylinidae: Pselaphinae), Polydesmida (Chelodesmidae; Pyrgodesmidae), Spirostreptida (Pseudonannolenidae), Geophilomorpha (Geophilidae: *Schizonampa* sp.), Scolopendromorpha (Scolopendridae: *Cryptops* sp.), Scutigeromorpha (Pselliodidae: *Sphendononema guildingii*) e Symphyla (Scutigerellidae: Hanseniella sp.).

Desta forma, no total foram encontrados 180 morfoespécies. Dentre essas, seis foram consideradas troglomórficas: Gastropoda (Systrophiidae), Araneae (Tetrablemmidae: *Matta* sp.), Collembola (Cyphoderidae; Isotomidae), Coleoptera (Staphylinidae: Pselaphinae) e Polydesmida (Pyrgodesmidae). Alguns organismos encontrados nesta caverna são mostrados na Figura 5.86.

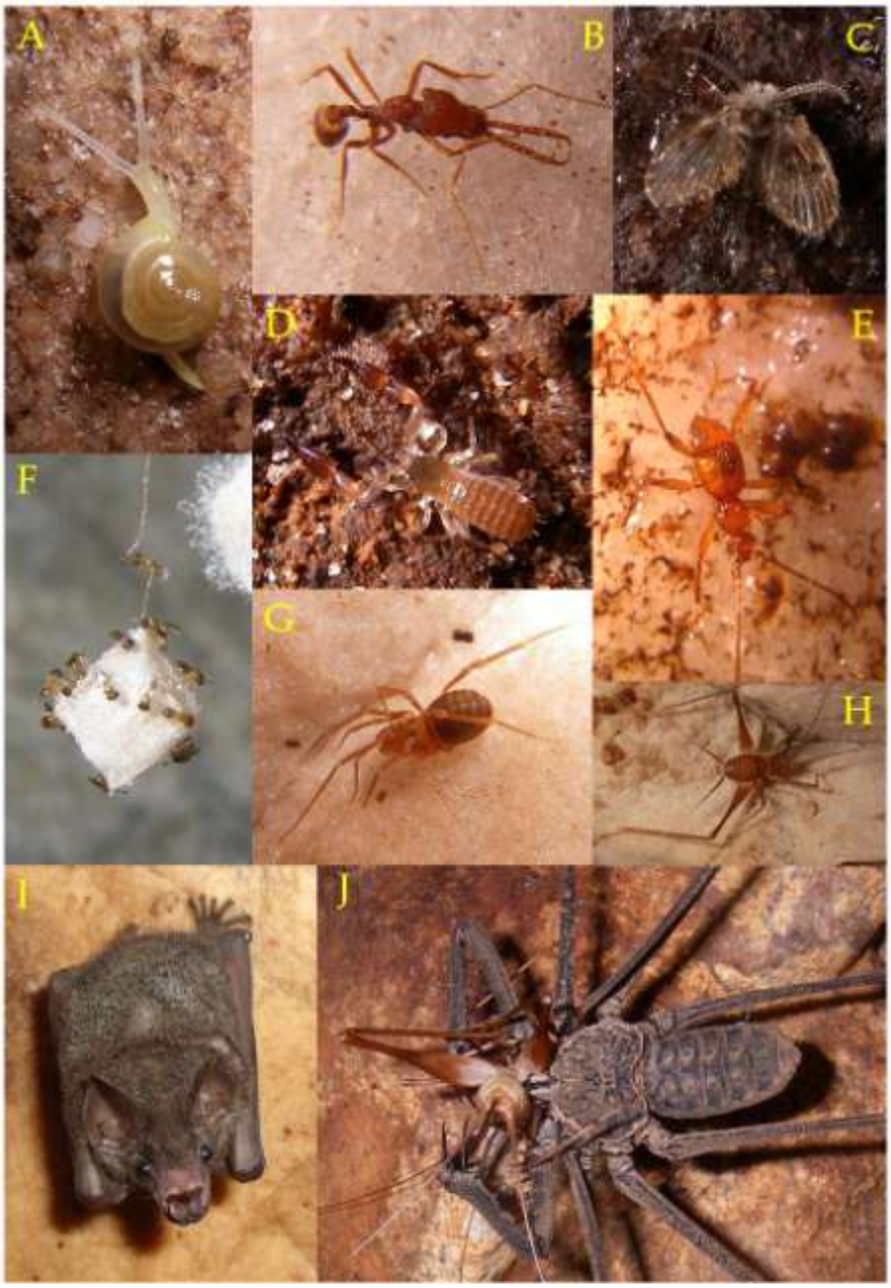

Figura 5.86 - A) Gastropoda: Systrophiidae; B) Formicidae: *Strumigenys* sp.; C) Diptera: Psychodidae; D) Pseudoscorpiones:Olpiidae; E) Coleoptera: Scydmaenidae; F) Hymenoptera: Scelionidae; G) Opiliones: Escadabiidae; H) Orthoptera: Phalangopsidae (*Phalangopsis* sp.); I) Chiroptera: Phyllostomidae (Carollia perspicillata); J) Amblypygi: Phrinidae (*Heterophrynus longicornis*) predando um grilo, *Phalangopsis* sp.

CAVIDADE SL-102

Cavidade com 20m de projeção horizontal, formado em quartzito e localizado no centro da encosta da Serra do Sereno. O entorno é composto por vegetação arbustiva com poucas árvores de grande porte e com sub-bosque predominantemente formado por gramíneas e algumas lianas. A cavidade possui duas entradas, sendo a principal de teto baixo e com pouca serrapilheira e a segunda, muito pequena, com aproximadamente 20 cm de altura e quase toda coberta por vegetação rasteira e arbustiva. A cavidade não apresenta zona afótica, porém pode-se observar uma pequena região de penumbra clara próxima a entrada menor, e de

penumbra escura em uma pequena área à direita da entrada principal. De forma geral, trata-se de uma cavidade seca, sem pontos de gotejamento ativos. O piso é predominantemente plano, coberto por sedimento de granulometria fina, com poucos seixos esparsos e poucos blocos abatidos. O sistema radicular é bem desenvolvido, sendo este composto por raízes de médio porte na entrada e no salão principal e micro-raízes dispostas nas regiões internas do abrigo. A serrapilheira é escassa e restrita a linha d'água e no salão de entrada. Alguns brotos são observados no interior da cavidade próximos a entrada menor. Pequenos depósitos de guano insetívoro antigos foram observados no interior da cavidade, porém não foram encontrados morcegos no seu interior. De forma geral trata-se de uma cavidade pobre em recursos alimentares e com elevada influência das condições ambientais epígeas.

Caracterização faunística no período de seca

Foi observado na caverna, um total de 15 morfoespécies de invertebrados de pelo menos 13 famílias dos Taxa: Isopoda (Armadillidae), Pseudoscorpiones (Chthoniidae), Araneae (Pholcidae: Ninetinae; Scytodidae: *Scytodes itapevi*), Blattodea (Polyphagidae), Isoptera (Termitidae: *Nasutitermes* sp.), Heteroptera (Cydnidae), Homoptera (Cixiidae: *Pintalia* sp.), Lepidoptera (Tineidae), Diptera (Drosophilidae), Hymenoptera (Formicidae: *Pachycondyla* sp.) e Coleoptera (Dermestidae; Staphylinidae).

Caracterização faunística no período de chuva

Foi observado na caverna, um total de 41 morfoespécies de invertebrados de pelo menos 28 famílias dos Taxa: Isopoda (Armadillidae), Acari (Oribatida), Pseudoscorpiones (Chthoniidae), Araneae (Pholcidae: *Mesabolivar* sp., *Metagonia* sp., Ninetinae; Salticidae: *Noegus* sp.; Scytodidae: *Scytodes itapevi*; Uloboridae), Thysanura (Nicoletiidae: Nicoletiinae), Collembola (Entomobryidae), Orthoptera (Phalangopsidae: *Phalangopsis* sp., *Uvaroviella* sp.), Blattodea (Polyphagidae), Isoptera (Termitidae: *Nasutitermes* sp.), Psocoptera (Pachytroctidae), Heteroptera (Cydnidae; Lygaeidae; Reduviidae: Emesinae), Homoptera (Cixiidae: *Pintalia* sp.; Derbidae), Lepidoptera (Noctuidae; Tineidae), Diptera (Chloropidae; Culicidae; Drosophilidae; Psychodidae: *Lutzomyia* sp.; Syrphidae), Hymenoptera (Formicidae: *Pachycondyla* sp., *Tapinoma* sp.; Braconidae; Vespidae: Polybiinae) e Coleoptera (Chrysomelidae).

Caracterização geral da fauna da cavidade

Foi observado na caverna, um total de 46 morfoespécies de invertebrados de pelo menos 30 famílias dos Taxa: Isopoda (Armadillidae), Acari (Oribatida), Pseudoscorpiones (Chthoniidae), Araneae (Pholcidae: *Mesabolivar* sp., *Metagonia* sp., Ninetinae; Salticidae: *Noegus* sp.; Scytodidae: *Scytodes itapevi*; Uloboridae), Thysanura (Nicoletiidae: Nicoletiinae), Collembola (Entomobryidae), Orthoptera (Phalangopsidae: *Phalangopsis* sp., *Uvaroviella* sp.), Blattodea (Polyphagidae), Isoptera (Termitidae: *Nasutitermes* spp.), Psocoptera (Pachytroctidae), Heteroptera (Cydnidae; Lygaeidae; Reduviidae: Emesinae), Homoptera (Cixiidae: *Pintalia* sp.; Derbidae), Lepidoptera (Noctuidae; Tineidae), Diptera (Chloropidae; Culicidae; Drosophilidae; Psychodidae: *Lutzomyia* sp.; Syrphidae), Hymenoptera (Formicidae: *Pachycondyla* sp., *Tapinoma* sp.; Braconidae; Vespidae: Polybiinae) e Coleoptera (Chrysomelidae; Dermestidae; Staphylinidae).

CAVIDADE SL-103

Cavidade em canga detrítica com 23m de projeção horizontal localizada em área de mata ciliar, próxima a uma drenagem ativa. O entorno apresenta vegetação arbórea com árvores de médio porte, dossel inferior a 6m, sub-bosque de baixa densidade composto por vegetação arbustiva além de lianas e pteridófitas. A caverna possui apenas uma entrada, sendo esta baixa e com piso em aclive suave. A entrada é sombreada, com muitos liquens, briófitas e pteridófitas revestindo as paredes e pisos. Nesta cavidade puderam-se observar, no período úmido, alguns pontos de gotejamento e áreas de percolação, principalmente na porção final da cavidade. Já na campanha seca não foram encontrados pontos de gotejamento e percolação. O piso é predominantemente plano, em grande parte coberto por sedimento fino, grânulos, alguns seixos e calhaus. O sistema radicular é bem desenvolvido, sendo este composto por raízes de médio porte na entrada e muitas micro-raízes amplamente distribuídas pelo restante da cavidade. A serrapilheira está restrita a linha d'água. Há uma colônia de morcegos frugívoros formada por dezenas de indivíduos, responsáveis pela produção elevada de guano, sendo este, um importante recurso trófico no sistema, estando presente em quase toda área do piso. Quase toda cavidade é em penumbra escura, devido ao teto rebaixado, com áreas de penumbra clara na região da entrada e sem zona afótica. Em frente à cavidade há uma drenagem ativa (riacho) a cerca de 5m. Na outra margem desta drenagem, em frente a esta cavidade, localiza-se a caverna SL-105. Quanto aos vertebrados, registrou-se a ocorrência de um espécime de Epicrates sp.. De forma geral trata-se de uma cavidade com alta diversidade de recursos alimentares e com pouca influência das condições ambientais externas devido à entrada em teto baixo, porém já é possível observar atividade mineradora em um raio inferior a 250 metros da caverna.

Caracterização faunística no período de seca

Foi observado na caverna, um total de 33 morfoespécies de invertebrados de pelo menos 22 famílias dos Taxa: Oligochaeta, Gastropoda (Subulinidae), Isopoda (Platyarthridae: *Trichorhina* sp.), Acari (Ixodida; Mesostigmata; Oribatida), Amblypygi (Phrynidae: *Heterophrynus longicornis*), Pseudoscorpiones (Chernetidae), Araneae (Corinnidae; Ctenidae; Theridiosomatidae: *Plato* sp.), Diplura (Campodeidae), Orthoptera (Phalangopsidae: *Phalangopsis* sp.), Blattodea (Blaberidae: *Blaberus* sp.), Isoptera (Termitidae: *Nasutitermes* sp.), Psocoptera, Heteroptera (Cydnidae; Reduviidae: *Zelurus* sp.), Lepidoptera (Tineidae), Diptera (Tipulidae), Hymenoptera (Formicidae: *Basiceros* sp., *Carebara* sp., *Ectatomma* sp., *Pachycondyla* sp.), Coleoptera (Carabidae; Elateridae: Agrypninae; Scydmaenidae; Staphylinidae), Spirostreptida (Pseudonannolenidae), e Scolopendromorpha (Newportiidae: *Dinocryptops* sp.).

Dentre os vertebrados, foi encontrada uma espécie de Anura (Strabomantidae: *Pristimantis* sp.) e uma de Chiroptera (Furipteridae: *Furipterus horrens).* Desta forma, no total foram encontrados 35 morfoespécies.

Caracterização faunística no período de chuva

Foi observado na caverna, um total de 82 morfoespécies de invertebrados de pelo menos 52 famílias dos Taxa: Oligochaeta, Gastropoda (Subulinidae; Systrophiidae), Platyhelminthes (Tricladida), Isopoda (Platyarthridae: *Trichorhina* sp.; Philosciidae), Acari (Mesostigmata:

Podocinidae, Uropodina; Oribatida; Prostigmata: Bdelloidea; Anystidae: *Eritracarus* sp.), Decapoda (Pseudothelphusidae: *Microthelphusa somanni*), Amblypygi (Phrynidae: *Heterophrynus longicornis*), Pseudoscorpiones (Cheliferidae; Chthoniidae), Opiliones (Sclerosomatidae), Araneae (Araneidae; Corinnidae: *Corinna* sp.; Ctenidae sp.; Dipluridae; Pholcidae: *Mesabolivar* sp.; Prodidomidae: *Lygromma* sp.; Theridiosomatidae: *Plato* sp.), Thysanura (Nicoletiidae: Nicoletiinae), Diplura (Campodeidae), Collembola (Cyphoderidae; Entomobryidae), Neuroptera (Myrmeleontidae), Orthoptera (Phalangopsidae: *Phalangopsis* sp.), Blattodea (Blaberidae: *Blaberus* spp.), Isoptera (Termitidae: *Nasutitermes* sp.), Heteroptera (Cydnidae; Nabidae; Reduviidae: Triatominae), Homoptera (Cercopidae; Cixiidae: *Pintalia* sp.; Derbidae), Lepidoptera (Noctuidae; Tineidae), Diptera (Drosophilidae; Keroplatidae; Psychodidae: *Lutzomyia* sp.; Simuliidae), Hymenoptera (Formicidae: *Acromyrmex* sp., *Cyphomyrmex* sp., *Ectatomma* sp., *Octostruma* sp., *Pachycondyla* sp., *Pseudomyrmex* sp., *Rogeria* sp., *Strumigenys* sp.), Coleoptera (Carabidae; Chrysomelidae: Alticinae; Elateridae: Agrypninae; Scydmaenidae; Staphylinidae), Polydesmida (Chelodesmidae; Pyrgodesmidae), Spirostreptida (Pseudonannolenidae), Scolopendromorpha (Newportiidae: *Newportia* sp.; Scolopendridae: *Cryptops* sp.) e Symphyla (Scutigerellidae: *Hanseniella* sp.).

Dentre os vertebrados, foram encontrados duas espécies de Chiroptera (Furipteridae: *Furipterus horrens;* Phyllostomidae: *Carollia perspicillata*).

Desta forma, no total foram encontrados 84 morfoespécies. Dentre essas, duas foram consideradas troglomórficas: Gastropoda (Systrophiidae) e Collembola (Cyphoderidae).

Caracterização geral da fauna da cavidade

Foi observado na caverna, um total de 96 morfoespécies de invertebrados de pelo menos 53 famílias dos Taxa: Oligochaeta, Gastropoda (Subulinidae; Systrophiidae), Platyhelminthes (Tricladida), Isopoda (Platyarthridae: *Trichorhina* sp.; Philosciidae), Acari (Ixodida; Mesostigmata (Podocinidae, Uropodina; Oribatida; Prostigmata: Bdelloidea; Anystidae: *Eritracarus* sp.), Decapoda (Pseudothelphusidae: *Microthelphusa somanni*), Amblypygi (Phrynidae: *Heterophrynus longicornis*), Pseudoscorpiones (Cheliferidae; Chernetidae; Chthoniidae), Opiliones (Sclerosomatidae), Araneae (Araneidae; Corinnidae: *Corinna* sp.; Ctenidae sp.; Dipluridae; Pholcidae: *Mesabolivar* sp.; Prodidomidae: *Lygromma* sp.; Theridiosomatidae: *Plato* sp.), Thysanura (Nicoletiidae: Nicoletiinae), Diplura (Campodeidae), Collembola (Cyphoderidae; Entomobryidae), Neuroptera (Myrmeleontidae), Orthoptera (Phalangopsidae: *Phalangopsis* sp.), Blattodea (Blaberidae: *Blaberus* spp.), Isoptera (Termitidae: *Nasutitermes* sp.), Psocoptera, Heteroptera (Cydnidae; Nabidae; Reduviidae: Triatominae, *Zelurus* sp.), Homoptera (Cercopidae; Cixiidae: *Pintalia* sp.; Derbidae), Lepidoptera (Noctuidae; Tineidae), Diptera (Drosophilidae; Keroplatidae; Psychodidae: *Lutzomyia* sp.; Simuliidae; Tipulidae), Hymenoptera (Formicidae: *Acromyrmex* sp., *Basiceros* sp., *Carebara* sp., *Cyphomyrmex* sp., *Ectatomma* sp., *Octostruma* sp., *Pachycondyla* sp., *Pseudomyrmex* sp., *Rogeria* sp., *Strumigenys* sp.), Coleoptera (Carabidae; Chrysomelidae: Alticinae; Elateridae: Agrypninae; Scydmaenidae; Staphylinidae), Polydesmida (Chelodesmidae; Pyrgodesmidae), Spirostreptida (Pseudonannolenidae), Scolopendromorpha (Newportiidae: *Dinocryptops* sp., *Newportia* sp.; Scolopendridae: *Cryptops* sp.) e Symphyla (Scutigerellidae: *Hanseniella* sp.).

Dentre os vertebrados, foi encontrada uma espécie de Anura (Strabomantidae: *Pristimantis* sp.) e duas espécies de Chiroptera (Furipteridae: *Furipterus horrens;* Phyllostomidae: *Carollia perspicillata*).

Desta forma, no total foram encontrados 99 morfoespécies. Dentre essas, duas foram consideradas troglomórficas: Gastropoda (Systrophiidae) e Collembola (Cyphoderidae). Alguns organismos encontrados nesta caverna são mostrados na Figura 5.87.

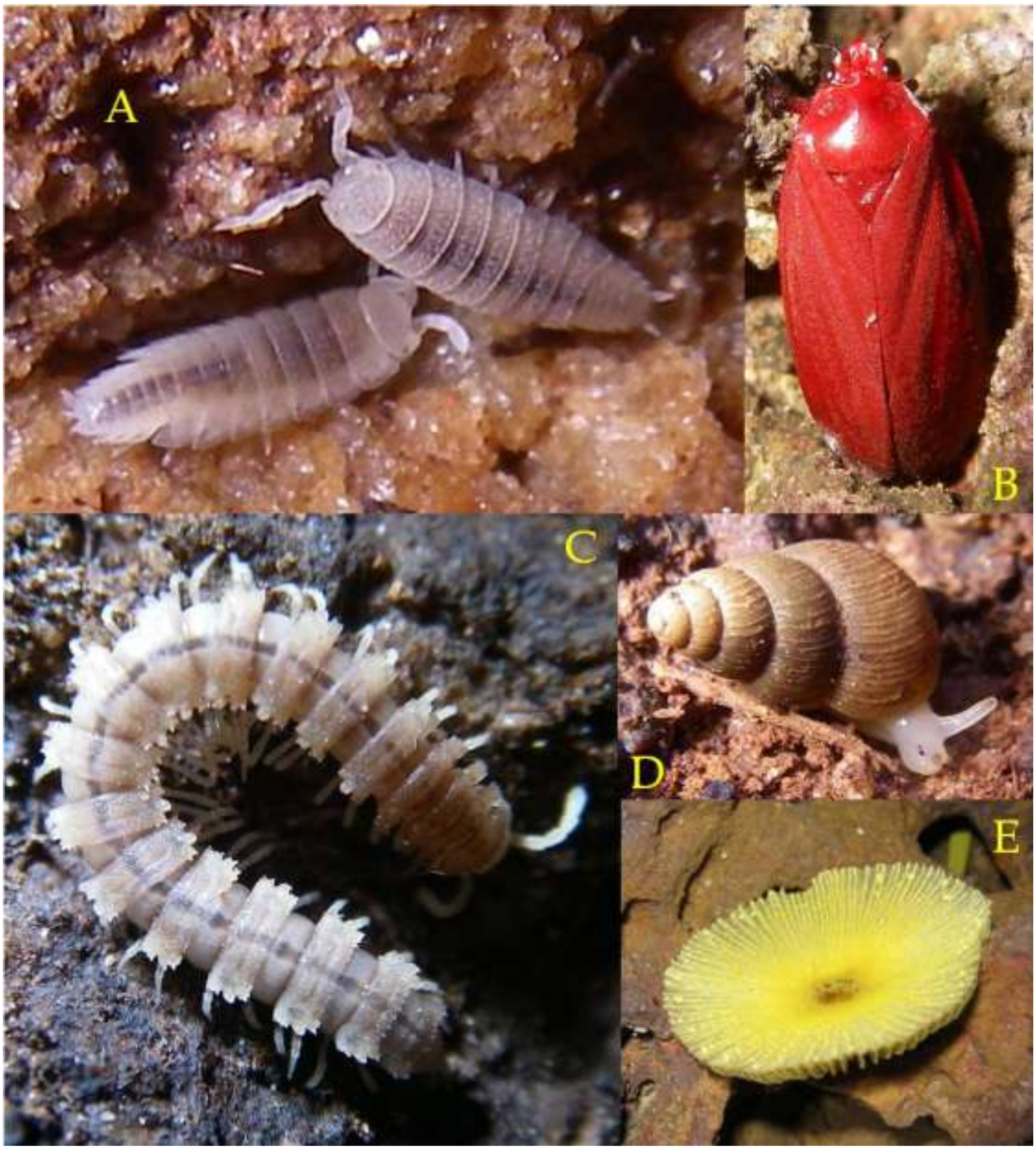

Figura 5.87 - A) Isopoda: Platyarthridae (Trichorhina sp.); B) Homoptera: Cercopidae.; C) Polydesmida: Chelodesmidae; D) Gastropoda: Subulinidae; E) Basidiomycota.

CAVIDADE SL-104

Cavidade com 13m de projeção horizontal, formado em quartzito e localizado no topo da encosta da Serra do Sereno. O entorno é composto por vegetação arbórea com várias árvores

de grande porte e com sub-bosque predominantemente formado por arbustos, gramíneas e muitas lianas. A cavidade possui duas entradas, sendo a principal ampla, de piso muito descendente com paredes e teto cobertos por raízes, liquens, musgos, briófitas e pteridófitas, e a segunda, menor e em desnível. A cavidade não apresenta zona afótica, porém pode-se observar uma pequena região de penumbra clara à direita. Na campanha chuvosa, a cavidade encontrava-se muito úmida, com pontos de gotejamento, além de um grande fluxo de água saindo da parede na região mais a direita do abrigo. Já no período seco a cavidade encontra-se sem pontos de gotejamento e sem o fluxo d'água observado na campanha anterior. O piso é predominantemente plano no interior da cavidade, coberto por alguns seixos esparsos e vários blocos abatidos. O sistema radicular é bem desenvolvido, sendo este composto por muitas raízes de médio e grande porte na entrada, e no local do fluxo d'água, observam-se raízes de pequeno calibre, porém de grande comprimento. A serrapilheira é restrita a linha d'água e no início do salão de entrada. Foram observados alguns depósitos de guano de morcego frugívoro antigos. De forma geral trata-se de uma cavidade rica em recursos alimentares, com grande aporte de recursos, devido ao formato descendente da entrada e do fluxo de água (período úmido), para o interior da cavidade, e com elevada influência das condições ambientais epígeas.

Caracterização faunística no período de seca

Foi observado na caverna, um total de 16 morfoespécies de invertebrados de pelo menos 11 famílias dos Taxa: Acari (Oribatida), Araneae (Pholcidae: *Metagonia* sp.), Collembola (Entomobryidae), Embiidina, Isoptera (Termitidae: *Nasutitermes* sp.), Psocoptera (Archipsocidae; Pachytroctidae; Ptiloneuridae), Lepidoptera (Noctuidae; Tineidae), Diptera (Milichiidae), Hymenoptera (Formicidae: *Camponotus* sp., *Cremagaster* sp., *Pachycondyla* sp., *Pseudomyrmex* sp.) e Glomeridesmida (Glomeridesmidae).

Caracterização faunística no período de chuva

Foi observado na caverna, um total de 41 morfoespécies de invertebrados de pelo menos 27 famílias dos Taxa: Gastropoda (Subulinidae; Systrophiidae), Acari (Oribatida), Opiliones (Escadabiidae), Araneae (Araneidae: *Alpaida* sp.; Pholcidae: *Mesabolivar* sp.; *Metagonia* sp., Ninetinae; Salticidae; Scytodidae: *Scytodes itapevi*; Theridiosomatidae: *Plato* sp.), Thysanura (Meinertellidae), Collembola (Bourletiellidae; Entomobryidae), Blattodea (Blattidae), Isoptera (Termitidae: *Nasutitermes* sp., *Velocitermes* sp.), Psocoptera, Heteroptera (Cydnidae), Homoptera (Derbidae), Lepidoptera (Noctuidae; Tineidae), Diptera (Chloropidae; Culicidae; Drosophilidae; Muscidae; Psychodidae: *Lutzomyia* spp.), Hymenoptera (Formicidae: *Camponotus* sp., *Cardiocondyla* sp., *Cremagaster* sp., *Octostruma* sp., *Solenopsis* sp.), Coleoptera (Carabidae; Staphylinidae: Pselaphinae), Glomeridesmida (Glomeridesmidae) e Polydesmida (Polydesmidae).

Dentre os vertebrados, foi encontrada apenas uma espécie de Chiroptera (Emballorunidae: *Peropteryx* sp.).

Desta forma, no total foram encontrados 42 morfoespécies. Dentre essas, apenas Gastropoda (Systrophiidae) foi considerada troglomórfica.

Caracterização geral da fauna da cavidade

Foi observado na caverna, um total de 42 morfoespécies de invertebrados de pelo menos 31 famílias dos Taxa: Gastropoda (Subulinidae; Systrophiidae), Acari (Oribatida), Opiliones (Escadabiidae), Araneae (Araneidae: *Alpaida* sp.; Pholcidae: *Mesabolivar* sp.; *Metagonia* sp., Ninetinae; Salticidae; Scytodidae: *Scytodes itapevi*; Theridiosomatidae: *Plato* sp.), Thysanura (Meinertellidae), Collembola (Bourletiellidae; Entomobryidae), Blattodea (Blattidae), Embiidina, Isoptera (Termitidae: *Nasutitermes* sp., *Velocitermes* sp.), Psocoptera (Archipsocidae; Pachytroctidae; Ptiloneuridae), Heteroptera (Cydnidae), Homoptera (Derbidae), Lepidoptera (Noctuidae; Tineidae), Diptera (Chloropidae; Culicidae; Drosophilidae; Milichiidae; Muscidae; Psychodidae: *Lutzomyia* spp.), Hymenoptera (Formicidae: *Camponotus* sp., *Cardiocondyla* sp., *Cremagaster* sp., *Octostruma* sp., *Pachycondyla* sp., *Pseudomyrmex* sp., *Solenopsis* sp.), Coleoptera (Carabidae; Staphylinidae: Pselaphinae), Glomeridesmida (Glomeridesmidae) e Polydesmida (Polydesmidae)

Dentre os vertebrados, foi encontrada apenas uma espécie de Chiroptera (Emballorunidae: Peropteryx sp.). Desta forma, no total foram encontrados 52 morfoespécies. Dentre essas, apenas Gastropoda (Systrophiidae) foi considerada troglomórfica. Alguns aspectos biológicos desta caverna são mostrados na Figura 5.88.

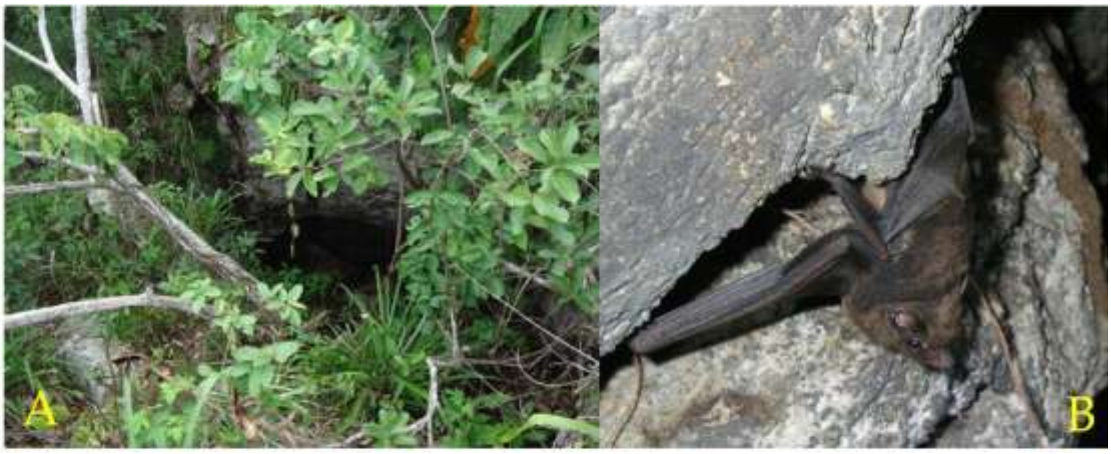

Figura 5.88 - A) Vista externa da entrada da cavidade; B) Chiroptera: Emballonuridae: Peropteryx sp.

CAVIDADE SL-105

Cavidade em canga detrítica com 23m de projeção horizontal localizada em área de mata ciliar, próxima a uma drenagem ativa. O entorno apresenta vegetação arbórea com árvores de médio porte, dossel inferior a 6m, sub-bosque de baixa densidade composto por vegetação arbustiva além de lianas e pteridófitas. A caverna possui duas entradas opostas, sendo a principal ampla e com piso em aclive. A entrada é pouco sombreada, com liquens, briófitas e pteridófitas revestindo as paredes e pisos. Trata-se de uma cavidade úmida, porém com pontos de gotejamento observados apenas na campanha úmida. O piso é predominantemente plano, em grande parte coberto por sedimento fino, grânulos e alguns seixos. O sistema radicular é bem desenvolvido, sendo este composto por raízes de pequeno e médio porte na entrada e micro-raízes distribuídas pelo restante da cavidade. A serrapilheira está restrita a linha d’água. Havia na campanha úmida, um depósito recente de guano de morcegos

frugívoros e nectarívoros no centro da cavidade, porém estes se encontram agora exauridos. Toda cavidade é em penumbra clara, devido à entrada ampla e pouco sombreada. Em frente à cavidade há uma drenagem ativa (riacho) a cerca de 5m. Na outra margem desta drenagem, em frente a esta cavidade, localiza-se a caverna SL-103. De forma geral trata-se de uma cavidade com pouca diversidade de recursos alimentares e com alta influência das condições ambientais externas. Além disso, já é possível observar atividade mineradora em um raio inferior a 250 metros da caverna.

Caracterização faunística no período de seca

Foi observado na caverna, um total de 31 morfoespécies de invertebrados de pelo menos 24 famílias dos Taxa: Isopoda (Philosciidae), Acari (Mesostigmata), Decapoda (Pseudothelphusidae: *Microthelphusa somanni*), Amblypygi (Phrynidae: *Heterophrynus longicornis*), Pseudoscorpiones (Chernetidae; Chthoniidae), Opiliones (Cosmetidae), Araneae (Pholcidae: *Mesabolivar* sp., *Metagonia* sp.; Theridiosomatidae: *Plato* sp.), Diplura (Campodeidae), Collembola (Bourletiellidae; Entomobryidae), Orthoptera (Phalangopsidae: *Phalangopsis* sp.), Psocoptera, Heteroptera (Cydnidae), Homoptera (Cixiidae), Lepidoptera (Tineidae), Diptera (Psychodidae: *Lutzomyia* sp.; Sciaridae; Tipulidae), Hymenoptera (Formicidae: *Dolichoderus* sp., *Pachycondyla* sp., *Solenopsis* sp.), Coleoptera (Elateridae; Staphylinidae: Pselaphinae), Scolopendromorpha (Scolopendridae: *Cryptops* sp.) e Symphyla (Scutigerellidae: *Hanseniella* sp.).

Dentre os vertebrados, foi encontrada uma espécie de Anura (Strabomantidae: *Pristimantis* sp.) e uma espécie de Chiroptera (Phyllostomidae: *Glossophaga soricina*). Desta forma, no total foram encontrados 33 morfoespécies.

Caracterização faunística no período de chuva

Foi observado na caverna, um total de 52 morfoespécies de invertebrados de pelo menos 35 famílias dos Taxa: Oligochaeta, Gastropoda (Systrophiidae), Isopoda (Philosciidae), Acari (Mesostigmata: Uropodina; Oribatida), Amblypygi (Phrynidae: *Heterophrynus longicornis*), Pseudoscorpiones (Chernetidae; Chthoniidae), Opiliones (Cosmetidae; Phalangiidae; Sclerosomatidae), Araneae (Ctenidae; Ochyroceratidae: *Speocera* sp.; Pholcidae: *Mesabolivar* sp.; Scytodidae: *Scytodes itapevi*; Theridiosomatidae: *Plato* sp.), Diplura (Campodeidae), Collembola (Bourletiellidae; Entomobryidae), Orthoptera (Phalangopsidae: *Phalangopsis* sp., *Uvaroviella* sp.), Isoptera, Thysanoptera (Phlaeothripidae), Psocoptera, Heteroptera (Cydnidae; Nabidae; Pyrrhocoridae), Homoptera (Cixiidae: *Pintalia* sp.), Lepidoptera (Noctuidae; Tineidae), Diptera (Ceratopogonidae; Drosophilidae; Psychodidae: *Lutzomyia* sp.), Hymenoptera (Formicidae: *Cardiocondyla* sp., *Cremagaster* sp., *Pachycondyla* sp., *Pheidole* sp., *Solenopsis* sp., *Stegomyrmex* sp.), Coleoptera (Carabidae; Chrysomelidae: Alticinae; Curculionidae; Scydmaenidae; Staphylinidae: Pselaphinae), Polydesmida (Polydesmidae) e Scolopendromorpha (Newportiidae: *Newportia* sp.). Dentre essas, apenas Gastropoda (Systrophiidae) foi considerada troglomórfica.

Caracterização geral da fauna da cavidade

Foi observado na caverna, um total de 63 morfoespécies de invertebrados de pelo menos 44 famílias dos Taxa: Oligochaeta, Gastropoda (Systrophiidae), Isopoda (Philosciidae), Acari (Mesostigmata: Uropodina; Oribatida), Decapoda (Pseudothelphusidae: *Microthelphusa*

somanni), Amblypygi (Phrynidae: *Heterophrynus longicornis*), Pseudoscorpiones (Chernetidae; Chthoniidae), Opiliones (Cosmetidae; Phalangiidae; Sclerosomatidae), Araneae (Ctenidae; Ochyroceratidae: *Speocera* sp.; Pholcidae: *Mesabolivar* sp., *Metagonia* sp.; Scytodidae: *Scytodes itapevi*; Theridiosomatidae: *Plato* sp.), Diplura (Campodeidae), Collembola (Bourletiellidae; Entomobryidae), Orthoptera (Phalangopsidae: *Phalangopsis* sp., *Uvaroviella* sp.), Isoptera, Thysanoptera (Phlaeothripidae), Psocoptera, Heteroptera (Cydnidae; Nabidae; Pyrrhocoridae), Homoptera (Cixiidae: *Pintalia* sp.), Lepidoptera (Noctuidae; Tineidae), Diptera (Ceratopogonidae; Drosophilidae; Psychodidae: *Lutzomyia* sp.; Sciaridae; Tipulidae), Hymenoptera (Formicidae: *Cardiocondyla* sp., *Cremagaster* sp., *Dolichoderus* sp., *Pachycondyla* sp., *Pheidole* sp., *Solenopsis* spp., *Stegomyrmex* sp.), Coleoptera (Carabidae; Chrysomelidae: Alticinae; Curculionidae; Elateridae; Scydmaenidae; Staphylinidae: Pselaphinae), Polydesmida (Polydesmidae), Scolopendromorpha (Newportiidae: *Newportia* sp.; Scolopendridae: *Cryptops* sp.) e Symphyla (Scutigerellidae: *Hanseniella* sp.).

Dentre os vertebrados, foi encontrada uma espécie de Anura (Strabomantidae: *Pristimantis* sp.) e uma espécie de Chiroptera (Phyllostomidae: *Glossophaga soricina*). Desta forma, no total foram encontrados 64 morfoespécies. Dentre essas, apenas Gastropoda (Systrophiidae) foi considerada troglomórfica. Alguns organismos encontrados nesta caverna são mostrados na Figura 5.89.

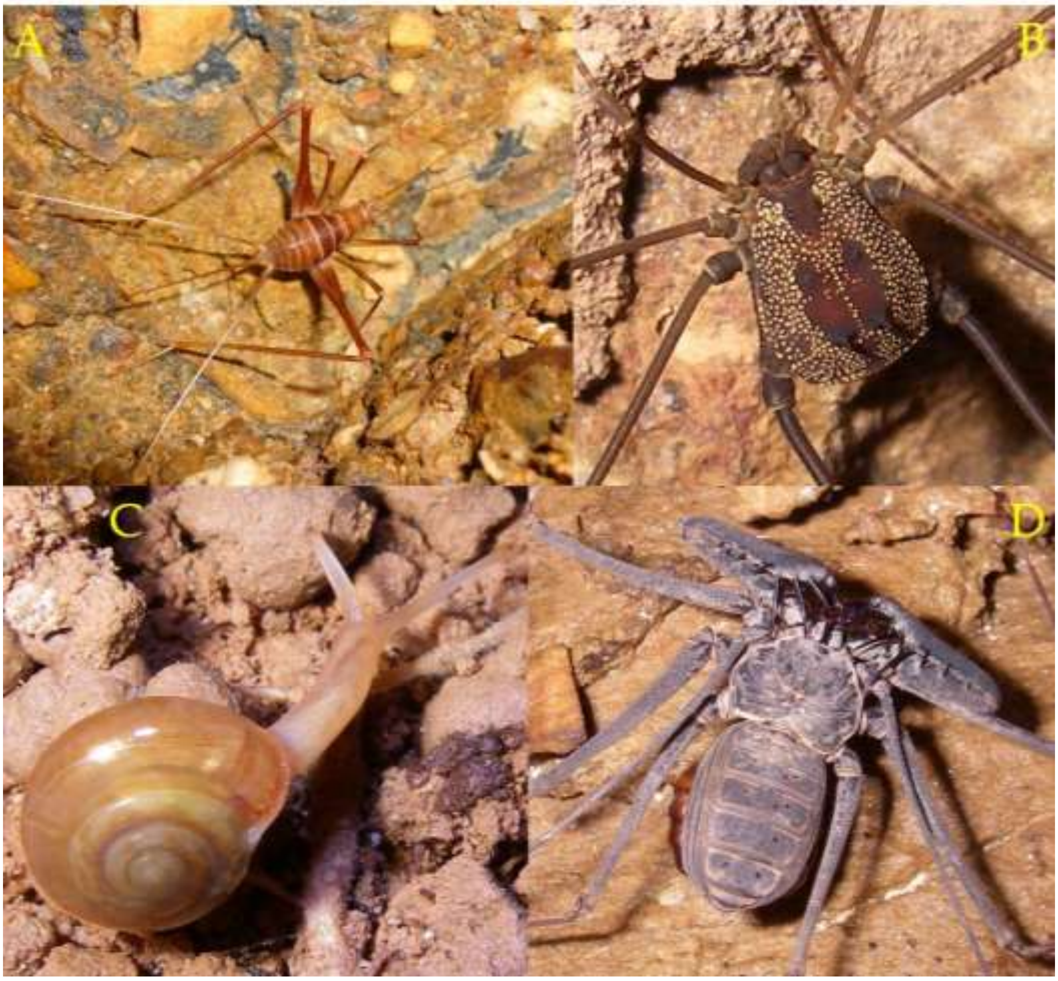

Figura 5.89 - A) Orthoptera: Phalangopsidae (*Phalangopsis* sp.); B) Opiliones: Cosmetidae; C) Gastropoda: Systrophiidae; D) Amblypygi: Phrinidae (*Heterophrynus longicornis*).

CAVIDADE SL-106

Pequena cavidade em canga detrítica com 8,5m de projeção horizontal localizada em área de mata ciliar, próxima a uma drenagem ativa. O entorno apresenta vegetação arbórea com árvores de médio porte, dossel inferior a 6m, sub-bosque de baixa densidade composto por vegetação arbustiva além de lianas e pteridófitas. A caverna apenas uma entrada, sendo esta em teto baixo, e com piso predominantemente plano. A entrada é sombreada, com alguns liquens, briófitas e fungos revestindo as paredes e pisos. Quanto à umidade, trata-se de uma cavidade seca, sem pontos de gotejamento ou percolação. O piso é predominantemente plano, em grande parte coberto por sedimento fino, grânulos e poucos seixos. O sistema radicular é pouco desenvolvido, sendo este composto por raízes de pequeno e médio porte restritas a entrada. A serrapilheira apenas na região da entrada, próximo à linha d'água. Não foram observados fezes ou guano no interior da cavidade. Toda cavidade é em penumbra clara, devido à pequena extensão da mesma. Próximo à cavidade há uma drenagem ativa (riacho), aproximadamente a 15m. De forma geral trata-se de uma cavidade com pouco recurso trófico e com alta influência das condições ambientais epígeas. Além disso, já é possível observar atividade mineradora em um raio inferior a 250 metros da caverna.

Caracterização faunística no período de seca

Foi observado na caverna, um total de 13 morfoespécies de invertebrados de pelo menos 12 famílias dos Taxa: Amblypygi (Phrynidae: *Heterophrynus longicornis*), Pseudoscorpiones (Chernetidae), Opiliones (Cosmetidae), Araneae (Ochyroceratidae: *Ochyrocera* sp.; Pholcidae: *Mesabolivar* sp.), Orthoptera (Phalangopsidae: *Phalangopsis* sp.), Heteroptera (Reduviidae: *Zelurus* sp.), Lepidoptera (Tineidae), Diptera (Tipulidae), Hymenoptera (Formicidae: *Pachycondyla* sp.), Coleoptera (Chrysomelidae: Alticinae) e Scolopendromorpha (Scolopendridae: *Cryptops* sp.).

Caracterização faunística no período de chuva

Foi observado na caverna, um total de 28 morfoespécies de invertebrados de pelo menos 20 famílias dos Taxa: Oligochaeta, Gastropoda (Systrophiidae), Isopoda (Platyarthridae: *Trichorhina* sp.), Acari (Actinedida: Parasitengonina; Oribatida), Amblypygi (Phrynidae: *Heterophrynus longicornis*), Pseudoscorpiones (Chernetidae; Chthoniidae), Opiliones (Cosmetidae), Araneae (Ochyroceratidae: *Ochyrocera* sp.; Pholcidae: *Mesabolivar* sp., Ninetinae; Salticidae; Theraphosidae), Thysanura (Nicoletiidae: Nicoletiinae), Collembola (Entomobryidae; Tomoceridae), Orthoptera (Phalangopsidae: *Phalangopsis* sp.), Isoptera (Rhinotermitidae: *Coptotermes* sp.), Heteroptera (Cydnidae; Reduviidae: *Zelurus* sp.), Lepidoptera (Tineidae), Diptera (Psychodidae: *Lutzomyia* sp.) e Hymenoptera (Formicidae: *Prionopelta* sp.).

Dentre os vertebrados, foi encontrada apenas uma espécie de Chiroptera (Phyllostomidae: Glossophaginae). Desta forma, no total foram encontrados 29 morfoespécies.

Caracterização geral da fauna da cavidade

Foi observado na caverna, um total de 32 morfoespécies de invertebrados de pelo menos 23 famílias dos Taxa: Oligochaeta, Gastropoda (Systrophiidae), Isopoda (Platyarthridae: *Trichorhina* sp.), Acari (Actinedida: Parasitengonina; Oribatida), Amblypygi (Phrynidae: *Heterophrynus longicornis*), Pseudoscorpiones (Chernetidae; Chthoniidae), Opiliones

(Cosmetidae), Araneae (Ochyroceratidae: *Ochyrocera* sp.; Pholcidae: *Mesabolivar* sp., Ninetinae; Salticidae; Theraphosidae), Thysanura (Nicoletiidae: Nicoletiinae), Collembola (Entomobryidae; Tomoceridae), Orthoptera (Phalangopsidae: *Phalangopsis* sp.), Isoptera (Rhinotermitidae: *Coptotermes* sp.), Heteroptera (Cydnidae; Reduviidae: *Zelurus* sp.), Lepidoptera (Tineidae), Diptera (Psychodidae: *Lutzomyia* sp.; Tipulidae), Hymenoptera (Formicidae: *Pachycondyla* sp., *Prionopelta* sp.), Coleoptera (Chrysomelidae: Alticinae) e Scolopendromorpha (Scolopendridae: *Cryptops* sp.).

Dentre os vertebrados, foi encontrada apenas uma espécie de Chiroptera (Phyllostomidae: Glossophaginae). Desta forma, no total foram encontrados 33 morfoespécies. Alguns organismos encontrados nesta caverna são mostrados na Figura 5.90.

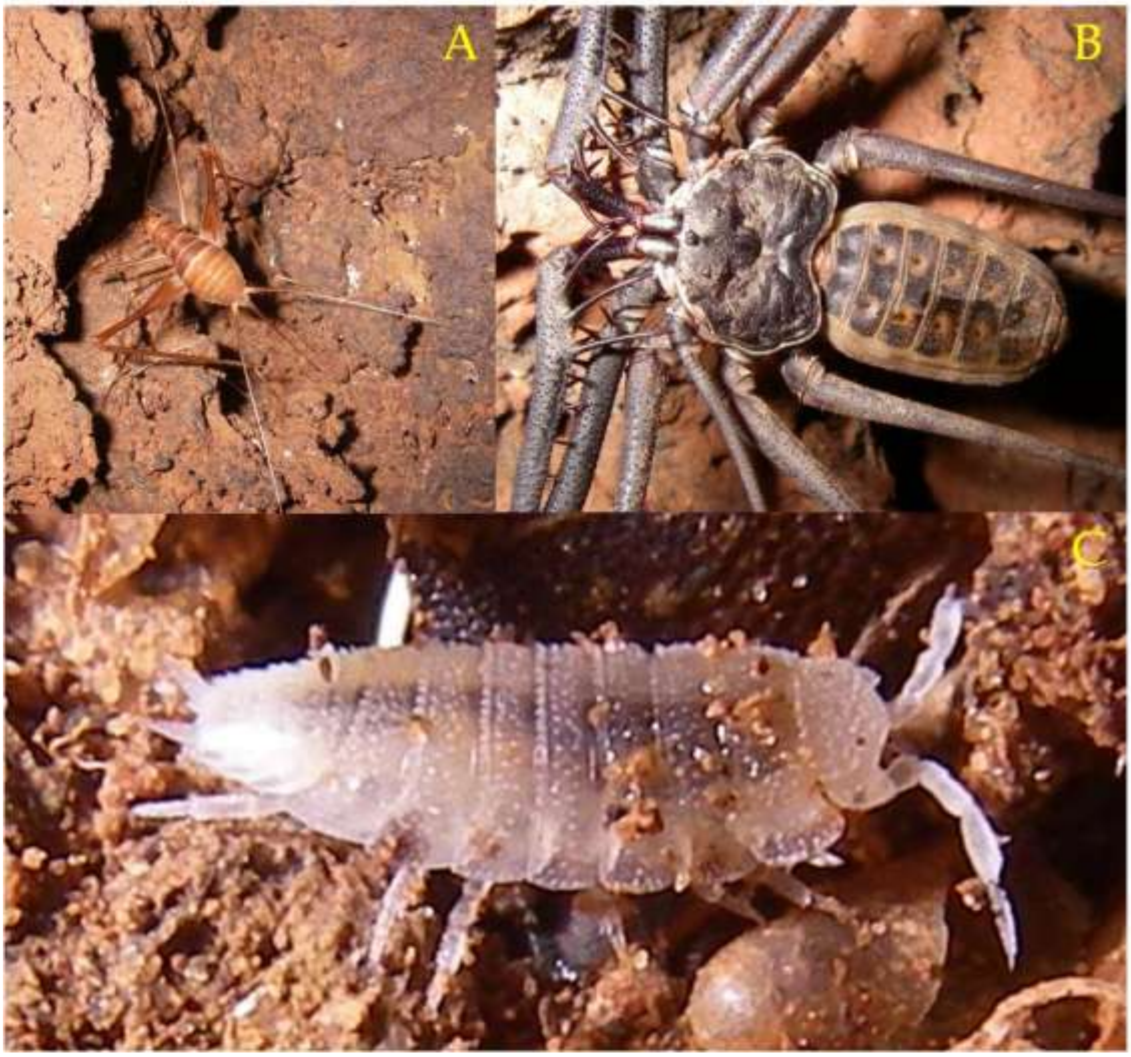

Figura 5.90 - A) Orthoptera: Phalangopsidae (*Phalangopsis* sp.); B) Amblypygi: Phrinidae (*Heterophrynus longicornis*).; C) Isopoda: Platyarthridae (*Trichorhina* sp.).

CAVIDADE SL-107

Caverna com 39,5m de projeção horizontal, formada em quartzito e localizada na região inferior da encosta da Serra do Sereno. O entorno é composto por vegetação arbórea com algumas árvores de grande porte e com sub-bosque predominantemente formado por arbustos, gramíneas e muitas lianas. A caverna pode ser divida em duas áreas, sendo a região

principal, mais alta em relação à encosta da Serra. Ela apresenta duas entradas, sendo a principal, pequena, com um desnível de aproximadamente 1,5m, e presença de liquens e musgos. A segunda entrada é de teto baixo, coberta por vegetação arbustiva e rasteira, com muitos liquens, briófitas, pteridófitas e alguns brotos. A cavidade não apresenta zona afótica, porém pode-se observar uma pequena região de penumbra escura de piso descendente, inicialmente de teto baixo e mais internamente inacessível (ligação entre um lado e outro da caverna), com predominância de sedimento arenoso, com alguns seixos esparsos e fluxo de água no sentido da segunda entrada. É possível visualizar penumbra clara em ambos os salões principais existentes. De forma geral, trata-se de uma cavidade úmida, com pontos de gotejamento esparsos por toda a caverna. Estes pontos de gotejamento são escassos no período de seca. O piso do salão da entrada principal é descendente, coberto por seixos, calhaus e muitos blocos abatidos. O piso deste local encontrava-se com baixíssima umidade no período seco. O segundo salão é descendente na entrada e ascendente em direção ao interior (região de empoçamentos e fluxo d'água advindo do setor mais alto da caverna, observáveis nas duas campanhas de coleta), com piso coberto por sedimento arenoso e com poucos seixos, além de alguns pontos empoçados. O sistema radicular é bem desenvolvido, sendo o salão principal composto por raízes de pequeno e médio porte na entrada e no seu interior algumas raízes são encontradas sobre o piso e no teto. No salão da segunda entrada, o piso é quase todo coberto por raízes de pequeno calibre e principalmente micro-raízes. A serrapilheira é restrita a linha d'água de ambas as entradas e esparsa no salão secundário. Foi observado um grande depósito de guano frugívoro fresco no salão principal e alguns depósitos no segundo salão. O carreamento, pela água, de guano da região principal da caverna para a outra região, é visível apenas na campanha úmida. De forma geral trata-se de uma cavidade rica em recursos alimentares, com elevada influência das condições ambientais epígeas.

Caracterização faunística no período de seca

Foi observado na caverna, um total de 45 morfoespécies de invertebrados de pelo menos 35 famílias dos Taxa: Isopoda (Philosciidae), Acari (Mesostigmata: Uropodina; Oribatida; Acaridae: *Tyrophagus* sp.), Pseudoscorpiones (Chthoniidae), Opiliones (Sclerosomatidae), Araneae (Araneidae; Corinnidae; Drymusidae; Ochyroceratidae: *Ochyrocera* sp.; Pholcidae: *Mesabolivar* sp., Ninetinae; Theridiosomatidae: *Plato* sp.), Diplura (Campodeidae), Collembola (Entomobryidae; Hypogastruridae), Orthoptera (Phalangopsidae: *Uvaroviella* sp.), Isoptera (Termitidae: *Nasutitermes* sp.), Psocoptera (Ptiloneuridae), Heteroptera (Cydnidae; Veliidae: *Paravelia* sp.), Homoptera (Cixiidae), Lepidoptera (Noctuidae; Tineidae; Tortricidae), Diptera (Culicidae; Drosophilidae; Psychodidae; Sciaridae; Tipulidae), Hymenoptera (Formicidae: *Acromyrmex* sp., *Atta* sp., *Hypoponera* sp., *Pachycondyla* sp., *Pheidole* sp.), Coleoptera (Carabidae; Curculionidae; Leiodidae; Scydmaenidae) e Symphyla (Scutigerellidae: *Hanseniella* sp.).

Dentre os vertebrados, foram encontradas duas espécies de Anura (Strabomantidae: *Pristimantis* sp. e um indeterminado), e três de Chiroptera (Emballorunidae: *Peropteryx kappleri*; Phyllostomidae: *Carollia perspicillata*, *Glossophaga soricina*). Desta forma, no total foram encontrados 50 morfoespécies.

Caracterização faunística no período de chuva

Foi observado na caverna, um total de 100 morfoespécies de invertebrados de pelo menos 62 famílias dos Taxa: Gastropoda (Subulinidae; Systrophiidae), Platyhelminthes (Tricladida), Isopoda (Scleropactidae), Acari (Mesostigmata: Uropodina), Amblypygi (Phrynidae: *Heterophrynus longicornis*), Pseudoscorpiones (Chthoniidae), Opiliones (Sclerosomatidae), Araneae (Araneidae; Ctenidae; Pholcidae: *Mesabolivar* sp., *Metagonia* sp.; Salticidae; Tetrablemmidae: *Matta* sp.; Theridiosomatidae: *Plato* sp.), Thysanura (Meinertellidae), Diplura (Campodeidae), Collembola (Bourletiellidae; Entomobryidae), Orthoptera (Phalangopsidae: *Phalangopsis* sp., *Uvaroviella* sp., Phalangopsinae), Blattodea (Blattidae), Psocoptera (Lepidopsocidae; Psyllipsocidae; Ptiloneuridae; Trogiidae), Heteroptera (Cydnidae; Dipsocoridae; Hebridae; Veliidae: *Paravelia* sp.), Homoptera (Cercopidae; Cixiidae: *Pintalia* sp.; Derbidae), Lepidoptera (Noctuidae; Tineidae; Tortricidae), Diptera (Calliphoridae; Cecidomyiidae; Ceratopogonidae; Culicidae; Drosophilidae; Fanniidae; Keroplatidae; Psychodidae: *Lutzomyia* sp.; Sciaridae; Streblidae; Tipulidae), Hymenoptera (Formicidae: *Acromyrmex* sp., *Apterostigma* sp., *Atta* sp., *Lachnomyrmex* sp., *Pachycondyla* sp., *Solenopsis* sp., *Strumigenys* sp.; Diapriidae; Dryinidae?), Coleoptera (Carabidae; Chrysomelidae: Alticinae; Curculionidae; Dytiscidae; Hydrophilidae; Scydmaenidae; Staphylinidae: Pselaphinae), Polydesmida (Chelodesmidae; Pyrgodesmidae), Spirostreptida (Pseudonannolenidae), Lithobiomorpha (Henicopidae: *Lamyctes* sp.), Scolopendromorpha (Scolopendridae: *Cryptops* sp.), Scutigeromorpha (Pselliodidae: *Sphendononema guildingii*) e Symphyla (Scutigerellidae: *Hanseniella* sp.).

Dentre os vertebrados, apenas uma espécie de Chiroptera (Phyllostomidae: *Carollia perspicillata*). Desta forma, no total foram encontrados 101 morfoespécies. Dentre essas, duas espécies foram consideradas troglomórficas Gastropoda (Systrophiidae) e Araneae (Tetrablemmidae: *Matta* sp.).

Caracterização geral da fauna da cavidade

Foi observado na caverna, um total de 119 morfoespécies de invertebrados de pelo menos 69 famílias dos Taxa: Gastropoda (Subulinidae; Systrophiidae), Platyhelminthes (Tricladida), Isopoda (Scleropactidae), Acari (Mesostigmata: Uropodina; Oribatida; Acaridae: *Tyrophagus* sp.), Amblypygi (Phrynidae: *Heterophrynus longicornis*), Pseudoscorpiones (Chthoniidae), Opiliones (Sclerosomatidae), Araneae (Araneidae; Corinnidae; Ctenidae; Drymusidae; Ochyroceratidae: *Ochyrocera* sp.; Pholcidae: *Mesabolivar* sp., *Metagonia* sp., Ninetinae; Salticidae; Tetrablemmidae: *Matta* sp.; Theridiosomatidae: *Plato* sp.), Thysanura (Meinertellidae), Diplura (Campodeidae), Collembola (Bourletiellidae; Entomobryidae; Hypogastruridae), Orthoptera (Phalangopsidae: *Phalangopsis* sp., *Uvaroviella* sp., Phalangopsinae), Blattodea (Blattidae), Psocoptera (Lepidopsocidae; Psyllipsocidae; Ptiloneuridae; Trogiidae), Heteroptera (Cydnidae; Dipsocoridae; Hebridae; Veliidae: *Paravelia* sp.), Homoptera (Cercopidae; Cixiidae: *Pintalia* sp.; Derbidae), Lepidoptera (Noctuidae; Tineidae; Tortricidae), Diptera (Calliphoridae; Cecidomyiidae; Ceratopogonidae; Culicidae; Drosophilidae; Fanniidae; Keroplatidae; Psychodidae: *Lutzomyia* sp.; Sciaridae; Streblidae; Tipulidae), Hymenoptera (Formicidae: *Acromyrmex* sp., *Apterostigma* sp., *Atta* sp., *Hypoponera* sp., *Lachnomyrmex* sp., *Pachycondyla* sp., *Pheidole* sp., *Solenopsis* sp., *Strumigenys* sp.; Diapriidae; Dryinidae?), Coleoptera (Carabidae; Chrysomelidae: Alticinae;

Curculionidae; Dytiscidae; Hydrophilidae; Scydmaenidae; Staphylinidae: Pselaphinae), Polydesmida (Chelodesmidae; Pyrgodesmidae), Spirostreptida (Pseudonannolenidae), Lithobiomorpha (Henicopidae: *Lamyctes* sp.), Scolopendromorpha (Scolopendridae: *Cryptops* sp.), Scutigeromorpha (Pselliodidae: *Sphendononema guildingii*) e Symphyla (Scutigerellidae: *Hanseniella* sp.).

Dentre os vertebrados, foram encontradas duas espécies de Anura (Strabomantidae: *Pristimantis* sp. e um indeterminado), e três de Chiroptera (Emballorunidae: *Peropteryx kappleri*; Phyllostomidae: *Carollia perspicillata*, *Glossophaga soricina*). Desta forma, no total foram encontrados 124 morfoespécies. Dentre essas, duas espécies foram consideradas troglomórficas Gastropoda (Systrophiidae) e Araneae (Tetrablemmidae: *Matta* sp.). Alguns aspectos biológicos associados a esta caverna são mostrados na Figura 5.91.

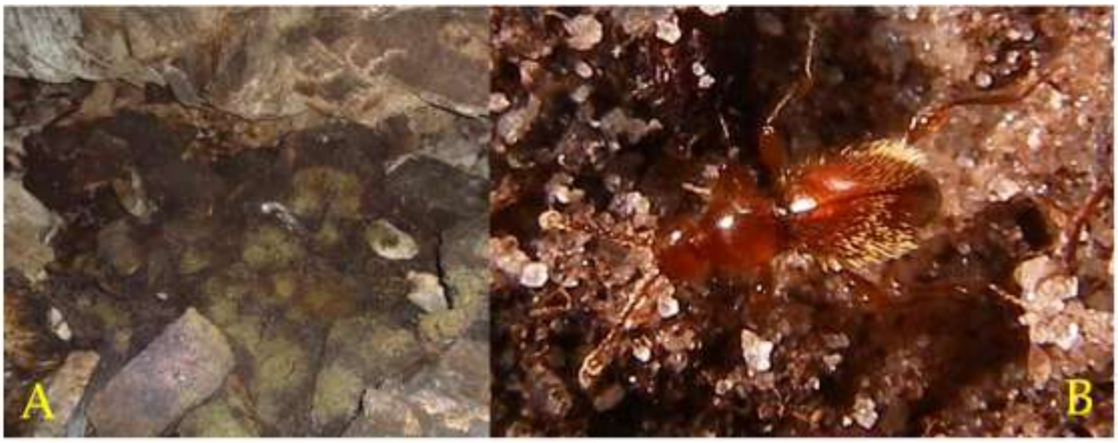

Figura 5.91 - A) Vista interna destacando as manchas de guano associadas ao piso da cavidade; B) Coleoptera: Staphylinidae (Pselaphinae).

CAVIDADE SL-108

Cavidade em canga detrítica com 24m de projeção horizontal localizada em área de mata ciliar, próxima a uma drenagem ativa. O entorno apresenta vegetação arbórea com árvores de médio porte, dossel inferior a 6m, sub-bosque de baixa densidade composto por vegetação arbustiva além de lianas e pteridófitas. A caverna possui apenas uma entrada, sendo esta ampla e com piso predominantemente plano. A entrada é sombreada, com muitos liquens, briófitas e pteridófitas revestindo as paredes e teto. Trata-se de uma cavidade úmida, com alguns pontos de gotejamento e áreas de percolação, principalmente na região central da cavidade. Estes pontos de gotejamento são visíveis apenas no período úmido. O piso é predominantemente plano, em grande parte coberto por sedimento fino, grânulos, seixos e alguns calhaus. O sistema radicular é pouco desenvolvido, sendo este composto por raízes de pequeno porte na entrada e rede sub-superficial de micro-raízes distribuídas pelo restante da cavidade. A serrapilheira está restrita a linha d'água. Há uma colônia de morcegos frugívoros, formada por dezenas de indivíduos, responsáveis pela produção elevada de guano, sendo este, um importante recurso trófico no sistema, estando presente em quase toda área central da cavidade. Além deste, também foram visualizadas fezes de anuros esparsas pelo piso da cavidade e um ninho de roedor não identificado. Quase toda cavidade é em penumbra escura, com áreas de penumbra clara na região da entrada e sem zona afótica. Em frente à cavidade

há uma drenagem ativa (riacho) a cerca de 3m. De forma geral trata-se de uma cavidade com alta diversidade de recursos alimentares e com pouca influência das condições ambientais externas devido à morfologia sinuosa do seu conduto principal, porém já é possível observar atividade mineradora em um raio inferior a 250 metros da caverna.

Caracterização faunística no período de seca

Foi observado na caverna, um total de 47 morfoespécies de invertebrados de pelo menos 31 famílias dos Taxa: Gastropoda (Systrophiidae), Isopoda (Platyarthridae: *Trichorhina* sp.; Philosciidae), Acari (Mesostigmata; Acaridae: *Tyrophagus* sp.), Amblypygi (Phrynidae: *Heterophrynus longicornis*), Pseudoscorpiones (Chernetidae), Opiliones (Cosmetidae; Sclerosomatidae), Araneae (Ctenidae; Ochyroceratidae: *Speocera* sp.; Pholcidae; Theridiidae: *Nesticodes rufipes*; Theridiosomatidae: *Plato* sp.), Thysanura (Nicoletiidae: Atelurinae), Diplura (Campodeidae), Collembola (Cyphoderidae; Entomobryidae), Orthoptera (Phalangopsidae: *Phalangopsis* sp., *Uvaroviella* sp.), Blattodea (Blaberidae: *Blaberus* sp.), Heteroptera (Cydnidae), Lepidoptera (Noctuidae; Tineidae), Diptera (Culicidae; Milichiidae; Phoridae; Psychodidae: *Lutzomyia* sp.; Sciaridae), Hymenoptera (Formicidae: *Ectatomma* sp., *Pachycondyla* sp., *Proceratium* sp., *Solenopsis* sp.), Coleoptera (Carabidae; Chrysomelidae: Alticinae; Elateridae; Scydmaenidae) e Spirostreptida (Pseudonannolenidae).

Dentre os vertebrados, foram encontradas duas espécies de Anura (Leptodactylidae: *Leptodactylus labyrinthicus*; Strabomantidae: *Pristimantis* sp.) e três de Chiroptera (Emballorunidae: *Peropteryx kappleri*; Phyllostomidae: *Carollia perspicillata*, *Glossophaga soricina*). Desta forma, no total foram encontrados 52 morfoespécies. Dentre essas, duas espécies foram consideradas troglomórficas Gastropoda (Systrophiidae) e Collembola (Cyphoderidae).

Caracterização faunística no período de chuva

Foi observado na caverna, um total de 85 morfoespécies de invertebrados de pelo menos 54 famílias dos Taxa: Oligochaeta, Gastropoda (Subulinidae), Isopoda (Dubioniscidae; Platyarthridae: *Trichorhina* sp.; Philosciidae), Acari (Actinedida: Parasitengonina; Mesostigmata; Oribatida; Anystidae: *Eritracarus* sp.), Amblypygi (Phrynidae: *Heterophrynus longicornis*), Pseudoscorpiones (Chernetidae; Chthoniidae), Opiliones (Sclerosomatidae), Araneae (Araneidae; Corinnidae: *Corinna* sp.; Ctenidae: *Enoploctenus* sp.; Ochyroceratidae: *Speocera* sp.; Oonopidae: Gamasomorphinae; Pholcidae: *Mesabolivar* sp.; Prodidomidae: *Lygromma* sp.; Scytodidae: *Scytodes itapevi*; Theraphosidae; Theridiosomatidae: *Plato* sp.), Thysanura (Nicoletiidae: Nicoletiinae, Atelurinae), Diplura (Campodeidae), Collembola (Bourletiellidae; Cyphoderidae; Entomobryidae; Tomoceridae), Orthoptera (Phalangopsidae: *Phalangopsis* sp., *Uvaroviella* sp.), Blattodea (Blaberidae: *Blaberus* sp.), Heteroptera (Cydnidae; Lygaeidae; Nabidae), Homoptera (Derbidae), Lepidoptera (Noctuidae; Tineidae; Tortricidae), Diptera (Cecidomyiidae; Ceratopogonidae; Drosophilidae; Milichiidae; Phoridae; Psychodidae: *Lutzomyia* sp.; Sciaridae), Hymenoptera (Formicidae: *Apterostigma* sp., *Atta* sp., *Azteca* sp., *Cardiocondyla* sp., *Ectatomma* sp., *Pachycondyla* spp., *Pheidole* sp.), Coleoptera (Carabidae; Chrysomelidae: Alticinae; Leiodidae; Scydmaenidae; Staphylinidae), Polydesmida (Cyrtodesmidae; Pyrgodesmidae), Spirostreptida (Pseudonannolenidae), Scolopendromorpha (Newportiidae: *Newportia* sp.; Scolopendridae: *Otostigmus* sp.) e Symphyla (Scutigerellidae: *Hanseniella* sp.).

Dentre os vertebrados, foram encontradas três espécies de Chiroptera (Emballorunidae: *Peropteryx kappleri*; Phyllostomidae: *Carollia perspicillata*, *Glossophaga soricina*) e um Squamata (Gekkonidae: *Thecadactylus rapicauda*).

Desta forma, no total foram encontrados 89 morfoespécies. Dentre essas, duas espécies foram consideradas troglomórficas Collembola (Cyphoderidae) e Polydesmida (Polydesmidae).

Caracterização geral da fauna da cavidade

Foi observado na caverna, um total de 106 morfoespécies de invertebrados de pelo menos 59 famílias dos Taxa: Oligochaeta, Gastropoda (Subulinidae; Systrophiidae), Isopoda (Dubioniscidae; Platyarthridae: *Trichorhina* sp.; Philosciidae), Acari (Actinedida: Parasitengonina; Mesostigmata; Oribatida; Acaridae: *Tyrophagus* sp.; Anystidae: *Eritracarus* sp.), Amblypygi (Phrynidae: *Heterophrynus longicornis*), Pseudoscorpiones (Chernetidae; Chthoniidae), Opiliones (Cosmetidae; Sclerosomatidae), Araneae (Araneidae; Corinnidae: *Corinna* sp.; Ctenidae: *Enoploctenus* sp.; Ochyroceratidae: *Speocera* sp.; Oonopidae: Gamasomorphinae; Pholcidae: *Mesabolivar* sp.; Prodidomidae: *Lygromma* sp.; Scytodidae: *Scytodes itapevi*; Theraphosidae; Theridiidae: *Nesticodes rufipes*; Theridiosomatidae: *Plato* sp.), Thysanura (Nicoletiidae: Nicoletiinae, Atelurinae), Diplura (Campodeidae), Collembola (Bourletiellidae; Cyphoderidae; Entomobryidae; Tomoceridae), Orthoptera (Phalangopsidae: *Phalangopsis* sp., *Uvaroviella* sp.), Blattodea (Blaberidae: *Blaberus* sp.), Heteroptera (Cydnidae; Lygaeidae; Nabidae), Homoptera (Derbidae), Lepidoptera (Noctuidae; Tineidae; Tortricidae), Diptera (Cecidomyiidae; Ceratopogonidae; Culicidae; Drosophilidae; Milichiidae; Phoridae; Psychodidae: *Lutzomyia* sp.; Sciaridae), Hymenoptera (Formicidae: *Apterostigma* sp., *Atta* sp., *Azteca* sp., *Cardiocondyla* sp., *Ectatomma* sp., *Pachycondyla* spp., *Pheidole* sp., *Proceratium* sp., *Solenopsis* sp.), Coleoptera (Carabidae; Chrysomelidae: Alticinae; Elateridae; Leiodidae; Scydmaenidae; Staphylinidae), Polydesmida (Cyrtodesmidae; Pyrgodesmidae), Spirostreptida (Pseudonannolenidae), Scolopendromorpha (Newportiidae: *Newportia* sp.; Scolopendridae: *Otostigmus* sp.) e Symphyla (Scutigerellidae: *Hanseniella* sp.).

Dentre os vertebrados, foram encontradas duas espécies de Anura (Leptodactylidae: *Leptodactylus labyrinthicus*; Strabomantidae: *Pristimantis* sp.), três espécies de Chiroptera (Emballorunidae: *Peropteryx kappleri*; Phyllostomidae: *Carollia perspicillata*, *Glossophaga soricina*) e um Squamata (Gekkonidae: *Thecadactylus rapicauda*).

Desta forma, no total foram encontrados 112 morfoespécies. Dentre essas, três espécies foram consideradas troglomórficas Gastropoda (Systrophiidae), Collembola (Cyphoderidae) e Polydesmida (Polydesmidae). Alguns organismos encontrados nesta caverna são mostrados na Figura 5.92.

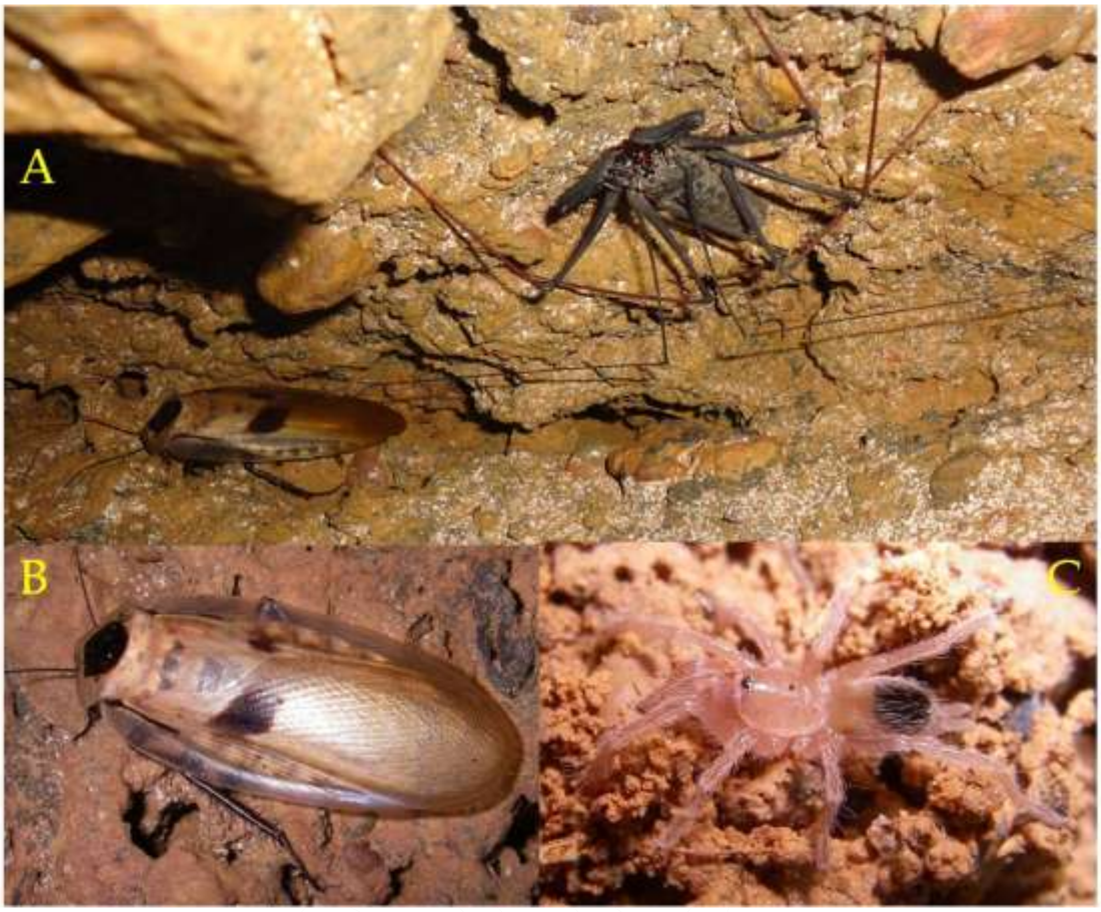

Figura 5.92 - A) Amblypygi: Phrinidae (*Heterophrynus longicornis*); B) Blattodea: Blaberidae (*Blaberus* sp.); C) Araneae: Theraphosidae.

CAVIDADE SL-109

Cavidade em canga detrítica com 15m de projeção horizontal localizada em área de mata ciliar, próxima a uma drenagem ativa. O entorno apresenta vegetação arbórea com árvores de médio porte, dossel inferior a 6m, sub-bosque de baixa densidade composto por vegetação arbustiva além de lianas e pteridófitas. A caverna possui apenas uma entrada, sendo esta baixa, porém ampla, e com piso reto. A entrada é sombreada, com muitos liquens, briófitas e pteridófitas revestindo as paredes e teto. Trata-se de uma cavidade úmida, porém pontos de gotejamento e áreas de percolação, principalmente a partir da região central da cavidade, são visualizados apenas no período chuvoso. O piso é predominantemente plano, com sedimento fino, grânulos e alguns seixos. O sistema radicular é pouco desenvolvido, sendo este composto por raízes de pequeno e médio porte na entrada e rede sub-superficial de micro-raízes. A serrapilheira está restrita a regiões próximas a linha d'água. Há uma colônia de morcegos na região central da cavidade. Estes são responsáveis pela produção elevada de guano. Quase toda cavidade é em penumbra clara, devido à entrada ampla, com áreas de penumbra escura nas regiões mais distais. Em frente à cavidade há uma drenagem ativa (riacho) a cerca de 3m. De forma geral trata-se de uma cavidade com elevada influência das condições ambientais externas, devido à entrada ampla e a proximidade com a drenagem, que provavelmente

promove eventos de inundação nesta cavidade. Além disso, já é possível observar atividade mineradora em um raio inferior a 250 metros da caverna.

Caracterização faunística no período de seca

Foi observado na caverna, um total de 38 morfoespécies de invertebrados de pelo menos 25 famílias dos Taxa: Gastropoda (Systrophiidae), Isopoda (Platyarthridae: *Trichorhina* sp.; Philosciidae; Scleropactidae), Acari (Mesostigmata: Macrochelidae; Sarcoptiformes: Arthronotina), Amblypygi (Phrynidae: *Heterophrynus longicornis*), Opiliones (Cosmetidae; Sclerosomatidae), Collembola (Cyphoderidae), Orthoptera (Phalangopsidae: *Phalangopsis* sp., *Uvaroviella* sp.), Isoptera (Termitidae: *Cornitermes* sp.), Heteroptera (Cydnidae), Lepidoptera (Tineidae), Diptera (Cecidomyiidae; Drosophilidae; Platypezidae aff.; Sciaridae), Hymenoptera (Formicidae: *Carebara* sp., *Ectatomma* sp., *Linepithema* sp., *Pachycondyla* spp., *Pheidole* sp.), Coleoptera (Carabidae; Curculionidae; Staphylinidae), Polydesmida (Cyrtodesmidae), Lithobiomorpha (Henicopidae: *Lamyctes* sp.) e Symphyla (Scutigerellidae: *Hanseniella* sp.).

Dentre os vertebrados, foi encontrada uma espécie de Anura (Strabomantidae: *Pristimantis* sp.) e duas espécies de Chiroptera (Phyllostomidae: *Carollia perspicillata*, *Glossophaga soricina*).

Desta forma, no total foram encontrados 41 morfoespécies. Dentre essas, duas espécies foram consideradas troglomórficas Gastropoda (Systrophiidae) e Collembola (Cyphoderidae).

Caracterização faunística no período de chuva

Foi observado na caverna, um total de 85 morfoespécies de invertebrados de pelo menos 53 famílias dos Taxa: Oligochaeta, Gastropoda (Systrophiidae), Isopoda (Platyarthridae: *Trichorhina* sp.; Philosciidae), Acari (Mesostigmata: Uropodina; Oribatida; Trombidiforme: Tydaidae), Decapoda (Pseudothelphusidae: *Microthelphusa somanni*), Amblypygi (Phrynidae: *Heterophrynus longicornis*), Scorpiones (Buthidae: *Ananteris luciae*), Pseudoscorpiones (Chernetidae), Opiliones (Escadabiidae; Phalangiidae; Sclerosomatidae), Araneae (Ctenidae; Dipluridae; Ochyroceratidae: *Speocera* sp.; Oonopidae: *Oonops* sp.; Pholcidae: *Mesabolivar* sp.; Salticidae; Theridiidae), Thysanura (Nicoletiidae: Atelurinae), Diplura (Campodeidae), Collembola (Bourletiellidae; Cyphoderidae; Entomobryidae; Tomoceridae), Orthoptera (Phalangopsidae: *Phalangopsis* sp., *Uvaroviella* sp., Phalangopsinae), Isoptera, Heteroptera (Cydnidae; Dipsocoridae; Lygaeidae; Nabidae; Ortheziidae; Pyrrhocoridae), Homoptera (Cixiidae), Lepidoptera (Tineidae), Diptera (Cecidomyiidae; Drosophilidae; Fanniidae; Phoridae; Platypezidae aff.; Psychodidae: *Lutzomyia* spp.; Sciaridae; Sphaeroceridae), Hymenoptera (Formicidae: *Acanthostichus* sp., *Anochetus* sp., *Azteca* sp., *Carebara* spp., *Hypoponera* sp., *Leptogenys* sp., *Linepithema* sp., *Pachycondyla* spp., *Solenopsis* sp.), Coleoptera (Carabidae: Clivinina; Curculionidae; Scydmaenidae; Staphylinidae), Polydesmida (Chelodesmidae; Cyrtodesmidae; Pyrgodesmidae), Lithobiomorpha (Henicopidae: *Lamyctes* sp.), Scolopendromorpha (Newportiidae: *Newportia* sp.), Scutigeromorpha (Pselliodidae: *Sphendononema guildingii*) e Symphyla (Scutigerellidae: *Hanseniella* sp.).

Dentre os vertebrados, foram encontradas duas espécies de Chiroptera (Phyllostomidae: *Carollia perspicillata*, *Glossophaga soricina*).

Desta forma, no total foram encontrados 87 morfoespécies. Dentre essas, três espécies foram consideradas troglomórficas Gastropoda (Systrophiidae), Collembola (Cyphoderidae) e Polydesmida (Pyrgodesmidae).

Caracterização geral da fauna da cavidade

Foi observado na caverna, um total de 102 morfoespécies de invertebrados de pelo menos 58 famílias dos Taxa: Oligochaeta, Gastropoda (Systrophiidae), Isopoda (Platyarthridae: *Trichorhina* sp.; Philosciidae; Scleropactidae), Acari (Mesostigmata: Macrochelidae, Uropodina; Oribatida; Sarcoptiformes: Arthronotina; Trombidiforme: Tydaidae), Decapoda (Pseudothelphusidae: *Microthelphusa somanni*), Amblypygi (Phrynidae: *Heterophrynus longicornis*), Scorpiones (Buthidae: *Ananteris luciae*), Pseudoscorpiones (Chernetidae), Opiliones (Cosmetidae; Escadabiidae; Phalangiidae; Sclerosomatidae), Araneae (Ctenidae; Dipluridae; Ochyroceratidae: *Speocera* sp.; Oonopidae: *Oonops* sp.; Pholcidae: *Mesabolivar* sp.; Salticidae; Theridiidae), Thysanura (Nicoletiidae: Atelurinae), Diplura (Campodeidae), Collembola (Bourletiellidae; Cyphoderidae; Entomobryidae; Tomoceridae), Orthoptera (Phalangopsidae: *Phalangopsis* sp., *Uvaroviella* sp., Phalangopsinae), Isoptera (Termitidae: *Cornitermes* sp.), Heteroptera (Cydnidae; Dipsocoridae; Lygaeidae; Nabidae; Ortheziidae; Pyrrhocoridae), Homoptera (Cixiidae), Lepidoptera (Tineidae), Diptera (Cecidomyiidae; Drosophilidae; Fanniidae; Phoridae; Platypezidae aff.; Psychodidae: *Lutzomyia* spp.; Sciaridae; Sphaeroceridae), Hymenoptera (Formicidae: *Acanthostichus* sp., *Anochetus* sp., *Azteca* sp., *Carebara* spp., *Hypoponera* sp., *Leptogenys* sp., *Linepithema* sp., *Pachycondyla* spp., *Solenopsis* sp.), Coleoptera (Carabidae: Clivinina; Curculionidae; Scydmaenidae; Staphylinidae), Polydesmida (Chelodesmidae; Cyrtodesmidae; Pyrgodesmidae), Lithobiomorpha (Henicopidae: *Lamyctes* sp.), Scolopendromorpha (Newportiidae: *Newportia* sp.), Scutigeromorpha (Pselliodidae: *Sphendononema guildingii*) e Symphyla (Scutigerellidae: *Hanseniella* sp.).

Dentre os vertebrados, foi encontrada uma espécie de Anura (Strabomantidae: *Pristimantis* sp.) e duas espécies de Chiroptera (Phyllostomidae: *Carollia perspicillata*, *Glossophaga soricina*).

Desta forma, no total foram encontrados 105 morfoespécies. Dentre essas, três espécies foram consideradas troglomórficas Gastropoda (Systrophiidae), Collembola (Cyphoderidae) e Polydesmida (Pyrgodesmidae).

CAVIDADE SL-110

Cavidade em canga detrítica com 32m de projeção horizontal localizada em área de mata ciliar, próxima a uma drenagem ativa. O entorno apresenta vegetação arbórea com árvores de médio e grande porte, dossel inferior a 10m, sub-bosque de baixa densidade composto por vegetação arbustiva além de lianas e pteridófitas. A caverna possui apenas uma entrada, sendo esta baixa, porém ampla, e com piso em declive. A entrada é sombreada, com muitos liquens, briófitas e pteridófitas revestindo as paredes e teto. Trata-se de uma cavidade úmida, porém pontos de gotejamento e áreas de percolação são visíveis apenas no período chuvoso. O piso é predominantemente plano, úmido apenas na campanha chuvosa, com sedimento fino, grânulos, alguns seixos e calhaus. O sistema radicular é pouco desenvolvido, sendo este

composto por raízes de pequeno e médio porte na entrada. A serrapilheira está restrita a regiões próximas a linha d'água. Há uma grande colônia de morcegos, com alta diversidade de espécies, formada por centenas de indivíduos, sendo estes responsáveis pela produção elevada de guano, importante recurso trófico da cavidade, estando presente em quase toda área do piso, recobrindo-o. Quase toda cavidade é em penumbra escura, devido à entrada em teto baixo, com áreas de penumbra clara próxima a mesma. De forma geral trata-se de uma cavidade em que o guano predomina como recurso trófico. Assim, há uma abundância elevada de alguns táxons especializados neste tipo de recurso, promovendo uma baixa equitabilidade da comunidade. A cavidade apresenta baixa influência das condições ambientais externas, devido à entrada em teto baixo, porém já é possível observar atividade mineradora em um raio inferior a 250 metros da cavidade.

Caracterização faunística no período de seca

Foi observado na caverna, um total de 66 morfoespécies de invertebrados de pelo menos 39 famílias dos Taxa: Isopoda (Philosciidae), Acari (Actinedida: Parasitengonina; Mesostigmata; Oribatida; Acaridae: *Tyrophagus* sp.), Amblypygi (Phrynidae: *Heterophrynus longicornis*), Pseudoscorpiones (Chernetidae; Chthoniidae), Opiliones (Cosmetidae; Sclerosomatidae), Araneae (Ctenidae; Pholcidae: *Mesabolivar* sp.; Salticidae; Theridiidae: *Nesticodes rufipes*), Thysanura (Nicoletiidae: Nicoletiinae, Atelurinae), Collembola (Cyphoderidae; Entomobryidae), Orthoptera (Phalangopsidae: *Phalangopsis* sp., *Uvaroviella* sp., Phalangopsinae), Blattodea (Blaberidae: *Blaberus* spp.), Isoptera (Termitidae: *Nasutitermes* sp.), Heteroptera (Cydnidae; Reduviidae: *Zelurus* sp.), Lepidoptera (Tineidae; Tortricidae), Diptera (Culicidae; Keroplatidae; Milichiidae; Mycetophilidae; Psychodidae: *Lutzomyia* sp.; Streblidae), Hymenoptera (Formicidae: *Azteca* sp., *Camponotus* spp., *Cardiocondyla* sp., *Cyphomyrmex* sp., *Odontomachus* sp., *Pachycondyla* sp., *Pheidole* sp., *Rogeria* sp., *Solenopsis* spp.; Bethilidae; Chalcididae; Cynipidae; Diapriidae), Coleoptera (Carabidae; Curculionidae; Elateridae: Cardiophorinae; Histeridae; Staphylinidae: Pselaphinae) e Symphyla (Scutigerellidae: *Hanseniella* sp.).

Dentre os vertebrados, foram encontradas duas espécies de Anura (Bufonidae: *Rhinella* sp.; Strabomantidae: *Pristimantis* sp.) e cinco espécies de Chiroptera (Furipteridae: *Furipterus horrens*; Phyllostomidae: *Carollia perspicillata*, *Lonchorhina aurita*; *Lionycteris spurrelli*; *Glossophaga soricina*).

Desta forma, no total foram encontrados 74 morfoespécies. Dentre essas, apenas Collembola (Cyphoderidae) foi considerada troglomórfica.

Caracterização faunística no período de chuva

Foi observado na caverna, um total de 105 morfoespécies de invertebrados de pelo menos 69 famílias dos Taxa: Gastropoda (Subulinidae; Systrophiidae), Isopoda (Dubioniscidae; Platyarthridae: *Trichorhina* sp.; Philosciidae), Acari (Actinedida: Rhagidiidae, Parasitengonina; Ixodida; Mesostigmata: Ameroseiidae; Oribatida; Acaridae: *Tyrophagus* sp.), Amblypygi (Phrynidae: *Heterophrynus longicornis*), Pseudoscorpiones (Chernetidae; Chthoniidae), Opiliones (Phalangiidae; Stygnommatidae), Araneae (Amaurobiidae; Corinnidae: *Corinna* sp.; Ctenidae; Oonopidae; Pholcidae: *Mesabolivar* sp.; Salticidae; Scytodidae: *Scytodes itapevi*; Theraphosidae; Theridiidae: *Nesticodes rufipes*), Thysanura (Nicoletiidae: Nicoletiinae), Diplura

(Campodeidae), Collembola (Entomobryidae; Isotomidae), Orthoptera (Phalangopsidae: *Phalangopsis* sp., *Uvaroviella* sp., Phalangopsinae), Blattodea (Blaberidae: *Blaberus* sp.; Blattidae), Isoptera (Termitidae: *Nasutitermes* sp.; Rhinotermitidae: *Heterotermes* sp.), Psocoptera (Myopsocidae), Heteroptera (Cydnidae; Lygaeidae; Nabidae; Reduviidae: Emesinae), Lepidoptera (Noctuidae; Tineidae; Tortricidae), Diptera (Calliphoridae; Culicidae; Drosophilidae; Fanniidae; Keroplatidae; Milichiidae; Muscidae; Sciaridae; Streblidae), Hymenoptera (Formicidae: *Acromyrmex* sp., *Apterostigma* sp., *Atta* sp., *Camponotus* sp., *Carebara* sp., *Odontomachus* sp., *Pachycondyla* spp., *Pheidole* sp., *Solenopsis* sp.; Bethilidae; Chalcididae; Diapriidae; Eulophidae), Coleoptera (Carabidae; Elateridae: Cardiophorinae; Eucnemidae; Leiodidae; Scydmaenidae; Staphylinidae), Polydesmida (Chelodesmidae; Pyrgodesmidae), Spirostreptida (Pseudonannolenidae), Stemmiulida (Stemmiulidae), Scolopendromorpha (Newportiidae: *Newportia* sp.; Scolopendridae: *Otostigmus* sp.) e Symphyla (Scutigerellidae: *Scutigerella* sp).

Dentre os vertebrados, foram encontradas duas espécies de Anura (Strabomantidae: *Pristimantis* sp.; e um indeterminado) e cinco espécies de Chiroptera (Furipteridae: *Furipterus horrens*; Mormoopidae: *Pteronotus parnellii*; Phyllostomidae: *Carollia perspicillata*, *Lionycteris spurrelli, Lonchorhina sp.*; *Glossophaga soricina*).

Desta forma, no total foram encontrados 113 morfoespécies. Dentre essas, duas espécies foram consideradas troglomórficas Gastropoda (Systrophiidae) e Coleoptera (Eucnemidae).

Caracterização geral da fauna da cavidade

Foi observado na caverna, um total de 139 morfoespécies de invertebrados de pelo menos 74 famílias dos Taxa: Gastropoda (Subulinidae; Systrophiidae), Isopoda (Dubioniscidae; Platyarthridae: *Trichorhina* sp.; Philosciidae), Acari (Actinedida: Rhagidiidae, Parasitengonina; Ixodida; Mesostigmata: Ameroseiidae; Oribatida; Acaridae: *Tyrophagus* sp.), Amblypygi (Phrynidae: *Heterophrynus longicornis*), Pseudoscorpiones (Chernetidae; Chthoniidae), Opiliones (Cosmetidae; Phalangiidae; Sclerosomatidae; Stygnommatidae), Araneae (Amaurobiidae; Corinnidae: *Corinna* sp.; Ctenidae; Oonopidae; Pholcidae: *Mesabolivar* sp.; Salticidae; Scytodidae: *Scytodes itapevi*; Theraphosidae; Theridiidae: *Nesticodes rufipes*), Thysanura (Nicoletiidae: Nicoletiinae, Atelurinae), Diplura (Campodeidae), Collembola (Cyphoderidae; Entomobryidae; Isotomidae), Orthoptera (Phalangopsidae: *Phalangopsis* sp., *Uvaroviella* sp., Phalangopsinae), Blattodea (Blaberidae: *Blaberus* spp.; Blattidae), Isoptera (Termitidae: *Nasutitermes* sp.; Rhinotermitidae: *Heterotermes* sp.), Psocoptera (Myopsocidae), Heteroptera (Cydnidae; Lygaeidae; Nabidae; Reduviidae: Emesinae, *Zelurus* sp.), Lepidoptera (Noctuidae; Tineidae; Tortricidae), Diptera (Calliphoridae; Culicidae; Drosophilidae; Fanniidae; Keroplatidae; Milichiidae; Muscidae; Mycetophilidae; Psychodidae: *Lutzomyia* sp.; Sciaridae; Streblidae), Hymenoptera (Formicidae: *Acromyrmex* sp., *Apterostigma* sp., *Atta* sp., *Azteca* sp.; *Camponotus* spp., *Carebara* sp., *Cyphomyrmex* sp., *Odontomachus* sp., *Pachycondyla* spp., *Pheidole* spp., *Rogeria* sp., *Solenopsis* spp.; Bethilidae; Chalcididae; Cynipidae; Diapriidae; Eulophidae), Coleoptera (Carabidae; Elateridae: Cardiophorinae; Eucnemidae; Histeridae; Leiodidae; Scydmaenidae; Staphylinidae: Pselaphinae), Polydesmida (Chelodesmidae; Pyrgodesmidae), Spirostreptida (Pseudonannolenidae), Stemmiulida (Stemmiulidae), Scolopendromorpha (Newportiidae: *Newportia* sp.; Scolopendridae: *Otostigmus* sp.) e Symphyla (Scutigerellidae: *Hanseniella* sp., *Scutigerella* sp).

Dentre os vertebrados, foram encontradas três espécies de Anura (Bufonidae: *Rhinella* sp.; Strabomantidae: *Pristimantis* sp.; e um indeterminado) e cinco espécies de Chiroptera (Furipteridae: *Furipterus horrens*; Mormoopidae: *Pteronotus parnellii*; Phyllostomidae: *Carollia perspicillata*, *Lionycteris spurrelli, Lonchorhina aurita*; *Lonchorhina sp.*; *Glossophaga soricina*).

Desta forma, no total foram encontrados 149 morfoespécies. Dentre essas, três espécies foram consideradas troglomórficas Gastropoda (Systrophiidae), Collembola (Cyphoderidae) e Coleoptera (Eucnemidae). Alguns organismos encontrados nesta caverna são mostrados na Figura 5.93 e na Figura 5.94.

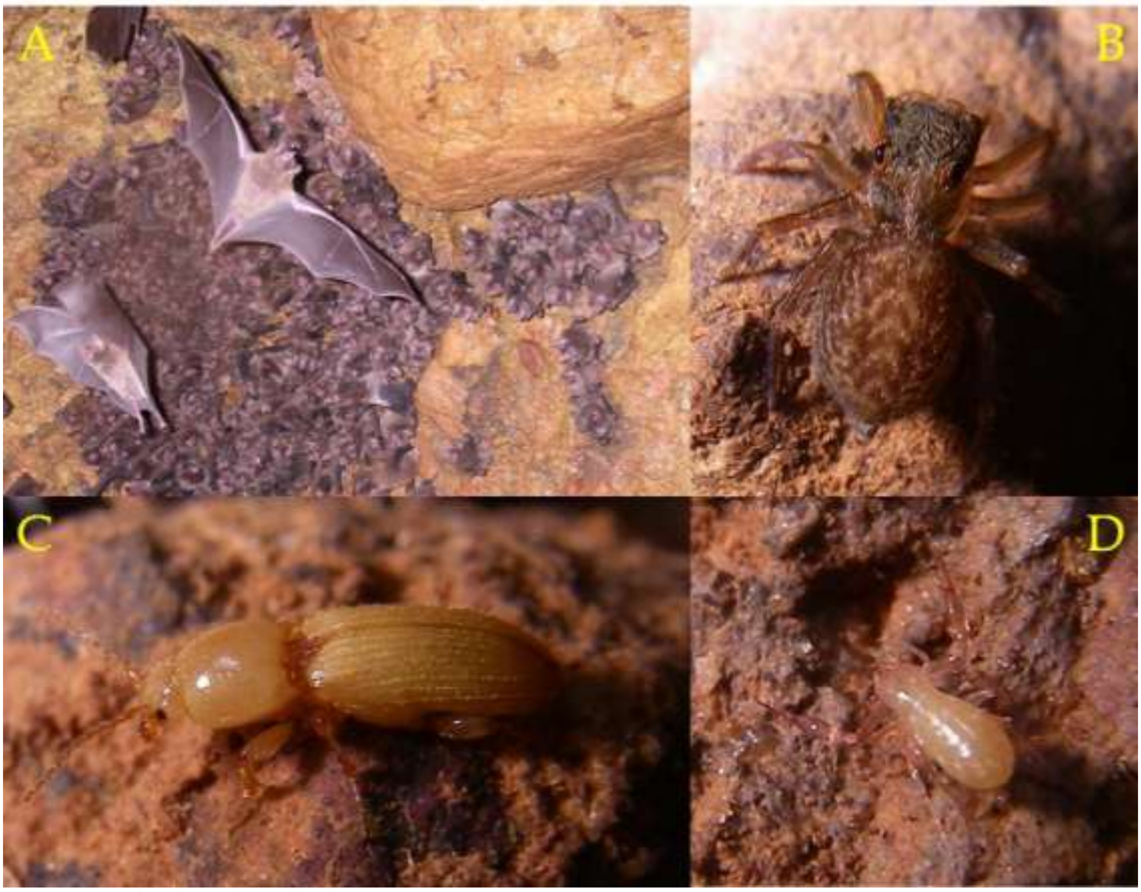

Figura 5.93 - A) Colônia mista de Chiroptera:; B) Araneae: Salticidae; C) Coleoptera: Eucnemidae; D) Pseudoscorpiones: Chthoniidae; E) Decapoda: Pseudothelphusidae (*Microthelphusa somanni*).

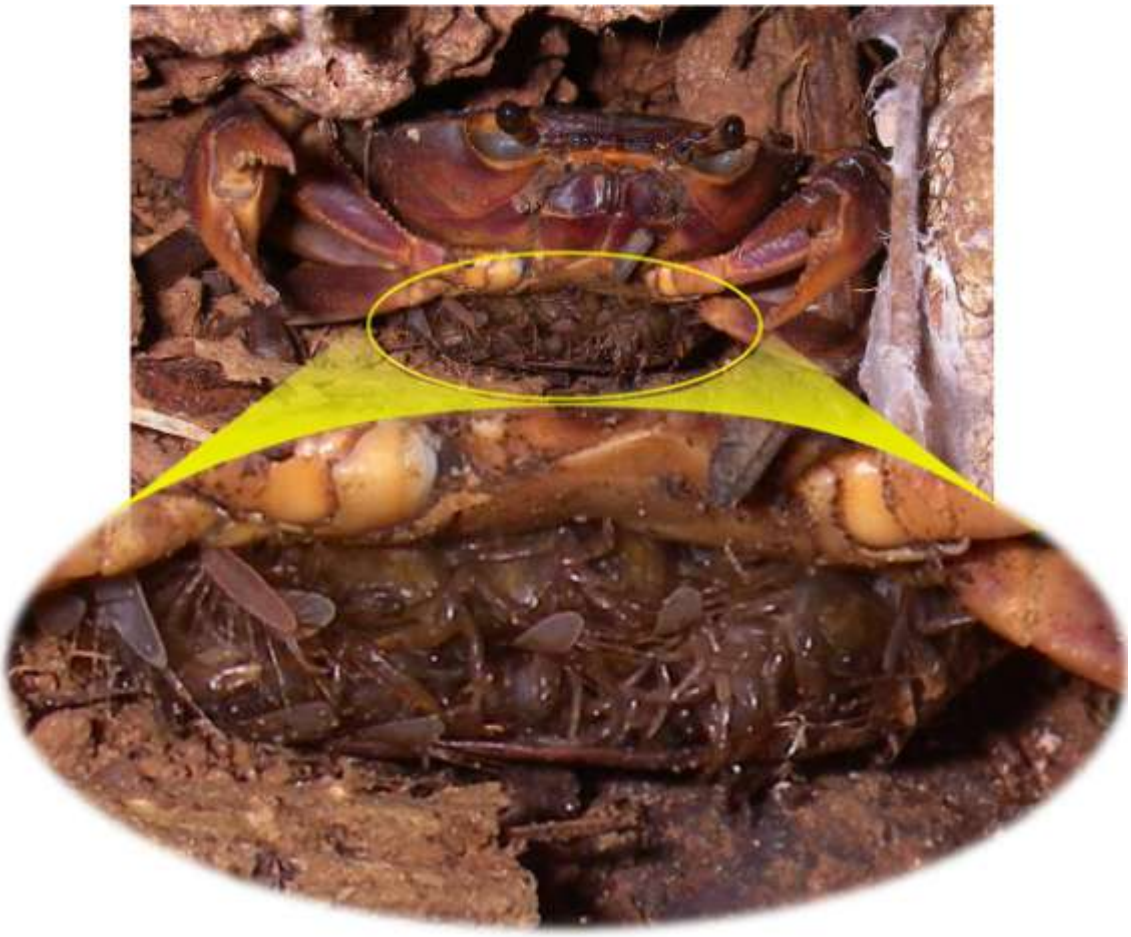

Figura 5.94 - Decapoda: Pseudothelphusidae (*Microthelphusa somanni*). Em detalhe, filhotes guardados sob o abdômen.

CAVIDADE SL-111

Cavidade com 8,0m de projeção horizontal, formada em canga detrítica e localizada junto a uma drenagem ativa, com um lago a sua frente. A vegetação de entorno é composta por mata ciliar, com árvores de troncos finos, dossel inferior a seis metros de altura, com arbustos e lianas associadas ao sub-bosque e serrapilheira pouco densa acumulada junto ao substrato. Não há cobertura vegetal em sua zona de entrada. Entrada ampla e alta, com grande concentração de musgos, fungos, liquens, algas, pteridófitas, brotos e alguns troncos por toda parede e piso da cavidade. A cavidade apresenta um pequeno desnível inicial, sendo seu salão principal plano. De forma geral, o piso apresenta-se úmido, composto por sedimento fino e alguns grânulos e seixos, sendo o mesmo recoberto, em grande parte, por matéria orgânica vegetal em decomposição, a qual foi carreada pela água do riacho localizado à frente do abrigo. Foram encontrados restos de coco, chinelos e recipientes de plásticos em seu interior, o que indica que a região de entorno é utilizada pela comunidade de Serra Pelada como área de lazer. A cavidade apresenta somente zona de entrada e seus canalículos são pouco desenvolvidos. O sistema radicular é composto por raízes de pequeno e médio porte. A presença de gotejamento é visível apenas no período chuvoso. Um cupinzeiro ativo encontra-se no interior do abrigo. Ausência de depósitos de guano e espécies de Chiroptera. De forma geral, trata-se de uma cavidade com elevada influência das condições ambientais epígeas.

Além disso, já é possível observar atividade mineradora em um raio inferior a 250 metros do abrigo.

Caracterização faunística no período de seca

Foi observado na caverna, um total de 18 morfoespécies de invertebrados de pelo menos 12 famílias dos Taxa: Oligochaeta, Decapoda (Pseudothelphusidae: *Microthelphusa somanni*), Actinedida (Parasitengonina), Trombidiforme (Cheyletidae), Araneae (Ctenidae, Pholcidae: *Mesabolivar* sp., Salticidae, Theridiosomatidae: *Plato* sp.), Diplura (Campodeidae), Collembola (Entomobryidae), Isoptera, Psocoptera, Hymenoptera (Formicidae: *Pachycondyla* sp.; *Solenopsis* sp.), Coleoptera (Staphylinidae), Scutigeromorpha (Pselliodidae: *Sphendononema guildingii*), Symphyla (Scutigerellidae: *Hanseniella* sp.).

Caracterização faunística no período de chuva

Foi observado na caverna, um total de 46 morfoespécies de invertebrados de pelo menos 30 famílias dos Taxa: Oligochaeta, Platyhelminthes (Tricladida), Isopoda (Dubioniscidae), Pseudoscorpiones (Syarinidae), Opiliones (Sclerosomatidae), Acari, Actinedida (Parasitengonina), Araneae (Araneidae, Ctenidae: *Enoploctenus* sp., Pholcidae: *Mesabolivar* sp., Salticidae, Scytodidae: *Scytodes itapevi*, Symphytognathidae: *Anapistula* sp.), Thysanura (Meinertellidae), Diplura (Campodeidae), Collembola (Entomobryidae, Sminthuridae), Neuroptera (Myrmeleontidae), Orthoptera (Phalangopsidae: *Uvaroviella* sp.), Blattodea (Blattidae), Isoptera (Termitidae: *Nasutitermes* sp.; *Velocitermes* sp.), Psocoptera (Archipsocidae), Lepidoptera (Tineidae), Diptera (Milichiidae), Hymenoptera (Formicidae: *Atta* sp.; *Pachycondyla* sp.; *Rogeria* sp.; *Solenopsis* sp., Bethilidae), Coleoptera (Carabidae: Clivinina, Chrysomelidae: Alticinae, Staphylinidae: Pselaphinae), Polydesmida (Polydesmidae), Siphonophorida (Siphonophoridae), Stemmiulida (Stemmiulidae), Lithobiomorpha (Henicopidae: *Lamyctes* sp.), Symphyla (Scutigerellidae: *Hanseniella* sp.; *Scutigerella* sp.).

Caracterização geral da fauna da cavidade

Foi observado na caverna, um total de 54 morfoespécies de invertebrados de pelo menos 34 famílias dos Taxa: Oligochaeta, Platyhelminthes (Tricladida), Isopoda (Dubioniscidae), Decapoda (Pseudothelphusidae: *Microthelphusa somanni*), Pseudoscorpiones (Syarinidae), Opiliones (Sclerosomatidae), Acari, Actinedida (Parasitengonina), Trombidiforme (Cheyletidae), Araneae (Araneidae, Ctenidae: *Enoploctenus* sp., Pholcidae: *Mesabolivar* sp., Salticidae, Scytodidae: *Scytodes itapevi*, Symphytognathidae: *Anapistula* sp., Theridiosomatidae: *Plato* sp.), Thysanura (Meinertellidae), Diplura (Campodeidae), Collembola (Entomobryidae, Sminthuridae), Neuroptera (Myrmeleontidae), Orthoptera (Phalangopsidae: *Uvaroviella* sp.), Blattodea (Blattidae), Isoptera (Termitidae: *Nasutitermes* sp.; *Velocitermes* sp.), Psocoptera (Archipsocidae), Lepidoptera (Tineidae), Diptera (Milichiidae), Hymenoptera (Formicidae: *Atta* sp.; *Pachycondyla* sp.; *Rogeria* sp.; *Solenopsis* sp., Bethilidae), Coleoptera (Carabidae: Clivinina, Chrysomelidae: Alticinae, Staphylinidae: Pselaphinae), Polydesmia (Polydesmidae), Siphonophorida(Siphonophoridae), Stemmiulida (Stemmiulidae), Lithobiomorpha (Henicopidae: *Lamyctes* sp.), Scutigeromorpha (Pselliodidae: *Sphendononema guildingii*), Symphyla (Scutigerellidae: *Hanseniella* sp.; *Scutigerella* sp.). Alguns organismos encontrados nesta caverna são mostrados na figura 22.

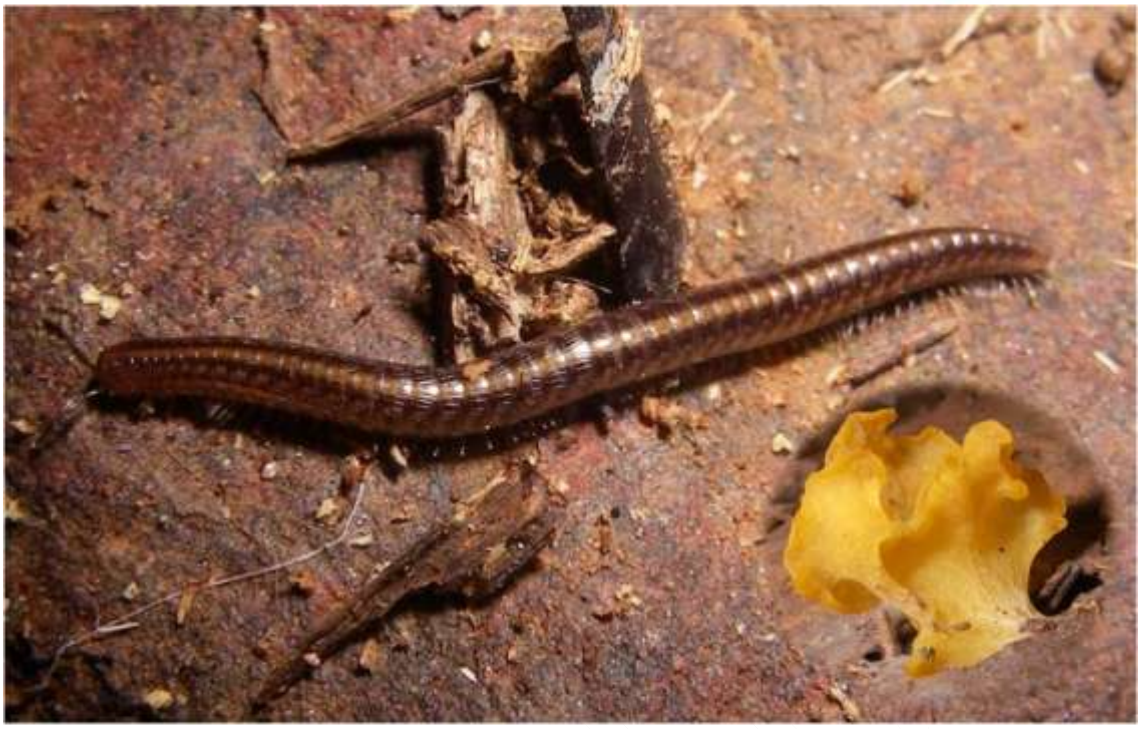

Figura 5.95 - Stemmiulida: Stemmiulidae. E, detalhe, um fungo Basidiomyceto.

CAVIDADE SL-112

Cavidade com 21,0m de projeção horizontal, formada em canga detrítica, localizada próxima a uma drenagem ativa (cerca de 5m). A vegetação de entorno é composta por mata ciliar, com árvores de troncos finos, dossel inferior a seis metros de altura, com arbustos e lianas associadas ao sub-bosque e serrapilheira pouco densa acumulada junto ao substrato. A entrada é ampla e sombreada, com musgos e pteridófitas pelas paredes, fungos pelo teto, alguns brotos esparsos e presença de serrapilheira restrita a linha d'água. O piso é levemente ascendente e composto por sedimento fino e granulado, com alguns seixos e calhaus por toda sua extensão. De forma geral, a cavidade é seca. A presença de pontos de gotejamento é registrada apenas durante o período úmido. O sistema radicular é composto por raízes de pequeno, médio e grande porte, além de micro-raízes esparsas e presença de rizotemas. A cavidade apresenta canalículos pouco desenvolvidos. Ausência de depósitos de guano. Há um cupinzeiro desativado no interior do abrigo. A cavidade apresenta somente penumbra clara. De forma geral, trata-se de uma cavidade pobre em recursos tróficos e com elevada influência das condições ambientais externas. Além disso, já é possível observar atividade mineradora em um raio inferior a 250 metros da cavidade.

Caracterização faunística no período de seca

Foi observado na caverna, um total de 55 morfoespécies de invertebrados de pelo menos 39 famílias dos Taxa: Isopoda (Dubioniscidae, Platyarthridae: *Trichorhina* sp., Philosciidae), Pseudoscorpiones (Chernetidae, Chthoniidae), Acari, Oribatiba, Araneae (Ochyroceratidae: *Speocera* sp., Oonopidae: *Oonops* sp., Pholcidae: *Metagonia* sp.; Ninetinae, Theridiidae, Theridiosomatidae: *Plato* sp., Uloboridae), Diplura (Campodeidae), Collembola (Entomobryidae), Neuroptera (Myrmeleontidae), Orthoptera (Phalangopsidae: *Phalangopsis*

sp.; *Uvaroviella* sp.), Blattodea (Blaberidae: *Blaberus* sp., Blattidae), Isoptera (Termitidae: *Nasutitermes* sp.), Siphonaptera (Pulicidae), Psocoptera (Epipsocidae), Heteroptera (Enicocephalidae, Reduviidae: *Zelurus* sp.; Emesinae), Homoptera (Cixiidae: *Pintalia* sp.), Lepidoptera (Tineidae, Tortricidae), Diptera (Culicidae, Drosophilidae, Fanniidae, Keroplatidae, Milichiidae, Psychodidae: *Lutzomyia* sp., Tipulidae), Hymenoptera (Formicidae: *Camponotus* sp.; *Pachycondyla* sp.; *Pheidole* sp.; *Rogeria* sp.), Coleoptera (Carabidae, Leiodidae, Staphylinidae, Elateridae: Cardiophorinae), Scolopendromorpha (Scolopendridae: *Cryptops* sp.), Symphyla (Scutigerellidae: *Hanseniella* sp.).

Dentre os vertebrados, foram encontradas duas espécies das ordens: Anura (Strabomantidae: *Pristimantis* sp.) e Chiroptera (Phyllostomidae: Glossophaginae). Desta forma, no total foram encontrados 57 morfoespécies.

Caracterização faunística no período de chuva

Foi observado na caverna, um total de 85 morfoespécies de invertebrados de pelo menos 50 famílias dos Taxa: Oligochaeta, Gastropoda (Systrophiidae), Isopoda (Dubioniscidae, Platyarthridae: *Trichorhina* sp., Philosciidae), Amblypygi (Phrinidae: *Heterophrynus longicornis*), Pseudoscorpiones (Chernetidae, Chthoniidae), Opiliones (Sclerosomatidae), Actinedida (Parasitengonina), Mesostigmata, Oribatida, Araneae (Amaurobiidae, Ctenidae: *Enoploctenus* sp., Ochyroceratidae: *Speocera* sp., Oonopidae: Gamasophorphinae, Pholcidae: *Mesabolivar* sp., Prodidomidae: *Lygromma* sp., Salticidae, Scytodidae: *Scytodes itapevi*, Theridiidae, Theridiosomatidae: *Plato* sp.), Thysanura (Nicoletiidae: Atelurinae, Meinertellidae), Diplura (Campodeidae), Collembola (Entomobryidae, Tomoceridae), Neuroptera (Myrmeleontidae), Orthoptera (Phalangopsidae: *Uvaroviella* sp.), Blattodea (Blaberidae: *Blaberus* sp., Blattidae), Isoptera (Termitidae: *Nasutitermes* sp.), Psocoptera (Lepidopsocidae, Myopsocidae, Pachytroctidae; Trogiidae), Heteroptera (Alydidae, Cydnidae, Lygaeidae, Reduviidae: *Zelurus* sp.), Lepidoptera (Noctuidae, Tineidae), Diptera (Cecidomyiidae, Ceratopogonidae, Drosophilidae, Keroplatidae, Phoridae), Hymenoptera (Formicidae: *Apterostigma* sp.; *Atta* spp.; *Camponotus* sp.; *Carebara* sp.; *Crematogaster* sp.; *Ectatomma* sp.; *Linepithema* sp.; *Octostruma* sp.; *Pachycondyla* sp.; *Pheidole* spp.; *Solenopsis* sp.; *Strumigenys* sp.), Coleoptera (Carabidae: Clivinina, Chrysomelidae, Lampyridae, Staphylinidae: Pselaphinae, Scydmaenidae), Scolopendromorpha (Scolopendridae: *Cryptops* sp.).

Dentre os vertebrados, foi encontrada uma espécie da ordem Chiroptera (Phyllostomidae: *Glossophaga soricina*).

Desta forma, no total foram encontrados 86 morfoespécies. Dentre essas, duas espécies de invertebrados foram consideradas troglomórficas: Gastropoda (Systrophiidae) e Araneae (Oonopidae).

Caracterização geral da fauna da cavidade

Foi observado na caverna, um total de 122 morfoespécies de invertebrados de pelo menos 64 famílias dos Taxa: Oligochaeta, Gastropoda (Systrophiidae), Isopoda (Dubioniscidae, Platyarthridae: *Trichorhina* sp., Philosciidae), Amblypygi (Phrinidae: *Heterophrynus longicornis*), Pseudoscorpiones (Chernetidae, Chthoniidae), Opiliones (Sclerosomatidae), Acari, Actinedida (Parasitengonina), Mesostigmata, Oribatida, Araneae (Amaurobiidae, Ctenidae:

Enoploctenus sp., Ochyroceratidae: *Speocera* sp., Oonopidae: Gamasophorphinae; *Oonops* sp., Pholcidae: *Mesabolivar* sp.; *Metagonia* sp.; Ninetinae, Prodidomidae: *Lygromma* sp., Salticidae, Scytodidae: *Scytodes itapevi*, Theridiidae, Theridiosomatidae: *Plato* sp., Uloboridae), Thysanura (Nicoletiidae: Atelurinae, Meinertellidae), Diplura (Campodeidae), Collembola (Entomobryidae, Tomoceridae), Neuroptera (Myrmeleontidae), Orthoptera (Phalangopsidae: *Phalangopsis* sp.; *Uvaroviella* sp.), Blattodea (Blaberidae: *Blaberus* spp., Blattidae), Isoptera (Termitidae: *Nasutitermes* sp.), Psocoptera (Epipsocidae, Lepidopsocidae, Myopsocidae, Pachytroctidae; Trogiidae), Heteroptera (Alydidae, Cydnidae, Enicocephalidae, Lygaeidae, Reduviidae: *Zelurus* sp.; Emesinae), Homoptera (Cixiidae: *Pintalia* sp.), Lepidoptera (Noctuidae, Tineidae, Tortricidae), Diptera (Cecidomyiidae, Ceratopogonidae, Culicidae, Drosophilidae, Fanniidae, Keroplatidae, Milichiidae, Phoridae, Psychodidae, Tipulidae), Hymenoptera (Formicidae: *Apterostigma* sp.; *Atta* spp.; *Camponotus* sp.; *Carebara* sp.; *Crematogaster* sp.; *Ectatomma* sp.; *Linepithema* sp.; *Octostruma* sp.; *Pachycondyla* sp.; *Pheidole* spp.; *Rogeria* sp.; *Solenopsis* sp.; *Strumigenys* sp.), Coleoptera (Carabidae: Clivinina, Chrysomelidae, Elateridae: Cardiophorinae, Lampyridae, Leiodidae, Staphylinidae: Pselaphinae, Scydmaenidae), Scolopendromorpha (Scolopendridae: *Cryptops* spp.), Symphyla (Scutigerellidae: *Hanseniella* spp.).

Dentre os vertebrados, foram encontradas três espécies das ordens: Anura (Strabomantidae: *Pristimantis* sp.), Chiroptera (Phyllostomidae: Glossophaginae, *Glossophaga soricina*).

Desta forma, no total foram encontrados 125 morfoespécies. Dentre essas, duas espécies de invertebrados foram consideradas troglomórficas: Gastropoda (Systrophiidae) e Araneae (Oonopidae). Alguns organismos encontrados nesta caverna são mostrados na Figura 5.95.

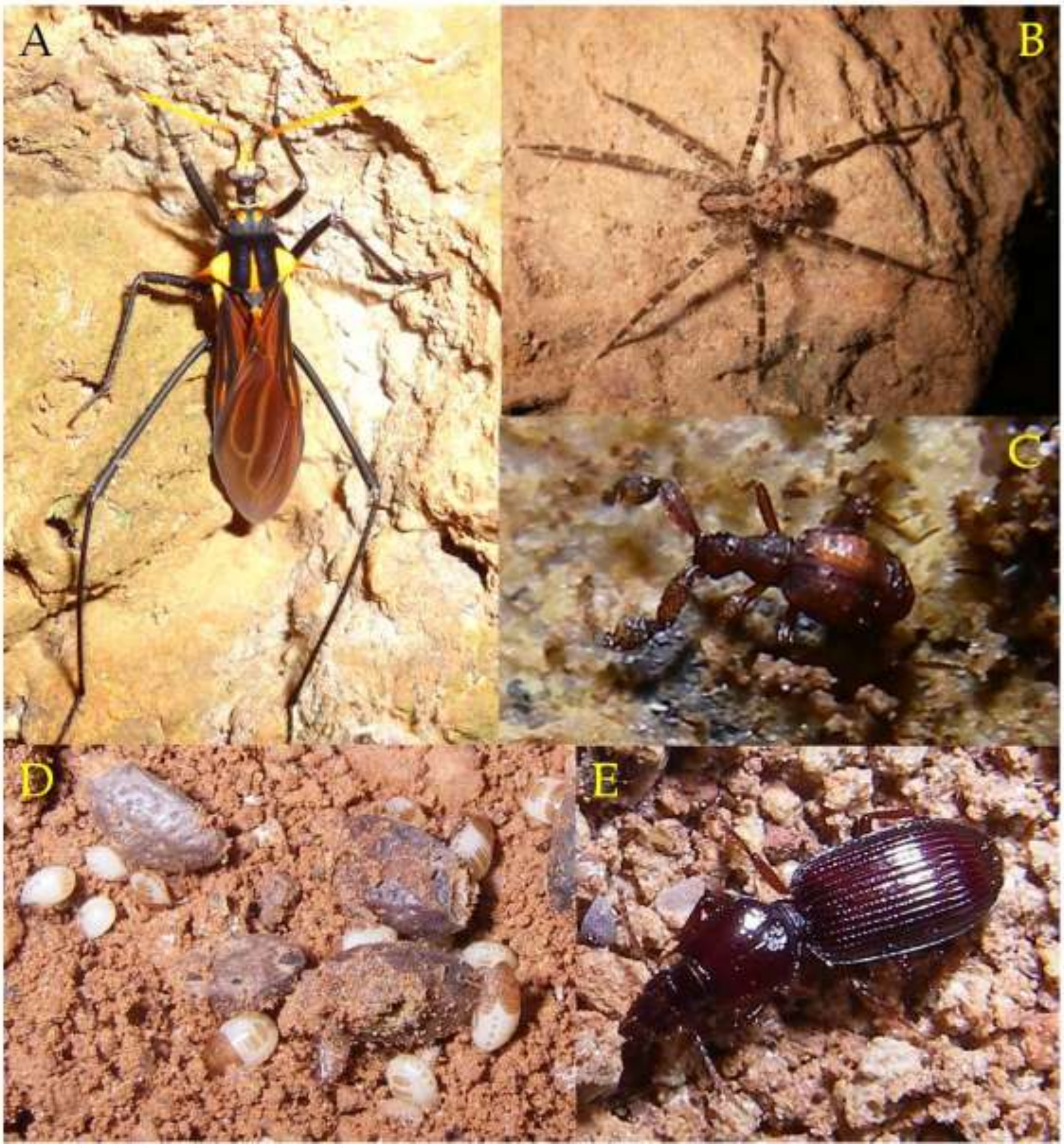

Figura 5.96 - A) Heteroptera: Reduviidae (*Zelurus* sp.); B) Araneae: Ctenidae; C) Coleoptera: Scydmaenidae; D) Ninfas de Cydnidae associadas ao recurso trófico; E) Coleoptera: Carabidae (Clivinina).

CAVIDADE SL-113

Cavidade com 5,5m de projeção horizontal, formado em quartzito e localizado na região inferior da encosta da Serra do Sereno, inserida logo abaixo e a direita da entrada inferior (secundária) da caverna SL-107. O entorno é composto por vegetação arbórea com algumas árvores de grande porte e com sub-bosque predominantemente formado por arbustos, gramíneas e muitas lianas. A cavidade possui apenas uma entrada, sendo esta, pequena e de piso descendente, com paredes e teto cobertos por raízes, liquens, musgos, briófitas, pteridófitas e uma pequena angiosperma (Melastomataceae). A cavidade não apresenta zona afótica, porém pode-se observar uma pequena região de penumbra clara. De forma geral, trata-se de uma cavidade úmida, porém pontos de gotejamento e um empoçamento são observados apenas no período chuvoso. Neste mesmo período foi possível visualizar um fluxo

de água advindo de uma surgência na região mais interna e superior do abrigo. O piso é predominantemente ascendente, de sedimento arenoso e com a presença de alguns seixos esparsos. O sistema radicular é pouco desenvolvido, sendo este composto por algumas raízes de pequeno porte na entrada e micro-raízes no interior da cavidade. A serrapilheira é pouca e restrita a linha d'água. Não foram observados depósitos de guano. De forma geral trata-se de uma cavidade pobre em recursos alimentares, principalmente pelo fluxo d'água e com elevada influência das condições ambientais epígeas devido ao seu pequeno tamanho.

Caracterização faunística no período de seca

Foi observado na caverna, um total de 19 morfoespécies de invertebrados de pelo menos 14 famílias dos Taxa: Gastropoda (Systrophiidae), Opiliones (Cosmetidae), Mesostigmata (Uropodina), Araneae (Ochyroceratidae: *Speocera* sp., Pholcidae, Salticidae: *Noegus* sp., Theridiosomatidae: *Plato* sp.), Collembola (Entomobryidae), Orthoptera (Phalangopsidae: *Uvaroviella* sp.), Isoptera (Termitidae: *Cylindrotermes* sp.), Homoptera (Cixiidae), Lepidoptera (Noctuidae), Hymenoptera (Formicidae: *Linepithema* sp.; *Pachycondyla* spp.), Coleoptera (Carabidae), Symphyla (Scutigerellidae: *Hanseniella* sp.).

Dentre os vertebrados, foram encontradas duas espécies da ordem Anura (Dendrobatidae: *Ameerega* sp., Strabomantidae: *Pristimantis* sp.).

Desta forma, no total foram encontrados 21 morfoespécies. Dentre essas, uma espécie de invertebrado foi considerada troglomórfica: Gastropoda (Systrophiidae).

Caracterização faunística no período de chuva

Foi observado na caverna, um total de 17 morfoespécies de invertebrados de pelo menos 17 famílias dos Taxa: Amblypygi (Phrinidae: *Heterophrynus longicornis*), Opiliones (Sclerosomatidae), Araneae (Ctenidae, Pholcidae: *Mesabolivar* sp., Salticidae), Thysanura (Meinertellidae), Collembola (Bourletiellidae, Entomobryidae), Blattodea (Blattidae), Psocoptera (Ptiloneuridae), Heteroptera (Hebridae, Veliidae: *Paravelia* sp., Dipsocoridae,), Homoptera (Derbidae), Lepidoptera (Tortricidae), Diptera (Sciaridae), Hymenoptera (Formicidae: *Linepithema* sp.).

Caracterização geral da fauna da cavidade

Foi observado na caverna, um total de 34 morfoespécies de invertebrados de pelo menos 27 famílias dos Taxa: Gastropoda (Systrophiidae), Amblypygi (Phrinidae: *Heterophrynus longicornis*), Opiliones (Cosmetidae, Sclerosomatidae), Mesostigmata (Uropodina), Araneae (Ctenidae, Ochyroceratidae: *Speocera* sp., Pholcidae: *Mesabolivar* sp., Salticidae: *Noegus* sp., Theridiosomatidae: *Plato* sp.), Thysanura (Meinertellidae), Collembola (Bourletiellidae, Entomobryidae), Orthoptera (Phalangopsidae: *Uvaroviella* sp.), Blattodea (Blattidae), Isoptera (Termitidae: *Cylindrotermes* sp.), Psocoptera (Ptiloneuridae), Heteroptera (Hebridae, Veliidae: *Paravelia* sp., Dipsocoridae), Homoptera (Cixiidae, Derbidae), Lepidoptera (Noctuidae, Tortricidae), Diptera (Sciaridae), Hymenoptera (Formicidae: *Linepithema* sp.; *Pachycondyla* spp.), Coleoptera (Carabidae), Symphyla (Scutigerellidae: *Hanseniella* sp.).

Dentre os vertebrados, foram encontradas duas espécies da ordem Anura (Dendrobatidae: *Ameerega* sp., Strabomantidae: *Pristimantis* sp.).

Desta forma, no total foram encontrados 29 morfoespécies. Dentre essas, uma espécie de invertebrado foi considerada troglomórfica: Gastropoda (Systrophiidae). Alguns organismos encontrados nesta caverna são mostrados na Figura 5.97.

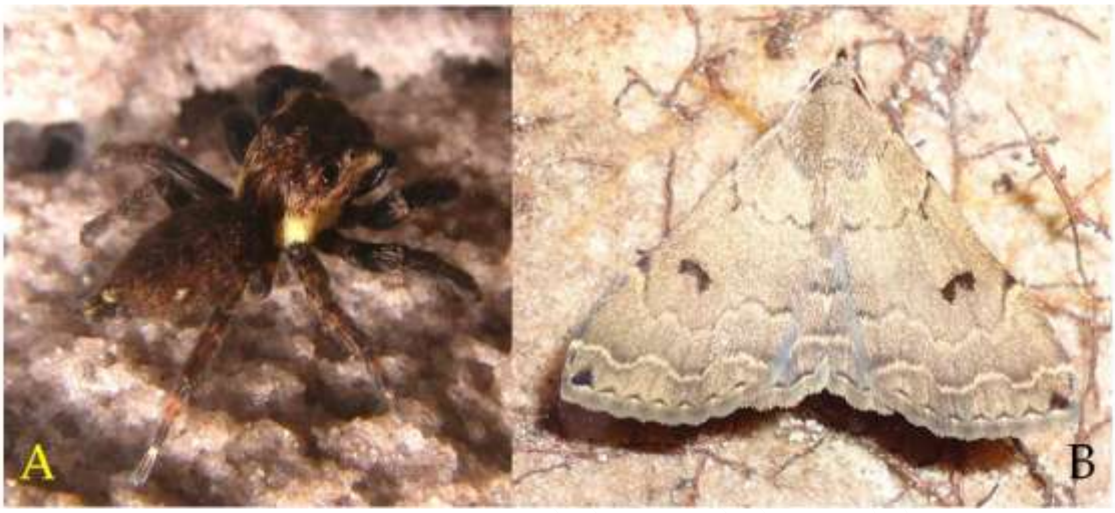

Figura 5.97 - A) Araneae: Salticidae; B) Lepidoptera: Noctuidae.

CAVIDADE SL-114

Cavidade com 25,5m de projeção horizontal, formada em canga detrítica e localizada próxima a uma estrada (5m). A vegetação de entorno é arbórea, com algumas árvores de médio porte, grande acúmulo de lianas associadas ao sub-bosque e serrapilheira pouco densa junto ao substrato. Já a vegetação na porção superior do abrigo é composta predominantemente por gramíneas (pastagem). A cavidade apresenta entrada sombreada e de teto baixo devido à grande deposição de rejeito proveniente de atividades antrópicas (construção da estrada). O piso é plano, composto por sedimento fino compactado, com poucos seixos associados ao mesmo. De forma geral, a cavidade é úmida, porém pontos de gotejamento são visíveis apenas no período chuvoso. Presença de musgos, serrapilheira e brotos restritos à linha d’água e fungos por todo o teto do abrigo. Existência de algumas raízes esparsas pelo piso da cavidade, porém as mesmas estão parcialmente ou totalmente recobertas pelo rejeito carreado por enxurradas. Presença de depósitos pontuais de guano frugívoro. A cavidade possui somente penumbra clara e seus canalículos são pouco desenvolvidos. De forma geral é uma cavidade com poucos recursos alimentares, com grande interferência do meio externo e que se encontra severamente ameaçado devido às contínuas vibrações causadas pelos veículos que transitam na estrada localizada acima do mesmo.

Caracterização faunística no período de seca

Foi observado na caverna, um total de 19 morfoespécies de invertebrados de pelo menos 16 famílias dos Taxa: Isopoda (Dubioniscidae), Opiliones (Sclerosomatidae), Collembola (Bourletiellidae, Entomobryidae), Orthoptera (Phalangopsidae: *Uvaroviella* sp.), Blattodea, Heteroptera (Cydnidae, Lygaeidae, Nabidae), Diptera (Cecidomyiidae, Sciaridae, Tipulidae),

Hymenoptera (Formicidae: *Acromyrmex* sp.; *Dolichoderus* sp.; *Solenopsis* sp.), Coleoptera (Alleculidae, Anthicidae, Carabidae), Symphyla (Scutigerellidae: *Hanseniella* sp.).

Dentre os vertebrados, foi encontrada uma espécie da ordem Chiroptera (Phyllostomidae: *Carollia perspicillata*). Desta forma, no total foram encontrados 20 morfoespécies.

Caracterização faunística no período de chuva

Foi observado na caverna, um total de 39 morfoespécies de invertebrados de pelo menos 25 famílias dos Taxa: Oligochaeta, Gastropoda (Systrophiidae), Amblypygi (Phrinidae: *Heterophrynus longicornis*), Opiliones (Sclerosomatidae), Acari, Mesostigmata (Uropodina), Araneae (Lyniphiidae), Collembola (Bourletiellidae, Cyphoderidae, Entomobryidae), Orthoptera (Phalangopsidae: *Uvaroviella* sp.), Embiidina, Heteroptera (Cydnidae), Lepidoptera (Noctuidae), Diptera (Culicidae, Drosophilidae, Phoridae, Sciaridae, Stratiomyidae), Hymenoptera (Formicidae: *Atta* sp.; *Pachycondyla* sp.; *Solenopsis* sp., Chalcididae, Cynipidae, Diapriidae), Coleoptera (Carabidae: Clivinina, Phalacridae, Staphylinidae), Symphyla (Scutigerellidae: *Scutigerella* sp.).

Dentre os vertebrados, foi encontrada uma espécie da ordem Chiroptera (Phyllostomidae: *Carollia* sp.).

Desta forma, no total foram encontrados 40 morfoespécies. Dentre essas, duas espécies de invertebrados foram consideradas troglomórficas: Gastropoda (Systrophiidae), Collembola (Cyphoderidae).

Caracterização geral da fauna da cavidade

Foi observado na caverna, um total de 53 morfoespécies de invertebrados de pelo menos 30 famílias dos Taxa: Oligochaeta, Gastropoda (Systrophiidae), Isopoda (Dubioniscidae), Amblypygi (Phrinidae: *Heterophrynus longicornis*), Opiliones (Sclerosomatidae), Acari, Mesostigmata (Uropodina), Araneae (Lyniphiidae), Collembola (Bourletiellidae, Cyphoderidae, Entomobryidae), Orthoptera (Phalangopsidae: *Uvaroviella* sp.), Blattodea, Embiidina, Heteroptera (Cydnidae, Lygaeidae, Nabidae), Lepidoptera (Noctuidae), Diptera (Cecidomyiidae, Culicidae, Drosophilidae, Phoridae, Sciaridae, Stratiomyidae, Tipulidae), Hymenoptera (Formicidae: *Acromyrmex* sp.; *Atta* sp.; *Dolichoderus* sp.; *Pachycondyla* sp.; *Solenopsis* sp., Chalcididae, Cynipidae, Diapriidae), Coleoptera (Alleculidae, Anthicidae, Carabidae: Clivinina, Phalacridae, Staphylinidae: Pselaphinae), Symphyla (Scutigerellidae: *Hanseniella* sp.; *Scutigerella* sp.).

Dentre os vertebrados, foram encontradas duas espécies da ordem Chiroptera (Phyllostomidae: *Carollia perspicillata*; *Carollia* sp.).

Desta forma, no total foram encontrados 55 morfoespécies. Dentre essas, duas espécies de invertebrados foram consideradas troglomórficas: Gastropoda (Systrophiidae), Collembola (Cyphoderidae).

CAVIDADE SL-115

Cavidade com 12,0m de projeção horizontal, formada em canga detrítica, localizada em área de savana metalófila. A vegetação na parte superior é arbórea, composta por árvores de médio porte, com arbustos e poucas lianas associadas ao sub-bosque e serrapilheira pouco densa acumulada junto ao substrato. A vegetação na região de entrada da cavidade é arbustiva, composta predominantemente por pteridófitas (samambaias). A entrada é baixa e sombreada, com pteridófitas, brotos, troncos, raízes e musgos por toda essa região, além da presença de serrapilheira restrita à linha d'água. O piso é levemente ascendente, composto por sedimento granulado com diversos seixos e calhaus esparsos por toda a sua extensão. No período chuvoso, o abrigo é bastante úmido, apresentando diversos pontos de gotejamento em seu interior, além de um escorrimento na porção inicial da parede do lado direito. Já no período seco, a cavidade se encontra com baixíssima umidade, e não são mais visíveis os pontos de gotejamento e escorrimento. A caverna apresenta sistema de canalículos bastante desenvolvido. Seu sistema radicular é bem desenvolvido, com raízes de pequeno porte e micro-raízes. Foi registrada a ocorrência de alguns depósitos pontuais de guano de morcegos frugívoros e nectarívoros durante os levantamentos bioespeleológicos. Ocorrência somente de penumbra clara e escura. De forma geral, trata-se de uma cavidade rica em recursos tróficos e com elevada influência das condições ambientais epígeas, devido a estar localizado em um nível inferior ao platô ao qual está inserido, com grande aporte de material orgânico proveniente de chuvas.

Caracterização faunística no período de seca

Foi observado na caverna, um total de 42 morfoespécies de invertebrados de pelo menos 28 famílias dos Taxa: Platyhelminthes (Tricladida), Amblypygi (Phrinidae: *Heterophrynus longicornis*, Charinidae: *Charinus* sp.), Opiliones (Manaosbiidae), Mesostigmata (Podocinidae, Uropodina), Oribatida, Araneae (Prodidomidae, Theridiidae: *Nesticodes rufipes*), Diplura (Campodeidae), Collembola (Bourletiellidae, Cyphoderidae, Entomobryidae, Isotomidae, Paronellidae), Orthoptera (Phalangopsidae: *Phalangopsis* sp.; *Uvaroviella* sp.; Phalangopsinae), Blattodea (Blattidae), Isoptera (Termitidae: *Nasutitermes* sp.), Psocoptera (Ptiloneuridae), Heteroptera (Cydnidae), Homoptera (Cixiidae: *Pintalia* sp.), Lepidoptera (Noctuidae), Diptera (Cecidomyiidae, Ceratopogonidae, Culicidae, Psychodidae: *Lutzomyia* spp.), Hymenoptera (Formicidae: *Apterostigma* sp.; *Pachycondyla* sp.; *Tapinoma* sp.; Myrmicinae), Coleoptera (Carabidae, Scydmaenidae), Scolopendromorpha (Scolopendridae: *Cryptops* sp.).

Dentre os vertebrados, foram encontradas três espécies das ordens: Anura (Strabomantidae: *Pristimantis* sp.), Chiroptera (Phyllostomidae: *Carollia perspicillata*; *Glossophaga soricina*).

Desta forma, no total foram encontrados 45 morfoespécies. Dentre essas, três espécies de invertebrados foram consideradas troglomórficas: Amblypygi (Charinidae: *Charinus* sp.), Collembola (Cyphoderidae, Isotomidae).

Caracterização faunística no período de chuva

Foi observado na caverna, um total de 77 morfoespécies de invertebrados de pelo menos 43 famílias dos Taxa: Oligochaeta, Gastropoda (Subulinidae, Systrophiidae), Pseudoscorpiones (Chthoniidae), Opiliones (Stygnidae), Mesostigmata (Uropodina), Oribatida, Sarcoptiformes

(Arthronotina), Araneae (Ctenidae: *Enoploctenus* sp., Corinnidae: *Corinna* sp., Pholcidae: *Metagonia* sp., Prodidomidae, Scytodidae: *Scytodes itapevi*, Theraphosidae, Theridiidae: *Nesticodes rufipes*), Diplura (Campodeidae, Japygidae), Collembola (Bourletiellidae, Cyphoderidae, Entomobryidae), Orthoptera (Phalangopsidae: *Phalangopsis* sp.; *Uvaroviella* sp.), Blattodea (Blattidae), Isoptera (Termitidae: *Nasutitermes* sp.), Heteroptera (Cydnidae), Homoptera (Cixiidae: *Pintalia* sp.), Lepidoptera (Noctuidae, Tineidae), Diptera (Culicidae, Drosophilidae, Keroplatidae, Milichiidae, Muscidae, Stratiomyidae, Tipulidae), Hymenoptera (Formicidae: *Apterostigma* sp.; *Hypoponera* sp.; *Pachycondyla* sp.; *Pseudomyrmex* sp.; *Rogeria* sp.; *Strumigenys* sp.; *Tapinoma* sp., Diapriidae), Coleoptera (Carabidae, Cerambycidae, Curculionidae, Dytiscidae, Staphylinidae: Pselaphinae), Polydesmida (Polydesmidae, Pyrgodesmidae), Spirostreptida (Pseudonannolenidae), Scolopendromorpha (Newportiidae: *Newportia* sp., Scolopendridae: *Cryptops* sp.).

Dentre os vertebrados, foram encontradas duas espécies da ordem Chiroptera (Phyllostomidae: *Carollia perspicillata; Glossophaga soricina*).

Desta forma, no total foram encontrados 79 morfoespécies. Dentre essas, quatro espécies de invertebrados foram consideradas troglomórficas: Gastropoda (Systrophiidae), Collembola (Cyphoderidae), Coleoptera (Dytiscidae), Polydesmida (Pyrgodesmidae).

Caracterização geral da fauna da cavidade

Foi observado na caverna, um total de 100 morfoespécies de invertebrados de pelo menos 53 famílias dos Taxa: Oligochaeta, Gastropoda (Subulinidae, Systrophiidae), Platyhelminthes (Tricladida), Amblypygi (Phrinidae: *Heterophrynus longicornis*, Charinidae: *Charinus* sp.), Pseudoscorpiones (Chthoniidae), Opiliones (Manaosbiidae, Stygnidae), Araneae (Ctenidae: *Enoploctenus* sp., Corinnidae: *Corinna* sp., Pholcidae: *Metagonia* sp., Prodidomidae, Scytodidae: *Scytodes itapevi*, Theraphosidae, Theridiidae: *Nesticodes rufipes*), Diplura (Campodeidae, Japygidae), Collembola (Bourletiellidae, Cyphoderidae, Entomobryidae, Isotomidae, Paronellidae), Orthoptera (Phalangopsidae: *Phalangopsis* sp.; *Uvaroviella* sp., Phalangopsinae), Blattodea (Blattidae), Isoptera (Termitidae: *Nasutitermes* spp.), Psocoptera (Ptiloneuridae), Heteroptera (Cydnidae), Homoptera (Cixiidae: *Pintalia* sp.), Lepidoptera (Noctuidae, Tineidae), Diptera (Cecidomyiidae, Ceratopogonidae, Culicidae, Drosophilidae, Keroplatidae, Milichiidae, Muscidae, Psychodidae: *Lutzomyia* spp., Sciaridae, Stratiomyidae, Tipulidae), Hymenoptera (Formicidae: *Apterostigma* sp.; *Hypoponera* sp.; *Pachycondyla* sp.; *Pseudomyrmex* sp.; *Rogeria* sp.; *Strumigenys* sp.; *Tapinoma* sp.; Myrmicinae, Diapriidae), Coleoptera (Carabidae, Cerambycidae, Curculionidae, Dytiscidae, Scydmaenidae, Staphylinidae: Pselaphinae), Polydesmida (Polydesmidae, Pyrgodesmidae), Spirostreptida (Pseudonannolenidae), Scolopendromorpha (Newportiidae: *Newportia* sp., Scolopendridae: *Cryptops* sp.).

Dentre os vertebrados, foram encontradas três espécies das ordens: Anura (Strabomantidae: *Pristimantis* sp.), Chiroptera (Phyllostomidae: *Carollia perspicillata; Glossophaga soricina*).

Desta forma, no total foram encontrados 103 morfoespécies. Dentre essas, seis espécies de invertebrados foram consideradas troglomórficas: Gastropoda (Systrophiidae), Amblypygi (Charinidae: *Charinus* sp.), Collembola (Cyphoderidae, Isotomidae), Coleoptera (Dytiscidae),

Polydesmida (Pyrgodesmidae). Alguns organismos encontrados nesta caverna são mostrados na Figura 5.98.

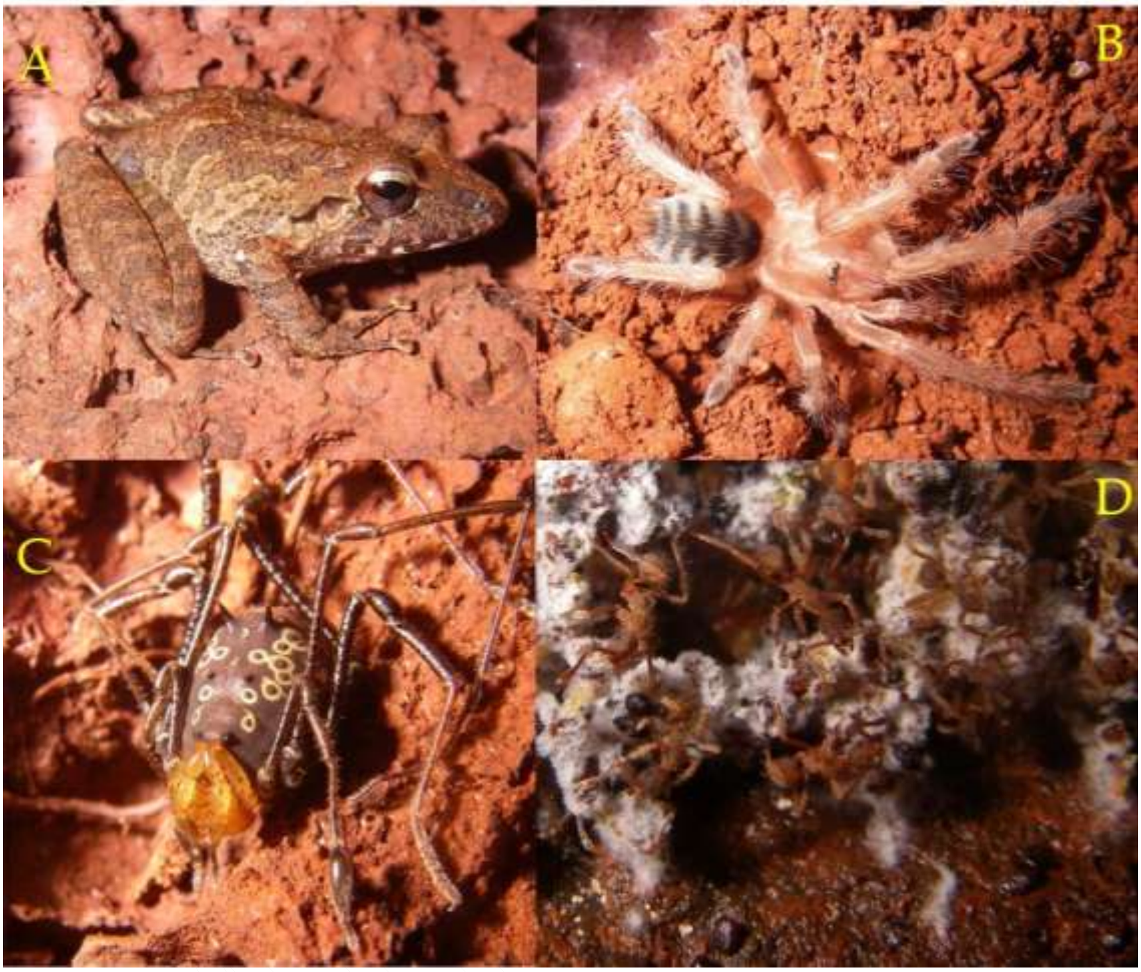

Figura 5.98 - A) Anura: Strabomantidae (*Pristimantis* sp.); B) Araneae: Theraphosidae; C) Opiliones: Manaosbiidae; D) Colônia de Hymenoptera: Formicidae *(Apterostigma* sp.).

CAVIDADE SL-116

Cavidade com 9,0m de projeção horizontal, formada em canga detrítica e localizada em área de savana metalófila. A vegetação de entorno é arbórea, composta por árvores de médio porte, com arbustos e poucas lianas associadas ao sub-bosque e serrapilheira pouco densa acumulada junto ao substrato. A entrada é baixa e sombreada, localizada a um nível inferior ao platô ao qual está inserida. Nela ocorrem musgos, liquens, fungos, brotos, raízes e serrapilheira. De forma geral o piso é levemente descendente, composto por sedimento fino com diversos seixos e calhaus esparsos por toda a sua extensão. O sistema radicular é bastante desenvolvido, com raízes de pequeno e médio porte, além de micro-raízes. Presença de detritos por toda a cavidade, devido à sua topografia a qual favorece o carreamento de material vegetal para o seu interior. Abrigo úmido em ambos os períodos (seco e chuvoso), porém a presença de gotejamento é visualizada apenas no período úmido. Ausência de depósitos de guano e espécies de Chiroptera durante o levantamento bioespeleológico. Presença somente de penumbra clara e canalículos pouco desenvolvidos. De forma geral trata-

se de uma pequena cavidade com baixa estabilidade ambiental devido à forte influência epígea por estar localizada em um nível inferior ao platô ao qual está inserida, com grande aporte de material orgânico proveniente de chuvas.

Caracterização faunística no período de seca

Foi observado na caverna, um total de 35 morfoespécies de invertebrados de pelo menos 25 famílias dos Taxa: Oligochaeta, Isopoda (Armadillidae, Philosciidae), Amblypygi (Phrinidae: *Heterophrynus longicornis*), Pseudoscorpiones (Chernetidae), Opiliones (Cosmetidae), Mesostigmata (Uropodina), Oribatida, Araneae (Ochyroceratidae: *Ochyrocera* sp., Prodidomidae), Thysanura (Nicoletiidae: Nicoletiinae), Diplura (Campodeidae), Collembola (Bourletiellidae, Entomobryidae, Isotomidae), Orthoptera (Phalangopsidae: *Phalangopsis* sp.; *Uvaroviella* sp.; Phalangopsinae), Heteroptera (Cydnidae), Homoptera (Cixiidae: *Pintalia* sp.), Lepidoptera (Noctuidae, Tineidae), Diptera (Ceratopogonidae, Psychodidae: *Lutzomyia* sp., Sciaridae), Hymenoptera (Formicidae: *Azteca* sp.; *Neivamyrmex* sp.; *Odontomachus* sp.; *Pachycondyla* sp.), Coleoptera (Carabidae, Elateridae, Staphylinidae: Pselaphinae), Scolopendromorpha (Newportiidae: *Newportia* sp.).

Dentre os vertebrados, foi encontrada uma espécie da ordem Anura (Strabomantidae: *Pristimantis* sp.).

Desta forma, no total foram encontrados 36 morfoespécies. Dentre essas, uma espécie de invertebrado foi considerada troglomórfica: Collembola (Isotomidae).

Caracterização faunística no período de chuva

Foi observado na caverna, um total de 46 morfoespécies de invertebrados de pelo menos 35 famílias dos Taxa: Oligochaeta, Gastropoda (Subulinidae, Systrophiidae), Isopoda (Armadillidae), Amblypygi (Phrinidae: *Heterophrynus longicornis*), Pseudoscorpiones (Olpiidae), Opiliones (Phalangiidae, Sclerosomatidae, Stygnidae), Mesostigmata, Oribatida, Araneae (Ochyroceratidae: *Ochyrocera* sp., Palpimanidae, Pholcidae: *Mesabolivar* sp., Scytodidae: *Scytodes itapevi*), Collembola (Bourletiellidae, Tomoceridae), Neuroptera (Chrysopidae), Orthoptera (Phalangopsidae: *Phalangopsis* sp.; *Uvaroviella* sp.),Embiidina, Heteroptera (Cydnidae, Pentatomidae), Homoptera (Cercopidae, Cixiidae), Lepidoptera (Noctuidae, Tineidae), Diptera (Cecidomyiidae, Psychodidae: *Lutzomyia* spp., Sciaridae), Hymenoptera (Formicidae: *Camponotus* sp.; *Pachycondyla* sp.), Coleoptera (Carabidae, Hydrophilidae, Staphylinidae: Pselaphinae), Polydesmida (Chelodesmidae, Polydesmidae, Pyrgodesmidae), Geophilomorpha (Ballophilidae: *Ballophilus* sp.), Scolopendromorpha (Newportiidae: *Newportia* sp., Scolopendridae: *Cryptops* sp.).

Dentre os vertebrados, foi encontrada uma espécie da ordem Chiroptera (Phyllostomidae: *Carollia* sp.).

Desta forma, no total foram encontrados 47 morfoespécies. Dentre essas, duas espécies de invertebrados foram consideradas troglomórficas: Gastropoda (Systrophiidae), Polydesmida (Pyrgodesmidae).

Caracterização geral da fauna da cavidade

Foi observado na caverna, um total de 67 morfoespécies de invertebrados de pelo menos 45 famílias dos Taxa: Oligochaeta, Gastropoda (Subulinidae, Systrophiidae), Isopoda

(Armadillidae, Philosciidae), Amblypygi (Phrinidae: *Heterophrynus longicornis*), Pseudoscorpiones (Chernetidae, Olpiidae), Opiliones (Cosmetidae, Phalangiidae, Sclerosomatidae, Stygnidae), Mesostigmata (Uropodina), Oribatida, Araneae (Ochyroceratidae: *Ochyrocera* sp., Palpimanidae, Pholcidae: *Mesabolivar* sp., Prodidomidae, Scytodidae: *Scytodes itapevi*), Thysanura (Nicoletiidae: Nicoletiinae), Diplura (Campodeidae), Collembola (Bourletiellidae, Entomobryidae, Isotomidae, Tomoceridae), Neuroptera (Chrysopidae), Orthoptera (Phalangopsidae: *Phalangopsis* sp.; *Uvaroviella* sp.; Phalangopsinae),Embiidina, Heteroptera (Cydnidae, Pentatomidae), Homoptera (Cercopidae, Cixiidae: *Pintalia* sp.), Lepidoptera (Noctuidae, Tineidae), Diptera (Cecidomyiidae, Ceratopogonidae, Psychodidae: *Lutzomyia* spp., Sciaridae), Hymenoptera (Formicidae: *Azteca* sp.; *Camponotus* sp.; *Neivamyrmex* sp.; *Odontomachus* sp.; *Pachycondyla* sp.), Coleoptera (Carabidae, Elateridae, Hydrophilidae, Staphylinidae: Pselaphinae), Polydesmida (Chelodesmidae, Polydesmidae, Pyrgodesmidae), Geophilomorpha (Ballophilidae: *Ballophilus* sp.), Scolopendromorpha (Newportiidae: *Newportia* sp., Scolopendridae: *Cryptops* sp.).

Dentre os vertebrados, foram encontradas duas espécies das ordens: Anura (Strabomantidae: *Pristimantis* sp.), Chiroptera (Phyllostomidae: *Carollia* sp.).

Desta forma, no total foram encontrados 69 morfoespécies. Dentre essas, três espécies de invertebrados foram consideradas troglomórficas: Gastropoda (Systrophiidae), Collembola (Isotomidae), Polydesmida (Pyrgodesmidae). Alguns organismos encontrados nesta caverna são mostrados na Figura 5.99.

www.carste.com.br - e-mail: contato@carste.com.br - Tel.: (31) 2552-9976

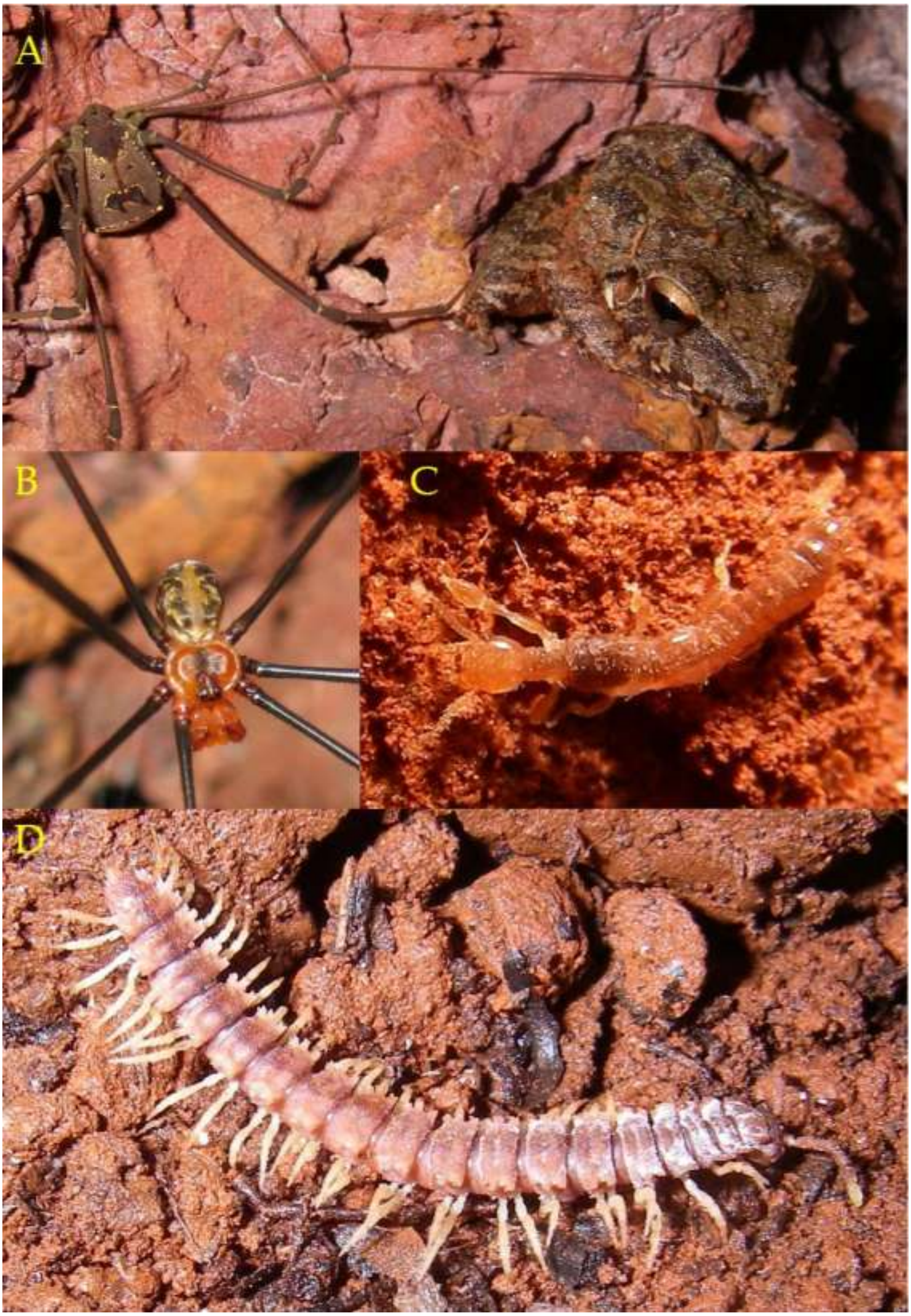

Figura 5.99 - A) Opiliones: Cosmetidae e Anura: Strabomantidae (*Pristimantis* sp.); B) Araneae: Pholcidae (*Mesabolivar* sp.); C) Embiidina; D) Polydesmida: Chelodesmidae.

CAVIDADE SL-117

Cavidade com 9,5m de projeção horizontal, formada em quartzito e localizada na região inferior da encosta da Serra do Sereno. O entorno é composto por vegetação arbustiva com algumas árvores de médio porte e com sub-bosque predominantemente formado por gramíneas e lianas. A cavidade possui três entradas, sendo todas estas pequenas, com paredes e tetos cobertos por musgos, briófitas, algumas pteridófitas e gramíneas. A cavidade não apresenta zona afótica, porém pode-se observar uma pequena região de penumbra clara na região mais superior do salão. De forma geral, trata-se de uma cavidade úmida, onde pontos de gotejamento, um pequeno fluxo de água advindo da região mais interna e superior do abrigo, e um empoçamento próximo à entrada podem ser observados apenas no período chuvoso de coleta. O piso de uma das entradas é descendente em relação ao centro da caverna, com seixos esparsos. Em relação às outras duas entradas, ambas apresentam piso com sedimento arenoso, porém a entrada central não apresenta alguns seixos esparsos como a outra. Do centro ao interior da cavidade o piso é ascendente, composto pela rocha exposta e com alguns blocos abatidos. O sistema radicular é pouco desenvolvido, sendo este composto por algumas raízes de pequeno porte nas entradas. A serrapilheira é pouca e restrita a linha d'água. Foram observados depósitos antigos de guano frugívoro e fezes de anura. Também foi encontrado um ninho de ave (Trochilidae) na entrada da cavidade. De forma geral trata-se de uma cavidade pobre em recursos alimentares e com elevada influência das condições ambientais epígeas devido ao seu pequeno tamanho.

Caracterização faunística no período de seca

Foi observado na caverna, um total de 31 morfoespécies de invertebrados de pelo menos 19 famílias dos Taxa: Gastropoda (Subulinidae), Platyhelminthes (Tricladida), Pseudoscorpiones (Chthoniidae), Opiliones (Sclerosomatidae), Acari, Prostigmata (Cunaxidae: *Armascirus* sp.), Araneae (Salticidae: *Noegus* sp., Theridiidae), Collembola (Entomobryidae), Neuroptera (Myrmeleontidae), Orthoptera (Phalangopsidae: *Uvaroviella* sp.), Blattodea (Blattidae), Isoptera (Termitidae: *Nasutitermes* sp.), Dermaptera, Psocoptera (Psyllipsocidae), Heteroptera (Hebridae, Tingidae), Homoptera (Cicadellidae: Typhlocybinae), Diptera (Sciaridae), Hymenoptera (Formicidae: *Atta* sp.; *Cyphomyrmex* sp.; *Linepithema* sp.; *Pachycondyla* sp.; *Pseudomyrmex* sp.; *Solenopsis* sp.; *Tapinoma* sp.), Coleoptera (Staphylinidae: Pselaphinae), Symphyla (Scutigerellidae: *Hanseniella* sp.).

Dentre os vertebrados, foram encontradas duas espécies das ordens: Anura (Strabomantidae: *Pristimantis* sp.), Chiroptera (Phyllostomidae: Glossophaginae).

Desta forma, no total foram encontrados 33 morfoespécies.

Caracterização faunística no período de chuva

Foi observado na caverna, um total de 41 morfoespécies de invertebrados de pelo menos 25 famílias dos Taxa: Isopoda (Dubioniscidae), Amblypygi (Phrinidae: *Heterophrynus longicornis*), Pseudoscorpiones (Chthoniidae), Opiliones (Cosmetidae,Phalangiidae, Sclerosomatidae), Acari, Mesostigmata (Uropodina), Oribatida, Araneae (Ctenidae, Corinnidae: *Corinna* sp., Ochyroceratidae, Pholcidae: *Metagonia* sp., Tetrablemmidae: *Matta* sp.), Diplura (Campodeidae), Collembola (Cyphoderidae, Entomobryidae), Orthoptera (Phalangopsidae: *Phalangopsis* sp.; *Uvaroviella* sp.), Psocoptera (Archipsocidae), Heteroptera (Hebridae),

Homoptera (Derbidae), Lepidoptera (Noctuidae), Diptera (Chloropidae, Sciaridae), Hymenoptera (Formicidae: *Atta* sp.; *Cyphomyrmex* sp.; *Ectatomma* sp.; *Linepithema* sp.; *Solenopsis* sp.; *Strumigenys* sp.; *Tapinoma* sp.), Coleoptera (Carabidae, Staphylinidae: Pselaphinae), Symphyla (Scutigerellidae: *Hanseniella* sp.; *Symphyella* sp.).

Dentre os vertebrados, foram encontradas duas espécies da ordem Chiroptera (Phyllostomidae: *Carollia perspicillata; Glossophaga soricina*).

Desta forma, no total foram encontrados 43 morfoespécies. Dentre essas, duas espécies de invertebrados foram consideradas troglomórficas: Araneae (Tetrablemmidae: *Matta* sp.), Collembola (Cyphoderidae).

Caracterização geral da fauna da cavidade

Foi observado na caverna, um total de 62 morfoespécies de invertebrados de pelo menos 35 famílias dos Taxa: Gastropoda (Subulinidae), Platyhelminthes (Tricladida), Isopoda (Dubioniscidae), Amblypygi (Phrinidae: *Heterophrynus longicornis*), Pseudoscorpiones (Chthoniidae), Opiliones (Cosmetidae, Phalangiidae, Sclerosomatidae), Acari, Mesostigmata (Uropodina), Oribatida, Prostigmata (Cunaxidae: *Armascirus* sp.), Araneae (Ctenidae, Corinnidae: *Corinna* sp., Ochyroceratidae, Pholcidae: *Metagonia* sp., Salticidae: *Noegus* sp., Tetrablemmidae: *Matta* sp., Theridiidae), Diplura (Campodeidae), Collembola (Cyphoderidae, Entomobryidae), Neuroptera (Myrmeleontidae), Orthoptera (Phalangopsidae: *Phalangopsis* sp.; *Uvaroviella* sp.), Blattodea (Blattidae), Isoptera (Termitidae: *Nasutitermes* sp.), Dermaptera, Psocoptera (Archipsocidae, Psyllipsocidae), Heteroptera (Hebridae, Tingidae), Homoptera (Cicadellidae: Typhlocybinae,Derbidae), Lepidoptera (Noctuidae), Diptera (Chloropidae, Sciaridae), Hymenoptera (Formicidae: *Atta* sp.; *Cyphomyrmex* sp.; *Ectatomma* sp.; *Linepithema* sp.; *Pachycondyla* sp.; *Pseudomyrmex* sp.; *Solenopsis* spp.; *Strumigenys* sp.; *Tapinoma* sp.), Coleoptera (Carabidae, Staphylinidae: Pselaphinae), Symphyla (Scutigerellidae: *Hanseniella* sp.; *Symphyella* sp.).

Dentre os vertebrados, foram encontradas três espécies das Ordens: Anura (Strabomantidae: *Pristimantis* sp.), Chiroptera (Phyllostomidae: *Carollia perspicillata; Glossophaga soricina*).

Desta forma, no total foram encontrados 65 morfoespécies. Dentre essas, duas espécies de invertebrados foram consideradas troglomórficas: Araneae (Tetrablemmidae: *Matta* sp.), Collembola (Cyphoderidae).

CAVIDADE SL-121

Cavidade com 68,5m de projeção horizontal, formada em canga detrítica e localizada em área de savana metalófila. A vegetação de entorno é arbórea, composta por árvores de médio porte, com arbustos e lianas associadas ao sub-bosque e serrapilheira pouco densa acumulada junto ao substrato. A entrada é sombreada e em teto baixo, com musgos e serrapilheira associados à linha d’água. A caverna apresenta um pequeno trecho à parte, à esquerda, o qual não se conecta com o restante. Nesta seção foram encontrados brotos, pteridófitas, musgos, fungos, liquens, raízes e micro-raízes esparsas, além de uma carcaça de anura. De forma geral, a cavidade apresenta piso levemente ascendente, composto por sedimento fino com diversos seixos, calhaus e alguns matacões esparsos por toda sua extensão. O piso do salão de entrada

da caverna encontra-se muito seco quando comparado ao período chuvoso. Alguns pontos de gotejamento e percolação são visíveis tanto no período seco, quanto úmido. Seu sistema radicular é bem desenvolvido, composto por raízes de pequeno calibre, micro-raízes e alguns rizotemas em diversos trechos. A caverna apresenta penumbra clara, penumbra escura e zona afótica, além de apresentar canalículos bastante desenvolvidos. Após seu o primeiro trecho, há um quebra corpo e, em seguida, o salão principal. Este apresenta o piso totalmente recoberto por uma densa camada de guano proveniente de morcegos frugívoros e nectarívoros, além de pequenos depósitos esparsos de fezes de anura e roedor. De forma geral, esta caverna apresenta alta estabilidade ambiental devido a sua entrada restritiva e extensão.

Caracterização faunística no período de seca

Foi observado na caverna, um total de 74 morfoespécies de invertebrados de pelo menos 46 famílias dos Taxa: Pseudoscorpiones (Chernetidae), Acari, Isodida, Mesostigmata (Uropodina), Oribatida, Araneae (Araneidae, Ctenidae, Micropholcommatidae, Palpimanidae, Pholcidae: Ninetinae, Prodidomidae, Salticidae, Scytodidae: *Scytodes itapevi*, Theridiidae: *Nesticodes rufipes*, Theridiosomatidae: *Plato* sp.), Thysanura (Nicoletiidae: Nicoletiinae), Diplura (Campodeidae), Collembola (Cyphoderidae, Entomobryidae, Isotomidae), Orthoptera (Mogoplistidae, Phalangopsidae: Phalangopsis sp.; Phalangopsinae), Blattodea (Blattellidae, Polyphagidae), Isoptera (Termitidae: *Nasutitermes* sp.), Psocoptera, Heteroptera (Cydnidae, Lygaeidae, Nabidae), Homoptera (Cercopidae, Cixiidae: *Pintalia* sp.), Lepidoptera (Coleophoridae, Gelechidae, Noctuidae, Tineidae), Diptera (Cecidomyiidae, Ceratopogonidae, Dolichopodidae, Drosophilidae, Empididae, Milichiidae, Muscidae, Psychodidae: *Lutzomyia* spp.), Hymenoptera (Formicidae: *Camponotus* sp.; *Carebara* sp.; *Gnamptogenys* sp.; *Hypoponera* sp.; *Labidus* sp.; *Pachycondyla* sp.; *Rogeria* sp.; *Solenopsis* sp., Bethilidae, Braconidae, Diapriidae), Coleoptera (Carabidae, Elateridae, Scarabaeidae, Staphylinidae).

Dentre os vertebrados, foram encontradas quatro espécies das ordens: Anura (Strabomantidae: *Pristimantis* sp.), Chiroptera (Phyllostomidae: *Carollia perspicillata*; *Lonchorhina aurita*; *Diphylla ecaudata*).

Desta forma, no total foram encontrados 78 morfoespécies. Dentre essas, duas espécies de invertebrados foram consideradas troglomórficas: Collembola (Cyphoderidae, Isotomidae).

Caracterização faunística no período de chuva

Foi observado na caverna, um total de 95 morfoespécies de invertebrados de pelo menos 56 famílias dos Taxa: Oligochaeta, Gastropoda (Systrophiidae), Isopoda (Dubioniscidae), Amblypygi (Phrinidae: *Heterophrynus longicornis*, Charinidae: *Charinus* sp.), Pseudoscorpiones (Chernetidae, Chthoniidae, Olpiidae, Syarinidae), Opiliones (Escadabiidae), Acari, Actinedida (Rhagidiidae), Ixodida, Mesostigmata (Ameroseiidae, Podocinidae, Uropodina), Oribatida, Araneae (Araneidae, Ctenidae, Corinnidae, Ochyroceratidae: *Ochyrocera* sp., Pholcidae, Prodidomidae, Scytodidae: *Scytodes itapevi*, Theridiidae: *Nesticodes rufipes*, Theridiosomatidae: *Plato* sp.), Diplura (Campodeidae), Collembola (Cyphoderidae, Entomobryidae, Isotomidae, Tomoceridae), Orthoptera (Phalangopsidae: *Phalangopsis* sp.; *Uvaroviella* sp.; Phalangopsinae), Blattodea (Blaberidae: *Blaberus* sp., Polyphagidae), Psocoptera (Ptiloneuridae), Heteroptera (Cydnidae, Lygaeidae, Nabidae), Homoptera (Cercopidae, Cixiidae: *Pintalia* sp., Derbidae: *Mysidia* sp.), Lepidoptera (Coleophoridae,

Noctuidae, Tineidae, Arctiidae), Diptera (Cecidomyiidae, Ceratopogonidae, Chironomidae, Drosophilidae, Milichiidae, Muscidae, Psychodidae: *Lutzomyia* sp.), Hymenoptera (Formicidae: *Camponotus* sp.; *Gnamptogenys* sp.; *Hypoponera* sp.; *Pachycondyla* spp.; *Pheidole* sp.; *Solenopsis* spp., Bethilidae), Coleoptera (Carabidae, Elateridae, Staphylinidae, Scydmaenidae), Glomeridesmida (Glomeridesmidae), Polydesmida, Spirostreptida (Pseudonannolenidae), Scolopendromorpha (Scolopendridae: *Cryptops* sp.).

Dentre os vertebrados, foram encontradas duas espécies da ordem Chiroptera (Phyllostomidae: *Carollia perspicillata*; *Lonchorhina* sp.).

Desta forma, no total foram encontrados 97 morfoespécies. Dentre essas, cinco espécies de invertebrados foram consideradas troglomórficas: Gastropoda (Systrophiidae), Amblypygi (Charinidae: *Charinus* sp.), Collembola (Cyphoderidae, Isotomidae), Coleoptera (Scydmaenidae).

Caracterização geral da fauna da cavidade

Foi observado na caverna, um total de 129 morfoespécies de invertebrados de pelo menos 69 famílias dos Taxa: Oligochaeta, Gastropoda (Systrophiidae), Isopoda (Dubioniscidae), Amblypygi (Phrinidae: *Heterophrynus longicornis*, Charinidae: *Charinus* sp.), Pseudoscorpiones (Chernetidae, Chthoniidae, Olpiidae, Syarinidae), Opiliones (Escadabiidae), Acari, Actinedida (Rhagidiidae), Ixodida, Mesostigmata (Ameroseiidae, Podocinidae, Uropodina), Oribatida, Araneae (Araneidae, Ctenidae, Corinnidae, Micropholcommatidae, Ochyroceratidae: *Ochyrocera* sp., Palpimanidae, Pholcidae: Ninetinae, Prodidomidae, Salticidae, Scytodidae: *Scytodes itapevi*, Theridiidae: *Nesticodes rufipes*, Theridiosomatidae: *Plato* sp.), Thysanura (Nicoletiidae: Nicoletiinae), Diplura (Campodeidae), Collembola (Cyphoderidae, Entomobryidae, Isotomidae, Tomoceridae), Orthoptera (Mogoplistidae, Phalangopsidae: *Phalangopsis* sp.; *Uvaroviella* sp.; Phalangopsinae), Blattodea (Blaberidae: *Blaberus* sp., Blattellidae, Polyphagidae), Isoptera (Termitidae: *Nasutitermes* sp.), Psocoptera (Ptiloneuridae), Heteroptera (Cydnidae, Lygaeidae, Nabidae), Homoptera (Cercopidae, Cixiidae: *Pintalia* sp., Derbidae: *Mysidia* sp.), Lepidoptera (Coleophoridae, Gelechidae, Noctuidae, Tineidae, Arctiidae), Diptera (Cecidomyiidae, Ceratopogonidae, Chironomidae, Dolichopodidae, Drosophilidae, Empididae, Milichiidae, Muscidae, Psychodidae: *Lutzomyia* spp.), Hymenoptera (Formicidae: *Camponotus* sp.; *Carebara* sp.; *Gnamptogenys* sp.; *Hypoponera* spp.; *Labidus* sp.; *Pachycondyla* spp.; *Pheidole* sp.; *Rogeria* sp.; *Solenopsis* spp., Bethilidae, Braconidae, Diapriidae), Coleoptera (Carabidae, Elateridae, Scarabaeidae, Staphylinidae, Scydmaenidae), Glomeridesmida (Glomeridesmidae), Polydesmida, Spirostreptida (Pseudonannolenidae), Scolopendromorpha (Scolopendridae: *Cryptops* sp.).

Dentre os vertebrados, foram encontradas cinco espécies das ordens: Anura (Strabomantidae: *Pristimantis* sp.), Chiroptera (Phyllostomidae: *Carollia perspicillata*; *Lonchorhina aurita*; *Lonchorhina* sp.; *Diphylla ecaudata*).

Desta forma, no total foram encontrados 134 morfoespécies. Dentre essas, cinco espécies de invertebrados foram consideradas troglomórficas: Gastropoda (Systrophiidae), Amblypygi (Charinidae: *Charinus* sp.), Collembola (Cyphoderidae, Isotomidae), Coleoptera (Scydmaenidae). Alguns organismos encontrados na caverna são mostrados na Figura 5.100.

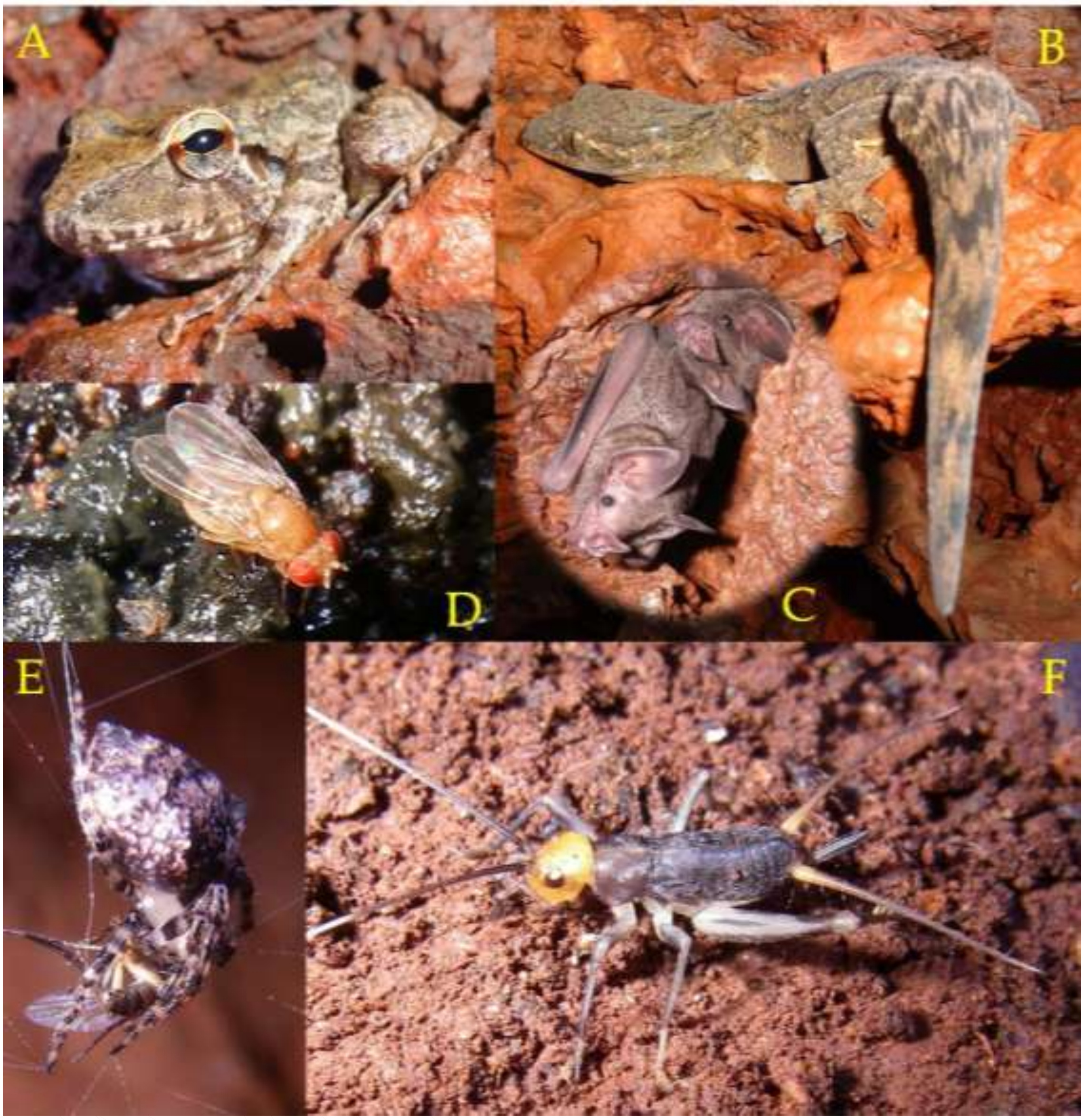

Figura 5.100 - A) Anura: Strabomantidae (*Pristimantis* sp.); B) Squamata: Gekkonidae (*Thecadactylus rapicauda*); C) Chiroptera: Phyllostomidae (*Carollia perspicillata*); D) Diptera: Drosophilidae; E) Araneae: Araneidae; F) Orthoptera: Phalangopsidae (Phalangopsinae).

CAVIDADE SL-122

Caverna com 34m de projeção horizontal, formada em canga detrítica, localizada na região de Serra Leste. Caverna localizada em uma drenagem. O entorno é composto por vegetação arbórea de mata ciliar, com árvores de grande porte e com sub-bosque predominantemente formado por arbustos, muitas gramíneas e lianas. Ela apresenta apenas uma entrada, sendo esta comprida e de teto baixo, coberta por vegetação arbustiva e rasteira, com muitos liquens, briófitas, pteridófitas e alguns brotos. A cavidade não apresenta zona afótica, porém é quase toda de penumbra clara com uma pequena região de penumbra escura à esquerda da entrada. De forma geral, trata-se de uma cavidade muito úmida. O piso da entrada da caverna é quase todo coberto por água, decorrente do grande volume de água que desce em forma de "cascata" da sua parte superior, formando um curso d'água com sentindo da direita para a

esquerda. Apresenta também uma surgência no seu interior, em que o fluxo segue em direção à entrada, e pontos de gotejamento esparsos por toda a cavidade. O piso da caverna é levemente ascendente, formado por sedimento fino, encharcado na entrada e muito úmido no seu interior. Apresenta alguns seixos e calhaus esparsos e um bloco abatido no centro do salão principal. O sistema radicular é bem desenvolvido, sendo este composto por raízes de médio e grande porte na entrada. A serrapilheira é restrita a linha d'água. Foram observados alguns pequenos depósitos de guano nectarívoro esparsos e lavados, além de um ninho de ave. De forma geral trata-se de uma cavidade pobre em recursos alimentares e com elevada influência das condições ambientais epígeas, por não apresentar grande profundidade e por sua área estar constantemente sendo lavada.

Caracterização faunística no período de seca

Foi observado na caverna, um total de 50 morfoespécies de invertebrados de pelo menos 33 famílias dos Taxa: Oligochaeta, Gastropoda (Subulinidae, Systrophiidae), Platyhelminthes (Tricladida), Amblypygi (Phrinidae: *Heterophrynus longicornis*), Opiliones (Sclerosomatidae), Mesostigmata (Laelapidae), Oribatida, Sarcoptiforme (Acaridae: *Tyrophagus* sp.), Araneae (Corinnidae, Pholcidae: *Mesabolivar* sp., Theridiosomatidae: *Plato* sp., Trechaleidae: *Enna* aff. *paraense*), Thysanura (Nicoletiidae: Nicoletiinae), Collembola (Bourletiellidae, Entomobryidae, Tomoceridae), Orthoptera (Gryllidae, Phalangopsidae: *Phalangopsis* sp.), Heteroptera (Cydnidae, Dipsocoridae, Veliidae: *Paravelia* sp.), Diptera (Cecidomyiidae, Culicidae: *Anopheles* sp.; *Culex* sp., Drosophilidae, Milichiidae, Phoridae, Sciaridae, Sphaeroceridae, Tipulidae), Hymenoptera (Formicidae: *Camponotus* sp.; *Odontomachus* sp.; *Pheidole* spp.; *Rogeria* sp.; *Solenopsis* spp.),Coleoptera (Carabidae, Hydrophilidae, Staphylinidae: Pselaphinae), Polydesmida (Pyrgodesmidae), Symphyla (Scutigerellidae: *Hanseniella* sp.).

Dentre os vertebrados, foram encontradas duas espécies das ordens: Anura (Strabomantidae: *Pristimantis* sp.), Chiroptera (Phyllostomidae).

Desta forma, no total foram encontrados 52 morfoespécies. Dentre essas, uma espécie de invertebrado foi considerada troglomórfica: Polydesmida (Pyrgodesmidae).

Caracterização faunística no período de chuva

Foi observado na caverna, um total de 75 morfoespécies de invertebrados de pelo menos 46 famílias dos Taxa: Oligochaeta, Gastropoda (Systrophiidae), Isopoda (Philosciidae), Decapoda (Pseudothelphusidae: *Microthelphusa somanni*), Amblypygi (Phrinidae: *Heterophrynus longicornis*), Opiliones (Sclerosomatidae), Acari, Actinedida (Parasitengonina), Mesostigmata (Uropodina), Oribatida, Prostigmata (Bdelloidea), Araneae (Ctenidae, Pholcidae: *Mesabolivar* sp., Salticidae, Theridiosomatidae: *Plato* sp., Trechaleidae: *Enna* aff. *paraense*), Thysanura (Nicoletiidae: Nicoletiinae; Atelurinae), Collembola (Bourletiellidae, Entomobryidae, Isotomidae, Tomoceridae), Orthoptera (Phalangopsidae: *Phalangopsis* sp.), Blattodea (Blattellidae, Blattidae), Ephemeroptera (Euthyplociidae: *Campylocia anceps*), Heteroptera (Cydnidae, Dipsocoridae, Hebridae, Mesoveliidae, Veliidae: *Paravelia* sp.; *Rhagovelia* sp), Homoptera (Cixiidae), Lepidoptera (Tineidae), Diptera (Ceratopogonidae, Chironomidae, Chloropidae, Culicidae: *Anopheles* spp.; *Culex* sp.; Drosophilidae, Milichiidae, Psychodidae, Sciaridae, Simuliidae, Streblidae), Hymenoptera (Formicidae: *Linepithema* sp.; *Pheidole* sp.; *Rogeria* sp.; *Solenopsis* spp.; *Strumigenys* sp., Scelionidae), Coleoptera (Hydrophilidae,

Staphylinidae: Pselaphinae, Scydmaenidae, Tenebrionidae), Polydesmida (Polydesmidae, Pyrgodesmidae), Scolopendromorpha (Newportiidae: *Dinocryptops* sp.), Symphyla (Scutigerellidae: *Hanseniella* sp.).

Dentre os vertebrados, foram encontradas duas espécies das ordens: Anura, Chiroptera (Phyllostomidae: Glossophaginae).

Desta forma, no total foram encontrados 77 morfoespécies. Dentre essas, quatro espécies de invertebrados foram consideradas troglomórficas: Gastropoda (Systrophiidae), Collembola (Isotomidae), Polydesmida (Pyrgodesmidae 2spp.).

Caracterização geral da fauna da cavidade

Foi observado na caverna, um total de 102 morfoespécies de invertebrados de pelo menos 56 famílias dos Taxa: Oligochaeta, Gastropoda (Subulinidae, Systrophiidae), Platyhelminthes (Tricladida), Isopoda (Philosciidae), Decapoda (Pseudothelphusidae: *Microthelphusa somanni*), Amblypygi (Phrinidae: *Heterophrynus longicornis*), Opiliones (Sclerosomatidae), Acari, Actinedida (Parasitengonina), Mesostigmata (Laelapidae, Uropodina), Oribatida, Prostigmata (Bdelloidea), Sarcoptiformes (Acaridae: *Tyrophagus* sp.), Araneae (Ctenidae, Corinnidae, Pholcidae: *Mesabolivar* sp., Salticidae, Theridiosomatidae: *Plato* sp., Trechaleidae: *Enna* aff. *paraense*), Thysanura (Nicoletiidae: Nicoletiinae; Atelurinae), Collembola (Bourletiellidae, Entomobryidae, Isotomidae, Tomoceridae), Orthoptera (Gryllidae, Phalangopsidae: *Phalangopsis* sp.), Blattodea (Blattellidae, Blattidae), Ephemeroptera (Euthyplociidae: *Campylocia anceps*), Heteroptera (Cydnidae, Dipsocoridae, Hebridae, Mesoveliidae, Veliidae: *Paravelia* sp.; *Rhagovelia* sp), Homoptera (Cixiidae), Lepidoptera (Tineidae), Diptera (Cecidomyiidae, Ceratopogonidae, Chironomidae, Chloropidae, Culicidae: *Anopheles* spp.; *Culex* spp.; Drosophilidae, Milichiidae, Phoridae, Psychodidae, Sciaridae, Simuliidae, Sphaeroceridae, Streblidae, Tipulidae), Hymenoptera (Formicidae: *Camponotus* sp.; *Linepithema* sp.; *Odontomachus* sp.; *Pheidole* spp.; *Rogeria* sp.; *Solenopsis* spp.; *Strumigenys* sp., Scelionidae), Coleoptera (Hydrophilidae, Staphylinidae: Pselaphinae, Scydmaenidae, Tenebrionidae), Polydesmida (Polydesmidae, Pyrgodesmidae), Scolopendromorpha (Newportiidae: *Dinocryptops* sp.), Symphyla (Scutigerellidae: *Hanseniella* spp.).

Dentre os vertebrados, foram encontradas três espécies das ordens: Anura (Anura indet, Strabomantidae: *Pristimantis* sp.), Chiroptera (Phyllostomidae: Glossophaginae, Phyllostomidae indet.).

Desta forma, no total foram encontrados 105 morfoespécies. Dentre essas, quatro espécies de invertebrados foram consideradas troglomórficas: Gastropoda (Systrophiidae), Collembola (Isotomidae), Polydesmida (Pyrgodesmidae 2spp.). Alguns organismos encontrados nesta caverna são mostrados na Figura 5.101.

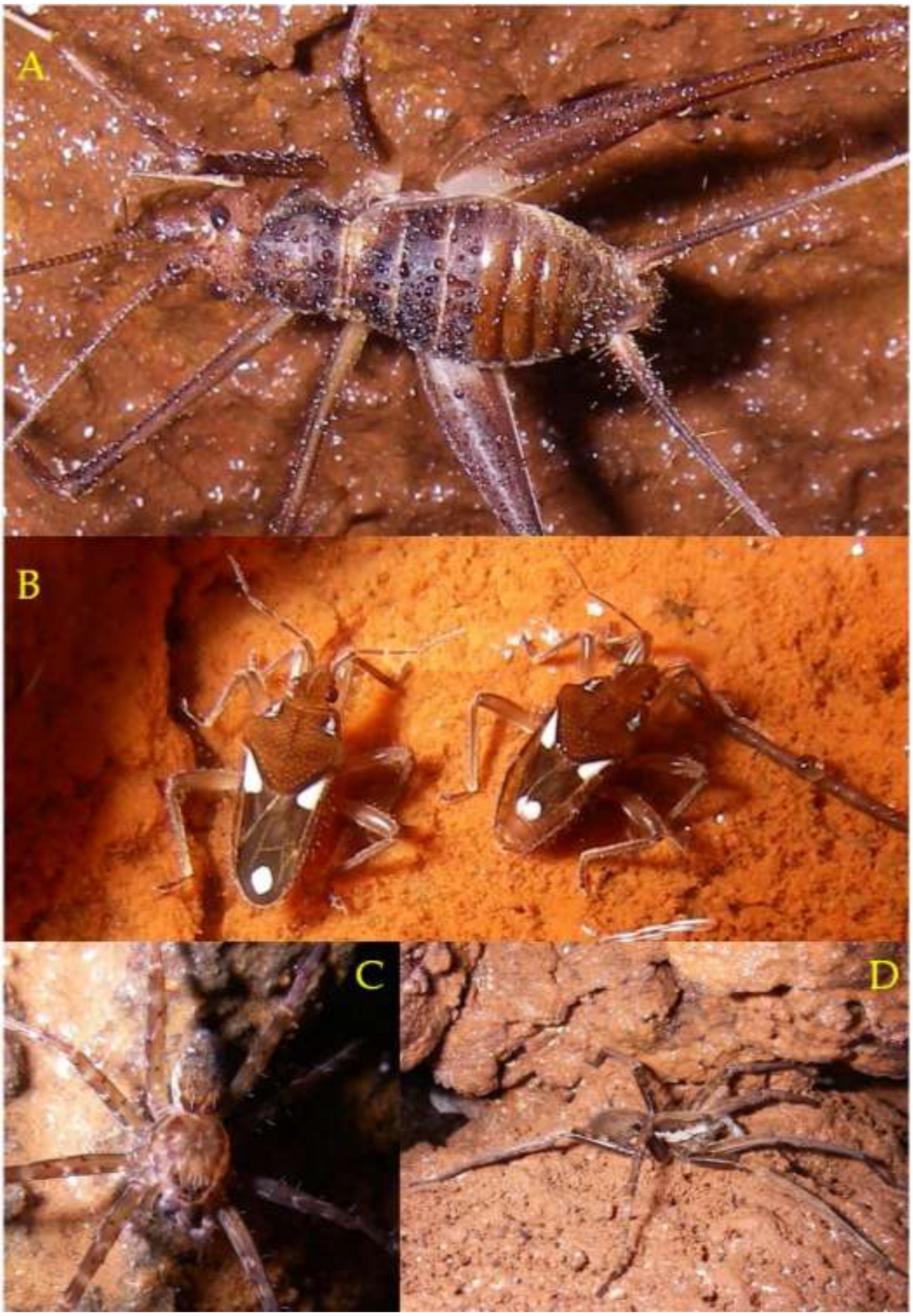

Figura 5.101 - A) Orthoptera: Phalangopsidae (*Phalangopsis* sp.); B) Heteroptera: Veliidae; C) Araneae: Trechaleidae (*Enna* aff. *paraense*); D) Araneae: Ctenidae.

CAVIDADE SL-130

Cavidade com 18,5m de projeção horizontal, formada em quartzito e localizada em área de encosta da Serra do Sereno. A vegetação de entorno é arbórea, composta por árvores de médio porte e sub-bosque pouco denso, com serrapilheira e gramíneas associadas ao substrato. A entrada é bastante baixa e sombreada, formada por blocos abatidos e de difícil acesso para o pequeno salão principal. Este é composto predominantemente por piso granulado com diversos seixos, calhaus e alguns matacões. Presença de musgos, fungos, serrapilheira, brotos e raízes próximos à zona de entrada e linha d'água. O sistema radicular é bastante desenvolvido, com muitas raízes de pequeno calibre e micro-raízes associadas à rocha. De forma geral a cavidade é úmida, com pontos de gotejamento visíveis apenas no período chuvoso. A caverna apresenta canalículos pouco desenvolvidos e penumbra escura. Não foram registrados depósitos de guano. Na campanha úmida observou-se um ninho de ave abandonado e um ninho ativo de Apodiformes (Beija-flor). Já no período seco, o ninho não se encontrava mais ativo. A cavidade apresenta entrada bastante restritiva, o que impede o aporte de recursos orgânicos provenientes do meio externo.

Caracterização faunística no período de seca

Foi observado na caverna, um total de 10 morfoespécies de invertebrados de pelo menos seis famílias dos Taxa: Pseudoscorpiones (Chernetidae), Actinedida (Parasitengonina), Oribatida, Araneae (Pholcidae: *Mesabolivar* sp.; Ninetinae, Prodidomidae), Orthoptera (Phalangopsidae: *Uvaroviella* sp.), Isoptera (Termitidae: *Nasutitermes* sp.), Hymenoptera (Formicidae: *Linepithema* sp.; *Pachycondyla* sp.)

Dentre os vertebrados, foi encontrada uma espécie da ordem Anura (Strabomantidae: *Pristimantis* sp.). Desta forma, no total foram encontrados 11 morfoespécies.

Caracterização faunística no período de chuva

Foi observado na caverna, um total de 45 morfoespécies de invertebrados de pelo menos 35 famílias dos Taxa: Gastropoda (Systrophiidae), Isopoda (Armadillidae, Dubioniscidae, Scleropactidae), Amblypygi (Phrinidae: *Heterophrynus longicornis*), Palpigradi (Eukoeneniidae: *Leptokoenenia* sp.), Pseudoscorpiones (Chernetidae), Opiliones (Escadabiidae), Actinedida (Parasitengonina), Oribatida, Araneae (Ochyroceratidae, Pholcidae: *Mesabolivar* sp.; *Metagonia* sp.; Ninetinae, Salticidae, Tetrablemmidae: *Matta* sp.), Diplura (Campodeidae), Collembola (Bourletiellidae, Cyphoderidae, Entomobryidae, Paronellidae), Orthoptera (Phalangopsidae: *Phalangopsis* sp.), Blattodea (Blattidae, Isoptera (Termitidae: *Nasutitermes* sp.), Heteroptera (Cydnidae), Homoptera (Cixiidae: *Pintalia* sp., Derbidae), Lepidoptera (Noctuidae), Diptera (Drosophilidae, Milichiidae, Psychodidae: *Lutzomyia* sp.), Hymenoptera (Formicidae: *Apterostigma* sp.; *Camponotus* sp.; *Linepithema* sp.; *Pachycondyla* sp., Vespidae: Polybiinae), Coleoptera (Carabidae, Elateridae, Rhizophagidae, Tenebrionidae), Geophilomorpha (Geophilidae: *Schizonampa* sp.), Scolopendromorpha (Scolopendridae: *Cryptops* sp.).

Dentre os vertebrados, foram encontradas quatro espécies das ordens: Apodiformes (Trochilidae), Chiroptera (Emballorunidae: *Peropteryx* sp.), Rodentia (Cricetidae: *Rhipidomys* sp.), Squamata (Gekkonidae: *Thecadactylus rapicauda*). Desta forma, no total foram encontrados 49 morfoespécies. Dentre essas, três espécies de invertebrados foram

consideradas troglomórficas: Gastropoda (Systrophiidae), Araneae (Tetrablemmidae: *Matta* sp.), Collembola (Cyphoderidae).

Caracterização geral da fauna da cavidade

Foi observado na caverna, um total de 49 morfoespécies de invertebrados de pelo menos 36 famílias dos Taxa: Gastropoda (Systrophiidae), Isopoda (Armadillidae, Dubioniscidae, Scleropactidae), Amblypygi (Phrinidae: *Heterophrynus longicornis*), Palpigradi (Eukoeneniidae: *Leptokoenenia* sp.), Pseudoscorpiones (Chernetidae), Opiliones (Escadabiidae), Actinedida (Parasitengonina), Oribatida, Araneae (Ochyroceratidae, Pholcidae: *Mesabolivar* sp.; *Metagonia* sp.; Ninetinae, Prodidomidae, Salticidae, Tetrablemmidae: *Matta* sp.), Diplura (Campodeidae), Collembola (Bourletiellidae, Cyphoderidae, Entomobryidae, Paronellidae), Orthoptera (Phalangopsidae: *Phalangopsis* sp.; *Uvaroviella* sp.), Blattodea (Blattidae), Isoptera (Termitidae: *Nasutitermes* sp.), Heteroptera (Cydnidae), Homoptera (Cixiidae: *Pintalia* sp., Derbidae), Lepidoptera (Noctuidae), Diptera (Drosophilidae, Milichiidae, Psychodidae: *Lutzomyia* sp.), Hymenoptera (Formicidae: *Apterostigma* sp.; *Camponotus* sp.; *Linepithema* sp.; *Pachycondyla* sp., Vespidae: Polybiinae), Coleoptera (Carabidae, Elateridae, Rhizophagidae, Tenebrionidae), Geophilomorpha (Geophilidae: *Schizonampa* sp.), Scolopendromorpha (Scolopendridae: *Cryptops* sp.).

Dentre os vertebrados, foram encontradas cinco espécies das ordens: Anura (Strabomantidae: *Pristimantis* sp.), Apodiformes (Trochilidae), Chiroptera (Emballorunidae: *Peropteryx* sp.), Rodentia (Cricetidae: *Rhipidomys* sp.), Squamata (Gekkonidae: *Thecadactylus rapicauda*).

Desta forma, no total foram encontrados 54 morfoespécies. Dentre essas, três espécies de invertebrados foram consideradas troglomórficas: Gastropoda (Systrophiidae), Araneae (Tetrablemmidae: *Matta* sp.), Collembola (Cyphoderidae). Alguns organismos encontrados nesta caverna são mostrados na Figura 5.102.

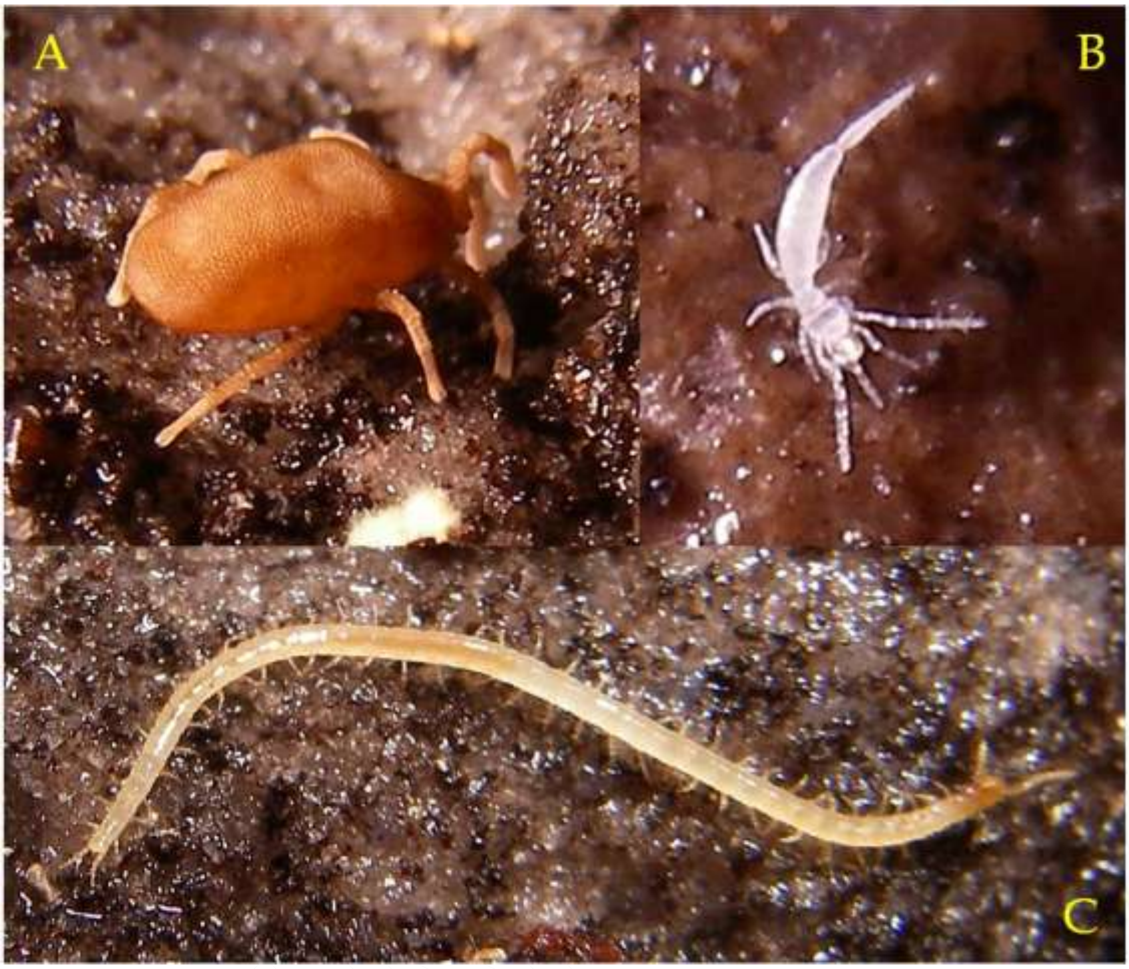

Figura 5.102 - A) Acari: Trombidiforme; B) Palpigradi: Eukoeneniidae (*Leptokoenenia* sp.); C) Geophilomorpha: Geophilidae (*Schizonampa* sp.).

CAVIDADE SL-131

Cavidade com 13m de projeção horizontal, formada em quartzito e localizada na região no topo da encosta da Serra do Sereno. O entorno é composto por vegetação arbustiva com algumas árvores de médio porte e com sub-bosque predominantemente formado por gramíneas e lianas. A cavidade possui apenas uma entrada, sendo esta comprida e de teto baixo com alguns pontos cobertos por briófitas. A cavidade não apresenta zona afótica, porém pode-se observar uma pequena região de penumbra clara na região mais à direita e ao centro da cavidade. Devido ao seu pequeno tamanho, a cavidade sofre grande influência externa, sendo a umidade do piso do abrigo constantemente alterada e tendo relação direta com a do ambiente externo. Portanto, na campanha seca observou-se um piso com baixíssima umidade, enquanto no período chuvoso, o piso encontra-se muito úmido. Pontos de gotejamento podem ser observados apenas no período chuvoso. O piso é plano ao centro, e levemente descendente à direita, ambos com vários seixos, calhaus e blocos abatidos. À esquerda o piso é descendente por ser formado apenas pela rocha nua, ao final pode ser observar um acúmulo de sedimento fino (solo). O piso de toda a cavidade é revestido por lianas. O sistema radicular é pouco desenvolvido, sendo este composto por algumas raízes de pequeno porte esparsas pela cavidade, e de médio calibre, que descem em direção a região à esquerda do abrigo.

Serrapilheira é ausente. Não foram observados depósitos de guano. De forma geral trata-se de uma cavidade com elevada influência das condições ambientais epígeas devido ao seu pequeno tamanho.

Caracterização faunística no período de seca

Foi observado na caverna, um total de nove morfoespécies de invertebrados de pelo menos oito famílias dos Taxa: Opilioacarida (Opiliacaridae), Araneae (Araneidae, Oonopidae, Scytodidae: *Scytodes itapevi*), Collembola (Entomobryidae), Isoptera (Termitidae: *Nasutitermes* sp.), Psocoptera (Myopsocidae), Homoptera (Cixiidae: *Pintalia* sp.).

Dentre os vertebrados, foram encontradas três espécies da ordem Chiroptera (Emballorunidae: *Peropteryx* sp., Furipteridae: *Furipterus horrens*, Phyllostomidae: *Lionycteris spurrelli*).

Desta forma, no total foram encontrados 12 morfoespécies.

Caracterização faunística no período de chuva

Foi observado na caverna, um total de 46 morfoespécies de invertebrados de pelo menos 28 famílias dos Taxa: Isopoda (Dubioniscidae), Oribatida, Prostigmata (Cunaxidae: *Armascirus* sp.), Araneae (Araneidae: *Alpaida* sp., Ctenidae: *Enoploctenus* sp., Pholcidae: *Metagonia* sp., Prodidomidae: *Lygromma* sp., Salticidae: *Noegus* sp., Scytodidae: *Scytodes itapevi; Scytodes* sp., Theridiidae: *Nesticodes rufipes*), Collembola (Cyphoderidae, Entomobryidae, Isotomidae), Isoptera (Termitidae: *Nasutitermes* sp.), Dermaptera, Psocoptera (Pachytroctidae, Ptiloneuridae), Homoptera (Cixiidae: *Pintalia* spp., Derbidae), Lepidoptera (Noctuidae, Tineidae, Tortricidae), Diptera (Cecidomyiidae, Chloropidae, Culicidae, Drosophilidae, Milichiidae, Psychodidae: *Lutzomyia* spp.), Hymenoptera (Formicidae: *Linepithema* sp.; *Pachycondyla* sp., Vespidae: Polybiinae), Coleoptera.

Dentre os vertebrados, foi encontrada uma espécie da ordem Chiroptera (Emballorunidae: *Peropteryx* sp.).

Desta forma, no total foram encontrados 47 morfoespécies. Dentre essas, uma espécie de invertebrado foi considerada troglomórfica: Collembola (Isotomidae).

Caracterização geral da fauna da cavidade

Foi observado na caverna, um total de 51 morfoespécies de invertebrados de pelo menos 31 famílias dos Taxa: Isopoda (Dubioniscidae), Opilioacarida (Opiliacaridae), Oribatida, Prostigmata (Cunaxidae: *Armascirus* sp.), Araneae (Araneidae: *Alpaida* sp., Ctenidae: *Enoploctenus* sp., Oonopidae, Pholcidae: *Metagonia* sp., Prodidomidae: *Lygromma* sp., Salticidae: *Noegus* sp., Scytodidae: *Scytodes itapevi; Scytodes* sp., Theridiidae: *Nesticodes rufipes*), Collembola (Cyphoderidae, Entomobryidae, Isotomidae), Isoptera (Termitidae: *Nasutitermes* sp.), Dermaptera, Psocoptera (Myopsocidae, Pachytroctidae, Ptiloneuridae), Homoptera (Cixiidae: *Pintalia* spp., Derbidae), Lepidoptera (Noctuidae, Tineidae, Tortricidae), Diptera (Cecidomyiidae, Chloropidae, Culicidae, Drosophilidae, Milichiidae, Psychodidae: *Lutzomyia* spp.), Hymenoptera (Formicidae: *Linepithema* sp.; *Pachycondyla* sp., Vespidae: Polybiinae), Coleoptera.

Dentre os vertebrados, foram encontradas três espécies da ordem Chiroptera (Emballorunidae: *Peropteryx* sp., Furipteridae: *Furipterus horrens*, Phyllostomidae: *Lionycteris spurrelli*). Desta forma, no total foram encontrados 54 morfoespécies. Dentre essas, uma espécie de invertebrado foi considerada troglomórfica: Collembola (Isotomidae).

5.2.4. Avaliação preliminar do patrimônio espeleológico

As cavernas vêm sendo objeto de uma valorização crescente, não só como patrimônio natural/cultural, mas como um ecossistema frágil e peculiar. Diante dos atributos selecionados para a valoração da caverna inventariada (científico, ambiental, paisagístico, religioso e econômico) foi possível efetuar uma avaliação preliminar do patrimônio espeleológico das cavernas presentes em Serra Leste.

Sob o ponto de vista científico, todas as cavernas que apresentaram espécies troglóbias apresentam elevado potencial para estudos bioespeleológicos, principalmente no que concerne a trabalhos sobre evolução regressiva, tendo em vista a ocorrência destas espécies que podem contribuir para um melhor entendimento da evolução destes grupos em cavernas.

Ambientalmente, as cavernas da área não apresentam fortes atributos de valoração física, tendo em vista a inexistência de sumidouros e mesmo suas desconexões com aqüíferos. A exceção a esta regra compreende algumas poucas cavidades associadas a pequenas nascentes de água. O valor paisagístico das cavernas é relativamente baixo, tendo em vista a presença de morfologias de baixo atrativo estético. Os valores religiosos são virtualmente inexistentes, já que nenhuma das cavidades apresentou indícios de uso para manifestações religiosas. O potencial para o uso turístico das cavernas é pequeno em função da baixa qualidade estética/paisagística e reduzido tamanho das cavidades.

5.2.5. Análises ecológicas

Padrões gerais de riqueza e diversidade

As cavidades mostraram valores de riqueza de espécies bastante variáveis. Além disso, a diversidade, equitabilidade e dominância também se mostraram variáveis entre estas cavernas (Tabela 5.2 - Valores de riqueza, dominância, diversidade e equitabilidade para as cavernas do estudo (estação seca).). Estas variações são esperadas, e decorrem das diferentes condições físicas, tróficas e dinâmicas de cada um dos sistemas estudados. Por mais parecidas que duas cavidades possam ser, dificilmente possuirão atributos biológicos semelhantes, em função da complexidade (e dinamismo) das interações que estruturam cada comunidade.

Tabela 5.2 - Valores de riqueza, dominância, diversidade e equitabilidade para as cavernas do estudo (estação seca).

Caverna	Riqueza			Dominância		Diversidade		Equitabilidade	
	Seca	Chuva	Total	Seca	Chuva	Seca	Chuva	Seca	Chuva
**SL-101**	110	138	180	0,09	0,15	3,13	2,23	0,67	0,45
**SL-102**	15	41	46	0,18	0,05	2,20	3,32	0,81	0,89
**SL-103**	35	84	99	0,32	0,66	1,74	1,05	0,49	0,24

**SL-104**	16	42	52	0,36	0,07	1,30	3,17	0,47	0,85
**SL-105**	33	52	65	0,17	0,17	2,45	2,59	0,70	0,66
**SL-106**	13	29	33	0,23	0,09	1,86	2,86	0,73	0,85
**SL-107**	50	101	124	0,08	0,21	3,10	2,41	0,79	0,52
**SL-108**	52	89	112	0,06	0,53	3,28	1,40	0,83	0,31
**SL-109**	41	87	105	0,12	0,09	2,53	3,01	0,68	0,67
**SL-110**	73	113	149	0,35	0,10	1,75	2,39	0,41	0,51
**SL-111**	18	46	54	0,13	0,15	2,46	2,84	0,85	0,74
**SL-112**	57	86	125	0,08	0,04	3,15	3,83	0,78	0,86
**SL-113**	21	17	36	0,07	0,14	2,87	2,35	0,94	0,83
**SL-114**	20	40	55	0,18	0,08	2,16	2,83	0,72	0,77
**SL-115**	45	79	103	0,07	0,25	3,11	2,42	0,82	0,55
**SL-116**	36	48	69	0,16	0,08	2,45	3,13	0,68	0,81
**SL-117**	33	43	66	0,12	0,10	2,76	2,82	0,79	0,75
**SL-121**	78	97	134	0,16	0,21	2,56	1,86	0,59	0,41
**SL-122**	52	77	106	0,08	0,13	3,03	2,67	0,77	0,61
**SL-130**	11	49	54	0,15	0,18	2,10	2,73	0,88	0,70
**SL-131**	12	47	54	0,15	0,06	2,15	3,25	0,87	0,84

Um fato realmente notável diz respeito à elevada riqueza observada nas cavernas da área. Um total de nove cavidades (42,7% do total inventariado) apresentou mais de 100 espécies, considerando-se as duas amostragens (Figura 30).

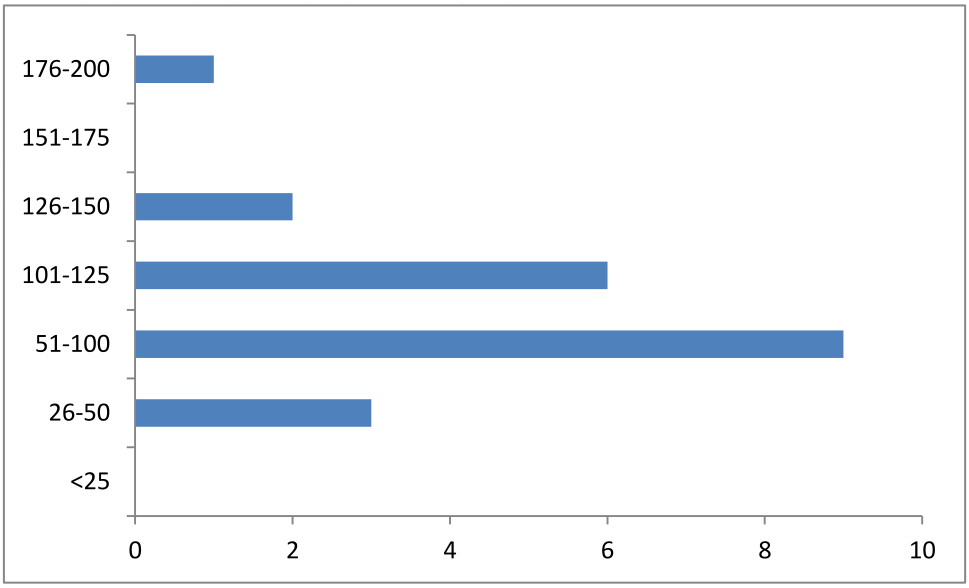

Figura 5.103 - Classes de riqueza total das cavernas da área. Os valores no eixo vertical indicam o número total de espécies, e no eixo horizontal, o número de cavernas em cada categoria.

Souza-Silva e colaboradores (2011) demonstraram as fortes relações existentes entre o tamanho das cavernas e a riqueza de espécies na Mata Atlântica brasileira, dentre as quais se

destacaram as cavernas ferruginosas. As cavernas ferruginosas inventariadas naquele estudo situam-se no Quadrilátero Ferrífero, em Minas Gerais. Tais autores encontraram uma riqueza média correspondente a 53 espécies (± 26.35) em cavernas carbonáticas, 45,4 espécies (±22.7) em cavernas siliciclásticas, 39,9 espécies (±19.27) em cavernas magmáticas e 37,5 espécies (±20.96) em cavernas ferruginosas. A média de riqueza encontrada nas 21 cavernas de Serra leste (considerando a média obtida entre os valores médios de riqueza do período de seca e do período chuvoso) correspondeu a 53 espécies, um número consideravelmente maior que o observado por Souza-Silva e colaboradores (2011) para as cavernas do quadrilátero ferrífero. A análise integrada com outras 98 cavernas de Serra Leste e 146 cavernas de Serra Norte (morro I e II) mostrou riqueza média de 52 espécies.

Estimadores de riqueza

As curvas de rarefação construídas (Figura 5.104) indicaram amostragens, a princípio, não satisfatórias do número potencial de espécies presente na área (considerando-se o total de espécies e as coletas referentes a cada estação, separadamente). Caso o total de espécies presente na região tivesse sido amostrado, seria observada uma estabilização nas curvas (assíntota – a linha se curvaria tornando-se paralela ao eixo horizontal do gráfico), indicando que o que existe já foi amostrado. O padrão obtido indica que se a amostragem fosse intensificada (através da inclusão de outras cavidades existentes nos arredores e que não foram amostradas), provavelmente seriam encontradas mais espécies que ainda não foram avistadas nas 21 cavernas amostradas.

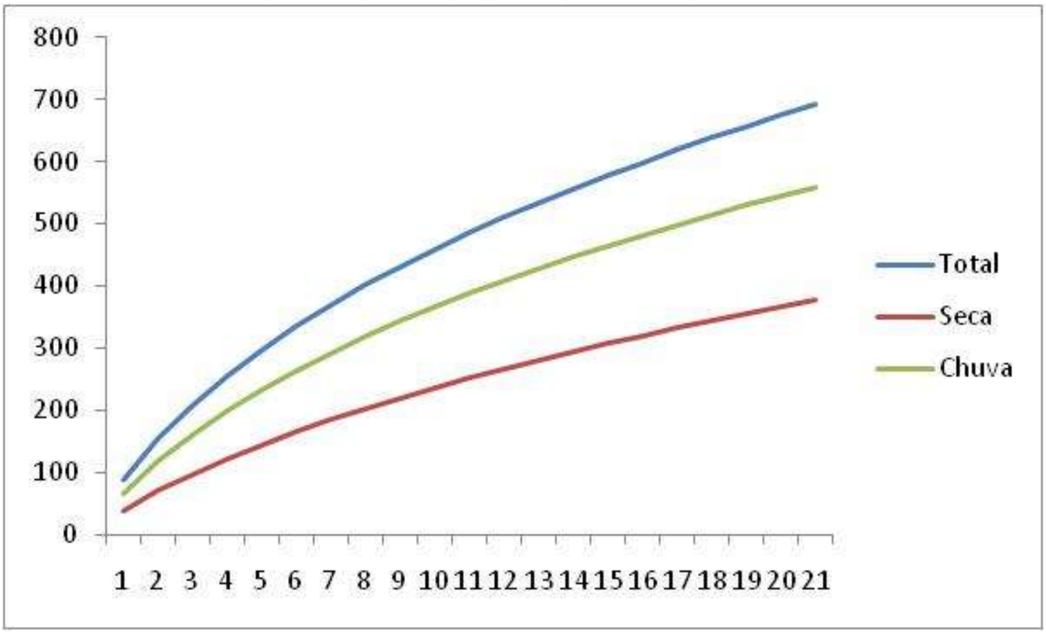

Figura 5.104 - Curvas de rarefação construídas para as cavernas da área.

Reitera-se que dificilmente atinge-se a assíntota nestas curvas do coletor, a não ser que o número de amostras (no caso, cavernas) seja muito grande. Mesmo assim, tendo em vista a constante contribuição de espécies acidentais ou mesmo “transientes”, é provável que, para certos sistemas, a assíntota nunca seja atingida, já que o “estoque” epígeo de grupos

acidentais é inimaginável (especialmente quando se considera a região Neotropical, conhecida pela megadiversiade epígea).

Padrões gerais de similaridade da fauna

Os dendrogramas de similaridade entre as diferentes cavernas são mostrados na Figura 5.105. De forma geral, as cavidades apresentaram baixos valores de similaridade entre as comunidades bióticas, sendo que a grande maioria das cavidades apresentou valores de similaridade inferiores a 0,5 (em geral, consideram-se valores elevados aqueles iguais ou superiores a 70% de similaridade – 0,7). A baixa similaridade entre as comunidades presentes nas diferentes cavernas evidencia a importância da estrutura física e trófica (com particularidades inerentes a cada caverna) para a determinação de quais espécies possuem viabilidade de colonização e permanência em cada cavidade.

Além das cavidades terem exibido baixos valores de similaridade, os mesmos aparentemente não estão relacionados à distribuição espacial das cavernas. Desta forma, as cavidades geograficamente mais próximas não apresentam necessariamente comunidades mais similares quando comparadas a cavernas mais distantes.

Tendo em vista a elevada dissimilaridade entre as cavernas da área, acredita-se que a destruição de cada uma das cavernas amostradas dificilmente acarretará em colonizações futuras que culminem com a instalação bem sucedida destas comunidades em outras cavernas da região.

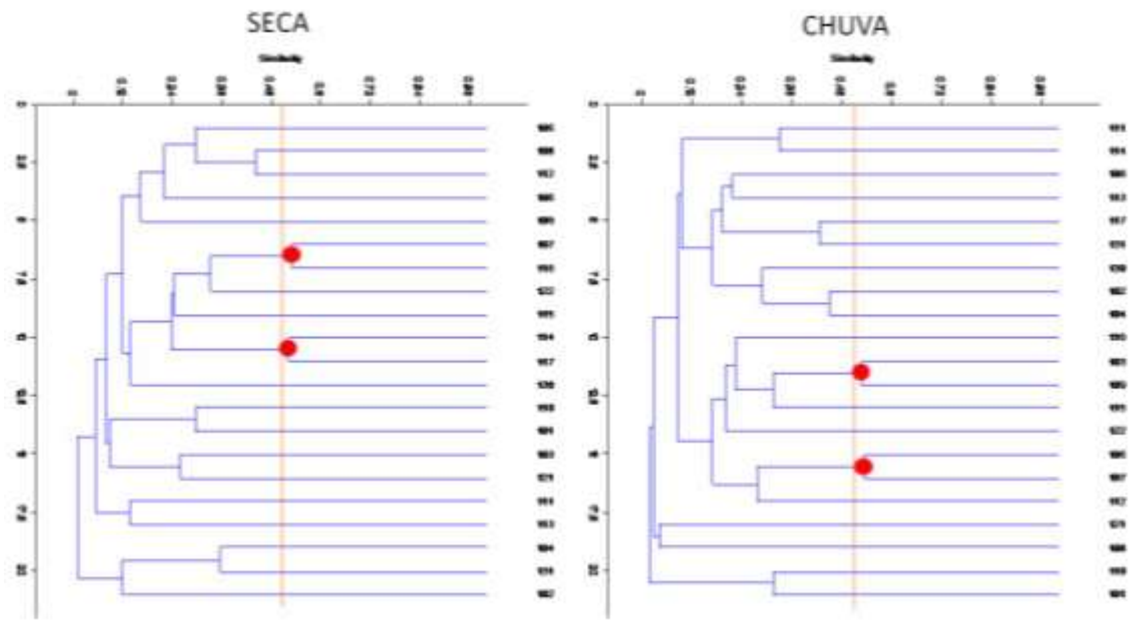

Figura 5.105 - Dendrogramas de similaridade (Bray-Curtis), evidenciando os padrões de similaridade entre as cavernas. A figura da esquerda representa o dendrograma referente à estação de seca e, a da direita, a estação chuvosa. Os pontos vermelhos representam valores de similaridade entre cavernas superiores a 60% e, os pontos verdes, valores superiores a 50%.

Relações entre variáveis bióticas e abióticas

O tamanho das cavernas mostrou-se positivamente relacionado à riqueza de espécies na estação seca (R =0,71; p<0,0003) e na estação chuvosa (R =0,60; p<0,004) (Figura 190 A, B).

Além disso, o tamanho das cavernas mostrou-se positivamente relacionado ao número total de espécies observado em cada caverna ($R = 0,602$; $p<0,004$).

Embora se tenha especulado durante décadas acerca da relação entre o tamanho de uma caverna e sua riqueza, esta relação foi pela primeira vez demonstrada empiricamente por Ferreira (2004). A relação positiva encontrada entre a projeção horizontal das cavidades e a riqueza das comunidades sugere a importância do espaço para a determinação do número de espécies presente em uma caverna. Segundo aquele autor, cavernas maiores tendem a ser geomorfologicamente mais heterogêneas, o que provavelmente resulta em aumento da quantidade de microhabitats. Consequentemente, um número maior de espécies pode se estabelecer nestas condições. Além disso, a quantidade de recursos orgânicos "estocada" pode ser eventualmente maior, uma vez que há maior quantidade de espaço disponível. Entretanto, a relação entre tamanho da cavidade e quantidade de recursos não deve ser sempre linear, pois também depende de outros fatores relacionados à produção externa de matéria orgânica (de acordo com o bioma em que a caverna se insere) e mesmo da estrutura geral das entradas (que podem possuir maior ou menor propensão a funcionar como "coletoras" de materiais externos).

Nesta perspectiva, novamente menciona-se aqui o trabalho realizado por Souza-Silva e colaboradores (2011), que demonstraram as fortes relações existentes entre o tamanho das cavernas e a riqueza de espécies para cavernas na Mata Atlântica brasileira. Dentre as diversas litologias testadas, se destacaram as cavernas ferruginosas.

Os autores do referido trabalho argumentaram que as cavernas ferruginosas mostraram a mais forte tendência de aumento de espécies com o aumento da projeção linear. Tal fato, segundo aqueles autores, pode dever-se às características micro-ambientais heterogêneas destas cavernas associadas à intricada malha de canalículos que são adicionados à extensão disponibilizada aos invertebrados nestas macrocavernas. Provavelmente, as macrocavernas ferruginosas atuam "condensando" ou acumulando uma maior quantidade de recursos orgânicos, além de potencialmente poderem se conectar a uma maior quantidade de canalículos (pelo aumento do volume subterrâneo). Tal condição faz com que as macro-cavernas possam funcionar como "atratores" de fauna (pela quantidade e qualidade de recursos alimentares), sendo que esta atração pode ocorrer de forma exponencial, isto é, pequenos aumentos no desenvolvimento linear podem levar a um aumento exponencial de atratividade, elevando de forma surpreendente a quantidade de espécies presentes, diferentemente do que ocorre para outras litologias, onde tais canalículos não são abundantes.

6. ANÁLISE DE RELEVÂNCIA DAS CAVERNAS DE SERRA LESTE

A análise de relevância foi efetuada através dos procedimentos definidos no Decreto nº 6640, de 07 de novembro de 2008. Este decreto alterou significativamente o status jurídico referente à proteção das cavernas brasileiras e passou a prever a classificação das cavernas segundo quatro graus de relevância: máximo, alto, médio e baixo. A determinação das cavernas de relevância máxima é realizada através de parâmetros definidos pelo próprio decreto. Os

parâmetros dos demais graus de relevância foram apenas esboçados, estando remetidos à Instrução Normativa nº 2 (IN 2), publicada no dia 20 de agosto de 2009 pelo Ministério do Meio Ambiente - MMA.

6.1. Metodologia

Com base no Decreto Federal nº 6640, as cavidades naturais subterrâneas deverão ser classificadas segundo graus de relevância determinados pela análise de atributos geológicos, hidrológicos, ecológicos, biológicos, paleontológicos, cênicos, histórico-culturais e socioeconômicos. As cavernas classificadas com o grau máximo de relevância devem ser integralmente conservadas. As cavidades classificadas como de relevância alta e média poderão ser impactadas parcial ou integralmente mediante compensação ambiental a ser definida pelo órgão ambiental. Cavidades de baixa relevância poderão ser impactadas sem compensação. Qualquer impacto em cavernas somente poderá ocorrer mediante o processo de licenciamento ambiental (Art. 4 do Decreto 6640).

6.1.1. Discriminação litológica

De acordo com o Decreto 6640 em seu Artigo 2º, § 1º, "A análise dos atributos geológicos, para a determinação do grau de relevância, deverá ser realizada comparando cavernas da mesma litologia". O primeiro passo envolve a determinação da litologia das cavernas. As cavidades de Serra Leste alojam-se em ferricrete, em variações de canga e em quartzito. O ferricrete e a canga são agrupados na classificação de rochas ferríferas, não havendo distinção entre elas. Neste grupo insere-se a maior parte das cavidades da Serra Leste, somando 106 cavernas, sendo 12 estudadas neste documento. Classificado como rocha siliciclástica, o quartzito que abriga nove cavernas da área, constitui uma segunda categoria litológica na área de estudo.

6.1.2. Escala de análise

A determinação da escala de análise foi feita com base na IN 2, que define que os atributos deverão ser avaliados sob o enfoque regional e local. O enfoque local, segundo a instrução normativa, será limitado à unidade geomorfológica que apresenta continuidade espacial, podendo abranger feições como serras, morrotes ou sistema cárstico, o que for mais restritivo em termos de área, desde que contemplada a área de influência da cavidade. O enfoque regional deverá compreender uma unidade espeleológica, definida por uma área que apresente homogeneidade fisiográfica.

A escala regional abrange toda a região da Província Mineral de Carajás, Pará. Valentim e Olivito (2011) estabeleceram, para a região sudeste deste estado, a unidade espeleológica Carajás composta por nove unidades geomorfológicas sob enfoque local, todas configuradas como serra: (i) Serra Norte; (ii) Serra Sul; (iii) Serra da Bocaina; (iv) Serra do Rabo – Estrela; (v) Serra Leste; (vi) Serra do Cinzento; (vii) Serra Aquiri; (viii) Serra de São Felix e (ix) Serra Arqueada (Figura 6.1).

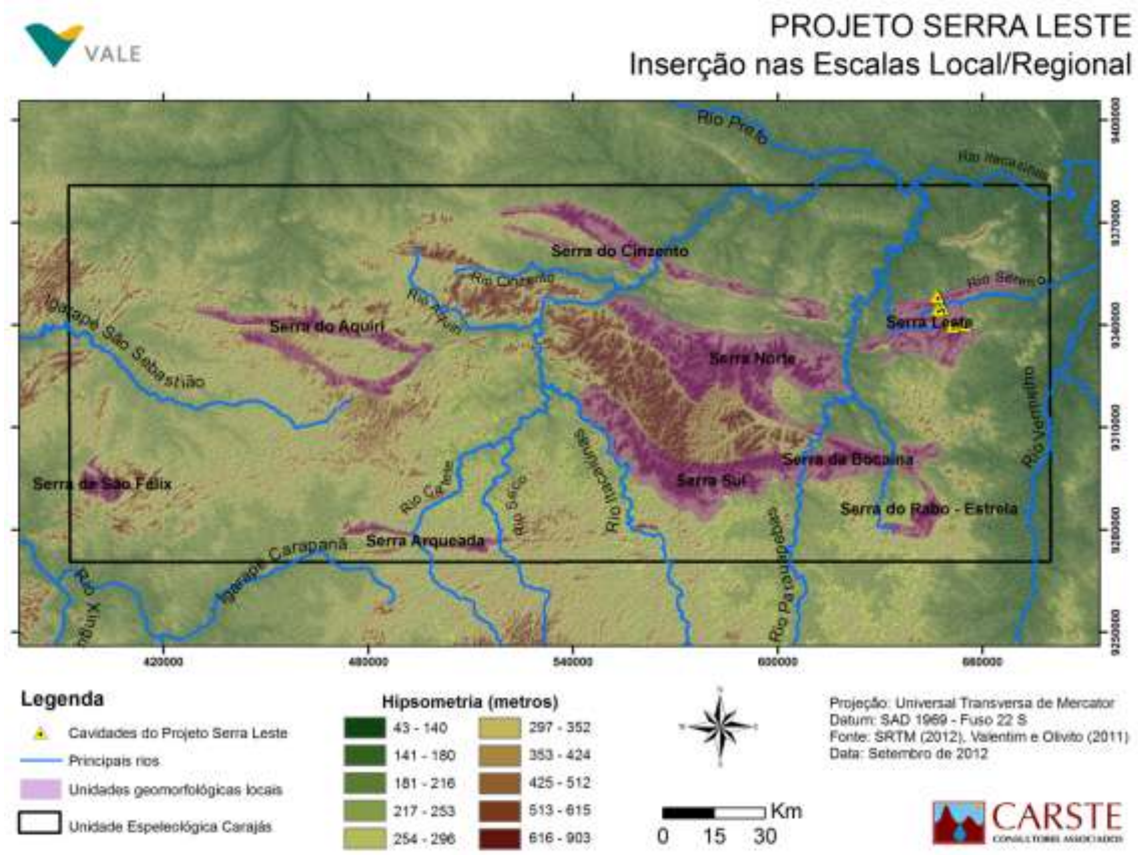

Figura 6.1 - Localização da unidade espeleológica Carajás composta por nove unidades geomorfológicas.

As cavidades que compõem a amostra local deste estudo englobam todas as cavidades de Serra Leste. Desta forma, para as cavernas alojadas em quartzito, a amostra local compreende apenas as nove cavernas analisadas e, para as cavernas em rochas ferríferas, a amostra contempla as 12 cavernas estudadas neste documento somadas a 94 cavernas analisadas em documento elaborado por CARSTE (2011a) totalizando 106 cavidades (Anexo IV).

A amostra regional, que abarca toda a unidade espeleológica de Carajás, é mais ampla: para as cavidades alojadas em rochas siliciclásticas, soma-se à amostra local outras cavidades inseridas na área do Projeto Alemão. Esta região, localizada entre as serras Norte e Sul, não foi inserida em nenhuma das unidades geomorfológicas que compõem a unidade espeleológica Carajás. Trata-se de uma porção rebaixada na paisagem, onde afloram rochas do embasamento representado pelo Complexo Xingu, rochas vulcânicas, além de arenitos da Formação Águas Claras, onde se desenvolvem feições espeleológicas.

Para as cavernas em rochas ferríferas, além das 106 cavernas que compõem a amostra local, somam-se outras 434 cavidades inseridas em projetos da Serra Norte (por exemplo, N1, N4E, N4WS, N5S, N5SM1, N5SM2, PESE, PESL), além de 148 grutas da Serra Sul (S11, S11D).

6.1.3. *Grau de relevância*

O grau de relevância **máximo** é aplicado às cavidades que apresentem ao menos um dos seguintes atributos: gênese única ou rara; morfologia rara; dimensões notáveis em extensão, área ou volume; espeleotemas únicos; isolamento geográfico; cavidade testemunho; ou destacada relevância histórico-cultural ou religiosa. Tais cavidades deverão ser conservadas integralmente, não podendo ser objeto de impactos adversos. A utilização dessas cavidades somente poderá ocorrer de modo que fique assegurada a manutenção de seu equilíbrio físico e biológico.

As cavidades com grau de relevância **alto** deverão apresentar atributos de importância acentuada sob enfoque local e regional; ou acentuada sob enfoque local e significativa sob enfoque regional. Essas cavidades poderão sofrer impactos irreversíveis, desde que compensados através da preservação de duas outras cavidades na mesma litologia, com atributos similares e com mesmo grau de relevância da caverna a ser impactada.

As cavidades com grau de relevância **médio** deverão englobar atributos de importância acentuada sob enfoque local e baixa sob enfoque regional, ou significativa sob enfoque local e regional. Impactos irreversíveis nessas cavidades deverão ser compensados com ações que contribuam para a conservação e uso adequado do patrimônio espeleológico nacional, a serem definidas pelo órgão ambiental competente.

As cavernas classificadas com grau de relevância **baixo** deverão conter atributos considerados de importância significativa sob enfoque local e baixa sob enfoque regional, ou baixa sob enfoque local e regional. Tais cavidades poderão sofrer impactos irreversíveis, não sendo necessária a compensação ambiental.

O fluxograma para a classificação do grau de relevância das cavidades é apresentado na Figura 6.2.

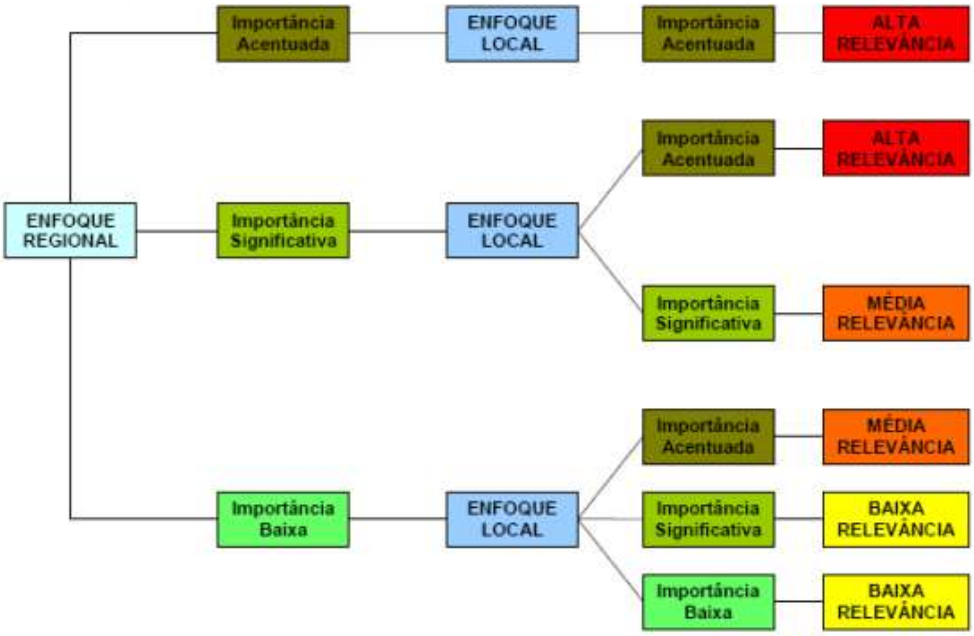

Figura 6.2 - Fluxograma de classificação do grau de relevância de cavidades naturais subterrâneas, segundo a Instrução Normativa nº 2, do MMA.

6.2.Resultados da análise de relevância das cavernas de Serra Leste

Dentre os atributos físicos que classificam as cavidades segundo os graus de relevância, encontram-se atributos espeleométricos, tais como projeção horizontal, desnível, área e volume. A Tabela 6.1 e a Tabela 6.2 apresentam os valores de corte a partir de atributos dimensionais para a definição da relevância das cavidades alojadas em rochas ferríferas e siliciclásticas, respectivamente, em escala regional, tendo como base a IN 2. As Tabela 6.3 e Tabela 6.4 demonstram os valores para a escala local. De acordo com o inciso III do Art. 3º, salienta-se que a legislação adota o termo “dimensões notáveis” para a classificação de máxima relevância que, dimensionalmente, é bastante subjetivo. Em função disto, é aqui adotado um valor numérico cinco vezes maior do que a média, interpretado como um valor apropriado, visto que permite a conservação de cavernas de grandes dimensões em relação ao conjunto regional amostrado em uma determinada litologia.

Tabela 6.1 - Parâmetros espeleométricos do enfoque regional para cavidades em rocha ferrífera de Serra Leste.

Escala Regional: 688 cavernas em rochas ferríferas	Projeção Horizontal (m)	Desnível (m)	Área (m^2)	Volume (m^3)
Média	31,72	4,33	123,01	243,49
Desvio Padrão	39,03	5,04	182,09	431,80
Alto (> μ +σ)	70,75	9,37	305,10	675,29
Médio - Intervalo entre (μ - σ) e (μ + σ)	-7,31 < x < 70,75		-59,08 < x < 305,10	-188,30 < x < 675,29
Baixo (< μ – σ)	-7,31		-59,08	-188,30
Máxima (5 x média regional)	158,61		615,04	1217,46

* valores negativos oriundos de erros estatísticos

Tabela 6.2 - Parâmetros espeleométricos do enfoque regional para cavidades em rocha siliciclástica de Serra Leste.

Escala Regional: 22 cavernas em rochas siliciclásticas	Projeção Horizontal (m)	Desnível (m)	Área (m2)	Volume (m3)
Média	35,11	4,00	118,94	157,87
Desvio Padrão	95,21	6,64	384,24	560,47
Alto (> μ +σ)	130,32	10,64	503,19	718,34
Médio - Intervalo entre (μ - σ) e (μ + σ)	-60,10 < x < 130,32		-265,30 < x < 503,19	-402,61 < x < 718,34
Baixo (< μ – σ)	-60,10		-265,30	-402,61
Máxima (5 x média regional)	175,53		594,71	789,33

* valores negativos oriundos de erros estatísticos

Tabela 6.3 - Parâmetros espeleométricos do enfoque local para cavidades em rocha ferrífera de Serra Leste.

Escala Local: 106 cavernas em rochas ferríferas	Projeção Horizontal (m)	Desnível (m)	Área (m2)	Volume (m3)

Média	21,34	2,26	80,30	139,14
Desvio Padrão	18,33	1,86	94,84	187,79
Alto (> μ +σ)	39,68	4,11	175,14	326,94
Médio - Intervalo entre (μ - σ) e (μ + σ)	3,01 < x < 39,68		-14,55 < x < 175,14	-48,65 < x < 326,94
Baixo (< μ – σ)	3,01		-14,55	-48,65
Máxima (5 x média regional)	106,72		401,48	695,71

* valores negativos oriundos de erros estatísticos

Tabela 6.4 - Parâmetros espeleométricos do enfoque local para cavidades em rocha siliciclástica de Serra Leste.

Escala Local: 9 cavernas em rochas siliciclásticas	Projeção Horizontal (m)	Desnível (m)	Área (m2)	Volume (m3)
Média	66,89	7,29	248,06	349,89
Desvio Padrão	147,74	9,63	596,15	868,14
Alto (> μ +σ)	214,63	16,92	844,21	1218,03
Médio - Intervalo entre (μ - σ) e (μ + σ)	-80,85 < x < 214,63		-348,10 < x < 844,21	-518,25 < x < 1218,03
Baixo (< μ – σ)	-80,85		-348,10	-518,25
Máxima (5 x média regional)	334,44		1240,28	1749,44

* valores negativos oriundos de erros estatísticos

Como pode ser observado no Quadro 6.1 apenas uma cavidade natural subterrânea de Serra Leste apresentou relevância máxima segundo os atributos físicos e/ou histórico-culturais, com base nos incisos dos Art. 3º: a caverna SL-101 apresenta dimensões notáveis em extensão, área e volume. Uma cavidade apenas têm atributos com importância acentuada sobre os enfoques local e regional, segundo o artigo 7º: a caverna SL-122 apresenta drenagem subterrânea perene que pode apresentar influência acentuada em pelo menos um dos atributos a seguir: presença de táxons novos; alta riqueza de espécies; alta abundância relativa de espécies; presença de composição singular da fauna; presença de troglóbios não raros endêmicos ou relictos; presença de espécies troglomórficas; presença de trogloxeno obrigatório; presença de população excepcional em tamanho e/ou presença de espécie rara.

Exceto a caverna SL-101, todas as cavidades deste estudo apresentam, em termos dimensionais, valores abaixo da média regional mais o desvio padrão, para projeção horizontal, área e volume, sendo, portanto, classificadas como de média relevância, com base nos incisos II, III e V do Art. 9º. Em relação à deposição química, não foi observada grande diversidade de tipos ou processos. Desse modo, 19 cavidades naturais se enquadram nos termos do inciso IX do Art. 10º. As duas demais (SL-113 e SL-114) não apresentam espeleotemas.

Em relação aos atributos biológicos, esclarecem-se os métodos utilizados para o cálculo de alguns dos atributos considerados na análise de relevância. A riqueza de espécies foi obtida por meio da média e o desvio padrão para o conjunto de dados obtidos. As cavernas que excederam o limite superior à média +/- desvio padrão foram consideradas de alta riqueza de espécies. Da mesma forma, as cavernas que obtiveram valores inferiores a este mesmo

intervalo foram consideradas de baixa riqueza de espécies. Cavernas com valores de riqueza dentro deste intervalo foram consideradas como de média riqueza de espécies. Tal procedimento foi realizado considerando-se somente as cavernas inventariadas (enfoque local), e as cavernas inventariadas em Serra Leste e Serra Norte (Morro I e Morro II), enfoque regional.

A abundância relativa de espécies foi obtida da seguinte forma: primeiramente extraiu-se a raiz quadrada dos valores de abundância de cada população. Este procedimento teve como objetivo reduzir as diferenças numéricas existentes entre certas populações. Tal procedimento poderia ter sido feito por meio do logaritmo natural dos valores de abundância, mas como muitas espécies foram representadas por somente um indivíduo, o grande número de “zeros” poderia levar a ruídos na análise. Os valores transformados (pela extração das raízes quadradas) foram então divididos em três categorias, tomando-se como base o maior valor obtido para cada caverna (que foi, desta forma, dividido por três, criando cada uma das categorias de tamanhos populacionais para cada caverna).

Com base no inciso VII do Art. 3 º, a presença de troglóbio raro em quatro cavidades classificou tais cavidades no grau de relevância máximo, sendo elas SL-101, SL-110, SL-115, SL-121. Neste caso, utilizou-se o conceito de raridade ligado principalmente à estrita distribuição, isto é, o elevado grau de endemismo, comparando as espécies coletadas com aquelas depositadas na coleção de invertebrados subterrâneos da Universidade Federal de Lavras (Serra Leste e Serra Norte).

Quadro 6.1 - Classificação de relevância das cavernas em Serra Leste.

GRAU DE IMPORTÂNCIA/ ENFOQUE	ATRIBUTO CLASSIFICATÓRIO	SL-101	SL-102	SL-103	SL-104	SL-105	SL-106	SL-107	SL-108	SL-109	SL-110	SL-111	SL-112	SL-113	SL-114	SL-115	SL-116	SL-117	SL-121	SL-122	SL-130	SL-131
	**Incisos do Art. 3º**																					
Relevância máxima	**I** - Gênese única ou rara;	Ausente	Ausente	Ausente	Ausente	Ausente	Ausente	Ausente	Ausente	Ausente	Ausente	Ausente	Ausente	Ausente	Ausente	Ausente	Ausente	Ausente	Ausente	Ausente	Ausente	Ausente
	**II** - Morfologia única;	Ausente	Ausente	Ausente	Ausente	Ausente	Ausente	Ausente	Ausente	Ausente	Ausente	Ausente	Ausente	Ausente	Ausente	Ausente	Ausente	Ausente	Ausente	Ausente	Ausente	Ausente
	**III** - Dimensões notáveis em extensão, área ou volume;	Presente	Ausente	Ausente	Ausente	Ausente	Ausente	Ausente	Ausente	Ausente	Ausente	Ausente	Ausente	Ausente	Ausente	Ausente	Ausente	Ausente	Ausente	Ausente	Ausente	Ausente
	**IV** - Espeleotemas únicos;	Ausente	Ausente	Ausente	Ausente	Ausente	Ausente	Ausente	Ausente	Ausente	Ausente	Ausente	Ausente	Ausente	Ausente	Ausente	Ausente	Ausente	Ausente	Ausente	Ausente	Ausente
	**V** - Isolamento geográfico;	Ausente	Ausente	Ausente	Ausente	Ausente	Ausente	Ausente	Ausente	Ausente	Ausente	Ausente	Ausente	Ausente	Ausente	Ausente	Ausente	Ausente	Ausente	Ausente	Ausente	Ausente
	**VI** - Abrigo essencial para a preservação de populações geneticamente viáveis de espécies de animais em risco de extinção, constantes de listas oficiais;	Ausente	Ausente	Ausente	Ausente	Ausente	Ausente	Ausente	Ausente	Ausente	Ausente	Ausente	Ausente	Ausente	Ausente	Ausente	Ausente	Ausente	Ausente	Ausente	Ausente	Ausente
	**VII** – Habitat para a preservação de populações geneticamente viáveis de relictos;	Ausente	Ausente	Ausente	Ausente	Ausente	Ausente	Ausente	Ausente	Ausente	Ausente	Ausente	Ausente	Ausente	Ausente	Ausente	Ausente	Ausente	Ausente	Ausente	Ausente	Ausente
	**VIII** – Habitat de troglóbio raro;	Presente	Ausente	Ausente	Ausente	Ausente	Ausente	Ausente	Ausente	Ausente	Presente	Ausente	Ausente	Ausente	Ausente	Presente	Ausente	Ausente	Presente	Ausente	Ausente	Ausente
	**IX** – Interações ecológicas únicas;	Ausente	Ausente	Ausente	Ausente	Ausente	Ausente	Ausente	Ausente	Ausente	Ausente	Ausente	Ausente	Ausente	Ausente	Ausente	Ausente	Ausente	Ausente	Ausente	Ausente	Ausente
	**X** - Cavidade testemunho; ou	Ausente	Ausente	Ausente	Ausente	Ausente	Ausente	Ausente	Ausente	Ausente	Ausente	Ausente	Ausente	Ausente	Ausente	Ausente	Ausente	Ausente	Ausente	Ausente	Ausente	Ausente
	**XI** - Destacada relevância histórico cultural ou religiosa.	Ausente	Ausente	Ausente	Ausente	Ausente	Ausente	Ausente	Ausente	Ausente	Ausente	Ausente	Ausente	Ausente	Ausente	Ausente	Ausente	Ausente	Ausente	Ausente	Ausente	Ausente
	**Incisos do Art. 7º**																					
Importância acentuada sob os enfoques local e regional	**I** - Localidade tipo;	Ausente	Ausente	Ausente	Ausente	Ausente	Ausente	Ausente	Ausente	Ausente	Ausente	Ausente	Ausente	Ausente	Ausente	Ausente	Ausente	Ausente	Ausente	Ausente	Ausente	Ausente
	**II** - Presença de espécies com função ecológica importante;	Ausente	Ausente	Ausente	Ausente	Ausente	Ausente	Ausente	Ausente	Ausente	Ausente	Ausente	Ausente	Ausente	Ausente	Ausente	Ausente	Ausente	Ausente	Ausente	Ausente	Ausente
	**III** - Presença de táxons novos;	Presente	Ausente	Presente	Presente	Presente	Ausente	Presente	Presente	Presente	Presente	Ausente	Presente	Presente	Presente	Presente	Presente	Presente	Presente	Presente	Presente	Presente
	**IV** - Alta riqueza de espécies;	Presente	Ausente	Ausente	Ausente	Ausente	Ausente	Presente	Presente	Ausente	Presente	Ausente	Presente	Ausente	Ausente	Ausente	Ausente	Ausente	Presente	Ausente	Ausente	Ausente
	**V** - Alta abundância relativa de espécies;	Ausente	Presente	Ausente	Presente	Ausente	Presente	Ausente	Ausente	Ausente	Ausente	Presente	Ausente	Presente	Presente	Ausente	Ausente	Presente	Ausente	Ausente	Presente	Presente
	**VI** - Presença de composição singular da fauna;	Ausente	Ausente	Ausente	Ausente	Ausente	Ausente	Ausente	Ausente	Ausente	Ausente	Ausente	Ausente	Ausente	Ausente	Ausente	Ausente	Ausente	Ausente	Ausente	Ausente	Ausente
	**VII** - Presença de troglóbios não raros endêmicos ou relictos;	Presente	Ausente	Presente	Presente	Presente	Ausente	Presente	Presente	Presente	Presente	Ausente	Presente	Presente	Presente	Presente	Presente	Presente	Presente	Presente	Presente	Presente
	**VIII** - Presença de espécies troglomórficas;	Presente	Ausente	Presente	Presente	Presente	Ausente	Presente	Presente	Presente	Presente	Ausente	Presente	Presente	Presente	Presente	Presente	Presente	Presente	Presente	Presente	Presente
	**IX** - Presença de troglóxeno obrigatório;	Ausente	Ausente	Ausente	Ausente	Ausente	Ausente	Ausente	Ausente	Ausente	Ausente	Ausente	Ausente	Ausente	Ausente	Ausente	Ausente	Ausente	Ausente	Ausente	Ausente	Ausente
	**X** - Presença de população excepcional em tamanho;	Ausente	Ausente	Ausente	Ausente	Ausente	Ausente	Ausente	Ausente	Ausente	Ausente	Ausente	Ausente	Ausente	Ausente	Ausente	Ausente	Ausente	Ausente	Ausente	Ausente	Ausente
	**XI** - Presença de espécie rara;	Ausente	Ausente	Ausente	Ausente	Ausente	Ausente	Ausente	Ausente	Ausente	Ausente	Ausente	Ausente	Ausente	Ausente	Ausente	Ausente	Ausente	Ausente	Ausente	Presente	Presente
	**XII** - Alta projeção horizontal da cavidade em relação às demais cavidades que se distribuem na mesma unidade espeleológica, conforme definido no § 3o do art. 14, desta Instrução Normativa;	Ausente	Ausente	Ausente	Ausente	Ausente	Ausente	Ausente	Ausente	Ausente	Ausente	Ausente	Ausente	Ausente	Ausente	Ausente	Ausente	Ausente	Ausente	Ausente	Ausente	Ausente
	**XIII** - Alta área da projeção horizontal da cavidade em relação às demais cavidades que se distribuem na mesma unidade espeleológica, conforme definido no § 3o do art. 14, desta Instrução Normativa;	Ausente	Ausente	Ausente	Ausente	Ausente	Ausente	Ausente	Ausente	Ausente	Ausente	Ausente	Ausente	Ausente	Ausente	Ausente	Ausente	Ausente	Ausente	Ausente	Ausente	Ausente
	**XIV** - Alto volume da cavidade em relação às demais cavidades que se distribuem na mesma unidade espeleológica, conforme definido no § 3o do art. 14, desta Instrução Normativa;	Ausente	Ausente	Ausente	Ausente	Ausente	Ausente	Ausente	Ausente	Ausente	Ausente	Ausente	Ausente	Ausente	Ausente	Ausente	Ausente	Ausente	Ausente	Ausente	Ausente	Ausente
	**XV** - Presença significativa de estruturas espeleogenéticas raras;	Ausente	Ausente	Ausente	Ausente	Ausente	Ausente	Ausente	Ausente	Ausente	Ausente	Ausente	Ausente	Ausente	Ausente	Ausente	Ausente	Ausente	Ausente	Ausente	Ausente	Ausente
	**XVI** - Lago ou drenagem subterrânea perene com influência acentuada sobre os atributos da cavidade que tenham as configurações relacionadas nos incisos deste artigo;	Ausente	Ausente	Ausente	Ausente	Ausente	Ausente	Ausente	Ausente	Ausente	Ausente	Ausente	Ausente	Ausente	Ausente	Ausente	Ausente	Ausente	Ausente	Presente	Ausente	Ausente
	**XVII** - Diversidade da sedimentação química com muitos tipos de espeleotemas e processos de deposição;	Ausente	Ausente	Ausente	Ausente	Ausente	Ausente	Ausente	Ausente	Ausente	Ausente	Ausente	Ausente	Ausente	Ausente	Ausente	Ausente	Ausente	Ausente	Ausente	Ausente	Ausente
	**XVIII** - Configuração notável dos espeleotemas;	Ausente	Ausente	Ausente	Ausente	Ausente	Ausente	Ausente	Ausente	Ausente	Ausente	Ausente	Ausente	Ausente	Ausente	Ausente	Ausente	Ausente	Ausente	Ausente	Ausente	Ausente
	**XIX** - Alta influência da cavidade sobre o sistema cárstico;	Ausente	Ausente	Ausente	Ausente	Ausente	Ausente	Ausente	Ausente	Ausente	Ausente	Ausente	Ausente	Ausente	Ausente	Ausente	Ausente	Ausente	Ausente	Ausente	Ausente	Ausente
	**XX** - Presença de inter-relação da cavidade com alguma de relevância máxima;	Ausente	Ausente	Ausente	Ausente	Ausente	Ausente	Ausente	Ausente	Ausente	Ausente	Ausente	Ausente	Ausente	Ausente	Ausente	Ausente	Ausente	Ausente	Ausente	Ausente	Ausente
	**XXI** - Reconhecimento nacional ou mundial do valor estético/cênico da cavidade;	Ausente	Ausente	Ausente	Ausente	Ausente	Ausente	Ausente	Ausente	Ausente	Ausente	Ausente	Ausente	Ausente	Ausente	Ausente	Ausente	Ausente	Ausente	Ausente	Ausente	Ausente
	**XXII** - Visitação pública sistemática na cavidade, com abrangência regional ou nacional.	Ausente	Ausente	Ausente	Ausente	Ausente	Ausente	Ausente	Ausente	Ausente	Ausente	Ausente	Ausente	Ausente	Ausente	Ausente	Ausente	Ausente	Ausente	Ausente	Ausente	Ausente
	**Incisos do Art. 8º**																					
Importância acentuada sob o enfoque local	**I**- Populações residentes de quiroptera;	Ausente	Ausente	Ausente	Ausente	Ausente	Ausente	Ausente	Ausente	Ausente	Ausente	Ausente	Ausente	Ausente	Ausente	Ausente	Ausente	Ausente	Ausente	Ausente	Ausente	Ausente
	**II** - Local de nidificação de aves silvestres;	Ausente	Ausente	Ausente	Ausente	Ausente	Ausente	Ausente	Ausente	Ausente	Ausente	Ausente	Ausente	Ausente	Ausente	Ausente	Ausente	Ausente	Ausente	Ausente	Ausente	Ausente
	**III** - Alta diversidade de substratos orgânicos;	Alta	Alta	Alta	Alta	Alta	Alta	Alta	Alta	Alta	Alta	Alta	Alta	Alta	Alta	Alta	Alta	Alta	Alta	Alta	Alta	Baixa
	**IV** - Média riqueza de espécies;	Ausente	Ausente	Ausente	Ausente	Ausente	Ausente	Ausente	Ausente	Ausente	Ausente	Ausente	Ausente	Ausente	Ausente	Ausente	Ausente	Ausente	Ausente	Ausente	Ausente	Ausente
	**V** - Média abundância relativa de espécies;	Presente	Ausente	Baixa	Ausente	Média	Ausente	Presente	Baixa	Baixa	Presente	Ausente	Baixa	Ausente	Ausente	Presente	Presente	Ausente	Presente	Presente	Ausente	Ausente
	**VI** - Constatação do uso da cavidade por espécies migratórias;	Ausente	Ausente	Ausente	Ausente	Ausente	Ausente	Ausente	Ausente	Ausente	Ausente	Ausente	Ausente	Ausente	Ausente	Ausente	Ausente	Ausente	Ausente	Ausente	Ausente	Ausente
	**VII** - Presença de singularidade dos elementos faunísticos da cavidade sob enfoque local;	Ausente	Ausente	Ausente	Ausente	Ausente	Ausente	Ausente	Ausente	Ausente	Ausente	Ausente	Ausente	Ausente	Ausente	Ausente	Ausente	Ausente	Ausente	Ausente	Ausente	Ausente
	**VIII** - Presença de estrutura geológica de interesse científico;	Ausente	Ausente	Ausente	Ausente	Ausente	Ausente	Ausente	Ausente	Ausente	Ausente	Ausente	Ausente	Ausente	Ausente	Ausente	Ausente	Ausente	Presente	Ausente	Ausente	Ausente
	**IX** - Presença de registros paleontológicos;	Ausente	Ausente	Ausente	Ausente	Ausente	Ausente	Ausente	Ausente	Ausente	Ausente	Ausente	Ausente	Ausente	Ausente	Ausente	Ausente	Ausente	Ausente	Ausente	Ausente	Ausente
	**X** - Reconhecimento local do valor estético/cênico da cavidade;	Ausente	Ausente	Ausente	Ausente	Ausente	Ausente	Ausente	Ausente	Ausente	Ausente	Ausente	Ausente	Ausente	Ausente	Ausente	Ausente	Ausente	Ausente	Ausente	Ausente	Ausente
	**XI** - Visitação pública sistemática na cavidade, com abrangência local;	Ausente	Ausente	Ausente	Ausente	Ausente	Ausente	Ausente	Ausente	Ausente	Ausente	Ausente	Ausente	Ausente	Ausente	Ausente	Ausente	Ausente	Ausente	Ausente	Ausente	Ausente
	**XII** - Presença de água de percolação ou condensação com influência acentuada sobre os atributos da cavidade que tenham as configurações relacionadas nos incisos deste artigo;	Ausente	Ausente	Ausente	Ausente	Ausente	Ausente	Ausente	Ausente	Ausente	Ausente	Ausente	Ausente	Ausente	Ausente	Ausente	Ausente	Ausente	Ausente	Ausente	Ausente	Ausente
	**XIII** - Lago ou drenagem subterrânea intermitente com influência acentuada sobre os atributos da cavidade que tenham as configurações relacionadas nos incisos deste artigo.	Ausente	Ausente	Ausente	Ausente	Ausente	Ausente	Ausente	Ausente	Ausente	Ausente	Ausente	Ausente	Ausente	Ausente	Ausente	Ausente	Ausente	Ausente	Ausente	Ausente	Ausente
	**Incisos do Art. 9º**																					
Importância significativa sob os enfoques local e regional	**I** - Singularidade dos elementos faunísticos sob enfoque regional;	Ausente	Ausente	Ausente	Ausente	Ausente	Ausente	Ausente	Ausente	Ausente	Ausente	Ausente	Ausente	Ausente	Ausente	Ausente	Ausente	Ausente	Ausente	Ausente	Ausente	Ausente
	**II** - Média projeção horizontal da cavidade em relação às demais cavidades que se distribuem na mesma unidade espeleológica, conforme definido no § 3o do art. 14, desta Instrução Normativa;	Ausente	Presente	Presente	Presente	Presente	Presente	Presente	Presente	Presente	Presente	Presente	Presente	Presente	Presente	Presente	Presente	Presente	Presente	Presente	Presente	Presente
	**III** - Média área da projeção horizontal da cavidade em relação às demais cavidades que se distribuem na mesma unidade espeleológica, conforme definido no § 3o do art. 14, desta Instrução Normativa;	Ausente	Presente	Presente	Presente	Presente	Presente	Presente	Presente	Presente	Presente	Presente	Presente	Presente	Presente	Presente	Presente	Presente	Presente	Presente	Presente	Presente
	**IV** - Alto desnível da cavidade em relação às demais cavidades que se distribuem na mesma unidade espeleológica, conforme definido no § 3º do art. 14, desta Instrução Normativa;	Presente	Ausente	Ausente	Ausente	Ausente	Ausente	Ausente	Ausente	Ausente	Ausente	Ausente	Ausente	Ausente	Ausente	Ausente	Ausente	Ausente	Ausente	Ausente	Ausente	Ausente
	**V** - Médio volume da cavidade em relação às demais cavidades que se distribuem na mesma unidade espeleológica, conforme definido no § 3º do art. 14, desta Instrução Normativa;	Ausente	Presente	Presente	Presente	Presente	Presente	Presente	Presente	Presente	Presente	Presente	Presente	Presente	Presente	Presente	Presente	Presente	Presente	Presente	Presente	Presente
	**VI** - Presença de estruturas espeleogenéticas raras;	Ausente	Ausente	Ausente	Presente	Ausente	Ausente	Ausente	Ausente	Ausente	Ausente	Ausente	Ausente	Ausente	Ausente	Ausente	Ausente	Ausente	Ausente	Ausente	Ausente	Ausente
	**VII** - Lago ou drenagem subterrânea intermitente com influência significativa sobre os atributos da cavidade que tenham as configurações relacionadas nos incisos deste artigo;	Ausente	Ausente	Ausente	Ausente	Ausente	Ausente	Ausente	Ausente	Ausente	Ausente	Ausente	Ausente	Ausente	Ausente	Ausente	Ausente	Ausente	Ausente	Ausente	Ausente	Ausente
	**VIII** - Diversidade da sedimentação química com muitos tipos de espeleotemas ou processos de deposição;	Ausente	Ausente	Ausente	Ausente	Ausente	Ausente	Ausente	Ausente	Ausente	Ausente	Ausente	Ausente	Ausente	Ausente	Ausente	Ausente	Ausente	Ausente	Ausente	Ausente	Ausente
	**IX** - Sedimentação clástica ou química com valor científico;	Ausente	Ausente	Ausente	Ausente	Ausente	Ausente	Ausente	Ausente	Ausente	Ausente	Ausente	Ausente	Ausente	Ausente	Ausente	Ausente	Ausente	Ausente	Ausente	Ausente	Ausente
	**X** - Reconhecimento regional do valor estético/cênico da cavidade;	Ausente	Ausente	Ausente	Ausente	Ausente	Ausente	Ausente	Ausente	Ausente	Ausente	Ausente	Ausente	Ausente	Ausente	Ausente	Ausente	Ausente	Ausente	Ausente	Ausente	Ausente
	**XI** - Uso constante, periódico ou sistemático para fins educacionais, recreativos ou esportivos.	Ausente	Ausente	Ausente	Ausente	Ausente	Ausente	Ausente	Ausente	Ausente	Ausente	Ausente	Ausente	Ausente	Ausente	Ausente	Ausente	Ausente	Ausente	Ausente	Ausente	Ausente
	**Incisos do Art. 10º**																					
Importância significativa sob o enfoque local	**I** - Baixa diversidade de substratos orgânicos;	Ausente	Ausente	Ausente	Ausente	Ausente	Ausente	Ausente	Ausente	Ausente	Ausente	Ausente	Ausente	Ausente	Ausente	Ausente	Ausente	Ausente	Ausente	Ausente	Ausente	Ausente
	**II** - Baixa riqueza de espécies;	Ausente	Presente	Presente	Presente	Presente	Presente	Ausente	Ausente	Presente	Ausente	Presente	Ausente	Presente	Presente	Presente	Presente	Presente	Ausente	Presente	Presente	Presente
	**III** - Baixa abundância relativa de espécies;	Ausente	Ausente	Ausente	Ausente	Ausente	Ausente	Ausente	Ausente	Ausente	Ausente	Ausente	Ausente	Ausente	Ausente	Ausente	Ausente	Ausente	Ausente	Ausente	Ausente	Ausente
	**IV** - Presença de singularidade dos elementos faunísticos sob enfoque local;	Ausente	Ausente	Ausente	Ausente	Ausente	Ausente	Ausente	Ausente	Ausente	Ausente	Ausente	Ausente	Ausente	Ausente	Ausente	Ausente	Ausente	Ausente	Ausente	Ausente	Ausente
	**V** - Média projeção horizontal da cavidade em relação às demais cavidades que se distribuem na mesma unidade geomorfológica, conforme definido no § 1o do art. 14, desta Instrução Normativa;	Ausente	Presente	Presente	Presente	Presente	Presente	Presente	Presente	Presente	Presente	Presente	Presente	Presente	Presente	Presente	Presente	Presente	Ausente	Presente	Presente	Presente
	**VI** - Média área da projeção horizontal da cavidade em relação às demais cavidades que se distribuem na mesma unidade geomorfológica, conforme definido no § 1o do art. 14, desta Instrução Normativa;	Ausente	Presente	Presente	Presente	Presente	Presente	Presente	Presente	Presente	Ausente	Presente	Presente	Presente	Presente	Presente	Presente	Presente	Presente	Presente	Presente	Presente
	**VII** - Alto desnível da cavidade em relação às demais cavidades que se distribuem na mesma unidade geomorfológica, conforme definido no § 1º do art. 14, desta Instrução Normativa;	Presente	Ausente	Ausente	Ausente	Ausente	Ausente	Ausente	Ausente	Ausente	Ausente	Ausente	Ausente	Ausente	Ausente	Ausente	Ausente	Ausente	Ausente	Ausente	Ausente	Ausente
	**VIII** - Médio volume da cavidade em relação às demais cavidades que se distribuem na mesma unidade geomorfológica, conforme definido no § 1º do art. 14, desta Instrução Normativa;	Ausente	Presente	Presente	Presente	Presente	Presente	Presente	Presente	Presente	Presente	Presente	Presente	Presente	Presente	Presente	Presente	Presente	Presente	Presente	Presente	Presente
	**IX** - Poucos tipos de espeleotemas e processos de deposição em termos de diversidade de sedimentação química;	Presente	Presente	Presente	Presente	Presente	Presente	Presente	Presente	Presente	Presente	Presente	Presente	Ausente	Ausente	Presente	Presente	Presente	Presente	Presente	Presente	Presente
	**X** – Uso esporádico ou casual para fins educacionais, recreativos ou esportivos;	Ausente	Ausente	Ausente	Ausente	Ausente	Ausente	Ausente	Ausente	Ausente	Ausente	Ausente	Ausente	Ausente	Ausente	Ausente	Ausente	Ausente	Ausente	Ausente	Ausente	Ausente
	**XI** - Visitação pública esporádica ou casual na cavidade;	Ausente	Ausente	Ausente	Ausente	Ausente	Ausente	Ausente	Ausente	Ausente	Ausente	Ausente	Ausente	Ausente	Ausente	Ausente	Ausente	Ausente	Ausente	Ausente	Ausente	Ausente
	**XII** - Presença de água de percolação ou condensação com influência sobre os atributos da cavidade que tenham as configurações relacionadas nos incisos deste artigo; e	Ausente	Ausente	Ausente	Ausente	Ausente	Ausente	Ausente	Ausente	Ausente	Ausente	Ausente	Ausente	Ausente	Ausente	Ausente	Ausente	Ausente	Ausente	Presente	Ausente	Ausente
	**XIII** - Lago ou drenagem subterrânea intermitente com influência sobre os atributos da cavidade que tenham as configurações relacionadas nos incisos deste artigo.	Ausente	Ausente	Ausente	Ausente	Ausente	Ausente	Ausente	Ausente	Ausente	Ausente	Ausente	Ausente	Ausente	Ausente	Ausente	Ausente	Ausente	Ausente	Ausente	Ausente	Ausente
	RELEVÂNCIA FINAL	Máxima	Alta	Alta	Alta	Alta	Alta	Alta	Alta	Alta	Máxima	Alta	Alta	Alta	Alta	Máxima	Alta	Alta	Máxima	Alta	Alta	Alta

Dentre as cavernas consideradas como de relevância máxima, duas cavernas destacaram-se. Tais cavernas apresentam espécies troglóbias aqui categorizada no tipo mais extremo de raridade (tipo III), isto é, espécie representada por um único indivíduo amostrado. Foram encontradas duas espécies troglóbias enquadradas nesta categoria de raridade (Eucnemidae sp3 e Scydmaenidae spH) (Quadro 6.2).

Quadro 6.2 - Relação das cavidades de Serra Leste (dentre 21 amostradas) que apresentam espécies troglóbias com raridade tipo I, II, III (I+II) e IV.

Ordem	Genero/specie	Tipo I	Tipo II	Tipo III (I +II)	Tipo IV	> pop
Gastropoda	Systrophiidae sp1					93,101
Amblypygi	Charinus sp1					121, 82
Araneae	Oonopidae sp9				112	
Araneae	Matta sp.					130
Collembola	Cyphoderidae sp2					74
Collembola	Cyphoderidae spA					110
Collembola	Isotomidae sp3					115, 116, 121
Coleoptera	Dytiscidae spA	115				115
Coleoptera	Eucnemidae sp3	95, 110,	110	110		
Coleoptera	Pselaphinae spG	101				
Coleoptera	Scydmaenidae spH	121	121	121		
Polydesmida	Pyrgodesmidae sp3					93
Polydesmida	Pyrgodesmidae spC					122

Dentre as cavernas consideradas como de relevância máxima, duas cavernas apresentaram espécies troglóbias aqui categorizadas na raridade tipo II, isto é, um único indivíduo observado por caverna, independentemente do número de cavernas em que a espécie ocorra. Foram encontradas duas espécies troglóbias enquadradas nesta categoria de raridade. Finalmente, quatro cavernas foram consideradas como de relevância máxima (como anteriormente mencionado), pelo fato de possuírem espécies troglóbias categorizadas na raridade tipo I, isto é, espécies distribuídas em até três cavernas. Foram encontradas quatro espécies troglóbias enquadradas nesta categoria de raridade.

Com base no Art. 7º, dezessete cavernas foram consideradas de relevância alta (SL-102, SL-103, SL-104, SL-105, SL-106, SL-107, SL 108, SL-109, SL-111, SL-112, SL-113, SL-114, SL-116, SL-117, SL-122, SL-130, SL-131) em função de atributos de valor acentuado sobre o enfoque local e regional. Os atributos das cavernas estão sumarizados no Quadro 6.1.

Outro aspecto que merece menção diz respeito às cavernas que abrigam populações de espécies troglóbias "não raras". A atual legislação não protege tais espécies, já que as cavernas que as abrigam são, do ponto de vista biológico, categorizadas como de relevância alta.

CARSTE
CONSULTORES ASSOCIADOS

Quadro 6.3 - Relação das cavidades de Serra Leste (dentre 117 amostradas) que apresentam espécies troglóbias com raridade tipo I, II, III (I+II) e IV.

		Raridade		Raridade		Raridade		Raridade		
**Ordem**	**Genero/specie**	**Tipo I**		**Tipo II**		**Tipo III (I + II)**		**Tipo IV**		> popul
Nematomorpha	Gordioidea sp1									28
Gastropoda	Systrophiidae sp1									93
Turbellaria	Geoplanidae sp6	1	26, 82							
Isopoda	Balloniscidae sp8									96
Isopoda	Balloniscidae sp9	1	93, 95							
Isopoda	Balloniscidae sp10	1	94	1	94	1	94	1	94	
Isopoda	Trichorhina sp1									
Isopoda	Trichorhina sp2	1	96	1	96	1	96	1	96	
Isopoda	Trichorhina sp4	1	33							
Isopoda	Styloniscidae sp1	1	7	1	7	1	7	1	7	
Trombidiforme	Rhagidiidae sp2	1	1, 35, 51							
Trombidiforme	Trombidiforme sp1	1	4, 16, 35							
Amblypygi	Charinus sp1									82
Amblypygi	Charinus sp2	1	24	1	24	1	24	1	24	
Amblypygi	Charinus sp3	1	35	1	35	1	35	1	35	
Schizomida	Hubbardiinae sp									31
Opiliones	Escadabiidae sp5	1	5							
Opiliones	Escadabiidae sp6	1	18	1	18	1	18	1	18	
Opiliones	Opiliones INDET.	1	4, 12							
Araneae	Prodidomidae sp1	1	25, 74							
Araneae	Ochiroceratidae sp8									31
Araneae	Ochiroceratidae sp9									35/31
Araneae	Ochiroceratidae sp10	1	4, 35, 36							
Araneae	Ochiroceratidae sp11	1	4, 30							
Araneae	Oonopinae sp9									87
Araneae	Oonopinae sp11	1	57	1	57	1	57	1	57	
Araneae	Prodidomidae sp1	1	31	1	31	1	31	1	31	
Thysanura	Atelurinae sp2									31
Thysanura	Atelurinae sp4	1	4, 80	1	4, 80	1	4, 80			
Diplura	Anajapygidae sp3	1	92	1	92	1	92	1	92	
Collembola	Isotomidae sp2									31
Collembola	Isotomidae sp3									115, 116, 121
Collembola	Tomoceridae sp3									93
Collembola	Cyphoderidae sp2									74
Collembola	Cyphoderidae sp3	1	65							
Collembola	Cyphoderidae spA	110								
Hemiptera	Dipsocoridae sp5									82
Hemiptera	Thyreocoridae sp1	1	18	1	18	1	18	1	18	
Hymenoptera	Hypoponera sp4			1	22, 44, 62, 74					
Hymenoptera	Hypoponera sp7	1	74							
Hymenoptera	Solenopsis sp7	1	97	1	97	1	97	1	97	
Coleoptera	Coarazuphium sp1									35
Coleoptera	Coarazuphium sp2	1	74	1	74	1	74	1	74	
Coleoptera	Dytiscidae sp4	1	45, 82							
Coleoptera	Eucnemidae sp3	1	96	1	96, 110	1	95	1	95	
Coleoptera	Pselaphidae sp9	1	6	1	6	1	6	1	6	
Coleoptera	Pselaphidae sp17	1	75	1	75	1	75	1	75	
Coleoptera	Ptylidae sp5			1	44, 52, 54, 94					
Coleoptera	Scydmaenidae sp17	1	32							
Coleoptera	Scydmaenidae sp20	1	75	1	75	1	75	1	75	
Coleoptera	Scydmaenidae sp21	1	1, 30							
Coleoptera	Scydmaenidae sp22	1	4	1	4	1	4	1	4	
Coleoptera	Staphylinidae sp62	1	72							
Coleoptera	Dytiscidae spA	115								
Coleoptera	Pselaphinae spG	101								
Coleoptera	Scydmaenidae spH	121		121						
Polydesmida	Pyrgodesmidae sp3									93
Polydesmida	Pyrgodesmidae sp7	1	74							
Polydesmida	Pyrgodesmidae sp8	1	74	1	74	1	74	1	74	
Polydesmida	Pyrgodesmidae spC									122
Polydesmida	Polydesmida sp5	1	52, 65, 67							
Polydesmida	Polydesmida sp6			1	12, 51, 52, 74					
Polydesmida	Polydesmida sp7	1	51, 54	1	51, 54	1	51, 54			
Glomerida	Glomeridesmus sp2									31
Polyxenida	Lophoproctidae sp1	1	42, 67							
Spirostreptida	Pseudonannolenidae sp2									69

Além disso, dentre estas cavidades, certamente merecem destaque as cavernas SL-103, SL-108 e SL-110. Estas cavidades concentraram as maiores populações de duas espécies troglóbias dentre as 21 amostradas. Desta forma, a preservação destas cavernas é essencial no intuito de se garantir a preservação destas espécies na área. Quatro cavernas destacaram-se, ainda, por apresentarem mais de quatro espécies troglóbias: SL-101, SL-115, SL-121 e SL-122. Estas cavernas também foram consideradas “hotspots” de diversidade subterrânea na área.

Desta forma, tais cavernas não devem sofrer quaisquer tipos de impactos irreversíveis (como as demais enquadradas em relevância máxima, obviamente). Além disso, recomenda-se que o entorno de 250 metros seja irrestritamente (e minimamente) preservado para estas cavernas.

6.3.Síntese da análise de relevância

Com base nos atributos físicos, todas as cavidades analisadas foram classificadas como de médio grau de relevância, exceto a SL-101 definida como de máxima relevância devido aos atributos espeleométricos. Os atributos biológicos classificaram quatro cavidades com grau máximo: SL-101, SL-110, SL-115, SL-121. O status de troglóbio raro das espécies poderá ser modificado com a continuação dos estudos taxonômicos e pareamento de espécimes conhecidos coletados anteriormente na área e em áreas alheias a este projeto.

O grau alto de relevância foi atribuído a outras 17 cavidades, todas segundo atributos biológicos. Não foram classificadas cavidades com grau de relevância médio e baixo. O Quadro 6.1 apresenta a síntese dos graus de relevância e dos atributos classificatórios para análise dos atributos físico-culturais e biológicos.

6.4.Considerações finais acerca da análise de relevância

Com exceção das quatro cavernas de relevância máxima, todas as demais poderão, de acordo com a legislação atual, sofrer impactos irreversíveis mediante ações de compensação e anuência do órgão ambiental no processo de licenciamento.

No caso de empreendimento que ocasione impacto negativo irreversível em cavidade natural subterrânea com grau de relevância alto, o empreendedor deverá compensar com duas cavidades naturais subterrâneas, com o mesmo grau de relevância, de mesma litologia e com atributos similares à que sofreu o impacto, que serão consideradas cavidades testemunho (Art. 4o, § 1o do Decreto 6.640).

Conforme previsto no artigo 4º § 3º, não havendo, na área do empreendimento, outras cavidades representativas que possam ser preservadas sob a forma de cavidades testemunho, o Instituto Chico Mendes poderá definir, de comum acordo com o empreendedor, outras formas de compensação, sendo os procedimentos administrativos e técnicos e as ações compensatórias definidos na Instrução Normativa Nº 30, de 19 de setembro de 2012.

De acordo com o artigo 19 da IN. 2, qualquer impacto negativo irreversível nas cavidades deverá ser precedido de registro e armazenamento cartográfico e fotográfico, bem como de

inventário e coleta de espeleotemas e elementos geológicos e biológicos representativos do ecossistema cavernícola, compreendendo o resgate, transporte adequado e a destinação a coleções científicas institucionais. Ressalta-se que é necessária a elaboração de planos de salvamento, bem como a aquisição de autorização junto aos órgãos competentes, antes que ocorra o resgate. O salvamento de cavidades pode ser proposto como medida compensatória, na medida em que seja oferecido suporte financeiro para o desenvolvimento das atividades, apoio à divulgação científica dos resultados e estabelecimento de parcerias com instituições de pesquisa.

7. ATUALIZAÇÃO DA ANÁLISE DE RELEVÂNCIA DAS CAVIDADES DE SERRA LESTE

No capítulo anterior, apresentou-se a análise de relevância para 21 cavidades localizadas na área do projeto Serra Leste. Entretanto, esclarece-se que após compilação de tal documento, foram realizados estudos complementares que influenciaram nos resultados anteriormente demonstrados, desencadeando a necessidade de alteração do grau de relevância.

Assim, atualização da análise de relevância inclui os novos resultados encontrados nos seguintes estudos *"Levantamento dos Invertebrados com Caracteres Troglomórficos no Entorno das Cavidades de Serra Leste"* e *"Pareamento de Espécies Troglomórficas"*, apresentados respectivamente nos anexos V e VI.

Destaca-se que esta revisão refere-se estritamente às alterações relacionadas aos atributos biológicos determinados no inciso VIII do art. 3° e nos incisos VII e VIII do art. 7° da IN 2/2009. Tais atributos classificatórios prognosticam sobre habitat de troglóbios raro; presença de troglóbios não raros, endêmicos ou relictos; presença de espécies troglomórficas, respectivamente.

Conforme o item 2.5.3 (Determinação de troglomorfismos) do presente relatório, a determinação de espécies potencialmente troglóbias do estudo espeleológico de Serra Leste foi realizada através da identificação de características morfológicas denominadas troglomorfismos. Tais características, como redução da pigmentação melânica, redução das estruturas oculares, alongamento de apêndices, dentre outras, foram utilizadas para grande parte dos grupos, uma vez que resultam de processos evolutivos ocorrentes após o isolamento de populações em cavernas. No entanto, as características utilizadas para estes diagnósticos, diferiram no caso de organismos pertencentes à *taxa* distintos. Ressalta-se que as espécies troglóbias foram assim definidas em função dos seus troglomorfismos. Desta forma, os termos "Troglóbios" e "Troglomórficos" foram considerados como coincidentes, conforme explicitado no item *2.5.3.*

No que se refere à determinação da raridade, no item *2.5.4.* do presente relatório, foram demonstrados distintos conceitos atribuídos às espécies troglóbias. Tais atribuições foram primordialmente ilustrativas, tendo sido realizadas no intuito de exemplificar a possibilidade de aplicação de diferentes abordagens, desde aquelas mais restritivas até as mais amplas. No entanto, para efeito da legislação vigente, foram utilizadas as duas abordagens propostas no

workshop técnico científico “Troglóbios raros”: incertezas e encaminhamentos, realizado em Belo Horizonte nos dias 03 e 04 de março de 2011.

Levando em consideração o atributo de distribuição geográfica, foi sugerido, no referido workshop, que uma espécie troglóbia encontrada em até três cavidades seja considerada rara. Este tipo de raridade foi chamado de raridade Tipo I. Já em referência ao atributo de abundância, foi sugerido pelos Profs. Dr. Marconi Souza Silva e Dr. Rodrigo Lopes Ferreira que deve ser considerada rara aquela espécie troglóbia com um exemplar por cavidade amostrada, não importando o número de cavidades em que ocorra e independentemente da distribuição geográfica. Esta abordagem também foi adotada, sendo chamada de raridade Tipo II, conforme descrito no item *2.5.4. (Determinação de Raridade).*

Além das abordagens utilizadas para a definição de troglóbios raros (aplicadas para a obtenção do grau de relevância das cavernas – raridades dos tipos I e II), foram também exemplificadas outras abordagens: espécies que compreenderam *uniques* (com a ocorrência de uma única espécie em todo o estudo – raridade Tipo III); e espécies que corresponderam à sobreposição dos critérios de distribuição (até três cavernas) e abundância (um exemplar por cavidade amostrada, não importando o número de cavidades em que ocorra), chamadas de raridade Tipo IV.

Dessa forma, levando em consideração o que foi apresentado item *5.2.2. (Espécies Troglomórficas)* do presente relatório, *Troglomórficas)* do presente relatório, destaca-se que 13 morfoespécies foram categorizadas como troglomórficas sendo que como troglomórficas sendo que destas, quatro foram consideradas “troglóbios raros” pertencentes à Raridade Tipo I. O

pertencentes à Raridade Tipo I. O

Quadro 7.1 especifica a distribuição dos troglomórficos encontrados no atual estudo, bem como no estudo espeleológico realizado em 96 cavidades de Serra Leste.

Quadro 7.1 - Táxons troglomórficos encontrados nos dois estudos já executados na área de Serra Leste.

Táxon		Serra Leste - 21 cavidades	Serra Leste - 96 cavidades (CARSTE, 2011a)
**Gastropoda**	Systrophiidae sp1	SL-101, SL-103, SL-104, SL-107, SL-108, SL-109, SL-110, SL-112, SL-113, SL-114, SL-115, SL-116, SL-121, SL-122, SL-130.	SL-01, SL-04, SL-05, SL-06, SL-08, SL-13, SL-15, SL-16, SL-22, SL-24, SL-26, SL-27, SL-29, SL-30, SL-31, SL-35, SL-42, SL-44, SL-51, SL-52, SL-53, SL-54, SL-56, SL-58, SL-60, SL-61, SL-62, SL-69, SL-74, SL-76, SL-77, SL-85, SL-87, SL-89, SL-93, SL-95, SL-97.
**Amblypygi**	Charinus sp1	SL-115, SL-121.	SL-06, SL-09, SL-14, SL-22, SL-23, SL-27, SL-31, SL-35, SL-42, SL-44, SL-45, SL-50, SL-57, SL-58, SL-61, SL-62, SL-64, SL-73, SL-74, SL-75, SL-76, SL-77, SL-82, SL-83, SL-87, SL-89, SL-90, SL-96, SL-97, SL-100.
**Araneae**	Oonopidae sp9	SL-112	SL-27, SL-49, SL-54, SL-74, SL-87.
**Araneae**	Matta sp.	SL-101, SL-107, SL-117, SL-130.	-
**Collembola**	Cyphoderidae sp2	SL-108	SL-01, SL-02, SL-27, SL-30, SL-35, SL-37, SL-51, SL-58, SL-69, SL-74, SL-79, SL-87, SL-97

**Collembola**	Cyphoderidae spA	SL-101, SL-103, SL-109, SL-110, SL-114, SL-115, SL-117, SL-121, SL-130.	-
**Collembola**	Isotomidae sp3	SL-101, SL-115, SL-116, SL-121, SL-122, SL-131.	SL-46, SL-50, SL-70, SL93.
**Coleoptera**	Dytiscidae spA	SL-115	-
**Coleoptera**	Eucnemidae sp3	SL-110	SL-95
**Coleoptera**	Pselaphinae spG	SL-101	-
**Coleoptera**	Scydmaenidae spH	SL-121	-
**Polydesmida**	Pyrgodesmidae sp3	SL-101, SL-115, SL-122.	SL-01, SL-31, SL-33, SL-35, SL-44, SL-45, SL-46, SL-51, SL-55, SL-56, SL-74, SL-75, SL-83, SL-87, SL-89, SL-93, SL-95, SL-97.
**Polydesmida**	Pyrgodesmidae spC	SL-105, SL-108, SL-109, SL-116, SL-122.	-

Ressalta-se que a presença dos organismos troglomórficos supracitados subsidiou a execução dos estudos complementares realizados na área do Projeto Serra Leste, conforme Anexos V e VI, base para a consolidação desta Análise de Relevância atualizada. Os atributos físicos determinados na IN 2/2009 não sofreram qualquer alteração e, aqui, serão reapresentados somente na recomposição da análise final.

7.1.Metodologia

7.1.1. Estudo de Coleta no Entorno de Cavidades

Os resultados encontrados no relatório do levantamento de invertebrados com características troglomórficas do entorno das 21 cavidades do Projeto Serra Leste (ANEXO V) foram um dos balizadores para a atualização da Análise de Relevância Espeleológica. A metodologia empregada em tal estudo abrangeu um inventário em áreas epígeas com o intuito de excluir a possibilidade de ocorrência de troglóbios/troglomórficos restrita, exclusivamente, às cavidades naturais subterrâneas. Assim, reavaliaram-se os espécimes determinados como troglóbios raros, endêmicos ou relictos, a presença de troglomórficos e a presença de troglóbios não raros.

Primeiramente, o estudo de coleta externa preconizou a subdivisão de três áreas (de acordo com sua a litologia, localização na região e inserção na paisagem) do Projeto Serra Leste . Cada área englobou cavidades distintas objetivando contemplar com coletas, o entorno de “grupos de cavernas”. Especificamente em cada área, foram escolhidos pontos que apresentavam características propícias à colonização dos animais alvo e nos quais as metodologias de coletas pudessem ser aplicadas eficientemente, conforme especificado no Anexo V.

Os procedimentos de coleta de invertebrados utilizados foram delineados de acordo com a lista preliminar dos animais indicados como potencialmente troglomórficos na primeira campanha de coleta bioespeleológica nas 21 cavidades de Serra Leste. Naquela ocasião, foram consideradas com possibilidade de serem consideradas troglomórficas, nove espécies

pertencentes aos grupos Isopoda, Coleoptera, Collembola, Araneae, Amblypygi, Annelida e Diplopoda (Anexo V).

Para o inventário desses táxons no ambiente epígeo, foi proposta a utilização de quatro métodos de coleta de invertebrados: coleta manual ativa noturna, winkler, revisão de folhiço de winkler e coleta aquática com rede de benton, distribuídos na área de estudo conforme demonstrado na Tabela 5.

Tabela 5 - Número de unidades amostrais obtidas para cada área: extrator de winkler; revisão de folhiço do winkler; coleta ativa noturna; coleta aquática.

Áreas	Winkler	Revisão de Winkler	Noturna	Aquática
Área 1	25	25	9	-
Área 2	50	50	9	-
Área 3	25	25	9	10

Descreveu-se relatório de coleta no entorno de cavidades (Anexo V), que no processamento das amostras coletadas em campo foi dada especial atenção aos grupos de interesse que apresentavam animais com características troglomórficas. Esses animais foram separados para comparação com aqueles capturados no interior das cavidades durante os estudos iniciais. De tal modo, os exemplares com caracteres troglomórficos coletados no entorno e a fauna troglomórfica coletada nas cavidades foram pareados pelos mesmos especialistas que identificaram o material das cavidades para evitar divergência nas identificações.

7.1.2. Estudo de Pareamento de Espécies Troglomórficas

Os resultados apresentados no estudo de pareamento de espécies foram também utilizados como balizador para a atualização da Análise de Relevância Espeleológica. O método aplicado em tal estudo buscou a comparação de troglomórficos encontrados em estudos anteriores já realizados na região de Carajás/PA, pela Carste Consultores Associados. Tais organismos foram comparados com os espécimes considerados “troglóbios raros” no item *6.2. (Resultados da Análise de Relevância das Cavernas de Serra Leste)* do presente relatório. A finalidade do estudo foi ampliar o quadro de distribuição dos espécimes troglomórficos e atualizar o *status* de troglóbios raro para as espécies pareadas.

Para tanto, somente as quatro morfoespécies de Colepptera pertencentes à Raridade Tipo I e II, determinadas no item 6.2. II, determinadas no item 6.2. foram contempladas com o pareamento, conforme exposto no

Quadro 7.1

Os coleópteros foram comparados pela MSc Daniela de Cássia Bená, com experiência na área de zoologia com a taxonomia dos grupos recentes, sistemática de Coleoptera Nitidulidae e também biologia, morfologia e sistemática de Coleoptera, com ênfase na família Curculionidae.

Assim, as morfoespécies Dystiscidae spA, Eucnemidae sp3, Pselaphinae spG e Sydmaenidae spH foram comparadas através da avaliação estritamente dos seus caracteres morfológicos

com outros organismos do mesmo *taxa* encontrados nos seguintes estudos já realizados na região, especificados no Quadro 7.2 e Figura 7.1.

Quadro 7.2 - Estudos utilizados para subsidiar o pareamento de espécies troglomórficas do Projeto Serra Leste

Estudo	Autores
Espeleologia do Projeto S11D Serra Sul, Região de Carajás, PA. 2010.	COELHO, A.; PILÓ, L.B.; AULER, A.S.; ANDRADE, R.; BESSI, R.; FRANCO, F.P.;
Estudos espeleológicos Morro I: geoespeleologia, bioespeleologia e análise de relevância. 2011.	AULER, A. S.; LENHARE, B.D.; FERREIRA, R.L.; SOUZA-SILVA, M.
Estudos espeleológicos Morro II: geoespeleologia, bioespeleologia e análise de relevância. 2011.	AULER, A. S.; LEÃO, M.R; FERREIRA, R.L.; SOUZA-SILVA, M.
Serra Leste – Espeleologia: diagnóstico geoespeleológico, diagnóstico bioespeleológico e análise de relevância. 2011.	CALUX, A.S.; AULER, A. S.; FERREIRA, R.L.; SOUZA-SILVA, M.
Estudos espeleológicos Serra Leste: geoespeleologia, bioespeleologia e análise de relevância. 2012.	LEÃO, M.R.; AULER, A. S.; FERREIRA, R.L. & SOUZA-SILVA, M.
Levantamento dos invertebrados com caracteres troglomórficos do entorno das cavidades de Serra Leste. 2012.	TAKANO, B.F.
Espeleologia N4/N5/PESE: Diagnóstico e Análise de Relevância de 180 cavidades. 2012.	COELHO, A.; AULER, A.S.; FRANCO, F.P.; ANDRADE, R.
Serra da Bocaina Região de Carajás, PA: Diagnóstico Espeleológico e Análise de Relevância das Cavernas. 2013.	PILÓ, L.B.; COELHO, A.; FRANCO, F.P.; SOUZA, C.A.

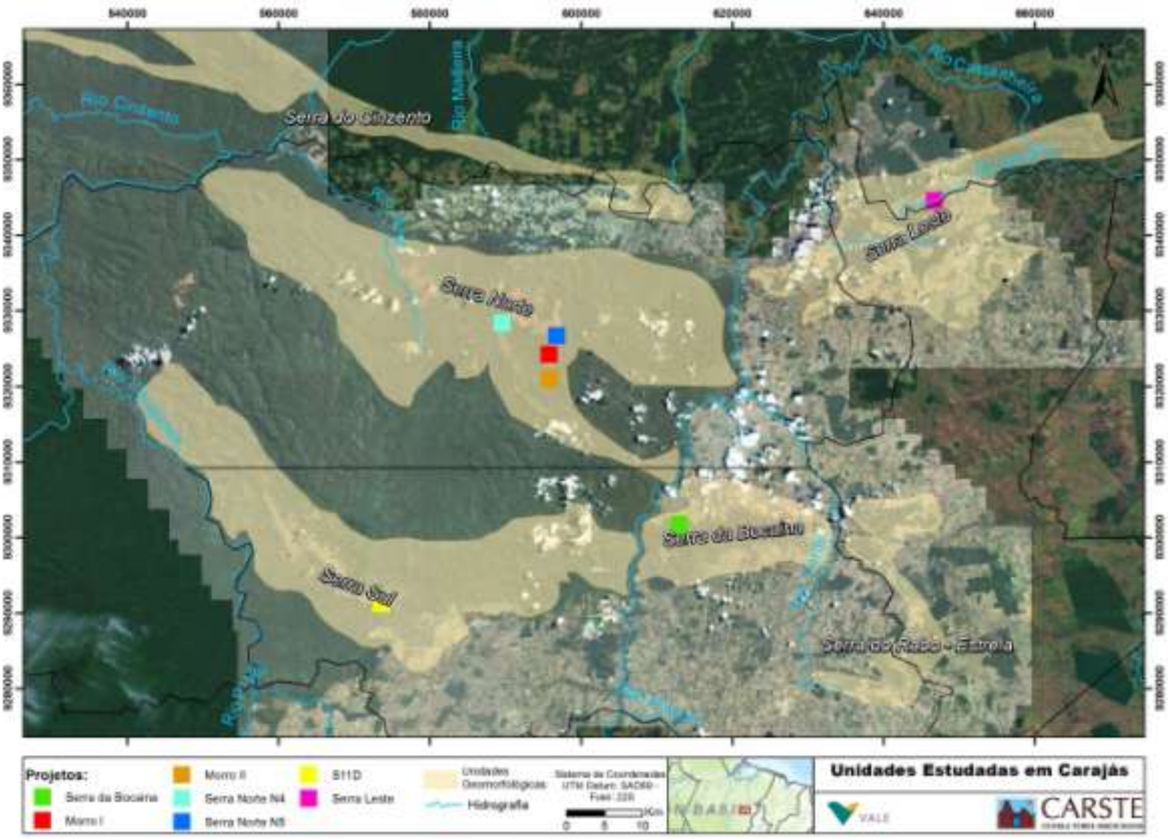

Figura 7.1 - Localização pontual dos projetos realizados pela Carste Consultores Associados inseridos nas Unidades Geomorfológicas em Carajás/PA.

7.2.Novos resultados para os atributos biológicos

Com base no inciso VII do Art. 3 º da IN 2/2009, quatro morfoespécies foram consideradas “troglóbios raros” (Coleoptera: Dytiscidae sp.A, Eucnemidae sp.3, Pselaphinae sp.G, Scydmaenidae sp.H) no item 6.2. do presente relatório. Tais espécies determinaram que quatro cavidades fossem categorizadas como de relevância máxima: SL-101, SL-110, SL-115 e SL-121.

Baseando-se nos resultados encontrados no *“Levantamento dos Invertebrados com Caracteres Troglomórficos no Entorno das Cavidades de Serra Leste”* (ANEXO V), verificou-se que foram coletados indivíduos com características troglomórficas pertencentes aos táxons Araneae, Collembola, Coleoptera e Diplopoda. Destas, apenas duas morfoespécies troglomórficas que foram coletadas no interior das cavidades também tiveram exemplares capturados no ambiente epígeo, no entorno das cavidades, a saber: Matta sp. e Scydmaenidae spH, ilustrados na Figura 7.2.

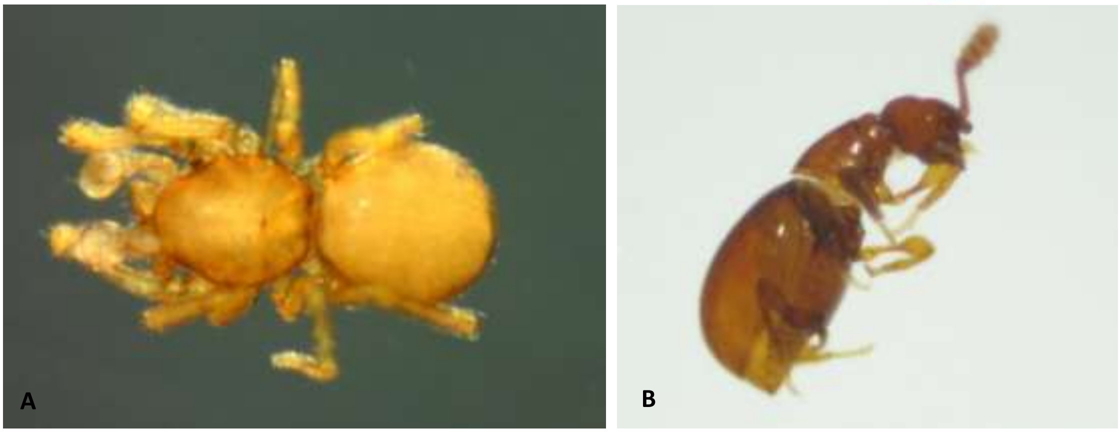

Figura 7.2 – (A) Matta sp. troglomórfico, semelhante aos capturados nas cavidades e (B) o coleóptero anoftálmico Scydmaenidae troglomórfico, semelhante ao capturado na cavidade de Serra Leste.

Ressalta-se que as duas morfoespécies encontradas no ambiente externo, foram coletadas através da aplicação metodologia Winkler. A morfoespécie Matta sp. foi encontrada no ambiente epígeo, representada por apenas um indivíduo. Esse indivíduo foi encontrado a uma distância média de 247 metros das cavidades SL-101, SL-107, SL-117 e SL-130 as quais foram evidenciadas originalmente pela presença da morfoespécie em questão. Já para a morfoespécie Scydmaenidae spH, destaca-se que foram localizados dois indivíduos em dois pontos distintos no ambiente epígeo. Ressalta-se que esses pontos distanciam-se em média de 7.355 quilômetros da cavidade SL-121, ambiente hipógeo onde a morfoespécie foi primeiramente encontrada. A localização das espécies e a distância entre as cavernas estão ilustradas na Figura 7.3.

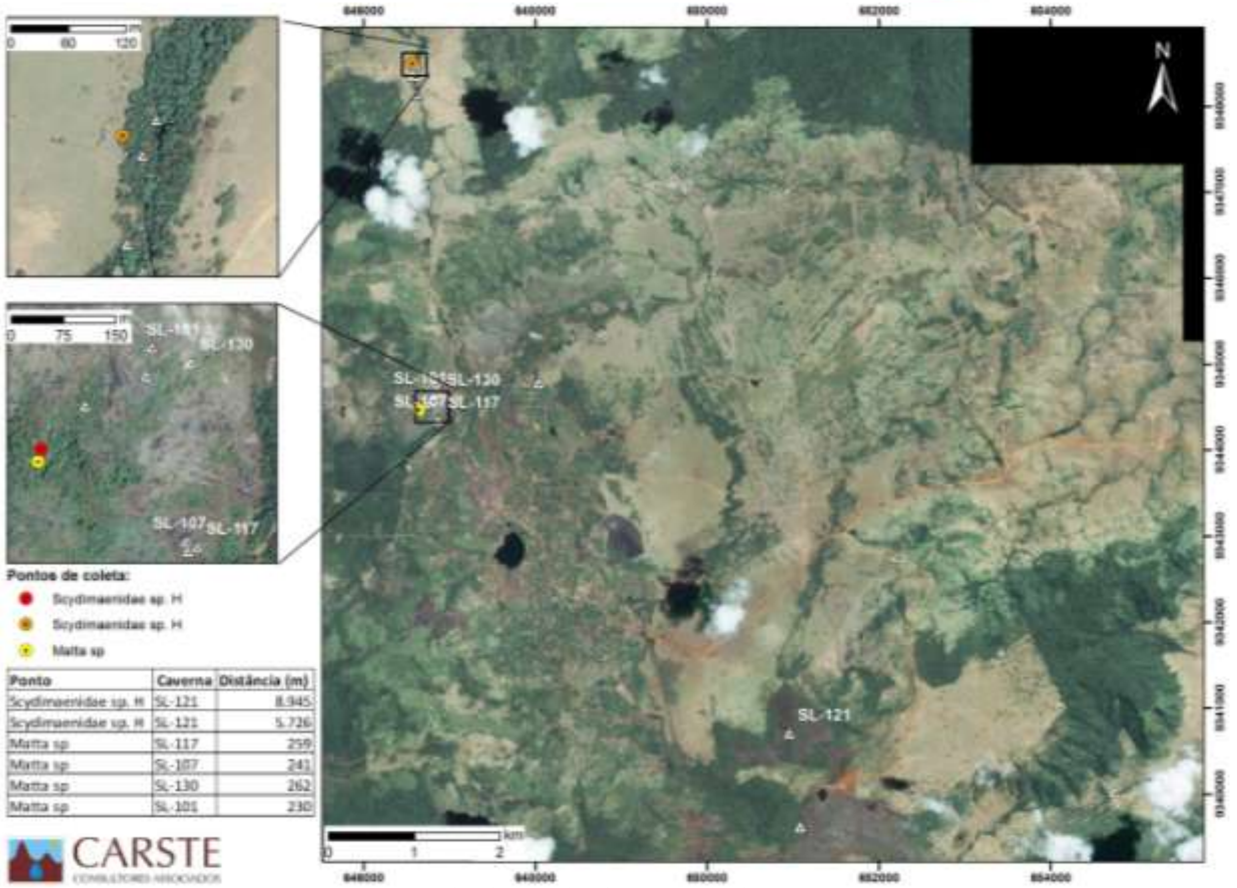

Ponto	Caverna	Distância (m)
Scydimaenidae sp. H	SL-121	8.945
Scydimaenidae sp. H	SL-121	5.726
Matta sp	SL-117	259
Matta sp	SL-107	241
Matta sp	SL-130	262
Matta sp	SL-101	230

Figura 7.3 – Localização das morfoespécies troglomórficas Matta sp. e Scydmaenidae spH no ambiente epígeo e a distância em relação às cavidades em que foram originalmente encontradas.

No entanto, é destacado no item *6.2. (Resultados da Análise de Relevância das cavernas de Serra Leste)*, que a morfoespécie Matta sp. não foi considerada como “troglóbio raro” devido ao fato de ter sido localizada em outras cavidades da área, a saber: SL-101, SL-107, SL-117, SL-130. Todavia, para a morfoespécie Scydmaenidae spH que foi localizada somente na cavidade SL-121, determinou-se o grau de relevância máximo devido à presença do atributo “troglóbio raro”.

A IN 2/2009 define que troglóbios são “animais de ocorrência restrita ao ambiente subterrâneo”. Conforme destacado no Anexo V, através da utilização desse conceito superficial, a simples coleta ou observação de indivíduos considerados troglóbios fora do ambiente cavernícola, altera seu *status* de troglóbio para troglófilo. Desta forma, a aranha de gênero *Matta* e o coleóptero Scydmaenidae passariam a ser consideradas espécies troglófilas. Especificamente para a morfoespécie Scydmaenidae spH, a sua localização em área externa conforme determinado no Anexo V, indica que a sua distribuição não seja restrita ao ambiente subterrâneo e o grau de relevância da cavidade pode ser alterado para alto.

Entretanto, no Anexo V o autor destaca que do ponto de vista biológico, é provável que alguns troglóbios amplamente distribuídos se dispersem através de rachaduras e fendas em habitats epicársticos acima de cavernas e até mesmo na serapilheira úmida ou solo profundo fora das áreas cársticas. Uma hipótese que não pode ser descartada é que os animais encontrados no

ambiente epígeo podem estar explorando o habitat-dreno e são provenientes da população-fonte dentro da cavidade. Dessa maneira, apesar de terem representantes no meio epígeo, podem ser considerados troglóbios originários da população-fonte troglóbia (TRAJANO, 2012). Assim, a mudança de *status* de troglóbio para troglófilo e, consequentemente, a mudança na condição da relevância das cavidades deu-se unicamente pela aplicação da legislação vigente e não está de acordo com a bibliografia atual considerada em BARR & HOLSINGER, 1985; HOLSINGER, 2012 e TRAJANO, 2012.

Por fim, o autor ressalta que, apesar de a cavidade SL-121 poder ser categorizada com relevância alta de acordo com a legislação, ela foi considerada como “hotspot” de diversidade subterrânea na área. Desta forma, caso a cavidade sofra impactos negativos, a região circunvizinha, incluindo ambientes epígeos e hipógeos, podem sofrer impactos negativos indiretos e irreversíveis. Sendo assim, sugere-se no ANEXO V que sejam realizados estudos mais aprofundados das dinâmicas populacionais e da estrutura da comunidade cavernícola da região para monitoramento da biota das cavidades que não sofrerão impactos negativos diretos.

Em outro âmbito, no que se refere ao estudo de “*Pareamento de Espécies Troglomórficas”* descrito no Anexo VI, as comparações morfológicas realizadas pela especialista MSc Daniela Bená, resultaram em dististas implicações para os coleópteros.

Destaca-se inicialmente que a morfoespécie de coleóptero Pselaphinae sp G (Figura 7.4 A) encontrada na cavidade SL-101 de Serra Leste é correspondente aos exemplares encontrados nas cavidades S11A_0014 (GEM-865); SB_0041 (GEM-1457); SB_0045 (GEM-1461); SB_0063 (GEM-1479); SB_0065 (GEM-1481); SB_0066 (GEM-1482) e SB_0110 (GEM-0110) do Projeto Serra da Bocaina (CARSTE, 2013), ampliando dessa forma, a sua distribuição.

Já a morfoespécie Scydmaenidae spH (Figura 7.4 B) encontrada somente na cavidade SL-121 é correspondente à morfoespécie encontrada nas cavidades N4E-86 e N4E-74 do Projeto Serra Norte (CARSTE, 2012).

O coleóptero Dytiscidae spA (Figura 7.4 C) localizado na cavidade SL-115 do Projeto Serra Leste pareia-se com exemplares de Dytiscidae encontrados na cavidade S11D-078 do Projeto Serra Sul (CARSTE, 2010) e nas cavidades N5S-63/64/65, N5S-68, N5S74, N5S-10 do Projeto Serra Norte (CARSTE, 2012).

Por fim, a morfoespécie Eucnemidae sp3 (Figura 7.4 D) encontrada na cavidade SL-110 de Serra Leste e na cavidade SL-95 também da área de Serra Leste (96 cavidades, CARSTE, 2011a) foi categorizada pela especialista como troglóbia. Trata-se na verdade, de um Elateridae, Cardiophorinae, sendo que tal espécie está associada a troncos caídos e raízes de plantas. Conforme a especialista, não existe nenhum registro bibliográfico para o Brasil de espécimes troglóbios e devido a esse fato, esse exemplar é único e exige muita atenção por se tratar de algo raro.

www.carste.com.br - e-mail: contato@carste.com.br - Tel.: (31) 2552-9976

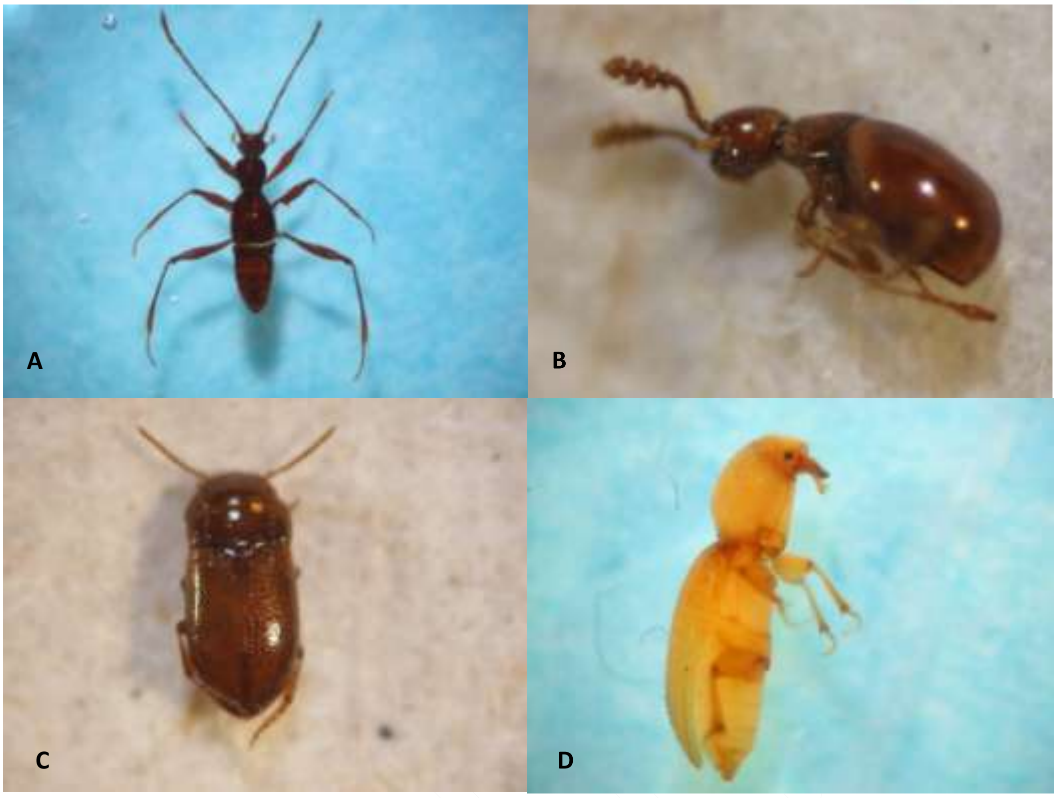

Figura 7.4 – (A) Vista dorsal Pselaphinae sp G; (B) vista lateral de Scydmaenidae spH; (C) vista dorsal de Dytiscidae spA: (D) vista lateral de Eucnemidae sp3;

Diante dos resultados do pareamento, as morfoespécies Pselaphinae sp G e Dytiscidae spA tiveram suas distribuições ampliadas o que permite a modificação do status de ”troglóbio raro” para “troglóbio não raro”. Dessa forma, as cavidades SL-101 e SL-115 têm o seu grau de relevância alterado de máximo para alto.

Além disso, o pareamento de Scydmaenidae spH, contribuiu para a ampliação da distribuição da morfoespécie (de uma para três cavidades). Conforme o atributo de distribuição geográfica adotado no item 6.2 *(Resultados da Análise de Relevância das cavernas de Serra Leste)* do presente relatório, uma espécie troglóbia encontrada em até três cavidades é considerada rara, sendo este tipo chamado de raridade Tipo I. Subsidiado somente deste conceito, o aumento na distribuição não influenciaria na alteração do grau de relevância. Entretanto, ressalta-se que a mesma morfoespécie foi localizada no ambiente epígeo no estudo que contemplou coletas no entorno de cavidade das cavidades de Serra Leste (ANEXO V). Conforme já destacado, de acordo com a aplicação da legislação vigente a simples coleta ou observação de indivíduos considerados troglóbios fora do ambiente cavernícola, altera seu *status* de troglóbio para troglófilo. Assim, mudança de *status* para morfoespécie Scydmaenidae spH, altera o grau de relevância da cavidade SL-121 de máximo para alto.

Por fim, a presença da morfoespécie Eucnemidae sp3, na verdade um Elateridae, Cardiophorinae na cavidade SL-110, mantém o seu grau de relevância como máximo, devido à confirmação do *status* de “troglóbio raro”, não havendo qualquer alteração diante dos resultados de pareamento de espécies. Neste caso, utilizou-se o conceito de raridade ligado principalmente à estrita distribuição, isto é, o elevado grau de endemismo assim como

descrito no item *6.2* do presente relatório. O Quadro 7.3 sintetiza os resultados atualizados referentes ao pareamento dos espécimes.

Quadro 7.3 - Relação de morfoespécies caracterizadas anteriormente como "troglóbios raros", modificação de status e ampliação de distribuição após o pareamento de espécies.

Ordem	Gênero/Espécie	Cavidades Projeto Serra Leste	Demais cavidades FLONA Carajás.
Coleoptera	Dytiscidae spA	SL-115	S11D-078, N5S-63/64/65, N5S-68, N5S74, N5S-10
Coleoptera	Eucnemidae sp3	SL-95, SL-110	Não foram localizadas.
Coleoptera	Pselaphinae spG	SL-101	S11A_0014; SB_0041; SB_0045; SB_0063; SB_0065; SB_0066; SB_0110
Coleoptera	Scydmaenidae spH	SL-121	N4E-86 e N4E-74

7.3.Síntese da análise de relevância

Com base nos atributos físicos, todas as cavidades analisadas foram classificadas como de médio grau de relevância, exceto a SL-101, definida como de máxima relevância devido aos atributos espeleométricos. Os atributos biológicos classificaram uma cavidade com grau máximo: SL-110 devido à presença de troglóbios raro.

Com base no Art. 7º, 19 cavidades foram consideradas de relevância alta (SL-102, SL-103, SL-104, SL-105, SL-106, SL-107, SL 108, SL-109, SL-111, SL-112, SL-113, SL-114, SL-115, SL-116, SL-117, SL-121, SL-122, SL-130, SL-131) em função de atributos de valor acentuado sobre o enfoque local e regional. O Quadro 7.4 apresenta a síntese dos graus de relevância e dos atributos classificatórios para análise dos atributos físico-culturais e biológicos.

Quadro 7.4 - Síntese da relevância das cavidades estudadas na área de Serra Leste, de acordo com a configuração de importância dos atributos físicos e biológicos.

Cavidade	Grau de relevância e atributos biológicos classificatórios	Grau de relevância e atributos físicos classificatórios	Grau de relevância final
**SL-101**	Alto - presença de táxons novos, alta riqueza, presença de troglóbios não raros endêmicos ou relictos, espécies troglomórficas.	Máximo - dimensões notáveis em extensão, área, volume.	**MÁXIMO**
**SL-102**	Alto - alta abundância de espécies.	Médio - média projeção horizontal, média área e médio volume.	**ALTO**
**SL-103**	Alto - presença de táxons novos, presença de troglóbios não raros endêmicos ou relictos, espécies troglomórficas.	Médio - média projeção horizontal, média área e médio volume.	**ALTO**
**SL-104**	Alto - presença de táxons novos, alta abundância relativa de espécies presença de troglóbios não raros endêmicos ou relictos, espécies	Médio - média projeção horizontal, média área e médio volume, presença de estruturas espeleogenéticas raras.	**ALTO**

	troglomórficas.		
**SL-105**	Alto - presença de táxons novos, presença de troglóbios não raros endêmicos ou relictos, espécies troglomórficas.	Médio - média projeção horizontal, média área e médio volume.	**ALTO**
**SL-106**	Alto - alta abundância relativa de espécies.	Médio - média projeção horizontal, média área e médio volume.	**ALTO**
**SL-107**	Alto - presença de táxons novos, alta riqueza de espécies, presença de troglóbios não raros endêmicos ou relictos, espécies troglomórficas.	Médio - média projeção horizontal, média área e médio volume.	**ALTO**
**SL-108**	Alto - presença de táxons novos, alta riqueza de espécies, presença de troglóbios não raros endêmicos ou relictos, espécies troglomórficas.	Médio - média projeção horizontal, média área e médio volume.	**ALTO**
**SL-109**	Alto - presença de táxons novos, presença de troglóbios não raros endêmicos ou relictos, espécies troglomórficas.	Médio - média projeção horizontal, média área e médio volume.	**ALTO**
**SL-110**	Máximo - habitat troglóbio raro.	Médio - média projeção horizontal, média área e médio volume.	**MÁXIMO**
**SL-111**	Alto - alta abundância relativa de espécies.	Médio - média projeção horizontal, média área e médio volume.	**ALTO**
**SL-112**	Alto - presença de táxons novos, alta riqueza de espécies, presença de troglóbios não raros endêmicos ou relictos, espécies troglomórficas.	Médio - média projeção horizontal, média área e médio volume.	**ALTO**
**SL-113**	Alto - presença de táxons novos, alta abundância relativa de espécies, presença de troglóbios não raros endêmicos ou relictos, espécies troglomórficas.	Médio - média projeção horizontal, média área e médio volume.	**ALTO**
**SL-114**	Alto - presença de táxons novos, alta abundância relativa de espécies, presença de troglóbios não raros endêmicos ou relictos, espécies troglomórficas.	Médio - média projeção horizontal, média área e médio volume.	**ALTO**
**SL-115**	Alto - presença de táxons novos, presença de troglóbios não raros endêmicos ou relictos, espécies troglomórficas.	Médio - média projeção horizontal, média área e médio volume.	**ALTO**

**SL-116**	Alto - presença de táxons novos, presença de troglóbios não raros endêmicos ou relictos, espécies troglomórficas.	Médio - média projeção horizontal, média área e médio volume.	**ALTO**
**SL-117**	Alto - presença de táxons novos, alta abundância relativa de espécies, presença de troglóbios não raros endêmicos ou relictos, espécies troglomórficas.	Médio - média projeção horizontal, média área e médio volume.	**ALTO**
**SL-121**	Alto - presença de táxons novos, alta riqueza, presença de troglóbios não raros endêmicos ou relictos, espécies troglomórficas .	Alto - presença de estrutura geológica de interesse cinetífico, média projeção horizontal, média área e médio volume	**ALTO**
**SL-122**	Alto - presença de táxons novos, presença de troglóbios não raros endêmicos ou relictos, espécies troglomórficas.	Alto - lago, ou drenagem subterrânea perene com influência acentuada sobre os atributos da cavidade.	**ALTO**
**SL-130**	Alto - presença de táxons novos, alta abundância relativa de espécies, presença de troglóbios não raros endêmicos ou relictos, espécies troglomórficas, presença de espécie rara.	Médio - média projeção horizontal, média área e médio volume.	**ALTO**
**SL-131**	Alto - presença de táxons novos, alta abundância relativa de espécies, presença de troglóbios não raros endêmicos ou relictos, espécies troglomórficas, presença de espécie rara.	Médio - média projeção horizontal, média área e médio volume.	**ALTO**

7.4. Considerações finais acerca da atualização da análise de relevância

Os estudos complementares realizados na área do Projeto Serra Leste auxiliaram na determinação final da análise de relevância do estudo das 21 cavidades. Levando em consideração os atributos biológicos, a princípio, quatro cavidades foram categorizadas com o grau de relevância máximo: SL-101, SL-110, SL-115 e SL-121, devido à presença de troglóbios raros. Os resultados encontrados nos estudos complementares proporcionaram a reavaliação dos espécimes ampliando a sua distribuição e/ou retirando o *status* de troglóbio. Assim, na reavaliação da análise somente uma cavidade, a SL-110, permaneceu através atributos biológicos com o grau de relevância máximo.

Destaca-se que devido aos constantes refinamentos dos estudos e incremento de informações pelos especialistas dos distintos grupos taxonômicos, há possibilidade de realização de outros pareamentos, contemplando grupos ainda não analisados no presente trabalho. Tais

pareamentos poderão refletir em mudanças de *status*, e consequentemente na necessidade de reavaliações das análises apresentadas.

É importante ressaltar que a mudança de *status* de troglóbio ou remoção do *status* de troglóbio raro e consequentemente, a mudança na condição da relevância das cavidades deram-se unicamente pela aplicação da legislação vigente, sendo destacada neste documento, a necessidade de estudos mais aprofundados a respeito das espécies avaliadas.

Com exceção das duas cavernas de relevância máxima, todas as demais poderão, de acordo com a legislação atual, sofrer impactos irreversíveis mediante ações de compensação e anuência do órgão ambiental no processo de licenciamento.

No caso de empreendimento que ocasione impacto negativo irreversível em cavidade natural subterrânea com grau de relevância alto, o empreendedor deverá compensar com duas cavidades naturais subterrâneas, com o mesmo grau de relevância, de mesma litologia e com atributos similares à que sofreu o impacto, que serão consideradas cavidades testemunho (Art. 4o, § 1o do Decreto 6.640).

Conforme previsto no artigo 4º § 3º, não havendo, na área do empreendimento, outras cavidades representativas que possam ser preservadas sob a forma de cavidades testemunho, o Instituto Chico Mendes poderá definir, de comum acordo com o empreendedor, outras formas de compensação, sendo os procedimentos administrativos e técnicos e as ações compensatórias definidos na Instrução Normativa Nº 30, de 19 de setembro de 2012.

De acordo com o artigo 19 da IN 2/2009, qualquer impacto negativo irreversível nas cavidades deverá ser precedido de registro e armazenamento cartográfico e fotográfico, bem como de inventário e coleta de espeleotemas e elementos geológicos e biológicos representativos do ecossistema cavernícola, compreendendo o resgate, transporte adequado e a destinação a coleções científicas institucionais. Ressalta-se que é necessária a elaboração de planos de salvamento, bem como a aquisição de autorização junto aos órgãos competentes, antes que ocorra o resgate. O salvamento de cavidades pode ser proposto como medida compensatória, na medida em que seja oferecido suporte financeiro para o desenvolvimento das atividades, apoio à divulgação científica dos resultados e estabelecimento de parcerias com instituições de pesquisa.

8. REFERÊNCIAS BIBLIOGRÁFICAS

8.1.Geoespeleologia

ARAÚJO O.J.B., MAIA R.G.N. 1991. Programa levantamentos geológicos básicos do Brasil. Projeto especial maps de recursos minerais, de solos e de vegetação para a área do Programa Grande Carajás. Subprojeto Recursos Minerais. Serra dos Carajás, Folha SB.22-Z-A. Brasília: DNPM/Companhia de Pesquisa e Recursos Minerais, 152p.

ATZINGEN, V. N.; CRESCÊNCIO, G. 1999. Estudos espeleológicos em Serra Pelada, Curionópolis – PA. Boletim Informativo da Fundação Casa da Cultura de Marabá, 1: 63-72.

AULER A. S. 2006. Relevância de cavidades naturais subterrâneas: contextualização, impactos ambientais e aspectos jurídicos. Relatório técnico, Ministério de Minas e Energia (MME) Brasília, 166 pp. http://www.mme.gov.br

AULER, A.S; PILÓ, L.B. 2005. Introdução às cavernas em minério de ferro e canga. O Carste 17(3): 70-72.

BALTAZAR, O.F.;BAARS F.J.; LOBATO, L.M.; REIS, L.B.; ACHTSCHIN, A.B.; BERNI, G.V.; SILVEIRA, V.D. 2005. Mapa Geológico...com nota explicativa em escala 1:50000. In: Projeto Geologia do Quadrilátero Ferrífero – Integração e Correção Cartográfica em SIG.

BEISIEGEL, V. de R.; BERNARDELLI, A.L.; DRUMMOND, N.F.; RUFF, A.W. e TREMAINE, J.W. 1973. Geologia e recursos minerais da Serra dos Carajás. Revista Brasileira de Geociências, 3, p. 215-242.

CARSTE CONSULTORES ASSOCIADOS LTDA. Belo Horizonte, 2011a. Serra leste – Espeleologia: diagnóstico geoespeleológico, diagnóstico bioespeleológico e análise de relevância. CALUX, A.S.; AULER, A. S.; FERREIRA, R.L. & SOUZA-SILVA, M. (Org.). Relatório inédito: 596p.

COELHO, M.R.; VIDAL-TORRADO, P. & LADEIRA, F.S.B. 2001. Macro e micromorfologia de ferricretes nodulares desenvolvidos de arenito do Grupo Bauru, Formação Adamantina. R. Bras. Ci. Solo, 25:371-385.

COSTA, L. P. 2007. Caracterização das Sequências Metavulcanossedimentares da Porção Leste da Província Mineral de Carajás (PA). Dissertação de Mestrado. Universidade Federal de Minas Gerais.

COSTA J.B.S., TEIXEIRA N.P., PINHEIRO R.V.L., BERMERGUY, R.L. 1990. Os sistemas estruturais transcorrentes do Cinturão Itacaiúnas na Região de Curionópolis, Leste do Estado do Pará. In: SBG, Cong. Bras. Geol., 35, Natal, Anais, 5:2345-2352.

COSTA, J.B.S., SIQUEIRA, J.B. 1990. Transtração e transpressão ao longo do Lineamento Cinzento (região da Serra dos Carajás). Rev. Bras. Geoc., 20(1-4): 234-238.

CORRÊA NETO, A.V., BAPTISTA FILHO, J. 1997. Espeleogênese em Quartzitos da Serra do Ibitipoca, sudeste de Minas Gerais. Anuário do Instituto de Geociências. Volume 20, p. 75-87.

DOCEGEO (Rio Doce Mineração S. A.). 1988. Revisão litoestratigráfica da Província Mineral de Carajás. Anexos XXX. Belém: Congresso Brasileiro de Geologia, p. 11- 54

DOERR, S.H. 1999. Karst-like landforms and hydrology in quartzites of the Venezuelan Guyana Shield: Pseudokarst or “real” Karst? Zeitschrift fur Geomorphologie, v. 43, p.1-17.

DORR, J.N. 1969. Physiographic, Stratigraphic and Structural Development of the Quadrilátero Ferrífero, Minas Gerais, Brazil. United States Geological Survey Professional Paper 641-A, 110p.

FERREIRA R. L. 2005. A vida subterrânea nos campos ferruginosos. O Carste. 3(17):106-115.

GEOEYE, 2012. Imagem retirada do Google Earth em 25 de setembro de 2012.

GALÁN, C. 1991. Disolución y gênesis del karst en rocas carbonáticas y rocas slíceas: unestudio comparado. Munibe, v.43, p. 43-72.

GALÁN, C., HERRERA, F.F., CARREÑO R. 2004. Geomorfologia e Hidrologia del Sistema Roraima Sur, Venezuela, La maior cavidad del en cuarcitas: 10,8 km. Bol. Soc. Venezoelana Espeleol. V. 38, p.2-16.

GILBERT, J., DANIELPOL, D. L. & STANFORD, J. A. 1994. Groundwater Ecology. Academic Press Limited, San Diego, California. 571 pp.

GUILD, P.W. 1957. Geology and Mineral Resouces of the Congonhas District. United States Geological Survey Professional Paper 290, 90 p.

HILL, C.; FORTI, P. 1997. Cave Minerals of the World. National Speleological Society, 463p.

Instrução Normativa MMA Nr. 02 de 2009 _ BRASIL. INSTRUÇÃO NORMATIVA MMA Nº 2, DE 20 DE AGOSTO DE 2009. Dispões sobre a metodologia e critérios para classificação de cavidades naturais subterrâneas e dá outras providências.

JENNINGS, J.N. Sanstone pseudokarst or karst? In: Aspects of Australian Sandstone Landscapes (Young, R. W.; Nanson, G.C. ed). Australian and New Zealand Geomorphology Group Special Publication. V. 1, p. 21-30, 1983.

MARTINI, J. Karst in the Black Reef Quartzite near Kaapsehoop, Transvaal. Ann. Geol. Surv. South Africa, v.13, p. 115-128, 1979.

MAURITY, C.W. & KOTSCHOUBEY, B. 1995. Evolução recente da cobertura de alteração no Platô N1 – Serra dos Carajás-PA. Degradação, pseudocarstificação, espeleotemas. Boletim do Museu Paraense Emilio Goeldi. Série Ciências da Terra 7: 331-362.

MAURITY, C.W.; KOTSCHOUBEY, B. 2005. Evolução recente da cobertura de alteração no Platô N1 – Serra dos Carajás-PA. Degradação, pseudocarstificação, espeleotemas. O Carste 17(3): 78-91.

PILÓ, L.B; ANDRADE, R. 2006. Estudos espeleológicos na área da Mina N5S, Serra dos Carajás. CVRD/PRCZ. 170p. (inédito).

PILÓ, L. B. & AULER, 2006. Estudos geoespeleológicos na mina do Pico do Itabirito, MG. Minerações Brasileiras Reunidas - MBR. Relatório Inédito, 380p.

PILÓ, L.B. & AULER, A.S. 2009. Geoespeleologia das cavernas em rochas ferríferas da região de Carajás, PA. XXX Congresso Brasileiro de Espeleologia. Montes Claros. Anais...Montes Claros: SBE, 2009. p. 181-186.

PINHEIRO, R.V.L.; MAURITY, C.W.; HENRIQUES, A.L.; SILVEIRA, L.T.; MOREIRA, J.R.A.; LOPES,P.R.C.; SILVEIRA, O.T.; PAIVA, R.S.; LINS, A.L.F.A.; VERÍSSIMO, C.U.V.; PINHEIRO, S.H.S.,HENRIQUES, R.V.L. 1985. Considerações Preliminares sobre a Espeleologia da Serra dos Carajás (PA). Grupo Espeleológico Paraense - GEP. Relatório inédito, 38p.

PINHEIRO, R.V., HOLDSWORTH, R. 2000. Evolução tectono-estratigráfica dos sistemas transcorrentes Carajás e Cinzento, Cinturão Itacaiúnas, borda leste do cráton Amazônico, Pará. Rev. Bras. Geoc., 30(4):597-606

PINHEIRO, R.V.L.; MAURITY, C.W. 1988. As cavernas em rochas intempéricas da Serra dos Carajás (PA) – Brasil. Anais 1º Congresso de Espeleologia da América Latina e do Caribe, Belo Horizonte, pp. 179-186.

PINTO-DA-ROCHA, R. 1995. Sinopse da fauna cavernícola do Brasil (1907 - 1994). Papéis Avulsos de Zoologia, 39(6), 61-163.

PISSIS, N.A. 1842. Mémoire sur la position géologique des terrains de la partie australe du Brésil et sur lês soulèvements qui, à diverses époques, ont changé le relief de cette contrée. Memoire de L' Institute de France 10: 353-413.

RADAMBRASIL. 1974 Levantamento de recursos naturais, v.4, Folha SB.22 - Araguaia. Ministério das Minas e Energia. Departamento Nacional da Produção Mineral. Rio de Janeiro, RJ.

ROSIÈRE,C.A.; RENGER,F.E.; PIUZANA,D.; SPIER,C.A. 2005. Pico de Itabira, MG - Marco estrutural, histórico e geográfico do Quadrilátero Ferrífero. In: Winge, M.; Schobbenhaus,C.; Berbert-Born,M.; Queiroz,E.T.; Campos,D.A.; Souza,C.R.G. & Fernandes,A.C.S. (Edit.) Sítios Geológicos e Paleontológicos do Brasil. Publicado na Internet em 21/6/2005 no endereço http://www.unb.br/ig/sigep/sitio042/sitio042.pdf

RUBBIOLI, E., MOURA, V. 2005. Mapeamento de cavernas: guia prático. São Paulo. Redespeleo Brasil. 92 p.

SIMMONS, G.C. 1963. Canga caves in the Quadrilátero Ferrífero, Minas Gerais, Brazil. The National Speleological Society Bulletin 25: 66-72.

SOUZA-SILVA, M. 2008. Ecologia e conservação das comunidades de invertebrados cavernícolas na mata atlântica brasileira. Tese de doutorado. Universidade Federal de Minas Gerais/Pós-Graduação em Ecologia Conservação e Manejo da Vida Silvestre, 217pp.

SZCZERBAN, E., URBANI, F. 1974. Carsos de Venezuela. Parte 4: Formas carsicas em areniscas precambrianas del territorio federal Amazonas y estado Bolivar. Boletin de la Sociedade Venezolana de Espeleologia, v. 5, p.27-54.

TALLARICO, F.H.B., MCNAUGHTON, N.J., GROVES, D.I., FLETCHER, I.R., FIGUEIREDO, B.R., CARVALHO, J.B., REGO, J.L., NUNES, A.R. 2003. Geological and SHRIMP II U-Pb constraints on the age and origin of the Breves Cu-Au-(W-Bi-Sn) deposit, Carajás, Brazil. Mineralium Deposita,39: 68-86.

TASSINARI C.C.G.,BETTENCOURT J.S.,GERALDES M.C.,MACAMBIRA M.J.B., LAFON J.M. 2000. The Amazonian Craton. In: Cordani J.G., Milani E.J., Campos D.A. (orgs.) Tectonic evolution of South America. In: International Geological Congress, 31st, Rio de Janeiro-RJ, p. 41-95.

TASSINI, R. 1947. Verdades Históricas e Pré-históricas de Belo Horizonte. Belo Horizonte, editora do autor.

TOLBERT, G.E.; TREMAINE, J.W.; MELCHER, G.C.; GOMES, C.B. 1971. The recently discovered Serra dos Carajás iron deposits, northern Brazil. Economic Geology, v. 66, p. 985-994.

URBANI, F. 1986. Una revision preliminar sobre el desarrollo de cavidades y otras formas relacionadas en las rocas cuarciferas del Grupo Roraima, Guayana Venezolana. Parte 2. Algunos comentarios sobre terminologia. Boletin de Geociencias, v. 7. p.10-14.

VENEZIANI P, SANTOS AR & PARADELLA WR. 2004. A evolução tectono-estratigráfica da Província Mineral de Carajás: um modelo com base em dados de sensores remotos orbitais (SAR-C RADARSAT-1, TM LANDSAT-5), aerogeofísica e dados de campo. Rev. Bras. Geoc., 34(1): 67-78.

VILLAS R.N. & SANTOS M.D. 2001. Gold deposits of the Carajás mineral province: deposit type and metallogenesis. Mineralium Deposita, 36:300-331.

WHITE, W.B., JEFFERSON, G.L., HAMAN, J.F. 1966. Quartzite Karst in southeastern Venezuela. International Journal of Speleology, v. 2.p. 309-314.

WIEGAND, J., FEY, M., HAUS, N., 2004. Geochemical and hydrochemical investigation onthe genesis of sandstone and quarzite karst of the Chapada Diamantina and the IronQuadrangle (Brazil). ZEITSCHRIFT-DEUTSCHEN GEOLOGISCHEN GESELLSCHAFT:61-90p.

WRAY, R.A.L. 1997. A global review of solutional weathering forms on quartz sandstones. Earth-Science Reviews, v. 42, p.137-160.

YOUNG, R.W. Quartz etching and sandstone Karst: Examples from the East Kimberleys, northwestern Australia. Zeitschrift fur Geomorphologie, v. 32, p. 409-423, 1988.

8.2.Bioespeleologia

ARECHAVALETA, M., L. L. SALA & P. OROMI, 1999. La fauna invertebada de la Cueva de Felipe Reventón (Icod de los Vinos, Tenerife, Islas Canarias). Vieraea 27:229-244.

AULER A. S. & L. B. PILÓ. 2005. Introdução às cavernas em minério de ferro e canga. O Carste, 17(3):70-72.

AULER A. S. 2006. Relevância de cavidades naturais subterrâneas: contextualização, impactos ambientais e aspectos jurídicos. Relatório técnico, Ministério de Minas e Energia (MME) Brasília, 166 pp. http://www.mme.gov.br

BAHIA, G. R. 2007. Sucessão ecológica em guano de morcegos insetívoros em cavernas. Dissertação apresentada ao Instituto de Ciências Biológicas da Universidade Federal de Minas Gerais, como requisito parcial para a obtenção do título de Mestre em Ecologia, Conservação e Manejo da Vida Silvestre. 117 pp.

BARR T. C. & J. R. HOLSINGER, 1985. Speciation in cave faunas. Annual Review of Ecology and Systematics, 16: 313-317.

CARSTE CONSULTORES ASSOCIADOS LTDA. Belo Horizonte, 2013. Serra da Bocaina Região de Carajás, PA: Diagnóstico Espeleológico e Análise de Relevância das Cavernas. PILÓ, L.B.; COELHO, A.; FRANCO, F.P.; SOUZA, C.A. (Org.). Relatório inédito. 237 p.

CARSTE CONSULTORES ASSOCIADOS LTDA. Belo Horizonte, 2012. Espeleologia N4/N5/PESE: Diagnóstico e Análise de Relevância de 180 cavidades. COELHO, A.; AULER, A.S.; FRANCO, F.P.; ANDRADE, R. (Org.). Relatório inédito. 217 p.

CARSTE CONSULTORES ASSOCIADOS LTDA. Belo Horizonte, 2011a. Serra leste – Espeleologia: diagnóstico geoespeleológico, diagnóstico bioespeleológico e análise de relevância. CALUX, A.S.; AULER, A. S.; FERREIRA, R.L. & SOUZA-SILVA, M. (Org.). Relatório inédito: 596p.

CARSTE CONSULTORES ASSOCIADOS LTDA. Belo Horizonte, 2011b. Estudos espeleológicos Morro I: geoespeleologia, bioespeleologia e análise de relevância. AULER, A. S.; LENHARE, B.D.; FERREIRA, R.L. & SOUZA-SILVA, M. (Org.). Relatório inédito. 306 p.

CARSTE CONSULTORES ASSOCIADOS LTDA. Belo Horizonte, 2011c. Estudos espeleológicos Morro II: geoespeleologia, bioespeleologia e análise de relevância. LEÃO, M.R.; AULER, A. S.; FERREIRA, R.L. & SOUZA-SILVA, M. (Org.). Relatório inédito. 504 p.

CARSTE CONSTULTORES ASSOCIADOS LTDA. Belo Horizonte, 2010. Espeleologia do Projeto S11D Serra Sul, Região de Carajás, PA. COELHO, A.; AULER, A.S.; PILÓ, L.B.; ANDRADE, R. (Org.). Relatório Inédito.

CHIVIAN, D., BRODIE, E. L., ALM E. J., CULLEY, D. E., DEHAL. P. S., DESANTIS, T. Z., GIHRING, T. M., LAPIDUS, A., LIN, L.H., LOWRY, S. R., MOSER D. P., RICHARDSON P. M., SOUTHAM , G., WANGER G., PRATT, L. M., ANDERSEN, G. L., HAZEN, T. C., BROCKMAN, F. J., ARKIN, A. P. AND T. C. ONSTOTT. 2008. Environmental Genomics Reveals a Single-Species Ecosystem Deep Within Earth, *Science* 10 (322):275 – 278

CULVER D. C. & W. B. WHITE 2004. Encyclopedia of caves, Publisher ElsevierAcademic Press, 654 pp.

CULVER DC, PIPAN T 2009. The biology of caves and other subterranean habitats. Library of Congress Cataloging in Publication Data, Oxford University Press, Oxford

CULVER, D. C. & B. SKET. 2002 Biological Monitoring in Caves, Acta carsologica, 31(1): 55-64

Culver, D. C. 1982. Cave Life. Evolution and Ecology. Harvard University Press. Cambridge, Massachussets and London, England. 189 pp.

DECU, V., JUBERTHIE C. & E, NITZU. 1998. Coleoptera (Varia). In: Juberthie, C. &Decu, V. (Org.). Encyclopaedia Biospeologica. 1 ed. França: Moulis: Société de Biospéologie, volume II, 113-1173pp.

FERREIRA R. L. 2004. A medida da complexidade ecológica e suas aplicações na conservação e manejo de ecossistemas subterrâneos. Tese apresentada ao programa de pós-graduação em Ecologia Conservação e Manejo da Vida Silvestre do Instituto de Ciências Biológicas da Universidade Federal de Minas Gerais, Belo Horizonte, Minas Gerais, Brasil, 158pp.

FERREIRA R. L. 2005. A vida subterrânea nos campos ferruginosos. O Carste. 3(17):106-115.

FERREIRA R. L. 2006. Caracterização de ecossistemas subterrâneos do Complexo Mina do Pico (Itabirito, MG), Minerações Brasileiras Reunidas, MBR. Relatório Técnico 123 pp. drops@ufla.br.

FERREIRA, R. L. 2000. Lixeiras de Formigueiros: Recursos Adicionais em Sistemas Cavernícolas ? O Carste, 3(12):154-158

FERREIRA, R. L., & MARTINS, R. P. 2001. Cavernas em risco de 'extinção'. Ciência Hoje, 29, p.20–28.

FERREIRA, R. L., SOUZA-SILVA, M. E BERNARDI, L. F. O. 2009. Diagnóstico do conhecimento da biodiversidade de invertebrados terrestres em Minas Gerais: contexto Bioespeleológico. Biota Minas: Diagnóstico do conhecimento sobre a biodiversidade no Estado de Minas Gerais - Subsídio ao programa biota minas. Glaucia Moreira Drumond, Cássio Soares Martins e Fábio Vieira Editores, Fundação biodiversitas

FERREIRA, R.L. & MARTINS, R.P. 1998. Diversity and distribution of spiders associated with bat guano piles in Morrinho cave (Bahia State, Brazil). Diversity and Distributions, 4:235-241.

FERREIRA, R.L. & MARTINS, R.P. 1999(a). Guano de morcegos: fonte de vida em cavernas. Ciência Hoje 25(146):34-40

FERREIRA, R.L. & MARTINS, R.P. 1999(a). Guano de morcegos: fonte de vida em cavernas. Ciência Hoje 25(146):34-40

FERREIRA, R.L. & MARTINS, R.P. 1999(b). "Trophic Structure and Natural History of Bat Guano Invertebrate Communities with Special Reference to Brazilian Caves". Tropical Zoology 12(2):231-259.

FERREIRA, R.L. & MARTINS, R.P. 1999(b). "Trophic Structure and Natural History of Bat Guano Invertebrate Communities with Special Reference to Brazilian Caves". Tropical Zoology 12(2):231-259.

FERREIRA, R.L. & POMPEU, P.S. 1997. Riqueza e diversidade da fauna associada a depósitos de guano na gruta Taboa, Sete Lagoas, Minas Gerais, Brasil. *O Carste*, 9(2): 30-33.

GILBERT, J., DANIELPOL, D. L. & STANFORD, J. A. 1994. Groundwater Ecology. Academic Press Limited, San Diego, California. 571 pp.

GOMES, F. T. M. C, R. L. FERREIRA & C. M. JACOBI 2000. Comunidade de artrópodes de uma caverna calcária em área de mineração: composição e estrutura, Revista Brasileira de Zoociências, 1(2):77-96.

HAMILTON-SMITH. E. 1965. Pselaphidae (Coleoptera) from Australian caves, South Australian Museum, Adelaide, S.A, 70-71

HARRISON S, ROSS SJ, LAWTON JH (1992) Beta diversity on geographic gradients in Britain. J Anim Ecol 61:151–158

HOLSINGER, J. R. 2012. Vicariance and dispersalist biogeography, In Culver, D. C. & W. B. White. Encyclopedia of caves. – 2nd ed.

HOLSINGER, R. & CULVER, D. C. 1988. The Invertebrate Cave Fauna of Virginia and a Part of Eastern Tennessee: Zoogeography and Ecology. *Brimleyana*, 14. 1-162.

HOSE, L. D., A. N. PALMER, M. V. PALMER, D. E. NORTHUP, P. J. BOSTON, AND H. R. DUCHENE. 2000. Microbiology and geochemistry in a hydrogen-sulphide-rich karst environment. Chemical Geology 169:399-423.

HOWARTH, F. G., JAMES, S. A., MCDOWELL, W., PRESTON D. J. & C. T. IMADA. 2007. Identification of roots in lava tube caves using molecular techniques: implications for conservation of cave arthropod faunas. Journal of Insect Conservation. 3(11): 251-261.

HOWARTH, F.G. 1983. Ecology of cave arthropods. Annual Rewiew of Entomology 28:365-389.

JASINSKA, E.J.; KNOTT, B. & MCCOMB, A.J. 1996. Hot mats in groundwater: a fauna-rich cave habitat. Journal of American Benthological Society 15(4):508-519.

KOLEFF P, GASTON KJ, LENNON JJ (2003) Measuring beta diversity for presence–absence data. J Anim Ecol 72:367–382

LAKE, P.S. 2000. Disturbance, patchiness, and diversity in streams. Journal of the North American Benthological Society 19, 573–592.

LEYS, R., WATTS, C. H. S., COOPER S. J. B., & W. F. 2003. Humphreys. Evolutionofsubterraneandivingbeetles (Coleoptera: Dytiscidae: Hydroporini, Bidessini) in thearid zone ofAustralia, Evolution, 57(12):2819-2834.

MAURIÈS, J.P. 2004 Myryapoda (centipedes and millipedes). In: GUNN, J. (editor) 2004. Encyclopedia of caves and karst science. Taylor & Francis Books Inc, New York, 1143-1146.

MARGULES C. R & R. L. PRESSEY. Systematic conservation planning, Nature 405:243-253.

MAURITY, C. W., KOTSCHOUBEY, B.. 2005. Evolução da cobertura de alteração no platô N1 Serra dos Carajás, Pa. Degradação, pseudocarstificação, espeleotemas. O Carste. Vol.17(3):78-91.

MOLDOVAN O. T. 2004. Beetles, In Encyclopedia of caves, Culver D. C. & W. B. White editors Publisher ElsevierAcademic Press, 45- 51pp.

PARK. O. 1960. -Cavernicolous Pselaphid beetles of the United States. Amer. Mid/. Nur. 64: 66-104

PECK S. B. 1976. The effect of cave entrances on the distribution of cave-inhabiting terrestrial arthropods. International Journal of Speleology. 8, 309–21.

PILÓ L. B. & A. S. AULER. 2005. Cavernas em minério de ferro e canga de capão Xavier, quadrilátero ferrífero, MG. O Carste. Vol.17(3):92-105.

PINTO-DA-ROCHA, R. 1995. Sinopse da fauna cavernícola do Brasil (1907 - 1994). Papéis Avulsos de Zoologia, 39(6), 61-163.

POGGI, R., DECU, V. & C. JUBERTHIE 1998. Coleoptera Peselaphidae. In: Juberthie, C. &Decu, V. (Org.). Encyclopaedia Biospeologica. 1 ed. França: Moulis: Société de Biospéologie, volume II, 1138-1146pp.

PROUS, X, FERREIRA, R. L. & R. P. MARTINS. 2004. Ecotone delimitation: epigean-hypogean transition in cave ecosystems Austral Ecology 29, 374–382

ROMERO A. & M. GREEN 2005. The end of regressive evolution: examining and interpreting the evidence from cave fishes. Journal of Fish Biology. 67:3-32.

SARBU, S.M; KANE, T.C. & KINKLE, B.K. 1996. A chemoautotrophically basead cave ecosystem. Science (272):1953-1955.

SCHMALFUSS, H. 2003. World catalog of terrestrial isopods (Isopoda, Oniscidea). Stuttgarter Beiträge zur Naturkunde A 654: 1- 341. Available in the World Wide Web at: http:// www.naturkundemuseum-bw.de/stuttgart/projekte/ oniscidea-catalog/Cat_terr_isop.pdf [Accessed in 20/VI/2007].

SCHNEIDER, K. CHRISTMAN, M. C., & W. F. FAGAN. 2011. The influence of resource subsidies on cave invertebrates: results from an ecosystem-level manipulation experimente, Ecology, 92(3):765–776.

SCHOTTE, C. B. BOYKO, N. L. BRUCE, J. MARKHAM, G. C. B. POORE, S. TAITI & G. D. F. WILSON. 2010 "World List of Marine, Freshwater and Terrestrial Isopod Crustaceans". World Register of Marine Species. http://www.marinespecies.org/isopoda/index.php.

SHARRATT N. J., M. PICKER AND M. SAMWAYS. (2000). The invertebrate fauna of the sandstone of the cave s of the Cape Penisula (South Africa): patterns of endemism and conservation priorities. Biodiversity and Conservation 9: 107-143.

SHEAR, W. A. 1969. A synopsis of the cave millipedes of the United States, with an illustrated key to genera. Psyche 76, 126–143.

SIMMONS G. C. 1963. Canga caves in quadrilátero ferrífero, Minas Gerais, Brasil. The National Speleological Society Bullettin 25: 66-72.

SIMON, K.S., T. PIPAN, & D.C. CULVER . 2007 A conceptual model of the flow and distribution of organic carbon in caves. Journal of Cave and Karst Studies, v. 69, no. 2, p. 279–284.

SOUZA-SILVA, M, MARTINS R. P. & R. L. FERREIRA 2011. Cave lithology determining the structure of the invertebrate communities in the Brazilian Atlantic Rain Forest. Biodiversity and Conservation DOI 10.1007/s10531-011-0057-5

SOUZA-SILVA, M. 2003. Dinâmica de disponibilidade de recursos alimentares em uma caverna calcária. Dissertação de mestrado. Universidade Federal de Minas Gerais/Pós-Graduação em Ecologia Conservação e Manejo da Vida Silvestre.76pp.

SOUZA-SILVA, M. 2008. Ecologia e conservação das comunidades de invertebrados cavernícolas na mata atlântica brasileira. Tese de doutorado. Universidade Federal de Minas Gerais/Pós-Graduação em Ecologia Conservação e Manejo da Vida Silvestre, 217pp.

SPANGLER, P.J. & V. DECU 1998. Coleoptera aquática. In: Juberthie, C. &Decu, V. (Org.). Encyclopaedia Biospeologica. 1 ed. França: Moulis: Société de Biospéologie, volume II, 1030-1046pp.

TRAJANO, E. 2012. Ecological Classification of subterranean organisms, In Encyclopedia of caves. Editors David C. Culver, William B. White – 2nd ed.

TRAJANO, E., GOLOVATCH S. I., GEOFFROY, J. J., PINTO-DA-ROCHA R. & C. S. FONTANETTI. 2000. Synopsis of Brazilian cave-dewelling millipides (diplopoda). Papéis Avulsos de Zoologia, 18(41):259-287.

TRAJANO, E. & M. E. BICHUETTE. 2010. Diversity of Brazilian subterranean invertebrates, with a list of troglomorphic taxa. Subterranean Biology 7: 1-16.

TRAJANO, E. & MOREIRA, J.R.A. 1991. Estudo da fauna de cavernas da Província Espeleológica Arenítica Altamira-Itaituba, Pará. Revista Brasileira de Biologia, 51(1):13-29.

VÍT, S. & P. HLAVÁ. 2005. New cavernicolous ant-like beetle of the genus *Euconnus* (subg. *tetramelus*) from Croatia (Coleoptera: Scydmaenidae), Nat. Croat. 1(14): 29-38.

WEINSTEIN, P. & SLANEY, D. 1995. Invertebrate faunal survey of Rope Ladder cave, Northern Queensland: a comparative study of sampling methods. Journal of the Australian Entomological Society 34: 233–236

WOLDA, H. 1981. Similarity indices, sample size and diversity. Oecologia 50(3): 296-302.

ZAMPAULO, R. A. 2010. Diversidade de invertebrados na província espeleológica de Arcos, Pains, Doresópolis (MG): Subsídios para a determinação de áreas prioritárias para a conservação. Dissertação apresentada ao Programa de Pós-graduação em Ecologia Aplicada da Universidade Federal de Lavras, MG 190 pp.

CARSTE
CONSULTORES ASSOCIADOS

Pareamento de Espécies Troglomórficas

Projeto Serra Leste

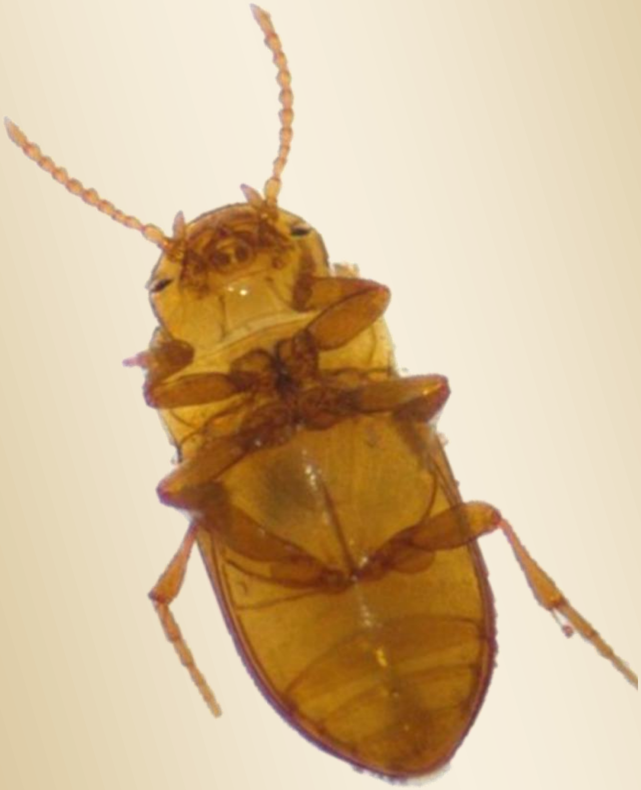

Junho de 2013

VALE

PROJETO SERRA LESTE

Pareamento de Espécies Troglomórficas

Ana Paula Bueno, MSc

Belo Horizonte, Junho de 2013

EMPRESA RESPONSÁVEL POR ESTE RELATÓRIO	
**Razão social:** Carste Consultores Associados Ltda<br>**CNPJ:** 08.000.418/0001-00<br>**Endereço:** Rua Brasópolis 139, Floresta<br>Belo Horizonte/ MG<br>CEP 30150-170	**http:** www.carste.com.br<br>**Telefone:** (31) 2552-9976

EQUIPE TÉCNICA DA CARSTE CONSULTORES ASSOCIADOS		
ESTA EQUIPE PARTICIPOU DA ELABORAÇÃO DESTE DOCUMENTO E RESPONSABILIZA-SE TECNICAMENTE POR SUAS ÁREAS		
**TÉCNICO**	**FORMAÇÃO / REGISTRO PROFISSIONAL**	**RESPONSABILIDADE NO PROJETO**
Ana Paula Bueno da Silva	Bióloga<br>CRBio 62303-04/D	Relatório Final.

EMPRESA RESPONSÁVEL PELO PROJETO	
**Razão social:** Vale S/A -Mina de Carajás<br>**CNPJ:** 33.592.510/0370-74<br>**Endereço:** Área Serra dos Carajás S/N - Serra dos Carajás / PA	**Contato:** Daniele Freitas<br>**Telefone:** (94)88019828 / 33275869<br>**E-mail:** daniele.freitas.goncalves@vale.com

SUMÁRIO

1. INTRODUÇÃO 1

2. METODOLOGIA APLICADA 3

3. RESULTADOS 3

4. ATUALIZAÇÃO DA RELEVÂNCIA 4

5. REFERÊNCIAS BIBLIOGRÁFICAS 7

ANEXO A – Parecer Técnico referente ao pareamento de Coleoptera.

ANEXO B – Currículo Lattes da especialista.

ANEXO C – Anotação de Responsabilidade Técnica.

LISTA DE TABELAS

Tabela 1: Relação das cavidades de Serra Leste (dentre 21 amostradas) que apresentam espécies troglóbias com raridade tipo I, II, III (I+II) e IV. Retirado de CARSTE, 2012a. 2

Tabela 2: Estudos utilizados para o pareamento de espécies troglomórficas do Projeto Serra Leste. 3

Tabela 3 - Síntese da relevância das cavidades estudadas em Serra Leste, de acordo com a configuração de importância dos atributos biológicos. Alterado de CARSTE, 2011b e CARSTE, 2012a. 5

1. INTRODUÇÃO

O presente documento tem como objetivo apresentar os resultados do pareamento de espécies que foram consideradas como "troglóbios raros" no relatório "Estudos Espeleológicos de Serra Leste" (CARSTE, 2012a). A comparação de espécies se deu através da análise morfológica de exemplares encontrados em estudos anteriores já realizados na região de Carajás, pela Carste Consultores Associados.

Através do pareamento de espécies, objetiva-se, portanto, reavaliar a distribuição dos espécimes determinados como "troglóbios raros", bem como propor nova análise de relevância baseada especificamente neste atributo, integrando tal resultado para os dois estudos (CARSTE, 2011b; CARSTE, 2012a) já realizados na área que engloba o Projeto Serra Leste.

CARSTE (2012a) indica nos Estudos Espeleológicos de Serra Leste que a determinação de espécies potencialmente troglóbias foi realizada através da identificação de características morfológicas denominadas troglomorfismos, nos espécimes encontrados. Segundo os autores, tais características, como redução da pigmentação melânica, redução das estruturas oculares, alongamento de apêndices, dentre outras, são utilizadas frequentemente para grande parte dos grupos, uma vez que resultam de processos evolutivos ocorrentes após o isolamento de populações em cavernas (CARSTE, 2012a). As características a serem utilizadas para estes diagnósticos, no entanto, diferem no caso de organismos pertencentes à taxa distintos. Certos grupos, por exemplo, possuem espécies sempre despigmentadas e anoftálmicas, mesmo no ambiente epígeo (e.g. Palpigradi). Desta forma, é necessário se conhecer a biologia de cada grupo no intuito de se diagnosticar efetivamente a existência ou não de troglomorfismos. Os autores enfatizam que somente a análise de despigmentação e anoftalmia muitas vezes não são diagnóstico de presença de troglomorfismos (CARSTE, 2012a).

Ainda no mesmo relatório, é destacado que as espécies troglóbias foram assim definidas em função dos seus troglomorfismos. Desta forma, os termos "Troglóbios" e "Troglomórficos" são coincidentes (CARSTE, 2012a).

Dessa forma, o Relatório Final que determinou a relevância das 21 cavidades de Serra Leste indicou 13 morfoespécies troglomórficas, a saber: Gastropoda (Systrophiidae), Amblypygi (Charinidae: *Charinus* sp.), Araneae (Oonopidae; Tetrablemmidae: *Matta* sp.), Collembola (Cyphoderidae 2spp.; Isotomidae), Coleoptera (Dytiscidae; Eucnemidae; Scydmaenidae; Staphylinidae: Pselaphinae) e Polydesmida (Pyrgodesmidae 2spp.) (CARSTE, 2012a).

No que se refere à determinação de raridade, CARSTE (2012a) demonstram distintos conceitos atribuídos às espécies troglóbias. Tais atribuições foram primordialmente ilustrativas, tendo sido realizadas no intuito de exemplificar a possibilidade de aplicação de diferentes abordagens, desde aquelas mais restritivas até as mais amplas. No entanto, para efeito da legislação vigente, utilizaram-se, em tal relatório, as duas abordagens propostas no workshop técnico científico "Troglóbios raros": incertezas e encaminhamentos, realizado em Belo Horizonte nos dias 03 e 04 de Março de 2011.

Levando em consideração o atributo de distribuição geográfica, foi sugerido, no referido workshop, que uma espécie troglóbia encontrada em até três cavidades seja considerada rara. Para CARSTE (2012a) este tipo de raridade é chamado de raridade Tipo I. Já

em referência ao atributo de abundância, foi sugerido pelos Profs. Dr. Marconi Souza Silva e Dr. Rodrigo Lopes Ferreira que deve ser considerada rara a espécie troglóbia com um exemplar por cavidade amostrada, não importando o número de cavidades em que ocorra, independentemente da distribuição geográfica. Esta abordagem também foi adotada nem tal relatório, sendo chamada de raridade Tipo II (CARSTE, 2012a).

Além das abordagens utilizadas para a definição de troglóbios raros (aplicadas para a obtenção do grau de relevância das cavernas – raridades dos tipos I e II), foram também exemplificadas outras abordagens: espécies que compreenderam *uniques* (com a ocorrência de uma única espécie em todo o estudo – raridade Tipo III); e espécies que corresponderam à sobreposição dos critérios de distribuição (até três cavernas) e abundância (um exemplar por cavidade amostrada, não importando o número de cavidades em que ocorra), chamadas de raridade Tipo IV.

De tal modo, das 13 morfoespécies categorizadas como troglomórficas nos Estudos Espeleológicos de Serra Leste, CARSTE (2012a) indicam que destas, quatro foram consideradas "troglóbios raros" (Coleoptera: Dytiscidae sp.A, Eucnemidae sp.3, Pselaphinae sp.G, Scydmaenidae sp.H). Tais espécies determinaram quatro cavidades como de relevância máxima: SL-101, SL-110, SL-115 e SL-121 conforme a Tabela 1 abaixo.

Tabela 1: Relação das cavidades de Serra Leste (dentre 21 amostradas) que apresentam espécies troglóbias com raridade tipo I, II, III (I+II) e IV**.** Retirado de CARSTE, 2012a.

Ordem	Gênero/espécie	Tipo I	Tipo II	Tipo III (I +II)	Tipo IV	> pop
Gastropoda	Systrophiidae sp1					93,101
Amblypygi	Charinus sp1					121, 82
Araneae	Oonopidae sp9				112	
Araneae	Matta sp.					130
Collembola	Cyphoderidae sp2					74
Collembola	Cyphoderidae spA					110
Collembola	Isotomidae sp3					115, 116, 121
Coleoptera	Dytiscidae spA	115				115
Coleoptera	Eucnemidae sp3	95, 110,	110	110		
Coleoptera	Pselaphinae spG	101				
Coleoptera	Scydmaenidae spH	121	121	121		
Polydesmida	Pyrgodesmidae sp3					93
Polydesmida	Pyrgodesmidae spC					122

Buscou-se através deste estudo complementar, a comparação das quatro espécies supracitadas com outros espécimes encontrados em estudos já realizados na região, de forma a atualizar a distribuição geográfica e reavaliar a aplicação do grau de relevância em toda a área que envolve o Projeto Serra Leste.

2. METODOLOGIA APLICADA

As espécies consideradas como "troglóbios raros" (CARSTE, 2012a) foram pareadas pela especialista MSc Daniela de Cássia Bená, com experiência na área de zoologia com a taxonomia dos grupos recentes, sistemática de Coleoptera Nitidulidae e também biologia, morfologia e sistemática de Coleoptera, com ênfase na família Curculionidae.

Para o presente estudo as quatro morfoespécies pertencentes à ordem Coleoptera foram comparadas através da avaliação estritamente dos seus caracteres morfológicos com outros organismos do mesmo taxa encontrados nos seguintes estudos já realizados na região, conforme Tabela 2:

Tabela 2: Estudos utilizados para o pareamento de espécies troglomórficas do Projeto Serra Leste.

Estudo	Autores
Espeleologia do Projeto S11D Serra Sul, Região de Carajás, PA. 2010.	COELHO, A.; PILÓ, L.B.; AULER, A.S.; ANDRADE, R.; BESSI, R.; FRANCO, F.P.;
Estudos espeleológicos Morro I: geoespeleologia, bioespeleologia e análise de relevância. 2011.	AULER, A. S.; LENHARE, B.D.; FERREIRA, R.L.; SOUZA-SILVA, M.
Estudos espeleológicos Morro II: geoespeleologia, bioespeleologia e análise de relevância. 2011.	AULER, A. S.; LENHARE, B.D.; FERREIRA, R.L.; SOUZA-SILVA, M.
Serra leste – Espeleologia: diagnóstico geoespeleológico, diagnóstico bioespeleológico e análise de relevância. 2011.	CALUX, A.S.; AULER, A. S.; FERREIRA, R.L.; SOUZA-SILVA, M.
Estudos espeleológicos Serra Leste: geoespeleologia, bioespeleologia e análise de relevância. 2012.	AULER, A. S.; LEÃO, M.R.; FERREIRA, R.L. & SOUZA-SILVA, M.
Levantamento dos invertebrados com caracteres troglomórficos do entorno das cavidades de Serra Leste. 2012.	TAKANO, B.F.
Espeleologia N4/N5/PESE: Diagnóstico e Análise de Relevância de 180 cavidades. 2012.	COELHO, A.; AULER, A.S.; FRANCO, F.P.; ANDRADE, R.
Serra da Bocaina Região de Carajás, PA: Diagnóstico Espeleológico e Análise de Relevância das Cavernas.2013.	PILÓ, L.B.; COELHO, A.; FRANCO, F.P.; SOUZA, C.A.

Ressalta-se que todos os estudos utilizados para o pareamento de espécies foram conduzidos em consonância com o Decreto Federal Nº 6.640 de 2008, e foram elaborados segundo as metodologias estabelecidas na Instrução Normativa MMA Nº 02 de 2009.

3. RESULTADOS

Considerando-se o pareamento realizado pela especialista MSc Daniela Bená, a morfoespécie Pselaphinae sp G encontrada na cavidade SL-101 de Serra Leste (CARSTE, 2012a) é correspondente aos exemplares encontrados nas cavidades GEM-865, GEM-1457, GEM-1461, GEM-1479, GEM-1481, GEM-1482, GEM-1523 do Projeto Serra da Bocaina (CARSTE, 2013), ampliando dessa forma, a sua distribuição.

Já a morfoespécie Scydmaenidae spH encontrada somente na cavidade SL-121 (CARSTE, 2012A) é correspondente à morfoespécie encontrada nas cavidades N4E-86 e N4E-74 do Projeto Serra Norte (CARSTE, 2012b).

O coleóptero Dytiscidae spA localizado na cavidade SL-115 do Projeto Serra Leste (CARSTE, 2012a) pareia-se com exemplares de Dytiscidae encontrados na cavidade S11D-078 do Projeto Serra Sul (CARSTE, 2010) e nas cavidades N5S-63/64/65, N5S-68, N5S74, N5S-10 do Projeto Serra Norte (CARSTE, 2012b).

Por fim, a morfoespécie Eucnemidae sp3, encontrada nas cavidades SL-95 e SL-110 (CARSTE, 2012a) foi categorizada pela especialista como troglóbia. Trata-se na verdade, de um Elateridae, Cardiophorinae, sendo que tal espécie está associada a troncos caídos e raízes de plantas. Conforme a especialista, não existe nenhum registro bibliográfico para o Brasil de espécimes troglóbios e devido a esse fato, esse exemplar é único e exige muita atenção por se tratar de algo raro.

O anexo II apresenta o resultado do pareamento realizado pela especialista MSc Daniela Bená.

4. ATUALIZAÇÃO DA RELEVÂNCIA

Das morfoespécies avaliadas, Pselaphinae sp G e Dytiscidae spA tivreram suas distribuições ampliadas o que permite a modificação do status de ”troglóbio raro” para “troglóbio não raro”. Dessa forma, as cavidades SL-101 e SL-115 têm o seu grau de relevância alterado de Máxima para Alta.

O pareamento de Scydmaenidae spH, contribuiu para a ampliação da distribuição da morfoespécie (de uma cavidade para três cavidades). Conforme o atributo de distribuição geográfica adotado por CARSTE (2012a), uma espécie troglóbia encontrada em até três cavidades é considerada rara, sendo este tipo chamado de raridade Tipo I. Neste caso, o aumento na distribuição não influenciou na alteração do grau de relevância.

A cavidade SL-110 mantém o seu grau de relevância como Máximo, devido a presença de “troglóbio raro”, não havendo qualquer alteração diante do pareamento de espécies.

Diante dos resultados encontrados, segue abaixo a atualização - no que se refere aos atributos biológicos do grau de relevância de todas as cavidades inseridas no Projeto Serra Leste.

Tabela 3 - Síntese da relevância das cavidades estudadas em Serra Leste, de acordo com a configuração de importância dos atributos biológicos. Alterado de CARSTE, 2011b e CARSTE, 2012a.

Caverna	Grau de relevância e atributos biológicos classificatórios	Grau de relevância biológica anterior	Grau de relevância biológica após pareamento
SL-101	Alto - presença de troglóbios não raros endêmicos ou relictos, espécies troglomórficas.	Máxima	Alto
SL-102	Alto - alta abundância de espécies.	Alto	Alto
SL-103	Alto - presença de táxons novos, presença de troglóbios não raros endêmicos ou relictos, espécies troglomórficas.	Alto	Alto
SL-104	Alto - presença de táxons novos, alta abundância relativa de espécies presença de troglóbios não raros endêmicos ou relictos, espécies troglomórficas.	Alto	Alto
SL-105	Alto - presença de táxons novos, presença de troglóbios não raros endêmicos ou relictos, espécies troglomórficas.	Alto	Alto
SL-106	Alto - alta abundância relativa de espécies.	Alto	Alto
SL-107	Alto - presença de táxons novos, alta riqueza de espécies, presença de troglóbios não raros endêmicos ou relictos, espécies troglomórficas.	Alto	Alto
SL-108	Alto - presença de táxons novos, alta riqueza de espécies, presença de troglóbios não raros endêmicos ou relictos, espécies troglomórficas.	Alto	Alto
SL-109	Alto - presença de táxons novos, presença de troglóbios não raros endêmicos ou relictos, espécies troglomórficas.	Alto	Alto

SL-110	Máximo - habita troglóbio raro.	Máxima	Máxima
SL-111	Alto - alta abundância relativa de espécies.	Alto	Alto
SL-112	Alto - presença de táxons novos, alta riqueza de espécies, presença de troglóbios não raros endêmicos ou relictos, espécies troglomórficas.	Alto	Alto
SL-113	Alto - presença de táxons novos, alta abundância relativa de espécies, presença de troglóbios não raros endêmicos ou relictos, espécies troglomórficas.	Alto	Alto
SL-114	Alto - presença de táxons novos, alta abundância relativa de espécies, presença de troglóbios não raros endêmicos ou relictos, espécies troglomórficas.	Alto	Alto
SL-115	Alto- presença de troglóbios não raros endêmicos ou relictos, espécies troglomórficas.	Máxima	Alto
SL-116	Alto - presença de táxons novos, presença de troglóbios não raros endêmicos ou relictos, espécies troglomórficas.	Alto	Alto
SL-117	Alto - presença de táxons novos, alta abundância relativa de espécies, presença de troglóbios não raros endêmicos ou relictos, espécies troglomórficas.	Alto	Alto
SL-121	Máximo - habitat troglóbio raro.	Máxima	Máxima
SL-122	Alto - presença de táxons novos, presença de troglóbios não raros endêmicos ou relictos, espécies troglomórficas.	Alto	Alto

SL-130	Alto - presença de táxons novos, alta abundância relativa de espécies, presença de troglóbios não raros endêmicos ou relictos, espécies troglomórficas, presença de espécie rara.	Alto	Alto
SL-131	Alto - presença de táxons novos, alta abundância relativa de espécies, presença de troglóbios não raros endêmicos ou relictos, espécies troglomórficas, presença de espécie rara.	Alto	Alto

5. REFERÊNCIAS BIBLIOGRÁFICAS

CARSTE CONSULTORES ASSOCIADOS LTDA. Belo Horizonte, 2012a. Estudos espeleológicos Serra Leste: geoespeleologia, bioespeleologia e análise de relevância. AULER, A. S.; LEÃO, M.R.; FERREIRA, R.L. & SOUZA-SILVA, M. (Org.). Relatório inédito. 209 p.

CARSTE CONSULTORES ASSOCIADOS LTDA. Belo Horizonte, 2011a. Estudos espeleológicos Morro I: geoespeleologia, bioespeleologia e análise de relevância. AULER, A. S.; LENHARE, B.D.; FERREIRA, R.L. & SOUZA-SILVA, M. (Org.). Relatório inédito. 306 p.

CARSTE CONSULTORES ASSOCIADOS LTDA. Belo Horizonte, 2011b. Serra leste – Espeleologia: diagnóstico geoespeleológico, diagnóstico bioespeleológico e análise de relevância. CALUX, A.S.; AULER, A. S.; FERREIRA, R.L. & SOUZA-SILVA, M. (Org.). Relatório inédito: 596p.

CARSTE CONSULTORES ASSOCIADOS LTDA. Belo Horizonte, 2012b. Espeleologia N4/N5/PESE: Diagnóstico e Análise de Relevância de 180 cavidades. COELHO, A.; AULER, A.S.; FRANCO, F.P.; ANDRADE, R. (Org.). Relatório inédito. 217 p.

CARSTE CONSULTORES ASSOCIADOS LTDA. Belo Horizonte, 2011c. Estudos espeleológicos Morro II: geoespeleologia, bioespeleologia e análise de relevância. LEÃO, M.R.; AULER, A. S.; FERREIRA, R.L. & SOUZA-SILVA, M. (Org.). Relatório inédito. 504 p.

CARSTE CONSULTORES ASSOCIADOS LTDA. Belo Horizonte, 2013. Serra da Bocaina Região de Carajás, PA: Diagnóstico Espeleológico e Análise de Relevância das Cavernas. PILÓ, L.B.; COELHO, A.; FRANCO, F.P.; SOUZA, C.A. (Org.). Relatório inédito. 237 p.

CARSTE CONSULTORES ASSOCIADOS LTDA. Belo Horizonte, 2012c. Levantamento dos invertebrados com caracteres troglomórficos do entorno das cavidades de Serra Leste. TAKANO, B.F. (Org.). Relatório inédito. 21 p.

CARSTE CONSTULTORES ASSOCIADOS LTDA. Belo Horizonte, 2010. Espeleologia do Projeto S11D Serra Sul, Região de Carajás, PA. COELHO, A.; AULER, A.S.; PILÓ, L.B.; ANDRADE, R. (Org.). Relatório Inédito.

ANEXO A

CARSTE
CONSULTORES ASSOCIADOS

São Paulo, 28 de junho de 2013.

Abaixo segue o resultado do pareamento efetuado a partir de exemplares de Coleoptera coletados em Serra Leste (região de Carajás, PA).

A morfoespécie Pselaphinae sp G encontrada na cavidade SL-101 de Serra Leste é correspondente a exemplares coletados nas cavidades GEM-865, GEM-1457, GEM-1461, GEM-1479, GEM-1481, GEM-1482, GEM-1523 do Projeto Serra da Bocaina.

A morfoespécie Scydmaenidae spH encontrada na cavidade SL-121 é correspondente a exemplares coletados nas cavidades N4E-86 e N4E-74 do Projeto Serra Norte.

O coleóptero Dytiscidae spA localizado na cavidade SL-115 do Projeto Serra Leste pareia-se com exemplares coletados na cavidade S11D-078 do Projeto Serra Sul bem como nas cavidades N5S-63/64/65, N5S-68, N5S74, N5S-10 do Projeto Serra Norte.

A morfoespécie Eucnemidae sp3, encontrada nas cavidades SL-95 e SL-110 é considerada troglóbia. Trata-se na verdade, de um Elateridae, Cardiophorinae, sendo que tal espécie está associada a troncos caídos e raízes de plantas. Não existe nenhum registro bibliográfico para o Brasil de espécimes troglóbios e devido a esse fato, esse exemplar é único e exige muita atenção por se tratar de algo raro.

Daniela de Cassia Bená
Bióloga
Mestre em Entomologia

Msc. Daniela de Cássia Bená

Projeto Serra Leste

Levantamento dos invertebrados com caracteres troglomórficos do entorno das cavidades de Serra Leste

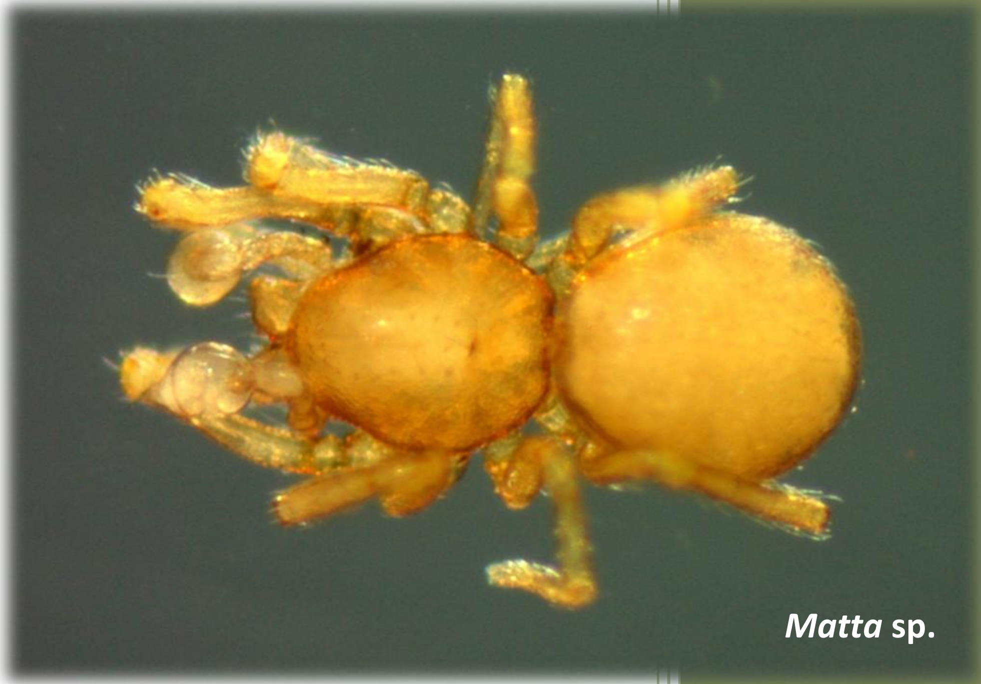

Matta sp.

Bruno Fernandes Takano
Carste Consultores Associados LTDA.

Novembro de 2012

PROJETO SERRA LESTE

Levantamento dos invertebrados com caracteres troglomórficos do entorno das cavidades de Serra Leste

Bioespeleologia

Relatório Final

VALE S/A

Bruno Fernandes Takano, Msc

Belo Horizonte, Novembro de 2012

EMPRESA RESPONSÁVEL POR ESTE RELATÓRIO	
**Razão social:** Carste Consultores Associados Ltda	**http:** www.carste.com.br
**CNPJ:** 08.000.418/0001-00	**Telefone:** (31) 2552-9976
**Endereço:** Rua Brasópolis 139, Floresta<br>Belo Horizonte/ MG - CEP 30150-170	**Email:** brunotakano@yahoo.com.br

EQUIPE TÉCNICA DA CARSTE CONSULTORES ASSOCIADOS		
**ESTA EQUIPE PARTICIPOU DA ELABORAÇÃO DESTE DOCUMENTO<br>E RESPONSABILIZA-SE TECNICAMENTE POR SUAS RESPECTIVAS ÁREAS**		
**TÉCNICO**	**FORMAÇÃO /<br>REGISTRO PROFISSIONAL**	**RESPONSABILIDADE<br>NO PROJETO**
Bruno Fernandes Takano	Biólogo<br>CRBio-01: 68783/01-D	Relatório Final

EMPRESA RESPONSÁVEL PELO PROJETO			
**Razão social:**	Vale S/A -Mina de Carajás	**Contato:**	Daniele Freitas
**CNPJ:**	33.592.510/0370-74	**Telefone:**	(94)88019828 / 33275869
**Endereço:**	Área Serra dos Carajás S/N - Serra dos Carajás / PA	**E-mail:**	daniele.freitas.goncalves@vale.com

SUMÁRIO

1. INTRODUÇÃO 1

2. MATERIAL E MÉTODOS 4

2.1. Descrição da área 4

2.2. Procedimentos de coleta 6

2.2.1. Coleta Noturna 6

2.2.2. Coleta Aquática 7

2.2.3. Extratores de Winkler e Revisão de Winkler 8

3. RESULTADOS 9

3.1. Amblypygi 12

3.2. Araneae 12

3.3. Collembola 14

3.4. Coleoptera 14

3.5. Diplopoda 15

4. CONCLUSÃO 16

4. REFERENÊNCIAS BIBLIOGRÁFICAS 19

ANEXO A – Quadro com classificação da relevância das cavidades de Serra Leste conforme apresentado no Estudo Espeleológico Serra Leste (CARSTE, 2012).

ANEXO B – Coordenadas em UTM SAD’69 dos pontos de coleta de acordo com o método de coleta empregado.

ANEXO C – Táxons capturados nos métodos de coleta. X = captura positiva; (X) = presença de troglomórficos.

ANEXO D – Anotação de Responsabilidade Técnica.

LISTA DE FIGURAS

Figura 1: Inserção das cavidades de Serra Leste nas escalas regional (Unidade Espeleológica de Carajás) e local (Unidade Geomorfológica de Serra Leste). 2
Figura 2: Detalhes da localização das 21 cavidades da UG de Serra Leste e divisão em áreas P1, P2 e P3. 5
Figura 3: Demarcação prévia de 10 metros para realização da coleta ativa noturna. 7
Figura 4: A - Pontos que foram realizadas as coletas aquáticas. Triângulos verdes: cavidades; ondas azuis: pontos de coleta aquática. B – Coleta aquática com peneira de benton em um riacho da área P3. 8
Figura 5: Recolhimento de um metro quadrado de folhiço para instalação em extrator de Winkler. 8
Figura 6: Amblypygi: *Charinus* sp. capturado na coleta ativa noturna na área P2. 12
Figura 7: Exemplar de Tetrablemmidae: *Matta* sp. troglomórfico, semelhante aos capturados na cavidade. 13
Figura 8: Exemplar da família Oonopidae sem troglomorfismos. 14
Figura 9: Exemplares de Collembola anoftálmicos e despigmentados. A – Cyphoderidae; B – Isotomidae. 14
Figura 10: Exemplares de coleópteros anoftálmicos. A – Scydmaenidae troglomórfico, semelhante aos capturados na cavidade de Serra Leste; B – Detalhe da região cefálica do Scydmaenidae troglomórfico. 15
Figura 11: Exemplar jovem de Polydesmidae anoftálmico. 16

LISTA DE FIGURAS

Tabela 1: Troglóbios coletados no interior das cavidades de Serra Leste. Tabela adaptada de do relatório de relevância das 21 cavidades de Serra Leste (CARSTE, 2012). 4

Tabela 2: Número de unidades amostrais obtidas para cada área. W = extrator de winkler; RW = revisão de folhiço do winkler; N = coleta ativa noturna; A = coleta aquática. 9

Tabela 3: Cronograma de coleta de invertebrados no entorno das cavidades de Serra Leste. ... 9

Tabela 4: Troglomórficos coletados no entorno das 21 cavidades de Serra Leste distribuídas de acordo com a metodologia e ponto de coleta, identificação, abundância (N), comparação positiva ou negativa com os troglóbios coletados nas cavidades e características que os igualam ou diferenciam dos troglóbios. (-): comparação negativa, indivíduos de morfoespécies diferentes; (+): comparação positiva; indivíduos da mesma morfoespécie. 11

Tabela 5: Status dos troglomórficos e relevância de suas respectivas cavidades antes (*Status* relevância) e após (*Status* entorno) a comparação com exemplares hipógeos com os epígeos. 18

1. INTRODUÇÃO

O presente relatório atua de forma conjunta e complementar aos "Estudos Espeleológicos de Serra Leste – Geoespeleologia, Bioespeleologia e Análise de Relevância" (CARSTE, 2012). O referido estudo avaliou as 21 cavidades localizadas na área do Projeto Serra Leste - pelo ponto de vista regional e local, e de acordo com características geológicas e biológicas - para determinação da relevância das cavidades, conforme determina o Decreto Federal nº 6.640 e a Instrução Normativa nº 2, do Ministério do Meio Ambiente – MMA. Do ponto de vista local, as 21 cavidades foram comparadas com outras 96 cavidades também inseridas na Unidade Geomorfológica de Serra Leste (CALUX *et al.*, 2011). Já do ponto de vista regional, essas cavidades foram analisadas em conjunto com as cavidades de Serra Norte (Morro I e Morro II) (AULER *et al.*, 2011; LEÃO *et al.*,2011). O quadro com o resumo da análise de relevância das 21 cavidades apresentado por Auler e colaboradores, 2012 naquele relatório, encontra-se no Anexo I (CARSTE, 2012).

Este relatório tem como objetivos apresentar os resultados do levantamento de invertebrados com características troglomórficas do entorno das 21 cavidades do Projeto Serra Leste, reavaliar os espécimes determinados como troglóbios no relatório "Estudos Espeleológicos de Serra Leste" e propor nova análise de relevância baseada nos atributos: presença de troglóbios raros, endêmicos ou relictos; presença de troglomórficos; e presença de troglóbios não raros.

Do ponto de vista regional, a área de estudo está inserida na Unidade Espeleológica (UE) de Carajás, situada no sudeste do estado do Pará. Observadas pela escala local, as cavidades estão inseridas na Unidade Geomorfológica (UG) Serra Leste, situada na porção leste da EU de Carajás (VALENTIM & OLIVITO, 2011) (**Figura 1**).

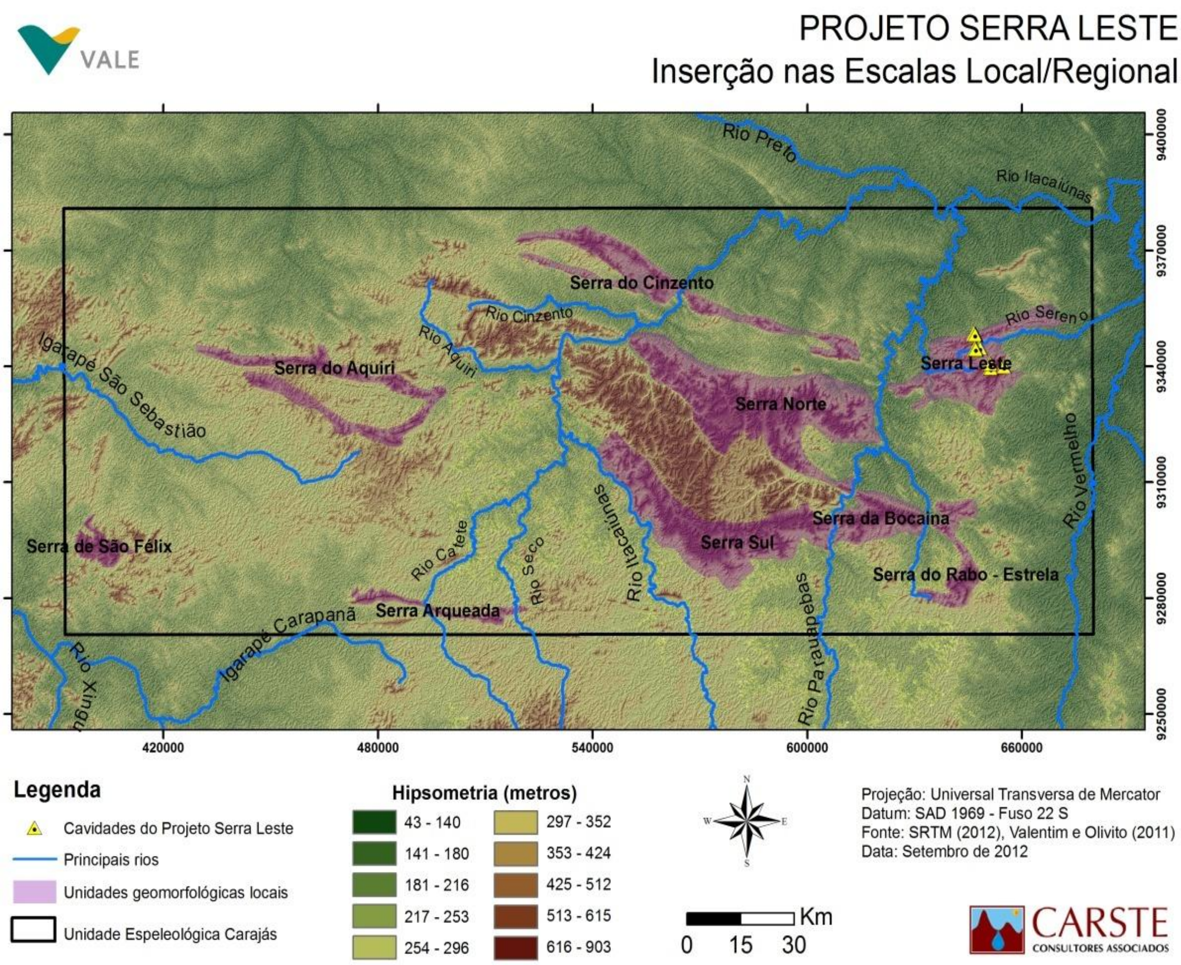

Figura 1: Inserção das cavidades de Serra Leste nas escalas regional (Unidade Espeleológica de Carajás) e local (Unidade Geomorfológica de Serra Leste).

O ambiente cavernícola (sistema hipógeo) apresenta características peculiares que o torna um ambiente único por sua estrutura física e função biológica e, consequentemente, diferente do ambiente externo (sistema epígeo). O sistema hipógeo apresenta ausência permanente de luz, que impede o desenvolvimento de organismos fotossintetizantes e, em decorrência desse fenômeno, geralmente constitui ambiente oligotrófico que depende do aporte de recursos alimentares do meio epígeo (CULVER, 1982). É comum que as cavernas apresentem temperaturas constantes e próximas à média anual do ambiente externo e umidade próxima à saturação. Desta forma, os organismos que habitam o ambiente hipógeo devem ser minimamente pré-adaptados às limitações físicas e alimentares existentes nas cavidades (CULVER, 1982).

A clássica divisão baseada na distribuição e ecologia dos organismos cavernícolas em troglóxenos, troglófilos e troglóbios foi proposta por Schiner (1854) e modificada por Racovitza (2006). Recentemente, Trajano (2012) propôs a aplicação do conceito de populações-fonte

(*source populations*) e populações-dreno (*sink populations*) (PULLIAM, 1988; FONG, 2004) à classificação de Schiner-Racovitza.

Em muitas populações, uma parte dos indivíduos pode ocorrer em habitat-dreno, que propicia à população um sucesso reprodutivo que é insuficiente para equilibrar a taxa de mortalidade local; entretanto, a população do habitat-dreno consegue manter-se devido à constante imigração proveniente de áreas denominadas habitat-fonte, cujo sucesso reprodutivo é maior que a taxa de mortalidade e, consequentemente, onde é gerado um excedente populacional (PULLIAM, 1988).

Segundo Trajano (2012), a conexão de conceitos de metapopulações às classificações criadas por Schiner-Racovitza conduz a definições claras e biologicamente significativas: troglóbios correspondem a populações-fonte exclusivamente subterrâneas, entretanto, populações-dreno podem ser encontradas no ambiente epígeo; troglófilos possuem população-fonte tanto em ambiente hipógeo quanto epígeo, com indivíduos transitando regularmente entre esses dois habitat; trogloxenos são casos de populações-fonte em ambiente epígeo, mas que utilizam os recursos subterrâneos (TRAJANO, 2012).

Os troglóbios geralmente apresentam adaptações morfológicas (troglomorfismos) provenientes de modificações evolutivas na morfologia das linhagens dos organismos associados ao modo de vida hipógeo (CHRISTIANSEN, 2012). Essas adaptações podem ser regressivas ou progressivas: as primeiras basicamente envolvem a redução ou perda de estruturas comuns aos organismos epígeos, as segundas estão relacionadas ao alargamento, modificação ou desenvolvimento de estruturas que não ocorrem em organismos epígeos (PECK, 1998; CHRISTIANSEN, 2012). Entretanto, nem todo indivíduo que apresenta características troglomórficas são considerados troglóbios. Existem organismos que apresentam “troglomorfismos” como despigmentação e anoftalmia, e são comumente encontrados em ambientes epígeos.

O Relatório Final que determinou a relevância das 21 cavidades de Serra Leste indicou 13 espécies troglomórficas. Quatro delas foram consideradas troglóbios raros (Coleoptera: Dytiscidae sp.A, Eucnemidae sp.3, Pselaphinae sp.G e Scydmaenidae sp.H) que por sua vez determinaram quatro cavidades como de relevância máxima (SL-101, SL-110, SL-115 e SL-121) devido à presença desse atributo. Essas morfoespécies foram definidas como raras devido ao fato de serem capturadas em até três cavidades, conforme definição proposta no workshop técnico científico **“Troglóbios raros”: incertezas e encaminhamentos**, realizado em Belo Horizonte nos dias 03 e 04 de Março de 2011.

Outros troglomórficos foram categorizados naquele relatório como troglóbios não raros. São eles: Gastropoda: Systrophiidae sp.1; Amblypygi: Charinus sp.1; Araneae: Oonopidae sp.9 e Matta sp.; Collembola: Cyphoderidae sp.2, Cyphoderidae sp.A, Isotomidae sp.3; Polydesmida: Pyrgodesmidae sp.3 e Pyrgodesmidae sp.C.

A **Tabela 1** abaixo lista os troglóbios apresentados no Relatório Final que determinou a relevância das 21 cavidades de Serra Leste (CARSTE, 2012).

Tabela 1: Troglóbios coletados no interior das cavidades de Serra Leste. Tabela adaptada do relatório de relevância das 21 cavidades de Serra Leste (CARSTE, 2012). SL-74, SL-82, SL-93 e SL-95 referem-se a cavidades amostradas em estudo anterior (Callux et al., 2011)

Ordem	Gênero/Espécie	Raridade tipo I	Raridade tipo IV	> pop.
Gastropoda	Systrophiidae sp.1			93, 101
Amblypygi	Charinus sp.1			82, 121
Araneae	Oonopidae sp.9		112	
Araneae	Matta sp.			130
Collembola	Cyphoderidae sp.2			74
Collembola	Cyphoderidae sp.A			110
Collembola	Isotomidae sp.3			115, 116, 121
Coleoptera	Dytiscidae sp.A	115		
Coleoptera	Eucnemidae sp.3	95, 110		
Coleoptera	Pselaphinae sp.G	101		
Coleoptera	Scydmaenidae sp.H	121		
Polydesmida	Pyrgodesmidae sp.3			93
Polydesmida	Pyrgodesmidae sp.C			122

2. MATERIAL E MÉTODOS

2.1. Descrição da área

As 21 cavidades da UG de Serra Leste foram agrupadas em três áreas distintas, nomeadas de P1, P2 e P3, de acordo com sua a litologia, localização na região e inserção na

paisagem. A área P1 englobou as cavidades SL-103, SL-105, SL-106, SL-108, SL-109, SL-110, SL-111 e SL-112; na área P2 foram incluídas as cavidades SL-101, SL-102, SL-104, SL-107, SL-113, SL-114, SL-117, SL-130 e SL-131; e na área P3 inseridas as cavidades SL-115, SL-116, SL-121 e SL-122. Em cada área, foram escolhidos pontos que apresentavam características propícias à colonização dos animais buscados e nos quais as metodologias de coletas pudessem ser aplicadas eficientemente. A visão geral e o detalhamento das áreas de coleta encontram-se na **Figura 2** e as coordenadas dos pontos de coleta no Anexo II.

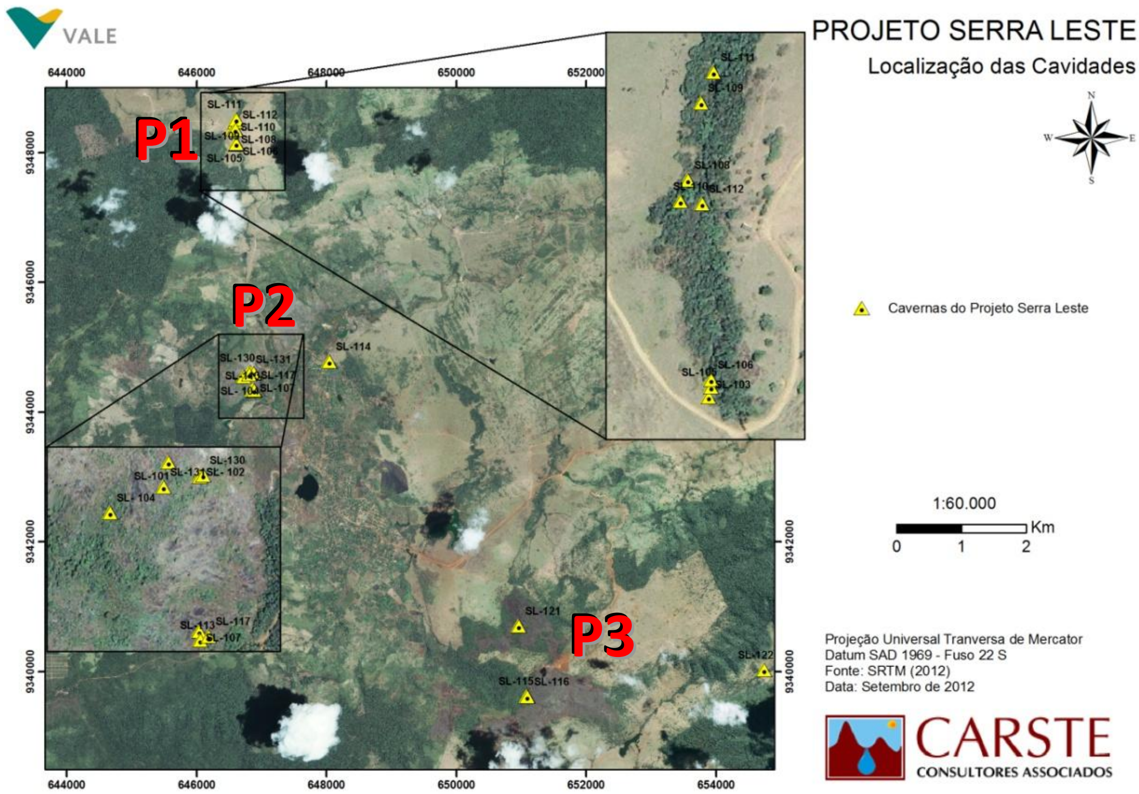

Figura 2: Detalhes da localização das 21 cavidades da UG de Serra Leste e divisão em áreas P1, P2 e P3.

A área P1 está localizada ao norte da região estudada. Compreende cavidades localizadas no sistema fluvial da borda externa da serra e cujas cavernas estão inseridas em litologia de ferricrete. O entorno dessas cavidades apresenta vegetação arbórea com árvores de médio porte, dossel inferior a 6m, sub-bosque de baixa densidade composto por vegetação arbustiva além de lianas e pteridófitas. Está inserida em um fragmento de mata ciliar que circunda um riacho de pequeno porte. A região circunvizinha desse fragmento possui grande influencia antrópica, com presença de pastagens, residências, estradas e estruturas minerárias.

A área P2 é formada pelas cavidades da Serra do Sereno, localizada na região central da área de estudo. Suas cavidades estão inseridas em rocha quartzíticas na vertente inclinada da serra. Essa área foi subdividida em duas regiões: a primeira formada pelas cavidades SL-

107, SL-113 e SL-117, localizadas próximas à base da Serra e, desta forma, mais próximas à drenagem; e a segunda formada pelas cavidades SL-101, SL-102, SL-104, SL-114, SL-130 e SL-131, localizadas na encosta e platô da Serra do Sereno. A vegetação é formada por poucas árvores de grande porte com sub-bosque de baixa densidade com vegetação arbustiva e muitas gramíneas, lianas e pteridófitas. Havia indícios de queimada por toda a encosta e platô da Serra. Na face nordeste da Serra do Sereno encontra-se uma pedreira desativada de quartzito, a poucos metros das cavidades.

A Área P3 está localizada ao sul da região estudada. Suas cavidades estão inseridas em canga na vertente inclinada da serra. A vegetação na parte superior é arbórea, composta por árvores de médio porte, com arbustos e poucas lianas associadas ao sub-bosque e serrapilheira pouco densa acumulada junto ao substrato. A vegetação na região de entrada das cavidades é arbustiva, composta predominantemente por pteridófitas. Essa área sofre grande influencia das atividades minerarias presentes no seu entorno, onde grande parte da área encontra-se degradada. Foi notada a presença de estradas de acesso e sondagem, lago artificial, edificações, etc.

2.2. Procedimentos de coleta

Os procedimentos de coleta de invertebrados do entorno de Serra Leste foram delineados de acordo com a lista preliminar dos animais indicados como troglomórficos na primeira campanha de coleta bioespeleológica nas 21 cavidades de Serra Leste. Naquela ocasião, foram consideradas como troglomórficas nove espécies: Isopoda: Plathyarthridae; Collembola: Entomobryomorpha, Isotomidae; Araneae: Ochyroceratidae (?), Oonopidae (?); Anellida; Polydesmida: Pyrgodesmidae; Coleoptera: Dytiscidae e Amblypygi: Charinidae.

Para coleta desses sete táxons no ambiente epígeo, foi proposta a utilização de quatro métodos de coleta de invertebrados: coleta manual ativa noturna (N), winkler (W), revisão de folhiço de winkler (RW) e coleta aquática com rede de benton (A). Abaixo estão detalhadas as metodologias.

2.2.1.Coleta Noturna

Foram extraídas 27 amostras de coleta ativa manual no período noturno, sendo nove em cada uma das três áreas. Nesta coleta buscou-se pelos locais mais propícios para a colonização de fauna, sendo procurados ambientes crípticos embaixo de rochas, em meio ao folhiço e dentro de troncos caídos. Para a extração de cada amostra, vasculhou-se uma área de

dez metros de comprimento por um metro de largura, previamente demarcada (**Figura 3**), durante 30 minutos. Os animais encontrados foram capturados com o auxílio de pinças e pincéis e acondicionados em recipientes com álcool 70%. A busca foi focada na captura dos grupos alvo, entretanto, foram coletados todos os exemplares que foram observados e puderam ser capturados. As coletas tiveram início às 19 horas, após o pôr do sol, e contou com o apoio da Segurança Florestal da Vale.

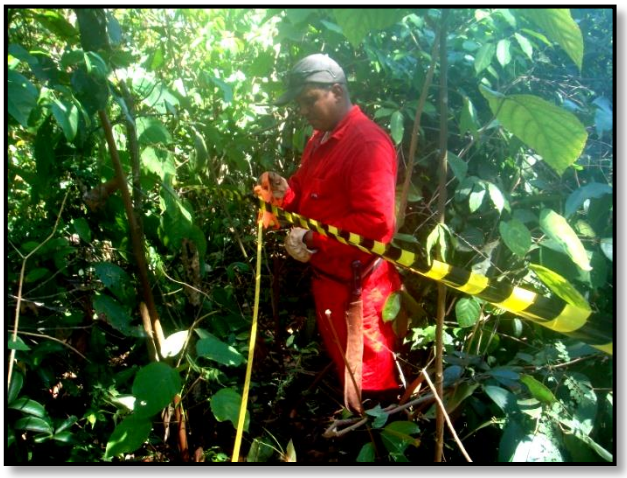

Figura 3: Demarcação prévia de 10 metros para realização da coleta ativa noturna.

2.2.2.Coleta Aquática

As dez amostras de coleta aquáticas foram obtidas com o auxílio de peneiras de benton em dois pontos da área P3 (**Figura 4A**). A coleta aquática foi realizada apenas na área P3 devido à proximidade da cavidade SL-115, onde foram encontrados os coleópteros aquáticos da família Dytiscidae. Buscaram-se corpos d'água com mata ciliar e que tivessem condições de serem colonizados por invertebrados aquáticos. Para a extração de cada amostra, a peneira foi posicionada contra o fluxo de água do riacho enquanto um metro quadrado de substrato a sua frente era revirado de modo que o substrato entrasse na peneira (**Figura 4B**). Ainda em campo, os animais recolhido pela peneira foram separados do substrato com o auxílio de bandejas e os exemplares avistados foram fixados em recipientes com álcool 70%.

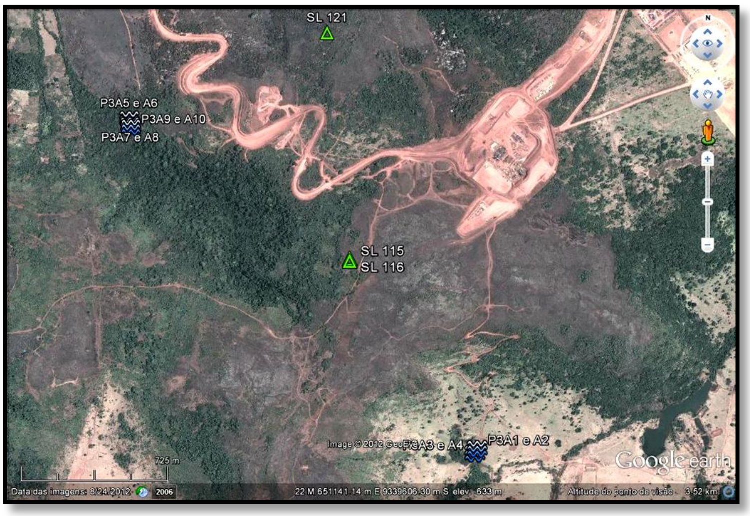

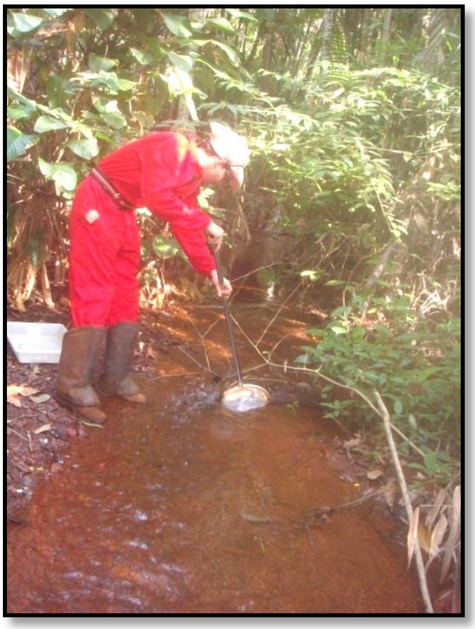

Figura 4: A - Pontos que foram realizadas as coletas aquáticas. Triângulos verdes: cavidades; ondas azuis: pontos de coleta aquática. B – Coleta aquática com peneira de benton em um riacho da área P3.

2.2.3.Extratores de Winkler e Revisão de Winkler

Foram coletadas 100 amostras aleatórias de $1m^2$ de folhiço (**Figura 5**). Cada amostra de folhiço ficou exposta em um extrator de Winkler durante três dias. Ao final desse período, as amostras com álcool contendo os animais foram retiradas do extrator e etiquetadas. O folhiço remanescente foi triado em bandejas logo após a retirada das amostras. Os animais com baixa mobilidade ou que eram grandes o suficiente para não passarem pela rede do extrator foram coletados e fixados em recipientes com álcool 70%.

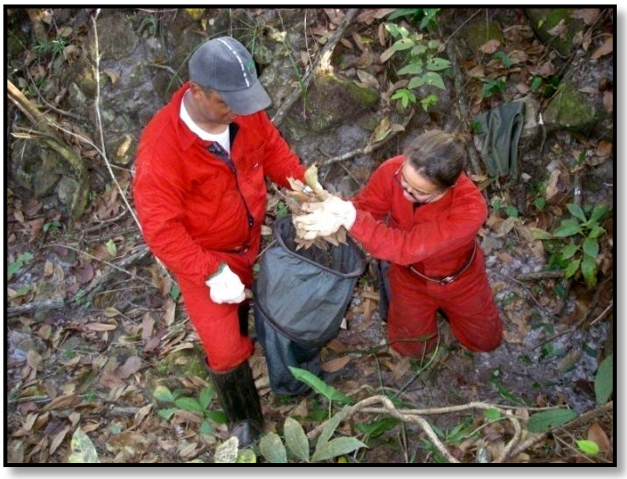

Figura 5: Recolhimento de um metro quadrado de folhiço para instalação em extrator de Winkler.

Em síntese, as coletas foram realizadas nas três áreas pré-determinadas de acordo com a distribuição exposta na **Tabela 2** abaixo:

Tabela 2: Número de unidades amostrais obtidas para cada área. W = extrator de winkler; RW = revisão de folhiço do winkler; N = coleta ativa noturna; A = coleta aquática.

	Métodos de coleta			
	**W**	**RW**	**N**	**A**
Área P1	25	25	9	-
Área P2	50	50	9	-
Área P3	25	25	9	10

Em laboratório as amostras coletadas foram triadas ao nível taxonômico de ordem com o auxílio de lupas. Foi dada especial atenção aos grupos de interesse que apresentavam animais com características troglomórficas no Relatório Preliminar de Campo: Isopoda, Coleoptera, Collembola, Araneae, Amblypygi, Annelida e Diplopoda. Os animais com características troglomórficas foram separados para comparação com aqueles capturados no interior das cavidades nas duas camapnhas realizadas para a obtenção da relevância das 21 cavidades. Os exemplares com caracteres troglomórficos coletados no entorno e a fauna troglomórfica coletada nas cavidades foram pareados pelos mesmos especialistas que identificaram o material das cavidades para evitar divergência nas identificações.

Todos os troglomórficos coletados no entorno das cavidades, serão depositados no Laboratório de Ecologia Subterrânea da Universidade Federal de Lavras (UFLA).

3. RESULTADOS

A metodologia foi executada conforme cronograma abaixo (**Tabela 3**).

Tabela 3: Cronograma de coleta de invertebrados no entorno das cavidades de Serra Leste.

	Atividades		
**Data**	**Área P1**	**Área P2**	**Área P3**
01/07/2012	Vistoria técnica da área	Vistoria técnica da área	Vistoria técnica da área
02/07/2012		Coleta de amostras de folhiço para Winkler<br>Montagem de extratores de Winkler	
03/07/2012	Coleta de amostras de folhiço para Winkler<br>Montagem de extratores de Winkler<br>Coleta ativa noturna		Coleta de amostras de folhiço para Winkler<br>Montagem de extratores de Winkler
04/07/2012			Coleta aquática<br>Coleta ativa noturna
05/07/2012		Coleta ativa noturna	Coleta aquática

06/07/2012		Retirada dos extratores de Winkler e revisão de folhiço de Winkler	
07/07/2012	Retirada dos extratores de Winkler e revisão de folhiço de Winkler		Retirada dos extratores de Winkler e revisão de folhiço de Winkler

Analisando os quatro métodos de coleta em conjunto, foram coletados 42 táxons, dispostos no **Anexo III**. Winkler e coleta ativa noturna foram os métodos que resultaram na maior variedade de táxons: 33 (78,6%) e 32 (76,2%), respectivamente. Acari, Araneae, Coleoptera e Hymenoptera foram coletados pelos quatro métodos. Alguns táxons, entretanto, foram coletados exclusivamente por determinado método. Neuroptera, Plecoptera, Thysanura, Pauropoda, Gastropoda e Onychophora foram capturados apenas no Winkler. Amblypygi, Scorpiones, Lepidoptera, Mantodea e Odonata foram capturados apenas na coleta ativa noturna. Hirudinea, Trichoptera e Crustacea foram capturados apenas na coleta aquática. Todos os táxons capturados na revisão de Winkler foram também coletados em outros métodos.

Considerando apenas os grupos alvo, Araneae foi capturada em 91% das armadilhas de Winkler e em 85,2% das amostras da coleta noturna. Outro táxon que se destacou foi Coleoptera que foi capturado em 90% das armadilhas de Winkler e 90% das coletas aquáticas. Das armadilhas de Winkler, 82% continha pelo menos um exemplar de colêmbolo e 63% pelo menos um Diplopoda. O único exemplar de Amblypygi foi coletado na coleta ativa noturna. O **Anexo III** apresenta a frequência dos táxons capturados por armadilha. Nota-se que o método de Winkler foi o mais eficiente para a captura de exemplares dos grupos alvo, entretanto, a coleta ativa noturna foi essencial para o objetivo do estudo, uma vez que capturou animais de hábito noturno e pouca mobilidade como os Amblypygi.

Neste levantamento faunístico, foram coletados indivíduos com características troglomórficas de Araneae: *Matta*; Collembola: Isotomidae e Cyphoderidae; Coleoptera: Rhizophagidae, Curculionidae, Pselaphidae e Scydmaenidae; e Polydesmidae: Siphonophoridae. Dentre esses, Coleoptera: Rhizophagidae e Curculionidae; e Polydesmidae: Siphonophoridae apesar de apresentarem troglomorfismos, não foram coletados no interior das cavidades (**Tabela 4**).

CARSTE CONSULTORES ASSOCIADOS

Tabela 4: Troglomórficos coletados no entorno das 21 cavidades de Serra Leste distribuídas de acordo com a metodologia e ponto de coleta, identificação, abundância (N), comparação positiva ou negativa com os troglóbios coletados nas cavidades e características que os igualam ou diferenciam dos troglóbios. (-): comparação negativa, indivíduos de morfoespécies diferentes; (+): comparação positiva; indivíduos da mesma morfoespécie.

Metodologia	Ponto	Filo	Classe	Subclasse/Ordem	Família/Espécie	N	Comparação com troglóbio	Características
Winkler	P1W02	Arthropoda	Entognatha	Collembola	Isotomidae	3	(-)	Troglomórficos são 2x maiores, com unguis mais alongadas, antenas mais alongadas
Winkler	P1W08	Arthropoda	Entognatha	Collembola	Isotomidae	5	(-)	Troglomórficos são 2x maiores, com unguis mais alongadas, antenas mais alongadas
Winkler	P1W09	Arthropoda	Entognatha	Collembola	Cyphoderidae	1	(-)	Os indivíduos troglomórficos são maiores, com unguis mais alongadas
Winkler	P1W11	Arthropoda	Entognatha	Collembola	Cyphoderidae	1	(-)	Os indivíduos troglomórficos são maiores, com unguis mais alongadas
Winkler	P2W44	Arthropoda	Entognatha	Collembola	Isotomidae	2	(-)	Troglomórficos são 2x maiores, com unguis mais alongadas, antenas mais alongadas
Winkler	P2W60	Arthropoda	Entognatha	Collembola	Isotomidae	5	(-)	Troglomórficos são 2x maiores, com unguis mais alongadas, antenas mais alongadas
Winkler	P2W63	Arthropoda	Entognatha	Collembola	Isotomidae	1	(-)	Troglomórficos são 2x maiores, com unguis mais alongadas, antenas mais alongadas
Winkler	P2W64	Arthropoda	Entognatha	Collembola	Isotomidae	1	(-)	Troglomórficos são 2x maiores, com unguis mais alongadas, antenas mais alongadas
Winkler	P2W65	Arthropoda	Entognatha	Collembola	Isotomidae	1	(-)	Troglomórficos são 2x maiores, com unguis mais alongadas, antenas mais alongadas
Winkler	P3W92	Arthropoda	Entognatha	Collembola	Isotomidae	1	(-)	Troglomórficos são 2x maiores, com unguis mais alongadas, antenas mais alongadas
Winkler	P3W92	Arthropoda	Entognatha	Collembola	Cyphoderidae	1	(-)	Os indivíduos troglomórficos são maiores, com unguis mais alongadas
Winkler	P3W96	Arthropoda	Entognatha	Collembola	Isotomidae	1	(-)	Troglomórficos são 2x maiores, com unguis mais alongadas, antenas mais alongadas
Noturna	P2N27	Arthropoda	Entognatha	Collembola	Cyphoderidae	1	(-)	Os indivíduos troglomórficos são maiores, com unguis mais alongadas
Winkler	P2W27	Arthropoda	Arachnida	Araneae	Tetrablemmidae / Matta sp.	1	(+)	Espécime semelhante a *Matta* sp. troglomórfica coletada nas cavernas do Projeto Serra Leste (21 Cav.).
Winkler	P2W65	Arthropoda	Diplopoda	Siphonophorida	Siphonophoridae	1	(-)	A despigmentação e ausência de estruturas oculares não são troglomorfismos neste grupo.
Winkler	P2W44	Arthropoda	Insecta	Coleoptera	Rhizophagidae	1	(-)	Espécimes frequentemente encontrados no ambiente externo apresentam caracteríscas similares.
Winkler	P2W30	Arthropoda	Insecta	Coleoptera	Curculionidae	2	?	Apesar de apresentar algumas características troglomórficas, estas provavelmente são adaptações ao modo de vida endógeo
Winkler	P2W54	Arthropoda	Insecta	Coleoptera	Pselaphinae	1	?	Apresenta olhos reduzidos e despigmentação. É necessário consultar um especialista do grupo para comprovação.
Winkler	P2W42	Arthropoda	Insecta	Coleoptera	Scydimaenidae sp. H	1	(+)	Espécime semelhante ao Scydmaenidae sp.H troglomórfico coletado nas cavernas do Projeto Serra Leste (21 Cav.).
Winkler	P1W02	Arthropoda	Insecta	Coleoptera	Scydimaenidae sp. H	1	(+)	Espécime semelhante ao Scydmaenidae sp.H troglomórfico coletado nas cavernas do Projeto Serra Leste (21 Cav.).

Dos táxons Araneae e Coleoptera, foram coletados indivíduos pertencentes às mesmas famílias dos troglóbios, entretanto, esses não apresentavam troglomorfismos.

Abaixo são detalhados os grupos alvo.

3.1. Amblypygi

Foi coletado apenas um exemplar de Amblypygi, do gênero *Heterophrynus*, que não apresentava características troglomórficas (**Figura 6**). Espécies cavernícolas ocorrem em todas as famílias e virtualmente todas as espécies encontradas em regiões cársticas também habitam cavernas (REDDELL, 2012). Não foram coletados exemplares de *Charinus*, o gênero do exemplar troglóbio de Amblypygi coletado na cavidade SL-121. Os organismos desse gênero dificilmente são observados e capturados na natureza em ambientes epígeos devido ao seu hábito críptico. O gênero inclui 28 espécies na região neotropical (JOCQUE & GIUPPONI, 2012). Nove dessas espécies são conhecidas no Brasil (Miranda & Giupponi, 2011), sendo duas troglóbias *C. troglobius* (BAPTISTA & GIUPPONI, 2002) e *C. eleonorae* (BAPTISTA & GIUPPONI, 2003).

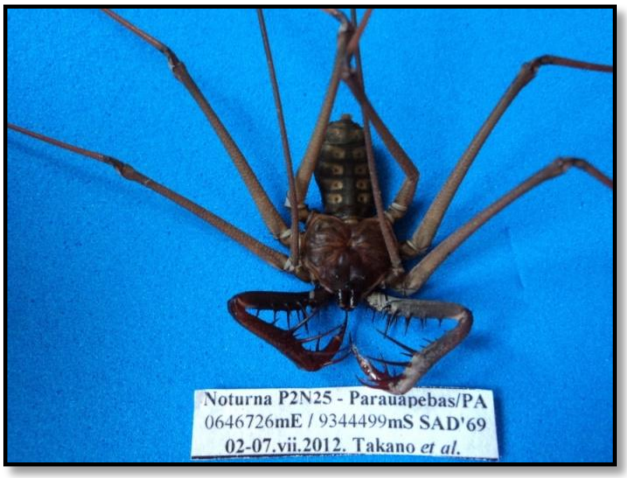

Figura 6: Amblypygi: *Charinus* sp. capturado na coleta ativa noturna na área P2.

3.2. Araneae

O gênero *Matta* inclui apenas três espécies, sendo duas com ocorrência no Brasil: *M. hambletoni* (CROSBY, 1934), a espécie-tipo, de Minas Gerais e *M. angelomachadoi* (BRESCOVIT, 2005) em Alagoas e Bahia. A terceira espécie ocorre no México (SHEAR, 1978). As duas espécies brasileiras apresentam dois olhos enquanto a mexicana é uma espécie cavernícola anoftálmica. O exemplar de *Matta* coletado neste estudo (**Figura 7**), por meio de Winkler na área P2, é anoftálmico e com fraca esclerotização do exoesqueleto e redução da pigmentação assim como o exemplar coletado na cavidade. Após comparação morfológica dos

exemplares epígeos e hipógeos, constatou-se que são da mesma morfoespécie. Trata-se de uma espécie nova, ainda não descrita pela ciência. A redução ocular é uma característica comum entre os Tetrablemmidae e, apesar de a maioria das espécies apresentarem seis olhos, espécies com quatro, dois ou apenas um olho ocorrem em diferentes gêneros (BURGER ET AL., 2010). A perda total de olhos, entretanto, é rara e limitada a espécies troglóbias da Tailândia, México e Austrália (SHEAR, 1978, DEELEMAN-REINHOLD, 1993 E BURGER ET AL., 2010).

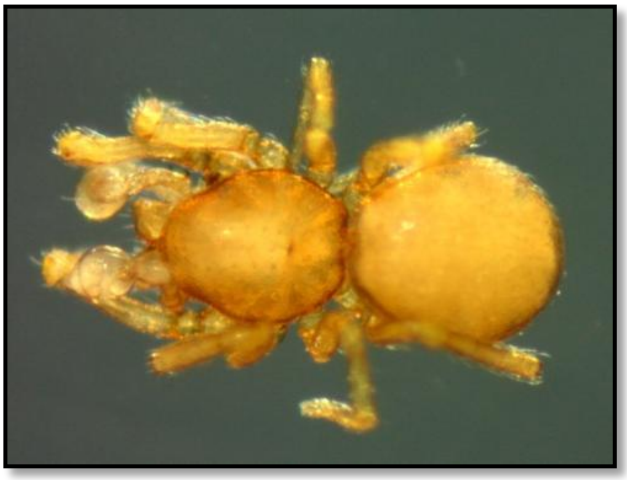

Figura 7: Exemplar de Tetrablemmidae: *Matta* sp. troglomórfico, semelhante aos capturados na cavidade.

Da família Oonopidae, foram coletados apenas indivíduos sem características troglomórficas evidentes sendo que todos os exemplares possuíam olhos desenvolvidos (**Figura 8**). Animais dessa família são encontrados facilmente na serrapilheira, casca de árvores, vegetação arbustiva e embaixo de rochas (OTT, 2003). Muitas espécies dessa família estão associadas a cavernas, incluindo vários troglóbios como *Wanops* no México, *Oonopsides* em Cuba, *Gamasomorpha* no Equador e Ceilão, e *Dysderoides* na Índia (REDDELL, 2012).

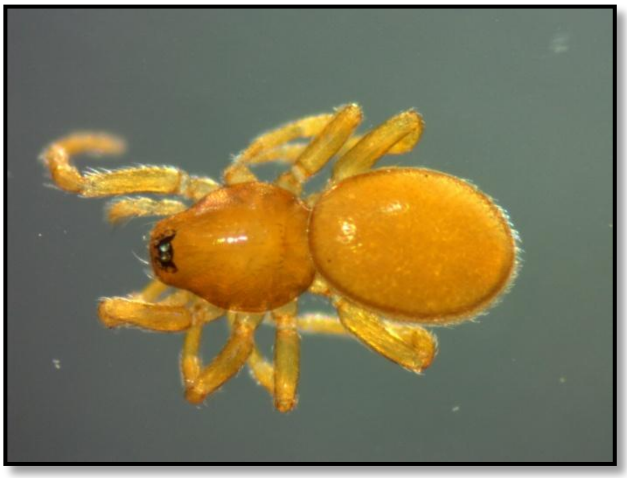

Figura 8: Exemplar da família Oonopidae sem troglomorfismos.

3.3. Collembola

Foram coletados exemplares de Collembola anoftálmicos das duas famílias alvo (Cyphoderidae e Isotomidae) em amostras de Winkler das três áreas P1, P2 e P3 (**Figura 9**). Entretanto, nenhum dos exemplares é semelhante aos coletados nas cavidades. Os Cyphoderidae do entorno das cavidades diferiram dos troglóbios por estes últimos apresentarem exemplares maiores e com unguis mais alongadas. Os Isotomidae troglóbios também são cerca de duas vezes maiores e com unguis e antenas mais alongadas que os exemplares anoftálmicos capturados no entorno das cavidades. A família Isotomidae, com centenas de espécies epígeas despigmentadas e com redução ou ausência de olhos, tem apenas duas espécies de troglóbios (CHRISTIANSEN, 2012).

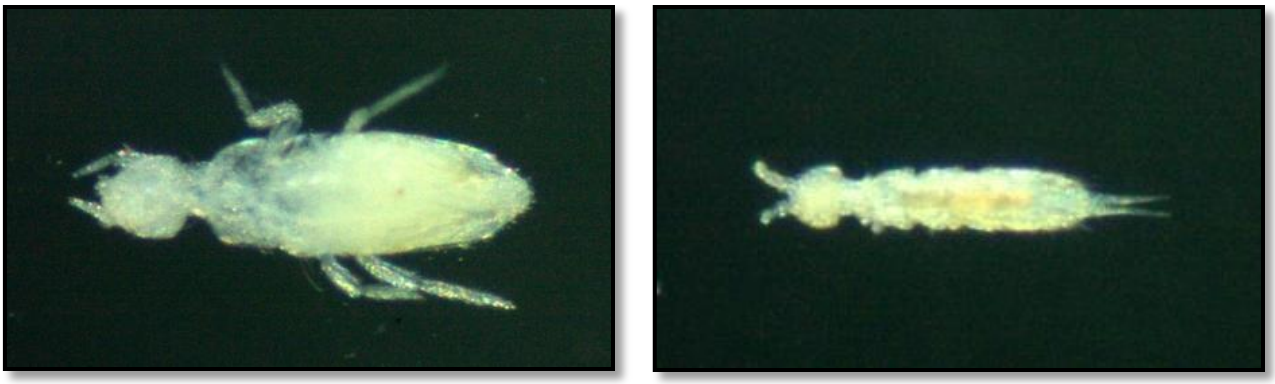

Figura 9: Exemplares de Collembola anoftálmicos e despigmentados. A – Cyphoderidae; B – Isotomidae.

3.4. Coleoptera

Das 166 famílias da ordem Coleoptera, apenas algumas possuem representantes nos sistemas subterrâneos, entre eles os aquáticos Dytiscidae e os terrestres Pselaphinae e Scydmaenidae, e ainda assim representam o grupo mais diverso, com mais de 2000 espécies

(MOLDOVAN, 2012). Características que geralmente ocorrem nos coleópteros troglóbios são a perda de pigmentação e a cutícula mais fina, além de apresentarem falta ou redução dos olhos e alongamento do corpo e antenas (MOLDOVAN, 2012).

Foram coletados dois exemplares de Curculionidae anoftálmicos, entretanto, esse tipo de adaptação para essa família está relacionado ao modo de vida especializado ao solo profundo (JUBERTHIE & DECOU, 1998). Mais de 150 taxa de Pselaphinae são habitantes subterrâneos (MOLDOVAN, 2012). Aproximadamente trinta gêneros de Pselaphidae têm representantes troglóbios no mundo (PARK, 1960; HAMILTON-SMITH, 1965; POGGI *et al.,* 1998; MOLDOVAM, 2004). Existe a citaçao de uma espécie de Pselaphidae troglomórfico para a caverna Santana em Iporanga, São Paulo (PINTO-DA-ROCHA, 1995). Entretanto, ainda não existem espécies troglóbias desta família descritas para o Brasil. Também não existem espécies troglóbias Eucnemidae descritas no mundo. Por outro lado, a família Dytiscidae possui 99 espécies de estigóbios distribuídas em quatro gêneros na Austrália (WATTS & HUMPHREYS, 2009). Foram coletados exemplares de Scydmaenidae troglomórficos do mesmo morfotipo dos capturados no interior das cavidades (**Figura 10**).

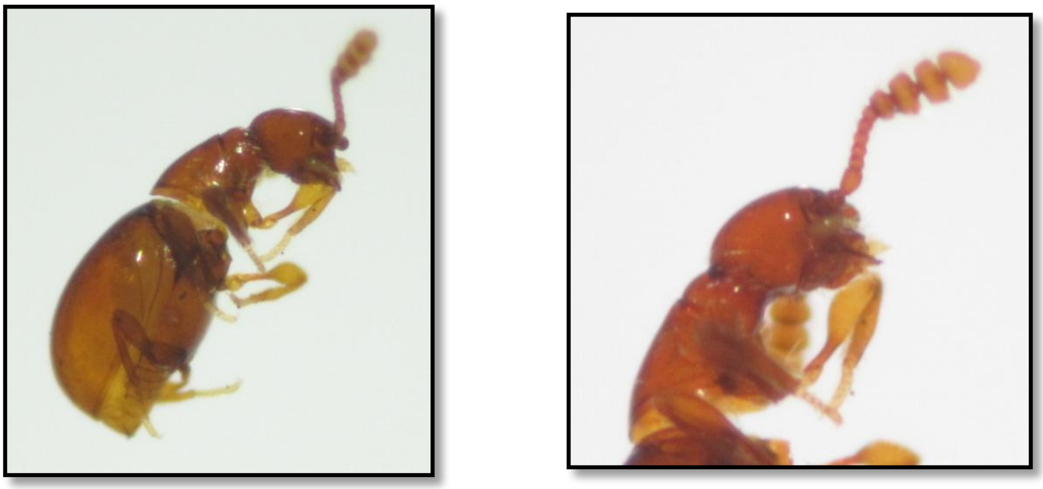

Figura 10: Exemplares de coleópteros anoftálmicos. A – Scydmaenidae troglomórfico, semelhante aos capturados na cavidade de Serra Leste; B – Detalhe da região cefálica do Scydmaenidae troglomórfico.

3.5. Diplopoda

Das quatro classes de Myriapoda existentes, três possuem espécies troglóbias. Os Diplopoda são frequentemente os detritívoros mais abundantes das comunidades terrestres das cavernas (CULVER & SHEAR, 212). A ordem Polydesmida é a que contém o maior número de espécies descritas, sendo que todas as espécies dessa ordem são anoftálmicas (CULVER & SHEAR, 2012). A família Pyrgodesmidae é pouco registrada em cavernas brasileiras, com apenas uma espécies troglóbia descrita (*Yporangiella stygius* Schubart, 1946) (TRAJANO *et al.*,

2000). Os diplópodes coletados no entorno das cavidades pertencem às ordens Polydesmida (famílias Polydesmidae ou Fuhrmannodesmidae) e Polyxenida, entretanto, a ausência de estruturas oculares e a despigmentação não são considerados troglomorfismos para os exemplares capturados (**Figura 11**).

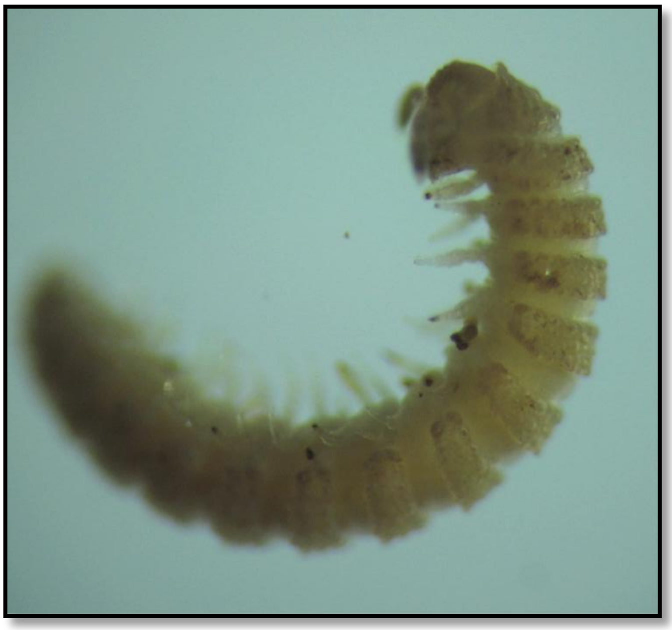

Figura 11: Exemplar jovem de Polydesmidae anoftálmico.

4. CONCLUSÃO

Foram coletados indivíduos com características troglomórficas nos táxons Araneae, Collembola, Coleoptera e Diplopoda. Apenas duas morfoespécies troglomórficas que foram coletadas no interior das cavidades também tiveram exemplares capturados no ambiente epígeo, no entorno das cavidades: *Matta* sp. e Scydmaenidae sp.H. Não foram capturados exemplares das ordens Opilioacarida e Palpigradi, considerados raros no ambiente hipógeo (CARSTE, 2012).

Segundo a IN-02/2009, troglóbios são "animais de ocorrência restrita ao ambiente subterrâneo". Através da utilização desse conceito superficial, a simples coleta ou observação de indivíduos considerados troglóbios fora do ambiente cavernícola, altera seu *status* de troglóbio para troglófilo. Desta forma, a aranha de gênero *Matta* e o coleóptero Scydmaenidae passariam a ser consideradas espécies troglófilas.

Do ponto de vista biológico, é provável que alguns troglóbios amplamente distribuídos se dispersem através de rachaduras e fendas em habitats epicársticos acima

cavernas e até mesmo na serapilheira úmida ou solo profundo fora das áreas cársticas (BARR & HOLSINGER, 1985 E HOLSINGER, 2012). *Matta* compõe população bem estabelecida a se julgar pelo seu tamanho populacional na cavidade e foi pouco amostrada no ambiente epígeo. Uma hipótese que não pode ser descartada é que os animais encontrados no ambiente epígeo podem estar explorando o habitat-dreno e são provenientes da população-fonte dentro da cavidade. Dessa maneira, apesar de terem representantes no meio epígeo, podem ser considerados troglóbios originários da população-fonte troglóbia (TRAJANO, 2012).

No caso das cavidades da área P2, que sofre com eventuais queimadas, as populações cavernícolas poderiam servir de populações-fonte para a recolonização da Serra do Sereno em períodos pós-queimadas. Desta forma, as cavidades funcionariam como um refúgio para o epicentro de recolonização do ambiente externo às cavidades. Algumas espécies parecem ter sua distribuição limitada a áreas que compreendem apenas cavernas interligadas em uma área cárstica contígua (Barr & Holsinger, 1985) e, desta forma, fornecem evidências que a distribuição desses troglóbios é geralmente definida pelos limites da área cárstica (Holsinger, 2012).

Mesmo aceitando o fato de que o exemplar de *Matta* sp. não seja troglóbio, o cenário geral da relevância da cavidade que ocorre não muda (**Tabela 5**). *Matta* sp., que ocorre apenas na cavidade SL-130 na área P2, não se enquadraria mais nos Incisos VII e VIII do Artigo 7º (espécie troglóbia não rara e espécie troglomórfica, respectivamente). Entretanto, essa espécie não deixa de ser considerada rara (Inciso XI do referido Artigo) e não descrita, constituindo um táxon novo (Inciso III do referido Artigo). Esses dois atributos, somados ao fato que a cavidade apresenta alta abundância relativa de espécies (Inciso V do referido Artigo), conferem à cavidade o status de Alta Relevância.

Scydmaenidae sp.H deixa de ser considerada espécie troglóbia rara e espécie troglomórfica pelos Incisos VII e VIII do Artigo 7º. A cavidade SL-121 na qual o coleóptero foi coletado, abriga mais dois troglóbios não raros (*Charinus* sp.1; Collembola: Isotomidae sp.3). Desta forma, por não ter mais indivíduos considerados troglóbios pela legislação em vigor, a cavidade passa a ser considerada de Alta Relevância (**Tabela 5**). De acordo com o Decreto Nº 6.640, caso o empreendimento pretenda ocasionar impacto negativo irreversível nessa cavidade, deverá atender às condicionantes do licenciamento ambiental: adotar medidas e ações para assegurar a preservação, em caráter permanente, de duas cavidades naturais subterrâneas com mesmo grau de relevância, de mesma litologia e com atributos similares à que sofreu impacto.

Tabela 5: Status dos troglomórficos e relevância de suas respectivas cavidades antes (*Status* relevância) e após (*Status* entorno) a comparação com exemplares hipógeos com os epígeos.

		**Matta sp. / SL-130**	**Scydmaenidae sp.H / SL-121**
Exemplar	*Status* relevância	Troglóbio não raro	Troglóbio não raro
	Status entorno	Troglófilo	Troglófilo
Cavidade	*Status* relevância	Alta	Máxima
	Status entorno	Alta	Alta

Cabe ressaltar que, apesar da cavidade SL-121 poder ser caracterizada com de relevância Alta de acordo com a legislação, ela foi considerada como “hotspot” de diversidade subterrânea na área (CARSTE, 2012). Desta forma, caso a cavidade sofra impactos negativos, a região circunvizinha, incluindo ambientes epígeos e hipógeos, podem sofrer impactos negativos indiretos e irreversíveis. Nesse caso, sugere-se que sejam realizados estudos mais aprofundados das dinâmicas populacionais e da estrutura da comunidade cavernícola da região para monitoramento da biota das cavidades que não sofrerão impactos negativos diretos.

Destaca-se, ainda, conforme explicitado nos parágrafos anteriores, que a mudança de *status* de troglóbio para troglófilo e, consequentemente, a mudança na condição da relevância das cavidades deram-se unicamente pela aplicação da legislação vigente e não está de acordo com a bibliografia atual.

Para evitar eventuais dificuldades nos próximos estudos de levantamento de fauna do entorno de cavidades, sugere-se que as coletas sejam realizadas após a definição dos táxons troglóbios. Para este relatório foi realizada coleta da fauna epígea no entorno das cavidades sincronicamente com a segunda campanha da coleta da fauna hipógea nas cavidades. Esse fato dificultou o delineamento da metodologia de coleta, pois os troglóbios que determinaram os grupos alvo na coleta da primeira campanha ainda não tinham o *status* definitivo.

Sugere-se ainda que sejam realizadas no mínimo duas campanhas de coleta (verão e inverno) do entorno das cavidades, uma vez que a sazonalidade na ocorrência de algumas espécies é evidente tanto para ambientes hipógeos como epígeos. Ressalta-se que um banco de dados expressivo tanto da fauna cavernícola quanto da fauna externa proporcionaria uma determinação mais precisa da taxonomia e ecologia desses animais, e consequentemente, forneceria melhores subsídios para uma determinação mais sólida da relevância das cavidades.

4. REFERÊNCIAS BIBLIOGRÁFICAS

AULER, A. S.; LENHARE, B.D.; FERREIRA, R.L. & SOUZA-SILVA, M. 2011. Estudos espeleológicos Morro I: geoespeleologia, bioespeleologia e análise de relevância. Relatório inédito. 306 pp.

BAPTISTA, R. L. C. & A. P. L. GIUPPONI, 2002. A new troglomorphic *Charinus* from Brazil (Arachnida: Amblypygi: Charinidae). Revista Ibérica de Aracnologia, Vol. 6, pp. 105-110.

BAPTISTA, R. L. C. & A. P. L. GIUPPONI, 2003. A new troglomorphic *Charinus* from Minas Gerais State, Brazil (Arachnida: Amblypygi: Charinidae). Revista Ibérica de Aracnologia, Vol. 7, pp. 79-84.

BARR T. C. & J. R. HOLSINGER, 1985. Speciation in cave faunas. Annual Review of Ecology and Systematics, 16: 313-317.

BARR, T. C. 1968. Cave ecology and the evolution of troglobites. Evolutionary Biology 2: 35-102.

BRESCOVIT, A. 2005. A new species of the spider genus *Matta* Crosby from Brazil (Araneae: Tetrablemmidae). Lundiana, 6: 49-54.

BURGER, M., M. S. HARVEY, AND N. STEVENS. 2010. A new species of blind subterranean Tetrablemma (Araneae: Tetrablemmidae) from Australia. The Journal of Arachnology 38: 146–149.

CALUX, A.S.; AULER, A. S.; FERREIRA, R.L. & SOUZA-SILVA, M. 2011. Serra leste – Espeleologia: diagnóstico geoespeleológico, diagnóstico bioespeleológico e análise de relevância. Relatório inédito: 596pp.

CARSTE CONSULTORES ASSOCIADOS, 2012. AULER, A. S.; LEÃO, M.R.; FERREIRA, R.L. & SOUZA-SILVA, M. 2012. Estudos espeleológicos Serra Leste: geoespeleologia, bioespeleologia e análise de relevância. Relatório inédito. 209 pp.

CHRISTIANSEN, K. 2012. Morphological adaptations, In Encyclopedia of caves. Editors David C. Culver, William B. White – 2nd ed.

Crosby, C. R. 1934. An interesting two-eyed spider from Brazil (Tetrablemmidae). Bulletin of Brooklyn Entomological Society, 29: 19-23.

CULVER D. C. & W. A. SHEAR, 2012. Myriapoda, In Encyclopedia of caves. Editors David C. Culver, William B. White – 2nd ed.

CULVER, D. C. (1982). Cave life: Evolution and ecology. Cambridge, MA: Harvard University Press.

BRASIL. Decreto nº 6.640, de 7 de novembro de 2008. Dá nova redação aos arts. 1o, 2o, 3o, 4o e 5o e acrescenta os arts. 5-A e 5-B ao Decreto no 99.556, de 1o de outubro de 1990, que dispõe sobre a proteção das cavidades naturais subterrâneas existentes no território nacional.

DEELEMAN-REINHOLD, C.L. 1993. A remarkable troglobitic tetrablemmid spider from a cave in Thailand (Arachnida: Araneae: Tetrablemmidae). Natural History Bulletin of the Siam Society 41:99–103.

FONG, D. W. (2004). Intermittent pools at headwaters of subterranean drainage basins as sampling sites for epikarst fauna. In Jones, W. K., W. V. Coord, D. C. Culver & J. S. Herman, Epikarst. pp.114-188.

HOLSINGER, J. R. 2012. Vicariance and dispersalist biogeography, In Culver, D. C. & W. B. White. Encyclopedia of caves. – 2nd ed.

BRASIL. Instrução normativa mma nº 2, de 20 de agosto de 2009. Dispõe sobre a metodologia e critérios para classificação de cavidades naturais subterrâneas e dá outras providências.

JUBERTHIE, C., & V. DECOU. 1998. Encyclopaedia biospeologica (Vol. II). Paris: Socièté de Biospeologie.

LEÃO, M.R.; AULER, A. S.; FERREIRA, R.L. & SOUZA-SILVA, M. 2011. Estudos espeleológicos Morro II: geoespeleologia, bioespeleologia e análise de relevância. Relatório inédito. 504 pp.

LIN, Y; S. LI ; P Jäger. 2012. Two new species of the family Tetrablemmidae (Araneae) from Laos and Malaysia. Zootaxa, 3475: 55-64.

MIRANDA, G. S. & A. P. L. GIUPPONI, 2011. A new synanthropic species of *Charinus* Simon ,1892 from Brazilian Amazonia and notes on the genus (Arachnida: Amblypygi: Charinidae). Zootaxa 2980: 61-68.

MOLDOVAN, O. T. 2012. Beetles, In Encyclopedia of caves. Editors David C. Culver, William B. White – 2nd ed.

OTT, R. 2003. Descrição de duas espécies novas de *Opopaea* do sul do Brasil (Oonopidae, Araneae). Iheringia, 93(2): 177-182.

PECK, S. B. 1998. Cladistic biogeography of cavernicolous *Ptomaphagus* beetles (Leiodidae, Cholevinae: Ptomaphagini) in the United States. In: Proceedings of the 20th International Congress of Entomology (pp. 235-260). Torino, Italy: Museo Regionale di Scienze Naturali.

PINTO-DA-ROCHA, R. 1995. Sinopse da fauna cavernícola do Brasil (1907 - 1994). Papéis Avulsos De Zoologia, 39(6): 61-163.

PULLIAM, H. R. 1988. Sources, sinks, and population regulation. The American Naturalist, 132(5): 652-661.

RACOVITZA, E. G. 2006. Essay on biospeleological problems: French, English, Romanian. [Facsimile of the publication Essai sur les problèmes biospéologiques (1907), translated by D. C. Culver & O. Moldovan.] Cluj-Napoca, Romania: Institul de Speologie “Emil Racovitza.”

REDDELL, J. R. 2012. Spiders and related groups, In Encyclopedia of caves. Editors David C. Culver, William B. White – 2nd ed.

SCHINER J. R., 1854 - Fauna der Adelsberger, Lueger und Magdalener-Grotte. Verhandlungen der Zoologisch-Botanischen Gesellschaft in Wien, 3: 1-40.

SHEAR, W. A. 1978. Taxonomic notes on the armored spiders of the families Tetrablemmidae and Pacullidae. The American Museum of Natural History, 2650, pp. 1-46.

TRAJANO, E. 2012. Ecological Classification of subterranean organisms, In Encyclopedia of caves. Editors David C. Culver, William B. White – 2nd ed.

TRAJANO, E., S. I. GOLOVATCH, J. J. GEOFFROY, R. PINTO-DA-ROCHA & C. S. FONTANETTI. 2000. Synopsis of Brazilian cave-dewelling millipides (Diplopoda). Papéis Avulsos de Zoologia, 18(41):259-287.

WATTS, C. H. S. & W. F. HUMPHREYS. 2009**.** Fourteen New Dytiscidae (Coleoptera) of the Genera *Limbodessus* Guignot, *Paroster* Sharp, and *Exocelina* Broun from Underground Waters in Australia. Transactions of the Royal Society of South Australia, Volume 133, Number 1, May 2009 , pp. 62-107(46)

ANEXO A

Anexo I: Quadro com classificação da relevância das cavidades de Serra Leste conforme apresentado no Estudo Espeleológico Serra Leste (AULER *et al.*, 2012).

GRAU DE IMPORTÂNCIA/ ENFOQUE	ATRIBUTO CLASSIFICATÓRIO	SL-101	SL-102	SL-103	SL-104	SL-105	SL-106	SL-107	SL-108	SL-109	SL-110	SL-111	SL-112	SL-113	SL-114	SL-115	SL-116	SL-117	SL-121	SL-122	SL-130	SL-131
	**Incisos do Art. 3º**																					
Relevância máxima	**I** - Gênese única ou rara;	Ausente	Ausente	Ausente	Ausente	Ausente	Ausente	Ausente	Ausente	Ausente	Ausente	Ausente	Ausente	Ausente	Ausente	Ausente	Ausente	Ausente	Ausente	Ausente	Ausente	Ausente
	**II** - Morfologia única;	Ausente	Ausente	Ausente	Ausente	Ausente	Ausente	Ausente	Ausente	Ausente	Ausente	Ausente	Ausente	Ausente	Ausente	Ausente	Ausente	Ausente	Ausente	Ausente	Ausente	Ausente
	**III** - Dimensões notáveis em extensão, área ou volume;	Presente	Ausente	Ausente	Ausente	Ausente	Ausente	Ausente	Ausente	Ausente	Ausente	Ausente	Ausente	Ausente	Ausente	Ausente	Ausente	Ausente	Ausente	Ausente	Ausente	Ausente
	**IV** - Espeleotemas únicos;	Ausente	Ausente	Ausente	Ausente	Ausente	Ausente	Ausente	Ausente	Ausente	Ausente	Ausente	Ausente	Ausente	Ausente	Ausente	Ausente	Ausente	Ausente	Ausente	Ausente	Ausente
	**V** - Isolamento geográfico;	Ausente	Ausente	Ausente	Ausente	Ausente	Ausente	Ausente	Ausente	Ausente	Ausente	Ausente	Ausente	Ausente	Ausente	Ausente	Ausente	Ausente	Ausente	Ausente	Ausente	Ausente
	**VI** - Abrigo essencial para a preservação de populações geneticamente viáveis de espécies de animais em risco de extinção, constantes de listas oficiais;	Ausente	Ausente	Ausente	Ausente	Ausente	Ausente	Ausente	Ausente	Ausente	Ausente	Ausente	Ausente	Ausente	Ausente	Ausente	Ausente	Ausente	Ausente	Ausente	Ausente	Ausente
	**VII** – Habitat para a preservação de populações geneticamente viáveis de relictos;	Ausente	Ausente	Ausente	Ausente	Ausente	Ausente	Ausente	Ausente	Ausente	Ausente	Ausente	Ausente	Ausente	Ausente	Ausente	Ausente	Ausente	Ausente	Ausente	Ausente	Ausente
	**VIII** – Habitat de troglóbio raro;	Presente	Ausente	Ausente	Ausente	Ausente	Ausente	Ausente	Ausente	Ausente	Presente	Ausente	Ausente	Ausente	Ausente	Presente	Ausente	Ausente	Presente	Ausente	Ausente	Ausente
	**IX** – Interações ecológicas únicas;	Ausente	Ausente	Ausente	Ausente	Ausente	Ausente	Ausente	Ausente	Ausente	Ausente	Ausente	Ausente	Ausente	Ausente	Ausente	Ausente	Ausente	Ausente	Ausente	Ausente	Ausente
	**X** - Cavidade testemunho; ou	Ausente	Ausente	Ausente	Ausente	Ausente	Ausente	Ausente	Ausente	Ausente	Ausente	Ausente	Ausente	Ausente	Ausente	Ausente	Ausente	Ausente	Ausente	Ausente	Ausente	Ausente
	**XI** - Destacada relevância histórico cultural ou religiosa.	Ausente	Ausente	Ausente	Ausente	Ausente	Ausente	Ausente	Ausente	Ausente	Ausente	Ausente	Ausente	Ausente	Ausente	Ausente	Ausente	Ausente	Ausente	Ausente	Ausente	Ausente
	**Incisos do Art. 7º**																					
Importância acentuada sob os enfoques local e regional	**I** - Localidade tipo;	Ausente	Ausente	Ausente	Ausente	Ausente	Ausente	Ausente	Ausente	Ausente	Ausente	Ausente	Ausente	Ausente	Ausente	Ausente	Ausente	Ausente	Ausente	Ausente	Ausente	Ausente
	**II** - Presença de espécies com função ecológica importante;	Ausente	Ausente	Ausente	Ausente	Ausente	Ausente	Ausente	Ausente	Ausente	Ausente	Ausente	Ausente	Ausente	Ausente	Ausente	Ausente	Ausente	Ausente	Ausente	Ausente	Ausente
	**III** - Presença de táxons novos;	Presente	Ausente	Presente	Presente	Presente	Ausente	Presente	Presente	Presente	Presente	Ausente	Presente	Presente	Presente	Presente	Presente	Presente	Presente	Presente	Presente	Presente
	**IV** - Alta riqueza de espécies;	Presente	Ausente	Ausente	Ausente	Ausente	Ausente	Presente	Presente	Ausente	Presente	Ausente	Presente	Ausente	Ausente	Ausente	Ausente	Ausente	Presente	Ausente	Ausente	Ausente
	**V** - Alta abundância relativa de espécies;	Ausente	Presente	Ausente	Presente	Ausente	Presente	Ausente	Ausente	Ausente	Ausente	Presente	Ausente	Presente	Presente	Ausente	Ausente	Presente	Ausente	Ausente	Presente	Presente
	**VI** - Presença de composição singular da fauna;	Ausente	Ausente	Ausente	Ausente	Ausente	Ausente	Ausente	Ausente	Ausente	Ausente	Ausente	Ausente	Ausente	Ausente	Ausente	Ausente	Ausente	Ausente	Ausente	Ausente	Ausente
	**VII** - Presença de troglóbios não raros endêmicos ou relictos;	Presente	Ausente	Presente	Presente	Presente	Ausente	Presente	Presente	Presente	Presente	Ausente	Presente	Presente	Presente	Presente	Presente	Presente	Presente	Presente	Presente	Presente
	**VIII** - Presença de espécies troglomórficas;	Presente	Ausente	Presente	Presente	Presente	Ausente	Presente	Presente	Presente	Presente	Ausente	Presente	Presente	Presente	Presente	Presente	Presente	Presente	Presente	Presente	Presente
	**IX** - Presença de trogloxeno obrigatório;	Ausente	Ausente	Ausente	Ausente	Ausente	Ausente	Ausente	Ausente	Ausente	Ausente	Ausente	Ausente	Ausente	Ausente	Ausente	Ausente	Ausente	Ausente	Ausente	Ausente	Ausente
	**X** - Presença de população excepcional em tamanho;	Ausente	Ausente	Ausente	Ausente	Ausente	Ausente	Ausente	Ausente	Ausente	Ausente	Ausente	Ausente	Ausente	Ausente	Ausente	Ausente	Ausente	Ausente	Ausente	Ausente	Ausente
	**XI** - Presença de espécie rara;	Ausente	Ausente	Ausente	Ausente	Ausente	Ausente	Ausente	Ausente	Ausente	Ausente	Ausente	Ausente	Ausente	Ausente	Ausente	Ausente	Ausente	Ausente	Ausente	Presente	Presente
	**XII** - Alta projeção horizontal da cavidade em relação às demais cavidades que se distribuem na mesma unidade espeleológica, conforme definido no § 3o do art. 14, desta Instrução Normativa;	Ausente	Ausente	Ausente	Ausente	Ausente	Ausente	Ausente	Ausente	Ausente	Ausente	Ausente	Ausente	Ausente	Ausente	Ausente	Ausente	Ausente	Ausente	Ausente	Ausente	Ausente
	**XIII** - Alta área da projeção horizontal da cavidade em relação às demais cavidades que se distribuem na mesma unidade espeleológica, conforme definido no § 3o do art. 14, desta Instrução Normativa;	Ausente	Ausente	Ausente	Ausente	Ausente	Ausente	Ausente	Ausente	Ausente	Ausente	Ausente	Ausente	Ausente	Ausente	Ausente	Ausente	Ausente	Ausente	Ausente	Ausente	Ausente
	**XIV** - Alto volume da cavidade em relação às demais cavidades que se distribuem na mesma unidade espeleológica, conforme definido no § 3o do art. 14, desta Instrução Normativa;	Ausente	Ausente	Ausente	Ausente	Ausente	Ausente	Ausente	Ausente	Ausente	Ausente	Ausente	Ausente	Ausente	Ausente	Ausente	Ausente	Ausente	Ausente	Ausente	Ausente	Ausente
	**XV** - Presença significativa de estruturas espeleogenéticas raras;	Ausente	Ausente	Ausente	Ausente	Ausente	Ausente	Ausente	Ausente	Ausente	Ausente	Ausente	Ausente	Ausente	Ausente	Ausente	Ausente	Ausente	Ausente	Ausente	Ausente	Ausente
	**XVI** - Lago ou drenagem subterrânea perene com influência acentuada sobre os atributos da cavidade que tenham as configurações relacionadas nos incisos deste artigo;	Ausente	Ausente	Ausente	Ausente	Ausente	Ausente	Ausente	Ausente	Ausente	Ausente	Ausente	Ausente	Ausente	Ausente	Ausente	Ausente	Ausente	Ausente	Presente	Ausente	Ausente
	**XVII** - Diversidade da sedimentação química com muitos tipos de espeleotemas e processos de deposição;	Ausente	Ausente	Ausente	Ausente	Ausente	Ausente	Ausente	Ausente	Ausente	Ausente	Ausente	Ausente	Ausente	Ausente	Ausente	Ausente	Ausente	Ausente	Ausente	Ausente	Ausente
	**XVIII** - Configuração notável dos espeleotemas;	Ausente	Ausente	Ausente	Ausente	Ausente	Ausente	Ausente	Ausente	Ausente	Ausente	Ausente	Ausente	Ausente	Ausente	Ausente	Ausente	Ausente	Ausente	Ausente	Ausente	Ausente
	**XIX** - Alta influência da cavidade sobre o sistema cárstico;	Ausente	Ausente	Ausente	Ausente	Ausente	Ausente	Ausente	Ausente	Ausente	Ausente	Ausente	Ausente	Ausente	Ausente	Ausente	Ausente	Ausente	Ausente	Ausente	Ausente	Ausente
	**XX** - Presença de inter-relação da cavidade com alguma de relevância máxima;	Ausente	Ausente	Ausente	Ausente	Ausente	Ausente	Ausente	Ausente	Ausente	Ausente	Ausente	Ausente	Ausente	Ausente	Ausente	Ausente	Ausente	Ausente	Ausente	Ausente	Ausente
	**XXI** - Reconhecimento nacional ou mundial do valor estético/cênico da cavidade;	Ausente	Ausente	Ausente	Ausente	Ausente	Ausente	Ausente	Ausente	Ausente	Ausente	Ausente	Ausente	Ausente	Ausente	Ausente	Ausente	Ausente	Ausente	Ausente	Ausente	Ausente
	**XXII** - Visitação pública sistemática na cavidade, com abrangência regional ou nacional.	Ausente	Ausente	Ausente	Ausente	Ausente	Ausente	Ausente	Ausente	Ausente	Ausente	Ausente	Ausente	Ausente	Ausente	Ausente	Ausente	Ausente	Ausente	Ausente	Ausente	Ausente
	**Incisos do Art. 8º**																					
Importância acentuada sob o enfoque local	**I**- Populações residentes de quiroptera;	Ausente	Ausente	Ausente	Ausente	Ausente	Ausente	Ausente	Ausente	Ausente	Ausente	Ausente	Ausente	Ausente	Ausente	Ausente	Ausente	Ausente	Ausente	Ausente	Ausente	Ausente
	**II** - Local de nidificação de aves silvestres;	Ausente	Ausente	Ausente	Ausente	Ausente	Ausente	Ausente	Ausente	Ausente	Ausente	Ausente	Ausente	Ausente	Ausente	Ausente	Ausente	Ausente	Ausente	Ausente	Ausente	Ausente
	**III** - Alta diversidade de substratos orgânicos;	Alta	Alta	Alta	Alta	Alta	Alta	Alta	Alta	Alta	Alta	Alta	Alta	Alta	Alta	Alta	Alta	Alta	Alta	Alta	Alta	Baixa
	**IV** - Média riqueza de espécies;	Ausente	Ausente	Ausente	Ausente	Ausente	Ausente	Ausente	Ausente	Ausente	Ausente	Ausente	Ausente	Ausente	Ausente	Ausente	Ausente	Ausente	Ausente	Ausente	Ausente	Ausente
	**V** - Média abundância relativa de espécies;	Presente	Ausente	Baixa	Ausente	Média	Ausente	Presente	Baixa	Baixa	Presente	Ausente	Baixa	Ausente	Ausente	Presente	Presente	Ausente	Presente	Presente	Ausente	Ausente
	**VI** - Constatação do uso da cavidade por espécies migratórias;	Ausente	Ausente	Ausente	Ausente	Ausente	Ausente	Ausente	Ausente	Ausente	Ausente	Ausente	Ausente	Ausente	Ausente	Ausente	Ausente	Ausente	Ausente	Ausente	Ausente	Ausente
	**VII** - Presença de singularidade dos elementos faunísticos da cavidade sob enfoque local;	Ausente	Ausente	Ausente	Ausente	Ausente	Ausente	Ausente	Ausente	Ausente	Ausente	Ausente	Ausente	Ausente	Ausente	Ausente	Ausente	Ausente	Ausente	Ausente	Ausente	Ausente
	**VIII** - Presença de estrutura geológica de interesse científico;	Ausente	Ausente	Ausente	Ausente	Ausente	Ausente	Ausente	Ausente	Ausente	Ausente	Ausente	Ausente	Ausente	Ausente	Ausente	Ausente	Ausente	Presente	Ausente	Ausente	Ausente
	**IX** - Presença de registros paleontológicos;	Ausente	Ausente	Ausente	Ausente	Ausente	Ausente	Ausente	Ausente	Ausente	Ausente	Ausente	Ausente	Ausente	Ausente	Ausente	Ausente	Ausente	Ausente	Ausente	Ausente	Ausente
	**X** - Reconhecimento local do valor estético/cênico da cavidade;	Ausente	Ausente	Ausente	Ausente	Ausente	Ausente	Ausente	Ausente	Ausente	Ausente	Ausente	Ausente	Ausente	Ausente	Ausente	Ausente	Ausente	Ausente	Ausente	Ausente	Ausente
	**XI** - Visitação pública sistemática na cavidade, com abrangência local;	Ausente	Ausente	Ausente	Ausente	Ausente	Ausente	Ausente	Ausente	Ausente	Ausente	Ausente	Ausente	Ausente	Ausente	Ausente	Ausente	Ausente	Ausente	Ausente	Ausente	Ausente
	**XII** - Presença de água de percolação ou condensação com influência acentuada sobre os atributos da cavidade que tenham as configurações relacionadas nos incisos deste artigo;	Ausente	Ausente	Ausente	Ausente	Ausente	Ausente	Ausente	Ausente	Ausente	Ausente	Ausente	Ausente	Ausente	Ausente	Ausente	Ausente	Ausente	Ausente	Ausente	Ausente	Ausente
	**XIII** - Lago ou drenagem subterrânea intermitente com influência acentuada sobre os atributos da cavidade que tenham as configurações relacionadas nos incisos deste artigo.	Ausente	Ausente	Ausente	Ausente	Ausente	Ausente	Ausente	Ausente	Ausente	Ausente	Ausente	Ausente	Ausente	Ausente	Ausente	Ausente	Ausente	Ausente	Ausente	Ausente	Ausente
	**Incisos do Art. 9º**																					
Importância significativa sob os enfoques local e regional	**I** - Singularidade dos elementos faunísticos sob enfoque regional;	Ausente	Ausente	Ausente	Ausente	Ausente	Ausente	Ausente	Ausente	Ausente	Ausente	Ausente	Ausente	Ausente	Ausente	Ausente	Ausente	Ausente	Ausente	Ausente	Ausente	Ausente
	**II** - Média projeção horizontal da cavidade em relação às demais cavidades que se distribuem na mesma unidade espeleológica, conforme definido no § 3o do art. 14, desta Instrução Normativa;	Ausente	Presente	Presente	Presente	Presente	Presente	Presente	Presente	Presente	Presente	Presente	Presente	Presente	Presente	Presente	Presente	Presente	Presente	Presente	Presente	Presente
	**III** - Média área da projeção horizontal da cavidade em relação às demais cavidades que se distribuem na mesma unidade espeleológica, conforme definido no § 3o do art. 14, desta Instrução Normativa;	Ausente	Presente	Presente	Presente	Presente	Presente	Presente	Presente	Presente	Presente	Presente	Presente	Presente	Presente	Presente	Presente	Presente	Presente	Presente	Presente	Presente
	**IV** - Alto desnível da cavidade em relação às demais cavidades que se distribuem na mesma unidade espeleológica, conforme definido no § 3º do art. 14, desta Instrução Normativa;	Presente	Ausente	Ausente	Ausente	Ausente	Ausente	Ausente	Ausente	Ausente	Ausente	Ausente	Ausente	Ausente	Ausente	Ausente	Ausente	Ausente	Ausente	Ausente	Ausente	Ausente
	**V** - Médio volume da cavidade em relação às demais cavidades que se distribuem na mesma unidade espeleológica, conforme definido no § 3º do art. 14, desta Instrução Normativa;	Ausente	Presente	Presente	Presente	Presente	Presente	Presente	Presente	Presente	Presente	Presente	Presente	Presente	Presente	Presente	Presente	Presente	Presente	Presente	Presente	Presente
	**VI** - Presença de estruturas espeleogenéticas raras;	Ausente	Ausente	Ausente	Presente	Ausente	Ausente	Ausente	Ausente	Ausente	Ausente	Ausente	Ausente	Ausente	Ausente	Ausente	Ausente	Ausente	Ausente	Ausente	Ausente	Ausente
	**VII** - Lago ou drenagem subterrânea intermitente com influência significativa sobre os atributos da cavidade que tenham as configurações relacionadas nos incisos deste artigo;	Ausente	Ausente	Ausente	Ausente	Ausente	Ausente	Ausente	Ausente	Ausente	Ausente	Ausente	Ausente	Ausente	Ausente	Ausente	Ausente	Ausente	Ausente	Ausente	Ausente	Ausente
	**VIII** - Diversidade da sedimentação química com muitos tipos de espeleotemas ou processos de deposição;	Ausente	Ausente	Ausente	Ausente	Ausente	Ausente	Ausente	Ausente	Ausente	Ausente	Ausente	Ausente	Ausente	Ausente	Ausente	Ausente	Ausente	Ausente	Ausente	Ausente	Ausente
	**IX** - Sedimentação clástica ou química com valor científico;	Ausente	Ausente	Ausente	Ausente	Ausente	Ausente	Ausente	Ausente	Ausente	Ausente	Ausente	Ausente	Ausente	Ausente	Ausente	Ausente	Ausente	Ausente	Ausente	Ausente	Ausente
	**X** - Reconhecimento regional do valor estético/cênico da cavidade;	Ausente	Ausente	Ausente	Ausente	Ausente	Ausente	Ausente	Ausente	Ausente	Ausente	Ausente	Ausente	Ausente	Ausente	Ausente	Ausente	Ausente	Ausente	Ausente	Ausente	Ausente
	**XI** - Uso constante, periódico ou sistemático para fins educacionais, recreativos ou esportivos.	Ausente	Ausente	Ausente	Ausente	Ausente	Ausente	Ausente	Ausente	Ausente	Ausente	Ausente	Ausente	Ausente	Ausente	Ausente	Ausente	Ausente	Ausente	Ausente	Ausente	Ausente
	**Incisos do Art. 10º**																					
Importância significativa sob o enfoque local	**I** - Baixa diversidade de substratos orgânicos;	Ausente	Ausente	Ausente	Ausente	Ausente	Ausente	Ausente	Ausente	Ausente	Ausente	Ausente	Ausente	Ausente	Ausente	Ausente	Ausente	Ausente	Ausente	Ausente	Ausente	Ausente
	**II** - Baixa riqueza de espécies;	Ausente	Presente	Presente	Presente	Presente	Presente	Ausente	Ausente	Presente	Ausente	Presente	Ausente	Presente	Presente	Presente	Presente	Presente	Ausente	Presente	Presente	Presente
	**III** - Baixa abundância relativa de espécies;	Ausente	Ausente	Ausente	Ausente	Ausente	Ausente	Ausente	Ausente	Ausente	Ausente	Ausente	Ausente	Ausente	Ausente	Ausente	Ausente	Ausente	Ausente	Ausente	Ausente	Ausente
	**IV** - Presença de singularidade dos elementos faunísticos sob enfoque local;	Ausente	Ausente	Ausente	Ausente	Ausente	Ausente	Ausente	Ausente	Ausente	Ausente	Ausente	Ausente	Ausente	Ausente	Ausente	Ausente	Ausente	Ausente	Ausente	Ausente	Ausente
	**V** - Média projeção horizontal da cavidade em relação às demais cavidades que se distribuem na mesma unidade geomorfológica, conforme definido no § 1o do art. 14, desta Instrução Normativa;	Ausente	Presente	Presente	Presente	Presente	Presente	Presente	Presente	Presente	Presente	Presente	Presente	Presente	Presente	Presente	Presente	Presente	Ausente	Presente	Presente	Presente
	**VI** - Média área da projeção horizontal da cavidade em relação às demais cavidades que se distribuem na mesma unidade geomorfológica, conforme definido no § 1o do art. 14, desta Instrução Normativa;	Ausente	Presente	Presente	Presente	Presente	Presente	Presente	Presente	Presente	Ausente	Presente	Presente	Presente	Presente	Presente	Presente	Presente	Presente	Presente	Presente	Presente
	**VII** - Alto desnível da cavidade em relação às demais cavidades que se distribuem na mesma unidade geomorfológica, conforme definido no § 1º do art. 14, desta Instrução Normativa;	Presente	Ausente	Ausente	Ausente	Ausente	Ausente	Ausente	Ausente	Ausente	Ausente	Ausente	Ausente	Ausente	Ausente	Ausente	Ausente	Ausente	Ausente	Ausente	Ausente	Ausente
	**VIII** - Médio volume da cavidade em relação às demais cavidades que se distribuem na mesma unidade geomorfológica, conforme definido no § 1º do art. 14, desta Instrução Normativa;	Ausente	Presente	Presente	Presente	Presente	Presente	Presente	Presente	Presente	Presente	Presente	Presente	Presente	Presente	Presente	Presente	Presente	Presente	Presente	Presente	Presente
	**IX** - Poucos tipos de espeleotemas e processos de deposição em termos de diversidade de sedimentação química;	Presente	Presente	Presente	Presente	Presente	Presente	Presente	Presente	Presente	Presente	Presente	Presente	Ausente	Ausente	Presente	Presente	Presente	Presente	Presente	Presente	Presente
	**X** – Uso esporádico ou casual para fins educacionais, recreativos ou esportivos;	Ausente	Ausente	Ausente	Ausente	Ausente	Ausente	Ausente	Ausente	Ausente	Ausente	Ausente	Ausente	Ausente	Ausente	Ausente	Ausente	Ausente	Ausente	Ausente	Ausente	Ausente
	**XI** - Visitação pública esporádica ou casual na cavidade;	Ausente	Ausente	Ausente	Ausente	Ausente	Ausente	Ausente	Ausente	Ausente	Ausente	Ausente	Ausente	Ausente	Ausente	Ausente	Ausente	Ausente	Ausente	Ausente	Ausente	Ausente
	**XII** - Presença de água de percolação ou condensação com influência sobre os atributos da cavidade que tenham as configurações relacionadas nos incisos deste artigo; e	Ausente	Ausente	Ausente	Ausente	Ausente	Ausente	Ausente	Ausente	Ausente	Ausente	Ausente	Ausente	Ausente	Ausente	Ausente	Ausente	Ausente	Ausente	Presente	Ausente	Ausente
	**XIII** - Lago ou drenagem subterrânea intermitente com influência sobre os atributos da cavidade que tenham as configurações relacionadas nos incisos deste artigo.	Ausente	Ausente	Ausente	Ausente	Ausente	Ausente	Ausente	Ausente	Ausente	Ausente	Ausente	Ausente	Ausente	Ausente	Ausente	Ausente	Ausente	Ausente	Ausente	Ausente	Ausente
	RELEVÂNCIA FINAL	Máxima	Alta	Alta	Alta	Alta	Alta	Alta	Alta	Alta	Máxima	Alta	Alta	Alta	Alta	Máxima	Alta	Alta	Máxima	Alta	Alta	Alta

NA - Não avaliado

ANEXO B

Coordenadas em UTM SAD'69 dos pontos de coleta de acordo com o método de coleta empregado.

Winkler e Revisão de Winkler			
**Ponto**	**mE**	**mS**	**Altitude (m)**
P1W01	0646572	9348498	229
P1W02	0646569	9348500	229
P1W03	0646578	9348508	230
P1W04	0646578	9348507	230
P1W05	0646573	9348511	226
P1W06	0646577	9348516	226
P1W07	0646573	9348506	227
P1W08	0646583	9348497	231
P1W09	0646585	9348503	229
P1W10	0646580	9348504	229
P1W11	0646580	9348498	229
P1W12	0646583	9348497	227
P1W13	0646583	9348497	228
P1W14	0646576	9348492	227
P1W15	0646579	9348483	227
P1W16	0646533	9348409	222
P1W17	0646531	9348407	224
P1W18	0646531	9348407	224
P1W19	0646531	9348407	223
P1W20	0646532	9348409	223
P1W21	0646526	9348427	226
P1W22	0646526	9348427	225
P1W23	0646526	9348428	226
P1W24	0646527	9348434	223
P1W25	0646528	9348437	227
P2W26	0646646	9344461	341
P2W27	0646648	9344463	342
P2W28	0646648	9344464	342
P2W29	0646648	9344465	343
P2W30	0646649	9344465	343
P2W31	0646649	9344466	343
P2W32	0646648	9344466	342

Winkler e Revisão de Winkler			
**Ponto**	**mE**	**mS**	**Altitude (m)**
P2W51	0646737	9344517	350
P2W52	0646740	9344515	349
P2W53	0646740	9344515	347
P2W54	0646739	9344515	348
P2W55	0646739	9344515	350
P2W56	0646857	9344289	264
P2W57	0646856	9344292	263
P2W58	0646852	9344294	264
P2W59	0646853	9344298	263
P2W60	0646856	9344297	263
P2W61	0646859	9344295	263
P2W62	0646866	9344298	261
P2W63	0646868	9344297	261
P2W64	0646868	9344297	260
P2W65	0646868	9344299	259
P2W66	0646872	9344297	259
P2W67	0646871	9344295	258
P2W68	0646871	9344291	258
P2W69	0646863	9344287	258
P2W70	0646865	9344282	258
P2W71	0646854	9344279	258
P2W72	0646843	9344270	258
P2W73	0646844	9344270	258
P2W74	0646842	9344265	258
P2W75	0646861	9344291	259
P3W76	0650991	9339556	624
P3W77	0650995	9339559	624
P3W78	0650994	9339562	623
P3W79	0650992	9339566	623
P3W80	0650994	9339568	623
P3W81	0650996	9339569	622
P3W82	0651001	9339573	621

P2W33	0646645	9344469	345	P3W83	0651002	9339584	621
P2W34	0646642	9344472	346	P3W84	0651005	9339586	621
P2W35	0646641	9344476	346	P3W85	0651006	9339591	620
P2W36	0646644	9344476	346	P3W86	0651006	9339591	620
P2W37	0646644	9344476	346	P3W87	0651004	9339593	619
P2W38	0646646	9344473	345	P3W88	0651002	9339596	618
P2W39	0646649	9344473	344	P3W89	0651001	9339600	618
P2W40	0646653	9344481	348	P3W90	0651000	9339601	618
P2W41	0646652	9344481	350	P3W91	0651128	9339892	589
P2W42	0646652	9344481	349	P3W92	0651130	9339894	589
P2W43	0646653	9344480	350	P3W93	0651130	9339896	589
P2W44	0646655	9344481	349	P3W94	0651130	9339895	590
P2W45	0646656	9344481	351	P3W95	0651147	9339890	595
P2W46	0646728	9344493	357	P3W96	0651142	9339886	595
P2W47	0646726	9344495	356	P3W97	0651146	9339885	596
P2W48	0646722	9344501	357	P3W98	0651146	9339886	595
P2W49	0646726	9344508	356	P3W99	0651151	9339894	597
P2W50	0646732	9344505	355	P3W100	0651157	9339897	598

Noturna			
**Ponto**	**mE**	**mS**	**Altitude**
P1N01	0646587	9348496	213
P1N02	0646576	9348498	213
P1N03	0646570	9348484	208
P1N04	0646551	9348409	221
P1N05	0646552	9348412	225
P1N06	0646564	9348409	228
P1N07	0646536	9348435	226
P1N08	0646537	9348425	226
P1N09	0646543	9348410	225
P3N10	0651001	9339603	612
P3N11	0650999	9339572	614
P3N12	0651003	9339592	614
P3N13	0651015	9339725	600
P3N14	0651015	9339725	600
P3N15	0651015	9339725	600
P3N16	0651128	9339897	579
P3N17	0651132	9339890	586
P3N18	0651137	9339892	586
P2N19	0646654	9344478	329
P2N20	0646650	9344467	324
P2N21	0646654	9344462	326
P2N22	0646684	9344515	352
P2N23	0646684	9344515	351
P2N24	0646684	9344515	350
P2N25	0646726	9344499	345
P2N26	0646726	9344499	345
P2N27	0646739	9344517	343

Aquática			
**Ponto**	**mE**	**mS**	**Altitude**
P3A01	0651650	9338653	509
P3A02	0651650	9338653	509
P3A03	0651639	9338630	506
P3A04	0651639	9338630	506
P3A05	0649920	9340216	358
P3A06	0649920	9340216	358
P3A07	0649925	9340185	360
P3A08	0649925	9340185	360
P3A09	0649930	9340174	359
P3A10	0649930	9340174	359

ANEXO C

Táxons capturados nos métodos de coleta. X = captura positiva; (X) = presença de troglomórficos.

Táxons	Aquática	Winkler	R. Winkler	Noturna
Annelida				
Clitellata				
Oligochaeta		X		X
Hirudinea	X			
Arthropoda				
Arachnida				
Acari	X	X	X	X
AMBLYPYGI				X
ARANEAE	X	(X)	X	X
Carrapato		X		X
Opiliones		X		X
Pseudoscorpiones		X	X	X
Scorpiones				X
Entognatha				
COLLEMBOLA	X	(X)		(X)
Diplura		X		X
Insecta				
Archaeognatha		X		X
Blattodea	X	X		X
COLEOPTERA	X	(X)	X	X
Dermaptera		X		X
Diptera		X		X
Embioptera		X		X
Ephemeroptera	X			X
Hemiptera	X	X		X
Hymenoptera	X	X	X	X
Isoptera		X		X
Larva	X	X	X	X
Lepidoptera				X
Mantodea				X

Neuroptera		X		
Odonata				X
Orthoptera		X		X
Plecoptera		X		
Psocoptera		X		X
Thysanoptera	X	X		
Thysanura		X		
Trichoptera	X			
Myriapoda				
Chilopoda		X	X	X
DIPLOPODA		(X)	X	X
Pauropoda		X		X
Symphyla		X		X
Crustacea	X			
Isopoda		X		X
Mollusca				
Gastropoda				
PULMONATA		X		
Onychophora		X		
