# Supplementary material for: Reconciling Mining with the Conservation of Cave Biodiversity: A Quantitative Baseline to Help Establish Conservation Priorities
Source: PLoS One. 2016 Dec 20;11(12):e0168348. doi: 10.1371/journal.pone.0168348 (PMC5173368; doi:10.1371/journal.pone.0168348)
Supplement: S1 Dataset — (ZIP) [file pone.0168348.s002.zip › Taxa/Serra da Bocaina/Taxons inventariados_ Parte 1.pdf]

SB-0001					
**TÁXONS**				**Seca**	**Úmida**
Arachnida	Araneae	Anapidae	Anapidae sp.1	-	X
		Ctenidae	Ctenidae jovem	-	X
		Filistatidae	Filistatidae jovem	-	X
		Oonopidae	Oonopidae sp.5	-	X
		Pholcidae	*Leptopholcu* s sp.1	X	-
			Mesabolivar eberhardi	-	X
		Salticidae	Salticidae sp.3	X	-
		Scytodidae	Scytodidae jovem	X	-
		Theridiidae	Theridiidae jovem	X	-
	Pseudoscorpiones	Chernetidae	*Spelaeochernes* sp.1	-	X
	Scorpiones	Buthidae	*Ananteris luciae*	-	X
Diplopoda	Glomeridesmida	Glomeridesmidae	Glomeridesmida jovem	-	X
	Polydesmida	Fuhrmanodesmidae	Fuhrmanodesmidae sp.1	-	X
	Spirostreptida	Pseudonannolenidae	Pseudonannolenidae sp.1	X	X
Insecta	Hymenoptera	Formicidae	*Camponotus atriceps*	X	-
			Crematogaster limata	X	-
			Pachycondyla constricta	X	-
	Orthoptera	Phalangopsidae	*Paraclodes* sp.1	X	X
	Psocoptera		Psocomorpha jovem	X	-
Symphyla		Scutigerellidae	*Hanseniella* jovem	-	X
Amphibia	Anura	Strabomantidae	*Pristimantis* cf. *fenestratus*	X	X

SB-0002					
TÁXONS				Seca	Úmida
Arachnida	Acari	Ixodidae	*Amblyomma* sp.1	X	-
		Trombiculidae	Trombiculidae sp.1	-	X
			Trombiculidae sp.2	X	-
			Acariformes sp.1	-	X
			Acariformes sp.3	X	-
			Mesostigmata sp.4	-	X
			Oribatida sp.2	-	X
	Amblypygi	Charinidae	*Charinus* sp.1	-	1
		Phrynidae	*Heterophrinus longicornis*	X	X
	Araneae	Anapidae	Anapidae jovem	-	X
		Corinnidae	*Abapeba* sp.1	-	X
			Corinnidae jovem	-	X
		Ctenidae	Ctenidae jovem	-	X
		Filistatidae	Filistatidae jovem	X	-
			Filistatidae sp.1	-	X
		Ochyroceratidae	*Ochyrocera* sp.1	X	-
			Speocera sp.1	X	X
		Oonopidae	Oonopidae sp.3	X	-
			Oonopidae sp.13	-	X
		Palpimanidae	Palpimanidae sp.1	-	X
		Pholcidae	*Leptopholcus* sp.1	X	X
			Mesabolivar eberhardi	-	X
		Prodidomidae	Prodidomidae jovem	-	X
		Salticidae	Salticidae sp.1	X	-
			Salticidae sp.11	-	X
		Scytodidae	*Scytodes* sp.1	X	-
	Opiliones	Cosmetidae	Cosmetidae sp.2	X	-
			Roquettea carajas	-	X
		Gonyleptidae	Gonyleptidae sp.1	-	X
		Stygnidae	*Protimesus* sp.2	X	-
	Pseudoscorpiones	Chernetidae	*Spelaeochernes* sp.1	X	X
		Olpiidae	Olpiidae sp.1	-	X
Chilopoda	Scolopendromorpha	Scolopocryptopidae	*Newportia* jovem	-	X
	Scutigeromorpha	Pselliodidae	*Sphendononema* jovem	-	X
Diplopoda	Polydesmida	Fuhrmanodesmidae	Fuhrmanodesmidae sp.2	-	X
			Fuhrmanodesmidae sp.4	-	X
		Paradoxosomatidae	Paradoxosomatidae sp.2	-	X
			Paradoxosomatidae sp.3	-	X
		Pyrgodesmidae	Pyrgodesmidae sp.1	-	X
	Spirostreptida	Pseudonannolenidae	Pseudonannolenidae jovem	X	-
			Spirostreptida jovem	-	X
Entognatha	Collembola	Paronellidae	Paronellidae sp.1	X	-
	Diplura	Campodeidae	Campodeidae sp.1	-	X
		Japygidae	Japygidae sp.1	-	X
Insecta	Blattodea	Blaberidae	Blaberidae jovem	-	X
		Blattidae	Blattidae sp.2	X	-
		Polyphagidae	Polyphagidae jovem	X	X
			Polyphagidae sp.1	X	-
			Polyphagidae sp.2	X	-
	Coleoptera	Leiodidae	Leiodidae sp.2	-	X
		Scarabaeidae	*Canthon* sp.1	-	X
			Scybalocanthon sp.1	-	X
		Staphylinidae	Staphylininae sp.2	-	X
			Coleoptera jovem	X	X
	Diptera	Drosophilidae	Drosophilidae sp.	-	X
		Muscidae	Muscidae jovem	-	X
		Psychodidae	Phlebotominae sp.	X	-

		Tipulidae	Tipulidae sp.	-	X
	Hemiptera	Reduviidae	Reduviinae jovem	X	X
	Hymenoptera	Formicidae	*Crematogaster brasiliensis*	X	X
			Dolichoderus bispinosus	X	-
			Odontomachus meinerti	X	-
			Pachycondyla constricta	-	X
			Rogeria cf. *belti*	-	X
		Vespidae	Vespidae sp.1	X	-
	Isoptera	Termitidae	*Nasutitermes* sp.1	X	X
			Nasutitermes sp.2	X	-
			Nasutitermes sp.3	X	X
	Lepidoptera	Noctuoidea	Noctouidea sp. 1	-	X
	Neuroptera	Mantispidae	*Plega* sp.1	-	X
	Orthoptera	Phalangopsidae	*Paraclodes* sp.1	X	X
			Phalangopsis sp.1	X	X
	Psocoptera		Psocomorpha jovem	-	X
	Thysanura	Nicoletiidae	Nicoletiinae sp.1	-	X
Malacostraca	Isopoda	Armadillidae	Armadillidae sp.1	-	X
		Philosciidae	Philosciidae sp.1	-	X
Symphyla		Scolopendrellidae	*Symphylella* sp.1	-	X
Gastropoda	Pulmonata	Subulinidae	*Leptinaria* sp.1	-	X
			Leptinaria sp.3	X	-
		Systrophiidae	*Happia* sp.1	X	X
			Happia sp.4	X	-
Amphibia	Anura	Strabomantidae	*Pristimantis* cf. *fenestratus*	X	X
Mammalia	Chiroptera	Phyllostomidae	*Carollia perspicillata*	X	X
	Rodentia	Cricetidae	*Rhipidomys* sp.	-	X
Reptilia	Squamata	Gekkonidae	*Thecadactylus rapicauda*	X	-

SB-0003					
**TÁXONS**				**Seca**	**Úmida**
Arachnida	Acari	Trombiculidae	Trombiculidae sp.1	X	-
			Trombiculidae sp.2	X	-
			Acariformes sp.3	X	-
			Oribatida sp.2	X	-
	Amblypygi	Phrynidae	*Heterophrinus longicornis*	-	X
	Araneae	Ochyroceratidae	*Speocera* sp.1	-	X
		Oonopidae	Oonopidae jovem	X	-
			Oonopidae sp.5	-	X
		Pholcidae	*Mesabolivar eberhardi*	X	-
		Pisauridae	Pisauridae jovem	1	-
		Theraphosidae	Theraphosidae jovem	-	X
		Theridiosomatidae	*Plato* sp.1	X	X
		Trechaleidae	Trechaleidae jovem	-	X
	Opiliones	Cosmetidae	*Roquettea carajas*	X	X
		Sclerosomatidae	Prionostema sp.1	X	X
	Pseudoscorpiones	Chernetidae	*Spelaeochernes* sp.1	X	X
Entognatha	Collembola	Paronellidae	Paronellidae sp.1	X	X
	Diplura	Campodeidae	Campodeidae sp.1	X	-
Insecta	Blattodea	Blaberidae	Blaberidae jovem	-	X
		Blattellidae	Blattellidae sp.1	X	-
	Coleoptera	Byrrhidae	Byrrhidae sp.1	-	X
		Carabidae	Carabidae sp.1	-	X
		Staphylinidae	Pselaphinae sp.2	-	X
			Staphylininae sp.1	X	-
	Diptera	Chironomidae	Chironomidae jovem	X	-
		Drosophilidae	Drosophilidae sp.	X	-
		Psychodidae	Phlebotominae sp.	X	-
	Hemiptera	Naucoridae	Naucorinae jovem	X	-
		Reduviidae	Reduviinae jovem	-	X
		Veliidae	*Rhagovelia* sp.2	X	-
	Hymenoptera	Formicidae	*Acropyga* cf. *smithii*	X	X
			Pachycondyla constricta	X	X
	Lepidoptera	Hesperiidae	Hesperiidae sp. 1	X	-
	Orthoptera	Phalangopsidae	*Paraclodes* sp.1	X	-
			Phalangopsis sp.1	-	X
	Trichoptera		Trichoptera jovem	-	X
	Thysanura	Nicoletiidae	Nicoletiinae sp.1	X	-
Malacostraca	Isopoda	Philosciidae	Philosciidae sp.1	X	X
		Platyarthridae	Platyarthridae sp.1	X	-
			Platyarthridae sp.2	X	-
Gastropoda	Pulmonata	Systrophiidae	*Happia* sp.1	X	-
Amphibia	Anura	Leptodactylidae	*Leptodactylus* cf. *vastus*	-	X
		Strabomantidae	*Pristimantis* cf. *fenestratus*	X	-
Mammalia	Chiroptera	Furipteridae	*Furipterus horrens*	-	X
		Phyllostomidae	*Carollia perspicillata*	X	X
			Glossophaga soricina	-	X
	Rodentia	Cricetidae	*Rhipidomys* sp.	-	X
Reptilia	Squamata	Gymnophthalmidae	*Neusticurus* sp.	X	-

SB-0004					
TÁXONS				Seca	Úmida
Annelida	Haplotaxida		Haplotaxida sp.1	X	-
	Rhynchobdellida		Rhynchobdellida sp.2	-	X
Arachnida	Acari	Opilioacaridae	Opilioacaridae sp.1	-	X
			Astigmata sp.4	X	-
			Mesostigmata sp.1	X	X
			Oribatida sp.2	-	X
	Araneae	Barychelidae	Barychelidae jovem	-	X
		Corinnidae	*Abapeba* sp.1	X	X
			Corinnidae jovem	X	X
		Ctenidae	Ctenidae jovem	X	X
		Pholcidae	Pholcidae jovem	X	X
		Salticidae	Salticidae jovem	-	X
		Scytodidae	Scytodidae jovem	X	X
		Theraphosidae	Theraphosidae jovem	X	-
		Theridiidae	*Achaearanea* sp.1	-	X
			Nesticodes rufipes	X	X
	Opiliones	Manaosbiidae	Manaosbiidae sp.1	X	-
	Pseudoscorpiones	Chthoniidae	Chthoniidae sp.1	-	X
Chilopoda	Geophilomorpha	Schendylidae	*Schendylops* sp.1	-	X
	Scolopendromorpha	Scolopocryptopidae	*Tidops* sp.1	X	X
Diplopoda	Polydesmida	Fuhrmanodesmidae	Fuhrmanodesmidae sp.1	-	X
			Fuhrmanodesmidae sp.2	X	-
	Spirostreptida	Pseudonannolenidae	Pseudonannolenidae jovem	-	X
			Pseudonannolenidae sp.1	X	-
		Spirostreptidae	Spirostreptidae sp.2	X	-
Entognatha	Collembola	Cyphoderidae	Cyphoderidae sp.1	X	-
		Entomobryidae	Entomobryidae sp.5	-	X
		Paronellidae	Paronellidae sp.1	X	X
	Diplura	Campodeidae	Campodeidae sp.1	X	X
Insecta	Archaeognatha	Meinertellidae	Meinertellidae sp.1	-	X
	Blattodea	Blaberidae	Blaberidae jovem	X	X
	Coleoptera	Carabidae	Carabidae sp.1	X	-
			Carabidae sp.3	X	-
		Staphylinidae	Staphylininae sp.2	X	-
			Staphylininae sp.4	-	X
	Diptera	Drosophilidae	Drosophilidae sp.	X	X
		Psychodidae	Phlebotominae sp.	-	X
			Psychodidae sp.	X	X
		Tipulidae	Tipulidae sp.	X	-
	Hemiptera	Cixiidae	Cixiidae sp.3	X	-
		Cydnidae	Cydnidae sp.1	X	X
		Reduviidae	Reduviinae jovem	X	-
		Veliidae	Veliidae jovem	-	X
	Hymenoptera	Formicidae	*Acromyrmex octopinosus*	-	X
			Acropyga cf. *smithii*	-	X
			Camponotus atriceps	X	X
			Crematogaster erecta	X	X
			Leptogenys sp.1	-	X
			Pachycondyla constricta	X	X
			Pheidole sp.12	-	X
			Pheidole sp.3	X	-
		Vespidae	Vespidae sp.1	X	-
	Isoptera	Termitidae	*Nasutitermes* sp.1	X	-
	Lepidoptera	Tineoidea	Tineoidea sp. 8	-	X
	Orthoptera	Phalangopsidae	*Paraclodes* sp.1	X	-
			Phalangopsis sp.1	X	X
	Thysanura	Nicoletiidae	Nicoletiinae sp.1	X	-

Malacostraca	Isopoda	Armadillidae	Armadillidae sp.1	-	X
		Dubioniscidae	Dubioniscidae sp.1	-	X
		Philosciidae	Philosciidae sp.2	X	X
		Platyarthridae	Platyarthridae sp.2	-	X
			Platyarthridae sp.3	-	X
Symphyla		Scutigerellidae	*Hanseniella* sp.1	X	X
Amphibia	Anura	Bufonidae	*Rhinella* sp.	X	-
		Strabomantidae	*Pristimantis* cf. *fenestratus*	X	-
Mammalia	Chiroptera	Mormoopidae	*Pteronotus gymnonotus*	X	-
			Pteronotus parnellii	X	X
		Phyllostomidae	*Carollia perspicillata*	X	X
Reptilia	Squamata	Gekkonidae	*Thecadactylus rapicauda*	X	-

SB-0005					
TÁXONS				**Seca**	**Úmida**
Annelida	Haplotaxida	Glossoscolecidae	Rhinodrilus sp.1	-	X
			Haplotaxida sp.5	X	X
Arachnida	Acari		Mesostigmata sp.1	X	-
	Amblypygi	Phrynidae	*Heterophrinus longicornis*	X	X
	Araneae	Pholcidae	*Mesabolivar eberhardi*	-	X
	Pseudoscorpiones	Chthoniidae	Chthoniidae sp.1	X	-
Entognatha	Collembola	Entomobryidae	Entomobryidae sp.3	X	-
		Paronellidae	Paronellidae sp.1	X	X
	Diplura	Campodeidae	Campodeidae sp.1	X	-
Insecta	Blattodea	Blaberidae	Blaberidae jovem	-	X
	Coleoptera	Melyridae	Melyridae jovem	X	-
		Staphylinidae	Staphylininae sp.3	X	-
		Tenebrionidae	Tenebrionidae jovem	X	-
	Diptera	Ceratopogonidae	Ceratopogonidae jovem	X	-
		Tipulidae	Tipulidae sp.	X	X
	Hemiptera		Heteroptera jovem	X	-
	Hymenoptera	Formicidae	*Camponotus atriceps*	-	X
			Pachycondyla constricta	-	X
	Isoptera	Termitidae	*Nasutitermes* sp.2	-	X
	Lepidoptera		Lepidoptera jovem	X	-
	Orthoptera	Phalangopsidae	*Paraclodes* sp.1	X	-
	Psocoptera	Troctopsocidae	Troctopsocidae sp.1	X	-
			Trogiomorpha jovem	X	-
	Thysanura	Nicoletiidae	Nicoletiinae jovem	-	X
Malacostraca	Isopoda	Platyarthridae	Platyarthridae sp.1	X	-
Symphyla		Scutigerellidae	*Hanseniella* sp.1	-	X
Amphibia	Anura	Strabomantidae	*Pristimantis* cf. *fenestratus*	X	-
Mammalia	Chiroptera	Phyllostomidae	*Carollia perspicillata*	X	X

SB-0006					
TÁXONS				**Seca**	**Úmida**
Arachnida	Acari		Acariformes sp.4	X	-
	Amblypygi	Phrynidae	*Heterophrinus longicornis*	X	X
	Araneae	Oonopidae	Oonopidae sp.6	X	-
		Pholcidae	Pholcidae jovem	X	-
		Pisauridae	Pisauridae sp.1	X	X
		Scytodidae	Scytodidae jovem	X	-
		Theraphosidae	*Guyruita cerrado*	X	-
			Theraphosidae jovem	-	X
		Theridiosomatidae	*Plato* sp.1	X	X
	Opiliones	Cosmetidae	Cosmetidae sp.1	X	-
		Sclerosomatidae	*Prionostema* sp.1	X	-
			Sclerosomatidae jovem	X	-
		Stygnidae	*Protimesus* sp.1	X	-
	Pseudoscorpiones	Chernetidae	*Spelaeochernes* sp.1	X	X
Chilopoda	Scolopendromorpha	Scolopocryptopidae	*Dinocryptops miersii*	X	-
Entognatha	Collembola	Cyphoderidae	Cyphoderidae sp.1	X	-
		Paronellidae	Paronellidae sp.1	X	-
	Diplura	Campodeidae	Campodeidae sp.1	X	X
Insecta	Blattodea	Blaberidae	Blaberidae jovem	X	-
	Coleoptera	Staphylinidae	Pselaphinae sp.5	X	-
			Staphylininae sp.1	X	-
	Diptera	Acroceridae	Acroceridae sp.	X	-
		Cecidomyiidae	Cecidomyiidae sp.	X	-
		Ceratopogonidae	Ceratopogonidae sp.	X	-
		Chaoboridae	Chaoboridae sp.	X	-
		Dolichopodidae	Dolichopodidae sp.	-	X
		Phoridae	Phoridae sp.	X	-
		Psychodidae	Phlebotominae sp.	X	-
		Tipulidae	Tipulidae sp.	X	-
	Hemiptera	Cercopidae	Cercopidae jovem	X	-
		Cixiidae	Cixiidae sp.3	X	-
	Hymenoptera	Formicidae	*Apterostigma collare*	X	-
			Camponotus atriceps	X	-
			Dolichoderus bispinosus	X	-
			Pachycondyla constricta	X	X
			Paratrechina sp.1	X	-
			Strumigenys sp.2	-	X
	Isoptera	Termitidae	*Nasutitermes* sp.1	X	-
	Lepidoptera	Noctuoidea	Noctouidea sp. 2	X	-
	Orthoptera	Phalangopsidae	*Phalangopsis* sp.1	X	X
	Psocoptera		Psocomorpha jovem	X	-
	Thysanura	Nicoletiidae	Nicoletiinae sp.1	X	-
Malacostraca	Isopoda	Philosciidae	Philosciidae sp.1	-	X
			Philosciidae sp.2	X	-
		Scleropactidae	Scleropactidae sp.1	X	-
Symphyla		Scutigerellidae	*Hanseniella* sp.1	X	-
Amphibia	Anura	Strabomantidae	*Pristimantis* cf. *fenestratus*	X	-
Reptilia	Squamata	Gymnophthalmidae	*Neusticurus* sp.	X	-

SB-0007					
TÁXONS				Seca	Úmida
Arachnida	Acari	Argasidae	*Ornithodoros* sp.1	X	X
		Trombiculidae	Trombiculidae sp.1	-	X
			Trombiculidae sp.2	-	X
			Acariformes sp.4	-	X
			Astigmata sp.2	-	X
			Mesostigmata sp.1	X	X
			Oribatida sp.5	X	X
	Amblypygi	Phrynidae	*Heterophrinus longicornis*	X	X
	Araneae	Corinnidae	Corinnidae jovem	X	-
		Ctenidae	Ctenidae jovem	X	X
		Ochyroceratidae	Speocera sp.1	X	X
		Oonopidae	Oonopidae sp.4	X	-
		Pholcidae	*Mesabolivar aurantiacus*	X	-
			Mesabolivar eberhardi	X	-
		Pisauridae	Pisauridae jovem	-	X
		Theridiosomatidae	*Plato* sp.1	X	X
		Trechaleidae	Trechaleidae jovem	-	X
	Opiliones	Neogoveidae	*Canga renatae*	X	X
		Sclerosomatidae	*Prionostema* sp.1	X	-
			Sclerosomatidae jovem	-	X
		Stygnidae	Stygnidae sp.1	X	X
	Pseudoscorpiones	Chernetidae	*Spelaeochernes* sp.1	X	X
Chilopoda	Scutigeromorpha	Pselliodidae	*Sphendononema guildingii*	-	1
Diplopoda	Polydesmida	Fuhrmanodesmidae	Fuhrmanodesmidae jovem	X	X
		Pyrgodesmidae	Pyrgodesmidae sp.1	X	X
Entognatha	Collembola	Cyphoderidae	Cyphoderidae sp.1	X	X
		Isotomidae	Isotomidae sp.1	X	X
		Paronellidae	Paronellidae sp.4	-	X
		Sminthuroidea	*Pararrhopalites* sp.n.6	-	X
			Sminthuroidea sp.2	X	X
Insecta	Blattodea	Blattidae	Blattidae jovem	-	X
	Coleoptera	Carabidae	*Chlaenius* sp.1	X	-
		Staphylinidae	Pselaphinae sp.5	X	X
			Coproporus sp.1	X	-
			Staphylininae sp.1	-	X
			Staphylininae sp.2	-	X
	Diptera	Ceratopogonidae	Ceratopogonidae jovem	-	X
		Drosophilidae	Drosophilidae sp.	X	-
		Psychodidae	Phlebotominae sp.	-	X
			Psychodidae sp.	X	-
		Streblidae	Streblidae sp.	X	X
		Tipulidae	Tipulidae sp.	X	-
	Hemiptera	Cixiidae	Cixiidae sp.1	-	X
		Cydnidae	Cydnidae sp.1	X	X
			Cydnidae sp.2	X	X
		Reduviidae	Emesinae sp.1	X	-
			Emesinae sp.2	X	-
			Emesinae sp.6	-	X
		Veliidae	Veliidae jovem	X	X
	Hymenoptera	Formicidae	*Camponotus* sp.2	X	X
			Camponotus sp.3	X	-
			Hypoponera sp.6	X	-
			Pheidole sp.11	X	-
			Pheidole sp.3	X	X
			Solenopsis invicta	-	X
			Solenopsis sp.2	-	X
			Strumigenys calamita	-	X

	Lepidoptera	Tineoidea	Tineoidea sp. 3	X	X
			Lepidoptera jovem	X	X
	Orthoptera	Phalangopsidae	*Phalangopsis* sp.1	X	X
Malacostraca	Decapoda	Pseudothelphusidae	Pseudothelphusidae sp.1	-	X
	Isopoda	Platyarthridae	Platyarthridae sp.2	X	-
			Platyarthridae sp.3	X	X
		Scleropactidae	Scleropactidae sp.2	-	X
Gastropoda	Pulmonata	Subulinidae	*Lamellaxis* sp.1	X	X
		Systrophiidae	*Happia* sp.4	-	X
			Systrophiidae jovem	-	X
Amphibia	Anura	Bufonidae	*Rhaebo* sp.	X	X
		Strabomantidae	*Pristimantis* cf. *fenestratus*	X	-
Mammalia	Chiroptera	Furipteridae	*Furipterus horrens*	-	X
		Phyllostomidae	*Carollia perspicillata*	X	X
			Phyllostomus latifolius	X	X

SB-0008					
TÁXONS				Seca	Úmida
Arachnida	Acari	Argasidae	*Ornithodoros* sp.1	X	-
			Oribatida sp.2	-	X
			Oribatida sp.4	X	-
	Amblypygi	Phrynidae	*Heterophrinus longicornis*	X	-
	Araneae	Ctenidae	Ctenidae jovem	-	X
		Filistatidae	Filistatidae jovem	X	-
		Ochyroceratidae	Ochyroceratidae jovem	X	X
		Salticidae	Salticidae jovem	-	X
		Scytodidae	*Scytodes* sp.1	X	-
	Opiliones	Gonyleptidae	Gonyleptidae jovem	-	X
	Pseudoscorpiones	Chernetidae	*Spelaeochernes* sp.1	X	X
		Tridenchthoniidae	Tridenchthoniidae sp.2	-	X
Chilopoda	Geophilomorpha	Schendylidae	*Schendylops* sp.1	-	X
Diplopoda	Stemmiulida	Stemmiulidae	Stemmiulidae jovem	X	-
			Stemmiulidae sp.1	-	X
Entognatha	Collembola	Paronellidae	Paronellidae sp.1	X	X
Insecta	Coleoptera	Chrysomelinae	Alticini sp.2	X	-
		Staphylinidae	Pselaphinae sp.5	X	-
			Coleoptera jovem	X	X
	Diptera	Drosophilidae	Drosophilidae sp.	X	-
	Hemiptera	Cixiidae	Cixiidae jovem	-	X
			Cixiidae sp.3	X	-
	Hymenoptera	Formicidae	*Apterostigma collare*	X	-
			Pheidole sp.1	X	-
			Rogeria cf. *belti*	X	-
	Isoptera	Termitidae	*Nasutitermes* sp.1	X	-
	Lepidoptera	Tineoidea	Tineoidea sp. 8	-	X
			Lepidoptera jovem	X	-
	Orthoptera	Phalangopsidae	*Paraclodes* sp.1	X	X
			Phalangopsis sp.1	X	X
Amphibia	Anura	Strabomantidae	*Pristimantis* cf. *fenestratus*	-	X
Mammalia	Chiroptera	Furipteridae	*Furipterus horrens*	X	-

SB-0009					
TÁXONS				Seca	Úmida
Arachnida	Acari	Trombiculidae	Trombiculidae sp.2	X	-
			Acariformes sp.4	X	-
			Oribatida sp.2	X	-
	Amblypygi	Phrynidae	*Heterophrinus longicornis*	X	-
	Araneae	Araneidae	Araneidae jovem	-	X
		Corinnidae	Corinnidae jovem	-	X
		Oonopidae	Oonopidae sp.5	-	X
			Oonopidae sp.11	X	-
		Pholcidae	*Mesabolivar cambridgei*	X	-
			Mesabolivar eberhardi	-	X
	Opiliones	Cosmetidae	*Roquettea carajas*	X	-
		Stygnidae	*Protimesus* sp.1	X	-
	Pseudoscorpiones	Chernetidae	*Spelaeochernes* sp.1	X	X
		Chthoniidae	Chthoniidae sp.1	X	-
Diplopoda	Polydesmida	Fuhrmanodesmidae	Fuhrmanodesmidae jovem	X	-
Entognatha	Collembola	Cyphoderidae	Cyphoderidae sp.1	X	-
		Paronellidae	Paronellidae sp.1	X	X
	Diplura	Campodeidae	Campodeidae sp.1	X	-
Insecta	Blattodea	Blaberidae	Blaberidae jovem	X	-
	Coleoptera	Curculionidae	Scolytinae sp.3	-	X
		Staphylinidae	Staphylinidae sp.6	X	-
			Staphylininae sp.5	-	X
		Tenebrionidae	Tenebrionidae jovem	X	-
	Diptera	Cecidomyiidae	Cecidomyiidae sp.	X	-
		Conopidae	Conopidae sp.	X	-
		Drosophilidae	Drosophilidae sp.	X	X
		Psychodidae	Phlebotominae sp.	X	X
		Sarcophagidae	Sarcophagidae jovem	-	X
		Tipulidae	Tipulidae sp.	-	X
	Hemiptera	Cydnidae	Cydnidae sp.1	X	X
			Cydnidae sp.2	X	-
		Fulgoridae	Fulgoridae sp.2	-	X
		Lygaeidae	Lygaeidae jovem	X	-
		Schizopteridae	Schizopteridae sp.3	X	-
		Tingidae	Tingidae sp.1	X	-
	Hymenoptera	Diapriidae	Diapriidae sp.5	X	-
		Formicidae	*Camponotus atriceps*	X	-
			Camponotus sp.3	X	-
			Ectatomma tuberculatum	X	-
			Ochetomyrmex neopolitus	X	X
			Pachycondyla constricta	X	X
			Rogeria blanda	X	-
			Rogeria cf. *belti*	X	-
			Solenopsis invicta	X	-
			Trachymyrmex sp.1	X	-
			Wasmannia sp.1	X	-
			Wasmannia sp.3	X	-
	Isoptera	Termitidae	*Nasutitermes* sp.1	X	-
	Lepidoptera	Noctuoidea	Noctouidea sp. 2	-	X
			Noctouidea sp. 7	X	-
	Orthoptera	Phalangopsidae	*Eidmanacris* sp.1	X	-
			Paraclodes sp.1	X	X
			Phalangopsis sp.1	X	X
	Thysanura	Nicoletiidae	Nicoletiinae sp.1	X	-
Malacostraca	Isopoda	Dubioniscidae	Dubioniscidae sp.1	X	-
		Philosciidae	Philosciidae sp.1	X	-
			Philosciidae sp.2	X	-

Mammalia	Chiroptera	Phyllostomidae	*Carollia perspicillata*	X	X
			Glossophaga soricina	X	X

SB-0010					
TÁXONS				Seca	Úmida
Arachnida	Acari	Argasidae	*Ornithodoros* sp.1	X	X
		Ixodidae	*Amblyomma* sp.1	-	X
		Trombiculidae	Trombiculidae sp.1	X	X
			Trombiculidae sp.2	X	X
			Astigmata jovem	X	-
			Mesostigmata sp.1	X	X
			Oribatida sp.2	-	X
	Amblypygi	Phrynidae	*Heterophrinus longicornis*	X	X
	Araneae	Corinnidae	Corinnidae jovem	X	X
		Ochyroceratidae	Speocera sp.1	X	X
		Oonopidae	Oonopidae jovem	X	-
			Oonopidae sp.5	-	X
		Pholcidae	*Leptopholcus* sp.1	X	X
			Mesabolivar sp.1	-	X
			Ninetinae sp.1	-	X
		Salticidae	Salticidae sp.15	X	-
		Theraphosidae	*Guyruita cerrado*	X	-
			Theraphosidae jovem	-	X
		Theridiosomatidae	*Plato* sp.1	-	X
	Opiliones	Cosmetidae	*Roquettea carajas*	X	-
		Escadabiidae	Escadabiidae jovem	-	X
			Escadabiidae sp.1	X	-
		Manaosbiidae	Manaosbiidae sp.1	X	-
		Stygnidae	Stygnidae jovem	-	X
	Pseudoscorpiones	Chernetidae	*Spelaeochernes* sp.1	X	X
		Chthoniidae	Chthoniidae sp.1	X	X
Chilopoda	Geophilomorpha	Ballophilidae	*Ityphilus* sp.2	X	-
		Schendylidae	*Schendylops* sp.1	-	X
	Lithobiomorpha	Henicopidae	*Lamyctes* p.1	-	X
	Scolopendromorpha	Scolopocryptopidae	*Newportia* sp.2	-	X
	Scutigeromorpha	Pselliodidae	*Sphendononema* jovem	-	X
Diplopoda	Glomeridesmida	Glomeridesmidae	Glomeridesmida sp.1	-	X
	Polydesmida	Chelodesmidae	Chelodesmidae jovem	X	-
		Fuhrmanodesmidae	Fuhrmanodesmidae sp.1	X	-
Entognatha	Collembola	Cyphoderidae	Cyphoderidae sp.1	-	X
		Paronellidae	Paronellidae sp.1	X	X
		Sminthuroidea	Sminthuroidea sp.2	X	X
	Diplura	Campodeidae	Campodeidae sp.1	X	X
		Projapygidae	Projapygidae sp.1	X	X
Insecta	Blattodea	Blaberidae	*Blaberus* sp.1	-	X
		Polyphagidae	Polyphagidae jovem	X	X
			Polyphagidae sp.1	X	-
	Coleoptera	Carabidae	Carabidae sp.1	-	X
			Notibia sp.1	X	X
	Dermaptera	Diplatydae	Diplatydae sp.1	X	-
	Diptera	Ceratopogonidae	Ceratopogonidae sp.	X	X
		Dolichopodidae	Dolichopodidae sp.	X	-
		Drosophilidae	Drosophilidae sp.	-	X
		Psychodidae	Phlebotominae sp.	-	X
			Psychodidae sp.	X	-
	Hemiptera	Cixiidae	Cixiidae jovem	X	-
		Cydnidae	Cydnidae sp.1	X	X
			Cydnidae sp.2	-	X
		Reduviidae	*Zelurus* sp.	-	X
	Hymenoptera	Formicidae	*Camponotus* sp.2	X	X
			Crematogaster brasiliensis	X	-
			Gnamptogenys sp.1	X	X

			Pachycondyla constricta	X	X
	Isoptera		Isoptera jovem	X	X
	Lepidoptera	Noctuoidea	Noctouidea sp. 3	-	X
		Tineoidea	Tineoidea sp. 1	-	X
			Tineoidea sp. 2	-	X
			Tineoidea sp. 3	-	X
			Tineoidea sp. 4	-	X
			Lepidoptera jovem	X	X
	Orthoptera	Phalangopsidae	*Eidmanacris* sp.1	X	X
			Paraclodes sp.1	X	X
			Phalangopsis sp.1	X	X
	Psocoptera	Epipsocidae	Epipsocidae sp.1	X	-
			Psocomorpha jovem	-	X
			Trogiomorpha jovem	-	X
	Thysanura	Nicoletiidae	Atelurinae sp.1	-	X
			Nicoletiinae sp.1	X	X
Malacostraca	Isopoda	Armadillidae	Armadillidae sp.1	X	X
		Philosciidae	Philosciidae sp.2	X	X
Gastropoda	Pulmonata	Subulinidae	*Lamellaxis* sp.1	X	X
		Systrophiidae	Systrophiidae jovem	-	X
Amphibia	Anura	Strabomantidae	*Pristimantis* cf. *fenestratus*	X	X
Aves	Accipitriformes	Cathartidae	*Coragyps* sp.	X	-
Mammalia	Chiroptera	Emballonuridae	*Peropteryx kappleri*	X	X
		Phyllostomidae	*Carollia perspicillata*	X	X
			Glossophaga soricina	X	X

SB-0011					
TÁXONS				Seca	Úmida
Arachnida	Acari	Ixodidae	*Amblyomma* sp.4	X	-
		Trombiculidae	Trombiculidae sp.1	X	-
			Acariformes sp.3	-	X
			Holothyrida sp.2	X	-
			Mesostigmata sp.1	X	X
			Oribatida sp.2	-	X
	Amblypygi	Phrynidae	*Heterophrinus longicornis*	-	X
	Araneae	Corinnidae	Corinnidae jovem	X	X
		Hahniidae	Hahniidae sp.1	X	-
		Ochyroceratidae	*Speocera* sp.1	X	X
		Oonopidae	Oonopidae sp.4	X	X
		Pholcidae	*Mesabolivar cambridgei*	X	-
			Mesabolivar eberhardi	X	-
			Mesabolivar sp.1	X	X
			Ninetinae sp.1	X	-
		Salticidae	Salticidae jovem	-	X
			Salticidae sp.1	X	-
		Scytodidae	Scytodidae jovem	X	-
		Theraphosidae	Theraphosidae jovem	X	X
		Theridiidae	*Thymoites* sp.1	X	-
		Theridiosomatidae	*Plato* sp.1	-	X
	Opiliones	Cosmetidae	Cosmetidae sp.2	X	-
			Cosmetidae sp.4	X	-
			Roquettea carajas	X	-
		Gonyleptidae	Gonyleptidae sp.1	X	-
		Manaosbiidae	Manaosbiidae sp.1	X	-
		Sclerosomatidae	Sclerosomatidae jovem	X	-
		Stygnidae	*Protimesus* sp.1	-	X
	Pseudoscorpiones	Chernetidae	*Spelaeochernes* sp.1	X	X
		Chthoniidae	Chthoniidae sp.1	X	X
		Ideoroncidae	Ideoroncidae sp.1	-	X
Chilopoda	Geophilomorpha	Schendylidae	*Schendylops* sp.1	X	-
	Scutigeromorpha	Pselliodidae	Sphendononema jovem	-	X
Diplopoda	Polydesmida	Fuhrmanodesmidae	Fuhrmanodesmidae jovem	-	X
		Pyrgodesmidae	Pyrgodesmidae sp.2	-	X
	Spirostreptida	Pseudonannolenidae	Pseudonannolenidae sp.1	X	X
Entognatha	Collembola	Cyphoderidae	Cyphoderidae sp.1	X	-
		Paronellidae	Paronellidae sp.1	X	X
		Poduromorpha	Poduromorpha sp.1	-	X
		Sminthuroidea	Sminthuroidea sp.2	-	X
	Diplura	Campodeidae	Campodeidae sp.1	X	X
Insecta	Blattodea	Blattellidae	Blattellidae sp.1	-	X
	Coleoptera	Dytiscidae	*Platynectes* sp.1	-	X
	Diptera	Cecidomyiidae	Cecidomyiidae sp.	X	-
		Drosophilidae	Drosophilidae sp.	X	X
		Psychodidae	Phlebotominae sp.	-	X
		Tipulidae	Tipulidae sp.	X	-
	Hemiptera	Cixiidae	Cixiidae jovem	X	X
		Cydnidae	Cydnidae sp.1	X	X
			Cydnidae sp.2	-	X
		Ochteridae	Ochteridae jovem	-	X
		Veliidae	Veliidae jovem	-	X
	Hymenoptera	Formicidae	*Brachymyrmex* sp.1	X	-
			Camponotus sp.2	X	X
			Pachycondyla constricta	-	X
			Solenopsis invicta	X	-
	Isoptera		Isoptera jovem	X	-

	Lepidoptera	Noctuoidea	Noctouidea sp. 5	X	-
	Orthoptera	Phalangopsidae	*Eidmanacris* sp.1	X	-
			Paraclodes sp.1	X	X
			Phalangopsis sp.1	X	X
	Psocoptera	Epipsocidae	Epipsocidae sp.1	X	-
			Epipsocidae sp.2	-	X
		Troctopsocidae	Troctopsocidae sp.1	-	X
	Thysanura	Nicoletiidae	Atelurinae sp.1	X	-
			Nicoletiinae sp.1	-	X
Malacostraca	Isopoda	Armadillidae	Armadillidae sp.1	X	X
		Philosciidae	Philosciidae sp.1	X	X
			Philosciidae sp.2	X	X
Gastropoda	Pulmonata	Subulinidae	*Lamellaxis* sp.1	-	X
		Systrophiidae	*Happia* sp.1	X	-
Amphibia	Anura	Strabomantidae	*Pristimantis* cf. *fenestratus*	X	X
Mammalia	Chiroptera	Emballonuridae	*Peropteryx kappleri*	X	-
		Phyllostomidae	*Carollia perspicillata*	X	X
			Glossophaga soricina	X	X

SB-0012					
TÁXONS				Seca	Úmida
Annelida	Haplotaxida		Haplotaxida sp.2	-	X
			Haplotaxida sp.4	-	X
			Haplotaxida sp.5	X	-
Arachnida	Acari	Ixodidae	*Amblyomma* sp.1	X	X
		Trombiculidae	Trombiculidae sp.1	X	-
			Trombiculidae sp.2	X	X
			Acariformes sp.1	-	X
			Acariformes sp.4	X	-
			Holothyrida sp.1	X	X
			Mesostigmata sp.1	-	X
			Mesostigmata sp.2	X	-
			Mesostigmata sp.3	-	X
			Oribatida sp.5	X	-
	Amblypygi	Phrynidae	*Heterophrinus longicornis*	X	X
	Araneae	Araneidae	*Alpaida* sp.1	X	-
		Corinnidae	*Abapeba* sp.1	-	X
			Corinnidae jovem	X	X
		Ctenidae	Ctenidae jovem	X	X
		Ochyroceratidae	*Ochyrocera* sp.1	X	-
			Speocera sp.1	X	X
		Oonopidae	gr. *Xycarpphy* sp.1	X	-
			Oonopidae jovem	X	X
			Oonopidae sp.1	X	-
			Oonopidae sp.2	X	-
		Pholcidae	*Mesabolivar aurantiacus*	X	-
			Mesabolivar eberhardi	X	-
			Pholcidae jovem	X	X
		Salticidae	Salticidae jovem	X	-
		Scytodidae	Scytodidae jovem	X	-
		Theraphosidae	Theraphosidae jovem	-	X
		Theridiosomatidae	*Plato* sp.1	X	X
			Theridiosomatidae jovem	X	-
	Opiliones	Cosmetidae	Cosmetidae jovem	-	X
			Roquettea carajas	X	-
		Escadabiidae	Escadabiidae sp.1	X	X
		Neogoveidae	*Canga renatae*	X	X
		Sclerosomatidae	*Prionostema* sp.1	X	-
		Stygnidae	*Protimesus* sp.1	X	-
			Stygnidae sp.1	-	X
	Pseudoscorpiones	Bochicidae	Bochicidae sp.1	-	X
		Chernetidae	*Spelaeochernes* sp.1	X	X
		Chthoniidae	Chthoniidae sp.1	X	X
	Scorpiones	Buthidae	*Ananteris* jovem	X	-
Chilopoda	Scolopendromorpha	Scolopocryptopidae	*Newportia* jovem	X	X
Diplopoda	Glomeridesmida	Glomeridesmidae	Glomeridesmida sp.1	X	X
	Polydesmida	Chelodesmidae	Chelodesmidae sp.1	X	X
		Pyrgodesmidae	Pyrgodesmidae sp.1	X	X
	Spirostreptida	Pseudonannolenidae	Pseudonannolenidae sp.1	-	X
Entognatha	Collembola	Paronellidae	Paronellidae sp.1	X	X
	Diplura	Campodeidae	Campodeidae sp.1	X	X
Insecta	Blattodea	Blaberidae	*Blaberus* sp.1	X	-
			Blaberus sp.3	-	X
		Blattidae	Blattidae jovem	X	X
	Coleoptera	Chrysomelidae	Chrysomelinae sp.1	-	X
		Hydrophilidae	Hydrophilidae sp.5	-	X
		Staphylinidae	*Coproporus* sp.1	X	X
			Coproporus sp.2	-	X

			Scydmaeninae sp.2	X	X
			Staphylinidae sp.10	-	X
			Staphylinidae sp.11	-	X
			Staphylinidae sp.8	-	X
			Staphylinidae sp.9	-	X
	Diptera	Ceratopogonidae	Ceratopogonidae jovem	-	X
		Drosophilidae	Drosophilidae sp.	-	X
		Phoridae	Phoridae sp.	-	X
		Psychodidae	Phlebotominae sp.	X	X
			Psychodidae sp.	X	-
		Sciaridae	Sciaridae sp.	X	-
		Tipulidae	Tipulidae sp.	X	-
	Hemiptera	Cixiidae	Cixiidae jovem	-	X
		Cydnidae	Cydnidae sp.1	X	X
			Cydnidae sp.2	-	X
		Reduviidae	Reduviinae jovem	X	X
			Homoptera jovem	X	-
	Hymenoptera	Apidae	Apidae sp.2	-	X
		Formicidae	*Apterostigma collare*	-	X
			Brachymyrmex sp.1	X	-
			Camponotus sp.7	-	X
			Crematogaster brasiliensis	X	X
			Dolichoderus bispinosus	X	-
			Gnamptogenys sp.1	X	-
			Labidus coecus	X	-
			Pachycondyla constricta	X	X
			Paratrechina sp.1	X	X
			Pheidole sp.1	X	-
			Pheidole sp.3	-	X
			Solenopsis sp.7	X	X
			Wasmannia auropunctata	X	-
		Scelionidae	Scelionidae sp.1	-	X
		Vespidae	Vespidae sp.4	-	X
	Isoptera	Termitidae	*Nasutitermes* sp.1	X	X
			Nasutitermes sp.3	-	X
	Lepidoptera	Tineoidea	Tineoidea sp. 1	X	-
			Tineoidea sp. 3	-	X
	Orthoptera	Phalangopsidae	*Eidmanacris* sp.1	X	-
			Paraclodes sp.1	X	X
			Phalangopsis sp.1	X	X
		Tettigoniidae	*Choeroparnops* sp.1	-	X
	Psocoptera	Epipsocidae	Epipsocidae sp.1	X	-
	Thysanura	Nicoletiidae	Atelurinae sp.1	X	-
			Nicoletiinae sp.1	X	-
Malacostraca	Decapoda	Pseudothelphusidae	Pseudothelphusidae sp.1	X	X
	Isopoda	Armadillidae	Armadillidae sp.1	X	X
		Dubioniscidae	Dubioniscidae sp.1	-	X
		Philosciidae	Philosciidae sp.1	-	X
			Philosciidae sp.2	X	X
		Scleropactidae	Scleropactidae sp.2	-	X
Gastropoda	Pulmonata	Subulinidae	*Lamellaxis* sp.1	-	X
		Systrophiidae	*Happia* sp.1	X	X
Nematoda	Rhabditida		Rhabditia sp.2	X	-
Amphibia	Anura	Bufonidae	*Rhinella* sp.	X	X
		Leptodactylidae	*Leptodactylus* cf. *vastus*	-	X
		Strabomantidae	Pristimantis cf. fenestratus	X	-
			Anura sp.1	-	X
Mammalia	Chiroptera	Emballonuridae	*Peropteryx kappleri*	X	X
		Phyllostomidae	*Carollia perspicillata*	X	X

			Diphylla ecaudata	X	X
			Glossophaga soricina	X	-
			Lonchorhina aurita	X	X
			Phyllostomus latifolius	X	X
	Rodentia	Cricetidae	*Rhipidomys* sp.	-	X

SB-0013					
**TÁXONS**				**Seca**	**Úmida**
Arachnida	Acari	Ixodidae	*Amblyomma* sp.3	X	-
	Amblypygi	Phrynidae	*Heterophrinus longicornis*	-	X
	Araneae	Anyphaenidae	Anyphaenidae jovem	X	-
		Araneidae	*Alpaida* sp.1	X	-
		Barychelidae	Barychaelidae sp.1	X	-
		Corinnidae	Corinnidae jovem	X	-
		Pholcidae	*Mesabolivar eberhardi*	X	-
		Salticidae	Salticidae jovem	X	-
		Scytodidae	Scytodidae jovem	-	X
		Theraphosidae	Theraphosidae jovem	X	-
	Opiliones	Cosmetidae	*Roquettea carajas*	X	-
		Sclerosomatidae	*Prionostema* sp.1	X	X
	Pseudoscorpiones	Chernetidae	*Spelaeochernes* sp.1	X	X
		Chthoniidae	Chthoniidae sp.1	X	-
Entognatha	Diplura	Campodeidae	Campodeidae sp.1	-	X
Insecta	Coleoptera		Coleoptera jovem	-	X
	Diptera	Psychodidae	Phlebotominae sp.	X	X
	Embioptera		Embioptera jovem	X	-
	Hemiptera	Reduviidae	Reduviinae jovem	X	X
	Hymenoptera	Formicidae	*Apterostigma collare*	-	X
			Camponotus atriceps	X	-
			Crematogaster brasiliensis	-	X
			Dolichoderus bispinosus	X	-
			Hypoponera sp.1	X	-
	Lepidoptera	Tineoidea	Tineoidea sp. 7	-	X
	Orthoptera	Phalangopsidae	*Paraclodes* sp.1	X	-
Malacostraca	Isopoda	Armadillidae	Armadillidae sp.1	X	-
		Philosciidae	Philosciidae sp.1	X	-
Amphibia	Anura	Strabomantidae	*Pristimantis* cf. *fenestratus*	-	X
			Anura sp.1	-	X
Mammalia	Chiroptera	Emballonuridae	*Peropteryx kappleri*	-	X
		Phyllostomidae	*Carollia* sp.	X	-

SB-0014					
TÁXONS				Seca	Úmida
Annelida	Haplotaxida		Haplotaxida sp.4	-	X
Arachnida	Acari		Acari jovem	X	-
	Amblypygi	Phrynidae	*Heterophrinus longicornis*	-	X
	Araneae	Barychelidae	Barychaelidae sp.1	X	-
		Corinnidae	Corinnidae sp.1	X	-
		Ctenidae	Ctenidae jovem	X	X
		Ochyroceratidae	*Speocera* sp.1	-	X
		Paratropididae	Paratropididae jovem	X	-
		Pholcidae	*Mesabolivar eberhardi*	X	X
		Salticidae	Salticidae jovem	X	-
		Theraphosidae	Theraphosidae jovem	X	-
		Theridiidae	Theridiidae jovem	X	-
	Opiliones	Escadabiidae	Escadabiidae sp.1	X	-
		Neogoveidae	*Canga renatae*	-	X
	Pseudoscorpiones	Chernetidae	Chernetidae jovem	-	X
		Chthoniidae	Chthoniidae sp.1	X	-
Diplopoda	Glomeridesmida	Glomeridesmidae	Glomeridesmida jovem	X	-
Entognatha	Collembola	Sminthuroidea	Sminthuroidea sp.2	X	-
Insecta	Coleoptera		Coleoptera jovem	X	-
	Hemiptera	Reduviidae	Reduviinae jovem	X	X
	Hymenoptera	Formicidae	*Crematogaster brasiliensis*	-	X
	Orthoptera	Phalangopsidae	*Paraclodes* sp.1	X	-
			Phalangopsis sp.1	X	-
Malacostraca	Isopoda	Philosciidae	Philosciidae sp.1	X	-
			Philosciidae sp.2	-	X
Amphibia	Anura	Leptodactylidae	*Leptodactylus* cf. *vastus*	X	-
			Anura sp.1	-	X

SB-0015					
**TÁXONS**				**Seca**	**Úmida**
Arachnida	Acari	Opilioacaridae	Opilioacaridae sp.1	-	X
			Acari jovem	X	-
	Araneae	Araneidae	*Alpaida* sp.3	-	X
		Corinnidae	Corinnidae jovem	-	X
		Ctenidae	Ctenidae jovem	X	X
		Ochyroceratidae	*Speocera* sp.1	-	X
		Oonopidae	Oonopidae jovem	X	-
			Scaphiella sp.1	-	X
		Pholcidae	*Mesabolivar aurantiacus*	-	X
			Pholcidae jovem	X	-
		Symphytognathidae	*Anapistula* sp.1	X	-
		Theraphosidae	Theraphosidae jovem	X	X
	Opiliones	Sclerosomatidae	Sclerosomatidae jovem	-	X
	Pseudoscorpiones	Chernetidae	*Spelaeochernes* sp.1	-	X
		Chthoniidae	Chthoniidae jovem	X	-
Chilopoda	Scolopendromorpha	Scolopocryptopidae	*Dinocryptops miersii*	-	X
	Scutigeromorpha	Pselliodidae	*Sphendononema guildingii*	X	-
Entognatha	Collembola	Paronellidae	Paronellidae sp.1	X	-
	Diplura	Campodeidae	Campodeidae sp.1	X	-
Insecta	Blattodea	Blattidae	Blattidae jovem	-	X
	Coleoptera	Staphylinidae	Scydmaeninae sp.1	-	X
			Coleoptera jovem	X	-
	Diptera		Diptera jovem	-	X
	Hemiptera	Cixiidae	Cixiidae jovem	-	X
		Reduviidae	Reduviinae jovem	X	-
	Hymenoptera	Formicidae	*Pachycondyla constricta*	X	-
			Pachycondyla verenae	-	X
	Lepidoptera		Lepidoptera jovem	-	X
	Orthoptera	Phalangopsidae	*Paraclodes* sp.1	X	X
			Phalangopsis sp.1	X	X
	Psocoptera	Troctopsocidae	Troctopsocidae sp.1	-	X
			Psocomorpha jovem	X	X
Malacostraca	Isopoda	Armadillidae	Armadillidae sp.1	-	X
		Philosciidae	Philosciidae sp.1	X	-
Gastropoda	Pulmonata	Systrophiidae	*Happia* sp.1	-	X
Amphibia	Anura	Strabomantidae	*Pristimantis* cf. *fenestratus*	X	X
Mammalia	Chiroptera	Phyllostomidae	*Carollia perspicillata*	X	X
			Glossophaga soricina	X	X
	Rodentia	Cricetidae	*Rhipidomys* sp.	-	X

SB-0016					
TÁXONS				Seca	Úmida
Annelida	Haplotaxida		Haplotaxida sp.3	-	X
			Tubificina sp.1	-	X
Arachnida	Acari	Trombiculidae	Trombiculidae sp.1	X	X
			Trombiculidae sp.2	X	X
			Holothyrida sp.1	-	X
			Mesostigmata sp.1	-	X
	Amblypygi	Charinidae	*Charinus* sp.1	X	X
		Phrynidae	*Heterophrinus longicornis*	X	X
	Araneae	Araneidae	Araneidae jovem	X	-
		Corinnidae	*Abapeba* sp.1	-	X
			Corinnidae jovem	X	X
		Ctenidae	Ctenidae jovem	-	X
		Ochyroceratidae	*Ochyrocera* sp.1	-	X
			Speocera sp.1	X	X
		Oonopidae	Oonopidae jovem	-	X
			Oonopidae sp.4	X	-
		Paratropididae	*Paratrops* sp.1	X	-
		Pholcidae	Pholcidae jovem	X	X
		Prodidomidae	Prodidomidae jovem	-	X
		Theridiidae	Theridiidae jovem	-	X
		Theridiosomatidae	*Plato* sp.1	X	X
	Opiliones	Cosmetidae	Cosmetidae jovem	X	-
			Roquettea carajas	X	-
		Escadabiidae	Escadabiidae sp.2	X	X
		Gonyleptidae	Gonyleptidae jovem	-	X
		Neogoveidae	*Canga renatae*	X	X
		Sclerosomatidae	*Prionostema* sp.1	X	X
		Stygnidae	Stygnidae sp.1	X	-
	Pseudoscorpiones	Bochicidae	Bochicidae sp.1	X	X
		Chernetidae	*Spelaeochernes* sp.1	X	X
		Chthoniidae	Chthoniidae sp.1	X	X
		Olpiidae	Olpiidae sp.1	-	X
	Ricinulei	Ricinoididae	*Cryptocellus tarsilae*	X	-
Chilopoda	Geophilomorpha	Ballophilidae	Ityphilus sp.2	-	X
		Geophilidae	*Schizonampa* sp.1	X	X
	Scolopendromorpha	Cryptopidae	*Cryptops* sp.1	-	X
		Scolopendridae	*Otostigmus* sp.1	X	-
		Scolopocryptopidae	*Dinocryptops miersii*	-	X
			Newportia sp.2	-	X
			Newportia sp.3	X	-
			Newportia sp.5	-	X
	Scutigeromorpha	Pselliodidae	*Sphendononema guildingii*	X	X
Diplopoda	Glomeridesmida	Glomeridesmidae	Glomeridesmida sp.1	X	X
	Polydesmida	Chelodesmidae	Chelodesmidae sp.1	-	X
			Chelodesmidae sp.3	-	X
		Cyrtodesmidae	Cyrtodesmidae sp.1	-	X
		Fuhrmanodesmidae	Fuhrmanodesmidae sp.1	X	X
		Paradoxosomatidae	Paradoxosomatidae jovem	-	X
		Pyrgodesmidae	Pyrgodesmidae sp.1	X	X
	Spirostreptida	Pseudonannolenidae	Pseudonannolene sp.1	X	X
			Pseudonannolenidae sp.1	X	X
			Spirostreptida jovem	X	X
Entognatha	Collembola	Cyphoderidae	Cyphoderidae sp.1	X	X
		Entomobryidae	Entomobryidae sp.3	X	-
		Paronellidae	Paronellidae sp.1	X	X
			Paronellidae sp.4	X	X
		Sminthuroidea	Sminthuroidea sp.2	X	X

	Diplura	Campodeidae	Campodeidae sp.1	X	X
		Japygidae	Japygidae sp.1	-	X
		Projapygidae	Projapygidae sp.1	X	-
Insecta	Blattodea	Blaberidae	Blaberidae jovem	X	-
		Blattellidae	Blattellidae jovem	-	X
		Blattidae	Blattidae jovem	-	X
	Coleoptera	Carabidae	*Chlaenius* sp.1	X	-
		Elateridae	Elateridae jovem	-	X
		Scarabaeidae	Scarabaeidae jovem	-	X
		Staphylinidae	Staphylininae sp.2	X	-
			Staphylininae sp.3	-	X
		Tenebrionidae	Tenebrionidae jovem	X	X
	Diptera	Dolichopodidae	Dolichopodidae sp.	-	X
		Drosophilidae	Drosophilidae sp.	X	X
		Psychodidae	Phlebotominae sp.	X	X
			Psychodidae sp.	X	X
		Sciaridae	Sciaridae sp.	X	X
	Hemiptera	Cixiidae	Cixiidae jovem	X	X
		Cydnidae	Cydnidae sp.1	X	X
			Cydnidae sp.2	-	X
		Fulgoridae	Fulgoridae sp.3	-	X
		Reduviidae	Reduviinae jovem	-	X
	Hymenoptera	Diapriidae	Diapriidae sp.1	-	X
		Eurytomidae	Eurytomidae sp.1	-	X
		Formicidae	*Acromyrmex octopinosus*	-	X
			Aphaenogaster sp.1	-	X
			Carebara sp.11	X	X
			Crematogaster brasiliensis	-	X
			Gnamptogenys minuta	X	-
			Labidus coecus	-	X
			Octostruma iheringi	X	-
			Pachycondyla constricta	X	X
			Paratrechina sp.1	X	X
			Pheidole sp.13	-	X
			Prionopelta modesta	-	X
			Rogeria cf. *belti*	-	X
			Solenopsis invicta	X	X
			Stegomyrmex sp.1	-	X
			Strumigenys calamita	-	X
	Isoptera	Termitidae	*Nasutitermes* sp.1	-	X
	Lepidoptera		Lepidoptera jovem	-	X
	Orthoptera	Phalangopsidae	*Paraclodes* sp.1	X	-
			Phalangopsis sp.1	X	X
	Thysanura	Nicoletiidae	Atelurinae sp.1	X	X
			Nicoletiinae sp.1	X	-
Malacostraca	Decapoda	Palaemonidae	*Macrobrachium* sp.1	X	-
	Isopoda	Dubioniscidae	Dubioniscidae sp.1	X	-
		Philosciidae	Philosciidae sp.1	-	X
			Philosciidae sp.3	-	X
		Platyarthridae	Platyarthridae sp.2	X	X
			Platyarthridae sp.3	-	X
		Scleropactidae	Scleropactidae sp.1	X	X
Symphyla		Scutigerellidae	Hanseniella sp.1	X	X
Gastropoda	Pulmonata	Subulinidae	*Lamellaxis* sp.1	X	X
			Lamellaxis sp.2	X	-
			Leptinaria sp.1	X	-
		Systrophiidae	*Happia* sp.1	X	X
Amphibia	Anura	Bufonidae	*Rhinella* sp.	X	X
		Strabomantidae	*Pristimantis* cf. *fenestratus*	X	-

Mammalia	Chiroptera	Phyllostomidae	*Carollia perspicillata*	X	X
			Lonchorhina aurita	X	-

SB-0017					
TÁXONS				**Seca**	**Úmida**
Arachnida	Amblypygi	Phrynidae	*Heterophrinus longicornis*	X	X
	Araneae	Araneidae	*Alpaida* sp.4	X	-
			Araneidae jovem	X	X
		Corinnidae	*Abapeba* sp.1	-	X
		Ctenidae	Ctenidae jovem	-	X
		Ochyroceratidae	*Speocera* sp.1	X	X
		Oonopidae	Oonopidae jovem	X	-
		Pholcidae	*Mesabolivar aurantiacus*	X	-
			Mesabolivar cambridgei	X	-
			Mesabolivar eberhardi	X	-
		Salticidae	Salticidae jovem	X	-
		Scytodidae	Scytodidae jovem	X	X
		Theridiidae	*Achaearanea* sp.1	-	X
		Theridiosomatidae	*Plato* sp.1	X	X
		Uloboridae	Uloboridae jovem	X	-
	Opiliones	Cosmetidae	*Roquettea carajas*	X	X
		Sclerosomatidae	*Prionostema* sp.1	-	X
	Pseudoscorpiones	Chernetidae	*Spelaeochernes* sp.1	X	-
		Chthoniidae	Chthoniidae sp.1	X	-
Chilopoda	Scolopendromorpha	Scolopocryptopidae	*Dinocryptops miersii*	-	X
Diplopoda	Polydesmida	Chelodesmidae	Chelodesmidae sp.1	-	X
			Chelodesmidae sp.3	-	X
	Spirostreptida	Pseudonannolenidae	Pseudonannolenidae sp.1	X	X
			Spirostreptida jovem	-	X
Entognatha	Collembola	Paronellidae	Paronellidae sp.1	X	X
	Diplura	Campodeidae	Campodeidae sp.1	X	-
Insecta	Coleoptera	Staphylinidae	Staphylininae sp.1	-	X
			Coleoptera jovem	X	-
	Diptera	Ceratopogonidae	Ceratopogonidae jovem	-	X
		Phoridae	Phoridae sp.	X	X
	Hemiptera	Cixiidae	Cixiidae sp.3	X	X
		Cydnidae	Cydnidae sp.1	X	X
		Fulgoridae	Fulgoridae sp.2	-	X
	Hymenoptera	Formicidae	*Acromyrmex octopinosus*	-	X
			Crematogaster limata	-	X
			Pachycondyla constricta	X	-
			Paratrechina sp.1	-	X
			Pheidole sp.5	-	X
			Solenopsis invicta	X	-
	Isoptera	Termitidae	*Nasutitermes* sp.1	X	X
			Nasutitermes sp.2	X	-
	Orthoptera	Phalangopsidae	*Paraclodes* sp.1	X	-
			Phalangopsis sp.1	X	X
	Psocoptera	Epipsocidae	Epipsocidae sp.1	X	-
	Trichoptera	Philopotamidae	Philopotamidae sp.1	-	X
	Thysanura	Nicoletiidae	Nicoletiinae sp.1	X	X
Malacostraca	Isopoda	Philosciidae	Philosciidae sp.1	-	X
			Philosciidae sp.2	X	-
		Platyarthridae	Platyarthridae sp.2	-	X
Symphyla		Scutigerellidae	*Hanseniella* sp.1	-	X
Amphibia	Anura	Strabomantidae	*Pristimantis* cf. *fenestratus*	X	-
Mammalia	Chiroptera	Phyllostomidae	*Carollia perspicillata*	X	X
			Glossophaga soricina	X	-

SB-0018					
TÁXONS				**Seca**	**Úmida**
Arachnida	Acari	Trombiculidae	Trombiculidae sp.1	X	-
			Mesostigmata sp.1	-	X
	Araneae	Pholcidae	Pholcidae jovem	X	-
		Pisauridae	Pisauridae sp.1	X	X
		Salticidae	Salticidae sp.4	X	-
		Theridiosomatidae	*Plato* sp.1	X	-
		Trechaleidae	Trechaleidae jovem	-	X
	Opiliones	Neogoveidae	Neogoveidae jovem	-	X
		Sclerosomatidae	Sclerosomatidae jovem	X	-
Diplopoda	Polydesmida	Chelodesmidae	Chelodesmidae sp.1	-	X
			Chelodesmidae sp.3	-	X
		Fuhrmanodesmidae	Fuhrmanodesmidae sp.1	-	X
Entognatha	Collembola	Cyphoderidae	Cyphoderidae sp.1	X	-
Insecta	Coleoptera	Byrrhidae	Byrrhidae sp.1	X	-
		Hydrophilidae	Hydrophilidae sp.6	X	-
		Staphylinidae	Pselaphinae sp.2	X	-
			Staphylinidae sp.5	X	-
			Staphylininae sp.1	X	-
			Staphylininae sp.3	X	-
			Coleoptera jovem	-	X
	Diptera	Drosophilidae	Drosophilidae sp.	X	-
	Hemiptera	Cixiidae	Cixiidae jovem	X	-
		Cydnidae	Cydnidae sp.1	-	X
	Hymenoptera	Formicidae	*Acromyrmex octopinosus*	X	-
			Paratrechina sp.1	X	-
			Solenopsis invicta	X	-
	Orthoptera	Phalangopsidae	*Phalangopsis* sp.1	X	X
	Trichoptera	Philopotamidae	Philopotamidae sp.1	X	-
Malacostraca	Decapoda	Palaemonidae	*Macrobrachium* sp.1	X	X
	Isopoda	Platyarthridae	Platyarthridae sp.2	-	X
Symphyla		Scolopendrellidae	*Symphylella* sp.1	X	-
Gastropoda	Caenogastropoda	Ampullariidae	*Pomacea* sp.1	-	X
	Pulmonata	Subulinidae	*Leptinaria* sp.2	-	X
Amphibia	Anura	Bufonidae	*Rhinella* sp.	-	X
		Strabomantidae	*Pristimantis* cf. *fenestratus*	X	-
Mammalia	Chiroptera	Phyllostomidae	*Carollia perspicillata*	X	X
			Glossophaga soricina	X	-

SB-0019					
TÁXONS				Seca	Úmida
Arachnida	Acari		Astigmata sp.4	X	-
	Amblypygi	Phrynidae	*Heterophrinus longicornis*	X	X
	Araneae	Amaurobiidae	Amaurobiidae jovem	-	X
		Araneidae	*Alpaida* sp.1	-	X
		Ochyroceratidae	*Speocera* sp.1	-	X
		Pholcidae	*Mesabolivar aurantiacus*	-	X
			Pholcidae jovem	X	-
		Theridiosomatidae	*Plato* sp.1	-	X
	Opiliones	Cosmetidae	*Roquettea carajas*	X	X
	Pseudoscorpiones	Chernetidae	*Spelaeochernes* sp.1	X	-
		Chthoniidae	Chthoniidae sp.1	-	X
Diplopoda	Polydesmida	Chelodesmidae	Chelodesmidae sp.3	-	X
	Spirostreptida	Pseudonannolenidae	Pseudonannolenidae jovem	X	-
Entognatha	Collembola	Paronellidae	Paronellidae sp.1	X	X
Insecta	Coleoptera	Staphylinidae	Staphylininae sp.3	X	-
	Diptera	Cecidomyiidae	Cecidomyiidae sp.	X	-
		Dolichopodidae	Dolichopodidae sp.	-	X
		Phoridae	Phoridae sp.	X	-
	Hemiptera	Cixiidae	Cixiidae jovem	-	X
		Fulgoridae	Fulgoridae sp.1	-	X
			Fulgoridae sp.3	-	X
		Reduviidae	Reduviinae jovem	-	1
	Hymenoptera	Formicidae	*Apterostigma auriculatum*	X	-
			Camponotus sp.8	-	X
			Crematogaster brasiliensis	X	-
			Pachycondyla constricta	X	-
			Paratrechina sp.1	-	X
			Pheidole sp.1	X	-
	Lepidoptera		Lepidoptera jovem	X	X
	Orthoptera	Phalangopsidae	*Phalangopsis* sp.1	X	X
Amphibia	Anura	Bufonidae	*Rhinella* sp.	-	X
		Strabomantidae	*Pristimantis* cf. *fenestratus*	X	X
Mammalia	Chiroptera	Phyllostomidae	*Carollia perspicillata*	X	-

SB-0020					
TÁXONS				Seca	Úmida
Annelida	Haplotaxida	Glossoscolecidae	*Rhinodrilus* sp.1	-	X
Arachnida	Acari	Trombiculidae	Trombiculidae sp.1	-	X
			Acariformes sp.3	-	X
			Acariformes sp.4	-	X
			Astigmata sp.1	-	X
			Mesostigmata sp.1	-	X
			Mesostigmata sp.2	X	-
			Mesostigmata sp.5	-	X
	Amblypygi	Phrynidae	*Heterophrinus longicornis*	X	X
	Araneae	Araneidae	*Alpaida* sp.1	X	X
			Araneidae jovem	X	X
		Corinnidae	Corinnidae jovem	-	X
		Ctenidae	Ctenidae jovem	-	X
		Pholcidae	*Leptopholcus* sp.1	-	X
			Mesabolivar aurantiacus	-	X
			Mesabolivar eberhardi	X	-
		Salticidae	Salticidae sp.1	-	X
		Scytodidae	Scytodidae jovem	-	X
		Theraphosidae	*Guyruita cerrado*	X	X
		Theridiidae	Theridiidae sp.3	X	-
		Theridiosomatidae	*Plato* sp.1	-	X
	Opiliones	Gonyleptidae	Gonyleptidae jovem	-	X
	Pseudoscorpiones	Chernetidae	*Spelaeochernes* sp.1	X	X
		Chthoniidae	Chthoniidae sp.1	X	X
Entognatha	Collembola	Paronellidae	Paronellidae sp.1	-	X
	Diplura	Campodeidae	Campodeidae sp.1	-	X
Insecta	Coleoptera	Tenebrionidae	Tenebrionidae jovem	-	X
	Diptera	Cecidomyiidae	Cecidomyiidae sp.	X	X
		Ceratopogonidae	Ceratopogonidae jovem	-	X
		Psychodidae	Phlebotominae sp.	X	X
	Hemiptera	Cixiidae	Cixiidae jovem	X	X
		Cydnidae	Cydnidae sp.1	X	X
		Reduviidae	Reduviinae jovem	X	-
	Hymenoptera	Formicidae	*Camponotus* sp.2	-	X
			Camponotus sp.3	X	X
			Crematogaster limata	X	X
			Pachycondyla constricta	-	X
			Pheidole sp.4	-	X
			Pheidole sp.5	X	X
			Pogonomyrmex sp.1	X	-
	Isoptera	Termitidae	*Nasutitermes* sp.1	X	X
	Orthoptera	Phalangopsidae	*Paraclodes* sp.1	X	-
			Phalangopsis sp.1	X	X
	Psocoptera		Psocomorpha jovem	X	X
Malacostraca	Decapoda	Pseudothelphusidae	Pseudothelphusidae sp.1	-	X
	Isopoda	Philosciidae	Philosciidae sp.1	-	X
Gastropoda	Pulmonata	Systrophiidae	*Happia* sp.4	X	-
Amphibia	Anura	Strabomantidae	*Pristimantis* cf. *fenestratus*	X	-
Mammalia	Chiroptera	Emballonuridae	*Peropteryx kappleri*	X	X
		Phyllostomidae	*Carollia perspicillata*	-	X
Reptilia	Squamata	Gymnophthalmidae	*Neusticurus* sp.	X	-

SB-0021					
TÁXONS				Seca	Úmida
Annelida	Haplotaxida		Haplotaxida jovem	-	X
Arachnida	Acari		Mesostigmata sp.1	X	-
	Amblypygi	Phrynidae	*Heterophrinus longicornis*	-	X
	Araneae	Araneidae	Araneidae jovem	X	X
		Corinnidae	Corinnidae jovem	-	X
		Ctenidae	Ctenidae jovem	X	-
		Linyphiidae	Linyphiidae sp.1	X	-
		Ochyroceratidae	*Speocera* sp.1	X	-
		Pholcidae	*Mesabolivar aurantiacus*	-	X
			Ninetinae sp.1	X	-
		Salticidae	Salticidae jovem	X	X
			Salticidae sp.3	X	-
			Salticidae sp.4	X	-
			Salticidae sp.6	X	-
		Scytodidae	*Scytodes* sp.1	X	X
		Theraphosidae	Theraphosidae jovem	-	X
		Theridiidae	Theridiidae jovem	-	X
	Opiliones	Cosmetidae	Cosmetidae sp.2	X	-
		Escadabiidae	Escadabiidae sp.3	X	-
		Sclerosomatidae	Sclerosomatidae jovem	-	X
		Stygnidae	*Protimesus* sp.2	X	-
	Pseudoscorpiones	Chernetidae	*Spelaeochernes* sp.1	X	X
		Chthoniidae	Chthoniidae sp.1	X	-
Entognatha	Diplura	Campodeidae	Campodeidae sp.1	-	X
Insecta	Blattodea	Polyphagidae	Polyphagidae sp.1	X	X
	Coleoptera	Elateridae	Elateridae jovem	-	X
		Staphylinidae	Scydmaeninae sp.2	X	-
	Diptera	Ceratopogonidae	Ceratopogonidae jovem	-	X
		Drosophilidae	Drosophilidae sp.	X	-
		Psychodidae	Phlebotominae sp.	-	X
		Tipulidae	Tipulidae sp.	-	X
	Hemiptera	Cixiidae	Cixiidae jovem	-	X
		Reduviidae	Reduviinae jovem	X	X
	Hymenoptera	Formicidae	*Pachycondyla constricta*	X	-
			Pheidole sp.1	X	-
	Isoptera	Rhinotermitidae	*Heterotermes* sp.1	-	X
	Lepidoptera	Hesperiidae	Hesperiidae sp. 1	-	X
		Noctuoidea	Noctouidea sp. 1	-	X
			Lepidoptera jovem	-	X
	Neuroptera	Myrmeleontidae	Myrmeleontidae sp.1	X	X
	Orthoptera	Phalangopsidae	*Paraclodes* sp.1	X	X
			Phalangopsis sp.1	-	X
	Psocoptera	Archipsocidae	Archipsocidae sp.2	X	-
		Psyllipsocidae	Psyllipsocidae sp.4	-	X
			Psocomorpha jovem	X	X
Malacostraca	Isopoda	Armadillidae	Armadillidae sp.1	X	X
		Dubioniscidae	Dubioniscidae sp.1	X	-
		Platyarthridae	Platyarthridae sp.3	-	X
Mammalia	Chiroptera	Emballonuridae	*Peropteryx* sp.	-	X
	Rodentia	Cricetidae	*Rhipidomys* sp.	-	X

SB-0022					
**TÁXONS**				**Seca**	**Úmida**
Arachnida	Acari	Opilioacaridae	Opilioacaridae sp.1	-	X
			Astigmata sp.1	X	X
			Oribatida sp.2	-	X
	Amblypygi	Phrynidae	*Heterophrinus longicornis*	X	-
	Araneae	Corinnidae	Corinnidae jovem	-	X
		Ctenidae	Ctenidae jovem	-	X
		Ochyroceratidae	Ochyroceratidae jovem	X	X
		Oonopidae	Oonopidae sp.8	X	-
			Oonopidae sp.11	-	X
		Pholcidae	*Mesabolivar cambridgei*	X	-
			Pholcidae jovem	X	X
		Salticidae	Salticidae jovem	X	-
		Symphytognathidae	Symphytognathidae jovem	-	X
		Theraphosidae	Theraphosidae jovem	X	-
		Theridiosomatidae	*Plato* sp.1	-	X
	Opiliones	Cosmetidae	*Roquettea carajas*	X	-
	Pseudoscorpiones	Chernetidae	*Spelaeochernes* sp.1	-	X
		Chthoniidae	Chthoniidae sp.1	X	-
Diplopoda	Polydesmida	Fuhrmanodesmidae	Fuhrmanodesmidae sp.4	-	X
Entognatha	Collembola	Paronellidae	Paronellidae sp.1	-	X
	Diplura	Campodeidae	Campodeidae sp.1	X	-
		Projapygidae	Projapygidae sp.1	-	X
Insecta	Blattodea	Blaberidae	Blaberidae jovem	X	-
	Diptera	Psychodidae	Phlebotominae sp.	X	-
	Hemiptera	Cydnidae	Cydnidae sp.1	X	X
		Fulgoridae	Fulgoridae sp.3	-	X
		Schizopteridae	Schizopteridae sp.3	X	-
		Tingidae	Tingidae sp.1	X	-
	Hymenoptera	Formicidae	*Camponotus melanoticus*	X	X
			Pachycondyla constricta	-	X
	Isoptera	Termitidae	*Coatitermes* sp.1	-	X
			Nasutitermes sp.1	-	X
			Nasutitermes sp.2	X	-
			Nasutitermes sp.3	X	-
	Lepidoptera	Noctuoidea	Noctouidea sp. 2	X	-
			Lepidoptera jovem	-	X
	Orthoptera	Phalangopsidae	*Paraclodes* sp.1	X	-
			Phalangopsis sp.1	-	X
	Psocoptera		Psocomorpha jovem	X	-
Malacostraca	Isopoda	Dubioniscidae	Dubioniscidae sp.1	X	-
		Philosciidae	Philosciidae sp.1	-	X
			Philosciidae sp.2	X	-
		Platyarthridae	Platyarthridae sp.2	X	-
Gastropoda	Pulmonata	Systrophiidae	Systrophiidae jovem	-	X
Amphibia	Anura	Strabomantidae	*Pristimantis* cf. *fenestratus*	X	-
Mammalia	Rodentia	Cricetidae	*Rhipidomys* sp.	X	-

SB-0023					
TÁXONS				Seca	Úmida
Arachnida	Acari	Argasidae	*Ornithodoros* sp.1	X	-
		Trombiculidae	Trombiculidae sp.1	-	X
			Acari jovem	X	-
			Oribatida sp.2	-	X
			Oribatida sp.5	-	X
	Amblypygi	Phrynidae	*Heterophrinus longicornis*	X	-
	Araneae	Ctenidae	Ctenidae jovem	X	-
		Ochyroceratidae	Ochyroceratidae jovem	X	-
			Speocera sp.1	-	X
		Pholcidae	*Leptopholcus* sp.1	-	X
		Salticidae	Salticidae jovem	X	-
		Theraphosidae	Theraphosidae sp.1	-	X
	Opiliones	Stygnidae	*Protimesus* sp.2	X	-
	Pseudoscorpiones	Chernetidae	*Spelaeochernes* sp.1	-	X
Diplopoda	Glomeridesmida	Glomeridesmidae	Glomeridesmida sp.1	-	X
	Polydesmida	Pyrgodesmidae	Pyrgodesmidae sp.1	X	X
Entognatha	Collembola	Paronellidae	Paronellidae sp.1	X	-
Insecta	Diptera	Cecidomyiidae	Cecidomyiidae sp.	-	X
		Drosophilidae	Drosophilidae sp.	X	X
	Hymenoptera	Formicidae	*Camponotus melanoticus*	X	X
			Dolichoderus bispinosus	-	X
			Pachycondyla constricta	X	-
	Isoptera	Termitidae	*Nasutitermes* sp.3	X	X
	Orthoptera	Phalangopsidae	*Eidmanacris* sp.1	X	-
			Paraclodes sp.1	X	-
			Phalangopsis sp.1	X	X
	Psocoptera	Psyllipsocidae	Psyllipsocidae sp.6	-	X
Malacostraca	Isopoda	Armadillidae	Armadillidae sp.1	X	-
		Philosciidae	Philosciidae sp.2	X	X
		Platyarthridae	Platyarthridae sp.3	-	X
Amphibia	Anura	Strabomantidae	*Pristimantis* cf. *fenestratus*	X	-

SB-0024					
TÁXONS				Seca	Úmida
Arachnida	Acari		Astigmata sp.1	X	-
	Araneae	Ochyroceratidae	Ochyroceratidae jovem	-	X
		Oonopidae	Oonopidae sp.9	X	-
		Philodromidae	Philodromidae jovem	X	-
		Pholcidae	*Leptopholcus* sp.1	-	X
		Salticidae	Salticidae jovem	X	X
		Scytodidae	Scytodidae jovem	X	X
		Theraphosidae	Theraphosidae jovem	X	-
		Theridiidae	*Nesticodes rufipes*	-	X
	Opiliones	Neogoveidae	*Canga renatae*	-	X
	Pseudoscorpiones	Chernetidae	*Spelaeochernes* sp.1	X	X
		Chthoniidae	Chthoniidae sp.1	X	-
Chilopoda	Scolopendromorpha	Scolopocryptopidae	*Newportia* sp.2	X	-
Entognatha	Collembola	Paronellidae	Paronellidae sp.1	X	-
	Diplura	Campodeidae	Campodeidae sp.1	-	X
Insecta	Blattodea	Polyphagidae	Polyphagidae jovem	X	-
	Coleoptera		Coleoptera jovem	-	X
	Diptera	Drosophilidae	Drosophilidae sp.	-	X
	Hemiptera	Cydnidae	Cydnidae sp.1	-	X
	Hymenoptera	Formicidae	*Gnamptogenys* sp.1	-	X
			Pheidole sp.4	X	-
			Pheidole sp.5	-	X
	Lepidoptera	Tineoidea	Tineoidea sp. 5	-	X
			Tineoidea sp. 8	X	-
	Neuroptera	Myrmeleontidae	Myrmeleontidae sp.1	-	X
	Orthoptera	Phalangopsidae	*Paraclodes* sp.1	X	-
			Phalangopsis sp.1	X	X
Mammalia	Chiroptera	Emballonuridae	*Peropteryx* sp.	-	X
		Phyllostomidae	*Carollia* sp.	-	X

SB-0025					
TÁXONS				Seca	Úmida
Annelida	Haplotaxida		Haplotaxida sp.4	X	-
			Haplotaxida sp.7	-	X
Arachnida	Acari	Argasidae	*Ornithodoros* sp.1	X	X
		Ixodidae	*Amblyomma* sp.1	-	X
		Trombiculidae	Trombiculidae sp.1	X	X
			Trombiculidae sp.2	X	-
			Acari jovem	X	-
			Astigmata sp.2	X	X
			Astigmata sp.4	X	-
			Holothyrida sp.2	X	X
			Mesostigmata sp.1	X	X
			Mesostigmata sp.2	X	-
			Mesostigmata sp.5	-	X
			Oribatida sp.2	X	X
	Amblypygi	Phrynidae	*Heterophrinus longicornis*	X	X
	Araneae	Araneidae	Araneidae jovem	-	X
		Corinnidae	Corinnidae jovem	X	X
		Ctenidae	Ctenidae jovem	-	X
		Drymusidae	Drymusidae jovem	-	X
		Mysmenidae	Mysmenidae jovem	-	X
			Mysmenidae sp.2	X	-
		Ochyroceratidae	*Ochyrocera* sp.3	-	X
			Speocera sp.1	X	X
		Oonopidae	Oonopidae sp.3	X	-
		Pholcidae	*Leptopholcus* sp.1	-	X
			Pholcidae jovem	X	-
		Salticidae	Salticidae jovem	-	X
			Salticidae sp.4	X	-
		Scytodidae	*Scytodes* sp.1	X	X
		Theridiosomatidae	Theridiosomatidae jovem	X	X
	Opiliones	Manaosbiidae	Manaosbiidae sp.1	X	-
		Neogoveidae	*Canga renatae*	X	X
		Stygnidae	*Protimesus* sp.2	X	-
	Pseudoscorpiones	Chernetidae	*Spelaeochernes* sp.1	X	X
		Chthoniidae	Chthoniidae sp.1	X	X
Diplopoda	Glomeridesmida	Glomeridesmidae	Glomeridesmida sp.1	X	X
	Polydesmida	Pyrgodesmidae	Pyrgodesmidae sp.1	X	X
Entognatha	Collembola	Cyphoderidae	Cyphoderidae sp.1	X	X
		Entomobryidae	Entomobryidae sp.6	-	X
		Isotomidae	Isotomidae sp.1	X	-
		Paronellidae	Paronellidae sp.1	X	X
		Sminthuroidea	Sminthuroidea sp.2	X	X
	Diplura	Campodeidae	Campodeidae sp.1	X	X
Insecta	Blattodea	Blaberidae	Blaberidae jovem	X	X
		Polyphagidae	Polyphagidae jovem	-	X
	Coleoptera	Curculionidae	Scolytinae sp.1	X	-
		Tenebrionidae	Tenebrionidae jovem	-	X
	Diptera	Acroceridae	Acroceridae sp.	X	-
		Ceratopogonidae	Ceratopogonidae jovem	-	X
		Chironomidae	Chironomidae jovem	-	X
		Culicidae	Culicinae jovem	-	X
		Dolichopodidae	Dolichopodidae sp.	X	-
		Drosophilidae	Drosophilidae sp.	X	X
		Psychodidae	Phlebotominae sp.	X	X
			Psychodidae sp.	X	X
		Sciaridae	Sciaridae sp.	X	-
		Streblidae	Streblidae sp.	-	X

	Hemiptera	Cydnidae	Cydnidae sp.1	X	X
		Veliidae	*Paravelia* sp.1	X	X
	Hymenoptera	Formicidae	*Camponotus* sp.3	X	X
			Dolichoderus bispinosus	X	X
			Labidus sp.1	X	-
			Myrmicocrypta spinosa	-	X
			Pachycondyla constricta	X	X
			Pheidole sp.7	X	X
			Rogeria cf. *belti*	-	X
			Solenopsis invicta	X	X
			Solenopsis sp.2	X	-
	Isoptera	Rhinotermitidae	*Heterotermes* sp.1	-	X
		Termitidae	*Nasutitermes* sp.1	-	X
	Lepidoptera	Noctuoidea	Noctouidea sp. 10	-	X
		Tineoidea	Tineoidea sp. 3	X	-
			Tineoidea sp. 5	-	X
			Tineoidea sp. 6	X	-
	Orthoptera	Phalangopsidae	*Paraclodes* sp.1	X	-
			Phalangopsis sp.1	X	X
	Psocoptera	Pseudocaeciliidae	Pseudocaeciliidae sp.3	-	X
		Psyllipsocidae	Psyllipsocidae sp.3	-	X
		Troctopsocidae	Troctopsocidae sp.1	-	X
			Psocomorpha jovem	X	X
	Thysanura	Nicoletiidae	Nicoletiinae sp.1	X	X
Malacostraca	Isopoda	Armadillidae	Armadillidae sp.1	X	X
		Philosciidae	Philosciidae sp.1	X	X
			Philosciidae sp.2	X	-
		Scleropactidae	Scleropactidae sp.1	-	X
Amphibia	Anura	Bufonidae	*Rhinella* sp.	X	X
		Leptodactylidae	*Leptodactylus* cf. *vastus*	X	X
		Strabomantidae	*Pristimantis* cf. *fenestratus*	X	-
Mammalia	Chiroptera	Emballonuridae	*Peropteryx kappleri*	X	X
		Furipteridae	*Furipterus horrens*	X	-
		Phyllostomidae	*Carollia perspicillata*	X	X
			Glossophaga soricina	X	X
			Phyllostomus latifolius	X	-
Reptilia	Squamata	Boidae	*Epicrates* sp.	X	-

SB-0026					
TÁXONS				Seca	Úmida
Annelida	Haplotaxida		Haplotaxida sp.2	X	X
			Haplotaxida sp.3	-	X
			Haplotaxida sp.4	X	X
			Haplotaxida sp.5	X	-
			Haplotaxida sp.8	-	X
			Tubificina sp.1	X	-
			Tubificina sp.3	-	X
Arachnida	Acari	Argasidae	*Ornithodoros* sp.1	X	-
		Ixodidae	*Amblyomma* sp.1	X	-
		Trombiculidae	Trombiculidae sp.1	-	X
			Trombiculidae sp.2	X	-
			Acariformes sp.4	X	X
			Astigmata sp.1	X	X
			Astigmata sp.4	X	-
			Holothyrida sp.1	X	-
			Holothyrida sp.2	X	X
			Holothyrida sp.6	-	X
			Mesostigmata sp.1	X	X
			Mesostigmata sp.5	-	X
	Amblypygi	Phrynidae	*Heterophrinus longicornis*	X	X
	Araneae	Araneidae	*Alpaida* sp.1	X	-
			Alpaida sp.2	X	-
			Araneidae jovem	X	X
		Corinnidae	*Abapeba* sp.1	X	X
			Corinnidae jovem	X	X
		Ctenidae	Ctenidae jovem	X	X
		Ochyroceratidae	*Speocera* sp.1	X	-
		Oonopidae	Oonopidae jovem	X	-
			Oonopidae sp.1	-	X
		Pholcidae	*Mesabolivar aurantiacus*	X	X
			Mesabolivar cambridgei	X	-
		Pisauridae	Pisauridae sp.1	X	X
		Salticidae	Salticidae sp.4	X	-
		Theraphosidae	Theraphosidae jovem	-	X
		Theridiidae	*Episinus* sp.1	X	-
		Theridiosomatidae	*Plato* sp.1	X	X
		Trechaleidae	Rhoicinus sp.1	X	-
			Trechaleidae jovem	-	X
	Opiliones	Cosmetidae	Cosmetidae sp.3	X	-
			Roquettea carajas	X	X
		Escadabiidae	Escadabiidae sp.1	X	-
		Gonyleptidae	Gonyleptidae sp.1	X	-
		Neogoveidae	*Canga renatae*	X	X
		Sclerosomatidae	Sclerosomatidae jovem	X	-
	Pseudoscorpiones	Chernetidae	*Spelaeochernes* sp.1	X	X
		Chthoniidae	Chthoniidae sp.1	X	X
	Ricinulei	Ricinoididae	Ricinoididae jovem	-	X
Chilopoda	Geophilomorpha	Ballophilidae	*Ityphilus* sp.1	X	-
	Scolopendromorpha	Scolopocryptopidae	*Newportia* sp.2	X	X
Diplopoda	Glomeridesmida	Glomeridesmidae	Glomeridesmida sp.1	X	X
	Polydesmida	Chelodesmidae	Chelodesmidae sp.3	-	X
		Fuhrmanodesmidae	Fuhrmanodesmidae sp.1	X	X
		Pyrgodesmidae	Pyrgodesmidae sp.1	-	X
	Spirostreptida	Pseudonannolenidae	Pseudonannolenidae sp.1	X	X
Entognatha	Collembola	Cyphoderidae	Cyphoderidae sp.1	X	X
		Isotomidae	Isotomidae sp.1	X	X
		Paronellidae	Paronellidae sp.1	X	X

		Poduromorpha	Poduromorpha sp.1	X	-
	Diplura	Campodeidae	Campodeidae sp.1	X	-
Insecta	Blattodea	Blattidae	Blattidae jovem	-	X
	Coleoptera	Carabidae	Carabidae sp.1	X	-
			Lelis sp.1	X	-
		Curculionidae	Scolytinae sp.1	X	-
			Scolytinae sp.2	X	-
		Elateridae	Elateridae jovem	-	X
			Elateridae sp.3	X	-
		Staphylinidae	*Coproporus* sp.1	X	X
			Coproporus sp.2	X	-
			Staphylininae sp.1	-	X
		Tenebrionidae	Tenebrionidae jovem	-	X
	Diptera	Cecidomyiidae	Cecidomyiidae sp.	X	-
		Ceratopogonidae	Ceratopogonidae sp.	-	X
		Dolichopodidae	Dolichopodidae sp.	-	X
		Drosophilidae	Drosophilidae sp.	X	X
		Limoniidae	Limoniidae sp.	X	-
		Muscidae	Muscidae jovem	-	X
		Phoridae	Phoridae sp.	-	X
		Psychodidae	Psychodidae sp.	-	X
		Sciaridae	Sciaridae sp.	X	X
		Streblidae	Streblidae sp.	X	X
		Tipulidae	Tipulidae jovem	-	X
	Hemiptera	Cydnidae	Cydnidae sp.1	X	X
			Cydnidae sp.2	X	X
		Fulgoridae	Fulgoridae sp.3	-	X
		Tingidae	Tingidae sp.1	X	-
		Veliidae	*Rhagovelia* jovem	X	X
	Hymenoptera	Diapriidae	Diapriidae sp.1	X	-
			Diapriidae sp.5	X	X
			Diapriidae sp.8	X	-
		Eurytomidae	Eurytomidae sp.1	-	X
		Formicidae	*Acanthostichus* sp.1	X	-
			Acromyrmex octopinosus	X	-
			Apterostigma collare	X	-
			Brachymyrmex sp.1	X	-
			Camponotus renggeri	X	-
			Carebara sp.2	X	-
			Crematogaster brasiliensis	-	X
			Gnamptogenys haenschi	X	X
			Hypoponera sp.5	-	X
			Labidus coecus	X	-
			Pachycondyla constricta	X	X
			Paratrechina sp.1	X	X
			Rogeria cf. *belti*	-	X
			Solenopsis invicta	X	X
			Solenopsis sp.2	-	X
			Solenopsis sp.11	-	X
			Strumigenys calamita	-	X
	Isoptera	Termitidae	*Nasutitermes* sp.1	X	-
			Nasutitermes sp.2	X	-
			Nasutitermes sp.3	X	-
	Lepidoptera	Tineoidea	Tineoidea sp. 1	-	X
			Tineoidea sp. 3	X	X
	Orthoptera	Phalangopsidae	*Paraclodes* sp.1	X	-
			Phalangopsis sp.1	X	X
	Thysanura	Nicoletiidae	Atelurinae sp.1	-	X
			Nicoletiinae sp.1	X	-

Malacostraca	Decapoda	Palaemonidae	*Macrobrachium* sp.1	X	X
	Isopoda	Dubioniscidae	Dubioniscidae sp.1	X	-
		Philosciidae	Philosciidae sp.1	-	X
			Philosciidae sp.2	X	X
		Platyarthridae	Platyarthridae sp.3	-	X
		Scleropactidae	Scleropactidae sp.1	-	X
Symphyla		Scutigerellidae	*Hanseniella* sp.1	X	X
			Scutigerellidae jovem	X	X
Gastropoda	Caenogastropoda	Ampullariidae	*Pomacea* sp.1	X	X
	Pulmonata	Subulinidae	*Lamellaxis* sp.1	X	X
			Leptinaria sp.1	-	X
		Systrophiidae	*Happia* sp.1	X	X
Turbellaria	Tricladida		Continenticola sp.1	-	X
Amphibia	Anura	Bufonidae	*Rhinella* sp.	-	X
		Strabomantidae	*Pristimantis* cf. *fenestratus*	X	X
Mammalia	Chiroptera	Mormoopidae	*Pteronotus parnellii*	X	-
		Phyllostomidae	*Carollia perspicillata*	X	X
			Lionycteris spurrelli	-	X
			Lonchorhina aurita	X	X
			Phyllostomus latifolius	X	X
			Trachops cirrhosus	X	-

SB-0027					
TÁXONS				Seca	Úmida
Arachnida	Araneae	Pholcidae	*Mesabolivar aurantiacus*	-	X
			Mesabolivar eberhardi	X	-
		Pisauridae	Pisauridae sp.1	-	X
		Theraphosidae	Theraphosidae sp.5	-	X
		Theridiosomatidae	*Plato* sp.1	-	X
	Opiliones	Cosmetidae	*Roquettea carajas*	-	X
Diplopoda	Polydesmida	Chelodesmidae	Chelodesmidae sp.3	-	X
Insecta	Hemiptera	Cixiidae	Cixiidae sp.4	X	-
		Veliidae	*Rhagovelia* sp.4	-	X
			Veliidae jovem	X	-
	Orthoptera	Phalangopsidae	*Phalangopsis* sp.1	-	X
Malacostraca	Decapoda	Palaemonidae	*Macrobrachium* sp.1	X	-

SB-0028					
TÁXONS				Seca	Úmida
Arachnida	Acari	Opilioacaridae	Opilioacaridae sp.1	-	X
			Acariformes sp.1	-	X
			Holothyrida sp.1	-	X
			Oribatida sp.2	-	X
	Araneae	Araneidae	Araneidae jovem	-	X
		Ochyroceratidae	Ochyroceratidae jovem	X	-
		Oonopidae	Oonopidae jovem	-	X
		Pholcidae	*Mesabolivar aurantiacus*	X	-
		Pisauridae	Pisauridae jovem	X	-
		Salticidae	Salticidae jovem	-	X
		Scytodidae	Scytodidae jovem	X	X
	Opiliones	Cosmetidae	Cosmetidae sp.3	X	-
			Roquettea carajas	X	X
		Escadabiidae	Escadabiidae sp.2	X	-
	Pseudoscorpiones	Chernetidae	*Spelaeochernes* sp.1	X	X
		Chthoniidae	Chthoniidae jovem	-	X
Chilopoda	Scolopendromorpha	Cryptopidae	*Cryptops* sp.1	X	-
Diplopoda	Polydesmida	Chelodesmidae	Chelodesmidae sp.3	-	X
Entognatha	Collembola	Paronellidae	Paronellidae sp.1	-	X
	Diplura	Campodeidae	Campodeidae sp.1	X	X
Insecta	Diptera	Psychodidae	Phlebotominae sp.	X	-
			Diptera jovem	-	X
	Hymenoptera	Formicidae	*Apterostigma collare*	-	X
			Pachycondyla constricta	-	X
			Pheidole sp.13	-	X
			Rogeria cf. *belti*	X	-
	Isoptera	Termitidae	*Nasutitermes* sp.1	X	-
	Orthoptera	Phalangopsidae	*Paraclodes* sp.1	X	X
	Psocoptera	Pseudocaeciliidae	Pseudocaeciliidae sp.2	-	X
Malacostraca	Isopoda	Philosciidae	Philosciidae sp.1	X	-
Symphyla		Scutigerellidae	*Hanseniella* sp.1	-	X
Gastropoda	Pulmonata	Systrophiidae	*Happia* sp.1	-	X
Amphibia	Anura	Strabomantidae	*Pristimantis* cf. *fenestratus*	X	X

SB-0029					
TÁXONS				Seca	Úmida
Arachnida	Acari	Ixodidae	*Amblyomma* sp.1	X	-
		Trombiculidae	Trombiculidae sp.2	X	-
			Acariformes sp.4	X	-
			Mesostigmata sp.1	X	-
			Oribatida sp.2	X	X
	Amblypygi	Phrynidae	*Heterophrinus longicornis*	X	-
	Araneae	Araneidae	*Alpaida* sp.1	X	-
		Ochyroceratidae	*Speocera* sp.1	X	X
		Pholcidae	*Mesabolivar eberhardi*	X	X
		Theraphosidae	Theraphosidae jovem	X	X
		Theridiidae	Theridiidae jovem	X	-
		Theridiosomatidae	Theridiosomatidae jovem	X	-
	Opiliones	Escadabiidae	Escadabiidae jovem	X	-
		Sclerosomatidae	Sclerosomatidae jovem	X	-
		Stygnidae	Stygnidae jovem	-	X
	Pseudoscorpiones	Chernetidae	*Spelaeochernes* sp.1	X	-
		Chthoniidae	Chthoniidae sp.1	-	X
	Ricinulei	Ricinoididae	*Cryptocellus tarsilae*	-	X
Chilopoda	Scutigeromorpha	Pselliodidae	*Sphendononema* jovem	X	-
Diplopoda	Polydesmida	Chelodesmidae	Chelodesmidae jovem	-	X
	Spirostreptida	Pseudonannolenidae	Pseudonannolenidae sp.1	X	-
Entognatha	Collembola	Entomobryidae	Entomobryidae sp.5	X	-
		Isotomidae	Isotomidae sp.1	X	-
		Onychiuridae	Onychiuridae sp.1	X	-
		Paronellidae	Paronellidae sp.1	X	X
	Diplura	Campodeidae	Campodeidae sp.1	X	X
		Projapygidae	Projapygidae sp.1	X	-
Insecta	Coleoptera	Carabidae	Carabidae sp.1	X	-
	Diptera	Culicidae	Culicidae sp.	-	X
		Psychodidae	Phlebotominae sp.	X	X
		Tabanidae	Tabanidae sp.	X	-
	Hemiptera	Cixiidae	Cixiidae sp.3	X	X
		Cydnidae	Cydnidae jovem	-	X
			Cydnidae sp.1	X	-
		Lygaeidae	Lygaeidae jovem	X	-
	Hymenoptera	Braconidae	Braconidae sp.1	X	-
		Formicidae	*Brachymyrmex* sp.1	X	-
			Camponotus sp.2	X	-
			Carebara urichii	X	-
			Eurhopalothrix sp.1	X	-
			Pachycondyla constricta	X	X
			Paratrechina sp.1	-	X
			Stegomyrmex sp.1	-	X
	Lepidoptera	Noctuoidea	Noctouidea sp. 2	-	X
		Noctuoidea	Noctouidea sp. 3	-	X
	Orthoptera	Phalangopsidae	*Paraclodes* sp.1	X	X
			Phalangopsis sp.1	X	X
	Psocoptera		Psocomorpha jovem	X	-
Malacostraca	Isopoda	Armadillidae	Armadillidae sp.1	X	X
		Philosciidae	Philosciidae sp.2	X	-
Amphibia	Anura	Leptodactylidae	*Leptodactylus* cf. *vastus*	-	X
		Strabomantidae	*Pristimantis* cf. *fenestratus*	X	-
Mammalia	Chiroptera	Emballonuridae	*Peropteryx kappleri*	X	X
		Furipteridae	*Furipterus horrens*	X	-
		Phyllostomidae	*Carollia perspicillata*	X	-
			Glossophaga soricina	X	X
			Phyllostomus latifolius	X	X

SB-0030					
TÁXONS				Seca	Úmida
Arachnida	Araneae	Ctenidae	Ctenidae jovem	X	-
		Pholcidae	Pholcidae jovem	X	-
		Scytodidae	*Scytodes* sp.1	-	X
		Theridiidae	Theridiidae sp.3	-	X
		Theridiosomatidae	*Plato* sp.1	-	X
		Trechaleidae	Trechaleidae sp.1	X	-
	Opiliones	Sclerosomatidae	*Prionostema* sp.1	-	X
	Pseudoscorpiones	Chernetidae	*Spelaeochernes* sp.1	-	X
		Chthoniidae	Chthoniidae sp.1	X	-
Diplopoda	Polydesmida	Fuhrmanodesmidae	Fuhrmanodesmidae sp.1	X	X
Entognatha	Collembola	Cyphoderidae	Cyphoderidae sp.1	-	X
		Paronellidae	Paronellidae sp.1	X	X
		Poduromorpha	Poduromorpha sp.1	-	X
		Sminthuroidea	Sminthuroidea sp.2	-	X
Insecta	Coleoptera		Coleoptera jovem	X	-
	Diptera	Cecidomyiidae	Cecidomyiidae sp.	X	-
		Psychodidae	Phlebotominae sp.	-	X
		Sciaridae	Sciaridae sp.	-	X
	Hemiptera	Cydnidae	Cydnidae jovem	-	X
		Veliidae	*Rhagovelia* sp.3	-	X
	Hymenoptera	Formicidae	*Apterostigma collare*	-	X
			Gnamptogenys regularis	-	X
			Paratrechina sp.1	-	X
			Prionopelta modesta	X	-
			Solenopsis invicta	-	X
	Isoptera	Termitidae	*Nasutitermes* sp.1	-	X
			Nasutitermes sp.2	X	-
	Orthoptera	Phalangopsidae	*Paraclodes* sp.1	X	-
			Phalangopsis sp.1	-	X
Malacostraca	Decapoda	Palaemonidae	*Macrobrachium* sp.1	-	X
	Isopoda	Philosciidae	Philosciidae sp.1	X	X
Symphyla		Scutigerellidae	*Hanseniella* sp.1	-	X
Gastropoda	Caenogastropoda	Ampullariidae	*Pomacea* sp.1	X	-

SB-0031					
TÁXONS				**Seca**	**Úmida**
Arachnida	Amblypygi	Phrynidae	*Heterophrinus longicornis*	-	X
	Araneae	Araneidae	*Alpaida* sp.2	X	-
			Araneidae jovem	X	-
		Corinnidae	Corinnidae jovem	X	X
		Ctenidae	Ctenidae jovem	-	X
			Enoploctenus sp.	-	X
		Nesticidae	Nesticidae sp.1	-	X
		Ochyroceratidae	Ochyroceratidae jovem	-	X
		Oonopidae	Oonopidae sp.5	X	-
		Pholcidae	*Mesabolivar aurantiacus*	X	X
			Mesabolivar cambridgei	X	-
		Scytodidae	Scytodidae jovem	X	-
		Theraphosidae	Theraphosidae jovem	-	X
		Theridiidae	Theridiidae jovem	-	X
		Theridiosomatidae	*Plato* sp.1	-	X
	Opiliones	Cosmetidae	Cosmetidae jovem	X	-
			Roquettea carajas	X	X
		Gonyleptidae	Gonyleptidae jovem	-	X
		Stygnidae	Stygnidae jovem	-	X
	Pseudoscorpiones	Chernetidae	*Spelaeochernes* sp.1	X	X
		Chthoniidae	Chthoniidae jovem	X	X
			Chthoniidae sp.1	X	X
Chilopoda	Geophilomorpha	Geophilidae	Geophilidae sp.1	-	X
	Scolopendromorpha	Scolopocryptopidae	*Newportia* jovem	-	X
Diplopoda	Polydesmida	Chelodesmidae	Chelodesmidae sp.3	-	X
		Fuhrmanodesmidae	Fuhrmanodesmidae jovem	-	X
	Polyxenida	Hypogexenidae	Hypogexenidae sp.1	-	X
Entognatha	Collembola	Cyphoderidae	Cyphoderidae sp.1	-	X
		Entomobryidae	Entomobryidae sp.6	X	-
		Paronellidae	Paronellidae sp.1	X	X
		Sminthuroidea	Sminthuroidea sp.2	-	X
Insecta	Coleoptera	Staphylinidae	Pselaphinae sp.2	-	X
			Staphylininae sp.1	-	X
			Staphylininae sp.3	-	X
		Tenebrionidae	Tenebrionidae jovem	X	-
	Diptera	Drosophilidae	Drosophilidae sp.	-	X
		Psychodidae	Phlebotominae sp.	X	X
	Hemiptera	Coccoidea	Coccoidea jovem	X	-
		Cydnidae	Cydnidae sp.1	-	X
			Cydnidae sp.2	-	X
		Reduviidae	Emesinae jovem	-	X
		Schizopteridae	Schizopteridae sp.2	-	X
	Hymenoptera	Formicidae	*Atta* sp.1	X	-
			Crematogaster erecta	X	-
			Pachycondyla constricta	X	X
			Pachycondyla striata	X	X
			Paratrechina sp.1	X	-
			Rogeria foreli	X	-
			Solenopsis invicta	X	-
	Isoptera	Termitidae	*Nasutitermes* sp.1	-	X
	Orthoptera	Phalangopsidae	*Paraclodes* sp.1	X	X
			Phalangopsis sp.1	X	X
	Psocoptera	Psyllipsocidae	Psyllipsocidae sp.5	X	-
			Psocomorpha jovem	X	-
Malacostraca	Decapoda	Pseudothelphusidae	Pseudothelphusidae sp.1	-	X
	Isopoda	Armadillidae	Armadillidae sp.1	X	X
		Philosciidae	Philosciidae sp.1	X	X

			Philosciidae sp.2	X	X
		Platyarthridae	Platyarthridae sp.2	X	X
Symphyla		Scutigerellidae	*Hanseniella* sp.1	-	X
Gastropoda	Pulmonata	Subulinidae	*Lamellaxis* sp.2	X	X
			Leptinaria sp.2	X	X
		Systrophiidae	*Happia* sp.1	-	X
Amphibia	Anura	Bufonidae	*Rhinella* sp.	-	X
		Strabomantidae	*Pristimantis* cf. *fenestratus*	-	X

SB-0032					
TÁXONS				Seca	Úmida
Annelida	Haplotaxida		Haplotaxida sp.4	-	X
Arachnida	Acari	Ixodidae	*Amblyomma* sp.1	X	-
		Opilioacaridae	Opilioacaridae sp.1	-	X
			Astigmata sp.4	-	X
			Mesostigmata jovem	-	X
			Oribatida sp.2	X	X
	Amblypygi	Phrynidae	*Heterophrinus longicornis*	X	X
	Araneae	Araneidae	Araneidae jovem	X	X
		Barychelidae	Barychelidae jovem	X	X
		Corinnidae	*Abapeba* sp.1	-	X
			Corinnidae jovem	-	X
		Ctenidae	Ctenidae jovem	-	X
		Oonopidae	Oonopidae jovem	X	-
		Palpimanidae	Palpimanidae jovem	X	-
		Paratropididae	Paratropididae jovem	X	X
		Pholcidae	*Mesabolivar aurantiacus*	-	X
			Pholcidae sp.1	X	-
		Salticidae	Salticidae sp.1	X	X
		Theraphosidae	*Guyruita cerrado*	-	X
		Theridiosomatidae	*Plato* sp.1	-	X
		Zodariidae	Zodariidae jovem	-	X
	Opiliones	Cosmetidae	Cosmetidae sp.1	X	-
			Roquettea carajas	-	X
		Gonyleptidae	Gonyleptidae jovem	-	X
		Sclerosomatidae	*Prionostema* sp.1	X	X
		Stygnidae	*Protimesus* sp.1	X	X
			Protimesus sp.2	X	-
	Pseudoscorpiones	Chernetidae	Chernetidae jovem	X	-
			Spelaeochernes sp.1	-	X
		Chthoniidae	Chthoniidae sp.1	X	-
Chilopoda	Scolopendromorpha	Scolopocryptopidae	*Newportia* sp.2	-	X
	Scutigeromorpha	Pselliodidae	*Sphendononema guildingii*	X	-
Diplopoda	Polydesmida	Chelodesmidae	Chelodesmidae jovem	X	-
			Chelodesmidae sp.1	-	X
		Paradoxosomatidae	Paradoxosomatidae sp.4	-	X
	Stemmiulida	Stemmiulidae	Stemmiulidae sp.1	X	-
Entognatha	Collembola	Paronellidae	Paronellidae sp.1	X	X
	Diplura	Projapygidae	Projapygidae sp.1	X	-
Insecta	Blattodea	Blaberidae	Blaberus sp.1	-	X
		Blattidae	Blattidae jovem	-	X
	Coleoptera	Ptilidae	Ptilidae sp.1	-	X
		Staphylinidae	Pselaphinae sp.5	-	X
		Tenebrionidae	Tenebrionidae sp.1	X	-
			Coleoptera jovem	-	X
	Diptera	Cecidomyiidae	Cecidomyiidae sp.	-	X
		Phoridae	Phoridae sp.	-	X
		Psychodidae	Phlebotominae sp.	X	-
		Tipulidae	Tipulidae sp.	-	X
	Hemiptera	Cixiidae	Cixiidae sp.1	X	X
		Ochteridae	Ochteridae sp.1	X	X
		Reduviidae	*Zelurus* sp.1	X	X
	Hymenoptera	Formicidae	*Brachymyrmex* sp.1	X	-
			Camponotus sp.5	X	-
			Dolichoderus bispinosus	-	X
			Dolichoderus lutosus	X	-
			Gnamptogenys sp.1	X	-
			Pachycondyla constricta	X	-

			Paratrechina sp.1	-	X
			Pheidole sp.3	X	-
			Prionopelta modesta	-	X
			Solenopsis sp.2	-	X
		Vespidae	Vespidae sp.1	X	-
	Lepidoptera	Gelechioidea	Gelechioidea sp.1	-	X
		Noctuoidea	Noctouidea sp. 7	X	-
	Neuroptera	Myrmeleontidae	Myrmeleontidae sp.1	X	-
	Orthoptera	Phalangopsidae	*Paraclodes* sp.1	X	X
			Phalangopsis sp.1	X	X
	Psocoptera	Archipsocidae	Archipsocidae sp.2	X	-
		Epipsocidae	Epipsocidae sp.2	X	-
		Troctopsocidae	Troctopsocidae sp.1	X	-
			Psocomorpha jovem	X	X
	Thysanura	Nicoletiidae	Nicoletiinae sp.1	-	X
Malacostraca	Isopoda	Philosciidae	Philosciidae sp.1	X	X
		Platyarthridae	Platyarthridae sp.1	-	X
Gastropoda	Pulmonata	Systrophiidae	*Happia* sp.1	-	X
Amphibia	Anura	Strabomantidae	*Pristimantis* cf. *fenestratus*	X	-
			Anura sp.1	-	X
			Anura sp.5	-	X
Aves	Passeriforme	Turdidae	*Turdus* sp.	-	X
Mammalia	Chiroptera	Emballonuridae	*Peropteryx kappleri*	X	X
		Phyllostomidae	*Carollia perspicillata*	X	-
			Glossophaga soricina	X	X

SB-0033					
**TÁXONS**				**Seca**	**Úmida**
Arachnida	Acari	Trombiculidae	Trombiculidae sp.2	X	-
			Holothyrida sp.1	-	X
	Amblypygi	Phrynidae	*Heterophrinus longicornis*	-	X
	Araneae	Ochyroceratidae	Ochyroceratidae jovem	-	X
			Speocera sp.1	X	-
		Oonopidae	Oonopidae jovem	X	-
		Pholcidae	*Mesabolivar eberhardi*	-	X
			Pholcidae jovem	X	-
		Prodidomidae	Prodidomidae sp.1	X	-
		Salticidae	Salticidae sp.1	X	-
	Pseudoscorpiones	Chernetidae	*Spelaeochernes* sp.1	X	X
Entognatha	Collembola	Paronellidae	Paronellidae sp.1	X	-
	Diplura	Campodeidae	Campodeidae sp.1	X	X
Insecta	Archaeognatha	Meinertellidae	Meinertellidae sp.1	-	X
	Coleoptera	Curculionidae	Scolytinae sp.1	X	-
			Coleoptera jovem	-	X
	Diptera	Ceratopogonidae	Ceratopogonidae jovem	-	X
		Drosophilidae	Drosophilidae sp.	X	-
		Psychodidae	Phlebotominae sp.	-	X
	Hemiptera	Cixiidae	Cixiidae jovem	X	-
		Cydnidae	Cydnidae sp.1	-	X
			Heteroptera jovem	X	-
	Hymenoptera	Formicidae	*Camponotus* sp.2	X	-
			Carebara urichii	X	-
			Pachycondyla constricta	X	-
			Pachycondyla impressa	X	-
			Rogeria cf. *belti*	X	-
	Isoptera		Isoptera jovem	-	X
	Lepidoptera	Hesperiidae	Hesperiidae sp. 1	X	-
		Noctuoidea	Noctouidea sp. 1	-	X
	Orthoptera	Phalangopsidae	*Paraclodes* sp.1	X	X
			Phalangopsis sp.1	-	X
	Psocoptera	Epipsocidae	Epipsocidae sp.1	X	-
Malacostraca	Isopoda	Philosciidae	Philosciidae sp.1	X	X
			Philosciidae sp.2	X	-
Gastropoda	Pulmonata	Systrophiidae	*Happia* sp.1	X	-
Mammalia	Chiroptera	Emballonuridae	*Peropteryx kappleri*	-	X

SB-0034					
TÁXONS				Seca	Úmida
Arachnida	Acari		Mesostigmata sp.1	X	-
	Araneae	Corinnidae	Corinnidae jovem	X	X
		Pholcidae	*Mesabolivar eberhardi*	X	X
			Pholcidae jovem	-	X
		Salticidae	Salticidae jovem	X	-
	Opiliones	Cosmetidae	*Roquettea carajas*	-	X
		Sclerosomatidae	*Prionostema* sp.1	X	X
Entognatha	Collembola	Paronellidae	Paronellidae sp.1	X	-
Insecta	Coleoptera	Carabidae	Carabidae sp.1	X	-
		Staphylinidae	Staphylininae sp.1	X	-
			Staphylininae sp.2	X	-
	Diptera	Cecidomyiidae	Cecidomyiidae sp.	-	X
		Drosophilidae	Drosophilidae sp.	X	-
	Hemiptera	Cydnidae	Cydnidae sp.1	X	-
		Largidae	Largidae sp.1	X	-
	Hymenoptera	Formicidae	*Carebara urichii*	X	-
			Gnamptogenys sp.1	X	X
			Odontomachus meinerti	X	X
			Pachycondyla constricta	X	-
			Paratrechina sp.1	X	-
			Pheidole sp.12	X	X
			Pheidole sp.3	X	-
			Wasmannia sp.1	X	-
		Ichneumonidae	Ichneumonidae sp.1	X	-
	Isoptera	Termitidae	*Nasutitermes* sp.4	X	-
	Orthoptera	Phalangopsidae	*Paraclodes* sp.1	X	X
	Psocoptera		Psocomorpha jovem	X	-
Amphibia	Anura	Strabomantidae	*Pristimantis* cf. *fenestratus*	X	-

SB-0035					
**TÁXONS**				**Seca**	**Úmida**
Arachnida	Amblypygi	Phrynidae	*Heterophrinus longicornis*	X	-
	Araneae	Araneidae	*Alpaida* sp.1	X	-
			Araneidae jovem	-	X
		Pholcidae	*Mesabolivar eberhardi*	X	-
			Pholcidae jovem	-	X
		Scytodidae	*Scytodes* sp.1	X	X
	Opiliones	Cosmetidae	*Roquettea carajas*	X	-
		Sclerosomatidae	*Prionostema* sp.1	X	-
			Sclerosomatidae jovem	-	X
Insecta	Diptera	Cecidomyiidae	Cecidomyiidae sp.	-	X
		Culicidae	Culicidae sp.	-	X
		Phoridae	Phoridae sp.	X	-
		Tipulidae	Tipulidae sp.	-	X
	Hemiptera	Cixiidae	Cixiidae sp.1	X	X
		Cydnidae	Cydnidae sp.1	X	X
	Hymenoptera	Formicidae	*Pachycondyla verenae*	X	-
	Lepidoptera	Hesperiidae	Hesperiidae sp. 1	-	X
		Noctuoidea	Noctouidea sp. 2	-	X
	Orthoptera	Phalangopsidae	*Paraclodes* sp.1	X	X
			Phalangopsis sp.1	X	X
Amphibia	Anura		Anura sp.6	X	-
			Anura sp.8	X	-

SB-0036					
TÁXONS				**Seca**	**Úmida**
Annelida	Haplotaxida		Haplotaxida sp.3	-	X
Arachnida	Acari		Astigmata sp.2	X	-
	Amblypygi	Phrynidae	*Heterophrinus longicornis*	-	X
	Araneae	Ctenidae	Ctenidae jovem	X	-
			Phoneutria sp.	-	X
		Pholcidae	*Mesabolivar aurantiacus*	X	-
		Pisauridae	Pisauridae jovem	-	X
		Theridiosomatidae	*Plato* sp.1	X	-
	Pseudoscorpiones	Chernetidae	*Spelaeochernes* sp.1	-	X
Diplopoda	Polydesmida	Chelodesmidae	Chelodesmidae jovem	-	X
		Fuhrmanodesmidae	Fuhrmanodesmidae sp.1	-	X
			Polydesmida jovem	X	X
Entognatha	Collembola	Paronellidae	Paronellidae sp.1	-	X
		Poduromorpha	Poduromorpha sp.1	X	-
		Sminthuroidea	Sminthuroidea sp.2	X	-
Insecta	Coleoptera	Staphylinidae	Staphylininae sp.2	-	X
	Hemiptera	Cydnidae	Cydnidae sp.1	-	X
		Notonectidae	Notonectidae jovem	X	-
	Hymenoptera	Formicidae	*Hypoponera* sp.1	-	X
			Solenopsis invicta	-	X
	Odonata	Anisoptera	Anisoptera jovem	X	-
	Orthoptera	Phalangopsidae	*Phalangopsis* sp.1	X	X
Malacostraca	Decapoda	Palaemonidae	*Macrobrachium* sp.1	-	X
		Pseudothelphusidae	Pseudothelphusidae sp.1	X	X
	Isopoda	Philosciidae	Philosciidae sp.1	-	X
		Platyarthridae	Platyarthridae sp.2	X	-
Mammalia	Chiroptera	Phyllostomidae	*Carollia perspicillata*	-	X
	Rodentia	Cricetidae	*Rhipidomys* sp.	-	X

SB-0037					
TÁXONS				Seca	Úmida
Arachnida	Acari	Opilioacaridae	Opilioacaridae sp.1	-	X
			Holothyrida sp.6	-	X
			Oribatida sp.2	X	X
	Araneae	Corinnidae	Corinnidae jovem	-	X
		Ochyroceratidae	Ochyroceratidae jovem	-	X
			Speocera sp.1	X	-
		Pholcidae	Ninetinae sp.1	X	-
			Pholcidae jovem	X	X
		Salticidae	Salticidae jovem	-	X
			Salticidae sp.1	X	-
			Salticidae sp.3	X	-
		Scytodidae	*Scytodes* sp.1	X	X
		Theridiosomatidae	Theridiosomatidae jovem	X	-
	Pseudoscorpiones	Chernetidae	*Spelaeochernes* sp.1	X	X
		Chthoniidae	Chthoniidae sp.1	X	X
	Scorpiones	Buthidae	*Ananteris* jovem	-	X
Entognatha	Collembola	Paronellidae	Paronellidae sp.1	X	X
	Diplura	Campodeidae	Campodeidae sp.1	X	-
Insecta	Coleoptera	Staphylinidae	Pselaphinae sp.5	X	-
			Coleoptera jovem	-	X
	Diptera	Phoridae	Phoridae sp.	-	X
		Psychodidae	Phlebotominae sp.	X	-
	Hemiptera	Cixiidae	Cixiidae jovem	-	X
		Cydnidae	Cydnidae sp.1	-	X
		Veliidae	*Paravelia* sp.1	X	-
	Hymenoptera	Formicidae	*Gnamptogenys* sp.1	-	X
			Pachycondyla constricta	-	X
			Solenopsis invicta	-	X
	Isoptera	Termitidae	*Nasutitermes* sp.1	-	X
			Nasutitermes sp.2	X	-
	Lepidoptera	Noctuoidea	Noctouidea sp. 3	-	X
			Noctouidea sp. 8	X	-
		Tineoidea	Tineoidea sp. 6	X	X
	Orthoptera	Phalangopsidae	*Paraclodes* sp.1	X	X
	Psocoptera	Troctopsocidae	Troctopsocidae sp.1	X	X
			Psocomorpha jovem	X	X
Mammalia	Chiroptera	Emballonuridae	*Peropteryx* sp.	X	-

SB-0038					
TÁXONS				Seca	Úmida
Arachnida	Araneae	Pholcidae	Ninetinae sp.1	X	-
			Pholcidae jovem	X	X
		Scytodidae	Scytodidae jovem	-	X
	Pseudoscorpiones	Chernetidae	*Spelaeochernes* sp.1	X	X
		Chthoniidae	Chthoniidae sp.1	-	X
Chilopoda	Scutigeromorpha	Pselliodidae	*Sphendononema* jovem	X	-
Entognatha	Collembola	Paronellidae	Paronellidae sp.1	X	-
Insecta	Coleoptera		Coleoptera jovem	X	X
	Diptera	Cecidomyiidae	Cecidomyiidae sp.	X	-
		Psychodidae	Phlebotominae sp.	X	-
	Hemiptera	Reduviidae	Reduviinae jovem	-	X
	Hymenoptera	Formicidae	*Apterostigma pilosum*	X	X
			Camponotus crassus	-	X
			Camponotus melanoticus	X	-
			Pachycondyla constricta	X	-
		Vespidae	Vespidae sp.4	-	X
	Isoptera	Termitidae	*Nasutitermes* sp.2	X	-
	Lepidoptera	Gelechioidea	Gelechioidea sp.3	-	X
	Orthoptera	Phalangopsidae	*Paraclodes* sp.1	X	-
			Phalangopsis sp.1	X	X
	Psocoptera	Psyllipsocidae	Psyllipsocidae sp.4	X	-
			Psocomorpha jovem	X	-
Malacostraca	Isopoda	Armadillidae	Armadillidae sp.1	-	X
Gastropoda	Pulmonata	Systrophiidae	*Happia* sp.1	-	X
Onychophora	Euonychophora	Peripatidae	Peripatidae sp.1	X	-
Mammalia	Chiroptera	Phyllostomidae	*Carollia perspicillata*	X	-

SB-0039					
TÁXONS				Seca	Úmida
Arachnida	Acari	Trombiculidae	Trombiculidae sp.2	X	-
			Astigmata sp.1	-	X
			Astigmata sp.2	-	X
			Astigmata sp.4	-	X
	Amblypygi	Phrynidae	*Heterophrinus longicornis*	X	X
	Araneae	Araneidae	Araneidae jovem	X	-
		Ctenidae	Ctenidae jovem	X	X
		Ochyroceratidae	*Speocera* sp.1	X	X
		Pholcidae	*Mesabolivar aurantiacus*	X	-
			Mesabolivar eberhardi	-	X
			Mesabolivar sp.2	X	-
		Salticidae	Salticidae jovem	X	X
		Theraphosidae	Theraphosidae jovem	-	X
		Theridiosomatidae	*Plato* sp.1	X	X
	Opiliones	Sclerosomatidae	*Prionostema* sp.1	-	X
	Palpigradi	Eukoeneniidae	*Eukoenenia* sp.1	X	-
	Pseudoscorpiones	Chernetidae	*Spelaeochernes* sp.1	X	X
Diplopoda	Polydesmida	Pyrgodesmidae	Pyrgodesmidae sp.1	X	-
Entognatha	Collembola	Isotomidae	Isotomidae sp.1	-	X
		Paronellidae	Paronellidae sp.1	X	-
Insecta	Blattodea	Blaberidae	Blaberidae jovem	X	-
		Blattidae	Blattidae jovem	X	X
	Coleoptera	Scarabaeidae	*Gama* sp.1	X	-
		Staphylinidae	*Coproporus* sp.2	X	-
	Diptera	Psychodidae	Phlebotominae sp.	X	X
		Tipulidae	Tipulidae sp.	-	X
	Hemiptera	Cixiidae	Cixiidae jovem	-	X
		Cydnidae	Cydnidae sp.1	-	X
		Reduviidae	Emesinae sp.4	X	-
			Reduviinae jovem	X	X
		Schizopteridae	Schizopteridae jovem	-	X
	Hymenoptera	Diapriidae	Diapriidae sp.9	-	X
		Formicidae	*Crematogaster brasiliensis*	X	-
			Pachycondyla constricta	-	X
			Paratrechina sp.1	X	-
	Isoptera	Termitidae	*Nasutitermes* sp.3	X	-
	Neuroptera	Myrmeleontidae	Myrmeleontidae sp.1	X	-
	Orthoptera	Phalangopsidae	*Paraclodes* sp.1	X	X
			Phalangopsis sp.1	X	X
	Psocoptera		Psocoptera jovem	X	-
	Thysanura	Nicoletiidae	Nicoletiinae sp.1	X	-
Malacostraca	Isopoda	Armadillidae	Armadillidae sp.1	X	X
		Philosciidae	Philosciidae sp.2	-	X
Gastropoda	Pulmonata	Subulinidae	*Leptinaria* sp.1	-	X
		Systrophiidae	Systrophiidae jovem	X	-
Mammalia	Chiroptera	Emballonuridae	*Peropteryx kappleri*	-	X
		Phyllostomidae	*Carollia perspicillata*	-	X

SB-0040					
TÁXONS				**Seca**	**Úmida**
Arachnida	Acari	Trombiculidae	Trombiculidae sp.1	-	X
			Astigmata sp.2	-	X
			Astigmata sp.4	X	-
			Holothyrida sp.2	X	-
			Mesostigmata sp.1	X	-
			Mesostigmata sp.2	X	-
			Oribatida sp.2	X	-
	Amblypygi	Phrynidae	*Heterophrinus longicornis*	-	X
	Araneae	Ctenidae	Ctenidae jovem	-	X
		Oonopidae	gr. *Xycarpphy* sp.1	X	-
			Oonopidae jovem	-	X
		Pholcidae	*Mesabolivar eberhardi*	X	X
		Theridiosomatidae	*Plato* sp.1	-	X
	Opiliones	Sclerosomatidae	Sclerosomatidae jovem	X	-
	Pseudoscorpiones	Bochicidae	Bochicidae sp.1	X	X
Entognatha	Collembola	Cyphoderidae	Cyphoderidae sp.1	X	-
		Isotomidae	Isotomidae sp.1	-	X
	Diplura	Campodeidae	Campodeidae sp.1	X	-
		Japygidae	Japygidae sp.1	X	-
Insecta	Coleoptera	Gyrinidae	*Gyretes* sp.1	X	-
		Staphylinidae	Pselaphinae sp.2	-	X
			Staphylininae sp.2	-	X
	Diptera	Phoridae	Phoridae sp.	-	X
		Psychodidae	Psychodidae sp.	X	-
		Tipulidae	Tipulidae sp.	X	-
	Hemiptera	Cydnidae	Cydnidae sp.1	X	X
		Reduviidae	Reduviidae jovem	X	-
		Veliidae	*Rhagovelia* sp.2	X	-
	Hymenoptera	Formicidae	*Acromyrmex octopinosus*	-	X
			Paratrechina sp.1	X	X
			Pheidole sp.12	X	-
			Strumigenys elongata	X	X
	Lepidoptera		Lepidoptera jovem	X	-
	Orthoptera	Phalangopsidae	*Phalangopsis* sp.1	X	X
	Trichoptera	Philopotamidae	Philopotamidae sp.1	-	X
	Thysanura	Nicoletiidae	Nicoletiinae sp.1	X	X
Malacostraca	Decapoda	Palaemonidae	*Macrobrachium* sp.1	X	-
		Pseudothelphusidae	Pseudothelphusidae sp.1	X	-
	Isopoda	Philosciidae	Philosciidae sp.1	X	-
Gastropoda	Pulmonata	Subulinidae	*Lamellaxis* sp.1	X	-
Mammalia	Chiroptera	Phyllostomidae	*Carollia perspicillata*	X	X

SB-0041					
TÁXONS				Seca	Úmida
Arachnida	Acari		Mesostigmata sp.1	X	-
	Araneae	Ctenidae	*Isoctenus* sp.1	X	X
		Pholcidae	*Mesabolivar aurantiacus*	X	-
			Mesabolivar eberhardi	X	-
			Modisimus sp.1	-	X
		Salticidae	Salticidae jovem	X	-
		Scytodidae	Scytodidae jovem	X	X
		Theridiidae	Theridiidae jovem	-	X
		Theridiosomatidae	*Plato* sp.1	-	X
	Opiliones	Cosmetidae	*Roquettea carajas*	-	X
		Escadabiidae	Escadabiidae jovem	X	-
		Sclerosomatidae	*Prionostema* sp.1	-	X
	Pseudoscorpiones	Bochicidae	Bochicidae sp.1	-	X
		Chernetidae	*Spelaeochernes* sp.1	-	X
		Chthoniidae	Chthoniidae sp.1	X	X
	Schizomida	Hubbardiidae	*Rowlandius* sp.1	-	X
Chilopoda	Scolopendromorpha	Cryptopidae	*Cryptops* sp.1	X	-
		Scolopocryptopidae	*Newportia* jovem	X	-
Diplopoda	Glomeridesmida	Glomeridesmidae	Glomeridesmida sp.1	-	X
	Polydesmida	Pyrgodesmidae	Pyrgodesmidae sp.1	-	X
Entognatha	Collembola	Entomobryidae	Entomobryidae sp.5	-	X
		Paronellidae	Paronellidae sp.1	X	X
Insecta	Blattodea	Blaberidae	Blaberidae jovem	X	X
		Blattidae	Blattidae jovem	X	X
			Periplaneta sp.1	-	X
	Coleoptera	Carabidae	Carabidae sp.4	X	-
		Staphylinidae	Aleocarinae sp.1	X	-
			Staphylinidae sp.5	X	-
			Coleoptera jovem	X	X
	Diptera	Psychodidae	Phlebotominae sp.	-	X
		Tipulidae	Tipulidae sp.	-	X
	Hemiptera	Cydnidae	Cydnidae jovem	X	-
			Cydnidae sp.2	-	X
		Dipsocoroidea	Dipsocoroidea jovem	X	-
		Reduviidae	Emesinae jovem	-	X
			Reduviinae jovem	X	-
	Hymenoptera	Formicidae	*Acromyrmex octopinosus*	X	-
			Crematogaster brasiliensis	X	-
			Gnamptogenys sp.1	-	X
			Pachycondyla verenae	X	-
			Paratrechina sp.1	X	-
		Vespidae	Vespidae sp.1	X	-
	Neuroptera	Myrmeleontidae	Myrmeleontidae sp.1	X	-
	Orthoptera	Phalangopsidae	*Eidmanacris* sp.1	X	-
			Paraclodes sp.1	X	-
			Phalangopsis sp.1	X	X
	Thysanura	Nicoletiidae	Nicoletiinae sp.1	X	-
Malacostraca	Isopoda	Scleropactidae	Scleropactidae sp.2	-	X
Symphyla		Scutigerellidae	*Hanseniella* sp.1	X	-
Gastropoda	Pulmonata	Subulinidae	*Lamellaxis* sp.1	X	-
		Systrophiidae	*Happia* sp.1	X	-
Amphibia	Anura		Anura sp.3	X	-
Mammalia	Chiroptera	Emballonuridae	*Peropteryx kappleri*	X	-
		Phyllostomidae	*Carollia perspicillata*	-	X

SB-0042					
TÁXONS				**Seca**	**Úmida**
Arachnida	Acari	Trombiculidae	Trombiculidae sp.1	-	X
			Trombiculidae sp.2	X	-
	Amblypygi	Phrynidae	*Heterophrinus longicornis*	X	X
	Araneae	Araneidae	Araneidae jovem	X	-
		Corinnidae	Corinnidae jovem	-	X
		Ctenidae	Ctenidae jovem	-	X
		Ochyroceratidae	Ochyroceratidae jovem	X	-
			Speocera sp.1	-	X
		Oonopidae	Oonopidae sp.1	-	X
		Paratropididae	Paratropididae jovem	X	-
		Pholcidae	*Leptopholcus* sp.1	X	-
			Mesabolivar sp.2	X	-
			Modisimus sp.1	X	-
	Pseudoscorpiones	Chernetidae	*Spelaeochernes* sp.1	X	X
		Chthoniidae	Chthoniidae sp.1	X	X
Chilopoda	Scolopendromorpha	Scolopocryptopidae	*Tidops* sp.1	-	X
Diplopoda	Glomeridesmida	Glomeridesmidae	Glomeridesmida jovem	-	X
Entognatha	Collembola	Entomobryidae	Entomobryidae sp.3	X	-
		Paronellidae	Paronellidae sp.1	-	X
	Diplura	Campodeidae	Campodeidae sp.1	X	-
Insecta	Hemiptera	Cixiidae	Cixiidae jovem	X	-
	Hymenoptera	Formicidae	*Camponotus* sp.7	X	-
			Carebara urichii	-	X
			Cyphomyrmex peltatus	-	X
			Pachycondyla constricta	-	X
			Paratrechina sp.1	X	-
	Isoptera	Termitidae	*Nasutitermes* sp.3	X	-
	Orthoptera	Phalangopsidae	*Phalangopsis* sp.1	-	X
	Thysanura	Nicoletiidae	Nicoletiinae sp.1	X	-
Malacostraca	Isopoda	Philosciidae	Philosciidae sp.1	X	-
Symphyla		Scutigerellidae	*Hanseniella* sp.1	-	X
Gastropoda	Pulmonata	Subulinidae	*Leptinaria* sp.1	X	-
		Systrophiidae	*Happia* sp.1	X	-
Mammalia	Chiroptera	Emballonuridae	*Peropteryx kappleri*	X	-
		Phyllostomidae	*Micronycteris megalotis*	-	X

SB-0043					
**TÁXONS**				**Seca**	**Úmida**
Arachnida	Acari		Mesostigmata sp.1	X	-
	Amblypygi	Phrynidae	*Heterophrinus longicornis*	-	X
	Araneae	Ctenidae	Ctenidae jovem	-	X
		Pholcidae	*Mesabolivar eberhardi*	X	X
		Scytodidae	*Scytodes* sp.1	X	-
			Scytodidae jovem	-	X
		Theridiosomatidae	*Plato* sp.1	-	X
	Opiliones	Cosmetidae	*Roquettea carajas*	X	-
		Sclerosomatidae	*Prionostema* sp.1	-	X
			Sclerosomatidae jovem	X	X
		Stygnidae	*Protimesus* sp.1	X	X
	Pseudoscorpiones	Chernetidae	Chernetidae jovem	-	X
			Spelaeochernes sp.1	X	-
		Chthoniidae	Chthoniidae sp.2	X	-
		Ideoroncidae	Ideoroncidae sp.1	X	-
Chilopoda	Scolopendromorpha	Scolopocryptopidae	*Newportia* sp.1	X	-
Diplopoda	Glomeridesmida	Glomeridesmidae	Glomeridesmida jovem	-	X
	Polydesmida	Fuhrmanodesmidae	Fuhrmanodesmidae jovem	-	X
			Fuhrmanodesmidae sp.1	X	-
		Pyrgodesmidae	Pyrgodesmidae sp.1	X	-
Entognatha	Collembola	Isotomidae	Isotomidae sp.1	X	-
		Sminthuroidea	Sminthuroidea sp.2	-	X
Insecta	Coleoptera	Carabidae	Carabidae sp.1	X	-
		Erotylidae	Erotylidae sp.1	X	-
	Diptera	Psychodidae	Phlebotominae sp.	X	X
		Tipulidae	Tipulidae sp.	X	X
	Hemiptera	Cixiidae	Cixiidae jovem	-	X
		Ochteridae	Ochteridae sp.2	X	-
		Reduviidae	Emesinae sp.6	X	-
			Reduviinae jovem	-	X
		Veliidae	Veliidae jovem	X	-
	Hymenoptera	Formicidae	*Apterostigma collare*	X	-
			Dolichoderus bispinosus	X	-
			Paratrechina sp.1	X	X
	Orthoptera	Phalangopsidae	*Paraclodes* sp.1	X	-
			Phalangopsis sp.1	X	X
Malacostraca	Isopoda	Armadillidae	Armadillidae sp.1	X	-
		Philosciidae	Philosciidae sp.1	X	X
Symphyla			Symphyla jovem	-	X
Gastropoda	Pulmonata	Subulinidae	*Lamellaxis* sp.2	X	X
Amphibia	Anura	Strabomantidae	*Pristimantis* cf. *fenestratus*	X	-
			Anura sp.3	X	-
			Anura sp.7	X	-
Reptilia	Squamata	Gekkonidae	*Thecadactylus rapicauda*	X	-

SB-0044					
TÁXONS				**Seca**	**Úmida**
Arachnida	Araneae	Araneidae	Araneidae jovem	-	X
		Pholcidae	*Mesabolivar eberhardi*	-	X
			Modisimus sp.1	-	X
		Salticidae	Salticidae jovem	-	X
		Theridiidae	Theridiidae jovem	-	X
		Theridiosomatidae	*Plato* sp.1	-	X
	Opiliones	Manaosbiidae	Manaosbiidae sp.1	X	-
		Sclerosomatidae	*Prionostema* sp.1	-	X
Entognatha	Collembola	Paronellidae	Paronellidae sp.1	X	-
Insecta	Diptera	Cecidomyiidae	Cecidomyiidae sp.	-	X
		Ceratopogonidae	Ceratopogonidae jovem	-	X
		Psychodidae	Phlebotominae sp.	-	X
	Hymenoptera	Formicidae	*Dolichoderus bispinosus*	X	-
			Pheidole sp.4	X	-
Mammalia	Chiroptera	Phyllostomidae	*Carollia perspicillata*	X	-
			Glossophaga soricina	X	-

SB-0045					
TÁXONS				Seca	Úmida
Arachnida	Acari	Trombiculidae	Trombiculidae sp.2	X	-
			Acari jovem	-	X
			Acariformes sp.3	-	X
			Mesostigmata sp.1	-	X
	Amblypygi	Phrynidae	*Heterophrinus longicornis*	X	X
	Araneae	Araneidae	Araneidae jovem	X	-
		Corinnidae	Corinnidae jovem	X	X
		Ctenidae	Ctenidae jovem	X	X
		Drymusidae	Drymusidae jovem	X	X
		Ochyroceratidae	Ochyroceratidae jovem	X	X
		Pholcidae	Pholcidae jovem	X	X
		Salticidae	Salticidae jovem	X	-
		Theraphosidae	Theraphosidae jovem	-	X
		Theridiosomatidae	*Plato* sp.1	X	X
	Opiliones	Cosmetidae	*Roquettea carajas*	-	1
		Escadabiidae	Escadabiidae jovem	-	X
		Sclerosomatidae	Sclerosomatidae jovem	-	X
	Pseudoscorpiones	Chernetidae	*Spelaeochernes* sp.1	-	X
Chilopoda	Scolopendromorpha	Scolopocryptopidae	*Newportia* sp.4	X	-
Diplopoda	Polydesmida	Chelodesmidae	Chelodesmidae sp.1	-	X
		Paradoxosomatidae	Paradoxosomatidae sp.1	-	X
		Pyrgodesmidae	Pyrgodesmidae sp.1	-	X
Entognatha	Collembola	Entomobryidae	Entomobryidae sp.3	-	X
		Paronellidae	Paronellidae sp.1	X	X
		Sminthuroidea	Sminthuroidea sp.2	X	-
	Diplura	Campodeidae	Campodeidae sp.1	X	-
Insecta	Blattodea	Blaberidae	Blaberidae jovem	X	-
	Coleoptera	Staphylinidae	Pselaphinae sp.7	X	-
			Coleoptera jovem	-	X
	Diptera	Cecidomyiidae	Cecidomyiidae sp.	-	X
		Dolichopodidae	Dolichopodidae sp.	X	-
	Hemiptera	Cydnidae	Cydnidae jovem	X	-
			Cydnidae sp.1	-	X
		Reduviidae	Reduviidae jovem	-	X
	Hymenoptera	Eurytomidae	Eurytomidae sp.1	-	X
		Formicidae	*Camponotus* sp.2	-	X
			Dolichoderus bispinosus	X	-
			Paratrechina sp.1	X	X
			Solenopsis sp.2	X	-
			Strumigenys elongata	-	X
		Vespidae	Vespidae sp.1	X	X
	Isoptera	Termitidae	*Nasutitermes* sp.1	X	X
			Nasutitermes sp.3	-	X
	Lepidoptera	Noctuoidea	Noctouidea sp. 1	X	-
			Lepidoptera jovem	-	X
	Orthoptera	Phalangopsidae	*Phalangopsis* sp.1	X	X
	Psocoptera		Psocomorpha jovem	-	X
	Thysanura	Nicoletiidae	Nicoletiinae sp.1	X	-
Malacostraca	Isopoda	Armadillidae	Armadillidae sp.1	X	X
		Philosciidae	Philosciidae sp.1	X	X
Mammalia	Chiroptera	Emballonuridae	*Peropteryx kappleri*	-	X

SB-0046					
TÁXONS				Seca	Úmida
Annelida	Haplotaxida		Haplotaxida sp.4	-	X
			Haplotaxida sp.7	X	-
Arachnida	Acari		Astigmata sp.4	X	-
			Mesostigmata sp.1	X	X
			Mesostigmata sp.2	X	X
			Oribatida sp.2	X	-
	Amblypygi	Phrynidae	*Heterophrinus longicornis*	X	-
	Araneae	Corinnidae	*Abapeba* sp.1	X	-
			Corinnidae jovem	X	X
		Ctenidae	Ctenidae jovem	X	X
		Ochyroceratidae	*Speocera* sp.1	X	X
		Pholcidae	*Mesabolivar aurantiacus*	X	X
			Mesabolivar eberhardi	X	-
		Theridiosomatidae	*Plato* sp.1	X	X
		Trechaleidae	Trechaleidae jovem	-	X
	Opiliones	Escadabiidae	Escadabiidae jovem	-	X
		Sclerosomatidae	*Prionostema* sp.1	-	X
	Pseudoscorpiones	Bochicidae	Bochicidae sp.1	-	X
		Chernetidae	*Spelaeochernes* sp.1	-	X
		Chthoniidae	Chthoniidae jovem	-	X
			Pseudochthonius sp.4	X	-
Chilopoda	Scolopendromorpha	Scolopocryptopidae	*Dinocryptops miersii*	X	-
			Newportia sp.1	X	-
			Newportia sp.4	X	-
	Scutigeromorpha	Pselliodidae	*Sphendononema guildingii*	X	-
Diplopoda	Glomeridesmida	Glomeridesmidae	Glomeridesmida jovem	-	X
	Polydesmida	Chelodesmidae	Chelodesmidae sp.1	X	-
		Fuhrmanodesmidae	Fuhrmanodesmidae sp.1	-	X
			Fuhrmanodesmidae sp.2	X	-
			Fuhrmanodesmidae sp.4	X	-
		Pyrgodesmidae	Pyrgodesmidae sp.1	-	X
	Spirostreptida	Pseudonannolenidae	Pseudonannolenidae jovem	X	-
Entognatha	Collembola	Cyphoderidae	Cyphoderidae sp.1	-	X
		Paronellidae	Paronellidae sp.1	X	-
		Sminthuroidea	Sminthuroidea sp.2	X	X
	Diplura	Campodeidae	Campodeidae sp.1	-	X
Insecta	Blattodea	Blattellidae	Blattellidae jovem	-	X
	Coleoptera	Carabidae	*Acupalpus* sp.1	-	X
			Carabidae sp.1	-	X
		Curculionidae	Scolytinae sp.1	X	-
		Gyrinidae	*Gyretes* sp.1	X	-
		Hydrophilidae	Hydrophilidae sp.4	X	-
		Staphylinidae	Pselaphinae sp.1	X	X
			Pselaphinae sp.2	X	X
	Diptera	Culicidae	Culicidae sp.	X	-
		Drosophilidae	Drosophilidae sp.	-	X
		Psychodidae	Phlebotominae sp.	X	-
			Psychodidae sp.	X	X
		Tipulidae	Tipulidae jovem	X	-
	Hemiptera	Cixiidae	Cixiidae jovem	X	-
		Cydnidae	Cydnidae sp.1	X	X
		Veliidae	Veliidae jovem	X	-
	Hymenoptera	Eurytomidae	Eurytomidae sp.1	-	X
		Formicidae	*Carebara* sp.10	X	-
			Crematogaster brasiliensis	-	X
			Gnamptogenys regularis	-	X
			Hypoponera sp.5	-	X

			Paratrechina sp.1	X	X
			Solenopsis invicta	X	X
			Solenopsis sp.7	-	X
	Lepidoptera	Noctuoidea	Noctouidea sp. 2	-	X
	Orthoptera	Phalangopsidae	*Paraclodes* sp.1	X	-
			Phalangopsis sp.1	X	X
	Thysanura	Nicoletiidae	Atelurinae sp.1	-	X
			Nicoletiinae sp.1	X	-
Malacostraca	Decapoda	Palaemonidae	*Macrobrachium* sp.1	X	-
		Pseudothelphusidae	Pseudothelphusidae sp.1	X	X
	Isopoda	Philosciidae	Philosciidae sp.1	-	X
			Philosciidae sp.2	X	-
Symphyla		Scolopendrellidae	*Symphylella* sp.1	-	X
		Scutigerellidae	*Hanseniella* sp.1	X	X
Gastropoda	Pulmonata	Subulinidae	*Lamellaxis* sp.1	X	X
			Lamellaxis sp.2	-	X
			Leptinaria sp.2	-	X
		Systrophiidae	*Happia* sp.1	X	X
Mammalia	Chiroptera	Phyllostomidae	*Carollia perspicillata*	X	X
			Glossophaga soricina	X	-
			Phyllostomus latifolius	X	X

SB-0047					
**TÁXONS**				**Seca**	**Úmida**
Annelida	Haplotaxida		Haplotaxida sp.4	-	X
Arachnida	Acari	Trombiculidae	Trombiculidae sp.2	X	-
			Acari jovem	-	X
			Astigmata sp.3	-	X
			Mesostigmata sp.1	-	X
			Oribatida sp.2	-	X
			Oribatida sp.5	-	X
	Amblypygi	Phrynidae	*Heterophrinus longicornis*	X	X
	Araneae	Anapidae	Anapidae sp.1	-	X
		Araneidae	Alpaida sp.1	-	X
		Dipluridae	Dipluridae jovem	-	X
		Ochyroceratidae	Ochyroceratidae jovem	X	-
			Speocera sp.1	-	X
		Paratropididae	Paratropididae jovem	-	X
		Pholcidae	*Mesabolivar aurantiacus*	X	X
			Mesabolivar eberhardi	-	X
		Salticidae	Salticidae sp.1	-	X
		Scytodidae	Scytodidae jovem	-	X
		Theridiosomatidae	*Plato* sp.1	X	-
	Opiliones	Cosmetidae	*Roquettea carajas*	-	X
		Stygnidae	Stygnidae sp.1	X	-
	Pseudoscorpiones	Chthoniidae	*Pseudochthonius* sp.4	-	X
	Scorpiones	Buthidae	*Ananteris luciae*	X	-
Chilopoda	Scutigeromorpha	Pselliodidae	*Sphendononema* jovem	X	X
Diplopoda	Spirostreptida		Spirostreptida jovem	-	X
Entognatha	Collembola	Isotomidae	Isotomidae sp.1	-	X
		Paronellidae	Paronellidae sp.1	-	X
		Sminthuroidea	Sminthuroidea sp.2	-	X
	Diplura	Campodeidae	Campodeidae sp.1	-	X
Insecta	Coleoptera	Curculionidae	Scolytinae sp.1	X	-
			Coleoptera jovem	-	X
	Diptera	Ceratopogonidae	Ceratopogonidae jovem	-	X
		Psychodidae	Phlebotominae sp.	X	X
	Hemiptera	Cixiidae	Cixiidae jovem	X	X
		Cydnidae	Cydnidae jovem	X	-
		Reduviidae	Emesinae jovem	X	-
			Reduviinae jovem	X	X
		Schizopteridae	Schizopteridae sp.1	-	X
	Hymenoptera	Formicidae	*Apterostigma collare*	-	X
			Pachycondyla constricta	-	X
			Paratrechina sp.1	X	X
			Pheidole sp.4	-	X
	Isoptera	Termitidae	*Nasutitermes* sp.1	X	-
			Nasutitermes sp.3	-	X
	Lepidoptera	Noctuoidea	Noctouidea sp. 1	X	X
			Noctouidea sp. 2	-	X
	Orthoptera	Phalangopsidae	*Paraclodes* sp.1	-	X
			Phalangopsis sp.1	X	X
	Psocoptera		Psocomorpha jovem	X	-
Malacostraca	Isopoda	Armadillidae	Armadillidae sp.1	-	X
Symphyla		Scutigerellidae	*Hanseniella* sp.1	-	X
Gastropoda	Pulmonata	Subulinidae	*Lamellaxis* sp.1	X	X
Mammalia	Chiroptera	Emballonuridae	*Peropteryx kappleri*	-	X
		Phyllostomidae	*Carollia perspicillata*	-	X
			Glossophaga soricina	-	X

SB-0048					
TÁXONS				Seca	Úmida
Annelida	Haplotaxida		Haplotaxida sp.1	X	-
Arachnida	Acari	Astigmata	Astigmata sp.1	X	-
			Astigmata sp.2	-	X
		Eupodidae	*Linopodes* sp.1	X	X
		Opilioacaridae	Opilioacaridae sp.1	X	-
	Amblypygi	Charinidae	*Charinus* sp.1	-	X
		Phrynidae	*Heterophrinus longicornis*	-	X
	Araneae	Barychelidae	Barychelidae jovem	X	-
		Corinnidae	*Abapeba* sp.1	-	X
		Drymusidae	Drymusidae jovem	X	-
		Pholcidae	Pholcidae jovem	X	-
		Salticidae	Salticidae jovem	-	X
		Scytodidae	*Scytodes* sp.1	-	X
		Theraphosidae	Theraphosidae jovem	X	-
		Theridiidae	*Achaearanea* sp.1	X	-
	Opiliones	Cosmetidae	*Roquettea carajas*	X	X
		Escadabiidae	Escadabiidae sp.1	X	-
		Sclerosomatidae	*Prionostema* sp.1	-	X
			Sclerosomatidae jovem	X	-
		Stygnidae	Stygnidae jovem	-	X
	Pseudoscorpiones	Chernetidae	*Spelaeochernes* sp.1	X	X
		Chthoniidae	Chthoniidae sp.1	X	-
	Schizomida	Hubbardiidae	*Rowlandius* sp.1	-	X
Chilopoda	Scolopendromorpha	Scolopocryptopidae	*Dinocryptops* sp.1	X	-
			Newportia jovem	X	-
	Scutigeromorpha	Pselliodidae	*Sphendononema* jovem	X	X
Diplopoda	Glomeridesmida	Glomeridesmidae	Glomeridesmida sp.1	-	X
Entognatha	Collembola	Paronellidae	Paronellidae sp.1	X	-
		Sminthuroidea	Sminthuroidea sp.2	-	X
Insecta	Coleoptera	Carabidae	Carabidae sp.1	X	-
		Elateridae	Elateridae sp.1	X	-
		Gyrinidae	*Gyretes* sp.1	X	-
			Coleoptera jovem	X	X
	Diptera	Cecidomyiidae	Cecidomyiidae sp.	-	X
		Tipulidae	Tipulidae sp.	-	X
	Hemiptera	Cixiidae	Cixiidae sp.1	-	X
		Cydnidae	Cydnidae jovem	X	-
			Cydnidae sp.1	-	X
		Diaspididae	Diaspididae sp.1	-	X
		Reduviidae	Reduviinae jovem	X	X
	Hymenoptera	Formicidae	*Acromyrmex octopinosus*	X	-
			Crematogaster brasiliensis	X	-
			Myrcidris epicharis	-	X
			Pachycondyla constricta	X	-
			Paratrechina sp.1	X	X
	Isoptera		Isoptera jovem	-	X
	Neuroptera	Myrmeleontidae	Myrmeleontidae sp.1	X	-
	Orthoptera	Phalangopsidae	*Phalangopsis* sp.1	X	X
	Psocoptera		Psocomorpha jovem	X	-
Malacostraca	Isopoda	Dubioniscidae	Dubioniscidae sp.1	X	-
		Philosciidae	Philosciidae sp.1	-	X
		Platyarthridae	Platyarthridae sp.2	X	-
Gastropoda	Pulmonata	Subulinidae	*Lamellaxis* sp.1	X	X
		Systrophiidae	*Happia* sp.1	X	-
Mammalia	Chiroptera	Emballonuridae	*Peropteryx kappleri*	-	X
		Phyllostomidae	*Carollia perspicillata*	X	X
			Glossophaga soricina	-	X

Reptilia	Squamata	Gymnophthalmidae	*Neusticurus* sp.	-	X

SB-0049					
TÁXONS				Seca	Úmida
Arachnida	Acari	Ixodidae	*Amblyomma* sp.1	X	-
		Trombiculidae	Trombiculidae sp.1	-	X
			Trombiculidae sp.2	X	-
			Astigmata sp.2	X	X
			Astigmata sp.5	X	-
			Holothyrida sp.1	-	X
			Mesostigmata sp.1	X	X
			Mesostigmata sp.2	X	-
			Oribatida sp.1	-	X
	Amblypygi	Charinidae	*Charinus* sp.1	-	X
		Phrynidae	*Heterophrinus longicornis*	X	X
	Araneae	Corinnidae	*Abapeba* sp.1	X	-
			Corinnidae jovem	X	X
		Ctenidae	Ctenidae jovem	X	X
		Drymusidae	Drymusidae jovem	X	-
		Ochyroceratidae	Ochyroceratidae jovem	X	-
			Speocera sp.1	-	X
		Oonopidae	Oonopidae jovem	X	-
		Pholcidae	*Mesabolivar aurantiacus*	X	X
		Theraphosidae	Theraphosidae jovem	-	X
		Theridiidae	*Achaearanea* sp.1	-	X
		Theridiosomatidae	*Plato* sp.1	X	X
	Opiliones	Cosmetidae	*Roquettea carajas*	X	-
		Escadabiidae	Escadabiidae sp.1	X	-
			Escadabiidae sp.2	X	X
		Gonyleptidae	Gonyleptidae jovem	-	X
	Pseudoscorpiones	Chernetidae	Chernetidae jovem	X	X
			Spelaeochernes sp.1	-	X
		Chthoniidae	Chthoniidae jovem	X	-
	Scorpiones	Buthidae	*Ananteris* jovem	X	-
Chilopoda	Geophilomorpha	Ballophilidae	*Ityphilus* sp.1	X	-
	Scolopendromorpha	Scolopocryptopidae	*Tidops* sp.1	X	-
	Scutigeromorpha	Pselliodidae	*Sphendononema* jovem	X	-
Diplopoda	Glomeridesmida	Glomeridesmidae	Glomeridesmida sp.1	X	X
	Polydesmida	Fuhrmanodesmidae	Fuhrmanodesmidae sp.1	X	-
		Pyrgodesmidae	Pyrgodesmidae sp.1	X	X
Entognatha	Collembola	Isotomidae	Isotomidae sp.1	-	X
		Paronellidae	Paronellidae sp.1	X	-
Insecta	Coleoptera	Carabidae	*Chlaenius* sp.1	-	X
		Curculionidae	Scolytinae sp.4	X	-
		Hydrophilidae	Hydrophilidae sp.5	X	X
		Staphylinidae	*Coproporus* sp.1	X	-
			Scydmaeninae sp.1	-	X
			Scydmaeninae sp.2	X	X
			Spedophilus sp.1	X	-
			Staphylininae sp.2	-	X
	Diptera	Chironomidae	Chironomidae jovem	X	X
		Drosophilidae	Drosophilidae sp.	X	X
		Psychodidae	Phlebotominae sp.	X	X
			Psychodidae sp.	X	X
		Tipulidae	Tipulidae sp.	X	-
	Hemiptera	Cixiidae	Cixiidae jovem	X	X
		Cydnidae	Cydnidae sp.1	X	X
			Cydnidae sp.2	-	X
		Reduviidae	Emesinae jovem	-	X
			Reduviinae jovem	X	X
	Hymenoptera	Formicidae	*Camponotus melanoticus*	X	-

			Camponotus sp.8	-	X
			Carebara sp.10	X	-
			Crematogaster brasiliensis	X	-
			Eurhopalothrix sp.1	-	X
			Odontomachus meinerti	X	-
			Pachycondyla constricta	-	X
			Pachycondyla impressa	-	X
			Pachycondyla striata	X	-
			Pachycondyla verenae	X	-
			Paratrechina sp.1	X	-
			Pheidole sp.1	X	-
			Pheidole sp.4	-	X
			Pheidole sp.5	X	-
			Solenopsis sp.7	X	-
			Strumigenys calamita	X	X
	Isoptera		Isoptera jovem	-	X
	Lepidoptera	Noctuoidea	Noctouidea sp. 2	-	X
			Lepidoptera jovem	X	-
	Orthoptera	Phalangopsidae	*Paraclodes* sp.1	-	X
			Phalangopsis sp.1	X	X
	Thysanura	Nicoletiidae	Atelurinae sp.1	-	X
Malacostraca	Isopoda	Armadillidae	Armadillidae sp.1	X	X
		Philosciidae	Philosciidae sp.1	X	X
			Philosciidae sp.2	X	X
		Platyarthridae	Platyarthridae sp.2	X	X
		Scleropactidae	Scleropactidae sp.2	X	X
Gastropoda	Pulmonata	Subulinidae	*Lamellaxis* sp.1	X	X
Mammalia	Chiroptera	Phyllostomidae	*Carollia perspicillata*	X	X
			Diphylla ecaudata	X	X
			Phyllostomus latifolius	-	X

SB-0050					
TÁXONS				**Seca**	**Úmida**
Arachnida	Acari		Oribatida sp.2	-	X
	Araneae	Ochyroceratidae	*Speocera* sp.1	-	X
		Oonopidae	Oonopidae sp.3	X	-
		Segestriidae	Segestriidae jovem	X	-
		Theridiosomatidae	Theridiosomatidae jovem	-	X
	Opiliones	Cosmetidae	Cosmetidae sp.4	-	X
		Escadabiidae	Escadabiidae jovem	-	X
			Escadabiidae sp.1	X	-
	Palpigradi	Eukoeneniidae	*Eukoenenia* sp.1	-	X
	Pseudoscorpiones	Chernetidae	*Spelaeochernes* sp.1	X	X
		Olpiidae	Olpiidae sp.1	X	-
	Scorpiones	Buthidae	*Ananteris luciae*	X	-
Chilopoda	Scolopendromorpha	Cryptopidae	*Cryptops* sp.1	-	X
	Scutigeromorpha	Pselliodidae	*Sphendononema* jovem	X	-
Diplopoda	Glomeridesmida	Glomeridesmidae	Glomeridesmida sp.1	X	X
	Polydesmida	Chelodesmidae	Chelodesmidae jovem	X	-
		Pyrgodesmidae	Pyrgodesmidae sp.1	X	X
	Stemmiulida	Stemmiulidae	Stemmiulidae sp.1	X	-
Entognatha	Collembola	Entomobryidae	Entomobryidae sp.4	-	X
		Paronellidae	Paronellidae sp.1	X	X
		Sminthuroidea	Sminthuroidea sp.2	-	X
	Diplura	Projapygidae	Projapygidae sp.1	X	X
Insecta	Coleoptera	Carabidae	Carabidae sp.1	-	X
		Staphylinidae	Pselaphinae sp.1	-	X
	Diptera	Cecidomyiidae	Cecidomyiidae sp.	-	X
		Psychodidae	Phlebotominae sp.	-	X
	Hemiptera	Cixiidae	Cixiidae jovem	X	X
	Hymenoptera	Formicidae	*Camponotus crassus*	X	-
			Cephalotes cristatus	X	-
			Rogeria blanda	X	-
	Orthoptera	Phalangopsidae	*Paraclodes* sp.1	X	X
			Phalangopsis sp.1	-	X
Malacostraca	Isopoda	Armadillidae	Armadillidae sp.1	X	-
		Philosciidae	Philosciidae sp.1	X	X
			Philosciidae sp.2	X	X
Symphyla		Scolopendrellidae	*Scolopendrellopsis* sp.1	X	-
Gastropoda	Pulmonata	Subulinidae	*Lamellaxis* sp.1	X	X
Amphibia	Anura	Strabomantidae	*Pristimantis* cf. *fenestratus*	X	-
Mammalia	Chiroptera	Emballonuridae	*Peropteryx* sp.	-	X

SB-0051					
TÁXONS				Seca	Úmida
Arachnida	Acari	Argasidae	*Ornithodoros* sp.1	-	X
		Ixodidae	*Amblyomma* sp.1	X	-
			Amblyomma sp.2	-	X
		Trombiculidae	Trombiculidae sp.1	X	X
			Trombiculidae sp.2	X	X
			Acariformes sp.4	X	-
			Holothyrida sp.2	X	X
			Mesostigmata sp.1	X	-
			Oribatida sp.2	X	-
			Oribatida sp.4	-	X
	Amblypygi	Phrynidae	*Heterophrinus longicornis*	X	X
	Araneae	Corinnidae	*Abapeba* sp.1	-	X
			Corinnidae jovem	X	-
		Ctenidae	Ctenidae jovem	X	X
		Ochyroceratidae	*Ochyrocera* sp.1	X	-
			Speocera sp.1	X	X
		Oonopidae	Oonopidae jovem	X	X
			Oonopidae sp.4	X	-
		Pholcidae	*Mesabolivar aurantiacus*	X	X
		Scytodidae	*Scytodes* sp.1	X	X
		Theridiosomatidae	*Plato* sp.1	X	X
	Opiliones	Escadabiidae	Escadabiidae sp.1	X	-
			Escadabiidae sp.2	X	-
		Neogoveidae	Neogoveidae jovem	X	-
		Sclerosomatidae	Sclerosomatidae jovem	X	-
	Pseudoscorpiones	Bochicidae	Bochicidae sp.1	-	X
		Chernetidae	*Spelaeochernes* sp.1	X	X
		Chthoniidae	Chthoniidae sp.1	X	X
Chilopoda	Scutigeromorpha	Pselliodidae	*Sphendononema guildingii*	X	-
Diplopoda	Polydesmida	Pyrgodesmidae	Pyrgodesmidae sp.1	X	X
	Siphonophorida	Siphonophoridae	Siphonophoridae sp.1	-	X
Entognatha	Collembola	Paronellidae	Paronellidae sp.1	X	-
	Diplura	Campodeidae	Campodeidae sp.1	X	-
		Japygidae	Japygidae sp.1	-	X
Insecta	Blattodea	Blaberidae	*Blaberus* sp.1	X	X
		Blattidae	Blattidae jovem	X	-
	Coleoptera	Carabidae	*Lelis* sp.1	X	-
		Chrysomelinae	Chrysomelinae sp.2	X	-
		Staphylinidae	*Coproporus* sp.1	-	X
			Scydmaeninae sp.2	X	X
	Diptera	Ceratopogonidae	Ceratopogonidae jovem	-	X
		Culicidae	Culicidae sp.	X	-
		Drosophilidae	Drosophilidae sp.	-	X
		Phoridae	Phoridae sp.	X	-
		Psychodidae	Phlebotominae sp.	X	X
			Psychodidae sp.	-	X
		Tipulidae	Tipulidae sp.	X	X
	Hemiptera	Cixiidae	Cixiidae jovem	X	-
			Cixiidae sp.5	-	X
		Cydnidae	Cydnidae sp.1	-	X
		Reduviidae	Emesinae jovem	-	X
			Reduviinae jovem	X	-
			Zelurus sp.1	-	X
	Hymenoptera	Formicidae	*Pachycondyla constricta*	-	X
	Isoptera	Termitidae	*Nasutitermes* sp.1	X	-
	Lepidoptera	Noctuoidea	Noctouidea sp. 1	X	X
		Tineoidea	Tineoidea sp. 6	X	-

	Orthoptera	Phalangopsidae	*Paraclodes* sp.1	X	X
			Phalangopsis sp.1	X	X
	Psocoptera		Psocomorpha jovem	X	-
			Trogiomorpha jovem	X	-
Malacostraca	Isopoda	Armadillidae	Armadillidae sp.1	X	X
		Philosciidae	Philosciidae sp.1	X	-
Symphyla		Scutigerellidae	*Hanseniella* sp.1	X	-
Gastropoda	Pulmonata	Subulinidae	*Lamellaxis* sp.1	X	-
		Systrophiidae	*Happia* sp.1	X	X
Mammalia	Chiroptera	Emballonuridae	*Peropteryx kappleri*	X	X
		Furipteridae	*Furipterus horrens*	-	X
		Phyllostomidae	*Carollia perspicillata*	-	X
			Diphylla ecaudata	-	X
			Phyllostomus latifolius	-	X
			Trachops cirrhosus	X	X

SB-0052					
TÁXONS				**Seca**	**Úmida**
Annelida	Haplotaxida		Tubificina sp.1	-	X
Arachnida	Acari		Mesostigmata sp.4	-	X
			Oribatida sp.2	X	X
			Oribatida sp.3	-	X
	Amblypygi	Phrynidae	*Heterophrinus longicornis*	-	X
	Araneae	Araneidae	*Alpaida* sp.1	-	X
		Corinnidae	*Tupirina* sp.1	X	-
		Drymusidae	Drymusa sp.1	X	X
		Mysmenidae	Mysmenidae jovem	X	-
		Ochyroceratidae	*Ochyrocera* sp.2	X	-
			Ochyroceratidae jovem	-	X
		Pholcidae	*Mesabolivar aurantiacus*	-	X
			Pholcidae jovem	X	X
		Theridiosomatidae	*Plato* sp.1	-	X
		Trechaleidae	Trechaleidae jovem	-	X
	Opiliones	Cosmetidae	Cosmetidae jovem	X	-
		Escadabiidae	Escadabiidae jovem	-	X
		Neogoveidae	*Canga renatae*	-	X
Chilopoda	Scolopendromorpha	Scolopendridae	*Otostigmus* sp.1	X	X
Diplopoda	Polydesmida	Fuhrmanodesmidae	Fuhrmanodesmidae sp.4	-	X
Entognatha	Collembola	Paronellidae	Paronellidae sp.1	X	-
			Paronellidae sp.4	-	X
Insecta	Coleoptera	Chrysomelidae	Alticini sp.1	-	X
	Diptera	Cecidomyiidae	Cecidomyiidae sp.	-	X
		Ceratopogonidae	Ceratopogonidae sp.	X	-
		Psychodidae	Phlebotominae sp.	X	X
			Psychodidae sp.	X	-
		Tipulidae	Tipulidae sp.	X	X
	Hemiptera	cf. Nabidae	cf. Nabidae jovem	-	X
		Cixiidae	Cixiidae jovem	X	-
		Fulgoridae	Fulgoridae sp.3	-	X
		Reduviidae	Reduviinae jovem	-	X
	Hymenoptera	Formicidae	*Carebara* sp.2	-	X
			Pachycondyla constricta	X	-
			Paratrechina sp.1	-	X
	Lepidoptera	Noctuoidea	Noctouidea sp. 2	X	X
			Noctouidea sp. 6	X	-
	Orthoptera	Phalangopsidae	*Paraclodes* sp.1	X	-
			Phalangopsis sp.1	X	X
	Psocoptera		Psocomorpha jovem	X	X
Pauropoda	Tetramerocerata		Tetramerocerata sp.1	-	X

SB-0053					
TÁXONS				Seca	Úmida
Arachnida	Acari	Ixodidae	*Amblyomma* sp.1	X	-
			Astigmata sp.6	X	-
	Amblypygi	Phrynidae	*Heterophrinus longicornis*	X	X
	Araneae	Drymusidae	Drymusidae jovem	-	X
		Ochyroceratidae	*Speocera* sp.1	X	-
		Oonopidae	Oonopidae jovem	X	-
		Pholcidae	*Mesabolivar aurantiacus*	-	X
		Theridiosomatidae	*Plato* sp.1	X	X
	Opiliones	Cosmetidae	*Roquettea carajas*	-	X
		Sclerosomatidae	*Prionostema* sp.1	-	X
	Pseudoscorpiones	Chernetidae	*Spelaeochernes* sp.1	X	-
Diplopoda	Polyzoniida	Siphonotidae	Siphonotidae sp.1	X	X
	Siphonophorida	Siphonophoridae	Siphonophoridae sp.1	X	-
Entognatha	Collembola	Paronellidae	Paronellidae sp.1	X	-
		Sminthuroidea	Sminthuroidea sp.2	X	X
	Diplura	Campodeidae	Campodeidae sp.1	X	-
Insecta	Coleoptera	Leiodidae	Leiodidae sp.2	-	X
		Staphylinidae	Scydmaeninae sp.1	X	-
	Diptera	Culicidae	Culicinae jovem	-	X
		Tipulidae	Tipulidae sp.	-	X
			Diptera jovem	X	-
	Hemiptera	Reduviidae	Emesinae jovem	-	X
	Hymenoptera	Braconidae	Braconidae sp.1	-	X
		Formicidae	*Crematogaster brasiliensis*	-	X
			Dolichoderus bispinosus	X	-
			Gnamptogenys haenschi	X	-
			Pheidole sp.12	-	X
	Lepidoptera	Noctuoidea	Noctouidea sp. 6	X	-
	Psocoptera	Ectopsocidae	Ectopsocidae sp.1	X	-
Malacostraca	Isopoda	Philosciidae	Philosciidae sp.2	X	-
		Platyarthridae	Platyarthridae sp.3	X	-
Gastropoda	Pulmonata	Subulinidae	*Lamellaxis* sp.3	X	-
Amphibia	Anura	Strabomantidae	*Pristimantis* cf. *fenestratus*	-	X

SB-0054					
TÁXONS				Seca	Úmida
Arachnida	Acari		Astigmata sp.4	-	X
			Holothyrida sp.2	-	X
			Oribatida sp.2	-	X
	Amblypygi	Phrynidae	*Heterophrinus longicornis*	-	X
	Araneae	Araneidae	*Alpaida* sp.1	X	X
		Ctenidae	Ctenidae jovem	X	-
		Drymusidae	Drymusa sp.1	X	X
		Ochyroceratidae	*Ochyrocera* sp.1	X	-
			Ochyroceratidae jovem	-	X
		Oonopidae	gr. *Xycarpphy* sp.1	-	X
		Pholcidae	*Leptopholcus* sp.1	-	X
			Mesabolivar eberhardi	-	X
			Modisimus sp.1	X	X
		Theraphosidae	*Guyruita cerrado*	-	X
		Theridiosomatidae	Theridiosomatidae jovem	-	X
	Opiliones	Cosmetidae	Cosmetidae jovem	X	X
		Escadabiidae	Escadabiidae sp.2	-	X
		Sclerosomatidae	*Prionostema* sp.1	X	X
	Pseudoscorpiones	Cheiridiidae	Cheiridiidae sp.1	-	X
		Chernetidae	*Spelaeochernes* sp.1	-	X
		Chthoniidae	Chthoniidae sp.1	-	X
Chilopoda	Geophilomorpha	Schendylidae	*Schendylops* sp.1	-	X
	Scolopendromorpha	Scolopocryptopidae	*Newportia* jovem	-	X
			Newportia sp.3	X	-
Diplopoda	Polydesmida	Pyrgodesmidae	Pyrgodesmidae sp.1	X	X
	Siphonophorida	Siphonophoridae	Siphonophoridae sp.1	-	X
Entognatha	Collembola	Cyphoderidae	Cyphoderidae sp.1	-	X
		Paronellidae	Paronellidae sp.1	X	X
	Diplura	Campodeidae	Campodeidae sp.1	-	X
Insecta	Blattodea	Blattidae	Blattidae jovem	X	X
		Polyphagidae	Polyphagidae jovem	-	X
	Coleoptera	Scarabaeidae	*Ataenus* sp.1	-	X
	Diptera	Cecidomyiidae	Cecidomyiidae sp.	-	X
		Drosophilidae	Drosophilidae sp.	X	-
		Phoridae	Phoridae sp.	X	X
		Psychodidae	Phlebotominae sp.	-	X
		Tipulidae	Tipulidae sp.	-	X
	Hemiptera	cf. Nabidae	cf. Nabidae sp.1	-	X
		Cixiidae	Cixiidae jovem	-	X
			Cixiidae sp.1	X	-
		Cydnidae	Cydnidae jovem	X	-
		Fulgoridae	Fulgoridae sp.2	-	X
		Reduviidae	Emesinae jovem	X	-
			Reduviinae jovem	X	-
	Hymenoptera	Formicidae	*Crematogaster limata*	-	X
			Dolichoderus bispinosus	X	-
			Hypoponera opacior	-	X
			Paratrechina sp.1	-	X
			Pheidole sp.12	-	X
			Pheidole sp.4	-	X
			Pheidole sp.5	-	X
			Pheidole sp.6	-	X
	Isoptera	Termitidae	*Nasutitermes* sp.1	X	X
	Lepidoptera	Noctuoidea	Noctouidea sp. 2	-	X
	Orthoptera	Phalangopsidae	*Paraclodes* sp.1	-	X
			Phalangopsis sp.1	-	X
	Psocoptera		Psocomorpha jovem	-	X

Malacostraca	Isopoda	Armadillidae	Armadillidae sp.1	-	X
		Philosciidae	Philosciidae sp.1	X	X
Gastropoda	Pulmonata	Subulinidae	*Leptinaria* sp.1	X	-
		Systrophiidae	*Happia* sp.4	X	-
Turbellaria	Tricladida	Geoplanidae	Geoplanidae sp.1	-	X
Amphibia	Anura		Anura sp.3	X	-
Mammalia	Chiroptera	Emballonuridae	*Peropteryx kappleri*	-	X

SB-0055					
TÁXONS				Seca	Úmida
Arachnida	Amblypygi	Phrynidae	*Heterophrinus longicornis*	-	X
	Araneae	Araneidae	*Alpaida* sp.1	X	-
			Araneidae jovem	-	X
		Ctenidae	Ctenidae jovem	X	X
		Mysmenidae	*Microdipoena* sp.1	-	X
		Ochyroceratidae	Ochyroceratidae jovem	X	-
		Pholcidae	Pholcidae jovem	X	-
		Salticidae	Salticidae jovem	X	X
		Symphytognathidae	*Anapistula* sp.1	-	X
		Theraphosidae	Theraphosidae jovem	-	X
		Theridiosomatidae	*Plato* sp.1	-	X
			Theridiosomatidae jovem	X	X
	Pseudoscorpiones	Chernetidae	*Spelaeochernes* sp.1	X	-
	Scorpiones	Buthidae	*Ananteris* jovem	X	-
Diplopoda	Polydesmida	Pyrgodesmidae	Pyrgodesmidae sp.1	-	X
Entognatha	Diplura	Campodeidae	Campodeidae sp.1	-	X
Insecta	Coleoptera	Staphylinidae	Pselaphinae sp.5	-	X
			Staphylinidae jovem	X	-
		Tenebrionidae	Tenebrionidae jovem	X	-
	Diptera	Cecidomyiidae	Cecidomyiidae sp.	X	X
		Ceratopogonidae	Ceratopogonidae jovem	X	X
		Culicidae	Culicidae sp.	X	-
		Muscidae	Muscidae jovem	X	-
		Phoridae	Phoridae sp.	X	-
		Tipulidae	Tipulidae sp.	-	X
	Hemiptera	cf. Nabidae	cf. Nabidae jovem	X	-
		Cydnidae	Cydnidae sp.1	X	-
		Reduviidae	Reduviinae jovem	X	X
	Hymenoptera	Formicidae	*Dolichoderus bispinosus*	-	X
			Pheidole sp.4	-	X
	Isoptera	Termitidae	*Nasutitermes* sp.1	-	X
			Nasutitermes sp.2	X	X
	Lepidoptera	Noctuoidea	Noctouidea sp. 2	X	X
		Tineoidea	Tineoidea sp. 8	X	-
	Neuroptera	Myrmeleontidae	Myrmeleontidae sp.1	X	-
	Orthoptera	Phalangopsidae	*Paraclodes* sp.1	X	-
			Phalangopsis sp.1	X	-
Malacostraca	Isopoda	Armadillidae	Armadillidae sp.1	-	X
Mammalia	Chiroptera	Emballonuridae	*Peropteryx kappleri*	X	-

SB-0056					
TÁXONS				**Seca**	**Úmida**
Arachnida	Acari	Ixodidae	*Amblyomma* sp.1	X	-
			Oribatida sp.2	X	-
	Araneae	Araneidae	*Alpaida* sp.1	X	X
		Ctenidae	Ctenidae jovem	-	X
		Linyphiidae	Linyphiidae sp.3	X	-
		Pholcidae	*Mesabolivar aurantiacus*	-	X
			Mesabolivar cambridgei	X	-
			Mesabolivar eberhardi	-	X
		Pisauridae	Pisauridae jovem	X	-
		Salticidae	Salticidae sp.10	X	-
		Tetragnathidae	Tetragnathidae jovem	X	-
	Opiliones	Sclerosomatidae	*Prionostema* sp.1	X	X
Diplopoda	Polydesmida	Pyrgodesmidae	Pyrgodesmidae sp.2	X	-
Entognatha	Collembola	Entomobryidae	Entomobryidae sp.3	X	-
Insecta	Coleoptera	Staphylinidae	Staphylininae sp.2	X	-
	Diptera	Acroceridae	Acroceridae sp.	X	-
		Tabanidae	Tabanidae sp.	X	-
	Hemiptera	Cydnidae	Cydnidae jovem	X	-
		Gerridae	*Rheumatobates* sp.1	X	-
			Trepobates sp.1	-	X
	Hymenoptera	Formicidae	*Dolichoderus bispinosus*	X	-
			Gnamptogenys sp.1	X	-
			Myrcidris epicharis	X	-
			Neivamyrmex sp.2	-	X
			Pachycondyla arhuaca	X	-
			Pheidole sp.12	X	-
			Pyramica sp.1	X	-
		Vespidae	Vespidae sp.1	X	-
	Isoptera		Isoptera jovem	X	-
	Odonata	Anisoptera	Anisoptera jovem	-	X
Gastropoda	Pulmonata	Systrophiidae	*Happia* sp.2	X	-
Amphibia	Anura		Anura sp.3	X	-
			Anura sp.4	-	X
Mammalia	Chiroptera	Emballonuridae	*Peropteryx* sp.	-	X

SB-0057					
TÁXONS				Seca	Úmida
Annelida	Haplotaxida		Haplotaxida sp.4	-	X
Arachnida	Araneae	Araneidae	*Cyclosa* sp.1	-	X
		Corinnidae	Corinnidae jovem	-	X
		Ctenidae	Ctenidae jovem	X	X
		Drymusidae	Drymusidae jovem	-	X
		Oonopidae	Oonopidae sp.4	X	-
			Oonopidae sp.13	X	X
		Pholcidae	*Mesabolivar eberhardi*	X	-
			Pholcidae jovem	X	X
Entognatha	Collembola	Entomobryidae	Entomobryidae sp.3	-	X
		Paronellidae	Paronellidae sp.1	-	X
Insecta	Diptera	Culicidae	Culicidae sp.	X	-
		Tipulidae	Tipulidae sp.	-	X
	Hymenoptera	Formicidae	*Acropyga* cf. *smithii*	-	X
			Camponotus atriceps	-	X
			Crematogaster limata	X	-
			Ochetomyrmex neopolitus	X	-
			Pheidole sp.8	-	X
			Pseudomyrmex lizeri	-	X
		Vespidae	Vespidae sp.1	-	X
	Isoptera	Termitidae	*Nasutitermes* sp.2	-	X
	Lepidoptera	Noctuoidea	Noctouidea sp. 2	-	X
			Noctouidea sp. 6	X	-
Malacostraca	Decapoda	Pseudothelphusidae	Pseudothelphusidae sp.1	-	X
	Isopoda	Armadillidae	Armadillidae sp.1	-	X
Reptilia	Squamata	Gymnophthalmidae	*Neusticurus* sp.	-	X

SB-0058					
TÁXONS				**Seca**	**Úmida**
Arachnida	Amblypygi	Phrynidae	*Heterophrinus longicornis*	X	-
	Araneae	Drymusidae	Drymusidae jovem	X	-
		Oonopidae	Oonopidae jovem	X	-
		Pholcidae	*Mesabolivar aurantiacus*	-	X
			Pholcidae jovem	X	-
		Scytodidae	Scytodidae jovem	X	-
		Tetrablemmidae	*Matta* sp.1	X	-
		Theraphosidae	Theraphosidae jovem	X	-
		Theridiosomatidae	Theridiosomatidae jovem	-	X
	Pseudoscorpiones	Chernetidae	*Spelaeochernes* sp.1	X	X
		Chthoniidae	Chthoniidae sp.1	X	-
Chilopoda	Scolopendromorpha	Cryptopidae	*Cryptops* jovem	X	-
Entognatha	Collembola	Paronellidae	Paronellidae sp.1	X	-
Insecta	Blattodea	Blaberidae	Blaberidae jovem	-	X
	Diptera	Acroceridae	Acroceridae sp.	X	-
		Ceratopogonidae	Ceratopogonidae sp.	X	-
		Drosophilidae	Drosophilidae sp.	X	-
		Tipulidae	Tipulidae sp.	-	X
	Hemiptera	Delphacidae	Delphacidae jovem	X	-
		Ochteridae	Ochteridae sp.1	X	-
		Reduviidae	Reduviinae jovem	X	X
		Schizopteridae	Schizopteridae sp.1	X	-
	Hymenoptera	Formicidae	*Gnamptogenys strigata*	-	X
			Pachycondyla striata	-	X
			Paratrechina sp.1	X	-
			Pheidole sp.1	X	-
		Vespidae	Vespidae sp.1	X	-
	Isoptera	Termitidae	*Nasutitermes* sp.2	X	-
	Lepidoptera	Noctuoidea	Noctouidea sp. 2	-	X
	Orthoptera	Phalangopsidae	*Paraclodes* sp.1	-	X
			Phalangopsis sp.1	X	-
	Thysanura	Nicoletiidae	Nicoletiinae sp.1	X	-
Malacostraca	Decapoda	Pseudothelphusidae	Pseudothelphusidae sp.1	X	-
	Isopoda	Armadillidae	Armadillidae sp.1	X	-
		Philosciidae	Philosciidae sp.1	-	X
			Philosciidae sp.2	X	-
Amphibia	Anura	Strabomantidae	*Pristimantis* cf. *fenestratus*	-	X
			Anura sp.3	X	-

SB-0059					
TÁXONS				Seca	Úmida
Arachnida	Araneae	Pholcidae	*Mesabolivar eberhardi*	-	X
			Pholcidae jovem	X	-
		Theridiosomatidae	*Plato* sp.1	-	X
		Trechaleidae	Trechaleidae jovem	X	X
	Opiliones	Sclerosomatidae	*Prionostema* sp.1	-	X
	Scorpiones	Buthidae	*Tityus tucurui*	X	-
Insecta	Blattodea	Blaberidae	Blaberidae jovem	X	-
	Coleoptera	Gyrinidae	*Gyretes* sp.1	X	X
		Staphylinidae	Staphylinidae sp.6	X	-
	Diptera	Tipulidae	Tipulidae sp.	X	-
	Hemiptera	Veliidae	*Rhagovelia* sp.1	X	X
			Rhagovelia sp.2	X	-
	Hymenoptera	Formicidae	*Camponotus atriceps*	X	-
			Camponotus sp.2	-	X
			Crematogaster brasiliensis	X	-
			Dolichoderus bispinosus	X	-
			Pheidole sp.5	-	X
			Wasmannia auropunctata	X	-
			Wasmannia sp.1	X	-
	Isoptera	Termitidae	*Nasutitermes* sp.1	-	X
	Orthoptera	Phalangopsidae	*Paraclodes* sp.1	X	X
Malacostraca	Decapoda	Palaemonidae	*Macrobrachium* sp.1	X	X
		Pseudothelphusidae	Pseudothelphusidae sp.1	X	X
Amphibia	Anura	Strabomantidae	*Pristimantis* cf. *fenestratus*	X	-

SB-0060					
TÁXONS				Seca	Úmida
Arachnida	Acari	Trombiculidae	Trombiculidae sp.2	X	-
	Amblypygi	Phrynidae	*Heterophrinus longicornis*	X	-
	Araneae	Araneidae	*Alpaida* sp.1	-	X
			Araneidae jovem	X	X
		Linyphiidae	Linyphiidae sp.4	X	-
		Oonopidae	gr. *Xycarpphy* sp.1	X	-
		Pholcidae	*Mesabolivar eberhardi*	-	X
			Pholcidae jovem	X	X
		Scytodidae	Scytodidae jovem	X	-
		Theridiosomatidae	*Plato* sp.1	X	-
			Theridiosomatidae sp.1	-	X
	Opiliones	Cosmetidae	Cosmetidae jovem	X	-
		Escadabiidae	Escadabiidae jovem	X	-
	Pseudoscorpiones	Chernetidae	*Spelaeochernes* sp.1	X	-
Diplopoda	Polydesmida	Paradoxosomatidae	Paradoxosomatidae jovem	X	-
	Spirostreptida	Pseudonannolenidae	Pseudonannolenidae sp.1	X	-
Entognatha	Diplura	Campodeidae	Campodeidae sp.1	X	-
Insecta	Coleoptera	Byrrhidae	Byrrhidae sp.2	X	-
		Carabidae	Carabidae sp.1	X	-
			Carabidae sp.3	X	-
		Staphylinidae	Pselaphinae sp.2	X	-
			Staphylininae sp.2	X	-
	Diptera	Cecidomyiidae	Cecidomyiidae sp.	X	-
		Culicidae	*Anopheles* jovem	-	X
		Psychodidae	Phlebotominae sp.	X	-
		Sciaridae	Sciaridae sp.	X	-
		Tipulidae	Tipulidae sp.	-	X
	Hemiptera	Cixiidae	Cixiidae jovem	X	-
		Cydnidae	Cydnidae jovem	X	-
			Cydnidae sp.1	-	X
		Veliidae	*Paravelia* sp.1	-	X
	Hymenoptera	Formicidae	*Dolichoderus bispinosus*	X	-
	Lepidoptera		Lepidoptera jovem	-	X
	Odonata	Anisoptera	Anisoptera jovem	-	X
	Orthoptera	Phalangopsidae	*Paraclodes* sp.1	X	-
			Phalangopsis sp.1	X	X
	Thysanura	Nicoletiidae	Nicoletiinae sp.1	-	X
Malacostraca	Decapoda	Pseudothelphusidae	Pseudothelphusidae sp.1	-	X
	Isopoda	Armadillidae	Armadillidae sp.1	X	-
		Philosciidae	Philosciidae sp.1	X	-
Symphyla		Scutigerellidae	*Hanseniella* sp.1	X	-
Turbellaria	Tricladida		Continenticola sp.1	X	-
Amphibia	Anura	Strabomantidae	*Pristimantis* cf. *fenestratus*	X	-
Mammalia	Chiroptera	Phyllostomidae	*Carollia perspicillata*	-	X
			Glossophaga soricina	-	X

SB-0061					
TÁXONS				**Seca**	**Úmida**
Annelida	Haplotaxida		Haplotaxida sp.2	-	X
Arachnida	Amblypygi	Phrynidae	*Heterophrinus longicornis*	X	-
	Araneae	Araneidae	Araneidae jovem	-	X
		Corinnidae	*Tupirina* sp.1	X	-
		Mysmenidae	Mysmenidae sp.1	-	X
		Pholcidae	*Mesabolivar eberhardi*	X	X
			Pholcidae jovem	X	X
		Scytodidae	*Scytodes* sp.1	X	-
		Theridiidae	Theridiidae jovem	-	X
		Theridiosomatidae	Theridiosomatidae sp.1	-	X
	Opiliones	Cosmetidae	*Roquettea carajas*	X	X
	Pseudoscorpiones	Chernetidae	*Spelaeochernes* sp.1	-	X
Diplopoda	Spirostreptida		Spirostreptida jovem	-	X
Entognatha	Collembola	Sminthuroidea	Sminthuroidea sp.2	-	X
	Diplura	Campodeidae	Campodeidae sp.1	-	X
Insecta	Coleoptera	Carabidae	Carabidae sp.2	X	-
	Diptera	Cecidomyiidae	Cecidomyiidae sp.	-	X
		Psychodidae	Psychodidae sp.	-	X
	Hemiptera	Cydnidae	Cydnidae sp.1	X	X
	Hymenoptera	Formicidae	*Acromyrmex octopinosus*	X	-
			Camponotus sp.2	-	X
			Crematogaster brasiliensis	X	-
			Pachycondyla constricta	-	X
			Pheidole sp.1	X	-
			Pheidole sp.11	-	X
	Isoptera	Termitidae	*Nasutitermes* sp.1	X	-
			Nasutitermes sp.3	X	-
	Lepidoptera		Lepidoptera jovem	X	-
	Orthoptera	Phalangopsidae	*Paraclodes* sp.1	X	-
			Phalangopsis sp.1	-	X
	Psocoptera		Troctomorpha jovem	X	-
Malacostraca	Isopoda	Armadillidae	Armadillidae sp.1	-	X
		Dubioniscidae	Dubioniscidae sp.1	-	X
		Scleropactidae	Scleropactidae sp.2	-	X
Amphibia	Anura	Strabomantidae	*Pristimantis* cf. *fenestratus*	-	X
Mammalia	Chiroptera	Phyllostomidae	*Carollia perspicillata*	X	X
	Rodentia	Cricetidae	*Rhipidomys* sp.	X	-
Reptilia	Squamata	Gekkonidae	*Thecadactylus rapicauda*	X	-
			Sauria sp.3	-	X

SB-0063					
**TÁXONS**				**Seca**	**Úmida**
Arachnida	Acari		Holothyrida sp.2	-	X
	Amblypygi	Charinidae	*Charinus* sp.1	X	-
	Araneae	Araneidae	*Alpaida* sp.1	X	-
			Araneidae jovem	X	X
		Ctenidae	Ctenidae jovem	X	-
		Oonopidae	Oonopidae jovem	X	-
		Paratropididae	*Paratrops* sp.1	X	X
		Pholcidae	*Mesabolivar eberhardi*	X	X
		Scytodidae	*Scytodes* sp.1	-	X
	Opiliones	Sclerosomatidae	*Prionostema* sp.1	X	X
Entognatha	Collembola	Paronellidae	Paronellidae sp.1	X	-
		Sminthuroidea	Sminthuroidea sp.2	-	X
Insecta	Diptera	Cecidomyiidae	Cecidomyiidae sp.	X	-
		Drosophilidae	Drosophilidae sp.	X	X
		Muscidae	Muscidae sp.	-	X
		Psychodidae	Phlebotominae sp.	X	-
	Hemiptera	Ochteridae	Ochteridae jovem	X	X
	Hymenoptera	Formicidae	*Forelius* sp.1	-	X
			Hypoponera sp.5	-	X
			Neivamyrmex sp.1	X	-
			Neivamyrmex sp.2	-	X
			Paratrechina sp.1	X	-
	Isoptera	Termitidae	*Nasutitermes* sp.1	-	X
	Lepidoptera	Noctuoidea	Noctouidea sp. 1	-	X
	Orthoptera	Phalangopsidae	*Paraclodes* sp.1	X	-
			Phalangopsis sp.1	X	-
Amphibia	Anura	Leptodactylidae	*Leptodactylus* cf. *vastus*	X	-
		Strabomantidae	*Pristimantis* cf. *fenestratus*	X	-
Mammalia	Chiroptera	Phyllostomidae	*Carollia perspicillata*	X	-
Reptilia	Crocodylia	Alligatoridae	Alligatoridae sp.	-	X
	Squamata		Sauria sp.2	X	-

SB-0064					
TÁXONS				Seca	Úmida
Arachnida	Acari	Argasidae	*Ornithodoros* sp.1	X	X
	Amblypygi	Phrynidae	*Heterophrinus longicornis*	-	X
	Araneae	Araneidae	Araneidae jovem	X	-
		Corinnidae	Corinnidae jovem	X	X
		Ctenidae	Ctenidae jovem	X	-
			Enoploctenus sp.	X	X
		Filistatidae	Filistatidae jovem	X	-
			Filistatidae sp.1	-	X
		Oonopidae	Oonopidae sp.3	X	-
		Pholcidae	*Leptopholcus* sp.1	X	-
			Mesabolivar aurantiacus	-	X
			Mesabolivar eberhardi	X	-
			Modisimus sp.1	X	-
			Ninetinae sp.1	X	-
		Salticidae	Salticidae jovem	X	X
		Scytodidae	Scytodidae jovem	X	X
	Opiliones	Cosmetidae	Cosmetidae sp.1	X	-
			Cosmetidae sp.3	X	-
		Stygnidae	Stygnidae jovem	-	X
	Pseudoscorpiones	Chernetidae	*Spelaeochernes* sp.1	X	X
		Chthoniidae	Chthoniidae sp.1	-	X
Chilopoda	Scutigeromorpha	Pselliodidae	*Sphendononema* jovem	X	-
Diplopoda	Polydesmida	Paradoxosomatidae	Paradoxosomatidae sp.2	-	X
Entognatha	Diplura	Campodeidae	Campodeidae sp.1	-	X
Insecta	Blattodea	Polyphagidae	Polyphagidae jovem	X	X
	Coleoptera	Staphylinidae	*Coproporus* sp.1	-	X
	Diptera	Dolichopodidae	Dolichopodidae sp.	X	-
		Drosophilidae	Drosophilidae sp.	X	-
		Psychodidae	Phlebotominae sp.	X	-
	Hemiptera	Cydnidae	Cydnidae jovem	X	-
		Reduviidae	Emesinae jovem	-	X
	Hymenoptera	Formicidae	*Dolichoderus bispinosus*	X	X
			Neivamyrmex sp.1	-	X
			Pachycondyla constricta	X	X
			Paratrechina sp.1	X	-
	Isoptera	Termitidae	*Nasutitermes* sp.1	X	X
			Nasutitermes sp.2	-	X
	Lepidoptera	Noctuoidea	Noctouidea sp. 3	X	-
	Orthoptera	Phalangopsidae	*Paraclodes* sp.1	X	X
			Phalangopsis sp.1	X	X
	Psocoptera		Psocomorpha jovem	-	X
			Trogiomorpha jovem	-	X
	Thysanura	Nicoletiidae	Nicoletiinae sp.1	-	X
Malacostraca	Isopoda	Armadillidae	Armadillidae sp.1	X	X
		Platyarthridae	Platyarthridae sp.1	-	X
Amphibia	Anura	Strabomantidae	*Pristimantis* cf. *fenestratus*	X	-
Mammalia	Chiroptera	Emballonuridae	*Peropteryx* sp.	-	X
		Phyllostomidae	*Glossophaga soricina*	X	X
	Rodentia	Cricetidae	*Rhipidomys* sp.	-	X

SB-0065					
TÁXONS				Seca	Úmida
Annelida	Haplotaxida		Haplotaxida sp.4	-	X
Arachnida	Acari	Trombiculidae	Trombiculidae sp.1	-	X
			Trombiculidae sp.2	X	-
			Astigmata sp.4	X	-
	Amblypygi	Phrynidae	*Heterophrinus longicornis*	-	X
	Araneae	Araneidae	*Alpaida* sp.1	-	X
			Araneidae jovem	-	X
		Corinnidae	Corinnidae jovem	-	X
		Ctenidae	Ctenidae jovem	X	-
		Filistatidae	Filistatidae sp.1	-	X
		Mysmenidae	*Microdipoena* sp.1	-	X
		Ochyroceratidae	*Speocera* sp.1	X	X
		Pholcidae	*Mesabolivar aurantiacus*	-	X
			Pholcidae jovem	X	X
		Salticidae	Salticidae jovem	X	-
		Theraphosidae	*Guyruita cerrado*	-	X
		Theridiidae	*Argyrodes* sp.1	-	X
		Theridiosomatidae	*Plato* sp.1	-	X
		Trechaleidae	*Rhoicinus* sp.1	X	-
	Opiliones	Cosmetidae	Cosmetidae jovem	X	-
		Sclerosomatidae	*Prionostema* sp.1	X	-
			Sclerosomatidae jovem	X	-
	Pseudoscorpiones	Chernetidae	*Spelaeochernes* sp.1	X	X
Chilopoda	Scolopendromorpha	Scolopocryptopidae	*Dinocryptops miersii*	-	X
Diplopoda	Polydesmida	Chelodesmidae	Chelodesmidae sp.1	X	X
		Pyrgodesmidae	Pyrgodesmidae sp.2	-	X
	Siphonophorida	Siphonophoridae	Siphonophoridae sp.1	-	X
Entognatha	Collembola	Isotomidae	Isotomidae sp.1	-	X
		Paronellidae	Paronellidae sp.1	X	X
		Sminthuroidea	Sminthuroidea sp.2	X	X
	Diplura	Campodeidae	Campodeidae sp.1	X	-
Insecta	Blattodea	Blaberidae	Blaberidae jovem	X	X
	Coleoptera	Carabidae	*Acupalpus* sp.1	-	X
		Staphylinidae	Scydmaeninae sp.5	X	-
			Staphylininae sp.1	X	-
	Diptera	Cecidomyiidae	Cecidomyiidae sp.	-	X
		Phoridae	Phoridae sp.	X	-
		Psychodidae	Phlebotominae sp.	X	X
	Hemiptera	cf. Nabidae	cf. Nabidae jovem	X	-
		Cydnidae	Cydnidae jovem	X	-
			Cydnidae sp.1	-	X
			Cydnidae sp.2	-	X
		Reduviidae	Reduviinae jovem	X	-
	Hymenoptera	Formicidae	*Forelius* sp.1	-	X
			Gnamptogenys sp.1	-	X
			Pachycondyla sp.1	-	X
			Paratrechina sp.1	-	X
			Solenopsis sp.11	X	-
			Wasmannia auropunctata	X	-
	Isoptera	Termitidae	*Nasutitermes* sp.3	X	-
	Orthoptera	Phalangopsidae	*Phalangopsis* sp.1	X	X
	Psocoptera	Epipsocidae	Epipsocidae sp.1	X	-
		Troctopsocidae	Troctopsocidae sp.1	X	-
	Thysanura	Nicoletiidae	Nicoletiinae sp.1	X	X
Malacostraca	Isopoda	Armadillidae	Armadillidae sp.1	X	-
Amphibia	Anura		Anura sp.1	X	-
Mammalia	Chiroptera	Emballonuridae	*Peropteryx kappleri*	X	X

		Phyllostomidae	*Carollia perspicillata*	X	X
			Glossophaga soricina	X	X
Reptilia	Squamata	Gymnophthalmidae	*Neusticurus* sp.	X	-

SB-0066					
**TÁXONS**				**Seca**	**Úmida**
Arachnida	Araneae	Araneidae	*Alpaida* sp.1	-	X
		Ctenidae	Ctenidae jovem	X	X
		Ochyroceratidae	Ochyroceratidae jovem	X	-
		Pholcidae	*Mesabolivar eberhardi*	-	X
			Mesabolivar sp.2	X	-
			Modisimus sp.1	-	X
		Salticidae	Salticidae sp.8	X	-
		Theraphosidae	Theraphosidae jovem	X	-
	Opiliones	Manaosbiidae	Manaosbiidae sp.1	X	-
		Sclerosomatidae	*Prionostema* sp.1	-	X
Entognatha	Collembola	Paronellidae	Paronellidae sp.1	-	X
Insecta	Coleoptera		Coleoptera jovem	X	-
	Diptera		Diptera jovem	-	X
	Hymenoptera	Formicidae	*Hypoponera* sp.1	-	X
			Pachycondyla striata	X	-
			Pheidole sp.1	X	-
			Pheidole sp.13	-	X
	Orthoptera	Phalangopsidae	*Phalangopsis* sp.1	-	X
	Psocoptera	Psyllipsocidae	Psyllipsocidae sp.6	X	-
Pauropoda	Hexamerocerata		Hexamerocerata sp.1	X	-
Amphibia	Anura		Anura sp.3	-	X
Mammalia	Chiroptera	Emballonuridae	*Peropteryx kappleri*	X	-

SB-0067					
TÁXONS				**Seca**	**Úmida**
Arachnida	Acari	Ixodidae	*Amblyomma* sp.1	X	-
			Amblyomma sp.2	-	X
	Amblypygi	Phrynidae	*Heterophrinus longicornis*	X	X
	Araneae	Araneidae	Araneidae jovem	X	X
		Ctenidae	Ctenidae jovem	-	X
		Drymusidae	Drymusidae jovem	-	X
		Ochyroceratidae	*Speocera* sp.1	X	X
		Pholcidae	Pholcidae jovem	X	-
		Salticidae	Salticidae jovem	-	X
		Theraphosidae	*Guyruita cerrado*	-	X
		Theridiosomatidae	*Plato* sp.1	-	X
Diplopoda	Glomeridesmida	Glomeridesmidae	Glomeridesmida sp.1	-	X
	Polydesmida	Chelodesmidae	Chelodesmidae jovem	X	-
			Chelodesmidae sp.1	-	X
Entognatha	Collembola	Entomobryidae	Entomobryidae sp.4	-	X
		Paronellidae	Paronellidae sp.1	X	X
	Diplura	Campodeidae	Campodeidae sp.1	X	X
Insecta	Blattodea	Blaberidae	Blaberidae jovem	X	X
	Diptera	Drosophilidae	Drosophilidae sp.	X	-
		Psychodidae	Phlebotominae sp.	X	X
	Hemiptera	Cixiidae	Cixiidae sp.1	X	X
	Hymenoptera	Formicidae	*Pachycondyla constricta*	-	X
	Lepidoptera	Noctuoidea	Noctouidea sp. 2	-	X
			Noctouidea sp. 3	-	X
			Lepidoptera jovem	X	-
	Orthoptera	Phalangopsidae	*Paraclodes* sp.1	X	-
			Phalangopsis sp.1	X	X
Malacostraca	Isopoda	Armadillidae	Armadillidae sp.1	-	X
		Philosciidae	Philosciidae sp.1	-	X
Gastropoda	Pulmonata	Subulinidae	*Lamellaxis* sp.2	-	X
Amphibia	Anura	Leptodactylidae	*Leptodactylus* cf. *vastus*	X	X
		Strabomantidae	*Pristimantis* cf. *fenestratus*	X	-
			Anura sp.1	X	-
			Anura sp.2	X	-
Mammalia	Rodentia	Cricetidae	*Rhipidomys* sp.	X	-

SB-0068					
TÁXONS				**Seca**	**Úmida**
Arachnida	Acari		Astigmata sp.2	-	X
			Mesostigmata sp.4	-	X
	Araneae	Hahniidae	Hahniidae jovem	-	X
		Oonopidae	Oonopidae jovem	-	X
		Theridiosomatidae	*Plato* sp.1	X	X
		Trechaleidae	Trechaleidae jovem	X	-
	Opiliones	Neogoveidae	*Canga renatae*	-	X
	Pseudoscorpiones	Bochicidae	Bochicidae sp.1	-	X
Diplopoda	Glomeridesmida	Glomeridesmidae	Glomeridesmida sp.1	X	-
	Polydesmida	Fuhrmanodesmidae	Fuhrmanodesmidae sp.1	-	X
Entognatha	Collembola	Paronellidae	Paronellidae sp.1	-	X
		Sminthuroidea	Sminthuroidea sp.2	X	-
	Diplura	Campodeidae	Campodeidae sp.1	-	X
Insecta	Coleoptera	Byrrhidae	Byrrhidae sp.2	X	-
		Carabidae	Carabidae sp.3	X	-
		Staphylinidae	Pselaphinae sp.1	X	-
			Staphylininae sp.1	X	-
			Coleoptera jovem	X	X
	Diptera	Cecidomyiidae	Cecidomyiidae sp.	-	X
		Tipulidae	Tipulidae sp.	X	-
	Hemiptera	Veliidae	Veliidae jovem	X	-
	Hymenoptera	Formicidae	*Acropyga* cf. *smithii*	X	X
			Hypoponera opacior	-	X
			Paratrechina sp.1	X	-
	Lepidoptera	Hesperiidae	Hesperiidae sp. 1	X	-
	Orthoptera	Phalangopsidae	*Paraclodes* sp.1	X	-
Malacostraca	Isopoda	Philosciidae	Philosciidae sp.1	-	X
		Platyarthridae	Platyarthridae sp.1	-	X
Symphyla		Scutigerellidae	*Hanseniella* sp.1	X	-

SB-0069					
TÁXONS				Seca	Úmida
Arachnida	Acari	Argasidae	*Ornithodoros* sp.1	X	X
		Trombiculidae	Trombiculidae sp.1	X	X
			Trombiculidae sp.2	X	-
			Acariformes sp.4	X	-
			Astigmata sp.4	-	X
			Holothyrida sp.2	X	X
			Mesostigmata sp.1	X	X
			Mesostigmata sp.2	X	-
			Oribatida sp.2	X	-
	Amblypygi	Phrynidae	*Heterophrinus longicornis*	-	X
	Araneae	Araneidae	Araneidae jovem	-	X
		Corinnidae	Corinnidae jovem	X	X
		Ctenidae	Ctenidae jovem	-	X
		Filistatidae	Filistatidae jovem	X	X
		Ochyroceratidae	Ochyroceratidae jovem	-	X
		Oonopidae	Oonopidae jovem	X	-
			Oonopidae sp.3	-	X
		Pholcidae	*Leptopholcus* sp.1	X	X
			Mesabolivar eberhardi	-	X
			Ninetinae sp.1	-	X
		Salticidae	Salticidae jovem	-	X
		Scytodidae	*Scytodes* sp.1	X	X
		Theridiidae	*Nesticodes rufipes*	X	X
	Opiliones	Escadabiidae	Escadabiidae jovem	-	X
	Pseudoscorpiones	Chernetidae	*Spelaeochernes* sp.1	X	X
		Chthoniidae	Chthoniidae sp.1	X	X
Chilopoda	Scutigeromorpha	Pselliodidae	*Sphendononema guildingii*	X	-
Diplopoda	Glomeridesmida	Glomeridesmidae	Glomeridesmida jovem	-	X
	Spirostreptida	Pseudonannolenidae	Pseudonannolenidae sp.1	X	X
		Spirostreptidae	Spirostreptidae sp.1	X	-
Entognatha	Collembola	Cyphoderidae	Cyphoderidae sp.1	X	X
	Diplura	Campodeidae	Campodeidae sp.1	X	X
Insecta	Blattodea	Blaberidae	Blaberidae jovem	X	X
		Polyphagidae	Polyphagidae jovem	X	X
			Polyphagidae sp.1	X	-
	Coleoptera	Carabidae	*Notibia* sp.1	-	X
		Chrysomelidae	*Bruchinae* sp.1	X	-
		Staphylinidae	Staphylinidae sp.5	X	-
		Zopheridae	Momommatinae sp.1	-	X
	Diptera	Ceratopogonidae	Ceratopogonidae jovem	X	-
		Drosophilidae	Drosophilidae sp.	X	X
		Muscidae	Muscidae jovem	X	X
			Muscidae sp.	X	-
		Psychodidae	Phlebotominae sp.	X	X
	Hemiptera	Cixiidae	Cixiidae jovem	-	X
		Cydnidae	Cydnidae sp.1	X	X
		Diaspididae	Diaspididae sp.1	X	-
		Lygaeidae	Lygaeidae sp.1	X	X
	Hymenoptera	Eucharitidae	Eucharitidae sp.1	X	-
		Figitidae	Figitidae sp.1	X	-
		Formicidae	*Acromyrmex octopinosus*	X	-
			Carebara sp.2	-	X
			Pachycondyla constricta	X	X
			Pheidole sp.5	X	X
			Solenopsis invicta	X	X
			Solenopsis sp.2	X	-
	Isoptera	Termitidae	*Nasutitermes* sp.1	X	-

	Lepidoptera	Gelechioidea	Gelechioidea sp.1	X	-
		Tineoidea	Tineoidea sp. 1	X	-
			Tineoidea sp. 3	X	-
			Tineoidea sp. 5	X	X
	Orthoptera	Phalangopsidae	*Paraclodes* sp.1	X	-
			Phalangopsis sp.1	X	X
Malacostraca	Isopoda	Armadillidae	Armadillidae sp.1	X	X
		Dubioniscidae	Dubioniscidae sp.3	-	X
		Philosciidae	Philosciidae sp.1	X	X
		Platyarthridae	Platyarthridae sp.2	-	X
Gastropoda	Pulmonata	Systrophiidae	*Happia* sp.1	-	X
			Happia sp.4	-	X
Amphibia	Anura	Leptodactylidae	*Leptodactylus* cf. *vastus*	X	X
		Strabomantidae	*Pristimantis* cf. *fenestratus*	X	-
Mammalia	Chiroptera	Emballonuridae	*Peropteryx kappleri*	X	X
		Phyllostomidae	*Carollia perspicillata*	X	X
			Glossophaga soricina	X	X

SB-0070					
TÁXONS				Seca	Úmida
Annelida	Haplotaxida		Haplotaxida sp.2	-	X
			Haplotaxida sp.6	X	-
			Haplotaxida sp.7	X	-
Arachnida	Acari	Trombiculidae	Trombiculidae sp.1	X	X
			Trombiculidae sp.2	X	X
			Mesostigmata sp.2	-	X
			Mesostigmata sp.4	X	-
	Amblypygi	Phrynidae	*Heterophrinus longicornis*	X	X
	Araneae	Ctenidae	Ctenidae jovem	-	X
		Ochyroceratidae	*Speocera* sp.1	X	X
		Pholcidae	*Leptopholcus* sp.1	-	X
			Mesabolivar eberhardi	X	X
		Salticidae	Salticidae jovem	X	X
		Scytodidae	*Scytodes* sp.1	-	X
		Segestriidae	Segestriidae jovem	X	X
		Tetragnathidae	Tetragnathidae jovem	-	X
		Theraphosidae	Theraphosidae jovem	-	X
		Theridiosomatidae	*Plato* sp.1	-	X
	Opiliones	Cosmetidae	Cosmetidae jovem	X	-
		Escadabiidae	Escadabiidae sp.1	X	X
		Neogoveidae	Neogoveidae jovem	X	X
		Stygnidae	Stygnidae sp.1	X	-
	Pseudoscorpiones	Chernetidae	*Spelaeochernes* sp.1	X	X
		Chthoniidae	Chthoniidae sp.1	X	X
	Scorpiones	Buthidae	*Ananteris luciae*	X	X
Chilopoda	Geophilomorpha	Ballophilidae	*Ityphilus* sp.2	X	-
	Scolopendromorpha	Scolopocryptopidae	*Newportia* sp.2	X	-
	Scutigeromorpha	Pselliodidae	*Sphendononema* jovem	X	X
Diplopoda	Glomeridesmida	Glomeridesmidae	Glomeridesmida sp.1	X	X
	Polydesmida	Chelodesmidae	Chelodesmidae sp.1	-	X
		Fuhrmanodesmidae	Fuhrmanodesmidae sp.1	X	-
		Pyrgodesmidae	Pyrgodesmidae sp.1	X	X
			Pyrgodesmidae sp.2	X	-
	Spirostreptida	Pseudonannolenidae	Pseudonannolenidae sp.1	-	X
Entognatha	Collembola	Isotomidae	Isotomidae sp.1	-	X
		Paronellidae	Paronellidae sp.1	X	X
			Paronellidae sp.4	X	-
		Sminthuroidea	Sminthuroidea sp.2	X	-
	Diplura	Campodeidae	Campodeidae sp.1	X	-
		Projapygidae	Projapygidae sp.1	X	-
Insecta	Blattodea		Blattodea jovem	X	-
	Coleoptera	Dytiscidae	*Copelatus* sp.1	-	X
		Staphylinidae	Pselaphinae sp.1	X	X
			Pselaphinae sp.5	X	X
			Pselaphinae sp.6	X	X
			Staphylininae sp.1	-	X
			Staphylininae sp.2	-	X
		Tenebrionidae	Tenebrionidae jovem	X	-
	Diptera	Cecidomyiidae	Cecidomyiidae sp.	-	X
		Drosophilidae	Drosophilidae sp.	X	X
		Psychodidae	Psychodidae sp.	X	-
		Sciaridae	Sciaridae sp.	X	-
	Hemiptera	Cixiidae	Cixiidae jovem	X	X
		Cydnidae	Cydnidae sp.1	X	X
		Reduviidae	Emesinae jovem	-	X
	Hymenoptera	Formicidae	*Camponotus crassus*	X	-
			Camponotus melanoticus	X	-

			Camponotus sp.2	X	-
			Gnamptogenys sp.1	X	-
			Pachycondyla constricta	X	-
			Pseudomyrmex lizeri	X	-
			Solenopsis invicta	-	X
		Sphecidae	Sphecidae sp.1	-	X
		Vespidae	Vespidae sp.2	X	-
	Isoptera		Isoptera jovem	-	X
	Orthoptera	Phalangopsidae	*Eidmanacris* sp.1	X	-
			Phalangopsis sp.1	X	X
	Psocoptera		Psocomorpha jovem	X	-
	Thysanura	Nicoletiidae	Atelurinae sp.1	X	X
Malacostraca	Isopoda	Armadillidae	Armadillidae sp.1	X	X
		Philosciidae	Philosciidae sp.1	X	-
		Scleropactidae	Scleropactidae sp.1	X	X
			Scleropactidae sp.2	X	-
Gastropoda	Pulmonata	Subulinidae	*Lamellaxis* sp.1	-	X
Nematoda	Rhabditida		Rhabditia sp.1	-	X
			Rhabditia sp.2	-	X
Amphibia	Anura	Strabomantidae	*Pristimantis* cf. *fenestratus*	X	X
			Anura sp.4	-	X
Mammalia	Chiroptera	Emballonuridae	*Peropteryx kappleri*	X	X
		Phyllostomidae	*Carollia perspicillata*	X	X
			Lionycteris spurrelli	X	-

SB-0071					
TÁXONS				Seca	Úmida
Arachnida	Acari	Argasidae	*Ornithodoros* sp.1	-	X
		Ixodidae	*Amblyomma* sp.1	X	-
		Trombiculidae	Trombiculidae sp.1	-	X
			Trombiculidae sp.2	X	-
			Astigmata sp.4	-	X
			Mesostigmata sp.1	X	X
			Mesostigmata sp.2	X	X
	Amblypygi	Phrynidae	*Heterophrinus longicornis*	X	X
	Araneae	Araneidae	*Eustala* sp.1	-	X
		Corinnidae	Corinnidae jovem	X	X
		Ochyroceratidae	*Ochyrocera* sp.1	-	X
			Speocera sp.1	X	X
		Oonopidae	Oonopidae jovem	X	-
			Oonopidae sp.1	X	-
		Pholcidae	*Mesabolivar eberhardi*	-	X
		Salticidae	Salticidae jovem	-	X
		Segestriidae	Segestriidae jovem	X	X
		Theridiosomatidae	*Plato* sp.1	-	X
	Opiliones	Cosmetidae	Cosmetidae jovem	X	-
		Escadabiidae	Escadabiidae sp.1	X	X
			Escadabiidae sp.2	-	X
		Neogoveidae	*Canga renatae*	X	X
		Stygnidae	*Protimesus* sp.2	X	-
	Pseudoscorpiones	Bochicidae	Bochicidae sp.1	-	X
		Chernetidae	*Spelaeochernes* sp.1	X	X
		Chthoniidae	Chthoniidae sp.1	-	X
	Scorpiones	Buthidae	*Ananteris* jovem	X	-
Chilopoda	Scolopendromorpha	Cryptopidae	*Cryptops* sp.2	-	X
		Scolopocryptopidae	*Dinocryptops miersii*	X	-
			Newportia sp.2	X	X
Diplopoda	Polydesmida	Chelodesmidae	Chelodesmidae jovem	-	X
	Spirostreptida	Pseudonannolenidae	Pseudonannolene sp.1	X	-
Entognatha	Collembola	Cyphoderidae	Cyphoderidae sp.1	-	X
		Isotomidae	Isotomidae sp.1	-	X
		Paronellidae	Paronellidae sp.1	X	X
		Sminthuroidea	Sminthuroidea sp.2	X	X
	Diplura	Campodeidae	Campodeidae sp.1	X	X
		Japygidae	Japygidae sp.1	X	-
Insecta	Blattodea	Blaberidae	Blaberidae jovem	X	X
		Blattidae	Blattidae jovem	X	-
			Periplaneta sp.1	X	-
	Coleoptera	Chrysomelinae	Chrysomelinae sp.1	X	-
		Dytiscidae	*Copelatus* sp.1	-	X
		Staphylinidae	Pselaphinae sp.1	X	X
			Scydmaeninae sp.2	X	X
		Tenebrionidae	Tenebrionidae jovem	X	-
	Diptera	Cecidomyiidae	Cecidomyiidae sp.	-	X
		Ceratopogonidae	Ceratopogonidae sp.	X	-
		Culicidae	Culicidae sp.	-	X
		Drosophilidae	Drosophilidae sp.	X	X
		Psychodidae	Phlebotominae sp.	X	-
			Psychodidae sp.	X	X
		Streblidae	Streblidae sp.	X	X
	Hemiptera	Cydnidae	Cydnidae sp.1	X	X
			Cydnidae sp.2	-	X
	Hymenoptera	Braconidae	Braconidae sp.1	X	X
		Diapriidae	Diapriidae sp.4	X	-

		Formicidae	*Apterostigma collare*	-	X
			Camponotus atriceps	-	X
			Camponotus sp.2	X	X
			Carebara sp.10	-	X
			Gnamptogenys sp.1	X	X
			Hypoponera sp.6	-	X
			Pachycondyla constricta	X	-
			Pheidole sp.1	-	X
			Pseudomyrmex lizeri	X	-
			Solenopsis sp.2	-	X
			Solenopsis sp.7	X	X
			Strumigenys elongata	X	-
		Platygastridae	*Baeus* sp.1	-	X
	Isoptera	Termitidae	*Nasutitermes* sp.2	X	-
	Lepidoptera	Tineoidea	Tineoidea sp. 1	X	-
			Tineoidea sp. 2	-	X
			Tineoidea sp. 3	X	X
	Orthoptera	Phalangopsidae	*Paraclodes* sp.1	-	X
			Phalangopsis sp.1	X	X
	Thysanura	Nicoletiidae	Atelurinae sp.1	X	X
			Nicoletiinae sp.1	X	X
Malacostraca	Isopoda	Armadillidae	Armadillidae sp.1	X	X
		Philosciidae	Philosciidae sp.1	-	X
			Philosciidae sp.2	X	X
		Scleropactidae	Scleropactidae sp.1	-	X
Gastropoda	Pulmonata	Subulinidae	*Lamellaxis* sp.1	-	X
			Leptinaria sp.1	X	-
		Systrophiidae	*Happia* sp.1	X	-
Amphibia	Anura	Leptodactylidae	*Leptodactylus* cf. *vastus*	-	X
		Strabomantidae	*Pristimantis* cf. *fenestratus*	X	-
Mammalia	Chiroptera	Furipteridae	*Furipterus horrens*	-	X
		Phyllostomidae	*Anoura geoffroyi*	X	X
			Carollia perspicillata	X	X
			Lionycteris spurrelli	X	X

SB-0072					
TÁXONS				Seca	Úmida
Arachnida	Acari	Trombiculidae	Trombiculidae sp.2	X	-
			Astigmata sp.4	X	-
	Amblypygi	Phrynidae	*Heterophrinus longicornis*	-	X
	Araneae	Araneidae	*Alpaida* sp.1	-	X
			Araneidae jovem	X	-
		Ctenidae	Ctenidae jovem	X	X
		Filistatidae	Filistatidae jovem	-	X
			Filistatidae sp.1	X	-
		Oonopidae	Oonopidae sp.3	X	-
		Pholcidae	*Mesabolivar cambridgei*	X	-
			Mesabolivar eberhardi	X	X
			Ninetinae sp.1	X	-
		Salticidae	Salticidae sp.11	X	-
		Scytodidae	*Scytodes* sp.1	X	X
		Theraphosidae	Theraphosidae jovem	X	-
		Theridiidae	*Nesticodes rufipes*	X	-
			Thymoites sp.1	X	-
		Theridiosomatidae	*Plato* sp.1	-	X
	Pseudoscorpiones	Cheliferidae	Cheliferidae sp.1	X	X
		Chernetidae	*Spelaeochernes* sp.1	X	X
	Scorpiones	Buthidae	*Ananteris luciae*	X	-
Chilopoda	Scolopendromorpha	Cryptopidae	*Cryptops* sp.2	X	-
Insecta	Blattodea	Blaberidae	*Blaberus* sp.1	X	X
			Blaberus sp.2	-	X
			Blaberus sp.3	X	-
		Polyphagidae	Polyphagidae jovem	-	X
	Coleoptera	Carabidae	*Notibia* sp.1	X	-
		Coccinellidae	Coccinellidae sp.1	-	X
		Staphylinidae	*Scaphidium* sp.1	X	-
			Staphylininae sp.2	X	-
	Dermaptera	Labiduridae	Labiduridae sp.1	-	X
	Diptera	Ceratopogonidae	Ceratopogonidae jovem	-	X
		Chironomidae	Chironomidae jovem	-	X
		Drosophilidae	Drosophilidae sp.	X	X
		Muscidae	Muscidae jovem	-	X
		Psychodidae	Phlebotominae sp.	X	X
		Tipulidae	Tipulidae sp.	-	X
	Hemiptera	Cydnidae	Cydnidae sp.1	X	X
		Lygaeidae	Lygaeidae jovem	X	-
		Reduviidae	Reduviidae sp.1	X	X
			Reduviinae jovem	X	X
	Hymenoptera	Eulophidae	Eulophidae sp.1	-	X
		Formicidae	*Dolichoderus bispinosus*	X	-
			Hypoponera sp.1	X	-
			Hypoponera sp.6	-	X
			Labidus coecus	X	-
			Odontomachus meinerti	X	X
			Pachycondyla constricta	-	X
			Pheidole sp.7	X	-
			Rogeria foreli	-	X
			Solenopsis sp.11	X	-
	Lepidoptera	Hesperiidae	Hesperiidae sp. 1	-	X
		Lycaenidae	Lycaenidae sp. 1	-	X
		Tineoidea	Tineoidea sp. 5	-	X
			Tineoidea sp. 6	X	-
			Tineoidea sp. 8	X	-
	Orthoptera	Phalangopsidae	*Paraclodes* sp.1	X	X

			Phalangopsis sp.1	X	X
	Psocoptera	Psyllipsocidae	Psyllipsocidae sp.3	X	-
		Troctopsocidae	Troctopsocidae sp.1	-	X
			Trogiomorpha jovem	X	-
	Trichoptera	Philopotamidae	Philopotamidae sp.1	X	-
Malacostraca	Isopoda	Philosciidae	Philosciidae sp.2	X	-
Symphyla		Scutigerellidae	*Hanseniella* sp.1	X	-
Amphibia	Anura	Bufonidae	*Rhinella* sp.	-	X
Mammalia	Chiroptera	Mormoopidae	*Pteronotus parnellii*	X	X
		Phyllostomidae	*Carollia perspicillata*	X	X
			Glossophaga soricina	X	X
Reptilia	Squamata	Gekkonidae	*Thecadactylus rapicauda*	X	-

SB-0073					
**TÁXONS**				**Seca**	**Úmida**
Arachnida	Acari	Argasidae	*Ornithodoros* sp.1	-	X
		Opilioacaridae	Opilioacaridae sp.1	X	-
			Acariformes sp.3	-	X
			Mesostigmata sp.1	-	X
	Araneae	Araneidae	Araneidae jovem	-	X
		Oonopidae	Oonopidae jovem	X	X
		Pholcidae	Pholcidae jovem	-	X
		Prodidomidae	Prodidomidae jovem	-	X
		Salticidae	Salticidae jovem	-	X
		Scytodidae	Scytodidae jovem	-	X
		Segestriidae	*Ariadna* sp.2	-	X
		Theridiosomatidae	Theridiosomatidae jovem	-	X
	Opiliones	Sclerosomatidae	*Prionostema* sp.1	X	X
	Pseudoscorpiones	Bochicidae	Bochicidae sp.1	X	-
		Chernetidae	*Spelaeochernes* sp.1	-	X
Chilopoda	Scolopendromorpha	Scolopendridae	*Otostigmus* sp.1	-	X
	Scutigeromorpha	Pselliodidae	*Sphendononema guildingii*	X	-
			Sphendononema jovem	-	X
Entognatha	Collembola	Paronellidae	Paronellidae sp.1	X	-
	Diplura	Campodeidae	Campodeidae sp.1	X	-
Insecta	Coleoptera	Staphylinidae	Pselaphinae sp.5	-	X
			Coproporus sp.2	-	X
			Staphylininae sp.1	X	-
	Diptera	Psychodidae	Phlebotominae sp.	-	X
		Tipulidae	Tipulidae sp.	-	X
	Hemiptera	Cydnidae	Cydnidae jovem	X	-
	Hymenoptera	Formicidae	Pachycondyla striata	-	X
	Isoptera	Termitidae	*Nasutitermes* sp.1	X	-
	Lepidoptera	Noctuoidea	Noctouidea sp. 1	X	-
			Noctouidea sp. 4	X	-
		Tineoidea	Tineoidea sp. 6	-	X
	Orthoptera	Phalangopsidae	*Paraclodes* sp.1	-	X
	Psocoptera	Archipsocidae	Archipsocidae sp.2	-	X
		Troctopsocidae	Troctopsocidae sp.1	-	X
Malacostraca	Isopoda	Dubioniscidae	Dubioniscidae sp.1	-	X
		Philosciidae	Philosciidae sp.1	-	X
Symphyla		Scolopendrellidae	*Symphylella* sp.1	-	X
		Scutigerellidae	*Hanseniella* sp.1	-	X
Amphibia	Anura		Anura sp.5	X	-

SB-0074					
TÁXONS				Seca	Úmida
Arachnida	Acari	Argasidae	*Ornithodoros* sp.1	X	-
		Ixodidae	*Amblyomma* sp.1	X	-
		Trombiculidae	Trombiculidae sp.1	-	X
			Trombiculidae sp.2	X	-
			Astigmata sp.1	X	-
			Mesostigmata sp.2	-	X
	Amblypygi	Phrynidae	*Heterophrinus longicornis*	-	X
	Araneae	Araneidae	*Alpaida* sp.1	-	X
		Barychelidae	Barychelidae jovem	-	X
		Ctenidae	Ctenidae jovem	-	X
		Ochyroceratidae	*Speocera* sp.1	-	X
		Oonopidae	Oonopidae jovem	-	X
		Paratropididae	Paratropididae jovem	-	X
		Pholcidae	*Mesabolivar eberhardi*	-	X
			Modisimus sp.1	-	X
			Pholcidae jovem	X	X
		Salticidae	Salticidae jovem	X	X
		Scytodidae	*Scytodes* sp.1	X	-
			Scytodidae jovem	-	X
		Segestriidae	*Ariadna* sp.2	X	-
		Theridiosomatidae	*Plato* sp.1	-	X
	Opiliones	Escadabiidae	Escadabiidae sp.1	-	X
		Sclerosomatidae	*Prionostema* sp.1	X	-
	Pseudoscorpiones	Chernetidae	*Spelaeochernes* sp.1	X	X
		Chthoniidae	Chthoniidae jovem	-	X
	Scorpiones	Buthidae	*Ananteris* jovem	-	X
Chilopoda	Scolopendromorpha	Scolopocryptopidae	*Newportia* jovem	-	X
	Scutigeromorpha	Pselliodidae	*Sphendononema* jovem	-	X
Diplopoda	Polydesmida	Chelodesmidae	Chelodesmidae jovem	-	X
Entognatha	Collembola	Isotomidae	Isotomidae sp.1	-	X
		Paronellidae	Paronellidae sp.1	-	X
	Diplura	Campodeidae	Campodeidae sp.1	-	X
Insecta	Coleoptera	Chrysomelidae	Chrysomelinae sp.1	X	-
		Hydrophilidae	Hydrophilidae sp.3	-	X
	Diptera	Ceratopogonidae	Ceratopogonidae jovem	-	X
		Psychodidae	Psychodidae jovem	-	X
		Tipulidae	Tipulidae sp.	X	X
	Hemiptera		Heteroptera jovem	-	X
	Hymenoptera	Formicidae	*Camponotus* sp.2	X	-
			Crematogaster brasiliensis	X	-
			Pachycondyla constricta	X	X
			Pachycondyla striata	-	X
			Solenopsis invicta	X	-
	Lepidoptera	Tineoidea	Tineoidea sp. 3	-	X
	Orthoptera	Phalangopsidae	*Phalangopsis* sp.1	-	X
	Psocoptera	Liposcelidae	Liposcelidae sp.4	-	X
	Thysanura	Nicoletiidae	Nicoletiinae jovem	X	-
Gastropoda	Pulmonata	Subulinidae	*Lamellaxis* sp.1	X	-
		Systrophiidae	*Happia* sp.1	X	-
Amphibia	Anura	Strabomantidae	*Pristimantis* cf. *fenestratus*	-	X
			Anura sp.3	X	-
Mammalia	Chiroptera	Emballonuridae	*Peropteryx kappleri*	X	X
		Phyllostomidae	*Glossophaga soricina*	-	X

SB-0075					
TÁXONS				Seca	Úmida
Arachnida	Acari	Argasidae	*Ornithodoros* sp.1	X	-
	Amblypygi	Phrynidae	*Heterophrinus longicornis*	-	X
	Araneae	Araneidae	*Alpaida* sp.1	X	X
		Ctenidae	Ctenidae jovem	-	X
		Oonopidae	Oonopidae sp.3	-	X
		Pholcidae	*Leptopholcus* sp.1	-	X
			Mesabolivar eberhardi	-	X
			Modisimus sp.1	-	X
			Pholcidae jovem	X	X
		Salticidae	Salticidae sp.4	-	X
		Scytodidae	Scytodidae jovem	-	X
		Theridiosomatidae	*Plato* sp.1	-	X
	Opiliones	Cosmetidae	Cosmetidae sp.1	X	-
			Roquettea carajas	-	X
		Sclerosomatidae	*Prionostema* sp.1	-	X
		Stygnidae	*Protimesus* sp.2	X	-
	Pseudoscorpiones	Chernetidae	*Spelaeochernes* sp.1	X	X
Chilopoda	Scolopendromorpha	Scolopocryptopidae	*Newportia* sp.2	-	X
Insecta	Blattodea	Polyphagidae	Polyphagidae jovem	X	-
	Coleoptera		Coleoptera jovem	X	X
	Diptera	Tipulidae	Tipulidae sp.	-	X
			Diptera jovem	X	-
	Hymenoptera	Eurytomidae	Eurytomidae sp.1	X	-
		Formicidae	*Pachycondyla constricta*	X	-
	Neuroptera	Myrmeleontidae	Myrmeleontidae sp.1	X	-
	Orthoptera	Phalangopsidae	*Paraclodes* sp.1	X	X
			Phalangopsis sp.1	-	X
	Psocoptera		Psocomorpha jovem	X	-
			Trogiomorpha jovem	X	-
Amphibia	Anura	Strabomantidae	*Pristimantis* cf. *fenestratus*	-	X

SB-0076					
TÁXONS				Seca	Úmida
Annelida	Haplotaxida		Haplotaxida sp.2	X	-
			Haplotaxida sp.3	X	-
			Haplotaxida sp.4	X	X
			Tubificina sp.1	X	-
Arachnida	Acari	Trombiculidae	Trombiculidae sp.1	X	X
			Trombiculidae sp.2	X	-
			Acariformes sp.2	-	X
			Acariformes sp.3	-	X
			Astigmata sp.1	-	X
			Astigmata sp.5	X	-
			Holothyrida sp.1	X	X
			Mesostigmata sp.1	X	-
			Mesostigmata sp.4	X	-
			Oribatida sp.5	X	-
	Amblypygi	Charinidae	*Charinus* sp.1	-	X
		Phrynidae	*Heterophrinus longicornis*	X	X
	Araneae	Araneidae	*Alpaida* sp.1	X	-
		Corinnidae	*Abapeba* sp.1	-	X
			Corinidae jovem	-	X
		Ochyroceratidae	Ochyroceratidae jovem	-	X
		Pholcidae	*Mesabolivar eberhardi*	X	-
			Mesabolivar sp.1	X	X
		Salticidae	Salticidae sp.3	X	-
		Segestriidae	Segestriidae jovem	X	X
		Theraphosidae	Theraphosidae jovem	-	X
		Theridiosomatidae	*Plato* sp.1	X	X
	Opiliones	Escadabiidae	Escadabiidae sp.1	X	X
		Neogoveidae	*Canga renatae*	X	-
		Sclerosomatidae	Sclerosomatidae jovem	X	-
	Pseudoscorpiones	Chernetidae	*Spelaeochernes* sp.1	X	X
		Chthoniidae	Chthoniidae sp.1	X	X
			Pseudochthonius sp.4	X	-
	Scorpiones	Buthidae	*Ananteris* jovem	X	X
Chilopoda	Scolopendromorpha	Scolopocryptopidae	*Newportia* sp.2	X	-
Diplopoda	Glomeridesmida	Glomeridesmidae	Glomeridesmida sp.1	X	X
	Polydesmida	Fuhrmanodesmidae	Fuhrmanodesmidae sp.1	-	X
		Pyrgodesmidae	Pyrgodesmidae sp.1	X	X
			Pyrgodesmidae sp.2	X	-
Entognatha	Collembola	Cyphoderidae	Cyphoderidae sp.1	-	X
		Paronellidae	Paronellidae sp.1	X	X
		Sminthuroidea	Sminthuroidea sp.2	-	X
	Diplura	Japygidae	Japygidae sp.1	-	X
Insecta	Blattodea	Blattellidae	Blattellidae jovem	X	X
	Coleoptera	Carabidae	*Notibia* sp.1	X	-
		Chrysomelidae	Alticini sp.1	-	X
		Histeridae	Histeridae sp.1	X	-
		Tenebrionidae	Tenebrionidae jovem	X	-
	Dermaptera	Forficuloidea	Forficuloidea sp.1	-	X
		Labiduridae	Labiduridae sp.1	X	X
	Diptera	Cecidomyiidae	Cecidomyiidae sp.	X	-
		Drosophilidae	Drosophilidae sp.	X	X
		Muscidae	Muscidae jovem	-	X
		Phoridae	Phoridae sp.	X	X
		Psychodidae	Phlebotominae sp.	X	-
			Psychodidae sp.	X	X
	Hemiptera	Cixiidae	Cixiidae jovem	X	-
		Cydnidae	Cydnidae sp.1	X	X

		Mesoveliidae	Mesoveliidae jovem	-	X
		Veliidae	*Paravelia* sp.1	-	X
			Veliidae jovem	X	X
	Hymenoptera	Bethylidae	Bethylidae sp.1	X	-
		Diapriidae	Diapriidae sp.3	X	X
		Eurytomidae	Eurytomidae sp.1	X	-
		Formicidae	*Acromyrmex octopinosus*	X	-
			Apterostigma collare	X	-
			Camponotus sp.2	X	-
			Gnamptogenys sp.1	-	X
			Labidus sp.1	X	-
			Pachycondyla constricta	X	X
			Pheidole sp.3	X	-
			Rogeria cf. *belti*	X	-
			Solenopsis invicta	X	X
			Strumigenys cordovensis	X	-
	Lepidoptera	Tineoidea	Tineoidea sp. 2	X	X
			Tineoidea sp. 3	X	X
	Orthoptera	Phalangopsidae	*Paraclodes* sp.1	X	-
			Phalangopsis sp.1	X	X
	Thysanura	Nicoletiidae	Atelurinae sp.1	-	X
			Nicoletiinae sp.1	X	X
Malacostraca	Decapoda	Pseudothelphusidae	Pseudothelphusidae sp.1	X	X
	Isopoda	Armadillidae	Armadillidae sp.1	X	-
		Philosciidae	Philosciidae sp.2	-	X
		Scleropactidae	Scleropactidae sp.1	X	X
			Scleropactidae sp.2	X	X
Symphyla		Scutigerellidae	*Hanseniella* sp.1	-	X
			Scutigerellidae jovem	-	X
Gastropoda	Pulmonata	Subulinidae	*Lamellaxis* sp.1	X	X
		Systrophiidae	*Happia* sp.1	X	-
Turbellaria	Tricladida		Continenticola sp.2	-	X
Amphibia	Anura	Bufonidae	*Rhinella* sp.	X	X
		Leptodactylidae	*Leptodactylus* cf. *vastus*	X	X
		Strabomantidae	*Pristimantis* cf. *fenestratus*	X	X
			Anura sp.5	-	X
Mammalia	Chiroptera	Emballonuridae	*Peropteryx kappleri*	X	X
		Phyllostomidae	*Carollia perspicillata*	X	X
			Desmodus rotundus	-	X
			Lonchorhina aurita	X	X
			Trachops cirrhosus	-	X

SB-0077					
TÁXONS				**Seca**	**Úmida**
Annelida	Haplotaxida		Haplotaxida sp.4	-	X
Arachnida	Acari	Ixodidae	*Amblyomma* sp.1	-	X
			Holothyrida sp.2	X	X
			Mesostigmata sp.1	-	X
	Amblypygi	Phrynidae	*Heterophrinus longicornis*	X	X
	Araneae	Araneidae	*Alpaida* sp.1	X	X
		Corinnidae	Corinnidae jovem	X	X
		Ctenidae	Ctenidae jovem	X	X
		Ochyroceratidae	Ochyroceratidae jovem	-	X
		Pholcidae	*Mesabolivar eberhardi*	X	X
		Scytodidae	*Scytodes* sp.1	X	X
		Theridiidae	Theridiidae jovem	X	-
		Theridiosomatidae	*Plato* sp.1	X	X
	Opiliones	Cosmetidae	*Roquettea carajas*	X	-
		Escadabiidae	Escadabiidae sp.1	X	-
		Stygnidae	*Protimesus* sp.1	X	X
			Protimesus sp.2	X	-
	Pseudoscorpiones	Cheliferidae	Cheliferidae sp.2	-	X
		Chernetidae	*Spelaeochernes* sp.1	X	X
	Ricinulei	Ricinoididae	Ricinoididae jovem	-	X
	Schizomida	Hubbardiidae	*Rowlandius* sp.1	X	X
Chilopoda	Geophilomorpha	Shendylidae	*Schendylops* sp.1	X	-
	Scolopendromorpha	Scolopocryptopidae	*Newportia* sp.2	X	-
Diplopoda	Polydesmida	Chelodesmidae	Chelodesmidae sp.1	X	-
		Paradoxosomatidae	Paradoxosomatidae sp.1	-	X
		Pyrgodesmidae	Pyrgodesmidae jovem	X	-
			Pyrgodesmidae sp.2	-	X
Entognatha	Collembola	Paronellidae	Paronellidae sp.1	-	X
			Paronellidae sp.4	-	X
	Diplura	Campodeidae	Campodeidae sp.1	X	X
Insecta	Blattodea	Blaberidae	*Blaberus* sp.1	X	X
		Blattellidae	Blattellidae sp.1	-	X
		Blattidae	Blattidae jovem	X	X
		Polyphagidae	Polyphagidae sp.1	X	-
	Coleoptera	Carabidae	*Acupalpus* sp.2	X	-
		Chrysomelidae	Chrysomelinae sp.1	X	X
		Elateridae	Elateridae sp.2	X	-
		Hydrophilidae	Hydrophilidae sp.5	X	X
		Staphylinidae	Scydmaeninae sp.2	-	X
			Staphylinidae sp.6	-	X
		Tenebrionidae	Tenebrionidae sp.2	X	X
	Diptera	Ceratopogonidae	Ceratopogonidae sp.	X	X
		Drosophilidae	Drosophilidae sp.	X	X
		Muscidae	Muscidae jovem	-	X
		Psychodidae	Phlebotominae sp.	X	X
		Tipulidae	Tipulidae sp.	X	X
	Hemiptera	Cydnidae	Cydnidae sp.1	-	X
		Schizopteridae	Schizopteridae sp.1	-	X
	Hymenoptera	Apidae	*Apis mellifera*	X	-
		Formicidae	*Camponotus atriceps*	X	-
			Dolichoderus bispinosus	X	-
			Odontomachus meinerti	X	-
			Pachycondyla constricta	X	X
			Paratrechina sp.1	X	-
			Pheidole sp.3	X	-
			Wasmannia auropunctata	-	X
	Isoptera	Termitidae	*Nasutitermes* sp.1	X	X

			Nasutitermes sp.3	-	X
	Lepidoptera	Noctuoidea	Noctouidea sp. 2	X	-
	Orthoptera	Phalangopsidae	*Paraclodes* sp.1	X	X
			Phalangopsis sp.1	X	X
Malacostraca	Isopoda	Armadillidae	Armadillidae sp.1	X	X
		Philosciidae	Philosciidae sp.1	X	X
			Philosciidae sp.2	X	-
Onychophora	Euonychophora	Peripatidae	Peripatidae sp.1	X	-
Amphibia	Anura	Strabomantidae	*Pristimantis* cf. *fenestratus*	X	-
			Anura sp.1	X	X
			Anura sp.2	X	-
Mammalia	Chiroptera	Emballonuridae	*Peropteryx kappleri*	X	X
		Phyllostomidae	*Carollia perspicillata*	X	X
			Phyllostomus latifolius	X	X
	Rodentia	Cricetidae	*Rhipidomys* sp.	-	X

SB-0078					
TÁXONS				**Seca**	**Úmida**
Arachnida	Acari	Argasidae	*Ornithodoros* sp.1	X	X
		Trombiculidae	Trombiculidae sp.1	X	X
	Araneae	Ochyroceratidae	Ochyroceratidae jovem	X	-
		Pholcidae	*Leptopholcus* sp.1	X	X
			Mesabolivar cambridgei	X	-
			Mesabolivar eberhardi	-	X
			Mesabolivar sp.1	X	-
		Salticidae	Salticidae jovem	X	X
		Theridiosomatidae	*Plato* sp.1	-	X
	Opiliones	Escadabiidae	Escadabiidae jovem	-	X
			Escadabiidae sp.1	X	-
	Pseudoscorpiones	Bochicidae	Bochicidae sp.1	-	X
		Chernetidae	*Spelaeochernes* sp.1	X	-
		Chthoniidae	Chthoniidae sp.1	X	X
Diplopoda	Polydesmida	Fuhrmanodesmidae	Fuhrmanodesmidae sp.1	X	-
		Pyrgodesmidae	Pyrgodesmidae sp.1	-	X
Entognatha	Collembola	Isotomidae	Isotomidae sp.1	X	-
		Paronellidae	Paronellidae sp.1	-	X
Insecta	Blattodea	Polyphagidae	Polyphagidae jovem	X	-
	Coleoptera		Coleoptera jovem	X	-
	Diptera	Drosophilidae	Drosophilidae sp.	X	-
		Psychodidae	Phlebotominae sp.	X	X
	Hemiptera	Cydnidae	Cydnidae sp.1	-	X
	Hymenoptera	Formicidae	*Acromyrmex octopinosus*	X	-
			Dolichoderus bispinosus	X	-
	Lepidoptera	Noctuoidea	Noctouidea sp. 2	-	X
			Noctouidea sp. 3	-	X
		Tineoidea	Tineoidea sp. 4	X	-
	Orthoptera	Phalangopsidae	*Paraclodes* sp.1	1	-
			Phalangopsis sp.1	X	X
	Psocoptera		Trogiomorpha jovem	X	-
Malacostraca	Isopoda	Armadillidae	Armadillidae sp.1	X	-
		Philosciidae	Philosciidae sp.1	X	X
			Philosciidae sp.2	X	-
Symphyla		Scutigerellidae	*Hanseniella* sp.1	X	-
Gastropoda	Pulmonata	Subulinidae	*Lamellaxis* sp.1	X	-
			Subulinidae jovem	-	X
		Systrophiidae	Systrophiidae jovem	-	X
Amphibia	Anura	Strabomantidae	*Pristimantis* cf. *fenestratus*	X	-
Mammalia	Chiroptera	Emballonuridae	*Peropteryx* sp.	-	X
		Phyllostomidae	*Carollia* sp.	-	X

SB-0079					
TÁXONS				Seca	Úmida
Arachnida	Araneae	Araneidae	*Alpaida* sp.1	X	X
		Ctenidae	Ctenidae jovem	X	-
		Ochyroceratidae	Ochyroceratidae jovem	-	X
		Oonopidae	Oonopidae jovem	X	-
		Pholcidae	*Mesabolivar eberhardi*	-	X
			Pholcidae jovem	X	-
		Prodidomidae	Prodidomidae jovem	X	-
		Salticidae	Salticidae jovem	X	-
			Salticidae sp.1	-	X
		Scytodidae	*Scytodes* sp.1	-	X
			Scytodidae jovem	X	-
		Symphytognathidae	Symphytognathidae jovem	X	-
		Theridiosomatidae	Theridiosomatidae jovem	X	X
	Opiliones	Sclerosomatidae	Sclerosomatidae jovem	X	-
		Stygnidae	*Protimesus* sp.1	X	-
	Pseudoscorpiones	Chernetidae	*Spelaeochernes* sp.1	X	X
		Chthoniidae	Chthoniidae sp.1	X	-
Chilopoda	Scolopendromorpha	Scolopocryptopidae	*Newportia* jovem	-	X
Diplopoda	Glomeridesmida	Glomeridesmidae	Glomeridesmida sp.1	-	X
	Polydesmida	Fuhrmanodesmidae	Fuhrmanodesmidae sp.1	-	X
Entognatha	Collembola	Paronellidae	Paronellidae sp.1	X	X
	Diplura	Campodeidae	Campodeidae sp.1	X	X
Insecta	Blattodea		Blattodea jovem	X	-
	Coleoptera	Curculionidae	Scolytinae sp.1	-	X
	Diptera	Cecidomyiidae	Cecidomyiidae sp.	X	X
		Drosophilidae	Drosophilidae sp.	X	-
		Psychodidae	Phlebotominae sp.	-	X
	Hymenoptera	Formicidae	*Camponotus atriceps*	-	X
			Dolichoderus bispinosus	X	-
			Hypoponera opacior	X	-
			Pachycondyla constricta	X	X
	Lepidoptera		Lepidoptera jovem	X	X
	Orthoptera	Phalangopsidae	*Paraclodes* sp.1	X	X
	Psocoptera		Psocomorpha jovem	X	X
Mammalia	Chiroptera	Emballonuridae	*Peropteryx* sp.	-	X
		Phyllostomidae	*Glossophaga soricina*	X	-

SB-0080					
TÁXONS				**Seca**	**Úmida**
Annelida	Haplotaxida		Haplotaxida sp.2	-	X
Arachnida	Acari		Astigmata sp.5	X	-
			Mesostigmata sp.1	X	-
	Araneae	Pholcidae	*Mesabolivar aurantiacus*	X	-
			Pholcidae jovem	-	X
		Salticidae	Salticidae jovem	-	X
	Opiliones	Cosmetidae	Cosmetidae jovem	X	-
		Sclerosomatidae	Sclerosomatidae jovem	-	X
		Stygnidae	Stygnidae jovem	-	X
Entognatha	Collembola	Paronellidae	Paronellidae sp.1	-	X
		Poduromorpha	Poduromorpha sp.1	X	-
Insecta	Coleoptera	Carabidae	Carabidae sp.3	X	-
		Staphylinidae	Staphylininae sp.1	X	X
	Diptera	Cecidomyiidae	Cecidomyiidae sp.	X	-
		Drosophilidae	Drosophilidae sp.	-	X
		Psychodidae	Psychodidae jovem	-	X
	Hemiptera	Veliidae	*Rhagovelia* jovem	X	-
			Rhagovelia sp.1	-	X
			Rhagovelia sp.2	-	X
	Hymenoptera	Formicidae	*Crematogaster brasiliensis*	-	X
			Solenopsis invicta	X	-
	Isoptera	Termitidae	*Nasutitermes* sp.1	X	-
	Lepidoptera		Lepidoptera jovem	X	-
	Odonata	Anisoptera	Anisoptera jovem	X	-
	Orthoptera	Phalangopsidae	*Phalangopsis* sp.1	X	-
	Psocoptera		Psocomorpha jovem	-	X
	Trichoptera	Philopotamidae	Philopotamidae sp.1	X	-
Malacostraca	Decapoda	Palaemonidae	*Macrobrachium* sp.1	X	X
Symphyla		Scutigerellidae	*Hanseniella* sp.1	X	-
Amphibia	Anura	Strabomantidae	*Pristimantis* cf. *fenestratus*	X	-
Mammalia	Chiroptera	Phyllostomidae	*Carollia perspicillata*	X	X

SB-0081					
TÁXONS				Seca	Úmida
Arachnida	Acari	Ixodidae	*Amblyomma* sp.4	X	-
		Trombiculidae	Trombiculidae sp.2	-	X
			Acariformes sp.2	-	X
			Astigmata sp.4	-	X
			Mesostigmata sp.2	-	X
			Oribatida sp.2	-	X
	Araneae	Araneidae	*Alpaida* sp.1	-	X
			Araneidae jovem	X	-
		Corinnidae	Corinnidae jovem	X	X
		Ctenidae	Ctenidae jovem	-	X
		Ochyroceratidae	Ochyroceratidae jovem	-	X
		Pholcidae	*Leptopholcus* sp.1	-	X
			Mesabolivar eberhardi	-	X
			Pholcidae jovem	X	X
		Salticidae	Salticidae jovem	X	X
		Scytodidae	*Scytodes* sp.1	-	X
			Scytodidae jovem	X	-
		Theraphosidae	*Guyruita cerrado*	X	-
			Theraphosa blondi	-	X
		Theridiidae	*Achaearanea* sp.1	-	X
			Theridiidae jovem	X	-
		Theridiosomatidae	Theridiosomatidae jovem	-	X
	Opiliones	Cosmetidae	Cosmetidae jovem	X	-
		Escadabiidae	Escadabiidae jovem	-	X
			Escadabiidae sp.1	X	-
	Pseudoscorpiones	Chernetidae	*Spelaeochernes* sp.1	X	X
		Chthoniidae	Chthoniidae sp.1	-	X
Chilopoda	Scolopendromorpha	Scolopocryptopidae	*Scolopocryptops* sp.1	X	-
Diplopoda	Glomeridesmida	Glomeridesmidae	Glomeridesmida jovem	-	X
	Polydesmida	Fuhrmanodesmidae	Fuhrmanodesmidae sp.4	-	X
Entognatha	Collembola	Cyphoderidae	Cyphoderidae sp.1	-	X
		Entomobryidae	Entomobryidae sp.5	X	-
		Paronellidae	Paronellidae sp.1	-	X
	Diplura	Campodeidae	Campodeidae sp.1	-	X
Insecta	Blattodea	Blaberidae	Blaberidae jovem	X	-
		Blattidae	Blattidae jovem	X	-
		Polyphagidae	Polyphagidae sp.1	X	-
	Coleoptera	Nitidulidae	*Colopterus* sp.1	X	-
	Dermaptera	Labiduridae	Labiduridae sp.2	X	-
	Diptera	Ceratopogonidae	Ceratopogonidae jovem	-	X
		Dolichopodidae	Dolichopodidae sp.	X	-
		Drosophilidae	Drosophilidae sp.	X	-
		Psychodidae	Phlebotominae sp.	-	X
		Tipulidae	Tipulidae sp.	-	X
	Hemiptera	Cydnidae	Cydnidae sp.1	X	X
			Cydnidae sp.2	X	-
	Hymenoptera	Diapriidae	Diapriidae sp.2	X	-
		Formicidae	*Camponotus* sp.6	X	-
			Cephalotes clypeatus	X	-
			Forelius sp.1	X	-
			Hypoponera opacior	X	X
			Odontomachus meinerti	X	-
			Solenopsis invicta	X	-
			Solenopsis sp.7	X	-
	Isoptera	Termitidae	*Nasutitermes* sp.1	X	-
			Isoptera jovem	-	X
	Lepidoptera		Lepidoptera jovem	X	-

	Orthoptera	Phalangopsidae	*Paraclodes* sp.1	X	-
			Phalangopsis sp.1	-	X
	Psocoptera		Psocomorpha jovem	-	X
			Trogiomorpha jovem	X	-
	Thysanura	Nicoletiidae	Nicoletiinae sp.1	X	X
Malacostraca	Isopoda	Philosciidae	Philosciidae sp.1	-	X
			Philosciidae sp.2	-	X
		Platyarthridae	Platyarthridae sp.2	X	-
Amphibia	Anura	Strabomantidae	*Pristimantis* cf. *fenestratus*	-	X
			Anura sp.6	-	X
Mammalia	Chiroptera	Phyllostomidae	*Carollia* sp.	-	X

SB-0082					
TÁXONS				**Seca**	**Úmida**
Arachnida	Araneae	Ctenidae	Ctenidae jovem	X	-
		Ochyroceratidae	*Speocera* sp.1	X	-
		Pholcidae	Pholcidae jovem	X	X
		Salticidae	Salticidae sp.4	X	-
		Trechaleidae	Trechaleidae jovem	X	-
	Opiliones	Sclerosomatidae	Sclerosomatidae jovem	X	-
Entognatha	Diplura	Campodeidae	Campodeidae sp.1	X	-
Insecta	Coleoptera	Gyrinidae	*Gyretes* sp.1	X	-
	Diptera	Sciaridae	Sciaridae sp.	-	X
	Hemiptera	Cixiidae	Cixiidae sp.4	X	X
		Veliidae	*Rhagovelia* jovem	-	X
			Rhagovelia sp.1	X	-
			Rhagovelia sp.3	X	-
	Hymenoptera	Formicidae	*Camponotus* sp.2	X	-
			Gnamptogenys haenschi	X	-
			Paratrechina sp.1	X	X
Symphyla		Scutigerellidae	*Hanseniella* sp.1	-	X

SB-0083					
TÁXONS				Seca	Úmida
Arachnida	Acari		Acariformes sp.3	X	X
			Astigmata sp.2	-	X
			Mesostigmata sp.1	X	-
	Amblypygi	Phrynidae	*Heterophrinus longicornis*	X	X
	Araneae	Corinnidae	Corinnidae jovem	X	X
		Ochyroceratidae	Ochyroceratidae jovem	-	X
			Speocera sp.1	X	-
		Pholcidae	*Mesabolivar eberhardi*	X	X
			Ninetinae sp.1	-	X
		Salticidae	Salticidae sp.3	-	X
			Salticidae sp.5	X	-
		Scytodidae	Scytodidae jovem	X	-
		Theraphosidae	Theraphosidae jovem	-	X
		Theridiidae	*Achaearanea* sp.1	-	X
			Theridiidae jovem	X	-
		Theridiosomatidae	*Plato* sp.1	-	X
	Opiliones	Sclerosomatidae	Sclerosomatidae jovem	-	X
		Stygnidae	*Protimesus* sp.2	X	-
	Pseudoscorpiones	Chernetidae	*Spelaeochernes* sp.1	X	X
Entognatha	Collembola	Paronellidae	Paronellidae sp.1	X	X
	Diplura	Campodeidae	Campodeidae sp.1	X	X
Insecta	Blattodea	Blaberidae	Blaberidae jovem	-	X
		Blattidae	Blattidae jovem	-	X
	Coleoptera	Tenebrionidae	Tenebrionidae jovem	X	-
	Diptera	Ceratopogonidae	Ceratopogonidae jovem	-	X
		Drosophilidae	Drosophilidae sp.	X	-
	Hemiptera	Cixiidae	Cixiidae jovem	-	X
		Cydnidae	Cydnidae sp.1	-	X
		Reduviidae	Reduviinae jovem	-	X
			Heteroptera jovem	X	-
	Hymenoptera	Formicidae	*Acromyrmex octopinosus*	-	X
			Crematogaster brasiliensis	X	-
			Pheidole sp.7	X	X
			Prionopelta modesta	X	-
			Solenopsis invicta	X	-
	Lepidoptera	Noctuoidea	Noctouidea sp. 9	X	-
		Tineoidea	Tineoidea sp. 9	-	X
	Orthoptera	Phalangopsidae	*Eidmanacris* sp.1	X	-
			Paraclodes sp.1	X	-
			Phalangopsis sp.1	X	X
	Psocoptera		Psocomorpha jovem	X	-
	Thysanura	Nicoletiidae	Nicoletiinae sp.1	X	X
Amphibia	Anura	Strabomantidae	*Pristimantis* cf. *fenestratus*	X	-
Mammalia	Chiroptera	Emballonuridae	*Peropteryx kappleri*	X	X
		Phyllostomidae	*Trachops cirrhosus*	X	-

SB-0084					
TÁXONS				Seca	Úmida
Arachnida	Acari	Trombiculidae	Trombiculidae sp.1	-	X
			Mesostigmata sp.1	-	X
	Amblypygi	Phrynidae	*Heterophrinus longicornis*	X	X
	Araneae	Araneidae	Araneidae jovem	-	X
		Corinnidae	Corinnidae jovem	X	-
		Ctenidae	Ctenidae jovem	X	X
		Oonopidae	Oonopidae sp.3	X	X
		Pholcidae	*Mesabolivar eberhardi*	X	-
			Pholcidae jovem	X	X
		Salticidae	Salticidae sp.4	X	-
		Scytodidae	Scytodidae jovem	X	X
	Opiliones	Escadabiidae	Escadabiidae jovem	-	X
		Sclerosomatidae	*Prionostema* sp.1	-	X
	Pseudoscorpiones	Chernetidae	*Spelaeochernes* sp.1	X	X
Chilopoda	Scutigeromorpha	Pselliodidae	*Sphendononema guildingii*	-	X
Diplopoda	Siphonophorida	Siphonophoridae	Siphonophoridae sp.1	-	X
Entognatha	Collembola	Entomobryidae	Entomobryidae sp.2	-	X
	Diplura	Campodeidae	Campodeidae sp.1	X	-
Insecta	Blattodea	Blaberidae	Blaberidae jovem	-	X
		Polyphagidae	Polyphagidae jovem	X	X
			Polyphagidae sp.1	-	X
	Coleoptera		Coleoptera jovem	-	X
	Diptera	Ceratopogonidae	Ceratopogonidae jovem	-	X
		Psychodidae	Phlebotominae sp.	X	X
	Hemiptera	Fulgoridae	Fulgoridae sp.3	-	X
		Reduviidae	Emesinae sp.5	-	X
			Reduviinae jovem	-	X
	Hymenoptera	Diapriidae	Diapriidae sp.3	X	-
		Formicidae	*Cephalotes atratus*	X	X
			Cephalotes minutus	X	-
			Pachycondyla constricta	X	X
	Isoptera	Termitidae	*Nasutitermes* sp.1	-	X
	Lepidoptera	Tineoidea	Tineoidea sp. 5	-	X
			Tineoidea sp. 6	-	X
	Neuroptera	Myrmeleontidae	Myrmeleontidae sp.1	X	-
	Orthoptera	Phalangopsidae	*Eidmanacris* sp.1	X	-
			Paraclodes sp.1	X	-
			Phalangopsis sp.1	X	X
	Psocoptera	Psyllipsocidae	Psyllipsocidae sp.3	X	X
			Psocomorpha jovem	-	X
Malacostraca	Isopoda	Armadillidae	Armadillidae sp.1	X	X
		Philosciidae	Philosciidae sp.2	X	-
Gastropoda	Pulmonata	Systrophiidae	*Happia* sp.1	-	X
Amphibia	Anura	Strabomantidae	*Pristimantis* cf. *fenestratus*	X	-
Mammalia	Chiroptera	Emballonuridae	*Peropteryx kappleri*	X	X
		Furipteridae	*Furipterus horrens*	X	-
		Phyllostomidae	*Carollia* sp.	X	-
			Glossophaga soricina	-	X

SB-0085					
TÁXONS				Seca	Úmida
Annelida	Haplotaxida		Haplotaxida sp.3	X	X
			Haplotaxida sp.4	X	X
			Tubificina sp.1	X	-
Arachnida	Acari	Trombiculidae	Trombiculidae sp.1	-	X
			Astigmata sp.4	X	-
			Astigmata sp.5	-	X
			Holothyrida sp.2	X	X
			Mesostigmata sp.1	X	X
			Mesostigmata sp.4	-	X
			Mesostigmata sp.5	-	X
			Oribatida sp.2	X	-
	Amblypygi	Phrynidae	*Heterophrinus longicornis*	X	X
	Araneae	Corinnidae	Corinnidae jovem	X	X
		Oonopidae	Oonopidae sp.4	X	X
			Oonopidae sp.5	X	-
			Oonopidae sp.10	X	-
			Oonopidae sp.11	-	X
		Pholcidae	*Mesabolivar aurantiacus*	X	X
			Mesabolivar eberhardi	X	-
		Pisauridae	Pisauridae jovem	X	X
		Prodidomidae	Prodidomidae sp.2	X	-
		Salticidae	Salticidae sp.4	X	-
		Segestriidae	*Ariadna* sp.1	X	X
		Theraphosidae	Theraphosa blondi	X	-
		Theridiosomatidae	*Plato* sp.1	X	X
		Trechaleidae	Trechaleidae jovem	X	X
	Opiliones	Cosmetidae	*Roquettea carajas*	X	-
		Escadabiidae	Escadabiidae jovem	X	X
			Escadabiidae sp.1	X	-
			Escadabiidae sp.2	X	-
		Manaosbiidae	Manaosbiidae sp.1	X	-
		Sclerosomatidae	*Prionostema* sp.1	X	X
		Stygnidae	Stygnidae sp.1	X	-
	Pseudoscorpiones	Chernetidae	*Spelaeochernes* sp.1	X	X
Chilopoda	Scolopendromorpha	Scolopendridae	*Otostigmus* sp.1	X	-
		Scolopocryptopidae	*Dinocryptops miersii*	X	-
			Newportia sp.2	X	-
	Scutigeromorpha	Pselliodidae	*Sphendononema guildingii*	X	X
Diplopoda	Glomeridesmida	Glomeridesmidae	Glomeridesmida jovem	X	-
	Polydesmida	Chelodesmidae	Chelodesmidae SP.1	X	-
		Pyrgodesmidae	Pyrgodesmidae sp.1	X	X
	Polyzoniida	Siphonotidae	Siphonotidae sp.1	X	-
	Spirostreptida	Pseudonannolenidae	Pseudonannolenidae sp.1	X	-
Entognatha	Collembola	Cyphoderidae	Cyphoderidae sp.1	X	X
		Entomobryidae	Entomobryidae sp.3	-	X
			Entomobryidae sp.4	-	X
		Paronellidae	Paronellidae sp.1	X	X
	Diplura	Campodeidae	Campodeidae sp.1	-	X
Insecta	Blattodea	Blattellidae	Blattellidae jovem	X	X
	Coleoptera	Carabidae	*Chlaenius* sp.1	X	-
		Elmidae	Elmidae sp.1	-	X
		Histeridae	Histeridae sp.2	X	-
		Hydrophilidae	Hydrophilidae sp.1	X	X
			Hydrophilidae sp.4	-	X
		Staphylinidae	Pselaphinae sp.2	X	X
			Coproporus sp.1	X	-
			Scydmaeninae sp.2	X	X

			Scydmaeninae sp.4	-	X
			Staphylinidae sp.6	X	X
			Staphylinidae sp.7	X	-
			Staphylininae sp.1	X	-
			Staphylininae sp.2	X	X
			Staphylininae sp.3	X	-
		Tenebrionidae	Tenebrionidae sp.3	X	-
	Diptera	Cecidomyiidae	Cecidomyiidae sp.	X	X
		Ceratopogonidae	Ceratopogonidae sp.	X	-
		Culicidae	Culicidae sp.	X	-
		Drosophilidae	Drosophilidae sp.	X	X
		Phoridae	Phoridae sp.	X	-
		Psychodidae	Psychodidae sp.	X	X
		Sciaridae	Sciaridae sp.	X	-
		Tipulidae	Tipulidae sp.	X	X
	Hemiptera	Cydnidae	Cydnidae sp.1	X	X
		Hebridae	Hebridae jovem	-	X
			Hebridae sp.1	X	-
		Veliidae	Rhagovelia sp.1	X	-
			Veliidae jovem	X	X
			Homoptera jovem	-	X
	Hymenoptera	Braconidae	Braconidae sp.1	X	-
		Formicidae	*Acropyga* cf. *smithii*	X	-
			Camponotus atriceps	X	X
			Camponotus sp.2	X	X
			Gnamptogenys sp.3	X	-
			Hypoponera sp.5	-	X
			Hypoponera sp.6	X	-
			Neivamyrmex sp.2	-	X
			Odontomachus meinerti	X	X
			Pachycondyla constricta	X	X
			Pachycondyla impressa	X	-
			Pachycondyla striata	X	-
			Paratrechina sp.1	X	-
			Pheidole sp.3	X	X
			Platythyrea angusta	X	-
			Solenopsis invicta	X	-
			Solenopsis sp.2	X	X
		Vespidae	Vespidae sp.1	X	-
	Isoptera	Termitidae	*Nasutitermes* sp.2	X	-
	Lepidoptera	Noctuoidea	Noctouidea sp. 6	X	-
		Tineoidea	Tineoidea sp. 2	-	X
			Tineoidea sp. 3	X	X
	Orthoptera	Phalangopsidae	*Paraclodes* sp.1	-	X
			Phalangopsis sp.1	X	X
	Plecoptera	Perlidae	Perlidae jovem	-	X
	Trichoptera	Hydrobiosidae	Hydrobiosidae jovem	-	X
		Philopotamidae	Philopotamidae sp.1	X	-
			Trichoptera jovem	-	X
	Thysanura	Nicoletiidae	Atelurinae sp.1	X	X
			Nicoletiinae sp.1	X	X
Malacostraca	Decapoda	Palaemonidae	*Macrobrachium* sp.1	X	X
		Pseudothelphusidae	Pseudothelphusidae sp.1	X	X
	Isopoda	Philosciidae	Philosciidae sp.2	X	-
		Platyarthridae	Platyarthridae sp.1	-	X
		Scleropactidae	Scleropactidae sp.1	X	X
Symphyla		Scutigerellidae	*Hanseniella* sp.1	X	X
Gastropoda	Pulmonata	Subulinidae	*Lamellaxis* sp.1	X	X
			Leptinaria sp.2	X	-

		Systrophiidae	*Happia* sp.3	X	-
			Happia sp.4	X	-
			Systrophiidae jovem	X	X
Turbellaria	Tricladida		Continenticola sp.1	-	X
Amphibia	Anura	Leptodactylidae	*Leptodactylus* cf. *vastus*	X	-
		Strabomantidae	*Pristimantis* cf. *fenestratus*	X	X
Mammalia	Chiroptera	Emballonuridae	*Peropteryx kappleri*	X	-
		Mormoopidae	*Pteronotus gymnonotus*	X	-
			Pteronotus parnellii	X	X
		Phyllostomidae	*Anoura geoffroyi*	X	X
			Carollia perspicillata	X	X
			Lionycteris spurrelli	X	X
			Lonchorhina aurita	X	X
	Rodentia	Cuniculidae	*Cuniculus paca*	-	X
Reptilia	Squamata	Gymnophthalmidae	*Neusticurus* sp.	X	X

SB-0086					
TÁXONS				**Seca**	**Úmida**
Arachnida	Araneae	Oonopidae	Oonopidae jovem	X	-
		Pholcidae	*Mesabolivar aurantiacus*	-	X
			Mesabolivar sp.1	X	-
		Theridiosomatidae	*Plato* sp.1	X	X
	Opiliones	Sclerosomatidae	*Prionostema* sp.1	X	X
	Pseudoscorpiones	Cheiridiidae	Cheiridiidae sp.1	-	X
Insecta	Blattodea		Blattodea jovem	X	-
	Coleoptera	Staphylinidae	Staphylininae sp.1	X	-
			Coleoptera jovem	-	X
	Diptera	Tipulidae	Tipulidae sp.	-	X
	Hymenoptera	Formicidae	*Camponotus atriceps*	-	X
			Camponotus sp.2	-	X
			Crematogaster brasiliensis	-	X
	Isoptera	Termitidae	*Nasutitermes* sp.1	-	X
	Lepidoptera	Noctuoidea	Noctouidea sp. 6	X	-
		Tineoidea	Tineoidea sp. 5	X	-
	Orthoptera	Phalangopsidae	*Paraclodes* sp.1	X	X
			Phalangopsis sp.1	-	X
	Psocoptera		Psocomorpha jovem	X	X
	Trichoptera		Trichoptera jovem	X	-
Symphyla		Scutigerellidae	*Hanseniella* sp.1	X	-
Mammalia	Chiroptera	Phyllostomidae	*Micronycteris* sp.	-	X

SB-0087					
**TÁXONS**				**Seca**	**Úmida**
Annelida	Haplotaxida		Haplotaxida sp.1	-	X
Arachnida	Acari		Holothyrida sp.1	-	X
			Mesostigmata sp.1	-	X
	Amblypygi	Phrynidae	*Heterophrinus longicornis*	X	X
	Araneae	Araneidae	Araneidae jovem	X	-
		Corinnidae	*Abapeba* sp.1	X	X
		Ctenidae	Ctenidae jovem	X	X
		Ochyroceratidae	*Speocera* sp.1	X	X
		Pholcidae	*Mesabolivar aurantiacus*	X	-
			Pholcidae jovem	-	X
		Theraphosidae	Theraphosidae jovem	X	-
		Theridiidae	Theridiidae jovem	X	X
		Theridiosomatidae	*Plato* sp.1	X	X
	Opiliones	Cosmetidae	*Roquettea carajas*	X	X
		Escadabiidae	Escadabiidae jovem	-	X
		Stygnidae	Stygnidae jovem	-	X
	Pseudoscorpiones	Chernetidae	*Spelaeochernes* sp.1	X	-
		Chthoniidae	Chthoniidae sp.1	-	X
Chilopoda	Scolopendromorpha	Scolopocryptopidae	*Dinocryptops miersii*	X	-
	Scutigeromorpha	Pselliodidae	*Sphendononema* jovem	-	X
Diplopoda	Polydesmida	Cryptodesmidae	Cryptodesmidae sp.1	-	X
		Pyrgodesmidae	Pyrgodesmidae sp.1	-	X
Entognatha	Collembola	Isotomidae	Isotomidae sp.1	-	X
		Paronellidae	Paronellidae sp.1	-	X
		Sminthuroidea	Sminthuroidea sp.2	-	X
	Diplura	Campodeidae	Campodeidae sp.1	-	X
Insecta	Blattodea	Blaberidae	Blaberidae jovem	X	-
		Polyphagidae	Polyphagidae jovem	X	-
	Coleoptera	Endomychidae	Endomychidae sp.1	X	-
		Staphylinidae	Pselaphinae sp.2	X	-
			Staphylinidae sp.2	X	-
	Diptera	Drosophilidae	Drosophilidae sp.	-	X
		Phoridae	Phoridae sp.	X	-
		Psychodidae	Phlebotominae sp.	-	X
	Hemiptera	Cixiidae	Cixiidae jovem	X	-
		Cydnidae	Cydnidae sp.1	-	X
	Hymenoptera	Formicidae	*Apterostigma collare*	-	X
			Camponotus sp.2	X	X
			Crematogaster brasiliensis	X	-
			Pachycondyla constricta	X	-
			Strumigenys precava	-	X
	Isoptera	Termitidae	*Coatitermes* sp.1	-	X
			Nasutitermes sp.2	X	X
	Lepidoptera	Noctuoidea	Noctouidea sp. 1	X	-
			Lepidoptera jovem	-	X
	Orthoptera	Phalangopsidae	*Eidmanacris* sp.1	X	-
			Paraclodes sp.1	X	X
			Phalangopsis sp.1	X	X
Malacostraca	Isopoda	Philosciidae	Philosciidae sp.1	-	X
			Philosciidae sp.2	X	-
Symphyla		Scutigerellidae	*Hanseniella* sp.1	X	-
			Scutigerellidae jovem	-	X
Gastropoda	Pulmonata	Subulinidae	*Lamellaxis* sp.1	-	X
Amphibia	Anura	Strabomantidae	*Pristimantis* cf. *fenestratus*	X	-
Mammalia	Chiroptera	Phyllostomidae	*Carollia perspicillata*	-	X
			Desmodus rotundus	-	X
			Glossophaga soricina	X	X

SB-0088					
TÁXONS				Seca	Úmida
Annelida	Haplotaxida		Haplotaxida sp.2	-	X
			Haplotaxida sp.7	X	-
Arachnida	Acari		Astigmata sp.2	-	X
			Holothyrida sp.2	-	X
			Mesostigmata sp.1	-	X
			Oribatida sp.2	-	X
	Amblypygi	Phrynidae	*Heterophrinus longicornis*	X	X
	Araneae	Corinnidae	*Abapeba* sp.1	X	-
			Corinnidae jovem	X	-
		Ochyroceratidae	*Speocera* sp.1	X	-
		Pholcidae	*Mesabolivar aurantiacus*	X	-
			Pholcidae jovem	X	-
		Salticidae	Salticidae sp.11	X	-
		Theridiosomatidae	*Plato* sp.1	X	X
	Opiliones	Cosmetidae	*Roquettea carajas*	X	-
		Escadabiidae	Escadabiidae sp.1	X	-
			Escadabiidae sp.2	X	-
	Pseudoscorpiones	Cheliferidae	Cheliferidae sp.1	X	-
		Chernetidae	*Spelaeochernes* sp.1	X	-
		Olpiidae	Olpiidae sp.1	-	X
Diplopoda	Polydesmida	Fuhrmanodesmidae	Fuhrmanodesmidae sp.1	-	X
Entognatha	Collembola	Cyphoderidae	Cyphoderidae sp.1	-	X
		Paronellidae	Paronellidae sp.1	X	-
Insecta	Blattodea	Blaberidae	Blaberidae jovem	X	-
		Polyphagidae	Polyphagidae jovem	X	-
	Coleoptera	Carabidae	Carabidae sp.1	-	X
			Lelis sp.1	X	-
			Notibia sp.1	X	X
		Noteridae	*Notomicrus* sp.1	X	-
	Diptera	Asilidae	Asilidae sp.	X	-
		Cecidomyiidae	Cecidomyiidae sp.	X	X
		Drosophilidae	Drosophilidae sp.	X	-
		Muscidae	Muscidae sp.	-	X
		Phoridae	Phoridae sp.	-	X
		Psychodidae	Phlebotominae sp.	-	X
			Psychodidae sp.	X	X
	Hemiptera	Cydnidae	Cydnidae sp.1	X	X
		Reduviidae	Emesinae jovem	X	-
			Reduviidae jovem	X	-
	Hymenoptera	Diapriidae	Diapriidae sp.6	X	-
			Diapriidae sp.7	-	X
		Figitidae	Figitidae sp.1	X	-
		Formicidae	*Odontomachus bauri*	-	X
			Paratrechina sp.1	-	X
	Isoptera		Isoptera jovem	-	X
	Lepidoptera	Tineoidea	Tineoidea sp. 2	X	-
			Tineoidea sp. 3	X	-
	Orthoptera	Phalangopsidae	*Paraclodes* sp.1	X	-
			Phalangopsis sp.1	X	X
Malacostraca	Isopoda	Philosciidae	Philosciidae sp.1	-	X
			Philosciidae sp.2	X	X
		Platyarthridae	Platyarthridae sp.2	X	-
Symphyla		Scutigerellidae	*Hanseniella* sp.1	-	X
Gastropoda	Pulmonata	Subulinidae	*Lamellaxis* sp.1	X	-
		Systrophiidae	*Happia* sp.1	X	-
			Systrophiidae jovem	X	-
Turbellaria	Tricladida	Planariidae	Planariidae sp.1	X	-

Amphibia	Anura	Bufonidae	*Rhinella* sp.	X	-
		Strabomantidae	*Pristimantis* cf. *fenestratus*	X	-
Mammalia	Chiroptera	Phyllostomidae	*Anoura geoffroyi*	X	-
			Carollia perspicillata	X	X
			Glossophaga soricina	X	X
			Lonchorhina aurita	X	-

SB-0089					
**TÁXONS**				**Seca**	**Úmida**
Arachnida	Acari		Oribatida sp.5	-	X
	Amblypygi	Phrynidae	*Heterophrinus longicornis*	X	X
	Araneae	Ctenidae	Ctenidae jovem	-	X
		Oonopidae	Oonopidae jovem	-	X
		Paratropididae	Paratropididae jovem	-	X
		Pholcidae	*Mesabolivar aurantiacus*	X	-
			Mesabolivar eberhardi	-	X
		Theraphosidae	Theraphosidae jovem	-	X
		Theridiosomatidae	Theridiosomatidae jovem	-	X
	Opiliones	Cosmetidae	Cosmetidae jovem	X	-
		Sclerosomatidae	*Prionostema* sp.1	X	-
	Pseudoscorpiones	Chernetidae	*Spelaeochernes* sp.1	-	X
Diplopoda	Glomeridesmida	Glomeridesmidae	Glomeridesmida jovem	-	X
Entognatha	Collembola	Paronellidae	Paronellidae sp.1	X	X
	Diplura	Campodeidae	Campodeidae sp.1	-	X
		Projapygidae	Projapygidae sp.1	-	X
Insecta	Archaeognatha	Meinertellidae	Meinertellidae sp.1	-	X
	Diptera	Ceratopogonidae	Ceratopogonidae jovem	-	X
		Tipulidae	Tipulidae sp.	X	X
	Hemiptera	Cixiidae	Cixiidae jovem	-	X
		Cydnidae	Cydnidae sp.1	-	X
		Reduviidae	Reduviidae jovem	X	-
		Schizopteridae	Schizopteridae jovem	-	X
	Hymenoptera	Formicidae	*Camponotus atriceps*	X	-
			Dolichoderus bispinosus	X	-
			Ectatomma edentatum	X	-
			Paratrechina sp.1	X	X
	Isoptera	Termitidae	*Nasutitermes* sp.1	X	-
	Lepidoptera		Lepidoptera jovem	-	X
	Neuroptera	Myrmeleontidae	Myrmeleontidae sp.1	X	-
	Orthoptera	Phalangopsidae	*Paraclodes* sp.1	X	X
			Phalangopsis sp.1	-	X
	Psocoptera	Pseudocaeciliidae	Pseudocaeciliidae sp.1	X	-
			Psocomorpha jovem	X	X
			Troctomorpha jovem	-	X
Malacostraca	Isopoda	Armadillidae	Armadillidae sp.1	X	X
		Philosciidae	Philosciidae sp.1	X	X
Gastropoda	Pulmonata	Systrophiidae	Systrophiidae jovem	X	-
Amphibia	Anura	Strabomantidae	*Pristimantis* cf. *fenestratus*	X	-
Mammalia	Chiroptera	Emballonuridae	*Peropteryx* sp.	-	X
		Phyllostomidae	*Micronycteris megalotis*	-	X

SB-0090					
TÁXONS				Seca	Úmida
Arachnida	Acari	Ixodidae	*Amblyomma* sp.3	X	-
			Acariformes sp.4	X	-
			Astigmata sp.1	-	X
			Astigmata sp.2	-	X
			Astigmata sp.4	-	X
			Mesostigmata sp.1	-	X
			Oribatida sp.1	-	X
	Amblypygi	Phrynidae	*Heterophrinus longicornis*	X	X
	Araneae	Araneidae	*Alpaida* sp.1	X	X
		Corinnidae	Corinnidae jovem	X	-
		Ctenidae	Ctenidae jovem	-	X
		Dipluridae	Dipluridae jovem	-	X
		Ochyroceratidae	Ochyroceratidae jovem	X	-
			Speocera sp.1	-	X
		Oonopidae	Oonopidae sp.2	X	-
		Pholcidae	*Mesabolivar aurantiacus*	-	X
			Mesabolivar cambridgei	X	-
			Mesabolivar eberhardi	-	X
		Salticidae	Salticidae jovem	X	-
		Scytodidae	*Scytodes* sp.1	X	-
			Scytodidae jovem	-	X
		Tetrablemmidae	*Matta* sp.1	X	-
		Tetragnathidae	Tetragnathidae jovem	X	X
		Theraphosidae	*Guyruita cerrado*	X	X
		Theridiidae	*Thymoites* sp.2	X	-
		Theridiosomatidae	*Plato* sp.1	X	X
		Trechaleidae	Trechaleidae jovem	-	X
	Opiliones	Cosmetidae	Cosmetidae sp.3	X	-
			Roquettea carajas	X	X
		Sclerosomatidae	*Prionostema* sp.1	X	X
		Stygnidae	*Protimesus* sp.2	X	-
			Stygnidae jovem	-	X
	Palpigradi	Eukoeneniidae	*Eukoenenia* sp.1	-	X
	Pseudoscorpiones	Chernetidae	*Spelaeochernes* sp.1	X	X
		Chthoniidae	Chthoniidae sp.1	X	-
Chilopoda	Lithobiomorpha	Henicopidae	*Lamyctes* p.2	-	X
	Scolopendromorpha	Cryptopidae	*Cryptops* sp.2	X	-
		Scolopocryptopidae	*Newportia* sp.2	X	-
			Newportia sp.3	X	-
			Tidops sp.1	-	X
	Scutigeromorpha	Pselliodidae	*Sphendononema* jovem	X	X
Diplopoda	Glomeridesmida	Glomeridesmidae	Glomeridesmida sp.1	-	X
	Polydesmida	Chelodesmidae	Chelodesmidae sp.1	-	X
		Fuhrmanodesmidae	Fuhrmanodesmidae jovem	-	X
		Pyrgodesmidae	Pyrgodesmidae sp.1	-	X
	Spirostreptida		Spirostreptida jovem	-	X
Entognatha	Collembola	Cyphoderidae	Cyphoderidae sp.1	X	-
		Entomobryidae	Entomobryidae sp.4	-	X
		Paronellidae	Paronellidae sp.1	X	X
		Poduromorpha	Poduromorpha sp.1	-	X
			Onychiuridae sp.1	-	X
	Diplura	Campodeidae	Campodeidae sp.1	X	X
Insecta	Blattodea	Blattellidae	Blattellidae sp.1	-	X
		Blattidae	Blattidae jovem	X	-
	Coleoptera	Noteridae	*Stelidoda* sp.1	-	X
		Staphylinidae	Scydmaeninae sp.2	X	-
			Staphylinidae sp.5	-	X

			Staphylininae sp.1	X	-
			Staphylininae sp.3	X	-
	Diptera	Cecidomyiidae	Cecidomyiidae sp.	-	X
		Muscidae	Muscidae jovem	-	X
		Psychodidae	Phlebotominae sp.	X	X
		Sciaridae	Sciaridae sp.	X	-
		Tipulidae	Tipulidae sp.	X	X
	Hemiptera	Cixiidae	Cixiidae jovem	X	X
			Cixiidae sp.1	-	X
			Cixiidae sp.3	-	X
		Cydnidae	Cydnidae sp.1	X	X
			Cydnidae sp.2	-	X
		Hebridae	Hebridae sp.1	X	-
		Ochteridae	Ochteridae sp.2	X	-
		Reduviidae	Emesinae jovem	-	X
		Schizopteridae	Schizopteridae sp.1	-	X
	Hymenoptera	Formicidae	*Acromyrmex octopinosus*	X	-
			Camponotus sp.2	X	X
			Carebara urichii	X	X
			Crematogaster erecta	X	X
			Eurhopalothrix sp.1	-	X
			Myrmicinae sp.1	-	X
			Octostruma iheringi	-	X
			Pachycondyla constricta	X	X
			Pachycondyla harpax	-	X
			Paratrechina sp.1	X	X
			Solenopsis sp.7	-	X
			Strumigenys precava	-	X
	Isoptera	Termitidae	*Nasutitermes* sp.1	X	-
	Lepidoptera	Hesperiidae	Hesperiidae sp. 1	X	X
		Noctuoidea	Noctouidea sp. 1	-	X
	Orthoptera	Phalangopsidae	*Paraclodes* sp.1	X	X
			Phalangopsis sp.1	X	X
	Psocoptera		Psocomorpha jovem	X	-
Malacostraca	Isopoda	Armadillidae	Armadillidae sp.1	X	-
		Philosciidae	Philosciidae sp.1	-	X
			Philosciidae sp.2	X	X
		Scleropactidae	Scleropactidae sp.1	-	X
Symphyla		Scutigerellidae	*Hanseniella* sp.1	X	-
Gastropoda	Pulmonata	Subulinidae	*Lamellaxis* sp.1	X	X
			Leptinaria sp.2	X	X
		Systrophiidae	*Happia* sp.1	X	X
Amphibia	Anura	Strabomantidae	*Pristimantis* cf. *fenestratus*	X	-
			Anura sp.5	X	-
Mammalia	Chiroptera	Emballonuridae	*Peropteryx kappleri*	X	X
		Phyllostomidae	*Carollia perspicillata*	X	X
			Glossophaga soricina	X	X

SB-0091					
TÁXONS				Seca	Úmida
Arachnida	Amblypygi	Phrynidae	*Heterophrinus longicornis*	X	-
	Araneae	Araneidae	*Alpaida* sp.1	-	X
			Araneidae jovem	X	-
		Corinnidae	*Abapeba* sp.1	-	X
		Pholcidae	*Mesabolivar aurantiacus*	X	-
			Mesabolivar eberhardi	-	X
		Salticidae	Salticidae sp.3	-	X
		Scytodidae	*Scytodes* sp.1	-	X
		Theridiosomatidae	*Plato* sp.1	-	X
	Opiliones	Sclerosomatidae	*Prionostema* sp.1	X	X
	Pseudoscorpiones	Chernetidae	Chernetidae jovem	X	-
Entognatha	Collembola	Paronellidae	Paronellidae sp.1	X	-
Insecta	Blattodea		Blattodea jovem	X	-
	Coleoptera	Byrrhidae	Byrrhidae sp.1	X	-
	Diptera	Ceratopogonidae	Ceratopogonidae jovem	-	X
		Psychodidae	Phlebotominae sp.	X	-
		Tipulidae	Tipulidae sp.	X	-
	Hemiptera	Cixiidae	Cixiidae jovem	X	-
		Cydnidae	Cydnidae sp.1	-	X
	Hymenoptera	Formicidae	*Apterostigma collare*	-	X
			Camponotus sp.2	X	X
			Crematogaster erecta	X	-
			Gnamptogenys sp.2	X	-
			Pheidole sp.1	-	X
	Lepidoptera	Hesperiidae	Hesperiidae sp. 1	X	-
		Noctuoidea	Noctouidea sp. 1	X	-
	Orthoptera	Phalangopsidae	*Paraclodes* sp.1	X	X
			Phalangopsis sp.1	X	X
	Psocoptera		Psocomorpha jovem	X	-
Malacostraca	Isopoda	Philosciidae	Philosciidae sp.1	X	-
			Philosciidae sp.2	X	X
Amphibia	Anura	Strabomantidae	*Pristimantis* cf. *fenestratus*	X	-
Mammalia	Chiroptera	Emballonuridae	*Peropteryx kappleri*	X	-
		Phyllostomidae	*Carollia perspicillata*	X	-
			Glossophaga soricina	X	-

SB-0092					
TÁXONS				Seca	Úmida
Arachnida	Acari		Holothyrida sp.2	X	-
			Mesostigmata sp.1	X	-
	Amblypygi	Phrynidae	*Heterophrinus longicornis*	X	X
	Araneae	Araneidae	Araneidae jovem	X	-
		Corinnidae	Corinnidae jovem	X	X
		Pholcidae	*Mesabolivar aurantiacus*	X	X
		Pisauridae	Pisauridae jovem	X	-
		Salticidae	Salticidae sp.7	X	-
		Theridiosomatidae	Theridiosomatidae jovem	X	-
		Thomisidae	Thomisidae jovem	X	-
	Opiliones	Neogoveidae	Neogoveidae jovem	-	X
		Sclerosomatidae	*Prionostema* sp.1	X	-
	Pseudoscorpiones	Chernetidae	Chernetidae jovem	-	X
		Chthoniidae	Chthoniidae sp.1	X	-
Diplopoda	Polydesmida	Fuhrmanodesmidae	Fuhrmanodesmidae sp.1	X	-
			Fuhrmanodesmidae sp.2	X	-
Entognatha	Collembola	Cyphoderidae	Cyphoderidae sp.1	X	-
		Onychiuridae	Onychiuridae sp.1	-	X
		Paronellidae	Paronellidae sp.1	X	-
Insecta	Blattodea		Blattodea jovem	X	-
	Coleoptera	Carabidae	*Notibia* sp.1	-	X
		Hydrophilidae	Hydrophilidae sp.2	X	-
		Latridiidae	Latridiidae sp.1	X	-
		Staphylinidae	Pselaphinae sp.2	-	X
			Scydmaeninae sp.2	X	-
			Scydmaeninae sp.4	-	X
	Diptera	Cecidomyiidae	Cecidomyiidae sp.	X	-
		Drosophilidae	Drosophilidae sp.	X	-
		Muscidae	Muscidae jovem	-	X
		Psychodidae	Psychodidae sp.	X	-
		Sciaridae	Sciaridae sp.	X	-
		Tipulidae	Tipulidae sp.	X	-
	Hemiptera	Cydnidae	Cydnidae sp.1	X	X
			Cydnidae sp.2	X	-
		Fulgoridae	Fulgoridae sp.1	X	-
		Veliidae	Veliidae jovem	X	-
	Hymenoptera	Diapriidae	Diapriidae sp.7	X	X
		Formicidae	*Acromyrmex octopinosus*	X	-
			Camponotus sp.2	X	-
			Crematogaster brasiliensis	X	-
			Cyphomyrmex peltatus	X	-
			Dolichoderus bispinosus	X	-
			Gnamptogenys sp.1	-	X
			Paratrechina sp.1	-	X
			Pheidole sp.3	X	-
			Solenopsis invicta	X	-
			Strumigenys calamita	-	X
		Sphecidae	Sphecidae sp.1	-	X
	Isoptera	Termitidae	*Crepititermes* sp.1	X	-
			Nasutitermes sp.1	X	-
	Orthoptera	Phalangopsidae	*Eidmanacris* sp.1	X	-
			Paraclodes sp.1	X	X
			Phalangopsis sp.1	X	X
	Trichoptera	Philopotamidae	Philopotamidae sp.1	X	-
Malacostraca	Decapoda	Pseudothelphusidae	Pseudothelphusidae sp.1	X	X
	Isopoda	Philosciidae	Philosciidae sp.2	X	X
		Platyarthridae	Platyarthridae sp.2	X	-

Symphyla		Scutigerellidae	*Hanseniella* sp.1	X	X
Gastropoda	Pulmonata	Subulinidae	*Lamellaxis* sp.1	X	-
Amphibia	Anura	Leptodactylidae	*Leptodactylus* cf. *vastus*	X	-
Mammalia	Chiroptera	Phyllostomidae	*Carollia perspicillata*	X	X
			Glossophaga soricina	X	X
Reptilia	Squamata	Viperidae	*Bothrops* sp.	-	X
			Sauria sp.2	-	X

SB-0094					
TÁXONS				**Seca**	**Úmida**
Arachnida	Acari	Opilioacaridae	Opilioacaridae sp.1	-	X
		Trombiculidae	Trombiculidae sp.1	-	X
			Trombiculidae sp.2	X	-
			Acariformes sp.4	-	X
			Oribatida sp.2	-	X
	Amblypygi	Phrynidae	*Heterophrinus longicornis*	-	X
	Araneae	Araneidae	*Alpaida* sp.1	-	X
			Araneidae jovem	X	X
		Corinnidae	Corinnidae sp.3	-	X
		Oonopidae	gr. *Xycarpphy* sp.1	-	X
			Oonopidae jovem	X	X
			Oonopidae sp.3	-	X
			Oonopidae sp.12	-	X
		Pholcidae	*Mesabolivar aurantiacus*	X	-
			Ninetinae sp.1	X	X
		Salticidae	Salticidae sp.2	-	X
			Salticidae sp.3	-	X
			Salticidae sp.5	X	X
		Scytodidae	Scytodidae jovem	X	X
		Theridiidae	Theridiidae jovem	X	X
	Opiliones	Cosmetidae	Cosmetidae sp.1	X	-
			Roquettea carajas	X	-
		Sclerosomatidae	*Prionostema* sp.1	-	X
	Pseudoscorpiones	Chernetidae	*Spelaeochernes* sp.1	X	X
Chilopoda	Scolopendromorpha	Cryptopidae	*Cryptops* sp.2	-	X
Diplopoda	Polydesmida	Fuhrmanodesmidae	Fuhrmanodesmidae sp.1	-	X
	Spirostreptida	Pseudonannolenidae	Pseudonannolenidae sp.1	-	X
	Stemmiulida	Stemmiulidae	Stemmiulidae sp.1	-	X
Entognatha	Collembola	Entomobryidae	Entomobryidae sp.2	-	X
		Paronellidae	Paronellidae sp.1	X	X
	Diplura	Campodeidae	Campodeidae sp.1	-	X
Insecta	Blattodea	Blaberidae	Blaberidae jovem	X	X
		Polyphagidae	Polyphagidae sp.1	X	-
	Coleoptera	Elateridae	Elateridae jovem	-	X
		Tenebrionidae	Tenebrionidae jovem	-	X
			Coleoptera jovem	X	X
	Diptera	Cecidomyiidae	Cecidomyiidae sp.	-	X
		Ceratopogonidae	Ceratopogonidae jovem	-	X
		Drosophilidae	Drosophilidae sp.	X	X
		Psychodidae	Phlebotominae sp.	-	X
	Hemiptera	Cixiidae	Cixiidae jovem	-	X
		Cydnidae	Cydnidae sp.1	-	X
	Hymenoptera	Formicidae	*Apterostigma collare*	-	X
			Cephalotes atratus	-	X
			Dolichoderus bispinosus	-	X
			Gnamptogenys sp.1	X	X
			Hypoponera sp.6	-	X
			Pachycondyla constricta	X	X
			Pheidole sp.1	-	X
			Pheidole sp.7	-	X
			Pheidole sp.13	-	X
			Rogeria foreli	X	-
			Solenopsis invicta	X	X
			Wasmannia sp.1	X	-
	Isoptera	Termitidae	*Coatitermes* sp.2	-	X
			Nasutitermes sp.1	X	-
	Lepidoptera	Noctuoidea	Noctouidea sp. 1	-	X

		Tineoidea	Tineoidea sp. 5	X	-
			Tineoidea sp. 6	X	X
	Neuroptera	Myrmeleontidae	Myrmeleontidae sp.1	-	X
	Orthoptera	Phalangopsidae	*Paraclodes* sp.1	X	X
			Phalangopsis sp.1	X	X
	Psocoptera	Liposcelidae	Liposcelidae sp.1	-	X
		Troctopsocidae	Troctopsocidae sp.1	-	X
			Psocomorpha jovem	-	X
	Thysanura	Nicoletiidae	Nicoletiinae sp.1	X	X
Malacostraca	Isopoda	Armadillidae	Armadillidae sp.1	X	X
		Dubioniscidae	Dubioniscidae sp.1	-	X
		Philosciidae	Philosciidae sp.1	X	-
Mammalia	Chiroptera	Emballonuridae	*Peropteryx kappleri*	X	X

SB-0095					
TÁXONS				Seca	Úmida
Arachnida	Acari	Opilioacaridae	Opilioacaridae sp.1	-	X
		Trombiculidae	Trombiculidae sp.1	-	X
			Acariformes sp.1	-	X
			Acariformes sp.4	-	X
			Holothyrida sp.2	X	X
			Mesostigmata sp.1	-	X
			Mesostigmata sp.5	-	X
			Oribatida sp.2	-	X
	Amblypygi	Phrynidae	*Heterophrinus longicornis*	X	X
	Araneae	Corinnidae	*Abapeba* sp.1	X	X
			Corinnidae jovem	X	X
		Ctenidae	Ctenidae jovem	-	X
		Mimetidae	*Ero* sp.1	X	-
		Ochyroceratidae	*Ochyrocera* sp.1	X	-
			Speocera sp.1	-	X
		Oonopidae	Oonopidae jovem	-	X
			Oonopidae sp.12	X	-
		Pholcidae	Pholcidae jovem	X	-
		Salticidae	Salticidae jovem	X	X
		Scytodidae	*Scytodes* sp.1	X	X
		Theridiidae	*Achaearanea* sp.1	-	X
			Nesticodes rufipes	X	-
			Theridiidae sp.3	-	X
		Theridiosomatidae	*Plato* sp.1	-	X
	Opiliones	Cosmetidae	*Roquettea carajas*	-	X
		Escadabiidae	Escadabiidae sp.1	X	-
			Escadabiidae sp.2	X	X
	Pseudoscorpiones	Chernetidae	*Spelaeochernes* sp.1	X	X
		Chthoniidae	Chthoniidae sp.1	X	X
Chilopoda	Scutigeromorpha	Pselliodidae	*Sphendononema guildingii*	-	X
Diplopoda	Polydesmida	Chelodesmidae	Chelodesmidae sp.2	-	X
Entognatha	Collembola	Cyphoderidae	Cyphoderidae sp.1	-	X
		Paronellidae	Paronellidae sp.1	X	X
	Diplura	Campodeidae	Campodeidae sp.1	-	X
Insecta	Blattodea	Blaberidae	*Blaberus* sp.1	X	X
		Polyphagidae	Polyphagidae jovem	-	X
	Coleoptera	Carabidae	*Lelis* sp.1	X	-
		Elateridae	Elateridae jovem	-	X
		Staphylinidae	Scydmaeninae sp.2	X	-
	Diptera	Ceratopogonidae	Ceratopogonidae sp.	-	X
		Dolichopodidae	Dolichopodidae sp.	-	X
		Drosophilidae	Drosophilidae sp.	X	X
		Psychodidae	Phlebotominae sp.	-	X
			Psychodidae sp.	-	X
	Hemiptera	cf. Nabidae	cf. Nabidae jovem	-	X
		Cixiidae	Cixiidae jovem	-	X
		Cydnidae	Cydnidae sp.1	X	X
			Cydnidae sp.2	-	X
	Hymenoptera	Chalcicoidea	Chalcicoidea jovem	-	X
		Formicidae	*Apterostigma collare*	X	-
			Dolichoderus bispinosus	X	X
			Odontomachus meinerti	-	X
			Pachycondyla constricta	X	X
			Pheidole sp.4	-	X
			Solenopsis invicta	X	X
			Stegomyrmex sp.1	-	X
	Isoptera	Termitidae	*Nasutitermes* sp.1	-	X

	Lepidoptera	Gelechioidea	Gelechioidea sp.2	X	-
		Tineoidea	Tineoidea sp. 3	X	-
			Tineoidea sp. 6	-	X
			Tineoidea sp. 7	-	X
	Orthoptera	Phalangopsidae	*Paraclodes* sp.1	X	-
			Phalangopsis sp.1	X	X
	Psocoptera		Psocomorpha jovem	-	X
	Thysanura	Nicoletiidae	Nicoletiinae sp.1	X	X
Malacostraca	Isopoda	Armadillidae	Armadillidae sp.1	X	X
		Dubioniscidae	Dubioniscidae sp.2	-	X
		Philosciidae	Philosciidae sp.1	-	X
		Platyarthridae	*Trichorhina* sp.1	-	X
Symphyla		Scutigerellidae	*Hanseniella* sp.1	-	X
Gastropoda	Pulmonata	Subulinidae	*Lamellaxis* sp.1	-	X
		Systrophiidae	*Happia* sp.1	-	X
Amphibia	Anura	Bufonidae	*Rhinella* sp.	-	X
		Leptodactylidae	*Leptodactylus* cf. *vastus*	-	X
			Anura sp.2	X	-
Mammalia	Chiroptera	Furipteridae	*Furipterus horrens*	X	X
		Phyllostomidae	*Carollia perspicillata*	X	X
			Desmodus rotundus	X	X
			Diphylla ecaudata	-	X
			Glossophaga soricina	X	X
			Lionycteris spurrelli	-	X
			Lonchorhina aurita	X	X
	Rodentia	Cricetidae	*Rhipidomys* sp.	-	X

SB-0096					
TÁXONS				Seca	Úmida
Annelida	Rhynchobdellida		Rhynchobdellida sp.1	-	X
			Rhynchobdellida sp.2	-	X
Arachnida	Acari		Acari jovem	X	-
			Holothyrida sp.1	-	X
			Holothyrida sp.2	-	X
			Mesostigmata sp.1	-	X
	Amblypygi	Charinidae	*Charinus* sp.1	X	-
		Phrynidae	*Heterophrinus longicornis*	X	X
	Araneae	Corinnidae	Corinnidae jovem	X	X
		Ctenidae	Ctenidae jovem	-	X
		Oonopidae	Oonopidae jovem	X	X
			Oonopidae sp.5	X	-
		Scytodidae	*Scytodes* sp.2	X	-
		Theraphosidae	Theraphosidae jovem	X	-
			Theraphosidae sp.5	X	X
		Theridiosomatidae	*Plato* sp.1	X	X
			Theridiosomatidae jovem	-	X
	Opiliones	Cosmetidae	Cosmetidae jovem	X	-
			Cosmetidae sp.2	-	X
		Escadabiidae	Escadabiidae sp.1	X	-
			Escadabiidae sp.2	-	X
			Escadabiidae sp.6	-	X
		Stygnidae	Stygnidae sp.1	-	X
	Pseudoscorpiones	Chernetidae	*Spelaeochernes* sp.1	X	X
		Chthoniidae	Chthoniidae sp.1	X	-
Chilopoda	Geophilomorpha	Ballophilidae	*Ityphilus* sp.1	X	-
	Scolopendromorpha	Cryptopidae	*Cryptops* sp.1	X	-
	Scutigeromorpha	Pselliodidae	*Sphendononema* jovem	X	-
Diplopoda	Glomeridesmida	Glomeridesmidae	Glomeridesmida jovem	-	X
	Polydesmida	Chelodesmidae	Chelodesmidae sp.1	-	X
		Paradoxosomatidae	Paradoxosomatidae sp.2	X	X
Entognatha	Collembola	Cyphoderidae	Cyphoderidae sp.1	-	X
		Paronellidae	Paronellidae sp.1	X	X
	Diplura	Campodeidae	Campodeidae sp.1	X	X
Insecta	Blattodea	Blattidae	Blattidae jovem	X	-
	Coleoptera	Carabidae	*Notibia* sp.1	X	-
		Endomychidae	Endomychidae sp.1	X	-
	Diptera	Cecidomyiidae	Cecidomyiidae sp.	X	-
		Ceratopogonidae	Ceratopogonidae jovem	-	X
		Drosophilidae	Drosophilidae sp.	X	X
		Psychodidae	Phlebotominae sp.	X	X
	Hemiptera	Cydnidae	Cydnidae sp.1	X	X
	Hymenoptera	Formicidae	*Crematogaster brasiliensis*	-	X
			Eurhopalothrix sp.1	X	-
			Labidus coecus	-	X
			Octostruma iheringi	X	-
			Pachycondyla constricta	X	-
			Rogeria cf. *belti*	-	X
			Solenopsis invicta	X	X
	Isoptera	Termitidae	*Nasutitermes* sp.1	X	X
	Lepidoptera	Pyralidae	Pyralidae jovem	X	-
		Tineoidea	Tineoidea sp. 3	X	-
			Tineoidea sp. 6	X	X
	Orthoptera	Phalangopsidae	*Paraclodes* sp.1	X	-
			Phalangopsis sp.1	X	X
Malacostraca	Isopoda	Armadillidae	Armadillidae sp.1	X	X
		Dubioniscidae	Dubioniscidae sp.2	-	X

		Philosciidae	Philosciidae sp.1	X	X
		Platyarthridae	Platyarthridae sp.2	-	X
			Platyarthridae sp.3	-	X
		Scleropactidae	Scleropactidae sp.1	-	X
			Scleropactidae sp.3	-	X
Symphyla		Scutigerellidae	*Hanseniella* sp.1	X	X
Gastropoda	Pulmonata	Subulinidae	*Lamellaxis* sp.2	-	X
			Leptinaria sp.2	X	X
		Systrophiidae	Systrophiidae jovem	X	-
Amphibia	Anura	Bufonidae	*Rhinella* sp.	-	X
		Strabomantidae	*Pristimantis* cf. *fenestratus*	X	X
Mammalia	Chiroptera	Phyllostomidae	*Carollia perspicillata*	X	X
			Glossophaga soricina	X	X
			Micronycteris megalotis	-	X
			Phyllostomus latifolius	-	X

SB-0097					
TÁXONS				Seca	Úmida
Arachnida	Acari	Trombiculidae	Trombiculidae sp.1	-	X
			Astigmata sp.1	-	X
			Oribatida sp.3	-	X
	Amblypygi	Phrynidae	*Heterophrinus longicornis*	-	X
	Araneae	Corinnidae	Corinnidae jovem	X	-
		Filistatidae	Filistatidae sp.1	X	-
		Ochyroceratidae	Ochyroceratidae jovem	-	X
		Oonopidae	Oonopidae jovem	-	X
		Pholcidae	Pholcidae jovem	X	-
		Scytodidae	*Scytodes* sp.1	-	X
			Scytodidae jovem	X	-
		Theridiidae	Theridiidae jovem	-	X
		Theridiosomatidae	Theridiosomatidae jovem	X	-
	Opiliones	Stygnidae	*Protimesus* sp.1	X	-
	Pseudoscorpiones	Chernetidae	*Spelaeochernes* sp.1	X	X
		Chthoniidae	Chthoniidae sp.1	X	X
Diplopoda	Polydesmida	Chelodesmidae	Chelodesmidae sp.1	-	X
Entognatha	Collembola	Paronellidae	Paronellidae sp.1	-	X
Insecta	Blattodea	Blaberidae	Blaberidae jovem	-	X
	Diptera	Drosophilidae	Drosophilidae sp.	-	X
		Psychodidae	Phlebotominae sp.	-	X
	Hemiptera	Reduviidae	Reduviinae jovem	X	X
	Hymenoptera	Formicidae	*Pachycondyla constricta*	-	X
			Pheidole sp.4	-	X
			Solenopsis invicta	-	X
	Isoptera	Termitidae	*Nasutitermes* sp.1	X	-
	Orthoptera	Phalangopsidae	*Paraclodes* sp.1	X	-
			Phalangopsis sp.1	X	X
	Psocoptera	Troctopsocidae	Troctopsocidae sp.1	-	X
			Psocomorpha jovem	X	X
	Thysanura	Nicoletiidae	Nicoletiinae sp.1	X	-
Malacostraca	Isopoda	Armadillidae	Armadillidae sp.1	X	X
Mammalia	Chiroptera	Emballonuridae	*Peropteryx kappleri*	-	X

SB-0098					
TÁXONS				Seca	Úmida
Arachnida	Acari	Ixodidae	*Amblyomma* sp.4	-	X
		Opilioacaridae	Opilioacaridae sp.1	-	X
			Holothyrida sp.1	-	X
			Mesostigmata sp.1	X	-
	Amblypygi	Phrynidae	*Heterophrinus longicornis*	-	X
	Araneae	Araneidae	*Alpaida* sp.1	X	-
		Corinnidae	Corinnidae jovem	-	X
		Ctenidae	Ctenidae jovem	-	X
		Oonopidae	gr. *Xycarpphy* sp.1	X	-
			Oonopidae sp.3	-	X
			Oonopidae sp.5	X	-
			Oonopidae sp.6	X	-
		Pholcidae	*Mesabolivar aurantiacus*	X	-
		Salticidae	Salticidae sp.5	X	-
			Salticidae sp.9	X	-
		Scytodidae	Scytodidae jovem	X	X
		Theraphosidae	Theraphosidae jovem	-	X
		Theridiosomatidae	*Plato* sp.1	-	X
	Opiliones	Cosmetidae	*Roquettea carajas*	-	X
		Escadabiidae	Escadabiidae sp.2	-	X
			Escadabiidae sp.3	-	X
		Sclerosomatidae	Sclerosomatidae jovem	-	X
	Pseudoscorpiones	Chernetidae	*Spelaeochernes* sp.1	X	X
		Chthoniidae	Chthoniidae sp.1	X	-
Chilopoda	Geophilomorpha	Ballophilidae	*Ityphilus* sp.2	-	X
		Schendylidae	*Schendylops* sp.1	X	-
	Scolopendromorpha	Scolopocryptopidae	*Newportia* sp.2	X	-
	Scutigeromorpha	Pselliodidae	*Sphendononema* jovem	X	-
Diplopoda	Polydesmida	Chelodesmidae	Chelodesmidae sp.1	X	-
		Paradoxosomatidae	Paradoxosomatidae sp.1	-	X
			Paradoxosomatidae sp.3	-	X
			Polydesmida sp.1	-	X
	Polyxenida		Polyxenida jovem	-	X
Entognatha	Collembola	Isotomidae	Isotomidae sp.1	X	-
		Paronellidae	Paronellidae sp.1	X	X
			Paronellidae sp.3	X	-
	Diplura	Campodeidae	Campodeidae sp.1	X	X
Insecta	Blattodea	Polyphagidae	Polyphagidae jovem	X	-
	Coleoptera	Endomychidae	Endomychidae sp.1	X	-
		Staphylinidae	Staphylininae sp.2	X	-
	Diptera	Cecidomyiidae	Cecidomyiidae sp.	X	X
		Ceratopogonidae	Ceratopogonidae sp.	X	X
		Culicidae	Culicidae sp.	X	-
		Dolichopodidae	Dolichopodidae sp.	X	-
		Drosophilidae	Drosophilidae sp.	X	X
		Psychodidae	Phlebotominae sp.	X	X
		Sciaridae	Sciaridae sp.	X	-
		Tipulidae	Tipulidae sp.	X	-
	Hemiptera	Cercopidae	Cercopidae jovem	-	X
		Cixiidae	Cixiidae jovem	X	X
		Cydnidae	Cydnidae sp.1	X	X
		Reduviidae	Reduviinae jovem	X	-
	Hymenoptera	Formicidae	*Acromyrmex octopinosus*	X	-
			Atta sp.1	-	X
			Camponotus renggeri	X	-
			Camponotus sp.2	X	-
			Pachycondyla constricta	X	X

			Paratrechina sp.1	X	-
			Pheidole sp.1	-	X
			Pheidole sp.3	-	X
			Rogeria cf. *belti*	X	-
			Solenopsis invicta	-	X
	Isoptera	Termitidae	*Nasutitermes* sp.1	X	X
	Lepidoptera		Lepidoptera jovem	X	-
	Orthoptera	Phalangopsidae	*Eidmanacris* sp.1	X	-
			Paraclodes sp.1	X	-
			Phalangopsis sp.1	X	X
	Psocoptera	Troctopsocidae	Troctopsocidae sp.1	-	X
			Psocomorpha jovem	X	X
Malacostraca	Isopoda	Dubioniscidae	Dubioniscidae sp.1	X	X
		Philosciidae	Philosciidae sp.1	X	-
			Philosciidae sp.2	X	X
		Platyarthridae	Platyarthridae sp.3	-	X
Symphyla		Scutigerellidae	*Hanseniella* sp.1	X	-
Gastropoda	Pulmonata	Subulinidae	*Lamellaxis* sp.1	X	-
		Systrophiidae	*Entodina* sp.1	X	-
			Happia sp.1	X	-
Amphibia	Anura	Strabomantidae	*Pristimantis* cf. *fenestratus*	X	X
			Anura sp.1	-	X
Mammalia	Chiroptera	Phyllostomidae	*Carollia perspicillata*	-	X
			Glossophaga soricina	-	X

SB-0099					
TÁXONS				Seca	Úmida
Arachnida	Acari	Opilioacaridae	Opilioacaridae sp.1	-	X
			Holothyrida sp.1	-	X
			Mesostigmata sp.1	-	X
	Amblypygi	Phrynidae	*Heterophrinus longicornis*	-	X
	Araneae	Anyphaenidae	Anyphaenidae jovem	X	-
		Corinnidae	Corinnidae jovem	-	X
		Ctenidae	Ctenidae jovem	-	X
		Gnaphosidae	Gnaphosidae jovem	X	-
		Ochyroceratidae	Ochyroceratidae sp.2	X	X
		Oonopidae	Oonopidae jovem	-	X
			Oonopidae sp.1	-	X
		Pholcidae	*Mesabolivar aurantiacus*	X	-
		Salticidae	Salticidae jovem	X	-
			Salticidae sp.3	-	X
		Scytodidae	*Scytodes* sp.1	X	X
		Theridiidae	*Achaearanea* sp.1	-	X
			Theridiidae sp.3	X	-
		Theridiosomatidae	*Plato* sp.1	X	-
	Opiliones	Cosmetidae	Cosmetidae jovem	-	X
			Roquettea carajas	X	-
		Escadabiidae	Escadabiidae sp.2	X	-
			Escadabiidae sp.5	X	-
	Pseudoscorpiones	Chernetidae	*Spelaeochernes* sp.1	X	X
		Chthoniidae	Chthoniidae sp.1	X	-
Diplopoda	Polydesmida	Cyrtodesmidae	Cyrtodesmidae sp.1	X	-
		Pyrgodesmidae	Pyrgodesmidae sp.2	-	X
	Spirostreptida	Pseudonannolenidae	Pseudonannolenidae jovem	X	-
Entognatha	Collembola	Entomobryidae	Entomobryidae sp.5	-	X
		Paronellidae	Paronellidae sp.1	X	X
			Paronellidae sp.4	-	X
	Diplura	Campodeidae	Campodeidae sp.1	X	X
Insecta	Blattodea		Blattodea jovem	X	-
	Coleoptera	Staphylinidae	Staphylininae sp.2	-	X
	Diptera	Cecidomyiidae	Cecidomyiidae sp.	X	-
		Ceratopogonidae	Ceratopogonidae jovem	-	X
		Dolichopodidae	Dolichopodidae sp.	-	X
		Phoridae	Phoridae sp.	-	X
		Psychodidae	Phlebotominae sp.	X	X
		Tipulidae	Tipulidae sp.	X	-
	Hemiptera	Cixiidae	Cixiidae jovem	X	-
		Cydnidae	Cydnidae sp.1	-	X
		Reduviidae	Emesinae jovem	-	X
		Schizopteridae	Schizopteridae sp.3	X	X
	Hymenoptera	Formicidae	*Pachycondyla constricta*	X	-
			Pheidole sp.3	-	X
			Solenopsis invicta	-	X
			Solenopsis sp.7	-	X
	Isoptera	Termitidae	*Nasutitermes* sp.1	X	X
	Lepidoptera	Noctuoidea	Noctouidea sp. 2	X	-
	Orthoptera	Phalangopsidae	*Eidmanacris* sp.1	X	-
			Paraclodes sp.1	X	-
			Phalangopsis sp.1	X	X
	Psocoptera		Psocomorpha jovem	X	X
Malacostraca	Isopoda	Armadillidae	Armadillidae sp.1	-	X
		Dubioniscidae	Dubioniscidae sp.1	X	-
		Philosciidae	Philosciidae sp.2	-	X
Symphyla		Scutigerellidae	*Hanseniella* sp.1	-	X

Amphibia	Anura	Leptodactylidae	*Leptodactylus* cf. *vastus*	X	-
		Strabomantidae	*Pristimantis* cf. *fenestratus*	X	-
			Anura sp.6	X	-
Mammalia	Chiroptera	Furipteridae	*Furipterus horrens*	X	-
		Phyllostomidae	*Carollia perspicillata*	-	X
			Glossophaga soricina	-	X
	Rodentia	Cricetidae	*Rhipidomys* sp.	X	-

SB-0100					
TÁXONS				Seca	Úmida
Arachnida	Acari	Ixodidae	*Amblyomma* sp.1	X	-
			Oribatida sp.2	X	-
	Amblypygi	Phrynidae	*Heterophrinus longicornis*	X	-
	Araneae	Araneidae	Araneidae jovem	X	-
		Corinnidae	Corinnidae jovem	-	X
		Pholcidae	Ninetinae sp.1	X	-
			Pholcidae jovem	-	X
		Salticidae	Salticidae sp.4	X	-
		Scytodidae	*Scytodes* sp.1	X	X
		Theridiidae	*Achaearanea* sp.1	-	X
	Opiliones	Cosmetidae	Cosmetidae sp.1	X	-
			Roquettea carajas	X	-
	Pseudoscorpiones	Chernetidae	Chernetidae jovem	X	-
Chilopoda	Geophilomorpha	Micronicophilidae	*Micronicophilus* sp.1	-	X
Diplopoda	Polydesmida	Paradoxosomatidae	Paradoxosomatidae sp.2	-	X
			Paradoxosomatidae sp.3	-	X
Entognatha	Collembola	Cyphoderidae	Cyphoderidae sp.1	-	X
		Paronellidae	Paronellidae sp.1	-	X
Insecta	Coleoptera	Endomychidae	Endomychidae sp.1	X	-
	Diptera	Culicidae	Culicidae sp.	-	X
		Drosophilidae	Drosophilidae sp.	X	-
		Psychodidae	Phlebotominae sp.	-	X
	Hemiptera	Cercopidae	Cercopidae jovem	-	X
		Cicadellidae	Cicadellidae sp.1	X	-
		Reduviidae	Reduviinae jovem	X	X
		Schizopteridae	Schizopteridae sp.2	X	-
	Hymenoptera	Formicidae	*Camponotus atriceps*	-	X
			Ectatomma brunneum	X	X
			Pachycondyla constricta	X	-
			Pheidole sp.4	X	-
			Rogeria tonduzi	X	-
	Isoptera	Termitidae	*Nasutitermes* sp.1	-	X
			Nasutitermes sp.3	X	-
	Neuroptera	Myrmeleontidae	Myrmeleontidae sp.1	X	X
	Orthoptera	Phalangopsidae	*Paraclodes* sp.1	X	-
			Phalangopsis sp.1	-	X
	Psocoptera	Troctopsocidae	Troctopsocidae sp.1	X	-
Malacostraca	Isopoda	Philosciidae	Philosciidae sp.1	-	X
Symphyla		Scutigerellidae	*Hanseniella* sp.1	-	X
Amphibia	Anura	Strabomantidae	*Pristimantis* cf. *fenestratus*	X	-
			Anura sp.1	-	X
Reptilia	Squamata	Gekkonidae	*Thecadactylus rapicauda*	-	X
	Testudinata	Testudinidae	Chelonoides sp.	X	-

SB-0101					
**TÁXONS**				**Seca**	**Úmida**
Annelida	Arhynchobdellida		Arhynchobdellida sp.	X	-
	Haplotaxida		Haplotaxida sp.4	X	-
			Haplotaxida sp.5	X	-
Arachnida	Amblypygi	Phrynidae	*Heterophrinus longicornis*	X	-
	Araneae	Araneidae	Araneidae jovem	X	-
		Corinnidae	Corinnidae jovem	X	X
		Ctenidae	Ctenidae jovem	-	X
		Ochyroceratidae	*Ochyrocera* sp.2	-	X
		Pholcidae	*Mesabolivar aurantiacus*	X	-
	Opiliones	Cosmetidae	*Roquettea carajas*	-	X
		Escadabiidae	Escadabiidae sp.4	-	X
		Sclerosomatidae	*Prionostema* sp.1	X	-
Chilopoda	Geophilomorpha	Schendylidae	Schendylidae jovem	X	-
	Scolopendromorpha	Scolopocryptopidae	*Dinocryptops miersii*	X	-
			Tidops sp.1	-	X
Entognatha	Collembola	Cyphoderidae	Cyphoderidae sp.1	X	X
		Paronellidae	Paronellidae sp.1	-	X
Insecta	Coleoptera	Carabidae	*Notibia* sp.1	X	-
		Staphylinidae	Staphylinidae sp.7	X	-
	Diptera	Drosophilidae	Drosophilidae sp.	X	-
		Tipulidae	Tipulidae sp.	X	-
	Hemiptera	cf. Nabidae	cf. Nabidae jovem	X	-
		Cydnidae	Cydnidae sp.1	X	-
		Veliidae	*Rhagovelia* sp.2	X	-
	Hymenoptera	Formicidae	*Pachycondyla constricta*	X	-
			Paratrechina sp.1	-	X
	Isoptera	Termitidae	*Nasutitermes* sp.1	X	-
	Lepidoptera	Hesperiidae	Hesperiidae sp. 1	X	-
		Noctuoidea	Noctouidea sp. 6	X	-
		Tineoidea	Tineoidea sp. 3	X	-
	Orthoptera	Phalangopsidae	*Paraclodes* sp.1	X	-
			Phalangopsis sp.1	X	-
	Trichoptera	Philopotamidae	Philopotamidae sp.1	X	-
	Thysanura	Nicoletiidae	Nicoletiinae sp.1	X	X
Malacostraca	Decapoda	Pseudothelphusidae	Pseudothelphusidae sp.1	-	X
	Isopoda	Philosciidae	Philosciidae sp.2	X	-
Amphibia	Anura	Strabomantidae	*Pristimantis* cf. *fenestratus*	X	-
Reptilia	Squamata	Gekkonidae	*Thecadactylus rapicauda*	X	-
Mammalia	Chiroptera	Phyllostomidae	*Carollia perspicillata*	X	-

SB-0102					
TÁXONS				**Seca**	**Úmida**
Annelida	Haplotaxida		Haplotaxida sp.2	-	X
Arachnida	Acari	Ixodidae	*Amblyomma* sp.1	-	X
		Opilioacaridae	Opilioacaridae sp.1	-	X
			Oribatida sp.2	-	X
	Araneae	Araneidae	*Alpaida* sp.1	X	-
			Araneidae jovem	-	X
		Drymusidae	*Drymusa* sp.1	-	X
			Drymusidae jovem	X	X
		Ochyroceratidae	Ochyroceratidae jovem	X	X
		Oonopidae	Oonopidae jovem	X	-
		Scytodidae	*Scytodes* sp.1	-	X
		Theraphosidae	Theraphosidae jovem	-	X
		Theridiidae	*Achaearanea* sp.1	-	X
		Theridiosomatidae	Theridiosomatidae jovem	X	X
	Opiliones	Escadabiidae	Escadabiidae sp.2	-	X
	Pseudoscorpiones	Chernetidae	*Spelaeochernes* sp.1	-	X
	Ricinulei	Ricinoididae	Ricinoididae jovem	-	X
Chilopoda	Lithobiomorpha	Henicopidae	*Lamyctes* p.2	-	X
	Scutigeromorpha	Pselliodidae	*Sphendononema* jovem	-	X
Entognatha	Collembola	Cyphoderidae	Cyphoderidae sp.1	-	X
		Paronellidae	Paronellidae sp.1	X	X
	Diplura	Campodeidae	Campodeidae sp.1	-	X
Insecta	Blattodea	Blaberidae	Blaberidae jovem	X	-
		Blattidae	Blattidae jovem	X	-
			Blattodea jovem	-	X
	Coleoptera	Carabidae	Carabidae sp.1	X	-
			Coleoptera jovem	X	X
	Diptera	Drosophilidae	Drosophilidae sp.	X	-
		Streblidae	Streblidae sp.	-	X
	Hemiptera	Cixiidae	Cixiidae jovem	-	X
		Cydnidae	Cydnidae jovem	X	X
			Cydnidae sp.1	-	X
		Reduviidae	Emesinae jovem	-	X
	Hymenoptera	Formicidae	*Acromyrmex octopinosus*	X	-
			Carebara urichii	-	X
			Crematogaster brasiliensis	X	X
			Paratrechina sp.1	-	X
			Pheidole sp.3	X	X
	Orthoptera	Phalangopsidae	*Paraclodes* sp.1	X	-
			Phalangopsis sp.1	X	X
	Thysanura	Nicoletiidae	Nicoletiinae sp.1	X	-
Malacostraca	Isopoda	Armadillidae	Armadillidae sp.1	X	X
		Philosciidae	Philosciidae sp.1	X	-
			Philosciidae sp.2	-	X
		Platyarthridae	Platyarthridae sp.3	-	X
Gastropoda	Pulmonata	Systrophiidae	*Happia* sp.1	-	X
Amphibia	Anura	Strabomantidae	*Pristimantis* cf. *fenestratus*	X	-
Mammalia	Chiroptera	Emballonuridae	*Peropteryx kappleri*	X	X
		Phyllostomidae	*Carollia perspicillata*	X	X
			Glossophaga soricina	X	-

SB-0103					
TÁXONS				Seca	Úmida
Arachnida	Acari		Mesostigmata sp.1	-	X
	Amblypygi	Phrynidae	*Heterophrinus longicornis*	-	X
	Araneae	Corinnidae	Corinnidae jovem	X	-
		Ctenidae	Ctenidae jovem	X	-
		Filistatidae	Filistatidae jovem	X	-
		Oonopidae	Oonopidae sp.3	X	-
			Oonopidae sp.12	-	X
		Pholcidae	Pholcidae jovem	-	X
		Salticidae	Salticidae sp.4	X	-
		Scytodidae	*Scytodes* sp.1	-	X
			Scytodidae jovem	X	-
		Theridiidae	Theridiidae jovem	X	-
		Theridiosomatidae	*Plato* sp.1	-	X
	Opiliones	Sclerosomatidae	*Prionostema* sp.1	-	X
			Sclerosomatidae jovem	-	X
	Pseudoscorpiones	Chernetidae	*Spelaeochernes* sp.1	X	X
		Chthoniidae	Chthoniidae sp.1	X	-
Entognatha	Collembola	Paronellidae	Paronellidae sp.1	X	-
Insecta	Blattodea	Blaberidae	Blaberidae jovem	-	X
		Blattidae	Blattidae jovem	X	-
	Coleoptera	Curculionidae	Curculionidae sp.1	-	X
	Diptera	Cecidomyiidae	Cecidomyiidae sp.	-	X
		Ceratopogonidae	Ceratopogonidae sp.	-	X
		Ceratopogonidae	Ceratopogonidae jovem	-	X
		Drosophilidae	Drosophilidae sp.	-	X
		Muscidae	Muscidae jovem	-	X
		Phoridae	Phoridae sp.	-	X
			Diptera jovem	X	-
	Hemiptera	Cydnidae	Cydnidae jovem	-	X
		Reduviidae	Reduviinae jovem	X	-
	Hymenoptera	Formicidae	*Camponotus renggeri*	X	-
			Crematogaster brasiliensis	-	X
			Gnamptogenys sp.1	-	X
			Pachycondyla constricta	-	X
		Mymaridae	Mymaridae sp.1	X	-
	Orthoptera	Phalangopsidae	*Paraclodes* sp.1	X	-
			Phalangopsis sp.1	X	X
	Psocoptera		Psocomorpha jovem	-	X
Malacostraca	Isopoda	Philosciidae	Philosciidae sp.1	-	X
Symphyla		Scutigerellidae	Scutigerellidae jovem	X	-
Mammalia	Chiroptera	Emballonuridae	*Peropteryx kappleri*	X	-

SB-0104					
TÁXONS				**Seca**	**Úmida**
Arachnida	Amblypygi	Phrynidae	*Heterophrinus longicornis*	-	X
	Araneae	Corinnidae	*Tupirina* sp.1	X	-
		Pholcidae	*Mesabolivar cambridgei*	-	X
			Mesabolivar eberhardi	X	X
		Scytodidae	Scytodidae jovem	-	X
		Theridiosomatidae	*Plato* sp.1	-	X
	Opiliones	Sclerosomatidae	*Prionostema* sp.1	X	X
	Pseudoscorpiones	Chernetidae	*Spelaeochernes* sp.1	-	X
Entognatha	Collembola	Cyphoderidae	Cyphoderidae sp.1	X	X
		Paronellidae	Paronellidae sp.1	X	-
Insecta	Coleoptera	Staphylinidae	Staphylinidae sp.3	X	-
			Staphylininae sp.1	X	X
	Diptera	Cecidomyiidae	Cecidomyiidae sp.	X	-
		Ceratopogonidae	Ceratopogonidae sp.	X	-
		Conopidae	Conopidae sp.	X	-
		Phoridae	Phoridae sp.	X	-
		Psychodidae	Psychodidae sp.	X	-
		Tipulidae	Tipulidae sp.	-	X
	Hemiptera	Cydnidae	Cydnidae jovem	X	-
		Reduviidae	Reduviinae jovem	X	-
		Veliidae	*Rhagovelia* sp.4	X	-
	Hymenoptera	Formicidae	*Acropyga* cf. *smithii*	-	X
			Hypoponera sp.6	-	X
			Paratrechina sp.1	X	X
	Isoptera	Termitidae	*Nasutitermes* sp.1	X	-
			Nasutitermes sp.2	X	-
	Lepidoptera	Noctuoidea	Noctouidea sp. 10	-	X
			Noctouidea sp. 6	X	-
	Orthoptera	Phalangopsidae	*Paraclodes* sp.1	X	-
			Phalangopsis sp.1	X	X
	Trichoptera	Philopotamidae	Philopotamidae sp.1	X	-
	Thysanura	Nicoletiidae	Nicoletiinae sp.1	X	X

SB-0106					
TÁXONS				Seca	Úmida
Arachnida	Acari	Argasidae	*Ornithodoros* sp.1	-	X
			Acariformes sp.4	-	X
			Mesostigmata sp.1	-	X
			Oribatida sp.5	-	X
	Amblypygi	Phrynidae	*Heterophrinus longicornis*	-	X
	Araneae	Araneidae	Araneidae jovem	-	X
		Ctenidae	Ctenidae jovem	-	X
		Filistatidae	Filistatidae sp.1	-	X
		Ochyroceratidae	*Speocera* sp.1	-	X
		Pholcidae	*Mesabolivar eberhardi*	-	X
		Salticidae	Salticidae sp.16	-	X
		Theraphosidae	Theraphosidae jovem	X	-
		Theridiosomatidae	*Plato* sp.1	-	X
	Opiliones	Escadabiidae	Escadabiidae sp.3	-	X
		Stygnidae	Stygnidae jovem	-	X
Diplopoda	Spirostreptida	Pseudonannolenidae	Pseudonannolenidae jovem	-	X
Entognatha	Collembola	Cyphoderidae	Cyphoderidae sp.1	-	X
		Entomobryidae	Entomobryidae sp.2	-	X
		Paronellidae	Paronellidae sp.1	-	X
Insecta	Coleoptera	Staphylinidae	Staphylininae sp.1	-	X
			Staphylininae sp.4	-	X
	Diptera	Culicidae	Culicidae sp.	-	X
		Dolichopodidae	Dolichopodidae sp.	-	X
		Drosophilidae	Drosophilidae sp.	-	X
		Psychodidae	Phlebotominae sp.	-	X
		Tipulidae	Tipulidae sp.	-	X
	Hemiptera	Cixiidae	Cixiidae jovem	-	X
		Cydnidae	Cydnidae jovem	-	X
		Hebridae	Hebridae jovem	-	X
		Reduviidae	Emesinae jovem	-	X
	Hymenoptera	Chrysidoidea	Chrysidoidea sp.1	-	X
		Formicidae	*Carebara* sp.11	-	X
			Crematogaster brasiliensis	-	X
			Dolichoderus bispinosus	-	X
			Hypoponera opacior	-	X
			Hypoponera sp.1	-	X
			Hypoponera sp.4	-	X
			Hypoponera sp.5	-	X
			Pachycondyla constricta	-	X
			Solenopsis invicta	-	X
			Strumigenys calamita	-	X
	Isoptera	Termitidae	*Coatitermes* sp.1	-	X
			Nasutitermes sp.1	-	X
	Orthoptera	Phalangopsidae	*Paraclodes* sp.1	X	-
			Phalangopsis sp.1	X	-
	Psocoptera	Troctopsocidae	Troctopsocidae sp.1	-	X
	Thysanura	Nicoletiidae	Nicoletiinae jovem	-	X
Malacostraca	Isopoda	Philosciidae	Philosciidae sp.2	-	X
		Platyarthridae	Platyarthridae sp.2	-	X
			Platyarthridae sp.3	-	X
Symphyla		Scutigerellidae	*Hanseniella* sp.1	-	X
Amphibia	Anura	Bufonidae	*Rhinella* sp.	X	-
		Strabomantidae	*Pristimantis* cf. *fenestratus*	X	-
Mammalia	Chiroptera	Emballonuridae	*Peropteryx kappleri*	X	X
		Phyllostomidae	*Carollia perspicillata*	X	X
			Trachops cirrhosus	-	X
Reptilia	Squamata	Gymnophthalmidae	*Neusticurus* sp.	X	-

SB-0107					
TÁXONS				Seca	Úmida
Arachnida	Acari	Ixodidae	*Amblyomma* sp.3	-	X
		Trombiculidae	Trombiculidae sp.1	-	X
			Trombiculidae sp.2	X	-
	Amblypygi	Phrynidae	*Heterophrinus longicornis*	-	X
	Araneae	Araneidae	Araneidae jovem	X	-
			Micrathena sp.1	-	X
		Ctenidae	Ctenidae jovem	X	X
		Ochyroceratidae	Ochyroceratidae jovem	X	-
		Oonopidae	gr. *Xycarpphy* sp.1	X	-
			Oonopidae sp.7	X	-
		Pholcidae	*Mesabolivar eberhardi*	-	X
			Ninetinae sp.1	X	-
		Salticidae	Salticidae jovem	X	-
		Scytodidae	Scytodidae jovem	X	X
		Theraphosidae	Theraphosidae jovem	X	-
		Theridiosomatidae	*Plato* sp.1	X	X
	Opiliones	Cosmetidae	Cosmetidae sp.2	-	X
		Escadabiidae	Escadabiidae sp.2	X	-
	Pseudoscorpiones	Chernetidae	*Spelaeochernes* sp.1	X	X
		Ideoroncidae	Ideoroncidae sp.1	-	X
Chilopoda	Scolopendromorpha	Cryptopidae	*Cryptops* sp.1	X	-
Diplopoda	Polydesmida	Pyrgodesmidae	Pyrgodesmidae sp.1	-	X
	Spirostreptida		Spirostreptida jovem	-	X
Entognatha	Collembola	Paronellidae	Paronellidae sp.1	X	-
	Diplura	Campodeidae	Campodeidae sp.1	X	-
Insecta	Blattodea	Blaberidae	Blaberidae jovem	-	X
		Blattidae	Blattidae jovem	-	X
			Blattodea jovem	X	-
	Coleoptera	Staphylinidae	*Coproporus* sp.1	X	-
	Diptera	Cecidomyiidae	Cecidomyiidae sp.	X	-
		Psychodidae	Phlebotominae sp.	-	X
	Hemiptera	Cydnidae	Cydnidae jovem	-	X
	Hymenoptera	Formicidae	*Cyphomyrmex peltatus*	-	X
			Pachycondyla constricta	X	-
			Rogeria tonduzi	X	-
	Lepidoptera	Noctuoidea	Noctouidea sp. 1	-	X
			Noctouidea sp. 3	-	X
	Orthoptera	Phalangopsidae	*Eidmanacris* sp.1	X	-
			Paraclodes sp.1	X	X
			Phalangopsis sp.1	X	X
	Psocoptera	Troctopsocidae	Troctopsocidae sp.1	X	-
			Psocomorpha jovem	X	-
			Troctomorpha jovem	-	X
Malacostraca	Isopoda	Armadillidae	Armadillidae sp.1	X	X
		Philosciidae	Philosciidae sp.2	-	X
Amphibia	Anura	Bufonidae	*Rhinella* sp.	X	-
		Strabomantidae	*Pristimantis* cf. *fenestratus*	X	-
Mammalia	Chiroptera	Emballonuridae	*Peropteryx kappleri*	X	X
		Phyllostomidae	*Carollia perspicillata*	X	X
			Glossophaga soricina	X	X

SB-0115					
TÁXONS				**Seca**	**Úmida**
Arachnida	Araneae	Ctenidae	Ctenidae jovem	X	-
		Pholcidae	*Mesabolivar aurantiacus*	X	-
		Pisauridae	Pisauridae jovem	X	-
		Salticidae	Salticidae sp.10	X	-
		Scytodidae	*Scytodes* sp.1	X	-
		Theridiidae	Theridiidae sp.3	X	-
		Theridiosomatidae	*Plato* sp.1	X	X
	Opiliones	Cosmetidae	Cosmetidae sp.1	X	-
		Sclerosomatidae	*Prionostema* sp.1	X	-
			Sclerosomatidae jovem	X	X
Chilopoda	Lithobiomorpha	Henicopidae	*Lamyctes* p.1	-	X
Diplopoda	Polydesmida	Paradoxosomatidae	Paradoxosomatidae jovem	-	X
Entognatha	Collembola	Paronellidae	Paronellidae sp.1	X	X
		Sminthuroidea	Sminthuroidea sp.2	X	-
	Diplura	Campodeidae	Campodeidae sp.1	-	X
Insecta	Blattodea	Blattellidae	Blattellidae jovem	X	-
	Coleoptera	Leiodidae	Leiodidae sp.1	X	-
	Diptera	Cecidomyiidae	Cecidomyiidae sp.	X	-
		Culicidae	Culicidae sp.	X	-
		Drosophilidae	Drosophilidae sp.	X	-
		Psychodidae	Phlebotominae sp.	X	X
		Tipulidae	Tipulidae sp.	X	-
	Hemiptera	Cixiidae	Cixiidae jovem	X	-
		Cydnidae	Cydnidae jovem	X	-
		Reduviidae	Emesinae sp.2	X	-
	Hymenoptera	Braconidae	Braconidae sp.1	X	-
		Diapriidae	Diapriidae sp.6	X	-
		Formicidae	*Eurhopalothrix* sp.1	X	-
			Gnamptogenys sp.2	X	-
			Pachycondyla constricta	-	X
			Paratrechina sp.1	X	X
			Pheidole sp.7	X	X
			Prionopelta modesta	X	-
	Isoptera	Termitidae	*Nasutitermes* sp.3	X	-
	Lepidoptera	Noctuoidea	Noctouidea sp. 1	X	-
	Orthoptera	Phalangopsidae	*Paraclodes* sp.1	X	-
	Psocoptera		Troctomorpha jovem	X	-
Malacostraca	Isopoda	Philosciidae	Philosciidae sp.2	-	X
Symphyla		Scolopendrellidae	*Symphylella* sp.1	-	X
		Scutigerellidae	*Hanseniella* sp.1	-	X
Mammalia	Chiroptera	Emballonuridae	*Peropteryx kappleri*	-	X
		Phyllostomidae	*Carollia perspicillata*	-	X
			Glossophaga soricina	-	X

SB-0116					
**TÁXONS**				**Seca**	**Úmida**
Annelida	Haplotaxida		Tubificina sp.1	X	-
Arachnida	Amblypygi	Phrynidae	*Heterophrinus longicornis*	-	X
	Araneae	Araneidae	Araneidae jovem	X	-
		Linyphiidae	Linyphiidae jovem	-	X
		Oonopidae	gr. *Xycarpphy* sp.1	-	X
		Pholcidae	Pholcidae jovem	X	X
		Salticidae	Salticidae jovem	-	X
		Theridiosomatidae	Theridiosomatidae jovem	-	X
	Opiliones	Sclerosomatidae	Sclerosomatidae jovem	X	X
	Pseudoscorpiones	Chernetidae	Chernetidae jovem	-	X
Chilopoda	Geophilomorpha		Geophilomorpha jovem	-	X
	Scolopendromorpha	Scolopendridae	*Otostigmus* sp.1	-	X
Diplopoda	Polydesmida	Chelodesmidae	Chelodesmidae sp.1	X	-
		Paradoxosomatidae	Paradoxosomatidae sp.4	-	X
		Pyrgodesmidae	Pyrgodesmidae jovem	X	-
Entognatha	Collembola	Entomobryidae	Entomobryidae sp.5	X	-
		Paronellidae	Paronellidae sp.1	-	X
		Sminthuroidea	Sminthuroidea sp.2	-	X
Insecta	Blattodea		Blattodea jovem	-	X
	Coleoptera	Hydrophilidae	Hydrophilidae sp.4	X	-
		Staphylinidae	Pselaphinae sp.2	-	X
			Pselaphinae sp.3	X	-
	Diptera	Cecidomyiidae	Cecidomyiidae sp.	-	X
		Ceratopogonidae	Ceratopogonidae jovem	-	X
		Psychodidae	Phlebotominae sp.	-	X
	Hymenoptera	Braconidae	Braconidae sp.1	X	-
		Formicidae	*Acanthognathus* sp.1	-	X
			Acropyga cf. *smithii*	X	X
			Hypoponera sp.3	-	X
			Paratrechina sp.1	X	-
			Pheidole sp.1	-	X
			Pheidole sp.5	-	X
			Rogeria cf. *belti*	X	-
			Trachymyrmex sp.2	-	X
	Isoptera	Termitidae	*Nasutitermes* sp.1	X	-
	Lepidoptera	Noctuoidea	Noctouidea sp. 2	-	X
	Orthoptera	Phalangopsidae	*Paraclodes* sp.1	X	-
			Phalangopsis sp.1	-	X
Malacostraca	Decapoda	Palaemonidae	*Macrobrachium* sp.1	-	X
	Isopoda	Armadillidae	Armadillidae sp.1	-	X
Amphibia	Anura	Bufonidae	*Rhinella* sp.	X	-
Aves	Passeriforme	Turdidae	*Turdus* sp.	X	-

SB-0117					
TÁXONS				**Seca**	**Úmida**
Arachnida	Acari	Argasidae	*Ornithodoros* sp.1	X	-
		Ixodidae	*Amblyomma* sp.1	X	-
		Trombiculidae	Trombiculidae sp.1	-	X
			Mesostigmata sp.1	-	X
	Amblypygi	Phrynidae	*Heterophrinus longicornis*	X	X
	Araneae	Araneidae	*Alpaida* sp.1	X	-
		Corinnidae	Corinnidae jovem	-	X
		Ctenidae	Ctenidae jovem	-	X
		Ochyroceratidae	Ochyroceratidae jovem	X	X
		Pholcidae	*Mesabolivar eberhardi*	-	X
			Pholcidae jovem	X	-
		Salticidae	Salticidae jovem	X	X
			Salticidae sp.3	-	X
		Theridiosomatidae	*Plato* sp.1	X	X
		Uloboridae	Uloboridae sp.1	-	X
	Opiliones	Cosmetidae	Cosmetidae jovem	X	-
			Roquettea carajas	-	X
		Escadabiidae	Escadabiidae jovem	X	-
		Sclerosomatidae	*Prionostema* sp.1	-	X
	Pseudoscorpiones	Bochicidae	Bochicidae sp.1	-	X
		Chernetidae	*Spelaeochernes* sp.1	X	-
	Scorpiones	Buthidae	*Ananteris* jovem	X	-
Diplopoda	Spirostreptida	Pseudonannolenidae	Pseudonannolenidae sp.1	-	X
			Pseudonannolenidae sp.2	X	X
Entognatha	Collembola	Isotomidae	Isotomidae sp.1	-	X
		Paronellidae	Paronellidae sp.1	X	X
Insecta	Coleoptera	Leiodidae	Leiodidae sp.2	X	-
		Staphylinidae	Staphylinidae sp.5	X	-
			Staphylininae sp.3	-	X
	Diptera	Drosophilidae	Drosophilidae sp.	X	-
		Phoridae	Phoridae sp.	X	-
		Tipulidae	Tipulidae sp.	X	-
	Hemiptera	Cixiidae	Cixiidae jovem	-	X
		Cydnidae	Cydnidae jovem	-	X
		Fulgoridae	Fulgoridae sp.2	-	X
		Reduviidae	Emesinae jovem	X	-
			Reduviinae jovem	X	X
	Hymenoptera	Formicidae	*Apterostigma collare*	-	X
			Cephalotes cristatus	X	-
			Neivamyrmex sp.1	X	-
			Pachycondyla constricta	X	-
			Pheidole sp.1	X	-
			Pheidole sp.6	-	X
			Pheidole sp.7	X	X
			Prionopelta modesta	X	-
		Vespidae	Vespidae sp.1	-	X
	Isoptera	Termitidae	*Nasutitermes* sp.1	X	X
			Nasutitermes sp.2	X	-
	Lepidoptera	Hesperiidae	Hesperiidae sp. 1	X	X
		Noctuoidea	Noctouidea sp. 1	X	-
	Neuroptera	Mantispidae	*Plega* sp.1	X	-
	Orthoptera	Phalangopsidae	*Paraclodes* sp.1	X	-
			Phalangopsis sp.1	X	X
	Psocoptera		Psocomorpha jovem	X	-
Malacostraca	Isopoda	Philosciidae	Philosciidae sp.2	-	X
Symphyla		Scutigerellidae	*Hanseniella* sp.1	-	X
Gastropoda	Pulmonata	Subulinidae	*Lamellaxis* sp.1	X	X

			Lamellaxis sp.2	-	X
		Systrophiidae	*Happia* sp.2	-	X
			Happia sp.4	X	-
Amphibia	Anura	Strabomantidae	*Pristimantis* cf. *fenestratus*	-	X
Reptilia	Squamata		Sauria sp.2	-	X
Mammalia	Chiroptera	Emballonuridae	*Peropteryx* sp.	-	X
		Phyllostomidae	*Carollia perspicillata*	-	X

SB-0133					
**TÁXONS**				**Seca**	**Úmida**
Arachnida	Amblypygi	Phrynidae	*Heterophrinus longicornis*	X	X
	Araneae	Corinnidae	Corinnidae jovem	-	X
		Ctenidae	Ctenidae jovem	-	X
		Ochyroceratidae	*Speocera* sp.1	-	X
		Pholcidae	Pholcidae jovem	X	-
		Salticidae	Salticidae jovem	X	X
		Scytodidae	*Scytodes* sp.1	-	X
		Theridiidae	Theridiidae jovem	X	X
		Theridiosomatidae	*Plato* sp.1	-	X
	Opiliones	Escadabiidae	Escadabiidae jovem	-	X
Diplopoda	Spirostreptida	Pseudonannolenidae	Pseudonannolenidae jovem	X	-
Entognatha	Collembola	Entomobryidae	Entomobryidae sp.2	-	X
		Paronellidae	Paronellidae sp.1	X	X
Insecta	Diptera	Ceratopogonidae	Ceratopogonidae jovem	-	X
			Diptera jovem	X	-
	Hemiptera	Cixiidae	Cixiidae jovem	X	X
		Cydnidae	Cydnidae jovem	X	X
	Hymenoptera	Formicidae	*Camponotus* sp.2	X	X
			Paratrechina sp.1	X	-
			Pheidole sp.4	X	X
	Lepidoptera		Lepidoptera jovem	X	-
	Orthoptera	Phalangopsidae	*Paraclodes* sp.1	X	-
			Phalangopsis sp.1	X	X
	Psocoptera		Psocomorpha jovem	X	X
Malacostraca	Isopoda	Armadillidae	Armadillidae sp.1	X	X
Mammalia	Chiroptera	Emballonuridae	*Peropteryx* sp.	-	X
		Phyllostomidae	*Carollia* sp.	-	X

SB-0134					
TÁXONS				Seca	Úmida
Arachnida	Acari	Argasidae	*Ornithodoros* sp.1	X	X
		Trombiculidae	Trombiculidae sp.2	X	-
			Mesostigmata sp.1	-	X
			Oribatida sp.3	-	X
	Amblypygi	Phrynidae	*Heterophrinus longicornis*	X	X
	Araneae	Araneidae	Araneidae jovem	-	X
		Corinnidae	*Abapeba* sp.1	X	X
			Corinnidae jovem	X	X
		Ctenidae	Ctenidae jovem	X	X
		Mimetidae	*Gelanor* sp.1	-	X
		Oonopidae	Oonopidae sp.4	-	X
			Oonopidae sp.6	X	-
		Pholcidae	*Mesabolivar cambridgei*	X	-
			Mesabolivar eberhardi	X	-
		Salticidae	Salticidae jovem	X	-
		Scytodidae	Scytodidae jovem	X	X
		Theraphosidae	*Guyruita cerrado*	X	-
		Theridiidae	*Achaearanea* sp.1	X	-
			Episinus sp.2	-	X
			Theridiidae jovem	-	X
		Theridiosomatidae	*Plato* sp.1	-	X
	Opiliones	Cosmetidae	Cosmetidae jovem	-	X
	Pseudoscorpiones	Chernetidae	*Spelaeochernes* sp.1	X	X
		Chthoniidae	Chthoniidae sp.1	-	X
Entognatha	Collembola	Paronellidae	Paronellidae sp.1	X	X
	Diplura	Campodeidae	Campodeidae sp.1	-	X
Insecta	Archaeognatha	Meinertellidae	Meinertellidae sp.1	-	X
	Blattodea	Blaberidae	*Blaberus* sp.1	X	X
		Blattidae	Blattidae jovem	X	X
		Polyphagidae	Polyphagidae jovem	X	X
			Polyphagidae sp.1	X	-
	Coleoptera	Curculionidae	Scolytinae sp.3	X	-
		Noteridae	*Stelidoda* sp.1	X	-
		Staphylinidae	Staphylininae sp.1	X	-
		Tenebrionidae	Tenebrionidae jovem	-	X
	Diptera	Ceratopogonidae	Ceratopogonidae jovem	-	X
		Conopidae	Conopidae sp.	-	X
		Drosophilidae	Drosophilidae sp.	X	-
		Muscidae	Muscidae jovem	-	X
		Psychodidae	Phlebotominae sp.	-	X
		Streblidae	Streblidae sp.	X	X
	Hemiptera	Cydnidae	Cydnidae sp.1	X	X
			Cydnidae sp.2	X	-
		Delphacidae	Delphacidae sp.1	X	-
		Reduviidae	Reduviinae jovem	X	X
	Hymenoptera	Formicidae	*Brachymyrmex* sp.1	-	X
			Camponotus sp.2	X	X
			Dolichoderus bispinosus	X	-
			Gnamptogenys sp.1	X	-
			Odontomachus bauri	-	X
			Pachycondyla constricta	-	X
			Pheidole sp.3	X	-
			Prionopelta modesta	X	-
	Isoptera	Termitidae	*Nasutitermes* sp.1	X	X
			Nasutitermes sp.2	X	X
			Nasutitermes sp.3	X	-
	Lepidoptera	Tineoidea	Tineoidea sp. 3	-	X

			Tineoidea sp. 9	X	-
	Orthoptera	Phalangopsidae	*Phalangopsis* sp.1	X	X
	Psocoptera	Psyllipsocidae	Psyllipsocidae sp.3	X	X
			Psocomorpha jovem	-	X
Malacostraca	Isopoda	Armadillidae	Armadillidae sp.1	X	X
		Philosciidae	Philosciidae sp.1	-	X
Amphibia	Anura	Strabomantidae	*Pristimantis* cf. *fenestratus*	X	-
Mammalia	Chiroptera	Emballonuridae	*Peropteryx* sp.	X	X
		Phyllostomidae	*Carollia perspicillata*	X	X
			Glossophaga soricina	X	-
Reptilia	Squamata	Gekkonidae	*Thecadactylus rapicauda*	-	X

Sb-0135					
**TÁXONS**				**Seca**	**Úmida**
Arachnida	Acari	Ixodidae	*Amblyomma* sp.2	X	-
	Amblypygi	Phrynidae	*Heterophrinus longicornis*	X	X
	Araneae	Ochyroceratidae	Ochyroceratidae jovem	X	X
		Pholcidae	*Mesabolivar cambridgei*	X	-
		Theridiosomatidae	*Plato* sp.1	X	X
	Opiliones	Sclerosomatidae	*Prionostema* sp.1	X	-
	Pseudoscorpiones	Chernetidae	Chernetidae jovem	X	-
			Spelaeochernes sp.1	-	X
Chilopoda	Geophilomorpha	Ballophilidae	*Ityphilus* sp.1	X	-
Diplopoda	Polydesmida	Chelodesmidae	Chelodesmidae jovem	X	X
	Polyzoniida	Siphonotidae	Siphonotidae sp.1	X	-
	Spirostreptida	Pseudonannolenidae	Pseudonannolenidae sp.1	X	-
Entognatha	Collembola	Paronellidae	Paronellidae sp.1	X	X
	Diplura	Campodeidae	Campodeidae sp.1	X	X
Insecta	Coleoptera	Carabidae	*Galerita* sp.1	X	-
		Tenebrionidae	Tenebrionidae jovem	-	X
	Diptera	Psychodidae	Phlebotominae sp.	X	-
		Tipulidae	Tipulidae sp.	X	-
	Hemiptera	Cixiidae	Cixiidae jovem	X	-
	Hymenoptera	Formicidae	*Pachycondyla constricta*	X	X
	Isoptera	Termitidae	*Nasutitermes* sp.1	X	-
	Orthoptera	Phalangopsidae	*Eidmanacris* sp.1	X	-
			Paraclodes sp.1	X	X
			Phalangopsis sp.1	X	X
	Thysanura	Nicoletiidae	Nicoletiinae sp.1	X	-
Malacostraca	Isopoda	Armadillidae	Armadillidae sp.1	X	-
		Philosciidae	Philosciidae sp.1	X	X
			Philosciidae sp.2	-	X
Mammalia	Chiroptera	Phyllostomidae	*Carollia* sp.	X	-
			Glossophaga soricina	-	X
	Rodentia	Cricetidae	*Rhipidomys* sp.	X	X

SB-0136					
TÁXONS				**Seca**	**Úmida**
Arachnida	Acari	Opilioacaridae	Opilioacaridae sp.1	X	-
			Oribatida sp.2	X	-
	Amblypygi	Phrynidae	*Heterophrinus longicornis*	X	-
	Araneae	Araneidae	*Alpaida* sp.1	X	-
		Corinnidae	Corinnidae jovem	X	-
		Ctenidae	Ctenidae jovem	X	X
		Dipluridae	Dipluridae jovem	X	-
		Mysmenidae	Mysmenidae sp.3	X	-
		Ochyroceratidae	Ochyroceratidae jovem	X	X
		Oonopidae	gr. *Xycarpphy* sp.1	X	-
			Oonopidae sp.5	X	-
		Palpimanidae	Palpimanidae jovem	X	-
		Pholcidae	*Mesabolivar cambridgei*	X	X
			Mesabolivar eberhardi	X	X
			Ninetinae sp.1	X	-
		Prodidomidae	Prodidomidae jovem	X	-
		Salticidae	Salticidae sp.13	X	-
			Salticidae sp.9	X	-
		Scytodidae	*Scytodes* sp.2	X	-
		Theridiidae	Theridiidae jovem	X	-
			Theridion sp.1	-	X
		Theridiosomatidae	Theridiosomatidae jovem	X	-
	Opiliones	Sclerosomatidae	*Prionostema* sp.1	X	-
			Sclerosomatidae jovem	-	X
	Pseudoscorpiones	Chernetidae	*Spelaeochernes* sp.1	X	-
		Chthoniidae	Chthoniidae jovem	X	-
			Chthoniidae sp.1	-	X
Chilopoda	Scolopendromorpha	Scolopocryptopidae	*Newportia* sp.2	X	-
	Scutigeromorpha	Pselliodidae	*Sphendononema* jovem	X	-
Entognatha	Collembola	Entomobryidae	Entomobryidae sp.1	X	-
		Paronellidae	Paronellidae sp.1	X	-
			Paronellidae sp.3	-	X
Insecta	Diptera	Acroceridae	Acroceridae sp.	X	-
		Psychodidae	Phlebotominae sp.	-	X
		Tipulidae	Tipulidae sp.	X	-
	Hemiptera	Cixiidae	Cixiidae jovem	-	X
			Cixiidae sp.3	X	-
		Reduviidae	Reduviinae jovem	X	-
	Hymenoptera	Formicidae	*Camponotus* sp.2	X	X
			Odontomachus meinerti	X	-
			Paratrechina sp.1	X	-
			Pheidole sp.5	X	-
			Rogeria blanda	X	-
			Strumigenys smithii	X	-
			Trachymyrmex sp.1	X	-
			Wasmannia auropunctata	X	-
	Lepidoptera	Noctuoidea	Noctouidea sp. 1	-	X
	Neuroptera	Myrmeleontidae	Myrmeleontidae sp.2	X	-
	Orthoptera	Phalangopsidae	*Paraclodes* sp.1	X	-
			Phalangopsis sp.1	X	X
	Psocoptera		Psocomorpha jovem	X	-
	Thysanura	Nicoletiidae	Nicoletiinae sp.1	-	X
Malacostraca	Isopoda	Armadillidae	Armadillidae sp.1	X	-
		Philosciidae	Philosciidae sp.1	-	X
			Philosciidae sp.2	X	-
Symphyla		Scolopendrellidae	*Symphylella* sp.1	-	X
Amphibia	Anura	Strabomantidae	*Pristimantis* cf. *fenestratus*	X	-

SB-0137					
TÁXONS				Seca	Úmida
Annelida	Haplotaxida		Haplotaxida sp.7	X	-
			Tubificina sp.1	X	-
Arachnida	Acari	Trombiculidae	Trombiculidae sp.1	X	X
			Trombiculidae sp.2	X	X
			Acariformes sp.4	X	-
			Acariformes sp.5	X	-
			Astigmata sp.1	X	-
	Araneae	Corinnidae	Corinnidae jovem	X	X
		Ochyroceratidae	*Speocera* sp.1	X	X
		Oonopidae	Oonopidae jovem	X	X
		Pholcidae	*Mesabolivar eberhardi*	X	X
		Salticidae	Salticidae jovem	X	X
			Salticidae sp.1	-	X
			Salticidae sp.2	-	X
		Theridiidae	Theridiidae jovem	-	X
		Theridiosomatidae	*Plato* sp.1	X	X
	Opiliones	Escadabiidae	Escadabiidae jovem	X	-
		Neogoveidae	*Canga renatae*	X	-
		Sclerosomatidae	*Prionostema* sp.1	-	X
			Sclerosomatidae jovem	X	X
		Stygnidae	*Protimesus* sp.1	-	X
	Pseudoscorpiones	Chernetidae	Chernetidae sp.1	X	X
			Spelaeochernes sp.1	X	X
		Chthoniidae	Chthoniidae sp.2	X	-
Chilopoda	Geophilomorpha		Geophilomorpha jovem	X	-
Diplopoda	Glomeridesmida	Glomeridesmidae	Glomeridesmida jovem	X	-
	Polydesmida	Fuhrmanodesmidae	Fuhrmanodesmidae sp.1	X	-
		Pyrgodesmidae	Pyrgodesmidae sp.1	-	X
	Spirostreptida	Pseudonannolenidae	Pseudonannolenidae jovem	X	-
Entognatha	Collembola	Cyphoderidae	Cyphoderidae sp.1	X	-
		Paronellidae	Paronellidae sp.1	X	X
		Sminthuroidea	Sminthuroidea sp.2	X	X
	Diplura	Campodeidae	Campodeidae sp.1	X	X
		Japygidae	Japygidae sp.1	X	-
Insecta	Archaeognatha	Meinertellidae	Meinertellidae sp.1	-	X
	Blattodea	Blaberidae	Blaberidae jovem	X	X
		Blattellidae	Blattellidae jovem	X	X
	Coleoptera	Cantharidae	Cantharidae jovem	X	-
		Carabidae	*Acupalpus* sp.1	X	-
		Curculionidae	Scolytinae sp.3	X	-
		Histeridae	Histeridae sp.4	X	-
		Melyridae	Melyridae jovem	X	-
		Staphylinidae	Pselaphinae sp.2	X	-
			Scydmaeninae sp.2	X	-
			Staphylinidae sp.4	X	-
		Tenebrionidae	Tenebrionidae jovem	X	-
			Coleoptera jovem	X	X
	Diptera	Ceratopogonidae	Ceratopogonidae jovem	X	-
		Dolichopodidae	Dolichopodidae sp.	-	X
		Drosophilidae	Drosophilidae sp.	X	X
		Phoridae	Phoridae sp.	X	-
		Psychodidae	Phlebotominae sp.	-	X
			Psychodidae sp.	-	X
		Streblidae	Streblidae sp.	X	X
	Hemiptera	cf. Nabidae	cf. Nabidae jovem	-	X
		Cydnidae	Cydnidae sp.1	X	X
			Cydnidae sp.2	X	X

			Cydnidae sp.3	-	X
		Reduviidae	*Zelurus* sp.1	X	X
	Hymenoptera	Braconidae	Braconidae sp.1	X	-
		Diapriidae	Diapriidae sp.1	X	-
		Eurytomidae	Eurytomidae sp.1	X	-
		Formicidae	*Acanthognathus* sp.1	X	-
			Camponotus sp.2	X	X
			Pachycondyla constricta	X	X
			Pheidole sp.5	-	X
			Solenopsis invicta	X	-
			Solenopsis sp.7	X	-
			Solenopsis sp.11	-	X
			Wasmannia sp.1	X	X
	Lepidoptera	Tineoidea	Tineoidea sp. 3	X	X
	Orthoptera	Phalangopsidae	*Paraclodes* sp.1	X	X
			Phalangopsis sp.1	X	X
	Psocoptera	Epipsocidae	Epipsocidae sp.2	X	-
		Psyllipsocidae	Psyllipsocidae sp.3	X	-
	Thysanura	Nicoletiidae	Atelurinae sp.1	X	-
			Nicoletiinae sp.1	X	X
Malacostraca	Isopoda	Armadillidae	Armadillidae sp.1	X	X
		Philosciidae	Philosciidae sp.1	X	-
			Philosciidae sp.2	X	X
Gastropoda	Pulmonata	Subulinidae	Subulinidae jovem	-	X
		Systrophiidae	Systrophiidae jovem	-	X
Amphibia	Anura	Leptodactylidae	*Leptodactylus* cf. *vastus*	X	-
		Strabomantidae	*Pristimantis* cf. *fenestratus*	X	-
Mammalia	Chiroptera	Emballonuridae	*Peropteryx kappleri*	-	X
		Furipteridae	*Furipterus horrens*	X	X
		Phyllostomidae	*Carollia perspicillata*	X	X
			Glossophaga soricina	X	X
			Lonchorhina aurita	X	X
			Phyllostomus latifolius	X	X
			Trachops cirrhosus	-	X
	Rodentia	Cricetidae	*Rhipidomys* sp.	X	X

SB-0138					
TÁXONS				Seca	Úmida
Arachnida	Acari	Trombiculidae	Trombiculidae sp.1	X	-
			Oribatida sp.2	X	-
	Amblypygi	Phrynidae	*Heterophrinus longicornis*	-	X
	Araneae	Araneidae	*Alpaida* sp.1	-	X
		Corinnidae	Corinnidae jovem	X	X
		Ctenidae	Ctenidae jovem	-	X
		Ochyroceratidae	*Speocera* sp.1	X	X
		Oonopidae	Oonopidae sp.3	X	-
		Pholcidae	*Mesabolivar eberhardi*	X	X
			Ninetinae sp.1	X	-
		Salticidae	Salticidae jovem	X	-
			Salticidae sp.9	-	X
		Scytodidae	*Scytodes* sp.1	-	X
			Scytodidae jovem	X	-
		Theridiosomatidae	*Plato* sp.1	X	-
			Theridiosomatidae jovem	-	X
	Opiliones	Escadabiidae	Escadabiidae jovem	-	X
			Escadabiidae sp.1	X	-
		Sclerosomatidae	*Prionostema* sp.1	X	X
	Pseudoscorpiones	Chernetidae	*Spelaeochernes* sp.1	X	-
Entognatha	Collembola	Sminthuroidea	Sminthuroidea sp.2	-	X
Insecta	Blattodea		Blattodea jovem	X	-
	Coleoptera	Staphylinidae	Staphylininae sp.2	-	X
	Diptera	Cecidomyiidae	Cecidomyiidae sp.	X	-
		Ceratopogonidae	Ceratopogonidae jovem	-	X
		Dolichopodidae	Dolichopodidae sp.	-	X
		Drosophilidae	Drosophilidae sp.	X	X
		Phoridae	Phoridae sp.	X	-
		Psychodidae	Phlebotominae sp.	X	-
		Sciaridae	Sciaridae sp.	X	-
	Hemiptera	Cixiidae	Cixiidae jovem	-	X
		Cydnidae	Cydnidae sp.1	X	X
		Ochteridae	Ochteridae sp.1	X	-
		Reduviidae	Emesinae jovem	-	X
			Reduviinae jovem	X	-
			Zelurus sp.1	-	X
	Hymenoptera	Formicidae	*Rogeria* cf. *belti*	X	X
	Isoptera		Isoptera jovem	-	X
	Lepidoptera	Noctuoidea	Noctouidea sp. 2	-	X
	Orthoptera	Phalangopsidae	*Paraclodes* sp.1	-	X
			Phalangopsis sp.1	X	X
	Psocoptera		Psocomorpha jovem	X	-
Malacostraca	Isopoda	Armadillidae	Armadillidae sp.1	X	-
Gastropoda	Pulmonata	Subulinidae	*Lamellaxis* sp.1	X	-

SB-0139					
TÁXONS				Seca	Úmida
Arachnida	Acari		Acariformes sp.4	X	-
			Astigmata sp.1	X	-
			Mesostigmata sp.1	X	-
	Amblypygi	Phrynidae	*Heterophrinus longicornis*	-	X
	Araneae	Corinnidae	*Corinna ducke*	X	X
		Ctenidae	Ctenidae jovem	-	X
		Ochyroceratidae	*Speocera* sp.1	X	X
			Speocera sp.3	-	X
		Pholcidae	*Mesabolivar eberhardi*	-	X
			Ninetinae sp.1	X	-
		Salticidae	Salticidae sp.1	-	X
		Scytodidae	*Scytodes* sp.1	X	X
		Theridiosomatidae	*Plato* sp.1	X	X
	Opiliones	Escadabiidae	Escadabiidae sp.1	-	X
		Sclerosomatidae	*Prionostema* sp.1	X	X
	Pseudoscorpiones	Chernetidae	*Spelaeochernes* sp.1	X	X
		Chthoniidae	Chthoniidae sp.1	X	X
Diplopoda	Polydesmida	Pyrgodesmidae	Pyrgodesmidae sp.1	-	X
	Siphonophorida	Siphonophoridae	Siphonophoridae sp.1	X	-
	Spirostreptida		Spirostreptida jovem	-	X
Entognatha	Collembola	Paronellidae	Paronellidae sp.1	X	X
		Sminthuroidea	Sminthuroidea sp.2	-	X
Insecta	Coleoptera	Anthribidae	Anthribidae sp.1	-	X
		Curculionidae	Curculionidae sp.4	X	-
			Scolytinae sp.1	X	-
	Diptera	Culicidae	Culicidae sp.	X	-
		Drosophilidae	Drosophilidae sp.	X	X
		Phoridae	Phoridae sp.	X	-
		Psychodidae	Phlebotominae sp.	X	X
	Hemiptera	Reduviidae	Reduviinae jovem	X	X
	Hymenoptera	Formicidae	*Odontomachus meinerti*	X	X
			Pachycondyla constricta	X	X
	Lepidoptera	Noctuoidea	Noctouidea sp. 2	X	-
		Tineoidea	Tineoidea sp. 4	-	X
	Orthoptera	Phalangopsidae	*Paraclodes* sp.1	X	X
			Phalangopsis sp.1	X	X
Malacostraca	Isopoda	Armadillidae	Armadillidae sp.1	X	X
		Philosciidae	Philosciidae sp.2	-	X
Gastropoda	Pulmonata	Subulinidae	*Lamellaxis* sp.1	X	-
			Leptinaria sp.1	X	-
			Leptinaria sp.2	X	-

SB-0140					
**TÁXONS**				**Seca**	**Úmida**
Arachnida	Acari	Argasidae	*Ornithodoros* sp.1	X	X
			Astigmata sp.1	-	X
			Oribatida sp.5	-	X
	Araneae	Ctenidae	Ctenidae jovem	X	X
		Filistatidae	Filistatidae jovem	X	X
			Filistatidae sp.1	-	X
		Oonopidae	Oonopidae sp.3	X	-
			Oonopidae sp.14	-	X
		Pholcidae	*Mesabolivar cambridgei*	X	-
			Physocyclus globosus	-	X
		Salticidae	Salticidae jovem	-	X
		Scytodidae	*Scytodes* sp.1	-	X
		Theridiidae	*Nesticodes rufipes*	-	X
			Theridiidae jovem	X	-
	Pseudoscorpiones	Chernetidae	*Spelaeochernes* sp.1	X	X
		Chthoniidae	Chthoniidae sp.1	-	X
Chilopoda	Scutigeromorpha	Pselliodidae	*Sphendononema* jovem	-	X
Diplopoda	Polydesmida	Paradoxosomatidae	Paradoxosomatidae sp.1	-	X
Entognatha	Collembola	Entomobryidae	Entomobryidae sp.2	-	X
Insecta	Blattodea	Blaberidae	*Blaberus* sp.1	X	X
			Blaberus sp.3	-	X
	Coleoptera	Elateridae	Elateridae sp.2	-	X
	Diptera	Cecidomyiidae	Cecidomyiidae sp.	-	X
		Ceratopogonidae	Ceratopogonidae jovem	-	X
		Psychodidae	Phlebotominae sp.	-	X
	Hemiptera	Cydnidae	Cydnidae sp.1	-	X
	Hymenoptera	Formicidae	*Camponotus renggeri*	X	-
			Labidus sp.1	X	-
			Solenopsis invicta	X	X
		Vespidae	Vespidae sp.3	-	X
	Lepidoptera	Tineoidea	Tineoidea sp. 5	-	X
			Tineoidea sp. 6	-	X
			Lepidoptera jovem	X	-
	Neuroptera	Myrmeleontidae	Myrmeleontidae sp.1	X	X
	Orthoptera	Phalangopsidae	*Phalangopsis* sp.1	X	X
	Psocoptera	Psyllipsocidae	Psyllipsocidae sp.1	X	-
		Troctopsocidae	Troctopsocidae sp.1	X	-
			Psocomorpha jovem	-	X
Malacostraca	Isopoda	Philosciidae	Philosciidae sp.2	-	X
Amphibia	Anura	Bufonidae	*Rhinella* sp.	X	-
		Strabomantidae	*Pristimantis* cf. *fenestratus*	X	-
Aves	Strigiformes	Tytonidae	*Tyto alba*	X	-
Mammalia	Chiroptera	Emballonuridae	*Peropteryx kappleri*	X	X
		Furipteridae	*Furipterus horrens*	X	X
		Phyllostomidae	*Carollia perspicillata*	X	X
			Glossophaga soricina	X	X
			Phyllostomus latifolius	X	-
			Vampyrum spectrum	X	-
Reptilia	Squamata		Sauria sp.1	X	-

SB-0141					
TÁXONS				**Seca**	**Úmida**
Arachnida	Acari	Ixodidae	*Amblyomma* sp.5	-	X
	Araneae	Theridiosomatidae	Theridiosomatidae jovem	-	X
	Opiliones	Cosmetidae	*Roquettea carajas*	-	X
Diplopoda	Polydesmida	Chelodesmidae	Chelodesmidae sp.1	-	X
Insecta	Diptera	Cecidomyiidae	Cecidomyiidae sp.	-	X
		Psychodidae	Phlebotominae sp.	-	X
	Hemiptera	Reduviidae	Reduviinae jovem	-	X
	Hymenoptera	Formicidae	*Pachycondyla constricta*	-	X
			Rogeria cf. *belti*	-	X
			Solenopsis invicta	X	X
	Isoptera	Termitidae	*Nasutitermes* sp.1	-	X
	Lepidoptera	Noctuoidea	Noctouidea sp. 2	-	X
		Tineoidea	Tineoidea sp. 8	-	X
	Neuroptera	Myrmeleontidae	Myrmeleontidae sp.1	X	-
	Orthoptera	Phalangopsidae	*Paraclodes* sp.1	-	X
Malacostraca	Isopoda	Platyarthridae	Platyarthridae sp.3	-	X
Mammalia	Chiroptera	Phyllostomidae	*Carollia* sp.	-	X

SB-0142					
**TÁXONS**				**Seca**	**Úmida**
Annelida	Haplotaxida		Tubificina sp.1	-	X
Arachnida	Acari		Acariformes sp.3	-	X
			Holothyrida sp.1	-	X
			Mesostigmata jovem	X	-
			Mesostigmata sp.1	-	X
	Araneae	Corinnidae	Corinnidae jovem	-	X
		Linyphiidae	Linyphiidae jovem	X	X
			Linyphiidae sp.2	X	-
		Philodromidae	Philodromidae jovem	X	-
		Pholcidae	*Modisimus* sp.1	X	X
		Salticidae	Salticidae jovem	X	X
			Salticidae sp.2	X	-
		Scytodidae	Scytodidae jovem	-	X
		Theridiidae	*Nesticodes rufipes*	-	X
	Pseudoscorpiones	Chernetidae	*Spelaeochernes* sp.1	X	-
		Olpiidae	Olpiidae sp.1	X	-
		Tridenchthoniidae	Tridenchthoniidae sp.1	-	X
	Scorpiones	Buthidae	*Ananteris luciae*	-	X
Chilopoda	Lithobiomorpha	Henicopidae	*Lamyctes* p.2	-	X
	Scolopendromorpha	Cryptopidae	*Cryptops* jovem	X	-
		Scolopocryptopidae	*Dinocryptops* sp.1	X	-
	Scutigeromorpha	Pselliodidae	*Sphendononema guildingii*	-	X
Diplopoda	Polydesmida	Paradoxosomatidae	Paradoxosomatidae sp.1	-	X
			Paradoxosomatidae sp.3	-	X
	Spirostreptida		Spirostreptida jovem	X	-
	Stemmiulida	Stemmiulidae	Stemmiulidae jovem	-	X
Entognatha	Collembola	Entomobryidae	Entomobryidae sp.2	-	X
		Paronellidae	Paronellidae sp.1	X	-
	Diplura	Campodeidae	Campodeidae sp.1	-	X
Insecta	Blattodea	Blaberidae	Blaberidae jovem	X	X
	Coleoptera	Carabidae	Pterostichini sp.1	X	-
		Dermestidae	*Attagenus* sp.1	X	-
			Coleoptera jovem	-	X
	Diptera	Cecidomyiidae	Cecidomyiidae sp.	X	-
		Drosophilidae	Drosophilidae sp.	X	-
		Psychodidae	Phlebotominae sp.	X	X
		Sciaridae	Sciaridae sp.	-	X
	Hemiptera	Cydnidae	Cydnidae sp.1	-	X
		Reduviidae	Emesinae sp.3	X	-
	Hymenoptera	Formicidae	*Acromyrmex octopinosus*	X	X
			Pachycondyla constricta	X	X
		Vespidae	Vespidae sp.1	-	X
	Lepidoptera	Tineoidea	Tineoidea sp. 5	X	-
			Tineoidea sp. 6	X	X
	Orthoptera	Phalangopsidae	*Phalangopsis* sp.1	X	-
	Psocoptera	Psyllipsocidae	Psyllipsocidae sp.1	X	X
			Psyllipsocidae sp.2	X	-
			Psocomorpha jovem	X	X
Malacostraca	Isopoda	Armadillidae	Armadillidae sp.1	X	X
		Dubioniscidae	Dubioniscidae sp.1	X	X
		Scleropactidae	Scleropactidae sp.3	-	X
Nematoda	Rhabditida		Rhabditia sp.2	-	X
Amphibia	Anura	Strabomantidae	*Pristimantis* cf. *fenestratus*	X	-
Mammalia	Chiroptera	Emballonuridae	*Peropteryx kappleri*	X	X
		Phyllostomidae	*Carollia perspicillata*	X	X
			Glossophaga sp.	-	X

SB-0143					
**TÁXONS**				**Seca**	**Úmida**
Arachnida	Amblypygi	Phrynidae	*Heterophrinus longicornis*	X	-
	Araneae	Linyphiidae	Linyphiidae jovem	X	-
		Pholcidae	Pholcidae jovem	-	X
		Prodidomidae	Prodidomidae jovem	-	X
		Theridiidae	Theridiidae jovem	-	X
	Opiliones	Cosmetidae	*Roquettea carajas*	X	-
	Pseudoscorpiones	Chernetidae	*Spelaeochernes* sp.1	X	X
		Chthoniidae	Chthoniidae sp.1	-	X
Diplopoda	Polydesmida	Paradoxosomatidae	Paradoxosomatidae jovem	-	X
			Paradoxosomatidae sp.1	-	X
			Paradoxosomatidae sp.3	-	X
Entognatha	Collembola	Paronellidae	Paronellidae sp.1	X	X
		Sminthuroidea	Sminthuroidea sp.2	-	X
	Diplura	Campodeidae	Campodeidae sp.1	-	X
Insecta	Coleoptera		Coleoptera jovem	X	-
	Hemiptera	Reduviidae	Reduviinae jovem	-	X
	Hymenoptera	Formicidae	*Hypoponera* sp.2	-	X
			Pachycondyla constricta	X	-
			Solenopsis invicta	X	-
		Vespidae	Vespidae sp.3	X	-
	Orthoptera	Phalangopsidae	*Phalangopsis* sp.1	X	X
	Psocoptera	Liposcelidae	Liposcelidae sp.2	X	-
			Trogiomorpha jovem	X	-
Malacostraca	Isopoda	Dubioniscidae	Dubioniscidae sp.1	X	-
			Dubioniscidae sp.2	-	X
Symphyla		Scutigerellidae	*Hanseniella* sp.1	-	X
Amphibia	Anura	Leptodactylidae	*Leptodactylus* cf. *vastus*	-	X
		Strabomantidae	*Pristimantis* cf. *fenestratus*	X	-
Mammalia	Chiroptera	Emballonuridae	*Peropteryx kappleri*	-	X

SB-0144					
TÁXONS				**Seca**	**Úmida**
Arachnida	Acari		Oribatida sp.2	-	X
	Amblypygi	Phrynidae	*Heterophrinus longicornis*	X	-
	Araneae	Corinnidae	Corinnidae jovem	-	X
		Oonopidae	Oonopidae sp.4	X	-
		Salticidae	Salticidae sp.1	-	X
		Theridiidae	*Nesticodes rufipes*	-	X
	Opiliones	Cosmetidae	Cosmetidae sp.2	-	X
			Roquettea carajas	X	-
	Pseudoscorpiones	Chernetidae	*Spelaeochernes* sp.1	-	X
		Chthoniidae	Chthoniidae sp.1	X	-
Diplopoda	Polydesmida	Fuhrmanodesmidae	Fuhrmanodesmidae sp.1	-	X
		Paradoxosomatidae	Paradoxosomatidae jovem	-	X
	Siphonophorida	Siphonophoridae	Siphonophoridae jovem	-	X
Insecta	Hemiptera	Cixiidae	Cixiidae jovem	-	X
	Hymenoptera	Formicidae	*Solenopsis invicta*	X	X
	Lepidoptera	Tineoidea	Tineoidea sp. 8	X	-
	Neuroptera	Myrmeleontidae	Myrmeleontidae sp.1	X	X
	Orthoptera	Phalangopsidae	*Paraclodes* sp.1	X	-
			Phalangopsis sp.1	X	X
	Psocoptera		Psocomorpha jovem	-	X
Malacostraca	Isopoda	Armadillidae	Armadillidae sp.1	-	X
		Dubioniscidae	Dubioniscidae sp.1	X	X
Amphibia	Anura	Strabomantidae	*Pristimantis* cf. *fenestratus*	X	-

SB-0145					
TÁXONS				Seca	Úmida
Arachnida	Acari		Acariformes sp.3	-	X
			Oribatida sp.2	X	X
	Amblypygi	Phrynidae	*Heterophrinus longicornis*	X	X
	Araneae	Corinnidae	Corinnidae jovem	X	X
		Oonopidae	Oonopidae sp.3	X	-
			Oonopidae sp.9	X	-
			Oonopidae sp.11	X	-
		Pholcidae	*Leptopholcus* sp.1	-	X
		Salticidae	Salticidae jovem	X	-
			Salticidae sp.1	-	X
			Salticidae sp.4	-	X
		Scytodidae	Scytodidae jovem	X	X
		Theraphosidae	Theraphosidae jovem	X	-
		Theridiidae	*Nesticodes rufipes*	-	X
	Opiliones	Cosmetidae	Cosmetidae sp.2	-	X
	Pseudoscorpiones	Chernetidae	*Spelaeochernes* sp.1	X	X
Chilopoda	Scutigeromorpha	Pselliodidae	*Sphendononema guildingii*	X	X
Insecta	Blattodea	Blaberidae	*Blaberus* sp.1	X	X
	Coleoptera	Carabidae	*Lelis* sp.1	X	-
		Staphylinidae	Scydmaeninae sp.1	X	-
			Staphylininae sp.3	X	-
			Coleoptera jovem	X	X
	Diptera	Ceratopogonidae	Ceratopogonidae sp.	-	X
		Muscidae	Muscidae sp.	-	X
		Phoridae	Phoridae sp.	X	-
		Psychodidae	Phlebotominae sp.	-	X
	Hemiptera	Cydnidae	Cydnidae sp.1	X	X
	Hymenoptera	Formicidae	*Acromyrmex octopinosus*	X	X
			Camponotus renggeri	-	X
			Solenopsis invicta	X	X
	Isoptera	Termitidae	*Nasutitermes* sp.1	X	-
	Lepidoptera	Tineoidea	Tineoidea sp. 3	X	-
			Tineoidea sp. 5	-	X
	Neuroptera	Myrmeleontidae	Myrmeleontidae sp.1	X	X
	Orthoptera	Phalangopsidae	*Phalangopsis* sp.1	X	X
	Psocoptera	Elipsocidae	Elipsocidae sp.1	-	X
		Lepidopsocidae	Lepidopsocidae sp.1	-	X
		Liposcelidae	Liposcelidae sp.4	-	X
	Thysanura	Nicoletiidae	Nicoletiinae sp.1	X	X
Malacostraca	Isopoda	Armadillidae	Armadillidae sp.1	-	X
		Dubioniscidae	Dubioniscidae sp.1	X	X
		Philosciidae	Philosciidae sp.1	X	-
Amphibia	Anura	Strabomantidae	*Pristimantis* cf. *fenestratus*	X	-
Mammalia	Chiroptera	Emballonuridae	*Peropteryx kappleri*	X	X
		Phyllostomidae	*Carollia perspicillata*	X	X
			Glossophaga soricina	X	X

SB-0146					
TÁXONS				Seca	Úmida
Arachnida	Acari		Oribatida sp.2	-	X
	Araneae	Corinnidae	Corinnidae jovem	X	-
		Oonopidae	Oonopidae sp.3	-	X
		Pholcidae	*Leptopholcus* sp.1	-	X
			Modisimus sp.1	-	X
			Pholcidae jovem	X	X
		Theridiidae	Theridiidae jovem	X	-
Diplopoda	Polydesmida	Chelodesmidae	Chelodesmidae sp.3	-	X
Entognatha	Collembola	Paronellidae	Paronellidae sp.1	-	X
Insecta	Archaeognatha	Meinertellidae	Meinertellidae jovem	-	X
	Coleoptera		Coleoptera jovem	X	-
	Diptera	Cecidomyiidae	Cecidomyiidae sp.	-	X
	Hemiptera	Cixiidae	Cixiidae jovem	-	X
		Cydnidae	Cydnidae sp.1	-	X
		Fulgoridae	Fulgoridae sp.3	-	X
	Hymenoptera	Formicidae	*Acanthostichus bentoni*	X	-
			Pachycondyla constricta	-	X
			Solenopsis invicta	X	-
			Strumigenys elongata	X	-
	Lepidoptera		Lepidoptera jovem	-	X
	Neuroptera	Myrmeleontidae	Myrmeleontidae sp.1	X	-
	Orthoptera	Phalangopsidae	*Phalangopsis* sp.1	X	X
	Thysanura	Nicoletiidae	Atelurinae sp.1	X	-
Malacostraca	Isopoda	Armadillidae	Armadillidae sp.1	-	X
		Dubioniscidae	Dubioniscidae sp.1	X	-
			Dubioniscidae sp.2	-	X
Symphyla		Scutigerellidae	*Hanseniella* jovem	-	X
Amphibia	Anura	Leptodactylidae	*Leptodactylus* cf. *vastus*	X	-
		Strabomantidae	*Pristimantis* cf. *fenestratus*	X	-
Mammalia	Chiroptera	Emballonuridae	*Peropteryx kappleri*	X	X

SB-0147					
TÁXONS				Seca	Úmida
Annelida	Haplotaxida		Haplotaxida jovem	-	X
			Haplotaxida sp.7	-	X
			Haplotaxida sp.9	-	X
Arachnida	Acari	Opilioacaridae	Opilioacaridae sp.1	X	X
		Trombiculidae	Trombiculidae sp.1	-	X
			Astigmata sp.2	X	-
			Holothyrida sp.2	X	X
			Mesostigmata sp.1	X	-
			Mesostigmata sp.2	X	-
			Oribatida sp.1	-	X
			Oribatida sp.2	X	X
			Oribatida sp.4	X	-
			Oribatida sp.5	X	X
	Amblypygi	Phrynidae	*Heterophrinus longicornis*	X	X
	Araneae	Corinnidae	*Abapeba* sp.1	X	-
			Corinnidae jovem	X	X
		Nesticidae	Nesticidae sp.1	X	-
		Oonopidae	Oonopidae sp.3	-	X
			Oonopidae sp.4	X	-
			Oonopidae sp.5	X	-
			Oonopidae sp.8	X	-
		Salticidae	Salticidae sp.1	-	X
			Salticidae sp.2	X	-
			Salticidae sp.3	-	X
		Scytodidae	Scytodidae jovem	X	X
		Theraphosidae	Theraphosidae sp.3	-	X
		Theridiidae	*Nesticodes rufipes*	X	X
			Theridiidae sp.1	-	X
		Theridiosomatidae	*Plato* sp.1	X	-
	Opiliones	Cosmetidae	Cosmetidae jovem	X	X
		Escadabiidae	Escadabiidae sp.1	X	X
	Pseudoscorpiones	Chernetidae	*Spelaeochernes* sp.1	X	X
		Chthoniidae	Chthoniidae sp.1	X	X
	Ricinulei	Ricinoididae	Ricinoididae jovem	-	X
Chilopoda	Lithobiomorpha	Henicopidae	*Lamyctes* p.1	-	X
	Scolopendromorpha	Cryptopidae	*Cryptops* sp.1	X	-
			Cryptops sp.2	X	X
			Cryptops sp.3	-	X
	Scutigeromorpha	Pselliodidae	*Sphendononema guildingii*	X	X
Diplopoda	Glomeridesmida	Glomeridesmidae	Glomeridesmida sp.1	-	X
	Polydesmida	Fuhrmanodesmidae	Fuhrmanodesmidae sp.1	X	X
		Pyrgodesmidae	Pyrgodesmidae sp.1	X	X
			Pyrgodesmidae sp.2	X	X
	Spirostreptida	Pseudonannolenidae	Pseudonannolenidae sp.1	X	-
Entognatha	Collembola	Cyphoderidae	Cyphoderidae sp.1	X	-
		Entomobryidae	Entomobryidae sp.6	-	X
		Isotomidae	Isotomidae sp.1	X	X
		Paronellidae	Paronellidae sp.1	X	X
		Sminthuroidea	Sminthuroidea sp.2	X	X
	Diplura	Campodeidae	Campodeidae sp.1	-	X
Insecta	Blattodea	Blaberidae	*Blaberus* sp.1	-	X
	Coleoptera	Dytiscidae	Dytiscidae sp.2	-	X
			Dytiscidae sp.3	-	X
		Endomychidae	Endomychidae sp.1	X	-
		Staphylinidae	Scydmaeninae sp.1	X	-
			Scydmaeninae sp.2	X	-
			Staphylininae sp.2	X	-

	Diptera	Cecidomyiidae	Cecidomyiidae sp.	X	-
		Ceratopogonidae	Ceratopogonidae sp.	-	X
		Chironomidae	Chironomidae sp.	-	X
		Culicidae	Culicidae sp.	X	X
		Drosophilidae	Drosophilidae sp.	X	-
		Psychodidae	Phlebotominae sp.	X	-
			Psychodidae sp.	X	-
	Hemiptera	Cydnidae	Cydnidae sp.1	X	X
			Cydnidae sp.2	-	X
	Hymenoptera	Braconidae	Braconidae sp.1	X	-
		Diapriidae	Diapriidae sp.2	X	X
		Formicidae	*Acanthostichus* sp.1	-	X
			Crematogaster abstinens	X	X
			Gnamptogenys sp.1	-	X
			Hypoponera opacior	X	X
			Pachycondyla arhuaca	X	-
			Pachycondyla constricta	X	X
			Pheidole sp.4	-	X
			Solenopsis invicta	X	X
	Isoptera		Isoptera jovem	-	X
	Lepidoptera	Tineoidea	Tineoidea sp. 3	X	-
			Lepidoptera jovem	X	X
	Orthoptera	Phalangopsidae	*Phalangopsis* sp.1	X	X
	Psocoptera	Archipsocidae	Archipsocidae sp.1	-	X
		Liposcelidae	Liposcelidae sp.3	X	-
		Psocidae	Psocidae sp.1	-	X
		Psyllipsocidae	Psyllipsocidae sp.3	X	-
		Troctopsocidae	Troctopsocidae sp.1	X	X
	Thysanura	Nicoletiidae	Atelurinae sp.1	X	-
			Nicoletiinae sp.1	X	-
Malacostraca	Isopoda	Dubioniscidae	Dubioniscidae sp.1	X	X
			Dubioniscidae sp.2	-	X
		Philosciidae	Philosciidae sp.2	X	-
		Platyarthridae	Platyarthridae sp.1	X	-
		Scleropactidae	Scleropactidae sp.1	X	X
			Scleropactidae sp.3	-	X
Symphyla		Scutigerellidae	*Hanseniella* sp.1	X	X
Gastropoda	Pulmonata	Subulinidae	*Lamellaxis* sp.1	X	X
			Leptinaria sp.1	-	X
			Leptinaria sp.2	X	X
		Systrophiidae	Systrophiidae jovem	X	X
Turbellaria	Tricladida		Continenticola sp.1	-	X
Amphibia	Anura	Bufonidae	*Rhinella* sp.	X	X
		Leptodactylidae	*Leptodactylus* cf. *vastus*	X	X
		Strabomantidae	*Pristimantis* cf. *fenestratus*	X	-
Mammalia	Chiroptera	Phyllostomidae	*Carollia perspicillata*	X	X
			Desmodus rotundus	-	X
			Diphylla ecaudata	X	-
			Glossophaga soricina	X	X
			Lionycteris spurrelli	X	X
			Lonchorhina aurita	X	-

SB-0149					
TÁXONS				Seca	Úmida
Arachnida	Acari	Argasidae	*Ornithodoros* sp.1	-	X
		Trombiculidae	Trombiculidae sp.1	-	X
			Acariformes sp.3	-	X
			Acariformes sp.4	X	X
			Astigmata sp.2	-	X
			Mesostigmata sp.1	X	-
			Oribatida sp.5	-	X
	Amblypygi	Charinidae	*Charinus* sp.1	-	X
		Phrynidae	*Heterophrinus longicornis*	X	-
	Araneae	Araneidae	*Alpaida* sp.1	X	-
		Corinnidae	*Abapeba* sp.1	X	X
		Ctenidae	Ctenidae jovem	X	X
		Filistatidae	Filistatidae jovem	X	-
			Filistatidae sp.1	-	X
		Ochyroceratidae	Ochyroceratidae sp.1	X	-
			Ochyroceratidae sp.2	-	X
		Pholcidae	*Leptopholcus* sp.1	-	X
			Ninetinae sp.1	X	-
		Salticidae	Salticidae jovem	X	X
			Salticidae sp.2	-	X
		Scytodidae	*Scytodes* sp.1	X	X
		Segestriidae	*Ariadna* sp.2	X	-
		Theraphosidae	Theraphosidae jovem	X	X
			Theraphosidae sp.5	-	X
		Theridiidae	Theridiidae jovem	-	X
		Theridiosomatidae	Theridiosomatidae jovem	X	-
	Opiliones	Escadabiidae	Escadabiidae sp.1	X	-
			Escadabiidae sp.2	-	X
	Pseudoscorpiones	Chernetidae	*Spelaeochernes* sp.1	X	X
	Scorpiones	Buthidae	*Ananteris luciae*	X	X
Chilopoda	Scolopendromorpha	Scolopocryptopidae	*Newportia* jovem	X	-
			Tidops sp.2	-	X
Entognatha	Collembola	Cyphoderidae	Cyphoderidae sp.1	X	-
		Paronellidae	Paronellidae sp.1	X	X
		Sminthuroidea	Sminthuroidea sp.2	-	X
	Diplura	Campodeidae	Campodeidae sp.1	-	X
Insecta	Archaeognatha	Meinertellidae	Meinertellidae sp.1	-	X
	Blattodea	Blaberidae	Blaberidae jovem	X	X
	Coleoptera	Elateridae	Elateridae jovem	-	X
		Staphylinidae	Scydmaeninae sp.3	X	-
	Diptera	Ceratopogonidae	Ceratopogonidae jovem	-	X
		Dolichopodidae	Dolichopodidae sp.	-	X
		Drosophilidae	Drosophilidae sp.	X	-
	Hemiptera	Cercopidae	Cercopidae jovem	X	-
		Cixiidae	Cixiidae jovem	-	X
		Cydnidae	Cydnidae sp.1	X	X
		Reduviidae	Reduviidae jovem	X	-
	Hymenoptera	Formicidae	*Carebara* sp.2	X	-
			Pachycondyla constricta	X	X
			Pheidole sp.3	X	X
	Lepidoptera	Noctuoidea	Noctouidea sp. 3	-	X
		Tineoidea	Tineoidea sp. 9	X	-
	Orthoptera	Phalangopsidae	*Eidmanacris* sp.1	X	-
			Paraclodes sp.1	X	X
			Phalangopsis sp.1	X	X
	Psocoptera	Psyllipsocidae	Psyllipsocidae sp.1	-	X
			Psocomorpha jovem	X	-

Malacostraca	Isopoda	Armadillidae	Armadillidae sp.1	X	X
		Dubioniscidae	Dubioniscidae sp.1	X	-
		Platyarthridae	Platyarthridae sp.2	X	X
			Platyarthridae sp.3	-	X
		Scleropactidae	Scleropactidae sp.1	-	X
Gastropoda	Pulmonata	Helicinidae	*Helicina* sp.1	-	X
		Systrophiidae	*Happia* sp.1	-	X
			Happia sp.4	-	X
Amphibia	Anura	Leptodactylidae	*Leptodactylus* cf. *vastus*	X	-
Mammalia	Chiroptera	Emballonuridae	*Peropteryx kappleri*	X	X
		Phyllostomidae	*Desmodus rotundus*	X	X
			Lonchorhina aurita	-	X
	Rodentia	Cricetidae	*Rhipidomys* sp.	-	X

SB-0150					
TÁXONS				**Seca**	**Úmida**
Arachnida	Acari	Opilioacaridae	Opilioacaridae sp.1	-	X
		Trombiculidae	Trombiculidae sp.1	-	X
			Acari jovem	X	-
	Araneae	Ctenidae	Ctenidae jovem	X	X
		Filistatidae	Filistatidae jovem	X	X
			Filistatidae sp.1	-	X
		Linyphiidae	Linyphiidae jovem	X	-
		Nemesiidae	Nemesiidae sp.1	-	X
		Oonopidae	Oonopidae sp.13	X	-
		Pholcidae	*Mesabolivar eberhardi*	X	-
			Pholcidae jovem	X	X
		Salticidae	Salticidae jovem	X	-
		Scytodidae	*Scytodes* sp.1	-	X
		Theraphosidae	*Guyruita cerrado*	X	-
			Theraphosidae sp.2	X	-
			Theraphosidae sp.4	-	X
		Theridiidae	Theridiidae jovem	-	X
	Opiliones	Cosmetidae	*Roquettea carajas*	X	-
		Escadabiidae	Escadabiidae jovem	-	X
	Pseudoscorpiones	Chernetidae	*Spelaeochernes* sp.1	X	X
	Ricinulei	Ricinoididae	*Cryptocellus tarsilae*	-	X
Chilopoda	Scolopendromorpha	Scolopocryptopidae	*Newportia* sp.5	X	-
			Scolopendromorpha jovem	-	X
	Scutigeromorpha	Pselliodidae	*Sphendononema guildingii*	-	X
Diplopoda	Polydesmida	Fuhrmanodesmidae	Fuhrmanodesmidae sp.1	-	X
Entognatha	Collembola	Entomobryidae	Entomobryidae sp.2	-	X
		Paronellidae	Paronellidae sp.1	X	X
Insecta	Archaeognatha	Meinertellidae	Meinertellidae sp.1	-	X
	Blattodea	Polyphagidae	Polyphagidae jovem	X	X
	Diptera	Drosophilidae	Drosophilidae sp.	X	-
		Psychodidae	Phlebotominae sp.	-	X
	Hemiptera	Reduviidae	Emesinae jovem	X	-
	Hymenoptera	Diapriidae	Diapriidae sp.10	-	X
		Formicidae	*Acromyrmex octopinosus*	-	X
			Gnamptogenys haenschi	-	X
			Neivamyrmex sp.2	X	-
			Pachycondyla striata	-	X
			Solenopsis invicta	-	X
	Lepidoptera	Tineoidea	Tineoidea sp. 5	X	X
			Tineoidea sp. 6	X	X
			Tineoidea sp. 8	X	-
			Tineoidea sp. 11	-	X
	Orthoptera	Phalangopsidae	Phalangopsis sp.1	X	-
	Psocoptera	Psyllipsocidae	Psyllipsocidae sp.1	-	X
			Psyllipsocidae sp.2	-	X
			Psocomorpha jovem	X	X
Malacostraca	Isopoda	Armadillidae	Armadillidae sp.1	X	X
Gastropoda	Pulmonata	Systrophiidae	*Happia* sp.1	-	X
Mammalia	Chiroptera	Emballonuridae	*Peropteryx kappleri*	X	X

SB-0151					
**TÁXONS**				**Seca**	**Úmida**
Arachnida	Acari	Argasidae	Ornithodoros sp.1	-	X
		Opilioacaridae	Opilioacaridae sp.1	-	X
	Araneae	Corinnidae	*Abapeba* sp.1	-	X
			Corinnidae jovem	X	-
		Ctenidae	Ctenidae jovem	X	X
		Filistatidae	Filistatidae jovem	-	X
		Pholcidae	Ninetinae sp.1	X	-
			Pholcidae jovem	-	X
		Salticidae	Salticidae sp.9	X	-
		Scytodidae	*Scytodes* sp.2	X	-
		Theraphosidae	Theraphosidae jovem	X	-
		Theridiidae	Theridiidae jovem	-	X
	Pseudoscorpiones	Chernetidae	*Spelaeochernes* sp.1	-	X
Diplopoda	Stemmiulida	Stemmiulidae	Stemmiulidae sp.1	-	X
Insecta	Blattodea	Blaberidae	Blaberidae jovem	X	-
		Blattidae	Blattidae jovem	X	-
		Polyphagidae	Polyphagidae jovem	-	X
	Diptera	Psychodidae	Phlebotominae sp.	-	X
	Hemiptera	Reduviidae	Reduviinae jovem	X	2
	Hymenoptera	Formicidae	*Solenopsis invicta*	-	X
	Lepidoptera		Lepidoptera jovem	-	X
	Orthoptera	Phalangopsidae	*Eidmanacris* sp.1	X	X
	Psocoptera	Liposcelidae	Liposcelidae sp.2	-	X
		Pachytroctidae	Pachytroctidae sp.1	-	X
Amphibia	Anura	Leptodactylidae	*Leptodactylus* cf. *vastus*	-	X
Mammalia	Chiroptera	Phyllostomidae	*Micronycteris* sp.	X	X

SB-0152					
TÁXONS				**Seca**	**Úmida**
Annelida	Haplotaxida		Haplotaxida sp.1	-	X
Arachnida	Acari	Argasidae	*Ornithodoros* sp.1	X	X
		Trombiculidae	Trombiculidae sp.1	-	X
			Trombiculidae sp.2	X	X
			Acariformes sp.4	X	X
			Astigmata sp.4	X	-
			Holothyrida sp.1	-	X
			Holothyrida sp.2	X	-
			Holothyrida sp.4	X	X
			Holothyrida sp.5	X	-
			Mesostigmata sp.1	X	X
			Oribatida sp.1	-	X
			Oribatida sp.2	-	X
	Amblypygi	Phrynidae	*Heterophrinus longicornis*	X	X
	Araneae	Amaurobiidae	Amaurobiidae jovem	X	X
		Araneidae	*Alpaida* sp.1	X	-
		Corinnidae	Corinnidae jovem	X	X
			Corinnidae sp.2	-	X
		Ctenidae	Ctenidae jovem	X	-
		Pholcidae	*Mesabolivar aurantiacus*	X	X
			Mesabolivar eberhardi	X	-
			Ninetinae sp.1	X	-
		Salticidae	Salticidae sp.3	X	X
			Salticidae sp.4	X	-
		Scytodidae	*Scytodes* sp.1	-	X
			Scytodidae jovem	X	X
		Theraphosidae	*Guyruita cerrado*	-	X
			Theraphosidae jovem	X	-
			Theraphosidae sp.5	-	X
		Theridiosomatidae	*Plato* sp.1	-	X
	Opiliones	Cosmetidae	Cosmetidae sp.1	X	X
			Roquettea carajas	X	X
		Escadabiidae	Escadabiidae jovem	X	X
			Escadabiidae sp.1	-	X
	Pseudoscorpiones	Chernetidae	*Spelaeochernes* sp.1	X	X
		Chthoniidae	Chthoniidae sp.1	X	X
	Scorpiones	Buthidae	*Ananteris* jovem	-	X
Chilopoda	Lithobiomorpha	Henicopidae	Henicopidae jovem	-	X
	Scolopendromorpha	Cryptopidae	*Cryptops* sp.1	-	X
		Scolopocryptopidae	*Newportia* sp.2	X	-
	Scutigeromorpha	Pselliodidae	*Sphendononema guildingii*	X	-
Diplopoda	Polydesmida	Fuhrmanodesmidae	Fuhrmanodesmidae sp.1	X	X
		Paradoxosomatidae	Paradoxosomatidae sp.2	-	X
		Pyrgodesmidae	Pyrgodesmidae sp.1	-	X
	Spirostreptida		Spirostreptida jovem	X	-
Entognatha	Collembola	Cyphoderidae	Cyphoderidae sp.1	X	-
		Entomobryidae	Entomobryidae sp.4	-	X
			Entomobryidae sp.5	-	X
		Paronellidae	Paronellidae sp.1	X	X
	Diplura	Campodeidae	Campodeidae sp.1	X	X
		Japygidae	Japygidae sp.1	X	-
Insecta	Archaeognatha	Meinertellidae	Meinertellidae jovem	-	X
	Blattodea	Blaberidae	Blaberidae jovem	X	X
		Blattidae	Blattidae jovem	X	X
	Coleoptera	Curculionidae	Scolytinae sp.1	X	-
		Elateridae	Elateridae jovem	-	X
		Staphylinidae	*Coproporus* sp.1	X	X

			Scydmaeninae sp.2	X	-
			Vatesus sp.1	X	-
		Tenebrionidae	Tenebrionidae jovem	-	X
	Diptera	Cecidomyiidae	Cecidomyiidae sp.	X	-
		Ceratopogonidae	Ceratopogonidae jovem	-	X
		Dolichopodidae	Dolichopodidae sp.	-	X
		Drosophilidae	Drosophilidae sp.	X	X
		Muscidae	Muscidae sp.	-	X
		Psychodidae	Phlebotominae sp.	X	X
			Psychodidae sp.	X	-
		Sciaridae	Sciaridae sp.	X	X
	Hemiptera	Cydnidae	Cydnidae sp.1	X	X
			Cydnidae sp.2	X	X
		Dictyopharidae	Dictyopharidae jovem	-	X
		Reduviidae	Reduviinae jovem	X	X
	Hymenoptera	Chalcicoidea	Chalcicoidea sp.1	-	X
		Diapriidae	Diapriidae sp.2	-	X
		Formicidae	*Acromyrmex octopinosus*	X	X
			Carebara sp.10	X	-
			Dolichoderus bispinosus	X	-
			Dolichoderus doloniger	X	-
			Gnamptogenys haenschi	X	X
			Odontomachus bauri	-	X
			Odontomachus meinerti	-	X
			Pachycondyla constricta	X	X
			Pachycondyla impressa	-	X
			Pachycondyla striata	-	X
			Pheidole sp.5	-	X
			Pheidole sp.7	X	-
			Probolomyrmex sp.1	X	-
			Solenopsis invicta	X	-
	Isoptera		Isoptera jovem	-	X
	Lepidoptera	Tineoidea	Tineoidea sp. 2	X	-
			Tineoidea sp. 3	X	-
			Lepidoptera jovem	X	X
	Orthoptera	Phalangopsidae	*Eidmanacris* sp.1	X	-
			Paraclodes sp.1	X	-
			Phalangopsis sp.1	X	X
	Psocoptera		Psocomorpha jovem	-	X
	Thysanura	Nicoletiidae	Atelurinae sp.1	X	-
			Nicoletiinae sp.1	X	-
Malacostraca	Isopoda	Armadillidae	Armadillidae sp.1	X	X
		Philosciidae	Philosciidae sp.1	-	X
			Philosciidae sp.2	X	X
		Platyarthridae	Platyarthridae sp.2	-	X
Gastropoda	Pulmonata	Subulinidae	*Lamellaxis* sp.2	-	X
			Leptinaria sp.1	-	X
			Leptinaria sp.2	X	X
		Systrophiidae	*Happia* sp.1	-	X
Amphibia	Anura	Bufonidae	*Rhinella* sp.	X	X
		Leptodactylidae	*Leptodactylus* cf. *vastus*	X	X
			Anura sp.1	X	X
Mammalia	Chiroptera	Emballonuridae	*Peropteryx kappleri*	-	X
		Furipteridae	*Furipterus horrens*	X	-
		Phyllostomidae	*Carollia perspicillata*	X	X
			Desmodus rotundus	X	X
			Diphylla ecaudata	-	X
			Lonchorhina aurita	X	X
	Rodentia	Cricetidae	*Rhipidomys* sp.	X	-

Reptilia	Squamata		Sauria sp.1	X	X

SB-0153					
TÁXONS				**Seca**	**Úmida**
Arachnida	Acari	Argasidae	*Ornithodoros* sp.1	X	X
		Ixodidae	*Amblyomma* sp.3	X	-
		Trombiculidae	Trombiculidae sp.1	-	X
			Oribatida sp.2	-	X
			Oribatida sp.6	-	X
	Amblypygi	Phrynidae	*Heterophrinus longicornis*	-	X
	Araneae	Corinnidae	Corinnidae jovem	-	X
		Ctenidae	Ctenidae jovem	-	X
			Enoploctenus sp.	-	X
		Ochyroceratidae	Ochyroceratidae jovem	-	X
		Oonopidae	Oonopidae sp.13	-	X
		Oxyopidae	Oxyopidae jovem	X	-
		Pholcidae	Pholcidae jovem	X	-
		Salticidae	Salticidae jovem	-	X
		Scytodidae	*Scytodes* sp.1	-	X
			Scytodidae jovem	X	-
		Theridiosomatidae	Theridiosomatidae jovem	X	-
	Opiliones	Cosmetidae	Cosmetidae sp.3	X	-
			Roquettea carajas	X	-
		Sclerosomatidae	*Prionostema* sp.1	-	X
	Pseudoscorpiones	Chernetidae	*Spelaeochernes* sp.1	X	-
Diplopoda	Polydesmida	Fuhrmanodesmidae	Fuhrmanodesmidae sp.1	X	-
Entognatha	Collembola	Paronellidae	Paronellidae sp.1	X	-
	Diplura	Campodeidae	Campodeidae sp.1	-	X
Insecta	Diptera	Psychodidae	Phlebotominae sp.	X	X
		Sciaridae	Sciaridae sp.	-	X
	Hemiptera	Cydnidae	Cydnidae jovem	X	-
			Cydnidae sp.1	-	X
	Hymenoptera	Formicidae	*Pachycondyla striata*	-	X
			Pheidole sp.3	-	X
			Pheidole sp.5	X	X
			Strumigenys elongata	-	X
	Isoptera	Termitidae	*Nasutitermes* sp.1	X	-
			Nasutitermes sp.2	X	X
	Orthoptera	Phalangopsidae	*Paraclodes* sp.1	X	-
			Phalangopsis sp.1	X	X
	Psocoptera	Archipsocidae	Archipsocidae sp.1	-	X
Malacostraca	Isopoda	Philosciidae	Philosciidae sp.1	X	-
			Philosciidae sp.2	-	X
Gastropoda	Pulmonata	Subulinidae	*Leptinaria* sp.1	X	-
Mammalia	Chiroptera	Emballonuridae	*Peropteryx* sp.	-	X
Reptilia	Squamata	Colubridae	*Chironius* sp.	X	-

SB-0154					
TÁXONS				Seca	Úmida
Arachnida	Araneae	Ctenidae	Ctenidae jovem	-	X
		Filistatidae	Filistatidae jovem	X	-
		Oonopidae	gr. *Xycarpphy* sp.1	X	-
			Oonopidae sp.13	-	X
		Salticidae	Salticidae sp.2	X	-
		Scytodidae	Scytodidae jovem	X	X
	Pseudoscorpiones	Chernetidae	Chernetidae jovem	X	-
Chilopoda	Scutigeromorpha	Pselliodidae	*Sphendononema guildingii*	X	-
Entognatha	Collembola	Paronellidae	Paronellidae sp.1	X	-
Insecta	Blattodea	Blaberidae	Blaberidae jovem	-	X
	Diptera	Drosophilidae	Drosophilidae sp.	X	-
		Stratiomyidae	Stratiomyidae jovem	-	X
	Hemiptera	Cydnidae	Cydnidae sp.1	X	-
		Lygaeidae	Lygaeidae jovem	X	-
		Reduviidae	Emesinae jovem	-	X
	Hymenoptera	Formicidae	*Crematogaster brasiliensis*	X	-
			Pachycondyla constricta	X	-
	Lepidoptera	Noctuoidea	Noctouidea sp. 1	-	X
			Lepidoptera jovem	X	-
	Neuroptera	Myrmeleontidae	Myrmeleontidae sp.1	-	X
			Myrmeleontidae sp.2	X	-
	Orthoptera	Phalangopsidae	*Paraclodes* sp.1	X	-
	Psocoptera	Archipsocidae	Archipsocidae sp.2	X	-
Mammalia	Chiroptera	Emballonuridae	*Peropteryx* sp.	-	X

SB-0155					
TÁXONS				Seca	Úmida
Arachnida	Acari	Argasidae	*Ornithodoros* sp.1	X	X
		Trombiculidae	Trombiculidae sp.1	-	X
			Trombiculidae sp.2	X	-
			Mesostigmata sp.1	X	-
	Amblypygi	Phrynidae	*Heterophrinus longicornis*	X	-
	Araneae	Araneidae	*Alpaida* sp.1	X	-
		Ctenidae	Ctenidae jovem	-	X
		Ochyroceratidae	*Speocera* sp.1	X	X
		Pholcidae	*Mesabolivar eberhardi*	-	X
			Pholcidae jovem	X	-
		Salticidae	Salticidae jovem	X	-
		Theridiosomatidae	*Plato* sp.1	X	X
	Opiliones	Escadabiidae	Escadabiidae jovem	X	X
	Pseudoscorpiones	Chernetidae	*Spelaeochernes* sp.1	X	-
		Chthoniidae	Chthoniidae jovem	X	-
Chilopoda	Scutigeromorpha	Scolopocryptopidae	*Newportia* sp.3	-	X
Diplopoda	Polydesmida	Pyrgodesmidae	Pyrgodesmidae sp.1	-	X
Entognatha	Collembola	Cyphoderidae	Cyphoderidae sp.1	X	X
	Diplura	Japygidae	Japygidae sp.1	X	-
Insecta	Coleoptera	Carabidae	Carabidae sp.1	X	-
		Staphylinidae	Pselaphinae sp.4	X	-
			Staphylininae sp.2	X	-
	Diptera	Phoridae	Phoridae sp.	X	-
		Psychodidae	Phlebotominae sp.	-	X
		Sciaridae	Sciaridae sp.	X	-
	Hemiptera	Cercopidae	Cercopidae jovem	-	X
		Cixiidae	Cixiidae jovem	X	X
		Dipsocoroidea	Dipsocoroidea jovem	X	-
			Heteroptera jovem	-	X
	Hymenoptera	Formicidae	*Odontomachus meinerti*	X	X
			Pheidole sp.7	X	-
	Orthoptera	Phalangopsidae	*Paraclodes* sp.1	X	-
			Phalangopsis sp.1	X	X
	Thysanura	Nicoletiidae	Nicoletiinae sp.1	X	-
Malacostraca	Isopoda	Philosciidae	Philosciidae sp.1	-	X
		Platyarthridae	Platyarthridae sp.2	X	-
Amphibia	Anura	Strabomantidae	*Pristimantis* cf. *fenestratus*	X	-

SB-0156					
TÁXONS				Seca	Úmida
Arachnida	Acari	Trombiculidae	Trombiculidae sp.1	-	X
			Acariformes sp.1	-	X
			Acariformes sp.4	X	-
	Araneae	Araneidae	*Alpaida* sp.1	X	X
		Ctenidae	Ctenidae jovem	X	X
		Pholcidae	*Leptopholcus* sp.1	X	X
			Mesabolivar aurantiacus	X	-
		Salticidae	Salticidae sp.1	X	-
		Theridiosomatidae	*Plato* sp.1	-	X
			Theridiosomatidae jovem	X	-
	Opiliones	Cosmetidae	*Roquettea carajas*	X	X
		Escadabiidae	Escadabiidae jovem	-	X
		Stygnidae	*Protimesus* sp.1	X	-
	Pseudoscorpiones	Chernetidae	*Spelaeochernes* sp.1	X	-
		Chthoniidae	Chthoniidae sp.1	-	X
Chilopoda	Scutigeromorpha	Pselliodidae	*Sphendononema guildingii*	X	-
Diplopoda	Polydesmida	Fuhrmanodesmidae	Fuhrmanodesmidae sp.1	X	-
Entognatha	Collembola	Paronellidae	Paronellidae sp.1	-	X
Insecta	Blattodea	Blaberidae	Blaberidae jovem	X	X
		Blattidae	Blattidae sp.3	X	X
	Coleoptera	Histeridae	Histeridae sp.3	X	-
	Diptera	Cecidomyiidae	Cecidomyiidae sp.	X	-
		Dolichopodidae	Dolichopodidae sp.	X	-
		Drosophilidae	Drosophilidae sp.	-	X
		Phoridae	Phoridae sp.	-	X
		Psychodidae	Phlebotominae sp.	-	X
		Sciaridae	Sciaridae sp.	X	-
		Tipulidae	Tipulidae sp.	X	X
	Hemiptera	Cydnidae	Cydnidae sp.1	X	X
	Hymenoptera	Formicidae	*Pachycondyla constricta*	X	-
			Pachycondyla striata	-	X
			Pheidole sp.7	X	-
		Mymaridae	Mymaridae sp.1	X	-
	Isoptera	Termitidae	*Nasutitermes* sp.3	X	-
	Lepidoptera	Tineoidea	Tineoidea sp. 6	X	X
	Orthoptera	Phalangopsidae	*Paraclodes* sp.1	X	-
			Phalangopsis sp.1	-	X
	Psocoptera		Psocomorpha jovem	-	X
	Thysanura	Nicoletiidae	Atelurinae sp.1	X	X
			Nicoletiinae sp.1	X	X
Malacostraca	Isopoda	Philosciidae	Philosciidae sp.1	-	X
			Philosciidae sp.2	X	-
Amphibia	Anura		Anura sp.3	X	-
Mammalia	Chiroptera	Phyllostomidae	*Carollia perspicillata*	-	X
			Desmodus rotundus	-	X
			Lonchorhina aurita	-	X
			Phyllostomus latifolius	-	X

SB-0157					
TÁXONS				Seca	Úmida
Arachnida	Acari		Acari jovem	X	-
			Holothyrida sp.7	-	X
	Araneae	Ctenidae	Ctenidae jovem	-	X
		Oonopidae	Oonopidae sp.13	-	X
		Pholcidae	*Mesabolivar eberhardi*	X	-
			Pholcidae jovem	-	X
		Salticidae	Salticidae jovem	X	-
		Theridiidae	Theridiidae jovem	-	X
	Opiliones	Sclerosomatidae	*Prionostema* sp.1	-	X
Chilopoda	Scolopendromorpha	Cryptopidae	*Cryptops* sp.3	-	X
Entognatha	Collembola	Paronellidae	Paronellidae sp.1	X	-
	Diplura	Campodeidae	Campodeidae sp.1	X	-
Insecta	Coleoptera		Coleoptera jovem	X	-
	Diptera	Ceratopogonidae	Ceratopogonidae jovem	-	X
		Psychodidae	Phlebotominae sp.	-	X
	Hemiptera	Reduviidae	Reduviinae jovem	-	X
	Hymenoptera	Formicidae	*Pachycondyla constricta*	X	X
	Lepidoptera	Tineoidea	Tineoidea sp. 6	X	-
	Orthoptera	Phalangopsidae	*Paraclodes* sp.1	X	-
	Psocoptera		Psocomorpha jovem	-	X
	Thysanura	Nicoletiidae	Nicoletiinae sp.1	-	X
Malacostraca	Isopoda	Philosciidae	Philosciidae sp.1	-	X
Mammalia	Chiroptera	Emballonuridae	*Peropteryx kappleri*	X	-

SB-0159					
TÁXONS				Seca	Úmida
Arachnida	Acari		Mesostigmata sp.1	-	X
	Amblypygi	Phrynidae	*Heterophrinus longicornis*	X	-
	Araneae	Ctenidae	Ctenidae jovem	X	X
		Pisauridae	Pisauridae jovem	X	-
		Salticidae	Salticidae sp.17	X	-
		Theraphosidae	Theraphosidae jovem	X	-
		Theridiosomatidae	*Plato* sp.1	X	-
	Opiliones	Cosmetidae	Cosmetidae sp.1	X	-
			Roquettea carajas	X	-
		Sclerosomatidae	*Prionostema* sp.1	-	X
Chilopoda	Scolopendromorpha	Scolopendridae	*Otostigmus* sp.1	X	-
Diplopoda	Polydesmida	Fuhrmanodesmidae	Fuhrmanodesmidae jovem	X	-
		Pyrgodesmidae	Pyrgodesmidae sp.1	-	X
Entognatha	Collembola	Paronellidae	Paronellidae sp.1	X	X
		Sminthuroidea	Sminthuroidea sp.2	X	X
	Diplura	Campodeidae	Campodeidae sp.1	-	X
Insecta	Coleoptera	Hydrophilidae	Hydrophilidae sp.7	X	-
	Diptera	Ceratopogonidae	Ceratopogonidae jovem	-	X
		Drosophilidae	Drosophilidae sp.	-	X
		Phoridae	Phoridae sp.	-	X
		Psychodidae	Phlebotominae sp.	-	X
		Tipulidae	Tipulidae sp.	X	-
	Hemiptera	Cydnidae	Cydnidae jovem	-	X
			Cydnidae sp.1	X	-
		Veliidae	Veliidae jovem	X	X
	Hymenoptera	Diapriidae	Diapriidae sp.9	X	-
		Formicidae	*Pachycondyla constricta*	X	-
			Pyramica sp.2	X	-
			Solenopsis invicta	X	X
	Orthoptera	Phalangopsidae	*Paraclodes* sp.1	X	-
			Phalangopsis sp.1	X	X
Malacostraca	Decapoda	Pseudothelphusidae	Pseudothelphusidae sp.1	X	X
	Isopoda	Dubioniscidae	Dubioniscidae sp.1	X	-
Amphibia	Anura	Strabomantidae	*Pristimantis* cf. *fenestratus*	X	-
Mammalia	Chiroptera	Furipteridae	*Furipterus horrens*	-	X
		Phyllostomidae	*Carollia perspicillata*	X	X
			Micronycteris sp.	-	X

SB-0160					
TÁXONS				Seca	Úmida
Arachnida	Acari	Ixodidae	*Amblyomma* sp.1	X	-
		Opilioacaridae	Opilioacaridae sp.1	-	X
			Acariformes sp.3	-	X
			Mesostigmata sp.4	X	-
	Araneae	Corinnidae	Corinnidae jovem	X	X
		Ctenidae	Ctenidae jovem	-	X
			Enoploctenus sp.	-	X
		Ochyroceratidae	Ochyroceratidae jovem	-	X
		Pholcidae	*Leptopholcus* sp.1	-	X
		Prodidomidae	Prodidomidae jovem	-	X
		Salticidae	Salticidae sp.2	X	-
		Scytodidae	*Scytodes* sp.2	-	X
		Segestriidae	Segestriidae jovem	-	X
		Theraphosidae	Theraphosidae jovem	-	X
		Theridiidae	*Achaearanea* sp.1	-	X
		Theridiosomatidae	*Plato* sp.1	-	X
	Pseudoscorpiones	Chernetidae	*Spelaeochernes* sp.1	X	X
	Solifugae	Mummuciidae	*Mummucia* sp.1	X	-
Insecta	Blattodea	Blaberidae	Blaberidae jovem	X	X
	Coleoptera	Elateridae	Elateridae jovem	-	X
		Tenebrionidae	Tenebrionidae jovem	X	X
	Diptera	Psychodidae	Phlebotominae sp.	X	X
	Hemiptera	Cydnidae	Cydnidae sp.1	X	-
		Reduviidae	Reduviinae jovem	X	X
	Hymenoptera	Apidae	*Trigona truculenta*	X	-
		Formicidae	*Cyphomyrmex peltatus*	X	-
			Pachycondyla constricta	-	X
			Pheidole sp.5	-	X
	Isoptera	Rhinotermitidae	*Heterotermes* sp.1	-	X
	Lepidoptera	Noctuoidea	Noctouidea sp. 10	-	X
		Tineoidea	Tineoidea sp. 4	X	-
			Tineoidea sp. 7	-	X
	Neuroptera	Myrmeleontidae	Myrmeleontidae sp.2	X	-
	Orthoptera	Phalangopsidae	*Paraclodes* sp.1	X	X
			Phalangopsis sp.1	X	X
Malacostraca	Isopoda	Armadillidae	Armadillidae sp.1	X	X
Amphibia	Anura	Leptodactylidae	*Leptodactylus* cf. *vastus*	-	X
		Strabomantidae	*Pristimantis* cf. *fenestratus*	X	X
Mammalia	Chiroptera	Phyllostomidae	*Micronycteris megalotis*	X	X

SB-0161					
TÁXONS				Seca	Úmida
Arachnida	Acari	Ixodidae	*Amblyomma* sp.1	X	-
			Amblyomma sp.4	-	X
		Trombiculidae	Trombiculidae sp.1	X	-
			Astigmata sp.4	X	-
			Holothyrida sp.1	-	X
			Mesostigmata sp.2	X	-
			Oribatida sp.1	X	-
	Amblypygi	Phrynidae	*Heterophrinus longicornis*	X	-
	Araneae	Araneidae	*Alpaida* sp.1	X	-
		Corinnidae	*Abapeba sp.1*	X	-
		Ctenidae	Ctenidae jovem	X	X
		Ochyroceratidae	Ochyroceratidae sp.1	X	X
			Speocera sp.1	X	-
		Oonopidae	Oonopidae jovem	-	X
			Oonopidae sp.4	X	-
			Oonopidae sp.7	X	-
		Pholcidae	*Mesabolivar cambridgei*	X	-
			Mesabolivar eberhardi	X	-
			Pholcidae jovem	X	X
		Salticidae	Salticidae sp.1	X	X
			Salticidae sp.14	X	-
			Salticidae sp.3	X	-
		Segestriidae	Segestriidae jovem	-	X
		Theraphosidae	Theraphosidae jovem	-	X
		Theridiidae	Theridiidae jovem	-	X
		Theridiosomatidae	*Plato* sp.1	X	X
	Opiliones	Escadabiidae	Escadabiidae jovem	-	X
	Pseudoscorpiones	Chernetidae	*Spelaeochernes* sp.1	X	X
		Olpiidae	Olpiidae sp.1	X	-
Chilopoda	Scolopendromorpha	Scolopocryptopidae	*Newportia* jovem	-	X
			Newportia sp.2	X	-
Diplopoda	Glomeridesmida	Glomeridesmidae	Glomeridesmida sp.1	X	X
	Polydesmida	Chelodesmidae	Chelodesmidae sp.1	X	X
		Paradoxosomatidae	Paradoxosomatidae sp.1	-	X
	Spirostreptida	Pseudonannolenidae	Pseudonannolenidae sp.1	X	-
	Stemmiulida	Stemmiulidae	Stemmiulidae sp.1	-	X
Entognatha	Collembola	Cyphoderidae	Cyphoderidae sp.1	X	-
		Paronellidae	Paronellidae sp.1	X	X
			Paronellidae sp.3	X	X
		Sminthuroidea	Sminthuroidea sp.2	X	X
	Diplura	Japygidae	Japygidae sp.1	-	X
Insecta	Archaeognatha	Meinertellidae	Meinertellidae sp.1	-	X
	Coleoptera	Curculionidae	Curculionidae sp.2	X	-
		Dytiscidae	Dytiscidae sp.1	X	-
		Tenebrionidae	Tenebrionidae jovem	X	-
	Diptera	Ceratopogonidae	Ceratopogonidae jovem	-	X
		Psychodidae	Phlebotominae sp.	X	X
		Sciaridae	Sciaridae jovem	X	-
	Hemiptera	Cydnidae	Cydnidae sp.1	X	X
		Reduviidae	Reduviinae jovem	X	-
		Veliidae	*Paravelia* sp.1	X	-
	Hymenoptera	Formicidae	*Acromyrmex octopinosus*	-	X
			Camponotus atriceps	-	X
			Camponotus sp.2	X	-
			Carebara sp.10	X	-
			Cyphomyrmex peltatus	X	-
			Neivamyrmex sp.2	-	X

			Pachycondyla constricta	X	X
			Pheidole sp.5	X	-
			Wasmannia auropunctata	X	X
		Mymaridae	Mymaridae sp.2	-	X
	Isoptera	Termitidae	Nasutitermes sp.1	X	X
			Nasutitermes sp.2	X	-
			Nasutitermes sp.3	X	-
	Orthoptera	Phalangopsidae	*Paraclodes* sp.1	X	-
			Phalangopsis sp.1	X	X
		Tettigoniidae	Listroscelidinae sp.1	-	X
	Psocoptera	Archipsocidae	Archipsocidae sp.2	-	X
		Troctopsocidae	Troctopsocidae sp.1	X	-
			Psocomorpha jovem	X	-
Malacostraca	Isopoda	Armadillidae	Armadillidae sp.1	X	X
		Philosciidae	Philosciidae sp.1	X	X
Amphibia	Anura	Strabomantidae	*Pristimantis* cf. *fenestratus*	X	-
Mammalia	Chiroptera	Emballonuridae	*Peropteryx kappleri*	X	X
		Phyllostomidae	*Carollia perspicillata*	X	X
			Glossophaga soricina	X	-
	Rodentia	Cricetidae	*Rhipidomys* sp.	X	-

SB-0162					
TÁXONS				**Seca**	**Úmida**
Arachnida	Araneae	Pholcidae	Pholcidae jovem	X	-
		Salticidae	Salticidae jovem	X	X
			Salticidae sp.4	-	X
		Tetragnathidae	Tetragnathidae jovem	-	X
		Theridiosomatidae	Theridiosomatidae jovem	-	X
	Opiliones	Cosmetidae	*Roquettea carajas*	X	-
		Manaosbiidae	Manaosbiidae sp.1	X	-
	Pseudoscorpiones	Chernetidae	*Spelaeochernes* sp.1	-	X
Entognatha	Collembola	Paronellidae	Paronellidae sp.1	-	X
	Diplura	Campodeidae	Campodeidae sp.1	-	X
Insecta	Hymenoptera	Formicidae	*Camponotus melanoticus*	-	X
	Isoptera	Termitidae	*Nasutitermes* sp.1	-	X
	Lepidoptera	Tineoidea	Tineoidea sp. 3	-	X
			Lepidoptera jovem	X	-
	Orthoptera	Phalangopsidae	*Paraclodes* sp.1	X	-
			Phalangopsis sp.1	-	X
	Psocoptera	Archipsocidae	Archipsocidae sp.2	-	X
		Liposcelidae	Liposcelidae sp.2	X	-
Malacostraca	Isopoda	Platyarthridae	Platyarthridae sp.3	-	X
Amphibia	Anura	Strabomantidae	*Pristimantis* cf. *fenestratus*	X	X
