# Supplementary material for: Reconciling Mining with the Conservation of Cave Biodiversity: A Quantitative Baseline to Help Establish Conservation Priorities
Source: PLoS One. 2016 Dec 20;11(12):e0168348. doi: 10.1371/journal.pone.0168348 (PMC5173368; doi:10.1371/journal.pone.0168348)
Supplement: S1 Dataset — (ZIP) [file pone.0168348.s002.zip › Taxa/Serra da Bocaina/Taxons inventariados_ Parte 2.pdf]

SB-0110					
**TÁXONS**				**Seco**	**Úmido**
Arachnida	Araneae	Corinnidae	Corinnidae Jovem	X	-
		Ctenidae	Ctenidae Jovem	X	X
		Drymusidae	Drymusidae Jovem	X	-
		Nemesiidae	Nemesiidae Jovem	-	X
		Oonopidae	Oonopidae sp.13	-	X
		Pholcidae	Pholcidae Jovem	-	X
			Mesabolivar aurantiacus	X	-
		Salticidae	Salticidae sp.8	X	-
		Scytodidae	Scytodidae Jovem	X	X
			Scytodes eleonorae	-	X
		Tetragnathidae	Tetragnathidae Jovem	X	-
		Theraphosidae	Theraphosidae Jovem	X	X
		Theridiidae	Theridiidae Jovem	X	X
			Achaearanea sp.1	X	-
	Opiliones	Sclerosomatidae	Jovem	X	X
		Cosmetidae	sp.1	X	-
Acari			Acari Jovem	-	X
	Mesostigmata		Mesostigmata sp.2	X	-
	Trombidiforme	Trombidiidae	Trombidiidae sp.1	X	-
Entognatha	Collembola	Entomobryidae	Entomobryidae sp.5	-	X
		Paronellidae	Paronellidae sp.1	X	X
Insecta	Diptera	Psychodidae	Phlebotominae sp.1	X	X
		Tipulidae	Tipulidae sp.1	X	-

SB-0111					
TÁXONS				**Seco**	**Úmido**
Arachnida	Amblypygi	Phrynidae	*Heterophrynus longicornis*	X	-
	Araneae	Araneidae	Araneidae Jovem	X	-
		Ctenidae	Ctenidae Jovem	-	X
		Oonopidae	Oonopidae Jovem	-	X
		Pholcidae	Pholcidae Jovem	X	X
		Salticidae	Salticidae Jovem	X	-
			Salticidae sp.3	-	X
		Scytodidae	Scytodidae Jovem	X	X
			Scytodes eleonorae	X	X
		Theridiidae	Theridiidae Jovem	X	X
			Nesticodes rufipes	X	X
	Opiliones	Cosmetidae	Cosmetidae Jovem	-	X
			Roquettea carajas	-	X
Acari			Acari Jovem	X	-
	Mesostigmata		Mesostigmata sp.3	X	-
	Sarcoptiforme	Oribatida	*Oribatida* sp.1	X	X
Entognatha	Collembola	Paronellidae	Paronellidae sp.1	-	X
Insecta	Diptera	Psychodidae	Phlebotominae sp.1	X	X
		Tipulidae	Tipulidae sp.1	X	-
	Lepidoptera	Noctuoidea	Noctuoidea sp.1	X	-
			Noctuoidea sp.8	-	X

SB-0112				
TÁXONS				**Seco**
Arachnida	Araneae	Corinnidae	Corinnidae Jovem	X
		Drymusidae	Drymusidae Jovem	X
		Ochyroceratidae	Ochyroceratidae Jovem	X
		Pholcidae	Pholcidae Jovem	X
			Mesabolivar aurantiacus	X
		Segestriidae	*Ariadna boliviana*	X
		Tetrablemmidae	Tetrablemmidae sp.1	X
		Theraphosidae	Theraphosidae Jovem	X
		Theridiidae	Theridiidae sp.2	X
			Theridion sp.2	X
	Opiliones	Cosmetidae	*Roquettea carajas*	X
	Pseudoscorpiones	Chernetidae	*Spelaeochernes* sp.1	X
		Chthoniidae	*Pseudochthonius* sp.2	X
	Scorpiones	Buthidae	*Ananteris luciae*	X
Acari	Sarcoptiforme	Oribatida	Oribatida sp.1	X
Diplopoda	Polydesmida	Fuhrmannodesmidae	Fuhrmannodesmidae Jovem	X
Entognatha	Collembola	Paronellidae	Paronellidae sp.1	X
	Diplura	Campodeidae	Campodeidae sp.1	X
Insecta	Coleoptera	Elateridae	Elateridae Jovem	X
		Tenebrionidae	Tenebrionidae Jovem	X
	Diptera	Drosophilidae	Drosophilidae sp.4	X
		Sciaridae	Sciaridae sp.1	X
			Sciaridae sp.2	X
		Psychodidae	Phlebotominae sp.1	X
		Tipulidae	Tipulidae sp.1	X
	Hemiptera	Reduviidae	*Zelurus* Jovem	X
			Reduviidae Jovem	X
	Homoptera	Cixiidae	Cixiidae Jovem	X
	Hymenoptera	Bethylidae	Bethylidae sp.3	X
		Figitidae	Figitidae sp.3	X
		Formicidae	*Apterostigma pilosum*	X
			Camponotus cingulatus	X
			Cyphomyrmex rimosus	X
			Pachycondyla constricta	X
			Pheidole sp.4	X
			Rogeria belti	X
		Scelionidae	Scelionidae sp.1	X
	Isoptera	Termitidae	*Nasutitermes* sp.2	X
	Orthoptera	Phalangopsidae	*Paraclodes* sp.1	X
			Phalangopsis sp.1	X
	Zygentoma	Nicoletiidae	Nicoletiinae sp.1	X
Malacostraca	Isopoda	Philosciidae	Philosciidae Jovem	X
			Philosciidae sp.2	X
Gastropoda	Pulmonata	Subulinidae	Subulinidae Jovem	X

SB-0113					
TÁXONS				**Seco**	**Úmido**
Arachnida	Amblypygi	Phrynidae	*Heterophrynus longicornis*	X	-
	Araneae	Araneidae	Araneidae Jovem	-	X
			Alpaida antonio	X	-
		Drymusidae	*Drymusa spelunca*	X	X
		Ochyroceratidae	Ochyroceratidae Jovem	-	X
		Pholcidae	Pholcidae Jovem	X	-
			Mesabolivar aurantiacus	X	-
		Prodidomidae	Prodidomidae Jovem	-	X
		Theridiidae	Theridiidae Jovem	X	X
	Opiliones	Sclerosomatidae	*Prionostema* sp.1	-	X
		Cosmetidae	Cosmetidae Jovem	X	-
			Roquettea carajas	X	-
		Stygnidae	*Protimesius laevis*	X	-
Acari			Acari Jovem	X	-
	Mesostigmata		Mesostigmata sp.3	-	X
	Trombidiforme	Trombiculidae	Trombiculidae sp.1	-	X
			Trombiculidae sp.2	-	X
Entognatha	Collembola	Paronellidae	Paronellidae sp.1	-	X
Insecta	Diptera	Cecidomyiidae	Cecidomyiidae sp.1	X	-
		Ceratopogonidae	Ceratopogonidae sp.1	X	-
		Psychodidae	Phlebotominae sp.1	-	X
		Tipulidae	Tipulidae sp.1	X	-
	Lepidoptera	Tineoidea	Tineoidea sp.7	X	-

SB-0114					
TÁXONS				Seco	Úmida
Clitellata	Haplotaxida		Haplotaxida sp.8	X	-
Arachnida	Araneae	Araneidae	*Alpaida antonio*	X	-
		Corinnidae	Corinnidae Jovem	X	-
			Abapeba hoeferi	X	-
		Ctenidae	Ctenidae Jovem	X	-
			Ctenus sp.1	X	-
		Drymusidae	Drymusidae Jovem	X	-
		Nemesiidae	Nemesiidae Jovem	X	-
		Ochyroceratidae	Ochyroceratidae Jovem	X	-
		Oonopidae	Oonopidae Jovem	X	-
			Oonopidae sp.	X	-
			Oonopidae sp.2	X	-
			gr. *Xycarphhy* sp.1	X	-
		Pholcidae	Pholcidae Jovem	X	-
			Mesabolivar aurantiacus	X	-
		Salticidae	Salticidae Jovem	X	-
		Scytodidae	Scytodidae Jovem	X	-
			Scytodes eleonorae	X	-
		Theridiidae	*Theridion* sp.2	X	-
		Theridiosomatidae	Theridiosomatidae Jovem	X	-
			Plato sp.1	X	-
	Opiliones	Cosmetidae	Cosmetidae sp.1	X	-
			Roquettea carajas	X	X
		Escadabiidae	Escadabiidae Jovem	X	-
			Escadabiidae sp.1	X	-
		Sclerosomatidae	Sclerosomatidae Jovem	X	-
		Stygnidae	Stygnidae Jovem	X	-
			Protimesius laevis	X	-
		Sclerosomatidae	*Prionostema* sp.1	-	X
		Escadabiidae	Escadabiidae sp.2	-	X
	Pseudoscorpiones	Chernetidae	Chernetidae Jovem	X	-
			Spelaeochernes sp.1	X	-
		Chthoniidae	Chthoniidae Jovem	X	-
			Pseudochthonius sp.2	X	-
			Pseudochthonius sp.4	X	-
	Schizomida	Hubardiidae	Hubardiidae Jovem	X	-
			Rowlandius sp.1	X	-
	Scorpiones	Buthidae	*Ananteris* Jovem	X	-
Acari	Astigmata		Astigmata sp.1	X	X
	Ixodida	Argasidae	Ornithodoros sp.1	X	X
		Ixodidae	*Amblyomma goeldii*	X	-
			Amblyomma rotundatum	-	X
	Mesostigmata		Mesostigmata sp.1	X	X
			Mesostigmata sp.3	X	X
		Uropodoidea	Uropodoidea sp.1	X	-
	Sarcoptiforme	Oribatida	Oribatida sp.1	X	X
			Oribatida sp.3	-	X
	Trombidiforme	Trombiculidae	Trombiculidae sp.1	X	X
			Trombiculidae sp.2	-	X
		Trombidiidae	Trombidiidae sp.1	X	X
Chilopoda	Geophilomorpha		Geophilomorpha sp.1	X	-
	Scolopendromorpha	Cryptopidae	*Cryptops* sp.1	X	-
Diplopoda	Glomeridesmida	Glomeridesmidae	Glomeridesmidae Jovem	X	-
			Glomeridesmidae sp.1	X	-
	Polydesmida	Fuhrmannodesmidae	Fuhrmannodesmidae Jovem	X	-
			Fuhrmannodesmidae sp.1	X	-
		Pyrgodesmidae	Pyrgodesmidae sp.1	X	-

			Pyrgodesmidae Jovem	X	-
	Spirostreptida	Pseudonannolenidae	*Pseudonannolene* sp.1	X	-
Entognatha	Collembola	Isotomidae	Isotomidae sp.1	X	-
		Paronellidae	Paronellidae sp.1	X	-
			Cyphoderus javanus	X	-
		Symphypleona	Symphypleona sp.2	X	-
	Diplura	Campodeidae	Campodeidae sp.1	X	-
		Projapygidae	Projapygidae sp.1	X	-
Insecta	Blattaria	Blattidae	Blattidae Jovem	X	-
	Coleoptera	Chrysomelidae	Chrysomelidae sp.4	X	-
			Chrysomelidae sp.5	X	-
		Elateridae	Elateridae Jovem	X	-
		Hydrophilidae	Hydrophilidae sp.2	X	-
		Scarabaeidae	Scarabaeidae sp.2	X	-
		Scydmaenidae	Scydmaeninae sp.3	X	-
			Scydmaeninae sp.4	X	-
			Scydmaeninae sp.7	X	-
		Staphylinidae	Staphylinidae sp.10	X	-
			Staphylinidae sp.11	X	-
			Staphylinidae sp.17	X	-
			Staphylinidae sp.19	X	-
			Staphylinidae sp.2	X	-
			Pselaphinae sp.7	X	-
		Tenebrionidae	Tenebrionidae Jovem	X	-
	Diptera	Phoridae	Phoridae sp.1	X	-
		Stratiomyidae	Stratiomyidae sp.1	X	-
		Cecidomyiidae	Cecidomyiidae sp.1	X	-
		Keroplatidae	Keroplatidae Jovem	X	-
		Sciaridae	Sciaridae Jovem	X	-
			Sciaridae sp.1	X	-
			Sciaridae sp.2	X	-
		Psychodidae	*Phlebotominae* sp.1	X	-
	Hemiptera	Cydnidae	*Pangaeus* sp.1	X	-
		Nabidae	Nabidae Jovem	X	-
		Reduviidae	Emesinae Jovem	X	-
			Zelurus Jovem	X	-
			Panstrongylus geniculatus	X	-
			Triatoma Jovem	X	-
	Homoptera	Cixiidae	Cixiidae Jovem	X	-
	Hymenoptera	Bethylidae	Bethylidae sp.5	X	-
		Formicidae	*Carebara* pr. *urichi*	X	-
			Cerapachys sp.1	X	-
			Gnamptogenys striatula	X	-
			Hypoponera pr. *opacior*	X	-
			Pachycondyla constricta	X	-
			Pachycondyla harpax	X	-
			Pheidole sp.3	X	-
			Pheidole sp.4	X	-
			Rogeria belti	X	-
			Solenopsis invicta	X	-
			Strumigenys calamita	X	-
			Strumigenys elongata	X	-
	Isoptera	Termitidae	Termitidae sp.	X	-
			Cornitermes sp.1	X	-
			Nasutitermes sp.2	X	-
			Neocapritermes sp.1	X	-
	Lepidoptera		Lepidoptera Jovem	X	-
		Noctuoidea	Noctuoidea sp.1	X	-
	Neuroptera	Mantispidae	*Plega* sp.1	X	-

	Orthoptera	Phalangopsidae	Phalangopsidae sp.1	X	-
			Paraclodes sp.1	X	-
			Phalangopsis sp.1	X	-
	Psocoptera	Psocomorpha	Psocomorpha Jovem	X	-
	Zygentoma	Nicoletiidae	*Atelurinae* sp.1	X	-
			Nicoletiina e sp.1	X	-
Malacostraca	Isopoda	Armadillidae	Armadillidae Jovem	X	X
			Armadillidae sp.1	X	X
		Dubioniscidae	Dubioniscidae sp.1	-	X
		Philosciidae	Philosciidae Jovem	X	-
			Philosciidae sp.1	X	X
			Philosciidae sp.2	X	X
		Platyarthridae	Platyarthridae sp.5	X	-
		Scleropactidae	Scleropactidae sp.1	X	-
Symphyla		Scolopendrellidae	*Scolopendrellopsis* sp.1	X	-
		Scutigerellidae	*Hanseniella* sp.1	X	-
Gastropoda	Pulmonata	Subulinidae	Subulinidae Jovem	X	-
			Lamellaxis sp.1	X	-
			Leptinaria sp.1	X	-
		Systrophiidae	Systrophiidae Jovem	X	-
			Happia sp.1	X	-
			Happia sp.2	X	-
Turbellaria	Tricladida	Contineticola	Contineticola sp.2	X	-

SB-0118					
TÁXONS				**Seco**	**Úmido**
Arachnida	Araneae	Ochyroceratidae	Ochyroceratidae Jovem	X	-
		Pholcidae	Pholcidae Jovem	X	-
		Pisauridae	Pisauridae Jovem	X	-
		Theridiidae	*Achaearanea* sp.1	X	-
			Dipoena sp.1	X	-
		Theridiosomatidae	Theridiosomatidae Jovem	X	-
	Pseudoscorpiones	Chernetidae	*Spelaeochernes* sp.1	X	-
Acari	Trombidiforme	Trombidiidae	Trombidiidae sp.1	X	-
Entognatha	Collembola	Paronellidae	Paronellidae sp.2	X	-
			Cyphoderus javanus	X	-
Insecta	Blattaria	Blattelidae	Blattelidae Jovem	X	-
	Coleoptera	Staphylinidae	Staphylinidae sp.4	X	-
	Diptera	Tipulidae	Tipulidae sp.1	X	-
	Heteroptera	Mesoveliidae	Madeoveliinae sp.1	X	-
		Reduviidae	Emesinae Jovem	X	-
	Hymenoptera	Formicidae	*Nylanderia* sp.1	X	-
	Isoptera	Termitidae	Termitidae sp.	X	-
	Lepidoptera		Lepidoptera Jovem	X	-
		Noctuoidea	Noctuoidea sp.2		X
	Psocoptera		Psocoptera Jovem	X	-
Malacostraca	Decapoda	Pseudothelphusidae	Pseudothelphusidae sp.1	X	-
Turbellaria	Tricladida	Planariidae	Planariidae sp.1	X	-

SB-0119					
TÁXONS				Seco	Úmido
Arachnida	Araneae	Drymusidae	Drymusidae Jovem	X	-
		Oonopidae	Oonopidae Jovem	X	-
			Oonopidae sp.8	X	-
		Pholcidae	Pholcidae Jovem	X	-
		Prodidomidae	Prodidomidae Jovem	X	-
		Salticidae	Salticidae Jovem	X	-
		Scytodidae	*Scytodes eleonorae*	X	-
		Theridiidae	Theridiidae Jovem	X	-
	Opiliones	Cosmetidae	*Roquettea carajas*	-	X
	Pseudoscorpiones	Chernetidae	*Spelaeochernes* sp.1	X	-
		Olpiidae	Olpiidae Jovem	X	-
Acari	Mesostigmata		Mesostigmata sp.1	-	X
	Trombidiforme	Trombiculidae	Trombiculidae sp.1	-	X
Chilopoda	Scolopendromorpha	Cryptopidae	*Cryptops* sp.1	X	-
Entognatha	Collembola	Entomobryidae	Entomobryidae sp.2	-	X
			Entomobryidae sp.5	X	-
		Paronellidae	Paronellidae sp.1	X	X
			Cyphoderus arlei	X	X
			Cyphoderus javanus	X	-
	Diplura	Campodeidae	Campodeidae sp.1	X	-
Insecta	Blattaria	Polyphagidae	Polyphagidae Jovem	X	-
	Coleoptera		Coleoptera Jovem	X	-
		Curculionidae	Scolytinae sp.1	X	-
		Elateridae	Elateridae Jovem	X	-
		Ptilidae	Ptilidae sp.4	X	-
		Scydmaenidae	Scydmaeninae sp.1	X	-
		Staphylinidae	Staphylinidae sp.3	X	-
		Tenebrionidae	Staphylininae Jovem	X	-
	Diptera	Drosophilidae	Drosophilidae sp.1	X	-
	Heteroptera	Reduviidae	*Zelurus* Jovem	X	-
	Homoptera	Cixiidae	Cixiidae Jovem	X	-
	Hymenoptera	Formicidae	*Crematogaster limata*	X	-
			Crematogaster sp.1	X	-
			Pheidole sp.8	X	-
	Isoptera	Termitidae	*Nasutitermes* sp.1	X	-
			Nasutitermes sp.2	X	-
	Lepidoptera	Noctuoidea	Noctuoidea sp.2	X	-
	Orthoptera	Phalangopsidae	*Paraclodes* sp.1	X	-
			Phalangopsis sp.1	X	-
	Psocoptera	Epipsocidae	Epipsocidae sp.4	X	-
	Zygentoma	Nicoletiidae	Nicoletiinae sp.1	X	-
Malacostraca	Isopoda	Armadillidae	Armadillidae sp.1	X	X
		Philosciidae	Philosciidae sp.2	X	-
		Platyarthridae	Platyarthridae sp.4	-	X
		Scleropactidae	Scleropactidae sp.1	-	X
Symphyla		Scutigerellidae	*Hanseniella* sp.1	X	-

SB-0120					
**TÁXONS**				**Seco**	**Úmido**
Arachnida	Araneae	Corinnidae	Corinnidae Jovem	X	-
		Drymusidae	*Drymusa spelunca*	X	-
		Oonopidae	Oonopidae Jovem	X	-
		Prodidomidae	Prodidomidae Jovem	X	-
		Salticidae	Salticidae Jovem	X	-
		Scytodidae	Scytodidae Jovem	X	-
		Theridiidae	Theridiidae Jovem	X	-
			Achaearanea sp.1	X	-
	Pseudoscorpiones	Chernetidae	*Spelaeochernes* sp.1	X	-
		Chthoniidae	*Pseudochthonius* sp.2	X	-
	Scorpiones	Buthidae	*Ananteris* Jovem	X	-
Acari	Ixodida	Ixodidae	*Amblyomma ajennense*	-	X
	Mesostigmata		Mesostigmata sp.3	-	X
	Sarcoptiforme	Oribatida	Oribatida sp.6	-	X
Chilopoda	Scolopendromorpha	Cryptopidae	*Cryptops* sp.	X	-
		Scolopendridae	*Otostigmu* s sp.2	X	-
Diplopoda	Polydesmida	Chelodesmidae	Chelodesmidae Jovem	X	-
Entognatha	Collembola	Entomobryidae	*Pseudosinella* sp.1	X	-
		Paronellidae	*Paronellidae* sp.1	X	X
	Diplura	Campodeidae	Campodeidae sp.1	X	-
Insecta	Blattaria	Blattidae	Blattidae Jovem	X	-
	Diptera	Keroplatidae	Keroplatidae Jovem	X	-
	Heteroptera	Reduviidae	*Zelurus* Jovem	X	-
		Schizopteridae	Schizopteridae Jovem	X	-
	Hymenoptera	Diapriidae	Diapriidae sp.1	X	-
		Formicidae	*Cerapachys* sp.1	X	-
			Hypoponera sp.8	X	-
			Pachycondyla constricta	X	-
			Pheidole sp.4	X	-
	Isoptera	Termitidae	*Coatitermes* sp.1	X	-
			Nasutitermes sp.2	X	-
	Psocoptera	Psocomorpha	Psocomorpha Jovem	X	-
		Psyllipsocidae	Psyllipsocidae sp.7	X	-
Malacostraca	Isopoda	Armadillidae	Armadillidae Jovem	-	X
			Armadillidae sp.1	X	X
		Philosciidae	Philosciidae Jovem	X	-
			Philosciidae sp.1	X	-
		Platyarthridae	Platyarthridae Jovem	-	X
Symphyla		Scutigerellidae	*Hanseniella* sp.1	X	-

SB-0122					
**TÁXONS**				**Seco**	**Úmido**
Arachnida	Araneae	Drymusidae	*Drymusa spelunca*	X	-
		Pholcidae	Pholcidae Jovem	X	-
			Mesabolivar aurantiacus	X	-
		Scytodidae	Scytodidae Jovem	X	-
		Theraphosidae	Theraphosidae Jovem	X	-
		Uloboridae	Uloboridae sp.1	X	-
	Opiliones	Cosmetidae	Cosmetidae sp.1	X	-
		Cosmetidae	*Roquettea carajas*	-	X
		Escadabiidae	Escadabiidae sp.4	-	X
Acari	Opilioacarida	Opilioacaridae	Opilioacaridae sp.1	-	X
	Trombidiforme	Trombiculidae	Trombiculidae sp.2	X	-
Chilopoda	Scutigeromorpha	Pselliodidae	*Sphendononema* Jovem	X	-
Entognatha	Collembola	Entomobryidae	*Pseudosinella* sp.1	-	X
		Paronellidae	Paronellidae sp.1	X	X
	Diplura	Campodeidae	Campodeidae sp.1	X	-
		Projapygidae	Projapygidae sp.1	X	-
Insecta	Coleoptera		Coleoptera Jovem	X	-
	Diptera	Sciaridae	Sciaridae sp.1	X	-
		Psychodidae	Phlebotominae sp.1	X	-
		Tipulidae	Tipulidae sp.1	X	-
	Heteroptera	Reduviidae	*Zelurus* Jovem	X	-
	Homoptera	Cixiidae	Cixiidae Jovem	X	-
	Hymenoptera	Bethylidae	Bethylidae sp.4	X	-
		Eulophidae	Eulophidae sp.2	X	-
		Formicidae	*Acropyga smithii*	X	-
			Pachycondyla constricta	X	-
			Solenopsis invicta	X	-
	Isoptera	Termitidae	*Cornitermes* sp.1	X	-
			Nasutitermes sp.5	X	-
	Lepidoptera	Geometridae	Geometridae Jovem	X	-
		Noctuoidea	Noctuoidea sp.1	X	-
			Noctuoidea sp.7	X	-
		Tineoidea	Tineoidea sp.8	X	-
	Neuroptera	Mantispidae	*Plega* sp.1	X	-
	Psocoptera	Psocomorpha	Psocomorpha Jovem	X	-
		Psyllipsocidae	Psyllipsocidae sp.7	X	-
Malacostraca	Isopoda	Philosciidae	Philosciidae Jovem	X	-
			Philosciidae sp.2	X	-
		Platyarthridae	Platyarthridae sp.3	-	X

SB-0123					
TÁXONS				Seco	Úmido
Arachnida	Araneae	Araneidae	Araneidae Jovem	X	-
		Corinnidae	Corinnidae Jovem	X	-
		Nemesiidae	Nemesiidae Jovem	X	-
		Oonopidae	Oonopidae Jovem	X	-
		Pholcidae	Pholcidae Jovem	X	-
		Scytodidae	Scytodidae Jovem	X	-
		Tetragnathidae	*Leucauge* sp.1	X	-
		Theridiidae	Theridiidae Jovem	X	-
	Opiliones	Sclerosomatidae	Sclerosomatidae Jovem	X	-
		Sclerosomatidae	*Prionostema* sp.1	-	X
	Pseudoscorpiones	Chernetidae	*Spelaeochernes* sp.1	X	-
		Chthoniidae	*Pseudochthonius* sp.3	X	-
	Schizomida	Hubardiidae	Hubardiidae Jovem	X	-
			Rowlandius sp.1	X	-
Acari	Mesostigmata		Mesostigmata sp.1	X	-
	Trombidiforme	Trombiculidae	Trombiculidae sp.2	-	X
Chilopoda	Scolopendromorpha	Scolopocryptopidae	*Newportia* sp.	X	-
Diplopoda	Polydesmida	Fuhrmannodesmidae	Fuhrmannodesmidae Jovem	X	-
	Stemmiulida	Stemmiulidae	Stemmiulidae Jovem	X	-
Entognatha	Collembola	Entomobryidae	Entomobryidae sp.5	-	X
		Paronellidae	Paronellidae sp.1	X	-
	Diplura	Campodeidae	Campodeidae sp.1	X	-
Insecta	Coleoptera		Coleoptera Jovem	X	-
		Carabidae	*Notiobia* sp.1	X	-
		Curculionidae	Scolytinae sp.	X	-
			Scolytinae sp.1	X	-
	Diptera	Psychodidae	Phlebotominae sp.1	X	-
		Tipulidae	Tipulidae sp.1	X	-
	Heteroptera	Cydnidae	Cydnidae Jovem	X	-
			Pangaeus sp.1	X	-
		Lygaeidae	Lygaeidae sp.2	X	-
		Nabidae	Prostemmatinae sp.1	X	-
	Homoptera	Cixiidae	Cixiidae sp.6	X	-
	Hymenoptera	Formicidae	*Acropyga* sp.1	X	-
			Apterostigma pilosum	X	-
			Camponotus sp.3	X	-
			Crematogaster limata	X	-
			Nylanderia sp.1	X	-
			Prionopelta modesta	X	-
	Isoptera	Termitidae	Termitidae sp.	X	-
			Nasutitermes sp.2	X	-
	Lepidoptera	Noctuoidea	Noctuoidea sp.7	X	-
	Orthoptera	Phalangopsidae	Phalangopsidae sp.1	X	-
Malacostraca	Isopoda	Armadillidae	Armadillidae sp.1	-	X
		Philosciidae	Philosciidae Jovem	X	-
			Philosciidae sp.2	X	-
Symphyla		Scutigerellidae	*Hanseniella* sp.1	X	-

SB-0124					
TÁXONS				Seco	Úmido
Arachnida	Amblypygi	Charinidae	Charinidae Jovem	X	-
			Charinus sp.1	-	X
	Araneae	Araneidae	Araneidae Jovem	X	-
		Corinnidae	Corinnidae Jovem	X	-
		Ctenidae	Ctenidae Jovem	X	-
		Dipluridae	*Linothele* sp.1	X	-
		Ochyroceratidae	*Speocera* sp.1	X	-
		Pholcidae	Pholcidae Jovem	X	-
			Ninetinae sp.1	X	-
		Theridiidae	Theridiidae Jovem	X	-
			Theridion sp.2	X	-
		Theridiosomatidae	Theridiosomatidae Jovem	X	-
	Opiliones	Cosmetidae	Cosmetidae sp.1	X	-
		Escadabiidae	Escadabiidae Jovem	X	-
			Escadabiidae sp.1	X	-
		Escadabiidae	Escadabiidae sp.2	-	X
		Stygnidae	*Paraphareus* sp.1	-	X
	Pseudoscorpiones	Chernetidae	Chernetidae Jovem	X	-
			Spelaeochernes sp.1	X	-
		Chthoniidae	*Pseudochthonius* sp.4	X	-
Acari	Astigmata		Astigmata sp.2	-	X
	Holothryda		Holothryda sp.1	-	X
	Mesostigmata		Mesostigmata sp.3	X	X
	Trombidiforme	Anystidae	Anystidae sp.1	-	X
		Trombiculidae	Trombiculidae sp.1	-	X
		Trombidiidae	Trombidiidae sp.1	X	-
Chilopoda	Scutigeromorpha	Pselliodidae	*Sphendononema* Jovem	X	-
Diplopoda	Polydesmida	Paradoxosomatidae	Paradoxosomatidae sp.1	X	-
		Pyrgodesmidae	Pyrgodesmidae sp.1	X	-
Entognatha	Collembola	Isotomidae	Isotomidae sp.1	X	-
		Paronellidae	Paronellidae sp.1	X	X
		Symphypleona	Symphypleona sp.2	-	X
	Diplura	Campodeidae	Campodeidae sp.1	X	-
Insecta	Blattaria	Blattidae	Blattidae Jovem	X	-
		Polyphagidae	Polyphagidae sp.1	X	-
	Coleoptera	Tenebrionidae	Tenebrionidae Jovem	X	-
	Diptera	Ceratopogonidae	Ceratopogonidae Jovem	X	-
			Ceratopogonidae sp.3	X	-
		Psychodidae	Phlebotominae sp.1	X	-
		Tipulidae	Tipulidae sp.1	X	-
	Heteroptera	Reduviidae	*Zelurus* Jovem	X	-
		Schizopteridae	Schizopteridae Jovem	X	-
	Hymenoptera	Formicidae	*Camponotus* sp.2	X	-
			Camponotus sp.3	X	-
			Crematogaster limata	X	-
			Eurhopalothrix sp.1	X	-
			Nylanderia sp.1	X	-
			Pachycondyla constricta	X	-
			Rogeria belti	X	-
	Isoptera	Termitidae	*Nasutitermes* sp.1	X	-
			Nasutitermes sp.2	X	-
	Lepidoptera	Tineidae	Tineidae Jovem	X	-
	Psocoptera	Psocomorpha	Psocomorpha Jovem	X	-
Malacostraca	Isopoda	Armadillidae	Armadillidae Jovem	-	X
			Armadillidae sp.1	X	X
		Philosciidae	Philosciidae sp.1	X	-
			Philosciidae sp.2	X	X

Symphyla		Scutigerellidae	*Hanseniella* sp.1	X	-
Gastropoda	Pulmonata	Subulinidae	*Lamellaxis* sp.1	X	-

SB-0125					
**TÁXONS**				**Seco**	**Úmido**
Arachnida	Araneae	Araneidae	Araneidae Jovem	X	-
			Alpaida antonio	X	-
		Corinnidae	Corinnidae Jovem	X	-
		Ctenidae	*Ctenus* sp.1	X	-
		Oonopidae	Oonopidae Jovem	X	-
		Pholcidae	Ninetinae sp.1	X	-
		Salticidae	Salticidae Jovem	X	-
		Symphytognathidae	*Anapistula* sp.1	X	-
		Theraphosidae	Theraphosidae Jovem	X	-
		Theridiosomatidae	*Plato* sp.1	X	-
	Opiliones	Sclerosomatidae	*Prionostema* sp.1	-	X
	Pseudoscorpiones	Chernetidae	*Spelaeochernes* sp.1	X	-
	Scorpiones	Buthidae	*Ananteris luciae*	X	-
Acari	Ixodida	Ixodidae	*Ixodes* sp.1	X	-
	Trombidiforme	Trombiculidae	Trombiculidae sp.1	X	-
Chilopoda	Scolopendromorpha	Cryptopidae	*Cryptops* sp.2	X	-
Entognatha	Collembola	Paronellidae	Paronellidae sp.1	X	-
	Diplura	Campodeidae	Campodeidae sp.1	X	-
Insecta	Blattaria	Blattidae	Blattidae Jovem	X	-
	Coleoptera		Coleoptera Jovem	X	-
	Heteroptera	Reduviidae	*Zelurus* Jovem	X	-
	Hymenoptera	Formicidae	*Carebara* pr. *urichi*	X	-
			Dolichoderus bispinosus	X	-
			Pachycondyla constricta	X	-
			Rogeria belti	X	-
	Psocoptera	Psocomorpha	Psocomorpha Jovem	X	-
Malacostraca	Isopoda	Armadillidae	Armadillidae sp.1	X	-
		Philosciidae	Philosciidae sp.1	X	-
			Philosciidae sp.2	X	-
Symphyla		Scutigerellidae	*Hanseniella* sp.1	X	-

SB-0126					
**TÁXONS**				**Seco**	**Úmido**
Arachnida	Araneae	Araneidae	Araneidae Jovem	X	-
			Alpaida negro	X	X
		Ctenidae	*Ctenus* sp.1	X	-
		Dipluridae	Dipluridae Jovem	X	-
			Linothele sp.1	-	X
		Pholcidae	*Mesabolivar aurantiacus*	X	-
			Ninetinae sp.1		X
		Salticidae	Salticidae Jovem	X	-
		Scytodidae	Scytodidae Jovem	X	-
			Scytodes eleonorae	X	X
	Opiliones	Stygnidae	*Paraphareus* sp.1	X	-
	Pseudoscorpiones	Chernetidae	*Spelaeochernes* sp.1	X	-
Acari	Opilioacarida	Opilioacaridae	Opilioacaridae sp.1	-	X
	Trombidiforme	Anystidae	Anystidae sp.1	-	X
Chilopoda	Scolopendromorpha	Scolopocryptopidae	*Newportia* sp.1	X	-
	Scutigeromorpha	Pselliodidae	*Sphendononema guildingii*	X	-
Diplopoda	Siphonophorida	Siphonophoridae	Siphonophoridae sp.1	X	-
Entognatha	Collembola	Isotomidae	Isotomidae sp.1	X	-
		Paronellidae	Paronellidae sp.1	-	X
	Diplura	Campodeidae	Campodeidae sp.1	X	-
Insecta	Diptera	Keroplatidae	Keroplatidae Jovem	X	-
		Sciaridae	Sciaridae sp.2	X	-
	Heteroptera	Reduviidae	Emesinae sp.8	X	-
			Zelurus Jovem	X	-
	Hymenoptera	Formicidae	*Dolichoderus bispinosus*	X	-
Malacostraca	Isopoda	Armadillidae	Armadillidae Jovem	-	X
			Armadillidae sp.1	X	X
		Philosciidae	Philosciidae Jovem	-	X
			Philosciidae sp.1	-	X
			Philosciidae sp.2	X	-
Turbellaria	Tricladida	Geoplanidae	Geoplanidae sp.2	X	-

SB-0127					
TÁXONS				**Seco**	**Úmido**
Arachnida	Araneae	Corinnidae	*Abapeba hoeferi*	X	-
		Ochyroceratidae	Ochyroceratidae Jovem	X	-
		Pholcidae	Pholcidae Jovem	X	-
		Theraphosidae	*Guyruita cerrado*	X	-
		Theridiosomatidae	Theridiosomatidae Jovem	X	-
			Plato sp.1	X	-
	Opiliones	Sclerosomatidae	Sclerosomatidae Jovem	X	-
			Prionostema sp.1	X	-
	Pseudoscorpiones	Chernetidae	Chernetidae Jovem	X	-
			Spelaeochernes sp.1	X	-
		Chthoniidae	Chthoniidae Jovem	X	-
			Pseudochthonius sp.2	X	-
Acari	Astigmata		Astigmata sp.2	X	X
	Mesostigmata		Mesostigmata sp.1	-	X
			Mesostigmatasp.3	-	X
	Trombidiforme	Trombiculidae	Trombiculidae sp.1	X	X
Chilopoda	Scolopendromorpha	Scolopendridae	*Otostigmus* sp.1	X	-
Diplopoda	Polydesmida		Polydesmida Jovem	X	-
Entognatha	Collembola	Isotomidae	Isotomidae sp.1	X	-
		Paronellidae	Paronellidae sp.1	X	X
			Cyphoderus agnotus	-	X
			Cyphoderus javanus	-	X
		Symphypleona	Symphypleona sp.2	X	X
Insecta	Coleoptera		Coleoptera Jovem	X	-
		Elateridae	Elateridae Jovem	X	-
		Staphylinidae	Staphylinidae sp.18	X	-
		Tenebrionidae	Tenebrionidae Jovem	X	-
	Diptera	Cecidomyiidae	Cecidomyiidae sp.1	X	-
		Keroplatidae	Keroplatidae Jovem	X	-
		Tipulidae	Tipulidae sp.1	X	-
	Heteroptera	Cydnidae	*Pangaeus* sp.1	X	-
	Hymenoptera	Formicidae	*Pheidole* sp.14	X	-
			Solenopsis invicta	X	-
			Tranopelta gilva	X	-
	Orthoptera	Phalangopsidae	*Phalangopsis* sp.1	X	-
Malacostraca	Isopoda	Philosciidae	Philosciidae Jovem	X	-
Gastropoda	Pulmonata	Subulinidae	Subulinidae Jovem	X	-

SB-0128					
TÁXONS				**Seco**	**Úmido**
Clitellata	Haplotaxida		Haplotaxida sp.11	X	-
Arachnida	Araneae	Araneidae	Araneidae Jovem	X	-
		Ctenidae	Ctenidae sp.1	-	X
		Dipluridae	Dipluridae Jovem	X	-
		Pholcidae	Pholcidae Jovem	X	-
			Ninetinae sp.1	-	X
		Salticidae	Salticidae Jovem	X	-
		Scytodidae	*Scytodes eleonorae*	-	X
		Theraphosidae	Theraphosidae Jovem	X	-
		Theridiidae	*Echinotheridion* sp.1	X	-
			Theridion sp.2	X	-
	Opiliones	Sclerosomatidae	*Prionostema* sp.1	-	X
	Pseudoscorpiones	Chernetidae	*Spelaeochernes* sp.1	X	-
		Chthoniidae	*Pseudochthonius* sp.2	X	-
	Scorpiones	Chactidae	Chactidae Jovem	X	-
Acari	Astigmata		Astigmata sp.2	X	-
	Opilioacarida	Opilioacaridae	Opilioacaridae sp.1	-	X
	Trombidiforme	Trombidiidae	Trombidiidae sp.1	-	X
Entognatha	Collembola	Entomobryidae	Entomobryidae sp.5	X	-
		Paronellidae	Paronellidae sp.1	X	X
Insecta	Coleoptera	Staphylinidae	Staphylinidae sp.18	X	-
	Diptera	Tipulidae	Tipulidae sp.1	X	-
	Heteroptera	Reduviidae	*Zelurus* Jovem	X	-
	Hymenoptera	Bethylidae	Bethylidae sp.3	X	-
		Formicidae	*Camponotus* sp.2	X	-
			Eurhopalothrix sp.1	X	-
			Rogeria belti	X	-
	Isoptera	Termitidae	*Nasutitermes* sp.2	X	-
			Nasutitermes sp.5	X	-
	Psocoptera	Psocomorpha	Psocomorpha Jovem	X	-
		Epipsocidae	Epipsocidae sp.4	X	-
Malacostraca	Isopoda	Armadillidae	Armadillidae Jovem	X	-
			Armadillidae sp.1	-	X
		Philosciidae	Philosciidae Jovem	X	-
			Philosciidae sp.2	X	-
Symphyla		Scutigerellidae	*Hanseniella* sp.1	X	-

SB-0129					
**TÁXONS**				**Seco**	**Úmido**
Clitellata	Haplotaxida		Haplotaxida sp.11	X	-
Arachnida	Amblypygi	Phrynidae	*Heterophrynus longicornis*	X	-
	Araneae	Araneidae	Araneidae Jovem	X	-
			Alpaida antonio	X	-
		Corinnidae	Corinnidae Jovem	X	-
			Abapeba hoeferi	-	X
		Ctenidae	Ctenidae Jovem	X	-
		Dipluridae	*Linothele* sp.1	X	-
		Ochyroceratidae	Ochyroceratidae Jovem	X	-
			Speocera sp.1	X	X
		Oonopidae	Oonopidae Jovem	X	-
		Pholcidae	Pholcidae Jovem	X	-
			Pholcidae sp.	X	-
			Mesabolivar aurantiacus	X	X
		Scytodidae	*Scytodes eleonorae*	X	-
		Theraphosidae	Theraphosidae Jovem	X	-
		Theridiidae	*Achaearanea* sp.1	X	X
	Opiliones	Escadabiidae	Escadabiidae Jovem	X	-
			Escadabiidae sp.1	X	-
		Sclerosomatidae	Sclerosomatidae Jovem	X	-
			Prionostema sp.1	X	-
		Escadabiidae	Escadabiidae sp.2	-	X
	Pseudoscorpiones	Chernetidae	Chernetidae Jovem	X	-
			Spelaeochernes sp.1	X	-
		Chthoniidae	*Pseudochthonius* sp.2	X	-
	Scorpiones	Buthidae	*Ananteris* Jovem	X	-
Acari	Astigmata		Astigmata sp.1	X	-
			Astigmata sp.2	X	-
	Holothryda		Holothryda sp.7	X	-
	Ixodida	Argasidae	Ornithodoros sp.1	X	X
	Mesostigmata		Mesostigmata sp.1	X	X
			Mesostigmata sp.2	X	-
			Mesostigmata sp.3	X	X
	Sarcoptiforme	Oribatida	Oribatida sp.1	X	X
			Oribatida sp.5	-	X
	Trombidiforme	Trombiculidae	Trombiculidae sp.1	-	X
			Trombiculidae sp.2	-	X
		Trombidiidae	Trombidiidae sp.1	X	X
Diplopoda	Glomeridesmida	Glomeridesmidae	Glomeridesmidae Jovem	X	-
			Glomeridesmidae sp.1	X	-
	Polydesmida		Polydesmida Jovem	X	-
		Fuhrmannodesmidae	Fuhrmannodesmidae Jovem	X	-
		Pyrgodesmidae	Pyrgodesmidae sp.1	X	-
	Spirostreptida	Spirostreptidae	Spirostreptidae sp.3	X	-
Entognatha	Collembola	Entomobryidae	Entomobryidae sp.6	X	X
		Isotomidae	Isotomidae sp.1	X	-
		Paronellidae	Paronellidae sp.1	X	X
			Cyphoderus agnotus	-	X
		Symphypleona	Symphypleona sp.2	X	X
Insecta	Blattaria	Blaberidae	Blaberidae Jovem	X	-
			Blaberidae sp.3	X	-
		Blattidae	Blattidae Jovem	X	-
		Polyphagidae	Polyphagidae sp.1	X	-
	Coleoptera		Coleoptera Jovem	X	-
		Carabidae	Carabidae sp.5	X	-
		Elateridae	Elateridae Jovem	X	-
		Ptilidae	Ptilidae sp.1	X	-

			Ptilidae sp.4	X	-
		Scydmaenidae	Scydmaeninae sp.2	X	-
			Scydmaeninae sp.6	X	-
		Staphylinidae	Staphylinidae sp.11	X	-
			Staphylininae sp.	X	-
		Tenebrionidae	Tenebrionidae Jovem	X	-
	Diptera		Diptera Jovem	X	-
		Conopidae	Conopidae sp.1	X	-
		Drosophilidae	Drosophilidae sp.1	X	-
		Sciaridae	Sciaridae sp.2	X	-
		Ceratopogonidae	Ceratopogonidae Jovem	X	-
		Tipulidae	Tipulidae sp.1	X	-
	Heteroptera	Cydnidae	Cydnidae Jovem	X	-
			Pangaeus sp.1	X	-
		Reduviidae	*Zelurus* Jovem	X	-
			Triatoma Jovem	X	-
	Hymenoptera	Bethylidae	Bethylidae sp.4	X	-
			Bethylidae sp.5	X	-
		Figitidae	Figitidae sp.1	X	-
		Formicidae	*Camponotus atriceps*	X	-
			Camponotus sp.2	X	-
			Carebara sp.12	X	-
			Neivamyrmex sp.3	X	-
			Pachycondyla constricta	X	-
			Pachycondyla impressa	X	-
			Pheidole sp.3	X	-
			Rogeria belti	X	-
			Solenopsis invicta	X	-
	Isoptera	Termitidae	*Nasutitermes* sp.3	X	-
	Lepidoptera		Lepidoptera Jovem	X	-
		Geometridae	Geometridae Jovem	X	-
		Tineoidea	Tineoidea sp.1	-	X
	Neuroptera	Myrmeleontidae	Myrmeleontidae sp.5	X	-
	Orthoptera	Phalangopsidae	*Phalangopsis* sp.1	X	-
	Psocoptera	Psocomorpha	Psocomorpha Jovem	X	-
			Psocomorpha sp.3	-	X
			Psocomorpha sp.6	-	X
Malacostraca	Isopoda	Armadillidae	Armadillidae Jovem	X	X
			Armadillidae sp.1	X	X
		Philosciidae	Philosciidae sp.1	X	-
			Philosciidae sp.2	-	X
Gastropoda	Pulmonata	Subulinidae	Subulinidae Jovem	X	-
			Lamellaxis sp.1	X	-
		Systrophiidae	*Happia* sp.1	X	-
Secernentea	Diplogasteria		*Diplogasteria* sp.1	X	-
			Diplogasteria sp.3	X	-

SB-0130					
TÁXONS				Seco	Úmido
Arachnida	Araneae	Oonopidae	Oonopidae sp.9	-	X
	Opiliones	Sclerosomatidae	*Prionostema* sp.1	X	X
	Pseudoscorpiones	Chernetidae	*Spelaeochernes* sp.1	X	-
		Chthoniidae	*Pseudochthonius* sp.2	X	-
Acari	Trombidiforme	Trombiculidae	Trombiculidae sp.1	-	X
Diplopoda	Polydesmida	Fuhrmannodesmidae	Fuhrmannodesmidae Jovem	X	-
Entognatha	Collembola	Entomobryidae	Entomobryidae sp.3	-	X
		Isotomidae	Isotomidae sp.1	X	-
		Paronellidae	Paronellidae sp.1	X	X
		Symphypleona	Symphypleona sp.2	X	X
Insecta	Coleoptera	Curculionidae	Scolytinae sp.1	X	-
			Scolytinae sp.5	X	-
		Tenebrionidae	Tenebrionidae Jovem	X	-
	Diptera	Chaoboridae	Chaoboridae sp.1	X	-
	Hemiptera	Enicocephalidae	Enicocephalidae Jovem	X	-
	Hymenoptera	Ceraphronidae	Ceraphronidae sp.1	X	-
		Formicidae	*Camponotus atriceps*	X	-
			Solenopsis invicta	X	-
	Psocoptera	Psocomorpha	Psocomorpha Jovem	X	-
		Psyllipsocidae	Psyllipsocidae sp.4	X	-
Malacostraca	Isopoda	Armadillidae	Armadillidae Jovem	-	X
			Armadillidae sp.1	X	-
		Platyarthridae	Platyarthridae sp.5	-	X

SB-0131					
**TÁXONS**				**Seco**	**Úmido**
Arachnida	Araneae	Araneidae	Araneidae Jovem	X	-
		Nemesiidae	Nemesiidae Jovem	X	-
		Ochyroceratidae	Ochyroceratidae Jovem	X	-
			Speocera sp.1	X	-
		Oonopidae	Oonopidae Jovem	X	-
			Oonopidae sp.3	-	X
			gr. *Xycarpphy* sp.1	-	X
		Pholcidae	Pholcidae Jovem	X	-
		Salticidae	Salticidae Jovem	X	-
			Salticidae sp.12	-	X
		Scytodidae	Scytodidae Jovem	X	-
	Opiliones	Sclerosomatidae	*Prionostema* sp.1	-	X
	Pseudoscorpiones	Chthoniidae	*Pseudochthonius* sp.3	X	-
	Scorpiones	Buthidae	*Tityus* Jovem	X	-
Acari	Mesostigmata		Mesostigmata sp.1	-	X
	Opilioacarida	Opilioacaridae	Opilioacaridae sp.1	-	X
	Sarcoptiforme	Oribatida	Oribatida sp.1	-	X
			Oribatida sp.4	-	X
Chilopoda	Scolopendromorpha	Scolopocryptopidae	*Newportia* sp.2	X	-
Diplopoda	Spirostreptida		Spirostreptida Jovem	X	-
Entognatha	Collembola	Isotomidae	Isotomidae sp.1	X	-
		Paronellidae	Paronellidae sp.1	-	X
	Diplura	Campodeidae	Campodeidae sp.1	X	-
Insecta	Blattaria	Blattidae	Blattidae Jovem	X	-
	Heteroptera	Reduviidae	*Zelurus* Jovem	X	-
	Hymenoptera	Formicidae	*Camponotus* sp.2	X	-
	Isoptera	Termitidae	*Nasutitermes* sp.2	X	-
	Orthoptera	Phalangopsidae	Phalangopsidae sp.1	X	-
Malacostraca	Isopoda	Armadillidae	Armadillidae sp.1	-	X
Symphyla		Scutigerellidae	*Hanseniella* sp.1	X	-

SB-0132					
TÁXONS				**Seco**	**Úmido**
Arachnida	Araneae	Oonopidae	Oonopidae Jovem	X	-
		Pholcidae	Pholcidae Jovem	X	-
			Ninetinae sp.1	-	X
		Theridiidae	Theridiidae Jovem	X	-
	Opiliones	Stygnidae	*Protimesius gracilis*	X	-
		Cosmetidae	*Roquettea carajas*	-	X
		Stygnidae	*Paraphareus* sp.1	-	X
	Pseudoscorpiones	Chernetidae	*Spelaeochernes* sp.1	X	-
		Chthoniidae	*Pseudochthonius* sp.2	X	-
	Ricinulei	Ricinoididae	*Cryptocellus tarsilae*	-	X
Acari	Astigmata		Astigmata sp.1	-	X
	Ixodida	Ixodidae	*Amblyomma rotundatum*	-	X
	Mesostigmata		Mesostigmata sp.1	X	-
	Opilioacarida	Opilioacaridae	Opilioacaridae sp.1	-	X
Entognatha	Collembola	Paronellidae	Paronellidae sp.1	X	X
			Paronellidae sp.3	X	-
	Diplura	Campodeidae	Campodeidae sp.1	X	-
Insecta	Diptera	Sciaridae	Sciaridae sp.3	X	-
		Psychodidae	Phlebotominae sp.1	X	-
	Heteroptera	Reduviidae	*Zelurus* Jovem	X	-
		Schizopteridae	Schizopteridae Jovem	X	-
	Homoptera	Cixiidae	Cixiidae Jovem	X	-
	Hymenoptera	Formicidae	*Acromyrmex* sp.1	X	-
			Carebara pr. *urichi*	X	-
	Isoptera	Termitidae	*Crepititermes* sp.1	X	-
	Psocoptera	Psocomorpha	Psocomorpha Jovem	X	-
Malacostraca	Isopoda	Armadillidae	Armadillidae sp.1	-	X
		Philosciidae	Philosciidae Jovem	X	-
			Philosciidae sp.2	X	-

SB-0163					
**TÁXONS**				**Seco**	**Úmido**
Arachnida	Amblypygi	Phrynidae	*Heterophrynus longicornis*	X	X
	Araneae	Corinnidae	Corinnidae Jovem	X	-
		Ochyroceratidae	Ochyroceratidae Jovem	X	X
		Oonopidae	Oonopidae sp.8	X	-
		Pholcidae	*Mesabolivar eberhardi*	X	-
		Salticidae	Salticidae Jovem	-	X
			Salticidae sp.1	X	-
		Theridiidae	Theridiidae Jovem	X	-
		Theridiosomatidae	*Plato* sp.1	X	X
	Pseudoscorpiones	Chernetidae	Chernetidae Jovem	X	-
			Spelaeochernes sp.1	X	-
		Chthoniidae	*Pseudochthonius* sp.2	-	X
Acari	Trombidiforme	Anystidae	Anystidae sp.1	X	-
		Trombiculidae	Trombiculidae sp.1	X	X
Diplopoda	Spirostreptida		Spirostreptida Jovem	-	X
Entognatha	Collembola	Paronellidae	Paronellidae sp.1	X	X
			Paronellidae sp.3	X	-
			Trogolaphysa sp.2	-	X
		Symphypleona	Symphypleona sp.2	X	-
	Diplura	Campodeidae	Campodeidae sp.1	-	X
Insecta	Blattaria	Blaberidae	Blaberidae Jovem	-	X
	Coleoptera		Coleoptera Jovem	-	X
		Elateridae	Elateridae Jovem	X	-
	Diptera	Empididae	Empididae sp.1	-	X
		Psychodidae	Phlebotominae sp.1	-	X
		Tipulidae	Tipulidae sp.1	-	X
	Heteroptera	Reduviidae	*Zelurus* Jovem	X	X
			Zelurus sp.1	-	X
	Homoptera	Cixiidae	Cixiidae sp.6	X	-
	Hymenoptera	Formicidae	*Camponotus cingulatus*	X	X
			Nylanderia sp.1	X	-
			Nylanderia sp.2	X	-
			Solenopsis invicta	-	X
			Wasmannia auropunctata	-	X
	Isoptera	Termitidae	*Nasutitermes* sp.2	X	X
	Lepidoptera	Tineidae	Tineidae Jovem	X	-
	Orthoptera	Phalangopsidae	*Eidmanacris* sp.1	X	X
	Psocoptera	Psocomorpha	Psocomorpha Jovem	X	X
		Liposcelidae	Liposcelidae sp.6	X	-
	Zygentoma	Nicoletiidae	Nicoletiinae sp.1	-	X
Malacostraca	Isopoda	Philosciidae	Philosciidae Jovem	X	-
			Philosciidae sp.2	X	-
Amphibia	Anura	Craugastoridae	*Pristimantis* cf. *fenestratus*	X	X
		Leptodactylidae	*Physalaemus* gr. *cuvieri*	X	-
Mammalia	Chiroptera	Emballonuridae	*Peropteryx* sp.	-	X
		Phyllostomidae	*Carollia* sp.	X	-
Gastropoda	Pulmonata	Spiraxidae	*Euglandina* sp.1	-	X
		Subulinidae	*Leptinaria* sp.2	-	X

SB-0164					
TÁXONS				Seco	Úmido
Arachnida	Amblypygi	Charinidae	Charinidae Jovem	-	X
	Araneae	Ctenidae	Ctenidae Jovem	X	-
		Drymusidae	Drymusidae Jovem	X	-
		Nemesiidae	Nemesiidae Jovem	-	X
		Ochyroceratidae	*Ochyrocera* sp.2	-	X
			Speocera sp.1	X	-
		Paratropididae	Paratropididae Jovem	X	-
		Pholcidae	Pholcidae Jovem	X	-
			Mesabolivar cambridgei	-	X
		Theridiosomatidae	*Plato* sp.1	-	X
		Trechaleidae	Trechaleidae Jovem	-	X
	Opiliones	Cosmetidae	Cosmetidae Jovem	X	-
		Escadabiidae	Escadabiidae Jovem	X	-
		Sclerosomatidae	*Prionostema* sp.1	-	X
		Stygnidae	*Paraphareus* sp.1	X	-
	Pseudoscorpiones	Chernetidae	Chernetidae Jovem	X	-
			Spelaeochernes sp.1	X	X
		Chthoniidae	*Pseudochthonius* sp.4	X	-
Acari	Acariforme		Acariforme sp.1	X	-
	Sarcoptiforme		Oribatida sp.1	X	-
			Sarcoptiforme sp.7	X	-
	Trombidiforme	Trombidiidae	Trombidiidae sp.1	X	-
Chilopoda	Scolopendromorpha	Scolopendridae	*Otostigmus* sp.1	-	X
	Scutigeromorpha	Pselliodidae	*Sphendononema* Jovem	-	X
Diplopoda	Polydesmida		Polydesmida Jovem	X	X
		Fuhrmannodesmidae	Fuhrmannodesmidae sp.1	X	-
		Pyrgodesmidae	Pyrgodesmidae sp.1	X	X
Entognatha	Collembola		Collembola sp.	X	-
		Entomobryidae	Entomobryidae sp.5	X	-
		Paronellidae	Paronellidae sp.1	X	-
		Symphypleona	Symphypleona sp.2	X	-
	Diplura	Campodeidae	Campodeidae sp.1	X	-
Insecta	Blattaria	Blattelidae	Blattelidae Jovem	X	-
		Blattidae	Blattidae Jovem	X	-
		Pseudophyllodromiidae	Pseudophyllodromiidae Jovem	-	X
	Coleoptera		Coleoptera Jovem	X	-
		Curculionidae	Curculionidae sp.5	X	-
		Staphylinidae	Staphylinidae sp.12	X	-
			Staphylinidae sp.14	-	X
			Pselaphinae sp.8	-	X
	Diptera		Diptera Jovem	X	-
		Ceratopogonidae	Ceratopogonidae Jovem	X	-
		Tipulidae	Tipulidae sp.1	-	X
	Heteroptera	Cydnidae	*Pangaeus* sp.1	X	-
	Hymenoptera	Formicidae	*Nylanderia* sp.2	X	-
			Pheidole sp.3	X	-
			Pheidole sp.6	X	-
			Strumigenys elongata	X	-
	Isoptera	Termitidae	*Nasutitermes* sp.1	-	X
			Nasutitermes sp.2	X	X
	Lepidoptera		Lepidoptera sp.	X	X
	Orthoptera		Orthoptera sp.	-	X
		Phalangopsidae	*Eidmanacris* sp.1	X	X
			Paraclodes sp.1	X	-
			Phalangopsis sp.1	X	X
Malacostraca	Isopoda	Philosciidae	Philosciidae Jovem	-	X
			Philosciidae sp.2	X	X

		Scleropactidae	Scleropactidae sp.2	X	-
Amphibia	Anura	Craugastoridae	*Pristimantis* cf. *fenestratus*	X	X
Mammalia	Chiroptera	Emballonuridae	*Peropteryx kappleri*	X	-
			Peropteryx sp.	-	X
		Phyllostomidae	*Carollia* sp.	X	-
Gastropoda	Pulmonata	Solaropsidae	*Solaropsidae* sp.1	-	X
		Subulinidae	*Leptinaria* sp.1	-	X

SB-0165					
**TÁXONS**				**Seco**	**Úmido**
Clitellata	Haplotaxida		Haplotaxida Jovem	X	-
			Haplotaxida sp.1	X	-
	Lumbriculida	Lumbriculidae	Lumbriculidae sp.1	X	X
Arachnida	Amblypygi	Phrynidae	*Heterophrynus longicornis*	X	X
	Araneae	Anapidae	Anapidae sp.2	-	X
		Corinnidae	Corinnidae Jovem	X	-
		Ctenidae	*Phoneutria* sp.	-	X
		Pholcidae	Pholcidae Jovem	-	X
		Pisauridae	Pisauridae Jovem	-	X
			Pisauridae sp.	-	X
		Trechaleidae	Trechaleidae Jovem	X	-
			Rhoicinus sp.1	-	X
	Opiliones	Sclerosomatidae	*Prionostema* sp.1	-	X
	Pseudoscorpiones	Chernetidae	Chernetidae Jovem	-	X
			Spelaeochernes sp.1	X	-
Diplopoda	Polydesmida	Chelodesmidae	Chelodesmidae Jovem	-	X
		Fuhrmannodesmidae	Fuhrmannodesmidae Jovem	-	X
		Pyrgodesmidae	Pyrgodesmidae sp.2	-	X
	Siphonophorida	Siphonophoridae	Siphonophoridae sp.1	-	X
Insecta			Insecta Jovem	-	X
	Blattaria	Blattidae	Blattidae Jovem	-	X
			Blattidae sp.3	-	X
	Coleoptera		Coleoptera Jovem	-	X
		Hydrophilidae	Hydrophilidae sp.4	-	X
		Scydmaenidae	Scydmaeninae sp.6	X	-
		Staphylinidae	Staphylinidae sp.14	-	X
			Staphylininae sp.14	X	-
			Staphylininae sp.8	X	-
	Diptera		Diptera Jovem	X	X
			Diptera sp.	X	-
		Brachycera	Brachycera Jovem	-	X
		Sciaridae	Sciaridae sp.1	-	X
		Tipulidae	Tipulidae Jovem	X	-
			Tipulidae sp.1	-	X
	Heteroptera	Belostomatidae	Belostoma Jovem	-	X
		Cydnidae	Cydnidae sp.	-	X
		Veliidae	*Microvelia* Jovem	X	-
			Rhagovelia sp.4	X	-
			Paravelia Jovem	X	-
			Paravelia sp.1	-	X
	Hymenoptera	Formicidae	*Nylanderia* sp.1	-	X
			Odontomachus bauri	X	-
			Odontomachus meinerti	-	X
			Pheidole sp.3	X	-
			Pheidole sp.7	-	X
			Solenopsis invicta	X	X
			Solenopsis sp.2	X	X
	Isoptera	Termitidae	*Nasutitermes* sp.1	-	X
			Nasutitermes sp.2	X	X
	Odonata		Odonata Jovem	-	X
	Orthoptera		Orthoptera sp.	X	X
		Phalangopsidae	*Paraclodes* sp.1	X	-
	Plecoptera	Perlidae	*Perlidae* sp.1	X	-
Malacostraca	Decapoda	Pseudothelphusidae	Pseudothelphusidae Jovem	-	X
			Pseudothelphusidae sp.	X	-
Amphibia	Anura		Anura Jovem	X	-
Mammalia	Chiroptera		Chiroptera sp.	X	-

		Phyllostomidae	*Carollia* sp.	-	X
Reptilia	Squamata	Gymnophthalmidae	*Neusticurus ecpleopus*	-	X

SB-0166					
TÁXONS				**Seco**	**Úmido**
Arachnida	Araneae	Corinnidae	Corinnidae Jovem	-	X
		Ochyroceratidae	Ochyroceratidae Jovem	X	X
		Pholcidae	Pholcidae Jovem	X	-
		Theridiidae	Theridiidae Jovem	-	X
		Theridiosomatidae	*Plato* sp.1	X	X
	Opiliones	Escadabiidae	Escadabiidae Jovem	X	-
		Sclerosomatidae	Sclerosomatidae Jovem	X	-
			Prionostema sp.1	-	X
		Zalmoxidae	Zalmoxidae sp.1	-	X
	Pseudoscorpiones	Chernetidae	*Spelaeochernes* sp.1	-	X
Diplopoda	Polydesmida	Fuhrmannodesmidae	Fuhrmannodesmidae Jovem	-	X
Insecta	Coleoptera	Scydmaenidae	Scydmaeninae sp.1	X	-
	Diptera	Tipulidae	Tipulidae sp.3	-	X
	Heteroptera	Cydnidae	Cydnidae sp.1	-	X
	Hymenoptera	Formicidae	*Hypoponera* sp.1	-	X
	Isoptera	Termitidae	*Nasutitermes* sp.1	X	-
			Nasutitermes sp.7	-	X
	Lepidoptera	Noctuoidea	Noctuoidea sp.7	X	-
		Tineoidea	Tineoidea sp.5	X	-
	Orthoptera		Orthoptera sp.	X	X

SB-0167					
TÁXONS				Seco	Úmido
Clitellata	Haplotaxida		Haplotaxida sp.11	-	X
Arachnida	Amblypygi	Phrynidae	*Heterophrynus longicornis*	X	X
	Araneae	Ctenidae	*Ancylometes rufus*	X	X
		Ochyroceratidae	Ochyroceratidae Jovem	-	X
		Pholcidae	*Mesabolivar aurantiacus*	X	X
		Theridiosomatidae	*Plato* sp.1	X	X
		Trechaleidae	*Rhoicinus* sp.1	-	X
	Opiliones	Sclerosomatidae	*Prionostema* sp.1	-	X
	Pseudoscorpiones	Chernetidae	Chernetidae Jovem	-	X
			Spelaeochernes sp.1	X	-
Diplopoda	Polydesmida	Fuhrmannodesmidae	Fuhrmannodesmidae Jovem	-	X
Entognatha	Collembola	Entomobryidae	Entomobryidae sp.5	X	-
		Paronellidae	*Cyphoderus agnotus*	X	X
Insecta	Blattaria	Blattidae	Blattidae Jovem	-	X
	Coleoptera		Coleoptera Jovem	X	-
		Curculionidae	Scolytinae sp.1	X	-
		Scirtidae	Scirtidae Jovem	-	X
	Diptera		Diptera Jovem	X	-
		Drosophilidae	Drosophilidae sp.3	-	X
		Chironomidae	Chironomidae sp.1	-	X
		Tipulidae	Tipulidae sp.1	-	X
	Heteroptera	Cydnidae	*Pangaeus* sp.1	X	X
	Homoptera	Cixiidae	Cixiidae sp.6	X	-
	Hymenoptera	Formicidae	*Camponotus* sp.2	X	-
			Pheidole sp.15	-	X
			Pheidole sp.4	-	X
			Pheidole sp.5	-	X
			Solenopsis invicta	X	-
	Isoptera	Termitidae	*Nasutitermes* sp.2	-	X
	Orthoptera		Orthoptera sp.	X	X
	Trichoptera		Trichoptera Jovem	-	X
		Philopotamidae	Philopotamidae Jovem	X	-
	Zygentoma	Nicoletiidae	Atelurinae sp.1	-	X
Amphibia	Anura	Craugastoridae	*Pristimantis* cf. *fenestratus*	-	X
Mammalia	Chiroptera		Chiroptera sp.	-	X
		Furipteridae	*Furipterus horrens*	X	-
Gastropoda	Pulmonata	Subulinidae	Subulinidae Jovem	X	-
			Lamellaxis sp.1	-	X

SB-0168					
TÁXONS				Seco	Úmido
Arachnida	Amblypygi	Phrynidae	*Heterophrynus longicornis*	X	X
	Araneae	Ctenidae	Ctenidae Jovem	-	X
		Ochyroceratidae	Ochyroceratidae Jovem	X	X
			Speocera sp.1	X	X
		Oonopidae	Oonopidae Jovem	X	-
			gr. *Xycarphhy* sp.1	X	-
		Pholcidae	Pholcidae Jovem	X	-
			Mesabolivar aurantiacus	X	X
		Pisauridae	Pisauridae Jovem	X	-
			Pisauridae sp.	X	-
		Theridiosomatidae	*Plato* sp.1	X	X
		Trechaleidae	Trechaleidae Jovem	X	X
			Rhoicinus sp.1	X	-
	Opiliones	Escadabiidae	Escadabiidae Jovem	-	X
			Escadabiidae sp.1	X	-
		Manaosbiidae	Manaosbiidae Jovem	X	-
		Neogoveidae	*Canga renatae*	X	X
		Sclerosomatidae	*Prionostema* sp.1	X	-
		Stygnidae	Stygnidae Jovem	X	-
	Pseudoscorpiones	Chernetidae	Chernetidae Jovem	X	-
			Spelaeochernes sp.1	X	X
		Chthoniidae	*Pseudochthonius* sp.4	X	-
		Syarinidae	*Nannobisium* sp.1	X	X
Acari	Mesostigmata		Mesostigmata sp.2	X	-
		Uropodoidea	Uropodoidea sp.1	-	X
	Sarcoptiforme	Oribatida	Oribatida sp.4	X	-
			Oribatida sp.5	-	X
	Trombidiforme	Trombiculidae	Trombiculidae sp.1	X	-
Chilopoda	Scolopendromorpha	Cryptopidae	*Cryptops* Jovem	-	X
			Cryptops sp.	-	X
Diplopoda	Glomeridesmida	Glomeridesmidae	Glomeridesmidae sp.1	X	X
	Polydesmida	Fuhrmannodesmidae	Fuhrmannodesmidae Jovem	X	-
		Pyrgodesmidae	Pyrgodesmidae sp.2	X	-
Entognatha	Collembola	Entomobryidae	*Pseudosinella* sp.1	X	-
		Paronellidae	*Cyphoderus agnotus*	X	X
			Cyphoderus arlei	X	-
			Cyphoderus javanus	X	-
	Diplura	Campodeidae	Campodeidae sp.1	X	-
Insecta	Blattaria	Blattidae	Blattidae Jovem	X	X
	Coleoptera		Coleoptera Jovem	X	-
		Carabidae	Carabidae sp.1	X	-
		Scirtidae	Scirtidae sp.1	-	X
		Tenebrionidae	Tenebrionidae Jovem	X	-
	Diptera		Diptera Jovem	X	X
		Sciaridae	Sciaridae sp.1	-	X
		Psychodidae	Psychodidae sp.1	-	X
		Tipulidae	Tipulidae Jovem	X	-
			Tipulidae sp.4	-	X
	Heteroptera	Cydnidae	Cydnidae Jovem	X	-
			Pangaeus sp.1	-	X
		Mesoveliidae	Madeoveliinae Jovem	-	X
		Reduviidae	Emesinae sp.6	X	-
		Veliidae	*Rhagovelia* sp.4	X	-
			Paravelia Jovem	X	-
			Paravelia sp.1	-	X
	Hymenoptera	Formicidae	*Nylanderia* sp.1	X	X
			Nylanderia sp.2	X	-

			Odontomachus bauri	X	X
			Odontomachus meinerti	-	X
			Pachycondyla sp.1	X	-
			Pheidole sp.1	X	-
			Pheidole sp.16	X	-
			Pheidole sp.4	-	X
			Solenopsis invicta	X	X
			Solenopsis sp.1	X	-
	Isoptera	Termitidae	*Nasutitermes* sp.1	X	-
			Nasutitermes sp.2	X	-
	Lepidoptera		Lepidoptera Jovem	X	-
	Orthoptera	Phalangopsidae	*Phalangopsis* sp.1	X	X
	Trichoptera		Trichoptera Jovem	-	X
		Philopotamidae	Philopotamidae Jovem	X	-
			Philopotamidae sp.1	X	-
	Zygentoma	Nicoletiidae	Nicoletiidae sp.1	X	X
Malacostraca	Decapoda		Decapoda sp.	X	X
		Palaemonidae	*Macrobrachium* sp.1	X	X
		Pseudothelphusidae	Pseudothelphusidae sp.1	X	X
	Isopoda	Philosciidae	Philosciidae Jovem	-	X
			Philosciidae sp.1	X	-
			Philosciidae sp.2	X	-
		Platyarthridae	Platyarthridae Jovem	-	X
			Platyarthridae sp.5	X	-
Mammalia	Chiroptera	Phyllostomidae	*Carollia perspicillata*	X	-
			Carollia sp.	-	X
			Anoura geoffroyi	X	-
			Glossophaginae sp.	-	X
			Lionycteris sp.	X	-
			Lonchophylla sp.	-	X
			Phyllostomus sp.	X	X
Reptilia	Pleurodonta	Tropiduridae	Tropiduridae sp.	X	-
Gastropoda	Pulmonata	Subulinidae	*Lamellaxis* sp.1	X	-
Secernentea		Diplogasteria	*Diplogasteria* sp.1	X	X

SB-0169					
TÁXONS				Seco	Úmido
Arachnida	Amblypygi	Phrynidae	*Heterophrynus longicornis*	X	-
	Araneae	Corinnidae	Corinnidae Jovem	X	-
			Creugas sp.2	X	-
		Ochyroceratidae	Ochyroceratidae Jovem	X	X
		Pholcidae	Pholcidae Jovem	-	X
			aff. *Ibotyporanga* sp.1	-	X
			Mesabolivar aurantiacus	-	X
		Salticidae	Salticidae Jovem	-	X
			Salticidae sp.1	-	X
		Theridiidae	Theridiidae Jovem	-	X
			Theridiidae sp.1	X	-
			Echinotheridion sp.1	-	X
		Theridiosomatidae	Theridiosomatidae Jovem	X	-
			Plato sp.1	X	X
		Trechaleidae	Trechaleidae Jovem	X	-
	Opiliones		Opiliones Jovem	X	-
		Cosmetidae	*Roquettea carajas*	X	-
		Escadabiidae	Escadabiidae Jovem	X	X
			Escadabiidae sp.1	X	X
			Escadabiidae sp.nov.7	X	-
		Sclerosomatidae	Sclerosomatidae Jovem	-	X
		Stygnidae	Stygnidae Jovem	-	X
			Paraphareus sp.1	X	-
			Protimesius gracilis	-	X
	Palpigradi	Eukoeneniidae	Eukoeneniidae Jovem	-	X
	Pseudoscorpiones	Chernetidae	*Spelaeochernes* sp.1	X	X
		Chthoniidae	Chthoniidae Jovem	X	-
			Pseudochthonius sp.4	X	-
Acari	Acariforme		Acariforme sp.1	X	-
	Ixodida	Ixodidae	*Amblyomma cajennense*	-	X
	Sarcoptiforme	Oribatida	Oribatida sp.12	X	-
	Trombidiforme	Trombidiidae	Trombidiidae sp.1	X	X
Chilopoda	Scutigeromorpha		Scutigeromorpha Jovem	X	-
Diplopoda	Polydesmida		Polydesmida Jovem	X	-
		Fuhrmannodesmidae	Fuhrmannodesmidae Jovem	X	X
			Fuhrmannodesmidae sp.1	X	-
		Pyrgodesmidae	Pyrgodesmidae Jovem	X	-
			Pyrgodesmidae sp.1	X	X
Entognatha	Collembola	Entomobryidae	Entomobryidae sp.5	-	X
		Isotomidae	Isotomidae sp.1	-	X
		Paronellidae	Paronellidae sp.1	X	X
			Cyphoderus ca. *arlei*	-	X
			Cyphoderus sp.nov.5	X	-
	Diplura	Campodeidae	Campodeidae sp.1	-	X
Insecta	Archaeognatha	Meinertellidae	Meinertellidae sp.1	-	X
	Blattaria	Blattelidae	Blattelidae Jovem	X	-
		Blattidae	Blattidae Jovem	-	X
	Coleoptera		Coleoptera Jovem	X	-
		Staphylinidae	Pselaphinae sp.14	X	-
		Tenebrionidae	Tenebrionidae sp.7	X	-
	Diptera		Diptera Jovem	-	X
		Ceratopogonidae	Ceratopogonidae Jovem	X	-
		Psychodidae	Phlebotominae sp.1	-	X
		Tipulidae	Tipulidae sp.1	-	X
	Heteroptera	Ceratocombidae	Ceratocombidae sp.2	X	-
		Cydnidae	Cydnidae Jovem	-	X
			Cydnidae sp.4	-	X

			Pangaeus sp.1	-	X
	Hymenoptera	Formicidae	*Camponotus* sp.2	X	X
			Camponotus sp.3	X	-
			Crematogaster erecta	X	-
			Crematogaster limata	-	X
			Nylanderia sp.2	X	X
			Pheidole sp.1	X	-
			Solenopsis sp.4	-	X
	Lepidoptera		Lepidoptera Jovem	-	X
	Orthoptera	Phalangopsidae	*Phalangopsis* sp.1	X	X
	Psocoptera	Psocomorpha	Psocomorpha Jovem	-	X
			Psocomorpha sp.	-	X
		Pseudocaeciliidae	Pseudocaeciliidae sp.2	X	-
		Psyllipsocidae	Psyllipsocidae sp.7	-	X
Malacostraca	Isopoda	Armadillidae	Armadillidae Jovem	-	X
		Scleropactidae	Scleropactidae Jovem	-	X
			Scleropactidae sp.2	X	X
Amphibia	Anura	Craugastoridae	*Pristimantis* cf. *fenestratus*	X	X
Mammalia	Chiroptera	Emballonuridae	*Peropteryx kappleri*	X	-
			Peropteryx sp.	-	X
		Phyllostomidae	*Carollia perspicillata*	X	-
			Carollia sp.	-	X
Gastropoda	Pulmonata	Subulinidae	Subulinidae Jovem	X	-
Nematoda	Diplogasteria		*Diplogasteria* sp.2	-	X
			Diplogasteria sp.3	-	X

SB-0170					
TÁXONS				Seco	Úmido
Clitellata	Haplotaxida		Haplotaxida sp.	-	X
			Haplotaxida sp.1	-	X
			Haplotaxida sp.10	-	X
			Haplotaxida sp.11	-	X
			Haplotaxida sp.8	-	X
			Haplotaxida sp.9	-	X
Arachnida	Amblypygi	Phrynidae	*Heterophrynus longicornis*	X	X
	Araneae	Anapidae	Anapidae sp.3	-	X
		Araneidae	*Micrathena plana*	-	X
		Corinnidae	Corinnidae Jovem	-	X
		Ctenidae	*Ctenus* sp.1	-	X
		Drymusidae	Drymusidae Jovem	-	X
		Ochyroceratidae	Ochyroceratidae Jovem	X	X
			Speocera sp.1	X	X
		Oonopidae	Oonopidae sp.4	-	X
		Pholcidae	Pholcidae Jovem	X	X
			Mesabolivar aurantiacus	-	X
			Mesabolivar eberhardi	X	X
		Prodidomidae	Prodidomidae Jovem	X	-
		Salticidae	Salticidae Jovem	X	X
		Scytodidae	*Scytodes eleonorae*	X	X
		Theraphosidae	Theraphosidae Jovem	X	X
		Theridiidae	Theridiidae Jovem	X	X
			Theridiidae sp.1	-	X
			Theridion sp.2	X	-
			Thymoites sp.1	-	X
	Opiliones		Opiliones sp.	-	X
		Cosmetidae	Cosmetidae Jovem	X	-
			Cosmetidae sp.	X	-
			Cosmetidae sp.1	X	X
			Roquettea carajas	-	X
		Sclerosomatidae	Sclerosomatidae Jovem	-	X
			Prionostema sp.1	-	X
		Stygnidae	*Protimesius laevis*	-	X
	Pseudoscorpiones	Chernetidae	Chernetidae Jovem	X	-
			Spelaeochernes sp.1	X	X
		Chthoniidae	*Pseudochthonius* sp.2	X	X
	Scorpiones	Buthidae	*Ananteris* Jovem	X	-
			Ananteris luciae	-	X
Acari	Astigmata		Astigmata sp.2	-	X
	Opilioacarida	Opilioacaridae	Opilioacaridae sp.1	-	X
	Sarcoptiforme	Oribatida	Oribatida sp.1	-	X
	Trombidiforme	Trombiculidae	Trombiculidae sp.1	-	X
			Trombiculidae sp.3	X	-
		Trombidiidae	Trombidiidae sp.1	-	X
Chilopoda	Geophilomorpha		Geophilomorpha Jovem	X	-
	Scolopendromorpha	Cryptopidae	*Cryptops* sp.2	X	-
		Scolopocryptopidae	*Newportia* sp.3	-	X
Diplopoda	Glomeridesmida	Glomeridesmidae	Glomeridesmidae Jovem	-	X
	Polydesmida	Chelodesmidae	Chelodesmidae Jovem	-	X
			Chelodesmidae sp.3	-	X
		Cyrtodesmidae	Cyrtodesmidae Jovem	-	X
		Fuhrmannodesmidae	Fuhrmannodesmidae Jovem	X	-
			Fuhrmannodesmidae sp.2	-	X
			Fuhrmannodesmidae sp.4	X	X
		Paradoxosomatidae	Paradoxosomatidae Jovem	-	X
			Paradoxosomatidae sp.1	-	X

			Paradoxosomatidae sp.2	X	-
Entognatha	Collembola	Entomobryidae	Entomobryidae sp.2	-	X
		Paronellidae	Paronellidae sp.1	X	X
		Tomoceridae	Tomoceridae sp.1	-	X
		Symphypleona	Symphypleona sp.2	-	X
	Diplura	Campodeidae	Campodeidae sp.1	X	X
		Japygidae	Japygidae sp.1	-	X
Insecta	Coleoptera		Coleoptera Jovem	-	X
		Carabidae	*Lelis* sp.1	-	X
		Curculionidae	Curculionidae sp.5	-	X
		Scydmaenidae	Scydmaenidae sp.2	-	X
			Scydmaenidae sp.6	-	X
	Diptera		Diptera Jovem	X	X
		Dolichopodidae	Dolichopodidae sp.1	-	X
		Cecidomyiidae	Cecidomyiidae sp.2	-	X
		Sciaridae	Sciaridae sp.	-	X
		Ceratopogonidae	Ceratopogonidae Jovem	X	-
		Culicidae	Culicinae sp.1	-	X
		Psychodidae	Phlebotominae sp.1	-	X
	Heteroptera		Heteroptera sp.	X	-
		Cydnidae	*Pangaeus* sp.1	-	X
		Nabidae	Nabidae Jovem	-	X
		Reduviidae	*Zelurus* Jovem	X	X
			Zelurus sp.	X	-
	Homoptera	Coccoidea	Coccoidea sp.	X	-
		Cicadellidae	Cicadellidae Jovem	-	X
	Hymenoptera	Formicidae	*Acanthostichus bentoni*	-	X
			Apterostigma pilosum	-	X
			Brachymyrmex sp.1	X	-
			Dolichoderus bispinosus	X	X
			Hypoponera sp.7	-	X
			Pachycondyla constricta	X	X
			Prionopelta modesta	-	X
			Pyramica sp.1	X	-
			Rogeria blanda	-	X
			Rogeria pr. *belti*	-	X
			Rogeria tonduzi	X	-
			Solenopsis invicta	X	X
	Isoptera	Termitidae	Termitidae sp.	X	X
			Cornitermes sp.1	X	-
			Nasutitermes sp.	-	X
			Nasutitermes sp.1	-	X
			Nasutitermes sp.2	X	-
	Lepidoptera	Noctuoidea	Noctuoidea sp.7	-	X
	Orthoptera	Phalangopsidae	Phalangopsidae sp.	X	X
			Phalangopsidae sp.5	-	X
			Phalangopsidae sp.6	-	X
			Eidmanacris sp.1	X	-
			Paraclodes sp.1	X	X
			Phalangopsis sp.1	X	X
	Psocoptera	Psocomorpha	Psocomorpha Jovem	X	-
	Zygentoma	Nicoletiidae	Nicoletiinae sp.1	-	X
Malacostraca	Isopoda	Armadillidae	Armadillidae Jovem	X	X
			Armadillidae sp.1	X	X
		Philosciidae	Philosciidae sp.1	-	X
			Philosciidae sp.2	X	X
		Platyarthridae	Platyarthridae sp.1	-	X
Symphyla		Scolopendrellidae	*Symphylella* sp.1	-	X
		Scutigerellidae	*Hanseniella* sp.1	X	X

Amphibia	Anura	Aromobatidae	*Allobates* gr. *marchesianus*	-	X
		Craugastoridae	*Pristimantis* cf. *fenestratus*	X	X
Mammalia	Chiroptera	Phyllostomidae	*Carollia* sp.	X	-
			Glossophaga sp.	X	-
Gastropoda	Pulmonata	Streptaxidae	Streptaxidae sp.1	X	-
		Subulinidae	*Leptinaria* sp.1	-	X
		Systrophiidae	*Happia* sp.3	-	X
		Veronicellidae	*Veronicellidae* sp.1	-	X

SB-0171					
TÁXONS				**Seco**	**Úmido**
Arachnida	Amblypygi	Charinidae	Charinidae Jovem	X	-
			Charinidae sp.	X	X
	Araneae	Araneidae	Araneidae Jovem	X	-
		Ctenidae	Ctenidae Jovem	X	-
		Ochyroceratidae	Ochyroceratidae Jovem	X	-
		Pholcidae	Pholcidae Jovem	-	X
			Mesabolivar aurantiacus	-	X
		Salticidae	Salticidae Jovem	-	X
		Theridiidae	Theridiidae Jovem	-	X
		Theridiosomatidae	Theridiosomatidae Jovem	-	X
	Opiliones	Cosmetidae	*Roquettea carajas*	X	-
		Stygnidae	*Protimesius laevis*	-	X
	Pseudoscorpiones	Chthoniidae	*Pseudochthonius* sp.4	X	-
	Schizomida	Hubardiidae	Hubardiidae Jovem	X	-
Acari	Trombidiforme	Trombiculidae	Trombiculidae sp.1	X	-
Diplopoda	Glomeridesmida	Glomeridesmidae	Glomeridesmidae Jovem	-	X
Entognatha	Collembola	Paronellidae	Paronellidae sp.1	X	-
		Symphypleona	Symphypleona sp.2	X	X
Insecta	Coleoptera		Coleoptera Jovem	X	X
			Coleoptera sp.1	-	X
		Tenebrionidae	Tenebrionidae sp.8	X	-
	Diptera		Diptera Jovem	-	X
		Ceratopogonidae	Ceratopogonidae Jovem	X	-
	Heteroptera		Heteroptera Jovem	X	-
		Cydnidae	Cydnidae Jovem	X	-
			Pangaeus sp.1	-	X
		Lygaeidae	Lygaeidae Jovem	X	-
		Reduviidae	*Zelurus* Jovem	-	X
	Hymenoptera	Formicidae	*Tranopelta gilva*	X	-
	Isoptera	Termitidae	*Nasutitermes* sp.5	-	X
	Orthoptera	Phalangopsidae	Phalangopsidae sp.	X	X
			Eidmanacris sp.1	X	-
			Paraclodes sp.1	X	X
			Phalangopsis sp.1	X	-
	Psocoptera	Psocomorpha	Psocomorpha Jovem	-	X
	Zygentoma	Nicoletiidae	Nicoletiinae sp.1	-	X
Mammalia	Chiroptera	Phyllostomidae	*Carollia* sp.	X	X
			Glossophaga sp.	-	X
Gastropoda	Pulmonata	Subulinidae	*Lamellaxis* sp.1	X	-
		Systrophiidae	*Happia* sp.1	-	X

SB-0172					
TÁXONS				Seco	Úmido
Clitellata	Haplotaxida		Haplotaxida sp.11	-	X
Arachnida	Amblypygi	Phrynidae	*Heterophrynus longicornis*	X	-
	Araneae	Ochyroceratidae	Ochyroceratidae Jovem	X	-
			Speocera sp.1	X	-
		Pholcidae	Pholcidae Jovem	X	X
		Symphytognathidae	*Anapistula* sp.1	X	-
		Theraphosidae	Theraphosidae Jovem	-	X
		Theridiosomatidae	*Plato* sp.1	X	-
		Trechaleidae	Trechaleidae Jovem	X	-
	Opiliones	Escadabiidae	Escadabiidae sp.2	X	-
		Manaosbiidae	Manaosbiidae Jovem	X	-
		Sclerosomatidae	Sclerosomatidae Jovem	X	-
	Pseudoscorpiones	Chernetidae	*Spelaeochernes* sp.1	X	-
Chilopoda	Scolopendromorpha	Scolopendridae	*Otostigmus* sp.1	X	-
		Scolopocryptopidae	*Newportia* sp.4	X	-
Diplopoda	Polydesmida	Pyrgodesmidae	Pyrgodesmidae Jovem	X	-
Entognatha	Collembola	Entomobryidae	Entomobryidae sp.3	-	X
		Isotomidae	Isotomidae sp.1	X	-
		Paronellidae	Paronellidae sp.1	X	-
		Symphypleona	Symphypleona sp.2	X	X
Insecta	Coleoptera		Coleoptera Jovem	X	X
		Staphylinidae	Pselaphinae sp.8	X	-
			Staphylininae sp.3	X	-
	Diptera		Diptera Jovem	X	X
		Sciaridae	Sciaridae Jovem	X	-
		Ceratopogonidae	Ceratopogonidae Jovem	X	-
		Tipulidae	Tipulidae sp.1	-	X
	Heteroptera	Ceratocombidae	Ceratocombidae sp.1	X	-
		Cydnidae	*Pangaeus* sp.1	X	-
	Homoptera	Cixiidae	Cixiidae Jovem	X	X
	Hymenoptera	Apidae	Apidae sp.3	X	-
		Formicidae	*Azteca* sp.1	-	X
			Camponotus sp.2	-	X
			Crematogaster limata	-	X
			Nylanderia sp.1	X	-
			Nylanderia sp.2	-	X
			Pachycondyla harpax	-	X
			Pheidole sp.8	-	X
			Solenopsis invicta	-	X
	Isoptera	Termitidae	Termitidae sp.	-	X
			Nasutitermes sp.6	-	X
	Orthoptera		Orthoptera sp.	X	X
		Phalangopsidae	*Paraclodes* sp.1	-	X
	Psocoptera	Psocomorpha	Psocomorpha Jovem	X	-
	Zygentoma	Nicoletiidae	Nicoletiinae sp.1	-	X
Malacostraca	Decapoda		Decapoda sp.	-	X
	Isopoda	Philosciidae	Philosciidae Jovem	X	-
			Philosciidae sp.1	X	-
Amphibia	Anura		Anura sp.1	-	X
		Bufonidae	*Rhinella* gr. *marina*	-	X
Mammalia	Chiroptera	Phyllostomidae	*Carollia perspicillata*	X	-
			Carollia sp.	-	X
			Glossophaga sp.	X	-
Reptilia	Squamata	Gymnophthalmidae	*Neusticurus ecpleopus*	-	X

SB-0173					
TÁXONS				**Seco**	**Úmido**
Arachnida	Amblypygi	Phrynidae	*Heterophrynus longicornis*	X	X
	Araneae	Ctenidae	*Ancylometes rufus*	-	X
		Theridiosomatidae	*Plato* sp.1	X	X
Acari	Mesostigmata		Mesostigmata sp.2	-	X
Entognatha	Collembola	Paronellidae	*Cyphoderus agnotus*	-	X
			Cyphoderus arlei	-	X
Insecta	Coleoptera	Gyrinidae	*Gyretes* sp.1	X	-
			Gyrinus sp.1	X	-
		Nitidulidae	Stelidota sp.1	-	X
		Staphylinidae	Staphylininae sp.12	X	-
	Diptera		Diptera Jovem	-	X
		Drosophilidae	Drosophilidae sp.1	-	X
		Psychodidae	Psychodidae sp.1	-	X
	Orthoptera	Phalangopsidae	*Phalangopsis* sp.1	-	X
Malacostraca	Decapoda		Decapoda sp.	-	X
Mammalia	Chiroptera		Chiroptera sp.	X	-
		Phyllostomidae	*Carollia* sp.	-	X
			Phyllostomus sp.	-	X

SB-0174					
TÁXONS				**Seco**	**Úmido**
Clitellata	Haplotaxida		Haplotaxida sp.11	-	X
Arachnida	Amblypygi	Phrynidae	*Heterophrynus longicornis*	-	X
	Araneae	Corinnidae	Corinnidae Jovem	-	X
		Ctenidae	Ctenidae Jovem	X	-
		Mysmenidae	*Microdipoena* sp.1	-	X
		Ochyroceratidae	Ochyroceratidae Jovem	X	X
			Speocera sp.1	-	X
		Pholcidae	Pholcidae Jovem	-	X
			Mesabolivar eberhardi	X	-
		Scytodidae	Scytodidae Jovem	-	X
		Symphytognathidae	*Anapistula* sp.1	-	X
		Tetrablemmidae	Tetrablemmidae Jovem	X	-
		Theridiosomatidae	Theridiosomatidae Jovem	-	X
			Plato sp.1	-	X
	Opiliones	Escadabiidae	Escadabiidae Jovem	-	X
	Pseudoscorpiones	Chernetidae	*Spelaeochernes* sp.1	X	-
Acari	Trombidiforme	Trombidiidae	Trombidiidae sp.1	-	X
Diplopoda	Glomeridesmida	Glomeridesmidae	Glomeridesmidae sp.1	-	X
	Polydesmida	Pyrgodesmidae	Pyrgodesmidae sp.1	X	-
Entognatha	Diplura	Campodeidae	Campodeidae sp.1	-	X
Insecta	Blattaria	Blattidae	Blattidae Jovem	-	X
	Coleoptera		Coleoptera Jovem	-	X
	Diptera		Diptera Jovem	-	X
		Cecidomyiidae	Cecidomyiidae sp.1	-	X
		Psychodidae	Phlebotominae sp.1	-	X
		Tipulidae	Tipulidae sp.1	X	X
	Heteroptera	Reduviidae	*Zelurus* Jovem	X	-
	Hymenoptera	Formicidae	*Dolichoderus bispinosus*	X	X
	Isoptera	Termitidae	*Nasutitermes* sp.1	X	-
			Nasutitermes sp.2	-	X
	Orthoptera	Phalangopsidae	*Phalangopsinae* sp.	-	X
			Paraclodes sp.1	X	-
			Phalangopsis sp.1	-	X
	Psocoptera	Psocomorpha	Psocomorpha Jovem	-	X
Malacostraca	Isopoda	Philosciidae	Philosciidae sp.2	X	-
Amphibia	Anura	Craugastoridae	*Pristimantis* cf. *fenestratus*	-	X
Mammalia	Chiroptera	Emballonuridae	*Peropteryx kappleri*	X	-
Gastropoda	Pulmonata	Subulinidae	*Lamellaxis* sp.2	-	X

SB-0175					
TÁXONS				**Seco**	**Úmido**
Arachnida	Amblypygi		Amblypygi sp.	-	X
	Araneae	Araneidae	Araneidae Jovem	X	-
		Corinnidae	Corinnidae Jovem	X	-
		Ochyroceratidae	Ochyroceratidae Jovem	X	X
		Oonopidae	Oonopidae Jovem	X	-
		Pholcidae	Pholcidae Jovem	X	X
			Mesabolivar eberhardi	-	X
		Salticidae	Salticidae Jovem	X	X
		Scytodidae	*Scytodes* sp.2	-	X
		Theridiosomatidae	*Plato* sp.1	-	X
	Opiliones	Sclerosomatidae	Sclerosomatidae Jovem	-	X
	Pseudoscorpiones	Chernetidae	Chernetidae Jovem	X	-
			Spelaeochernes sp.1	X	X
	Scorpiones	Buthidae	*Ananteris* Jovem	-	X
Acari	Ixodida	Ixodidae	*Ixodes* sp.1	-	X
	Trombidiforme	Trombiculidae	Trombiculidae sp.1	-	X
Diplopoda	Polydesmida	Fuhrmannodesmidae	Fuhrmannodesmidae Jovem	-	X
Entognatha	Collembola	Entomobryidae	Entomobryidae sp.1	X	-
		Paronellidae	Paronellidae sp.1	X	X
Insecta	Blattaria	Blattidae	Blattidae Jovem	X	-
		Polyphagidae	Polyphagidae Jovem	X	-
			Polyphagidae sp.1	X	-
	Coleoptera		Coleoptera Jovem	-	X
		Curculionidae	Curculionidae sp.	X	-
		Lampyridae	Lampyridae sp.1	-	X
	Diptera		Diptera Jovem	-	X
		Cecidomyiidae	Cecidomyiidae sp.1	-	X
		Ceratopogonidae	Ceratopogonidae Jovem	X	-
		Psychodidae	Phlebotominae sp.1	-	X
		Tipulidae	Tipulidae sp.1	-	X
			Tipulidae sp.3	-	X
	Heteroptera	Reduviidae	Triatoma Jovem	X	-
	Homoptera	Cixiidae	Cixiidae sp.6	X	-
		Cicadellidae	Cicadellidae sp.1	-	X
	Hymenoptera	Formicidae	*Camponotus cingulatus*	X	-
			Dolichoderus bispinosus	X	X
			Pachycondyla constricta	X	X
	Isoptera	Termitidae	*Cornitermes* sp.1	X	-
			Nasutitermes sp.1	-	X
			Nasutitermes sp.2	X	X
			Nasutitermes sp.3	X	-
	Lepidoptera	Noctuoidea	Noctuoidea sp.7	X	X
	Neuroptera	Myrmeleontidae	Myrmeleontidae sp.5	-	X
	Orthoptera		Orthoptera sp.	X	X
		Phalangopsidae	*Phalangopsis* sp.1	X	-
	Psocoptera	Psocomorpha	Psocomorpha Jovem	X	-
		Ptiloneuridae	*Ptiloneura* sp.1	X	-
Malacostraca	Isopoda	Armadillidae	Armadillidae Jovem	X	X
		Philosciidae	Philosciidae Jovem	X	X
			Philosciidae sp.1	-	X
			Philosciidae sp.2	X	X
Mammalia	Chiroptera	Emballonuridae	*Peropteryx* sp.	X	-
		Phyllostomidae	*Carollia* sp.	X	-
Gastropoda	Pulmonata	Systrophiidae	*Happia* sp.1	-	X

SB-0176					
TÁXONS				Seco	Úmido
Arachnida	Amblypygi	Phrynidae	*Heterophrynus longicornis*	X	X
	Araneae	Araneidae	*Alpaida antonio*	X	-
		Corinnidae	Corinnidae Jovem	X	X
		Ctenidae	Ctenidae Jovem	-	X
		Ochyroceratidae	Ochyroceratidae Jovem	X	X
		Pholcidae	Pholcidae Jovem	X	X
			Pholcidae sp.	-	X
		Tetrablemmidae	Tetrablemmidae sp.1	X	-
		Theridiosomatidae	*Plato* sp.	-	X
			Plato sp.1	X	X
	Opiliones	Cosmetidae	*Roquettea* sp.1	X	-
		Sclerosomatidae	Sclerosomatidae Jovem	-	X
			Prionostema sp.1	X	-
	Pseudoscorpiones	Chernetidae	*Spelaeochernes* sp.1	X	X
		Chthoniidae	*Pseudochthonius* sp.2	X	X
	Ricinulei	Ricinoididae	*Cryptocellus canga*	X	-
	Schizomida	Hubardiidae	*Rowlandius* sp.1	X	-
Acari	Acariforme		Acariforme sp.1	-	X
Chilopoda	Geophilomorpha	Macronicophilidae	*Macronicophilus* sp.1	-	X
	Scolopendromorpha	Scolopocryptopidae	Scolopocryptopidae sp.	X	X
			Newportia sp.6	X	-
			Tidops sp.1	X	-
Diplopoda	Glomeridesmida	Glomeridesmidae	Glomeridesmidae sp.1	X	-
	Polydesmida	Chelodesmidae	Chelodesmidae Jovem	-	X
		Fuhrmannodesmidae	Fuhrmannodesmidae Jovem	-	X
			Fuhrmannodesmidae sp.1	X	-
			Fuhrmannodesmidae sp.3	-	X
		Pyrgodesmidae	Pyrgodesmidae sp.1	X	X
Entognatha	Collembola	Paronellidae	*Cyphoderus agnotus*	-	X
			Cyphoderus javanus	-	X
	Diplura	Campodeidae	Campodeidae sp.1	X	X
Insecta	Coleoptera	Staphylinidae	Pselaphinae sp.2	X	-
		Tenebrionidae	Tenebrionidae Jovem	X	-
	Diptera	Cecidomyiidae	Cecidomyiidae sp.3	-	X
		Psychodidae	Phlebotominae sp.1	-	X
	Homoptera	Cixiidae	Cixiidae Jovem	X	-
	Hymenoptera	Formicidae	*Apterostigma pilosum*	-	X
			Nylanderia sp.2	-	X
			Pachycondyla constricta	X	X
			Pachycondyla impressa	-	X
	Isoptera	Rhinotermitidae	*Heterotermes* sp.1	X	-
		Termitidae	*Nasutitermes* sp.2	-	X
	Lepidoptera		Lepidoptera sp.	-	X
	Orthoptera	Phalangopsidae	Phalangopsidae sp.	-	X
			Phalangopsidae sp.1	-	X
			Paraclodes sp.1	-	X
			Phalangopsis sp.1	X	-
Malacostraca	Isopoda	Armadillidae	Armadillidae sp.1	-	X
		Philosciidae	Philosciidae Jovem	X	X
Gastropoda	Pulmonata	Subulinidae	*Lamellaxis* sp.2	X	-
		Systrophiidae	*Happia* sp.1	X	-

SB-0177					
TÁXONS				Seco	Úmido
Arachnida	Amblypygi		Amblypygi sp.	-	X
		Phrynidae	*Heterophrynus longicornis*	-	X
	Araneae	Ctenidae	Ctenidae Jovem	-	X
		Drymusidae	Drymusidae Jovem	X	X
		Ochyroceratidae	Ochyroceratidae Jovem	-	X
		Oonopidae	Oonopidae sp.2	-	X
		Palpimanidae	Palpimanidae Jovem	-	X
		Pholcidae	Pholcidae Jovem	X	X
			Mesabolivar aurantiacus	X	X
		Pisauridae	Pisauridae Jovem	-	X
		Theraphosidae	Theraphosidae Jovem	-	X
		Theridiidae	*Achaearanea* sp.1	X	-
		Theridiosomatidae	Theridiosomatidae Jovem	X	X
			Plato sp.	-	X
		Trechaleidae	Trechaleidae Jovem	X	X
	Opiliones	Cosmetidae	Cosmetidae Jovem	X	-
		Escadabiidae	Escadabiidae sp.2	X	X
	Pseudoscorpiones	Chernetidae	*Spelaeochernes* sp.1	X	X
		Chthoniidae	*Pseudochthonius* sp.3	-	X
			Pseudochthonius sp.4	X	-
Acari	Mesostigmata		Mesostigmata sp.2	X	-
	Sarcoptiforme	Oribatida	Oribatida sp.10	-	X
			Oribatida sp.6	X	-
Chilopoda	Geophilomorpha		Geophilomorpha sp.	-	X
		Ballophilidae	Ballophilidae sp.1	X	-
	Scolopendromorpha	Cryptopidae	*Cryptops* Jovem	-	X
Diplopoda	Polydesmida		Polydesmida Jovem	X	-
		Fuhrmannodesmidae	Fuhrmannodesmidae Jovem	-	X
			Fuhrmannodesmidae sp.1	-	X
	Siphoniulida	Siphoniulidae	Siphoniulidae sp.1	X	-
Entognatha	Collembola	Paronellidae	Paronellidae sp.1	X	X
			Paronellidae sp.3	X	-
	Diplura	Campodeidae	Campodeidae sp.1	X	X
Insecta	Blattaria	Blattidae	Blattidae Jovem	X	X
	Coleoptera		Coleoptera Jovem	X	X
		Chrysomelidae	Chrysomelidae sp.2	X	-
		Hydrophilidae	Hydrophilidae sp.5	-	X
		Ptilidae	Ptilidae sp.3	X	-
		Staphylinidae	Pselaphinae sp.7	-	X
	Diptera		Diptera Jovem	X	X
		Drosophilidae	Drosophilidae sp.1	-	X
		Mycetophilidae	Mycetophilidae sp.2	-	X
		Psychodidae	Psychodidae Jovem	X	-
			Psychodidae sp.1	-	X
		Tipulidae	Tipulidae sp.1	-	X
	Heteroptera	Cydnidae	Cydnidae Jovem	X	-
			Pangaeus sp.1	X	X
		Reduviidae	*Zelurus* Jovem	X	X
		Schizopteridae	Schizopteridae sp.3	X	-
		Veliidae	*Microvelia* Jovem	X	-
			Paravelia Jovem	X	-
			Paravelia sp.1	-	X
	Homoptera	Cixiidae	Cixiidae Jovem	X	-
	Hymenoptera	Formicidae	*Camponotus cingulatus*	X	X
			Carebara pr. *urichi*	-	X
			Eurhopalothrix sp.1	X	-
			Gnamptogenys regularis	X	-

			Heteroponera sp.1	-	X
			Nylanderia sp.1	X	X
			Nylanderia sp.2	X	-
			Pachycondyla constricta	X	-
			Pheidole sp.1	X	-
			Pheidole sp.4	-	X
			Pheidole sp.5	X	-
			Pheidole sp.7	-	X
			Wasmannia auropunctata	X	X
	Isoptera	Termitidae	*Nasutitermes* sp.1	-	X
			Nasutitermes sp.2	X	X
	Lepidoptera		Lepidoptera Jovem	X	-
			Lepidoptera sp.	-	X
	Orthoptera		Orthoptera sp.1	X	X
		Phalangopsidae	*Phalangopsis* sp.1	X	X
	Psocoptera	Psocomorpha	Psocomorpha Jovem	X	X
		Epipsocidae	Epipsocidae sp.4	-	X
	Trichoptera		Trichoptera Jovem	-	X
	Zygentoma	Nicoletiidae	Nicoletiinae sp.1	X	X
Malacostraca	Decapoda	Pseudothelphusidae	Pseudothelphusidae Jovem	-	X
			Pseudothelphusidae sp.	X	-
	Isopoda	Armadillidae	Armadillidae Jovem	X	X
			Armadillidae sp.1	-	X
		Philosciidae	Philosciidae Jovem	X	-
			Philosciidae sp.1	-	X
			Philosciidae sp.2	-	X
Symphyla		Scutigerellidae	Hanseniella sp.1	X	-
Amphibia	Anura		Anura Jovem	-	X
		Craugastoridae	*Pristimantis* cf. *fenestratus*	-	X
Mammalia	Chiroptera	Emballonuridae	*Peropteryx* sp.	X	X
		Phyllostomidae	*Carollia perspicillata*	X	-
			Carollia sp.	-	X
			Glossophaga sp.	X	-
			Phyllostomus sp.	X	X
Reptilia	Squamata	Dipsadidae	*Erythrolamprus* cf. *reginae*	X	-
		Gymnophthalmidae	*Neusticurus* cf. *ecpleopus*	-	X
			Neusticurus ecpleopus	-	X
Gastropoda	Pulmonata	Subulinidae	*Lamellaxis* sp.3	X	-
			Leptinaria sp.2	-	X
		Systrophiidae	*Happia* sp.1	-	X

SB-0178					
TÁXONS				**Seco**	**Úmido**
Arachnida	Amblypygi		Amblypygi sp.	-	X
		Phrynidae	*Heterophrynus longicornis*	X	-
	Araneae	Corinnidae	Corinnidae Jovem	X	-
		Drymusidae	Drymusidae Jovem	X	X
			Drymusa spelunca	-	X
		Ochyroceratidae	Ochyroceratidae Jovem	X	X
		Pholcidae	Pholcidae Jovem	X	-
		Tetrablemmidae	Tetrablemmidae sp.1	X	-
		Theridiosomatidae	*Plato* sp.1	-	X
	Opiliones	Escadabiidae	Escadabiidae sp.1	X	X
	Pseudoscorpiones	Chernetidae	*Spelaeochernes* sp.1	X	-
		Chthoniidae	Chthoniidae Jovem	X	-
Acari	Ixodida	Ixodidae	*Amblyomma cajennense*	-	X
Diplopoda	Glomeridesmida	Glomeridesmidae	Glomeridesmidae sp.1	-	X
	Polydesmida	Fuhrmannodesmidae	Fuhrmannodesmidae sp.1	-	X
Entognatha	Diplura	Campodeidae	Campodeidae sp.1	X	-
Insecta	Coleoptera		Coleoptera Jovem	X	X
	Diptera	Psychodidae	Phlebotominae sp.1	-	X
		Tipulidae	Tipulidae sp.1	-	X
			Tipulidae sp.3	-	X
	Heteroptera	Reduviidae	*Zelurus* Jovem	-	X
		Tingidae	Tingidae Jovem	X	-
	Homoptera	Cixiidae	Cixiidae Jovem	-	X
			Cixiidae sp.1	X	-
	Hymenoptera	Formicidae	*Gnamptogenys minuta*	X	-
			Pheidole sp.1	-	X
			Pheidole sp.4	-	X
			Pheidole sp.5	X	-
	Isoptera	Termitidae	*Nasutitermes* sp.	-	X
			Nasutitermes sp.2	X	-
			Nasutitermes sp.3	-	X
	Orthoptera	Phalangopsidae	*Phalangopsis* sp.1	X	X
Malacostraca	Isopoda	Philosciidae	Philosciidae Jovem	X	-
			Philosciidae sp.2	X	-
Amphibia	Anura	Craugastoridae	*Pristimantis* cf. *fenestratus*	X	-
Mammalia	Chiroptera	Phyllostomidae	*Carollia* sp.	X	X
Gastropoda	Pulmonata	Subulinidae	Subulinidae Jovem	X	-
			sp.2	X	-

SB-0179					
TÁXONS				**Seco**	**Úmido**
Arachnida	Amblypygi	Charinidae	Charinidae Jovem	X	-
		Phrynidae	*Heterophrynus longicornis*	X	X
	Araneae	Ochyroceratidae	*Speocera* sp.1	X	-
		Psauridae	Psauridae sp.	X	-
		Theridiosomatidae	*Plato* sp.	X	-
			Plato sp.1	-	X
		Trechaleidae	Trechaleidae Jovem	-	X
			Rhoicinus sp.1	-	X
	Opiliones	Escadabiidae	Escadabiidae Jovem	X	-
	Pseudoscorpiones	Chthoniidae	*Pseudochthonius* sp.4	X	-
Acari	Astigmata		Astigmata sp.1	X	-
Diplopoda	Glomeridesmida	Glomeridesmidae	Glomeridesmidae sp.1	X	X
	Polydesmida	Fuhrmannodesmidae	Fuhrmannodesmidae sp.1	X	-
Entognatha	Collembola	Isotomidae	Isotomidae sp.1	X	-
		Paronellidae	Paronellidae sp.1	X	-
		Symphypleona	Symphypleona sp.2	X	-
Insecta	Coleoptera	Dytiscidae	*Laccophilus* sp.3	X	X
	Diptera	Tipulidae	Tipulidae sp.1	-	X
	Heteroptera	Veliidae	*Rhagovelia* sp.2	-	X
			Rhagovelia sp.3	X	X
	Hymenoptera	Formicidae	*Nylanderia* sp.1	X	-
			Nylanderia sp.2	-	X
	Orthoptera	Phalangopsidae	*Phalangopsis* sp.1	X	X
Malacostraca	Decapoda		Decapoda sp.	X	-
Mammalia	Chiroptera	Phyllostomidae	*Carollia* sp.	X	-
Gastropoda	Pulmonata	Subulinidae	*Lamellaxis* sp.1	X	-
Nematoda	Diplogasteria		*Diplogasteria* sp.3	-	X

SB-0180					
TÁXONS				**Seco**	**Úmido**
Arachnida	Amblypygi		Amblypygi sp.	-	X
		Phrynidae	*Heterophrynus longicornis*	X	-
	Araneae	Pholcidae	Pholcidae Jovem	X	-
			Mesabolivar aurantiacus	X	-
		Trechaleidae	Trechaleidae Jovem	X	X
	Opiliones	Stygnidae	*Protimesius gracilis*	X	-
Insecta	Diptera	Tipulidae	Tipulidae sp.1	-	X
	Lepidoptera		Lepidoptera sp.	-	X
	Orthoptera	Phalangopsidae	*Phalangopsis* sp.1	X	X

SB-0181					
**TÁXONS**				**Seco**	**Úmido**
Arachnida	Amblypygi		Amblypygi sp.	-	X
	Araneae		Araneae sp.	X	-
		Araneidae	Araneidae Jovem	X	-
		Pholcidae	Pholcidae Jovem	X	-
			Mesabolivar eberhardi	-	X
	Opiliones	Sclerosomatidae	*Prionostema* sp.1	X	-
	Schizomida	Hubardiidae	Hubardiidae Jovem	X	-
Entognatha	Collembola	Paronellidae	Paronellidae sp.1	X	-
Insecta	Coleoptera	Carabidae	Carabidae sp.4	-	X
		Nitidulidae	Stelidota sp.1	X	-
		Staphylinidae	Staphylininae sp.12	X	-
			Staphylininae sp.8	X	-
	Diptera		Diptera Jovem	X	-
		Ceratopogonidae	Ceratopogonidae Jovem	X	-
	Heteroptera	Cydnidae	*Pangaeus* sp.1	X	-
		Veliidae	*Paravelia* sp.2	-	X
	Homoptera	Cixiidae	Cixiidae Jovem	X	-
	Hymenoptera	Formicidae	*Camponotus* sp.2	X	-
			Camponotus sp.3	X	-
			Megalomyrmex sp.1	X	-
			Nylanderia sp.2	X	-
			Pheidole sp.16	X	-
			Pheidole sp.7	X	-
			Lepidoptera Jovem	X	-
	Orthoptera		Orthoptera sp.	X	-
		Phalangopsidae	*Phalangopsis* sp.1	-	X
	Psocoptera	Psocomorpha	Psocomorpha Jovem	X	-
Mammalia	Chiroptera	Emballonuridae	*Peropteryx kappleri*	X	-
			Peropteryx sp.	-	X
		Phyllostomidae	*Carollia perspicillata*	X	-
			Glossophaga sp.	X	-

SB-0182					
TÁXONS				**Seco**	**Úmido**
Arachnida	Amblypygi		Amblypygi sp.	-	X
	Araneae		Araneae sp.	-	X
		Ctenidae	Ctenidae Jovem	X	-
		Pholcidae	*Mesabolivar aurantiacus*	-	X
		Pisauridae	Pisauridae Jovem	-	X
		Prodidomidae	Prodidomidae sp.4	X	-
	Opiliones	Stygnidae	*Protimesius gracilis*	-	X
Entognatha	Collembola	Entomobryidae	Entomobryidae sp.3	X	-
Insecta	Coleoptera	Gyrinidae	*Gyretes* sp.1	X	-
			Gyrinus sp.1	-	X
	Diptera	Culicidae	Culicinae sp.1	-	X
		Tipulidae	Tipulidae sp.1	-	X
	Heteroptera		Heteroptera Jovem	-	X
		Veliidae	*Rhagovelia* Jovem	X	-
			Rhagovelia sp.2	X	-
			Rhagovelia sp.3	X	-
	Hymenoptera	Formicidae	*Camponotus* sp.2	-	X
			Gnamptogenys strigata	X	-
			Nylanderia sp.1	-	X
	Isoptera	Termitidae	*Nasutitermes* sp.1	X	-
	Lepidoptera		Lepidoptera sp.	-	X
	Odonata	Coenagrionidae	Coenagrionidae sp.	X	-
	Orthoptera	Phalangopsidae	*Paraclodes* sp.1	-	X
Malacostraca	Decapoda	Pseudothelphusidae	Pseudothelphusidae sp.1	-	X
	Isopoda	Philosciidae	Philosciidae sp.1	X	-
Amphibia	Anura		Anura Jovem	X	X
		Craugastoridae	*Pristimantis* cf. *fenestratus*	-	X
		Leptodactylidae	*Adenomera* cf. *andreae*	X	-
Mammalia	Chiroptera	Furipteridae	*Furipterus horrens*	-	X
		Phyllostomidae	*Carollia* sp.	-	X
			Glossophaga sp.	X	X
	Rodentia	Cricetidae	*Rhipidomys* sp.	-	X
Reptilia	Squamata	Gymnophthalmidae	*Neusticurus ecpleopus*	X	-

SB-0183					
TÁXONS				Seco	Úmido
Arachnida	Araneae		Araneae sp.	-	X
		Corinnidae	Corinnidae Jovem	X	X
		Pholcidae	Pholcidae Jovem	X	X
		Salticidae	Salticidae Jovem	X	-
		Scytodidae	Scytodidae Jovem	X	-
			Scytodes eleonorae	-	X
		Theridiidae	*Achaearanea* sp.1	-	X
		Theridiosomatidae	*Plato* sp.1	-	X
	Opiliones	Sclerosomatidae	Sclerosomatidae Jovem	-	X
			Prionostema sp.1	X	-
	Pseudoscorpiones	Chernetidae	Chernetidae Jovem	X	-
			Spelaeochernes sp.1	X	X
Acari	Mesostigmata		Mesostigmata sp.1	-	X
Chilopoda	Scutigeromorpha		Scutigeromorpha Jovem	X	-
Diplopoda	Polydesmida	Fuhrmannodesmidae	Fuhrmannodesmidae sp.1	X	-
		Pyrgodesmidae	Pyrgodesmidae sp.2	-	X
Entognatha	Collembola	Paronellidae	Paronellidae sp.1	X	-
			Cyphoderus agnotus	X	-
			Cyphoderus javanus	X	-
	Diplura	Campodeidae	Campodeidae sp.1	X	-
Insecta	Blattaria	Blaberidae	Blaberidae Jovem	-	X
		Blattidae	Blattidae Jovem	-	X
	Coleoptera	Staphylinidae	Staphylinidae sp.6	-	X
			Staphylininae sp.12	X	-
	Diptera		Diptera Jovem	X	X
		Tipulidae	Tipulidae sp.1	-	X
	Heteroptera		Heteroptera Jovem	-	X
		Cydnidae	Cydnidae Jovem	X	-
			Pangaeus sp.1	X	-
		Miridae	Mirinae Jovem	-	X
		Nabidae	Nabidae Jovem	-	X
	Hymenoptera	Bethylidae	Bethylidae sp.5	-	X
		Diapriidae	Diapriidae sp.12	X	-
		Formicidae	*Camponotus cingulatus*	X	-
			Camponotus sp.2	X	-
			Camponotus sp.3	X	X
			Gnamptogenys striatula	X	X
			Nylanderia sp.1	X	-
			Solenopsis invicta	-	X
			Solenopsis sp.3	X	-
	Isoptera	Termitidae	*Nasutitermes* sp.1	-	X
			Nasutitermes sp.2	X	-
	Lepidoptera	Noctuoidea	Noctuoidea sp.1	-	X
	Orthoptera		Orthoptera sp.	X	X
		Phalangopsidae	*Paraclodes* sp.1	-	X
	Psocoptera	Psyllipsocidae	Psyllipsocidae sp.4	X	-
	Zygentoma	Nicoletiidae	Nicoletiinae sp.1	-	X
Malacostraca	Isopoda	Armadillidae	Armadillidae sp.1	X	-
Amphibia	Anura	Craugastoridae	*Pristimantis* cf. *fenestratus*	-	X
Mammalia	Chiroptera	Emballonuridae	*Peropteryx* sp.	X	-
		Phyllostomidae	*Carollia perspicillata*	X	-
			Carollia sp.	-	X
			Glossophaga sp.	-	X
			Phyllostomus sp.	-	X

SB-0184					
TÁXONS				Seco	Úmido
Arachnida	Amblypygi	Phrynidae	*Heterophrynus longicornis*	X	X
	Araneae	Araneidae	Araneidae Jovem	-	X
			Alpaida antonio	X	-
		Corinnidae	*Abapeba hoeferi*	X	-
		Ctenidae	Ctenidae Jovem	X	X
			Ctenidae sp.	-	X
		Nemesiidae	Nemesiidae Jovem	X	-
		Ochyroceratidae	Ochyroceratidae Jovem	X	-
		Oonopidae	Oonopidae sp.2	X	-
		Pholcidae	Pholcidae Jovem	X	-
			Mesabolivar aurantiacus	X	X
		Salticidae	Salticidae sp.2	X	-
		Theridiidae	*Achaearanea* sp.1	X	X
		Trechaleidae	*Rhoicinus* sp.1	X	-
	Opiliones	Escadabiidae	Escadabiidae sp.1	-	X
		Stygnidae	*Protimesius gracilis*	-	X
	Pseudoscorpiones	Chernetidae	*Spelaeochernes* sp.1	X	-
		Chthoniidae	Chthoniidae Jovem	X	-
			Pseudochthonius sp.2	-	X
	Schizomida	Hubardiidae	Hubardiidae Jovem	X	-
			Rowlandius sp.1	-	X
Chilopoda	Scolopendromorpha	Cryptopidae	*Cryptops* sp.1	X	-
		Scolopocryptopidae	*Newportia* sp.6	X	-
Entognatha	Collembola	Isotomidae	Isotomidae sp.1	-	X
		Paronellidae	Paronellidae sp.1	X	-
		Symphypleona	Symphypleona sp.2	-	X
	Diplura	Campodeidae	Campodeidae sp.1	X	-
Insecta	Blattaria	Blattidae	Blattidae Jovem	X	-
	Coleoptera		Coleoptera Jovem	X	X
		Curculionidae	Curculionidae sp.3	X	-
	Diptera	Sciaridae	Sciaridae sp.2	-	X
		Ceratopogonidae	Ceratopogonidae sp.3	X	X
		Psychodidae	Phlebotominae sp.1	X	X
		Tipulidae	Tipulidae sp.1	X	X
	Heteroptera		Heteroptera Jovem	-	X
		Cydnidae	*Pangaeus* sp.1	X	-
		Reduviidae	Emesinae Jovem	-	X
			Emesinae sp.6	-	X
			Zelurus Jovem	X	X
			Triatoma Jovem	X	-
	Homoptera	Cixiidae	Cixiidae Jovem	-	X
	Hymenoptera	Formicidae	Formicidae sp.	-	X
			Apterostigma pilosum	-	X
			Camponotus sp.2	X	X
			Dolichoderus bispinosus	X	-
			Gnamptogenys striatula	-	X
			Pachycondyla constricta	-	X
			Prionopelta modesta	-	X
			Solenopsis sp.2	X	-
	Isoptera	Termitidae	*Nasutitermes* sp.2	X	-
	Lepidoptera	Noctuoidea	*Noctuoidea* sp.1	-	X
	Neuroptera	Mantispidae	*Plega* sp.1	X	-
	Orthoptera		Orthoptera sp.	X	-
		Phalangopsidae	*Phalangopsis* sp.1	X	X
	Psocoptera	Psocomorpha	Psocomorpha Jovem	X	-
		Psyllipsocidae	Psyllipsocidae sp.4	X	-
	Zygentoma	Nicoletiidae	Nicoletiinae sp.1	-	X

Malacostraca	Isopoda	Philosciidae	Philosciidae sp.1	-	X
			Philosciidae sp.2	X	-
Symphyla			Symphyla Jovem	-	X
Amphibia	Anura	Craugastoridae	*Pristimantis* cf. *fenestratus*	-	X
Mammalia	Chiroptera	Phyllostomidae	*Carollia* sp.	-	X
			Glossophaga sp.	X	X
Gastropoda	Pulmonata	Systrophiidae	*Happia* sp.1	X	-
Nematoda	Diplogasteria		*Diplogasteria* sp.2	-	X

SB-0185					
**TÁXON**				**Seco**	**Úmido**
Arachnida	Amblypygi		Amblypygi sp.	-	X
	Araneae	Corinnidae	Corinnidae Jovem	-	X
		Pholcidae	Pholcidae Jovem	-	X
			Mesabolivar aurantiacus	X	X
		Theridiidae	Theridiidae sp.1	-	X
			Theridion sp.2	-	X
		Trechaleidae	Trechaleidae Jovem	-	X
	Opiliones	Sclerosomatidae	Sclerosomatidae Jovem	-	X
			Prionostema sp.1	X	-
Acari	Ixodida	Ixodidae	*Amblyomma goeldii*	-	X
			Amblyomma rotundatum	-	X
Diplopoda	Polydesmida	Paradoxosomatidae	Paradoxosomatidae Jovem	-	X
Insecta	Coleoptera	Leiodidae	Leiodidae sp.1	-	X
		Staphylinidae	Pselaphinae sp.5	-	X
	Diptera		Diptera Jovem	-	X
		Dixidae	Dixidae sp.1	-	X
		Tipulidae	Tipulidae sp.1	-	X
	Heteroptera	Mesoveliidae	Madeoveliinae Jovem	-	X
		Veliidae	*Microvelia* sp.2	X	-
			Paravelia Jovem	X	-
			Paravelia sp.1	-	X
	Hymenoptera	Formicidae	*Nylanderia* sp.2	X	-
		Vespidae	Vespidae sp.1	X	-
	Isoptera	Termitidae	*Coatitermes* sp.1	-	X
			Nasutitermes sp.1	-	X
			Nasutitermes sp.2	X	X
	Orthoptera		Orthoptera sp.	-	X
		Phalangopsidae	Phalangopsidae sp.1	-	X
	Zygentoma	Nicoletiidae	Nicoletiinae sp.1	X	-
Malacostraca	Decapoda		Decapoda sp.	-	X
		Palaemonidae	Palaemonidae Jovem	-	X
			Macrobrachium sp.1	X	X
		Pseudothelphusidae	Pseudothelphusidae sp.1	X	X
Amphibia	Anura	Craugastoridae	*Pristimantis* cf. *fenestratus*	-	X
Mammalia	Chiroptera	Emballonuridae	*Peropteryx* sp.	-	X
		Phyllostomidae	*Carollia perspicillata*	X	-
			Carollia sp.	-	X
			Glossophaga sp.	X	X
			Phyllostomus sp.	X	X

SB-0186					
TÁXONS				Seco	Úmido
Clitellata	Haplotaxida		Haplotaxida sp.11	-	X
Arachnida	Amblypygi	Phrynidae	*Heterophrynus longicornis*	X	X
	Araneae	Drymusidae	Drymusidae Jovem	-	X
		Oonopidae	Oonopidae sp.3	X	-
		Pholcidae	Pholcidae Jovem	X	X
			Ninetinae sp.1	-	X
		Salticidae	Salticidae Jovem	-	X
		Theridiidae	*Episinus* sp.2	-	X
		Theridiosomatidae	*Ogulnius* sp.1	-	X
			Plato sp.1	-	X
		Trechaleidae	Trechaleidae Jovem	-	X
	Opiliones	Cosmetidae	Cosmetidae sp.1	-	X
			Roquettea carajas	X	X
		Neogoveidae	*Neogoveidae Jovem*	-	X
		Sclerosomatidae	*Prionostema* sp.1	-	X
		Stygnidae	*Protimesius gracilis*	-	X
	Pseudoscorpiones	Chernetidae	*Spelaeochernes* sp.1	X	X
		Chthoniidae	Chthoniidae Jovem	X	-
		Olpiidae	Olpiidae Jovem	-	X
		Syarinidae	*Nannobisium* sp.1	X	-
	Schizomida	Hubardiidae	Hubardiidae Jovem	X	-
	Scorpiones	Buthidae	*Tityus tucurui*	X	-
Acari	Astigmata		Astigmata sp.1	-	X
	Holothryda		Holothryda sp.1	-	X
	Sarcoptiforme	Oribatida	Oribatida sp.1	-	X
	Trombidiforme	Trombiculidae	Trombiculidae sp.2	-	X
		Trombidiidae	Trombidiidae sp.1	X	-
Chilopoda	Scolopendromorpha	Cryptopidae	*Cryptops* sp.	-	X
Diplopoda	Glomeridesmida	Glomeridesmidae	Glomeridesmidae Jovem	-	X
	Polydesmida	Fuhrmannodesmidae	Fuhrmannodesmidae Jovem	-	X
			Fuhrmannodesmidae sp.1	X	-
	Stemmiulida	Stemmiulidae	Stemmiulidae sp.2	X	-
Entognatha	Collembola	Isotomidae	Isotomidae sp.1	X	-
		Paronellidae	Paronellidae sp.1	X	X
		Onychiuridae	*Onychiurus* sp.2	-	X
		Symphypleona	Symphypleona sp.2	-	X
Insecta	Coleoptera		Coleoptera Jovem	-	X
		Scydmaenidae	Scydmaenidae sp.3	-	X
		Staphylinidae	Pselaphinae sp.3	-	X
	Diptera		Diptera Jovem	-	X
		Tipulidae	Tipulidae sp.1	X	X
	Heteroptera	Reduviidae	*Zelurus* Jovem	X	-
		Schizopteridae	Schizopteridae sp.1	-	X
	Homoptera	Cixiidae	Cixiidae Jovem	X	-
		Fulgoridae	Fulgoridae sp.2	-	X
	Hymenoptera	Formicidae	*Apterostigma pilosum*	-	X
			Carebara sp.11	-	X
			Crematogaster limata	X	-
			Pachycondyla constricta	X	X
	Isoptera	Termitidae	*Nasutitermes* sp.	X	-
			Nasutitermes sp.1	X	X
			Nasutitermes sp.2	-	X
	Lepidoptera		Lepidoptera Jovem	X	-
		Hesperiidae	Hesperiidae sp.1	X	-
		Noctuoidea	Noctuoidea sp.7	-	X
	Orthoptera		Orthoptera sp.	X	X
		Phalangopsidae	*Phalangopsis* sp.1	-	X

	Psocoptera	Psocomorpha	Psocomorpha Jovem	X	-
		Epipsocidae	Epipsocidae sp.3	-	X
Malacostraca	Isopoda	Philosciidae	Philosciidae sp.2	X	X
		Scleropactidae	Scleropactidae Jovem	-	X
			Scleropactidae sp.1	X	-
Amphibia	Anura	Craugastoridae	*Pristimantis* cf. *fenestratus*	X	-
Mammalia	Chiroptera	Phyllostomidae	*Carollia* sp.	X	-
Reptilia	Squamata	Tropiduridae	*Plica plica*	X	-
Gastropoda	Pulmonata	Spiraxidae	*Euglandina* sp.1	X	-
		Systrophiidae	Systrophiidae Jovem	-	X

SB-0187					
TÁXONS				**Seco**	**Úmido**
Arachnida	Amblypygi	Phrynidae	*Heterophrynus longicornis*	X	X
	Araneae	Drymusidae	Drymusidae Jovem	-	X
		Nemesiidae	Nemesiidae Jovem	-	X
		Ochyroceratidae	*Ochyrocera* sp.1	-	X
		Oonopidae	Oonopidae sp.2	-	X
		Pholcidae	Pholcidae Jovem	X	-
			Mesabolivar aurantiacus	-	X
			Ninetinae sp.1	-	X
		Salticidae	Salticidae sp.3	X	-
		Scytodidae	Scytodidae Jovem	X	X
			Scytodes eleonorae	X	X
		Theraphosidae	Theraphosidae Jovem	-	X
		Theridiidae	Theridiidae Jovem	-	X
		Theridiosomatidae	Theridiosomatidae Jovem	-	X
			Plato sp.1	-	X
	Opiliones	Cosmetidae	Cosmetidae sp.1	-	X
			Roquettea sp.1	X	-
	Pseudoscorpiones	Chernetidae	*Spelaeochernes* sp.1	X	X
	Scorpiones	Buthidae	*Ananteris* Jovem	-	X
Acari	Trombidiforme	Trombiculidae	Trombiculidae sp.3	-	X
Chilopoda	Geophilomorpha	Ballophilidae	Ballophilidae sp.	-	X
		Schendylidae	*Schendyylops* sp.1	-	X
Entognatha	Collembola	Paronellidae	Paronellidae sp.1	X	X
Insecta	Blattaria	Blaberidae	Blaberidae Jovem	-	X
		Polyphagidae	Polyphagidae Jovem	X	X
	Coleoptera		Coleoptera Jovem	-	X
	Diptera		Diptera Jovem	X	X
		Keroplatidae	Keroplatidae Jovem	-	X
		Tipulidae	Tipulidae sp.1	X	X
	Heteroptera	Reduviidae	*Zelurus* Jovem	X	X
			Triatoma Jovem	-	X
	Hymenoptera	Formicidae	*Acromyrmex* sp.1	-	X
			Apterostigma pilosum	-	X
			Camponotus atriceps	-	X
			Camponotus sp.2	X	-
			Cardiocondyla sp.1	-	X
			Pheidole sp.3	-	X
			Pheidole sp.4	-	X
			Pheidole sp.5	-	X
	Isoptera	Termitidae	*Nasutitermes* sp.1	-	X
			Nasutitermes sp.2	X	-
	Lepidoptera	Noctuoidea	Noctuoidea sp.7	X	-
		Tineoidea	Tineoidea sp.4	-	X
	Orthoptera		Orthoptera sp.	X	X
	Psocoptera	Psocomorpha	Psocomorpha Jovem	X	-
	Zygentoma	Nicoletiidae	Nicoletiinae sp.1	-	X
Malacostraca	Isopoda	Philosciidae	Philosciidae Jovem	X	-
			Philosciidae sp.2	X	X
Amphibia	Anura	Craugastoridae	*Pristimantis* cf. *fenestratus*	X	X
Mammalia	Chiroptera	Phyllostomidae	Glossophaginae sp.	X	-
			Glossophaga sp.	-	X
Turbellaria	Tricladida	Geoplanidae	Geoplanidae sp.2	X	-

SB-0188					
TÁXONS				**Seco**	**Úmido**
Arachnida	Araneae	Corinnidae	Corinnidae Jovem	X	X
		Pholcidae	*Mesabolivar eberhardi*		X
		Salticidae	Salticidae sp.2	X	
		Scytodidae	Scytodidae Jovem	X	
		Theridiidae	Theridiidae Jovem		X
			Theridion sp.3	X	
		Theridiosomatidae	*Plato* sp.1		X
	Pseudoscorpiones	Chernetidae	*Spelaeochernes* sp.1		X
		Chthoniidae	Chthoniidae Jovem	X	
			Pseudochthonius sp.2		X
Acari	Mesostigmata		Mesostigmata sp.7		X
	Sarcoptiforme		Sarcoptiforme sp.1	X	
Entognatha	Collembola	Paronellidae	Paronellidae sp.1	X	X
			Cyphoderus agnotus		X
			Cyphoderus javanus		X
		Symphypleona	Symphypleona sp.2		X
Insecta	Coleoptera	Staphylinidae	Pselaphinae sp.2	X	
	Heteroptera	Reduviidae	Emesinae sp.5		X
			Zelurus sp.1		X
	Hymenoptera	Formicidae	*Nylanderia* sp.2		X
			Pachycondyla sp.1		X
			Pheidole sp.5	X	
			Solenopsis sp.1	X	
	Lepidoptera		Lepidoptera Jovem	X	
			Lepidoptera sp.		X
	Orthoptera		Orthoptera sp.		X
	Psocoptera	Psocomorpha	Psocomorpha Jovem	X	
Malacostraca	Isopoda	Philosciidae	Philosciidae sp.2		X
Pauropoda	Tetramerocerata		*Tetramerocerata* Jovem		X
Amphibia	Anura	Leptodactylidae	*Leptodactylus* sp.1		X
Mammalia	Chiroptera		Chiroptera sp.	X	
		Emballonuridae	*Peropteryx* sp.		X

SB-0189					
TÁXONS				Seco	Úmido
Arachnida	Amblypygi	Phrynidae	*Heterophrynus longicornis*	X	-
	Araneae		Araneae sp.	-	X
		Corinnidae	Corinnidae Jovem	X	X
		Ctenidae	Ctenidae sp.	-	X
		Drymusidae	Drymusidae Jovem	-	X
		Ochyroceratidae	Ochyroceratidae Jovem	-	X
			Speocera sp.1	X	X
		Pholcidae	Pholcidae Jovem	-	X
		Theridiosomatidae	Theridiosomatidae Jovem	X	-
	Opiliones	Escadabiidae	Escadabiidae Jovem	X	-
		Stygnidae	Stygnidae Jovem	-	X
	Pseudoscorpiones	Chernetidae	*Spelaeochernes* sp.1	X	-
	Schizomida	Hubardiidae	*Rowlandius* sp.1	X	-
	Scorpiones	Buthidae	*Ananteris* sp.2	-	X
Chilopoda	Scolopendromorpha	Cryptopidae	*Cryptops* Jovem	-	X
			Cryptops sp.1	X	-
Diplopoda	Polydesmida	Pyrgodesmidae	Pyrgodesmidae sp.1	-	X
Entognatha	Collembola	Isotomidae	Isotomidae sp.1	X	-
		Paronellidae	Paronellidae sp.1	X	X
		Symphypleona	Symphypleona sp.2	X	-
	Diplura	Campodeidae	Campodeidae sp.1	X	X
Insecta	Heteroptera	Cydnidae	Cydnidae sp.1	-	X
		Reduviidae	Emesinae sp.5	-	X
	Homoptera	Cixiidae	Cixiidae Jovem	X	X
	Hymenoptera	Formicidae	*Pheidole* sp.2	-	X
			Rogeria tonduzi	X	-
		Scelionidae	Scelionidae sp.1	-	X
	Isoptera	Rhinotermitidae	*Heterotermes* sp.1	-	X
		Termitidae	Termitidae sp.	X	-
			Nasutitermes sp.2	-	X
	Orthoptera		Orthoptera sp.	X	X
Malacostraca	Decapoda	Palaemonidae	*Macrobrachium* sp.1	X	-
	Isopoda	Philosciidae	Philosciidae Jovem	X	X
			Philosciidae sp.2	X	X
Gastropoda	Pulmonata	Subulinidae	Subulinidae Jovem	X	-
			Lamellaxis sp.1	X	-

SB-0190					
TÁXONS				Seco	Úmido
Arachnida	Araneae	Ochyroceratidae	Ochyroceratidae Jovem	X	X
		Theridiidae	Theridiidae Jovem	X	-
			Achaearanea sp.1	X	-
	Opiliones	Escadabiidae	Escadabiidae sp.3	X	-
	Palpigradi	Eukoeneniidae	Eukoeneniidae Jovem	-	X
	Pseudoscorpiones	Chernetidae	*Spelaeochernes* sp.1	-	X
		Chthoniidae	*Pseudochthonius* sp.2	X	X
Acari	Ixodida	Argasidae	*Ornithodoros* sp.1	-	X
	Sarcoptiforme	Oribatida	Oribatida sp.1	-	X
	Trombidiforme	Bdellidae	Bdellidae sp.1	-	X
		Trombiculidae	Trombiculidae sp.1	-	X
Chilopoda	Scolopendromorpha	Cryptopidae	*Cryptops* sp.3	X	-
	Scutigeromorpha		Scutigeromorpha sp.	X	-
Entognatha	Collembola	Isotomidae	Isotomidae sp.1	-	X
		Paronellidae	*Cyphoderus javanus*	X	-
		Symphypleona	Symphypleona sp.2	X	-
Insecta	Blattaria	Polyphagidae	Polyphagidae Jovem	-	X
	Diptera	Psychodidae	Phlebotominae sp.1	-	X
	Heteroptera	Reduviidae	*Zelurus* Jovem	X	-
			Zelurus sp.1	-	X
	Homoptera	Coccoidea	Coccoidea sp.	-	X
		Cixiidae	Cixiidae Jovem	-	X
	Hymenoptera	Diapriidae	Diapriidae sp.5	X	-
		Formicidae	*Camponotus* sp.1	-	X
			Crematogaster sp.1	-	X
	Isoptera	Termitidae	*Nasutitermes* sp.1	X	-
	Lepidoptera		Lepidoptera Jovem	-	X
			Lepidoptera sp.	-	X
	Orthoptera		Orthoptera sp.	X	X
	Psocoptera	Pseudocaeciliidae	Pseudocaeciliidae sp.2	X	-
Amphibia	Anura	Craugastoridae	*Pristimantis* cf. *fenestratus*	X	-
Mammalia	Chiroptera		Chiroptera sp.	X	-
		Emballonuridae	*Peropteryx* sp.	-	X

SB-0191					
TÁXONS				**Seco**	**Úmido**
Arachnida	Amblypygi	Phrynidae	*Heterophrynus longicornis*	X	-
	Araneae	Caponiidae	*Nops* sp.2	-	X
		Corinnidae	Corinnidae Jovem	-	X
			Creugas sp.1	X	-
		Ctenidae	Ctenidae Jovem	-	X
		Oonopidae	Oonopidae Jovem	-	X
		Pholcidae	*Mesabolivar eberhardi*	-	X
		Salticidae	Salticidae Jovem	-	X
			Salticidae sp.2	-	X
		Theridiidae	Theridiidae Jovem	-	X
	Opiliones	Stygnidae	Stygnidae Jovem	-	X
	Pseudoscorpiones	Chernetidae	*Spelaeochernes* sp.1	-	X
Acari	Opilioacarida	Opilioacaridae	Opilioacaridae sp.1	-	X
	Trombidiforme	Trombiculidae	Trombiculidae sp.3	-	X
Diplopoda	Spirostreptida	Pseudonannolenidae	Pseudonannolenidae Jovem	-	X
Entognatha	Collembola	Paronellidae	Paronellidae sp.1	X	X
Insecta	Blattaria	Blaberidae	Blaberidae Jovem	-	X
		Polyphagidae	Polyphagidae Jovem	-	X
	Coleoptera		Coleoptera Jovem	-	X
	Diptera	Psychodidae	Phlebotominae sp.1	-	X
		Tipulidae	Tipulidae sp.1	-	X
	Heteroptera	Reduviidae	*Zelurus* sp.1	X	X
	Hymenoptera	Formicidae	*Camponotus cingulatus*	-	X
			Cyphomyrmex sp.1	-	X
			Dolichoderus bispinosus	X	-
			Pheidole sp.2	-	X
			Pheidole sp.5	X	-
	Isoptera	Termitidae	*Nasutitermes* sp.1	X	-
	Lepidoptera	Tineoidea	Tineoidea sp.5	X	-
	Orthoptera		Orthoptera sp.	X	-
Malacostraca	Isopoda	Philosciidae	Philosciidae sp.2	X	-
		Platyarthridae	Platyarthridae Jovem	X	-
Amphibia	Anura	Craugastoridae	*Pristimantis* cf. *fenestratus*	X	-
Mammalia	Chiroptera	Emballonuridae	*Peropteryx* sp.	X	-
		Furipteridae	*Furipterus horrens*	X	-

SB-0192					
TÁXONS				Seco	Úmido
Arachnida	Amblypygi	Phrynidae	*Heterophrynus longicornis*	-	X
	Araneae	Araneidae	Araneidae Jovem	-	X
			Alpaida antonio	X	-
		Ctenidae	Ctenidae Jovem	-	X
		Dipluridae	Dipluridae Jovem	X	-
		Ochyroceratidae	Ochyroceratidae Jovem	-	X
			Ochyrocera sp.3	X	-
		Oonopidae	Oonopidae Jovem	-	X
		Oxyopidae	Oxyopidae Jovem	X	-
		Pholcidae	Pholcidae Jovem	X	X
		Pholcidae	Pholcidae sp.	-	X
		Plato	Plato sp.	-	X
		Salticidae	Salticidae Jovem	X	X
			Salticidae sp.1	X	-
		Theridiosomatidae	*Plato* sp.1	-	X
	Opiliones	Sclerosomatidae	*Prionostema* sp.1	X	-
	Pseudoscorpiones	Cheiridiidae	*Neocheiridium* sp.1	X	-
		Chernetidae	Chernetidae Jovem	X	-
			Spelaeochernes sp.1	-	X
		Chthoniidae	*Pseudochthonius* sp.2	-	X
Acari	Trombidiforme	Trombiculidae	Trombiculidae sp.1	-	X
Diplopoda	Polydesmida	Fuhrmannodesmidae	Fuhrmannodesmidae Jovem	-	X
Entognatha	Collembola	Entomobryidae	Entomobryidae sp.	X	-
			Entomobryidae sp.5	-	X
		Paronellidae	Paronellidae sp.1	X	-
			Paronellidae sp.3	X	-
	Diplura	Campodeidae	Campodeidae sp.1	X	X
		Japygidae	Japygidae sp.1	-	X
Insecta	Blattaria	Blaberidae	Blaberidae Jovem	X	X
			Blaberus sp.1	-	X
	Coleoptera		Coleoptera Jovem	-	X
	Diptera	Ceratopogonidae	Ceratopogonidae Jovem	X	-
		Tipulidae	Tipulidae sp.1	-	X
	Heteroptera		Heteroptera sp.	X	-
		Cydnidae	Cydnidae Jovem	-	X
			Pangaeus sp.1	-	X
		Ochteridae	Ochteridae sp.1	X	-
		Reduviidae	*Zelurus* Jovem	X	X
			Triatoma Jovem	-	X
	Hymenoptera	Diapriidae	Diapriidae sp.13	-	X
		Formicidae	*Apterostigma pilosum*	-	X
			Dolichoderus bispinosus	X	X
			Pachycondyla constricta	-	X
			Pheidole sp.5	X	-
	Isoptera	Termitidae	Termitidae sp.	-	X
			Embriatermes sp.1	X	X
	Lepidoptera		Lepidoptera sp.	X	-
	Neuroptera	Myrmeleontidae	Myrmeleontidae sp.1	X	X
	Orthoptera		Orthoptera sp.	X	X
		Phalangopsidae	*Eidmanacris* sp.1	X	X
	Phasmatodea		Phasmatodea sp.	-	X
	Psocoptera	Psocomorpha	Psocomorpha Jovem	X	-
Malacostraca	Isopoda	Armadillidae	Armadillidae sp.1	X	-
		Philosciidae	Philosciidae Jovem	-	X
			Philosciidae sp.2	-	X
Amphibia	Anura	Craugastoridae	*Pristimantis* cf. *fenestratus*	X	-
Mammalia	Chiroptera	Emballonuridae	*Peropteryx* sp.	-	X

		Phyllostomidae	*Glossophaga* sp.	-	X
Platyhelminthes	Tricladida	Geoplanidae	Geoplanidae sp.3	-	X

SB-0193					
TÁXONS				Seco	Úmido
Annelida	Lumbriculida	Lumbriculidae	Lumbriculidae sp.1	X	-
Arachnida	Amblypygi	Phrynidae	*Heterophrynus longicornis*	-	X
	Araneae	Araneidae	Araneidae Jovem	-	X
		Corinnidae	Corinnidae Jovem	-	X
		Drymusidae	Drymusidae Jovem	X	-
		Nemesiidae	Nemesiidae Jovem	-	X
		Oonopidae	Oonopidae sp.6	X	-
		Pholcidae	Pholcidae Jovem	X	X
			Mesabolivar aurantiacus	X	-
		Salticidae	Salticidae Jovem	-	X
			Salticidae sp.2	X	-
		Scytodidae	Scytodidae Jovem	X	X
		Theraphosidae	Theraphosidae sp.	X	-
		Theridiidae	Theridiidae Jovem	-	X
		Theridiosomatidae	Theridiosomatidae Jovem	-	X
			Plato sp.1	X	X
		Trechaleidae	Trechaleidae Jovem	-	X
			Rhoicinus sp.1	X	-
	Opiliones	Manaosbiidae	Manaosbiidae sp.1	X	-
		Sclerosomatidae	Sclerosomatidae Jovem	-	X
			Prionostema sp.1	X	X
		Stygnidae	*Protimesius gracilis*	-	X
	Pseudoscorpiones	Chernetidae	Chernetidae Jovem	X	-
		Chthoniidae	Chthoniidae Jovem	-	X
			Pseudochthonius sp.2	-	X
Acari	Mesostigmata		Mesostigmata sp.2	X	-
	Sarcoptiforme	Oribatida	Oribatida sp.1	X	X
	Trombidiforme	Trombiculidae	Trombiculidae sp.3	X	-
Entognatha	Collembola	Entomobryidae	Entomobryidae sp.5	-	X
		Paronellidae	Paronellidae sp.1	X	-
Insecta	Blattaria	Blattidae	Blattidae Jovem	-	X
	Coleoptera	Elateridae	Elateridae Jovem	X	-
		Hydrophilidae	Hydrophilidae sp.8	-	X
		Scydmaenidae	Scydmaenidae sp.2	-	X
		Staphylinidae	Pselaphinae sp.2	X	-
			Pselaphinae sp.5	X	-
			Staphylininae sp.20	X	-
	Diptera		Diptera Jovem	X	-
		Phoridae	Phoridae sp.1	-	X
		Tipulidae	Tipulidae sp.1	-	X
			Tipulidae sp.2	-	X
	Heteroptera	Ceratocombidae	Ceratocombidae sp.1	-	X
		Cydnidae	Cydnidae Jovem	X	-
			Pangaeus sp.1	X	-
		Mesoveliidae	Madeoveliinae Jovem	-	X
		Ochteridae	Ochteridae sp.1	-	X
		Schizopteridae	Schizopteridae Jovem	X	-
		Veliidae	*Stridulivelia* sp.1	X	-
	Hymenoptera	Formicidae	*Camponotus cingulatus*	-	X
			Crematogaster erecta	X	-
			Hypoponera opacior	X	-
			Hypoponera sp.5	-	X
			Hypoponera sp.7	X	-
			Nylanderia sp.1	-	X
			Nylanderia sp.2	-	X
			Pachycondyla constricta	X	X
			Pheidole sp.8	X	-

			Solenopsis invicta	-	X
			Solenopsis sp.1	X	X
	Isoptera	Termitidae	Termitidae sp.	-	X
			Nasutitermes sp.1	X	-
	Lepidoptera	Tineidae	Tineidae Jovem	X	-
		Noctuoidea	Noctuoidea sp.1	-	X
			Noctuoidea sp.11	-	X
			Noctuoidea sp.12	-	X
	Neuroptera	Myrmeleontidae	Myrmeleontidae sp.5	X	-
	Orthoptera		Orthoptera sp.	X	-
		Myrmecophilidae	Myrmecophilidae sp.1	X	-
		Phalangopsidae	*Paraclodes* sp.1	-	X
	Psocoptera	Psocomorpha	Psocomorpha Jovem	-	X
		Psyllipsocidae	Psyllipsocidae sp.7	-	X
	Trichoptera	Calamoceratidae	Calamoceratidae Jovem	X	-
		Hydriopsychidae	Hydriopsychidae Jovem	X	-
	Zygentoma	Nicoletiidae	Nicoletiinae sp.1	X	-
Malacostraca	Isopoda	Armadillidae	Armadillidae Jovem	X	X
			Armadillidae sp.1	X	X
Amphibia	Anura	Craugastoridae	*Pristimantis* cf. *fenestratus*	X	X
Mammalia	Chiroptera	Emballonuridae	*Peropteryx kappleri*	X	-
		Phyllostomidae	*Carollia perspicillata*	X	-
			Carollia sp.	-	X
			Glossophaga sp.	-	X
Reptilia	Squamata	Gymnophthalmidae	*Neusticurus* cf. *ecpleopus*	X	-
			Neusticurus sp.	X	-
Gastropoda	Pulmonata		Pulmonata sp.	-	X
Platyhelminthes	Tricladida	Dugesiidae	Dugesiidae sp.1	X	-

SB-0194					
TÁXONS				Seco	Úmido
Annelida	Haplotaxida		Haplotaxida sp.11	X	-
Arachnida	Araneae		Araneae sp.	X	-
		Actinopodidae	Actinopodidae Jovem	X	-
			Actinopus sp.1	X	-
		Corinnidae	Corinnidae Jovem	-	X
		Ctenidae	Ctenidae Jovem	X	-
		Drymusidae	Drymusidae Jovem	X	X
		Linyphiidae	*Meioneta* sp.1	-	X
		Ochyroceratidae	Ochyroceratidae Jovem	-	X
		Oonopidae	Oonopidae Jovem	-	X
			Oonopidae sp.3	-	X
			Oonopidae sp.8	-	X
		Pholcidae	Pholcidae Jovem	-	X
			Ninetinae sp.2	-	X
		Salticidae	Salticidae Jovem	-	X
		Theraphosidae	Theraphosidae Jovem	-	X
		Theridiidae	Theridiidae Jovem	X	X
		Theridiosomatidae	*Plato* sp.1	-	X
			Plato sp.2	-	X
	Opiliones	Sclerosomatidae	Sclerosomatidae Jovem	X	-
			Prionostema sp.1	X	X
	Pseudoscorpiones	Chernetidae	Spelaeochernes sp.1	-	X
	Schizomida	Hubardiidae	Hubardiidae Jovem	-	X
Acari	Sarcoptiforme	Oribatida	Oribatida sp.1	-	X
Chilopoda	Geophilomorpha	Geophilidae	Geophilidae Jovem	-	X
	Scolopendromorpha	Scolopocryptopidae	*Dinocryptops* Jovem	-	X
			Newportia sp.1	X	-
Diplopoda	Polydesmida	Chelodesmidae	Chelodesmidae Jovem	-	X
			Chelodesmidae sp.1	X	X
		Fuhrmannodesmidae	Fuhrmannodesmidae Jovem	X	X
			Fuhrmannodesmidae sp.1	X	-
	Polyxenida		Polyxenida Jovem	X	-
	Stemmiulida	Stemmiulidae	Stemmiulidae Jovem	-	X
Entognatha	Collembola	Isotomidae	Isotomidae sp.1	-	X
		Paronellidae	Paronellidae sp.1	-	X
	Diplura	Campodeidae	Campodeidae sp.1	X	X
		Japygidae	Japygidae sp.1	-	X
Insecta	Blattaria		Blattaria sp.	-	X
		Blaberidae	Blaberidae Jovem	-	X
		Blattidae	Blattidae Jovem	X	X
	Coleoptera		Coleoptera Jovem	-	X
		Carabidae	Carabidae sp.3	X	-
			Acupalpus sp.1	X	X
		Gyrinidae	*Gyretes* sp.1	-	X
		Staphylinidae	Staphylininae sp.1	-	X
			Staphylininae sp.13	-	X
			Staphylininae sp.2	X	-
			Staphylininae sp.22	X	-
			Staphylininae sp.3	X	-
	Diptera		Diptera Jovem	X	X
		Phoridae	Phoridae sp.4	X	-
		Cecidomyiidae	Cecidomyiidae sp.2	X	-
		Sciaridae	Sciaridae sp.2	X	-
		Culicidae	Culicinae sp.3	-	X
		Psychodidae	Phlebotominae sp.1	-	X
		Tipulidae	Tipulidae sp.1	X	-
	Heteroptera	Cydnidae	Cydnidae Jovem	X	-

			Cydnidae sp.1	-	X
			Pangaeus sp.1	X	-
		Reduviidae	*Zelurus* sp.1	-	X
		Veliidae	*Rhagovelia* sp.1	-	X
	Homoptera	Coccoidea	Coccoidea sp.	X	X
	Hymenoptera	Formicidae	*Acropyga smithii*	X	-
			Acropyga sp.1	-	X
			Anochetus sp.1	X	X
			Gnamptogenys striatula	X	-
			Hypoponera sp.1	-	X
			Hypoponera sp.7	X	-
			Nylanderia sp.1	X	-
			Pachycondyla sp.2	-	X
			Pheidole sp.	X	-
			Pheidole sp.1	X	-
			Pheidole sp.16	-	X
			Pheidole sp.7	X	-
			Prionopelta modesta	X	-
			Prionopelta sp.1	X	-
			Solenopsis sp.1	X	X
		Scelionidae	Scelionidae sp.1	X	-
			Scelionidae sp.6	X	-
	Isoptera	Termitidae	Termitidae sp.	X	X
			Cornitermes sp.1	X	-
			Nasutitermes sp.2	-	X
	Orthoptera		Orthoptera sp.	X	X
	Psocoptera	Psocomorpha	Psocomorpha Jovem	X	X
	Trichoptera		Trichoptera Jovem	X	-
Malacostraca	Decapoda	Palaemonidae	Palaemonidae Jovem	-	X
			Palaemonidae sp.	X	-
	Isopoda	Dubioniscidae	Dubioniscidae sp.1	-	X
		Philosciidae	Philosciidae sp.1	X	X
			Philosciidae sp.2	-	X
		Scleropactidae	Scleropactidae sp.4	-	X
Symphyla		Scolopendrellidae	*Symphylella* sp.1	X	-
Amphibia	Anura	Dendrobatidae	*Adelphobates* cf. *galactonotus*	X	-
Mammalia	Chiroptera	Emballonuridae	*Peropteryx* sp.	X	X
		Furipteridae	*Furipterus horrens*	X	X
		Phyllostomidae	*Carollia* sp.	X	X
			Glossophaga sp.	X	X
			Lionycteris sp.	X	X
			Lonchophylla sp.	X	-
Reptilia	Squamata	Phyllodactylidae	*Thecadactylus rapicauda*	X	-
Gastropoda	Pulmonata	Systrophiidae	*Happia* sp.2	-	X

SB-0195					
TÁXONS				Seco	Úmido
Annelida	Haplotaxida		Haplotaxida sp.4	-	X
	Lumbriculida	Lumbriculidae	Lumbriculidae sp.1	X	-
Arachnida	Araneae	Linyphiidae	Linyphiidae sp.4	X	-
		Pholcidae	Pholcidae Jovem	X	-
			Mesabolivar aurantiacus	X	-
		Trechaleidae	Trechaleidae Jovem	-	X
	Opiliones	Cosmetidae	Cosmetidae Jovem	X	-
		Sclerosomatidae	Sclerosomatidae Jovem	X	-
			Prionostema sp.1	-	X
Acari	Astigmata		Astigmata sp.1	X	-
	Mesostigmata	Macrochelidae	Macrochelidae sp.1	-	X
		Uropodoidea	Uropodoidea sp.2	X	-
	Sarcoptiforme	Oribatida	Oribatida sp.1	X	-
Entognatha	Collembola	Entomobryidae	Entomobryidae sp.	X	-
		Paronellidae	Paronellidae sp.1	X	-
			Cyphoderus agnotus	X	-
			Cyphoderus javanus	X	-
	Diplura	Campodeidae	Campodeidae sp.1	X	-
		Japygidae	Japygidae sp.1	X	-
Insecta	Blattaria	Blattidae	Blattidae Jovem	X	X
	Coleoptera		Coleoptera Jovem	X	X
		Gyrinidae	*Gyretes* sp.1	X	-
		Staphylinidae	Pselaphinae sp.12	-	X
			Staphylininae sp.12	-	X
			Staphylininae sp.3	X	-
	Diptera		Diptera Jovem	-	X
		Conopidae	Conopidae sp.1	X	-
		Drosophilidae	Drosophilidae sp.1	X	-
		Phoridae	Phoridae sp.4	X	-
	Heteroptera	Cydnidae	Cydnidae Jovem	X	X
			Pangaeus sp.1	X	-
		Veliidae	*Rhagovelia* sp.2	X	-
	Hymenoptera	Formicidae	*Camponotus* sp.2	X	-
			Carebara urichi	X	-
			Ectatomma tuberculatum	X	-
			Hypoponera sp.1	-	X
			Odontomachus bauri	X	-
			Pachycondyla constricta	X	-
			Rogeria tonduzi	X	-
			Solenopsis invicta	X	-
			Solenopsis sp.1	X	X
			Wasmannia auropunctata	X	-
	Orthoptera		Orthoptera sp.	X	X
	Trichoptera		Trichoptera Jovem	X	-
Malacostraca	Isopoda	Philosciidae	Philosciidae Jovem	X	-
			Philosciidae sp.1	X	X
		Platyarthridae	Platyarthridae Jovem	X	-
Symphyla		Scolopendrellidae	*Symphylella* sp.1	X	-
Amphibia	Anura	Bufonidae	*Rhinella* gr. *marina*	X	-
Mammalia	Chiroptera	Phyllostomidae	*Carollia* sp.	X	-
			Glossophaga sp.	X	-
			Lonchophylla sp.	X	-
Gastropoda	Pulmonata	Subulinidae	Subulinidae Jovem	X	-

SB-0196					
**TÁXONS**				**Seco**	**Úmido**
Arachnida	Amblypygi	Phrynidae	*Heterophrynus longicornis*	-	X
	Araneae	Pholcidae	Pholcidae Jovem	X	-
			Mesabolivar aurantiacus	X	X
	Opiliones	Sclerosomatidae	*Prionostema* sp.1	X	-
Acari	Mesostigmata	Uropodoidea	Uropodoidea sp.2	X	-
	Sarcoptiforme	Oribatida	Oribatida sp.13	-	X
Entognatha	Collembola	Paronellidae	Paronellidae sp.1	X	-
	Diplura	Campodeidae	Campodeidae sp.1	X	-
Insecta	Coleoptera	Staphylinidae	Staphylininae sp.2	X	-
			Staphylininae sp.3	X	-
	Diptera	Chironomidae	Chironomidae sp.1	X	-
		Tipulidae	Tipulidae sp.1	X	-
			Tipulidae sp.3	-	X
	Hymenoptera	Formicidae	*Solenopsis invicta*	X	-
	Orthoptera		Orthoptera sp.	X	X
	Psocoptera	Psocomorpha	Psocomorpha Jovem	X	-
Malacostraca	Decapoda		Decapoda sp.	X	-
	Isopoda	Philosciidae	Philosciidae sp.1	X	-
Mammalia	Chiroptera	Phyllostomidae	Phyllostomidae sp.	X	-

SB-0197					
**TÁXONS**				**Seco**	**Úmido**
Annelida	Haplotaxida		Haplotaxida sp.11	X	-
			Haplotaxida sp.4	-	X
Arachnida	Amblypygi	Phrynidae	*Heterophrynus longicornis*	X	X
	Araneae	Ochyroceratidae	Ochyroceratidae Jovem	-	X
		Pholcidae	Pholcidae Jovem	-	X
			Mesabolivar eberhardi	X	-
		Theridiosomatidae	*Plato* sp.1	X	-
	Opiliones	Sclerosomatidae	*Prionostema* sp.1	X	-
		Stygnidae	Stygnidae Jovem	-	X
	Pseudoscorpiones	Chernetidae	Chernetidae Jovem	X	-
			Spelaeochernes sp.1	X	X
		Chthoniidae	*Pseudochthonius* sp.4	X	-
Acari	Mesostigmata		Mesostigmata sp.1	X	-
			Mesostigmata sp.3	-	X
Diplopoda	Polydesmida	Pyrgodesmidae	Pyrgodesmidae sp.1	-	X
	Spirostreptida	Pseudonannolenidae	Pseudonannolenidae sp.1	X	-
Entognatha	Collembola	Paronellidae	Paronellidae sp.1	X	X
		Symphypleona	Symphypleona sp.2	X	X
	Diplura	Campodeidae	Campodeidae sp.1	X	-
		Japygidae	Japygidae sp.1	-	X
Insecta	Blattaria	Blattidae	Blattidae Jovem	-	X
	Coleoptera	Staphylinidae	Pselaphinae sp.5	-	X
	Diptera		Diptera Jovem	X	-
	Heteroptera	Veliidae	*Microvelia* Jovem	-	X
			Paravelia Jovem	X	-
	Homoptera	Cixiidae	Cixiidae Jovem	X	X
		Fulgoridae	Fulgoridae Jovem	-	X
	Hymenoptera	Formicidae	*Acropyga* sp.1	-	X
			Camponotus sp.1	-	X
			Nylanderia sp.1	X	X
			Solenopsis sp.1	-	X
		Vespidae	Vespidae sp.6	X	-
	Isoptera	Termitidae	*Nasutitermes* sp.2	X	-
	Lepidoptera		Lepidoptera Jovem	-	X
	Orthoptera	Phalangopsidae	*Phalangopsis* sp.1	X	X
	Psocoptera	Psocomorpha	Psocomorpha Jovem	X	-
Malacostraca	Decapoda	Pseudothelphusidae	Pseudothelphusidae sp.1	X	-
	Isopoda	Philosciidae	Philosciidae Jovem	X	X
			Philosciidae sp.2	X	-
Amphibia	Anura	Aromobatidae	*Allobates* gr. *marchesianus*	-	X
Mammalia	Chiroptera		Chiroptera sp.	X	-
		Emballonuridae	*Peropteryx kappleri*	X	-
			Peropteryx sp.	-	X
Gastropoda	Pulmonata	Subulinidae	Subulinidae sp.1	X	-
		Systrophiidae	*Happia* sp.2	-	X

SB-0198					
TÁXONS				**Seco**	**Úmido**
Arachnida	Amblypygi	Phrynidae	*Heterophrynus longicornis*	X	X
	Araneae	Corinnidae	Corinnidae Jovem	-	X
		Drymusidae	Drymusidae Jovem	-	X
		Nemesiidae	Nemesiidae Jovem	X	-
		Ochyroceratidae	Ochyroceratidae Jovem	X	X
		Oonopidae	Oonopidae Jovem	-	X
			Oonopidae sp.2	X	-
		Pholcidae	Pholcidae Jovem	X	X
			Mesabolivar aurantiacus	-	X
			Mesabolivar eberhardi	X	-
		Scytodidae	Scytodidae Jovem	-	X
			Scytodes eleonorae	X	X
		Theridiidae	Theridiidae Jovem	-	X
	Opiliones	Cosmetidae	*Roquettea* sp.1	X	-
		Escadabiidae	Escadabiidae sp.2	-	X
			Escadabiidae sp.3	X	-
	Pseudoscorpiones	Chernetidae	*Spelaeochernes* sp.1	X	X
	Schizomida	Hubardiidae	*Rowlandius* sp.1	-	X
Acari	Astigmata		Astigmata sp.1	-	X
	Ixodida	Argasidae	Ornithodoros sp.1	X	-
Diplopoda	Polydesmida	Chelodesmidae	Chelodesmidae Jovem	-	X
		Paradoxosomatidae	Paradoxosomatidae sp.3	X	-
Entognatha	Collembola	Paronellidae	Paronellidae sp.1	-	X
	Diplura	Campodeidae	Campodeidae sp.1	X	X
Insecta	Blattaria	Blaberidae	Blaberidae Jovem	-	X
	Diptera	Cecidomyiidae	Cecidomyiidae sp.1	X	-
		Psychodidae	Phlebotominae sp.1	X	X
	Heteroptera	Reduviidae	Reduviidae Jovem	-	X
			Zelurus sp.1	-	X
			Triatoma Jovem	X	-
	Homoptera	Cixiidae	Cixiidae Jovem	X	-
	Hymenoptera	Formicidae	Apterostigma sp.1	-	X
			Camponotus sp.1	-	X
			Camponotus sp.2	X	-
			Hypoponera sp.1	-	X
			Hypoponera sp.8	X	-
			Pachycondyla sp.1	-	X
	Isoptera	Termitidae	*Nasutitermes* sp.2	-	X
	Orthoptera		Orthoptera sp.	X	-
		Phalangopsidae	*Phalangopsis* sp.1	-	X
	Psocoptera	Psyllipsocidae	Psyllipsocidae sp.7	-	X
	Zygentoma	Nicoletiidae	Atelurinae sp.1	X	-
Malacostraca	Isopoda	Philosciidae	Philosciidae Jovem	X	X
			Philosciidae sp.2	-	X
		Platyarthridae	Platyarthridae sp.5	X	-
Amphibia	Anura	Leptodactylidae	*Physalaemus* gr. *cuvieri*	-	X
Mammalia	Chiroptera		Chiroptera sp.	X	X
	Rodentia	Cricetidae	*Rhipidomys* sp.	X	-

SB-0199					
TÁXONS				Seco	Úmido
Arachnida	Amblypygi	Phrynidae	*Heterophrynus longicornis*	X	X
	Araneae	Corinnidae	Corinnidae Jovem	X	X
			Abapeba hoeferi	X	X
		Ctenidae	Ctenidae Jovem	X	X
		Ochyroceratidae	Ochyroceratidae Jovem	-	X
		Palpimanidae	Palpimanidae Jovem	-	X
		Pholcidae	Pholcidae Jovem	X	X
			Mesabolivar aurantiacus	X	-
			Mesabolivar eberhardi	X	X
		Scytodidae	Scytodidae Jovem	X	X
			Scytodes eleonorae	X	X
		Theridiidae	Theridiidae Jovem	-	X
			Achaearanea sp.1	X	X
		Theridiosomatidae	Theridiosomatidae Jovem	X	-
			Plato sp.1	X	-
	Opiliones	Neogoveidae	Neogoveidae Jovem	X	-
		Sclerosomatidae	Sclerosomatidae Jovem	X	X
			Prionostema sp.1	-	X
	Pseudoscorpiones	Chernetidae	Chernetidae Jovem	X	X
			Spelaeochernes sp.1	X	X
		Chthoniidae	Chthoniidae Jovem	X	-
			Pseudochthonius sp.4	X	X
Acari	Acariforme		Acariforme sp.2	-	X
	Ixodida	Argasidae	*Ornithodoros* sp.1	X	-
		Ixodidae	*Amblyomma rotundatum*	-	X
	Mesostigmata		Mesostigmata sp.1	X	-
			Mesostigmata sp.11	-	X
			Mesostigmata sp.12	-	X
			Mesostigmata sp.2	X	X
			Mesostigmata sp.3	X	X
			Mesostigmata sp.6	-	X
		Macrochelidae	Macrochelidae sp.1	-	X
			Macrochelidae sp.2	-	X
	Sarcoptiforme	Oribatida	Oribatida sp.13	-	X
			Oribatida sp.3	-	X
			Oribatida sp.5	-	X
	Trombidiforme	Trombiculidae	Trombiculidae sp.3	-	X
Diplopoda	Polydesmida	Pyrgodesmidae	Pyrgodesmidae sp.1	X	-
	Spirostreptida	Pseudonannolenidae	Pseudonannolenidae sp.1	X	-
Entognatha	Collembola	Paronellidae	Paronellidae sp.1	X	X
			Cyphoderus arlei	-	X
		Symphypleona	Symphypleona sp.2	X	X
Insecta	Blattaria		Blattaria sp.	X	X
		Blaberidae	Blaberidae Jovem	X	X
		Blattelidae	Blattelidae Jovem	X	X
		Blattidae	Blattidae Jovem	X	X
		Polyphagidae	Polyphagidae sp.1	X	-
	Coleoptera		Coleoptera Jovem	X	X
		Carabidae	Carabidae sp.1	-	X
			Carabidae sp.5	X	X
			Lelis sp.1	-	X
		Elateridae	Elateridae sp.3	-	X
		Gyrinidae	*Gyretes* sp.1	-	X
		Histeridae	Histeridae sp.2	X	-
			Histeridae sp.3	X	X
			Histeridae sp.5	-	X
		Scydmaenidae	Scydmaeninae sp.1	-	X

		Staphylinidae	Pselaphinae	sp.10	-	X
			Staphylininae	sp.5	X	-
			Staphylininae	sp.8	-	X
		Tenebrionidae	Tenebrionidae	sp.4	-	X
	Diptera		Diptera	Jovem	-	X
		Brachycera	Brachycera	sp.	-	X
		Chloropidae	Chloropidae	sp.1	X	X
		Drosophilidae	Drosophilidae	sp.1	-	X
		Streblidae	Streblidae	sp.1	X	X
		Phoridae	Phoridae	sp.2	X	-
		Sphaeroceridae	Sphaeroceridae	sp.1	-	X
		Ceratopogonidae	Ceratopogonidae	sp.1	X	-
		Culicidae	Culicinae	sp.1	X	-
		Psychodidae	Psychodidae	sp.1	X	X
			Phlebotominae	sp.1	-	X
		Tipulidae	Tipulidae	sp.1	-	X
	Ephemeroptera	Leptophlebiidae	Leptophlebiidae	sp.1	-	X
	Heteroptera	Cydnidae	Cydnidae	Jovem	X	-
			Cydnidae	sp.1	-	X
			Pangaeus	sp.1	X	-
		Nabidae	Nabidae	sp.1	-	X
			Prostemmatinae	sp.1	X	-
		Reduviidae	*Zelurus*	Jovem	-	X
		Veliidae	*Microvelia*	Jovem	-	X
	Homoptera	Cixiidae	Cixiidae	sp.6	-	X
		Cicadellidae	Cicadellidae	sp.2	-	X
	Hymenoptera	Apidae	Apidae	sp.3	-	X
		Bethylidae	Bethylidae	sp.3	X	-
		Braconidae	Braconidae	sp.5	X	-
		Diapriidae	Diapriidae	sp.11	-	X
		Formicidae	*Camponotus*	*cingulatus*	-	X
			Camponotus	sp.2	X	-
			Cephalotes	sp.1	X	-
			Crematogaster	*limata*	X	-
			Gnamptogenys	*regularis*	X	-
			Linepithema	sp.1	X	-
			Pachycondyla	*constricta*	X	-
			Pheidole	sp.16	-	X
			Pheidole	sp.5	X	-
			Pheidole	sp.7	X	-
			Prionopelta	*modesta*	X	-
			Solenopsis	*invicta*	X	-
			Solenopsis	sp.1	X	X
			Solenopsis	sp.4	-	X
			Strumigenys	sp.1	-	X
		Scelionidae	Scelionidae	sp.1	X	X
			Scelionidae	sp.3	-	X
	Isoptera	Termitidae	Termitidae	sp.	-	X
			Nasutitermes	sp.1	X	-
			Nasutitermes	sp.2	X	-
			Nasutitermes	sp.5	-	X
			Nasutitermes	sp.6	X	X
			Nasutitermes	sp.7	-	X
	Lepidoptera		Lepidoptera	Jovem	X	X
		Tineoidea	Tineoidea	sp.2	X	-
	Orthoptera	Phalangopsidae	Phalangopsidae	Jovem	-	X
			Phalangopsidae	sp.	X	X
			Phalangopsidae	sp.7	-	X
			Phalangopsidae	sp.8	-	X

			Paraclodes sp.1	-	X
			Phalangopsis sp.1	-	X
	Plecoptera	Perlidae	Perlidae sp.1	-	X
	Psocoptera	Psocomorpha	Psocomorpha Jovem	X	X
		Psyllipsocidae	Psyllipsocidae sp.7	-	X
	Siphonaptera	Pulicidae	Pulicidae sp.1	-	X
	Zygentoma	Nicoletiidae	Atelurinae sp.1	X	X
			Nicoletiinae sp.1	X	X
Malacostraca	Decapoda	Palaemonidae	Palaemonidae Jovem	-	X
			Palaemonidae sp.	-	X
Mammalia	Chiroptera	Mormoopidae	*Pteronotus gymnonotus*	-	X
			Pteronotus parnellii	X	X
		Phyllostomidae	*Carollia* sp.	X	X
			Diphylla ecaudata	X	-
			Lonchorhina aurita	X	-
Reptilia	Pleurodonta	Tropiduridae	Tropiduridae sp.	X	-
Gastropoda	Pulmonata	Ampullariidae	*Pomacea* sp.1	-	X
Platyhelminthes	Tricladida	Dugesiidae	Dugesiidae sp.1	-	X

SB-0200						
TÁXONS					Seco	Úmido
Arachnida	Amblypygi	Phrynidae	*Heterophrynus*	*longicornis*	X	X
	Araneae	Araneidae	Araneidae	Jovem	-	X
			Alpaida	*antonio*	X	-
		Corinnidae	Corinnidae	Jovem	X	-
		Ctenidae	Ctenidae	Jovem	-	X
		Ochyroceratidae	Ochyroceratidae	Jovem	-	X
		Oonopidae	Oonopidae	Jovem	-	X
		Pholcidae	*Leptopholcus*	sp.1	-	X
			Mesabolivar	*eberhardi*	-	X
		Salticidae	Salticidae	Jovem	-	X
		Theridiidae	Theridiidae	Jovem	-	X
			Episinus	sp.2	-	X
		Theridiosomatidae	*Plato*	sp.1	X	X
	Opiliones	Cosmetidae	Cosmetidae	Jovem	-	X
		Escadabiidae	Escadabiidae	Jovem	X	-
	Pseudoscorpiones	Chernetidae	Chernetidae	Jovem	X	-
			Spelaeochernes	sp.1	X	X
		Chthoniidae	*Pseudochthonius*	sp.2	-	X
	Scorpiones	Buthidae	*Ananteris*	sp.	X	-
Acari	Trombidiforme	Bdellidae	Bdellidae	sp.1	X	-
		Trombiculidae	Trombiculidae	sp.2	-	X
Diplopoda	Glomeridesmida	Glomeridesmidae	Glomeridesmidae	sp.1	X	-
	Polydesmida		Polydesmida	Jovem	X	-
	Spirostreptida		Spirostreptida	Jovem	X	-
		Pseudonannolenidae	Pseudonannolenidae	Jovem	-	X
Entognatha	Collembola	Paronellidae	Paronellidae	sp.1	X	X
	Diplura	Campodeidae	Campodeidae	sp.1	-	X
Insecta	Blattaria	Blaberidae	Blaberidae	Jovem	-	X
	Coleoptera		Coleoptera	Jovem	X	-
		Scydmaenidae	Scydmaeninae	sp.7	X	-
		Staphylinidae	Pselaphinae	sp.5	X	-
	Diptera	Psychodidae	Phlebotominae	sp.1	-	X
	Heteroptera	Cydnidae	Cydnidae	Jovem	X	-
			Cydnidae	sp.1	-	X
		Reduviidae	*Zelurus*	Jovem	X	-
	Homoptera	Cixiidae	Cixiidae	Jovem	X	X
	Hymenoptera	Formicidae	*Acromyrmex*	sp.1	X	-
			Cardiocondyla	sp.1	-	X
			Crematogaster	*abstinens*	X	-
			Nylanderia	sp.2	-	X
			Pheidole	sp.5	X	-
	Isoptera	Termitidae	*Nasutitermes*	sp.1	X	-
			Nasutitermes	sp.7	-	X
	Orthoptera	Phalangopsidae	Phalangopsidae	sp.	X	X
			Phalangopsidae	sp.5	-	X
			Phalangopsis	sp.1	-	X
	Psocoptera	Psocomorpha	Psocomorpha	Jovem	X	-
Malacostraca	Isopoda	Armadillidae	Armadillidae	Jovem	X	X
		Philosciidae	Philosciidae	Jovem	X	-
			Philosciidae	sp.2	X	-
Amphibia	Anura	Craugastoridae	*Pristimantis*	*cf. fenestratus*	X	-
Mammalia	Chiroptera	Emballonuridae	*Peropteryx*	sp.	-	X
		Phyllostomidae	*Carollia*	sp.	X	X
Gastropoda	Pulmonata	Spiraxidae	*Euglandina*	sp.1	-	X

SB-0201					
TÁXONS				Seco	Úmido
Arachnida	Amblypygi	Phrynidae	*Heterophrynus* *longicornis*	-	X
	Araneae	Araneidae	Araneidae Jovem	X	-
		Ctenidae	Ctenidae Jovem	-	X
			Ctenidae sp.	-	X
			Ctenus sp.1	X	-
		Ochyroceratidae	Ochyroceratidae Jovem	-	X
		Pholcidae	*Mesabolivar* *eberhardi*	X	-
		Symphytognathidae	*Anapistula* sp.1	-	X
		Theraphosidae	Theraphosidae Jovem	-	X
		Theridiidae	Theridiidae Jovem	-	X
			Thymoites sp.1	-	X
		Theridiosomatidae	*Plato* sp.1	-	X
	Pseudoscorpiones	Chthoniidae	*Pseudochthonius* sp.2	-	X
Acari	Sarcoptiforme	Oribatida	Oribatida sp.1	X	-
Entognatha	Collembola	Paronellidae	Paronellidae sp.1	X	X
Insecta	Blattaria	Blattidae	Blattidae Jovem	-	X
	Coleoptera	Staphylinidae	Staphylininae sp.5	-	X
	Diptera		Diptera Jovem	-	X
		Cecidomyiidae	Cecidomyiidae sp.2	-	X
		Psychodidae	Phlebotominae sp.1	-	X
	Heteroptera	Cydnidae	*Pangaeus* sp.1	X	-
		Reduviidae	Emesinae Jovem	X	-
			Emesinae sp.5	-	X
			Zelurus Jovem	X	-
			Zelurus sp.1	-	X
	Homoptera	Cixiidae	Cixiidae Jovem	-	X
	Hymenoptera	Formicidae	*Camponotus* *cingulatus*	-	X
			Camponotus sp.2	X	-
			Octostruma sp.1	X	X
			Pheidole sp.2	-	X
			Pheidole sp.5	X	-
	Isoptera	Termitidae	Termitidae sp.	X	X
	Lepidoptera	Noctuoidea	Noctuoidea sp.7	X	-
	Orthoptera		Orthoptera sp.	X	X
		Phalangopsidae	*Paraclodes* sp.1	-	X
	Psocoptera	Psocomorpha	Psocomorpha Jovem	-	X
Malacostraca	Isopoda	Philosciidae	Philosciidae sp.2	-	X
		Platyarthridae	Platyarthridae Jovem	X	-
Mammalia	Chiroptera	Phyllostomidae	*Glossophaga* sp.	-	X

SB-0202					
TÁXONS				**Seco**	**Úmido**
Arachnida	Araneae	Ochyroceratidae	Ochyroceratidae Jovem	X	X
		Tetrablemmidae	Tetrablemmidae sp.1	X	-
		Theraphosidae	Theraphosidae Jovem	-	X
	Pseudoscorpiones	Chernetidae	*Spelaeochernes* sp.1	X	-
Diplopoda	Polydesmida	Pyrgodesmidae	Pyrgodesmidae Jovem	-	X
Entognatha	Collembola	Isotomidae	Isotomidae sp.1	X	-
		Paronellidae	Paronellidae sp.1	X	-
			Cyphoderus sp.nov.2	X	-
	Diplura	Projapygidae	Projapygidae sp.1	-	X
Insecta	Coleoptera		Coleoptera Jovem	X	-
	Diptera	Simuliidae	Simuliidae sp.1	-	X
		Psychodidae	Phlebotominae sp.1	-	X
	Homoptera	Membracidae	Membracidae sp.1	X	-
	Hymenoptera	Formicidae	*Camponotus* sp.2	X	-
			Pachycondyla constricta	X	-
			Pheidole sp.4	-	X
			Pheidole sp.5	X	-
			Pheidole sp.7	X	-
	Isoptera	Termitidae	Termitidae sp.	X	-
			Nasutitermes sp.2	X	-
	Lepidoptera	Noctuoidea	Noctuoidea sp.1	-	X
	Orthoptera		Orthoptera sp.	X	-
		Phalangopsidae	*Eidmanacris* sp.1	X	X
Malacostraca	Isopoda	Philosciidae	Philosciidae Jovem	X	-
			Philosciidae sp.2	X	-
Amphibia	Anura	Craugastoridae	*Pristimantis cf. fenestratus*	X	X
		Leptodactylidae	*Adenomera cf. andreae*	X	-
Mammalia	Chiroptera	Phyllostomidae	*Carollia* sp.	X	-
	Rodentia	Cricetidae	*Rhipidomys* sp.	X	-

SB-0203					
TÁXONS				**Seco**	**Úmido**
Arachnida	Araneae	Araneidae	Araneidae Jovem	-	X
		Drymusidae	Drymusidae Jovem	-	X
		Oonopidae	Oonopidae Jovem	-	X
		Pholcidae	Pholcidae Jovem	X	X
		Salticidae	Salticidae Jovem	X	-
		Scytodidae	Scytodidae Jovem	-	X
		Symphytognathidae	*Anapistula* sp.1	X	-
		Tetragnathidae	Tetragnathidae Jovem	X	-
	Opiliones	Manaosbiidae	Manaosbiidae sp.1	X	-
	Pseudoscorpiones	Chernetidae	*Spelaeochernes* sp.1	X	X
Chilopoda	Scolopendromorpha	Scolopocryptopidae	*Newportia* sp.2	X	-
Diplopoda	Polydesmida		Polydesmida Jovem	-	X
Entognatha	Collembola	Paronellidae	Paronellidae sp.1	X	-
	Diplura	Campodeidae	Campodeidae sp.1	X	X
		Projapygidae	Projapygidae sp.1	-	X
Insecta	Blattaria	Blattelidae	Blattelidae Jovem	X	-
	Coleoptera		Coleoptera Jovem	-	X
	Diptera	Chloropidae	Chloropidae sp.1	-	X
	Heteroptera	Cydnidae	Cydnidae Jovem	X	-
			Pangaeus sp.1	-	X
	Hymenoptera	Formicidae	*Acanthostichus bentoni*	-	X
			Camponotus sp.2	X	-
			Pachycondyla constricta	X	X
	Isoptera	Termitidae	Termitidae sp.	X	-
	Orthoptera	Phalangopsidae	Phalangopsidae sp.1	X	X
Malacostraca	Isopoda	Philosciidae	Philosciidae sp.2	-	X
		Platyarthridae	Platyarthridae sp.1	-	X
Amphibia	Anura	Leptodactylidae	*Adenomera cf. andreae*	X	-
Mammalia	Chiroptera	Emballonuridae	*Peropteryx* sp.	X	X
		Phyllostomidae	*Carollia* sp.	-	X

SB-0204					
TÁXONS				**Seco**	**Úmido**
Arachnida	Amblypygi	Phrynidae	*Heterophrynus longicornis*	X	-
	Araneae	Araneidae	Araneidae Jovem	-	X
		Ctenidae	Ctenidae Jovem	X	-
		Ochyroceratidae	Ochyroceratidae Jovem	X	X
			Speocera sp.1	X	-
		Theraphosidae	Theraphosidae Jovem	X	X
	Palpigradi	Eukoeneniidae	Eukoeneniidae Jovem	-	X
	Pseudoscorpiones	Chernetidae	*Spelaeochernes* sp.1	X	-
Entognatha	Collembola	Symphypleona	Symphypleona sp.2	X	-
	Diplura	Projapygidae	Projapygidae sp.1	-	X
Insecta	Diptera	Muscidae	Muscidae sp.1	-	X
		Psychodidae	Phlebotominae sp.1	-	X
	Hymenoptera	Formicidae	*Pachycondyla constricta*	-	X
	Isoptera	Termitidae	*Cornitermes* sp.1	X	-
	Orthoptera	Phalangopsidae	Phalangopsidae sp.	X	-
			Eidmanacris sp.1	X	-
			Paraclodes sp.1	X	-
			Phalangopsis sp.1	-	X
Malacostraca	Isopoda	Philosciidae	Philosciidae Jovem	X	-
			Philosciidae sp.2	X	-
Amphibia	Anura	Craugastoridae	*Pristimantis cf. fenestratus*	X	X
		Leptodactylidae	*Adenomera cf. andreae*	-	X
Mammalia	Chiroptera	Phyllostomidae	*Carollia perspicillata*	X	-
			Micronycteris sp.	-	X

SB-0206					
TÁXONS				Seco	Úmido
Arachnida	Amblypygi	Phrynidae	*Heterophrynus longicornis*	X	X
	Araneae	Araneidae	Araneidae Jovem	-	X
		Corinnidae	Corinnidae Jovem	X	X
		Ochyroceratidae	Ochyroceratidae Jovem	X	X
		Oonopidae	Oonopidae Jovem	-	X
			Oonopidae sp.2	-	X
		Salticidae	Salticidae Jovem	-	X
		Scytodidae	*Scytodes* sp.2	X	-
		Theridiidae	*Dipoena* sp.1	-	X
		Theridiosomatidae	*Plato* sp.1	X	X
	Opiliones	Cosmetidae	Cosmetidae Jovem	-	X
			Roquettea carajas	X	X
		Escadabiidae	Escadabiidae sp.3	-	X
	Pseudoscorpiones	Chernetidae	*Spelaeochernes* sp.1	X	X
		Chthoniidae	*Pseudochthonius* sp.2	X	-
Acari	Mesostigmata		Mesostigmata sp.1	X	-
			Mesostigmata sp.13	-	X
	Opilioacarida	Opilioacaridae	Opilioacaridae sp.1	-	X
	Sarcoptiforme	Oribatida	Oribatida sp.1	X	-
			Oribatida sp.4	-	X
Diplopoda	Polydesmida	Chelodesmidae	Chelodesmidae Jovem	-	X
	Siphonophorida	Siphonophoridae	Siphonophoridae sp.1	-	X
	Spirostreptida		Spirostreptida sp.	X	-
Entognatha	Collembola	Paronellidae	Paronellidae sp.1	-	X
		Symphypleona	Symphypleona sp.2	X	X
	Diplura	Campodeidae	Campodeidae sp.1	-	X
Insecta	Blattaria	Blaberidae	Blaberidae sp.	-	X
	Coleoptera		Coleoptera Jovem	-	X
		Elateridae	Elateridae sp.3	X	-
		Scydmaenidae	Scydmaenidae sp.7	-	X
		Staphylinidae	Staphylininae sp.1	-	X
			Staphylininae sp.3	X	-
			Staphylininae sp.8	-	X
	Diptera		Diptera Jovem	-	X
		Brachycera	Brachycera sp.	-	X
		Drosophilidae	Drosophilidae sp.1	-	X
		Sciaridae	Sciaridae sp.2	X	X
		Psychodidae	Psychodidae sp.1	-	X
			Phlebotominae sp.1	X	X
	Heteroptera	Cydnidae	Cydnidae Jovem	X	-
			Cydnidae sp.1	-	X
		Reduviidae	*Triatoma* Jovem	X	-
	Hymenoptera	Diapriidae	Diapriidae sp.14	X	-
		Formicidae	*Atta* sp.1	X	X
			Pachycondyla constricta	X	-
			Pachycondyla sp.1	-	X
			Pheidole sp.15	-	X
			Pheidole sp.16	-	X
	Isoptera	Termitidae	Termitidae sp.	X	-
			Cornitermes sp.1	-	X
	Orthoptera		Orthoptera sp.	X	-
		Phalangopsidae	*Phalangopsis* sp.1	X	X
	Psocoptera	Psocomorpha	Psocomorpha Jovem	-	X
	Zygentoma	Nicoletiidae	Nicoletiinae sp.1	-	X
Malacostraca	Isopoda	Armadillidae	Armadillidae Jovem	-	X
			Armadillidae sp.1	-	X
		Dubioniscidae	Dubioniscidae sp.1	-	X

		Philosciidae	Philosciidae sp.2	X	X
Symphyla		Scutigerellidae	*Hanseniella* sp.1	-	X
Amphibia	Anura	Bufonidae	*Rhinella gr. marina*	X	-
		Craugastoridae	*Pristimantis cf. fenestratus*	X	-
Mammalia	Chiroptera	Phyllostomidae	*Carollia* sp.	-	X
			Glossophaga sp.	-	X
Gastropoda	Pulmonata	Systrophiidae	*Happia* sp.1	X	-

SB-0207					
TÁXONS				Seco	Úmido
Arachnida	Amblypygi	Phrynidae	*Heterophrynus longicornis*	X	-
	Araneae		Araneae sp.	X	-
		Araneidae	*Alpaida antonio*	X	-
		Corinnidae	Corinnidae Jovem	X	X
		Linyphiidae	Linyphiidae Jovem	-	X
		Nesticidae	Nesticidae Jovem	X	-
		Ochyroceratidae	Ochyroceratidae Jovem	X	X
			Speocera sp.1	-	X
		Oonopidae	Oonopidae sp.1	X	-
			Oonopidae sp.2	-	X
		Pholcidae	Pholcidae Jovem	X	X
			Mesabolivar sp.1	-	X
			Ninetinae sp.1	X	-
		Salticidae	Salticidae Jovem	X	X
		Scytodidae	Scytodidae Jovem	X	X
		Theridiosomatidae	Theridiosomatidae Jovem	-	X
		Trechaleidae	Trechaleidae Jovem	-	X
	Opiliones	Manaosbiidae	Manaosbiidae sp.1	X	-
		Neogoveidae	Neogoveidae Jovem	-	X
		Stygnidae	Stygnidae Jovem	-	X
	Pseudoscorpiones	Chernetidae	Chernetidae Jovem	-	X
			Spelaeochernes sp.1	X	X
		Chthoniidae	*Pseudochthonius* sp.2	-	X
Acari	Holothryda		Holothryda sp.4	-	X
	Ixodida	Argasidae	*Ornithodoros* sp.1	X	X
	Mesostigmata		Mesostigmata sp.3	-	X
	Opilioacarida	Opilioacaridae	Opilioacaridae sp.1	X	-
	Sarcoptiforme	Oribatida	Oribatida sp.1	X	X
			Oribatida sp.4	X	-
	Trombidiforme	Trombiculidae	Trombiculidae sp.1	-	X
Chilopoda	Geophilomorpha	Geophilidae	*Hyphydrophilus* sp.1	-	X
	Scutigeromorpha		Scutigeromorpha Jovem	X	-
		Pselliodidae	*Sphendononema guildingii*	X	-
Diplopoda	Glomeridesmida	Glomeridesmidae	Glomeridesmidae Jovem	-	X
	Polydesmida		Polydesmida Jovem	-	X
		Chelodesmidae	Chelodesmidae sp.1	X	X
		Fuhrmannodesmidae	Fuhrmannodesmidae Jovem	-	X
			Fuhrmannodesmidae sp.	X	-
		Pyrgodesmidae	Pyrgodesmidae Jovem	-	X
	Spirostreptida		Spirostreptida Jovem	X	X
		Pseudonannolenidae	Pseudonannolenidae sp.1	X	-
Entognatha	Collembola	Paronellidae	Paronellidae sp.1	X	X
			Cyphoderus javanus	X	X
		Symphypleona	Symphypleona sp.2	X	X
	Diplura	Campodeidae	Campodeidae sp.1	X	X
Insecta	Blattaria		Blattaria sp.	-	X
		Blaberidae	Blaberidae Jovem	X	X
			Blaberidae sp.	X	-
			Blaberidae sp.3	-	X
			Blaberidae sp.4	X	-
		Polyphagidae	Polyphagidae Jovem	X	X
			Polyphagidae sp.1	X	-
	Coleoptera		Coleoptera Jovem	-	X
		Elateridae	Elateridae Jovem	X	-
		Nitidulidae	Nitidulidae sp.1	-	X
		Tenebrionidae	Tenebrionidae Jovem	X	-
	Diptera		Diptera Jovem	X	X

		Dolichopodidae	Dolichopodidae	sp.1	-	X
		Muscidae	Muscidae	sp.1	-	X
		Sciaridae	Sciaridae	sp.1	-	X
		Ceratopogonidae	Ceratopogonidae	Jovem	X	-
		Psychodidae	Phlebotominae	sp.1	-	X
	Heteroptera	Cydnidae	Cydnidae	Jovem	X	X
			Pangaeus	sp.1	X	X
		Lygaeidae	Lygaeidae	Jovem	-	X
			Lygaeidae	sp.1	X	X
		Nabidae	Nabidae	Jovem	-	X
			Prostemmatinae	sp.1	X	X
		Reduviidae	*Zelurus*	Jovem	X	X
		Schizopteridae	Schizopteridae	Jovem	X	-
	Homoptera	Cixiidae	Cixiidae	Jovem	X	X
			Cixiidae	sp.6	X	-
	Hymenoptera	Formicidae	*Acromyrmex*	*octopinosus*	X	-
			Apterostigma	*collare*	X	-
			Brachymyrmex	sp.1	-	X
			Camponotus	*cingulatus*	X	X
			Camponotus	sp.10	-	X
			Cephalotes	sp.1	-	X
			Cyphomyrmex	*rimosus*	X	-
			Pachycondyla	*constricta*	X	X
			Pachycondyla	sp.1	X	-
			Solenopsis	*invicta*	X	-
			Solenopsis	sp.3	X	X
	Isoptera	Termitidae	*Coatitermes*	sp.1	-	X
			Nasutitermes	sp.1	X	X
			Nasutitermes	sp.2	X	-
			Nasutitermes	sp.5	-	X
			Termes	sp.1	X	-
	Lepidoptera	Noctuoidea	Noctuoidea	sp.14	X	-
		Tineoidea	Tineoidea	sp.4	X	-
			Tineoidea	sp.6	X	-
	Orthoptera		Orthoptera	sp.	X	X
		Phalangopsidae	Phalangopsidae	sp.	-	X
			Phalangopsidae	sp.5	-	X
			Paraclodes	sp.1	-	X
			Phalangopsis	sp.1	X	X
		Tettigoniidae	Listroscelidinae	sp.1	X	-
	Psocoptera	Psocomorpha	Psocomorpha	Jovem	X	-
	Zygentoma	Nicoletiidae	Nicoletiidae	sp.1	X	-
Malacostraca	Isopoda	Armadillidae	Armadillidae	Jovem	X	X
			Armadillidae	sp.1	X	X
		Philosciidae	Philosciidae	Jovem	X	X
			Philosciidae	sp.1	X	X
			Philosciidae	sp.2	X	X
		Platyarthridae	Platyarthridae	sp.1	-	X
		Scleropactidae	Scleropactidae	Jovem	-	X
			Scleropactidae	sp.2	X	X
Symphyla		Scutigerellidae	Scutigerellidae	sp.1	-	X
			Hanseniella	sp.1	X	-
Amphibia	Anura	Craugastoridae	*Pristimantis*	*cf. fenestratus*	X	X
		Leptodactylidae	*Adenomera*	*cf. andreae*	-	X
Mammalia	Chiroptera	Emballonuridae	*Peropteryx*	*kappleri*	X	-
			Peropteryx	sp.	-	X
		Phyllostomidae	*Carollia*	*perspicillata*	X	-
			Carollia	sp.	-	X
			Glossophaga	sp.	X	X

Gastropoda	Pulmonata	Subulinidae	*Glossophaga* Jovem	X	-
			Lamellaxis sp.2	X	X
		Systrophiidae	*Happia* sp.1	X	X

SB-0208						
TÁXONS					Seco	Úmido
Arachnida	Araneae	Corinnidae	Corinnidae	Jovem	-	X
		Ctenidae	Ctenidae	Jovem	-	X
		Ochyroceratidae	Ochyroceratidae	Jovem	-	X
			Ochyrocera	sp.3	X	-
			Speocera	sp.1	X	-
		Salticidae	Salticidae	Jovem	-	X
		Theraphosidae	Theraphosidae	Jovem	-	X
		Theridiosomatidae	*Plato*	sp.1	-	X
	Pseudoscorpiones	Chernetidae	*Spelaeochernes*	sp.1	-	X
		Chthoniidae	*Pseudochthonius*	sp.2	X	-
	Schizomida	Hubardiidae	Hubardiidae	Jovem	-	X
Acari	Ixodida	Argasidae	*Ornithodoros*	sp.1	-	X
	Trombidiforme	Trombiculidae	Trombiculidae	sp.1	-	X
		Trombidiidae	Trombidiidae	sp.1	-	X
Insecta	Heteroptera	Reduviidae	*Zelurus*	Jovem	X	-
	Hymenoptera	Formicidae	*Camponotus*	sp.2	X	X
			Cyphomyrmex	*pr. costatus*	X	-
	Orthoptera		Orthoptera	sp.	X	X
Malacostraca	Isopoda	Armadillidae	Armadillidae	Jovem	-	X
		Philosciidae	Philosciidae	sp.1	X	-
Mammalia	Chiroptera	Emballonuridae	*Peropteryx*	sp.	X	-

SB-0209					
TÁXONS				Seco	Úmido
Annelida	Haplotaxida		Haplotaxida sp.11	-	X
Arachnida	Amblypygi	Phrynidae	*Heterophrynus longicornis*	X	X
	Araneae	Corinnidae	Corinnidae Jovem	X	-
		Ctenidae	Ctenidae Jovem	-	X
		Dipluridae	Dipluridae sp.	-	X
		Oonopidae	Oonopidae sp.7	-	X
			gr. Xycarphhy sp.1	-	X
		Pholcidae	Pholcidae Jovem	-	X
		Salticidae	Salticidae Jovem	-	X
		Theridiosomatidae	*Plato* sp.1	-	X
	Opiliones	Stygnidae	Stygnidae Jovem	-	X
			Protimesius laevis	-	X
	Pseudoscorpiones	Chernetidae	*Spelaeochernes* sp.1	X	X
Acari	Ixodida	Argasidae	*Ornithodoros* sp.1	X	-
Diplopoda	Spirostreptida		Spirostreptida Jovem	-	X
		Pseudonannolenidae	Pseudonannolenidae sp.1	X	-
Entognatha	Collembola	Paronellidae	Paronellidae sp.1	-	X
			Cyphoderus javanus	X	-
Insecta	Blattaria	Polyphagidae	Polyphagidae sp.1	X	-
	Coleoptera		Coleoptera Jovem	-	X
		Staphylinidae	Pselaphinae sp.5	-	X
			Pselaphinae sp.6	X	-
	Diptera	Phoridae	Phoridae sp.2	-	X
	Heteroptera	Reduviidae	Emesinae Jovem	-	X
			Triatoma Jovem	X	X
			Triatoma sp.	X	-
	Homoptera	Cixiidae	Cixiidae Jovem	-	X
	Hymenoptera	Formicidae	*Camponotus* sp.2	X	X
			Nylanderia sp.2	-	X
			Pachycondyla constricta	-	X
	Isoptera	Termitidae	*Nasutitermes* sp.1	-	X
			Nasutitermes sp.2	X	-
			Nasutitermes sp.5	-	X
	Lepidoptera		Lepidoptera Jovem	-	X
	Orthoptera		Orthoptera sp.	X	X
		Phalangopsidae	*Eidmanacris* sp.1	-	X
			Paraclodes sp.1	X	-
	Psocoptera	Psocomorpha	Psocomorpha Jovem	-	X
Malacostraca	Isopoda	Philosciidae	Philosciidae sp.2	X	-
Mammalia	Chiroptera	Emballonuridae	*Peropteryx* sp.	X	-
Gastropoda	Pulmonata	Systrophiidae	*Happia* sp.1	X	-

SB-0210					
TÁXONS				Seco	Úmido
Arachnida	Amblypygi	Phrynidae	*Heterophrynus longicornis*	X	X
	Araneae	Filistatidae	Filistatidae sp.1	X	-
		Gnaphosidae	Gnaphosidae Jovem	X	X
		Mimetidae	Mimetidae Jovem	X	-
		Ochyroceratidae	Ochyroceratidae Jovem	-	X
		Pholcidae	Pholcidae Jovem	X	X
			Mesabolivar cambridgei	X	-
		Theridiidae	Theridiidae Jovem	X	-
		Theridiosomatidae	*Plato* sp.1	X	X
	Pseudoscorpiones	Chernetidae	*Spelaeochernes* sp.1	-	X
		Chthoniidae	Chthoniidae Jovem	-	X
			Pseudochthonius sp.4	X	-
Acari	Ixodida	Argasidae	Ornithodoros sp.1	X	X
	Trombidiforme	Trombiculidae	Trombiculidae sp.1	X	-
Entognatha	Collembola	Entomobryidae	Entomobryidae sp.2	-	X
		Paronellidae	Paronellidae sp.1	X	-
			Cyphoderus agnotus	-	X
	Diplura	Japygidae	Japygidae sp.1	-	X
Insecta	Blattaria	Blaberidae	Blaberidae Jovem	X	X
		Polyphagidae	Polyphagidae Jovem	X	X
	Coleoptera		Coleoptera Jovem	X	X
		Elateridae	Elateridae Jovem	X	-
	Diptera	Rangomaramidae	Rangomaramidae sp.2	-	X
		Psychodidae	Phlebotominae sp.1	-	X
	Heteroptera	Cydnidae	Cydnidae Jovem	-	X
		Reduviidae	*Zelurus* Jovem	X	X
	Homoptera	Derbidae	Derbidae sp.	X	-
	Hymenoptera	Formicidae	*Camponotus* sp.2	X	X
	Isoptera	Termitidae	*Nasutitermes* sp.2	-	X
			Nasutitermes sp.6	X	-
			Termes sp.1	X	-
	Lepidoptera	Hesperiidae	Hesperiidae sp.1	X	-
		Noctuoidea	Noctuoidea sp.13	-	X
			Noctuoidea sp.7	X	-
	Neuroptera	Myrmeleontidae	Myrmeleontidae sp.1	X	X
			Myrmeleontidae sp.5	X	-
	Orthoptera		Orthoptera sp.	X	X
		Phalangopsidae	*Eidmanacris* sp.1	X	-
Amphibia	Anura	Craugastoridae	*Pristimantis* cf. *fenestratus*	X	-
Mammalia	Chiroptera	Emballonuridae	*Peropteryx* sp.	X	-

SB-0211					
**TÁXONS**				**Seco**	**Úmido**
Arachnida	Amblypygi	Phrynidae	*Heterophrynus longicornis*	-	X
	Araneae	Araneidae	Araneidae Jovem	-	X
		Corinnidae	*Corinna ducke*	-	X
		Ctenidae	Ctenidae Jovem	-	X
		Oonopidae	Oonopidae Jovem	-	X
		Pholcidae	Pholcidae Jovem	X	X
			Mesabolivar eberhardi	-	X
		Salticidae	Salticidae Jovem	X	X
		Tetragnathidae	Tetragnathidae Jovem	-	X
		Theraphosidae	Theraphosidae Jovem	X	-
		Theridiidae	Theridiidae Jovem	X	X
			Achaearanea sp.1	-	X
		Theridiosomatidae	*Plato* sp.1	-	X
	Opiliones	Stygnidae	Stygnidae Jovem	-	X
	Pseudoscorpiones	Chernetidae	*Spelaeochernes* sp.1	X	X
		Chthoniidae	*Pseudochthonius* sp.2	X	X
	Scorpiones	Buthidae	*Ananteris* sp.3	-	X
		Liochelidae	*Opisthacanthus* Jovem	-	X
Entognatha	Collembola	Entomobryidae	Entomobryidae sp.1	X	-
	Diplura	Projapygidae	Projapygidae sp.2	-	X
Insecta	Coleoptera	Elateridae	Elateridae Jovem	X	-
	Diptera	Psychodidae	Phlebotominae sp.1	-	X
	Heteroptera	Reduviidae	Reduviidae Jovem	-	X
			Zelurus Jovem	X	-
			Zelurus sp.1	-	X
	Hymenoptera	Formicidae	*Camponotus* sp.1	-	X
			Camponotus sp.2	X	-
			Camponotus sp.5	X	-
			Dolichoderus bispinosus	X	-
			Dolichoderus sp.1	-	X
			Nylanderia sp.2	-	X
			Pachycondyla sp.1	-	X
		Icheneumonidae	Icheneumonidae sp.1	-	X
	Isoptera	Termitidae	*Embriatermes* sp.1	-	X
			Nasutitermes sp.1	-	X
	Lepidoptera		Lepidoptera sp.	-	X
	Psocoptera	Psocomorpha	Psocomorpha Jovem	X	X
Malacostraca	Isopoda	Armadillidae	Armadillidae sp.1	-	X
		Philosciidae	Philosciidae sp.2	X	X
		Platyarthridae	Platyarthridae sp.2	-	X
Mammalia	Chiroptera	Emballonuridae	*Peropteryx* sp.	X	X
Onychophora		Peripatidae	Peripatidae sp.1	X	-

SB-0212					
TÁXONS				Seco	Úmido
Annelida	Haplotaxida		Haplotaxida sp.7	-	X
	Lumbriculida	Lumbriculidae	Lumbriculidae sp.2	X	-
Arachnida	Amblypygi		Amblypygi sp.	-	X
		Phrynidae	*Heterophrynus longicornis*	X	X
	Araneae	Araneidae	Araneidae Jovem	X	X
		Corinnidae	Corinnidae Jovem	X	X
			Abapeba hoeferi	X	-
		Filistatidae	Filistatidae Jovem	X	X
		Ochyroceratidae	Ochyroceratidae Jovem	X	X
		Pholcidae	Pholcidae Jovem	X	X
			Pholcidae sp.	X	-
			Mesabolivar sp.1	X	X
		Pholcidae	Pholcidae sp.	X	-
		Plato	Plato sp.	X	-
		Salticidae	Salticidae Jovem	X	X
			Salticidae sp.1	-	X
		Scytodidae	*Scytodes eleonorae*	X	-
		Tetrablemmidae	Tetrablemmidae sp.1	-	X
		Theraphosidae	Theraphosidae Jovem	X	X
		Theridiosomatidae	Theridiosomatidae Jovem	X	X
			Plato sp.1	X	X
	Opiliones	Escadabiidae	Escadabiidae sp.1	X	-
		Neogoveidae	Neogoveidae Jovem	X	X
		Stygnidae	Stygnidae Jovem	X	-
			Paraphareus sp.1	-	X
	Pseudoscorpiones	Chernetidae	*Spelaeochernes* sp.1	X	X
		Chthoniidae	Chthoniidae Jovem	X	-
		Olpiidae	Olpiidae Jovem	-	X
	Ricinulei	Ricinoididae	*Cryptocellus tarsilae*	X	-
	Schizomida	Hubardiidae	Hubardiidae Jovem	X	-
	Scorpiones	Buthidae	*Ananteris luciae*	-	X
Acari	Astigmata		Astigmata sp.1	X	-
	Ixodida	Ixodidae	*Amblyomma cajennense*	-	X
	Mesostigmata		Mesostigmata sp.2	X	X
			Mesostigmata sp.3	X	-
	Sarcoptiforme	Oribatida	Oribatida sp.1	X	X
			Oribatida sp.11	X	-
	Trombidiforme	Trombidiidae	Trombidiidae sp.1	X	-
Chilopoda	Geophilomorpha	Ballophilidae	Ballophilidae sp.2	X	-
	Scolopendromorpha	Scolopocryptopidae	*Dinocryptops miersii*	X	-
			Newportia sp.6	X	-
	Scutigeromorpha		Scutigeromorpha Jovem	X	-
			Scutigeromorpha sp.	-	X
Diplopoda	Polydesmida		Polydesmida Jovem	X	-
			Polydesmida sp.	X	-
		Chelodesmidae	Chelodesmidae sp.1	X	-
		Cryptodesmidae	Cryptodesmidae sp.2	X	-
	Spirostreptida	Spirostreptidae	Spirostreptidae sp.3	-	X
Entognatha	Collembola	Isotomidae	Isotomidae sp.1	X	X
		Paronellidae	Paronellidae sp.1	X	X
			Cyphoderus agnotus	-	X
			Cyphoderus innominatus	X	-
		Sminturidae	*Songhaica* sp.	X	-
Insecta			Insecta Jovem	-	X
	Blattaria	Blaberidae	Blaberidae Jovem	X	-
		Polyphagidae	Polyphagidae Jovem	X	X
	Coleoptera		Coleoptera Jovem	X	X

		Carabidae	Carabidae	sp.1	-	X
		Dytiscidae	Dytiscidae	Jovem	X	-
			Laccophilus	sp.1	X	X
			Laccophilus	sp.2	X	X
		Staphylinidae	Pselaphinae	sp.9	-	X
	Diptera		Diptera	Jovem	X	X
		Conopidae	Conopidae	sp.1	-	X
		Muscidae	Muscidae	sp.1	-	X
		Cecidomyiidae	Cecidomyiidae	sp.1	-	X
		Ceratopogonidae	Ceratopogonidae	Jovem	X	-
			Ceratopogonidae	sp.1	-	X
			Ceratopogonidae	sp.2	-	X
		Chironomidae	Chironomidae	Jovem	X	-
			Chironomidae	sp.1	-	X
		Culicidae	Culicidae	Jovem	X	X
		Psychodidae	Psychodidae	Jovem	X	-
			Psychodidae	sp.1	-	X
	Heteroptera	Lygaeidae	Lygaeidae	sp.1	X	X
		Veliidae	*Microvelia*	sp.1	X	X
			Microvelia	sp.2	X	-
			Paravelia	sp.1	X	X
			Paravelia	sp.2	-	X
			Paravelia	sp.3	X	-
	Homoptera	Cixiidae	Cixiidae	sp.6	-	X
	Hymenoptera	Bethylidae	Bethylidae	sp.3	X	-
		Diapriidae	Diapriidae	sp.13	-	X
		Figitidae	Figitidae	sp.3	-	X
		Formicidae	*Brachymyrmex*	sp.1	X	-
			Camponotus	sp.2	X	X
			Camponotus	sp.5	X	-
			Dolichoderus	*bispinosus*	X	X
			Gnamptogenys	sp.1	X	-
			Gnamptogenys	*striatula*	X	X
			Odontomachus	*bauri*	X	X
			Pachycondyla	*constricta*	X	-
			Pheidole	sp.1	X	-
			Pheidole	sp.5	X	-
			Solenopsis	sp.11	X	-
			Strumigenys	*elongata*	X	-
	Isoptera	Termitidae	*Coatitermes*	sp.1	-	X
			Nasutitermes	sp.1	X	X
			Nasutitermes	sp.2	X	-
	Lepidoptera		Lepidoptera	Jovem	X	-
		Tineidae	Tineidae	Jovem	X	-
	Neuroptera	Myrmeleontidae	Myrmeleontidae	sp.1	X	X
	Orthoptera		Orthoptera	sp.	X	X
		Phalangopsidae	Phalangopsidae	Jovem	-	X
			Phalangopsidae	sp.	-	X
			Eidmanacris	sp.1	-	X
			Paraclodes	sp.1	-	X
			Phalangopsis	sp.1	X	X
	Psocoptera	Psocomorpha	Psocomorpha	Jovem	X	X
		Troctomorpha	Troctomorpha	Jovem	X	-
		Liposcelidae	Liposcelidae	sp.6	X	-
		Psyllipsocidae	Psyllipsocidae	sp.1	-	X
			Psyllipsocidae	sp.8	-	X
	Zygentoma	Nicoletiidae	Nicoletiinae	sp.1	X	-
Malacostraca	Isopoda	Armadillidae	Armadillidae	Jovem	-	X
			Armadillidae	sp.1	X	-

		Scleropactidae	Scleropactidae	Jovem	X	-
			Scleropactidae	sp.2	-	X
Symphyla		Scolopendrellidae	*Symphylella*	sp.1	X	-
Amphibia	Anura	Bufonidae	*Rhinella*	*gr. marina*	X	X
		Craugastoridae	*Pristimantis*	*cf. fenestratus*	X	-
		Leptodactylidae	*leptodactylus*	sp.1	X	-
			leptodactylus	sp.2	-	X
Mammalia	Chiroptera	Emballonuridae	*Peropteryx*	*kappleri*	X	-
			Peropteryx	sp.	-	X
		Furipteridae	*Furipterus*	*horrens*	X	X
		Phyllostomidae	*Carollia*	*perspicillata*	X	-
			Carollia	sp.	-	X
			Diphylla	*ecaudata*	X	X
			Glossophaga	sp.	X	-
			Lionycteris	sp.	-	X
			Lonchorhina	sp.	-	X
			Phyllostomus	sp.	X	X
			Trachops	*cirrhosus*	-	X

SB-0213						
TÁXONS					Seco	Úmido
Annelida	Haplotaxida		Haplotaxida	sp.12	-	X
			Haplotaxida	sp.7	-	X
Arachnida	Amblypygi	Phrynidae	*Heterophrynus*	*longicornis*	-	X
	Araneae	Araneidae	Araneidae	Jovem	-	X
			Alpaida	*antonio*	X	-
		Caponiidae	*Nops*	sp.2	X	-
		Corinnidae	Corinnidae	Jovem	X	-
		Ctenidae	Ctenidae	sp.	X	-
		Ochyroceratidae	Ochyroceratidae	Jovem	-	X
		Oonopidae	Oonopidae	Jovem	-	X
			Oonopidae	sp.1	-	X
		Pholcidae	Pholcidae	Jovem	X	X
			Ninetinae	sp.1	X	-
		Scytodidae	Scytodidae	Jovem	X	X
			Scytodes	*eleonorae*	X	X
		Symphytognathidae	*Anapistula*	sp.2	X	-
		Theraphosidae	Theraphosidae	Jovem	-	X
			Theraphosidae	sp.	-	X
			Guyruita	*cerrado*	X	-
		Theridiidae	Theridiidae	Jovem	X	X
			Achaearanea	sp.1	-	X
		Theridiosomatidae	*Plato*	sp.1	-	X
	Opiliones	Sclerosomatidae	Sclerosomatidae	Jovem	-	X
		Stygnidae	Stygnidae	Jovem	-	X
	Pseudoscorpiones	Chernetidae	*Spelaeochernes*	sp.1	X	X
		Chthoniidae	Chthoniidae	Jovem	X	-
Acari	Trombidiforme	Anystidae	Anystidae	sp.1	-	X
Chilopoda	Scolopendromorpha	Scolopendridae	*Otostigmus*	Jovem	-	X
	Scutigeromorpha		Scutigeromorpha	Jovem	X	-
Diplopoda			Diplopoda	Jovem	-	X
	Polydesmida	Chelodesmidae	Chelodesmidae	sp.1	X	-
		Fuhrmannodesmidae	Fuhrmannodesmidae	sp.1	-	X
	Spirostreptida	Pseudonannolenidae	Pseudonannolenidae	Jovem	-	X
Entognatha	Collembola	Paronellidae	Paronellidae	sp.1	X	X
			Cyphoderus	*agnotus*	-	X
			Cyphoderus	*javanus*	-	X
		Poduromorpha	Poduromorpha	sp.1	X	-
	Diplura	Campodeidae	Campodeidae	sp.1	X	X
Insecta	Blattaria	Blaberidae	Blaberidae	sp.	-	X
		Polyphagidae	Polyphagidae	Jovem	-	X
	Coleoptera		Coleoptera	Jovem	-	X
		Scydmaenidae	Scydmaeninae	sp.3	X	-
			Scydmaeninae	sp.7	X	-
		Staphylinidae	Pselaphinae	sp.2	X	-
	Diptera	Cecidomyiidae	Cecidomyiidae	sp.1	-	X
		Psychodidae	Phlebotominae	sp.	-	X
			Phlebotominae	sp.1	-	X
	Heteroptera	Cydnidae	Cydnidae	sp.1	-	X
			Cydnidae	sp.2	-	X
		Reduviidae	*Zelurus*	Jovem	X	-
			Zelurus	sp.	X	-
			Zelurus	sp.1	-	X
	Homoptera	Cixiidae	Cixiidae	Jovem	-	X
	Hymenoptera	Figitidae	Figitidae	sp.2	X	-
		Formicidae	*Apterostigma*	*pilosum*	X	-
			Camponotus	sp.1	-	X
			Camponotus	sp.2	X	-

			Cardiocondyla	sp.1	-	X
			Dolichoderus	*bispinosus*	X	-
			Hypoponera	sp.7	X	-
			Nylanderia	sp.2	-	X
			Pachycondyla	sp.1	-	X
			Solenopsis	sp.11	X	-
	Isoptera	Termitidae	*Nasutitermes*	sp.1	X	X
			Nasutitermes	sp.2	-	X
			Nasutitermes	sp.3	-	X
			Nasutitermes	sp.5	X	X
	Lepidoptera		Lepidoptera	Jovem	X	-
			Lepidoptera	sp.	-	X
	Neuroptera	Myrmeleontidae	Myrmeleontidae	sp.1	X	X
	Orthoptera		Orthoptera	sp.	X	X
		Phalangopsidae	Phalangopsidae	Jovem	-	X
			Eidmanacris	sp.1	X	-
			Paraclodes	sp.1	X	-
	Psocoptera	Psocomorpha	Psocomorpha	Jovem	X	X
		Psyllipsocidae	Psyllipsocidae	sp.4	X	-
Malacostraca	Isopoda	Armadillidae	Armadillidae	Jovem	-	X
			Armadillidae	sp.1	X	X
		Philosciidae	Philosciidae	Jovem	-	X
			Philosciidae	sp.2	X	X
Amphibia	Anura	Craugastoridae	*Pristimantis*	*cf. fenestratus*	X	X
Mammalia	Chiroptera	Emballonuridae	*Peropteryx*	*kappleri*	X	-
			Peropteryx	sp.	-	X
		Phyllostomidae	*Carollia*	sp.	-	X
			Anoura	sp.	-	X
			Glossophaga	sp.	X	-

SB-0214					
TÁXONS				Seco	Úmido
Annelida	Haplotaxida		Haplotaxida sp.1	-	X
			Haplotaxida sp.10	-	X
			Haplotaxida sp.11	X	X
			Haplotaxida sp.8	-	X
			Haplotaxida sp.9	-	X
Arachnida	Amblypygi		*Amblypygi* sp.	-	X
		Phrynidae	Heterophrynus longicornis	X	X
	Araneae	Corinnidae	Corinnidae Jovem	X	X
		Ctenidae	Ctenidae Jovem	-	X
			Ctenus sp.1	-	X
		Ochyroceratidae	Ochyroceratidae Jovem	X	X
			Speocera sp.1	X	-
		Oonopidae	Oonopidae Jovem	-	X
			Oonopidae sp.2	X	-
			Oonopidae sp.5	-	X
		Pholcidae	Pholcidae Jovem	X	X
			Mesabolivar aurantiacus	-	X
		Salticidae	Salticidae Jovem	X	-
		Symphytognathidae	*Anapistula* sp.1	-	X
		Theridiidae	*Achaearanea* sp.1	X	X
		Theridiosomatidae	Theridiosomatidae Jovem	-	X
			Plato sp.1	-	X
	Opiliones	Escadabiidae	Escadabiidae sp.1	X	X
		Neogoveidae	Neogoveidae Jovem	-	X
			Canga renatae	X	-
	Palpigradi	Eukoeneniidae	Eukoeneniidae Jovem	-	X
	Pseudoscorpiones	Chernetidae	Chernetidae Jovem	X	-
			Spelaeochernes sp.1	X	X
		Chthoniidae	Chthoniidae Jovem	X	-
			Pseudochthonius sp.2	X	-
			Pseudochthonius sp.4	X	-
		Syarinidae	Syarinidae Jovem	X	-
Acari	Mesostigmata		Mesostigmata sp.3	X	-
	Sarcoptiforme	Oribatida	Oribatida sp.4	X	-
			Oribatida sp.7	X	-
	Trombidiforme	Trombidiidae	Trombidiidae sp.1	X	X
Chilopoda	Geophilomorpha		Geophilomorpha sp.	X	-
		Schendylidae	*Schendyylops* sp.2	-	X
	Scolopendromorpha	Scolopocryptopidae	Scolopocryptopidae sp.	-	X
	Scutigeromorpha		Scutigeromorpha Jovem	X	-
Diplopoda	Glomeridesmida	Glomeridesmidae	Glomeridesmidae Jovem	-	X
			Glomeridesmidae sp.	-	X
	Polydesmida	Cyrtodesmidae	Cyrtodesmidae sp.1	X	-
		Fuhrmannodesmidae	Fuhrmannodesmidae Jovem	-	X
		Pyrgodesmidae	Pyrgodesmidae Jovem	-	X
			Pyrgodesmidae sp.1	X	-
	Spirostreptida	Pseudonannolenidae	Pseudonannolenidae sp.1	X	-
Entognatha	Collembola	Isotomidae	Isotomidae sp.1	X	-
		Paronellidae	Paronellidae sp.1	X	-
			Cyphoderus agnotus	-	X
			Trogolaphysa sp.2	X	X
		Symphypleona	Symphypleona sp.2	X	-
	Diplura	Campodeidae	Campodeidae sp.1	X	X
Insecta	Blattaria	Blaberidae	Blaberidae Jovem	X	-
	Coleoptera		Coleoptera Jovem	X	X
		Carabidae	Carabidae sp.5	X	-
		Chrysomelidae	Chrysomelidae sp.3	-	X

		Staphylinidae	Staphylinidae	sp.16	-	X
			Staphylininae	sp.16	X	-
	Diptera		Diptera	Jovem	X	-
		Psychodidae	Phlebotominae	sp.1	-	X
	Heteroptera	Cydnidae	*Pangaeus*	sp.1	-	X
		Reduviidae	*Zelurus*	Jovem	-	X
			Triatoma	Jovem	-	X
		Schizopteridae	Schizopteridae	sp.2	-	X
	Homoptera	Coccoidea	Coccoidea	sp.	-	X
		Cixiidae	Cixiidae	Jovem	X	-
	Hymenoptera	Braconidae	Braconidae	sp.6	X	-
		Formicidae	*Anochetus*	sp.1	X	-
			Apterostigma	*pilosum*	-	X
			Centromyrmex	sp.1	-	X
			Crematogaster	*limata*	-	X
			Labidus	*coecus*	X	-
			Pachycondyla	*constricta*	-	X
			Rogeria	pr. *belti*	-	X
	Isoptera	Termitidae	Termitidae	sp.	-	X
			Nasutitermes	sp.1	-	X
	Orthoptera	Phalangopsidae	*Phalangopsis*	sp.1	X	X
	Zygentoma	Nicoletiidae	Nicoletiinae	sp.1	-	X
Malacostraca	Isopoda	Armadillidae	Armadillidae	Jovem	X	X
			Armadillidae	sp.1	X	X
		Philosciidae	Philosciidae	Jovem	X	X
			Philosciidae	sp.2	X	X
		Platyarthridae	Platyarthridae	Jovem	X	-
			Platyarthridae	sp.2	X	-
			Platyarthridae	sp.5	X	X
Symphyla			Symphyla	Jovem	X	-
Amphibia	Anura		Anura	sp.	X	-
		Leptodactylidae	*Adenomera*	cf. *andreae*	X	-
Mammalia	Chiroptera	Phyllostomidae	*Carollia*	sp.	X	X
			Desmodus	*rotundus*	X	X
			Glossophaginae	sp.	-	X
	Rodentia	Cricetidae	*Rhipidomys*	sp.	X	-
Gastropoda	Pulmonata	Systrophiidae	Systrophiidae	Jovem	-	X
			Happia	sp.1	X	X
Onychophora		Peripatidae	Peripatidae	Jovem	-	X

SB-0215					
TÁXONS				Seco	Úmido
Clitellata	Haplotaxida		Haplotaxida sp.2	-	X
Arachnida	Amblypygi		*Amblypygi* sp.	-	X
	Araneae	Corinnidae	Corinnidae Jovem	-	X
		Salticidae	Salticidae Jovem	X	-
		Scytodidae	Scytodidae Jovem	X	-
		Theraphosidae	Theraphosidae Jovem	-	X
		Theridiidae	Theridiidae Jovem	X	X
	Opiliones	Cosmetidae	*Roquettea carajas*	-	X
		Stygnidae	*Protimesius laevis*	-	X
	Pseudoscorpiones	Cheliferoidea	Cheliferoidea Jovem	-	X
		Chernetidae	*Spelaeochernes* sp.1	-	X
	Scorpiones	Buthidae	*Ananteris* Jovem	X	-
Acari	Ixodida	Ixodidae	*Amblyomma cajennense*	X	-
	Sarcoptiforme		Sarcoptiforme sp.1X	-	X
Chilopoda	Geophilomorpha	Ballophilidae	*Ityphilus* sp.1	-	X
Diplopoda	Spirostreptida		Spirostreptida Jovem	-	X
Entognatha	Collembola	Entomobryidae	Entomobryidae sp.5	-	X
Insecta	Blattaria	Blaberidae	Blaberidae Jovem	-	X
	Coleoptera		Coleoptera Jovem	X	-
		Endomychidae	Endomychidae sp.1	-	X
	Diptera		Diptera Jovem	X	X
		Psychodidae	Phlebotominae sp.1	-	X
	Hemiptera	Cydnidae	*Pangaeus* sp.1	-	X
		Reduviidae	Reduviinae sp.1	-	X
			Zelurus Jovem	X	-
		Cixiidae	Cixiidae Jovem	-	X
	Hymenoptera	Formicidae	*Acromyrmex octopinosus*	X	-
			Eurhopalothrix sp.1	-	X
			Hypoponera sp.1	X	-
			Pachycondyla constricta	X	X
	Isoptera	Termitidae	*Nasutitermes* sp.1	-	X
			Nasutitermes sp.2	X	-
	Orthoptera		Orthoptera sp.	-	X
		Phalangopsidae	*Paraclodes* sp.1	-	X
			Phalangopsis sp.1	-	X
	Zygentoma	Nicoletiidae	Nicoletiidae sp.1	X	-
Malacostraca	Isopoda	Philosciidae	Philosciidae sp.2	-	X
Mammalia	Chiroptera	Phyllostomidae	*Carollia* sp.	X	-
Gastropoda	Pulmonata	Systrophiidae	*Happia* sp.1	X	X

SB-0216						
TÁXONS					Seco	Úmido
Annelida	Haplotaxida		Haplotaxida	sp.2	-	X
Arachnida	Amblypygi	Phrynidae	*Heterophrynus*	longicornis	X	X
	Araneae	Araneidae	Araneidae	Jovem	X	-
		Corinnidae	Corinnidae	Jovem	X	-
		Ctenidae	Ctenidae	Jovem	X	-
		Ochyroceratidae	Ochyroceratidae	Jovem	-	X
			Speocera	sp.1	-	X
		Pholcidae	*Mesabolivar*	aurantiacus	X	X
		Theraphosidae	Theraphosidae	Jovem	X	-
		Theridiosomatidae	*Plato*	sp.1	-	X
	Opiliones	Escadabiidae	Escadabiidae	sp.1	X	-
		Neogoveidae	Neogoveidae	Jovem	-	X
	Pseudoscorpiones	Chernetidae	*Spelaeochernes*	sp.1	X	X
Chilopoda	Scolopendromorpha	Scolopocryptopidae	*Newportia*	sp.3	-	X
	Scutigeromorpha		Scutigeromorpha	Jovem	X	-
Diplopoda	Polydesmida	Fuhrmannodesmidae	Fuhrmannodesmidae	Jovem	-	X
	Spirostreptida		Spirostreptida	Jovem	-	X
		Pseudonannolenidae	Pseudonannolenidae	sp.1	X	-
Entognatha	Collembola	Paronellidae	*Trogolaphysa*	sp.2	X	-
	Diplura	Campodeidae	Campodeidae	sp.1	-	X
Insecta	Coleoptera		Coleoptera	Jovem	X	-
		Staphylinidae	Staphylininae	sp.8	X	-
	Diptera		Diptera	Jovem	X	-
		Sciaridae	Sciaridae	Jovem	X	-
	Heteroptera	Cydnidae	*Pangaeus*	sp.1	-	X
		Reduviidae	*Triatoma*	Jovem	X	-
	Homoptera	Cixiidae	Cixiidae	Jovem	-	X
			Cixiidae	sp.6	-	X
	Hymenoptera	Formicidae	*Eurhopalothrix*	sp.1	-	X
			Pachycondyla	*constricta*	X	X
			Pheidole	sp.4	-	X
	Isoptera	Termitidae	Termitidae	sp.	X	-
	Lepidoptera	Tineoidea	Tineoidea	sp.4	-	X
	Orthoptera		Orthoptera	sp.	X	-
		Phalangopsidae	*Phalangopsis*	sp.1	-	X
	Zygentoma	Nicoletiidae	Nicoletiinae	sp.1	-	X
Malacostraca	Isopoda	Armadillidae	Armadillidae	sp.1	-	X
		Philosciidae	Philosciidae	Jovem	-	X
			Philosciidae	sp.2	-	X
Amphibia	Anura	Craugastoridae	*Pristimantis*	cf. *fenestratus*	X	-
		Leptodactylidae	*Physalaemus*	gr. *cuvieri*	X	X
Mammalia	Chiroptera	Furipteridae	*Furipterus*	*horrens*	-	X
		Phyllostomidae	*Carollia*	*perspicillata*	X	-
			Carollia	sp.	-	X
			Glossophaga	sp.	X	-
Gastropoda	Pulmonata	Systrophiidae	*Happia*	sp.1	X	-
			Happia	sp.4	-	X

SB-0217						
TÁXONS					Seco	Úmido
Arachnida	Amblypygi	Phrynidae	*Heterophrynus*	*longicornis*	X	X
	Araneae	Anapidae	Anapidae	sp.2	-	X
		Ochyroceratidae	Ochyroceratidae	Jovem	X	-
		Psauridae	Psauridae	sp.	X	-
		Theraphosidae	Theraphosidae	sp.	-	X
		Theridiosomatidae	*Plato*	sp.1	X	X
		Trechaleidae	Trechaleidae	Jovem	X	X
	Opiliones	Sclerosomatidae	*Prionostema*	sp.1	-	X
Diplopoda	Glomeridesmida	Glomeridesmidae	Glomeridesmidae	sp.1	X	-
	Polydesmida	Fuhrmannodesmidae	Fuhrmannodesmidae	Jovem	X	-
		Pyrgodesmidae	Pyrgodesmidae	Jovem	-	X
Entognatha	Collembola	Paronellidae	Paronellidae	sp.1	X	-
		Symphypleona	Symphypleona	sp.2	-	X
Insecta	Coleoptera		Coleoptera	Jovem	-	X
		Staphylinidae	Pselaphinae	sp.12	-	X
	Diptera	Chironomidae	Chironomidae	sp.1	X	-
		Tipulidae	Tipulidae	sp.1	-	X
	Heteroptera	Reduviidae	Emesinae	sp.6	-	X
	Hymenoptera	Formicidae	*Acropyga*	sp.1	-	X
			Basiceros	sp.1	-	X
			Nylanderia	sp.1	X	-
			Pachycondyla	*constricta*	-	X
			Solenopsis	sp.1	X	-
	Orthoptera	Phalangopsidae	*Phalangopsis*	sp.1	X	X
Malacostraca	Decapoda		Decapoda	sp.	X	X
		Pseudothelphusidae	Pseudothelphusidae	Jovem	X	-
Amphibia	Anura	Craugastoridae	*Pristimantis*	cf. *fenestratus*	-	X
Mammalia	Chiroptera	Phyllostomidae	*Carollia*	sp.	X	-

SB-0218						
TÁXONS					Seco	Úmido
Annelida	Haplotaxida		Haplotaxida	sp.3	X	X
Arachnida	Amblypygi	Phrynidae	*Heterophrynus*	*longicornis*	X	X
	Araneae	Ochyroceratidae	*Speocera*	sp.1	X	-
		Oonopidae	Oonopidae	Jovem	-	X
		Pholcidae	*Mesabolivar*	*aurantiacus*	-	X
		Pisauridae	Pisauridae	Jovem	X	-
			Pisauridae	sp.	X	-
		Theridiosomatidae	*Plato*	sp.1	X	-
		Trechaleidae	Trechaleidae	Jovem	X	X
	Opiliones	Sclerosomatidae	Sclerosomatidae	Jovem	-	X
	Pseudoscorpiones	Chernetidae	Chernetidae	Jovem	-	X
			Spelaeochernes	sp.1	-	X
Acari	Holothryda		Holothryda	sp.1	-	X
			Holothryda	sp.6	-	X
	Mesostigmata		Mesostigmata	sp.7	-	X
			Mesostigmata	sp.9	-	X
	Sarcoptiforme	Oribatida	Oribatida	sp.1	-	X
			Oribatida	sp.10	-	X
	Trombidiforme	Trombidiidae	Trombidiidae	sp.1	X	-
Chilopoda	Geophilomorpha		Geophilomorpha	Jovem	X	-
	Scolopendromorpha	Scolopendridae	*Otostigmus*	sp.1	-	X
Diplopoda	Glomeridesmida	Glomeridesmidae	Glomeridesmidae	Jovem	-	X
			Glomeridesmidae	sp.1	X	-
	Polydesmida	Chelodesmidae	Chelodesmidae	sp.1	X	-
		Fuhrmannodesmidae	Fuhrmannodesmidae	Jovem	-	X
			Fuhrmannodesmidae	sp.4	-	X
		Pyrgodesmidae	Pyrgodesmidae	sp.1	X	X
Entognatha	Collembola	Paronellidae	Paronellidae	sp.1	X	X
			Cyphoderus	*agnotus*	-	X
			Cyphoderus	sp.1	X	-
	Diplura	Campodeidae	Campodeidae	sp.1	X	X
Insecta	Coleoptera		Campodeidae	Jovem	X	X
		Curculionidae	Scolytinae	sp.1	-	X
		Nitidulidae	Nitidulidae	sp.2	-	X
		Ptilodactylidae	Ptilodactylidae	sp.1	-	X
		Staphylinidae	Pselaphinae	sp.10	-	X
			Pselaphinae	sp.12	-	X
			Staphylininae	sp.2	X	-
	Diptera		Diptera	Jovem	X	X
		Culicidae	Culicinae	sp.1	X	-
		Tipulidae	Tipulidae	sp.1	X	X
	Heteroptera		Heteroptera	Jovem	-	X
		Cydnidae	*Pangaeus*	sp.1	X	-
		Veliidae	*Rhagovelia*	Jovem	X	-
			Rhagovelia	sp.2	X	-
	Homoptera	Cixiidae	Cixiidae	Jovem	X	X
	Hymenoptera	Formicidae	*Acropyga*	sp.1	-	X
			Camponotus	sp.2	X	X
			Eurhopalothrix	sp.1	X	-
			Leptogenys	sp.1	-	X
			Nylanderia	sp.1	X	X
			Odontomachus	*bauri*	-	X
			Solenopsis	*invicta*	-	X
			Solenopsis	sp.1	X	-
			Strumigenys	*calamita*	X	X
			Tranopelta	*gilva*	-	X
	Lepidoptera		Lepidoptera	Jovem	-	X

			Lepidoptera sp.	X	-
	Orthoptera	Phalangopsidae	*Paraclodes* sp.1	-	X
			Phalangopsis sp.1	X	X
	Plecoptera	Perlidae	Perlidae sp.1	X	-
Malacostraca	Decapoda		Decapoda sp.	X	X
	Isopoda	Philosciidae	Philosciidae sp.2	X	-
		Scleropactidae	Scleropactidae Jovem	-	X
Amphibia	Anura	Craugastoridae	*Pristimantis* cf. *fenestratus*	-	X
Mammalia	Chiroptera	Phyllostomidae	*Carollia* sp.	X	-
Gastropoda	Pulmonata	Systrophiidae	*Happia* sp.4	-	X

SB-0219					
TÁXONS				Seco	Úmido
Annelida	Haplotaxida		Haplotaxida sp.	-	X
			Haplotaxida sp.1	-	X
			Haplotaxida sp.11	-	X
			Haplotaxida sp.2	-	X
			Haplotaxida sp.3	-	X
			Haplotaxida sp.4	-	X
			Haplotaxida sp.8	X	X
Arachnida	Amblypygi		Amblypygi sp.	-	X
		Phrynidae	*Heterophrynus longicornis*	X	-
	Araneae	Corinnidae	Corinnidae Jovem	X	X
			Abapeba hoeferi	X	X
		Ctenidae	Ctenidae sp.	-	X
		Mysmenidae	Mysmenidae Jovem	X	-
		Nesticidae	*Nesticus* sp.1	-	X
		Ochyroceratidae	Ochyroceratidae Jovem	X	X
			Speocera sp.1	-	X
		Oonopidae	Oonopidae Jovem	X	X
			Oonopidae sp.2	X	-
		Prodidomidae	Prodidomidae Jovem	X	-
		Salticidae	Salticidae Jovem	X	-
			Salticidae sp.1	-	X
		Scytodidae	*Scytodes* sp.2	-	X
		Theraphosidae	Theraphosidae Jovem	X	-
		Theridiidae	Theridiidae Jovem	X	X
			Coleosoma sp.1	-	X
		Theridiosomatidae	Theridiosomatidae Jovem	-	X
		Trechaleidae	Trechaleidae Jovem	-	X
	Opiliones	Cosmetidae	*Roquettea carajas*	-	X
		Escadabiidae	Escadabiidae sp.1	X	X
			Escadabiidae sp.3	-	X
		Neogoveidae	Neogoveidae Jovem	-	X
			Canga renatae	-	X
		Stygnidae	Stygnidae Jovem	X	-
	Pseudoscorpiones	Chernetidae	Chernetidae Jovem	X	X
			Spelaeochernes sp.1	X	X
		Chthoniidae	Chthoniidae Jovem	X	X
			Pseudochthonius sp.2	-	X
			Pseudochthonius sp.3	X	-
	Scorpiones	Buthidae	*Ananteris* Jovem	X	-
Acari	Holothryda		Holothryda sp.1	-	X
	Mesostigmata		Mesostigmata sp.6	-	X
		Uropodoidea	Uropodoidea sp.1	-	X
			Uropodoidea sp.2	X	-
	Opilioacarida	Opilioacaridae	Opilioacaridae sp.1	-	X
	Sarcoptiforme	Oribatida	Oribatida sp.1	X	X
			Oribatida sp.10	-	X
			Oribatida sp.4	X	X
Chilopoda			Chilopoda Jovem	X	-
			Chilopoda sp.	X	X
	Geophilomorpha	Geophilidae	*Schizonampa* sp.1	-	X
	Scolopendromorpha	Scolopocryptopidae	*Newportia* sp.4	X	-
	Scutigeromorpha	Pselliodidae	*Sphendononema guildingii*	-	X
Diplopoda			Diplopoda Jovem	X	-
	Glomeridesmida	Glomeridesmidae	Glomeridesmidae Jovem	-	X
			Glomeridesmidae sp.1	-	X
	Polydesmida	Chelodesmidae	Chelodesmidae Jovem	-	X
		Fuhrmannodesmidae	Fuhrmannodesmidae Jovem	-	X

		Paradoxosomatidae	Paradoxosomatidae sp.1	-	X
		Pyrgodesmidae	Pyrgodesmidae sp.1	-	X
	Spirostreptida		Spirostreptida Jovem	X	X
		Pseudonannolenidae	Pseudonannolenidae Jovem	X	-
		Spirostreptidae	Spirostreptidae Jovem	-	X
Entognatha	Collembola	Isotomidae	Isotomidae sp.1	X	-
		Paronellidae	Paronellidae sp.1	X	X
			Cyphoderus agnotus	X	X
			Cyphoderus javanus	X	-
			Trogolaphysa sp.2	-	X
	Diplura	Campodeidae	Campodeidae sp.1	X	X
		Projapygidae	Projapygidae sp.1	X	-
Insecta	Archaeognatha	Meinertellidae	Meinertellidae sp.1	-	X
	Coleoptera		Coleoptera Jovem	X	X
		Carabidae	*Notiobia* sp.1	X	X
		Elateridae	Elateridae sp.3	-	X
		Scarabaeidae	Scarabaeidae sp.1	-	X
		Scydmaenidae	Scydmaenidae sp.5	-	X
		Staphylinidae	Staphylinidae sp.12	-	X
			Staphylininae sp.15	-	X
	Diptera		Diptera Jovem	X	X
		Phoridae	Phoridae sp.2	-	X
		Syrphidae	Syrphidae sp.1	-	X
		Mycetophilidae	Mycetophilidae sp.1	-	X
		Tipulidae	Tipulidae sp.4	-	X
	Heteroptera		Heteroptera Jovem	-	X
		Cydnidae	Cydnidae Jovem	X	X
			Pangaeus sp.1	X	X
		Reduviidae	Emesinae Jovem	-	X
			Zelurus Jovem	-	X
			Triatoma Jovem	X	X
		Schizopteridae	Schizopteridae sp.2	-	X
			Schizopteridae sp.3	X	-
		Tingidae	Tingidae Jovem	X	-
	Homoptera	Cixiidae	Cixiidae Jovem	X	X
		Fulgoridae	Fulgoridae sp.1	-	X
			Fulgoridae sp.3	-	X
	Hymenoptera	Bethylidae	Bethylidae sp.3	-	X
		Formicidae	*Acromyrmex octopinosus*	X	-
			Acromyrmex sp.1	X	-
			Crematogaster sp.1	-	X
			Eurhopalothrix sp.1	-	X
			Gnamptogenys striatula	X	-
			Hypoponera sp.8	X	-
			Pachycondyla constricta	X	X
			Pheidole sp.4	-	X
			Rogeria pr. belti	-	X
			Solenopsis invicta	X	X
			Solenopsis sp.1	X	-
			Solenopsis sp.7	-	X
		Scelionidae	Scelionidae sp.4	-	X
	Isoptera	Termitidae	*Nasutitermes* sp.1	X	X
			Nasutitermes sp.2	X	X
	Lepidoptera		Lepidoptera Jovem	X	X
			Lepidoptera sp.	-	X
	Orthoptera	Phalangopsidae	Phalangopsidae Jovem	-	X
			Phalangopsidae sp.5	-	X
			Paraclodes sp.1	X	X
			Phalangopsis sp.1	X	X

	Psocoptera	Psocomorpha	Psocomorpha	Jovem	X	-
	Zygentoma	Nicoletiidae	Nicoletiinae	sp.1	-	X
Malacostraca	Isopoda	Armadillidae	Armadillidae	Jovem	-	X
			Armadillidae	sp.1	-	X
		Philosciidae	Philosciidae	Jovem	X	X
			Philosciidae	sp.1	-	X
			Philosciidae	sp.2	X	X
		Platyarthridae	Platyarthridae	Jovem	X	X
			Platyarthridae	sp.2	X	X
			Platyarthridae	sp.5	X	X
		Scleropactidae	Scleropactidae	sp.1	X	-
Symphyla		Scutigerellidae	*Hanseniella*	sp.1	X	X
Amphibia	Anura	Craugastoridae	*Pristimantis*	*cf. fenestratus*	X	-
Mammalia	Chiroptera	Phyllostomidae	*Carollia*	*perspicillata*	X	-
			Carollia	sp.	-	X
			Glossophaga	sp.	-	X
			Phyllostomus	sp.	X	-
Gastropoda	Pulmonata	Subulinidae	*Lamellaxis*	sp.1	X	-
			Leptinaria	sp.2	X	X
		Systrophiidae	Systrophiidae	Jovem	X	X

SB-0220						
TÁXONS					Seco	Úmido
Arachnida	Amblypygi	Phrynidae	*Heterophrynus*	*longicornis*	X	X
	Araneae	Araneidae	*Alpaida*	*antonio*	-	X
		Ctenidae	Ctenidae	Jovem	X	-
		Pholcidae	Pholcidae	Jovem	-	X
			Mesabolivar	*aurantiacus*	X	X
		Salticidae	Salticidae	Jovem	X	-
		Scytodidae	Scytodidae	Jovem	X	X
		Theridiidae	Theridiidae	sp.1	-	X
	Opiliones		Opiliones	sp.	-	X
		Cosmetidae	Cosmetidae	sp.1	-	X
			Roquettea	*carajas*	X	-
			Roquettea	*peba*	-	X
		Escadabiidae	Escadabiidae	Jovem	-	X
		Stygnidae	*Protimesius*	*laevis*	-	X
	Pseudoscorpiones	Chthoniidae	*Pseudochthonius*	sp.2	-	X
Diplopoda	Polydesmida		Polydesmida	Jovem	-	X
Entognatha	Collembola	Paronellidae	Paronellidae	sp.1	-	X
	Diplura	Campodeidae	Campodeidae	sp.1	-	X
Insecta	Blattaria	Blattidae	Blattidae	Jovem	-	X
	Coleoptera	Staphylinidae	Pselaphinae	sp.11	-	X
	Diptera	Chaoboridae	Chaoboridae	sp.1	-	X
		Psychodidae	Phlebotominae	sp.1	-	X
	Hymenoptera	Formicidae	*Acanthostichus*	*bentoni*	-	X
			Acromyrmex	*octopinosus*	X	-
			Pachycondyla	*constricta*	X	X
	Lepidoptera	Noctuoidea	Noctuoidea	sp.1	-	X
	Orthoptera		Orthoptera	sp.	X	X
		Phalangopsidae	*Eidmanacris*	sp.1	X	-
Malacostraca	Isopoda	Armadillidae	Armadillidae	Jovem	-	X
		Philosciidae	Philosciidae	Jovem	-	X
			Philosciidae	sp.2	X	-
Amphibia	Anura	Craugastoridae	*Pristimantis*	*cf. fenestratus*	-	X
		Leptodactylidae	*Physalaemus*	*gr. cuvieri*	-	X

SB-0221					
TÁXONS				**Seco**	**Úmido**
Arachnida	Amblypygi	Phrynidae	*Heterophrynus longicornis*	-	X
	Araneae		Araneae sp.	-	X
		Pholcidae	Pholcidae Jovem	X	-
			Mesabolivar aurantiacus	X	-
		Pisauridae	Pisauridae Jovem	-	X
		Theraphosidae	Theraphosidae sp.	X	-
		Theridiosomatidae	Theridiosomatidae Jovem	-	X
		Trechaleidae	Trechaleidae Jovem	-	X
	Opiliones	Sclerosomatidae	Sclerosomatidae Jovem	X	-
Insecta	Coleoptera	Carabidae	Carabidae sp.6	X	-
	Diptera		Diptera Jovem	X	-
		Drosophilidae	Drosophilidae sp.1	X	-
		Sciaridae	Sciaridae sp.2	X	-
		Psychodidae	Phlebotominae sp.1	-	X
	Heteroptera	Cydnidae	Cydnidae Jovem	X	-
		Veliidae	*Rhagovelia* sp.5	X	-
	Hymenoptera	Formicidae	*Camponotus* sp.3	X	X
	Isoptera	Termitidae	*Nasutitermes* sp.1	-	X
			Nasutitermes sp.2	-	X
	Orthoptera	Phalangopsidae	*Phalangopsis* sp.1	X	X
Malacostraca	Decapoda		Decapoda sp.	X	-
Mammalia	Chiroptera	Phyllostomidae	*Carollia perspicillata*	X	-
			Desmodontinae sp.	X	-
			Trachops cirrhosus	X	-

SB-0222					
TÁXONS				Seco	Úmido
Annelida	Haplotaxida		Haplotaxida sp.11	-	X
			Haplotaxida sp.7	-	X
Arachnida	Amblypygi		Amblypygi sp.	-	X
		Phrynidae	*Heterophrynus longicornis*	X	X
	Araneae	Anapidae	Anapidae Jovem	-	X
		Ctenidae	Ctenidae Jovem	-	X
		Ochyroceratidae	Ochyroceratidae Jovem	-	X
		Pholcidae	Pholcidae Jovem	X	X
			Mesabolivar aurantiacus	X	-
		Prodidomidae	Prodidomidae Jovem	-	X
		Salticidae	Salticidae Jovem	X	X
		Scytodidae	Scytodidae Jovem	-	X
			Scytodes eleonorae	X	-
		Tetrablemmidae	Tetrablemmidae Jovem	X	-
		Theridiidae	Theridiidae Jovem	X	X
			Achaearanea sp.1	X	-
		Theridiosomatidae	*Plato* sp.1	-	X
	Opiliones	Cosmetidae	*Roquettea* sp.1	X	-
		Sclerosomatidae	*Prionostema* sp.1	X	X
	Pseudoscorpiones	Chernetidae	Chernetidae Jovem	X	-
			Spelaeochernes sp.1	X	X
		Chthoniidae	*Pseudochthonius* sp.2	X	-
Acari	Astigmata		Astigmata sp.2	-	X
	Opilioacarida	Opilioacaridae	Opilioacaridae sp.1	-	X
Chilopoda	Scolopendromorpha	Cryptopidae	*Cryptops* sp.2	X	-
		Scolopendridae	*Otostigmus* sp.1	-	X
		Scolopocryptopidae	*Newportia* sp.	-	X
	Scutigeromorpha	Pselliodidae	*Sphendononema* Jovem	-	X
Diplopoda	Polydesmida	Pyrgodesmidae	Pyrgodesmidae Jovem	-	X
			Pyrgodesmidae sp.1	-	X
	Spirostreptida	Pseudonannolenidae	Pseudonannolenidae sp.2	X	-
Entognatha	Collembola	Entomobryidae	Entomobryidae sp.3	-	X
		Isotomidae	Isotomidae sp.1	X	-
		Symphypleona	Symphypleona sp.2	X	-
	Diplura	Campodeidae	Campodeidae sp.1	-	X
		Projapygidae	Projapygidae sp.1	-	X
Insecta	Blattaria	Polyphagidae	Polyphagidae sp.1	X	-
	Coleoptera		Coleoptera Jovem	X	-
		Scydmaenidae	Scydmaeninae sp.2	X	-
		Staphylinidae	Pselaphinae sp.11	X	-
	Diptera		Diptera Jovem	X	X
		Chloropidae	Chloropidae sp.1	-	X
		Fanniidae	Fanniidae sp.1	-	X
		Cecidomyiidae	Cecidomyiidae sp.1	-	X
		Simuliidae	Simuliidae sp.1	-	X
		Tipulidae	Tipulidae sp.1	X	X
	Heteroptera	Ceratocombidae	Ceratocombidae sp.2	X	-
		Cydnidae	Cydnidae Jovem	X	-
		Reduviidae	*Zelurus* Jovem	X	X
	Homoptera	Coccoidea	Coccoidea sp.	X	-
		Cixiidae	Cixiidae Jovem	X	X
		Delphacidae	Delphacidae Jovem	-	X
	Hymenoptera	Formicidae	*Acropyga smithii*	X	-
			Acropyga sp.1	-	X
			Apterostigma pilosum	-	X
			Camponotus sp.2	X	X
			Carebara sp.1X	-	X

			Crematogaster	*limata*	-	X
			Daceton	sp.1	X	-
			Hypoponera	sp.7	X	-
			Nylanderia	sp.1	-	X
			Pachycondyla	*constricta*	X	X
			Pachycondyla	sp.2	-	X
			Pheidole	sp.16	X	-
			Pheidole	sp.17	-	X
			Pheidole	sp.3	-	X
			Pheidole	sp.7	-	X
			Rogeria	*tonduzi*	-	X
			Solenopsis	sp.2	X	-
	Isoptera	Termitidae	*Nasutitermes*	sp.2	X	-
	Lepidoptera		Lepidoptera	Jovem	X	-
		Noctuoidea	Noctuoidea	sp.1	-	X
	Neuroptera	Myrmeleontidae	Myrmeleontidae	sp.5	-	X
	Orthoptera		Orthoptera	sp.	X	X
		Phalangopsidae	Phalangopsidae	sp.1	-	X
	Psocoptera	Psocomorpha	Psocomorpha	Jovem	X	X
	Zygentoma	Nicoletiidae	Atelurinae	sp.1	-	X
Malacostraca	Isopoda	Armadillidae	Armadillidae	Jovem	X	-
			Armadillidae	sp.1	-	X
		Philosciidae	Philosciidae	Jovem	-	X
			Philosciidae	sp.1	X	-
			Philosciidae	sp.2	-	X
Symphyla		Scutigerellidae	*Hanseniella*	sp.1	X	-
Amphibia	Anura	Craugastoridae	*Pristimantis*	*cf. fenestratus*	X	X
		Leptodactylidae	*Physalaemus*	*gr. cuvieri*	X	X
Mammalia	Chiroptera	Emballonuridae	*Peropteryx*	sp.	X	-
		Phyllostomidae	Phyllostomidae	sp.	X	-
			Glossophaga	sp.	-	X
Reptilia	Squamata	Gymnophthalmidae	*Neusticurus*	*ecpleopus*	-	X
Gastropoda	Pulmonata	Systrophiidae	*Happia*	sp.1		X

SB-0223						
TÁXONS					**Seco**	**Úmido**
Annelida	Haplotaxida		Haplotaxida	sp.11	-	X
Arachnida	Amblypygi	Phrynidae	*Heterophrynus*	*longicornis*	X	-
	Araneae	Corinnidae	Corinnidae	Jovem	-	X
		Ctenidae	Ctenidae	Jovem	-	X
		Drymusidae	Drymusidae	Jovem	X	X
		Oonopidae	Oonopidae	Jovem	X	-
		Salticidae	Salticidae	Jovem	-	X
		Scytodidae	Scytodidae	Jovem	-	X
		Tetragnathidae	Tetragnathidae	Jovem	-	X
		Theraphosidae	Theraphosidae	Jovem	-	X
		Theridiidae	Theridiidae	Jovem	X	-
			Thymoites	sp.2	-	X
	Opiliones	Cosmetidae	*Roquettea*	*carajas*	-	X
Diplopoda	Polydesmida	Cyrtodesmidae	Cyrtodesmidae	sp.1	X	-
Entognatha	Collembola	Poduromorpha	Poduromorpha	sp.1	X	-
Insecta	Heteroptera	Cydnidae	Cydnidae	Jovem	X	-
		Reduviidae	*Triatoma*	Jovem	-	X
			Vesciinae	sp.1	-	X
		Schizopteridae	Schizopteridae	Jovem	X	-
	Homoptera	Coccoidea	Coccoidea	sp.	X	-
	Hymenoptera	Formicidae	*Linepithema*	sp.1	X	-
			Nylanderia	sp.1	X	-
			Pachycondyla	*impressa*	-	X
			Pheidole	sp.2	-	X
			Rogeria	*belti*	-	X
	Isoptera	Termitidae	*Nasutitermes*	sp.1	X	X
			Nasutitermes	sp.2	X	-
			Nasutitermes	sp.5	-	X
	Orthoptera		Orthoptera	sp.	X	X
	Psocoptera	Psocomorpha	Psocomorpha	Jovem	X	-
Malacostraca	Isopoda	Philosciidae	Philosciidae	Jovem	-	X
			Philosciidae	sp.1	X	-
Symphyla		Scutigerellidae	*Hanseniella*	sp.1	X	-
Amphibia	Anura	Craugastoridae	*Pristimantis*	cf. *fenestratus*	X	-
Mammalia	Chiroptera	Emballonuridae	*Peropteryx*	sp.	X	-
		Phyllostomidae	*Glossophaga*	sp.	-	X

SB-0224					
TÁXONS				**Seco**	**Úmido**
Arachnida	Amblypygi		Amblypygi sp.	X	-
	Araneae	Araneidae	Araneidae Jovem	X	-
		Ctenidae	Ctenidae sp.	X	-
		Drymusidae	Drymusidae Jovem	-	X
			Drymusa spelunca	X	-
		Pholcidae	Pholcidae Jovem	X	X
			Mesabolivar aurantiacus	-	X
		Theraphosidae	Theraphosidae Jovem	X	-
		Theridiidae	Theridiidae Jovem	-	X
		Theridiosomatidae	*Plato* sp.1	X	X
	Pseudoscorpiones	Chernetidae	*Spelaeochernes* sp.1	-	X
	Scorpiones	Buthidae	*Ananteris* sp.	-	X
Entognatha	Diplura	Campodeidae	Campodeidae sp.1	-	X
Insecta	Coleoptera		Coleoptera Jovem	X	-
	Diptera	Tipulidae	Tipulidae sp.1	-	X
	Heteroptera	Ceratocombidae	Ceratocombidae Jovem	X	-
		Reduviidae	Reduviidae sp.	X	-
			Zelurus Jovem	-	X
			Panstrongylus geniculatus	-	X
	Isoptera	Termitidae	*Nasutitermes* sp.1	-	X
			Nasutitermes sp.2	X	-
			Nasutitermes sp.5	-	X
	Orthoptera		Orthoptera sp.	X	-
		Proscopidae	Proscopidae sp.1	X	X
	Psocoptera	Psocomorpha	Psocomorpha Jovem	X	-
Mammalia	Chiroptera	Emballonuridae	*Peropteryx* sp.	X	-
		Phyllostomidae	*Glossophaga* sp.	-	X

SB-0225					
**Classe**	**Ordem**	**Família**	**Espécie**	**Seco**	**Úmido**
Arachnida	Amblypygi	Phrynidae	*Heterophrynus longicornis*	X	X
	Araneae		Araneae sp.	-	X
		Ctenidae	Ctenidae Jovem	-	X
			Phoneutria reidyi	-	X
		Pholcidae	Pholcidae Jovem	X	-
			Mesabolivar aurantiacus	X	-
		Salticidae	Salticidae Jovem	-	X
			Salticidae sp.3	X	-
		Theraphosidae	Theraphosidae Jovem	X	X
		Theridiidae	Theridiidae Jovem	X	-
			Achaearanea sp.1	-	X
		Trechaleidae	Trechaleidae Jovem	X	-
	Pseudoscorpiones	Chernetidae	*Spelaeochernes* sp.1	X	-
		Chthoniidae	*Pseudochthonius* sp.2	-	X
	Scorpiones	Buthidae	*Ananteris* Jovem	X	-
Chilopoda	Geophilomorpha	Schendylidae	*Schendyylops* sp.1	-	X
Insecta	Blattaria		Blattaria sp.	-	X
		Blaberidae	Blaberidae Jovem	-	X
			Blaberidae sp.3	X	-
		Blattelidae	Blattelidae Jovem	X	-
		Blattidae	Blattidae Jovem	-	X
			Blattidae sp.3	X	-
	Coleoptera		Coleoptera Jovem	X	X
		Carabidae	*Notiobia* sp.1	X	-
		Chrysomelidae	Chrysomelidae sp.4	-	X
	Diptera		Diptera Jovem	-	X
		Cecidomyiidae	Cecidomyiidae sp.2	X	-
		Culicidae	Culicinae sp.1	-	X
		Psychodidae	Phlebotominae sp.1	X	X
		Tipulidae	Tipulidae sp.1	X	X
	Heteroptera	Nabidae	Prostemmatinae sp.1	X	-
		Reduviidae	*Zelurus* Jovem	X	X
	Hymenoptera	Formicidae	*Camponotus cingulatus*	-	X
			Camponotus sp.2	X	X
			Odontomachus bauri	X	-
		Vespidae	Vespidae sp.	-	X
	Isoptera	Termitidae	*Nasutitermes* sp.1	-	X
			Nasutitermes sp.6	X	-
	Neuroptera	Mantispidae	*Plega* sp.1	X	-
	Orthoptera		Orthoptera sp.	X	X
		Phalangopsidae	*Eidmanacris* sp.1	-	X
	Psocoptera	Psocomorpha	Psocomorpha Jovem	-	X
Malacostraca	Isopoda	Armadillidae	Armadillidae sp.1	X	-
		Dubioniscidae	Dubioniscidae sp.1	-	X
		Scleropactidae	Scleropactidae sp.1	X	-
Amphibia	Anura	Craugastoridae	*Pristimantis* cf. *fenestratus*	X	X

SB-0226					
TÁXONS				Seco	Úmido
Annelida	Haplotaxida		Haplotaxida sp.1	X	-
			Haplotaxida sp.11	-	X
		Glossoscolecidae	*Rhinodrilus* sp.1	-	X
Arachnida	Amblypygi	Phrynidae	*Heterophrynus longicornis*	X	X
	Araneae	Araneidae	Araneidae Jovem	-	X
		Ctenidae	Ctenidae sp.	-	X
			Ctenus sp.1	-	X
		Dipluridae	Dipluridae Jovem	-	X
		Drymusidae	Drymusidae Jovem	X	-
		Ochyroceratidae	Ochyroceratidae sp.2	-	X
		Oonopidae	Oonopidae sp.2	X	-
		Palpimanidae	Palpimanidae Jovem	X	-
		Pholcidae	*Mesabolivar aurantiacus*	X	-
		Prodidomidae	Prodidomidae sp.3	-	X
		Theraphosidae	Theraphosidae Jovem	X	X
		Theridiidae	Theridiidae Jovem	X	X
			Theridion sp.1	X	X
		Theridiosomatidae	Theridiosomatidae Jovem	X	-
	Opiliones	Sclerosomatidae	Sclerosomatidae Jovem	X	-
			Prionostema sp.1	X	-
		Stygnidae	Stygnidae Jovem	X	X
	Pseudoscorpiones	Chernetidae	*Spelaeochernes* sp.1	-	X
		Chthoniidae	*Pseudochthonius* sp.2	-	X
	Schizomida	Hubardiidae	*Rowlandius* sp.1	-	X
Acari	Astigmata		Astigmata sp.1	-	X
	Mesostigmata		Mesostigmata sp.3	X	X
	Sarcoptiforme	Oribatida	Oribatida sp.1	X	-
	Trombidiforme	Trombiculidae	Trombiculidae sp.1	X	-
Chilopoda	Geophilomorpha	Ballophilidae	*Ityphilus* sp.1	-	X
Diplopoda	Polydesmida	Chelodesmidae	Chelodesmidae Jovem	-	X
		Fuhrmannodesmidae	Fuhrmannodesmidae sp.1	-	X
			Fuhrmannodesmidae sp.3	-	X
		Paradoxosomatidae	Paradoxosomatidae Jovem	-	X
			Paradoxosomatidae sp.1	-	X
		Pyrgodesmidae	Pyrgodesmidae sp.1	X	X
	Siphonophorida	Siphonophoridae	Siphonophoridae Jovem	-	X
	Spirostreptida		Spirostreptida Jovem	-	X
Entognatha	Collembola	Entomobryidae	Entomobryidae sp.5	-	X
		Paronellidae	Paronellidae sp.1	-	X
			Cyphoderus javanus	X	-
Insecta	Blattaria	Blaberidae	Blaberidae Jovem	X	X
	Coleoptera		Coleoptera Jovem	-	X
		Ptilidae	Ptilidae sp.1	X	-
			Ptilidae sp.2	X	-
		Staphylinidae	Pselaphinae sp.1	-	X
	Diptera		Diptera Jovem	X	X
		Drosophilidae	Drosophilidae sp.1	X	-
		Cecidomyiidae	Cecidomyiidae sp.1	X	-
		Psychodidae	Phlebotominae sp.1	X	-
	Heteroptera		Heteroptera sp.	X	-
		Reduviidae	Emesinae Jovem	X	-
			Emesinae sp.6	-	X
			Zelurus Jovem	X	-
			Triatoma Jovem	X	X
		Schizopteridae	Schizopteridae sp.1	X	-
			Schizopteridae sp.3	X	-
	Hymenoptera	Formicidae	*Labidus praedator*	X	-

			Octostruma sp.1	-	X
			Pachycondyla constricta	X	X
			Prionopelta modesta	-	X
			Solenopsis invicta	X	-
	Isoptera	Termitidae	Coatitermes sp.1	X	-
			Nasutitermes sp.	-	X
			Nasutitermes sp.1	X	X
			Nasutitermes sp.2	-	X
			Nasutitermes sp.5	-	X
	Lepidoptera		Lepidoptera Jovem	X	-
	Orthoptera	Phalangopsidae	*Paraclodes* sp.1	X	X
			Phalangopsis sp.1	X	X
	Psocoptera	Psocomorpha	Psocomorpha Jovem	X	X
	Zygentoma	Nicoletiidae	Nicoletiinae sp.1	X	X
Malacostraca	Isopoda	Armadillidae	Armadillidae sp.1	X	-
		Philosciidae	Philosciidae Jovem	X	-
			Philosciidae sp.2	X	X
		Platyarthridae	Platyarthridae Jovem	-	X
		Scleropactidae	Scleropactidae sp.1	X	-
Symphyla			Scleropactidae Jovem	-	X
		Scutigerellidae	*Hanseniella* sp.1	-	X
Amphibia	Anura		Anura sp.	X	-
		Craugastoridae	*Pristimantis* cf. *fenestratus*	X	X
		Leptodactylidae	*Physalaemus* gr. *cuvieri*	X	-
Mammalia	Chiroptera	Emballonuridae	*Peropteryx* sp.	X	X
		Phyllostomidae	*Carollia perspicillata*	X	-
			Desmodus rotundus	X	-
			Desmodus sp.	-	X
Gastropoda	Pulmonata	Subulinidae	*Lamellaxis* sp.3	X	-
			Lamellaxis sp.1	X	-

SB-0227					
TÁXONS				**Seco**	**Úmido**
Annelida	Haplotaxida		Haplotaxida sp.12	X	-
Arachnida	Amblypygi	Phrynidae	*Heterophrynus longicornis*	X	X
	Araneae	Araneidae	Araneidae Jovem	-	X
		Ctenidae	Ctenidae sp.	-	X
		Salticidae	Salticidae Jovem	-	X
		Scytodidae	*Scytodes eleonorae*	-	X
		Theraphosidae	Theraphosidae Jovem	X	-
		Theridiidae	Theridiidae Jovem	-	X
		Theridiosomatidae	Plato sp.1	-	X
Acari	Mesostigmata		Mesostigmata sp.5	X	-
Entognatha	Collembola	Paronellidae	Paronellidae sp.1	-	X
Insecta	Blattaria		Blattaria sp.	-	X
		Blaberidae	Blaberidae Jovem	X	X
		Blattidae	Blattidae Jovem	-	X
	Coleoptera		Coleoptera Jovem	-	X
		Scarabaeidae	Scarabaeidae sp.3	X	-
	Diptera		Diptera Jovem	X	X
		Phoridae	Phoridae sp.1	-	X
		Psychodidae	Phlebotominae sp.1	-	X
		Tipulidae	Tipulidae sp.1	X	-
	Heteroptera	Reduviidae	*Zelurus* Jovem	X	X
	Hymenoptera	Figitidae	Figitidae sp.4	X	-
		Formicidae	*Camponotus* sp.2	-	X
			Labidus praedator	X	-
	Isoptera	Termitidae	*Nasutitermes* sp.	-	X
			Nasutitermes sp.2	X	X
	Lepidoptera	Tineoidea	Tineoidea sp.1	X	-
	Neuroptera	Myrmeleontidae	Myrmeleontidae sp.5	X	X
	Orthoptera		Orthoptera sp.	X	-
	Psocoptera	Psocomorpha	Psocomorpha Jovem	X	-
	Zygentoma	Nicoletiidae	Nicoletiinae sp.1	-	X
Malacostraca	Isopoda	Armadillidae	Armadillidae sp.1	X	-
		Philosciidae	Philosciidae sp.1	-	X
Symphyla		Scutigerellidae	Scutigerellidae sp.	-	X
Amphibia	Anura	Hylidae	Hylidae sp.1	X	-
		Leptodactylidae	*Physalaemus gr. cuvieri*	X	-
Mammalia	Chiroptera	Emballonuridae	*Peropteryx kappleri*	X	-
			Peropteryx sp.	-	X
		Phyllostomidae	*Glossophaga* sp.	X	-

SB-0228					
TÁXONS				Seco	Úmido
Arachnida	Amblypygi		Amblypygi sp.	-	X
	Araneae	Linyphiidae	Linyphiidae sp.1	-	X
		Ochyroceratidae	Ochyroceratidae Jovem	-	X
		Oonopidae	Oonopidae Jovem	-	X
			Oonopidae sp.2	X	-
		Pholcidae	Pholcidae Jovem	-	X
			Mesabolivar aurantiacus	X	X
		Salticidae	Salticidae Jovem	-	X
		Scytodidae	Scytodidae Jovem	X	-
			Scytodes eleonorae	-	X
		Theridiidae	Theridiidae Jovem	-	X
			Episinus sp.1	-	X
			Thymoites sp.1	X	X
	Opiliones	Cosmetidae	Cosmetidae Jovem	X	-
			Roquettea carajas	-	X
		Stygnidae	*Protimesius laevis*	-	X
	Pseudoscorpiones	Chernetidae	*Spelaeochernes* sp.1	-	X
		Chthoniidae	Chthoniidae Jovem	X	-
			Tyrannochthonius sp.1	X	-
	Schizomida	Hubardiidae	Hubardiidae Jovem	X	-
Acari	Mesostigmata		Mesostigmata sp.5	X	-
			Mesostigmata sp.7	-	X
			Mesostigmata sp.8	-	X
	Opilioacarida	Opilioacaridae	Opilioacaridae sp.1	-	X
	Sarcoptiforme	Oribatida	Oribatida sp.1	X	X
			Oribatida sp.10	-	X
			Oribatida sp.11	-	X
Diplopoda	Polydesmida	Fuhrmannodesmidae	Fuhrmannodesmidae Jovem	-	X
	Polyxenida		Polyxenida Jovem	-	X
	Spirostreptida	Pseudonannolenidae	Pseudonannolenidae sp.2	X	-
Entognatha	Collembola	Entomobryidae	Entomobryidae sp.5	-	X
		Paronellidae	Paronellidae sp.1	-	X
	Diplura	Campodeidae	Campodeidae sp.1	X	X
Insecta	Archaeognatha	Meinertellidae	Meinertellidae sp.1	X	-
	Blattaria	Blaberidae	Blaberidae Jovem	X	X
	Coleoptera		Coleoptera Jovem	X	X
		Ptilidae	Ptilidae sp.1	X	-
	Diptera		Diptera Jovem	-	X
		Cecidomyiidae	Cecidomyiidae sp.1	-	X
		Psychodidae	Phlebotominae sp.1	-	X
		Tipulidae	Tipulidae sp.1	X	-
	Hymenoptera	Formicidae	*Acropyga smithii*	X	-
			Acropyga sp.1	-	X
			Camponotus sp.2	-	X
			Nylanderia sp.1	-	X
			Odontomachus bauri	-	X
			Pheidole sp.4	-	X
	Isoptera	Termitidae	*Nasutitermes* sp.2	-	X
	Orthoptera		Orthoptera sp.	X	X
		Phalangopsidae	Phalangopsidae sp.1	-	X
	Psocoptera	Psocomorpha	Psocomorpha Jovem	X	-
		Epipsocidae	Epipsocidae sp.4	X	-
Malacostraca	Isopoda	Philosciidae	Philosciidae Jovem	-	X
			Philosciidae sp.1	-	X
			Philosciidae sp.2	X	-
		Styloniscidae	Styloniscidae Jovem	X	-
Amphibia	Anura	Hylidae	Hylidae sp.1	X	-

Mammalia	Chiroptera	Emballonuridae	*Peropteryx* sp.	X	X
		Phyllostomidae	*Glossophaga* sp.	X	X
Reptilia	Squamata	Phyllodactylidae	*Thecadactylus rapicauda*	-	X

SB-0229					
TÁXONS				Seco	Úmido
Arachnida	Amblypygi	Charinidae	Charinidae Jovem	-	X
		Phrynidae	*Heterophrynus longicornis*	X	X
	Araneae	Araneidae	*Alpaida truncata*	X	-
		Corinnidae	Corinnidae Jovem	X	X
			Corinnidae sp.	X	-
			Abapeba hoeferi	X	X
			Abapeba sp.	-	X
		Ctenidae	Ctenidae Jovem	-	X
			Ctenidae sp.	-	X
		Drymusidae	Drymusidae Jovem	-	X
		Linyphiidae	*Meioneta* sp.1	-	X
		Ochyroceratidae	Ochyroceratidae Jovem	X	X
			Ochyrocera sp.1	X	-
			Speocera sp.1	X	X
		Oonopidae	Oonopidae Jovem	-	X
			Oonopidae sp.2	-	X
		Palpimanidae	Palpimanidae Jovem	-	X
		Pholcidae	Pholcidae Jovem	-	X
			Mesabolivar aurantiacus	X	-
		Prodidomidae	Prodidomidae Jovem	X	-
		Scytodidae	Scytodidae Jovem	X	-
		Theraphosidae	Theraphosidae Jovem	X	X
		Theridiidae	Theridiidae Jovem	-	X
			Achaearanea sp.1	X	X
	Opiliones	Escadabiidae	Escadabiidae Jovem	X	-
			Escadabiidae sp.1	X	X
		Neogoveidae	Neogoveidae Jovem	X	-
			Canga renatae	X	X
		Stygnidae	Stygnidae Jovem	-	X
	Pseudoscorpiones	Chernetidae	Chernetidae Jovem	X	X
			Spelaeochernes sp.1	X	X
		Chthoniidae	Chthoniidae Jovem	X	-
			Pseudochthonius sp.2	X	X
	Schizomida	Hubardiidae	Hubardiidae Jovem	X	-
			Rowlandius sp.1	X	X
Acari	Astigmata		Astigmata sp.1	X	X
	Holothryda		Holothryda sp.7	-	X
	Ixodida	Ixodidae	*Amblyomma cajennense*	X	-
	Mesostigmata		Mesostigmata sp.1	X	X
			Mesostigmata sp.10	-	X
			Mesostigmata sp.3	X	X
			Mesostigmata sp.7	-	X
			Mesostigmata sp.9	-	X
		Macrochelidae	Macrochelidae sp.1	-	X
		Uropodoidea	Uropodoidea sp.1	X	X
			Uropodoidea sp.2	X	-
	Sarcoptiforme	Oribatida	Oribatida sp.1	X	-
	Trombidiforme	Trombiculidae	Trombiculidae sp.2	-	X
		Trombidiidae	Trombidiidae sp.1	X	X
Chilopoda	Geophilomorpha		Geophilomorpha Jovem	X	X
			Geophilomorpha sp.2	-	X
			Geophilomorpha sp.3	-	X
	Scolopendromorpha	Scolopocryptopidae	Dinocryptops Jovem	-	X
			Newportia sp.6	X	-
	Scutigeromorpha	Pselliodidae	*Sphendononema guildingii*	-	X
Diplopoda	Glomeridesmida	Glomeridesmidae	Glomeridesmidae sp.3	-	X
	Polydesmida	Fuhrmannodesmidae	Fuhrmannodesmidae Jovem	X	-

		Paradoxosomatidae	Paradoxosomatidae Jovem	-	X
		Pyrgodesmidae	Pyrgodesmidae Jovem	X	-
			Pyrgodesmidae sp.1	X	X
	Spirostreptida	Pseudonannolenidae	Pseudonannolenidae Jovem	-	X
			Pseudonannolenidae sp.1	X	-
	Stemmiulida	Stemmiulidae	Stemmiulidae sp.2	X	-
Entognatha	Collembola		Collembola sp.	X	-
		Isotomidae	Isotomidae sp.1	X	X
		Paronellidae	Paronellidae sp.1	X	X
			Cyphoderus agnotus	X	X
			Cyphoderus arlei	X	-
			Cyphoderus javanus	X	X
			Trogolaphysa sp.2	X	X
		Symphypleona	Symphypleona sp.2	X	X
	Diplura	Campodeidae	Campodeidaesp.1	X	X
Insecta	Blattaria	Blaberidae	Blaberidae Jovem	-	X
			Blaberidae sp.3	-	X
			Blaberus sp.1	-	X
			Blaberus sp.4	X	X
		Blattelidae	Blattelidae Jovem	-	X
			Blattelidae sp.	-	X
		Blattidae	Blattidae Jovem	X	-
			Blattidae sp.1	X	-
	Coleoptera		Coleoptera Jovem	X	X
		Carabidae	Carabidae sp.5	X	-
			Carabidae sp.7	-	X
			Lelis sp.1	-	X
		Chrysomelidae	Chrysomelidae sp.6	X	-
		Elateridae	Elateridae sp.3	-	X
		Histeridae	Histeridae sp.1	-	X
		Ptilodactylidae	Ptilodactylidae sp.1	-	X
		Scydmaenidae	Scydmaenidae sp.2	X	X
			Scydmaeninae sp.2	X	-
		Staphylinidae	Pselaphinae sp.2	-	X
		Tenebrionidae	Tenebrionidae sp.9	X	-
	Diptera		Diptera Jovem	X	X
		Chloropidae	Chloropidae sp.1	-	X
		Drosophilidae	Drosophilidae sp.1	X	X
			Drosophilidae sp.2	X	-
			Drosophilidae sp.3	X	-
		Streblidae	Streblidae sp.1	X	-
		Phoridae	Phoridae sp.1	-	X
		Mycetophilidae	Mycetophilidae sp.1	-	X
		Sciaridae	Sciaridae sp.2	X	X
			Sciaridae sp.3	X	-
		Psychodidae	Phlebotominae sp.1	X	X
			Phlebotominae sp.2	-	X
	Heteroptera	Cydnidae	Cydnidae Jovem	X	-
			Cydnidae sp.1	-	X
			Pangaeus sp.1	X	-
		Nabidae	Nabidae Jovem	X	-
		Reduviidae	Reduviidae Jovem	-	X
			Zelurus Jovem	X	-
			Triatoma Jovem	X	-
	Homoptera	Cixiidae	Cixiidae Jovem	X	X
		Fulgoridae	Fulgoridae sp.3	-	X
	Hymenoptera	Formicidae	Formicidae Jovem	-	X
			Camponotus sp.1	X	X
			Cephalotes sp.2	X	-

			Dolichoderus sp.1	X	-
			Nylanderia sp.1	-	X
			Odontomachus bauri	X	-
			Pachycondyla constricta	X	-
			Pheidole sp.16	-	X
			Pheidole sp.2	X	-
			Solenopsis sp.1	X	-
			Tranopelta gilva	-	X
		Scelionidae	Scelionidae sp.1	X	X
			Scelionidae sp.2	-	X
			Scelionidae sp.5	X	-
	Isoptera	Termitidae	Termitidae sp.	X	X
			Nasutitermes sp.1	X	-
			Nasutitermes sp.7	-	X
	Lepidoptera		Lepidoptera Jovem	-	X
		Tineoidea	Tineoidea sp.2	X	-
	Orthoptera		Orthoptera sp.	X	-
		Phalangopsidae	Phalangopsidae Jovem	-	X
			Phalangopsis sp.1	X	X
	Psocoptera	Psocomorpha	Psocomorpha Jovem	-	X
	Zygentoma	Nicoletiidae	Atelurinae sp.1	X	X
			Nicoletiinae sp.1	X	X
Malacostraca	Isopoda	Armadillidae	Armadillidae sp.1	X	X
		Philosciidae	Philosciidae Jovem	-	X
			Philosciidae sp.1	X	X
			Philosciidae sp.2	X	X
		Platyarthridae	Platyarthridae sp.5	-	X
		Scleropactidae	Scleropactidae sp.2	X	X
			Scleropactidae sp.4	-	X
Symphyla		Scutigerellidae	*Hanseniella* Jovem	-	X
Amphibia	Anura	Bufonidae	*Rhaebo gutatus*	X	-
		Craugastoridae	*Pristimantis* cf. *fenestratus*	X	-
Mammalia	Chiroptera	Furipteridae	*Furipterus horrens*	X	X
		Phyllostomidae	*Carollia* sp.	X	X
			Desmodus rotundus	X	X
			Glossophaga sp.	X	X
			Phyllostomus sp.	X	X
			Trachops cirrhosus	X	X
Gastropoda	Pulmonata	Subulinidae	*Subulinidae* Jovem	X	-
			Lamellaxis sp.1	X	X
		Systrophiidae	*Happia* sp.1	X	X
			Happia sp.4	-	X
Nematoda		Diplogasteria	Diplogasteria sp.1	-	X
			Diplogasteria sp.2	-	X

SB-0230					
TÁXONS				Seco	Úmido
Arachnida	Amblypygi	Phrynidae	*Heterophrynus longicornis*	X	X
	Araneae	Corinnidae	Corinnidae Jovem	X	-
		Ctenidae	Ctenidae Jovem	-	X
		Ochyroceratidae	Ochyroceratidae Jovem	-	X
			Speocera sp.1	X	X
		Pholcidae	Pholcidae Jovem	X	-
			Mesabolivar aurantiacus	-	X
		Theridiidae	Theridiidae Jovem	X	X
		Theridiosomatidae	Theridiosomatidae sp.1	-	X
	Opiliones	Sclerosomatidae	Sclerosomatidae Jovem	X	X
	Palpigradi	Eukoeneniidae	Eukoeneniidae Jovem	X	-
			Eukoenenia sp.1	X	-
	Pseudoscorpiones	Chernetidae	*Spelaeochernes* sp.1	X	X
		Chthoniidae	*Pseudochthonius* sp.2	X	-
	Schizomida	Hubardiidae	*Rowlandius* sp.1	-	X
Acari	Sarcoptiforme	Oribatida	Oribatida sp.13	-	X
	Trombidiforme	Trombiculidae	Trombiculidae sp.1	X	X
Chilopoda	Scolopendromorpha	Scolopocryptopidae	*Newportia* sp.6	X	-
Diplopoda	Polydesmida	Fuhrmannodesmidae	Fuhrmannodesmidae sp.2	X	-
Entognatha	Collembola	Isotomidae	Isotomidae sp.1	X	-
		Paronellidae	Paronellidae sp.1	-	X
	Diplura	Japygidae	Japygidae sp.1	X	-
Insecta	Coleoptera		Coleoptera Jovem	-	X
		Cerambycidae	Cerambycidae sp.1	X	-
		Hydrophilidae	Hydrophilidae sp.1	X	-
		Scydmaenidae	Scydmaeninae sp.1	-	X
	Diptera		Diptera Jovem	-	X
		Cecidomyiidae	Cecidomyiidae sp.1	X	-
		Tipulidae	Tipulidae sp.1	X	-
	Heteroptera	Ceratocombidae	Ceratocombidae sp.2	X	-
		Cydnidae	Cydnidae Jovem	X	-
		Reduviidae	Reduviidae Jovem	-	X
			Emesinae Jovem	-	X
			Zelurus Jovem	X	-
	Homoptera	Cixiidae	Cixiidae Jovem	-	X
			Cixiidae sp.6	-	X
	Hymenoptera	Formicidae	*Apterostigma* sp.1	-	X
			Camponotus sp.1	-	X
			Pheidole sp.16	-	X
			Pheidole sp.17	X	-
		Scelionidae	Scelionidae sp.1	X	X
	Isoptera	Termitidae	*Nasutitermes* sp.2	X	X
	Lepidoptera		Lepidoptera Jovem	X	-
			Lepidoptera sp.	X	X
		Noctuoidea	Noctuoidea sp.15	X	-
	Neuroptera	Mantispidae	*Plega* sp.1	-	X
	Orthoptera		Orthoptera sp.	X	X
	Psocoptera	Psocomorpha	Psocomorpha Jovem	X	X
		Psyllipsocidae	Psyllipsocidae sp.7	-	X
	Zygentoma	Nicoletiidae	Nicoletiinae sp.1	X	-
Malacostraca	Isopoda	Philosciidae	Philosciidae sp.2	-	X
		Platyarthridae	Platyarthridae sp.5	-	X

SB-0231					
TÁXONS				Seco	Úmido
Arachnida	Amblypygi	Phrynidae	*Heterophrynus longicornis*	X	X
	Araneae	Corinnidae	Corinnidae Jovem	-	X
		Ctenidae	Ctenidae Jovem	-	X
			Ctenidae sp.	-	X
			Ctenus sp.1	X	-
		Drymusidae	Drymusidae Jovem	-	X
		Ochyroceratidae	Ochyroceratidae Jovem	-	X
			Speocera sp.1	X	X
		Oonopidae	Oonopidae sp.2	X	-
		Palpimanidae	Palpimanidae Jovem	-	X
		Pholcidae	*Mesabolivar* sp.3	X	-
		Theraphosidae	Theraphosidae Jovem	X	X
			Theraphosidae sp.	-	X
		Theridiidae	Theridiidae Jovem	-	X
			Theridion sp.2	-	X
	Opiliones	Cosmetidae	Cosmetidae Jovem	X	-
		Escadabiidae	Escadabiidae Jovem	X	X
			Escadabiidae sp.1	-	X
		Neogoveidae	*Canga renatae*	X	-
		Stygnidae	Stygnidae Jovem	X	-
	Palpigradi	Eukoeneniidae	*Eukoenenia* sp.1	-	X
	Pseudoscorpiones	Chernetidae	*Spelaeochernes* sp.1	X	X
		Chthoniidae	Chthoniidae Jovem	-	X
			Pseudochthonius sp.2	-	X
			Pseudochthonius sp.4	X	-
	Ricinulei	Ricinoididae	*Cryptocellus tarsilae*	X	X
Acari	Mesostigmata		Mesostigmata sp.10	-	X
			Mesostigmata sp.3	-	X
	Sarcoptiforme	Oribatida	Oribatida sp.10	X	-
			Oribatida sp.7	-	X
	Trombidiforme	Anystidae	Anystidae sp.1	-	X
		Trombiculidae	Trombiculidae sp.1	-	X
		Trombidiidae	Trombidiidae sp.1	X	X
Diplopoda	Glomeridesmida	Glomeridesmidae	Glomeridesmidae sp.1	X	-
	Polydesmida	Chelodesmidae	Chelodesmidae Jovem	-	X
			Chelodesmidae sp.1	X	-
		Fuhrmannodesmidae	Fuhrmannodesmidae Jovem	X	-
	Spirostreptida	Pseudonannolenidae	Pseudonannolenidae Jovem	-	X
			Pseudonannolenidae sp.1	X	-
Entognatha	Collembola	Paronellidae	Paronellidae sp.1	-	X
		Symphypleona	Symphypleona sp.2	X	X
	Diplura	Campodeidae	Campodeidae sp.1	X	X
Insecta	Blattaria	Blattidae	Blattidae Jovem	X	X
	Coleoptera		Coleoptera Jovem	X	X
		Chrysomelidae	Chrysomelidae sp.2	X	-
		Ptilidae	Ptilidae sp.3	X	-
		Scydmaenidae	Scydmaeninae sp.7	-	X
		Staphylinidae	Staphylininae sp.3	X	-
	Diptera		Diptera Jovem	X	X
		Sciaridae	Sciaridae sp.2	X	X
		Ceratopogonidae	Ceratopogonidae sp.3	X	-
		Psychodidae	Phlebotominae sp.1	-	X
		Tipulidae	Tipulidae sp.1	X	-
	Heteroptera	Reduviidae	Reduviidae Jovem	-	X
			Reduviidae sp.	X	-
			Emesinae sp.6	X	-
			Zelurus Jovem	X	-

			Panstrongylus geniculatus	X	-
			Triatoma Jovem	X	-
	Homoptera	Cixiidae	Cixiidae Jovem	X	-
	Hymenoptera	Formicidae	*Acropyga smithii*	X	-
			Camponotus cingulatus	X	-
			Carebara urichi	X	-
			Crematogaster limata	X	-
			Linepithema sp.1	-	X
			Pheidole sp.16	-	X
			Solenopsis invicta	X	-
			Solenopsis sp.2	X	-
	Isoptera	Rhinotermitidae	Heterotermes sp.1	X	-
		Termitidae	Nasutitermes sp.2	-	X
	Lepidoptera		Lepidoptera Jovem	-	X
	Neuroptera	Myrmeleontidae	Myrmeleontidae sp.5	-	X
	Orthoptera		Orthoptera sp.	X	-
		Phalangopsidae	Phalangopsis sp.1	X	X
	Psocoptera	Psocomorpha	Psocomorpha Jovem	-	X
	Zygentoma	Nicoletiidae	Nicoletiinae sp.1	-	X
Malacostraca	Isopoda	Philosciidae	Philosciidae sp.2	X	X
Pauropoda	Tetramerocerata		Tetramerocerata Jovem	-	X
Amphibia	Anura	Bufonidae	*Rhinella* gr. *marina*	X	-
		Craugastoridae	*Pristimantis* cf. *fenestratus*	X	-
Mammalia	Chiroptera	Furipteridae	*Furipterus horrens*	-	X
		Phyllostomidae	*Carollia perspicillata*	X	-
			Carollia sp.	-	X
			Glossophaga sp.	X	X
			Micronycteris sp.	X	X
Gastropoda	Pulmonata	Subulinidae	*Lamellaxis* sp.4	X	X
			Leptinaria sp.2	X	X
		Systrophiidae	*Happia* sp.1	X	-

SB-0232					
TÁXONS				Seco	Úmido
Annelida	Haplotaxida		Haplotaxida sp.10	-	X
Arachnida	Amblypygi	Phrynidae	*Heterophrynus longicornis*	X	X
	Araneae	Ctenidae	Ctenidae Jovem	-	X
		Drymusidae	Drymusidae Jovem	-	X
		Ochyroceratidae	*Speocera* sp.1	X	X
		Pholcidae	*Mesabolivar aurantiacus*	X	X
		Salticidae	Salticidae Jovem	-	X
			Salticidae sp.3	X	-
		Theridiidae	Theridiidae Jovem	-	X
		Theridiosomatidae	Theridiosomatidae Jovem	X	-
	Opiliones	Cosmetidae	*Roquettea carajas*	-	X
			Roquettea peba	-	X
		Stygnidae	Stygnidae Jovem	X	X
	Pseudoscorpiones	Chernetidae	*Spelaeochernes* sp.1	X	X
		Chthoniidae	*Pseudochthonius* sp.2	X	-
Acari	Acariforme		Acariforme sp.1	X	-
	Opilioacarida	Opilioacaridae	Opilioacaridae sp.1	X	X
	Sarcoptiforme	Oribatida	Oribatida sp.1	X	-
	Trombidiforme	Trombiculidae	Trombiculidae sp.1	X	-
		Trombidiidae	Trombidiidae sp.1	X	X
Diplopoda	Polydesmida	Fuhrmannodesmidae	Fuhrmannodesmidae sp.2	-	X
		Pyrgodesmidae	Pyrgodesmidae sp.1	-	X
Entognatha	Collembola	Paronellidae	Paronellidae sp.1	X	X
		Symphypleona	Symphypleona sp.2	X	X
	Diplura	Campodeidae	Campodeidae sp.1	X	-
		Projapygidae	Projapygidae sp.1	X	-
Insecta	Blattaria	Blattidae	Blattidae Jovem	-	X
	Coleoptera	Chrysomelidae	Altimicini sp.1	X	-
		Staphylinidae	Staphylininae sp.1	X	-
	Diptera		Diptera Jovem	X	-
		Sciaridae	Sciaridae sp.2	X	-
		Psychodidae	Phlebotominae sp.1	X	X
		Tipulidae	Tipulidae sp.1	X	-
	Heteroptera	Cydnidae	Cydnidae sp.1	-	X
		Reduviidae	*Zelurus* Jovem	X	-
			Zelurus sp.1	-	X
	Homoptera	Cixiidae	Cixiidae Jovem	X	-
			Cixiidae sp.7	-	X
	Hymenoptera	Formicidae	*Carebara* sp.1	-	X
			Eurhopalothrix sp.1	X	-
			Pheidole sp.1	X	-
			Pheidole sp.13	X	-
			Rogeria tonduzi	X	-
			Solenopsis invicta	X	-
	Isoptera	Termitidae	Termitidae sp.	X	X
	Lepidoptera		Lepidoptera Jovem	X	-
		Noctuoidea	Noctuoidea sp.5	X	-
	Neuroptera	Mantispidae	*Plega* sp.1	-	X
	Orthoptera	Phalangopsidae	*Paraclodes* sp.1	-	X
			Phalangopsis sp.1	X	X
	Psocoptera	Psocomorpha	*Psocomorpha* Jovem	X	X
Malacostraca	Isopoda	Philosciidae	Philosciidae Jovem	X	-
			Philosciidae sp.2	X	X
		Platyarthridae	Platyarthridae sp.5	X	-
Amphibia	Anura	Dendrobatidae	*Adelphobates* cf. *galactonotu*	X	-
Mammalia	Chiroptera	Phyllostomidae	*Carollia* sp.	X	X
Gastropoda	Pulmonata	Systrophiidae	*Happia* sp.1	X	-

SB-0233					
TÁXONS				Seco	Úmido
Annelida	Haplotaxida		Haplotaxida sp.11	X	-
			Haplotaxida sp.2	X	-
Arachnida	Amblypygi	Phrynidae	*Heterophrynus longicornis*	X	X
	Araneae	Araneidae	Araneidae Jovem	X	-
		Ctenidae	Ctenidae sp.	-	X
		Ochyroceratidae	Ochyroceratidae Jovem	-	X
		Oonopidae	Oonopidae Jovem	X	-
			gr. *Xycarphhy* sp.1	-	X
		Pholcidae	Pholcidae Jovem	-	X
			Mesabolivar aurantiacus	X	X
			Ninetinae sp.1	X	-
		Pisauridae	Pisauridae Jovem	X	-
		Salticidae	Salticidae sp.1	X	-
			Salticidae sp.3	X	-
		Scytodidae	*Scytodes eleonorae*	-	X
		Tetragnathidae	Tetragnathidae Jovem	X	-
		Theridiidae	Theridiidae Jovem	-	X
		Theridiosomatidae	Theridiosomatidae Jovem	X	X
			Plato sp.1	-	X
	Opiliones	Cosmetidae	Cosmetidae Jovem	X	-
		Escadabiidae	Escadabiidae sp.2	-	X
		Neogoveidae	Neogoveidae Jovem	X	-
		Sclerosomatidae	Sclerosomatidae Jovem	X	X
			Prionostema sp.1	X	X
		Stygnidae	Stygnidae Jovem	X	-
	Pseudoscorpiones	Chernetidae	Chernetidae Jovem	X	X
			Spelaeochernes sp.1	X	X
		Chthoniidae	Chthoniidae Jovem	-	X
			Pseudochthonius sp.2	X	-
Acari	Astigmata		Astigmata sp.1	X	-
			Astigmata sp.2	X	X
	Ixodida	Argasidae	*Ornithodoros* sp.1	X	-
	Trombidiforme	Trombiculidae	Trombiculidae sp.1	-	X
Chilopoda	Geophilomorpha		Geophilomorpha sp.2	-	X
		Geophilidae	Geophilidae Jovem	-	X
	Scolopendromorpha		Scolopendromorpha Jovem	X	-
		Scolopendridae	*Otostigmus* sp.1	X	-
		Scolopocryptopidae	*Dinocryptops miersii*	X	-
Diplopoda	Polydesmida	Pyrgodesmidae	Pyrgodesmidae Jovem	-	X
			Pyrgodesmidae sp.1	-	X
	Polyxenida		Polyxenida Jovem	X	-
	Siphonophorida	Siphonophoridae	Siphonophoridae sp.1	-	X
Entognatha	Collembola	Paronellidae	Paronellidae sp.1	X	X
		Symphypleona	Symphypleona sp.2	-	X
Insecta	Blattaria		Blattaria sp.	X	-
		Blaberidae	Blaberidae Jovem	-	X
			Blaberidae sp.3	X	-
		Blattidae	Blattidae Jovem	X	X
	Coleoptera		Coleoptera Jovem	X	-
		Gyrinidae	*Gyretes* sp.1	-	X
		Scydmaenidae	*Scydmaeninae* sp.2	X	-
			Scydmaeninae sp.5	-	X
		Staphylinidae	*Pselaphinae* sp.2	X	-
			Pselaphinae sp.4	X	-
	Diptera		Diptera Jovem	X	X
		Cecidomyiidae	Cecidomyiidae sp.1	X	-
			Cecidomyiidae sp.2	-	X

		Psychodidae	Phlebotominae sp.1	X	X
		Tipulidae	Tipulidae sp.1	X	-
	Heteroptera	Cydnidae	Cydnidae sp.1	-	X
		Notonectidae	*Martarega* sp.1	-	X
	Hymenoptera	Eucharitidae	Eucharitidae sp.3	-	X
		Formicidae	Formicidae Jovem	-	X
			Camponotus sp.1	-	X
			Camponotus sp.2	X	-
			Hypoponera sp.7	X	-
			Nylanderia sp.1	X	-
			Nylanderia sp.3	X	-
			Pachycondyla constricta	X	-
			Pachycondyla sp.1	-	X
			Solenopsis sp.1	X	-
			Strumigenys sp.1	-	X
			Tranopelta gilva	-	X
		Vespidae	Vespidae sp.5	-	X
			Vespidae sp.6	-	X
	Isoptera	Termitidae	Termitidae sp.	X	X
			Nasutitermes sp.2	X	-
			Nasutitermes sp.6	X	X
	Lepidoptera		Lepidoptera Jovem	X	X
		Noctuoidea	Noctuoidea sp.9	X	-
	Odonata	Gomphidae	Gomphidae sp.	-	X
	Orthoptera		Orthoptera sp.	X	-
		Phalangopsidae	Phalangopsidae Jovem	-	X
			Phalangopsis sp.1	X	X
	Psocoptera	Psocomorpha	Psocomorpha Jovem	X	X
		Epipsocidae	Epipsocidae sp.4	X	X
		Psyllipsocidae	Psyllipsocidae sp.6	X	-
			Psyllipsocidae sp.7	-	X
	Zygentoma	Nicoletiidae	Nicoletiinae sp.1	-	X
Malacostraca	Decapoda		Decapoda sp.	X	X
		Palaemonidae	Palaemonidae Jovem	-	X
		Pseudothelphusidae	Pseudothelphusidae Jovem	X	X
	Isopoda	Armadillidae	Armadillidae Jovem	X	-
			Armadillidae sp.1	X	-
		Philosciidae	Philosciidae Jovem	-	X
		Platyarthridae	Platyarthridae Jovem	-	X
			Platyarthridae sp.5	X	X
Mammalia	Chiroptera	Phyllostomidae	*Carollia* sp.	X	X
Reptilia	Squamata	Tropiduridae	*Plica plica*	X	-
Gastropoda	Pulmonata	Systrophiidae	*Systrophia* sp.1	-	X

SB-0234					
**TÁXONS**				**Seco**	**Úmido**
Arachnida	Amblypygi	Phrynidae	*Heterophrynus longicornis*	X	X
	Araneae	Araneidae	Araneidae Jovem	-	X
			Alpaida antonio	X	X
		Corinnidae	Corinnidae Jovem	-	X
		Nesticidae	Nesticidae Jovem	X	-
		Pholcidae	Pholcidae Jovem	X	X
		Salticidae	Salticidae Jovem	X	-
		Theridiidae	*Theridion* sp.2	X	-
		Theridiosomatidae	Theridiosomatidae Jovem	X	X
		Mygalomorphae	Mygalomorphae sp.	X	-
	Opiliones	Sclerosomatidae	*Prionostema* sp.1	-	X
	Pseudoscorpiones	Chernetidae	*Spelaeochernes* sp.1	-	X
		Lechytiidae	*Lechytia* sp.1	-	X
Acari	Mesostigmata	Laelapidae	Laelapidae sp.2	-	X
	Trombidiforme	Anystidae	Anystidae sp.1	-	X
Entognatha	Collembola	Entomobryidae	Entomobryidae sp.5	X	-
		Paronellidae	Paronellidae sp.1	-	X
	Diplura	Campodeidae	Campodeidae sp.1	-	X
Insecta	Blattaria	Polyphagidae	Polyphagidae Jovem	X	-
			Polyphagidae sp.	X	-
	Coleoptera		Coleoptera Jovem	X	X
	Diptera	Syrphidae	Syrphidae sp.1	X	-
		Chaoboridae	Chaoboridae sp.1	-	X
		Psychodidae	Phlebotominae sp.1	X	X
		Tipulidae	Tipulidae sp.1	X	-
	Hymenoptera	Formicidae	*Camponotus* sp.1	-	X
			Crematogaster limata	X	-
			Crematogaster sp.1	-	X
			Gnamptogenys striatula	X	-
			Nylanderia sp.1	X	-
			Pachycondyla sp.1	-	X
	Isoptera	Rhinotermitidae	*Coptotermes* sp.1	-	X
		Termitidae	*Nasutitermes* sp.2	X	X
	Lepidoptera		Lepidoptera Jovem	-	X
			Lepidoptera sp.	X	-
	Neuroptera	Mantispidae	*Plega* sp.1	-	X
	Orthoptera		Orthoptera sp.	X	X
	Psocoptera	Psocomorpha	Psocomorpha Jovem	-	X
		Ptiloneuridae	*Ptiloneura* sp.2	X	-
		Troctomorpha	Troctomorpha Jovem	-	X
		Psyllipsocidae	Psyllipsocidae sp.7	-	X
Amphibia	Anura	Craugastoridae	*Pristimantis* cf. *fenestratus*	-	X
		Dendrobatidae	*Adelphobates* cf. *galactonotus*	-	X
Mammalia	Chiroptera	Phyllostomidae	*Carollia* sp.	X	X
Reptilia	Squamata	Tropiduridae	*Plica plica*	X	-

SB-0235					
TÁXONS				**Seco**	**Úmido**
Arachnida	Amblypygi	Phrynidae	*Heterophrynus longicornis*	X	X
	Araneae	Ctenidae	Ctenidae Jovem	-	X
			Ctenidae sp.	-	X
		Pholcidae	Pholcidae Jovem	X	X
		Salticidae	Salticidae sp.1	X	-
			Salticidae sp.3	X	-
		Scytodidae	Scytodidae Jovem	-	X
			Scytodes eleonorae	X	-
		Theraphosidae	Theraphosidae Jovem	X	-
		Theridiidae	Theridiidae Jovem	-	X
		Theridiosomatidae	*Plato* sp.1	-	X
	Opiliones	Cosmetidae	Cosmetidae Jovem	-	X
			Roquettea carajas	X	-
		Sclerosomatidae	Sclerosomatidae Jovem	X	-
			Prionostema sp.1	X	-
	Pseudoscorpiones	Chernetidae	*Spelaeochernes* sp.1	X	X
Acari	Ixodida	Argasidae	*Ornithodoros* sp.1	X	-
		Ixodidae	*Amblyomma rotundatum*	X	-
Chilopoda	Geophilomorpha		Geophilomorpha Jovem	X	-
Diplopoda	Polydesmida	Paradoxosomatidae	Paradoxosomatidae Jovem	X	-
Entognatha	Collembola	Paronellidae	Paronellidae sp.1	X	X
	Diplura	Campodeidae	Campodeidae sp.1	-	X
Insecta	Blattaria	Blaberidae	Blaberidae Jovem	X	X
		Blattidae	Blattidae Jovem	X	-
	Coleoptera		Coleoptera Jovem	-	X
		Curculionidae	Scolytinae sp.6	X	-
		Staphylinidae	Staphylininae sp.21	X	-
		Tenebrionidae	Tenebrionidae sp.6	X	-
	Diptera	Psychodidae	Phlebotominae sp.1	-	X
		Tipulidae	Tipulidae sp.1	X	-
	Heteroptera	Reduviidae	*Zelurus* Jovem	X	-
			Zelurus sp.1	-	X
	Homoptera	Cixiidae	Cixiidae Jovem	-	X
	Hymenoptera	Formicidae	*Odontomachus* sp.1	-	X
			Pachycondyla sp.2	-	X
	Isoptera	Termitidae	Termitidae sp.	X	-
			Nasutitermes sp.2	X	X
			Neocapritermes sp.1	-	X
	Lepidoptera	Hesperiidae	Hesperiidae sp.1	X	-
	Neuroptera	Myrmeleontidae	Myrmeleontidae sp.5	X	X
		Mantispidae	*Plega* sp.1	X	-
	Orthoptera		Orthoptera sp.	X	X
	Psocoptera	Psocomorpha	Psocomorpha Jovem	X	X
	Zygentoma	Nicoletiidae	Atelurinae sp.1	-	X
Malacostraca	Isopoda	Armadillidae	Armadillidae sp.1	X	-

SB-0236					
TÁXONS				Seco	Úmido
Annelida	Haplotaxida		Haplotaxida sp.11	X	X
			Haplotaxida sp.8	-	X
Arachnida	Amblypygi	Phrynidae	*Heterophrynus longicornis*	X	X
	Araneae	Corinnidae	*Abapeba hoeferi*	X	-
		Ctenidae	Ctenidae Jovem	X	-
		Drymusidae	Drymusidae Jovem	-	X
		Ochyroceratidae	Ochyroceratidae Jovem	-	X
			Speocera sp.1	X	X
		Pholcidae	Pholcidae Jovem	X	-
	Opiliones	Escadabiidae	Escadabiidae Jovem	-	X
	Pseudoscorpiones	Chernetidae	Chernetidae Jovem	-	X
			Spelaeochernes sp.1	X	X
		Chthoniidae	Chthoniidae Jovem	-	X
			Pseudochthonius sp.2	X	-
			Pseudochthonius sp.4	X	-
	Ricinulei	Ricinoididae	*Cryptocellus tarsilae*	-	X
	Schizomida	Hubardiidae	Hubardiidae Jovem	X	X
Acari	Mesostigmata		Mesostigmata sp.1	X	-
			Mesostigmata sp.2	-	X
			Mesostigmata sp.3	X	-
			Mesostigmata sp.6	-	X
Diplopoda	Glomeridesmida	Glomeridesmidae	Glomeridesmidae Jovem	-	X
			Glomeridesmidae sp.1	X	X
			Glomeridesmidae sp.3	-	X
	Polydesmida		Polydesmida Jovem	-	X
		Chelodesmidae	Chelodesmidae Jovem	X	X
		Fuhrmannodesmidae	Fuhrmannodesmidae Jovem	X	-
		Pyrgodesmidae	Pyrgodesmidae Jovem	-	X
	Spirostreptida		Spirostreptida sp.3	-	X
		Pseudonannolenidae	Pseudonannolenidae Jovem	-	X
Entognatha	Collembola		Collembola Jovem	-	X
		Paronellidae	Paronellidae sp.1	X	X
			Cyphoderus agnotus	X	-
			Cyphoderus javanus	X	-
Insecta	Coleoptera	Carabidae	Carabidae sp.4	-	X
		Chrysomelidae	Chrysomelidae sp.7	X	-
		Hydrophilidae	Hydrophilidae sp.7	-	X
		Scydmaenidae	Scydmaeninae sp.4	X	-
		Staphylinidae	Staphylinidae sp.12	-	X
	Diptera		Diptera Jovem	X	X
		Cecidomyiidae	Cecidomyiidae sp.1	-	X
		Culicidae	Culicinae sp.1	-	X
	Hymenoptera	Figitidae	Figitidae sp.3	X	-
		Formicidae	*Octostruma* sp.1	-	X
			Pachycondyla constricta	X	-
			Tranopelta gilva	X	X
	Isoptera	Termitidae	*Nasutitermes* sp.1	X	-
			Nasutitermes sp.2	X	-
	Lepidoptera		Lepidoptera Jovem	X	-
	Orthoptera		Orthoptera sp.	X	X
	Zygentoma	Nicoletiidae	Nicoletiinae sp.1	X	-
Malacostraca	Isopoda	Philosciidae	Philosciidae sp.2	X	X
		Platyarthridae	Platyarthridae sp.5	X	X
Mammalia	Chiroptera	Phyllostomidae	*Carollia* sp.	X	X
	Rodentia		Rodentia sp.	-	X
Gastropoda	Pulmonata	Subulinidae	*Lamellaxis* sp.1	-	X
			Lamellaxis sp.2	-	X

			Leptinaria sp.1	-	X
			Leptinaria sp.2	-	X
		Systrophiidae	*Happia* sp.1	-	X

SB-0237					
TÁXONS				Seco	Úmido
Annelida	Haplotaxida		Haplotaxida sp.4	-	X
Arachnida	Amblypygi	Phrynidae	*Heterophrynus longicornis*	X	-
	Araneae	Araneidae	Araneidae Jovem	X	-
		Ctenidae	Ctenidae Jovem	X	X
			Ctenidae sp.	-	X
			Ctenus sp.1	X	-
		Pholcidae	Pholcidae Jovem	-	X
			Ninetinae sp.2	X	X
		Scytodidae	Scytodidae Jovem	X	-
		Theraphosidae	Theraphosidae Jovem	-	X
		Theridiidae	Theridiidae Jovem	-	X
	Opiliones	Cosmetidae	Cosmetidae sp.1	X	-
	Pseudoscorpiones	Chernetidae	*Spelaeochernes* sp.1	-	X
		Chthoniidae	*Pseudochthonius* sp.2	-	X
	Scorpiones	Buthidae	*Ananteris* Jovem	X	-
Acari	Acariforme		Acariforme sp.1	-	X
	Sarcoptiforme	Oribatida	Oribatida sp.1	-	X
Diplopoda			Diplopoda Jovem	-	X
	Polydesmida	Paradoxosomatidae	Paradoxosomatidae sp.1	-	X
			Paradoxosomatidae sp.3	-	X
	Spirostreptida	Pseudonannolenidae	Pseudonannolenidae sp.1	X	-
Entognatha	Collembola	Paronellidae	Paronellidae sp.1	-	X
	Diplura	Campodeidae	Campodeidae sp.1	X	-
Insecta	Blattaria	Blattidae	Blattidae Jovem	X	X
		Polyphagidae	Polyphagidae Jovem	X	-
	Coleoptera		Coleoptera Jovem	-	X
		Staphylinidae	Staphylininae sp.3	-	X
	Diptera		Diptera Jovem	X	X
	Hymenoptera	Formicidae	*Heteroponera* sp.1	-	X
			Octostruma sp.1	-	X
			Pachycondyla sp.1	-	X
			Solenopsis sp.1	-	X
	Isoptera	Termitidae	*Nasutitermes* sp.2	X	X
	Lepidoptera		Lepidoptera Jovem	-	X
	Neuroptera	Myrmeleontidae	Myrmeleontidae sp.5	X	-
	Orthoptera	Phalangopsidae	*Phalangopsis* sp.1	X	X
	Psocoptera	Psocomorpha	Psocomorpha Jovem	X	X
Amphibia	Anura	Craugastoridae	*Pristimantis* cf. *fenestratus*	X	-
Mammalia	Chiroptera	Emballonuridae	*Peropteryx* sp.	X	X
		Phyllostomidae	*Desmodus rotundus*	-	X
			Desmodus sp.	X	-
Reptilia	Squamata	Phyllodactylidae	*Thecadactylus rapicauda*	X	-

SB-0238					
TÁXONS				Seco	Úmido
Arachnida	Araneae	Araneidae	Araneidae Jovem	-	X
			Alpaida antonio	X	-
		Ctenidae	Ctenidae sp.	-	X
		Pholcidae	Pholcidae Jovem	X	-
		Salticidae	Salticidae sp.2	X	-
		Scytodidae	Scytodidae Jovem	X	-
		Theridiosomatidae	Theridiosomatidae Jovem	-	X
	Opiliones	Sclerosomatidae	*Prionostema* sp.1	-	X
	Pseudoscorpiones	Chernetidae	*Spelaeochernes* sp.1	X	-
		Chthoniidae	*Pseudochthonius* sp.2	-	X
Acari	Trombidiforme	Trombiculidae	Trombiculidae sp.3	X	-
Entognatha	Collembola	Paronellidae	Paronellidae sp.1	-	X
		Symphypleona	Symphypleona sp.2	-	X
Insecta	Blattaria	Blaberidae	Blaberidae Jovem	X	-
		Polyphagidae	Polyphagidae sp.1	X	-
	Coleoptera		Coleoptera Jovem	X	X
	Diptera		Diptera Jovem	-	X
		Cecidomyiidae	Cecidomyiidae sp.2	-	X
	Heteroptera	Reduviidae	*Zelurus* Jovem	X	-
	Hymenoptera	Formicidae	*Cardiocondyla* sp.1	-	X
			Eciton sp.1	-	X
			Nylanderia sp.1	-	X
			Nylanderia sp.3	X	-
			Pachycondyla sp.1	-	X
			Pheidole sp.16	-	X
	Isoptera	Termitidae	*Nasutitermes* sp.2	-	X
			Termes sp.1	-	X
	Lepidoptera	Gelechioidea	Gelechioidea sp.3	X	-
	Neuroptera	Mantispidae	*Plega* sp.1	-	X
	Orthoptera		*Plega* sp.	X	-
Malacostraca	Isopoda	Armadillidae	Armadillidae Jovem	-	X
			Armadillidae sp.1	X	-
		Philosciidae	Philosciidae sp.1	X	-
Mammalia	Chiroptera	Emballonuridae	*Peropteryx* sp.	-	X
Gastropoda	Pulmonata	Systrophiidae	*Happia* sp.1	X	-

SB-0239					
**TÁXONS**				**Seco**	**Úmido**
Annelida	Haplotaxida		Haplotaxida sp.4	-	X
			Haplotaxida sp.5	X	-
Arachnida	Amblypygi		Amblypygi sp.	X	-
		Charinidae	*Charinu* s sp.1	-	X
		Phrynidae	*Heterophrynus longicornis*	-	X
	Araneae	Araneidae	Araneidae Jovem	X	-
		Corinnidae	Corinnidae Jovem	-	X
		Ctenidae	Ctenidae Jovem	X	X
		Ochyroceratidae	Ochyroceratidae Jovem	-	X
			Speocera sp.1	X	X
		Oonopidae	Oonopidae Jovem	-	X
			Oonopidae sp.2	X	X
			gr. *Xycarphhy* sp.1	X	-
		Pholcidae	Pholcidae Jovem	-	X
			Mesabolivar aurantiacus	X	X
		Salticidae	Salticidae Jovem	X	-
			Salticidae sp.1	-	X
		Theraphosidae	*Guyruita cerrado*	X	-
		Theridiidae	Theridiidae Jovem	X	X
		Theridiosomatidae	Theridiosomatidae Jovem	-	X
			Plato sp.1	X	X
	Opiliones	Cosmetidae	Cosmetidae Jovem	X	-
		Neogoveidae	*Canga renatae*	-	X
	Pseudoscorpiones	Chernetidae	Chernetidae Jovem	-	X
			Spelaeochernes sp.1	-	X
		Chthoniidae	*Pseudochthonius* sp.2	X	X
		Syarinidae	*Nannobisium* sp.1	X	-
	Schizomida	Hubardiidae	*Rowlandius* sp.1	X	-
Acari	Astigmata		*Rowlandius* sp.2	-	X
	Ixodida	Argasidae	*Ornithodoros* sp.1	-	X
		Ixodidae	*Amblyomma rotundatum*	-	X
	Mesostigmata		Mesostigmata sp.2	X	X
			Mesostigmata sp.3	X	X
			Mesostigmata sp.5	X	-
			Mesostigmata sp.7	-	X
		Laelapidae	Laelapidae sp.1	X	-
			Laelapidae sp.2	-	X
		Macrochelidae	Macrochelidae sp.2	-	X
	Sarcoptiforme	Oribatida	Oribatida sp.1	-	X
			Oribatida sp.10	X	-
			Oribatida sp.3	X	-
	Trombidiforme	Trombiculidae	Trombiculidae sp.	-	X
			Trombiculidae sp.1	X	-
		Trombidiidae	Trombidiidae sp.	-	X
			Trombidiidae sp.1	X	X
Chilopoda	Scolopendromorpha	Cryptopidae	*Cryptops* Jovem	-	X
			Cryptops sp.3	-	X
		Scolopocryptopidae	*Newportia* sp.1	X	-
Diplopoda	Glomeridesmida	Glomeridesmidae	Glomeridesmidae Jovem	-	X
			Glomeridesmidae sp.1	X	-
	Polydesmida	Chelodesmidae	Chelodesmidae sp.1	X	X
		Fuhrmannodesmidae	Fuhrmannodesmidae Jovem	X	X
		Paradoxosomatidae	Paradoxosomatidae Jovem	X	-
		Pyrgodesmidae	Pyrgodesmidae sp.1	-	X
	Spirostreptida	Pseudonannolenidae	Pseudonannolenidae Jovem	-	X
			Pseudonannolenidae sp.1	X	-
			Pseudonannolenidae sp.2	X	-

Entognatha	Collembola	Isotomidae	Isotomidae sp.1	-	X
		Paronellidae	Paronellidae sp.1	X	X
			Cyphoderus agnotus	X	-
			Cyphoderus javanus	X	-
		Symphypleona	Symphypleona sp.	-	X
			Symphypleona sp.2	X	X
	Diplura	Campodeidae	Campodeidae sp.1	X	X
Insecta	Blattaria		Blattaria sp.	-	X
		Blaberidae	Blaberidae Jovem	X	X
		Blattelidae	Blattelidae sp.3	X	-
		Blattidae	Blattidae Jovem	X	X
	Coleoptera		Coleoptera Jovem	X	X
		Carabidae	Carabidae sp.1	X	-
		Chrysomelidae	Chrysomelidae sp.2	-	X
			Chrysomelidae sp.6	X	-
		Desmetidae	*Attagenus* sp.1	X	-
		Histeridae	Histeridae sp.6	X	-
		Phalacridae	Phalacridae sp.1	X	-
		Staphylinidae	Staphylininae sp.3	X	-
	Diptera		Diptera Jovem	X	X
		Dolichopodidae	Dolichopodidae sp.2	-	X
		Drosophilidae	Drosophilidae sp.1	X	-
			Drosophilidae sp.3	X	-
		Sphaeroceridae	Sphaeroceridae sp.1	X	-
		Psychodidae	Phlebotominae sp.1	X	X
			Phlebotominae sp.2	-	X
		Tipulidae	Tipulidae sp.1	X	-
	Heteroptera	Cydnidae	Cydnidae sp.1	-	X
			Pangaeus sp.1	X	-
		Reduviidae	Emesinae sp.5	-	X
			Zelurus Jovem	X	-
		Veliidae	*Microvelia* Jovem	-	X
			Paravelia Jovem	X	-
	Homoptera		*Paravelia* Jovem	-	X
		Cixiidae	Cixiidae Jovem	X	X
			Cixiidae sp.6	-	X
	Hymenoptera	Diapriidae	Diapriidae sp.14	X	-
			Diapriidae sp.15	X	-
		Formicidae	*Apterostigma* sp.1	-	X
			Camponotus renggeri	X	-
			Daceton sp.1	-	X
			Leptogenys sp.1	X	-
			Nylanderia sp.1	X	X
			Nylanderia sp.2	X	-
			Pachycondyla constricta	X	-
			Pachycondyla impressa	X	-
			Pachycondyla sp.2	-	X
			Pheidole sp.2	-	X
			Solenopsis sp.1	X	-
			Wasmannia auropunctata	X	-
	Isoptera	Termitidae	*Nasutitermes* sp.2	X	X
	Lepidoptera		Lepidoptera Jovem	-	X
			Lepidoptera sp.	X	-
	Orthoptera		Orthoptera sp.	X	-
		Phalangopsidae	*Phalangopsis* sp.1	-	X
	Psocoptera	Psocomorpha	Psocomorpha Jovem	X	-
	Zygentoma	Nicoletiidae	Nicoletiinae sp.1	X	X
Malacostraca	Isopoda	Armadillidae	Armadillidae sp.1	X	X
		Philosciidae	Philosciidae Jovem	X	X

			Philosciidae sp.	X	-
			Philosciidae sp.1	X	X
			Philosciidae sp.2	-	X
		Scleropactidae	Scleropactidae sp.4	-	X
Amphibia	Anura	Craugastoridae	*Pristimantis* cf. *fenestratus*	X	-
Mammalia	Chiroptera	Emballonuridae	*Peropteryx* sp.	X	X
		Furipteridae	*Furipterus horrens*	-	X
		Phyllostomidae	*Desmodus rotundus*	X	X
			Diphylla ecaudata	X	X
			Glossophaga sp.	X	X
			Phyllostomus sp.	X	-
	Rodentia	Cricetidae	*Rhipidomys* sp.	X	-
Reptilia	Squamata	Phyllodactylidae	*Thecadactylus rapicauda*	X	-
Gastropoda	Pulmonata	Subulinidae	*Leptinaria* sp.1	X	X
		Systrophiidae	*Happia* sp.1	X	X
			Happia sp.4	-	X

SB-0240					
TÁXONS				**Seco**	**Úmido**
Annelida	Haplotaxida		Haplotaxida sp.11	X	-
Arachnida	Amblypygi	Phrynidae	*Heterophrynus longicornis*	-	X
	Araneae	Araneidae	Araneidae Jovem	X	-
		Corinnidae	Corinnidae Jovem	X	X
		Ochyroceratidae	Ochyroceratidae Jovem	X	-
		Oonopidae	Oonopidae sp.2	-	X
			Oonopidae sp.8	-	X
		Salticidae	Salticidae Jovem	X	-
		Theridiosomatidae	*Plato* sp.1	-	X
	Opiliones	Escadabiidae	Escadabiidae Jovem	-	X
	Pseudoscorpiones	Chernetidae	Chernetidae Jovem	X	X
			Spelaeochernes sp.1	X	X
		Chthoniidae	*Pseudochthonius* sp.2	-	X
		Syarinidae	*Nannobisium* sp.1	-	X
	Schizomida	Hubardiidae	*Rowlandius* sp.1	-	X
Acari	Astigmata		Astigmata sp.1	X	X
			Astigmata sp.2	X	X
	Mesostigmata		Mesostigmata sp.3	-	X
	Trombidiforme	Trombiculidae	Trombiculidae sp.1	X	-
		Trombidiidae	Trombidiidae sp.1	-	X
Chilopoda	Scolopendromorpha	Newpotiidae	Newpotiidae Jovem	X	-
Diplopoda	Polydesmida	Chelodesmidae	Chelodesmidae sp.	X	-
		Fuhrmannodesmidae	Fuhrmannodesmidae Jovem	-	X
		Pyrgodesmidae	Pyrgodesmidae sp.1	-	X
Entognatha	Collembola	Paronellidae	Paronellidae sp.1	X	X
	Diplura	Campodeidae	Campodeidae sp.1	-	X
Insecta	Blattaria	Polyphagidae	Polyphagidae Jovem	-	X
			Polyphagidae sp.	X	-
	Coleoptera		Coleoptera Jovem	X	-
		Staphylinidae	Pselaphinae sp.1	-	X
	Diptera		Diptera Jovem	-	X
		Psychodidae	Phlebotominae sp.1	-	X
		Tipulidae	Tipulidae sp.1	X	-
	Homoptera	Issidae	Issidae Jovem	-	X
	Hymenoptera	Formicidae	*Nylanderia* sp.1	-	X
			Nylanderia sp.2	X	-
			Pheidole sp.1	X	-
			Pheidole sp.16	-	X
	Isoptera	Termitidae	Termitidae sp.	-	X
			Nasutitermes sp.1	X	-
			Nasutitermes sp.2	X	-
	Lepidoptera		Lepidoptera sp.	-	X
		Hesperiidae	Hesperiidae sp.1	X	-
	Orthoptera		Orthoptera sp.	X	X
		Phalangopsidae	*Phalangopsis* sp.1	-	X
	Psocoptera	Psocomorpha	Psocomorpha Jovem	X	X
Malacostraca	Isopoda	Philosciidae	Philosciidae Jovem	-	X
			Philosciidae sp.1	-	X
Mammalia	Chiroptera	Furipteridae	*Furipterus horrens*	-	X
Gastropoda	Pulmonata	Subulinidae	*Lamellaxis* sp.2	-	X
			Leptinaria sp.1	-	X
		Systrophiidae	*Happia* sp.1	-	X

SB-0241					
TÁXONS				Seco	Úmido
Annelida	Haplotaxida		Haplotaxida sp.1	-	X
			Haplotaxida sp.4	-	X
			Haplotaxida sp.8	-	X
Arachnida	Amblypygi	Charinidae	Charinidae Jovem	X	-
			Charinus sp.1	-	X
		Phrynidae	*Heterophrynus longicornis*	X	X
	Araneae		*Heterophrynus* sp.	-	X
		Araneidae	Araneidae Jovem	-	X
			Alpaida antonio	X	-
			Alpaida negro	X	-
		Corinnidae	Corinnidae Jovem	X	X
			Abapeba hoeferi	-	X
		Drymusidae	Drymusidae Jovem	X	-
		Ochyroceratidae	Ochyroceratidae Jovem	X	X
		Oonopidae	Oonopidae Jovem	-	X
		Salticidae	Salticidae Jovem	-	X
			Salticidae sp.3	X	-
		Scytodidae	Scytodidae Jovem	X	-
		Theridiosomatidae	Theridiosomatidae Jovem	X	-
			Plato sp.1	X	X
	Opiliones	Cosmetidae	Cosmetidae Jovem	X	-
			Cosmetidae sp.1	X	-
			Roquettea peba	-	X
			Roquettea sp.1	X	-
		Escadabiidae	Escadabiidae Jovem	X	X
			Escadabiidae sp.1	-	X
			Escadabiidae sp.2	-	X
		Sclerosomatidae	*Prionostema* sp.1	-	X
		Stygnidae	Stygnidae sp.1	-	X
			Protimesius laevis	X	-
	Palpigradi	Eukoeneniidae	Eukoeneniidae Jovem	X	-
	Pseudoscorpiones	Chernetidae	*Spelaeochernes* sp.1	X	X
		Chthoniidae	*Pseudochthonius* sp.2	X	-
Acari	Opilioacarida	Opilioacaridae	Opilioacaridae sp.1	-	X
	Sarcoptiforme		Oribatida sp.1	X	-
Chilopoda	Lithobiomorpha		Lithobiomorpha Jovem	-	X
	Scolopendromorpha		Scolopendromorpha Jovem	X	-
		Cryptopidae	*Cryptops* sp.1	X	-
	Scutigeromorpha	Pselliodidae	*Sphendononema guildingii*	-	X
Diplopoda	Glomeridesmida	Glomeridesmidae	Glomeridesmidae Jovem	-	X
	Polydesmida		Polydesmida Jovem	-	X
		Chelodesmidae	Chelodesmidae sp.1	-	X
		Fuhrmannodesmidae	Fuhrmannodesmidae sp.2	X	-
		Paradoxosomatidae	Paradoxosomatidae Jovem	-	X
			Paradoxosomatidae sp.3	X	-
	Polyxenida		Polyxenida Jovem	X	-
	Spirostreptida	Pseudonannolenidae	Pseudonannolenidae Jovem	-	X
			Pseudonannolenidae sp.1	X	-
	Stemmiulida	Stemmiulidae	Stemmiulidae Jovem	-	X
Entognatha	Collembola	Paronellidae	Paronellidae sp.1	X	X
			Poduromorpha sp.1	-	X
			Symphypleona sp.2	X	X
	Diplura	Campodeidae	Campodeidae sp.1	X	X
		Japygidae	Japygidae sp.1	X	-
Insecta	Blattaria	Blaberidae	Blaberidae Jovem	X	-
		Blattelidae	Blattelidae Jovem	X	-
	Coleoptera		Coleoptera Jovem	X	X

		Chrysomelidae	Chrysomelidae sp.8	-	X
		Scydmaenidae	Scydmaeninae sp.7	-	X
	Diptera		Diptera Jovem	-	X
		Psychodidae	Phlebotominae sp.1	X	-
	Heteroptera	Reduviidae	Reduviidae Jovem	-	X
			Zelurus Jovem	X	-
			Zelurus sp.1	-	X
			Triatoma Jovem	X	-
	Homoptera	Cixiidae	Cixiidae Jovem	X	X
		Cicadellidae	Cicadellidae Jovem	X	-
	Hymenoptera	Formicidae	*Camponotus cingulatus*	X	X
			Cyphomyrmex rimosus	X	-
			Eurhopalothrix sp.1	X	-
			Octostruma sp.1	-	X
			Pachycondyla sp.2	-	X
			Pheidole sp.16	-	X
			Pheidole sp.2	-	X
			Pheidole sp.5	X	-
	Isoptera	Termitidae	Termitidae sp.	X	X
	Lepidoptera	Noctuoidea	Noctuoidea sp.7	X	-
		Noctuidae	*Latebraria* sp.1	X	-
	Neuroptera	Myrmeleontidae	*Myrmeleontidae* sp.5	X	-
		Mantispidae	*Plega* sp.1	-	X
	Orthoptera		Orthoptera sp.	X	-
		Phalangopsidae	Phalangopsidae Jovem	-	X
			Phalangopsis sp.1	X	X
	Psocoptera	Psocomorpha	Psocomorpha Jovem	X	-
		Psyllipsocidae	Psyllipsocidae sp.6	X	-
	Zygentoma	Nicoletiidae	Nicoletiinae sp.1	X	-
Malacostraca	Isopoda	Armadillidae	Armadillidae sp.	-	X
			Armadillidae sp.1	X	-
		Philosciidae	Philosciidae Jovem	X	X
			Philosciidae sp.1	X	X
			Philosciidae sp.2	X	X
Amphibia	Anura	Craugastoridae	*Pristimantis* cf. *fenestratus*	X	-
		Leptodactylidae	*Physalaemus* sp.1	X	-
Mammalia	Chiroptera	Furipteridae	*Furipterus horrens*	-	X
		Phyllostomidae	*Glossophaga* sp.	X	X
	Rodentia	Cricetidae	*Rhipidomys* sp.	X	-
Gastropoda	Pulmonata	Subulinidae	*Leptinaria* sp.1	-	X
			Leptinaria sp.2	-	X
		Systrophiidae	*Happia* sp.1	-	X
			Happia sp.4	-	X

SB-0242					
TÁXONS				Seco	Úmido
Annelida	Haplotaxida		Haplotaxida sp.4	-	X
Arachnida	Amblypygi	Phrynidae	*Heterophrynus longicornis*	X	
	Araneae	Pholcidae	Pholcidae Jovem	-	-
			Mesabolivar aurantiacus	-	X
			Mesabolivar eberhardi	-	X
		Pisauridae	*Thaumasia velox*	X	-
		Theridiidae	Theridiidae Jovem	-	X
	Opiliones	Sclerosomatidae	Prionostema sp.1	-	X
Acari	Acariforme		Acariforme sp.1	-	X
	Mesostigmata		Mesostigmata sp.3	X	-
		Macrochelidae	Macrochelidae sp.1	-	X
	Sarcoptiforme	Oribatida	Oribatida sp.1	X	X
			Oribatida sp.10	-	X
			Oribatida sp.12	-	X
			Oribatida sp.5	-	X
Chilopoda	Scolopendromorpha	Cryptopidae	*Cryptops* sp.1	X	-
Diplopoda	Polydesmida	Fuhrmannodesmidae	Fuhrmannodesmidae Jovem	X	-
	Siphonophorida	Siphonophoridae	Siphonophoridae Jovem	-	X
Entognatha	Collembola	Paronellidae	Paronellidae sp.1	X	X
		Symphypleona	Symphypleona sp.2	-	X
	Diplura	Campodeidae	Campodeidae sp.1	-	X
Insecta	Coleoptera		Coleoptera Jovem	X	-
		Curculionidae	Scolytinae sp.1	X	-
		Gyrinidae	*Gyretes* sp.1	X	X
		Ptilidae	Ptilidae sp.3	X	-
		Staphylinidae	Staphylininae sp.3	X	X
	Diptera		Diptera Jovem	-	X
		Drosophilidae	Drosophilidae sp.1	X	-
		Sciaridae	Sciaridae sp.2	-	X
	Heteroptera	Cydnidae	Cydnidae sp.1	-	X
		Nabidae	Nabidae Jovem	-	X
		Reduviidae	*Zelurus* Jovem	X	-
		Veliidae	Rhagovelia sp.1	-	X
			Rhagovelia sp.2	X	-
			Veliidae sp.3	X	-
	Homoptera	Cixiidae	Cixiidae Jovem	-	X
	Hymenoptera	Formicidae	*Apterostigma collare*	X	-
			Apterostigma sp.1	-	X
			Camponotus cingulatus	-	X
			Carebara sp.1	-	X
			Pheidole sp.16	-	X
			Solenopsis sp.1	X	X
			Solenopsis sp.2	X	-
	Isoptera	Termitidae	*Nasutitermes* sp.2	X	X
	Orthoptera		Orthoptera sp.	X	-
		Phalangopsidae	*Phalangopsis* sp.1	-	X
	Psocoptera	Psyllipsocidae	Psyllipsocidae sp.6	X	-
Malacostraca	Decapoda	Palaemonidae	*Macrobrachium* sp.1	X	X
	Isopoda	Philosciidae	Philosciidae sp.2	X	-
		Platyarthridae	Platyarthridae sp.5	X	-
		Styloniscidae	Styloniscidae Jovem	-	X
			Styloniscidae sp.1	X	X
Mammalia	Chiroptera	Phyllostomidae	*Carollia perspicillata*	X	-
			Carollia sp.	-	X
		Glossophaginae	*Glossophaga* sp.	-	X
		Phyllostominae	*Phyllostomus* sp.	X	-
Reptilia	Squamata	Tropiduridae	*Plica* cf. *plica*	X	-

Gastropoda	Pulmonata	Subulinidae	*Lamellaxis* sp.1	X	X

SB-0243					
TÁXONS				Seco	Úmido
Annelida	Lumbriculida	Lumbriculidae	Lumbriculidae sp.1	-	X
Arachnida	Amblypygi	Phrynidae	*Heterophrynus longicornis*	X	X
	Araneae	Ctenidae	Ctenidae Jovem	-	X
		Pholcidae	Pholcidae Jovem	-	X
		Theridiidae	Theridiidae Jovem	-	X
		Theridiosomatidae	*Plato* sp.1	X	-
		Trechaleidae	Trechaleidae Jovem	-	X
	Opiliones		Opiliones sp.	X	-
		Cosmetidae	Cosmetidae sp.1	X	-
			Roquettea peba	X	-
		Sclerosomatidae	*Prionostema* sp.1	-	X
		Stygnidae	*Protimesius laevis*	X	-
Chilopoda	Geophilomorpha		Geophilomorpha Jovem	X	-
Diplopoda	Polydesmida	Chelodesmidae	Chelodesmidae sp.1	X	-
Entognatha	Collembola	Isotomidae	Isotomidae sp.1	X	-
	Diplura	Campodeidae	Campodeidae sp.1	-	X
Insecta	Coleoptera	Carabidae	Carabidae sp.1	-	X
		Gyrinidae	*Gyretes* sp.1	-	X
		Scydmaenidae	Scydmaeninae sp.4	-	X
		Staphylinidae	Pselaphinae sp.2	X	-
			Staphylininae sp.2	-	X
	Diptera		Diptera Jovem	X	X
		Cecidomyiidae	Cecidomyiidae sp.2	-	X
		Tipulidae	Tipulidae sp.1	-	X
			Tipulidae sp.3	-	X
	Heteroptera	Cydnidae	Cydnidae Jovem	X	-
			Cydnidae sp.1	-	X
			Pangaeus sp.1	X	-
		Veliidae	*Rhagovelia* sp.1	-	X
			Rhagovelia sp.2	X	-
	Hymenoptera	Eucharitidae	Eucharitidae sp.4	-	X
		Formicidae	*Camponotus cingulatus*	X	-
			Nylanderia sp.1	X	-
			Nylanderia sp.2	-	X
			Pachycondyla sp.1	-	X
			Solenopsis invicta	X	-
			Solenopsis sp.1	-	X
	Isoptera	Termitidae	Termitidae sp.	X	-
			Nasutitermes sp.2	-	X
	Orthoptera		Orthoptera sp.	X	X
		Gryllotalpidae	Gryllotalpidae sp.1	-	X
Malacostraca	Isopoda	Philosciidae	Philosciidae sp.1	-	X
			Philosciidae sp.2	X	-
		Platyarthridae	Platyarthridae sp.5	-	X
Symphyla		Scolopendrellidae	*Symphylella* sp.1	X	-
Mammalia	Chiroptera	Phyllostomidae	*Carollia perspicillata*	X	-
			Carollia sp.	-	X

SB-0244					
**TÁXONS**				**Seco**	**Úmido**
Arachnida	Araneae	Corinnidae	Corinnidae Jovem	-	X
		Pholcidae	Pholcidae Jovem	-	X
		Scytodidae	Scytodidae Jovem	-	X
			Scytodes eleonorae	-	X
		Theraphosidae	Theraphosidae Jovem	-	X
		Theridiidae	Theridiidae Jovem	-	X
		Theridiosomatidae	*Plato* sp.1	-	X
	Opiliones	Escadabiidae	Escadabiidae Jovem	-	X
			Escadabiidae sp.1	-	X
			Escadabiidae sp.2	X	-
	Palpigradi	Eukoeneniidae	*Eukoenenia* sp.1	X	-
	Pseudoscorpiones	Chernetidae	*Spelaeochernes* sp.1	-	X
		Chthoniidae	*Pseudochthonius* sp.2	-	X
			Chthoniidae sp.4	-	X
Acari	Holothryda		Holothryda sp.1	X	-
	Ixodida	Argasidae	*Ornithodoros* sp.1	-	X
	Mesostigmata		Mesostigmata sp.1	X	-
	Trombidiforme	Trombidiidae	Trombidiidae sp.1	X	-
Chilopoda	Scutigeromorpha	Pselliodidae	*Sphendononema* Jovem	-	X
Diplopoda	Polydesmida	Fuhrmannodesmidae	Fuhrmannodesmidae Jovem	-	X
			Fuhrmannodesmidae sp.1	-	X
		Pyrgodesmidae	Pyrgodesmidae sp.1	-	X
Entognatha	Collembola	Paronellidae	Paronellidae sp.1	X	X
		Symphypleona	Symphypleona sp.2	-	X
	Diplura	Campodeidae	Campodeidae sp.1	-	X
Insecta	Blattaria	Blaberidae	Blaberidae Jovem	-	X
	Coleoptera	Elateridae	Elateridae Jovem	-	X
		Tenebrionidae	Tenebrionidae Jovem	-	X
	Diptera	Sciaridae	Sciaridae sp.2	-	X
		Psychodidae	Phlebotominae sp.1	-	X
		Tipulidae	sp.1	-	X
	Heteroptera	Reduviidae	*Zelurus* Jovem	-	X
	Hymenoptera	Bethylidae	Bethylidae sp.5	-	X
		Braconidae	Braconidae sp.2	-	X
		Formicidae	*Camponotus atriceps*	-	X
			Camponotus sp.2	-	X
			Pheidole sp.16	-	X
			Solenopsis invicta	-	X
	Isoptera	Termitidae	*Nasutitermes* sp.1	-	X
	Neuroptera	Myrmeleontidae	Myrmeleontidae sp.5	-	X
	Zygentoma	Nicoletiidae	Nicoletiidae sp.1	-	X
	Lepidoptera	Noctuoidea	Noctuoidea sp.2	X	-
Malacostraca	Isopoda	Armadillidae	Armadillidae sp.1	X	X
		Philosciidae	Philosciidae sp.1	X	X
Symphyla		Scolopendrellidae	*Scolopendrellopsis* sp.1	-	X
		Scutigerellidae	*Hanseniella* sp.1	-	X
