# Supplementary material for: Reconciling Mining with the Conservation of Cave Biodiversity: A Quantitative Baseline to Help Establish Conservation Priorities
Source: PLoS One. 2016 Dec 20;11(12):e0168348. doi: 10.1371/journal.pone.0168348 (PMC5173368; doi:10.1371/journal.pone.0168348)
Supplement: S1 Dataset — (ZIP) [file pone.0168348.s002.zip › Taxa/Serra Norte/SN_2007/Lista N5E-01.pdf]

CAVIDADE N5E-0001

Classe	Ordem	Fam/Outros	Gên/Outros	Espécie	Única
Arachnida	Acari			sp.6	X
Arachnida	Araneae	Drymusidae	*Drymusa*	*spelunca*	X
Arachnida	Araneae	Pholcidae	*Mesabolivar*	*eberhard*	X
Arachnida	Araneae	Theridiosomatidae	*Plato*	sp.	X
Arachnida	Opiliones	Escadabiidae		sp.n.2	X
Arachnida	Pseudoscorpiones	Chernetidae		sp.	X
Chilopoda	Scutigeromorpha			sp.	X
Entognatha	Collembola			sp.6	X
Insecta	Diptera	Cecidomyiidae		sp.	X
Insecta	Diptera	Culicidae	*Anopheles (Nyssorhynchus)*	*triannulatus*	X
Insecta	Diptera	Culicidae	*Culex (Aedinus)*	sp.	X
Insecta	Diptera	Culicidae	*Culex (Mel.*	sp.	X
Insecta	Diptera	Keroplatidae		jovem	X
Insecta	Homoptera	Cixiidae		jovem	X
Insecta	Hymenoptera	Formicidae		sp.1	X
Insecta	Orthoptera	Phalangopsidae	*Paraclodes*	sp.	X
Insecta	Orthoptera	Phalangopsidae	*Phalangopsis*	sp.	X
Mammalia	Chiroptera	Emballonuridae	*Cormura*	*brevirostris*	X
