# Supplementary material for: Reconciling Mining with the Conservation of Cave Biodiversity: A Quantitative Baseline to Help Establish Conservation Priorities
Source: PLoS One. 2016 Dec 20;11(12):e0168348. doi: 10.1371/journal.pone.0168348 (PMC5173368; doi:10.1371/journal.pone.0168348)
Supplement: S1 Dataset — (ZIP) [file pone.0168348.s002.zip › Taxa/Serra Norte/SN_2007/Lista N5E-02.pdf]

CAVIDADE N5E-0002

Classe	Ordem	Fam/Outros	Gên/Outros	Espécie	Única
Annelida	Oligochaeta			sp.	X
Arachnida	Acari			sp.1	X
Arachnida	Acari			sp.7	X
Arachnida	Amblypygi	Phrynidae	*Heterophrynus*	*longicornis*	X
Arachnida	Araneae	Ctenidae		sp.	X
Arachnida	Araneae	Ochyroceratidae	*Ochyrocera*	sp.1	X
Arachnida	Araneae	Pholcidae	*Mesabolivar*	*eberhard*	X
Arachnida	Araneae	Theridiosomatidae	*Plato*	sp.	X
Arachnida	Pseudoscorpiones	Chernetidae		sp.	X
Arachnida	Pseudoscorpiones	Chthoniidae		sp.	X
Entognatha	Collembola			sp.4	X
Entognatha	Collembola			sp.7	X
Entognatha	Diplura	Campodeidae		sp.	X
Entognatha	Diplura	Japygidae		sp.	X
Insecta	Blattodea	Blattellidae		sp.2	X
Insecta	Coleoptera	Scydmaenidae		sp.	X
Insecta	Diptera	Nematocera		sp.	X
Insecta	Diptera	Psychodidae	*Pifanomyia*	*gruta*	X
Insecta	Heteroptera	Cydnidae		sp.	X
Insecta	Heteroptera	Reduviidae	Emesinae	sp.1	X
Insecta	Heteroptera	Reduviidae	Reduviinae	sp.1	X
Insecta	Homoptera	Cixiidae		jovem	X
Insecta	Hymenoptera	Formicidae		sp.18	X
Insecta	Hymenoptera	Formicidae		sp.19	X
Insecta	Hymenoptera	Platygastridae		sp.	X
Insecta	Lepidoptera			sp.2	X
Insecta	Orthoptera	Phalangopsidae	*Paraclodes*	sp.	X
Insecta	Orthoptera	Phalangopsidae	*Phalangopsis*	sp.	X
Gastropoda	Pulmonata	Subulinidae	*Pseudopeas*	sp.	X
Malacostraca	Isopoda	Philosciidae		sp.	X
Nematoda				sp.	X
Amphibia	Anura	Leptodactylidae	*Eleutherodactylus*	cf. *fenestratus*	X
Mammalia	Chiroptera	Emballonuridae	*Cormura*	*brevirostris*	X
Mammalia	Chiroptera	Phyllostomidae	*Carollia*	sp.	X
