# Supplementary material for: Reconciling Mining with the Conservation of Cave Biodiversity: A Quantitative Baseline to Help Establish Conservation Priorities
Source: PLoS One. 2016 Dec 20;11(12):e0168348. doi: 10.1371/journal.pone.0168348 (PMC5173368; doi:10.1371/journal.pone.0168348)
Supplement: S1 Dataset — (ZIP) [file pone.0168348.s002.zip › Taxa/Serra Norte/SN_2007/Lista N5E-03.pdf]

CAVIDADE N5E-0003

Classe	Ordem	Fam/Outros	Gên/Outros	Espécie	Única
Arachnida	Amblypygi	Phrynidae	*Heterophrynus*	*longicornis*	X
Arachnida	Araneae	Ochyroceratidae		jovem	X
Arachnida	Araneae	Pholcidae	*Mesabolivar*	*eberhard*	X
Arachnida	Araneae	Salticidae	*Freya*	*infuscata*	X
Arachnida	Araneae	Theridiosomatidae	*Plato*	sp.	X
Insecta	Blattodea	Blattellidae		sp.1	X
Insecta	Heteroptera	Reduviidae	Reduviinae	sp.1	X
Insecta	Hymenoptera	Formicidae		sp.1	X
Insecta	Hymenoptera	Formicidae		sp.20	X
Insecta	Hymenoptera	Formicidae		sp.9	X
Insecta	Isoptera	Termitidae		sp.	X
Insecta	Lepidoptera			sp.1	X
Insecta	Orthoptera	Phalangopsidae	*Paraclodes*	sp.	X
Insecta	Thysanura	Nicoletiidae	Nicoletiinae	sp.	X
