# Supplementary material for: Reconciling Mining with the Conservation of Cave Biodiversity: A Quantitative Baseline to Help Establish Conservation Priorities
Source: PLoS One. 2016 Dec 20;11(12):e0168348. doi: 10.1371/journal.pone.0168348 (PMC5173368; doi:10.1371/journal.pone.0168348)
Supplement: S1 Dataset — (ZIP) [file pone.0168348.s002.zip › Taxa/Serra Norte/SN_2007/Lista N5E-04.pdf]

CAVIDADE N5E-0004

Classe	Ordem	Fam/Outros	Gên/Outros	Espécie	Única
Arachnida	Acari	Metastigmata		sp.	X
Arachnida	Amblypygi	Phrynidae	*Heterophrynus*	*longicornis*	X
Arachnida	Araneae	Araneidae	*Alpaida*	sp.1	X
Arachnida	Araneae	Ochyroceratidae	*Ochyrocera*	sp.1	X
Arachnida	Araneae	Pholcidae	*Mesabolivar*	sp.	X
Arachnida	Araneae	Salticidae	*Freya*	*infuscata*	X
Arachnida	Araneae	Theridiosomatidae	*Plato*	sp.	X
Diplopoda	Polydesmida	Pyrgodesmidae		sp.	X
Entognatha	Collembola			sp.3	X
Insecta	Blattodea			sp.3	X
Insecta	Coleoptera	Dytiscidae	Copelatinae	sp.	X
Insecta	Coleoptera	Elateridae		jovem	X
Insecta	Coleoptera	Staphylinidae	Goniacerini	sp.	X
Insecta	Heteroptera	Reduviidae	Emesinae	sp.1	X
Insecta	Homoptera	Cixiidae		jovem	X
Insecta	Hymenoptera	Formicidae		sp.10	X
Insecta	Hymenoptera	Formicidae		sp.11	X
Insecta	Hymenoptera	Formicidae		sp.8	X
Insecta	Orthoptera	Phalangopsidae	*Paraclodes*	sp.	X
Insecta	Orthoptera	Phalangopsidae	*Phalangopsis*	sp.	X
Insecta	Psocoptera			sp.	X
Gastropoda	Pulmonata	Systrophiidae	*Happia*	sp.	X
Amphibia	Anura	Leptodactylidae	*Eleutherodactylus*	cf. *fenestratus*	X
Mammalia	Marsupialia	Didelphidae	*Monodelphis*	cf. *domestica*	X
