# Supplementary material for: Reconciling Mining with the Conservation of Cave Biodiversity: A Quantitative Baseline to Help Establish Conservation Priorities
Source: PLoS One. 2016 Dec 20;11(12):e0168348. doi: 10.1371/journal.pone.0168348 (PMC5173368; doi:10.1371/journal.pone.0168348)
Supplement: S1 Dataset — (ZIP) [file pone.0168348.s002.zip › Taxa/Serra Norte/SN_2007/Lista N5E-05.pdf]

CAVIDADE N5E-0005

Classe	Ordem	Fam/Outros	Gên/Outros	Espécie	Única
Annelida	Oligochaeta			sp.	X
Arachnida	Acari			sp.1	X
Arachnida	Acari			sp.2	X
Arachnida	Acari			sp.4	X
Arachnida	Acari			sp.5	X
Arachnida	Amblypygi	Phrynidae	*Heterophrynus*	*longicornis*	X
Arachnida	Araneae	Corinnidae		sp.	X
Arachnida	Araneae	Ctenidae		sp.	X
Arachnida	Araneae	Ochyroceratidae	*Ochyrocera*	sp.1	X
Arachnida	Araneae	Pholcidae	*Mesabolivar*	*aurantiacus*	X
Arachnida	Araneae	Pholcidae	*Mesabolivar*	*eberhard*	X
Arachnida	Araneae	Theridiosomatidae	*Plato*	sp.	X
Arachnida	Opiliones	Escadabiidae		sp.n.2	X
Arachnida	Pseudoscorpiones	Chernetidae		sp.	X
Arachnida	Pseudoscorpiones	Chthoniidae		sp.	X
Chilopoda	Scolopendromorpha	Scolopocryptopidae	*Dinocryptops*	*miersii*	X
Chilopoda	Scutigeromorpha	Pselliodidae	*Sphendononema*	*guildingii*	X
Diplopoda	Polydesmida	Fuhrmannodesmidae		sp.	X
Diplopoda	Polydesmida	Pyrgodesmidae		sp.	X
Entognatha	Collembola	Sminthuroidea		sp.	X
Entognatha	Collembola			sp.4	X
Entognatha	Collembola			sp.5	X
Insecta	Blattodea	Blaberidae		sp.	X
Insecta	Blattodea	Blattellidae		sp.1	X
Insecta	Blattodea			sp.2	X
Insecta	Blattodea			sp.4	X
Insecta	Coleoptera	Carabidae	*Coarazuphium*	sp.n.2	X
Insecta	Coleoptera	Nitidulidae		sp.	X
Insecta	Coleoptera	Scydmaenidae		sp.	X
Insecta	Coleoptera	Tenebrionidae	Alleculinae	jovem	X
Insecta	Diptera	Cecidomyiidae		sp.	X
Insecta	Diptera	Phoridae		sp.	X
Insecta	Diptera	Psychodidae	*Sciopemyia*	*sordellii*	X
Insecta	Heteroptera	Cydnidae		sp.	X
Insecta	Heteroptera	Reduviidae	Emesinae	sp.1	X
Insecta	Hymenoptera	Formicidae		sp.12	X
Insecta	Hymenoptera	Formicidae		sp.13	X
Insecta	Hymenoptera	Formicidae		sp.14	X
Insecta	Hymenoptera	Formicidae		sp.15	X
Insecta	Hymenoptera	Formicidae		sp.16	X
Insecta	Hymenoptera	Formicidae		sp.17	X
Insecta	Hymenoptera	Formicidae		sp.7	X
Insecta	Hymenoptera	Formicidae		sp.8	X
Insecta	Hymenoptera	Scelionidae		sp.	X
Insecta	Orthoptera	Phalangopsidae	*Phalangopsis*	sp.	X
Insecta	Thysanura	Ateluridae		sp.	X
Insecta	Thysanura	Nicoletiidae	Nicoletiinae	sp.	X
Mammalia	Chiroptera	Emballonuridae	*Cormura*	*brevirostris*	X
Mammalia	Chiroptera	Furipteridae	*Furipterus*	*horrens*	X

Mammalia	Chiroptera	Phyllostomidae	*Anoura*	*caudifer*	X
Mammalia	Chiroptera	Phyllostomidae	*Anoura*	*geoffroyi*	X
