# Supplementary material for: Reconciling Mining with the Conservation of Cave Biodiversity: A Quantitative Baseline to Help Establish Conservation Priorities
Source: PLoS One. 2016 Dec 20;11(12):e0168348. doi: 10.1371/journal.pone.0168348 (PMC5173368; doi:10.1371/journal.pone.0168348)
Supplement: S1 Dataset — (ZIP) [file pone.0168348.s002.zip › Taxa/Serra Norte/SN_2007/Lista N5E-06.pdf]

CAVIDADE N5E-0006

Classe	Ordem	Fam/Outros	Gên/Outros	Espécie	Única
Annelida	Oligochaeta			sp.	X
Arachnida	Acari			sp.1	X
Arachnida	Amblypygi	Phrynidae	*Heterophrynus*	*longicornis*	X
Arachnida	Araneae	Ctenidae	*Isoctenus*	sp.	X
Arachnida	Araneae	Ochyroceratidae	*Gen*.n.	sp.1	X
Arachnida	Araneae	Ochyroceratidae	*Ochyrocera*	sp.1	X
Arachnida	Araneae	Scytodidae	*Scytodes*	*itapevi*	X
Arachnida	Araneae	Theridiosomatidae	*Plato*	sp.	X
Arachnida	Opiliones	Escadabiidae		sp.n.1	X
Arachnida	Opiliones	Escadabiidae		sp.n.2	X
Arachnida	Pseudoscorpiones	Bochicidae		sp.	X
Diplopoda	Polydesmida	Pyrgodesmidae		sp.	X
Entognatha	Collembola			sp.2	X
Entognatha	Diplura	Campodeidae		sp.	X
Insecta	Blattodea			sp.1	X
Insecta	Coleoptera	Scydmaenidae		sp.	X
Insecta	Coleoptera	Staphylinidae		jovem	X
Insecta	Diptera	Brachycera		sp.	X
Insecta	Diptera	Culicidae		jovem	X
Insecta	Diptera	Keroplatidae		jovem	X
Insecta	Diptera	Nematocera		sp.	X
Insecta	Diptera	Psychodidae	*Sciopemyia*	*sordellii*	X
Insecta	Heteroptera	Reduviidae	Emesinae	sp.1	X
Insecta	Homoptera	Cixiidae		jovem	X
Insecta	Hymenoptera	Formicidae		sp.1	X
Insecta	Hymenoptera	Formicidae		sp.8	X
Insecta	Hymenoptera	Formicidae		sp.9	X
Insecta	Isoptera	Termitidae		sp.	X
Insecta	Lepidoptera	Tineoidea		sp.1	X
Insecta	Lepidoptera			sp.1	X
Insecta	Orthoptera	Phalangopsidae	*Phalangopsis*	sp.	X
Insecta	Thysanura	Ateluridae		sp.	X
Insecta	Thysanura	Nicoletiidae	Nicoletiinae	sp.	X
Gastropoda	Pulmonata	Subulinidae	*Lamellaxis*	sp.	X
Mammalia	Rodentia			sp.	X
