# Supplementary material for: Reconciling Mining with the Conservation of Cave Biodiversity: A Quantitative Baseline to Help Establish Conservation Priorities
Source: PLoS One. 2016 Dec 20;11(12):e0168348. doi: 10.1371/journal.pone.0168348 (PMC5173368; doi:10.1371/journal.pone.0168348)
Supplement: S1 Dataset — (ZIP) [file pone.0168348.s002.zip › Taxa/Serra Norte/SN_2007/Lista N5E-07.pdf]

CAVIDADE N5E-0007

Classe	Ordem	Fam/Outros	Gên/Outros	Espécie	Única
Arachnida	Acari	Metastigmata		sp.	X
Arachnida	Acari			sp.3	X
Arachnida	Amblypygi	Phrynidae	*Heterophrynus*	*longicornis*	X
Arachnida	Araneae	Ochyroceratidae	*Gen*.n.	sp.1	X
Arachnida	Araneae	Ochyroceratidae	*Ochyrocera*	sp.1	X
Arachnida	Araneae	Pholcidae	*Mesabolivar*	aff. *togatus*	X
Arachnida	Opiliones	Cosmetidae	Cosmetinae	sp.	X
Arachnida	Opiliones	Escadabiidae		sp.n.2	X
Arachnida	Opiliones	Gonyleptidae	Pachylinae	sp.	X
Arachnida	Opiliones	Stygnidae	*Gen*.n.	sp.	X
Arachnida	Opiliones	Stygnidae	*Protimesius*	sp.	X
Arachnida	Pseudoscorpiones	Chthoniidae		sp.	X
Chilopoda	Scolopendromorpha	Scolopocryptopidae	*Newportia*	sp.	X
Diplopoda	Polydesmida	Pyrgodesmidae		sp.	X
Entognatha	Collembola			sp.1	X
Entognatha	Diplura	Campodeidae		sp.	X
Insecta	Coleoptera	Carabidae		sp.	X
Insecta	Coleoptera	Elateridae		sp.	X
Insecta	Diptera	Phoridae		sp.	X
Insecta	Homoptera	Cixiidae		sp.1	X
Insecta	Hymenoptera	Formicidae		sp.1	X
Insecta	Hymenoptera	Formicidae		sp.6	X
Insecta	Hymenoptera	Formicidae		sp.7	X
Insecta	Lepidoptera	Tineoidea		jovem	X
Insecta	Lepidoptera			sp.2	X
Insecta	Orthoptera	Phalangopsidae	*Phalangopsis*	sp.	X
Insecta	Thysanura	Nicoletiidae	Nicoletiinae	sp.	X
Nematoda				sp.	X
