# Supplementary material for: Reconciling Mining with the Conservation of Cave Biodiversity: A Quantitative Baseline to Help Establish Conservation Priorities
Source: PLoS One. 2016 Dec 20;11(12):e0168348. doi: 10.1371/journal.pone.0168348 (PMC5173368; doi:10.1371/journal.pone.0168348)
Supplement: S1 Dataset — (ZIP) [file pone.0168348.s002.zip › Taxa/Serra Norte/SN_2007/Lista N5E-08.pdf]

CAVIDADE N5E-0008

Classe	Ordem	Fam/Outros	Gên/Outros	Espécie	Única
Arachnida	Acari	Metastigmata		sp.	X
Arachnida	Acari			sp.1	X
Arachnida	Acari			sp.2	X
Arachnida	Amblypygi	Phrynidae	*Heterophrynus*	*longicornis*	X
Arachnida	Araneae	Ochyroceratidae	*Ochyrocera*	sp.1	X
Arachnida	Araneae	Pholcidae	*Mesabolivar*	*eberhard*	X
Arachnida	Araneae	Prodidomidae		sp.	X
Arachnida	Araneae	Scytodidae	*Scytodes*	*itapevi*	X
Arachnida	Araneae	Theraphosidae		sp.	X
Arachnida	Opiliones	Escadabiidae		sp.n.2	X
Chilopoda	Scutigeromorpha	Pselliodidae	*Sphendononema*	*guildingii*	X
Diplopoda	Polydesmida	Pyrgodesmidae		sp.	X
Entognatha	Collembola	Sminthuroidea		sp.	X
Insecta	Coleoptera	Carabidae	Scaritinae	sp.	X
Insecta	Coleoptera	Scydmaenidae		sp.	X
Insecta	Diptera	Phoridae		sp.	X
Insecta	Diptera	Psychodidae	*Pifanomyia*	*gruta*	X
Insecta	Homoptera	Cixiidae		sp.2	X
Insecta	Hymenoptera	Formicidae		sp.4	X
Insecta	Hymenoptera	Formicidae		sp.5	X
Insecta	Hymenoptera	Scelionidae		sp.	X
Insecta	Lepidoptera	Tineoidea		jovem	X
Insecta	Orthoptera	Phalangopsidae	*Phalangopsis*	sp.	X
Malacostraca	Isopoda	Armadillidae		sp.	X
Malacostraca	Isopoda	Scleropactidae		sp.	X
Symphyla		Scutigerellidae		sp.	X
Mammalia	Chiroptera	Emballonuridae	*Cormura*	*brevirostris*	X
Mammalia	Chiroptera	Phyllostomidae	*Trachops*	*cirrhosus*	X
