# Supplementary material for: Reconciling Mining with the Conservation of Cave Biodiversity: A Quantitative Baseline to Help Establish Conservation Priorities
Source: PLoS One. 2016 Dec 20;11(12):e0168348. doi: 10.1371/journal.pone.0168348 (PMC5173368; doi:10.1371/journal.pone.0168348)
Supplement: S1 Dataset — (ZIP) [file pone.0168348.s002.zip › Taxa/Serra Norte/SN_2007/Lista N5E-09.pdf]

CAVIDADE N5E-0009

Classe	Ordem	Fam/Outros	Gên/Outros	Espécie	Única
Arachnida	Acari	Metastigmata		sp.	X
Arachnida	Acari			sp.1	X
Arachnida	Amblypygi	Phrynidae	*Heterophrynus*	*longicornis*	X
Arachnida	Araneae	Ctenidae		sp.	X
Arachnida	Araneae	Ochyroceratidae	*Ochyrocera*	sp.1	X
Arachnida	Araneae	Pholcidae	*Mesabolivar*	*eberhard*	X
Arachnida	Araneae	Pholcidae	*Metagonia*	sp.	X
Arachnida	Araneae	Pholcidae	Ninetinae	sp.	X
Arachnida	Araneae	Scytodidae	*Scytodes*	*itapevi*	X
Arachnida	Araneae	Theridiosomatidae	*Plato*	sp.	X
Arachnida	Opiliones	Escadabiidae		sp.n.2	X
Arachnida	Opiliones	Stygnidae	*Protimesius*	sp.	X
Arachnida	Ricinulei	Ricinoididae	*Cryptocellus*	*tarsilae*	X
Diplopoda	Polydesmida	Pyrgodesmidae		sp.	X
Entognatha	Collembola	Sminthuroidea		sp.	X
Insecta	Coleoptera	Nitidulidae		sp.	X
Insecta	Coleoptera	Staphylinidae	Brachyglutini	sp.	X
Insecta	Coleoptera			sp.	X
Insecta	Diptera	Fanniidae		sp.	X
Insecta	Diptera	Muscidae		sp.	X
Insecta	Diptera	Phoridae		sp.	X
Insecta	Diptera	Psychodidae	*Sciopemyia*	*sordellii*	X
Insecta	Heteroptera	Reduviidae	Emesinae	sp.1	X
Insecta	Hymenoptera	Formicidae		sp.1	X
Insecta	Hymenoptera	Formicidae		sp.2	X
Insecta	Hymenoptera	Formicidae		sp.3	X
Insecta	Isoptera	Termitidae		sp.	X
Insecta	Lepidoptera	Tineoidea		sp.1	X
Insecta	Orthoptera	Phalangopsidae	*Phalangopsis*	sp.	X
Gastropoda	Pulmonata	Subulinidae	*Lamellaxis*	sp.	X
Gastropoda	Pulmonata	Systrophiidae	*Happia*	sp.	X
Symphyla		Scutigerellidae		sp.	X
Mammalia	Chiroptera	Emballonuridae	*Cormura*	*brevirostris*	X
