# Supplementary material for: Reconciling Mining with the Conservation of Cave Biodiversity: A Quantitative Baseline to Help Establish Conservation Priorities
Source: PLoS One. 2016 Dec 20;11(12):e0168348. doi: 10.1371/journal.pone.0168348 (PMC5173368; doi:10.1371/journal.pone.0168348)
Supplement: S1 Dataset — (ZIP) [file pone.0168348.s002.zip › Taxa/Serra Sul/SS_2012/taxons_103.pdf]

| | S11D-103 | | | |
|---|---|---|---|---|
| | Seco | | Úmido | |
| | col / obs | ab rel | col / obs | ab rel |
| **Filo Arthropoda** | | | | |
| **Classe Arachnida** | | | | |
| **Acari** | | | | |
| O. Opilioacarida - *Neoacarus* sp1 | **1** | | | |
| **Ordem Araneae** | | | | |
| Fam. Araneidae | | | | |
| *Alpaida* sp1 | | | **2** | |
| Fam. Corinnidae | | | | |
| *Tupirina* sp1 | **1** | **0,17** | | |
| Fam. Ctenidae | | | | |
| Ctenidae (jovens) | | | **4** | **0,16** |
| *Isoctenus* sp1 | | | | |
| Fam. Oonopidae | | | | |
| Oonopidae (jovem) | **1** | | | |
| gr. *Xycarphius sp1* | | | **1** | |
| Fam. Pholcidae | | | | |
| Pholcidae (jovens) | **8** | | | |
| Ninetinae sp1 | **1** | | | |
| *Mesabolivar cambridgei* | | | **1** | |
| Fam. Salticidae | | | | |
| Salticidae (jovens) | **1** | | | |
| Fam. Scytodidae | | | | |
| Scytodidae (jovens) | | | **1** | **0,04** |
| *Scytodes eleonorae* | **1** | **0,17** | | |
| Fam. Theridiidae | | | | |
| Theridiidae (jovens) | | | **1** | |
| *Theridion* sp2 | | | **1** | |
| **Ordem Opiliones** | | | | |
| Fam. Stygnidae | | | | |
| Stygnidae (jovens) | | | **3** | **0,12** |
| Stygnidae sp1 | | | | |
| **Ordem Pseudoscorpiones** | | | | |
| Fam. Olpiidae | | | | |
| Olpiidae sp1 | **1** | | | |
| **Classe Hexapoda** | | | | |
| **Ordem Blattodea** | | | | |
| Fam. Blaberidae (jovens) | | | **1** | **0,04** |
| Fam. Blattidae | | | | |
| Blattidae (jovens) | | | **1** | **0,04** |
| **Ordem Coleoptera** | | | | |
| Fam. Chrysomelidae | | | | |
| Chrysomelidae sp16 | | | **1** | |
| Coleoptera (larvas) | | | **2** | |
| **Ordem Collembola** | | | | |
| Fam. Paronellidae | | | | |
| Paronellidae sp1 | | | **3** | |
| **Ordem Dermaptera** (jovem) | **1** | | **1** | |
| **Ordem Hemiptera** | | | | |
| Subordem Homoptera | | | | |
| Fam. Cixiidae | | | | |
| Cixiidae (jovem) | **1** | | | |
| Fam. Reduviidae | | | | |
| Subfam. Reduviinae (jovens) | **2** | **0,33** | **10** | **0,4** |
| **Ordem Hymenoptera** | | | | |
| Superfam. Chalcidoidea - Chalcidoidea sp1 | **1** | | | |
| Fam. Formicidae | | | | |

| | | | | |
|---|---|---|---|---|
| *Camponotus* sp1 | **1** | | | |
| *Hypoponera* sp1 | | | **1** | |
| *Pheidole* sp1 | **1** | | | |
| *Pheidole* sp2 | | | **1** | |
| **Ordem Isoptera** | | | | |
| Fam. Termitidae | | | | |
| *Nasutitermes* sp | **2** | | **3** | |
| **Ordem Lepidoptera** | | | | |
| Superfam. Noctuoidea | | | | |
| Noctuoidea sp1 | | | **2** | **0,08** |
| **Ordem Orthoptera** | | | | |
| Fam. Phalangopsidae | | | | |
| *Paraclodes* sp1 | **2** | **0,33** | | |
| *Phalangopsis* sp1 | | | **3** | **0,12** |
| **Ordem Psocoptera** | | | | |
| Subordem Psocomorpha | | | | |
| Psocomorpha (jovens) | | | **2** | |
| **Diplopoda** | | | | |
| Fam. Fuhrmanodesmidae | | | | |
| Fuhrmanodesmidae (jovem) | | | **1** | |
| **Classe Crustacea** | | | | |
| **Ordem Isopoda** | | | | |
| Fam. Dubioniscidae - Dubioniscidae sp1 | | | **1** | |
