# Supplementary material for: Reconciling Mining with the Conservation of Cave Biodiversity: A Quantitative Baseline to Help Establish Conservation Priorities
Source: PLoS One. 2016 Dec 20;11(12):e0168348. doi: 10.1371/journal.pone.0168348 (PMC5173368; doi:10.1371/journal.pone.0168348)
Supplement: S1 Dataset — (ZIP) [file pone.0168348.s002.zip › Taxa/Serra Sul/SS_2012/taxons_103_com legenda.pdf]

Anexo IV. Táxons inventariados nas cavidades estudadas, com a quantidade de indivíduos coletados ou observados (no caso de maiores de 1 cm, com respectiva abundância relativa).

	S11D-103			
	Seco		Úmido	
	col / obs	ab rel	col / obs	ab rel
**Filo Arthropoda**				
**Classe Arachnida**				
**Acari**				
O. Opilioacarida - *Neoacarus* sp1	1			
**Ordem Araneae**				
Fam. Araneidae				
Alpaida sp1			2	
Fam. Corinnidae				
Tupirina sp1	1	0,17		
Fam. Ctenidae				
Ctenidae (jovens)			4	0,16
Isoctenus sp1				
Fam. Oonopidae				
Oonopidae (jovem)	1			
gr. *Xycarphius sp1*			1	
Fam. Pholcidae				
Pholcidae (jovens)	8			
Ninetinae sp1	1			
Mesabolivar cambridgei			1	
Fam. Salticidae				
Salticidae (jovens)	1			
Fam. Scytodidae				
Scytodidae (jovens)			1	0,04
Scytodes eleonorae	1	0,17		
Fam. Theridiidae				
Theridiidae (jovens)			1	
Theridion sp2			1	
**Ordem Opiliones**				
Fam. Stygnidae				
Stygnidae (jovens)			3	0,12
Stygnidae sp1				
**Ordem Pseudoscorpiones**				
Fam. Olpiidae				
Olpiidae sp1	1			
**Classe Hexapoda**				
**Ordem Blattodea**				
Fam. Blaberidae (jovens)			1	0,04
Fam. Blattidae				
Blattidae (jovens)			1	0,04
**Ordem Coleoptera**				
Fam. Chrysomelidae				
Chrysomelidae sp16			1	
Coleoptera (larvas)			2	
**Ordem Collembola**				
Fam. Paronellidae				
Paronellidae sp1			3	
**Ordem Dermaptera** (jovem)	1		1	
**Ordem Hemiptera**				
Subordem Homoptera				
Fam. Cixiidae				
Cixiidae (jovem)	1			
Fam. Reduviidae				
Subfam. Reduviinae (jovens)	2	0,33	10	0,4
**Ordem Hymenoptera**				

Superfam. Chalcidoidea - Chalcidoidea sp1	**1**			
Fam. Formicidae				
Camponotus sp1	**1**			
Hypoponera sp1			**1**	
Pheidole sp1	**1**			
Pheidole sp2			**1**	
**Ordem Isoptera**				
Fam. Termitidae				
Nasutitermes sp	**2**		**3**	
**Ordem Lepidoptera**				
Superfam. Noctuoidea				
Noctuoidea sp1			**2**	**0,08**
**Ordem Orthoptera**				
Fam. Phalangopsidae				
Paraclodes sp1	**2**	**0,33**		
Phalangopsis sp1			**3**	**0,12**
**Ordem Psocoptera**				
Subordem Psocomorpha				
Psocomorpha (jovens)			**2**	
**Diplopoda**				
Fam. Fuhrmanodesmidae				
Fuhrmanodesmidae (jovem)			**1**	
**Classe Crustacea**				
**Ordem Isopoda**				
Fam. Dubioniscidae - Dubioniscidae sp1			**1**	
