# Supplementary material for: Reconciling Mining with the Conservation of Cave Biodiversity: A Quantitative Baseline to Help Establish Conservation Priorities
Source: PLoS One. 2016 Dec 20;11(12):e0168348. doi: 10.1371/journal.pone.0168348 (PMC5173368; doi:10.1371/journal.pone.0168348)
Supplement: S1 Dataset — (ZIP) [file pone.0168348.s002.zip › Taxa/Serra Sul/SS_2012/taxons_104.pdf]

	S11D-104			
	Seco		Úmido	
	col / obs	ab rel	col / obs	ab rel
**Filo Arthropoda**				
**Classe Arachnida**				
**Acari**				
O. Ixodida				
Fam. Argasidae - *Ornithodorus* sp			**6**	
Fam. Ixodidae - *Amblyomma* sp	**1**			
O. Opilioacarida - *Neoacarus* sp1	**4**			
O. Sarcoptiforme				
Galumnidae sp1	**5**		**2**	
Mesoplophoridae sp1			**2**	
O. Trombidiforme				
Fam. Anystidae - *Erythracarus nasutus*	**1**			
**Ordem Amblypygi**				
Heterophrynus sp.			**4**	**0,01**
**Ordem Araneae**				
Fam. Araneidae				
Araneidae (jovens)	**2**			
Alpaida sp1			**1**	
Fam. Corinnidae				
Creugas sp1			**2**	**0,01**
Fam. Nesticidae (jovem)	**1**			
Fam. Ochyroceratidae				
Ochyroceratidae (jovem)	**4**		**3**	
Ochyrocera sp1	**3**		**5**	
Speocera sp1	**1**		**1**	
Fam. Oonopidae				
gr. *Xycarphius sp1*	**1**			
Fam. Pholcidae				
Pholcidae (jovens)			**1**	
Ninetinae sp1	**1**		**1**	
Mesabolivar cambridgei			**1**	
Fam. Scytodidae				
Scytodidae (jovens)	**2**	**0,01**	**1**	**0,004**
Scytodes eleonorae	**1**	**0,01**	**1**	**0,004**
Fam. Segestriidae				
Segestriidae (jovens)	**1**			
Ariadna sp1	**2**		**3**	
Fam. Theraphosidae				
aff. Holothele sp1			**1**	**0,004**
Fam. Theridiosomatidae				
Theridiosomatidae (jovens)	**3**		**3**	
Plato sp1	**5**		**2**	
**Ordem Opiliones**				
Fam. Stygnidae				
Stygnidae (jovens)			**8**	**0,03**
Stygnidae sp1	**4**	**0,02**		
**Ordem Schizomida**				
Hubbardiidae (jovem)	**1**		**2**	
Hubbardiidae - *Rowlandius* sp1	**3**		**3**	
**Classe Hexapoda**				
**Ordem Blattodea**				
Fam. Blaberidae (jovens)	**1**	**0,01**		
Fam. Blattellidae				
Blattellidae sp1	**1**			
**Ordem Coleoptera**				
Fam. Carabidae				

Carabidae sp10			**1**	
Carabidae sp21	**1**			
Fam. Dytiscidae - Hydroporinae sp1	**2**			
Fam. Staphylinidae				
Staphylinidae sp22			**1**	
Coleoptera (larvas)	**1**			
**Ordem Collembola**				
Fam. Cyphoderidae - Cyphoderidae sp2			**1**	
Fam. Paronellidae				
Paronellidae sp1	**3**		**1**	
Superfam. Sminthuroidea - Sminthuroidea sp2			**3**	
**Ordem Diplura**				
Fam. Campodeidae - Campodeidae sp1	**1**		**2**	
**Ordem Diptera**				
Fam. Culicidae	**1**		**12**	
Fam. Drosophilidae - *Drosophila eleonorae*	**3**			
Fam. Psychodidae - Phlebotominae sp.	**2**		**2**	
Fam. Tipulidae	**1**			
Diptera (larvas)	**2**		**3**	
**Ordem Hemiptera**				
Subordem Homoptera				
Fam. Cixiidae				
Cixiidae (jovem)	**2**		**1**	
Subordem Heteroptera				
Fam. Cydnidae				
Cydnidae (jovens)			**1**	
Cydninae sp1			**1**	
Fam. Reduviidae				
Subfam. Reduviinae (jovens)	**2**	**0,02**		
Fam. Tingidae				
Vianaidinae (jovem)			**1**	
Fam. Vellidae - *Paravelia* sp2	**3**			
**Ordem Hymenoptera**				
Fam. Formicidae				
Camponotus atriceps	**3**		**6**	
Hypoponera striata			**3**	
Pachycondyla constricta			**1**	**0,004**
Pachycondyla striata	**1**			
Solenopsis sp1			**1**	
**Ordem Isoptera**				
Fam. Termitidae				
Nasutitermes sp	**8**		**6**	
**Ordem Lepidoptera**				
Superfam. Noctuoidea				
Noctuoidea sp3			**1**	**0,004**
Lepidoptera (larvas)			**4**	
**Ordem Orthoptera**				
Fam. Phalangopsidae				
Paraclodes sp1	**5**	**0,03**		
Phalangopsis sp1	**103**	**0,64**	**142**	**0,52**
**Ordem Thysanura**				
Nicoletiidae sp1	**3**		**1**	
**Chilopoda**				
Ordem Scolopendromorpha				
Newportia sp1			**1**	**0,004**
Ordem Scutigeromorpha - Fam. Pselliodidae			**1**	**0,004**
**Diplopoda**				
Ordem Polydesmida				
Fam. Chelodesmidae - Chelodesmidae sp4			**1**	**0,004**
Fam. Fuhrmanodesmidae				

Fuhrmanodesmidae sp1			**2**	
Ordem Spirostreptida - Fam. Pseudonannolenidae				
Pseudonannolenidae (jovem)	**2**	**0,01**		
**Classe Crustacea**				
**Ordem Isopoda**				
Fam. Dubioniscidae - Dubioniscidae sp1			**1**	
Fam. Philosciidae - Philosciidae sp1			**3**	
Fam. Scleropactidae - Scleropactidae sp1			**1**	
**Filo Mollusca - Gastropoda**				
Fam. Systrophiidae - *Happia* sp1			**1**	
**Filo Annelida -** Oligochaeta (jovens)	**19**	**0,12**	**90**	**0,33**
**Filo Chordata**				
**Ordem Anura**				
Leptodactylus sp.			**2**	**0,01**
Pristimantis fenestratus	**3**	**0,02**		
**Ordem Chiroptera**				
Carollia perspicillata			**10**	**0,04**
Peropteryx sp.	**20**	**0,12**	**5**	**0,02**
