# Supplementary material for: Reconciling Mining with the Conservation of Cave Biodiversity: A Quantitative Baseline to Help Establish Conservation Priorities
Source: PLoS One. 2016 Dec 20;11(12):e0168348. doi: 10.1371/journal.pone.0168348 (PMC5173368; doi:10.1371/journal.pone.0168348)
Supplement: S1 Dataset — (ZIP) [file pone.0168348.s002.zip › Taxa/Serra Sul/SS_2012/taxons_105.pdf]

	S11D-105			
	Seco		Úmido	
	col / obs	ab rel	col / obs	ab rel
**Filo Arthropoda**				
**Classe Arachnida**				
**Acari**				
O. Ixodida				
Fam. Argasidae - *Ornithodorus* sp	6		5	
O. Mesostigmata				
Mesostigmata sp1			2	
Laelapidae sp1			2	
O. Opilioacarida - *Neoacarus* sp1	1		2	
**Ordem Amblypygi**				
Heterophrynus sp.	4	0,15	3	0,04
**Ordem Araneae**				
Fam. Corinnidae				
Creugas sp1			1	0,01
Fam. Ctenidae				
Isoctenus sp1	1	0,04		
Fam. Ochyroceratidae (jovem)			2	
Fam. Pholcidae				
Pholcidae (jovens)	3			
Ninetinae sp1	7		10	
Fam. Scytodidae				
Scytodidae (jovens)	2	0,07	3	0,04
Scytodes sp1			1	
Fam. Segestriidae				
Segestriidae (jovens)	4			
Ariadna sp1			2	
**Ordem Opiliones**				
Fam. Cosmetidae				
Cosmetidae (jovens)			1	0,01
Roquetea singularis	1	0,04		
Fam. Escadabiidae - Escadabiidae sp2			4	
Fam. Stygnidae				
Stygnidae sp1	5	0,19	1	0,01
**Ordem Palpigradi**				
Fam. Eukoeneniidae - *Allokoenenia* sp1			2	
**Ordem Pseudoscorpiones**				
Fam. Chernetidae				
Spelaeochernes sp1	1		3	
Fam. Chthoniidae				
Pseudochthonius sp1	2			
**Classe Hexapoda**				
**Ordem Blattodea**				
Fam. Blaberidae (jovens)	3	0,11	3	0,04
Fam. Blattellidae				
Blattellidae (jovens)			1	0,01
Fam. Blattidae				
Blattidae (jovens)			2	0,03
Blattidae sp1	2	0,07		
Fam. Polyphagidae				
Polyphagidae (jovem)			5	
Polyphagidae sp2	2		4	
**Ordem Coleoptera**				
Coleoptera (larvas)			1	
**Ordem Collembola**				
Fam. Paronellidae				
Paronellidae sp1			1	

**Ordem Diptera**				
Fam. Cecidomyiidae			**1**	
Fam. Psychodidae - Phlebotominae sp.	**1**		**5**	
Diptera (larvas)	**1**		**2**	
**Ordem Hemiptera**				
Subordem Homoptera				
Fam. Cixiidae				
Cixiidae (jovem)	**2**			
Subordem Heteroptera				
Fam. Reduviidae				
Subfam. Reduviinae (jovens)	**3**	**0,11**	**6**	**0,08**
Zelurus sp1				
**Ordem Isoptera**				
Fam. Termitidae				
Nasutitermes sp	**8**		**9**	
**Ordem Lepidoptera**				
Superfam. Gelechioidea sp1			**3**	
Superfam. Noctuoidea				
Noctuoidea sp1	**1**	**0,04**	**2**	**0,03**
Noctuoidea sp2			**1**	**0,01**
Noctuoidea sp3			**1**	
Superfam. Tineoidea - Tineidae sp1			**1**	
Lepidoptera (larvas)			**2**	
**Ordem Mantodea** - Acromantinae sp2			**1**	
**Ordem Orthoptera**				
Fam. Phalangopsidae				
Phalangopsis sp1	**1**	**0,04**	**47**	**0,62**
**Ordem Psocoptera**				
Subordem Troctomorpha				
Fam. Manicapsocidae - *Nothoentomum* sp1			**1**	
**Ordem Thysanura**				
Nicoletiidae sp1			**1**	
**Diplopoda**				
Ordem Spirostreptida - Fam. Pseudonannolenidae				
Pseudonannolenidae (jovem)	**2**	**0,07**		
Pseudonannolene sp6			**4**	**0,05**
**Filo Chordata**				
**Ordem Anura**				
Pristimantis fenestratus	**2**	**0,07**		
**Ordem Chiroptera**				
Peropteryx sp.			**1**	**0,01**
