# Supplementary material for: Reconciling Mining with the Conservation of Cave Biodiversity: A Quantitative Baseline to Help Establish Conservation Priorities
Source: PLoS One. 2016 Dec 20;11(12):e0168348. doi: 10.1371/journal.pone.0168348 (PMC5173368; doi:10.1371/journal.pone.0168348)
Supplement: S1 Dataset — (ZIP) [file pone.0168348.s002.zip › Taxa/Serra Sul/SS_2012/taxons_106.pdf]

	S11D-106			
	Seco		Úmido	
	col / obs	ab rel	col / obs	ab rel
**Filo Arthropoda**				
**Classe Arachnida**				
**Acari**				
O. Mesostigmata				
Mesostigmata sp1	1			
O. Opilioacarida - *Neoacarus* sp1	1		1	
O. Trombidiforme				
Trombidiidae sp6	1			
**Ordem Amblypygi**				
Heterophrynus sp.	2	0,14		
**Ordem Araneae**				
Fam. Araneidae				
Alpaida sp1			2	
Fam. Ctenidae				
Ctenidae (jovens)			3	0,12
Fam. Dipluridae (jovem)			2	0,08
Fam. Oonopidae				
Oonopidae sp2	1			
Fam. Pholcidae				
Pholcidae (jovens)	3			
Mesabolivar cambridgei	1		1	
Fam. Salticidae				
Salticidae (jovens)	4			
Salticidae sp1	1			
Fam. Scytodidae				
Scytodidae (jovens)			2	0,08
Scytodes eleonorae				
Fam. Trechaleidae (jovem)	1	0,07		
Fam. Uloboridae				
Uloboridae (jovem)	1			
**Ordem Opiliones**				
Fam. Cosmetidae				
Cosmetidae (jovens)			2	0,08
Roquetea singularis				
Fam. Stygnidae				
Stygnidae (jovens)			3	0,12
**Ordem Pseudoscorpiones**				
Fam. Chernetidae				
Spelaeochernes sp1			2	
Fam. Chthoniidae				
Chthoniidae (jovem)			1	
**Classe Hexapoda**				
**Ordem Blattodea**				
Blattodea (jovens)			1	
Fam. Polyphagidae				
Polyphagidae (jovem)	2			
**Ordem Coleoptera**				
Fam. Chrysomelidae				
Chrysomelidae sp3			1	
Coleoptera (larvas)	1			
**Ordem Collembola**				
Fam. Paronellidae				
Paronellidae sp1	3		3	
Paronellidae sp7			1	
**Ordem Diptera**				
Fam. Ceratopogonidae	1			

Fam. Psychodidae - Phlebotominae sp.	**3**		**2**	
**Ordem Hemiptera**				
Subordem Homoptera				
Fam. Cixiidae				
Cixiidae sp1			**1**	
Subordem Heteroptera				
Fam. Reduviidae				
Subfam. Reduviinae (jovens)	**2**	**0,14**	**5**	**0,2**
Fam. Schizopteridae				
Schizopteridae (jovem)	**2**			
**Ordem Hymenoptera**				
Fam. Formicidae				
Apterostigma sp1			**1**	
Crematogaster sp1	**1**			
Dolichoderus bispinosus	**2**		**1**	
Odontomachus sp1			**1**	**0,04**
**Ordem Isoptera**				
Fam. Termitidae				
Nasutitermes sp	**4**			
**Ordem Lepidoptera**				
Lepidoptera (larvas)			**2**	
**Ordem Orthoptera**				
Fam. Phalangopsidae				
Paraclodes sp1	**8**	**0,57**		
Phalangopsis sp1	**1**	**0,07**	**6**	**0,24**
**Ordem Psocoptera**				
Subordem Psocomorpha				
Psocomorpha (jovens)	**2**			
**Diplopoda**				
Fam. Pyrgodesmidae - Pyrgodesmidae sp5			**1**	**0,04**
**Classe Crustacea**				
**Ordem Isopoda**				
Fam. Dubioniscidae - Dubioniscidae sp1	**1**			
