# Supplementary material for: Reconciling Mining with the Conservation of Cave Biodiversity: A Quantitative Baseline to Help Establish Conservation Priorities
Source: PLoS One. 2016 Dec 20;11(12):e0168348. doi: 10.1371/journal.pone.0168348 (PMC5173368; doi:10.1371/journal.pone.0168348)
Supplement: S1 Dataset — (ZIP) [file pone.0168348.s002.zip › Taxa/Serra Sul/SS_2012/taxons_107.pdf]

	S11D-107			
	Seco		Úmido	
	col / obs	ab rel	col / obs	ab rel
**Filo Arthropoda**				
**Classe Arachnida**				
**Acari**				
O. Ixodida				
Fam. Argasidae - *Ornithodorus* sp	**1**			
O. Mesostigmata				
Mesostigmata sp1	**3**			
**Ordem Amblypygi**				
Heterophrynus sp.			**1**	**0,06**
**Ordem Araneae**				
Fam. Ctenidae				
Ctenidae (jovens)			**3**	**0,19**
Fam. Filistatidae - Filistatidae sp1	**1**			
Fam. Pholcidae				
Pholcidae (jovens)	**2**			
Ninetinae sp1	**1**		**5**	
Leptopholcus sp1			**1**	
aff. *Ibityporanga* sp1			**2**	
Fam. Salticidae				
Salticidae (jovens)	**1**			
Fam. Scytodidae				
Scytodidae (jovens)	**3**	**0,17**	**2**	**0,13**
**Ordem Opiliones**				
Fam. Gonyleptidae - Gonyleptidae sp1	**1**			
Fam. Stygnidae				
Stygnidae sp1	**2**	**0,11**	**2**	**0,13**
**Ordem Pseudoscorpiones**				
Fam. Chernetidae				
Spelaeochernes sp1			**1**	
Fam. Chthoniidae				
Pseudochthonius sp1			**1**	
Fam. Olpiidae				
Olpiidae sp1			**3**	
**Classe Hexapoda**				
**Ordem Blattodea**				
Fam. Blattidae				
Blattidae (jovens)			**1**	**0,06**
Fam. Polyphagidae				
Polyphagidae (jovem)	**1**			
**Ordem Coleoptera**				
Fam. Chrysomelidae				
Chrysomelidae sp3			**1**	
Fam. Endomychidae - Endomychidae sp1	**1**			
Fam. Scydmaenidae				
Scydmaenidae sp3			**1**	
Coleoptera (larvas)			**1**	
**Ordem Diptera**				
Fam. Psychodidae - Phlebotominae sp.			**3**	
**Ordem Hemiptera**				
Subordem Homoptera				
Fam. Cixiidae				
Cixiidae sp3	**1**			
Subordem Heteroptera				
Fam. Reduviidae				
Subfam. Emesinae (jovem)	**1**			
Subfam. Reduviinae (jovens)	**2**	**0,11**	**1**	**0,06**

**Ordem Hymenoptera**				
Fam. Formicidae				
Camponotus sp1	**3**		**3**	
Neivamyrmex sp1	**1**			
**Ordem Isoptera**				
Fam. Termitidae				
Nasutitermes sp			**5**	
**Ordem Lepidoptera**				
Superfam. Noctuoidea				
Noctuoidea sp9			**4**	**0,25**
**Ordem Neuroptera**				
Fam. Myrmeleontidae				
Myrmeleontidae (jovens)	**1**		**1**	
Myrmeleontidae sp1	**1**			
**Ordem Orthoptera**				
Fam. Phalangopsidae				
Paraclodes sp1	**3**	**0,17**	**1**	**0,06**
Phalangopsis sp1	**5**	**0,28**		
**Ordem Psocoptera**				
Subordem Psocomorpha				
Psocomorpha (jovens)	**2**			
**Chilopoda**				
Ordem Scutigeromorpha - Fam. Pselliodidae			**2**	
**Classe Crustacea**				
**Ordem Isopoda**				
Fam. Dubioniscidae - Dubioniscidae sp1			**1**	
**Filo Chordata**				
**Ordem Anura**				
Pristimantis fenestratus	**1**	**0,06**		
**Ordem Squamata** - *Thecadactylus rapicauda*	**1**	**0,06**	**1**	**0,06**
**Ordem Chiroptera**				
Peropteryx sp.	**1**	**0,06**		
