# Supplementary material for: Reconciling Mining with the Conservation of Cave Biodiversity: A Quantitative Baseline to Help Establish Conservation Priorities
Source: PLoS One. 2016 Dec 20;11(12):e0168348. doi: 10.1371/journal.pone.0168348 (PMC5173368; doi:10.1371/journal.pone.0168348)
Supplement: S1 Dataset — (ZIP) [file pone.0168348.s002.zip › Taxa/Serra Sul/SS_2012/taxons_108.pdf]

	S11D-108			
	Seco		Úmido	
	col / obs	ab rel	col / obs	ab rel
**Filo Arthropoda**				
**Classe Arachnida**				
**Acari**				
O. Opilioacarida - *Neoacarus* sp1			3	
O. Sarcoptiforme				
Oribatida sp20			3	
Acaridae sp1			3	
**Ordem Amblypygi**				
Heterophrynus sp.			1	0,05
**Ordem Araneae**				
Fam. Barychaelidae (jovem)	1	0,08		
Fam. Corinnidae				
Corinnidae (jovens)			5	0,24
Fam. Ctenidae				
Ctenidae (jovens)			1	0,05
Ctenidae sp4	1	0,08		
Fam. Ochyroceratidae				
Ochyrocera sp1			2	
Fam. Pholcidae				
Ninetinae sp1	2			
Leptopholcus sp1			2	
Fam. Scytodidae				
Scytodes eleonorae			1	0,05
Scytodes globula			1	0,05
Fam. Segestriidae				
Segestriidae (jovens)			1	
**Ordem Opiliones**				
Fam. Stygnidae				
Stygnidae (jovens)			1	0,05
Stygnidae sp1	2	0,15		
**Ordem Pseudoscorpiones**				
Fam. Olpiidae				
Olpiidae (jovens)	1			
**Ordem Ricinulei -** Ricinoididae (jovens)			1	
**Classe Hexapoda**				
**Ordem Collembola**				
Fam. Entomobryidae				
Entomobryidae sp4			5	
Fam. Paronellidae				
Paronellidae sp1			5	
**Ordem Diplura**				
Fam. Campodeidae - Campodeidae sp1			1	
**Ordem Diptera**				
Fam. Psychodidae - Phlebotominae sp.			3	
Diptera (larvas)			1	
**Ordem Hemiptera**				
Subordem Homoptera				
Fam. Cixiidae				
Cixiidae sp1			1	
Cixiidae sp3	1			
Subordem Heteroptera				
Fam. Lygaeidae				
Lygaeidae (jovem)			1	
Lygaeidae sp1			1	
Fam. Reduviidae				
Subfam. Reduviinae (jovens)	1	0,08	2	0,1

**Ordem Hymenoptera**				
Fam. Formicidae				
Carebara sp2	**4**			
Dolichoderus bispinosus	**4**			
**Ordem Isoptera**				
Fam. Termitidae				
Atlantitermes sp	**3**			
**Ordem Lepidoptera**				
Superfam. Noctuoidea				
Noctuoidea sp1			**1**	**0,05**
Noctuoidea sp3			**1**	
**Ordem Orthoptera**				
Fam. Phalangopsidae				
Paraclodes sp1	**8**	**0,62**	**2**	**0,1**
Phalangopsis sp1			**3**	**0,14**
**Ordem Psocoptera**				
Subordem Troctomorpha				
Fam. Psyllipsocidae (jovem)	**1**			
**Diplopoda**				
Ordem Polydesmida				
Fam. Aphelidesmidae - Aphelidesmidae sp3			**2**	**0,1**
Ordem Polyxenida - Hypogexenidae sp1	**1**			
**Filo Chordata**				
**Ordem Anura**				
Pristimantis fenestratus			**1**	**0,05**
