# Supplementary material for: Reconciling Mining with the Conservation of Cave Biodiversity: A Quantitative Baseline to Help Establish Conservation Priorities
Source: PLoS One. 2016 Dec 20;11(12):e0168348. doi: 10.1371/journal.pone.0168348 (PMC5173368; doi:10.1371/journal.pone.0168348)
Supplement: S1 Dataset — (ZIP) [file pone.0168348.s002.zip › Taxa/Serra Sul/SS_2012/taxons_109.pdf]

	S11D-109			
	Seco		Úmido	
	col / obs	ab rel	col / obs	ab rel
**Filo Arthropoda**				
**Classe Arachnida**				
**Acari**				
O. Ixodida				
Fam. Ixodidae - *Amblyomma* sp	**2**			
O. Mesostigmata				
Mesostigmata sp1	**2**			
O. Sarcoptiforme				
Oribatida sp20			**2**	
**Ordem Araneae**				
Fam. Ctenidae				
Ctenidae (jovens)	**1**	**0,2**	**2**	**0,22**
Fam. Oonopidae				
Oonopidae (jovem)	**1**			
Fam. Pholcidae				
Pholcidae (jovens)	**2**		**2**	
Ninetinae sp1	**1**		**3**	
Leptopholcus sp1			**1**	
Fam. Salticidae				
Salticidae (jovens)	**1**			
Fam. Scytodidae				
Scytodidae (jovens)	**1**	**0,2**		
Scytodes sp1			**1**	
**Ordem Opiliones**				
Fam. Stygnidae				
Stygnidae sp1	**3**	**0,6**		
**Ordem Pseudoscorpiones**				
Fam. Olpiidae				
Olpiidae sp1	**1**			
**Classe Hexapoda**				
**Ordem Archaeognatha -** Meinertellidae sp2			**1**	**0,11**
**Ordem Coleoptera**				
Fam. Chrysomelidae				
Chrysomelidae sp17			**1**	
Fam. Endomychidae - Endomychidae sp1	**1**			
**Ordem Collembola**				
Fam. Entomobryidae				
Entomobryidae sp4			**2**	
Fam. Paronellidae				
Paronellidae sp1			**2**	
**Ordem Diptera**				
Fam. Psychodidae - Phlebotominae sp.			**1**	
**Ordem Hemiptera**				
Subordem Homoptera				
Fam. Cixiidae				
Cixiidae sp3	**1**			
**Ordem Hymenoptera**				
Fam. Formicidae				
Camponotus atriceps			**2**	
Gnamptogenys striatula	**1**			
Hypoponera sp1	**1**			
**Ordem Isoptera**				
Fam. Termitidae				
Velocitermes sp1	**1**			
**Ordem Lepidoptera**				
Superfam. Noctuoidea				

Noctuoidea sp9			**3**	**0,33**
**Ordem Neuroptera**				
Fam. Myrmeleontidae				
Myrmeleontidae (jovens)	**3**		**2**	
**Ordem Orthoptera**				
Fam. Phalangopsidae sp2			**2**	**0,22**
**Ordem Psocoptera**				
Subordem Psocomorpha				
Psocomorpha (jovens)	**3**			
**Diplopoda**				
Ordem Spirostreptida - Fam. Pseudonannolenidae				
Pseudonannolene sp5			**1**	**0,11**
**Classe Symphyla**				
Fam. Scutigerellidae - *Hanseniella* sp1			**1**	
**Classe Crustacea**				
**Ordem Isopoda**				
Fam. Dubioniscidae - Dubioniscidae sp1			**2**	
