# Supplementary material for: Reconciling Mining with the Conservation of Cave Biodiversity: A Quantitative Baseline to Help Establish Conservation Priorities
Source: PLoS One. 2016 Dec 20;11(12):e0168348. doi: 10.1371/journal.pone.0168348 (PMC5173368; doi:10.1371/journal.pone.0168348)
Supplement: S1 Dataset — (ZIP) [file pone.0168348.s002.zip › Taxa/Serra Sul/SS_2012/taxons_110.pdf]

	S11D-110			
	Seco		Úmido	
	col / obs	ab rel	col / obs	ab rel
**Filo Arthropoda**				
**Classe Arachnida**				
**Acari**				
O. Mesostigmata				
Ameroseiidae sp1			1	
O. Opilioacarida - *Neoacarus* sp1	1			
O. Sarcoptiforme				
Oribatida sp2			1	
O. Trombidiforme				
Tydeidae sp1			3	
**Ordem Amblypygi**				
Charinus (jovens)			1	0,002
Charinus sp1	1	0,005		
Heterophrynus sp.	4	0,02	4	0,01
**Ordem Araneae**				
Fam. Araneidae				
Alpaida sp1	1			
Fam. Ctenidae				
Ctenidae (jovens)			1	0,002
Fam. Ochyroceratidae				
Ochyroceratidae (jovem)			1	
Ochyrocera sp1	1			
Fam. Pholcidae				
Mesabolivar cambridgei			1	
Fam. Salticidae				
Salticidae (jovens)	1			
Fam. Scytodidae				
Scytodidae (jovens)<br>*Scytodes eleonorae*	5	0,02	4	0,01
Fam. Tetrablemmidae - *Matta* sp1			2	
Fam. Theridiidae				
Theridiidae (jovens)	1			
Fam. Theridiosomatidae				
Theridiosomatidae (jovens)			1	
**Ordem Opiliones**				
Fam. Sclerosomatidae - Sclerosomatidae sp1	1	0,005		
Fam. Stygnidae				
Stygnidae sp1	53	0,26		
**Ordem Palpigradi**				
Fam. Eukoeneniidae - *Allokoenenia* sp1			1	
**Ordem Pseudoscorpiones**				
Fam. Chernetidae				
Chernetidae (jovens)			2	
Spelaeochernes sp1	1			
**Classe Hexapoda**				
**Ordem Blattodea**				
Fam. Blattidae				
Blattidae (jovens)			1	0,002
**Ordem Coleoptera**				
Fam. Carabidae				
Carabidae sp4	1		1	
Fam. Chrysomelidae				
Chrysomelidae sp18			1	
Fam. Scydmaenidae				
Scydmaenidae sp11			2	
Coleoptera (larvas)	1			
**Ordem Collembola**				

Fam. Cyphoderidae - Cyphoderidae sp2			**3**	
Fam. Paronellidae				
Paronellidae sp1			**3**	
Superfam. Sminthuroidea - Sminthuroidea sp2			**1**	
**Ordem Dermaptera** (jovem)			**1**	
**Ordem Diptera**				
Fam. Cecidomyiidae			**3**	
Fam. Chironomidae	**1**			
Fam. Dolichopodidae	**1**			
Fam. Psychodidae - Phlebotominae sp.			**1**	
Fam. Tipulidae	**1**			
**Ordem Hemiptera**				
Subordem Homoptera				
Fam. Cixiidae				
Cixiidae (jovem)			**1**	
Subordem Heteroptera				
Fam. Cydnidae				
Cydninae sp1			**1**	
Cydninae sp2	**1**			
Fam. Reduviidae				
Subfam. Reduviinae (jovens)	**1**	**0,01**		
Fam. Tingidae				
Vianaidinae sp1			**1**	
**Ordem Hymenoptera**				
Fam. Formicidae				
Camponotus sp2	**1**			
Dolichoderus bispinosus	**4**		**4**	
Pachycondyla constricta			**3**	**0,007**
Pachycondyla striata	**1**			
Pheidole sp1	**3**			
Solenopsis sp1	**1**			
Solenopsis sp3			**6**	
Hymenoptera (larvas)			**2**	
**Ordem Isoptera**				
Fam. Termitidae				
Cortaritermes silvestrii			**6**	
Nasutitermes sp	**4**			
**Ordem Lepidoptera**				
Superfam. Noctuoidea				
Noctuoidea sp1			**2**	**0,005**
Lepidoptera (larvas)			**2**	
**Ordem Orthoptera**				
Fam. Phalangopsidae				
Paraclodes sp1	**8**	**0,04**		
Phalangopsis sp1	**130**	**0,64**	**400**	**0,96**
**Ordem Psocoptera**				
Subordem Psocomorpha				
Psocomorpha (jovens)			**1**	
**Ordem Thysanura**				
Nicoletiidae sp1	**2**		**1**	
**Chilopoda**				
Ordem Scutigeromorpha - Fam. Pselliodidae			**1**	
**Diplopoda**				
Ordem Polydesmida				
Fam. Chelodesmidae - Chelodesmidae sp4			**1**	**0,002**
**Classe Symphyla**				
Fam. Scolopendrellidae - *Symphylella* sp1			**2**	
**Classe Crustacea**				
**Ordem Isopoda**				
Fam. Dubioniscidae - Dubioniscidae sp1	**3**		**2**	
