# Supplementary material for: Reconciling Mining with the Conservation of Cave Biodiversity: A Quantitative Baseline to Help Establish Conservation Priorities
Source: PLoS One. 2016 Dec 20;11(12):e0168348. doi: 10.1371/journal.pone.0168348 (PMC5173368; doi:10.1371/journal.pone.0168348)
Supplement: S1 Dataset — (ZIP) [file pone.0168348.s002.zip › Taxa/Serra Sul/SS_2012/taxons_111.pdf]

	S11D-111			
	Seco		Úmido	
	col / obs	ab rel	col / obs	ab rel
**Filo Arthropoda**				
**Classe Arachnida**				
**Ordem Amblypygi**				
Charinus (jovens)	**1**	**0,02**	**1**	**0,02**
**Ordem Araneae**				
Fam. Ochyroceratidae				
Ochyroceratidae (jovem)	**6**			
Ochyrocera sp1	**4**		**5**	
Fam. Oonopidae				
Oonopidae (jovem)	**2**		**1**	
Fam. Scytodidae				
Scytodidae (jovens)			**3**	**0,05**
Fam. Tetrablemmidae - *Matta* sp1	**2**			
Fam. Theridiidae				
Theridiidae (jovens)	**1**			
Fam. Theridiosomatidae				
Plato sp1			**1**	
**Ordem Opiliones**				
Fam. Cosmetidae				
Roquetea singularis	**1**	**0,02**		
Fam. Stygnidae				
Stygnidae (jovens)			**3**	**0,05**
Stygnidae sp1	**3**	**0,05**		
**Ordem Pseudoscorpiones**				
Fam. Chernetidae				
Spelaeochernes sp1	**3**		**4**	
Fam. Chthoniidae				
Chthoniidae (jovem)			**1**	
Pseudochthonius sp1			**3**	
**Classe Hexapoda**				
**Ordem Coleoptera**				
Fam. Carabidae				
Coarazuphium sp1	**1**			
**Ordem Collembola**				
Superfam. Sminthuroidea - Sminthuroidea sp2			**1**	
**Ordem Diplura**				
Fam. Campodeidae - Campodeidae sp1	**2**			
**Ordem Diptera**				
Fam. Psychodidae - Phlebotominae sp.	**1**		**1**	
**Ordem Hemiptera**				
Subordem Homoptera				
Superfam. Coccoidea (jovem)			**1**	
Fam. Cixiidae				
Cixiidae (jovem)	**2**		**5**	
**Ordem Hymenoptera**				
Fam. Formicidae				
Pachycondyla constricta			**2**	**0,03**
Pachycondyla striata	**3**			
Solenopsis sp1			**5**	
Solenopsis sp3			**1**	
Trachymyrmex sp1	**1**			
**Ordem Lepidoptera**				
Superfam. Noctuoidea				
Noctuoidea sp9			**1**	
**Ordem Orthoptera**				
Fam. Phalangopsidae				

Paraclodes sp1	**3**	**0,05**		
Phalangopsis sp1	**42**	**0,72**	**53**	**0,83**
**Ordem Thysanura**				
Ateluridae sp1			**1**	
**Diplopoda**				
Fam. Paradoxomatidae - Paradoxomatidae sp1	**1**	**0,02**		
**Filo Mollusca - Gastropoda**				
Fam. Systrophiidae - *Happia* sp1	**1**			
**Filo Chordata**				
**Ordem Anura**				
Pristimantis fenestratus	**5**	**0,09**		
**Ordem Chiroptera**				
Peropteryx sp.	**2**	**0,03**	**2**	**0,03**
