# Supplementary material for: Reconciling Mining with the Conservation of Cave Biodiversity: A Quantitative Baseline to Help Establish Conservation Priorities
Source: PLoS One. 2016 Dec 20;11(12):e0168348. doi: 10.1371/journal.pone.0168348 (PMC5173368; doi:10.1371/journal.pone.0168348)
Supplement: S1 Dataset — (ZIP) [file pone.0168348.s002.zip › Taxa/Serra Sul/SS_2012/taxons_112.pdf]

	S11D-112			
	Seco		Úmido	
	col / obs	ab rel	col / obs	ab rel
**Filo Arthropoda**				
**Classe Arachnida**				
**Ordem Amblypygi**				
Heterophrynus sp.	2	0,08		
**Ordem Araneae**				
Fam. Ochyroceratidae				
Ochyroceratidae (jovem)	3		3	
Ochyrocera sp1	1		4	
Speocera sp2	1			
Fam. Pholcidae				
Mesabolivar cambridgei			1	
aff. *Ibityporanga* sp1			1	
Fam. Scytodidae				
Scytodes eleonorae			1	0,06
Fam. Theraphosidae				
Theraphosidae (jovens)	1	0,04		
Fam. Theridiosomatidae				
Theridiosomatidae (jovens)	1			
Plato sp1	1		1	
**Ordem Opiliones**				
Fam. Cosmetidae				
Cosmetidae (jovens)	3	0,13		
Roquetea singularis				
Fam. Stygnidae				
Stygnidae (jovens)			1	0,06
Stygnidae sp1	2	0,08		
**Ordem Pseudoscorpiones**				
Fam. Chernetidae				
Chernetidae (jovens)			2	
Spelaeochernes sp1	2			
Fam. Chthoniidae				
Pseudochthonius sp1	2		2	
**Ordem Ricinulei -** Ricinoididae (jovens)			1	
**Ordem Schizomida**				
Hubbardiidae - *Rowlandius* sp1			1	
**Classe Hexapoda**				
**Ordem Blattodea**				
Fam. Blaberidae (jovens)	2	0,08	1	0,06
Fam. Blattellidae				
Blattellidae sp4	1	0,04		
**Ordem Coleoptera**				
Fam. Curculionidae				
Scolytinae sp2	1			
Fam. Staphylinidae				
Staphylinidae sp6			1	
**Ordem Collembola**				
Fam. Paronellidae				
Paronellidae sp1	4			
**Ordem Diptera**				
Fam. Cecidomyiidae			1	
Fam. Phoridae			1	
Fam. Psychodidae - Phlebotominae sp.	1		5	
Fam. Tipulidae	5		2	
Diptera (larvas)			2	
**Ordem Hemiptera**				
Subordem Homoptera				

Superfam. Coccoidea (jovem)			**3**	
Fam. Cixiidae				
Cixiidae (jovem)	**1**			
Fam. Lygaeidae				
Lygaeidae sp8			**1**	
Fam. Reduviidae				
Subfam. Reduviinae (jovens)	**1**	**0,04**	**1**	**0,06**
Fam. Schizopteridae				
Schizopteridae sp1	**1**			
**Ordem Hymenoptera**				
Fam. Formicidae				
Acanthognathus sp1	**1**			
Acropyga sp1	**4**		**1**	
Camponotus sp1	**1**		**1**	
Carebara sp1	**1**			
Strumygenys sp1			**1**	
Trachymyrmex sp1			**1**	
**Ordem Lepidoptera**				
Superfam. Noctuoidea				
Noctuoidea sp5			**1**	
Noctuoidea sp9			**1**	**0,06**
**Ordem Orthoptera**				
Fam. Phalangopsidae				
Paraclodes sp1	**7**	**0,29**		
Phalangopsis sp1			**10**	**0,63**
**Ordem Psocoptera**				
Subordem Psocomorpha				
Psocomorpha (jovens)			**2**	
**Ordem Thysanoptera** - Phlaeothripidae sp2	**3**	**0,13**		
**Diplopoda**				
Ordem Polyxenida - Hypogexenidae sp1			**1**	
**Filo Mollusca - Gastropoda**				
Fam. Systrophiidae - *Happia* sp1			**1**	
**Filo Chordata**				
**Ordem Anura**				
Pristimantis fenestratus	**2**	**0,08**		
**Ordem Chiroptera**				
Glossophaga soricina			**1**	**0,07**
