# Supplementary material for: Reconciling Mining with the Conservation of Cave Biodiversity: A Quantitative Baseline to Help Establish Conservation Priorities
Source: PLoS One. 2016 Dec 20;11(12):e0168348. doi: 10.1371/journal.pone.0168348 (PMC5173368; doi:10.1371/journal.pone.0168348)
Supplement: S1 Dataset — (ZIP) [file pone.0168348.s002.zip › Taxa/Serra Sul/SS_2012/taxons_113.pdf]

	S11D-113			
	Seco		Úmido	
	col / obs	ab rel	col / obs	ab rel
**Filo Arthropoda**				
**Classe Arachnida**				
Fam. Oonopidae				
Oonopidae sp1			1	
Fam. Prodidomidae (jovens)			3	
Fam. Scytodidae				
Scytodidae (jovens)	1	0,13		
Fam. Theraphosidae				
Theraphosidae (jovens)	3	0,37	5	0,19
**Ordem Opiliones**				
Fam. Stygnidae				
Stygnidae sp1	1	0,13		
**Ordem Pseudoscorpiones**				
Fam. Chthoniidae				
Chthoniidae (jovem)			1	
**Classe Hexapoda**				
**Ordem Blattodea**				
Fam. Polyphagidae				
Polyphagidae sp2			1	
**Ordem Diptera**				
Diptera (larvas)			2	
**Ordem Hemiptera**				
Subordem Homoptera				
Fam. Cixiidae				
Cixiidae (jovem)	1			
Subordem Heteroptera				
Fam. Cydnidae				
Cydnidae (jovens)			1	
**Ordem Hymenoptera**				
Fam. Formicidae				
Camponotus sp1	1		1	
Pheidole sp2			1	
Solenopsis sp1			1	
**Ordem Isoptera**				
Fam. Termitidae				
Embiratermes sp			2	
**Ordem Lepidoptera**				
Lepidoptera sp	3	0,37		
Superfam. Noctuoidea				
Noctuoidea sp9			2	0,07
Lepidoptera (larvas)			3	
**Ordem Psocoptera**				
Subordem Psocomorpha				
Psocomorpha (jovens)	2			
**Ordem Thysanura**				
Nicoletiidae sp1	1			
**Classe Crustacea**				
**Ordem Isopoda**				
Fam. Dubioniscidae - Dubioniscidae sp1			1	
**Filo Chordata**				
**Ordem Chiroptera**				
Carollia perspicillata			20	0,74
