# Supplementary material for: Reconciling Mining with the Conservation of Cave Biodiversity: A Quantitative Baseline to Help Establish Conservation Priorities
Source: PLoS One. 2016 Dec 20;11(12):e0168348. doi: 10.1371/journal.pone.0168348 (PMC5173368; doi:10.1371/journal.pone.0168348)
Supplement: S1 Dataset — (ZIP) [file pone.0168348.s002.zip › Taxa/Serra Sul/SS_2012/taxons_114.pdf]

	S11D-114			
	Seco		Úmido	
	col / obs	ab rel	col / obs	ab rel
**Filo Arthropoda**				
**Classe Arachnida**				
**Acari**				
O. Ixodida				
Fam. Ixodidae - *Amblyomma* sp	**3**			
O. Sarcoptiforme				
Oribatida sp2			**1**	
Galumnidae sp1	**5**			
**Ordem Amblypygi**				
Heterophrynus sp.	**1**	**0,01**	**3**	**0,04**
**Ordem Araneae**				
Fam. Araneidae				
Alpaida sp1			**1**	
Fam. Ochyroceratidae				
Ochyroceratidae (jovem)	**2**		**5**	
Ochyrocera sp1			**2**	
Speocera sp1			**3**	
Fam. Oonopidae				
Oonopidae (jovem)	**1**		**1**	
Fam. Pholcidae				
Pholcidae (jovens)	**1**		**2**	
Mesabolivar cambridgei			**1**	
Fam. Scytodidae				
Scytodidae (jovens)	**1**	**0,01**	**3**	**0,04**
Fam. Uloboridae				
Uloborus sp1	**1**			
**Ordem Opiliones**				
Fam. Stygnidae				
Stygnidae sp1	**2**	**0,03**	**1**	**0,01**
**Ordem Pseudoscorpiones**				
Fam. Chernetidae				
Spelaeochernes sp1	**3**			
Fam. Chthoniidae				
Chthoniidae (jovem)	**1**			
Pseudochthonius sp1	**1**		**2**	
Fam. Olpiidae				
Olpiidae sp1			**4**	
**Classe Hexapoda**				
**Ordem Blattodea**				
Fam. Blaberidae (jovens)			**1**	**0,01**
**Ordem Coleoptera**				
Fam. Chrysomelidae				
Chrysomelidae sp19			**1**	
Fam. Curculionidae				
Scolytinae sp3	**2**			
Superfamília Tenebrionoidea				
Tenebrionoidea sp1			**1**	**0,01**
Tenebrionoidea sp2			**1**	
**Ordem Diplura**				
Fam. Campodeidae - Campodeidae sp1	**4**		**2**	
**Ordem Diptera**				
Fam. Dolichopodidae	**2**			
Fam. Psychodidae - Phlebotominae sp.	**1**		**2**	
Fam. Tipulidae			**1**	
Diptera (larvas)			**2**	
**Ordem Hemiptera**				

Subordem Homoptera				
Fam. Cixiidae				
Cixiidae (jovem)	**2**		**1**	
Subordem Heteroptera				
Fam. Cydnidae				
Cydninae sp1			**1**	
Fam. Lygaeidae				
Lygaeidae sp7	**1**			
**Ordem Hymenoptera**				
Fam. Formicidae				
Apterostigma sp1			**3**	
Camponotus sp1	**1**		**2**	
Camponotus atriceps	**2**			
Gnamptogenys striatula	**1**			
Pachycondyla constricta			**1**	**0,01**
**Ordem Lepidoptera**				
Superfam. Gelechioidea sp1			**1**	
Superfam. Noctuoidea				
Noctuoidea sp3	**1**		**1**	
Noctuoidea sp9			**1**	**0,01**
Lepidoptera (larvas)			**1**	
**Ordem Orthoptera**				
Fam. Phalangopsidae				
Paraclodes sp1	**4**	**0,05**		
Phalangopsis sp1	**55**	**0,71**	**68**	**0,82**
**Ordem Psocoptera**				
Subordem Psocomorpha				
Psocomorpha (jovens)			**1**	
Fam. Ptiloneuridae - *Triplocania* sp7	**1**			
**Chilopoda**				
Ordem Scutigeromorpha - Fam. Pselliodidae	**1**			
**Diplopoda**				
Ordem Glomeridesmida - Glomeridesmidae sp1			**2**	
Ordem Spirostreptida - Fam. Pseudonannolenidae				
Pseudonannolene sp6			**1**	**0,01**
**Filo Mollusca - Gastropoda**				
Gastropoda (jovem)	**1**			
**Filo Chordata**				
**Ordem Anura**				
Pristimantis fenestratus	**3**	**0,04**		
**Ordem Chiroptera**				
Glossophaga soricina	**5**	**0,06**	**3**	**0,04**
Peropteryx sp.	**6**	**0,08**		
