# Supplementary material for: Reconciling Mining with the Conservation of Cave Biodiversity: A Quantitative Baseline to Help Establish Conservation Priorities
Source: PLoS One. 2016 Dec 20;11(12):e0168348. doi: 10.1371/journal.pone.0168348 (PMC5173368; doi:10.1371/journal.pone.0168348)
Supplement: S1 Dataset — (ZIP) [file pone.0168348.s002.zip › Taxa/Serra Sul/SS_2012/taxons_115.pdf]

	S11D-115			
	Seco		Úmido	
	col / obs	ab rel	col / obs	ab rel
**Filo Arthropoda**				
**Classe Arachnida**				
**Ordem Araneae**				
Fam. Pholcidae				
Pholcidae (jovens)	**2**		**1**	
Ninetinae sp1			**7**	
Mesabolivar cambridgei	**1**		**1**	
Fam. Scytodidae				
Scytodes eleonorae			**1**	**0,04**
Fam. Theraphosidae				
Theraphosidae (jovens)	**1**	**0,07**		
Fam. Theridiidae				
Theridiidae (jovens)			**1**	
Theridion sp1			**1**	
**Ordem Opiliones**				
Fam. Stygnidae				
Stygnidae (jovens)			**1**	**0,04**
**Ordem Pseudoscorpiones**				
Fam. Chernetidae				
Chernetidae (jovens)			**1**	
Fam. Chthoniidae				
Pseudochthonius sp1			**1**	
Fam. Olpiidae				
Olpiidae (jovens)			**1**	
Olpiidae sp1			**1**	
**Classe Hexapoda**				
**Ordem Blattodea**				
Fam. Blaberidae (jovens)			**3**	**0,12**
Fam. Blattidae				
Blattidae (jovens)	**1**	**0,07**	**2**	**0,08**
**Ordem Collembola**				
Fam. Entomobryidae				
Entomobryidae sp6	**1**			
Fam. Paronellidae				
Paronellidae sp1			**1**	
**Ordem Diptera**				
Fam. Dolichopodidae	**1**			
Fam. Psychodidae - Phlebotominae sp.			**1**	
Fam. Streblidae - *Trichobius* sp1	**1**			
Fam. Tabanidae			**1**	
Fam. Tipulidae			**1**	
**Ordem Hemiptera**				
Fam. Reduviidae				
Subfam. Reduviinae (jovens)	**1**	**0,07**		
Zelurus sp1			**1**	**0,04**
**Ordem Hymenoptera**				
Fam. Formicidae				
Apterostigma sp1			**1**	
Camponotus sp1	**3**		**4**	
Camponotus atriceps	**2**			
Odontomachus sp1	**1**	**0,07**		
Pachycondyla constricta			**1**	**0,04**
**Ordem Isoptera**				
Fam. Termitidae				
Embiratermes sp			**1**	
Nasutitermes sp	**2**		**7**	

**Ordem Lepidoptera**				
Superfam. Noctuoidea				
Noctuoidea sp9			**3**	**0,12**
**Ordem Neuroptera**				
Fam. Myrmeleontidae				
Myrmeleontidae (jovens)	**1**		**1**	
**Ordem Orthoptera**				
Fam. Phalangopsidae				
Phalangopsis sp1			**7**	**0,28**
**Filo Chordata**				
**Ordem Anura**				
Pristimantis fenestratus	**1**	**0,07**		
**Ordem Squamata** - *Thecadactylus rapicauda*			**1**	**0,04**
**Ordem Chiroptera**				
Glossophaga soricina	**3**	**0,2**		
Peropteryx sp.	**7**	**0,47**	**5**	**0,2**
