# Supplementary material for: Reconciling Mining with the Conservation of Cave Biodiversity: A Quantitative Baseline to Help Establish Conservation Priorities
Source: PLoS One. 2016 Dec 20;11(12):e0168348. doi: 10.1371/journal.pone.0168348 (PMC5173368; doi:10.1371/journal.pone.0168348)
Supplement: S1 Dataset — (ZIP) [file pone.0168348.s002.zip › Taxa/Serra Sul/SS_2012/taxons_116.pdf]

| | S11D-116 | | | |
|---|---|---|---|---|
| | Seco | | Úmido | |
| | col / obs | ab rel | col / obs | ab rel |
| **Filo Arthropoda** | | | | |
| **Classe Arachnida** | | | | |
| **Acari** | | | | |
| O. Mesostigmata | | | | |
| Uropodina sp2 | | | **2** | |
| Macronyssidae sp1 | | | **1** | |
| O. Sarcoptiforme | | | | |
| Oribatida sp22 | | | **6** | |
| Galumnidae sp1 | **3** | | **19** | |
| O. Trombidiforme | | | | |
| Parasitengonina sp1 | **2** | | | |
| Bdellidae sp1 | | | **1** | |
| Rhagidiidae sp1 | **1** | | | |
| **Ordem Amblypygi** | | | | |
| *Heterophrynus* sp. | **1** | **0,01** | **2** | **0,01** |
| **Ordem Araneae** | | | | |
| Fam. Corinnidae | | | | |
| Corinnidae (jovens) | | | **1** | **0,004** |
| *Creugas* sp1 | | | **4** | **0,02** |
| Fam. Ochyroceratidae | | | | |
| Ochyroceratidae (jovem) | **1** | | | |
| *Speocera* sp1 | **3** | | **1** | |
| Fam. Oonopidae | | | | |
| Oonopidae (jovem) | **5** | | **1** | |
| gr. *Xycarphius sp1* | **1** | | **1** | |
| Fam. Theridiidae | | | | |
| Theridiidae (jovens) | **1** | | | |
| Fam. Theridiosomatidae | | | | |
| Theridiosomatidae (jovens) | **2** | | | |
| *Plato* sp1 | **3** | | **1** | |
| **Ordem Opiliones** | | | | |
| Fam. Stygnidae | | | | |
| Stygnidae (jovens)<br>Stygnidae sp1 | **3** | **0,02** | **2** | **0,01** |
| **Ordem Pseudoscorpiones** | | | | |
| Fam. Olpiidae | | | | |
| Olpiidae sp1 | | | **1** | |
| **Ordem Schizomida** | | | | |
| Hubbardiidae (jovem) | | | **1** | |
| Hubbardiidae - *Rowlandius* sp1 | **1** | | **2** | |
| **Classe Hexapoda** | | | | |
| **Ordem Blattodea** | | | | |
| Blattodea (jovens) | | | **1** | |
| Fam. Polyphagidae | | | | |
| Polyphagidae (jovem) | **1** | | | |
| **Ordem Coleoptera** | | | | |
| Fam. Scydmaenidae | | | | |
| Scydmaenidae sp5 | | | **1** | |
| Scydmaenidae sp12 | | | **1** | |
| Fam. Staphylinidae | | | | |
| Staphylinidae sp9 | **3** | | | |
| Staphylinidae sp11 | | | **2** | |
| Staphylinidae sp52 | **1** | | **2** | |
| Coleoptera (larvas) | **1** | | **1** | |
| **Ordem Collembola** | | | | |
| Fam. Cyphoderidae - Cyphoderidae sp2 | | | **11** | |

| | | | | |
|---|---|---|---|---|
| Fam. Isotomidae - Isotomidae sp1 | **1** | | | |
| Fam. Paronellidae | | | | |
| Paronellidae sp1 | **2** | | **1** | |
| **Ordem Diplura** | | | | |
| Fam. Campodeidae - Campodeidae sp1 | **8** | | **8** | |
| **Ordem Diptera** | | | | |
| Fam. Drosophilidae - *Drosophila eleonorae* | **1** | | | |
| Fam. Keroplatidae - *Keroplatus* sp1 | **2** | | | |
| Fam. Psychodidae - Phlebotominae sp. | **1** | | **2** | |
| Fam. Tipulidae | **2** | | **1** | |
| Diptera (larvas) | **10** | | **4** | |
| **Ordem Hemiptera** | | | | |
| Subordem Heteroptera | | | | |
| Fam. Cydnidae | | | | |
| Cydnidae (jovens) | **6** | | **4** | |
| Cydninae sp1 | **6** | | **4** | |
| **Ordem Hymenoptera** | | | | |
| Fam. Formicidae | | | | |
| *Camponotus atriceps* | **5** | | **1** | |
| *Gnamptogenys striatula* | | | **5** | |
| *Solenopsis* sp1 | **1** | | | |
| **Ordem Lepidoptera** | | | | |
| Superfam. Noctuoidea | | | | |
| Noctuoidea sp3 | **1** | | **1** | |
| Lepidoptera (larvas) | | | **1** | |
| **Ordem Orthoptera** | | | | |
| Fam. Phalangopsidae | | | | |
| *Phalangopsis* sp1 | **120** | **0,75** | **150** | **0,68** |
| **Ordem Thysanura** | | | | |
| Nicoletiidae sp1 | **3** | | **1** | |
| **Chilopoda** | | | | |
| Ordem Geophilomorpha - Geophilidae sp1 | | | **1** | |
| Ordem Scolopendromorpha | | | | |
| *Dinocryptops miersii* | | | **1** | **0,004** |
| *Newportia* sp1 | | | **1** | **0,004** |
| *Tidops* sp1 | | | **1** | **0,004** |
| **Diplopoda** | | | | |
| Ordem Polydesmida | | | | |
| Fam. Chelodesmidae - Chelodesmidae sp4 | | | **1** | **0,004** |
| Fam. Fuhrmanodesmidae | | | | |
| Fuhrmanodesmidae (jovem) | **2** | | **1** | |
| Fuhrmanodesmidae sp6 | | | **2** | |
| Ordem Spirostreptida - Fam. Pseudonannolenidae | | | | |
| Pseudonannolenidae (jovem) | **3** | | | |
| *Pseudonannolene* sp6 | | | **15** | **0,07** |
| **Classe Crustacea** | | | | |
| **Ordem Isopoda** | | | | |
| Fam. Philosciidae - Philosciidae sp1 | **7** | | **8** | |
| **Filo Mollusca - Gastropoda** | | | | |
| Fam. Subulinidae - *Lamellaxis* sp.1 | | | **1** | |
| Fam. Systrophiidae - *Happia* sp1 | **3** | | | |
| **Filo Chordata** | | | | |
| **Ordem Anura** | | | | |
| *Pristimantis fenestratus* | **4** | **0,02** | **2** | **0,01** |
| **Ordem Chiroptera** | | | | |
| *Carollia perspicillata* | **30** | **0,19** | **40** | **0,18** |
| **Ordem Rodentia** | | | **1** | **0,004** |
