# Supplementary material for: Reconciling Mining with the Conservation of Cave Biodiversity: A Quantitative Baseline to Help Establish Conservation Priorities
Source: PLoS One. 2016 Dec 20;11(12):e0168348. doi: 10.1371/journal.pone.0168348 (PMC5173368; doi:10.1371/journal.pone.0168348)
Supplement: S1 Dataset — (ZIP) [file pone.0168348.s002.zip › Taxa/Serra Sul/SS_2010/CAV_01.pdf]

CAV-01			1ª	AB	2ª	AB	ZON
Arthropoda							
Arachnida							
Acari							
Macronyssidae		sp.1	1				P
Otopheidomenidae		sp.1	1				P
Podocinidae		sp.1			1		P
Mesostigmata		sp.1	1				P
Opilioacaridae		sp.1	1				P
Oribatida		sp.13	1				P
Anoetidae		sp.2	1				P
Anystidae							
	Erythracarus	*nasutus*			1		P
Rhagidiidae		sp.2			1		E
Tydeidae		sp.1	1				P
		sp.4			1		E
		sp.6	2				E P
Amblypygi							
Charinidae							
	Charinus	sp.2	1	0,012			P
Phrynidae							
	Heterophrynus	sp.1	8	0,098	5	0,074	P
Araneae							
Corinnidae		jovens	2	0,037			P
	Creugas	sp.1	1				P
Ctenidae							
	Ctenus	sp.1	1	0,012	1	0,015	P
Ochyroceratidae		jovens	1		1		P
	Ochyrocera	sp.1	2		1		P
	Speocera	sp.1	1				P
Salticidae		jovens					
	Amphidraus	sp.1			1		E
Scytodidae		jovens	1	0,012			P
	Scytodes	sp.			3	0,044	P
Tetrablemmidae		jovens	1				P
	Matta	sp.1			2		P
Theraphosidae		jovens	2	0,025			P
Theridiidae		jovens			1		P
	Theridion	sp.1	2				P
Theridiosomatidae		jovens					
	Plato	sp.1	3		3		E P
Opiliones							
Neogoveidae	*Canga*	*renatae*					
Sclerosomatidae		jovens			1		P
Cosmetidae		jovens			1	0,015	P
Stygnidae		jovens			1	0,073	P
		sp.1	1	0,012	4		E P
Pseudoscorpiones							
Chernetidae		sp.2	2				P
	Spelaeochernes	sp.1	3		3		E P
Chilopoda							
Pleurostigmophora							
Scolopocryptopidae		jovens	2	0,025			E
Diplopoda		jovens			4	0,059	P
Polydesmida							
Fuhrmannodesmidae		sp.1	1				E
Pyrgodesmidae		sp.2	2	0,025			P
Spirostreptida							
	Pseudonannolene	sp.1			1	0,015	P
Spirostreptidae		jovens	2				P
Entognatha							
Diplura							

Campodeidae	sp.1	1					P
Insecta							
Blattodea		10	0,123				P
Blaberidae	jovens	3	0,037	2	0,029		P
Blattellidae	sp.2	1	0,012				P
Polyphagidae	jovens	1	0,025				P
	sp.1	1					P
Coleoptera							
Scydmaenidae	sp.7	1					P
Staphylinidae	sp.32			1		E	
Trogidae	sp.			1			P
Collembola							
Isotomidae	sp.1	1					P
Paronellidae	sp.1	1					P
	sp.4	3				E	P
	sp.6			2		E	P
Diptera							
Dolichopodidae	sp.			2		E	P
Phoridae							
Metopininae	sp.	2					P
Phorinae	sp.	1		1		E	P
Mycetophilidae							
Macrocera	sp.	1					P
Psychodidae							
Pintomyia	*gruta*	1					P
Sciopemyia	*sordellii*	2		3		E	P
Sciaridae	sp.	1				E	
Tipulidae		2					P
Tipulinae	sp.	1		1		E	P
Hemiptera							
Dipsocoroidea	jovens	1					P
Reduviidae							
Reduviinae	sp.	2	0,025	7	0,103		P
Schizopteridae							
Schizopterinae	sp.1			2			P
	sp.3			1		E	
Cixiidae	jovens	1					P
Hymenoptera							
Formicidae							
Apterostigma	sp.1			1		E	
Camponotus	sp.1	2		2		E	P
Pachycondyla	*striata*	3				E	P
Isoptera							
Termitidae							
Nasutitermes	sp.	3		2			P
Lepidoptera							
Noctuidae	jovens	2	0,025	1	0,015	E	
Orthoptera	jovens	1		1			P
Phalangopsidae							
Paraclodes	sp.1	4	0,049	3	0,044	E	
Phalangopsis	sp.1	35	0,432	30	0,441		P
Thysanura							
Ateluridae	sp.1	1					P
Nicoletiidae	jovens	1					P
	sp.1	2		2		E	P
Malacostraca							
Isopoda							
Dubioniscidae	sp.1	1		1			P
Philosciidae	sp.1	1					P
Chordata							
Amphibia							
Anura							

Leptodactylidae					
Leptodactylus sp.	1	0,012	4	0,059	E P
Strabomantidae					
Pristimantis fenestratus			1	0,015	P
Mollusca					
Gastropoda					
Systrophiidae					
Happia sp.	1				P
