# Supplementary material for: Reconciling Mining with the Conservation of Cave Biodiversity: A Quantitative Baseline to Help Establish Conservation Priorities
Source: PLoS One. 2016 Dec 20;11(12):e0168348. doi: 10.1371/journal.pone.0168348 (PMC5173368; doi:10.1371/journal.pone.0168348)
Supplement: S1 Dataset — (ZIP) [file pone.0168348.s002.zip › Taxa/Serra Sul/SS_2010/CAV_02.pdf]

CAV-02		1ª	AB	2ª	AB	ZON
Arthropoda						
Arachnida						
Acari						
Ixodida						
Mesostigmata	sp.4	1				E
Rhagidiidae	sp.1	1				E
Araneae		4	0,286			
Araneidae	jovens	1				E
Alpaida	*negro*			1		E
	sp.2	1				E
Ochyroceratidae	jovens	1				E
Pholcidae	jovens	1		1		E
Salticidae						
Freya	sp.1			1		E
Scytodidae	jovens			1		E
Scytodes	*eleonorae*	1	0,071			E
Theraphosidae	jovens			1	0,100	E
Theridiosomatidae	jovens	1		1		E
Plato	sp.1	1				E
Opiliones						
Stygnidae	sp.1	1	0,071			E
Pseudoscorpiones						
Chernetidae						E
Spelaeochernes	sp.1	1				E
Chthonidae						E
Pseudochthonius	sp.1			1		E
Coleoptera	jovens	1				E
Collembola						
Entomobryidae	sp.10			1		E
Paronellidae	sp.1	1		1		E
	sp.6			1		E
Diptera						
Ceratopogonidae	sp.			1		E
Sciopemyia	*sordellii*	1		1		E
Hemiptera						
Cixiidae	jovens	1		1		E
Hymenoptera						
Formicidae						
Dolichoderus	*bispinosus*			1		E
Octostruma	sp.1	1				E
Isoptera						
Termitidae						
Nasutitermes	sp.	1				E
Orthoptera						
Phalangopsidae	jovens	1	0,285			E
Paraclodes	sp.	3		3	0,300	E
Psocoptera						
Asiopsocidae						
Asiopsocus	sp.1	1				E
Epipsocidae	jovens	1				E
Epipsocus	sp.2	1				E
	jovens			1		E
Chordata						
Amphibia						
Anura						
Strabomantidae						
Pristimantis	*fenestratus*	1	0,071	2	0,200	E
Mammalia						
Chiroptera						
Emballonuridae						
Peropteryx	sp.	1	0,071			E
Phyllostomidae						E
Glossophaginae	sp.	1	0,071	4	0,400	E
Reptilia						
Squamata						
Gekkonidae						
Thecadactylus	*rapicauda*	1	0,071			E
