# Supplementary material for: Reconciling Mining with the Conservation of Cave Biodiversity: A Quantitative Baseline to Help Establish Conservation Priorities
Source: PLoS One. 2016 Dec 20;11(12):e0168348. doi: 10.1371/journal.pone.0168348 (PMC5173368; doi:10.1371/journal.pone.0168348)
Supplement: S1 Dataset — (ZIP) [file pone.0168348.s002.zip › Taxa/Serra Sul/SS_2010/CAV_03.pdf]

CAV-03		1ª	AB	2ª	AB	ZON
Arthropoda						
Arachnida						
Acari						
Parasitiformes						
Mesostigmata	sp.2			1		E
Opilioacarida						
Opilioacaridae	sp.1	2		1		E
Trombidiformes						
Scutacaridae	sp.1			1		E
Amblypygi						
Phrynidae						
Heterophrynus	sp.	4	0,093			E
Araneae						
Araneidae						
Alpaida	*septemmammata*	2		1		E
Ochyroceratidae						
Ochyrocera	sp.1	2				E
Speocera	sp.1	1				E
Pholcidae						
Mesabolivar	sp.1	1				E
Ninetinae	sp.1	2				E
Salticidae						
Amphidraus	sp.1	1				E
Scytodidae	jovens	3				E
Theraphosidae	jovens			1	0,143	E
aff. *Holothele*	sp.1	1	0,023			E
Theridiosomatidae						
Plato	sp.1	3				E
Opiliones						
Laniatores						
Stygnidae	sp.1	1	0,023			E
Pseudoscorpiones						
Bochicidae	sp.1	2				E
Spelaeochernes	sp.1	1				E
Schizomida						
Hubbardiidae						
Rowlandius	sp.	1				E
Chilopoda						E
Geophilomorpha						
Geophilidae	sp.1	1	0,023		0,286	E
Diplopoda						E
Polyxenidae					0,143	
Hypogexenidae	sp.1	1				E
Entognatha						E
Diplura						
Campodeidae	sp.1	2				E
Insecta						E
Blattodea	jovens			2	0,429	E
Blaberidae	jovens	1	0,023	1		E
Coleoptera						
Endomychidae	sp.1	1				E
Staphylinidae						
Pselaphinae	sp.9	1				E
Collembola						
Arthropleona						
Entomobryoidea						
Entomobryidae	sp.1	1				E
	sp.6			1		E
Paronellidae	sp.4	1				E
Diptera						
Nematocera						
Cecidomyiidae						
Cecidomyiinae	sp.			1		E
Chironomidae	sp.	1				E
Culicidae						
Culicini	sp.	1				E

Mycetophilidae						
Lygistorrhina	sp.			1		E
Psychodidae						
Sciopemyia	*sordellii*	3				E
Sciaridae						
Bradysia	sp.	1				E
Tipulidae	jovens	1				E
Hemiptera						
Heteroptera						
Pyrrhocoroidea	jovens			1		E
Homoptera						
Cixiidae	sp.4	1				E
	sp.1	1	0,023			E
Hymenoptera						
Ichneumonoidea						
Braconidae	sp.1			1		E
Vespoidea						
Formicidae						
Acromyrmex	sp.1	1		1		E
Apterostigma	sp.1	2		1		E
Crematogaster	sp.1			1		E
Dinoponera	*quadriceps*	1	0,023			E
Hypoponera	sp.1	1				E
Nylanderia	sp.1			1		E
Octostruma	sp.1	1				E
	striata	2		2		E
Wasmania	*auropunctata*	1				E
Isoptera						
Termitidae						
Termes	sp.	1				E
	operários	1		1		E
Lepidoptera						
Tineoidea	jovens			1		E
Orthoptera						
Ensifera						
Phalangopsidae						
Paraclodes	sp.1	11	0,256	2	0,333	E
Phalangopsis	sp.1	12	0,279			E
Psocoptera						
Psocomorpha	jovens	1		2		E
Thysanura						
Ateluridae	jovens	1				E
Symphyla						
Scutigerellidae	jovens	1				E
Chordata						
Amphibia						
Anura						
Neobatrachia						
Strabomantidae						
Pristimantis	*fenestratus*	2	0,047			E
Mammalia						
Chiroptera						
Emballonuridae						
Peropteryx	*kappleri*	2	0,047	1		E
Phyllostomidae						
Glossophaginae	sp.	4	0,093			E
Mollusca						
Gastropoda						
Subulinidae						
Lamellaxis	sp.	1				E
