# Supplementary material for: Reconciling Mining with the Conservation of Cave Biodiversity: A Quantitative Baseline to Help Establish Conservation Priorities
Source: PLoS One. 2016 Dec 20;11(12):e0168348. doi: 10.1371/journal.pone.0168348 (PMC5173368; doi:10.1371/journal.pone.0168348)
Supplement: S1 Dataset — (ZIP) [file pone.0168348.s002.zip › Taxa/Serra Sul/SS_2010/CAV_04.pdf]

CAV-04			1ª	AB	2ª	AB	ZON
Arthropoda							
Arachnida							
Araneae							
	Ctenidae	jovens	1	0,04			E
	Salticidae	jovens	1				E
	Scytodidae	jovens			1		E
	Theridiidae						
	Theridion	sp.1			1		E
Pseudoscorpiones							
	Spelaeochernes	sp.1	1				E
Chilopoda							
Scutigeromorpha							
	Pselliodidae						
	Sphendononema	*guildingii*	1				E
Scolopendromorpha							
	Scolopocryptopidae *Tidops*	sp.1	1	0,04	1	0,09	E
Scutigeromorpha		jovens	1	0,04			E
Insecta							
Blattodea		jovens	2	0,08			E
Coleoptera		jovens			1		E
Collembola							
Arthropleona							
	Paronellidae	sp.1	1				E
		sp.9	1				E
Dermaptera		jovens			1		E
Diptera		jovens			1		E
Brachycera							
	Phoridae						
	Metopininae	sp.			1		E
Nematocera							
	Psychodidae						
	Bruchomyiinae	sp.			1		E
	Phlebotominae	sp.			1		E
	Tipulidae						
	Tipulinae	sp.	1				E
Hemiptera							
Heteroptera							
	Reduviidae						
	Reduviinae	jovens	4	0,16	2	0,18	E
Hymenoptera							
	Formicidae						
	Camponotus	sp.1	1		1		E
	Crematogaster	sp.1			1		E
	Pheidole	sp.2	1				E
Isoptera		sp.	1				E
Lepidoptera							
	Limacodidae	sp.2	1	0,04			E
	Noctuidae	jovens	2	0,08			E
Neuroptera							
	Mantispidae	sp.			1	0,09	E
	Myrmeleonthidae	jovens			1		E
Orthoptera							
Ensifera							
	Phalangopsidae						E
	Paraclodes	sp.	11	0,44	5	0,45	E
Chordata							
Amphibia							
Anura							
Neobatrachia							
	Strabomantidae						
	Pristimantis	*fenestratus*	1	0,04			E
Mammalia							
Chiroptera							
	Emballonuridae						
	Peropteryx	sp.	1	0,04	2	0,18	E
