# Supplementary material for: Reconciling Mining with the Conservation of Cave Biodiversity: A Quantitative Baseline to Help Establish Conservation Priorities
Source: PLoS One. 2016 Dec 20;11(12):e0168348. doi: 10.1371/journal.pone.0168348 (PMC5173368; doi:10.1371/journal.pone.0168348)
Supplement: S1 Dataset — (ZIP) [file pone.0168348.s002.zip › Taxa/Serra Sul/SS_2010/CAV_05.pdf]

CAV-05			1ª	AB	2ª	AB	ZON
Arthropoda							
Arachnida							
Acari							
Sarcoptiformes		sp.2	1				E
Araneae							
Araneidae		jovens	1		1		E P
		sp.2	1				E
Ochyroceratidae		jovens	1				P
Pholcidae							
	Mesabolivar	sp.1	1				E
Scytodidae		jovens	3				E P
	Scytodes	sp.	1	0,0952			
Theridiidae							
	Theridion	sp.1			1		E
Theridiosomatidae							
	Plato	sp.1	2		1		P
Zodariidae							
	Tenedos	sp.1			1	0,0294	E
Opiliones							
Eupnoi							
Sclerosomatidae		jovens			1		P
Laniatores							
Stygnidae		sp.1			1	0,0294	P
Pseudoscorpiones							
	Spelaeochernes	sp.1	2				P
Diplopoda							
Polydesmida							
Chelodesmidae		sp.4	1	0,0238			E
Entognatha							
Diplura							
Campodeidae		sp.1	1				E
Insecta							
Blattodea		jovens	1	0,0238			P
Coleoptera							
Chrysomelidae		jovens	1				P
		sp.13	1				E
Diptera							
Brachycera							
Drosophilidae							
	Drosophila	*eleonore*			1		E
Phoridae							
	Metopininae	sp.	1				P
Nematocera							
Ceratopogonidae		sp.			1		P
Culicidae							
	Culicini	sp.			1		E
Psychodidae							
	Phlebotominae	sp.	1				P
	Pintomyia	*gruta*	1		1		P
	Pintomyia	*sordellii*	1		1		P
Tipulidae		jovens			1		P
	Tipulinae	sp.	2		1		E P
Hemiptera							
Heteroptera							P
Reduviidae		jovens	10	0,238	1	0,0294	E P
Hymenoptera							
Ichneumonoidea							
Braconidae		sp.3	1				E
Vespoidea							
Formicidae							
	Camponotus	sp.1	2		1		E P
	Hypoponera	sp.1	1				E
	Pheidole	sp.2			2		E P
Isoptera							
Termitidae							
	Nasutitermes	sp.	1		1		E P

Lepidoptera							
Cossoidea							
Limacodidae		sp.1	1	0,0238			P
Noctuoidea		sp.2			1		P
Tineoidea	jovens		2				E P
		sp.1			1		P
Neuroptera							
Myrmeleonthidae	jovens				1		E
Orthoptera							
Phalangopsidae							
	Paraclodes	sp.1	5	0,119	6	0,1765	E
	Phalangopsis	sp.1	20	0,4762	25	0,7353	P
Malacostraca							
Isopoda							
Philosciidae		sp.1	1				P
Pauropoda							
Tetramerocerata		sp.	1				P
