# Supplementary material for: Reconciling Mining with the Conservation of Cave Biodiversity: A Quantitative Baseline to Help Establish Conservation Priorities
Source: PLoS One. 2016 Dec 20;11(12):e0168348. doi: 10.1371/journal.pone.0168348 (PMC5173368; doi:10.1371/journal.pone.0168348)
Supplement: S1 Dataset — (ZIP) [file pone.0168348.s002.zip › Taxa/Serra Sul/SS_2010/CAV_06.pdf]

CAV-06		1ª	AB	2ª	AB	ZON
Arthropoda						
Arachnida						
Acari						
Trombidiformes	sp.2	1				E
Amblypygi						
Phrynidae						
Heterophrynus	sp.	1	0,0263	1	0,0159	P
Araneae						
Ochyroceratidae						
Ochyrocera	sp.1			1		E
Speocera	sp.1	1		1		P
Salticidae *Freya*	sp.1			1		E
Scytodidae	jovens	1		1		E P
Theridiosomatidae	jovens			1		E
Plato	sp.1	1		1		E P
Trechaleidae	jovens			1	0,0159	E
Opiliones	jovens					E
Eupnoi						
Sclerosomatidae	sp.1			1		E
Laniatores	jovens					
Cosmetidae						
Roquettea	*singularis*			1	0,0159	E
Stygnidae	sp.1	3	0,0789			E
Pseudoscorpiones						
Spelaeochernes	sp.1	1		1		P
Chthoniidae						
Pseudochthonius	sp.1	2				P
Ricinulei						
Ricinoididae	jovens	1				E
Chilopoda						
Notostigmophora						
Scutigeromorpha						E
Pselliodidae		1				E
Pleurostigmophora						
Scolopendromorpha						
Scolopocryptopidae						
Dinocryptops	*miersii*	1	0,0263			P
Scutigeromorpha	jovens	1	0,0263			P
Diplopoda						
Polydesmida						
Pyrgodesmidae	sp.2	1	0,0263			E
Insecta						
Coleoptera	jovens	1				P
	sp.5			1		P
Diptera						
Brachycera						
Phoridae						
Metopininae	sp.			1		E
Nematocera						
Cecidomyiidae						
Cecidomyiinae	sp.			1		E
Ceratopogonidae	sp.			1		P
Psychodidae						
Sciopemyia	*sordellii*	1		1		P
Tipulidae						
Tipulinae	sp.	1				E
	jovens	2		1		E P
Hemiptera						
Heteroptera						
Hebridae	sp.1	1				E
Homoptera						
Cixiidae	jovens			1		P
Hymenoptera						
Vespoidea						
Formicidae						
Nylanderia	sp.1			2		E P

Lepidoptera							
Noctuoidea							
Noctuidae		sp.2			1		P
		sp.1			1	0,0159	E
Orthoptera							
Phalangopsidae							
	Paraclodes	sp.1			25	0,3968	E
	Phalangopsis	sp.1	30	0,7895	30	0,4762	P
Psocoptera							
Psocomorpha		jovens	1				E
Malacostraca							
Isopoda							
Philosciidae		sp.1	1				P
Chordata							
Amphibia							
Anura							
Neobatrachia							
Leptodactylidae							
	Leptodactylus	sp.			1	0,0159	E
Strabomantidae							
	Pristimantis	*fenestratus*			3	0,0476	P
Mollusca							
Gastropoda							
Subulinidae							
	Lamellaxis	sp.	1				P
