# Supplementary material for: Reconciling Mining with the Conservation of Cave Biodiversity: A Quantitative Baseline to Help Establish Conservation Priorities
Source: PLoS One. 2016 Dec 20;11(12):e0168348. doi: 10.1371/journal.pone.0168348 (PMC5173368; doi:10.1371/journal.pone.0168348)
Supplement: S1 Dataset — (ZIP) [file pone.0168348.s002.zip › Taxa/Serra Sul/SS_2010/CAV_07.pdf]

CAV-07			1ª	AB	2ª	AB	ZON
Annelida							
Clitellata							
Oligochaeta		jovens	1	0,25			E
Arthropoda							
Arachnida							
Acari							
Ixodida							
	Ixodidae						
	Ixodes	sp.	1				E
Sarcoptiformes		sp.1			1		E
Oribatida		sp.2			1		E
Amblypygi							
	Phrynidae						
	Heterophrynus	sp.	1	0,25			E
Araneae							
	Araneidae	jovens	1				E
	Oonopidae						
	gr. Xycarphius	sp.3			1		E
	gr. Xycarphius	sp.5			1		E
	Pholcidae	jovens	1				E
	Salticidae	jovens	1				E
	Scytodidae	jovens			1		E
	Theridiosomatidae	jovens	1				E
Pseudoscorpiones							
	Bochicidae	sp.1	2				E
	Spelaeochernes	sp.1	1				E
Chilopoda							
Pleurostigmophora							
Scolopendromorpha							
	Scolopocryptopidae						
	Newportia	sp.1			1		E
Entognatha							
Diplura							
	Campodeidae	sp.1			1		E
Insecta							
Blattodea		jovens	1	0,25			E
Coleoptera							
	Curculionidae						
	Scolytinae	sp.2	1				E
	Staphylinidae						
	Pselaphinae	sp.1	1				E
Collembola							
Arthropleona							
Entomobryoidea							
	Paronellidae	sp.1	1		1		E
Diptera							
Brachycera							
	Camillidae	sp.			1		E
Hemiptera							
Heteroptera							
aff. Pyrrhocoroidea							
	Cydnidae						
	Cydninae	sp.1			1		E
	Hebridae	sp.1	1				1
Hymenoptera							
Vespoidea							
	Formicidae						
	Solenopsis	sp.2			1		E
Lepidoptera							
Castnioidea							
	Castniidae	jovens	1		1		E
Orthoptera							
Ensifera							
	Phalangopsidae						
	Paraclodes	sp.1			1	0,1667	E
Psocoptera							

Psocomorpha								
	Epipsocidae							
		Epipsocus	sp.2	1				E
		jovens		1		2		E
Thysanura								
	Nicoletiidae		sp.1			1		E
Chordata								
Amphibia								
Anura								
Neobatrachia								
	Leptodactylidae							
		Leptodactylus	sp.	1	0,25			E
Mammalia								
Chiroptera								
	Phyllostomidae	*Carollia*	sp.			5	0,8333	E
