# Supplementary material for: Reconciling Mining with the Conservation of Cave Biodiversity: A Quantitative Baseline to Help Establish Conservation Priorities
Source: PLoS One. 2016 Dec 20;11(12):e0168348. doi: 10.1371/journal.pone.0168348 (PMC5173368; doi:10.1371/journal.pone.0168348)
Supplement: S1 Dataset — (ZIP) [file pone.0168348.s002.zip › Taxa/Serra Sul/SS_2010/CAV_08.pdf]

CAV-08			1ª	AB	2ª	AB	ZON
Clitellata							
Oligochaeta		sp.1	1	0,0667			E
Arthropoda							
Arachnida							
Acari							
Ixodida							
Ixodidae							
Amblyomma		sp.	1				E
Trombidiformes		sp.5	1				E
Araneae							
Araneidae	jovens		2				E
Caponiidae	jovens		1	0,0667			E
Corinnidae	jovens		1	0,0667			E
Ctenidae	jovens		2	0,1333			E
Cyrtaucheniidae	jovens				1	0,25	E
Mysmenidae	jovens		1				E
Pholcidae	jovens		1				E
aff. Ibityporanga		sp.1			1		E
Mesabolivar		sp.1	1				E
Ninetinae		sp.1			1		E
Salticidae	jovens		2		1		E
Scytodidae	jovens		2				E
Theridiidae	jovens				1		E
Theridion		sp.1	1				E
Theridiosomatidae	jovens		2				E
Opiliones							
Cyphophthalmi							
Neogoveidae							
Canga		*renatae*	1				E
Stygnidae		sp.1	1	0,133			E
Stygnidae	jovens		1				E
Pseudoscorpiones							
Spelaeochernes		sp.1	1				E
Olpiidae		sp.1	2		2		E
Insecta							
Blattodea	jovens		1	0,0667			E
Polyphagidae	jovens				1	0,25	E
Coleoptera							
Staphylinidae		sp.33	1				E
Diptera							
Nematocera							
Psychodidae							
Pintomyia		*gruta*	1				E
Simuliidae							
Simulium		sp.	1				E
Hemiptera							
Heteroptera							
Lygaeidae		sp.5			1		E
Reduviidae	jovens		2	0,2	2	0,5	E
Reduviinae		sp.	1				E
Homoptera							
Cixiidae	jovens		1				E
Hymenoptera							
Vespoidea							
Formicidae							
Acromyrmex		sp.1			1		E
Camponotus		sp.1	2		1		E
Gnamptogenys		*striatula*			1		E
Pheidole		sp.2			1		E
Solenopsis		sp.1	1				E
Isoptera							
Termitidae							
Nasutitermes		sp.	1				E
Neuroptera							
Myrmeleonthidae	jovens		1		2		E
Orthoptera							

Phalangopsidae							
	Paraclodes	sp.1	1	0,0667			E
Psocoptera							
Psocomorpha	jovens		1		1		E
Trogiomorpha							
Lepidopsocidae							
	Psocathropos	sp.1	2		2		E
	Psyllipsocus	sp.1	1		1		E
Thysanoptera							
Thripidae		sp.1			1		E
Malacostraca							
Isopoda							
Dubioniscidae		sp.1	1				E
Chordata							
Mammalia							
Chiroptera							
Emballonuridae							
	Peropteryx	*kappleri*	2	0,1333			E
Reptilia							
Squamata							
Sauria		sp.	1	0,0667			E
