# Supplementary material for: Reconciling Mining with the Conservation of Cave Biodiversity: A Quantitative Baseline to Help Establish Conservation Priorities
Source: PLoS One. 2016 Dec 20;11(12):e0168348. doi: 10.1371/journal.pone.0168348 (PMC5173368; doi:10.1371/journal.pone.0168348)
Supplement: S1 Dataset — (ZIP) [file pone.0168348.s002.zip › Taxa/Serra Sul/SS_2010/CAV_09.pdf]

CAV-09			1ª	AB	2ª	AB	ZON
Annelida							
Clitellata							
Oligochaeta		jovens			1	0,0118	E
Arthropoda							
Arachnida							
Acari							
Ixodida							
Ixodidae							
	Amblyomma	sp.			1		E
Parasitiformes							
Mesostigmata		sp.5	1				P
Laelapidae		sp.1			1		E
		sp.1			2		E P
Sarcoptiformes							
Oribatida							
		sp.4			1		P
Trombidiformes							
Tydeoidea		sp.6			1		E
Cunaxidae		sp.1			1		E
Rhagidiidae		sp.1			1		P
Amblypygi							
Phrynidae							
	Heterophrynus	sp.	1	0,0313			P
Araneae							
Ctenidae							
	Phoneutria	*reidyi*			1	0,0118	E
Pholcidae							
	Mesabolivar	sp.1			1		E
Scytodidae		jovens	1		1		E P
	Scytodes	sp.			3	0,0471	
	Scytodes	*eleonorae*			1		E
Theridiidae							
	Theridion	sp.2			1		E
Theridiosomatidae							
	Plato	sp.1	2		2		E P
Trechaleidae		jovens	1	0,0313			E
Opiliones			3	0,0938			
Eupnoi					21	0,2824	
Sclerosomatidae		jovens	1	0,0313	3		E P
		sp.1	1		2		E
Laniatores		jovens	1	0,062			P
Stygnidae		sp.1	1				E
Coleoptera							
Ptilodactylidae		sp.1	1				E
	Pselaphinae	sp.1			1		E
		sp.3			2		E P
		sp.5			1		E
Collembola							
Arthropleona							
Entomobryoidea							
Entomobryidae		sp.1			1		E
Isotomidae		sp.1			1		E
Symphypleona							
Sminthuroidea		sp.2			1		E
Diptera							
Nematocera							
Ceratopogonidae		sp.	1		1		E P
Chironomidae		sp.			1		E
Mycetophilidae							
	Keroplatus	sp.			1		E
Psychodidae							
	Sciopemyia	*sordellii*			1		E
Sciaridae							
	Bradysia	sp.			2		E P
		jovens	1		3		E P
Hemiptera							

Heteroptera							
Veliidae	jovens				1		E
	Rhagovelia	sp.1	1				E
Vespoidea							
Formicidae							
		sp.1	2		2		E P
		sp.2			1		P
Isoptera							
Termitidae							
	Nasutitermes	sp.			3		E P
Lepidoptera							
Hesperioidea							
Hesperiidae		sp.1			1		E
Noctuoidea		sp.2	1				E
Noctuidae	jovens		3	0,0938			E
Orthoptera							
Caelifera							
Phalangopsidae							
	Paraclodes	sp.1	1	0,0313			E
	Phalangopsis	sp.1	8	0,25	10	0,1176	P
Psocoptera							
Psocomorpha							
Epipsocidae	jovens						
	Epipsocus	sp.2			1		E
Malacostraca							
Decapoda							
Astacidea		sp.			15	0,1765	P
Palaemonidae	jovens		1		1		E
Chordata							
Amphibia							
Anura							
Neobatrachia							
Strabomantidae							
	Pristimantis	*fenestratus*			8	0,0941	P
Mammalia							
Chiroptera							
Furipteridae							
	Furipterus	*horrens*	2	0,0625	5	0,0588	P
Phyllostomidae							
	Carollia	*perspicillata*	6	0,1875	15	0,1765	P
Reptilia							
Squamata							
Gekkonidae							
	Thecadactylus	*rapicauda*			2	0,0235	E
Gymnophthalmidae							
	Neusticurus	sp.	3	0,0938			P
Mollusca							
Gastropoda							
Systrophiidae							
	Happia	sp.	1		1		P
Nemathelminthes		sp.	1	0,0313			E
