# Supplementary material for: Reconciling Mining with the Conservation of Cave Biodiversity: A Quantitative Baseline to Help Establish Conservation Priorities
Source: PLoS One. 2016 Dec 20;11(12):e0168348. doi: 10.1371/journal.pone.0168348 (PMC5173368; doi:10.1371/journal.pone.0168348)
Supplement: S1 Dataset — (ZIP) [file pone.0168348.s002.zip › Taxa/Serra Sul/SS_2010/CAV_10.pdf]

CAV-10		1ª	AB	2ª	AB	ZON
Arthropoda						
Arachnida						
Amblypygi						
Phrynidae						
Heterophrynus	sp.	1	0,04	2	0,0444	P
Araneae						
Ochyroceratidae	jovens	1				P
Scytodidae	jovens	3				P
Theridiosomatidae	jovens	1				P
Plato	sp.1	1		1		P
Opiliones						
Laniatores						
Stygnidae	sp.1	2	0,08	1		P
	jovens			2	0,066	E
Pseudoscorpiones						
Bochicidae	sp.1	2				P
Diplopoda						
Glomeridesmida						
Glomeridesmidae	sp.5			1		P
Insecta						
Blattodea						
Blaberidae	jovens	1	0,04			P
Coleoptera	jovens	1		1		E P
Collembola						
Arthropleona						
Entomobryoidea						
Paronellidae	sp.4			1		P
Diptera						
Nematocera						
Ceratopogonidae	sp.	2				P
Psychodidae						
Sciopemyia	*sordellii*	2		1		P
Tipulidae						
Tipulinae	sp.	1		1		P
Hemiptera						
Heteroptera						
Dipsocoroidea	jovens	1				P
Pyrrhocoroidea						
Reduviidae	jovens	1	0,04	2	0,0444	E P
Homoptera						
Cixiidae	jovens			1		P
Hymenoptera						
Vespoidea						
Formicidae						
Acromyrmex	sp.1			1		P
Brachymyrmex	sp.1			1		E
Camponotus	*atriceps*			1		P
	sp.1	2		1		E P
Crematogaster	sp.1	1				P
	sp.2	1		2		E P
Wasmania	*auropunctata*			1		P
Isoptera						
Termitidae						
Nasutitermes	sp.	2		1		P
Orthoptera						
Ensifera						
Phalangopsidae						
Phalangopsis	sp.1	1	0,04	16	0,3556	P
Paraclodes	sp.1	14	0,56	14	0,3111	E P
Psocoptera						
Psocomorpha	jovens	1		1		E P
Chordata						
Amphibia						
Anura						
Strabomantidae						
Pristimantis	*fenestratus*	1	0,04	1	0,0222	E

Mammalia							
Chiroptera							
	Micronycteris	sp.	4	0,16	7	0,1556	P
							E
