# Supplementary material for: Reconciling Mining with the Conservation of Cave Biodiversity: A Quantitative Baseline to Help Establish Conservation Priorities
Source: PLoS One. 2016 Dec 20;11(12):e0168348. doi: 10.1371/journal.pone.0168348 (PMC5173368; doi:10.1371/journal.pone.0168348)
Supplement: S1 Dataset — (ZIP) [file pone.0168348.s002.zip › Taxa/Serra Sul/SS_2010/CAV_11.pdf]

| CAV-11 | | | 1ª | AB | 2ª | AB | ZON |
|---|---|---|---|---|---|---|---|
| Arthropoda | | | | | | | |
| Arachnida | | | | | | | |
| Acari | | | | | | | |
| Ixodida | | | | | | | |
| Opilioacarida | | | | | | | |
| Opilioacaridae | | sp.1 | | | 1 | | E |
| Amblypygi | | | | | | | |
| Phrynidae | | | | | | | |
| | *Heterophrynus* | sp. | 1 | 0,0909 | | | E P |
| Araneae | | | | | | | |
| Pholcidae | jovens | | | | 1 | | E |
| Tetrablemmidae | jovens | | | | 1 | | E |
| Opiliones | | | | | | | |
| Eupnoi | | | | | | | |
| Sclerosomatidae | jovens | | | | 1 | | E |
| Laniatores | | | | | | | |
| Stygnidae | jovens | | 1 | | | | E P |
| Pseudoscorpiones | | | | | | | |
| Chernetidae | jovens | | | | 2 | | E |
| | *Spelaeochernes* | sp.1 | 1 | | | | E |
| Diplopoda | | | | | | | |
| Polydesmida | | | | | | | |
| Pyrgodesmidae | | sp.4 | 1 | | | | E P |
| Spirostreptida | | | | | | | |
| | *Pseudonannolene* | sp.1 | | | 1 | | E P |
| | jovens | | | | 1 | 0,0833 | E P |
| Insecta | | | | | | | |
| Blattodea | | | | | | | |
| | jovens | | 1 | | | | E P |
| Coleoptera | | | | | | | |
| Staphylinidae | | sp.34 | 1 | | | | E |
| Collembola | | | | | | | |
| Arthropleona | | | | | | | |
| Entomobryoidea | | | | | | | |
| Paronellidae | | sp.1 | | | 1 | | E |
| | | sp.6 | | | 1 | | E |
| Symphypleona | | | | | | | |
| Sminthuroidea | | sp.2 | 1 | | | | E |
| Diptera | | | | | | | |
| Nematocera | | | | | | | |
| Psychodidae | | | | | | | |
| | *Sciopemyia* | *sordellii* | 1 | | 1 | | E |
| Tipulidae | | | | | | | |
| | jovens | | 1 | | 1 | | E |
| Hemiptera | | | | | | | |
| Pyrrhocoroidea | | | | | | | |
| Schizopteridae | | | | | | | |
| | *Schizopterinae* | sp.1 | | | 1 | | E |
| Hymenoptera | | | | | | | |
| Vespoidea | | | | | | | |
| Formicidae | | | | | | | |
| | *Nylanderia* | sp.1 | 1 | | | | E |
| Isoptera | | | | | | | |
| Termitidae | | | | | | | |
| | *Nasutitermes* | sp. | | | 1 | | E |
| Orthoptera | | | | | | | |
| Ensifera | | | | | | | |
| Phalangopsidae | | | | | | | |
| | *Paraclodes* | sp.1 | 7 | 0,6364 | 3 | 0,25 | E P |
| | *Phalangopsis* | sp.1 | 2 | 0,1818 | 8 | 0,6667 | E P |
| Psocoptera | | | | | | | |
| Psocomorpha | jovens | | 1 | | 1 | | E |
| Trogiomorpha | | | | | | | |
| Psyllipsocidae | jovens | | | | 1 | | E |
| Malacostraca | | | | | | | |
| Isopoda | | | | | | | |

| | | | | | | |
|---|---|---|---|---|---|---|
| Dubioniscidae | sp.1 | | | 1 | | E |
| Mammalia | | | | | | |
| Chiroptera | sp. | 1 | 0,0909 | | | E P |
