# Supplementary material for: Reconciling Mining with the Conservation of Cave Biodiversity: A Quantitative Baseline to Help Establish Conservation Priorities
Source: PLoS One. 2016 Dec 20;11(12):e0168348. doi: 10.1371/journal.pone.0168348 (PMC5173368; doi:10.1371/journal.pone.0168348)
Supplement: S1 Dataset — (ZIP) [file pone.0168348.s002.zip › Taxa/Serra Sul/SS_2010/CAV_12.pdf]

| CAV-12 | | 1ª | AB | 2ª | AB | ZON |
|---|---|---|---|---|---|---|
| Arthropoda | | | | | | |
| Arachnida | | | | | | |
| Acari | | | | | | |
| Ixodida | | | | | | |
| Ixodidae | | | | | | |
| *Amblyomma* | sp. | 1 | | 1 | | E |
| Parasitiformes | | | | | | |
| Mesostigmata | | | | | | |
| Ascidae | sp.1 | 1 | | | | P |
| Sarcoptiformes | | | | | | |
| Oribatida | | | | | | |
| Acaridae | sp.1 | 1 | | | | P |
| Trombidiformes | | | | | | |
| Tydeoidea | | | | | | |
| Labdostomatidae | sp.1 | 1 | | | | E |
| Rhagidiidae | sp.1 | 1 | | | | P |
| Rhagidiidae | sp.4 | 1 | | | | P |
| Amblypygi | | | | | | |
| Phrynidae | | | | | | |
| *Heterophrynus* | sp. | 3 | 0,0968 | 3 | 0,0714 | P |
| Araneae | | | | | | |
| Araneidae | jovens | 1 | | | | P |
| Araneidae | sp.2 | | | 1 | | P |
| Mysmenidae | | | | | | |
| *Microdipoena* | sp.1 | | | 1 | | E |
| Ochyroceratidae | | | | | | |
| *Speocera* | sp.1 | 1 | | | | P |
| Oonopidae | | | | | | |
| gr. *Xycarphius* | sp.3 | | | 1 | | P |
| Pholcidae | jovens | 1 | | 2 | | E P |
| Salticidae | jovens | | | 1 | | E |
| Scytodidae | jovens | | | 1 | | E |
| Tetrablemmidae | | | | | | |
| *Matta* | sp.1 | 1 | | 1 | | P |
| Theridiidae | | | | | | |
| *Dipoena* | sp.1 | 1 | | | | P |
| Theridiosomatidae | | | | | | |
| *Plato* | sp.1 | 2 | | 1 | | E P |
| Opiliones | | 3 | 0,0968 | 9 | 0,2143 | |
| Laniatores | | | | | | |
| Cosmetidae | jovens | 1 | 0,0323 | 2 | 0,071 | E P |
| Cosmetidae | sp.4 | | | 1 | | E |
| Stygnidae | sp.1 | 2 | 0,0645 | 2 | 0,0476 | E P |
| Pseudoscorpiones | | | | | | |
| *Spelaeochernes* | sp.1 | 2 | | 1 | | E P |
| *Pseudochthonius* | sp.1 | 1 | | 1 | | E P |
| Chilopoda | | | | | | |
| Notostigmophora | | | | | | |
| Scutigeromorpha | | | | | | |
| Pselliodidae | jovens | | | 1 | | E |
| Pleurostigmophora | | | | | | |
| Geophilomorpha | | | | | | |
| Geophilidae | sp.1 | 1 | 0,0323 | | | E |
| Diplopoda | | | | | | |
| Spirostreptida | | | | | | |
| Pseudonannolenidae | jovens | 1 | 0,0323 | | | E |
| Entognatha | | | | | | |
| Diplura | | | | | | |
| Campodeidae | sp.1 | 1 | | | | P |
| Insecta | | | | | | |
| Coleoptera | | | | | | |
| Scydmaenidae | sp.9 | 1 | | | | P |
| Staphylinidae | jovens | 1 | | 2 | | E P |
| Collembola | | | | | | |
| Arthropleona | | | | | | |
| Entomobryoidea | | | | | | |

| | | | | | | |
|---|---|---|---|---|---|---|
| Entomobryidae | sp.1 | | | 1 | | E |
| Entomobryidae | sp.10 | | | 1 | | P |
| Entomobryidae | sp.6 | | | 1 | | E |
| Entomobryidae | sp.8 | | | 2 | | E P |
| Isotomidae | sp.1 | 1 | | | | E |
| Paronellidae | sp.1 | 1 | | 1 | | E P |
| Paronellidae | sp.4 | 1 | | 1 | | P |
| Paronellidae | sp.6 | | | 1 | | E |
| Diptera | | | | | | |
| Brachycera | | | | | | |
| Sarcophagidae | sp. | | | 1 | | P |
| Streblidae | | | | | | |
| *Trichobius* | sp. | | | 1 | | E |
| Nematocera | | | | | | |
| Ceratopogonidae | sp. | 1 | | | | E |
| Mycetophilidae | | | | | | |
| Sciophilinae | sp. | 1 | | | | E |
| Psychodidae | | | | | | |
| *Pintomyia* | *gruta* | 1 | | | | P |
| *Sciopemyia* | *sordellii* | 1 | | 1 | | P |
| Tipulidae | jovens | 1 | | | | E |
| *Tipulinae* | sp. | 2 | | 1 | | E P |
| Hemiptera | | | | | | |
| Heteroptera | | | | | | |
| Dipsocoroidea | jovens | 1 | | | | P |
| Homoptera | | | | | | |
| Cixiidae | jovens | | | 1 | | P |
| Cixiidae | sp.3 | 1 | | | | P |
| Cixiidae | sp.4 | | | 1 | | P |
| Diaspididae | jovens | | | 1 | | P |
| Hymenoptera | | | | | | |
| Vespoidea | | | | | | |
| Formicidae | | | | | | |
| *Crematogaster* | sp.1 | 1 | | 2 | | E P |
| *Nylanderia* | sp.1 | | | 1 | | E |
| *Pachycondyla* | *constricta* | | | | | |
| *Pachycondyla* | *striata* | 2 | | 1 | | E P |
| *Pheidole* | sp.1 | | | | | |
| *Pheidole* | sp.2 | 1 | | | | P |
| *Solenopsis* | sp.1 | | | | | |
| *Solenopsis* | sp.2 | 1 | | | | P |
| Isoptera | | | | | | |
| Termitidae | | | | | | |
| *Atlantitermes* | sp. | 1 | | | | E |
| *Nasutitermes* | sp. | 2 | | 1 | | E P |
| Lepidoptera | jovens | 1 | | 1 | | P |
| Noctuoidea | | | | | | |
| Noctuidae | jovens | | | 1 | 0,0238 | E |
| Noctuidae | sp.2 | | | 1 | | P |
| Orthoptera | | | | | | |
| Ensifera | | | | | | |
| Phalangopsidae | jovens | | | | | P |
| *Paraclodes* | sp.1 | 15 | 0,4839 | 8 | 0,1905 | E P |
| *Phalangopsis* | sp.1 | 1 | 0,0323 | 7 | 0,1667 | P |
| Psocoptera | | | | | | |
| Psocomorpha | jovens | | | 1 | | P |
| Malacostraca | | | | | | |
| Isopoda | | | | | | |
| Philosciidae | sp.1 | 2 | | 1 | | E P |
| Chordata | | | | | | |
| Amphibia | | | | | | |
| Anura | | | | | | |
| Neobatrachia | | | | | | |
| Strabomantidae | | | | | | |
| *Pristimantis* | *fenestratus* | 1 | 0,0323 | 3 | 0,0714 | E P |
| Mammalia | | | | | | |
| Chiroptera | | | | 5 | 0,119 | |

| | | | | | |
|---|---|---|---|---|---|
| Phyllostomidae | | | | | |
| *Glossophaginae* sp. | 2 | 0,0645 | | | P |
