# Supplementary material for: Reconciling Mining with the Conservation of Cave Biodiversity: A Quantitative Baseline to Help Establish Conservation Priorities
Source: PLoS One. 2016 Dec 20;11(12):e0168348. doi: 10.1371/journal.pone.0168348 (PMC5173368; doi:10.1371/journal.pone.0168348)
Supplement: S1 Dataset — (ZIP) [file pone.0168348.s002.zip › Taxa/Serra Sul/SS_2010/CAV_13.pdf]

CAV-13			1ª	AB	2ª	AB	ZON
Arthropoda							
Arachnida							
Acari							
Sarcoptiformes							
Oribatida		sp.3	1				E
Amblypygi							
Phrynidae							
	Heterophrynus	sp.	2	0,0253			E
Araneae							
Corinnidae	jovens		1	0,0127			E
Ochyroceratidae							
	Ochyrocera	sp.3			1		E
Oonopidae	jovens		1				E
Pholcidae							
	Mesabolivar	sp.1			1		E
Salticidae	jovens		1				E
	Amphidraus	sp.1			1		E
Scytodidae	jovens		2				E
	Scytodes	sp.	6	0,0759			E
Tetrablemmidae	jovens				1		E
Theridiosomatidae	jovens				1		E
Opiliones	jovens		12	0,1519			E
Laniatores							
Cosmetidae	jovens				1	0,0556	E
Stygnidae		sp.1	2	0,0253	1	0,0556	E
Pseudoscorpiones							
Bochicidae		sp.1	2				E
	Spelaeochernes	sp.1	2				E
	Pseudochthonius	sp.1	1				E
Scorpiones							
Buthidae	jovens		1	0,0127			E
Polyxenida							
Hypogexenidae		sp.1			1		E
Entognatha							
Diplura							
Campodeidae		sp.1	1				E
Insecta							
Blattodea	jovens		1	0,0127			E
Coleoptera	jovens		1				E
Staphylinidae		sp.24	1				E
		sp.35	1				E
		sp.36			1		E
Collembola							
Arthropleona							
Entomobryoidea							
Entomobryidae		sp.4	1				E
		sp.6			1		E
Diptera							
Brachycera							
Phoridae							
	Metopininae	sp.	2				E
Nematocera							
Culicidae							
	Wyeomyia	sp.	1				E
Psychodidae		sp.					
	Edentomyia	*piauensis*			1		E
	Pintomyia	*gruta*	1				E
	Sciopemyia	*sordellii*	1				E
Tipulidae							
	Tipulinae	sp.	1		2		E
Hemiptera							
Heteroptera							
aff. Pyrrhocoroidea							
aff. Lygaeidae	jovens		1				E
Reduviidae	jovens		1	0,0127			E
Homoptera							

Cixiidae	jovens		1		2		E
Vespoidea							
Formicidae							
	Nylanderia	sp.1	1		1		E
	Odontomachus	*bauri*	1		2		E
	Wasmania	*auropunctata*			1		E
Isoptera							
Termitidae							
	Nasutitermes	sp.	1		2		E
Lepidoptera	jovens				2		E
Orthoptera							
Ensifera							
Phalangopsidae							
	Paraclodes	sp.1	50	0,6329	15	0,8333	E
	Phalangopsis	sp.1	2	0,0253	1	0,0556	E
Malacostraca							
Isopoda							
Philosciidae		sp.1	1				E
Chordata							
Amphibia							
Anura							
Neobatrachia							
Strabomantidae							
	Pristimantis	*fenestratus*	1	0,0127			E
