# Supplementary material for: Reconciling Mining with the Conservation of Cave Biodiversity: A Quantitative Baseline to Help Establish Conservation Priorities
Source: PLoS One. 2016 Dec 20;11(12):e0168348. doi: 10.1371/journal.pone.0168348 (PMC5173368; doi:10.1371/journal.pone.0168348)
Supplement: S1 Dataset — (ZIP) [file pone.0168348.s002.zip › Taxa/Serra Sul/SS_2010/CAV_14.pdf]

CAV-14			1ª	AB	2ª	AB	ZON
Arthropoda							
Parasitiformes							
Mesostigmata		sp.5	1				E
Sarcoptiformes							
Sarcoptiformes		sp.1	1				E
Araneae							
Ochyroceratidae							
	Ochyrocera	sp.2			1		E
Pholcidae							
	Mesabolivar	sp.1	1		2		E
Scytodidae							
	Scytodes	*globula*			6	0,2	E
Theraphosidae	jovens		1	0,0159			E
Opiliones			5	0,0794			
Laniatores							
Stygnidae	jovens		1	0,031			E
		sp.1	1				E
Insecta							
Blattodea	jovens				2	0,069	E
Coleoptera	jovens		1				E
Carabidae		sp.13			1		E
Carabidae		sp.4			1		E
Diptera							
Phoridae							
	Metopininae	sp.			1		E
	Tipulinae	sp.	1		1		E
Hemiptera							
Homoptera							
Cixiidae	jovens				1		E
Cixiidae		sp.5			1		E
Hymenoptera							
Chalcidoidea		sp.3	1				E
Vespoidea							
Formicidae							
	Carebara	sp.1	1		1		E
	Crematogaster	sp.1	1		2		E
	Cyphomyrmex	sp.1			1		E
	Pheidole	sp.2			1		E
	Solenopsis	sp.1	1				E
Isoptera							
Termitidae							
	Cavitermes	sp.			1		E
	Cortaritermes	*silvestrii*			1		E
	Embiratermes	sp.	1				E
Isoptera	jovens				1		E
Lepidoptera							
Noctuoidea							
	jovens		1				E
Noctuidae							
		sp.2			1	0,0345	E
Orthoptera							
Caeliphera	jovens				2	0,069	E
Ensifera							
Phalangopsidae							
	jovens				1	0,551	E
	Paraclodes	sp.1	50	0,7937	15		E
Psocoptera							
Psocomorpha							
	jovens		1				E
Siphonaptera							
Tungidae							
	Tunga	sp.1			1		E
Chordata							
Amphibia							
Anura							
Neobatrachia							

Strabomantidae								
	Pristimantis	*fenestratus*	4	0,0635	1	0,0345	E
Mammalia								
Chiroptera								
Emballonuridae								
	Peropteryx	*kappleri*			1	0,0345	E
	Glossophaginae	sp.	1	0,0159			E
