# Supplementary material for: Reconciling Mining with the Conservation of Cave Biodiversity: A Quantitative Baseline to Help Establish Conservation Priorities
Source: PLoS One. 2016 Dec 20;11(12):e0168348. doi: 10.1371/journal.pone.0168348 (PMC5173368; doi:10.1371/journal.pone.0168348)
Supplement: S1 Dataset — (ZIP) [file pone.0168348.s002.zip › Taxa/Serra Sul/SS_2010/CAV_15.pdf]

CAV-15			1ª	AB	2ª	AB	ZON
Arthropoda							
Arachnida							
Araneae							
Ctenidae							
	Ctenus	sp.4	1	0,2			E
Scytodidae	jovens		1				E
Segestriidae	jovens				1		E
Tetragnathidae							
	Azilia	*histrio*	1	0,2			E
Theridiidae	jovens		1				E
Opiliones							
Cyphophthalmi							
Neogoveidae							
	Canga	*renatae*	2				E
Insecta							
Blattodea							
Blaberidae	jovens		1	0,2			E
Coleoptera							
Carabidae		sp.4			1		E
Collembola							
Arthropleona							
Entomobryoidea							
Entomobryidae		sp.9			1		E
Paronellidae		sp.1	1				E
Paronellidae		sp.4	1				E
Diptera							
Nematocera							
Ceratopogonidae		sp.			1		E
Tipulidae							
	Tipulinae	sp.	1				E
Hymenoptera							
Vespoidea							
Formicidae							
	Gnamptogenys	*striatula*			1		E
	Nylanderia	sp.1			1		E
Lepidoptera							
Noctuoidea							
Noctuidae		sp.2			1		E
Orthoptera							
Ensifera							
Gryllidae	jovens		1	0,2			E
Mogoplistidae	jovens				1	1	E
Psocoptera							
Psocomorpha	jovens		1				E
Chordata							
Amphibia							
Anura							
Neobatrachia							
Strabomantidae							
	Pristimantis	*fenestratus*	1	0,2			E
