# Supplementary material for: Reconciling Mining with the Conservation of Cave Biodiversity: A Quantitative Baseline to Help Establish Conservation Priorities
Source: PLoS One. 2016 Dec 20;11(12):e0168348. doi: 10.1371/journal.pone.0168348 (PMC5173368; doi:10.1371/journal.pone.0168348)
Supplement: S1 Dataset — (ZIP) [file pone.0168348.s002.zip › Taxa/Serra Sul/SS_2010/CAV_16.pdf]

CAV-16			1ª	AB	2ª	AB	ZON
Annelida							
Clitellata							
Oligochaeta		jovens	1	0,0476			E
Arthropoda							
Arachnida							
Acari							
Parasitiformes							
Mesostigmata							
	Ascidae	sp.3			1		E
	Ascidae	sp.16	1				E
Opilioacarida							
	Opilioacaridae	sp.1			1		E
Sarcoptiformes							
Oribatida		sp.1	1				E
Oribatida		sp.2	1		1		E
Oribatida		sp.3			1		E
Oribatida		sp.8	1				E
Oribatida		sp.9	1				E
Oribatida		sp.10	1				E
Oribatida		sp.15			1		E
	Anoetidae	sp.1	1				E
Trombidiformes							
Tydeoidea		sp.3			1		E
Amblypygi							
	Phrynidae						
	Heterophrynus	sp.			1	0,1	E
Araneae							
	Linyphiidae	jovens	1				E
	Oonopidae						
	gr. *Xycarphius*	sp.3			1		E
	Pholcidae	jovens	1		1		E
	Mesabolivar	*aurantiacus*			1		E
	Salticidae	jovens	1				E
	Tetragnathidae	jovens	1				E
	Theridiidae						
	Theridion	sp.3	1				E
	Theridiosomatidae	jovens			1		E
Opiliones							
Cyphophthalmi							
	Neogoveidae						
	Canga	*renatae*	1				E
Eupnoi							
	Sclerosomatidae	sp.1			1		E
Pseudoscorpiones							
	Spelaeochernes	sp.1	1				E
Entognatha							
Diplura							
	Campodeidae	sp.1	1				E
Coleoptera							
	Carabidae	sp.14	1				E
	Scydmaenidae	sp.5			1		E
	Scydmaenidae	sp.6	1				E
	Staphylinidae	sp.30	1				E
Collembola							
Arthropleona							
Entomobryoidea							
	Isotomidae	sp.1			1		E
	Paronellidae	sp.1	1				E
	Paronellidae	sp.2	1				E
	Paronellidae	sp.7	1				E
Diptera							
Brachycera							
	Drosophilidae						
	Drosophila	*eleonore*	1		1		E
	Phoridae						
	Phorinae	sp.	1				E

Nematocera	jovens	2		1		E
Cecidomyiidae						
Cecidomyiinae	sp.			1		E
Psychodidae						
Sciopemyia	*sordellii*	1				E
Sciaridae						
Odontosciara	sp.			1		E
Phytosciara	sp.	1				E
Tipulidae						
Tipulinae	sp.	1				E
Hemiptera						
Heteroptera						
Cydnidae						
Cydninae	sp.1	1				E
Lygaeidae	sp.4			1		E
Reduviidae						
Emesinae	sp.2			1		E
Rhopalidae	jovens			1		E
Hymenoptera						
Vespoidea						
Formicidae						
Camponotus	sp.1	1				E
Pheidole	sp.2	1		2		E
Isoptera	jovens	1				E
Lepidoptera						
Castnioidea	jovens	1				E
Orthoptera						
Ensifera						
Phalangopsidae						
Paraclodes	sp.1			3	0,3	E
Phalangopsis	sp.1	6	0,2857	5	0,5	E
Malacostraca						
Isopoda						
Philosciidae	sp.1	1		1		E
Chordata						
Amphibia						
Anura						
Neobatrachia						
Strabomantidae						
Pristimantis	*fenestratus*	1	0,0476	1	0,1	E
Mammalia						
Chiroptera						
Phyllostomidae						
Carollia	*perspicillata*	10	0,4762			E
Glossophaginae	sp.	2	0,0952			E
Nemathelminthes	sp.	1	0,0476			E
