# Supplementary material for: Reconciling Mining with the Conservation of Cave Biodiversity: A Quantitative Baseline to Help Establish Conservation Priorities
Source: PLoS One. 2016 Dec 20;11(12):e0168348. doi: 10.1371/journal.pone.0168348 (PMC5173368; doi:10.1371/journal.pone.0168348)
Supplement: S1 Dataset — (ZIP) [file pone.0168348.s002.zip › Taxa/Serra Sul/SS_2010/CAV_17.pdf]

CAV-17		1ª	AB	2ª	AB	ZON
Arthropoda						
Arachnida						
Amblypygi						
Phrynidae						
Heterophrynus	sp.	1	0,2			E
Araneae						
Ochyroceratidae						
Ochyrocera	sp.1	1				E
Pholcidae						
Ninetinae	sp.1	1				E
Segestriidae	jovens	1				E
Theraphosidae	jovens	1	0,2			E
Opiliones						
Eupnoi						
Sclerosomatidae	sp.1	1				E
Pseudoscorpiones						
Spelaeochernes	sp.1	1				E
Insecta						
Coleoptera	jovens	1				E
Diptera						
Nematocera						
Culicidae						
Culicini	sp.	1				E
Tipulidae						
Tipulinae	sp.	1				E
Hymenoptera						
Vespoidea						
Formicidae						
Camponotus	sp.1	1				E
Dolichoderus	*bispinosus*			1		E
Isoptera						
Termitidae						
Nasutitermes	sp.	1				E
Lepidoptera	jovens	1				E
Orthoptera						
Ensifera						
Phalangopsidae						
Paraclodes	sp.1	1	0,2			E
Phalangopsis	sp.1	1	0,2			E
Psocoptera						
Psocomorpha						
Epipsocidae						
Epipsocus	sp.2	1				E
Mammalia						
Chiroptera						
Emballonuridae						
Peropteryx	*kappleri*	1	0,2			E
