# Supplementary material for: Reconciling Mining with the Conservation of Cave Biodiversity: A Quantitative Baseline to Help Establish Conservation Priorities
Source: PLoS One. 2016 Dec 20;11(12):e0168348. doi: 10.1371/journal.pone.0168348 (PMC5173368; doi:10.1371/journal.pone.0168348)
Supplement: S1 Dataset — (ZIP) [file pone.0168348.s002.zip › Taxa/Serra Sul/SS_2010/CAV_18.pdf]

| CAV-18 | | | 1ª | AB | 2ª | AB | ZON |
|---|---|---|---|---|---|---|---|
| Arthropoda | | | | | | | |
| Arachnida | | | | | | | |
| Acari | | | | | | | |
| Sarcoptiformes | | sp.1 | 2 | | | | P |
| Oribatida | | sp.3 | 1 | | 1 | | P |
| Oribatida | | sp.10 | 1 | | | | P |
| Trombidiformes | | | | | | | |
| Tydeoidea | | | | | | | |
| Rhagidiidae | | sp.1 | | | 1 | | P |
| Amblypygi | | | | | | | |
| Phrynidae | | | | | | | |
| | *Heterophrynus* | sp. | 1 | 0,0114 | | | P |
| Araneae | | | | | | | |
| Ctenidae | jovens | | 3 | 0,0341 | | | E P |
| Nesticidae | | | | | | | |
| | *Nesticus* | sp.2 | 1 | | | | E |
| Ochyroceratidae | jovens | | | | 1 | | E |
| | *Ochyrocera* | sp.1 | 2 | | 1 | | P |
| | *Speocera* | sp.1 | 1 | | 1 | | P |
| Oonopidae | jovens | | 1 | | | | P |
| | Oonopinae | sp.1 | | | 1 | | P |
| Pholcidae | jovens | | | | 1 | | E |
| | *Mesabolivar* | *aurantiacus* | 1 | | 1 | | E |
| Prodidomidae | jovens | | | | 1 | | P |
| Symphytognathidae | | | | | | | |
| | *Symphytognatha* | sp.1 | | | 2 | | E P |
| Theraphosidae | jovens | | 2 | 0,0227 | 1 | 0,0417 | E P |
| Theridiidae | jovens | | | | 1 | | E |
| | *Theridion* | sp.1 | 1 | | 1 | | P |
| Theridiosomatidae | jovens | | 1 | | | | P |
| | *Plato* | sp.1 | | | 1 | | P |
| Opiliones | jovens | | 1 | 0,0114 | 2 | 0,0833 | E |
| Cyphophthalmi | | | | | | | |
| Neogoveidae | | | | | | | |
| | *Canga* | *renatae* | 2 | | | | P |
| Eupnoi | | | | | | | |
| Sclerosomatidae | | sp.1 | | | 1 | | E |
| Laniatores | | | | | | | |
| Escadabiidae | jovens | | | | 1 | | P |
| Escadabiidae | | sp.2 | 1 | | | | E |
| Stygnidae | | sp.1 | 1 | 0,0114 | 1 | 0,0417 | P |
| Pseudoscorpiones | | | | | | | |
| | *Spelaeochernes* | sp.1 | 2 | | 1 | | P |
| Chthoniidae | jovens | | | | 2 | | P |
| | *Pseudochthonius* | sp.1 | 1 | | 1 | | E P |
| Schizomida | | | | | | | |
| Hubbardiidae | jovens | | | | 1 | | P |
| Scorpiones | | | | | | | |
| Buthidae | | | | | | | |
| | *Ananteris* | *balzanii* | 1 | 0,0114 | | | E P |
| Pleurostigmophora | | | | | | | |
| Geophilomorpha | | | | | | | |
| Ballophilidae | | sp.1 | | | 1 | 0,0417 | P |
| Geophilidae | | sp.1 | | | 1 | 0,0417 | P |
| Scolopendromorpha | | | | | | | |
| Cryptopidae | | | | | | | |
| | *Cryptops* | sp.1 | 1 | 0,0114 | | | P |
| Diplopoda | jovens | | 1 | | | | E |
| Polydesmida | jovens | | 7 | 0,09 | | | P |
| Chelodesmidae | | sp.4 | 1 | 0,0114 | | | P |
| Pyrgodesmidae | | sp.2 | 3 | 0,0341 | 1 | 0,0417 | E P |
| Spirostreptida | jovens | | 1 | | 1 | | P |
| Entognatha | | | | | | | |
| Diplura | | | | | | | |
| Projapygidae | | sp.1 | 2 | | 1 | | E P |
| Projapygidae | | sp.2 | 1 | | | | P |

| | | | | | | | |
|---|---|---|---|---|---|---|---|
| Insecta | | | | | | | |
| Coleoptera | | | | | | | |
| Carabidae | | sp.3 | 1 | | | | E |
| Staphylinidae | | sp.37 | 1 | | | | P |
| | Pselaphinae | sp. | 1 | | | | P |
| Collembola | | | | | | | |
| Arthropleona | | | | | | | |
| Entomobryoidea | | | | | | | |
| Isotomidae | | sp.1 | 2 | | | | E P |
| Paronellidae | | sp.1 | | | 1 | | P |
| Paronellidae | | sp.4 | 2 | | 1 | | E P |
| Paronellidae | | sp.9 | 1 | | | | E |
| Neelipleona | | | | | | | |
| Neelidae | | sp.1 | 1 | | | | P |
| Diptera | jovens | | 3 | | 1 | | E P |
| Nematocera | | | | | | | |
| Psychodidae | | | | | | | |
| | *Sciopemyia* | *sordellii* | 1 | | 2 | | P |
| Tipulidae | | | | | | | |
| | Tipulinae | sp. | 2 | | | | E |
| Hemiptera | | | | | | | |
| Homoptera | | | | | | | |
| Cicadellidae | jovens | | | | 1 | | P |
| Cixiidae | jovens | | 2 | | | | P |
| Cixiidae | | sp.4 | | | 1 | | P |
| Hymenoptera | | | | | | | |
| Vespoidea | | | | | | | |
| Formicidae | | | | | | | |
| | *Camponotus* | sp.1 | 3 | | 2 | | E P |
| | *Hypoponera* | sp.1 | 3 | | | | E P |
| | *Nylanderia* | sp.1 | | | 2 | | P |
| | *Pheidole* | sp.1 | | | | | |
| | *Pheidole* | sp.2 | 1 | | | | E |
| | *Solenopsis* | sp.1 | | | | | |
| | *Solenopsis* | sp.2 | 1 | | | | P |
| Isoptera | operários | | 1 | | | | E |
| Termitidae | | | | | | | |
| | *Nasutitermes* | sp. | | | 1 | | P |
| Lepidoptera | | | | | | | |
| Noctuoidea | | sp.2 | | | 1 | | P |
| Orthoptera | | | | | | | |
| Ensifera | | | | | | | |
| Gryllidae | jovens | | 1 | 0,0114 | | | E |
| Phalangopsidae | | | | | | | |
| | *Paraclodes* | sp.1 | 2 | 0,0227 | 5 | 0,2083 | E |
| | *Phalangopsis* | sp.1 | 50 | 0,5682 | 7 | 0,2917 | P |
| Psocoptera | | | | | | | |
| Psocomorpha | jovens | | 1 | | | | P |
| Thysanoptera | | | | | | | |
| Phlaeothripidae | | sp.1 | | | 1 | | P |
| Malacostraca | | | | | | | |
| Isopoda | | | | | | | |
| Dubioniscidae | | sp.2 | 2 | | 2 | | E P |
| Philosciidae | | sp.1 | 2 | | | | P |
| Scleropactidae | | sp. | 1 | | | | P |
| Pauropoda | | | | | | | |
| Tetramerocerata | | sp. | 1 | | | | P |
| Symphyla | | | | | | | |
| Scutigerellidae | | | | | | | |
| | *Hanseniella* | sp.1 | 1 | | 1 | | P |
| Chordata | | | | | | | |
| Amphibia | | | | | | | |
| Anura | | | | | | | |
| Neobatrachia | | | | | | | |
| Strabomantidae | | | | | | | |
| | *Pristimantis* | *fenestratus* | 3 | 0,0341 | 2 | 0,0833 | P |
| Mammalia | | | | | | | |

| | | | | | | | |
|---|---|---|---|---|---|---|---|
| Chiroptera | | | | | | | |
| Emballonuridae | | | | | | | |
| | *Peropteryx* | *kappleri* | 10 | 0,1136 | 3 | 0,125 | E |
| Mollusca | | | | | | | |
| Gastropoda | | | | | | | |
| Bulimulidae | | | | | | | |
| | *Happia* | sp. | | | 1 | | P |
| Nemathelminthes | | sp. | 1 | | | | P |
