# Supplementary material for: Reconciling Mining with the Conservation of Cave Biodiversity: A Quantitative Baseline to Help Establish Conservation Priorities
Source: PLoS One. 2016 Dec 20;11(12):e0168348. doi: 10.1371/journal.pone.0168348 (PMC5173368; doi:10.1371/journal.pone.0168348)
Supplement: S1 Dataset — (ZIP) [file pone.0168348.s002.zip › Taxa/Serra Sul/SS_2010/CAV_19.pdf]

CAV-19		1ª	AB	2ª	AB	ZON
Arthropoda						
Arachnida						
Acari						
Sarcoptiformes						
Oribatida	sp.1	1				P
Trombidiformes	sp.1	1				P
Araneae						
Ctenidae	jovens			1	0,0556	P
Ochyroceratidae	jovens	2		2		P
Speocera	sp.1			1		P
Oonopidae	jovens	1		2		P
Prodidomidae	jovens	1				P
Symphytognathidae	jovens			1		P
Theridiosomatidae						
Plato	sp.1			1		P
Opiliones						
Cyphophthalmi						
Neogoveidae						
Canga	*renatae*			2		P
Laniatores	jovens			1		E
Cosmetidae						
Roquettea	*singularis*			3	0,17	P
Escadabiidae	sp.2	1		2		P
Stygnidae	sp.1	1	0,0714	1	0,0556	P
Pseudoscorpiones						
Bochicidae	sp.1			2		P
Spelaeochernes	sp.1	2				P
Pseudochthonius	sp.1			1		P
Ricinulei						
Ricinoididae	jovens			1		P
Cryptocellus	sp.	1				P
Diplopoda						
Chelodesmidae						
Glomeridesmidae	sp.5	1				P
Polydesmida						
Pyrgodesmidae	sp.2	2	0,1429			P
Coleoptera						
Staphylinidae						
Pselaphinae	sp.1			1		P
	sp.6	1				P
Collembola						
Entomobryidae	sp.4			1		P
Isotomidae	sp.1	1				P
Paronellidae	sp.4	1				P
Diptera	jovens	1				P
Nematocera	jovens	1				P
Psychodidae						
Sciopemyia	*sordellii*			1		P
Hemiptera						
Heteroptera						
Cydnidae	jovens			1		P
Reduviidae						
Emesinae	sp.2			1		P
Homoptera						
Cixiidae	jovens	1		1		P
Hymenoptera						
Formicidae						
Brachymyrmex	sp.1	1				P
Nylanderia	sp.1			1		P
Wasmania	*auropunctata*	1				P
Orthoptera						
Phalangopsidae						
Paraclodes	sp.1	2	0,1429			E
Phalangopsis	sp.1	5	0,3571	11	0,6111	P
Psocoptera	jovens			1		P
Epipsocidae						

	Mesepipsocus	sp.2			1		P
Isopoda							
Philosciidae		sp.1	1				P
Chordata							
Amphibia							
Anura							
Neobatrachia							
Strabomantidae							
	Pristimantis	*fenestratus*	1	0,0714	1	0,0556	E P
Mammalia							
Chiroptera							
Emballonuridae							
	Peropteryx	*kappleri*	3	0,2143	1	0,0556	E
Mollusca							
Gastropoda							
Systrophiidae							
	Happia	sp.	1				P
