# Supplementary material for: Reconciling Mining with the Conservation of Cave Biodiversity: A Quantitative Baseline to Help Establish Conservation Priorities
Source: PLoS One. 2016 Dec 20;11(12):e0168348. doi: 10.1371/journal.pone.0168348 (PMC5173368; doi:10.1371/journal.pone.0168348)
Supplement: S1 Dataset — (ZIP) [file pone.0168348.s002.zip › Taxa/Serra Sul/SS_2010/CAV_20.pdf]

CAV-20			1ª	AB	2ª	AB	ZON
Arthropoda							
Arachnida							
Acari							
Ixodida							
Opilioacarida							
Opilioacaridae		sp.1			1		P
Oribatida		sp.3	2				P
Trombidiformes							
Tydeoidea							
Bdellidae		sp.1			1		P
Amblypygi							
Phrynidae							
	Heterophrynus	sp.	1	0,0769			P
Araneae							
Ochyroceratidae	jovens		1				P
	Ochyrocera	sp.1	1		1		P
Oonopidae							
	Coxapopha	sp.1	1				P
Paratropididae	jovens		1	0,0769			P
Pholcidae	jovens		1		1		P
	Mesabolivar	aurantiacus	1				P
Segestriidae							
	Ariadna	sp.1	1				P
Theraphosidae	jovens		2	0,1538			P
Theridiidae	jovens				2		P
	Theridion	sp.3	2				P
Theridiosomatidae	jovens		1				P
Zodariidae	jovens		1	0,0769			E
Opiliones			1	0,0769	3	0,1875	
Eupnoi							
Sclerosomatidae	jovens		1		1		P
Sclerosomatidae		sp.1			1		P
Laniatores							
Escadabiidae	jovens		1				P
Stygnidae	jovens		1	0,0769	1	0,0625	P
		sp.1	1				P
Pseudoscorpiones							
	Spelaeochernes	sp.1	2				P
	Pseudochthonius	sp.1	2				P
Diplopoda							
Polydesmida							
Chelodesmidae		sp.4	1	0,0769			P
	jovens		1				P
Spirostreptida	jovens		2				P
Collembola							
Arthropleona							
Entomobryoidea							
Cyphoderidae		sp.1	1				P
Entomobryidae		sp.10			1		P
		sp.8			1		P
Isotomidae		sp.1	2				P
Paronellidae		sp.2	1				P
		sp.6			1		P
Diptera							
Nematocera							
Sciaridae							
	Bradysia	sp.			1		P
Hemiptera							
Heteroptera							
Cydnidae							
	Cydninae	sp.1	2				P
Homoptera							
Cixiidae	jovens		1				P
Hymenoptera							
Vespoidea							
Formicidae							

		Anochetus	sp.1	1				P
		Nylanderia	sp.1	1		1		P
		Pheidole	sp.2	1		2		P
		Strumygenys	sp.1	1				P
		Wasmania	*auropunctata*	1				P
Lepidoptera								
Tineoidea								
	Noctuidae		sp.			1	0,0625	E
		jovens		1		1		P
Orthoptera								
Ensifera								
	Phalangopsidae							
		Paraclodes	sp.1			5	0,3125	P
		Phalangopsis	sp.1	2	0,1538			P
Malacostraca								
Isopoda								
	Scleropactidae		sp.	1				P
Chordata								
Amphibia								
Anura								
Neot	Strabomantidae							
		Pristimantis	*fenestratus*	1	0,0769	2	0,125	P
Mammalia								
Chiroptera								
	Emballonuridae							
		Peropteryx	sp.			4	0,25	E
Reptilia								
Squamata								
Serpentes								
			sp.	1	0,0769			E
