# Supplementary material for: Reconciling Mining with the Conservation of Cave Biodiversity: A Quantitative Baseline to Help Establish Conservation Priorities
Source: PLoS One. 2016 Dec 20;11(12):e0168348. doi: 10.1371/journal.pone.0168348 (PMC5173368; doi:10.1371/journal.pone.0168348)
Supplement: S1 Dataset — (ZIP) [file pone.0168348.s002.zip › Taxa/Serra Sul/SS_2010/CAV_21.pdf]

CAV-21				1ª	AB	2ª	AB	ZON
Arthropoda								
Arachnida								
Acari								
Sarcoptiformes								
Oribatida			sp.3	2				P
Trombidiformes								
Tydeoidea			sp.2			1		P
Amblypygi								
	Phrynidae							
	Heterophrynus		sp.			1	0,05	P
Araneae								
	Ctenidae	jovens		1	0,0294			P
	Dipluridae	jovens				1		P
	Ochyroceratidae	jovens						P
	Ochyrocera		sp.1	1		2		P
	Speocera		sp.1	1		1		P
	Pholcidae							
	Mesabolivar		*aurantiacus*			1		P
	Scytodidae							
	Scytodes		sp.	1	0,0294			E
	Theraphosidae	jovens		1	0,58			P
			sp.3	1		1	0,05	P
	Theridiosomatidae	jovens		1				P
Opiliones				5	0,1471			
Laniatores								
	Cosmetidae							
	Roquettea		*singularis*	1	0,0294			P
	Escadabiidae	jovens		2				P
Pseudoscorpiones								
	Spelaeochernes		sp.1			1		P
	Chthoniidae							P
	Pseudochthonius		sp.4	2		2		P
Diplopoda						1	0,05	
Polydesmida								
	Chelodesmidae		sp.4	1	0,0294			P
	Pyrgodesmidae	jovens						
			sp.2	1	0,0294	1	0,05	P
Siphonophorida								
	Siphonophoridae		sp.2			1		P
Spirostreptida		jovens				2	0,1	P
Entognatha								
Diplura								
	Projapygidae		sp.1			1		P
Insecta								
Blattodea								
	Blattidae		sp.3	1	0,0294			P
Coleoptera		jovens		1				P
	Carabidae		sp.15			1		P
	Ptiliidae		sp.1	1				P
	Staphilinidae							
	Pselaphinae		sp.6	1				P
Collembola								
Arthropleona								
Entomobryoidea								
	Isotomidae		sp.1	1				P
	Paronellidae		sp.1			1		P
			sp.4	1				P
			sp.9	2				P
Diptera								
Nematocera		jovens		1				P
	Psychodidae							
	Pintomyia		*gruta*	1				P
	Sciopemyia		*sordellii*			1		P
Hemiptera								
Heteroptera								
Dipsocoroidea		jovens		1				P

Homoptera							
Cixiidae	jovens				1		P
Vespoidea							
Formicidae							
	Pheidole	sp.2	2				P
	Wasmania	*auropunctata*	1				P
Isoptera		sp.	1				P
Termitidae							
	Armitermes	sp.	1				P
	Termes	sp.	1				P
Orthoptera							
Ensifera							
Phalangopsidae							
	Paraclodes	sp.1	2	0,0588			E
	Phalangopsis	sp.1	15	0,4412	7	0,35	P
Malacostraca							
Isopoda							
Philosciidae		sp.1	2				P
Chordata							
Amphibia							
Anura							
Neobatrachia							
Strabomantidae							
	Pristimantis	*fenestratus*	1	0,0294	7	0,35	P
Mammalia							
Chiroptera							
Emballonuridae							
	Peropteryx	kappleri	3	0,0882			P
Mollusca							
Gastropoda							
Systrophiidae							
	Happia	sp.			1		P
