# Supplementary material for: Reconciling Mining with the Conservation of Cave Biodiversity: A Quantitative Baseline to Help Establish Conservation Priorities
Source: PLoS One. 2016 Dec 20;11(12):e0168348. doi: 10.1371/journal.pone.0168348 (PMC5173368; doi:10.1371/journal.pone.0168348)
Supplement: S1 Dataset — (ZIP) [file pone.0168348.s002.zip › Taxa/Serra Sul/SS_2010/CAV_22.pdf]

CAV-22			1ª	AB	2ª	AB	ZON
Annelida							
Clitellata							
Oligochaeta	jovens		2	0,0377			E
Arthropoda							
Arachnida							
Acari							
Ixodida	jovens		1				E
Parasitiformes							
Mesostigmata							
Heterozerconidae		sp.1	1				E
Macronyssidae		sp.1			1		E
		sp.3	1		2		E
		sp.6	1				E
Sarcoptiformes							
Oribatida		sp.12	1				E
		sp.3	1				E
		sp.6	1				E
Sarcoptiformes		sp.2	1				E
Anystidae							
	Erythracarus	*nasutus*	1				E
Rhagidiidae		sp.1	1				E
Amblypygi							
Phrynidae							
	Heterophrynus	sp.	2	0,0377	1	0,0167	E
Araneae							
Araneidae	jovens		1				E
Ctenidae	jovens		2	0,0377			E
	Ctenus	sp.3			1	0,0167	E
Paratropididae	jovens		1	0,0189			E
Pholcidae	jovens		1		1		E
	Mesabolivar	*aurantiacus*	1				E
Theridiidae							
	Theridion	sp.3	1				E
Theridiosomatidae	jovens				1		E
	Plato	sp.1	1				E
Opiliones	jovens		1				E
Cyphophthalmi							
Neogoveidae							
	Canga	*renatae*			1		E
Stygnidae		sp.1	1	0,0189			E
Eupnoi			2	0,0377			
Laniatores							
Cosmetidae	jovens				1	0,0167	E
	Roquettea	*singularis*	1	0,0189			E
Pseudoscorpiones							
Bochicidae		sp.1	2				E
Chthoniidae	jovens		2				E
Diplopoda							
Polydesmida							
Fuhrmannodesmidae		sp.1	1		1		E
		sp.5			1		E
Pyrgodesmidae		sp.2	1	0,0189			E
Spirostreptida							
Pseudonannolenidae							
	Pseudonannolene	sp.2	1	0,0189			E
	jovens		1				E
Entognatha							
Diplura							
Campodeidae		sp.1	1		1		E
Insecta							
Coleoptera	jovens		1				E
Curculionidae		sp.2			1		E
Ptiliidae		sp.1	1		1		E
Staphilinidae							
	Pselaphinae	sp.3	1				E
Collembola							

Arthropleona							
Entomobryoidea							
Entomobryidae		sp.1			1		E
		sp.7	1				E
Paronellidae		sp.1	1				E
		sp.9	1				E
Diptera	jovens		2		1		E
Brachycera							
Phoridae							
	Phorinae	sp.			1		E
Nematocera							
Culicidae							
	Culicini	sp.	1				E
Psychodidae							
	Edentomyia	*piauensis*			1		E
	Pintomyia	*gruta*			1		E
	Scyopemyia	*sordellii*	1		1		E
Sciaridae							
	Bradysia	sp.			1		E
Tipulidae							
	Tipulinae	sp.	1				E
Hemiptera							
Homoptera	jovens		1				E
Cixiidae	jovens		1				E
Hymenoptera							
Vespoidea							
Formicidae							
	Camponotus	sp.1	1		2		E
	Odontomachus	*bauri*	1				E
	Pheidole	sp.2	1				E
	Solenopsis	sp.3			2		E
Isoptera		sp.	2				E
Lepidoptera							
Noctuoidea		sp.2	1				E
Noctuoidea	jovens				1		E
Noctuidae		sp.1	1	0,0189			E
Orthoptera							
Ensifera							
Gryllidae	jovens		1	0,0189			E
Phalangopsidae							
	Paraclodes	sp.1	17	0,3208	3	0,05	E
	Phalangopsis	sp.1	15	0,283			E
Psocoptera							
Psocomorpha	jovens		1				E
Epipsocidae							
	Mesepipsocus	sp.1	1				E
Malacostraca							
Isopoda							
Philosciidae		sp.1	1				E
Chordata							
Amphibia							
Anura							
Neobatrachia							
Strabomantidae							
	Pristimantis	*fenestratus*	2	0,0377	4	0,0667	E
Mammalia							
Chiroptera							
Emballonuridae							
	Peropteryx	*kappleri*	4	0,0755			E
Phyllostomidae							
	Carollia	sp.			45	0,75	E
	Glossophaginae	sp.			5	0,0833	E
