# Supplementary material for: Reconciling Mining with the Conservation of Cave Biodiversity: A Quantitative Baseline to Help Establish Conservation Priorities
Source: PLoS One. 2016 Dec 20;11(12):e0168348. doi: 10.1371/journal.pone.0168348 (PMC5173368; doi:10.1371/journal.pone.0168348)
Supplement: S1 Dataset — (ZIP) [file pone.0168348.s002.zip › Taxa/Serra Sul/SS_2010/CAV_23.pdf]

CAV-23			1ª	AB	2ª	AB	ZON
Arthropoda							
Arachnida							
Acari							
Parasitiformes							
Mesostigmata							
Macronyssidae		sp.1	1		1		E
Sarcoptiformes							
Oribatida		sp.3			1		E
Trombidiformes							
Tydeoidea		sp.5	1				E
Amblypygi							
Phrynidae	jovens						
	Heterophrynus	sp.	2	0,0741			E
Araneae							
Araneidae	jovens		1				E
Ochyroceratidae	jovens		1				E
Oonopidae	jovens		1				E
Theridiosomatidae	jovens						
	Plato	sp.1	1				E
Pseudoscorpiones							
	Spelaeochernes	sp.1	1		1		E
Chilopoda							
Notostigmophora							
Scutigeromorpha							
Pselliodidae							
	Sphendononema	*guildingii*	1				E
Diplopoda							
Polydesmida	jovens		2	0,111			E
Chelodesmidae		sp.1	1				E
Fuhrmannodesmidae		sp.1	1				E
Insecta							
Coleoptera							
Anthicidae		sp.3			1		E
Ptiliidae		sp.1			1		E
Collembola							
Arthropleona							
Entomobryoidea							
Cyphoderidae		sp.1			1		E
Isotomidae		sp.1	1				E
Paronellidae		sp.5	1				E
Diptera							
Brachycera							
Chloropidae		sp.			1		E
Sphaeroceridae		sp.			1		E
Nematocera	jovens		2		3		E
Psychodidae		sp.					
	Sciopemyia	*sordellii*	1				E
Sciaridae							
	Bradysia	sp.			1		E
Tipulidae							
	Tipulinae	sp.	1		1		E
Hemiptera							
Heteroptera							
Cydnidae	jovens		1		1		E
	Cydninae	sp.1	2		1		E
Schizopteridae							
	Schizopterinae	sp.2			1		E
Homoptera							
Cixiidae	jovens		1		1		E
Hymenoptera							
Vespoidea							
Formicidae							
	Nylanderia	sp.1			2		E
	Pachycondyla	*striata*	1				E
	Solenopsis	sp.2			1		E
Isoptera							

Termitidae							
	Nasutitermes	sp.	1				E
Orthoptera							
Phalangopsidae							
	Paraclodes	sp.1	6	0,2222	3	0,15	E
	Phalangopsis	sp.1	9	0,3333			E
Psocoptera							
Psocomorpha	jovens		1				E
Malacostraca							
Isopoda							
Philosciidae		sp.1	3		1		E
Symphyla							
Scutigerellidae							
	Hanseniella	sp.1	1				E
Chordata							
Amphibia							
Anura							
Neobatrachia							
Strabomantidae							
	Pristimantis	*fenestratus*	1	0,037	9	0,45	E
Mammalia							
Chiroptera							
	Glossophaginae	sp.	5	0,1852	8	0,4	E
Mollusca							
Gastropoda							
Bulimulidae							
	Naesiotus	sp.	1		1		E
