# Supplementary material for: Reconciling Mining with the Conservation of Cave Biodiversity: A Quantitative Baseline to Help Establish Conservation Priorities
Source: PLoS One. 2016 Dec 20;11(12):e0168348. doi: 10.1371/journal.pone.0168348 (PMC5173368; doi:10.1371/journal.pone.0168348)
Supplement: S1 Dataset — (ZIP) [file pone.0168348.s002.zip › Taxa/Serra Sul/SS_2010/CAV_24.pdf]

CAV-24			1ª	AB	2ª	AB	ZON
Arthropoda							
Arachnida							
Acari							
Parasitiformes							
Mesostigmata							
Macronyssidae		sp.1			1		P
Opilioacarida							
Opilioacaridae		sp.1	2				P
Sarcoptiformes		sp.1	1		1		P
Oribatida		sp.2			1		P
Acaridae		sp.1	1				P
Trombidiformes							
Tydeoidea		sp.1			1		P
Bdellidae		sp.1	1				P
Amblypygi							
Phrynidae	jovens						
	Heterophrynus	sp.	2	0,0435			P
Araneae							
Araneidae	jovens				1		E
Corinnidae							
	Falconina	sp.2			1	0,0133	P
Ctenidae	jovens		2	0,0435	1	0,0133	E P
Ochyroceratidae	jovens		1		1		P
	Ochyrocera	sp.3	1		1		P
	Speocera	sp.1	1				P
Oonopidae	jovens		1				P
	gr. *Xycarphius*	sp.3			2		P
Pholcidae	jovens				1		E
	Mesabolivar	sp.1			1		E
	Ninetinae	sp.1			1		P
Scytodidae	jovens		2				E P
	Scytodes	*globula*			1	0,0133	E
		sp.			1	0,0133	E
Symphytognathidae							
	Anapistula	sp.1			1		P
Theridiosomatidae	jovens		1				P
	Plato	sp.1	1				P
Opiliones							
Cyphophthalmi							
Neogoveidae	*Canga*	*renatae*	1				P
Eupnoi							
Sclerosomatidae	jovens				1		P
Laniatores							
Cosmetidae	jovens				1	0,0133	E
Escadabiidae		sp.3			1		P
Stygnidae		sp.1	1	0,0217	1	0,0133	E
Pseudoscorpiones							
	Spelaeochernes	sp.1	3		2		P
	Pseudochthonius	sp.1	1		2		P
Ricinulei							
Ricinoididae	jovens		1				P
Schizomida							
Hubbardiidae	jovens		1				P
	Rowlandius	sp.	1		1		P
Chilopoda							
Pleurostigmophora	jovens				1	0,0133	P
Scolopendromorpha							
Scolopocryptopidae							
	Scolopocryptops	*guacharensis*			1	0,0133	P
	Tidops	sp.1	1	0,0217			E
Diplopoda							
Polyxenida							
Hypogexenidae		sp.1	2		1		P
Spirostreptida							
Pseudonannolenidae							
	Pseudonannolene	sp.4			1	0,0133	P

Spirobolida								
	Spirobolidae		sp.			2	0,0267	P
Entognatha								
Diplura								
	Campodeidae		sp.1	2		1		P
	Projapygidae		sp.1	1		1		P
Insecta								
Coleoptera		jovens				1		P
	Ptiliidae		sp.1	1				P
	Scydmaenidae		sp.10	1				P
			sp.5			1		P
			sp.7	2		1		P
Collembola								
Entomobryoidea			sp.1	1				P
	Entomobryidae		sp.1			1		P
	Isotomidae		sp.1	1				P
	Paronellidae		sp.1			1		P
			sp.2	1				P
			sp.5	1				P
Diptera								
Nematocera								
	Cecidomyiidae							
		Cecidomyiinae	sp.			1		P
	Culicidae							
		Culicini	sp.	1				E
	Psychodidae							
		Edentomyia	*piauensis*	1				P
		Sciopemyia	*sordellii*	2		2		P
	Sciaridae		sp.					
		Phytosciara	sp.	1				P
	Tipulidae		sp.					
		Tipulinae	sp.	1		2		E P
Hemiptera								
Heteroptera								
Dipsocoroidea		jovens		2				P
Pentatomoidea								
	Cydnidae	jovens		1		1		P
		Cydninae	sp.1			1		P
	Reduviidae	jovens				1	0,0133	E
	Schizopteridae							
		Schizopterinae	sp.1			1		P
Homoptera								
	Cixiidae	jovens		1				P
Hymenoptera								
Vespoidea								
	Formicidae							
		Acromyrmex	sp.1			1		P
		Camponotus	*atriceps*			1		P
			sp.1	1		1		P
		Hypoponera	sp.1	1				P
		Solenopsis	sp.2	1		1		P
Isoptera								
	Termitidae							
		Atlantitermes	sp.	1				P
		Nasutitermes	sp.	2		1		E P
Orthoptera								
	Phalangopsidae	jovens						
		Paraclodes	sp.1	1	0,0217	2	0,0267	E
		Phalangopsis	sp.1	22	0,4783	30	0,4	P
Thysanoptera								
	Thripidae		sp.1			1		P
Thysanura								
	Ateluridae		sp.1	1				P
Malacostraca								
Isopoda								
	Dubioniscidae		sp.1	1		3		P
	Philosciidae		sp.1	1				P

Symphyla						
Scutigerellidae						
	Hanseniella sp.1	1				P
	Scutigerella sp.1			1		P
Chordata						
Amphibia						
Anura						
Neobatrachia						
Strabomantidae						
	Pristimantis fenestratus			1	0,0133	P
Mammalia						
Chiroptera						
Phyllostomidae						
	Carollia perspicillata	15	0,3261	30	0,4	P
	Glossophaginae sp.	2	0,0435			P
