# Supplementary material for: Reconciling Mining with the Conservation of Cave Biodiversity: A Quantitative Baseline to Help Establish Conservation Priorities
Source: PLoS One. 2016 Dec 20;11(12):e0168348. doi: 10.1371/journal.pone.0168348 (PMC5173368; doi:10.1371/journal.pone.0168348)
Supplement: S1 Dataset — (ZIP) [file pone.0168348.s002.zip › Taxa/Serra Sul/SS_2010/CAV_25.pdf]

CAV-25			1ª	AB	2ª	AB	ZON
Annelida							
Clitellata							
Oligochaeta		jovens	2	0,019			E
Arthropoda							
Arachnida							
Acari							
Ixodida		jovens	1				E
Parasitiformes							
Mesostigmata							
	Laelapidae						
	Stratiolaelaps	sp.1			1		E
		sp.2	1				E
Sarcoptiformes							
Oribatida		sp.3	1				E
		sp.1	2				E
Trombidiformes							
Tydeoidea							
	Rhagidiidae	sp.1	1				E
		sp.5	1				E
Amblypygi							
	Phrynidae						
	Heterophrynus	sp.	1	0,0095			E
Araneae							
	Araneidae	jovens	1				E
	Ctenidae	jovens	1	0,0095			E
	Ochyroceratidae	jovens	1				E
	Pholcidae	jovens			1		E
	Leptopholcus	sp.1	1				E
	Mesabolivar	*aurantiacus*			1		E
	Scytodidae	jovens	1				E
		globula			1	0,0227	E
		sp.	1	0,0095	1	0,0227	E
	Theridiidae						
	Theridion	sp.1	1				E
		sp.2	1				E
	Theridiosomatidae	jovens			1		E
	Plato	sp.1	1				E
Opiliones			17	0,1619	3	0,0682	
Laniatores		jovens	2	0,019	1	0,0227	E
	Cosmetidae						
	Roquettea	*singularis*	1	0,0095	1	0,0227	E
	Stygnidae	sp.1	1	0,0095	1	0,0227	E
Pseudoscorpiones							
	Chernetidae	jovens			2		E
Schizomida							
	Hubbardiidae	jovens			1		E
Chilopoda							
Pleurostigmophora							
Scolopendromorpha							
	Cryptopidae						
	Cryptops	sp.1	1	0,0095			E
	Scolopocryptopidae						
	Dinocryptops	*miersii*	2	0,019			E
Diplopoda							
Polydesmida							
		jovens			1		E
	Chelodesmidae	jovens	1	0,009			E
	Chelodesmidae	sp.4	1				E
Spirostreptida							
	Pseudonannolenidae	jovens	1	0,0095			E
Spirobolida							
	Spirobolidae	sp.			4	0,0909	E
Insecta							
Coleoptera							
	Carabidae	sp.3			1		E
	Staphylinidae	sp.1					

		Pselaphinae	sp.10			1		E
			sp.11			1		E
Collembola								
Arthropleona								
Entomobryoidea								
	Isotomidae		sp.1	1				E
	Paronellidae		sp.1	1				E
Diptera								
Brachycera								
	Phoridae							
		Metopininae	sp.			1		E
Nematocera		jovens		2		1		E
	Cecidomyiidae							
		Cecidomyiinae	sp.			1		E
	Psychodidae							
		Pintomyia	*gruta*			1		E
		Sciopemyia	*sordellii*	1		1		E
	Sciaridae							
		Bradysia	sp.			1		E
	Tipulidae							
		Tipulinae	sp.	1		1		E
Hemiptera								
Homoptera								
	Cixiidae	jovens		1		1		E
			sp.3	1				E
Hymenoptera								
Chalcidoidea			sp.5			1		E
Vespoidea								
	Formicidae							
		Brachymyrmex	sp.1			1		E
		Camponotus	*atriceps*	1				E
		Nylanderia	sp.1	1		1		E
		Pachycondyla	*striata*	2		1		E
		Pheidole	sp.2			1		E
Isoptera								
	Termitidae							
		Nasutitermes	sp.	1		1		E
Lepidoptera								
Noctuoidea								
	Noctuidae		sp.1	2	0,019			E
Orthoptera								
Ensifera								
	Phalangopsidae							
		Paraclodes	sp.1	17	0,1619	7	0,1591	E
		Phalangopsis	sp.1	33	0,3143	17	0,3864	E
Malacostraca								
Isopoda								
	Philosciidae		sp.1	1				E
Chordata								
Amphibia								
Anura								
Neobatrachia								
	Strabomantidae							
		Pristimantis	*fenestratus*	4	0,0381	3	0,0682	E
Mammalia								
Chiroptera						4	0,0909	
	Emballonuridae							
		Peropteryx	*kappleri*	17	0,1619	1	0,0227	E
