# Supplementary material for: Reconciling Mining with the Conservation of Cave Biodiversity: A Quantitative Baseline to Help Establish Conservation Priorities
Source: PLoS One. 2016 Dec 20;11(12):e0168348. doi: 10.1371/journal.pone.0168348 (PMC5173368; doi:10.1371/journal.pone.0168348)
Supplement: S1 Dataset — (ZIP) [file pone.0168348.s002.zip › Taxa/Serra Sul/SS_2010/CAV_26.pdf]

CAV-26			1ª	AB	2ª	AB	ZON
Annelida							
Clitellata							
Oligochaeta	jovens		2	0,1333			E
Arthropoda							
Arachnida							
Acari							
Ixodida	*Amblyomma*	sp.			1		E
Sarcoptiformes							
Oribatida		sp.6	1				E
Araneae							
Araneidae	jovens		1				E
	Alpaida	sp.2	1				E
Corinnidae	jovens		1	0,0667			E
Ctenidae	jovens		2	0,1333	2	0,069	E
Mysmenidae	gen.1	sp.1	1				E
	Microdipoena	sp.1	1				E
Pholcidae	jovens				1		E
Salticidae	jovens				1		E
Scytodidae	jovens		1		1		E
	Scytodes	sp.			2	0,069	E
Theridiosomatidae	jovens		1				E
Opiliones					2		
Laniatores	jovens				1		E
Cosmetidae	jovens				1	0,068	E
	Roquettea	*singularis*	1	0,0667	1		E
Stygnidae		sp.1			1	0,0345	E
Pseudoscorpiones							
Chernetidae	jovens		2				E
	Spelaeochernes	sp.1	1		1		E
Entognatha							
Diplura							
Campodeidae		sp.1	1		1		E
Insecta							
Blattodea	jovens		1	0,0667			E
Coleoptera							
Carabidae		sp.3			1		E
Staphylinidae		sp.28	1	0,0667			E
		sp.38	1				E
		sp.9	1				E
Collembola							
Arthropleona							
Entomobryoidea							
Entomobryidae		sp.1	1				E
Paronellidae		sp.1	1		1		E
		sp.6			2		E
Diptera							
Brachycera							
Phoridae							
	Metopininae	sp.			1		E
Nematocera	jovens		1				E
Ceratopogonidae		sp.	1				E
Culicidae							
	Culicini	sp.	1				E
Mycetophilidae							
	Euceroplatus	sp.			1		E
Psychodidae							
	Psychodopygus	Série *chagasi*	1				E
	Sciopemyia	*sordellii*	1		1		E
Sciaridae							
	Bradysia	sp.	1				E
Tipulidae							
	Tipulinae	sp.	2		1		E
Hemiptera							
Heteroptera							
Cydnidae	jovens				1		E
Pentatomidae	jovens				1		E

Reduviidae							E
	Reduviinae	jovens	4	0,2667			E
Homoptera							
Cixiidae	jovens		1				E
Hymenoptera							
Vespoidea							
Formicidae							
	Cyphomyrmex	sp.1			1		E
	Myrmicocrypta	sp.1	1				E
	Pheidole	sp.2	3		2		E
	Solenopsis	sp.2			1		E
Isoptera							
Termitidae							
	Embiratermes	sp.	1				E
	Nasutitermes	sp.	3		1		E
	Velocitermes	sp.			2		E
Lepidoptera							
Cossoidea							
Limacodidae		sp.1			1	0,0345	E
Noctuidae		sp.	1	0,0667			E
	jovens				1		E
Orthoptera							
Ensifera							
Phalangopsidae							
	Paraclodes	sp.1	18	0,0667	17	0,5862	E
Psocoptera							
Psocomorpha	jovens		1				E
Asiopsocidae							
	Asiopsocus	sp.1			1		E
Psyllipsocidae	jovens				1		E
Thysanoptera	jovens		1				E
Malacostraca							
Isopoda							
Philosciidae		sp.1			1		E
Chordata							
Amphibia							
Anura							
Neobatrachia							
Strabomantidae							
	Pristimantis	*fenestratus*	6	0,0667	2	0,069	E
Mammalia							
Chiroptera							
Emballonuridae							
	Peropteryx	sp.			1	0,0345	E
Reptilia							
Squamata							
Serpentes		sp.			1	0,0345	E
