# Supplementary material for: Reconciling Mining with the Conservation of Cave Biodiversity: A Quantitative Baseline to Help Establish Conservation Priorities
Source: PLoS One. 2016 Dec 20;11(12):e0168348. doi: 10.1371/journal.pone.0168348 (PMC5173368; doi:10.1371/journal.pone.0168348)
Supplement: S1 Dataset — (ZIP) [file pone.0168348.s002.zip › Taxa/Serra Sul/SS_2010/CAV_27.pdf]

CAV-27			1ª	AB	2ª	AB	ZON
Arthropoda							
Arachnida							
Acari							
Ixodida	*Amblyomma*	sp.1			1		E
Parasitiformes							
Mesostigmata		sp.2	1				P
Sarcoptiformes							
Oribatida		sp.6	1				E
Amblypygi							
Phrynidae							
	Heterophrynus	sp.	1	0,0385			P
Araneae		jovens	2				E
Barychaelidae		jovens	1	0,0385			E
Corinnidae		jovens	1	0,0385			P
Ctenidae		jovens	1	0,076			E
	Ctenus	sp.1	1				E
Ochyroceratidae		jovens	1				P
Pholcidae		jovens	1				E
	Leptopholcus	sp.1			2		E P
Scytodidae		jovens	1				P
Theraphosidae		jovens	1	0,0385			E
Theridiosomatidae		jovens	1				P
Opiliones			1	0,0385	1	0,0345	
Laniatores							
		jovens	1	0,0385	1	0,0345	E
Escadabiidae		jovens	1				E
Stygnidae		sp.1	1	0,0385	1	0,0345	E
Pseudoscorpiones							
	Spelaeochernes	sp.1	1				E
Diplopoda							
Polyxenida							
Hypogexenidae		sp.1	1				P
Entognatha							
Diplura							
Campodeidae		sp.1	1		1		P
Insecta							
Blattodea		jovens	2	0,0769			E P
Blaberidae		jovens			1	0,0345	E
Coleoptera							
Carabidae		sp.3	1				P
Collembola							
Arthropleona							
Entomobryoidea							
Isotomidae		sp.1	1				P
Paronellidae		sp.1	1				E
		sp.4	1				E
		sp.9	1				E
Diptera							
Nematocera							
Psychodidae							
	Sciopemyia	*sordellii*	1		1		P
		sp.			1		P
Tipulidae							
	Tipulinae	sp.	1				E
Hemiptera							
Heteroptera							
aff. Pyrrhocoroidea							
Reduviidae							
	Reduviinae	jovens	1	0,0385	3	0,1034	E P
Homoptera							
Cixiidae		jovens	1				P
Hymenoptera							
Proctotrupoidea							
Diapriidae		sp.2			1		E
Vespoidea							
Formicidae							

Camponotus	sp.1	2		1		E P
Crematogaster	sp.1	1				E
Cyphomyrmex	sp.1	1				E
Pheidole	sp.2	2		1		E P
Isoptera						
Termitidae						
Armitermes	sp.			2		E P
Nasutitermes	sp.	1		2		E P
Lepidoptera						
Noctuoidea	jovens	1				P
Noctuidae	sp.2	2				E
Orthoptera						
Ensifera						
Phalangopsidae						
Paraclodes	sp.1	6	0,2308	20	0,6897	E
Phalangopsis	sp.1	4	0,1538	2	0,069	P
Psocoptera						
Psocomorpha						
Epipsocidae	jovens			1		P
Mesepipsocus	sp.1	1				P
	sp.2	1				E
Trogiomorpha	jovens			1		P
Chordata						
Amphibia						
Anura						
Neobatrachia						
Strabomantidae						
Pristimantis	*fenestratus*	1	0,0385			E
Mammalia						
Chiroptera						
Phyllostomidae						
Glossophaginae	sp.	3	0,1154			E
Nemathelminthes	sp.	1				P
