# Supplementary material for: Reconciling Mining with the Conservation of Cave Biodiversity: A Quantitative Baseline to Help Establish Conservation Priorities
Source: PLoS One. 2016 Dec 20;11(12):e0168348. doi: 10.1371/journal.pone.0168348 (PMC5173368; doi:10.1371/journal.pone.0168348)
Supplement: S1 Dataset — (ZIP) [file pone.0168348.s002.zip › Taxa/Serra Sul/SS_2010/CAV_28.pdf]

CAV-28				1ª	AB	2ª	AB	ZON
Annelida								
Clitellata								
Oligochaeta		jovens		1	0,1429			E
Arthropoda								
Arachnida								
Acari								
Ixodida								
	Ixodidae							
		Amblyomma	sp.			2		E
Parasitiformes								
Mesostigmata								
	Ascidae		sp.3			1		E
Trombidiformes								
Tydeoidea								
	Bdellidae		sp.1			1		E
			sp.7	1				E
Araneae								
	Araneidae							
		Alpaida	*septemmammata*			1		E
	Corinnidae	jovens		1	0,1429			E
		Tupirina	sp.1			1	0,100	E
	Ctenidae	jovens				1	0,100	E
	Pholcidae							
		Mesabolivar	*aurantiacus*			1		E
	Theridiidae	jovens				1		E
		Theridion	sp.2	1				E
	Theridiosomatidae							
		Natlo	sp.1			1		E
	Trechaleidae	jovens				1	0,100	E
Opiliones				1				
Eupnoi								
	Sclerosomatidae	jovens				1		E
Laniatores								
		jovens				1	0,100	E
	Escadabiidae		sp.3	1				E
			sp.6	1				E
Pseudoscorpiones								
	Bochicidae		sp.1	2				E
		Spelaeochernes	sp.1	1				E
		Pseudochthonius	sp.1			1		E
	Tridenchthoniidae		sp.1			2		E
Chilopoda								
Pleurostigmophora								
Geophilomorpha								
	Geophilidae		sp.1			1	0,100	E
Scolopendromorpha		jovens				2		
	Scolopocryptopidae							
		Scolopocryptops	*guacharensis*			1	0,100	E
Diplopoda								
Polydesmida								
	Chelodesmidae		sp.1	1	0,1429			E
	Pyrgodesmidae		sp.2	1	0,1429			E
		jovens		1				E
Entognatha								
Diplura								
	Campodeidae		sp.1	2				E
Insecta								
Coleoptera								
	Staphylinidae		sp.39			1		E
			sp.40	1				E
		jovens		1				E
Collembola								
Arthropleona								
Entomobryoidea								
	Entomobryidae		sp.1			1		E
	Paronellidae		sp.1	1		2		E

		sp.9	1				E
Diptera							
Brachycera							
Phoridae							
	aff. *Trophodeinus*	sp.	1				E
	Metopininae	sp.			1		E
Nematocera							
	jovens		2				E
Tipulidae							
	Tipulinae	sp.	2				E
Hemiptera							
Heteroptera							
Dipsocoroidea	jovens		1				E
Homoptera							
Cicadellidae	jovens		1				E
Cixiidae	jovens		2		1		E
Hymenoptera							
Chrysidoidea							
Bethylidae		sp.1	1				E
Vespoidea							
Formicidae							
	Brachymyrmex	sp.1			1		E
	Camponotus	sp.1	1		1		E
	Nylanderia	sp.1	1				E
	Octostruma	sp.1	1				E
	Pachycondyla	*striata*	1				E
	Pheidole	sp.1			1		E
		sp.2	1				E
Isoptera							
Termitidae							
	Diversitermes	sp.			1		E
	Nasutitermes	sp.	1		1		E
Lepidoptera							
Castnioidea	jovens		1				E
Orthoptera							
Ensifera							
Phalangopsidae	jovens						
	Paraclodes	sp.			2	0,200	E
	Phalangopsis	sp.	2	0,2857			E
Psocoptera							
Psocomorpha	jovens				1		E
Malacostraca							
Isopoda							
Dubioniscidae		sp.2			1		E
Philosciidae		sp.1	1				E
Chordata							
Amphibia							
Anura							
Neobatrachia							
Strabomantidae							
	Pristimantis	*fenestratus*			1	0,100	E
Mammalia							
Chiroptera			2				
Emballonuridae							
	Peropteryx	sp.	2	0,1429	1	0,100	E
Nemathelminthes		sp.	1				E
