# Supplementary material for: Reconciling Mining with the Conservation of Cave Biodiversity: A Quantitative Baseline to Help Establish Conservation Priorities
Source: PLoS One. 2016 Dec 20;11(12):e0168348. doi: 10.1371/journal.pone.0168348 (PMC5173368; doi:10.1371/journal.pone.0168348)
Supplement: S1 Dataset — (ZIP) [file pone.0168348.s002.zip › Taxa/Serra Sul/SS_2010/CAV_29.pdf]

CAV-29		1ª	AB	2ª	AB	ZON
Annelida						
Clitellata						
Oligochaeta	jovens	1				P
Arthropoda						
Arachnida						
Acari						
Ixodida	jovens	1				P
Parasitiformes						
Mesostigmata						
Macrochelidae	sp.1	1				P
Sarcoptiformes						
Oribatida	sp.3					P
	sp.9	1				P
	sp.2	1				P
Trombidiformes						
Tydeoidea	sp.7	2				P
Araneae						
Corinnidae						
Creugas	sp.1			1	0,14	P
Scytodidae	jovens	1				P
Scytodes	sp.	3	0,06			E
Opiliones						
Laniatores	jovens					
Stygnidae	sp.1	2	0,04			P
Pseudoscorpiones						
Spelaeochernes	sp.1			1	0,14	P
Entognatha						
Diplura						
Campodeidae	sp.1	1				P
Insecta						
Coleoptera	jovens			1		P
Collembola						
Arthropleona						
Entomobryoidea						
Entomobryidae	sp.1			1		P
	sp.8			1		P
Isotomidae	sp.1	1				P
Paronellidae	sp.1	1				P
Diptera						
Brachycera						
Phoridae						
Metopininae	sp.	2				P
Nematocera						
Psychodidae	sp.					
Sciopemyia	*sordellii*	1		1		P
Tipulidae						
Tipulinae	sp.	1		1		E P
	jovens	1		1		P
Hemiptera						
Heteroptera						
aff. Pyrrhocoroidea						
Reduviidae	jovens			1	0,14	P
Hymenoptera						
Vespoidea						
Formicidae						
Apterostigma	sp.1			1		P
Camponotus	*atriceps*	1				P
	sp.1	1				P
Crematogaster	sp.1	2		1		P
Nylanderia	sp.1	1				P
Octostruma	sp.1	1				P
Pheidole	sp.2	2				P
Solenopsis	sp.1			1		P
	sp.2	2				P
Isoptera						
Termitidae						

Nasutitermes	sp.	2				P
Orthoptera						
Ensifera						
Paraclodes	sp.1	20	0,41	3	0,43	E
Phalangopsis	sp.1	15	0,31			P
Thysanoptera						
Thripidae	sp.1	1	0,02	1	0,14	P
Malacostraca						
Isopoda						
Scleropactidae	sp.	1				P
Chordata						
Amphibia						
Anura						
Neobatrachia						
Strabomantidae						
Pristimantis	*fenestratus*	3	0,06			P
Mammalia						
Chiroptera						
Phyllostomidae						
Carollia	*perspicillata*	10	0,2			P
Glossophaginae	sp.	2	0,04			P
Micronycteris	sp.	2	0,04			P
