# Supplementary material for: Reconciling Mining with the Conservation of Cave Biodiversity: A Quantitative Baseline to Help Establish Conservation Priorities
Source: PLoS One. 2016 Dec 20;11(12):e0168348. doi: 10.1371/journal.pone.0168348 (PMC5173368; doi:10.1371/journal.pone.0168348)
Supplement: S1 Dataset — (ZIP) [file pone.0168348.s002.zip › Taxa/Serra Sul/SS_2010/CAV_30.pdf]

CAV-30		1ª	AB	2ª	AB	ZON
Annelida						
Clitellata						
Oligochaeta	jovens	1	0,0417			E
Arachnida						
Acari						
Sarcoptiformes						
Oribatida	sp.8	1				E
Acaridae	sp.1	1				E
	sp.1	1				E
	sp.19	1				E
Araneae						
Scytodidae						
Scytodes	*eleonorae*			1	0,0667	E
	sp.			1	0,0667	E
Trechaleidae ens		1	0,0417			E
Opiliones						
Eupnoi	jovens			6	0,4	E
Sclerosomatidae	sp.1	1		1		E
Insecta						
Blattodea	jovens	1				E
Coleoptera						
Hydrophilidae						
Sphaeridiinae	sp.2	1				E
Staphylinidae	sp.41			1		E
	sp.42			1		E
	sp.43			1		E
Pselaphinae	sp.3	1				E
Diptera						
Nematocera	jovens	1		1		E
Tipulidae						
Tipulinae	sp.	1		1		E
Hemiptera						
Heteroptera						
aff. Pyrrhocoroidea						
Veliidae ens				1		E
Hymenoptera						
Vespoidea						
Formicidae						
Dolichoderus	*bispinosus*			1		E
Pheidole	sp.1	1		1		E
Isoptera						
Termitidae						
Nasutitermes	sp.	1				E
Orthoptera						
Ensifera						
Phalangopsidae						
Paraclodes	sp.1	3	0,125			E
Malacostraca						
Decapoda						
Astacidea	sp.	10	0,4167			E
Palaemonidae ens		1				E
Symphyla						
Scutigerellidae						
Hanseniella	sp.1	1				E
Amphibia						
Anura						
Neobatrachia						
Strabomantidae						
Pristimantis	*fenestratus*	2	0,0833	2	0,1333	E
Mammalia						
Chiroptera	sp.			5	0,3333	E
Phyllostomidae						
Anoura	sp.	2	0,0833			E
Carollia	*perspicillata*	3	0,125			E
Reptilia						
Squamata						

Cryptodira					
Gymnophthalmidae					
Neusticurus sp.	2	0,0833			E
