# Supplementary material for: Reconciling Mining with the Conservation of Cave Biodiversity: A Quantitative Baseline to Help Establish Conservation Priorities
Source: PLoS One. 2016 Dec 20;11(12):e0168348. doi: 10.1371/journal.pone.0168348 (PMC5173368; doi:10.1371/journal.pone.0168348)
Supplement: S1 Dataset — (ZIP) [file pone.0168348.s002.zip › Taxa/Serra Sul/SS_2010/CAV_31.pdf]

CAV-31				1ª	AB	2ª	AB	ZON
Arthropoda								
Arachnida								
Acari								
Parasitiformes								
Mesostigmata								
	Podocinidae		sp.1	1				E
Trombidiformes								
	Anystidae	*rythracarus*	*nasutus*	2				E
	Bdellidae		sp.1	1				E
	Eupodidae		sp.1	1				E
	Tydeidae		sp.5	1				E
Amblypygi								
	Phrynidae							
		Heterophrynus	sp.	3	0,0588			E
Araneae				1				
		jovens		1	0,0196			E
	Araneidae	jovens		1				E
		Alpaida	sp.2	1				E
	Corinnidae	jovens		2	0,0392			E
	Ochyroceratidae	jovens		1				E
	Oonopidae	jovens		2				E
	Pholcidae	jovens						
		Mesabolivar	sp.1	1				E
	Salticidae	jovens		1				E
	Scytodidae	jovens		2	0,078			E
		Scytodes	sp.	2				E
	Theridiidae	jovens		1				E
Opiliones				2				
		jovens		1	0,0196			E
Laniatores								
	Escadabiidae		sp.3	1				E
	Stygnidae							
			sp.1	2	0,0392			E
Pseudoscorpiones								
		Spelaeochernes	sp.1	2				E
Chilopoda								
Notostigmophora								
Scutigeromorpha								
	Pselliodidae	jovens		1				E
Spirostreptida		jovens		2				E
Entognatha								
Diplura								
	Campodeidae							
			sp.1	1				E
Insecta								
Blattodea		jovens		2	0,0392			E
Coleoptera								
		jovens		2				E
	Carabidae		sp.3	1				E
	Staphylinidae		sp.43	1				E
		Pselaphinae	sp.5	1				E
Collembola								
Arthropleona								
Entomobryoidea								
	Entomobryidae		sp.7	1				E
	Paronellidae		sp.1	1				E
Diptera								
Nematocera								
	Psychodidae							
		Pintomyia	*gruta*	1				E
		Sciopemyia	*sordellii*	1				E
	Tipulidae							
		Tipulinae	sp.	2				E
Hemiptera								
Heteroptera								
Dipsocoroidea		jovens		1				E

Reduviidae	jovens		2	0,058			E
	Reduviinae	jovens	1				E
Homoptera							
Cixiidae	jovens		1				E
Hymenoptera							
Proctotrupoidea							
Scelionidae		sp.1	1				E
Vespoidea							
Formicidae							
	Eciton	cf. *burchelii*			1		E
	Pachycondyla	*striata*	1				E
	Wasmania	*auropunctata*	1				E
Isoptera							
Termitidae							
	Embiratermes	sp.	1				E
Lepidoptera							
	jovens		1				E
Noctuoidea		sp.2	1				E
Noctuidae		sp.1	1	0,0196			E
Orthoptera							
Ensifera							
Phalangopsidae							
	Paraclodes	sp.1	20	0,3922			E
	Phalangopsis	sp.1	8	0,1569			E
Psocoptera							
Psocomorpha	jovens		1				E
Malacostraca							
Isopoda							
Philosciidae		sp.1	2				E
Chordata							
Amphibia							
Anura							
Neobatrachia							
Strabomantidae							
	Pristimantis	*fenestratus*	2	0,0392			E
Mammalia							
Chiroptera							
Phyllostomidae							
	Glossophaginae	sp.	1	0,0196			E
Mollusca							
Gastropoda							
Systrophiidae							
	Happia	sp.	1				E
Nemathelminthes							
		sp.	1	0,0196			E
