# Supplementary material for: Reconciling Mining with the Conservation of Cave Biodiversity: A Quantitative Baseline to Help Establish Conservation Priorities
Source: PLoS One. 2016 Dec 20;11(12):e0168348. doi: 10.1371/journal.pone.0168348 (PMC5173368; doi:10.1371/journal.pone.0168348)
Supplement: S1 Dataset — (ZIP) [file pone.0168348.s002.zip › Taxa/Serra Sul/SS_2010/CAV_32.pdf]

CAV-32				1ª	AB	2ª	AB	ZON
Arthropoda								
Arachnida								
Acari								
Ixodida		jovens		2				E
Parasitiformes								
Mesostigmata								
	Laelapidae		sp.3	1				E
Sarcoptiformes								
			sp.1	1				E
			sp.2	1				E
Oribatida								
	Anoetidae		sp.1	1				E
Trombidiformes								
			sp.5	1				E
	Anystidae							
		Erythracarus	*nasutus*	1				E
Amblypygi								
	Phrynidae							
		Heterophrynus	sp.	7	0,12	3	0,1071	E
Araneae								
	Anapidae	jovens		1				E
	Araneidae	jovens		3				E
		Alpaida	sp.2			1		E
		Eriophora	*fuliginea*	1	0,0182			E
	Corinnidae	jovens		1	0,0182			E
	Ctenidae	jovens		1	0,0182			E
	Ochyroceratidae							
		Ochyrocera	sp.3			1		E
	Oonopidae	jovens		1				E
	Pholcidae	jovens		1		1		E
		Leptopholcus	sp.1			1		E
		Mesabolivar	*aurantiacus*	1		1		E
			cambridgei			1		E
	Prodidomidae	jovens				1		E
		Lygromma	sp.3	1				E
	Salticidae	jovens		1		1		E
	Scytodidae	jovens		2				E
		Scytodes	*globula*			1	0,0357	E
		Scytodes	sp.			3	0,1071	E
	Theridiidae	jovens		1				E
		Theridion	sp.1			2		E
			sp.2	1				E
	Theridiosomatidae							
		Plato	sp.1	1				E
	Trechaleidae	jovens				1	0,0357	E
Opiliones								
Laniatores								
	Cosmetidae		sp.1			1	0,0357	E
		Roquettea	*singularis*	1	0,0182			E
Pseudoscorpiones								
	Chernetidae	jovens				2		E
		Pseudochthonius	sp.1	1		1		E
Schizomida								
	Hubbardiidae	jovens		1		1		E
Chilopoda								
Notostigmophora								
Scutigeromorpha		jovens				2	0,0714	E
	Pselliodidae	jovens				1		E
Pleurostigmophora								
Scolopendromorpha								
	Scolopocryptopidae							
		Newportia	sp.1	1	0,0182			E
Diplopoda								
		jovens		1				E
Polydesmida								
	Chelodesmidae		sp.3	1	0,0182			E

Spirostreptida		jovens			1		E
	Pseudonannolenidae	jovens	1	0,0182			E
Entognatha							
Diplura							
	Campodeidae	sp.1	2				E
Insecta							
Blattodea		jovens	2	0,0364		0,0357	E
	Blaberidae	jovens	2	0,0364	2	0,07	E
	Blattellidae	sp.2			1	0,0357	E
Coleoptera							
		jovens	3		1		E
	Leiodidae	sp.3	1				E
	Staphylinidae	sp.44	1				E
	Staphylinidae	sp.45	1				E
	Staphylinidae	sp.46	1				E
Collembola							
Arthropleona							
Entomobryoidea							
	Cyphoderidae	sp.1			1		E
	Paronellidae	sp.6			1		E
Diptera							
		jovens	1				E
Brachycera							
	Phoridae						
	Metopininae	sp.	1				E
	Tabanidae	sp.			1		E
Nematocera							
	Ceratopogonidae	sp.	1				E
	Psychodidae						
	Sciopemyia	*sordellii*	1				E
	Sciaridae	sp.	1				E
	Tipulidae						
	Tipulinae	sp.	1				E
Hemiptera							
Heteroptera							
	Reduviidae	jovens	1		1	0,0357	E
	Panstrongylus	sp.1	1	0,036			E
Homoptera							
	Cixiidae	sp.3	1				E
Hymenoptera							
Vespoidea							
	Formicidae						
	Gnamptogenys	*striatula*	1		2		E
	Nylanderia	sp.1			1		E
	Pachycondyla	*harpax*	2				E
Isoptera							
	Termitidae						
	Nasutitermes	sp.	2		2		E
Orthoptera							
Ensifera							
	Phalangopsidae						
	Paraclodes	sp.1	20	0,3636	10	0,3571	E
	Phalangopsis	sp.1	7	0,1273			E
Psocoptera							
Psocomorpha		jovens	2		1		E
	Epipsocidae						
	Epipsocus	sp.2			1		E
Thysanoptera							
	Phlaeothripidae	sp.2	1				E
Thysanura							
	Nicoletiidae	sp.1	1				E
Malacostraca							
Isopoda							
	Philosciidae	sp.1	2				E
Chordata							
Amphibia							
Anura							

Neobatrachia							
Strabomantidae							
Pristimantis	*fenestratus*	3	0,0545	2	0,0714	E
Leiuperidae							
Physalaemus	sp.	1	0,0182			E
Mammalia							
Chiroptera							
Phyllostomidae							
Glossophaginae	sp.	3	0,0545			E
Reptilia							
Squamata							
Gekkonidae							
Thecadactylus	*rapicauda*			1	0,0357	E
Platyhelminthes							
Turbellaria	sp.5	1	0,0182			E
