# Supplementary material for: Reconciling Mining with the Conservation of Cave Biodiversity: A Quantitative Baseline to Help Establish Conservation Priorities
Source: PLoS One. 2016 Dec 20;11(12):e0168348. doi: 10.1371/journal.pone.0168348 (PMC5173368; doi:10.1371/journal.pone.0168348)
Supplement: S1 Dataset — (ZIP) [file pone.0168348.s002.zip › Taxa/Serra Sul/SS_2010/CAV_33.pdf]

CAV-33		2ª	AB	ZON
Arthropoda				
Arachnida				
Acari				
Parasitiformes				
Mesostigmata				
Ascidae	sp.1	1		E
Trombidiformes	sp.1	1		E
Amblypygi				
Phrynidae				
Heterophrynus	sp.	6	0,14	P
Araneae				
Corinnidae	jovens	1	0,02	E
Pholcidae				
Mesabolivar	sp.1	1		E
Scytodidae				
Scytodes	*eleonorae*	1		P
Scytodes	sp.	5	0,14	E
Opiliones		3		
	jovens	1	0,02	P
Cyphophthalmi				
Neogoveidae				
Canga	*renatae*	1		P
Eupnoi				
	jovens	1		E
Laniatores				
Cosmetidae	jovens	1	0,02	P
Stygnidae	sp.1	1	0,02	P
Polyxenida				
	jovens	1		E
Insecta				
Coleoptera				
Staphylinidae				
Pselaphinae	sp.12	1		E
Nematocera				
Cecidomyiidae				
Cecidomyiinae	sp.	2		E P
Tipulidae				
Tipulinae	sp.	1		E
Hemiptera				
Heteroptera				
Cydninae	sp.1	1		E
Reduviidae	jovens	1	0,02	E
Emesinae	sp.4	1		P
Reduviinae	sp.	5	0,1	E
Homoptera				
Cixiidae	sp.4	1		P
Hymenoptera				
Vespoidea				
Formicidae				
Camponotus	sp.1	1		P
Carebara	sp.1	2		E P
Ectatomma	*tuberculatum*	1		E
Pheidole	sp.2	1		E
Isoptera				
Termitidae				
Embiratermes	sp.	1		P
Nasutitermes	sp.	1		P
Lepidoptera				
	jovens	1		P
Noctuoidea	sp.2	1		E
	sp.4	1		E

Orthoptera						
Ensifera						
Phalangopsidae						
	Paraclodes	sp.1		14	0,29	E
	Phalangopsis	sp.		5	0,1	P
Psocomorpha						
jovens			1			
Mammalia						
Chiroptera		sp.		1	0,02	P
Rodentia		sp.		1	0,02	P
Reptilia						
Squamata						
Gekkonidae						
	Thecadactylus	*rapicauda*		1	0,02	E
