# Supplementary material for: Reconciling Mining with the Conservation of Cave Biodiversity: A Quantitative Baseline to Help Establish Conservation Priorities
Source: PLoS One. 2016 Dec 20;11(12):e0168348. doi: 10.1371/journal.pone.0168348 (PMC5173368; doi:10.1371/journal.pone.0168348)
Supplement: S1 Dataset — (ZIP) [file pone.0168348.s002.zip › Taxa/Serra Sul/SS_2010/CAV_34.pdf]

CAV-34		1ª	AB	2ª	AB	ZON
Arthropoda						
Arachnida						
Acari						
Ixodida	jovens	1				E
Argasidae						3
Ornithodoros	sp.			5		P
Ixodidae						
Amblyomma	sp.	1				P
Ixodidae	sp.1	1				P
Ornithodoros	sp.1	3				E P
Mesostigmata						
	sp.4			1		P
	sp.5			1		P
Laelapidae						
Stratiolaelaps	sp.1	1		1		E P
Opilioacarida						
Opilioacaridae						
	sp.1	2		5		E P
Sarcoptiformes						
	sp.1	2				E P
	sp.2	3		1		E P
Oribatida						
	sp.1			1		P
	sp.2	2		4		P
	sp.3	4		1		P
	sp.5			1		P
Trombidiformes						
	sp.1	1				P
	sp.6	1				P
Tydeoidea						
Cheyletidae	sp.3	1				E
Rhagidiidae	sp.1	1				E
	sp.2	1				P
Scutacaridae	sp.1	1				P
Amblypygi						
Phrynidae						
Heterophrynus	sp.	2	0,0084			P
Araneae						
Barychaelidae	jovens	1	0,0042			P
gen.1	sp.1			1	0,0026	E
Corinnidae						
Creugas	sp.1	1	0,0042			P
Tupirina	sp.1			2	0,0051	E
Ctenidae	jovens	1	0,0042	1	0,0026	P
Ochyroceratidae	jovens	4		5		E P
Ochyrocera	sp.1	6				E P
	sp.2	1				E
Speocera	sp.1	3		1		E P
Oonopidae	jovens	1		2		E
Pholcidae	jovens	1		1		E P
Mesabolivar	sp.1			1		E
Ninetinae	sp.1	7		10		E P
Salticidae	jovens	1				E
Scytodidae	jovens	4		4		E P
Scytodes	*eleonorae*			4	0,01	P
Segestriidae	jovens	6		5		E P
Ariadna	sp.1	3		4		E P
Symphytognathidae						
Anapistula	sp.1	1				P
Tetrablemmidae	jovens	3				E P
Matta	sp.1	1				P
Tetragnathidae	jovens	1				P
Theraphosidae	jovens	3	0,0126	1	0,0026	E P
Theridiosomatidae	jovens	1				E
Plato	sp.1			1		E
Opiliones						

Cyphophthalmi							
Neogoveidae							
	Canga	*renatae*	4				E P
Laniatores							
Escadabiidae	jovens		1				P
		sp.2	2		1		P
Stygnidae	jovens		1				P
		sp.1	2	0,012	1	0,0026	E P
Palpigradi							
Eukoeneniidae	jovens		1				E
	Allokoenenia	sp.1			1		P
Pseudoscorpiones							
aff. Olpiidae		sp.2			1		P
Bochicidae		sp.1	4		4		P
	Spelaeochernes	sp.1	6		3		E P
Chthoniidae	jovens				2		E
	Pseudochthonius	sp.1	5		2		E P
Olpiidae		sp.1	8		10		E P
		sp.2			1		P
Schizomida							
Hubbardiidae	jovens		1				E
Chilopoda							
Notostigmophora							
Scutigeromorpha	jovens		1	0,0042	2	0,0051	P
Pselliodidae	jovens				3		E P
	Sphendononema	*guildingii*	1				P
Pleurostigmophora							
Geophilomorpha							
Ballophilidae		sp.1	2	0,0084			P
Scolopendromorpha							
Cryptopidae							
	Cryptops	sp.1	3	0,0126			E P
Diplopoda							
Polydesmida							
Pyrgodesmidae		sp.2	1	0,0042	1	0,0026	P
Spirostreptida							
Pseudonannolenidae	jovens				1		E
	Pseudonannolene	sp.1	2	0,0084	1	0,005	P
Entognatha							
Diplura							
Campodeidae		sp.1	1				E
Insecta							
Blattodea	jovens		35	0,1464	37	0,0944	E P
Blaberidae	jovens		4	0,0167	4		E P
Blaberidae		sp.4			1	0,012	P
Blattellidae		sp.1	1	0,0042			E
Blattidae	jovens		2	0,0084	3	0,0077	P
Coleoptera	jovens		3		3		E P
		sp.3	1				P
Carabidae		sp.11	1		1		P
Chrysomelidae		sp.2			1		P
Ptiliidae		sp.1	1				P
Scydmaenidae		sp.10	1				P
		sp.2	1		1		P
		sp.5	1				E
Staphylinidae							
	Pselaphinae	sp.13			1		P
		sp.5			1		P
Collembola							
Arthropleona							
Entomobryoidea							
Cyphoderidae		sp.1	1				E
Entomobryidae		sp.1	1				P
		sp.4			1		P
		sp.6			2		E P
Paronellidae		sp.1	1		1		E P
		sp.4	2				P

Entomobryoidea	sp.1	1				P
Neelipleona						
Neelidae	sp.1	1				P
Symphypleona						
Sminthuroidea	sp.1	1				P
	sp.2	2				P
Dermaptera	jovens	1				P
Diptera	jovens	4		1		E P
Brachycera						
Camillidae	sp.	3				E P
Streblidae						
Trichobius	sp.			1		P
Nematocera						
Cecidomyiidae						
Cecidomyiinae	sp.	2				E P
Psychodidae						
Edentomyia	*piauensis*	2		1		P
Pintomyia	*gruta*	1				E
Sciaridae						
Epidapus	sp.	2				P
Hemiptera						
Heteroptera						
Reduviidae	jovens	4	0,0167	6		E P
Reduviinae	sp.			36	0,107	P
Schizopteridae						
Hypselosomatinae	sp.1			1		P
Schizopterinae	sp.3	1		1		P
Homoptera						
Aleyrodidae	sp.1			1		P
Cicadellidae	sp.3			1		E
Cixiidae	jovens	6		4		E P
Hymenoptera						
Chrysidoidea						
Bethylidae	sp.1			1		P
Vespoidea						
Formicidae						
Acromyrmex	sp.1			2		E P
Apterostigma	sp.1	1				P
Camponotus	sp.1	5		10		E P
Crematogaster	sp.1			1		E
Gnamptogenys	*striatula*	1				P
Hypoponera	sp.1	1		1		P
Pachycondyla	*striata*	3		2		E P
Solenopsis	sp.2			1		P
	sp.3	3				P
Isoptera						
operários				1		P
Lepidoptera	jovens	6		4		E P
Gelechioidea	sp.3			1		E
Tineoidea	sp.1	2		3		E P
Noctuidae	sp.	1	0,0042			P
Neuroptera						
Myrmeleonthidae	jovens			3		E P
Orthoptera						
Ensifera						
Phalangopsidae						
Phalangopsis	sp.1	1	0,0042			P
Paraclodes	sp.	1	0,0042	16	0,0408	P
Phalangopsis	sp.	80	0,3347	57	0,1454	P
Psocoptera						
Psocomorpha	jovens	1		3		E P
Ectopsocidae						
Ectopsocus	sp.1			1		P
Epipsocidae						
Epipsocus	sp.2			1		P
Mesepipsocus	sp.1			1		P
Ptiloneuridae						

	Triplocania	sp.4	1				E
Troctomorpha	jovens		2				E P
Liposcelididae	jovens	sp.			1		P
Manicapsocidae	*entomum*	sp.1	2				P
Trogiomorpha							
Psyllipsocidae	jovens		2		3		P
	Psyllipsocus	sp.1	2		10		E P
Thysanura							
Nicoletiidae	jovens		2				P
		sp.1	2		1		E P
Malacostraca							
Isopoda							
Dubioniscidae		sp.1	9		8		E P
Pauropoda							
Tetramerocerata		sp.	1		1		E
Symphyla							
Scutigerellidae	jovens		2				P
	Hanseniella	sp.1	3				E P
	Scutigerella	sp.1			1		P
Chordata							
Amphibia							
Anura							
Neobatrachia							
Strabomantidae							
	Pristimantis	*fenestratus*	7	0,0293	4	0,0102	P
Mammalia							
Chiroptera							
Emballonuridae							
	Peropteryx	*kappleri*	28	0,1172	10	0,0255	P
Furipteridae							
	Furipterus	*horrens*	20	0,0837	7	0,0179	P
Phyllostomidae							
	Anoura	sp.			150	0,3827	P
	Carollia	sp.			5	0,0128	P
	Glossophaginae	sp.	33	0,1381	40	0,102	P
Reptilia							
Squamata							
Serpentes							
Colubridae		sp.			1	0,0026	E
Mollusca							
Gastropoda							
Systrophiidae							
	Happia	sp.	2				E
Nemathelminthes							
		sp.	2	0,0084			P
