# Supplementary material for: Reconciling Mining with the Conservation of Cave Biodiversity: A Quantitative Baseline to Help Establish Conservation Priorities
Source: PLoS One. 2016 Dec 20;11(12):e0168348. doi: 10.1371/journal.pone.0168348 (PMC5173368; doi:10.1371/journal.pone.0168348)
Supplement: S1 Dataset — (ZIP) [file pone.0168348.s002.zip › Taxa/Serra Sul/SS_2010/CAV_35.pdf]

CAV-35		1ª	AB	2ª	AB	ZON
Annelida						
Clitellata						
Oligochaeta	jovens			1	0,0127	P
Arthropoda						
Arachnida						
Acari						
Parasitiformes						
Ixodi Ixodidae						
Ornithodoros	sp.1			1		P
Sarcoptiformes						
	sp.1	1				P
Oribatida						
	sp.3	1				E
	sp.5			1		P
	sp.9	1				E
Trombidiformes						
	sp.11	1				P
	sp.4	1				E
	sp.5			1		P
	sp.6			1		P
Tydeoidea						
Eupodidae	sp.1	1				P
Amblypygi						
Phrynidae						
Heterophrynus	sp.	9	0,1636	10	0,1266	P
Araneae						
Anapidae						
	sp.1	1		1		P
Araneidae	jovens			1		P
Alpaida	sp.2	1				P
Ctenidae	jovens	1	0,0182			P
Nesticidae	jovens	1				P
Ochyroceratidae	jovens	1				P
Speocera	sp.1	1		1		P
Oonopidae	jovens	1		1		E P
Capitato	sp.2			1		P
gr. Xycarphius	sp.1			1		E
Pholcidae	jovens			2		P
Scytodidae	*jovens*	1	0,0182			P
Scytodes	*eleonorae*			1	0,0127	P
Theridiidae	jovens					
Theridion	sp.3			1		P
Theridiosomatidae	jovens					
Plato	sp.1	3		2		P
Trechaleidae	jovens	2	0,0364	3	0,038	E P
Opiliones		1		43		
	jovens	1	0,0182	1	0,0127	P
Laniatores						
Cosmetidae	jovens					E
	sp.1			1	0,0127	P
	sp.2			1	0,0127	P
Roquettea	*singularis*	1	0,0182	2	0,0253	P
Escadabiidae	jovens	1		1		P
	sp.2	1				P
Gonyleptidae	sp.1			1	0,0127	P
Stygnidae	jovens					P
	sp.1	3	0,0545	2	0,024	P
Pseudoscorpiones						
Chthoniidae	jovens			2		P
Tridenchthoniidae	sp.1			2		E
Ricinulei						
Ricinoididae						
Cryptocellus	sp.			1		P
	jovens			1		P
Schizomida						
Hubbardiidae						

Rowlandius		sp.			1		P
Chilopoda							
Notostigmophora							
Scutigeromorpha							
Pselliodidae	jovens				1		P
Pleurostigmophora							
Scolopendromorpha							
Scolopocryptopidae	jovens				1	0,0127	P
Diplopoda	jovens	1	1		4		E P
Polydesmida							
Aphelidesmidae		sp.1			1	0,0127	E
		sp.2			1	0,0127	P
Cyrtodesmidae		sp.1	1				E
Fuhrmannodesmidae		sp.1	1				E
Spirostreptida	jovens				1		P
Pseudonannolenidae	jovens		1	0,0182			P
Pseudonannolene		sp.1			1	0,0127	P
Insecta							
Blattodea	jovens		2	0,0364	1	0,0127	P
Blaberidae	jovens				1	0,0127	E
Coleoptera							
	jovens		3		2		E P
Carabidae		sp.17			1		E
Chrysomelidae		sp.2	1				P
Curculionidae		sp.2			1	0,0127	E
Protocucujidae		sp.1			1		E
Staphylinidae		sp.33			1		E
		sp.47			1		E
		sp.48	1				P
		sp.49	1				E
		sp.50	1				E
Pselaphinae		sp.3	1		2		P
Collembola							
Arthropleona							
Entomobryoidea							
Entomobryidae		sp.1	1				E
Paronellidae		sp.1	1		1		P
Diptera							
	jovens		1		3		E P
Brachycera							
Phoridae							
Metopininae		sp.	3		1		E P
Nematocera							
Cecidomyiidae							
Lestremiinae		sp.	1				P
Ceratopogonidae		sp.	1				P
Chironomidae		sp.	2				P
Mycetophilidae							
Euceroplatus		sp.			1		E
Psychodidae							
Pericoma		sp.			1		E
Sciopemyia		*sordellii*			3		P
Tipulidae							
Tipulinae		sp.	3		2		P
Ephemeroptera							
Euthyplociidae	jovens		1				P
Hemiptera							
Heteroptera							
Ceratocombidae							
Ceratocombinae		sp.1			1		P
Reduviidae	jovens		1	0,0182	1	0,0127	E P
Veliidae							
Paravelia		sp.1	1		1		P
Rhagovelia		sp.1	1				P
Homoptera							
Cicadellidae		sp.4			1		E
Cixiidae	jovens		3		2		E P

Hymenoptera						
Vespoidea						
Formicidae	jovens			2	0,0253	E
Camponotus	*atriceps*			2	0,0253	P
Crematogaster	sp.1	1		1		E
Dolichoderus	sp.1			1		P
Hypoponera	sp.1	1		1		P
Nylanderia	sp.1			2		E P
Pachycondyla	*harpax*			1	0,0127	P
Pheidole	sp.2	1				E
Solenopsis	sp.2	1		1		E
Isoptera						
Termitidae						
Nasutitermes	sp.	2				E
Lepidoptera						
	jovens	1				P
Noctuoidea	sp.2			1		P
Orthoptera						
Ensifera						
Phalangopsidae	jovens			1	0,0127	P
	sp.3			2	0,0253	E P
Phalangopsis	sp.1	10	0,18	21	0,2658	P
Paraclodes	sp.	6	0,1091	8	0,1013	P
Psocoptera						
Psocomorpha	jovens			2		P
Epipsocidae						
Epipsocus	sp.2			1		P
Trichoptera	jovens	1				P
Malacostraca	sp.					P
Decapoda						
Astacidea						
Palaemonidae						
Macrobrachium	sp.1	2	0,036			P
Pseudothelphusidae	jovens			1		P
Isopoda						
Philosciidae	sp.1	2		1		E P
	sp.2			1		E
Platyarthridae	sp.			2		E P
Scleropactidae	sp.	1				E
Chordata						
Amphibia						
Anura						
Neobatrachia						
Strabomantidae						
Pristimantis	*fenestratus*	14	0,2545	6	0,0759	P
Mammalia						
Chiroptera	sp.			3	0,038	P
Reptilia						
Squamata						
Gekkonidae						
Thecadactylus	*rapicauda*	1	0,0182			P
