# Supplementary material for: Reconciling Mining with the Conservation of Cave Biodiversity: A Quantitative Baseline to Help Establish Conservation Priorities
Source: PLoS One. 2016 Dec 20;11(12):e0168348. doi: 10.1371/journal.pone.0168348 (PMC5173368; doi:10.1371/journal.pone.0168348)
Supplement: S1 Dataset — (ZIP) [file pone.0168348.s002.zip › Taxa/Serra Sul/SS_2010/CAV_36.pdf]

CAV-36			1ª	AB	2ª	AB	ZON
Arthropoda							
Arachnida							
Acari							
Ixodida							
Ixodidae	*Amblyomma*	sp.			1		E
Ixodida	jovens		1				E
Parasitiformes							
Holothyrida							
Diplothyridae							
	Diplothyrus	*scubarti*	1				E
Sarcoptiformes							
Oribatida		sp.2			1		E
		sp.3			1		E
Sarcoptiformes		sp.1	1				E
		sp.2	1				E
Trombidiformes							
Tydeoidea							
Tydeidae		sp.1			1		E
Amblypygi							
Phrynidae							
	Heterophrynus	sp.	1	0,0667			E
Araneae							
Araneidae	jovens		1				E
	Alpaida	*smila*			1		E
Ctenidae	jovens				1	0,0313	E
Ochyroceratidae	jovens		1				E
Oonopidae	jovens						
	gr. Xycarphius	sp.5	1				E
Pholcidae	jovens				1		E
Theridiidae	jovens				1		E
	Dipoena	sp.1			1		E
	Theridion	sp.3			1		E
Theridiosomatidae	jovens						
	Plato	sp.1			1		E
Opiliones	jovens				6	0,1875	E
Eupnoi							
Sclerosomatidae	jovens				1		E
		sp.1			1		E
Laniatores							
Cosmetidae							
	Roquettea	*singularis*			1	0,0313	E
Escadabiidae		sp.2	1				E
Stygnidae		sp.1			2	0,0625	E
Pseudoscorpiones							
	Spelaeochernes	sp.1	2		1		E
	Pseudochthonius	sp.1	1		1		E
Ricinulei							
Ricinoididae	jovens		1				E
Schizomida							
Hubbardiidae							
	Rowlandius	sp.			1		E
Diplopoda							
Polydesmida							
Chelodesmidae		sp.4			1	0,0313	E
		sp.6	1	0,0667			E
Fuhrmannodesmidae		sp.1	1		1		E
Spirostreptida							
Pseudonannolenidae							
	Pseudonannolene	sp.1			1	0,0313	E
Entognatha							
Diplura							
Campodeidae		sp.1	2		2		E
Insecta							
Coleoptera							
Staphylinidae		sp.1					
	Pselaphinae	sp.2	1				E

Coleoptera	jovens		1				E
Collembola							
Arthropleona							
Entomobryoidea							
Entomobryidae		sp.1			1		E
Diptera							
Nematocera							
Mycetophilidae							
	Sciophilinae	sp.	1				E
Psychodidae							
	Sciopemyia	*sordellii*	1		1		E
Sciaridae							
	Bradysia	sp.			1		E
	Epidapus	sp.			2		E
Diptera	jovens		2		1		E
Hemiptera							
Heteroptera							
Reduviidae		sp.			1		E
Schizopteridae							
	Schizopterinae	sp.1			1		E
Homoptera							
Cixiidae	jovens				2		E
Hymenoptera							
Vespoidea							
Formicidae							
	Brachymyrmex	sp.1	1		1		E
	Pachycondyla	*striata*			2		E
	Pheidole	sp.2			1		E
	Wasmania	*auropunctata*			1		E
Isoptera							
Termitidae							
	Nasutitermes	sp.	1				E
Isoptera	operários				1		E
Lepidoptera	jovens		2		1		E
Orthoptera							
Ensifera							
Phalangopsidae							
	Phalangopsis	sp.	6	0,4	12	0,375	E
Thysanura							
Ateluridae	jovens		1				E
Malacostraca							
Isopoda							
Dubioniscidae		sp.2			2		E
Philosciidae		sp.1	1				E
Chordata							
Amphibia							
Anura							
Neobatrachia							
Strabomantidae							
	Pristimantis	*fenestratus*	2	0,1333	8	0,25	E
Chiroptera		sp.	5	0,3333			E
Mollusca							
Gastropoda							
Systrophiidae							
	Happia	sp.	1		1		E
Nemathelminthes		sp.			1		E
