# Supplementary material for: Reconciling Mining with the Conservation of Cave Biodiversity: A Quantitative Baseline to Help Establish Conservation Priorities
Source: PLoS One. 2016 Dec 20;11(12):e0168348. doi: 10.1371/journal.pone.0168348 (PMC5173368; doi:10.1371/journal.pone.0168348)
Supplement: S1 Dataset — (ZIP) [file pone.0168348.s002.zip › Taxa/Serra Sul/SS_2010/CAV_37.pdf]

CAV-37			2ª	AB	ZON
Arthropoda					
Arachnida					
Araneae					
Ctenidae	jovens		1	0,3333	E
Trechaleidae	jovens		1	0,3333	E
Insecta					
Diptera	jovens		1		E
Nematocera					
Culicidae					
	Culicini	sp.	1		E
Hemiptera					
Heteroptera					
Pyrrhocoroidea					
Pyrrhocoridae	jovens		1		E
Veliidae	jovens		1		E
	Paravelia	sp.1	1		E
Vespoidea					
Formicidae					
	Carebara	sp.1	1		E
	Crematogaster	sp.1	1		E
	Pheidole	sp.2	1		E
Mammalia					
Chiroptera		sp.	1	0,3333	E
