# Supplementary material for: Reconciling Mining with the Conservation of Cave Biodiversity: A Quantitative Baseline to Help Establish Conservation Priorities
Source: PLoS One. 2016 Dec 20;11(12):e0168348. doi: 10.1371/journal.pone.0168348 (PMC5173368; doi:10.1371/journal.pone.0168348)
Supplement: S1 Dataset — (ZIP) [file pone.0168348.s002.zip › Taxa/Serra Sul/SS_2010/CAV_38.pdf]

CAV-38		1ª	AB	2ª	AB	ZON
Arthropoda						
Arachnida						
Acari						
Ixodida						
Ixodidae						
Amblyomma	sp.			1		P
Mesostigmata						
Ologamasidae	sp.1	1				E
Otopheidomenidae	sp.1	1				P
Trombidiformes						
Anystidae						
Erythracarus	*nasutus*	1				E
Eupodidae	sp.1	1				P
Tydeidae	sp.1	1				E
Amblypygi						
Phrynidae						
Heterophrynus	sp.	2	0,0333	5	0,07	E P
Araneae						
Araneidae	jovens	3		1		E P
Corinnidae	jovens	1	0,0167			P
Ctenidae	jovens	1	0,0167			P
Ochyroceratidae	jovens	2		1		E P
Oonopidae						
gr. *Xycarphius*	sp.5	1		1		E P
Pholcidae	jovens			2		E
Prodidomidae						
Lygromma	sp.4			1		P
Scytodidae						
Scytodes	*globula*	1	0,0167			E
Theraphosidae						
gen.1	sp.1			1	0,0149	P
Trechaleidae	jovens			1	0,0149	E
Opiliones						
Laniatores						
Cosmetidae	jovens			2	0,0299	E
Roquettea	*singularis*	1	0,0167			E
Escadabiidae	sp.2	1				P
Stygnidae	jovens	1				P
Stygnidae	sp.1	1	0,033			E
Pseudoscorpiones						
Chernetidae	jovens	2				E
Spelaeochernes	sp.1	1		1		P
Pseudochthonius	sp.1	1				E
Pseudochthonius	sp.4	2				P
Olpiidae	sp.1			2		E
Scorpiones						
Buthidae						
Ananteris	*balzanii*	1	0,0167			P
Chilopoda						
Notostigmophora						
Scolopendromorpha	jovens	2	0,0333			E
Diplopoda						
Polydesmida	jovens	1				P
Polyxenida						
Hypogexenidae	sp.1			1		P
Entognatha						
Diplura	jovens	1				E
Campodeidae	sp.1	2				E P
Insecta						
Blattodea	jovens			1	0,0149	E
Blattellidae	sp.2			2	0,0299	E P
Polyphagidae	jovens	2	0,0333	2	0,0299	E P
Coleoptera	jovens	1		1		E P
Pselaphinae	sp.5	1				E
Collembola						
Arthropleona						

Entomobryoidea							
Paronellidae		sp.4	1				P
Diptera		jovens	2				E P
Brachycera							
Phoridae							
	Metopininae	sp.	1				E
Nematocera							
Ceratopogonidae		sp.	1				E
Psychodidae							
	Pintomyia	*gruta*			1		E
	Pintomyia	*sordellii*	1		2		E P
Sciaridae							
	Epidapus	sp.			1		P
Tipulidae							
	Tipulinae	sp.	1				E
Hemiptera							
Heteroptera							
Reduviidae		jovens	2	0,0333	2		E P
	Reduviinae	sp.			5	0,104	E
Homoptera							
Cixiidae		jovens	1				E
Hymenoptera							
Vespoidea							
Formicidae							
	Camponotus	sp.1	1		1		P
	Crematogaster	sp.1	1		2		E
	Cyphomyrmex	sp.1	1				P
	Gnamptogenys	*striatula*	1				E
	Hypoponera	sp.1			1		P
	Pachycondyla	*harpax*	1				E
	Pachycondyla	*striata*			1		E
	Pheidole	sp.2	1				E
Isoptera							
Termitidae							
	Atlantitermes	sp.	1				P
	Nasutitermes	sp.	2		3		E P
Lepidoptera							
Cossoidea		jovens	2				E P
Limacodidae		sp.1	1	0,0167			P
Orthoptera							
Ensifera							
Phalangopsidae							
	Paraclodes	sp.	20	0,3333	13	0,194	E P
	Phalangopsis	sp.	10	0,1667	30	0,4478	P
Psocoptera							
Trogiomorpha							
Psyllipsocidae							
	Psyllipsocus	sp.1			1		E
Malacostraca							
Isopoda							
Dubioniscidae		jovens			1		E
Dubioniscidae		sp.1			1		P
Philosciidae		sp.1	2				E P
Symphyla							
Scutigerellidae		jovens	1				P
Chordata							
Amphibia							
Anura							
Neobatrachia							
Strabomantidae							
	Pristimantis	*fenestratus*	1	0,0167	1	0,0149	E
Mammalia							
Chiroptera							
Phyllostomidae							
	Carollia	*perspicillata*	1	0,0167			E
	Glossophaginae	sp.	8	0,1333	2	0,0299	E P
Rodentia		sp.	2	0,0333			E

Reptilia					
Squamata					
Cryptodira					
Testudinidae					
Geochelone sp.	1	0,0167			E
Mollusca					
Gastropoda					
Systrophiidae					
Happia sp.	1		1		P
