# Supplementary material for: Reconciling Mining with the Conservation of Cave Biodiversity: A Quantitative Baseline to Help Establish Conservation Priorities
Source: PLoS One. 2016 Dec 20;11(12):e0168348. doi: 10.1371/journal.pone.0168348 (PMC5173368; doi:10.1371/journal.pone.0168348)
Supplement: S1 Dataset — (ZIP) [file pone.0168348.s002.zip › Taxa/Serra Sul/SS_2010/CAV_39.pdf]

CAV-39			1ª	AB	2ª	AB	ZON
Arthropoda							
Arachnida							
Acari							
Parasitiformes							
Mesostigmata							
Otopheidomenidae		sp.1			1		E
Sarcoptiformes							
Oribatida		sp.15			1		E
Amblypygi							
Phrynidae							
Heterophrynus		sp.	2	0,0625	15	0,5556	E
Araneae							
Araneidae	jovens		1				E
Corinnidae	jovens		1	0,0313			E
Filistatidae	jovens		1		1		E
Filistatidae		sp.1	1		1		E
Salticidae							
Freya		sp.1	1				E
Scytodidae							E
Scytodes		*eleonorae*			1	0,08	E
Scytodes		*globula*	2	0,062			E
Theridiidae	jovens						
Theridion		sp.1	1		1		E
Opiliones							
Laniatores							
Stygnidae		sp.1	1	0,0313			E
Pseudoscorpiones							
Chernetidae		sp.1	1				E
Olpiidae		sp.1	2				E
Chilopoda							
Notostigmophora							
Scutigeromorpha							
Pselliodidae	jovens				1		E
Geophilomorpha							
Geophilidae		sp.1	1	0,0313			E
Insecta							
Blattodea							
Blaberidae	jovens		1	0,0313	1	0,037	E
Collembola							
Arthropleona							
Entomobryoidea							
Paronellidae		sp.1	1				E
Dermaptera	jovens		1				E
Dermaptera		sp.2			1	0,037	E
Diptera	jovens		1				E
Nematocera							
Mycetophilidae							
Hesperodes		sp.			1		E
Psychodidae							
Sciopemyia		*sordellii*			1		E
Sciaridae		sp.	1				E
Tipulidae							
Tipulinae		sp.	2				E
Hemiptera							
Heteroptera							
Reduviidae	jovens		2	0,0625	2		E
Reduviinae		sp.			2	0,0741	E
Hymenoptera							
Vespoidea							
Formicidae							
Pheidole		sp.2	2				E
Isoptera							
Termitidae							
Atlantitermes		sp.	1				E
Nasutitermes		sp.	2		2		E
Lepidoptera							

Cossoidea						
Limacodidae	sp.1	1	0,0313			E
Hesperioidea						
Hesperiidae	sp.1			1		E
Noctuoidea	sp.2	1				E
Noctuidae	sp.	3	0,0938			E
Neuroptera						
Myrmeleonthidae	jovens			1		E
Orthoptera						
Ensifera						
Phalangopsidae						
Paraclodes	sp.	14	0,437	3	0,1111	E
Phalangopsis	sp.	2	0,0625			E
Psocoptera						
Psocomorpha	jovens	1				E
Chordata						
Amphibia						
Anura						
Neobatrachia						
Strabomantidae						
Pristimantis	*fenestratus*	1	0,0313	1	0,037	E
Mammalia						
Chiroptera						
Emballonuridae				1		E
Peropteryx	sp.	1	0,0313	2	0,037	
Reptilia						
Squamata						
Cryptodira						
Gekkonidae						
Thecadactylus	*rapicauda*			1	0,037	E
