# Supplementary material for: Reconciling Mining with the Conservation of Cave Biodiversity: A Quantitative Baseline to Help Establish Conservation Priorities
Source: PLoS One. 2016 Dec 20;11(12):e0168348. doi: 10.1371/journal.pone.0168348 (PMC5173368; doi:10.1371/journal.pone.0168348)
Supplement: S1 Dataset — (ZIP) [file pone.0168348.s002.zip › Taxa/Serra Sul/SS_2010/CAV_40.pdf]

CAV-40		1ª	AB	2ª	AB	ZON
Arthropoda						
Arachnida						
Acari						
Parasitiformes						
Ixodida						
Ixodidae						
Ornithodoros	sp.1	1				E
Sarcoptiformes	sp.1	1				P
Sarcoptiformes	sp.2	1				E
Trombidiformes	sp.5			1		P
Trombidiformes	sp.6	1				E
Tydeoidea						
Rhagidiidae	sp.2			1		P
Amblypygi						
Phrynidae						
Heterophrynus	sp.	1	0,05			E
Araneae						
Araneidae						
Alpaida	*septemmammata*	1				E
Mysmenidae						
Microdipoena	sp.1			1		E
Oonopidae						
Capitato	sp.2			1		E
Gamasomorpha	sp.1	1		1		E
Pholcidae jovens		1		1		E
Scytodidae jovens		1				E
Theridiosomatidae jovens		1				E
Plato	sp.1	1				P
Trechaleidae jovens		1	0,05	1	0,0588	E
Opiliones jovens				1	0,0588	P
Eupnoi jovens				5	0,2941	E
Sclerosomatidae jovens				1		E
Sclerosomatidae	sp.1	1		1		E
Laniatores						
Cosmetidae						
Roquettea	*singularis*			1	0,0588	P
Pseudoscorpiones						
Chernetidae jovens				2		P
Spelaeochernes	sp.1	1				P
Pseudochthonius	sp.1	1		1		E P
Diplopoda						
Polydesmida jovens		1				E
Chelodesmidae	sp.4	1	0,05			P
Insecta						
Blattodea jovens				1	0,0588	E
Coleoptera jovens		1		1		E
Carabidae	sp.3	1				P
Chrysomelidae	sp.14			1		E
Elateridae	sp.3	1	0,05			E
Staphylinidae	sp.31	1				E
Collembola						
Arthropleona						
Entomobryoidea						
Paronellidae	sp.1			1		P
Diptera jovens		1		1		E P
Nematocera						
Sciaridae *Bradysia*	sp.			1		P
Tipulidae						
Tipulinae	sp.	1		2		E P
Hemiptera						
Heteroptera						
Dipsocoroidea jovens				1		P
Gerridae						
Tachygerris	sp.			1		E
Veliidae jovens				1		E
Homoptera jovens		1				E

Cixiidae	jovens			1		E
Hymenoptera						
Vespoidea						
Formicidae						
Dolichoderus	*bispinosus*	2		2		E
Octostruma	sp.1	1		1		P
Pachycondyla	*striata*	1				P
Pheidole	sp.2	2		2		E P
Wasmania	*auropunctata*	1				E
Isoptera						
Termitidae						
Nasutitermes	sp.	2		2		E
Lepidoptera	jovens			1		E
Gelechioidea	sp.2			2		E
Noctuoidea						
Noctuidae	sp.	1	0,05			E
Noctuidae	sp.2			1	0,0588	E
Orthoptera						
Ensifera						
Phalangopsidae						
Paraclodes	sp.1	1	0,05	2	0,1176	E P
Phalangopsis	sp.1	3	0,15	1	0,0588	P
Psocoptera						
Psocomorpha						
Epipsocidae						
Epipsocus	sp.1	1				E
Troctomorpha						
Manicapsocidae						
Nothoentomum	sp.1			1		E
Malacostraca						
Isopoda						
Scleropactidae	sp.	1				E
Symphyla						
Scutigerellidae						
Hanseniella	sp.1	1				P
Chordata						
Amphibia						
Anura						
Neobatrachia						
Strabomantidae						
Pristimantis	*fenestratus*	2	0,1	1	0,0588	E
Mammalia						
Chiroptera						
Emballonuridae						
Peropteryx	sp.	6	0,3	1	0,0588	E P
Furipteridae						
Furipterus	*horrens*	1	0,05	1	0,0588	E
Phyllostomidae						
Glossophaginae	sp.	2	0,1			E
Reptilia						
Squamata						
Cryptodira						
Gymnophthalmidae						
Neusticurus	sp.			1	0,0588	E
Mollusca						
Gastropoda						
Subulinidae						
Lamellaxis	sp.			1		P
Systrophiidae	jovens			1		P
