# Supplementary material for: Reconciling Mining with the Conservation of Cave Biodiversity: A Quantitative Baseline to Help Establish Conservation Priorities
Source: PLoS One. 2016 Dec 20;11(12):e0168348. doi: 10.1371/journal.pone.0168348 (PMC5173368; doi:10.1371/journal.pone.0168348)
Supplement: S1 Dataset — (ZIP) [file pone.0168348.s002.zip › Taxa/Serra Sul/SS_2010/CAV_41.pdf]

CAV-41		1ª	AB	2ª	AB	ZON
Annelida						
Clitellata						
Oligochaeta	jovens	2	0,0032	1	0,0014	P
Arthropoda						
Arachnida						
Acari						
Parasitiformes						
Mesostigmata	sp.2	2		2		P
Mesostigmata	sp.3			1		P
Mesostigmata	sp.4			1		P
Mesostigmata	sp.5	1		1		P
Laelapidae						
Stratiolaelaps	sp.1			1		P
Macronyssidae	sp.1			1		P
Otopheidomenidae	sp.1	1				P
Sarcoptiformes	sp.1	4				P
Oribatida	sp.2			1		P
Oribatida	sp.3	2		4		P
Oribatida	sp.5	1				P
Oribatida	sp.6			1		P
Oribatida	sp.7			2		P
Oribatida	sp.8	2		2		P
Oribatida	sp.11	1				P
Trombidiformes	sp.5			1		P
Cheyletidae	sp.2	1				P
Cunaxidae	sp.1	1				P
Rhagidiidae	sp.2			1		P
Tydeidae	sp.1	1				P
Amblypygi						
Phrynidae						
Heterophrynus	sp.	2	0,0032			P
Araneae						
Araneidae						
Alpaida	sp.2	1				P
Corinnidae	jovens	1	0,003	2	0,0058	P
Creugas	sp.1	1		2		P
Ctenidae	jovens	1	0,0016	2	0,0029	P
Ochyroceratidae						
Speocera	sp.1	1		1		P
Oonopidae						
gr. *Xycarphius*	sp.3	1				P
Pholcidae	jovens	1		1		P
Mesabolivar	*aurantiacus*			1		P
Mesabolivar	sp.1	1				P
Scytodidae	jovens	4				P
Scytodes	*eleonorae*	1	0,0016	3	0,0043	P
Scytodes	*globula*			1	0,0014	P
Scytodes	sp.	1	0,0016	6	0,0087	P
Theridiosomatidae						
Plato	sp.1	2				P
Trechaleidae	sp.1	2	0,0032			P
Opiliones						
Cyphophthalmi						
Neogoveidae						
Canga	*renatae*	1		1		P
Eupnoi	jovens			4	0,0058	P
Sclerosomatidae	jovens			1		P
Sclerosomatidae	sp.1			2		P
Laniatores						
Escadabiidae	sp.2			1		P
Stygnidae	sp.1	2	0,0032			P
Pseudoscorpiones						
Chernetidae	jovens	2				P
Spelaeochernes	sp.1	1		1		P
Ricinulei						
Ricinoididae	jovens	1				P

Diplopoda							
Polydesmida							
Chelodesmidae		sp.4			1	0,0014	P
Pyrgodesmidae		sp.2	1	0,0016			P
Insecta							
Coleoptera	jovens		4		2		P
Curculionidae		sp.3			1	0,0014	P
Hydrophilidae							
	Sphaeridiinae	sp.2	1				P
Scydmaenidae		sp.2			1		P
Collembola							
Arthropleona							
Entomobryoidea							
Cyphoderidae		sp.1	1				P
Cyphoderidae		sp.2	1		1		P
Paronellidae		sp.1	1		1		P
Paronellidae		sp.4	1				P
Poduroidea		sp.1	2		2		P
Diptera	jovens		3		4		P
Brachycera							
Conopidae		sp.	1				P
Drosophilidae							
	Drosophila	*eleonore*	3				P
Streblidae							
	Trichobius	sp.	1				P
Nematocera							
Ceratopogonidae		sp.	1				P
Psychodidae							
	Evandromyia	*monstruosa*	1				P
	Psathyromyia	*lutziana*			1		P
Sciaridae					1		P
	Epidapus	sp.			1		P
Tipulidae							
	Tipulinae	sp.	4		6		P
Hemiptera							
Heteroptera							
Dipsocoroidea	jovens		2		1		P
aff. Mesoveliidae	jovens				1		P
Ceratocombidae	*combinae*	sp.1			1		P
Cydnidae	jovens				1		P
	Cydninae	sp.1	4		3		P
Mesoveliidae	jovens				1		P
Reduviidae	jovens		1	0,0016			P
Tingidae	jovens				1		P
Veliidae	jovens		1				P
Homoptera							
Cixiidae	jovens		1		2		P
Hymenoptera							
Diaprioidea							
Diapriidae		sp.1	1				P
Diapriidae		sp.2			2		P
Vespoidea							
Formicidae							
	Brachymyrmex	sp.1	2		1		P
	Camponotus	sp.1	2				P
	Nylanderia	sp.1	1		2		P
	Pachycondyla	striata	3		4		P
	Solenopsis	sp.2	2		4		P
	Strumygenys	sp.1	1		1		P
Isoptera							
Termitidae							
	Nasutitermes	sp.	1		1		P
Lepidoptera	jovens		2		3		P
Cossoidea							
Limacodidae		sp.1			2	0,0029	P
Tineoidea		sp.1			1		P
Tineoidea		sp.2	2				P

Orthoptera							
Ensifera		jovens	1	0,0016			P
Phalangopsidae							
	Phalangopsis	sp.1	244	0,3892	246	0,355	P
Psocoptera							
Troctomorpha							
Liposcelididae			1				P
Thysanura							
Ateluridae		sp.1			1		P
Nicoletiidae		sp.1	1		2		P
Malacostraca							
Decapoda							
Pleocyemata		sp.			4	0,0058	P
Pseudothelphusidae		jovens			1		P
Pseudothelphusidae		sp.1			1	0,0014	P
Isopoda							
Philosciidae		sp.1	2				P
Scleropactidae		sp.	4		4		P
Chordata							
Amphibia							
Anura							
Bufonidae							
	Rhinella	cf. *marina*	2	0,0032	6	0,0087	P
Mammalia							
Chiroptera							
Phyllostomidae							
	Carollia	*perspicillata*			100	0,1443	P
	Glossophaginae	sp.	350	0,5582	300	0,4329	P
	Lampronycteris	sp.	5	0,008	3	0,0043	P
	Phylloderma	sp.	7	0,0112	6	0,0087	P
Reptilia							
Squamata							
Gymnophthalmidae							
	Neusticurus	sp.	3	0,0048	2	0,0029	P
Mollusca							
Gastropoda							
Subulinidae							
	Lamellaxis	sp.	2		3		P
