# Supplementary material for: Reconciling Mining with the Conservation of Cave Biodiversity: A Quantitative Baseline to Help Establish Conservation Priorities
Source: PLoS One. 2016 Dec 20;11(12):e0168348. doi: 10.1371/journal.pone.0168348 (PMC5173368; doi:10.1371/journal.pone.0168348)
Supplement: S1 Dataset — (ZIP) [file pone.0168348.s002.zip › Taxa/Serra Sul/SS_2010/S11-01.pdf]

S11-01		1ª	AB	2ª	AB	ZON
Annelida						
Clitellata						
Oligochaeta	jovens	2	0,5			E
Arthropoda						
Arachnida						
Araneae						
Oonopidae						
Neoxyphinus	sp.1	1				E
Salticidae	jovens			1		E
Theridiosomatidae						
Plato	sp.1	2				E
Opiliones						
Eupnoi						
Sclerosomatidae	jovens	1				E
Laniatores						
Stygnidae	sp.1	2	0,5			E
Insecta						
Collembola						
Arthropleona						
Entomobryoidea						
Paronellidae	sp.1	1				E
Diptera						
Nematocera						
Chaoboridae	sp.	1				E
Tipulidae						
Tipulinae	sp.	1				E
Hymenoptera						
Vespoidea						
Formicidae						
Nylanderia	sp.1	1				E
Isoptera	sp.	1				E
Termitidae						
Cortaritermes	*silvestrii*			1		E
Orthoptera						
Ensifera						
Phalangopsidae						
Paraclodes	sp.1			4	1	E
Mollusca						
Gastropoda						
Systrophiidae						
Happia	sp.	1				E
