# Supplementary material for: Reconciling Mining with the Conservation of Cave Biodiversity: A Quantitative Baseline to Help Establish Conservation Priorities
Source: PLoS One. 2016 Dec 20;11(12):e0168348. doi: 10.1371/journal.pone.0168348 (PMC5173368; doi:10.1371/journal.pone.0168348)
Supplement: S1 Dataset — (ZIP) [file pone.0168348.s002.zip › Taxa/Serra Sul/SS_2010/S11-02.pdf]

| S11-02 | | 1ª | AB | 2ª | AB | ZON |
|---|---|---|---|---|---|---|
| Arthropoda | | | | | | |
| Arachnida | | | | | | |
| Acari | | | | | | |
| Parasitiformes | | | | | | |
| Mesostigmata | | | | | | |
| Ascidae | sp.8 | 1 | | | | E |
| Araneae | | | | | | |
| Filistatidae | sp.1 | 2 | | 2 | | E |
| Pholcidae | | | | | | |
| Leptopholcus | sp.1 | 2 | | | | E |
| *Mesabolivar* | *aurantiacus* | 1 | | | | E |
| | sp.1 | 1 | | | | E |
| Scytodidae | jovens | 1 | | 1 | 0,0702 | E |
| *Scytodes* | sp. | | | 3 | | |
| Opiliones | | | | | | |
| Laniatores | | | | | | |
| Stygnidae | sp.1 | 3 | | | | E |
| Diplopoda | | | | | | |
| Spirobolida | | | | | | |
| Rhinocricidae | sp.1 | 1 | | | | E |
| Insecta | | | | | | |
| Blattodea | | | | | | |
| Blaberidae | jovens | 2 | | 4 | 0,0702 | E |
| Coleoptera | jovens | 1 | | | | E |
| Cerambycidae | sp.1 | | | 1 | 0,0175 | |
| Diptera | | | | | | |
| Nematocera | | | | 1 | | E |
| Psychodidae | | | | | | |
| *Sciopemyia* | *sordellii* | 1 | | | | E |
| Tipulidae | | | | | | |
| Tipulinae | sp. | 1 | | | | E |
| Hemiptera | | | | | | |
| Heteroptera | | | | | | |
| aff. Pyrrhocoroidea | | | | | | |
| Reduviidae | jovens | 7 | | | | E |
| Emesinae | sp.4 | 1 | 0,0455 | | | E |
| Reduviinae | sp. | 13 | 0,5909 | 29 | 0,4561 | E |
| *Zelurus* | sp.1 | 3 | 0,1364 | | | E |
| Hymenoptera | | | | | | |
| Vespoidea | | | | | | |
| Formicidae | | | | | | |
| *Camponotus* | sp.1 | 1 | | | | E |
| Lepidoptera | | 12 | | 16 | 0,2807 | |
| Noctuoidea | | | | | | |
| Noctuidae | sp.2 | 5 | | | | E |
| Tineoidea | sp.1 | 1 | | 1 | | E |
| | jovens | 1 | | 3 | 0,0526 | E |
| Orthoptera | | | | | | |
| Ensifera | | | | | | |
| Phalangopsidae | jovens | 4 | 0,1818 | | | E |
| Mammalia | | | | | | |
| Chiroptera | | | | | | |
| Emballonuridae | | | | | | |
| *Peropteryx* | sp. | 1 | 0,0455 | | | E |
