# Supplementary material for: Reconciling Mining with the Conservation of Cave Biodiversity: A Quantitative Baseline to Help Establish Conservation Priorities
Source: PLoS One. 2016 Dec 20;11(12):e0168348. doi: 10.1371/journal.pone.0168348 (PMC5173368; doi:10.1371/journal.pone.0168348)
Supplement: S1 Dataset — (ZIP) [file pone.0168348.s002.zip › Taxa/Serra Sul/SS_2010/S11-03.pdf]

S11-03			1ª	AB	2ª	AB	ZON
Annelida							
Clitellata							
Oligochaeta	jovens		2	0,0455			E
Arthropoda							
Arachnida							
Acari							
Sarcoptiformes							
Oribatida							
Anoetidae		sp.1	1				E
Araneae							
Corinnidae	jovens				3	0,12	E
Ctenidae	jovens		2	0,0455			E
Ochyroceratidae	jovens		1				E
	Ochyrocera	sp.1	1				E
Pholcidae							
	aff. Ibityporanga	sp.1	1				E
	Ninetinae	sp.1	1		1		E
Salticidae	jovens		1				E
Scytodidae	*jovens*		1	0,0227	1	0,04	E
	Scytodes	*eleonorae*	11	0,25			E
Theraphosidae	jovens		2	0,0455			E
Theridiosomatidae	jovens		1				E
Opiliones							
Laniatores							
Escadabiidae	jovens		1				E
Stygnidae	jovens		2	0,0455			E
Pseudoscorpiones							
Chernetidae							
	Spelaeochernes	sp.1			1		E
Chthoniidae							
	Pseudochthonius	sp.1	3				E
Chilopoda							
Pleurostigmophora							
Scolopendromorpha							
Scolopocryptopidae							
	Newportia	sp.1	2	0,0455	2	0,08	E
Diplopoda							
Polyxenida							
Hypogexenidae		sp.1	1				E
Insecta							
Collembola							
Arthropleona							
Entomobryoidea							
Entomobryidae		sp.9			1		E
Diptera							
Brachycera							
Conopidae		sp.	1				E
Nematocera							
Psychodidae							
	Pintomyia	*gruta*	1				E
	Sciopemyia	*sordellii*	1		1		E
Hemiptera							
Heteroptera							
aff. Pyrrhocoroidea							
Miridae	jovens		1				E
Homoptera							
Cixiidae	jovens				1		E
		sp.1	1				E
Hymenoptera							
Vespoidea							
Formicidae							
	Camponotus	*atriceps*			1		E
	Camponotus	sp.1	2				E
Termitidae							
	Nasutitermes	sp.	2				E
Lepidoptera	jovem				3	0,12	E

Cossoidea						
Limacodidae	sp.1	4	0,09			E
Orthoptera						
Ensifera						
Phalangopsidae						
Paraclodes	sp.1	17	0,3864	16	0,64	E
Psocoptera						
Psocomorpha	jovens	2				E
Trogiomorpha						
Psyllipsocidae						
Psyllipsocus	sp.1			1		E
Malacostraca						
Isopoda						
Philosciidae	sp.1	1				E
Mammalia						
Chiroptera	sp.	1	0,0227			
