# Supplementary material for: Reconciling Mining with the Conservation of Cave Biodiversity: A Quantitative Baseline to Help Establish Conservation Priorities
Source: PLoS One. 2016 Dec 20;11(12):e0168348. doi: 10.1371/journal.pone.0168348 (PMC5173368; doi:10.1371/journal.pone.0168348)
Supplement: S1 Dataset — (ZIP) [file pone.0168348.s002.zip › Taxa/Serra Sul/SS_2010/S11-04.pdf]

S11-04		1ª	AB	2ª	AB	ZON
Arthropoda						
Arachnida						
Acari						
Sarcoptiformes						
Oribatida	sp.3	1				E
	sp.1			1		E
Araneae						
Corinnidae jovens		2	0,0526			E
Ctenidae jovens		2	0,0526			E
Filistatidae jovens		1				E
Pholcidae						
Leptopholcus	sp.1	2				E
Mesabolivar	sp.1	1		1		E
Ninetinae	sp.1	2				E
Scytodidae *jovens*						E
Scytodes	*eleonorae*	8	0,21	2	0,0328	E
Opiliones						
Laniatores						
Cosmetidae						
Roquettea	*singularis*			4	0,0656	E
Pseudoscorpiones						
Olpiidae	sp.1	2				E
Chilopoda						
Pleurostigmophora						
Geophilomorpha						
Geophilidae	sp.1	2	0,0526			E
Insecta						
Blattodea jovens		3	0,0789			E
Blaberidae jovens				8	0,1311	E P
Polyphagidae	sp.	7	0,1842			E
	sp.1	3	0,0789			E
Coleoptera jovens		1				E
chrysomelidae	sp.11	1				E
Collembola						
Arthropleona						
Entomobryoidea	sp.1	1				E
Diptera						
Nematocera jovens		1				E
Psychodidae						
Edentomyia	*piauensis*	1				E
Hemiptera						
Heteroptera						
aff. Pyrrhocoroidea						
Cydnidae						
Cydninae	sp.1	1				E
Homoptera						
Cixiidae	sp.3			1		E
Hymenoptera						
Vespoidea						
Formicidae						
Acromyrmex	sp.1			1		E
Anochetus	sp.1			1		E
Apterostigma	sp.1	1				E
Camponotus	*atriceps*			1		E
	sp.1	1		1		E
Crematogaster	sp.1	1		1		E
Gnamptogenys	*striatula*	1				E
Odontomachus	*bauri*	2	0,0526			E
Pachycondyla	*striata*	1				E
Isoptera						
Termitidae						
Atlantitermes	sp.	1				E
Embiratermes	sp.	2				E
Microcerotermes	sp.	1				E
Nasutitermes	sp.	1		1		E
Lepidoptera						

Cossoidea						
Limacodidae	sp.1	2	0,0526			E
Orthoptera						
Ensifera						
Phalangopsidae	jovens					E
Paraclodes	sp.1	3	0,0789	31	0,5082	E
Phalangopsis	sp.1	2	0,0526	16	0,2623	E P
Psocoptera						
Psocomorpha	jovens	1				E
Isopoda						
Dubioniscidae	sp.1	2				E
