# Supplementary material for: Reconciling Mining with the Conservation of Cave Biodiversity: A Quantitative Baseline to Help Establish Conservation Priorities
Source: PLoS One. 2016 Dec 20;11(12):e0168348. doi: 10.1371/journal.pone.0168348 (PMC5173368; doi:10.1371/journal.pone.0168348)
Supplement: S1 Dataset — (ZIP) [file pone.0168348.s002.zip › Taxa/Serra Sul/SS_2010/S11-05.pdf]

| S11-05 | | | 1ª | AB | 2ª | AB | ZON |
|---|---|---|---|---|---|---|---|
| Annelida | | | | | | | |
| Clitellata | | | | | | | |
| Oligochaeta | jovens | | 2 | | | | E |
| Arthropoda | | | | | | | |
| Arachnida | | | | | | | |
| Amblypygi | | | | | | | |
| Phrynidae | | | | | | | |
| | *Heterophrynus* | sp. | 2 | 0,125 | | | E |
| Araneae | | | | | | | |
| Araneidae | jovens | | | | 1 | | E |
| | *Alpaida* | *septemmammata* | 1 | | | | E |
| Ochyroceratid | jovens | | 1 | | 1 | | E |
| | *Ochyrocera* | sp.1 | 1 | | | | E |
| Pholcidae | jovens | | | | 2 | | E |
| | | sp.1 | | | 1 | | E |
| Scytodidae | *jovens* | | | | 1 | | E |
| | *Scytodes* | *eleonorae* | 4 | 0,25 | 2 | 0,087 | E |
| Opiliones | *jovens* | | 7 | | | | E |
| Laniatores | | | | | | | |
| Stygnidae | | sp.1 | 5 | | | | E |
| Pseudoscorpiones | | | | | | | |
| Chernetidae | | | | | | | |
| | *Spelaeochernes* | sp.1 | 1 | | | | E |
| Chthoniidae | jovens | | | | 2 | | E |
| | *Pseudochthonius* | sp.1 | 1 | | | | E |
| Schizomida | | | | | | | |
| Hubbardiidae | jovens | | 1 | | | | E |
| Chilopoda | jovens | | 2 | | | | E |
| Diplopoda | | | | | | | |
| Spirostreptida | jovens | | 1 | | | | E |
| Collembola | | | | | | | |
| Arthropleona | | | | | | | |
| Entomobryoidea | | | | | | | |
| Entomobryidae | | sp.1 | | | | | |
| | | sp.6 | | | 1 | | E |
| | | sp.9 | | | 1 | | E |
| Paronellidae | | sp.1 | 2 | | | | E |
| | | sp.2 | | | 1 | | E |
| Dermaptera | jovens | | | | 1 | | E |
| Diptera | jovens | | 1 | | | | E |
| Nematocera | | | | | | | |
| Chironomidae | | sp. | 1 | | | | E |
| Tipulidae | | sp. | | | | | |
| | Tipulinae | sp. | 1 | | | | E |
| Hemiptera | | | | | | | |
| Heteroptera | | | | | | | |
| aff. Pyrrhocoroid | jovens | | | | | | |
| Lygaeidae | | sp.2 | 1 | | | | E |
| Homoptera | | | | | | | |
| Aleyrodidae | | sp.1 | | | | | |
| Cixiidae | | sp.1 | 1 | | | | E |
| | | sp.3 | | | 1 | | E |
| Hymenoptera | | | | | | | |
| Vespoidea | | | | | | | |
| Formicidae | | | | | | | |
| | *Apterostigma* | sp.1 | 1 | | | | E |
| | *Brachymyrmex* | sp.1 | 1 | | 2 | | E |
| | *Carebara* | sp.1 | 1 | | | | E |
| | *Crematogaster* | sp.1 | 1 | | | | E |
| | *Dolichoderus* | *bispinosus* | | | 2 | | E |
| | *Pachycondyla* | *striata* | 1 | | | | E |
| Isoptera | | | | | | | |
| Termitidae | | | | | | | |
| | *Nasutitermes* | sp. | | | 1 | | E |
| Lepidoptera | jovens | | | | 2 | | E |
| Orthoptera | | | | | | | |

| | | | | | | | |
|---|---|---|---|---|---|---|---|
| Ensifera | | | | | | | |
| Phalangopsidae | | | | | | | |
| *Paraclodes* | | sp.1 | 3 | 0,1875 | 6 | 0,2609 | E |
| *Phalangopsis* | | sp.1 | 5 | 0,3125 | | | E |
| Psocoptera | | | | | | | |
| Psocomorpha | jovens | | 1 | | 2 | | E |
| Epipsocidae | | | | | | | |
| *Mesepipsocus* | | sp.1 | 1 | | | | E |
| Chordata | | | | | | | |
| Amphibia | | | | | | | |
| Anura | | | | | | | |
| Neobatrachia | | | | | | | |
| Strabomantidae | | | | | | | |
| *Pristimantis* | | *fenestratus* | | | 3 | | E |
| Mammalia | | | | | | | |
| Chiroptera | | sp. | | | 2 | | E |
| Emballonuridae | | | | | | | |
| *Peropteryx* | | sp. | 2 | 0,125 | | | |
| Glossophaginae | | | | | 15 | 0,6522 | |
| Reptilia | | | | | | | |
| Squamata | | | | | | | |
| Cryptodira | | | | | | | |
| Gekkonidae | | | | | | | |
| *Thecadactylus* | | *rapicauda* | | | 1 | | E |
