# Supplementary material for: Reconciling Mining with the Conservation of Cave Biodiversity: A Quantitative Baseline to Help Establish Conservation Priorities
Source: PLoS One. 2016 Dec 20;11(12):e0168348. doi: 10.1371/journal.pone.0168348 (PMC5173368; doi:10.1371/journal.pone.0168348)
Supplement: S1 Dataset — (ZIP) [file pone.0168348.s002.zip › Taxa/Serra Sul/SS_2010/S11-06.pdf]

S11-06			1ª	AB	2ª	AB	ZON
Arthropoda							
Arachnida							
Acari							
Parasitiformes							
Mesostigmata							
Podocinidae		sp.1	1				E
Araneae							
Araneidae							
	Alpaida	sp.2	1				E
Ochyroceratidae	jovens		2				E
Oonopidae							
	Gamasomorpha	sp.1	1				E
Pholcidae							
	Mesabolivar	*cambridgei*			1		E
Salticidae	jovens		1		1		E
Scytodidae							
	Scytodes	*globula*	2	0,0714			E
Theridiosomatidae							
	Plato	sp.1	1				E
Opiliones							
Laniatores							
Stygnidae	jovens		2	0,0714			E
		sp.1	3	0,1071	2	0,0645	E
Pseudoscorpiones							
Chernetidae							
	Spelaeochernes	sp.1	1				E
Chthoniidae							
	Pseudochthonius	sp.1	1		1		E
Ricinulei							
Ricinoididae	jovens		1				E
Scolopendromorpha							
Scolopocryptopidae							
	Newportia	sp.1			2	0,0645	E
Diplopoda							
Polydesmida	jovens		1				E
Insecta							
Blattodea							
Blaberidae	jovens		2	0,0714			E
Blattellidae		sp.2			2	0,0645	E
Coleoptera	jovens				1		E
Staphylinidae							
	Pselaphinae	sp.1	1				E
Collembola							
Arthropleona							
Entomobryoidea							
Paronellidae		sp.1	1				E
		sp.6	1				E
Diptera	jovens		1				E
Brachycera							
Dolichopodidae		sp.			1		E
Nematocera							
Psychodidae							
	Sciopemyia	*sordellii*	1				E
Hemiptera							
Heteroptera							
aff. Pyrrhocoroidea							
Reduviidae	jovens		5	0,1786			E
Homoptera							
Cixiidae	jovens				1		E
Hymenoptera							
Vespoidea							
Formicidae		sp.1	2		1		E
	Pachycondyla	*striata*			1		E
Lepidoptera	jovens		1	0,0357	1	0,0323	E
Orthoptera							
Ensifera							

Phalangopsidae	jovens						E
	Paraclodes	sp.1	7	0,25	24	0,7742	E
	Phalangopsis	sp.1	4	0,1429			E
Psocoptera							
Psocomorpha	jovens		2				E
Malacostraca							
Isopoda							
Dubioniscidae		sp.1	1				E
Philosciidae		sp.1			1		E
Symphyla							
Scutigerellidae							
	Scutigerella	sp.1			1		E
Chordata							
Mammalia							
Chiroptera							
Emballonuridae							
	Peropteryx	sp.	2	0,0714			E
Mollusca							
Gastropoda							
Systrophiidae							
	Happia	sp.	1				E
