# Supplementary material for: Reconciling Mining with the Conservation of Cave Biodiversity: A Quantitative Baseline to Help Establish Conservation Priorities
Source: PLoS One. 2016 Dec 20;11(12):e0168348. doi: 10.1371/journal.pone.0168348 (PMC5173368; doi:10.1371/journal.pone.0168348)
Supplement: S1 Dataset — (ZIP) [file pone.0168348.s002.zip › Taxa/Serra Sul/SS_2010/S11-07.pdf]

S11-07	1ª	AB	2ª	AB	ZON
Annelida					
Oligochaeta					
Gen. sp.1 (jovens)	x				E, P, A
Gen. sp.2	1	0,0001			
Arthropoda					
Arachnida					
Acari	x		x		E, P, A
Opiliocaridae sp.			x		E
Ixodida					
Ixodidae					
Ixodes sp.	x				A
Oribatida sp.	x		x		E, P, A
Amblypygi					
Charinidae					
Charinus sp.	13	0,0024	1	0,0002	A
Phrynidae					
Heterophrynus sp.	30	0,0054	20	0,0045	A
Araneae					
Araneidae (jovens)			x		E
Corinnidae					
Creugas sp.1	7	0,0013	17	0,0038	E, P, A
Falconina sp.1	1	0,0002			A
Linyphiidae - *Meioneta* sp.1	x				E
Nesticidae					
Jovens	x		x		E, P, A
Nesticus sp.3	x		x		E, P, A
Ochyroceratidae					
Jovens	x		x		A
Speocera sp.1	x				A
Oonopidae					
Gen. sp.3			x		P
Gen. sp.4			x		A
Prodidomidae					
Jovens	x		x		P, A
Lygromma sp.3	x		x		P, A
Salticidae (jovens)			x		E
Scytodidae					
Jovens	5	0,0013			E, P
Scytodes eleonorae	2		3	0,0007	E, P, A
Theridiosomatidae					
Jovens	x		x		E, P, A
Plato sp.1	x		x		P, A
Opiliones					
Cyphophthalmi					
Canga renatae	x				A
Laniatores					
Cosmetidae - *Roquettea singularis*	16	0,0029			E, P
Escadabiidae					
Jovens	x		x		P, A
Gen. sp.2	x		x		P, A
Gen. sp.3	x		x		P, A
Stygnidae					
Jovens	10	0,0038	1	0,0018	E, P, A
Protimesius sp.1	11		7		E, P, A
Pseudoscorpiones					
Chernetidae - *Spelaeochernes* sp.1	x		x		E, P, A
Chthoniidae - *Pseudochthonius* sp.4	x		x		E, P, A
Schizomida					

Hubbardiidae - *Rowlandius* sp.			x		A
Scorpiones					
Buthidae					
Jovens	2	0,0009	1	0,0007	E, P
Ananteris balzanii	3		2		E, P
Chilopoda					
Geophilomorpha					
Geophilidae sp.2	1	0,0002			A
Scolopendromorpha					
Scolopocryptopidae					
Newportia sp.1			1	0,0002	E
Scutigeromorpha					
Pselliodidae					
Jovens	3	0,0018	2	0,0016	E, P, A
Sphendononema guildingii	7		5		
Diplopoda					
Glomeridesmida					
Glomeridesmidae sp.	x		x		P, A
Polydesmida					
Chelodesmidae sp.	x				A
Fuhrmanodesmidae sp.	x		x		E, P, A
Paradoxosomatidae sp.	x				E, P
Pyrgodesmidae	x		x		P, A
Spirostreptida					
Pseudonannolenidae					
Pseudonannolene sp.1	4	0,0008	4	0,0009	E, P, A
Pseudonannolene sp.3	4	0,0008	3	0,0007	P, A
Entognatha					
Collembola					
Entomobryoidea					
Cyphoderidae					
Gen. sp.1	x		x		A
Gen. sp.2	x				A
Entomobryidae sp.9			x		A
Isotomidae sp.1	x		x		A
Paronellidae					
Gen. sp.2			x		A
Gen. sp.4			x		A
Symphypleona					
Sminthuroidea					
Gen. sp.1	x				A
Gen. sp.2	x		x		A
Diplura					
Campodeidae sp.1	x				A
Japygidae sp.1			x		A
Projapygidae sp.1			x		A
Insecta					
Blattodea (jovens)			x		E, A
Coleoptera					
Gen. sp.	x				A
Carabidae					
Gen. sp.1	x				A
Gen. sp.2			x		A
Elateridae - Pyrophirini (larvas)	496	0,0901	337	0,0759	P, A
Ptiliidae sp.	x		x		A
Staphilinidae					
Gen. sp.1	x		x		A
Gen. sp.2			x		A
Diptera					

Brachycera					
Chloropidae sp.	x				A
Dolichopodidae sp.			x		A
Drosophilidae					
Drosophila eleonorae	x				E, P, A
Phoridae					
Metopininae - *Metopina* sp.	x				A
Phorinae sp.	x				P, A
Sphaeroceridae sp.	x				P, A
Streblidae - *Trichobius* sp.	x				P
Nematocera					
Psychodidae					
Phlebotominae sp.	x		x		E, P, A
Psychodinae					
aff. *Telmatoscopus* sp.	x				A
Sciaridae sp.	x		x		E, A
Hemiptera					
Heteroptera					
Dipsocoroidea sp.			x		E
Cydnidae sp.	x		x		P, A
Reduviidae					
Reduviinae (jovem)	1	0,0002			E
Triatominae (jovens)	4	0,0008	4	0,0009	E, P, A
Homoptera					
Cixiidae					
Jovens	x		x		E, P, A
Gen. sp.1	x				E, P, A
Gen. sp.2	x				P
Gen. sp.3	x				P
Hymenoptera					
Chrysidoidea					
Bethylidae sp.1			x		A
Diaprioidea					
Diapriidae	x				A
Vespoidea					
Formicidae					
Ecitoninae					
Labidus sp.1	x		x		A
Neivamyrmex sp.1	3				E
Formicinae					
Brachymyrmex sp.1			x		E
Camponotus sp.3	x				P
Myrmicinae					
Pheidole sp.3	x				A
Solenopsis sp.3	x		x		E, P, A
Ponerinae					
Hypoponera sp.1	x				A
Pachycondyla sp.2	x		x		E, P, A
Isoptera (operários)	x		x		E, P
Lepidoptera					
Tineidae					
Jovens	x		x		A
Gen. sp.1	x		x		A
Gen. sp.2			x		A
Orthoptera					
Ensifera					
Phalangopsidae					
Phalangopsis sp.1	4628	0,8403	3811	0,8583	P, A
Gen. sp.1			4	0,0009	E, P, A

Psocoptera					
Trogiomorpha					
Psyllipsocidae (jovens)			x		E
Thysanura					
Ateluridae sp.	x		x		P, A
Nicoletiidae sp.	x		x		P, A
Malacostraca					
Isopoda					
Dubioniscidae sp.			x		P, A
Philosciidae sp.	x		x		E
Scleropactidae					
Circoniscus sp.1	x		x		E, P
Symphyla					
Scutigerellidae					
Hanseniella sp.	x		x		E, P, A
Mollusca					
Gastropoda					
Happia sp.1	x		x		P, A
Lamellaxis sp.1	x		x		P, A
Leptinaria sp.1	x		x		P, A
Naesiotus sp.1	x				P, A
Platyhelminthes					
Tricladida					
Gen. sp.1	4	0,0008			A
Gen. sp.2			1	0,0002	A
Amphibia					
Anura					
Leptodactylus sp.	2	0,0004	2	0,0005	E, A
Pristimantis cf. f*enestratus*			24	0,0054	
Mammalia					
Chiroptera					
Phyllostomidae					
Carollia perspicillata	180	0,0328	120	0,027	P, A
Desmodus rotundus	5	0,0009	2	0,0005	P
Diphylla ecaudata	5	0,0009	3	0,0007	P
Glossophaginae sp.	30	0,0054	35	0,0079	P, A
Lonchorhina sp.	30	0,0054	30	0,0067	A
