# Supplementary material for: Reconciling Mining with the Conservation of Cave Biodiversity: A Quantitative Baseline to Help Establish Conservation Priorities
Source: PLoS One. 2016 Dec 20;11(12):e0168348. doi: 10.1371/journal.pone.0168348 (PMC5173368; doi:10.1371/journal.pone.0168348)
Supplement: S1 Dataset — (ZIP) [file pone.0168348.s002.zip › Taxa/Serra Sul/SS_2010/S11-08.pdf]

S11-08			1ª	AB	2ª	AB	ZON
Arthropoda							
Arachnida							
Acari							
Trombidiformes							
Tydeoidea							
Rhagidiidae		sp.1	1				P
Araneae							
Araneidae	jovens				1		P
Ochyroceratidae	jovens		1				P
Pholcidae							
	Mesabolivar	sp.1			1		P
Scytodidae	jovens		45	0,5056			P
Theridiidae							
	Theridion	sp.2			1		P
Theridiosomatidae	jovens		1				P
	Plato	sp.1	1		1		P
Trechaleidae	jovens		7	0,0787			P
Opiliones							
Eupnoi							
Sclerosomatidae		sp.1			1		P
Diplopoda							
Polydesmida							
Fuhrmannodesmidae		sp.1	1				P
Spirostreptida	jovens				1		P
Insecta							
Coleoptera							
Gyrinidae							
	Gyretes	sp.1			1		P
Collembola							
Arthropleona							
Entomobryoidea							
Paronellidae		sp.8			1		P
Diptera							
Nematocera							
Tipulidae							
	Tipulinae	sp.	1		1		P
Hymenoptera							
Diaprioidea							
Diapriidae		sp.2			1		P
Vespoidea							
Formicidae							
	Crematogaster	sp.1	1				P
Isoptera							
Termitidae							
	Nasutitermes	sp.			1		P
Orthoptera							
Ensifera	jovens		2	0,0225			P
Phalangopsidae							
	Phalangopsis	sp.1	32	0,3596	7	0,53	P
Chordata							
Mammalia							
Chiroptera		sp.	2	0,0337			P
	Carollia	sp.			8	0,6111	P
