# Supplementary material for: Reconciling Mining with the Conservation of Cave Biodiversity: A Quantitative Baseline to Help Establish Conservation Priorities
Source: PLoS One. 2016 Dec 20;11(12):e0168348. doi: 10.1371/journal.pone.0168348 (PMC5173368; doi:10.1371/journal.pone.0168348)
Supplement: S1 Dataset — (ZIP) [file pone.0168348.s002.zip › Taxa/Serra Sul/SS_2010/S11-09.pdf]

S11-09				1ª	AB	2ª	AB	ZON
Arthropoda								
Arachnida								
Araneae								
Araneidae								
		Alpaida	*septemmammata*	1				E
	Filistatidae	jovens		1		1		E
	Oonopidae							
		Oonopinae	sp.2	1				E
	Pholcidae	jovens				2		E
		aff. *Ibityporanga*	sp.1	1				E
		Leptopholcus	sp.1	1				E
		Ninetinae	sp.1	2				E
	Scytodidae	jovens		2	0,33	2	0,3333	E
	Theridiidae	jovens		1				E
Insecta								
Blattodea		jovens				2	0,3333	E
	Polyphagidae	jovens		2	0,33			E
Collembola								
Arthropleona								
Entomobryoidea								
	Entomobryidae		sp.5	1				E
Diptera								
Nematocera								
	Psychodidae							
		Micropygomyia	Série oswaldoi	1				E
Hemiptera								
Heteroptera								
aff. Pyrrhocoroidea								
	Lygaeidae		sp.1	1				E
Hymenoptera								
Vespoidea								
	Formicidae							
		Camponotus	sp.1	1				E
		Crematogaster	sp.1			1		E
		Dolichoderus	*bispinosus*	2		2		E
		Nylanderia	sp.1			1		E
		Pheidole	sp.1	1				E
Isoptera								
	Termitidae							
		Nasutitermes	sp.	1		2		E
Psocoptera								
Psocomorpha		jovens		1		1		E
Trogiomorpha								
	Psyllipsocidae							
		Psocathropos	sp.1	1				E
Chordata								
Mammalia								
Chiroptera								
	Emballonuridae							
		Peropteryx	sp.	2	0,33			E
Reptilia								
Squamata								
	Gekkonidae							
		Thecadactylus	*rapicauda*			2	0,3333	E
