# Supplementary material for: Reconciling Mining with the Conservation of Cave Biodiversity: A Quantitative Baseline to Help Establish Conservation Priorities
Source: PLoS One. 2016 Dec 20;11(12):e0168348. doi: 10.1371/journal.pone.0168348 (PMC5173368; doi:10.1371/journal.pone.0168348)
Supplement: S1 Dataset — (ZIP) [file pone.0168348.s002.zip › Taxa/Serra Sul/SS_2010/S11-10.pdf]

S11-10			1ª	AB	2ª	AB	ZON
Arthropoda							
Arachnida							
Acari							
Sarcoptiformes							
Oribatida		sp.3	1				E
Amblypygi							
Phrynidae							
	Heterophrynus	sp.	3	0,068			E
Araneae							
Araneidae	jovens				1		E
	Alpaida	sp.2	2				E
Corinnidae	jovens		4	0,091			E
Ctenidae	jovens		5	0,114			E
Ochyroceratidae	jovens		1				E
Pholcidae	jovens		1		1		E
Salticidae	jovens		1				E
	Marma	sp.1	1		1		E
Scytodidae	jovens		2	0,045			E
Insecta							
Blattodea			2	0,045			
Blaberidae	jovens		2				E
Coleoptera	jovens		1		1		E
Collembola							
Arthropleona							
Entomobryoidea							
Entomobryidae		sp.2	1				E
		sp.9			1		E
Diptera							
Nematocera	jovens		1				E
Psychodidae							
	Sciopemyia	*sordellii*	1		1		E
Hemiptera							
Homoptera							
Cixiidae		sp.3			1		E
Hymenoptera							
Vespoidea							
Formicidae							
	Camponotus	sp.1	1				E
Isoptera							
Termitidae	Nasutitermes	sp.	2		1		E
Neuroptera							
Myrmeleonthidae	jovens		1		1		E
Orthoptera							
Ensifera							
Phalangopsidae	jovens						E
	Paraclodes	sp.	2	0,045	21	0,8	E
	Phalangopsis	sp.	21	0,47			E
Psocoptera							
Psocomorpha	jovens		1				E
Trogiomorpha							
Psyllipsocidae							
	Psocathropos	sp.1			1		E
Malacostraca							
Isopoda							
Dubioniscidae		sp.1	1				E
Ave							
Cathartiformes							
Cathartidae		sp.			4	0,1	E
Mammalia							
Chiroptera							
Emballonuridae							

Peropteryx sp.			1	0,1	E
Phyllostomidae					
Glossophaginae sp.	2	0,068			
