# Supplementary material for: Reconciling Mining with the Conservation of Cave Biodiversity: A Quantitative Baseline to Help Establish Conservation Priorities
Source: PLoS One. 2016 Dec 20;11(12):e0168348. doi: 10.1371/journal.pone.0168348 (PMC5173368; doi:10.1371/journal.pone.0168348)
Supplement: S1 Dataset — (ZIP) [file pone.0168348.s002.zip › Taxa/Serra Sul/SS_2010/S11-11.pdf]

S11-11			1ª	AB	2ª	AB	ZON
Arthropoda							
Arachnida							
Acari							
Parasitiformes							
Mesostigmata							
Laelapidae		sp.2	1				E
		sp.3	1				E
Araneae							
Barychaelidae	jovens		2	0,045			E
Corinnidae	jovens		2	0,045			P
Ochyroceratidae	jovens		1				P
	Ochyrocera	sp.1	1		1		E
	Ninetinae	sp.1	1		2		E P
Prodidomidae	jovens		1				P
Scytodidae	jovens		1	0,023	2	0,02	E P
Segestriidae	jovens		1				E
Opiliones							
Laniatores							
Stygnidae		sp.1			2	0,02	E
Pseudoscorpiones							
Bochicidae		sp.1	2				P
Olpiidae		sp.1	2				E
Ricinulei							
Ricinoididae	jovens		1				P
Schizomida							
Hubbardiidae	jovens		1				P
	Rowlandius	sp.	1				E
Chilopoda							
Pleurostigmophora							
Scolopendromorpha							
Cryptopidae							
	Cryptops	sp.1	2	0,045			E
Scolopocryptopidae							
	Tidops	sp.1	2	0,045			E
Diplopoda							
Polyxenida							
Hypogexenidae		sp.1	1				E
Entognatha							
Diplura							
Campodeidae		sp.1	2		1		E P
Projapygidae		sp.1	1				P
Insecta							
Blattodea							
Blaberidae	jovens		7	0,159	2		E P
Blaberidae		sp.1			3	0,051	E
Coleoptera	jovens		1		2		E P
Scydmaenidae		sp.10	1				E
Collembola							
Arthropleona							
Entomobryoidea							
Entomobryidae		sp.9			2		E P
Paronellidae		sp.1			1		E
Paronellidae		sp.4	1				P
Diptera	jovens		2				E P
Nematocera							
Ceratopogonidae		sp.	1				E
Psychodidae							
	Pintomyia	*gruta*	1				E
		sordellii	2				E P
Hemiptera							
Heteroptera							

Reduviidae		jovens	2	0,045	2	0,02	E	P
Homoptera								
Cixiidae		jovens	2		2		E	P
Cixiidae		sp.2	1				E	
Cixiidae		sp.3			1		E	
Hymenoptera								
Vespoidea								
Formicidae								
	Camponotus	*atriceps*	1				E	
	Cyphomyrmex	sp.1			1		E	
	Myrmicocrypta	sp.1			1			P
		striata	2		2		E	P
	Wasmania	*auropunctata*	1					P
Isoptera								
Termitidae								
	Armitermes	sp.			1		E	
	Nasutitermes	sp.	1				E	
		sp.			1			P
Neuroptera								
Myrmeleonthidae		jovens	1				E	
Orthoptera								
Ensifera								
Phalangopsidae								
	Paraclodes	sp.			36	0,364		P
	Phalangopsis	sp.	10	0,227	31	0,313		P
Psocoptera								
Troctomorpha								
Liposcelididae								
	Liposcelis	sp.2			1			P
Trogiomorpha								
Psyllipsocidae		jovens			2		E	P
Psyllipsocidae								
	Psyllipsocus	sp.1			1		E	
Malacostraca								
Isopoda								
Armadillidae		sp.	1				E	
Dubioniscidae		sp.1	1					P
Chordata								
Mammalia								
Chiroptera								
Phyllostomidae								
	Carollia	sp.			21	0,212		P
	Glossophaginae	sp.	16	0,364				
Platyhelminthes								
Turbellaria		sp.3	1				E	
