# Supplementary material for: Reconciling Mining with the Conservation of Cave Biodiversity: A Quantitative Baseline to Help Establish Conservation Priorities
Source: PLoS One. 2016 Dec 20;11(12):e0168348. doi: 10.1371/journal.pone.0168348 (PMC5173368; doi:10.1371/journal.pone.0168348)
Supplement: S1 Dataset — (ZIP) [file pone.0168348.s002.zip › Taxa/Serra Sul/SS_2010/S11-12.pdf]

S11-12		1ª	AB	2ª	AB	ZON
Annelida						
Clitellata						
Oligochaeta	jovens	9	0,22			E
Arthropoda						
Arachnida						
Acari						
Ixodida						
Ixodidae						
Amblyomma	sp.			1		E
Parasitiformes						
Mesostigmata						
Laelapidae	sp.3	1				E
Macronyssidae	sp.1			1		E
Mesostigmata	sp.10	1				E
Mesostigmata	sp.9	1				E
Opilioacarida						
Opilioacaridae	sp.1	1				E
Sarcoptiformes						
Oribatida	sp.3	1				E
Araneae						
Araneidae	jovens	2				E
Corinnidae	jovens	7	0,17			E
Salticidae	jovens	1				E
Scytodidae	*jovens*	2	0,1			E
Scytodes	sp.	2				E
Theridiosomatidae						
Plato	sp.1	1				E
Opiliones						
Cyphophthalmi						
Neogoveidae						
Canga	*renatae*	1				E
Laniatores						
Escadabiidae	jovens	1				E
Escadabiidae	sp.3	1				E
	sp.4	1				E
Stygnidae	sp.1	2	0,05			E
Pseudoscorpiones						
Chernetidae	sp.2	2				E
Spelaeochernes	sp.1	3				E
Chthoniidae						
Pseudochthonius	sp.1	2				E
Ricinulei						
Ricinoididae	jovens	1				E
Chilopoda						
Pleurostigmophora						
Scolopendromorpha						
Cryptopidae						
Cryptops	sp.1	3	0,19	2	0,0952	E
Diplopoda	jovens	5				E
Glomeridesmida						
Glomeridesmidae	sp.1	1				E
Spirostreptida	jovens	2				E
Pseudonannolenidae						
Pseudonannolene	sp.1	2	0,05			E
Entognatha						
Diplura						
Campodeidae	sp.1	5				E
Blattodea	jovens	3	0,07			E
Blaberidae	jovens			4	0,1905	E
Coleoptera	jovens	1				E
Carabidae	sp.3	1				E
Carabidae	sp.4	1				E
Carabidae	sp.5	1				E
Scydmaenidae	sp.8	1				E
Staphylinidae	sp.1					
Pselaphinae	sp.14	1				E

Collembola							
Arthropleona							
Entomobryoidea							
Entomobryidae		sp.4	1				E
		sp.6	1		1		E
Paronellidae		sp.1	1				E
Diptera	jovens		3				E
Brachycera							
Phoridae							
	Metopininae	sp.	2				E
Nematocera							
Psychodidae		sp.					
	Micropygomyia	Série *oswaldoi*	1				E
	Pintomyia	*gruta*			1		E
	Sciopemyia	*sordellii*	1				E
Hemiptera							
Heteroptera							
Reduviidae	jovens		2	0,05			E
Schizopteridae							
	Hypselosomatinae	sp.1			1		E
Tingidae							
	Thaumamannia	sp.1	2				E
Homoptera							
Cicadellidae		sp.1	1				E
Cixiidae	jovens		3				E
Hymenoptera	jovens		1				E
Chalcidoidea		sp.3	1				E
Vespoidea							
Formicidae							
	Cyphomyrmex	sp.1			1		E
	Hypoponera	sp.1	1				E
	Nylanderia	sp.1			1		E
	Octostruma	sp.1	1				E
	Pachycondyla	*striata*	4				E
	Solenopsis	sp.1	1				E
		sp.2	1				E
	Wasmania	*auropunctata*	1				E
Isoptera		sp.	2				E
Termitidae							
	Nasutitermes	sp.	3				E
	Termes	sp.	1				E
Lepidoptera	jovens		1				E
Noctuoidea		sp.2	1				E
Tineoidea		sp.1	1		1		E
Mantodea	jovens				2	0,0952	E
Orthoptera							
Ensifera							
Phalangopsidae	jovens		3	0,07			E
	Phalangopsis	sp.1	2	0,05			E
	Paraclodes	sp.			10	0,4762	E
Psocoptera							
Psocomorpha	jovens		3		1		E
Trogiomorpha							
Psyllipsocidae	jovens				1		E
	Psocathropos	sp.1			1		E
Thysanura							
Ateluridae		sp.1	1				E
Malacostraca							
Isopoda							
Philosciidae		sp.1	2				E
Symphyla							
Scutigerellidae							
	Hanseniella	sp.1	1				E
Chordata							
Amphibia							
Anura							
Neobatrachia							

Strabomantidae						
Pristimantis	*fenestratus*			3	0,1429	E
Mammalia						
Chiroptera						
Phyllostomidae						
Carollia	sp.	2	0,05			
Glossophaginae	sp.	3	0,07			
Mollusca						
Gastropoda						
Systrophiidae						
Happia	sp.	1				E
