# Supplementary material for: Reconciling Mining with the Conservation of Cave Biodiversity: A Quantitative Baseline to Help Establish Conservation Priorities
Source: PLoS One. 2016 Dec 20;11(12):e0168348. doi: 10.1371/journal.pone.0168348 (PMC5173368; doi:10.1371/journal.pone.0168348)
Supplement: S1 Dataset — (ZIP) [file pone.0168348.s002.zip › Taxa/Serra Sul/SS_2010/S11-13.pdf]

S11-13			1ª	AB	2ª	AB	ZON
Arthropoda							
Arachnida							
Acari							
Sarcoptiformes							
Oribatida		sp.3	1				E
Amblypygi							
Charinidae							
Charinus		sp.	2	0,048			E
Araneae							
Corinnidae	jovens		10	0,238	1	0,027	E
Pholcidae	jovens		1				E
Leptopholcus		sp.1	1		1		E
Scytodidae	jovens		1	0,095	1	0,027	E
		globula	3				E
Theridiosomatidae							
Plato		sp.1	1				E
Opiliones							
Laniatores							
Escadabiidae		sp.3	1				E
Pseudoscorpiones							
Chernetidae							
Spelaeochernes		sp.1	2				E
Chthoniidae							
Pseudochthonius		sp.1	2				E
Chilopoda							
Pleurostigmophora							
Scolopendromorpha							
Scolopocryptopidae							
Tidops		sp.1	2	0,048			E
Entognatha							
Diplura							
Campodeidae		sp.1	1				E
Insecta							
Blattodea	jovens		2	0,143	11	0,378	E
Blaberidae	jovens		4		3		E
Coleoptera	jovens		2				E
Collembola							
Arthropleona							
Entomobryoidea							
Cyphoderidae		sp.1	1				E
		sp.2	1				E
Diptera							
Brachycera							
Chloropidae		sp.	1				E
Nematocera							
Cecidomyiidae							
Cecidomyiinae		sp.	1				E
Psychodidae							
Sciopemyia		*sordellii*	1		1		E
Hemiptera							
Heteroptera							
Cydnidae							
Cydninae		sp.1	3		1		E
		sp.2			1		E
Hymenoptera							
Vespoidea							
Formicidae							
Dolichoderus		*bispinosus*			1		E
Pachycondyla		*striata*	3		1		E
Solenopsis		sp.1	1				E
		sp.3	1				E

Isoptera						
Termitidae						
Nasutitermes	sp.			1		E
Lepidoptera						
Tineoidea	sp.1	2				E
Orthoptera						
Ensifera						
Phalangopsidae jovens		2	0,048			E
Paraclodes	sp.1			7	0,189	E
Phalangopsis	sp.1	8	0,19	14	0,378	E
Psocoptera						
Trogiomorpha						
Psyllipsocidae						
Psyllipsocus	sp.3	1				E
Malacostraca						
Isopoda	sp.1	2				E
Dubioniscidae jovens		1				E
Chordata						
Strabomantidae						
Pristimantis	*fenestratus*	2	0,048			E
Mammalia						
Chiroptera						
Phyllostomidae						
Carollia	sp.	3	0,071			
Glossophaginae	sp.	3	0,071			
