# Supplementary material for: Reconciling Mining with the Conservation of Cave Biodiversity: A Quantitative Baseline to Help Establish Conservation Priorities
Source: PLoS One. 2016 Dec 20;11(12):e0168348. doi: 10.1371/journal.pone.0168348 (PMC5173368; doi:10.1371/journal.pone.0168348)
Supplement: S1 Dataset — (ZIP) [file pone.0168348.s002.zip › Taxa/Serra Sul/SS_2010/S11-14.pdf]

S11-14		1ª	AB	2ª	AB	ZON
Arthropoda						
Arachnida						
Acari						
Parasitiformes						
Mesostigmata						
Laelapidae						
Stratiolaelaps	sp.1	1				P
Macronyssidae	sp.1	1				P
	sp.2	1				P
Opilioacarida						
Opilioacaridae	sp.1	1				P
Sarcoptiformes						
Oribatida						
	sp.13	1				P
	sp.2	1				P
	sp.3	1				P
Araneae						
Ochyroceratidae						
Speocera	sp.1	1				P
Oonopidae	sp.4	1				P
Pholcidae						
Leptopholcus	sp.1	2		1		P
Scytodidae	jovens			1		P
Theridiosomatidae						
Plato	sp.1	1				P
Opiliones						
Laniatores						
Escadabiidae	jovens	1				P
	sp.1	1				P
Palpigradi						
Eukoeneniidae						
Eukoenenia	sp.1	1				P
Pseudoscorpiones						
Chernetidae						
Spelaeochernes	sp.1	2				P
Chthoniidae						
Pseudochthonius	sp.1	1				P
Ricinulei						
Ricinoididae	jovens	1				P
Diplopoda	jovens	1				P
Glomeridesmida						
Glomeridesmidae	sp.1	1				P
Polydesmida						
Paradoxosomatidae	sp.1	3	0,176			P
Spirostreptida						
Pseudonannolenidae						
Pseudonannolene	sp.1	2	0,118			P
Insecta						
Blattodea	jovens	4	0,235	3	0,214	E P
Blaberidae	jovens			2	0,143	P
Blattellidae	sp.2			2	0,143	P
Polyphagidae	jovens	2	0,118			P
Coleoptera						
Anthicidae	jovens	2				P
Collembola						
Arthropleona						
Entomobryoidea	sp.1					
Isotomidae	sp.1	1				P
Paronellidae	sp.4			1		P
Diptera						
Brachycera	jovens	2				P

Hemiptera						
Heteroptera						
Cydnidae	sp.1					
Cydninae	sp.2	1				P
Homoptera						
Cixiidae	jovens	1				P
Hymenoptera						
Vespoidea						
Formicidae						
Camponotus	*atriceps*			1		P
Pachycondyla	*striata*	3		1		P
Lepidoptera	jovens	1				P
Orthoptera						
Ensifera						
Phalangopsidae						
Paraclodes	sp.1			2	0,143	E
Psocoptera						
Psocomorpha	jovens	1		1		P
Trogiomorpha						
Psyllipsocidae						
Psyllipsocus	sp.3			1		P
Malacostraca						
Isopoda						
Dubioniscidae	sp.1			1		P
Philosciidae	sp.1	1				P
Symphyla						
Scutigerellidae						
Hanseniella	sp.1	1				P
Chordata						
Amphibia						
Anura						
Neobatrachia						
Strabomantidae						
Pristimantis	*fenestratus*			3	0,214	P
Mammalia						
Chiroptera						
Phyllostomidae						
Carollia	sp.	6	0,353			
Platyhelminthes						
Turbellaria	jovens			2	0,143	P
