# Supplementary material for: Reconciling Mining with the Conservation of Cave Biodiversity: A Quantitative Baseline to Help Establish Conservation Priorities
Source: PLoS One. 2016 Dec 20;11(12):e0168348. doi: 10.1371/journal.pone.0168348 (PMC5173368; doi:10.1371/journal.pone.0168348)
Supplement: S1 Dataset — (ZIP) [file pone.0168348.s002.zip › Taxa/Serra Sul/SS_2010/S11-15.pdf]

| S11-15 | | | 1ª | AB | 2ª | AB | ZON |
|---|---|---|---|---|---|---|---|
| Annelida | | | | | | | |
| Clitellata | | | | | | | |
| Oligochaeta | jovens | | 4 | 0,0244 | 2 | 0,0196 | E |
| Arthropoda | | | | | | | |
| Arachnida | | | | | | | |
| Acari | | | | | | | |
| Ixodida | | | | | | | |
| Argasidae | | | | | | | |
| | *Ixodes* | sp. | 1 | | | | E |
| Amblypygi | | | | | | | |
| Phrynidae | | | | | | | |
| | *Heterophrynus* | sp. | | | 4 | 0,0392 | E |
| Araneae | | | | | | | |
| Araneidae | jovens | | | | 1 | | E |
| | *Alpaida* | *septemmammata* | 1 | | | | E |
| | | sp.2 | 1 | | | | E |
| Ctenidae | jovens | | 2 | 0,0122 | | | E |
| Scytodidae | *jovens* | | 1 | 0,0061 | | | E |
| | *Scytodes* | *eleonorae* | 3 | 0,0183 | 3 | 0,0294 | E |
| | | *globula* | 2 | 0,0122 | | | E |
| Theridiosomatidae | | | | | | | |
| | *Plato* | sp.1 | 2 | | 1 | | E |
| Opiliones | jovens | | 1 | 0,0061 | | | E |
| Laniatores | | | | | | | |
| Cosmetidae | | sp.3 | | | 6 | 0,0588 | E |
| Stygnidae | | sp.1 | 6 | 0,0366 | | | E |
| Pleurostigmophora | jovens | | 2 | 0,0122 | | | E |
| Diplopoda | | | | | | | |
| Polydesmida | | | | | | | |
| Chelodesmidae | | sp.4 | | | 2 | 0,0196 | E |
| Fuhrmannodesmidae | | sp.1 | 2 | | | | E |
| | | sp.2 | 1 | | | | E |
| Entognatha | | | | | | | |
| Diplura | | | | | | | |
| Campodeidae | | sp.1 | 1 | | | | E |
| Coleoptera | jovens | | 1 | | | | E |
| Carabidae | | sp.18 | 2 | | | | E |
| | | sp.20 | 1 | | | | E |
| | | sp.3 | | | 1 | | E |
| Staphylinidae | | sp.11 | 1 | | | | E |
| | | sp.20 | 1 | | | | E |
| | | sp.21 | | | 1 | | E |
| | | sp.22 | 1 | | 1 | | E |
| | | sp.23 | 1 | | | | E |
| Collembola | | | | | | | |
| Arthropleona | | | | | | | |
| Entomobryoidea | | | | | | | |
| Paronellidae | | sp.1 | 3 | | | | E |
| Poduroidea | | sp.2 | | | 1 | | E |
| Diptera | jovens | 1 | | | 1 | | |
| Brachycera | | | | | | | |
| Drosophilidae | | | | | | | |
| | *Drosophila* | *eleonore* | 1 | | 1 | | E |
| Nematocera | | | | | | | |
| Chironomidae | | sp. | 1 | | | | E |
| Psychodidae | | sp. | | | | | |
| | *Sciopemyia* | *sordellii* | | | 1 | | E |
| Sciaridae | | sp. | 1 | | | | E |
| Tipulidae | | sp. | 1 | | | | E |
| Hemiptera | | | | | | | |
| Heteroptera | | | | | | | |
| Pyrrhocoroidea | | | | | | | |
| aff. Lygaeidae | jovens | | 1 | | | | E |
| aff. Lygaeidae | | sp. | 1 | | | | E |
| Cydnidae | jovens | | | | 1 | | E |
| | Cydninae | sp.1 | 3 | | | | E |

| | | | | | | | |
|---|---|---|---|---|---|---|---|
| Lygaeidae | jovens | | 1 | | | | E |
| Mesoveliidae | jovens | | 1 | | | | E |
| Nabidae | | sp. | 1 | | | | E |
| Ochteridae | jovens | | 3 | | | | E |
| Veliidae | jovens | | 2 | | | | E |
| Homoptera | | | | | | | |
| Cixiidae | jovens | | 2 | | 1 | | E |
| Hymenoptera | | | | | | | |
| Vespoidea | | | | | | | |
| Formicidae | | | | | | | |
| | *Carebara* | sp.1 | 2 | | | | E |
| | *Solenopsis* | sp.2 | 2 | | | | E |
| Isoptera | | | | | | | |
| Termitidae | | | | | | | |
| | *Nasutitermes* | sp. | | | 1 | | E |
| | | sp. | 1 | | | | E |
| Orthoptera | | | | | | | |
| Ensifera | | | | | | | |
| Phalangopsidae | jovens | | | | | | E |
| | *Phalangopsis* | sp.1 | 32 | 0,1951 | | | E |
| | *Paraclodes* | sp. | | | 7 | 0,0686 | E |
| Psocoptera | | | | | | | |
| Troctomorpha | | | | | | | |
| Manicapsocidae | | | | | | | |
| | *Nothoentomum* | sp.1 | 1 | | | | E |
| Chordata | | | | | | | |
| Amphibia | | | | | | | |
| Anura | | | | | | | |
| Neobatrachia | | | | | | | |
| Leptodactylidae | | | | | | | |
| | *Leptodactylus* | sp. | 2 | 0,0122 | | | E |
| Strabomantidae | | | | | | | |
| | *Pristimantis* | *fenestratus* | 2 | 0,0122 | 6 | 0,0588 | E |
| Mammalia | | | | | | | |
| Chiroptera | | | | | | | |
| Phyllostomidae | | | | | | | |
| | *Carollia* | sp. | 31 | 0,189 | 31 | 0,3039 | E |
| | Glossophaginae | sp. | 71 | 0,4329 | 41 | 0,402 | E |
| | *Phyllostomus* | sp. | 3 | 0,0183 | | | |
| Mollusca | | | | | | | |
| Gastropoda | | | | | | | |
| Streptaxidae | | | | | | | |
| | *Streptaxis* | sp. | 1 | | | | E |
