# Supplementary material for: Reconciling Mining with the Conservation of Cave Biodiversity: A Quantitative Baseline to Help Establish Conservation Priorities
Source: PLoS One. 2016 Dec 20;11(12):e0168348. doi: 10.1371/journal.pone.0168348 (PMC5173368; doi:10.1371/journal.pone.0168348)
Supplement: S1 Dataset — (ZIP) [file pone.0168348.s002.zip › Taxa/Serra Sul/SS_2010/S11-16.pdf]

S11-16			1ª	AB	2ª	AB	ZON
Annelida							
Oligochaeta	jovens		2	0,0031			E
Arthropoda							
Arachnida							
Acari							
Parasitiformes							
Mesostigmata							
Laelapidae		sp.3	1				E
Sarcoptiformes							
Oribatida		sp.3	1				E
Araneae							
Filistatidae	jovens		1				E
		sp.1	1		2		E
Pholcidae	jovens		1		2		E
	Mesabolivar	sp.1	1		1		E
Salticidae	jovens		1				E
Scytodidae	jovens		2				E
	Scytodes	*globula*	4	0,0122	3	0,0222	E
		sp.	12		21		E
Theridiidae	jovens				1		E
	Theridion	sp.1	1				E
Pseudoscorpiones							
Chernetidae							
	Spelaeochernes	sp.1	2				E
Chthoniidae	jovens						
	Pseudochthonius	sp.1	1				E
Insecta							
Blattodea	jovens		4	0,0061			E
Blaberidae	jovens		10	0,0153			E
Blattidae	jovens		13	0,0199			E
Coleoptera	jovens		2				E
		sp.11			1		E
Staphylinidae		sp.21			1		E
	Pselaphinae	sp.1	1				E
Diptera							
Brachycera							
Sphaeroceridae		sp.			1		E
Nematocera	jovens		2				E
Psychodidae							
	Edentomyia	*piauensis*	1				E
	Pericoma	sp.	1				E
	Philosepedon	sp.			1		E
Hemiptera							
Heteroptera							
Cydnidae							
	Cydninae	sp.1	1				E
Gerridae	jovens				1		E
Lygaeidae		sp.3	1				E
Mesoveliidae	jovens				1		E
Reduviidae	jovens						
	Reduviinae	sp.			2	0,0037	E
	Triatominae	sp.1			2	0,0037	E
Hymenoptera							
Vespoidea							
Formicidae							
	Hypoponera	sp.1			1		E
	Pachycondyla	*striata*	1		1		E
	Pheidole	sp.2			3		E
	Solenopsis	sp.1			1		E
Mutillidae		sp.1	1				E
Isoptera		sp.	1				E
Termitidae							
	Nasutitermes	sp.	2		1		E
Lepidoptera							
Tineoidea		sp.1	2				E
Orthoptera							

Ensifera							
Phalangopsidae							
	Paraclodes sp.		3	0,0046	2	0,0037	E
Psocoptera							
Psocomorpha							
Archipsocidae		jovens	1		1		E
Chordata							
Amphibia							
Anura							
Neobatrachia							
Strabomantidae							
	Pristimantis fenestratus				3	0,0055	E
Hylidae							
	Scinax sp.		2	0,0031			E
Mammalia							
Chiroptera							
Furipteridae							
	Furipterus horrens		36	0,055	6	0,0111	E
Phyllostomidae							
	Carollia sp.		100	0,1544	100	0,1867	E
	Glossophaginae sp.		450	0,6896	400	0,7412	E
	Phyllostomus sp.		16	0,0245			
