# Supplementary material for: Reconciling Mining with the Conservation of Cave Biodiversity: A Quantitative Baseline to Help Establish Conservation Priorities
Source: PLoS One. 2016 Dec 20;11(12):e0168348. doi: 10.1371/journal.pone.0168348 (PMC5173368; doi:10.1371/journal.pone.0168348)
Supplement: S1 Dataset — (ZIP) [file pone.0168348.s002.zip › Taxa/Serra Sul/SS_2010/S11-17.pdf]

S11-17			1ª	AB	2ª	AB	ZON
Annelida							
Clitellata							
Oligochaeta		sp.	3	0,1			E
Arthropoda							
Arachnida							
Araneae							
Ochyroceratidae	jovens		1				E
Pholcidae	jovens						
	Leptopholcus	sp.1	1				E
Salticidae	jovens				1		E
	Soesilarishius	sp.1	1				E
Scytodidae	*jovens*				1	0,0714	E
	Scytodes	*eleonorae*	3	0,1			E
		sp.	2	0,0667	2	0,1429	E
Theridiosomatidae	jovens		1				E
Opiliones							
Laniatores							
Stygnidae		sp.1	2	0,0667			E
Chilopoda							
Pleurostigmophora							
Geophilomorpha							
Geophilidae		sp.1	2	0,0667			E
Diplopoda							
Spirostreptida	jovens		1				E
Entognatha							
Diplura							
Campodeidae		sp.1	1				E
Coleoptera	jovens		1				E
Collembola							
Arthropleona							
Entomobryoidea							
Entomobryidae		sp.6			1		E
		sp.9			1		E
Isotomidae		sp.1	1				E
Diptera							
Nematocera	jovens		2				E
Ceratopogonidae		sp.	1				E
Chironomidae		sp.	1				E
Mycetophilidae		sp.	1				E
Psychodidae		sp.					
	Pintomyia	*gruta*			1		E
Hemiptera							
Homoptera	jovens		1				E
Cixiidae		sp.1	1				E
		sp.3			1		E
Hymenoptera							
Vespoidea							
Formicidae							
	Acromyrmex	sp.1			1		E
	Camponotus	sp.1	1		1		E
	Crematogaster	sp.1			1		E
	Odontomachus	*bauri*			1		E
	Pachycondyla	striata	1		1		E
	Pheidole	sp.2	1				E
Isoptera		sp.	1		1		E
Lepidoptera							
Tineoidea		sp.1	1				E
Orthoptera							
Ensifera							
Phalangopsidae							
	Paraclodes	sp.			4	0,2857	E
	Phalangopsis	sp.	6	0,2			E
Psocoptera							
Trogiomorpha							
Psyllipsocidae							
	Psyllipsocus	sp.1			1		E

Malacostraca							
Isopoda							
Philosciidae		sp.1	1				E
Chordata							
Amphibia							
Anura							
Neobatrachia							
Strabomantidae							
	Pristimantis	*fenestratus*	2	0,0667			E
Mammalia							
Chiroptera							
Emballonuridae							
	Peropteryx	*kappleri*	8	0,2667			
		sp.			4	0,2857	E
Phyllostomidae							
	Glossophaginae	sp.	2	0,0667			
Reptilia							
Squamata							
Gekkonidae							
	Thecadactylus	*rapicauda*			3	0,2143	E
