# Supplementary material for: Reconciling Mining with the Conservation of Cave Biodiversity: A Quantitative Baseline to Help Establish Conservation Priorities
Source: PLoS One. 2016 Dec 20;11(12):e0168348. doi: 10.1371/journal.pone.0168348 (PMC5173368; doi:10.1371/journal.pone.0168348)
Supplement: S1 Dataset — (ZIP) [file pone.0168348.s002.zip › Taxa/Serra Sul/SS_2010/S11-18.pdf]

S11-18		1ª	AB	2ª	AB	ZON
Annelida						
Clitellata						
Oligochaeta	jovens	2	0,011			P
Arthropoda						
Arachnida						
Acari						
Sarcoptiformes						
Oribatida	sp.3	1				P
Amblypygi						
Phrynidae						
Heterophrynus	sp.	6	0,034			P
Araneae						
Corinnidae	jovens	2	0,011			P
Creugas	sp.1			2	0,02	P
Ctenidae	jovens	8	0,046			P
Ochyroceratidae	jovens			1		P
Speocera	sp.1	1				P
Prodidomidae	jovens	1				P
Theridiidae						
Episinus	sp.1			1		P
Theridiosomatidae						
Plato	sp.1	2				P
Opiliones	jovens			8	0,078	P
Cyphophthalmi						
Neogoveidae						
Canga	*renatae*	1				P
Laniatores						
Cosmetidae						
Roquettea	*singularis*			4	0,039	P
Stygnidae	sp.1	4	0,023	4	0,039	P
Pseudoscorpiones						
Chernetidae						
Spelaeochernes	sp.1	3		1		P
Chthoniidae						
Pseudochthonius	sp.1	2		1		P
Ricinulei						
Ricinoididae	jovens	1				P
Schizomida						
Hubbardiidae						
Rowlandius	sp.	1				P
Chilopoda						
Notostigmophora						
Scutigeromorpha						
Pselliodidae	jovens			1		P
Pleurostigmophora						
Geophilomorpha						
Geophilidae	sp.2	2	0,011			P
Scolopendromorpha	jovens	6	0,034			P
Diplopoda						
Glomeridesmida						
Glomeridesmidae	sp.1	2				P
Polydesmida	jovens	2				P
Chelodesmidae	sp.4	4	0,023			P
Fuhrmannodesmidae	sp.1	1				P
Polyxenida						
Hypogexenidae	sp.1	1		1		P
Spirostreptida	jovens	1				P
Entognatha						
Diplura						
Campodeidae	sp.1	1				P
Insecta						

Coleoptera	jovens	1				P
Scydmaenidae	sp.10			1		P
Collembola						
Arthropleona						
Entomobryoidea						
Paronellidae	sp.1	1				P
Symphypleona						
Sminthuroidea	sp.2	1				P
Diptera						
Nematocera						
Psychodidae						
Phlebotominae	sp.			1		P
Sciopemyia	*sordellii*	4		1		P
Sciaridae	sp.	1				P
Tipulidae						
Tipulinae	sp.			1		P
Hemiptera						
Heteroptera						
Dipsocoroidea						
Schizopteridae						
Schizopterinae	sp.3			1		P
Homoptera						
Cixiidae	jovens	2		2		P
	sp.1	1				P
Hymenoptera						
Vespoidea						
Formicidae						
Apterostigma	sp.1	1				P
Camponotus	*atriceps*	1				P
	sp.1	1		3		P
Cyphomyrmex	sp.1	1				P
Myrmicocrypta	sp.1	2				P
Nylanderia	sp.1	1				P
Odontomachus	*bauri*	2	0,011			P
Pachycondyla	*striata*	2				P
Pheidole	sp.2	1		1		P
Isoptera	jovens			2		P
Termitidae						
Diversitermes	sp.	1				P
Nasutitermes	sp.			3		P
Neuroptera						
Chrysopidae	jovens	1				P
Orthoptera						
Caelifera						
Acrididae	sp.	3	0,017			P
Ensifera	jovens			2	0,02	P
Phalangopsidae						
Paraclodes	sp.1			24	0,235	P
Phalangopsis	sp.1	124	0,713	44	0,431	P
Malacostraca						
Isopoda						
Dubioniscidae	sp.1			1		P
Philosciidae	sp.1	3		1		P
Scleropactidae	sp.	1				P
Chordata						
Amphibia						
Anura						
Neobatrachia						
Leptodactylidae						
Leptodactylus	sp.	4	0,023			P
Bufonidae						
Rhinella	cf. *marina*			4	0,039	P

Mammalia							
Chiroptera							
Phyllostomidae	sp.						P
Micronycteris	sp.		7	0,04	10	0,098	
Mollusca							
Gastropoda		jovens	1				P
Bulimulidae							
Naesiotus	sp.		2				P
Systrophiidae							
Happia	sp.		1				P
