# Supplementary material for: Reconciling Mining with the Conservation of Cave Biodiversity: A Quantitative Baseline to Help Establish Conservation Priorities
Source: PLoS One. 2016 Dec 20;11(12):e0168348. doi: 10.1371/journal.pone.0168348 (PMC5173368; doi:10.1371/journal.pone.0168348)
Supplement: S1 Dataset — (ZIP) [file pone.0168348.s002.zip › Taxa/Serra Sul/SS_2010/S11-19.pdf]

S11-19		1ª	AB	2ª	AB	ZON
Arthropoda						
Arachnida						
Amblypygi						
Phrynidae						
Heterophrynus	sp.	2	0,0488			E
Araneae						
Ctenidae	jovens	5	0,122			E
Ochyroceratidae						
Ochyrocera	sp.1	2				E
Pholcidae	jovens	1				E
Ninetinae	sp.1			1		E
Prodidomidae	jovens			1		E
Salticidae	jovens	1		1		E
Scytodidae	*jovens*			1	0,0333	E
Scytodes	*eleonorae*	6	0,1463			E
	sp.	4	0,0976			E
Theridiidae						
Theridion	sp.1			1		E
Theridiosomatidae	jovens	1				E
Plato	sp.1	1				E
Pseudoscorpiones						
Chernetidae						
Spelaeochernes	sp.1	2		1		E
Chthoniidae						
Pseudochthonius	sp.1	2				E
Chilopoda						
Pleurostigmophora						
Geophilomorpha						
Ballophilidae	sp.2	2	0,0488			E
Insecta						
Collembola						
Arthropleona						
Entomobryoidea						
Paronellidae	sp.1	1				E
	sp.2			1		E
Diptera						
Brachycera						
Conopidae	sp.	1				E
Streblidae		1				E
Strebla	sp.					
Nematocera	jovens	1				E
Cecidomyiidae						
Cecidomyiinae	sp.	1				E
Chironomidae	sp.	1				E
Psychodidae						
Micropygomyia	*Série oswaldoi*			1		E
Hemiptera						
Homoptera						
Cixiidae	jovens	1				E
	sp.1	1				E
Hymenoptera						
Vespoidea						
Formicidae						
Apterostigma	sp.1	1				E
Camponotus	sp.1	2		2		E
Labidus	*coecus*	2				E
Pachycondyla	*striata*	2		2		E
Isoptera						

		sp.	1				E
Lepidoptera	jovens		2	0,0488			E
Tineoidea							
Orthoptera							
Ensifera							
Phalangopsidae	jovens						E
	Paraclodes	sp.	3	0,0732	11	0,3667	E
	Phalangopsis	sp.	10	0,2439			E
Psocoptera							
Psocomorpha							
	jovens				1		E
Symphyla							
Scutigerellidae							
	Hanseniella	sp.1	1				E
Chordata							
Amphibia							
Anura							
Neobatrachia							
Strabomantidae							
	Pristimantis	*fenestratus*	2	0,0488			E
Mammalia							
Chiroptera							
Emballonuridae							
	Peropteryx	sp.	3	0,0732			
Phyllostomidae							
	Glossophaginae	sp.			18	0,6	E
Mollusca							
Gastropoda							
Systrophiidae							
	Happia	sp.	2				E
