# Supplementary material for: Reconciling Mining with the Conservation of Cave Biodiversity: A Quantitative Baseline to Help Establish Conservation Priorities
Source: PLoS One. 2016 Dec 20;11(12):e0168348. doi: 10.1371/journal.pone.0168348 (PMC5173368; doi:10.1371/journal.pone.0168348)
Supplement: S1 Dataset — (ZIP) [file pone.0168348.s002.zip › Taxa/Serra Sul/SS_2010/S11-20.pdf]

S11-20		1ª	AB	2ª	AB	ZON
Arthropoda						
Arachnida						
Acari						
Ixodida						
Argasidae						
Ornithodoros	sp.	1		1		P
Parasitiformes						
Opilioacarida						
Opilioacaridae	sp.1	1				P
Trombidiformes						
Tydeoidea						
Eupodidae	sp.1	1				P
Araneae						
Ochyroceratidae	jovens	1		1		E P
Ochyrocera	sp.1	1		1		P
Oonopidae	jovens					P
gr. *Xycarphius*	sp.3	1				E P
Pholcidae	jovens					P
Ninetinae	sp.1	1		1		E P
Salticidae	jovens			1		P
Theridiidae	jovens	1				P
Theridiosomatidae						
Plato	sp.1	1				P
Opiliones						P
Laniatores						P
Stygnidae	jovens	3	0,207			P
	sp.1	3		2	0,182	E P
Pseudoscorpiones						
Chernetidae						
Spelaeochernes	sp.1	1				P
Chilopoda						
Pleurostigmophora						
Scolopendromorpha						
Scolopocryptopidae						
Newportia	sp.1	2	0,069			P
Diplopoda						
Polyxenida						
Hypogexenidae	jovens	1				P
Entognatha						
Diplura						
Campodeidae	sp.1	1				P
Insecta						
Coleoptera	jovens	1				P
Dytiscidae	sp.4	1				P
Collembola						
Arthropleona						
Entomobryoidea						
Paronellidae	sp.1	1				E P
	sp.4	1				P
Diptera	jovens	1				E P
Nematocera						
Cecidomyiidae						
Cecidomyiinae	sp.	1				E P
Ceratopogonidae	sp.	1				E P
Chironomidae	sp.	1		1		E P
Psychodidae						
Sciopemyia	*sordellii*			1		P
Hemiptera						
Heteroptera						

Enicocephalidae	jovens		1					P
Hebridae		sp.1	1		1		E	P
Homoptera								
Cixiidae	jovens		1					P
		sp.1	1				E	P
Hymenoptera								
Vespoidea								
Formicidae								
	Hypoponera	sp.1	1					P
	Pachycondyla	*harpax*	1				E	P
	Pheidole	sp.1			1		E	P
Lepidoptera	jovens		1	0,034	2	0,182	E	P
Noctuoidea		sp.7	1				E	P
Orthoptera								
Ensifera								
Phalangopsidae	jovens						E	P
	Paraclodes	sp.1	16	0,552	2	0,182	E	P
	Phalangopsis	sp.1			2	0,182		P
Psocoptera								
Psocomorpha	jovens				1		E	P
Malacostraca								
Isopoda								
Philosciidae		sp.1			1		E	P
Chordata								
Amphibia								
Anura								
Neobatrachia								
Strabomantidae								
	Pristimantis	*fenestratus*	2	0,069	3	0,273	E	P
