# Supplementary material for: Reconciling Mining with the Conservation of Cave Biodiversity: A Quantitative Baseline to Help Establish Conservation Priorities
Source: PLoS One. 2016 Dec 20;11(12):e0168348. doi: 10.1371/journal.pone.0168348 (PMC5173368; doi:10.1371/journal.pone.0168348)
Supplement: S1 Dataset — (ZIP) [file pone.0168348.s002.zip › Taxa/Serra Sul/SS_2010/S11-21.pdf]

S11-21		1ª	AB	2ª	AB	ZON
Arthropoda						
Arachnida						
Acari						
Ixodida						
Ixodidae						
Amblyomma	sp.	1				P
	sp.1	1				E
Parasitiformes						
Mesostigmata						
Laelapidae	sp.2			1		E
Stratiolaelaps	sp.1	5		2		P
Macronyssidae	sp.1			2		P
	sp.2			2		P
	sp.4			1		P
	sp.5	1		1		P
Sarcoptiformes						
Oribatida	sp.1			1		P
	sp.16	1				P
	sp.3	1		3		P
	sp.7			1		P
Trombidiformes						
Tydeoidea	sp.1			2		P
	sp.2	1		1		P
	sp.6			1		P
	sp.7			3		P
Cunaxidae	sp.2	1				E
Araneae						
Araneidae	jovens			1		P
Alpaida	*septemmammata*	1				E
Corinnidae	jovens	26	0,0613	6	0,0124	E P
Ctenidae	jovens	2	0,0047			E
Ochyroceratidae	jovens	1				P
Ochyrocera	sp.1	1				E
Oonopidae	jovens	1				E
Pholcidae	jovens	1				P
Mesabolivar	sp.1	1		1		E P
Ninetinae	sp.1	2		1		E P
Salticidae	jovens	1				P
Freya	sp.1	1				E
Marma	sp.1	1				E
Scytodidae	jovens	3				E P
Theridiidae						
Theridion	sp.1	1				E
Theridiosomatidae						
Plato	sp.1	2		1		P
Opiliones						
Laniatores						
Stygnidae	sp.1	5	0,0118			E P
Pseudoscorpiones						
Chernetidae	jovens			2		P
Spelaeochernes	sp.1	3		1		P
Chthoniidae						
Pseudochthonius	sp.1	1				P
Olpiidae	sp.1	4				E P
Schizomida						
Hubbardiidae	jovens	1		1		P
Rowlandius	sp.	1		1		P
Chilopoda						
Notostigmophora						
Scutigeromorpha	jovens			2	0,0041	P
Pselliodidae	jovens			2		P
Diplopoda						
Glomeridesmida						

Glomeridesmidae	sp.1			1		P
Insecta						
Blattodea	jovens	3	0,0236	5	0,0103	E P
Blaberidae	jovens	7				E P
Coleoptera						
Curculionidae						
Scolytinae	sp.2			1		P
Ptiliidae	sp.1	1		3		P
Scydmaenidae	sp.2	1		1		P
	sp.5	1				P
	jovens	2		2		P
Collembola						
Arthropleona						
Entomobryoidea						
Cyphoderidae	sp.2			2		P
Paronellidae	sp.1			2		P
Symphypleona						
Sminthuroidea	sp.2	1				E
Diptera						
Brachycera						
Camillidae	sp.			1		E
Chloropidae	sp.			1		P
Conopidae	sp.	3				E P
Dolichopodidae	sp.			1		E
Nematocera						
Cecidomyiidae						
Cecidomyiinae	sp.	1				P
Chironomidae	sp.			2		E P
Psychodidae						
Edentomyia	*piauensis*	1				P
Nemopalpus	sp.	1				P
Pericoma	sp.	1		2		P
Sciopemyia	*sordellii*	2		2		E P
Sciaridae						
Bradysia	sp.			2		P
	jovens	2		4		E P
Hemiptera						
Heteroptera						
Cydnidae						
Cydninae	sp.1	4		4		P
Reduviidae	jovens	3	0,0071			E
Hymenoptera						
Apoidea						
Megachilidae	sp.1			1		E
Vespoidea						
Formicidae						
Camponotus	*atriceps*	1				P
	sp.1	2		2		E P
Crematogaster	sp.1			1		P
Pachycondyla	*striata*	5		4		E P
Solenopsis	sp.1	1		1		P
	sp.2			2		P
	sp.3			1		P
Isoptera						
Termitidae						
Nasutitermes	sp.	1		1		E
Lepidoptera						
Noctuoidea						
Noctuidae	sp.2	2	0,0047			E
Tineoidea	sp.1	2		1		E P
	jovens	1				P
Orthoptera						
Ensifera						
Phalangopsidae						

Phalangopsis	sp.1	204	0,4811	204	0,4215		P
Paraclodes	sp.1	7	0,0165	13	0,0269	E	P
Psocoptera							
Psocomorpha							
Archipsocidae							
Archipsocus	sp.1	1				E	
Trogiomorpha							
Psyllipsocidae	jovens			1		E	
Psocathropos	sp.1			1		E	
Thysanura							
Ateluridae	sp.1			1			P
Nicoletiidae	jovens			3		E	P
	sp.1	5		1		E	P
Malacostraca							
Isopoda							
Dubioniscidae	sp.1	1		1		E	
Philosciidae	sp.1	4		4			P
Chordata							
Amphibia							
Anura							
Neobatrachia							
Strabomantidae							
Pristimantis	*fenestratus*			7	0,0145		P
Ave							
Cathartiformes							
Cathartidae	sp.			3	0,0062	E	
Mammalia							
Chiroptera							
Furipteridae							
Furipterus	*horrens*	11	0,0259				
Phyllostomidae							
Carollia	sp.	31	0,0731	41	0,0847		P
Glossophaginae	sp.	121	0,2854	201	0,4153		P
Rodentia	sp.			2	0,0041	E	
Reptilia							
Squamata							
Gekkonidae							
Thecadactylus	*rapicauda*	2	0,0047			E	
Mollusca							
Gastropoda							
Bulimulidae							
Naesiotus	sp.			1			P
