# Supplementary material for: Reconciling Mining with the Conservation of Cave Biodiversity: A Quantitative Baseline to Help Establish Conservation Priorities
Source: PLoS One. 2016 Dec 20;11(12):e0168348. doi: 10.1371/journal.pone.0168348 (PMC5173368; doi:10.1371/journal.pone.0168348)
Supplement: S1 Dataset — (ZIP) [file pone.0168348.s002.zip › Taxa/Serra Sul/SS_2010/S11-22.pdf]

S11-22		1ª	AB	2ª	AB	ZON
Annelida						
Clitellata						
Oligochaeta	jovens	2	0,034			E
Arthropoda						
Arachnida						
Acari						
Ixodida						
Ixodidae						
Amblyomma	sp.1	1				E
Parasitiformes						
Mesostigmata	sp.1	1				E
Amblypygi						
Phrynidae						
Heterophrynus	sp.	2	0,034	2	0,029	E
Araneae						
Araneidae	jovens	2				E
Ctenidae	jovens	8	0,138			E
Phoneutria	*reidyi*			5	0,08	E
Pholcidae	jovens	2				E
Mesabolivar	*aurantiacus*			1		E
	sp.1			1		E
Salticidae						
Fluda	sp.1			1		E
Scytodidae	*jovens*			1	0,014	E
Theridiidae	jovens	1		1		E
Theridion	sp.2			1		E
Opiliones						
Eupnoi						
Sclerosomatidae	jovens	1				E
	sp.1	1		1		E
Laniatores						
Cosmetidae	sp.1			3	0,043	E
Escadabiidae	sp.5	1				E
Gonyleptidae	sp.1			3	0,043	E
Stygnidae	jovens	2	0,034			E
Pseudoscorpiones						
Chernetidae						
Spelaeochernes	sp.1	1				E
Diplopoda						
Polydesmida						
Fuhrmannodesmidae	sp.3	1				E
Paradoxosomatidae	sp.1	2				E
Spirostreptida						
Pseudonannolenidae						
Pseudonannolene	sp.1			2	0,029	E
Entognatha						
Diplura						
Campodeidae	sp.1	1				E
Japygidae	sp.1	1				E
Insecta						
Blattodea						
Blattidae	sp.3	4	0,069			E
Collembola						
Arthropleona						
Entomobryoidea						
Paronellidae	sp.1	2				E
	sp.5	1				E
Diptera	jovens	1				E
Nematocera						

Psychodidae						
Sciopemyia	*sordellii*	1				E
Sciaridae	sp.	1				E
Tipulidae						
Tipulinae	sp.			2		E
Hemiptera						
Heteroptera						
Cydnidae	jovens	1				E
Reduviidae	jovens	9		4	0,057	E
Reduviinae	sp.	11	0,345			E
Homoptera						
Cixiidae	jovens	1				E
Hymenoptera						
Vespoidea						
Formicidae						
Gnamptogenys	*striatula*	1				E
Paraponera	*clavata*			2	0,029	E
Pheidole	sp.2			1		E
Solenopsis	sp.2	1				E
Isoptera						
Termitidae						
Diversitermes	sp.			1		E
Nasutitermes	sp.	2		2		E
Lepidoptera	jovens			1		E
Cossoidea						
Limacodidae	sp.1	2	0,034			E
Noctuoidea						
Noctuidae	sp.2	2	0,034			E
Orthoptera						
Ensifera						
Phalangopsidae						
Paraclodes	sp.1	2	0,034	31	0,443	E
Psocoptera						
Psocomorpha						
Epipsocidae						
Hinduipsocus	sp.2	1				E
Malacostraca						
Isopoda						
Dubioniscidae	sp.1	1				E
Platyarthridae	sp.	1				E
Chordata						
Amphibia						
Anura						
Neobatrachia						
Strabomantidae						
Pristimantis	*fenestratus*	3	0,052	2	0,029	E
Mammalia						
Chiroptera						
Emballonuridae						
Peropteryx	sp.			3	0,043	E
Phyllostomidae						
Carollia	sp.	4	0,069	3	0,043	E
Glossophaginae	sp.	5	0,086	7	0,1	E
Reptilia						
Squamata						
Gekkonidae						
Thecadactylus	*rapicauda*			2	0,029	E
Mollusca						
Gastropoda						
Streptaxidae						

Streptaxis sp.			1		E
Systrophiidae					
Happia sp.	1				E
