# Supplementary material for: Reconciling Mining with the Conservation of Cave Biodiversity: A Quantitative Baseline to Help Establish Conservation Priorities
Source: PLoS One. 2016 Dec 20;11(12):e0168348. doi: 10.1371/journal.pone.0168348 (PMC5173368; doi:10.1371/journal.pone.0168348)
Supplement: S1 Dataset — (ZIP) [file pone.0168348.s002.zip › Taxa/Serra Sul/SS_2010/S11-23.pdf]

S11-23			1ª	AB	2ª	AB	ZON
Annelida							
Clitellata							
Oligochaeta	jovens		8	0,1633			P
Arthropoda							
Arachnida							
Acari							
Trombidiformes							
Tydeoidea							
Erythraeidae		sp.1	1				P
Labdostomatidae		sp.1			1		E P
Amblypygi							
Phrynidae							
	Heterophrynus	sp.	3	0,0612	6	0,2143	E P
Araneae							
Araneidae	jovens		2		1		E P
	Alpaida	sp.1	1				P
Mimetidae							
	Ero	sp.1	1				P
Ochyroceratidae							
	Speocera	sp.1	1		1		P
Pholcidae							
	Mesabolivar	sp.1	1				E P
Salticidae	jovens		1				E P
Scytodidae	jovens		2	0,0408			E P
	Scytodes	*eleonorae*			2	0,0714	P
	Scytodes	sp.			3	0,1071	P
Theridiidae	jovens		1		1		P
	Theridion	sp.2	1				E P
Theridiosomatidae	jovens		1				E P
	Plato	sp.1	2				E P
Opiliones							
Cyphophthalmi							
Neogoveidae							
	Canga	*renatae*	2				E P
Palpigradi							
Eukoeneniidae							
	Eukoenenia	sp.1	1				E P
Pseudoscorpiones							
Chernetidae		sp.1					
	Spelaeochernes	sp.1	1		1		E P
Chthoniidae							
	Pseudochthonius	sp.1	1				P
Schizomida							
Hubbardiidae							
	Rowlandius	sp.	1				P
Chilopoda	jovens		2	0,0408			P
Pleurostigmophora							
Scolopendromorpha							
Scolopocryptopidae							
	Newportia	sp.1	2	0,0408			P
Diplopoda	jovens		2	0,0408			P
Spirostreptida	jovens		1		1		E P
Pseudonannolenidae	jovens		2	0,0408			P
Entognatha							
Diplura							
Campodeidae		sp.1	1		1		E P
Insecta							
Blattodea	jovens				4	0,1429	E P
Coleoptera	jovens		1				P
Cerambycidae		sp.2			1		E P
Leiodidae		sp.3			2		E P
Ptiliidae		sp.1	1				E P
Scydmaenidae		sp.5	1				P

Diptera	jovens		1		1		E	P
Nematocera								
Chironomidae		sp.			1			P
Psychodidae								
	Phlebotominae	sp.			1			P
	Pintomyia	*gruta*	2				E	P
	Sciopemyia	*sordellii*	2		1		E	P
Tipulidae								
	Tipulinae	sp.			1			P
Hemiptera								
Heteroptera								
Cydnidae								
	Cydninae	sp.1	1					P
Homoptera								
Cixiidae	jovens		1		1		E	P
Hymenoptera								
Vespoidea								
Formicidae								
	Apterostigma	sp.1	2					P
	Camponotus	sp.1			1			P
	Crematogaster	sp.1	1				E	P
	Gnamptogenys	*striatula*	1					P
	Pachycondyla	*striata*	2				E	P
	Wasmania	*auropunctata*			2		E	P
Lepidoptera	jovens				1			P
Castnioidea								
Castniidae		sp.1	3	0,0612			E	P
Noctuoidea		sp.2	1				E	P
		sp.7			1		E	P
Orthoptera								
Ensifera								
Phalangopsidae								
	Phalangopsis	sp.1	25	0,5102				P
Psocoptera								
Psocomorpha	jovens		1		2		E	P
Thysanura								
Nicoletiidae		sp.1	1					P
Malacostraca								
Isopoda								
Philosciidae		sp.1	1		1		E	P
		sp.2			1			P
		sp.3	1				E	P
Chordata								
Amphibia								
Anura								
Neobatrachia								
Strabomantidae								
	Pristimantis	*fenestratus*			3	0,1071		P
Mammalia								
Chiroptera								
Phyllostomidae								
	Carollia	sp.			7	0,25		P
	Glossophaginae	sp.			3	0,1071		P
Mollusca								
Gastropoda								
Systrophiidae								
	Happia	sp.			1		E	P
