# Supplementary material for: Reconciling Mining with the Conservation of Cave Biodiversity: A Quantitative Baseline to Help Establish Conservation Priorities
Source: PLoS One. 2016 Dec 20;11(12):e0168348. doi: 10.1371/journal.pone.0168348 (PMC5173368; doi:10.1371/journal.pone.0168348)
Supplement: S1 Dataset — (ZIP) [file pone.0168348.s002.zip › Taxa/Serra Sul/SS_2010/S11-24.pdf]

S11-24			1ª	AB	2ª	AB	ZON
Annelida							
Clitellata							
Oligochaeta		sp.	2	0,0526			E
Arthropoda							
Arachnida							
Acari							
Trombidiformes		sp.7			1		E
Tydeoidea							
Labdostomatidae		sp.1	1				E
Amblypygi							
Phrynidae							
	Heterophrynus	sp.			2	0,1111	E
Araneae							
Ctenidae	jovens		2	0,0526			E
Pholcidae	jovens				1		E
	Mesabolivar	*aurantiacus*	1				E
Salticidae	jovens		1				E
Theridiosomatidae	jovens		1		1		E
	Plato	sp.1	1				E
Opiliones							
Eupnoi							
Sclerosomatidae		sp.1	1				E
Entognatha							
Diplura							
Campodeidae		sp.1	1				E
Insecta							
Blattodea	jovens		6	0,1579	4	0,2222	E
Coleoptera	jovens				1		E
Carabidae		sp.18	2				E
		sp.3			1		E
Staphylinidae		sp.25	1				E
	Pselaphinae	sp.3	2		1		E
Collembola							
Arthropleona							
Entomobryoidea							
Entomobryidae		sp.1	1		1		E
Paronellidae		sp.1	2				E
Diptera	jovens				1		E
Brachycera							
Dolichopodidae		sp.	1				E
Drosophilidae							
	Drosophila	sp.	1				E
Phoridae							
	Metopininae	sp.			1		E
Nematocera							
Sciaridae		sp.	1				E
	Bradysia	sp.			1		E
Tipulidae		sp.					
	Tipulinae	sp.	1				E
Hemiptera							
Heteroptera							
Dipsocoroidea							
Ceratocombidae							
	Ceratocombinae	sp.2			1		E
Hymenoptera							
Vespoidea							
Formicidae							
	Nylanderia	sp.1			1		E
	Pheidole	sp.2	1				E
Isoptera							
Termitidae							
	Armitermes	sp.			1		E
	Nasutitermes	sp.			1		E

Orthoptera							
Ensifera							
Phalangopsidae							
	Phalangopsis	sp.1	14	0,3684	2	0,1111	E
	Paraclodes	sp.1	2	0,0526			E
Chordata							
Amphibia							
Anura							
Neobatrachia							
Strabomantidae							
	Pristimantis	*fenestratus*			3	0,1667	E
Mammalia							
Chiroptera							
Emballonuridae							
	Peropteryx	sp.	3	0,0789			
Phyllostomidae							
	Carollia	sp.	5	0,1316	7	0,3889	E
	Glossophaginae	sp.	4	0,1053			
