# Supplementary material for: Reconciling Mining with the Conservation of Cave Biodiversity: A Quantitative Baseline to Help Establish Conservation Priorities
Source: PLoS One. 2016 Dec 20;11(12):e0168348. doi: 10.1371/journal.pone.0168348 (PMC5173368; doi:10.1371/journal.pone.0168348)
Supplement: S1 Dataset — (ZIP) [file pone.0168348.s002.zip › Taxa/Serra Sul/SS_2010/S11-25.pdf]

S11-25			1ª	AB	2ª	AB	ZON
Annelida							
Clitellata							
Oligochaeta	jovens		2	0,011			A
Arthropoda							
Arachnida							
Acari							
Ixodida							
Parasitiformes							
Mesostigmata							
Laelapidae		sp.3			1		A
Macrochelidae		sp.1	1				P
Sarcoptiformes							
Oribatida		sp.3	1				A
		sp.7			1		P
Trombidiformes							
Tydeoidea							
Scutacaridae		sp.1			1		A
Amblypygi							
Charinidae	jovens		2	0,011			A
Phrynidae							
	Heterophrynus	sp.	7	0,04	10	0,119	A
Araneae							
Corinnidae	jovens		8	0,069	5	0,06	E P A
	Creugas	sp.1	4				E
Ctenidae	jovens		4	0,023			A
Ochyroceratidae	jovens		2				E A
	Speocera	sp.1	1				E
Pholcidae	jovens				1		E
Scytodidae	jovens		1	0,006	1	0,012	E A
	Scytodes	*eleonorae*	4	0,023			E P
		sp.	3	0,017	3	0,036	P A
Theridiidae							
	Theridion	sp.2	1		1		E P
Theridiosomatidae							
	Plato	sp.1			1		A
Opiliones							
Cyphophthalmi							
Neogoveidae							
	Canga	*renatae*	2				P A
Pseudoscorpiones							
Bochicidae		sp.1	2		2		P
Chernetidae							E P A
	Spelaeochernes	sp.1	3		1		E P A
Chthoniidae							E A
	Pseudochthonius	sp.1	1		1		E A
		sp.4	2		2		P
Schizomida							
Hubbardiidae							
	Rowlandius	sp.	2				E A
Chilopoda	jovens		2	0,011			A
Diplopoda	jovens		11	0,063			A
Glomeridesmida							
Glomeridesmidae	jovens				1		A
		sp.1	2		1		P A
Polydesmida							
Chelodesmidae		sp.1	2	0,011			A
		sp.3	2	0,011			A
		sp.4	2	0,011	2	0,024	P A
Fuhrmannodesmida	jovens		1				E

		sp.1			1		P
		sp.3			1		P
Pyrgodesmidae		sp.2	2	0,011	2	0,024	P
Spirostreptida	jovens				1		A
Pseudonannolenida	jovens		4	0,023			A
Coleoptera	jovens				1		P
Scarabaeidae		sp.1	2	0,011			A
Staphylinidae		sp.26	2	0,011			A
	Pselaphinae	sp.3	1				E
Collembola							
Arthropleona							
Entomobryoidea							
Cyphoderidae		sp.1	1				A
Paronellidae		sp.1	2				E P
		sp.4			1		A
Dermaptera	jovens		1				P
Diptera	jovens		6		3		E P A
Brachycera							
Chloropidae		sp.			2		P A
Phoridae							
	Metopininae	sp.			1		A
Streblidae							
	Trichobius	sp.			1		P
Nematocera							
Chironomidae		sp.			1		A
Psychodidae							
	Pericoma	sp.	2				P A
	Phlebotominae	sp.			1		P
	Pintomyia	*gruta*	1		1		P
	Sciopemyia	*sordellii*	2				P
Sciaridae							
	Bradysia	sp.			2		P
Tipulidae							
	Tipulinae	sp.	1		2		E P A
Hemiptera							
Heteroptera							
Reduviidae	jovens		2	0,011			P
	Emesinae	sp.2			1		P
Homoptera							
Aphididae		sp.1			1		A
Cixiidae	jovens				1		P
Hymenoptera	jovens		2				E
Apoidea		sp.2			1		P
Vespoidea							
Formicidae							
	Acromyrmex	sp.1	2		2		P A
	Atta	sp.1			1		P
	Camponotus	sp.1			2		P A
	Gnamptogenys	*striatula*	1		1		E A
	Labidus	*coecus*			1		A
	Nylanderia	sp.1	1		2		P A
	Solenopsis	sp.2			3		P A
	Strumygenys	sp.1			1		A
Isoptera							
Termitidae							
	Nasutitermes	sp.			1		A
Lepidoptera	jovens		2	0,011			E
Noctuoidea		sp.2	1				E
Orthoptera							
Ensifera							

Phalangopsidae							
Phalangopsis	sp.1	103	0,592	61	0,726	P A
Psocoptera							
Psocomorpha	jovens		1				E
Thysanura							
Ateluridae	jovens				1		P
		sp.1	1				A
Malacostraca							
Isopoda							
Philosciidae		sp.1	3		3		E P A
Mammalia							
Chiroptera							
Emballonuridae							
Peropteryx		sp.	3	0,017			
Mollusca							
Gastropoda							
Bulimulidae							
Naesiotus		sp.	1				P
Subulinidae							
Lamellaxis		sp.	2		2		E P
Leptinaria		sp.	1				A
Systrophiidae							
Happia		sp.	2		2		E P A
