# Supplementary material for: Reconciling Mining with the Conservation of Cave Biodiversity: A Quantitative Baseline to Help Establish Conservation Priorities
Source: PLoS One. 2016 Dec 20;11(12):e0168348. doi: 10.1371/journal.pone.0168348 (PMC5173368; doi:10.1371/journal.pone.0168348)
Supplement: S1 Dataset — (ZIP) [file pone.0168348.s002.zip › Taxa/Serra Sul/SS_2010/S11-26.pdf]

S11-26			1ª	AB	2ª	AB	ZON
Arthropoda							
Arachnida							
Araneae							
Corinnidae		jovens	2	0,1538			P
Pholcidae		jovens	1				E
Segestriidae		jovens	1		1		E
Uloboridae		jovens			1		E
Opiliones							
Eupnoi							
Sclerosomatidae		sp.1	1		1		E
Chilopoda							
Pleurostigmophora							
Scolopendromorpha							
Scolopocryptopidae							
	Newportia	sp.1	2	0,1538			P
Diplopoda							
Polydesmida							
Chelodesmidae		sp.2	2	0,1538			E
Fuhrmannodesmidae		sp.1	2				E P
Insecta							
Blattodea		jovens	2	0,1538			P
Diptera							
Nematocera							
Sciaridae		sp.					
	Bradysia	sp.			1		E
Tipulidae		sp.	1				E
Hymenoptera							
Vespoidea							
Formicidae							
	Atta	sp.1	1		1		E P
	Camponotus	*atriceps*			1		P
		sp.1			1		E
	Nesomyrmex	sp.1			1		P
	Nylanderia	sp.1	1				E
	Platythyrea	*angusta*			1		P
Isoptera							
Termitidae							
	Nasutitermes	sp.	1		1		P
Lepidoptera							
Cossoidea							
Limacodidae		sp.1	2	0,1538			P
Psocoptera							
Psocomorpha							
Caeciliusidae							
	Paracaecilius	sp.1	1				E
Chordata							
Amphibia							
Anura							
Neobatrachia							
Strabomantidae							
	Pristimantis	*fenestratus*			2	0,5	P
Mammalia							
Chiroptera							
Emballonuridae							
	Peropteryx	sp.	1	0,0769	2	0,5	E
Platyhelminthes							
Turbellaria		sp.4	2	0,1538			E
