# Supplementary material for: Reconciling Mining with the Conservation of Cave Biodiversity: A Quantitative Baseline to Help Establish Conservation Priorities
Source: PLoS One. 2016 Dec 20;11(12):e0168348. doi: 10.1371/journal.pone.0168348 (PMC5173368; doi:10.1371/journal.pone.0168348)
Supplement: S1 Dataset — (ZIP) [file pone.0168348.s002.zip › Taxa/Serra Sul/SS_2010/S11-27.pdf]

S11-27		1ª	AB	2ª	AB	ZON
Arthropoda						
Arachnida						
Acari						
Ixodida						
Ixodidae						
Ixodes	sp.	1				P
Parasitiformes						
Holothyrida						
Diplothyridae						
Diplothyrus	*scubarti*	2				P
Mesostigmata						
Laelapidae	sp.3	1				P
Stratiolaelaps	sp.1	2				E P
Macronyssidae	sp.1	3		3		E P
Sarcoptiformes						
Oribatida	sp.2	1				P
Trombidiformes	sp.7	1				P
Amblypygi						
Charinidae	jovens	2	0,0141			E
Phrynidae						
Heterophrynus	sp.	3	0,0211			
Araneae						
Araneidae	jovens	1		2		E P
Alpaida	sp.2	1		1		E P
Corinnidae	jovens	7	0,0493	4	0,1176	E P
Ctenidae	jovens	9	0,0634	2	0,0588	E P
Ochyroceratidae	jovens	1				P
Speocera	sp.1	1		1		E
Pholcidae	jovens	1				E
Ninetinae	sp.1	1		1		P
Prodidomidae	jovens	1		1		E P
Scytodes	*eleonorae*	2	0,0141	2	0,0588	P
	sp.	7	0,0493			P
Segestriidae	jovens			3		E P
Ariadna	sp.1	1				E
Opiliones						
Laniatores						
Stygnidae	jovens	2	0,0141			E
Pseudoscorpiones						
Chernetidae						
Spelaeochernes	sp.1	2		3		E P
Chthoniidae						
Pseudochthonius	sp.1	4		2		E P
Scorpiones						
Buthidae						
Ananteris	*balzanii*	2	0,0141			P
Chilopoda	jovens	3	0,0211			E
Pleurostigmophora						
Scolopendromorpha	jovens	2	0,0141	1	0,0294	E
Scolopocryptopidae						
Tidops	sp.1	2	0,0141			E
Scutigeromorpha	jovens			2	0,0588	P
Diplopoda	jovens	6	0,0423			E
Polydesmida	jovens	2				E P
Chelodesmidae	sp.1	2	0,0141			P
	sp.4	2	0,0141			E
Spirostreptida	jovens	3		1		E P
Pseudonannolenidae	jovens	2	0,0141			E
Entognatha						
Diplura						
Campodeidae	sp.1	2		1		E P
Insecta						
Blattodea	jovens	2	0,0141			P

Blaberidae	jovens			2	0,0588	P
Coleoptera	jovens	1		1		P
Carabidae						
Coarazuphium	sp.1	1				E
Leiodidae	sp.3	1				P
Staphylinidae	sp.29	1				E
	sp.3	1				P
	sp.30	1				P
Collembola						
Arthropleona						
Entomobryoidea						
Cyphoderidae	sp.2	1				E
Paronellidae	sp.4	1				P
Diptera	jovens	3		1		E P
Brachycera						
Chloropidae	sp.			1		E
Phoridae						
Metopininae	sp.	3		1		E P
Phorinae	sp.	1				P
Sphaeroceridae	sp.	3				E P
Nematocera						
Culicidae						
Culicini	sp.			1		E
Psychodidae						
Pintomyia	*gruta*	1				P
Hemiptera						
Heteroptera						
Dipsocoroidea	jovens	1				P
Schizopteridae						
Schizopterinae	sp.3			1		P
Hymenoptera	jovens	1				P
Vespoidea						
Formicidae						
Camponotus	*atriceps*	4	0,0282			E
	sp.1			2		E
Hypoponera	sp.1	1		1		P
Octostruma	sp.1	2				E P
Pachycondyla	*harpax*			2		E
	striata	1				P
Solenopsis	sp.2	1				E
Isoptera	sp.			2		E P
Termitidae						
Nasutitermes	sp.	1		1		E P
Lepidoptera	jovens	2				E P
Orthoptera						
Ensifera						
Phalangopsidae						
Paraclodes	sp.1			2	0,0588	E
Phalangopsis	sp.1	66	0,4648	11	0,3235	P
Thysanura						
Ateluridae	sp.1			1		P
Nicoletiidae	sp.1	3				E P
Malacostraca						
Isopoda						
Philosciidae	sp.1	3				E P
Chordata						
Amphibia						
Anura						
Neobatrachia						
Bufonidae						
Rhinella	cf. *marina*			2	0,0588	P
Mammalia						
Chiroptera						
Phyllostomidae						

	Desmodus	rotundus			4	0,1176	
	Diphylla	*ecaudata*	5	0,0352			P
	Micronycteris	sp.			2	0,0588	P
	Trachops	*cirrhosus*	12	0,0845			
Mollusca							
Gastropoda							
Subulinidae							
	Lamellaxis	sp.	1				E
	Leptinaria	sp.	1				P
