# Supplementary material for: Reconciling Mining with the Conservation of Cave Biodiversity: A Quantitative Baseline to Help Establish Conservation Priorities
Source: PLoS One. 2016 Dec 20;11(12):e0168348. doi: 10.1371/journal.pone.0168348 (PMC5173368; doi:10.1371/journal.pone.0168348)
Supplement: S1 Dataset — (ZIP) [file pone.0168348.s002.zip › Taxa/Serra Sul/SS_2010/S11-28.pdf]

S11-28			1ª	AB	2ª	AB	ZON
Arthropoda							
Arachnida							
Araneae							
Araneidae	jovens		1				E
Opiliones							
Eupnoi							
Sclerosomatidae		sp.1			1		E
Laniatores							
Stygnidae	jovens		3	0,2			E
		sp.1			2	0,5	E
Pseudoscorpiones							
Chernetidae							
	Spelaeochernes	sp.1	1				E
Insecta							
Coleoptera	jovens		1		1		E
		sp.2	3				E
Staphylinidae		sp.21	1				E
		sp.27	2	0,1333			E
Trogidae		sp.1	2	0,1333			E
Collembola							
Arthropleona							
Entomobryoidea							
Cyphoderidae		sp.1			1		E
Entomobryidae		sp.7	1				E
Diptera	jovens				1		E
Nematocera							
Cecidomyiidae							
	Cecidomyiinae	sp.	1		1		E
Chironomidae		sp.	2				E
Tipulidae		sp.	1				E
Hemiptera							
Heteroptera							
Gerridae	jovens				1		E
Hebridae	jovens				1		E
Orthoptera							
Ensifera							
Phalangopsidae							
	Phalangopsis	sp.1	2	0,1333			E
Malacostraca							
Isopoda							
Philosciidae		sp.1			1		E
Chordata							
Reptilia							
Squamata							
Gekkonidae							
	Thecadactylus	*rapicauda*			2	0,5	E
Mollusca							
Gastropoda	jovens				1		E
Oleacinidae							
	Euglandina	sp.	2	0,1333			E
Platyhelminthes							
Turbellaria		sp.6	2	0,1333			E
