# Supplementary material for: Reconciling Mining with the Conservation of Cave Biodiversity: A Quantitative Baseline to Help Establish Conservation Priorities
Source: PLoS One. 2016 Dec 20;11(12):e0168348. doi: 10.1371/journal.pone.0168348 (PMC5173368; doi:10.1371/journal.pone.0168348)
Supplement: S1 Dataset — (ZIP) [file pone.0168348.s002.zip › Taxa/Serra Sul/SS_2010/S11-29.pdf]

S11-29			1ª	AB	2ª	AB	ZON
Annelida							
Clitellata							
Oligochaeta	jovens		6	0,2			E
Arthropoda							
Arachnida							
Acari							
Parasitiformes							
Mesostigmata							
Laelapidae							
	Stratiolaelaps	sp.1	2				E
Sarcoptiformes							
Oribatida		sp.3	1				E
Trombidiformes		sp.1	1				E
Tydeoidea							
Labdostomatidae		sp.1	1		1		E
Amblypygi							
Phrynidae							
	Heterophrynus	sp.	2	0,0667			E
Araneae							
Araneidae	jovens		2		1		E
	Alpaida	*septemmammata*	1				E
Ctenidae	jovens		2	0,0667			E
Ochyroceratidae							
	Theotima	sp.1	1				E
Salticidae	jovens		1				E
Scytodidae	jovens		1	0,1			E
	Scytodes	*globula*	2				E
Theridiosomatidae							
	Plato	sp.1	1				E
Pseudoscorpiones							
Chernetidae							E
	Spelaeochernes	sp.1	2				E
Ricinulei							
Ricinoididae	jovens		1				E
Chilopoda							
Notostigmophora							
Scutigeromorpha							
Pselliodidae	jovens				1		E
Diplopoda	jovens				2	0,0286	E
Spirostreptida	jovens				1		E
Entognatha							
Diplura							
Campodeidae		sp.1	1				E
Insecta							
Blattodea	jovens		2	0,0667			E
Coleoptera							
Carabidae		sp.5	1				E
Collembola							
Arthropleona							
Entomobryoidea							
Entomobryidae		sp.1			1		E
		sp.4	1				E
Isotomidae		sp.2	1				E
Paronellidae		sp.1	2		2		E
Diptera	jovens		1		1		E
Brachycera							
Conopidae		sp.	1				E
Dolichopodidae		sp.			1		E
Nematocera							
Ceratopogonidae		sp.	1				E
Psychodidae							
	Sciopemyia	*sordellii*	1				E
Tipulidae							

	Tipulinae	sp.	1				E
Hemiptera							
Heteroptera							
Cydnidae							
	Cydninae	sp.1	2		2		E
Hymenoptera	jovens		1				E
Vespoidea							
Formicidae							
	Apterostigma	sp.1	1				E
	Camponotus	sp.1			1		E
	Gnamptogenys	*striatula*	1				E
	Hypoponera	sp.1	1				E
	Pachycondyla	*striata*	1		1		E
	Wasmania	*auropunctata*	1				E
Lepidoptera	jovens				2	0,0286	E
Cossoidea							
Limacodidae		sp.1	2	0,0667			E
Orthoptera							
Ensifera							
Gryllidae	jovens		2	0,0667			E
Phalangopsidae							
	Paraclodes	sp.1	2	0,0667	2	0,0286	E
	Phalangopsis	sp.1	7	0,2333			E
Psocoptera							
Psocomorpha	jovens				1		E
Malacostraca							
Isopoda							
Philosciidae		sp.1	2				E
Chordata							
Mammalia							
Chiroptera							
Emballonuridae							
	Peropteryx	sp.	2	0,0667			
Phyllostomidae							
	Carollia	sp.			36	0,5143	E
	Glossophaginae	sp.			26	0,3714	E
	Micronycteris	sp.			2	0,0286	E
