# Supplementary material for: Reconciling Mining with the Conservation of Cave Biodiversity: A Quantitative Baseline to Help Establish Conservation Priorities
Source: PLoS One. 2016 Dec 20;11(12):e0168348. doi: 10.1371/journal.pone.0168348 (PMC5173368; doi:10.1371/journal.pone.0168348)
Supplement: S1 Dataset — (ZIP) [file pone.0168348.s002.zip › Taxa/Serra Sul/SS_2010/S11-30.pdf]

S11-30			1ª	AB	2ª	AB	ZON
Arthropoda							
Arachnida							
Acari							
Parasitiformes							
Mesostigmata							
Rhodacaridae		sp.1	1				E
Amblypygi							
Phrynidae							
	Heterophrynus	sp.	4	0,2222			E
Araneae							
Ochyrocerati	jovens		1				E
Pholcidae	jovens				1		E
	Leptopholcus	sp.1			1		E
Scytodidae	jovens				1	0,0667	E
Pseudoscorpiones							
Chernetidae							
	Spelaeochernes	sp.1	1				E
Schizomida							
Hubbardiidae							
	Rowlandius	sp.	1				E
Entognatha							
Diplura							
Campodeidae		sp.1	1				E
Insecta							
Blattodea	jovens				3	0,3333	E
Blaberidae	jovens				2		E
Collembola							
Arthropleona							
Entomobryoidea							
Entomobryidae		sp.1			1		E
Isotomidae		sp.1	1				E
Paronellidae		sp.1	1		1		E
Diptera	jovens		1		1		E
Nematocera							
Psychodidae							
	Sciopemyia	*sordellii*			1		E
Homoptera							
Cixiidae	jovens		1		1		E
		sp.3			1		E
Hymenoptera							
Vespoidea							
Formicidae							
	Camponotus	sp.1	1				E
	Pheidole	sp.1			1		E
	Wasmania	*auropunctata*	1		1		E
Orthoptera							
Ensifera							
Phalangopsidae							
	Paraclodes	sp.1	3	0,1667	6	0,4	E
	Phalangopsis	sp.1	8	0,4444	3	0,2	E
Psocoptera							
Psocomorpha	jovens				1		E
Symphyla							
Scutigerellidae							
	Hanseniella	sp.1	1				E
Mammalia							
Chiroptera							
Emballonuridae							
	Peropteryx	sp.	1	0,0556			
Platyhelminthes		sp.1					
Turbellaria		sp.7	2	0,1111			E
