# Supplementary material for: Reconciling Mining with the Conservation of Cave Biodiversity: A Quantitative Baseline to Help Establish Conservation Priorities
Source: PLoS One. 2016 Dec 20;11(12):e0168348. doi: 10.1371/journal.pone.0168348 (PMC5173368; doi:10.1371/journal.pone.0168348)
Supplement: S1 Dataset — (ZIP) [file pone.0168348.s002.zip › Taxa/Serra Sul/SS_2010/S11-31.pdf]

S11-31		1ª	AB	2ª	AB	ZON
Annelida						
Clitellata						
Oligochaeta	jovens	2	0,087			E
Arthropoda						
Arachnida						
Araneae						
Filist	jovens	1		1		E
Oonopidae						
gr. *Xycarphius*	sp.2			1		E
Pholcidae						
aff. *Ibityporanga*	sp.1	1				E
Mesabolivar	sp.1	1				E
Modisimus	sp.1	1				E
Scyt	jovens	2	0,1739	1	0,0714	E
Scytodes	sp.	2				E
Theridiidae						
Theridion	sp.1			1		E
Pseudoscorpiones						
Chernetidae						
Spelaeochernes	sp.1			1		E
Chilopoda						
Notostigmophora						
Scutigeromorpha						
Pselli	jovens			1		E
Diplopoda	jovens			5	0,3571	E
Polydesmida						
Chelodesmidae	sp.4	2	0,087			E
Paradoxosomatidae	sp.3			2	0,1429	E
Spirostreptid	jovens			1		E
Insecta						
Coleoptera						
Staphylinidae	sp.31	1				E
Diptera						
Nematocera						
Psychodidae						
Pintomyia	*gruta*	1				E
Hemiptera						
Heteroptera						
Redu	jovens	10	0,4348	2	0,1429	E
Hymenoptera						
Ichneumonoidea						
Braconidae	sp.1	1				E
Vespoidea						
Formicidae						
Camponotus	sp.1	2				E
Cephalotes	sp.1	1				E
Dolichoderus	*bispinosus*			1		E
Eciton	cf. *burchelli*			1		E
Nylanderia	sp.1	1				E

Vespidae	sp.4	1				E
Isoptera						
Termitidae						
Nasutitermes	sp.			1		E
Lepidoptera						
Cossoidea						
Limacodidae	sp.1	3	0,1304			E
Hesperioidea						
Hesperiidae	sp.1			1		E
Orthoptera						
Ensifera						
Phalangopsidae	sp.1			2	0,2857	E
Paraclodes	sp.1	2	0,087	2		E
Psocoptera						
Trogiomorpha						
Psyllipsocidae						
Psyllipsocus	sp.1	1				E
