# Supplementary material for: Reconciling Mining with the Conservation of Cave Biodiversity: A Quantitative Baseline to Help Establish Conservation Priorities
Source: PLoS One. 2016 Dec 20;11(12):e0168348. doi: 10.1371/journal.pone.0168348 (PMC5173368; doi:10.1371/journal.pone.0168348)
Supplement: S1 Dataset — (ZIP) [file pone.0168348.s002.zip › Taxa/Serra Sul/SS_2010/S11D-01.pdf]

S11D-01			1ª	AB	2ª	AB	ZON
Annelida							
Clitellata							
Oligochaeta	jovens		13	0,0075	1	0,0019	P A
Arthropoda							
Arachnida							
Acari							
Parasitiformes							
Holothyrida							
Diplothyridae							
	Diplothyrus	*scubarti*	3		2		P A
Mesostigmata		sp.1	2				P A
		sp.2	10		3		P A
		sp.4	1		1		P A
		sp.5	1				P A
		sp.9	1				P A
Laelapidae		sp.3	1		3		P A
	Stratiolaelaps	sp.1	5		3		P A
Macronyssidae		sp.1	1				P A
Podocinidae		sp.1	1				P A
Veigaiidae		sp.1			1		P A
Opilioacarida							
Opilioacaridae		sp.1	1				E P A
Sarcoptiformes		sp.19	2		1		P A
		sp.20			1		E P A
Oribatida		sp.2	1				P A
		sp.3	10		8		P A
Trombidiformes							
Tydeoidea		sp.1	6		2		P A
		sp.2	3		3		P A
		sp.6			1		P A
		sp.7	8		2		P A
		sp.10	1				P A
Amblypygi							
Phrynidae							
	Heterophrynus	sp.	19	0,0109	1	0,0019	P A
Araneae			4				
Barychaelidae	jovens		2	0,0011	1	0,0038	E P A
	gen.1	sp.1			1		P A
Corinnidae	jovens		7	0,004	6	0,0173	P A
	Creugas	sp.1	2	0,0011	3		P A
Ochyroceratidae	jovens		5		5		E P A
	Ochyrocera	sp.1	2		3		P A
	Speocera	sp.1	9		3		E P A

Oonopidae	jovens		6				P A
	gr. *Xycarphius*	sp.5	2		1		P A
Pholcidae							
	Ninetinae	sp.1	1		1		E P A
Scytodidae	*jovens*		6	0,0034	3	0,0058	E P A
	Scytodes	*eleonorae*	10	0,0057	3	0,0058	E P A
		sp.	19	0,0109	17	0,0327	P A
Segestriidae	jovens		3				P A
Tetrablemmidae	jovens				2		P A
Theridiidae	jovens		1				P A
	Theridion	sp.1	2		1		E P A
Trechaleidae	jovens		1	0,0006	1	0,0019	E P A
		sp.1	1	0,0006			P A
Opiliones	jovens		29	0,0166	1	0,0019	P A
Laniatores							
Cosmetidae							
	Roquettea	*singularis*			1	0,0019	E P A
Stygnidae	jovens		8	0,0046	2	0,0038	E P A
		sp.1	3	0,0017	3	0,0058	E P A
Palpigradi							
Eukoeneniidae							
	Allokoenenia	sp.1	1				P A
Pseudoscorpiones							
Chernetidae	jovens				2		P A
	Spelaeochernes	sp.1	16		6		E P A
Chthoniidae							
	Pseudochthonius	sp.1	11		8		E P A
		sp.4	2				P A
Lechytiidae		sp.1			2		P A
Ricinulei							
Ricinoididae	jovens		1				P A
Chilopoda							
Pleurostigmophora							
Geophilomorpha							
Ballophilidae		sp.3			1	0,0019	P A
Geophilidae		sp.1	1	0,0006	2	0,0038	E P A
Scolopendromorpha	jovens		1	0,0006			P A
Cryptopidae							
	Cryptops	sp.1	2	0,0011			P A
		sp.2	1	0,0006	1	0,0019	P A
Scolopocryptopidae	jovens		1	0,0017			P A
	Dinocryptops	*miersii*	2				P A
Diplopoda							
Glomeridesmida							

Glomeridesmidae		sp.1	1		1		P A
Polydesmida							
Pyrgodesmidae		sp.2	1	0,0006			P A
Spirostreptida	jovens		2		2		E P A
Pseudonannolenidae	jovens		2	0,0017			P A
Pseudonannolene		sp.1	1				P A
Entognatha							
Diplura							
Campodeidae		sp.1	13		8		E P A
Japygidae		sp.1	4				P A
Insecta							
Blattodea	jovens		32	0,0184	20	0,0385	P A
Blaberidae	jovens		3	0,0017	1	0,0019	P A
		sp.1	1	0,0006			P A
		sp.2	2	0,0011			P A
Blattidae	jovens		3	0,0017	2	0,0038	P A
		sp.2	2	0,0011			P A
Coleoptera	jovens		11	0,0063	6	0,0115	P A
Carabidae		sp.2	1				P A
		sp.3			1		P A
		sp.16	1				P A
Chrysomelidae		sp.12	1				P A
Dytiscidae		sp.1	1				P A
Ptiliidae		sp.1	6		1		P A
Scydmaenidae		sp.1	16		6		P A
		sp.2	2		1		P A
		sp.7	2				P A
		sp.8	1				P A
Staphylinidae		sp.3	3		4		P A
Collembola							
Arthropleona							
Entomobryoidea							
Cyphoderidae		sp.1	14		6		P A
		sp.2			2		P A
Entomobryidae		sp.3			1		E P A
Isotomidae		sp.1	1		3		P A
Paronellidae		sp.1			1		E P A
		sp.4	3		1		P A
Symphypleona							
Sminthuroidea		sp.1			1		P A
		sp.2	10		7		E P A
Diptera	jovens		10		11		E P A
Brachycera		sp.	2		1		P A
Chloropidae		sp.	1				P A

		Drosophilidae							
			Drosophila	*eleonore*	2		1		P A
		Milichiidae		sp.			2		P A
		Muscidae		sp.	1				P A
		Phoridae							
			Metopininae	sp.	1		1		E P A
		Streblidae							
			Trichobius	sp.	1		5		E P A
	Nematocera								
		Cecidomyiidae							
			Cecidomyiinae	sp.	1				P A
		Ceratopogonidae		sp.			1		P A
		Chironomidae		sp.	1		2		P A
		Culicidae							
			Culicini	sp.	8		1		E P A
		Mycetophilidae							
			Euceroplatus	sp.			1		E P A
		Psychodidae							
			aff. *Brunettia*	sp.			1		P A
			Breviscapus	sp.			1		P A
			Pericoma	sp.	3				P A
			Telmatoscopus	sp.	3		5		P A
		Sciaridae		sp.	1				P A
			Bradysia	sp.			1		P A
Hemiptera									
	Heteroptera								
		Belostomatidae							
			Belostoma	sp.	1	0,0006			P A
		Cydnidae	jovens		1				P A
		Pentatomidae	jovens				1		E P A
		Reduviidae	jovens		1	0,0006			E P A
		Veliidae	jovens		1		2		P A
			Paravelia	sp.1	3		1		P A
	Homoptera								
		Cixiidae	jovens		6		5		E P A
Hymenoptera			jovens		2	0,0011			P A
	Cynipoidea								
		Eucoilidae		sp.1	2		1		P A
	Proctotrupoidea								
		Diapriidae		sp.2			2		P A
	Vespoidea								
		Formicidae							
			Acromyrmex	sp.1			2		E P A
			Anochetus	sp.1	1		1		P A

	Camponotus	*atriceps*	5	0,0029	2	0,0038	E P A
		sp.1			1		P A
	Cephalotes	sp.1			1		P A
	Dolichoderus	*bispinosus*			1		P A
	Gnamptogenys	sp.1	1		2		P A
		striatula	1		1		E P A
	Hypoponera	sp.1	1		1		E P A
	Nylanderia	sp.1	3		1		P A
	Odontomachus	*bauri*			1		P A
	Pachycondyla	*striata*	15		4		P A
	Pheidole	sp.1	2				P A
	Solenopsis	sp.2			2		P A
	Strumygenys	sp.1	1				P A
	Tranopelta	sp.1	8				P A
	Wasmania	*auropunctata*	1				P A
Isoptera		sp.	5		2		E P A
Termitidae							
	Nasutitermes	sp.			2		E P A
Lepidoptera	jovens		1		1		E P A
Orthoptera							
Ensifera	jovens		1	0,0006			P A
Phalangopsidae							
	Phalangopsis	sp.1	###	0,5737	10	0,0192	P A
	Paraclodes	sp.1			4	0,0077	E P A
Psocoptera							
Psocomorpha	jovens		1				P A
Trogiomorpha							
Psyllipsocidae	jovens				1		E P A
Thysanura							
Ateluridae	jovens		4				P A
		sp.1	5		5		P A
Malacostraca							
Isopoda							
Dubioniscidae		sp.1	2		1		E P A
Symphyla							
Scutigerellidae	jovens		1				P A
	Hanseniella	sp.1	2				E P A
Chordata							
Amphibia							
Anura							
Neobatrachia							
Leptodactylidae							
	Leptodactylus	sp.	2	0,0011	1	0,0019	P A
Strabomantidae							

	Pristimantis	*fenestratus*	9	0,0052	1	0,0019	P A
Mollusca							
Gastropoda							
Subulinidae							
	Lamellaxis	sp.	4		1		P A
Systrophiidae							
	Happia	sp.	2				P A
Nemathelminthes		sp.	1	0,0006	1	0,0019	P A
Secementea							
Strongylida							
Ancylostomatidae							
	Ancylostoma	sp.			1		P A
Platyhelminthes							
Turbellaria		sp.2	3				P A
		sp.7	1	0,0006			P
		sp.8	2	0,0011			P
Chordata							
Mammalia							
Chiroptera							
Emballonuridae							
	Peropteryx	sp.			4	0,0077	P A
Mormoopidae							
	Pteronotus	*parnellii*	300	0,1721	200	0,3846	P A
Natalidae							
	Natalus	cf. *stramineus*	1	0,0006			P A
Phyllostomidae		sp.1					P A
	Anoura	*geoffroyi*	200	0,1147	200	0,3846	P A
	Carollia	*perspicillata*	10	0,0057			P A
	Glossophaginae	sp.	20	0,0115	20	0,0385	P A
	Lonchophylla	*thomasi*	1	0,0006			P A
