# Supplementary material for: Reconciling Mining with the Conservation of Cave Biodiversity: A Quantitative Baseline to Help Establish Conservation Priorities
Source: PLoS One. 2016 Dec 20;11(12):e0168348. doi: 10.1371/journal.pone.0168348 (PMC5173368; doi:10.1371/journal.pone.0168348)
Supplement: S1 Dataset — (ZIP) [file pone.0168348.s002.zip › Taxa/Serra Sul/SS_2010/S11D-02.pdf]

S11D-02			1ª	AB	2ª	AB	ZON
Arthropoda							
Arachnida							
Acari							
Parasitiformes							
Ixodida							
Argasidae							
	Ornithodoros	sp.	1		2		P
Mesostigmata		sp.2			1		P
Trombidiformes							P
Tydeoidea		sp.1	1				P
Amblypygi							
Charinidae	jovens		1	0,0139			P
Araneae							
Araneidae	jovens				1		E P
	Alpaida	*septemmammata*	1				E P
Corinnidae	*Creugas*	sp.1	1	0,0139			E P
Ochyroceratidae	jovens				1		E P
Palpimanidae	jovens				1	0,0476	E P
Pholcidae							
	Leptopholcus	sp.1	1		1		E P
	Ninetinae	sp.1	1		3		E P
Scytodidae	jovens				1	0,0476	E P
	Scytodes	*eleonorae*	2	0,0278			E P
Tetrablemmidae							
	Matta	sp.1			1		E P
Theridiidae	jovens				1		E P
Opiliones	jovens				1	0,0476	E P
Laniatores							
Stygnidae		sp.1	1	0,0139	1	0,0476	E P
Pseudoscorpiones							
Bochicidae		sp.1			2		E P
Chthoniidae							
	Pseudochthonius	sp.1			1		E P
Olpiidae		sp.1			6		E P
Chilopoda							
Pleurostigmophora							
Scolopendromorpha							
Scolopocryptopidae							
	Dinocryptops	*miersii*	1	0,0139			E P
Diplopoda							
Polydesmida							
Pyrgodesmidae		sp.2	1	0,0139			E P
Polyxenida							
Hypogexenidae		sp.1	1		2		E P
Insecta							
Blattodea							
Polyphagidae	jovens		1	0,0139			E P
Coleoptera	jovens				1		E P
Diptera							
Nematocera							
Cecidomyiidae							
	Cecidomyiinae	sp.	1				E P
Psychodidae							
	Sciopemyia	*sordellii*	1				E P
Hemiptera							
Heteroptera							
Reduviidae	jovens				1	0,0476	E P
Homoptera							
Cixiidae	jovens		1		1		E P
Hymenoptera							
Apoidea		sp.1	1				E P
Vespoidea							
Formicidae							
	Camponotus	*atriceps*	2				E P
	Pheidole	sp.1			1		E P

	Wasmania	*auropunctata*	1				E P
Lepidoptera	jovens		1				E P
Noctuoidea		sp.2	1				E P
Tineoidea		sp.1			1		E P
Orthoptera							
Ensifera							
Phalangopsidae	jovens		2	0,0278			E P
	Paraclodes	sp.1			5	0,2381	E P
	Phalangopsis	sp.1	52	0,7222	7	0,3333	E P
Psocoptera							
Psocomorpha	jovens				1		E P
Troctomorpha							
Manicapsocidae							
	Nothoentomum	sp.1			1		E P
Thysanura							
Nicoletiidae	jovens		1				P
Symphyla							
Scutigerellidae							
	Hanseniella	sp.1	1				P
Chordata							
Amphibia							
Anura							
Neobatrachia							
Strabomantidae							
	Pristimantis	*fenestratus*	1	0,0139	2	0,0952	P
Ave							
Strigiformes							
Tytodidae							
	Tyto	*alba*	1	0,0139			P
Mammalia							
Chiroptera							
Emballonuridae							
	Peropteryx	*kappleri*	4	0,0556			P
Phyllostomidae		sp.1			2	0,0952	P
	Glossophaginae	sp.	3	0,0417			
Reptilia							
Squamata							
Gekkonidae							
	Thecadactylus	*rapicauda*	1	0,0139			
