# Supplementary material for: Reconciling Mining with the Conservation of Cave Biodiversity: A Quantitative Baseline to Help Establish Conservation Priorities
Source: PLoS One. 2016 Dec 20;11(12):e0168348. doi: 10.1371/journal.pone.0168348 (PMC5173368; doi:10.1371/journal.pone.0168348)
Supplement: S1 Dataset — (ZIP) [file pone.0168348.s002.zip › Taxa/Serra Sul/SS_2010/S11D-03.pdf]

S11D-03			1ª	AB	2ª	AB	ZON
Arthropoda							
Arachnida							
Acari							
Parasitiformes							
Ixodida							
Argasidae							
	Ornithodoros	sp.	2				E
Sarcoptiformes							
Oribatida		sp.3	1				E
Trombidiformes		sp.7	1				E
Araneae							
Araneidae							
	Alpaida	*septemmammata*	1				E
Corinnidae	*jovens*				1	0,0909	E
Ctenidae	*jovens*		2	0,0513			E
		sp.1			1	0,0909	E
Filistatidae		sp.1			1		E
Ochyroceratidae	*jovens*		2		1		E
Pholcidae							
	Leptopholcus	sp.1	3				E
	Ninetinae	sp.1	1		1		E
Salticidae	*jovens*		1				E
Scytodidae	*jovens*		1	0,0256			E
	Scytodes	*eleonorae*	1	0,0256			E
		sp.	3	0,0769			E
Tetrablemmidae	*Matta*	sp.1	1				E
Theridiidae							
	Dipoena	sp.1			1		E
Theridiosomatidae	*jovens*		1				E
	Plato	sp.1	1				E
Opiliones	*jovens*				2	0,1818	E
Laniatores							
Cosmetidae	*jovens*				2		E
	Roquettea	*singularis*			1	0,0273	E
Stygnidae	*jovens*		1	0,0256			E
		sp.1			1	0,0909	E
Pseudoscorpiones							
Chernetidae							
	Spelaeochernes	sp.1	1				E
Insecta							
Diptera	*jovens*		1				E
Brachycera							
Phoridae							
	Metopininae	sp.	1				E
Nematocera							
Cecidomyiidae							
	Cecidomyiinae	sp.	2				E
Chironomidae		sp.			1		E
Vespoidea							
Formicidae							
	Atta	sp.1			1		E
	Myrmicocrypta	sp.1	1				E
	Pachycondyla	*striata*	2				E
Isoptera	jovens		2				E
Termitidae							
	Armitermes	sp.			1		E
	Nasutitermes	sp.	1		1		E
Lepidoptera	*jovens*		14	0,359			E
Cossoidea							
Limacodidae		sp.1	1	0,0256			E
Noctuoidea		sp.2	1				E
Noctuidae		sp.2	1	0,0256			E
Tineoidea		sp.1	1				E
Neuroptera							
Myrmeleonthidae	jovens		1				E

Orthoptera							
Ensifera							
Phalangopsidae							
	Paraclodes	sp.1			2	0,1818	E
	Phalangopsis	sp.1	8	0,2051			E
Psocoptera							
Psocomorpha	*jovens*		2				E
Ptiloneuridae							
	Triplocania	sp.5	1				E
Troctomorpha							
Liposcelididae							
	Liposcelis	sp.1	1				E
Trogiomorpha							
Lepidopsocidae							
	Loxopholia	sp.1	1				E
Chordata							
Amphibia							
Anura							
Neobatrachia							
Strabomantidae							
	Pristimantis	*fenestratus*	4	0,1026			E
Mammalia							
Chiroptera							
Emballonuridae							
	Peropteryx	sp.	1	0,0256			E
Reptilia							
Squamata							
Gekkonidae							
	Thecadactylus	*rapicauda*	2	0,0513			E
Sauria							
Tropiduridae							
	Plica	*plica*			1	0,0909	E
