# Supplementary material for: Reconciling Mining with the Conservation of Cave Biodiversity: A Quantitative Baseline to Help Establish Conservation Priorities
Source: PLoS One. 2016 Dec 20;11(12):e0168348. doi: 10.1371/journal.pone.0168348 (PMC5173368; doi:10.1371/journal.pone.0168348)
Supplement: S1 Dataset — (ZIP) [file pone.0168348.s002.zip › Taxa/Serra Sul/SS_2010/S11D-05.pdf]

S11D-05		1ª	AB	2ª	AB	ZON
Annelida						
Clitellata						
Oligochaeta	jovens	1	0,0122			E
Arthropoda						
Arachnida						
Acari						
Parasitiformes						
Ixodida						
Argasidae						
Ornithodoros	sp.1	1				E
Ixodidae						
Amblyomma	sp.			1		E
Mesostigmata	sp.1			2		E
Amblypygi						
Phrynidae						
Heterophrynus	sp.	1	0,0122			
Araneae						
Araneidae	jovens	1				E
Alpaida	*septemmammata*	1		1		E
Ctenidae	jovens	1	0,0122			
Nesticidae	jovens	1				E
Ochyroceratidae						
Ochyrocera	sp.1	2		2		E
Speocera	sp.1	1				E
Pholcidae						
Leptopholcus	sp.1	1				E
Ninetinae	sp.1	2		3		E
Salticidae	jovens			1		E
Scytodidae	*jovens*	1	0,0244	3	0,0857	E
Scytodes	*eleonorae*	1				E
Theridiosomatidae	jovens			1		E
Plato	sp.1	1				E
Opiliones	jovens			12	0,3429	E
Laniatores						
Cosmetidae						
Roquettea	*singularis*			1	0,0286	E
Stygnidae	jovens	1	0,0244			E
	sp.1	1		3	0,0857	E
Pseudoscorpiones						
Bochicidae	sp.1			4		E
Chthoniidae						
Pseudochthonius	sp.1			1		E
Schizomida						
Hubbardiidae						
Rowlandius	sp.	1				E
Entognatha						
Diplura						
Campodeidae	sp.1	1				E
Insecta						
Blattodea	jovens	1	0,0122			E
Coleoptera	jovens	1				E
Staphylinidae						
Pselaphinae	sp.1			1		E
Collembola						
Arthropleona						
Entomobryoidea						
Isotomidae	sp.1			1		E
Paronellidae	sp.1	1		1		E
Symphypleona						
Sminthuroidea	sp.2	1		1		E
Diptera	jovens			1		E
Nematocera						
Cecidomyiidae						
Cecidomyiinae	sp.	1				E
Ceratopogonidae	sp.			1		E

Mycetophilidae							
Keroplatus		sp.			1		E
Psychodidae							
Sciopemyia		*sordellii*	1		1		E
Hemiptera							
Heteroptera							
Hebridae		sp.1	1				E
Homoptera	jovens		26	0,3171			
Cixiidae	jovens		2		1		E
		sp.1	1				E
		sp.3			1		E
Hymenoptera							
Vespoidea							
Formicidae							
Gnamptogenys		*striatula*	1		2		E
Hypoponera		sp.1	2		1		E
Odontomachus		*bauri*	1	0,0122	1	0,0286	E
Lepidoptera	jovens		2		1		E
Noctuoidea		sp.2	1				E
Tineoidea		sp.1	1		1		E
		sp.4			1		E
Orthoptera							
Ensifera							
Gryllidae							
Eneopterinae		sp.1	1	0,0122			E
Phalangopsidae							
Paraclodes		sp.1	40	0,4878	6	0,1714	E
Phalangopsis		sp.1			1	0,0286	E
Psocoptera							
Psocomorpha	jovens		1		2		E
Epipsocidae							
Epipsocus		sp.2			1		E
Ptiloneuridae							
Ptiloneura		sp.2			1		E
Isopoda							
Philosciidae		sp.1	1				E
Symphyla							
Scutigerellidae							
Hanseniella		sp.1	1				E
Chordata							
Amphibia							
Anura							
Neobatrachia							
Strabomantidae							
Pristimantis		*fenestratus*	1	0,0122	4	0,1143	E
Mammalia							
Chiroptera							
Emballonuridae							
Peropteryx		*kappleri*	5	0,061	4	0,1143	
		sp.					E
