# Supplementary material for: Reconciling Mining with the Conservation of Cave Biodiversity: A Quantitative Baseline to Help Establish Conservation Priorities
Source: PLoS One. 2016 Dec 20;11(12):e0168348. doi: 10.1371/journal.pone.0168348 (PMC5173368; doi:10.1371/journal.pone.0168348)
Supplement: S1 Dataset — (ZIP) [file pone.0168348.s002.zip › Taxa/Serra Sul/SS_2010/S11D-06.pdf]

S11D-06			1ª	AB	2ª	AB	ZON	
Arthropoda								
Arachnida								
Acari								
Parasitiformes								
Ixodida								
Argasidae								
	Ornithodoros	sp.	4		5		E	P
	Ornithodoros	sp.1	2		2		E	P
Mesostigmata		sp.2	1				E	P
		sp.4	1				E	P
Sarcoptiformes		sp.1	1				E	P
Oribatida		sp.1					E	P
		sp.2	2				E	P
		sp.3	3		5		E	P
		sp.7	1				E	P
Trombidiformes								
Tydeoidea								
Cunaxidae		sp.1	1				E	P
Amblypygi								
Charinidae	jovens		1	0,0035			E	P
Phrynidae								
	Heterophrynus	sp.	3	0,0105	2	0,0122	E	P
Araneae	jovens		4	0,014			E	P
Araneidae	jovens		1				E	P
Corinnidae	jovens		2	0,007	1	0,0061	E	P
Mysmenidae	jovens		1				E	P
Ochyroceratidae	jovens		2		2		E	P
	Ochyrocera	sp.1	5		2		E	P
	Speocera	sp.1	2		2		E	P
Oonopidae								
	gr. *Xycarphius*	sp.3	2				E	P
Pholcidae	jovens				1		E	P
	Leptopholcus	sp.1	2				E	P
	Ninetinae	sp.1	2				E	P
Prodidomidae	jovens		1				E	P
Salticidae	jovens		1				E	P
	Amphidraus	sp.1	1				E	P
Scytodidae	jovens		3		1		E	P
	Scytodes	*eleonorae*			1	0,0061	E	P
		sp.	11	0,0385	4	0,0244	E	P
Tetrablemmidae								
	Matta	sp.1	1				E	P
Opiliones	jovens		1	0,0035	31	0,189	E	P

Cyphophthalmi								
Neogoveidae								
	Canga	*renatae*	1				E	P
Laniatores								
Stygnidae		sp.1			4	0,0244	E	P
Palpigradi								
Eukoeneniidae	jovens		1				E	P
Pseudoscorpiones								
Bochicidae		sp.1	2				E	P
Chernetidae	jovens				2		E	P
	Spelaeochernes	sp.1	3		3		E	P
Chthoniidae								
	Pseudochthonius	sp.1	5		4		E	P
Olpiidae		sp.1	2				E	P
Ricinulei								
Ricinoididae	jovens		2				E	P
Chilopoda								
Notostigmophora								
Scutigeromorpha								
Pselliodidae	jovens				1		E	P
Pleurostigmophora								
Geophilomorpha								
Geophilidae		sp.1	2	0,007			E	P
Diplopoda	jovens		2		1		E	P
Polydesmida	jovens		1				E	P
Chelodesmidae		sp.4	1	0,0035			E	P
		sp.5	1	0,0035			E	P
Fuhrmannodesmidae		sp.1	1				E	P
Pyrgodesmidae		sp.2	1	0,0035			E	P
Polyxenida								
Hypogexenidae		sp.1	7		4		E	P
Spirostreptida								
Pseudonannolenidae	jovens		1	0,0035			E	P
Entognatha								
Diplura								
Campodeidae		sp.1	5		3		E	P
Japygidae		sp.1	2				E	P
Insecta								
Coleoptera	jovens		4		3		E	P
		sp.1	1				E	P
Scydmaenidae		sp.3	1				E	P
		sp.1	1				E	P
Collembola								
Arthropleona								

Entomobryoidea							
Cyphoderidae		sp.1	2				E P
Isotomidae		sp.1	2		1		E P
Paronellidae		sp.1	7		1		E P
Symphypleona							
Sminthuroidea		sp.2	2		1		E P
Dermaptera	jovens		1				E P
		sp.2	1	0,0035			E P
Diptera	jovens		3		2		E P
Nematocera							
Psychodidae							
	Pintomyia	*gruta*			1		E P
	Sciopemyia	*sordellii*	3				E P
Homoptera							
Cixiidae	jovens		5		5		E P
		sp.1	2				E P
Hymenoptera							E P
Chalcidoidea		sp.2	2				E P
Diaprioidea							E P
Diapriidae		sp.1	1				E P
Vespoidea							
Formicidae							
	Camponotus	*atriceps*	3		1		E P
		sp.1	1				E P
	Hypoponera	sp.1			1		E P
	Pachycondyla	*striata*	6		1		E P
	Pheidole	sp.1	1				E P
	Solenopsis	sp.1	1				E P
		sp.2	2				E P
Isoptera	jovens		2				E P
Rhinotermitidae							
	Heterotermes	sp.	1				E P
Termitidae							
	Cortaritermes	*silvestrii*			1		E P
	Diversitermes	sp.	1				E P
	Nasutitermes	sp.	1				E P
Lepidoptera	jovens		7	0,0245	2	0,0122	E P
Cossoidea							
Limacodidae		sp.1	1	0,0035			E P
Orthoptera							
Ensifera	jovens		1	0,0035			E P
Phalangopsidae							
	Paraclodes	sp.1			3	0,0183	E P
	Phalangopsis	sp.1	208	0,7273	86	0,5244	E P

Psocoptera						
Psocomorpha	jovens	2		1		E P
Psilopsocidae						
Psilopsocus	sp.1	1				E P
Ptiloneuridae						
Triplocania	sp.9	1				E P
Troctomorpha						
Manicapsocidae						
Nothoentomum	sp.1	3				E P
Trogiomorpha						
Psyllipsocidae						
Psyllipsocus	sp.1			1		E P
Thysanura						
Nicoletiidae	sp.1	3		2		E P
Malacostraca						
Isopoda						
Dubioniscidae	sp.1	1				E P
Symphyla						
Scutigerellidae						
Hanseniella	sp.1	2				P
Chordata						
Amphibia						
Anura						
Neobatrachia						
Leptodactylidae						
Leptodactylus	sp.			1	0,0061	P
Strabomantidae						
Pristimantis	*fenestratus*	1	0,0035	5	0,0305	P
Mammalia						
Chiroptera						
Emballonuridae						
Peropteryx	sp.	8	0,028			P
Phyllostomidae	sp.1			1	0,0061	P
Glossophaginae				23	0,1402	
Glossophaga	*soricina*	12	0,042			P
Reptilia						
Squamata						
Gekkonidae						
Thecadactylus	*rapicauda*	1	0,0035			P
Mollusca						
Gastropoda						
Systrophiidae						
Happia	sp.	3		2		P
