# Supplementary material for: Reconciling Mining with the Conservation of Cave Biodiversity: A Quantitative Baseline to Help Establish Conservation Priorities
Source: PLoS One. 2016 Dec 20;11(12):e0168348. doi: 10.1371/journal.pone.0168348 (PMC5173368; doi:10.1371/journal.pone.0168348)
Supplement: S1 Dataset — (ZIP) [file pone.0168348.s002.zip › Taxa/Serra Sul/SS_2010/S11D-07.pdf]

S11D-07			2ª	ZON
Arachnida				
Araneae				
Tetrablemmidae	jovens		1	E
Tetragnathidae	jovens		1	E
Insecta				
Diptera				
Nematocera				
Chironomidae		sp.	1	E
