# Supplementary material for: Reconciling Mining with the Conservation of Cave Biodiversity: A Quantitative Baseline to Help Establish Conservation Priorities
Source: PLoS One. 2016 Dec 20;11(12):e0168348. doi: 10.1371/journal.pone.0168348 (PMC5173368; doi:10.1371/journal.pone.0168348)
Supplement: S1 Dataset — (ZIP) [file pone.0168348.s002.zip › Taxa/Serra Sul/SS_2010/S11D-09.pdf]

S11D-09			2ª	AB	ZON
Arthropoda					
Insecta					
Blattodea		jovens	1	0,25	E
Lepidoptera					
Cossoidea					
Limacodidae		sp.2	1	0,25	E
Orthoptera					
Phalangopsidae					
	Paraclodes	sp.	1	0,25	E
Chordata					
Mammalia					
Chiroptera					
Emballonuridae					
	Peropteryx	sp.	1	0,25	E
