# Supplementary material for: Reconciling Mining with the Conservation of Cave Biodiversity: A Quantitative Baseline to Help Establish Conservation Priorities
Source: PLoS One. 2016 Dec 20;11(12):e0168348. doi: 10.1371/journal.pone.0168348 (PMC5173368; doi:10.1371/journal.pone.0168348)
Supplement: S1 Dataset — (ZIP) [file pone.0168348.s002.zip › Taxa/Serra Sul/SS_2010/S11D-10.pdf]

S11D-10			1ª	AB	2ª	AB	ZON
Annelida							
Clitellata							
Oligochaeta	jovens		4	0,0069			P A
Arthropoda							
Arachnida							
Acari							
Parasitiformes							
Holothyrida							
Diplothyridae							
	Diplothyrus	*scubarti*	2				P A
Ixodida							
Argasidae							
	Ornithodoros	sp.	8		4		E P A
	Ornithodoros	sp.1			2		E P A
Ixodidae							
	Amblyomma	sp.	2				E P A
Mesostigmata		sp.1			1		E P
		sp.4	1		1		E P A
		sp.9	1				E P
		sp.10	1				E P
Laelapidae							
	Stratiolaelaps	sp.1			1		E P
Macronyssidae		sp.1	1		1		E P A
Oribatida		sp.2	1				E P
		sp.3	4		4		E P A
		sp.7			1		E P A
Trombidiformes		sp.1	2				E P A
		sp.2	2				E P A
		sp.7	5		3		E P A
Bdellidae		sp.1	1				E P A
Cunaxidae		sp.2	1				E P
Eupodidae		sp.1	1				E P A
Amblypygi							
Phrynidae							
	Heterophrynus	sp.	3	0,0052	5	0,0357	E P A
Araneae	jovens		1	0,0017			E P
Corinnidae	jovens		1	0,0017	2	0,0143	E P A
Ochyroceratidae	jovens		1		1		E P
	Ochyrocera	sp.1	2		3		E P A
	Speocera	sp.1	1				E P A
Scytodidae	*jovens*		3	0,0052	2	0,0143	E P A
	Scytodes	*eleonorae*	1	0,0017	1	0,0071	E P
		globula	1	0,0017			E P
		sp.			3	0,0214	E P A
Segestriidae	jovens		1				E P
Tetrablemmidae							E P
	Matta	sp.1	3		2		E P A
Opiliones							
Laniatores							
Stygnidae		sp.1	2	0,0035	1	0,0071	E P
Pseudoscorpiones							
Bochicidae	jovens				2		E P A
		sp.1	4		2		E P
Chernetidae							
	Spelaeochernes	sp.1	6		6		E P A
Chthoniidae							
	Pseudochthonius	sp.1	6		3		E P A
		sp.4			4		E P A
Olpiidae		sp.1	2				E P
Chilopoda	jovens		30	0,0519			E P
Notostigmophora							
Scutigeromorpha							
Pselliodidae	jovens		2				E P
Pleurostigmophora							
Geophilomorpha	jovens		1	0,0035			E P

Ballophilidae		sp.1	1				E P
Scolopendromorpha							
Scolopocryptopidae							
	Dinocryptops	*miersii*	5	0,0087			E P A
Diplopoda	jovens		8	0,0138	3	0,0214	E P A
Glomeridesmida							
Glomeridesmidae	jovens		1				E P A
		sp.1	2		2		E P A
Polydesmida	jovens		1				E P
Chelodesmidae	jovens				1	0,0071	E P
		sp.4	1	0,0017			E P
Pyrgodesmidae		sp.2	6	0,0104			E P A
Spirostreptida	jovens		4		2		E P A
Pseudonannolenidae							
	Pseudonannolene	sp.1	2	0,0035			E P A
Entognatha							
Diplura							
Campodeidae		sp.1	6		2		E P A
Japygidae		sp.1	1				E P A
Insecta							
Blattodea	jovens		46	0,0796			E P A
Blaberidae	jovens		2	0,0035			E P A
Blattidae	jovens		3	0,0052	1	0,0071	E P A
Coleoptera	jovens		4		4		E P A
Anthicidae		sp.1	1				E P
Scydmaenidae		sp.1	2		1		E P A
Staphylinidae							
	Pselaphinae	sp.1	1				E P
Collembola							
Arthropleona							
Entomobryoidea							
Cyphoderidae		sp.1	2				E P A
Entomobryidae		sp.4	1				E P
Isotomidae		sp.1	2				E P A
Paronellidae		sp.1	1				E P
		sp.4	1				E P A
Symphypleona							
Sminthuroidea		sp.2	2		1		E P A
Diptera	jovens		2		2		E P A
Brachycera							
Drosophilidae							
	Drosophila	*eleonore*			1		E P
Phoridae							
	Phorinae	sp.	2				E P A
Nematocera							
Chironomidae		sp.	1				E P A
Culicidae							
	Culicini	sp.	1				E P A
Psychodidae							
	Edentomyia	*piauensis*			2		E P A
	Pintomyia	*gruta*			1		E P
	Sciopemyia	*sordellii*	2				E P
Hemiptera							
Heteroptera							
Cydnidae	jovens		2				E P
	Cydninae	sp.1	1		2		E P
Reduviidae	jovens		3	0,0052			E P A
Homoptera							
Cixiidae	jovens		5		4		E P A
Hymenoptera							
Chrysidoidea							
Bethylidae		sp.1	1				E P
Vespoidea							
Formicidae							
	Anochetus	sp.1	1				E P
	Camponotus	*atriceps*	6				E P A

Taxon							
		sp.1			5		E P A
	Nylanderia	sp.1	1				E P A
	Pachycondyla	*striata*	4		2		E P A
	Solenopsis	sp.1	2				E P A
		sp.2	1		3		E P A
Isoptera	jovens		1				E P A
Termitidae							
	Nasutitermes	sp.			1		E P
Lepidoptera	jovens		1		1		E P A
Cossoidea							
Limacodidae		sp.1	1	0,0017			E P
Neuroptera							
Myrmeleonthidae	jovens		1				E P
Orthoptera							
Ensifera							
Phalangopsidae							
	Phalangopsis	sp.1	391	0,6765	67	0,4786	E P
Psocoptera							
Psocomorpha	jovens		1				E P
Thysanura							
Ateluridae	jovens		3				E P A
		sp.1	3		3		E P A
Symphyla							
Scutigerellidae							
	Hanseniella	sp.1	3				E P A
Chordata							
Amphibia							
Anura							
Neobatrachia							
Strabomantidae							
	Pristimantis	*fenestratus*	1	0,0017	13	0,0929	E P
Mammalia							
Chiroptera							
Emballonuridae							
	Peropteryx	sp.	5	0,0087			E P
Furipteridae							
	Furipterus	*horrens*	15	0,026	10	0,0714	E P
Phyllostomidae		sp.1					E P
	Carollia	sp.	30	0,0519			E P
	Glossophaginae	sp.	10	0,0173	30	0,2143	E P
Rodentia		sp.	1	0,0017			E P
Reptilia							
Squamata							
Serpentes							
Colubridae							
	Mastigodryas	*boddaerti*			1	0,0071	E P
Mollusca							
Gastropoda	jovens				1		P A
Subulinidae							
	Lamellaxis	sp.	1				P A
Systrophiidae							
	Happia	sp.	1				P
