# Supplementary material for: Reconciling Mining with the Conservation of Cave Biodiversity: A Quantitative Baseline to Help Establish Conservation Priorities
Source: PLoS One. 2016 Dec 20;11(12):e0168348. doi: 10.1371/journal.pone.0168348 (PMC5173368; doi:10.1371/journal.pone.0168348)
Supplement: S1 Dataset — (ZIP) [file pone.0168348.s002.zip › Taxa/Serra Sul/SS_2010/S11D-11.pdf]

S11D-11		1ª	AB	2ª	AB	ZON
Arthropoda						
Arachnida						
Acari						
Ixodida						
Argasidae						
Ornithodoros	sp.	3		2		E
Ixodidae						
Amblyomma	sp.	1				E
Parasitiformes						
Opilioacarida						
Opilioacaridae	sp.1	1		1		E
Sarcoptiformes						
Oribatida	sp.3	1		1		E
Trombidiformes						
Tydeoidea						
Rhagidiidae	sp.1			1		E
Araneae						
Araneidae jovens		1				E
Ctenidae jovens		2	0,0588			E
Ochyroceratidae						
Ochyrocera	sp.1	1				E
Speocera	sp.1	2				E
Pholcidae						
Leptopholcus	sp.1	1				E
Ninetinae	sp.1	4		4		E
Scytodidae						
Scytodes	*eleonorae*			1	0,04	E
	globula	1	0,0294			E
	sp.			1	0,04	E
Tetrablemmidae						
Matta	sp.1	1		1		E
Uloboridae						
Uloborus	sp.1	1				E
Opiliones jovens				2	0,08	E
Laniatores						
Cosmetidae						
Roquettea	*singularis*	1	0,0294			E
Stygnidae jovens		1	0,0294			E
	sp.1	1	0,0294	1	0,04	E
Pseudoscorpiones						
Chthoniidae						
Pseudochthonius	sp.1	1				E
Olpiidae	sp.1	8		6		E
Schizomida						
Hubbardiidae jovens		1				E
Chilopoda						
Notostigmophora						
Scutigeromorpha						
Pselliodidae jovens		1				E
Diplopoda						
Polyxenida						
Hypogexenidae	sp.1	1		1		E
Spirostreptida jovens				1		E
Entognatha						
Diplura						
Campodeidae	sp.1	1				E
Japygidae	sp.1	1				E
Insecta						
Blattodea jovens				1	0,08	E
Blaberidae jovens		1	0,0294	1		E
Coleoptera jovens				1		E
Chrysomelidae	sp.3	1				E

Diptera							
Nematocera	jovens		3				E
Psychodidae		sp.					
	Sciopemyia	*sordellii*	1				E
Hemiptera							
Heteroptera							
aff. Pyrrhocoroidea							
Tingidae							
	Thaumamannia	sp.1	2				E
Homoptera							
Cixiidae	jovens		4				E
Hymenoptera	jovens		1				E
Vespoidea							
Formicidae							
	Camponotus	*atriceps*	1				E
		sp.1	2		2		E
	Pachycondyla	*striata*	2		1		E
	Solenopsis	sp.2			1		E
	Wasmania	*auropunctata*	3		1		E
Isoptera		sp.	1		1		E
Lepidoptera							
Cossoidea							
Limacodidae		sp.1	1	0,0294			E
Noctuoidea		sp.2	1				E
Tineoidea		sp.1			1		E
	jovens		1				E
Neuroptera							
Myrmeleonthidae	jovens		2		1		E
Orthoptera							
Ensifera							
Phalangopsidae	jovens						
	Phalangopsis	sp.1	1	0,6176	14	0,56	E
	jovens		20				
Psocoptera							
Psocomorpha	jovens		4				E
Trogiomorpha							
Psyllipsocidae	jovens				1		E
	Psyllipsocus	sp.1	2				E
Malacostraca							
Isopoda							
Dubioniscidae		sp.1	3		1		E
Symphyla							
Scutigerellidae							
	Hanseniella	sp.1	1				E
Chordata							
Amphibia							
Anura							
Neobatrachia							
Strabomantidae							
	Pristimantis	*fenestratus*			4	0,16	E
Mammalia							
Chiroptera							
Emballonuridae							
	Peropteryx	*kappleri*	3	0,0882			
Phyllostomidae		sp.					
	Glossophaginae	sp.	2	0,0588			
Nemathelminthes		sp.			1		E
