# Supplementary material for: Reconciling Mining with the Conservation of Cave Biodiversity: A Quantitative Baseline to Help Establish Conservation Priorities
Source: PLoS One. 2016 Dec 20;11(12):e0168348. doi: 10.1371/journal.pone.0168348 (PMC5173368; doi:10.1371/journal.pone.0168348)
Supplement: S1 Dataset — (ZIP) [file pone.0168348.s002.zip › Taxa/Serra Sul/SS_2010/S11D-12.pdf]

S11D-12		1ª	AB	2ª	AB	ZON
Annelida						
Clitellata						
Oligochaeta	sp.	1	0,0012			P
Arthropoda						
Arachnida						
Acari						
Ixodida						
Argasidae						
Ixodidae						
Amblyomma	sp.	6		1		P A
	sp.1	1				P
Parasitiformes						
Ixodida						
Ixodidae						
Ornithodoros	sp.1	19		9		E P A
Mesostigmata	sp.1			1		A
	sp.2	1		1		A
	sp.4	2		1		P
	sp.5	1				P
	sp.11	1				P
Laelapidae	sp.5	1				P
Stratiolaelaps	sp.1	3				P A
Macronyssidae	sp.1	1				P
Sarcoptiformes						
Oribatida	sp.1	2				P A
	sp.2	2		1		P A
	sp.3	10		3		P A
	sp.5			1		P
	sp.7	1				A
	sp.12	1				P
Trombidiformes						
Tydeoidea						
Cheyletidae	sp.1	1				P
	sp.2	1				P
	sp.7	4		1		P A
Amblypygi						
Charinidae jovens		1	0,0012			A
Phrynidae						
Heterophrynus	sp.	4	0,0049	1	0,0026	P
Araneae						
Araneidae jovens		2		1		E P
Alpaida	*septemmammata*	2				P A
	smila	1				P
Barychaelidae jovens		1	0,0012	1	0,0026	E P
Corinnidae jovens		13	0,0158	5	0,0131	P A
Creugas	sp.1			1	0,0026	A
Ctenidae jovens		8	0,0097	2	0,0052	E P A
Filistatidae jovens				2		E
Ochyroceratidae jovens		4		4		E P A
Ochyrocera	sp.1	15		6		E P A
Speocera	sp.1	9		3		P A
Oonopidae jovens		3				P A
Gamasomorpha	sp.1	4				P A
Oonopinae	sp.1	1				P
Pholcidae jovens		3				P A
Leptopholcus	sp.1	4				P
Ninetinae	sp.1	12		11		E P A
Prodidomidae jovens		1				A
Salticidae						
Amphidraus	sp.1	2				P
Marma	sp.1			1		E
Scytodidae jovens		11		2		P A

Taxon	Family / Genus	Species					
	Scytodes	*eleonorae*	2	0,0024			P A
		sp.			2	0,0052	E
	Segestriidae	jovens	3		2		E P A
	Ariadna	sp.1	2		2		P A
	Tetrablemmidae	jovens	1		1		P
	Matta	sp.1			1		P
Opiliones			49	0,0597			
Laniatores							
	Escadabiidae	jovens	2				A
		sp.1	2		1		P A
	Stygnidae	jovens	10	0,0122			P A
		sp.1	7	0,0085	8	0,0209	E P A
		sp.1	1	0,0012			
		jovens			1	0,0026	A
Palpigradi							
	Eukoeneniidae	jovens	3				P A
	Allokoenenia	sp.1	1				P
Pseudoscorpiones							
	Chernetidae	jovens	2				P
	Spelaeochernes	sp.1	28		11		E P A
	Chthoniidae	jovens	2				P
	Pseudochthonius	sp.1	8		5		P A
		sp.4	4		4		P A
	Olpiidae	sp.1			2		E
Ricinulei							
	Ricinoididae						
	Cryptocellus	sp.	5				P A
Schizomida							
	Hubbardiidae						
	Rowlandius	sp.			1		E
Chilopoda							
Notostigmophora							
Scutigeromorpha							
	Pselliodidae	jovens	4				P A
Pleurostigmophora							
Geophilomorpha							
	Ballophilidae	sp.1	1	0,0012			P
Scolopendromorpha							
	Cryptopidae						
	Cryptops	sp.1			2	0,0052	P A
	Scolopocryptopidae						
	Scolopocryptops	sp.2	1	0,0012			P
Diplopoda		jovens	17	0,0207			
Glomeridesmida							
	Glomeridesmidae	sp.1	4		2		P A
		sp.2	1				A
Polydesmida							
	Aphelidesmidae	sp.1	1	0,0012			P
	Chelodesmidae	jovens			1	0,0026	P
		sp.4	3	0,0037			P A
		sp.5	2	0,0024			P A
	Fuhrmannodesmidae	sp.3	1				A
		sp.4	1				A
	Pyrgodesmidae	sp.2	2	0,0024			P
	Hypogexenidae	sp.1			1		E
Spirostreptida							
	Pseudonannolenidae	jovens	2	0,0024			A
	Pseudonannolene	sp.1	1	0,0012			A
		sp.3	1	0,0012			A
Entognatha							
Diplura							
	Campodeidae	sp.1	13		5		E P A
Insecta							

Blattodea			jovens		82	0,0999	4	0,0105	E P A
	Blaberidae		jovens		4	0,0049	2	0,0052	P A
				sp.1			1	0,0026	A
	Blattellidae			sp.2	6	0,0073	3	0,0079	P A
	Blattidae		jovens		1	0,0012			A
	Polyphagidae		jovens		10	0,0122	2	0,0052	P A
Coleoptera			jovens		41	0,0499	3	0,0079	P A
	Anthicidae			sp.1	1				A
	Chrysomelidae			sp.1	2				P A
				sp.3	1				P
				sp.6	2				P
	Curculionidae								
		Scolytinae		sp.1	1				A
	Elateridae			sp.1	3				P A
	Ptiliidae			sp.1	3		1		P A
	Scydmaenidae			sp.2	3				P A
Collembola									
Arthropleona									
Entomobryoidea									
	Entomobryidae			sp.2	1				P
	Isotomidae			sp.1	5		3		P A
				sp.2	4				P A
	Paronellidae			sp.1	1		1		E P
				sp.4	6		3		P A
Symphypleona									
Sminthuroidea				sp.1	1				P
				sp.2	10				P A
Diptera									
Brachycera									
	Camillidae			sp.			1		E
	Drosophilidae								
		Drosophila		*eleonore*	1				P
	Empididae								
		Drapetis		sp.	1				A
	Milichiidae			sp.	1				P
	Phoridae								
		Phorinae		sp.	1				P
	Xylomyidae								
		Xylomya		sp.			1		P
Nematocera			jovens		15		5		E P A
	Cecidomyiidae								
		Cecidomyiinae		sp.	1				A
	Chironomidae			sp.	2				P A
	Culicidae								
		Culicini		sp.	3				P A
	Mycetophilidae								
		Orfelia		sp.	1				P
	Psychodidae								
		Pintomyia		*gruta*	2				P A
		Sciopemyia		*sordellii*	2		1		P
	Sciaridae			sp.	1				P
Hemiptera									
Heteroptera									
Dipsocoroidea			jovens				1		E
	Cydnidae		jovens		6				P A
		Cydninae		sp.1	16		4		P A
	aff. Reduviidae		jovens		1				P
	Reduviidae		jovens		5	0,0061	2	0,0052	E P
		Emesinae		sp.1	1				P
Homoptera			jovens		6	0,0073	3	0,0079	P A
	Cixiidae		jovens		16		7		P A
				sp.4	1				A
Hymenoptera									

Chrysidoidea						
Bethylidae	sp.1	1				P
Diaprioidea						
Diapriidae	sp.1	2				P A
Vespoidea						
Formicidae						
Apterostigma	sp.1	5				P A
Camponotus	*atriceps*	3		4		E P A
Carebara	sp.1	6		4		E P A
Cyphomyrmex	sp.1			1		A
Dolichoderus	*bispinosus*			1		E
Gnamptogenys	*striatula*	3		1		P A
Hypoponera	sp.1	5		3		E P A
Odontomachus	*bauri*			1		E
Pachycondyla	*striata*	4	0,0049	2	0,0052	E P A
Solenopsis	sp.2			1		P
Typhlomyrmex	sp.1	1				P
Wasmania	*auropunctata*	4				P
Isoptera	sp.	1		1		P A
Termitidae						
Nasutitermes	sp.	13		6		P A
Lepidoptera		1	0,0012			
Noctuoidea						
Noctuidae	sp.1	1	0,0012			P
Tineoidea	sp.1	1				A
jovens		8	0,0097	6	0,0157	E P A
Orthoptera						
Ensifera						
Phalangopsidae						
Paraclodes	sp.1	1	0,0012	13	0,034	E P
Phalangopsis	sp.1	448	0,5457	280	0,733	A
Psocoptera						
Psocomorpha jovens		6				P A
Hinduipsocus	sp.1	1				P
Ptiloneuridae						
Triplocania	sp.8	2				P
Trogiomorpha						
Psyllipsocidae jovens				1		E
Psocathropos	sp.1	1				P
Malacostraca						
Isopoda						
Dubioniscidae	sp.1	1				P
Philosciidae	sp.1	5				P A
Scleropactidae	sp.	9		3		P A
Pauropoda						
Tetramerocerata	sp.	1				P
Symphyla						
Scutigerellidae jovens		1				P
Hanseniella	sp.1	3		1		P A
Scutigerella	sp.1	1		1		P A
Chordata						
Amphibia						
Anura						
Neobatrachia						
Leptodactylidae						
Leptodactylus	sp.			1	0,0026	P
Strabomantidae						
Pristimantis	*fenestratus*			6	0,0157	P
Mammalia						
Chiroptera						
Emballonuridae						
Peropteryx	*kappleri*	8	0,0097			
Furipteridae						

Furipterus	*horrens*	20	0,0244			
Phyllostomidae						
Carollia	*perspicillata*	40	0,0487			
	sp.			25	0,0654	A
Diphylla	*ecaudata*	1	0,0012			
Phyllostomus	*latifolius*	6	0,0073			
Trachops	sp.			4	0,0105	A
Mollusca						
Gastropoda						
Subulinidae						
Lamellaxis	sp.	7				P A
Leptinaria	sp.	2				P A
Systrophiidae						
Happia	sp.	7				P A
Nemathelminthes	sp.			1	0,0026	A
