# Supplementary material for: Reconciling Mining with the Conservation of Cave Biodiversity: A Quantitative Baseline to Help Establish Conservation Priorities
Source: PLoS One. 2016 Dec 20;11(12):e0168348. doi: 10.1371/journal.pone.0168348 (PMC5173368; doi:10.1371/journal.pone.0168348)
Supplement: S1 Dataset — (ZIP) [file pone.0168348.s002.zip › Taxa/Serra Sul/SS_2010/S11D-13.pdf]

S11D-13		1ª	AB	2ª	AB	ZON
Annelida						
Clitellata						
Oligochaeta	jovens	1	0,0036			P
Arthropoda						
Arachnida						
Acari						
Ixodida						
Argasidae						
Ornithodoros	sp.	8		3		P A
Ornithodoros	sp.1	2				P
Mesostigmata						
Laelapidae	sp.1					
Stratiolaelaps	sp.1	1				P
Macronyssidae	sp.1			4		P A
	sp.2	1				P
	sp.3			1		A
Sarcoptiformes						
Oribatida	sp.3	4		2		P A
	sp.5			1		P
Trombidiformes						
Tydeoidea	sp.1	1		1		
	sp.2	2				P
	sp.6			1		P
	sp.7	4		1		P A
Rhagidiidae	sp.1	1				P
Amblypygi						
Charinidae	jovens	3		1	0,004	A
Charinus	sp.2	1	0,0146			A
Phrynidae						
Heterophrynus	sp.	2	0,0073	1	0,004	A
	sp.1	1	0,0036			P
Araneae						
Araneidae	jovens	1				E
Alpaida	*septemmammata*	1				E
Ctenidae	jovens	1	0,0036			
Nesticidae	jovens	1				P
Ochyroceratidae	jovens	4		1		E P A
Ochyrocera	sp.1	4		3		P A
Speocera	sp.1	3		2		P
Pholcidae	jovens	2		1		P
Leptopholcus	sp.1	3				P
Ninetinae	sp.1	2		1		E P
Prodidomidae	jovens	5		3		P A
Salticidae	jovens	1				E
Scytodidae	*jovens*	6		1	0,004	E P A
Scytodes	*eleonorae*	1	0,0255			P
Segestriidae	jovens	1				P
Ariadna	sp.1	1				P
Tetrablemmidae						
Matta	sp.1	4		5		P A
Opiliones				3	0,0119	
Laniatores	jovens			1	0,004	P
Escadabiidae	jovens			1		A
Stygnidae	jovens	1				P
	sp.1	2	0,0109	2	0,0079	P
Pseudoscorpiones						
Bochicidae	sp.1	2				A
Chernetidae	jovens	4		2		P A
Spelaeochernes	sp.1	5		5		P A
Chthoniidae	jovens	6		2		P A
Pseudochthonius	sp.1	4		3		E P A
	sp.4			6		P A

Ricinulei						
Ricinoididae						
Cryptocellus	sp.			1		P
Chilopoda						
Notostigmophora						
Scutigeromorpha						
Pselliodidae	jovens			2		P
Pleurostigmophora						
Geophilomorpha						
Ballophilidae	sp.1	3	0,0109			P A
Scolopendromorpha						
Cryptopidae						
Cryptops	sp.1			1	0,004	P
Diplopoda						
Polydesmida						
Chelodesmidae	sp.4	3	0,0109			P A
	sp.5	3	0,0109			A
Fuhrmannodesmidae	sp.3			1		A
	sp.4			2		A
Pyrgodesmidae	jovens			1		A
	sp.2	2	0,0073	1	0,004	A
	jovens	1		1		A
Spirostreptida	jovens	2		1		P A
Pseudonannolenidae	jovens	1	0,0036			P
Pseudonannolene	sp.1	2	0,0073			A
	sp.3			1	0,004	P
Entognatha						
Diplura	jovens	1				P
Campodeidae	sp.1	2				E P
Insecta						
Blattodea		3	0,0109			
Blaberidae	jovens	3	0,0109	1	0,004	P A
	sp.			1	0,004	A
	sp.2			1	0,004	A
Polyphagidae	jovens	1	0,0036			P
	jovens	4	0,0146	1	0,004	P A
Coleoptera	jovens	4		1		P
Anthicidae	sp.1	1				P
Chrysomelidae	sp.1	1				P
Scydmaenidae	sp.2			1		P
Staphylinidae	sp.4			1		P
Pselaphinae	sp.1	1				P
Collembola						
Arthropleona						
Entomobryoidea						
Isotomidae	sp.1	1				P
Paronellidae	sp.1	1				E
	sp.4	1		1		A
	sp.1			1		A
Symphypleona						
Sminthuroidea	sp.1			1		A
	sp.2	4		1		P A
Diptera						
Brachycera						
Camillidae	sp.	2				P A
Phoridae						
Phorinae	sp.			1		A
Nematocera	jovens			1		A
Chironomidae	sp.	2				P
Mycetophilidae						
Keroplatus	sp.	1				A
Psychodidae						
Pintomyia	*gruta*	2				P A

Sciopemyia	*sordellii*	1		2		P A
Sciaridae	sp.	3				P A
Hemiptera						
Heteroptera		1	0,0036			
aff. Pyrrhocoro	jovens	1	0,0036			
Cydnidae	jovens	1				A
Cydninae	sp.1			2		A
Reduviidae	jovens	1	0,0036			P
Emesinae	sp.1	1				E
Homoptera	jovens	1	0,0036			
Cixiidae	jovens	3		1		P A
	sp.1	2				P A
Hymenoptera						
Vespoidea						
Formicidae						
Camponotus	*atriceps*	1				P
Hypoponera	sp.1	2		1		P A
Labidus	*coecus*	1				A
Odontomachus	*bauri*	1				P
Pachycondyla	*harpax*	1				P
	striata	3		3		P A
Solenopsis	sp.2			1		A
Isoptera	sp.	1		1		P
Rhinotermitidae						
Heterotermes	sp.	1				P
Termitidae						
Nasutitermes	sp.	1		2		E A
Lepidoptera		5	0,0182			
Cossoidea						
Limacodidae	sp.1	1	0,0036			P
Noctuoidea						
Noctuidae	sp.1	1	0,0036			P
	sp.2	1	0,0036			E
	sp.2	1				P
Tineoidea	sp.1	2		1		P
	jovens	6	0,0219	2	0,0079	E P
Orthoptera						
Ensifera						
Gryllidae	sp.2	1	0,0036			E
Phalangopsidae						
Paraclodes	sp.1			6	0,0238	P
Phalangopsis	sp.1	161	0,5876	160	0,6349	P A
Psocoptera						
Psocomorpha	jovens	3				E P
Troctomorpha						
Liposcelididae						
Liposcelis	sp.3	1				P
Trogiomorpha						
Lepidopsocidae						
Loxopholia	sp.1	1				E
Psyllipsocidae	jovens			1		P
Psyllipsocus	sp.1			1		P
Thysanura						
Ateluridae	jovens			1		A
Malacostraca						
Isopoda						
Dubioniscidae	sp.1	1				P
Pauropoda						
Tetramerocerata	sp.	1				P
Symphyla						
Scutigerellidae	jovens	1				A
Chordata						
Amphibia						

Anura						
Neobatrachia						
Strabomantidae						
Pristimantis	*fenestratus*	1	0,0036	9	0,0357	P
Mammalia						
Chiroptera						
Emballonuridae						
Peropteryx	sp.			15	0,0595	E
Furipteridae						
Furipterus	*horrens*	20	0,073			
Phyllostomidae	sp.1			1	0,004	A
	sp.2			1	0,004	A
Carollia	*perspicillata*	15	0,0547			
Carollia	*sp.*			25	0,0992	
Glossophaga	*soricina*	1	0,0036			
Glossophaginae	sp.	10	0,0365	15	0,0595	
Reptilia						
Squamata						
Serpentes						
Colubridae						
Mastigodryas	*boddaerti*			1	0,004	P
Mollusca						
Gastropoda						
Bulimulidae						
Naesiotus	sp.	1				P
Systrophiidae						
Happia	sp.	1				P
Nemathelminthes	sp.	3	0,0109			P A
