# Supplementary material for: Reconciling Mining with the Conservation of Cave Biodiversity: A Quantitative Baseline to Help Establish Conservation Priorities
Source: PLoS One. 2016 Dec 20;11(12):e0168348. doi: 10.1371/journal.pone.0168348 (PMC5173368; doi:10.1371/journal.pone.0168348)
Supplement: S1 Dataset — (ZIP) [file pone.0168348.s002.zip › Taxa/Serra Sul/SS_2010/S11D-14.pdf]

S11D-14		1ª	AB	2ª	AB	ZON
Annelida						
Clitellata						
Oligochaeta	jovens	3	0,0405			E P A
Arthropoda						
Arachnida						
Acari						
Ixodida						
Argasidae						
Ornithodoros	sp.	2				P A
Parasitiformes						
Mesostigmata						
Macronyssidae	sp.1			1		P
	sp.9	1				P
Sarcoptiformes						
Oribatida	sp.3			1		P
Trombidiformes						
Tydeoidea	sp.1	1				P
Amblypygi						
Phrynidae						
Heterophrynus	sp.	1	0,0135			
Araneae						
Araneidae	jovens	1	0,0135			P
Alpaida	*septemmammata*	1				P
Ctenidae						
Ctenus	sp.1			1	0,0147	P
Ochyroceratidae	jovens	1				P
Ochyrocera	sp.1	4		4		E P A
Speocera	sp.1	2		2		E P
Oonopidae						
gr. *Xycarphius*	sp.3	1				P
Tetrablemmidae	jovens	1				
Matta	sp.1	2		1		E P A
Theridiidae	jovens	1				P
	jovens	1	0,0135			
Opiliones				26	0,3824	
Laniatores	jovens			1		
Escadabiidae	jovens	1				E
Stygnidae	sp.1	1	0,0135	3	0,0441	E P A
Pseudoscorpiones						
Bochicidae	sp.1	2		2		E P
Chernetidae	jovens			2		E
Spelaeochernes	sp.1	6		3		E P A
Chtoniidae						
Pseudochthonius	sp.1	3		1		P A
	sp.4			4		P
Ricinulei						
Ricinoididae	jovens	1				E
Chilopoda						
Notostigmophora						
Scutigeromorpha						
Pselliodidae	jovens			1		
Pleurostigmophora						
Geophilomorpha						
Ballophilidae	sp.1	4	0,0541			P A
Scolopendromorpha						
Cryptopidae						
Cryptops	sp.1	2	0,027			E P
Scutigeromorpha						
Pselliodidae						
Sphendononema	sp.			2	0,0294	
Polydesmida						
Fuhrmannodesmidae	sp.3	1				E

		sp.4			1		
Pyrgodesmidae		sp.2	3	0,0405	2	0,0294	E P A
	jovens				1		E
Hypogexenidae		sp.2			1		
Spirostreptida							
Spirostreptidae		sp.1	1				E
	jovens		2		2		E P A
Entognatha							
Diplura							
Campodeidae		sp.1	5		2		E P
Japygidae		sp.1	2				P A
Insecta							
Coleoptera	jovens		1		1		E P
Ptiliidae		sp.1	2				P A
Collembola							
Arthropleona							
Entomobryoidea							
Paronellidae		sp.1	2		1		E P
		sp.4	1		2		P
Symphypleona							
Sminthuroidea		sp.2	4				E P A
Diptera							
Nematocera	jovens				3		E P
Cecidomyiidae							
	Cecidomyiinae	sp.			1		P
Psychodidae							
	Phlebotominae	sp.			1		P
	Sciopemyia	*sordellii*			1		E
Hemiptera							
Heteroptera							
Dipsocoroidea	jovens				1		P
Homoptera							
Cixiidae	jovens		3		4		E P A
		sp.1	1				
Hymenoptera							
Vespoidea							
Formicidae							
	Apterostigma	sp.1			1		P
	Crematogaster	sp.1			1		P
	Gnamptogenys	*striatula*			1		P
	Hypoponera	sp.1	1		1		E P
	Pachycondyla	*striata*	5		1		E P A
	Wasmania	*auropunctata*	3		1		P A
Isoptera		sp.	2		1		E
Lepidoptera							
Castnioidea	jovens		1				P
Orthoptera							
Ensifera							
Phalangopsidae	jovens		1	0,0135			
	Paraclodes	sp.1			4	0,0588	P
	Phalangopsis	sp.1	55	0,7432	23	0,3382	
Psocoptera							
Psocomorpha	jovens				1		E
Symphyla							
Scutigerellidae							
	Hanseniella	sp.1	4		1		E P A
Chordata							
Amphibia							
Anura			1	0,0135	2	0,0294	
Neobatrachia							
Strabomantidae							
	Pristimantis	*fenestratus*			1	0,0147	P
		sp.	1	0,0135	2	0,0294	E P

Mammalia					
Chiroptera					
Emballonuridae					
Peropteryx sp.			1	0,0147	E
Reptilia					
Squamata					
Serpentes					
Colubridae					
Mastigodryas boddaerti			1	0,0147	P
Mollusca					
Gastropoda					
Subulinidae					
Lamellaxis sp.	1				E
Leptinaria sp.	1		1		
Systrophiidae					
Happia sp.	2				E
