# Supplementary material for: Reconciling Mining with the Conservation of Cave Biodiversity: A Quantitative Baseline to Help Establish Conservation Priorities
Source: PLoS One. 2016 Dec 20;11(12):e0168348. doi: 10.1371/journal.pone.0168348 (PMC5173368; doi:10.1371/journal.pone.0168348)
Supplement: S1 Dataset — (ZIP) [file pone.0168348.s002.zip › Taxa/Serra Sul/SS_2010/S11D-15.pdf]

S11D-15		1ª	AB	2ª	AB	ZON
Arthropoda						
Arachnida						
Acari						
Ixodida						
Argasidae						
Ornithodoros	sp.	2		1		P
Sarcoptiformes						
Oribatida	sp.3	1		1		P
Amblypygi						
Phrynidae						
Heterophrynus	sp.			1	0,0667	P
Araneae						
Araneidae	jovens	1				E
Corinnidae	jovens	1	0,0455			P
Ctenidae	jovens			1	0,0667	E
Ochyroceratidae	jovens	1				P
Ochyrocera	sp.1			2		E P
Pholcidae						
Ninetinae	sp.1	3		1		P
Prodidomidae	jovens	1				P
Salticidae	jovens	1		1		E P
Scytodidae	jovens	2	0,1364			P
	Scytodes	1				
Segestriidae	jovens	1		1		P
Ariadna	sp.1	2				P
Theridiosomatidae						
Plato	sp.1	1				E
Opiliones						
Laniatores						
Escadabiidae	jovens	1				P
Stygnidae	jovens	1	0,0909			P
	sp.1	1				P
Pseudoscorpiones						
Bochicidae	sp.1	2				P
Chilopoda						
Notostigmophora						
Scolopendromorpha						
Scolopocryptopidae	jovens	1	0,0455			P
Diplopoda						
Polyxenida						
Hypogexenidae	sp.1	2		1		P
Entognatha						
Diplura						
Campodeidae	sp.1	1				P
Japygidae	sp.1	1				P
Insecta						
Coleoptera	jovens	1				P
Staphylinidae						
Pselaphinae	sp.1	1				E
	sp.2	1				P
Collembola						
Arthropleona						
Entomobryoidea						
Paronellidae	sp.1	1				E
	sp.4	1				P
Diptera						
Nematocera						
Cecidomyiidae						
Cecidomyiinae	sp.	1				P
Ceratopogonidae	sp.			1		E
Psychodidae						
Sciopemyia	*sordellii*	1		1		P

Hemiptera							
Heteroptera							
aff. Pyrrhocoroidea							
Reduviidae	jovens		1	0,0909			E
	Zelurus	sp.1	1				E
Homoptera							
Cixiidae	jovens		2		1		E P
		sp.1	1				P
		sp.4	2				E P
Hymenoptera							
Vespoidea							
Formicidae							
	Crematogaster	sp.1	1				P
	Gnamptogenys	*striatula*	1				P
	Labidus	*coecus*	1				P
		praedators	1				E
	Nylanderia	sp.1	2				P
Isoptera							
Termitidae							
	Nasutitermes	sp.	2		1		P
Lepidoptera	jovens		3	0,1364			P
Orthoptera							
Ensifera							
Phalangopsidae	jovens		10				
	Paraclodes	sp.1			6	0,4	E
	Phalangopsis	sp.1			3	0,2	P
Psocoptera							
Psocomorpha							
Archipsocidae							
	Archipsocus	sp.1	1				E
	jovens		3				E P
Malacostraca							
Isopoda							
Philosciidae		sp.1	2		1		P
Chordata							
Amphibia							
Anura					3	0,2	
Neobatrachia		sp.			1	0,0667	P
