# Supplementary material for: Reconciling Mining with the Conservation of Cave Biodiversity: A Quantitative Baseline to Help Establish Conservation Priorities
Source: PLoS One. 2016 Dec 20;11(12):e0168348. doi: 10.1371/journal.pone.0168348 (PMC5173368; doi:10.1371/journal.pone.0168348)
Supplement: S1 Dataset — (ZIP) [file pone.0168348.s002.zip › Taxa/Serra Sul/SS_2010/S11D-66.pdf]

S11D-66			1ª	AB	2ª	AB	ZON
Arthropoda							
Arachnida							
Acari							
Ixodida							
Argasidae							
	Ornithodoros	sp.			1		E
Sarcoptiformes							
Oribatida		sp.7	1				E
Trombidiformes							
Tydeoidea							
Rhagidiidae		sp.1	1				E
Araneae							
Ochyroceratidae							
	Ochyrocera	sp.1	1				E
	Speocera	sp.1	1				E
Pholcidae	jovens		2		1		E
Scytodidae							
	Scytodes	*eleonorae*	3	0,1875			E
Diplopoda							
Polyxenida							
Hypogexenidae		sp.1	1				E
Entognatha							
Diplura							
Campodeidae		sp.1	1				E
Projapygidae		sp.1	1				E
Insecta							
Coleoptera							
Scydmaenidae		sp.7	1				E
Staphylinidae		sp.15	1				E
Collembola							
Arthropleona							
Entomobryoidea							
Cyphoderidae		sp.1					
Isotomidae		sp.1	1				E
Paronellidae		sp.1			1		E
Diptera							
Nematocera							
Chironomidae		sp.	1				E
Psychodidae							
	Sciopemyia	*sordellii*	1				E
Tipulidae		sp.			16	0,89	E
Hymenoptera							
Vespoidea							
Formicidae							
	Camponotus	*atriceps*	1		1		E
	Crematogaster	sp.1	1		1		E
	Dolichoderus	*bispinosus*	1				E
Isoptera							
Termitidae							
	Atlantitermes	sp.	1				E
		sp.			1		E
Lepidoptera	jovens		3	0,1875			
Noctuoidea		sp.2			1		E
Orthoptera							
Ensifera							
Phalangopsidae							
	Paraclodes	sp.1	2	0,125	2	0,11	E
	Phalangopsis	sp.1	6	0,375			
Psocoptera							
Psocomorpha	jovens				1		E
Malacostraca							
Isopoda							

	Dubioniscidae	sp.1	1		1		E
Mammalia							
Chiroptera							
	Phyllostomidae						
		Glossophaginae sp.	2	0,125			
