# Supplementary material for: Reconciling Mining with the Conservation of Cave Biodiversity: A Quantitative Baseline to Help Establish Conservation Priorities
Source: PLoS One. 2016 Dec 20;11(12):e0168348. doi: 10.1371/journal.pone.0168348 (PMC5173368; doi:10.1371/journal.pone.0168348)
Supplement: S1 Dataset — (ZIP) [file pone.0168348.s002.zip › Taxa/Serra Sul/SS_2010/S11D-67.pdf]

S11D-67	1ª	AB	2ª	AB	ZON
Arthropoda					
Arachnida					
Acari					
Ixodida					
Ixodidae					
Amblyomma sp.			1		E
Parasitiformes					
Mesostigmata					
Diploginiidae sp.4			1		P
Araneae jovens	6	0,122			
Araneidae jovens			1		E
Drymusidae jovens			1		P
Filistatidae sp.1	1				E
Ochyroceratidae					
Ochyrocera sp.1	1				P
Oonopidae					
gr. *Xycarphius* sp.2	1				E
Pholcidae jovens			1		E
aff. *Ibityporanga* sp.1	1				E
Mesabolivar sp.1			1		P
Ninetinae sp.1	2		1		E P
Salticidae					
Freya sp.1			1		E
Scytodidae jovens	2	0,041			E P
Tetrablemmidae jovens	1				P
Opiliones					
Laniatores					
Stygnidae sp.1			2	0,042	P
Pseudoscorpiones					
Chernetidae jovens			2		P
Chthoniidae jovens	2				P
Insecta					
Blattodea jovens			2	0,042	P
Polyphagidae jovens	2	0,041			E
Collembola					
Arthropleona					
Entomobryoidea					
Entomobryidae sp.1	1				P
Diptera					
Nematocera					
Cecidomyiidae					
Cecidomyiinae sp.	1				E
Psychodidae					
Sciopemyia sordellii	2				E P
Hemiptera					
Heteroptera					
Reduviidae jovens	2	0,041	4	0,083	E P
Zelurus sp.1	2	0,041			P
Homoptera					
Cixiidae jovens	1		1		P
Hymenoptera					
Vespoidea					
Formicidae					
Camponotus sp.1	1		2		E P
Carebara sp.1			1		P
Gnamptogenys striatula	1				P
Pheidole sp.1	2		1		E P

Solenopsis	sp.2			1		P
Isoptera	sp.	1		1		E P
Termitidae						
Embiratermes	sp.	1				E
Nasutitermes	sp.	1		1		E P
Lepidoptera						
Cossoidea						
Limacodidae	sp.1	4	0,082			E P
Noctuoidea						
Noctuidae	sp.2	2	0,041	2	0,042	E P
Orthoptera						
Ensifera						
Phalangopsidae						
Phalangopsis	sp.1	25	0,51	19	0,395	P
Paraclodes	sp.			17	0,354	P
Psocoptera						
Psocomorpha	jovens	1				E
Thysanura						
Nicoletiidae	sp.1	1				P
Chordata						
Mammalia						
Chiroptera						
Phyllostomidae						
Glossophaginae	sp.	4	0,082			
Reptilia						
Squamata						
Gekkonidae						
Thecadactylus	*rapicauda*			2	0,042	E
