# Supplementary material for: Reconciling Mining with the Conservation of Cave Biodiversity: A Quantitative Baseline to Help Establish Conservation Priorities
Source: PLoS One. 2016 Dec 20;11(12):e0168348. doi: 10.1371/journal.pone.0168348 (PMC5173368; doi:10.1371/journal.pone.0168348)
Supplement: S1 Dataset — (ZIP) [file pone.0168348.s002.zip › Taxa/Serra Sul/SS_2010/S11D-68.pdf]

S11D-68			1ª	AB	2ª	AB	ZON
Arthropoda							
Arachnida							
Acari							
Sarcoptiformes							
Oribatida		sp.3			1		E
Trombidiformes							
Tydeoidea							
Rhagidiidae		sp.1	1				E
Trombidiformes		sp.7	1				E
Araneae							
Drymusidae	joven		1				E
Ochyroceratidae	joven		1				E
Scytodidae	joven		2	0,333	1	0,091	E
Theridiidae	joven		1				E
Theridiosomatidae							
	Plato	sp.1	2				E
Opiliones							
Laniatores							
Cosmetidae		sp.1			2	0,182	E
Stygnidae	joven		4	0,666			E
Pseudoscorpiones							
Chthoniidae							
	Pseudochthonius	sp.1			1		E
Chilopoda							
Notostigmophora							
Scutigeromorpha							
Pselliodidae	joven		1				E
Entognatha							
Diplura							
Campodeidae		sp.1	1				E
Insecta							
Collembola							
Arthropleona							
Entomobryoidea							
Isotomidae		sp.1	1				E
Diptera							
Brachycera	joven		2				E
Hymenoptera							
Vespoidea							
Formicidae							
	Camponotus	*atriceps*	1				E
		sp.1			1		E
	Crematogaster	sp.1	1				E
Orthoptera							
Ensifera							
Phalangopsidae							
	Paraclodes	sp.			3	0,273	E
Psocoptera							
Psocomorpha	joven		1				E
Troctomorpha							
Manicapsocidae	*oentomum*	sp.1	1				E
Psyllipsocidae	joven				1		E
Chordata							
Amphibia							
Anura		sp.			4	0,364	E
Neobatrachia							
Mammalia							
Chiroptera					1	0,091	E
Mollusca							
Gastropoda							

Bulimulidae					
Naesiotus sp.	1				E
