# Supplementary material for: Reconciling Mining with the Conservation of Cave Biodiversity: A Quantitative Baseline to Help Establish Conservation Priorities
Source: PLoS One. 2016 Dec 20;11(12):e0168348. doi: 10.1371/journal.pone.0168348 (PMC5173368; doi:10.1371/journal.pone.0168348)
Supplement: S1 Dataset — (ZIP) [file pone.0168348.s002.zip › Taxa/Serra Sul/SS_2010/S11D-69.pdf]

S11D-69			1ª	AB	2ª	AB	ZON
Annelida							
Clitellata							
Oligochaeta	joven		5	0,064			E
Arthropoda							
Arachnida							
Acari							
Sarcoptiformes							
Oribatida		sp.1	1				P
Trombidiformes							
Tydeoidea							
Anystidae							
	Erythracarus	*nasutus*	1				E
		sp.1	1				P
Araneae							
Araneidae	joven				1		E
Barychaelidae	joven						
	gen.1	sp.1	2	0,026			E
Ctenidae	joven		3	0,039			
Ochyroceratidae	joven				1		E
	Speocera	sp.1	1		1		P
Oonopidae	joven				1		E
Scytodidae	joven				1	0,022	E
	Scytodes	*eleonorae*	3	0,039			E
	Anapistula	sp.1	1				P
Tetrablemmidae	joven		1				E
	Matta	sp.1	1		1		P
Theridiidae							
	Theridion	sp.1	1				E
Theridiosomatidae							
	Plato	sp.1	2		1		E P
Opiliones							
Laniatores	joven				2	0,044	E
Stygnidae		sp.1			2	0,044	E
Pseudoscorpiones							
Bochicidae		sp.1	4		2		E P
Chernetidae							
	Spelaeochernes	sp.1	2		2		E P
Chthoniidae							
	Pseudochthonius	sp.1	2		2		E P
Entognatha							
Diplura							
Japygidae		sp.1	1				E
Insecta							
Blattodea	joven				2	0,044	E
Coleoptera	joven		1				E
Collembola							
Arthropleona							
Entomobryoidea							
Isotomidae		sp.1	1				P
Isotomidae		sp.2	1				P
Paronellidae		sp.1			1		E
Diptera							
Brachycera							
Phoridae							
	Metopininae	sp.	1				E
Nematocera	joven				1		P
Chironomidae		sp.	1				E
Psychodidae							
	Phlebotominae	sp.			1		P
	Pintomyia	*gruta*	1		1		E P

Sciopemyia	*sordellii*			1		E
Hemiptera						
Heteroptera						
Dipsocoroidea	joven	1		1		P
Homoptera						
Cixiidae	joven	2		1		E P
Hymenoptera						
Vespoidea						
Formicidae						
Camponotus	*atriceps*	1				E
Camponotus	sp.1			1		E
Camponotus	*striata*	1				E
Lepidoptera						
Castnioidea	joven	1	0,013			
Orthoptera						
Ensifera						
Phalangopsidae						
Paraclodes	sp.1			8	0,177	E
Phalangopsis	sp.1	55	0,714	28	0,622	P
Psocoptera						
Psocomorpha	joven			1		E
Chordata						
Amphibia						
Anura						
Aromobatidae						
Allobates	sp.	2	0,026	2	0,044	E
Mammalia						
Chiroptera						
Emballonuridae						
Peropteryx	*macrotis*	5	0,078			
