# Supplementary material for: Reconciling Mining with the Conservation of Cave Biodiversity: A Quantitative Baseline to Help Establish Conservation Priorities
Source: PLoS One. 2016 Dec 20;11(12):e0168348. doi: 10.1371/journal.pone.0168348 (PMC5173368; doi:10.1371/journal.pone.0168348)
Supplement: S1 Dataset — (ZIP) [file pone.0168348.s002.zip › Taxa/Serra Sul/SS_2010/S11D-70.pdf]

S11D-70			1ª	AB	2ª	AB	ZON
Arthropoda							
Arachnida							
Araneae							
Ochyroceratidae	jovens				1		E
Scytodidae							
Scytodes	*eleonorae*		21	0,656			E
Theridiosomatidae							
Plato	sp.1		1				E
Trechaleidae	jovens		2	0,062			E
Insecta							
Coleoptera							
Staphylinidae		sp.2			1		E
		sp.9			1		E
Diptera							
Brachycera							
Dolichopodidae		sp.			1		E
Nematocera							
Ceratopogonidae		sp.			1		E
Hymenoptera							
Vespoidea							
Formicidae							
Nylanderia	sp.1				1		E
Orthoptera							
Ensifera							
Phalangopsidae							
Paraclodes	sp.1				5	0,454	E
Phalangopsis	sp.1		4	0,125			
Psocoptera							
Psocomorpha	jovens				1		E
Chordata							
Amphibia							
Anura							
Aromobatidae							
Allobates	sp.		2	0,062			
Mammalia							
Chiroptera		sp.			3	0,363	E
Phyllostomidae							
Glossophaginae	sp.		2	0,093			
Reptilia							
Squamata							
Gekkonidae							
Thecadactylus	*rapicauda*				2	0,181	E
