# Supplementary material for: Reconciling Mining with the Conservation of Cave Biodiversity: A Quantitative Baseline to Help Establish Conservation Priorities
Source: PLoS One. 2016 Dec 20;11(12):e0168348. doi: 10.1371/journal.pone.0168348 (PMC5173368; doi:10.1371/journal.pone.0168348)
Supplement: S1 Dataset — (ZIP) [file pone.0168348.s002.zip › Taxa/Serra Sul/SS_2010/S11D-71.pdf]

S11D-71		1ª	AB	2ª	AB	ZON
Annelida						
Clitellata						P
Oligochaeta	jovens	2	0,054			P
	sp.	2	0,054			P
Arthropoda						
Arachnida						
Acari						
Parasitiformes						
Mesostigmata						
Laelapidae	sp.1			1		P
Trombidiformes						
Tydeoidea	sp.7			1		E P
Amblypygi						
Phrynidae						
Heterophrynus	sp.			2	0,08	P
Araneae						
Ochyroceratidae	jovens	1		1		E P
Ochyrocera	sp.1	1				P
Pholcidae	jovens			1		E P
Salticidae	jovens	1		1		E P
Scytodidae	jovens	1	0,081			E P
Scytodes	sp.	2				P
Segestriidae	jovens			1		E P
Ariadna	sp.1	1				E P
Tetrablemmidae	jovens			1		E P
Matta	sp.1			1		P
Theridiosomatidae						
Plato	sp.1	1		1		E P
Opiliones						
Laniatores						
Stygnidae	sp.1	7	0,304			E P
Pseudoscorpiones						
Chernetidae						
Spelaeochernes	sp.1	1		1		E P
Chthoniidae						
Pseudochthonius	sp.1	2		1		E P
Schizomida						
Hubbardiidae	jovens			1		P
Chilopoda						
Notostigmophora						
Scutigeromorpha						
Pselliodidae	jovens			1		P
Pleurostigmophora						
Scolopendromorpha	jovens			2	0,08	P
Diplopoda						
Polydesmida						
Fuhrmannodesmidae	sp.1	1				P
Entognatha						
Diplura						
Campodeidae	sp.1	1				P
Japygidae	sp.1	1				P
Insecta						P
Coleoptera	jovens	1		1		P
Collembola						
Arthropleona						
Entomobryoidea						
Entomobryidae	sp.1			1		E P
	sp.6			1		E P
Isotomidae	sp.1			1		E P
Paronellidae	sp.1	1		1		E P
	sp.4	1				P
Diptera						

Brachycera						
Phoridae						
Metopininae	sp.			1		E P
Nematocera						
Ceratopogonidae	sp.			1		P
Psychodidae						
Pintomyia	*gruta*	1				P
Sciopemyia	*sordellii*			1		P
Sciaridae	sp.	1				P
Hemiptera						
Heteroptera						
Cydnidae						
Cydninae	sp.1	1				E P
Homoptera						
Cixiidae jovens		1		1		E P
Hymenoptera						
Vespoidea						
Formicidae						
Crematogaster	sp.1			1		E P
Hypoponera	sp.1			1		P
Nylanderia	sp.1	1				E P
Pachycondyla	*striata*			1		E P
Lepidoptera jovens		1		1		P
Tineoidea	sp.1	1				P
Orthoptera						
Ensifera						
Phalangopsidae						
Paraclodes	sp.1			3	0,12	P
Phalangopsis	sp.1	23	0,621	14	0,56	P
Malacostraca						
Isopoda						
Philosciidae	sp.1	2				P
Symphyla						
Scutigerellidae						
Hanseniella	sp.1	1		1		E P
Chordata						
Amphibia						
Anura	sp.			2	0,08	E P
Mammalia						
Chiroptera	sp.			1	0,08	P
