# Supplementary material for: Reconciling Mining with the Conservation of Cave Biodiversity: A Quantitative Baseline to Help Establish Conservation Priorities
Source: PLoS One. 2016 Dec 20;11(12):e0168348. doi: 10.1371/journal.pone.0168348 (PMC5173368; doi:10.1371/journal.pone.0168348)
Supplement: S1 Dataset — (ZIP) [file pone.0168348.s002.zip › Taxa/Serra Sul/SS_2010/S11D-72.pdf]

S11D-72			1ª	AB	2ª	AB	ZON
Arthropoda							
Arachnida							
Amblypygi							
Phrynidae							
	Heterophrynus	sp.	2	0,037			
Araneae							
Araneidae	jovens		1				E
	Alpaida	*septemmammata*	2				E
Corinnidae	jovens		4	0,075			E
Ctenidae	jovens		3	0,056			E
Filistatidae	jovens		1				E
		sp.1	1				E
Pholcidae	jovens		1				E
	aff. *Ibityporanga*	sp.1	2				E
		sp.1	1				E
	Ninetinae	sp.1	1				E
Salticidae	jovens		1				E
Scytodidae	jovens		2	0,037			E
	Scytodes	*eleonorae*	5	0,094	2	0,023	E
		globula			3	0,035	E
		sp.			12	0,141	E
Theridiosomatidae	jovens		1		1		E
Pseudoscorpiones							
Olpiidae		sp.1	4		2		E
Insecta							
Blattodea							
Blattellidae		sp.2	2	0,037			E
Polyphagidae	jovens		2	0,037			E
Coleoptera	jovens		1				E
Collembola							
Arthropleona							
Entomobryoidea							
Entomobryidae		sp.4	1				E
Diptera	jovens		1				E
Brachycera							
Milichiidae		sp.			1		E
Phoridae							
	Metopininae	sp.			1		E
Nematocera							
Cecidomyiidae							
	Cecidomyiinae	sp.	1				E
Ceratopogonidae		sp.			1		E
Hemiptera							
Heteroptera	jovens		1	0,018			
aff. Pyrrhocoroidea							
Reduviidae	jovens		4	0,075	2	0,023	E
	Reduviinae	sp.			11	0,129	E
Hymenoptera							
Ichneumonoidea							
Braconidae		sp.1			1		E
Vespoidea							
Formicidae							
	Camponotus	*atriceps*	1		1		E
	Gnamptogenys	*striatula*	1				E
	Hypoponera	sp.1	1				E
	Pachycondyla	*constricta*	2	0,037			E
Isoptera							
Termitidae							
	Nasutitermes	sp.	2		1		E
Lepidoptera							
Cossoidea							
Limacodidae		sp.1	8	0,15			E

Noctuoidea							
Noctuidae		sp.1	10	0,188			E
Neuroptera							
Myrmeleontidae	jovens		2		1		E
		sp.			51	0,6	E
Orthoptera							
Ensifera							
Phalangopsidae							
	Paraclodes	sp.			4	0,047	E
Psocoptera							
Psocomorpha	jovens		1				E
Thysanura							
Nicoletiidae		sp.1			1		E
Chordata							
Mammalia							
Chiroptera							
Emballonuridae							
	Peropteryx	sp.	5	0,113			
Phyllostomidae							
	Glossophaginae	sp.	1	0,037			
