# Supplementary material for: Reconciling Mining with the Conservation of Cave Biodiversity: A Quantitative Baseline to Help Establish Conservation Priorities
Source: PLoS One. 2016 Dec 20;11(12):e0168348. doi: 10.1371/journal.pone.0168348 (PMC5173368; doi:10.1371/journal.pone.0168348)
Supplement: S1 Dataset — (ZIP) [file pone.0168348.s002.zip › Taxa/Serra Sul/SS_2010/S11D-73.pdf]

S11D-73		1ª	AB	2ª	AB	ZON
Arthropoda						
Arachnida						
Acari						
Parasitiformes						
Mesostigmata						
Diploginiidae	sp.7			1		E
Araneae						
Araneidae	jovens	1		1		E
Pholcidae	jovens	1				E
Salticidae	jovens	1		1		E
Amphidraus	sp.1	1				E
Scytodidae						
Scytodes	sp.2			1		E
Theraphosidae	jovens			5	0,161	E
Theridiosomatidae						
Plato	sp.1	2				E
Opiliones						
Eupnoi						
Sclerosomatidae	jovens	1		1		E
Laniatores	jovens			4	0,129	E
Stygnidae	jovens	3	0,15			E
	sp.1			2	0,064	E
Pseudoscorpiones						
Chernetidae						
Spelaeochernes	sp.1			1		E
Scorpiones						
Buthidae	jovens	2	0,1			E
Diplopoda	jovens			2	0,064	E
Spirostreptida						
Pseudonannolenidae						
Pseudonannolene	sp.1			2	0,064	E
Entognatha						
Diplura						
Campodeidae	sp.1	1				E
Insecta						
Blattodea	jovens			2	0,064	E
Blattidae	sp.3	2	0,1			E
Coleoptera	jovens			2		E
	sp.7			1		E
Collembola						
Arthropleona						
Entomobryoidea						
Paronellidae	sp.1			1		E
	sp.6			1		E
Diptera	jovens			1		E
Brachycera						
Camillidae	sp.			1		E
Nematocera						
Cecidomyiidae						
Cecidomyiinae	sp.	1				E
Hemiptera						
Heteroptera						
aff. Pyrrhocoroidea						
Reduviidae	jovens	2	0,1	2	0,064	E
Reduviinae	sp.			8	0,258	E
Homoptera	jovens	1				E
Cixiidae	jovens	1		1		E
Hymenoptera						
Chalcidoidea	sp.3			1		E
Vespoidea						
Formicidae						
Gnamptogenys	*striatula*			1		E

Nylanderia	sp.1			1		E
Pachycondyla	*striata*			1		E
Pompilidae	sp.1			1		E
Isoptera	sp.			1		E
Lepidoptera						
Cossoidea						
Limacodidae	sp.1	2	0,1			E
Orthoptera						
Ensifera						
Phalangopsidae	jovens	3	0,15			
Paraclodes	sp.			2	0,064	E
Psocoptera						
Psocomorpha	jovens			2		E
Trogiomorpha						
Psyllipsocidae						
Psyllipsocus	sp.6			1		E
Chordata						
Amphibia						
Anura						
Neobatrachia						
Strabomantidae						
Pristimantis	*fenestratus*	2	0,1	2	0,064	E
Mammalia						
Chiroptera						
Emballonuridae						
Peropteryx	sp.	3	0,2			
Mollusca						
Gastropoda						
Systrophiidae						
Happia	sp.	2				E
		28		50		
