# Supplementary material for: Reconciling Mining with the Conservation of Cave Biodiversity: A Quantitative Baseline to Help Establish Conservation Priorities
Source: PLoS One. 2016 Dec 20;11(12):e0168348. doi: 10.1371/journal.pone.0168348 (PMC5173368; doi:10.1371/journal.pone.0168348)
Supplement: S1 Dataset — (ZIP) [file pone.0168348.s002.zip › Taxa/Serra Sul/SS_2010/S11D-74.pdf]

S11D-74		1ª	AB	2ª	AB	ZON
Annelida						
Clitellata						
Oligochaeta	jovens	2	0,0238			E
Arthropoda						
Arachnida						
Acari						
Ixodida						
Ixodidae						
Amblyomma	sp.			1		E
Parasitiformes						
Holothyrida						
Diplothyridae						
Diplothyrus	*scubarti*	1				E
Mesostigmata	sp.4	1				P
Laelapidae	sp.2			1		E
Macronyssidae	sp.1	1				P
Opilioacarida						
Opilioacaridae	sp.1	1				P
Sarcoptiformes						
Oribatida	sp.3	1				E
Trombidiformes						
Tydeoidea	sp.1	1				E
Amblypygi						
Phrynidae						
Heterophrynus	sp.			7	0,196	P
Araneae						
Corinnidae	jovens	3	0,0357			E
Ctenidae	jovens	3	0,0357			E
Ochyroceratidae	jovens			1		E
Oonopidae						
gr. *Xycarphius*	sp.5	1				E
Oonopinae	sp.1			1		E
Pholcidae	jovens	1				E
Scytodidae	jovens	1	0,0119			E
Scytodes	*eleonorae*	2	0,0238			E
Tetrablemmidae						
Matta	sp.1	1				P
Theridiidae						
Theridion	sp.2	1				E
Opiliones	jovens			11	0,204	P
Laniatores						
Cosmetidae	sp.1			2	0,037	E
Roquettea	*singularis*			2	0,037	E
Stygnidae	sp.1	2	0,0238	3	0,055	E P
Pseudoscorpiones						
Chernetidae						
Spelaeochernes	sp.1	3		1		E P
Chthoniidae						
Pseudochthonius	sp.1	1		1		E P
Chilopoda						
Notostigmophora						
Scutigeromorpha						
Pselliodidae	jovens			1		E
Sphendononema	*guildingii*	1				E
Pleurostigmophora						
Geophilomorpha						
Ballophilidae	sp.1	2	0,0238			E
Diplopoda						
Glomeridesmida						
Glomeridesmidae	sp.2	2				E
Polydesmida						
Pyrgodesmidae	sp.2			2	0,037	P

Spirostreptida						
Pseudonannolenidae	jovens	2	0,0238			E
Entognatha						
Diplura						
Campodeidae	sp.1	1		1		E
Insecta						
Coleoptera	jovens	1				E
Staphylinidae	sp.5			1		E
Collembola						
Arthropleona						
Entomobryoidea						
Entomobryidae	sp.1			1		E
Isotomidae	sp.1	1		1		E
Paronellidae	sp.1	2				E P
Diptera						
Nematocera						
Psychodidae						
Pintomyia	*gruta*	1				E
Sciopemyia	*sordellii*	1		2		E P
Sciaridae	sp.	1				P
Hemiptera						
Heteroptera						
Dipsocoroidea	jovens			2		E P
Reduviidae						
Emesinae	sp.2			1		E
Reduviinae	sp.			2	0,037	P
Schizopteridae						
Schizopterinae	sp.1	1				P
Homoptera						
Cixiidae	jovens	1		2		E P
	sp.4	1				E
Homoptera	jovens	11				
Hymenoptera						
Vespoidea						
Formicidae						
Apterostigma	sp.1	2				E
Pachycondyla	*striata*			1		E
Pheidole	sp.1	1				E
Solenopsis	sp.2			1		E
Isoptera						
Termitidae						
Nasutitermes	sp.			1		E
Lepidoptera	jovens			1		E
Orthoptera						
Ensifera						
Phalangopsidae	jovens	58	0,6905			
Paraclodes	sp.1	3	0,0357	12	0,222	E
Phalangopsis	sp.1			9	0,166	P
Thysanoptera						
Thripidae	sp.1			1		E
Malacostraca						
Isopoda						
Philosciidae	sp.1	1		1		E P
Pauropoda						
Tetramerocerata	sp.			1		E
Symphyla						
Scutigerellidae						
Hanseniella	sp.1	1				E
Chordata						
Amphibia						
Anura	sp.			1	0,0185	P
Neobatrachia						
Strabomantidae						

		Pristimantis	*fenestratus*	2	0,0238	3	0,055	E
Mammalia								
Chiroptera								
	Phyllostomidae							
		Glossophaga	*soricina*	3	0,0476			
Mollusca								
Gastropoda								
	Systrophiidae							
		Happia	sp.	2				E P
