# Supplementary material for: Reconciling Mining with the Conservation of Cave Biodiversity: A Quantitative Baseline to Help Establish Conservation Priorities
Source: PLoS One. 2016 Dec 20;11(12):e0168348. doi: 10.1371/journal.pone.0168348 (PMC5173368; doi:10.1371/journal.pone.0168348)
Supplement: S1 Dataset — (ZIP) [file pone.0168348.s002.zip › Taxa/Serra Sul/SS_2010/S11D-75.pdf]

S11D-75		1ª	AB	2ª	AB	ZON
Arthropoda						
Arachnida						
Acari						
Parasitiformes						
Opilioacarida						
Opilioacaridae	sp.1	1				E
Sarcoptiformes						
Oribatida	sp.7	1				E
Amblypygi						
Phrynidae						
Heterophrynus	sp.	3	0,13			
Araneae						
Ctenidae jovens		2	0,086			E
Pholcidae						
aff. *Ibityporanga*	sp.1	1				E
Ninetinae	sp.1			1		E
Salticidae jovens		1				E
Scytodidae						
Scytodes	sp.			3	0,15	E
Theridiosomatidae jovens		1				E
Opiliones						
Laniatores						
Stygnidae jovens		2	0,086			E
Pseudoscorpiones						
Chthoniidae						
Pseudochthonius	sp.1	1				E
Diplopoda						
Polyxenida						
Hypogexenidae	sp.1	1				E
Insecta						
Blattodea						
Blattellidae	sp.2			2	0,1	E
Polyphagidae jovens				2	0,1	E
Diptera						
Nematocera						
Ceratopogonidae	sp.			1		E
Psychodidae						
Sciopemyia	*sordellii*	1				E
Tipulidae						
Tipulinae	sp.	1				E
Hemiptera						
Heteroptera						
Reduviidae jovens		2	0,086			E
Hymenoptera						
Vespoidea						
Formicidae						
Camponotus	*atriceps*	1				E
Isoptera	sp.			1		E
Termitidae						
Nasutitermes	sp.	1				E
Lepidoptera						
Cossoidea						
Limacodidae	sp.1	2	0,086			E
Noctuoidea						
Noctuidae	sp.2	1				E
	sp.1	2	0,086			
Orthoptera						
Ensifera						
Tettigoniidae	sp.2	2	0,086			E
Phalangopsidae jovens		4	0,173			
Paraclodes	sp.			11	0,55	E
Psocoptera						

Psocomorpha	jovens	2				E
Troctomorpha						
Manicapsocidae						
Nothoentomum	sp.1	1				E
Chordata						
Amphibia						
Anura						
Neobatrachia						
Strabomantidae						
Pristimantis	*fenestratus*			2	0,1	E
Mammalia						
Chiroptera						
Emballonuridae						
Peropteryx	*kappleri*	1	0,086			
	macrotis	1	0,086			
