# Supplementary material for: Reconciling Mining with the Conservation of Cave Biodiversity: A Quantitative Baseline to Help Establish Conservation Priorities
Source: PLoS One. 2016 Dec 20;11(12):e0168348. doi: 10.1371/journal.pone.0168348 (PMC5173368; doi:10.1371/journal.pone.0168348)
Supplement: S1 Dataset — (ZIP) [file pone.0168348.s002.zip › Taxa/Serra Sul/SS_2010/S11D-76.pdf]

S11D-76			1ª	AB	2ª	AB	ZON
Annelida							
Clitellata							
Oligochaeta	jovens		4	0,071			E
Arthropoda							
Arachnida							
Acari							
Ixodida							
Argasidae							
	Ornithodoros	sp.	1				E
Sarcoptiformes		sp.1	1				E
Trombidiformes		sp.4	1				E
Amblypygi							
Phrynidae							
	Heterophrynus	sp.	2	0,036	3	0,071	E
Araneae							
Araneidae	jovens		1		1		E
Filistatidae	jovens		1				E
		sp.1	1				E
Ochyroceratidae	jovens		2		1		E
Oonopidae	jovens		1				E
Pholcidae	jovens		3		1		E
	Mesabolivar	sp.1	1				E
	Ninetinae	sp.1	1				E
Salticidae	jovens				1		E
	Amphidraus	sp.1	1				E
Scytodidae	jovens		2	0,036	1	0,023	E
	Scytodes	*eleonorae*			2	0,048	E
		sp.			6	0,142	E
Tetrablemmidae							
	Matta	sp.1	1				E
Tetragnathidae							
	Leucauge	sp.1	1				E
Theridiosomatidae							
	Plato	sp.1	2				E
Opiliones							
Laniatores							
Stygnidae	jovens		2	0,036			E
		sp.1	2	0,036			E
Palpigradi							
Eukoeneniidae							
	Allokoenenia	sp.1			1		E
Pseudoscorpiones							
Bochicidae	jovens		2				E
		sp.1	2				E
Chernetidae							
	Spelaeochernes	sp.1	1				E
Olpiidae		sp.1	2				E
Chilopoda							
Scolopendromorpha	jovens				2	0,048	E
Diplopoda							
Polydesmida	jovens				2	0,048	E
Chelodesmidae		sp.4			2	0,048	E
Polyxenida							
Hypogexenidae		sp.1	1				E
Entognatha							
Diplura							
Campodeidae		sp.1	2				E
Insecta							
Blattodea	jovens		2	0,036			E
Polyphagidae	jovens		3	0,053			E
Coleoptera							

Chrysomelidae		sp.15	2	0,036			E
Phalacridae		sp.1	1				E
Coleoptera	jovens		3		1		E
Diptera							
Brachycera							
Calliphoridae		sp.			1		E
Drosophilidae							
	Drosophila	*eleonore*			1		E
Nematocera							
Psychodidae							
	Sciopemyia	*sordellii*	1		2		E
Hemiptera							
Heteroptera							
Reduviidae	jovens		3	0,053			E
Homoptera							
Cixiidae	jovens		2		1		E
Hymenoptera							
Vespoidea							
Formicidae							
	Camponotus	*atriceps*	3		1		E
	Wasmania	*auropunctata*			1		E
Vespidae		sp.2			1		E
Isoptera		sp.	1				E
Termitidae							
	Nasutitermes	sp.	3		2		E
Lepidoptera	jovens		2	0,036	3	0,071	E
Noctuoidea							
Noctuidae		sp.4			1		E
Tineoidea		sp.1	1		1		E
Neuroptera							
Myrmeleontidae	jovens		1				E
Orthoptera							
Ensifera							
Phalangopsidae							
	Phalangopsis	sp.1	26	0,464	5	0,119	E
	Paraclodes	sp.1			3	0,071	E
Psocoptera							
Psocomorpha	jovens		1				E
Dolabellopsocidae							
	Isthmopsocus	sp.1	1				E
Ptiloneuridae							
	Triplocania	sp.8	1				E
Troctomorpha							
Liposcelididae							
	Liposcelis	sp.1	1				E
Trogiomorpha							
Psyllipsocidae	jovens				1		E
	Psocathropos	sp.1	1				E
	Psyllipsocus	sp.1	1				E
Thysanura							
Nicoletiidae	jovens		1				E
		sp.1	1				E
Chordata							
Amphibia							
Anura							
Neobatrachia							
Strabomantidae							
	Pristimantis	*fenestratus*			4	0,095	E
Mammalia							
Chiroptera							
Emballonuridae							
	Peropteryx	*kappleri*	3	0,071			
		sp.			8	0,214	E

Reptilia					
Squamata					
Gekkonidae					
Thecadactylus rapicauda	2	0,036			
