# Supplementary material for: Reconciling Mining with the Conservation of Cave Biodiversity: A Quantitative Baseline to Help Establish Conservation Priorities
Source: PLoS One. 2016 Dec 20;11(12):e0168348. doi: 10.1371/journal.pone.0168348 (PMC5173368; doi:10.1371/journal.pone.0168348)
Supplement: S1 Dataset — (ZIP) [file pone.0168348.s002.zip › Taxa/Serra Sul/SS_2010/S11D-77.pdf]

S11D-77		1ª	AB	2ª	AB	ZON
Annelida						
Clitellata						
Oligochaeta	jovens	2	0,006			E
Oligochaeta	sp.	2	0,006			P
Arthropoda						
Arachnida						
Acari						
Ixodida						
Argasidae						
Ornithodoros	sp.			1		P
Parasitiformes						
Mesostigmata	sp.2	1				P
Sarcoptiformes						
Sarcoptiformes	sp.19	1				P
Sarcoptiformes	sp.2	1				P
Acaridae	sp.1	1				E
Oribatida	sp.3	3		4		E P
Trombidiformes						
Trombidiformes	sp.1	1		1		P
Trombidiformes	sp.3			1		P
Trombidiformes	sp.7			1		P
Cunaxidae	sp.2			1		P
Amblypygi						
Phrynidae						
Heterophrynus	sp.	4	0,012	4	0,036	P
Araneae						
Araneidae	jovens	1		1		E
Corinnidae	jovens			2	0,018	P
Ctenidae	jovens	2	0,006			E
Ochyroceratidae	jovens	1				P
Ochyrocera	sp.1	1		5		E P
Speocera	sp.1	1				E
Oonopidae						
gr. *Xycarphius*	sp.5			1		E
Pholcidae	jovens			1		E
Mesabolivar	sp.1	1				E
Salticidae						
Amphidraus	sp.1	1				E
Scytodidae	jovens	1		2		E P
Scytodes	*eleonorae*	6	0,019			E P
Scytodes	sp.			3	0,027	P
Symphytognathidae						
Anapistula	sp.1			1		E
Tetrablemmidae	jovens	1				P
Theraphosidae	jovens	2	0,006			E
Theridiosomatidae	jovens	1				P
Plato	sp.1	1		1		P
Palpigradi						
Eukoeneniidae	jovens	1				E
Allokoenenia	sp.1	1				E
Pseudoscorpiones						
Chernetidae						
Spelaeochernes	sp.1	1		4		E P
Chthoniidae						
Pseudochthonius	sp.1	4		2		E P
Chilopoda						
Notostigmophora						
Scutigeromorpha						
Pselliodidae						
Sphendononema	*guildingii*			1		E
Pleurostigmophora						
Scolopendromorpha						
Cryptopidae						
Cryptops	sp.1			2	0,018	E

Diplopoda							
Polydesmida							
Fuhrmannodesmidae		sp.3			1		P
Pyrgodesmidae		sp.2	2	0,006			P
Spirostreptida	jovens				1		P
Pseudonannolenidae							
	Pseudonannolene	sp.1	2	0,006			P
Entognatha							
Diplura	jovens		1				P
Campodeidae		sp.1	1				E
Japygidae		sp.1	1		1		E
Insecta							
Blattodea	jovens		2	0,006	3	0,027	E
Blattidae	jovens		2	0,006	2	0,018	P
Coleoptera	jovens		2				E P
Carabidae		sp.3	1		2		E
Dytiscidae		sp.1	1		1		P
Dytiscidae		sp.2	1		1		P
Staphylinidae		sp.16			1		E
Collembola							
Arthropleona							
Entomobryoidea		sp.1			1		P
Isotomidae		sp.1	3		1		E P
Paronellidae		sp.1	1				E
Paronellidae		sp.4	1		1		P
Poduroidea		sp.1			1		E
Symphypleona							
Sminthuroidea		sp.2	3		3		E P
Diptera	jovens		3		3		E P
Brachycera							
Phoridae							
	Metopininae	sp.			1		E
Nematocera							
Ceratopogonidae		sp.			1		E
Chironomidae		sp.	2				E P
Culicidae							
	Culicini	sp.			1		P
Mycetophilidae							
	Boletina	sp.	1				E
Psychodidae							
	Sciopemyia	*sordellii*	3		2		E P
Hemiptera							
Heteroptera	jovens		156	0,492			
aff. Pyrrhocoroidea							
Cydnidae	jovens		1				P
	Cydninae	sp.1			1		P
Veliidae							
	Paravelia	sp.1	1		1		P
Homoptera	jovens		16				
Cixiidae	jovens		2		2		E
Hymenoptera							
Vespoidea							
Formicidae							
	Camponotus	*atriceps*	2				E P
	Dolichoderus	*bispinosus*	1				E
	Gnamptogenys	*striatula*			1		E
	Octostruma	sp.1			1		E
Lepidoptera	jovens		1		1		E
Orthoptera							
Ensifera							
Phalangopsidae							
	Phalangopsis	sp.1	107	0,337	72	0,648	E P
	Paraclodes	sp.1			10	0,09	E
Psocoptera							
Psocomorpha	jovens				1		E

Malacostraca							
Isopoda							
Dubioniscidae		sp.1	1				E
Philosciidae		sp.1			1		E
Scleropactidae		sp.	1				P
Chordata							
Amphibia							
Anura							
Neobatrachia							
Bufonidae							
	Rhinella	cf. *marina*			2	0,018	E
Mammalia							
Chiroptera							
Emballonuridae							
	Peropteryx	*kappleri*	2	0,009			
	Peropteryx	sp.			4	0,045	P
Phyllostomidae							
	Carollia	*perspicillata*	5	0,019	3	0,036	P
	Desmodus	*rotundus*	1	0,006	1	0,018	P
Nematoda	jovens		1	0,003			
