# Supplementary material for: Reconciling Mining with the Conservation of Cave Biodiversity: A Quantitative Baseline to Help Establish Conservation Priorities
Source: PLoS One. 2016 Dec 20;11(12):e0168348. doi: 10.1371/journal.pone.0168348 (PMC5173368; doi:10.1371/journal.pone.0168348)
Supplement: S1 Dataset — (ZIP) [file pone.0168348.s002.zip › Taxa/Serra Sul/SS_2010/S11D-78.pdf]

S11D-78	1ª	AB	2ª	AB	ZON
Annelida					
Clitellata					
Oligochaeta jovens	2	0,006	2	0,005	A
Arthropoda					
Arachnida					
Acari					
Ixodida					
Argasidae					
Ornithodoros sp.	1				P
Parasitiformes					
Ixodida					
Ixodidae					
Ornithodoros sp.1			1		A
Mesostigmata					
Mesostigmata sp.1			1		P
Laelapidae					
Stratiolaelaps sp.1	1				P
Sarcoptiformes					
Sarcoptiformes sp.1			1		P
Oribatida					
Anoetidae sp.1			1		A
Trombidiformes sp.1	1		5		A
Trombidiformes sp.2	1				E
Trombidiformes sp.6			2		A
Amblypygi					
Charinidae jovens	4	0,011	6	0,02	P A
Charinus sp.	22	0,064	7	0,02	A
Charinus sp.2	7	0,02			P A
Phrynidae					
Heterophrynus sp.	5	0,014	2	0,005	A
Araneae					
Araneidae jovens			1		E
Alpaida septemmammata	1				E
Corinnidae jovens	2	0,006			
Ochyroceratidae jovens	4		4		E P A
Ochyrocera sp.1	6		1		P A
Pholcidae jovens	1		1		P
Ninetinae sp.1	7		1		E P A
Salticidae					
Mago sp.1	1				E
Scytodidae jovens	4		2		E P A
Scytodes eleonorae	7	0,02	4	0,011	P A
Scytodes globula	15	0,043			E
Scytodes sp.			5	0,014	
Tetrablemmidae jovens	1				A
Matta sp.1	5		5		P A
Theraphosidae jovens	2	0,006			E
Theridiosomatidae jovens	2				P A
Plato sp.1	2				E P
Opiliones jovens	12	0,035	9	0,025	P
Cyphophthalmi					
Neogoveidae					
Canga renatae	6		3		P A
Laniatores					
Escadabiidae jovens	1		1		P
Escadabiidae sp.1	9		7		E P A
Stygnidae jovens	4	0,011			E
Stygnidae sp.1			3	0,008	P
Palpigradi					
Eukoeneniidae jovens	1				
Pseudoscorpiones					
Bochicidae sp.1	6		4		A
Chernetidae jovens			4		P
Spelaeochernes sp.1	10		8		E P A

Chthoniidae	*jovens*		2				A
	Pseudochthonius	sp.1			3		E P A
	Pseudochthonius	sp.4			2		A
Diplopoda							
Polydesmida							
Fuhrmannodesmidae		sp.1	1				A
Fuhrmannodesmidae		sp.3	3		1		P A
Pyrgodesmidae		sp.2	2	0,006	2	0,005	A
Spirostreptida							
Pseudonannolenidae	jovens		2	0,006			E
Entognatha							
Diplura							
Campodeidae		sp.1	2		1		A
Insecta							
Blattodea	jovens				2	0,005	P
Blaberidae	jovens		6	0,02	4	0,011	E P A
Blattidae	jovens				2	0,005	E
Coleoptera	jovens		7		5		E P A
Coleoptera		sp.8	1				P
Curculionidae							
	Platypodinae	sp.1			1		A
Dytiscidae		sp.6	1				A
Ptiliidae		sp.1			1		A
Staphylinidae		sp.17			1		A
Collembola							
Arthropleona							
Entomobryoidea							
Cyphoderidae		sp.1	1				A
Entomobryidae		sp.4	1				P
Isotomidae		sp.1			1		A
Paronellidae		sp.1	1				A
Paronellidae		sp.4	6		9		E P A
Symphypleona							
Sminthuroidea		sp.1	1		2		P A
Sminthuroidea		sp.2			4		A
Diptera	jovens		2		2		E A
Brachycera							
Camillidae		sp.	1				E
Milichiidae		sp.			1		A
Nematocera							
Culicidae							
	Wyeomyia	sp.	1				E
Mycetophilidae							
	Keroplatus	sp.	1				E
Psychodidae							
	Edentomyia	*piauensis*	3		5		E P A
	Pintomyia	*gruta*			2		P A
	Sciopemyia	*sordellii*	3				E P A
Hemiptera							
Homoptera	jovens		16				
Cixiidae		sp.8	1				P
Cixiidae		sp.9	2				P
Hymenoptera	jovens		1				E
Vespoidea							
Formicidae							
	Camponotus	*atriceps*			5		E P A
	Odontomachus	*bauri*	2	0,006			P
	Pachycondyla	*striata*	1				E
	Solenopsis	sp.2	1		5		P A
Vespidae		sp.1	1				E
Isoptera		sp.			2		P A
Termitidae							
	Cortaritermes	*silvestrii*			2		A
	Nasutitermes	sp.			1		E
Lepidoptera	jovens		3				E P

Cossoidea							
Limacodidae		sp.1	7	0,02			E P
Orthoptera							
Ensifera							
Oecanthidae		sp.1	3	0,008			E
Phalangopsidae	*jovens*						
	Phalangopsis	sp.1	195	0,565	163	0,464	A
Tettigoniidae		sp.			3	0,008	A
Trigoniidae	*jovens*		2	0,006			E
Thysanura							
Ateluridae	jovens				1		A
Nicoletiidae	jovens		1		1		E A
Nicoletiidae		sp.1	2				P A
Malacostraca							
Isopoda							
Scleropactidae		sp.	1		1		P A
Chordata							
Mammalia							
Chiroptera							
Emballonuridae							
	Peropteryx	*kappleri*	6	0,02			
	Peropteryx	sp.			15	0,05	A
Furipteridae							
	Furipterus	*horrens*	30	0,09	120	0,344	A
Phyllostomidae							
	Carollia	sp.	2	0,008			
	Diphylla	*ecaudata*	1	0,006			
Reptilia							
Squamata							
Serpentes							
Viperidae							
	Lachesis	*muta*	1	0,003			
