# Supplementary material for: Reconciling Mining with the Conservation of Cave Biodiversity: A Quantitative Baseline to Help Establish Conservation Priorities
Source: PLoS One. 2016 Dec 20;11(12):e0168348. doi: 10.1371/journal.pone.0168348 (PMC5173368; doi:10.1371/journal.pone.0168348)
Supplement: S1 Dataset — (ZIP) [file pone.0168348.s002.zip › Taxa/Serra Sul/SS_2010/S11D-79.pdf]

S11D-79		1ª	AB	2ª	AB	ZON
Annelida						
Clitellata						
Oligochaeta	jovens	4	0,069			P
Arthropoda						
Arachnida						
Acari						
Ixodida						
Argasidae						
Ornithodoros	sp.	2				E P
Parasitiformes						
Mesostigmata	sp.5			1		P
Sarcoptiformes	sp.1	1				P
Trombidiformes						
Trombidiformes	sp.7	1				E
Tydeoidea						
Rhagidiidae	sp.1			1		P
Amblypygi						
Phrynidae						
Heterophrynus	sp.			4	0,08	P
Araneae	jovens	8	0,135			
Araneidae	jovens			1		E
Ctenidae	jovens			2	0,04	P
Ctenus	sp.1			2	0,04	P
Filistatidae	sp.1			1		E
Ochyroceratidae	jovens	3				E P
Ochyrocera	sp.1	1		2		P
Speocera	sp.1	2		1		E P
Oonopidae	jovens	1				P
gr. *Xycarphius*	sp.5	1		1		P
Pholcidae						
Leptopholcus	sp.1			1		P
Mesabolivar	sp.1			2		E P
Ninetinae	sp.1	3		3		E P
Scytodidae	jovens	2		2		E P
Segestriidae	jovens			1		P
Ariadna	sp.1	2				E
Tetrablemmidae	jovens	3				E P
Matta	sp.1	1		1		P
Opiliones						
Laniatores						
Escadabiidae	sp.1	3		1		E P
Stygnidae	jovens	8	0,135			E P
Stygnidae	sp.1			2	0,04	E
Pseudoscorpiones						
Bochicidae	sp.1	2				P
Chernetidae	jovens			2		P
Spelaeochernes	sp.1			1		P
Chthoniidae	*jovens*			2		P
Pseudochthonius	sp.1	1				P
Olpiidae	sp.1			2		E
Ricinulei						
Ricinoididae	jovens	1				P
Cryptocellus	sp.	1				P
Schizomida						
Hubbardiidae	jovens	1				P
Rowlandius	sp.	1				P
Chilopoda						
Pleurostigmophora						
Geophilomorpha						
Geophilidae	sp.1	6	0,102			E P
Diplopoda						
Glomeridesmida						
Glomeridesmidae	sp.1	1				P

Polydesmida								
Pyrgodesmidae		sp.2			2	0,04		P
Polyxenida								
Hypogexenidae		sp.1	1		1		E	P
Spirostreptida	jovens		1					P
Entognatha								
Diplura								
Campodeidae		sp.1	3		1		E	P
Japygidae		sp.1	1					P
Insecta								
Coleoptera	jovens		3					P
Carabidae		sp.10	1					P
Collembola								
Arthropleona								
Entomobryoidea								
Entomobryidae		sp.4	1					
Paronellidae		sp.1			1			
Paronellidae		sp.4	1		1			
Symphypleona								
Sminthuroidea		sp.1	1					
Diptera	jovens				1			
Nematocera								
Culicidae								
	Culicini	sp.			1			
Psychodidae								
	Sciopemyia	*sordellii*	3				E	
Hemiptera								
Heteroptera								
aff. Pyrrhocoroidea								
Reduviidae	jovens		2	0,034	4	0,16	E	
	Reduviinae	sp.			4			
Homoptera	jovens		21					
Cixiidae	jovens		3		1		E	
Cixiidae		sp.2	1		1			
Hymenoptera								
Vespoidea								
Formicidae								
	Camponotus	*atriceps*			2			
	Gnamptogenys	*striatula*	4				E	
	Nylanderia	sp.1	4		3		E	
	Octostruma	sp.1			2			
	Pachycondyla	*harpax*	1					
	Solenopsis	sp.1	1					
	Wasmania	*auropunctata*			1			
Isoptera		sp.	2					
Termitidae								
	Cortaritermes	*silvestrii*			1		E	
	Diversitermes	sp.	1					
	Nasutitermes	sp.	2		2		E	
Lepidoptera	jovens		3				E	
Noctuoidea		sp.2	1				E	
Orthoptera								
Ensifera								
Phalangopsidae	jovens				2	0,04		
	Phalangopsis	sp.1	31	0,525	20	0,4	E	
	Paraclodes	sp.1			3	0,06		
Psocoptera								
Psocomorpha	jovens		1		3		E	
Thysanoptera								
Phlaeothripidae		sp.1	2					
Thysanura								
Nicoletiidae		sp.1	1					
Malacostraca								
Isopoda								
Philosciidae		sp.1			2			

Philosciidae		sp.2			1		
Pauropoda							
Tetramerocerata		sp.	1				
Chordata							
Amphibia							
Anura							
Neobatrachia							
Strabomantidae							
	Pristimantis	*fenestratus*			2	0,04	
Mammalia							
Chiroptera							
Phyllostomidae		sp.			2	0,06	
Mollusca							
Gastropoda							
Systrophiidae							
	Happia	sp.	2				
