# Supplementary material for: Reconciling Mining with the Conservation of Cave Biodiversity: A Quantitative Baseline to Help Establish Conservation Priorities
Source: PLoS One. 2016 Dec 20;11(12):e0168348. doi: 10.1371/journal.pone.0168348 (PMC5173368; doi:10.1371/journal.pone.0168348)
Supplement: S1 Dataset — (ZIP) [file pone.0168348.s002.zip › Taxa/Serra Sul/SS_2010/S11D-80.pdf]

S11D-80			1ª	AB	2ª	AB	ZON
Arthropoda							
Arachnida							
Acari							
Ixodida							
Argasidae							
Ornithodoros		sp.	2		2		E P
Parasitiformes							
Mesostigmata		sp.4	2				P
Trombidiformes							
Tydeoidea							
Eupodidae		sp.1	1				P
Eupodidae		sp.2	1				E
Amblypygi							
Charinidae	Jovem		4	0,045			P
Charinus		sp.	5	0,056	4	0,0889	P
Charinus		sp.2			3	0,0667	P
Phrynidae							
Heterophrynus		sp.	2	0,022			
Araneae							
Ctenidae	Jovem		2	0,022			E
Drymusidae	Jovem		1				E
Ochyroceratidae	Jovem		3		1		E P
Ochyrocera		sp.1	1		1		E P
Oonopidae	Jovem				1		P
gr. Xycarphius		sp.5	2				E P
Pholcidae	Jovem		1				P
Ninetinae		sp.1	1		1		E
Salticidae	Jovem		1		1		E P
Segestriidae	Jovem		1		1		E P
Ariadna		sp.1	2		2		E P
Tetrablemmidae	Jovem		1				E
Matta		sp.1	3		1		E P
Theridiosomatidae	Jovem		1				E
Plato		sp.1	1		1		P
Opiliones							
Laniatores							
Escadabiidae	Jovem		1				P
Escadabiidae		sp.7	1				P
Stygnidae	Jovem		5	0,056			E P
Stygnidae		sp.1			2	0,0444	P
Pseudoscorpiones							
Bochicidae		sp.1			2		E
Chernetidae	Jovem		2				P
Spelaeochernes		sp.1	1				E
Pseudochthonius		sp.1	1				P
Ricinulei							
Ricinoididae							
Cryptocellus		sp.	1				E
Schizomida							
Hubbardiidae							
Rowlandius		sp.	1				P
Scorpiones							
Buthidae	Jovem				2	0,0444	E
Chilopoda	Jovem		2	0,022			E
Pleurostigmophora							
Geophilomorpha							
Diplopoda							
Polydesmida							
Chelodesmidae		sp.5	2	0,022			P
Fuhrmannodesmidae		sp.1	1				P
Pyrgodesmidae	Jovem		1				E
		sp.2	2	0,022	2	0,0444	P

Polyxenida							
Hypogexenidae		sp.1	1				E
Spirostreptida							
Entognatha							
Diplura							
Campodeidae		sp.1	3		2		E P
Japygidae		sp.1	1				P
Insecta							
Coleoptera							
Carabidae	Jovem		2				E P
Carabidae		sp.10	1				E
Collembola							
Arthropleona							
Entomobryoidea							
Entomobryidae		sp.4	1				E
Paronellidae		sp.1	1				E
Paronellidae		sp.4	2				P
Diptera							
Nematocera							
Psychodidae							
	Breviscapus	sp.	1				P
	Sciopemyia	*sordellii*	1		1		E P
Sciaridae	Jovem		1				P
Sciaridae		sp.	1				E
Hemiptera							
Heteroptera							
aff. Pyrrhocoroidea							
Reduviidae	Jovem				2	0,0444	E
Schizopteridae							
	Schizopterinae	sp.1			1		P
Tingidae							
	Thaumamannia	sp.1	1				P
Homoptera							
Cixiidae	Jovem		5				E P
Cixiidae		sp.1	1				E
Cixiidae		sp.2	1				P
Hymenoptera							
Chalcidoidea		sp.1	1				E
Vespoidea							
Formicidae							
	Camponotus	*atriceps*	1				E
	Carebara	sp.1	1				E
	Crematogaster	sp.1			1		E
	Hypoponera	sp.1	1				E
	Nylanderia	sp.1	3				E P
	Solenopsis	sp.2	1				P
Isoptera							
Termitidae							
	Labiotermes	sp.			2		E
	Labiotermes	sp.			1		P
Lepidoptera							
Noctuoidea							
Noctuidae							
Noctuidae	Jovem		1		2		E P
Noctuidae		sp.2	1		1		E P
Noctuidae		sp.1			2	0,0444	E
Orthoptera							
Ensifera							
Phalangopsidae	Jovem		2	0,022			E
	Phalangopsis	sp.1	64	0,711	25	0,5556	E P
Thysanura							
Nicoletiidae		sp.1	1				P
Malacostraca							
Isopoda							
Dubioniscidae		sp.1	1				E

Philosciidae		sp.1	1				E
Pauropoda							
Tetramerocerata		sp.	1				P
Chordata							
Amphibia							
Anura							
Neobatrachia							
Strabomantidae							
	Pristimantis	*fenestratus*			1	0,0222	E
Mammalia							
Chiroptera							
Emballonuridae							
	Peropteryx	sp.			1	0,0444	E
Mollusca							
Gastropoda							
Bulimulidae							
	Naesiotus	sp.	1				P
Systrophiidae							
	Happia	sp.	1				P
