# Supplementary material for: Reconciling Mining with the Conservation of Cave Biodiversity: A Quantitative Baseline to Help Establish Conservation Priorities
Source: PLoS One. 2016 Dec 20;11(12):e0168348. doi: 10.1371/journal.pone.0168348 (PMC5173368; doi:10.1371/journal.pone.0168348)
Supplement: S1 Dataset — (ZIP) [file pone.0168348.s002.zip › Taxa/Serra Sul/SS_2010/S11D-81.pdf]

S11D-81				1ª	AB	2ª	AB	ZON
Annelida								
Clitellata								
Oligochaeta		jovens		2	0,012			A
Arthropoda								
Arachnida								
Acari								
Ixodida		jovens				1		P
	Argasidae							
		Ornithodoros	sp.	3				E P
	Ixodidae							
		Amblyomma	sp.			1		P
Parasitiformes								
Mesostigmata								
	Heterozerconidae		sp.1	1				P
	Heterozerconidae		sp.2			1		P
	Heterozerconidae		sp.4			1		P
Sarcoptiformes								
Oribatida			sp.12	1				E
Oribatida			sp.13			1		P
Oribatida			sp.3			1		P
Oribatida			sp.2	1				A
Amblypygi								
	Charinidae					2	0,021	A
	Phrynidae							
		Heterophrynus	sp.			2	0,021	P
Araneae								
	Araneidae			1		1		E
	Ctenidae			2	0,012	2	0,021	P A
	Drymusidae					1		P
	Ochyroceratidae		sp.1	1				A
		Ochyrocera	sp.1	5		5		E P A
		Speocera	sp.1	2		2		E P A
	Oonopidae	jovens		3		1		P A
		gr. Xycarphius	sp.3	1				E
			sp.4	2				P
			sp.5	3				P A
		Oonopinae	sp.1			1		P
	Pholcidae							
		Ninetinae	sp.1	1		1		E A
	Scytodidae	jovens		2	0,023	1	0,01	E P
		Scytodes	*globula*	2				E
	Segestriidae	jovens		1		1		E P
		Ariadna	sp.1	2		1		E P A
	Tetrablemmidae	joven		2				E
		Matta	sp.1	5		5		P A
	Theraphosidae	jovens		2	0,012			E
	Theridiosomatidae							
		Plato	sp.1	3				E A
Opiliones								
Laniatores		jovens		6	0,035	8	0,084	A
	Escadabiidae		sp.1	2		1		P A
	Stygnidae	jovens		12	0,088	10	0,168	E P A
			sp.1	3		6		P A
Pseudoscorpiones								
	Bochicidae		sp.1	2		2		P
	Chernetidae	jovens		2				A
		Spelaeochernes	sp.1	6		6		E P A
	Chthoniidae	jovens				2		A
		Pseudochthonius	sp.1	4		5		E P A
Ricinulei								
	Ricinoididae	jovens		1				A
Scorpiones								
	Buthidae	jovens		2	0,012			E
		Ananteris	*balzanii*			4	0,042	P A

Chilopoda							
Notostigmophora							
Scutigeromorpha							
Pselliodidae	jovens		3				P A
Pleurostigmophora							
Geophilomorpha							
Ballophilidae		sp.1	2	0,012			E
Geophilidae		sp.1	2	0,012			E
Scutigeromorpha	jovens		2	0,012	2	0,021	A
Diplopoda							
Glomeridesmida							
Chelodesmidae		sp.4	5	0,03			A
Pyrgodesmidae		sp.2	4	0,023			P A
Polyxenida	jovens		1				
Hypogexenidae		sp.1	3		2		E P A
Entognatha							
Diplura							
Campodeidae		sp.1	7		3		E P A
Insecta							
Blattodea	jovens		4	0,023			
Blaberidae	jovens		10	0,059	6	0,063	E P
Collembola							
Arthropleona							
Entomobryoidea							
Isotomidae		sp.1	2				E P
Isotomidae		sp.3	1				A
Paronellidae		sp.1	3		2		E P A
Paronellidae		sp.3	1				P
Paronellidae		sp.4	6		3		E P A
Symphypleona							
Sminthuroidea		sp.1	1		1		A
Diptera							
Brachycera							
Camillidae		sp.			1		P
Dolichopodidae		sp.			2		P
Phoridae	Metopininae	sp.	1				A
Nematocera							
Mycetophilidae							
	Exechiopsis	sp.	1				A
Psychodidae							
	Pintomyia	*gruta*	1				P
	Sciopemyia	*sordellii*	2		2		E P
Hemiptera							
Heteroptera							
Dipsocoroidea							
Cydnidae							
	Cydninae	sp.1	1				P
Reduviidae	jovens		2	0,012	6	0,084	E P
	Reduviinae	sp.			2		P
Tingidae							
	Thaumamannia	sp.1			1		P
Homoptera	jovens		30				
Cixiidae	jovens		7		6		E P A
Cixiidae		sp.1	1				A
Hymenoptera							
Chrysidoidea							
Bethylidae		sp.2			1		E
Vespoidea							
Formicidae	jovens		1				E
	Apterostigma	sp.1			1		P
	Brachymyrmex	sp.1	1		1		E P
	Camponotus	*atriceps*	4		4		E P A
	Camponotus	sp.1			2		E A
	Cephalotes	sp.1	1				E
	Gnamptogenys	*striatula*	1				E

Hypoponera	sp.1	3				E P
Myrmicocrypta	sp.1	1				A
Nylanderia	sp.1	9		6		E P A
Pachycondyla	*harpax*			1		P
Pachycondyla	*striata*	1		1		P A
Pseudomyrmex	sp.1	1				A
Wasmania	*auropunctata*			1		A
Isoptera						
Termitidae						
Cavitermes	sp.			1		A
Nasutitermes	sp.	1				E
Lepidoptera	sp.1					
Noctuoidea	jovens	4		2		E P A
Noctuidae	sp.2	2				E
Orthoptera						
Ensifera						
Phalangopsidae						
Paraclodes	sp.1			3	0,032	P
Phalangopsis	sp.1	89	0,523	35	0,37	P
Thysanura						
Nicoletiidae	jovens	1				A
Nicoletiidae	sp.1	5		4		E P A
Malacostraca						
Isopoda						
Dubioniscidae	sp.1	1				A
Philosciidae	sp.1	4		1		P A
Pauropoda						
Tetramerocerata	sp.			1		P
Symphyla						
Scutigerellidae						
Hanseniella	sp.1	1				E
Chordata						
Amphibia						
Anura						
Neobatrachia						
Leiuperidae						
Physalaemus	sp.	2	0,012			
Mammalia						
Chiroptera	sp.			5	0,063	P
Emballonuridae						
Peropteryx	*kappleri*	6	0,041			
Furipteridae						
Furipterus	*horrens*	8	0,047			
Mollusca						
Gastropoda						
Subulinidae						
Lamellaxis	sp.	2				P
Systrophiidae						
Happia	sp.	3				P A
