# Supplementary material for: Reconciling Mining with the Conservation of Cave Biodiversity: A Quantitative Baseline to Help Establish Conservation Priorities
Source: PLoS One. 2016 Dec 20;11(12):e0168348. doi: 10.1371/journal.pone.0168348 (PMC5173368; doi:10.1371/journal.pone.0168348)
Supplement: S1 Dataset — (ZIP) [file pone.0168348.s002.zip › Taxa/Serra Sul/SS_2010/S11D-82.pdf]

| S11D-82 | | 1ª | AB | 2ª | AB |
|---|---|---|---|---|---|
| Arthropoda | | | | | |
| Arachnida | | | | | |
| Acari | | | | | |
| Ixodida | | | | | |
| Argasidae | | | | | |
| *Ornithodoros* | sp. | 1 | | | |
| Araneae | | | | | |
| Araneidae | joven | | | 1 | |
| Pholcidae | | | | | |
| *Leptopholcus* | sp.1 | 1 | | 1 | |
| *Ninetinae* | sp.1 | | | 1 | |
| Segestriidae | joven | 1 | | | |
| Uloboridae | | | | | |
| *Uloborus* | sp.1 | | | 1 | |
| Opiliones | | | | | |
| Laniatores | | | | | |
| Stygnidae | joven | | | | |
| Stygnidae | sp.1 | 4 | 0,167 | | |
| Diplopoda | | | | | |
| Glomeridesmida | | | | | |
| Polyxenida | | | | | |
| Hypogexenidae | sp.1 | 1 | | | |
| Insecta | | | | | |
| Coleoptera | | | | | |
| Carabidae | sp.4 | 1 | | | |
| Collembola | | | | | |
| Arthropleona | | | | | |
| Entomobryoidea | | | | | |
| Paronellidae | sp.4 | 1 | | | |
| Diptera | | | | | |
| Brachycera | | | | | |
| Nematocera | | | | | |
| Cecidomyiidae | | | | | |
| Cecidomyiinae | sp. | 1 | | | |
| Culicidae | | | | | |
| *Culicini* | sp. | 1 | | | |
| Hemiptera | | | | | |
| Heteroptera | | | | | |
| Dipsocoroidea | | | | | |
| Lygaeidae | sp.1 | 1 | | | |
| *Thaumamannia* | sp.1 | 1 | | | |
| Homoptera | | | | | |
| Cixiidae | sp.3 | | | 1 | |
| Cixiidae | sp.4 | 1 | | | |
| Hymenoptera | | | | | |
| Vespoidea | | | | | |
| Formicidae | | | | | |
| *Dolichoderus* | *bispinosus* | 1 | | 1 | |
| Isoptera | sp. | | | 1 | |
| Lepidoptera | | | | | |
| Noctuoidea | sp.2 | | | | |
| Lepidoptera | joven | 21 | 0,833 | | |
| Orthoptera | | | | | |
| Ensifera | | | | | |
| Phalangopsidae | | | | | |
| *Paraclodes* | sp. | | | 2 | 1 |
| Psocoptera | | | | | |
| Psocomorpha | joven | 1 | | | |
