# Supplementary material for: Reconciling Mining with the Conservation of Cave Biodiversity: A Quantitative Baseline to Help Establish Conservation Priorities
Source: PLoS One. 2016 Dec 20;11(12):e0168348. doi: 10.1371/journal.pone.0168348 (PMC5173368; doi:10.1371/journal.pone.0168348)
Supplement: S1 Dataset — (ZIP) [file pone.0168348.s002.zip › Taxa/Serra Sul/SS_2010/S11D-83.pdf]

S11D-83		1ª	AB	2ª	AB	ZON
Arthropoda						
Arachnida						
Acari						
Ixodida						
Ixodidae						
Amblyomma	sp.	1				E
Ixodes	sp.			1		E
Parasitiformes						
Mesostigmata						
Laelapidae	sp.3	2		2		A
	sp.1	2				A
Parasitiformes	sp.7			1		A
Trombidiformes						
Tydeoidea	sp.1	1				A
Rhagidiidae	sp.2	1				E
Tydeidae	sp.1			2		A
Amblypygi						
Phrynidae						
Heterophrynus	sp.	3	0,001	4	0,006	A
Araneae						
Araneidae						
Alpaida	*septemmammata*	1				E
Metazygia	sp.1			1		A
Barychaelidae	joven	5	0,002	2	0,003	E
Filistatidae	joven	2		1		E A
	sp.1	1		2		E A
Ochyroceratidae						
Ochyrocera	sp.1	1				E
Oonopidae	joven	1				E
Pholcidae	joven	1		2		E P A
Leptopholcus	sp.1	1		1		E
Ninetinae	sp.1	7		4		E P A
Salticidae	joven	1				E
Scytodidae	joven	8		4		E P A
Scytodes	*eleonorae*	7	0,003	11	0,017	P A
Scytodes	*globula*	15	0,006	2	0,003	E P A
Scytodes	sp.	13	0,006	35	0,052	P
Segestriidae	joven	1				A
Ariadna	sp.1	3		3		A
Opiliones						
Laniatores						
Stygnidae	sp.1	4	0,002			E
Pseudoscorpiones						
Chernetidae	joven			4		P A
Spelaeochernes	sp.1	1		1		P
Chthoniidae						
Pseudochthonius	sp.1	1				E
Olpiidae	sp.1	2		2		E
Entognatha						
Diplura						
Campodeidae	sp.1	1				E
Insecta						
Blattodea						
Blattidae	joven			2	0,003	E
Coleoptera	joven	6		3		E P A
Carabidae	sp.10					
Dytiscidae	sp.3			1		P
Histeridae	sp.1	3		1		A
Hydrophilidae						
Sphaeridiinae	sp.3	1		1		A
Sphaeridiinae	sp.4	1				A
Collembola						
Arthropleona						
Entomobryoidea						

Paronellidae		sp.1	2		1		E		
Diptera									
Brachycera									
Chloropidae		sp.	6					P	A
Drosophilidae									
	Drosophila	*eleonore*	1				E		
Milichiidae		sp.	1		3				A
Muscidae		sp.	2					P	
Streblidae									
	Trichobius	sp.	1		3				A
Nematocera	joven		7		2		E	P	A
Cecidomyiidae									
	Cecidomyiinae	sp.	1				E		
Culicidae									
	Culicini	sp.	2				E	P	
Psychodidae									
	Lutzomyia	*longipalpis*	1		1		E		
	Sciopemyia	*sordellii*	1				E		
Hemiptera									
Heteroptera									
Dipsocoroidea	joven		28						
Cydnidae	joven		1				E		
Mesoveliidae	joven				1			P	
Veliidae	joven								
	Paravelia	sp.2	2					P	
Homoptera									
Cixiidae	joven		1				E		
Cixiidae		sp.1	1				E		
Hymenoptera									
Diaprioidea									
Diapriidae		sp.1	2					P	
Vespoidea									
Formicidae	joven				1		E		
	Acromyrmex								
	Camponotus	*atriceps*	2		2				A
	Dolichoderus	*bispinosus*			1		E		
	Pachycondyla	*striata*	1		1		E		
	Platythyrea	*angusta*	1		2		E		A
	Solenopsis	sp.2	2		1			P	A
	Strumygenys	sp.1	1				E		
Isoptera									
Termitidae									
	Nasutitermes	sp.	1				E		
Lepidoptera	joven		523	0,228	9	0,013	E	P	A
Noctuoidea		sp.2	1				E		
Noctuidae		sp.2	5	0,002			E		
Tineoidea		sp.1	4		2		E	P	A
Tineoidea		sp.2	6		1			P	A
Orthoptera									
Ensifera									
Phalangopsidae	joven		2	0,001			E		
	Phalangopsis	sp.1	###	0,441	186	0,28		P	A
	Paraclodes	sp.			3	0,005	E		
Psocoptera									
Psocomorpha	joven		1				E		
Trogiomorpha									
Psyllipsocidae	joven				2		E		A
	Psocathropos	sp.1	1				E		
	Psyllipsocus	sp.1	2				E		A
Thysanura									
Nicoletiidae	joven		1				E		
Nicoletiidae		sp.1	1				E		
Malacostraca									
Isopoda									
Dubioniscidae	joven		1				E		

Dubioniscidae	sp.1	3				E
Symphyla						
Scutigerellidae						
Hanseniella	sp.1			1		
Chordata						
Amphibia						
Anura						
Neobatrachia	sp.			2	0,003	P
Leptodactylidae						
Leptodactylus	sp.	3	0,001			
Mammalia						
Chiroptera						
Emballonuridae						
Peropteryx	sp.			10	0,015	P
Natalidae						
Natalus	*cf. stramineus*	200	0,087	100	0,15	
Phyllostomidae						
Glossophaga	*soricina*	500	0,22	300	0,45	
Mollusca						
Gastropoda						
Bulimulidae						
Naesiotus	sp.	1				E
