# Supplementary material for: Reconciling Mining with the Conservation of Cave Biodiversity: A Quantitative Baseline to Help Establish Conservation Priorities
Source: PLoS One. 2016 Dec 20;11(12):e0168348. doi: 10.1371/journal.pone.0168348 (PMC5173368; doi:10.1371/journal.pone.0168348)
Supplement: S1 Dataset — (ZIP) [file pone.0168348.s002.zip › Taxa/Serra Sul/SS_2010/S11D-84.pdf]

S11D-84		1ª	AB	2ª	AB	ZON
Arthropoda						
Arachnida						
Acari						
Ixodida						
Argasidae						
Ornithodoros	sp.	1				P
Sarcoptiformes						
Oribatida	sp.2			1		P
	sp.3	2				E P
Amblypygi						
Phrynidae						
Heterophrynus	sp.	4	0,15			P
Araneae						
Araneidae	jovens			1		P
Ochyroceratidae	jovens	1				P
Salticidae	jovens	1				E P
Symphytognathidae	jovens	1				E P
Theridiosomatidae	jovens			1		E P
Plato	sp.1	2				E P
Opiliones						
	jovens			6	0,16	P
Laniatores						
Escadabiidae	sp.1	1				P
Stygnidae	jovens	4	0,15			E P
	sp.1			5	0,13	E P
Pseudoscorpiones						
Chernetidae						
Spelaeochernes	sp.1	2				E P
Polyxenida						
Hypogexenidae	sp.1	1				P
Insecta						
Blattodea						
Blattellidae	sp.3			5	0,13	P
Coleoptera	jovens			1		P
Collembola						
Arthropleona						
Entomobryoidea						
Entomobryidae	sp.1			1		E P
Diptera						
Brachycera						
Dolichopodidae	sp.			1		E P
Nematocera						
Cecidomyiidae						
Cecidomyiinae	sp.			1		P
Culicidae						
Culicini	sp.	1		1		E P
Hemiptera						
Heteroptera						
Lygaeidae	sp.2	1				P
Reduviidae	jovens			2	0,05	E P
Tingidae	jovens	1				P
Dipsocoroidea	jovens			1		P
Homoptera						
Cixiidae	jovens			1		P
	sp.1	2				E P
Hymenoptera						
Vespoidea						
Formicidae						
Hypoponera	sp.1			1		P
Pachycondyla	*striata*	1		1		E P
Wasmania	*auropunctata*			1		E P
Isoptera						
	sp.	1				E P
Termitidae						
Nasutitermes	sp.			1		E P

Lepidoptera	jovens	2				E P
Orthoptera						
Ensifera						
Phalangopsidae						
Phalangopsis	sp.1	3	0,11	17	0,45	P
Paraclodes	sp.	16	0,59	3	0,08	E P
Psocoptera						
Psocomorpha	jovens	1				E P
Thysanura						
Nicoletiidae	sp.1	1				P
Mollusca						
Gastropoda						
Systrophiidae						
Happia	sp.	1				P
