# Supplementary material for: Reconciling Mining with the Conservation of Cave Biodiversity: A Quantitative Baseline to Help Establish Conservation Priorities
Source: PLoS One. 2016 Dec 20;11(12):e0168348. doi: 10.1371/journal.pone.0168348 (PMC5173368; doi:10.1371/journal.pone.0168348)
Supplement: S1 Dataset — (ZIP) [file pone.0168348.s002.zip › Taxa/Serra Sul/SS_2010/S11D-85.pdf]

S11D-85			1ª	AB	2ª	AB	ZON
Arthropoda							
Arachnida							
Acari							
Ixodida							
Ixodidae							
	Amblyomma	sp.	1				E
Araneae							
Araneidae	jovens		2		1		E
Barychaelidae	jovens		2	0,11			E
Filistatidae	jovens		1				E
		sp.1	1				E
Mysmenidae							
	Microdipoena	sp.1	1				E
Oonopidae							
	gr. Xycarphius	sp.2	1				E
Pholcidae							
	aff. Ibityporanga	sp.1	1				E
	Leptopholcus	sp.1	1				E
	Ninetinae	sp.1	1				E
Scytodidae	jovens		2	0,23			E
	Scytodes	sp.	2				
Pseudoscorpiones							
Olpiidae		sp.1	2				E
Insecta							
Blattodea							
Blattidae		sp.2	2	0,11			E
Coleoptera							
	jovens		1				E
		sp.6	1				E
Diptera							
Brachycera							
Camillidae		sp.	1				E
Drosophilidae	*Drosophila*	*eleonore*	1				E
Nematocera							
Cecidomyiidae	ecidomyiinae	sp.	1				E
Hymenoptera							
Vespoidea							
Formicidae							
	Camponotus	*atriceps*	1				E
Isoptera							
Termitidae							
	Nasutitermes	sp.	2		1		E
Lepidoptera							
Cossoidea							
Limacodidae		sp.1	5	0,27			E
Noctuoidea							
		sp.2	1				E
Noctuidae		sp.2	3	0,17			E
Neuroptera							
Myrmeleonthidae	jovens		1		1		E
Psocoptera							
Psocomorpha	jovens		1				E
Trogiomorpha							
Psyllipsocidae							
	Psocathropos	sp.1	1				E
Chordata							
Reptilia							
Squamata							
Gekkonidae							
	Thecadactylus	*rapicauda*	2	0,11			
