# Supplementary material for: Reconciling Mining with the Conservation of Cave Biodiversity: A Quantitative Baseline to Help Establish Conservation Priorities
Source: PLoS One. 2016 Dec 20;11(12):e0168348. doi: 10.1371/journal.pone.0168348 (PMC5173368; doi:10.1371/journal.pone.0168348)
Supplement: S1 Dataset — (ZIP) [file pone.0168348.s002.zip › Taxa/Serra Sul/SS_2010/S11D-86.pdf]

S11D-86			1ª	AB	2ª	AB	ZON
Arthropoda							
Arachnida							
Acari							
Ixodida							
Ixodidae							
	Amblyomma	sp.	2		1		E
Parasitiformes							
Opilioacarida							
Opilioacaridae		sp.1	1				E
Araneae							
Ctenidae		sp.3	2	0,07			E
Ochyroceratidae	jovens		1				E
	Ochyrocera	sp.1	1		1		E
	Speocera	sp.1	1				E
Oonopidae							
	Oonopinae	sp.1			1		E
Pholcidae	jovens				1		E
	Ninetinae	sp.1	3		1		E
Salticidae	jovens				1		E
Scytodidae							
	Scytodes	*eleonorae*	6	0,23			E
	Scytodes	sp.1	1	0,03			E
Theridiidae	jovens				1		E
	Theridion	sp.1	1				E
Araneae	jovens		2				
Opiliones							
Laniatores							
Escadabiidae	jovens		1				E
		sp.1	1				E
Stygnidae	jovens		4	0,14			E
		sp.1	2	0,07			E
Polyxenida							
Hypogexenidae		sp.1	1				E
Insecta							
Blattodea	jovens		1	0,03			E
Blattidae	jovens		1	0,03			E
Collembola							
Arthropleona							
Entomobryoidea							
Paronellidae		sp.1	1				E
Dermaptera	jovens		1				E
Diptera							
Brachycera							
Camillidae		sp.			1		E
Nematocera							
Cecidomyiidae							
	Cecidomyiinae	sp.	1				E
Psychodidae							
	Sciopemyia	*sordellii*	1				E
Homoptera							
Cixiidae	jovens		2		1		E
Hymenoptera							
	jovens		1				E
Vespoidea							
Formicidae							
	Camponotus	*atriceps*	1				E
		sp.1	2	0,07			E
	Crematogaster	sp.1	2		1		E
	Gnamptogenys	*striatula*	1				E
	Hypoponera	sp.1	1		1		E
	Solenopsis	sp.2			1		E
Isoptera		sp.	1				E
Lepidoptera							
	jovens				1		E

Cossoidea							
Limacodidae		sp.1	2	0,07			E
Noctuoidea							
Noctuidae		sp.2	4	0,14			E
Orthoptera							
Ensifera							
Phalangopsidae	jovens		3	0,1			
	Paraclodes	sp.			2	0,4	E
Psocoptera							
Psocomorpha	jovens		2				E
Trogiomorpha							
Psyllipsocidae	jovens				2		E
Malacostraca							
Isopoda							
Dubioniscidae		sp.1	1				E
Chordata							
Amphibia							
Anura		sp.			1	0,2	E
Neobatrachia							
Strabomantidae							
	Pristimantis	*fenestratus*	2	0,02			
Chiroptera		sp.			1	0,4	E
