# Supplementary material for: Reconciling Mining with the Conservation of Cave Biodiversity: A Quantitative Baseline to Help Establish Conservation Priorities
Source: PLoS One. 2016 Dec 20;11(12):e0168348. doi: 10.1371/journal.pone.0168348 (PMC5173368; doi:10.1371/journal.pone.0168348)
Supplement: S1 Dataset — (ZIP) [file pone.0168348.s002.zip › Taxa/Serra Sul/SS_2010/S11D-87.pdf]

S11D-87			1ª	AB	2ª	AB	ZON
Arthropoda							
Arachnida							
Acari							
Ixodida							
Argasidae							
	Ornithodoros	sp.	1		1		E P
Parasitiformes							
Mesostigmata		sp.2			1		P
Araneae	jovens		2	0,04			
Barychaelidae	jovens				2	0,125	E
Ochyroceratidae	jovens		2				E P
	Speocera	sp.1			1		P
Oonopidae							
	Capitato	sp.1			1		E
Pholcidae							
	Leptopholcus	sp.1	1		1		E P
	Ninetinae	sp.1	1		2		E P
Scytodidae	jovens		1				P
	Scytodes	*globula*	2	0,04			E
		sp.	2	0,04	3	0,1875	E
Segestriidae	jovens		1				P
Theridiosomatidae	jovens		1				E
	Plato	sp.1	1				P
Opiliones							
	jovens				4	0,25	P
Laniatores							
Stygnidae		sp.1			4	0,25	E P
Pseudoscorpiones							
Bochicidae		sp.1	2		2		P
Chthoniidae							
	Pseudochthonius	sp.1			1		P
Olpiidae		sp.1	2		2		E
Blattodea	jovens		8	0,2			E
Coleoptera							
Chrysomelidae		sp.8	1				E
Staphylinidae							
	Pselaphinae	sp.2	1				E
Collembola							
Arthropleona							
Entomobryoidea							
Entomobryidae		sp.1			1		P
		sp.4			1		P
Paronellidae		sp.1	2				E P
Diptera							
	jovens	(vazio)	1				E
Nematocera							
Cecidomyiidae	Cecidomyiinae	sp.	3				E P
Culicidae	Culicini	sp.	2				E
Psychodidae	*Breviscapus*	sp.					
	Sciopemyia	*sordellii*	1				E
Homoptera	jovens		16				
Cixiidae	jovens		2				E P
Cixiidae		sp.3			1		E
Hymenoptera							
Vespoidea							
Formicidae							
	Camponotus	*atriceps*	1				E
		sp.1			1		E
	Crematogaster	sp.1	1				E
	Octostruma	sp.1			1		P
	Pachycondyla	*striata*	1				E
Isoptera		sp.	1				E
Lepidoptera							
	jovens		2				E
Cossoidea							

Limacodidae		sp.1	4	0,1			E P
Noctuoidea		sp.2	1				E
Orthoptera							
Ensifera							
Phalangopsidae	jovens		23	0,58			
	Paraclodes	sp.			3	0,1875	P
Psocoptera							
Psocomorpha							
	jovens	(vazio)			2		E P
Epipsocidae							
	Epipsocus	sp.2			1		E
Symphyla							
Scutigerellidae							
	Hanseniella	sp.1	1		1		P
Mollusca							
Gastropoda							
Systrophiidae							
	Happia	sp.	1				E
