# Supplementary material for: Reconciling Mining with the Conservation of Cave Biodiversity: A Quantitative Baseline to Help Establish Conservation Priorities
Source: PLoS One. 2016 Dec 20;11(12):e0168348. doi: 10.1371/journal.pone.0168348 (PMC5173368; doi:10.1371/journal.pone.0168348)
Supplement: S1 Dataset — (ZIP) [file pone.0168348.s002.zip › Taxa/Serra Sul/SS_2010/S11D-88.pdf]

| S11D-88 | | 1ª | AB | 2ª | AB | ZON |
|---|---|---|---|---|---|---|
| Annelida | | | | | | |
| Clitellata | | | | | | |
| Oligochaeta | jovens | 2 | 0,01 | | | E |
| Arthropoda | | | | | | |
| Arachnida | | | | | | |
| Acari | | | | | | |
| Ixodida | | | | | | |
| Argasidae | | | | | | |
| *Ornithodoros* | sp. | 3 | | 1 | | P |
| Parasitiformes | | | | | | |
| Mesostigmata | | | | | | |
| Ologamasidae | sp.4 | | | 1 | | E |
| Sarcoptiformes | sp.1 | 1 | | | | E |
| Trombidiformes | sp.2 | 1 | | | | P |
| Amblypygi | | | | | | |
| Phrynidae | | | | | | |
| *Heterophrynus* | sp. | | | 4 | 0,04 | P |
| Araneae | | | | | | |
| Ochyroceratidae | jovens | 1 | | 1 | | P |
| *Ochyrocera* | sp.1 | 3 | | | | E P |
| *Speocera* | sp.1 | 3 | | 1 | | P |
| Pholcidae | | | | | | |
| Ninetinae | sp.1 | 5 | | 3 | | E P |
| Scytodidae | jovens | 4 | 0,02 | 2 | 0,02 | P |
| *Scytodes* | *eleonorae* | 2 | 0,01 | | | P |
| *Scytodes* | sp. | | | 2 | 0,02 | P |
| Tetrablemmidae | jovens | 1 | | | | P |
| *Matta* | sp.1 | | | 2 | | P |
| Theridiosomatidae | jovens | 1 | | | | E |
| *Plato* | sp.1 | 1 | | 2 | | P |
| Opiliones | | | | | | |
| Laniatores | | | | | | |
| Escadabiidae | jovens | | | 1 | | E |
| | sp.1 | 3 | | 1 | | E P |
| Stygnidae | jovens | 2 | 0,01 | | | E |
| | sp.1 | 8 | 0,05 | 12 | 0,12 | E P |
| Pseudoscorpiones | | | | | | |
| Bochicidae | sp.1 | 4 | | | | P |
| Chernetidae | jovens | | | 2 | | P |
| Chthoniidae | | | | | | |
| *Pseudochthonius* | sp.1 | 3 | | 2 | | E P |
| Schizomida | | | | | | |
| Hubbardiidae | | | | | | |
| *Rowlandius* | sp. | | | 1 | | P |
| Chilopoda | jovens | 3 | 0,02 | | | |
| Notostigmophora | | | | | | |
| Scutigeromorpha | | | | | | |
| Pselliodidae | jovens | 1 | | 2 | | E P |
| Pleurostigmophora | | | | | | |
| Scolopendromorpha | | | | | | |
| Scolopocryptopidae | | | | | | |
| *Dinocryptops* | *miersii* | 2 | 0,01 | | | P |
| *Newportia* | sp.1 | | | 2 | 0,02 | P |
| Polyxenida | | | | | | |
| Hypogexenidae | sp.1 | 1 | | 1 | | P |
| Insecta | | | | | | |
| Blattodea | jovens | 4 | 0,04 | | | P |
| Blaberidae | jovens | 2 | | 2 | 0,02 | P |
| Coleoptera | jovens | 2 | | | | P |
| Collembola | | | | | | |
| Arthropleona | | | | | | |

| | | | | | | | |
|---|---|---|---|---|---|---|---|
| Entomobryoidea | | | | | | | |
| Isotomidae | | sp.1 | 1 | | | | P |
| Paronellidae | | sp.1 | 1 | | | | E |
| | | sp.4 | | | 1 | | E |
| Symphypleona | | | | | | | |
| Sminthuroidea | | sp.2 | 1 | | | | P |
| Diptera | | | | | | | |
| Nematocera | | | | | | | |
| Psychodidae | | | | | | | |
| | *Pintomyia* | *gruta* | 1 | | | | P |
| | *Sciopemyia* | *sordellii* | 4 | | 3 | | E P |
| Hemiptera | | | | | | | |
| Heteroptera | | | | | | | |
| Dipsocoroidea | jovens | | | | 1 | | E |
| Homoptera | | | | | | | |
| Cixiidae | jovens | (vazio) | 4 | | 2 | | E P |
| Hymenoptera | | | | | | | |
| Vespoidea | | | | | | | |
| | *Gnamptogenys* | *striatula* | 1 | | | | P |
| | *Labidus* | *coecus* | 3 | | | | E P |
| Isoptera | | | | | | | |
| | | sp. | | | 1 | | P |
| Termitidae | | | | | | | |
| | *Cortaritermes* | *silvestrii* | 1 | | | | P |
| | *Nasutitermes* | sp. | 3 | | 2 | | E P |
| Lepidoptera | | | | | | | |
| Cossoidea | | | | | | | |
| Limacodidae | | sp.1 | 9 | 0,05 | | | P |
| Tineoidea | | sp.1 | 1 | | | | P |
| Lepidoptera | jovens | | 2 | | 2 | | E P |
| Orthoptera | | | | | | | |
| Ensifera | | | | | | | |
| Phalangopsidae | jovens | | 1 | 0,01 | | | P |
| | *Phalangopsis* | sp.1 | 115 | 0,68 | 66 | 0,67 | P |
| Psocoptera | | | | | | | |
| Psocomorpha | jovens | | 1 | | | | P |
| Trogiomorpha | | | | | | | |
| Psyllipsocidae | jovens | | | | 1 | | P |
| | *Psyllipsocus* | sp.1 | 1 | | | | P |
| Chordata | | | | | | | |
| Amphibia | | | | | | | |
| Anura | | | | | | | |
| Neobatrachia | | | | | | | |
| Strabomantidae | | | | | | | |
| | *Pristimantis* | *fenestratus* | 3 | 0,02 | 6 | 0,06 | P |
| Mammalia | | | | | | | |
| Chiroptera | | | | | | | |
| Emballonuridae | | | | | | | |
| | *Peropteryx* | *kappleri* | 4 | 0,03 | | | |
| Phyllostomidae | | sp.1 | | | | | |
| Glossophaginae | | sp. | 5 | 0,04 | 2 | 0,03 | |
