# Supplementary material for: Reconciling Mining with the Conservation of Cave Biodiversity: A Quantitative Baseline to Help Establish Conservation Priorities
Source: PLoS One. 2016 Dec 20;11(12):e0168348. doi: 10.1371/journal.pone.0168348 (PMC5173368; doi:10.1371/journal.pone.0168348)
Supplement: S1 Dataset — (ZIP) [file pone.0168348.s002.zip › Taxa/Serra Sul/SS_2010/S11D-89.pdf]

S11D-89		1ª	AB	2ª	AB	ZON
Arthropoda						
Arachnida						
Acari						
Parasitiformes						
Mesostigmata	sp.4	1				E P
Trombidiformes	sp.2	1				E P
Araneae						
Araneidae						
Alpaida	*septemmammata*			1		P
Corinnidae jovens				2	0,1	P
Ochyroceratidae jovens		1		2		E P
Ochyrocera	sp.3	1				P
Speocera	sp.1	1				P
Oonopidae						
gr. Xycarphius	sp.5	1				E P
Pholcidae						
Leptopholcus	sp.1			1		P
Scytodidae jovens		1				P
Tetrablemmidae jovens		1				P
Matta	sp.1			1		E P
Theridiosomatidae						
Plato	sp.1	2		1		E P
Opiliones						
Laniatores						
Escadabiidae	sp.1	1				P
Stygnidae	sp.1	4	0,2	2	0,1	E P
Pseudoscorpiones						
Bochicidae	sp.1	2		2		E P
Chernetidae						
Spelaeochernes	sp.1			1		E P
Chthoniidae jovens		2				P
Olpiidae	sp.1			2		P
Schizomida						
Hubbardiidae jovens		1				P
Rowlandius	sp.	1				E P
Chilopoda						
Pleurostigmophora						
Scolopendromorpha						
Cryptopidae						
Cryptops	sp.1	2	0,1			E P
Entognatha						
Diplura						
Campodeidae	sp.1	2				E P
Insecta						
Collembola						
Arthropleona						
Entomobryoidea						
Isotomidae	sp.1			1		E P
Paronellidae	sp.1	1				E P
	sp.2			1		P
Diptera						
jovens		1				P
Brachycera						
Camillidae	sp.			1		P
Dolichopodidae	sp.			1		P
Nematocera						
Cecidomyiidae :idomyiinae	sp.	1				E P
Culicidae						
Culicini	sp.			1		P
Hemiptera						
Heteroptera						
Dipsocoroidea jovens				1		E P
Schizopteridae						
Schizopterinae	sp.1	1				P
Hymenoptera						

Vespoidea							
Formicidae							
	Acromyrmex	sp.1			1		P
	Dolichoderus	*bispinosus*	1				E P
	Myrmicocrypta	sp.1			1		E P
	Pachycondyla	*striata*	1				P
Lepidoptera	jovens		4	0,2	2	0,1	E P
Noctuoidea		sp.2	1				E P
Orthoptera							
Ensifera							
Phalangopsidae							
	Paraclodes	sp.	5	0,25	5	0,25	P
	Phalangopsis	sp.			5	0,25	P
Pscoptera							
Psocomorpha	jovens		1		1		E P
Thysanura							
Nicoletiidae		sp.1	2				E P
Chordata							
Amphibia							
Anura							
Neobatrachia							
Dendrobatidae		sp.	1	0,05			P
Mammalia							
Chiroptera							
		sp.			2	0,1	P
Emballonuridae							
	Peropteryx	*kappleri*	3	0,2			P
		sp.			2	0,1	P
Mollusca							
Gastropoda							
Systrophiidae							
	Happia	sp.	1				E P
