# Supplementary material for: Reconciling Mining with the Conservation of Cave Biodiversity: A Quantitative Baseline to Help Establish Conservation Priorities
Source: PLoS One. 2016 Dec 20;11(12):e0168348. doi: 10.1371/journal.pone.0168348 (PMC5173368; doi:10.1371/journal.pone.0168348)
Supplement: S1 Dataset — (ZIP) [file pone.0168348.s002.zip › Taxa/Serra Sul/SS_2010/S11D-90.pdf]

S11D-90		1ª	AB	2ª	AB	ZON
Arthropoda						
Arachnida						
Amblypygi						
Phrynidae						
Heterophrynus	sp.	2	0,09			E
Araneae						
Araneidae	jovens					
Alpaida	*septemmammata*	1				E
Salticidae	jovens			1		E
Scytodidae	jovens			2	0,06	E
Scytodes	*eleonorae*	3	0,14			E
	sp.	2	0,09			E
Theridiidae						
Theridion	sp.1	1				E
Opiliones						
Laniatores						
Stygnidae	sp.1			9	0,26	E
Insecta						
Blattodea	jovens			2	0,06	E
Collembola						
Paronellidae	sp.1			1		E
Diptera						
Cecidomyiidae						
Cecidomyiinae	sp.	1				E
Mycetophilidae						
Orfelia	sp.	1				E
Psychodidae						
Pintomyia	*gruta*			1		E
Sciopemyia	*sordellii*			1		E
Hymenoptera						
Formicidae						
Acromyrmex	sp.1			2		E
Isoptera						
Termitidae						
Diversitermes	sp.			1		E
Lepidoptera						
Noctuidae	sp.1	2	0,09			E
Orthoptera						
Phalangopsidae	jovens					
Paraclodes	sp.1	11	0,5	8	0,24	E
Phalangopsis	sp.1			7	0,2	
Psocoptera	jovens			1		E
Chordata						
Amphibia						
Anura						
Strabomantidae						
Pristimantis	*fenestratus*			2	0,06	E
Mammalia						
Chiroptera	sp.			2	0,12	E
Emballonuridae						
Peropteryx	*kappleri*	1	0,09			
