# Supplementary material for: Reconciling Mining with the Conservation of Cave Biodiversity: A Quantitative Baseline to Help Establish Conservation Priorities
Source: PLoS One. 2016 Dec 20;11(12):e0168348. doi: 10.1371/journal.pone.0168348 (PMC5173368; doi:10.1371/journal.pone.0168348)
Supplement: S1 Dataset — (ZIP) [file pone.0168348.s002.zip › Taxa/Serra Sul/SS_2010/S11D-91.pdf]

S11D-91	1ª	AB	2ª	AB	ZON
Arthropoda					
Arachnida					
Acari					
Argasidae					
Ornithodoros sp.	1				E
Ologamasidae sp.1	1				E
Araneae					
Araneidae jovens	1		2		E
Alpaida septemmammata	2				E
Alpaida smila	1				E
Ctenidae jovens	2	0,04			E
Ctenus sp.1	2				E
Nephiliidae jovens	2	0,03			E
Ochyroceratidae jovens	1				E
Pholcidae					
aff. Ibityporanga sp.1	2				E
Mesabolivar sp.1	1				E
Ninetinae sp.1	2		2		E
Salticidae jovens	4				E
Amphidraus sp.1	1				E
Scytodidae jovens	4	0,13			E
Scytodes sp.	7		5	0,0909	E
Segestriidae jovens	1				E
Theraphosidae jovens	4	0,04	2		E
Theridiosomatidae					
Plato sp.1	1				E
Stygnidae jovens	1	0,01	7	0,3091	E
sp.1			10		E
Pseudoscorpiones					
Chthoniidae					
Pseudochthonius sp.1	2				E
Olpiidae sp.1	4				E
Diplopoda					
Polyxenida					
Hypogexenidae sp.1	1				E
Spirostreptida					
Pseudonannolenidae jovens	2	0,03			E
Insecta					
Archaeognatha					
Meinertellidae sp.1	2	0,03			E
Blattodea jovens	17	0,2	3	0,0545	E
Blattellidae sp.1			2	0,0364	E
Polyphagidae jovens	2	0,03	2	0,0364	E
Coleoptera jovens	1				E
Staphylinidae					
Pselaphinae sp.7	1				E
Collembola					
Entomobryoidea					
Paronellidae sp.1	1				E
Diptera					
Cecidomyiidae					
Cecidomyiinae sp.	2				E
Ceratopogonidae sp.			1		E
Culicidae					
Culicini sp.			1		E
Psychodidae					
Pintomyia gruta	1				E
Sciopemyia sordellii	1				E
Hemiptera					
Homoptera					
Cixiidae sp.1	1				E
Hymenoptera					
Formicidae					
Camponotus atriceps	1				E

		Dolichoderus	*bispinosus*	2				E
		Gnamptogenys	*striatula*	3				E
		Odontomachus	*bauri*	2	0,03			E
		Pachycondyla	*harpax*	1				E
		Pheidole	sp.1	1				E
		Platythyrea	*angusta*	1				E
Isoptera								
	Termitidae	*Armitermes*	sp.					
		Nasutitermes	sp.	1				E
		Termes	sp.	1		1		E
Lepidoptera								
Castnioidea								
	Castniidae		sp.1	2	0,03			E
Cossoidea								
	Limacodidae		sp.1	5	0,05			E
	Noctuidae		sp.2	2	0,03			E
	Noctuidae		sp.1	2	0,03			E
Orthoptera								
	Phalangopsidae	jovens		11	0,17			E
		Paraclodes	sp.	3		13	0,2364	E
Psocoptera		jovens		3				E
	Ptiloneuridae							
		Triplocania	sp.9	1				E
	Manicapsocidae							
		Nothoentomum	sp.1	1				E
Thysanura								
	Nicoletiidae		sp.1	1				E
Malacostraca								
Isopoda								
	Dubioniscidae		sp.1	1				E
Chordata								
Amphibia								
Anura			sp.			1	0,0182	E
Mammalia								
Chiroptera								
	Emballonuridae							
		Peropteryx	*kappleri*	1	0,03			E
	Phyllostomidae		sp.			5	0,1818	E
		Glossophaga	*soricina*	4	0,09			E
