# Supplementary material for: Reconciling Mining with the Conservation of Cave Biodiversity: A Quantitative Baseline to Help Establish Conservation Priorities
Source: PLoS One. 2016 Dec 20;11(12):e0168348. doi: 10.1371/journal.pone.0168348 (PMC5173368; doi:10.1371/journal.pone.0168348)
Supplement: S1 Dataset — (ZIP) [file pone.0168348.s002.zip › Taxa/Serra Sul/SS_2010/S11D-92.pdf]

S11D-92			1ª	AB	2ª	AB	ZON
Arthropoda							
Arachnida							
Acari							
Opilioacarida							
Opilioacaridae		sp.1	1				E
Rhagidiidae		sp.1	1				E
Trombidiformes		sp.10	1				E
Araneae							
Araneidae	jovens				1		E
	Alpaida	*septemmammata*	1				E
Ctenidae	jovens		4	0,2			E
Filistatidae	jovens		2				E
Oonopidae							
	Oonopinae	sp.1	2				E
Pholcidae							
	Ninetinae	sp.1	1		2		E
Salticidae	jovens		1		1		E
	Amphidraus	sp.1	1				E
Scytodidae	jovens		2		1		E
		sp.1	1				E
Pseudoscorpiones							
Bochicidae		sp.1	2				E
Olpiidae		sp.1	6		2		E
Diplopoda							
Polydesmida							
Pyrgodesmidae		sp.3	2	0,11			E
Polyxenida							
Hypogexenidae		sp.1	2				E
Insecta							
Blattodea	jovens		4	0,2			E
Blattidae	jovens		2	0,11			E
Polyphagidae	jovens		2	0,11			E
Coleoptera	jovens		1				E
Collembola							
Entomobryoidea							
Cyphoderidae	@	sp.1					
Isotomidae		sp.1	1				E
Paronellidae		sp.1	3				E
		sp.2	1				E
		sp.4	1				E
Diptera	jovens		1				E
Nematocera							
Cecidomyiidae							
	Cecidomyiinae	sp.	1				E
Embioptera		sp.1	1		1		E
Hymenoptera							
Formicidae							
	Camponotus	*atriceps*			1		E
	Gnamptogenys	*striatula*	3		1		E
	Pheidole	sp.1	1				E
Lepidoptera	jovens		1				E
Cossoidea							
Limacodidae		sp.1	3	0,16			E
Noctuoidea		sp.3			1		E
Orthoptera							
Phalangopsidae							
	Paraclodes	sp.			4	0,66	E
Psocoptera	jovens		2				E
Psocomorpha							
Archipsocidae							
	Archipsocus	sp.1			1		E
		sp.2			1		E
Troctomorpha							
Liposcelididae							

		Liposcelis	sp.1	1				E
			sp.2	1				E
		Psyllipsocidae	jovens			1		E
Malacostraca								
	Isopoda							
		Dubioniscidae	sp.1	2				E
Chordata								
Mammalia								
	Chiroptera							
		Emballonuridae						
		Peropteryx	*kappleri*		0,11			E
		Phyllostomidae	sp.1				0,34	E
