# Supplementary material for: Reconciling Mining with the Conservation of Cave Biodiversity: A Quantitative Baseline to Help Establish Conservation Priorities
Source: PLoS One. 2016 Dec 20;11(12):e0168348. doi: 10.1371/journal.pone.0168348 (PMC5173368; doi:10.1371/journal.pone.0168348)
Supplement: S1 Dataset — (ZIP) [file pone.0168348.s002.zip › Taxa/Serra Sul/SS_2010/S11D-93.pdf]

S11D-93			1ª	AB	2ª	AB	ZON
Arthropoda							
Arachnida							
Acari							
Ixodidae		sp.1	1				E
	Amblyomma	sp.1	1				P
Sarcoptiformes		sp.1			1		P
Rhagidiidae							
Amblypygi							
Phrynidae							
	Heterophrynus	sp.	1		4		P
Araneae							
Araneidae	*jovens*						
	Eustala	sp.1			1		E
Ctenidae	*jovens*		2				E
Filistatidae	*jovens*		3				E P
		sp.1			2		E
Ochyroceratidae	*jovens*		2		1		E P
	Speocera	sp.1	1		2		P
Oonopidae	*jovens*		1				P
	gr. Xycarphius	sp.2	1				P
	Oonopinae	sp.1	1				E
Pholcidae	*jovens*		1		2		E P
	Leptopholcus	sp.1	1				P
	Mesabolivar	sp.1	2				E
	Ninetinae	sp.1	1		2		E P
Salticidae	*jovens*		3		1		E P
Scytodidae	*jovens*		5		3		E P
	Scytodes	*eleonorae*			3		E
	Scytodes	*globula*	4	0,03			P
		sp.	10	0,08	18	0,09	E P
Tetrablemmidae							
	Matta	sp.1	1				E
Theridiosomatidae	*jovens*		1				P
Opiliones							
Laniatores							
Stygnidae	*jovens*		2	0,03	1	0,04	E P
		sp.1	2		8		P
Pseudoscorpiones							
Bochicidae		sp.1			2		P
Chernetidae							
	Spelaeochernes	sp.1	2		1		E P
Chthoniidae							
	Pseudochthonius	sp.1	1				E
Olpiidae		sp.1	4		2		E P
Schizomida							
Hubbardiidae	*jovens*		1		1		E P
Scolopendromorpha							
Cryptopidae							
	Cryptops	sp.1	2	0,02			E
Diplopoda	*jovens*		1				P
Glomeridesmida							
Pyrgodesmidae		sp.2	2	0,02			E
Polyxenida							
Hypogexenidae		sp.1	2				P
Spirostreptida							
Pseudonannolenidae							
	Pseudonannolene	sp.1	2	0,02			P
Insecta							
Blattodea	*jovens*		5	0,04	6	0,03	E P
Blattellidae		sp.1			2	0,01	E
		sp.2			2	0,01	E
Blattidae	*jovens*		2	0,02	2	0,01	P
Polyphagidae	*jovens*		9	0,08			E P
		sp.1			3	0,01	E

Coleoptera	*jovens*		1					P
Leiodidae		sp.4			1			P
Staphylinidae		sp.18			2	0,01		P
		sp.6	1					P
Collembola								
Entomobryoidea								
Paronellidae		sp.4			1			P
		sp.2	2		1		E	P
Dermaptera	*jovens*		1				E	
Diptera	*jovens*		2		3		E	P
Brachycera								
Phoridae								
	Metopininae	sp.	1		4		E	P
Nematocera								
Cecidomyiidae								
	Cecidomyiinae	sp.	2				E	P
Chironomidae		sp.	1					P
Mycetophilidae								
	Euceroplatus	sp.			1			P
	Orfelia	sp.	1					P
Psychodidae								
	Lutzomyia	*longipalpis*			1		E	
	Micropygomyia	*pilosa*	1					P
	Nemopalpus	sp.			1			P
	Pintomyia	*gruta*	1					P
	Sciopemyia	*sordellii*	2		2			P
Hemiptera								
Heteroptera	jovens		8	0,06			E	
Reduviidae	jovens		6	0,05	4	0,02	E	P
	Emesinae	sp.1	1					P
	Reduviinae	sp.			66	0,32		P
Homoptera								
Cixiidae	*jovens*		1				E	
Hymenoptera								
Apoidea		sp.2			1		E	
Vespoidea								
Formicidae		sp.1	1					P
	Brachymyrmex	*atriceps*	2				E	P
	Camponotus	sp.1	1					P
	Crematogaster	*striatula*	4		2		E	P
	Gnamptogenys	sp.1			1			P
	Nylanderia	sp.2	1					P
	Pheidole	sp.1			1		E	
	Pseudomyrmex	*auropunctata*	1				E	
	Wasmania							
Isoptera								
Termitidae		sp.	5		3		E	P
	Nasutitermes	sp.			1			P
Lepidoptera	jovens		4	0,03	2	0,01	E	P
Limacodidae		sp.2	10	0,08			E	P
Noctuidae		sp.1	3	0,02			E	
Tineoidea		sp.1	3				E	
Neuroptera								
Myrmeleonthidae	jovens		2		2		E	P
Orthoptera								
Ensifera								
Gryllidae	jovens		2	0,02				P
Oecanthidae		sp.1	2	0,02				P
Phalangopsidae								
	Paraclodes	sp.1	30	0,23	11	0,05		P
	Phalangopsis	sp.1			11	0,05	E	P
Psocoptera	*jovens*							

Psyllipsocidae		sp.1	2				E P
	Psocathropos	sp.1	2		1		P
Malacostraca							
Isopoda							
Dubioniscidae		sp.1	3		1		E P
Mollusca							
Gastropoda							
Subulinidae		sp.	1		2		P
	Lamellaxis	sp.			1		P
Nemathelminthes		sp.	1				E
Chordata							
Mammalia							
Chiroptera							
Emballonuridae							
	Peropteryx	sp.			3	0,03	P
	Peropteryx	*kappleri*	5	0,08			E
Furipteridae							
	Furipterus	*horrens*			4	0,02	P
Phyllostomidae							
	Glossophaga	sp.			30	0,29	E P
	Glossophaginae	sp.	3	0,05			E P
Rodentia		sp.	2	0,02			E
