# Supplementary material for: Reconciling Mining with the Conservation of Cave Biodiversity: A Quantitative Baseline to Help Establish Conservation Priorities
Source: PLoS One. 2016 Dec 20;11(12):e0168348. doi: 10.1371/journal.pone.0168348 (PMC5173368; doi:10.1371/journal.pone.0168348)
Supplement: S1 Dataset — (ZIP) [file pone.0168348.s002.zip › Taxa/Serra Sul/SS_2010/S11D-94.pdf]

S11D-94		1ª	AB	2ª	AB	ZON
Annelida						
Clitellata						
Oligochaeta	jovens	17	0,12			E P
Parasitiformes						
Mesostigmata						
Laelapidae						
Stratiolaelaps	sp.1			1		P
Sarcoptiformes						
Oribatida	sp.3	1		1		P
Trombidiformes						
Tydeoidea						
Anystidae						
Erythracarus	*nasutus*	2				E P
Eupodidae	sp.1	1				P
Rhagidiidae	sp.1	1				E
Amblypygi						
Phrynidae						
Heterophrynus	sp.	5	0,03	4	0,04	P
Araneae						
Araneidae	jovens	1				E
Corinnidae	jovens	4	0,03			E P
Creugas	sp.1			4	0,04	P
Ochyroceratidae	jovens	2		1		P
Ochyrocera	sp.1	3				E P
	sp.3	1				P
Speocera	sp.1			2		P
Pholcidae	jovens	1				E
	sp.1	1				E
Scytodidae	jovens	2	0,02	1	0,05	E P
	sp.			4		P
Theridiosomatidae	jovens			1		P
Plato	sp.1	2				E
Opiliones						
Laniatores						
Stygnidae	jovens	4	0,06			E P
	sp.1	5		20	0,2	E P
Pseudoscorpiones						
Bochicidae	sp.1			2		E
Chernetidae						
Spelaeochernes	sp.1	3		3		E P
Schizomida						
Hubbardiidae	jovens	1				P
Chilopoda	jovens	2	0,02			
Pleurostigmophora						
Geophilomorpha						
Geophilidae	sp.1	4	0,03			E P
Scolopendromorpha						
Scolopocryptopidae						
Newportia	sp.1	2	0,02			P
Diplopoda	jovens	7	0,06			E
Polyxenida						
Hypogexenidae	sp.1	2				E P
Spirostreptida	jovens	1				P
Pseudonannolenidae						
Pseudonannolene	sp.1	2	0,02			P
Entognatha						
Diplura						
Campodeidae	sp.1	2				E P
Insecta						
Blattodea	jovens	2	0,02			
Coleoptera	jovens	1		1		E P
Staphylinidae	sp.19	1				P

Collembola						
Arthropleona						
Entomobryoidea						
Cyphoderidae	sp.1	1				P
Isotomidae	sp.1	2				P
Paronellidae	sp.1	2		1		E P
	sp.4			1		P
Symphypleona						
Sminthuroidea	sp.2			1		E
Diptera jovens		2		1		E P
Nematocera						
Cecidomyiidae						
Cecidomyiinae	sp.	2		1		E P
Chironomidae	sp.	1				E
Psychodidae						
Evandromyia	*saulensis*	1				P
Pintomyia	*gruta*			1		P
Sciopemyia	*sordellii*	5		2		E P
Sciaridae	sp.	1				P
Tipulidae						
Tipulinae	sp.	1		1		E P
Hemiptera						
Heteroptera jovens		1	0,01	2	0,02	E
Dipsocoroidea jovens		1				P
aff. Alydidae jovens		1				P
aff. Lygaeidae jovens				1		P
Cydnidae jovens		1				E
Cydninae	sp.1	1				P
Reduviidae jovens		3	0,02			P
Emesinae	sp.2			1		E
Homoptera jovens		1				
Cixiidae jovens		2		1		P
Hymenoptera						
Vespoidea						
Formicidae						
Acromyrmex	sp.1			1		P
Camponotus	*atriceps*	5	0,03			E P
Gnamptogenys	*striatula*			2		E P
Hypoponera	sp.1	1				P
Nylanderia	sp.1	4		3		E P
Odontomachus	*bauri*	2	0,02			E
Trachymyrmex	sp.1	1				P
Wasmania	*auropunctata*	1				E
Isoptera						
Rhinotermitidae						
Dolichorhinotermes	sp.	1				E
Termitidae						
Nasutitermes	sp.	3		2		E P
Lepidoptera jovens		3		2		E P
Noctuoidea	sp.2	1				E
Noctuidae	sp.2	2	0,02			E
	sp.1	4	0,03			
Neuroptera						
Mantispidae	sp.	2	0,02			E
Orthoptera						
Ensifera						
Gryllidae jovens		2	0,02			P
Phalangopsidae						
Paraclodes	sp.1			11	0,1	P
Phalangopsis	sp.1	49	0,36	46	0,45	P
Psocoptera						
Psocomorpha jovens		2		1		E
Epipsocidae						

	Epipsocus	sp.3			1		E
Trogiomorpha							
Psyllipsocidae							
	Psyllipsocus	sp.1	1				P
Malacostraca							
Isopoda							
Philosciidae		sp.1	1		2		E P
Scleropactidae		sp.	1				P
Chordata							
Amphibia							
Anura		sp.			1	0,01	P
Neobatrachia							
Strabomantidae							
	Pristimantis	*fenestratus*	2	0,02			
Mammalia							
Chiroptera							
Phyllostomidae		sp.1					
Glossophaginae		sp.			9	0,09	P
Rodentia		sp.	2	0,02			
Mollusca							
Gastropoda							
Systrophiidae							
	Happia	sp.	2				P
Platyhelminthes							
Turbellaria		sp.2	1				E
