# Supplementary material for: Reconciling Mining with the Conservation of Cave Biodiversity: A Quantitative Baseline to Help Establish Conservation Priorities
Source: PLoS One. 2016 Dec 20;11(12):e0168348. doi: 10.1371/journal.pone.0168348 (PMC5173368; doi:10.1371/journal.pone.0168348)
Supplement: S1 Dataset — (ZIP) [file pone.0168348.s002.zip › Taxa/Serra Sul/SS_2010/S11D-95.pdf]

S11D-95		1ª	AB	2ª	AB	ZON
Arthropoda						
Arachnida						
Acari						
Parasitiformes						
Mesostigmata	sp.4	1				E
Amblypygi						
Phrynidae						
Heterophrynus	sp.	3	0,12			
Araneae						
Barychaelidae	joven	2	0,08			E
Ctenidae	joven	2	0,08			E
Mysmenidae						
Microdipoena	sp.1			1		E
Ochyroceratidae						
Speocera	sp.1	1		1		E
Salticidae	joven	2		1		E
Scytodidae						
Scytodes	*eleonorae*	2	0,08			E
Theridiosomatidae	joven	2				E
Opiliones						
Laniatores						
Stygnidae	joven	3	0,12			E
Stygnidae	sp.1			2	0,34	E
Pseudoscorpiones						
Bochicidae	sp.1			2		E
Chernetidae						
Spelaeochernes	sp.1	2				E
Chthoniidae						
Pseudochthonius	sp.1			1		E
Olpiidae	sp.1			2		E
Chilopoda						
Pleurostigmophora						
Scolopendromorpha						
Cryptopidae						
Cryptops	sp.2	2	0,08			E
Entognatha						
Diplura						
Campodeidae	sp.1	1				E
Insecta						
Collembola						
Arthropleona						
Entomobryoidea						
Paronellidae	sp.1			1		E
Diptera						
Brachycera						
Camillidae	sp.			1		E
Dolichopodidae	sp.			1		E
Nematocera						
Ceratopogonidae	sp.			1		E
Psychodidae						
Evandromyia	*saulensis*	1				E
Lutzomyia	*longipalpis*			1		E
Psychodopygus	Série *chagas*	1				E
Sciopemyia	*sordellii*	2				E
Tipulidae						
Limoniinae	sp.	1				E
Hemiptera						
Heteroptera						
Dipsocoroidea						
Reduviidae	joven	2		2		E

Homoptera									
Cixiidae		joven				1		E
Hymenoptera									
Vespoidea									
Formicidae									
	Camponotus	sp.1		1				E
Isoptera									
Termitidae									
	Nasutitermes	sp.		1				E
Lepidoptera									
Noctuoidea		joven		1				E
		sp.2		2				E
Orthoptera									
Ensifera									
Phalangopsidae		joven		11	0,44			
	Paraclodes	sp.				4	0,66	E
Psocoptera									
Psocomorpha									
Ptiloneuridae									
	Ptiloneura	sp.2				1		E

RQ
RQ TOTAL
