# Supplementary material for: Reconciling Mining with the Conservation of Cave Biodiversity: A Quantitative Baseline to Help Establish Conservation Priorities
Source: PLoS One. 2016 Dec 20;11(12):e0168348. doi: 10.1371/journal.pone.0168348 (PMC5173368; doi:10.1371/journal.pone.0168348)
Supplement: S1 Dataset — (ZIP) [file pone.0168348.s002.zip › Taxa/Serra Sul/SS_2010/S11D-96.pdf]

S11D-96		1ª	AB	2ª	AB	ZON
Acanthocephala	sp.			1		P
Arthropoda						
Arachnida						
Acari						
Ixodida jovens				1		A
Argasidae						
Ornithodoros	sp.	2		4		E P A
Ixodidae						
Amblyomma	sp.	1				E
Parasitiformes						
Mesostigmata						
Laelapidae						
Stratiolaelaps	sp.1	1				P
Ologamasidae	sp.2	2		1		P
	sp.3			1		A
Sarcoptiformes						
Oribatida	sp.14	1				P
	sp.3	3		3		P A
	sp.5			1		A
	sp.7	1				A
Trombidiformes						
Tydeoidea	sp.1			1		A
	sp.2	2				P
	sp.7	1		1		E
Amblypygi						
Charinidae jovens		2	0,00			P
Phrynidae						
Heterophrynus	sp.	6	0,01	6	0,02	P
Araneae jovens		3		9		P
Araneidae jovens				2		P
Alpaida	sp.1			1		A
	sp.2	2				E P
Corinnidae jovens		2	0,00			P
Ctenidae jovens		9	0,02	10	0,03	E P
Ochyroceratidae jovens		1				E
	sp.1	1				P
Ochyrocera	sp.1	1		1		A
	sp.5	1				P
Speocera	sp.1	2		3		P A
Pholcidae						
Mesabolivar	sp.1	1				P
Ninetinae	sp.1	2		2		P
Scytodidae jovens		8		3		E P A
Scytodes	*eleonorae*	5	0,01	2	0,01	P A
	sp.			9	0,03	A
Segestriidae jovens		3		3		E P A
Ariadna	sp.1	1				A
Tetrablemmidae jovens				1		A
Theraphosidae jovens		2	0,01	4	0,01	P
aff. *Holothele*	sp.1	2				P
Theridiosomatidae jovens		1		2		P A
Plato	sp.1	5		4		E P A
Opiliones						
Laniatores						
Escadabiidae jovens		2		1		P A
	sp.1	3		1		P A
Stygnidae jovens		5	0,01			E A

		sp.1	2	0,01	9	0,03	E P A
Pseudoscorpiones							
Chernetidae		jovens	2				A
	Spelaeochernes	sp.1	3		5		E P A
Chthoniidae							
	Pseudochthonius	sp.1	2		3		E P A
Olpiidae		sp.1			2		A
Chilopoda							
Notostigmophora							
Scutigeromorpha							
Pselliodidae		jovens			1		A
Scutigeromorpha							
Pselliodidae							
	Sphendononema	sp.			2	0,01	A
Diplopoda							
Polydesmida							
Chelodesmidae		jovens			2		A
		sp.5	2	0,00			P
Pyrgodesmidae		sp.2	11	0,03	4	0,01	E P A
Spirostreptida		jovens	2				E P
Pseudonannolenidae							
	Pseudonannolene	sp.1	2	0,00			E
Entognatha							
Diplura					1		P
Campodeidae		sp.1	1		1		A
Insecta							
Blattodea		jovens	2	0,00	2	0,01	P
Blaberidae		jovens	6	0,01			P
Blattidae		jovens	3	0,01	4	0,01	E P
Polyphagidae		jovens	4	0,01	2	0,01	E P
Coleoptera		jovens	3		3		E P
Chrysomelidae		sp.4			1		P
Curculionidae							
	Scolytinae	sp.4			1		P
Elateridae		sp.1	1				P
Ptiliidae		sp.1			1		A
Staphylinidae		sp.6	1		1		A
		sp.8			1		A
Collembola							
Arthropleona							
Entomobryoidea							
Cyphoderidae		sp.1	1				A
		sp.2			1		P
Isotomidae		sp.1			2		A
Paronellidae		sp.1	3		2		E P A
		sp.4	2		2		P A
Symphypleona							
Sminthuroidea		sp.2	4		3		P A
Diptera		jovens	5		4		E P A
Brachycera							
Camillidae		sp.	4		1		P A
Conopidae		sp.	1				E
Drosophilidae							
	Drosophila	*eleonore*	1				A
Phoridae							
	Metopininae	sp.			1		E
	Phorinae	sp.			1		A
Streblidae							

Trichobius	sp.			1		P
Nematocera						
Ceratopogonidae	sp.	1		2		P A
Chironomidae	sp.	1		1		P A
Culicidae						
Culicini	sp.	3		2		E P A
Psychodidae						
Edentomyia	*piauensis*	1		1		E
Pintomyia	*gruta*	1		1		E P
Sciopemyia	*sordellii*	5		7		E P A
Hemiptera						
Heteroptera						
aff. Lygaeidae	jovens	1				P
Cydnidae	jovens					
Cydninae	sp.1	1		1		A
Reduviidae	jovens	5	0,01	4	0,01	E P
Panstrongylus	sp.1			2	0,01	P
Reduviinae	sp.			2	0,01	P
Veliidae	jovens			1		A
Homoptera						
Cixiidae	jovens	4		2		P A
Hymenoptera						
Chrysidoidea						
Bethylidae	sp.1	1				P
Vespoidea						
Formicidae						
Gnamptogenys	*striatula*	2				P A
Hypoponera	sp.1			1		P
Nylanderia	sp.1	1				A
Procryptocerus	sp.1			1		P
Solenopsis	sp.1			1		A
	sp.2	1				P
Isoptera	sp.	1		1		P
Termitidae						
Nasutitermes	sp.	4		4		E P
Lepidoptera	jovens	1		1		A
Cossoidea	sp.2			1		E
	sp.4	1				P
Limacodidae	sp.1	5	0,01			E P
	sp.2	2	0,00			E
Tineoidea	sp.1			1		P
Orthoptera						
Ensifera						
Phalangopsidae						
Phalangopsis	sp.1	226	0,46	122	0,35	P A
Paraclodes	sp.1			11	0,03	P
Psocoptera						
Psocomorpha	jovens	1				P
Archipsocidae						
Archipsocus	sp.2			1		A
Trogiomorpha						
Lepidopsocidae						
Psyllipsocus	sp.1	1				P
Thysanura						
Nicoletiidae	jovens	1				A
	sp.1	2		4		P A
Malacostraca						
Isopoda						

Philosciidae	sp.1			1		A
Chordata						
Amphibia						
Anura						
Neobatrachia						
Leptodactylidae						
Leptodactylus	sp.			2	0,01	P
Strabomantidae						
Pristimantis	*fenestratus*			3	0,01	P
Bufonidae						
Rhinella	cf. *marina*			13	0,04	A
Pipidae						
Pipa	*arrabali*	41	0,08	41	0,12	A
Mammalia						
Chiroptera						
Emballonuridae						
Peropteryx	*kappleri*	5	0,01			
Furipteridae						
Furipterus	*horrens*	10	0,06	30	0,03	A
Phyllostomidae						
Glossophaginae				30	0,09	A
Carollia	*perspicillata*	80	0,16	30		
Diphylla	*ecaudata*			3	0,01	A
Glossophaga	*soricina*	30	0,06			
Lonchophylla	*thomasi*	1	0,00			
Phyllostomus	*latifolius*	10	0,03			
Trachops	*cirrhosus*			3	0,01	A
Mollusca						
Gastropoda						
Systrophiidae						
Happia	sp.	1		1		P
Nemathelminthes	sp.			1	0,0	P
