# Supplementary material for: Reconciling Mining with the Conservation of Cave Biodiversity: A Quantitative Baseline to Help Establish Conservation Priorities
Source: PLoS One. 2016 Dec 20;11(12):e0168348. doi: 10.1371/journal.pone.0168348 (PMC5173368; doi:10.1371/journal.pone.0168348)
Supplement: S1 Dataset — (ZIP) [file pone.0168348.s002.zip › Taxa/Serra Sul/SS_2010/S11D-97.pdf]

S11D-97			1ª	AB	2ª	AB	ZON
Arthropoda							
Arachnida							
Acari							
Ixodida							
Ixodidae							
Amblyomma		sp.	1				E
Araneae							
Filistatidae	jovens				1		E
Pholcidae	jovens		1				E
Scytodidae	jovens		1	1			E
Pseudoscorpiones							
Olpiidae		sp.1	2				E
Insecta							
Coleoptera	jovens		1				E
Nematocera							
Psychodidae							
Pintomyia		*gruta*	1				E
Diptera	jovens		1				E
Hemiptera							
Homoptera							
Cixiidae		sp.3			1		E
Hymenoptera							
Vespoidea							
Formicidae							
Pheidole		sp.1			1		E
		sp.2	2				E
Vespidae		sp.3			1		E
Isoptera							
Termitidae							
Cornitermes		sp.	2				E
Cortaritermes		*silvestrii*			1		E
Nasutitermes		sp.	2				E
Velocitermes		sp.			1		E
Lepidoptera	jovens		1				E
Neuroptera							
Myrmeleonthidae	jovens		2		1		E
Psocoptera							
Psocomorpha	jovens		1				E
Trogiomorpha							
Lepidopsocidae							
Psocathropos		sp.1	1				E
Chordata							
Mammalia							
Chiroptera		sp.			1	1	E
