# Supplementary material for: Reconciling Mining with the Conservation of Cave Biodiversity: A Quantitative Baseline to Help Establish Conservation Priorities
Source: PLoS One. 2016 Dec 20;11(12):e0168348. doi: 10.1371/journal.pone.0168348 (PMC5173368; doi:10.1371/journal.pone.0168348)
Supplement: S1 Dataset — (ZIP) [file pone.0168348.s002.zip › Taxa/Serra Sul/SS_2010/S11D-98.pdf]

S11D-98		1ª	AB	2ª	AB	ZON
Arthropoda						
Arachnida						
Acari						
Parasitiformes						
Mesostigmata	sp.4	1				E
Sarcoptiformes	sp.1	1				E
Amblypygi						
Phrynidae						
Heterophrynus	sp.	2	0,05			
Araneae						
Araneidae	jovens	2				E
Corinnidae	jovens	5	0,14			E
Ctenidae	jovens	2	0,05			E
Filistatidae	sp.1			1		E
Nesticidae	jovens	1				E
Pholcidae	jovens			1		E
aff. *Ibityporanga*	sp.1	1				E
Mesabolivar	sp.1	1				E
Salticidae	jovens			1		E
Scytodidae	jovens	1				E
Theridiidae	jovens	1				E
Opiliones						
Laniatores						
Stygnidae	sp.1	2	0,05			E
Pseudoscorpiones						
Olpiidae	sp.1	2		2		E
Chilopoda						
Notostigmophora						
Scutigeromorpha						
Pselliodidae						
Sphendononema	*guildingii*	1				E
Diplopoda						
Polyxenida						
Hypogexenidae	sp.1			1		E
Entognatha						
Diplura						
Campodeidae	sp.1	1				E
Insecta						
Blattodea						
Blaberidae	jovens	2	0,05			E
Polyphagidae	jovens	5	0,14			E
Coleoptera	jovens	1				E
Diptera						
Brachycera						
Camillidae	sp.			1		E
Dolichopodidae	sp.			1		E
Nematocera						
Cecidomyiidae						
Cecidomyiinae	sp.	1				E
Chironomidae	sp.			1		E
Tipulidae						
Tipulinae	sp.			1		E
Hemiptera						
Heteroptera						
Reduviidae	jovens	4	0,11			E
Homoptera						
Cixiidae	sp.4	1				E
Hymenoptera						

Vespoidea						
Formicidae						
Camponotus	*atriceps*	4	0,11			E
Crematogaster	sp.1	2				E
Isoptera						
Termitidae						
Nasutitermes	sp.	1				E
Termes	sp.	1		1		E
Lepidoptera						
Cossoidea						
Limacodidae	sp.1	5	0,14			E
Noctuoidea	sp.1			1		E
	sp.2	1				E
Neuroptera						
Myrmeleonthidae	jovens	1				E
Orthoptera						
Ensifera						
Phalangopsidae						
Paraclodes	sp.1	4	0,11	9	1	E
Psocoptera						
Psocomorpha	jovens	2				E
Troctomorpha						
Liposcelididae						
Liposcelis	sp.1	1				E
Trogiomorpha						
Lepidopsocidae						
Loxopholia	sp.1	1				E
Thysanura						
Nicoletiidae	jovens	1				E
Malacostraca						
Isopoda						
Philosciidae	sp.1			1		E
Chordata						
Reptilia						
Squamata						
Gekkonidae						
Thecadactylus	*rapicauda*	2	0,05			
