# Supplementary material for: Reconciling Mining with the Conservation of Cave Biodiversity: A Quantitative Baseline to Help Establish Conservation Priorities
Source: PLoS One. 2016 Dec 20;11(12):e0168348. doi: 10.1371/journal.pone.0168348 (PMC5173368; doi:10.1371/journal.pone.0168348)
Supplement: S1 Dataset — (ZIP) [file pone.0168348.s002.zip › Taxa/Serra Sul/SS_2010/S11D-99.pdf]

S11D-99			1ª	AB	2ª	AB	ZON
Arthropoda							
Arachnida							
Acari							
Ixodida							
Argasidae							
	Ornithodoros	sp.	2				E
Ixodidae							
	Amblyomma	sp.	1				E
Parasitiformes							
Mesostigmata							
Laelapidae		sp.3	1				E
Mesostigmata		sp.2	1				E
		sp.4	1				E
Sarcoptiformes		sp.1	1				E
Oribatida		sp.3	2				E
		sp.7	1				E
		sp.18	1				E
		sp.2			1		E
Amblypygi							
Phrynidae							
	Heterophrynus	sp.	6	0,07	2	0,07	E
Araneae							
Araneidae	jovens		1				E
	Alpaida	*septemmammata*	2				E
Ctenidae	jovens		2	0,02			E
Ochyroceratidae	jovens		1				E
	Ochyrocera	sp.1			2		E
	Speocera	sp.1			1		E
Pholcidae	jovens		2				E
	Ninetinae	sp.1	1		1		E
Salticidae	jovens		1		1		E
Scytodidae	jovens		1				E
	Scytodes	*eleonorae*	2	0,02	3	0,11	E
		globula	3	0,04			E
Segestriidae	jovens		1		1		E
	Ariadna	sp.1	1		2		E
Theraphosidae	jovens		2	0,02			E
Theridiidae	jovens		1				E
Theridiosomatidae	jovens		4				E
	Plato	sp.1	1				E
Opiliones	jovens		10	0,13			
Eupnoi							
Sclerosomatidae	jovens		1				E
		sp.1	1		1		E
Laniatores							
Escadabiidae	jovens		1				E
Stygnidae	jovens		7	0,12			E
Stygnidae		sp.1	3				E
Pseudoscorpiones							
Bochicidae		sp.1	2				E
Olpiidae		sp.1	2				E
Schizomida							
Hubbardiidae	jovens		1				E
Chilopoda							
Pleurostigmophora							
Scolopendromorpha							
Cryptopidae							
	Cryptops	sp.1	2	0,02			E
Scutigeromorpha	jovens		2	0,02			
Diplopoda			2				
Polydesmida							
Pyrgodesmidae		sp.1	1	0,01			E
Polyxenida							
Hypogexenidae		sp.1	4		2		E

Spirostreptida							
Pseudonannolenidae							
Pseudonannolene		sp.1			2	0,07	E
Entognatha							
Diplura							
Campodeidae		sp.1	2				E
Projapygidae		sp.1	2				E
Insecta							
Archaeognatha							
Meinertellidae	jovens	sp.1	2	0,02			E
Blattodea	jovens		2	0,05			
Blattidae		sp.3	2				E
Coleoptera							
Chrysomelidae		sp.9	1				E
Scydmaenidae		sp.5	1				E
Staphylinidae		sp.18					
Pselaphinae		sp.9			1		E
Collembola							
Arthropleona							
Entomobryoidea							
Cyphoderidae		sp.1	2				E
Paronellidae		sp.1	5		1		E
Diptera	jovens		3				E
Brachycera							
Sarcophagidae		sp.	1				E
Nematocera							
Cecidomyiidae							
Cecidomyiinae		sp.			1		E
Mycetophilidae							
Keroplatus		sp.	1				E
Psychodidae							
Sciopemyia		*sordellii*	1		1		E
Sciaridae		sp.	1				E
Hemiptera							
Heteroptera							
Dipsocoroidea							
Tingidae							
Thaumamannia		sp.1	1				E
Hymenoptera							
Vespoidea							
Formicidae							
Crematogaster		sp.1			1		E
Nylanderia		sp.1	1				E
Octostruma		sp.1	1				E
Pachycondyla		*harpax*	1				E
		striata	4				E
		sp.2	1				E
Solenopsis		sp.1	1				E
		sp.2	1				E
Strumygenys		sp.1	1				E
Wasmania		*auropunctata*	1				E
Hymenoptera	jovens		1				E
Isoptera							
Rhinotermitidae							
Dolichorhinotermes		sp.	1				E
Termitidae *Armitermes*		sp.			1		E
Embiratermes		sp.	2		2		E
Nasutitermes		sp.	4		1		E
Subulitermes		sp.			1		E
Lepidoptera	jovens		5	0,06			E
Noctuoidea							
Noctuidae		sp.2	2	0,02			E
Neuroptera							
Mantispidae		sp.	2	0,02			E
Orthoptera							

Ensifera							
	Gryllidae	jovens	4	0,05			E
	Phalangopsidae	jovens	2	0,02			E
	Paraclodes	sp.	16	0,19	4	0,15	E
	Phalangopsis	sp.			2	0,07	E
Psocoptera							
Psocomorpha		jovens	2		1		E
	Ptiloneuridae						
	Triplocania	sp.3	1				E
Thysanura							
	Nicoletiidae	sp.1	2				E
Malacostraca							
Isopoda							
	Dubioniscidae	sp.1	2				E
	Philosciidae	sp.2	1				E
Chordata							
Amphibia							
Anura							
Neobatrachia							
	Strabomantidae						
	Pristimantis	*fenestratus*	3	0,04	5	0,19	E
Mammalia							
Chiroptera							
	Phyllostomidae						
	Glossophaga	*soricina*	4	0,06			
	Glossophaginae	sp.			8	0,34	E
Mollusca							
Gastropoda							
	Systrophiidae						
	Happia	sp.	1				E
