# Supplementary material for: Reconciling Mining with the Conservation of Cave Biodiversity: A Quantitative Baseline to Help Establish Conservation Priorities
Source: PLoS One. 2016 Dec 20;11(12):e0168348. doi: 10.1371/journal.pone.0168348 (PMC5173368; doi:10.1371/journal.pone.0168348)
Supplement: S1 Dataset — (ZIP) [file pone.0168348.s002.zip › Taxa/Serra Sul/SS_2010/S11D-100.pdf]

S11D-100		1ª	AB	2ª	AB	ZON
Arthropoda						
Arachnida						
Acari						
Argasidae						
Ornithodoros	sp.	1				E
Oribatida	sp.2	1				E
	sp.3	1				E
	sp.19	1				E
Trombidiformes	sp.1	1				E
Amblypygi						
Phrynidae						
Heterophrynus	sp.	5	0,03			
Araneae						
Araneidae						
Alpaida	*septemmammata*	1				E
Corinnidae jovens		2	0,01			P
Filistatidae jovens		1				E
Pholcidae jovens		1		1		P
Ninetinae	sp.1	1		2		E P
Scytodidae jovens		1	0,01	2	0,03	E P
Scytodes	*eleonorae*	4	0,03			E
	globula	5	0,03			E
Segestriidae jovens		1		1		E
Tetrablemmidae jovens		1				P
Theraphosidae jovens		2	0,01			P
Theridiosomatidae						
Plato	sp.1	2				E P
Opiliones						
Eupnoi						
Sclerosomatidae	sp.1	2				E
Laniatores						
Escadabiidae	sp.1	3				P
Palpigradi						
Eukoeneniidae jovens		1				E
Pseudoscorpiones						
Bochicidae	sp.1	2				P
Chernetidae						
Spelaeochernes	sp.1	2				P
Chthoniidae jovens				2		P
Olpiidae	sp.1			2		E
Schizomida						
Hubbardiidae jovens		1				P
Rowlandius	sp.	1				P
Chilopoda						
Pleurostigmophora						
Scolopendromorpha						
Cryptopidae						
Cryptops	sp.1	2	0,01			P
Scolopocryptopidae jovens		2	0,01			P
Dinocryptops	*miersii*	2	0,01			P
Diplopoda						
Polyxenida						
Hypogexenidae	sp.1	3				P
Spirostreptida						
Pseudonannolenidae jovens		2	0,01			P
Insecta						
Blattodea jovens		53	0,23			E P
Blaberidae jovens		2	0,01			P
Blattidae jovens		3	0,02	3	0,05	E
Polyphagidae jovens		2	0,01			E
Coleoptera jovens		2				E P
Carabidae	sp.12	2				E P
	sp.9			1		E
Collembola						

Arthropleona							
Entomobryoidea							
Cyphoderidae		sp.1	1				P
Isotomidae		sp.1	2				P
Paronellidae		sp.1	2				E P
Diptera	jovens		2				P
Brachycera							
Dolichopodidae		sp.			1		E
Hemiptera							
Heteroptera							
Reduviidae	jovens		4	0,03	4	0,07	E P
Tingidae	jovens		1				P
Veliidae	jovens						
	Paravelia	sp.2			1		E
Homoptera							
Cixiidae	jovens		2				P
		sp.1	1				P
Hymenoptera							
Vespoidea							
Formicidae							
	Acromyrmex	sp.1					
	Pachycondyla	*striata*	2				P
	Pheidole	sp.2	1		1		E
Isoptera							
Termitidae							
	Diversitermes	sp.	1				P
	Nasutitermes	sp.	4		3		E P
Lepidoptera	jovens		1				P
Cossoidea							
Limacodidae		sp.1	5	0,03			E P
Noctuoidea		sp.3			1		E
Orthoptera							
Ensifera							
Phalangopsidae	jovens		2	0,01			E
	Phalangopsis	sp.1	3	0,02			P
	Paraclodes	sp.1	55	0,32	3	0,05	E
Psocoptera							
Psocomorpha	jovens		1				E
Troctomorpha							
Liposcelididae							
	Liposcelis	sp.1	1				E
Mollusca							
Gastropoda							
Systrophiidae							
	Happia	sp.	2				P
Chordata							
Amphibia							
Anura							
Neobatrachia							
Leptodactylidae							
	Leptodactylus	sp.	2	0,01			
Mammalia							
Chiroptera							
Emballonuridae							
	Peropteryx	sp.	3	0,02	5	0,08	P
Phyllostomidae							
	Glossophaginae	sp.	21	0,12	42	0,72	E
Rodentia		sp.	2	0,01			
