# Supplementary material for: Reconciling Mining with the Conservation of Cave Biodiversity: A Quantitative Baseline to Help Establish Conservation Priorities
Source: PLoS One. 2016 Dec 20;11(12):e0168348. doi: 10.1371/journal.pone.0168348 (PMC5173368; doi:10.1371/journal.pone.0168348)
Supplement: S1 Dataset — (ZIP) [file pone.0168348.s002.zip › Taxa/Serra Sul/SS_2010/S11D-101.pdf]

S11D-101		1ª	AB	2ª	AB	ZON
Annelida						
Clitellata						
Oligochaeta	jovens	23	0,04	4	0,01	E P
Arthropoda						
Arachnida						
Acari						
Parasitiformes						
Mesostigmata						
Laelapidae						
Stratiolaelaps	sp.1	1		1		P
	sp.1	1				P
Sarcoptiformes						
Oribatida	sp.12	1				P
	sp.1			2		E P
Trombidiformes						
Tydeoidea	sp.1	1				E
Amblypygi						
Phrynidae						
Heterophrynus	sp.	10	0,01	6	0,01	P
Araneae	jovens			3		P
Araneidae	jovens	1				P
Ctenidae	jovens	4	0,01			P
Ochyroceratidae	jovens	2				E P
Ochyrocera	sp.3			1		P
Speocera	sp.1	3		4		E P
Oonopidae	jovens			2		E P
gr. Xycarphius	sp.3	1				P
Pholcidae	jovens	1		1		E
Mesabolivar	sp.1	1				E
Scytodidae	jovens	1	0,00			P
Scytodes	*eleonorae*	2	0,00	3	0,01	P
	globula	5	0,01			P
	sp.			15	0,04	P
Theraphosidae	jovens	7	0,01			E P
Theridiidae						
Dipoena	sp.1	1				P
Theridiosomatidae	jovens	1		1		E P
Plato	sp.1	3				E P
Trechaleidae	jovens	2	0,00			P
Opiliones						
Eupnoi						
Sclerosomatidae	jovens	3		1		P
	sp.1	1		1		E
Laniatores						
Cosmetidae	sp.3			2	0,00	E
	sp.2	1				P
Stygnidae	jovens	2	0,00			P
	sp.1			2	0,00	P
Pseudoscorpiones						
Chernetidae						
Spelaeochernes	sp.1	5		3		E P
Chthoniidae	jovens					
Pseudochthonius	sp.1	2		2		E P
Schizomida						
Hubbardiidae	jovens	2				P
Chilopoda						
Notostigmophora						
Scutigeromorpha						
Pselliodidae	jovens	1				P
Pleurostigmophora						
Scolopendromorpha						
Scolopocryptopidae						
Dinocryptops	*miersii*	2	0,00			P
Newportia	sp.1			2	0,00	E

Diplopoda								
Polydesmida								
	Chelodesmidae		jovens			2	0,00	P
	Fuhrmannodesmidae		jovens					
			sp.1	2				P
			sp.2	1				P
Spirostreptida			jovens	1				P
Entognatha								
Diplura								
	Campodeidae		sp.1	3				P
	Japygidae		sp.1			1		E
Insecta								
Blattodea			jovens	3	0,00			P
Coleoptera			jovens	2		1		E P
	Ptiliidae		sp.1	2				P
	Staphylinidae							
		Pselaphinae	sp.1			1		E
			sp.3	1		1		E P
Collembola								
Arthropleona								
Entomobryoidea								
	Isotomidae		sp.1	2		1		E P
	Paronellidae		sp.1	3		1		E P
	Tomoceridae		sp.1	1				P
Poduroidea			sp.1	1				P
Symphypleona								
Sminthuroidea			sp.2	6		2		E P
Diptera			jovens	4		3		E P
Brachycera								
	Phoridae							
		Metopininae	sp.			1		E
Nematocera								
	Cecidomyiidae							
		Cecidomyiinae	sp.	1				P
	Chironomidae		sp.	1		1		E P
	Psychodidae							
		Phlebotominae	sp.			2		E P
		Sciopemyia	*sordellii*	2		1		P
	Sciaridae		sp.	1				P
		Bradysia	sp.			2		E P
	Tipulidae							
		Tipulinae	sp.			3		E P
Hemiptera								
Heteroptera								
aff. Pyrrhocoroidea								
	Cydnidae							
		Cydninae	sp.1	5		3		P
	Reduviidae		jovens	3	0,00	2	0,00	P
		Emesinae	sp.2	1				P
	Veliidae							
		Paravelia	sp.1	1		1		E P
Homoptera								
	Cixiidae		jovens	1		2		E P
Hymenoptera								
Vespoidea								
	Formicidae							
		Acromyrmex	sp.1	2				P
		Carebara	sp.1	1				P
		Hypoponera	sp.1	1				P
		Myrmicocrypta	sp.1	1				P
		Octostruma	sp.1	1				P
		Pachycondyla	*striata*	5		3		P
		Solenopsis	sp.1	1				P
Isoptera								
	Termitidae		sp.	1		1		E

Convexitermes	sp.	1					P
Lepidoptera	jovens			1		E	
Castnioidea							
Castniidae	sp.1	2	0,00				P
Noctuoidea							
Noctuidae	sp.1	5	0,01	5	0,01		P
Orthoptera							
Ensifera	jovens	3	0,00			E	
Gryllidae	jovens	2	0,00				P
Phalangopsidae							
Phalangopsis	sp.1	597	0,87	263	0,61	E	P
Psocoptera							
Troctomorpha							
Manicapsocidae							
Nothoentomum	sp.1	1					P
Thysanura							
Nicoletiidae	sp.1	1		1			P
Malacostraca							
Isopoda							
Philosciidae	sp.1	5					P
	sp.2	1					P
Mollusca							
Gastropoda							
Streptaxidae							
Streptaxis	sp.			1			P
Systrophiidae							
Happia	sp.	1					P
Platyhelminthes							
Turbellaria	sp.7	2	0,00				P
Chordata							
Amphibia							
Anura							
Neobatrachia							
Leptodactylidae							
Leptodactylus	sp.	2	0,00				
Strabomantidae							
Pristimantis	*fenestratus*			79	0,19		P
Dendrobatidae							
Epipedobates	cf. *flavopictus*	4	0,01	36	0,09		P
Mammalia							
Chiroptera	sp.			11	0,03		P
Phyllostomidae							
Carollia	sp.	2	0,01				
Micronycteris	*microtis*	2	0,01				
Rodentia	sp.	5	0,01	2	0,00		P
Reptilia							
Squamata							
Serpentes							
Gymnophthalmidae							
Neusticurus	sp.			2	0,00	E	
