# Supplementary material for: Reconciling Mining with the Conservation of Cave Biodiversity: A Quantitative Baseline to Help Establish Conservation Priorities
Source: PLoS One. 2016 Dec 20;11(12):e0168348. doi: 10.1371/journal.pone.0168348 (PMC5173368; doi:10.1371/journal.pone.0168348)
Supplement: S1 Dataset — (ZIP) [file pone.0168348.s002.zip › Taxa/Serra Sul/SS_2010/S11D-102.pdf]

S11D-102		1ª	AB	2ª	AB	ZON
Arthropoda						
Arachnida						
Acari						
Parasitiformes						
Mesostigmata						
Podocinidae	sp.5	1				E
Trombidiformes						
Tydeoidea						
Labdostomatidae	sp.1	1				E
Amblypygi						
Phrynidae						
Heterophrynus	sp.			2	0,06	E
Araneae						
Araneidae						
Alpaida	*smila*	1				E
Oonopidae						
Oonopinae	sp.1			1		E
Pholcidae	jovens			1		E
Mesabolivar	*aurantiacus*			1		E
Theraphosidae	jovens	3	0,25			E
Theridiidae						
Theridion	sp.4			1		E
Opiliones						
Eupnoi	jovens			4	0,13	E
Sclerosomatidae	jovens	1				E
	sp.2			1		E
Laniatores						
Stygnidae	jovens	3	0,25			E
Entognatha						
Diplura						
Campodeidae	sp.1	1				E
Insecta						
Blattodea	jovens			2	0,06	E
Coleoptera	jovens			1		E
Chrysomelidae	sp.10	1				E
Dytiscidae	sp.4	1				E
Collembola						
Arthropleona						
Entomobryoidea						
Entomobryidae	sp.1	1				E
Diptera						
Nematocera						
Psychodidae						
Sciopemyia	*sordellii*	1				E
Sciaridae	sp.			1		E
Tipulidae						
Tipulinae	sp.			1		E
Hemiptera						
Heteroptera						
Reduviidae	jovens			2	0,06	E
Homoptera						
Cixiidae	jovens	1				E
Hymenoptera						
Vespoidea						
Formicidae						
Carebara	sp.1			1		E
Nylanderia	sp.1			1		E
Pachycondyla	*harpax*			1		E
Pachycondyla	*striata*	1				E
Pheidole	sp.1	1				E

Isoptera						
Termitidae						
Nasutitermes	sp.	1		1		E
Lepidoptera						
Noctuoidea						
Noctuidae	sp.			2	0,06	E
	sp.1	2	0,17			E
Tineoidea	sp.1	1				E
Orthoptera						
Ensifera						
Phalangopsidae	jovens	2	0,17			E
Paraclodes	sp.1			5	0,17	E
Phalangopsis	sp.1			10	0,33	E
Psocoptera						
Psocomorpha	jovens	1		1		E
Malacostraca						
Isopoda						
Philosciidae	sp.1	1				E
Chordata						
Amphibia						
Anura						
Neobatrachia						
Strabomantidae						
Pristimantis	*fenestratus*			4	0,13	E
Aromobatidae						
Allobates	sp.	2	0,17			E
