# Supplementary material for: Reconciling Mining with the Conservation of Cave Biodiversity: A Quantitative Baseline to Help Establish Conservation Priorities
Source: PLoS One. 2016 Dec 20;11(12):e0168348. doi: 10.1371/journal.pone.0168348 (PMC5173368; doi:10.1371/journal.pone.0168348)
Supplement: S1 Dataset — (ZIP) [file pone.0168348.s002.zip › Taxa/Serra Sul/SS_2010/S11D_16.pdf]

S11D-16		1ª	AB	2ª	AB	ZON
Arthropoda						
Arachnida						
Acari						
Parasitiformes						
Mesostigmata						
Diploginiidae	sp.4	1				E
Trombidiformes						
Tydeoidea						
Bdellidae	sp.1	1				E
Amblypygi						
Phrynidae						
Heterophrynus	sp.	2	0,1			
Araneae						
Araneidae						
Alpaida	*septemmammata*	1				E
Barychaelidae	jovens	1	0,05	1	0,0625	E
Ctenidae	jovens	1	0,05			E
Ochyroceratidae						
Ochyrocera	sp.1	1				E
Pholcidae						
Leptopholcus	sp.1	1				E
	sp.1	1				E
Ninetinae	sp.1			1		E
Scytodidae	jovens	1	0,05			E
Segestriidae	jovens	1				E
Theridiidae						
Theridion	sp.1	1				E
Opiliones						
Laniatores						
Cosmetidae						
Roquettea	*singularis*			1	0,0625	E
Stygnidae	sp.1	1	0,05			E
Pseudoscorpiones						
Pseudochthonius	sp.1	1				E
Chilopoda						
Pleurostigmophora						
Geophilomorpha						
Ballophilidae	sp.3	1	0,05			E
Entognatha						
Diplura						
Campodeidae	sp.1	1				E
Insecta						
Blattodea	jovens	1	0,05			E
Collembola						
Arthropleona						
Entomobryoidea						
Entomobryidae	sp.2	1				E
Paronellidae	sp.1	2				E
Dermaptera	jovens	1				E
Diptera						
Brachycera						
Camillidae	sp.			1		E
Nematocera	jovens	1				E
Mycetophilidae						
Epicypta	sp.			1		E
Euceroplatus	sp.			1		E
Lygistorrhina	sp.			1		E
Psychodidae						
Sciopemyia	*sordellii*			1		E
Hemiptera						
Homoptera						
Cixiidae	jovens	1				E

		sp.3			1		E
Hymenoptera							
Vespoidea							
Formicidae							
	Cephalotes	sp.1			2		E
	Crematogaster	sp.1	1				E
	Dolichoderus	*bispinosus*	1		2		E
	Nylanderia	sp.1	1				E
	Pheidole	sp.2	1				E
Vespidae		sp.2	1				E
Isoptera		sp.	1				E
Termitidae							
	Nasutitermes	sp.	1				E
Lepidoptera	jovens		1	0,05	2	0,125	E
Noctuoidea							
Noctuidae		sp.1	1	0,05			E
		sp.2	1		1		E
		sp.6	1				E
Orthoptera							
Ensifera							
Phalangopsidae	jovens		1	0,05			E
	Paraclodes	sp.1			8	0,5	E
Psocoptera							
Psocomorpha	jovens		2				E
Trogiomorpha							
Psyllipsocidae	jovens				1		E
Malacostraca							
Isopoda							
Dubioniscidae		sp.1	1				E
Chordata							
Amphibia							
Anura							
Neobatrachia		sp.			1	0,0625	E
Mammalia							
Chiroptera							
Emballonuridae							
	Peropteryx	kappleri	8	0,4			
		sp.			2	0,125	E
Reptilia							
Squamata							
Gekkonidae							
	Thecadactylus	*rapicauda*	1	0,05	1	0,0625	E
