# Supplementary material for: Reconciling Mining with the Conservation of Cave Biodiversity: A Quantitative Baseline to Help Establish Conservation Priorities
Source: PLoS One. 2016 Dec 20;11(12):e0168348. doi: 10.1371/journal.pone.0168348 (PMC5173368; doi:10.1371/journal.pone.0168348)
Supplement: S1 Dataset — (ZIP) [file pone.0168348.s002.zip › Taxa/Serra Sul/SS_2010/S11D_17.pdf]

S11D-17		1ª	AB	2ª	AB	ZON
Annelida						
Clitellata						
Oligochaeta	jovens	1				A
	sp.	1	0,0714			P
Arthropoda						
Arachnida						
Acari						
Ixodida						
Ixodidae						
Amblyomma	sp.1	1				P
Parasitiformes						
Holothyrida						
Diplothyridae						
Diplothyrus	*scubarti*	1				E
Opilioacarida						
Opilioacaridae	sp.1			1		E
Sarcoptiformes						
Oribatida	sp.3	1				A
Trombidiformes						
Tydeoidea						
Cunaxidae	sp.1			1		A
Amblypygi						
Charinidae	jovens	2	0,0714	1		P A
Charinus	sp.			2	0,1579	P
Phrynidae						
Heterophrynus	sp.	1	0,0357	1	0,0526	P
Araneae						
Araneidae						
Alpaida	*septemmammata*	1				E
Ochyroceratidae						
Ochyrocera	sp.1	3		2		E P A
Speocera	sp.1	2		2		P A
Oonopidae	jovens	2				P A
	sp.5	1				E
Pholcidae						
Leptopholcus	sp.1	1				E
Ninetinae	sp.1	2				E P
Prodidomidae	jovens			1		A
Lygromma	sp.2	1				P
Segestriidae	jovens	1				E
Tetrablemmidae						
Matta	sp.1	2		1		P A
Opiliones				1	0,0526	
Cyphophthalmi						
Neogoveidae						
Canga	*renatae*	2				P A
Laniatores	jovens			1	0,0526	P
Escadabiidae	jovens	1				A
	sp.1			1		A
Stygnidae	jovens	1	0,0357			E
	sp.1			1	0,0526	P
Pseudoscorpiones						
Bochicidae	sp.1	2				A
Chthoniidae						
Pseudochthonius	sp.4	4		2		P A
Schizomida						
Hubbardiidae						
Rowlandius	sp.	1				E
Chilopoda						
Notostigmophora						
Scutigeromorpha						
Pselliodidae	jovens	1				A

Diplopoda						
Glomeridesmida						
Glomeridesmidae	sp.1	1				P
Polydesmida	jovens	1				P
Chelodesmidae	sp.6	1	0,0357			A
Fuhrmannodesmidae	sp.3	1				P
Pyrgodesmidae	sp.2	1	0,0357	1	0,0526	P A
Hypogexenidae	sp.1	3		2		E P A
Spirostreptida	jovens			2		P A
Pseudonannolenidae						
Pseudonannolene	sp.3			1	0,0526	P
Entognatha						
Diplura						
Campodeidae	sp.1	2		3		E P A
Insecta						
Blattodea						
Blaberidae	jovens			1	0,0526	P
Coleoptera						
Carabidae	sp.2	2				P A
Scydmaenidae	sp.4	1		1		P
Staphylinidae						
Pselaphinae	sp.1	3				E P A
	sp.2	1				E
Collembola						
Arthropleona						
Entomobryoidea						
Cyphoderidae	sp.1	1				A
Paronellidae	sp.4	1		2		P A
Diptera						
Brachycera						
Streblidae						
Strebla	sp.	1				P
Nematocera	jovens			1		P
Psychodidae						
Sciopemyia	*sordellii*	1				P
Hemiptera						
Homoptera						
Cixiidae	jovens	2		1		E P
Hymenoptera						
Vespoidea						
Formicidae						
Acromyrmex	sp.1			1		E
Camponotus	*atriceps*	1		1		E P
Gnamptogenys	*striatula*	1				A
Hypoponera	sp.1	2		1		P A
Nylanderia	sp.1	2				E P
Octostruma	sp.1	1				P
Pachycondyla	*striata*	1				E
Strumygenys	sp.1			1		P
Isoptera	sp.	1		1		E
Lepidoptera	jovens	1				E
Noctuoidea	sp.2	1				E
Orthoptera						
Ensifera						
Phalangopsidae						
Paraclodes	sp.1			1	0,0526	E
Phalangopsis	sp.1	20	0,7143	6	0,3158	A
Psocoptera						
Psocomorpha	jovens	1		1		E
Pauropoda						
Tetramerocerata	sp.	1				A
Symphyla						
Scutigerellidae						

Hanseniella	sp.1	2				E A
Mammalia						
Chiroptera						
Emballonuridae						
Peropteryx	sp.			2	0,1053	P
Mollusca						
Gastropoda						
Systrophiidae						
Happia	sp.	2		2		P A
Nemathelminthes	sp.			1		P
