# Supplementary material for: Reconciling Mining with the Conservation of Cave Biodiversity: A Quantitative Baseline to Help Establish Conservation Priorities
Source: PLoS One. 2016 Dec 20;11(12):e0168348. doi: 10.1371/journal.pone.0168348 (PMC5173368; doi:10.1371/journal.pone.0168348)
Supplement: S1 Dataset — (ZIP) [file pone.0168348.s002.zip › Taxa/Serra Sul/SS_2010/S11D_18.pdf]

S11D-18			1ª	AB	2ª	AB	ZON
Arthropoda							
Arachnida							
Acari							
Parasitiformes							
Mesostigmata							
Heterozerconidae		sp.1	1				E
Macrochelidae		sp.1	1				E
Ologamasidae		sp.1	1				E
		sp.4	1				E
Opilioacarida							
Opilioacaridae		sp.1	2				E
Araneae							
Araneidae	jovens		1		1		E
	Alpaida	*septemmammata*	1				E
Caponiidae	jovens		1	0,0435			E
Corinnidae	jovens		3	0,1304			E
	Tupirina	sp.1			1	0,0345	E
Ctenidae	jovens		1				E
	Isoctenus	sp.1	1	0,087			E
Drymusidae	jovens		1				E
Pholcidae							
	aff. *Ibityporanga*	sp.1	2				E
		sp.1	1		1		E
	Ninetinae	sp.1	2		2		E
Salticidae	jovens		2				E
	Freya	sp.1	1				E
Scytodidae	*jovens*		2	0,087	1	0,0345	E
Segestriidae							
	Ariadna	sp.1	1				E
Theridiosomatidae							
	Plato	sp.1	1				E
Opiliones							
Laniatores							
Cosmetidae							
	Roquettea	*singularis*			1	0,0345	E
		sp.1	1	0,0435	1	0,0345	E
Stygnidae		sp.1	1	0,0435	5	0,1724	E
Pseudoscorpiones							
Chthoniidae							
	Pseudochthonius	sp.1	1				E
Olpiidae		sp.1	2		2		E
Chilopoda							
Notostigmophora							
Scutigeromorpha							
Pselliodidae	jovens		1				E
Diplopoda							
Polydesmida							
Pyrgodesmidae		sp.2	1	0,0435			E
Hypogexenidae		sp.1	1				E
Insecta							
Blattodea	jovens		2	0,087			E
Blattidae	jovens				1	0,0345	E
Coleoptera	jovens		1	0,0435			
Carabidae		sp.4			1		E
Elateridae		sp.2	1				E
Endomychidae		sp.1			1		E
Phalacridae		sp.2	1				E
Collembola							
Arthropleona							
Entomobryoidea							
Paronellidae		sp.1	1				E

Diptera							
Nematocera	jovens		1				E
Culicidae							
Culicini	sp.		1				E
Psychodidae							
Sciopemyia	*sordellii*		1				E
Hemiptera							
Heteroptera							
aff. Pyrrhocoroidea							
Reduviidae	jovens		5	0,2174	2	0,069	E
Homoptera							
Cixiidae	jovens		1				E
Hymenoptera							
Vespoidea							
Formicidae							
Camponotus	sp.1		1		1		E
Crematogaster	sp.1		1		1		E
Gnamptogenys	*striatula*		1				E
Hypoponera	sp.1		1				E
Isoptera	sp.				1		E
Lepidoptera	jovens		1		1		E
Cossoidea							
Limacodidae	sp.1		2	0,087			E
Hesperioidea							
Hesperiidae	sp.1				1		E
Noctuoidea							
Noctuidae	sp.1		1	0,0435			E
Orthoptera							
Ensifera							
Phalangopsidae							
Paraclodes	sp.1				17	0,5862	E
Psocoptera							
Psocomorpha	jovens		3				E
Troctomorpha							
Liposcelididae							
Liposcelis	sp.1		1				E
Malacostraca							
Isopoda							
Dubioniscidae	sp.1		2				E
Chordata							
Amphibia							
Anura							
Neobatrachia							
Strabomantidae							
Pristimantis	*fenestratus*		1	0,0435			
