# Supplementary material for: Reconciling Mining with the Conservation of Cave Biodiversity: A Quantitative Baseline to Help Establish Conservation Priorities
Source: PLoS One. 2016 Dec 20;11(12):e0168348. doi: 10.1371/journal.pone.0168348 (PMC5173368; doi:10.1371/journal.pone.0168348)
Supplement: S1 Dataset — (ZIP) [file pone.0168348.s002.zip › Taxa/Serra Sul/SS_2010/S11D_19.pdf]

S11D-19		1ª	AB	2ª	AB	ZON
Arthropoda						
Arachnida						
Acari						
Ixodida						
Ixodidae						
Amblyomma	sp.	1				E
Opilioacarida						
Opilioacaridae	sp.1			1		E
Sarcoptiformes						
Oribatida	sp.3			1		E
Trombidiformes	sp.11			1		E
Tydeoidea						
Tydeidae	sp.1			1		E
Amblypygi						
Phrynidae						
Heterophrynus	sp.	1	0,0833	2	0,1176	E
Araneae						
Araneidae jovens		1		1		E
Alpaida	*septemmamn*	1				E
Pholcidae						
Mesabolivar	sp.1	2		1		E
Salticidae jovens		1		1		E
Amphidraus	sp.1			1		E
Scytodidae *jovens*		1		2		E
Scytodes	*eleonorae*	1	0,1667	1	0,1765	E
Theridiosomatidae jovens		1				E
Plato	sp.1	1				E
Opiliones						
Laniatores						
Stygnidae	sp.1	4	0,3333	2	0,1176	E
Pseudoscorpiones						
Chernetidae	sp.1	1				E
Chernetidae	sp.3			1		E
Spelaeochernes	sp.1	1		1		E
Chilopoda jovens		1	0,0833			
Pleurostigmophora						
Scolopendromorpha jovens		1	0,0833			E
Entognatha						
Diplura						
Campodeidae	sp.1	1		1		E
Projapygidae	sp.1	1				E
Insecta						
Coleoptera jovens		1				E
Collembola						
Arthropleona						
Entomobryoidea						
Paronellidae	sp.1	1				E
Diptera						
Nematocera						
Chironomidae	spp.			1		E
Hemiptera						
Homoptera						
Cixiidae jovens		1		1		E
	sp.4	1				E
Hymenoptera						
Vespoidea						
Formicidae						
Brachymyrmex	sp.1	2				E
Camponotus	sp.1	1		1		E
Crematogaster	sp.1			2		E
Octostruma	sp.1	1				E
Pachycondyla	*striata*	1		1		E

		Pheidole		sp.2	1				E
		Wasmania		*auropunctata*	1				E
Isoptera				sp.	1				E
	Termitidae								
		Nasutitermes		sp.	1				E
Lepidoptera			jovens		1	0,0833			
Cossoidea									
	Limacodidae			sp.1	1	0,0833			E
Noctuoidea									
	Noctuidae								
				sp.2	1				E
Orthoptera									
Ensifera									
	Gryllidae		jovens		1	0,0833			E
	Tettigoniidae			sp.			1	0,0588	E
	Phalangopsidae								
		Paraclodes		sp.1			9	0,5294	E
Psocoptera									
Psocomorpha			jovens				1		E
