# Supplementary material for: Reconciling Mining with the Conservation of Cave Biodiversity: A Quantitative Baseline to Help Establish Conservation Priorities
Source: PLoS One. 2016 Dec 20;11(12):e0168348. doi: 10.1371/journal.pone.0168348 (PMC5173368; doi:10.1371/journal.pone.0168348)
Supplement: S1 Dataset — (ZIP) [file pone.0168348.s002.zip › Taxa/Serra Sul/SS_2010/S11D_20.pdf]

S11D-20		1ª	AB	2ª	AB	ZON
Annelida						
Clitellata						
Oligochaeta	jovens	1	0,0172			E
Arthropoda						
Arachnida						
Acari						
Parasitiformes						
Mesostigmata						
Macronyssidae	sp.1	1				E
Trombidiformes						
Tydeoidea						
Eupodidae	sp.1	1				E
Amblypygi						
Phrynidae						
Heterophrynus	sp.	1	0,0172	1	0,0909	E
Araneae						
Araneidae	jovens	2				E
Pholcidae	jovens	1				E
Salticidae	jovens	1				E
Scytodidae	*jovens*	2	0,0345			E
Scytodes	*eleonorae*	1	0,0172			E
	sp.	3	0,0517			
Symphytognathidae						
Anapistula	sp.1	1				E
Tetragnathidae	jovens	1				E
Theridiosomatidae	jovens	2				E
Plato	sp.1	1				E
Opiliones						
Laniatores						
Stygnidae	sp.1	1	0,0172			E
Pseudoscorpiones						
Chernetidae						
Spelaeochernes	sp.1	2		1		E
Scorpiones						
Buthidae						
Ananteris	*balzanii*			1	0,0909	E
Entognatha						
Diplura						
Campodeidae	sp.1	2				E
Insecta						
Blattodea	jovens			1	0,0909	E
Blattidae	sp.3	1	0,0172			E
Coleoptera	jovens	1				E
Carabidae	sp.4	1		1		E
Collembola						
Arthropleona						
Entomobryoidea						
Entomobryidae	sp.10			1		E
Isotomidae	sp.1	1				E
Paronellidae	sp.1	2				E
Dermaptera	jovens	1	0,0172			E
Diptera						
Nematocera	jovens	1				E
Cecidomyiidae						
Cecidomyiinae	sp.	1		1		E
Culicidae						
Culicini	sp.	1				E
Psychodidae	sp.	1				E
Sciopemyia	*sordellii*	2				E
Tipulidae						
Tipulinae	sp.	1		1		E
Hemiptera						

Homoptera						
Cixiidae	jovens	3		1		E
Hymenoptera						
Vespoidea						
Formicidae						
Brachymyrmex	sp.1	1				E
Carebara	sp.1	1				E
Nylanderia	sp.1	1		1		E
Pachycondyla	*striata*	1				E
Solenopsis	sp.1	1		1		E
Isoptera	sp.	1				E
Lepidoptera	jovens	10	0,1724	1	0,0909	E
Noctuoidea	sp.2	1		2		E
Orthoptera						
Ensifera						
Phalangopsidae	jovens	1	0,0172			E
Phalangopsis	sp.1	20	0,3448			E
Paraclodes	sp.1	6	0,1034	7	0,6364	E
Psocoptera						
Psocomorpha	jovens	1				E
Epipsocidae						
Epipsocus	sp.2			1		E
Chordata						
Mammalia						
Chiroptera						
Emballonuridae						
Peropteryx	*kappleri*	1	0,0172			
Phyllostomidae						
Glossophaga	*soricina*	1	0,0172			
Glossophaginae	sp.	8	0,1379			
