# Supplementary material for: Reconciling Mining with the Conservation of Cave Biodiversity: A Quantitative Baseline to Help Establish Conservation Priorities
Source: PLoS One. 2016 Dec 20;11(12):e0168348. doi: 10.1371/journal.pone.0168348 (PMC5173368; doi:10.1371/journal.pone.0168348)
Supplement: S1 Dataset — (ZIP) [file pone.0168348.s002.zip › Taxa/Serra Sul/SS_2010/S11D_21.pdf]

S11D-21			1ª	AB	2ª	AB	ZON
Annelida							
Clitellata							
Arthropoda							
Arachnida							
Acari							
Sarcoptiformes							
Oribatida		sp.3	1				E
Amblypygi							
Phrynidae							
Araneidae							
Alpaida		*septemmamn*	1				E
Mysmenidae							
Microdipoena		sp.1	1				E
Ochyroceratidae							
Speocera		sp.1	1				E
Pholcidae	jovens		1				E
Salticidae	jovens		2		1		E
Theridiosomatidae	jovens		1		1		E
Plato		sp.1	1				E
Opiliones							
Eupnoi							
Sclerosomatidae		sp.1			1		E
Laniatores							
Pseudoscorpiones							
Chernetidae							
Spelaeochernes		sp.1	2		1		E
Chthoniidae							
Pseudochthonius		sp.1	1				E
Ricinulei							
Ricinoididae	jovens		1				E
Schizomida							
Hubbardiidae							
Rowlandius		sp.	1				E
Chilopoda							
Notostigmophora							
Scutigeromorpha							
Pselliodidae	jovens		1				E
Scolopendromorpha							
Scolopocryptopidae							
Entognatha							
Diplura							
Campodeidae		sp.1	2				E
Insecta							
Blattodea							
Coleoptera	jovens		1				E
Collembola							
Arthropleona							
Entomobryoidea							
Paronellidae		sp.1	2		1		E
Diptera	jovens		2				E
Brachycera							
Chloropidae		sp.	1				E
Neriidae							
Odontoloxozus		sp.	1				E
Nematocera							
Psychodidae							
Sciopemyia		*sordellii*			1		E
Sciaridae		sp.	1				E
Hemiptera							
Heteroptera							
aff. Pyrrhocoroidea							
Ochteridae	jovens		1				E

Homoptera							
Cixiidae	jovens		1		1		E
		sp.6	1				E
Diaspididae		sp.1			1		E
Hymenoptera							
Vespoidea							
Formicidae							
	Brachymyrmex	sp.1	2		1		E
	Carebara	sp.1	1				E
	Nylanderia	sp.1	2				E
		sp.2			1		E
Pompilidae		sp.1	1				E
Lepidoptera	jovens		1				E
Noctuoidea		sp.2	1				E
Orthoptera							
Ensifera							
Phalangopsidae							
Psocoptera							
Psocomorpha							
Epipsocidae							
	Epipsocus	sp.2			1		E
Malacostraca							
Isopoda							
Philosciidae		sp.1	1				E
Chordata							
Amphibia							
Anura							
Neobatrachia							
Strabomantidae							
Mammalia							
Chiroptera							
Emballonuridae							
Phyllostomidae							
Mollusca							
Gastropoda							
Systrophiidae							
	Happia	sp.	1				E
