# Supplementary material for: Reconciling Mining with the Conservation of Cave Biodiversity: A Quantitative Baseline to Help Establish Conservation Priorities
Source: PLoS One. 2016 Dec 20;11(12):e0168348. doi: 10.1371/journal.pone.0168348 (PMC5173368; doi:10.1371/journal.pone.0168348)
Supplement: S1 Dataset — (ZIP) [file pone.0168348.s002.zip › Taxa/Serra Sul/SS_2010/S11D_22.pdf]

S11D-22		1ª		2ª	AB	ZON
Annelida						
Clitellata						
Oligochaeta	jovens	6	0,0201	1	0,0068	P A
Arthropoda						
Arachnida						
Acari						
Ixodida						
Argasidae						
Ornithodoros	sp.	1		2		E P
Mesostigmata						
Diploginiidae	sp.2			2		A
Sarcoptiformes	sp.1	1				P
Oribatida						
	sp.13			1		A
	sp.2			2		P A
	sp.3	1				E
Trombidiformes						
Tydeoidea						
	sp.1	2		2		E P A
	sp.2	1				P
	sp.6			1		P
	sp.7			2		E P
Amblypygi						
Charinidae	jovens	3	0,01	3	0,0205	P A
Charinus	sp.			2	0,0137	P
	sp.2	1	0,0033			E
Phrynidae						
Heterophrynus	sp.	1	0,0033			
	sp.1	1	0,0033			P
Araneae		1	0,0033			
Araneidae	jovens	2	0,0067			E
Ochyroceratidae	jovens	1		1		E P
Ochyrocera	sp.1	3		1		E P
Speocera	sp.1	2				E
Pholcidae	jovens	1				P
Scytodidae						
Scytodes	*eleonorae*	1	0,0033			E
Tetrablemmidae						
Matta	sp.1	2		1		P A
Theridiosomatidae	jovens			1		E
Plato	sp.1	4		1		E P
Opiliones	jovens	6	0,0201			
Laniatores						
Cosmetidae	sp.1			1	0,0068	A
Escadabiidae	jovens	3				E P
	sp.1	7		3		E P A
Stygnidae	jovens	1	0,0033			E
Palpigradi						
Eukoeneniidae						
Allokoenenia	sp.1	1				E
Pseudoscorpiones						
Chernetidae	jovens	2				P
Spelaeochernes	sp.1	7		4		E P A
Pseudochthonius	sp.1	3		1		E P A
Schizomida						
Hubbardiidae						
Rowlandius	sp.	2		1		P A
Scorpiones						
Buthidae	jovens	1	0,0033			E
Chilopoda						
Notostigmophora						
Scutigeromorpha						

Pselliodidae		jovens	6		1		E P A
Pleurostigmophora							
Scolopendromorpha							
Cryptopidae							
	Cryptops	sp.1	2	0,0067	1	0,0068	E A
Diplopoda							
Glomeridesmida							
Glomeridesmidae		sp.1	2				A
Polydesmida							
Pyrgodesmidae		sp.1	1	0,0033			P
		sp.2	4	0,0134	3	0,0205	E P A
Spirostreptida		jovens	2		1		E P
Diplopoda		jovens	55	0,1839			
Entognatha							
Diplura							
Campodeidae		sp.1	3				E P
Insecta							
Blattodea		jovens	1	0,0033			P
Blattidae		jovens					
		sp.3	1	0,0033			E
Coleoptera		jovens			1		A
Curculionidae							
	Platypodinae	sp.1	1				E
Ptiliidae		sp.1			1		A
Collembola							
Arthropleona							
Entomobryoidea							
Entomobryidae		sp.2			1		P
Paronellidae		sp.1	1		1		E
		sp.4			1		A
Poduroidea		sp.1			1		E
Symphypleona							
Sminthuroidea		sp.2	3		3		E P A
Diptera		jovens	5		4		E P A
Brachycera							
aff. Muscidae		sp.	1				E
Conopidae		sp.	1				P
Drosophilidae							
	Drosophila	*eleonore*	3				E A
Muscidae		sp.	1				E
Phoridae							
	Metopininae	sp.	1				P
	Phorinae	sp.	1		1		P A
Streblidae							
	Strebla	sp.	1				E
Nematocera							
Chironomidae		sp.	1		1		A
Psychodidae							
	Pericoma	sp.	4				E P A
	Pintomyia	*gruta*	1				P
	Sciopemyia	*sordellii*	4		1		E P
Sciaridae							
	Schwenkfeldina	sp.			1		A
Hemiptera							
Heteroptera							
Dipsocoroidea		jovens	3				P A
Lygaeidae		jovens	1				E
Reduviidae		jovens	1	0,0033	1	0,0068	E
Schizopteridae							
	Schizopterinae	sp.1	1		3		E P A
Homoptera		jovens	2	0,0067			
Cixiidae		jovens	3		1		E P
Hymenoptera							

Vespoidea						
Formicidae						
Carebara	sp.1	1				P
Hypoponera	sp.1	1				P
Octostruma	sp.1	1		1		P
Odontomachus	*bauri*	1		2		A
Solenopsis	sp.2	2		2		P A
Wasmania	*auropunctata*	1				E
Isoptera	sp.			1		P
Termitidae						
Nasutitermes	sp.	6		2		E P A
Lepidoptera	jovens	3		1		E P
Noctuoidea	sp.2	2				E
Noctuidae	sp.1	6	0,0201			
Orthoptera						
Ensifera						
Phalangopsidae						
Phalangopsis	sp.1	172	0,5753	99	0,6781	E P A
Thysanura						
Nicoletiidae	sp.1	1				E
Malacostraca						
Isopoda						
Philosciidae	sp.1	6		1		E P A
Scleropactidae	sp.	1				A
Chordata						
Amphibia						
Anura						
Neobatrachia						
Leptodactylidae						
Leptodactylus	sp.			1	0,0068	E
Strabomantidae						
Pristimantis	*fenestratus*			4	0,0274	E
Mammalia						
Chiroptera						
Emballonuridae						
Peropteryx	*kappleri*	2	0,0067			
	sp.	5	0,0167			
Phyllostomidae						
Carollia	*brevicauda*	2	0,0067			
	sp.	20	0,0669	20	0,137	A
Glossophaga	*soricina*	1	0,0033			
Glossophaginae	sp.			10	0,0685	A
Mollusca						
Gastropoda						
Subulinidae						
Lamellaxis	sp.	8		2		E P A
Systrophiidae						
Happia	sp.	2				E A
