# Supplementary material for: Reconciling Mining with the Conservation of Cave Biodiversity: A Quantitative Baseline to Help Establish Conservation Priorities
Source: PLoS One. 2016 Dec 20;11(12):e0168348. doi: 10.1371/journal.pone.0168348 (PMC5173368; doi:10.1371/journal.pone.0168348)
Supplement: S1 Dataset — (ZIP) [file pone.0168348.s002.zip › Taxa/Serra Sul/SS_2010/S11D_23.pdf]

S11D-23		1ª	AB	2ª	AB	ZON
Arthropoda						
Arachnida						
Amblypygi						
Charinidae	jovens	1	0,0333			E
Phrynidae						
Heterophrynus	sp.	1	0,0333	1	0,0909	E
Araneae						
Araneidae	jovens	1				E
Ochyroceratidae						
Ochyrocera	sp.1	1				E
Speocera	sp.1	2				E P
Pholcidae						
Mesabolivar	sp.1	1				E
Salticidae	jovens			1		E
Theridiidae						
Theridion	sp.1	1				E
Theridiosomatidae	jovens			1		P
Plato	sp.1	1				E
Opiliones						
Laniatores						
Escadabiidae	sp.1	1				P
Pseudoscorpiones						
Chernetidae						
Spelaeochernes	sp.1	1				E
Schizomida						
Hubbardiidae	jovens	1				P
Chilopoda						
Pleurostigmophora						
Geophilomorpha	jovens	1	0,0333			P
Scolopendromorpha						
Scolopocryptopidae						
Newportia	sp.1	1	0,0333			E
Diplopoda	jovens			2	0,1818	P
Polydesmida						
Pyrgodesmidae	sp.2	1	0,0333			P
Spirostreptida	jovens	1		1		P
Entognatha						
Diplura						
Campodeidae	sp.1			1		P
Insecta						
Coleoptera	jovens	1				P
Collembola						
Arthropleona						
Entomobryoidea						
Entomobryidae	sp.10			1		E
Isotomidae	sp.1	1				P
Paronellidae	sp.3	1				P
Symphypleona						
Sminthuroidea	sp.2	1				P
Diptera	jovens	1				E
Nematocera						
Cecidomyiidae						
Cecidomyiinae	sp.	1				E
Psychodidae						
Sciopemyia	*sordellii*			1		E
Tipulidae						
Tipulinae	sp.	1		1		E
Hemiptera						
Heteroptera						
Dipsocoroidea	jovens			1		E
Homoptera	jovens	2	0,0667			E
Cixiidae	jovens	1		1		E

		sp.3	1				E
Hymenoptera							
Vespoidea							
Formicidae							
	Odontomachus	*bauri*	1	0,0333			E
	Pachycondyla	*striata*	1		1		E P
Lepidoptera	jovens		10	0,3333	1	0,0909	P
Noctuoidea							
Noctuidae		sp.2	1				E
Orthoptera							
Ensifera							
Phalangopsidae							
	Paraclodes	sp.			4	0,3636	E
	Phalangopsis	sp.	4	0,1333	2	0,1818	P
Psocoptera							
Psocomorpha	jovens				1		E
Chordata							
Amphibia							
Anura							
Neobatrachia							
Strabomantidae							
	Pristimantis	*fenestratus*	1	0,0333	1	0,0909	E
Mammalia							
Chiroptera							
Emballonuridae							
	Peropteryx	*kappleri*	1	0,0333			
		sp.	5	0,1667			
Phyllostomidae							
	Glossophaga	*soricina*	1	0,0333			
