# Supplementary material for: Reconciling Mining with the Conservation of Cave Biodiversity: A Quantitative Baseline to Help Establish Conservation Priorities
Source: PLoS One. 2016 Dec 20;11(12):e0168348. doi: 10.1371/journal.pone.0168348 (PMC5173368; doi:10.1371/journal.pone.0168348)
Supplement: S1 Dataset — (ZIP) [file pone.0168348.s002.zip › Taxa/Serra Sul/SS_2010/S11D_24.pdf]

S11D-24		1ª	AB	2ª		ZON
Arthropoda						
Arachnida						
Acari						
Sarcoptiformes						
Oribatida	sp.3			1		E
Trombidiformes						
Tydeoidea						
Labdostomatidae	sp.1	1				P
Amblypygi						
Phrynidae						
Heterophrynus	sp.			2	0,0267	E
Araneae						
Araneidae						
Alpaida	*septemmammata*	1				E
Ochyroceratidae						
Ochyrocera	sp.1	4				E P
Speocera	sp.1	4		2		E P
Salticidae jovens		1				E
Scytodidae	jovens	1	0,0545	1	0,0133	E
Scytodes	*eleonorae*	2				E P
Theridiosomatidae						
Plato	sp.1	2		2		E P
Opiliones						
Laniatores						
Cosmetidae	jovens	1	0,0182			E
Stygnidae	sp.1	1	0,0182	3	0,04	E P
Pseudoscorpiones						
Chernetidae						
Spelaeochernes	sp.1	3		2		E P
Chtoniidae						
Pseudochthonius	sp.1	1		2		E P
Ricinulei						
Ricinoididae	jovens	1		1		E
Cryptocellus	sp.			1		P
Schizomida						
Hubbardiidae	jovens	1				P
Rowlandius	sp.	2				E
Diplopoda						
Glomeridesmida						
Glomeridesmidae	sp.1	1				E
Polydesmida						
Pyrgodesmidae	sp.2	2	0,0364			E
Polyxenida						
Hypogexenidae	sp.1			1		P
Entognatha						
Diplura						
Campodeidae	sp.1	4		1		E P
Insecta						
Blattodea	jovens	2	0,0364			E
Blaberidae	jovens	2	0,0364			P
Coleoptera	jovens			1		E
Curculionidae						
Scolytinae	sp.2			1		P
Ptiliidae	sp.1	1				P
Collembola						
Symphypleona						
Sminthuroidea	sp.2	1				E
Diptera	jovens	1				E
Brachycera						
Dolichopodidae	sp.			1		P
Phoridae						
Phorinae	sp.	1				E

Nematocera						
Psychodidae						
Pintomyia	*gruta*	2				E
Sciopemyia	*sordellii*	2		1		E P
Tipulidae						
Tipulinae	sp.			1		P
Hemiptera						
Heteroptera						
aff. Pyrrhocoroidea						
Cydnidae						
Cydninae	sp.1	3				E P
Reduviidae	jovens	1	0,0182	3	0,1067	E P
Reduviinae	sp.			5		E
Schizopteridae						
Schizopterinae	sp.1			1		E
	sp.2			1		E
Homoptera						
Cixiidae	jovens	4		2		E P
Hymenoptera	jovens	1				P
Vespoidea						
Formicidae						
Camponotus	*atriceps*	2				E
	sp.1	1		2		E P
Hypoponera	sp.1			1		P
Pachycondyla	*striata*	2				E P
Wasmania	*auropunctata*	1				E
Isoptera	sp.	1				E
Termitidae						
Nasutitermes	sp.	2		2		E P
Lepidoptera	jovens	1		1		E P
Noctuoidea						
Noctuidae	sp.2	1				E
	sp.4	1				E
	sp.1	1	0,0182			
Orthoptera						
Ensifera						
Phalangopsidae						
Phalangopsis	sp.1	1	0,0182	37	0,4933	E P
Paraclodes	sp.1	27	0,4909	7	0,0933	E
Psocoptera						
Psocomorpha	jovens	1				E
Siphonaptera						
Leptopsyllidae						
Leptopsylla	sp.1	1				E
Thysanura						
Nicoletiidae	sp.1	1		2		E P
Malacostraca						
Isopoda						
Philosciidae	sp.1	4		1		E P
Chordata						
Amphibia						
Anura						
Neobatrachia						
Strabomantidae						
Pristimantis	*fenestratus*			1	0,0133	P
Mammalia						
Chiroptera						
Emballonuridae						
Peropteryx	sp.	2	0,0364			
Phyllostomidae						
Carollia	*perspicillata*			10	0,1333	P
	sp.	12	0,2182			
Micronycteris	sp.			6	0,08	P

Mollusca					
Gastropoda					
Subulinidae					
Lamellaxis sp.	1		1		P
Systrophiidae					
Happia sp.	1				E
