# Supplementary material for: Reconciling Mining with the Conservation of Cave Biodiversity: A Quantitative Baseline to Help Establish Conservation Priorities
Source: PLoS One. 2016 Dec 20;11(12):e0168348. doi: 10.1371/journal.pone.0168348 (PMC5173368; doi:10.1371/journal.pone.0168348)
Supplement: S1 Dataset — (ZIP) [file pone.0168348.s002.zip › Taxa/Serra Sul/SS_2010/S11D_25.pdf]

S11D-25		1ª	AB	2ª	AB	ZON
Annelida						
Clitellata						
Oligochaeta	sp.	1	0,04			P
Arthropoda						
Arachnida						
Acari						
Parasitiformes						
Mesostigmata	sp.8	1				P
Sarcoptiformes						
Oribatida	sp.2	1				P
Amblypygi						
Phrynidae						
Heterophrynus	sp.	1	0,04	2	0,1667	P
Araneae						
Ctenidae	jovens			1		P
Ochyroceratidae	jovens	1				P
Pholcidae						
Mesabolivar	*aurantiacus*	1				P
Ninetinae	sp.1			1		P
Scytodidae						
Scytodes	*eleonorae*			1		P
Theridiidae	jovens	1		1		P
Theridiosomatidae	jovens	1				P
Plato	sp.1	1				P
Opiliones	jovens	2	0,08			
Eupnoi						
Sclerosomatidae	jovens	1				P
	sp.1	1		1		P
Pseudoscorpiones						
Pseudochthonius	sp.1	1				P
Diplopoda						
Glomeridesmida						
Glomeridesmidae	sp.4	1				P
Spirobolida						
Rhinocricidae	sp.1	1				P
Spirostreptida	jovens	1				P
Pseudonannolenidae	jovens	1	0,04			P
Diplopoda	jovens	4	0,17			
Entognatha						
Diplura						
Campodeidae	sp.1	2		1		P
Insecta						
Blattodea	jovens	1	0,04			P
Blattidae	sp.1			1	0,0833	P
Coleoptera						
Carabidae	sp.4			1		P
	sp.5	1				P
Staphylinidae	sp.5	1				P
Collembola						
Arthropleona						
Entomobryoidea						
Entomobryidae	sp.11			1		P
Paronellidae	sp.1	2				P
Diptera						
Nematocera						
Chironomidae	sp.			1		P
Culicidae						
Wyeomyia	sp.			1		P
Tipulidae						
Tipulinae	sp.	1		1		P
Hemiptera						

Heteroptera							
	Cydnidae	jovens	1				P
Hymenoptera							
Chalcidoidea		sp.4			1		P
Vespoidea							
	Formicidae						
	Acromyrmex	sp.1	1				P
	Apterostigma	sp.1			1		P
	Carebara	sp.1	1				P
	Cephalotes	sp.1	2				P
	Crematogaster	sp.1	2				P
	Dolichoderus	*bispinosus*			1		P
	Platythyrea	*angusta*	1				P
	Wasmania	*auropunctata*	1				P
Isoptera							
	Termitidae						
	Nasutitermes	sp.	1		1		P
Lepidoptera		jovens	4	0,17			
Gelechioidea		sp.2			1		P
Noctuoidea		sp.2	2				P
	Noctuidae	sp.1					
		sp.			1	0,0833	P
Orthoptera							
Ensifera							
	Phalangopsidae	jovens	10	0,42			
	Paraclodes	sp.			3	0,25	P
Psocoptera							
Psocomorpha		jovens	2		1		P
Trogiomorpha							
	Psyllipsocidae	jovens			1		P
Malacostraca							
Isopoda							
	Dubioniscidae	sp.1	1				P
	Platyarthridae	sp.	1				P
Chordata							
Amphibia							
Anura							
Neobatrachia							
	Strabomantidae						
	Pristimantis	*fenestratus*			5	0,4167	P

=
