# Supplementary material for: Reconciling Mining with the Conservation of Cave Biodiversity: A Quantitative Baseline to Help Establish Conservation Priorities
Source: PLoS One. 2016 Dec 20;11(12):e0168348. doi: 10.1371/journal.pone.0168348 (PMC5173368; doi:10.1371/journal.pone.0168348)
Supplement: S1 Dataset — (ZIP) [file pone.0168348.s002.zip › Taxa/Serra Sul/SS_2010/S11D_26.pdf]

S11D-26		1ª	AB	2ª	AB	ZON
Arthropoda						
Arachnida						
Acari						
Sarcoptiformes						
Oribatida	sp.1			1		E
Trombidiformes						
Tydeoidea	sp.1	1				E
Amblypygi						
Phrynidae						
Heterophrynus	sp.	1	0,0217	1	0,0667	E
Araneae						
Araneidae	jovens	1				E
Mangora	*mitu*	1				E
Ochyroceratidae	jovens	1				E
Ochyrocera	sp.1	2		2		E
	sp.3	1				E
Speocera	sp.1	1				E
Oonopidae	jovens	1				E
Pholcidae	jovens	1				E
Mesabolivar	sp.1	1		1		E
Salticidae	jovens	1				E
Scytodidae	jovens	2	0,0435			E
Scytodes		1	0,0217			
	sp.	1	0,0217			
Theraphosidae	jovens	1	0,0217	1	0,0667	E
Theridiosomatidae						
Plato	sp.1	1		1		E
Opiliones						
Laniatores						
Cosmetidae						
Roquettea	*singularis*	1	0,0217			E
Stygnidae	jovens	1	0,0217			E
	sp.1	3	0,0652	2	0,1333	E
Pseudoscorpiones						
Spelaeochernes	sp.1	1		2		E
Schizomida						
Hubbardiidae						
Rowlandius	sp.	1				E
Diplopoda						
Spirostreptida						
Pseudonannolenidae	jovens	1	0,0217			E
Entognatha						
Diplura						
Campodeidae	sp.1	1				E
Insecta						
Collembola						
Arthropleona						
Entomobryoidea						
Entomobryidae	sp.4	1				E
Diptera	jovens			1		E
Nematocera						
Chironomidae	sp.			1		E
Psychodidae						
Phlebotominae	sp.			1		E
Pintomyia	*gruta*			1		E
Sciopemyia	*sordellii*	2		1		E
Hemiptera						
Homoptera	jovens	1				
Cicadellidae	sp.1			1		E
Cixiidae	sp.4	2				E
Hymenoptera						
Vespoidea						

Formicidae							
	Camponotus	*atriceps*	3				E
		sp.1			1		E
	Hypoponera	sp.1			1		E
	Nylanderia	sp.1	2				E
	Octostruma	sp.1	2				E
	Solenopsis	sp.1	1				E
Isoptera		sp.	1				E
Termitidae							
	Nasutitermes	sp.	1				E
Lepidoptera		jovens	2	0,0435			E
Cossoidea							
Limacodidae		sp.1	1	0,0217			E
Orthoptera							
Ensifera							
Gryllidae		jovens	1	0,0217			E
Phalangopsidae							
	Paraclodes	sp.1			5	0,3333	E
	Phalangopsis	sp.1	26	0,5652	4	0,2667	E
Psocoptera							
Psocomorpha		jovens			2		E
Epipsocidae							
	Mesepipsocus	sp.1			1		E
Symphyla							
Scutigerellidae		jovens			1		E
	Hanseniella	sp.1			1		E
Chordata							
Amphibia							
Anura							
Neobatrachia							
Strabomantidae							
	Pristimantis	*fenestratus*	1	0,0217			
Mammalia							
Chiroptera		sp.			2	0,1333	E
Phyllostomidae							
	Carollia	sp.	3	0,0652			
