# Supplementary material for: Reconciling Mining with the Conservation of Cave Biodiversity: A Quantitative Baseline to Help Establish Conservation Priorities
Source: PLoS One. 2016 Dec 20;11(12):e0168348. doi: 10.1371/journal.pone.0168348 (PMC5173368; doi:10.1371/journal.pone.0168348)
Supplement: S1 Dataset — (ZIP) [file pone.0168348.s002.zip › Taxa/Serra Sul/SS_2010/S11D_27.pdf]

S11D-27			1ª	AB	2ª	AB	ZON
Annelida							
Clitellata							
Oligochaeta		jovens	3	0,0283			E
Arthropoda							
Arachnida							
Acari							
Parasitiformes							
Mesostigmata		sp.5	1				E
	Macronyssidae	sp.1	2				E
Sarcoptiformes		sp.1	1		1		E
Oribatida		sp.5			1		E
		sp.7	1				E
	Acaridae	sp.1			1		E
		sp.2			1		E
Trombidiformes							
Tydeoidea							
	Rhagidiidae	sp.1	1		1		E
Araneae							
	Ctenidae	jovens	1	0,0094			E
	Ochyroceratidae	jovens			1		E
	Ochyrocera	sp.1	1		2		E
	Pholcidae	jovens	2				E
	Scytodidae						
	Scytodes	*eleonorae*	1	0,0094			E
		sp.	1	0,0094			
	Theridiidae	jovens	1		1		E
	Thymoites	sp.1			1		E
	Theridiosomatidae	jovens			1		E
	Plato	sp.1	3				E
Opiliones							
Laniatores							
	Stygnidae	jovens	1	0,0094			E
Pseudoscorpiones							
	Chernetidae						
	Spelaeochernes	sp.1	1		1		E
	Chthoniidae						
	Pseudochthonius	sp.1			1		E
Chilopoda							
Pleurostigmophora							
Scutigeromorpha							
	Pselliodidae						
	Sphendononema	sp.			1	0,0345	E
Insecta							
Blattodea							
	Blattidae	sp.3	1	0,0094			E
Coleoptera		jovens	1				E
	Leiodidae	sp.1	4				E
	Ptiliidae	sp.1			1		E
		sp.6	2				E
Collembola							
Arthropleona							
Entomobryoidea							
	Isotomidae	sp.1	2				E
		sp.2	3				E
	Paronellidae	sp.1	1		1		E
		sp.4	1				E
Symphypleona							
Sminthuroidea		sp.2	4		3		E
Diptera		jovens			1		E
Brachycera							
	Camillidae	sp.	1		1		E
	Chloropidae	sp.	1				E

Conopidae	sp.	1		1		E
Dolichopodidae	sp.			1		E
Drosophilidae						
Drosophila	*eleonore*	1				E
Phoridae						
Metopininae	sp.	1				E
Phorinae	sp.	1				E
Nematocera						
Chironomidae	sp.	1		1		E
Psychodidae						
Pintomyia	*gruta*	1		1		E
Sciopemyia	*sordellii*	2				E
Tipulidae						
Tipulinae	sp.	1		1		E
Hemiptera						
Heteroptera						
Cydnidae jovens		1		2		E
Cydninae	sp.1	3				E
Homoptera						
Cixiidae jovens		4		2		E
Hymenoptera						
Vespoidea						
Formicidae						
Camponotus	*atriceps*	2				E
Pachycondyla	*harpax*	1				E
Solenopsis	sp.2			1		E
Lepidoptera jovens		1				E
Noctuoidea						
Noctuidae	sp.2	1				E
	sp.1	1	0,0094			
Orthoptera						
Ensifera						
Phalangopsidae jovens		1	0,0094			E
Paraclodes	sp.1			6	0,2069	E
Phalangopsis	sp.1	68	0,6415			
Psocoptera						
Psocomorpha jovens				1		E
Chordata						
Amphibia						
Anura						
Neobatrachia						
Strabomantidae						
Pristimantis	*fenestratus*	1	0,0094	6	0,2069	E
Mammalia						
Chiroptera						
Emballonuridae						
Peropteryx	*kappleri*	3	0,0283			
	sp.			2	0,069	E
Phyllostomidae						
Carollia	*perspicillata*	20	0,1887			
	sp.			4	0,1379	E
Glossophaginae	sp.			10	0,3448	E
Rodentia	sp.	4	0,0377			
Mollusca						
Gastropoda						
Systrophiidae						
Happia	sp.	1				E
