# Supplementary material for: Reconciling Mining with the Conservation of Cave Biodiversity: A Quantitative Baseline to Help Establish Conservation Priorities
Source: PLoS One. 2016 Dec 20;11(12):e0168348. doi: 10.1371/journal.pone.0168348 (PMC5173368; doi:10.1371/journal.pone.0168348)
Supplement: S1 Dataset — (ZIP) [file pone.0168348.s002.zip › Taxa/Serra Sul/SS_2010/S11D_28.pdf]

S11D-28			1ª	AB	2ª	AB	ZON
Annelida							
Clitellata							
Oligochaeta		jovens	1	0,0112			P
Arthropoda							
Arachnida							
Acari							
Parasitiformes							
Mesostigmata		sp.1			1		P
Sarcoptiformes							
Oribatida		sp.2	1				P
		sp.5			1		P
	Acaridae	sp.1	1				P
Amblypygi							
	Phrynidae						
	Heterophrynus	sp.	2	0,0225	3	0,1	P
Araneae					1	0	
	Araneidae	jovens	1				E
	Corinnidae	jovens			1	0	P
	Ctenidae	jovens	1	0,0112	1	0	E
	Mysmenidae						
	Microdipoena	sp.1	1				E
	Ochyroceratidae	jovens	1				P
	Ochyrocera	sp.1			1		P
	Speocera	sp.1	1				E
	Pholcidae						
	Mesabolivar	sp.1	2		1		E
	Prodidomidae						
	Lygromma	sp.3			1		P
	Scytodidae	jovens	1	0,0112	1	0	P
	Theridiidae						
	Theridion	sp.1			1		P
	Theridiosomatidae	jovens	1				P
	Plato	sp.1	1				E
Opiliones			1	0,0112			
Eupnoi							
	Sclerosomatidae	sp.1			1		E
Laniatores		jovens	1	0,0112			
	Stygnidae	sp.1	1	0,0112	13		E
		sp.1			1	0,3	P
Pseudoscorpiones							
	Spelaeochernes	sp.1	1				E
Chilopoda							
Notostigmophora							
Scutigeromorpha							
	Pselliodidae						
	Sphendononema	*guildingii*			1		P
Pleurostigmophora							
Scolopendromorpha							
	Scolopocryptopidae						
	Tidops	sp.1	1	0,0112			P
Entognatha							
Diplura							
	Campodeidae	sp.1	3		1		E P
Insecta							
Coleoptera		jovens	1				P
	Carabidae	sp.6			1		P
	Leiodidae	sp.2	1				E
Collembola							
Arthropleona							
Entomobryoidea							
	Paronellidae	sp.1	2				E P
Symphypleona							

Sminthuroidea	sp.2	1				P
Diptera						
Brachycera						
Dolichopodidae	sp.			1		P
Nematocera						
Cecidomyiidae						
Cecidomyiinae	sp.	1				P
Chironomidae	sp.			1		P
Psychodidae						
Sciopemyia	*sordellii*	1				E
Tipulidae						
Tipulinae	sp.			1		E
Hemiptera						
Heteroptera	jovens	2	0,0225			
aff. Pyrrhocoroidea						
Reduviidae	jovens	1	0,0112	1	0	P
Homoptera						
Cixiidae	jovens	1				P
Hymenoptera	jovens	1				E
Ichneumonoidea						
Braconidae	sp.1			1		P
Vespoidea						
Formicidae						
Carebara	sp.1	1				E
Gnamptogenys	*striatula*	1				P
Octostruma	sp.1	1				E
Lepidoptera						
Noctuoidea						
Noctuidae	sp.2	1	0,0112			E
	sp.			3	0,1	P
Orthoptera						
Ensifera						
Phalangopsidae						
Paraclodes	sp.1	3	0,0337	8	0,2	P
Phalangopsis	sp.1	66	0,7416	10	0,2	P
Thysanura						
Nicoletiidae	jovens	1				E
	sp.1	1				P
Malacostraca						
Isopoda						
Philosciidae	sp.1	2		1		E P
Symphyla						
Scutigerellidae						
Hanseniella	sp.1	1				E
Chordata						
Amphibia						
Anura	sp.			1	0	P
Neobatrachia						
Bufonidae						
Rhinella	cf. *marina*	1	0,0112			
Mammalia						
Chiroptera						
Emballonuridae						
Peropteryx	sp.			2	0	P
Phyllostomidae						
Carollia	sp.	6	0,0674			
Mollusca						
Gastropoda						
Bulimulidae						
Naesiotus	sp.	1				P
Systrophiidae						
Happia	sp.	1				P
