# Supplementary material for: Reconciling Mining with the Conservation of Cave Biodiversity: A Quantitative Baseline to Help Establish Conservation Priorities
Source: PLoS One. 2016 Dec 20;11(12):e0168348. doi: 10.1371/journal.pone.0168348 (PMC5173368; doi:10.1371/journal.pone.0168348)
Supplement: S1 Dataset — (ZIP) [file pone.0168348.s002.zip › Taxa/Serra Sul/SS_2010/S11D_29.pdf]

S11D-29			1ª	AB	2ª	AB	ZON
Arthropoda							
Arachnida							
Acari				0,0932		0,1196	
Ixodida				0,0085			
Ixodidae							
Amblyomma		sp.			1		P
Parasitiformes							
Mesostigmata							
Diploginiidae		sp.4	1				P
Sarcoptiformes							
Oribatida		sp.1	2				E P
Trombidiformes							
Tydeoidea		sp.6		0,0254	2		P
Amblypygi				0,0169			
Charinidae	jovens			0,0169	1		P
Phrynidae				0,0254			
Heterophrynus		sp.	11		5		P
Araneae	jovens		1				P
Araneidae	jovens		1				E P
Ctenidae	jovens				1		P
Ochyroceratidae	jovens		1	0,0085			P
Ochyrocera		sp.1	4		1		E P
Speocera		sp.1	3		3		P
Pholcidae	jovens		2	0,0085			P
aff. *Ibityporanga*		sp.1	1	0,0169			E P
Mesabolivar		sp.1	1	0,0169		0,0217	E P
Ninetinae		sp.1			1		E P
Salticidae	jovens		1	0,0254		0,0326	E P
Scytodidae	jovens		3	0,0254		0,0326	E P
Scytodes		*eleonorae*	2	0,0169		0,0217	E P
		globula	2				E P
		sp.	3				P
Segestriidae							
Ariadna		sp.1	2				E P
Theridiosomatidae	jovens		2		1		E P
Plato		sp.1	2				P
Opiliones							
Laniatores							
Escadabiidae		sp.1	3		3		P
Stygnidae	jovens		1	0,0085			E P
Pseudoscorpiones							
Bochicidae		sp.1			2		P
Chernetidae				0,0254		0,0326	
Spelaeochernes		sp.1	2		2		E P
Chthoniidae							
Pseudochthonius		sp.1	3		2		P
Chilopoda							
Pleurostigmophora							
Scolopendromorpha							
Cryptopidae							
Cryptops		sp.1	1				P
Scolopocryptopidae							
Dinocryptops		*miersii*	2				E P
Diplopoda				0,0254			
Polydesmida				0,0424			
Pyrgodesmidae		sp.2	2	0,0169	1	0,0217	P
Entognatha				0,5593		0,7174	
Diplura							
Campodeidae		sp.1	1		1		P
Insecta							
Blattodea	jovens		3		1		E P
Blaberidae	jovens		3		1		P

Blattidae	jovens		2		2		P
Coleoptera	jovens		3	0,0169	1		E P
Staphylinidae		sp.7	1				E P
Collembola							
Arthropleona							
Entomobryoidea							
Cyphoderidae		sp.1	1				P
Paronellidae		sp.1	2				E P
Diptera	jovens		5		2		E P
Brachycera							
Drosophilidae							
Drosophila		*eleonore*	2				P
Phoridae							
Acontistoptera		sp.			1		P
Nematocera							
Chironomidae		sp.	1				P
Psychodidae							
Deanemyia		*ramirezi*			1		P
Pintomyia		*gruta*	5				E P
Sciopemyia		*sordellii*	3				E P
Hemiptera							
Heteroptera	jovens		1				P
Dipsocoroidea	jovens				1		P
Ceratocombidae							
Trichotonanninae		sp.1	1				E P
Cydnidae							
Cydninae		sp.1	1				P
Reduviidae	jovens		3		1		E P
Homoptera							
Cixiidae	jovens		4		2		E P
		sp.4	1				P
Hymenoptera							
Ichneumonoidea							
Braconidae		sp.1	1				P
Vespoidea							
Formicidae							
Acromyrmex		sp.1			1		P
Camponotus		*atriceps*	2				P
Gnamptogenys		*striatula*	1				E P
Hypoponera		sp.1	2		1		E P
Pachycondyla		*striata*	1				P
Pheidole		sp.2	1				E P
Solenopsis		sp.1	1				P
		sp.2			1		P
Wasmania		*auropunctata*			1		P
Lepidoptera	jovens		2		1		P
Cossoidea							
Limacodidae		sp.1	3				E P
Noctuoidea							
Noctuidae		sp.1	5				P
		sp.2	2		1		E P
Tineoidea		sp.1			1		E P
Orthoptera							
Ensifera							
Phalangopsidae							
Phalangopsis		sp.1	66		32		P
Paraclodes		sp.1			3		P
Psocoptera							
Psocomorpha	jovens		1				E P
Ptiloneuridae							
Triplocania		sp.8	1				P
Thysanura							
Ateluridae	jovens				1		P

			sp.1	1				P
Malacostraca								
Isopoda								
	Philosciidae		sp.1	1				P
Symphyla								
	Scutigerellidae							
		Hanseniella	sp.1	1				P
		Scutigerella	sp.1	1				P
Chordata								
Mammalia								
Chiroptera								
	Emballonuridae							
		Peropteryx	sp.	2				P
