# Supplementary material for: Reconciling Mining with the Conservation of Cave Biodiversity: A Quantitative Baseline to Help Establish Conservation Priorities
Source: PLoS One. 2016 Dec 20;11(12):e0168348. doi: 10.1371/journal.pone.0168348 (PMC5173368; doi:10.1371/journal.pone.0168348)
Supplement: S1 Dataset — (ZIP) [file pone.0168348.s002.zip › Taxa/Serra Sul/SS_2010/S11D_30.pdf]

S11D-30		1ª	AB	2ª	AB	ZON
Arthropoda						
Arachnida						
Acari						
Parasitiformes						
Opilioacarida						
Opilioacaridae	sp.1	1				E
Sarcoptiformes						
Acaridae	sp.1	1				E
Araneae						
Araneidae	jovens	1		1		E
Ctenidae	jovens	1	0,0526			E
Ochyroceratidae	jovens	1				E
Pholcidae	jovens	2				E
	sp.1	1				E
Ninetinae	sp.1			1		E
Salticidae						
Amphidraus	sp.1	1				E
Scytodidae	jovens	1	0,0526	1	0,125	E
Theridiidae	jovens			1		E
Opiliones						
Eupnoi						
Sclerosomatidae	jovens	1				E
Laniatores	jovens	1				E
Stygnidae	jovens	1	0,0526			E
	sp.1	1	0,0526			E
Pseudoscorpiones						
Chernetidae						
Spelaeochernes	sp.1	1		1		E
Chthoniidae						
Pseudochthonius	sp.1	1				E
Chilopoda						
Notostigmophora						
Scutigeromorpha						
Pselliodidae	jovens			1		E
Entognatha						
Diplura						
Campodeidae	sp.1	1				E
Insecta						
Blattodea		3	0,1579			
Polyphagidae	sp.1			1	0,125	E
	jovens	1	0,0526	1	0,125	E
Collembola						
Arthropleona						
Entomobryoidea						
Cyphoderidae	sp.1			1		E
Entomobryidae	sp.1			1		E
	sp.8			1		E
Paronellidae	sp.1	1				E
Diptera						
Nematocera	jovens	1				E
Tipulidae						
Tipulinae	sp.	1				E
Hemiptera						
Heteroptera						
aff. Pyrrhocoroidea						
Reduviidae	jovens			1	0,125	E
Homoptera						
Cixiidae	jovens	1		1		E
Hymenoptera						
Vespoidea						
Formicidae						

Hypoponera	sp.1	1				E
Nylanderia	sp.1	1		1		E
Odontomachus	*bauri*	1	0,0526	1	0,125	E
Pachycondyla	*striata*	1				E
Pheidole	sp.1	2				E
Solenopsis	sp.3	1		1		E
Isoptera	sp.	1				E
Termitidae						
Labiotermes	sp.	1				E
Nasutitermes	sp.			1		E
Lepidoptera	jovens	2	0,1053			
Cossoidea						
Limacodidae	sp.1	1	0,0526			E
Orthoptera						
Ensifera						
Gryllidae	jovens	1	0,0526			E
Phalangopsidae	jovens	1	0,0526			E
Paraclodes	sp.1	2	0,1053			
Psocoptera						
Psocomorpha	jovens	1				E
Troctomorpha						
Manicapsocidae						
Nothoentomum	sp.1	1		1		E
Mammalia						
Chiroptera		2	0,1053	2	0,25	
Emballonuridae	sp.	1	0,0526	1	0,125	E
