# Supplementary material for: Reconciling Mining with the Conservation of Cave Biodiversity: A Quantitative Baseline to Help Establish Conservation Priorities
Source: PLoS One. 2016 Dec 20;11(12):e0168348. doi: 10.1371/journal.pone.0168348 (PMC5173368; doi:10.1371/journal.pone.0168348)
Supplement: S1 Dataset — (ZIP) [file pone.0168348.s002.zip › Taxa/Serra Sul/SS_2010/S11D_31.pdf]

S11D-31		1ª	AB	2ª	AB	ZON
Annelida						
Clitellata						
Oligochaeta	sp.	1	0,0122			E
Arthropoda						
Arachnida						
Acari						
Parasitiformes						
Mesostigmata						
Macronyssidae	sp.1	1				P
Sarcoptiformes	sp.1	1				P
Amblypygi						
Charinidae						
Charinus	sp.2	2	0,0244			P
Phrynidae						
Heterophrynus	sp.	8	0,0976	3	0,2308	P
Araneae		1	0,0122			
Ctenidae	jovens	1	0,0122			P
Ochyroceratidae	jovens	1				P
Ochyrocera	sp.1	2				P
	sp.3			1		P
Oonopidae	jovens	1				P
Paratropididae	jovens	1	0,0122			E
Pholcidae	sp.1	1				P
Prodidomidae	jovens	1				E
Salticidae	jovens	1				E
Scytodidae	jovens	1	0,0122			P
Scytodes	*eleonorae*	1	0,0122			P
Tetrablemmidae	jovens			1		P
Theridiosomatidae	jovens	1				P
Opiliones						
Laniatores						
Escadabiidae	jovens	1				P
Stygnidae	sp.1	1	0,0122	1	0,0769	P
Pseudoscorpiones						
Chernetidae						
Spelaeochernes	sp.1	3				E P
Schizomida						
Hubbardiidae						
Rowlandius	sp.	1		1		E P
Diplopoda	jovens	6	0,0732			
Polydesmida						
Pyrgodesmidae	sp.1	1	0,0122			E
	sp.2	2	0,0244	1	0,0769	P
Spirostreptida						
Pseudonannolenidae	jovens	1	0,0122			P
	jovens	1		1		E
Insecta						
Coleoptera	jovens	1				E
Carabidae	sp.3	1				P
Staphylinidae	sp.8	1				P
Collembola						
Arthropleona						
Entomobryoidea						
Paronellidae	sp.1	1		1		P
	sp.4	2		1		P
Diptera						
Nematocera						
Psychodidae						
Sciopemyia	*sordellii*	3		1		E P
	jovens	1		1		P
Hemiptera						
Homoptera						

Cixiidae	jovens	3		1		E	P
Hymenoptera							
Chrysidoidea							
Bethylidae	sp.1	1				E	
Vespoidea							
Formicidae							
Labidus	*coecus*	1				E	
Nylanderia	sp.1			1			P
Octostruma	sp.1	2				E	P
Isoptera	sp.			1			P
Termitidae							
Atlantitermes	sp.	1					P
Lepidoptera							
Noctuoidea	sp.2			1		E	
	jovens	4					P
Orthoptera							
Ensifera							
Phalangopsidae							
Phalangopsis	sp.1	1	0,0122			E	
Paraclodes	sp.1			2	0,1538		P
Phalangopsis	sp.1	49	0,5976	5	0,3846		P
Malacostraca							
Isopoda							
Philosciidae	sp.1	2		1		E	P
Symphyla							
Scutigerellidae							
Hanseniella	sp.1	1				E	
Chordata							
Amphibia							
Anura							
Nematocera	sp.			1	0,0769		P
Mammalia							
Chiroptera							
Emballonuridae							
Peropteryx	*kappleri*	1	0,0122				
Phyllostomidae							
Glossophaginae	sp.	2	0,0244				
Mollusca							
Gastropoda							
Subulinidae							
Lamellaxis	sp.	1					P
Nemathelminthes	sp.	1	0,0122				P
