# Supplementary material for: Reconciling Mining with the Conservation of Cave Biodiversity: A Quantitative Baseline to Help Establish Conservation Priorities
Source: PLoS One. 2016 Dec 20;11(12):e0168348. doi: 10.1371/journal.pone.0168348 (PMC5173368; doi:10.1371/journal.pone.0168348)
Supplement: S1 Dataset — (ZIP) [file pone.0168348.s002.zip › Taxa/Serra Sul/SS_2010/S11D_32.pdf]

| S11D-32 | | 1ª | AB | 2ª | AB | ZON |
|---|---|---|---|---|---|---|
| Arthropoda | | | | | | |
| Arachnida | | | | | | |
| Acari | | | | | | |
| Trombidiformes | | | | | | |
| Tydeoidea | | | | | | |
| Cunaxidae | sp.1 | 1 | | | | P |
| Labdostomatidae | sp.1 | | | 1 | | E |
| Amblypygi | | | | | | |
| Phrynidae | | | | | | |
| *Heterophrynus* | sp. | | | 1 | 0,0588 | P |
| Araneae | | | | | | |
| Araneidae | | | | | | |
| *Alpaida* | *septemmammata* | 1 | | | | E |
| Ctenidae | jovens | 1 | 0,0833 | | | E |
| Ochyroceratidae | jovens | 1 | | | | E |
| *Ochyrocera* | sp.1 | 1 | | | | P |
| Oonopidae | jovens | 1 | | | | E |
| Pholcidae | jovens | 1 | | | | E |
| Tetrablemmidae | | | | | | |
| *Matta* | sp.1 | | | 1 | | P |
| Theridiidae | jovens | 1 | | | | E |
| Opiliones | | | | | | |
| Eupnoi | | | | | | |
| Sclerosomatidae | sp.1 | 1 | | | | E |
| Laniatores | | | | | | |
| Escadabiidae | jovens | 1 | | | | P |
| | sp.2 | | | 1 | | P |
| Stygnidae | sp.1 | | | 1 | 0,0588 | P |
| Pseudoscorpiones | | | | | | |
| Chernetidae | | | | | | |
| *Spelaeochernes* | sp.1 | 1 | | 1 | | E |
| Olpiidae | sp.1 | 2 | | | | E |
| Diplopoda | | | | | | |
| Polydesmida | | | | | | |
| Pyrgodesmidae | sp.2 | 1 | 0,0833 | | | P |
| Entognatha | | | | | | |
| Diplura | | | | | | |
| Campodeidae | sp.1 | 1 | | | | P |
| Insecta | | | | | | |
| Coleoptera | | | | | | |
| Staphylinidae | | | | | | |
| Pselaphinae | sp.1 | 1 | | | | E |
| Collembola | | | | | | |
| Arthropleona | | | | | | |
| Entomobryoidea | | | | | | |
| Paronellidae | sp.1 | 1 | | | | E |
| Symphypleona | | | | | | |
| Sminthuroidea | sp.2 | 1 | | | | P |
| Dermaptera | sp.1 | 1 | 0,0833 | | | P |
| Diptera | | | | | | |
| Nematocera | | | | | | |
| Cecidomyiidae | | | | | | |
| Cecidomyiinae | sp. | 1 | | | | E |
| Psychodidae | | | | | | |
| *Sciopemyia* | *sordellii* | 1 | | | | P |
| Hemiptera | | | | | | |
| Heteroptera | | | | | | |
| aff. Pyrrhocoroidea | | | | | | |
| Reduviidae | jovens | | | 1 | 0,0588 | E |
| Homoptera | | | | | | |
| Cixiidae | jovens | 1 | | 1 | | E P |
| | sp.4 | 1 | | | | P |

| | | | | | | |
|---|---|---|---|---|---|---|
| Hymenoptera | | | | | | |
| Vespoidea | | | | | | |
| Formicidae | | | | | | |
| *Dolichoderus* | *bispinosus* | | | 1 | | E |
| *Nylanderia* | sp.1 | | | 1 | | P |
| Isoptera | | | | | | |
| Termitidae | | | | | | |
| *Nasutitermes* | sp. | 1 | | 2 | | E P |
| Lepidoptera | jovens | 1 | | | | P |
| Noctuoidea | sp.2 | 1 | | | | E |
| Orthoptera | | | | | | |
| Ensifera | | | | | | |
| Phalangopsidae | jovens | 2 | 0,1667 | | | E |
| *Paraclodes* | sp.1 | | | 8 | 0,4706 | P |
| *Phalangopsis* | sp.1 | 6 | 0,5 | 5 | 0,2941 | P |
| Psocoptera | | | | | | |
| Psocomorpha | | | | | | |
| | jovens | 1 | | 1 | | E |
| Thysanura | | | | | | |
| Nicoletiidae | sp.1 | 1 | | | | P |
| Pauropoda | | | | | | |
| Tetramerocerata | sp. | | | 1 | | E |
| Symphyla | | | | | | |
| Scutigerellidae | | | | | | |
| *Hanseniella* | sp.1 | 1 | | | | P |
| Chordata | | | | | | |
| Amphibia | | | | | | |
| Anura | | | | | | |
| Neobatrachia | | | | | | |
| Strabomantidae | | | | | | |
| *Pristimantis* | *fenestratus* | 1 | 0,0833 | | | |
| Mammalia | | | | | | |
| Chiroptera | | | | | | |
| Furipteridae | | | | | | |
| *Furipterus* | *horrens* | | | 1 | 0,0588 | P |
| Mollusca | | | | | | |
| Gastropoda | | | | | | |
| Bulimulidae | | | | | | |
| *Naesiotus* | sp. | 1 | | | | E |
| Subulinidae | | | | | | |
| *Lamellaxis* | sp. | 1 | | | | P |
