# Supplementary material for: Reconciling Mining with the Conservation of Cave Biodiversity: A Quantitative Baseline to Help Establish Conservation Priorities
Source: PLoS One. 2016 Dec 20;11(12):e0168348. doi: 10.1371/journal.pone.0168348 (PMC5173368; doi:10.1371/journal.pone.0168348)
Supplement: S1 Dataset — (ZIP) [file pone.0168348.s002.zip › Taxa/Serra Sul/SS_2010/S11D_33.pdf]

S11D-33			1ª	AB	2ª	AB	ZON
Annelida							
Clitellata							
Hirudinea	jovens		1	0,003			A
Oligochaeta	jovens		4	0,012	1	0,0064	E P
		sp.	2	0,006			E
Arthropoda							
Arachnida							
Acari							
Ixodida							
Ixodidae							
	Amblyomma	sp.			1		P
Parasitiformes							
Mesostigmata							
Macronyssidae		sp.1	2		3		E P
Ologamasidae		sp.1	1				E
		sp.2	1				E
		sp.5			2		P
		sp.6	1				P
Sarcoptiformes							
Oribatida		sp.12	1				E
		sp.3			1		P
		sp.19	1				E
Trombidiformes							
Tydeoidea		sp.1	1				E
Bdellidae		sp.1	2		1		E P
Labdostomatidae		sp.1	3				E
Rhagidiidae		sp.1	1		1		E P
		sp.2	3		1		E
		sp.6			1		E
		sp.7			1		P
Amblypygi							
Charinidae							
	Charinus	sp.	1	0,003			
Phrynidae							
	Heterophrynus	sp.	7	0,021	4	0,0256	P
Araneae							
Araneidae	jovens				1		E
	Alpaida	*septemmammata*	1				P
Corinnidae	jovens		1	0,003	1	0,0064	E
Ctenidae	jovens		2	0,006			E
Filistatidae	jovens				1		E
Linyphiidae	jovens				1		P
Ochyroceratidae	jovens		3		2		E P
	Ochyrocera	sp.1	6		5		E P
	Speocera	sp.1	1				E
Oonopidae	jovens		2				P
Pholcidae	jovens		5		3		E P
		sp.1	4		3		E P
Prodidomidae	jovens		2				E P
Salticidae	jovens		1		1		E
	Amphidraus	sp.1	1				E
	Soesilarishius	sp.2	1				E
Scytodidae	*jovens*		4	0,012	2	0,0128	E P
	Scytodes	*eleonorae*	5	0,015	1	0,0064	E P
		globula	1	0,003			E
		sp.			4	0,0256	E
Tetrablemmidae							
	Matta	sp.1			3		E
Theraphosidae	jovens		1	0,003			P
Theridiidae	jovens		2		2		E
	Theridion	sp.1			2		P
Theridiosomatidae	jovens		2		1		P

	Plato	sp.1	4		3		E P
Opiliones							
Eupnoi							
Sclerosomatidae	jovens		4		3		E P
		sp.1	3		1		E P
Laniatores							
Escadabiidae	jovens		3				E P
		sp.2	1		1		P A
Stygnidae	jovens		4				E P
		sp.1	3	0,021	1	0,0064	E P
Palpigradi							
Eukoeneniidae	jovens				2		P
Pseudoscorpiones							
Bochicidae		sp.1	2				E
Chernetidae	jovens		2				P
	Spelaeochernes	sp.1	2		2		E
Chthoniidae							
	Pseudochthonius	sp.1	3		4		E P
Ricinulei							
Ricinoididae	jovens		1				E
Schizomida							
Hubbardiidae	jovens		1		2		E P
	Rowlandius	sp.	1				P
Chilopoda			8	0,024			
Pleurostigmophora	jovens		1	0,003			
Scolopendromorpha	jovens		1	0,003			E
Scolopocryptopidae							
	Dinocryptops	*miersii*	3	0,009			E
	Newportia	sp.1	1	0,003			E
Diplopoda							
Glomeridesmida							
Glomeridesmidae		sp.1	1				P
Polydesmida	jovens		1				P
Chelodesmidae		sp.6			1	0,0064	P
Fuhrmannodesmidae		sp.1	1				E
Pyrgodesmidae		sp.1	1	0,003			P
		sp.2	1	0,003			E
		sp.3			1	0,0064	P
Hypogexenidae		sp.1	1				P
Spirostreptida							
	jovens		4		1		E P
Entognatha							
Diplura							
Campodeidae		sp.1	3				E P
Japygidae		sp.1	1		1		P
Insecta							
Blattodea	jovens		1				E
Blaberidae	jovens		1	0,006			P
Coleoptera	jovens		9		1		E P
Carabidae		sp.3	2				P
		sp.7	3				E
Dytiscidae		sp.1	1				P
Ptiliidae		sp.1	3		5		E P
Scydmaenidae		sp.7			1		E
		sp.9	1				E
Staphylinidae		sp.11	1				E
		sp.6	1				E
		sp.9	1				P
	Pselaphinae	sp.1	2		2		E
		sp.4	1				E
		sp.5			1		E
Collembola							
Arthropleona							

Entomobryoidea							
Cyphoderidae		sp.1	1		2		E P
Entomobryidae		sp.3			1		P
Isotomidae		sp.1	1		1		P
Paronellidae		sp.1	8		5		E P
		sp.4	1		2		E P
		sp.5	1				E
Symphypleona							
Sminthuroidea		sp.2	11		5		E P
Diptera							
Brachycera							
Camillidae		sp.	3				E
Nematocera	jovens		5		3		E P A
Culicidae							
	Culicini	sp.			1		E
Psychodidae							
	Pericoma	sp.	1				E
	Sciopemyia	*sordellii*	3		1		E P
Sciaridae							
	Bradysia	sp.			1		P
Tipulidae							
	Tipulinae	sp.	2				E P
Hemiptera							
Heteroptera							
Dipsocoroidea	jovens		1		1		E P
Cydnidae	jovens		1				E
	Cydninae	sp.1	2				E P
Enicocephalidae	jovens		1		1		E P
Veliidae	jovens		1				P
	Paravelia	sp.1	3				E A
Homoptera							
Cixiidae	jovens		1				P
Hymenoptera	jovens		1				E
Ichneumonoidea							
Braconidae		sp.1			1		E
Vespoidea							
Formicidae							
	Camponotus	sp.1	1				P
	Crematogaster	sp.1	3		3		E
	Gnamptogenys	*striatula*	3		1		E
	Hypoponera	sp.1	5				E P
	Labidus	*coecus*	1				P
	Nylanderia	sp.1	5		2		E P
	Octostruma	sp.1	3				P
	Odontomachus	*bauri*	1	0,003			E
	Pachycondyla	*harpax*	1				E
		striata	2		3		E P
	Pheidole	sp.2	1				E
	Solenopsis	sp.1	1		1		E P
		sp.2	2				E
	Strumygenys	sp.1	1				P
	Wasmania	*auropunctata*	2				E
Isoptera							
Termitidae							
	Nasutitermes	sp.	3		3		E P
		sp.	1				P
Lepidoptera					1	0,0064	
Cossoidea							
Limacodidae		sp.1	1	0,003	1	0,0064	P
Noctuoidea		sp.5	1				E
	Noctuidea	sp.1	12	0,0359			
Tineoidea		sp.1	1	0,003			
	jovens		1	0,003	4	0,0256	E P

Orthoptera							
Ensifera							
Gryllidae		jovens	2	0,006			E P
Phalangopsidae		jovens	2	0,006			E
	Paraclodes	sp.1			8	0,0513	P
	Phalangopsis	sp.1	127	0,3802	69	0,4423	E P
Psocoptera							
Psocomorpha		jovens			4		E P
Epipsocidae							
	Epipsocus	sp.2			1		P
Ptiloneuridae							
	Triplocania	sp.1			1		P
Trogiomorpha							
Lepidopsocidae							
	Psyllipsocus	sp.1	1				E
Thysanura							
Nicoletiidae		jovens	1				E
		sp.1	3		1		E P
Malacostraca							
Isopoda							
Philosciidae		sp.1	2		1		E P
		sp.2	5				E P
Scleropactidae		sp.	1		2		P
Chordata							
Amphibia							
Anura							
Neobatrachia							
Strabomantidae							
	Pristimantis	*fenestratus*			2	0,0128	P
Dendrobatidae							
	Epipedobates	cf. *flavopictus*			1	0,0064	E
Pipidae							
	Pipa	*arrabali*	65	0,1946	18	0,1154	P
Mammalia							
Chiroptera							
Emballonuridae							
	Peropteryx	*kappleri*	15	0,0449			
		sp.			10	0,0641	P
Furipteridae							
	Furipterus	*horrens*	10	0,0299	10	0,0641	P
Phyllostomidae							
	Carollia	*perspicillata*	30	0,0898			
	Diphylla	*ecaudata*	1	0,003			
	Glossophaginae	sp.	3	0,009			
	Trachops	*cirrhosus*	8	0,024	15	0,0962	
		sp.			1	0,0064	P
Mollusca							
Gastropoda							
Subulinidae							
	Lamellaxis	sp.	4		1		E P
Systrophiidae							
	Happia	sp.	2				E
Platyhelminthes							
Turbellaria		sp.2	1				P
		sp.8	1	0,003			P
