# Supplementary material for: Reconciling Mining with the Conservation of Cave Biodiversity: A Quantitative Baseline to Help Establish Conservation Priorities
Source: PLoS One. 2016 Dec 20;11(12):e0168348. doi: 10.1371/journal.pone.0168348 (PMC5173368; doi:10.1371/journal.pone.0168348)
Supplement: S1 Dataset — (ZIP) [file pone.0168348.s002.zip › Taxa/Serra Sul/SS_2010/S11D_34.pdf]

S11D-34				1ª	AB	2ª	AB	ZON
Annelida								
Clitellata								
Oligochaeta	jovens			1	0,0172			E
Arthropoda								
Arachnida								
Acari								
Parasitiformes								
Mesostigmata			sp.1	1				E
Trombidiformes								
Tydeoidea								
Anystidae								
		Erythracarus	*nasutus*	1				E
Scutacaridae			sp.1			1		E
			sp.7	1				E
Araneae								
Araneidae	jovens			1		1		E
		Alpaida	*septemmammata*	1				E
			sp.2	1				E
Corinnidae	jovens			2	0,0345			E
Ochyroceratidae	jovens			1				E
		Ochyrocera	sp.1	1				E
Pholcidae	jovens			2				E
			sp.1	1				E
Salticidae	jovens			1		1		E
Scytodidae	jovens			3	0,0517	1	0,0294	E
			globula	2	0,0345			E
			sp.	1	0,0172	2	0,0588	E
Tetragnathidae	jovens			1				E
Theraphosidae	jovens			4	0,069	1	0,0294	E
Theridiosomatidae	jovens			1				E
		Plato	sp.1	2				E
Opiliones								
Laniatores								
Stygnidae	jovens			2	0,0345			E
			sp.1	1	0,0172			E
Pseudoscorpiones								
Chernetidae								
		Spelaeochernes	sp.1	1		1		E
Chthoniidae								
		Pseudochthonius	sp.1	1				E
Olpiidae			sp.1			2		E
Diplopoda								
Spirostreptida	jovens			1				E
Pseudonannolenidae	jovens			1	0,0172			E
Insecta								
Blattodea	jovens			1	0,0172			
Blaberidae	jovens					1	0,0294	E
		Blattellidae	sp.2			1	0,0294	E
Blattidae			sp.3	1	0,0172			E
Polyphagidae	jovens			1	0,0172			E
Coleoptera	jovens			2				E
Melyridae			sp.1	1				E
Ptiliidae			sp.1	2				E
Staphylinidae			sp.11	1				E
Collembola								
Arthropleona								
Entomobryoidea								
Paronellidae			sp.1	2				E
Diptera	jovens			3		1		E
Nematocera								
Cecidomyiidae								
		Cecidomyiinae	sp.	1				E

Mycetophilidae							
	Synapha	sp.	1				E
Psychodidae							
	Pintomyia	*gruta*	1				E
	Sciopemyia	*sordellii*	2				E
Tipulidae							
Hemiptera							
Heteroptera							
aff. Pyrrhocoroidea							
Reduviidae	jovens		2	0,0345	1		E
	Reduviinae	sp.			20	0,6176	E
Hymenoptera	jovens		1	0,0172			
Vespoidea							
Formicidae							
	Camponotus	sp.1	2		1		E
	Carebara	sp.1	1				E
	Crematogaster	sp.1	1				E
	Labidus	*coecus*	1				E
	Pachycondyla	*harpax*	1				E
	Wasmania	*auropunctata*	1		1		E
Pompilidae		sp.1			1		E
Isoptera		sp.	1		1		E
Termitidae							
	Cavitermes	sp.	1				E
	Nasutitermes	sp.	1				E
Lepidoptera							
Cossoidea							
Limacodidae		sp.1	4	0,069			E
Noctuoidea							
Noctuidae		sp.			1	0,0294	E
		sp.1	25	0,431			E
Tineoidea	jovens				1		E
		sp.1	1	0,0172			
		sp.2	1				E
Neuroptera							
Mantispidae		sp.	2	0,0345			E
Myrmeleonthidae	jovens		1		1		E
Psocoptera							
Psocomorpha	jovens		3		1		E
Ptiloneuridae							
	Triplocania	sp.10	1				E
Malacostraca							
Isopoda							
Philosciidae		sp.1	1				E
Symphyla							
Scutigerellidae							
	Hanseniella	sp.1	1				E
	Scutigerella	sp.1	1				E
Chordata							
Amphibia							
Anura							
Neobatrachia							
Strabomantidae							
	Pristimantis	*fenestratus*	1	0,0172			
Mammalia							
Chiroptera							
Emballonuridae							
	Peropteryx	*kappleri*	2	0,0345	3	0,0882	
		sp.			3	0,0882	E
