# Supplementary material for: Reconciling Mining with the Conservation of Cave Biodiversity: A Quantitative Baseline to Help Establish Conservation Priorities
Source: PLoS One. 2016 Dec 20;11(12):e0168348. doi: 10.1371/journal.pone.0168348 (PMC5173368; doi:10.1371/journal.pone.0168348)
Supplement: S1 Dataset — (ZIP) [file pone.0168348.s002.zip › Taxa/Serra Sul/SS_2010/S11D_35.pdf]

S11D-35			1ª	AB	2ª	AB	ZON
Annelida							
Clitellata							
Oligochaeta	jovens		1	0,0167			E
Arthropoda							
Arachnida							
Acari							
Ixodida							
Argasidae							
	Ornithodoros	sp.			1		E
Ixodidae							
	Amblyomma	sp.			1		E
Parasitiformes							
Mesostigmata							
Diploginiidae		sp.2	1				P
Sarcoptiformes							
Oribatida		sp.1	1				E
Trombidiformes							
Tydeoidea							
Bdellidae		sp.1	1				P
Eupodidae		sp.1	1				P
		sp.2	1				P
Amblypygi							
Phrynidae							
	Heterophrynus	sp.			2	0,0274	P
Araneae			1	0,0167			
Araneidae	jovens				1		E
	Alpaida	*septemmammata*	2				E
Corinnidae	jovens				1	0,0137	E
Ctenidae	jovens		1	0,0167			E
Linyphiidae	gen.1	sp.1	1				P
Ochyroceratidae	jovens		1		1		E
	Ochyrocera	sp.1	2		1		P
		sp.4			1		P
Oonopidae							
	Oonopinae	sp.1	1				P
Pholcidae	jovens				1		E
		sp.1	1				E
	Ninetinae	sp.1	1		1		E
Scytodidae	*jovens*		1	0,0167	1	0,0137	E P
	Scytodes	*eleonorae*			1	0,0137	P
		sp.			1	0,0137	E
Symphytognathidae							
	Anapistula	sp.1	1				P
Tetrablemmidae	jovens		1				P
	Matta	sp.1	3		1		E P
Tetragnathidae	jovens		1				E
Theraphosidae	jovens		1	0,0167	1	0,0137	E P
Theridiosomatidae	jovens		1		1		E P
	Plato	sp.1	3				E P
	jovens		1	0,0167			
Opiliones							
Laniatores							
Escadabiidae	jovens		1				E
		sp.2	1				P
Stygnidae	jovens		2	0,0333			E P
		sp.1	1	0,0167			P
	jovens		1				E
Palpigradi							
Eukoeneniidae							
	Allokoenenia	sp.1	1				P
Pseudoscorpiones							
Bochicidae		sp.1			2		P

Chernetidae		jovens	2				P
	Spelaeochernes	sp.1	2		2		E P
Chthoniidae							
	Pseudochthonius	sp.1	1		2		E P
Schizomida							
Hubbardiidae							
	Rowlandius	sp.	1				P
Scorpiones							
Buthidae		jovens			1	0,0137	P
Chilopoda							
Pleurostigmophora							
Geophilomorpha							
Geophilidae		sp.1	1	0,0167			E
Scolopendromorpha							
Scolopocryptopidae							
	Newportia	sp.1	1	0,0167			E
Scutigeromorpha							
Pselliodidae							
	Sphendononema	sp.			1	0,0137	E
Diplopoda							
Spirostreptida							
Pseudonannolenidae		jovens	1	0,0167			P
Spirostreptidae		sp.1	1				E
		jovens			1		P
Ento Diplura Campodeidae		sp.1	3		1		E P
Insecta							
Blattodea		jovens	1	0,0167			E
Blattidae		sp.3	1	0,0167			E
Coleoptera		jovens			2		E P
Chrysomelidae		sp.5	1				E
Staphylinidae		sp.12			1		E
		sp.51	1				P
Collembola							
Arthropleona							
Entomobryoidea							
Entomobryidae		sp.6			1		E
Isotomidae		sp.1	2				E P
Paronellidae		sp.4	1				P
Symphypleona							
Sminthuroidea		sp.2	1				P
Diptera							
Brachycera							
Phoridae							
	Metopininae	sp.	1				P
		sp.	1				P
Nematocera		jovens	2				E
Psychodidae							
	Bruchomyiinae	sp.			1		P
	Edentomyia	*piauensis*			1		P
	Phlebotominae	sp.			1		P
Simuliidae		sp.	1				P
Hemiptera							
Heteroptera							
Dipsocoroidea		jovens	1		2		E P
Reduviidae		jovens	2	0,0333			E P
Homoptera							
Cixiidae		jovens	4		2		E P
Hymenoptera							
Vespoidea							
Formicidae							
	Camponotus	*atriceps*	2				E
		sp.1	3	0,05	4	0,0548	E P
	Crematogaster	sp.1	1				E

Hypoponera	sp.1	1		1		P
Labidus	*coecus*	2				E
Nylanderia	sp.1	4		1		E P
Odontomachus	*bauri*	1				E
Isoptera	sp.	4				E P
Termitidae						
Labiotermes	sp.	1				E
Nasutitermes	sp.			1		E
Lepidoptera	jovens	2		1		E P
Cossoidea						
Limacodidae	sp.1	3	0,05			E P
Noctuoidea						
Noctuidae	sp.1	1	0,0167			E
Orthoptera						
Ensifera						
Phalangopsidae	jovens	36	0,6			
Paraclodes	sp.1			5	0,0685	E P
Phalangopsis	sp.1			2	0,0274	P
Phalangopsis	sp.			46	0,6301	
Psocoptera						
Psocomorpha	jovens			2		E P
Epipsocidae						
Mesepipsocus	sp.1			1		P
Trogiomorpha						
Psyllipsocidae	jovens			1		E
Thysanura						
Ateluridae	sp.1	2				E P
Nicoletiidae	sp.1	2				E P
Malacostraca						
Isopoda						
Dubioniscidae	jovens			1		P
Philosciidae	sp.1	1		3		E P
Pauropoda						
Tetramerocerata	sp.	1				P
Symphyla Scutigerellidae	jovens			1		E
Hanseniella	sp.1	1				P
Chordata						
Amphibia						
Anura						
Neobatrachia						
Strabomantidae						
Pristimantis	*fenestratus*	1	0,0167			
Mammalia						
Chiroptera						
Emballonuridae						
Peropteryx	sp.			2	0,0274	P
Furipteridae						
Furipterus	*horrens*			4	0,0548	P
Trachops	sirrhosus			1	0,0137	P
Mollusca						
Gastropoda						
Systrophiidae						
Happia	sp.	3				E P
