# Supplementary material for: Reconciling Mining with the Conservation of Cave Biodiversity: A Quantitative Baseline to Help Establish Conservation Priorities
Source: PLoS One. 2016 Dec 20;11(12):e0168348. doi: 10.1371/journal.pone.0168348 (PMC5173368; doi:10.1371/journal.pone.0168348)
Supplement: S1 Dataset — (ZIP) [file pone.0168348.s002.zip › Taxa/Serra Sul/SS_2010/S11D_36.pdf]

S11D-36		1ª	AB	2ª	AB	ZON
Annelida						
Clitellata						
Oligochaeta	jovens	2	0,0556			E
Arthropoda						
Arachnida						
Acari						
Parasitiformes						
Opilioacarida						
Opilioacaridae	sp.1	1				E
Sarcoptiformes						
Oribatida	sp.3			1		E
Trombidiformes						
Tydeoidea						
Rhagidiidae	sp.1	1				E
	sp.9			1		E
Amblypygi						
Phrynidae						
Heterophrynus	sp.	1	0,0278			
Araneae						
Araneidae	jovens	1				E
Corinnidae	jovens			2	0,0741	E
Myrmecium	sp.1	1	0,0278			E
Ctenidae	jovens	3	0,0833	3	0,1111	E
Nesticidae	jovens	1				E
Ochyroceratidae	jovens			1		E
Ochyrocera	sp.1	1				E
Oonopidae	jovens	1		1		E
Pholcidae	jovens	1		1		E
	sp.1	1				E
Salticidae	jovens	2		1		E
Scytodidae						
Scytodes	*eleonorae*	1	0,0278			E
Tetrablemmidae						
Matta	sp.1	1				E
Theridiosomatidae	jovens	1				E
Plato	sp.1	1				E
Opiliones						
Laniatores						
Escadabiidae	sp.3	1				E
Stygnidae	jovens	2	0,0556			E
Pseudoscorpiones						
Chernetidae						
Spelaeochernes	sp.1	2		2		E
Chthoniidae						
Pseudochthonius	sp.1	1				E
Chilopoda						
Notostigmophora						
Scutigeromorpha						
Pselliodidae	jovens	1		1		E
Diplopoda						
Glomeridesmida						
Glomeridesmidae	sp.2	1				E
Polydesmida						
Pyrgodesmidae	sp.2	1	0,0278			E
Spirostreptida	jovens	1				E
Pseudonannolenidae	jovens	1	0,0278			E
Pseudonannolene	sp.1			1	0,037	E
Entognatha						
Diplura						
Campodeidae	sp.1	2		2		E
Projapygidae	sp.1	1		1		E
Insecta						

Blattodea			1	0,0278			
	Polyphagidae	jovens	1	0,0278			E
		jovens	1	0,0278			
Coleoptera		jovens	2				E
	Carabidae	sp.8	1				E
	Staphylinidae	sp.4	1				E
Collembola							
Arthropleona							
Entomobryoidea							
	Entomobryidae	sp.1	2				E
		sp.10			2		E
	Isotomidae	sp.1	1				E
		sp.2	1				E
	Paronellidae	sp.6			1		E
Dermaptera		sp.1			1	0,037	E
Diptera							
Brachycera							
	Dolichopodidae	sp.			1		E
Nematocera		jovens	1				E
	Psychodidae						
	Pintomyia	*gruta*			1		E
	Sciopemyia	*sordellii*	2				E
Hemiptera							
Heteroptera							
aff. Pyrrhocoroidea							
	Reduviidae	jovens	5	0,1389	2	0,0741	E
Homoptera							
	Cixiidae	jovens	1		2		E
		sp.1	1				E
Hymenoptera							
Vespoidea							
	Formicidae						
	Camponotus	sp.1			2		E
	Hypoponera	sp.1			1		E
	Nylanderia	sp.1	2				E
	Odontomachus	*bauri*			1		E
	Pachycondyla	*striata*			1		E
	Solenopsis	sp.2	1		1		E
	Mutillidae	sp.2	1				E
Isoptera		sp.			1		E
	Termitidae						
	Embiratermes	sp.	1				E
	Nasutitermes	sp.	2				E
Lepidoptera		jovens	3	0,0833			
Cossoidea							
	Limacodidae	sp.1	1	0,0278			E
Noctuoidea							
	Noctuidae	sp.1	1	0,0278			E
Orthoptera							
Ensifera							
	Gryllidae	jovens	1	0,0278			E
	Phalangopsidae	jovens	10	0,2778			
	Paraclodes	sp.1			18	0,6667	E
Psocoptera							
Psocomorpha		jovens	1				E
Symphyla							
	Scutigerellidae						
	Hanseniella	sp.1	2				E
