# Supplementary material for: Reconciling Mining with the Conservation of Cave Biodiversity: A Quantitative Baseline to Help Establish Conservation Priorities
Source: PLoS One. 2016 Dec 20;11(12):e0168348. doi: 10.1371/journal.pone.0168348 (PMC5173368; doi:10.1371/journal.pone.0168348)
Supplement: S1 Dataset — (ZIP) [file pone.0168348.s002.zip › Taxa/Serra Sul/SS_2010/S11D_37.pdf]

S11D-37			1ª	AB	2ª	AB	ZON
Annelida							
Clitellata							
Oligochaeta		jovens	3	0,0114			P
Arthropoda							
Arachnida							
Acari							
Sarcoptiformes							
Oribatida		sp.1	1				P
Trombidiformes		sp.6			1		P
Amblypygi							
Charinidae		jovens	1	0,0076	1	0,0149	P
		sp.2	1				P
Phrynidae							
	Heterophrynus	sp.			3	0,0448	P
Araneae							
Araneidae							
	Alpaida	*septemmammata*	1				E
Corinnidae							
	Creugas	sp.1	1	0,0038			P
Ctenidae		jovens	1	0,0076			
	Ctenus	sp.1	1				P
Ochyroceratidae		jovens			1		E
	Ochyrocera	sp.1	2				E P
	Speocera	sp.1	2		2		E P
Oonopidae		jovens			2		P
Prodidomidae		jovens	2				P
Salticidae		jovens			1		P
Theridiosomatidae		jovens			1		P
	Plato	sp.1	2				E P
Opiliones							
Cyphophthalmi							
Neogoveidae							
	Canga	*renatae*	1				P
Laniatores							
Escadabiidae		jovens	1		1		E P
		sp.1	1				P
Palpigradi							
Eukoeneniidae							
	Allokoenenia	sp.1	1				P
Pseudoscorpiones							
Chernetidae							
	Spelaeochernes	sp.1	6		1		E P
Chthoniidae							
	Pseudochthonius	sp.1	1		2		E P
Ricinulei							
Ricinoididae		jovens	2		1		P
	Cryptocellus	sp.	1				P
Scorpiones							
Buthidae		jovens	1	0,0076			P
	Ananteris	*balzanii*	1				P
Chilopoda			1	0,0038			
Notostigmophora							
Scutigeromorpha							
Pselliodidae		jovens	1				P
Pleurostigmophora		jovens	1	0,0038			
Geophilomorpha							
Ballophilidae		sp.3	1	0,0038			P
Diplopoda		jovens	20	0,076			
Glomeridesmida							
Glomeridesmidae		sp.1	5				E P
Polydesmida							
Pyrgodesmidae		sp.2	3	0,0114			P

Spirostreptida	jovens			1			P
Pseudonannolenidae	jovens	1	0,0038				P
Insecta							
Blattodea	jovens	1	0,0038				
Coleoptera	jovens			1			
Hydrophilidae							
Sphaeridiinae	sp.1			1			P
Staphylinidae							
Pselaphinae	sp.1	1				E	
	sp.6	3				E	P
Collembola							
Arthropleona							
Entomobryoidea							
Isotomidae	sp.1	1		1			P
	sp.2	1					P
Paronellidae	sp.1	3				E	P
	sp.4	6		1		E	P
Diptera							
Brachycera							
Camillidae	sp.	1				E	
Drosophilidae							
Drosophila	*eleonore*	3				E	P
Nematocera	jovens			2		E	P
Psychodidae							
Pintomyia	*gruta*	1					P
Sciopemyia	*sordellii*	2		2		E	P
Hemiptera							
Heteroptera		6	0,0228	7	0,1045		
aff. Pyrrhocoroidea	jovens	1	0,0038	1	0,0149		P
Cydnidae	jovens	1		1			P
Cydninae	sp.1	2					P
Reduviidae	jovens	2	0,0076	3	0,0746	E	P
Reduviinae	sp.			2			P
Schizopteridae							
Schizopterinae	sp.3	1				E	
Homoptera							
Cixiidae	jovens	5		1		E	P
Cixiidae	sp.4	1					P
Hymenoptera							
Vespoidea							
Formicidae							
Leptogenys	sp.1	1					P
Nylanderia	sp.1	1		1		E	P
Pachycondyla	*striata*	1		1			P
Solenopsis	sp.2	1					P
Isoptera							
Termitidae							
Nasutitermes	sp.	2		2		E	P
Lepidoptera	jovens	1		1		E	P
Noctuoidea	sp.2	2				E	P
Orthoptera							
Ensifera							
Phalangopsidae							
Phalangopsis	sp.1	187	0,711	30	0,4478		P
Paraclodes	sp.1	7	0,0266	2	0,0299		P
Malacostraca							
Isopoda							
Dubioniscidae	sp.1	1				E	
Philosciidae	sp.1	2		2		E	P
Scleropactidae	sp.	1					P
Symphyla							
Scutigerellidae							
Hanseniella	sp.1	1		1		E	P

Chordata							
Amphibia							
Anura							
Neobatrachia							
Leptodactylidae							
	Leptodactylus	sp.			1	0,0149	P
Strabomantidae							
	Pristimantis	*fenestratus*			6	0,0896	P
Bufonidae							
	Rhinella	cf. *marina*			2	0,0299	P
Mammalia							
Chiroptera							
Emballonuridae							
	Peropteryx	*macrotis*	1	0,0038			
		sp.	5	0,019			
Furipteridae							
	Furipterus	*horrens*	15	0,057			
Phyllostomidae							
	Carollia	*perspicillata*	1	0,0038			
		sp.			9	0,1343	P
Mollusca							
Gastropoda							
Subulinidae							
	Lamellaxis	sp.	1				P
Systrophiidae							
	Happia	sp.	1		1		P
