# Supplementary material for: Reconciling Mining with the Conservation of Cave Biodiversity: A Quantitative Baseline to Help Establish Conservation Priorities
Source: PLoS One. 2016 Dec 20;11(12):e0168348. doi: 10.1371/journal.pone.0168348 (PMC5173368; doi:10.1371/journal.pone.0168348)
Supplement: S1 Dataset — (ZIP) [file pone.0168348.s002.zip › Taxa/Serra Sul/SS_2010/S11D_38.pdf]

S11D-38			1ª	AB	2ª	AB	ZON
Arthropoda							
Arachnida							
Acari							
Parasitiformes							
Mesostigmata		sp.2	1		1		P
Sarcoptiformes							
Oribatida		sp.1	1				P
Trombidiformes							
Tydeoidea		sp.1	1				P
Amblypygi							
Phrynidae							
	Heterophrynus	sp.	8	0,0851	1	0,0068	P
Araneae							
Araneidae	jovens				1		P
Corinnidae	jovens		1	0,0106			P
Ochyroceratidae							
	Speocera	sp.1	1		1		P
Oonopidae		sp.5	1				P
Scytodidae	*jovens*		4	0,0426	1	0,0068	P
	Scytodes	*eleonorae*			2	0,0136	P
		globula	1	0,0106			P
		sp.	7	0,0745	1	0,0068	P
Tetrablemmidae							
	Matta	sp.1	1		1		P
Theridiosomatidae	jovens				1		P
	Plato	sp.1	1				P
Opiliones							
Eupnoi	jovens				12	0,0816	P
Laniatores					53	0,3605	
Cosmetidae							
	Roquettea	*singularis*			1	0,0068	P
Stygnidae		sp.1	1	0,0106	2	0,0136	P
		sp.			17	0,1156	P
Palpigradi							
Eukoeneniidae	jovens		1				P
Pseudoscorpiones							
Chernetidae							
	Spelaeochernes	sp.1	2				P
Chthoniidae	jovens		2				P
	Pseudochthonius	sp.1	1				P
Schizomida							
Hubbardiidae	jovens		2				P
Chilopoda							
Pleurostigmophora	jovens		1	0,0106			
Scolopendromorpha							
Cryptopidae							
	Cryptops	sp.1	1	0,0106			P
Diplopoda							
Polydesmida							
Pyrgodesmidae		sp.2	1	0,0106			P
Polyxenida							
Hypogexenidae		sp.1	4				P
Spirostreptida							
Pseudonannolenidae	jovens		1	0,0106			P
Insecta	jovens						
Blattodea	jovens		10	0,1064	2	0,0136	P
Blaberidae	jovens		3	0,0319			P
Blattidae	jovens				1	0,0068	P
Coleoptera							
Curculionidae		sp.1			1	0,0068	P
	jovens		1				P
Collembola							

Arthropleona						
Entomobryoidea						
Entomobryidae	sp.1	1				P
Paronellidae	sp.1	1				P
Diptera						
Nematocera	jovens	2		1		P
Chironomidae	sp.			1		P
Psychodidae						
Pintomyia	*gruta*			1		P
Sciaridae						
Bradysia	sp.			1		P
Tipulidae						
Tipulinae	sp.			1		P
Hemiptera						
Heteroptera						
aff. Pyrrhocoroidea						
Reduviidae	jovens	1	0,0106	1	0,0068	P
Veliidae						
Paravelia	sp.2			1		P
Homoptera						
Cicadellidae						
Cixiidae	jovens	3				P
Hymenoptera						
Vespoidea						
Formicidae						
Hypoponera	sp.1			1		P
Odontomachus	*bauri*	1				P
Pachycondyla	*striata*	3				P
Solenopsis	sp.2			1		P
Isoptera	sp.	1				P
Lepidoptera	jovens			3	0,0204	P
	sp.1	1	0,0106			
Cossoidea						
Limacodidae	sp.1	1	0,0106			P
Noctuoidea	sp.2			2		P
Noctuidae	sp.1	2	0,0213			
Noctuidae	sp.2			1	0,0068	P
Mantodea						
Hymenopodidae						
Acromantinae	sp.1			1	0,0068	P
Orthoptera						
Ensifera						
Phalangopsidae						
Paraclodes	sp.1			3	0,0204	P
Phalangopsis	sp.1	50	0,5319	3	0,0204	P
Tettigoniidae	sp.1			1	0,0068	P
Psocoptera						
Psocomorpha	jovens	1				P
Malacostraca						
Isopoda						
Philosciidae	sp.1	1		1		P
Mammalia						
Chiroptera						
Phyllostomidae						
Carollia	*perspicillata*			20	0,1361	P
Glossophaginae	*anoura*			20	0,1361	P
Mollusca						
Gastropoda						
Subulinidae						
Lamellaxis	sp.	1				P
