# Supplementary material for: Reconciling Mining with the Conservation of Cave Biodiversity: A Quantitative Baseline to Help Establish Conservation Priorities
Source: PLoS One. 2016 Dec 20;11(12):e0168348. doi: 10.1371/journal.pone.0168348 (PMC5173368; doi:10.1371/journal.pone.0168348)
Supplement: S1 Dataset — (ZIP) [file pone.0168348.s002.zip › Taxa/Serra Sul/SS_2010/S11D_39.pdf]

S11D-39			1ª	AB	2ª	AB	ZON
Annelida							
Clitellata							
Oligochaeta	jovens		7	0			P A
Arthropoda							
Arachnida							
Acari							
Ixodida							
Ixodidae							
	Amblyomma	sp.			1		P
Parasitiformes							
Holothyrida							
Diplothyridae							
	Diplothyrus	*scubarti*	1				P
Mesostigmata							
Laelapidae							
	Stratiolaelaps	sp.1	2				P
Macrochelidae		sp.1	1				P
Macronyssidae		sp.1	2				P A
Podocinidae		sp.1	2				P A
		sp.2	4				P
		sp.5	1		1		P
Sarcoptiformes		sp.1	1		1		P
Oribatida		sp.2	1				P
		sp.3	12		4		P A
		sp.7	1				P
Acaridae		sp.1			1		A
		sp.19	1				P
Trombidiformes							
Tydeoidea		sp.1	12		4		E P A
		sp.11	1				A
		sp.2	4				P
		sp.6			2		P A
		sp.7	11				P A
Eupodidae		sp.1	1				A
Rhagidiidae		sp.1			2		P A
Amblypygi							
Charinidae	jovens		5	0	2	0	A
	Charinus	sp.	54	0,1	12	0	A
		sp.2	6	0	1	0	P A
Phrynidae							
	Heterophrynus	sp.	8	0	7	0	A
Araneae							
Ctenidae	jovens		3	0			P A
Ochyroceratidae	jovens		3		1		P A
	Ochyrocera	sp.1	7		3		P A
		sp.3	4		2		P A
	Speocera	sp.1	3		4		P A
Oonopidae	jovens		1		1		P
		sp.5	1				A
Prodidomidae	jovens		3				P
Scytodidae	*jovens*		1	0	1		P A
	Scytodes	*eleonorae*			1	0	P
Segestriidae	jovens		1				A
Tetrablemmidae	jovens		1		2		A
	Matta	sp.1	3		1		P A
Theridiosomatidae	jovens		2				P A
	Plato	sp.1	4		1		E P A
Opiliones			6	0			
Laniatores	jovens		1	0			
Cosmetidae							
	Roquettea	*singularis*	1	0			P
Escadabiidae	jovens		7		1		P A

			sp.1	9		7		P A
	Stygnidae	jovens		3	0			E P
			sp.1	1	0	20	0	P A
Palpigradi								
	Eukoeneniidae	jovens		2		1		P A
		Allokoenenia	sp.1	3		1		P A
Pseudoscorpiones								
	Chernetidae	jovens		2				P
		Spelaeochernes	sp.1	11		5		P A
	Chthoniidae							
			sp.1	6		2		P A
		Pseudochthonius	sp.1	7		3		P A
			sp.4	6		2		P A
Schizomida								
	Hubbardiidae	jovens		4		2		P A
		Rowlandius	sp.	8		1		P A
Scorpiones		jovens		6	0			
	Buthidae	jovens		2	0			P
		Ananteris	*balzanii*	2	0	2	0	P A
Chilopoda		jovens		1	0			
Notostigmophora								
Scutigeromorpha		jovens		8	0	3	0	E P A
	Pselliodidae							
		Sphendononema	*guildingii*			4	0	P
Pleurostigmophora		jovens		1	0			
Scolopendromorpha		jovens		1	0			P
	Cryptopidae							
		Cryptops	sp.1	1	0			P
Diplopoda				5	0			
Polydesmida		jovens				1		A
	Chelodesmidae		sp.5	5	0			P A
	Fuhrmannodesmidae		sp.1	4		1		P A
			sp.3	6		3		P A
	Pyrgodesmidae		sp.2	9	0	4	0	P A
Polyxenida								
	Hypogexenidae		sp.1	5		1		P A
Spirostreptida		jovens		1		2		P A
	Pseudonannolenidae							
		Pseudonannolene	sp.	42	0,1	1	0	P
			sp.1	1	0	2	0	A
Entognatha								
Diplura								
	Campodeidae		sp.1	7		2		E P A
	Japygidae		sp.1	3				P
Insecta								
Blattodea		jovens		1	0			P
Coleoptera		jovens		5		1		E P A
			sp.1			1		E
	Carabidae		sp.2	1				A
			sp.9	1		2		A
	Curculionidae							
		Scolytinae	sp.2			2		P A
			sp.3	1				P
	Ptiliidae		sp.1	3		3		P A
	Scydmaenidae		sp.4			1		P
			sp.8	2		1		P A
	Staphylinidae		sp.3	1		1		P
			sp.51	1				P
		Pselaphinae	sp.6	1				P
Collembola								
Arthropleona								
Entomobryoidea								
	Isotomidae		sp.1	2		1		P A

		sp.2	1				P
Paronellidae		sp.1	6		4		E P A
		sp.4	18		6		P A
Neelipleona							
Neelidae		sp.1	2		2		P A
Symphypleona							
Sminthuroidea		sp.1			1		A
		sp.2	12		2		P A
Diptera							
Brachycera		sp.	1				P
Camillidae		sp.	8				A
Drosophilidae							
	Drosophila	*eleonore*	1		1		E
Phoridae							
	Phorinae	sp.	1		1		P A
Nematocera	jovens		13		5		E P A
		sp.	1				P
Cecidomyiidae							
	Cecidomyiinae	sp.	1				P
Chironomidae		sp.			2		P A
Psychodidae		sp.	1				P
	Pericoma	sp.	1				P
	Pintomyia	*gruta*	2		1		P A
	Sciopemyia	*sordellii*	6				E P A
Sciaridae		sp.	3				P A
Hemiptera							
Heteroptera							
Dipsocoroidea	jovens		1				P
Cydnidae	jovens		3				P
	Cydninae	sp.1	7				P
Schizopteridae							
	Schizopterinae	sp.2	1				P
Homoptera	jovens		1				P
Cixiidae	jovens		10		4		P A
		sp.3	1				P
Hymenoptera							
Vespoidea							
Formicidae							
	Carebara	sp.1	1		1		P A
	Gnamptogenys	*striatula*	1				E
	Hypoponera	sp.1	3		2		E P
	Nylanderia	sp.1	5				P A
	Octostruma	sp.1	1				E
	Pachycondyla	*striata*	5		1		E P
	Solenopsis	sp.2	1		1		P
	Tapinoma	sp.1	1				P
	Wasmania	*auropunctata*	2				A
Isoptera		sp.	1				A
Rhinotermitidae							
	Heterotermes	sp.	1		1		P A
Termitidae							
	Nasutitermes	sp.	17		5		E P A
	Termes	sp.	1		1		E P
Lepidoptera	jovens		2		1		P A
Cossoidea							
Limacodidae		sp.1	1	0			P
Orthoptera							
Ensifera	jovens		3	0			A
Phalangopsidae							
	Phalangopsis	sp.1	432	0,6	448	0,8	A
Thysanura							
Ateluridae	jovens				1		P
Nicoletiidae	jovens		1				P

		sp.1	3				E P
Malacostraca							
Isopoda							
Philosciidae		sp.1	7				P
Scleropactidae		sp.	1		1		P A
Pauropoda							
Tetramerocerata		sp.			1		A
Chordata							
Amphibia							
Anura							
Neobatrachia							
Leptodactylidae							
	Leptodactylus	sp.			1	0	P
Strabomantidae							
	Pristimantis	*fenestratus*			6	0	P
Bufonidae							
	Rhinella	cf. *marina*	2	0			
Mammalia							
Chiroptera					1	0	
Furipteridae							
	Furipterus	*horrens*	5	0	40	0,1	A
Phyllostomidae							
	Carollia	*perspicillata*	5	0			
		sp.	50	0,1	1	0	A
	Diphylla	*ecaudata*			1	0	P
	Glossophaga	*soricina*	1	0			
	Glossophaga	*sp.*	100	0,1			
	Glossophaginae	sp.			1	0	A
	Lonchophylla	*thomasi*	1	0			
		sp.			1	0	P
Mollusca							
Gastropoda							
Bulimulidae							
	Naesiotus	sp.	1				A
Subulinidae							
	Lamellaxis	sp.	2				E A
	Leptinaria	sp.	2				P
Systrophiidae							
	Happia	sp.			1		A
Nemathelminthes		sp.	1	0	1	0	P A
