# Supplementary material for: Reconciling Mining with the Conservation of Cave Biodiversity: A Quantitative Baseline to Help Establish Conservation Priorities
Source: PLoS One. 2016 Dec 20;11(12):e0168348. doi: 10.1371/journal.pone.0168348 (PMC5173368; doi:10.1371/journal.pone.0168348)
Supplement: S1 Dataset — (ZIP) [file pone.0168348.s002.zip › Taxa/Serra Sul/SS_2010/S11D_40.pdf]

| S11D-40 | | | 1ª | AB | 2ª | AB | ZON |
|---|---|---|---|---|---|---|---|
| Annelida | | | | | | | |
| Clitellata | | | | | | | |
| Oligochaeta | jovens | | 3 | 0,0035 | | | P |
| Oligochaeta | | sp. | 1 | 0,0012 | | | P |
| Arthropoda | | | | | | | |
| Arachnida | | | | | | | |
| Acari | | | | | | | |
| Parasitiformes | | | | | | | |
| Mesostigmata | | | | | | | |
| Mesostigmata | | sp.2 | 1 | | | | A |
| Laelapidae | | sp.4 | | | 1 | | P |
| Sarcoptiformes | | | | | | | |
| Sarcoptiformes | | sp.19 | 1 | | | | A |
| Oribatida | | sp.13 | | | 1 | | P |
| Oribatida | | sp.2 | | | 1 | | P |
| Oribatida | | sp.3 | 5 | | 2 | | P A |
| Trombidiformes | | | | | | | |
| Trombidiformes | | sp.1 | 4 | | 2 | | P A |
| Tydeoidea | | | | | | | |
| Rhagidiidae | | sp.1 | 1 | | 2 | | E A |
| Amblypygi | | | | | | | |
| Charinidae | jovens | | 1 | 0,0012 | 2 | 0,0025 | P A |
| | *Charinus* | sp. | | | 2 | 0,0025 | A |
| Phrynidae | | | | | | | |
| | *Heterophrynus* | sp. | 10 | 0,0116 | 4 | 0,005 | P |
| | *Heterophrynus* | sp.1 | 1 | 0,0012 | | | P |
| Araneae | | | | | | | |
| Araneidae | jovens | | | | 1 | | P |
| | *Alpaida* | sp.2 | 2 | | 1 | | E P |
| Corinnidae | jovens | | 7 | 0,0081 | 4 | 0,005 | E P A |
| | *Creugas* | sp.1 | 4 | 0,0046 | 3 | 0,0038 | P A |
| Ctenidae | jovens | | | | 1 | 0,0013 | E |
| Nesticidae | jovens | | 1 | | | | P |
| Ochyroceratidae | jovens | | 2 | | | | P |
| | *Ochyrocera* | sp.1 | 1 | | 3 | | E P |
| | *Speocera* | sp.1 | 2 | | 2 | | P |
| Pholcidae | | | | | | | |
| | *Mesabolivar* | sp.1 | | | 1 | | E |
| Salticidae | jovens | | | | 1 | | E |
| | *Amphidraus* | sp.1 | 1 | | | | E |
| Scytodidae | jovens | | 2 | 0,0023 | 2 | 0,0025 | E P |
| Theridiosomatidae | jovens | | 1 | | 1 | | P |
| | *Plato* | sp.1 | 2 | | | | E P |
| Opiliones | | | | | | | |
| Cyphophthalmi | | | | | | | |
| Neogoveidae | | | | | | | |
| | *Canga* | *renatae* | 9 | | 3 | | P A |
| Laniatores | | | | | | | |
| Stygnidae | | sp.1 | 1 | 0,0012 | 1 | 0,0013 | E P |
| Pseudoscorpiones | | | | | | | |
| Chernetidae | | | | | | | |
| | *Spelaeochernes* | sp.1 | 10 | | 4 | | E P A |
| Chthoniidae | | | | | | | |
| | *Pseudochthonius* | sp.1 | 2 | | 1 | | E P |
| | *Pseudochthonius* | sp.4 | 2 | | 2 | | P |
| Ricinulei | | | | | | | |
| Ricinoididae | jovens | | | | 1 | | E |
| | *Cryptocellus* | sp. | 2 | | 1 | | P |
| Scorpiones | | | | | | | |
| Buthidae | jovens | | | | 2 | 0,0025 | P |
| Chilopoda | | | | | | | |
| Pleurostigmophora | | | | | | | |
| Scolopendromorpha | | | | | | | |
| Cryptopidae | | | | | | | |

| | | | | | | |
|---|---|---|---|---|---|---|
| *Cryptops* | sp.1 | 1 | 0,0012 | | | P |
| Scolopocryptopidae | | | | | | |
| *Newportia* | sp.1 | 1 | 0,0012 | | | P |
| Diplopoda | jovens | 1 | 0,0012 | | | |
| Glomeridesmida | | | | | | |
| Glomeridesmidae | sp.3 | 1 | | | | P |
| Polydesmida | jovens | 1 | | | | P |
| Chelodesmidae | sp.4 | | | 2 | 0,0025 | P |
| Pyrgodesmidae | sp.2 | | | 1 | 0,0013 | A |
| Spirostreptida | jovens | | | 2 | | E P |
| Pseudonannolenidae | | | | | | |
| *Pseudonannolene* | sp.1 | 2 | 0,0023 | | | P |
| *Pseudonannolene* | sp.3 | 1 | 0,0012 | | | P |
| Entognatha | | | | | | |
| Diplura | | | | | | |
| Campodeidae | sp.1 | 2 | | 1 | | E P |
| Insecta | | | | | | |
| Blattodea | jovens | 15 | 0,0174 | 15 | 0,0188 | P A |
| Blaberidae | jovens | 4 | 0,0046 | 2 | 0,0025 | P |
| Blaberidae | sp.2 | | | 1 | 0,0013 | P |
| Coleoptera | jovens | 5 | | 2 | | P A |
| Hydrophilidae | | | | | | |
| Sphaeridiinae | sp.2 | 3 | | | | P A |
| Staphylinidae | sp.11 | 4 | | | | P A |
| Collembola | | | | | | |
| Arthropleona | | | | | | |
| Entomobryoidea | | | | | | |
| Entomobryoidea | sp.1 | 3 | | | | P A |
| Cyphoderidae | sp.1 | 3 | | | | P |
| Cyphoderidae | sp.2 | | | 2 | | P A |
| Cyphoderidae | sp.3 | 2 | | | | P A |
| Isotomidae | sp.1 | | | 1 | | P |
| Paronellidae | sp.1 | 3 | | 2 | | E P |
| Paronellidae | sp.4 | | | 1 | | P |
| Symphypleona | | | | | | |
| Sminthuroidea | sp.2 | 2 | | 2 | | P |
| Dermaptera | jovens | | | 1 | 0,0013 | P |
| Diptera | jovens | 3 | | 2 | | E P A |
| Brachycera | | | | | | |
| Dolichopodidae | sp. | | | 1 | | E |
| Drosophilidae | | | | | | |
| *Drosophila* | *eleonore* | 7 | | | | P A |
| Nematocera | | | | | | |
| Cecidomyiidae | | | | | | |
| Cecidomyiinae | sp. | 1 | | | | E |
| Culicidae | | | | | | |
| Culicinae | sp. | 1 | | | | E |
| *Culicini* | sp. | 2 | | | | P A |
| Psychodidae | | | | | | |
| *Pericoma* | sp. | 3 | | 2 | | P A |
| Phlebotominae | sp. | | | 1 | | P |
| *Sciopemyia* | *sordellii* | | | 1 | | P |
| Hemiptera | | | | | | |
| Heteroptera | | | | | | |
| aff. Pyrrhocoroidea | | | | | | |
| Cydnidae | jovens | 1 | | | | P |
| Cydninae | sp.1 | 10 | | | | P A |
| Homoptera | | | | | | |
| Cixiidae | jovens | 1 | | 2 | | E P |
| Hymenoptera | jovens | 2 | | | | P |
| Diaprioidea | | | | | | |
| Diapriidae | sp.1 | 2 | | | | P |
| Proctotrupoidea | | | | | | |
| Diapriidae | sp.2 | | | 1 | | P |
| Vespoidea | | | | | | |

| | | | | | | | |
|---|---|---|---|---|---|---|---|
| Formicidae | | | | | | | |
| | *Camponotus* | sp.1 | 3 | | 1 | | P A |
| | *Dolichoderus* | *bispinosus* | | | 1 | | P |
| | *Labidus* | *coecus* | 4 | | | | P A |
| | *Nylanderia* | sp.1 | 1 | | | | P |
| | *Pachycondyla* | *striata* | 10 | | 3 | | E P A |
| | *Pheidole* | sp.1 | 1 | | | | P |
| | *Solenopsis* | sp.1 | 1 | | | | P |
| | *Wasmania* | *auropunctata* | 1 | | | | E |
| Isoptera | | sp. | | | 1 | | P |
| Termitidae | | | | | | | |
| | *Nasutitermes* | sp. | 3 | | 2 | | E P |
| Lepidoptera | jovens | | 3 | | | | P A |
| Orthoptera | | | | | | | |
| Ensifera | | | | | | | |
| Phalangopsidae | | | | | | | |
| | *Phalangopsis* | sp.1 | 500 | 0,5794 | 500 | 0,625 | P |
| Psocoptera | | | | | | | |
| Psocomorpha | jovens | | 1 | | 1 | | E |
| Thysanura | | | | | | | |
| Ateluridae | jovens | | 1 | | | | P |
| Nicoletiidae | jovens | | 1 | | | | E |
| Malacostraca | | | | | | | |
| Isopoda | | | | | | | |
| Philosciidae | | sp.1 | 4 | | 3 | | E P |
| Scleropactidae | | sp. | 1 | | | | P |
| Symphyla | | | | | | | |
| Scutigerellidae | | | | | | | |
| | *Hanseniella* | sp.1 | 4 | | 3 | | E P |
| Chordata | | | | | | | |
| Amphibia | | | | | | | |
| Anura | | | | | | | |
| Anura | | sp. | | | 3 | 0,0038 | E |
| Neobatrachia | | | | | | | |
| Strabomantidae | | | | | | | |
| | *Pristimantis* | *fenestratus* | | | 1 | 0,0013 | P |
| Mammalia | | | | | | | |
| Chiroptera | | | | | | | |
| Emballonuridae | | | | | | | |
| | *Peropteryx* | sp. | | | 3 | 0,0038 | E |
| Phyllostomidae | | | | | | | |
| | *Carollia* | *perspicillata* | 300 | 0,3476 | 250 | 0,3125 | |
| | *Glossophaga* | *soricina* | 5 | 0,0058 | | | |
| | *Lampronycteris* | *brachyotis* | 1 | 0,0012 | | | |
| | *Phyllostomus* | *latifolius* | 2 | 0,0023 | | | |
| Mollusca | | | | | | | |
| Gastropoda | | | | | | | |
| Bulimulidae | | | | | | | |
| | *Naesiotus* | sp. | 1 | | | | E |
| Subulinidae | | | | | | | |
| | *Lamellaxis* | sp. | 3 | | 1 | | P A |
| | *Leptinaria* | sp. | 1 | | 1 | | P |
