# Supplementary material for: Reconciling Mining with the Conservation of Cave Biodiversity: A Quantitative Baseline to Help Establish Conservation Priorities
Source: PLoS One. 2016 Dec 20;11(12):e0168348. doi: 10.1371/journal.pone.0168348 (PMC5173368; doi:10.1371/journal.pone.0168348)
Supplement: S1 Dataset — (ZIP) [file pone.0168348.s002.zip › Taxa/Serra Sul/SS_2010/S11D_41.pdf]

S11D-41			1ª	AB	2ª	AB	ZON
Annelida							
Clitellata							
Oligochaeta	jovens		3	0,0448			E P
Arthropoda							
Arachnida							
Acari							
Sarcoptiformes							
Oribatida		sp.2	1				E
Oribatida		sp.3			1		E
Trombidiformes		sp.6			1		P
Amblypygi							
Phrynidae							
	Heterophrynus	sp.	1	0,0149			
Araneae							
Araneidae	jovens				1		P
	Alpaida	*septemmammata*	1				E
Ochyroceratidae	jovens		1				E
	Ochyrocera	sp.1	1		1		E P
	Speocera	sp.1			1		E
Palpimanidae	jovens		1	0,0149			E
Scytodidae	jovens		1	0,0149	1	0,2	E P
Symphytognathidae	jovens		1				E
Theridiidae	jovens				1		P
Theridiosomatidae							
	Plato	sp.1			1		P
Opiliones							
Laniatores							
Escadabiidae		sp.2	3				E P
Pseudoscorpiones							
Chernetidae							
	Spelaeochernes	sp.1	1		1		E
Chthoniidae							
	Pseudochthonius	sp.1			1		E
Olpiidae		sp.1	2				E
Ricinulei							
Ricinoididae							
	Cryptocellus	sp.	1		1		E P
Diplopoda							
Polydesmida							
Chelodesmidae		sp.5	1	0,0149			P
Entognatha							
Diplura							
Campodeidae		sp.1	1				P
Insecta							
Blattodea	jovens		2	0,0299			
Coleoptera							
Carabidae		sp.3	1				E
Collembola							
Arthropleona							
Entomobryoidea							
Entomobryoidea		sp.1			2		E
Paronellidae		sp.1	2				E
Paronellidae		sp.4	1				E
Paronellidae		sp.6			1		E
Symphypleona							
Sminthuroidea		sp.2	1				E
Diptera	jovens				1		P
Nematocera							
Cecidomyiidae							
	Cecidomyiinae	sp.	1				E
Psychodidae							
	Sciopemyia	*sordellii*	2		2		E P
Homoptera	jovens		35	0,5224			
Cixiidae	jovens		2		1		E P

Cixiidae		sp.4	1				E	
Hymenoptera								
Vespoidea								
Formicidae								
	Apterostigma	sp.1			2		E	P
	Hypoponera	sp.1	1					P
	Nylanderia	sp.1	2		1		E	P
	Pachycondyla	*striata*	1				E	
	Wasmania	*auropunctata*			1		E	
Lepidoptera	jovens		1					P
Orthoptera								
Ensifera								
Phalangopsidae	jovens		1	0,0149				P
	Paraclodes	sp.1			1		E	
	Phalangopsis	sp.1	18	0,2687				
Psocoptera								
Psocomorpha	jovens				1			P
Symphyla								
Scutigerellidae								
	Hanseniella	sp.1	1		1		E	
Chordata								
Mammalia								
Chiroptera								
Emballonuridae								
	Peropteryx	sp.	2	0,0299	4	0,8		P
Phyllostomidae								
	Glossophaginae	sp.	1	0,0149				
Rodentia		sp.	1	0,0149				
Mollusca								
Gastropoda								
Systrophiidae								
	Happia	sp.	1				E	
