# Supplementary material for: Reconciling Mining with the Conservation of Cave Biodiversity: A Quantitative Baseline to Help Establish Conservation Priorities
Source: PLoS One. 2016 Dec 20;11(12):e0168348. doi: 10.1371/journal.pone.0168348 (PMC5173368; doi:10.1371/journal.pone.0168348)
Supplement: S1 Dataset — (ZIP) [file pone.0168348.s002.zip › Taxa/Serra Sul/SS_2010/S11D_42.pdf]

S11D-42			1ª	AB	2ª	AB	ZON
Annelida							
Clitellata							
Oligochaeta	jovens		1	0,0455			E
Arthropoda							
Arachnida							
Acari							
Ixodida							
Ixodidae							
	Amblyomma	sp.	1				E
Parasitiformes							
Opilioacarida							
Opilioacaridae		sp.1	1				E
Trombidiformes		sp.7			1		E
Araneae							
Araneidae							
	Alpaida	*septemmammata*	1				E
Ochyroceratidae	jovens		1				E
Pholcidae							
	Mesabolivar	sp.1	1				E
Salticidae	jovens		1				E
Scytodidae	jovens		1	0,1364	1	0,0833	E
	Scytodes	sp.	2		1	0,0833	E
Tetrablemmidae							
	Matta	sp.1	1				E
Opiliones							
Laniatores							
Stygnidae	jovens		1	0,0455			E
Pseudoscorpiones							
Chthoniidae							
	Pseudochthonius	sp.1			1		E
Diplopoda	jovens				2	0,1667	E
Spirostreptida	jovens				1		E
Insecta							
Collembola							
Arthropleona							
Entomobryoidea							
Entomobryidae		sp.3	1				E
Paronellidae		sp.1	1		1		E
Diptera							
Nematocera							
Psychodidae							
	Pintomyia	*gruta*	1				E
Tipulidae							
	Tipulinae	sp.	1		1		E
Hymenoptera							
Vespoidea							
Formicidae							
	Dolichoderus	*bispinosus*	2		1		E
	Gnamptogenys	*striatula*	1				E
	Nylanderia	sp.1	1				E
	Odontomachus	*bauri*	1	0,0455			E
	Wasmania	*auropunctata*			1		E
Isoptera							
Termitidae							
	Nasutitermes	sp.	1		1		E
Lepidoptera	jovens				1		E
Cossoidea							
Limacodidae		sp.1	1	0,0455			E
Noctuoidea							
Noctuidae		sp.2	1	0,0455			E
Orthoptera							
Ensifera							
Phalangopsidae	jovens		9	0,4545			
	Phalangopsis	sp.1	1				E

Psocoptera							
Psocomorpha	jovens				1		E
Chordata							
Mammalia							
Chiroptera							
Phyllostomidae							
	Glossophaginae	sp.	4	0,1818	8	0,6667	E
