# Supplementary material for: Reconciling Mining with the Conservation of Cave Biodiversity: A Quantitative Baseline to Help Establish Conservation Priorities
Source: PLoS One. 2016 Dec 20;11(12):e0168348. doi: 10.1371/journal.pone.0168348 (PMC5173368; doi:10.1371/journal.pone.0168348)
Supplement: S1 Dataset — (ZIP) [file pone.0168348.s002.zip › Taxa/Serra Sul/SS_2010/S11D_43.pdf]

S11D-43		1ª	AB	2ª	AB	ZON
Annelida						
Clitellata						
Oligochaeta	jovens	1				A
Oligochaeta	sp.	1	0,0032			A
Arthropoda						
Arachnida						
Acari						
Parasitiformes						
Mesostigmata						
Mesostigmata	sp.2	2				P
Mesostigmata	sp.6	1				A
Laelapidae						
Stratiolaelaps	sp.1	1				P
Sarcoptiformes						
Oribatida	sp.2	1		1		A
Oribatida	sp.3	5				P A
Oribatida	sp.5			1		A
Acaridae	sp.1			1		P
Trombidiformes	sp.1	1		1		P
Trombidiformes	sp.2	2				E
Trombidiformes	sp.6			1		P
Trombidiformes	sp.7	1				E
Amblypygi						
Charinidae						
Charinus	sp.	1	0,0032			
Phrynidae						
Heterophrynus	sp.	2	0,0063	2	0,0119	A
Araneae	jovens	1	0,0032			
Araneidae						
Alpaida	*septemmammata*	1				E
Alpaida	sp.2	1				P
Corinnidae	jovens	1	0,0032			E
Ochyroceratidae	jovens	2		4		E P A
Ochyrocera	sp.1	4		2		E P A
Speocera	sp.1	3		2		P A
Pholcidae						
aff. *Ibityporanga*	sp.1	1				E
Mesabolivar	*cambridgei*			1		E
Salticidae	jovens	1				E
Scytodidae	jovens	3	0,0095	1	0,006	E A
Scytodes	*eleonorae*	2	0,0063			P
Scytodes	*globula*	1	0,0032			P
Scytodes	sp.	1	0,0032			
Tetrablemmidae	jovens			1		A
Matta	sp.1	3		3		E P A
Theridiosomatidae	jovens	1				A
Plato	sp.1	3		3		E P
Uloboridae						
Miagrammopes	sp.1	1				E
Opiliones						
Cyphophthalmi						
Neogoveidae						
Canga	*renatae*	2				P A
Laniatores						
Escadabiidae	jovens	3		1		P A
Escadabiidae	sp.2	1		2		P A
Palpigradi						
Eukoeneniidae						
Allokoenenia	sp.1	1		1		A
Pseudoscorpiones						
Chernetidae	jovens	2				E
Chernetidae						
Spelaeochernes	sp.1	6		2		E P A
Chthoniidae	jovens	4				P A

Chthoniidae							
	Pseudochthonius	sp.1	2		1		E P A
	Pseudochthonius	sp.4	2				A
Schizomida							
Hubbardiidae							
	Rowlandius	sp.	1				E
Scorpiones							
Buthidae		jovens			1	0,006	A
Chilopoda							
Notostigmophora							
Scutigeromorpha							
Pselliodidae		jovens	1				P
Diplopoda							
Glomeridesmida							
Glomeridesmidae		jovens	1		1		P A
Glomeridesmidae		sp.1	3				E A
Polydesmida							
Pyrgodesmidae		sp.2	1	0,0032	1	0,006	P
Entognatha							
Diplura							
Campodeidae		sp.1	1		3		P A
Insecta							
Blattodea		jovens	1	0,0032	4	0,0238	E P
Blaberidae		jovens	1	0,0032	1	0,006	E P
Blaberidae		sp.1	1	0,0032			A
Blattidae		jovens	1	0,0032			P
Coleoptera		jovens	30	0,0952	1	0,006	P A
Carabidae							
	Coarazuphium	sp.1			1		A
Chrysomelidae		sp.7	1				P
Curculionidae							
	Scolytinae	sp.2	2				A
Ptiliidae		sp.1	5				E P A
Staphylinidae		sp.11	1				E
Staphylinidae		sp.13	1				A
Collembola							
Arthropleona							
Entomobryoidea							
Cyphoderidae		sp.1	1				E
Isotomidae		sp.1	1		1		A
Isotomidae		sp.2	2				P
Paronellidae		sp.4	3		1		P A
Symphypleona							
Sminthuroidea		sp.1	1		1		A
Sminthuroidea		sp.2	2				E A
Diptera		jovens	7		2		E P A
Brachycera							
Drosophilidae							
	Drosophila	*eleonore*	3		1		E P
Psychodidae							
	Drosophila	*eleonore*			1		A
Nematocera							
Chironomidae		sp.			1		E
Culicidae							
	Culicini	sp.	1				P
Psychodidae							
	Evandromyia	*saulensis*	1				E
	Evandromyia	*sordellii*	1				E
	Pericoma	sp.	2				P
	Phlebotominae	sp.			1		P
	Pintomyia	*gruta*	2				P A
	Sciopemyia	*sordellii*	5		2		E P A
Tipulidae							
	Tipulinae	sp.	1		1		E P
Hemiptera							

Heteroptera						
aff. Pyrrhocoroidea						
Cydnidae						
Cydninae	sp.1	4		3		P A
Reduviidae	jovens	1				A
Homoptera	jovens	1	0,0032			
Cixiidae	jovens	4		1		P A
Cixiidae	sp.4	1		1		E P
Hymenoptera						
Vespoidea						
Formicidae						
Acromyrmex	sp.1			1		P
Apterostigma	sp.1	1		1		E A
Camponotus	*atriceps*	1				P
Camponotus	sp.1	3		3		P A
Dolichoderus	*bispinosus*	1				E
Myrmicocrypta	sp.1	1				E
Nylanderia	sp.1	2		2		P A
Pachycondyla	*striata*	5				E P
Solenopsis	sp.2	1				A
Strumygenys	sp.1	1		1		A
Wasmania	*auropunctata*	1		1		A
Isoptera						
Termitidae						
Nasutitermes	sp.	7		1		E P
Lepidoptera	jovens	1		1		E P
Cossoidea						
Limacodidae	sp.1	2	0,0063			E
Noctuoidea	sp.2	1				P
Tineoidea	sp.1	1		1		P
Noctuidae	sp.1	4	0,0127			
Orthoptera						
Ensifera						
Phalangopsidae						
Paraclodes	sp.1	1	0,0032	4	0,0238	E
Phalangopsis	sp.1	183	0,581	69	0,4107	A
Psocoptera						
Psocomorpha	jovens	1				E
Thysanura						
Ateluridae	jovens			1		A
Ateluridae	sp.1	1				A
Malacostraca						
Isopoda						
Philosciidae	sp.1	7		4		E P A
Scleropactidae	sp.	1				E
Symphyla						
Scutigerellidae						
Hanseniella	sp.1	1				P
Chordata						
Amphibia						
Anura						
Neobatrachia						
Leptodactylidae						
Leptodactylus	sp.	2	0,0063			
Strabomantidae						
Pristimantis	*fenestratus*			28	0,1667	
Bufonidae						
Rhinella	cf. *marina*	1	0,0032			
Mammalia						
Chiroptera						
Emballonuridae						
Peropteryx	sp.	1	0,0032	6	0,0357	
Furipteridae						
Furipterus	*horrens*	20	0,0635	2	0,0119	
Phyllostomidae						

Carollia	*perspicillata*	30	0,0952	30	0,1786	
Diphylla	*ecaudata*	1	0,0032	1	0,006	
Glossophaga	*soricina*	5	0,0159			
Glossophaginae	*sp.*			16	0,0952	
Lampronycteris	*brachyotis*	4	0,0127			
Micronycteris	*microtis*	7	0,0222			
Phylloderma	sp.			1	0,006	
Pilosa						
Myrmecophagidae						
Tamandua	*tetradactyla*	1	0,0032			
Rodentia	*sp.*	1	0,0032			
Reptilia						
Squamata						
Gekkonidae						
Thecadactylus	*rapicauda*	1	0,0032			
Mollusca						
Gastropoda jovens		1				P
Subulinidae						
Lamellaxis	sp.	1		1		P A
Leptinaria	sp.	4		3		E P A
Systrophiidae						
Happia	sp.	1				A
Nemathelminthes	sp.	1	0,0032			P
Platyhelminthes						
Turbellaria	sp.2	1				A
