# Supplementary material for: Reconciling Mining with the Conservation of Cave Biodiversity: A Quantitative Baseline to Help Establish Conservation Priorities
Source: PLoS One. 2016 Dec 20;11(12):e0168348. doi: 10.1371/journal.pone.0168348 (PMC5173368; doi:10.1371/journal.pone.0168348)
Supplement: S1 Dataset — (ZIP) [file pone.0168348.s002.zip › Taxa/Serra Sul/SS_2010/S11D_44.pdf]

S11D-44			1ª	AB	2ª	AB	ZON
Arthropoda							
Arachnida							
Acari							
Parasitiformes							
Mesostigmata		sp.3			1		E
Sarcoptiformes							
Oribatida		sp.2			1		E
Trombidiformes		sp.4	1				E
Amblypygi							
Phrynidae							
	Heterophrynus	sp.			1	0,1111	E
Araneae							
Araneidae	jovens		1				E
Corinnidae	jovens		1	0,0769			E
Ctenidae		sp.2	1	0,0769			E
Ochyroceratidae							
	Speocera	sp.1	1				E
Pholcidae	jovens		1				E
	Leptopholcus	sp.1			1		E
	Mesabolivar	sp.1	2				E
Prodidomidae	jovens		1				E
Scytodidae	jovens		1	0,0769			E
Theridiosomatidae							
	Plato	sp.1	1				E
Opiliones							
Laniatores							
Stygnidae		sp.1			1	0,2222	E
Stygnidae		sp.1			1		E
Pseudoscorpiones							
Chernetidae							
	Spelaeochernes	sp.1	2		1		E
Chthoniidae							
	Pseudochthonius	sp.1	1				E
Chilopoda							
Pleurostigmophora							
Scolopendromorpha							
Scolopocryptopidae	jovens		1	0,0769			E
Entognatha							
Diplura							
Campodeidae		sp.1	2				E
Insecta							
Coleoptera	jovens		1				E
Collembola							
Arthropleona							
Entomobryoidea							
Cyphoderidae		sp.1	1				E
Entomobryidae		sp.1			1		E
Paronellidae		sp.1	2				E
Paronellidae		sp.6			1		E
Diptera							
Nematocera							
Psychodidae							
	Sciopemyia	*sordellii*	2		1		E
Tipulidae							
	Tipulinae	sp.	1		1		E
Hemiptera							
Heteroptera							
Dipsocoroidea	jovens				1		E
Homoptera							
Cixiidae	jovens		1		1		E
Hymenoptera							
Vespoidea							
Formicidae							
	Apterostigma	sp.1	1				E

					Camponotus	sp.1	2		1		E
					Cyphomyrmex	sp.1	1				E
					Gnamptogenys	*striatula*			1		E
					Hypoponera	sp.1	1				E
		Isoptera				sp.	1				E
		Lepidoptera									
			Castnioidea								
				Noctuidae		sp.1	1	0,0769			
		Orthoptera									
			Ensifera								
				Phalangopsidae	jovens		8	0,6154			
					Paraclodes	sp.1			3	0,3333	E
		Thysanura									
				Nicoletiidae		sp.1	1				E
	Malacostraca										
		Isopoda									
				Dubioniscidae		sp.1	1				E
				Philosciidae		sp.1	1				E
Chordata											
	Amphibia										
		Anura									
			Neobatrachia								
				Strabomantidae							
					Pristimantis	*fenestratus*			3	0,3333	E
Mollusca											
	Gastropoda										
				Systrophiidae							
					Happia	sp.	1				E
