# Supplementary material for: Reconciling Mining with the Conservation of Cave Biodiversity: A Quantitative Baseline to Help Establish Conservation Priorities
Source: PLoS One. 2016 Dec 20;11(12):e0168348. doi: 10.1371/journal.pone.0168348 (PMC5173368; doi:10.1371/journal.pone.0168348)
Supplement: S1 Dataset — (ZIP) [file pone.0168348.s002.zip › Taxa/Serra Sul/SS_2010/S11D_45.pdf]

S11D-45		1ª	AB	2ª	AB	ZON
Annelida						
Clitellata						
Oligochaeta	jovens	2	0,0081			E
Arthropoda						
Arachnida						
Acari						
Parasitiformes						
Mesostigmata						
Laelapidae						
Stratiolaelaps	sp.1	1				E
Ologamasidae	sp.1	1				E
Sarcoptiformes						
Sarcoptiformes	sp.2	1				E
Oribatida	sp.2			1		E
Oribatida	sp.3	2				E
Trombidiformes	sp.2	1				P
Trombidiformes	sp.6			1		P
Araneae						
Ochyroceratidae	jovens			1		P
Pholcidae	jovens	1		2		E P
Salticidae	jovens	1				E
Onofre	sp.1			1		E
Scytodidae	jovens			1	0,006	P
Scytodes	*eleonorae*	2	0,0081			E P
Scytodes	sp.	1	0,004			
Theraphosidae	jovens	1	0,004	3	0,0179	E
Theridiosomatidae	jovens			1		E
Plato	sp.1	2		1		E P
Trechaleidae	jovens			1	0,006	E
Opiliones						
Eupnoi						
Sclerosomatidae	jovens	1		1		E
Sclerosomatidae	sp.1	1		1		E
Laniatores						
Stygnidae	jovens	1	0,0161			P
Stygnidae	sp.1	3		1	0,006	E P
Pseudoscorpiones						
Bochicidae	sp.1	4				P
Chernetidae						
Spelaeochernes	sp.1	2		1		E
Schizomida						
Hubbardiidae						
Rowlandius	sp.	2		1		P
Diplopoda						
Polydesmida						
Pyrgodesmidae	sp.2	1	0,004			P
Spirostreptida	jovens			1		P
Pseudonannolenidae	jovens	1	0,004			P
Insecta						
Blattodea	jovens	1	0,004	1	0,006	E P
Blaberidae	jovens	1	0,004	1	0,006	E P
Blattidae	sp.3	3	0,0121			E P
Coleoptera	jovens			1		P
Chrysomelidae	sp.1	1				E
Ptiliidae	sp.1	1				P
Scydmaenidae	sp.5	1				P
Collembola						
Arthropleona						
Entomobryoidea						
Isotomidae	sp.1			1		E
Diptera						
Brachycera						
Dolichopodidae	sp.			2		E P
Phoridae						

Metopininae	sp.	1		1		E	
Nematocera							
Culicidae							
Culicini	sp.			1			P
Psychodidae							
Pintomyia	*gruta*	1					P
Sciopemyia	*sordellii*	2		1		E	P
Hemiptera							
Heteroptera	jovens	50	0,2016				
aff. Pyrrhocoroidea							
Cydnidae	jovens	1		2		E	P
Cydninae	sp.1	4				E	P
Reduviidae	jovens	1	0,004	1	0,0417	E	
Reduviinae	sp.			6		E	
Homoptera							
Cixiidae	jovens	1		2		E	P
Cixiidae	sp.7	1	0,004				P
Hymenoptera							
Ichneumonoidea							
Braconidae	sp.1	1					P
Isoptera	sp.	1				E	
Termitidae							
Nasutitermes	sp.	1		2		E	P
Lepidoptera	jovens			1		E	
Noctuoidea	sp.2	1				E	
Neuroptera							
Myrmeleonthidae	jovens	1		1		E	
Orthoptera							
Ensifera							
Phalangopsidae	jovens	140	0,5645				
Phalangopsis	sp.1	1	0,004				P
Paraclodes	sp.1			2	0,0119		P
Phalangopsis	sp.1			100	0,5952		P
Psocoptera							
Psocomorpha	jovens	1					P
Thysanura							
Nicoletiidae	jovens	1					P
Nicoletiidae	sp.1	1		1			P
Malacostraca							
Isopoda							
Philosciidae	sp.1	3				E	P
Scleropactidae	sp.	1		1		E	P
Chordata							
Amphibia							
Anura							
Neobatrachia							
Strabomantidae							
Pristimantis	*fenestratus*			5	0,0298		P
Mammalia							
Chiroptera							
Emballonuridae							
Peropteryx	*kappleri*	3	0,0121				
Peropteryx	sp.			6	0,0357		P
Furipteridae							
Furipterus	*horrens*	10	0,0403				
Phyllostomidae							
Carollia	*perspicillata*	5	0,0202	17	0,1012		
Carollia	sp.						P
Glossophaga	*soricina*	20	0,0806				
Glossophaginae	sp.			23	0,1369		P
Mollusca							
Gastropoda							
Subulinidae							
Lamellaxis	sp.	1					P
