# Supplementary material for: Reconciling Mining with the Conservation of Cave Biodiversity: A Quantitative Baseline to Help Establish Conservation Priorities
Source: PLoS One. 2016 Dec 20;11(12):e0168348. doi: 10.1371/journal.pone.0168348 (PMC5173368; doi:10.1371/journal.pone.0168348)
Supplement: S1 Dataset — (ZIP) [file pone.0168348.s002.zip › Taxa/Serra Sul/SS_2010/S11D_46.pdf]

S11D-46		1ª	AB	2ª	AB	ZON
Arthropoda						
Arachnida						
Acari						
Sarcoptiformes	sp.1	1				E
Trombidiformes						
Anystidae						
Erythracarus	*nasutus*	1				P
Amblypygi						
Phrynidae						
Heterophrynus	sp.			3	0,1	P
Araneae						
Araneidae	jovens			1		P
Alpaida	*septemmammata*	1				E
Alpaida	*smila*	1				P
Ochyroceratidae						
Ochyrocera	sp.1	1		2		E P
Speocera	sp.1	1		1		E P
Pholcidae	jovens	1				E
Mesabolivar	*aurantiacus*			1		P
Mesabolivar	sp.1	1				E
Scytodidae						
Scytodes	*globula*	1	0,025			E
Theridiosomatidae						
Plato	sp.1	2				E P
Opiliones						
Cyphophthalmi						
Neogoveidae						
Canga	*renatae*	1				P
Eupnoi						
Sclerosomatidae	jovens	2				E
Laniatores						
Stygnidae	jovens	1	0,05			E
Stygnidae	sp.1	1		1	0,0333	E P
Palpigradi						
Eukoeneniidae	jovens	1				P
Pseudoscorpiones						
Chernetidae						
Spelaeochernes	sp.1	1		1		E P
Chthoniidae						
Pseudochthonius	sp.1			1		E
Diplopoda						
Polydesmida						
Pyrgodesmidae	sp.2	1	0,025			P
Spirostreptida	jovens	1				P
Entognatha						
Diplura						
Campodeidae	sp.1			1		E
Insecta						
Blattodea	jovens			1	0,0333	P
Coleoptera	jovens	1				E
Scarabaeidae						
Melolonthinae	sp.1			1	0,0333	E
Collembola						

Arthropleona						
Entomobryoidea						
Isotomidae	sp.1			1		P
Paronellidae	sp.1	1				P
Symphypleona						
Sminthuroidea	sp.2	1				P
Diptera						
Brachycera						
Dolichopodidae	sp.			1		P
Nematocera						
Cecidomyiidae						
Cecidomyiinae	sp.	1				E
Psychodidae						
Sciopemyia	*sordellii*	1		1		E
Hemiptera						
Heteroptera						
aff. Pyrrhocoroidea						
Reduviidae						
Emesinae	sp.2			1		P
Panstrongylus	sp.1			1	0,0333	P
Homoptera						
Cixiidae	jovens			1		E
Hymenoptera						
Vespoidea						
Formicidae						
Camponotus	sp.1	1		1		E P
Octostruma	sp.1	1		1		E P
Solenopsis	sp.2			1		E
Solenopsis	sp.3	1				P
Wasmania	*auropunctata*	1				E
Isoptera						
Termitidae						
Armitermes	sp.	1				E
Nasutitermes	sp.	1				E
Lepidoptera	jovens	1	0,025	1	0,0333	E
Noctuoidea	sp.2	1				E
Orthoptera						
Ensifera						
Gryllidae	jovens			1	0,0333	P
Phalangopsidae	jovens	1	0,025	1	0,0333	E P
Paraclodes	sp.1			1	0,0333	E
Phalangopsis	sp.1	30	0,75	12	0,4	P
Chordata						
Amphibia						
Anura						
Neobatrachia						
Strabomantidae						
Pristimantis	*fenestratus*			2	0,0667	P
Mammalia						
Chiroptera						
Emballonuridae						
Peropteryx	*kappleri*	4	0,1	3	0,1	

	Peropteryx	sp.			1	0,0333	P
Rodentia		sp.			1	0,0333	E
