# Supplementary material for: Reconciling Mining with the Conservation of Cave Biodiversity: A Quantitative Baseline to Help Establish Conservation Priorities
Source: PLoS One. 2016 Dec 20;11(12):e0168348. doi: 10.1371/journal.pone.0168348 (PMC5173368; doi:10.1371/journal.pone.0168348)
Supplement: S1 Dataset — (ZIP) [file pone.0168348.s002.zip › Taxa/Serra Sul/SS_2010/S11D_47.pdf]

S11D-47			1ª	AB	2ª	AB	ZON
Annelida							
Clitellata							
Oligochaeta	jovens		1	0,0034			E
Arthropoda							
Arachnida							
Acari							
Ixodida							
Argasidae							
	Ornithodoros	sp.	5		3		P
Parasitiformes							
Mesostigmata							
Mesostigmata		sp.4	1				P
Laelapidae		sp.3	1				P
Macronyssidae		sp.1	1				P
Podocinidae		sp.1	1				P
Sarcoptiformes							
Oribatida		sp.3			1		P
Trombidiformes							
Trombidiformes		sp.1	1		2		P
Trombidiformes		sp.6			1		P
Anystidae							
	Erythracarus	*nasutus*	1				P
Rhagidiidae		sp.1			1		P
Amblypygi							
Phrynidae							
	Heterophrynus	sp.			3	0,0087	P
Araneae							
Corinnidae	jovens		1	0,0034	3	0,0116	P
	Creugas	sp.1			1		P
Ctenidae	jovens		2	0,0068			P
Ochyroceratic	jovens				1		P
	Speocera	sp.1	1		1		P
	Theotima	sp.1			1		P
Oonopidae							
	Oonopinae	sp.3			1		P
Pholcidae	jovens		1				E
	Mesabolivar	sp.1	1				E
Salticidae	jovens		1				E
Scytodidae	jovens		1	0,0034	1	0,0029	E P
	Scytodes	*eleonorae*	2	0,0068	1	0,0029	E P
	Scytodes	sp.			1	0,0029	P
Tetrablemmid	jovens		1		1		P
Theraphosida	jovens		2	0,0102			E P
	aff. *Holothele*	sp.1	1				E
Theridiosoma	jovens		2				E P
	Plato	sp.1	1		1		P
Trechaleidae	jovens		1	0,0034			P
Trechaleidae		sp.1	1	0,0034			P
Trechaleidae	gen.1	sp.1			1	0,0029	P
Opiliones							
Eupnoi							
Sclerosomatic	jovens		1				E
Sclerosomatidae		sp.1	2				E P
Laniatores							
Stygnidae	jovens		3	0,0102	1	0,0029	E P
Palpigradi							
Eukoeneniida	jovens		1				P
	Allokoenenia	sp.1	1				P
Pseudoscorpiones							
Chernetidae	jovens		3				P
Chernetidae							
	Spelaeochernes	sp.1	7		6		E P
Chthoniidae	jovens		4				E P
	Pseudochthonius	sp.1	2		4		P

Entognatha							
Diplura							
Campodeidae		sp.1	1				P
Japygidae		sp.1	1				P
Insecta							
Blattodea	jovens		3	0,0102	11	0,032	E P
Blaberidae	jovens		1	0,0034	4	0,0174	E P
Blaberidae		sp.4			2		P
Blattidae	jovens		1	0,0068			P
Blattidae		sp.3	1				P
Coleoptera	jovens		6		1		P
Elateridae		sp.2	1				P
Ptiliidae		sp.1	5		2		E P
Staphylinidae		sp.11	1				P
Collembola							
Arthropleona							
Entomobryoidea							
Entomobryidae		sp.2	1				P
Isotomidae		sp.1			1		P
Paronellidae		sp.1	5				E P
Symphypleona							
Sminthuroidea		sp.2	2		2		E P
Diptera	jovens		3				E P
Brachycera							
Camillidae		sp.	1		1		E P
Drosophilidae							
	Drosophila	*eleonore*	1				E
Nematocera							
Chironomidae		sp.	1				P
Culicidae							
	Culicini	sp.	1		1		E
Psychodidae							
	Bruchomyiinae	sp.			2		E P
	Phlebotominae	sp.			1		P
	Pintomyia	*gruta*	1				P
	Sciopemyia	*sordellii*	2		1		P
Sciaridae		sp.	1				P
Tipulidae							
	Tipulinae	sp.			1		P
Hemiptera							
Heteroptera							
aff. Pyrrhocoroidea							
Cydnidae	jovens		2				P
	Cydninae	sp.1	1		1		P
Reduviidae	jovens		2	0,0068	2	0,0058	P
	Emesinae	sp.3	1				P
	Reduviinae	sp.			6	0,0174	P
Homoptera							
Cixiidae	jovens		1				E
Hymenoptera							
Chalcidoidea		sp.1	1				P
Chalcidoidea		sp.2			1		P
Vespoidea							
Formicidae							
	Camponotus	sp.1	7		7		E P
	Carebara	sp.1	1				E
	Pachycondyla	*harpax*	1	0,0034			E
	Pachycondyla	*striata*	1				P
	Wasmania	*auropunctata*	1				E
Isoptera		sp.	1		1		P
Termitidae							
	Nasutitermes	sp.	7		5		E P
Lepidoptera	jovens		2				P
Cossoidea							
Limacodidae		sp.1	2	0,0068			E P

Orthoptera							
Ensifera							
Gryllidae		sp.1			1	0,0029	P
Phalangopsid jovens			1	0,0034			E
	Phalangopsis	sp.1	1	0,7543			E
	Phalangopsis	sp.1	220		239	0,6948	P
	Paraclodes	sp.1			2	0,0058	P
Chordata							
Amphibia							
Anura							
Neobatrachia							
Strabomantidae							
	Pristimantis	*fenestratus*	5	0,0171	28	0,0814	P
Mammalia							
Chiroptera							
Emballonuridae							
	Peropteryx	sp.			2	0,0058	E
	Carollia	*perspicillata*	20	0,0683			
Glossophaginae					35	0,1017	
	Glossophaga	*soricina*	20	0,0683			
