# Supplementary material for: Reconciling Mining with the Conservation of Cave Biodiversity: A Quantitative Baseline to Help Establish Conservation Priorities
Source: PLoS One. 2016 Dec 20;11(12):e0168348. doi: 10.1371/journal.pone.0168348 (PMC5173368; doi:10.1371/journal.pone.0168348)
Supplement: S1 Dataset — (ZIP) [file pone.0168348.s002.zip › Taxa/Serra Sul/SS_2010/S11D_48.pdf]

S11D-48			1ª	AB	2ª	AB	ZON
Arthropoda							
Arachnida							
Araneae							
Araneidae	jovens		1		1		E
Ochyroceratidae	jovens		1				E
Pholcidae	jovens		1				E
	Mesabolivar	sp.1	1		1		E
	Ninetinae	sp.1	1		1		E
Salticidae	jovens		1		1		E
	Amphidraus	sp.1	1				E
Scytodidae	jovens		1		1		E
	Scytodes	*globula*	1	0,0909			E
Theridiosomatidae							
	Plato	sp.1	2				E
Pseudoscorpiones							
Chernetidae							
	Spelaeochernes	sp.1	1				E
Entognatha							
Diplura							
Campodeidae		sp.1	2				E
Insecta							
Blattodea	jovens		1	0,0909			E
Polyphagidae	jovens		1	0,0909			E
Collembola							
Arthropleona							
Entomobryoidea							
Entomobryidae		sp.10			1		E
Paronellidae		sp.6			1		E
Diptera							
Nematocera							
Culicidae							
	Culicini	sp.	1				E
Tipulidae							
	Tipulinae	sp.			1		E
Hemiptera							
Heteroptera							
aff. Pyrrhocoroidea							
Reduviidae	jovens		1	0,0909	1	0,25	E
Hymenoptera							
Vespoidea							
Formicidae							
	Camponotus	sp.1			1		E
	Pachycondyla	*striata*	1				E
	Wasmania	*auropunctata*			1		E
Isoptera							
Termitidae							
	Cortaritermes	*silvestrii*			1		E
	Nasutitermes	sp.	1				E
Lepidoptera	jovens		3	0,2727	1	0,25	E
Cossoidea							
Limacodidae		sp.1	3	0,2727			E
Noctuoidea		sp.2	1				E
Psocoptera							
Psocomorpha	jovens		1				E
Malacostraca							
Isopoda							
Dubioniscidae		sp.1	1				E
Chordata							
Mammalia							
Chiroptera							
Chiroptera		sp.			2	0,5	E
Phyllostomidae							
	Glossophaga	*soricina*	1	0,0909			
Mollusca							

Gastropoda							
	Systrophiidae						
	Happia	sp.	1				E
