# Supplementary material for: Reconciling Mining with the Conservation of Cave Biodiversity: A Quantitative Baseline to Help Establish Conservation Priorities
Source: PLoS One. 2016 Dec 20;11(12):e0168348. doi: 10.1371/journal.pone.0168348 (PMC5173368; doi:10.1371/journal.pone.0168348)
Supplement: S1 Dataset — (ZIP) [file pone.0168348.s002.zip › Taxa/Serra Sul/SS_2010/S11D_49.pdf]

S11D-49				1ª	AB	2ª	AB	ZON
Arthropoda								
Arachnida								
Araneae								
	Filistatidae	jovens		1				E
	Filistatidae		sp.1	1				E
	Pholcidae	jovens		1				E
		Leptopholcus	sp.1			1		E
	Scytodidae	jovens				1		E
		Scytodes	sp.1	1				E
Pseudoscorpiones								
	Olpiidae		sp.1	2				E
Chilopoda								
Notostigmophora								
Scutigeromorpha								
	Pselliodidae	jovens		1				E
Insecta								
Dermaptera		jovens				1		E
Diptera								
Brachycera								
	Dolichopodidae		sp.			1		E
Hymenoptera								
Vespoidea								
	Formicidae							
		Crematogaster	sp.1			1		E
Isoptera			sp.	2				E
	Termitidae							
		Cornitermes	sp.			1		E
Lepidoptera								
Tineoidea			sp.1	1				E
Tineoidea			sp.3	1				E
Neuroptera								
	Myrmeleonthidae	jovens		2		1		E
Orthoptera								
Ensifera								
	Phalangopsidae							
		Paraclodes	sp.1			2	0,4	E
Psocoptera								
Psocomorpha		jovens				1		E
Trogiomorpha								
	Psyllipsocidae							
		Psyllipsocus	sp.1			1		E
Chordata								
Mammalia								
Chiroptera								
Chiroptera			sp.			3	0,6	E
	Phyllostomidae							
		Glossophaginae	sp.	3	1			
