# Supplementary material for: Reconciling Mining with the Conservation of Cave Biodiversity: A Quantitative Baseline to Help Establish Conservation Priorities
Source: PLoS One. 2016 Dec 20;11(12):e0168348. doi: 10.1371/journal.pone.0168348 (PMC5173368; doi:10.1371/journal.pone.0168348)
Supplement: S1 Dataset — (ZIP) [file pone.0168348.s002.zip › Taxa/Serra Sul/SS_2010/S11D_50.pdf]

S11D-50			1ª	AB	2ª	AB	ZON
Annelida							
Clitellata							
Oligochaeta	jovens		1	0,0556			E
Arthropoda							
Arachnida							
Amblypygi							
Charinidae	jovens		1	0,0556			E
Araneae							
Araneidae	jovens		1		1		E
Ctenidae	jovens		1	0,0556	1	0,25	E
	Ctenus	sp.2			1		E
Nesticidae	jovens				1		E
Ochyroceratidae							
	Ochyrocera	sp.1	1		1		E
	Speocera	sp.1	1				E
Pholcidae							
	Leptopholcus	sp.1	1				E
Salticidae	jovens		1		1		E
Scytodidae	jovens		2	0,1111			E
	Scytodes	*eleonorae*			1	0,125	E
		sp.			1	0,125	E
Opiliones							
Laniatores							
Stygnidae		sp.1	2	0,1111			E
Pseudoscorpiones							
Chthoniidae							
	Pseudochthonius	sp.1	2				E
Olpiidae		sp.1	2		4		E
Chilopoda							
Notostigmophora							
Scutigeromorpha							
Pselliodidae	jovens				1		E
Diplopoda							
Spirostreptida							
Pseudonannolenidae	jovens		2	0,1111			E
Insecta							
Coleoptera	jovens		1				E
Staphylinidae							
	Pselaphinae	sp.1	1				E
Collembola							
Arthropleona							
Entomobryoidea							
Paronellidae		sp.1			1		E
Diptera							
Brachycera							
Phoridae							
	Metopininae	sp.	1				E
Hemiptera							
Homoptera							
Cixiidae	jovens		1		1		E
		sp.3	1		1		E
Hymenoptera							
Vespoidea							
Formicidae							
	Hypoponera	sp.1			1		E
	Nylanderia	sp.1	1				E
	Pachycondyla	*striata*	1				E
	Pheidole	sp.1			1		E
		sp.2			1		E
	Wasmania	*auropunctata*	1				E
Isoptera							
Termitidae							

	Embiratermes	sp.			1		E	
	Nasutitermes	sp.	1				E	
	Velocitermes	sp.			1		E	
Lepidoptera		jovens	2	0,1111	1	0,125	E	P
Noctuoidea								
Noctuidae		sp.2	2	0,1111			E	
Neuroptera								
Myrmeleonthidae		jovens	1		1		E	
Orthoptera								
Ensifera								
Phalangopsidae		jovens	1	0,0556				P
	Paraclodes	sp.1			1	0,125	E	
	Phalangopsis	sp.1	2	0,1111	1	0,125	E	
Psocoptera								
Psocomorpha		jovens	1		2		E	
Trogiomorpha								
Lepidopsocidae								
	Loxopholia	sp.1			1		E	
Malacostraca								
Isopoda								
Dubioniscidae		sp.1	1				E	
Mammalia								
Chiroptera		sp.			1	0,125	E	
Phyllostomidae								
	Glossophaginae	sp.	2	0,1111				P
Mollusca								
Gastropoda								
Systrophiidae								
	Happia	sp.	1				E	
