# Supplementary material for: Reconciling Mining with the Conservation of Cave Biodiversity: A Quantitative Baseline to Help Establish Conservation Priorities
Source: PLoS One. 2016 Dec 20;11(12):e0168348. doi: 10.1371/journal.pone.0168348 (PMC5173368; doi:10.1371/journal.pone.0168348)
Supplement: S1 Dataset — (ZIP) [file pone.0168348.s002.zip › Taxa/Serra Sul/SS_2010/S11D_51.pdf]

S11D-51			1ª	AB	2ª	AB	ZON
Arthropoda							
Arachnida							
Acari							
Ixodida							
Argasidae							
	Ornithodoros	sp.			1		P
Araneae	jovens		4	0,3077			
Araneidae	jovens		1				P
Barychaelidae	jovens		1	0,0769	1	0,125	P
Ctenidae	jovens		2	0,1538			P
Ochyroceratidae							
	Ochyrocera	sp.1	1				P
Pholcidae							
	Ninetinae	sp.1	1		1		P
Salticidae	jovens		1				P
Scytodidae	jovens				1	0,125	P
Theridiosomatidae							
	Plato	sp.1	1				P
Opiliones							
Laniatores							
Stygnidae	jovens		1	0,0769			P
Pseudoscorpiones							
Olpiidae		sp.1	2		2		P
Diplopoda							
Spirostreptida	jovens				1		P
Insecta							
Blattodea	jovens				1	0,125	P
Blaberidae	jovens		1	0,0769			P
Coleoptera							
Staphylinidae							
	Pselaphinae	sp.1	1				P
Collembola							
Arthropleona							
Entomobryoidea							
Cyphoderidae		sp.1	1				P
Entomobryidae		sp.1			1		P
		sp.6			1		P
Paronellidae		sp.1	1				P
Diptera	jovens		1				P
Nematocera							
Cecidomyiidae							
	Cecidomyiinae	sp.	1				P
Hemiptera							
Heteroptera							
Reduviidae	jovens		1	0,0769			P
Veliidae							
	Paravelia	sp.2	1				P
Homoptera							
Cixiidae	jovens		1				P
Hymenoptera							
Vespoidea							
Formicidae							
	Pachycondyla	*striata*	1		1		P
	Wasmania	*auropunctata*	1		1		P
Isoptera		sp.	1				P
Termitidae							
	Nasutitermes	sp.			1		P
Lepidoptera							
Noctuoidea							
Noctuidae		sp.2	1	0,0769			P
Orthoptera							
Ensifera							

Phalangopsidae							
	Paraclodes	sp.1			2	0,25	P
Psocoptera							
Psocomorpha	jovens				1		P
Ptiloneuridae							
	Ptiloneura	sp.3			1		P
Trogiomorpha							
Psyllipsocidae							
	Psyllipsocus	sp.1			1		P
Chordata							
Amphibia							
Anura							
Neobatrachia							
Strabomantidae							
	Pristimantis	*fenestratus*			1	0,125	P
Mammalia							
Chiroptera		sp.			2	0,25	P
Phyllostomidae							
	Glossophaginae	sp.	2	0,1538			
