# Supplementary material for: Reconciling Mining with the Conservation of Cave Biodiversity: A Quantitative Baseline to Help Establish Conservation Priorities
Source: PLoS One. 2016 Dec 20;11(12):e0168348. doi: 10.1371/journal.pone.0168348 (PMC5173368; doi:10.1371/journal.pone.0168348)
Supplement: S1 Dataset — (ZIP) [file pone.0168348.s002.zip › Taxa/Serra Sul/SS_2010/S11D_52.pdf]

S11D-52			1ª	AB	2ª	AB	ZON
Arthropoda							
Arachnida							
Acari							
Parasitiformes							
Holothyrida							
Diplothyridae							
Diplothyrus		*scubarti*	1				E
Sarcoptiformes							
Oribatida		sp.3			1		E
Araneae	jovens		5	0,3571			
Barychaelidae	jovens		1	0,0714			E
Ctenidae	jovens		1	0,0714			
Ochyroceratidae							
Ochyrocera		sp.1	1				E
Pholcidae							
Leptopholcus		sp.1			1		E
Ninetinae		sp.1			1		E
Opiliones							
Laniatores							
Stygnidae	jovens		1	0,0714			E
Pseudoscorpiones							
Chernetidae							
Spelaeochernes		sp.1	1				E
Diplopoda							
Spirostreptida	jovens				1		E
Insecta							
Blattodea	jovens		1				
Blaberidae	jovens		1	0,1429	1	0,3333	E
Coleoptera							
Staphylinidae		sp.14	1				E
Collembola							
Arthropleona							
Entomobryoidea							
Entomobryidae		sp.6			1		E
		sp.9			1		E
Diptera							
Nematocera							
Psychodidae							
Sciopemyia		*sordellii*	1				E
Hemiptera							
Homoptera							
Cixiidae	jovens				1		E
		sp.1	1				E
Hymenoptera							
Vespoidea							
Formicidae							
Pachycondyla		*striata*	1		1		E
Solenopsis		sp.2			1		E
Wasmania		*auropunctata*	1				E
Isoptera		sp.	1				E
Orthoptera							
Ensifera							
Phalangopsidae							
Paraclodes		sp.1			1	0,3333	E
Psocoptera							
Psocomorpha							
Ptiloneuridae							
Ptiloneura		sp.2			1		E
Chordata							
Amphibia							
Anura							
Neobatrachia							

Strabomantidae							
	Pristimantis	*fenestratus*			1	0,3333	E
Anura		sp.	1	0,0714			
Mammalia							
Chiroptera							
Phyllostomidae							
	Glossophaginae	sp.	3	0,2143			
Mollusca							
Gastropoda							
Systrophiidae							
	Happia	sp.	1				E
