# Supplementary material for: Reconciling Mining with the Conservation of Cave Biodiversity: A Quantitative Baseline to Help Establish Conservation Priorities
Source: PLoS One. 2016 Dec 20;11(12):e0168348. doi: 10.1371/journal.pone.0168348 (PMC5173368; doi:10.1371/journal.pone.0168348)
Supplement: S1 Dataset — (ZIP) [file pone.0168348.s002.zip › Taxa/Serra Sul/SS_2010/S11D_53.pdf]

S11D-53		1ª	AB	2ª	AB	ZON
Annelida						
Clitellata						
Oligochaeta	jovens	2	0,041			E
	sp.	1				P
Arthropoda						
Arachnida						
Acari						
Ixodida						
Argasidae						
Ornithodoros	sp.	1				E
Parasitiformes						
Ixodida						
Ixodidae						
Ornithodoros	sp.1	1				E
Sarcoptiformes						
Oribatida	sp.3	2		1		E P
Trombidiformes	sp.2	1				E
Anystidae						
Erythracarus	*nasutus*	1				E
Amblypygi						
Phrynidae						
Heterophrynus	sp.	3	0,061	1	0,0102	P
Araneae	jovens	1	0,02			
Ctenidae	jovens	3	0,061	1	0,0102	E P
Ochyroceratidae	jovens	1				E
Ochyrocera	sp.1	4		3		E P
Oonopidae	jovens	3				E P
Pholcidae						
Leptopholcus	sp.1	1				E
Ninetinae	sp.1	1		3		E P
Scytodidae	jovens	1	0,02	3	0,0306	E P
Scytodes	eleonorae	1	0,02			P
	sp.			1	0,0102	E
	sp.1	1	0,02			E
Segestriidae	jovens	1				E
Tetrablemmidae						
Matta	sp.1	3		1		E P
Theridiosomatidae	jovens	1				E
Uloboridae	jovens	1				E
Opiliones						
Laniatores						
Stygnidae	jovens	3	0,102			E P
	sp.1	2				E
Pseudoscorpiones						
Chernetidae						
Spelaeochernes	sp.1	3				E P
Chthoniidae	jovens	2				E
Pseudochthonius	sp.1	1		1		E P
Olpiidae	sp.1	6				E
Schizomida						
Hubbardiidae	jovens	1				E
Chilopoda						
Pleurostigmophora						
Scolopendromorpha						
Scolopocryptopidae						
Newportia	sp.1	1				P
Diplopoda						
Polydesmida						
Chelodesmidae	sp.4	1		1		P
Siphonophorida						
Siphonophoridae	sp.1	1				P
Spirostreptida	jovens			2	0,0204	E P
Pseudonannolenidae	jovens	1				P
Entognatha						

Diplura						
Campodeidae	sp.1	3		1		E P
Japygidae	sp.1	1				E
Insecta						
Coleoptera	jovens	2		1		E
Carabidae	sp.3	1		2		E P
Dytiscidae	sp.5			1		P
Scydmaenidae	sp.7	1				P
Staphylinidae						
Pselaphinae	sp.1	1				E
Collembola						
Arthropleona						
Entomobryoidea						
Cyphoderidae	sp.1	1				P
Isotomidae	sp.1	1				E
Paronellidae	sp.1	3		1		E P
	sp.2			1		E
	sp.4	1				E
Symphypleona						
Sminthuroidea	sp.2	2		1		E P
Diptera	jovens	2				E
Nematocera						
Cecidomyiidae						
Cecidomyiinae	sp.	1				E
Psychodidae						
Sciopemyia	*sordellii*	1				P
Sciaridae	sp.	3				E P
Hemiptera						
Heteroptera						
aff. Pyrrhocoroidea	jovens	1				E
Lygaeidae	sp.1	1				E
Homoptera						
Cixiidae	jovens	4				E P
	sp.1	1				E
	sp.3			1		E
Hymenoptera						
Cynipoidea						
Eucoilidae	sp.1	1				E
Vespoidea						
Formicidae						
Acromyrmex	sp.1			2		E P
Apterostigma	sp.1	1				E
Hypoponera	sp.1	1				P
Pachycondyla	*striata*	3		1		E P
Pseudomyrmex	sp.1	1				E
Solenopsis	sp.1	1				P
	sp.2	2				E
Wasmania	*auropunctata*	1				E
Isoptera						
Termitidae						
Labiotermes	sp.			1		E
	sp.	1		1		E
Lepidoptera	jovens	2	0,041	1	0,0102	E
Cossoidea						
Limacodidae	sp.1	1	0,02			E
Noctuoidea						
Noctuidae	sp.2	1	0,02			E
Tineoidea	sp.1	1		1		E
Orthoptera						
Ensifera						
Phalangopsidae	jovens	23	0,469	32	0,3265	E
Paraclodes	sp.1			2	0,0204	E
Phalangopsis	sp.1			1		P
Psocoptera						
Psocomorpha	jovens	2		2		E P

Troctomorpha						
Manicapsocidae						
Nothoentomum	sp.1	1		1		E
Trogiomorpha						
Psyllipsocidae	jovens			1		P
Psyllipsocus	sp.1	2				E
Thysanura						
Nicoletiidae	jovens	1				E
Malacostraca						
Isopoda						
Dubioniscidae	sp.1	2				E
Scleropactidae	sp.	1				P
Chordata						
Amphibia						
Anura						
Neobatrachia						
Strabomantidae						
Pristimantis	*fenestratus*	1	0,02	3	0,0306	P
Mammalia						
Chiroptera						
Emballonuridae						
Peropteryx	sp.			14	0,1429	E
Glossophaga	*soricina*	4	0,082	38	0,3878	
Mollusca						
Gastropoda						
Bulimulidae						
Naesiotus	sp.	1				P
Subulinidae						
Lamellaxis	sp.	1				E
Systrophiidae						
Happia	sp.	1				E
