# Supplementary material for: Reconciling Mining with the Conservation of Cave Biodiversity: A Quantitative Baseline to Help Establish Conservation Priorities
Source: PLoS One. 2016 Dec 20;11(12):e0168348. doi: 10.1371/journal.pone.0168348 (PMC5173368; doi:10.1371/journal.pone.0168348)
Supplement: S1 Dataset — (ZIP) [file pone.0168348.s002.zip › Taxa/Serra Sul/SS_2010/S11D_54.pdf]

S11D-54			1ª	AB	2ª	AB	ZON
Arthropoda							
Arachnida							
Acari							
Ixodida							
Argasidae							
	Antricola	sp.	2				E
	Ornithodoros	sp.	1				E
Parasitiformes							
Mesostigmata							
Diploginiidae		sp.1	1				P
Laelapidae		sp.3	1				E
	Stratiolaelaps	sp.1	1				E
Macrochelidae		sp.1	2				E
		sp.2	1				P
		sp.3	1				P
		sp.3	1				P
		sp.7	1				P
Sarcoptiformes		sp.2	1				E
Trombidiformes							
Eupodidae		sp.1	1				P
		sp.10	1				P
		sp.4	1				E
Araneae							
Araneidae	jovens		2		1		E
	Alpaida	*septemmammata*	4		2		E
		sp.2	1				E
Barychaelidae							
	gen.1	sp.1	1	0,0009			E
Corinnidae	jovens		1	0,0009			E
Ctenidae	jovens		1	0,0009			E
Filistatidae	jovens		2				E
Ochyroceratidae	jovens		2				E
Pholcidae							
	Leptopholcus	sp.1			1		E
Salticidae	jovens		1				E
Scytodidae	*jovens*		1	0,0009	1	0,00096	E
	Scytodes	*eleonorae*	1	0,0009			E
		globula			2	0,00192	E
Segestriidae							
	Ariadna	sp.1	2		2		E
Theridiidae	jovens				1		P
	Coleosoma	*floridanum*	3				E
Opiliones							
Laniatores							
Stygnidae		sp.1	1	0,0009			E
Pseudoscorpiones							
Chernetidae							
	Spelaeochernes	sp.1	2		1		E P
Entognatha							
Diplura							
Campodeidae		sp.1	1				E
Insecta							
Blattodea							
Blaberidae	jovens		1	0,0009			E
Coleoptera	jovens		4				E P
Carabidae		sp.11	1				E
Histeridae		sp.1	4		1		E P
Hydrophilidae							
	Sphaeridiinae	sp.3	6		1		E P
		sp.4	1				E
Staphylinidae		sp.11	2				E P
Collembola							
Arthropleona							
Entomobryoidea							

Paronellidae		sp.1	2				E	
Diptera	jovens		5		1		E	P
Brachycera								
Camillidae		sp.	1				E	
Dolichopodidae		sp.			1			P
Drosophilidae								
	Drosophila	*eleonore*	3		1		E	
Milichiidae		sp.	4				E	
Muscidae		sp.	1				E	
Streblidae								
	Trichobius	sp.	7				E	P
Nematocera								
Chironomidae		sp.	2				E	
Psychodidae								
	Telmatoscopus	sp.	4		3		E	
Hemiptera								
Heteroptera								
Mesoveliidae	jovens		1				E	
Miridae	jovens		1		1		E	
		sp.1	2		1		E	P
Pyrrhocoridae	jovens		1				E	
Reduviidae	jovens				1	0,00096	E	
Homoptera								
Cixiidae	jovens		1				E	
Hymenoptera								
Apoidea								
Halictidae		sp.1			1		E	
Ichneumonoidea								
Braconidae		sp.2	1					P
Vespoidea								
Formicidae								
	Cephalotes	sp.1			1		E	
	Gnamptogenys	*striatula*	8				E	P
	Odontomachus	*bauri*	1					P
	Pheidole	sp.1	1				E	
	Solenopsis	sp.1	1					P
		sp.2	1				E	
	Wasmania	*auropunctata*	1					P
Isoptera								
Termitidae								
	Nasutitermes	sp.	1				E	
Lepidoptera	jovens		3				E	
Cossoidea								
Limacodidae		sp.1	3	0,0028			E	P
Noctuoidea								
Noctuidae		sp.2	1	0,0009			E	
Tineoidea		sp.2	3		1		E	P
Orthoptera								
Ensifera								
Phalangopsidae								
	Paraclodes	sp.1			2	0,00192	E	
	Phalangopsis	sp.1	500	0,4748	500	0,47985	E	
Thysanura								
Nicoletiidae		sp.1			1		E	
Chordata								
Mammalia								
Chiroptera								
Mormoopidae								
	Pteronotus	*gymnonotus*	20	0,019	20	0,01919		
		parnellii	500	0,4748	500	0,47985		
		personatus	20	0,019	20	0,01919		
Phyllostomidae		sp.			1	0,00096		
Reptilia								
Squamata								
Gekkonidae								

Thecadactylus	*rapicauda*	2	0,0019			
Mollusca						
Gastropoda						
Bulimulidae						
Naesiotus	sp.	1				E
Systrophiidae						
Happia	sp.	1				E
