# Supplementary material for: Reconciling Mining with the Conservation of Cave Biodiversity: A Quantitative Baseline to Help Establish Conservation Priorities
Source: PLoS One. 2016 Dec 20;11(12):e0168348. doi: 10.1371/journal.pone.0168348 (PMC5173368; doi:10.1371/journal.pone.0168348)
Supplement: S1 Dataset — (ZIP) [file pone.0168348.s002.zip › Taxa/Serra Sul/SS_2010/S11D_55.pdf]

S11D-55		1ª	AB	2ª	AB	ZON
Arthropoda						
Arachnida						
Acari						
Ixodida						
Argasidae						
Ornithodoros	sp.	3		1		P
Ixodidae						
Ixodes	sp.	1				P
Parasitiformes						
Ixodida						
Ixodidae						
Ornithodoros	sp.1			2		P
Mesostigmata	sp.2	2		1		P A
Laelapidae	sp.1	1				A
Macronyssidae	sp.1			1		P
	sp.4	1				P
Sarcoptiformes	sp.19	1				A
Oribatida	sp.2	1				P
	sp.3	3		5		P A
Trombidiformes	sp.1	2				P A
	sp.2	2		1		P
Amblypygi						
Charinidae jovens		1	0,0023			P
Charinus	sp.	1				
Phrynidae						
Heterophrynus	sp.	8	0,0092	8	0,0293	A
Araneae						
Corinnidae jovens		3	0,0046	4	0,0147	P A
Creugas	sp.1	1				P
Ctenidae jovens		1	0,0011	1	0,0037	E
Filistatidae jovens		2				E P
	sp.1			2		E P
Ochyroceratidae jovens				1		A
Ochyrocera	sp.1	4		2		P A
Speocera	sp.1	1		1		P
Pholcidae jovens		1				E
Leptopholcus	sp.1	1				P
Ninetinae	sp.1	3		3		P
Scytodidae *jovens*		6	0,0103	3	0,011	E P
Scytodes	*eleonorae*	3				E P
Tetrablemmidae						
Matta	sp.1			2		P
Theridiosomatidae						
Plato	sp.1	3		1		P A
Opiliones						
Laniatores						
Escadabiidae	sp.1	1				P
Stygnidae	sp.1	2	0,0023	2	0,0073	P
Pseudoscorpiones						
Chernetidae jovens		2		2		P
Spelaeochernes	sp.1	5		5		E P A
Pseudochthonius	sp.1	2		5		P A
Olpiidae	sp.1	4				E P
Chilopoda						
Notostigmophora						
Scutigeromorpha						
Pselliodidae jovens				1		P
Diplopoda jovens		1				P
Polydesmida						
Chelodesmidae	sp.4	1	0,0011			P
Spirostreptida jovens				1		P
Pseudonannolenidae						
Pseudonannolene	sp.1	1	0,0011	1	0,0037	P A
Entognatha						

Diplura							
Campodeidae		sp.1	3		1		P
Insecta							
Blattodea	jovens		98	0,1124	3	0,011	E P
Blaberidae	jovens		6	0,0069	6	0,022	E P A
Blattellidae		sp.2			1	0,0037	E
Coleoptera	jovens		1		4		E P
Elateridae		sp.1	2				P
Endomychidae		sp.1			1		E
Ptiliidae		sp.1	1				P
Collembola							
Arthropleona							
Entomobryoidea							
Cyphoderidae		sp.1	1				A
		sp.2	1		1		P A
Isotomidae		sp.1			1		P
Symphypleona							
Sminthuroidea		sp.2	3		1		P A
Diptera	jovens		5		2		E P
Brachycera							
Drosophilidae							
Drosophila		*eleonore*	1		1		E P
Phoridae							
Metopininae		sp.	1				P
Nematocera							
Cecidomyiidae							
Cecidomyiinae		sp.	1				E
Chironomidae		sp.	2				P A
Culicidae							
Culicini		sp.	1				E
Psychodidae							
Deanemyia		*ramirezi*			1		E
Edentomyia		*piauensis*	1				P
Nemopalpus		sp.	1				P
Pericoma		sp.	1				P
Sciopemyia		*sordellii*	1				E
Hemiptera							
Heteroptera	jovens		30	0,0344			
Cydnidae							
Cydninae		sp.1	4		2		P A
Reduviidae	jovens		2	0,0034	2	0,0073	E P
Zelurus		sp.1	1				E
Homoptera							
Cixiidae	jovens		1		1		P
Hymenoptera	jovens				1		P
Vespoidea							
Formicidae							
Crematogaster		sp.1	1		1		E P
Labidus		*coecus*	3				P
Pachycondyla		*striata*	7		5		P A
Solenopsis		sp.2			1		P
Isoptera							
Rhinotermitidae							
Heterotermes		sp.			1		P
Termitidae							
Atlantitermes		sp.	1				E
Nasutitermes		sp.	1		1		P
Lepidoptera	jovens		1		2		P
Cossoidea							
Limacodidae		sp.1	3	0,0034			P
Noctuoidea							
Noctuidae		sp.1	50	0,0573			
		sp.2	1	0,0011			P
Tineoidea		sp.1	3		1		P
Neuroptera							

Myrmeleonthidae	jovens	2		1		E P
Orthoptera						
Ensifera						
Phalangopsidae	jovens	1	0,0011			
	sp.2	1	0,0011			E
Phalangopsis	sp.1	573	0,6571	234	0,8571	P
Thysanura						
Nicoletiidae	jovens			1		A
	sp.1	2		2		P A
Malacostraca						
Isopoda						
Philosciidae	sp.1	2		2		P A
Chordata						
Amphibia						
Anura						
Neobatrachia						
Leptodactylidae						
Leptodactylus	sp.			1	0,0037	A
Mammalia						
Chiroptera						
Emballonuridae						
Peropteryx	*kappleri*	8	0,0092			
Furipteridae						
Furipterus	*horrens*	10	0,0115			
Phyllostomidae	sp.1			1	0,0037	P
Carollia	*perspicillata*	50	0,0573	4	0,0147	
	sp.			1	0,0037	P
Glossophaga	*soricina*	2	0,0023			
Glossophaginae	sp.	5	0,0057			
Lonchophylla	*thomasi*	1	0,0011			
Phyllostomus	*latifolius*	2	0,0023	1	0,0037	
