# Supplementary material for: Reconciling Mining with the Conservation of Cave Biodiversity: A Quantitative Baseline to Help Establish Conservation Priorities
Source: PLoS One. 2016 Dec 20;11(12):e0168348. doi: 10.1371/journal.pone.0168348 (PMC5173368; doi:10.1371/journal.pone.0168348)
Supplement: S1 Dataset — (ZIP) [file pone.0168348.s002.zip › Taxa/Serra Sul/SS_2010/S11D_56.pdf]

S11D-56	1ª	AB	2ª	AB	ZON
Arthropoda					
Arachnida					
Acari					
Ixodida					
Argasidae					
Ornithodoros sp.			1		E
Parasitiformes					
Mesostigmata					
Diploginiidae sp.4	1				E
Trombidiformes sp.2	1				P
Trombidiformes sp.7	1				P
Amblypygi					
Phrynidae					
Heterophrynus sp.	5	0,1316			
Araneae					
Araneidae jovens	1				E
Barychaelidae jovens	1	0,0263			E
Ctenidae jovens			1	0,1	E
Filistatidae jovens	1		1		E P
Ochyroceratidae					
Ochyrocera sp.1	1				P
Oonopidae					
gr. *Xycarphius* sp.2	1				E
Pholcidae jovens			1		P
Ninetinae sp.1	1		1		E P
Salticidae					
Noegus sp.1			1		P
Scytodidae *jovens*	1				E
Scytodes eleonorae	1	0,0526	1	0,1	P
Scytodes sp.1			1		P
Opiliones					
Laniatores					
Stygnidae jovens	3				E P
Stygnidae sp.1	1	0,1053			E
Pseudoscorpiones					
Olpiidae sp.1	2		2		E P
Insecta					
Blattodea jovens	11	0,2895			
Blaberidae jovens	2	0,0526			E P
Coleoptera jovens	1				P
Collembola					
Arthropleona					
Entomobryoidea					
Cyphoderidae sp.1	1				P
Diptera					
Brachycera					
Camillidae sp.			1		E
Nematocera			1		P
Psychodidae					
Evandromyia termitophila			1		E
Sciopemyia sordellii	1				P
Hemiptera					
Heteroptera					
Reduviidae jovens			1	0,1	P
Homoptera					
Cixiidae jovens	3				E P
Cixiidae sp.3			1		P
Hymenoptera					
Vespoidea					
Formicidae					
Cephalotes sp.1			1		P
Solenopsis sp.1	1				P
Wasmania auropunctata	2				E P
Isoptera sp.			1		P

Rhinotermitidae						
Heterotermes	sp.	1				P
Termitidae						
Cornitermes	sp.	2		1		E P
Lepidoptera	jovens	1	0,0263			
Noctuoidea						
Noctuidea	*sp.2*	2	0,0526			
Tineoidea	sp.1			1		P
Tineoidea	sp.2			1	0,1	P
Neuroptera						
Myrmeleonthidae	jovens	1		1		E P
Orthoptera						
Ensifera						
Phalangopsidae						
Phalangopsis	sp.			2	0,2	P
Psocoptera						
Psocomorpha						
Ptiloneuridae						
Ptiloneura	sp.3			1		P
Malacostraca						
Isopoda						
Dubioniscidae	sp.1	1				P
Pauropoda						
Tetramerocerata	sp.	1				P
Symphyla						
Scutigerellidae						
Hanseniella	sp.1	1				P
Chordata						
Amphibia						
Anura						
Neobatrachia						
Strabomantidae						
Pristimantis	*fenestratus*	2	0,0526			
Mammalia						
Chiroptera						
Emballonuridae						
Peropteryx	*kappleri*	8	0,2105	3	0,3	
Peropteryx	sp.			1	0,1	P
