# Supplementary material for: Reconciling Mining with the Conservation of Cave Biodiversity: A Quantitative Baseline to Help Establish Conservation Priorities
Source: PLoS One. 2016 Dec 20;11(12):e0168348. doi: 10.1371/journal.pone.0168348 (PMC5173368; doi:10.1371/journal.pone.0168348)
Supplement: S1 Dataset — (ZIP) [file pone.0168348.s002.zip › Taxa/Serra Sul/SS_2010/S11D_57.pdf]

S11D-57			1ª	AB	2ª	AB	ZON
Arthropoda							
Arachnida							
Acari							
Ixodida							
Ornithodoros		sp.	2		1		P A
Parasitiformes							
Holothyrida							
Diplothyridae							
Diplothyrus		*scubarti*	1				A
Opilioacarida							
Opilioacaridae		sp.1	1				P
Trombidiformes		sp.1	1		1		A
Amblypygi							
Charinidae			1	0,0123			A
Phrynidae							
Heterophrynus		sp.	1	0,0123	2	0,0426	P
Araneae							
Ctenidae	jovens		2	0,0247	1	0,0213	P
Ochyroceratidae	jovens		3		1		P A
Prodidomidae	jovens				1		A
Salticidae							
Amphidraus		sp.1	1				P
Scytodidae	jovens		1	0,0123			P
Tetrablemmidae							
Matta		sp.1	1		2		P A
Opiliones	jovens				19	0,4043	P
Laniatores							
Escadabiidae		sp.1	1		2		P A
Stygnidae	jovens		1	0,0123			A
Stygnidae		sp.1			1	0,0213	P
Pseudoscorpiones							
Spelaeochernes		sp.1	3		2		P A
Chthoniidae	jovens		2				A
Pseudochthonius		sp.1	1				P
Pseudochthonius		sp.4			2		A
Chilopoda							
Notostigmophora							
Scutigeromorpha							
Pselliodidae	jovens		1				P
Pleurostigmophora							
Geophilomorpha							
Ballophilidae		sp.3	1	0,0123			P
Diplopoda							
Polydesmida							
Fuhrmannodesmidae		sp.3	1				A
Spirostreptida	jovens				2		P A
Entognatha							
Diplura							
Campodeidae		sp.1	2		1		P A
Insecta	jovens		1	0,0123			
Blattodea			40	0,4938			
Blaberidae	jovens				1	0,0213	P
Polyphagidae	jovens		1	0,0123			P
Coleoptera	jovens		1				P
Collembola							
Arthropleona							
Entomobryoidea							
Paronellidae		sp.4			1		A
Diptera							
Nematocera							
Cecidomyiidae							
Cecidomyiinae		sp.	1				P
Psychodidae							
Pintomyia		*gruta*	1				A

Hemiptera							
Heteroptera							
Lygaeidae		sp.2	1				P
Homoptera							
Cixiidae	jovens		2		2		P A
Hymenoptera							
Vespoidea							
Formicidae							
	Crematogaster	sp.1			1		P
	Cyphomyrmex	sp.1	1				P
	Pachycondyla	*striata*	2				P
	Wasmania	*auropunctata*			1		P
Isoptera							
Termitidae							
	Termes	sp.	1				P
Lepidoptera							
Noctuoidea	jovens		3				P
Noctuidae		sp.1	2	0,0247			P
Orthoptera							
Ensifera							
Phalangopsidae	jovens		28	0,3457			
	Paraclodes	sp.1			1	0,0213	P
	Phalangopsis	sp.1			10	0,2128	A
Psocoptera							
Psocomorpha	jovens				1		P
Malacostraca							
Isopoda							
Philosciidae		sp.1	2				P
Scleropactidae		sp.	1				P
Chordata							
Amphibia							
Anura							
Neobatrachia							
Strabomantidae							
	Pristimantis	*fenestratus*			4	0,0851	P
Mammalia							
Chiroptera	jovens				8	0,1702	P
Phyllostomidae							
	Glossophaginae	sp.	2	0,0247			
Mollusca							
Gastropoda							
Subulinidae							
	Lamellaxis	sp.	1				P
Systrophiidae							
	Happia	sp.	2				P
