# Supplementary material for: Reconciling Mining with the Conservation of Cave Biodiversity: A Quantitative Baseline to Help Establish Conservation Priorities
Source: PLoS One. 2016 Dec 20;11(12):e0168348. doi: 10.1371/journal.pone.0168348 (PMC5173368; doi:10.1371/journal.pone.0168348)
Supplement: S1 Dataset — (ZIP) [file pone.0168348.s002.zip › Taxa/Serra Sul/SS_2010/S11D_58.pdf]

S11D-58		1ª	AB	2ª	AB	ZON
Annelida						
Clitellata						
Oligochaeta	jovens	1	0,0058			E
Arthropoda						
Arachnida						
Acari						
Ixodida						
Argasidae						
Ornithodoros	sp.	1		1		P
Parasitiformes						
Mesostigmata						
Diploginiidae	sp.4	2				P
Trombidiformes						
Tydeoidea						
Rhagidiidae	sp.1	1				P
Amblypygi						
Phrynidae						
Heterophrynus	sp.	6	0,0347	2	0,0364	A
Araneae						
Araneidae	jovens			1		E
Alpaida	*septemmammata*	1				P
Barychaelidae	jovens	1	0,0058			E
Ctenidae	jovens	1	0,0058			
Nesticidae	jovens	1				E
Ochyroceratidae						
Ochyrocera	sp.1	2		1		E
Pholcidae	jovens	1		1		P
Ninetinae	sp.1	4		3		E P
Salticidae	jovens			1		E
Scytodidae	*jovens*	3	0,0173	1	0,0182	E P
Scytodes	*eleonorae*	1	0,0058			P
Scytodes	*globula*	2	0,0116			E P
Scytodes	sp.1	1				E
Segestriidae	jovens	1		3		E P
Ariadna	sp.1	2				P
Opiliones	jovens			25	0,4545	A
Laniatores						
Gonyleptidae	sp.1			1	0,0182	E
Stygnidae	jovens	3	0,0289	1	0,0727	E P A
Stygnidae	sp.1	2		3		E P
Pseudoscorpiones						
Spelaeochernes	sp.1	3		4		E P
Pseudochthonius	sp.1	2				E P
Ricinulei						
Ricinoididae	jovens	1				E
Chilopoda						
Notostigmophora						
Scutigeromorpha						
Pselliodidae	jovens	1				P
Polyxenida						
Hypogexenidae	sp.1	1				P
Entognatha						
Diplura						
Campodeidae	sp.1	2				E P
Insecta	jovens	2	0,0116	2	0,0364	E P A
Blattodea		6	0,0347	1	0,0182	
Blaberidae	jovens	1	0,0058	1	0,0182	E P
Coleoptera	jovens	1				P
Carabidae	sp.3	1				E
Endomychidae	sp.1			1		E
Collembola						
Arthropleona						
Entomobryoidea						
Paronellidae	sp.1	3				E P

Diptera						
Nematocera						
Cecidomyiidae						
Cecidomyiinae	sp.	1				E
Psychodidae						
Sciopemyia	*sordellii*	1				E
Sciaridae						
Bradysia	sp.			1		P
Hemiptera						
Heteroptera						
Reduviidae	jovens	2	0,0116	1	0,0182	P
Homoptera						
Cixiidae	jovens	2		3		E P
Hymenoptera						
Vespoidea						
Formicidae						
Gnamptogenys	*striatula*	1		1		E P
Pachycondyla	*striata*	1				E
Solenopsis	sp.2	1		1		P
Isoptera						
Termitidae						
Cortaritermes	*silvestrii*			1		E
Nasutitermes	sp.	4		1		P
Lepidoptera	jovens			3		P
Cossoidea						
Limacodidae	sp.1	1	0,0058			E
Tineoidea	sp.1	1				P
Noctuidae	sp.1	1	0,0058			
Orthoptera						
Ensifera						
Phalangopsidae						
Paraclodes	sp.1			11	0,2	P
Phalangopsis	sp.1	137	0,7919	2	0,0364	P A
Psocoptera						
Psocomorpha						
Ptiloneuridae						
Ptiloneura	sp.1			1		E
Thysanura						
Nicoletiidae	sp.1	1				P
Chordata						
Amphibia						
Anura						
Neobatrachia						
Strabomantidae						
Pristimantis	*fenestratus*	3	0,0173	4	0,0727	P
