# Supplementary material for: Reconciling Mining with the Conservation of Cave Biodiversity: A Quantitative Baseline to Help Establish Conservation Priorities
Source: PLoS One. 2016 Dec 20;11(12):e0168348. doi: 10.1371/journal.pone.0168348 (PMC5173368; doi:10.1371/journal.pone.0168348)
Supplement: S1 Dataset — (ZIP) [file pone.0168348.s002.zip › Taxa/Serra Sul/SS_2010/S11D_59.pdf]

S11D-59	1ª	AB	2ª	AB	ZON
Arthropoda					
Arachnida					
Acari					
Ixodida					
Ixodidae					
Amblyomma sp.			1		E
Parasitiformes					
Mesostigmata					
Laelapidae					
Stratiolaelaps sp.1	1				E
Stratiolaelaps sp.4			1		E
Amblypygi					
Phrynidae					
Heterophrynus sp.	2	0,1			
Araneae					
Araneidae					
Alpaida smila			1		E
Corinnidae jovens			1	0	E
Ochyroceratidae jovens			1		E
Pholcidae					
Leptopholcus sp.1	1				E
Mesabolivar aurantiacus	1				E
Mesabolivar sp.1	2		1		E
Ninetinae sp.1			1		E
Scytodidae					
Scytodes eleonorae	1	0	2	0,1	E
Scytodes globula	1	0			E
Scytodes sp.			1	0	P
Symphytognathidae					
Anapistula sp.1			1		E
Tetragnathidae jovens	1				E
Theraphosidae jovens	1	0			E
Theridiidae					
gen. sp.1			1		E
Theridiosomatidae jovens			2		E
Plato sp.1	1				E
Opiliones					
Eupnoi					
Sclerosomatidae jovens	1				E
Sclerosomatidae sp.1	1				E
Laniatores					
Escadabiidae sp.1			1		E
Stygnidae jovens	2				E
Stygnidae sp.1	1	0,1	2	0,1	E
Pseudoscorpiones					
Spelaeochernes sp.1	1				E
Chthoniidae jovens			2		E
Pseudochthonius sp.1	1		1		E
Schizomida					
Hubbardiidae jovens	1				E
Chilopoda					
Notostigmophora					
Scutigeromorpha					
Pselliodidae jovens					
Pleurostigmophora					
Scolopendromorpha jovens			1	0	P
Diplopoda					
Polydesmida					
Chelodesmidae sp.4	1	0			E
Entognatha					
Diplura					
Campodeidae sp.1	2				E
Insecta					
Blattodea					

Blattidae	jovens	1	0	1	0	E
Coleoptera	jovens			2		E
Staphylinidae	sp.6	1				E
Collembola						
Arthropleona						
Entomobryoidea						
Cyphoderidae	sp.1	1				E
Paronellidae	sp.1	4		1		E
Symphypleona						
Sminthuroidea	sp.1	1				E
Diptera	jovens	1		1		E
Nematocera						
Cecidomyiidae						
Cecidomyiinae	sp.	1				E
Mycetophilidae						
Euceroplatus	sp.			1		E
Psychodidae						
Sciopemyia	*sordellii*	1				E
Sciaridae						
Bradysia	sp.			1		E
Tipulidae						
Tipulinae	sp.	1				E
Hemiptera						
Heteroptera						
Veliidae	jovens	1				E
Paravelia	sp.2	1				E
Homoptera						
Cixiidae	jovens	1		1		E
Hymenoptera						
Vespoidea						
Formicidae						
Camponotus	sp.1	1		1		E
Crematogaster	sp.1			1		E
Dolichoderus	*bispinosus*	3		2		E
Gnamptogenys	*striatula*	1				E
Nylanderia	sp.1			1		E
Pachycondyla	*striata*	2		3		E
Wasmania	*auropunctata*	1				E
Isoptera	sp.			1		E
Termitidae						
Nasutitermes	sp.	1		2		E
Lepidoptera	jovens			1		E
Noctuoidea	sp.2	1				E
Orthoptera						
Ensifera						
Phalangopsidae	jovens	13	0,3			
Paraclodes	sp.1			15	0,4	E P
Phalangopsis	sp.1	1	0	2	0,1	E P
Psocoptera						
Psocomorpha	jovens	2		2		E
Epipsocidae						
Mesepipsocus	sp.1			1		E
Troctomorpha						
Manicapsocidae						
Nothoentomum	sp.1			1		E
Thysanura						
Nicoletiidae	sp.1	1		1		E
Malacostraca						
Isopoda						
Dubioniscidae	sp.1	1		1		E
Philosciidae	sp.1	2		3		E
Chordata						
Amphibia						
Anura						
Neobatrachia						

Strabomantidae						
Pristimantis	*fenestratus*	3	0,1	6	0,2	P
Mammalia						
Chiroptera	sp.			2	0,1	P
Emballonuridae						
Peropteryx	*kappleri*	6	0,2			
Peropteryx	sp.			1	0	P
Phyllostomidae	sp.					
Carollia	*brevicauda*					
Carollia	sp.	2	0,1			
Glossophaga	*soricina*	5	0,1			
Mollusca						
Gastropoda						
Systrophiidae						
Happia	sp.	1				E
