# Supplementary material for: Reconciling Mining with the Conservation of Cave Biodiversity: A Quantitative Baseline to Help Establish Conservation Priorities
Source: PLoS One. 2016 Dec 20;11(12):e0168348. doi: 10.1371/journal.pone.0168348 (PMC5173368; doi:10.1371/journal.pone.0168348)
Supplement: S1 Dataset — (ZIP) [file pone.0168348.s002.zip › Taxa/Serra Sul/SS_2010/S11D_60.pdf]

S11D-60			1ª	AB	2ª	AB	ZON
Annelida							
Clitellata							
Oligochaeta	jovens		1	0,0101			P
Arthropoda							
Arachnida							
Acari							
Parasitiformes							
Holothyrida							
Laelapidae		sp.3			1		E
Sarcoptiformes		sp.1	1				P
Oribatida		sp.3	1				E
Trombidiformes							
Tydeoidea							
Rhagidiidae		sp.2			1		P
Amblypygi							
Phrynidae							
	Heterophrynus	sp.	2	0,0202			
Araneae							
Araneidae	jovens				1		E
	Alpaida	*truncata*	1				E
Corinnidae	jovens		1	0,0101			P
Ctenidae	jovens		2	0,0202	1	0,0244	E P
Linyphiidae	jovens				1		E
	gen.2	sp.1	1				E
Oonopidae	jovens				1		P
Pholcidae	jovens		1				E
Symphytognathidae	jovens						
	Anapistula	sp.1	1				P
Tetrablemmidae	jovens		1				P
	Matta	sp.1	1		2		E P
Tetragnathidae	jovens		1				E
Theraphosidae	jovens		2	0,0202	2	0,0488	E P
Theridiidae	jovens						
	Theridion	sp.1	1				E
Theridiosomatidae	jovens						
	Plato	sp.1	2		1		E P
Opiliones							
Laniatores							
Stygnidae		sp.1	1	0,0101	1	0,0244	P
Pseudoscorpiones							
Bochicidae		sp.1			2		E
Chernetidae							
	Spelaeochernes	sp.1	3		2		E P
Chthoniidae							
	Pseudochthonius	sp.1	1		1		E P
Chilopoda							
Pleurostigmophora							
Geophilomorpha	jovens		1	0,0101			P
Diplopoda							
Polydesmida							
Fuhrmannodesmidae	jovens		1				E
Polyxenida	jovens		1				P
Entognatha							
Diplura							
Campodeidae		sp.1	4		2		E P
Insecta							
Blattodea	jovens		2	0,0202	1	0,0244	E P
Coleoptera	jovens		2				E P
Carabidae		sp.2	1				P
Collembola							
Arthropleona							
Entomobryoidea		sp.1			1		E

Entomobryidae		sp.1	1				E
Isotomidae		sp.3	1				E
Paronellidae		sp.1	2				E
		sp.4	1		1		P
Diptera	jovens		1		1		E
Nematocera							
Ceratopogonidae		sp.	1				E
Psychodidae							
	Pintomyia	*gruta*			1		P
	Sciopemyia	*sordellii*	3		2		E P
Hemiptera							
Homoptera	jovens		30	0,303			
Cixiidae	jovens		4		2		E P
Hymenoptera							
Vespoidea							
Formicidae							
	Crematogaster	sp.1			1		P
	Pachycondyla	*striata*	2		2		E P
	Solenopsis	sp.1	1				P
Isoptera							
Termitidae							
	Nasutitermes	sp.	2		3		E P
Lepidoptera	jovens		1				E
Tineoidea		sp.1	1				E
Orthoptera							
Ensifera							
Phalangopsidae	jovens		1		1		E
	Phalangopsis	sp.1	48	0,4949	31	0,7805	P
Psocoptera							
Psocomorpha	jovens		1		1		E
Thysanura							
Nicoletiidae		sp.1	3		1		E P
Malacostraca							
Isopoda							
Philosciidae		sp.2	1				E
Symphyla							
Scutigerellidae							
	Hanseniella	sp.1	2		1		E P
Chordata							
Amphibia							
Anura							
Neobatrachia							
Strabomantidae							
	Pristimantis	*fenestratus*			4	0,0976	E
Mammalia							
Chiroptera							
Emballonuridae							
	Peropteryx	*kappleri*	3	0,0303			
Phyllostomidae							
	Glossophaga	*soricina*	4	0,0404			
Nemathelminthes		sp.	1	0,0101			E
