# Supplementary material for: Reconciling Mining with the Conservation of Cave Biodiversity: A Quantitative Baseline to Help Establish Conservation Priorities
Source: PLoS One. 2016 Dec 20;11(12):e0168348. doi: 10.1371/journal.pone.0168348 (PMC5173368; doi:10.1371/journal.pone.0168348)
Supplement: S1 Dataset — (ZIP) [file pone.0168348.s002.zip › Taxa/Serra Sul/SS_2010/S11D_61.pdf]

S11D-61			1ª	AB	2ª	AB	ZON
Arthropoda							
Arachnida							
Acari							
Ixodida							
Argasidae							
	Ornithodoros	sp.	4		3		P
Parasitiformes							
Ixodida							
Ixodidae							
Mesostigmata		sp.2	1				P
Trombidiformes		sp.1	1				P
Amblypygi							
Phrynidae							
	Heterophrynus	sp.	8	0,0152	11	0,0598	P
Araneae							
Araneidae	jovens		1				E
Filistatidae	jovens		6				E P
		sp.1	1		2		P
Ochyroceratidae							
	Ochyrocera	sp.1	3		1		P
Pholcidae	jovens		2				P
		sp.1	1				E
	Ninetinae	sp.1	1		1		P
Salticidae	jovens				1		E
Scytodidae	*jovens*		5	0,0095	3	0,0163	E P
	Scytodes	*eleonorae*	5	0,0095	2	0,0109	P
		globula	1	0,0019	1	0,0054	P
		sp.	47	0,0894	39	0,212	P
		sp.1	1				P
Opiliones							
Laniatores							
Escadabiidae		sp.1	3		1		P
		sp.8			1		P
Pseudoscorpiones							
Chernetidae	jovens		2		4		P
	Spelaeochernes	sp.1	3		5		E P
Chthoniidae							
	Pseudochthonius	sp.1			2		P
Chilopoda							
Notostigmophora							
Scutigeromorpha							
Pselliodidae	jovens		2				P
Pleurostigmophora							
Scolopendromorpha							
Scolopocryptopidae							
	Dinocryptops	*miersii*			1	0,0054	P
Diplopoda	jovens		11	0,0209			
Polydesmida							
Pyrgodesmidae		sp.2	1	0,0019	3	0,0163	P
Insecta							
Blattodea							
Blaberidae	jovens		1	0,0019	1	0,0054	P
Blattellidae		sp.1			1	0,0054	E
		sp.3	1	0,0019			E
Blattidae	jovens		3	0,0057			P
Coleoptera	jovens		2		3		E P
Diptera	jovens		2				P
Brachycera							
Calliphoridae		sp.	1				E
Drosophilidae							
	Drosophila	*eleonore*	1				P
Nematocera							

Cecidomyiidae								
	Cecidomyiinae	sp.	1		1			P
Culicidae								
	Culicini	sp.	1					P
Psychodidae								
	Edentomyia	*piauensis*	2		1			P
	Phlebotominae	sp.			2			P
	Sciopemyia	*sordellii*	3		1			P
Sciaridae		sp.	1				E	
	Pseudosciara	sp.			1			P
Hemiptera								
Heteroptera			40	0,076				
aff. Pyrrhocoroidea		jovens	1	0,0019				
Reduviidae		jovens	6	0,0114	4	0,0217	E	P
	Emesinae	sp.1	2					P
	Reduviinae	sp.			1	0,0054		P
	Zelurus	sp.1	1	0,0019				P
Tingidae		sp.1	1					P
Hymenoptera								
Apoidea								
Pemphredonidae		sp.1			1			P
Vespoidea								
Formicidae								
	Gnamptogenys	*striatula*	1		1		E	P
	Odontomachus	*bauri*	3	0,0057			E	P
	Pheidole	sp.1	3		2		E	P
	Solenopsis	sp.1	5		3			P
		sp.2	1					P
Lepidoptera		jovens	3	0,0057			E	P
Cossoidea								
Limacodidae		sp.1	4	0,0076				P
Noctuoidea								
Noctuidae		sp.2	1	0,0019			E	
Tineoidea		sp.1	1		3		E	P
Neuroptera								
Myrmeleonthidae		jovens	2					P
Orthoptera								
Ensifera								
Phalangopsidae								
	Paraclodes	sp.1			1	0,0054		P
	Phalangopsis	sp.1	262	0,4981	63	0,3424		P
Psocoptera								
Psocomorpha		jovens	1				E	
Epipsocidae								
	Epipsocus	sp.1			1			P
Trogiomorpha								
Psyllipsocidae								
	Psocathropos	sp.1	1					P
Thysanura								
Nicoletiidae		jovens	1					P
		sp.1	2		5			P
Chordata								
Amphibia								
Anura		sp.			1	0,0054		P
Ave								
Strigiformes								
Tytodidae								
	Tyto	*alba*	1	0,0019				
Mammalia								
Chiroptera								
Emballonuridae								
	Peropteryx	sp.	20	0,038	10	0,0543		P
Phyllostomidae		sp.			1	0,0054		P

	Glossophaga	*soricina*	100	0,1901	40	0,2174	
Rodentia		sp.			1	0,0054	P
Nemathelminthes		sp.	1	0,0019			P
