# Supplementary material for: Reconciling Mining with the Conservation of Cave Biodiversity: A Quantitative Baseline to Help Establish Conservation Priorities
Source: PLoS One. 2016 Dec 20;11(12):e0168348. doi: 10.1371/journal.pone.0168348 (PMC5173368; doi:10.1371/journal.pone.0168348)
Supplement: S1 Dataset — (ZIP) [file pone.0168348.s002.zip › Taxa/Serra Sul/SS_2010/S11D_62.pdf]

S11D-62			1ª	AB	2ª	AB	ZON
Arthropoda							
Arachnida							
Acari							
Parasitiformes							
Mesostigmata		sp.2			1		P
Trombidiformes		sp.8			1		P
Amblypygi							
Charinidae	jovens		1	0,0455			E
Phrynidae							
Heterophrynus		sp.			1	0,2	P
Araneae	jovens		1	0,0455			
Araneidae	jovens		1				E
Ctenidae	jovens		1	0,0455			E
Ochyroceratidae	jovens		1				E
Ochyrocera		sp.1	1				P
Pholcidae							
Leptopholcus		sp.1	1				E
		sp.1	1				P
Theridiidae							
Theridion		sp.1			1		E
Theridiosomatidae	jovens		1				E
Plato		sp.1	1				P
Opiliones							
Laniatores							
Stygnidae		sp.1			1	0,2	P
Pseudoscorpiones							
Chthoniidae							
Pseudochthonius		sp.1			1		P
Diplopoda							
Polyxenida	jovens				1		E
Hypogexenidae		sp.1			1		P
Entognatha							
Diplura							
Projapygidae		sp.1	1				E
Insecta							
Archaeognatha							
Meinertellidae		sp.2	1	0,0455			E
Blattodea							
Blaberidae	jovens		2	0,0909			E P
Polyphagidae	jovens				1	0,2	P
Collembola							
Arthropleona							
Entomobryoidea							
Cyphoderidae		sp.1			1		P
Entomobryidae		sp.1	1				E
		sp.2			1		E
Paronellidae		sp.1	1				E
Diptera							
Nematocera							
Culicidae							
Culicini		sp.	1				P
Psychodidae							
Edentomyia		*piauensis*			1		P
Pintomyia		*gruta*	1				P
Sciopemyia		*sordellii*			1		P
Tipulidae							
Limoniinae		sp.	1				E
Tipulinae		sp.	1				P
Hymenoptera							
Vespoidea							
Formicidae							
Camponotus		*atriceps*	1				P

		sp.1	1		2		E P
	Gnamptogenys	*striatula*	1				E
	Odontomachus	*bauri*			1		E
	Pachycondyla	*striata*			1		P
	Pheidole	sp.2			1		E
Isoptera							
Termitidae							
	Diversitermes	sp.	1				E
	Nasutitermes	sp.			1		E
Lepidoptera	jovens		1				E
Cossoidea							
Limacodidae		sp.1	2	0,0909			E P
Noctuoidea		sp.2			1		E
		sp.1	6	0,2727			
Orthoptera							
Ensifera							
Phalangopsidae							
	Phalangopsis	sp.1	8	0,3636	2	0,4	P
Psocoptera							
Psocomorpha	jovens		1		1		E
