# Supplementary material for: Reconciling Mining with the Conservation of Cave Biodiversity: A Quantitative Baseline to Help Establish Conservation Priorities
Source: PLoS One. 2016 Dec 20;11(12):e0168348. doi: 10.1371/journal.pone.0168348 (PMC5173368; doi:10.1371/journal.pone.0168348)
Supplement: S1 Dataset — (ZIP) [file pone.0168348.s002.zip › Taxa/Serra Sul/SS_2010/S11D_63.pdf]

S11D-63			1ª	AB	2ª	AB	ZON
Arthropoda							
Arachnida							
Acari							
Sarcoptiformes							
Oribatida		sp.2	1				E
		sp.7	1				E
Amblypygi							
Phrynidae							
	Heterophrynus	sp.	2	0,08			
Araneae							
Araneidae	jovens				2		E P
	Alpaida	*septemmammata*	1				P
Ctenidae	jovens		2	0,08			P
Filistatidae	jovens				1		E
Ochyroceratidae	jovens		1				P
Oonopidae	jovens		1				E
Pholcidae							
	Leptopholcus	sp.1	2				E P
	Ninetinae	sp.1	1		2		E P
Salticidae	jovens		1				E
Scytodidae	*jovens*				1	0,0625	E
	Scytodes	*eleonorae*			1	0,0625	P
		globula	2	0,08			E P
Segestriidae	jovens		1		1		P
Tetrablemmidae	jovens		1				P
Theraphosidae	jovens		1	0,04			E
Opiliones							
Eupnoi							
Sclerosomatidae	jovens		1				P
Laniatores							
Cosmetidae	jovens		1	0,04			P
Stygnidae	jovens		1	0,04			P
Pseudoscorpiones							
Bochicidae							
Bochicidae		sp.1			2		P
Chernetidae	jovens		2				P
Chthoniidae							
	Pseudochthonius	sp.1	2				E P
Olpiidae		sp.1	2				E
Diplopoda							
Glomeridesmida							
Glomeridesmidae		sp.1	1				E
Insecta							
Blattodea	jovens		1	0,04			
Blaberidae	jovens		1	0,04			P
Blattidae	jovens				1	0,0625	P
Coleoptera							
Scydmaenidae		sp.7	1				P
Collembola							
Arthropleona							
Entomobryoidea							
Cyphoderidae		sp.1			1		E
Entomobryidae		sp.1	1				E
Diptera							
Nematocera							
Culicidae							
	Culicini	sp.	1				P
Tipulidae							
	Tipulinae	sp.			1		P
Hemiptera							
Heteroptera							
aff. Pyrrhocoroidea							

Reduviidae	jovens			1		P
Reduviinae	sp.			3	0,25	P
Hymenoptera						
Apoidea	sp.2			1		E
Proctotrupoidea						
Diapriidae	sp.2			1		E
Vespoidea						
Formicidae						
Camponotus	*atriceps*	1				P
	sp.1			1		P
Crematogaster	sp.1	1		1		E
Gnamptogenys	*striatula*	1				P
Octostruma	sp.1	1				P
Pheidole	sp.1	1				E
Trachymyrmex	sp.1	1				E
Wasmania	*auropunctata*	1				E
Isoptera	sp.	1				E
Lepidoptera	jovens	1				E
Noctuoidea	sp.2	1		1		P
Noctuidae	sp.1	1	0,04			
	sp.2			1	0,0625	P
Orthoptera						
Ensifera	jovens	1	0,04			E
Phalangopsidae	jovens					
Paraclodes	sp.1			1	0,0625	P
Phalangopsis	sp.1	6	0,24	4	0,25	P
Psocoptera						
Psocomorpha	jovens			1		P
Chordata						
Mammalia						
Chiroptera						
Emballonuridae						
Peropteryx	*kappleri*	5	0,2			
	sp.			2	0,125	P
Phyllostomidae						
Glossophaginae	sp.	1	0,04	1	0,0625	P
